# KLF8: so different in ovarian and breast cancer

**DOI:** 10.18632/oncoscience.34

**Published:** 2014-04-30

**Authors:** Jihe Zhao

**Affiliations:** Burnett School of Biomedical Sciences, College of Medicine, University of Central Florida, Orlando, FL, USA.

At the time when KLF8 was cloned as a FAK-induced gene, nothing but a partial mRNA sequence of it was known. During the study into the role of KLF8 in FAK-promoted cell cycle progression [1], we noticed that KLF8 expression is upregulated in some human cancer cell lines particularly breast and ovarian cancer types as demonstrated by the Stanford University cDNA microarray database for the NCI-60 human cancer cell line panel. Meanwhile, we also obtained unpublished data showing that ectopic overexpression of KLF8 prolongs survival of various cell types in nutrient-deprived or over-consumed medium. These observations pointed to a potential role of KLF8 in cancer.

Can KLF8 transform cells? Indeed, when overexpressed, KLF8 transforms NIH3T3 cells, albeit partially, and cyclin D1 expression downstream of it plays an important role [2]. Does KLF8 transform human epithelial cells? After confirming the aberrant overexpression of KLF8 in a panel of human ovarian and breast cancer cell lines and patient tumor tissues [2, 3], we tested if KLF8 could transform non-tumorigenic human ovarian surface epithelial cell line T80 and mammary epithelial cell line MCF10A. Interestingly, the T80 stably overexpressing KLF8 became tumorigenic although the tumors are not disseminated [4]. The MCF10A overexpressing KLF8 alone, however, failed to form tumors [5], but unexpectedly underwent the epithelial to mesenchymal transition or EMT [3] along with the induction of cancer stem-like cell traits [5]. When KLF8 was knocked down from the MDA-MB-231 human breast cancer cells, the cells formed much less metastases [6].

Why does KLF8 regulate the two types of cancer differently? KLF8-target gene expression profiling offers some answers to the questions [Fig.1 and Supplemental Table 1 & 2]. Obviously, KLF8 targets one set of genes in T80 but a distinct set of genes in MCF10A. Interestingly, the cell cycle regulating genes associated with tumor growth such as cyclin D1 and USP44 are regulated by KLF8 in T80 [4] but not in MCF10A, whereas the EMT and metastasis associated genes such as E-cadherin, MMPs, and EPSTI1 are regulated by KLF8 in MCF10A [3, 7, 8] but not in T80 [4]. The differential gene regulation by KLF8 could be attributed to the histological difference between the origins of the two cell lines. T80 was derived from human ovarian surface epithelium- the origin of the vast majority of ovarian carcinomas- that is contiguous with peritoneal mesothelium, as is well known. In contrast, MCF10A was derived from the mammary glandular epithelium of a very different form. Indeed, there is a huge difference in gene expression pattern between T80 and MCF10A. For example, the E-cadherin expresses more than 200-times higher in MCF10A than in T80. It is unlikely that this difference is solely due to the different ways the two cell lines were established. More interestingly, some genes such as KLF4 shown here can be upregulated in T80 but downregulated in MCF10A by the same KLF8. This could serve as a clue as to why KLF17 inhibits EMT in one cancer type but promotes EMT instead in another cancer type. Therefore, just as the histological feature of an epithelium determines what type of cancer (squamous carcinoma or adenocarcinoma) it may form, it may as well determine how a transcription factor regulates its targets. Needless to say, expression pattern of targets (genes and microRNAs alike) of a transcription factor can likely change even in the same cell in response to a change in the cell's status of proliferation, migration, or interaction with the surrounding environment. The tissue- or cell-context dependent change in target expression pattern of a transcription factor may likely govern the physiological or pathological outcome it regulates.

**Fig 1:**
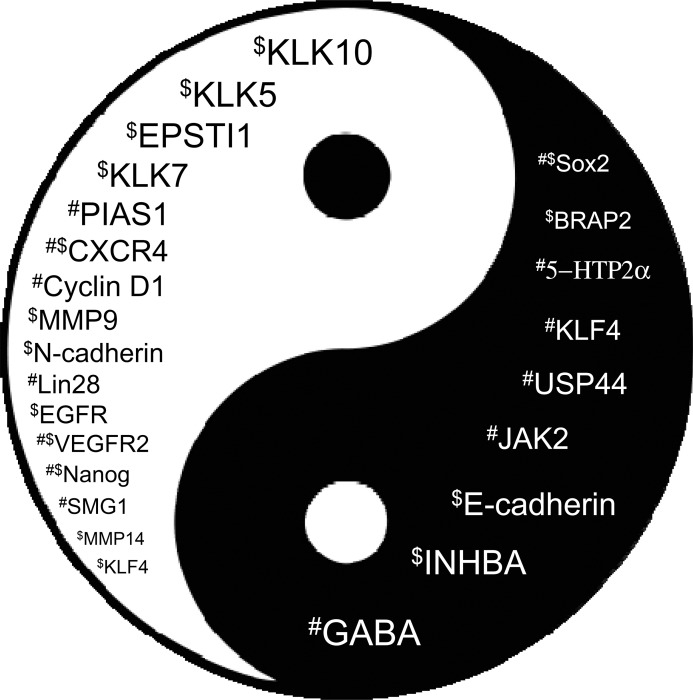
KLF8 regulated genes related to cancer Shown are significantly upregulated (white background on the left with KLK10 being the most highly upregulated) and downregulated (black background on the right with GABA being the most significantly downregulated) genes with an expression change > 2 and a P-value < .05. #, T80 cells over-expressing KLF8; $, MCF10A over-expressing KLF8. The cDNA array results were obtained from triplicate experiments using Human Genome U133 Plus 2.0 arrays (Affymetrix) and GeneSpring software (Silicon Genetics) (see Supplemental Table 1 & 2 for more details).

## SUPPLEMENTARY TABLES



## References

[1] Zhao J (2003). Mol Cell.

[2] Wang X (2007). Oncogene.

[3] Wang X (2007). Cancer Res.

[4] Lu H (2014). Oncogene.

[5] Wang X (2013). Am J Cancer Res.

[6] Wang X (2011). Oncogene.

[7] Li T (2013). Oncogene.

[8] Lu H (2013). Oncogene.

