# Supplementary material for: KLF8: so different in ovarian and breast cancer
Source: Oncoscience. 2014 Apr 30;1(4):248–9. doi: 10.18632/oncoscience.34 (PMC4256715; doi:10.18632/oncoscience.34)
Supplement: Supplementary file 1 [file oncoscience-01-0248-s001.pdf]

Transcript	Control Group Signal	Exp Group Signal	Est Log Ratio	Raw Est Fold Change	Affy PA Calls Cntl Grp-Exp Grp		Delta (PADE)	Set (PADE)	FDR for Accumulated Sets (PADE)	FDR for Specific Set (PADE)		Individual Transcript p-value
**Group Size**	**3.0**	**3.0**										
1562918_at	3.7	47.6	3.70	13.02	AAA-AAP		2.00	1	0.143	0.143		0.0014
243753_at	4.9	62.4	3.67	12.72	AAA-PAA		2.00	1	0.143	0.143		0.0005
240292_x_at	3.7	43.3	3.55	11.68	AAA-MPP		2.00	1	0.143	0.143		0.0000
205464_at	18.2	196.2	3.43	10.78	AAA-PMP		2.00	1	0.143	0.143		0.0002
1560962_at	3.5	27.4	2.95	7.74	AAA-AAP		2.00	1	0.143	0.143		0.0002
1569850_at	8.4	57.4	2.78	6.85	AAA-PAA		2.00	1	0.143	0.143		0.0005
1562576_at	12.8	74.1	2.53	5.77	AAA-PMA		2.00	1	0.143	0.143		0.0000
1556491_at	5.9	33.3	2.49	5.60	AAA-APA		2.00	1	0.143	0.143		0.0003
216280_s_at	11.7	64.6	2.47	5.54	AAA-APA		2.00	1	0.143	0.143		0.0010
242658_at	12.5	68.4	2.45	5.47	AAA-APP		2.00	1	0.143	0.143		0.0008
1562133_x_at	8.7	36.2	2.06	4.17	AAA-APP		2.00	1	0.143	0.143		0.0008
207781_s_at	22.2	79.4	1.84	3.58	AAA-PPP		2.00	1	0.143	0.143		0.0004
232324_x_at	18.5	56.0	1.60	3.03	AAA-PAP		2.00	1	0.143	0.143		0.0003
227949_at	387.2	958.1	1.31	2.47	PPP-PPP		2.00	1	0.143	0.143		0.0001
AFFX-LysX-3_at	5.5	29.5	2.42	5.36	AAA-AAP		1.80	2	0.167	0.500		0.0014
215474_at	6.9	83.7	3.59	12.05	AAA-AAP		1.60	3	0.182	0.214		0.0026
201374_x_at	8.9	65.9	2.89	7.40	AAA-APA		1.60	3	0.182	0.214		0.0023
205498_at	128.0	390.6	1.61	3.05	APP-PPP		1.60	3	0.182	0.214		0.0010
220317_at	75.4	218.3	1.53	2.89	PPA-PPP		1.60	3	0.182	0.214		0.0011
202869_at	164.1	416.5	1.34	2.54	PPP-PPP		1.60	3	0.182	0.214		0.0008
160020_at	372.5	901.5	1.29	2.42	PPP-PPP		1.60	3	0.182	0.214		0.0009
227475_at	909.9	2050.2	1.17	2.25	PPP-PPP		1.60	3	0.182	0.214		0.0006
201426_s_at	11166.8	25125.3	1.17	2.25	PPP-PPP		1.60	3	0.182	0.214		0.0027
203638_s_at	115.8	245.1	1.08	2.12	MPP-APP		1.60	3	0.182	0.214		0.0006
1557475_at	18.4	49.6	1.43	2.69	AAA-AAP		1.50	4	0.188	0.250		0.0013
47560_at	319.3	509.6	0.67	1.60	PPP-PPP		1.50	4	0.188	0.250		0.0001
243784_s_at	2.1	29.1	3.81	14.01	AAA-PAA		1.40	5	0.200	0.250		0.0042
225275_at	5.5	48.9	3.15	8.90	AAA-MPA		1.40	5	0.200	0.250		0.0039
1561969_at	23.6	74.6	1.66	3.16	AAA-PPP		1.40	5	0.200	0.250		0.0022
1560443_at	77.5	171.9	1.15	2.22	AAA-APM		1.40	5	0.200	0.250		0.0012
244462_at	52.9	103.1	0.96	1.95	PAP-PPP		1.40	5	0.200	0.250		0.0006
231830_x_at	247.2	386.4	0.64	1.56	PMP-PPP		1.40	5	0.200	0.250		0.0001
203936_s_at	103.0	159.6	0.63	1.55	PPM-PPP		1.40	5	0.200	0.250		0.0005
241770_x_at	10.1	60.5	2.59	6.01	AAM-PAP		1.20	6	0.211	0.250		0.0039
209613_s_at	15.5	65.7	2.08	4.23	AAA-MPA		1.20	6	0.211	0.250		0.0039
203139_at	222.2	511.5	1.20	2.30	PPP-PPP		1.20	6	0.211	0.250		0.0018
209909_s_at	74.9	157.6	1.07	2.10	PPP-PPP		1.20	6	0.211	0.250		0.0015
207408_at	105.9	210.8	0.99	1.99	AAA-PPM		1.20	6	0.211	0.250		0.0013

218806_s_at	313.0	556.3	0.83	1.78	PPP-PPP	1.20	6	0.211	0.250	0.0006
242696_at	199.4	349.7	0.81	1.75	APP-PPP	1.20	6	0.211	0.250	0.0005
227525_at	770.4	1292.6	0.75	1.68	PPP-PPP	1.20	6	0.211	0.250	0.0005
205778_at	11.9	177.1	3.90	14.88	AAA-AAP	1.10	7	0.218	0.235	0.0075
235183_at	2.3	23.8	3.36	10.29	AAA-PAA	1.10	7	0.218	0.235	0.0066
1560999_a_at	3.7	31.6	3.08	8.43	AAA-PAA	1.10	7	0.218	0.235	0.0066
1558743_at	3.7	29.8	3.02	8.13	AAA-AAP	1.10	7	0.218	0.235	0.0064
217065_at	8.1	57.5	2.82	7.06	AAA-APA	1.10	7	0.218	0.235	0.0057
232411_at	3.5	24.1	2.81	7.00	AAA-PAA	1.10	7	0.218	0.235	0.0064
1558859_at	20.6	88.1	2.10	4.28	AAA-PAM	1.10	7	0.218	0.235	0.0059
234081_at	9.7	37.5	1.95	3.88	AAA-APA	1.10	7	0.218	0.235	0.0052
219115_s_at	100.9	262.8	1.38	2.61	PPA-PPP	1.10	7	0.218	0.235	0.0033
1565762_at	51.6	133.7	1.37	2.59	AAP-PPP	1.10	7	0.218	0.235	0.0031
220167_s_at	68.7	165.6	1.27	2.41	AAA-PAM	1.10	7	0.218	0.235	0.0025
220586_at	50.8	106.8	1.07	2.10	PAA-PPP	1.10	7	0.218	0.235	0.0022
206880_at	77.8	155.0	0.99	1.99	AAA-APA	1.10	7	0.218	0.235	0.0025
230076_at	451.2	824.7	0.87	1.83	PPP-PPP	1.10	7	0.218	0.235	0.0014
228256_s_at	613.8	1074.0	0.81	1.75	PPP-PPP	1.10	7	0.218	0.235	0.0014
215446_s_at	751.9	1184.6	0.66	1.58	PPP-PPP	1.10	7	0.218	0.235	0.0007
223194_s_at	331.0	502.9	0.60	1.52	PPP-PPP	1.10	7	0.218	0.235	0.0004
244382_at	11.0	59.4	2.44	5.41	AAA-PAA	1.00	8	0.254	1.000	0.0076
206125_s_at	168.0	379.6	1.18	2.26	AAA-APP	1.00	8	0.254	1.000	0.0038
205350_at	6.4	48.2	2.93	7.59	AAA-PAA	0.90	9	0.265	0.318	0.0103
243245_at	14.7	67.4	2.20	4.59	AAA-APA	0.90	9	0.265	0.318	0.0074
244697_at	65.3	207.3	1.67	3.18	AAA-AAP	0.90	9	0.265	0.318	0.0061
225540_at	91.3	257.6	1.50	2.82	PMP-PPP	0.90	9	0.265	0.318	0.0067
221872_at	139.6	379.3	1.44	2.72	AAA-PPP	0.90	9	0.265	0.318	0.0062
202827_s_at	168.3	403.9	1.27	2.40	AMP-PMP	0.90	9	0.265	0.318	0.0052
233087_at	33.8	66.9	0.99	1.98	AAA-AAP	0.90	9	0.265	0.318	0.0037
226066_at	142.8	258.2	0.85	1.81	PPP-PPP	0.90	9	0.265	0.318	0.0029
1560493_a_at	54.6	96.1	0.82	1.76	PAM-MPP	0.90	9	0.265	0.318	0.0021
216933_x_at	55.0	92.0	0.74	1.67	PPP-APP	0.90	9	0.265	0.318	0.0018
238610_s_at	164.0	264.1	0.69	1.61	PPP-PPP	0.90	9	0.265	0.318	0.0015
232527_at	192.1	307.0	0.68	1.60	PPP-PPP	0.90	9	0.265	0.318	0.0014
204389_at	693.4	1619.0	1.22	2.34	PPP-PPP	0.80	10	0.307	1.000	0.0056
242671_at	55.7	103.4	0.89	1.86	AAA-APA	0.80	10	0.307	1.000	0.0035
209309_at	34.5	258.6	2.91	7.49	AAP-PPP	0.70	11	0.337	0.397	0.0133
234021_at	10.0	74.0	2.89	7.43	AAA-APA	0.70	11	0.337	0.397	0.0155
233799_at	6.2	43.7	2.81	7.01	AAA-APP	0.70	11	0.337	0.397	0.0115
1562406_at	4.5	29.5	2.72	6.60	AAA-AAP	0.70	11	0.337	0.397	0.0128
219645_at	8.0	52.2	2.71	6.52	AAA-AAP	0.70	11	0.337	0.397	0.0156
1559403_at	15.7	75.5	2.27	4.82	AAA-PAA	0.70	11	0.337	0.397	0.0126

222242_s_at	154.2	685.2	2.15	4.44	AAP-PPP	0.70	11	0.337	0.397	0.0143
244265_at	19.9	78.1	1.97	3.92	AAA-AAP	0.70	11	0.337	0.397	0.0105
221013_s_at	18.2	69.9	1.95	3.85	AAA-APA	0.70	11	0.337	0.397	0.0129
239381_at	139.0	523.3	1.91	3.76	APA-PPP	0.70	11	0.337	0.397	0.0113
242830_at	21.6	80.5	1.90	3.73	AAA-AAP	0.70	11	0.337	0.397	0.0131
239200_at	10.3	38.2	1.89	3.72	AAA-AAP	0.70	11	0.337	0.397	0.0124
224138_at	23.1	82.1	1.83	3.55	AAA-APA	0.70	11	0.337	0.397	0.0107
224369_s_at	647.4	2239.6	1.79	3.46	PPP-PPP	0.70	11	0.337	0.397	0.0096
237418_at	40.4	115.9	1.52	2.87	AAA-AAP	0.70	11	0.337	0.397	0.0096
210198_s_at	23.7	65.5	1.47	2.76	AAA-PAA	0.70	11	0.337	0.397	0.0093
208792_s_at	510.0	1329.0	1.38	2.61	PPP-PPP	0.70	11	0.337	0.397	0.0091
209324_s_at	76.3	179.2	1.23	2.35	AAM-PPP	0.70	11	0.337	0.397	0.0079
242077_x_at	190.3	399.1	1.07	2.10	PAA-PPP	0.70	11	0.337	0.397	0.0065
206315_at	81.0	168.5	1.06	2.08	AAA-APP	0.70	11	0.337	0.397	0.0076
209732_at	842.1	1671.3	0.99	1.99	PPP-PPP	0.70	11	0.337	0.397	0.0061
218394_at	173.4	312.8	0.85	1.80	PAP-PPP	0.70	11	0.337	0.397	0.0053
1558807_at	28.4	51.1	0.85	1.80	AAA-AMP	0.70	11	0.337	0.397	0.0034
227007_at	198.8	354.6	0.84	1.78	PAP-PPP	0.70	11	0.337	0.397	0.0042
244107_at	387.0	652.5	0.75	1.69	PPP-PPP	0.70	11	0.337	0.397	0.0032
1559362_at	52.8	88.9	0.75	1.68	PAA-PPP	0.70	11	0.337	0.397	0.0036
205832_at	347.3	578.3	0.74	1.67	PPP-PPP	0.70	11	0.337	0.397	0.0042
239300_at	271.7	433.3	0.67	1.60	PPP-PPP	0.70	11	0.337	0.397	0.0030
1554757_a_at	114.5	181.5	0.66	1.59	APP-PPP	0.70	11	0.337	0.397	0.0032
200921_s_at	4673.9	7229.1	0.63	1.55	PPP-PPP	0.70	11	0.337	0.397	0.0017
239600_at	92.5	139.5	0.59	1.51	PPP-PPP	0.70	11	0.337	0.397	0.0020
223324_s_at	327.0	479.2	0.55	1.47	PPP-PPP	0.70	11	0.337	0.397	0.0016
218261_at	1131.6	1607.5	0.51	1.42	APP-PPP	0.70	11	0.337	0.397	0.0006
222258_s_at	1487.4	2109.9	0.50	1.42	PPP-PPP	0.70	11	0.337	0.397	0.0011
209792_s_at	106.1	1921.8	4.18	18.11	APA-PPP	0.60	12	0.358	0.398	0.0204
1563396_x_at	3.8	35.2	3.20	9.18	AAA-APP	0.60	12	0.358	0.398	0.0224
1560066_at	3.3	25.1	2.95	7.73	AAA-PAA	0.60	12	0.358	0.398	0.0202
1554812_at	9.6	48.0	2.33	5.01	AAA-PAP	0.60	12	0.358	0.398	0.0196
1569365_a_at	14.4	57.7	2.00	4.01	AAA-AAP	0.60	12	0.358	0.398	0.0145
219360_s_at	31.3	110.8	1.83	3.54	AAA-AAP	0.60	12	0.358	0.398	0.0153
244677_at	64.5	221.2	1.78	3.43	APP-PPP	0.60	12	0.358	0.398	0.0149
240869_at	131.8	417.4	1.66	3.17	APP-PPP	0.60	12	0.358	0.398	0.0146
210261_at	42.9	117.2	1.45	2.73	AAA-PAM	0.60	12	0.358	0.398	0.0129
206392_s_at	77.5	190.1	1.30	2.45	AAA-PAP	0.60	12	0.358	0.398	0.0113
1561195_at	45.7	112.0	1.29	2.45	AAA-AAP	0.60	12	0.358	0.398	0.0148
215377_at	41.4	100.6	1.28	2.43	AAA-PAP	0.60	12	0.358	0.398	0.0152
238474_at	68.0	162.5	1.26	2.39	AAA-PAA	0.60	12	0.358	0.398	0.0111
1555578_at	21.8	51.4	1.23	2.35	AAA-APP	0.60	12	0.358	0.398	0.0151

208523_x_at	72.2	162.5	1.17	2.25	AAA-PPP	0.60	12	0.358	0.398	0.0113
242051_at	38.1	85.6	1.17	2.25	AAM-APP	0.60	12	0.358	0.398	0.0143
208791_at	620.6	1374.5	1.15	2.22	PPP-PPP	0.60	12	0.358	0.398	0.0090
1556436_at	38.2	82.2	1.11	2.15	AAA-AAP	0.60	12	0.358	0.398	0.0124
225645_at	503.2	1080.0	1.10	2.15	PPP-PPP	0.60	12	0.358	0.398	0.0125
211896_s_at	307.7	645.2	1.07	2.10	PPP-PPP	0.60	12	0.358	0.398	0.0105
217202_s_at	3368.8	6887.0	1.03	2.04	PPP-PPP	0.60	12	0.358	0.398	0.0080
244551_at	37.7	76.3	1.02	2.03	PAP-PPP	0.60	12	0.358	0.398	0.0080
219799_s_at	89.7	179.7	1.00	2.00	PAA-MAA	0.60	12	0.358	0.398	0.0106
234435_at	75.4	148.1	0.98	1.97	AAA-APA	0.60	12	0.358	0.398	0.0090
222301_at	62.2	118.3	0.93	1.90	AAP-PPA	0.60	12	0.358	0.398	0.0102
226044_at	154.3	292.4	0.92	1.90	PAP-APA	0.60	12	0.358	0.398	0.0087
1557252_at	57.1	107.1	0.91	1.88	AAP-PPP	0.60	12	0.358	0.398	0.0109
210239_at	632.8	1183.2	0.90	1.87	PPP-PPP	0.60	12	0.358	0.398	0.0093
1554264_at	96.9	176.3	0.86	1.82	PPP-PPP	0.60	12	0.358	0.398	0.0099
232997_at	82.1	149.0	0.86	1.82	PAP-PPP	0.60	12	0.358	0.398	0.0102
203889_at	94.9	171.0	0.85	1.80	APP-PPP	0.60	12	0.358	0.398	0.0100
1570366_x_at	30.9	55.2	0.84	1.79	AAP-MPP	0.60	12	0.358	0.398	0.0061
212741_at	3316.4	5879.1	0.83	1.77	PPP-PPP	0.60	12	0.358	0.398	0.0071
214782_at	83.9	146.4	0.80	1.75	AAM-PPP	0.60	12	0.358	0.398	0.0058
233270_x_at	107.5	184.3	0.78	1.72	PPP-PPP	0.60	12	0.358	0.398	0.0079
217637_at	113.6	194.7	0.78	1.71	PAP-PAP	0.60	12	0.358	0.398	0.0080
1559128_at	43.5	73.1	0.75	1.68	AAA-APP	0.60	12	0.358	0.398	0.0049
242726_at	93.4	153.7	0.72	1.65	PAP-PPP	0.60	12	0.358	0.398	0.0060
242146_at	127.0	206.1	0.70	1.62	PPP-PPP	0.60	12	0.358	0.398	0.0062
203866_at	115.0	184.3	0.68	1.60	AAA-PAA	0.60	12	0.358	0.398	0.0066
218122_s_at	267.9	427.4	0.67	1.60	PPP-PPP	0.60	12	0.358	0.398	0.0061
1557375_at	105.4	164.9	0.65	1.57	PPP-PPP	0.60	12	0.358	0.398	0.0043
211540_s_at	169.0	250.6	0.57	1.48	PPP-PPP	0.60	12	0.358	0.398	0.0041
1556090_at	37.4	55.3	0.57	1.48	AAA-PPA	0.60	12	0.358	0.398	0.0038
1557217_a_at	58.4	85.2	0.54	1.46	PPM-PPP	0.60	12	0.358	0.398	0.0034
209794_at	703.9	1018.1	0.53	1.45	PPP-PPP	0.60	12	0.358	0.398	0.0026
203789_s_at	2896.9	4155.8	0.52	1.44	PPP-PPP	0.60	12	0.358	0.398	0.0021
239830_at	87.2	124.0	0.51	1.42	PPP-PPP	0.60	12	0.358	0.398	0.0020
221085_at	56.0	79.0	0.50	1.41	PPM-PPP	0.60	12	0.358	0.398	0.0023
201598_s_at	362.2	508.4	0.49	1.40	PPP-PPP	0.60	12	0.358	0.398	0.0017
224797_at	522.0	728.0	0.48	1.40	PPP-PPP	0.60	12	0.358	0.398	0.0020
239379_at	129.5	175.3	0.44	1.35	PPP-PPP	0.60	12	0.358	0.398	0.0013
2028_s_at	269.9	358.4	0.41	1.33	PPP-PPP	0.60	12	0.358	0.398	0.0006
213895_at	860.2	1120.4	0.38	1.30	PPP-PPP	0.60	12	0.358	0.398	0.0005
1554657_a_at	8.0	69.7	3.13	8.74	AAA-AAP	0.50	13	0.384	0.431	0.0263
207731_at	8.1	69.0	3.09	8.53	AAA-APA	0.50	13	0.384	0.431	0.0344

221212_x_at	7.3	50.8	2.80	6.96	AAA-APA	0.50	13	0.384	0.431	0.0313
216258_s_at	7.3	43.3	2.58	5.98	AAA-AAP	0.50	13	0.384	0.431	0.0275
231961_at	13.4	77.9	2.54	5.79	AAA-AMP	0.50	13	0.384	0.431	0.0236
233284_at	9.1	49.7	2.46	5.49	AAA-APA	0.50	13	0.384	0.431	0.0288
244840_x_at	12.0	63.8	2.41	5.30	AAA-MPA	0.50	13	0.384	0.431	0.0312
227243_s_at	10.5	55.3	2.41	5.30	AAA-APA	0.50	13	0.384	0.431	0.0319
1556160_a_at	5.9	30.3	2.36	5.12	AAA-PAA	0.50	13	0.384	0.431	0.0339
238492_at	13.0	56.0	2.10	4.30	AAA-AAP	0.50	13	0.384	0.431	0.0296
219534_x_at	79.1	298.4	1.92	3.77	AAA-AAP	0.50	13	0.384	0.431	0.0250
1556136_at	13.9	47.0	1.76	3.39	AAA-AAP	0.50	13	0.384	0.431	0.0259
240206_at	22.7	69.2	1.61	3.05	AAA-PPP	0.50	13	0.384	0.431	0.0250
232152_at	23.8	62.0	1.38	2.61	AAA-PAP	0.50	13	0.384	0.431	0.0176
229996_s_at	32.5	82.5	1.34	2.54	AAA-PAP	0.50	13	0.384	0.431	0.0248
219995_s_at	74.0	174.0	1.23	2.35	APP-PPP	0.50	13	0.384	0.431	0.0237
235629_at	22.6	51.4	1.18	2.27	AAA-APP	0.50	13	0.384	0.431	0.0203
239928_at	43.7	97.5	1.16	2.23	PAA-AAA	0.50	13	0.384	0.431	0.0227
209656_s_at	84.6	186.5	1.14	2.20	PAP-PPP	0.50	13	0.384	0.431	0.0232
238607_at	86.0	186.8	1.12	2.17	AAA-APP	0.50	13	0.384	0.431	0.0220
241756_at	202.3	434.4	1.10	2.15	PPP-PPP	0.50	13	0.384	0.431	0.0189
241704_x_at	103.3	219.4	1.09	2.12	PPP-PPP	0.50	13	0.384	0.431	0.0179
208154_at	33.6	69.2	1.04	2.06	AAP-APA	0.50	13	0.384	0.431	0.0214
202388_at	1601.9	3295.3	1.04	2.06	PPP-PPP	0.50	13	0.384	0.431	0.0216
1558410_s_at	87.3	178.2	1.03	2.04	PPP-PPP	0.50	13	0.384	0.431	0.0142
205960_at	133.6	272.3	1.03	2.04	PPP-PPP	0.50	13	0.384	0.431	0.0143
1566294_at	21.4	43.5	1.03	2.04	AAA-AAP	0.50	13	0.384	0.431	0.0188
1559171_at	54.3	110.1	1.02	2.03	AAA-PPA	0.50	13	0.384	0.431	0.0149
204650_s_at	110.5	223.8	1.02	2.03	PAP-PPP	0.50	13	0.384	0.431	0.0160
213948_x_at	89.2	180.4	1.02	2.02	APA-PPP	0.50	13	0.384	0.431	0.0209
210469_at	94.5	189.6	1.01	2.01	AAA-PPP	0.50	13	0.384	0.431	0.0149
206932_at	42.0	84.1	1.00	2.00	AAA-AAP	0.50	13	0.384	0.431	0.0210
223468_s_at	157.9	315.6	1.00	2.00	AAA-PPP	0.50	13	0.384	0.431	0.0156
238569_at	84.2	167.2	0.99	1.99	AAA-PPP	0.50	13	0.384	0.431	0.0184
239633_at	58.2	112.9	0.96	1.94	AAP-MAP	0.50	13	0.384	0.431	0.0129
205608_s_at	98.6	185.8	0.92	1.89	MPP-PPP	0.50	13	0.384	0.431	0.0170
232034_at	60.4	113.8	0.92	1.89	PAA-AAP	0.50	13	0.384	0.431	0.0118
202936_s_at	309.9	582.5	0.91	1.88	PPP-PPP	0.50	13	0.384	0.431	0.0155
241685_x_at	29.0	54.3	0.90	1.87	AAP-PMP	0.50	13	0.384	0.431	0.0171
217889_s_at	351.4	652.5	0.89	1.86	PPP-PPP	0.50	13	0.384	0.431	0.0118
215587_x_at	98.7	182.4	0.89	1.85	AAA-PAP	0.50	13	0.384	0.431	0.0141
244489_at	40.2	73.2	0.86	1.82	AAA-MPA	0.50	13	0.384	0.431	0.0177
206548_at	80.4	144.7	0.85	1.80	PAA-PPP	0.50	13	0.384	0.431	0.0158
222853_at	1821.8	3271.9	0.85	1.80	PPP-PPP	0.50	13	0.384	0.431	0.0117

230077_at	175.9	306.7	0.80	1.74	PPP-PPP	0.50	13	0.384	0.431	0.0124
200648_s_at	5223.8	9080.2	0.80	1.74	PPP-PPP	0.50	13	0.384	0.431	0.0158
1563162_at	27.0	46.1	0.77	1.71	AAA-PPP	0.50	13	0.384	0.431	0.0130
226411_at	122.3	208.7	0.77	1.71	AAA-MPP	0.50	13	0.384	0.431	0.0106
238029_s_at	239.3	407.8	0.77	1.70	PPP-PPP	0.50	13	0.384	0.431	0.0121
227987_at	329.0	560.4	0.77	1.70	PPP-PPP	0.50	13	0.384	0.431	0.0149
241436_at	918.2	1555.8	0.76	1.69	PPP-PPP	0.50	13	0.384	0.431	0.0155
241036_at	62.9	105.6	0.75	1.68	APP-PAP	0.50	13	0.384	0.431	0.0097
227170_at	108.5	182.0	0.75	1.68	APM-APP	0.50	13	0.384	0.431	0.0133
211721_s_at	241.2	403.7	0.74	1.67	PPP-PPP	0.50	13	0.384	0.431	0.0123
202489_s_at	7413.6	12325.4	0.73	1.66	PPP-PPP	0.50	13	0.384	0.431	0.0102
202970_at	143.5	237.5	0.73	1.66	PPP-PPP	0.50	13	0.384	0.431	0.0119
244358_at	78.5	129.5	0.72	1.65	AAA-APP	0.50	13	0.384	0.431	0.0131
215577_at	109.0	178.3	0.71	1.64	PPP-PPP	0.50	13	0.384	0.431	0.0102
212136_at	493.3	801.3	0.70	1.62	PPP-PPP	0.50	13	0.384	0.431	0.0087
203961_at	1217.3	1976.0	0.70	1.62	PPP-PPP	0.50	13	0.384	0.431	0.0073
235258_at	67.9	106.0	0.64	1.56	APP-PPP	0.50	13	0.384	0.431	0.0066
1570165_at	59.6	91.8	0.63	1.54	PPP-PPP	0.50	13	0.384	0.431	0.0108
241706_at	315.6	486.0	0.62	1.54	PAP-PPP	0.50	13	0.384	0.431	0.0110
242554_at	174.9	268.6	0.62	1.54	PPP-PPP	0.50	13	0.384	0.431	0.0103
200831_s_at	3157.2	4841.8	0.62	1.53	PPP-PPP	0.50	13	0.384	0.431	0.0059
212567_s_at	294.2	448.8	0.61	1.53	PPP-PPP	0.50	13	0.384	0.431	0.0084
204691_x_at	236.1	359.9	0.61	1.52	PAP-PPP	0.50	13	0.384	0.431	0.0083
203962_s_at	1703.4	2588.3	0.60	1.52	PPP-PPP	0.50	13	0.384	0.431	0.0098
213558_at	162.5	245.7	0.60	1.51	PPP-PPP	0.50	13	0.384	0.431	0.0084
226675_s_at	1906.9	2834.0	0.57	1.49	PPP-PPP	0.50	13	0.384	0.431	0.0094
235620_x_at	127.8	187.7	0.56	1.47	PPP-PPP	0.50	13	0.384	0.431	0.0080
215551_at	78.7	115.4	0.55	1.47	AAA-APA	0.50	13	0.384	0.431	0.0045
211631_x_at	125.8	182.2	0.53	1.45	PAP-PPP	0.50	13	0.384	0.431	0.0063
230270_at	301.1	435.5	0.53	1.45	PPP-PPP	0.50	13	0.384	0.431	0.0050
222157_s_at	309.0	444.4	0.52	1.44	PPP-PPP	0.50	13	0.384	0.431	0.0063
228854_at	2724.3	3880.0	0.51	1.42	PPP-PPP	0.50	13	0.384	0.431	0.0036
229101_at	525.8	739.3	0.49	1.41	PPP-PPP	0.50	13	0.384	0.431	0.0051
203092_at	655.0	892.1	0.45	1.36	PPP-PPP	0.50	13	0.384	0.431	0.0045
222749_at	158.5	215.3	0.44	1.36	MPP-APP	0.50	13	0.384	0.431	0.0028
211916_s_at	78.4	106.5	0.44	1.36	AMA-APP	0.50	13	0.384	0.431	0.0027
209185_s_at	3681.7	4993.7	0.44	1.36	PPP-PPP	0.50	13	0.384	0.431	0.0040
204871_at	370.0	501.6	0.44	1.36	PAP-APA	0.50	13	0.384	0.431	0.0018
219027_s_at	498.4	667.7	0.42	1.34	PPP-PPP	0.50	13	0.384	0.431	0.0024
233613_x_at	119.9	159.7	0.41	1.33	PPP-PPP	0.50	13	0.384	0.431	0.0034
228834_at	1712.4	2263.5	0.40	1.32	PPP-PPP	0.50	13	0.384	0.431	0.0012
204602_at	8684.8	11447.6	0.40	1.32	PPP-PPP	0.50	13	0.384	0.431	0.0014

1552931_a_at	363.1	473.2	0.38	1.30	PPP-PPP	0.50	13	0.384	0.431	0.0012
225016_at	6.2	71.7	3.54	11.63	AAA-APM	0.40	14	0.446	0.490	0.0497
240876_x_at	2.8	22.9	3.01	8.07	AAA-APA	0.40	14	0.446	0.490	0.0553
213240_s_at	14.3	115.2	3.01	8.06	AAA-AAP	0.40	14	0.446	0.490	0.0414
241340_at	5.6	42.8	2.93	7.59	AAA-APA	0.40	14	0.446	0.490	0.0449
1562463_at	9.2	66.9	2.86	7.24	AAA-PAP	0.40	14	0.446	0.490	0.0635
242047_at	3.3	22.7	2.80	6.98	AAA-AAP	0.40	14	0.446	0.490	0.0484
244586_x_at	4.9	34.2	2.80	6.96	AAA-AAP	0.40	14	0.446	0.490	0.0365
232916_at	11.6	79.2	2.77	6.80	AAA-APP	0.40	14	0.446	0.490	0.0402
1555001_at	10.3	66.7	2.70	6.49	AAA-PPA	0.40	14	0.446	0.490	0.0493
217014_s_at	29.7	191.0	2.69	6.43	AAA-PPA	0.40	14	0.446	0.490	0.0416
240630_at	18.7	112.5	2.59	6.03	AAA-AAP	0.40	14	0.446	0.490	0.0476
1565685_at	3.1	17.8	2.50	5.67	AAA-APA	0.40	14	0.446	0.490	0.0434
239714_at	11.9	64.6	2.44	5.41	AAA-PAA	0.40	14	0.446	0.490	0.0554
1563595_at	19.8	105.5	2.42	5.35	AAA-AAP	0.40	14	0.446	0.490	0.0494
241832_at	13.5	71.7	2.41	5.31	AAA-APA	0.40	14	0.446	0.490	0.0611
221625_at	7.6	40.0	2.40	5.28	AAA-AAP	0.40	14	0.446	0.490	0.0596
231588_at	9.2	47.1	2.36	5.12	AAA-PAA	0.40	14	0.446	0.490	0.0696
241287_x_at	6.7	34.0	2.35	5.11	AAA-PAA	0.40	14	0.446	0.490	0.0484
1560903_at	5.6	28.2	2.32	5.00	AAA-APA	0.40	14	0.446	0.490	0.0543
234259_at	4.6	22.5	2.28	4.85	AAA-PAA	0.40	14	0.446	0.490	0.0514
214362_at	18.7	90.8	2.28	4.85	AAA-PAA	0.40	14	0.446	0.490	0.0451
213001_at	22.8	106.3	2.22	4.66	AAA-PAP	0.40	14	0.446	0.490	0.0645
237955_at	7.0	32.5	2.21	4.61	AAA-PAA	0.40	14	0.446	0.490	0.0427
243059_at	12.0	55.2	2.20	4.60	AAA-AAP	0.40	14	0.446	0.490	0.0447
234678_at	11.4	51.8	2.18	4.55	AAA-AAP	0.40	14	0.446	0.490	0.0350
1555123_at	10.2	42.7	2.07	4.19	AAA-APA	0.40	14	0.446	0.490	0.0571
215078_at	8.2	32.5	1.99	3.96	AAA-PAA	0.40	14	0.446	0.490	0.0578
1569834_at	45.6	179.1	1.97	3.92	AAA-PAA	0.40	14	0.446	0.490	0.0372
243310_at	26.3	103.1	1.97	3.92	AAA-AAP	0.40	14	0.446	0.490	0.0479
205625_s_at	664.8	2560.3	1.95	3.85	PPP-PPP	0.40	14	0.446	0.490	0.0508
235739_at	28.0	107.3	1.94	3.83	AAA-APM	0.40	14	0.446	0.490	0.0573
220247_at	5.0	18.8	1.92	3.79	AAA-AAP	0.40	14	0.446	0.490	0.0389
205511_at	25.1	94.9	1.92	3.77	AAA-PPP	0.40	14	0.446	0.490	0.0348
243909_x_at	25.1	94.5	1.91	3.77	AAA-MPA	0.40	14	0.446	0.490	0.0653
243839_s_at	13.3	47.8	1.85	3.59	AAA-APA	0.40	14	0.446	0.490	0.0345
206311_s_at	17.0	61.2	1.84	3.59	AAA-PPA	0.40	14	0.446	0.490	0.0456
210364_at	23.6	84.3	1.84	3.57	AAA-PAA	0.40	14	0.446	0.490	0.0404
239702_x_at	35.0	124.1	1.83	3.55	AAA-APA	0.40	14	0.446	0.490	0.0486
244873_s_at	10.7	37.7	1.82	3.52	AAA-AAP	0.40	14	0.446	0.490	0.0461
239653_at	60.0	210.1	1.81	3.50	AAA-AAP	0.40	14	0.446	0.490	0.0505
1564760_at	22.0	76.1	1.79	3.46	AAA-APP	0.40	14	0.446	0.490	0.0516

1554844_at	12.9	44.2	1.78	3.44	AAA-PAA	0.40	14	0.446	0.490	0.0443
230348_at	25.6	82.4	1.69	3.22	AAA-PAA	0.40	14	0.446	0.490	0.0557
1561300_at	12.6	40.6	1.68	3.21	AAA-AAP	0.40	14	0.446	0.490	0.0304
1560342_at	23.1	73.9	1.68	3.21	AAA-AAP	0.40	14	0.446	0.490	0.0524
1559285_at	11.9	37.5	1.66	3.15	AAA-PPA	0.40	14	0.446	0.490	0.0526
228295_at	48.4	151.2	1.64	3.12	PAA-PPP	0.40	14	0.446	0.490	0.0611
222655_s_at	27.3	83.0	1.61	3.05	AAA-APA	0.40	14	0.446	0.490	0.0621
233599_at	50.0	149.1	1.58	2.98	AAA-AAP	0.40	14	0.446	0.490	0.0432
233081_at	24.5	72.7	1.57	2.97	AAM-APA	0.40	14	0.446	0.490	0.0459
1554310_a_at	47.4	140.5	1.57	2.96	PAA-AAA	0.40	14	0.446	0.490	0.0490
212841_s_at	103.6	305.1	1.56	2.95	AAA-MAP	0.40	14	0.446	0.490	0.0617
243658_at	16.8	46.4	1.46	2.76	AAA-APA	0.40	14	0.446	0.490	0.0574
232705_at	22.8	61.4	1.43	2.69	AAA-PAA	0.40	14	0.446	0.490	0.0281
217028_at	151.9	401.8	1.40	2.65	PPP-PPP	0.40	14	0.446	0.490	0.0292
233417_at	48.2	125.7	1.38	2.61	AAA-PAP	0.40	14	0.446	0.490	0.0419
234731_at	84.9	220.6	1.38	2.60	APP-PPA	0.40	14	0.446	0.490	0.0511
233222_at	35.3	88.8	1.33	2.52	AAA-PAP	0.40	14	0.446	0.490	0.0474
221155_x_at	49.9	125.1	1.33	2.51	AAA-AAP	0.40	14	0.446	0.490	0.0377
234628_at	33.8	84.5	1.32	2.50	AAA-APP	0.40	14	0.446	0.490	0.0452
243586_at	34.4	84.8	1.30	2.46	AAA-APP	0.40	14	0.446	0.490	0.0394
244674_at	69.9	171.9	1.30	2.46	APP-PPP	0.40	14	0.446	0.490	0.0482
214982_at	24.7	60.7	1.30	2.45	AAA-PAM	0.40	14	0.446	0.490	0.0523
1561529_at	17.4	42.7	1.29	2.45	AAP-AAM	0.40	14	0.446	0.490	0.0511
235658_at	26.1	63.7	1.29	2.44	AAA-PPP	0.40	14	0.446	0.490	0.0436
243327_at	34.5	83.3	1.27	2.42	AAM-PPP	0.40	14	0.446	0.490	0.0340
210809_s_at	47.5	113.8	1.26	2.40	AAA-PPM	0.40	14	0.446	0.490	0.0534
220158_at	42.4	101.4	1.26	2.39	AAA-AAP	0.40	14	0.446	0.490	0.0289
240174_at	23.9	57.0	1.26	2.39	PAA-PAP	0.40	14	0.446	0.490	0.0283
210096_at	61.1	145.3	1.25	2.38	PAP-PPP	0.40	14	0.446	0.490	0.0305
229385_s_at	285.3	673.8	1.24	2.36	PPP-PPP	0.40	14	0.446	0.490	0.0419
242203_at	36.8	86.8	1.24	2.36	AAA-AAP	0.40	14	0.446	0.490	0.0467
204351_at	4096.5	9646.0	1.24	2.36	PPP-PPP	0.40	14	0.446	0.490	0.0279
239487_at	28.1	66.0	1.23	2.35	AAA-MPP	0.40	14	0.446	0.490	0.0325
241751_at	76.3	176.9	1.21	2.32	PPP-PPP	0.40	14	0.446	0.490	0.0288
211813_x_at	282.1	651.6	1.21	2.31	PPP-PPP	0.40	14	0.446	0.490	0.0339
1559731_x_at	51.8	118.2	1.19	2.28	AAA-PAP	0.40	14	0.446	0.490	0.0358
1569241_a_at	28.1	62.4	1.15	2.22	AAA-PAP	0.40	14	0.446	0.490	0.0245
224964_s_at	27.5	60.7	1.15	2.21	PAA-AAM	0.40	14	0.446	0.490	0.0534
228758_at	271.9	593.6	1.13	2.18	PPP-PPP	0.40	14	0.446	0.490	0.0399
230768_at	45.7	99.0	1.12	2.17	AAA-PPP	0.40	14	0.446	0.490	0.0534
215342_s_at	52.9	114.6	1.12	2.17	PAP-PAP	0.40	14	0.446	0.490	0.0310
212172_at	84.9	183.5	1.11	2.16	PPP-PPP	0.40	14	0.446	0.490	0.0240

209800_at	343.4	740.2	1.11	2.16	PPP-PPP	0.40	14	0.446	0.490	0.0446
241990_at	85.4	182.8	1.10	2.14	AAA-APA	0.40	14	0.446	0.490	0.0288
214605_x_at	96.9	205.2	1.08	2.12	APP-PPP	0.40	14	0.446	0.490	0.0318
241875_at	21.2	44.8	1.08	2.11	AAA-PAA	0.40	14	0.446	0.490	0.0290
241788_x_at	86.3	182.2	1.08	2.11	AAA-MAP	0.40	14	0.446	0.490	0.0342
243708_at	39.1	81.9	1.07	2.09	AAA-PAA	0.40	14	0.446	0.490	0.0470
224696_s_at	157.8	328.6	1.06	2.08	PPM-PPP	0.40	14	0.446	0.490	0.0392
214748_at	60.1	125.1	1.06	2.08	APP-PPP	0.40	14	0.446	0.490	0.0440
210360_s_at	53.2	110.3	1.05	2.07	PMA-PPP	0.40	14	0.446	0.490	0.0279
238611_at	82.1	169.0	1.04	2.06	PPP-PPP	0.40	14	0.446	0.490	0.0392
242281_at	228.2	467.5	1.03	2.05	PPP-PPP	0.40	14	0.446	0.490	0.0259
1561324_at	33.8	69.0	1.03	2.04	AAA-APA	0.40	14	0.446	0.490	0.0332
231038_s_at	37.4	76.1	1.03	2.03	AAA-PAP	0.40	14	0.446	0.490	0.0399
235138_at	156.2	316.6	1.02	2.03	PPP-PPP	0.40	14	0.446	0.490	0.0310
241745_at	15.3	31.0	1.02	2.02	AAA-PAA	0.40	14	0.446	0.490	0.0372
225706_at	374.2	754.6	1.01	2.02	PPP-PPP	0.40	14	0.446	0.490	0.0373
241944_x_at	44.8	89.9	1.01	2.01	AAA-AAP	0.40	14	0.446	0.490	0.0316
225207_at	961.7	1925.1	1.00	2.00	PPP-PPP	0.40	14	0.446	0.490	0.0382
219250_s_at	1128.7	2244.8	0.99	1.99	PPP-PPP	0.40	14	0.446	0.490	0.0388
1560069_at	44.3	88.1	0.99	1.99	MAP-MPA	0.40	14	0.446	0.490	0.0258
205319_at	177.3	352.4	0.99	1.99	PPP-PPP	0.40	14	0.446	0.490	0.0493
214688_at	40.2	79.7	0.99	1.98	APP-APP	0.40	14	0.446	0.490	0.0460
234011_at	54.3	106.7	0.98	1.97	AAM-PPP	0.40	14	0.446	0.490	0.0361
242325_at	37.1	72.9	0.98	1.97	AAA-MPA	0.40	14	0.446	0.490	0.0328
1555497_a_at	128.1	249.2	0.96	1.95	AAA-AMP	0.40	14	0.446	0.490	0.0488
209839_at	43.1	83.5	0.96	1.94	AAA-APP	0.40	14	0.446	0.490	0.0458
233152_x_at	40.8	79.0	0.95	1.94	AAA-APP	0.40	14	0.446	0.490	0.0317
244341_at	153.6	296.6	0.95	1.93	PPP-PPP	0.40	14	0.446	0.490	0.0281
225250_at	57.1	109.3	0.94	1.92	AAA-PPP	0.40	14	0.446	0.490	0.0476
1566507_a_at	57.4	109.4	0.93	1.91	AAA-PPP	0.40	14	0.446	0.490	0.0244
1569577_x_at	20.0	38.1	0.93	1.91	AAA-PAA	0.40	14	0.446	0.490	0.0442
228278_at	128.5	244.7	0.93	1.91	PAP-PPM	0.40	14	0.446	0.490	0.0364
217461_x_at	178.3	339.3	0.93	1.90	MPP-PPP	0.40	14	0.446	0.490	0.0280
204449_at	348.8	661.0	0.92	1.90	PPP-PPP	0.40	14	0.446	0.490	0.0364
229795_at	65.8	124.5	0.92	1.89	AAA-APP	0.40	14	0.446	0.490	0.0229
204437_s_at	153.8	290.8	0.92	1.89	AAA-APP	0.40	14	0.446	0.490	0.0447
1566764_at	40.5	76.5	0.92	1.89	APA-PAP	0.40	14	0.446	0.490	0.0423
244347_at	82.1	155.1	0.92	1.89	PAP-PPP	0.40	14	0.446	0.490	0.0243
236610_at	48.4	91.3	0.92	1.89	PMA-AAP	0.40	14	0.446	0.490	0.0358
244502_at	96.7	180.9	0.90	1.87	MPP-PPP	0.40	14	0.446	0.490	0.0270
215992_s_at	98.2	183.3	0.90	1.87	APM-PAP	0.40	14	0.446	0.490	0.0314
238894_at	168.8	314.3	0.90	1.86	AAA-AAP	0.40	14	0.446	0.490	0.0402

1553216_at	44.5	82.7	0.89	1.86	AAA-APP	0.40	14	0.446	0.490	0.0286
204388_s_at	1680.5	3103.4	0.89	1.85	PPP-PPP	0.40	14	0.446	0.490	0.0257
238888_at	66.3	122.1	0.88	1.84	MPA-PAP	0.40	14	0.446	0.490	0.0286
224340_at	61.2	112.0	0.87	1.83	APA-MPP	0.40	14	0.446	0.490	0.0330
232635_at	114.8	209.6	0.87	1.83	MMP-PMP	0.40	14	0.446	0.490	0.0262
213402_at	178.2	325.2	0.87	1.83	APP-PPP	0.40	14	0.446	0.490	0.0423
221748_s_at	69.4	126.4	0.87	1.82	APP-PPP	0.40	14	0.446	0.490	0.0376
241860_at	17.0	30.9	0.86	1.82	AAA-AAP	0.40	14	0.446	0.490	0.0199
238134_at	71.4	130.0	0.86	1.82	PPP-PPP	0.40	14	0.446	0.490	0.0321
1566482_at	35.5	64.5	0.86	1.82	AAA-APP	0.40	14	0.446	0.490	0.0272
233587_s_at	146.8	266.7	0.86	1.82	PPP-PPP	0.40	14	0.446	0.490	0.0439
203913_s_at	270.6	489.6	0.86	1.81	PPP-PPP	0.40	14	0.446	0.490	0.0194
236966_at	52.1	94.1	0.85	1.81	AAA-AAP	0.40	14	0.446	0.490	0.0376
225123_at	57.6	104.0	0.85	1.81	APA-PPP	0.40	14	0.446	0.490	0.0338
236166_at	36.2	65.2	0.85	1.80	APA-APP	0.40	14	0.446	0.490	0.0298
209106_at	125.8	226.4	0.85	1.80	PPP-PPP	0.40	14	0.446	0.490	0.0276
203140_at	1926.9	3468.3	0.85	1.80	PPP-PPP	0.40	14	0.446	0.490	0.0211
240314_at	82.1	147.6	0.85	1.80	PAP-PPP	0.40	14	0.446	0.490	0.0417
204235_s_at	84.6	151.2	0.84	1.79	PPP-PPP	0.40	14	0.446	0.490	0.0416
201893_x_at	533.4	952.3	0.84	1.79	PPP-PPP	0.40	14	0.446	0.490	0.0276
214185_at	65.7	117.0	0.83	1.78	PAA-AAA	0.40	14	0.446	0.490	0.0331
219934_s_at	96.6	171.8	0.83	1.78	PPP-PPP	0.40	14	0.446	0.490	0.0359
206704_at	43.0	76.2	0.83	1.77	AAP-PPP	0.40	14	0.446	0.490	0.0409
229092_at	62.0	109.2	0.82	1.76	PPP-PPP	0.40	14	0.446	0.490	0.0427
209184_s_at	641.0	1129.5	0.82	1.76	PPP-PPP	0.40	14	0.446	0.490	0.0410
213430_at	43.0	75.6	0.81	1.76	AAA-AAP	0.40	14	0.446	0.490	0.0317
243512_x_at	39.3	68.9	0.81	1.75	PAP-PPP	0.40	14	0.446	0.490	0.0389
221537_at	276.7	484.7	0.81	1.75	PPP-PPP	0.40	14	0.446	0.490	0.0410
233068_at	130.5	228.5	0.81	1.75	PAP-PPP	0.40	14	0.446	0.490	0.0423
241885_at	68.4	119.8	0.81	1.75	PPP-PPP	0.40	14	0.446	0.490	0.0210
224724_at	1193.7	2084.2	0.80	1.75	PPP-PPP	0.40	14	0.446	0.490	0.0313
225728_at	74.9	130.3	0.80	1.74	PAP-PPP	0.40	14	0.446	0.490	0.0428
205609_at	73.3	127.3	0.80	1.74	AAA-PPP	0.40	14	0.446	0.490	0.0270
222341_x_at	48.4	83.8	0.79	1.73	MAP-PPA	0.40	14	0.446	0.490	0.0390
222120_at	79.3	137.4	0.79	1.73	APA-PPP	0.40	14	0.446	0.490	0.0178
209006_s_at	298.4	516.8	0.79	1.73	PPP-PPP	0.40	14	0.446	0.490	0.0363
225955_at	277.4	480.3	0.79	1.73	PPP-PPP	0.40	14	0.446	0.490	0.0201
243993_at	65.7	112.9	0.78	1.72	APP-PPP	0.40	14	0.446	0.490	0.0267
1558304_s_at	668.7	1143.3	0.77	1.71	PMP-PPP	0.40	14	0.446	0.490	0.0286
216628_at	52.8	90.1	0.77	1.71	AAA-AMP	0.40	14	0.446	0.490	0.0347
231952_at	77.8	132.9	0.77	1.71	AAA-AMP	0.40	14	0.446	0.490	0.0220
230769_at	292.8	499.4	0.77	1.71	PPP-PPP	0.40	14	0.446	0.490	0.0232

1557521_a_at	197.2	336.1	0.77	1.70	PPP-PPP	0.40	14	0.446	0.490	0.0205
1568850_at	49.1	83.3	0.76	1.70	AAA-APA	0.40	14	0.446	0.490	0.0259
240451_at	68.5	116.2	0.76	1.70	PPP-PPP	0.40	14	0.446	0.490	0.0185
1566760_at	61.3	103.8	0.76	1.69	AAA-APP	0.40	14	0.446	0.490	0.0163
239227_at	132.0	223.5	0.76	1.69	PPP-PPP	0.40	14	0.446	0.490	0.0290
234562_x_at	83.6	141.4	0.76	1.69	PAP-PPP	0.40	14	0.446	0.490	0.0336
215694_at	50.8	86.0	0.76	1.69	PPA-PPP	0.40	14	0.446	0.490	0.0274
223168_at	202.3	342.0	0.76	1.69	PMP-PPP	0.40	14	0.446	0.490	0.0280
224566_at	1827.7	3084.3	0.76	1.69	PPP-PPP	0.40	14	0.446	0.490	0.0263
207980_s_at	533.8	899.4	0.75	1.69	PPP-PPP	0.40	14	0.446	0.490	0.0357
233007_at	55.7	93.7	0.75	1.68	AAA-APP	0.40	14	0.446	0.490	0.0322
201427_s_at	467.8	785.6	0.75	1.68	PPP-PPP	0.40	14	0.446	0.490	0.0380
227539_at	335.7	558.9	0.74	1.67	PPP-PPP	0.40	14	0.446	0.490	0.0203
228214_at	123.6	205.7	0.74	1.67	AAP-PPP	0.40	14	0.446	0.490	0.0352
222830_at	183.3	304.8	0.73	1.66	PPP-PPP	0.40	14	0.446	0.490	0.0401
219650_at	391.4	649.6	0.73	1.66	PPP-PPP	0.40	14	0.446	0.490	0.0194
1563531_at	39.6	65.6	0.73	1.66	AAA-PAP	0.40	14	0.446	0.490	0.0185
217104_at	46.6	77.2	0.73	1.66	PPA-PPP	0.40	14	0.446	0.490	0.0356
224658_x_at	319.4	528.7	0.73	1.66	PPP-PPP	0.40	14	0.446	0.490	0.0380
224325_at	65.1	107.1	0.72	1.65	PAP-PPP	0.40	14	0.446	0.490	0.0235
225178_at	183.4	299.8	0.71	1.63	AAA-MAP	0.40	14	0.446	0.490	0.0365
211834_s_at	72.5	118.4	0.71	1.63	AAA-AAP	0.40	14	0.446	0.490	0.0310
203932_at	82.1	134.1	0.71	1.63	PPP-PPP	0.40	14	0.446	0.490	0.0244
238988_at	131.2	214.0	0.71	1.63	PAP-PPA	0.40	14	0.446	0.490	0.0175
210959_s_at	467.4	758.1	0.70	1.62	PPP-PPP	0.40	14	0.446	0.490	0.0337
220236_at	58.2	94.3	0.70	1.62	PAP-PPA	0.40	14	0.446	0.490	0.0356
242467_at	148.2	239.4	0.69	1.62	PPP-PPP	0.40	14	0.446	0.490	0.0163
234604_at	99.7	160.5	0.69	1.61	APP-PPP	0.40	14	0.446	0.490	0.0277
235095_at	126.5	203.7	0.69	1.61	AAA-PPP	0.40	14	0.446	0.490	0.0140
243768_at	237.4	381.1	0.68	1.61	PPP-PPP	0.40	14	0.446	0.490	0.0273
1553679_s_at	268.5	430.4	0.68	1.60	PPP-PPP	0.40	14	0.446	0.490	0.0216
1559524_at	67.3	107.8	0.68	1.60	PAA-APA	0.40	14	0.446	0.490	0.0158
238951_at	77.6	124.2	0.68	1.60	PPA-PPP	0.40	14	0.446	0.490	0.0359
229692_at	179.4	287.1	0.68	1.60	AAP-PPP	0.40	14	0.446	0.490	0.0145
214816_x_at	181.0	289.1	0.68	1.60	PAP-PPP	0.40	14	0.446	0.490	0.0290
232890_at	171.0	273.1	0.68	1.60	AAP-PPP	0.40	14	0.446	0.490	0.0194
208650_s_at	4097.5	6531.0	0.67	1.59	PPP-PPP	0.40	14	0.446	0.490	0.0335
230708_at	125.2	199.0	0.67	1.59	PPP-PPP	0.40	14	0.446	0.490	0.0337
201933_at	824.6	1311.0	0.67	1.59	PPP-PPP	0.40	14	0.446	0.490	0.0142
209682_at	580.5	921.0	0.67	1.59	PPP-PPP	0.40	14	0.446	0.490	0.0139
240243_at	41.2	65.3	0.67	1.59	AAA-PAA	0.40	14	0.446	0.490	0.0326
206033_s_at	1912.2	3029.0	0.66	1.58	PPP-PPP	0.40	14	0.446	0.490	0.0345

227576_at	175.6	277.8	0.66	1.58	PAP-PPP	0.40	14	0.446	0.490	0.0317
215990_s_at	236.1	372.7	0.66	1.58	AAA-PMA	0.40	14	0.446	0.490	0.0138
236124_at	95.5	150.5	0.66	1.58	AMA-MAP	0.40	14	0.446	0.490	0.0139
217618_x_at	374.1	588.6	0.65	1.57	PPP-PPP	0.40	14	0.446	0.490	0.0198
214751_at	179.2	281.1	0.65	1.57	PPP-PPP	0.40	14	0.446	0.490	0.0263
244185_at	166.3	260.4	0.65	1.57	PPP-PMP	0.40	14	0.446	0.490	0.0295
204859_s_at	198.4	310.4	0.65	1.56	PPP-PPP	0.40	14	0.446	0.490	0.0126
207279_s_at	170.9	267.3	0.65	1.56	APP-PPP	0.40	14	0.446	0.490	0.0120
224565_at	3766.6	5883.3	0.64	1.56	PPP-PPP	0.40	14	0.446	0.490	0.0249
241906_at	137.5	214.4	0.64	1.56	PPP-PPP	0.40	14	0.446	0.490	0.0260
208186_s_at	149.9	233.4	0.64	1.56	PAP-AMP	0.40	14	0.446	0.490	0.0253
200920_s_at	2619.5	4079.5	0.64	1.56	PPP-PPP	0.40	14	0.446	0.490	0.0129
220740_s_at	101.6	157.6	0.63	1.55	PPP-PPP	0.40	14	0.446	0.490	0.0290
218182_s_at	2022.4	3136.1	0.63	1.55	PPP-PPP	0.40	14	0.446	0.490	0.0153
233914_s_at	116.4	180.4	0.63	1.55	PPP-PPP	0.40	14	0.446	0.490	0.0273
225855_at	115.8	178.8	0.63	1.54	PPP-PPP	0.40	14	0.446	0.490	0.0236
227330_x_at	136.9	211.2	0.63	1.54	PAA-PPP	0.40	14	0.446	0.490	0.0330
242111_at	111.4	171.8	0.63	1.54	PAP-PPP	0.40	14	0.446	0.490	0.0316
239711_at	119.5	184.3	0.63	1.54	PPP-PMP	0.40	14	0.446	0.490	0.0139
243527_at	50.4	77.6	0.62	1.54	PPA-PPP	0.40	14	0.446	0.490	0.0199
1559005_s_at	122.8	188.6	0.62	1.54	PPP-PPP	0.40	14	0.446	0.490	0.0277
208185_x_at	156.9	240.5	0.62	1.53	PPP-PPP	0.40	14	0.446	0.490	0.0124
1569433_at	195.5	299.3	0.61	1.53	PPP-PPP	0.40	14	0.446	0.490	0.0226
205728_at	229.4	350.7	0.61	1.53	PPP-PPP	0.40	14	0.446	0.490	0.0131
219613_s_at	355.4	542.2	0.61	1.53	PPP-PPP	0.40	14	0.446	0.490	0.0323
226899_at	369.2	561.0	0.60	1.52	PPP-PPP	0.40	14	0.446	0.490	0.0235
1554726_at	224.5	340.1	0.60	1.52	PPP-PPP	0.40	14	0.446	0.490	0.0113
1552976_at	144.2	217.7	0.59	1.51	PPA-PPP	0.40	14	0.446	0.490	0.0270
230886_at	89.2	134.3	0.59	1.51	AMA-PPP	0.40	14	0.446	0.490	0.0125
236129_at	34.5	51.8	0.59	1.50	APP-PAA	0.40	14	0.446	0.490	0.0141
230040_at	49.3	74.1	0.59	1.50	APA-PMA	0.40	14	0.446	0.490	0.0110
209568_s_at	199.9	299.8	0.59	1.50	PAP-MPP	0.40	14	0.446	0.490	0.0286
231929_at	457.7	685.4	0.58	1.50	PPP-PPP	0.40	14	0.446	0.490	0.0279
228203_at	99.0	148.3	0.58	1.50	AAP-MAP	0.40	14	0.446	0.490	0.0120
231234_at	322.7	478.0	0.57	1.48	PPP-PPP	0.40	14	0.446	0.490	0.0184
202459_s_at	214.3	317.5	0.57	1.48	PPP-PPP	0.40	14	0.446	0.490	0.0175
221766_s_at	266.5	394.5	0.57	1.48	MPP-PPP	0.40	14	0.446	0.490	0.0183
219565_at	272.7	403.3	0.56	1.48	PPP-PPP	0.40	14	0.446	0.490	0.0238
215359_x_at	558.4	825.0	0.56	1.48	PPP-PPP	0.40	14	0.446	0.490	0.0269
202820_at	1235.9	1824.0	0.56	1.48	PPP-PPP	0.40	14	0.446	0.490	0.0284
206032_at	2906.8	4282.0	0.56	1.47	PPP-PPP	0.40	14	0.446	0.490	0.0192
242932_at	147.1	216.4	0.56	1.47	PPP-PPP	0.40	14	0.446	0.490	0.0279

243309_at	295.8	433.5	0.55	1.47	PPP-PPP	0.40	14	0.446	0.490	0.0281
203021_at	14874.5	21780.6	0.55	1.46	PPP-PPP	0.40	14	0.446	0.490	0.0267
227762_at	1341.3	1961.8	0.55	1.46	PPP-PPP	0.40	14	0.446	0.490	0.0158
215465_at	647.9	945.4	0.55	1.46	PPP-PPP	0.40	14	0.446	0.490	0.0123
228156_at	314.1	458.1	0.54	1.46	PPP-PPP	0.40	14	0.446	0.490	0.0116
1558695_at	225.0	327.7	0.54	1.46	PPP-PPP	0.40	14	0.446	0.490	0.0140
225078_at	1385.2	2017.5	0.54	1.46	PPP-PPP	0.40	14	0.446	0.490	0.0130
241448_at	129.5	188.6	0.54	1.46	AAA-PPP	0.40	14	0.446	0.490	0.0086
221974_at	190.3	276.4	0.54	1.45	PPP-PPP	0.40	14	0.446	0.490	0.0156
1554921_a_at	390.4	563.8	0.53	1.44	PPP-PPP	0.40	14	0.446	0.490	0.0254
204477_at	342.1	492.5	0.53	1.44	PPP-PPP	0.40	14	0.446	0.490	0.0120
236721_at	68.9	99.1	0.53	1.44	PAA-PPP	0.40	14	0.446	0.490	0.0262
226460_at	273.0	392.5	0.52	1.44	PPP-PPP	0.40	14	0.446	0.490	0.0213
226596_x_at	546.2	784.1	0.52	1.44	PPP-PPP	0.40	14	0.446	0.490	0.0109
1554972_at	63.7	91.1	0.52	1.43	AAA-AAP	0.40	14	0.446	0.490	0.0202
228506_at	148.4	212.2	0.52	1.43	PAA-APP	0.40	14	0.446	0.490	0.0137
202504_at	13090.3	18704.7	0.52	1.43	PPP-PPP	0.40	14	0.446	0.490	0.0108
1568815_a_at	96.1	137.1	0.51	1.43	PPP-APP	0.40	14	0.446	0.490	0.0220
1557996_at	375.6	535.6	0.51	1.43	PPP-PPP	0.40	14	0.446	0.490	0.0200
208502_s_at	632.8	897.6	0.50	1.42	PPP-PPP	0.40	14	0.446	0.490	0.0085
239348_at	184.5	261.2	0.50	1.42	PPP-PPP	0.40	14	0.446	0.490	0.0096
228115_at	757.3	1071.2	0.50	1.41	PPP-PPP	0.40	14	0.446	0.490	0.0194
1565651_at	149.7	211.3	0.50	1.41	PPP-PPP	0.40	14	0.446	0.490	0.0105
229638_at	2505.5	3535.6	0.50	1.41	PPP-PPP	0.40	14	0.446	0.490	0.0069
211002_s_at	2118.1	2985.9	0.50	1.41	PPP-PPP	0.40	14	0.446	0.490	0.0172
222721_at	136.8	191.8	0.49	1.40	PPP-PPP	0.40	14	0.446	0.490	0.0110
225299_at	144.9	203.1	0.49	1.40	PPP-PPP	0.40	14	0.446	0.490	0.0106
239007_at	41.2	57.6	0.48	1.40	PAP-PPP	0.40	14	0.446	0.490	0.0139
220338_at	84.8	118.4	0.48	1.40	PPP-PPP	0.40	14	0.446	0.490	0.0179
205466_s_at	94.8	132.3	0.48	1.40	AAP-PAM	0.40	14	0.446	0.490	0.0089
215001_s_at	18387.7	25624.0	0.48	1.39	PPP-PPP	0.40	14	0.446	0.490	0.0146
204742_s_at	160.4	223.0	0.48	1.39	PPP-PPP	0.40	14	0.446	0.490	0.0220
218807_at	1069.0	1486.7	0.48	1.39	PPP-PPP	0.40	14	0.446	0.490	0.0169
209414_at	204.5	284.0	0.47	1.39	PPP-PPP	0.40	14	0.446	0.490	0.0097
210251_s_at	216.8	298.8	0.46	1.38	PPP-PPP	0.40	14	0.446	0.490	0.0106
208770_s_at	507.0	698.6	0.46	1.38	PPP-PPP	0.40	14	0.446	0.490	0.0097
213164_at	1367.3	1878.3	0.46	1.37	PPP-PPP	0.40	14	0.446	0.490	0.0061
50221_at	241.5	329.1	0.45	1.36	PPP-PPP	0.40	14	0.446	0.490	0.0139
210655_s_at	257.0	350.0	0.45	1.36	PPM-PPP	0.40	14	0.446	0.490	0.0152
219278_at	546.2	743.9	0.45	1.36	PPP-PPP	0.40	14	0.446	0.490	0.0132
214600_at	321.8	438.5	0.45	1.36	PPP-PPP	0.40	14	0.446	0.490	0.0113
212850_s_at	171.6	233.4	0.44	1.36	PPP-MPP	0.40	14	0.446	0.490	0.0097

212240_s_at	2052.9	2791.4	0.44	1.36	PPP-PPP	0.40	14	0.446	0.490	0.0178
207023_x_at	2773.7	3754.1	0.44	1.35	PPP-PPP	0.40	14	0.446	0.490	0.0184
207564_x_at	488.4	661.4	0.44	1.35	PPP-PPP	0.40	14	0.446	0.490	0.0068
226338_at	274.8	370.9	0.43	1.35	PPP-PPP	0.40	14	0.446	0.490	0.0070
1558699_a_at	471.2	634.6	0.43	1.35	PPP-PPP	0.40	14	0.446	0.490	0.0170
221564_at	730.2	977.1	0.42	1.34	PPP-PPP	0.40	14	0.446	0.490	0.0092
238096_at	269.2	358.5	0.41	1.33	PPP-PPP	0.40	14	0.446	0.490	0.0134
236905_at	163.0	217.1	0.41	1.33	AAA-APP	0.40	14	0.446	0.490	0.0125
225533_at	510.3	678.9	0.41	1.33	PPP-PPP	0.40	14	0.446	0.490	0.0088
222414_at	863.7	1147.8	0.41	1.33	PPP-PPP	0.40	14	0.446	0.490	0.0095
238633_at	112.1	148.9	0.41	1.33	APP-PPP	0.40	14	0.446	0.490	0.0121
32209_at	1029.8	1366.3	0.41	1.33	PPP-PPP	0.40	14	0.446	0.490	0.0116
227207_x_at	301.3	399.6	0.41	1.33	PPP-PPP	0.40	14	0.446	0.490	0.0111
37254_at	499.3	662.2	0.41	1.33	PPP-PPP	0.40	14	0.446	0.490	0.0068
202355_s_at	1252.8	1658.4	0.41	1.32	PPP-PPP	0.40	14	0.446	0.490	0.0144
210716_s_at	604.9	800.4	0.40	1.32	PPP-PPP	0.40	14	0.446	0.490	0.0147
212559_at	444.6	587.8	0.40	1.32	PPP-PPP	0.40	14	0.446	0.490	0.0109
212373_at	641.0	845.9	0.40	1.32	PPP-PPP	0.40	14	0.446	0.490	0.0092
227806_at	481.6	632.6	0.39	1.31	PPP-PPP	0.40	14	0.446	0.490	0.0111
203917_at	546.2	717.5	0.39	1.31	PPP-PPP	0.40	14	0.446	0.490	0.0032
223364_s_at	232.0	304.6	0.39	1.31	APP-MPP	0.40	14	0.446	0.490	0.0027
225067_at	342.1	448.2	0.39	1.31	PPP-PPP	0.40	14	0.446	0.490	0.0075
202219_at	703.9	918.1	0.38	1.30	PPP-PPP	0.40	14	0.446	0.490	0.0116
244235_at	82.0	106.8	0.38	1.30	PMP-PPP	0.40	14	0.446	0.490	0.0121
1558896_at	352.3	457.5	0.38	1.30	PPP-PPP	0.40	14	0.446	0.490	0.0045
233748_x_at	1477.4	1916.2	0.38	1.30	PPP-PPP	0.40	14	0.446	0.490	0.0058
209832_s_at	429.7	556.9	0.37	1.30	PPP-PPP	0.40	14	0.446	0.490	0.0111
1553768_a_at	360.3	464.9	0.37	1.29	PPP-PPP	0.40	14	0.446	0.490	0.0078
215075_s_at	517.9	668.2	0.37	1.29	PPP-PPP	0.40	14	0.446	0.490	0.0112
217975_at	5869.7	7571.5	0.37	1.29	PPP-PPP	0.40	14	0.446	0.490	0.0088
235444_at	115.0	148.3	0.37	1.29	PAP-PAP	0.40	14	0.446	0.490	0.0021
207717_s_at	1348.1	1730.6	0.36	1.28	PPP-PPP	0.40	14	0.446	0.490	0.0109
202247_s_at	235.8	302.7	0.36	1.28	PPP-PPP	0.40	14	0.446	0.490	0.0097
229395_at	159.0	204.0	0.36	1.28	PPP-PPP	0.40	14	0.446	0.490	0.0060
228385_at	569.4	728.2	0.36	1.28	PPP-PPP	0.40	14	0.446	0.490	0.0037
204253_s_at	301.2	384.6	0.35	1.28	PPP-PPP	0.40	14	0.446	0.490	0.0065
221565_s_at	355.7	454.2	0.35	1.28	PPP-PPP	0.40	14	0.446	0.490	0.0057
212930_at	293.3	374.6	0.35	1.28	PPP-PPP	0.40	14	0.446	0.490	0.0056
226203_at	452.8	576.9	0.35	1.27	PPP-PPP	0.40	14	0.446	0.490	0.0025
225787_at	3912.1	4975.9	0.35	1.27	PPP-PPP	0.40	14	0.446	0.490	0.0083
202356_s_at	1009.2	1280.3	0.34	1.27	PPP-PPP	0.40	14	0.446	0.490	0.0065
208960_s_at	1618.5	2039.5	0.33	1.26	PPP-PPP	0.40	14	0.446	0.490	0.0044

210480_s_at	518.3	652.2	0.33	1.26	PPP-PPP	0.40	14	0.446	0.490	0.0066
218143_s_at	947.6	1190.4	0.33	1.26	PPP-PPP	0.40	14	0.446	0.490	0.0014
208961_s_at	2790.8	3499.2	0.33	1.25	PPP-PPP	0.40	14	0.446	0.490	0.0038
1559062_at	161.7	201.6	0.32	1.25	PPP-PPP	0.40	14	0.446	0.490	0.0052
244653_at	80.1	98.9	0.31	1.24	AAP-APP	0.40	14	0.446	0.490	0.0009
225517_at	1931.8	2375.8	0.30	1.23	PPP-PPP	0.40	14	0.446	0.490	0.0009
230734_x_at	1022.7	1256.7	0.30	1.23	PPP-PPP	0.40	14	0.446	0.490	0.0027
218210_at	1221.6	1501.0	0.30	1.23	PPP-PPP	0.40	14	0.446	0.490	0.0010
212867_at	905.1	1109.2	0.29	1.23	PPP-PPP	0.40	14	0.446	0.490	0.0012
200759_x_at	2529.4	3085.7	0.29	1.22	PPP-PPP	0.40	14	0.446	0.490	0.0036
226202_at	510.1	617.7	0.28	1.21	PPP-PPP	0.40	14	0.446	0.490	0.0002
205856_at	26.1	2.6	-3.32	-9.97	PAA-AAA	0.30	15	0.551	0.662	0.0087
244125_at	1.8	13.0	2.86	7.25	AAA-APA	0.30	15	0.551	0.662	0.1164
1556529_a_at	4.3	30.1	2.82	7.06	AAA-PAA	0.30	15	0.551	0.662	0.0960
1557354_at	32.6	4.7	-2.81	-7.02	APP-AAA	0.30	15	0.551	0.662	0.0020
244640_at	10.7	74.2	2.80	6.97	AAA-PAP	0.30	15	0.551	0.662	0.0923
226830_x_at	56.8	8.2	-2.79	-6.94	PAA-AAA	0.30	15	0.551	0.662	0.0097
230469_at	6.0	41.6	2.79	6.94	AAA-PPM	0.30	15	0.551	0.662	0.1142
204932_at	7.2	47.8	2.74	6.67	AAA-APA	0.30	15	0.551	0.662	0.1206
224261_at	21.0	124.4	2.56	5.91	AAA-PAA	0.30	15	0.551	0.662	0.0775
237240_at	7.7	45.3	2.56	5.91	AAA-APP	0.30	15	0.551	0.662	0.0741
222912_at	9.0	52.7	2.55	5.85	AMA-APA	0.30	15	0.551	0.662	0.1102
213796_at	131.2	746.4	2.51	5.69	APP-PPP	0.30	15	0.551	0.662	0.1101
240472_at	5.0	28.0	2.49	5.63	AAA-PAA	0.30	15	0.551	0.662	0.0944
1562639_at	10.7	59.7	2.48	5.59	AAA-AAP	0.30	15	0.551	0.662	0.0859
237330_at	18.8	104.9	2.48	5.58	APA-PPP	0.30	15	0.551	0.662	0.1144
231380_at	53.7	9.7	-2.47	-5.52	APP-AAA	0.30	15	0.551	0.662	0.0104
230647_at	15.5	84.5	2.45	5.46	AAA-PAM	0.30	15	0.551	0.662	0.1146
1569344_a_at	6.1	32.4	2.40	5.28	AAA-APA	0.30	15	0.551	0.662	0.1150
1553618_at	9.2	48.1	2.38	5.21	AAA-AAP	0.30	15	0.551	0.662	0.0978
239605_x_at	4.6	24.2	2.38	5.21	AAA-APA	0.30	15	0.551	0.662	0.1219
240602_at	10.1	50.9	2.33	5.02	AAA-PAA	0.30	15	0.551	0.662	0.0763
219195_at	16.3	80.8	2.31	4.97	AAA-AMP	0.30	15	0.551	0.662	0.1212
228432_at	8.1	38.4	2.25	4.76	AAA-PAA	0.30	15	0.551	0.662	0.1123
228907_at	13.5	64.1	2.25	4.75	AAA-AAP	0.30	15	0.551	0.662	0.0967
227140_at	1195.0	253.7	-2.24	-4.71	PPP-PPP	0.30	15	0.551	0.662	0.0075
1559410_at	17.5	82.0	2.23	4.70	AAA-PAP	0.30	15	0.551	0.662	0.0700
243488_at	9.4	44.2	2.23	4.69	APA-AAP	0.30	15	0.551	0.662	0.0702
1561269_at	13.6	62.8	2.20	4.60	AAA-APA	0.30	15	0.551	0.662	0.0931
238188_at	11.6	49.8	2.10	4.28	AAA-APA	0.30	15	0.551	0.662	0.0892
227759_at	49.7	11.7	-2.09	-4.25	PAA-AAA	0.30	15	0.551	0.662	0.0067
1558327_at	32.7	135.5	2.05	4.15	AAA-APP	0.30	15	0.551	0.662	0.0845

1552765_x_at	10.6	43.5	2.04	4.12	AAA-AAP	0.30	15	0.551	0.662	0.0884
205626_s_at	783.4	3221.3	2.04	4.11	PPP-PPP	0.30	15	0.551	0.662	0.0747
1565949_x_at	12.8	51.5	2.01	4.01	AAA-APA	0.30	15	0.551	0.662	0.0903
1565582_at	11.1	44.4	2.00	4.00	PAA-AAA	0.30	15	0.551	0.662	0.1094
228647_at	24.4	97.4	2.00	4.00	AAA-PPP	0.30	15	0.551	0.662	0.1069
1560723_at	46.1	11.7	-1.98	-3.93	APA-AAA	0.30	15	0.551	0.662	0.0022
234517_at	19.4	75.4	1.96	3.90	AAA-APP	0.30	15	0.551	0.662	0.1030
239533_at	9.8	37.6	1.95	3.85	AAA-PAP	0.30	15	0.551	0.662	0.0980
241562_x_at	14.7	56.1	1.94	3.83	AAA-APA	0.30	15	0.551	0.662	0.0947
230372_at	24.2	90.7	1.91	3.75	AAA-APP	0.30	15	0.551	0.662	0.1004
230462_at	33.7	122.7	1.86	3.64	AAA-APP	0.30	15	0.551	0.662	0.1170
210511_s_at	482.5	132.8	-1.86	-3.63	PPP-AAA	0.30	15	0.551	0.662	0.0014
215797_at	25.3	92.0	1.86	3.63	AAA-AAP	0.30	15	0.551	0.662	0.0904
1563754_at	7.2	25.9	1.86	3.62	AAA-AAP	0.30	15	0.551	0.662	0.0950
1566465_at	17.9	64.6	1.85	3.61	AAA-APA	0.30	15	0.551	0.662	0.0836
211006_s_at	114.6	412.0	1.85	3.59	APP-PPP	0.30	15	0.551	0.662	0.1063
229339_at	23.6	83.0	1.81	3.52	AAA-AAP	0.30	15	0.551	0.662	0.0816
241365_at	14.4	50.4	1.81	3.51	AAA-PPA	0.30	15	0.551	0.662	0.1066
204580_at	17.8	62.5	1.81	3.50	PAA-PPA	0.30	15	0.551	0.662	0.1110
1559520_at	14.7	51.1	1.80	3.49	AAA-PMA	0.30	15	0.551	0.662	0.0895
232822_x_at	18.6	64.0	1.78	3.44	AAA-PAP	0.30	15	0.551	0.662	0.0690
1559936_at	12.7	43.4	1.77	3.42	AAA-PAA	0.30	15	0.551	0.662	0.0926
1553998_at	41.2	139.1	1.75	3.37	AAM-APP	0.30	15	0.551	0.662	0.1073
232497_at	11.7	38.8	1.73	3.32	AAA-APA	0.30	15	0.551	0.662	0.1005
242163_at	15.5	50.2	1.70	3.25	APA-PPP	0.30	15	0.551	0.662	0.0804
207485_x_at	24.0	77.5	1.69	3.23	AAA-APA	0.30	15	0.551	0.662	0.1002
205067_at	909.6	281.9	-1.69	-3.23	PPP-AAA	0.30	15	0.551	0.662	0.0038
207728_at	15.7	50.3	1.68	3.20	AAP-AAA	0.30	15	0.551	0.662	0.1002
244485_at	19.8	63.3	1.68	3.20	APA-APP	0.30	15	0.551	0.662	0.1033
201131_s_at	5721.0	1787.8	-1.68	-3.20	PPP-PPP	0.30	15	0.551	0.662	0.0075
224140_at	24.1	76.8	1.67	3.19	AAA-AAP	0.30	15	0.551	0.662	0.1074
219160_s_at	80.4	25.5	-1.66	-3.15	AAP-AAA	0.30	15	0.551	0.662	0.0005
1570373_at	111.0	35.3	-1.65	-3.15	AAP-AAP	0.30	15	0.551	0.662	0.0057
243655_x_at	17.4	54.4	1.65	3.13	AAA-AAP	0.30	15	0.551	0.662	0.0948
229480_at	14.4	44.7	1.64	3.11	AAA-APP	0.30	15	0.551	0.662	0.1030
227474_at	21.7	67.3	1.63	3.10	AAA-AAP	0.30	15	0.551	0.662	0.0896
1553080_at	29.8	92.1	1.63	3.09	APA-APA	0.30	15	0.551	0.662	0.0640
240952_at	11.6	35.6	1.62	3.08	AAA-AAP	0.30	15	0.551	0.662	0.0755
232304_at	20.4	62.7	1.62	3.07	AAA-APP	0.30	15	0.551	0.662	0.0954
205170_at	34.3	105.0	1.61	3.06	AAA-APP	0.30	15	0.551	0.662	0.0849
239230_at	16.0	48.7	1.60	3.04	AAA-AAP	0.30	15	0.551	0.662	0.1121
215920_s_at	37.0	112.1	1.60	3.03	AAA-AAP	0.30	15	0.551	0.662	0.0986

236512_at	20.0	60.6	1.60	3.02	AAA-AAP	0.30	15	0.551	0.662	0.1049
237946_at	20.7	62.4	1.59	3.01	AAP-AAM	0.30	15	0.551	0.662	0.0925
236634_at	22.5	66.5	1.56	2.95	AAA-APP	0.30	15	0.551	0.662	0.0699
242055_at	33.8	99.6	1.56	2.95	AAA-PAP	0.30	15	0.551	0.662	0.0998
81737_at	40.5	118.7	1.55	2.93	PAA-APP	0.30	15	0.551	0.662	0.0833
222887_s_at	77.0	224.9	1.55	2.92	AAA-APP	0.30	15	0.551	0.662	0.0921
214235_at	68.2	23.6	-1.53	-2.89	AAP-AAA	0.30	15	0.551	0.662	0.0034
233011_at	26.6	76.4	1.53	2.88	AAA-APA	0.30	15	0.551	0.662	0.0797
237511_at	19.5	55.9	1.52	2.87	AAA-PAA	0.30	15	0.551	0.662	0.0670
243695_at	69.9	200.1	1.52	2.86	AAA-AAP	0.30	15	0.551	0.662	0.0678
216662_at	27.7	78.5	1.50	2.83	AAA-APA	0.30	15	0.551	0.662	0.0850
242573_at	23.3	65.6	1.49	2.81	AAP-PPP	0.30	15	0.551	0.662	0.0759
205064_at	2402.2	6720.8	1.48	2.80	PPP-PPP	0.30	15	0.551	0.662	0.0926
232000_at	27.7	76.7	1.47	2.77	AAA-AAP	0.30	15	0.551	0.662	0.1071
239942_at	19.6	54.4	1.47	2.77	AAM-PAA	0.30	15	0.551	0.662	0.0868
41469_at	3187.8	8798.3	1.47	2.76	PPP-PPP	0.30	15	0.551	0.662	0.1088
207154_at	28.2	77.6	1.46	2.75	AAA-APA	0.30	15	0.551	0.662	0.0762
230481_at	47.6	130.7	1.46	2.75	AAA-APP	0.30	15	0.551	0.662	0.0738
236080_at	43.2	118.4	1.46	2.74	AAA-APA	0.30	15	0.551	0.662	0.0636
215816_at	32.8	88.4	1.43	2.70	AAA-AAP	0.30	15	0.551	0.662	0.1023
202998_s_at	708.1	262.9	-1.43	-2.69	PPP-PPP	0.30	15	0.551	0.662	0.0053
242868_at	67.2	181.2	1.43	2.70	APP-PMP	0.30	15	0.551	0.662	0.0893
237157_at	26.9	72.2	1.43	2.69	AAA-AAP	0.30	15	0.551	0.662	0.0966
1563253_s_at	74.7	200.5	1.43	2.69	AAA-APP	0.30	15	0.551	0.662	0.0940
207684_at	47.3	126.0	1.42	2.67	AAA-PAA	0.30	15	0.551	0.662	0.1019
233440_at	22.3	59.0	1.41	2.65	AAA-AAP	0.30	15	0.551	0.662	0.0921
39402_at	984.8	378.3	-1.38	-2.60	PPP-PPP	0.30	15	0.551	0.662	0.0020
235844_at	27.8	72.0	1.37	2.59	APA-AAA	0.30	15	0.551	0.662	0.1037
230417_at	29.3	75.4	1.37	2.58	AAA-APA	0.30	15	0.551	0.662	0.0971
243805_at	218.0	84.7	-1.36	-2.57	PPP-AMA	0.30	15	0.551	0.662	0.0044
211361_s_at	52.6	135.0	1.36	2.57	APP-MPP	0.30	15	0.551	0.662	0.0894
204470_at	1792.6	704.7	-1.35	-2.54	PPP-PPP	0.30	15	0.551	0.662	0.0019
1559049_a_at	28.1	70.9	1.34	2.53	AAA-PAP	0.30	15	0.551	0.662	0.0925
210869_s_at	299.7	119.2	-1.33	-2.51	PPP-PPP	0.30	15	0.551	0.662	0.0055
229960_at	20.3	50.9	1.33	2.51	AAA-APA	0.30	15	0.551	0.662	0.1018
232882_at	84.9	212.1	1.32	2.50	AAA-PAP	0.30	15	0.551	0.662	0.0860
205775_at	110.8	273.2	1.30	2.47	AAM-AMP	0.30	15	0.551	0.662	0.0909
1558846_at	219.5	528.6	1.27	2.41	PPP-PPP	0.30	15	0.551	0.662	0.0710
1558815_at	21.6	51.9	1.27	2.41	AAA-APA	0.30	15	0.551	0.662	0.0671
1554250_s_at	38.7	92.4	1.26	2.39	PAA-PMP	0.30	15	0.551	0.662	0.0864
230610_at	106.1	252.7	1.25	2.38	PAA-PPP	0.30	15	0.551	0.662	0.1034
217274_x_at	62.3	147.8	1.25	2.38	AAA-PPA	0.30	15	0.551	0.662	0.0869

1554072_s_at	30.9	13.1	-1.24	-2.36	APA-AAA	0.30	15	0.551	0.662	0.0003
1569503_at	20.7	48.6	1.23	2.35	AAA-AAP	0.30	15	0.551	0.662	0.0851
224189_x_at	48.1	112.9	1.23	2.35	AAA-PPP	0.30	15	0.551	0.662	0.0753
1552427_at	26.0	61.0	1.23	2.34	APP-APP	0.30	15	0.551	0.662	0.0664
1564423_a_at	25.4	59.5	1.23	2.34	AAA-APP	0.30	15	0.551	0.662	0.0604
206467_x_at	1214.0	519.4	-1.23	-2.34	PPP-PPP	0.30	15	0.551	0.662	0.0060
223484_at	146.6	339.5	1.21	2.32	PPP-PPP	0.30	15	0.551	0.662	0.1057
201042_at	360.3	155.8	-1.21	-2.31	PMA-AAA	0.30	15	0.551	0.662	0.0054
1555926_a_at	30.3	70.1	1.21	2.31	AAM-APP	0.30	15	0.551	0.662	0.0951
244822_at	36.0	83.0	1.21	2.31	PAA-PPA	0.30	15	0.551	0.662	0.0871
1555632_at	36.9	84.9	1.20	2.30	AAA-PAA	0.30	15	0.551	0.662	0.0981
240865_at	24.7	56.9	1.20	2.30	AAA-PAA	0.30	15	0.551	0.662	0.0744
220234_at	47.0	107.9	1.20	2.30	AAA-PAP	0.30	15	0.551	0.662	0.0674
233106_at	32.8	74.9	1.19	2.29	AAA-APP	0.30	15	0.551	0.662	0.0993
239773_at	45.2	103.1	1.19	2.28	AAP-PPP	0.30	15	0.551	0.662	0.0945
242248_at	17.8	40.6	1.19	2.28	AAA-PAP	0.30	15	0.551	0.662	0.0846
240510_at	82.0	186.9	1.19	2.28	AAM-APP	0.30	15	0.551	0.662	0.0562
1562162_at	87.5	38.5	-1.19	-2.28	APA-AAA	0.30	15	0.551	0.662	0.0019
238207_at	70.6	159.0	1.17	2.25	AAA-APP	0.30	15	0.551	0.662	0.0931
207434_s_at	26.7	59.8	1.17	2.24	AAA-APA	0.30	15	0.551	0.662	0.1034
212233_at	120.3	53.7	-1.16	-2.24	PPP-AAA	0.30	15	0.551	0.662	0.0043
1569573_at	35.4	79.1	1.16	2.23	AAA-PAP	0.30	15	0.551	0.662	0.0888
1558710_at	67.5	150.7	1.16	2.23	PPP-PPP	0.30	15	0.551	0.662	0.0572
213156_at	55.1	122.4	1.15	2.22	PAA-PPP	0.30	15	0.551	0.662	0.0725
243158_at	33.4	73.7	1.14	2.21	AAA-AAP	0.30	15	0.551	0.662	0.0606
244219_at	55.1	121.4	1.14	2.21	PPP-PPP	0.30	15	0.551	0.662	0.0699
228469_at	56.6	124.0	1.13	2.19	PAM-PPP	0.30	15	0.551	0.662	0.0818
237669_at	24.2	53.0	1.13	2.19	AAA-PPA	0.30	15	0.551	0.662	0.0554
210377_at	68.1	31.1	-1.13	-2.19	AAP-APA	0.30	15	0.551	0.662	0.0041
209145_s_at	67.4	145.9	1.11	2.16	PPA-AMP	0.30	15	0.551	0.662	0.0900
214549_x_at	737.2	1581.6	1.10	2.15	PPP-PPP	0.30	15	0.551	0.662	0.0794
1562488_at	55.8	119.5	1.10	2.14	AAA-APA	0.30	15	0.551	0.662	0.0680
236865_at	37.0	78.9	1.09	2.13	AAA-APA	0.30	15	0.551	0.662	0.0735
206002_at	64.7	136.4	1.08	2.11	APA-PPP	0.30	15	0.551	0.662	0.0700
210874_s_at	188.2	90.0	-1.06	-2.09	AAP-AAA	0.30	15	0.551	0.662	0.0014
215163_at	51.5	107.2	1.06	2.08	PPM-APP	0.30	15	0.551	0.662	0.0628
243508_at	34.3	71.3	1.05	2.08	AAA-AAP	0.30	15	0.551	0.662	0.0928
220908_at	31.0	64.4	1.05	2.08	AAA-PAA	0.30	15	0.551	0.662	0.0727
236181_at	29.5	61.2	1.05	2.08	AAA-APA	0.30	15	0.551	0.662	0.0928
231449_at	49.8	102.5	1.04	2.06	AAP-PPM	0.30	15	0.551	0.662	0.0631
220330_s_at	64.9	31.6	-1.04	-2.05	APP-AAA	0.30	15	0.551	0.662	0.0050
1557293_at	151.4	310.8	1.04	2.05	PPP-PPP	0.30	15	0.551	0.662	0.0773

217713_x_at	165.9	337.9	1.03	2.04	PPP-PPP	0.30	15	0.551	0.662	0.0887
231779_at	269.2	132.8	-1.02	-2.03	PPP-PPP	0.30	15	0.551	0.662	0.0042
1555194_at	38.9	78.2	1.01	2.01	PAP-APP	0.30	15	0.551	0.662	0.0913
237451_x_at	66.3	133.2	1.01	2.01	PPA-APP	0.30	15	0.551	0.662	0.0627
1557488_at	23.0	46.1	1.00	2.01	AAA-PPA	0.30	15	0.551	0.662	0.0860
210356_x_at	41.2	82.7	1.00	2.01	AAA-APA	0.30	15	0.551	0.662	0.0515
244471_x_at	66.6	133.3	1.00	2.00	PAP-APM	0.30	15	0.551	0.662	0.0513
201130_s_at	210.6	105.3	-1.00	-2.00	PMP-PPP	0.30	15	0.551	0.662	0.0060
240076_at	43.7	87.3	1.00	2.00	AAA-PMA	0.30	15	0.551	0.662	0.0648
244665_at	117.9	234.5	0.99	1.99	PPA-PPP	0.30	15	0.551	0.662	0.0580
231033_at	239.8	476.8	0.99	1.99	MPP-PPP	0.30	15	0.551	0.662	0.0596
1552867_at	88.3	174.8	0.99	1.98	PAP-PPP	0.30	15	0.551	0.662	0.0871
238972_at	46.2	91.4	0.99	1.98	AAA-PAP	0.30	15	0.551	0.662	0.0788
213182_x_at	89.2	176.3	0.98	1.98	AAA-PAP	0.30	15	0.551	0.662	0.0843
1558794_at	70.1	137.9	0.98	1.97	PPP-PPP	0.30	15	0.551	0.662	0.0620
213348_at	389.1	761.4	0.97	1.96	PPP-PPP	0.30	15	0.551	0.662	0.0646
229398_at	60.1	117.3	0.97	1.95	PAA-PMP	0.30	15	0.551	0.662	0.0755
241838_at	62.4	121.1	0.96	1.94	PAP-PPP	0.30	15	0.551	0.662	0.0539
37802_r_at	99.7	193.1	0.95	1.94	PPP-PPP	0.30	15	0.551	0.662	0.0586
1555606_a_at	38.1	73.4	0.95	1.93	AAA-MAP	0.30	15	0.551	0.662	0.0705
215231_at	47.0	90.6	0.95	1.93	PAM-PPP	0.30	15	0.551	0.662	0.0595
235608_at	96.1	185.0	0.95	1.93	PPA-PPP	0.30	15	0.551	0.662	0.0958
235419_at	396.9	764.2	0.95	1.93	PPP-PPP	0.30	15	0.551	0.662	0.0838
216109_at	58.1	111.8	0.94	1.92	PAP-PPP	0.30	15	0.551	0.662	0.0631
235879_at	139.8	267.8	0.94	1.92	PPP-PPP	0.30	15	0.551	0.662	0.0715
209147_s_at	214.0	409.9	0.94	1.92	PPP-PPP	0.30	15	0.551	0.662	0.0505
1553973_a_at	160.8	304.5	0.92	1.89	AAP-PPP	0.30	15	0.551	0.662	0.0859
208371_s_at	369.4	695.4	0.91	1.88	AAP-MPP	0.30	15	0.551	0.662	0.0502
1561749_at	42.4	79.7	0.91	1.88	AAA-PAA	0.30	15	0.551	0.662	0.0542
228570_at	378.1	201.9	-0.91	-1.87	PPP-PPP	0.30	15	0.551	0.662	0.0033
238140_at	99.8	186.9	0.91	1.87	AAA-MPP	0.30	15	0.551	0.662	0.0820
1564776_at	30.6	57.1	0.90	1.86	APA-PAA	0.30	15	0.551	0.662	0.0932
1559490_at	69.6	129.0	0.89	1.85	PPM-PPP	0.30	15	0.551	0.662	0.0807
217164_at	70.8	131.1	0.89	1.85	PAP-PPP	0.30	15	0.551	0.662	0.0508
211548_s_at	226.1	416.2	0.88	1.84	PPP-PPP	0.30	15	0.551	0.662	0.0780
1554670_at	66.9	123.0	0.88	1.84	APA-PPP	0.30	15	0.551	0.662	0.0475
228040_at	159.2	292.4	0.88	1.84	APA-PPP	0.30	15	0.551	0.662	0.0808
234193_at	57.5	105.7	0.88	1.84	PMA-PAP	0.30	15	0.551	0.662	0.0502
219487_at	479.0	262.3	-0.87	-1.83	PPP-PPP	0.30	15	0.551	0.662	0.0018
1558652_at	43.5	79.2	0.87	1.82	AAP-PPP	0.30	15	0.551	0.662	0.0524
220694_at	78.6	143.0	0.86	1.82	AAA-APA	0.30	15	0.551	0.662	0.0881
50965_at	78.4	141.5	0.85	1.81	AAA-AAP	0.30	15	0.551	0.662	0.0777

1560661_x_at	96.6	173.8	0.85	1.80	AAP-PPP	0.30	15	0.551	0.662	0.0472
1565863_at	56.4	101.3	0.85	1.80	PPA-APA	0.30	15	0.551	0.662	0.0697
218980_at	610.3	340.0	-0.84	-1.80	PPP-PPP	0.30	15	0.551	0.662	0.0021
223551_at	120.6	216.1	0.84	1.79	PPP-PPP	0.30	15	0.551	0.662	0.0854
223517_at	84.8	152.0	0.84	1.79	AMA-MPM	0.30	15	0.551	0.662	0.0764
242968_at	100.8	180.5	0.84	1.79	AAA-MAP	0.30	15	0.551	0.662	0.0482
215833_s_at	106.4	190.6	0.84	1.79	AAA-AMP	0.30	15	0.551	0.662	0.0755
232964_at	91.2	163.2	0.84	1.79	PAA-PPP	0.30	15	0.551	0.662	0.0848
207233_s_at	159.6	284.4	0.83	1.78	PAP-PPP	0.30	15	0.551	0.662	0.0519
229657_at	113.8	202.8	0.83	1.78	PPP-PPP	0.30	15	0.551	0.662	0.0472
225066_at	72.6	128.8	0.83	1.77	AAP-MPA	0.30	15	0.551	0.662	0.0854
213737_x_at	421.7	747.6	0.83	1.77	PPP-PPP	0.30	15	0.551	0.662	0.0556
238883_at	92.2	163.4	0.83	1.77	PPP-PPP	0.30	15	0.551	0.662	0.0781
216453_at	61.2	108.3	0.82	1.77	AAM-APM	0.30	15	0.551	0.662	0.0879
1553349_at	86.0	151.9	0.82	1.77	PPM-APP	0.30	15	0.551	0.662	0.0720
216723_at	24.9	44.0	0.82	1.76	AAA-PAA	0.30	15	0.551	0.662	0.0622
43427_at	76.1	134.1	0.82	1.76	AAA-APP	0.30	15	0.551	0.662	0.0499
242240_at	171.7	301.8	0.81	1.76	PPP-PPP	0.30	15	0.551	0.662	0.0877
1566491_at	54.0	94.9	0.81	1.76	PAA-PPP	0.30	15	0.551	0.662	0.0587
231180_at	42.1	73.7	0.81	1.75	AAA-APA	0.30	15	0.551	0.662	0.0610
232740_at	42.2	73.7	0.81	1.75	PAP-PPP	0.30	15	0.551	0.662	0.0491
212848_s_at	333.3	581.7	0.80	1.75	PAP-PPP	0.30	15	0.551	0.662	0.0731
1557527_at	146.8	255.7	0.80	1.74	PPP-PPP	0.30	15	0.551	0.662	0.0573
229276_at	179.8	313.1	0.80	1.74	AAM-APP	0.30	15	0.551	0.662	0.0495
215526_at	39.2	68.1	0.80	1.74	AAA-APA	0.30	15	0.551	0.662	0.0492
231644_at	78.9	136.8	0.80	1.74	PMP-PMP	0.30	15	0.551	0.662	0.0487
239698_at	40.2	69.7	0.79	1.73	AAA-PAP	0.30	15	0.551	0.662	0.0456
243005_at	45.7	79.2	0.79	1.73	AAA-PMM	0.30	15	0.551	0.662	0.0773
229483_at	179.7	310.7	0.79	1.73	PPP-PPP	0.30	15	0.551	0.662	0.0435
222492_at	229.6	395.2	0.78	1.72	PPP-PPP	0.30	15	0.551	0.662	0.0461
218785_s_at	141.0	242.6	0.78	1.72	MPP-PPP	0.30	15	0.551	0.662	0.0534
220992_s_at	211.8	363.8	0.78	1.72	PPP-PPP	0.30	15	0.551	0.662	0.0698
1560754_at	88.1	151.2	0.78	1.72	PAA-PPP	0.30	15	0.551	0.662	0.0583
225721_at	114.4	195.8	0.78	1.71	AAM-PPM	0.30	15	0.551	0.662	0.0705
215501_s_at	111.2	190.2	0.78	1.71	PPP-PPP	0.30	15	0.551	0.662	0.0667
230922_x_at	98.9	169.0	0.77	1.71	AAA-AAP	0.30	15	0.551	0.662	0.0570
205689_at	107.5	183.0	0.77	1.70	PAA-APP	0.30	15	0.551	0.662	0.0823
227197_at	99.7	169.6	0.77	1.70	PPA-PPP	0.30	15	0.551	0.662	0.0569
1566426_at	43.6	73.9	0.76	1.70	AAA-APP	0.30	15	0.551	0.662	0.0815
243395_at	215.4	365.4	0.76	1.70	APP-PPP	0.30	15	0.551	0.662	0.0839
219012_s_at	141.5	239.7	0.76	1.70	AAP-PPP	0.30	15	0.551	0.662	0.0848
1560386_at	58.8	99.3	0.76	1.69	MAP-PPP	0.30	15	0.551	0.662	0.0755

243841_at	228.7	386.5	0.76	1.69	PPP-PPP	0.30	15	0.551	0.662	0.0463
236956_at	80.0	135.1	0.76	1.69	PAA-MAA	0.30	15	0.551	0.662	0.0816
244788_at	49.8	84.1	0.76	1.69	AAA-PAA	0.30	15	0.551	0.662	0.0621
1563478_at	67.9	114.6	0.76	1.69	AMA-APP	0.30	15	0.551	0.662	0.0860
226791_at	309.7	522.4	0.75	1.69	PAA-PPP	0.30	15	0.551	0.662	0.0749
236953_s_at	98.9	166.6	0.75	1.68	PPP-PPP	0.30	15	0.551	0.662	0.0582
229674_at	182.5	307.3	0.75	1.68	MPP-PPP	0.30	15	0.551	0.662	0.0508
236128_at	108.5	182.4	0.75	1.68	PPP-PPP	0.30	15	0.551	0.662	0.0488
239496_at	53.9	90.4	0.75	1.68	APA-PAP	0.30	15	0.551	0.662	0.0760
231875_at	85.5	143.5	0.75	1.68	PPP-PPP	0.30	15	0.551	0.662	0.0731
228988_at	255.9	429.6	0.75	1.68	PPP-PPP	0.30	15	0.551	0.662	0.0490
230127_at	111.1	185.9	0.74	1.67	PPP-PPP	0.30	15	0.551	0.662	0.0598
225175_s_at	113.4	189.5	0.74	1.67	APA-AAA	0.30	15	0.551	0.662	0.0750
1570566_at	45.8	76.4	0.74	1.67	PPP-PPP	0.30	15	0.551	0.662	0.0692
216755_at	93.8	156.2	0.74	1.67	MPA-PPP	0.30	15	0.551	0.662	0.0697
235772_at	60.6	100.9	0.74	1.66	AAA-PPP	0.30	15	0.551	0.662	0.0846
206783_at	102.7	170.8	0.73	1.66	AAP-PPA	0.30	15	0.551	0.662	0.0763
222835_at	1402.4	847.2	-0.73	-1.66	PPP-PPP	0.30	15	0.551	0.662	0.0024
1557238_s_at	63.7	105.5	0.73	1.66	PPP-PPP	0.30	15	0.551	0.662	0.0704
233555_s_at	414.1	685.5	0.73	1.66	PPP-PPP	0.30	15	0.551	0.662	0.0621
208438_s_at	74.4	123.1	0.73	1.66	AAA-PAA	0.30	15	0.551	0.662	0.0478
1557813_at	148.1	243.9	0.72	1.65	PAM-PPP	0.30	15	0.551	0.662	0.0816
215063_x_at	183.5	302.3	0.72	1.65	PPA-PAP	0.30	15	0.551	0.662	0.0470
235729_at	80.0	131.5	0.72	1.64	PPP-PPP	0.30	15	0.551	0.662	0.0458
1568938_at	66.5	109.1	0.71	1.64	AAA-APA	0.30	15	0.551	0.662	0.0589
208455_at	36.7	60.1	0.71	1.64	AAA-APP	0.30	15	0.551	0.662	0.0534
220682_s_at	161.3	98.6	-0.71	-1.64	PAP-PAA	0.30	15	0.551	0.662	0.0019
201117_s_at	107.6	176.1	0.71	1.64	PPP-PPP	0.30	15	0.551	0.662	0.0594
1561518_at	92.8	151.7	0.71	1.64	APA-PPP	0.30	15	0.551	0.662	0.0749
1557540_at	48.8	79.8	0.71	1.63	PAA-APA	0.30	15	0.551	0.662	0.0434
203157_s_at	342.5	558.8	0.71	1.63	PPP-PPP	0.30	15	0.551	0.662	0.0530
238774_at	210.2	342.4	0.70	1.63	PPP-PPP	0.30	15	0.551	0.662	0.0435
237690_at	59.7	97.1	0.70	1.63	APP-PPP	0.30	15	0.551	0.662	0.0588
221841_s_at	796.0	1294.2	0.70	1.63	PPP-PPP	0.30	15	0.551	0.662	0.0387
227663_at	181.4	293.8	0.70	1.62	PPP-PPP	0.30	15	0.551	0.662	0.0460
222207_x_at	507.2	821.7	0.70	1.62	PPP-PPP	0.30	15	0.551	0.662	0.0386
239654_at	138.9	224.8	0.70	1.62	PPP-PPP	0.30	15	0.551	0.662	0.0786
1562391_at	111.8	180.6	0.69	1.62	PAP-PPP	0.30	15	0.551	0.662	0.0755
216092_s_at	121.5	196.2	0.69	1.62	AAP-PPP	0.30	15	0.551	0.662	0.0531
244753_at	240.5	387.9	0.69	1.61	PPP-PPP	0.30	15	0.551	0.662	0.0691
231770_x_at	175.7	283.1	0.69	1.61	PPP-PPP	0.30	15	0.551	0.662	0.0423
216470_x_at	64.2	103.3	0.69	1.61	AAA-AMP	0.30	15	0.551	0.662	0.0655

237006_at	79.6	127.7	0.68	1.61	PAA-PPP	0.30	15	0.551	0.662	0.0504
200766_at	830.0	1330.8	0.68	1.60	PPP-PPP	0.30	15	0.551	0.662	0.0752
210210_at	165.9	265.9	0.68	1.60	PPM-PPP	0.30	15	0.551	0.662	0.0483
1557286_at	58.8	94.1	0.68	1.60	APP-PPP	0.30	15	0.551	0.662	0.0465
242442_x_at	234.5	374.9	0.68	1.60	PPP-PPP	0.30	15	0.551	0.662	0.0560
235604_x_at	73.5	117.3	0.68	1.60	APP-PPP	0.30	15	0.551	0.662	0.0710
1563075_s_at	46.7	74.5	0.68	1.60	PPP-PPP	0.30	15	0.551	0.662	0.0618
226069_at	101.5	162.0	0.68	1.60	PPP-PPP	0.30	15	0.551	0.662	0.0615
230143_at	61.6	98.2	0.67	1.60	AAP-APP	0.30	15	0.551	0.662	0.0676
1555677_s_at	124.4	198.4	0.67	1.59	AAP-MPP	0.30	15	0.551	0.662	0.0447
205883_at	1341.9	2136.1	0.67	1.59	PPP-PPP	0.30	15	0.551	0.662	0.0378
240222_at	120.8	192.0	0.67	1.59	AAP-PMP	0.30	15	0.551	0.662	0.0537
202488_s_at	1807.3	2873.0	0.67	1.59	PPP-PPP	0.30	15	0.551	0.662	0.0471
235550_at	402.9	253.7	-0.67	-1.59	PPP-PPP	0.30	15	0.551	0.662	0.0006
212911_at	172.4	273.8	0.67	1.59	PPP-PPP	0.30	15	0.551	0.662	0.0368
230791_at	266.0	421.7	0.66	1.59	PPP-PPP	0.30	15	0.551	0.662	0.0409
243233_at	71.4	113.0	0.66	1.58	AAP-PPP	0.30	15	0.551	0.662	0.0520
215783_s_at	37.6	59.5	0.66	1.58	AAA-AAP	0.30	15	0.551	0.662	0.0430
226494_at	89.5	141.7	0.66	1.58	AAA-MAP	0.30	15	0.551	0.662	0.0790
212497_at	200.9	317.8	0.66	1.58	MPP-PPP	0.30	15	0.551	0.662	0.0418
213593_s_at	183.3	289.9	0.66	1.58	PPP-PPP	0.30	15	0.551	0.662	0.0503
1553726_s_at	33.4	52.8	0.66	1.58	AAP-AAA	0.30	15	0.551	0.662	0.0401
229396_at	252.4	398.4	0.66	1.58	AMM-PPP	0.30	15	0.551	0.662	0.0726
236924_at	54.1	85.4	0.66	1.58	APP-PPP	0.30	15	0.551	0.662	0.0526
236961_at	83.4	131.5	0.66	1.58	PPP-PPP	0.30	15	0.551	0.662	0.0690
242865_at	53.8	84.8	0.66	1.58	APA-PPP	0.30	15	0.551	0.662	0.0451
237464_at	94.2	148.3	0.66	1.57	PPP-PPP	0.30	15	0.551	0.662	0.0557
1557666_s_at	78.9	123.8	0.65	1.57	APP-PPP	0.30	15	0.551	0.662	0.0399
241789_at	166.9	261.1	0.65	1.56	PPP-PPP	0.30	15	0.551	0.662	0.0553
1559103_s_at	39.8	62.0	0.64	1.56	PPA-PPP	0.30	15	0.551	0.662	0.0525
233807_at	53.5	83.4	0.64	1.56	AAA-AAP	0.30	15	0.551	0.662	0.0637
242448_at	111.3	172.7	0.64	1.55	MPP-PMP	0.30	15	0.551	0.662	0.0492
238501_at	147.3	228.8	0.64	1.55	PPP-PPP	0.30	15	0.551	0.662	0.0382
212365_at	6130.1	3960.1	-0.63	-1.55	PPP-PPP	0.30	15	0.551	0.662	0.0012
226637_at	55.1	85.3	0.63	1.55	AAA-PAM	0.30	15	0.551	0.662	0.0719
235123_at	209.1	323.4	0.63	1.55	PPP-PPP	0.30	15	0.551	0.662	0.0731
230009_at	181.3	280.4	0.63	1.55	PPP-PPP	0.30	15	0.551	0.662	0.0730
209925_at	220.6	341.1	0.63	1.55	PPP-PPP	0.30	15	0.551	0.662	0.0582
236243_at	156.8	242.6	0.63	1.55	PPP-PPP	0.30	15	0.551	0.662	0.0415
1570354_s_at	30.1	46.6	0.63	1.55	AAA-AAP	0.30	15	0.551	0.662	0.0413
1558755_x_at	54.4	84.1	0.63	1.55	AAA-MPP	0.30	15	0.551	0.662	0.0629
236974_at	89.1	137.6	0.63	1.54	PAP-PPP	0.30	15	0.551	0.662	0.0368

206864_s_at	163.7	252.5	0.63	1.54	PPP-PPP	0.30	15	0.551	0.662	0.0517
240214_at	93.7	144.3	0.62	1.54	PPP-PPP	0.30	15	0.551	0.662	0.0653
215260_s_at	342.2	527.4	0.62	1.54	PPP-PPP	0.30	15	0.551	0.662	0.0405
209587_at	187.3	288.1	0.62	1.54	AAA-APP	0.30	15	0.551	0.662	0.0616
220467_at	165.9	254.5	0.62	1.53	PPP-PPP	0.30	15	0.551	0.662	0.0573
213974_at	37.1	56.8	0.62	1.53	PAA-PAP	0.30	15	0.551	0.662	0.0487
235075_at	4127.2	6320.5	0.62	1.53	PPP-PPP	0.30	15	0.551	0.662	0.0615
243450_at	57.3	87.7	0.61	1.53	AAA-APA	0.30	15	0.551	0.662	0.0375
230020_at	98.5	150.6	0.61	1.53	AMP-MPA	0.30	15	0.551	0.662	0.0576
209964_s_at	129.7	197.8	0.61	1.53	APP-PPP	0.30	15	0.551	0.662	0.0668
1438_at	219.5	334.7	0.61	1.53	APA-PPM	0.30	15	0.551	0.662	0.0636
219517_at	349.0	531.5	0.61	1.52	PPP-PPP	0.30	15	0.551	0.662	0.0613
1558330_x_at	212.7	324.0	0.61	1.52	AAA-AAP	0.30	15	0.551	0.662	0.0612
1556144_at	80.8	123.0	0.61	1.52	APP-PPP	0.30	15	0.551	0.662	0.0546
233219_at	140.3	212.5	0.60	1.52	APP-PMP	0.30	15	0.551	0.662	0.0709
235285_at	86.4	130.8	0.60	1.52	PPP-PPP	0.30	15	0.551	0.662	0.0659
231003_at	77.8	117.5	0.60	1.51	PMA-AAP	0.30	15	0.551	0.662	0.0654
1569477_at	60.0	90.6	0.59	1.51	PAP-PPP	0.30	15	0.551	0.662	0.0416
204950_at	81.4	122.5	0.59	1.51	PPA-PPP	0.30	15	0.551	0.662	0.0716
218419_s_at	227.4	342.2	0.59	1.51	PAP-PPP	0.30	15	0.551	0.662	0.0525
235000_at	181.6	273.2	0.59	1.51	PPP-PPP	0.30	15	0.551	0.662	0.0382
243593_s_at	95.6	143.6	0.59	1.50	AAP-PPP	0.30	15	0.551	0.662	0.0680
222183_x_at	163.0	244.7	0.59	1.50	PPM-PPP	0.30	15	0.551	0.662	0.0611
229111_at	141.1	211.5	0.58	1.50	PPP-PPP	0.30	15	0.551	0.662	0.0566
207661_s_at	96.1	144.1	0.58	1.50	PPP-PPP	0.30	15	0.551	0.662	0.0514
1569808_at	37.8	56.5	0.58	1.50	AAA-PPP	0.30	15	0.551	0.662	0.0553
209169_at	232.0	346.5	0.58	1.49	PPP-PPP	0.30	15	0.551	0.662	0.0603
212890_at	302.0	451.2	0.58	1.49	AAP-APP	0.30	15	0.551	0.662	0.0405
232600_at	77.8	115.8	0.58	1.49	PPP-PPP	0.30	15	0.551	0.662	0.0505
233678_at	41.1	61.2	0.57	1.49	AAA-PAP	0.30	15	0.551	0.662	0.0403
243759_at	82.8	123.1	0.57	1.49	PAP-PPP	0.30	15	0.551	0.662	0.0417
34478_at	155.5	230.9	0.57	1.49	PPP-PPP	0.30	15	0.551	0.662	0.0632
231199_at	163.1	242.1	0.57	1.48	PPP-PPP	0.30	15	0.551	0.662	0.0608
244418_at	45.1	66.7	0.56	1.48	AAP-PAA	0.30	15	0.551	0.662	0.0674
235493_at	77.7	114.8	0.56	1.48	PPP-PPP	0.30	15	0.551	0.662	0.0605
1557553_at	66.0	97.5	0.56	1.48	PPP-PPM	0.30	15	0.551	0.662	0.0435
233017_x_at	196.5	289.7	0.56	1.47	PPP-PPP	0.30	15	0.551	0.662	0.0301
213420_at	196.1	288.6	0.56	1.47	PPP-PPP	0.30	15	0.551	0.662	0.0459
219304_s_at	336.3	494.5	0.56	1.47	PPP-PPP	0.30	15	0.551	0.662	0.0387
225692_at	5835.8	3973.5	-0.56	-1.47	PPP-PPP	0.30	15	0.551	0.662	0.0006
244703_x_at	301.2	442.6	0.56	1.47	PPP-PPP	0.30	15	0.551	0.662	0.0622
202206_at	396.5	270.1	-0.55	-1.47	PPP-PMP	0.30	15	0.551	0.662	0.0001

223082_at	5191.6	3535.1	-0.55	-1.47	PPP-PPP	0.30	15	0.551	0.662	0.0008
236488_s_at	404.4	592.9	0.55	1.47	PPP-PPP	0.30	15	0.551	0.662	0.0514
226871_s_at	271.6	398.3	0.55	1.47	PPP-PPP	0.30	15	0.551	0.662	0.0506
227492_at	1399.1	2050.7	0.55	1.47	PPP-PPP	0.30	15	0.551	0.662	0.0310
227810_at	187.3	274.5	0.55	1.47	PAP-PPP	0.30	15	0.551	0.662	0.0553
217622_at	97.3	142.5	0.55	1.46	AAA-APA	0.30	15	0.551	0.662	0.0347
219239_s_at	239.5	350.2	0.55	1.46	PPP-PPP	0.30	15	0.551	0.662	0.0456
205346_at	74.7	109.1	0.55	1.46	PPP-PPP	0.30	15	0.551	0.662	0.0400
223131_s_at	92.6	135.3	0.55	1.46	APP-PPP	0.30	15	0.551	0.662	0.0399
228950_s_at	380.8	556.0	0.55	1.46	PPP-PPP	0.30	15	0.551	0.662	0.0608
236241_at	90.6	132.3	0.55	1.46	PPP-PPP	0.30	15	0.551	0.662	0.0412
1561526_at	51.1	74.6	0.55	1.46	AAA-PAA	0.30	15	0.551	0.662	0.0475
221899_at	919.9	1340.7	0.54	1.46	PPP-PPP	0.30	15	0.551	0.662	0.0411
235094_at	267.8	390.3	0.54	1.46	PPP-PPP	0.30	15	0.551	0.662	0.0404
212436_at	448.9	653.0	0.54	1.46	PPP-PPP	0.30	15	0.551	0.662	0.0391
214691_x_at	217.6	316.1	0.54	1.45	PPP-PPP	0.30	15	0.551	0.662	0.0653
1555842_at	464.9	675.1	0.54	1.45	PPP-PPP	0.30	15	0.551	0.662	0.0656
221860_at	196.7	285.4	0.54	1.45	PPP-PPP	0.30	15	0.551	0.662	0.0581
221679_s_at	101.3	146.9	0.54	1.45	PPP-PMP	0.30	15	0.551	0.662	0.0565
1557118_a_at	80.3	116.4	0.54	1.45	PAP-PAP	0.30	15	0.551	0.662	0.0358
205157_s_at	20011.7	28984.6	0.53	1.45	PPP-PPP	0.30	15	0.551	0.662	0.0444
219836_at	1694.4	1174.8	-0.53	-1.44	PPP-PPP	0.30	15	0.551	0.662	0.0006
243303_at	127.8	184.4	0.53	1.44	PPA-PPP	0.30	15	0.551	0.662	0.0606
233788_at	48.4	69.8	0.53	1.44	AAP-PMP	0.30	15	0.551	0.662	0.0371
201044_x_at	596.7	858.6	0.53	1.44	PPP-PPP	0.30	15	0.551	0.662	0.0383
1556202_at	90.7	130.5	0.52	1.44	APA-AAP	0.30	15	0.551	0.662	0.0331
1565436_s_at	96.6	138.7	0.52	1.44	PPP-MMP	0.30	15	0.551	0.662	0.0503
1555847_a_at	614.3	882.9	0.52	1.44	PPP-PPP	0.30	15	0.551	0.662	0.0490
39549_at	126.6	181.9	0.52	1.44	PPP-PPP	0.30	15	0.551	0.662	0.0447
205794_s_at	54.2	77.7	0.52	1.44	AAA-AAP	0.30	15	0.551	0.662	0.0609
203134_at	233.8	335.2	0.52	1.43	PPP-PPP	0.30	15	0.551	0.662	0.0265
225956_at	306.4	439.1	0.52	1.43	PPP-PPP	0.30	15	0.551	0.662	0.0289
232471_at	175.3	251.1	0.52	1.43	APP-PPP	0.30	15	0.551	0.662	0.0286
1553227_s_at	146.4	209.3	0.52	1.43	PPP-PPP	0.30	15	0.551	0.662	0.0586
209212_s_at	7431.4	10623.1	0.52	1.43	PPP-PPP	0.30	15	0.551	0.662	0.0457
227406_at	206.6	294.8	0.51	1.43	PMM-PPP	0.30	15	0.551	0.662	0.0352
225656_at	170.8	243.3	0.51	1.43	PPP-PPP	0.30	15	0.551	0.662	0.0371
226672_s_at	164.0	233.7	0.51	1.43	PAA-AMA	0.30	15	0.551	0.662	0.0332
215068_s_at	283.5	403.9	0.51	1.43	PPP-PPP	0.30	15	0.551	0.662	0.0303
238185_at	55.0	78.2	0.51	1.42	AAA-AAP	0.30	15	0.551	0.662	0.0329
219377_at	1298.9	1846.6	0.51	1.42	PPP-PPP	0.30	15	0.551	0.662	0.0533
225937_at	257.3	365.5	0.51	1.42	APP-MPP	0.30	15	0.551	0.662	0.0557

226817_at	2006.6	2851.0	0.51	1.42	PPP-PPP	0.30	15	0.551	0.662	0.0311
242875_at	76.8	109.0	0.51	1.42	AAM-AAP	0.30	15	0.551	0.662	0.0500
219581_at	616.3	874.3	0.50	1.42	PPP-PPP	0.30	15	0.551	0.662	0.0462
216250_s_at	416.9	294.1	-0.50	-1.42	PPP-PPP	0.30	15	0.551	0.662	0.0004
243134_at	312.3	442.6	0.50	1.42	PPP-PPP	0.30	15	0.551	0.662	0.0544
215287_at	337.8	478.0	0.50	1.42	PPP-PPP	0.30	15	0.551	0.662	0.0369
214929_s_at	80.1	113.3	0.50	1.42	PAP-PPP	0.30	15	0.551	0.662	0.0548
219341_at	137.6	194.4	0.50	1.41	AAA-APA	0.30	15	0.551	0.662	0.0344
231988_x_at	152.1	214.8	0.50	1.41	AAP-PPP	0.30	15	0.551	0.662	0.0460
202724_s_at	1162.8	1638.2	0.50	1.41	PPP-PPP	0.30	15	0.551	0.662	0.0304
243995_at	97.6	137.2	0.49	1.41	AAA-PAP	0.30	15	0.551	0.662	0.0533
201838_s_at	136.5	191.8	0.49	1.41	PPP-PPP	0.30	15	0.551	0.662	0.0322
212276_at	1172.5	1647.2	0.49	1.41	PPP-PPP	0.30	15	0.551	0.662	0.0245
236429_at	377.1	529.0	0.49	1.40	PPP-PPP	0.30	15	0.551	0.662	0.0377
215991_s_at	95.4	133.8	0.49	1.40	PAA-AAA	0.30	15	0.551	0.662	0.0327
240326_at	95.2	133.4	0.49	1.40	PPP-PPP	0.30	15	0.551	0.662	0.0276
229426_at	279.1	390.6	0.49	1.40	PPP-PPP	0.30	15	0.551	0.662	0.0394
226805_at	460.3	644.0	0.48	1.40	PPP-PPP	0.30	15	0.551	0.662	0.0338
215253_s_at	136.0	190.0	0.48	1.40	AAA-PAP	0.30	15	0.551	0.662	0.0510
222133_s_at	258.0	359.9	0.48	1.40	PPP-PPP	0.30	15	0.551	0.662	0.0316
225916_at	388.5	541.9	0.48	1.40	PPP-PPP	0.30	15	0.551	0.662	0.0252
215046_at	61.1	85.0	0.48	1.39	PAA-MPP	0.30	15	0.551	0.662	0.0541
205595_at	3263.5	4540.1	0.48	1.39	PPP-PPP	0.30	15	0.551	0.662	0.0443
229779_at	123.0	170.8	0.47	1.39	PPP-PPP	0.30	15	0.551	0.662	0.0510
232064_at	233.4	324.2	0.47	1.39	PPP-PPP	0.30	15	0.551	0.662	0.0492
231718_at	1111.1	1541.7	0.47	1.39	PPP-PPP	0.30	15	0.551	0.662	0.0417
219189_at	387.8	537.4	0.47	1.39	PPP-PPP	0.30	15	0.551	0.662	0.0565
228361_at	294.5	408.0	0.47	1.39	PPP-PPP	0.30	15	0.551	0.662	0.0343
223658_at	99.4	137.5	0.47	1.38	PAM-MPP	0.30	15	0.551	0.662	0.0373
230490_x_at	784.8	1080.5	0.46	1.38	PPP-PPP	0.30	15	0.551	0.662	0.0283
1558365_at	189.3	260.2	0.46	1.38	PPP-PPP	0.30	15	0.551	0.662	0.0331
228910_at	441.2	605.4	0.46	1.37	PPP-PPP	0.30	15	0.551	0.662	0.0484
213386_at	431.1	591.8	0.46	1.37	PPP-PPP	0.30	15	0.551	0.662	0.0441
209189_at	2433.8	3334.8	0.45	1.37	PPP-PPP	0.30	15	0.551	0.662	0.0328
204011_at	1874.8	2567.5	0.45	1.37	PPP-PPP	0.30	15	0.551	0.662	0.0267
224435_at	6022.7	8242.3	0.45	1.37	PPP-PPP	0.30	15	0.551	0.662	0.0504
210540_s_at	522.0	714.6	0.45	1.37	PPP-PPP	0.30	15	0.551	0.662	0.0213
229054_at	111.3	152.2	0.45	1.37	APA-AMM	0.30	15	0.551	0.662	0.0382
204858_s_at	149.3	203.7	0.45	1.37	PAP-PPP	0.30	15	0.551	0.662	0.0276
221794_at	267.8	365.0	0.45	1.36	PPP-PPP	0.30	15	0.551	0.662	0.0512
1552612_at	251.2	342.5	0.45	1.36	PPP-PPP	0.30	15	0.551	0.662	0.0207
212291_at	358.2	487.9	0.45	1.36	PPP-PPP	0.30	15	0.551	0.662	0.0347

223500_at	86.9	118.2	0.44	1.36	PAP-APP	0.30	15	0.551	0.662	0.0464
219484_at	184.1	250.2	0.44	1.36	PPP-PPP	0.30	15	0.551	0.662	0.0423
213786_at	854.9	1161.6	0.44	1.36	PPP-PPP	0.30	15	0.551	0.662	0.0265
209328_x_at	417.2	565.1	0.44	1.36	PPP-PPP	0.30	15	0.551	0.662	0.0496
214482_at	125.9	170.4	0.44	1.35	PPP-PPP	0.30	15	0.551	0.662	0.0519
214881_s_at	189.3	256.1	0.44	1.35	APP-MPP	0.30	15	0.551	0.662	0.0338
230085_at	83.4	112.9	0.44	1.35	PPA-PPP	0.30	15	0.551	0.662	0.0223
213213_at	300.9	406.9	0.44	1.35	PPP-PPP	0.30	15	0.551	0.662	0.0440
241198_s_at	138.7	187.5	0.44	1.35	PPP-PMP	0.30	15	0.551	0.662	0.0282
209107_x_at	455.7	615.4	0.43	1.35	AAA-APP	0.30	15	0.551	0.662	0.0288
227250_at	364.5	491.9	0.43	1.35	PPP-PPP	0.30	15	0.551	0.662	0.0196
202734_at	1400.8	1889.4	0.43	1.35	PPP-PPP	0.30	15	0.551	0.662	0.0440
233350_s_at	457.5	616.9	0.43	1.35	PPP-PPP	0.30	15	0.551	0.662	0.0476
206833_s_at	375.6	506.4	0.43	1.35	PPP-PPP	0.30	15	0.551	0.662	0.0203
1555325_s_at	69.6	93.6	0.43	1.35	MPP-PPP	0.30	15	0.551	0.662	0.0390
228736_at	263.4	354.0	0.43	1.34	PPP-PPP	0.30	15	0.551	0.662	0.0262
231863_at	266.1	357.4	0.43	1.34	PPP-PPP	0.30	15	0.551	0.662	0.0224
242463_x_at	424.0	567.7	0.42	1.34	PPP-PPP	0.30	15	0.551	0.662	0.0304
228744_at	396.4	530.2	0.42	1.34	PPP-PPP	0.30	15	0.551	0.662	0.0360
217703_x_at	666.8	892.3	0.42	1.34	PPP-PPP	0.30	15	0.551	0.662	0.0196
225401_at	440.2	588.7	0.42	1.34	PPP-PPP	0.30	15	0.551	0.662	0.0416
237059_at	123.8	165.5	0.42	1.34	PAP-PPP	0.30	15	0.551	0.662	0.0317
230559_x_at	276.6	368.9	0.42	1.33	PPP-PPP	0.30	15	0.551	0.662	0.0407
1570138_at	63.2	84.2	0.41	1.33	AAP-PMP	0.30	15	0.551	0.662	0.0388
222549_at	7674.8	10209.3	0.41	1.33	PPP-PPP	0.30	15	0.551	0.662	0.0309
91816_f_at	568.2	753.9	0.41	1.33	PPP-PPP	0.30	15	0.551	0.662	0.0275
233264_at	104.6	138.7	0.41	1.33	PPP-PPP	0.30	15	0.551	0.662	0.0297
214686_at	638.5	844.7	0.40	1.32	PPP-PPP	0.30	15	0.551	0.662	0.0372
239866_at	137.3	180.9	0.40	1.32	PPP-PPP	0.30	15	0.551	0.662	0.0373
229881_at	218.0	286.9	0.40	1.32	PAM-PPP	0.30	15	0.551	0.662	0.0209
223125_s_at	1346.3	1771.6	0.40	1.32	PPP-PPP	0.30	15	0.551	0.662	0.0199
201679_at	327.6	430.5	0.39	1.31	PPP-PPP	0.30	15	0.551	0.662	0.0414
205415_s_at	346.8	455.6	0.39	1.31	PPP-PPP	0.30	15	0.551	0.662	0.0357
244563_at	707.1	929.1	0.39	1.31	PPP-PPP	0.30	15	0.551	0.662	0.0288
236832_at	76.3	100.1	0.39	1.31	PAA-PPM	0.30	15	0.551	0.662	0.0424
242293_at	199.4	261.1	0.39	1.31	MMP-PPP	0.30	15	0.551	0.662	0.0434
219538_at	170.1	222.7	0.39	1.31	PPP-PPP	0.30	15	0.551	0.662	0.0374
226557_at	206.2	269.8	0.39	1.31	PMA-PPP	0.30	15	0.551	0.662	0.0169
65438_at	329.3	430.3	0.39	1.31	PPP-PPP	0.30	15	0.551	0.662	0.0328
220915_s_at	143.3	186.3	0.38	1.30	PPM-PPP	0.30	15	0.551	0.662	0.0298
214902_x_at	565.4	734.8	0.38	1.30	PPP-PPP	0.30	15	0.551	0.662	0.0273
236192_at	473.1	614.7	0.38	1.30	PPP-PPP	0.30	15	0.551	0.662	0.0264

202286_s_at	14623.6	18985.8	0.38	1.30	PPP-PPP	0.30	15	0.551	0.662	0.0171
228331_at	71.3	92.5	0.38	1.30	PMP-PPP	0.30	15	0.551	0.662	0.0175
202098_s_at	423.6	548.4	0.37	1.30	PPP-PAP	0.30	15	0.551	0.662	0.0146
225120_at	1246.8	1610.7	0.37	1.29	PPP-PPP	0.30	15	0.551	0.662	0.0387
237435_at	480.6	620.2	0.37	1.29	PPP-PPP	0.30	15	0.551	0.662	0.0377
1556373_a_at	137.2	177.2	0.37	1.29	PAM-PPP	0.30	15	0.551	0.662	0.0333
207303_at	50.7	65.5	0.37	1.29	AAA-PAA	0.30	15	0.551	0.662	0.0198
217744_s_at	13113.3	16896.7	0.37	1.29	PPP-PPP	0.30	15	0.551	0.662	0.0177
230281_at	240.6	309.7	0.37	1.29	PPP-PPP	0.30	15	0.551	0.662	0.0370
215535_s_at	905.0	1165.5	0.37	1.29	PPP-PPP	0.30	15	0.551	0.662	0.0293
216272_x_at	179.3	230.9	0.37	1.29	PPP-PPP	0.30	15	0.551	0.662	0.0264
213029_at	2603.4	3350.8	0.36	1.29	PPP-PPP	0.30	15	0.551	0.662	0.0193
221139_s_at	151.6	195.2	0.36	1.29	PPP-PPP	0.30	15	0.551	0.662	0.0152
201150_s_at	4440.8	5685.3	0.36	1.28	PPP-PPP	0.30	15	0.551	0.662	0.0187
203600_s_at	617.7	790.8	0.36	1.28	PPP-PPP	0.30	15	0.551	0.662	0.0159
234068_s_at	295.7	378.1	0.36	1.28	PPP-PPP	0.30	15	0.551	0.662	0.0164
225205_at	1270.0	1621.0	0.35	1.28	PPP-PPP	0.30	15	0.551	0.662	0.0240
233734_s_at	376.1	479.3	0.35	1.27	PPP-PPP	0.30	15	0.551	0.662	0.0193
230229_at	240.6	306.5	0.35	1.27	PPP-PPP	0.30	15	0.551	0.662	0.0136
202044_at	159.2	202.7	0.35	1.27	APP-AMP	0.30	15	0.551	0.662	0.0177
228158_at	561.6	714.2	0.35	1.27	PPP-PPP	0.30	15	0.551	0.662	0.0117
209351_at	27468.3	34879.9	0.35	1.27	PPP-PPP	0.30	15	0.551	0.662	0.0250
218518_at	386.0	490.1	0.34	1.27	PPP-PPP	0.30	15	0.551	0.662	0.0341
227443_at	226.6	287.5	0.34	1.27	PPP-PPP	0.30	15	0.551	0.662	0.0303
222436_s_at	1836.3	2328.1	0.34	1.27	PPP-PPP	0.30	15	0.551	0.662	0.0229
200951_s_at	940.7	1189.6	0.34	1.27	PPP-PPP	0.30	15	0.551	0.662	0.0288
1553993_s_at	2595.2	3280.7	0.34	1.26	PPP-PPP	0.30	15	0.551	0.662	0.0251
224857_s_at	3925.3	4951.2	0.34	1.26	PPP-PPP	0.30	15	0.551	0.662	0.0108
200852_x_at	2840.6	3580.5	0.33	1.26	PPP-PPP	0.30	15	0.551	0.662	0.0221
226213_at	511.8	645.2	0.33	1.26	PPP-PPP	0.30	15	0.551	0.662	0.0209
209170_s_at	1692.4	2131.1	0.33	1.26	PPP-PPP	0.30	15	0.551	0.662	0.0140
235296_at	237.8	298.1	0.33	1.25	PPP-PPP	0.30	15	0.551	0.662	0.0139
238567_at	287.7	360.4	0.33	1.25	PPP-PPP	0.30	15	0.551	0.662	0.0094
212387_at	228.7	286.3	0.32	1.25	MAA-APP	0.30	15	0.551	0.662	0.0108
206200_s_at	5668.9	7068.4	0.32	1.25	PPP-PPP	0.30	15	0.551	0.662	0.0182
223412_at	392.7	488.5	0.32	1.24	PPP-PPP	0.30	15	0.551	0.662	0.0274
210685_s_at	325.7	405.1	0.32	1.24	PPP-PPP	0.30	15	0.551	0.662	0.0144
222263_at	670.0	832.0	0.31	1.24	PPP-PPP	0.30	15	0.551	0.662	0.0155
200824_at	29425.0	36540.5	0.31	1.24	PPP-PPP	0.30	15	0.551	0.662	0.0090
203963_at	2130.3	2642.8	0.31	1.24	PPP-PPP	0.30	15	0.551	0.662	0.0201
229841_at	857.5	1062.8	0.31	1.24	PPP-PPP	0.30	15	0.551	0.662	0.0232
242214_at	449.5	556.4	0.31	1.24	PPP-PPP	0.30	15	0.551	0.662	0.0103

201244_s_at	1636.5	2020.1	0.30	1.23	PPP-PPP	0.30	15	0.551	0.662	0.0076
238536_at	107.1	132.0	0.30	1.23	PPP-PMP	0.30	15	0.551	0.662	0.0177
220052_s_at	296.6	363.9	0.30	1.23	PPP-PPP	0.30	15	0.551	0.662	0.0193
209217_s_at	510.6	626.2	0.29	1.23	PPP-PPP	0.30	15	0.551	0.662	0.0209
223126_s_at	285.0	349.5	0.29	1.23	PPP-PPP	0.30	15	0.551	0.662	0.0096
203448_s_at	1203.0	1468.4	0.29	1.22	PPP-PPP	0.30	15	0.551	0.662	0.0177
202704_at	2793.6	3409.3	0.29	1.22	PPP-PPP	0.30	15	0.551	0.662	0.0181
206295_at	7580.7	9245.2	0.29	1.22	PPP-PPP	0.30	15	0.551	0.662	0.0080
207821_s_at	2466.5	3007.0	0.29	1.22	PPP-PPP	0.30	15	0.551	0.662	0.0059
200762_at	9627.9	11707.6	0.28	1.22	PPP-PPP	0.30	15	0.551	0.662	0.0125
217973_at	1901.2	2312.1	0.28	1.22	PPP-PPP	0.30	15	0.551	0.662	0.0058
49329_at	312.9	379.3	0.28	1.21	PPP-PPP	0.30	15	0.551	0.662	0.0092
224999_at	4512.6	10292.3	0.27	2.29	PPP-PPP	0.30	15	0.551	0.662	0.0084
215693_x_at	2925.3	3528.5	0.27	1.21	PPP-PPP	0.30	15	0.551	0.662	0.0057
211713_x_at	1735.7	2091.6	0.27	1.21	PPP-PPP	0.30	15	0.551	0.662	0.0115
238513_at	1831.9	2203.5	0.27	1.20	PPP-PPP	0.30	15	0.551	0.662	0.0117
225806_at	5638.9	6777.6	0.27	1.20	PPP-PPP	0.30	15	0.551	0.662	0.0051
216060_s_at	5331.4	6390.4	0.26	1.20	PPP-PPP	0.30	15	0.551	0.662	0.0134
235253_at	359.6	430.3	0.26	1.20	PPP-PPP	0.30	15	0.551	0.662	0.0101
37872_at	357.3	426.3	0.26	1.19	PPP-PPP	0.30	15	0.551	0.662	0.0130
37424_at	285.0	337.9	0.25	1.19	PPP-PPP	0.30	15	0.551	0.662	0.0051
222621_at	1293.8	1534.7	0.25	1.19	PPP-PPP	0.30	15	0.551	0.662	0.0043
208820_at	7093.8	8395.6	0.24	1.18	PPP-PPP	0.30	15	0.551	0.662	0.0099
226118_at	350.7	414.7	0.24	1.18	PPP-PPP	0.30	15	0.551	0.662	0.0082
215691_x_at	5351.9	6325.0	0.24	1.18	PPP-PPP	0.30	15	0.551	0.662	0.0028
222392_x_at	25346.4	29927.0	0.24	1.18	PPP-PPP	0.30	15	0.551	0.662	0.0097
227001_at	2704.0	3182.7	0.24	1.18	PPP-PPP	0.30	15	0.551	0.662	0.0079
200932_s_at	3279.3	3857.0	0.23	1.18	PPP-PPP	0.30	15	0.551	0.662	0.0019
232349_x_at	1242.3	1456.3	0.23	1.17	PPP-PPP	0.30	15	0.551	0.662	0.0077
238022_at	1258.5	1472.8	0.23	1.17	PPP-PPP	0.30	15	0.551	0.662	0.0028
212096_s_at	1992.9	2310.3	0.21	1.16	PPP-PPP	0.30	15	0.551	0.662	0.0015
226399_at	1136.1	1301.1	0.20	1.15	PPP-PPP	0.30	15	0.551	0.662	0.0020
1564405_at	33.2	3.1	-3.44	-10.89	AAP-AAA	0.20	16	0.682	0.765	0.0157
221154_at	32.0	3.1	-3.37	-10.35	AAP-AAA	0.20	16	0.682	0.765	0.0177
1564301_a_at	49.1	4.9	-3.33	-10.02	PAA-AAA	0.20	16	0.682	0.765	0.0179
220666_at	3.6	29.3	3.03	8.14	APA-APA	0.20	16	0.682	0.765	0.1284
237566_at	4.8	37.8	2.99	7.94	APA-APA	0.20	16	0.682	0.765	0.1504
238919_at	8.0	62.3	2.96	7.76	APA-AMP	0.20	16	0.682	0.765	0.1592
221805_at	1.5	11.4	2.93	7.63	AAA-APA	0.20	16	0.682	0.765	0.1455
232030_at	8.0	57.2	2.84	7.17	AAA-APP	0.20	16	0.682	0.765	0.1549
230248_x_at	4.3	28.3	2.72	6.59	AAA-PAP	0.20	16	0.682	0.765	0.2064
217058_at	14.6	92.3	2.67	6.34	AAA-APA	0.20	16	0.682	0.765	0.1344

202015_x_at	8.9	55.4	2.63	6.20	AAA-PAP	0.20	16	0.682	0.765	0.1967
224137_at	8.6	51.9	2.59	6.00	AAA-PAA	0.20	16	0.682	0.765	0.1322
214618_at	9.9	56.9	2.52	5.73	AAA-PAA	0.20	16	0.682	0.765	0.1441
240806_at	24.5	138.8	2.50	5.66	AAA-PAA	0.20	16	0.682	0.765	0.1304
238874_at	7.3	40.7	2.48	5.57	AAA-MAP	0.20	16	0.682	0.765	0.1580
230245_s_at	11.3	62.2	2.46	5.49	AAA-PAA	0.20	16	0.682	0.765	0.1920
1558331_at	12.0	65.7	2.45	5.46	AAA-PAA	0.20	16	0.682	0.765	0.1875
233351_at	4.2	22.9	2.44	5.44	AAA-AAP	0.20	16	0.682	0.765	0.1803
234260_at	17.9	93.1	2.38	5.21	AAA-PPP	0.20	16	0.682	0.765	0.2254
1564950_at	9.5	49.2	2.38	5.21	AAA-MPP	0.20	16	0.682	0.765	0.2032
232692_at	11.7	58.4	2.32	4.98	AAA-AAP	0.20	16	0.682	0.765	0.1288
1570633_at	3.7	18.3	2.29	4.91	AAA-AAP	0.20	16	0.682	0.765	0.2154
1553654_at	2.7	13.3	2.29	4.89	AAA-APA	0.20	16	0.682	0.765	0.1786
232864_s_at	17.1	83.3	2.28	4.86	AAP-AAP	0.20	16	0.682	0.765	0.1250
237361_at	16.3	78.8	2.27	4.84	PAA-APP	0.20	16	0.682	0.765	0.2368
216456_at	6.6	31.6	2.25	4.76	AAA-AAP	0.20	16	0.682	0.765	0.1761
203772_at	7.9	36.9	2.22	4.66	AAA-AAP	0.20	16	0.682	0.765	0.1711
1552318_at	5.0	23.3	2.22	4.66	AAA-AAP	0.20	16	0.682	0.765	0.1625
232746_at	11.8	54.9	2.22	4.65	AAA-PPP	0.20	16	0.682	0.765	0.1403
228848_at	18.2	84.8	2.22	4.65	AAA-APA	0.20	16	0.682	0.765	0.1349
233937_at	9.6	44.1	2.19	4.57	AAA-PAP	0.20	16	0.682	0.765	0.2061
210521_s_at	3.3	15.0	2.17	4.51	AAA-AAP	0.20	16	0.682	0.765	0.2464
228333_at	8.3	37.6	2.17	4.51	AAM-AAP	0.20	16	0.682	0.765	0.2098
1557155_a_at	18.3	81.7	2.16	4.47	PMA-PAP	0.20	16	0.682	0.765	0.2498
237081_at	12.1	54.1	2.16	4.47	AAP-AAA	0.20	16	0.682	0.765	0.1592
235226_at	9.5	42.4	2.16	4.46	AAA-AAP	0.20	16	0.682	0.765	0.1365
242947_at	7.2	32.0	2.15	4.43	AAP-AAA	0.20	16	0.682	0.765	0.1756
1566959_at	4.0	17.5	2.13	4.37	AAA-AAP	0.20	16	0.682	0.765	0.2604
236197_at	7.6	33.0	2.12	4.35	AAA-APA	0.20	16	0.682	0.765	0.1677
236782_at	11.6	50.0	2.10	4.30	AAA-PAA	0.20	16	0.682	0.765	0.1421
215575_at	10.3	44.3	2.10	4.29	AAA-AAP	0.20	16	0.682	0.765	0.1323
1562280_at	8.8	37.7	2.09	4.27	AAA-APP	0.20	16	0.682	0.765	0.1287
214070_s_at	30.8	131.5	2.09	4.27	AAP-AAA	0.20	16	0.682	0.765	0.2456
1552727_s_at	7.6	32.5	2.09	4.27	AAA-PAA	0.20	16	0.682	0.765	0.1588
1558385_at	18.5	78.8	2.09	4.25	AAA-AAP	0.20	16	0.682	0.765	0.1619
233393_at	7.5	31.7	2.09	4.25	AAA-AAP	0.20	16	0.682	0.765	0.1952
244359_s_at	21.5	91.3	2.09	4.24	AAA-PAP	0.20	16	0.682	0.765	0.1581
1558934_a_at	13.1	53.8	2.04	4.12	AAA-PAA	0.20	16	0.682	0.765	0.1408
224007_at	9.5	39.0	2.04	4.11	AAM-PAA	0.20	16	0.682	0.765	0.1426
239614_x_at	14.4	59.1	2.04	4.11	AAA-AAP	0.20	16	0.682	0.765	0.2569
219574_at	12.9	53.0	2.03	4.09	AAA-PAA	0.20	16	0.682	0.765	0.1962
1561328_at	7.9	31.9	2.02	4.05	AAA-PAP	0.20	16	0.682	0.765	0.1986

219040_at	35.1	141.7	2.01	4.03	AAA-APM	0.20	16	0.682	0.765	0.1681
1554157_a_at	10.1	40.6	2.01	4.02	AAA-APA	0.20	16	0.682	0.765	0.2177
221755_at	51.4	205.9	2.00	4.00	AAA-AAP	0.20	16	0.682	0.765	0.1929
220124_at	18.8	74.8	1.99	3.98	AAA-APA	0.20	16	0.682	0.765	0.1317
227209_at	6.6	26.0	1.97	3.91	AAA-AAP	0.20	16	0.682	0.765	0.1873
230224_at	14.8	57.5	1.96	3.89	AAA-PPA	0.20	16	0.682	0.765	0.1807
231804_at	11.2	43.5	1.95	3.87	AAA-PAA	0.20	16	0.682	0.765	0.1448
214409_at	5.2	20.3	1.95	3.87	AAA-AAP	0.20	16	0.682	0.765	0.1305
209822_s_at	12.3	47.2	1.94	3.84	PAA-PAM	0.20	16	0.682	0.765	0.2557
241865_at	49.5	190.0	1.94	3.84	PAP-MAP	0.20	16	0.682	0.765	0.2521
215610_at	29.5	112.5	1.93	3.81	AAA-AAP	0.20	16	0.682	0.765	0.2372
215400_x_at	15.0	57.2	1.93	3.81	AMA-AAP	0.20	16	0.682	0.765	0.2197
1552947_x_at	13.4	50.7	1.92	3.79	AAP-AAA	0.20	16	0.682	0.765	0.1527
238537_at	5.9	22.1	1.92	3.78	AAA-PAA	0.20	16	0.682	0.765	0.1370
237961_at	24.9	6.7	-1.90	-3.74	PAA-AAA	0.20	16	0.682	0.765	0.0114
210703_at	8.3	31.0	1.90	3.74	AAA-APA	0.20	16	0.682	0.765	0.2191
238712_at	43.9	164.1	1.90	3.73	AAP-PPP	0.20	16	0.682	0.765	0.1607
213904_at	10.1	37.4	1.89	3.70	AAA-APA	0.20	16	0.682	0.765	0.2054
211795_s_at	29.4	108.8	1.89	3.70	AAA-APP	0.20	16	0.682	0.765	0.1572
224454_at	18.1	66.7	1.88	3.69	MAA-APP	0.20	16	0.682	0.765	0.1434
236113_at	29.5	108.5	1.88	3.68	AAA-AAP	0.20	16	0.682	0.765	0.1403
232795_at	9.0	33.2	1.88	3.68	AAA-PAA	0.20	16	0.682	0.765	0.1342
214072_x_at	29.1	106.3	1.87	3.65	PAA-AAA	0.20	16	0.682	0.765	0.1824
244044_at	12.7	46.4	1.87	3.64	AAA-PAA	0.20	16	0.682	0.765	0.1462
236955_at	18.3	66.7	1.86	3.64	AAP-AMP	0.20	16	0.682	0.765	0.1852
243193_at	4.7	17.0	1.86	3.63	AAA-APA	0.20	16	0.682	0.765	0.1882
242708_at	17.0	61.5	1.86	3.63	AAA-AAP	0.20	16	0.682	0.765	0.1397
243346_at	8.6	30.8	1.85	3.60	AAA-APA	0.20	16	0.682	0.765	0.1228
242204_at	22.5	80.9	1.85	3.60	APA-AAP	0.20	16	0.682	0.765	0.2264
234170_at	22.8	82.0	1.85	3.59	AAA-PAP	0.20	16	0.682	0.765	0.1700
243872_at	6.1	21.9	1.84	3.59	AAA-PAA	0.20	16	0.682	0.765	0.1443
244042_x_at	14.9	53.0	1.83	3.56	AAA-PAA	0.20	16	0.682	0.765	0.1329
234437_at	13.3	47.5	1.83	3.56	AAA-PAA	0.20	16	0.682	0.765	0.1600
1569443_s_at	9.5	33.8	1.83	3.56	AAA-APA	0.20	16	0.682	0.765	0.1513
242886_at	22.2	78.9	1.83	3.55	AAA-PAP	0.20	16	0.682	0.765	0.1524
214676_x_at	26.5	93.8	1.82	3.54	PAA-AAA	0.20	16	0.682	0.765	0.1332
203691_at	2240.3	7918.6	1.82	3.54	PPP-PPP	0.20	16	0.682	0.765	0.1366
201667_at	10.2	35.7	1.81	3.51	APA-AAA	0.20	16	0.682	0.765	0.2371
207538_at	14.1	49.5	1.81	3.51	AAA-APA	0.20	16	0.682	0.765	0.2370
1553288_a_at	22.1	77.1	1.81	3.50	AAA-AAP	0.20	16	0.682	0.765	0.2078
240964_at	11.5	39.8	1.79	3.46	AAA-APA	0.20	16	0.682	0.765	0.1760
240448_at	12.3	42.5	1.79	3.46	AAA-AAP	0.20	16	0.682	0.765	0.1480

1563471_at	19.8	68.5	1.79	3.45	AAA-APA	0.20	16	0.682	0.765	0.1964
205236_x_at	30.7	106.0	1.79	3.45	AAA-AAP	0.20	16	0.682	0.765	0.1889
1566438_at	24.5	84.5	1.79	3.45	AAP-AAA	0.20	16	0.682	0.765	0.2099
244579_at	59.2	203.5	1.78	3.44	AAA-PPP	0.20	16	0.682	0.765	0.1281
1568665_at	20.1	68.9	1.78	3.44	AAA-AAP	0.20	16	0.682	0.765	0.1399
219685_at	13.4	45.9	1.78	3.43	APA-AAA	0.20	16	0.682	0.765	0.1914
217563_at	14.4	49.2	1.78	3.43	AAA-PAA	0.20	16	0.682	0.765	0.1206
205659_at	26.2	89.6	1.78	3.42	AAA-MAP	0.20	16	0.682	0.765	0.1938
241336_at	20.9	71.1	1.77	3.41	AAA-AAP	0.20	16	0.682	0.765	0.1400
1562454_at	23.1	78.5	1.76	3.40	AAA-PAP	0.20	16	0.682	0.765	0.1276
207487_at	15.8	53.3	1.76	3.38	AAA-PAP	0.20	16	0.682	0.765	0.1681
220774_at	23.2	78.3	1.75	3.37	APA-APP	0.20	16	0.682	0.765	0.2203
241692_at	22.4	75.4	1.75	3.37	AAA-AAP	0.20	16	0.682	0.765	0.1209
223605_at	17.4	58.5	1.75	3.36	APA-AAA	0.20	16	0.682	0.765	0.1434
233815_at	13.8	46.4	1.75	3.36	AAA-AAP	0.20	16	0.682	0.765	0.1969
1552975_x_at	7.2	24.1	1.75	3.36	AAA-AAP	0.20	16	0.682	0.765	0.2396
241826_x_at	17.4	58.1	1.74	3.35	AAA-PAP	0.20	16	0.682	0.765	0.2080
1570585_at	20.1	67.1	1.74	3.34	AAA-AAP	0.20	16	0.682	0.765	0.2403
236791_at	7.9	26.5	1.74	3.34	AAA-APA	0.20	16	0.682	0.765	0.2012
241195_at	13.1	43.6	1.74	3.33	AAA-AAP	0.20	16	0.682	0.765	0.2183
237189_at	21.5	71.4	1.73	3.33	AAA-APA	0.20	16	0.682	0.765	0.1264
1564640_at	10.4	34.4	1.73	3.32	AAA-APA	0.20	16	0.682	0.765	0.2389
1556607_at	12.8	42.2	1.72	3.29	PAA-AAA	0.20	16	0.682	0.765	0.2533
206058_at	26.9	88.3	1.71	3.28	AAA-AAP	0.20	16	0.682	0.765	0.1559
235880_at	14.5	47.6	1.71	3.28	AAA-PAA	0.20	16	0.682	0.765	0.2212
242168_at	31.2	102.2	1.71	3.27	AAA-AAP	0.20	16	0.682	0.765	0.2137
206773_at	23.4	75.6	1.70	3.24	PAA-PPA	0.20	16	0.682	0.765	0.1718
230718_at	9.4	30.4	1.69	3.23	AAA-PAA	0.20	16	0.682	0.765	0.1841
1557044_at	23.3	75.3	1.69	3.23	PAP-PPA	0.20	16	0.682	0.765	0.1676
242340_at	13.1	41.9	1.67	3.19	AAA-APP	0.20	16	0.682	0.765	0.2077
1567697_at	11.2	35.7	1.67	3.19	AAA-APA	0.20	16	0.682	0.765	0.1744
211521_s_at	25.9	82.1	1.67	3.18	AAA-AAP	0.20	16	0.682	0.765	0.1734
203003_at	52.2	165.4	1.67	3.17	PAA-PPP	0.20	16	0.682	0.765	0.1550
211388_s_at	22.2	70.3	1.66	3.17	AAP-MAP	0.20	16	0.682	0.765	0.1482
243325_at	10.6	33.6	1.66	3.17	AAA-APA	0.20	16	0.682	0.765	0.2444
220667_at	13.7	43.2	1.66	3.17	AAP-APA	0.20	16	0.682	0.765	0.1850
224803_s_at	25.4	80.2	1.66	3.16	AAA-AAP	0.20	16	0.682	0.765	0.2157
222166_at	15.9	50.3	1.66	3.16	AAA-PAM	0.20	16	0.682	0.765	0.2283
1564039_at	17.1	53.7	1.66	3.15	AAA-PAA	0.20	16	0.682	0.765	0.2132
230211_at	26.9	84.8	1.66	3.15	AAP-PAP	0.20	16	0.682	0.765	0.1151
239758_at	18.8	58.9	1.65	3.13	AAA-MAP	0.20	16	0.682	0.765	0.2213
244181_at	30.6	95.8	1.65	3.13	APP-PPP	0.20	16	0.682	0.765	0.2133

231969_at	10.4	32.6	1.65	3.13	AAA-AAP	0.20	16	0.682	0.765	0.1356
1557661_at	13.1	41.0	1.64	3.13	AAA-APA	0.20	16	0.682	0.765	0.1264
242173_at	14.2	44.5	1.64	3.12	AAA-AAP	0.20	16	0.682	0.765	0.2443
240299_at	12.9	39.8	1.63	3.10	AAA-AAP	0.20	16	0.682	0.765	0.2067
208181_at	8.8	27.1	1.62	3.08	AAA-PAA	0.20	16	0.682	0.765	0.1504
222972_at	18.6	57.3	1.62	3.08	AAA-PAA	0.20	16	0.682	0.765	0.1585
240904_at	15.1	46.2	1.61	3.06	AAA-PPA	0.20	16	0.682	0.765	0.1745
1559640_at	9.6	29.2	1.60	3.04	AAA-APA	0.20	16	0.682	0.765	0.2383
224044_at	17.6	53.3	1.60	3.04	PAA-PAA	0.20	16	0.682	0.765	0.1878
242428_at	30.5	92.2	1.60	3.03	PAP-PPP	0.20	16	0.682	0.765	0.1982
215439_x_at	25.7	77.0	1.59	3.00	AAA-PPP	0.20	16	0.682	0.765	0.2149
233104_at	33.0	98.9	1.58	2.99	AAA-MPA	0.20	16	0.682	0.765	0.2240
239646_at	24.7	73.8	1.58	2.98	APA-MMP	0.20	16	0.682	0.765	0.1222
243178_at	33.4	99.1	1.57	2.97	AAA-AAP	0.20	16	0.682	0.765	0.1765
1569930_at	21.5	63.9	1.57	2.97	APA-APA	0.20	16	0.682	0.765	0.2125
1560129_at	33.2	97.6	1.56	2.94	AAA-PPP	0.20	16	0.682	0.765	0.1300
1562953_s_at	16.7	49.1	1.56	2.94	AAA-AAP	0.20	16	0.682	0.765	0.1681
207415_at	14.7	43.0	1.55	2.94	AAA-PAA	0.20	16	0.682	0.765	0.2010
236700_at	18.7	54.5	1.54	2.91	AAA-PPA	0.20	16	0.682	0.765	0.1702
235116_at	20.9	61.0	1.54	2.91	PAA-AMA	0.20	16	0.682	0.765	0.1625
217687_at	41.4	120.4	1.54	2.91	AAA-APA	0.20	16	0.682	0.765	0.1196
233229_at	20.3	58.9	1.54	2.90	AAP-AAA	0.20	16	0.682	0.765	0.1933
1554519_at	15.8	45.5	1.53	2.88	AAA-APA	0.20	16	0.682	0.765	0.1377
235362_at	23.6	67.3	1.52	2.86	PPA-AAP	0.20	16	0.682	0.765	0.2441
231566_at	21.2	60.6	1.52	2.86	AAA-AAP	0.20	16	0.682	0.765	0.1500
1552957_at	10.9	31.1	1.51	2.85	AAA-PAA	0.20	16	0.682	0.765	0.2508
232653_at	37.0	105.2	1.51	2.84	AAA-PAP	0.20	16	0.682	0.765	0.1554
1557167_at	18.3	52.0	1.51	2.84	AAA-AAP	0.20	16	0.682	0.765	0.2376
1570227_at	18.1	51.4	1.50	2.84	AAA-PAM	0.20	16	0.682	0.765	0.1614
242824_at	74.8	211.6	1.50	2.83	PAA-AAM	0.20	16	0.682	0.765	0.1898
238450_at	31.0	87.3	1.50	2.82	AAA-AAP	0.20	16	0.682	0.765	0.1866
240443_at	31.6	88.6	1.49	2.80	AAA-AAP	0.20	16	0.682	0.765	0.1959
239576_at	28.3	78.8	1.48	2.79	AAA-AAP	0.20	16	0.682	0.765	0.2104
214757_at	55.1	153.6	1.48	2.79	AAA-APA	0.20	16	0.682	0.765	0.1814
210873_x_at	19.8	55.0	1.47	2.78	AAA-APP	0.20	16	0.682	0.765	0.2058
233118_at	10.3	28.7	1.47	2.77	AAA-AAP	0.20	16	0.682	0.765	0.2182
1555778_a_at	19.8	54.9	1.47	2.77	PAA-PPP	0.20	16	0.682	0.765	0.2484
213172_at	16.0	44.3	1.47	2.76	AAA-MAP	0.20	16	0.682	0.765	0.1776
214631_at	24.6	67.9	1.46	2.76	AAA-PAA	0.20	16	0.682	0.765	0.2209
232963_at	34.1	93.7	1.46	2.75	APP-PPP	0.20	16	0.682	0.765	0.1272
239297_at	53.0	145.6	1.46	2.75	MAM-APP	0.20	16	0.682	0.765	0.1827
207370_at	11.1	30.3	1.45	2.73	AAA-AAP	0.20	16	0.682	0.765	0.1692

229687_s_at	14.4	39.2	1.45	2.73	AAA-APA	0.20	16	0.682	0.765	0.1277
243719_at	31.6	85.9	1.44	2.72	AAA-PMP	0.20	16	0.682	0.765	0.2406
244369_at	10.6	28.7	1.44	2.71	APA-AAA	0.20	16	0.682	0.765	0.2309
241478_at	17.5	47.4	1.44	2.71	AAA-APA	0.20	16	0.682	0.765	0.2140
206951_at	36.3	97.6	1.43	2.69	AAA-AAP	0.20	16	0.682	0.765	0.1604
239385_at	24.2	65.1	1.43	2.69	AAA-PAM	0.20	16	0.682	0.765	0.1145
205051_s_at	27.7	74.3	1.42	2.68	AAA-PPA	0.20	16	0.682	0.765	0.1489
203812_at	42.2	113.1	1.42	2.68	AAA-PAA	0.20	16	0.682	0.765	0.1487
243996_at	19.2	51.1	1.41	2.66	AAA-APP	0.20	16	0.682	0.765	0.2407
204164_at	56.8	151.0	1.41	2.66	AAA-AAP	0.20	16	0.682	0.765	0.1464
232410_at	39.4	104.6	1.41	2.66	AAP-PPP	0.20	16	0.682	0.765	0.2255
243762_at	36.3	96.5	1.41	2.66	AAA-PAA	0.20	16	0.682	0.765	0.1413
231009_at	29.8	79.1	1.41	2.66	APA-AAA	0.20	16	0.682	0.765	0.1686
206868_at	39.6	104.9	1.40	2.65	AAP-PAP	0.20	16	0.682	0.765	0.1639
230171_at	42.6	112.2	1.40	2.64	AAP-AAA	0.20	16	0.682	0.765	0.2439
205386_s_at	21.2	55.8	1.40	2.63	AAA-AAP	0.20	16	0.682	0.765	0.2454
227004_at	35.8	92.9	1.37	2.59	AAA-PAP	0.20	16	0.682	0.765	0.2454
1558406_a_at	19.5	50.5	1.37	2.59	AAA-APA	0.20	16	0.682	0.765	0.1262
1561726_s_at	23.6	61.1	1.37	2.59	AAA-PAP	0.20	16	0.682	0.765	0.1438
205093_at	57.7	148.6	1.36	2.57	AAA-AAP	0.20	16	0.682	0.765	0.1834
217616_at	25.5	65.6	1.36	2.58	PAA-AAA	0.20	16	0.682	0.765	0.1760
243357_at	30.3	78.0	1.36	2.57	AAA-PAA	0.20	16	0.682	0.765	0.2239
244530_at	30.5	78.2	1.36	2.57	AAA-AAP	0.20	16	0.682	0.765	0.1840
244352_at	35.6	91.3	1.36	2.56	AAA-PPA	0.20	16	0.682	0.765	0.1109
238728_at	51.9	132.4	1.35	2.55	AAP-APP	0.20	16	0.682	0.765	0.1795
234278_at	37.2	94.9	1.35	2.55	AAA-PPP	0.20	16	0.682	0.765	0.1988
216114_at	40.4	103.0	1.35	2.55	AAA-AAP	0.20	16	0.682	0.765	0.1231
201565_s_at	163.3	416.0	1.35	2.55	APA-APP	0.20	16	0.682	0.765	0.2164
236714_at	18.5	47.0	1.35	2.55	AAA-PAA	0.20	16	0.682	0.765	0.1251
229560_at	27.1	69.0	1.35	2.54	AAP-AAA	0.20	16	0.682	0.765	0.2358
1556704_s_at	18.1	46.1	1.35	2.54	AAP-PAP	0.20	16	0.682	0.765	0.2419
1559696_at	38.1	96.5	1.34	2.54	PAA-AAA	0.20	16	0.682	0.765	0.1875
1562161_at	28.3	71.8	1.34	2.53	PAM-PPP	0.20	16	0.682	0.765	0.1891
222142_at	22.2	56.0	1.34	2.53	AAA-PPP	0.20	16	0.682	0.765	0.1979
1555938_x_at	18.3	46.1	1.33	2.52	AAP-APA	0.20	16	0.682	0.765	0.1392
1559066_at	19.8	49.9	1.33	2.52	AAA-APA	0.20	16	0.682	0.765	0.2211
234573_at	39.6	99.4	1.33	2.51	AAA-MAP	0.20	16	0.682	0.765	0.1336
235055_x_at	25.1	62.6	1.32	2.50	AAA-PAA	0.20	16	0.682	0.765	0.2133
203440_at	62.5	156.3	1.32	2.50	PAP-PPP	0.20	16	0.682	0.765	0.0085
1557080_s_at	19.4	48.4	1.32	2.50	AAA-APA	0.20	16	0.682	0.765	0.1835
231407_s_at	26.0	64.5	1.31	2.48	AAP-AAA	0.20	16	0.682	0.765	0.2057
201431_s_at	26.5	65.9	1.31	2.48	AAA-APA	0.20	16	0.682	0.765	0.1897

227410_at	99.2	246.2	1.31	2.48	AAA-PPP	0.20	16	0.682	0.765	0.1838
233352_at	29.8	73.7	1.31	2.47	AAA-APP	0.20	16	0.682	0.765	0.2133
1559139_at	53.6	132.4	1.31	2.47	AAA-AAP	0.20	16	0.682	0.765	0.1091
223966_at	19.0	46.9	1.30	2.47	PAA-AMA	0.20	16	0.682	0.765	0.1332
1555681_at	42.2	104.0	1.30	2.46	AAA-PAA	0.20	16	0.682	0.765	0.1088
243170_at	36.2	88.8	1.30	2.46	APA-APM	0.20	16	0.682	0.765	0.2077
1563840_at	27.7	68.0	1.29	2.45	AAP-PAM	0.20	16	0.682	0.765	0.1585
1569369_at	23.5	57.7	1.29	2.45	AAA-APA	0.20	16	0.682	0.765	0.1243
1560340_s_at	22.7	55.3	1.29	2.44	AAP-AAA	0.20	16	0.682	0.765	0.2395
239245_at	24.5	59.8	1.29	2.44	AAA-APA	0.20	16	0.682	0.765	0.2220
221316_at	22.3	54.0	1.28	2.42	AAA-AAP	0.20	16	0.682	0.765	0.1704
208585_at	39.1	94.5	1.27	2.42	AAA-APA	0.20	16	0.682	0.765	0.1972
233164_x_at	41.0	99.0	1.27	2.41	PAA-APA	0.20	16	0.682	0.765	0.2168
242472_x_at	85.3	205.7	1.27	2.41	PAP-PPP	0.20	16	0.682	0.765	0.1791
1554274_a_at	52.5	126.8	1.27	2.41	MAA-PAP	0.20	16	0.682	0.765	0.1157
231065_at	32.3	77.4	1.26	2.39	AAA-APP	0.20	16	0.682	0.765	0.1969
229794_at	40.9	97.8	1.26	2.39	APP-PPP	0.20	16	0.682	0.765	0.1362
240457_at	27.1	64.7	1.26	2.39	PAP-APA	0.20	16	0.682	0.765	0.2285
242479_s_at	20.1	47.7	1.25	2.38	AAA-APA	0.20	16	0.682	0.765	0.1975
204734_at	487.0	1157.7	1.25	2.38	APP-PPP	0.20	16	0.682	0.765	0.1079
235364_at	18.7	44.3	1.25	2.37	AAP-AAP	0.20	16	0.682	0.765	0.1874
210486_at	55.5	131.1	1.24	2.37	PAA-AAA	0.20	16	0.682	0.765	0.1626
204614_at	691.3	1634.8	1.24	2.37	PPP-PPP	0.20	16	0.682	0.765	0.1404
219211_at	41.5	97.8	1.24	2.36	AAA-AAP	0.20	16	0.682	0.765	0.1341
235392_at	69.9	164.3	1.23	2.35	AAA-APA	0.20	16	0.682	0.765	0.2057
203969_at	40.1	94.1	1.23	2.34	AAP-PAP	0.20	16	0.682	0.765	0.2411
240465_at	36.9	86.3	1.23	2.34	PAA-AMP	0.20	16	0.682	0.765	0.1688
1558830_at	32.2	75.3	1.22	2.34	AAA-APA	0.20	16	0.682	0.765	0.1817
1559500_at	31.1	72.5	1.22	2.33	AAA-PPA	0.20	16	0.682	0.765	0.1619
237114_at	30.0	69.9	1.22	2.33	AAP-AAA	0.20	16	0.682	0.765	0.1279
206368_at	13.2	30.9	1.22	2.33	AAA-AAP	0.20	16	0.682	0.765	0.1442
211267_at	28.7	66.9	1.22	2.33	AAA-PAA	0.20	16	0.682	0.765	0.2458
213202_at	38.7	89.9	1.22	2.32	AAA-APP	0.20	16	0.682	0.765	0.2090
222715_s_at	92.5	215.0	1.22	2.32	AMP-PPP	0.20	16	0.682	0.765	0.1345
223860_at	20.1	46.5	1.21	2.31	AAA-PPA	0.20	16	0.682	0.765	0.1629
222882_s_at	43.7	100.6	1.20	2.30	AAA-APA	0.20	16	0.682	0.765	0.1477
230942_at	125.7	54.6	-1.20	-2.30	PMA-AAA	0.20	16	0.682	0.765	0.0086
212657_s_at	108.4	248.2	1.20	2.29	APP-PPP	0.20	16	0.682	0.765	0.1374
231074_at	29.0	66.3	1.19	2.29	AAA-PPA	0.20	16	0.682	0.765	0.1892
241707_at	29.4	67.3	1.19	2.29	APA-AAA	0.20	16	0.682	0.765	0.1501
222940_at	118.0	269.5	1.19	2.28	APP-PPP	0.20	16	0.682	0.765	0.1222
207987_s_at	21.2	48.4	1.19	2.28	AAA-AAP	0.20	16	0.682	0.765	0.1350

237216_at	39.6	90.0	1.19	2.27	AAP-PAP	0.20	16	0.682	0.765	0.1472
244761_at	43.8	99.1	1.18	2.27	AAP-APP	0.20	16	0.682	0.765	0.1970
241617_x_at	16.9	38.3	1.18	2.26	AAA-APA	0.20	16	0.682	0.765	0.1682
232210_at	34.2	77.0	1.17	2.26	AMA-APA	0.20	16	0.682	0.765	0.1492
229103_at	40.4	90.9	1.17	2.25	AAP-AAA	0.20	16	0.682	0.765	0.2125
210730_s_at	24.1	54.3	1.17	2.25	AAA-APA	0.20	16	0.682	0.765	0.2167
242508_at	47.0	105.7	1.17	2.25	AAA-PAA	0.20	16	0.682	0.765	0.1543
204750_s_at	127.0	284.4	1.16	2.24	APA-PPP	0.20	16	0.682	0.765	0.1528
241417_at	44.2	98.9	1.16	2.24	APP-AAP	0.20	16	0.682	0.765	0.2048
242359_at	34.1	76.2	1.16	2.24	AAA-APP	0.20	16	0.682	0.765	0.2362
214433_s_at	63.5	141.8	1.16	2.24	AAP-APP	0.20	16	0.682	0.765	0.1634
1562681_at	37.7	83.9	1.16	2.23	PAA-PPP	0.20	16	0.682	0.765	0.1914
214674_at	85.0	188.8	1.15	2.22	PAA-PPP	0.20	16	0.682	0.765	0.2377
211414_at	25.1	55.7	1.15	2.22	AAP-MAP	0.20	16	0.682	0.765	0.2032
239612_at	25.4	56.3	1.15	2.22	AAA-PAA	0.20	16	0.682	0.765	0.1784
242230_at	34.5	76.6	1.15	2.22	AAA-PAA	0.20	16	0.682	0.765	0.2191
209691_s_at	61.5	136.2	1.15	2.22	AAA-AAP	0.20	16	0.682	0.765	0.1762
1555323_at	41.6	91.5	1.14	2.20	AAA-APA	0.20	16	0.682	0.765	0.2207
212225_at	77.2	169.5	1.13	2.20	MAA-PPP	0.20	16	0.682	0.765	0.1133
236784_s_at	87.4	191.5	1.13	2.19	AAA-AAP	0.20	16	0.682	0.765	0.1822
217712_at	31.8	69.6	1.13	2.19	AAA-PAP	0.20	16	0.682	0.765	0.1812
243696_at	18.3	40.0	1.13	2.19	AAA-PAA	0.20	16	0.682	0.765	0.2269
236883_at	59.3	129.6	1.13	2.19	PAP-PAP	0.20	16	0.682	0.765	0.1345
222162_s_at	34.2	74.6	1.13	2.18	AAA-PPP	0.20	16	0.682	0.765	0.1882
1562794_at	45.2	98.4	1.12	2.18	PAA-PPP	0.20	16	0.682	0.765	0.2236
240431_at	24.5	53.3	1.12	2.18	AAA-AAP	0.20	16	0.682	0.765	0.1701
209459_s_at	34.4	74.9	1.12	2.18	AAP-PAP	0.20	16	0.682	0.765	0.1364
1562236_at	13.3	28.9	1.12	2.18	AAA-AAP	0.20	16	0.682	0.765	0.2338
1569936_a_at	23.5	51.1	1.12	2.17	AAA-PAA	0.20	16	0.682	0.765	0.1832
235370_at	55.0	119.4	1.12	2.17	AAA-AAP	0.20	16	0.682	0.765	0.1577
222313_at	58.7	127.2	1.12	2.17	PAA-PPP	0.20	16	0.682	0.765	0.1939
209617_s_at	35.7	77.2	1.11	2.16	MAA-APA	0.20	16	0.682	0.765	0.1152
239511_s_at	55.4	119.6	1.11	2.16	APP-PPP	0.20	16	0.682	0.765	0.1462
238932_at	63.0	135.6	1.11	2.15	AAP-APP	0.20	16	0.682	0.765	0.2174
232235_at	67.6	145.4	1.11	2.15	APP-PPP	0.20	16	0.682	0.765	0.1443
238525_at	38.7	83.0	1.10	2.15	AAA-AAP	0.20	16	0.682	0.765	0.2191
208797_s_at	42.4	90.7	1.10	2.14	AAP-PPP	0.20	16	0.682	0.765	0.2078
204304_s_at	20.1	43.1	1.10	2.14	AAA-AAP	0.20	16	0.682	0.765	0.1696
239505_at	39.7	84.9	1.10	2.14	AAA-APA	0.20	16	0.682	0.765	0.2276
243682_at	59.1	126.2	1.10	2.14	AAA-APA	0.20	16	0.682	0.765	0.2342
221667_s_at	38.8	82.8	1.10	2.14	AAA-PPA	0.20	16	0.682	0.765	0.2249
215611_at	25.4	53.8	1.09	2.12	AAA-APP	0.20	16	0.682	0.765	0.1865

236671_at	95.3	202.0	1.08	2.12	PAA-PAP	0.20	16	0.682	0.765	0.1191
234697_x_at	43.0	90.9	1.08	2.11	APA-PPP	0.20	16	0.682	0.765	0.1756
232529_at	46.4	97.6	1.07	2.10	APP-PPP	0.20	16	0.682	0.765	0.1060
236427_at	59.0	123.4	1.06	2.09	AAA-PAA	0.20	16	0.682	0.765	0.1776
237239_at	75.4	157.4	1.06	2.09	AAA-PAP	0.20	16	0.682	0.765	0.1307
230928_at	25.1	52.3	1.06	2.09	AAA-PAA	0.20	16	0.682	0.765	0.1697
215054_at	65.6	136.2	1.05	2.08	AAP-PPP	0.20	16	0.682	0.765	0.1420
1559119_at	54.0	111.9	1.05	2.07	AAA-AAP	0.20	16	0.682	0.765	0.1664
1559952_x_at	45.0	93.0	1.05	2.07	APA-PPP	0.20	16	0.682	0.765	0.1201
232145_at	36.8	76.1	1.05	2.07	AAA-AAP	0.20	16	0.682	0.765	0.1529
207227_x_at	48.7	100.4	1.04	2.06	AAA-PPA	0.20	16	0.682	0.765	0.1776
228387_at	58.4	120.4	1.04	2.06	MAP-APA	0.20	16	0.682	0.765	0.1279
1566403_at	32.0	65.7	1.04	2.05	PPA-APP	0.20	16	0.682	0.765	0.2370
232905_at	14.9	30.6	1.04	2.05	AAA-PAA	0.20	16	0.682	0.765	0.1363
242854_x_at	60.9	125.0	1.04	2.05	PAM-APP	0.20	16	0.682	0.765	0.1013
1564295_at	32.6	66.7	1.03	2.05	AAA-APA	0.20	16	0.682	0.765	0.2041
1558578_a_at	15.2	31.1	1.03	2.05	AAA-AAP	0.20	16	0.682	0.765	0.1891
244186_at	61.7	125.5	1.02	2.03	PPA-PAP	0.20	16	0.682	0.765	0.2154
1559949_at	23.8	48.3	1.02	2.03	AAA-AAP	0.20	16	0.682	0.765	0.1738
240801_at	32.5	65.8	1.02	2.03	PPA-PAA	0.20	16	0.682	0.765	0.2332
240991_at	160.1	324.6	1.02	2.03	AAA-AAP	0.20	16	0.682	0.765	0.1739
1564796_at	99.0	200.6	1.02	2.03	MAA-PPP	0.20	16	0.682	0.765	0.1366
1560172_at	39.4	79.9	1.02	2.03	PAA-AAP	0.20	16	0.682	0.765	0.1237
1561398_at	33.1	67.0	1.02	2.02	AAA-APA	0.20	16	0.682	0.765	0.1871
237814_at	29.0	58.7	1.02	2.02	AAA-MAP	0.20	16	0.682	0.765	0.2281
215800_at	73.0	147.6	1.02	2.02	APP-PPP	0.20	16	0.682	0.765	0.1001
AFFX-r2-Bs-lys-3_at	21.0	42.5	1.01	2.02	AAA-PAA	0.20	16	0.682	0.765	0.2117
243705_at	62.4	125.4	1.01	2.01	APP-APP	0.20	16	0.682	0.765	0.1388
1555595_at	39.0	78.1	1.00	2.00	AAA-AAP	0.20	16	0.682	0.765	0.1071
244646_at	57.3	114.8	1.00	2.00	AAP-AMP	0.20	16	0.682	0.765	0.1439
1560932_at	26.9	53.8	1.00	2.00	AAA-APA	0.20	16	0.682	0.765	0.1934
234331_s_at	50.8	101.4	1.00	2.00	APA-PPA	0.20	16	0.682	0.765	0.1572
223948_s_at	79.3	158.3	1.00	2.00	AAA-PPP	0.20	16	0.682	0.765	0.1136
239410_at	61.0	121.8	1.00	2.00	APP-PPP	0.20	16	0.682	0.765	0.2267
222368_at	85.3	169.6	0.99	1.99	AAA-PAP	0.20	16	0.682	0.765	0.2316
236569_at	79.0	157.1	0.99	1.99	PAP-APP	0.20	16	0.682	0.765	0.1575
235475_at	38.1	75.8	0.99	1.99	AAA-PPP	0.20	16	0.682	0.765	0.1417
227366_at	152.5	302.9	0.99	1.99	AAP-AAA	0.20	16	0.682	0.765	0.1316
221009_s_at	1551.4	781.8	-0.99	-1.98	PPP-PPP	0.20	16	0.682	0.765	0.0059
1561242_at	16.3	32.4	0.99	1.99	AAA-APA	0.20	16	0.682	0.765	0.1608
242836_at	58.0	115.1	0.99	1.99	PPA-PPP	0.20	16	0.682	0.765	0.1574
216882_s_at	81.3	161.1	0.99	1.98	AAA-PAP	0.20	16	0.682	0.765	0.2362

232012_at	136.7	270.5	0.99	1.98	AAM-APP	0.20	16	0.682	0.765	0.1888
240332_at	31.0	61.2	0.98	1.98	APA-AAA	0.20	16	0.682	0.765	0.2262
243875_at	26.9	53.2	0.98	1.98	AAA-APP	0.20	16	0.682	0.765	0.1190
236439_at	184.6	364.3	0.98	1.97	AAP-PPP	0.20	16	0.682	0.765	0.1016
209301_at	3796.9	7488.5	0.98	1.97	PPP-PPP	0.20	16	0.682	0.765	0.0984
213668_s_at	135.4	266.7	0.98	1.97	AAP-PAP	0.20	16	0.682	0.765	0.1450
216814_at	23.4	46.1	0.98	1.97	AAA-APA	0.20	16	0.682	0.765	0.1722
1557236_at	58.5	29.8	-0.97	-1.96	PPP-PAA	0.20	16	0.682	0.765	0.0050
233202_at	37.2	72.5	0.96	1.95	AAA-PPA	0.20	16	0.682	0.765	0.2240
230786_at	73.2	142.5	0.96	1.95	MAA-APA	0.20	16	0.682	0.765	0.1703
225758_s_at	96.3	187.4	0.96	1.95	PAP-PPP	0.20	16	0.682	0.765	0.1482
241400_at	52.4	101.9	0.96	1.95	APP-PAP	0.20	16	0.682	0.765	0.2282
227526_at	58.0	112.8	0.96	1.95	MAA-MPA	0.20	16	0.682	0.765	0.1460
204628_s_at	56.5	109.8	0.96	1.94	AAP-AAA	0.20	16	0.682	0.765	0.2014
208578_at	43.9	85.1	0.95	1.94	APA-MAA	0.20	16	0.682	0.765	0.2026
232084_at	53.4	103.3	0.95	1.94	AMA-APP	0.20	16	0.682	0.765	0.1093
218487_at	167.8	324.1	0.95	1.93	APP-PPP	0.20	16	0.682	0.765	0.1568
238651_at	66.2	127.9	0.95	1.93	PAA-APP	0.20	16	0.682	0.765	0.1688
216067_at	28.2	54.4	0.95	1.93	AAA-APM	0.20	16	0.682	0.765	0.1124
1557578_at	32.4	62.4	0.95	1.93	AAA-PAP	0.20	16	0.682	0.765	0.1815
221511_x_at	135.3	260.2	0.94	1.92	APA-PMP	0.20	16	0.682	0.765	0.1180
1556393_at	34.8	66.9	0.94	1.92	APP-PAP	0.20	16	0.682	0.765	0.1694
237514_at	33.6	64.5	0.94	1.92	AAM-PPA	0.20	16	0.682	0.765	0.0969
232935_at	26.7	51.1	0.94	1.91	AAA-PAP	0.20	16	0.682	0.765	0.1960
222043_at	48.7	92.9	0.93	1.91	APA-PPP	0.20	16	0.682	0.765	0.1824
1570511_at	81.7	155.5	0.93	1.90	AAA-APP	0.20	16	0.682	0.765	0.1741
234093_at	40.8	77.5	0.92	1.90	AAA-APA	0.20	16	0.682	0.765	0.1630
1554866_at	21.2	40.2	0.92	1.90	AAA-PAP	0.20	16	0.682	0.765	0.1452
228857_at	71.0	134.5	0.92	1.90	AAP-PPP	0.20	16	0.682	0.765	0.1365
238913_at	82.3	155.6	0.92	1.89	PPP-PPP	0.20	16	0.682	0.765	0.1578
1569320_at	102.1	192.6	0.92	1.89	PPP-PPP	0.20	16	0.682	0.765	0.1487
242784_at	44.5	83.7	0.91	1.88	AAP-PPA	0.20	16	0.682	0.765	0.2061
221973_at	150.9	284.0	0.91	1.88	PPP-PPP	0.20	16	0.682	0.765	0.1694
1561048_at	65.0	122.1	0.91	1.88	AAA-PPM	0.20	16	0.682	0.765	0.2227
223204_at	230.8	123.2	-0.91	-1.87	PPP-PPP	0.20	16	0.682	0.765	0.0074
242790_at	85.9	160.6	0.90	1.87	AAA-APP	0.20	16	0.682	0.765	0.1340
231172_at	51.6	96.4	0.90	1.87	AAA-MAP	0.20	16	0.682	0.765	0.0970
214735_at	29.4	54.9	0.90	1.87	APA-AAA	0.20	16	0.682	0.765	0.1590
240815_at	126.2	234.6	0.89	1.86	PPP-PAP	0.20	16	0.682	0.765	0.1724
239185_at	20.4	37.8	0.89	1.86	APA-AAA	0.20	16	0.682	0.765	0.2212
231448_at	77.1	143.0	0.89	1.86	PAA-AAP	0.20	16	0.682	0.765	0.1002
216991_at	33.9	62.9	0.89	1.86	AAA-APA	0.20	16	0.682	0.765	0.1546

230358_at	94.0	174.1	0.89	1.85	AAA-APA	0.20	16	0.682	0.765	0.1602
236881_at	24.5	45.4	0.89	1.85	PAA-AAA	0.20	16	0.682	0.765	0.2239
229240_at	61.6	114.0	0.89	1.85	PPP-APP	0.20	16	0.682	0.765	0.1290
1558220_at	126.2	233.5	0.89	1.85	MAP-PPP	0.20	16	0.682	0.765	0.1674
244633_at	59.0	109.1	0.89	1.85	APP-PPP	0.20	16	0.682	0.765	0.1111
236796_at	43.3	79.8	0.88	1.84	AMA-PAP	0.20	16	0.682	0.765	0.1414
220838_at	117.3	216.3	0.88	1.84	AAP-PAP	0.20	16	0.682	0.765	0.1015
226444_at	56.3	103.6	0.88	1.84	AAA-PAP	0.20	16	0.682	0.765	0.1668
1569714_at	64.4	118.1	0.87	1.83	AAP-AAP	0.20	16	0.682	0.765	0.1208
230123_at	97.8	179.2	0.87	1.83	PAP-PAP	0.20	16	0.682	0.765	0.2067
224982_at	936.6	1715.0	0.87	1.83	PPP-PPP	0.20	16	0.682	0.765	0.1555
204775_at	312.3	571.1	0.87	1.83	AAP-PPP	0.20	16	0.682	0.765	0.0965
1569600_at	38.4	70.2	0.87	1.83	PAP-PPP	0.20	16	0.682	0.765	0.1150
232291_at	117.2	213.5	0.87	1.82	PAP-APP	0.20	16	0.682	0.765	0.1688
220572_at	35.5	64.7	0.87	1.82	AAM-AAP	0.20	16	0.682	0.765	0.1151
210718_s_at	54.6	99.2	0.86	1.82	APA-PAA	0.20	16	0.682	0.765	0.1539
1557672_s_at	21.2	38.6	0.86	1.82	AAA-PAA	0.20	16	0.682	0.765	0.2300
235337_at	51.0	92.6	0.86	1.82	AAP-PPP	0.20	16	0.682	0.765	0.1108
240516_at	89.6	162.5	0.86	1.81	APA-PPP	0.20	16	0.682	0.765	0.1361
232865_at	117.1	212.4	0.86	1.81	PPP-PPP	0.20	16	0.682	0.765	0.1144
211445_x_at	4388.4	7950.5	0.86	1.81	PPP-PPP	0.20	16	0.682	0.765	0.1774
215318_at	58.7	106.2	0.86	1.81	AMA-PPP	0.20	16	0.682	0.765	0.1145
219764_at	41.1	74.3	0.86	1.81	AMA-PAP	0.20	16	0.682	0.765	0.1338
236314_at	46.8	84.5	0.85	1.81	PAA-AMP	0.20	16	0.682	0.765	0.1664
225323_at	126.7	228.6	0.85	1.81	AAA-APA	0.20	16	0.682	0.765	0.2192
231124_x_at	82.8	149.2	0.85	1.80	PMM-PAP	0.20	16	0.682	0.765	0.1802
206996_x_at	60.4	108.8	0.85	1.80	AAA-APA	0.20	16	0.682	0.765	0.1647
228823_at	56.5	101.8	0.85	1.80	AAA-AAP	0.20	16	0.682	0.765	0.1513
232151_at	46.8	83.9	0.84	1.79	AAP-APP	0.20	16	0.682	0.765	0.2298
226947_at	148.1	265.5	0.84	1.79	PPP-PPP	0.20	16	0.682	0.765	0.1176
215314_at	38.9	69.6	0.84	1.79	AAA-AAP	0.20	16	0.682	0.765	0.1383
236622_at	76.5	136.8	0.84	1.79	PPP-PPP	0.20	16	0.682	0.765	0.1225
215447_at	113.5	203.0	0.84	1.79	PPA-PPP	0.20	16	0.682	0.765	0.1503
210086_at	104.2	186.2	0.84	1.79	AAA-AAP	0.20	16	0.682	0.765	0.1837
223546_x_at	206.5	368.7	0.84	1.79	PAP-PPP	0.20	16	0.682	0.765	0.1543
1558050_at	49.4	88.2	0.84	1.79	AAA-APA	0.20	16	0.682	0.765	0.0930
232295_at	30.6	54.4	0.83	1.78	APP-PPP	0.20	16	0.682	0.765	0.1109
232049_at	29.2	51.9	0.83	1.78	APA-PAA	0.20	16	0.682	0.765	0.1330
228254_at	256.1	454.5	0.83	1.78	APP-APP	0.20	16	0.682	0.765	0.1444
1557543_at	50.0	88.6	0.83	1.77	AAP-AAP	0.20	16	0.682	0.765	0.1206
239331_at	165.9	294.1	0.83	1.77	PPA-PPP	0.20	16	0.682	0.765	0.2133
1563010_at	95.4	168.9	0.82	1.77	PAA-APM	0.20	16	0.682	0.765	0.2086

240861_at	46.6	82.5	0.82	1.77	AAA-PPA	0.20	16	0.682	0.765	0.1968
217340_at	126.5	224.0	0.82	1.77	PPP-PPP	0.20	16	0.682	0.765	0.1350
215607_x_at	80.3	142.0	0.82	1.77	AAA-APA	0.20	16	0.682	0.765	0.2084
237013_at	59.2	104.4	0.82	1.77	AAP-AAA	0.20	16	0.682	0.765	0.1153
215521_at	64.9	114.5	0.82	1.76	PPP-PPP	0.20	16	0.682	0.765	0.1203
232597_x_at	221.8	391.0	0.82	1.76	PPP-PPP	0.20	16	0.682	0.765	0.1532
233473_x_at	130.8	230.3	0.82	1.76	PAP-AMP	0.20	16	0.682	0.765	0.2135
1552558_a_at	93.4	164.0	0.81	1.76	AAA-PAA	0.20	16	0.682	0.765	0.1423
231650_s_at	32.9	57.6	0.81	1.75	AAA-APP	0.20	16	0.682	0.765	0.1970
233845_at	14.7	25.7	0.81	1.75	AAA-AAP	0.20	16	0.682	0.765	0.1877
214242_at	37.2	65.1	0.81	1.75	AAP-AAA	0.20	16	0.682	0.765	0.1436
1566735_at	53.2	93.0	0.81	1.75	AAP-MAM	0.20	16	0.682	0.765	0.1315
237586_at	40.8	71.2	0.80	1.74	AAP-AMP	0.20	16	0.682	0.765	0.1231
206792_x_at	1863.2	3243.5	0.80	1.74	PPP-PPP	0.20	16	0.682	0.765	0.1095
234650_at	42.3	73.5	0.80	1.74	PAP-PPP	0.20	16	0.682	0.765	0.1591
238548_at	56.8	98.9	0.80	1.74	AAP-AAP	0.20	16	0.682	0.765	0.1482
237147_at	33.5	58.3	0.80	1.74	PAP-APP	0.20	16	0.682	0.765	0.1216
221291_at	180.6	314.2	0.80	1.74	AAP-AAM	0.20	16	0.682	0.765	0.1199
231909_x_at	57.5	100.1	0.80	1.74	APP-PPP	0.20	16	0.682	0.765	0.1003
234266_at	38.5	66.9	0.80	1.74	AAP-PPP	0.20	16	0.682	0.765	0.2055
238782_at	57.4	99.7	0.80	1.74	AAP-APP	0.20	16	0.682	0.765	0.1376
236821_at	66.9	116.3	0.80	1.74	APA-MPP	0.20	16	0.682	0.765	0.1163
239845_at	44.9	77.8	0.79	1.73	APA-PPA	0.20	16	0.682	0.765	0.1486
232775_at	64.3	111.5	0.79	1.73	AAA-AAP	0.20	16	0.682	0.765	0.1230
231931_at	86.0	149.2	0.79	1.73	PAM-PPP	0.20	16	0.682	0.765	0.1026
230873_at	88.9	154.2	0.79	1.73	APA-PPP	0.20	16	0.682	0.765	0.0930
231660_at	93.6	162.2	0.79	1.73	AAA-PMA	0.20	16	0.682	0.765	0.2088
205688_at	92.9	160.9	0.79	1.73	AAA-PAA	0.20	16	0.682	0.765	0.1804
205172_x_at	1598.0	2769.5	0.79	1.73	PPP-PPP	0.20	16	0.682	0.765	0.1152
217027_x_at	730.1	1263.6	0.79	1.73	PPP-PPP	0.20	16	0.682	0.765	0.2229
214638_s_at	93.1	161.2	0.79	1.73	PPP-PPP	0.20	16	0.682	0.765	0.0916
207474_at	38.8	67.1	0.79	1.73	PAA-APA	0.20	16	0.682	0.765	0.1609
232013_at	53.6	92.6	0.79	1.73	APA-PAP	0.20	16	0.682	0.765	0.0962
1556657_at	70.3	121.4	0.79	1.73	AAA-PPP	0.20	16	0.682	0.765	0.1960
63305_at	64.4	111.2	0.79	1.73	APA-PPP	0.20	16	0.682	0.765	0.1961
219499_at	72.4	125.0	0.79	1.73	AAP-PAP	0.20	16	0.682	0.765	0.2133
219858_s_at	240.7	414.9	0.79	1.72	PPP-PPP	0.20	16	0.682	0.765	0.1573
217446_x_at	105.0	181.0	0.79	1.72	PPM-PPP	0.20	16	0.682	0.765	0.2080
229156_s_at	84.9	146.3	0.79	1.72	AAP-PAP	0.20	16	0.682	0.765	0.1911
236244_at	124.3	214.1	0.79	1.72	PAA-PPP	0.20	16	0.682	0.765	0.1257
1556277_a_at	110.3	189.9	0.78	1.72	PPA-PAP	0.20	16	0.682	0.765	0.2053
241401_at	21.7	37.4	0.78	1.72	AAP-AAA	0.20	16	0.682	0.765	0.1283

217542_at	96.1	165.5	0.78	1.72	AAA-PPM	0.20	16	0.682	0.765	0.0924
1562719_at	89.8	154.5	0.78	1.72	APP-PPP	0.20	16	0.682	0.765	0.2143
1570571_at	51.0	87.8	0.78	1.72	APP-PAP	0.20	16	0.682	0.765	0.1604
242413_at	63.3	108.7	0.78	1.72	PAA-PAP	0.20	16	0.682	0.765	0.1227
210528_at	38.4	66.0	0.78	1.72	AAA-APP	0.20	16	0.682	0.765	0.1554
240515_at	58.5	100.2	0.78	1.71	AAP-APA	0.20	16	0.682	0.765	0.2129
1553553_at	40.1	68.7	0.78	1.71	AAA-PAA	0.20	16	0.682	0.765	0.0996
236552_at	66.6	113.8	0.77	1.71	PMP-PPP	0.20	16	0.682	0.765	0.1357
209105_at	109.5	187.3	0.77	1.71	PPP-PPP	0.20	16	0.682	0.765	0.1084
222354_at	241.8	413.0	0.77	1.71	APP-PPP	0.20	16	0.682	0.765	0.1028
1562928_at	43.2	73.8	0.77	1.71	APA-PPP	0.20	16	0.682	0.765	0.2029
228157_at	208.6	356.2	0.77	1.71	PPP-PPP	0.20	16	0.682	0.765	0.1048
232925_at	207.6	354.7	0.77	1.71	PPP-PPP	0.20	16	0.682	0.765	0.0973
226702_at	40.6	69.2	0.77	1.71	AAP-PPP	0.20	16	0.682	0.765	0.0997
1554852_a_at	44.4	75.5	0.77	1.70	PAA-PPP	0.20	16	0.682	0.765	0.2173
240205_x_at	172.0	292.8	0.77	1.70	MPP-PPP	0.20	16	0.682	0.765	0.1261
208441_at	49.5	84.1	0.77	1.70	APA-MAA	0.20	16	0.682	0.765	0.1497
239476_at	81.9	138.8	0.76	1.70	PPA-PAP	0.20	16	0.682	0.765	0.1961
223504_at	89.7	152.1	0.76	1.70	AAP-MAA	0.20	16	0.682	0.765	0.1949
1555551_at	116.2	197.2	0.76	1.70	PPP-PPP	0.20	16	0.682	0.765	0.1319
1559471_s_at	60.4	102.4	0.76	1.69	AAA-APM	0.20	16	0.682	0.765	0.1949
235154_at	100.5	170.3	0.76	1.69	AAA-APP	0.20	16	0.682	0.765	0.1569
1566527_at	42.7	72.2	0.76	1.69	AAA-PPP	0.20	16	0.682	0.765	0.1539
228982_s_at	37.1	62.6	0.76	1.69	AAM-PAA	0.20	16	0.682	0.765	0.0959
1566901_at	109.1	183.9	0.75	1.69	PPA-PPP	0.20	16	0.682	0.765	0.1531
235676_at	66.5	111.9	0.75	1.68	AAA-PAP	0.20	16	0.682	0.765	0.1861
227448_at	103.6	174.4	0.75	1.68	PPP-PPP	0.20	16	0.682	0.765	0.1032
1566191_at	59.5	100.0	0.75	1.68	MAP-APP	0.20	16	0.682	0.765	0.0862
239102_s_at	79.3	133.4	0.75	1.68	PPA-PPP	0.20	16	0.682	0.765	0.1080
1556318_s_at	74.5	125.3	0.75	1.68	PPP-PPP	0.20	16	0.682	0.765	0.1011
238908_at	110.2	185.1	0.75	1.68	APP-APP	0.20	16	0.682	0.765	0.1925
220444_at	184.0	309.2	0.75	1.68	PMP-PPP	0.20	16	0.682	0.765	0.1229
239091_at	40.6	68.2	0.75	1.68	PAA-PPP	0.20	16	0.682	0.765	0.2207
1560068_a_at	59.7	100.1	0.75	1.68	AAP-PPA	0.20	16	0.682	0.765	0.1783
217438_at	34.8	58.3	0.75	1.68	AAA-PAA	0.20	16	0.682	0.765	0.1532
209772_s_at	2118.4	3549.4	0.75	1.68	PPP-PPP	0.20	16	0.682	0.765	0.1458
207655_s_at	27.5	46.0	0.74	1.67	AAA-PAA	0.20	16	0.682	0.765	0.1125
234553_at	70.1	117.5	0.74	1.68	AAA-PAM	0.20	16	0.682	0.765	0.0952
1556352_at	184.1	308.1	0.74	1.67	PMP-PPP	0.20	16	0.682	0.765	0.0996
205793_x_at	174.0	290.9	0.74	1.67	PPA-APP	0.20	16	0.682	0.765	0.1829
239096_at	111.8	186.9	0.74	1.67	PPP-PPP	0.20	16	0.682	0.765	0.1614
243413_at	54.3	90.7	0.74	1.67	APP-PPP	0.20	16	0.682	0.765	0.1244

220965_s_at	63.8	106.5	0.74	1.67	AMA-PPA	0.20	16	0.682	0.765	0.0859
207432_at	109.3	182.5	0.74	1.67	AAA-PAA	0.20	16	0.682	0.765	0.1244
201645_at	179.4	107.6	-0.74	-1.67	PPP-PPA	0.20	16	0.682	0.765	0.0044
241700_at	39.5	65.9	0.74	1.67	AAP-PAA	0.20	16	0.682	0.765	0.2010
1553129_at	40.8	68.1	0.74	1.67	AAA-APA	0.20	16	0.682	0.765	0.1708
239717_at	70.0	116.8	0.74	1.67	AAA-APP	0.20	16	0.682	0.765	0.1107
227399_at	1496.2	2494.2	0.74	1.67	PPP-PPP	0.20	16	0.682	0.765	0.1032
213748_at	115.5	192.3	0.74	1.67	PMP-PPP	0.20	16	0.682	0.765	0.0912
230711_at	58.0	96.6	0.74	1.67	PPA-AAP	0.20	16	0.682	0.765	0.1895
236470_at	53.9	89.7	0.74	1.66	APA-PPP	0.20	16	0.682	0.765	0.1660
238563_at	125.7	208.9	0.73	1.66	PPP-PPP	0.20	16	0.682	0.765	0.2192
242100_at	98.5	163.7	0.73	1.66	PPP-PPA	0.20	16	0.682	0.765	0.1139
232360_at	43.6	72.4	0.73	1.66	AAA-APP	0.20	16	0.682	0.765	0.1924
207018_s_at	129.0	214.2	0.73	1.66	PPP-PPP	0.20	16	0.682	0.765	0.1871
225372_at	125.7	208.8	0.73	1.66	APP-PPP	0.20	16	0.682	0.765	0.1137
1552870_s_at	22.5	37.4	0.73	1.66	APA-APA	0.20	16	0.682	0.765	0.2223
1565823_at	103.4	171.6	0.73	1.66	PPP-PPP	0.20	16	0.682	0.765	0.1035
233056_x_at	282.0	467.8	0.73	1.66	PAP-PAP	0.20	16	0.682	0.765	0.0921
1561389_at	53.3	88.2	0.73	1.66	AAP-PPP	0.20	16	0.682	0.765	0.1135
202935_s_at	362.0	598.9	0.73	1.66	PPP-PPP	0.20	16	0.682	0.765	0.1089
1553750_a_at	84.5	139.8	0.73	1.66	APP-APP	0.20	16	0.682	0.765	0.0880
243729_at	44.3	73.2	0.73	1.65	PPP-PPP	0.20	16	0.682	0.765	0.1042
241774_at	97.8	161.5	0.72	1.65	PPP-PPP	0.20	16	0.682	0.765	0.1976
236322_at	211.8	349.8	0.72	1.65	PPP-PPP	0.20	16	0.682	0.765	0.0892
203713_s_at	232.0	382.8	0.72	1.65	PPP-PPP	0.20	16	0.682	0.765	0.2065
240593_x_at	60.8	100.3	0.72	1.65	AAA-PPA	0.20	16	0.682	0.765	0.1997
242627_at	47.4	78.1	0.72	1.65	APP-PPP	0.20	16	0.682	0.765	0.1700
207001_x_at	205.2	337.8	0.72	1.65	PPP-PPP	0.20	16	0.682	0.765	0.1857
1555068_at	47.0	77.3	0.72	1.65	PAM-PPP	0.20	16	0.682	0.765	0.1546
232356_at	82.4	135.6	0.72	1.65	PPP-PPP	0.20	16	0.682	0.765	0.1446
219883_at	145.0	238.2	0.72	1.64	PPP-PPP	0.20	16	0.682	0.765	0.2127
239635_at	120.1	197.3	0.72	1.64	AAP-APP	0.20	16	0.682	0.765	0.1913
1554039_at	125.5	206.2	0.72	1.64	MAA-PAP	0.20	16	0.682	0.765	0.1072
229457_at	111.9	183.6	0.72	1.64	PAP-PPP	0.20	16	0.682	0.765	0.2185
241775_at	74.8	122.8	0.71	1.64	PPP-PPP	0.20	16	0.682	0.765	0.2057
216229_x_at	150.2	246.5	0.71	1.64	PAP-PPP	0.20	16	0.682	0.765	0.1877
213183_s_at	84.6	138.7	0.71	1.64	APA-PPP	0.20	16	0.682	0.765	0.1412
240202_x_at	92.4	151.7	0.71	1.64	AAA-APP	0.20	16	0.682	0.765	0.1098
1553145_at	82.2	134.8	0.71	1.64	AAA-APP	0.20	16	0.682	0.765	0.0847
225524_at	667.7	407.4	-0.71	-1.64	PPP-PPP	0.20	16	0.682	0.765	0.0033
238909_at	315.9	517.4	0.71	1.64	PPP-PPP	0.20	16	0.682	0.765	0.1128
215712_s_at	82.7	135.3	0.71	1.64	PMA-PAP	0.20	16	0.682	0.765	0.1108

206971_at	126.8	207.2	0.71	1.63	PAP-AAP	0.20	16	0.682	0.765	0.2068
219795_at	38.0	61.9	0.71	1.63	PPP-APP	0.20	16	0.682	0.765	0.2068
238078_at	56.2	91.7	0.71	1.63	AAA-AAP	0.20	16	0.682	0.765	0.1837
1557129_a_at	201.0	327.8	0.71	1.63	PPP-PPP	0.20	16	0.682	0.765	0.1186
1566142_at	47.1	76.8	0.71	1.63	APA-AAP	0.20	16	0.682	0.765	0.1819
219264_s_at	114.9	187.3	0.71	1.63	APA-PPP	0.20	16	0.682	0.765	0.1562
242811_x_at	23.3	37.9	0.71	1.63	AAA-AAP	0.20	16	0.682	0.765	0.1319
241588_at	57.8	94.2	0.71	1.63	AAA-APA	0.20	16	0.682	0.765	0.1069
227303_at	139.9	228.0	0.70	1.63	AAP-APP	0.20	16	0.682	0.765	0.1377
214888_at	125.5	204.5	0.70	1.63	APP-PPP	0.20	16	0.682	0.765	0.0879
239796_x_at	97.0	157.7	0.70	1.63	PPA-PPP	0.20	16	0.682	0.765	0.2022
237383_at	118.2	192.3	0.70	1.63	PPP-PPP	0.20	16	0.682	0.765	0.1048
207273_at	31.4	51.0	0.70	1.63	PAA-PPA	0.20	16	0.682	0.765	0.2004
215854_at	52.7	85.6	0.70	1.63	PPM-MPP	0.20	16	0.682	0.765	0.1229
244565_at	41.9	68.1	0.70	1.62	AMP-AAP	0.20	16	0.682	0.765	0.2030
231356_at	27.7	45.0	0.70	1.62	PAA-APA	0.20	16	0.682	0.765	0.1238
1554737_at	49.1	79.7	0.70	1.62	AAA-AAP	0.20	16	0.682	0.765	0.1962
228865_at	1138.6	1847.1	0.70	1.62	PPP-PPP	0.20	16	0.682	0.765	0.1205
227062_at	2005.7	3253.0	0.70	1.62	PPP-PPP	0.20	16	0.682	0.765	0.1013
210985_s_at	78.7	127.7	0.70	1.62	MPP-PPP	0.20	16	0.682	0.765	0.0882
204995_at	192.5	311.8	0.70	1.62	PPP-PPP	0.20	16	0.682	0.765	0.1103
231247_s_at	45.8	74.1	0.69	1.62	AMA-AAP	0.20	16	0.682	0.765	0.1871
238967_at	313.4	506.9	0.69	1.62	PPP-PPP	0.20	16	0.682	0.765	0.1826
244356_at	56.7	91.8	0.69	1.62	PPA-APP	0.20	16	0.682	0.765	0.1031
224973_at	62.8	101.7	0.69	1.62	PAP-PPP	0.20	16	0.682	0.765	0.0892
1560981_a_at	67.9	109.7	0.69	1.62	PPP-PPP	0.20	16	0.682	0.765	0.1829
1560872_at	46.2	74.7	0.69	1.62	AAA-APA	0.20	16	0.682	0.765	0.1055
1556339_a_at	55.3	89.3	0.69	1.62	MMA-APP	0.20	16	0.682	0.765	0.1746
1569052_at	77.6	125.4	0.69	1.62	AAP-APP	0.20	16	0.682	0.765	0.1275
231933_at	68.8	111.1	0.69	1.62	APA-APP	0.20	16	0.682	0.765	0.1557
227505_at	90.8	146.5	0.69	1.61	AAA-AAP	0.20	16	0.682	0.765	0.0888
228297_at	1800.1	1115.7	-0.69	-1.61	PPP-PPP	0.20	16	0.682	0.765	0.0049
202708_s_at	62.5	100.7	0.69	1.61	PPA-PPP	0.20	16	0.682	0.765	0.1452
1556744_a_at	111.3	179.2	0.69	1.61	PPP-PPP	0.20	16	0.682	0.765	0.0860
216527_at	73.5	118.3	0.69	1.61	MPP-PPP	0.20	16	0.682	0.765	0.1358
1561079_at	77.4	124.5	0.69	1.61	PPP-PPP	0.20	16	0.682	0.765	0.1371
213017_at	984.7	1583.1	0.69	1.61	PPP-PPP	0.20	16	0.682	0.765	0.0965
1564231_at	36.4	58.6	0.68	1.61	APP-APP	0.20	16	0.682	0.765	0.1992
231026_at	84.3	135.4	0.68	1.61	AAA-PPA	0.20	16	0.682	0.765	0.0838
229787_s_at	81.6	130.9	0.68	1.60	PPA-PPP	0.20	16	0.682	0.765	0.1969
214915_at	24.9	39.9	0.68	1.60	AAA-APA	0.20	16	0.682	0.765	0.1545
1558202_at	52.9	84.8	0.68	1.61	PAP-PPM	0.20	16	0.682	0.765	0.1227

240331_at	56.4	90.5	0.68	1.60	APP-PPP	0.20	16	0.682	0.765	0.2128
228100_at	42.4	67.9	0.68	1.60	AAA-APA	0.20	16	0.682	0.765	0.1302
215608_at	102.1	163.6	0.68	1.60	MPP-MPP	0.20	16	0.682	0.765	0.1208
207976_at	75.0	120.2	0.68	1.60	PPP-PAP	0.20	16	0.682	0.765	0.0849
1559658_at	64.2	102.7	0.68	1.60	MAM-PAP	0.20	16	0.682	0.765	0.1180
243792_x_at	151.2	242.1	0.68	1.60	PPP-PPP	0.20	16	0.682	0.765	0.1042
216813_at	39.0	62.3	0.68	1.60	AAA-AAP	0.20	16	0.682	0.765	0.1910
1560071_a_at	79.2	126.6	0.68	1.60	PMP-PPP	0.20	16	0.682	0.765	0.1010
244523_at	84.1	134.4	0.68	1.60	PPA-APP	0.20	16	0.682	0.765	0.1014
1568594_s_at	106.3	169.9	0.68	1.60	PPP-PPP	0.20	16	0.682	0.765	0.0977
1570143_at	114.2	182.4	0.68	1.60	PPP-PPP	0.20	16	0.682	0.765	0.1718
1558922_at	84.3	134.5	0.67	1.60	PPP-PPP	0.20	16	0.682	0.765	0.2168
221778_at	215.1	342.9	0.67	1.59	PMP-PPP	0.20	16	0.682	0.765	0.1986
235662_at	83.4	132.9	0.67	1.60	AAA-AAP	0.20	16	0.682	0.765	0.1384
215343_at	95.6	152.3	0.67	1.59	APA-PPA	0.20	16	0.682	0.765	0.1987
215387_x_at	105.1	167.4	0.67	1.59	APP-APP	0.20	16	0.682	0.765	0.1069
233254_x_at	108.9	173.4	0.67	1.59	AMA-AAP	0.20	16	0.682	0.765	0.1059
1557580_at	57.3	91.0	0.67	1.59	PAP-PPP	0.20	16	0.682	0.765	0.1713
235866_at	53.4	84.7	0.67	1.59	APP-PPA	0.20	16	0.682	0.765	0.2148
244289_at	37.5	59.6	0.67	1.59	APA-AAA	0.20	16	0.682	0.765	0.1717
214048_at	124.8	198.0	0.67	1.59	PPP-PPP	0.20	16	0.682	0.765	0.0860
1570490_at	79.5	50.2	-0.67	-1.59	APP-AAP	0.20	16	0.682	0.765	0.0046
236116_at	66.5	105.4	0.67	1.59	AAA-PMA	0.20	16	0.682	0.765	0.1440
1564362_x_at	191.2	303.2	0.67	1.59	PPP-PPP	0.20	16	0.682	0.765	0.1412
225847_at	584.6	369.0	-0.66	-1.58	PPP-PPP	0.20	16	0.682	0.765	0.0031
1569912_at	32.9	52.0	0.66	1.58	AAA-AAP	0.20	16	0.682	0.765	0.0919
243410_at	67.8	107.2	0.66	1.58	PAP-PPP	0.20	16	0.682	0.765	0.2147
235165_at	213.4	337.4	0.66	1.58	PPP-PPP	0.20	16	0.682	0.765	0.2050
223237_x_at	200.1	316.3	0.66	1.58	AAM-APA	0.20	16	0.682	0.765	0.1689
218869_at	214.9	339.8	0.66	1.58	PPP-PPP	0.20	16	0.682	0.765	0.1675
220163_s_at	97.9	154.7	0.66	1.58	AAA-AAP	0.20	16	0.682	0.765	0.1638
231071_at	77.9	123.0	0.66	1.58	AAA-AAP	0.20	16	0.682	0.765	0.2136
236437_at	44.5	70.4	0.66	1.58	APA-PAP	0.20	16	0.682	0.765	0.1177
215536_at	51.7	81.6	0.66	1.58	AAP-AAA	0.20	16	0.682	0.765	0.1555
224316_at	194.3	306.6	0.66	1.58	PPP-PPP	0.20	16	0.682	0.765	0.1258
241897_at	67.5	106.4	0.66	1.58	AAA-AAP	0.20	16	0.682	0.765	0.2062
236752_at	117.8	185.8	0.66	1.58	PPP-PPP	0.20	16	0.682	0.765	0.1765
236000_s_at	95.3	150.2	0.66	1.58	AAA-PPP	0.20	16	0.682	0.765	0.1098
213501_at	139.6	220.0	0.66	1.58	PPP-PPP	0.20	16	0.682	0.765	0.1977
231858_x_at	255.0	401.9	0.66	1.58	PPP-PPP	0.20	16	0.682	0.765	0.1322
241345_at	57.4	90.4	0.66	1.58	PAP-PPP	0.20	16	0.682	0.765	0.1224
218404_at	1348.4	856.0	-0.66	-1.58	PPP-PPP	0.20	16	0.682	0.765	0.0042

230703_at	103.6	162.8	0.65	1.57	PPM-PPP	0.20	16	0.682	0.765	0.1814
215291_at	71.4	112.0	0.65	1.57	AAA-PAP	0.20	16	0.682	0.765	0.1693
244264_at	87.3	137.0	0.65	1.57	AAA-AAP	0.20	16	0.682	0.765	0.1430
229470_at	118.4	185.9	0.65	1.57	MAA-PPP	0.20	16	0.682	0.765	0.1255
243751_at	81.3	127.7	0.65	1.57	PPP-PPP	0.20	16	0.682	0.765	0.0997
221850_x_at	403.9	634.1	0.65	1.57	PAP-PPP	0.20	16	0.682	0.765	0.0799
224221_s_at	137.4	215.6	0.65	1.57	PPP-PPP	0.20	16	0.682	0.765	0.0940
221628_s_at	130.9	205.3	0.65	1.57	PPM-PPP	0.20	16	0.682	0.765	0.2038
218858_at	105.1	164.6	0.65	1.57	APP-PAP	0.20	16	0.682	0.765	0.1456
243219_x_at	51.6	80.6	0.65	1.56	PAA-PAP	0.20	16	0.682	0.765	0.0980
232288_at	53.7	84.0	0.65	1.56	PMP-PPP	0.20	16	0.682	0.765	0.1784
236034_at	49.3	77.2	0.65	1.56	AAA-APA	0.20	16	0.682	0.765	0.1637
1557270_at	106.0	165.8	0.65	1.56	PPP-PPP	0.20	16	0.682	0.765	0.1371
215204_at	137.0	214.3	0.65	1.56	PPP-PPP	0.20	16	0.682	0.765	0.1354
239432_at	103.9	162.5	0.65	1.56	PPP-PPP	0.20	16	0.682	0.765	0.0827
232058_at	204.3	319.3	0.64	1.56	PPP-PPP	0.20	16	0.682	0.765	0.1012
230742_at	72.9	113.9	0.64	1.56	PPP-PPP	0.20	16	0.682	0.765	0.1602
243312_at	69.3	108.1	0.64	1.56	AAA-AAP	0.20	16	0.682	0.765	0.0937
205218_at	422.9	660.0	0.64	1.56	PPP-PPP	0.20	16	0.682	0.765	0.1380
239233_at	101.1	157.7	0.64	1.56	PPP-PPP	0.20	16	0.682	0.765	0.1200
1560596_at	45.8	71.3	0.64	1.56	AAP-PPP	0.20	16	0.682	0.765	0.0951
235993_at	197.8	308.2	0.64	1.56	PPP-PPP	0.20	16	0.682	0.765	0.1421
235926_at	216.4	337.3	0.64	1.56	PPP-PPP	0.20	16	0.682	0.765	0.1142
238446_at	80.2	125.0	0.64	1.56	AAA-APP	0.20	16	0.682	0.765	0.1098
223763_at	62.5	97.3	0.64	1.56	AAA-AAP	0.20	16	0.682	0.765	0.2001
243194_at	72.3	112.5	0.64	1.56	AAA-APP	0.20	16	0.682	0.765	0.1524
231821_x_at	176.0	274.1	0.64	1.56	PPP-PPP	0.20	16	0.682	0.765	0.1328
214657_s_at	153.3	238.5	0.64	1.56	PPM-PPP	0.20	16	0.682	0.765	0.1876
217353_at	188.9	293.9	0.64	1.56	PPP-PPP	0.20	16	0.682	0.765	0.1845
231825_x_at	692.1	1076.8	0.64	1.56	PPP-PPP	0.20	16	0.682	0.765	0.1616
228723_at	166.1	258.2	0.64	1.56	PPP-PPP	0.20	16	0.682	0.765	0.1060
241845_at	48.4	75.2	0.64	1.55	MPP-PPP	0.20	16	0.682	0.765	0.1863
244728_at	81.9	127.2	0.64	1.55	AAA-PPA	0.20	16	0.682	0.765	0.1835
1554948_at	174.0	270.4	0.64	1.55	PAP-PPP	0.20	16	0.682	0.765	0.1730
205092_x_at	112.0	174.1	0.64	1.55	PPP-PPP	0.20	16	0.682	0.765	0.0941
214487_s_at	587.6	912.7	0.64	1.55	PPP-PPP	0.20	16	0.682	0.765	0.2016
238619_at	132.9	206.4	0.64	1.55	PMA-PPP	0.20	16	0.682	0.765	0.1155
232772_at	58.9	91.5	0.64	1.55	AAM-APP	0.20	16	0.682	0.765	0.0786
221925_s_at	237.5	368.3	0.63	1.55	PPP-PPP	0.20	16	0.682	0.765	0.2036
237005_at	95.7	148.5	0.63	1.55	APP-APP	0.20	16	0.682	0.765	0.1221
206848_at	120.7	187.0	0.63	1.55	PPP-PPP	0.20	16	0.682	0.765	0.1784
241821_at	33.9	52.5	0.63	1.55	APA-PAM	0.20	16	0.682	0.765	0.1949

1561206_at	45.0	69.6	0.63	1.55	PAA-PPP	0.20	16	0.682	0.765	0.1855
1569041_at	55.6	86.0	0.63	1.55	PAP-PPP	0.20	16	0.682	0.765	0.2113
215330_at	52.8	81.7	0.63	1.55	AAA-MAP	0.20	16	0.682	0.765	0.1613
225912_at	278.8	431.5	0.63	1.55	PPP-PPP	0.20	16	0.682	0.765	0.0800
227497_at	72.0	111.4	0.63	1.55	AAA-APP	0.20	16	0.682	0.765	0.1926
1557875_at	55.4	85.6	0.63	1.55	APA-PAP	0.20	16	0.682	0.765	0.1967
243031_at	67.8	104.7	0.63	1.55	APP-PPP	0.20	16	0.682	0.765	0.0896
221618_s_at	72.3	111.5	0.63	1.54	PPP-PMP	0.20	16	0.682	0.765	0.1639
214017_s_at	272.9	420.6	0.62	1.54	AAP-PPP	0.20	16	0.682	0.765	0.1796
1557772_at	24.7	38.1	0.62	1.54	AAP-AAP	0.20	16	0.682	0.765	0.1263
1560145_at	156.6	241.2	0.62	1.54	PAP-PMP	0.20	16	0.682	0.765	0.1626
1560204_at	36.3	55.8	0.62	1.54	AAA-MPP	0.20	16	0.682	0.765	0.0795
1553185_at	388.6	598.1	0.62	1.54	PPP-PPP	0.20	16	0.682	0.765	0.1783
232172_at	127.5	196.2	0.62	1.54	AAP-PPP	0.20	16	0.682	0.765	0.1448
1562063_x_at	273.7	421.2	0.62	1.54	PPP-PPP	0.20	16	0.682	0.765	0.1081
243591_at	170.8	262.7	0.62	1.54	MPP-PPP	0.20	16	0.682	0.765	0.0759
230156_x_at	37.4	57.5	0.62	1.54	PAA-PPP	0.20	16	0.682	0.765	0.1850
204182_s_at	213.8	328.5	0.62	1.54	MAP-PPP	0.20	16	0.682	0.765	0.2088
240262_at	73.6	113.0	0.62	1.54	PPP-PPP	0.20	16	0.682	0.765	0.1089
208763_s_at	2571.1	3943.7	0.62	1.53	PPP-PPP	0.20	16	0.682	0.765	0.1025
210176_at	92.3	141.5	0.62	1.53	PPP-PPP	0.20	16	0.682	0.765	0.0931
230820_at	1599.1	1043.2	-0.62	-1.53	PPP-PPP	0.20	16	0.682	0.765	0.0022
227070_at	40.8	62.5	0.62	1.53	APA-MPA	0.20	16	0.682	0.765	0.1192
242417_at	340.3	521.1	0.62	1.53	APP-PPP	0.20	16	0.682	0.765	0.1743
1567319_at	87.0	133.3	0.62	1.53	AAA-PPP	0.20	16	0.682	0.765	0.1597
218775_s_at	300.9	460.6	0.61	1.53	PPP-PPP	0.20	16	0.682	0.765	0.1986
233736_at	81.5	124.7	0.61	1.53	AAA-APM	0.20	16	0.682	0.765	0.1926
244826_at	253.6	388.1	0.61	1.53	PPP-PPP	0.20	16	0.682	0.765	0.1032
1558686_at	23.9	36.5	0.61	1.53	AAA-PPA	0.20	16	0.682	0.765	0.0979
212598_at	130.2	199.2	0.61	1.53	AAP-APP	0.20	16	0.682	0.765	0.1810
234991_at	154.5	236.2	0.61	1.53	PPP-PPP	0.20	16	0.682	0.765	0.0839
211071_s_at	1459.1	955.0	-0.61	-1.53	PPP-PPP	0.20	16	0.682	0.765	0.0024
207746_at	118.4	181.0	0.61	1.53	PPP-PMP	0.20	16	0.682	0.765	0.1028
242123_at	86.9	132.8	0.61	1.53	PPP-PPP	0.20	16	0.682	0.765	0.0973
227903_x_at	160.6	245.4	0.61	1.53	PAM-AAP	0.20	16	0.682	0.765	0.1854
1560509_at	129.9	198.4	0.61	1.53	PPP-PPP	0.20	16	0.682	0.765	0.1485
237187_at	136.5	208.4	0.61	1.53	PPP-PPP	0.20	16	0.682	0.765	0.1042
208995_s_at	551.4	841.6	0.61	1.53	PPP-PPP	0.20	16	0.682	0.765	0.1509
234322_at	83.4	127.3	0.61	1.53	PMA-APP	0.20	16	0.682	0.765	0.1023
205857_at	39.1	59.6	0.61	1.53	AAA-PAA	0.20	16	0.682	0.765	0.1319
231997_at	98.3	149.8	0.61	1.53	PAA-PPP	0.20	16	0.682	0.765	0.0952
209824_s_at	2111.0	3212.4	0.61	1.52	PPP-PPP	0.20	16	0.682	0.765	0.2079

235345_at	48.7	74.2	0.61	1.52	PAP-PAA	0.20	16	0.682	0.765	0.1283
215553_x_at	245.8	373.7	0.60	1.52	PPP-PPP	0.20	16	0.682	0.765	0.1567
1559529_at	94.6	143.9	0.60	1.52	PPP-PPP	0.20	16	0.682	0.765	0.1243
233887_at	54.4	82.6	0.60	1.52	AAP-PPP	0.20	16	0.682	0.765	0.1499
244165_at	106.2	161.3	0.60	1.52	PPP-PPP	0.20	16	0.682	0.765	0.1061
214098_at	56.4	85.7	0.60	1.52	PPP-PPP	0.20	16	0.682	0.765	0.1303
33148_at	238.4	361.7	0.60	1.52	PPP-PPP	0.20	16	0.682	0.765	0.1302
222305_at	69.9	106.0	0.60	1.52	AAP-AAM	0.20	16	0.682	0.765	0.1177
243276_at	263.7	399.6	0.60	1.52	AAM-MPP	0.20	16	0.682	0.765	0.0901
1569730_at	71.1	107.7	0.60	1.52	PAA-PPA	0.20	16	0.682	0.765	0.2090
235203_at	375.7	569.0	0.60	1.52	PPP-PPP	0.20	16	0.682	0.765	0.0781
243343_at	76.8	116.3	0.60	1.51	AAA-PPP	0.20	16	0.682	0.765	0.1888
1558678_s_at	7075.2	10707.5	0.60	1.51	PPP-PPP	0.20	16	0.682	0.765	0.1507
1555829_at	125.5	189.9	0.60	1.51	APP-PPP	0.20	16	0.682	0.765	0.1429
227581_at	155.9	236.0	0.60	1.51	PPP-PPP	0.20	16	0.682	0.765	0.1196
1559409_a_at	58.2	88.1	0.60	1.51	AAP-AMP	0.20	16	0.682	0.765	0.1016
238114_at	81.7	123.7	0.60	1.51	PAA-PPP	0.20	16	0.682	0.765	0.0765
219283_at	3537.2	2338.2	-0.60	-1.51	PPP-PPP	0.20	16	0.682	0.765	0.0015
240062_at	60.9	92.2	0.60	1.51	APP-PPP	0.20	16	0.682	0.765	0.2070
243515_at	119.5	180.7	0.60	1.51	PPP-PPP	0.20	16	0.682	0.765	0.1691
216012_at	67.8	102.5	0.60	1.51	APA-PAP	0.20	16	0.682	0.765	0.0908
1554638_at	73.2	110.8	0.60	1.51	PPP-PPP	0.20	16	0.682	0.765	0.0905
213121_at	56.6	85.5	0.60	1.51	AAA-APA	0.20	16	0.682	0.765	0.2043
228062_at	60.4	91.3	0.60	1.51	PAP-PPP	0.20	16	0.682	0.765	0.1528
232633_at	61.8	93.4	0.60	1.51	AAP-APP	0.20	16	0.682	0.765	0.1395
225999_at	161.0	243.4	0.60	1.51	PPP-PPP	0.20	16	0.682	0.765	0.1124
231295_at	45.7	69.1	0.60	1.51	PAA-AAA	0.20	16	0.682	0.765	0.0973
236725_at	218.5	330.1	0.60	1.51	PPP-PPP	0.20	16	0.682	0.765	0.1114
238575_at	380.3	574.2	0.59	1.51	MPP-PPP	0.20	16	0.682	0.765	0.1246
220288_at	56.6	85.5	0.59	1.51	APP-AAP	0.20	16	0.682	0.765	0.1244
240149_at	43.9	66.2	0.59	1.51	AAP-PPP	0.20	16	0.682	0.765	0.1147
229878_at	285.4	430.8	0.59	1.51	PPP-PPP	0.20	16	0.682	0.765	0.1090
226357_at	653.9	987.0	0.59	1.51	PPP-PPP	0.20	16	0.682	0.765	0.0822
1556049_at	83.4	125.9	0.59	1.51	AAA-APA	0.20	16	0.682	0.765	0.1824
205310_at	554.2	835.2	0.59	1.51	PPP-PPP	0.20	16	0.682	0.765	0.1534
207954_at	129.4	195.0	0.59	1.51	APA-AAP	0.20	16	0.682	0.765	0.1147
240268_at	42.2	63.6	0.59	1.51	AAP-PPA	0.20	16	0.682	0.765	0.1030
1557828_a_at	203.8	307.0	0.59	1.51	PPP-PPP	0.20	16	0.682	0.765	0.1883
32029_at	336.7	507.3	0.59	1.51	PAP-PPP	0.20	16	0.682	0.765	0.1264
242688_at	104.9	158.0	0.59	1.51	PAA-PAP	0.20	16	0.682	0.765	0.1393
220580_at	95.9	144.4	0.59	1.51	PPP-PPP	0.20	16	0.682	0.765	0.1280
219352_at	229.4	344.9	0.59	1.50	PPP-PPP	0.20	16	0.682	0.765	0.1628

238946_at	47.3	71.1	0.59	1.50	APA-APP	0.20	16	0.682	0.765	0.1426
204105_s_at	564.8	375.8	-0.59	-1.50	PPP-PPP	0.20	16	0.682	0.765	0.0026
225786_at	711.0	1068.7	0.59	1.50	PPP-PPP	0.20	16	0.682	0.765	0.1488
242645_at	83.8	125.9	0.59	1.50	PPP-PPP	0.20	16	0.682	0.765	0.1063
233388_at	136.7	205.4	0.59	1.50	PPP-PPP	0.20	16	0.682	0.765	0.0833
203914_x_at	247.2	371.2	0.59	1.50	PPP-PPP	0.20	16	0.682	0.765	0.1393
236986_at	94.3	141.7	0.59	1.50	APP-PPP	0.20	16	0.682	0.765	0.1275
229814_at	74.7	112.2	0.59	1.50	AAA-AAP	0.20	16	0.682	0.765	0.1223
215852_x_at	94.7	142.3	0.59	1.50	AAA-PPA	0.20	16	0.682	0.765	0.1061
239469_at	131.7	197.6	0.59	1.50	PPP-PPP	0.20	16	0.682	0.765	0.1300
240831_at	86.2	129.3	0.59	1.50	AAA-PPP	0.20	16	0.682	0.765	0.1960
239001_at	170.9	256.3	0.59	1.50	PAP-MPP	0.20	16	0.682	0.765	0.1362
1560622_at	213.4	319.7	0.58	1.50	PPP-PPP	0.20	16	0.682	0.765	0.1696
209211_at	4584.0	6872.4	0.58	1.50	PPP-PPP	0.20	16	0.682	0.765	0.1077
209766_at	76.6	114.9	0.58	1.50	PAM-PPA	0.20	16	0.682	0.765	0.0873
238549_at	270.6	405.3	0.58	1.50	PPP-PPP	0.20	16	0.682	0.765	0.0915
208472_at	72.4	108.4	0.58	1.50	APA-PPP	0.20	16	0.682	0.765	0.1995
224203_at	99.4	148.7	0.58	1.50	AAA-PPP	0.20	16	0.682	0.765	0.1988
221187_s_at	158.8	237.5	0.58	1.50	AAP-AAP	0.20	16	0.682	0.765	0.1594
242225_at	128.7	192.6	0.58	1.50	PPP-PPP	0.20	16	0.682	0.765	0.1439
221683_s_at	79.4	118.9	0.58	1.50	PAP-PPP	0.20	16	0.682	0.765	0.0796
231433_at	48.9	73.2	0.58	1.50	AAP-AAA	0.20	16	0.682	0.765	0.1479
223388_s_at	161.5	241.4	0.58	1.50	APA-MAA	0.20	16	0.682	0.765	0.0900
222453_at	1439.6	2151.8	0.58	1.50	PPP-PPP	0.20	16	0.682	0.765	0.0807
219737_s_at	225.4	336.7	0.58	1.49	PPP-PPP	0.20	16	0.682	0.765	0.1452
239314_at	140.1	209.0	0.58	1.49	MAA-MPP	0.20	16	0.682	0.765	0.1876
219327_s_at	126.9	189.5	0.58	1.49	AAA-PAA	0.20	16	0.682	0.765	0.0832
243367_at	79.3	118.4	0.58	1.49	PPP-PPP	0.20	16	0.682	0.765	0.1994
242438_at	301.4	449.3	0.58	1.49	PPP-PPP	0.20	16	0.682	0.765	0.0880
217497_at	83.8	124.8	0.57	1.49	APA-MPP	0.20	16	0.682	0.765	0.1559
220500_s_at	117.5	175.0	0.57	1.49	PPP-PPP	0.20	16	0.682	0.765	0.1518
1555982_at	95.4	141.9	0.57	1.49	PAP-APA	0.20	16	0.682	0.765	0.1345
244569_at	129.5	192.7	0.57	1.49	PAP-PPP	0.20	16	0.682	0.765	0.1225
226808_at	181.0	269.3	0.57	1.49	PPP-PPP	0.20	16	0.682	0.765	0.0740
1556820_a_at	264.7	393.6	0.57	1.49	PPP-PPP	0.20	16	0.682	0.765	0.0732
201081_s_at	203.1	136.7	-0.57	-1.49	PPP-PPP	0.20	16	0.682	0.765	0.0015
239284_at	98.2	145.9	0.57	1.49	APA-PPP	0.20	16	0.682	0.765	0.1720
227884_at	287.7	427.3	0.57	1.49	PPP-PPP	0.20	16	0.682	0.765	0.1073
219025_at	132.3	196.5	0.57	1.49	PAP-PAP	0.20	16	0.682	0.765	0.0853
203185_at	48.8	72.4	0.57	1.49	AAA-PAA	0.20	16	0.682	0.765	0.0773
229981_at	256.6	380.6	0.57	1.48	PPP-PPP	0.20	16	0.682	0.765	0.2011
242512_at	46.2	68.5	0.57	1.48	AAP-PAA	0.20	16	0.682	0.765	0.1916

232098_at	193.5	286.8	0.57	1.48	MAA-PAP	0.20	16	0.682	0.765	0.1498
205783_at	75.8	112.4	0.57	1.48	AAP-AAP	0.20	16	0.682	0.765	0.1465
209951_s_at	115.2	170.6	0.57	1.48	PPP-PPP	0.20	16	0.682	0.765	0.1991
242052_at	282.4	418.5	0.57	1.48	PPP-PPP	0.20	16	0.682	0.765	0.1361
219966_x_at	856.6	1269.3	0.57	1.48	PPP-PPP	0.20	16	0.682	0.765	0.1109
225239_at	7629.9	11306.5	0.57	1.48	PPP-PPP	0.20	16	0.682	0.765	0.0916
207283_at	283.2	419.5	0.57	1.48	PPP-PPP	0.20	16	0.682	0.765	0.0752
203407_at	1127.2	1670.5	0.57	1.48	PPP-PPP	0.20	16	0.682	0.765	0.0725
1559530_at	176.6	261.4	0.57	1.48	PAP-AAP	0.20	16	0.682	0.765	0.1890
1559121_s_at	153.1	226.7	0.57	1.48	PPP-PPP	0.20	16	0.682	0.765	0.1775
221224_s_at	80.2	118.6	0.57	1.48	PPA-AAA	0.20	16	0.682	0.765	0.1570
230333_at	1238.9	1832.9	0.57	1.48	PPP-PPP	0.20	16	0.682	0.765	0.1169
215909_x_at	269.1	397.9	0.56	1.48	PPP-PPP	0.20	16	0.682	0.765	0.1240
1558700_s_at	437.4	646.5	0.56	1.48	PPP-PPP	0.20	16	0.682	0.765	0.1097
218500_at	399.6	590.8	0.56	1.48	PPP-PPP	0.20	16	0.682	0.765	0.0791
232337_at	127.1	187.8	0.56	1.48	PPP-PPM	0.20	16	0.682	0.765	0.1859
210222_s_at	131.4	194.1	0.56	1.48	PPA-PPP	0.20	16	0.682	0.765	0.0829
243553_x_at	91.1	134.5	0.56	1.48	APP-APP	0.20	16	0.682	0.765	0.1769
206213_at	142.4	210.0	0.56	1.47	PAA-AMA	0.20	16	0.682	0.765	0.1855
235809_at	65.3	96.2	0.56	1.47	AMP-PPP	0.20	16	0.682	0.765	0.1310
215425_at	50.5	74.5	0.56	1.47	APP-PPP	0.20	16	0.682	0.765	0.0858
215018_at	41.1	60.6	0.56	1.47	AAA-AAP	0.20	16	0.682	0.765	0.0771
207859_s_at	46.2	68.1	0.56	1.47	AAM-PAA	0.20	16	0.682	0.765	0.1947
202490_at	56.5	83.2	0.56	1.47	PAP-PPA	0.20	16	0.682	0.765	0.1762
227266_s_at	79.0	116.3	0.56	1.47	PPP-PPP	0.20	16	0.682	0.765	0.1911
227751_at	211.9	312.0	0.56	1.47	PPP-PPP	0.20	16	0.682	0.765	0.1808
1558280_s_at	158.4	232.8	0.56	1.47	PMP-MAP	0.20	16	0.682	0.765	0.1797
221962_s_at	682.2	1002.6	0.56	1.47	PPP-PPP	0.20	16	0.682	0.765	0.1950
1556081_at	54.9	80.6	0.56	1.47	AAP-PPP	0.20	16	0.682	0.765	0.1523
240155_x_at	136.2	200.1	0.56	1.47	APP-PPP	0.20	16	0.682	0.765	0.1431
203532_x_at	360.2	529.3	0.56	1.47	PPP-PPP	0.20	16	0.682	0.765	0.0959
241459_at	93.9	137.9	0.55	1.47	PPP-PPP	0.20	16	0.682	0.765	0.0757
239435_x_at	858.5	585.1	-0.55	-1.47	PPP-PPP	0.20	16	0.682	0.765	0.0026
221997_s_at	107.1	157.2	0.55	1.47	APP-PPP	0.20	16	0.682	0.765	0.1929
240499_at	106.2	155.8	0.55	1.47	PPP-PPP	0.20	16	0.682	0.765	0.0850
210203_at	85.8	125.8	0.55	1.47	AAP-AAP	0.20	16	0.682	0.765	0.1812
219208_at	127.5	186.9	0.55	1.47	PAP-PPP	0.20	16	0.682	0.765	0.1067
1561166_a_at	69.3	101.4	0.55	1.47	APP-PPP	0.20	16	0.682	0.765	0.0882
231735_s_at	632.6	925.8	0.55	1.46	PPP-PPP	0.20	16	0.682	0.765	0.0796
238239_at	56.6	82.8	0.55	1.46	PAA-PPA	0.20	16	0.682	0.765	0.0710
229673_at	138.5	202.7	0.55	1.46	PPP-PPP	0.20	16	0.682	0.765	0.1493
244481_at	105.6	154.4	0.55	1.46	AAP-AMA	0.20	16	0.682	0.765	0.1418

233321_x_at	86.4	126.3	0.55	1.46	PPP-MPP	0.20	16	0.682	0.765	0.1376
213359_at	450.4	658.8	0.55	1.46	PAP-PPP	0.20	16	0.682	0.765	0.1302
203141_s_at	348.5	509.8	0.55	1.46	PPP-PPP	0.20	16	0.682	0.765	0.0774
1554712_a_at	62.6	91.5	0.55	1.46	AAA-PAP	0.20	16	0.682	0.765	0.1197
242722_at	1125.7	1644.4	0.55	1.46	PPP-PPP	0.20	16	0.682	0.765	0.0894
235647_at	154.6	225.9	0.55	1.46	AAP-APP	0.20	16	0.682	0.765	0.0706
205289_at	162.7	237.5	0.55	1.46	APP-PPA	0.20	16	0.682	0.765	0.1346
218301_at	221.2	323.0	0.55	1.46	PPP-PPP	0.20	16	0.682	0.765	0.1181
1559753_at	52.0	75.9	0.55	1.46	AAP-PAP	0.20	16	0.682	0.765	0.1002
228716_at	226.2	330.3	0.55	1.46	PPP-PPP	0.20	16	0.682	0.765	0.0935
1558996_at	96.3	140.5	0.55	1.46	APA-PPP	0.20	16	0.682	0.765	0.0927
220735_s_at	57.0	83.1	0.54	1.46	MPA-PPP	0.20	16	0.682	0.765	0.1094
243904_at	142.3	207.5	0.54	1.46	PPP-PPP	0.20	16	0.682	0.765	0.0696
229123_at	73.9	107.6	0.54	1.46	PPA-AMP	0.20	16	0.682	0.765	0.1816
222318_at	108.9	158.5	0.54	1.46	APA-AAA	0.20	16	0.682	0.765	0.1746
223835_x_at	60.7	88.3	0.54	1.46	APP-PAP	0.20	16	0.682	0.765	0.1448
217081_at	37.0	53.8	0.54	1.46	APA-PAA	0.20	16	0.682	0.765	0.1007
202801_at	444.0	646.0	0.54	1.46	PPP-PPP	0.20	16	0.682	0.765	0.0812
202714_s_at	188.7	274.5	0.54	1.46	PAP-AAA	0.20	16	0.682	0.765	0.0782
232138_at	209.1	304.1	0.54	1.45	PPP-PPP	0.20	16	0.682	0.765	0.1174
1554262_s_at	86.0	124.9	0.54	1.45	MAA-APA	0.20	16	0.682	0.765	0.1866
226657_at	91.9	133.5	0.54	1.45	PAP-APP	0.20	16	0.682	0.765	0.1650
238736_at	106.3	154.5	0.54	1.45	MPP-PAP	0.20	16	0.682	0.765	0.1378
1558466_at	66.0	95.8	0.54	1.45	APA-APP	0.20	16	0.682	0.765	0.1533
229946_at	131.2	190.5	0.54	1.45	PAM-PPP	0.20	16	0.682	0.765	0.1529
236466_at	49.8	72.3	0.54	1.45	AAA-APA	0.20	16	0.682	0.765	0.1024
238614_x_at	273.7	397.4	0.54	1.45	PPP-PPP	0.20	16	0.682	0.765	0.0891
205552_s_at	170.8	248.0	0.54	1.45	PPP-PPP	0.20	16	0.682	0.765	0.0788
242003_at	85.8	124.6	0.54	1.45	PPP-PPP	0.20	16	0.682	0.765	0.1223
241887_at	80.7	117.0	0.54	1.45	PPP-PPP	0.20	16	0.682	0.765	0.1195
222164_at	224.1	325.0	0.54	1.45	APP-PPP	0.20	16	0.682	0.765	0.1068
60815_at	120.6	174.8	0.54	1.45	PPP-PPP	0.20	16	0.682	0.765	0.1776
239597_at	297.0	430.1	0.54	1.45	PPP-PPP	0.20	16	0.682	0.765	0.0730
228664_at	278.8	403.7	0.53	1.45	PAP-PPP	0.20	16	0.682	0.765	0.1671
222316_at	96.7	140.0	0.53	1.45	PPP-PPP	0.20	16	0.682	0.765	0.1358
1570259_at	38.6	55.9	0.53	1.45	AAP-APP	0.20	16	0.682	0.765	0.1223
236350_at	154.8	224.1	0.53	1.45	PPP-PPP	0.20	16	0.682	0.765	0.1173
229546_at	99.7	144.4	0.53	1.45	PPP-PPP	0.20	16	0.682	0.765	0.0713
203562_at	1015.4	701.6	-0.53	-1.45	PPP-PPP	0.20	16	0.682	0.765	0.0020
243431_at	82.5	119.3	0.53	1.45	AAP-PPP	0.20	16	0.682	0.765	0.1909
1569961_at	60.4	87.4	0.53	1.45	AAP-PAP	0.20	16	0.682	0.765	0.1577
1567242_at	58.1	84.0	0.53	1.45	MAA-PPP	0.20	16	0.682	0.765	0.1354

214442_s_at	271.8	392.9	0.53	1.45	APM-PPP	0.20	16	0.682	0.765	0.1117
216025_x_at	52.9	76.4	0.53	1.45	PAA-PAP	0.20	16	0.682	0.765	0.1890
213158_at	88.7	128.2	0.53	1.45	PPP-PPP	0.20	16	0.682	0.765	0.1299
213653_at	282.9	408.8	0.53	1.45	PPP-PPP	0.20	16	0.682	0.765	0.1269
230106_at	151.8	219.1	0.53	1.44	AAP-PAP	0.20	16	0.682	0.765	0.0889
233061_at	125.2	180.8	0.53	1.44	PPP-PPP	0.20	16	0.682	0.765	0.0745
1558381_a_at	115.3	166.4	0.53	1.44	PAA-APA	0.20	16	0.682	0.765	0.1683
1553852_at	59.2	85.4	0.53	1.44	AAA-APA	0.20	16	0.682	0.765	0.1272
243543_at	140.4	202.4	0.53	1.44	PPP-PPP	0.20	16	0.682	0.765	0.0990
205928_at	292.2	421.4	0.53	1.44	PPP-PPP	0.20	16	0.682	0.765	0.0700
229689_s_at	554.5	799.3	0.53	1.44	PPP-PPP	0.20	16	0.682	0.765	0.0656
241348_at	288.7	415.8	0.53	1.44	PPP-PPP	0.20	16	0.682	0.765	0.1667
202363_at	234.9	338.4	0.53	1.44	AAA-APM	0.20	16	0.682	0.765	0.1159
1569472_s_at	221.3	318.5	0.53	1.44	PPP-PPP	0.20	16	0.682	0.765	0.1858
236923_x_at	172.7	248.6	0.53	1.44	AAP-PPP	0.20	16	0.682	0.765	0.1092
203378_at	414.5	596.9	0.53	1.44	PPP-PPP	0.20	16	0.682	0.765	0.0891
226869_at	431.0	620.2	0.53	1.44	PPP-PPP	0.20	16	0.682	0.765	0.1551
228817_at	261.4	376.1	0.53	1.44	PPP-PPP	0.20	16	0.682	0.765	0.0756
244164_at	57.3	82.4	0.53	1.44	AAP-PAP	0.20	16	0.682	0.765	0.0743
223355_at	383.4	551.3	0.52	1.44	APP-PPP	0.20	16	0.682	0.765	0.1074
1557285_at	98.4	141.4	0.52	1.44	PPP-PPP	0.20	16	0.682	0.765	0.0843
233614_at	39.9	57.3	0.52	1.44	PAA-AAP	0.20	16	0.682	0.765	0.1490
1564254_at	126.7	182.0	0.52	1.44	AAA-APA	0.20	16	0.682	0.765	0.1364
203908_at	34.6	49.6	0.52	1.44	APA-APA	0.20	16	0.682	0.765	0.1545
212525_s_at	253.8	363.9	0.52	1.43	PAP-PPP	0.20	16	0.682	0.765	0.1529
230104_s_at	85.9	123.2	0.52	1.43	AAA-APP	0.20	16	0.682	0.765	0.1499
234989_at	8022.2	11502.8	0.52	1.43	PPP-PPP	0.20	16	0.682	0.765	0.1192
32259_at	408.9	586.3	0.52	1.43	PPP-PPP	0.20	16	0.682	0.765	0.1064
231928_at	459.2	658.2	0.52	1.43	PPP-PPP	0.20	16	0.682	0.765	0.0684
239333_x_at	128.3	183.9	0.52	1.43	PPA-PPP	0.20	16	0.682	0.765	0.1916
211085_s_at	165.4	237.0	0.52	1.43	PPP-PPP	0.20	16	0.682	0.765	0.1375
238937_at	56.4	80.8	0.52	1.43	APA-AAA	0.20	16	0.682	0.765	0.1351
226849_at	182.5	261.5	0.52	1.43	PPP-PPP	0.20	16	0.682	0.765	0.1104
216486_x_at	229.9	329.4	0.52	1.43	APP-PPP	0.20	16	0.682	0.765	0.1065
219393_s_at	135.4	193.8	0.52	1.43	PPP-PPP	0.20	16	0.682	0.765	0.1413
233702_x_at	1437.3	2057.5	0.52	1.43	PPP-PPP	0.20	16	0.682	0.765	0.1343
1557409_at	295.0	422.4	0.52	1.43	AAA-PAP	0.20	16	0.682	0.765	0.0833
230651_at	67.0	95.9	0.52	1.43	PPP-PPP	0.20	16	0.682	0.765	0.1805
1558847_at	59.1	84.5	0.52	1.43	AAP-PAP	0.20	16	0.682	0.765	0.1678
1553466_at	73.9	105.7	0.52	1.43	AAA-PMA	0.20	16	0.682	0.765	0.1266
229899_s_at	291.2	416.5	0.52	1.43	PPP-PPP	0.20	16	0.682	0.765	0.0865
32723_at	532.0	760.7	0.52	1.43	PPP-PPP	0.20	16	0.682	0.765	0.0661

228250_at	207.8	296.9	0.52	1.43	PPP-PPP	0.20	16	0.682	0.765	0.0936
235667_at	68.6	98.1	0.52	1.43	APP-APP	0.20	16	0.682	0.765	0.0874
217657_at	92.6	132.2	0.51	1.43	PPP-PPP	0.20	16	0.682	0.765	0.1801
236274_at	133.0	190.0	0.51	1.43	PPP-PPP	0.20	16	0.682	0.765	0.1704
233832_at	97.3	139.0	0.51	1.43	AAA-APM	0.20	16	0.682	0.765	0.1402
218060_s_at	190.1	271.5	0.51	1.43	MAP-PAP	0.20	16	0.682	0.765	0.1331
242450_at	48.5	69.3	0.51	1.43	APA-AMP	0.20	16	0.682	0.765	0.1135
206825_at	66.5	94.9	0.51	1.43	AAP-APP	0.20	16	0.682	0.765	0.1062
225869_s_at	618.4	883.0	0.51	1.43	PPP-PPP	0.20	16	0.682	0.765	0.0659
1565786_x_at	151.7	216.3	0.51	1.43	PPP-PPP	0.20	16	0.682	0.765	0.1348
235065_at	476.1	678.9	0.51	1.43	PPP-PPP	0.20	16	0.682	0.765	0.1076
218757_s_at	553.1	788.8	0.51	1.43	PPP-PPP	0.20	16	0.682	0.765	0.0890
235497_at	79.3	113.0	0.51	1.43	AAP-PPP	0.20	16	0.682	0.765	0.1710
224673_at	268.3	382.2	0.51	1.43	PPP-PPP	0.20	16	0.682	0.765	0.1316
204357_s_at	101.3	144.4	0.51	1.43	APP-APP	0.20	16	0.682	0.765	0.0919
1558111_at	134.9	192.1	0.51	1.42	PPP-PPP	0.20	16	0.682	0.765	0.1838
235079_at	201.7	287.2	0.51	1.42	PPP-PPP	0.20	16	0.682	0.765	0.1693
224486_s_at	312.4	444.9	0.51	1.42	PPP-PPP	0.20	16	0.682	0.765	0.1118
219697_at	71.1	101.3	0.51	1.42	AAA-APP	0.20	16	0.682	0.765	0.1766
1559214_at	76.8	109.4	0.51	1.42	PPP-PPP	0.20	16	0.682	0.765	0.1641
1558924_s_at	330.9	470.8	0.51	1.42	PPP-PPP	0.20	16	0.682	0.765	0.0718
234248_at	133.8	190.3	0.51	1.42	PPP-PPP	0.20	16	0.682	0.765	0.1258
201787_at	962.0	1368.1	0.51	1.42	PPP-PPP	0.20	16	0.682	0.765	0.0942
1552401_a_at	51.2	72.8	0.51	1.42	APA-PAA	0.20	16	0.682	0.765	0.1778
222788_s_at	144.9	205.8	0.51	1.42	AAP-PPP	0.20	16	0.682	0.765	0.1374
227900_at	89.5	127.0	0.51	1.42	AAA-AMP	0.20	16	0.682	0.765	0.1452
221894_at	514.2	729.7	0.51	1.42	PPP-PPP	0.20	16	0.682	0.765	0.1417
223322_at	651.0	923.8	0.51	1.42	PPP-PPP	0.20	16	0.682	0.765	0.1339
1565628_at	59.3	84.1	0.51	1.42	AAA-PPA	0.20	16	0.682	0.765	0.1040
223366_at	281.4	399.2	0.50	1.42	PPP-PPP	0.20	16	0.682	0.765	0.1814
222540_s_at	552.9	783.8	0.50	1.42	PPP-PPP	0.20	16	0.682	0.765	0.1763
1554780_a_at	354.0	501.7	0.50	1.42	PPP-PPP	0.20	16	0.682	0.765	0.1917
210499_s_at	110.7	156.9	0.50	1.42	APP-PAP	0.20	16	0.682	0.765	0.0843
220352_x_at	204.7	290.0	0.50	1.42	PPP-PPP	0.20	16	0.682	0.765	0.0808
238826_x_at	163.9	232.2	0.50	1.42	PPP-MPP	0.20	16	0.682	0.765	0.0705
1554168_a_at	5330.9	3765.4	-0.50	-1.42	PPP-PPP	0.20	16	0.682	0.765	0.0016
1569601_at	120.4	170.5	0.50	1.42	APP-PPP	0.20	16	0.682	0.765	0.1944
1556183_at	92.6	131.2	0.50	1.42	AAA-PPP	0.20	16	0.682	0.765	0.0765
237145_at	87.5	123.8	0.50	1.42	PAM-PPP	0.20	16	0.682	0.765	0.1816
205232_s_at	157.1	222.2	0.50	1.41	PPP-PPP	0.20	16	0.682	0.765	0.1491
235347_at	208.0	293.9	0.50	1.41	PPP-PMP	0.20	16	0.682	0.765	0.1081
235453_at	56.2	79.3	0.50	1.41	PAP-PPP	0.20	16	0.682	0.765	0.1822

229928_at	33.5	47.2	0.50	1.41	AAA-APA	0.20	16	0.682	0.765	0.1661
218503_at	432.9	611.0	0.50	1.41	PAP-PPP	0.20	16	0.682	0.765	0.1595
240156_at	92.0	129.8	0.50	1.41	PPP-PPP	0.20	16	0.682	0.765	0.1236
204483_at	67.4	95.1	0.50	1.41	AAA-APP	0.20	16	0.682	0.765	0.1887
236022_at	193.0	272.2	0.50	1.41	PPP-PPP	0.20	16	0.682	0.765	0.1626
1569311_at	84.7	119.4	0.50	1.41	PPP-PPP	0.20	16	0.682	0.765	0.1366
201015_s_at	3409.1	4807.3	0.50	1.41	PPP-PPP	0.20	16	0.682	0.765	0.0746
226246_at	1898.4	2675.4	0.50	1.41	PPP-PPP	0.20	16	0.682	0.765	0.0666
207847_s_at	1419.4	1998.5	0.49	1.41	PPP-PPP	0.20	16	0.682	0.765	0.1504
228249_at	496.2	698.9	0.49	1.41	PPP-PPP	0.20	16	0.682	0.765	0.1262
232431_at	102.2	143.7	0.49	1.41	PPA-PPP	0.20	16	0.682	0.765	0.1431
230097_at	227.3	319.6	0.49	1.41	PPP-PPP	0.20	16	0.682	0.765	0.0999
211996_s_at	765.4	1076.5	0.49	1.41	PPP-PPP	0.20	16	0.682	0.765	0.0730
214715_x_at	1328.4	1865.7	0.49	1.41	PPP-PPP	0.20	16	0.682	0.765	0.1383
78383_at	260.2	365.4	0.49	1.41	PPP-PPP	0.20	16	0.682	0.765	0.1234
39248_at	2658.2	3731.3	0.49	1.40	PPP-PPP	0.20	16	0.682	0.765	0.1251
222417_s_at	6452.9	9054.4	0.49	1.40	PPP-PPP	0.20	16	0.682	0.765	0.1023
204118_at	65.0	91.1	0.49	1.40	AAA-APA	0.20	16	0.682	0.765	0.1680
212759_s_at	256.1	359.2	0.49	1.40	PPM-PPP	0.20	16	0.682	0.765	0.1590
223548_at	121.7	170.6	0.49	1.40	PPA-APP	0.20	16	0.682	0.765	0.1475
1566885_at	114.5	160.5	0.49	1.40	PPA-PPP	0.20	16	0.682	0.765	0.1398
203991_s_at	189.4	265.6	0.49	1.40	PPP-PPP	0.20	16	0.682	0.765	0.1193
1564336_at	55.2	77.5	0.49	1.40	AAA-PAA	0.20	16	0.682	0.765	0.0878
1570567_at	88.3	123.7	0.49	1.40	PPP-PPP	0.20	16	0.682	0.765	0.0955
1563468_at	72.4	101.5	0.49	1.40	PAA-PPP	0.20	16	0.682	0.765	0.0759
1563063_at	102.9	144.1	0.49	1.40	AAA-PPM	0.20	16	0.682	0.765	0.1084
1564886_at	122.3	171.3	0.49	1.40	APA-PPP	0.20	16	0.682	0.765	0.1073
209815_at	222.4	311.2	0.49	1.40	PPP-PPP	0.20	16	0.682	0.765	0.1368
227169_at	109.5	153.3	0.49	1.40	AAA-PAA	0.20	16	0.682	0.765	0.0924
235882_at	146.7	205.2	0.48	1.40	PPP-PPP	0.20	16	0.682	0.765	0.1870
213882_at	76.1	106.4	0.48	1.40	PPP-PPP	0.20	16	0.682	0.765	0.1396
220032_at	103.2	144.4	0.48	1.40	PAM-PAP	0.20	16	0.682	0.765	0.1016
1568923_at	77.4	108.2	0.48	1.40	APM-PAA	0.20	16	0.682	0.765	0.1732
1552613_s_at	350.8	490.2	0.48	1.40	PPP-PPP	0.20	16	0.682	0.765	0.0842
202860_at	604.3	844.8	0.48	1.40	PPP-PPP	0.20	16	0.682	0.765	0.0760
219088_s_at	302.2	421.9	0.48	1.40	PPA-APP	0.20	16	0.682	0.765	0.1414
217310_s_at	107.2	149.7	0.48	1.40	PAP-MPP	0.20	16	0.682	0.765	0.1239
220014_at	44.9	62.7	0.48	1.40	PPA-PPP	0.20	16	0.682	0.765	0.0807
232387_at	82.2	114.7	0.48	1.40	PMP-PAA	0.20	16	0.682	0.765	0.1550
224496_s_at	122.1	170.4	0.48	1.40	PPP-PPP	0.20	16	0.682	0.765	0.1356
219518_s_at	169.1	235.8	0.48	1.39	PPP-PPP	0.20	16	0.682	0.765	0.1069
225160_x_at	2092.7	1501.9	-0.48	-1.39	PPP-PPP	0.20	16	0.682	0.765	0.0006

1562546_at	45.3	63.2	0.48	1.39	PAP-PPP	0.20	16	0.682	0.765	0.1655
218880_at	266.9	372.0	0.48	1.39	PAP-PPP	0.20	16	0.682	0.765	0.1479
205525_at	137.7	191.9	0.48	1.39	PPP-APP	0.20	16	0.682	0.765	0.1355
241014_at	44.3	61.7	0.48	1.39	AAA-PPA	0.20	16	0.682	0.765	0.1301
202903_at	304.5	424.2	0.48	1.39	PPP-PPP	0.20	16	0.682	0.765	0.0930
204921_at	165.4	230.3	0.48	1.39	APA-PPM	0.20	16	0.682	0.765	0.0630
236527_at	163.2	227.2	0.48	1.39	PAP-MPP	0.20	16	0.682	0.765	0.1321
235045_at	343.4	478.0	0.48	1.39	PPP-PPP	0.20	16	0.682	0.765	0.1231
243188_at	145.1	201.9	0.48	1.39	PPP-PPP	0.20	16	0.682	0.765	0.1021
211094_s_at	191.2	266.0	0.48	1.39	PPP-PPP	0.20	16	0.682	0.765	0.1462
243924_at	145.4	202.2	0.48	1.39	AAP-PAM	0.20	16	0.682	0.765	0.1167
219446_at	324.9	452.0	0.48	1.39	PPP-PPP	0.20	16	0.682	0.765	0.0831
229586_at	540.4	751.1	0.48	1.39	PPP-PPP	0.20	16	0.682	0.765	0.0627
209717_at	671.2	932.0	0.47	1.39	PPP-PPP	0.20	16	0.682	0.765	0.1535
238853_at	194.9	270.7	0.47	1.39	PAP-PPP	0.20	16	0.682	0.765	0.1372
240106_at	121.4	168.6	0.47	1.39	PPP-PPP	0.20	16	0.682	0.765	0.1061
201693_s_at	369.2	512.5	0.47	1.39	PPP-PPP	0.20	16	0.682	0.765	0.1878
227098_at	199.6	277.0	0.47	1.39	PPP-APP	0.20	16	0.682	0.765	0.0763
232627_at	78.6	109.1	0.47	1.39	AAA-AMP	0.20	16	0.682	0.765	0.1571
209713_s_at	149.9	207.9	0.47	1.39	PPP-PPP	0.20	16	0.682	0.765	0.1357
211584_s_at	566.6	785.1	0.47	1.39	PPP-PPP	0.20	16	0.682	0.765	0.1078
230937_at	105.4	146.2	0.47	1.39	APM-AAA	0.20	16	0.682	0.765	0.0802
221314_at	92.3	127.6	0.47	1.38	AAA-PAM	0.20	16	0.682	0.765	0.1816
208670_s_at	1354.9	1874.1	0.47	1.38	PPP-PPP	0.20	16	0.682	0.765	0.0930
225923_at	318.3	440.2	0.47	1.38	PPP-PPP	0.20	16	0.682	0.765	0.0920
232541_at	1256.1	1737.5	0.47	1.38	PPP-PPP	0.20	16	0.682	0.765	0.0884
1563031_at	72.0	99.6	0.47	1.38	PAP-PPP	0.20	16	0.682	0.765	0.0864
239989_at	90.8	125.4	0.47	1.38	PAA-PPP	0.20	16	0.682	0.765	0.1841
1558522_at	190.0	262.7	0.47	1.38	PPP-PPP	0.20	16	0.682	0.765	0.1603
235114_x_at	232.6	321.5	0.47	1.38	PPP-PPP	0.20	16	0.682	0.765	0.1443
227717_at	333.9	461.2	0.47	1.38	PPP-PPP	0.20	16	0.682	0.765	0.1452
228613_at	240.8	332.5	0.47	1.38	PPP-PPP	0.20	16	0.682	0.765	0.1009
236711_at	41.4	57.2	0.47	1.38	APA-AAA	0.20	16	0.682	0.765	0.0771
236506_at	153.6	212.2	0.47	1.38	PPP-PPP	0.20	16	0.682	0.765	0.0655
1554538_at	105.5	145.6	0.47	1.38	PPA-APP	0.20	16	0.682	0.765	0.1231
223444_at	135.8	187.3	0.46	1.38	PAP-PPP	0.20	16	0.682	0.765	0.1636
238320_at	1050.8	1449.8	0.46	1.38	PPP-PPP	0.20	16	0.682	0.765	0.1132
1554987_at	92.5	127.6	0.46	1.38	PPP-PPP	0.20	16	0.682	0.765	0.0979
1569590_at	98.9	136.4	0.46	1.38	APA-APP	0.20	16	0.682	0.765	0.0967
230713_at	184.3	254.2	0.46	1.38	PPP-PPP	0.20	16	0.682	0.765	0.0919
33768_at	339.2	467.2	0.46	1.38	PPP-PPP	0.20	16	0.682	0.765	0.0785
242195_x_at	1478.4	2035.4	0.46	1.38	PPP-PPP	0.20	16	0.682	0.765	0.1033

225271_at	218.4	300.7	0.46	1.38	PPA-PAP	0.20	16	0.682	0.765	0.0954
209640_at	94.9	130.6	0.46	1.38	PPA-AAM	0.20	16	0.682	0.765	0.0870
213236_at	383.8	528.1	0.46	1.38	AMA-APP	0.20	16	0.682	0.765	0.0838
219409_at	226.6	311.7	0.46	1.38	PMP-PPP	0.20	16	0.682	0.765	0.1821
227102_at	512.1	704.3	0.46	1.38	PPP-PPP	0.20	16	0.682	0.765	0.1478
1556035_s_at	467.2	642.8	0.46	1.38	PPP-PPP	0.20	16	0.682	0.765	0.1338
210630_s_at	88.9	122.3	0.46	1.38	PPP-PPP	0.20	16	0.682	0.765	0.1141
1557786_s_at	64.6	88.8	0.46	1.38	APA-MMP	0.20	16	0.682	0.765	0.1360
214703_s_at	217.1	298.3	0.46	1.37	PPP-PPP	0.20	16	0.682	0.765	0.1662
222647_at	476.3	653.7	0.46	1.37	PPP-PPP	0.20	16	0.682	0.765	0.1703
242492_at	243.7	334.5	0.46	1.37	APA-MMP	0.20	16	0.682	0.765	0.1027
236215_at	99.6	136.7	0.46	1.37	PAP-PPP	0.20	16	0.682	0.765	0.0560
212809_at	600.5	823.7	0.46	1.37	PPP-PPP	0.20	16	0.682	0.765	0.1276
233995_at	114.3	156.8	0.46	1.37	PAP-PMP	0.20	16	0.682	0.765	0.0756
202335_s_at	77.1	105.8	0.46	1.37	PPP-PPP	0.20	16	0.682	0.765	0.0724
239122_at	77.9	106.7	0.46	1.37	APM-PMP	0.20	16	0.682	0.765	0.1091
242969_at	51.3	70.3	0.46	1.37	AAP-PPP	0.20	16	0.682	0.765	0.1017
237097_at	196.5	269.2	0.45	1.37	PPP-PPP	0.20	16	0.682	0.765	0.0870
225056_at	302.0	413.6	0.45	1.37	PPP-PPP	0.20	16	0.682	0.765	0.0597
227563_at	143.5	196.5	0.45	1.37	PPP-PPP	0.20	16	0.682	0.765	0.1650
220311_at	323.8	443.0	0.45	1.37	PPP-PPP	0.20	16	0.682	0.765	0.0893
1552314_a_at	86.1	117.7	0.45	1.37	PPP-PPP	0.20	16	0.682	0.765	0.1723
222006_at	455.1	622.1	0.45	1.37	PPP-PPP	0.20	16	0.682	0.765	0.1397
237262_at	479.3	655.3	0.45	1.37	PPP-PPP	0.20	16	0.682	0.765	0.0998
228743_at	187.7	256.6	0.45	1.37	MPP-PPA	0.20	16	0.682	0.765	0.0671
218650_at	297.0	405.8	0.45	1.37	PPP-PPP	0.20	16	0.682	0.765	0.1286
218572_at	998.4	1364.1	0.45	1.37	PPP-PPP	0.20	16	0.682	0.765	0.1204
219703_at	169.9	231.9	0.45	1.37	PPP-PPP	0.20	16	0.682	0.765	0.0792
219150_s_at	354.3	483.5	0.45	1.37	PPP-PPP	0.20	16	0.682	0.765	0.0758
211986_at	3120.9	4256.5	0.45	1.36	PPP-PPP	0.20	16	0.682	0.765	0.1517
237553_at	100.8	137.4	0.45	1.36	PPP-PPP	0.20	16	0.682	0.765	0.1255
204861_s_at	97.3	132.6	0.45	1.36	AAA-AAP	0.20	16	0.682	0.765	0.1403
233532_x_at	146.0	198.9	0.45	1.36	PAP-PPP	0.20	16	0.682	0.765	0.0858
227642_at	2382.5	3242.6	0.45	1.36	PPP-PPP	0.20	16	0.682	0.765	0.1218
234762_x_at	844.1	1148.8	0.45	1.36	PPP-PPP	0.20	16	0.682	0.765	0.0734
1556385_at	180.2	245.1	0.44	1.36	PPP-PPP	0.20	16	0.682	0.765	0.1650
231145_at	117.6	160.0	0.44	1.36	PPP-PPP	0.20	16	0.682	0.765	0.1623
217504_at	337.8	459.6	0.44	1.36	PPP-PPP	0.20	16	0.682	0.765	0.1563
228180_at	122.7	166.9	0.44	1.36	PPP-PPP	0.20	16	0.682	0.765	0.0829
1556555_at	105.8	143.8	0.44	1.36	PMP-PPM	0.20	16	0.682	0.765	0.0904
221621_at	78.6	106.8	0.44	1.36	PPP-PPP	0.20	16	0.682	0.765	0.0794
223805_at	1107.9	1505.5	0.44	1.36	PPP-PPP	0.20	16	0.682	0.765	0.0790

229460_at	343.6	466.5	0.44	1.36	PPP-PPP	0.20	16	0.682	0.765	0.1374
229804_x_at	714.6	970.3	0.44	1.36	PPP-PPP	0.20	16	0.682	0.765	0.1080
1569057_s_at	479.4	651.0	0.44	1.36	PAP-PPP	0.20	16	0.682	0.765	0.0937
214291_at	253.1	343.4	0.44	1.36	PPP-PPP	0.20	16	0.682	0.765	0.1020
207382_at	106.0	143.8	0.44	1.36	AAA-APP	0.20	16	0.682	0.765	0.0912
243827_at	93.4	126.6	0.44	1.36	PPP-APP	0.20	16	0.682	0.765	0.1670
35617_at	151.5	205.3	0.44	1.36	MPP-PPP	0.20	16	0.682	0.765	0.1817
214093_s_at	395.8	536.2	0.44	1.36	PPP-PPP	0.20	16	0.682	0.765	0.1802
236907_at	470.6	637.4	0.44	1.36	PPP-PPP	0.20	16	0.682	0.765	0.1543
213739_at	216.0	292.5	0.44	1.35	PAM-AAP	0.20	16	0.682	0.765	0.1264
239198_at	89.2	120.8	0.44	1.36	PPA-APP	0.20	16	0.682	0.765	0.0595
1555437_at	74.0	100.1	0.44	1.35	AAP-PAA	0.20	16	0.682	0.765	0.1347
221025_x_at	179.4	242.6	0.44	1.35	APA-APA	0.20	16	0.682	0.765	0.1677
238635_at	86.8	117.3	0.44	1.35	PPP-PPP	0.20	16	0.682	0.765	0.1611
210180_s_at	490.6	663.1	0.44	1.35	PPP-PPP	0.20	16	0.682	0.765	0.0718
205411_at	82.8	111.9	0.43	1.35	APA-PAM	0.20	16	0.682	0.765	0.1777
241596_at	69.8	94.3	0.43	1.35	PAA-AAM	0.20	16	0.682	0.765	0.1766
241708_at	43.2	58.4	0.43	1.35	AAA-AAP	0.20	16	0.682	0.765	0.1669
224589_at	440.4	594.5	0.43	1.35	PPP-PPP	0.20	16	0.682	0.765	0.1603
226939_at	272.7	368.2	0.43	1.35	PPP-PPP	0.20	16	0.682	0.765	0.0837
201841_s_at	8503.0	11475.9	0.43	1.35	PPP-PPP	0.20	16	0.682	0.765	0.0614
242377_x_at	180.7	243.5	0.43	1.35	PAP-PPM	0.20	16	0.682	0.765	0.1768
224185_at	141.4	190.6	0.43	1.35	PPP-PPP	0.20	16	0.682	0.765	0.1476
207319_s_at	335.6	452.4	0.43	1.35	PPP-PPP	0.20	16	0.682	0.765	0.1305
213353_at	183.5	247.4	0.43	1.35	PPP-PPP	0.20	16	0.682	0.765	0.1192
233224_at	81.2	109.5	0.43	1.35	PPP-PPP	0.20	16	0.682	0.765	0.0931
224567_x_at	6867.2	9256.2	0.43	1.35	PPP-PPP	0.20	16	0.682	0.765	0.0778
211152_s_at	853.0	633.3	-0.43	-1.35	PPP-PPP	0.20	16	0.682	0.765	0.0007
237652_at	88.0	118.6	0.43	1.35	PMA-PPP	0.20	16	0.682	0.765	0.1755
229766_at	163.2	219.8	0.43	1.35	PPP-PPP	0.20	16	0.682	0.765	0.1477
1570087_at	83.0	111.8	0.43	1.35	PAA-AMA	0.20	16	0.682	0.765	0.1446
219500_at	250.0	336.8	0.43	1.35	APP-PPP	0.20	16	0.682	0.765	0.1032
203805_s_at	391.3	526.7	0.43	1.35	PPP-PPP	0.20	16	0.682	0.765	0.1734
227594_at	280.2	377.4	0.43	1.35	PPP-PPP	0.20	16	0.682	0.765	0.1047
225023_at	332.5	447.3	0.43	1.35	PPP-PPP	0.20	16	0.682	0.765	0.1722
1555929_s_at	91.3	122.9	0.43	1.35	PPP-PPP	0.20	16	0.682	0.765	0.0993
204364_s_at	80.8	108.7	0.43	1.35	AAP-PPP	0.20	16	0.682	0.765	0.0706
227196_at	467.9	629.1	0.43	1.35	PPP-PPP	0.20	16	0.682	0.765	0.0647
222131_x_at	367.3	493.7	0.43	1.34	PPP-PPP	0.20	16	0.682	0.765	0.0529
218219_s_at	680.9	914.6	0.43	1.34	PPP-PPP	0.20	16	0.682	0.765	0.1191
227408_s_at	429.7	577.4	0.43	1.34	PPP-PPP	0.20	16	0.682	0.765	0.0859
219476_at	1546.4	2075.7	0.43	1.34	PPP-PPP	0.20	16	0.682	0.765	0.1601

227798_at	846.6	1136.4	0.43	1.34	PPP-PPP	0.20	16	0.682	0.765	0.1516
229571_at	152.0	204.1	0.43	1.34	PAP-PAA	0.20	16	0.682	0.765	0.0883
229518_at	713.4	957.4	0.42	1.34	PPP-PPP	0.20	16	0.682	0.765	0.0867
235652_at	121.3	162.7	0.42	1.34	PAP-AAP	0.20	16	0.682	0.765	0.1435
214212_x_at	242.8	325.6	0.42	1.34	PPP-PPP	0.20	16	0.682	0.765	0.1300
220458_at	123.0	164.6	0.42	1.34	PPP-PPP	0.20	16	0.682	0.765	0.1577
220225_at	1012.1	1354.7	0.42	1.34	PPP-PPP	0.20	16	0.682	0.765	0.0744
221963_x_at	617.5	826.5	0.42	1.34	PPP-PPP	0.20	16	0.682	0.765	0.0551
221986_s_at	324.0	433.6	0.42	1.34	PPP-PPP	0.20	16	0.682	0.765	0.1089
203837_at	1047.4	1401.1	0.42	1.34	PPP-PPP	0.20	16	0.682	0.765	0.1026
222286_at	88.0	117.8	0.42	1.34	PPP-PPP	0.20	16	0.682	0.765	0.0934
243524_at	126.0	168.5	0.42	1.34	PPP-PPP	0.20	16	0.682	0.765	0.0609
AFFX-BioB-3_at	346.4	463.1	0.42	1.34	PPP-PPP	0.20	16	0.682	0.765	0.1699
214581_x_at	373.0	498.6	0.42	1.34	PPP-PPP	0.20	16	0.682	0.765	0.1403
201087_at	996.9	1331.7	0.42	1.34	PPP-PPP	0.20	16	0.682	0.765	0.1542
204298_s_at	577.1	770.9	0.42	1.34	PPP-PPP	0.20	16	0.682	0.765	0.1141
232493_at	113.6	151.8	0.42	1.34	PAM-PPP	0.20	16	0.682	0.765	0.1664
226010_at	580.6	775.4	0.42	1.34	PPP-PPP	0.20	16	0.682	0.765	0.1446
1557352_at	187.7	250.6	0.42	1.34	PPP-PPP	0.20	16	0.682	0.765	0.0830
1554036_at	107.0	142.9	0.42	1.34	PPP-PPP	0.20	16	0.682	0.765	0.0818
222802_at	530.0	707.5	0.42	1.34	PPP-PPP	0.20	16	0.682	0.765	0.0817
232094_at	110.3	147.2	0.42	1.34	PPP-PPP	0.20	16	0.682	0.765	0.0699
203555_at	426.4	568.9	0.42	1.33	PPP-PPP	0.20	16	0.682	0.765	0.1535
1558143_a_at	272.7	363.8	0.42	1.33	PPP-PPP	0.20	16	0.682	0.765	0.1474
243159_x_at	27.5	36.7	0.42	1.33	AAA-AMP	0.20	16	0.682	0.765	0.1638
59433_at	200.3	267.1	0.42	1.33	PPP-PPP	0.20	16	0.682	0.765	0.1512
1557224_at	120.8	160.9	0.41	1.33	PPP-PPP	0.20	16	0.682	0.765	0.1032
238295_at	62.7	83.6	0.41	1.33	AAA-AAP	0.20	16	0.682	0.765	0.0750
216525_x_at	1243.0	1655.4	0.41	1.33	PPP-PPP	0.20	16	0.682	0.765	0.1379
205121_at	103.0	137.1	0.41	1.33	PPP-PPP	0.20	16	0.682	0.765	0.1371
232569_at	324.1	431.6	0.41	1.33	PPP-PPP	0.20	16	0.682	0.765	0.1190
219990_at	507.3	675.5	0.41	1.33	PPP-PPP	0.20	16	0.682	0.765	0.0750
239888_at	119.6	159.3	0.41	1.33	PPP-PPP	0.20	16	0.682	0.765	0.0725
65472_at	101.6	135.3	0.41	1.33	PPP-PPP	0.20	16	0.682	0.765	0.0664
217523_at	443.1	589.6	0.41	1.33	PPP-PPP	0.20	16	0.682	0.765	0.1756
226485_at	207.7	276.3	0.41	1.33	PPP-PPP	0.20	16	0.682	0.765	0.1529
211514_at	76.4	101.6	0.41	1.33	APP-PPP	0.20	16	0.682	0.765	0.1393
214923_at	116.6	155.2	0.41	1.33	PPM-PPP	0.20	16	0.682	0.765	0.1263
212182_at	60.4	80.3	0.41	1.33	AMA-PPP	0.20	16	0.682	0.765	0.1069
1559360_at	205.3	273.0	0.41	1.33	PPP-PPP	0.20	16	0.682	0.765	0.1264
205296_at	398.5	529.7	0.41	1.33	PPP-PPP	0.20	16	0.682	0.765	0.1704
217844_at	927.0	1231.7	0.41	1.33	PPP-PPP	0.20	16	0.682	0.765	0.1159

238666_at	231.3	307.3	0.41	1.33	MPP-PPP	0.20	16	0.682	0.765	0.0933
222265_at	765.8	1017.7	0.41	1.33	PPP-PPP	0.20	16	0.682	0.765	0.0735
242121_at	536.5	712.6	0.41	1.33	PPP-PPP	0.20	16	0.682	0.765	0.0704
226769_at	80.0	106.3	0.41	1.33	AAA-AAP	0.20	16	0.682	0.765	0.0504
239164_at	92.2	122.4	0.41	1.33	APP-PPP	0.20	16	0.682	0.765	0.1576
213596_at	104.1	138.3	0.41	1.33	PMP-PAP	0.20	16	0.682	0.765	0.1565
218998_at	1200.8	1592.3	0.41	1.33	PPP-PPP	0.20	16	0.682	0.765	0.1333
204601_at	224.8	298.1	0.41	1.33	PMP-PPP	0.20	16	0.682	0.765	0.0971
1553122_s_at	171.3	227.2	0.41	1.33	PPP-PPP	0.20	16	0.682	0.765	0.0817
218433_at	405.9	538.0	0.41	1.33	PPP-PPP	0.20	16	0.682	0.765	0.1660
1562442_a_at	158.1	209.5	0.41	1.33	PPP-PPP	0.20	16	0.682	0.765	0.1343
222018_at	235.5	312.2	0.41	1.33	PPP-PAP	0.20	16	0.682	0.765	0.1331
1559347_at	98.0	129.8	0.41	1.32	PPP-PPP	0.20	16	0.682	0.765	0.1655
205345_at	504.4	667.7	0.41	1.32	PPP-PPP	0.20	16	0.682	0.765	0.1519
228990_at	428.5	567.5	0.41	1.32	PPP-PPP	0.20	16	0.682	0.765	0.0664
210120_s_at	251.2	332.6	0.41	1.32	PPP-APP	0.20	16	0.682	0.765	0.0512
235693_at	86.9	115.0	0.40	1.32	MPA-PPP	0.20	16	0.682	0.765	0.1609
208664_s_at	265.1	350.6	0.40	1.32	PPP-MAP	0.20	16	0.682	0.765	0.1457
213178_s_at	253.9	335.5	0.40	1.32	AAA-APM	0.20	16	0.682	0.765	0.1338
1555243_x_at	637.7	842.8	0.40	1.32	PPP-PPP	0.20	16	0.682	0.765	0.1245
213812_s_at	4556.3	3450.6	-0.40	-1.32	PPP-PPP	0.20	16	0.682	0.765	0.0004
215123_at	361.0	476.7	0.40	1.32	PPP-PPP	0.20	16	0.682	0.765	0.1506
224958_at	483.7	638.6	0.40	1.32	PPP-PPP	0.20	16	0.682	0.765	0.1327
211019_s_at	250.2	330.5	0.40	1.32	PPP-PPP	0.20	16	0.682	0.765	0.0643
221466_at	69.7	92.0	0.40	1.32	PPP-PPP	0.20	16	0.682	0.765	0.0829
238049_at	104.2	137.4	0.40	1.32	PPP-MPP	0.20	16	0.682	0.765	0.1724
201712_s_at	421.2	555.4	0.40	1.32	PPP-PPP	0.20	16	0.682	0.765	0.1546
206284_x_at	5655.7	7455.9	0.40	1.32	PPP-PPP	0.20	16	0.682	0.765	0.0832
212760_at	742.0	977.4	0.40	1.32	PPP-PPP	0.20	16	0.682	0.765	0.0684
242008_at	97.3	128.1	0.40	1.32	PPP-PMP	0.20	16	0.682	0.765	0.1653
211677_x_at	172.4	227.0	0.40	1.32	APA-PPP	0.20	16	0.682	0.765	0.1302
232533_at	108.2	142.4	0.40	1.32	PPP-PPP	0.20	16	0.682	0.765	0.0694
200946_x_at	4053.5	5337.2	0.40	1.32	PPP-PPP	0.20	16	0.682	0.765	0.0536
203822_s_at	585.0	769.3	0.40	1.32	PPP-PPP	0.20	16	0.682	0.765	0.0966
225504_at	665.0	874.6	0.40	1.32	PPP-PPP	0.20	16	0.682	0.765	0.0915
243907_at	105.8	139.1	0.39	1.31	APP-PPP	0.20	16	0.682	0.765	0.1290
237172_at	58.9	77.4	0.39	1.31	PPM-AAP	0.20	16	0.682	0.765	0.0899
240494_at	197.0	258.8	0.39	1.31	APA-PPP	0.20	16	0.682	0.765	0.0814
208540_x_at	19199.0	25225.0	0.39	1.31	PPP-PPP	0.20	16	0.682	0.765	0.0447
243462_s_at	220.8	289.9	0.39	1.31	PPP-PPP	0.20	16	0.682	0.765	0.1322
224453_s_at	104.0	136.6	0.39	1.31	PPP-PPP	0.20	16	0.682	0.765	0.1157
220287_at	105.8	138.9	0.39	1.31	PPP-PPP	0.20	16	0.682	0.765	0.0845

209534_x_at	359.2	471.8	0.39	1.31	PPP-PPP	0.20	16	0.682	0.765	0.0677
230146_s_at	168.3	220.9	0.39	1.31	AAA-APA	0.20	16	0.682	0.765	0.1443
232269_x_at	798.9	1047.5	0.39	1.31	PPP-PPP	0.20	16	0.682	0.765	0.1321
215588_x_at	770.6	1010.3	0.39	1.31	PPP-PPP	0.20	16	0.682	0.765	0.1310
1552777_a_at	601.6	788.8	0.39	1.31	PPP-PPP	0.20	16	0.682	0.765	0.1196
224606_at	4090.2	5364.7	0.39	1.31	PPP-PPP	0.20	16	0.682	0.765	0.0902
204270_at	235.8	309.1	0.39	1.31	PPP-PPP	0.20	16	0.682	0.765	0.0597
220119_at	120.3	157.6	0.39	1.31	AMP-PPP	0.20	16	0.682	0.765	0.1584
219444_at	296.2	388.2	0.39	1.31	PPP-PPP	0.20	16	0.682	0.765	0.1518
238142_at	108.4	142.0	0.39	1.31	PPP-PPP	0.20	16	0.682	0.765	0.1317
214731_at	152.6	199.9	0.39	1.31	PPA-PPP	0.20	16	0.682	0.765	0.0750
236026_at	174.0	228.0	0.39	1.31	PPP-PPP	0.20	16	0.682	0.765	0.1397
223300_s_at	670.9	878.1	0.39	1.31	PPP-PPP	0.20	16	0.682	0.765	0.1464
212161_at	196.5	257.1	0.39	1.31	PPP-PPP	0.20	16	0.682	0.765	0.1298
219735_s_at	966.0	1264.4	0.39	1.31	PPP-PPP	0.20	16	0.682	0.765	0.0767
222657_s_at	739.0	966.7	0.39	1.31	PMP-PPP	0.20	16	0.682	0.765	0.1321
204373_s_at	1423.3	1860.9	0.39	1.31	PPP-PPP	0.20	16	0.682	0.765	0.0911
36545_s_at	171.9	224.9	0.39	1.31	PPP-PPP	0.20	16	0.682	0.765	0.0817
203317_at	114.8	150.0	0.39	1.31	PPP-PPP	0.20	16	0.682	0.765	0.1375
222338_x_at	352.8	460.7	0.39	1.31	PPP-PPP	0.20	16	0.682	0.765	0.1608
234394_at	33.5	43.7	0.39	1.31	PAA-AAA	0.20	16	0.682	0.765	0.1364
211815_s_at	117.9	153.9	0.39	1.31	AAP-PAA	0.20	16	0.682	0.765	0.0789
230058_at	298.5	389.8	0.39	1.31	PPP-PPP	0.20	16	0.682	0.765	0.0553
226088_at	687.8	898.2	0.39	1.31	AMP-PAP	0.20	16	0.682	0.765	0.0445
1559078_at	150.9	196.9	0.38	1.31	PPP-PPP	0.20	16	0.682	0.765	0.1665
222718_at	319.7	417.1	0.38	1.31	AAA-AMP	0.20	16	0.682	0.765	0.1245
201701_s_at	837.5	1093.3	0.38	1.31	PPP-PPP	0.20	16	0.682	0.765	0.0519
209358_at	1212.5	1582.7	0.38	1.31	PPP-PPP	0.20	16	0.682	0.765	0.0472
213805_at	397.6	518.7	0.38	1.30	PPP-PPP	0.20	16	0.682	0.765	0.0991
1568997_at	258.5	337.2	0.38	1.30	PPP-PPP	0.20	16	0.682	0.765	0.0518
223119_s_at	317.0	413.4	0.38	1.30	APM-AMP	0.20	16	0.682	0.765	0.0462
1555310_a_at	343.4	447.5	0.38	1.30	PPP-PPP	0.20	16	0.682	0.765	0.1506
209569_x_at	198.3	258.2	0.38	1.30	PPP-PPP	0.20	16	0.682	0.765	0.1417
203990_s_at	169.9	221.2	0.38	1.30	PPP-PPP	0.20	16	0.682	0.765	0.0515
1554408_a_at	1324.8	1723.7	0.38	1.30	PPP-PPP	0.20	16	0.682	0.765	0.1543
1554145_a_at	190.9	248.4	0.38	1.30	PPP-PPP	0.20	16	0.682	0.765	0.1001
242604_at	40.3	52.4	0.38	1.30	APP-MPP	0.20	16	0.682	0.765	0.0965
225437_s_at	536.6	698.4	0.38	1.30	PPP-PPP	0.20	16	0.682	0.765	0.0834
221549_at	1200.8	1563.0	0.38	1.30	PPP-PPP	0.20	16	0.682	0.765	0.0782
235806_at	108.1	140.6	0.38	1.30	PAA-AAM	0.20	16	0.682	0.765	0.0672
1555734_x_at	381.7	496.3	0.38	1.30	PPP-PPP	0.20	16	0.682	0.765	0.1019
228441_s_at	163.9	213.0	0.38	1.30	PPP-PPP	0.20	16	0.682	0.765	0.1571

206169_x_at	291.4	378.6	0.38	1.30	AAP-APM	0.20	16	0.682	0.765	0.1565
1560136_at	93.2	121.2	0.38	1.30	AAM-APA	0.20	16	0.682	0.765	0.1465
223376_s_at	6404.8	8325.3	0.38	1.30	PPP-PPP	0.20	16	0.682	0.765	0.1072
221673_s_at	131.3	170.6	0.38	1.30	PPP-PPP	0.20	16	0.682	0.765	0.0692
205140_at	635.5	824.7	0.38	1.30	PPP-PPP	0.20	16	0.682	0.765	0.1242
205109_s_at	439.8	570.6	0.38	1.30	PPP-PPP	0.20	16	0.682	0.765	0.0776
217804_s_at	923.9	1199.0	0.38	1.30	PPP-PPP	0.20	16	0.682	0.765	0.0737
207469_s_at	1444.9	1874.5	0.38	1.30	PPP-PPP	0.20	16	0.682	0.765	0.0638
204840_s_at	152.5	197.9	0.38	1.30	PPP-PPP	0.20	16	0.682	0.765	0.0540
227304_at	222.5	288.7	0.38	1.30	PPP-PPP	0.20	16	0.682	0.765	0.0438
226973_at	360.5	467.2	0.37	1.30	APP-MPP	0.20	16	0.682	0.765	0.1216
222237_s_at	190.3	246.6	0.37	1.30	PPP-PPP	0.20	16	0.682	0.765	0.1114
242879_x_at	363.3	470.8	0.37	1.30	PPP-PPP	0.20	16	0.682	0.765	0.0694
238813_at	139.4	180.4	0.37	1.29	PPP-PPP	0.20	16	0.682	0.765	0.1324
203933_at	369.8	478.6	0.37	1.29	PPP-PPP	0.20	16	0.682	0.765	0.1082
212529_at	578.0	748.2	0.37	1.29	PPP-PPP	0.20	16	0.682	0.765	0.1061
202437_s_at	787.0	1018.3	0.37	1.29	PPP-PPP	0.20	16	0.682	0.765	0.0930
224322_at	45.5	58.8	0.37	1.29	AAP-PPM	0.20	16	0.682	0.765	0.0812
214739_at	330.4	426.6	0.37	1.29	PPP-PPP	0.20	16	0.682	0.765	0.1108
235924_at	127.7	164.8	0.37	1.29	PPP-PPP	0.20	16	0.682	0.765	0.1439
226761_at	124.7	160.9	0.37	1.29	PPP-PPP	0.20	16	0.682	0.765	0.1323
1569349_at	243.5	314.0	0.37	1.29	PPP-PPP	0.20	16	0.682	0.765	0.1139
204201_s_at	1823.3	2351.9	0.37	1.29	PPP-PPP	0.20	16	0.682	0.765	0.0794
218418_s_at	658.2	848.1	0.37	1.29	PPP-PPP	0.20	16	0.682	0.765	0.1620
223354_x_at	885.1	1139.9	0.37	1.29	PPP-PPP	0.20	16	0.682	0.765	0.0795
206565_x_at	65.4	84.2	0.36	1.29	AAP-AAP	0.20	16	0.682	0.765	0.1442
241702_at	122.0	157.0	0.36	1.29	AAA-PAP	0.20	16	0.682	0.765	0.0876
227285_at	145.9	187.7	0.36	1.29	APA-AAA	0.20	16	0.682	0.765	0.0510
222159_at	146.5	188.6	0.36	1.29	PPP-PPP	0.20	16	0.682	0.765	0.0459
225659_at	307.5	395.3	0.36	1.29	PPP-PPP	0.20	16	0.682	0.765	0.1488
207625_s_at	465.1	598.1	0.36	1.29	PPP-PPP	0.20	16	0.682	0.765	0.1382
204453_at	195.4	251.0	0.36	1.29	PPP-PPP	0.20	16	0.682	0.765	0.1551
200664_s_at	1852.2	2381.2	0.36	1.29	PPP-PPP	0.20	16	0.682	0.765	0.0514
238929_at	315.4	405.4	0.36	1.29	PPP-PPP	0.20	16	0.682	0.765	0.0422
221669_s_at	547.8	703.3	0.36	1.28	PPP-PPP	0.20	16	0.682	0.765	0.1408
215170_s_at	110.1	141.3	0.36	1.28	PPP-PPP	0.20	16	0.682	0.765	0.1466
217329_x_at	1042.8	1338.6	0.36	1.28	PPP-PPP	0.20	16	0.682	0.765	0.1445
225529_at	310.9	399.1	0.36	1.28	PPP-PMP	0.20	16	0.682	0.765	0.1427
214945_at	58.3	74.9	0.36	1.28	PPP-APP	0.20	16	0.682	0.765	0.0856
227741_at	1996.3	2561.3	0.36	1.28	PPP-PPP	0.20	16	0.682	0.765	0.0770
203825_at	1189.5	1527.1	0.36	1.28	PPP-PPP	0.20	16	0.682	0.765	0.0673
211091_s_at	142.6	182.9	0.36	1.28	PPP-PPP	0.20	16	0.682	0.765	0.1604

207722_s_at	519.1	665.9	0.36	1.28	PPP-PPP	0.20	16	0.682	0.765	0.1540
49452_at	105.5	135.4	0.36	1.28	AMM-PMA	0.20	16	0.682	0.765	0.1476
206098_at	111.6	143.1	0.36	1.28	PPP-PPP	0.20	16	0.682	0.765	0.0807
221220_s_at	562.3	721.3	0.36	1.28	PPP-PPP	0.20	16	0.682	0.765	0.0432
215848_at	93.7	120.0	0.36	1.28	APA-PPP	0.20	16	0.682	0.765	0.1173
228164_at	696.7	892.7	0.36	1.28	PPP-PPP	0.20	16	0.682	0.765	0.1063
213650_at	837.5	1073.0	0.36	1.28	PPP-PPP	0.20	16	0.682	0.765	0.0451
239433_at	414.5	531.1	0.36	1.28	PPP-PPP	0.20	16	0.682	0.765	0.0415
239093_at	90.5	115.9	0.36	1.28	PPA-PPP	0.20	16	0.682	0.765	0.1380
1558487_a_at	687.6	880.9	0.36	1.28	PPP-PPP	0.20	16	0.682	0.765	0.0922
218762_at	414.5	530.8	0.36	1.28	PPP-PPP	0.20	16	0.682	0.765	0.0489
229366_at	265.3	339.6	0.36	1.28	PPP-PPP	0.20	16	0.682	0.765	0.0939
215983_s_at	1915.4	2451.0	0.36	1.28	PPP-PPP	0.20	16	0.682	0.765	0.0500
209667_at	930.6	1190.6	0.36	1.28	PPP-PPP	0.20	16	0.682	0.765	0.1549
236492_at	292.2	373.8	0.36	1.28	PPP-PPP	0.20	16	0.682	0.765	0.0931
225316_at	531.0	679.1	0.36	1.28	PPP-PPP	0.20	16	0.682	0.765	0.0696
200947_s_at	4352.1	5567.3	0.36	1.28	PPP-PPP	0.20	16	0.682	0.765	0.0570
205383_s_at	177.2	226.5	0.35	1.28	PAP-PAP	0.20	16	0.682	0.765	0.1333
227467_at	551.0	704.2	0.35	1.28	PPP-PPP	0.20	16	0.682	0.765	0.1190
211475_s_at	1527.1	1952.0	0.35	1.28	PPP-PPP	0.20	16	0.682	0.765	0.0873
222881_at	179.8	229.6	0.35	1.28	PPP-PAP	0.20	16	0.682	0.765	0.1433
203103_s_at	2836.4	3622.7	0.35	1.28	PPP-PPP	0.20	16	0.682	0.765	0.0991
235365_at	85.0	108.5	0.35	1.28	APM-PPP	0.20	16	0.682	0.765	0.0565
212236_x_at	27875.0	35600.7	0.35	1.28	PPP-PPP	0.20	16	0.682	0.765	0.0426
218958_at	308.5	393.7	0.35	1.28	PPP-APP	0.20	16	0.682	0.765	0.1045
226187_at	567.4	724.0	0.35	1.28	PPP-PPP	0.20	16	0.682	0.765	0.0680
208503_s_at	996.6	1271.4	0.35	1.28	PPP-PPP	0.20	16	0.682	0.765	0.1552
208735_s_at	496.1	633.0	0.35	1.28	PPP-PPP	0.20	16	0.682	0.765	0.1365
212992_at	1709.2	2179.8	0.35	1.28	PPP-PPP	0.20	16	0.682	0.765	0.0598
225373_at	470.7	600.4	0.35	1.28	PPP-PPP	0.20	16	0.682	0.765	0.0487
226108_at	519.0	661.7	0.35	1.28	PPP-PPP	0.20	16	0.682	0.765	0.1462
235232_at	265.1	337.9	0.35	1.28	PPP-PPP	0.20	16	0.682	0.765	0.0829
226797_at	655.4	835.3	0.35	1.28	PPP-PPP	0.20	16	0.682	0.765	0.0669
205707_at	534.0	680.7	0.35	1.28	PPP-PPP	0.20	16	0.682	0.765	0.0528
204462_s_at	535.9	683.2	0.35	1.28	PPP-PPP	0.20	16	0.682	0.765	0.0522
1558622_a_at	97.2	123.8	0.35	1.27	PAA-PPP	0.20	16	0.682	0.765	0.1360
240551_at	139.0	177.0	0.35	1.27	PAP-PPP	0.20	16	0.682	0.765	0.1230
1559054_a_at	63.3	80.6	0.35	1.27	PAA-PAA	0.20	16	0.682	0.765	0.1398
215820_x_at	224.6	286.0	0.35	1.27	PMP-PPP	0.20	16	0.682	0.765	0.1100
203481_at	283.5	360.9	0.35	1.27	PPP-PPP	0.20	16	0.682	0.765	0.0858
212094_at	2341.3	2979.7	0.35	1.27	PPP-PPP	0.20	16	0.682	0.765	0.0824
203409_at	1745.3	1371.8	-0.35	-1.27	PPP-PPP	0.20	16	0.682	0.765	0.0001

236978_at	147.3	187.4	0.35	1.27	PPP-PPP	0.20	16	0.682	0.765	0.1565
221616_s_at	85.8	109.1	0.35	1.27	AMA-PAP	0.20	16	0.682	0.765	0.1444
204027_s_at	499.9	635.8	0.35	1.27	PPP-PPP	0.20	16	0.682	0.765	0.1283
1558508_a_at	851.7	1083.2	0.35	1.27	PPP-PPP	0.20	16	0.682	0.765	0.1264
204512_at	378.2	481.1	0.35	1.27	PPP-PPP	0.20	16	0.682	0.765	0.1171
207291_at	1144.0	1454.1	0.35	1.27	PPP-PPP	0.20	16	0.682	0.765	0.0787
213209_at	171.4	217.9	0.35	1.27	PAP-PPP	0.20	16	0.682	0.765	0.0768
58994_at	450.0	571.8	0.35	1.27	PPP-PPP	0.20	16	0.682	0.765	0.0502
216360_x_at	270.9	344.3	0.35	1.27	PPP-MPP	0.20	16	0.682	0.765	0.0485
205530_at	427.9	543.4	0.35	1.27	PPP-PPP	0.20	16	0.682	0.765	0.0653
226379_s_at	204.7	259.9	0.34	1.27	APA-MMP	0.20	16	0.682	0.765	0.1093
1558142_at	775.0	983.9	0.34	1.27	PPP-PPP	0.20	16	0.682	0.765	0.0932
205829_at	961.0	1219.7	0.34	1.27	PPP-PPP	0.20	16	0.682	0.765	0.0919
230256_at	1255.9	1594.5	0.34	1.27	PPP-PPP	0.20	16	0.682	0.765	0.0523
230860_at	144.0	182.7	0.34	1.27	PPP-PPP	0.20	16	0.682	0.765	0.1404
228612_at	555.7	704.7	0.34	1.27	PPP-PPP	0.20	16	0.682	0.765	0.1123
226733_at	429.9	545.1	0.34	1.27	PPP-PPP	0.20	16	0.682	0.765	0.0914
204202_at	295.2	374.1	0.34	1.27	PPP-PPP	0.20	16	0.682	0.765	0.0837
204797_s_at	337.3	427.7	0.34	1.27	PPP-PPP	0.20	16	0.682	0.765	0.0476
203099_s_at	276.3	350.0	0.34	1.27	AAA-AAP	0.20	16	0.682	0.765	0.1378
203701_s_at	1177.9	1491.8	0.34	1.27	PPP-PPP	0.20	16	0.682	0.765	0.0458
238793_at	76.6	96.9	0.34	1.27	AAP-AAA	0.20	16	0.682	0.765	0.0917
1568617_a_at	195.5	247.1	0.34	1.26	AAA-AMP	0.20	16	0.682	0.765	0.1285
238418_at	396.8	501.5	0.34	1.26	PPP-PPP	0.20	16	0.682	0.765	0.0846
221851_at	239.5	302.5	0.34	1.26	PPP-PPP	0.20	16	0.682	0.765	0.0760
233230_s_at	276.9	349.6	0.34	1.26	PPP-PPP	0.20	16	0.682	0.765	0.0733
218031_s_at	412.6	521.0	0.34	1.26	PPP-PPP	0.20	16	0.682	0.765	0.0485
202215_s_at	564.4	712.9	0.34	1.26	PPP-PPP	0.20	16	0.682	0.765	0.0335
1569181_x_at	68.8	86.8	0.34	1.26	MPP-PPP	0.20	16	0.682	0.765	0.1496
1553088_a_at	166.4	209.9	0.34	1.26	PPP-PPP	0.20	16	0.682	0.765	0.1484
226036_x_at	220.3	278.0	0.34	1.26	PAP-PPP	0.20	16	0.682	0.765	0.1468
223418_x_at	512.1	645.9	0.34	1.26	PPP-PPP	0.20	16	0.682	0.765	0.1353
238800_s_at	105.2	132.7	0.34	1.26	PAP-PPP	0.20	16	0.682	0.765	0.1077
220669_at	110.3	139.2	0.34	1.26	PPP-PPP	0.20	16	0.682	0.765	0.1042
242358_at	74.0	93.3	0.34	1.26	PPP-PPP	0.20	16	0.682	0.765	0.0650
225231_at	1584.3	1998.4	0.34	1.26	PPP-PPP	0.20	16	0.682	0.765	0.0626
239930_at	102.8	129.6	0.33	1.26	APP-PAP	0.20	16	0.682	0.765	0.1125
243816_at	91.5	115.2	0.33	1.26	PMA-AAA	0.20	16	0.682	0.765	0.1498
221750_at	2276.7	2868.6	0.33	1.26	PPP-PPP	0.20	16	0.682	0.765	0.1495
1566144_at	159.6	201.0	0.33	1.26	PPP-APP	0.20	16	0.682	0.765	0.1236
229887_at	939.2	1183.0	0.33	1.26	PPP-PPP	0.20	16	0.682	0.765	0.0852
227972_at	224.9	283.2	0.33	1.26	PPP-PPP	0.20	16	0.682	0.765	0.0806

1559776_at	615.2	775.1	0.33	1.26	PPP-PPP	0.20	16	0.682	0.765	0.0619
204866_at	344.7	434.1	0.33	1.26	PPP-PPP	0.20	16	0.682	0.765	0.1505
227203_at	549.3	691.0	0.33	1.26	PPP-PPP	0.20	16	0.682	0.765	0.1318
213287_s_at	5131.2	6455.3	0.33	1.26	PPP-PPP	0.20	16	0.682	0.765	0.1111
238807_at	381.1	479.3	0.33	1.26	AAA-APP	0.20	16	0.682	0.765	0.0654
227367_at	522.6	657.6	0.33	1.26	PPP-PPP	0.20	16	0.682	0.765	0.0502
212090_at	1849.5	2324.3	0.33	1.26	PPP-PPP	0.20	16	0.682	0.765	0.1317
231840_x_at	805.0	1011.8	0.33	1.26	PPP-PPP	0.20	16	0.682	0.765	0.1054
206900_x_at	105.8	133.1	0.33	1.26	PPA-MMP	0.20	16	0.682	0.765	0.0979
223218_s_at	1535.2	1928.6	0.33	1.26	PPP-PPP	0.20	16	0.682	0.765	0.1393
219427_at	158.6	199.3	0.33	1.26	PPP-PPP	0.20	16	0.682	0.765	0.0510
211391_s_at	161.5	202.9	0.33	1.26	PPP-APP	0.20	16	0.682	0.765	0.0484
202761_s_at	3151.3	3956.1	0.33	1.26	PPP-PPP	0.20	16	0.682	0.765	0.0318
242408_at	128.9	161.7	0.33	1.26	PMP-PPP	0.20	16	0.682	0.765	0.0884
1554415_at	293.4	368.2	0.33	1.26	PPP-PPP	0.20	16	0.682	0.765	0.0493
204922_at	456.9	573.2	0.33	1.26	PPP-PPP	0.20	16	0.682	0.765	0.0399
227940_at	127.4	159.8	0.33	1.25	PPP-PPP	0.20	16	0.682	0.765	0.0366
222481_at	627.2	785.5	0.33	1.25	PPP-PPP	0.20	16	0.682	0.765	0.1025
224590_at	633.3	793.5	0.33	1.25	PPP-PPP	0.20	16	0.682	0.765	0.0692
212338_at	796.9	998.0	0.33	1.25	PPP-PPP	0.20	16	0.682	0.765	0.0664
236772_s_at	271.0	339.5	0.33	1.25	PPP-PPP	0.20	16	0.682	0.765	0.0557
225601_at	1189.5	1489.7	0.33	1.25	PPP-PPP	0.20	16	0.682	0.765	0.0374
235204_at	140.7	176.2	0.32	1.25	PPP-PPP	0.20	16	0.682	0.765	0.1398
202898_at	322.0	403.0	0.32	1.25	APA-MPP	0.20	16	0.682	0.765	0.1013
205014_at	9349.9	11704.0	0.32	1.25	PPP-PPP	0.20	16	0.682	0.765	0.0720
238199_x_at	4110.2	5144.1	0.32	1.25	PPP-PPP	0.20	16	0.682	0.765	0.0581
203904_x_at	1612.2	2018.9	0.32	1.25	PPP-PPP	0.20	16	0.682	0.765	0.0569
211326_x_at	140.0	175.2	0.32	1.25	PAP-PPP	0.20	16	0.682	0.765	0.0327
223400_s_at	200.4	250.7	0.32	1.25	PPP-PPP	0.20	16	0.682	0.765	0.1138
204538_x_at	1984.0	2481.4	0.32	1.25	PPP-PPP	0.20	16	0.682	0.765	0.0856
239973_at	347.2	434.4	0.32	1.25	PPP-PPP	0.20	16	0.682	0.765	0.0848
228483_s_at	391.2	489.4	0.32	1.25	PPP-PPP	0.20	16	0.682	0.765	0.0682
222625_s_at	319.9	400.0	0.32	1.25	PPP-PPP	0.20	16	0.682	0.765	0.0491
203742_s_at	409.5	512.3	0.32	1.25	PPP-PPP	0.20	16	0.682	0.765	0.0423
216918_s_at	2232.6	2790.4	0.32	1.25	PPP-PPP	0.20	16	0.682	0.765	0.1070
231932_at	411.7	514.7	0.32	1.25	PPP-PPP	0.20	16	0.682	0.765	0.0989
211317_s_at	593.4	741.8	0.32	1.25	PPP-PPP	0.20	16	0.682	0.765	0.0841
236072_at	139.1	173.9	0.32	1.25	PPP-PPP	0.20	16	0.682	0.765	0.0561
226873_at	548.7	686.1	0.32	1.25	PPP-PPP	0.20	16	0.682	0.765	0.0490
219800_s_at	80.0	100.0	0.32	1.25	PPP-PPP	0.20	16	0.682	0.765	0.0439
204524_at	481.0	600.8	0.32	1.25	PPP-PPP	0.20	16	0.682	0.765	0.1426
202790_at	2443.8	3053.1	0.32	1.25	PPP-PPP	0.20	16	0.682	0.765	0.1201

238506_at	281.8	352.0	0.32	1.25	PPP-PPP	0.20	16	0.682	0.765	0.0535
200965_s_at	3711.2	4636.5	0.32	1.25	PPP-PPP	0.20	16	0.682	0.765	0.0323
225939_at	63.5	79.2	0.32	1.25	PPP-PPP	0.20	16	0.682	0.765	0.1209
224338_s_at	308.3	384.9	0.32	1.25	APP-PPP	0.20	16	0.682	0.765	0.0709
228840_at	327.1	408.3	0.32	1.25	AAP-MPP	0.20	16	0.682	0.765	0.0395
214182_at	2171.8	2709.4	0.32	1.25	PPP-PPP	0.20	16	0.682	0.765	0.1400
1555004_a_at	299.9	374.2	0.32	1.25	PPP-PPP	0.20	16	0.682	0.765	0.1355
222129_at	290.3	362.1	0.32	1.25	PPP-PPP	0.20	16	0.682	0.765	0.1230
231851_at	405.4	505.5	0.32	1.25	PPP-PPP	0.20	16	0.682	0.765	0.1061
201539_s_at	236.9	295.5	0.32	1.25	PPP-PPP	0.20	16	0.682	0.765	0.0329
238945_at	117.5	146.3	0.32	1.25	PPP-PPP	0.20	16	0.682	0.765	0.1366
221728_x_at	2151.9	2680.7	0.32	1.25	PPP-PPP	0.20	16	0.682	0.765	0.1213
228453_at	184.7	230.1	0.32	1.25	PPP-PPP	0.20	16	0.682	0.765	0.0695
228575_at	4192.1	5217.1	0.32	1.24	PPP-PPP	0.20	16	0.682	0.765	0.1197
208587_s_at	79.0	98.4	0.32	1.25	PPA-PAP	0.20	16	0.682	0.765	0.0345
223790_at	240.8	299.5	0.32	1.24	PPP-PPP	0.20	16	0.682	0.765	0.1410
212132_at	3318.4	4128.3	0.32	1.24	PPP-PPP	0.20	16	0.682	0.765	0.1396
204975_at	1362.9	1695.6	0.32	1.24	PPP-PPP	0.20	16	0.682	0.765	0.1279
222653_at	1099.7	1368.0	0.32	1.24	PPP-PPP	0.20	16	0.682	0.765	0.1121
225019_at	830.1	1032.4	0.32	1.24	PPP-PPP	0.20	16	0.682	0.765	0.0841
204369_at	816.0	1014.9	0.32	1.24	PPP-PPP	0.20	16	0.682	0.765	0.0534
219540_at	378.4	470.6	0.32	1.24	PPP-PPP	0.20	16	0.682	0.765	0.0315
219022_at	569.3	707.8	0.31	1.24	PPP-PPP	0.20	16	0.682	0.765	0.0987
224588_at	4383.3	5443.6	0.31	1.24	PPP-PPP	0.20	16	0.682	0.765	0.0871
202518_at	727.7	904.0	0.31	1.24	PPP-PPP	0.20	16	0.682	0.765	0.0852
204526_s_at	1316.6	1635.0	0.31	1.24	PPP-PPP	0.20	16	0.682	0.765	0.0847
212530_at	4158.9	5161.8	0.31	1.24	PPP-PPP	0.20	16	0.682	0.765	0.1115
214154_s_at	125.5	155.8	0.31	1.24	PPP-PMM	0.20	16	0.682	0.765	0.0955
219392_x_at	3858.4	4789.2	0.31	1.24	PPP-PPP	0.20	16	0.682	0.765	0.0735
212386_at	700.7	869.9	0.31	1.24	PPP-PPP	0.20	16	0.682	0.765	0.0679
206708_at	131.8	163.6	0.31	1.24	PPA-PPP	0.20	16	0.682	0.765	0.0673
201904_s_at	1967.5	2442.3	0.31	1.24	PPP-PPP	0.20	16	0.682	0.765	0.0589
223501_at	66.7	82.8	0.31	1.24	PAA-PAP	0.20	16	0.682	0.765	0.0472
212830_at	415.1	515.2	0.31	1.24	PPP-PPP	0.20	16	0.682	0.765	0.0460
227516_at	587.8	729.7	0.31	1.24	PPP-PPP	0.20	16	0.682	0.765	0.0392
225777_at	1151.1	1428.4	0.31	1.24	PPP-PPP	0.20	16	0.682	0.765	0.0913
224901_at	2217.9	2749.4	0.31	1.24	PPP-PPP	0.20	16	0.682	0.765	0.0988
1554155_at	231.0	286.4	0.31	1.24	PPP-PPP	0.20	16	0.682	0.765	0.0293
219751_at	505.6	626.4	0.31	1.24	PPP-PPP	0.20	16	0.682	0.765	0.1262
1569302_at	298.4	369.7	0.31	1.24	PPP-PPP	0.20	16	0.682	0.765	0.1098
227461_at	374.9	464.3	0.31	1.24	PPP-PPP	0.20	16	0.682	0.765	0.1333
238994_at	99.9	123.6	0.31	1.24	APP-PPP	0.20	16	0.682	0.765	0.0770

202526_at	88.8	109.8	0.31	1.24	PPP-PPP	0.20	16	0.682	0.765	0.1369
225321_s_at	1123.0	1388.9	0.31	1.24	PPP-PPP	0.20	16	0.682	0.765	0.1244
202818_s_at	664.6	822.2	0.31	1.24	PPP-PPP	0.20	16	0.682	0.765	0.1172
211665_s_at	197.5	244.4	0.31	1.24	PAP-PAP	0.20	16	0.682	0.765	0.0914
207992_s_at	571.1	706.3	0.31	1.24	PPP-PPP	0.20	16	0.682	0.765	0.0700
1552656_s_at	264.0	326.5	0.31	1.24	MAA-PPP	0.20	16	0.682	0.765	0.0685
235585_at	123.4	152.6	0.31	1.24	PPP-PPP	0.20	16	0.682	0.765	0.1130
227948_at	569.0	703.3	0.31	1.24	PPP-PPP	0.20	16	0.682	0.765	0.0844
212640_at	4504.9	5569.2	0.31	1.24	PPP-PPP	0.20	16	0.682	0.765	0.0683
205260_s_at	1109.8	1371.0	0.31	1.24	PPP-PPP	0.20	16	0.682	0.765	0.1101
204004_at	2616.1	3231.9	0.31	1.24	PPP-PPP	0.20	16	0.682	0.765	0.0798
1555833_a_at	831.1	1026.5	0.31	1.24	PPP-PPP	0.20	16	0.682	0.765	0.0629
212322_at	1217.4	1503.8	0.31	1.24	PPP-PPP	0.20	16	0.682	0.765	0.0562
220760_x_at	373.3	460.8	0.30	1.23	PPP-PPP	0.20	16	0.682	0.765	0.0815
221752_at	373.4	460.7	0.30	1.23	PPP-PPP	0.20	16	0.682	0.765	0.0783
1555009_a_at	101.5	125.3	0.30	1.23	AAP-AAA	0.20	16	0.682	0.765	0.0289
224793_s_at	1441.7	1777.7	0.30	1.23	PPP-PPP	0.20	16	0.682	0.765	0.0574
204658_at	1354.3	1669.4	0.30	1.23	PPP-PPP	0.20	16	0.682	0.765	0.0422
210240_s_at	175.8	216.6	0.30	1.23	PAP-PPP	0.20	16	0.682	0.765	0.1198
235363_at	535.4	659.3	0.30	1.23	PPP-PPP	0.20	16	0.682	0.765	0.0830
227276_at	497.1	612.0	0.30	1.23	PPP-PPP	0.20	16	0.682	0.765	0.0606
234512_x_at	16733.2	20597.4	0.30	1.23	PPP-PPP	0.20	16	0.682	0.765	0.0305
220796_x_at	1677.4	2064.0	0.30	1.23	PPP-PPP	0.20	16	0.682	0.765	0.1324
221761_at	5039.9	6200.5	0.30	1.23	PPP-PPP	0.20	16	0.682	0.765	0.1168
217122_s_at	2113.6	2600.3	0.30	1.23	PPP-PPP	0.20	16	0.682	0.765	0.0443
222750_s_at	860.7	1058.5	0.30	1.23	PPP-PPP	0.20	16	0.682	0.765	0.0291
201036_s_at	3222.2	3961.4	0.30	1.23	PPP-PPP	0.20	16	0.682	0.765	0.1352
217501_at	702.3	863.4	0.30	1.23	PPP-PPP	0.20	16	0.682	0.765	0.1258
213154_s_at	2025.2	2489.6	0.30	1.23	PPP-PPP	0.20	16	0.682	0.765	0.1000
221839_s_at	1139.3	1400.9	0.30	1.23	PPP-PPP	0.20	16	0.682	0.765	0.0640
238465_at	501.9	617.2	0.30	1.23	PPP-PPP	0.20	16	0.682	0.765	0.0555
224624_at	4625.9	5684.8	0.30	1.23	PPP-PPP	0.20	16	0.682	0.765	0.1001
223748_at	222.7	273.5	0.30	1.23	PPP-PMP	0.20	16	0.682	0.765	0.1250
223577_x_at	441.1	541.7	0.30	1.23	PPP-PPP	0.20	16	0.682	0.765	0.1161
230363_s_at	400.9	492.1	0.30	1.23	PPP-PPP	0.20	16	0.682	0.765	0.0708
201906_s_at	3465.2	4254.1	0.30	1.23	PPP-PPP	0.20	16	0.682	0.765	0.0536
221227_x_at	873.0	1071.1	0.30	1.23	PPP-PPP	0.20	16	0.682	0.765	0.1261
216570_x_at	14863.5	18242.1	0.30	1.23	PPP-PPP	0.20	16	0.682	0.765	0.0988
220128_s_at	394.2	483.2	0.29	1.23	AAA-MAP	0.20	16	0.682	0.765	0.0816
227227_at	484.5	594.0	0.29	1.23	PPP-PPP	0.20	16	0.682	0.765	0.0412
1560020_at	68.4	83.9	0.29	1.23	PAP-PPP	0.20	16	0.682	0.765	0.0942
228944_at	176.0	215.5	0.29	1.22	PPP-PPP	0.20	16	0.682	0.765	0.1114

217047_s_at	776.6	951.0	0.29	1.23	PPP-PPP	0.20	16	0.682	0.765	0.1086
225060_at	4173.2	5110.9	0.29	1.23	PPP-PPP	0.20	16	0.682	0.765	0.0342
209553_at	456.4	558.2	0.29	1.22	PPP-PPP	0.20	16	0.682	0.765	0.0983
202826_at	1140.8	1395.7	0.29	1.22	PPP-PPP	0.20	16	0.682	0.765	0.0348
218739_at	1339.2	1636.9	0.29	1.22	PPP-PPP	0.20	16	0.682	0.765	0.1032
209805_at	432.8	529.2	0.29	1.22	PPP-PPP	0.20	16	0.682	0.765	0.0826
226721_at	1526.3	1866.1	0.29	1.22	PPP-PPP	0.20	16	0.682	0.765	0.0819
207727_s_at	392.9	480.1	0.29	1.22	PPP-PPP	0.20	16	0.682	0.765	0.0825
212356_at	497.4	607.9	0.29	1.22	PAM-MPP	0.20	16	0.682	0.765	0.0486
202872_at	1654.1	2021.4	0.29	1.22	PPP-PPP	0.20	16	0.682	0.765	0.0479
205181_at	197.3	240.8	0.29	1.22	PPP-PPP	0.20	16	0.682	0.765	0.1212
226710_at	1639.7	2001.9	0.29	1.22	PPP-PPP	0.20	16	0.682	0.765	0.0783
232592_at	145.8	177.9	0.29	1.22	PPA-AAP	0.20	16	0.682	0.765	0.0512
32099_at	1030.4	1257.9	0.29	1.22	PPP-PPP	0.20	16	0.682	0.765	0.0444
224715_at	1257.5	1534.8	0.29	1.22	PPP-PPP	0.20	16	0.682	0.765	0.1278
218010_x_at	1080.6	1318.4	0.29	1.22	PPP-PPP	0.20	16	0.682	0.765	0.0757
1554471_a_at	263.0	320.9	0.29	1.22	PPP-PPP	0.20	16	0.682	0.765	0.0523
226565_at	932.7	1137.7	0.29	1.22	PPP-PPP	0.20	16	0.682	0.765	0.0272
220758_s_at	108.4	132.1	0.29	1.22	AAM-APA	0.20	16	0.682	0.765	0.1227
223165_s_at	1478.3	1802.6	0.29	1.22	PPP-PPP	0.20	16	0.682	0.765	0.0909
224598_at	7170.9	8743.9	0.29	1.22	PPP-PPP	0.20	16	0.682	0.765	0.0229
216677_at	102.2	124.5	0.29	1.22	AAM-PAA	0.20	16	0.682	0.765	0.1010
209367_at	585.2	712.8	0.29	1.22	PPP-PPP	0.20	16	0.682	0.765	0.0653
234972_at	424.9	517.6	0.29	1.22	PPP-PPP	0.20	16	0.682	0.765	0.0425
226021_at	1095.4	1333.8	0.28	1.22	PPP-PPP	0.20	16	0.682	0.765	0.1034
1560402_at	119.8	145.8	0.28	1.22	PPP-PPP	0.20	16	0.682	0.765	0.0356
225514_at	442.1	537.9	0.28	1.22	PPP-PPP	0.20	16	0.682	0.765	0.1198
209341_s_at	906.6	1102.8	0.28	1.22	PPP-PPP	0.20	16	0.682	0.765	0.0865
231171_at	107.6	131.0	0.28	1.22	PPP-PPP	0.20	16	0.682	0.765	0.0803
210971_s_at	1670.2	2032.1	0.28	1.22	PPP-PPP	0.20	16	0.682	0.765	0.0360
201535_at	1208.0	1469.7	0.28	1.22	PPP-PPP	0.20	16	0.682	0.765	0.0306
202967_at	553.7	673.1	0.28	1.22	PPP-PPP	0.20	16	0.682	0.765	0.0383
226509_at	217.0	263.8	0.28	1.22	MAP-AAA	0.20	16	0.682	0.765	0.0214
206884_s_at	1457.5	1769.6	0.28	1.21	PPP-PPP	0.20	16	0.682	0.765	0.0284
204132_s_at	587.8	713.4	0.28	1.21	PPP-PPP	0.20	16	0.682	0.765	0.0731
224604_at	1353.6	1641.9	0.28	1.21	PPP-PPP	0.20	16	0.682	0.765	0.0695
223088_x_at	2032.1	2465.0	0.28	1.21	PPP-PPP	0.20	16	0.682	0.765	0.0337
203997_at	851.3	1032.1	0.28	1.21	PPP-PPP	0.20	16	0.682	0.765	0.1106
226170_at	1023.6	1241.0	0.28	1.21	PPP-PPP	0.20	16	0.682	0.765	0.0596
202245_at	3039.9	3682.8	0.28	1.21	PPP-PPP	0.20	16	0.682	0.765	0.0686
1552927_at	56.4	68.3	0.28	1.21	AAP-PAP	0.20	16	0.682	0.765	0.0779
225190_x_at	25919.8	31374.7	0.28	1.21	PPP-PPP	0.20	16	0.682	0.765	0.0773

218643_s_at	724.8	877.8	0.28	1.21	PPP-PPP	0.20	16	0.682	0.765	0.0528
34408_at	386.8	468.5	0.28	1.21	PPP-PPP	0.20	16	0.682	0.765	0.0448
215954_s_at	454.9	550.5	0.28	1.21	PPP-PPP	0.20	16	0.682	0.765	0.1215
213093_at	435.1	526.5	0.28	1.21	PPP-PPP	0.20	16	0.682	0.765	0.0711
223433_at	1019.5	1233.7	0.28	1.21	PPP-PPP	0.20	16	0.682	0.765	0.0641
224722_at	566.5	685.3	0.28	1.21	PPP-PPP	0.20	16	0.682	0.765	0.0211
218270_at	3589.2	4339.3	0.27	1.21	PPP-PPP	0.20	16	0.682	0.765	0.0921
227466_at	1057.1	1277.9	0.27	1.21	PPP-PPP	0.20	16	0.682	0.765	0.0299
225267_at	4033.9	4876.4	0.27	1.21	PPP-PPP	0.20	16	0.682	0.765	0.0270
227802_at	277.1	334.9	0.27	1.21	PPP-PPP	0.20	16	0.682	0.765	0.0821
210175_at	1152.8	1393.4	0.27	1.21	PPP-PPP	0.20	16	0.682	0.765	0.0574
227354_at	54.5	65.9	0.27	1.21	APA-APP	0.20	16	0.682	0.765	0.0367
209394_at	346.6	418.6	0.27	1.21	PPA-PPP	0.20	16	0.682	0.765	0.1188
210788_s_at	3764.7	4545.5	0.27	1.21	PPP-PPP	0.20	16	0.682	0.765	0.0837
213792_s_at	419.3	506.3	0.27	1.21	PPP-PPP	0.20	16	0.682	0.765	0.0685
219265_at	343.9	414.8	0.27	1.21	MAP-AAM	0.20	16	0.682	0.765	0.1211
203725_at	2029.6	2448.5	0.27	1.21	PPP-PPP	0.20	16	0.682	0.765	0.0943
219646_at	1736.1	2095.5	0.27	1.21	PPP-PPP	0.20	16	0.682	0.765	0.0939
234206_at	68.4	82.5	0.27	1.21	AAA-PPP	0.20	16	0.682	0.765	0.0542
212272_at	312.4	376.9	0.27	1.21	PPP-PPP	0.20	16	0.682	0.765	0.0472
1554478_a_at	441.7	532.5	0.27	1.21	PPP-PPP	0.20	16	0.682	0.765	0.0742
215978_x_at	1410.9	1699.5	0.27	1.21	PPP-PPP	0.20	16	0.682	0.765	0.0882
228829_at	998.2	1203.1	0.27	1.21	PPP-PPP	0.20	16	0.682	0.765	0.0868
202572_s_at	311.6	375.4	0.27	1.21	PPP-PPP	0.20	16	0.682	0.765	0.0793
226503_at	827.0	996.7	0.27	1.21	PPP-PPP	0.20	16	0.682	0.765	0.0409
231252_at	227.7	274.4	0.27	1.21	PPP-PPP	0.20	16	0.682	0.765	0.0404
212289_at	270.3	325.4	0.27	1.20	PPP-PPP	0.20	16	0.682	0.765	0.1023
229267_at	603.2	726.4	0.27	1.20	PPP-PPP	0.20	16	0.682	0.765	0.0803
201686_x_at	897.0	1080.1	0.27	1.20	PPP-PPP	0.20	16	0.682	0.765	0.0396
232921_at	267.7	322.3	0.27	1.20	PPP-PPP	0.20	16	0.682	0.765	0.0582
217208_s_at	298.6	359.4	0.27	1.20	PPP-PPP	0.20	16	0.682	0.765	0.0240
243910_x_at	1011.6	1216.4	0.27	1.20	PPP-PPP	0.20	16	0.682	0.765	0.0604
218278_at	1878.0	2256.4	0.27	1.20	PPP-PPP	0.20	16	0.682	0.765	0.0604
234723_x_at	228.9	274.9	0.27	1.20	PPP-PPP	0.20	16	0.682	0.765	0.0408
201975_at	1516.5	1820.9	0.26	1.20	PPP-PPP	0.20	16	0.682	0.765	0.0320
229016_s_at	111.7	134.0	0.26	1.20	PPA-PPP	0.20	16	0.682	0.765	0.1092
232148_at	134.1	161.0	0.26	1.20	APP-APP	0.20	16	0.682	0.765	0.1060
216242_x_at	886.0	1062.8	0.26	1.20	PPP-PPP	0.20	16	0.682	0.765	0.1021
223301_s_at	406.3	487.5	0.26	1.20	PPP-PPP	0.20	16	0.682	0.765	0.0667
226987_at	521.8	626.1	0.26	1.20	PPP-PPP	0.20	16	0.682	0.765	0.0427
236831_at	340.4	408.3	0.26	1.20	PPP-PPP	0.20	16	0.682	0.765	0.0418
226650_at	970.7	1164.2	0.26	1.20	PPP-PPP	0.20	16	0.682	0.765	0.0547

215600_x_at	908.8	1088.5	0.26	1.20	PPP-PPP	0.20	16	0.682	0.765	0.0605
201926_s_at	4020.2	4813.1	0.26	1.20	PPP-PPP	0.20	16	0.682	0.765	0.0408
208319_s_at	6926.6	8297.3	0.26	1.20	PPP-PPP	0.20	16	0.682	0.765	0.0334
204610_s_at	993.4	1189.5	0.26	1.20	PPP-PPP	0.20	16	0.682	0.765	0.0303
218492_s_at	584.1	699.4	0.26	1.20	PPP-PPP	0.20	16	0.682	0.765	0.0205
228846_at	269.4	322.4	0.26	1.20	PPP-PPP	0.20	16	0.682	0.765	0.0986
218432_at	1576.0	1886.0	0.26	1.20	PPP-PPP	0.20	16	0.682	0.765	0.0203
227312_at	747.4	893.8	0.26	1.20	PPP-PPP	0.20	16	0.682	0.765	0.0645
218109_s_at	2539.1	3033.5	0.26	1.20	PPP-PPP	0.20	16	0.682	0.765	0.1034
239474_at	107.6	128.5	0.26	1.20	PAA-PPA	0.20	16	0.682	0.765	0.1024
224650_at	15944.1	19049.8	0.26	1.20	PPP-PPP	0.20	16	0.682	0.765	0.0275
204686_at	371.5	443.9	0.26	1.20	PPP-PPP	0.20	16	0.682	0.765	0.0210
228462_at	3360.7	4014.3	0.26	1.19	PPP-PPP	0.20	16	0.682	0.765	0.0821
236004_at	467.4	558.3	0.26	1.20	PPP-PPP	0.20	16	0.682	0.765	0.0662
200901_s_at	1483.0	1771.4	0.26	1.19	PPP-PPP	0.20	16	0.682	0.765	0.0525
208611_s_at	1901.9	2269.4	0.26	1.19	PPP-PPP	0.20	16	0.682	0.765	0.0626
227268_at	212.8	253.8	0.25	1.19	PPP-PPP	0.20	16	0.682	0.765	0.0789
218344_s_at	363.9	433.9	0.25	1.19	PPP-PPP	0.20	16	0.682	0.765	0.0490
244669_at	244.6	291.7	0.25	1.19	PPP-PPP	0.20	16	0.682	0.765	0.0269
225827_at	2359.9	2811.5	0.25	1.19	PPP-PPP	0.20	16	0.682	0.765	0.1098
223694_at	3680.6	4387.1	0.25	1.19	PPP-PPP	0.20	16	0.682	0.765	0.0906
202281_at	627.5	747.8	0.25	1.19	PPP-PPP	0.20	16	0.682	0.765	0.0504
1553968_a_at	538.9	641.8	0.25	1.19	PPP-PPP	0.20	16	0.682	0.765	0.1065
222317_at	133.3	158.8	0.25	1.19	PPP-PPP	0.20	16	0.682	0.765	0.0275
223206_s_at	706.0	840.7	0.25	1.19	PPP-PPP	0.20	16	0.682	0.765	0.0252
212093_s_at	529.7	630.4	0.25	1.19	PPP-PPP	0.20	16	0.682	0.765	0.0421
202447_at	7274.3	8659.0	0.25	1.19	PPP-PPP	0.20	16	0.682	0.765	0.0383
213295_at	257.4	306.2	0.25	1.19	PPP-PPP	0.20	16	0.682	0.765	0.0205
234942_s_at	1461.7	1738.8	0.25	1.19	PPP-PPP	0.20	16	0.682	0.765	0.1034
228552_s_at	122.5	145.6	0.25	1.19	APP-PPP	0.20	16	0.682	0.765	0.0687
218562_s_at	521.1	619.0	0.25	1.19	PPP-PPP	0.20	16	0.682	0.765	0.0448
216037_x_at	1023.0	1214.9	0.25	1.19	PPP-PPP	0.20	16	0.682	0.765	0.0135
210859_x_at	704.1	835.7	0.25	1.19	PPP-PPP	0.20	16	0.682	0.765	0.0840
203641_s_at	370.6	439.8	0.25	1.19	PPP-PPP	0.20	16	0.682	0.765	0.0807
222403_at	1443.5	1713.1	0.25	1.19	PPP-PPP	0.20	16	0.682	0.765	0.0497
202552_s_at	1861.7	2207.9	0.25	1.19	PPP-PPP	0.20	16	0.682	0.765	0.0986
235551_at	304.3	360.8	0.25	1.19	PPP-PPP	0.20	16	0.682	0.765	0.0857
223527_s_at	260.3	308.8	0.25	1.19	PPP-PPP	0.20	16	0.682	0.765	0.0695
215016_x_at	2456.9	2912.8	0.25	1.19	PPP-PPP	0.20	16	0.682	0.765	0.0642
218388_at	2361.6	2801.0	0.25	1.19	PPP-PPP	0.20	16	0.682	0.765	0.0357
214665_s_at	5541.2	6569.5	0.25	1.19	PPP-PPP	0.20	16	0.682	0.765	0.0207
212516_at	298.8	353.5	0.24	1.18	PAM-PPP	0.20	16	0.682	0.765	0.1028

222111_at	665.4	787.6	0.24	1.18	PPP-PPP	0.20	16	0.682	0.765	0.0948
38892_at	324.5	384.0	0.24	1.18	PPP-PPP	0.20	16	0.682	0.765	0.0899
231793_s_at	247.3	292.6	0.24	1.18	PPP-PPP	0.20	16	0.682	0.765	0.0827
201657_at	713.8	844.6	0.24	1.18	PPP-PPP	0.20	16	0.682	0.765	0.0433
220553_s_at	562.4	664.9	0.24	1.18	PPP-PPP	0.20	16	0.682	0.765	0.0300
202609_at	1647.2	1947.8	0.24	1.18	PPP-PPP	0.20	16	0.682	0.765	0.0118
32042_at	266.1	314.4	0.24	1.18	PPP-PPP	0.20	16	0.682	0.765	0.0817
202043_s_at	4618.7	5453.1	0.24	1.18	PPP-PPP	0.20	16	0.682	0.765	0.0170
218867_s_at	524.0	618.2	0.24	1.18	PPP-PPP	0.20	16	0.682	0.765	0.0895
235152_at	197.5	233.1	0.24	1.18	PPP-PPP	0.20	16	0.682	0.765	0.0686
222451_s_at	1224.7	1445.7	0.24	1.18	PPP-PPP	0.20	16	0.682	0.765	0.0623
201321_s_at	830.7	979.3	0.24	1.18	PPP-PPP	0.20	16	0.682	0.765	0.0863
1555639_a_at	258.5	304.6	0.24	1.18	PPP-PPP	0.20	16	0.682	0.765	0.0715
201329_s_at	783.1	922.5	0.24	1.18	PPP-PPP	0.20	16	0.682	0.765	0.0271
217185_s_at	2139.4	2518.9	0.24	1.18	PPP-PPP	0.20	16	0.682	0.765	0.0155
212200_at	933.8	1099.0	0.24	1.18	PPP-PPP	0.20	16	0.682	0.765	0.0797
226630_at	727.6	856.4	0.24	1.18	PPP-PPP	0.20	16	0.682	0.765	0.0299
240180_at	285.6	335.9	0.23	1.18	PPP-PPP	0.20	16	0.682	0.765	0.0931
218677_at	11134.2	13099.2	0.23	1.18	PPP-PPP	0.20	16	0.682	0.765	0.0397
230002_at	980.0	1152.4	0.23	1.18	PPP-PPP	0.20	16	0.682	0.765	0.0232
1559954_s_at	701.2	824.3	0.23	1.18	PPP-PPP	0.20	16	0.682	0.765	0.0155
220933_s_at	1082.9	1272.3	0.23	1.18	PPP-PPP	0.20	16	0.682	0.765	0.0923
203585_at	1002.1	1177.0	0.23	1.18	PPP-PPP	0.20	16	0.682	0.765	0.0537
205773_at	68.7	80.7	0.23	1.18	PPA-PMP	0.20	16	0.682	0.765	0.0310
205178_s_at	790.6	928.0	0.23	1.17	PPP-PPP	0.20	16	0.682	0.765	0.0793
204045_at	1016.1	1191.3	0.23	1.17	PPP-PPP	0.20	16	0.682	0.765	0.0704
1555832_s_at	5069.1	5940.0	0.23	1.17	PPP-PPP	0.20	16	0.682	0.765	0.0275
224891_at	3151.0	3693.3	0.23	1.17	PPP-PPP	0.20	16	0.682	0.765	0.0172
218642_s_at	1000.7	1171.1	0.23	1.17	PPP-PPP	0.20	16	0.682	0.765	0.0689
224789_at	1224.6	1433.3	0.23	1.17	PPP-PPP	0.20	16	0.682	0.765	0.0170
235046_at	1104.1	1291.1	0.23	1.17	PPP-PPP	0.20	16	0.682	0.765	0.0409
222228_s_at	248.5	290.5	0.23	1.17	PPP-PPP	0.20	16	0.682	0.765	0.0431
227224_at	1619.0	1892.5	0.23	1.17	PPP-PPP	0.20	16	0.682	0.765	0.0132
226931_at	1388.7	1622.2	0.22	1.17	PPP-PPP	0.20	16	0.682	0.765	0.0472
201286_at	2440.8	2850.2	0.22	1.17	PPP-PPP	0.20	16	0.682	0.765	0.0404
235032_at	1251.9	1462.1	0.22	1.17	PPP-PPP	0.20	16	0.682	0.765	0.0183
204971_at	16033.3	18720.4	0.22	1.17	PPP-PPP	0.20	16	0.682	0.765	0.0142
226322_at	2899.8	3384.5	0.22	1.17	PPP-PPP	0.20	16	0.682	0.765	0.0860
227313_at	327.2	381.9	0.22	1.17	PPP-PPP	0.20	16	0.682	0.765	0.0568
212025_s_at	1074.1	1253.6	0.22	1.17	PPP-PPP	0.20	16	0.682	0.765	0.0405
219281_at	1649.0	1923.2	0.22	1.17	PPP-PPP	0.20	16	0.682	0.765	0.0490
217256_x_at	7750.6	9039.3	0.22	1.17	PPP-PPP	0.20	16	0.682	0.765	0.0259

204863_s_at	188.1	219.4	0.22	1.17	PPP-PPP	0.20	16	0.682	0.765	0.0242
201041_s_at	20354.6	23712.7	0.22	1.17	PPP-PPP	0.20	16	0.682	0.765	0.0830
225390_s_at	1779.5	2072.0	0.22	1.16	PPP-PPP	0.20	16	0.682	0.765	0.0806
227481_at	435.2	506.7	0.22	1.16	PPP-PPP	0.20	16	0.682	0.765	0.0438
210592_s_at	10813.6	12593.4	0.22	1.17	PPP-PPP	0.20	16	0.682	0.765	0.0132
1553979_at	3574.2	4160.3	0.22	1.16	PPP-PPP	0.20	16	0.682	0.765	0.0746
209168_at	447.1	520.3	0.22	1.16	PPP-PPP	0.20	16	0.682	0.765	0.0375
219069_at	1230.2	1431.9	0.22	1.16	PPP-PPP	0.20	16	0.682	0.765	0.0147
203243_s_at	4353.2	5063.0	0.22	1.16	PPP-PPP	0.20	16	0.682	0.765	0.0168
214258_x_at	868.0	1009.3	0.22	1.16	PPP-PPP	0.20	16	0.682	0.765	0.0557
227964_at	2259.7	2625.8	0.22	1.16	PPP-PPP	0.20	16	0.682	0.765	0.0498
213789_at	1091.6	1267.1	0.22	1.16	PPP-PPP	0.20	16	0.682	0.765	0.0735
225062_at	984.4	1142.4	0.22	1.16	PPP-PPP	0.20	16	0.682	0.765	0.0701
1554628_at	487.2	565.6	0.22	1.16	PPP-PPP	0.20	16	0.682	0.765	0.0691
201925_s_at	5284.9	6134.7	0.22	1.16	PPP-PPP	0.20	16	0.682	0.765	0.0383
223209_s_at	4196.8	4871.6	0.22	1.16	PPP-PPP	0.20	16	0.682	0.765	0.0265
238469_at	388.2	450.4	0.21	1.16	PPP-PPP	0.20	16	0.682	0.765	0.0725
225313_at	1468.1	1702.7	0.21	1.16	PPP-PPP	0.20	16	0.682	0.765	0.0335
212274_at	1962.6	2275.4	0.21	1.16	PPP-PPP	0.20	16	0.682	0.765	0.0704
224151_s_at	3292.2	3815.8	0.21	1.16	PPP-PPP	0.20	16	0.682	0.765	0.0588
201325_s_at	4628.6	5364.1	0.21	1.16	PPP-PPP	0.20	16	0.682	0.765	0.0366
201889_at	9358.7	10840.4	0.21	1.16	PPP-PPP	0.20	16	0.682	0.765	0.0681
200810_s_at	3594.8	4162.8	0.21	1.16	PPP-PPP	0.20	16	0.682	0.765	0.0431
214464_at	1189.9	1377.6	0.21	1.16	PPP-PPP	0.20	16	0.682	0.765	0.0618
208119_s_at	219.2	253.8	0.21	1.16	APP-PPP	0.20	16	0.682	0.765	0.0199
201739_at	3267.9	3782.7	0.21	1.16	PPP-PPP	0.20	16	0.682	0.765	0.0097
202420_s_at	2269.2	2625.4	0.21	1.16	PPP-PPP	0.20	16	0.682	0.765	0.0704
213079_at	386.6	446.2	0.21	1.15	PPP-PPP	0.20	16	0.682	0.765	0.0306
214743_at	1818.6	2099.3	0.21	1.15	PPP-PPP	0.20	16	0.682	0.765	0.0172
204050_s_at	6146.7	7089.1	0.21	1.15	PPP-PPP	0.20	16	0.682	0.765	0.0067
227954_at	630.3	726.0	0.20	1.15	PPP-PPP	0.20	16	0.682	0.765	0.0730
203655_at	663.0	763.6	0.20	1.15	PPP-PPP	0.20	16	0.682	0.765	0.0581
217987_at	3101.1	3571.5	0.20	1.15	PPP-PPP	0.20	16	0.682	0.765	0.0523
227517_s_at	3296.4	3796.5	0.20	1.15	PPP-PPP	0.20	16	0.682	0.765	0.0486
208882_s_at	1641.5	1891.0	0.20	1.15	PPP-PPP	0.20	16	0.682	0.765	0.0262
226853_at	353.3	407.1	0.20	1.15	PPP-PPP	0.20	16	0.682	0.765	0.0039
207388_s_at	1633.0	1880.0	0.20	1.15	PPP-PPP	0.20	16	0.682	0.765	0.0437
225435_at	800.8	921.0	0.20	1.15	PPP-PPP	0.20	16	0.682	0.765	0.0650
222982_x_at	18495.4	21281.9	0.20	1.15	PPP-PPP	0.20	16	0.682	0.765	0.0247
201820_at	27754.1	31930.8	0.20	1.15	PPP-PPP	0.20	16	0.682	0.765	0.0243
210367_s_at	10125.5	11648.7	0.20	1.15	PPP-PPP	0.20	16	0.682	0.765	0.0179
222415_at	1528.1	1757.8	0.20	1.15	PPP-PPP	0.20	16	0.682	0.765	0.0062

202371_at	4498.8	5170.2	0.20	1.15	PPP-PPP	0.20	16	0.682	0.765	0.0603
212050_at	829.8	953.6	0.20	1.15	PPP-PPP	0.20	16	0.682	0.765	0.0234
226831_at	1132.2	1300.9	0.20	1.15	PPP-PPP	0.20	16	0.682	0.765	0.0669
203047_at	414.3	475.5	0.20	1.15	PAP-MMP	0.20	16	0.682	0.765	0.0261
230291_s_at	2195.2	2519.7	0.20	1.15	PPP-PPP	0.20	16	0.682	0.765	0.0204
212918_at	3367.8	3860.7	0.20	1.15	PPP-PPP	0.20	16	0.682	0.765	0.0222
204605_at	449.4	514.7	0.20	1.15	PPP-PPP	0.20	16	0.682	0.765	0.0532
200608_s_at	11882.2	13610.9	0.20	1.15	PPP-PPP	0.20	16	0.682	0.765	0.0371
212751_at	1527.5	1749.7	0.20	1.15	PPP-PPP	0.20	16	0.682	0.765	0.0141
202907_s_at	2841.0	3251.8	0.20	1.15	PPP-PPP	0.20	16	0.682	0.765	0.0486
219130_at	717.5	820.6	0.19	1.14	PPP-PPP	0.20	16	0.682	0.765	0.0536
227019_at	192.2	219.8	0.19	1.14	MPP-PPP	0.20	16	0.682	0.765	0.0301
209234_at	677.4	775.1	0.19	1.14	PPP-PPP	0.20	16	0.682	0.765	0.0201
216342_x_at	27495.2	31446.3	0.19	1.14	PPP-PPP	0.20	16	0.682	0.765	0.0153
221196_x_at	1226.3	1399.9	0.19	1.14	PPP-PPP	0.20	16	0.682	0.765	0.0425
223287_s_at	1598.6	1824.0	0.19	1.14	PPP-PPP	0.20	16	0.682	0.765	0.0609
218041_x_at	17132.3	19533.3	0.19	1.14	PPP-PPP	0.20	16	0.682	0.765	0.0608
208949_s_at	15307.5	17450.8	0.19	1.14	PPP-PPP	0.20	16	0.682	0.765	0.0436
209218_at	11899.4	13566.1	0.19	1.14	PPP-PPP	0.20	16	0.682	0.765	0.0355
228070_at	597.9	681.8	0.19	1.14	PPP-PPP	0.20	16	0.682	0.765	0.0044
209163_at	947.8	1079.7	0.19	1.14	PPP-PPP	0.20	16	0.682	0.765	0.0533
225132_at	3664.2	4173.2	0.19	1.14	PPP-PPP	0.20	16	0.682	0.765	0.0492
211953_s_at	4702.0	5356.1	0.19	1.14	PPP-PPP	0.20	16	0.682	0.765	0.0076
209075_s_at	9146.3	10395.0	0.19	1.14	PPP-PPP	0.20	16	0.682	0.765	0.0516
204547_at	572.0	650.0	0.19	1.14	PPP-PPP	0.20	16	0.682	0.765	0.0513
225848_at	1025.9	1166.3	0.19	1.14	PPP-PPP	0.20	16	0.682	0.765	0.0138
211763_s_at	1777.3	2019.5	0.18	1.14	PPP-PPP	0.20	16	0.682	0.765	0.0442
208898_at	3889.2	4419.2	0.18	1.14	PPP-PPP	0.20	16	0.682	0.765	0.0435
200937_s_at	32925.4	37398.4	0.18	1.14	PPP-PPP	0.20	16	0.682	0.765	0.0424
204391_x_at	939.2	1066.3	0.18	1.14	PPP-PPP	0.20	16	0.682	0.765	0.0391
202548_s_at	2303.4	2615.7	0.18	1.14	PPP-PPP	0.20	16	0.682	0.765	0.0382
223356_s_at	2012.5	2284.6	0.18	1.14	PPP-PPP	0.20	16	0.682	0.765	0.0329
224799_at	4210.7	4778.4	0.18	1.14	PPP-PPP	0.20	16	0.682	0.765	0.0396
206023_at	5921.3	6712.1	0.18	1.13	PPP-PPP	0.20	16	0.682	0.765	0.0448
230205_at	339.7	385.1	0.18	1.13	PPP-PPP	0.20	16	0.682	0.765	0.0343
202386_s_at	779.6	883.8	0.18	1.13	PPP-PPP	0.20	16	0.682	0.765	0.0112
209492_x_at	14994.5	16999.3	0.18	1.13	PPP-PPP	0.20	16	0.682	0.765	0.0046
201339_s_at	4331.5	4903.1	0.18	1.13	PPP-PPP	0.20	16	0.682	0.765	0.0473
201454_s_at	2444.1	2762.7	0.18	1.13	PPP-PPP	0.20	16	0.682	0.765	0.0438
236388_at	462.5	522.6	0.18	1.13	PPP-PPP	0.20	16	0.682	0.765	0.0034
211257_x_at	1700.8	1918.4	0.17	1.13	PPP-PPP	0.20	16	0.682	0.765	0.0276
202757_at	1596.5	1801.5	0.17	1.13	PPP-PPP	0.20	16	0.682	0.765	0.0189

212704_at	1228.3	1385.8	0.17	1.13	PPP-PPP	0.20	16	0.682	0.765	0.0126
226763_at	1388.5	1566.7	0.17	1.13	PPP-PPP	0.20	16	0.682	0.765	0.0017
1555950_a_at	5123.5	5774.1	0.17	1.13	PPP-PPP	0.20	16	0.682	0.765	0.0017
201597_at	18915.9	21238.8	0.17	1.12	PPP-PPP	0.20	16	0.682	0.765	0.0172
201312_s_at	3574.7	4013.2	0.17	1.12	PPP-PPP	0.20	16	0.682	0.765	0.0014
218965_s_at	187.2	210.1	0.17	1.12	AAA-APM	0.20	16	0.682	0.765	0.0265
214047_s_at	1356.0	1520.0	0.17	1.12	PPP-PPP	0.20	16	0.682	0.765	0.0133
213594_x_at	2531.7	2834.8	0.16	1.12	PPP-PPP	0.20	16	0.682	0.765	0.0134
208835_s_at	3476.8	3884.9	0.16	1.12	PPP-PPP	0.20	16	0.682	0.765	0.0274
220547_s_at	3429.4	3831.2	0.16	1.12	PPP-PPP	0.20	16	0.682	0.765	0.0160
223402_at	3067.1	3424.7	0.16	1.12	PPP-PPP	0.20	16	0.682	0.765	0.0083
226868_at	434.6	484.6	0.16	1.12	PPP-PPP	0.20	16	0.682	0.765	0.0141
224955_at	5706.7	6362.6	0.16	1.12	PPP-PPP	0.20	16	0.682	0.765	0.0092
200080_s_at	27264.3	30369.4	0.16	1.11	PPP-PPP	0.20	16	0.682	0.765	0.0111
218237_s_at	7884.6	8777.5	0.16	1.11	PPP-PPP	0.20	16	0.682	0.765	0.0124
202428_x_at	15054.6	16744.6	0.15	1.11	PPP-PPP	0.20	16	0.682	0.765	0.0008
205854_at	879.5	977.0	0.15	1.11	PPP-PPP	0.20	16	0.682	0.765	0.0110
200858_s_at	27789.9	30831.7	0.15	1.11	PPP-PPP	0.20	16	0.682	0.765	0.0123
209153_s_at	2789.4	3090.3	0.15	1.11	PPP-PPP	0.20	16	0.682	0.765	0.0068
204175_at	1997.4	2212.2	0.15	1.11	PPP-PPP	0.20	16	0.682	0.765	0.0148
210616_s_at	2321.5	2571.2	0.15	1.11	PPP-PPP	0.20	16	0.682	0.765	0.0021
217758_s_at	4319.3	4768.0	0.14	1.10	PPP-PPP	0.20	16	0.682	0.765	0.0099
212920_at	868.1	956.6	0.14	1.10	PPP-PPP	0.20	16	0.682	0.765	0.0069
208718_at	8853.3	9757.4	0.14	1.10	PPP-PPP	0.20	16	0.682	0.765	0.0048
222442_s_at	1211.1	1332.4	0.14	1.10	PPP-PPP	0.20	16	0.682	0.765	0.0079
225312_at	10844.6	11930.2	0.14	1.10	PPP-PPP	0.20	16	0.682	0.765	0.0034
200764_s_at	4689.6	5141.0	0.13	1.10	PPP-PPP	0.20	16	0.682	0.765	0.0034
201037_at	8019.8	8767.0	0.13	1.09	PPP-PPP	0.20	16	0.682	0.765	0.0041
1555560_at	43.1	3.3	-3.72	-13.14	APA-AAA	0.10	17	0.868	0.955	0.0351
237939_at	30.4	3.1	-3.29	-9.80	APA-AAA	0.10	17	0.868	0.955	0.0302
206481_s_at	44.1	4.8	-3.19	-9.13	AAP-AAA	0.10	17	0.868	0.955	0.0324
1553402_a_at	71.6	10.9	-2.72	-6.57	AAP-AAA	0.10	17	0.868	0.955	0.0263
1555573_at	44.9	7.2	-2.64	-6.25	AAP-AAA	0.10	17	0.868	0.955	0.0247
1552323_s_at	64.2	10.9	-2.56	-5.91	PAA-AAA	0.10	17	0.868	0.955	0.0300
1554295_x_at	97.3	17.6	-2.47	-5.54	AAP-AAA	0.10	17	0.868	0.955	0.0296
241485_at	5.0	24.9	2.31	4.97	AAP-AAA	0.10	17	0.868	0.955	0.2712
233039_at	5.0	22.2	2.15	4.45	AAA-AAP	0.10	17	0.868	0.955	0.2714
1564215_at	10.0	41.8	2.07	4.19	AAA-APP	0.10	17	0.868	0.955	0.2785
239554_at	54.2	13.0	-2.06	-4.17	AAP-AAA	0.10	17	0.868	0.955	0.0217
1564836_at	7.5	29.9	1.99	3.97	APA-AAA	0.10	17	0.868	0.955	0.2789
206521_s_at	45.0	173.4	1.95	3.85	AAA-AAP	0.10	17	0.868	0.955	0.2938
237131_at	74.6	19.5	-1.94	-3.83	AAP-AAA	0.10	17	0.868	0.955	0.0245

239726_at	17.6	65.8	1.90	3.74	AAA-PAP	0.10	17	0.868	0.955	0.2927
240113_at	30.6	113.3	1.89	3.70	AAP-AAP	0.10	17	0.868	0.955	0.2840
237982_at	68.2	18.4	-1.89	-3.70	PPA-AAA	0.10	17	0.868	0.955	0.0251
225714_s_at	20.1	74.4	1.89	3.70	PAA-PAA	0.10	17	0.868	0.955	0.2652
1570207_at	5.5	20.3	1.88	3.68	AAA-APA	0.10	17	0.868	0.955	0.3283
241147_at	6.9	25.5	1.88	3.68	AAA-PAA	0.10	17	0.868	0.955	0.2656
1557636_a_at	3.9	14.2	1.87	3.66	AAA-PAA	0.10	17	0.868	0.955	0.3616
231434_at	64.2	17.6	-1.87	-3.65	PAA-AAA	0.10	17	0.868	0.955	0.0229
243799_x_at	4.1	14.7	1.86	3.62	AAA-PAA	0.10	17	0.868	0.955	0.4181
220853_at	33.3	118.1	1.83	3.55	PAA-PAM	0.10	17	0.868	0.955	0.3233
231235_at	25.2	88.5	1.81	3.51	AAP-MPP	0.10	17	0.868	0.955	0.2860
1569569_x_at	6.8	23.6	1.80	3.48	AAA-APA	0.10	17	0.868	0.955	0.2800
216669_at	7.0	24.0	1.77	3.41	AAA-AAP	0.10	17	0.868	0.955	0.2735
1564072_at	13.8	47.1	1.77	3.41	AAA-PAA	0.10	17	0.868	0.955	0.2694
1553658_at	26.5	90.0	1.77	3.40	APA-APM	0.10	17	0.868	0.955	0.3015
232096_x_at	40.3	136.0	1.75	3.37	PPA-APP	0.10	17	0.868	0.955	0.3292
210661_at	5.9	19.7	1.74	3.33	AAA-PAA	0.10	17	0.868	0.955	0.2797
234758_at	15.1	49.9	1.73	3.31	AAP-AAP	0.10	17	0.868	0.955	0.2982
205978_at	10.9	35.7	1.71	3.28	PAA-AAA	0.10	17	0.868	0.955	0.3882
1566695_at	16.5	53.1	1.69	3.22	AAP-PAP	0.10	17	0.868	0.955	0.2656
1561780_at	4.1	13.3	1.68	3.21	AAA-AAP	0.10	17	0.868	0.955	0.3726
215065_at	18.5	58.9	1.67	3.19	AAA-AAP	0.10	17	0.868	0.955	0.3407
244272_s_at	10.9	34.7	1.67	3.19	AAP-AMA	0.10	17	0.868	0.955	0.2836
209591_s_at	9.3	29.4	1.67	3.18	AAA-AAP	0.10	17	0.868	0.955	0.2586
217659_at	13.1	41.6	1.67	3.17	AAA-MAP	0.10	17	0.868	0.955	0.3643
222031_at	18.0	57.0	1.66	3.16	PAA-PPP	0.10	17	0.868	0.955	0.3634
238234_at	4.7	14.7	1.65	3.14	AAA-PAA	0.10	17	0.868	0.955	0.4421
230038_at	24.0	75.2	1.65	3.14	AAA-AAP	0.10	17	0.868	0.955	0.2840
210800_at	9.6	30.1	1.65	3.13	AAA-AAP	0.10	17	0.868	0.955	0.2680
232363_at	19.8	61.7	1.64	3.12	AAA-AAP	0.10	17	0.868	0.955	0.3208
1562230_at	20.3	63.1	1.63	3.10	AAA-PAA	0.10	17	0.868	0.955	0.2616
236255_at	12.8	39.5	1.62	3.08	AAA-PAA	0.10	17	0.868	0.955	0.3021
242221_at	9.5	29.0	1.61	3.05	AAA-APA	0.10	17	0.868	0.955	0.3008
1567862_at	6.2	18.8	1.60	3.04	AAA-PAA	0.10	17	0.868	0.955	0.4011
216147_at	31.9	96.5	1.60	3.03	PAP-PPP	0.10	17	0.868	0.955	0.3799
1553847_a_at	33.0	98.7	1.58	3.00	PAA-PAA	0.10	17	0.868	0.955	0.2573
214650_x_at	17.4	52.0	1.58	2.99	AAP-AMA	0.10	17	0.868	0.955	0.3838
229354_at	38.3	114.7	1.58	2.99	AAA-PPP	0.10	17	0.868	0.955	0.3037
216074_x_at	24.1	71.9	1.58	2.99	AAA-AAP	0.10	17	0.868	0.955	0.4363
244680_at	19.9	59.2	1.57	2.98	PAA-AAM	0.10	17	0.868	0.955	0.4139
239757_at	18.0	53.5	1.57	2.98	APA-APP	0.10	17	0.868	0.955	0.3606
235547_at	31.2	92.8	1.57	2.98	PAP-PPP	0.10	17	0.868	0.955	0.2715

240176_at	55.0	163.4	1.57	2.97	PAA-AAA	0.10	17	0.868	0.955	0.3304
235701_at	30.7	90.7	1.56	2.95	AAA-AAP	0.10	17	0.868	0.955	0.3952
1562106_at	19.2	56.5	1.56	2.95	APA-PAP	0.10	17	0.868	0.955	0.3660
1561396_at	9.1	26.9	1.56	2.94	AAA-PAA	0.10	17	0.868	0.955	0.3382
205470_s_at	31.9	93.3	1.55	2.93	PAA-MAA	0.10	17	0.868	0.955	0.3098
207207_at	8.4	24.4	1.54	2.92	AAA-MAP	0.10	17	0.868	0.955	0.4996
244770_at	18.2	52.9	1.54	2.91	AAA-PAP	0.10	17	0.868	0.955	0.2787
211512_s_at	75.7	220.3	1.54	2.91	PAP-PPP	0.10	17	0.868	0.955	0.3105
1559114_a_at	7.9	22.9	1.54	2.91	AAA-PAA	0.10	17	0.868	0.955	0.2715
233636_at	3.7	10.7	1.53	2.90	AAA-AAP	0.10	17	0.868	0.955	0.3646
216029_at	27.1	78.3	1.53	2.90	AAA-APA	0.10	17	0.868	0.955	0.3217
220564_at	6.6	19.0	1.53	2.89	APA-AAA	0.10	17	0.868	0.955	0.4149
228547_at	12.3	35.5	1.52	2.88	APA-AAA	0.10	17	0.868	0.955	0.3449
231726_at	18.4	53.0	1.52	2.88	AAA-AAP	0.10	17	0.868	0.955	0.2696
226718_at	15.8	45.1	1.52	2.86	PAA-AAA	0.10	17	0.868	0.955	0.4472
240759_at	8.3	23.7	1.51	2.85	AAA-PAP	0.10	17	0.868	0.955	0.3710
233297_s_at	8.4	23.9	1.51	2.85	AAA-APA	0.10	17	0.868	0.955	0.2837
237577_at	17.9	51.1	1.51	2.85	AAP-APP	0.10	17	0.868	0.955	0.3763
244312_at	22.3	63.6	1.51	2.85	APA-AAM	0.10	17	0.868	0.955	0.3675
237628_at	12.3	35.1	1.51	2.85	AAA-AAP	0.10	17	0.868	0.955	0.3909
1558599_at	12.2	34.7	1.51	2.85	AAP-AAA	0.10	17	0.868	0.955	0.3137
241750_x_at	9.0	25.6	1.50	2.83	AAA-PAA	0.10	17	0.868	0.955	0.3199
233934_at	13.5	38.2	1.50	2.82	AAA-APA	0.10	17	0.868	0.955	0.3759
243019_at	39.4	14.0	-1.50	-2.82	MPA-AAA	0.10	17	0.868	0.955	0.0188
1564331_at	9.6	27.0	1.49	2.80	APA-AAA	0.10	17	0.868	0.955	0.3936
236943_at	19.7	55.2	1.49	2.80	AAA-PPA	0.10	17	0.868	0.955	0.3704
241614_at	26.1	73.2	1.49	2.80	APA-AAA	0.10	17	0.868	0.955	0.3178
1556542_a_at	19.6	54.7	1.48	2.80	AAA-AAP	0.10	17	0.868	0.955	0.2803
1560712_at	14.5	40.6	1.48	2.79	APA-PAA	0.10	17	0.868	0.955	0.3189
219285_s_at	14.5	40.4	1.48	2.79	PAA-AAA	0.10	17	0.868	0.955	0.2798
229809_at	12.7	35.2	1.47	2.77	PAA-AAA	0.10	17	0.868	0.955	0.3071
1559515_at	16.0	44.1	1.46	2.76	APA-PAA	0.10	17	0.868	0.955	0.3825
240788_at	27.9	76.9	1.46	2.76	AAA-AAP	0.10	17	0.868	0.955	0.2982
239759_at	19.7	54.2	1.46	2.75	PAA-MAP	0.10	17	0.868	0.955	0.2543
208348_s_at	6.4	17.6	1.46	2.75	AAA-APA	0.10	17	0.868	0.955	0.3281
1557805_at	14.1	38.4	1.45	2.73	AAA-PAM	0.10	17	0.868	0.955	0.2786
242952_at	6.1	16.6	1.45	2.72	PAA-AAA	0.10	17	0.868	0.955	0.4808
237100_at	32.7	89.0	1.44	2.72	AAA-PPA	0.10	17	0.868	0.955	0.2720
242135_at	30.8	83.4	1.44	2.71	APA-PAA	0.10	17	0.868	0.955	0.4028
231647_s_at	1.7	4.7	1.44	2.71	AAA-PAA	0.10	17	0.868	0.955	0.4100
211100_x_at	19.4	52.4	1.44	2.71	AAA-APA	0.10	17	0.868	0.955	0.2768
207175_at	19.6	52.9	1.44	2.71	APP-PPP	0.10	17	0.868	0.955	0.3307

217193_x_at	40.6	109.7	1.44	2.70	AAA-AAP	0.10	17	0.868	0.955	0.2886
207417_s_at	14.2	38.2	1.43	2.70	PAA-AAA	0.10	17	0.868	0.955	0.4471
233720_at	26.1	70.4	1.43	2.70	APP-PAA	0.10	17	0.868	0.955	0.3286
220197_at	155.0	57.6	-1.43	-2.69	APP-AAA	0.10	17	0.868	0.955	0.0162
234851_at	24.2	65.0	1.43	2.69	APA-AAA	0.10	17	0.868	0.955	0.4288
230861_at	4.4	11.7	1.42	2.67	AAA-AAP	0.10	17	0.868	0.955	0.3835
243997_x_at	15.3	40.6	1.41	2.66	PAA-PAA	0.10	17	0.868	0.955	0.2676
1557277_a_at	10.8	28.5	1.40	2.65	AAA-AAP	0.10	17	0.868	0.955	0.3787
216759_at	27.3	72.0	1.40	2.64	PAA-AAA	0.10	17	0.868	0.955	0.4078
232771_at	4.4	11.6	1.40	2.64	AAA-APA	0.10	17	0.868	0.955	0.3424
243846_x_at	18.4	48.5	1.40	2.64	AAA-PAP	0.10	17	0.868	0.955	0.3235
201428_at	40.9	107.8	1.40	2.63	AAA-PAA	0.10	17	0.868	0.955	0.3299
206631_at	29.6	77.9	1.40	2.63	APA-AAA	0.10	17	0.868	0.955	0.2648
216595_at	8.8	23.0	1.39	2.63	APA-PAA	0.10	17	0.868	0.955	0.4687
211170_s_at	15.8	41.6	1.39	2.63	PAA-AAP	0.10	17	0.868	0.955	0.3787
211429_s_at	389.4	149.1	-1.39	-2.61	PPP-PMP	0.10	17	0.868	0.955	0.0214
1553749_at	349.5	133.9	-1.38	-2.61	PPP-PPP	0.10	17	0.868	0.955	0.0150
237837_at	9.6	25.1	1.38	2.61	AAA-PAA	0.10	17	0.868	0.955	0.3987
238406_x_at	60.3	157.3	1.38	2.61	AAP-APP	0.10	17	0.868	0.955	0.2974
216020_at	13.0	33.8	1.38	2.61	PAA-AAA	0.10	17	0.868	0.955	0.3517
1561762_s_at	15.7	40.8	1.38	2.60	AAP-AAA	0.10	17	0.868	0.955	0.2644
239752_at	14.1	36.7	1.38	2.60	AAA-APA	0.10	17	0.868	0.955	0.2759
209928_s_at	23.0	59.5	1.37	2.59	AAP-MAP	0.10	17	0.868	0.955	0.2785
210491_at	23.5	60.8	1.37	2.59	APP-AAP	0.10	17	0.868	0.955	0.4043
232525_at	76.3	197.3	1.37	2.59	AAA-AAP	0.10	17	0.868	0.955	0.2830
216931_at	20.4	52.7	1.37	2.59	PAA-AAA	0.10	17	0.868	0.955	0.4700
1560251_at	5.0	12.8	1.37	2.58	AAA-APA	0.10	17	0.868	0.955	0.4655
1561847_at	41.0	105.6	1.37	2.58	AAA-AMP	0.10	17	0.868	0.955	0.2744
233192_s_at	17.6	45.1	1.36	2.56	APA-AAA	0.10	17	0.868	0.955	0.2820
244440_at	30.0	76.9	1.36	2.56	AAA-PAA	0.10	17	0.868	0.955	0.3852
211819_s_at	23.0	58.7	1.35	2.55	AAP-PAA	0.10	17	0.868	0.955	0.4221
220545_s_at	6.1	15.6	1.35	2.55	AAA-PAA	0.10	17	0.868	0.955	0.4201
1556493_a_at	27.3	69.8	1.35	2.55	AAA-PAM	0.10	17	0.868	0.955	0.4744
244275_at	18.4	46.7	1.35	2.55	PAA-AAA	0.10	17	0.868	0.955	0.3014
213832_at	25.6	65.2	1.35	2.55	MAM-MPA	0.10	17	0.868	0.955	0.2861
240830_at	24.7	62.8	1.35	2.54	AAP-AAA	0.10	17	0.868	0.955	0.3864
1558881_at	18.3	46.6	1.35	2.54	AAA-APA	0.10	17	0.868	0.955	0.3294
233553_at	15.3	38.9	1.35	2.54	AAA-AAP	0.10	17	0.868	0.955	0.3188
226051_at	421.8	166.0	-1.35	-2.54	PPP-PPP	0.10	17	0.868	0.955	0.0198
215858_at	28.8	73.1	1.35	2.54	AAA-PAA	0.10	17	0.868	0.955	0.3313
221065_s_at	42.3	107.4	1.35	2.54	AAP-AAA	0.10	17	0.868	0.955	0.3279
231001_at	15.8	40.2	1.34	2.54	AAA-PAA	0.10	17	0.868	0.955	0.2868

1554608_at	41.8	105.9	1.34	2.54	MAA-PPP	0.10	17	0.868	0.955	0.2577
226828_s_at	14.6	36.9	1.34	2.53	AAA-APA	0.10	17	0.868	0.955	0.3667
216314_at	9.1	23.1	1.34	2.53	AAA-AAP	0.10	17	0.868	0.955	0.3080
1558647_at	64.9	164.3	1.34	2.53	AAM-PAA	0.10	17	0.868	0.955	0.2887
213087_s_at	50.9	128.9	1.34	2.53	AAA-PAA	0.10	17	0.868	0.955	0.3611
240568_at	15.4	39.0	1.34	2.52	AAA-PAP	0.10	17	0.868	0.955	0.3208
231981_at	20.9	52.7	1.33	2.52	AAP-PAP	0.10	17	0.868	0.955	0.2518
235378_at	30.5	76.7	1.33	2.52	AAA-APA	0.10	17	0.868	0.955	0.2999
237716_at	27.7	69.3	1.32	2.50	PAA-AAA	0.10	17	0.868	0.955	0.2561
230300_at	39.1	97.7	1.32	2.50	PAP-PPP	0.10	17	0.868	0.955	0.3326
1570496_at	18.9	47.2	1.32	2.50	PAA-PAA	0.10	17	0.868	0.955	0.2486
229788_s_at	18.5	46.0	1.32	2.49	PAA-AAA	0.10	17	0.868	0.955	0.3064
216291_at	12.5	31.1	1.31	2.49	AAA-AAP	0.10	17	0.868	0.955	0.3910
232901_at	18.5	46.0	1.31	2.48	AAA-APA	0.10	17	0.868	0.955	0.2548
244105_at	16.0	39.6	1.31	2.48	APA-AAP	0.10	17	0.868	0.955	0.3464
244375_at	12.2	30.0	1.30	2.47	AAA-APA	0.10	17	0.868	0.955	0.4439
210409_at	24.1	59.3	1.30	2.46	PAA-APA	0.10	17	0.868	0.955	0.3694
205643_s_at	23.3	57.3	1.30	2.46	AAM-APA	0.10	17	0.868	0.955	0.3799
236237_at	28.8	70.8	1.30	2.46	APA-AAA	0.10	17	0.868	0.955	0.2594
211086_x_at	66.0	161.6	1.29	2.45	PAP-PPP	0.10	17	0.868	0.955	0.3238
207267_s_at	24.2	59.0	1.29	2.44	PAA-AAP	0.10	17	0.868	0.955	0.5221
243271_at	31.6	77.0	1.29	2.44	PAA-AAP	0.10	17	0.868	0.955	0.2671
214472_at	18.3	44.5	1.28	2.43	AAA-AAP	0.10	17	0.868	0.955	0.2681
240728_at	15.5	37.6	1.28	2.43	AMA-PAA	0.10	17	0.868	0.955	0.4830
240947_at	26.4	64.0	1.28	2.43	AAM-APA	0.10	17	0.868	0.955	0.3094
1553284_s_at	27.0	65.6	1.28	2.43	AAA-PPA	0.10	17	0.868	0.955	0.2978
239219_at	39.7	96.3	1.28	2.43	AMA-AAP	0.10	17	0.868	0.955	0.3613
1553021_s_at	63.3	153.4	1.28	2.43	AAA-AAP	0.10	17	0.868	0.955	0.4012
205245_at	60.6	146.2	1.27	2.41	PAA-PAA	0.10	17	0.868	0.955	0.3033
202663_at	18.2	43.8	1.27	2.41	AAA-AAP	0.10	17	0.868	0.955	0.3648
242170_at	7.8	18.7	1.27	2.41	AAA-AAP	0.10	17	0.868	0.955	0.4996
1566653_at	40.0	96.2	1.27	2.41	AAA-APA	0.10	17	0.868	0.955	0.2638
241099_at	11.7	28.0	1.27	2.40	PAP-AAP	0.10	17	0.868	0.955	0.4156
1561770_at	32.7	78.7	1.27	2.40	AAP-AAP	0.10	17	0.868	0.955	0.3512
216200_at	24.8	59.6	1.27	2.40	AAA-PAP	0.10	17	0.868	0.955	0.2795
219677_at	38.5	92.5	1.26	2.40	PAP-AMA	0.10	17	0.868	0.955	0.4884
244710_at	22.6	54.3	1.26	2.40	PAA-AAA	0.10	17	0.868	0.955	0.2741
214159_at	14.7	34.9	1.25	2.38	AAA-AAP	0.10	17	0.868	0.955	0.2939
211258_s_at	234.3	98.7	-1.25	-2.37	PPP-AAA	0.10	17	0.868	0.955	0.0208
1560638_a_at	16.7	39.5	1.25	2.37	AAA-APA	0.10	17	0.868	0.955	0.3900
205871_at	11.4	27.1	1.25	2.37	AAP-AAP	0.10	17	0.868	0.955	0.5014
225709_at	58.0	137.2	1.24	2.37	APA-MAA	0.10	17	0.868	0.955	0.3831

242576_x_at	43.0	101.6	1.24	2.37	MAA-PPP	0.10	17	0.868	0.955	0.3109
241938_at	35.5	83.8	1.24	2.36	AAP-AAA	0.10	17	0.868	0.955	0.3091
1560449_at	40.6	95.7	1.24	2.36	AAP-PAP	0.10	17	0.868	0.955	0.3577
1570228_at	12.9	30.4	1.24	2.36	AAA-PAP	0.10	17	0.868	0.955	0.3491
1558394_s_at	28.0	65.8	1.23	2.35	APA-APA	0.10	17	0.868	0.955	0.3536
232849_at	63.0	148.2	1.23	2.35	PPA-PPP	0.10	17	0.868	0.955	0.2584
1565635_at	21.7	50.9	1.23	2.35	AAA-PAA	0.10	17	0.868	0.955	0.2613
244377_at	33.1	77.7	1.23	2.35	AAA-APA	0.10	17	0.868	0.955	0.2783
1565566_a_at	34.3	80.5	1.23	2.35	AAP-PPP	0.10	17	0.868	0.955	0.2657
229543_at	57.0	24.4	-1.23	-2.34	PAA-AAA	0.10	17	0.868	0.955	0.0157
1566852_at	26.3	61.3	1.22	2.33	AAA-PAA	0.10	17	0.868	0.955	0.4398
238586_at	16.3	38.1	1.22	2.33	AAA-AAP	0.10	17	0.868	0.955	0.2537
242399_at	34.2	79.5	1.22	2.33	AAP-PPA	0.10	17	0.868	0.955	0.4087
204595_s_at	215.6	92.8	-1.22	-2.32	PPP-AMA	0.10	17	0.868	0.955	0.0196
242999_at	17.9	41.5	1.22	2.32	AAA-PAA	0.10	17	0.868	0.955	0.3427
216110_x_at	13.6	31.6	1.21	2.32	PAA-AAA	0.10	17	0.868	0.955	0.4988
232928_at	37.8	87.7	1.21	2.32	AAA-PAM	0.10	17	0.868	0.955	0.3593
1553711_a_at	16.2	37.5	1.21	2.32	APA-AAA	0.10	17	0.868	0.955	0.5290
243692_at	9.7	22.3	1.21	2.31	AAA-PAA	0.10	17	0.868	0.955	0.3544
237387_at	42.7	98.7	1.21	2.31	APP-APP	0.10	17	0.868	0.955	0.3462
232461_at	17.1	39.4	1.20	2.30	AAA-APA	0.10	17	0.868	0.955	0.2688
233906_at	21.3	49.1	1.20	2.30	AAA-AAP	0.10	17	0.868	0.955	0.4817
213751_at	35.2	80.9	1.20	2.30	APA-PAA	0.10	17	0.868	0.955	0.2995
222948_s_at	19.3	44.2	1.20	2.30	AAA-PAA	0.10	17	0.868	0.955	0.2544
239661_at	17.8	40.7	1.20	2.29	AAA-AAP	0.10	17	0.868	0.955	0.2586
232911_at	17.6	40.2	1.20	2.29	AAP-AAA	0.10	17	0.868	0.955	0.2742
237084_at	32.6	74.6	1.19	2.29	AAP-AAP	0.10	17	0.868	0.955	0.4663
1553363_at	15.0	34.4	1.19	2.29	AAP-AAA	0.10	17	0.868	0.955	0.4154
220663_at	28.3	64.7	1.19	2.28	AAA-AAP	0.10	17	0.868	0.955	0.2570
222336_at	15.8	36.1	1.19	2.28	AAP-AAA	0.10	17	0.868	0.955	0.5384
228691_at	60.9	138.5	1.19	2.27	AAA-PAA	0.10	17	0.868	0.955	0.2919
1553315_at	15.7	35.6	1.18	2.27	AAP-AAA	0.10	17	0.868	0.955	0.3741
234094_x_at	21.2	48.1	1.18	2.27	AAA-APA	0.10	17	0.868	0.955	0.3893
236879_at	16.8	38.1	1.18	2.27	AAP-AAM	0.10	17	0.868	0.955	0.2704
236757_at	19.3	43.6	1.18	2.26	AAP-APA	0.10	17	0.868	0.955	0.3877
239718_at	36.0	81.4	1.18	2.26	AAA-APA	0.10	17	0.868	0.955	0.3030
228906_at	24.2	54.8	1.18	2.26	APA-PPA	0.10	17	0.868	0.955	0.3612
234330_at	11.7	26.4	1.18	2.26	AAA-APA	0.10	17	0.868	0.955	0.3727
237798_at	18.4	41.5	1.18	2.26	AAA-PAA	0.10	17	0.868	0.955	0.3145
238861_at	33.8	76.2	1.17	2.25	AAA-AAP	0.10	17	0.868	0.955	0.3261
236902_at	7.5	16.9	1.17	2.25	PAA-AAA	0.10	17	0.868	0.955	0.5032
239849_at	16.7	37.5	1.17	2.25	AAA-APP	0.10	17	0.868	0.955	0.3874

238644_at	32.4	72.6	1.17	2.24	PAA-APP	0.10	17	0.868	0.955	0.4818
207536_s_at	20.0	44.8	1.16	2.24	APM-AAA	0.10	17	0.868	0.955	0.4293
1554233_at	26.3	58.7	1.16	2.24	AAP-PAP	0.10	17	0.868	0.955	0.2556
230292_at	56.3	125.7	1.16	2.24	APA-PMP	0.10	17	0.868	0.955	0.3700
218881_s_at	64.5	144.1	1.16	2.24	AAA-APA	0.10	17	0.868	0.955	0.2646
1557754_at	22.8	50.9	1.16	2.23	AAA-APA	0.10	17	0.868	0.955	0.3901
232100_at	25.6	57.0	1.16	2.23	AAA-APA	0.10	17	0.868	0.955	0.3648
206537_at	45.1	100.5	1.16	2.23	AAA-MPA	0.10	17	0.868	0.955	0.2647
1556763_at	13.2	29.3	1.15	2.22	AAA-PAA	0.10	17	0.868	0.955	0.2489
223941_at	31.7	70.5	1.15	2.22	APA-AAA	0.10	17	0.868	0.955	0.2465
233479_at	30.0	66.8	1.15	2.22	AMA-APA	0.10	17	0.868	0.955	0.3985
1556153_s_at	19.4	43.0	1.15	2.22	AMA-APP	0.10	17	0.868	0.955	0.3472
1555113_at	18.3	40.7	1.15	2.22	APA-AAA	0.10	17	0.868	0.955	0.3726
214912_at	27.7	61.6	1.15	2.22	AAA-MAP	0.10	17	0.868	0.955	0.3076
220092_s_at	40.2	89.1	1.15	2.22	PAA-AAP	0.10	17	0.868	0.955	0.3955
1562145_at	31.1	68.9	1.15	2.22	APA-AAA	0.10	17	0.868	0.955	0.3232
219728_at	11.2	24.8	1.15	2.22	AAA-AAP	0.10	17	0.868	0.955	0.3783
207558_s_at	18.9	41.7	1.15	2.21	PAP-PAP	0.10	17	0.868	0.955	0.4987
1559712_at	26.5	58.7	1.15	2.22	AAA-PAA	0.10	17	0.868	0.955	0.3159
226972_s_at	34.8	76.9	1.15	2.21	AAA-AAP	0.10	17	0.868	0.955	0.3764
224459_at	11.7	25.8	1.15	2.21	AAA-AAP	0.10	17	0.868	0.955	0.3557
240818_at	29.6	65.4	1.14	2.21	AAP-MAA	0.10	17	0.868	0.955	0.4377
243547_at	10.8	23.8	1.14	2.20	AAA-APA	0.10	17	0.868	0.955	0.5806
244835_at	26.4	58.1	1.14	2.20	AAP-APP	0.10	17	0.868	0.955	0.2981
217143_s_at	19.7	43.2	1.14	2.20	AAA-PAA	0.10	17	0.868	0.955	0.3365
213672_at	31.5	69.2	1.14	2.20	PAA-AAM	0.10	17	0.868	0.955	0.3861
1569669_at	17.6	38.6	1.13	2.19	APA-AAP	0.10	17	0.868	0.955	0.4376
242562_at	20.5	45.0	1.13	2.19	AAA-APP	0.10	17	0.868	0.955	0.3891
1552520_at	26.6	58.4	1.13	2.19	PAP-APP	0.10	17	0.868	0.955	0.4612
233556_at	74.2	33.9	-1.13	-2.19	MPA-AAA	0.10	17	0.868	0.955	0.0183
216766_at	29.2	63.9	1.13	2.19	PAA-PAP	0.10	17	0.868	0.955	0.5014
1555217_at	17.5	38.3	1.13	2.19	APA-MAA	0.10	17	0.868	0.955	0.3584
1564467_at	39.2	85.7	1.13	2.19	AAA-PAP	0.10	17	0.868	0.955	0.3063
220898_at	25.1	54.8	1.13	2.19	AAA-AAP	0.10	17	0.868	0.955	0.2561
239642_at	23.4	51.1	1.13	2.18	APA-AAP	0.10	17	0.868	0.955	0.3671
1561018_at	20.0	43.7	1.13	2.18	PAA-AAA	0.10	17	0.868	0.955	0.3438
1569788_at	7.2	15.6	1.13	2.18	AAA-APA	0.10	17	0.868	0.955	0.5676
1556414_at	15.8	34.5	1.12	2.18	AAM-PAA	0.10	17	0.868	0.955	0.2914
217619_x_at	140.0	305.3	1.12	2.18	PAP-PMA	0.10	17	0.868	0.955	0.2615
222080_s_at	16.1	34.7	1.11	2.16	AAP-AAA	0.10	17	0.868	0.955	0.4846
239999_at	15.3	33.1	1.11	2.16	AAA-PPP	0.10	17	0.868	0.955	0.4179
1557132_at	18.7	40.4	1.11	2.16	PAA-APA	0.10	17	0.868	0.955	0.5829

238451_at	8.8	19.0	1.11	2.16	AAA-PAA	0.10	17	0.868	0.955	0.5360
1553686_at	35.1	75.6	1.11	2.15	PAP-AAP	0.10	17	0.868	0.955	0.3163
242058_at	24.9	53.6	1.11	2.15	AAA-APA	0.10	17	0.868	0.955	0.2967
242986_at	47.0	21.8	-1.11	-2.15	MPP-AAA	0.10	17	0.868	0.955	0.0147
216926_s_at	13.8	29.7	1.11	2.15	AAA-AAP	0.10	17	0.868	0.955	0.4250
243565_at	33.2	71.4	1.11	2.15	AAP-AAP	0.10	17	0.868	0.955	0.4014
220668_s_at	202.1	94.2	-1.10	-2.15	PAP-AAA	0.10	17	0.868	0.955	0.0156
207867_at	13.6	29.2	1.10	2.14	AAA-AAP	0.10	17	0.868	0.955	0.4989
211641_x_at	18.9	40.5	1.10	2.14	AAA-PAA	0.10	17	0.868	0.955	0.4602
1559929_at	37.7	80.9	1.10	2.14	AAA-APA	0.10	17	0.868	0.955	0.2546
232271_at	18.4	39.3	1.10	2.14	PAA-AAP	0.10	17	0.868	0.955	0.3389
1559036_at	22.0	47.0	1.10	2.14	APA-AAA	0.10	17	0.868	0.955	0.4647
212210_at	35.0	74.7	1.10	2.14	PAA-PAA	0.10	17	0.868	0.955	0.4044
242158_at	18.7	39.9	1.10	2.14	PAA-AAA	0.10	17	0.868	0.955	0.3042
1555248_a_at	28.3	60.3	1.09	2.13	AAP-PPP	0.10	17	0.868	0.955	0.3023
238024_at	44.0	93.9	1.09	2.13	AAA-AAP	0.10	17	0.868	0.955	0.4328
205347_s_at	30.5	65.0	1.09	2.13	AAA-AAP	0.10	17	0.868	0.955	0.2810
219118_at	27.9	59.3	1.09	2.13	APA-APP	0.10	17	0.868	0.955	0.3901
237839_at	36.1	76.7	1.09	2.13	PAA-AMP	0.10	17	0.868	0.955	0.2885
228503_at	10.3	21.8	1.09	2.12	PAA-AAA	0.10	17	0.868	0.955	0.5246
230470_at	39.5	83.9	1.09	2.12	AAA-APA	0.10	17	0.868	0.955	0.2749
216749_at	12.5	26.5	1.08	2.12	AAA-AAP	0.10	17	0.868	0.955	0.4021
227108_at	53.8	114.1	1.08	2.12	MAA-PPA	0.10	17	0.868	0.955	0.3950
234091_at	53.1	112.4	1.08	2.12	AAA-AAP	0.10	17	0.868	0.955	0.3873
238922_at	16.4	34.7	1.08	2.12	AAA-PAA	0.10	17	0.868	0.955	0.4779
236416_at	36.3	76.7	1.08	2.11	AAA-PAA	0.10	17	0.868	0.955	0.3484
237198_at	32.7	69.0	1.08	2.11	AAA-PAA	0.10	17	0.868	0.955	0.2501
226818_at	10.3	21.8	1.07	2.11	AAA-PAP	0.10	17	0.868	0.955	0.5123
231165_at	20.3	42.8	1.07	2.11	APA-PAA	0.10	17	0.868	0.955	0.4563
231956_at	39.9	84.1	1.07	2.11	PAA-AAA	0.10	17	0.868	0.955	0.3750
230130_at	594.1	282.5	-1.07	-2.10	PPP-PPP	0.10	17	0.868	0.955	0.0153
1564598_a_at	25.5	53.6	1.07	2.10	AAP-APA	0.10	17	0.868	0.955	0.3990
207662_at	83.1	39.6	-1.07	-2.10	APP-AAP	0.10	17	0.868	0.955	0.0175
228447_at	39.7	83.3	1.07	2.10	AAA-AAP	0.10	17	0.868	0.955	0.3280
215777_at	26.9	56.6	1.07	2.10	AAA-PAA	0.10	17	0.868	0.955	0.2740
237043_at	29.8	62.4	1.07	2.10	AAA-AAP	0.10	17	0.868	0.955	0.4364
243088_at	75.7	158.6	1.07	2.10	AAP-PAP	0.10	17	0.868	0.955	0.2568
244791_at	38.7	81.0	1.07	2.09	APA-PPP	0.10	17	0.868	0.955	0.4226
1554672_at	28.0	58.6	1.06	2.09	APA-PPM	0.10	17	0.868	0.955	0.3890
239751_at	31.0	64.9	1.06	2.09	PPA-AAP	0.10	17	0.868	0.955	0.5360
232127_at	15.8	33.0	1.06	2.09	AAA-APA	0.10	17	0.868	0.955	0.4109
215238_s_at	69.3	144.7	1.06	2.09	AAP-APP	0.10	17	0.868	0.955	0.2798

242805_at	19.6	40.8	1.06	2.08	AAA-PPA	0.10	17	0.868	0.955	0.3935
1556661_at	16.4	34.1	1.06	2.08	AAA-APA	0.10	17	0.868	0.955	0.4178
1566484_at	32.3	67.3	1.06	2.08	AAA-APA	0.10	17	0.868	0.955	0.2945
235002_at	40.8	85.0	1.06	2.08	AAA-PAM	0.10	17	0.868	0.955	0.2452
238493_at	18.6	38.8	1.06	2.08	AMP-AMP	0.10	17	0.868	0.955	0.4695
234826_at	25.1	52.3	1.06	2.08	AAA-PAA	0.10	17	0.868	0.955	0.3598
1561273_at	17.8	37.0	1.06	2.08	AAP-AAA	0.10	17	0.868	0.955	0.3435
1554621_at	16.3	33.8	1.06	2.08	APA-AAA	0.10	17	0.868	0.955	0.3139
241728_at	23.3	48.3	1.06	2.08	AAP-AAA	0.10	17	0.868	0.955	0.5723
1563511_at	45.5	94.6	1.06	2.08	AAP-MAP	0.10	17	0.868	0.955	0.3375
223693_s_at	47.7	99.0	1.05	2.08	PAA-AAA	0.10	17	0.868	0.955	0.3468
236239_at	44.9	93.0	1.05	2.07	AAA-PAA	0.10	17	0.868	0.955	0.4748
227233_at	9.5	19.8	1.05	2.07	AAA-PAA	0.10	17	0.868	0.955	0.4239
205247_at	41.0	85.1	1.05	2.07	PAA-AAM	0.10	17	0.868	0.955	0.3953
232371_at	22.9	47.4	1.05	2.07	AAA-PAP	0.10	17	0.868	0.955	0.3809
1561690_at	15.5	32.0	1.05	2.07	AAA-AAP	0.10	17	0.868	0.955	0.3967
229800_at	15.3	31.7	1.05	2.07	AAA-APA	0.10	17	0.868	0.955	0.3386
229768_at	23.6	48.8	1.05	2.07	AAA-PAA	0.10	17	0.868	0.955	0.5210
1553401_at	12.3	25.3	1.05	2.07	AAA-AAP	0.10	17	0.868	0.955	0.4238
1557169_x_at	38.2	78.8	1.04	2.06	AAP-PAP	0.10	17	0.868	0.955	0.3510
1559420_x_at	16.1	33.2	1.04	2.06	AAM-PAA	0.10	17	0.868	0.955	0.5414
240385_at	39.9	82.2	1.04	2.06	PPA-APP	0.10	17	0.868	0.955	0.4353
209348_s_at	15.5	31.8	1.04	2.06	APA-AAA	0.10	17	0.868	0.955	0.5191
244150_at	29.0	59.6	1.04	2.05	AAP-APP	0.10	17	0.868	0.955	0.6020
216344_at	34.6	70.9	1.04	2.05	AAA-PAA	0.10	17	0.868	0.955	0.4034
238393_at	11.2	23.0	1.04	2.05	AAA-APA	0.10	17	0.868	0.955	0.3643
235960_at	28.4	58.2	1.04	2.05	APP-AAP	0.10	17	0.868	0.955	0.4494
244469_at	24.6	50.5	1.04	2.05	AAA-PAP	0.10	17	0.868	0.955	0.3767
215698_at	168.0	344.2	1.04	2.05	PPP-PPP	0.10	17	0.868	0.955	0.4925
1556240_at	30.2	61.7	1.03	2.04	APA-AAA	0.10	17	0.868	0.955	0.4574
223890_at	31.7	64.7	1.03	2.04	AAA-APA	0.10	17	0.868	0.955	0.2584
1556378_a_at	18.0	36.7	1.03	2.04	AAA-PAA	0.10	17	0.868	0.955	0.3327
1563127_at	36.3	73.9	1.03	2.04	APA-AAA	0.10	17	0.868	0.955	0.3566
236805_at	15.1	30.7	1.03	2.04	APA-AAA	0.10	17	0.868	0.955	0.5998
201566_x_at	76.4	155.3	1.02	2.03	AAP-PPP	0.10	17	0.868	0.955	0.3239
227965_at	69.7	141.8	1.02	2.03	MAA-APP	0.10	17	0.868	0.955	0.2590
244729_at	24.2	49.1	1.02	2.03	AAA-APA	0.10	17	0.868	0.955	0.4320
224697_at	127.4	62.7	-1.02	-2.03	APA-AAA	0.10	17	0.868	0.955	0.0199
210882_s_at	28.1	57.0	1.02	2.03	AAA-APA	0.10	17	0.868	0.955	0.3715
1561073_at	38.6	78.4	1.02	2.03	AAA-APA	0.10	17	0.868	0.955	0.3167
219687_at	67.4	136.6	1.02	2.03	APA-AAA	0.10	17	0.868	0.955	0.3675
216758_at	13.7	27.7	1.02	2.02	AAA-PAA	0.10	17	0.868	0.955	0.3546

212555_at	299.5	605.4	1.02	2.02	APA-APA	0.10	17	0.868	0.955	0.2606
1563166_at	28.5	57.6	1.01	2.02	PAA-PAP	0.10	17	0.868	0.955	0.4173
239372_at	19.2	38.8	1.01	2.02	AAA-PMA	0.10	17	0.868	0.955	0.3639
238052_at	56.8	114.7	1.01	2.02	AAA-PPA	0.10	17	0.868	0.955	0.2505
242201_at	64.3	129.7	1.01	2.02	PPA-PAP	0.10	17	0.868	0.955	0.4219
1558841_at	33.3	67.0	1.01	2.01	AAA-APA	0.10	17	0.868	0.955	0.3917
238553_at	29.8	60.1	1.01	2.01	AAA-APA	0.10	17	0.868	0.955	0.3440
224759_s_at	1913.4	952.1	-1.01	-2.01	PPP-PPP	0.10	17	0.868	0.955	0.0135
1553126_a_at	7.9	15.8	1.01	2.01	AAA-APA	0.10	17	0.868	0.955	0.5694
233705_at	26.0	52.2	1.01	2.01	AAP-AMA	0.10	17	0.868	0.955	0.3511
232054_at	26.0	52.2	1.01	2.01	PAA-AAA	0.10	17	0.868	0.955	0.3943
230519_at	33.1	66.4	1.00	2.01	AAA-APA	0.10	17	0.868	0.955	0.4631
204942_s_at	24.5	49.1	1.00	2.01	APA-AAA	0.10	17	0.868	0.955	0.3794
1561887_at	34.1	68.3	1.00	2.00	AAA-PAA	0.10	17	0.868	0.955	0.3749
235798_at	13.9	27.7	1.00	2.00	AAP-AAA	0.10	17	0.868	0.955	0.4701
244071_at	52.7	105.5	1.00	2.00	AAA-PAA	0.10	17	0.868	0.955	0.3040
225387_at	508.0	254.0	-1.00	-2.00	PPP-PPP	0.10	17	0.868	0.955	0.0113
242628_at	25.8	51.6	1.00	2.00	PAA-AAP	0.10	17	0.868	0.955	0.3069
243182_at	51.6	103.0	1.00	2.00	AMA-APA	0.10	17	0.868	0.955	0.3007
1558388_a_at	10.1	20.1	1.00	2.00	AAP-AAA	0.10	17	0.868	0.955	0.5763
229552_at	25.4	50.5	0.99	1.99	AAA-AAP	0.10	17	0.868	0.955	0.5397
1565974_at	58.7	116.8	0.99	1.99	MAP-PAP	0.10	17	0.868	0.955	0.4529
238372_s_at	18.2	36.3	0.99	1.99	AAA-AAP	0.10	17	0.868	0.955	0.4035
1560570_a_at	7.4	14.6	0.99	1.99	AAA-AAP	0.10	17	0.868	0.955	0.5598
1562527_at	20.8	41.3	0.99	1.99	AAA-PPA	0.10	17	0.868	0.955	0.3017
242960_at	28.0	55.7	0.99	1.99	AAA-PPP	0.10	17	0.868	0.955	0.2421
211349_at	38.7	76.8	0.99	1.99	AAA-AAP	0.10	17	0.868	0.955	0.3228
1559755_at	39.8	78.8	0.99	1.98	AAA-APA	0.10	17	0.868	0.955	0.2961
220289_s_at	74.1	146.6	0.99	1.98	AMA-PPA	0.10	17	0.868	0.955	0.4181
219256_s_at	130.8	66.2	-0.98	-1.98	PPM-APA	0.10	17	0.868	0.955	0.0161
223814_at	33.2	65.5	0.98	1.98	AAP-PAA	0.10	17	0.868	0.955	0.4872
1552690_a_at	41.2	81.5	0.98	1.98	AAA-PAA	0.10	17	0.868	0.955	0.2747
240204_at	18.7	36.9	0.98	1.97	AAP-APA	0.10	17	0.868	0.955	0.2744
234424_at	21.5	42.4	0.98	1.97	AAA-AAP	0.10	17	0.868	0.955	0.2460
227686_at	457.1	900.2	0.98	1.97	PAP-PPP	0.10	17	0.868	0.955	0.3070
1563104_at	49.6	97.5	0.98	1.97	AAA-MAP	0.10	17	0.868	0.955	0.2905
242843_at	48.8	96.0	0.98	1.97	PAP-AAP	0.10	17	0.868	0.955	0.2659
1556423_at	9.5	18.6	0.98	1.97	AAA-APA	0.10	17	0.868	0.955	0.5419
210020_x_at	61.7	121.2	0.97	1.96	AAA-AAP	0.10	17	0.868	0.955	0.4644
216809_at	25.6	50.4	0.97	1.96	AAA-APA	0.10	17	0.868	0.955	0.2734
213900_at	55.5	108.9	0.97	1.96	AAA-PPA	0.10	17	0.868	0.955	0.3224
239655_at	26.4	51.8	0.97	1.96	PAA-APP	0.10	17	0.868	0.955	0.4173

244269_at	34.2	67.0	0.97	1.96	APA-AAA	0.10	17	0.868	0.955	0.3985
1552952_at	30.3	59.5	0.97	1.96	APA-AAA	0.10	17	0.868	0.955	0.3507
236946_at	35.1	68.5	0.97	1.95	AAA-AAP	0.10	17	0.868	0.955	0.2498
239129_at	6.6	12.8	0.97	1.95	AAA-AAP	0.10	17	0.868	0.955	0.5594
1561894_at	24.1	47.1	0.97	1.95	AAA-APA	0.10	17	0.868	0.955	0.3477
239778_x_at	19.2	37.4	0.96	1.95	MAA-MPA	0.10	17	0.868	0.955	0.3943
239430_at	324.9	633.3	0.96	1.95	APP-PPP	0.10	17	0.868	0.955	0.2590
236002_at	6.2	12.0	0.96	1.95	AAA-AAP	0.10	17	0.868	0.955	0.5331
221209_s_at	17.0	33.2	0.96	1.95	AAP-AAA	0.10	17	0.868	0.955	0.4178
1554997_a_at	20.1	39.1	0.96	1.95	AAP-PAA	0.10	17	0.868	0.955	0.3794
243640_x_at	31.0	60.4	0.96	1.95	PAA-PPA	0.10	17	0.868	0.955	0.4622
206816_s_at	28.7	55.8	0.96	1.95	AAP-AAA	0.10	17	0.868	0.955	0.3758
243649_at	35.6	69.2	0.96	1.94	AAA-PAP	0.10	17	0.868	0.955	0.4116
220134_x_at	115.7	224.7	0.96	1.94	PAA-PAA	0.10	17	0.868	0.955	0.4357
238210_at	36.9	71.5	0.96	1.94	PAA-AAP	0.10	17	0.868	0.955	0.3845
210961_s_at	19.5	37.7	0.96	1.94	AAA-AAP	0.10	17	0.868	0.955	0.4850
1556938_a_at	26.3	50.9	0.95	1.94	MPA-AAP	0.10	17	0.868	0.955	0.4706
205974_at	9.7	18.8	0.95	1.94	PAA-AAA	0.10	17	0.868	0.955	0.4762
204365_s_at	58.4	113.0	0.95	1.93	AAA-APA	0.10	17	0.868	0.955	0.3918
204611_s_at	187.6	362.2	0.95	1.93	AAP-AMA	0.10	17	0.868	0.955	0.2717
40524_at	36.2	69.9	0.95	1.93	AAP-APA	0.10	17	0.868	0.955	0.2569
239561_at	43.4	83.7	0.95	1.93	AAA-APP	0.10	17	0.868	0.955	0.3793
1556294_at	19.6	37.8	0.95	1.93	AAA-APA	0.10	17	0.868	0.955	0.4827
234643_x_at	41.4	79.6	0.94	1.92	AAA-PAA	0.10	17	0.868	0.955	0.4075
230960_at	23.7	45.6	0.94	1.92	AAA-AAP	0.10	17	0.868	0.955	0.4200
219724_s_at	43.6	83.8	0.94	1.92	AAP-AAP	0.10	17	0.868	0.955	0.3376
210780_at	44.6	85.7	0.94	1.92	PAA-APP	0.10	17	0.868	0.955	0.2812
1552955_at	22.6	43.4	0.94	1.92	AAA-PAA	0.10	17	0.868	0.955	0.5397
233151_s_at	28.7	54.9	0.94	1.92	AAA-APA	0.10	17	0.868	0.955	0.4896
1555176_at	21.7	41.6	0.94	1.92	AAA-AAP	0.10	17	0.868	0.955	0.3878
233608_at	49.5	94.9	0.94	1.92	AAA-PAP	0.10	17	0.868	0.955	0.2608
210969_at	17.1	32.6	0.94	1.91	AAA-AAP	0.10	17	0.868	0.955	0.2532
219983_at	40.6	77.4	0.93	1.91	AAP-APA	0.10	17	0.868	0.955	0.3344
241569_at	19.7	37.6	0.93	1.91	AAA-APP	0.10	17	0.868	0.955	0.3881
241495_at	22.6	43.1	0.93	1.91	AAA-PAA	0.10	17	0.868	0.955	0.3032
223643_at	23.1	43.9	0.93	1.90	AAA-APA	0.10	17	0.868	0.955	0.3992
233777_at	32.8	62.2	0.92	1.90	PAA-AAP	0.10	17	0.868	0.955	0.3457
240967_at	22.0	41.8	0.92	1.90	AAA-APA	0.10	17	0.868	0.955	0.3213
222868_s_at	42.2	79.9	0.92	1.90	AAA-PAA	0.10	17	0.868	0.955	0.3784
1568012_at	10.3	19.6	0.92	1.89	AAA-APA	0.10	17	0.868	0.955	0.5735
234118_at	10.0	18.9	0.92	1.89	AAA-AAP	0.10	17	0.868	0.955	0.4911
237375_at	20.7	39.2	0.92	1.89	PAA-AAA	0.10	17	0.868	0.955	0.4000

1556518_at	20.3	38.4	0.92	1.89	PAM-AAP	0.10	17	0.868	0.955	0.3947
205317_s_at	39.5	74.8	0.92	1.89	AAA-AAP	0.10	17	0.868	0.955	0.4467
216703_at	25.3	47.8	0.92	1.89	AAA-APA	0.10	17	0.868	0.955	0.3926
236818_at	29.5	55.7	0.92	1.89	PAA-PPP	0.10	17	0.868	0.955	0.4810
242993_at	27.2	51.4	0.92	1.89	APP-AAP	0.10	17	0.868	0.955	0.3862
206965_at	28.9	54.6	0.92	1.89	APA-PPP	0.10	17	0.868	0.955	0.2999
227253_at	24.5	46.2	0.91	1.88	AAA-PMA	0.10	17	0.868	0.955	0.4199
228800_x_at	83.6	157.3	0.91	1.88	APP-PPP	0.10	17	0.868	0.955	0.4881
216946_at	25.6	48.2	0.91	1.88	AAP-AAA	0.10	17	0.868	0.955	0.3879
239611_at	23.7	44.5	0.91	1.88	APA-AAA	0.10	17	0.868	0.955	0.3586
241752_at	14.0	26.1	0.91	1.87	AAA-PAA	0.10	17	0.868	0.955	0.5521
215796_at	11.8	22.1	0.91	1.87	PAA-AAA	0.10	17	0.868	0.955	0.5302
1565887_at	17.5	32.8	0.91	1.87	AAA-PAA	0.10	17	0.868	0.955	0.2713
242713_at	108.5	58.0	-0.90	-1.87	PAA-AAA	0.10	17	0.868	0.955	0.0165
233662_at	2.0	3.8	0.90	1.87	AAA-APA	0.10	17	0.868	0.955	0.5837
234034_at	22.6	42.3	0.90	1.87	AAA-AAP	0.10	17	0.868	0.955	0.2834
1553585_a_at	44.4	83.0	0.90	1.87	PAA-AAA	0.10	17	0.868	0.955	0.5146
232913_at	57.2	106.8	0.90	1.87	AAA-PAP	0.10	17	0.868	0.955	0.3279
226418_at	46.3	86.5	0.90	1.87	AAA-APA	0.10	17	0.868	0.955	0.3410
244492_at	41.6	77.5	0.90	1.87	AAP-AAA	0.10	17	0.868	0.955	0.3201
1562387_at	19.9	37.0	0.90	1.86	AMA-AAP	0.10	17	0.868	0.955	0.4955
216895_at	26.6	49.5	0.90	1.86	PAP-PAA	0.10	17	0.868	0.955	0.4334
1566599_at	29.2	54.4	0.90	1.86	AAA-APA	0.10	17	0.868	0.955	0.3855
239175_at	8.9	16.6	0.90	1.86	AAA-AAP	0.10	17	0.868	0.955	0.6350
236365_at	29.5	54.9	0.90	1.86	PAA-PPP	0.10	17	0.868	0.955	0.5559
206449_s_at	23.2	43.1	0.90	1.86	APA-AMM	0.10	17	0.868	0.955	0.4877
205630_at	25.0	46.4	0.90	1.86	PAA-AAA	0.10	17	0.868	0.955	0.4466
221451_s_at	17.7	33.0	0.89	1.86	AAA-PAA	0.10	17	0.868	0.955	0.3260
1556903_at	19.8	36.7	0.89	1.86	AAA-AAP	0.10	17	0.868	0.955	0.2994
206005_s_at	81.1	150.6	0.89	1.86	PPA-APP	0.10	17	0.868	0.955	0.3927
220823_at	35.2	65.4	0.89	1.86	APA-AAA	0.10	17	0.868	0.955	0.3568
240456_at	36.4	67.4	0.89	1.85	APA-AAA	0.10	17	0.868	0.955	0.3915
236404_at	37.6	69.6	0.89	1.85	AAA-APP	0.10	17	0.868	0.955	0.2368
231939_s_at	71.6	132.5	0.89	1.85	PAP-PPA	0.10	17	0.868	0.955	0.3291
236931_at	33.1	61.3	0.89	1.85	PAA-AMA	0.10	17	0.868	0.955	0.3198
1570441_at	47.8	88.5	0.89	1.85	AAA-AAP	0.10	17	0.868	0.955	0.3025
243652_at	5.4	10.0	0.89	1.85	AAA-AAP	0.10	17	0.868	0.955	0.5220
236457_at	31.9	59.0	0.89	1.85	AAP-AAP	0.10	17	0.868	0.955	0.4501
1564960_at	45.1	83.4	0.89	1.85	AAA-PAP	0.10	17	0.868	0.955	0.3982
206084_at	9.7	18.0	0.89	1.85	AAA-AAP	0.10	17	0.868	0.955	0.6122
1569108_a_at	24.3	44.8	0.89	1.85	APA-PAP	0.10	17	0.868	0.955	0.4366
1561418_at	42.0	77.6	0.88	1.85	AAA-PPA	0.10	17	0.868	0.955	0.2973

236446_at	40.9	75.2	0.88	1.84	AAA-PPA	0.10	17	0.868	0.955	0.2504
237102_at	6.3	11.6	0.88	1.84	AAP-AAA	0.10	17	0.868	0.955	0.6189
213307_at	19.0	35.0	0.88	1.84	AAA-APA	0.10	17	0.868	0.955	0.3830
244611_at	21.4	39.3	0.88	1.84	AAA-PAA	0.10	17	0.868	0.955	0.6073
240634_x_at	19.2	35.4	0.88	1.84	APP-APA	0.10	17	0.868	0.955	0.5704
214802_at	46.0	84.5	0.88	1.84	AAA-AAP	0.10	17	0.868	0.955	0.3693
1559607_s_at	23.3	42.8	0.88	1.84	APA-AAA	0.10	17	0.868	0.955	0.5305
1561449_at	28.1	51.5	0.88	1.83	AAP-AAA	0.10	17	0.868	0.955	0.3820
235719_at	19.6	35.9	0.87	1.83	APA-AAA	0.10	17	0.868	0.955	0.4867
236862_at	43.9	80.5	0.87	1.83	AAP-AAA	0.10	17	0.868	0.955	0.2355
226497_s_at	26.9	49.2	0.87	1.83	AAP-AAA	0.10	17	0.868	0.955	0.3856
238292_at	22.4	40.9	0.87	1.83	AAA-PAA	0.10	17	0.868	0.955	0.5447
217051_s_at	43.9	80.3	0.87	1.83	PAA-AAA	0.10	17	0.868	0.955	0.4458
230500_at	36.7	67.1	0.87	1.83	AAA-APA	0.10	17	0.868	0.955	0.3417
207973_x_at	41.6	76.0	0.87	1.83	MAA-PPA	0.10	17	0.868	0.955	0.3305
213382_at	8.1	14.7	0.87	1.83	AAA-APA	0.10	17	0.868	0.955	0.5482
229037_at	56.9	103.9	0.87	1.83	AMA-PAA	0.10	17	0.868	0.955	0.4667
234362_s_at	27.1	49.3	0.87	1.82	AAA-APA	0.10	17	0.868	0.955	0.3830
244098_at	19.4	35.2	0.86	1.82	PAA-MAA	0.10	17	0.868	0.955	0.3679
1570107_at	62.9	114.2	0.86	1.82	AAP-AAA	0.10	17	0.868	0.955	0.2402
209829_at	24.3	44.2	0.86	1.81	APA-AAA	0.10	17	0.868	0.955	0.4525
236389_x_at	61.2	111.0	0.86	1.82	PMA-AAA	0.10	17	0.868	0.955	0.3646
1555117_at	19.9	36.1	0.86	1.81	AAA-PAA	0.10	17	0.868	0.955	0.6148
1556896_at	29.6	53.6	0.86	1.81	AAA-AMP	0.10	17	0.868	0.955	0.5294
1570022_at	32.4	58.7	0.86	1.81	PAA-AAA	0.10	17	0.868	0.955	0.4555
214131_at	22.4	40.5	0.86	1.81	AAA-PAA	0.10	17	0.868	0.955	0.4205
211590_x_at	90.9	164.7	0.86	1.81	AAA-AAP	0.10	17	0.868	0.955	0.2997
1570183_at	14.0	25.3	0.86	1.81	AAA-APA	0.10	17	0.868	0.955	0.3647
1562472_at	25.7	46.4	0.86	1.81	AMA-PPP	0.10	17	0.868	0.955	0.4610
237569_at	24.5	44.4	0.86	1.81	AAA-PAA	0.10	17	0.868	0.955	0.3604
211340_s_at	453.7	250.8	-0.86	-1.81	PPP-PPP	0.10	17	0.868	0.955	0.0111
211516_at	12.9	23.3	0.86	1.81	AAA-APA	0.10	17	0.868	0.955	0.5672
240654_at	41.9	75.8	0.86	1.81	AAA-AAP	0.10	17	0.868	0.955	0.3769
244111_at	35.3	63.7	0.85	1.81	AMA-APP	0.10	17	0.868	0.955	0.2736
213411_at	36.2	65.3	0.85	1.81	AAP-PAA	0.10	17	0.868	0.955	0.3935
204666_s_at	155.2	86.0	-0.85	-1.80	PPP-APP	0.10	17	0.868	0.955	0.0096
231465_at	37.1	66.9	0.85	1.80	AAP-AAA	0.10	17	0.868	0.955	0.4008
1559503_a_at	20.2	36.4	0.85	1.80	PAA-AMA	0.10	17	0.868	0.955	0.3416
229561_at	57.2	103.0	0.85	1.80	AAA-PPP	0.10	17	0.868	0.955	0.3861
227386_s_at	49.0	88.2	0.85	1.80	AAA-AAP	0.10	17	0.868	0.955	0.3640
1558822_at	75.9	136.4	0.85	1.80	PAA-PAP	0.10	17	0.868	0.955	0.3688
232413_at	49.2	88.4	0.85	1.80	PAP-PAP	0.10	17	0.868	0.955	0.3292

1565917_at	24.4	43.8	0.85	1.80	APA-AAP	0.10	17	0.868	0.955	0.5243
1553876_at	17.4	31.2	0.84	1.79	AAA-PAA	0.10	17	0.868	0.955	0.5906
240926_at	48.8	87.3	0.84	1.79	AAA-AAP	0.10	17	0.868	0.955	0.4661
235047_x_at	35.1	62.8	0.84	1.79	PAA-AAA	0.10	17	0.868	0.955	0.5482
217920_at	137.0	245.2	0.84	1.79	PAP-PPP	0.10	17	0.868	0.955	0.4462
1555261_at	49.7	88.9	0.84	1.79	AAA-APA	0.10	17	0.868	0.955	0.2305
1552372_at	6.0	10.8	0.84	1.79	AAP-AAA	0.10	17	0.868	0.955	0.6058
229273_at	11.8	21.1	0.84	1.79	AAA-APM	0.10	17	0.868	0.955	0.4940
240694_at	17.5	31.3	0.84	1.79	AAA-APA	0.10	17	0.868	0.955	0.4432
244668_at	20.3	36.3	0.84	1.79	AAA-APA	0.10	17	0.868	0.955	0.5762
229155_at	69.8	124.7	0.84	1.79	PAA-PAA	0.10	17	0.868	0.955	0.5438
218033_s_at	39.8	71.1	0.84	1.79	APP-APA	0.10	17	0.868	0.955	0.3011
210366_at	13.5	24.1	0.84	1.79	APA-AAA	0.10	17	0.868	0.955	0.5766
216735_x_at	29.0	51.7	0.84	1.79	AAA-PAA	0.10	17	0.868	0.955	0.5375
240769_at	55.2	31.0	-0.83	-1.78	MPA-AAA	0.10	17	0.868	0.955	0.0137
218759_at	77.4	137.9	0.83	1.78	AAP-AAA	0.10	17	0.868	0.955	0.5080
1553237_x_at	52.0	92.7	0.83	1.78	AAA-AAP	0.10	17	0.868	0.955	0.2595
209598_at	24.2	43.0	0.83	1.78	PAA-AAA	0.10	17	0.868	0.955	0.5214
244056_at	19.5	34.6	0.83	1.78	APA-AAA	0.10	17	0.868	0.955	0.5421
211097_s_at	59.6	105.9	0.83	1.78	AAP-PPM	0.10	17	0.868	0.955	0.4353
205540_s_at	73.6	130.8	0.83	1.78	PAA-APP	0.10	17	0.868	0.955	0.2785
1563536_at	23.3	41.4	0.83	1.78	AAP-AAA	0.10	17	0.868	0.955	0.2372
240892_at	17.7	31.3	0.83	1.77	AAP-AAP	0.10	17	0.868	0.955	0.5679
238311_at	30.3	53.7	0.83	1.77	AAP-PPP	0.10	17	0.868	0.955	0.3464
238270_x_at	34.0	60.3	0.83	1.77	PAA-AAA	0.10	17	0.868	0.955	0.3853
237849_at	21.4	37.8	0.82	1.77	AAA-APA	0.10	17	0.868	0.955	0.5237
1563949_at	49.6	87.7	0.82	1.77	AAP-PPA	0.10	17	0.868	0.955	0.5163
1568974_at	48.7	86.2	0.82	1.77	APA-PAA	0.10	17	0.868	0.955	0.3707
240604_at	27.6	48.8	0.82	1.77	AAP-PAA	0.10	17	0.868	0.955	0.3403
230005_at	168.9	298.5	0.82	1.77	PPP-PPP	0.10	17	0.868	0.955	0.2704
1566697_at	30.5	53.8	0.82	1.77	AAA-APA	0.10	17	0.868	0.955	0.4728
213678_at	43.6	76.9	0.82	1.77	AAP-PPA	0.10	17	0.868	0.955	0.4521
1562736_at	14.1	24.9	0.82	1.77	APA-AAA	0.10	17	0.868	0.955	0.5924
220509_at	41.2	72.8	0.82	1.77	PAP-PAP	0.10	17	0.868	0.955	0.4417
222925_at	23.0	40.6	0.82	1.76	AAA-PAA	0.10	17	0.868	0.955	0.2547
1553301_a_at	24.2	42.5	0.82	1.76	AAA-AAP	0.10	17	0.868	0.955	0.4131
215506_s_at	22.1	38.8	0.81	1.76	AAA-AAP	0.10	17	0.868	0.955	0.2923
239004_at	102.0	179.2	0.81	1.76	APA-AAP	0.10	17	0.868	0.955	0.5274
1557446_x_at	8.0	14.0	0.81	1.76	AAA-PAA	0.10	17	0.868	0.955	0.5907
240606_at	50.4	88.3	0.81	1.75	AAA-AMP	0.10	17	0.868	0.955	0.3143
1557659_a_at	31.0	54.4	0.81	1.75	APA-AAA	0.10	17	0.868	0.955	0.4992
223103_at	766.6	437.7	-0.81	-1.75	PPP-PPP	0.10	17	0.868	0.955	0.0121

216690_at	21.9	38.3	0.81	1.75	PAA-AAM	0.10	17	0.868	0.955	0.4918
207097_s_at	4.7	8.1	0.81	1.75	AAA-AAP	0.10	17	0.868	0.955	0.5026
234395_at	37.3	65.2	0.81	1.75	AAA-AAP	0.10	17	0.868	0.955	0.3154
243003_at	82.9	145.0	0.81	1.75	PAA-PPP	0.10	17	0.868	0.955	0.2619
235802_at	21.5	37.5	0.81	1.75	APA-AAA	0.10	17	0.868	0.955	0.5861
1559018_at	41.7	72.9	0.81	1.75	PAA-PPM	0.10	17	0.868	0.955	0.2632
223817_at	12.1	21.1	0.80	1.75	AAA-PAA	0.10	17	0.868	0.955	0.5055
207301_at	26.7	46.7	0.80	1.75	AAA-PAA	0.10	17	0.868	0.955	0.4951
242943_at	20.0	35.0	0.80	1.75	AAA-AAP	0.10	17	0.868	0.955	0.5401
213772_s_at	68.2	119.1	0.80	1.75	AAA-APP	0.10	17	0.868	0.955	0.4769
117_at	47.6	83.0	0.80	1.74	AAA-PAA	0.10	17	0.868	0.955	0.4254
1564932_at	20.8	36.3	0.80	1.74	APA-AAA	0.10	17	0.868	0.955	0.5087
241913_at	27.9	48.6	0.80	1.74	AAP-PAP	0.10	17	0.868	0.955	0.3008
202768_at	114.5	199.2	0.80	1.74	AAA-AAP	0.10	17	0.868	0.955	0.3575
213364_s_at	157.4	273.9	0.80	1.74	PPP-PPP	0.10	17	0.868	0.955	0.3519
207160_at	26.1	45.3	0.80	1.74	PAP-AAA	0.10	17	0.868	0.955	0.5486
201006_at	169.2	293.9	0.80	1.74	PAP-PMP	0.10	17	0.868	0.955	0.2706
206576_s_at	55.6	96.6	0.80	1.74	AAA-APA	0.10	17	0.868	0.955	0.2445
1558561_at	34.8	60.3	0.79	1.73	AAM-APA	0.10	17	0.868	0.955	0.5582
210525_x_at	32.8	56.8	0.79	1.73	AAA-PAA	0.10	17	0.868	0.955	0.4176
235839_at	29.2	50.6	0.79	1.73	AAA-PAA	0.10	17	0.868	0.955	0.5543
220523_at	9.6	16.6	0.79	1.73	AAA-AAP	0.10	17	0.868	0.955	0.5491
228833_s_at	15.1	26.1	0.79	1.73	APA-AAA	0.10	17	0.868	0.955	0.5903
1494_f_at	77.9	134.8	0.79	1.73	PAA-PAA	0.10	17	0.868	0.955	0.4557
236019_at	89.7	155.2	0.79	1.73	PAA-APP	0.10	17	0.868	0.955	0.3127
214603_at	14.3	24.7	0.79	1.73	PAA-AAA	0.10	17	0.868	0.955	0.5422
239903_at	43.3	74.9	0.79	1.73	AAA-MAP	0.10	17	0.868	0.955	0.4741
225054_x_at	33.1	57.3	0.79	1.73	AAP-AAP	0.10	17	0.868	0.955	0.4115
217579_x_at	178.7	308.9	0.79	1.73	APA-AMP	0.10	17	0.868	0.955	0.2560
236257_at	26.8	46.3	0.79	1.73	AAA-APA	0.10	17	0.868	0.955	0.2452
1568720_at	22.3	38.5	0.79	1.73	AAP-APM	0.10	17	0.868	0.955	0.3886
215875_at	45.1	77.8	0.79	1.73	APA-AAA	0.10	17	0.868	0.955	0.3785
203587_at	17.4	30.0	0.79	1.72	AAA-APA	0.10	17	0.868	0.955	0.5243
242609_x_at	124.5	214.6	0.79	1.72	PAA-AMA	0.10	17	0.868	0.955	0.2715
237001_at	67.9	117.2	0.79	1.73	PPP-PPP	0.10	17	0.868	0.955	0.2305
1560349_at	15.3	26.3	0.79	1.72	PAA-PAP	0.10	17	0.868	0.955	0.6478
216057_at	22.5	38.7	0.78	1.72	AAP-AAP	0.10	17	0.868	0.955	0.6064
1556336_at	46.4	79.8	0.78	1.72	AMP-AAP	0.10	17	0.868	0.955	0.3951
212654_at	37.2	64.0	0.78	1.72	APP-PPA	0.10	17	0.868	0.955	0.6463
1564707_x_at	26.5	45.5	0.78	1.72	AAP-PPA	0.10	17	0.868	0.955	0.2996
212364_at	8718.3	5073.6	-0.78	-1.72	PPP-PPP	0.10	17	0.868	0.955	0.0091
1559848_at	17.8	30.6	0.78	1.72	AAA-PAP	0.10	17	0.868	0.955	0.6420

232081_at	57.3	98.5	0.78	1.72	MPA-AAP	0.10	17	0.868	0.955	0.6076
220575_at	22.6	38.8	0.78	1.72	APA-AAP	0.10	17	0.868	0.955	0.6338
239629_at	127.7	219.2	0.78	1.72	PPA-PPP	0.10	17	0.868	0.955	0.2949
239723_at	12.2	20.9	0.78	1.72	AAA-AAP	0.10	17	0.868	0.955	0.6103
242280_x_at	39.5	67.8	0.78	1.72	PMA-AAM	0.10	17	0.868	0.955	0.4547
1565660_at	74.7	128.2	0.78	1.72	AAA-AAP	0.10	17	0.868	0.955	0.4878
215468_at	14.0	23.9	0.78	1.72	AAA-AAP	0.10	17	0.868	0.955	0.3968
228347_at	78.9	135.2	0.78	1.71	PPP-PPP	0.10	17	0.868	0.955	0.2746
243435_at	70.7	120.8	0.77	1.71	AAA-APA	0.10	17	0.868	0.955	0.3477
235984_at	83.7	143.0	0.77	1.71	PAA-AAA	0.10	17	0.868	0.955	0.3032
1564736_a_at	36.1	61.6	0.77	1.71	AAA-APA	0.10	17	0.868	0.955	0.3910
210932_s_at	156.0	266.4	0.77	1.71	PPP-MPP	0.10	17	0.868	0.955	0.3263
229892_at	204.7	349.4	0.77	1.71	PAP-MMP	0.10	17	0.868	0.955	0.2539
1561225_at	37.2	63.5	0.77	1.71	AAA-AAP	0.10	17	0.868	0.955	0.5422
243951_at	23.0	39.2	0.77	1.71	PAA-AAA	0.10	17	0.868	0.955	0.4916
1557505_a_at	40.1	68.3	0.77	1.71	AAA-PPP	0.10	17	0.868	0.955	0.2403
229796_at	618.2	362.9	-0.77	-1.70	PPP-PPP	0.10	17	0.868	0.955	0.0120
217142_at	221.0	376.2	0.77	1.70	AAA-PPP	0.10	17	0.868	0.955	0.4196
239209_at	86.8	147.9	0.77	1.70	PPP-PPP	0.10	17	0.868	0.955	0.2782
232126_at	39.9	68.0	0.77	1.70	PAA-AAA	0.10	17	0.868	0.955	0.2538
1559975_at	22.3	37.9	0.77	1.70	APA-AMP	0.10	17	0.868	0.955	0.4489
239419_at	54.1	91.9	0.77	1.70	AAA-PAP	0.10	17	0.868	0.955	0.3517
232002_at	52.1	88.6	0.77	1.70	AAP-AAA	0.10	17	0.868	0.955	0.5250
227842_at	18.6	31.6	0.77	1.70	AAA-AAP	0.10	17	0.868	0.955	0.3205
214609_at	49.5	84.1	0.76	1.70	APA-AAA	0.10	17	0.868	0.955	0.6239
241017_at	26.2	44.6	0.76	1.70	PAA-AAM	0.10	17	0.868	0.955	0.5942
235804_at	117.6	199.6	0.76	1.70	APA-MPP	0.10	17	0.868	0.955	0.2509
234509_at	24.9	42.2	0.76	1.70	APA-PPA	0.10	17	0.868	0.955	0.5967
1562398_at	29.1	49.2	0.76	1.69	AAA-PPA	0.10	17	0.868	0.955	0.4419
239567_at	30.5	51.7	0.76	1.70	AAP-PAA	0.10	17	0.868	0.955	0.3481
240366_at	7.2	12.2	0.76	1.69	AAA-PAA	0.10	17	0.868	0.955	0.6310
236760_at	50.5	85.5	0.76	1.69	AAM-AAP	0.10	17	0.868	0.955	0.4942
227888_at	32.6	55.1	0.76	1.69	AAA-PAA	0.10	17	0.868	0.955	0.6459
1569973_at	40.6	68.6	0.76	1.69	APA-AAA	0.10	17	0.868	0.955	0.5704
237095_at	50.7	85.7	0.76	1.69	PAP-APP	0.10	17	0.868	0.955	0.3568
206887_at	92.0	155.6	0.76	1.69	AAA-APA	0.10	17	0.868	0.955	0.3301
231791_at	41.9	70.8	0.76	1.69	AAA-PAA	0.10	17	0.868	0.955	0.3554
1559746_a_at	46.6	78.8	0.76	1.69	APA-PPP	0.10	17	0.868	0.955	0.3216
210595_at	37.1	62.6	0.76	1.69	AAA-APA	0.10	17	0.868	0.955	0.2367
221323_at	47.9	80.8	0.76	1.69	AAP-PPA	0.10	17	0.868	0.955	0.5078
1561917_at	35.1	59.2	0.76	1.69	AAA-APA	0.10	17	0.868	0.955	0.4474
201171_at	246.1	145.9	-0.75	-1.69	PPP-PPP	0.10	17	0.868	0.955	0.0117

232082_x_at	52.4	88.4	0.75	1.69	AAA-AAP	0.10	17	0.868	0.955	0.4658
217068_at	30.5	51.4	0.75	1.69	AAA-APA	0.10	17	0.868	0.955	0.4622
1561050_a_at	20.6	34.8	0.75	1.68	AAA-PAA	0.10	17	0.868	0.955	0.5500
237218_at	40.5	68.3	0.75	1.69	PAA-AAP	0.10	17	0.868	0.955	0.3451
209098_s_at	295.7	175.7	-0.75	-1.68	PPP-PPP	0.10	17	0.868	0.955	0.0132
211329_x_at	106.8	179.8	0.75	1.68	APP-PPP	0.10	17	0.868	0.955	0.5510
244087_at	42.6	71.7	0.75	1.68	PAA-AAA	0.10	17	0.868	0.955	0.3877
208015_at	50.7	85.3	0.75	1.68	AAA-PAA	0.10	17	0.868	0.955	0.2295
238430_x_at	597.5	355.5	-0.75	-1.68	PPP-PPP	0.10	17	0.868	0.955	0.0072
230415_at	47.4	79.6	0.75	1.68	AAP-AAP	0.10	17	0.868	0.955	0.5209
1557620_a_at	41.2	69.2	0.75	1.68	AAA-PAA	0.10	17	0.868	0.955	0.4997
205923_at	37.4	62.8	0.75	1.68	PAA-AAA	0.10	17	0.868	0.955	0.4597
207755_at	40.0	67.1	0.75	1.68	PAM-PAP	0.10	17	0.868	0.955	0.4453
228197_at	69.2	116.2	0.75	1.68	PAA-PAA	0.10	17	0.868	0.955	0.2555
231495_at	26.3	44.3	0.75	1.68	AAA-MAP	0.10	17	0.868	0.955	0.2480
244443_at	46.4	77.9	0.75	1.68	PPA-APP	0.10	17	0.868	0.955	0.4436
223709_s_at	60.4	101.4	0.75	1.68	APP-APP	0.10	17	0.868	0.955	0.2443
230783_at	11.6	19.5	0.75	1.68	APA-AAA	0.10	17	0.868	0.955	0.6062
220429_at	22.8	38.2	0.75	1.68	APA-AAA	0.10	17	0.868	0.955	0.5880
233193_x_at	218.8	366.7	0.75	1.68	PPP-PPP	0.10	17	0.868	0.955	0.4423
213952_s_at	25.2	42.2	0.75	1.68	AAA-AAP	0.10	17	0.868	0.955	0.4279
232580_x_at	78.8	132.0	0.75	1.68	PAA-AAP	0.10	17	0.868	0.955	0.3061
240046_at	51.4	86.1	0.75	1.68	PAA-APP	0.10	17	0.868	0.955	0.2513
214763_at	332.8	198.7	-0.74	-1.67	MPP-APA	0.10	17	0.868	0.955	0.0100
207123_s_at	54.3	91.0	0.74	1.68	PMA-MAA	0.10	17	0.868	0.955	0.4632
1556063_s_at	43.2	72.3	0.74	1.67	AAP-AAP	0.10	17	0.868	0.955	0.5837
241455_at	54.5	91.0	0.74	1.67	AAA-MAP	0.10	17	0.868	0.955	0.2984
241824_at	36.8	61.5	0.74	1.67	APA-AMP	0.10	17	0.868	0.955	0.2743
230921_s_at	92.2	153.7	0.74	1.67	APA-AAA	0.10	17	0.868	0.955	0.4543
240130_at	31.7	52.8	0.74	1.67	MAA-AAP	0.10	17	0.868	0.955	0.3957
233280_at	40.2	67.0	0.74	1.67	AAA-AAP	0.10	17	0.868	0.955	0.3009
1569668_at	33.5	55.8	0.74	1.66	PAA-PPA	0.10	17	0.868	0.955	0.5008
1562116_at	33.0	54.9	0.74	1.66	PAA-AAA	0.10	17	0.868	0.955	0.4734
236380_at	62.3	103.7	0.73	1.66	AAM-AMP	0.10	17	0.868	0.955	0.3332
1555319_at	38.6	64.3	0.73	1.66	AAA-PAA	0.10	17	0.868	0.955	0.2408
1570507_at	24.2	40.3	0.73	1.66	AAA-AAP	0.10	17	0.868	0.955	0.5996
203249_at	158.6	263.5	0.73	1.66	MAA-PAP	0.10	17	0.868	0.955	0.4671
237971_at	25.7	42.6	0.73	1.66	APA-AAP	0.10	17	0.868	0.955	0.6361
216588_at	12.7	21.2	0.73	1.66	AAA-AAP	0.10	17	0.868	0.955	0.6239
243324_x_at	28.3	47.1	0.73	1.66	APA-PPP	0.10	17	0.868	0.955	0.3773
218990_s_at	85.8	142.6	0.73	1.66	AAA-AAP	0.10	17	0.868	0.955	0.3309
232242_at	6.4	10.6	0.73	1.66	AAA-APA	0.10	17	0.868	0.955	0.5309

244679_at	41.5	68.9	0.73	1.66	PPA-PMP	0.10	17	0.868	0.955	0.4571
211241_at	210.6	349.6	0.73	1.66	APA-APP	0.10	17	0.868	0.955	0.3919
238448_at	142.2	235.9	0.73	1.66	PPP-PAP	0.10	17	0.868	0.955	0.3196
1563228_x_at	19.9	33.0	0.73	1.66	AAA-PAP	0.10	17	0.868	0.955	0.5546
1552622_s_at	331.3	549.2	0.73	1.66	PPP-PPP	0.10	17	0.868	0.955	0.2501
205528_s_at	13.5	22.4	0.73	1.66	AAA-AAP	0.10	17	0.868	0.955	0.4413
220266_s_at	77.3	127.8	0.73	1.65	AAA-AAP	0.10	17	0.868	0.955	0.4218
1553983_at	344.0	568.9	0.73	1.65	PAP-PMP	0.10	17	0.868	0.955	0.3256
232991_at	81.4	134.6	0.73	1.66	APP-APP	0.10	17	0.868	0.955	0.2855
230757_at	41.9	69.3	0.73	1.65	APA-APA	0.10	17	0.868	0.955	0.4786
221964_at	160.8	265.8	0.73	1.65	AAP-PPP	0.10	17	0.868	0.955	0.2586
1570130_at	34.2	56.6	0.73	1.65	AAA-APP	0.10	17	0.868	0.955	0.2372
236154_at	27.0	44.6	0.72	1.65	AAP-PAA	0.10	17	0.868	0.955	0.5104
235251_at	54.0	89.2	0.72	1.65	AAA-AAP	0.10	17	0.868	0.955	0.4311
1558409_at	46.4	76.6	0.72	1.65	APP-PAP	0.10	17	0.868	0.955	0.4261
227326_at	307.4	186.4	-0.72	-1.65	PPP-PPP	0.10	17	0.868	0.955	0.0060
242172_at	22.3	36.7	0.72	1.65	AAA-PAA	0.10	17	0.868	0.955	0.5618
211287_x_at	19.4	31.9	0.72	1.65	AAP-AAA	0.10	17	0.868	0.955	0.5191
242488_at	20.6	34.0	0.72	1.65	AAA-AAP	0.10	17	0.868	0.955	0.5991
1554983_at	22.5	37.0	0.72	1.65	AAA-PAA	0.10	17	0.868	0.955	0.5250
244294_at	48.2	79.4	0.72	1.65	PAP-AAM	0.10	17	0.868	0.955	0.4070
1563165_at	36.7	60.4	0.72	1.65	PAA-AAP	0.10	17	0.868	0.955	0.4413
216239_at	51.1	84.1	0.72	1.65	APA-AAA	0.10	17	0.868	0.955	0.3992
228223_at	63.1	103.8	0.72	1.65	AAA-APA	0.10	17	0.868	0.955	0.2550
241076_at	48.0	78.8	0.72	1.64	AAA-AAP	0.10	17	0.868	0.955	0.4508
243922_at	61.8	101.6	0.72	1.64	APA-AAP	0.10	17	0.868	0.955	0.2993
235021_at	41.7	68.5	0.72	1.64	AAP-APP	0.10	17	0.868	0.955	0.5221
226306_at	207.0	339.6	0.71	1.64	PAA-PPP	0.10	17	0.868	0.955	0.3243
222406_s_at	397.9	652.1	0.71	1.64	PPP-PPP	0.10	17	0.868	0.955	0.4028
217328_at	37.3	61.2	0.71	1.64	APA-AAA	0.10	17	0.868	0.955	0.3682
242284_at	38.9	63.7	0.71	1.64	PAA-AAA	0.10	17	0.868	0.955	0.3068
206498_at	29.5	48.2	0.71	1.64	AAA-PAA	0.10	17	0.868	0.955	0.2709
210687_at	46.9	76.9	0.71	1.64	AAP-AAP	0.10	17	0.868	0.955	0.2391
206078_at	42.3	69.2	0.71	1.64	APA-AAA	0.10	17	0.868	0.955	0.4687
1553821_at	40.5	66.2	0.71	1.64	APA-MAA	0.10	17	0.868	0.955	0.4676
1555133_at	18.0	29.4	0.71	1.64	AAA-PAA	0.10	17	0.868	0.955	0.4365
232447_at	83.0	136.0	0.71	1.64	PAA-PAP	0.10	17	0.868	0.955	0.3716
240442_at	23.3	38.2	0.71	1.64	AAP-AAA	0.10	17	0.868	0.955	0.2879
205767_at	561.8	343.8	-0.71	-1.63	PPP-PPP	0.10	17	0.868	0.955	0.0112
1562352_at	19.1	31.3	0.71	1.64	APA-AAA	0.10	17	0.868	0.955	0.5919
211980_at	1905.0	1166.4	-0.71	-1.63	PPP-PPP	0.10	17	0.868	0.955	0.0064
236568_at	34.6	56.5	0.71	1.63	AAA-PAA	0.10	17	0.868	0.955	0.6225

1563259_at	33.5	54.7	0.71	1.63	AAP-APP	0.10	17	0.868	0.955	0.3377
215249_at	23.1	37.6	0.71	1.63	AAA-APA	0.10	17	0.868	0.955	0.4635
207178_s_at	32.5	52.9	0.71	1.63	AAA-APA	0.10	17	0.868	0.955	0.3344
236668_at	70.6	115.1	0.71	1.63	APP-APP	0.10	17	0.868	0.955	0.2465
216115_at	43.7	71.1	0.70	1.63	PAA-AAA	0.10	17	0.868	0.955	0.4936
212443_at	180.6	294.3	0.70	1.63	APP-PAP	0.10	17	0.868	0.955	0.2258
244152_at	43.1	70.2	0.70	1.63	PAA-AAA	0.10	17	0.868	0.955	0.6128
242919_at	51.9	84.5	0.70	1.63	PAP-PPP	0.10	17	0.868	0.955	0.2687
1560297_at	73.6	119.7	0.70	1.63	AAA-APP	0.10	17	0.868	0.955	0.3019
234492_at	20.1	32.6	0.70	1.63	AAP-AAA	0.10	17	0.868	0.955	0.5786
244798_at	49.9	81.0	0.70	1.63	AAA-MPA	0.10	17	0.868	0.955	0.5733
222173_s_at	577.3	937.4	0.70	1.62	PPP-PPP	0.10	17	0.868	0.955	0.2231
1560647_at	28.5	46.2	0.70	1.62	PAP-PPA	0.10	17	0.868	0.955	0.4099
210070_s_at	67.2	108.9	0.70	1.62	AAA-PAA	0.10	17	0.868	0.955	0.3210
1557145_at	31.5	51.0	0.70	1.62	APA-APA	0.10	17	0.868	0.955	0.2890
216629_at	35.2	57.1	0.70	1.62	AAA-APA	0.10	17	0.868	0.955	0.5033
213613_s_at	35.2	57.0	0.70	1.62	AAP-AAA	0.10	17	0.868	0.955	0.5025
206088_at	81.3	131.6	0.69	1.62	PPA-PAP	0.10	17	0.868	0.955	0.2985
207394_at	55.1	89.0	0.69	1.62	MPP-PPP	0.10	17	0.868	0.955	0.2206
213081_at	87.1	140.7	0.69	1.62	PAA-AAA	0.10	17	0.868	0.955	0.4298
1558969_a_at	39.2	63.4	0.69	1.62	PAA-AAA	0.10	17	0.868	0.955	0.3932
222306_at	47.2	76.4	0.69	1.62	AAA-PAA	0.10	17	0.868	0.955	0.3875
217610_at	163.6	264.4	0.69	1.62	PPA-PAP	0.10	17	0.868	0.955	0.2233
222297_x_at	1635.6	2639.9	0.69	1.61	PPP-PPP	0.10	17	0.868	0.955	0.4970
1564125_at	23.5	37.9	0.69	1.61	AAP-AAA	0.10	17	0.868	0.955	0.4894
238509_at	58.0	93.6	0.69	1.61	PAP-APA	0.10	17	0.868	0.955	0.4412
1568887_at	46.6	75.1	0.69	1.61	APA-PMP	0.10	17	0.868	0.955	0.2238
1569588_x_at	29.2	47.1	0.69	1.61	AMA-APA	0.10	17	0.868	0.955	0.6377
216769_x_at	58.2	93.8	0.69	1.61	APA-APP	0.10	17	0.868	0.955	0.2820
238279_x_at	44.5	71.7	0.69	1.61	PAP-PAM	0.10	17	0.868	0.955	0.3121
240405_at	62.2	100.3	0.69	1.61	MAA-PPA	0.10	17	0.868	0.955	0.2868
216493_s_at	78.9	126.9	0.69	1.61	PAP-PAP	0.10	17	0.868	0.955	0.5007
233911_s_at	24.8	39.9	0.69	1.61	APA-AAA	0.10	17	0.868	0.955	0.4118
1555447_at	66.7	107.3	0.69	1.61	AAP-MPA	0.10	17	0.868	0.955	0.4796
227969_at	42.6	68.5	0.69	1.61	AAA-PPA	0.10	17	0.868	0.955	0.4634
1552887_at	52.9	85.0	0.69	1.61	AAA-PAA	0.10	17	0.868	0.955	0.4119
232542_at	77.4	124.5	0.69	1.61	AAA-APP	0.10	17	0.868	0.955	0.3999
1561288_at	32.1	51.6	0.68	1.61	AAA-PAA	0.10	17	0.868	0.955	0.5255
240835_at	35.4	56.8	0.68	1.61	PAA-AAP	0.10	17	0.868	0.955	0.4784
238738_at	120.6	193.6	0.68	1.61	PAP-PPP	0.10	17	0.868	0.955	0.2769
1565696_at	55.2	88.6	0.68	1.60	APA-PPA	0.10	17	0.868	0.955	0.2948
216499_at	67.9	109.0	0.68	1.60	AAA-PAM	0.10	17	0.868	0.955	0.2628

1552991_at	35.1	56.3	0.68	1.60	APA-AAA	0.10	17	0.868	0.955	0.3458
229948_at	55.3	88.6	0.68	1.60	AAA-PAP	0.10	17	0.868	0.955	0.4269
1561445_at	24.5	39.2	0.68	1.60	PAA-AAA	0.10	17	0.868	0.955	0.4573
237377_at	43.3	69.4	0.68	1.60	PAA-APA	0.10	17	0.868	0.955	0.3131
1552609_s_at	38.3	61.3	0.68	1.60	AAP-AAP	0.10	17	0.868	0.955	0.2501
242756_at	47.4	75.9	0.68	1.60	PAA-PPP	0.10	17	0.868	0.955	0.2436
240049_at	16.6	26.5	0.68	1.60	AAA-PAA	0.10	17	0.868	0.955	0.5831
242489_at	44.8	71.6	0.68	1.60	PPA-AAA	0.10	17	0.868	0.955	0.5103
220372_at	21.8	34.8	0.68	1.60	MAA-APA	0.10	17	0.868	0.955	0.4602
1564344_at	58.2	93.0	0.68	1.60	PAA-PAP	0.10	17	0.868	0.955	0.4299
1555407_s_at	30.5	48.6	0.68	1.60	AAA-AAP	0.10	17	0.868	0.955	0.4053
207286_at	29.7	47.3	0.68	1.60	AAA-MPP	0.10	17	0.868	0.955	0.2194
1557706_at	22.9	36.6	0.67	1.60	AAA-PAA	0.10	17	0.868	0.955	0.5386
239788_at	33.8	54.0	0.67	1.60	PAP-APP	0.10	17	0.868	0.955	0.3886
227995_at	73.3	116.9	0.67	1.60	PAA-PPP	0.10	17	0.868	0.955	0.2395
242241_x_at	39.7	63.3	0.67	1.59	PAA-PPP	0.10	17	0.868	0.955	0.2982
216178_x_at	67.5	107.3	0.67	1.59	APA-PPA	0.10	17	0.868	0.955	0.4609
242106_at	28.9	45.9	0.67	1.59	PAA-AAP	0.10	17	0.868	0.955	0.5933
221642_at	60.2	95.6	0.67	1.59	AAA-APA	0.10	17	0.868	0.955	0.3256
232220_at	89.9	142.8	0.67	1.59	AAA-PMA	0.10	17	0.868	0.955	0.2874
206652_at	78.1	124.1	0.67	1.59	PPP-PMP	0.10	17	0.868	0.955	0.2550
214972_at	81.4	129.3	0.67	1.59	PPA-PAP	0.10	17	0.868	0.955	0.4483
228958_at	124.3	197.3	0.67	1.59	APA-PAP	0.10	17	0.868	0.955	0.2402
212651_at	41.2	65.4	0.67	1.59	PAA-MAP	0.10	17	0.868	0.955	0.4276
200795_at	14.4	22.9	0.67	1.59	AAA-AAP	0.10	17	0.868	0.955	0.5619
233890_at	41.7	66.1	0.67	1.59	AAA-PAA	0.10	17	0.868	0.955	0.5393
235156_at	58.5	92.8	0.67	1.59	APA-AAA	0.10	17	0.868	0.955	0.3592
228831_s_at	61.3	97.2	0.67	1.59	AAA-PPA	0.10	17	0.868	0.955	0.3226
218835_at	29.5	46.8	0.66	1.58	APA-AAA	0.10	17	0.868	0.955	0.4362
1560282_at	25.9	41.0	0.66	1.59	PAA-PPA	0.10	17	0.868	0.955	0.3961
242024_at	115.6	183.2	0.66	1.58	PPM-PAP	0.10	17	0.868	0.955	0.3142
243718_at	86.8	137.5	0.66	1.59	APA-MAP	0.10	17	0.868	0.955	0.2446
244891_x_at	11.0	17.5	0.66	1.58	APA-AAA	0.10	17	0.868	0.955	0.6030
1562650_at	11.9	18.8	0.66	1.58	AAA-AAP	0.10	17	0.868	0.955	0.5314
237388_at	66.5	105.2	0.66	1.58	AAP-PAP	0.10	17	0.868	0.955	0.3500
216021_s_at	29.2	46.3	0.66	1.58	AAA-APA	0.10	17	0.868	0.955	0.3415
1555042_at	38.6	61.1	0.66	1.58	PAA-PAA	0.10	17	0.868	0.955	0.3116
233369_at	60.6	96.0	0.66	1.58	PAA-PAP	0.10	17	0.868	0.955	0.2464
227501_at	82.0	129.9	0.66	1.58	PAA-PPP	0.10	17	0.868	0.955	0.2191
207141_s_at	43.3	68.5	0.66	1.58	PAP-MAA	0.10	17	0.868	0.955	0.5242
1559376_at	45.0	71.3	0.66	1.58	APA-AAA	0.10	17	0.868	0.955	0.3768
235939_at	52.3	82.8	0.66	1.58	PAP-AAP	0.10	17	0.868	0.955	0.4849

233415_at	34.7	54.8	0.66	1.58	APA-AAP	0.10	17	0.868	0.955	0.3544
244059_at	41.6	65.7	0.66	1.58	PAA-MAP	0.10	17	0.868	0.955	0.3414
213072_at	101.6	160.4	0.66	1.58	PAP-AAA	0.10	17	0.868	0.955	0.5890
1552884_at	59.4	93.7	0.66	1.58	AAP-AAP	0.10	17	0.868	0.955	0.3491
1558430_at	101.8	160.7	0.66	1.58	PAA-PMP	0.10	17	0.868	0.955	0.2973
1555685_at	50.0	78.9	0.66	1.58	AAA-PAA	0.10	17	0.868	0.955	0.2938
217763_s_at	481.3	305.1	-0.66	-1.58	PPP-PPP	0.10	17	0.868	0.955	0.0099
236031_x_at	19.9	31.4	0.66	1.58	AAP-AAA	0.10	17	0.868	0.955	0.5247
242006_at	30.3	47.9	0.66	1.58	AAP-PAA	0.10	17	0.868	0.955	0.5111
209151_x_at	120.2	189.6	0.66	1.58	PAP-AMM	0.10	17	0.868	0.955	0.3772
226268_at	101.8	160.4	0.66	1.58	PPP-PPP	0.10	17	0.868	0.955	0.3647
1566079_at	106.0	167.2	0.66	1.58	PAP-PPP	0.10	17	0.868	0.955	0.2828
210827_s_at	328.5	517.9	0.66	1.58	PPP-PPP	0.10	17	0.868	0.955	0.2275
1557047_at	409.9	260.1	-0.66	-1.58	PPP-PPP	0.10	17	0.868	0.955	0.0081
236363_at	32.4	51.0	0.66	1.58	AAA-APA	0.10	17	0.868	0.955	0.3971
217484_at	48.0	75.7	0.66	1.58	AAM-APA	0.10	17	0.868	0.955	0.3749
233248_at	56.1	88.3	0.66	1.57	PAA-APP	0.10	17	0.868	0.955	0.2974
222180_at	54.6	85.9	0.66	1.57	PPP-PPP	0.10	17	0.868	0.955	0.2571
206006_s_at	31.6	49.7	0.65	1.57	AAA-AAP	0.10	17	0.868	0.955	0.5520
1552735_at	41.0	64.5	0.65	1.57	AAA-PPA	0.10	17	0.868	0.955	0.2520
215855_s_at	74.3	116.8	0.65	1.57	PAP-MMP	0.10	17	0.868	0.955	0.2253
60528_at	447.1	284.4	-0.65	-1.57	PPP-PPP	0.10	17	0.868	0.955	0.0055
1556595_at	42.5	66.9	0.65	1.57	APA-APP	0.10	17	0.868	0.955	0.2947
1564632_at	17.5	27.5	0.65	1.57	AAP-AAA	0.10	17	0.868	0.955	0.5405
235034_at	151.7	238.5	0.65	1.57	AAA-APA	0.10	17	0.868	0.955	0.3260
215135_at	38.7	60.9	0.65	1.57	AAA-PAM	0.10	17	0.868	0.955	0.2564
235518_at	23.7	37.3	0.65	1.57	PAA-AAP	0.10	17	0.868	0.955	0.2520
211781_x_at	64.7	101.6	0.65	1.57	AAP-PPA	0.10	17	0.868	0.955	0.3023
238172_at	78.8	123.6	0.65	1.57	AAP-AAP	0.10	17	0.868	0.955	0.4470
220096_at	68.0	106.7	0.65	1.57	AAP-AAA	0.10	17	0.868	0.955	0.5031
241364_at	139.7	219.0	0.65	1.57	PPP-PPP	0.10	17	0.868	0.955	0.3023
204622_x_at	78.2	122.5	0.65	1.57	APP-APP	0.10	17	0.868	0.955	0.2501
216288_at	29.8	46.6	0.65	1.57	PAA-AAP	0.10	17	0.868	0.955	0.3305
215591_at	35.6	55.7	0.65	1.57	PAA-PMP	0.10	17	0.868	0.955	0.2716
243893_at	37.0	57.9	0.65	1.56	APA-AAP	0.10	17	0.868	0.955	0.3596
216773_at	41.1	64.3	0.65	1.56	AAA-PAA	0.10	17	0.868	0.955	0.3332
239867_at	50.7	79.4	0.65	1.57	AAA-PMP	0.10	17	0.868	0.955	0.2161
1553125_x_at	40.0	62.6	0.65	1.56	AAP-PAA	0.10	17	0.868	0.955	0.4643
229474_at	67.4	105.3	0.64	1.56	AAA-PAP	0.10	17	0.868	0.955	0.3757
220432_s_at	63.5	99.3	0.64	1.56	PAP-PPP	0.10	17	0.868	0.955	0.2204
1559007_s_at	107.5	167.9	0.64	1.56	PPP-PPP	0.10	17	0.868	0.955	0.2726
1569111_at	32.2	50.2	0.64	1.56	AAA-APA	0.10	17	0.868	0.955	0.5796

1555571_at	36.3	56.7	0.64	1.56	APA-AAP	0.10	17	0.868	0.955	0.3732
233692_at	41.0	63.9	0.64	1.56	PAA-APP	0.10	17	0.868	0.955	0.3105
1562947_x_at	30.0	46.7	0.64	1.56	APA-AAM	0.10	17	0.868	0.955	0.3458
1564822_at	49.2	76.6	0.64	1.56	PAA-PAP	0.10	17	0.868	0.955	0.4769
1552586_at	53.3	82.9	0.64	1.56	AAA-APA	0.10	17	0.868	0.955	0.4067
214485_at	43.6	67.8	0.64	1.56	AAA-AAP	0.10	17	0.868	0.955	0.3154
1567997_x_at	23.6	36.7	0.64	1.56	AAA-PAA	0.10	17	0.868	0.955	0.6190
233333_x_at	84.6	131.5	0.64	1.55	APA-AAA	0.10	17	0.868	0.955	0.4892
215882_at	55.8	86.7	0.64	1.55	AAA-AAP	0.10	17	0.868	0.955	0.4279
230173_at	31.9	49.5	0.64	1.55	AAA-AAP	0.10	17	0.868	0.955	0.5346
221344_at	56.8	88.1	0.63	1.55	AAP-AAM	0.10	17	0.868	0.955	0.2923
1560836_at	26.6	41.2	0.63	1.55	APA-AAA	0.10	17	0.868	0.955	0.5903
216096_s_at	20.3	31.5	0.63	1.55	AAA-PAA	0.10	17	0.868	0.955	0.5121
220791_x_at	34.7	53.8	0.63	1.55	AMP-PAP	0.10	17	0.868	0.955	0.2450
1564063_a_at	188.2	291.7	0.63	1.55	PPP-PPP	0.10	17	0.868	0.955	0.2207
216081_at	40.1	62.1	0.63	1.55	AAA-PAA	0.10	17	0.868	0.955	0.5380
1559921_at	130.7	202.6	0.63	1.55	PPA-PPP	0.10	17	0.868	0.955	0.2658
234087_at	23.9	37.0	0.63	1.55	AAP-AAA	0.10	17	0.868	0.955	0.5305
244190_at	17.3	26.8	0.63	1.55	AAP-AAA	0.10	17	0.868	0.955	0.6347
219095_at	76.8	118.9	0.63	1.55	AAA-APP	0.10	17	0.868	0.955	0.2205
235716_at	507.6	785.5	0.63	1.55	PPP-PPP	0.10	17	0.868	0.955	0.2169
230894_s_at	49.4	76.3	0.63	1.55	APA-AAA	0.10	17	0.868	0.955	0.4806
215467_x_at	142.0	219.7	0.63	1.55	PPP-PPP	0.10	17	0.868	0.955	0.2279
1565577_s_at	99.7	154.0	0.63	1.55	PPP-PPP	0.10	17	0.868	0.955	0.5515
1555668_a_at	46.0	71.1	0.63	1.55	PAA-AAA	0.10	17	0.868	0.955	0.4771
241069_at	82.9	128.0	0.63	1.55	AAA-AAP	0.10	17	0.868	0.955	0.2833
222328_x_at	29.1	44.9	0.63	1.54	AAA-PAA	0.10	17	0.868	0.955	0.6157
243598_at	38.4	59.2	0.63	1.54	PAA-PPP	0.10	17	0.868	0.955	0.4701
238412_at	160.1	246.8	0.63	1.54	AAP-AAP	0.10	17	0.868	0.955	0.2662
242821_at	28.5	44.0	0.62	1.54	PAA-AAA	0.10	17	0.868	0.955	0.5795
214222_at	39.5	60.8	0.62	1.54	AAA-APA	0.10	17	0.868	0.955	0.4101
239251_at	293.6	452.5	0.62	1.54	PPP-PPP	0.10	17	0.868	0.955	0.3412
224181_at	33.2	51.1	0.62	1.54	PAA-AAA	0.10	17	0.868	0.955	0.5407
226961_at	182.1	280.5	0.62	1.54	PPP-PPP	0.10	17	0.868	0.955	0.2253
240073_at	21.4	32.9	0.62	1.54	AAA-APA	0.10	17	0.868	0.955	0.2942
206571_s_at	1432.0	931.1	-0.62	-1.54	PPP-PPP	0.10	17	0.868	0.955	0.0090
216054_x_at	79.1	121.7	0.62	1.54	PMM-PPP	0.10	17	0.868	0.955	0.3011
225746_at	44.3	68.2	0.62	1.54	AAA-APA	0.10	17	0.868	0.955	0.2686
236035_at	21.0	32.3	0.62	1.54	PAA-AAA	0.10	17	0.868	0.955	0.3559
220175_s_at	1582.2	2431.3	0.62	1.54	PPP-PPP	0.10	17	0.868	0.955	0.2811
217369_at	79.3	122.0	0.62	1.54	AMP-PPP	0.10	17	0.868	0.955	0.2750
232052_at	75.5	116.1	0.62	1.54	MAP-PPP	0.10	17	0.868	0.955	0.2172

1569253_at	30.2	46.3	0.62	1.54	AAP-AAA	0.10	17	0.868	0.955	0.3793
225280_x_at	69.1	106.0	0.62	1.53	AAA-APA	0.10	17	0.868	0.955	0.5886
214826_at	42.5	65.2	0.62	1.53	AAA-APA	0.10	17	0.868	0.955	0.5371
219828_at	228.2	350.0	0.62	1.53	PAP-PPP	0.10	17	0.868	0.955	0.5130
222953_at	15.5	23.8	0.62	1.53	AAA-APA	0.10	17	0.868	0.955	0.6035
242059_at	75.1	115.0	0.62	1.53	PPM-PPP	0.10	17	0.868	0.955	0.3357
233984_at	66.5	102.0	0.62	1.53	AAP-MPA	0.10	17	0.868	0.955	0.3351
209538_at	74.2	113.7	0.62	1.53	APA-APP	0.10	17	0.868	0.955	0.3034
242028_at	49.3	75.5	0.62	1.53	PAA-PAA	0.10	17	0.868	0.955	0.3560
241534_at	56.8	87.1	0.62	1.53	PPP-PPP	0.10	17	0.868	0.955	0.2392
234526_at	64.1	98.2	0.61	1.53	APP-AAP	0.10	17	0.868	0.955	0.4871
207790_at	35.4	54.2	0.61	1.53	AAA-AAP	0.10	17	0.868	0.955	0.4382
233156_at	37.5	57.4	0.61	1.53	AAA-AAP	0.10	17	0.868	0.955	0.2990
233077_at	76.1	116.3	0.61	1.53	AAA-PAA	0.10	17	0.868	0.955	0.3467
216657_at	39.5	60.4	0.61	1.53	PAP-PAP	0.10	17	0.868	0.955	0.4481
222670_s_at	561.6	857.8	0.61	1.53	PPP-PPP	0.10	17	0.868	0.955	0.2639
1552375_at	42.4	64.6	0.61	1.53	AAA-APA	0.10	17	0.868	0.955	0.4847
235654_at	115.3	75.6	-0.61	-1.53	AAA-APP	0.10	17	0.868	0.955	0.0094
236251_at	123.2	187.9	0.61	1.52	PMP-PPP	0.10	17	0.868	0.955	0.2352
1563364_at	50.2	76.5	0.61	1.52	APP-PPP	0.10	17	0.868	0.955	0.2117
1553627_s_at	13.1	19.9	0.61	1.52	PAA-AAA	0.10	17	0.868	0.955	0.5483
232931_at	187.1	284.9	0.61	1.52	PPP-PPP	0.10	17	0.868	0.955	0.2557
203144_s_at	183.2	279.0	0.61	1.52	AAA-APM	0.10	17	0.868	0.955	0.5501
202149_at	60.6	92.2	0.61	1.52	APA-PPP	0.10	17	0.868	0.955	0.2657
232466_at	121.9	185.4	0.61	1.52	PPP-PPP	0.10	17	0.868	0.955	0.2647
1558719_s_at	64.1	97.5	0.61	1.52	APP-PPP	0.10	17	0.868	0.955	0.2158
236947_at	111.9	170.1	0.60	1.52	APP-PAM	0.10	17	0.868	0.955	0.2756
225042_s_at	194.0	294.9	0.60	1.52	PPP-PPP	0.10	17	0.868	0.955	0.2641
212177_at	411.8	625.8	0.60	1.52	PPP-PPP	0.10	17	0.868	0.955	0.2560
230064_at	63.8	97.0	0.60	1.52	AAM-PAP	0.10	17	0.868	0.955	0.2286
1562827_at	51.6	78.4	0.60	1.52	APA-PPA	0.10	17	0.868	0.955	0.2861
223487_x_at	215.4	327.1	0.60	1.52	PPP-PPP	0.10	17	0.868	0.955	0.2760
1565132_at	36.2	55.0	0.60	1.52	AAA-PAA	0.10	17	0.868	0.955	0.2376
1568873_at	56.1	85.1	0.60	1.52	PAP-PAP	0.10	17	0.868	0.955	0.3573
235279_at	204.0	309.1	0.60	1.52	PAP-MAA	0.10	17	0.868	0.955	0.3638
1554784_at	26.5	40.1	0.60	1.52	PAA-AMP	0.10	17	0.868	0.955	0.4750
218413_s_at	47.2	71.6	0.60	1.52	AMA-PAP	0.10	17	0.868	0.955	0.2508
241731_x_at	58.2	88.1	0.60	1.51	PPA-PPP	0.10	17	0.868	0.955	0.5213
232843_s_at	51.8	78.4	0.60	1.51	PAA-PPP	0.10	17	0.868	0.955	0.3469
242723_at	47.7	72.2	0.60	1.51	AAA-AAP	0.10	17	0.868	0.955	0.2573
234906_at	32.0	48.4	0.60	1.51	PAA-AAM	0.10	17	0.868	0.955	0.5085
243558_at	31.5	47.7	0.60	1.51	APA-AAA	0.10	17	0.868	0.955	0.3844

232632_at	62.1	93.9	0.60	1.51	AAA-PAP	0.10	17	0.868	0.955	0.3396
235289_at	47.0	71.2	0.60	1.51	AAP-AAP	0.10	17	0.868	0.955	0.2975
242232_at	54.9	83.0	0.60	1.51	PAA-PPP	0.10	17	0.868	0.955	0.2846
1565830_at	83.9	126.8	0.60	1.51	PAP-PPP	0.10	17	0.868	0.955	0.2830
242424_at	75.5	114.1	0.60	1.51	AAM-PAP	0.10	17	0.868	0.955	0.3000
220315_at	42.5	64.2	0.60	1.51	AAA-PAA	0.10	17	0.868	0.955	0.2501
227234_at	38.8	58.6	0.59	1.51	AAP-APM	0.10	17	0.868	0.955	0.6424
240978_at	23.5	35.5	0.59	1.51	AAA-APA	0.10	17	0.868	0.955	0.6052
213837_at	62.4	94.1	0.59	1.51	APP-PPM	0.10	17	0.868	0.955	0.2870
240170_at	65.5	98.9	0.59	1.51	AAA-PPP	0.10	17	0.868	0.955	0.2225
1565620_at	118.8	179.1	0.59	1.51	PPP-PPP	0.10	17	0.868	0.955	0.2973
231903_x_at	72.3	108.9	0.59	1.51	AAA-APP	0.10	17	0.868	0.955	0.5203
1559025_at	87.2	131.3	0.59	1.51	PAP-PAP	0.10	17	0.868	0.955	0.3744
240594_at	59.9	90.2	0.59	1.51	MPP-PAP	0.10	17	0.868	0.955	0.3435
1559573_at	41.8	63.0	0.59	1.51	PPA-PMP	0.10	17	0.868	0.955	0.2701
236605_at	66.5	100.1	0.59	1.51	APP-AAA	0.10	17	0.868	0.955	0.2359
241905_at	128.2	193.2	0.59	1.51	PAP-PPP	0.10	17	0.868	0.955	0.2342
1570299_at	53.4	80.4	0.59	1.51	AAA-AAP	0.10	17	0.868	0.955	0.2224
232163_at	58.0	87.3	0.59	1.51	PAA-AAM	0.10	17	0.868	0.955	0.3437
234923_at	58.9	88.7	0.59	1.51	APP-PPP	0.10	17	0.868	0.955	0.2558
230890_at	36.9	55.5	0.59	1.50	AAP-PAA	0.10	17	0.868	0.955	0.5312
1564093_at	36.2	54.5	0.59	1.51	AAP-PAP	0.10	17	0.868	0.955	0.3847
1554804_a_at	214.0	321.9	0.59	1.50	PPA-PAP	0.10	17	0.868	0.955	0.3026
224240_s_at	105.1	158.0	0.59	1.50	APA-PPP	0.10	17	0.868	0.955	0.2632
241722_x_at	459.6	690.9	0.59	1.50	PPP-PPP	0.10	17	0.868	0.955	0.2373
214216_s_at	96.1	144.4	0.59	1.50	PAA-APP	0.10	17	0.868	0.955	0.3743
242995_at	61.8	92.8	0.59	1.50	AAP-AMM	0.10	17	0.868	0.955	0.2885
240372_at	88.8	133.3	0.59	1.50	APA-AAA	0.10	17	0.868	0.955	0.6289
230662_at	78.6	118.0	0.59	1.50	AAA-AAP	0.10	17	0.868	0.955	0.5065
64899_at	267.0	400.7	0.59	1.50	AAM-AAP	0.10	17	0.868	0.955	0.3104
216716_at	47.1	70.6	0.59	1.50	PPA-PAA	0.10	17	0.868	0.955	0.5080
242685_at	43.4	65.1	0.58	1.50	AAP-PAA	0.10	17	0.868	0.955	0.5525
1558588_at	34.1	51.1	0.58	1.50	AAA-APA	0.10	17	0.868	0.955	0.5665
227566_at	40.4	60.6	0.58	1.50	PPA-APP	0.10	17	0.868	0.955	0.4773
1553438_at	150.0	224.5	0.58	1.50	PAA-AMP	0.10	17	0.868	0.955	0.3812
209816_at	33.0	49.3	0.58	1.50	PAA-AAA	0.10	17	0.868	0.955	0.3678
204584_at	183.3	122.5	-0.58	-1.50	APP-AAP	0.10	17	0.868	0.955	0.0058
243906_at	26.7	40.0	0.58	1.50	APA-AAA	0.10	17	0.868	0.955	0.5935
239669_at	17.4	26.1	0.58	1.50	AAA-AAP	0.10	17	0.868	0.955	0.5557
237484_at	30.9	46.2	0.58	1.50	AAP-AAA	0.10	17	0.868	0.955	0.2751
217695_x_at	60.8	90.9	0.58	1.50	AAA-AAP	0.10	17	0.868	0.955	0.2713
1560002_at	43.3	64.8	0.58	1.49	AAM-APA	0.10	17	0.868	0.955	0.5207

1563398_at	42.0	62.7	0.58	1.49	PAA-AAA	0.10	17	0.868	0.955	0.2831
227351_at	89.0	132.9	0.58	1.49	PPP-PAP	0.10	17	0.868	0.955	0.3962
242563_at	41.6	62.0	0.58	1.49	MPP-PPA	0.10	17	0.868	0.955	0.2645
217539_at	35.7	53.3	0.58	1.49	AAA-APP	0.10	17	0.868	0.955	0.2393
219441_s_at	201.6	300.5	0.58	1.49	PPP-PPP	0.10	17	0.868	0.955	0.3963
237246_at	60.4	90.0	0.58	1.49	AAM-PAP	0.10	17	0.868	0.955	0.2611
205539_at	37.1	55.2	0.57	1.49	APA-AAP	0.10	17	0.868	0.955	0.3843
223797_at	82.5	122.9	0.57	1.49	APA-PPP	0.10	17	0.868	0.955	0.2385
234020_x_at	88.4	131.6	0.57	1.49	APA-PAP	0.10	17	0.868	0.955	0.2146
209322_s_at	160.1	238.3	0.57	1.49	PAM-PPP	0.10	17	0.868	0.955	0.3947
233657_at	154.8	230.2	0.57	1.49	PPP-PPP	0.10	17	0.868	0.955	0.2888
232927_at	32.5	48.2	0.57	1.49	AAP-AAP	0.10	17	0.868	0.955	0.3778
229871_at	176.6	261.9	0.57	1.48	APA-PPP	0.10	17	0.868	0.955	0.2184
1559393_at	48.9	72.4	0.57	1.48	APP-AMA	0.10	17	0.868	0.955	0.4879
237272_at	61.6	91.3	0.57	1.48	AAA-AAP	0.10	17	0.868	0.955	0.2172
210704_at	31.3	46.4	0.57	1.48	PAA-MAA	0.10	17	0.868	0.955	0.6368
1557697_at	61.6	91.2	0.57	1.48	PAP-PAA	0.10	17	0.868	0.955	0.5180
239022_at	125.6	186.0	0.57	1.48	PPP-PPP	0.10	17	0.868	0.955	0.4301
227099_s_at	53.0	78.6	0.57	1.48	AAA-APA	0.10	17	0.868	0.955	0.2824
209782_s_at	85.8	127.1	0.57	1.48	APA-PAP	0.10	17	0.868	0.955	0.2701
1561090_at	26.6	39.5	0.57	1.48	PAA-APP	0.10	17	0.868	0.955	0.2404
236229_at	248.6	368.0	0.57	1.48	PMP-PPP	0.10	17	0.868	0.955	0.3898
240655_at	106.7	157.9	0.57	1.48	AAP-PMP	0.10	17	0.868	0.955	0.3125
242373_at	76.9	113.8	0.57	1.48	APA-AAA	0.10	17	0.868	0.955	0.2950
234258_at	59.3	87.7	0.57	1.48	PAA-AAP	0.10	17	0.868	0.955	0.2900
1558452_at	39.9	59.0	0.57	1.48	AAA-PAP	0.10	17	0.868	0.955	0.4098
32402_s_at	123.8	183.0	0.56	1.48	PAP-APP	0.10	17	0.868	0.955	0.5753
202390_s_at	94.2	139.3	0.56	1.48	APA-AAA	0.10	17	0.868	0.955	0.5274
205318_at	55.5	82.1	0.56	1.48	APA-AAA	0.10	17	0.868	0.955	0.4121
1553947_at	88.4	130.6	0.56	1.48	AAA-APP	0.10	17	0.868	0.955	0.3054
236738_at	22.2	32.9	0.56	1.48	PAA-AAA	0.10	17	0.868	0.955	0.2589
239130_at	41.9	61.8	0.56	1.48	AAA-APA	0.10	17	0.868	0.955	0.5423
237888_at	31.1	45.9	0.56	1.48	AAA-PAA	0.10	17	0.868	0.955	0.2316
229283_at	45.2	66.7	0.56	1.48	AAA-AAP	0.10	17	0.868	0.955	0.3608
213744_at	38.1	56.1	0.56	1.48	AAP-MAA	0.10	17	0.868	0.955	0.3084
239238_at	136.9	202.0	0.56	1.48	AAA-MAP	0.10	17	0.868	0.955	0.2724
242889_x_at	127.0	187.1	0.56	1.47	PAA-APA	0.10	17	0.868	0.955	0.3364
39313_at	71.7	105.7	0.56	1.47	APA-PPP	0.10	17	0.868	0.955	0.3148
204379_s_at	1594.8	2351.3	0.56	1.47	PPP-PPP	0.10	17	0.868	0.955	0.2164
237334_at	37.0	54.5	0.56	1.47	APA-AAA	0.10	17	0.868	0.955	0.4776
239963_at	33.3	49.0	0.56	1.47	PAA-APA	0.10	17	0.868	0.955	0.4481
1563272_at	55.2	81.4	0.56	1.47	AAA-PAA	0.10	17	0.868	0.955	0.2484

1560625_s_at	26.3	38.7	0.56	1.47	AAA-PAA	0.10	17	0.868	0.955	0.6391
1553357_at	14.8	21.8	0.56	1.47	AAP-AAA	0.10	17	0.868	0.955	0.5747
208446_s_at	79.0	116.3	0.56	1.47	PMA-APM	0.10	17	0.868	0.955	0.2847
228652_at	330.8	486.5	0.56	1.47	PPP-PPP	0.10	17	0.868	0.955	0.2269
1558277_at	41.2	60.6	0.56	1.47	PPA-AMP	0.10	17	0.868	0.955	0.5057
1569077_x_at	212.5	312.5	0.56	1.47	PPP-PPA	0.10	17	0.868	0.955	0.4587
233964_at	35.0	51.5	0.56	1.47	AAA-PAA	0.10	17	0.868	0.955	0.4115
209522_s_at	264.7	389.1	0.56	1.47	PPP-PPP	0.10	17	0.868	0.955	0.2395
202871_at	295.7	434.8	0.56	1.47	PPP-PPP	0.10	17	0.868	0.955	0.2061
242680_at	23.9	35.1	0.56	1.47	AAA-AAP	0.10	17	0.868	0.955	0.5160
206724_at	134.0	196.8	0.55	1.47	APP-PPP	0.10	17	0.868	0.955	0.3130
241769_at	69.5	102.0	0.55	1.47	APA-AAP	0.10	17	0.868	0.955	0.2226
231136_at	57.3	84.1	0.55	1.47	AAA-APA	0.10	17	0.868	0.955	0.2218
232251_at	29.1	42.7	0.55	1.47	AAA-MPP	0.10	17	0.868	0.955	0.2198
1553651_at	27.2	39.9	0.55	1.47	AAA-AAP	0.10	17	0.868	0.955	0.4281
215368_at	34.5	50.6	0.55	1.47	AAA-AAP	0.10	17	0.868	0.955	0.3926
213680_at	875.2	1284.4	0.55	1.47	PPP-PPP	0.10	17	0.868	0.955	0.2630
215213_at	10.4	15.2	0.55	1.47	AAA-AAP	0.10	17	0.868	0.955	0.6170
222381_at	50.7	74.3	0.55	1.47	AAA-PAA	0.10	17	0.868	0.955	0.4112
1557852_at	132.5	194.3	0.55	1.47	MAP-AAP	0.10	17	0.868	0.955	0.2180
201348_at	32.7	47.9	0.55	1.47	APA-AMA	0.10	17	0.868	0.955	0.6088
239149_at	66.0	96.8	0.55	1.47	AAA-PAA	0.10	17	0.868	0.955	0.4897
223788_at	36.9	54.0	0.55	1.46	AAP-AAA	0.10	17	0.868	0.955	0.5615
213089_at	249.8	365.7	0.55	1.46	PPP-PPP	0.10	17	0.868	0.955	0.2642
235730_at	103.1	151.0	0.55	1.46	PPP-PPP	0.10	17	0.868	0.955	0.2391
203235_at	1220.0	1785.6	0.55	1.46	PPP-PPP	0.10	17	0.868	0.955	0.2190
1555812_a_at	3148.3	2151.4	-0.55	-1.46	PPP-PPP	0.10	17	0.868	0.955	0.0075
227757_at	94.9	138.8	0.55	1.46	AAA-PAA	0.10	17	0.868	0.955	0.4482
227464_at	127.2	186.0	0.55	1.46	APA-AAP	0.10	17	0.868	0.955	0.4121
202875_s_at	91.6	134.0	0.55	1.46	AAP-MPP	0.10	17	0.868	0.955	0.3055
1554703_at	164.8	241.0	0.55	1.46	AMA-AAP	0.10	17	0.868	0.955	0.2537
1560755_at	35.1	51.3	0.55	1.46	APA-AAA	0.10	17	0.868	0.955	0.4471
242333_at	29.3	42.9	0.55	1.46	PAA-AAA	0.10	17	0.868	0.955	0.3173
225273_at	409.3	598.3	0.55	1.46	PPP-PPP	0.10	17	0.868	0.955	0.2166
236106_at	57.9	84.6	0.55	1.46	AAP-APP	0.10	17	0.868	0.955	0.5172
1562056_at	83.6	122.1	0.55	1.46	AAA-PAP	0.10	17	0.868	0.955	0.3494
232835_at	63.9	93.4	0.55	1.46	AAA-AMP	0.10	17	0.868	0.955	0.3283
242233_at	139.0	203.0	0.55	1.46	PPP-PPP	0.10	17	0.868	0.955	0.3121
233719_s_at	93.2	136.1	0.55	1.46	AAP-AAP	0.10	17	0.868	0.955	0.2494
203682_s_at	541.2	370.6	-0.55	-1.46	PPP-PPP	0.10	17	0.868	0.955	0.0057
234036_x_at	28.2	41.2	0.55	1.46	AAA-APA	0.10	17	0.868	0.955	0.5598
216673_at	37.0	53.9	0.55	1.46	PAA-AAA	0.10	17	0.868	0.955	0.5439

223884_at	103.4	150.8	0.55	1.46	APP-AAP	0.10	17	0.868	0.955	0.5405
235027_at	120.5	175.8	0.55	1.46	PPP-PPP	0.10	17	0.868	0.955	0.3951
239678_at	41.8	61.0	0.55	1.46	AAA-APA	0.10	17	0.868	0.955	0.2643
1560112_at	58.9	85.9	0.55	1.46	AAA-AAP	0.10	17	0.868	0.955	0.2389
232016_at	48.3	70.5	0.54	1.46	AAA-AAP	0.10	17	0.868	0.955	0.4940
240829_at	20.6	30.1	0.54	1.46	AAP-APA	0.10	17	0.868	0.955	0.3881
242033_at	46.8	68.3	0.54	1.46	PAA-PPP	0.10	17	0.868	0.955	0.3850
228457_at	70.2	102.4	0.54	1.46	AAA-AAP	0.10	17	0.868	0.955	0.3241
215206_at	192.2	280.1	0.54	1.46	APP-MAP	0.10	17	0.868	0.955	0.2183
244786_at	51.0	74.4	0.54	1.46	PAA-AAM	0.10	17	0.868	0.955	0.4099
217297_s_at	282.0	411.0	0.54	1.46	PPP-MPP	0.10	17	0.868	0.955	0.2659
239072_at	161.8	235.8	0.54	1.46	AAP-APP	0.10	17	0.868	0.955	0.2655
227858_at	194.7	283.7	0.54	1.46	AAA-AAP	0.10	17	0.868	0.955	0.2148
228465_at	45.7	66.6	0.54	1.46	PAA-AAA	0.10	17	0.868	0.955	0.5865
238890_at	171.6	249.8	0.54	1.46	PPP-PPP	0.10	17	0.868	0.955	0.3717
1554443_s_at	34.5	50.2	0.54	1.46	AAP-AAA	0.10	17	0.868	0.955	0.6140
240228_at	50.7	73.7	0.54	1.46	APP-PPP	0.10	17	0.868	0.955	0.2768
229642_at	128.4	186.8	0.54	1.46	AAM-PPP	0.10	17	0.868	0.955	0.2252
218559_s_at	1140.7	1659.6	0.54	1.46	PPP-PPP	0.10	17	0.868	0.955	0.2230
207951_at	25.9	37.7	0.54	1.45	AAP-AAA	0.10	17	0.868	0.955	0.6187
244771_at	31.3	45.6	0.54	1.45	AAP-AAA	0.10	17	0.868	0.955	0.5680
1561503_at	58.7	85.3	0.54	1.45	PAA-PPA	0.10	17	0.868	0.955	0.4624
220178_at	210.2	305.6	0.54	1.45	APP-APP	0.10	17	0.868	0.955	0.2638
226008_at	178.5	259.6	0.54	1.45	PPP-PPP	0.10	17	0.868	0.955	0.2060
243162_at	28.3	41.2	0.54	1.45	PAA-AAA	0.10	17	0.868	0.955	0.5109
1553208_s_at	31.8	46.1	0.54	1.45	AAA-APP	0.10	17	0.868	0.955	0.4262
238315_s_at	129.3	187.8	0.54	1.45	PPP-PPP	0.10	17	0.868	0.955	0.2041
224549_x_at	380.3	552.3	0.54	1.45	PPP-PPP	0.10	17	0.868	0.955	0.3154
242194_at	100.8	146.4	0.54	1.45	AAP-APP	0.10	17	0.868	0.955	0.2374
1566700_at	87.0	126.3	0.54	1.45	PAA-MPP	0.10	17	0.868	0.955	0.4531
239451_at	89.5	129.8	0.54	1.45	AAP-AAA	0.10	17	0.868	0.955	0.2976
242255_at	51.9	75.2	0.54	1.45	PPA-APA	0.10	17	0.868	0.955	0.2753
1569516_at	50.2	72.8	0.54	1.45	PAA-PAP	0.10	17	0.868	0.955	0.4076
211440_x_at	57.5	83.3	0.54	1.45	AAA-APA	0.10	17	0.868	0.955	0.3846
238620_at	51.6	74.7	0.54	1.45	APP-PPP	0.10	17	0.868	0.955	0.3703
1557788_a_at	54.5	78.9	0.54	1.45	AAA-APA	0.10	17	0.868	0.955	0.3011
1563014_at	95.7	138.7	0.54	1.45	PPP-PPP	0.10	17	0.868	0.955	0.2337
210841_s_at	151.6	219.4	0.53	1.45	AAP-PPA	0.10	17	0.868	0.955	0.4194
235904_at	40.1	58.0	0.53	1.45	AAP-APA	0.10	17	0.868	0.955	0.3174
228307_at	178.3	258.0	0.53	1.45	PAA-PPP	0.10	17	0.868	0.955	0.3003
1566243_at	68.6	99.3	0.53	1.45	PPP-PPP	0.10	17	0.868	0.955	0.2998
1568777_at	22.3	32.3	0.53	1.45	APA-APP	0.10	17	0.868	0.955	0.4977

215648_at	117.8	170.5	0.53	1.45	PPP-PPP	0.10	17	0.868	0.955	0.3822
216129_at	63.0	91.1	0.53	1.45	PAA-PPP	0.10	17	0.868	0.955	0.3540
242959_at	87.7	126.9	0.53	1.45	PAA-APA	0.10	17	0.868	0.955	0.3119
222366_at	174.6	252.3	0.53	1.45	PPP-PPP	0.10	17	0.868	0.955	0.2797
232209_x_at	1212.7	1753.4	0.53	1.45	PPP-PPP	0.10	17	0.868	0.955	0.2778
242068_at	86.4	125.0	0.53	1.45	PPP-PPP	0.10	17	0.868	0.955	0.2603
232974_at	63.7	92.1	0.53	1.45	PPA-APP	0.10	17	0.868	0.955	0.4555
232097_at	68.6	99.2	0.53	1.45	APA-AAA	0.10	17	0.868	0.955	0.4316
244511_at	41.3	59.7	0.53	1.45	APA-PAA	0.10	17	0.868	0.955	0.4153
1553967_at	135.1	195.3	0.53	1.45	PPP-PAP	0.10	17	0.868	0.955	0.2976
223615_at	100.8	145.5	0.53	1.44	AAA-AAP	0.10	17	0.868	0.955	0.4793
226274_at	113.0	163.2	0.53	1.44	AAM-APP	0.10	17	0.868	0.955	0.2562
1558759_s_at	43.1	62.2	0.53	1.44	AAA-AAP	0.10	17	0.868	0.955	0.5865
1569393_at	71.6	103.4	0.53	1.44	APA-AAA	0.10	17	0.868	0.955	0.4994
206022_at	29.7	42.9	0.53	1.44	AAA-APA	0.10	17	0.868	0.955	0.3750
1554294_s_at	47.8	69.0	0.53	1.44	PAA-PPM	0.10	17	0.868	0.955	0.3640
1554906_a_at	83.2	120.0	0.53	1.44	PPP-PPP	0.10	17	0.868	0.955	0.3226
226413_at	295.9	427.0	0.53	1.44	PPP-PPP	0.10	17	0.868	0.955	0.2457
225775_at	135.4	195.4	0.53	1.44	PAP-PAA	0.10	17	0.868	0.955	0.2402
203170_at	66.5	95.9	0.53	1.44	APP-PPP	0.10	17	0.868	0.955	0.3027
1565920_at	42.3	61.0	0.53	1.44	AAP-AAM	0.10	17	0.868	0.955	0.3000
229523_at	31.4	45.2	0.53	1.44	AAA-APP	0.10	17	0.868	0.955	0.6182
241499_at	45.2	65.0	0.53	1.44	APP-PPA	0.10	17	0.868	0.955	0.5683
226564_at	150.9	217.4	0.53	1.44	PPP-PPP	0.10	17	0.868	0.955	0.4403
210641_at	57.6	82.8	0.53	1.44	PAA-PAA	0.10	17	0.868	0.955	0.4199
208297_s_at	65.5	94.2	0.52	1.44	PAP-PPA	0.10	17	0.868	0.955	0.3189
241716_at	139.9	201.2	0.52	1.44	PPP-PPP	0.10	17	0.868	0.955	0.2616
1559964_at	56.5	81.2	0.52	1.44	PAA-PPP	0.10	17	0.868	0.955	0.4409
203840_at	602.4	865.8	0.52	1.44	PPP-PPP	0.10	17	0.868	0.955	0.3570
222375_at	59.1	84.8	0.52	1.44	APP-PPP	0.10	17	0.868	0.955	0.4944
242482_at	57.2	82.1	0.52	1.44	AAA-MAP	0.10	17	0.868	0.955	0.4504
236232_at	46.5	66.7	0.52	1.44	AAM-APA	0.10	17	0.868	0.955	0.4482
229362_at	85.9	123.4	0.52	1.44	PPA-APA	0.10	17	0.868	0.955	0.4354
230737_s_at	47.0	67.5	0.52	1.44	AAP-PPA	0.10	17	0.868	0.955	0.4092
1554372_at	97.5	140.0	0.52	1.44	APP-PPA	0.10	17	0.868	0.955	0.3567
242535_at	101.0	144.9	0.52	1.44	AAP-APA	0.10	17	0.868	0.955	0.3094
235618_at	196.3	281.8	0.52	1.44	PAM-AAA	0.10	17	0.868	0.955	0.2910
237632_at	42.8	61.4	0.52	1.44	AMA-APA	0.10	17	0.868	0.955	0.3243
209305_s_at	246.7	354.0	0.52	1.44	PPP-PPP	0.10	17	0.868	0.955	0.2329
231024_at	102.3	146.7	0.52	1.43	APP-PPM	0.10	17	0.868	0.955	0.4354
221633_at	71.8	102.9	0.52	1.43	AAM-PAA	0.10	17	0.868	0.955	0.4268
208228_s_at	96.9	139.0	0.52	1.43	AAP-AAA	0.10	17	0.868	0.955	0.2560

244248_at	127.1	182.3	0.52	1.43	MAA-MAP	0.10	17	0.868	0.955	0.2518
239043_at	21.2	30.3	0.52	1.43	AAA-PAA	0.10	17	0.868	0.955	0.4972
242270_at	71.6	102.6	0.52	1.43	PPA-PMP	0.10	17	0.868	0.955	0.4952
213684_s_at	70.6	101.1	0.52	1.43	PAP-APP	0.10	17	0.868	0.955	0.2556
241899_at	58.2	83.3	0.52	1.43	APP-APA	0.10	17	0.868	0.955	0.5821
203408_s_at	124.8	178.6	0.52	1.43	PAA-PAP	0.10	17	0.868	0.955	0.5579
203272_s_at	590.1	844.2	0.52	1.43	PMA-PPP	0.10	17	0.868	0.955	0.3898
216494_at	28.4	40.7	0.52	1.43	AAA-PAA	0.10	17	0.868	0.955	0.4388
236011_at	58.6	83.7	0.52	1.43	APA-AAA	0.10	17	0.868	0.955	0.2685
239196_at	105.0	150.0	0.52	1.43	APA-MPP	0.10	17	0.868	0.955	0.4183
233726_at	99.0	141.5	0.52	1.43	PPA-PPP	0.10	17	0.868	0.955	0.2633
215538_at	53.1	75.9	0.52	1.43	AAP-AAA	0.10	17	0.868	0.955	0.2609
232978_at	72.1	103.1	0.52	1.43	MPP-PPP	0.10	17	0.868	0.955	0.2517
240436_at	63.6	90.9	0.52	1.43	PPA-PPP	0.10	17	0.868	0.955	0.2348
1552510_at	267.1	381.4	0.51	1.43	AAA-APA	0.10	17	0.868	0.955	0.3576
204719_at	39.2	56.0	0.51	1.43	APA-PPP	0.10	17	0.868	0.955	0.2026
1552430_at	35.1	50.1	0.51	1.43	AAM-MPA	0.10	17	0.868	0.955	0.2009
240843_at	85.9	122.5	0.51	1.43	PAP-AMP	0.10	17	0.868	0.955	0.4112
233575_s_at	35.7	51.0	0.51	1.43	AMA-PAP	0.10	17	0.868	0.955	0.3494
231860_at	112.7	160.8	0.51	1.43	AAA-PAA	0.10	17	0.868	0.955	0.2459
1565741_at	24.3	34.6	0.51	1.43	AAA-AAP	0.10	17	0.868	0.955	0.6285
1556787_s_at	21.3	30.3	0.51	1.43	AAA-APA	0.10	17	0.868	0.955	0.6201
207231_at	43.9	62.6	0.51	1.43	AAA-PAA	0.10	17	0.868	0.955	0.5438
200928_s_at	46.7	66.6	0.51	1.43	MAP-PPP	0.10	17	0.868	0.955	0.4680
1569512_at	76.1	108.5	0.51	1.43	APP-PAP	0.10	17	0.868	0.955	0.4664
228124_at	250.2	356.8	0.51	1.43	PPP-PPP	0.10	17	0.868	0.955	0.3783
204008_at	173.5	247.3	0.51	1.43	AMM-PAA	0.10	17	0.868	0.955	0.3191
239660_at	89.3	127.4	0.51	1.43	PPP-PPP	0.10	17	0.868	0.955	0.2976
227912_s_at	145.1	206.9	0.51	1.43	AAM-MAP	0.10	17	0.868	0.955	0.2235
222371_at	128.6	183.4	0.51	1.43	PPP-PPP	0.10	17	0.868	0.955	0.2159
243284_at	34.6	49.3	0.51	1.43	APA-AAA	0.10	17	0.868	0.955	0.5639
231012_at	106.4	151.7	0.51	1.43	AAA-PAA	0.10	17	0.868	0.955	0.5528
217049_x_at	54.7	77.9	0.51	1.43	PAA-AAP	0.10	17	0.868	0.955	0.4606
215086_at	29.7	42.3	0.51	1.43	APP-AAP	0.10	17	0.868	0.955	0.4477
1557866_at	87.3	124.5	0.51	1.43	AAP-PPP	0.10	17	0.868	0.955	0.4141
1563321_s_at	215.0	306.3	0.51	1.43	PPP-PPP	0.10	17	0.868	0.955	0.3821
207712_at	30.8	43.9	0.51	1.43	AAA-APA	0.10	17	0.868	0.955	0.3583
217849_s_at	432.8	617.0	0.51	1.43	PPP-PPP	0.10	17	0.868	0.955	0.2351
1556345_s_at	87.9	125.2	0.51	1.42	AAA-AAP	0.10	17	0.868	0.955	0.4252
222563_s_at	118.6	169.0	0.51	1.42	PPP-PPP	0.10	17	0.868	0.955	0.3825
1557302_at	145.8	207.7	0.51	1.42	PPP-PPP	0.10	17	0.868	0.955	0.2771
238518_x_at	107.7	153.3	0.51	1.42	APA-AMA	0.10	17	0.868	0.955	0.2627

1569782_at	35.6	50.6	0.51	1.42	PAA-APA	0.10	17	0.868	0.955	0.3418
225034_at	85.1	121.1	0.51	1.42	PPA-APP	0.10	17	0.868	0.955	0.3347
239252_at	171.3	243.9	0.51	1.42	PPP-PPP	0.10	17	0.868	0.955	0.3185
238342_at	170.7	242.9	0.51	1.42	PPP-PPP	0.10	17	0.868	0.955	0.3148
1560070_at	52.1	74.1	0.51	1.42	APA-AAP	0.10	17	0.868	0.955	0.3119
217953_at	36.7	52.3	0.51	1.42	PAA-APP	0.10	17	0.868	0.955	0.3104
201295_s_at	48.0	68.3	0.51	1.42	PAP-PPP	0.10	17	0.868	0.955	0.4957
1564257_at	49.9	70.9	0.51	1.42	APA-AAP	0.10	17	0.868	0.955	0.2479
239577_at	74.3	105.6	0.51	1.42	PAP-PPP	0.10	17	0.868	0.955	0.2416
210524_x_at	3471.0	2442.1	-0.51	-1.42	PPP-PPP	0.10	17	0.868	0.955	0.0039
209936_at	53.3	75.8	0.51	1.42	AAP-AAP	0.10	17	0.868	0.955	0.4138
237803_x_at	36.3	51.6	0.51	1.42	APA-AAA	0.10	17	0.868	0.955	0.4135
210701_at	93.5	132.8	0.51	1.42	AAA-AAP	0.10	17	0.868	0.955	0.2649
1558467_a_at	145.5	206.7	0.51	1.42	APP-APP	0.10	17	0.868	0.955	0.2162
244093_at	49.8	70.6	0.51	1.42	APP-PPP	0.10	17	0.868	0.955	0.5099
229697_at	98.6	140.0	0.51	1.42	AAA-PPM	0.10	17	0.868	0.955	0.3314
228268_at	70.5	100.2	0.51	1.42	PAA-PPA	0.10	17	0.868	0.955	0.3217
211866_x_at	224.1	157.9	-0.51	-1.42	APP-MAA	0.10	17	0.868	0.955	0.0062
217270_s_at	84.5	119.8	0.51	1.42	APA-PAA	0.10	17	0.868	0.955	0.6032
1554388_at	104.6	148.5	0.51	1.42	APP-APM	0.10	17	0.868	0.955	0.3065
233808_at	59.5	84.4	0.51	1.42	AAA-PPA	0.10	17	0.868	0.955	0.2699
232708_at	46.8	66.3	0.50	1.42	APP-AAP	0.10	17	0.868	0.955	0.5090
1559129_a_at	39.8	56.4	0.50	1.42	AAP-AAP	0.10	17	0.868	0.955	0.4929
214378_at	78.3	111.0	0.50	1.42	AAM-MPM	0.10	17	0.868	0.955	0.2517
228144_at	192.8	273.5	0.50	1.42	PPP-PPP	0.10	17	0.868	0.955	0.2275
226365_at	131.4	186.2	0.50	1.42	AAA-PAP	0.10	17	0.868	0.955	0.2246
203178_at	30.0	42.5	0.50	1.42	AAP-PPA	0.10	17	0.868	0.955	0.5091
244384_at	110.8	157.0	0.50	1.42	PAA-PMP	0.10	17	0.868	0.955	0.2629
215781_s_at	83.3	118.0	0.50	1.42	AAA-PAP	0.10	17	0.868	0.955	0.2362
241520_x_at	110.0	155.8	0.50	1.42	AAM-MAP	0.10	17	0.868	0.955	0.2298
1554291_at	27.2	38.6	0.50	1.42	AAA-PPA	0.10	17	0.868	0.955	0.5537
213249_at	65.9	93.3	0.50	1.42	AAA-APA	0.10	17	0.868	0.955	0.3665
232560_at	53.0	75.1	0.50	1.42	AAP-AAA	0.10	17	0.868	0.955	0.3306
242161_at	34.1	48.3	0.50	1.42	AAP-MPA	0.10	17	0.868	0.955	0.2291
1554594_at	74.2	105.0	0.50	1.42	AAM-APP	0.10	17	0.868	0.955	0.3260
240111_at	61.3	86.7	0.50	1.42	AAA-PAP	0.10	17	0.868	0.955	0.2736
232551_at	158.6	224.4	0.50	1.42	PAP-PPP	0.10	17	0.868	0.955	0.2325
208651_x_at	3541.8	5010.7	0.50	1.42	PPP-PPP	0.10	17	0.868	0.955	0.2087
1556989_at	43.4	61.4	0.50	1.41	AAP-AMP	0.10	17	0.868	0.955	0.4171
235617_x_at	27.9	39.5	0.50	1.42	APA-PAP	0.10	17	0.868	0.955	0.3971
207095_at	31.2	44.1	0.50	1.41	AAA-APP	0.10	17	0.868	0.955	0.3705
1558251_a_at	58.5	82.8	0.50	1.41	APP-PPP	0.10	17	0.868	0.955	0.3208

210033_s_at	41.0	58.0	0.50	1.41	AAA-PAA	0.10	17	0.868	0.955	0.3151
240081_at	78.1	110.4	0.50	1.41	PAA-APP	0.10	17	0.868	0.955	0.2995
231355_at	91.9	129.9	0.50	1.41	AAA-PAA	0.10	17	0.868	0.955	0.2534
229736_at	58.9	83.3	0.50	1.41	AAA-APA	0.10	17	0.868	0.955	0.2316
230572_at	52.7	74.6	0.50	1.41	PAA-PPP	0.10	17	0.868	0.955	0.2210
1553991_s_at	88.6	125.2	0.50	1.41	PAA-APP	0.10	17	0.868	0.955	0.3598
219298_at	898.0	1268.8	0.50	1.41	PPP-PPP	0.10	17	0.868	0.955	0.3034
226714_at	276.7	391.1	0.50	1.41	AAA-APM	0.10	17	0.868	0.955	0.2934
241966_at	91.6	129.5	0.50	1.41	AAP-PAP	0.10	17	0.868	0.955	0.2904
224042_at	68.4	96.6	0.50	1.41	MPP-PMP	0.10	17	0.868	0.955	0.3932
1553177_at	30.3	42.8	0.50	1.41	AAA-AAP	0.10	17	0.868	0.955	0.3004
217602_at	106.2	149.9	0.50	1.41	PPP-PPP	0.10	17	0.868	0.955	0.2621
224618_at	721.6	1019.0	0.50	1.41	PPP-PPP	0.10	17	0.868	0.955	0.2308
211572_s_at	88.5	124.8	0.50	1.41	APP-PPP	0.10	17	0.868	0.955	0.3363
213977_s_at	518.9	732.1	0.50	1.41	PPP-PPP	0.10	17	0.868	0.955	0.2208
222340_at	33.0	46.5	0.50	1.41	APA-AAP	0.10	17	0.868	0.955	0.4887
207780_at	27.8	39.2	0.50	1.41	AAA-AAP	0.10	17	0.868	0.955	0.4030
1563745_a_at	73.5	103.7	0.50	1.41	PPA-PAA	0.10	17	0.868	0.955	0.2450
217042_at	64.8	91.4	0.50	1.41	APP-PPP	0.10	17	0.868	0.955	0.2255
213716_s_at	284.4	400.7	0.50	1.41	APP-PPP	0.10	17	0.868	0.955	0.5638
1559883_s_at	64.1	90.3	0.50	1.41	PAA-AAM	0.10	17	0.868	0.955	0.3812
215295_at	35.5	50.0	0.50	1.41	AAA-APA	0.10	17	0.868	0.955	0.2950
214329_x_at	91.7	129.2	0.50	1.41	PPP-PPP	0.10	17	0.868	0.955	0.2339
238735_at	49.0	68.9	0.49	1.41	AAA-AAP	0.10	17	0.868	0.955	0.4645
212384_at	96.3	135.6	0.49	1.41	PPP-AAP	0.10	17	0.868	0.955	0.4557
1560048_at	57.8	81.3	0.49	1.41	PAA-AMP	0.10	17	0.868	0.955	0.4410
233976_at	81.5	114.8	0.49	1.41	AAP-PPP	0.10	17	0.868	0.955	0.3160
1556347_at	37.9	53.4	0.49	1.41	PAP-PAP	0.10	17	0.868	0.955	0.2875
214428_x_at	98.1	138.1	0.49	1.41	APA-AAA	0.10	17	0.868	0.955	0.2439
1554086_at	123.1	173.4	0.49	1.41	PPP-PPP	0.10	17	0.868	0.955	0.2149
1554542_at	89.4	126.0	0.49	1.41	APA-AAA	0.10	17	0.868	0.955	0.1992
227776_at	1823.2	1295.4	-0.49	-1.41	PPP-PPP	0.10	17	0.868	0.955	0.0052
213486_at	77.4	108.9	0.49	1.41	APA-PPP	0.10	17	0.868	0.955	0.5919
206888_s_at	68.2	95.9	0.49	1.41	APA-AAA	0.10	17	0.868	0.955	0.4431
241387_at	143.8	202.4	0.49	1.41	PPP-PPP	0.10	17	0.868	0.955	0.3811
217212_s_at	70.1	98.7	0.49	1.41	APA-APA	0.10	17	0.868	0.955	0.3383
235864_at	65.7	92.5	0.49	1.41	AAP-PPA	0.10	17	0.868	0.955	0.2510
1559804_at	43.5	61.1	0.49	1.41	APA-AAA	0.10	17	0.868	0.955	0.5236
216683_at	29.9	42.0	0.49	1.41	AAA-AAP	0.10	17	0.868	0.955	0.4770
219225_at	469.5	660.2	0.49	1.41	APP-PPP	0.10	17	0.868	0.955	0.4368
232063_x_at	58.3	82.0	0.49	1.41	MAP-PPP	0.10	17	0.868	0.955	0.3129
1554834_a_at	107.5	151.1	0.49	1.41	AAA-PAA	0.10	17	0.868	0.955	0.5699

216479_at	101.8	143.0	0.49	1.41	APP-PPA	0.10	17	0.868	0.955	0.4642
1554889_at	52.1	73.1	0.49	1.40	PAA-PAP	0.10	17	0.868	0.955	0.3913
234645_at	60.4	84.9	0.49	1.41	AAP-AAA	0.10	17	0.868	0.955	0.3870
231834_at	70.4	99.0	0.49	1.41	AAA-AAP	0.10	17	0.868	0.955	0.3601
224997_x_at	68.5	96.2	0.49	1.41	PPA-PPM	0.10	17	0.868	0.955	0.3170
226533_at	168.0	236.0	0.49	1.41	PPP-PPP	0.10	17	0.868	0.955	0.3097
216374_at	64.8	91.0	0.49	1.40	APA-PPP	0.10	17	0.868	0.955	0.2851
229968_at	89.2	125.3	0.49	1.40	MPP-PAP	0.10	17	0.868	0.955	0.2345
237436_at	22.2	31.1	0.49	1.40	PAA-AAA	0.10	17	0.868	0.955	0.5883
217272_s_at	207.0	290.5	0.49	1.40	APP-PPP	0.10	17	0.868	0.955	0.4404
217632_at	51.7	72.6	0.49	1.40	MPP-PPA	0.10	17	0.868	0.955	0.4283
1561486_at	33.6	47.2	0.49	1.40	PAP-APA	0.10	17	0.868	0.955	0.3091
214135_at	58.5	82.1	0.49	1.40	AAA-APA	0.10	17	0.868	0.955	0.2892
223771_at	25.7	36.1	0.49	1.40	AAP-AAA	0.10	17	0.868	0.955	0.6034
237355_at	46.4	65.1	0.49	1.40	AAA-AAP	0.10	17	0.868	0.955	0.5699
234929_s_at	166.2	233.1	0.49	1.40	PAP-PPP	0.10	17	0.868	0.955	0.2488
210500_at	36.9	51.8	0.49	1.40	AAP-APA	0.10	17	0.868	0.955	0.2447
230034_x_at	54.1	75.8	0.49	1.40	AAA-PAA	0.10	17	0.868	0.955	0.5111
1557529_at	42.8	59.9	0.49	1.40	AAA-APA	0.10	17	0.868	0.955	0.4936
1564333_a_at	101.9	142.8	0.49	1.40	APP-PPA	0.10	17	0.868	0.955	0.4700
1554514_at	39.4	55.2	0.49	1.40	APA-AAA	0.10	17	0.868	0.955	0.4426
233294_at	137.7	192.9	0.49	1.40	PPP-APP	0.10	17	0.868	0.955	0.2612
221614_s_at	37.1	52.0	0.49	1.40	AAA-AAP	0.10	17	0.868	0.955	0.6121
204902_s_at	85.3	119.5	0.49	1.40	PAA-AAA	0.10	17	0.868	0.955	0.5987
214008_at	80.2	112.3	0.49	1.40	APP-PAA	0.10	17	0.868	0.955	0.5589
232879_at	52.6	73.6	0.49	1.40	APA-AAP	0.10	17	0.868	0.955	0.4203
239623_at	159.0	222.6	0.49	1.40	PPP-PAP	0.10	17	0.868	0.955	0.4196
242898_at	75.3	105.6	0.49	1.40	AAA-PAA	0.10	17	0.868	0.955	0.3368
232759_at	90.5	126.7	0.49	1.40	MAP-PPP	0.10	17	0.868	0.955	0.3135
1557751_at	100.0	140.0	0.49	1.40	PPP-MPP	0.10	17	0.868	0.955	0.2895
219856_at	446.2	625.0	0.49	1.40	PPP-PPP	0.10	17	0.868	0.955	0.2607
214738_s_at	163.5	228.8	0.49	1.40	APA-AAA	0.10	17	0.868	0.955	0.6011
230000_at	122.0	170.8	0.49	1.40	PPP-PPP	0.10	17	0.868	0.955	0.5115
232797_at	71.2	99.6	0.49	1.40	PPA-PPP	0.10	17	0.868	0.955	0.4278
204159_at	645.8	903.8	0.49	1.40	PPP-PPP	0.10	17	0.868	0.955	0.2767
214754_at	81.5	114.1	0.49	1.40	PAA-AAP	0.10	17	0.868	0.955	0.2750
222720_x_at	101.4	141.9	0.49	1.40	APA-AMP	0.10	17	0.868	0.955	0.2147
206566_at	115.4	161.4	0.48	1.40	MPP-PPP	0.10	17	0.868	0.955	0.2966
228883_at	67.5	94.5	0.48	1.40	AAA-APA	0.10	17	0.868	0.955	0.2402
1556672_a_at	42.9	60.0	0.48	1.40	AAA-AMP	0.10	17	0.868	0.955	0.5764
1563489_at	79.9	111.7	0.48	1.40	AAP-PMP	0.10	17	0.868	0.955	0.4363
223153_x_at	235.4	328.9	0.48	1.40	PAA-AAA	0.10	17	0.868	0.955	0.4209

217157_x_at	47.1	65.9	0.48	1.40	AAA-AAP	0.10	17	0.868	0.955	0.2827
222822_s_at	71.1	99.4	0.48	1.40	AAA-APA	0.10	17	0.868	0.955	0.2689
1558682_at	56.5	78.9	0.48	1.40	PPA-APA	0.10	17	0.868	0.955	0.6385
239954_at	52.2	72.9	0.48	1.40	PAA-APP	0.10	17	0.868	0.955	0.4593
213216_at	64.6	90.2	0.48	1.40	AAA-AAP	0.10	17	0.868	0.955	0.4348
244026_at	67.9	94.8	0.48	1.40	MPA-PPP	0.10	17	0.868	0.955	0.3686
241294_at	38.5	53.8	0.48	1.40	PPA-AAA	0.10	17	0.868	0.955	0.5871
230887_at	70.8	98.8	0.48	1.40	APP-AAA	0.10	17	0.868	0.955	0.4666
1554367_at	91.4	127.5	0.48	1.40	PPA-PPP	0.10	17	0.868	0.955	0.2519
226275_at	310.2	433.0	0.48	1.40	PPP-PPP	0.10	17	0.868	0.955	0.2371
243135_x_at	56.7	79.1	0.48	1.40	AAA-APA	0.10	17	0.868	0.955	0.2118
232166_at	51.2	71.4	0.48	1.39	PAA-MPA	0.10	17	0.868	0.955	0.4930
215169_at	46.2	64.4	0.48	1.39	AAP-PAM	0.10	17	0.868	0.955	0.3952
201278_at	64.1	89.4	0.48	1.39	PAA-APA	0.10	17	0.868	0.955	0.3460
204328_at	85.6	119.3	0.48	1.39	PAA-AMA	0.10	17	0.868	0.955	0.2775
234608_at	158.1	220.3	0.48	1.39	PPP-PPP	0.10	17	0.868	0.955	0.2321
1555372_at	185.9	259.1	0.48	1.39	APA-PPP	0.10	17	0.868	0.955	0.2186
1552546_a_at	760.3	545.8	-0.48	-1.39	PPP-PPP	0.10	17	0.868	0.955	0.0040
1553430_a_at	49.9	69.6	0.48	1.39	AAA-AAP	0.10	17	0.868	0.955	0.4090
242434_at	75.0	104.5	0.48	1.39	AAA-PMA	0.10	17	0.868	0.955	0.4001
239804_at	86.6	120.6	0.48	1.39	PAA-APP	0.10	17	0.868	0.955	0.3889
204479_at	443.6	617.9	0.48	1.39	PPP-PPP	0.10	17	0.868	0.955	0.3665
228801_at	171.5	239.0	0.48	1.39	PPP-PPP	0.10	17	0.868	0.955	0.2870
201450_s_at	877.2	1222.1	0.48	1.39	PPP-PPP	0.10	17	0.868	0.955	0.2536
1565743_at	77.1	107.3	0.48	1.39	PPP-PPP	0.10	17	0.868	0.955	0.4873
210944_s_at	62.8	87.5	0.48	1.39	APA-PPP	0.10	17	0.868	0.955	0.4120
236665_at	69.3	96.5	0.48	1.39	PPP-PPA	0.10	17	0.868	0.955	0.3936
207374_at	44.6	62.0	0.48	1.39	AAA-PAA	0.10	17	0.868	0.955	0.3410
219504_s_at	115.5	160.8	0.48	1.39	PPP-MAP	0.10	17	0.868	0.955	0.3225
228630_at	151.9	211.3	0.48	1.39	PPP-PPP	0.10	17	0.868	0.955	0.2799
238775_at	81.7	113.7	0.48	1.39	PPP-PPP	0.10	17	0.868	0.955	0.2783
236723_at	184.1	256.1	0.48	1.39	PPP-PPP	0.10	17	0.868	0.955	0.2594
229842_at	112.4	156.4	0.48	1.39	MPP-PAP	0.10	17	0.868	0.955	0.2405
208345_s_at	24.4	34.0	0.48	1.39	AAA-APA	0.10	17	0.868	0.955	0.5992
1560271_at	131.0	182.2	0.48	1.39	PPP-PPP	0.10	17	0.868	0.955	0.5323
1552283_s_at	60.2	83.6	0.48	1.39	APA-AAA	0.10	17	0.868	0.955	0.4069
1553693_s_at	100.0	139.0	0.48	1.39	PPP-MAP	0.10	17	0.868	0.955	0.2475
229541_at	166.4	231.2	0.47	1.39	MPP-PPP	0.10	17	0.868	0.955	0.4578
1555547_at	33.5	46.6	0.47	1.39	AAA-PAA	0.10	17	0.868	0.955	0.4185
1555775_a_at	81.7	113.4	0.47	1.39	PPP-PPP	0.10	17	0.868	0.955	0.3986
1565939_at	41.3	57.4	0.47	1.39	APP-AMP	0.10	17	0.868	0.955	0.3176
231567_s_at	81.9	113.7	0.47	1.39	AAA-AAP	0.10	17	0.868	0.955	0.1978

215246_at	44.3	61.5	0.47	1.39	APA-AAA	0.10	17	0.868	0.955	0.6263
231709_x_at	16.1	22.4	0.47	1.39	APA-AAA	0.10	17	0.868	0.955	0.5253
239068_at	73.2	101.6	0.47	1.39	MAP-PPA	0.10	17	0.868	0.955	0.3639
235846_at	126.8	176.0	0.47	1.39	PPP-PPP	0.10	17	0.868	0.955	0.3182
228902_at	158.2	219.6	0.47	1.39	PAP-PPP	0.10	17	0.868	0.955	0.2269
1555933_at	124.5	172.7	0.47	1.39	AAA-PAP	0.10	17	0.868	0.955	0.3648
224027_at	48.4	67.1	0.47	1.39	AAA-AAP	0.10	17	0.868	0.955	0.3428
1563077_at	157.2	218.0	0.47	1.39	PPP-PPP	0.10	17	0.868	0.955	0.2367
243933_at	198.8	275.7	0.47	1.39	PPP-PPP	0.10	17	0.868	0.955	0.2119
212849_at	194.6	269.8	0.47	1.39	PAA-APA	0.10	17	0.868	0.955	0.5161
233539_at	80.6	111.7	0.47	1.39	PAA-PPP	0.10	17	0.868	0.955	0.3757
209275_s_at	472.6	654.8	0.47	1.39	PPP-PPP	0.10	17	0.868	0.955	0.3654
243963_at	167.1	231.7	0.47	1.39	PPP-PPP	0.10	17	0.868	0.955	0.3209
232500_at	128.0	177.3	0.47	1.39	PPA-PPP	0.10	17	0.868	0.955	0.2067
233025_at	45.1	62.4	0.47	1.38	AAA-AAP	0.10	17	0.868	0.955	0.5544
203653_s_at	243.6	337.2	0.47	1.38	PPP-PPP	0.10	17	0.868	0.955	0.3757
241782_at	88.3	122.2	0.47	1.38	PPP-PPP	0.10	17	0.868	0.955	0.3291
242874_at	91.6	126.8	0.47	1.38	AAA-AAP	0.10	17	0.868	0.955	0.3202
223595_at	40.8	56.5	0.47	1.38	AAA-PPA	0.10	17	0.868	0.955	0.3152
214868_at	38.2	52.9	0.47	1.38	AAA-APA	0.10	17	0.868	0.955	0.2375
238299_at	237.1	328.2	0.47	1.38	PPP-PPP	0.10	17	0.868	0.955	0.2232
229765_at	251.6	348.0	0.47	1.38	PPP-PPP	0.10	17	0.868	0.955	0.4767
218933_at	437.9	605.8	0.47	1.38	PPP-PPP	0.10	17	0.868	0.955	0.4643
237856_at	47.7	66.0	0.47	1.38	PAP-AAA	0.10	17	0.868	0.955	0.4447
243045_at	72.5	100.4	0.47	1.38	AAA-PAA	0.10	17	0.868	0.955	0.3611
223862_at	59.7	82.5	0.47	1.38	AAP-AAA	0.10	17	0.868	0.955	0.3297
242663_at	79.0	109.3	0.47	1.38	PAP-PPP	0.10	17	0.868	0.955	0.2806
234620_at	47.2	65.2	0.47	1.38	APA-APA	0.10	17	0.868	0.955	0.6228
1559069_at	33.5	46.3	0.47	1.38	PAA-AAP	0.10	17	0.868	0.955	0.4828
210395_x_at	179.6	248.2	0.47	1.38	PPP-PPP	0.10	17	0.868	0.955	0.4017
216550_x_at	173.8	240.0	0.47	1.38	PPP-PPP	0.10	17	0.868	0.955	0.5207
215398_at	112.7	155.7	0.47	1.38	AAA-PAP	0.10	17	0.868	0.955	0.3980
235424_at	107.1	148.0	0.47	1.38	PPP-PMP	0.10	17	0.868	0.955	0.2133
242057_at	52.8	72.9	0.47	1.38	APA-PPA	0.10	17	0.868	0.955	0.6111
203923_s_at	37.2	51.4	0.47	1.38	AAA-APA	0.10	17	0.868	0.955	0.5262
234357_at	40.0	55.2	0.47	1.38	AAA-PMA	0.10	17	0.868	0.955	0.3955
230535_s_at	139.7	192.8	0.47	1.38	PPP-PPP	0.10	17	0.868	0.955	0.2676
222165_x_at	260.2	359.1	0.47	1.38	PPP-PPP	0.10	17	0.868	0.955	0.2426
229461_x_at	44.5	61.4	0.47	1.38	AAA-PPP	0.10	17	0.868	0.955	0.2134
1562771_at	46.0	63.5	0.46	1.38	AAP-AAA	0.10	17	0.868	0.955	0.5668
238108_at	66.3	91.4	0.46	1.38	APA-APP	0.10	17	0.868	0.955	0.4826
1565975_at	64.1	88.4	0.46	1.38	PAA-PPP	0.10	17	0.868	0.955	0.3920

235956_at	82.1	113.3	0.46	1.38	PPP-PPP	0.10	17	0.868	0.955	0.3817
217487_x_at	68.6	94.6	0.46	1.38	APP-PAP	0.10	17	0.868	0.955	0.3739
232086_at	74.4	102.6	0.46	1.38	PAP-PAP	0.10	17	0.868	0.955	0.3679
239243_at	92.8	128.1	0.46	1.38	PPP-PPP	0.10	17	0.868	0.955	0.3263
205441_at	264.2	364.3	0.46	1.38	PPP-PPP	0.10	17	0.868	0.955	0.2878
221044_s_at	130.3	179.7	0.46	1.38	PPP-PPP	0.10	17	0.868	0.955	0.2618
231868_at	48.7	67.1	0.46	1.38	APA-PAP	0.10	17	0.868	0.955	0.2522
1568854_at	30.7	42.3	0.46	1.38	AAA-PPA	0.10	17	0.868	0.955	0.2125
237968_at	55.5	76.5	0.46	1.38	MAA-PAP	0.10	17	0.868	0.955	0.4139
218276_s_at	670.5	924.1	0.46	1.38	PPP-PPP	0.10	17	0.868	0.955	0.3004
235190_at	411.1	566.7	0.46	1.38	PPP-PPP	0.10	17	0.868	0.955	0.2476
244597_at	136.1	187.6	0.46	1.38	PAP-PPP	0.10	17	0.868	0.955	0.2446
241626_at	64.2	88.5	0.46	1.38	MAP-PPP	0.10	17	0.868	0.955	0.2307
225542_at	64.3	88.6	0.46	1.38	APA-AAA	0.10	17	0.868	0.955	0.4335
222895_s_at	102.9	141.7	0.46	1.38	APP-PAP	0.10	17	0.868	0.955	0.2946
212162_at	354.9	489.0	0.46	1.38	PPP-PPP	0.10	17	0.868	0.955	0.2219
1564798_at	68.2	93.9	0.46	1.38	AAA-AMP	0.10	17	0.868	0.955	0.5068
217671_at	66.0	90.8	0.46	1.38	APA-AAP	0.10	17	0.868	0.955	0.3481
223728_at	163.2	224.6	0.46	1.38	APP-PPP	0.10	17	0.868	0.955	0.3120
1555370_a_at	51.5	70.8	0.46	1.38	APA-PPP	0.10	17	0.868	0.955	0.2981
212991_at	76.8	105.7	0.46	1.38	PPA-APP	0.10	17	0.868	0.955	0.2903
1556088_at	32.3	44.4	0.46	1.38	PAP-APM	0.10	17	0.868	0.955	0.4978
232816_s_at	177.2	243.7	0.46	1.38	PPP-PPP	0.10	17	0.868	0.955	0.3921
232161_x_at	49.1	67.5	0.46	1.38	APA-PPP	0.10	17	0.868	0.955	0.3701
239680_at	165.7	227.9	0.46	1.38	PPP-PPP	0.10	17	0.868	0.955	0.2690
230213_at	142.6	196.1	0.46	1.38	PAP-PAP	0.10	17	0.868	0.955	0.2237
236194_at	355.0	488.4	0.46	1.38	PPP-PPP	0.10	17	0.868	0.955	0.1979
205571_at	493.9	359.3	-0.46	-1.37	PPP-PPP	0.10	17	0.868	0.955	0.0019
1558624_at	52.9	72.7	0.46	1.38	AAA-AAP	0.10	17	0.868	0.955	0.5160
240107_at	28.1	38.7	0.46	1.38	APA-APA	0.10	17	0.868	0.955	0.5079
211549_s_at	97.2	133.6	0.46	1.38	MAA-APP	0.10	17	0.868	0.955	0.4229
226783_at	139.3	191.5	0.46	1.37	PAP-MPP	0.10	17	0.868	0.955	0.4174
212803_at	224.8	309.1	0.46	1.38	APP-AAP	0.10	17	0.868	0.955	0.3587
235462_at	25.2	34.6	0.46	1.37	PAA-PPA	0.10	17	0.868	0.955	0.3276
235028_at	478.4	657.6	0.46	1.38	PPP-PPP	0.10	17	0.868	0.955	0.2583
228718_at	120.8	166.1	0.46	1.37	PPP-PPP	0.10	17	0.868	0.955	0.2129
222860_s_at	50.5	69.4	0.46	1.37	AAA-PAA	0.10	17	0.868	0.955	0.5466
218939_at	224.3	308.2	0.46	1.37	PPP-PPP	0.10	17	0.868	0.955	0.5084
238248_at	41.6	57.1	0.46	1.37	AAA-AAP	0.10	17	0.868	0.955	0.4463
219551_at	103.8	142.6	0.46	1.37	PPP-APP	0.10	17	0.868	0.955	0.2631
1562940_at	78.8	108.3	0.46	1.37	AAA-APP	0.10	17	0.868	0.955	0.2352
203560_at	2769.0	2017.4	-0.46	-1.37	PPP-PPP	0.10	17	0.868	0.955	0.0034

240251_at	19.5	26.7	0.46	1.37	AAP-AAA	0.10	17	0.868	0.955	0.5819
242853_at	121.6	166.9	0.46	1.37	PAP-PPP	0.10	17	0.868	0.955	0.3934
235611_at	195.8	268.7	0.46	1.37	PPP-PPP	0.10	17	0.868	0.955	0.3237
239489_at	109.4	150.1	0.46	1.37	PAM-PAP	0.10	17	0.868	0.955	0.1958
242091_at	34.4	47.2	0.46	1.37	AAA-AMP	0.10	17	0.868	0.955	0.3703
221213_s_at	140.0	192.1	0.46	1.37	PPP-PPP	0.10	17	0.868	0.955	0.3280
235757_at	123.8	169.7	0.46	1.37	AMM-PPP	0.10	17	0.868	0.955	0.3228
216858_x_at	307.3	421.4	0.46	1.37	APP-APP	0.10	17	0.868	0.955	0.2692
219713_at	173.7	238.2	0.46	1.37	PAP-PAP	0.10	17	0.868	0.955	0.2083
220121_at	58.8	80.6	0.46	1.37	PAP-PMA	0.10	17	0.868	0.955	0.4191
243509_at	61.1	83.7	0.46	1.37	APA-PPP	0.10	17	0.868	0.955	0.3466
240778_at	68.6	93.9	0.46	1.37	PPA-AAA	0.10	17	0.868	0.955	0.3340
235646_at	94.6	129.7	0.46	1.37	AAP-PAP	0.10	17	0.868	0.955	0.3186
205409_at	133.5	183.0	0.46	1.37	APA-AAP	0.10	17	0.868	0.955	0.3163
233203_at	62.3	85.4	0.45	1.37	AMA-AAP	0.10	17	0.868	0.955	0.4898
242311_x_at	275.3	377.2	0.45	1.37	PPP-PPP	0.10	17	0.868	0.955	0.4823
220609_at	77.4	106.1	0.45	1.37	AAA-PAA	0.10	17	0.868	0.955	0.3315
228566_at	142.8	195.6	0.45	1.37	AAA-AAP	0.10	17	0.868	0.955	0.3000
1556750_at	29.3	40.1	0.45	1.37	AAA-AAP	0.10	17	0.868	0.955	0.2398
1560144_at	28.3	38.7	0.45	1.37	APA-PPA	0.10	17	0.868	0.955	0.5934
1555729_a_at	49.4	67.6	0.45	1.37	AAA-PPA	0.10	17	0.868	0.955	0.5260
235059_at	94.1	128.8	0.45	1.37	AAP-APA	0.10	17	0.868	0.955	0.4367
214808_at	333.1	455.9	0.45	1.37	PPP-PPP	0.10	17	0.868	0.955	0.4063
213703_at	119.0	162.9	0.45	1.37	PPP-PPP	0.10	17	0.868	0.955	0.4044
231078_at	145.2	198.8	0.45	1.37	AMA-PAM	0.10	17	0.868	0.955	0.3389
244864_at	65.2	89.2	0.45	1.37	AAP-PAA	0.10	17	0.868	0.955	0.3326
211993_at	304.6	417.0	0.45	1.37	PPP-PPP	0.10	17	0.868	0.955	0.2526
203451_at	270.7	370.4	0.45	1.37	PAM-AAM	0.10	17	0.868	0.955	0.2038
224004_at	48.7	66.6	0.45	1.37	APA-AAA	0.10	17	0.868	0.955	0.6306
215639_at	31.5	43.1	0.45	1.37	AAA-PAA	0.10	17	0.868	0.955	0.5752
238417_at	113.6	155.5	0.45	1.37	MAA-APP	0.10	17	0.868	0.955	0.4737
1569310_at	70.6	96.5	0.45	1.37	AAA-PAP	0.10	17	0.868	0.955	0.2856
221648_s_at	44.6	61.0	0.45	1.37	APA-PPA	0.10	17	0.868	0.955	0.2809
242881_x_at	677.8	927.3	0.45	1.37	PPP-PPP	0.10	17	0.868	0.955	0.2016
1555049_at	39.2	53.5	0.45	1.37	AAA-PAA	0.10	17	0.868	0.955	0.6357
240061_at	31.4	43.0	0.45	1.37	AAA-PAM	0.10	17	0.868	0.955	0.5290
1565495_at	110.5	151.1	0.45	1.37	PAA-APM	0.10	17	0.868	0.955	0.2212
244349_at	88.0	120.3	0.45	1.37	PAA-AAA	0.10	17	0.868	0.955	0.2049
232180_at	70.7	96.6	0.45	1.37	PPP-PPP	0.10	17	0.868	0.955	0.3418
223620_at	32.0	43.7	0.45	1.37	PAA-AAA	0.10	17	0.868	0.955	0.3080
240263_at	89.0	121.6	0.45	1.37	AAA-AAP	0.10	17	0.868	0.955	0.2896
227478_at	199.1	272.1	0.45	1.37	PPP-PPP	0.10	17	0.868	0.955	0.2049

242992_at	85.9	117.3	0.45	1.37	PPA-PAA	0.10	17	0.868	0.955	0.5998
1556047_s_at	41.4	56.5	0.45	1.37	APA-APA	0.10	17	0.868	0.955	0.4799
242976_at	35.5	48.4	0.45	1.37	AAA-APP	0.10	17	0.868	0.955	0.3905
244297_at	199.2	272.0	0.45	1.37	PPP-PPP	0.10	17	0.868	0.955	0.3588
235011_at	65.4	89.3	0.45	1.37	PPP-PPP	0.10	17	0.868	0.955	0.3093
233078_at	216.5	295.5	0.45	1.37	PPP-PPP	0.10	17	0.868	0.955	0.2633
216488_s_at	181.1	247.1	0.45	1.36	AAP-PAP	0.10	17	0.868	0.955	0.5506
236379_at	90.8	123.9	0.45	1.36	PAP-PPP	0.10	17	0.868	0.955	0.5321
243444_at	97.0	132.3	0.45	1.36	AAP-AAP	0.10	17	0.868	0.955	0.4815
216678_at	239.3	326.4	0.45	1.36	PPP-PPP	0.10	17	0.868	0.955	0.2501
214087_s_at	46.6	63.5	0.45	1.36	AAA-MAP	0.10	17	0.868	0.955	0.2143
241755_at	67.9	92.6	0.45	1.36	PPP-PPP	0.10	17	0.868	0.955	0.1868
214588_s_at	81.5	111.1	0.45	1.36	AAP-AMA	0.10	17	0.868	0.955	0.6036
222861_x_at	69.5	94.8	0.45	1.36	PAA-AAA	0.10	17	0.868	0.955	0.4742
228173_at	173.3	236.2	0.45	1.36	PPP-PPP	0.10	17	0.868	0.955	0.4234
243358_at	116.8	159.3	0.45	1.36	AAA-APP	0.10	17	0.868	0.955	0.3969
1555996_s_at	120.1	163.8	0.45	1.36	PPP-PPP	0.10	17	0.868	0.955	0.3379
205668_at	27.2	37.1	0.45	1.36	PAP-PAA	0.10	17	0.868	0.955	0.5867
239907_at	54.1	73.7	0.45	1.36	AAA-PAA	0.10	17	0.868	0.955	0.3770
1553275_s_at	58.0	79.0	0.45	1.36	PPP-PPP	0.10	17	0.868	0.955	0.3465
1557149_at	57.5	78.3	0.45	1.36	AAP-APA	0.10	17	0.868	0.955	0.3447
235208_at	126.1	171.7	0.45	1.36	PPA-PPP	0.10	17	0.868	0.955	0.2326
1569024_at	96.3	131.2	0.45	1.36	PPP-PPP	0.10	17	0.868	0.955	0.2246
206393_at	392.1	534.2	0.45	1.36	PPP-PPP	0.10	17	0.868	0.955	0.1878
1569607_s_at	209.7	285.4	0.45	1.36	PPP-PPP	0.10	17	0.868	0.955	0.5667
215850_s_at	32.0	43.6	0.45	1.36	PAA-AAA	0.10	17	0.868	0.955	0.4310
1561303_at	42.7	58.1	0.45	1.36	AAA-PAA	0.10	17	0.868	0.955	0.3886
223357_s_at	38.9	53.0	0.45	1.36	PAA-PAA	0.10	17	0.868	0.955	0.3732
238231_at	47.3	64.4	0.45	1.36	PAA-AAM	0.10	17	0.868	0.955	0.3379
233364_s_at	173.6	236.2	0.45	1.36	PPP-PPP	0.10	17	0.868	0.955	0.2367
1552414_at	49.6	67.4	0.44	1.36	AAA-AAP	0.10	17	0.868	0.955	0.6192
1569251_a_at	81.2	110.5	0.44	1.36	AAM-PAA	0.10	17	0.868	0.955	0.4819
214163_at	47.0	63.9	0.44	1.36	PAA-PAP	0.10	17	0.868	0.955	0.4780
216805_at	50.0	68.0	0.44	1.36	PAP-PAP	0.10	17	0.868	0.955	0.4727
215513_at	57.9	78.8	0.44	1.36	AAA-PAA	0.10	17	0.868	0.955	0.4406
232200_at	44.1	60.0	0.44	1.36	PAA-APP	0.10	17	0.868	0.955	0.4334
211362_s_at	67.1	91.3	0.44	1.36	PPA-PAP	0.10	17	0.868	0.955	0.4311
227290_at	77.9	105.9	0.44	1.36	AAA-PAP	0.10	17	0.868	0.955	0.2902
210531_at	101.7	138.3	0.44	1.36	PAP-PPP	0.10	17	0.868	0.955	0.2650
228397_at	190.8	259.4	0.44	1.36	PPP-PPP	0.10	17	0.868	0.955	0.2644
235559_at	200.0	272.1	0.44	1.36	PPP-PPP	0.10	17	0.868	0.955	0.2129
204349_at	943.4	1283.1	0.44	1.36	PPP-PPP	0.10	17	0.868	0.955	0.1949

238550_at	43.0	58.4	0.44	1.36	APP-PPP	0.10	17	0.868	0.955	0.5000
215945_s_at	202.2	274.9	0.44	1.36	PPP-PPP	0.10	17	0.868	0.955	0.3439
203469_s_at	148.9	202.4	0.44	1.36	PAP-APP	0.10	17	0.868	0.955	0.2908
242586_at	80.1	108.9	0.44	1.36	PPP-PPP	0.10	17	0.868	0.955	0.2686
244290_at	60.6	82.4	0.44	1.36	AAP-AAP	0.10	17	0.868	0.955	0.2546
227973_at	833.0	1132.8	0.44	1.36	PPP-PPP	0.10	17	0.868	0.955	0.2200
239693_at	33.0	44.8	0.44	1.36	AAA-PAA	0.10	17	0.868	0.955	0.6306
1562467_at	43.6	59.3	0.44	1.36	MAP-AAP	0.10	17	0.868	0.955	0.3867
1552309_a_at	53.1	72.1	0.44	1.36	AAP-PPP	0.10	17	0.868	0.955	0.3444
215203_at	161.7	219.7	0.44	1.36	PPP-PPP	0.10	17	0.868	0.955	0.2291
207005_s_at	38.5	52.3	0.44	1.36	PAA-PAA	0.10	17	0.868	0.955	0.6040
234667_at	71.8	97.4	0.44	1.36	APA-PPP	0.10	17	0.868	0.955	0.5596
1552897_a_at	28.6	38.8	0.44	1.36	AAA-AAP	0.10	17	0.868	0.955	0.5047
218614_at	405.5	550.3	0.44	1.36	PPP-PPP	0.10	17	0.868	0.955	0.5045
240971_x_at	90.8	123.3	0.44	1.36	AAA-APP	0.10	17	0.868	0.955	0.4583
238970_at	54.4	73.8	0.44	1.36	PPM-PPP	0.10	17	0.868	0.955	0.3452
227647_at	55.0	74.7	0.44	1.36	AAA-PAA	0.10	17	0.868	0.955	0.2985
241816_at	183.4	249.1	0.44	1.36	PPP-PPP	0.10	17	0.868	0.955	0.1933
203851_at	7451.9	5491.3	-0.44	-1.36	PPP-PPP	0.10	17	0.868	0.955	0.0031
1555489_at	25.4	34.5	0.44	1.36	AAA-PAA	0.10	17	0.868	0.955	0.5415
232048_at	137.8	187.0	0.44	1.36	PAP-PPP	0.10	17	0.868	0.955	0.4362
233252_s_at	350.3	475.1	0.44	1.36	PPP-PPP	0.10	17	0.868	0.955	0.1958
216187_x_at	1767.3	2397.6	0.44	1.36	PPP-PPP	0.10	17	0.868	0.955	0.1908
226667_x_at	193.9	262.9	0.44	1.36	APP-PPP	0.10	17	0.868	0.955	0.4770
1557309_at	49.6	67.2	0.44	1.36	APP-PPP	0.10	17	0.868	0.955	0.3032
229574_at	245.3	332.5	0.44	1.36	PPP-PPP	0.10	17	0.868	0.955	0.2127
213693_s_at	2480.3	3363.4	0.44	1.36	PPP-PPP	0.10	17	0.868	0.955	0.1968
1552794_a_at	36.3	49.1	0.44	1.36	AAA-PAA	0.10	17	0.868	0.955	0.6221
238289_at	48.5	65.7	0.44	1.35	APA-AAA	0.10	17	0.868	0.955	0.5228
216983_s_at	75.2	101.9	0.44	1.36	PAP-PAP	0.10	17	0.868	0.955	0.4047
226587_at	116.1	157.3	0.44	1.36	PAP-MPP	0.10	17	0.868	0.955	0.2466
220243_at	96.7	130.9	0.44	1.35	AAA-APP	0.10	17	0.868	0.955	0.2214
1558515_at	236.3	320.1	0.44	1.35	PPP-PPP	0.10	17	0.868	0.955	0.2133
222903_s_at	72.0	97.5	0.44	1.35	PPP-PPP	0.10	17	0.868	0.955	0.2080
220140_s_at	243.4	329.5	0.44	1.35	PPP-PPP	0.10	17	0.868	0.955	0.3143
1556186_s_at	541.6	733.2	0.44	1.35	PPP-PPP	0.10	17	0.868	0.955	0.2773
1558783_at	109.8	148.6	0.44	1.35	PPP-PPP	0.10	17	0.868	0.955	0.5440
221874_at	30.1	40.8	0.44	1.35	AAP-PAA	0.10	17	0.868	0.955	0.4696
1557986_s_at	70.6	95.5	0.44	1.35	AAA-PAM	0.10	17	0.868	0.955	0.3562
1560785_at	75.4	102.1	0.44	1.35	AAA-APA	0.10	17	0.868	0.955	0.2978
230733_at	158.0	213.7	0.44	1.35	PPP-PPP	0.10	17	0.868	0.955	0.2540
1554869_at	126.9	171.7	0.44	1.35	PAP-PPP	0.10	17	0.868	0.955	0.1840

1563528_at	34.9	47.2	0.44	1.35	APA-PAA	0.10	17	0.868	0.955	0.5452
235675_at	81.8	110.6	0.44	1.35	PPP-PPP	0.10	17	0.868	0.955	0.3794
207624_s_at	358.9	485.0	0.44	1.35	PPP-PPP	0.10	17	0.868	0.955	0.3371
234135_x_at	161.5	218.3	0.44	1.35	PPP-PPP	0.10	17	0.868	0.955	0.2670
203106_s_at	120.4	162.7	0.44	1.35	PPP-PPP	0.10	17	0.868	0.955	0.1827
240625_at	47.7	64.4	0.43	1.35	PAA-AAA	0.10	17	0.868	0.955	0.4426
241798_at	225.3	304.4	0.43	1.35	PPP-PPP	0.10	17	0.868	0.955	0.4114
1555860_x_at	129.4	174.8	0.43	1.35	PPP-PPP	0.10	17	0.868	0.955	0.3108
238130_at	120.7	163.1	0.43	1.35	AAA-MAP	0.10	17	0.868	0.955	0.2743
223882_at	76.3	103.1	0.43	1.35	PPA-PAP	0.10	17	0.868	0.955	0.2713
202132_at	308.3	416.6	0.43	1.35	PPP-PPP	0.10	17	0.868	0.955	0.2502
213584_s_at	154.5	208.7	0.43	1.35	PAM-PPP	0.10	17	0.868	0.955	0.1874
202032_s_at	305.7	412.6	0.43	1.35	PPP-PPP	0.10	17	0.868	0.955	0.4868
235084_x_at	259.2	349.9	0.43	1.35	PPP-PPP	0.10	17	0.868	0.955	0.4230
244716_x_at	171.4	231.4	0.43	1.35	AAP-AMA	0.10	17	0.868	0.955	0.3229
218382_s_at	491.3	663.3	0.43	1.35	PPP-PPP	0.10	17	0.868	0.955	0.2572
234082_at	184.5	248.9	0.43	1.35	PPP-PPP	0.10	17	0.868	0.955	0.4235
238545_at	87.3	117.7	0.43	1.35	PAA-AAA	0.10	17	0.868	0.955	0.3656
212148_at	143.3	193.3	0.43	1.35	PAP-PPP	0.10	17	0.868	0.955	0.3352
235506_at	44.6	60.2	0.43	1.35	AAA-AAP	0.10	17	0.868	0.955	0.3161
1559332_at	54.1	72.9	0.43	1.35	PAA-PAP	0.10	17	0.868	0.955	0.5726
242256_x_at	153.9	207.5	0.43	1.35	PAP-PAP	0.10	17	0.868	0.955	0.3022
237085_x_at	154.6	208.4	0.43	1.35	AAP-PAP	0.10	17	0.868	0.955	0.2328
240877_x_at	70.9	95.6	0.43	1.35	APP-APP	0.10	17	0.868	0.955	0.2068
224051_at	35.9	48.4	0.43	1.35	AAA-AAP	0.10	17	0.868	0.955	0.3111
228676_at	105.5	142.1	0.43	1.35	AAA-PPA	0.10	17	0.868	0.955	0.2708
207559_s_at	433.0	321.6	-0.43	-1.35	PPP-PPP	0.10	17	0.868	0.955	0.0021
1569597_at	69.2	93.2	0.43	1.35	AAP-AAA	0.10	17	0.868	0.955	0.6247
232715_at	67.6	91.0	0.43	1.35	PAP-PPP	0.10	17	0.868	0.955	0.4114
242931_at	67.0	90.2	0.43	1.35	AAP-PPP	0.10	17	0.868	0.955	0.3457
243253_at	71.2	95.8	0.43	1.35	PPP-PPP	0.10	17	0.868	0.955	0.3054
1554411_at	60.0	80.8	0.43	1.35	AAP-PPA	0.10	17	0.868	0.955	0.2902
220474_at	78.3	105.4	0.43	1.35	PPP-PPP	0.10	17	0.868	0.955	0.2317
219608_s_at	212.4	286.1	0.43	1.35	PPP-PPP	0.10	17	0.868	0.955	0.2184
232156_at	212.8	286.5	0.43	1.35	PPP-PPP	0.10	17	0.868	0.955	0.2025
216379_x_at	11251.7	15149.2	0.43	1.35	PPP-PPP	0.10	17	0.868	0.955	0.1926
207130_at	111.9	150.6	0.43	1.35	PPP-PAP	0.10	17	0.868	0.955	0.4993
234541_s_at	41.2	55.5	0.43	1.35	PAA-AAA	0.10	17	0.868	0.955	0.4574
242822_at	79.7	107.2	0.43	1.35	PAA-PPA	0.10	17	0.868	0.955	0.3797
241799_x_at	236.9	318.8	0.43	1.35	PPP-PMP	0.10	17	0.868	0.955	0.3442
213173_at	122.1	164.3	0.43	1.35	PAP-PPP	0.10	17	0.868	0.955	0.3385
205112_at	37.5	50.5	0.43	1.35	AAA-APA	0.10	17	0.868	0.955	0.3282

52651_at	65.7	88.5	0.43	1.35	PMA-PPP	0.10	17	0.868	0.955	0.3067
207425_s_at	130.9	176.2	0.43	1.35	APP-PPP	0.10	17	0.868	0.955	0.2510
242371_x_at	296.1	398.5	0.43	1.35	PPP-PPP	0.10	17	0.868	0.955	0.1942
228675_at	45.4	61.0	0.43	1.35	AAP-AAP	0.10	17	0.868	0.955	0.5401
221957_at	108.4	145.7	0.43	1.35	PPP-PPP	0.10	17	0.868	0.955	0.2624
240269_at	40.3	54.2	0.43	1.34	PAM-APP	0.10	17	0.868	0.955	0.2584
227270_at	255.7	343.9	0.43	1.35	PPP-PPP	0.10	17	0.868	0.955	0.2313
202182_at	792.5	1065.2	0.43	1.34	PPP-PPP	0.10	17	0.868	0.955	0.1849
243644_at	34.9	46.9	0.43	1.34	APA-PAP	0.10	17	0.868	0.955	0.6291
1555159_at	68.2	91.6	0.43	1.34	AMA-PAP	0.10	17	0.868	0.955	0.5991
241491_at	61.9	83.0	0.43	1.34	APA-APP	0.10	17	0.868	0.955	0.4949
227703_s_at	72.3	97.0	0.43	1.34	AAP-PAA	0.10	17	0.868	0.955	0.3147
229926_at	141.6	190.1	0.43	1.34	PPP-PPP	0.10	17	0.868	0.955	0.2144
233684_at	36.2	48.6	0.42	1.34	APA-PAA	0.10	17	0.868	0.955	0.5213
1556722_a_at	117.4	157.5	0.42	1.34	PAA-AAA	0.10	17	0.868	0.955	0.4748
239571_at	65.0	87.2	0.42	1.34	APP-PPP	0.10	17	0.868	0.955	0.4493
202687_s_at	91.9	123.3	0.42	1.34	PPA-PPP	0.10	17	0.868	0.955	0.4090
200785_s_at	128.3	172.0	0.42	1.34	PAM-PPP	0.10	17	0.868	0.955	0.3037
211077_s_at	96.7	129.8	0.42	1.34	PPP-APP	0.10	17	0.868	0.955	0.2787
237650_at	55.6	74.6	0.42	1.34	AAA-APP	0.10	17	0.868	0.955	0.2765
1558154_at	84.9	113.9	0.42	1.34	AAA-PPA	0.10	17	0.868	0.955	0.2634
235273_at	103.7	139.1	0.42	1.34	PMP-APP	0.10	17	0.868	0.955	0.2144
232463_at	83.9	112.5	0.42	1.34	PAP-AAA	0.10	17	0.868	0.955	0.4478
237032_x_at	117.9	158.0	0.42	1.34	PPP-PPP	0.10	17	0.868	0.955	0.4252
226212_s_at	111.5	149.5	0.42	1.34	PPA-PPP	0.10	17	0.868	0.955	0.3929
224259_at	57.9	77.6	0.42	1.34	AAA-APA	0.10	17	0.868	0.955	0.2827
228142_at	370.4	496.8	0.42	1.34	APA-PPP	0.10	17	0.868	0.955	0.1861
1553133_at	109.4	146.5	0.42	1.34	PPP-PPP	0.10	17	0.868	0.955	0.5326
226334_s_at	414.6	555.5	0.42	1.34	PPP-PPP	0.10	17	0.868	0.955	0.3785
244425_at	75.7	101.5	0.42	1.34	PAP-AAP	0.10	17	0.868	0.955	0.3270
237626_at	184.8	247.7	0.42	1.34	PPP-PPP	0.10	17	0.868	0.955	0.2615
242110_at	166.9	223.4	0.42	1.34	PPP-PPP	0.10	17	0.868	0.955	0.4353
1569665_at	64.5	86.3	0.42	1.34	MPA-APP	0.10	17	0.868	0.955	0.3779
230180_at	337.2	451.4	0.42	1.34	PPP-PPP	0.10	17	0.868	0.955	0.3630
205705_at	70.3	94.1	0.42	1.34	AAA-AAP	0.10	17	0.868	0.955	0.3372
203892_at	186.1	249.1	0.42	1.34	PPP-PPP	0.10	17	0.868	0.955	0.3252
232150_at	107.9	144.4	0.42	1.34	APP-PPP	0.10	17	0.868	0.955	0.2642
205158_at	263.9	353.2	0.42	1.34	PPP-PPP	0.10	17	0.868	0.955	0.2306
234442_at	71.8	96.1	0.42	1.34	APA-APP	0.10	17	0.868	0.955	0.5159
235274_at	101.9	136.4	0.42	1.34	PPA-PPP	0.10	17	0.868	0.955	0.3912
239478_x_at	51.4	68.8	0.42	1.34	PPP-MMP	0.10	17	0.868	0.955	0.3865
226111_s_at	125.6	168.0	0.42	1.34	APP-PPA	0.10	17	0.868	0.955	0.3211

208883_at	519.2	694.7	0.42	1.34	PPP-PPP	0.10	17	0.868	0.955	0.2137
210598_at	83.5	111.7	0.42	1.34	PPM-PPP	0.10	17	0.868	0.955	0.2121
215711_s_at	488.4	653.4	0.42	1.34	AAM-AAP	0.10	17	0.868	0.955	0.1825
230842_at	55.2	73.8	0.42	1.34	APA-AAP	0.10	17	0.868	0.955	0.5608
233413_at	176.4	235.8	0.42	1.34	PPP-PPP	0.10	17	0.868	0.955	0.3422
227498_at	149.7	200.2	0.42	1.34	PPP-PPP	0.10	17	0.868	0.955	0.2361
1553781_at	32.1	42.9	0.42	1.34	AAA-PPP	0.10	17	0.868	0.955	0.2137
203787_at	688.9	921.1	0.42	1.34	PPP-PPP	0.10	17	0.868	0.955	0.1856
1569323_at	84.4	112.8	0.42	1.34	APP-PAP	0.10	17	0.868	0.955	0.6048
230516_at	47.5	63.4	0.42	1.34	AAA-AAP	0.10	17	0.868	0.955	0.4854
239872_at	64.6	86.3	0.42	1.34	PAA-APP	0.10	17	0.868	0.955	0.4388
208452_x_at	136.2	181.9	0.42	1.34	AAA-PAA	0.10	17	0.868	0.955	0.3646
212070_at	224.7	300.1	0.42	1.34	PPP-PPP	0.10	17	0.868	0.955	0.2978
238161_at	85.6	114.3	0.42	1.34	PAP-PPP	0.10	17	0.868	0.955	0.5075
206008_at	643.9	859.6	0.42	1.34	PPP-PPP	0.10	17	0.868	0.955	0.4282
214969_at	138.9	185.4	0.42	1.34	AAP-APP	0.10	17	0.868	0.955	0.3675
241613_at	117.1	156.4	0.42	1.34	AMP-PAP	0.10	17	0.868	0.955	0.2950
1567079_at	142.3	190.0	0.42	1.34	MPP-PAP	0.10	17	0.868	0.955	0.2589
239893_at	104.5	139.5	0.42	1.34	PPP-PPP	0.10	17	0.868	0.955	0.2385
1555448_at	50.1	66.9	0.42	1.34	AAM-PAA	0.10	17	0.868	0.955	0.1947
1570506_at	23.4	31.2	0.42	1.33	AAA-AAP	0.10	17	0.868	0.955	0.6253
216049_at	35.3	47.1	0.42	1.33	PAA-APP	0.10	17	0.868	0.955	0.4171
204867_at	239.1	318.9	0.42	1.33	PPP-PPP	0.10	17	0.868	0.955	0.3191
1557400_at	77.5	103.4	0.42	1.33	APA-PPP	0.10	17	0.868	0.955	0.2736
226239_at	166.7	222.4	0.42	1.33	PPP-PPA	0.10	17	0.868	0.955	0.2694
238535_at	77.9	103.9	0.42	1.33	PPM-APM	0.10	17	0.868	0.955	0.2401
238490_at	189.7	253.0	0.42	1.33	AMP-PPP	0.10	17	0.868	0.955	0.2089
238093_at	50.6	67.5	0.42	1.33	AAP-AAA	0.10	17	0.868	0.955	0.5825
1566446_at	45.6	60.8	0.42	1.33	AAA-AAP	0.10	17	0.868	0.955	0.5027
241075_at	29.5	39.4	0.42	1.33	AAA-PAA	0.10	17	0.868	0.955	0.4536
1558698_at	108.1	144.1	0.42	1.33	AAP-PPP	0.10	17	0.868	0.955	0.3146
215604_x_at	437.0	582.6	0.42	1.33	PPP-PPP	0.10	17	0.868	0.955	0.3016
243316_x_at	32.4	43.2	0.42	1.33	AAA-AAP	0.10	17	0.868	0.955	0.2462
240847_at	22.8	30.3	0.41	1.33	APA-AAA	0.10	17	0.868	0.955	0.6259
223478_at	953.6	1270.6	0.41	1.33	PPP-PPP	0.10	17	0.868	0.955	0.3828
239127_at	104.8	139.7	0.41	1.33	AAA-APA	0.10	17	0.868	0.955	0.3537
207711_at	124.9	166.4	0.41	1.33	MAP-PPP	0.10	17	0.868	0.955	0.3810
226872_at	97.2	129.3	0.41	1.33	MAA-PPP	0.10	17	0.868	0.955	0.3443
1566597_at	40.1	53.4	0.41	1.33	APA-PAP	0.10	17	0.868	0.955	0.3062
207176_s_at	90.7	120.8	0.41	1.33	AAA-PAA	0.10	17	0.868	0.955	0.3026
223608_at	739.7	984.8	0.41	1.33	PPP-PPP	0.10	17	0.868	0.955	0.2760
243414_at	111.8	148.8	0.41	1.33	APP-PAP	0.10	17	0.868	0.955	0.2449

236985_at	117.1	156.0	0.41	1.33	PAP-PPP	0.10	17	0.868	0.955	0.2061
223640_at	108.9	144.8	0.41	1.33	PPA-PAA	0.10	17	0.868	0.955	0.5555
232071_at	186.7	248.4	0.41	1.33	PPP-PPP	0.10	17	0.868	0.955	0.4201
208858_s_at	1298.5	1727.2	0.41	1.33	PPP-PPP	0.10	17	0.868	0.955	0.2881
220999_s_at	144.4	192.2	0.41	1.33	PMP-PPP	0.10	17	0.868	0.955	0.2821
227611_at	217.1	288.9	0.41	1.33	PPP-PPP	0.10	17	0.868	0.955	0.2766
242331_x_at	33.7	44.9	0.41	1.33	AAP-PAP	0.10	17	0.868	0.955	0.2427
215412_x_at	174.9	232.7	0.41	1.33	PPP-PMP	0.10	17	0.868	0.955	0.1938
220451_s_at	91.3	121.4	0.41	1.33	PAA-APA	0.10	17	0.868	0.955	0.5734
1569342_at	60.2	80.0	0.41	1.33	APA-PAA	0.10	17	0.868	0.955	0.5566
236649_at	95.5	127.0	0.41	1.33	PAP-PPP	0.10	17	0.868	0.955	0.4477
205881_at	110.8	147.2	0.41	1.33	PPP-PMP	0.10	17	0.868	0.955	0.3772
203427_at	664.4	883.3	0.41	1.33	PPP-PPP	0.10	17	0.868	0.955	0.3771
206931_at	67.8	90.2	0.41	1.33	PAP-PPP	0.10	17	0.868	0.955	0.3154
1554106_at	277.2	368.6	0.41	1.33	PPP-PPP	0.10	17	0.868	0.955	0.2368
223714_at	61.5	81.7	0.41	1.33	APM-PAP	0.10	17	0.868	0.955	0.4575
1569864_at	33.9	45.0	0.41	1.33	AAA-PAP	0.10	17	0.868	0.955	0.4486
212503_s_at	58.3	77.5	0.41	1.33	PAM-PPM	0.10	17	0.868	0.955	0.4080
1570122_at	49.2	65.4	0.41	1.33	APA-AAP	0.10	17	0.868	0.955	0.3941
1566785_x_at	209.5	278.4	0.41	1.33	PPP-PPP	0.10	17	0.868	0.955	0.3118
225565_at	437.6	581.5	0.41	1.33	PPP-PPP	0.10	17	0.868	0.955	0.3059
223940_x_at	1447.2	1923.0	0.41	1.33	PPP-PPP	0.10	17	0.868	0.955	0.2928
215802_at	47.4	63.0	0.41	1.33	AAA-APA	0.10	17	0.868	0.955	0.2788
213205_s_at	119.5	158.7	0.41	1.33	PAP-APP	0.10	17	0.868	0.955	0.2748
1562755_at	25.2	33.5	0.41	1.33	APA-APA	0.10	17	0.868	0.955	0.5864
234066_at	126.1	167.4	0.41	1.33	AAA-PAA	0.10	17	0.868	0.955	0.4716
234902_s_at	66.2	87.9	0.41	1.33	AAA-AAP	0.10	17	0.868	0.955	0.3251
1552738_a_at	80.9	107.4	0.41	1.33	PAM-PPP	0.10	17	0.868	0.955	0.3038
242074_at	123.3	163.6	0.41	1.33	PPP-PPP	0.10	17	0.868	0.955	0.2736
1556051_a_at	167.3	222.1	0.41	1.33	PPP-PPP	0.10	17	0.868	0.955	0.2381
208092_s_at	135.5	179.9	0.41	1.33	APA-AAA	0.10	17	0.868	0.955	0.1842
241413_at	74.6	99.0	0.41	1.33	AAP-PPP	0.10	17	0.868	0.955	0.4759
208094_s_at	254.9	338.2	0.41	1.33	PMP-PPP	0.10	17	0.868	0.955	0.4407
208653_s_at	1208.3	1603.2	0.41	1.33	PPP-PPP	0.10	17	0.868	0.955	0.3608
235220_at	93.1	123.5	0.41	1.33	PPP-PPP	0.10	17	0.868	0.955	0.2257
1555865_at	98.6	130.8	0.41	1.33	PAA-PPA	0.10	17	0.868	0.955	0.2099
237923_at	31.8	42.2	0.41	1.33	PAA-PAP	0.10	17	0.868	0.955	0.6291
232009_at	46.8	62.0	0.41	1.33	AAP-PAP	0.10	17	0.868	0.955	0.5299
240435_at	92.9	123.2	0.41	1.33	APA-PMP	0.10	17	0.868	0.955	0.3673
229013_at	165.6	219.6	0.41	1.33	AAP-APP	0.10	17	0.868	0.955	0.3455
210829_s_at	408.8	542.0	0.41	1.33	PPP-PPP	0.10	17	0.868	0.955	0.2379
239074_at	90.9	120.5	0.41	1.33	PAA-PPP	0.10	17	0.868	0.955	0.2023

240929_at	92.3	122.4	0.41	1.33	PAA-AAA	0.10	17	0.868	0.955	0.6155
1558748_at	45.4	60.1	0.41	1.33	AAA-AAP	0.10	17	0.868	0.955	0.5595
222575_at	136.1	180.3	0.41	1.33	APP-PAP	0.10	17	0.868	0.955	0.4276
221869_at	160.6	212.7	0.41	1.33	APM-AAA	0.10	17	0.868	0.955	0.3510
209126_x_at	11483.6	15218.2	0.41	1.33	PPP-PPP	0.10	17	0.868	0.955	0.2480
236664_at	103.3	136.9	0.41	1.33	AAA-PAA	0.10	17	0.868	0.955	0.2453
238443_at	178.2	236.1	0.41	1.33	PPP-PPP	0.10	17	0.868	0.955	0.2076
229665_at	306.1	405.8	0.41	1.33	PPP-PPP	0.10	17	0.868	0.955	0.1767
212645_x_at	620.5	468.7	-0.41	-1.32	MPP-PPA	0.10	17	0.868	0.955	0.0019
221680_s_at	92.2	122.0	0.41	1.32	PAP-PPP	0.10	17	0.868	0.955	0.5615
1565852_at	105.8	140.0	0.41	1.32	AAP-PPP	0.10	17	0.868	0.955	0.3874
1552633_at	187.5	248.3	0.41	1.32	PPP-PPP	0.10	17	0.868	0.955	0.3466
242431_at	106.5	140.9	0.41	1.32	PPP-PPP	0.10	17	0.868	0.955	0.3430
233462_at	23.8	31.5	0.40	1.32	AAA-APA	0.10	17	0.868	0.955	0.6192
216126_at	103.8	137.3	0.40	1.32	AAA-MPA	0.10	17	0.868	0.955	0.5820
241773_at	32.2	42.6	0.40	1.32	APA-PAA	0.10	17	0.868	0.955	0.5725
215073_s_at	294.1	389.3	0.40	1.32	PPP-PPP	0.10	17	0.868	0.955	0.3947
1563945_at	143.1	189.2	0.40	1.32	PAM-APM	0.10	17	0.868	0.955	0.3575
1554741_s_at	124.3	164.5	0.40	1.32	PPP-PPP	0.10	17	0.868	0.955	0.2834
219312_s_at	188.3	249.2	0.40	1.32	PPP-PPP	0.10	17	0.868	0.955	0.2345
230387_at	160.7	212.7	0.40	1.32	PPP-PPP	0.10	17	0.868	0.955	0.2298
238235_at	61.3	81.1	0.40	1.32	APA-AAA	0.10	17	0.868	0.955	0.6319
211466_at	56.7	75.0	0.40	1.32	AAA-APA	0.10	17	0.868	0.955	0.5790
216509_x_at	63.5	84.0	0.40	1.32	PAP-PPM	0.10	17	0.868	0.955	0.5258
237952_at	45.4	60.1	0.40	1.32	AAA-APA	0.10	17	0.868	0.955	0.5177
219587_at	191.4	253.1	0.40	1.32	PPP-PMP	0.10	17	0.868	0.955	0.5170
228023_x_at	319.8	422.9	0.40	1.32	PPP-PPP	0.10	17	0.868	0.955	0.3032
228460_at	302.7	400.4	0.40	1.32	PPP-PPP	0.10	17	0.868	0.955	0.2239
214917_at	68.0	89.8	0.40	1.32	PAP-PPP	0.10	17	0.868	0.955	0.4695
229365_at	98.7	130.4	0.40	1.32	APP-APP	0.10	17	0.868	0.955	0.3911
234949_at	47.7	63.0	0.40	1.32	AAA-PAP	0.10	17	0.868	0.955	0.3037
241940_at	50.3	66.5	0.40	1.32	APP-APP	0.10	17	0.868	0.955	0.3026
227793_at	280.6	370.8	0.40	1.32	AAP-PPP	0.10	17	0.868	0.955	0.2155
239229_at	129.2	170.6	0.40	1.32	PPP-PPP	0.10	17	0.868	0.955	0.2012
218083_at	510.8	674.9	0.40	1.32	PPP-PPP	0.10	17	0.868	0.955	0.1981
225651_at	1609.3	2126.9	0.40	1.32	PPP-PPP	0.10	17	0.868	0.955	0.1960
237779_at	132.1	174.2	0.40	1.32	APA-AAA	0.10	17	0.868	0.955	0.5615
1566761_a_at	69.1	91.1	0.40	1.32	AAM-PAA	0.10	17	0.868	0.955	0.5592
218841_at	174.5	230.2	0.40	1.32	PPP-PPP	0.10	17	0.868	0.955	0.5021
233757_x_at	785.4	1036.2	0.40	1.32	PAM-PPM	0.10	17	0.868	0.955	0.4852
232620_at	92.5	122.0	0.40	1.32	AAA-PMA	0.10	17	0.868	0.955	0.2514
215294_s_at	58.0	76.5	0.40	1.32	AAM-PPP	0.10	17	0.868	0.955	0.2315

266_s_at	4313.1	5689.7	0.40	1.32	PPP-PPP	0.10	17	0.868	0.955	0.1847
241280_at	31.2	41.1	0.40	1.32	AAA-PAA	0.10	17	0.868	0.955	0.5761
225762_x_at	57.2	75.4	0.40	1.32	AAA-AAP	0.10	17	0.868	0.955	0.4217
1559835_at	50.6	66.7	0.40	1.32	AAA-APA	0.10	17	0.868	0.955	0.3578
239393_at	171.2	225.8	0.40	1.32	PPP-PPP	0.10	17	0.868	0.955	0.2158
1558742_at	67.5	88.9	0.40	1.32	PAA-AMP	0.10	17	0.868	0.955	0.1951
208314_at	25.1	33.1	0.40	1.32	APA-AAP	0.10	17	0.868	0.955	0.5691
216197_at	90.3	119.0	0.40	1.32	PAP-PPP	0.10	17	0.868	0.955	0.5494
1556348_at	210.1	276.9	0.40	1.32	PPP-PPP	0.10	17	0.868	0.955	0.4006
225724_at	298.8	393.8	0.40	1.32	PPP-PPP	0.10	17	0.868	0.955	0.3692
201685_s_at	363.2	478.5	0.40	1.32	PPP-PPP	0.10	17	0.868	0.955	0.2893
211460_at	85.3	112.4	0.40	1.32	PAP-PPP	0.10	17	0.868	0.955	0.2387
235989_at	163.7	215.7	0.40	1.32	AAA-APA	0.10	17	0.868	0.955	0.2185
241993_x_at	285.5	376.3	0.40	1.32	PPP-PPP	0.10	17	0.868	0.955	0.1878
238704_at	94.1	123.9	0.40	1.32	PMM-APP	0.10	17	0.868	0.955	0.6163
216859_x_at	93.7	123.4	0.40	1.32	APA-AAP	0.10	17	0.868	0.955	0.5932
202756_s_at	319.1	420.3	0.40	1.32	APP-PPP	0.10	17	0.868	0.955	0.4289
238449_at	489.9	645.2	0.40	1.32	PPP-PPP	0.10	17	0.868	0.955	0.3775
207513_s_at	521.5	686.5	0.40	1.32	PPP-PPP	0.10	17	0.868	0.955	0.3583
233605_x_at	141.3	186.0	0.40	1.32	PPP-PPP	0.10	17	0.868	0.955	0.3198
242861_at	79.2	104.2	0.40	1.32	PAP-PAA	0.10	17	0.868	0.955	0.2402
216711_s_at	128.3	169.0	0.40	1.32	APA-AAP	0.10	17	0.868	0.955	0.1833
1557350_at	153.8	202.5	0.40	1.32	PAA-APP	0.10	17	0.868	0.955	0.4394
226572_at	591.6	778.3	0.40	1.32	PPP-PPP	0.10	17	0.868	0.955	0.3995
239848_at	87.9	115.7	0.40	1.32	PPP-PPP	0.10	17	0.868	0.955	0.3875
220346_at	472.3	621.4	0.40	1.32	PPP-PPP	0.10	17	0.868	0.955	0.3699
216745_x_at	205.1	269.9	0.40	1.32	MAA-PMP	0.10	17	0.868	0.955	0.2451
205301_s_at	187.0	246.1	0.40	1.32	PPP-PPP	0.10	17	0.868	0.955	0.2182
243166_at	265.7	349.6	0.40	1.32	PPP-PPP	0.10	17	0.868	0.955	0.1895
215874_at	49.1	64.6	0.40	1.32	PAA-AAM	0.10	17	0.868	0.955	0.5067
1557953_at	640.5	842.0	0.40	1.32	PPP-PPP	0.10	17	0.868	0.955	0.4751
1570042_a_at	50.7	66.6	0.40	1.32	AAP-PAP	0.10	17	0.868	0.955	0.4464
242577_at	37.7	49.5	0.40	1.32	PAA-MAA	0.10	17	0.868	0.955	0.4055
213448_at	204.0	268.2	0.40	1.32	PPP-APP	0.10	17	0.868	0.955	0.2262
222616_s_at	1063.5	1398.3	0.40	1.32	PPP-PPP	0.10	17	0.868	0.955	0.2064
211078_s_at	101.6	133.6	0.39	1.31	PMP-PPP	0.10	17	0.868	0.955	0.5678
234182_at	72.6	95.3	0.39	1.31	PAA-PAM	0.10	17	0.868	0.955	0.5496
244457_at	59.6	78.3	0.39	1.31	APA-PPA	0.10	17	0.868	0.955	0.4382
209325_s_at	77.9	102.4	0.39	1.31	AAP-APP	0.10	17	0.868	0.955	0.4214
237703_at	97.6	128.2	0.39	1.31	APA-PPP	0.10	17	0.868	0.955	0.3880
239948_at	64.2	84.3	0.39	1.31	PPP-PPP	0.10	17	0.868	0.955	0.3654
216048_s_at	108.4	142.5	0.39	1.31	PAP-PAP	0.10	17	0.868	0.955	0.2937

234347_s_at	71.2	93.6	0.39	1.31	PPP-PPA	0.10	17	0.868	0.955	0.2673
1554176_a_at	51.8	68.1	0.39	1.31	APP-PPP	0.10	17	0.868	0.955	0.1864
217441_at	80.2	105.3	0.39	1.31	PAM-PPP	0.10	17	0.868	0.955	0.3690
213858_at	346.5	454.9	0.39	1.31	AAA-PAP	0.10	17	0.868	0.955	0.2195
215791_at	74.1	97.3	0.39	1.31	PAP-PAP	0.10	17	0.868	0.955	0.1795
214130_s_at	424.6	557.6	0.39	1.31	PPP-PPP	0.10	17	0.868	0.955	0.1749
205994_at	48.0	63.0	0.39	1.31	APP-AAA	0.10	17	0.868	0.955	0.5275
226018_at	156.8	205.7	0.39	1.31	PPP-PPP	0.10	17	0.868	0.955	0.4736
217586_x_at	253.8	333.1	0.39	1.31	PPP-PPP	0.10	17	0.868	0.955	0.4687
217544_at	59.3	77.9	0.39	1.31	APP-PPP	0.10	17	0.868	0.955	0.3317
234920_at	50.0	65.6	0.39	1.31	AAA-PAA	0.10	17	0.868	0.955	0.3246
227503_at	138.0	181.0	0.39	1.31	PPP-PPP	0.10	17	0.868	0.955	0.2756
1570210_x_at	281.2	368.9	0.39	1.31	AAP-APP	0.10	17	0.868	0.955	0.2089
1554606_at	182.8	239.6	0.39	1.31	PPP-PPP	0.10	17	0.868	0.955	0.3095
243121_x_at	188.1	246.6	0.39	1.31	AAP-PAP	0.10	17	0.868	0.955	0.3044
235990_at	337.3	442.4	0.39	1.31	PPP-PPP	0.10	17	0.868	0.955	0.2869
241366_at	143.8	188.6	0.39	1.31	PPP-PPP	0.10	17	0.868	0.955	0.2804
214140_at	135.8	178.1	0.39	1.31	PPP-PPA	0.10	17	0.868	0.955	0.2371
207857_at	29.7	38.9	0.39	1.31	PAA-AAA	0.10	17	0.868	0.955	0.5509
244049_at	49.6	65.0	0.39	1.31	AAA-APA	0.10	17	0.868	0.955	0.4630
215012_at	202.2	264.9	0.39	1.31	PPP-PPP	0.10	17	0.868	0.955	0.4311
1557987_at	887.5	1163.0	0.39	1.31	PPP-PPP	0.10	17	0.868	0.955	0.1968
1568638_a_at	34.7	45.4	0.39	1.31	APA-APA	0.10	17	0.868	0.955	0.5542
228582_x_at	638.9	836.8	0.39	1.31	PPP-PPP	0.10	17	0.868	0.955	0.4542
243604_at	80.2	105.0	0.39	1.31	APA-PAP	0.10	17	0.868	0.955	0.4158
242458_at	171.4	224.4	0.39	1.31	PPP-PPP	0.10	17	0.868	0.955	0.4079
235085_at	528.1	691.4	0.39	1.31	PPP-PPP	0.10	17	0.868	0.955	0.3797
243213_at	72.7	95.2	0.39	1.31	PPP-PAP	0.10	17	0.868	0.955	0.3603
239898_x_at	146.7	192.2	0.39	1.31	PAA-APA	0.10	17	0.868	0.955	0.2070
242030_at	83.7	109.5	0.39	1.31	PPP-PPA	0.10	17	0.868	0.955	0.4724
1557128_at	314.5	411.6	0.39	1.31	PPP-PPP	0.10	17	0.868	0.955	0.2774
1555900_at	179.2	234.6	0.39	1.31	MPP-APP	0.10	17	0.868	0.955	0.2399
206010_at	55.1	72.1	0.39	1.31	PAA-PAA	0.10	17	0.868	0.955	0.6259
239258_at	50.2	65.6	0.39	1.31	APA-PAP	0.10	17	0.868	0.955	0.4555
223936_s_at	66.1	86.4	0.39	1.31	AAP-AMP	0.10	17	0.868	0.955	0.4370
227701_at	457.9	598.9	0.39	1.31	PPP-PPP	0.10	17	0.868	0.955	0.3927
1560434_x_at	88.0	115.1	0.39	1.31	PPP-PPP	0.10	17	0.868	0.955	0.3663
205305_at	86.8	113.5	0.39	1.31	PAP-PAP	0.10	17	0.868	0.955	0.2054
209808_x_at	315.0	411.8	0.39	1.31	PMP-PPP	0.10	17	0.868	0.955	0.1856
206261_at	51.8	67.6	0.39	1.31	PAA-PAA	0.10	17	0.868	0.955	0.5597
216006_at	24.9	32.5	0.39	1.31	AAA-AAP	0.10	17	0.868	0.955	0.4834
230505_at	71.6	93.6	0.39	1.31	AAA-APA	0.10	17	0.868	0.955	0.4001

230192_at	190.5	248.9	0.39	1.31	PPP-PPP	0.10	17	0.868	0.955	0.3709
209811_at	108.9	142.3	0.39	1.31	PPP-PPP	0.10	17	0.868	0.955	0.3453
240365_at	106.9	139.7	0.39	1.31	PPP-PPP	0.10	17	0.868	0.955	0.3056
212382_at	129.2	168.9	0.39	1.31	PPP-PPP	0.10	17	0.868	0.955	0.2764
242772_x_at	111.3	145.4	0.39	1.31	PPP-PPP	0.10	17	0.868	0.955	0.1988
239259_at	48.8	63.7	0.39	1.31	AAA-APA	0.10	17	0.868	0.955	0.4848
214015_at	78.4	102.5	0.39	1.31	APM-PAA	0.10	17	0.868	0.955	0.4749
233070_at	86.4	112.9	0.39	1.31	PPP-PPP	0.10	17	0.868	0.955	0.4048
204075_s_at	148.2	193.5	0.39	1.31	PPP-PPP	0.10	17	0.868	0.955	0.2027
1556322_a_at	61.5	80.4	0.39	1.31	AAA-APP	0.10	17	0.868	0.955	0.1702
222169_x_at	179.6	234.5	0.39	1.31	PAA-PPP	0.10	17	0.868	0.955	0.1695
214716_at	36.2	47.2	0.38	1.31	PAA-AAA	0.10	17	0.868	0.955	0.5556
238673_at	460.3	600.8	0.38	1.31	PPP-PPP	0.10	17	0.868	0.955	0.5305
238421_at	93.6	122.1	0.38	1.31	PAP-PPP	0.10	17	0.868	0.955	0.3848
244561_at	170.3	222.2	0.38	1.31	APP-PAP	0.10	17	0.868	0.955	0.2513
205216_s_at	53.5	69.8	0.38	1.30	APA-AAA	0.10	17	0.868	0.955	0.5139
216556_x_at	81.2	105.9	0.38	1.30	AAA-MPA	0.10	17	0.868	0.955	0.4860
220399_at	46.5	60.6	0.38	1.30	AAP-PMP	0.10	17	0.868	0.955	0.4828
214395_x_at	179.8	234.5	0.38	1.30	PPP-PPP	0.10	17	0.868	0.955	0.4286
240125_at	85.2	111.1	0.38	1.30	AAP-APM	0.10	17	0.868	0.955	0.4024
1569519_at	113.2	147.7	0.38	1.30	PPP-PPP	0.10	17	0.868	0.955	0.3937
235638_at	122.8	160.1	0.38	1.30	PPP-PPP	0.10	17	0.868	0.955	0.2936
204237_at	106.1	138.3	0.38	1.30	PPP-PPP	0.10	17	0.868	0.955	0.2897
221790_s_at	421.6	549.8	0.38	1.30	PPP-PPP	0.10	17	0.868	0.955	0.2285
202435_s_at	440.8	574.9	0.38	1.30	PPP-PPP	0.10	17	0.868	0.955	0.2102
216349_at	256.8	334.7	0.38	1.30	PPA-AAP	0.10	17	0.868	0.955	0.5238
233908_x_at	70.4	91.8	0.38	1.30	AAP-APP	0.10	17	0.868	0.955	0.4258
218141_at	218.1	284.1	0.38	1.30	PPA-PPP	0.10	17	0.868	0.955	0.3436
217640_x_at	489.0	637.4	0.38	1.30	PPP-PPP	0.10	17	0.868	0.955	0.2718
244171_at	78.0	101.6	0.38	1.30	PAP-AAP	0.10	17	0.868	0.955	0.2697
214583_at	115.9	150.9	0.38	1.30	PPP-PPP	0.10	17	0.868	0.955	0.2622
228003_at	81.9	106.7	0.38	1.30	AAP-PPP	0.10	17	0.868	0.955	0.2561
1558569_at	153.7	200.1	0.38	1.30	PMP-PPP	0.10	17	0.868	0.955	0.4192
244852_at	53.7	69.9	0.38	1.30	APP-MPP	0.10	17	0.868	0.955	0.3140
237003_at	67.7	88.3	0.38	1.30	APP-PPP	0.10	17	0.868	0.955	0.2621
1568858_at	24.2	31.5	0.38	1.30	APP-PAP	0.10	17	0.868	0.955	0.6200
202876_s_at	153.1	199.2	0.38	1.30	PPA-PPA	0.10	17	0.868	0.955	0.5544
205586_x_at	112.9	146.9	0.38	1.30	APA-MAP	0.10	17	0.868	0.955	0.5411
242456_at	225.1	292.8	0.38	1.30	PPP-PPP	0.10	17	0.868	0.955	0.4567
235540_at	46.9	61.1	0.38	1.30	PPP-PAP	0.10	17	0.868	0.955	0.2439
218794_s_at	299.8	390.0	0.38	1.30	PPP-PPP	0.10	17	0.868	0.955	0.2262
227977_at	340.2	442.8	0.38	1.30	PPP-PPP	0.10	17	0.868	0.955	0.2196

232702_at	153.0	199.1	0.38	1.30	PPP-PPP	0.10	17	0.868	0.955	0.2071
235516_at	77.1	100.3	0.38	1.30	PAP-PPP	0.10	17	0.868	0.955	0.2025
225703_at	756.8	985.0	0.38	1.30	PPP-PPP	0.10	17	0.868	0.955	0.1865
204909_at	87.2	113.5	0.38	1.30	MPM-APP	0.10	17	0.868	0.955	0.1833
232658_at	152.9	198.9	0.38	1.30	MAA-PPP	0.10	17	0.868	0.955	0.1816
221867_at	173.3	225.5	0.38	1.30	PAP-PAA	0.10	17	0.868	0.955	0.5934
228076_s_at	114.6	149.0	0.38	1.30	AAA-AAP	0.10	17	0.868	0.955	0.5493
1564307_a_at	55.0	71.5	0.38	1.30	AAA-APP	0.10	17	0.868	0.955	0.5421
234759_at	64.0	83.3	0.38	1.30	PPP-APP	0.10	17	0.868	0.955	0.4742
227510_x_at	211.8	275.5	0.38	1.30	PPP-PPP	0.10	17	0.868	0.955	0.4603
235536_at	181.0	235.3	0.38	1.30	PPP-PPP	0.10	17	0.868	0.955	0.3560
202601_s_at	221.5	288.0	0.38	1.30	APP-PPP	0.10	17	0.868	0.955	0.3143
231399_at	132.3	172.0	0.38	1.30	PPP-MPP	0.10	17	0.868	0.955	0.3013
231616_at	49.8	64.7	0.38	1.30	AAP-PAM	0.10	17	0.868	0.955	0.2625
1559706_at	49.7	64.6	0.38	1.30	PAA-APA	0.10	17	0.868	0.955	0.2032
231458_at	42.0	54.6	0.38	1.30	AAA-AAP	0.10	17	0.868	0.955	0.5288
1567081_x_at	298.6	388.0	0.38	1.30	PPP-PPP	0.10	17	0.868	0.955	0.4499
235815_at	103.7	134.7	0.38	1.30	PAA-PPP	0.10	17	0.868	0.955	0.2353
215274_at	67.4	87.6	0.38	1.30	APA-AAA	0.10	17	0.868	0.955	0.5846
229315_at	71.9	93.4	0.38	1.30	PPA-PPP	0.10	17	0.868	0.955	0.4344
232060_at	145.2	188.6	0.38	1.30	PPP-PPP	0.10	17	0.868	0.955	0.3871
207595_s_at	352.3	457.4	0.38	1.30	PPP-PPP	0.10	17	0.868	0.955	0.3306
200688_at	337.5	438.4	0.38	1.30	PPA-PMP	0.10	17	0.868	0.955	0.3224
1556212_x_at	57.1	74.2	0.38	1.30	PPP-AAP	0.10	17	0.868	0.955	0.3064
225700_at	117.3	152.3	0.38	1.30	AAA-APP	0.10	17	0.868	0.955	0.2670
233101_at	113.1	146.9	0.38	1.30	AAA-APA	0.10	17	0.868	0.955	0.2514
238678_at	178.1	231.2	0.38	1.30	PMP-APP	0.10	17	0.868	0.955	0.1932
1557945_at	56.3	73.1	0.38	1.30	APP-PPP	0.10	17	0.868	0.955	0.1740
237062_at	190.5	247.5	0.38	1.30	PPP-PPP	0.10	17	0.868	0.955	0.1734
235874_at	28.0	36.3	0.38	1.30	PPA-PPA	0.10	17	0.868	0.955	0.5587
235043_at	89.3	115.9	0.38	1.30	AAA-AAP	0.10	17	0.868	0.955	0.5249
1557363_a_at	24.8	32.2	0.38	1.30	APA-PAM	0.10	17	0.868	0.955	0.4741
1560648_s_at	197.1	255.7	0.38	1.30	PAM-AAM	0.10	17	0.868	0.955	0.4085
229065_at	289.6	375.9	0.38	1.30	PPP-PPP	0.10	17	0.868	0.955	0.3949
232186_at	55.3	71.8	0.38	1.30	APA-AAP	0.10	17	0.868	0.955	0.3831
229213_at	84.5	109.7	0.38	1.30	AAA-AMP	0.10	17	0.868	0.955	0.2900
219615_s_at	338.7	439.4	0.38	1.30	PPP-PPP	0.10	17	0.868	0.955	0.2247
43934_at	209.4	271.8	0.38	1.30	PPP-PPP	0.10	17	0.868	0.955	0.1737
235073_at	106.5	138.2	0.38	1.30	AMA-PAP	0.10	17	0.868	0.955	0.1725
208273_at	53.8	69.8	0.38	1.30	PAA-PAP	0.10	17	0.868	0.955	0.6011
230461_s_at	143.3	185.8	0.38	1.30	PMP-PPP	0.10	17	0.868	0.955	0.5826
243125_x_at	49.2	63.8	0.38	1.30	PAA-PAA	0.10	17	0.868	0.955	0.5005

230637_at	105.0	136.2	0.38	1.30	AAA-AAP	0.10	17	0.868	0.955	0.4305
243579_at	235.0	304.7	0.38	1.30	PPP-PPP	0.10	17	0.868	0.955	0.1698
241262_at	58.3	75.6	0.37	1.30	APM-APM	0.10	17	0.868	0.955	0.4214
217666_at	115.7	149.9	0.37	1.30	PPP-PPP	0.10	17	0.868	0.955	0.3769
232044_at	101.5	131.5	0.37	1.30	PMP-MPP	0.10	17	0.868	0.955	0.3756
206738_at	92.2	119.4	0.37	1.30	PPP-PAP	0.10	17	0.868	0.955	0.2211
215281_x_at	108.5	140.6	0.37	1.30	PPP-PPP	0.10	17	0.868	0.955	0.5642
231233_at	88.2	114.2	0.37	1.30	PPA-AAP	0.10	17	0.868	0.955	0.5617
235486_at	88.8	115.0	0.37	1.30	PAP-AAM	0.10	17	0.868	0.955	0.4240
220590_at	85.6	110.8	0.37	1.30	AAP-AAP	0.10	17	0.868	0.955	0.4041
227876_at	263.5	341.2	0.37	1.30	PPP-PPP	0.10	17	0.868	0.955	0.2603
222635_s_at	378.6	490.2	0.37	1.30	PPP-PPP	0.10	17	0.868	0.955	0.2330
210713_at	45.5	58.9	0.37	1.30	AAA-AAP	0.10	17	0.868	0.955	0.5592
219421_at	369.1	477.7	0.37	1.29	PPP-PPP	0.10	17	0.868	0.955	0.5148
221663_x_at	73.1	94.5	0.37	1.29	APA-PAA	0.10	17	0.868	0.955	0.4991
1553386_at	116.1	150.2	0.37	1.29	PMA-PMP	0.10	17	0.868	0.955	0.4164
232338_at	263.3	340.7	0.37	1.29	PPP-PPP	0.10	17	0.868	0.955	0.4055
238341_at	131.2	169.8	0.37	1.29	PPP-PPP	0.10	17	0.868	0.955	0.2946
1565862_a_at	59.4	76.8	0.37	1.29	AAA-APP	0.10	17	0.868	0.955	0.2862
232331_at	128.3	166.1	0.37	1.29	PAP-PPP	0.10	17	0.868	0.955	0.1792
216692_at	93.7	121.2	0.37	1.29	AAA-PAA	0.10	17	0.868	0.955	0.5221
1554280_a_at	61.9	80.0	0.37	1.29	AAM-APA	0.10	17	0.868	0.955	0.4029
233770_at	58.7	75.9	0.37	1.29	APA-AAP	0.10	17	0.868	0.955	0.3871
210628_x_at	120.7	156.2	0.37	1.29	AAA-APA	0.10	17	0.868	0.955	0.3732
243386_at	152.2	196.8	0.37	1.29	PPP-PPP	0.10	17	0.868	0.955	0.3516
1553096_s_at	591.6	765.0	0.37	1.29	PPP-PPP	0.10	17	0.868	0.955	0.2466
202549_at	34.5	44.6	0.37	1.29	AAA-APA	0.10	17	0.868	0.955	0.3934
241815_at	95.5	123.5	0.37	1.29	PAP-PPP	0.10	17	0.868	0.955	0.2981
243790_at	48.1	62.2	0.37	1.29	PAA-PPM	0.10	17	0.868	0.955	0.2375
227359_at	103.5	133.8	0.37	1.29	AAA-MPP	0.10	17	0.868	0.955	0.2231
212637_s_at	752.7	972.4	0.37	1.29	PPP-PPP	0.10	17	0.868	0.955	0.2067
203347_s_at	385.8	498.7	0.37	1.29	PPP-PPP	0.10	17	0.868	0.955	0.2013
201741_x_at	159.9	206.7	0.37	1.29	AAA-PPM	0.10	17	0.868	0.955	0.1937
205865_at	90.4	116.9	0.37	1.29	AAA-APA	0.10	17	0.868	0.955	0.1853
220710_at	34.1	44.1	0.37	1.29	AAA-PAP	0.10	17	0.868	0.955	0.5271
240271_at	84.8	109.6	0.37	1.29	PPP-PPP	0.10	17	0.868	0.955	0.5255
204884_s_at	77.8	100.5	0.37	1.29	PPP-PPP	0.10	17	0.868	0.955	0.4818
206913_at	58.9	76.0	0.37	1.29	AAA-APP	0.10	17	0.868	0.955	0.4259
1559739_at	82.4	106.3	0.37	1.29	AAA-APP	0.10	17	0.868	0.955	0.2776
48808_at	1925.7	2487.4	0.37	1.29	PPP-PPP	0.10	17	0.868	0.955	0.2647
1559059_s_at	48.3	62.4	0.37	1.29	APA-APP	0.10	17	0.868	0.955	0.2425
1560014_s_at	64.3	83.0	0.37	1.29	AAP-PAP	0.10	17	0.868	0.955	0.5551

226603_at	58.6	75.7	0.37	1.29	APP-PPP	0.10	17	0.868	0.955	0.4903
229509_at	248.0	320.1	0.37	1.29	APM-PPP	0.10	17	0.868	0.955	0.3502
209350_s_at	1030.8	1330.1	0.37	1.29	PPP-PPP	0.10	17	0.868	0.955	0.2877
222146_s_at	210.3	271.4	0.37	1.29	PPP-PPP	0.10	17	0.868	0.955	0.2754
238875_at	65.4	84.4	0.37	1.29	AAA-APA	0.10	17	0.868	0.955	0.1743
1564323_at	42.1	54.3	0.37	1.29	AAA-PPA	0.10	17	0.868	0.955	0.6089
1554163_at	74.7	96.3	0.37	1.29	APP-PPP	0.10	17	0.868	0.955	0.5282
242761_s_at	121.0	156.0	0.37	1.29	PPA-APP	0.10	17	0.868	0.955	0.4279
1569723_a_at	121.0	156.1	0.37	1.29	PPP-PPP	0.10	17	0.868	0.955	0.3666
241418_at	87.1	112.3	0.37	1.29	PAP-AAA	0.10	17	0.868	0.955	0.2237
204367_at	791.3	1020.5	0.37	1.29	PPP-PPP	0.10	17	0.868	0.955	0.1978
1554549_a_at	82.4	106.3	0.37	1.29	MAA-APP	0.10	17	0.868	0.955	0.1847
227909_at	56.5	72.8	0.37	1.29	AAA-APA	0.10	17	0.868	0.955	0.3710
230888_at	58.8	75.8	0.37	1.29	PPA-PPP	0.10	17	0.868	0.955	0.3376
1554783_s_at	89.3	115.1	0.37	1.29	AAA-AAP	0.10	17	0.868	0.955	0.3239
215385_at	163.2	210.3	0.37	1.29	PPP-PPP	0.10	17	0.868	0.955	0.3075
1557103_a_at	84.8	109.3	0.37	1.29	APP-PPP	0.10	17	0.868	0.955	0.2833
1553186_x_at	407.5	525.2	0.37	1.29	PPP-PPP	0.10	17	0.868	0.955	0.2485
232405_at	52.2	67.3	0.37	1.29	AAA-PAA	0.10	17	0.868	0.955	0.5906
1556432_at	66.4	85.4	0.37	1.29	AAM-MAP	0.10	17	0.868	0.955	0.5589
240191_at	29.3	37.7	0.37	1.29	AAA-APA	0.10	17	0.868	0.955	0.4935
209446_s_at	265.4	341.9	0.37	1.29	PPP-PPP	0.10	17	0.868	0.955	0.4853
211753_s_at	56.3	72.5	0.37	1.29	PAP-APP	0.10	17	0.868	0.955	0.3648
236242_at	105.8	136.3	0.37	1.29	AMP-APP	0.10	17	0.868	0.955	0.3525
241867_at	87.3	112.4	0.37	1.29	PPP-PPP	0.10	17	0.868	0.955	0.3366
207730_x_at	980.3	1262.5	0.37	1.29	PPP-PPP	0.10	17	0.868	0.955	0.2546
222872_x_at	429.2	552.7	0.37	1.29	PPP-PPP	0.10	17	0.868	0.955	0.2412
217092_x_at	10827.9	13948.3	0.37	1.29	PPP-PPP	0.10	17	0.868	0.955	0.2389
232628_at	113.7	146.5	0.37	1.29	PAM-APP	0.10	17	0.868	0.955	0.1705
238696_at	91.6	117.9	0.37	1.29	PPP-PPP	0.10	17	0.868	0.955	0.1669
230904_at	71.6	92.2	0.36	1.29	PPP-PPP	0.10	17	0.868	0.955	0.4995
227859_at	80.0	103.0	0.36	1.29	PAP-PAP	0.10	17	0.868	0.955	0.3029
222025_s_at	220.6	284.0	0.36	1.29	PPP-PPP	0.10	17	0.868	0.955	0.2569
1556464_a_at	137.9	177.4	0.36	1.29	APP-PPP	0.10	17	0.868	0.955	0.1797
228975_at	80.5	103.6	0.36	1.29	PPA-PAM	0.10	17	0.868	0.955	0.4552
220605_s_at	603.9	776.6	0.36	1.29	PPP-PPP	0.10	17	0.868	0.955	0.3795
207436_x_at	873.9	1123.9	0.36	1.29	PPP-PPP	0.10	17	0.868	0.955	0.3254
1559382_at	186.0	239.2	0.36	1.29	PPP-PPP	0.10	17	0.868	0.955	0.3201
213686_at	275.2	353.9	0.36	1.29	PPP-PPP	0.10	17	0.868	0.955	0.3028
219917_at	174.5	224.5	0.36	1.29	MPP-PAP	0.10	17	0.868	0.955	0.2499
242751_at	162.6	209.2	0.36	1.29	PPP-PPP	0.10	17	0.868	0.955	0.2392
224518_s_at	160.3	206.2	0.36	1.29	APP-PPP	0.10	17	0.868	0.955	0.2351

216682_s_at	94.2	121.1	0.36	1.29	AAP-PAM	0.10	17	0.868	0.955	0.1855
235744_at	302.3	388.8	0.36	1.29	PPP-PPP	0.10	17	0.868	0.955	0.1852
219313_at	84.3	108.3	0.36	1.29	AAA-PAA	0.10	17	0.868	0.955	0.4670
244587_at	268.8	345.5	0.36	1.29	PPP-PPP	0.10	17	0.868	0.955	0.3898
213935_at	345.2	443.7	0.36	1.29	PPP-PPP	0.10	17	0.868	0.955	0.3694
222115_x_at	147.4	189.3	0.36	1.29	PMP-PPP	0.10	17	0.868	0.955	0.2937
206355_at	70.3	90.3	0.36	1.29	PPP-PPP	0.10	17	0.868	0.955	0.2671
1553639_a_at	200.9	258.1	0.36	1.29	AAA-APP	0.10	17	0.868	0.955	0.2219
241916_at	60.4	77.5	0.36	1.29	PAP-PAP	0.10	17	0.868	0.955	0.5658
1556221_a_at	49.1	63.0	0.36	1.29	PAP-PPA	0.10	17	0.868	0.955	0.5624
235556_at	181.8	233.5	0.36	1.28	PPP-PPP	0.10	17	0.868	0.955	0.2890
222644_s_at	339.7	436.1	0.36	1.28	PPM-PPP	0.10	17	0.868	0.955	0.1952
235275_at	70.6	90.6	0.36	1.28	PPA-APA	0.10	17	0.868	0.955	0.5928
233957_at	36.4	46.7	0.36	1.28	APA-AAA	0.10	17	0.868	0.955	0.5903
236468_at	45.3	58.2	0.36	1.28	AAA-PAA	0.10	17	0.868	0.955	0.5673
243992_at	82.6	106.1	0.36	1.28	PPP-PAP	0.10	17	0.868	0.955	0.5167
204687_at	93.6	120.2	0.36	1.28	PAA-AAP	0.10	17	0.868	0.955	0.3900
237768_x_at	295.9	379.6	0.36	1.28	PPP-PPP	0.10	17	0.868	0.955	0.3472
244535_at	111.3	142.8	0.36	1.28	APP-APA	0.10	17	0.868	0.955	0.3384
204225_at	230.1	295.4	0.36	1.28	PPP-PPP	0.10	17	0.868	0.955	0.3119
205887_x_at	653.9	839.5	0.36	1.28	PPP-PPP	0.10	17	0.868	0.955	0.2446
212621_at	818.4	1050.5	0.36	1.28	PPP-PPP	0.10	17	0.868	0.955	0.1928
1558839_at	63.3	81.1	0.36	1.28	AAA-AAP	0.10	17	0.868	0.955	0.4264
214294_at	55.2	70.9	0.36	1.28	APP-AAA	0.10	17	0.868	0.955	0.4201
202027_at	908.7	1165.6	0.36	1.28	PPP-PPP	0.10	17	0.868	0.955	0.3571
224534_at	134.3	172.2	0.36	1.28	PPP-PPP	0.10	17	0.868	0.955	0.1851
211279_at	82.7	106.0	0.36	1.28	PAP-PPP	0.10	17	0.868	0.955	0.5925
213169_at	60.5	77.5	0.36	1.28	AAP-AAA	0.10	17	0.868	0.955	0.4875
242369_x_at	107.9	138.4	0.36	1.28	PAP-PPP	0.10	17	0.868	0.955	0.4356
229507_at	286.6	367.3	0.36	1.28	PAP-PPP	0.10	17	0.868	0.955	0.3608
214423_x_at	125.2	160.5	0.36	1.28	AAA-PAA	0.10	17	0.868	0.955	0.3258
209182_s_at	128.7	165.0	0.36	1.28	APA-APA	0.10	17	0.868	0.955	0.3157
1566340_at	88.3	113.1	0.36	1.28	APP-PPP	0.10	17	0.868	0.955	0.2331
242297_at	170.2	218.1	0.36	1.28	PPP-PPP	0.10	17	0.868	0.955	0.2116
217534_at	111.0	142.1	0.36	1.28	APA-AAP	0.10	17	0.868	0.955	0.5796
232347_x_at	228.5	292.7	0.36	1.28	PPP-PPP	0.10	17	0.868	0.955	0.4826
224427_s_at	169.9	217.6	0.36	1.28	PPP-PPP	0.10	17	0.868	0.955	0.4277
220370_s_at	116.0	148.6	0.36	1.28	PPP-PPP	0.10	17	0.868	0.955	0.3255
224553_s_at	70.2	89.9	0.36	1.28	AAA-APP	0.10	17	0.868	0.955	0.3141
228121_at	282.9	362.4	0.36	1.28	PPP-PPP	0.10	17	0.868	0.955	0.2803
208082_x_at	972.4	1244.4	0.36	1.28	PPP-PPP	0.10	17	0.868	0.955	0.5295
243042_at	165.0	211.2	0.36	1.28	PPP-PPP	0.10	17	0.868	0.955	0.5141

204983_s_at	91.7	117.3	0.36	1.28	APA-AAA	0.10	17	0.868	0.955	0.4222
239370_at	220.5	282.1	0.36	1.28	PPP-PPP	0.10	17	0.868	0.955	0.3983
215883_at	109.0	139.5	0.36	1.28	APM-APP	0.10	17	0.868	0.955	0.3494
201452_at	102.8	131.6	0.36	1.28	PMA-AAA	0.10	17	0.868	0.955	0.3443
226687_at	258.2	330.6	0.36	1.28	PPP-PPP	0.10	17	0.868	0.955	0.3046
228556_at	112.0	143.3	0.36	1.28	APP-PPP	0.10	17	0.868	0.955	0.2610
1555974_a_at	503.6	644.4	0.36	1.28	PPP-PPP	0.10	17	0.868	0.955	0.2569
1560026_at	84.2	107.7	0.36	1.28	PPP-PPP	0.10	17	0.868	0.955	0.3822
242646_at	195.9	250.5	0.36	1.28	AAP-PPP	0.10	17	0.868	0.955	0.3603
219074_at	1017.0	1300.4	0.36	1.28	PPP-PPP	0.10	17	0.868	0.955	0.2876
214870_x_at	2004.6	2564.6	0.36	1.28	PPP-PPP	0.10	17	0.868	0.955	0.2698
225301_s_at	1295.4	1657.3	0.36	1.28	PPP-PPP	0.10	17	0.868	0.955	0.2103
229082_at	78.8	100.7	0.35	1.28	AMM-PAA	0.10	17	0.868	0.955	0.5373
201373_at	973.2	1243.5	0.35	1.28	PPP-PPP	0.10	17	0.868	0.955	0.5205
235167_at	279.3	356.9	0.35	1.28	PPP-PPP	0.10	17	0.868	0.955	0.4573
205366_s_at	164.6	210.4	0.35	1.28	PPP-PPP	0.10	17	0.868	0.955	0.3635
230302_at	91.5	116.9	0.35	1.28	APA-MAP	0.10	17	0.868	0.955	0.3623
243016_at	112.2	143.4	0.35	1.28	PPP-PPP	0.10	17	0.868	0.955	0.3455
238023_at	163.8	209.3	0.35	1.28	MPP-PPP	0.10	17	0.868	0.955	0.3444
223635_s_at	113.6	145.2	0.35	1.28	AAA-PAM	0.10	17	0.868	0.955	0.3240
204176_at	610.3	779.9	0.35	1.28	PPP-PPP	0.10	17	0.868	0.955	0.1936
205224_at	370.4	473.3	0.35	1.28	PPP-PPP	0.10	17	0.868	0.955	0.1639
243095_at	92.0	117.5	0.35	1.28	PPP-PPP	0.10	17	0.868	0.955	0.6237
236777_at	63.4	81.0	0.35	1.28	PAA-APP	0.10	17	0.868	0.955	0.5976
211692_s_at	164.0	209.5	0.35	1.28	PAP-AMA	0.10	17	0.868	0.955	0.3881
227758_at	85.5	109.3	0.35	1.28	AMA-APP	0.10	17	0.868	0.955	0.3557
242997_at	175.4	224.0	0.35	1.28	AAP-PPP	0.10	17	0.868	0.955	0.3244
206845_s_at	559.3	714.1	0.35	1.28	PPM-PPP	0.10	17	0.868	0.955	0.1955
1558613_at	85.9	109.7	0.35	1.28	PAP-APP	0.10	17	0.868	0.955	0.6093
216540_at	45.8	58.4	0.35	1.28	AAP-PAA	0.10	17	0.868	0.955	0.5468
1570447_at	123.1	157.1	0.35	1.28	PAA-MAA	0.10	17	0.868	0.955	0.5188
207754_at	88.4	112.9	0.35	1.28	PPA-PPM	0.10	17	0.868	0.955	0.4641
1562062_at	285.2	364.1	0.35	1.28	PPA-MAP	0.10	17	0.868	0.955	0.4611
235886_at	73.6	93.9	0.35	1.28	APP-PAP	0.10	17	0.868	0.955	0.4419
244490_at	42.9	54.7	0.35	1.28	AAA-APA	0.10	17	0.868	0.955	0.4382
232689_at	70.8	90.3	0.35	1.28	PPP-PPP	0.10	17	0.868	0.955	0.4187
238822_at	273.6	349.3	0.35	1.28	PPP-PPP	0.10	17	0.868	0.955	0.2899
1569189_at	129.8	165.7	0.35	1.28	PPP-PPP	0.10	17	0.868	0.955	0.2728
241360_at	137.8	175.9	0.35	1.28	PPP-PPP	0.10	17	0.868	0.955	0.2703
202334_s_at	1368.0	1746.0	0.35	1.28	PPP-PPP	0.10	17	0.868	0.955	0.2426
236009_at	837.4	1068.7	0.35	1.28	PPP-PPP	0.10	17	0.868	0.955	0.1929
236837_x_at	80.5	102.7	0.35	1.28	PMP-PPP	0.10	17	0.868	0.955	0.5896

235789_at	117.6	149.9	0.35	1.28	PPA-APP	0.10	17	0.868	0.955	0.5166
232023_at	58.5	74.6	0.35	1.28	PPA-PPP	0.10	17	0.868	0.955	0.4848
210281_s_at	87.1	111.2	0.35	1.28	PAM-PPP	0.10	17	0.868	0.955	0.4350
1557267_s_at	41.0	52.3	0.35	1.28	APP-APP	0.10	17	0.868	0.955	0.4049
220954_s_at	461.3	588.4	0.35	1.28	PPP-PPP	0.10	17	0.868	0.955	0.3347
217347_at	454.8	580.2	0.35	1.28	PPP-PPP	0.10	17	0.868	0.955	0.2496
1556951_at	68.0	86.7	0.35	1.28	AAA-PAA	0.10	17	0.868	0.955	0.2494
1562731_s_at	99.6	127.0	0.35	1.28	AAP-AAA	0.10	17	0.868	0.955	0.1922
238992_at	65.3	83.3	0.35	1.28	PAP-PPP	0.10	17	0.868	0.955	0.1624
1561042_at	64.3	82.0	0.35	1.28	PAA-AAA	0.10	17	0.868	0.955	0.5449
1566577_at	47.3	60.3	0.35	1.28	AAA-PAP	0.10	17	0.868	0.955	0.4935
204632_at	572.7	729.7	0.35	1.27	PPP-PPP	0.10	17	0.868	0.955	0.4168
1557197_a_at	82.6	105.3	0.35	1.28	AAA-AAP	0.10	17	0.868	0.955	0.3966
236841_at	286.4	364.8	0.35	1.27	PPM-PPP	0.10	17	0.868	0.955	0.3708
236577_at	81.5	103.8	0.35	1.27	PAM-PPP	0.10	17	0.868	0.955	0.3240
242176_at	82.3	104.8	0.35	1.27	PPP-PPA	0.10	17	0.868	0.955	0.3198
243589_at	103.0	131.1	0.35	1.27	PPP-PPP	0.10	17	0.868	0.955	0.3116
235903_at	201.7	256.8	0.35	1.27	PAP-PAP	0.10	17	0.868	0.955	0.2980
203309_s_at	85.7	109.2	0.35	1.27	PPP-PPP	0.10	17	0.868	0.955	0.2592
242626_at	381.6	486.0	0.35	1.27	PPP-PPP	0.10	17	0.868	0.955	0.2550
1555529_at	90.4	115.0	0.35	1.27	APM-PPP	0.10	17	0.868	0.955	0.5230
234276_at	52.5	66.8	0.35	1.27	PMP-APA	0.10	17	0.868	0.955	0.5020
229304_s_at	206.6	263.0	0.35	1.27	PPP-PPP	0.10	17	0.868	0.955	0.4625
235834_at	125.1	159.3	0.35	1.27	PPP-PAP	0.10	17	0.868	0.955	0.4372
203809_s_at	144.3	183.6	0.35	1.27	PPP-PPP	0.10	17	0.868	0.955	0.2737
216309_x_at	400.6	509.8	0.35	1.27	PPP-PPP	0.10	17	0.868	0.955	0.2370
213252_at	182.9	232.8	0.35	1.27	PPA-PPP	0.10	17	0.868	0.955	0.2025
203287_at	2774.3	3531.8	0.35	1.27	PPP-PPP	0.10	17	0.868	0.955	0.1689
239139_at	57.8	73.5	0.35	1.27	AAP-AAA	0.10	17	0.868	0.955	0.5629
234649_at	80.9	102.9	0.35	1.27	AAA-AAP	0.10	17	0.868	0.955	0.4558
243189_at	77.7	98.9	0.35	1.27	AAP-APA	0.10	17	0.868	0.955	0.4365
233149_at	139.6	177.6	0.35	1.27	PMP-PPA	0.10	17	0.868	0.955	0.4195
203117_s_at	258.6	328.9	0.35	1.27	PPP-PPP	0.10	17	0.868	0.955	0.3593
215032_at	102.9	130.8	0.35	1.27	PPP-PPP	0.10	17	0.868	0.955	0.3392
214474_at	80.5	102.4	0.35	1.27	PPP-PPP	0.10	17	0.868	0.955	0.3238
207064_s_at	40.5	51.4	0.35	1.27	AAA-PAA	0.10	17	0.868	0.955	0.2986
1569703_a_at	34.3	43.5	0.35	1.27	AAP-AAM	0.10	17	0.868	0.955	0.5597
1555718_x_at	28.9	36.7	0.35	1.27	AAP-APA	0.10	17	0.868	0.955	0.4700
232116_at	263.6	335.1	0.35	1.27	APA-PPP	0.10	17	0.868	0.955	0.3718
211014_s_at	195.5	248.4	0.35	1.27	PPP-PPP	0.10	17	0.868	0.955	0.3563
244182_at	70.6	89.7	0.35	1.27	AAP-AAA	0.10	17	0.868	0.955	0.3512
211905_s_at	634.3	806.3	0.35	1.27	PPP-PPP	0.10	17	0.868	0.955	0.2667

239910_at	73.5	93.4	0.35	1.27	AAA-PPM	0.10	17	0.868	0.955	0.2662
1554161_at	58.1	73.9	0.35	1.27	PPP-PAP	0.10	17	0.868	0.955	0.4972
227340_s_at	164.9	209.5	0.35	1.27	PPP-PPP	0.10	17	0.868	0.955	0.3970
206235_at	250.3	317.9	0.35	1.27	PPP-PPP	0.10	17	0.868	0.955	0.3833
237136_at	84.6	107.4	0.35	1.27	AAA-AAP	0.10	17	0.868	0.955	0.3557
242923_at	171.1	217.2	0.35	1.27	PPP-PPP	0.10	17	0.868	0.955	0.3342
221260_s_at	366.0	464.9	0.35	1.27	PPP-PPP	0.10	17	0.868	0.955	0.3215
226130_at	240.3	305.2	0.35	1.27	PAA-PPP	0.10	17	0.868	0.955	0.2772
243370_at	94.1	119.5	0.35	1.27	PPP-PPP	0.10	17	0.868	0.955	0.2414
201236_s_at	521.7	662.8	0.35	1.27	PPP-PPP	0.10	17	0.868	0.955	0.1827
222713_s_at	700.0	889.2	0.35	1.27	PPP-PPP	0.10	17	0.868	0.955	0.1810
1558250_s_at	436.1	554.1	0.35	1.27	PPP-PPP	0.10	17	0.868	0.955	0.1767
232198_at	114.7	145.6	0.34	1.27	APP-PPP	0.10	17	0.868	0.955	0.5881
243619_at	51.7	65.7	0.34	1.27	PMA-PAP	0.10	17	0.868	0.955	0.5356
204507_s_at	171.8	218.0	0.34	1.27	PAP-APP	0.10	17	0.868	0.955	0.4367
211693_at	116.6	147.9	0.34	1.27	AAP-PAP	0.10	17	0.868	0.955	0.3212
1554177_a_at	243.9	309.6	0.34	1.27	PPP-PPP	0.10	17	0.868	0.955	0.3068
1555942_a_at	966.5	1226.4	0.34	1.27	PPP-PPP	0.10	17	0.868	0.955	0.2656
211883_x_at	71.3	90.5	0.34	1.27	AAP-AAP	0.10	17	0.868	0.955	0.2116
201640_x_at	764.3	970.1	0.34	1.27	PPP-PPP	0.10	17	0.868	0.955	0.2104
242990_at	102.3	129.7	0.34	1.27	APP-PPP	0.10	17	0.868	0.955	0.5759
211513_s_at	177.7	225.5	0.34	1.27	PPA-PMP	0.10	17	0.868	0.955	0.5064
219597_s_at	205.5	260.6	0.34	1.27	PPP-PPP	0.10	17	0.868	0.955	0.4135
213605_s_at	312.8	396.7	0.34	1.27	PPP-PPP	0.10	17	0.868	0.955	0.3262
231697_s_at	101.9	129.3	0.34	1.27	PPP-PPP	0.10	17	0.868	0.955	0.2319
203450_at	429.0	544.2	0.34	1.27	MAP-PPP	0.10	17	0.868	0.955	0.2269
241635_at	28.4	36.0	0.34	1.27	PPA-PAA	0.10	17	0.868	0.955	0.5529
217605_at	31.1	39.5	0.34	1.27	PAA-AAA	0.10	17	0.868	0.955	0.5127
230934_at	126.8	160.8	0.34	1.27	AAP-PAM	0.10	17	0.868	0.955	0.4633
228813_at	90.7	114.9	0.34	1.27	PPP-PPP	0.10	17	0.868	0.955	0.4274
226040_at	443.9	562.8	0.34	1.27	PPP-PPP	0.10	17	0.868	0.955	0.2256
213760_s_at	485.6	615.6	0.34	1.27	PPP-PPP	0.10	17	0.868	0.955	0.2052
243857_at	120.3	152.6	0.34	1.27	PPP-PPP	0.10	17	0.868	0.955	0.1833
212092_at	616.9	782.2	0.34	1.27	PPP-PPP	0.10	17	0.868	0.955	0.1803
229418_at	253.4	321.3	0.34	1.27	PMP-PPP	0.10	17	0.868	0.955	0.1734
1558407_at	251.1	318.2	0.34	1.27	MPP-PMP	0.10	17	0.868	0.955	0.1634
221523_s_at	72.9	92.3	0.34	1.27	APA-PPM	0.10	17	0.868	0.955	0.4329
219091_s_at	65.6	83.1	0.34	1.27	APP-PPA	0.10	17	0.868	0.955	0.3841
236196_at	200.2	253.5	0.34	1.27	PPP-PPP	0.10	17	0.868	0.955	0.3741
214666_x_at	212.5	269.1	0.34	1.27	PAP-PPP	0.10	17	0.868	0.955	0.1708
236294_at	93.1	117.8	0.34	1.27	AAA-AAP	0.10	17	0.868	0.955	0.5312
226670_s_at	299.4	378.9	0.34	1.27	AAA-AAP	0.10	17	0.868	0.955	0.4973

240168_at	115.1	145.7	0.34	1.27	PAP-MAP	0.10	17	0.868	0.955	0.3973
233105_at	92.7	117.3	0.34	1.27	PPP-PPP	0.10	17	0.868	0.955	0.3558
244517_x_at	36.6	46.4	0.34	1.27	AAP-PPP	0.10	17	0.868	0.955	0.3493
225705_at	395.2	500.3	0.34	1.27	PPP-PPP	0.10	17	0.868	0.955	0.3193
226877_at	83.1	105.2	0.34	1.27	PAA-PPP	0.10	17	0.868	0.955	0.2598
203692_s_at	354.2	448.4	0.34	1.27	PPP-PPP	0.10	17	0.868	0.955	0.1546
227329_at	64.9	82.1	0.34	1.27	AAM-APA	0.10	17	0.868	0.955	0.4981
210965_x_at	99.8	126.3	0.34	1.27	APA-AAA	0.10	17	0.868	0.955	0.4368
239978_at	158.7	200.8	0.34	1.27	PAP-PPP	0.10	17	0.868	0.955	0.4162
1555500_s_at	233.4	295.2	0.34	1.27	PPP-PPP	0.10	17	0.868	0.955	0.3273
244193_at	132.7	167.9	0.34	1.27	PAP-PPP	0.10	17	0.868	0.955	0.3233
226768_at	256.2	324.0	0.34	1.27	PAA-AAA	0.10	17	0.868	0.955	0.3087
240690_at	152.8	193.2	0.34	1.26	PPP-PPP	0.10	17	0.868	0.955	0.3068
1557724_a_at	104.4	132.0	0.34	1.27	APA-MAA	0.10	17	0.868	0.955	0.2537
204968_at	163.4	206.6	0.34	1.26	AAP-APP	0.10	17	0.868	0.955	0.2503
227970_at	473.9	599.5	0.34	1.27	PPP-PPP	0.10	17	0.868	0.955	0.2179
212302_at	452.6	572.4	0.34	1.26	PPP-PPP	0.10	17	0.868	0.955	0.2009
223774_at	535.0	676.9	0.34	1.27	PPP-PPP	0.10	17	0.868	0.955	0.1683
212806_at	40.6	51.4	0.34	1.26	APA-AAA	0.10	17	0.868	0.955	0.6267
211133_x_at	67.0	84.7	0.34	1.26	AAA-AAP	0.10	17	0.868	0.955	0.5905
1570249_x_at	104.3	131.9	0.34	1.26	PPM-PPP	0.10	17	0.868	0.955	0.5497
217662_x_at	144.5	182.6	0.34	1.26	PPP-PPP	0.10	17	0.868	0.955	0.5457
1557755_at	44.0	55.6	0.34	1.26	PAA-PAP	0.10	17	0.868	0.955	0.4900
227959_at	215.5	272.3	0.34	1.26	PAP-PPP	0.10	17	0.868	0.955	0.4729
238831_at	138.3	174.9	0.34	1.26	PPP-PPP	0.10	17	0.868	0.955	0.3333
232174_at	399.7	505.0	0.34	1.26	PPP-PPP	0.10	17	0.868	0.955	0.3149
1555446_s_at	128.0	161.8	0.34	1.26	PPP-PPP	0.10	17	0.868	0.955	0.3101
210401_at	65.2	82.4	0.34	1.26	AAA-AAP	0.10	17	0.868	0.955	0.2794
229694_at	229.4	290.0	0.34	1.26	AAA-MPP	0.10	17	0.868	0.955	0.1767
205640_at	303.9	384.1	0.34	1.26	PPP-PPP	0.10	17	0.868	0.955	0.1681
1562612_at	61.9	78.1	0.34	1.26	APM-MAP	0.10	17	0.868	0.955	0.5091
238146_at	99.5	125.7	0.34	1.26	PPM-PPP	0.10	17	0.868	0.955	0.4260
242224_at	158.7	200.5	0.34	1.26	PPP-PPP	0.10	17	0.868	0.955	0.4224
222873_s_at	169.6	214.2	0.34	1.26	APP-PPP	0.10	17	0.868	0.955	0.3924
242129_at	103.5	130.7	0.34	1.26	APP-APA	0.10	17	0.868	0.955	0.3886
215354_s_at	484.9	612.4	0.34	1.26	PPP-PPP	0.10	17	0.868	0.955	0.3726
203506_s_at	223.9	282.8	0.34	1.26	PPP-PPP	0.10	17	0.868	0.955	0.3133
236715_x_at	384.7	485.8	0.34	1.26	PPP-PPP	0.10	17	0.868	0.955	0.1597
202450_s_at	153.5	193.9	0.34	1.26	PPP-PPP	0.10	17	0.868	0.955	0.1544
233779_x_at	161.7	204.0	0.34	1.26	PAP-PPP	0.10	17	0.868	0.955	0.5731
239512_at	151.5	191.3	0.34	1.26	PPP-PPP	0.10	17	0.868	0.955	0.3901
240513_at	64.9	81.9	0.34	1.26	PPP-MPP	0.10	17	0.868	0.955	0.3051

239378_at	77.6	98.0	0.34	1.26	AAA-AAP	0.10	17	0.868	0.955	0.2637
216497_at	296.3	374.0	0.34	1.26	PPP-PPP	0.10	17	0.868	0.955	0.1937
1561905_at	62.9	79.3	0.34	1.26	AAA-PPA	0.10	17	0.868	0.955	0.5740
242730_at	56.2	70.8	0.34	1.26	PMP-PPP	0.10	17	0.868	0.955	0.5570
204481_at	274.9	346.8	0.34	1.26	PPP-PPP	0.10	17	0.868	0.955	0.3667
204857_at	208.3	262.7	0.34	1.26	PPP-PPP	0.10	17	0.868	0.955	0.3151
229490_s_at	1221.0	1540.3	0.34	1.26	PPP-PPP	0.10	17	0.868	0.955	0.2756
216283_s_at	152.2	192.0	0.34	1.26	PAP-APP	0.10	17	0.868	0.955	0.2311
227568_at	259.1	326.8	0.34	1.26	PPP-PPP	0.10	17	0.868	0.955	0.1958
243895_x_at	87.4	110.3	0.34	1.26	PPP-AMP	0.10	17	0.868	0.955	0.1549
228919_at	432.3	544.8	0.33	1.26	PPP-PPP	0.10	17	0.868	0.955	0.5447
238574_at	260.5	328.4	0.33	1.26	PPP-PPP	0.10	17	0.868	0.955	0.2847
222502_s_at	535.5	674.8	0.33	1.26	PPP-PPP	0.10	17	0.868	0.955	0.1811
205414_s_at	46.9	59.1	0.33	1.26	PAA-AAA	0.10	17	0.868	0.955	0.5815
1555872_a_at	145.9	183.7	0.33	1.26	APP-PPA	0.10	17	0.868	0.955	0.5629
241818_at	77.6	97.8	0.33	1.26	PPA-APP	0.10	17	0.868	0.955	0.5313
241903_at	82.6	104.1	0.33	1.26	PPP-PPP	0.10	17	0.868	0.955	0.4943
1569392_at	64.3	81.0	0.33	1.26	AAA-AAP	0.10	17	0.868	0.955	0.4650
227088_at	58.7	74.0	0.33	1.26	PAP-APA	0.10	17	0.868	0.955	0.4370
203299_s_at	319.5	402.5	0.33	1.26	PAP-PPP	0.10	17	0.868	0.955	0.4058
205607_s_at	232.8	293.3	0.33	1.26	APP-PMP	0.10	17	0.868	0.955	0.3428
236816_at	127.6	160.7	0.33	1.26	PPP-PPP	0.10	17	0.868	0.955	0.3372
242407_at	101.3	127.5	0.33	1.26	PPP-AAP	0.10	17	0.868	0.955	0.2749
238467_at	143.4	180.6	0.33	1.26	MAP-APM	0.10	17	0.868	0.955	0.2379
244655_at	44.7	56.4	0.33	1.26	APA-AAP	0.10	17	0.868	0.955	0.2348
232141_at	171.6	216.2	0.33	1.26	PPP-PPP	0.10	17	0.868	0.955	0.2009
242693_at	158.9	200.1	0.33	1.26	PPP-PPP	0.10	17	0.868	0.955	0.2004
239289_x_at	271.6	342.1	0.33	1.26	PPP-PPP	0.10	17	0.868	0.955	0.1822
228643_at	102.5	129.1	0.33	1.26	PPP-PPP	0.10	17	0.868	0.955	0.1554
218786_at	182.8	230.2	0.33	1.26	APP-PAP	0.10	17	0.868	0.955	0.5157
207568_at	64.7	81.5	0.33	1.26	AAA-AMP	0.10	17	0.868	0.955	0.4320
232885_at	40.5	51.0	0.33	1.26	APP-APM	0.10	17	0.868	0.955	0.4258
242423_x_at	296.5	373.2	0.33	1.26	PPP-PPP	0.10	17	0.868	0.955	0.3687
237064_x_at	189.5	238.5	0.33	1.26	PPP-PPP	0.10	17	0.868	0.955	0.2778
226252_at	121.7	153.3	0.33	1.26	PAA-PAP	0.10	17	0.868	0.955	0.2713
241352_at	199.4	251.0	0.33	1.26	PPP-PPP	0.10	17	0.868	0.955	0.2692
1557895_at	146.7	184.7	0.33	1.26	PPP-PPP	0.10	17	0.868	0.955	0.1971
1552779_a_at	124.2	156.4	0.33	1.26	PMP-PPP	0.10	17	0.868	0.955	0.1661
211248_s_at	51.8	65.2	0.33	1.26	AAA-APA	0.10	17	0.868	0.955	0.6091
241293_x_at	71.1	89.5	0.33	1.26	APA-APA	0.10	17	0.868	0.955	0.5287
215504_x_at	685.5	862.4	0.33	1.26	AAP-AAP	0.10	17	0.868	0.955	0.5252
244778_x_at	85.3	107.3	0.33	1.26	APP-AAP	0.10	17	0.868	0.955	0.4685

235529_x_at	999.7	1257.4	0.33	1.26	PPP-PPP	0.10	17	0.868	0.955	0.3958
240241_at	75.8	95.3	0.33	1.26	MPA-AAA	0.10	17	0.868	0.955	0.3700
222562_s_at	174.4	219.3	0.33	1.26	PPP-PPP	0.10	17	0.868	0.955	0.3693
234941_s_at	354.2	445.5	0.33	1.26	PMP-PPP	0.10	17	0.868	0.955	0.2950
1556006_s_at	279.3	351.4	0.33	1.26	PPP-PPP	0.10	17	0.868	0.955	0.2385
230706_s_at	131.1	164.9	0.33	1.26	PAP-AMP	0.10	17	0.868	0.955	0.1878
1554015_a_at	235.9	296.5	0.33	1.26	PAP-PPP	0.10	17	0.868	0.955	0.5599
214241_at	124.4	156.4	0.33	1.26	PPP-PMP	0.10	17	0.868	0.955	0.5434
220265_at	71.5	89.8	0.33	1.26	AAA-PAA	0.10	17	0.868	0.955	0.5052
229007_at	156.2	196.3	0.33	1.26	PPP-PPP	0.10	17	0.868	0.955	0.4072
204139_x_at	313.4	393.8	0.33	1.26	PPP-PPP	0.10	17	0.868	0.955	0.3143
203289_s_at	244.6	307.5	0.33	1.26	PPP-PPP	0.10	17	0.868	0.955	0.2101
48031_r_at	87.7	110.2	0.33	1.26	PPP-PPA	0.10	17	0.868	0.955	0.1967
208042_at	553.7	695.9	0.33	1.26	PPP-PPP	0.10	17	0.868	0.955	0.1507
215024_at	101.8	127.9	0.33	1.26	PPA-AAP	0.10	17	0.868	0.955	0.5227
215069_at	68.0	85.3	0.33	1.26	APM-AAP	0.10	17	0.868	0.955	0.4880
232528_at	97.7	122.8	0.33	1.26	PPP-PAP	0.10	17	0.868	0.955	0.4796
219008_at	335.3	421.1	0.33	1.26	PPP-PPP	0.10	17	0.868	0.955	0.4442
229017_s_at	534.9	671.9	0.33	1.26	PPP-PPP	0.10	17	0.868	0.955	0.4133
215114_at	70.3	88.3	0.33	1.26	MPP-PPP	0.10	17	0.868	0.955	0.3973
201797_s_at	1293.7	1625.6	0.33	1.26	PPP-PPP	0.10	17	0.868	0.955	0.3717
203706_s_at	97.2	122.1	0.33	1.26	PPP-MPP	0.10	17	0.868	0.955	0.3612
213230_at	486.7	611.3	0.33	1.26	PAP-PMP	0.10	17	0.868	0.955	0.3169
238744_at	47.6	59.8	0.33	1.26	AAA-AAP	0.10	17	0.868	0.955	0.3084
218655_s_at	482.9	606.6	0.33	1.26	PPP-PPP	0.10	17	0.868	0.955	0.2252
212534_at	827.3	1039.3	0.33	1.26	PPP-PPP	0.10	17	0.868	0.955	0.2187
211089_s_at	131.6	165.3	0.33	1.26	PPP-PPP	0.10	17	0.868	0.955	0.2118
37793_r_at	91.4	114.8	0.33	1.26	PPP-PPP	0.10	17	0.868	0.955	0.1616
222762_x_at	623.1	782.2	0.33	1.26	PPP-PPP	0.10	17	0.868	0.955	0.6025
208241_at	95.6	120.0	0.33	1.26	PPP-PPP	0.10	17	0.868	0.955	0.5248
242842_at	93.4	117.3	0.33	1.26	APP-PAP	0.10	17	0.868	0.955	0.4778
235613_at	126.9	159.3	0.33	1.26	PPM-PPP	0.10	17	0.868	0.955	0.4671
242108_at	115.3	144.7	0.33	1.26	PPP-PPP	0.10	17	0.868	0.955	0.4292
210001_s_at	51.5	64.7	0.33	1.26	AAA-AAP	0.10	17	0.868	0.955	0.3959
205352_at	148.1	185.9	0.33	1.26	PPP-PPP	0.10	17	0.868	0.955	0.3865
243407_at	117.8	147.9	0.33	1.26	MPP-PPP	0.10	17	0.868	0.955	0.3813
1561883_at	101.0	126.7	0.33	1.26	AAA-PPP	0.10	17	0.868	0.955	0.3127
233732_at	259.5	325.7	0.33	1.26	PAP-PPP	0.10	17	0.868	0.955	0.2772
204451_at	293.5	368.5	0.33	1.26	PPP-PPP	0.10	17	0.868	0.955	0.2687
1564224_x_at	140.0	175.8	0.33	1.26	MAA-AAP	0.10	17	0.868	0.955	0.2674
202893_at	456.4	573.0	0.33	1.26	PPP-PPP	0.10	17	0.868	0.955	0.2672
237459_at	85.5	107.3	0.33	1.26	AAA-AAP	0.10	17	0.868	0.955	0.2525

212257_s_at	329.0	412.9	0.33	1.26	PPP-PPP	0.10	17	0.868	0.955	0.2233
243927_x_at	95.8	120.2	0.33	1.26	PPP-PPP	0.10	17	0.868	0.955	0.2146
223405_at	168.5	211.5	0.33	1.26	AAP-MAP	0.10	17	0.868	0.955	0.2047
205098_at	61.1	76.6	0.33	1.25	AAA-PAA	0.10	17	0.868	0.955	0.5912
235272_at	152.5	191.2	0.33	1.25	AAM-APP	0.10	17	0.868	0.955	0.5226
216363_at	61.6	77.3	0.33	1.25	APM-AAP	0.10	17	0.868	0.955	0.5110
241470_x_at	71.4	89.6	0.33	1.26	PPA-PPP	0.10	17	0.868	0.955	0.4644
244427_at	134.2	168.4	0.33	1.26	PPP-PPP	0.10	17	0.868	0.955	0.3516
1555882_at	57.3	71.9	0.33	1.26	AAA-MPA	0.10	17	0.868	0.955	0.3426
1557943_at	151.1	189.6	0.33	1.25	PMA-AAA	0.10	17	0.868	0.955	0.3239
219245_s_at	188.7	236.6	0.33	1.25	PPP-PPP	0.10	17	0.868	0.955	0.2518
240542_at	44.3	55.6	0.33	1.26	MAP-PAP	0.10	17	0.868	0.955	0.2521
201179_s_at	1606.1	2014.4	0.33	1.25	PPP-PPP	0.10	17	0.868	0.955	0.2129
202631_s_at	280.3	351.5	0.33	1.25	PPP-PPP	0.10	17	0.868	0.955	0.1530
231207_at	258.0	323.5	0.33	1.25	PAP-PPP	0.10	17	0.868	0.955	0.5642
1555878_at	375.7	471.0	0.33	1.25	PPP-PPP	0.10	17	0.868	0.955	0.5099
1557239_at	35.2	44.1	0.33	1.25	AAP-AAP	0.10	17	0.868	0.955	0.4937
232800_at	109.7	137.5	0.33	1.25	PAP-PAP	0.10	17	0.868	0.955	0.4654
233912_x_at	121.4	152.3	0.33	1.25	AAA-PPP	0.10	17	0.868	0.955	0.4264
203479_s_at	280.2	351.3	0.33	1.25	PPP-PPP	0.10	17	0.868	0.955	0.3595
227869_at	316.9	397.2	0.33	1.25	PPP-PPP	0.10	17	0.868	0.955	0.3334
213267_at	50.0	62.7	0.33	1.25	AAP-PPM	0.10	17	0.868	0.955	0.2874
240307_at	142.1	178.2	0.33	1.25	PPP-PPP	0.10	17	0.868	0.955	0.2605
227093_at	620.9	778.5	0.33	1.25	PPP-PPP	0.10	17	0.868	0.955	0.2543
209583_s_at	67.0	83.9	0.33	1.25	PAA-AAA	0.10	17	0.868	0.955	0.5803
240823_at	62.3	78.0	0.33	1.25	PAA-PAP	0.10	17	0.868	0.955	0.5392
203767_s_at	224.2	280.9	0.33	1.25	PPP-PPP	0.10	17	0.868	0.955	0.5122
1562948_at	91.0	114.0	0.33	1.25	PPP-PAP	0.10	17	0.868	0.955	0.4968
233487_s_at	439.3	550.4	0.33	1.25	PPP-PPP	0.10	17	0.868	0.955	0.4530
226211_at	71.1	89.1	0.33	1.25	AAA-APA	0.10	17	0.868	0.955	0.4034
224040_at	86.4	108.2	0.33	1.25	AAA-PPP	0.10	17	0.868	0.955	0.3715
1553698_a_at	131.5	164.8	0.33	1.25	MPP-AAP	0.10	17	0.868	0.955	0.3160
217474_at	55.8	69.9	0.33	1.25	AAA-APP	0.10	17	0.868	0.955	0.3013
216905_s_at	970.8	1215.9	0.33	1.25	PPP-PPP	0.10	17	0.868	0.955	0.3005
230188_at	211.6	265.1	0.33	1.25	AAP-PPP	0.10	17	0.868	0.955	0.2886
236531_at	98.3	123.1	0.33	1.25	PPP-PPP	0.10	17	0.868	0.955	0.2279
207735_at	350.2	438.7	0.33	1.25	PPP-PPP	0.10	17	0.868	0.955	0.1775
212699_at	155.4	194.6	0.33	1.25	PPP-PPP	0.10	17	0.868	0.955	0.1548
34846_at	188.1	235.5	0.32	1.25	PPA-MAA	0.10	17	0.868	0.955	0.5914
209765_at	114.4	143.1	0.32	1.25	PAP-PAM	0.10	17	0.868	0.955	0.5770
224568_x_at	787.8	986.1	0.32	1.25	PPP-PPP	0.10	17	0.868	0.955	0.5298
242673_at	278.6	348.8	0.32	1.25	PPP-PPP	0.10	17	0.868	0.955	0.5251

209726_at	98.0	122.6	0.32	1.25	PAA-PPP	0.10	17	0.868	0.955	0.5008
242759_at	106.9	133.8	0.32	1.25	PPP-PPP	0.10	17	0.868	0.955	0.4959
1562497_at	61.3	76.7	0.32	1.25	AAM-PAP	0.10	17	0.868	0.955	0.4492
239572_at	717.4	898.1	0.32	1.25	PPP-PPP	0.10	17	0.868	0.955	0.4015
228519_x_at	296.8	371.6	0.32	1.25	PAP-PPP	0.10	17	0.868	0.955	0.3319
203651_at	402.6	504.0	0.32	1.25	PPP-PPP	0.10	17	0.868	0.955	0.3265
214083_at	199.3	249.5	0.32	1.25	PPP-MMA	0.10	17	0.868	0.955	0.3018
224605_at	429.8	538.0	0.32	1.25	PPP-PPP	0.10	17	0.868	0.955	0.2841
1570269_at	55.1	68.9	0.32	1.25	PPP-MPP	0.10	17	0.868	0.955	0.5458
214599_at	81.9	102.5	0.32	1.25	APP-AAA	0.10	17	0.868	0.955	0.5430
204537_s_at	98.2	122.7	0.32	1.25	AMA-APP	0.10	17	0.868	0.955	0.4924
243663_at	64.7	80.9	0.32	1.25	AMA-APA	0.10	17	0.868	0.955	0.4866
214964_at	90.0	112.6	0.32	1.25	AAA-AAP	0.10	17	0.868	0.955	0.4607
240383_at	178.1	222.8	0.32	1.25	PPP-PPP	0.10	17	0.868	0.955	0.4459
243249_at	158.5	198.2	0.32	1.25	PPP-PPP	0.10	17	0.868	0.955	0.3957
1552935_at	96.4	120.6	0.32	1.25	PAA-PPP	0.10	17	0.868	0.955	0.3810
235406_x_at	67.1	83.9	0.32	1.25	AAA-AAP	0.10	17	0.868	0.955	0.3672
242112_at	115.7	144.8	0.32	1.25	PAP-PPP	0.10	17	0.868	0.955	0.3411
220998_s_at	357.3	446.9	0.32	1.25	AMP-PPP	0.10	17	0.868	0.955	0.3211
230533_at	224.6	281.0	0.32	1.25	PPP-PPP	0.10	17	0.868	0.955	0.3016
1558675_s_at	991.7	1240.1	0.32	1.25	PPP-PPP	0.10	17	0.868	0.955	0.2435
214683_s_at	775.7	970.4	0.32	1.25	PPP-PPP	0.10	17	0.868	0.955	0.2250
1554004_a_at	358.8	448.8	0.32	1.25	PPP-PPP	0.10	17	0.868	0.955	0.2242
212700_x_at	192.4	240.7	0.32	1.25	APA-PPP	0.10	17	0.868	0.955	0.1758
214778_at	91.0	113.7	0.32	1.25	PPP-PPP	0.10	17	0.868	0.955	0.5503
213058_at	75.5	94.3	0.32	1.25	PAA-PPA	0.10	17	0.868	0.955	0.4723
225843_at	247.6	309.6	0.32	1.25	PPP-PPP	0.10	17	0.868	0.955	0.4556
210069_at	92.2	115.2	0.32	1.25	APP-PAP	0.10	17	0.868	0.955	0.4091
235060_at	893.6	1116.8	0.32	1.25	PPP-PPP	0.10	17	0.868	0.955	0.3733
230759_at	146.1	182.6	0.32	1.25	PAP-PPP	0.10	17	0.868	0.955	0.3238
202781_s_at	302.6	378.2	0.32	1.25	PPP-PPP	0.10	17	0.868	0.955	0.2548
239311_at	125.2	156.4	0.32	1.25	APP-PPP	0.10	17	0.868	0.955	0.2443
1564157_at	82.6	103.3	0.32	1.25	APA-PPP	0.10	17	0.868	0.955	0.2180
243291_at	78.4	97.9	0.32	1.25	AAA-PAA	0.10	17	0.868	0.955	0.5993
224425_x_at	80.4	100.4	0.32	1.25	APA-PPA	0.10	17	0.868	0.955	0.5570
208165_s_at	178.4	222.8	0.32	1.25	PPA-PPP	0.10	17	0.868	0.955	0.5449
235513_at	92.4	115.4	0.32	1.25	MAA-AAP	0.10	17	0.868	0.955	0.4297
1554501_at	70.3	87.8	0.32	1.25	MPP-PMP	0.10	17	0.868	0.955	0.4083
226728_at	222.4	277.8	0.32	1.25	PPP-PPP	0.10	17	0.868	0.955	0.3444
234738_s_at	172.3	215.2	0.32	1.25	AAA-AAP	0.10	17	0.868	0.955	0.3235
1561631_at	74.1	92.5	0.32	1.25	PPP-MPP	0.10	17	0.868	0.955	0.3102
223742_at	610.1	762.1	0.32	1.25	PPP-PPP	0.10	17	0.868	0.955	0.3091

235344_at	148.7	185.8	0.32	1.25	PMP-PPP	0.10	17	0.868	0.955	0.2999
209771_x_at	11791.2	14729.5	0.32	1.25	PPP-PPP	0.10	17	0.868	0.955	0.2425
214747_at	100.4	125.3	0.32	1.25	PAP-AAA	0.10	17	0.868	0.955	0.2125
1562863_at	99.7	124.5	0.32	1.25	APA-AAP	0.10	17	0.868	0.955	0.6191
1563596_at	56.1	70.0	0.32	1.25	PAA-PAP	0.10	17	0.868	0.955	0.6172
235062_at	59.3	74.0	0.32	1.25	APP-PPP	0.10	17	0.868	0.955	0.5851
215375_x_at	83.2	103.9	0.32	1.25	PPA-AMA	0.10	17	0.868	0.955	0.5424
239842_x_at	126.3	157.6	0.32	1.25	APP-MPM	0.10	17	0.868	0.955	0.5205
1554785_at	75.7	94.4	0.32	1.25	APP-PMP	0.10	17	0.868	0.955	0.4898
1555436_a_at	148.9	185.9	0.32	1.25	PPP-PPP	0.10	17	0.868	0.955	0.3827
228757_at	123.7	154.4	0.32	1.25	AAA-MPA	0.10	17	0.868	0.955	0.3779
236170_x_at	149.0	186.1	0.32	1.25	PPP-PPP	0.10	17	0.868	0.955	0.3437
235109_at	263.4	328.8	0.32	1.25	PPP-MPP	0.10	17	0.868	0.955	0.3016
234617_at	81.8	102.1	0.32	1.25	AAA-APA	0.10	17	0.868	0.955	0.2912
227325_at	284.4	355.0	0.32	1.25	PPP-PPP	0.10	17	0.868	0.955	0.2857
223898_at	296.4	369.9	0.32	1.25	PPP-PPP	0.10	17	0.868	0.955	0.1909
211947_s_at	1591.8	1987.5	0.32	1.25	PPP-PPP	0.10	17	0.868	0.955	0.1895
213839_at	290.9	363.1	0.32	1.25	PPP-PPP	0.10	17	0.868	0.955	0.1725
1552445_a_at	27.2	33.9	0.32	1.25	AAA-APA	0.10	17	0.868	0.955	0.5942
1559979_at	42.3	52.8	0.32	1.25	AAA-AAP	0.10	17	0.868	0.955	0.5105
235348_at	133.9	167.0	0.32	1.25	PPP-PPP	0.10	17	0.868	0.955	0.5025
212127_at	525.4	655.6	0.32	1.25	PPP-PAP	0.10	17	0.868	0.955	0.3846
242847_at	147.0	183.3	0.32	1.25	PPP-PPP	0.10	17	0.868	0.955	0.3764
238122_at	179.6	224.1	0.32	1.25	PPP-PPP	0.10	17	0.868	0.955	0.3376
222482_at	214.2	267.2	0.32	1.25	PPP-PPP	0.10	17	0.868	0.955	0.2494
217704_x_at	200.4	250.0	0.32	1.25	PPP-PPP	0.10	17	0.868	0.955	0.1978
239071_at	159.0	198.2	0.32	1.25	AMP-AAP	0.10	17	0.868	0.955	0.5729
208232_x_at	82.3	102.6	0.32	1.25	AAA-PAM	0.10	17	0.868	0.955	0.4846
213582_at	110.8	138.2	0.32	1.25	PPP-PPP	0.10	17	0.868	0.955	0.4644
240105_at	123.7	154.1	0.32	1.25	PPP-PPP	0.10	17	0.868	0.955	0.4296
242828_at	212.5	264.9	0.32	1.25	PPP-PPP	0.10	17	0.868	0.955	0.4008
1554449_at	33.6	41.8	0.32	1.25	AAA-MAP	0.10	17	0.868	0.955	0.3959
1559307_s_at	84.0	104.7	0.32	1.25	PPP-PPP	0.10	17	0.868	0.955	0.3932
203801_at	245.5	306.0	0.32	1.25	PPP-PPP	0.10	17	0.868	0.955	0.3251
213022_s_at	143.7	179.1	0.32	1.25	PAP-AAA	0.10	17	0.868	0.955	0.2467
205617_at	157.7	196.7	0.32	1.25	APP-PPP	0.10	17	0.868	0.955	0.2434
1554178_a_at	180.8	225.4	0.32	1.25	PPP-PPP	0.10	17	0.868	0.955	0.2282
225569_at	1664.0	2074.5	0.32	1.25	PPP-PPP	0.10	17	0.868	0.955	0.2006
204142_at	953.7	1188.5	0.32	1.25	PPP-PPP	0.10	17	0.868	0.955	0.1901
225104_at	404.6	503.9	0.32	1.25	PPP-PPP	0.10	17	0.868	0.955	0.5363
217857_s_at	410.7	511.5	0.32	1.25	PAP-PAP	0.10	17	0.868	0.955	0.5267
227010_at	162.3	202.2	0.32	1.25	MAP-MAA	0.10	17	0.868	0.955	0.4669

203504_s_at	47.2	58.8	0.32	1.25	AAP-AAA	0.10	17	0.868	0.955	0.4514
200867_at	1578.7	1967.3	0.32	1.25	PPP-PPP	0.10	17	0.868	0.955	0.3219
240231_at	115.7	144.1	0.32	1.25	PAP-PPP	0.10	17	0.868	0.955	0.3076
231056_at	407.3	507.4	0.32	1.25	PPP-PPP	0.10	17	0.868	0.955	0.2862
226577_at	653.9	814.5	0.32	1.25	PPP-PPP	0.10	17	0.868	0.955	0.2712
212811_x_at	175.4	218.4	0.32	1.25	PPP-PPP	0.10	17	0.868	0.955	0.2194
242287_at	88.8	110.6	0.32	1.25	PAP-PPP	0.10	17	0.868	0.955	0.1759
226990_at	523.0	651.5	0.32	1.25	PPP-PPP	0.10	17	0.868	0.955	0.1739
232480_at	192.9	240.1	0.32	1.24	PAP-PPP	0.10	17	0.868	0.955	0.5809
227335_at	688.2	857.0	0.32	1.25	PPP-PPP	0.10	17	0.868	0.955	0.3213
219416_at	377.5	470.0	0.32	1.25	PPP-PPP	0.10	17	0.868	0.955	0.3099
201466_s_at	281.4	350.4	0.32	1.25	PPP-PPP	0.10	17	0.868	0.955	0.1629
1773_at	294.3	366.5	0.32	1.25	PAA-PPP	0.10	17	0.868	0.955	0.1553
228005_at	261.8	325.6	0.32	1.24	PPP-PPP	0.10	17	0.868	0.955	0.5646
232455_x_at	173.2	215.5	0.32	1.24	PAP-PPP	0.10	17	0.868	0.955	0.5531
215250_at	67.2	83.7	0.32	1.24	PAA-AAP	0.10	17	0.868	0.955	0.5256
215358_x_at	124.7	155.1	0.32	1.24	AAP-PPP	0.10	17	0.868	0.955	0.4253
241737_x_at	122.8	152.7	0.32	1.24	PAP-PPP	0.10	17	0.868	0.955	0.3488
241808_at	346.2	430.5	0.32	1.24	PPP-PPP	0.10	17	0.868	0.955	0.2918
216652_s_at	1185.8	1475.3	0.32	1.24	PPP-PPP	0.10	17	0.868	0.955	0.1849
1557053_s_at	718.8	894.4	0.32	1.24	PPP-PPP	0.10	17	0.868	0.955	0.1718
243020_at	90.0	112.0	0.32	1.24	PPA-PPP	0.10	17	0.868	0.955	0.1702
219972_s_at	563.9	701.5	0.32	1.24	PPP-PPP	0.10	17	0.868	0.955	0.1608
230454_at	263.2	327.5	0.32	1.24	PPP-PPP	0.10	17	0.868	0.955	0.1507
219941_at	56.6	70.3	0.31	1.24	PAA-AAA	0.10	17	0.868	0.955	0.5344
244334_at	107.7	133.9	0.31	1.24	PPA-PPP	0.10	17	0.868	0.955	0.5285
239816_at	157.8	196.1	0.31	1.24	AAP-PAP	0.10	17	0.868	0.955	0.5250
214313_s_at	70.2	87.2	0.31	1.24	PPP-PPP	0.10	17	0.868	0.955	0.4810
1569520_at	70.7	87.9	0.31	1.24	APP-PPP	0.10	17	0.868	0.955	0.4385
1563320_at	85.5	106.2	0.31	1.24	AAM-APP	0.10	17	0.868	0.955	0.4061
227994_x_at	1982.6	2464.7	0.31	1.24	PPP-PPP	0.10	17	0.868	0.955	0.3898
230136_at	192.1	238.8	0.31	1.24	PPP-MPM	0.10	17	0.868	0.955	0.3087
1558345_a_at	159.3	197.9	0.31	1.24	PPP-PPP	0.10	17	0.868	0.955	0.2612
215222_x_at	804.0	999.4	0.31	1.24	PPP-PPP	0.10	17	0.868	0.955	0.2607
209827_s_at	86.6	107.7	0.31	1.24	AAP-AAP	0.10	17	0.868	0.955	0.2482
227122_at	260.6	323.9	0.31	1.24	PPP-PPP	0.10	17	0.868	0.955	0.2117
226014_at	82.2	102.1	0.31	1.24	AAP-MPP	0.10	17	0.868	0.955	0.1935
238990_x_at	44.5	55.3	0.31	1.24	MPA-APP	0.10	17	0.868	0.955	0.5650
243908_at	53.8	66.9	0.31	1.24	PAP-PAP	0.10	17	0.868	0.955	0.5526
202210_x_at	165.6	205.8	0.31	1.24	AAA-PAA	0.10	17	0.868	0.955	0.5164
228464_at	222.8	276.7	0.31	1.24	PPP-PPP	0.10	17	0.868	0.955	0.4956
235143_at	198.9	247.1	0.31	1.24	PPP-PPP	0.10	17	0.868	0.955	0.4509

206108_s_at	435.7	541.1	0.31	1.24	PPP-PPP	0.10	17	0.868	0.955	0.4422
202430_s_at	1162.7	1444.8	0.31	1.24	PPP-PPP	0.10	17	0.868	0.955	0.3912
200884_at	236.4	293.7	0.31	1.24	PAA-PAA	0.10	17	0.868	0.955	0.3824
229886_at	470.1	583.9	0.31	1.24	PPP-PPP	0.10	17	0.868	0.955	0.3262
203122_at	593.3	737.2	0.31	1.24	PPP-PPP	0.10	17	0.868	0.955	0.2506
244842_x_at	244.3	303.6	0.31	1.24	PPP-PPP	0.10	17	0.868	0.955	0.2433
219741_x_at	378.1	469.8	0.31	1.24	PPP-PPP	0.10	17	0.868	0.955	0.2292
241983_at	116.3	144.4	0.31	1.24	AAP-PAP	0.10	17	0.868	0.955	0.1823
224009_x_at	98.3	122.2	0.31	1.24	AAA-APA	0.10	17	0.868	0.955	0.1672
239134_at	156.4	194.1	0.31	1.24	PPP-PPP	0.10	17	0.868	0.955	0.5452
215435_at	67.9	84.2	0.31	1.24	PPP-PAP	0.10	17	0.868	0.955	0.4561
224868_at	536.0	665.3	0.31	1.24	PPP-PPP	0.10	17	0.868	0.955	0.4136
1554001_at	146.3	181.7	0.31	1.24	PAP-PPP	0.10	17	0.868	0.955	0.4043
232489_at	124.1	154.1	0.31	1.24	PPP-PPP	0.10	17	0.868	0.955	0.3899
1553719_s_at	143.3	177.9	0.31	1.24	PPP-PPP	0.10	17	0.868	0.955	0.3784
214493_s_at	145.7	180.9	0.31	1.24	PPP-MPP	0.10	17	0.868	0.955	0.3675
235714_at	105.8	131.3	0.31	1.24	AAA-AAP	0.10	17	0.868	0.955	0.3112
240482_at	148.8	184.8	0.31	1.24	PPA-PAP	0.10	17	0.868	0.955	0.2816
1554609_at	200.9	249.5	0.31	1.24	PPP-PPP	0.10	17	0.868	0.955	0.1651
235430_at	57.9	71.8	0.31	1.24	AAA-APP	0.10	17	0.868	0.955	0.6245
212755_at	71.3	88.4	0.31	1.24	PMP-APA	0.10	17	0.868	0.955	0.5669
241174_at	63.9	79.3	0.31	1.24	APA-PPA	0.10	17	0.868	0.955	0.5355
204088_at	100.5	124.7	0.31	1.24	PAA-PAA	0.10	17	0.868	0.955	0.4082
230164_at	137.7	170.7	0.31	1.24	APP-APP	0.10	17	0.868	0.955	0.3455
242844_at	371.4	460.6	0.31	1.24	PPP-PPP	0.10	17	0.868	0.955	0.2995
202387_at	1232.0	1528.5	0.31	1.24	PPP-PPP	0.10	17	0.868	0.955	0.2794
1557873_at	90.0	111.6	0.31	1.24	APA-MAA	0.10	17	0.868	0.955	0.2155
235205_at	356.9	442.6	0.31	1.24	PPP-MPP	0.10	17	0.868	0.955	0.1971
217144_at	1216.4	1508.8	0.31	1.24	PPP-PPP	0.10	17	0.868	0.955	0.1471
227384_s_at	344.8	427.3	0.31	1.24	PPP-PPP	0.10	17	0.868	0.955	0.6103
239877_at	42.1	52.3	0.31	1.24	AAA-APA	0.10	17	0.868	0.955	0.5174
215178_x_at	158.4	196.5	0.31	1.24	PPP-PPP	0.10	17	0.868	0.955	0.5037
238551_at	156.2	193.7	0.31	1.24	APP-PPA	0.10	17	0.868	0.955	0.4819
232773_at	55.0	68.2	0.31	1.24	PPA-PPP	0.10	17	0.868	0.955	0.4689
239271_at	69.4	86.0	0.31	1.24	APM-PAP	0.10	17	0.868	0.955	0.4642
239840_at	95.5	118.4	0.31	1.24	AAM-APP	0.10	17	0.868	0.955	0.4085
221713_s_at	135.3	167.7	0.31	1.24	PAA-PMP	0.10	17	0.868	0.955	0.3939
226166_x_at	128.9	159.8	0.31	1.24	APP-PAP	0.10	17	0.868	0.955	0.3827
224470_at	151.3	187.6	0.31	1.24	AAP-APP	0.10	17	0.868	0.955	0.3447
221507_at	891.9	1105.5	0.31	1.24	PPP-PPP	0.10	17	0.868	0.955	0.2090
203485_at	127.3	157.8	0.31	1.24	PPP-PPP	0.10	17	0.868	0.955	0.2058
1566087_at	32.7	40.5	0.31	1.24	AAP-PAA	0.10	17	0.868	0.955	0.1694

229355_at	307.0	380.3	0.31	1.24	PPP-PPP	0.10	17	0.868	0.955	0.5587
239318_at	42.8	53.0	0.31	1.24	PAA-AAA	0.10	17	0.868	0.955	0.5507
219301_s_at	57.0	70.6	0.31	1.24	APA-AAA	0.10	17	0.868	0.955	0.5450
238526_at	78.4	97.2	0.31	1.24	AAP-APA	0.10	17	0.868	0.955	0.4313
232704_s_at	80.0	99.1	0.31	1.24	AAP-PPP	0.10	17	0.868	0.955	0.4223
203015_s_at	275.6	341.3	0.31	1.24	PPP-PPP	0.10	17	0.868	0.955	0.3845
206572_x_at	486.6	602.8	0.31	1.24	PPP-PPP	0.10	17	0.868	0.955	0.2622
239329_at	190.4	235.9	0.31	1.24	PPP-PPP	0.10	17	0.868	0.955	0.1574
201308_s_at	327.8	406.1	0.31	1.24	PPP-PPP	0.10	17	0.868	0.955	0.1561
233940_at	55.5	68.7	0.31	1.24	APA-APP	0.10	17	0.868	0.955	0.5848
1568964_x_at	169.1	209.3	0.31	1.24	PPP-PPP	0.10	17	0.868	0.955	0.5588
218779_x_at	296.1	366.7	0.31	1.24	APA-AMP	0.10	17	0.868	0.955	0.4947
235925_at	98.6	122.0	0.31	1.24	APP-APP	0.10	17	0.868	0.955	0.4832
238848_at	257.1	318.4	0.31	1.24	PPP-PPP	0.10	17	0.868	0.955	0.4660
215366_at	133.5	165.3	0.31	1.24	MAA-PPP	0.10	17	0.868	0.955	0.4649
1569706_at	50.0	61.9	0.31	1.24	APM-PPP	0.10	17	0.868	0.955	0.4613
205500_at	121.8	150.8	0.31	1.24	PPP-PPP	0.10	17	0.868	0.955	0.4053
213147_at	302.8	374.8	0.31	1.24	PPP-AMP	0.10	17	0.868	0.955	0.3796
227523_s_at	786.3	973.5	0.31	1.24	PPP-PPP	0.10	17	0.868	0.955	0.3113
236520_at	43.3	53.6	0.31	1.24	APP-AAA	0.10	17	0.868	0.955	0.3093
1558236_at	387.7	480.1	0.31	1.24	PPP-PPP	0.10	17	0.868	0.955	0.2938
241541_at	184.6	228.5	0.31	1.24	MPA-PAP	0.10	17	0.868	0.955	0.2316
222233_s_at	474.3	587.2	0.31	1.24	PPP-PPP	0.10	17	0.868	0.955	0.2209
213221_s_at	460.6	570.4	0.31	1.24	PPP-PPP	0.10	17	0.868	0.955	0.2108
241127_at	63.8	79.0	0.31	1.24	APA-APA	0.10	17	0.868	0.955	0.6111
208274_at	51.4	63.6	0.31	1.24	MAA-APA	0.10	17	0.868	0.955	0.5968
241164_at	47.5	58.7	0.31	1.24	PAA-PPP	0.10	17	0.868	0.955	0.5747
243629_x_at	97.6	120.7	0.31	1.24	AAP-APP	0.10	17	0.868	0.955	0.5637
1564856_s_at	56.0	69.2	0.31	1.24	APP-PPP	0.10	17	0.868	0.955	0.3842
244404_at	80.1	99.1	0.31	1.24	MPP-PPP	0.10	17	0.868	0.955	0.3819
1568742_at	202.4	250.4	0.31	1.24	PPP-PPP	0.10	17	0.868	0.955	0.3656
1556319_at	92.7	114.7	0.31	1.24	AAA-AAP	0.10	17	0.868	0.955	0.3573
211676_s_at	1096.1	1356.1	0.31	1.24	PPP-PPP	0.10	17	0.868	0.955	0.1872
225186_at	269.6	333.5	0.31	1.24	PPP-PPP	0.10	17	0.868	0.955	0.1486
1555259_at	264.6	327.2	0.31	1.24	PAP-PPP	0.10	17	0.868	0.955	0.5381
1559993_at	166.6	206.0	0.31	1.24	PPP-PPP	0.10	17	0.868	0.955	0.5152
233302_at	41.2	51.0	0.31	1.24	AAA-AAP	0.10	17	0.868	0.955	0.4613
216823_at	300.1	370.9	0.31	1.24	PPP-PPP	0.10	17	0.868	0.955	0.4598
238055_at	129.6	160.3	0.31	1.24	PPP-PAP	0.10	17	0.868	0.955	0.4337
221992_at	165.9	205.1	0.31	1.24	PPP-PPP	0.10	17	0.868	0.955	0.3618
227835_at	145.8	180.2	0.31	1.24	AAP-AAA	0.10	17	0.868	0.955	0.3490
1557938_s_at	1544.2	1908.9	0.31	1.24	PPP-PPP	0.10	17	0.868	0.955	0.3214

222456_s_at	111.0	137.2	0.31	1.24	MAA-PPP	0.10	17	0.868	0.955	0.3114
215873_x_at	428.4	529.6	0.31	1.24	PPP-PPP	0.10	17	0.868	0.955	0.1773
209003_at	615.4	760.6	0.31	1.24	PPP-PPP	0.10	17	0.868	0.955	0.1683
203301_s_at	959.2	1185.6	0.31	1.24	PPP-PPP	0.10	17	0.868	0.955	0.1650
240723_at	71.1	87.8	0.31	1.24	AMA-PMA	0.10	17	0.868	0.955	0.5359
239232_at	85.9	106.1	0.31	1.24	PAA-MAP	0.10	17	0.868	0.955	0.4355
1555288_s_at	118.1	145.9	0.31	1.24	APP-PAP	0.10	17	0.868	0.955	0.3776
230235_at	119.8	148.0	0.31	1.24	PAA-MPP	0.10	17	0.868	0.955	0.3574
1554249_a_at	256.3	316.6	0.31	1.24	PPP-PPP	0.10	17	0.868	0.955	0.3381
225521_at	246.0	303.9	0.31	1.24	PPP-PPP	0.10	17	0.868	0.955	0.3312
202472_at	197.9	244.5	0.31	1.24	PPP-PAP	0.10	17	0.868	0.955	0.3138
1561190_at	68.9	85.1	0.31	1.24	APA-APP	0.10	17	0.868	0.955	0.2457
1554887_at	66.5	82.1	0.30	1.24	PAP-PPA	0.10	17	0.868	0.955	0.5861
238155_at	49.7	61.4	0.30	1.24	AAA-APP	0.10	17	0.868	0.955	0.5002
1553155_x_at	91.9	113.4	0.30	1.23	PPA-AMP	0.10	17	0.868	0.955	0.4977
203898_at	743.6	918.2	0.30	1.24	PPP-PPP	0.10	17	0.868	0.955	0.4837
220842_at	50.7	62.6	0.30	1.24	APP-APA	0.10	17	0.868	0.955	0.4317
232613_at	79.8	98.5	0.30	1.24	APP-PAA	0.10	17	0.868	0.955	0.3865
242617_at	558.5	689.6	0.30	1.24	PPP-PPP	0.10	17	0.868	0.955	0.3674
204360_s_at	233.4	288.1	0.30	1.23	PPP-PPP	0.10	17	0.868	0.955	0.3407
239020_at	187.0	230.8	0.30	1.23	PAP-AMM	0.10	17	0.868	0.955	0.2694
211695_x_at	337.4	416.5	0.30	1.24	PPP-PPP	0.10	17	0.868	0.955	0.2519
227561_at	119.4	147.4	0.30	1.24	MAA-PPM	0.10	17	0.868	0.955	0.1534
240572_s_at	163.4	201.7	0.30	1.23	PMP-PPP	0.10	17	0.868	0.955	0.1414
214108_at	89.8	110.7	0.30	1.23	PPP-APA	0.10	17	0.868	0.955	0.5467
229793_at	180.9	223.2	0.30	1.23	PPP-PPP	0.10	17	0.868	0.955	0.4878
207078_at	66.5	82.1	0.30	1.23	PPP-AAP	0.10	17	0.868	0.955	0.3998
203920_at	183.6	226.5	0.30	1.23	PPP-PPP	0.10	17	0.868	0.955	0.3627
227470_at	301.5	372.0	0.30	1.23	PPP-PAP	0.10	17	0.868	0.955	0.3176
240146_at	107.8	133.1	0.30	1.23	PPP-PPP	0.10	17	0.868	0.955	0.3139
204818_at	885.5	1092.4	0.30	1.23	PPP-PPP	0.10	17	0.868	0.955	0.2729
205107_s_at	316.0	389.9	0.30	1.23	PPP-PPP	0.10	17	0.868	0.955	0.2390
235324_at	120.5	148.6	0.30	1.23	PPP-PPP	0.10	17	0.868	0.955	0.2094
212892_at	495.8	611.5	0.30	1.23	PPP-PAP	0.10	17	0.868	0.955	0.2075
235878_at	269.2	332.2	0.30	1.23	PPP-PPP	0.10	17	0.868	0.955	0.1727
227600_at	354.8	437.6	0.30	1.23	PPP-PPP	0.10	17	0.868	0.955	0.1501
236386_at	64.4	79.4	0.30	1.23	PAP-PPP	0.10	17	0.868	0.955	0.6188
242662_at	83.3	102.7	0.30	1.23	AAA-AAP	0.10	17	0.868	0.955	0.6023
231906_at	66.5	82.0	0.30	1.23	PAA-AAM	0.10	17	0.868	0.955	0.5693
226404_at	485.5	598.5	0.30	1.23	PPP-PPP	0.10	17	0.868	0.955	0.5191
222343_at	107.9	133.0	0.30	1.23	PPP-PPP	0.10	17	0.868	0.955	0.4725
1558486_at	134.9	166.4	0.30	1.23	MPP-PPP	0.10	17	0.868	0.955	0.4334

235573_at	203.0	250.3	0.30	1.23	PPP-PPP	0.10	17	0.868	0.955	0.3272
213046_at	259.0	319.3	0.30	1.23	PPP-PPP	0.10	17	0.868	0.955	0.2778
1565027_at	109.4	134.9	0.30	1.23	PAA-APA	0.10	17	0.868	0.955	0.2504
202342_s_at	380.1	468.6	0.30	1.23	PPP-PPP	0.10	17	0.868	0.955	0.2443
213393_at	93.8	115.6	0.30	1.23	PPP-PPP	0.10	17	0.868	0.955	0.2419
213286_at	351.9	433.8	0.30	1.23	PPP-PPP	0.10	17	0.868	0.955	0.2100
223576_at	865.9	1067.4	0.30	1.23	PPP-PPP	0.10	17	0.868	0.955	0.2090
227700_x_at	745.6	918.9	0.30	1.23	PPP-PPP	0.10	17	0.868	0.955	0.1621
242073_at	180.2	221.9	0.30	1.23	PPP-PPP	0.10	17	0.868	0.955	0.6121
215557_at	112.1	138.1	0.30	1.23	PAA-PPP	0.10	17	0.868	0.955	0.5516
212884_x_at	144.3	177.7	0.30	1.23	APA-AAM	0.10	17	0.868	0.955	0.5070
221813_at	391.1	481.9	0.30	1.23	AAP-AAP	0.10	17	0.868	0.955	0.4405
244504_x_at	95.1	117.1	0.30	1.23	AAP-AAP	0.10	17	0.868	0.955	0.4194
210172_at	203.6	250.8	0.30	1.23	PAP-PPP	0.10	17	0.868	0.955	0.3894
241680_at	324.4	399.6	0.30	1.23	PPP-PPP	0.10	17	0.868	0.955	0.3892
211595_s_at	1130.7	1392.6	0.30	1.23	PPP-PPP	0.10	17	0.868	0.955	0.2211
239246_at	178.7	220.1	0.30	1.23	PPP-PPP	0.10	17	0.868	0.955	0.1726
236007_at	152.0	187.2	0.30	1.23	PPP-PPP	0.10	17	0.868	0.955	0.6191
243078_at	92.2	113.5	0.30	1.23	APP-PPP	0.10	17	0.868	0.955	0.5355
243835_at	83.1	102.3	0.30	1.23	AAP-AMP	0.10	17	0.868	0.955	0.4037
205208_at	69.0	84.9	0.30	1.23	AAA-AAP	0.10	17	0.868	0.955	0.3964
221563_at	248.5	306.0	0.30	1.23	PPP-PPP	0.10	17	0.868	0.955	0.2248
204375_at	87.0	107.1	0.30	1.23	PAA-PMP	0.10	17	0.868	0.955	0.1648
213873_at	69.2	85.0	0.30	1.23	AAA-AAP	0.10	17	0.868	0.955	0.6009
1562836_at	178.1	219.1	0.30	1.23	APP-PPP	0.10	17	0.868	0.955	0.5155
240824_at	60.3	74.2	0.30	1.23	PPP-APP	0.10	17	0.868	0.955	0.4958
244783_at	68.6	84.3	0.30	1.23	PAP-PPP	0.10	17	0.868	0.955	0.4280
205198_s_at	66.7	82.1	0.30	1.23	PAA-MPA	0.10	17	0.868	0.955	0.4111
1555272_at	249.7	307.2	0.30	1.23	APM-PPM	0.10	17	0.868	0.955	0.4010
221079_s_at	750.2	922.7	0.30	1.23	PPP-PPP	0.10	17	0.868	0.955	0.3660
202152_x_at	817.6	1005.9	0.30	1.23	PPP-MPP	0.10	17	0.868	0.955	0.3569
202500_at	98.9	121.6	0.30	1.23	AAP-AAP	0.10	17	0.868	0.955	0.3081
1553831_at	115.0	141.4	0.30	1.23	AAA-MPA	0.10	17	0.868	0.955	0.2525
221501_x_at	2914.1	3585.8	0.30	1.23	PPP-PPP	0.10	17	0.868	0.955	0.1817
242113_at	88.1	108.3	0.30	1.23	PMP-PPP	0.10	17	0.868	0.955	0.5892
232113_at	282.5	347.3	0.30	1.23	PPP-PPP	0.10	17	0.868	0.955	0.5664
235168_at	229.1	281.8	0.30	1.23	PPP-PPP	0.10	17	0.868	0.955	0.5192
212807_s_at	181.6	223.2	0.30	1.23	PPP-PPP	0.10	17	0.868	0.955	0.4330
1565886_at	116.4	143.1	0.30	1.23	PPP-PPP	0.10	17	0.868	0.955	0.3441
1568680_s_at	146.1	179.7	0.30	1.23	MPM-MPP	0.10	17	0.868	0.955	0.3153
224418_x_at	69.1	84.9	0.30	1.23	MPA-AAA	0.10	17	0.868	0.955	0.3114
204135_at	79.4	97.7	0.30	1.23	PMM-PPP	0.10	17	0.868	0.955	0.2399

223392_s_at	97.4	119.8	0.30	1.23	APA-PAA	0.10	17	0.868	0.955	0.2085
1564360_a_at	32.0	39.3	0.30	1.23	PAA-AAP	0.10	17	0.868	0.955	0.5646
229334_at	312.7	384.2	0.30	1.23	PPP-PPP	0.10	17	0.868	0.955	0.4717
1560609_at	30.0	36.9	0.30	1.23	AAA-PAP	0.10	17	0.868	0.955	0.4696
1558877_at	75.8	93.1	0.30	1.23	MPA-APP	0.10	17	0.868	0.955	0.4656
241372_at	52.9	65.0	0.30	1.23	AMA-PAA	0.10	17	0.868	0.955	0.4521
239811_at	149.7	183.9	0.30	1.23	PPP-PPP	0.10	17	0.868	0.955	0.4023
206141_at	206.4	253.5	0.30	1.23	PPP-PPP	0.10	17	0.868	0.955	0.3929
234710_s_at	317.8	390.4	0.30	1.23	PPP-PPP	0.10	17	0.868	0.955	0.2946
238271_x_at	58.1	71.3	0.30	1.23	AAA-PAA	0.10	17	0.868	0.955	0.2798
227199_at	378.6	465.0	0.30	1.23	PPP-PPP	0.10	17	0.868	0.955	0.2706
222686_s_at	243.8	299.6	0.30	1.23	MAP-PAA	0.10	17	0.868	0.955	0.2280
209643_s_at	662.7	814.3	0.30	1.23	PPP-PPP	0.10	17	0.868	0.955	0.1995
231976_at	155.4	190.9	0.30	1.23	PPP-PPP	0.10	17	0.868	0.955	0.1958
228775_at	371.6	456.4	0.30	1.23	PPP-PPP	0.10	17	0.868	0.955	0.1841
230148_at	39.5	48.5	0.30	1.23	AAA-PAA	0.10	17	0.868	0.955	0.5802
244154_at	93.7	115.0	0.30	1.23	PAA-PPP	0.10	17	0.868	0.955	0.4005
227590_at	204.6	251.2	0.30	1.23	PAP-MPP	0.10	17	0.868	0.955	0.3916
206590_x_at	157.1	192.9	0.30	1.23	PPP-PMP	0.10	17	0.868	0.955	0.3739
1557121_s_at	112.4	138.0	0.30	1.23	PPP-PPP	0.10	17	0.868	0.955	0.2455
1566207_at	158.4	194.5	0.30	1.23	AAA-PPP	0.10	17	0.868	0.955	0.2029
232909_s_at	935.7	1149.2	0.30	1.23	PPP-PPP	0.10	17	0.868	0.955	0.1956
220174_at	459.1	563.6	0.30	1.23	PPP-PPP	0.10	17	0.868	0.955	0.1804
1568619_s_at	1092.8	1341.4	0.30	1.23	PPP-PPP	0.10	17	0.868	0.955	0.1636
227162_at	123.1	151.2	0.30	1.23	PPP-PPP	0.10	17	0.868	0.955	0.1449
238706_at	177.3	217.5	0.30	1.23	PPP-PPP	0.10	17	0.868	0.955	0.5953
205058_at	50.7	62.2	0.30	1.23	AAA-PAA	0.10	17	0.868	0.955	0.5192
215919_s_at	58.3	71.5	0.30	1.23	APA-PAP	0.10	17	0.868	0.955	0.4835
230294_at	97.2	119.2	0.30	1.23	AAA-APA	0.10	17	0.868	0.955	0.3149
220720_x_at	379.0	464.9	0.30	1.23	MPP-PPP	0.10	17	0.868	0.955	0.2848
219821_s_at	499.2	612.4	0.30	1.23	PPP-PPP	0.10	17	0.868	0.955	0.1870
222806_s_at	703.3	863.1	0.30	1.23	PPP-PPP	0.10	17	0.868	0.955	0.1729
230913_at	80.8	99.2	0.30	1.23	AAA-PPP	0.10	17	0.868	0.955	0.1485
41856_at	242.2	296.9	0.29	1.23	PPP-PPP	0.10	17	0.868	0.955	0.3305
232270_at	191.8	235.1	0.29	1.23	APP-PPP	0.10	17	0.868	0.955	0.3089
210425_x_at	1079.8	1323.8	0.29	1.23	PPP-PPP	0.10	17	0.868	0.955	0.2274
203788_s_at	512.0	627.9	0.29	1.23	PPP-PPP	0.10	17	0.868	0.955	0.1747
241408_at	168.7	206.7	0.29	1.23	PPP-PPP	0.10	17	0.868	0.955	0.5803
227145_at	78.3	95.9	0.29	1.23	AAA-APA	0.10	17	0.868	0.955	0.5290
214723_x_at	218.3	267.4	0.29	1.23	PPA-PPP	0.10	17	0.868	0.955	0.5082
238880_at	374.0	458.2	0.29	1.23	PPP-PMP	0.10	17	0.868	0.955	0.4715
232350_x_at	253.0	309.9	0.29	1.23	PPP-PPP	0.10	17	0.868	0.955	0.4496

231877_at	158.1	193.8	0.29	1.23	PPP-PPP	0.10	17	0.868	0.955	0.4193
212000_at	283.8	347.6	0.29	1.23	PPP-PPP	0.10	17	0.868	0.955	0.3746
224619_at	2831.3	3469.8	0.29	1.23	PPP-PPP	0.10	17	0.868	0.955	0.3237
1555846_a_at	203.0	248.8	0.29	1.23	PPA-PPP	0.10	17	0.868	0.955	0.2949
219338_s_at	189.6	232.2	0.29	1.23	PPP-PPP	0.10	17	0.868	0.955	0.2858
212253_x_at	366.0	448.5	0.29	1.23	APP-PPP	0.10	17	0.868	0.955	0.2816
1553992_s_at	215.0	263.3	0.29	1.23	PPP-PPP	0.10	17	0.868	0.955	0.2759
226594_at	285.7	350.0	0.29	1.23	PPP-PPP	0.10	17	0.868	0.955	0.2467
229341_at	597.5	732.3	0.29	1.23	PPP-PPP	0.10	17	0.868	0.955	0.2445
207610_s_at	98.7	120.8	0.29	1.23	APP-PAM	0.10	17	0.868	0.955	0.2171
209696_at	69.3	84.9	0.29	1.22	AAA-PAA	0.10	17	0.868	0.955	0.5995
226261_at	395.3	484.1	0.29	1.23	PPP-PPP	0.10	17	0.868	0.955	0.4554
204740_at	379.7	465.1	0.29	1.23	PPP-PPP	0.10	17	0.868	0.955	0.4530
215483_at	106.8	130.7	0.29	1.22	PPP-PPP	0.10	17	0.868	0.955	0.4010
233480_at	152.9	187.1	0.29	1.22	PPM-PPP	0.10	17	0.868	0.955	0.3407
202289_s_at	1481.9	1814.1	0.29	1.22	PPP-PPP	0.10	17	0.868	0.955	0.2984
219190_s_at	119.2	146.0	0.29	1.23	PPP-PPP	0.10	17	0.868	0.955	0.2635
211530_x_at	762.0	933.2	0.29	1.23	PPP-PPP	0.10	17	0.868	0.955	0.2578
203968_s_at	577.2	706.7	0.29	1.22	PPP-PPP	0.10	17	0.868	0.955	0.2539
206506_s_at	264.9	324.3	0.29	1.22	PPP-PPP	0.10	17	0.868	0.955	0.2162
225327_at	574.4	703.4	0.29	1.23	PPP-PPP	0.10	17	0.868	0.955	0.2100
203090_at	629.3	770.6	0.29	1.23	PPP-PPP	0.10	17	0.868	0.955	0.1864
229720_at	1519.6	1860.5	0.29	1.22	PPP-PPP	0.10	17	0.868	0.955	0.1820
AFFX-HUMISGF3A/M!	132.1	161.8	0.29	1.23	PPP-PPP	0.10	17	0.868	0.955	0.1822
226095_s_at	637.5	780.6	0.29	1.22	MMP-MAM	0.10	17	0.868	0.955	0.1410
239946_at	62.7	76.7	0.29	1.22	PAM-APA	0.10	17	0.868	0.955	0.5862
1552410_at	68.7	84.0	0.29	1.22	APP-AAA	0.10	17	0.868	0.955	0.4360
1552812_a_at	172.9	211.5	0.29	1.22	PPP-PPP	0.10	17	0.868	0.955	0.3250
215545_at	106.7	130.5	0.29	1.22	PMP-PPP	0.10	17	0.868	0.955	0.3141
235269_at	231.3	283.0	0.29	1.22	MPP-PPP	0.10	17	0.868	0.955	0.2558
1553718_at	634.1	775.8	0.29	1.22	PPP-PPP	0.10	17	0.868	0.955	0.2141
241840_at	136.3	166.7	0.29	1.22	PAP-AAA	0.10	17	0.868	0.955	0.1774
46947_at	247.4	302.7	0.29	1.22	PPP-PPP	0.10	17	0.868	0.955	0.1765
230039_at	125.3	153.3	0.29	1.22	PAP-PAP	0.10	17	0.868	0.955	0.1657
203836_s_at	621.0	759.5	0.29	1.22	PPP-PPP	0.10	17	0.868	0.955	0.5365
234970_at	107.8	131.8	0.29	1.22	PPP-PPP	0.10	17	0.868	0.955	0.5208
204090_at	228.0	278.7	0.29	1.22	MAM-PAP	0.10	17	0.868	0.955	0.5045
239425_at	146.8	179.5	0.29	1.22	PPP-PAP	0.10	17	0.868	0.955	0.4656
202971_s_at	316.1	386.4	0.29	1.22	PAP-PPP	0.10	17	0.868	0.955	0.4598
1559566_at	51.9	63.5	0.29	1.22	PPA-MAP	0.10	17	0.868	0.955	0.4270
213659_at	103.5	126.6	0.29	1.22	PPP-PPP	0.10	17	0.868	0.955	0.3270
1556162_at	161.7	197.6	0.29	1.22	AMP-AAM	0.10	17	0.868	0.955	0.2597

238694_at	201.0	245.7	0.29	1.22	PPP-PPP	0.10	17	0.868	0.955	0.2504
217627_at	266.9	326.3	0.29	1.22	PPP-PPP	0.10	17	0.868	0.955	0.1884
1558723_at	84.9	103.8	0.29	1.22	AAA-AAP	0.10	17	0.868	0.955	0.1857
241432_at	31.7	38.7	0.29	1.22	AAA-AAP	0.10	17	0.868	0.955	0.5511
228183_s_at	1010.0	1234.3	0.29	1.22	PPP-PPP	0.10	17	0.868	0.955	0.5457
240000_at	85.2	104.1	0.29	1.22	PPP-PAP	0.10	17	0.868	0.955	0.4740
207920_x_at	160.8	196.4	0.29	1.22	APP-APA	0.10	17	0.868	0.955	0.4625
230201_at	142.4	174.0	0.29	1.22	PPP-PPP	0.10	17	0.868	0.955	0.4308
229970_at	204.9	250.4	0.29	1.22	PPP-PPP	0.10	17	0.868	0.955	0.3515
224493_x_at	704.3	860.6	0.29	1.22	PPP-PPP	0.10	17	0.868	0.955	0.2544
1560082_at	74.6	91.1	0.29	1.22	AAA-APA	0.10	17	0.868	0.955	0.5380
241786_at	133.7	163.3	0.29	1.22	PPP-PPP	0.10	17	0.868	0.955	0.4732
221147_x_at	174.2	212.7	0.29	1.22	AAA-PAM	0.10	17	0.868	0.955	0.4157
200856_x_at	353.8	432.0	0.29	1.22	PPP-PPP	0.10	17	0.868	0.955	0.3753
227924_at	183.6	224.1	0.29	1.22	PPP-PPP	0.10	17	0.868	0.955	0.3608
202124_s_at	192.8	235.5	0.29	1.22	APP-PPP	0.10	17	0.868	0.955	0.2947
202269_x_at	239.4	292.2	0.29	1.22	PPP-PPP	0.10	17	0.868	0.955	0.2280
238895_at	53.8	65.7	0.29	1.22	AAM-PAP	0.10	17	0.868	0.955	0.2280
208930_s_at	2245.9	2741.7	0.29	1.22	PPP-PPP	0.10	17	0.868	0.955	0.1464
241356_at	54.5	66.5	0.29	1.22	AAP-MPA	0.10	17	0.868	0.955	0.6022
204935_at	242.5	295.8	0.29	1.22	PPP-PPP	0.10	17	0.868	0.955	0.5000
243490_at	81.7	99.7	0.29	1.22	PAP-PPP	0.10	17	0.868	0.955	0.4895
1553810_a_at	207.9	253.7	0.29	1.22	PPP-PPP	0.10	17	0.868	0.955	0.4552
1553030_a_at	116.7	142.4	0.29	1.22	AAA-PAP	0.10	17	0.868	0.955	0.4433
220963_s_at	100.8	122.9	0.29	1.22	PPA-PPP	0.10	17	0.868	0.955	0.4286
227715_at	186.0	226.9	0.29	1.22	AMA-MAP	0.10	17	0.868	0.955	0.4182
235096_at	829.5	1012.4	0.29	1.22	PPP-PPP	0.10	17	0.868	0.955	0.3847
230099_at	184.8	225.5	0.29	1.22	AAA-AAP	0.10	17	0.868	0.955	0.3433
212226_s_at	102.2	124.7	0.29	1.22	PPA-APP	0.10	17	0.868	0.955	0.3384
232056_at	840.8	1025.8	0.29	1.22	PPP-PPP	0.10	17	0.868	0.955	0.3385
210128_s_at	190.5	232.5	0.29	1.22	PPP-PPP	0.10	17	0.868	0.955	0.3285
211383_s_at	523.1	638.4	0.29	1.22	PPP-PPP	0.10	17	0.868	0.955	0.2703
225980_at	464.6	566.9	0.29	1.22	PPP-PPP	0.10	17	0.868	0.955	0.2561
229826_at	238.3	290.8	0.29	1.22	PPP-PPP	0.10	17	0.868	0.955	0.1728
223295_s_at	1099.3	1340.9	0.29	1.22	PPP-PPP	0.10	17	0.868	0.955	0.1312
242982_x_at	98.4	119.9	0.29	1.22	PAA-AAA	0.10	17	0.868	0.955	0.5761
233411_at	119.2	145.4	0.29	1.22	AAA-PAP	0.10	17	0.868	0.955	0.5321
232986_at	68.9	83.9	0.29	1.22	PPP-MAA	0.10	17	0.868	0.955	0.5157
233931_at	90.7	110.6	0.29	1.22	APM-AAP	0.10	17	0.868	0.955	0.5006
223248_at	248.6	303.0	0.29	1.22	PPP-PPP	0.10	17	0.868	0.955	0.4784
206044_s_at	123.0	149.9	0.29	1.22	PPA-PPP	0.10	17	0.868	0.955	0.4388
219657_s_at	138.5	168.8	0.29	1.22	AAA-PPA	0.10	17	0.868	0.955	0.4324

204029_at	399.8	487.4	0.29	1.22	PPP-PPP	0.10	17	0.868	0.955	0.3768
224525_s_at	187.4	228.5	0.29	1.22	PPP-PPP	0.10	17	0.868	0.955	0.3281
225965_at	208.0	253.5	0.29	1.22	PPP-PPP	0.10	17	0.868	0.955	0.3183
233354_at	258.3	315.1	0.29	1.22	PPP-PMP	0.10	17	0.868	0.955	0.2786
232266_x_at	1988.7	2425.2	0.29	1.22	PPP-PPP	0.10	17	0.868	0.955	0.2717
40020_at	133.0	162.1	0.29	1.22	PAP-PPP	0.10	17	0.868	0.955	0.2526
206411_s_at	134.7	164.2	0.29	1.22	PAA-MPA	0.10	17	0.868	0.955	0.1892
221729_at	108.9	132.7	0.29	1.22	PPP-PPP	0.10	17	0.868	0.955	0.1516
229594_at	533.8	650.1	0.29	1.22	PPP-PPP	0.10	17	0.868	0.955	0.5610
206959_s_at	201.7	245.6	0.29	1.22	PPP-PPP	0.10	17	0.868	0.955	0.3650
242376_at	51.9	63.2	0.29	1.22	AAA-APP	0.10	17	0.868	0.955	0.3544
227722_at	110.5	134.7	0.29	1.22	PAP-AAP	0.10	17	0.868	0.955	0.3428
233595_at	302.4	368.5	0.29	1.22	PPP-PPP	0.10	17	0.868	0.955	0.3259
228622_s_at	291.8	355.5	0.29	1.22	PAP-APP	0.10	17	0.868	0.955	0.3101
214707_x_at	409.5	499.1	0.29	1.22	PPP-PPP	0.10	17	0.868	0.955	0.2805
201370_s_at	480.9	585.7	0.29	1.22	PPP-PPP	0.10	17	0.868	0.955	0.2262
211043_s_at	7197.4	8767.0	0.29	1.22	PPP-PPP	0.10	17	0.868	0.955	0.1958
226554_at	1006.3	1225.9	0.29	1.22	PPP-PPP	0.10	17	0.868	0.955	0.1813
225989_at	680.6	828.8	0.28	1.22	PPP-PPP	0.10	17	0.868	0.955	0.4743
210876_at	388.3	472.8	0.28	1.22	PPP-PPP	0.10	17	0.868	0.955	0.4480
240310_at	163.9	199.6	0.28	1.22	PPP-PPP	0.10	17	0.868	0.955	0.3794
239841_at	252.0	306.8	0.28	1.22	PPP-PPP	0.10	17	0.868	0.955	0.3580
203797_at	1402.9	1707.9	0.28	1.22	PPP-PPP	0.10	17	0.868	0.955	0.2613
205917_at	496.0	604.0	0.28	1.22	PPP-PPP	0.10	17	0.868	0.955	0.2243
1563720_at	32.3	39.3	0.28	1.22	APA-AAA	0.10	17	0.868	0.955	0.5964
243673_at	103.0	125.3	0.28	1.22	PPP-PPP	0.10	17	0.868	0.955	0.5502
204458_at	103.8	126.4	0.28	1.22	PPP-PPP	0.10	17	0.868	0.955	0.5233
243001_at	81.8	99.6	0.28	1.22	PPA-APP	0.10	17	0.868	0.955	0.4957
209079_x_at	442.1	538.0	0.28	1.22	PPP-PPP	0.10	17	0.868	0.955	0.4845
226201_at	241.0	293.3	0.28	1.22	PPP-PPP	0.10	17	0.868	0.955	0.4247
203860_at	870.7	1059.2	0.28	1.22	PPP-PPP	0.10	17	0.868	0.955	0.3831
203442_x_at	617.2	750.8	0.28	1.22	PPP-PPP	0.10	17	0.868	0.955	0.3563
1553542_at	91.0	110.7	0.28	1.22	PPP-MAP	0.10	17	0.868	0.955	0.3447
205647_at	110.1	134.0	0.28	1.22	AAA-AAP	0.10	17	0.868	0.955	0.3414
218723_s_at	1348.8	1641.5	0.28	1.22	PPP-PPP	0.10	17	0.868	0.955	0.3175
215931_s_at	166.9	203.0	0.28	1.22	PPP-PPP	0.10	17	0.868	0.955	0.3165
202045_s_at	273.9	333.2	0.28	1.22	PPP-PPP	0.10	17	0.868	0.955	0.3135
1563012_x_at	71.0	86.4	0.28	1.22	AAM-PAP	0.10	17	0.868	0.955	0.3060
214692_s_at	229.2	278.9	0.28	1.22	PPP-APP	0.10	17	0.868	0.955	0.1837
222329_x_at	155.1	188.6	0.28	1.22	MAP-PPP	0.10	17	0.868	0.955	0.5253
1569999_at	87.9	106.9	0.28	1.22	MPA-AAP	0.10	17	0.868	0.955	0.5245
218681_s_at	708.8	862.1	0.28	1.22	PPP-PPP	0.10	17	0.868	0.955	0.5083

1565867_a_at	171.7	208.8	0.28	1.22	PPP-PPP	0.10	17	0.868	0.955	0.3536
242019_at	132.9	161.6	0.28	1.22	PPP-PPP	0.10	17	0.868	0.955	0.3168
203354_s_at	385.1	468.3	0.28	1.22	PPP-PPP	0.10	17	0.868	0.955	0.3004
217925_s_at	541.7	658.6	0.28	1.22	PPP-MPP	0.10	17	0.868	0.955	0.2839
211162_x_at	516.8	628.4	0.28	1.22	PAM-MPP	0.10	17	0.868	0.955	0.2802
1552575_a_at	335.1	407.4	0.28	1.22	PPA-AAM	0.10	17	0.868	0.955	0.2673
230016_at	164.8	200.4	0.28	1.22	MAP-PPA	0.10	17	0.868	0.955	0.2198
238479_at	249.3	303.1	0.28	1.22	PPP-PPP	0.10	17	0.868	0.955	0.2144
225605_at	203.1	246.9	0.28	1.22	PPP-PPP	0.10	17	0.868	0.955	0.2135
232274_at	164.3	199.8	0.28	1.22	PPA-MAA	0.10	17	0.868	0.955	0.1871
220940_at	557.2	676.9	0.28	1.22	PPP-PPP	0.10	17	0.868	0.955	0.5433
223665_at	166.8	202.8	0.28	1.22	APP-PPP	0.10	17	0.868	0.955	0.5403
210365_at	43.5	52.8	0.28	1.22	APA-AAP	0.10	17	0.868	0.955	0.5144
1557684_at	186.1	226.1	0.28	1.22	PPP-PPP	0.10	17	0.868	0.955	0.5014
202805_s_at	319.9	388.6	0.28	1.22	PPP-PPP	0.10	17	0.868	0.955	0.4026
212317_at	1060.4	1288.2	0.28	1.22	PPP-PPP	0.10	17	0.868	0.955	0.3522
217679_x_at	1041.5	1265.4	0.28	1.22	PPP-PPP	0.10	17	0.868	0.955	0.3469
204181_s_at	360.7	438.2	0.28	1.22	PPP-PPP	0.10	17	0.868	0.955	0.2838
213311_s_at	776.6	943.8	0.28	1.22	PPP-PPP	0.10	17	0.868	0.955	0.2193
218177_at	381.5	463.5	0.28	1.22	PPP-PPP	0.10	17	0.868	0.955	0.2111
220262_s_at	1169.7	1421.3	0.28	1.22	PPP-PPP	0.10	17	0.868	0.955	0.2079
212812_at	1520.0	1847.2	0.28	1.22	PPP-PPP	0.10	17	0.868	0.955	0.1967
203361_s_at	397.9	483.5	0.28	1.22	PPP-PPP	0.10	17	0.868	0.955	0.1928
221335_x_at	390.1	473.9	0.28	1.22	PPP-PPP	0.10	17	0.868	0.955	0.1669
233315_at	169.6	205.9	0.28	1.21	PPP-PPP	0.10	17	0.868	0.955	0.5594
220327_at	241.6	293.4	0.28	1.22	PPP-PPP	0.10	17	0.868	0.955	0.5559
203311_s_at	529.5	642.8	0.28	1.21	PPP-PPP	0.10	17	0.868	0.955	0.4253
1563483_at	124.7	151.4	0.28	1.21	PAP-PMP	0.10	17	0.868	0.955	0.4075
200997_at	2432.8	2954.5	0.28	1.21	PPP-PPP	0.10	17	0.868	0.955	0.3786
236619_at	105.7	128.4	0.28	1.22	PPP-PPP	0.10	17	0.868	0.955	0.3036
242981_at	200.0	242.9	0.28	1.21	PPP-PPP	0.10	17	0.868	0.955	0.2759
225995_x_at	448.1	544.2	0.28	1.22	PPP-PPP	0.10	17	0.868	0.955	0.2516
212079_s_at	226.7	275.3	0.28	1.22	PPP-PPP	0.10	17	0.868	0.955	0.2294
243299_at	228.9	278.0	0.28	1.21	PPP-PPP	0.10	17	0.868	0.955	0.2078
207186_s_at	1348.7	1637.4	0.28	1.21	PPP-PPP	0.10	17	0.868	0.955	0.1942
209357_at	1557.7	1890.8	0.28	1.21	PPP-PPP	0.10	17	0.868	0.955	0.1704
209066_x_at	12374.3	15021.1	0.28	1.21	PPP-PPP	0.10	17	0.868	0.955	0.1502
209450_at	451.4	548.0	0.28	1.21	PPP-PPP	0.10	17	0.868	0.955	0.1297
1561194_at	66.1	80.2	0.28	1.21	PAA-MPA	0.10	17	0.868	0.955	0.6150
207574_s_at	620.6	753.1	0.28	1.21	PPP-PPP	0.10	17	0.868	0.955	0.5746
204036_at	40.9	49.7	0.28	1.21	PAA-APA	0.10	17	0.868	0.955	0.5573
205434_s_at	453.0	549.6	0.28	1.21	PPP-PPP	0.10	17	0.868	0.955	0.4125

229705_at	255.4	310.0	0.28	1.21	PPP-PPP	0.10	17	0.868	0.955	0.4022
212349_at	474.9	576.4	0.28	1.21	PPP-PPP	0.10	17	0.868	0.955	0.3454
214349_at	146.5	177.8	0.28	1.21	AAA-PAM	0.10	17	0.868	0.955	0.2955
204099_at	151.3	183.6	0.28	1.21	PPP-PPP	0.10	17	0.868	0.955	0.2923
219254_at	166.9	202.5	0.28	1.21	PAP-APP	0.10	17	0.868	0.955	0.2743
232662_x_at	908.2	1101.8	0.28	1.21	PPP-PPP	0.10	17	0.868	0.955	0.1648
203794_at	835.5	1014.1	0.28	1.21	PPP-PPP	0.10	17	0.868	0.955	0.1336
238558_at	74.6	90.4	0.28	1.21	APP-AAP	0.10	17	0.868	0.955	0.6072
225026_at	208.9	253.3	0.28	1.21	PMP-PPP	0.10	17	0.868	0.955	0.5228
227383_at	327.7	397.3	0.28	1.21	PAP-PPP	0.10	17	0.868	0.955	0.4667
225495_x_at	88.5	107.4	0.28	1.21	AAP-APA	0.10	17	0.868	0.955	0.3845
1555786_s_at	692.7	839.8	0.28	1.21	PPP-PPP	0.10	17	0.868	0.955	0.3782
1555408_at	97.8	118.5	0.28	1.21	AAA-APA	0.10	17	0.868	0.955	0.3630
229115_at	1090.4	1322.1	0.28	1.21	PPP-MPP	0.10	17	0.868	0.955	0.3388
230766_at	143.0	173.4	0.28	1.21	PPP-PPP	0.10	17	0.868	0.955	0.3138
244271_at	124.0	150.3	0.28	1.21	PPP-PPP	0.10	17	0.868	0.955	0.3048
238164_at	127.5	154.6	0.28	1.21	PPP-PPP	0.10	17	0.868	0.955	0.2787
244031_at	66.4	80.5	0.28	1.21	PAP-PPP	0.10	17	0.868	0.955	0.2720
212619_at	140.7	170.7	0.28	1.21	PPP-PPP	0.10	17	0.868	0.955	0.2402
242873_at	278.1	337.2	0.28	1.21	PPP-PPP	0.10	17	0.868	0.955	0.1598
238012_at	261.2	316.7	0.28	1.21	PPP-PPP	0.10	17	0.868	0.955	0.1442
201167_x_at	565.2	685.5	0.28	1.21	PPP-PPP	0.10	17	0.868	0.955	0.1360
1552312_a_at	245.8	297.9	0.28	1.21	PPP-PPP	0.10	17	0.868	0.955	0.4470
211009_s_at	386.4	468.3	0.28	1.21	PPP-PPP	0.10	17	0.868	0.955	0.4402
1558983_at	40.2	48.7	0.28	1.21	APA-PAA	0.10	17	0.868	0.955	0.4067
1562505_at	64.8	78.5	0.28	1.21	PPP-APP	0.10	17	0.868	0.955	0.3930
218030_at	398.3	482.8	0.28	1.21	PPP-PPP	0.10	17	0.868	0.955	0.3416
219128_at	337.0	408.3	0.28	1.21	PPP-PPP	0.10	17	0.868	0.955	0.3363
232181_at	737.9	894.4	0.28	1.21	PPP-PPP	0.10	17	0.868	0.955	0.2025
213766_x_at	2544.9	3083.6	0.28	1.21	PPP-PPP	0.10	17	0.868	0.955	0.2017
1558401_at	294.1	356.4	0.28	1.21	PPP-PPP	0.10	17	0.868	0.955	0.1601
214759_at	86.9	105.2	0.28	1.21	PAP-PPP	0.10	17	0.868	0.955	0.4714
219381_at	78.4	94.9	0.28	1.21	AAM-PPA	0.10	17	0.868	0.955	0.4676
213323_s_at	297.1	359.6	0.28	1.21	MPA-APA	0.10	17	0.868	0.955	0.4584
219774_at	197.5	239.1	0.28	1.21	PPP-PPP	0.10	17	0.868	0.955	0.3972
221011_s_at	122.9	148.8	0.28	1.21	PAA-PPP	0.10	17	0.868	0.955	0.3899
222204_s_at	1468.3	1777.6	0.28	1.21	PPP-PPP	0.10	17	0.868	0.955	0.3658
200696_s_at	373.6	452.4	0.28	1.21	PPP-PPP	0.10	17	0.868	0.955	0.3394
215263_at	58.4	70.7	0.28	1.21	PAP-PPP	0.10	17	0.868	0.955	0.2830
218758_s_at	449.4	544.1	0.28	1.21	AMP-PPP	0.10	17	0.868	0.955	0.1834
215794_x_at	2026.4	2454.1	0.28	1.21	PPP-PPP	0.10	17	0.868	0.955	0.1452
230028_at	255.3	309.1	0.28	1.21	PPP-PPP	0.10	17	0.868	0.955	0.1394

1554690_a_at	217.7	263.5	0.28	1.21	PPP-MPP	0.10	17	0.868	0.955	0.1268
1561279_at	57.1	69.1	0.28	1.21	APA-APP	0.10	17	0.868	0.955	0.6050
230337_at	97.8	118.4	0.28	1.21	PAA-AAA	0.10	17	0.868	0.955	0.5686
216282_x_at	274.2	331.7	0.28	1.21	MPP-PPP	0.10	17	0.868	0.955	0.5189
229307_at	145.4	176.0	0.28	1.21	PPP-PPP	0.10	17	0.868	0.955	0.3979
242966_x_at	77.6	93.9	0.28	1.21	PPP-AMP	0.10	17	0.868	0.955	0.3681
204081_at	211.2	255.6	0.28	1.21	AAA-AAP	0.10	17	0.868	0.955	0.3510
219662_at	701.9	849.3	0.28	1.21	PPP-PPP	0.10	17	0.868	0.955	0.2414
242779_at	71.2	86.1	0.28	1.21	PAP-PPP	0.10	17	0.868	0.955	0.2407
218176_at	639.6	773.7	0.28	1.21	PPP-PPP	0.10	17	0.868	0.955	0.2128
242812_at	111.0	134.3	0.28	1.21	PPP-PPP	0.10	17	0.868	0.955	0.1855
206683_at	312.6	378.1	0.27	1.21	PPP-PPP	0.10	17	0.868	0.955	0.5371
207180_s_at	1292.3	1562.3	0.27	1.21	PPP-PPP	0.10	17	0.868	0.955	0.4912
226829_at	119.1	144.0	0.27	1.21	PPP-PPP	0.10	17	0.868	0.955	0.4401
213299_at	102.0	123.4	0.27	1.21	PPP-PAP	0.10	17	0.868	0.955	0.4387
213526_s_at	764.1	923.7	0.27	1.21	PPP-PPP	0.10	17	0.868	0.955	0.2983
220841_s_at	129.7	156.8	0.27	1.21	APA-AAA	0.10	17	0.868	0.955	0.2761
208957_at	157.7	190.7	0.27	1.21	PPP-PPP	0.10	17	0.868	0.955	0.2411
225195_at	1026.9	1241.6	0.27	1.21	PPP-PPP	0.10	17	0.868	0.955	0.1616
229050_s_at	1685.7	2038.6	0.27	1.21	PPP-PPP	0.10	17	0.868	0.955	0.1599
202973_x_at	1214.4	1468.3	0.27	1.21	PPP-PPP	0.10	17	0.868	0.955	0.1409
1559002_at	75.8	91.7	0.27	1.21	MPA-PAP	0.10	17	0.868	0.955	0.6170
231403_at	86.2	104.1	0.27	1.21	PAA-AAP	0.10	17	0.868	0.955	0.6075
216010_x_at	79.8	96.4	0.27	1.21	AAA-AAP	0.10	17	0.868	0.955	0.5568
239607_at	144.6	174.7	0.27	1.21	AAP-PAP	0.10	17	0.868	0.955	0.5156
235957_at	208.6	251.9	0.27	1.21	PPP-PPP	0.10	17	0.868	0.955	0.4868
1553796_at	57.1	69.0	0.27	1.21	PAA-AAA	0.10	17	0.868	0.955	0.4851
1564010_at	62.7	75.8	0.27	1.21	AAA-PAM	0.10	17	0.868	0.955	0.3979
215044_s_at	197.9	239.1	0.27	1.21	PPP-PPM	0.10	17	0.868	0.955	0.3207
232183_at	246.7	298.0	0.27	1.21	PPP-PPP	0.10	17	0.868	0.955	0.2689
243074_at	240.5	290.5	0.27	1.21	AAM-AAP	0.10	17	0.868	0.955	0.2587
237958_at	50.6	61.2	0.27	1.21	AAA-PAA	0.10	17	0.868	0.955	0.1980
65493_at	178.0	215.1	0.27	1.21	PPP-PPP	0.10	17	0.868	0.955	0.1978
1555943_at	386.0	466.3	0.27	1.21	PPP-PPP	0.10	17	0.868	0.955	0.1620
227515_at	267.0	322.7	0.27	1.21	PPP-PAP	0.10	17	0.868	0.955	0.1467
AFFX-BioDn-5_at	3252.3	3929.1	0.27	1.21	PPP-PPP	0.10	17	0.868	0.955	0.1459
232171_x_at	413.7	499.9	0.27	1.21	PPP-PPP	0.10	17	0.868	0.955	0.1428
218840_s_at	526.0	635.7	0.27	1.21	PPP-PPP	0.10	17	0.868	0.955	0.1374
209989_at	57.0	68.8	0.27	1.21	APP-PPP	0.10	17	0.868	0.955	0.5778
223958_s_at	118.9	143.6	0.27	1.21	PPP-PPP	0.10	17	0.868	0.955	0.5701
1553704_x_at	681.7	823.2	0.27	1.21	PPP-PPP	0.10	17	0.868	0.955	0.4734
228858_at	90.0	108.7	0.27	1.21	AAP-PPP	0.10	17	0.868	0.955	0.4496

228971_at	816.7	986.5	0.27	1.21	PPP-PPP	0.10	17	0.868	0.955	0.4087
214251_s_at	432.4	522.2	0.27	1.21	PPP-PPP	0.10	17	0.868	0.955	0.3802
229785_at	168.9	203.9	0.27	1.21	PPP-PPP	0.10	17	0.868	0.955	0.2546
201147_s_at	1616.1	1951.5	0.27	1.21	PPP-PPP	0.10	17	0.868	0.955	0.2475
201439_at	487.8	589.0	0.27	1.21	PPP-PPP	0.10	17	0.868	0.955	0.2348
241443_at	174.8	211.0	0.27	1.21	PPP-PPP	0.10	17	0.868	0.955	0.2330
214805_at	466.8	563.9	0.27	1.21	PPP-PPP	0.10	17	0.868	0.955	0.2197
226065_at	301.8	364.3	0.27	1.21	MPP-PPP	0.10	17	0.868	0.955	0.1880
220295_x_at	808.4	975.9	0.27	1.21	PPP-PPP	0.10	17	0.868	0.955	0.1837
1554220_a_at	69.0	83.3	0.27	1.21	AAM-AAP	0.10	17	0.868	0.955	0.1711
206968_s_at	362.8	438.0	0.27	1.21	PPP-PPP	0.10	17	0.868	0.955	0.1658
212983_at	3117.9	3763.5	0.27	1.21	PPP-PPP	0.10	17	0.868	0.955	0.1601
229498_at	718.4	867.1	0.27	1.21	PPP-PPP	0.10	17	0.868	0.955	0.1556
214820_at	149.0	179.9	0.27	1.21	PPP-PPP	0.10	17	0.868	0.955	0.1455
1557609_s_at	53.9	65.0	0.27	1.21	AAP-PAP	0.10	17	0.868	0.955	0.5889
217364_x_at	239.8	289.5	0.27	1.21	PPP-PPP	0.10	17	0.868	0.955	0.5488
242188_at	159.7	192.7	0.27	1.21	PPP-PPP	0.10	17	0.868	0.955	0.5399
230634_x_at	354.9	428.2	0.27	1.21	PPP-PPP	0.10	17	0.868	0.955	0.4868
212494_at	105.3	127.0	0.27	1.21	AAP-MPP	0.10	17	0.868	0.955	0.4351
228531_at	815.9	984.7	0.27	1.21	PPP-PPP	0.10	17	0.868	0.955	0.4016
207936_x_at	103.2	124.6	0.27	1.21	PPP-APP	0.10	17	0.868	0.955	0.3646
242887_at	114.3	137.9	0.27	1.21	PPP-PPP	0.10	17	0.868	0.955	0.3468
227932_at	730.1	881.0	0.27	1.21	PPP-PPP	0.10	17	0.868	0.955	0.3264
227671_at	2907.2	3507.6	0.27	1.21	PPP-PPP	0.10	17	0.868	0.955	0.2877
213116_at	247.6	298.8	0.27	1.21	PPP-PPP	0.10	17	0.868	0.955	0.2713
230624_at	122.7	148.1	0.27	1.21	PAA-PPP	0.10	17	0.868	0.955	0.2711
206178_at	76.3	92.1	0.27	1.21	MAA-AAP	0.10	17	0.868	0.955	0.2587
216606_x_at	575.9	694.9	0.27	1.21	PPP-PPP	0.10	17	0.868	0.955	0.2200
212064_x_at	1576.4	1901.5	0.27	1.21	PPP-PPP	0.10	17	0.868	0.955	0.2012
209007_s_at	1363.6	1645.9	0.27	1.21	PPP-PPP	0.10	17	0.868	0.955	0.1998
201671_x_at	1934.6	2334.3	0.27	1.21	PPP-PPP	0.10	17	0.868	0.955	0.1667
210981_s_at	490.6	591.9	0.27	1.21	PPP-PPP	0.10	17	0.868	0.955	0.1560
208876_s_at	442.5	534.1	0.27	1.21	PPP-PPP	0.10	17	0.868	0.955	0.1486
229975_at	563.0	679.3	0.27	1.21	PPP-PPP	0.10	17	0.868	0.955	0.1400
227601_at	436.5	526.5	0.27	1.21	PPP-PPP	0.10	17	0.868	0.955	0.5923
229415_at	264.4	318.8	0.27	1.21	PPP-PPP	0.10	17	0.868	0.955	0.5102
238332_at	91.7	110.5	0.27	1.21	PAM-PPP	0.10	17	0.868	0.955	0.5052
221159_at	68.8	82.9	0.27	1.21	APA-PAP	0.10	17	0.868	0.955	0.5013
204380_s_at	99.4	119.8	0.27	1.21	APP-PPP	0.10	17	0.868	0.955	0.3841
226716_at	238.2	287.2	0.27	1.21	AAA-APA	0.10	17	0.868	0.955	0.3720
213383_at	308.5	371.9	0.27	1.21	PPP-APP	0.10	17	0.868	0.955	0.3388
37547_at	41.5	50.1	0.27	1.21	PPA-AAM	0.10	17	0.868	0.955	0.3244

208328_s_at	154.4	186.1	0.27	1.21	PPP-PPP	0.10	17	0.868	0.955	0.2902
203543_s_at	564.8	681.2	0.27	1.21	PPP-PPP	0.10	17	0.868	0.955	0.2467
1568596_a_at	831.7	1002.9	0.27	1.21	PPP-PPP	0.10	17	0.868	0.955	0.1598
220229_s_at	51.7	62.3	0.27	1.21	AMA-APA	0.10	17	0.868	0.955	0.6053
239725_at	112.4	135.4	0.27	1.21	PPP-PPP	0.10	17	0.868	0.955	0.5961
238040_at	244.2	294.3	0.27	1.21	PPP-PPP	0.10	17	0.868	0.955	0.5801
238825_at	99.0	119.3	0.27	1.21	AAA-PMA	0.10	17	0.868	0.955	0.4372
243008_at	73.9	89.1	0.27	1.21	PPP-PAP	0.10	17	0.868	0.955	0.4194
214960_at	535.7	645.5	0.27	1.21	PPP-PPP	0.10	17	0.868	0.955	0.3954
218914_at	229.8	276.9	0.27	1.21	PPA-PAP	0.10	17	0.868	0.955	0.3375
227531_at	1272.6	1533.6	0.27	1.21	PPP-PPP	0.10	17	0.868	0.955	0.3270
241391_at	130.4	157.1	0.27	1.21	PPP-PPP	0.10	17	0.868	0.955	0.3107
214894_x_at	830.1	1000.2	0.27	1.21	PPP-PPP	0.10	17	0.868	0.955	0.1990
234997_x_at	731.8	881.9	0.27	1.21	PPP-PPP	0.10	17	0.868	0.955	0.1416
226231_at	714.4	860.9	0.27	1.21	PPP-PPP	0.10	17	0.868	0.955	0.1307
215071_s_at	272.5	328.3	0.27	1.21	PPP-PPP	0.10	17	0.868	0.955	0.1275
237741_at	161.0	193.8	0.27	1.20	PPP-PPP	0.10	17	0.868	0.955	0.5669
210918_at	55.0	66.2	0.27	1.20	AAA-APA	0.10	17	0.868	0.955	0.5541
229663_at	282.5	340.2	0.27	1.20	PPP-PPP	0.10	17	0.868	0.955	0.5425
1557357_at	44.5	53.5	0.27	1.20	AAA-AAP	0.10	17	0.868	0.955	0.5104
207061_at	116.6	140.4	0.27	1.20	AAP-PAP	0.10	17	0.868	0.955	0.4939
226776_at	130.1	156.6	0.27	1.20	PPP-PPP	0.10	17	0.868	0.955	0.4874
210255_at	99.1	119.3	0.27	1.20	APA-PAA	0.10	17	0.868	0.955	0.4599
214580_x_at	9338.5	11246.6	0.27	1.20	PPP-PPP	0.10	17	0.868	0.955	0.4541
1556588_at	93.4	112.5	0.27	1.20	PAP-PPA	0.10	17	0.868	0.955	0.4475
236372_at	196.9	237.1	0.27	1.20	PPP-PPP	0.10	17	0.868	0.955	0.4402
217620_s_at	138.4	166.7	0.27	1.20	PPP-PPP	0.10	17	0.868	0.955	0.4273
222270_at	106.1	127.8	0.27	1.20	PPP-PPP	0.10	17	0.868	0.955	0.3457
243495_s_at	232.2	279.6	0.27	1.20	PPP-PPP	0.10	17	0.868	0.955	0.3379
243612_at	121.0	145.7	0.27	1.20	PAP-PPP	0.10	17	0.868	0.955	0.2758
203598_s_at	560.7	675.3	0.27	1.20	PPP-PPP	0.10	17	0.868	0.955	0.2695
235837_at	214.8	258.6	0.27	1.20	PPP-PPP	0.10	17	0.868	0.955	0.2207
227864_s_at	1667.0	2007.3	0.27	1.20	PPP-PPP	0.10	17	0.868	0.955	0.2060
215903_s_at	606.1	729.9	0.27	1.20	PPP-PPP	0.10	17	0.868	0.955	0.1586
210825_s_at	8649.7	10412.4	0.27	1.20	PPP-PPP	0.10	17	0.868	0.955	0.1379
1567013_at	212.0	255.1	0.27	1.20	PPP-PPP	0.10	17	0.868	0.955	0.5836
240874_at	48.8	58.8	0.27	1.20	AAA-APA	0.10	17	0.868	0.955	0.5655
222807_at	347.4	417.8	0.27	1.20	PPP-PPP	0.10	17	0.868	0.955	0.4688
228686_at	146.9	176.7	0.27	1.20	AAP-PPP	0.10	17	0.868	0.955	0.4509
234687_x_at	191.7	230.7	0.27	1.20	PPP-PAP	0.10	17	0.868	0.955	0.4510
204104_at	216.0	260.0	0.27	1.20	MPP-PPP	0.10	17	0.868	0.955	0.3873
236261_at	309.7	372.5	0.27	1.20	PPP-PPP	0.10	17	0.868	0.955	0.3848

209011_at	375.9	452.2	0.27	1.20	PPP-PPP	0.10	17	0.868	0.955	0.3423
31807_at	1514.4	1822.5	0.27	1.20	PPP-PPP	0.10	17	0.868	0.955	0.3124
227433_at	485.9	584.8	0.27	1.20	PPP-PPP	0.10	17	0.868	0.955	0.2060
212847_at	659.2	793.4	0.27	1.20	PPP-PPP	0.10	17	0.868	0.955	0.1927
1553668_at	117.3	141.0	0.27	1.20	APP-PPP	0.10	17	0.868	0.955	0.4679
238672_at	249.0	299.3	0.27	1.20	PPP-PPP	0.10	17	0.868	0.955	0.3470
213473_at	243.3	292.5	0.27	1.20	PPP-PPP	0.10	17	0.868	0.955	0.2990
204520_x_at	483.1	580.7	0.27	1.20	PPP-PPP	0.10	17	0.868	0.955	0.2718
229211_at	252.7	304.0	0.27	1.20	MPP-APP	0.10	17	0.868	0.955	0.2108
221884_at	708.4	851.6	0.27	1.20	PPP-PPP	0.10	17	0.868	0.955	0.2046
226912_at	230.2	276.9	0.27	1.20	PPP-PPP	0.10	17	0.868	0.955	0.2011
230918_at	243.0	292.2	0.27	1.20	PPP-PPP	0.10	17	0.868	0.955	0.1877
238606_at	146.8	176.5	0.27	1.20	PAA-PPP	0.10	17	0.868	0.955	0.1507
203055_s_at	505.1	607.4	0.27	1.20	PPP-PPP	0.10	17	0.868	0.955	0.1214
1555078_at	141.6	170.1	0.27	1.20	APA-PPP	0.10	17	0.868	0.955	0.5564
238389_s_at	482.0	579.0	0.27	1.20	APP-PPP	0.10	17	0.868	0.955	0.4938
211424_x_at	119.0	142.9	0.27	1.20	PPP-PPP	0.10	17	0.868	0.955	0.4842
202795_x_at	806.7	969.7	0.27	1.20	PPP-PPP	0.10	17	0.868	0.955	0.4755
243606_at	89.8	108.0	0.27	1.20	APA-APA	0.10	17	0.868	0.955	0.4684
209516_at	575.6	691.9	0.27	1.20	PPP-PPP	0.10	17	0.868	0.955	0.3777
233445_at	100.1	120.3	0.27	1.20	AAP-PPA	0.10	17	0.868	0.955	0.3772
1559806_at	90.6	108.9	0.27	1.20	APA-AAA	0.10	17	0.868	0.955	0.3745
238472_at	134.4	161.5	0.27	1.20	PAP-AAP	0.10	17	0.868	0.955	0.3663
228450_at	257.2	309.2	0.27	1.20	PPP-PPP	0.10	17	0.868	0.955	0.3645
228859_at	155.3	186.7	0.27	1.20	PPP-PPP	0.10	17	0.868	0.955	0.3535
223238_s_at	1714.7	2060.5	0.27	1.20	PPP-PPP	0.10	17	0.868	0.955	0.3244
230398_at	4073.1	4894.9	0.27	1.20	PPP-PPP	0.10	17	0.868	0.955	0.1488
209335_at	85.7	103.0	0.26	1.20	MPA-PPP	0.10	17	0.868	0.955	0.6100
227229_at	270.8	325.2	0.26	1.20	PPP-PPP	0.10	17	0.868	0.955	0.5607
229522_at	174.8	209.9	0.26	1.20	APA-AAA	0.10	17	0.868	0.955	0.5589
242701_at	122.7	147.4	0.26	1.20	APP-AAP	0.10	17	0.868	0.955	0.5414
218240_at	377.9	453.7	0.26	1.20	PPP-PPP	0.10	17	0.868	0.955	0.4990
54037_at	146.0	175.2	0.26	1.20	PPP-PPP	0.10	17	0.868	0.955	0.4929
230446_at	240.7	289.1	0.26	1.20	AAP-MAP	0.10	17	0.868	0.955	0.4688
214314_s_at	1275.1	1530.8	0.26	1.20	PPP-PPP	0.10	17	0.868	0.955	0.4229
227554_at	52.1	62.6	0.26	1.20	PAA-PPA	0.10	17	0.868	0.955	0.4026
1558722_at	113.8	136.6	0.26	1.20	PPP-PPP	0.10	17	0.868	0.955	0.4020
222704_at	352.2	422.8	0.26	1.20	PPP-PPP	0.10	17	0.868	0.955	0.2280
205251_at	649.9	780.3	0.26	1.20	PPP-PPP	0.10	17	0.868	0.955	0.2124
223124_s_at	1335.2	1602.9	0.26	1.20	PPP-PPP	0.10	17	0.868	0.955	0.1993
204728_s_at	388.9	467.1	0.26	1.20	PPP-PPP	0.10	17	0.868	0.955	0.1983
239731_at	134.3	161.1	0.26	1.20	PPA-MAP	0.10	17	0.868	0.955	0.5882

1554311_a_at	99.1	119.0	0.26	1.20	PAP-PPP	0.10	17	0.868	0.955	0.5867
1569690_at	49.4	59.3	0.26	1.20	PAP-PPP	0.10	17	0.868	0.955	0.5346
224977_at	387.7	465.1	0.26	1.20	PPP-PPP	0.10	17	0.868	0.955	0.5309
235427_at	155.6	186.7	0.26	1.20	PPP-PPP	0.10	17	0.868	0.955	0.4453
238561_s_at	206.3	247.5	0.26	1.20	PPP-PPP	0.10	17	0.868	0.955	0.4260
222793_at	148.0	177.6	0.26	1.20	APA-PAP	0.10	17	0.868	0.955	0.3139
207159_x_at	191.9	230.3	0.26	1.20	PPP-PPP	0.10	17	0.868	0.955	0.2993
202654_x_at	1141.4	1369.6	0.26	1.20	PPP-PPP	0.10	17	0.868	0.955	0.2909
1556061_at	166.8	200.2	0.26	1.20	PPP-PPP	0.10	17	0.868	0.955	0.2907
214201_x_at	500.4	600.3	0.26	1.20	PPP-PPP	0.10	17	0.868	0.955	0.1993
211316_x_at	780.1	936.2	0.26	1.20	PPP-PPP	0.10	17	0.868	0.955	0.1874
234339_s_at	1783.3	2139.4	0.26	1.20	PPP-PPP	0.10	17	0.868	0.955	0.1712
226863_at	559.5	671.5	0.26	1.20	PPP-PPP	0.10	17	0.868	0.955	0.1637
216173_at	80.2	96.1	0.26	1.20	MPP-AAP	0.10	17	0.868	0.955	0.5952
231149_s_at	89.9	107.9	0.26	1.20	APP-PPP	0.10	17	0.868	0.955	0.5457
230561_s_at	71.3	85.4	0.26	1.20	PPP-PPP	0.10	17	0.868	0.955	0.5335
204217_s_at	137.7	165.1	0.26	1.20	APA-PAP	0.10	17	0.868	0.955	0.5306
215623_x_at	86.8	104.1	0.26	1.20	PPP-PPP	0.10	17	0.868	0.955	0.5234
225924_at	351.3	421.2	0.26	1.20	PPP-PPP	0.10	17	0.868	0.955	0.4681
241925_x_at	273.4	327.9	0.26	1.20	PPP-PPP	0.10	17	0.868	0.955	0.4334
239619_at	72.5	86.9	0.26	1.20	MAP-APA	0.10	17	0.868	0.955	0.4210
233992_x_at	225.1	270.0	0.26	1.20	PPP-PPP	0.10	17	0.868	0.955	0.4090
236254_at	373.9	448.5	0.26	1.20	PPP-PPP	0.10	17	0.868	0.955	0.3969
232120_at	233.2	279.7	0.26	1.20	PPP-PPP	0.10	17	0.868	0.955	0.3830
231809_x_at	2076.6	2490.4	0.26	1.20	PPP-PPP	0.10	17	0.868	0.955	0.2077
213939_s_at	338.5	405.9	0.26	1.20	PPP-PPP	0.10	17	0.868	0.955	0.1921
244476_at	100.1	119.9	0.26	1.20	APA-PPP	0.10	17	0.868	0.955	0.5585
207608_x_at	213.8	256.2	0.26	1.20	PAP-PPA	0.10	17	0.868	0.955	0.5276
225329_at	306.3	367.1	0.26	1.20	AAP-AAP	0.10	17	0.868	0.955	0.5229
228255_at	375.3	449.6	0.26	1.20	PPP-PPP	0.10	17	0.868	0.955	0.4522
223494_at	394.8	473.1	0.26	1.20	PPM-PPP	0.10	17	0.868	0.955	0.4377
226840_at	307.1	368.1	0.26	1.20	AAA-PAP	0.10	17	0.868	0.955	0.4366
232346_at	200.3	240.0	0.26	1.20	PAP-MPP	0.10	17	0.868	0.955	0.4248
227946_at	147.2	176.3	0.26	1.20	APP-PPP	0.10	17	0.868	0.955	0.4197
209284_s_at	1003.1	1201.7	0.26	1.20	PPP-PPP	0.10	17	0.868	0.955	0.3398
231848_x_at	529.7	634.6	0.26	1.20	PPP-PPP	0.10	17	0.868	0.955	0.3336
204635_at	206.6	247.5	0.26	1.20	PPP-PPP	0.10	17	0.868	0.955	0.2926
211536_x_at	552.0	661.4	0.26	1.20	PPP-PPP	0.10	17	0.868	0.955	0.2835
228239_at	166.0	198.8	0.26	1.20	PPP-PPP	0.10	17	0.868	0.955	0.2334
228029_at	533.4	639.2	0.26	1.20	PPP-PPP	0.10	17	0.868	0.955	0.2304
203222_s_at	210.8	252.5	0.26	1.20	PPP-PPP	0.10	17	0.868	0.955	0.2273
231225_at	144.0	172.6	0.26	1.20	PPM-PPP	0.10	17	0.868	0.955	0.2236

207495_at	96.5	115.7	0.26	1.20	PPA-PPP	0.10	17	0.868	0.955	0.1926
217604_at	238.2	285.4	0.26	1.20	PPP-PPP	0.10	17	0.868	0.955	0.1843
57532_at	646.9	775.0	0.26	1.20	PPP-PPP	0.10	17	0.868	0.955	0.1479
236381_s_at	685.4	821.2	0.26	1.20	PPP-PPP	0.10	17	0.868	0.955	0.1329
1552972_at	32.7	39.1	0.26	1.20	APA-APA	0.10	17	0.868	0.955	0.4917
222064_s_at	457.5	547.7	0.26	1.20	MPP-PPP	0.10	17	0.868	0.955	0.4686
208539_x_at	201.7	241.6	0.26	1.20	AAM-PAA	0.10	17	0.868	0.955	0.4559
220201_at	665.9	797.2	0.26	1.20	PPP-PPP	0.10	17	0.868	0.955	0.4440
1554043_a_at	72.7	87.1	0.26	1.20	AAP-AAA	0.10	17	0.868	0.955	0.4350
210282_at	84.5	101.2	0.26	1.20	PPP-PPP	0.10	17	0.868	0.955	0.4137
238495_at	56.8	68.1	0.26	1.20	PPP-PPP	0.10	17	0.868	0.955	0.4078
228641_at	421.3	504.4	0.26	1.20	PPP-PPP	0.10	17	0.868	0.955	0.3812
207826_s_at	5236.9	6272.5	0.26	1.20	PPP-PPP	0.10	17	0.868	0.955	0.3447
236012_at	163.0	195.1	0.26	1.20	PPP-PPP	0.10	17	0.868	0.955	0.2961
221892_at	195.1	233.5	0.26	1.20	PPP-PPP	0.10	17	0.868	0.955	0.2780
210735_s_at	828.6	992.4	0.26	1.20	PPP-PPP	0.10	17	0.868	0.955	0.2587
233898_s_at	477.1	571.3	0.26	1.20	PPP-PPP	0.10	17	0.868	0.955	0.2539
225839_at	199.3	238.7	0.26	1.20	MAP-PPP	0.10	17	0.868	0.955	0.2505
234372_at	69.6	83.3	0.26	1.20	AAA-APP	0.10	17	0.868	0.955	0.5285
242794_at	83.5	100.0	0.26	1.20	PPP-PPP	0.10	17	0.868	0.955	0.5177
222435_s_at	1240.7	1484.8	0.26	1.20	PPP-PPP	0.10	17	0.868	0.955	0.5148
235819_at	149.1	178.4	0.26	1.20	PPP-PPP	0.10	17	0.868	0.955	0.4458
232566_at	162.0	194.0	0.26	1.20	AAA-AAP	0.10	17	0.868	0.955	0.3782
218612_s_at	361.8	433.1	0.26	1.20	MPP-PPP	0.10	17	0.868	0.955	0.3699
229173_at	714.3	854.6	0.26	1.20	PPP-PPP	0.10	17	0.868	0.955	0.3602
239026_x_at	552.1	660.5	0.26	1.20	PPP-PPP	0.10	17	0.868	0.955	0.3406
217991_x_at	165.6	198.2	0.26	1.20	PPP-PAP	0.10	17	0.868	0.955	0.2597
228225_at	300.4	359.4	0.26	1.20	PPP-PPP	0.10	17	0.868	0.955	0.1977
206752_s_at	287.1	343.5	0.26	1.20	PPP-PPP	0.10	17	0.868	0.955	0.1713
218760_at	255.8	306.2	0.26	1.20	PPP-PPP	0.10	17	0.868	0.955	0.1689
231890_at	113.3	135.5	0.26	1.20	PAP-APP	0.10	17	0.868	0.955	0.5767
242983_at	62.3	74.5	0.26	1.20	PPA-PMP	0.10	17	0.868	0.955	0.5664
226802_s_at	538.6	644.1	0.26	1.20	PPP-PPP	0.10	17	0.868	0.955	0.5243
1556121_at	364.2	435.6	0.26	1.20	PPP-PPP	0.10	17	0.868	0.955	0.4528
243646_at	116.7	139.6	0.26	1.20	PMP-APP	0.10	17	0.868	0.955	0.4508
214833_at	142.1	169.8	0.26	1.20	PAA-PPP	0.10	17	0.868	0.955	0.4353
230871_at	444.0	530.8	0.26	1.20	PPP-PPP	0.10	17	0.868	0.955	0.4059
226778_at	662.5	792.3	0.26	1.20	PPP-PPP	0.10	17	0.868	0.955	0.3424
236346_at	543.4	649.7	0.26	1.20	PPP-PPP	0.10	17	0.868	0.955	0.2726
209039_x_at	1055.5	1261.8	0.26	1.20	PPP-PPP	0.10	17	0.868	0.955	0.2287
239050_s_at	169.9	203.1	0.26	1.20	PPP-PPP	0.10	17	0.868	0.955	0.2053
239376_at	296.4	354.5	0.26	1.20	PPP-PPP	0.10	17	0.868	0.955	0.1941

1552682_a_at	138.6	165.7	0.26	1.20	APP-PPP	0.10	17	0.868	0.955	0.1367
232011_s_at	53.4	63.8	0.26	1.20	PAA-AAP	0.10	17	0.868	0.955	0.6000
242261_at	111.6	133.4	0.26	1.20	PPP-PPP	0.10	17	0.868	0.955	0.5070
243983_at	102.1	122.0	0.26	1.20	PAP-PAA	0.10	17	0.868	0.955	0.4703
232319_at	55.9	66.8	0.26	1.20	AAP-PPP	0.10	17	0.868	0.955	0.4109
231016_s_at	247.2	295.3	0.26	1.20	PPP-PPP	0.10	17	0.868	0.955	0.3767
219904_at	168.5	201.2	0.26	1.20	APP-MPP	0.10	17	0.868	0.955	0.3306
223450_s_at	487.9	582.9	0.26	1.20	PPP-PPP	0.10	17	0.868	0.955	0.3000
235722_at	249.4	298.0	0.26	1.20	PPP-PPP	0.10	17	0.868	0.955	0.2942
230356_at	162.6	194.3	0.26	1.20	PMP-PMP	0.10	17	0.868	0.955	0.2829
232101_s_at	332.1	396.7	0.26	1.20	PPP-PPP	0.10	17	0.868	0.955	0.2533
237623_at	375.9	449.4	0.26	1.20	APP-PPP	0.10	17	0.868	0.955	0.2532
234208_at	91.8	109.7	0.26	1.20	AAA-AAP	0.10	17	0.868	0.955	0.2208
200836_s_at	989.8	1183.1	0.26	1.20	PPP-PPP	0.10	17	0.868	0.955	0.1377
203934_at	183.1	218.5	0.26	1.19	PPP-PPP	0.10	17	0.868	0.955	0.5853
213397_x_at	430.0	513.5	0.26	1.19	PPP-PPP	0.10	17	0.868	0.955	0.5504
210534_s_at	238.9	285.3	0.26	1.19	PPP-PPP	0.10	17	0.868	0.955	0.5372
243013_at	127.2	151.8	0.26	1.19	PPP-PPP	0.10	17	0.868	0.955	0.5240
231827_at	203.0	242.4	0.26	1.19	APP-APP	0.10	17	0.868	0.955	0.5229
217655_at	122.4	146.3	0.26	1.19	PAP-PPP	0.10	17	0.868	0.955	0.4925
216294_s_at	119.7	143.0	0.26	1.19	PAA-MAP	0.10	17	0.868	0.955	0.4304
223274_at	502.0	599.4	0.26	1.19	PPP-PPP	0.10	17	0.868	0.955	0.3827
219234_x_at	100.2	119.6	0.26	1.19	PPP-PPP	0.10	17	0.868	0.955	0.3737
218991_at	272.2	324.9	0.26	1.19	PPP-PPP	0.10	17	0.868	0.955	0.3324
204207_s_at	817.6	976.0	0.26	1.19	PPP-PPP	0.10	17	0.868	0.955	0.3275
243141_at	395.2	472.0	0.26	1.19	PPP-PPP	0.10	17	0.868	0.955	0.3093
236477_at	162.0	193.5	0.26	1.19	PPP-PPP	0.10	17	0.868	0.955	0.2801
228611_s_at	387.4	462.5	0.26	1.19	PPP-PPP	0.10	17	0.868	0.955	0.2572
202476_s_at	378.1	451.3	0.26	1.19	PAP-PPP	0.10	17	0.868	0.955	0.5733
241425_at	348.3	415.6	0.26	1.19	PPP-PPP	0.10	17	0.868	0.955	0.5140
210975_x_at	557.5	665.4	0.26	1.19	PPP-PPP	0.10	17	0.868	0.955	0.4735
1553219_a_at	183.6	219.1	0.26	1.19	PPA-MAP	0.10	17	0.868	0.955	0.4637
212286_at	425.3	507.7	0.26	1.19	PPP-PPP	0.10	17	0.868	0.955	0.4258
218130_at	312.4	372.8	0.26	1.19	PPP-PPP	0.10	17	0.868	0.955	0.3552
232216_at	174.4	208.1	0.26	1.19	PPP-PPP	0.10	17	0.868	0.955	0.3367
205739_x_at	187.1	223.3	0.26	1.19	PPP-PPP	0.10	17	0.868	0.955	0.3220
232481_s_at	335.4	400.4	0.26	1.19	PPP-PPP	0.10	17	0.868	0.955	0.3014
219291_at	466.6	556.8	0.26	1.19	PPP-PPP	0.10	17	0.868	0.955	0.2963
207723_s_at	139.9	167.0	0.26	1.19	PPP-PPP	0.10	17	0.868	0.955	0.2948
39650_s_at	365.4	436.0	0.26	1.19	PPP-PPP	0.10	17	0.868	0.955	0.2637
238637_at	208.5	248.8	0.26	1.19	PPP-PAP	0.10	17	0.868	0.955	0.2453
207960_at	98.9	118.1	0.26	1.19	PAM-AAA	0.10	17	0.868	0.955	0.2432

202750_s_at	416.3	496.6	0.26	1.19	PPP-PPP	0.10	17	0.868	0.955	0.2289
231882_at	1229.7	1467.5	0.26	1.19	PPP-PPP	0.10	17	0.868	0.955	0.2098
202816_s_at	1069.0	1275.7	0.26	1.19	PPP-PPP	0.10	17	0.868	0.955	0.1944
211064_at	39.8	47.5	0.25	1.19	PMP-AAP	0.10	17	0.868	0.955	0.4845
222335_at	189.8	226.3	0.25	1.19	PPP-PPP	0.10	17	0.868	0.955	0.4673
214088_s_at	119.9	142.9	0.25	1.19	PPP-APP	0.10	17	0.868	0.955	0.4406
217203_at	202.7	241.8	0.25	1.19	APP-PPP	0.10	17	0.868	0.955	0.4282
1554316_at	211.4	252.0	0.25	1.19	PPP-PPP	0.10	17	0.868	0.955	0.3810
211339_s_at	72.1	86.0	0.25	1.19	MAA-AAP	0.10	17	0.868	0.955	0.3624
1553252_a_at	223.1	266.2	0.25	1.19	PPP-PPP	0.10	17	0.868	0.955	0.3318
221206_at	534.6	637.6	0.25	1.19	PPP-PPP	0.10	17	0.868	0.955	0.2934
238597_at	137.1	163.6	0.25	1.19	PPP-PPP	0.10	17	0.868	0.955	0.2894
1557779_at	184.3	219.8	0.25	1.19	PPP-PPP	0.10	17	0.868	0.955	0.2745
229184_at	90.9	108.3	0.25	1.19	PPP-PPP	0.10	17	0.868	0.955	0.2653
202025_x_at	754.4	900.0	0.25	1.19	PPP-PPP	0.10	17	0.868	0.955	0.1922
214220_s_at	214.3	255.5	0.25	1.19	PPP-PPP	0.10	17	0.868	0.955	0.1801
233933_s_at	235.4	280.8	0.25	1.19	PPP-PPP	0.10	17	0.868	0.955	0.1316
233239_at	61.6	73.3	0.25	1.19	PPM-PPP	0.10	17	0.868	0.955	0.4686
238923_at	112.3	133.9	0.25	1.19	PPP-PPP	0.10	17	0.868	0.955	0.4610
232330_at	131.4	156.5	0.25	1.19	APP-AAP	0.10	17	0.868	0.955	0.4573
209448_at	4527.7	5396.9	0.25	1.19	PPP-PPP	0.10	17	0.868	0.955	0.3554
244663_at	163.2	194.4	0.25	1.19	PPP-PPP	0.10	17	0.868	0.955	0.3497
209913_x_at	213.7	254.7	0.25	1.19	PPP-PPP	0.10	17	0.868	0.955	0.2303
221877_at	559.4	666.5	0.25	1.19	PAP-APP	0.10	17	0.868	0.955	0.2103
1557174_a_at	311.7	371.4	0.25	1.19	PPP-PPP	0.10	17	0.868	0.955	0.1874
207953_at	100.4	119.6	0.25	1.19	PAP-PPP	0.10	17	0.868	0.955	0.6093
202082_s_at	274.2	326.5	0.25	1.19	PPP-PPP	0.10	17	0.868	0.955	0.5866
208868_s_at	173.9	207.1	0.25	1.19	PPP-PAP	0.10	17	0.868	0.955	0.5759
235512_at	217.1	258.6	0.25	1.19	PPP-PPP	0.10	17	0.868	0.955	0.5678
237347_at	74.2	88.4	0.25	1.19	AMP-PPA	0.10	17	0.868	0.955	0.5519
220566_at	118.2	140.7	0.25	1.19	PAA-APP	0.10	17	0.868	0.955	0.5018
225760_at	399.8	476.2	0.25	1.19	PPP-PPP	0.10	17	0.868	0.955	0.4799
219878_s_at	572.3	681.6	0.25	1.19	PPP-PPP	0.10	17	0.868	0.955	0.4041
223446_s_at	246.7	293.7	0.25	1.19	PAA-MAP	0.10	17	0.868	0.955	0.3905
225921_at	987.6	1176.4	0.25	1.19	PPP-PPP	0.10	17	0.868	0.955	0.3455
232140_at	188.1	224.0	0.25	1.19	PAA-PPP	0.10	17	0.868	0.955	0.3412
216228_s_at	393.2	468.3	0.25	1.19	PPP-PPP	0.10	17	0.868	0.955	0.2999
218126_at	749.7	892.8	0.25	1.19	PPP-PPP	0.10	17	0.868	0.955	0.2883
219203_at	514.1	612.1	0.25	1.19	PPP-PPP	0.10	17	0.868	0.955	0.2378
201158_at	423.6	504.6	0.25	1.19	PPP-PPP	0.10	17	0.868	0.955	0.2333
212189_s_at	608.9	725.3	0.25	1.19	PPP-PPP	0.10	17	0.868	0.955	0.2269
203798_s_at	465.9	554.9	0.25	1.19	PPP-PPP	0.10	17	0.868	0.955	0.1698

204053_x_at	2333.1	2778.6	0.25	1.19	PPP-PPP	0.10	17	0.868	0.955	0.1684
240452_at	148.9	177.2	0.25	1.19	PPM-PMP	0.10	17	0.868	0.955	0.5891
218923_at	274.3	326.5	0.25	1.19	PPP-PPP	0.10	17	0.868	0.955	0.4761
223602_at	279.0	332.1	0.25	1.19	PPP-PPP	0.10	17	0.868	0.955	0.4046
214848_at	283.2	336.9	0.25	1.19	PPP-PPP	0.10	17	0.868	0.955	0.4012
1552621_at	858.2	1021.6	0.25	1.19	PPP-PPP	0.10	17	0.868	0.955	0.3858
213528_at	381.6	453.9	0.25	1.19	PPP-PPP	0.10	17	0.868	0.955	0.3192
1556821_x_at	593.7	706.5	0.25	1.19	PPP-PPP	0.10	17	0.868	0.955	0.3142
201942_s_at	407.4	484.7	0.25	1.19	PPP-PPP	0.10	17	0.868	0.955	0.3063
227395_at	378.9	450.9	0.25	1.19	PPP-PPP	0.10	17	0.868	0.955	0.3002
213727_x_at	473.5	563.5	0.25	1.19	AMA-PAA	0.10	17	0.868	0.955	0.2131
219053_s_at	1190.0	1416.5	0.25	1.19	PPP-PPP	0.10	17	0.868	0.955	0.2031
222310_at	99.3	118.2	0.25	1.19	PAP-PPP	0.10	17	0.868	0.955	0.1536
222995_s_at	919.8	1094.7	0.25	1.19	PPP-PPP	0.10	17	0.868	0.955	0.1158
215469_at	70.4	83.7	0.25	1.19	AAP-PAP	0.10	17	0.868	0.955	0.5924
234690_at	34.8	41.4	0.25	1.19	AMA-AAP	0.10	17	0.868	0.955	0.5423
203474_at	60.8	72.3	0.25	1.19	AAP-AAA	0.10	17	0.868	0.955	0.5351
211610_at	129.8	154.3	0.25	1.19	PAA-PPP	0.10	17	0.868	0.955	0.5205
227025_at	183.7	218.5	0.25	1.19	PPP-PPP	0.10	17	0.868	0.955	0.4857
1555241_at	576.1	685.4	0.25	1.19	PPP-PPP	0.10	17	0.868	0.955	0.4809
212786_at	275.2	327.3	0.25	1.19	PPP-PPP	0.10	17	0.868	0.955	0.3413
227026_at	1318.3	1567.9	0.25	1.19	PPP-PPP	0.10	17	0.868	0.955	0.3380
218328_at	464.0	552.0	0.25	1.19	PPP-PPP	0.10	17	0.868	0.955	0.3285
1569112_at	313.2	372.3	0.25	1.19	PPP-PPP	0.10	17	0.868	0.955	0.2545
209297_at	657.8	782.1	0.25	1.19	PPP-PPP	0.10	17	0.868	0.955	0.2526
218584_at	500.3	595.0	0.25	1.19	PPP-PPP	0.10	17	0.868	0.955	0.1680
228155_at	11738.4	13962.0	0.25	1.19	PPP-PPP	0.10	17	0.868	0.955	0.1534
214729_at	119.4	141.9	0.25	1.19	PPP-PPP	0.10	17	0.868	0.955	0.1506
235410_at	203.1	241.4	0.25	1.19	PPP-PPP	0.10	17	0.868	0.955	0.5285
220334_at	118.9	141.3	0.25	1.19	PPP-PAP	0.10	17	0.868	0.955	0.5243
230660_at	161.8	192.3	0.25	1.19	PMM-APP	0.10	17	0.868	0.955	0.4789
205323_s_at	331.5	393.9	0.25	1.19	PPP-PPP	0.10	17	0.868	0.955	0.4666
1562408_at	129.6	154.0	0.25	1.19	PPP-PPP	0.10	17	0.868	0.955	0.4498
235764_at	59.5	70.8	0.25	1.19	PPP-PPP	0.10	17	0.868	0.955	0.4124
1560250_s_at	60.0	71.3	0.25	1.19	PAP-AAP	0.10	17	0.868	0.955	0.4067
213388_at	485.8	577.4	0.25	1.19	PPP-PPP	0.10	17	0.868	0.955	0.3422
232958_at	102.2	121.5	0.25	1.19	PPP-PPP	0.10	17	0.868	0.955	0.2995
210361_s_at	352.4	418.9	0.25	1.19	PPP-PPP	0.10	17	0.868	0.955	0.2897
228006_at	654.2	777.5	0.25	1.19	PPP-PPP	0.10	17	0.868	0.955	0.2232
214661_s_at	1947.3	2313.4	0.25	1.19	PPP-PPP	0.10	17	0.868	0.955	0.1965
227256_at	1063.5	1264.2	0.25	1.19	PPP-PPP	0.10	17	0.868	0.955	0.1928
223041_at	453.6	539.1	0.25	1.19	PPP-PPP	0.10	17	0.868	0.955	0.1897

241355_at	743.1	883.2	0.25	1.19	PPP-PPP	0.10	17	0.868	0.955	0.1243
213742_at	633.2	752.5	0.25	1.19	PPP-PPP	0.10	17	0.868	0.955	0.1136
233035_at	86.9	103.2	0.25	1.19	PPM-PPP	0.10	17	0.868	0.955	0.6071
225672_at	481.9	572.1	0.25	1.19	PPP-PPP	0.10	17	0.868	0.955	0.5030
209530_at	282.6	335.6	0.25	1.19	PPP-PPP	0.10	17	0.868	0.955	0.4950
215099_s_at	188.9	224.3	0.25	1.19	PPP-PPP	0.10	17	0.868	0.955	0.4568
1569894_at	80.2	95.2	0.25	1.19	PAA-PPP	0.10	17	0.868	0.955	0.4488
236829_at	66.6	79.1	0.25	1.19	AAA-PAM	0.10	17	0.868	0.955	0.3486
223787_s_at	157.3	186.7	0.25	1.19	APP-PPP	0.10	17	0.868	0.955	0.3462
209244_s_at	187.5	222.7	0.25	1.19	MPM-PPP	0.10	17	0.868	0.955	0.3456
1560738_at	71.2	84.5	0.25	1.19	PAP-PAP	0.10	17	0.868	0.955	0.3112
238015_at	738.5	876.8	0.25	1.19	PPP-PPP	0.10	17	0.868	0.955	0.2847
226796_at	215.8	256.3	0.25	1.19	PPP-APP	0.10	17	0.868	0.955	0.2649
202811_at	1512.4	1796.7	0.25	1.19	PPP-PPP	0.10	17	0.868	0.955	0.2308
212134_at	304.7	361.9	0.25	1.19	PAP-PPP	0.10	17	0.868	0.955	0.2073
204071_s_at	672.3	798.5	0.25	1.19	PPP-PPP	0.10	17	0.868	0.955	0.1907
228708_at	2630.8	3124.7	0.25	1.19	PPP-PPP	0.10	17	0.868	0.955	0.1778
208420_x_at	524.8	623.3	0.25	1.19	PPP-PPP	0.10	17	0.868	0.955	0.1749
232469_x_at	88.4	105.0	0.25	1.19	PMP-PPP	0.10	17	0.868	0.955	0.1553
201392_s_at	448.5	532.7	0.25	1.19	PPP-PPP	0.10	17	0.868	0.955	0.1522
229949_at	2313.1	2747.8	0.25	1.19	PPP-PPP	0.10	17	0.868	0.955	0.1353
1560224_at	220.6	261.8	0.25	1.19	AAP-PPA	0.10	17	0.868	0.955	0.5628
210034_s_at	69.9	83.0	0.25	1.19	PPA-PPP	0.10	17	0.868	0.955	0.5030
231892_at	106.7	126.7	0.25	1.19	APP-MPP	0.10	17	0.868	0.955	0.4989
220961_s_at	140.7	166.9	0.25	1.19	PPP-PPP	0.10	17	0.868	0.955	0.4662
210162_s_at	164.0	194.7	0.25	1.19	APP-MAA	0.10	17	0.868	0.955	0.4376
214107_x_at	1043.9	1239.0	0.25	1.19	PPP-PPP	0.10	17	0.868	0.955	0.3104
234084_x_at	216.1	256.4	0.25	1.19	PPP-PPP	0.10	17	0.868	0.955	0.2743
211987_at	4507.6	5349.6	0.25	1.19	PPP-PPP	0.10	17	0.868	0.955	0.2157
229520_s_at	220.6	261.9	0.25	1.19	PPP-PPP	0.10	17	0.868	0.955	0.1914
218294_s_at	611.3	725.3	0.25	1.19	PPP-PPP	0.10	17	0.868	0.955	0.1819
210074_at	837.6	994.0	0.25	1.19	PPP-PPP	0.10	17	0.868	0.955	0.1763
226625_at	830.1	985.3	0.25	1.19	PPP-PPP	0.10	17	0.868	0.955	0.1545
226129_at	4939.7	5860.3	0.25	1.19	PPP-PPP	0.10	17	0.868	0.955	0.1466
206665_s_at	257.1	305.1	0.25	1.19	PPP-PPP	0.10	17	0.868	0.955	0.1417
203577_at	300.9	356.9	0.25	1.19	PPP-PPP	0.10	17	0.868	0.955	0.5569
242809_at	244.0	289.4	0.25	1.19	MAA-PAA	0.10	17	0.868	0.955	0.5201
227177_at	528.5	626.6	0.25	1.19	PPP-PPP	0.10	17	0.868	0.955	0.4946
238824_at	101.0	119.7	0.25	1.19	MAP-AAA	0.10	17	0.868	0.955	0.4916
220750_s_at	424.2	503.2	0.25	1.19	PPP-PPP	0.10	17	0.868	0.955	0.4051
243065_at	202.4	240.0	0.25	1.19	PPP-PPP	0.10	17	0.868	0.955	0.3877
1558560_s_at	76.2	90.3	0.25	1.19	PPP-PPP	0.10	17	0.868	0.955	0.3049

230060_at	177.4	210.4	0.25	1.19	PPP-PPP	0.10	17	0.868	0.955	0.2675
202223_at	1902.4	2255.4	0.25	1.19	PPP-PPP	0.10	17	0.868	0.955	0.2597
209980_s_at	719.0	852.5	0.25	1.19	PAP-PPP	0.10	17	0.868	0.955	0.2351
237025_at	80.3	95.3	0.25	1.19	PPP-PPP	0.10	17	0.868	0.955	0.2297
202955_s_at	647.7	767.9	0.25	1.19	PPP-PPP	0.10	17	0.868	0.955	0.2122
244025_at	209.0	247.7	0.25	1.19	APM-PPP	0.10	17	0.868	0.955	0.6089
1557452_at	89.9	106.6	0.25	1.19	APA-AMP	0.10	17	0.868	0.955	0.5732
223438_s_at	142.8	169.1	0.25	1.19	APP-APP	0.10	17	0.868	0.955	0.5425
220398_at	86.4	102.5	0.25	1.19	APA-AAA	0.10	17	0.868	0.955	0.5321
240277_at	347.6	412.0	0.25	1.19	PPP-PPP	0.10	17	0.868	0.955	0.5221
201747_s_at	211.7	250.9	0.25	1.19	PPP-PPP	0.10	17	0.868	0.955	0.5165
241303_x_at	292.0	346.1	0.25	1.19	PAP-PPP	0.10	17	0.868	0.955	0.4812
203900_at	95.9	113.7	0.25	1.19	MPP-MAP	0.10	17	0.868	0.955	0.4586
215083_at	164.4	194.9	0.25	1.19	PPP-PPP	0.10	17	0.868	0.955	0.4426
1552334_at	412.5	489.0	0.25	1.19	PPP-PPP	0.10	17	0.868	0.955	0.4218
212705_x_at	198.5	235.3	0.25	1.19	AAA-APP	0.10	17	0.868	0.955	0.3622
219207_at	243.4	288.4	0.25	1.19	PPP-PPP	0.10	17	0.868	0.955	0.3581
218948_at	457.8	542.6	0.25	1.19	MPP-PPP	0.10	17	0.868	0.955	0.2398
236503_at	83.6	99.1	0.25	1.19	PPP-PPP	0.10	17	0.868	0.955	0.2343
217656_at	131.5	155.8	0.25	1.19	AAA-AAP	0.10	17	0.868	0.955	0.2097
209746_s_at	381.1	451.7	0.25	1.19	PPP-PPP	0.10	17	0.868	0.955	0.2057
204872_at	486.4	576.3	0.25	1.19	PPP-PPP	0.10	17	0.868	0.955	0.1958
225098_at	730.5	865.7	0.25	1.19	PPP-PPP	0.10	17	0.868	0.955	0.1603
209148_at	566.9	671.4	0.24	1.18	PPP-PPP	0.10	17	0.868	0.955	0.6080
213747_at	320.1	379.0	0.24	1.18	PPP-PPP	0.10	17	0.868	0.955	0.5196
1562257_x_at	112.4	133.1	0.24	1.18	PPA-PAP	0.10	17	0.868	0.955	0.5098
235264_at	67.4	79.8	0.24	1.19	PPA-PPP	0.10	17	0.868	0.955	0.4210
238069_at	101.0	119.7	0.24	1.19	PPP-PPP	0.10	17	0.868	0.955	0.4016
227720_at	287.0	340.0	0.24	1.18	PPP-PPP	0.10	17	0.868	0.955	0.3050
242558_at	156.7	185.5	0.24	1.18	AMP-PPP	0.10	17	0.868	0.955	0.2661
213757_at	2229.9	2640.1	0.24	1.18	PPP-PPP	0.10	17	0.868	0.955	0.2271
212819_at	113.0	133.9	0.24	1.18	PPP-PPP	0.10	17	0.868	0.955	0.2197
225031_at	313.4	371.1	0.24	1.18	PPP-PPP	0.10	17	0.868	0.955	0.2131
235952_at	383.9	454.8	0.24	1.19	PPP-PPP	0.10	17	0.868	0.955	0.2108
226730_s_at	204.5	242.3	0.24	1.19	PPP-PPP	0.10	17	0.868	0.955	0.1663
227415_at	1965.4	2326.9	0.24	1.18	PPP-PPP	0.10	17	0.868	0.955	0.1493
238649_at	145.7	172.6	0.24	1.19	PPP-PPP	0.10	17	0.868	0.955	0.1453
224889_at	830.6	983.7	0.24	1.18	PPP-PPP	0.10	17	0.868	0.955	0.1076
215067_x_at	297.4	351.9	0.24	1.18	PPP-MPP	0.10	17	0.868	0.955	0.4698
232683_s_at	553.0	654.5	0.24	1.18	PPP-PPP	0.10	17	0.868	0.955	0.4381
1565834_a_at	83.5	98.8	0.24	1.18	APP-PPP	0.10	17	0.868	0.955	0.3458
201639_s_at	1356.6	1605.1	0.24	1.18	PPP-PPP	0.10	17	0.868	0.955	0.3405

213260_at	371.0	439.1	0.24	1.18	PPP-PPP	0.10	17	0.868	0.955	0.2742
213314_at	99.4	117.6	0.24	1.18	PPP-PPP	0.10	17	0.868	0.955	0.2621
203530_s_at	838.9	992.6	0.24	1.18	PPP-PPP	0.10	17	0.868	0.955	0.2353
209125_at	26987.6	31944.8	0.24	1.18	PPP-PPP	0.10	17	0.868	0.955	0.1713
238609_at	296.1	350.4	0.24	1.18	PPP-PPP	0.10	17	0.868	0.955	0.1632
232112_at	171.3	202.7	0.24	1.18	PPP-PPA	0.10	17	0.868	0.955	0.1213
218816_at	1006.0	1190.6	0.24	1.18	PPP-PPP	0.10	17	0.868	0.955	0.1209
1557383_a_at	60.0	70.9	0.24	1.18	PPP-PPP	0.10	17	0.868	0.955	0.6002
210973_s_at	147.2	174.2	0.24	1.18	AAP-PPP	0.10	17	0.868	0.955	0.5962
244532_x_at	62.3	73.6	0.24	1.18	PAA-MAP	0.10	17	0.868	0.955	0.5887
213681_at	226.7	268.1	0.24	1.18	MPP-PPP	0.10	17	0.868	0.955	0.5880
216170_at	74.3	87.9	0.24	1.18	APP-MPP	0.10	17	0.868	0.955	0.5581
227172_at	189.9	224.5	0.24	1.18	PPP-PPP	0.10	17	0.868	0.955	0.4634
215834_x_at	188.0	222.4	0.24	1.18	PMM-AAM	0.10	17	0.868	0.955	0.4231
219063_at	922.5	1090.7	0.24	1.18	PPP-PPP	0.10	17	0.868	0.955	0.4195
209705_at	171.2	202.6	0.24	1.18	PPP-PPP	0.10	17	0.868	0.955	0.4145
209872_s_at	665.5	786.9	0.24	1.18	PPP-PPP	0.10	17	0.868	0.955	0.3672
1553703_at	915.4	1082.5	0.24	1.18	PPP-PPP	0.10	17	0.868	0.955	0.3148
232184_at	145.9	172.6	0.24	1.18	PPP-PPP	0.10	17	0.868	0.955	0.2885
203337_x_at	305.7	361.4	0.24	1.18	PPP-PPP	0.10	17	0.868	0.955	0.2751
1555294_a_at	333.3	394.2	0.24	1.18	PPP-PPP	0.10	17	0.868	0.955	0.2438
216111_x_at	427.9	506.1	0.24	1.18	PPP-PPP	0.10	17	0.868	0.955	0.2305
1552663_a_at	391.7	463.2	0.24	1.18	APP-PPP	0.10	17	0.868	0.955	0.2214
1554202_x_at	116.6	137.9	0.24	1.18	MAP-PPA	0.10	17	0.868	0.955	0.1288
227132_at	545.6	645.4	0.24	1.18	PPP-PPP	0.10	17	0.868	0.955	0.1228
232079_s_at	114.9	135.8	0.24	1.18	PAP-PMA	0.10	17	0.868	0.955	0.6066
239404_at	115.4	136.5	0.24	1.18	PPP-PPP	0.10	17	0.868	0.955	0.5877
229733_s_at	208.7	246.7	0.24	1.18	PAA-PAP	0.10	17	0.868	0.955	0.4951
227073_at	204.1	241.2	0.24	1.18	PPP-PPP	0.10	17	0.868	0.955	0.4728
202454_s_at	343.7	406.3	0.24	1.18	PPP-PPP	0.10	17	0.868	0.955	0.4138
224824_at	1589.0	1878.0	0.24	1.18	PPP-PPP	0.10	17	0.868	0.955	0.4069
212303_x_at	380.7	449.9	0.24	1.18	PPP-PPP	0.10	17	0.868	0.955	0.4034
205211_s_at	401.9	475.0	0.24	1.18	PPP-PPP	0.10	17	0.868	0.955	0.3634
206020_at	261.9	309.4	0.24	1.18	PPP-PPP	0.10	17	0.868	0.955	0.3124
213957_s_at	510.8	603.7	0.24	1.18	PPP-PPP	0.10	17	0.868	0.955	0.3089
223455_at	867.8	1025.3	0.24	1.18	PPP-PPP	0.10	17	0.868	0.955	0.2765
214892_x_at	367.5	434.3	0.24	1.18	AAM-APP	0.10	17	0.868	0.955	0.2677
227274_at	589.6	696.8	0.24	1.18	PPP-PPP	0.10	17	0.868	0.955	0.2538
1557681_s_at	52.1	61.6	0.24	1.18	APA-AAP	0.10	17	0.868	0.955	0.2047
201206_s_at	410.4	485.2	0.24	1.18	PAP-PPP	0.10	17	0.868	0.955	0.1912
203232_s_at	280.2	331.1	0.24	1.18	PPP-PPP	0.10	17	0.868	0.955	0.1843
241718_x_at	108.9	128.7	0.24	1.18	AAP-PPP	0.10	17	0.868	0.955	0.1554

244688_at	94.5	111.6	0.24	1.18	PPP-PMP	0.10	17	0.868	0.955	0.6044
232815_at	86.6	102.2	0.24	1.18	AAA-APP	0.10	17	0.868	0.955	0.5444
234788_x_at	464.5	548.6	0.24	1.18	PPP-PPP	0.10	17	0.868	0.955	0.5250
236240_at	248.6	293.6	0.24	1.18	PPP-PPP	0.10	17	0.868	0.955	0.5015
242471_at	384.6	454.4	0.24	1.18	PPP-PPP	0.10	17	0.868	0.955	0.4872
214403_x_at	186.2	219.8	0.24	1.18	APP-PAM	0.10	17	0.868	0.955	0.4601
223186_at	1273.5	1503.9	0.24	1.18	PPP-PPP	0.10	17	0.868	0.955	0.4229
234140_s_at	361.6	426.9	0.24	1.18	PPP-PPP	0.10	17	0.868	0.955	0.4031
231806_s_at	140.6	166.1	0.24	1.18	PAP-APA	0.10	17	0.868	0.955	0.3485
201411_s_at	1135.0	1340.2	0.24	1.18	PPP-PPP	0.10	17	0.868	0.955	0.2939
214857_at	175.1	206.9	0.24	1.18	PPP-PPP	0.10	17	0.868	0.955	0.2732
226032_at	2084.6	2462.5	0.24	1.18	PPP-PPP	0.10	17	0.868	0.955	0.1429
225157_at	653.7	772.2	0.24	1.18	PPP-PPP	0.10	17	0.868	0.955	0.1158
242676_at	52.1	61.5	0.24	1.18	PAP-AMA	0.10	17	0.868	0.955	0.5435
225940_at	344.3	406.4	0.24	1.18	PPP-PPP	0.10	17	0.868	0.955	0.4494
211467_s_at	467.2	551.4	0.24	1.18	PPP-PPP	0.10	17	0.868	0.955	0.3463
224961_at	1471.6	1737.3	0.24	1.18	PPP-PPP	0.10	17	0.868	0.955	0.3233
238087_at	256.9	303.3	0.24	1.18	AMA-AAP	0.10	17	0.868	0.955	0.2604
220734_s_at	353.0	416.7	0.24	1.18	PAA-PPP	0.10	17	0.868	0.955	0.2141
200980_s_at	5030.9	5935.8	0.24	1.18	PPP-PPP	0.10	17	0.868	0.955	0.2129
215948_x_at	280.6	331.1	0.24	1.18	PPP-PPP	0.10	17	0.868	0.955	0.2129
229069_at	352.8	416.5	0.24	1.18	PPP-PPP	0.10	17	0.868	0.955	0.2003
209018_s_at	1673.1	1974.4	0.24	1.18	PPP-PPP	0.10	17	0.868	0.955	0.1890
213618_at	664.9	784.9	0.24	1.18	PPP-PPP	0.10	17	0.868	0.955	0.1408
209711_at	984.1	1161.1	0.24	1.18	PPP-PPP	0.10	17	0.868	0.955	0.1234
226745_at	293.3	346.3	0.24	1.18	PPP-PPP	0.10	17	0.868	0.955	0.1057
241689_at	150.2	177.0	0.24	1.18	PPP-PPP	0.10	17	0.868	0.955	0.5530
210811_s_at	368.4	434.3	0.24	1.18	PPP-PPP	0.10	17	0.868	0.955	0.5154
205642_at	151.5	178.6	0.24	1.18	APP-PPP	0.10	17	0.868	0.955	0.5081
230780_at	104.9	123.7	0.24	1.18	AAA-PAA	0.10	17	0.868	0.955	0.4699
206092_x_at	209.9	247.6	0.24	1.18	PPP-PPP	0.10	17	0.868	0.955	0.4672
1557165_s_at	321.2	378.7	0.24	1.18	PPP-PPP	0.10	17	0.868	0.955	0.4218
202519_at	732.2	863.3	0.24	1.18	PPP-PPP	0.10	17	0.868	0.955	0.4092
226585_at	241.8	285.1	0.24	1.18	PMP-MPP	0.10	17	0.868	0.955	0.3718
207598_x_at	480.7	566.8	0.24	1.18	PPP-PPP	0.10	17	0.868	0.955	0.3684
227090_at	194.8	229.7	0.24	1.18	PAP-PPP	0.10	17	0.868	0.955	0.2909
219592_at	192.3	226.9	0.24	1.18	APA-AMP	0.10	17	0.868	0.955	0.2427
227943_at	159.1	187.6	0.24	1.18	PPP-MPP	0.10	17	0.868	0.955	0.1642
1553106_at	716.2	844.8	0.24	1.18	PPP-PPP	0.10	17	0.868	0.955	0.1516
236262_at	44.3	52.2	0.24	1.18	APA-PAP	0.10	17	0.868	0.955	0.6078
1559401_a_at	80.1	94.3	0.24	1.18	PAA-APP	0.10	17	0.868	0.955	0.5832
1557698_at	52.4	61.8	0.24	1.18	APP-PAP	0.10	17	0.868	0.955	0.4810

1554293_at	54.2	63.9	0.24	1.18	MPP-AAA	0.10	17	0.868	0.955	0.4687
238964_at	73.9	87.1	0.24	1.18	AAP-APA	0.10	17	0.868	0.955	0.4598
225963_at	516.1	608.1	0.24	1.18	PPP-PPP	0.10	17	0.868	0.955	0.4569
225191_at	123.4	145.4	0.24	1.18	PPP-PMA	0.10	17	0.868	0.955	0.4558
1560017_at	183.2	215.8	0.24	1.18	PAP-PPP	0.10	17	0.868	0.955	0.4367
202723_s_at	1766.0	2080.9	0.24	1.18	PPP-PPP	0.10	17	0.868	0.955	0.2678
232130_at	611.0	720.2	0.24	1.18	PPP-PPP	0.10	17	0.868	0.955	0.2660
228662_at	716.9	844.7	0.24	1.18	PPP-PPP	0.10	17	0.868	0.955	0.2360
228272_at	643.3	758.3	0.24	1.18	PPP-PPP	0.10	17	0.868	0.955	0.1216
224919_at	4991.6	5882.9	0.24	1.18	PPP-PPP	0.10	17	0.868	0.955	0.1059
212660_at	3249.4	3830.8	0.24	1.18	PPP-PPP	0.10	17	0.868	0.955	0.1055
203238_s_at	131.0	154.3	0.24	1.18	AAP-APP	0.10	17	0.868	0.955	0.6068
1556204_a_at	62.0	73.0	0.24	1.18	APP-PPP	0.10	17	0.868	0.955	0.5579
205967_at	7156.3	8426.5	0.24	1.18	PPP-PPP	0.10	17	0.868	0.955	0.4794
222267_at	123.4	145.4	0.24	1.18	PPP-APA	0.10	17	0.868	0.955	0.4795
217247_at	106.6	125.5	0.24	1.18	AAP-AAA	0.10	17	0.868	0.955	0.4759
222015_at	389.5	458.8	0.24	1.18	PAA-PAP	0.10	17	0.868	0.955	0.3673
217653_x_at	435.1	512.4	0.24	1.18	PPP-MPP	0.10	17	0.868	0.955	0.3513
200854_at	834.3	982.8	0.24	1.18	PPP-PPP	0.10	17	0.868	0.955	0.3147
209203_s_at	813.5	957.8	0.24	1.18	PPP-PPP	0.10	17	0.868	0.955	0.2529
223440_at	514.8	606.1	0.24	1.18	PPP-PPP	0.10	17	0.868	0.955	0.2009
211056_s_at	400.2	471.4	0.24	1.18	PPP-PPP	0.10	17	0.868	0.955	0.1904
212908_at	719.7	847.5	0.24	1.18	PPP-PPP	0.10	17	0.868	0.955	0.1654
225263_at	711.1	837.4	0.24	1.18	PPP-PPP	0.10	17	0.868	0.955	0.1537
208723_at	610.5	719.1	0.24	1.18	PPP-PPP	0.10	17	0.868	0.955	0.1152
223339_at	394.8	464.9	0.24	1.18	PPP-PPP	0.10	17	0.868	0.955	0.1033
215421_at	86.9	102.3	0.24	1.18	MPA-PAP	0.10	17	0.868	0.955	0.5566
213826_s_at	231.6	272.6	0.24	1.18	PAP-PPP	0.10	17	0.868	0.955	0.5006
223843_at	420.0	494.2	0.24	1.18	PPP-PPP	0.10	17	0.868	0.955	0.4661
202873_at	348.8	410.5	0.24	1.18	PPP-PPP	0.10	17	0.868	0.955	0.4061
201538_s_at	143.4	168.8	0.24	1.18	PAP-PPP	0.10	17	0.868	0.955	0.4027
213490_s_at	497.7	585.6	0.24	1.18	PPP-PPP	0.10	17	0.868	0.955	0.3978
220113_x_at	458.1	539.0	0.24	1.18	PPP-PPP	0.10	17	0.868	0.955	0.3218
1567014_s_at	711.2	837.0	0.24	1.18	PPP-PPP	0.10	17	0.868	0.955	0.2971
211320_s_at	612.5	721.0	0.24	1.18	PPP-PPP	0.10	17	0.868	0.955	0.2854
235581_at	230.7	271.5	0.24	1.18	PPP-PPP	0.10	17	0.868	0.955	0.1492
204455_at	6772.1	7971.4	0.24	1.18	PPP-PPP	0.10	17	0.868	0.955	0.1431
201561_s_at	2220.5	2614.2	0.24	1.18	PPP-PPP	0.10	17	0.868	0.955	0.1372
1554053_at	88.4	104.0	0.23	1.18	MPP-PPP	0.10	17	0.868	0.955	0.6103
215825_at	81.8	96.2	0.23	1.18	PAP-PPP	0.10	17	0.868	0.955	0.5618
237475_x_at	1233.3	1450.2	0.23	1.18	PPP-PPP	0.10	17	0.868	0.955	0.5448
227489_at	748.2	880.2	0.23	1.18	PPP-PPP	0.10	17	0.868	0.955	0.5267

239818_x_at	52.5	61.7	0.23	1.18	PAA-AAA	0.10	17	0.868	0.955	0.4881
213750_at	1112.1	1307.9	0.23	1.18	PPP-PPP	0.10	17	0.868	0.955	0.4426
209255_at	540.5	635.8	0.23	1.18	PPP-PPP	0.10	17	0.868	0.955	0.4364
203375_s_at	1220.4	1435.4	0.23	1.18	PPP-PPP	0.10	17	0.868	0.955	0.4236
231730_at	118.1	138.9	0.23	1.18	AMP-PPP	0.10	17	0.868	0.955	0.3083
221464_at	119.6	140.7	0.23	1.18	PPA-PPA	0.10	17	0.868	0.955	0.2628
232244_at	271.8	319.5	0.23	1.18	PPP-PPP	0.10	17	0.868	0.955	0.2543
230492_s_at	263.1	309.3	0.23	1.18	PPP-PPP	0.10	17	0.868	0.955	0.2433
1554537_at	129.7	152.6	0.23	1.18	PPP-PPP	0.10	17	0.868	0.955	0.2070
223921_s_at	437.6	514.8	0.23	1.18	PPP-PPP	0.10	17	0.868	0.955	0.1972
234983_at	1515.8	1782.1	0.23	1.18	PPP-PPP	0.10	17	0.868	0.955	0.1763
203419_at	497.6	585.2	0.23	1.18	PPP-PPP	0.10	17	0.868	0.955	0.1710
35436_at	591.8	695.9	0.23	1.18	PPP-PPP	0.10	17	0.868	0.955	0.1525
40149_at	389.3	457.8	0.23	1.18	PPP-PPP	0.10	17	0.868	0.955	0.1507
220202_s_at	784.8	923.0	0.23	1.18	PPP-PPP	0.10	17	0.868	0.955	0.1274
223641_at	563.4	662.5	0.23	1.18	PPP-PPP	0.10	17	0.868	0.955	0.1008
215201_at	40.2	47.2	0.23	1.18	PAA-AAA	0.10	17	0.868	0.955	0.6052
235092_at	133.3	156.6	0.23	1.18	PPP-PPP	0.10	17	0.868	0.955	0.5976
236125_at	82.8	97.3	0.23	1.18	PPA-PPP	0.10	17	0.868	0.955	0.5568
226570_at	96.6	113.6	0.23	1.18	PPP-MPP	0.10	17	0.868	0.955	0.5540
1554637_a_at	108.5	127.5	0.23	1.18	APP-MAA	0.10	17	0.868	0.955	0.4607
235502_at	118.6	139.4	0.23	1.18	PAP-APP	0.10	17	0.868	0.955	0.4192
215074_at	76.2	89.6	0.23	1.18	PPP-PPP	0.10	17	0.868	0.955	0.4151
220482_s_at	186.2	218.8	0.23	1.18	PAP-PPP	0.10	17	0.868	0.955	0.3699
212043_at	1829.1	2149.8	0.23	1.18	PPP-PPP	0.10	17	0.868	0.955	0.2875
214494_s_at	889.1	1044.7	0.23	1.18	PPP-PPP	0.10	17	0.868	0.955	0.2790
213654_at	715.1	840.2	0.23	1.18	PPP-PPP	0.10	17	0.868	0.955	0.1469
208132_x_at	515.8	606.1	0.23	1.18	PPP-PPP	0.10	17	0.868	0.955	0.1443
229888_at	127.6	149.9	0.23	1.18	PPP-PPP	0.10	17	0.868	0.955	0.5914
227891_s_at	299.4	351.5	0.23	1.17	PPP-PPP	0.10	17	0.868	0.955	0.5505
242048_at	168.2	197.6	0.23	1.17	PPP-PPP	0.10	17	0.868	0.955	0.5412
230465_at	54.2	63.7	0.23	1.17	AAP-PPP	0.10	17	0.868	0.955	0.5107
223652_at	56.9	66.9	0.23	1.18	AAA-AMP	0.10	17	0.868	0.955	0.4900
222769_at	114.8	134.8	0.23	1.17	PPP-PPP	0.10	17	0.868	0.955	0.4709
239683_at	102.9	120.9	0.23	1.18	APA-PPM	0.10	17	0.868	0.955	0.3494
226269_at	239.8	281.7	0.23	1.18	PPP-PPP	0.10	17	0.868	0.955	0.3485
1559132_at	309.7	363.7	0.23	1.17	PPP-PPP	0.10	17	0.868	0.955	0.2842
212288_at	498.1	585.0	0.23	1.17	PPP-PPP	0.10	17	0.868	0.955	0.2171
216036_x_at	283.5	332.9	0.23	1.17	PMP-PPP	0.10	17	0.868	0.955	0.1858
216101_at	137.8	161.7	0.23	1.17	PMA-AAP	0.10	17	0.868	0.955	0.5947
235195_at	141.3	165.8	0.23	1.17	PPP-PPP	0.10	17	0.868	0.955	0.5791
218630_at	100.5	117.9	0.23	1.17	PPA-PPP	0.10	17	0.868	0.955	0.5775

238056_at	262.6	308.2	0.23	1.17	PPP-PPP	0.10	17	0.868	0.955	0.5701
1552306_at	85.8	100.7	0.23	1.17	PPP-APP	0.10	17	0.868	0.955	0.5586
227616_at	537.3	630.5	0.23	1.17	PPP-PPP	0.10	17	0.868	0.955	0.5394
229256_at	152.8	179.4	0.23	1.17	PPP-PPP	0.10	17	0.868	0.955	0.4398
219501_at	156.1	183.2	0.23	1.17	PPP-PPP	0.10	17	0.868	0.955	0.4373
223437_at	251.4	295.1	0.23	1.17	PAP-PPA	0.10	17	0.868	0.955	0.3658
201668_x_at	344.0	403.8	0.23	1.17	PPP-PPP	0.10	17	0.868	0.955	0.3349
211773_s_at	193.5	227.1	0.23	1.17	PPM-PPP	0.10	17	0.868	0.955	0.2925
229287_at	494.0	579.7	0.23	1.17	PPP-PPP	0.10	17	0.868	0.955	0.2077
206139_at	287.5	337.2	0.23	1.17	AMM-APP	0.10	17	0.868	0.955	0.6057
230868_at	86.6	101.6	0.23	1.17	AMP-PPP	0.10	17	0.868	0.955	0.6045
219635_at	88.8	104.2	0.23	1.17	APP-PMP	0.10	17	0.868	0.955	0.6035
231090_s_at	240.1	281.6	0.23	1.17	PPP-PPP	0.10	17	0.868	0.955	0.5945
244610_x_at	69.7	81.7	0.23	1.17	PPP-APP	0.10	17	0.868	0.955	0.5838
242136_x_at	347.2	407.3	0.23	1.17	PPP-PPP	0.10	17	0.868	0.955	0.5806
40016_g_at	552.4	647.8	0.23	1.17	PPP-PPP	0.10	17	0.868	0.955	0.5223
207263_x_at	93.2	109.4	0.23	1.17	PMP-APP	0.10	17	0.868	0.955	0.5169
208798_x_at	1132.4	1328.6	0.23	1.17	PPP-PPP	0.10	17	0.868	0.955	0.4943
218658_s_at	124.0	145.4	0.23	1.17	APM-PPP	0.10	17	0.868	0.955	0.4233
234988_at	183.0	214.6	0.23	1.17	PPP-PPP	0.10	17	0.868	0.955	0.4055
226245_at	2036.1	2388.6	0.23	1.17	PPP-PPP	0.10	17	0.868	0.955	0.3366
220089_at	301.9	354.2	0.23	1.17	PPP-PPP	0.10	17	0.868	0.955	0.2995
221443_x_at	107.8	126.5	0.23	1.17	AAA-APP	0.10	17	0.868	0.955	0.2777
201481_s_at	5580.4	6543.6	0.23	1.17	PPP-PPP	0.10	17	0.868	0.955	0.2087
235283_at	251.1	294.5	0.23	1.17	PPP-PPP	0.10	17	0.868	0.955	0.1778
211374_x_at	92.4	108.3	0.23	1.17	PAA-PPP	0.10	17	0.868	0.955	0.5388
1554444_s_at	366.8	429.9	0.23	1.17	AAA-APP	0.10	17	0.868	0.955	0.5330
201369_s_at	579.4	679.1	0.23	1.17	PPP-PPP	0.10	17	0.868	0.955	0.5058
214746_s_at	133.8	156.9	0.23	1.17	PPA-PPP	0.10	17	0.868	0.955	0.4951
233867_at	99.2	116.3	0.23	1.17	MPA-AAA	0.10	17	0.868	0.955	0.4921
1562103_at	109.4	128.2	0.23	1.17	PPP-PPP	0.10	17	0.868	0.955	0.4800
1570153_at	52.4	61.4	0.23	1.17	APM-AAP	0.10	17	0.868	0.955	0.4773
223258_s_at	773.4	906.2	0.23	1.17	PPP-PPP	0.10	17	0.868	0.955	0.4526
215916_at	83.1	97.4	0.23	1.17	AAA-AAP	0.10	17	0.868	0.955	0.4004
237333_at	1070.9	1255.2	0.23	1.17	PPP-PPP	0.10	17	0.868	0.955	0.3748
238156_at	406.8	476.9	0.23	1.17	PPP-PPP	0.10	17	0.868	0.955	0.3716
226455_at	296.2	347.1	0.23	1.17	PPP-PPP	0.10	17	0.868	0.955	0.3587
205451_at	67.6	79.3	0.23	1.17	AAA-APP	0.10	17	0.868	0.955	0.3532
225675_at	461.9	541.2	0.23	1.17	PPP-PPP	0.10	17	0.868	0.955	0.3421
208736_at	8962.9	10508.3	0.23	1.17	PPP-PPP	0.10	17	0.868	0.955	0.2142
212929_s_at	662.0	776.1	0.23	1.17	PPP-PPP	0.10	17	0.868	0.955	0.1900
224479_s_at	1454.6	1705.1	0.23	1.17	PPP-PPP	0.10	17	0.868	0.955	0.1709

235919_at	602.7	706.3	0.23	1.17	PPP-PPP	0.10	17	0.868	0.955	0.1693
212624_s_at	725.6	850.2	0.23	1.17	PPP-PPP	0.10	17	0.868	0.955	0.1195
219988_s_at	838.2	982.4	0.23	1.17	PPP-PPP	0.10	17	0.868	0.955	0.1046
232369_at	186.1	217.9	0.23	1.17	PPP-PPP	0.10	17	0.868	0.955	0.5756
212517_at	781.6	915.3	0.23	1.17	PPP-PPP	0.10	17	0.868	0.955	0.5316
220137_at	161.3	189.0	0.23	1.17	PPP-PPP	0.10	17	0.868	0.955	0.5318
224884_at	213.8	250.5	0.23	1.17	PPP-PPP	0.10	17	0.868	0.955	0.4417
214784_x_at	1411.1	1652.3	0.23	1.17	PPP-PPP	0.10	17	0.868	0.955	0.4091
222768_s_at	2281.4	2671.4	0.23	1.17	PPP-PPP	0.10	17	0.868	0.955	0.4079
34449_at	95.1	111.4	0.23	1.17	PPP-PPP	0.10	17	0.868	0.955	0.3916
227747_at	241.5	282.7	0.23	1.17	PPP-PPP	0.10	17	0.868	0.955	0.3830
228099_at	255.2	298.9	0.23	1.17	PPP-PPP	0.10	17	0.868	0.955	0.3815
212193_s_at	3464.9	4057.3	0.23	1.17	PPP-PPP	0.10	17	0.868	0.955	0.3682
211082_x_at	168.4	197.2	0.23	1.17	MPA-APA	0.10	17	0.868	0.955	0.3618
215399_s_at	1029.1	1205.1	0.23	1.17	PPP-PPP	0.10	17	0.868	0.955	0.3251
243648_at	465.2	544.7	0.23	1.17	PPP-PPP	0.10	17	0.868	0.955	0.2328
242607_at	132.5	155.2	0.23	1.17	PPP-PPP	0.10	17	0.868	0.955	0.2245
202250_s_at	526.5	616.4	0.23	1.17	PPP-PPP	0.10	17	0.868	0.955	0.1368
201703_s_at	478.9	560.8	0.23	1.17	PAP-PPP	0.10	17	0.868	0.955	0.1145
222093_s_at	332.1	388.8	0.23	1.17	PPP-PPP	0.10	17	0.868	0.955	0.0949
244028_at	90.7	106.2	0.23	1.17	PPP-PPP	0.10	17	0.868	0.955	0.5889
220176_at	672.2	786.8	0.23	1.17	PPP-PPP	0.10	17	0.868	0.955	0.4902
219572_at	277.8	325.2	0.23	1.17	PPP-PPP	0.10	17	0.868	0.955	0.4660
215541_s_at	845.1	989.3	0.23	1.17	PPP-PPP	0.10	17	0.868	0.955	0.3690
208368_s_at	369.5	432.5	0.23	1.17	PPP-PPP	0.10	17	0.868	0.955	0.3496
204120_s_at	1362.4	1594.6	0.23	1.17	PPP-PPP	0.10	17	0.868	0.955	0.2812
210926_at	1044.1	1222.1	0.23	1.17	PPP-PPP	0.10	17	0.868	0.955	0.2075
222518_at	1034.0	1210.0	0.23	1.17	PPP-PPP	0.10	17	0.868	0.955	0.0980
236059_at	77.2	90.3	0.23	1.17	PPM-PPP	0.10	17	0.868	0.955	0.6029
228996_at	258.3	302.0	0.23	1.17	PPP-PPP	0.10	17	0.868	0.955	0.5749
1569129_s_at	259.8	303.8	0.23	1.17	PPP-PPP	0.10	17	0.868	0.955	0.5602
239161_at	464.1	543.0	0.23	1.17	PPP-PPP	0.10	17	0.868	0.955	0.5069
1556250_at	83.8	98.0	0.23	1.17	AAA-AAP	0.10	17	0.868	0.955	0.4846
239545_at	105.8	123.7	0.23	1.17	PPP-PPP	0.10	17	0.868	0.955	0.4681
48030_i_at	578.6	676.5	0.23	1.17	PPM-PMP	0.10	17	0.868	0.955	0.4154
231960_at	149.3	174.5	0.23	1.17	PPP-PPP	0.10	17	0.868	0.955	0.4082
208740_at	700.3	819.1	0.23	1.17	PPP-PPP	0.10	17	0.868	0.955	0.3910
225473_at	558.1	652.7	0.23	1.17	PPP-PPP	0.10	17	0.868	0.955	0.3398
1564654_at	65.9	77.1	0.23	1.17	AAA-APA	0.10	17	0.868	0.955	0.3093
226415_at	248.2	290.4	0.23	1.17	MAA-PAM	0.10	17	0.868	0.955	0.2943
213327_s_at	581.6	680.5	0.23	1.17	PPP-PPP	0.10	17	0.868	0.955	0.2833
230219_at	383.3	448.3	0.23	1.17	PPP-PPP	0.10	17	0.868	0.955	0.2280

229860_x_at	2026.2	2370.6	0.23	1.17	PPP-PPP	0.10	17	0.868	0.955	0.1992
225429_at	584.4	683.4	0.23	1.17	PPP-PPP	0.10	17	0.868	0.955	0.1851
228318_s_at	523.5	612.4	0.23	1.17	PPP-PPP	0.10	17	0.868	0.955	0.1753
236722_at	169.7	198.5	0.23	1.17	AAP-MAA	0.10	17	0.868	0.955	0.1368
205684_s_at	946.3	1106.8	0.23	1.17	PPP-PPP	0.10	17	0.868	0.955	0.1188
242935_at	100.6	117.6	0.23	1.17	PPA-AAP	0.10	17	0.868	0.955	0.5944
221572_s_at	483.6	565.1	0.23	1.17	PPP-PPP	0.10	17	0.868	0.955	0.5917
235707_at	201.2	235.2	0.23	1.17	PPP-PPP	0.10	17	0.868	0.955	0.5813
242354_at	289.4	338.1	0.23	1.17	PPP-PPP	0.10	17	0.868	0.955	0.5633
236132_at	121.8	142.4	0.23	1.17	PPP-APP	0.10	17	0.868	0.955	0.4939
212960_at	151.2	176.7	0.23	1.17	MAP-AAM	0.10	17	0.868	0.955	0.4885
235750_at	264.4	309.1	0.23	1.17	AAA-PPA	0.10	17	0.868	0.955	0.4712
1562144_at	247.2	288.9	0.23	1.17	PPP-PPP	0.10	17	0.868	0.955	0.4640
212401_s_at	1014.7	1186.0	0.23	1.17	PPP-PPP	0.10	17	0.868	0.955	0.4533
1556059_s_at	541.0	632.1	0.23	1.17	PPP-PPP	0.10	17	0.868	0.955	0.4243
232679_at	42.6	49.8	0.23	1.17	PPP-PAP	0.10	17	0.868	0.955	0.4104
217905_at	637.1	744.4	0.23	1.17	PPP-PPP	0.10	17	0.868	0.955	0.4075
216253_s_at	198.6	232.1	0.23	1.17	PPP-PPP	0.10	17	0.868	0.955	0.4044
225111_s_at	209.8	245.2	0.23	1.17	AMP-APA	0.10	17	0.868	0.955	0.4029
1552486_s_at	254.0	296.8	0.23	1.17	PPP-PPP	0.10	17	0.868	0.955	0.3629
211698_at	3467.7	4053.0	0.23	1.17	PPP-PPP	0.10	17	0.868	0.955	0.2645
202994_s_at	1097.2	1282.5	0.23	1.17	PPP-PPP	0.10	17	0.868	0.955	0.2477
211454_x_at	678.5	792.9	0.23	1.17	PPP-PPP	0.10	17	0.868	0.955	0.2206
241002_at	150.3	175.5	0.22	1.17	PAA-APP	0.10	17	0.868	0.955	0.6032
239163_at	84.8	99.1	0.22	1.17	PAA-AAP	0.10	17	0.868	0.955	0.5767
219543_at	57.2	66.8	0.22	1.17	PAP-PPP	0.10	17	0.868	0.955	0.5622
230154_at	76.5	89.4	0.22	1.17	PPP-PPP	0.10	17	0.868	0.955	0.5542
1566887_x_at	932.9	1089.3	0.22	1.17	PPP-PPP	0.10	17	0.868	0.955	0.5402
231055_at	223.5	261.0	0.22	1.17	PPP-PPP	0.10	17	0.868	0.955	0.5235
244475_at	104.4	122.0	0.22	1.17	MAP-APA	0.10	17	0.868	0.955	0.5068
235442_at	127.2	148.5	0.22	1.17	APM-APP	0.10	17	0.868	0.955	0.5000
223435_s_at	453.2	529.4	0.22	1.17	PPP-PPP	0.10	17	0.868	0.955	0.4971
238795_at	256.8	299.9	0.22	1.17	PPP-PPP	0.10	17	0.868	0.955	0.4459
212558_at	256.9	300.0	0.22	1.17	PPP-PPP	0.10	17	0.868	0.955	0.4432
227937_at	336.5	393.0	0.22	1.17	PPP-PPP	0.10	17	0.868	0.955	0.4202
219117_s_at	280.8	328.1	0.22	1.17	PPP-PPP	0.10	17	0.868	0.955	0.3934
240554_at	111.1	129.8	0.22	1.17	MAA-PPP	0.10	17	0.868	0.955	0.3512
1558739_at	58.5	68.4	0.22	1.17	AAP-PPP	0.10	17	0.868	0.955	0.3463
202712_s_at	6743.1	7875.0	0.22	1.17	PPP-PPP	0.10	17	0.868	0.955	0.2279
31837_at	1000.2	1167.8	0.22	1.17	PPP-PPP	0.10	17	0.868	0.955	0.1746
223283_s_at	851.1	993.9	0.22	1.17	PPP-PPP	0.10	17	0.868	0.955	0.1691
231716_at	878.8	1026.3	0.22	1.17	PPP-PPP	0.10	17	0.868	0.955	0.1217

222510_s_at	415.3	485.0	0.22	1.17	PPP-PPP	0.10	17	0.868	0.955	0.1134
200666_s_at	4031.3	4709.8	0.22	1.17	PPP-PPP	0.10	17	0.868	0.955	0.0977
208316_s_at	298.5	348.3	0.22	1.17	PAP-MPP	0.10	17	0.868	0.955	0.5867
1564424_at	144.0	168.0	0.22	1.17	PPP-PPP	0.10	17	0.868	0.955	0.5400
225457_s_at	502.7	586.9	0.22	1.17	PPP-PPP	0.10	17	0.868	0.955	0.5372
235180_at	95.9	111.9	0.22	1.17	PPP-PPP	0.10	17	0.868	0.955	0.4538
227500_at	254.4	297.1	0.22	1.17	PMP-PPP	0.10	17	0.868	0.955	0.3873
225506_at	421.2	491.7	0.22	1.17	PPP-PPP	0.10	17	0.868	0.955	0.3242
218061_at	2637.0	3077.7	0.22	1.17	PPP-PPP	0.10	17	0.868	0.955	0.2052
223299_at	1342.4	1567.3	0.22	1.17	PPP-PPP	0.10	17	0.868	0.955	0.1029
214727_at	252.2	294.2	0.22	1.17	PPP-PPP	0.10	17	0.868	0.955	0.5962
244756_at	191.3	223.1	0.22	1.17	PPA-PAP	0.10	17	0.868	0.955	0.5598
214838_at	372.0	433.8	0.22	1.17	APP-PPP	0.10	17	0.868	0.955	0.4975
219676_at	128.5	149.9	0.22	1.17	AMP-PAP	0.10	17	0.868	0.955	0.4812
222509_s_at	164.5	191.9	0.22	1.17	PMA-APP	0.10	17	0.868	0.955	0.4704
213591_at	207.9	242.4	0.22	1.17	PPP-PPP	0.10	17	0.868	0.955	0.4677
216267_s_at	608.5	709.8	0.22	1.17	PPP-PPP	0.10	17	0.868	0.955	0.4621
230296_at	651.9	760.6	0.22	1.17	PPP-PPP	0.10	17	0.868	0.955	0.4487
232160_s_at	376.1	438.7	0.22	1.17	APP-PPP	0.10	17	0.868	0.955	0.3155
242807_at	84.1	98.0	0.22	1.17	PPP-PPP	0.10	17	0.868	0.955	0.3125
227264_at	344.2	401.4	0.22	1.17	PPP-PPP	0.10	17	0.868	0.955	0.3055
204154_at	117.5	137.0	0.22	1.17	PPP-AAP	0.10	17	0.868	0.955	0.1956
225018_at	800.8	934.2	0.22	1.17	PPP-PPP	0.10	17	0.868	0.955	0.1597
224368_s_at	839.8	979.5	0.22	1.17	PPP-PPP	0.10	17	0.868	0.955	0.1249
AFFX-r2-Ec-bioD-5_at	10635.8	12402.4	0.22	1.17	PPP-PPP	0.10	17	0.868	0.955	0.1246
221825_at	1860.0	2169.4	0.22	1.17	PPP-PPP	0.10	17	0.868	0.955	0.1019
200847_s_at	11525.8	13440.2	0.22	1.17	PPP-PPP	0.10	17	0.868	0.955	0.1007
208978_at	682.6	796.3	0.22	1.17	PPP-PPP	0.10	17	0.868	0.955	0.0953
206081_at	126.6	147.5	0.22	1.17	PAP-PAA	0.10	17	0.868	0.955	0.5339
203938_s_at	223.9	260.9	0.22	1.17	AAA-APA	0.10	17	0.868	0.955	0.5156
1553644_at	98.4	114.7	0.22	1.17	PPP-PPP	0.10	17	0.868	0.955	0.4198
244197_x_at	649.0	756.2	0.22	1.17	PPP-PPP	0.10	17	0.868	0.955	0.4050
212103_at	810.8	944.9	0.22	1.17	PPP-PPP	0.10	17	0.868	0.955	0.4042
202978_s_at	1034.1	1205.1	0.22	1.17	PPP-PPP	0.10	17	0.868	0.955	0.3639
238542_at	289.1	336.9	0.22	1.17	PPP-PPP	0.10	17	0.868	0.955	0.3408
227894_at	380.9	443.9	0.22	1.17	PPP-PPP	0.10	17	0.868	0.955	0.3164
65635_at	529.7	617.2	0.22	1.17	PPP-PPP	0.10	17	0.868	0.955	0.2893
209565_at	794.7	926.4	0.22	1.17	PPP-PPP	0.10	17	0.868	0.955	0.2669
213333_at	867.5	1011.5	0.22	1.17	PPP-PPP	0.10	17	0.868	0.955	0.1813
222883_at	882.1	1028.3	0.22	1.17	PPP-PPP	0.10	17	0.868	0.955	0.1644
220946_s_at	199.6	232.6	0.22	1.17	PPP-PPP	0.10	17	0.868	0.955	0.0957
227752_at	169.2	197.0	0.22	1.16	PPP-PPP	0.10	17	0.868	0.955	0.5649

228067_at	275.4	320.8	0.22	1.17	MPP-PAM	0.10	17	0.868	0.955	0.4786
1557050_at	187.7	218.7	0.22	1.17	PPP-PPP	0.10	17	0.868	0.955	0.4618
212403_at	573.1	667.3	0.22	1.16	PPP-PPP	0.10	17	0.868	0.955	0.4374
204908_s_at	388.3	452.4	0.22	1.17	PPP-PPP	0.10	17	0.868	0.955	0.3809
218426_s_at	403.6	469.9	0.22	1.16	PPP-PPP	0.10	17	0.868	0.955	0.3777
1569399_at	31.9	37.2	0.22	1.17	AAM-APA	0.10	17	0.868	0.955	0.3619
239792_at	151.2	176.1	0.22	1.17	PPP-PPP	0.10	17	0.868	0.955	0.3592
222582_at	831.3	968.1	0.22	1.17	PPP-PPP	0.10	17	0.868	0.955	0.3319
200653_s_at	4025.8	4688.4	0.22	1.17	PPP-PPP	0.10	17	0.868	0.955	0.3255
219055_at	304.1	354.3	0.22	1.17	PPP-PPP	0.10	17	0.868	0.955	0.2824
240128_at	191.4	222.8	0.22	1.16	APP-PPP	0.10	17	0.868	0.955	0.2770
235713_at	387.4	451.1	0.22	1.16	PPM-PPP	0.10	17	0.868	0.955	0.2128
214499_s_at	991.8	1155.2	0.22	1.17	PPP-PPP	0.10	17	0.868	0.955	0.2067
239392_s_at	1119.6	1304.4	0.22	1.17	PPP-PPP	0.10	17	0.868	0.955	0.1672
222531_s_at	1737.4	2023.1	0.22	1.16	PPP-PPP	0.10	17	0.868	0.955	0.1629
226767_s_at	1055.0	1228.5	0.22	1.17	PPP-PPP	0.10	17	0.868	0.955	0.1187
1553684_at	73.4	85.5	0.22	1.16	PPP-PPP	0.10	17	0.868	0.955	0.5999
209486_at	1337.9	1557.7	0.22	1.16	PPP-PPP	0.10	17	0.868	0.955	0.5173
1556111_s_at	266.9	310.8	0.22	1.16	PPP-PPP	0.10	17	0.868	0.955	0.4916
206104_at	89.4	104.1	0.22	1.16	PAP-APA	0.10	17	0.868	0.955	0.4694
226949_at	357.4	416.1	0.22	1.16	PPP-PPP	0.10	17	0.868	0.955	0.4199
213546_at	445.0	518.0	0.22	1.16	PPP-PPP	0.10	17	0.868	0.955	0.3150
220040_x_at	234.8	273.2	0.22	1.16	PAP-PPP	0.10	17	0.868	0.955	0.2786
214196_s_at	330.6	384.8	0.22	1.16	PPP-PPP	0.10	17	0.868	0.955	0.2669
207275_s_at	1076.1	1252.7	0.22	1.16	PPP-PPP	0.10	17	0.868	0.955	0.2411
208760_at	312.2	363.3	0.22	1.16	PPP-PPP	0.10	17	0.868	0.955	0.2315
209595_at	1026.7	1194.9	0.22	1.16	PPP-PPP	0.10	17	0.868	0.955	0.2183
219461_at	222.8	259.4	0.22	1.16	PAP-PPP	0.10	17	0.868	0.955	0.2178
225306_s_at	433.5	504.5	0.22	1.16	AMP-APA	0.10	17	0.868	0.955	0.1759
203266_s_at	708.6	824.5	0.22	1.16	PPP-PPP	0.10	17	0.868	0.955	0.1500
229062_at	163.7	190.5	0.22	1.16	AAA-APM	0.10	17	0.868	0.955	0.5775
207068_at	87.8	102.2	0.22	1.16	APA-AAP	0.10	17	0.868	0.955	0.5043
219822_at	334.7	389.2	0.22	1.16	PPP-PPP	0.10	17	0.868	0.955	0.4759
239528_at	97.2	113.0	0.22	1.16	APA-APM	0.10	17	0.868	0.955	0.4671
235001_at	98.5	114.5	0.22	1.16	PAA-AMA	0.10	17	0.868	0.955	0.4639
202834_at	92.5	107.5	0.22	1.16	PPP-PAP	0.10	17	0.868	0.955	0.4454
218554_s_at	222.1	258.2	0.22	1.16	PPP-PPP	0.10	17	0.868	0.955	0.3950
241985_at	84.5	98.2	0.22	1.16	PPP-PPP	0.10	17	0.868	0.955	0.3924
208184_s_at	215.9	251.1	0.22	1.16	PPP-PPP	0.10	17	0.868	0.955	0.3599
238977_at	116.0	134.9	0.22	1.16	PPA-PPP	0.10	17	0.868	0.955	0.3273
202260_s_at	710.9	826.6	0.22	1.16	PPP-PPP	0.10	17	0.868	0.955	0.2808
223268_at	563.6	655.5	0.22	1.16	PPP-PPP	0.10	17	0.868	0.955	0.2638

232814_x_at	573.2	666.6	0.22	1.16	PPP-PPP	0.10	17	0.868	0.955	0.2544
218052_s_at	1355.5	1576.4	0.22	1.16	PPP-PPP	0.10	17	0.868	0.955	0.2144
222663_at	178.0	207.0	0.22	1.16	PPP-PPP	0.10	17	0.868	0.955	0.1928
234979_at	221.5	257.6	0.22	1.16	PPP-PPP	0.10	17	0.868	0.955	0.1920
236467_at	134.0	155.8	0.22	1.16	PAP-PPA	0.10	17	0.868	0.955	0.1676
200606_at	14365.6	16710.2	0.22	1.16	PPP-PPP	0.10	17	0.868	0.955	0.1550
203752_s_at	7992.1	9297.6	0.22	1.16	PPP-PPP	0.10	17	0.868	0.955	0.1432
235223_at	237.4	276.1	0.22	1.16	PPP-PPP	0.10	17	0.868	0.955	0.1414
228932_at	250.6	291.2	0.22	1.16	PPP-PPP	0.10	17	0.868	0.955	0.4588
226340_x_at	610.0	709.1	0.22	1.16	PPP-PPP	0.10	17	0.868	0.955	0.4212
228032_s_at	339.3	394.5	0.22	1.16	PPP-PPP	0.10	17	0.868	0.955	0.3891
1559096_x_at	104.2	121.1	0.22	1.16	AAP-PPP	0.10	17	0.868	0.955	0.3839
227061_at	165.0	191.8	0.22	1.16	PAA-PMP	0.10	17	0.868	0.955	0.3798
202866_at	1383.1	1607.6	0.22	1.16	PPP-PPP	0.10	17	0.868	0.955	0.3297
226845_s_at	4573.8	5316.1	0.22	1.16	PPP-PPP	0.10	17	0.868	0.955	0.3049
225507_at	1009.7	1173.9	0.22	1.16	PPP-PPP	0.10	17	0.868	0.955	0.2938
204503_at	224.8	261.2	0.22	1.16	PAA-AMA	0.10	17	0.868	0.955	0.2756
233013_x_at	1074.7	1249.0	0.22	1.16	PPP-PPP	0.10	17	0.868	0.955	0.2303
236058_at	394.3	458.2	0.22	1.16	PPP-PPP	0.10	17	0.868	0.955	0.2125
1562733_at	203.6	236.6	0.22	1.16	APP-PPP	0.10	17	0.868	0.955	0.1795
226638_at	1140.1	1324.9	0.22	1.16	PPP-PPP	0.10	17	0.868	0.955	0.1211
228646_at	107.3	124.7	0.22	1.16	PAA-PPP	0.10	17	0.868	0.955	0.5285
228960_at	168.9	196.1	0.22	1.16	PPP-PPP	0.10	17	0.868	0.955	0.4415
230379_x_at	489.2	568.1	0.22	1.16	PPP-PPP	0.10	17	0.868	0.955	0.4279
210620_s_at	392.9	456.4	0.22	1.16	PPP-PPP	0.10	17	0.868	0.955	0.3569
221638_s_at	144.3	167.6	0.22	1.16	APM-AAP	0.10	17	0.868	0.955	0.3479
217745_s_at	9240.7	10731.0	0.22	1.16	PPP-PPP	0.10	17	0.868	0.955	0.3439
221768_at	535.8	622.3	0.22	1.16	PPP-PPP	0.10	17	0.868	0.955	0.3186
203918_at	474.8	551.5	0.22	1.16	MPP-PPP	0.10	17	0.868	0.955	0.3175
1554132_a_at	304.5	353.7	0.22	1.16	PPP-PPP	0.10	17	0.868	0.955	0.2652
225805_at	861.2	1000.2	0.22	1.16	PPP-PPP	0.10	17	0.868	0.955	0.2553
244153_at	331.8	385.4	0.22	1.16	PPP-PPP	0.10	17	0.868	0.955	0.2095
200695_at	1595.6	1852.7	0.22	1.16	PPP-PPP	0.10	17	0.868	0.955	0.1865
224468_s_at	3067.4	3562.4	0.22	1.16	PPP-PPP	0.10	17	0.868	0.955	0.1797
220079_s_at	1231.6	1430.8	0.22	1.16	PPP-PPP	0.10	17	0.868	0.955	0.1158
213304_at	640.6	744.1	0.22	1.16	PPP-PPP	0.10	17	0.868	0.955	0.0958
218552_at	818.3	949.6	0.22	1.16	PPP-PPP	0.10	17	0.868	0.955	0.5858
204294_at	141.5	164.3	0.22	1.16	AMA-PAP	0.10	17	0.868	0.955	0.5857
1559675_at	97.2	112.9	0.22	1.16	PPP-PAP	0.10	17	0.868	0.955	0.5754
242903_at	67.5	78.4	0.22	1.16	PPP-PAP	0.10	17	0.868	0.955	0.5108
215985_at	80.1	93.0	0.22	1.16	PAA-PPA	0.10	17	0.868	0.955	0.5072
230413_s_at	503.5	584.4	0.22	1.16	PPP-PPP	0.10	17	0.868	0.955	0.4995

226216_at	193.3	224.4	0.22	1.16	PPP-PPP	0.10	17	0.868	0.955	0.4896
223622_s_at	138.7	161.0	0.22	1.16	AMP-APP	0.10	17	0.868	0.955	0.4529
209372_x_at	992.9	1152.8	0.22	1.16	PPP-PPP	0.10	17	0.868	0.955	0.4197
222413_s_at	850.0	986.5	0.22	1.16	PPP-PPP	0.10	17	0.868	0.955	0.4036
212916_at	253.0	293.7	0.22	1.16	PPP-PPP	0.10	17	0.868	0.955	0.3798
202725_at	470.0	545.5	0.22	1.16	PPP-AMP	0.10	17	0.868	0.955	0.3611
203467_at	474.7	550.9	0.22	1.16	PPP-PPP	0.10	17	0.868	0.955	0.3363
212512_s_at	1692.1	1964.4	0.22	1.16	PPP-PPP	0.10	17	0.868	0.955	0.2986
219520_s_at	640.6	743.5	0.22	1.16	PPP-PPP	0.10	17	0.868	0.955	0.2750
218521_s_at	364.6	423.3	0.22	1.16	PPP-PPP	0.10	17	0.868	0.955	0.2523
217124_at	276.8	321.3	0.22	1.16	PPP-PPP	0.10	17	0.868	0.955	0.2423
227156_at	373.3	433.2	0.22	1.16	PPP-PPP	0.10	17	0.868	0.955	0.2399
225410_at	993.6	1152.9	0.22	1.16	PPP-PPA	0.10	17	0.868	0.955	0.2001
211994_at	2216.5	2572.3	0.22	1.16	PPP-PPP	0.10	17	0.868	0.955	0.1917
1553955_at	492.2	571.2	0.22	1.16	PPP-PPP	0.10	17	0.868	0.955	0.1646
221519_at	679.9	789.4	0.22	1.16	PPP-PPP	0.10	17	0.868	0.955	0.1463
213980_s_at	2404.6	2790.6	0.22	1.16	PPP-PPP	0.10	17	0.868	0.955	0.1207
204651_at	414.1	480.5	0.22	1.16	PPP-PPP	0.10	17	0.868	0.955	0.1160
203711_s_at	713.2	828.1	0.22	1.16	PPP-PPP	0.10	17	0.868	0.955	0.1141
214690_at	476.0	552.7	0.22	1.16	PPP-PPP	0.10	17	0.868	0.955	0.0911
243964_at	54.3	62.9	0.21	1.16	PAP-PAP	0.10	17	0.868	0.955	0.5929
213481_at	93.6	108.6	0.21	1.16	PPA-PPP	0.10	17	0.868	0.955	0.5238
222280_at	570.1	661.5	0.21	1.16	PMP-PPP	0.10	17	0.868	0.955	0.4800
201822_at	153.6	178.1	0.21	1.16	APM-PAP	0.10	17	0.868	0.955	0.4420
41858_at	520.3	603.4	0.21	1.16	PPP-PPP	0.10	17	0.868	0.955	0.4410
1555021_a_at	91.6	106.3	0.21	1.16	PPA-PAP	0.10	17	0.868	0.955	0.3889
219472_at	138.5	160.6	0.21	1.16	PPP-PPP	0.10	17	0.868	0.955	0.3874
222475_at	244.6	283.7	0.21	1.16	MAP-PPP	0.10	17	0.868	0.955	0.3419
217588_at	83.7	97.1	0.21	1.16	AAA-APP	0.10	17	0.868	0.955	0.3178
212407_at	1065.2	1235.7	0.21	1.16	PPP-PPP	0.10	17	0.868	0.955	0.2337
225041_at	1472.2	1707.5	0.21	1.16	PPP-PPP	0.10	17	0.868	0.955	0.1783
1558953_s_at	1540.3	1786.8	0.21	1.16	PPP-PPP	0.10	17	0.868	0.955	0.1371
239223_s_at	97.2	112.8	0.21	1.16	AAA-APP	0.10	17	0.868	0.955	0.1089
217855_x_at	2134.7	2476.4	0.21	1.16	PPP-PPP	0.10	17	0.868	0.955	0.0930
233436_at	81.0	93.9	0.21	1.16	PPP-PPP	0.10	17	0.868	0.955	0.5974
239466_at	145.1	168.3	0.21	1.16	PPP-PPP	0.10	17	0.868	0.955	0.5838
218896_s_at	381.7	442.5	0.21	1.16	PPP-PPP	0.10	17	0.868	0.955	0.5694
231011_at	146.1	169.3	0.21	1.16	AAP-PPP	0.10	17	0.868	0.955	0.5493
227983_at	683.6	792.6	0.21	1.16	PPP-PPP	0.10	17	0.868	0.955	0.5452
228515_at	318.5	369.2	0.21	1.16	AAP-AAP	0.10	17	0.868	0.955	0.5278
218423_x_at	1028.2	1191.8	0.21	1.16	PPP-PPP	0.10	17	0.868	0.955	0.4612
202487_s_at	9363.2	10853.9	0.21	1.16	PPP-PPP	0.10	17	0.868	0.955	0.3429

222024_s_at	411.3	476.8	0.21	1.16	PPP-PPP	0.10	17	0.868	0.955	0.3389
223443_s_at	418.0	484.4	0.21	1.16	PPP-PPP	0.10	17	0.868	0.955	0.3347
210633_x_at	4991.0	5783.9	0.21	1.16	PPP-PPP	0.10	17	0.868	0.955	0.2683
201937_s_at	819.5	950.1	0.21	1.16	PPP-PPP	0.10	17	0.868	0.955	0.2219
213530_at	582.7	675.4	0.21	1.16	PPP-PPP	0.10	17	0.868	0.955	0.1549
212934_at	2687.0	3114.4	0.21	1.16	PPP-PPP	0.10	17	0.868	0.955	0.0973
212632_at	725.6	841.1	0.21	1.16	PPP-PPP	0.10	17	0.868	0.955	0.0841
236235_at	297.9	345.0	0.21	1.16	PPP-PPP	0.10	17	0.868	0.955	0.5892
207779_at	60.7	70.3	0.21	1.16	APA-AAP	0.10	17	0.868	0.955	0.5568
213263_s_at	403.9	467.7	0.21	1.16	PPP-PPP	0.10	17	0.868	0.955	0.5543
202507_s_at	131.4	152.2	0.21	1.16	MPA-AAA	0.10	17	0.868	0.955	0.5061
203597_s_at	509.0	589.8	0.21	1.16	PPP-PPP	0.10	17	0.868	0.955	0.4330
1554894_a_at	466.3	540.3	0.21	1.16	PPP-PPP	0.10	17	0.868	0.955	0.4241
232615_at	530.1	613.9	0.21	1.16	PPP-PPP	0.10	17	0.868	0.955	0.3846
228559_at	1400.9	1622.7	0.21	1.16	PPP-PPP	0.10	17	0.868	0.955	0.3789
204026_s_at	5032.2	5829.7	0.21	1.16	PPP-PPP	0.10	17	0.868	0.955	0.2978
228793_at	260.7	301.9	0.21	1.16	PPP-PPP	0.10	17	0.868	0.955	0.2816
235282_at	193.3	224.0	0.21	1.16	PPM-MPP	0.10	17	0.868	0.955	0.2683
224046_s_at	227.2	263.1	0.21	1.16	PPP-PAP	0.10	17	0.868	0.955	0.2041
201754_at	24592.1	28484.4	0.21	1.16	PPP-PPP	0.10	17	0.868	0.955	0.1113
209703_x_at	1718.6	1991.1	0.21	1.16	PPP-PPP	0.10	17	0.868	0.955	0.1109
201327_s_at	12464.7	14438.9	0.21	1.16	PPP-PPP	0.10	17	0.868	0.955	0.0974
240247_at	213.7	247.4	0.21	1.16	PPP-PPP	0.10	17	0.868	0.955	0.5803
235610_at	271.3	313.9	0.21	1.16	PPP-PPP	0.10	17	0.868	0.955	0.5748
229675_at	56.3	65.1	0.21	1.16	APA-AAP	0.10	17	0.868	0.955	0.5595
212630_at	357.8	414.2	0.21	1.16	PMP-PPP	0.10	17	0.868	0.955	0.5523
1560942_at	71.0	82.2	0.21	1.16	AAP-AAA	0.10	17	0.868	0.955	0.5285
203392_s_at	2189.4	2533.5	0.21	1.16	PPP-PPP	0.10	17	0.868	0.955	0.5222
1554445_at	236.3	273.4	0.21	1.16	PPP-PPP	0.10	17	0.868	0.955	0.5022
212399_s_at	890.0	1030.2	0.21	1.16	PPP-PPP	0.10	17	0.868	0.955	0.4447
225628_s_at	135.5	156.9	0.21	1.16	PAP-APA	0.10	17	0.868	0.955	0.3646
215009_s_at	1410.2	1631.8	0.21	1.16	PPP-PPP	0.10	17	0.868	0.955	0.3419
226735_at	194.8	225.5	0.21	1.16	AAA-AAP	0.10	17	0.868	0.955	0.3211
242616_at	128.3	148.5	0.21	1.16	PPP-PPP	0.10	17	0.868	0.955	0.2993
221509_at	4218.1	4884.0	0.21	1.16	PPP-PPP	0.10	17	0.868	0.955	0.2421
243000_at	187.4	216.9	0.21	1.16	PPP-PPP	0.10	17	0.868	0.955	0.2312
226741_at	1011.0	1169.9	0.21	1.16	PPP-PPP	0.10	17	0.868	0.955	0.2194
200898_s_at	609.7	705.8	0.21	1.16	PPP-PPP	0.10	17	0.868	0.955	0.2099
228653_at	822.0	951.7	0.21	1.16	PPP-PPP	0.10	17	0.868	0.955	0.1814
223439_at	815.9	944.8	0.21	1.16	PPP-PPP	0.10	17	0.868	0.955	0.1734
209065_at	1206.9	1396.9	0.21	1.16	PPP-PPP	0.10	17	0.868	0.955	0.1616
222137_at	103.0	119.1	0.21	1.16	APM-AAP	0.10	17	0.868	0.955	0.5794

228972_at	378.3	437.6	0.21	1.16	PPP-PPP	0.10	17	0.868	0.955	0.5685
236408_at	63.7	73.8	0.21	1.16	PAA-AAM	0.10	17	0.868	0.955	0.5563
213670_x_at	628.1	726.5	0.21	1.16	PPP-PPP	0.10	17	0.868	0.955	0.5386
227252_at	421.0	486.8	0.21	1.16	MAM-PAA	0.10	17	0.868	0.955	0.5326
227941_at	450.7	521.2	0.21	1.16	PPP-PPP	0.10	17	0.868	0.955	0.5197
216641_s_at	1624.6	1879.7	0.21	1.16	PPP-PPP	0.10	17	0.868	0.955	0.4897
218245_at	525.5	607.9	0.21	1.16	PPP-PPP	0.10	17	0.868	0.955	0.4133
1316_at	118.0	136.5	0.21	1.16	PPP-PPP	0.10	17	0.868	0.955	0.3894
223063_at	871.1	1007.5	0.21	1.16	PPP-PPP	0.10	17	0.868	0.955	0.3851
222438_at	959.2	1109.6	0.21	1.16	PPP-PPP	0.10	17	0.868	0.955	0.3487
235003_at	1049.5	1214.0	0.21	1.16	PPP-PPP	0.10	17	0.868	0.955	0.3444
202338_at	929.3	1074.7	0.21	1.16	PPP-PPP	0.10	17	0.868	0.955	0.3077
236526_x_at	211.4	244.6	0.21	1.16	PPP-PPP	0.10	17	0.868	0.955	0.2851
235566_at	315.3	364.6	0.21	1.16	PPP-PPP	0.10	17	0.868	0.955	0.1534
212456_at	1100.0	1272.1	0.21	1.16	PPP-PPP	0.10	17	0.868	0.955	0.1352
241065_x_at	130.1	150.4	0.21	1.16	PAP-PPP	0.10	17	0.868	0.955	0.5762
1558702_at	98.9	114.3	0.21	1.16	PPP-PPP	0.10	17	0.868	0.955	0.5518
1554455_at	104.8	121.1	0.21	1.16	PPP-PPP	0.10	17	0.868	0.955	0.5351
221272_s_at	116.8	135.1	0.21	1.16	AAP-AAP	0.10	17	0.868	0.955	0.4846
208989_s_at	208.0	240.4	0.21	1.16	APP-PPP	0.10	17	0.868	0.955	0.4762
242116_x_at	87.3	100.9	0.21	1.16	AAP-APP	0.10	17	0.868	0.955	0.4625
213208_at	242.1	279.9	0.21	1.16	PPP-PPP	0.10	17	0.868	0.955	0.3951
224723_x_at	1916.6	2215.4	0.21	1.16	PPP-PPP	0.10	17	0.868	0.955	0.3878
202534_x_at	3158.4	3650.4	0.21	1.16	PPP-PPP	0.10	17	0.868	0.955	0.2917
230057_at	286.9	331.7	0.21	1.16	APP-AMP	0.10	17	0.868	0.955	0.2897
223321_s_at	333.0	384.9	0.21	1.16	APA-APP	0.10	17	0.868	0.955	0.2803
209778_at	280.1	323.9	0.21	1.16	PPP-PPP	0.10	17	0.868	0.955	0.2609
217854_s_at	7365.8	8515.0	0.21	1.16	PPP-PPP	0.10	17	0.868	0.955	0.1940
214221_at	352.7	407.7	0.21	1.16	PPP-PPP	0.10	17	0.868	0.955	0.1751
212254_s_at	2237.6	2586.6	0.21	1.16	PPP-PPP	0.10	17	0.868	0.955	0.1133
244749_at	97.5	112.7	0.21	1.16	PAP-PPP	0.10	17	0.868	0.955	0.5779
231013_at	185.7	214.5	0.21	1.16	PPP-PPP	0.10	17	0.868	0.955	0.5743
238420_at	244.7	282.6	0.21	1.16	PPP-PPP	0.10	17	0.868	0.955	0.5605
242337_at	104.5	120.7	0.21	1.16	PAP-PAP	0.10	17	0.868	0.955	0.5274
222636_at	256.3	296.2	0.21	1.16	APM-AAP	0.10	17	0.868	0.955	0.4746
209384_at	2110.3	2438.2	0.21	1.16	PPP-PPP	0.10	17	0.868	0.955	0.4331
213998_s_at	228.9	264.4	0.21	1.16	PAP-PPP	0.10	17	0.868	0.955	0.4174
215529_x_at	565.2	653.0	0.21	1.16	PPP-MPP	0.10	17	0.868	0.955	0.3315
207891_s_at	307.6	355.2	0.21	1.16	PPP-PPP	0.10	17	0.868	0.955	0.3009
1553348_a_at	225.3	260.2	0.21	1.16	PPP-PPP	0.10	17	0.868	0.955	0.2756
233999_s_at	265.0	306.1	0.21	1.16	PPP-PPP	0.10	17	0.868	0.955	0.2694
219037_at	3380.8	3904.4	0.21	1.16	PPP-PPP	0.10	17	0.868	0.955	0.2635

212418_at	2524.6	2915.8	0.21	1.16	PPP-PPP	0.10	17	0.868	0.955	0.2193
220712_at	159.7	184.4	0.21	1.16	AAP-PPA	0.10	17	0.868	0.955	0.2149
228122_at	361.7	417.9	0.21	1.16	PPP-PPP	0.10	17	0.868	0.955	0.2017
238701_x_at	2713.1	3134.1	0.21	1.16	PPP-PPP	0.10	17	0.868	0.955	0.1956
229900_at	1257.2	1452.5	0.21	1.16	PPP-PPP	0.10	17	0.868	0.955	0.1826
215190_at	155.3	179.4	0.21	1.16	PPP-PPP	0.10	17	0.868	0.955	0.1404
235792_x_at	972.8	1123.9	0.21	1.16	PPP-PPP	0.10	17	0.868	0.955	0.1352
201419_at	638.6	737.6	0.21	1.16	PPP-PPP	0.10	17	0.868	0.955	0.1250
1557512_at	80.4	92.9	0.21	1.16	PPA-PPP	0.10	17	0.868	0.955	0.5579
210482_x_at	228.6	263.9	0.21	1.15	AAP-AAP	0.10	17	0.868	0.955	0.5219
212948_at	359.0	414.5	0.21	1.16	PPP-PPP	0.10	17	0.868	0.955	0.4713
226523_at	167.2	193.1	0.21	1.16	AAM-APM	0.10	17	0.868	0.955	0.4331
207620_s_at	162.2	187.3	0.21	1.15	PPP-PPP	0.10	17	0.868	0.955	0.4254
204527_at	451.1	520.7	0.21	1.15	PPP-PPP	0.10	17	0.868	0.955	0.3720
208927_at	763.6	881.6	0.21	1.16	PPP-PPP	0.10	17	0.868	0.955	0.3590
209326_at	770.9	889.6	0.21	1.15	PPP-PPP	0.10	17	0.868	0.955	0.3482
1555390_at	175.3	202.2	0.21	1.15	PPP-PPP	0.10	17	0.868	0.955	0.2941
233124_s_at	2017.5	2328.3	0.21	1.15	PPP-PPP	0.10	17	0.868	0.955	0.2155
225080_at	1295.2	1494.6	0.21	1.15	PPP-PPP	0.10	17	0.868	0.955	0.1472
241464_s_at	141.6	163.3	0.21	1.15	PAM-PAP	0.10	17	0.868	0.955	0.5384
208112_x_at	812.0	936.8	0.21	1.15	PPP-PPP	0.10	17	0.868	0.955	0.5141
202694_at	213.0	245.6	0.21	1.15	PAP-PPP	0.10	17	0.868	0.955	0.4659
212171_x_at	456.1	526.0	0.21	1.15	PPP-PPP	0.10	17	0.868	0.955	0.4575
1569791_at	66.0	76.1	0.21	1.15	PPM-PPP	0.10	17	0.868	0.955	0.4532
222784_at	616.0	710.6	0.21	1.15	PPP-PPP	0.10	17	0.868	0.955	0.4311
236050_at	146.8	169.4	0.21	1.15	APA-PPP	0.10	17	0.868	0.955	0.4236
222109_at	133.7	154.3	0.21	1.15	PPP-PAP	0.10	17	0.868	0.955	0.4077
230350_at	522.5	602.8	0.21	1.15	PPP-PPP	0.10	17	0.868	0.955	0.3859
212861_at	659.8	761.0	0.21	1.15	PPP-PPP	0.10	17	0.868	0.955	0.3617
217221_x_at	796.4	918.9	0.21	1.15	PPP-PPP	0.10	17	0.868	0.955	0.3414
232412_at	268.5	309.7	0.21	1.15	PPP-PPP	0.10	17	0.868	0.955	0.3380
214310_s_at	251.0	289.4	0.21	1.15	PPP-PPP	0.10	17	0.868	0.955	0.2786
221596_s_at	486.2	561.0	0.21	1.15	PPP-PPP	0.10	17	0.868	0.955	0.2397
219571_s_at	874.4	1008.7	0.21	1.15	PPP-PPP	0.10	17	0.868	0.955	0.1184
206829_x_at	768.7	886.5	0.21	1.15	PPP-PPP	0.10	17	0.868	0.955	0.1142
211285_s_at	2159.4	2490.8	0.21	1.15	PPP-PPP	0.10	17	0.868	0.955	0.0928
232287_at	179.5	206.9	0.21	1.15	MPP-PPP	0.10	17	0.868	0.955	0.5615
229089_at	104.7	120.7	0.21	1.15	AAP-PPP	0.10	17	0.868	0.955	0.5561
225346_at	311.1	358.6	0.21	1.15	PPP-PPP	0.10	17	0.868	0.955	0.5261
218275_at	263.4	303.5	0.21	1.15	PPP-PPP	0.10	17	0.868	0.955	0.4941
209997_x_at	270.2	311.5	0.21	1.15	PPP-PPP	0.10	17	0.868	0.955	0.4797
202602_s_at	1788.7	2061.3	0.21	1.15	PPP-PPP	0.10	17	0.868	0.955	0.4594

201905_s_at	760.2	876.1	0.21	1.15	PPP-PPP	0.10	17	0.868	0.955	0.4522
213460_x_at	941.1	1084.5	0.21	1.15	PPP-PPP	0.10	17	0.868	0.955	0.4515
218524_at	298.6	344.1	0.21	1.15	PPP-PPP	0.10	17	0.868	0.955	0.4356
202341_s_at	257.0	296.4	0.21	1.15	PAP-PPP	0.10	17	0.868	0.955	0.4238
211137_s_at	416.7	480.5	0.21	1.15	PPP-PPP	0.10	17	0.868	0.955	0.3641
205992_s_at	475.4	547.8	0.21	1.15	PPP-PPP	0.10	17	0.868	0.955	0.3248
242913_at	82.3	94.8	0.21	1.15	AAM-PAP	0.10	17	0.868	0.955	0.3157
226383_at	783.2	902.5	0.21	1.15	PPP-PPP	0.10	17	0.868	0.955	0.2918
234953_x_at	138.6	159.8	0.21	1.15	APM-AAA	0.10	17	0.868	0.955	0.2914
217937_s_at	219.1	252.7	0.21	1.15	PPP-PPP	0.10	17	0.868	0.955	0.2914
226648_at	673.5	776.0	0.21	1.15	PPP-PPP	0.10	17	0.868	0.955	0.1729
212808_at	407.6	469.9	0.21	1.15	PPP-PPP	0.10	17	0.868	0.955	0.1728
1554149_at	2549.0	2938.0	0.21	1.15	PPP-PPP	0.10	17	0.868	0.955	0.1392
202592_at	1371.4	1580.8	0.21	1.15	PPP-PPP	0.10	17	0.868	0.955	0.1128
207883_s_at	78.1	90.0	0.20	1.15	AAA-MAP	0.10	17	0.868	0.955	0.5676
1555742_at	1009.7	1162.9	0.20	1.15	PPP-PPP	0.10	17	0.868	0.955	0.5214
61297_at	102.3	117.9	0.20	1.15	PMA-APP	0.10	17	0.868	0.955	0.4990
214337_at	76.4	88.1	0.20	1.15	PAP-PAP	0.10	17	0.868	0.955	0.4008
227047_x_at	672.2	774.2	0.20	1.15	PPP-PPP	0.10	17	0.868	0.955	0.3956
213313_at	1228.7	1415.5	0.20	1.15	PPP-PPP	0.10	17	0.868	0.955	0.3899
225481_at	2840.4	3271.6	0.20	1.15	PPP-PPP	0.10	17	0.868	0.955	0.3852
225255_at	214.2	246.8	0.20	1.15	MPP-PPP	0.10	17	0.868	0.955	0.3646
221767_x_at	5185.8	5972.3	0.20	1.15	PPP-PPP	0.10	17	0.868	0.955	0.3267
224669_at	522.9	602.5	0.20	1.15	PPP-PPP	0.10	17	0.868	0.955	0.3049
219345_at	278.5	320.8	0.20	1.15	PPP-PMP	0.10	17	0.868	0.955	0.2899
225657_at	1020.9	1175.9	0.20	1.15	PPP-PPP	0.10	17	0.868	0.955	0.2792
242191_at	738.7	851.1	0.20	1.15	PPP-PPP	0.10	17	0.868	0.955	0.2465
39548_at	98.5	113.4	0.20	1.15	PPP-PPP	0.10	17	0.868	0.955	0.1886
213376_at	753.8	868.2	0.20	1.15	PPP-PPP	0.10	17	0.868	0.955	0.1723
203333_at	1504.1	1733.1	0.20	1.15	PPP-PPP	0.10	17	0.868	0.955	0.1567
220602_s_at	190.7	219.7	0.20	1.15	MAM-APP	0.10	17	0.868	0.955	0.1412
214718_at	1396.4	1608.7	0.20	1.15	PPP-PPP	0.10	17	0.868	0.955	0.0972
242727_at	63.4	73.0	0.20	1.15	PAP-APM	0.10	17	0.868	0.955	0.5235
231102_at	237.7	273.5	0.20	1.15	PPP-PPP	0.10	17	0.868	0.955	0.5121
221088_s_at	84.2	96.8	0.20	1.15	PPP-PPP	0.10	17	0.868	0.955	0.4819
225348_at	538.4	619.7	0.20	1.15	PPP-PPP	0.10	17	0.868	0.955	0.4616
244592_at	69.3	79.8	0.20	1.15	PAP-APP	0.10	17	0.868	0.955	0.4609
215617_at	177.4	204.1	0.20	1.15	PPP-PPP	0.10	17	0.868	0.955	0.3621
228717_at	92.2	106.1	0.20	1.15	MAP-PPA	0.10	17	0.868	0.955	0.3499
203802_x_at	1737.4	1999.9	0.20	1.15	PPP-PPP	0.10	17	0.868	0.955	0.2850
230569_at	204.3	235.1	0.20	1.15	PPP-PPP	0.10	17	0.868	0.955	0.2237
225572_at	893.4	1028.5	0.20	1.15	PPP-PPP	0.10	17	0.868	0.955	0.2086

202126_at	3074.9	3540.1	0.20	1.15	PPP-PPP	0.10	17	0.868	0.955	0.1691
209838_at	344.0	396.1	0.20	1.15	PPP-PPP	0.10	17	0.868	0.955	0.1552
227385_at	405.9	467.2	0.20	1.15	PPP-PPP	0.10	17	0.868	0.955	0.1328
232902_s_at	785.5	904.5	0.20	1.15	PPP-PPP	0.10	17	0.868	0.955	0.1064
220917_s_at	432.2	497.4	0.20	1.15	PPP-PPP	0.10	17	0.868	0.955	0.0903
242668_x_at	357.7	411.4	0.20	1.15	PPP-PPP	0.10	17	0.868	0.955	0.5669
209502_s_at	306.0	351.9	0.20	1.15	PPP-PPP	0.10	17	0.868	0.955	0.5178
218400_at	725.4	834.6	0.20	1.15	PPP-PPP	0.10	17	0.868	0.955	0.5084
203888_at	909.2	1045.8	0.20	1.15	PPP-PPP	0.10	17	0.868	0.955	0.4836
202912_at	1205.4	1386.5	0.20	1.15	PPP-PPP	0.10	17	0.868	0.955	0.4199
243552_at	126.0	144.9	0.20	1.15	PPP-PPP	0.10	17	0.868	0.955	0.4176
218772_x_at	747.7	859.8	0.20	1.15	PPP-PPP	0.10	17	0.868	0.955	0.4032
226144_at	172.4	198.3	0.20	1.15	AAA-PAA	0.10	17	0.868	0.955	0.3919
1553280_at	102.5	117.9	0.20	1.15	PPP-PPA	0.10	17	0.868	0.955	0.3033
218128_at	607.2	698.5	0.20	1.15	PPP-PPP	0.10	17	0.868	0.955	0.3008
217370_x_at	999.2	1149.5	0.20	1.15	PPP-PPP	0.10	17	0.868	0.955	0.2679
1554102_a_at	348.5	400.9	0.20	1.15	PPP-PPP	0.10	17	0.868	0.955	0.2598
226429_at	411.3	473.0	0.20	1.15	PPP-PPP	0.10	17	0.868	0.955	0.2588
45297_at	2279.7	2623.2	0.20	1.15	PPP-PPP	0.10	17	0.868	0.955	0.2246
203213_at	10614.9	12214.0	0.20	1.15	PPP-PPP	0.10	17	0.868	0.955	0.2083
226850_at	1293.5	1487.5	0.20	1.15	PPP-PPP	0.10	17	0.868	0.955	0.1833
226839_at	739.7	851.1	0.20	1.15	PPP-PPP	0.10	17	0.868	0.955	0.1469
AFFX-r2-Ec-bioD-3_at	11596.5	13339.7	0.20	1.15	PPP-PPP	0.10	17	0.868	0.955	0.0898
224152_s_at	151.9	174.6	0.20	1.15	AAP-PPM	0.10	17	0.868	0.955	0.5928
212767_at	389.0	447.1	0.20	1.15	MAA-PAP	0.10	17	0.868	0.955	0.5746
205110_s_at	56.6	65.1	0.20	1.15	PAP-PPP	0.10	17	0.868	0.955	0.5749
241701_at	113.2	130.1	0.20	1.15	PMP-APP	0.10	17	0.868	0.955	0.5677
231087_at	63.6	73.1	0.20	1.15	PPP-PPP	0.10	17	0.868	0.955	0.5574
224614_at	426.0	489.6	0.20	1.15	PPP-PPP	0.10	17	0.868	0.955	0.5327
242477_at	102.1	117.4	0.20	1.15	AMP-PAP	0.10	17	0.868	0.955	0.5210
228190_at	434.9	499.9	0.20	1.15	PPP-PPP	0.10	17	0.868	0.955	0.4668
227347_x_at	573.2	658.9	0.20	1.15	PPP-PPP	0.10	17	0.868	0.955	0.4108
226551_at	361.2	415.3	0.20	1.15	PPP-PPP	0.10	17	0.868	0.955	0.4027
213607_x_at	524.4	602.9	0.20	1.15	PPP-PPP	0.10	17	0.868	0.955	0.3748
224177_s_at	685.6	788.3	0.20	1.15	PPP-PPP	0.10	17	0.868	0.955	0.3506
40273_at	311.9	358.5	0.20	1.15	AAA-MPP	0.10	17	0.868	0.955	0.3286
218155_x_at	1289.1	1481.4	0.20	1.15	PPP-PPP	0.10	17	0.868	0.955	0.2568
214962_s_at	404.6	465.0	0.20	1.15	PPP-PPP	0.10	17	0.868	0.955	0.2532
228832_at	192.6	221.5	0.20	1.15	PPP-PPP	0.10	17	0.868	0.955	0.2037
204560_at	1988.8	2285.9	0.20	1.15	PPP-PPP	0.10	17	0.868	0.955	0.1648
208789_at	2587.1	2973.5	0.20	1.15	PPP-PPP	0.10	17	0.868	0.955	0.1477
53987_at	226.5	260.2	0.20	1.15	PPP-PPP	0.10	17	0.868	0.955	0.5950

208039_at	78.3	90.0	0.20	1.15	PAP-PAP	0.10	17	0.868	0.955	0.5896
242876_at	92.4	106.1	0.20	1.15	PAA-AAP	0.10	17	0.868	0.955	0.5792
214807_at	203.8	234.1	0.20	1.15	PPP-PPP	0.10	17	0.868	0.955	0.5548
241921_x_at	142.1	163.2	0.20	1.15	AAA-APA	0.10	17	0.868	0.955	0.5514
225254_at	210.7	242.0	0.20	1.15	AAA-PAA	0.10	17	0.868	0.955	0.5022
225767_at	195.1	224.0	0.20	1.15	PPP-PPP	0.10	17	0.868	0.955	0.4896
221245_s_at	328.3	377.2	0.20	1.15	PPP-PPP	0.10	17	0.868	0.955	0.4849
241385_at	77.9	89.5	0.20	1.15	PPP-PPP	0.10	17	0.868	0.955	0.4823
236297_at	137.2	157.6	0.20	1.15	PPP-PPP	0.10	17	0.868	0.955	0.3732
241720_at	72.2	82.9	0.20	1.15	PPP-PPP	0.10	17	0.868	0.955	0.3736
223661_at	245.8	282.4	0.20	1.15	PPP-PPP	0.10	17	0.868	0.955	0.3540
210057_at	215.6	247.6	0.20	1.15	PPP-PPP	0.10	17	0.868	0.955	0.3062
220223_at	192.9	221.6	0.20	1.15	PPP-PAP	0.10	17	0.868	0.955	0.2944
206936_x_at	450.5	517.6	0.20	1.15	PPP-PPP	0.10	17	0.868	0.955	0.2600
223208_at	636.0	730.5	0.20	1.15	PPP-PPP	0.10	17	0.868	0.955	0.2400
222816_s_at	457.2	525.4	0.20	1.15	PPP-PPP	0.10	17	0.868	0.955	0.2254
209418_s_at	1141.9	1311.8	0.20	1.15	PPP-PPP	0.10	17	0.868	0.955	0.2128
221542_s_at	397.5	456.6	0.20	1.15	PPP-PPP	0.10	17	0.868	0.955	0.1824
235037_at	618.1	709.9	0.20	1.15	PPP-PPP	0.10	17	0.868	0.955	0.1742
224801_at	1198.5	1376.9	0.20	1.15	PPP-PPP	0.10	17	0.868	0.955	0.1724
212956_at	856.8	984.0	0.20	1.15	PPP-PPP	0.10	17	0.868	0.955	0.1314
212469_at	630.1	723.6	0.20	1.15	PPP-PAP	0.10	17	0.868	0.955	0.1172
232395_x_at	70.6	81.1	0.20	1.15	MPP-PPA	0.10	17	0.868	0.955	0.5860
1556809_a_at	168.4	193.3	0.20	1.15	MAM-AAP	0.10	17	0.868	0.955	0.5781
219923_at	134.5	154.3	0.20	1.15	PPP-APP	0.10	17	0.868	0.955	0.5091
219048_at	620.9	712.6	0.20	1.15	PPP-PPP	0.10	17	0.868	0.955	0.4915
213384_x_at	472.4	542.4	0.20	1.15	PPP-PPP	0.10	17	0.868	0.955	0.4168
203273_s_at	431.8	495.8	0.20	1.15	PPP-PPP	0.10	17	0.868	0.955	0.4022
207551_s_at	615.4	706.3	0.20	1.15	PPP-PPP	0.10	17	0.868	0.955	0.3908
218652_s_at	1654.2	1899.4	0.20	1.15	PPP-PPP	0.10	17	0.868	0.955	0.3590
235433_at	549.0	630.5	0.20	1.15	PPP-PPP	0.10	17	0.868	0.955	0.3456
204281_at	1155.3	1326.2	0.20	1.15	PPP-PPP	0.10	17	0.868	0.955	0.3257
212194_s_at	1441.0	1654.7	0.20	1.15	PPP-PPP	0.10	17	0.868	0.955	0.3103
1554410_a_at	865.7	993.4	0.20	1.15	PPP-PPP	0.10	17	0.868	0.955	0.2733
235780_at	61.9	71.0	0.20	1.15	AAP-PMA	0.10	17	0.868	0.955	0.2622
214202_at	506.0	580.8	0.20	1.15	PPP-PPP	0.10	17	0.868	0.955	0.1815
219762_s_at	23352.6	26804.2	0.20	1.15	PPP-PPP	0.10	17	0.868	0.955	0.1306
201484_at	753.0	864.6	0.20	1.15	PPP-PPP	0.10	17	0.868	0.955	0.1068
208716_s_at	7105.5	8155.6	0.20	1.15	PPP-PPP	0.10	17	0.868	0.955	0.0930
212321_at	734.1	843.0	0.20	1.15	PPP-PPP	0.10	17	0.868	0.955	0.0717
1563389_at	54.4	62.4	0.20	1.15	AAP-AMA	0.10	17	0.868	0.955	0.5869
233089_at	90.5	103.8	0.20	1.15	AAA-AAP	0.10	17	0.868	0.955	0.5110

236545_at	115.5	132.6	0.20	1.15	PPP-PPP	0.10	17	0.868	0.955	0.4897
233713_at	337.4	386.9	0.20	1.15	PPP-PPP	0.10	17	0.868	0.955	0.4544
1553603_s_at	213.8	245.3	0.20	1.15	PPP-PPP	0.10	17	0.868	0.955	0.4204
228287_at	305.9	350.9	0.20	1.15	PPP-PPP	0.10	17	0.868	0.955	0.3478
221005_s_at	538.9	618.2	0.20	1.15	PPP-PPP	0.10	17	0.868	0.955	0.2320
209604_s_at	1274.7	1462.7	0.20	1.15	PPP-PPP	0.10	17	0.868	0.955	0.2203
202565_s_at	1785.3	2047.9	0.20	1.15	PPP-PPP	0.10	17	0.868	0.955	0.2004
205482_x_at	557.6	639.5	0.20	1.15	PPP-PPP	0.10	17	0.868	0.955	0.1898
228149_at	683.6	783.9	0.20	1.15	PPP-PPP	0.10	17	0.868	0.955	0.1081
201005_at	5120.8	5872.2	0.20	1.15	PPP-PPP	0.10	17	0.868	0.955	0.0707
228856_at	111.7	128.0	0.20	1.15	PAA-PPP	0.10	17	0.868	0.955	0.5741
1552627_a_at	291.2	333.9	0.20	1.15	PPP-PPP	0.10	17	0.868	0.955	0.5564
231954_at	127.3	146.0	0.20	1.15	PPP-PPP	0.10	17	0.868	0.955	0.5403
228714_at	150.2	172.1	0.20	1.15	MPP-APP	0.10	17	0.868	0.955	0.5293
235833_at	72.8	83.4	0.20	1.15	PPA-PPP	0.10	17	0.868	0.955	0.5265
224808_s_at	527.0	604.2	0.20	1.15	PPP-AAP	0.10	17	0.868	0.955	0.4608
212437_at	934.2	1070.8	0.20	1.15	PPP-PPP	0.10	17	0.868	0.955	0.4035
218225_at	1175.9	1347.6	0.20	1.15	PPP-PPP	0.10	17	0.868	0.955	0.3858
243750_x_at	647.5	742.1	0.20	1.15	PPP-PPP	0.10	17	0.868	0.955	0.3587
208003_s_at	405.2	464.7	0.20	1.15	PPP-PPP	0.10	17	0.868	0.955	0.3185
238675_x_at	229.1	262.7	0.20	1.15	PPP-PPP	0.10	17	0.868	0.955	0.3134
233058_at	270.0	309.6	0.20	1.15	PPP-PPP	0.10	17	0.868	0.955	0.2663
204835_at	678.9	778.1	0.20	1.15	PPP-PPP	0.10	17	0.868	0.955	0.2630
204371_s_at	236.9	271.7	0.20	1.15	PAP-PAM	0.10	17	0.868	0.955	0.2508
214764_at	766.8	879.1	0.20	1.15	PPP-PPP	0.10	17	0.868	0.955	0.2502
219109_at	693.3	794.6	0.20	1.15	PPP-PPP	0.10	17	0.868	0.955	0.1878
218248_at	1570.6	1801.0	0.20	1.15	PPP-PPP	0.10	17	0.868	0.955	0.1749
219546_at	244.4	279.9	0.20	1.15	PPP-PPP	0.10	17	0.868	0.955	0.5697
242462_at	128.3	147.0	0.20	1.15	AMM-APP	0.10	17	0.868	0.955	0.5128
213637_at	1033.5	1183.6	0.20	1.15	PPP-PPP	0.10	17	0.868	0.955	0.5067
222273_at	466.8	534.6	0.20	1.15	PPP-PPP	0.10	17	0.868	0.955	0.4894
217976_s_at	1224.4	1402.6	0.20	1.15	PPP-PPP	0.10	17	0.868	0.955	0.4169
227129_x_at	702.4	804.5	0.20	1.15	PPP-PPP	0.10	17	0.868	0.955	0.4105
235532_at	278.8	319.4	0.20	1.15	PPP-AMP	0.10	17	0.868	0.955	0.3765
237289_at	266.7	305.5	0.20	1.15	PPP-PPP	0.10	17	0.868	0.955	0.3628
244340_x_at	118.2	135.4	0.20	1.15	PPP-PPP	0.10	17	0.868	0.955	0.3598
244808_at	102.2	117.1	0.20	1.15	MPM-AAP	0.10	17	0.868	0.955	0.3572
219320_at	693.4	794.4	0.20	1.15	PPP-PPP	0.10	17	0.868	0.955	0.2359
211392_s_at	171.0	195.9	0.20	1.15	PAP-APP	0.10	17	0.868	0.955	0.1189
212579_at	868.8	995.1	0.20	1.15	PPP-PPP	0.10	17	0.868	0.955	0.1070
221758_at	666.6	763.3	0.20	1.15	PPP-PPP	0.10	17	0.868	0.955	0.1006
65517_at	656.1	751.5	0.20	1.15	PPP-PPP	0.10	17	0.868	0.955	0.0805

232264_at	172.7	197.7	0.20	1.15	PPP-PPP	0.10	17	0.868	0.955	0.5907
242476_at	506.0	579.3	0.20	1.15	PPP-PPP	0.10	17	0.868	0.955	0.5817
235492_at	141.3	161.8	0.20	1.15	PAP-AAP	0.10	17	0.868	0.955	0.5742
242561_at	238.5	273.0	0.20	1.15	PPP-PPP	0.10	17	0.868	0.955	0.5685
AFFX-BioB-5_at	463.1	530.1	0.20	1.15	PPP-PPP	0.10	17	0.868	0.955	0.5546
209019_s_at	1570.5	1797.4	0.20	1.14	PPP-PPP	0.10	17	0.868	0.955	0.5154
231592_at	75.0	85.9	0.20	1.15	PPP-AAP	0.10	17	0.868	0.955	0.5037
207105_s_at	257.0	294.2	0.20	1.15	PPP-PPP	0.10	17	0.868	0.955	0.4915
224334_s_at	889.3	1018.1	0.20	1.15	PPP-PPP	0.10	17	0.868	0.955	0.2795
228221_at	91.4	104.6	0.20	1.14	AAM-AAP	0.10	17	0.868	0.955	0.2120
219105_x_at	2108.3	2413.1	0.20	1.15	PPP-PPP	0.10	17	0.868	0.955	0.1727
224377_s_at	3527.9	4039.3	0.20	1.15	PPP-PPP	0.10	17	0.868	0.955	0.1195
1560327_at	454.7	520.1	0.19	1.14	PPP-MPP	0.10	17	0.868	0.955	0.5589
38447_at	314.3	359.4	0.19	1.14	PPP-APM	0.10	17	0.868	0.955	0.5150
1557432_at	69.4	79.4	0.19	1.14	APA-APP	0.10	17	0.868	0.955	0.4647
223454_at	434.0	496.3	0.19	1.14	PPP-PPP	0.10	17	0.868	0.955	0.4236
1555411_a_at	759.6	868.8	0.19	1.14	PPP-PPP	0.10	17	0.868	0.955	0.4089
208741_at	379.7	434.3	0.19	1.14	PPP-PPP	0.10	17	0.868	0.955	0.3908
1563497_at	130.6	149.4	0.19	1.14	PPP-PPP	0.10	17	0.868	0.955	0.3657
204817_at	1112.1	1271.8	0.19	1.14	PPP-PPP	0.10	17	0.868	0.955	0.3507
201188_s_at	1556.7	1781.2	0.19	1.14	PPP-PPP	0.10	17	0.868	0.955	0.3219
1554021_a_at	751.6	859.9	0.19	1.14	PPP-PPP	0.10	17	0.868	0.955	0.2998
213876_x_at	537.4	614.7	0.19	1.14	PPP-PPP	0.10	17	0.868	0.955	0.2893
225319_s_at	652.4	746.4	0.19	1.14	PPP-PPP	0.10	17	0.868	0.955	0.2614
211213_at	35.1	40.1	0.19	1.14	MPP-AMP	0.10	17	0.868	0.955	0.2424
225802_at	1204.3	1378.1	0.19	1.14	PPP-PPP	0.10	17	0.868	0.955	0.2293
222420_s_at	3225.7	3688.7	0.19	1.14	PPP-PPP	0.10	17	0.868	0.955	0.2174
221248_s_at	142.6	163.1	0.19	1.14	APP-MPP	0.10	17	0.868	0.955	0.1550
212927_at	1250.5	1430.3	0.19	1.14	PPP-PPP	0.10	17	0.868	0.955	0.0911
238716_at	181.8	207.9	0.19	1.14	PPP-PPP	0.10	17	0.868	0.955	0.5893
1557611_at	146.3	167.1	0.19	1.14	PAM-AAM	0.10	17	0.868	0.955	0.5769
202782_s_at	361.2	412.8	0.19	1.14	PPP-PPP	0.10	17	0.868	0.955	0.5684
1558117_s_at	205.3	234.7	0.19	1.14	PPP-PPP	0.10	17	0.868	0.955	0.5307
222605_at	805.2	920.2	0.19	1.14	PPP-PPP	0.10	17	0.868	0.955	0.5151
205953_at	211.7	242.0	0.19	1.14	PPP-PPP	0.10	17	0.868	0.955	0.4528
235572_at	692.3	791.2	0.19	1.14	PPP-PPP	0.10	17	0.868	0.955	0.4438
209204_at	76.2	87.1	0.19	1.14	PAA-AAA	0.10	17	0.868	0.955	0.4064
223448_x_at	1638.1	1872.4	0.19	1.14	PPP-PPP	0.10	17	0.868	0.955	0.3909
209193_at	324.3	370.7	0.19	1.14	PPP-PPP	0.10	17	0.868	0.955	0.3571
201670_s_at	1786.7	2043.1	0.19	1.14	PPP-PPP	0.10	17	0.868	0.955	0.3028
201218_at	5940.9	6790.9	0.19	1.14	PPP-PPP	0.10	17	0.868	0.955	0.2216
201083_s_at	1079.2	1233.7	0.19	1.14	PPP-PPP	0.10	17	0.868	0.955	0.1815

224615_x_at	1002.2	1145.9	0.19	1.14	PPP-PPP	0.10	17	0.868	0.955	0.1743
222400_s_at	8037.1	9185.6	0.19	1.14	PPP-PPP	0.10	17	0.868	0.955	0.1567
215269_at	117.3	133.9	0.19	1.14	PPP-PPP	0.10	17	0.868	0.955	0.5588
229513_at	153.3	175.1	0.19	1.14	PPP-PPP	0.10	17	0.868	0.955	0.5566
224704_at	540.5	617.3	0.19	1.14	PPP-PPP	0.10	17	0.868	0.955	0.4967
225196_s_at	3009.9	3438.5	0.19	1.14	PPP-PPP	0.10	17	0.868	0.955	0.4202
219412_at	4151.0	4742.6	0.19	1.14	PPP-PPP	0.10	17	0.868	0.955	0.4163
225690_at	614.6	702.3	0.19	1.14	PPP-PPP	0.10	17	0.868	0.955	0.4021
203745_at	576.0	658.0	0.19	1.14	PPP-PPP	0.10	17	0.868	0.955	0.3979
229305_at	263.9	301.4	0.19	1.14	PPP-PPP	0.10	17	0.868	0.955	0.3976
224837_at	2661.4	3039.8	0.19	1.14	PPP-PPP	0.10	17	0.868	0.955	0.2779
212507_at	2074.7	2370.0	0.19	1.14	PPP-PPP	0.10	17	0.868	0.955	0.2725
212546_s_at	1225.9	1400.8	0.19	1.14	PPP-PPP	0.10	17	0.868	0.955	0.2638
203431_s_at	1231.6	1407.0	0.19	1.14	PPP-PPP	0.10	17	0.868	0.955	0.2631
202137_s_at	404.7	462.3	0.19	1.14	PPP-PPP	0.10	17	0.868	0.955	0.2536
204418_x_at	221.3	252.9	0.19	1.14	PAA-PPP	0.10	17	0.868	0.955	0.1937
203126_at	1668.0	1905.6	0.19	1.14	PPP-PPP	0.10	17	0.868	0.955	0.1870
210112_at	375.8	428.9	0.19	1.14	PPP-PPP	0.10	17	0.868	0.955	0.5937
206115_at	121.2	138.3	0.19	1.14	PPP-PPP	0.10	17	0.868	0.955	0.5368
227898_s_at	237.1	270.6	0.19	1.14	AAA-AAP	0.10	17	0.868	0.955	0.5338
221703_at	178.0	203.2	0.19	1.14	PPP-PPP	0.10	17	0.868	0.955	0.4018
1556110_at	285.1	325.6	0.19	1.14	PPP-PPP	0.10	17	0.868	0.955	0.3959
229371_at	257.0	293.4	0.19	1.14	PPP-PPP	0.10	17	0.868	0.955	0.3928
211926_s_at	1979.3	2259.2	0.19	1.14	PPP-PPP	0.10	17	0.868	0.955	0.3442
218436_at	644.4	735.3	0.19	1.14	PPP-PPP	0.10	17	0.868	0.955	0.3243
227543_at	261.3	298.2	0.19	1.14	PPP-PPP	0.10	17	0.868	0.955	0.3139
218838_s_at	362.3	413.4	0.19	1.14	PPP-PPP	0.10	17	0.868	0.955	0.3118
203689_s_at	1575.6	1798.6	0.19	1.14	PPP-PPP	0.10	17	0.868	0.955	0.2931
223380_s_at	1569.1	1791.8	0.19	1.14	PPP-PPP	0.10	17	0.868	0.955	0.2830
244743_x_at	231.4	264.2	0.19	1.14	PPP-PPP	0.10	17	0.868	0.955	0.2473
212702_s_at	897.8	1024.6	0.19	1.14	PPP-PPP	0.10	17	0.868	0.955	0.2114
202293_at	1098.9	1254.5	0.19	1.14	PPP-PPP	0.10	17	0.868	0.955	0.1957
223423_at	347.2	396.4	0.19	1.14	PPP-PPP	0.10	17	0.868	0.955	0.1788
239735_at	245.5	280.0	0.19	1.14	PPP-PPP	0.10	17	0.868	0.955	0.5695
214136_at	127.4	145.3	0.19	1.14	PPP-PPP	0.10	17	0.868	0.955	0.4899
228162_at	413.5	471.9	0.19	1.14	PPP-PPP	0.10	17	0.868	0.955	0.4731
235286_at	141.4	161.3	0.19	1.14	PPP-PPP	0.10	17	0.868	0.955	0.4577
209312_x_at	123.4	140.8	0.19	1.14	PPP-PPP	0.10	17	0.868	0.955	0.4337
228528_at	222.3	253.6	0.19	1.14	PPP-PPP	0.10	17	0.868	0.955	0.4290
204346_s_at	401.4	458.0	0.19	1.14	PPA-APP	0.10	17	0.868	0.955	0.3907
208987_s_at	790.5	901.7	0.19	1.14	PPP-PPP	0.10	17	0.868	0.955	0.3349
225509_at	948.5	1082.3	0.19	1.14	PPP-PPP	0.10	17	0.868	0.955	0.2845

224873_s_at	150.3	171.4	0.19	1.14	PPP-PPP	0.10	17	0.868	0.955	0.2451
220189_s_at	2601.3	2968.4	0.19	1.14	PPP-PPP	0.10	17	0.868	0.955	0.1963
218823_s_at	2344.2	2674.7	0.19	1.14	PPP-PPP	0.10	17	0.868	0.955	0.1890
204873_at	474.2	540.8	0.19	1.14	PPP-PPP	0.10	17	0.868	0.955	0.1742
204299_at	1481.2	1689.4	0.19	1.14	PPP-PPP	0.10	17	0.868	0.955	0.1492
209061_at	1724.0	1967.1	0.19	1.14	PPP-PPP	0.10	17	0.868	0.955	0.1467
226907_at	1903.3	2171.7	0.19	1.14	PPP-PPP	0.10	17	0.868	0.955	0.1354
219399_at	831.5	948.6	0.19	1.14	PPP-PPP	0.10	17	0.868	0.955	0.1336
225592_at	1037.4	1183.8	0.19	1.14	PPP-PPP	0.10	17	0.868	0.955	0.1206
224702_at	1587.9	1811.4	0.19	1.14	PPP-PPP	0.10	17	0.868	0.955	0.1031
212655_at	902.7	1029.6	0.19	1.14	PPP-PPP	0.10	17	0.868	0.955	0.0958
1561640_at	108.0	123.2	0.19	1.14	PPP-PPP	0.10	17	0.868	0.955	0.0848
205574_x_at	453.1	516.4	0.19	1.14	PPP-PPP	0.10	17	0.868	0.955	0.5863
232811_x_at	182.1	207.6	0.19	1.14	PPP-PPP	0.10	17	0.868	0.955	0.5737
228105_at	208.5	237.7	0.19	1.14	APP-AAP	0.10	17	0.868	0.955	0.5464
221103_s_at	56.8	64.7	0.19	1.14	PPP-PPP	0.10	17	0.868	0.955	0.5278
233334_x_at	677.1	771.6	0.19	1.14	PPP-PPP	0.10	17	0.868	0.955	0.5092
209998_at	699.1	796.7	0.19	1.14	PPP-PPP	0.10	17	0.868	0.955	0.4874
212152_x_at	1283.0	1463.0	0.19	1.14	PPP-PPP	0.10	17	0.868	0.955	0.4213
242139_s_at	493.7	562.8	0.19	1.14	PPP-PPP	0.10	17	0.868	0.955	0.3922
227738_s_at	163.6	186.4	0.19	1.14	PPP-AAA	0.10	17	0.868	0.955	0.3760
230604_at	215.2	245.3	0.19	1.14	PPP-PPP	0.10	17	0.868	0.955	0.3215
219362_at	811.3	924.8	0.19	1.14	PPP-PPP	0.10	17	0.868	0.955	0.3216
230479_at	287.0	327.2	0.19	1.14	PPP-PPP	0.10	17	0.868	0.955	0.3202
1559881_s_at	433.9	494.7	0.19	1.14	PPP-PPP	0.10	17	0.868	0.955	0.3185
1558750_a_at	368.8	420.4	0.19	1.14	PPP-PPP	0.10	17	0.868	0.955	0.2953
218231_at	581.1	662.6	0.19	1.14	PPP-PPP	0.10	17	0.868	0.955	0.2650
1554721_a_at	663.6	756.4	0.19	1.14	PPP-PPP	0.10	17	0.868	0.955	0.2014
202379_s_at	1351.2	1540.7	0.19	1.14	PPP-PPP	0.10	17	0.868	0.955	0.1811
244287_at	905.6	1032.6	0.19	1.14	PPP-PPP	0.10	17	0.868	0.955	0.1271
206323_x_at	1650.9	1881.8	0.19	1.14	PPP-PPP	0.10	17	0.868	0.955	0.0824
1569887_a_at	62.9	71.6	0.19	1.14	PAP-AAA	0.10	17	0.868	0.955	0.5659
215807_s_at	478.2	544.8	0.19	1.14	APP-MAP	0.10	17	0.868	0.955	0.5245
203985_at	514.4	585.8	0.19	1.14	PPP-PPP	0.10	17	0.868	0.955	0.4997
202046_s_at	238.0	271.2	0.19	1.14	PPP-PPP	0.10	17	0.868	0.955	0.4921
229114_at	172.6	196.7	0.19	1.14	PPP-PPP	0.10	17	0.868	0.955	0.4799
208859_s_at	439.8	501.1	0.19	1.14	PPP-PPP	0.10	17	0.868	0.955	0.4577
222833_at	299.5	341.2	0.19	1.14	PPP-PPP	0.10	17	0.868	0.955	0.4519
231370_at	565.2	643.8	0.19	1.14	PPP-PPP	0.10	17	0.868	0.955	0.4379
219818_s_at	457.8	521.7	0.19	1.14	PPP-PPP	0.10	17	0.868	0.955	0.4224
212615_at	725.2	826.1	0.19	1.14	PPP-PPP	0.10	17	0.868	0.955	0.3724
202564_x_at	2439.6	2779.6	0.19	1.14	PPP-PPP	0.10	17	0.868	0.955	0.3667

225947_at	1305.3	1487.2	0.19	1.14	PPP-PPP	0.10	17	0.868	0.955	0.3561
224775_at	318.6	363.1	0.19	1.14	PPP-PMP	0.10	17	0.868	0.955	0.3536
1569952_x_at	454.2	517.6	0.19	1.14	PPP-PPP	0.10	17	0.868	0.955	0.3527
218460_at	3603.1	4104.3	0.19	1.14	PPP-PPP	0.10	17	0.868	0.955	0.2797
204066_s_at	1022.7	1164.8	0.19	1.14	PPP-PPP	0.10	17	0.868	0.955	0.2546
221972_s_at	902.9	1028.6	0.19	1.14	PPP-PPP	0.10	17	0.868	0.955	0.1823
202683_s_at	852.8	971.1	0.19	1.14	PPP-PPP	0.10	17	0.868	0.955	0.1725
1554450_s_at	195.8	223.1	0.19	1.14	PPP-PPP	0.10	17	0.868	0.955	0.1682
230305_at	217.2	247.5	0.19	1.14	PPP-PPP	0.10	17	0.868	0.955	0.1300
211563_s_at	2880.0	3280.6	0.19	1.14	PPP-PPP	0.10	17	0.868	0.955	0.1266
202160_at	1478.9	1684.4	0.19	1.14	PPP-PPP	0.10	17	0.868	0.955	0.0812
215891_s_at	213.0	242.5	0.19	1.14	PPP-PPP	0.10	17	0.868	0.955	0.5781
48106_at	337.5	384.3	0.19	1.14	PPP-PPP	0.10	17	0.868	0.955	0.5363
217120_s_at	87.8	99.9	0.19	1.14	AAP-PAP	0.10	17	0.868	0.955	0.5051
205160_at	279.7	318.5	0.19	1.14	PPP-PPP	0.10	17	0.868	0.955	0.4653
212487_at	784.3	893.1	0.19	1.14	PPP-PPP	0.10	17	0.868	0.955	0.4490
212231_at	1732.1	1971.9	0.19	1.14	PPP-PPP	0.10	17	0.868	0.955	0.4354
219172_at	198.7	226.2	0.19	1.14	PAP-PPP	0.10	17	0.868	0.955	0.4060
227279_at	1034.7	1177.6	0.19	1.14	PPP-PPP	0.10	17	0.868	0.955	0.3829
226003_at	1506.4	1715.3	0.19	1.14	PPP-PPP	0.10	17	0.868	0.955	0.3594
210543_s_at	2847.6	3242.3	0.19	1.14	PPP-PPP	0.10	17	0.868	0.955	0.1971
234578_at	115.4	131.4	0.19	1.14	MAP-APP	0.10	17	0.868	0.955	0.1586
218305_at	1249.3	1421.7	0.19	1.14	PPP-PPP	0.10	17	0.868	0.955	0.0915
214741_at	1460.4	1662.9	0.19	1.14	PPP-PPP	0.10	17	0.868	0.955	0.0835
208985_s_at	4984.9	5676.4	0.19	1.14	PPP-PPP	0.10	17	0.868	0.955	0.0621
1558517_s_at	182.0	207.1	0.19	1.14	PPP-PPP	0.10	17	0.868	0.955	0.5722
238806_at	168.0	191.1	0.19	1.14	PPP-PPA	0.10	17	0.868	0.955	0.5532
213623_at	637.2	725.0	0.19	1.14	PPP-PPP	0.10	17	0.868	0.955	0.5414
227853_at	553.9	630.1	0.19	1.14	PPP-PPP	0.10	17	0.868	0.955	0.4951
235178_x_at	257.0	292.4	0.19	1.14	PPP-PPP	0.10	17	0.868	0.955	0.4752
203901_at	165.2	187.9	0.19	1.14	PAP-APP	0.10	17	0.868	0.955	0.4608
226707_at	782.8	890.3	0.19	1.14	PPP-PPP	0.10	17	0.868	0.955	0.4227
1556123_a_at	392.9	447.0	0.19	1.14	PPP-PPP	0.10	17	0.868	0.955	0.3935
228830_s_at	573.7	652.6	0.19	1.14	PPP-PPP	0.10	17	0.868	0.955	0.3533
219402_s_at	5270.5	5996.7	0.19	1.14	PPP-PPP	0.10	17	0.868	0.955	0.3418
219833_s_at	286.6	326.0	0.19	1.14	PPP-PPP	0.10	17	0.868	0.955	0.3125
201287_s_at	5926.4	6742.3	0.19	1.14	PPP-PPP	0.10	17	0.868	0.955	0.2577
225585_at	3642.7	4144.0	0.19	1.14	PPP-PPP	0.10	17	0.868	0.955	0.1628
221771_s_at	1161.9	1321.5	0.19	1.14	PPP-PPP	0.10	17	0.868	0.955	0.1152
201324_at	10967.9	12477.4	0.19	1.14	PPP-PPP	0.10	17	0.868	0.955	0.1155
225633_at	811.3	923.2	0.19	1.14	PPP-PPP	0.10	17	0.868	0.955	0.0725
215489_x_at	1008.6	1146.3	0.19	1.14	PPP-PPP	0.10	17	0.868	0.955	0.5789

47608_at	1056.5	1201.2	0.19	1.14	PPP-PPP	0.10	17	0.868	0.955	0.4210
227641_at	4979.0	5661.1	0.19	1.14	PPP-PPP	0.10	17	0.868	0.955	0.4155
229348_at	523.1	594.6	0.19	1.14	PPP-PPP	0.10	17	0.868	0.955	0.2312
219226_at	658.5	748.4	0.19	1.14	PPP-PPP	0.10	17	0.868	0.955	0.2176
205809_s_at	727.0	826.7	0.19	1.14	PPP-PPP	0.10	17	0.868	0.955	0.1845
1557100_s_at	615.5	699.5	0.19	1.14	PPP-PPP	0.10	17	0.868	0.955	0.1725
218533_s_at	1476.1	1678.3	0.19	1.14	PPP-PPP	0.10	17	0.868	0.955	0.1036
212202_s_at	1229.3	1397.1	0.19	1.14	PPP-PPP	0.10	17	0.868	0.955	0.0911
204489_s_at	4435.7	5043.1	0.19	1.14	PPP-PPP	0.10	17	0.868	0.955	0.0833
235338_s_at	209.2	237.7	0.18	1.14	PPP-PPP	0.10	17	0.868	0.955	0.5844
1555808_a_at	218.4	248.1	0.18	1.14	PPP-PPP	0.10	17	0.868	0.955	0.4746
211979_at	306.7	348.5	0.18	1.14	PPM-PPP	0.10	17	0.868	0.955	0.4149
210212_x_at	847.1	962.6	0.18	1.14	PPP-PPP	0.10	17	0.868	0.955	0.2959
208922_s_at	1611.0	1829.7	0.18	1.14	PPP-PPP	0.10	17	0.868	0.955	0.2933
223531_x_at	2011.5	2285.2	0.18	1.14	PPP-PPP	0.10	17	0.868	0.955	0.2689
221487_s_at	543.3	617.2	0.18	1.14	PPP-PPP	0.10	17	0.868	0.955	0.2560
228452_at	660.9	750.6	0.18	1.14	PPP-PPP	0.10	17	0.868	0.955	0.2297
1552291_at	873.8	992.9	0.18	1.14	PPP-PPP	0.10	17	0.868	0.955	0.2105
234981_x_at	3165.7	3596.1	0.18	1.14	PPP-PPP	0.10	17	0.868	0.955	0.1935
224464_s_at	1311.3	1489.4	0.18	1.14	PPP-PPP	0.10	17	0.868	0.955	0.1231
32062_at	368.9	419.1	0.18	1.14	PPP-PPP	0.10	17	0.868	0.955	0.0801
213842_x_at	2464.4	2798.9	0.18	1.14	PPP-PPP	0.10	17	0.868	0.955	0.0752
217866_at	1754.7	1993.3	0.18	1.14	PPP-PPP	0.10	17	0.868	0.955	0.0750
201148_s_at	980.5	1112.9	0.18	1.14	PPP-PPP	0.10	17	0.868	0.955	0.5914
209556_at	199.0	226.0	0.18	1.14	PPM-PPP	0.10	17	0.868	0.955	0.5862
214484_s_at	2082.9	2363.8	0.18	1.14	PPP-PPP	0.10	17	0.868	0.955	0.5516
1566776_at	75.8	86.1	0.18	1.14	PAM-AAA	0.10	17	0.868	0.955	0.5075
210230_at	140.6	159.6	0.18	1.14	PPP-PPP	0.10	17	0.868	0.955	0.4966
226037_s_at	269.4	305.8	0.18	1.14	PPP-PPP	0.10	17	0.868	0.955	0.4457
223398_at	706.7	802.0	0.18	1.14	PPP-PPP	0.10	17	0.868	0.955	0.3917
230168_at	168.7	191.6	0.18	1.14	PPP-PPP	0.10	17	0.868	0.955	0.3924
214988_s_at	5592.8	6350.7	0.18	1.14	PPP-PPP	0.10	17	0.868	0.955	0.3800
224776_at	814.9	925.2	0.18	1.14	PPP-PPP	0.10	17	0.868	0.955	0.3622
212856_at	358.2	406.6	0.18	1.14	PAP-PPP	0.10	17	0.868	0.955	0.3347
1563549_a_at	194.5	220.9	0.18	1.14	PAA-AAP	0.10	17	0.868	0.955	0.3217
1554251_at	235.3	267.0	0.18	1.14	PMP-PPP	0.10	17	0.868	0.955	0.3156
205034_at	1119.1	1270.0	0.18	1.14	PPP-PPP	0.10	17	0.868	0.955	0.3009
232983_s_at	658.9	748.1	0.18	1.14	PPP-PPP	0.10	17	0.868	0.955	0.2712
218966_at	1083.6	1229.8	0.18	1.14	PPP-PPP	0.10	17	0.868	0.955	0.2559
1555948_s_at	3513.0	3987.5	0.18	1.14	PPP-PPP	0.10	17	0.868	0.955	0.1747
200710_at	2985.9	3389.2	0.18	1.14	PPP-PPP	0.10	17	0.868	0.955	0.1744
214035_x_at	1685.2	1913.1	0.18	1.14	PPP-PPP	0.10	17	0.868	0.955	0.1274

203973_s_at	11133.7	12641.6	0.18	1.14	PPP-PPP	0.10	17	0.868	0.955	0.1204
209649_at	555.5	630.0	0.18	1.13	PPP-PPP	0.10	17	0.868	0.955	0.5829
221645_s_at	890.6	1010.5	0.18	1.14	PPP-PPP	0.10	17	0.868	0.955	0.5083
243507_s_at	146.3	165.9	0.18	1.13	PPP-PPP	0.10	17	0.868	0.955	0.5069
1555783_x_at	310.9	352.7	0.18	1.14	AAA-APM	0.10	17	0.868	0.955	0.4225
51200_at	917.9	1041.2	0.18	1.13	PPP-PPP	0.10	17	0.868	0.955	0.3905
218298_s_at	400.2	453.9	0.18	1.13	PPP-PPP	0.10	17	0.868	0.955	0.3837
235800_at	125.1	142.0	0.18	1.14	PPP-PPP	0.10	17	0.868	0.955	0.3370
228037_at	152.8	173.4	0.18	1.13	PMP-AAA	0.10	17	0.868	0.955	0.3226
204291_at	206.8	234.6	0.18	1.13	PPP-PPP	0.10	17	0.868	0.955	0.3131
223773_s_at	1567.6	1778.7	0.18	1.14	PPP-PPP	0.10	17	0.868	0.955	0.2548
225704_at	244.1	276.9	0.18	1.13	PPP-PPP	0.10	17	0.868	0.955	0.2441
214144_at	289.5	328.5	0.18	1.14	PPP-PPP	0.10	17	0.868	0.955	0.2255
243808_at	117.7	133.6	0.18	1.14	PPP-PPP	0.10	17	0.868	0.955	0.2209
1554830_a_at	298.4	338.4	0.18	1.13	PPP-PPP	0.10	17	0.868	0.955	0.1931
202314_at	8024.8	9101.1	0.18	1.13	PPP-PPP	0.10	17	0.868	0.955	0.1274
1567080_s_at	529.3	600.5	0.18	1.14	PPP-PPP	0.10	17	0.868	0.955	0.1152
239042_at	522.7	592.5	0.18	1.13	PPP-PPP	0.10	17	0.868	0.955	0.5656
213761_at	263.1	298.3	0.18	1.13	PPP-PPP	0.10	17	0.868	0.955	0.5388
214668_at	295.0	334.5	0.18	1.13	PPP-PPP	0.10	17	0.868	0.955	0.5385
218741_at	651.6	738.5	0.18	1.13	APP-PPP	0.10	17	0.868	0.955	0.4989
205964_at	151.6	171.9	0.18	1.13	PPP-PPP	0.10	17	0.868	0.955	0.4882
203120_at	1221.2	1384.4	0.18	1.13	PPP-PPP	0.10	17	0.868	0.955	0.4469
225462_at	822.3	932.3	0.18	1.13	PPP-PPP	0.10	17	0.868	0.955	0.4379
227847_at	365.2	414.1	0.18	1.13	PPP-PPP	0.10	17	0.868	0.955	0.3633
218463_s_at	927.7	1051.7	0.18	1.13	PPP-PPP	0.10	17	0.868	0.955	0.3536
210793_s_at	1547.5	1754.4	0.18	1.13	PPP-PPP	0.10	17	0.868	0.955	0.3346
211136_s_at	1835.9	2081.5	0.18	1.13	PPP-PPP	0.10	17	0.868	0.955	0.2949
203353_s_at	1460.9	1656.3	0.18	1.13	PPP-PPP	0.10	17	0.868	0.955	0.2128
223656_s_at	354.2	401.6	0.18	1.13	PPP-PPP	0.10	17	0.868	0.955	0.1854
40359_at	1099.4	1246.5	0.18	1.13	PPP-PPP	0.10	17	0.868	0.955	0.1592
213098_at	368.6	417.8	0.18	1.13	PPP-PPP	0.10	17	0.868	0.955	0.1272
211924_s_at	417.6	473.1	0.18	1.13	PPP-PPP	0.10	17	0.868	0.955	0.5754
218264_at	230.7	261.4	0.18	1.13	PPP-PPP	0.10	17	0.868	0.955	0.5447
219257_s_at	235.5	266.8	0.18	1.13	PPP-PPP	0.10	17	0.868	0.955	0.5445
225725_at	1132.9	1283.4	0.18	1.13	PPP-PPP	0.10	17	0.868	0.955	0.5339
214218_s_at	1028.9	1166.0	0.18	1.13	PPP-PPP	0.10	17	0.868	0.955	0.5275
1556306_at	387.6	439.2	0.18	1.13	PPP-PPP	0.10	17	0.868	0.955	0.5001
224218_s_at	252.3	285.9	0.18	1.13	PPP-PPP	0.10	17	0.868	0.955	0.4965
218251_at	768.1	870.0	0.18	1.13	PPP-PPP	0.10	17	0.868	0.955	0.4485
223312_at	510.4	578.1	0.18	1.13	PPP-PPP	0.10	17	0.868	0.955	0.4348
215038_s_at	521.7	591.2	0.18	1.13	PPP-PPP	0.10	17	0.868	0.955	0.4330

218917_s_at	1755.5	1988.6	0.18	1.13	PPP-PPP	0.10	17	0.868	0.955	0.4084
216210_x_at	996.4	1128.5	0.18	1.13	PPP-PAP	0.10	17	0.868	0.955	0.2852
212521_s_at	761.1	862.5	0.18	1.13	PPP-PPP	0.10	17	0.868	0.955	0.2443
217898_at	4400.3	4985.2	0.18	1.13	PPP-PPP	0.10	17	0.868	0.955	0.2432
229945_at	102.0	115.6	0.18	1.13	AAP-AAA	0.10	17	0.868	0.955	0.2156
223160_s_at	905.7	1026.3	0.18	1.13	PPP-PPP	0.10	17	0.868	0.955	0.1887
238021_s_at	5750.2	6516.1	0.18	1.13	PPP-PPP	0.10	17	0.868	0.955	0.1300
201065_s_at	5065.1	5737.2	0.18	1.13	PPP-PPP	0.10	17	0.868	0.955	0.0812
235870_at	101.0	114.3	0.18	1.13	APM-AAM	0.10	17	0.868	0.955	0.5819
223833_at	269.8	305.5	0.18	1.13	PAP-AAP	0.10	17	0.868	0.955	0.5678
244659_at	139.6	158.1	0.18	1.13	PPP-PPP	0.10	17	0.868	0.955	0.5640
241240_at	69.6	78.9	0.18	1.13	PAP-PAP	0.10	17	0.868	0.955	0.5393
238447_at	759.9	860.0	0.18	1.13	PPP-PPP	0.10	17	0.868	0.955	0.5184
228435_at	210.9	238.8	0.18	1.13	PPP-PPP	0.10	17	0.868	0.955	0.5149
227041_at	516.6	584.8	0.18	1.13	PPP-PPP	0.10	17	0.868	0.955	0.4760
226157_at	467.4	529.2	0.18	1.13	PPP-PPP	0.10	17	0.868	0.955	0.4707
230416_at	1602.6	1814.7	0.18	1.13	PPP-PPP	0.10	17	0.868	0.955	0.4695
48825_at	221.8	251.2	0.18	1.13	PPP-PPP	0.10	17	0.868	0.955	0.4673
219698_s_at	532.5	602.8	0.18	1.13	PPP-PPP	0.10	17	0.868	0.955	0.4410
221718_s_at	535.0	605.6	0.18	1.13	PPP-PPP	0.10	17	0.868	0.955	0.4133
228348_at	361.0	408.7	0.18	1.13	PPP-PPP	0.10	17	0.868	0.955	0.3574
203515_s_at	1430.3	1619.4	0.18	1.13	PPP-PPP	0.10	17	0.868	0.955	0.2995
215867_x_at	2289.8	2592.4	0.18	1.13	PPP-PPP	0.10	17	0.868	0.955	0.2682
1557064_s_at	245.1	277.5	0.18	1.13	PPP-PPP	0.10	17	0.868	0.955	0.2467
1564378_a_at	264.9	299.9	0.18	1.13	PPP-PPP	0.10	17	0.868	0.955	0.2281
224229_s_at	191.0	216.3	0.18	1.13	PPP-PPP	0.10	17	0.868	0.955	0.2266
227382_at	217.7	246.4	0.18	1.13	PPP-PPP	0.10	17	0.868	0.955	0.1908
232028_at	429.0	485.5	0.18	1.13	PPP-PPP	0.10	17	0.868	0.955	0.1263
220076_at	150.7	170.6	0.18	1.13	APM-PMP	0.10	17	0.868	0.955	0.1252
236850_at	93.0	105.3	0.18	1.13	APA-MAA	0.10	17	0.868	0.955	0.1230
218267_at	472.3	534.6	0.18	1.13	PPP-PPP	0.10	17	0.868	0.955	0.1220
203324_s_at	15281.9	17301.9	0.18	1.13	PPP-PPP	0.10	17	0.868	0.955	0.0859
201060_x_at	8094.2	9164.8	0.18	1.13	PPP-PPP	0.10	17	0.868	0.955	0.0623
208886_at	389.8	441.3	0.18	1.13	PPP-PPP	0.10	17	0.868	0.955	0.0536
221954_at	480.2	543.4	0.18	1.13	PPP-PPP	0.10	17	0.868	0.955	0.5896
238917_s_at	213.9	242.1	0.18	1.13	PPP-PPP	0.10	17	0.868	0.955	0.5817
215336_at	134.5	152.2	0.18	1.13	PPP-PPP	0.10	17	0.868	0.955	0.5577
219875_s_at	281.7	318.7	0.18	1.13	PPP-PPP	0.10	17	0.868	0.955	0.5025
1554774_at	311.4	352.2	0.18	1.13	PMM-APP	0.10	17	0.868	0.955	0.4831
206003_at	451.4	510.6	0.18	1.13	PPP-PPP	0.10	17	0.868	0.955	0.4559
222439_s_at	1594.9	1804.5	0.18	1.13	PPP-PPP	0.10	17	0.868	0.955	0.4164
218114_at	429.8	486.4	0.18	1.13	PPP-PMP	0.10	17	0.868	0.955	0.4042

224859_at	693.3	784.5	0.18	1.13	PPP-PPP	0.10	17	0.868	0.955	0.3924
238599_at	445.9	504.4	0.18	1.13	PPP-PPP	0.10	17	0.868	0.955	0.3837
218037_at	630.0	712.7	0.18	1.13	PPP-PPP	0.10	17	0.868	0.955	0.2999
235767_x_at	1107.8	1253.0	0.18	1.13	PPP-PPP	0.10	17	0.868	0.955	0.2959
221277_s_at	860.1	972.7	0.18	1.13	PPP-PPP	0.10	17	0.868	0.955	0.2281
227018_at	283.7	320.9	0.18	1.13	PPP-PPP	0.10	17	0.868	0.955	0.1980
210778_s_at	409.4	463.3	0.18	1.13	PAM-PPP	0.10	17	0.868	0.955	0.1732
223308_s_at	631.7	714.0	0.18	1.13	PPP-PPP	0.10	17	0.868	0.955	0.5607
236285_at	53.5	60.5	0.18	1.13	AAA-APP	0.10	17	0.868	0.955	0.5475
231034_s_at	326.9	369.7	0.18	1.13	PPP-PPP	0.10	17	0.868	0.955	0.5427
241430_at	112.7	127.4	0.18	1.13	AAA-APA	0.10	17	0.868	0.955	0.5342
1568611_at	245.8	278.0	0.18	1.13	PPP-PPP	0.10	17	0.868	0.955	0.5023
201978_s_at	1200.2	1356.5	0.18	1.13	PMM-AMM	0.10	17	0.868	0.955	0.4926
235320_at	261.6	295.7	0.18	1.13	PPP-PPP	0.10	17	0.868	0.955	0.4721
225954_s_at	296.1	334.7	0.18	1.13	PPP-PPP	0.10	17	0.868	0.955	0.4510
1568600_at	113.3	128.1	0.18	1.13	PPP-PPP	0.10	17	0.868	0.955	0.4475
227602_at	110.5	124.9	0.18	1.13	PPP-APP	0.10	17	0.868	0.955	0.4419
233903_s_at	51.8	58.5	0.18	1.13	AAP-APP	0.10	17	0.868	0.955	0.4192
210023_s_at	982.3	1110.6	0.18	1.13	PPP-PPP	0.10	17	0.868	0.955	0.4163
202822_at	1506.8	1704.0	0.18	1.13	PPP-PPP	0.10	17	0.868	0.955	0.4066
200875_s_at	8126.9	9189.7	0.18	1.13	PPP-PPP	0.10	17	0.868	0.955	0.3927
206059_at	671.9	759.5	0.18	1.13	PPP-PPP	0.10	17	0.868	0.955	0.3872
205169_at	322.6	364.8	0.18	1.13	PPP-PPP	0.10	17	0.868	0.955	0.3749
202857_at	5120.6	5787.8	0.18	1.13	PPP-PPP	0.10	17	0.868	0.955	0.3491
215159_s_at	221.6	250.5	0.18	1.13	PPP-PPP	0.10	17	0.868	0.955	0.3469
229285_at	196.3	221.9	0.18	1.13	PPP-PPP	0.10	17	0.868	0.955	0.3425
121_at	533.7	603.4	0.18	1.13	PPP-PPP	0.10	17	0.868	0.955	0.3126
205231_s_at	199.9	226.1	0.18	1.13	PPP-PPP	0.10	17	0.868	0.955	0.2987
242140_at	468.5	529.8	0.18	1.13	PPP-PPP	0.10	17	0.868	0.955	0.2895
223179_at	396.6	448.5	0.18	1.13	PPP-PPP	0.10	17	0.868	0.955	0.2823
204948_s_at	696.7	787.5	0.18	1.13	PPP-PPP	0.10	17	0.868	0.955	0.2547
219806_s_at	824.1	931.6	0.18	1.13	PPP-PPP	0.10	17	0.868	0.955	0.2504
226247_at	3878.1	4385.2	0.18	1.13	PPP-PPP	0.10	17	0.868	0.955	0.1903
1555653_at	26612.8	30095.6	0.18	1.13	PPP-PPP	0.10	17	0.868	0.955	0.1592
1567458_s_at	9117.8	10308.9	0.18	1.13	PPP-PPP	0.10	17	0.868	0.955	0.1104
221263_s_at	6561.2	7417.8	0.18	1.13	PPP-PPP	0.10	17	0.868	0.955	0.0584
213391_at	1694.3	1915.6	0.18	1.13	PPP-PPP	0.10	17	0.868	0.955	0.0512
225425_s_at	954.4	1078.1	0.18	1.13	PPP-PPP	0.10	17	0.868	0.955	0.5738
1558027_s_at	361.3	408.0	0.18	1.13	PPP-PPP	0.10	17	0.868	0.955	0.5552
218113_at	156.3	176.5	0.18	1.13	AMP-PMP	0.10	17	0.868	0.955	0.5409
225210_s_at	1073.9	1213.0	0.18	1.13	PPP-PPP	0.10	17	0.868	0.955	0.4801
217526_at	1074.0	1213.0	0.18	1.13	PPP-PPP	0.10	17	0.868	0.955	0.4563

226451_at	666.7	753.3	0.18	1.13	PPP-PPP	0.10	17	0.868	0.955	0.4166
229333_at	490.7	554.3	0.18	1.13	PPP-PPP	0.10	17	0.868	0.955	0.4040
221629_x_at	653.8	738.8	0.18	1.13	PPP-PPP	0.10	17	0.868	0.955	0.3747
220214_at	310.3	350.5	0.18	1.13	PPP-PPP	0.10	17	0.868	0.955	0.3669
201856_s_at	564.6	637.9	0.18	1.13	PPP-PPP	0.10	17	0.868	0.955	0.3579
201250_s_at	807.8	912.9	0.18	1.13	PPP-PPP	0.10	17	0.868	0.955	0.2760
213521_at	543.8	614.2	0.18	1.13	PPP-PPP	0.10	17	0.868	0.955	0.2177
42361_g_at	200.5	226.5	0.18	1.13	PPP-PPP	0.10	17	0.868	0.955	0.2144
203216_s_at	1622.4	1833.3	0.18	1.13	PPP-PPP	0.10	17	0.868	0.955	0.1804
239413_at	743.6	839.9	0.18	1.13	PPP-PPP	0.10	17	0.868	0.955	0.1775
224866_at	3255.6	3678.1	0.18	1.13	PPP-PPP	0.10	17	0.868	0.955	0.1651
222130_s_at	971.0	1097.4	0.18	1.13	PPP-PPP	0.10	17	0.868	0.955	0.0910
240234_at	132.2	149.2	0.18	1.13	APA-PPP	0.10	17	0.868	0.955	0.5827
220725_x_at	601.3	678.9	0.18	1.13	PPP-PPP	0.10	17	0.868	0.955	0.5789
203870_at	387.2	437.0	0.18	1.13	PPP-PPP	0.10	17	0.868	0.955	0.5683
200917_s_at	1096.3	1237.5	0.18	1.13	PPP-PPP	0.10	17	0.868	0.955	0.5245
206451_at	365.1	412.2	0.18	1.13	PPP-PPP	0.10	17	0.868	0.955	0.5242
91703_at	325.2	367.0	0.18	1.13	PPP-PPP	0.10	17	0.868	0.955	0.5210
1556126_s_at	271.5	306.5	0.18	1.13	PPP-PPP	0.10	17	0.868	0.955	0.5147
243236_at	40.7	45.9	0.18	1.13	AAP-AAA	0.10	17	0.868	0.955	0.5006
222841_s_at	714.9	807.2	0.18	1.13	PPP-PPP	0.10	17	0.868	0.955	0.3396
206473_at	270.2	305.0	0.18	1.13	APM-AAA	0.10	17	0.868	0.955	0.2965
217367_s_at	318.0	359.0	0.18	1.13	PPP-PPP	0.10	17	0.868	0.955	0.2948
224521_s_at	283.7	320.3	0.18	1.13	PPP-PPP	0.10	17	0.868	0.955	0.2919
205273_s_at	2476.7	2795.8	0.18	1.13	PPP-PPP	0.10	17	0.868	0.955	0.2887
214113_s_at	2156.9	2435.0	0.18	1.13	PPP-PPP	0.10	17	0.868	0.955	0.2239
227031_at	565.2	638.3	0.18	1.13	PPP-PPP	0.10	17	0.868	0.955	0.2038
233563_s_at	607.6	686.2	0.18	1.13	PPP-PPP	0.10	17	0.868	0.955	0.1914
213506_at	4316.0	4872.1	0.18	1.13	PPP-PPP	0.10	17	0.868	0.955	0.1888
224304_x_at	459.2	518.5	0.18	1.13	PPP-PPP	0.10	17	0.868	0.955	0.1787
225780_at	946.6	1068.9	0.18	1.13	PPP-PPP	0.10	17	0.868	0.955	0.1639
1557918_s_at	815.9	920.9	0.18	1.13	PPP-PPP	0.10	17	0.868	0.955	0.1168
204576_s_at	373.2	421.4	0.18	1.13	PPP-PPP	0.10	17	0.868	0.955	0.0646
1555964_at	87.5	98.7	0.17	1.13	MPA-PPP	0.10	17	0.868	0.955	0.5784
202197_at	406.7	459.0	0.17	1.13	PPP-PPP	0.10	17	0.868	0.955	0.5487
205835_s_at	160.5	181.0	0.17	1.13	PPP-PPP	0.10	17	0.868	0.955	0.5323
211052_s_at	472.9	533.6	0.17	1.13	APP-MAP	0.10	17	0.868	0.955	0.5017
1566113_at	118.8	134.0	0.17	1.13	PPP-PPP	0.10	17	0.868	0.955	0.4954
240983_s_at	645.3	728.0	0.17	1.13	PPP-PPP	0.10	17	0.868	0.955	0.4946
1555358_a_at	252.6	285.0	0.17	1.13	PPP-PPP	0.10	17	0.868	0.955	0.4941
212518_at	434.8	490.5	0.17	1.13	PPP-PPP	0.10	17	0.868	0.955	0.4824
208733_at	227.9	257.2	0.17	1.13	PPP-PPP	0.10	17	0.868	0.955	0.4777

220643_s_at	1643.2	1853.2	0.17	1.13	PPP-PPP	0.10	17	0.868	0.955	0.4597
37433_at	114.3	128.9	0.17	1.13	PPP-PPP	0.10	17	0.868	0.955	0.4258
201085_s_at	1782.4	2010.8	0.17	1.13	PPP-PPP	0.10	17	0.868	0.955	0.3910
209954_x_at	488.4	551.2	0.17	1.13	PPP-PPP	0.10	17	0.868	0.955	0.3915
205227_at	641.2	723.3	0.17	1.13	PPP-PPP	0.10	17	0.868	0.955	0.3791
217916_s_at	3398.8	3835.4	0.17	1.13	PPP-PPP	0.10	17	0.868	0.955	0.3706
209519_at	171.3	193.3	0.17	1.13	PPP-PPP	0.10	17	0.868	0.955	0.3118
210249_s_at	424.3	478.7	0.17	1.13	APM-MPP	0.10	17	0.868	0.955	0.2977
215434_x_at	2116.6	2388.4	0.17	1.13	PPP-PPP	0.10	17	0.868	0.955	0.2422
201084_s_at	8919.4	10061.5	0.17	1.13	PPP-PPP	0.10	17	0.868	0.955	0.1713
212054_x_at	1516.6	1710.7	0.17	1.13	PPP-PPP	0.10	17	0.868	0.955	0.1023
224709_s_at	4603.6	5192.4	0.17	1.13	PPP-PPP	0.10	17	0.868	0.955	0.0496
227988_s_at	601.0	677.4	0.17	1.13	PPP-PPP	0.10	17	0.868	0.955	0.5666
202924_s_at	285.8	322.3	0.17	1.13	PPP-PPP	0.10	17	0.868	0.955	0.5649
226938_at	614.7	692.8	0.17	1.13	PPP-PPP	0.10	17	0.868	0.955	0.5185
203433_at	572.4	645.4	0.17	1.13	PPP-PPP	0.10	17	0.868	0.955	0.4898
236010_at	66.2	74.6	0.17	1.13	PAP-PMP	0.10	17	0.868	0.955	0.4866
207343_at	102.4	115.4	0.17	1.13	PAA-AAP	0.10	17	0.868	0.955	0.4242
226514_at	289.3	326.2	0.17	1.13	MMP-PPP	0.10	17	0.868	0.955	0.4162
210984_x_at	1087.7	1226.0	0.17	1.13	PPP-PPP	0.10	17	0.868	0.955	0.4103
202173_s_at	1089.1	1227.6	0.17	1.13	PPP-PPP	0.10	17	0.868	0.955	0.3359
1554470_s_at	596.7	672.6	0.17	1.13	PPP-PPP	0.10	17	0.868	0.955	0.3150
225334_at	1318.0	1486.3	0.17	1.13	PPP-PPP	0.10	17	0.868	0.955	0.2903
202033_s_at	3829.9	4318.6	0.17	1.13	PPP-PPP	0.10	17	0.868	0.955	0.2564
234947_s_at	586.4	661.2	0.17	1.13	PPP-PPP	0.10	17	0.868	0.955	0.2045
204387_x_at	974.3	1098.5	0.17	1.13	PPP-PPP	0.10	17	0.868	0.955	0.1287
220046_s_at	1166.4	1315.3	0.17	1.13	PPP-PPP	0.10	17	0.868	0.955	0.1254
218067_s_at	3090.8	3484.3	0.17	1.13	PPP-PPP	0.10	17	0.868	0.955	0.0963
233319_x_at	242.6	273.4	0.17	1.13	PPP-PPP	0.10	17	0.868	0.955	0.5812
228049_x_at	2982.5	3359.9	0.17	1.13	PPP-PPP	0.10	17	0.868	0.955	0.5687
212201_at	834.5	940.2	0.17	1.13	PPP-PPP	0.10	17	0.868	0.955	0.4967
215460_x_at	447.6	504.3	0.17	1.13	PPP-PPP	0.10	17	0.868	0.955	0.4460
37384_at	633.3	713.4	0.17	1.13	PPP-PPP	0.10	17	0.868	0.955	0.3954
221998_s_at	617.9	696.0	0.17	1.13	PPP-PPP	0.10	17	0.868	0.955	0.3766
209760_at	603.5	679.9	0.17	1.13	PPP-PPP	0.10	17	0.868	0.955	0.3396
212945_s_at	334.1	376.3	0.17	1.13	PPP-PPP	0.10	17	0.868	0.955	0.3254
206170_at	2330.7	2625.6	0.17	1.13	PPP-PPP	0.10	17	0.868	0.955	0.2715
214785_at	480.4	541.1	0.17	1.13	PPP-PPP	0.10	17	0.868	0.955	0.2662
1558028_x_at	801.8	903.2	0.17	1.13	PPP-PPP	0.10	17	0.868	0.955	0.2662
208836_at	16157.8	18206.6	0.17	1.13	PPP-PPP	0.10	17	0.868	0.955	0.2262
226033_at	456.1	514.0	0.17	1.13	PPP-PPP	0.10	17	0.868	0.955	0.2019
1555764_s_at	3866.8	4356.0	0.17	1.13	PPP-PPP	0.10	17	0.868	0.955	0.1945

212462_at	725.0	817.0	0.17	1.13	PPP-PPP	0.10	17	0.868	0.955	0.1650
209391_at	785.8	885.2	0.17	1.13	PPP-PPP	0.10	17	0.868	0.955	0.1483
239038_at	103.5	116.6	0.17	1.13	PAM-AAP	0.10	17	0.868	0.955	0.1412
231152_at	129.3	145.6	0.17	1.13	APP-PPP	0.10	17	0.868	0.955	0.5627
221762_s_at	338.7	381.4	0.17	1.13	PMP-PPP	0.10	17	0.868	0.955	0.5180
210791_s_at	639.0	719.6	0.17	1.13	PPP-PPP	0.10	17	0.868	0.955	0.4842
236588_at	157.5	177.4	0.17	1.13	PAP-MPP	0.10	17	0.868	0.955	0.4122
233167_at	348.5	392.4	0.17	1.13	PPP-PPP	0.10	17	0.868	0.955	0.3770
215785_s_at	653.0	735.1	0.17	1.13	PPP-PPP	0.10	17	0.868	0.955	0.3486
74694_s_at	401.5	452.0	0.17	1.13	PPP-PPP	0.10	17	0.868	0.955	0.3435
225355_at	642.4	723.3	0.17	1.13	PPP-PPP	0.10	17	0.868	0.955	0.3331
223556_at	1454.0	1636.4	0.17	1.13	PPP-PPP	0.10	17	0.868	0.955	0.3075
203169_at	444.2	500.2	0.17	1.13	PPP-PPP	0.10	17	0.868	0.955	0.2230
224750_at	671.8	756.4	0.17	1.13	AAP-PMM	0.10	17	0.868	0.955	0.1403
202773_s_at	368.3	414.6	0.17	1.13	PPP-PPP	0.10	17	0.868	0.955	0.1118
217730_at	2762.8	3108.1	0.17	1.13	PPP-PPP	0.10	17	0.868	0.955	0.5518
225622_at	104.7	117.8	0.17	1.13	PPP-PPP	0.10	17	0.868	0.955	0.5294
230951_at	195.8	220.3	0.17	1.13	PPP-PPP	0.10	17	0.868	0.955	0.5139
230930_at	431.6	485.4	0.17	1.13	PPP-PPP	0.10	17	0.868	0.955	0.4895
222364_at	148.6	167.2	0.17	1.13	PPP-AAP	0.10	17	0.868	0.955	0.4640
213073_at	211.6	238.1	0.17	1.13	PPP-PPP	0.10	17	0.868	0.955	0.4531
228765_at	1278.3	1438.3	0.17	1.13	PPP-PPP	0.10	17	0.868	0.955	0.4505
229906_at	99.3	111.8	0.17	1.13	AAP-PAP	0.10	17	0.868	0.955	0.4288
225079_at	1440.1	1620.6	0.17	1.13	PPP-PPP	0.10	17	0.868	0.955	0.4283
244647_at	139.1	156.6	0.17	1.13	PPP-PPP	0.10	17	0.868	0.955	0.3719
221744_at	1183.4	1331.8	0.17	1.13	PPP-PPP	0.10	17	0.868	0.955	0.3627
226395_at	956.1	1075.5	0.17	1.13	PPP-PPP	0.10	17	0.868	0.955	0.3549
1553681_a_at	122.5	137.8	0.17	1.13	AAA-APP	0.10	17	0.868	0.955	0.3526
234987_at	402.2	452.6	0.17	1.13	PPP-PPP	0.10	17	0.868	0.955	0.3266
211600_at	17519.0	19713.0	0.17	1.13	PPP-PPP	0.10	17	0.868	0.955	0.2940
225124_at	536.3	603.5	0.17	1.13	PPP-PPP	0.10	17	0.868	0.955	0.2028
222667_s_at	1902.8	2140.6	0.17	1.13	PPP-PPP	0.10	17	0.868	0.955	0.2001
224838_at	2097.1	2359.8	0.17	1.13	PPP-PPP	0.10	17	0.868	0.955	0.1822
219079_at	859.1	966.6	0.17	1.13	PPP-PPP	0.10	17	0.868	0.955	0.1579
229699_at	490.7	552.1	0.17	1.13	PPP-PPP	0.10	17	0.868	0.955	0.1445
225325_at	1601.0	1800.7	0.17	1.13	PPP-PPP	0.10	17	0.868	0.955	0.1274
202946_s_at	1392.2	1566.5	0.17	1.13	PPP-PPP	0.10	17	0.868	0.955	0.0430
214375_at	80.7	90.8	0.17	1.12	APA-PPP	0.10	17	0.868	0.955	0.5726
213554_s_at	1402.6	1577.3	0.17	1.13	PPP-PPP	0.10	17	0.868	0.955	0.5421
47571_at	337.1	379.0	0.17	1.12	PPP-PPP	0.10	17	0.868	0.955	0.5112
243004_at	160.4	180.4	0.17	1.13	PPP-PPP	0.10	17	0.868	0.955	0.4658
214462_at	240.5	270.4	0.17	1.12	PPP-PPP	0.10	17	0.868	0.955	0.4374

204756_at	251.7	283.1	0.17	1.13	PPP-AAP	0.10	17	0.868	0.955	0.4338
1554667_s_at	1556.2	1749.5	0.17	1.12	PPP-PPP	0.10	17	0.868	0.955	0.3328
217437_s_at	738.2	829.8	0.17	1.12	PPP-PPP	0.10	17	0.868	0.955	0.2527
224980_at	357.0	401.5	0.17	1.13	PPP-PPP	0.10	17	0.868	0.955	0.2212
57539_at	520.8	585.5	0.17	1.12	PPP-PPP	0.10	17	0.868	0.955	0.1895
219681_s_at	813.2	914.2	0.17	1.12	PPP-PPP	0.10	17	0.868	0.955	0.1589
209281_s_at	682.4	767.0	0.17	1.12	PPP-PPP	0.10	17	0.868	0.955	0.1504
218783_at	292.0	328.2	0.17	1.12	PPP-PPP	0.10	17	0.868	0.955	0.1404
208906_at	857.2	963.9	0.17	1.12	PPP-PPP	0.10	17	0.868	0.955	0.1209
210252_s_at	444.7	499.9	0.17	1.12	AAM-APM	0.10	17	0.868	0.955	0.0505
224831_at	517.1	581.3	0.17	1.12	PPP-PPP	0.10	17	0.868	0.955	0.0492
1554472_a_at	176.2	198.0	0.17	1.12	PPP-PPP	0.10	17	0.868	0.955	0.5705
202775_s_at	385.6	433.2	0.17	1.12	PPP-PPP	0.10	17	0.868	0.955	0.5581
231785_at	223.7	251.4	0.17	1.12	PAA-AAA	0.10	17	0.868	0.955	0.5347
227131_at	459.0	515.6	0.17	1.12	PPP-PPP	0.10	17	0.868	0.955	0.5271
238902_at	382.5	429.7	0.17	1.12	PPP-PPP	0.10	17	0.868	0.955	0.5263
1564002_a_at	94.9	106.6	0.17	1.12	PPP-PPP	0.10	17	0.868	0.955	0.5171
240445_at	44.0	49.5	0.17	1.12	AAA-PAA	0.10	17	0.868	0.955	0.5095
214277_at	150.4	169.0	0.17	1.12	PPP-PPP	0.10	17	0.868	0.955	0.5005
230012_at	266.3	299.1	0.17	1.12	PPP-PPP	0.10	17	0.868	0.955	0.4689
204991_s_at	183.9	206.6	0.17	1.12	PPP-APP	0.10	17	0.868	0.955	0.4483
202777_at	1838.2	2064.6	0.17	1.12	PPP-PPP	0.10	17	0.868	0.955	0.4477
227431_at	345.0	387.4	0.17	1.12	PPP-PPP	0.10	17	0.868	0.955	0.3998
226894_at	592.4	665.8	0.17	1.12	PPP-PPP	0.10	17	0.868	0.955	0.4002
210676_x_at	370.4	416.1	0.17	1.12	PPP-PPP	0.10	17	0.868	0.955	0.3660
210007_s_at	395.9	444.9	0.17	1.12	PPP-PPP	0.10	17	0.868	0.955	0.3402
223507_at	563.2	632.8	0.17	1.12	PPP-PPP	0.10	17	0.868	0.955	0.2766
206875_s_at	2939.8	3302.7	0.17	1.12	PPP-PPP	0.10	17	0.868	0.955	0.2364
218937_at	512.2	575.3	0.17	1.12	PPP-PPP	0.10	17	0.868	0.955	0.1572
209668_x_at	932.6	1047.8	0.17	1.12	PPP-PPP	0.10	17	0.868	0.955	0.1527
202614_at	2276.1	2556.6	0.17	1.12	PPP-PPP	0.10	17	0.868	0.955	0.1398
224842_at	1508.1	1694.3	0.17	1.12	PPP-PPP	0.10	17	0.868	0.955	0.1373
203544_s_at	1612.7	1811.9	0.17	1.12	PPP-PPP	0.10	17	0.868	0.955	0.1297
229223_at	397.9	446.9	0.17	1.12	PPP-PPP	0.10	17	0.868	0.955	0.1237
201416_at	863.4	969.7	0.17	1.12	PPP-PPP	0.10	17	0.868	0.955	0.5826
221760_at	116.8	131.2	0.17	1.12	PPP-APP	0.10	17	0.868	0.955	0.5593
219644_at	438.7	492.4	0.17	1.12	PPP-PPP	0.10	17	0.868	0.955	0.5191
227165_at	748.4	840.2	0.17	1.12	PPP-PPP	0.10	17	0.868	0.955	0.4915
216971_s_at	2157.6	2422.2	0.17	1.12	PPP-PPP	0.10	17	0.868	0.955	0.4864
203550_s_at	3554.3	3990.1	0.17	1.12	PPP-PPP	0.10	17	0.868	0.955	0.4597
227541_at	361.2	405.5	0.17	1.12	PPP-PPP	0.10	17	0.868	0.955	0.4550
228577_x_at	185.8	208.5	0.17	1.12	PPP-PPP	0.10	17	0.868	0.955	0.4346

209477_at	597.8	671.0	0.17	1.12	PPP-PPP	0.10	17	0.868	0.955	0.4165
209874_x_at	198.7	223.1	0.17	1.12	APA-AAA	0.10	17	0.868	0.955	0.4157
211358_s_at	561.6	630.4	0.17	1.12	PPP-PPP	0.10	17	0.868	0.955	0.4107
213682_at	2517.9	2826.1	0.17	1.12	PPP-PPP	0.10	17	0.868	0.955	0.3708
240449_at	229.7	258.0	0.17	1.12	PPP-PPP	0.10	17	0.868	0.955	0.3371
212454_x_at	3138.7	3524.1	0.17	1.12	PPP-PPP	0.10	17	0.868	0.955	0.3002
230376_at	241.1	270.7	0.17	1.12	PPP-PPP	0.10	17	0.868	0.955	0.2482
213378_s_at	551.7	619.4	0.17	1.12	PPP-PPP	0.10	17	0.868	0.955	0.2382
213041_s_at	4516.3	5069.5	0.17	1.12	PPP-PPP	0.10	17	0.868	0.955	0.1689
217742_s_at	2290.4	2571.4	0.17	1.12	PPP-PPP	0.10	17	0.868	0.955	0.1397
218396_at	1408.7	1581.8	0.17	1.12	PPP-PPP	0.10	17	0.868	0.955	0.0608
227889_at	809.1	908.4	0.17	1.12	PPP-PPP	0.10	17	0.868	0.955	0.0403
209605_at	1558.8	1748.5	0.17	1.12	PPP-PPP	0.10	17	0.868	0.955	0.5468
1554085_at	350.3	393.1	0.17	1.12	PPP-PPP	0.10	17	0.868	0.955	0.5397
235857_at	141.6	158.8	0.17	1.12	MAA-AAP	0.10	17	0.868	0.955	0.5361
204003_s_at	808.3	906.9	0.17	1.12	PPP-PPP	0.10	17	0.868	0.955	0.5317
204361_s_at	603.4	676.8	0.17	1.12	PPP-PPP	0.10	17	0.868	0.955	0.3764
210879_s_at	544.8	611.2	0.17	1.12	PPP-PPP	0.10	17	0.868	0.955	0.3673
225666_at	1311.2	1471.1	0.17	1.12	PPP-PPP	0.10	17	0.868	0.955	0.3017
209157_at	2045.2	2294.9	0.17	1.12	PPP-PPP	0.10	17	0.868	0.955	0.2939
200911_s_at	1814.3	2036.2	0.17	1.12	PPP-PPP	0.10	17	0.868	0.955	0.2454
225363_at	1871.4	2099.9	0.17	1.12	PPP-PPP	0.10	17	0.868	0.955	0.1897
236097_at	200.7	225.1	0.17	1.12	PAP-PPP	0.10	17	0.868	0.955	0.1886
202039_at	586.3	657.7	0.17	1.12	PPP-PPP	0.10	17	0.868	0.955	0.1483
201580_s_at	1519.0	1704.5	0.17	1.12	PPP-PPP	0.10	17	0.868	0.955	0.1280
210840_s_at	4285.6	4809.7	0.17	1.12	PPP-PPP	0.10	17	0.868	0.955	0.1241
226307_at	358.2	401.9	0.17	1.12	PPP-PPP	0.10	17	0.868	0.955	0.1235
219913_s_at	1037.5	1163.8	0.17	1.12	PPP-PPP	0.10	17	0.868	0.955	0.1038
235628_x_at	439.1	492.5	0.17	1.12	PPP-PPP	0.10	17	0.868	0.955	0.5510
228967_at	326.4	366.0	0.17	1.12	PPP-APP	0.10	17	0.868	0.955	0.5038
203223_at	175.0	196.1	0.17	1.12	PPP-PPP	0.10	17	0.868	0.955	0.4853
34726_at	364.9	409.0	0.17	1.12	PPP-PPP	0.10	17	0.868	0.955	0.4566
226922_at	458.6	514.1	0.17	1.12	PPP-PPP	0.10	17	0.868	0.955	0.4049
226502_at	882.6	989.8	0.17	1.12	PPP-PPP	0.10	17	0.868	0.955	0.3681
1558249_s_at	452.1	506.7	0.17	1.12	PPP-PPP	0.10	17	0.868	0.955	0.3192
1557522_x_at	444.4	498.3	0.17	1.12	PPP-PPP	0.10	17	0.868	0.955	0.2940
203068_at	680.9	763.6	0.17	1.12	PPP-PPP	0.10	17	0.868	0.955	0.2828
222999_s_at	1936.5	2171.5	0.17	1.12	PPP-PPP	0.10	17	0.868	0.955	0.2396
224719_s_at	3172.1	3556.6	0.17	1.12	PPP-PPP	0.10	17	0.868	0.955	0.2302
225951_s_at	2982.9	3344.5	0.17	1.12	PPP-PPP	0.10	17	0.868	0.955	0.1955
211702_s_at	434.2	486.8	0.17	1.12	PPP-PPP	0.10	17	0.868	0.955	0.1609
218458_at	1029.2	1153.9	0.17	1.12	PPP-PPP	0.10	17	0.868	0.955	0.0808

217882_at	2986.6	3348.9	0.17	1.12	PPP-PPP	0.10	17	0.868	0.955	0.0517
1554795_a_at	406.3	455.2	0.16	1.12	PAM-AMP	0.10	17	0.868	0.955	0.5571
212001_at	479.3	537.2	0.16	1.12	PPP-PPP	0.10	17	0.868	0.955	0.5503
203803_at	585.4	655.9	0.16	1.12	PPP-PPP	0.10	17	0.868	0.955	0.5308
224947_at	900.0	1008.5	0.16	1.12	PPP-PPP	0.10	17	0.868	0.955	0.4635
208751_at	847.0	948.8	0.16	1.12	PPP-PPP	0.10	17	0.868	0.955	0.4021
226221_at	402.3	450.8	0.16	1.12	PPP-PPP	0.10	17	0.868	0.955	0.3919
202874_s_at	2270.3	2542.8	0.16	1.12	PPP-PPP	0.10	17	0.868	0.955	0.2350
202904_s_at	1446.7	1620.6	0.16	1.12	PPP-PPP	0.10	17	0.868	0.955	0.2222
205880_at	292.1	327.2	0.16	1.12	PPP-PPP	0.10	17	0.868	0.955	0.2182
232680_at	235.0	263.4	0.16	1.12	AAP-PPP	0.10	17	0.868	0.955	0.2173
202890_at	1236.2	1384.8	0.16	1.12	PPP-PPP	0.10	17	0.868	0.955	0.1325
201095_at	835.2	935.6	0.16	1.12	PPP-PPP	0.10	17	0.868	0.955	0.1157
233893_s_at	380.4	426.1	0.16	1.12	PPP-PPP	0.10	17	0.868	0.955	0.1123
227840_at	325.5	364.6	0.16	1.12	PPP-PPP	0.10	17	0.868	0.955	0.0704
225358_at	876.9	982.6	0.16	1.12	PPP-PPP	0.10	17	0.868	0.955	0.0422
233851_s_at	332.6	372.4	0.16	1.12	PPP-PPP	0.10	17	0.868	0.955	0.5657
235902_at	116.9	130.8	0.16	1.12	PPP-PPP	0.10	17	0.868	0.955	0.5322
223264_at	1042.3	1167.3	0.16	1.12	PPP-PPP	0.10	17	0.868	0.955	0.5233
1552977_a_at	411.1	460.4	0.16	1.12	PPP-PPP	0.10	17	0.868	0.955	0.5122
213937_s_at	717.1	802.9	0.16	1.12	PPP-PPP	0.10	17	0.868	0.955	0.5004
229274_at	80.4	90.0	0.16	1.12	MPP-APA	0.10	17	0.868	0.955	0.4879
209907_s_at	393.9	440.9	0.16	1.12	PPP-PPP	0.10	17	0.868	0.955	0.4559
228968_at	183.8	205.8	0.16	1.12	AAP-APP	0.10	17	0.868	0.955	0.4524
204706_at	699.6	783.5	0.16	1.12	PPP-PPP	0.10	17	0.868	0.955	0.3929
238789_at	230.8	258.4	0.16	1.12	MPA-PPP	0.10	17	0.868	0.955	0.3868
219512_at	647.1	724.7	0.16	1.12	PPP-PPP	0.10	17	0.868	0.955	0.3864
224639_at	984.9	1102.9	0.16	1.12	PPP-PPP	0.10	17	0.868	0.955	0.3293
209931_s_at	219.5	245.8	0.16	1.12	PPP-PPP	0.10	17	0.868	0.955	0.3155
202105_at	1395.4	1561.9	0.16	1.12	PPP-PPP	0.10	17	0.868	0.955	0.2317
228204_at	517.5	579.3	0.16	1.12	PPP-PPP	0.10	17	0.868	0.955	0.2225
201731_s_at	3230.0	3617.3	0.16	1.12	PPP-PPP	0.10	17	0.868	0.955	0.1898
241820_at	590.1	660.7	0.16	1.12	PPP-PPP	0.10	17	0.868	0.955	0.1848
213514_s_at	1186.1	1328.3	0.16	1.12	PPP-PPP	0.10	17	0.868	0.955	0.1815
202130_at	4532.1	5075.0	0.16	1.12	PPP-PPP	0.10	17	0.868	0.955	0.1438
222154_s_at	8958.2	10029.6	0.16	1.12	PPP-PPP	0.10	17	0.868	0.955	0.0653
203020_at	885.7	991.4	0.16	1.12	PPP-PPP	0.10	17	0.868	0.955	0.0626
1569013_s_at	144.2	161.3	0.16	1.12	PPP-PPP	0.10	17	0.868	0.955	0.5663
204646_at	1440.9	1611.9	0.16	1.12	PPP-PPP	0.10	17	0.868	0.955	0.5492
227455_at	835.4	935.0	0.16	1.12	PPP-PPP	0.10	17	0.868	0.955	0.5075
200959_at	3928.3	4394.5	0.16	1.12	PPP-PPP	0.10	17	0.868	0.955	0.4981
1569003_at	478.0	534.9	0.16	1.12	PPP-PPP	0.10	17	0.868	0.955	0.4755

219348_at	547.6	612.7	0.16	1.12	PPP-PPP	0.10	17	0.868	0.955	0.4734
240528_s_at	344.4	385.4	0.16	1.12	PPP-PPP	0.10	17	0.868	0.955	0.4541
227306_at	121.4	135.8	0.16	1.12	APA-PMA	0.10	17	0.868	0.955	0.4526
205171_at	609.5	681.8	0.16	1.12	PPP-PPP	0.10	17	0.868	0.955	0.4117
203143_s_at	709.3	793.7	0.16	1.12	PPP-PPP	0.10	17	0.868	0.955	0.3677
202604_x_at	490.0	548.1	0.16	1.12	PPP-PPP	0.10	17	0.868	0.955	0.3042
212376_s_at	442.4	495.1	0.16	1.12	PPP-PPP	0.10	17	0.868	0.955	0.2817
230449_x_at	295.1	330.1	0.16	1.12	AAA-MPA	0.10	17	0.868	0.955	0.2776
226897_s_at	646.1	723.0	0.16	1.12	PPP-PPP	0.10	17	0.868	0.955	0.2621
233814_at	597.7	668.6	0.16	1.12	PPP-PPP	0.10	17	0.868	0.955	0.2155
221490_at	854.8	956.1	0.16	1.12	PMP-PPP	0.10	17	0.868	0.955	0.1632
214394_x_at	34871.3	39024.1	0.16	1.12	PPP-PPP	0.10	17	0.868	0.955	0.1588
226803_at	1928.9	2158.6	0.16	1.12	PPP-PPP	0.10	17	0.868	0.955	0.1276
200093_s_at	25318.2	28334.1	0.16	1.12	PPP-PPP	0.10	17	0.868	0.955	0.0787
200933_x_at	42967.3	48084.4	0.16	1.12	PPP-PPP	0.10	17	0.868	0.955	0.0720
220924_s_at	16164.0	18080.4	0.16	1.12	PPP-PPP	0.10	17	0.868	0.955	0.0613
205469_s_at	431.5	482.6	0.16	1.12	PAP-PPP	0.10	17	0.868	0.955	0.5774
219754_at	365.4	408.6	0.16	1.12	PPP-PPP	0.10	17	0.868	0.955	0.5683
35179_at	141.3	157.9	0.16	1.12	PPP-PPP	0.10	17	0.868	0.955	0.5635
222716_s_at	749.7	838.3	0.16	1.12	PPP-PPP	0.10	17	0.868	0.955	0.5515
213076_at	523.4	585.4	0.16	1.12	PPP-PPP	0.10	17	0.868	0.955	0.4316
220086_at	311.9	348.7	0.16	1.12	PMP-PPP	0.10	17	0.868	0.955	0.4105
1553986_at	329.6	368.4	0.16	1.12	PPP-PPP	0.10	17	0.868	0.955	0.3994
215606_s_at	715.1	799.6	0.16	1.12	PPP-PPP	0.10	17	0.868	0.955	0.3927
202516_s_at	478.8	535.3	0.16	1.12	PPP-PPP	0.10	17	0.868	0.955	0.3747
218224_at	1732.3	1936.4	0.16	1.12	PPP-PPP	0.10	17	0.868	0.955	0.3717
228842_at	107.4	120.1	0.16	1.12	AAA-AAP	0.10	17	0.868	0.955	0.3347
231975_s_at	290.0	324.3	0.16	1.12	PPP-PPP	0.10	17	0.868	0.955	0.3182
227016_at	878.8	982.7	0.16	1.12	PPP-PPP	0.10	17	0.868	0.955	0.3131
226770_at	304.3	340.4	0.16	1.12	PPP-PPP	0.10	17	0.868	0.955	0.2746
219000_s_at	2230.1	2492.7	0.16	1.12	PPP-PPP	0.10	17	0.868	0.955	0.2725
225339_at	810.6	906.2	0.16	1.12	PPP-PPP	0.10	17	0.868	0.955	0.2708
218397_at	1510.8	1688.8	0.16	1.12	PPP-PPP	0.10	17	0.868	0.955	0.2324
241472_at	76.8	85.9	0.16	1.12	PPP-PPP	0.10	17	0.868	0.955	0.2108
1553172_at	962.9	1076.5	0.16	1.12	PPP-PPP	0.10	17	0.868	0.955	0.2077
242979_at	154.2	172.4	0.16	1.12	PPP-MPP	0.10	17	0.868	0.955	0.1822
204215_at	2686.9	3003.4	0.16	1.12	PPP-PPP	0.10	17	0.868	0.955	0.1647
221742_at	1063.4	1189.1	0.16	1.12	PPP-PPP	0.10	17	0.868	0.955	0.1294
227204_at	895.6	1001.4	0.16	1.12	PPP-PPP	0.10	17	0.868	0.955	0.1174
209179_s_at	945.4	1057.1	0.16	1.12	PPP-PPP	0.10	17	0.868	0.955	0.1094
225525_at	3249.4	3632.9	0.16	1.12	PPP-PPP	0.10	17	0.868	0.955	0.0954
202325_s_at	12120.6	13552.2	0.16	1.12	PPP-PPP	0.10	17	0.868	0.955	0.0346

208626_s_at	1823.1	2037.1	0.16	1.12	PPP-PPP	0.10	17	0.868	0.955	0.5617
236047_at	92.4	103.3	0.16	1.12	PAP-MAP	0.10	17	0.868	0.955	0.5571
209454_s_at	208.6	233.1	0.16	1.12	PPP-PPP	0.10	17	0.868	0.955	0.5518
243686_at	53.3	59.5	0.16	1.12	APA-PMP	0.10	17	0.868	0.955	0.4915
207782_s_at	856.8	957.3	0.16	1.12	PPP-PPP	0.10	17	0.868	0.955	0.4522
224455_s_at	284.7	318.1	0.16	1.12	PPP-PPP	0.10	17	0.868	0.955	0.4512
AFFX-r2-Ec-bioB-M_at	861.2	962.4	0.16	1.12	PPP-PPP	0.10	17	0.868	0.955	0.4437
213940_s_at	344.4	384.7	0.16	1.12	PPP-PPP	0.10	17	0.868	0.955	0.4151
217188_s_at	952.6	1064.6	0.16	1.12	PPP-PPP	0.10	17	0.868	0.955	0.4138
1553994_at	1512.5	1690.4	0.16	1.12	PPP-PPP	0.10	17	0.868	0.955	0.3406
212540_at	1517.4	1695.6	0.16	1.12	PPP-PPP	0.10	17	0.868	0.955	0.3180
212763_at	1079.3	1206.0	0.16	1.12	PPP-PPP	0.10	17	0.868	0.955	0.3025
211452_x_at	7696.8	8598.2	0.16	1.12	PPP-PPP	0.10	17	0.868	0.955	0.2961
222632_s_at	334.6	373.9	0.16	1.12	PPP-PPP	0.10	17	0.868	0.955	0.2689
219979_s_at	2942.5	3286.5	0.16	1.12	PPP-PPP	0.10	17	0.868	0.955	0.2674
217806_s_at	1882.2	2102.3	0.16	1.12	PPP-PPP	0.10	17	0.868	0.955	0.2479
222851_at	273.7	305.8	0.16	1.12	PPP-PPP	0.10	17	0.868	0.955	0.2484
210093_s_at	2022.5	2259.0	0.16	1.12	PPP-PPP	0.10	17	0.868	0.955	0.2281
206095_s_at	4466.6	4990.3	0.16	1.12	PPP-PPP	0.10	17	0.868	0.955	0.1265
222266_at	1158.2	1294.0	0.16	1.12	PPP-PPP	0.10	17	0.868	0.955	0.0979
226159_at	916.6	1024.0	0.16	1.12	PPP-PPP	0.10	17	0.868	0.955	0.0797
238109_at	124.0	138.5	0.16	1.12	PAA-AAP	0.10	17	0.868	0.955	0.0734
202583_s_at	946.6	1057.4	0.16	1.12	PPP-PPP	0.10	17	0.868	0.955	0.0581
238686_at	600.2	670.0	0.16	1.12	PPP-PPP	0.10	17	0.868	0.955	0.5572
219358_s_at	235.3	262.8	0.16	1.12	PPP-PPP	0.10	17	0.868	0.955	0.5178
212176_at	886.7	990.0	0.16	1.12	PPP-PPP	0.10	17	0.868	0.955	0.5176
1569974_x_at	70.1	78.2	0.16	1.12	AAP-AAP	0.10	17	0.868	0.955	0.4943
219953_s_at	1770.8	1976.5	0.16	1.12	PPP-PPP	0.10	17	0.868	0.955	0.4674
233255_s_at	1074.7	1200.1	0.16	1.12	PPP-PPP	0.10	17	0.868	0.955	0.4093
242621_at	295.7	330.1	0.16	1.12	PPP-PPP	0.10	17	0.868	0.955	0.3986
213374_x_at	1445.5	1614.2	0.16	1.12	PPP-PPP	0.10	17	0.868	0.955	0.3942
219229_at	707.5	789.8	0.16	1.12	PPP-PPP	0.10	17	0.868	0.955	0.3763
217754_at	1743.7	1947.5	0.16	1.12	PPP-PPP	0.10	17	0.868	0.955	0.3236
215198_s_at	167.8	187.3	0.16	1.12	PPP-APP	0.10	17	0.868	0.955	0.2970
207170_s_at	1510.6	1686.5	0.16	1.12	PPP-PPP	0.10	17	0.868	0.955	0.2778
212163_at	888.9	992.3	0.16	1.12	PPP-PPP	0.10	17	0.868	0.955	0.2140
204765_at	1066.3	1190.7	0.16	1.12	PPP-PPP	0.10	17	0.868	0.955	0.2011
210006_at	568.9	635.1	0.16	1.12	PPP-PPP	0.10	17	0.868	0.955	0.1896
204544_at	2309.1	2579.0	0.16	1.12	PPP-PPP	0.10	17	0.868	0.955	0.0482
204319_s_at	2367.4	2640.8	0.16	1.12	PPP-PPP	0.10	17	0.868	0.955	0.5654
209334_s_at	174.7	195.0	0.16	1.12	APP-PPP	0.10	17	0.868	0.955	0.5615
1554213_at	175.4	195.7	0.16	1.12	APP-PPP	0.10	17	0.868	0.955	0.5609

226780_s_at	1059.8	1182.4	0.16	1.12	PPP-PPP	0.10	17	0.868	0.955	0.4883
219492_at	1234.3	1377.4	0.16	1.12	PPP-PPP	0.10	17	0.868	0.955	0.4441
218371_s_at	614.5	685.8	0.16	1.12	PPP-PPP	0.10	17	0.868	0.955	0.4355
201825_s_at	1010.9	1128.2	0.16	1.12	PPP-PPP	0.10	17	0.868	0.955	0.4293
220020_at	653.9	729.6	0.16	1.12	PPP-PPP	0.10	17	0.868	0.955	0.3576
225718_at	893.8	996.9	0.16	1.12	PPP-PPP	0.10	17	0.868	0.955	0.3417
219848_s_at	239.8	267.7	0.16	1.12	PPP-PPP	0.10	17	0.868	0.955	0.3150
211034_s_at	397.2	443.3	0.16	1.12	PPP-PPP	0.10	17	0.868	0.955	0.2993
209164_s_at	596.3	665.5	0.16	1.12	PPP-PPP	0.10	17	0.868	0.955	0.2954
228951_at	167.7	187.1	0.16	1.12	PPP-PPP	0.10	17	0.868	0.955	0.2674
208264_s_at	1111.0	1239.3	0.16	1.12	PPP-PPP	0.10	17	0.868	0.955	0.2146
227068_at	2476.8	2763.4	0.16	1.12	PPP-PPP	0.10	17	0.868	0.955	0.1995
224749_at	482.1	537.9	0.16	1.12	PPP-PPP	0.10	17	0.868	0.955	0.1807
220748_s_at	721.0	804.7	0.16	1.12	PPP-PPP	0.10	17	0.868	0.955	0.1740
226619_at	383.4	427.8	0.16	1.12	PPP-PPP	0.10	17	0.868	0.955	0.0815
204321_at	304.6	339.7	0.16	1.12	PPP-PPP	0.10	17	0.868	0.955	0.5702
214016_s_at	1136.7	1267.7	0.16	1.12	PPP-PPP	0.10	17	0.868	0.955	0.5662
241955_at	285.5	318.3	0.16	1.12	PPP-PPP	0.10	17	0.868	0.955	0.5254
222454_s_at	515.5	574.6	0.16	1.12	PPP-PPP	0.10	17	0.868	0.955	0.4979
226393_at	319.6	356.4	0.16	1.12	PPP-PPP	0.10	17	0.868	0.955	0.4955
221234_s_at	147.0	163.8	0.16	1.12	PPP-PPP	0.10	17	0.868	0.955	0.4630
242963_at	222.6	248.2	0.16	1.12	PPP-PPP	0.10	17	0.868	0.955	0.4158
203310_at	2268.8	2530.4	0.16	1.12	PPP-PPP	0.10	17	0.868	0.955	0.3733
202040_s_at	981.9	1095.0	0.16	1.12	PPP-PPP	0.10	17	0.868	0.955	0.3495
215758_x_at	428.4	477.5	0.16	1.12	PPP-PPP	0.10	17	0.868	0.955	0.3238
202224_at	1736.3	1936.4	0.16	1.12	PPP-PPP	0.10	17	0.868	0.955	0.1423
202119_s_at	7655.9	8533.4	0.16	1.12	PPP-PPP	0.10	17	0.868	0.955	0.0722
218158_s_at	1161.7	1294.8	0.16	1.12	PPP-PPP	0.10	17	0.868	0.955	0.0697
229310_at	1241.8	1384.2	0.16	1.12	PPP-PPP	0.10	17	0.868	0.955	0.0509
204434_at	256.6	285.9	0.16	1.11	PMP-APP	0.10	17	0.868	0.955	0.5465
242640_at	113.5	126.4	0.16	1.11	PPA-PPP	0.10	17	0.868	0.955	0.5198
223588_at	92.0	102.5	0.16	1.11	PPP-PPP	0.10	17	0.868	0.955	0.5038
231927_at	626.0	697.5	0.16	1.11	PPP-PPP	0.10	17	0.868	0.955	0.4589
211502_s_at	493.5	549.8	0.16	1.11	PPP-PPP	0.10	17	0.868	0.955	0.3870
230669_at	1052.8	1173.0	0.16	1.11	PPP-PPP	0.10	17	0.868	0.955	0.3677
217448_s_at	288.5	321.5	0.16	1.11	PPP-PPP	0.10	17	0.868	0.955	0.3649
209751_s_at	1473.0	1641.4	0.16	1.11	PPP-PPP	0.10	17	0.868	0.955	0.2528
207435_s_at	1024.9	1141.7	0.16	1.11	PPP-PPP	0.10	17	0.868	0.955	0.2281
227100_at	270.2	301.1	0.16	1.11	PPP-PPP	0.10	17	0.868	0.955	0.1843
202041_s_at	3162.4	3523.4	0.16	1.11	PPP-PPP	0.10	17	0.868	0.955	0.1243
200741_s_at	45214.6	50361.9	0.16	1.11	PPP-PPP	0.10	17	0.868	0.955	0.0982
204657_s_at	220.5	245.5	0.16	1.11	PPA-APM	0.10	17	0.868	0.955	0.5753

225910_at	832.7	927.2	0.16	1.11	PPP-PPP	0.10	17	0.868	0.955	0.4792
202528_at	980.3	1091.7	0.16	1.11	PPP-PPP	0.10	17	0.868	0.955	0.4454
243071_at	193.0	215.0	0.16	1.11	PPP-PPP	0.10	17	0.868	0.955	0.3777
201294_s_at	150.4	167.4	0.16	1.11	AMP-PPP	0.10	17	0.868	0.955	0.3742
203749_s_at	160.9	179.2	0.16	1.11	APP-PAA	0.10	17	0.868	0.955	0.3172
226686_at	985.7	1097.8	0.16	1.11	PPP-PPP	0.10	17	0.868	0.955	0.3074
202720_at	7970.0	8871.7	0.16	1.11	PPP-PPP	0.10	17	0.868	0.955	0.2580
208795_s_at	2874.3	3200.1	0.16	1.11	PPP-PPP	0.10	17	0.868	0.955	0.2367
227454_at	682.5	759.8	0.16	1.11	PPP-PPP	0.10	17	0.868	0.955	0.2234
235009_at	615.1	685.1	0.16	1.11	PPP-PPP	0.10	17	0.868	0.955	0.1970
201032_at	4930.8	5491.0	0.16	1.11	PPP-PPP	0.10	17	0.868	0.955	0.1676
218259_at	1200.0	1335.7	0.16	1.11	PPP-PPP	0.10	17	0.868	0.955	0.1619
202220_at	2736.0	3045.7	0.16	1.11	PPP-PPP	0.10	17	0.868	0.955	0.1502
203660_s_at	1107.5	1232.9	0.16	1.11	PPP-PPP	0.10	17	0.868	0.955	0.1461
209027_s_at	2598.7	2892.8	0.16	1.11	PPP-PPP	0.10	17	0.868	0.955	0.1118
200958_s_at	16022.5	17837.1	0.16	1.11	PPP-PPP	0.10	17	0.868	0.955	0.0669
204168_at	2861.6	3186.2	0.16	1.11	PPP-PPP	0.10	17	0.868	0.955	0.0622
213238_at	759.6	845.4	0.15	1.11	PPP-PPP	0.10	17	0.868	0.955	0.5717
220757_s_at	615.5	685.0	0.15	1.11	PPP-PPP	0.10	17	0.868	0.955	0.5581
1554438_at	144.6	160.9	0.15	1.11	PPP-PPP	0.10	17	0.868	0.955	0.5488
225595_at	81.8	91.0	0.15	1.11	PPP-PPP	0.10	17	0.868	0.955	0.5316
214835_s_at	2997.5	3336.1	0.15	1.11	PPP-PPP	0.10	17	0.868	0.955	0.4720
225296_at	835.2	929.5	0.15	1.11	PPP-PPP	0.10	17	0.868	0.955	0.3492
227413_at	1936.1	2154.2	0.15	1.11	PPP-PPP	0.10	17	0.868	0.955	0.3389
238437_at	115.0	128.0	0.15	1.11	PPP-PPP	0.10	17	0.868	0.955	0.3225
223050_s_at	577.8	642.7	0.15	1.11	PPP-PPP	0.10	17	0.868	0.955	0.3012
218437_s_at	496.6	552.7	0.15	1.11	PPP-PPP	0.10	17	0.868	0.955	0.2970
229105_at	398.4	443.3	0.15	1.11	PPP-PPP	0.10	17	0.868	0.955	0.2627
203007_x_at	8064.7	8971.5	0.15	1.11	PPP-PPP	0.10	17	0.868	0.955	0.2286
225180_at	854.8	951.0	0.15	1.11	PPP-PPP	0.10	17	0.868	0.955	0.1848
202235_at	662.0	736.7	0.15	1.11	PPP-PPP	0.10	17	0.868	0.955	0.1699
218078_s_at	1092.4	1215.3	0.15	1.11	PPP-PPP	0.10	17	0.868	0.955	0.1683
213944_x_at	1247.3	1388.2	0.15	1.11	PPP-PPP	0.10	17	0.868	0.955	0.1306
201611_s_at	1245.9	1386.5	0.15	1.11	PPP-PPP	0.10	17	0.868	0.955	0.1024
216295_s_at	8463.6	9418.5	0.15	1.11	PPP-PPP	0.10	17	0.868	0.955	0.0768
221473_x_at	2372.4	2639.2	0.15	1.11	PPP-PPP	0.10	17	0.868	0.955	0.0727
223049_at	1753.6	1951.5	0.15	1.11	PPP-PPP	0.10	17	0.868	0.955	0.0493
31845_at	1101.0	1225.4	0.15	1.11	PPP-PPP	0.10	17	0.868	0.955	0.0452
209389_x_at	7546.5	8393.9	0.15	1.11	PPP-PPP	0.10	17	0.868	0.955	0.0372
1552634_a_at	271.6	302.0	0.15	1.11	PPP-PPP	0.10	17	0.868	0.955	0.5711
243338_at	241.6	268.6	0.15	1.11	PPP-PPP	0.10	17	0.868	0.955	0.5711
214152_at	304.5	338.7	0.15	1.11	PPP-MPP	0.10	17	0.868	0.955	0.5433

212020_s_at	528.0	587.0	0.15	1.11	PPP-PPP	0.10	17	0.868	0.955	0.5266
223004_s_at	3215.0	3575.8	0.15	1.11	PPP-PPP	0.10	17	0.868	0.955	0.5123
222891_s_at	570.2	634.1	0.15	1.11	PPP-PPP	0.10	17	0.868	0.955	0.4838
207127_s_at	2084.7	2318.2	0.15	1.11	PPP-PPP	0.10	17	0.868	0.955	0.4539
225516_at	3708.3	4122.6	0.15	1.11	PPP-PPP	0.10	17	0.868	0.955	0.4513
214882_s_at	8483.7	9431.1	0.15	1.11	PPP-PPP	0.10	17	0.868	0.955	0.2882
212616_at	2107.3	2342.5	0.15	1.11	PPP-PPP	0.10	17	0.868	0.955	0.2329
226149_at	465.8	517.9	0.15	1.11	PPP-PPP	0.10	17	0.868	0.955	0.2188
238879_at	364.7	405.4	0.15	1.11	PPP-PPP	0.10	17	0.868	0.955	0.2133
223460_at	242.0	269.0	0.15	1.11	PPP-APP	0.10	17	0.868	0.955	0.1661
225426_at	558.2	620.5	0.15	1.11	PPP-PPP	0.10	17	0.868	0.955	0.1064
213491_x_at	11172.3	12423.2	0.15	1.11	PPP-PPP	0.10	17	0.868	0.955	0.0633
221524_s_at	240.3	267.0	0.15	1.11	APP-PPP	0.10	17	0.868	0.955	0.5725
214594_x_at	627.7	697.4	0.15	1.11	PPP-PPP	0.10	17	0.868	0.955	0.5552
225341_at	241.2	268.1	0.15	1.11	PPP-PPP	0.10	17	0.868	0.955	0.5066
225832_s_at	490.6	545.2	0.15	1.11	PPP-PPP	0.10	17	0.868	0.955	0.4409
212932_at	618.8	687.3	0.15	1.11	PPP-PPP	0.10	17	0.868	0.955	0.3844
226450_at	327.2	363.7	0.15	1.11	PPP-PPP	0.10	17	0.868	0.955	0.3574
219494_at	710.8	789.8	0.15	1.11	PPP-PPP	0.10	17	0.868	0.955	0.3467
219267_at	904.8	1005.6	0.15	1.11	PPP-PPP	0.10	17	0.868	0.955	0.3402
223202_s_at	832.3	924.5	0.15	1.11	PPP-PPP	0.10	17	0.868	0.955	0.3360
222843_at	1196.7	1329.5	0.15	1.11	PPP-PPP	0.10	17	0.868	0.955	0.3337
224858_at	3879.1	4311.5	0.15	1.11	PPP-PPP	0.10	17	0.868	0.955	0.2712
1559038_at	209.7	233.0	0.15	1.11	PAP-MPP	0.10	17	0.868	0.955	0.2567
212099_at	339.6	377.2	0.15	1.11	PPP-PPP	0.10	17	0.868	0.955	0.2546
201424_s_at	2930.8	3256.7	0.15	1.11	PPP-PPP	0.10	17	0.868	0.955	0.1899
212284_x_at	47542.0	52806.1	0.15	1.11	PPP-PPP	0.10	17	0.868	0.955	0.1230
205037_at	251.8	279.8	0.15	1.11	PPP-PPP	0.10	17	0.868	0.955	0.0800
211942_x_at	34446.8	38262.9	0.15	1.11	PPP-PPP	0.10	17	0.868	0.955	0.0614
209271_at	961.5	1067.6	0.15	1.11	PPP-PPP	0.10	17	0.868	0.955	0.5406
203629_s_at	660.6	733.6	0.15	1.11	PPP-PPP	0.10	17	0.868	0.955	0.5252
226579_at	917.3	1018.4	0.15	1.11	PPP-PPP	0.10	17	0.868	0.955	0.5225
203958_s_at	134.3	149.1	0.15	1.11	PPP-PPP	0.10	17	0.868	0.955	0.4920
201155_s_at	2081.2	2310.2	0.15	1.11	PPP-PPP	0.10	17	0.868	0.955	0.4889
203810_at	459.3	510.0	0.15	1.11	PPP-PPP	0.10	17	0.868	0.955	0.4794
213330_s_at	3123.3	3467.3	0.15	1.11	PPP-PPP	0.10	17	0.868	0.955	0.3899
205371_s_at	357.7	397.1	0.15	1.11	PPP-PPP	0.10	17	0.868	0.955	0.3565
218613_at	892.2	990.7	0.15	1.11	PPP-PPP	0.10	17	0.868	0.955	0.3427
222011_s_at	2020.8	2243.7	0.15	1.11	PPP-PPP	0.10	17	0.868	0.955	0.2596
244317_at	284.1	315.4	0.15	1.11	PPP-PPP	0.10	17	0.868	0.955	0.2306
222684_s_at	1650.9	1832.4	0.15	1.11	PPP-PPP	0.10	17	0.868	0.955	0.2080
210830_s_at	8615.6	9565.1	0.15	1.11	PPP-PPP	0.10	17	0.868	0.955	0.1108

218203_at	6482.1	7198.6	0.15	1.11	PPP-PPP	0.10	17	0.868	0.955	0.0920
227998_at	22083.4	24523.3	0.15	1.11	PPP-PPP	0.10	17	0.868	0.955	0.0466
209885_at	3850.8	4275.5	0.15	1.11	PPP-PPP	0.10	17	0.868	0.955	0.0424
217732_s_at	15463.5	17167.3	0.15	1.11	PPP-PPP	0.10	17	0.868	0.955	0.0414
212593_s_at	4146.4	4605.3	0.15	1.11	PPP-PPP	0.10	17	0.868	0.955	0.0400
238602_at	173.6	192.5	0.15	1.11	PPP-PPP	0.10	17	0.868	0.955	0.5651
221834_at	287.3	318.8	0.15	1.11	PPP-PPP	0.10	17	0.868	0.955	0.5594
243948_at	129.6	143.8	0.15	1.11	PPP-PAP	0.10	17	0.868	0.955	0.5562
1555858_at	98.8	109.6	0.15	1.11	PPP-PPP	0.10	17	0.868	0.955	0.5504
212570_at	522.9	580.3	0.15	1.11	PPP-PPP	0.10	17	0.868	0.955	0.5465
201080_at	795.8	883.3	0.15	1.11	PPP-PPP	0.10	17	0.868	0.955	0.5460
235954_at	100.2	111.2	0.15	1.11	PPP-PPP	0.10	17	0.868	0.955	0.5150
1562664_at	93.0	103.2	0.15	1.11	AAM-APP	0.10	17	0.868	0.955	0.3844
206920_s_at	545.1	604.9	0.15	1.11	PPP-PPP	0.10	17	0.868	0.955	0.3819
203025_at	1693.2	1878.8	0.15	1.11	PPP-PPP	0.10	17	0.868	0.955	0.3050
233041_x_at	522.6	579.7	0.15	1.11	PPP-PPP	0.10	17	0.868	0.955	0.2876
208824_x_at	953.1	1057.7	0.15	1.11	PPP-PPP	0.10	17	0.868	0.955	0.2723
220081_x_at	1986.6	2204.2	0.15	1.11	PPP-PPP	0.10	17	0.868	0.955	0.2024
217961_at	790.0	876.7	0.15	1.11	PPP-PPP	0.10	17	0.868	0.955	0.1904
222077_s_at	5810.4	6447.7	0.15	1.11	PPP-PPP	0.10	17	0.868	0.955	0.1389
218012_at	366.5	406.3	0.15	1.11	PPP-PPP	0.10	17	0.868	0.955	0.5469
209187_at	3275.3	3630.7	0.15	1.11	PPP-PPP	0.10	17	0.868	0.955	0.5004
202256_at	311.6	345.3	0.15	1.11	PPP-PPP	0.10	17	0.868	0.955	0.4654
34221_at	652.8	724.0	0.15	1.11	PPP-PPP	0.10	17	0.868	0.955	0.4219
225723_at	763.1	846.3	0.15	1.11	PPP-PPP	0.10	17	0.868	0.955	0.3953
200067_x_at	8835.6	9795.8	0.15	1.11	PPP-PPP	0.10	17	0.868	0.955	0.3741
223919_at	221.7	245.9	0.15	1.11	PPA-APM	0.10	17	0.868	0.955	0.3633
219683_at	695.8	771.4	0.15	1.11	PPP-PPP	0.10	17	0.868	0.955	0.3600
203142_s_at	1831.7	2030.7	0.15	1.11	PPP-PPP	0.10	17	0.868	0.955	0.3553
221158_at	624.3	692.4	0.15	1.11	PPP-PPP	0.10	17	0.868	0.955	0.3539
1554082_a_at	1418.4	1572.3	0.15	1.11	PPP-PPP	0.10	17	0.868	0.955	0.3171
202094_at	903.3	1001.9	0.15	1.11	PPP-PPP	0.10	17	0.868	0.955	0.3175
235233_s_at	428.0	474.6	0.15	1.11	PPP-PPP	0.10	17	0.868	0.955	0.2997
218071_s_at	716.2	793.9	0.15	1.11	PPP-PPP	0.10	17	0.868	0.955	0.2582
205184_at	478.7	530.9	0.15	1.11	PPP-PPP	0.10	17	0.868	0.955	0.2070
221569_at	329.7	365.7	0.15	1.11	PPP-PPP	0.10	17	0.868	0.955	0.1304
202799_at	3428.7	3803.1	0.15	1.11	PPP-PPP	0.10	17	0.868	0.955	0.0726
225679_at	931.2	1032.8	0.15	1.11	PPP-PPP	0.10	17	0.868	0.955	0.0382
212033_at	1247.2	1381.7	0.15	1.11	PPP-PPP	0.10	17	0.868	0.955	0.5235
205115_s_at	214.2	237.4	0.15	1.11	AAP-MPP	0.10	17	0.868	0.955	0.5218
1552370_at	223.2	247.3	0.15	1.11	PPP-PPP	0.10	17	0.868	0.955	0.4995
240044_x_at	757.9	839.6	0.15	1.11	PPP-PPP	0.10	17	0.868	0.955	0.4310

206788_s_at	499.8	554.0	0.15	1.11	PPP-PPP	0.10	17	0.868	0.955	0.4227
1555307_at	82.6	91.5	0.15	1.11	AMA-APM	0.10	17	0.868	0.955	0.4184
225561_at	731.7	811.0	0.15	1.11	PPP-PPP	0.10	17	0.868	0.955	0.4067
223273_at	1291.0	1430.5	0.15	1.11	PPP-PPP	0.10	17	0.868	0.955	0.3857
222566_at	400.3	443.4	0.15	1.11	PPP-PPP	0.10	17	0.868	0.955	0.3639
241836_x_at	135.2	149.8	0.15	1.11	APP-PPP	0.10	17	0.868	0.955	0.3162
201784_s_at	10800.2	11967.2	0.15	1.11	PPP-PPP	0.10	17	0.868	0.955	0.2639
228088_at	429.2	475.6	0.15	1.11	PPP-PPP	0.10	17	0.868	0.955	0.2137
36566_at	225.4	249.7	0.15	1.11	PPP-PPP	0.10	17	0.868	0.955	0.2081
AFFX-r2-P1-cre-5_at	31446.5	34841.2	0.15	1.11	PPP-PPP	0.10	17	0.868	0.955	0.1731
201149_s_at	1769.7	1961.1	0.15	1.11	PPP-PPP	0.10	17	0.868	0.955	0.1047
223361_at	4591.0	5087.4	0.15	1.11	PPP-PPP	0.10	17	0.868	0.955	0.0918
226908_at	669.9	742.4	0.15	1.11	PPP-PPP	0.10	17	0.868	0.955	0.0780
208873_s_at	4673.9	5180.4	0.15	1.11	PPP-PPP	0.10	17	0.868	0.955	0.0768
216271_x_at	148.3	164.3	0.15	1.11	APA-APA	0.10	17	0.868	0.955	0.5593
1560006_a_at	187.9	208.1	0.15	1.11	PPP-PPP	0.10	17	0.868	0.955	0.5263
222748_s_at	290.3	321.5	0.15	1.11	PPP-PPP	0.10	17	0.868	0.955	0.4491
225813_at	970.6	1074.6	0.15	1.11	PPP-PPP	0.10	17	0.868	0.955	0.4214
226885_at	612.0	677.7	0.15	1.11	PPP-PPP	0.10	17	0.868	0.955	0.3390
217591_at	167.8	185.8	0.15	1.11	PAP-PPP	0.10	17	0.868	0.955	0.3282
204699_s_at	1717.5	1902.3	0.15	1.11	PPP-PPP	0.10	17	0.868	0.955	0.3014
AFFX-CreX-3_at	28340.0	31379.8	0.15	1.11	PPP-PPP	0.10	17	0.868	0.955	0.2779
209489_at	2341.2	2591.5	0.15	1.11	PPP-PPP	0.10	17	0.868	0.955	0.2521
225308_s_at	2163.5	2395.0	0.15	1.11	PPP-PPP	0.10	17	0.868	0.955	0.1198
208033_s_at	175.7	194.5	0.15	1.11	PMP-PPP	0.10	17	0.868	0.955	0.1164
223173_at	595.0	658.6	0.15	1.11	PPP-PPP	0.10	17	0.868	0.955	0.0623
202606_s_at	1976.1	2188.0	0.15	1.11	PPP-PPP	0.10	17	0.868	0.955	0.0476
200909_s_at	41985.4	46489.6	0.15	1.11	PPP-PPP	0.10	17	0.868	0.955	0.0446
224484_s_at	314.1	347.6	0.15	1.11	PPP-PPP	0.10	17	0.868	0.955	0.5515
219678_x_at	917.9	1015.5	0.15	1.11	PPP-PPP	0.10	17	0.868	0.955	0.5370
202275_at	301.5	333.5	0.15	1.11	PAP-PPP	0.10	17	0.868	0.955	0.5346
223516_s_at	5670.4	6275.7	0.15	1.11	PPP-PPP	0.10	17	0.868	0.955	0.5203
211208_s_at	967.3	1070.6	0.15	1.11	PPP-PPP	0.10	17	0.868	0.955	0.4966
201939_at	1751.2	1937.1	0.15	1.11	PPP-PPP	0.10	17	0.868	0.955	0.4870
1555944_at	58.0	64.2	0.15	1.11	PAP-PAP	0.10	17	0.868	0.955	0.4794
223081_at	1331.3	1473.4	0.15	1.11	PPP-PPP	0.10	17	0.868	0.955	0.4792
235549_at	131.2	145.1	0.15	1.11	PPP-PPP	0.10	17	0.868	0.955	0.4757
225242_s_at	787.3	871.0	0.15	1.11	PPP-PPP	0.10	17	0.868	0.955	0.4657
206156_at	910.8	1007.5	0.15	1.11	PPP-PPP	0.10	17	0.868	0.955	0.4586
215737_x_at	552.3	611.1	0.15	1.11	PPP-PPP	0.10	17	0.868	0.955	0.4575
209452_s_at	2776.0	3071.0	0.15	1.11	PPP-PPP	0.10	17	0.868	0.955	0.3480
1559190_s_at	580.9	642.9	0.15	1.11	PPP-PPP	0.10	17	0.868	0.955	0.3282

217322_x_at	149.8	165.8	0.15	1.11	APA-PPP	0.10	17	0.868	0.955	0.2944
226979_at	851.9	942.7	0.15	1.11	PPP-PPP	0.10	17	0.868	0.955	0.2929
223397_s_at	1722.9	1905.9	0.15	1.11	PPP-PPP	0.10	17	0.868	0.955	0.2679
225406_at	3758.1	4157.8	0.15	1.11	PPP-PPP	0.10	17	0.868	0.955	0.2669
223023_at	890.2	985.2	0.15	1.11	PPP-PPP	0.10	17	0.868	0.955	0.2647
202852_s_at	1205.8	1333.8	0.15	1.11	PPP-PPP	0.10	17	0.868	0.955	0.2532
203569_s_at	2121.5	2346.9	0.15	1.11	PPP-PPP	0.10	17	0.868	0.955	0.2361
236922_at	204.3	226.0	0.15	1.11	PPP-PPP	0.10	17	0.868	0.955	0.1876
223112_s_at	10056.0	11126.4	0.15	1.11	PPP-PPP	0.10	17	0.868	0.955	0.1606
201061_s_at	8526.3	9435.4	0.15	1.11	PPP-PPP	0.10	17	0.868	0.955	0.1382
206621_s_at	11881.8	13147.5	0.15	1.11	PPP-PPP	0.10	17	0.868	0.955	0.1100
212543_at	3408.1	3769.9	0.15	1.11	PPP-PPP	0.10	17	0.868	0.955	0.0881
224504_s_at	886.8	981.2	0.15	1.11	PPP-PPP	0.10	17	0.868	0.955	0.0818
201172_x_at	9348.0	10342.3	0.15	1.11	PPP-PPP	0.10	17	0.868	0.955	0.0800
200660_at	27923.9	30904.7	0.15	1.11	PPP-PPP	0.10	17	0.868	0.955	0.0620
218373_at	530.0	586.2	0.15	1.11	PPP-PPP	0.10	17	0.868	0.955	0.5660
205896_at	521.4	576.4	0.15	1.11	PPP-PPP	0.10	17	0.868	0.955	0.5224
204693_at	649.8	718.3	0.15	1.11	PPP-PPP	0.10	17	0.868	0.955	0.5044
213927_at	517.5	572.2	0.15	1.11	PPP-PPP	0.10	17	0.868	0.955	0.4769
216070_at	38.2	42.2	0.15	1.11	APA-APA	0.10	17	0.868	0.955	0.4761
210564_x_at	496.4	548.7	0.15	1.11	PPP-PPP	0.10	17	0.868	0.955	0.4705
44696_at	306.5	339.0	0.15	1.11	PPP-PPP	0.10	17	0.868	0.955	0.4589
201589_at	6089.1	6733.7	0.15	1.11	PPP-PPP	0.10	17	0.868	0.955	0.4461
219749_at	654.0	723.1	0.15	1.11	PPP-PPP	0.10	17	0.868	0.955	0.4097
212022_s_at	3404.5	3765.2	0.15	1.11	PPP-PPP	0.10	17	0.868	0.955	0.3981
225391_at	967.0	1069.1	0.15	1.11	PPP-PPP	0.10	17	0.868	0.955	0.3788
203274_at	1158.9	1281.6	0.15	1.11	PPP-PPP	0.10	17	0.868	0.955	0.3003
219623_at	449.4	497.0	0.15	1.11	PPP-PPP	0.10	17	0.868	0.955	0.2969
200096_s_at	11088.2	12261.9	0.15	1.11	PPP-PPP	0.10	17	0.868	0.955	0.2720
206011_at	452.8	500.5	0.15	1.11	PPP-PPP	0.10	17	0.868	0.955	0.1845
212787_at	2053.7	2271.3	0.15	1.11	PPP-PPP	0.10	17	0.868	0.955	0.1784
203978_at	827.8	915.3	0.15	1.11	PPP-PPP	0.10	17	0.868	0.955	0.1693
201876_at	13826.2	15287.0	0.15	1.11	PPP-PPP	0.10	17	0.868	0.955	0.1469
202232_s_at	12636.8	13976.3	0.15	1.11	PPP-PPP	0.10	17	0.868	0.955	0.1358
204604_at	1345.1	1487.1	0.15	1.11	PPP-PPP	0.10	17	0.868	0.955	0.1252
202814_s_at	1706.4	1886.2	0.15	1.11	PPP-PPP	0.10	17	0.868	0.955	0.1192
212124_at	1189.4	1315.3	0.15	1.11	PPP-PPP	0.10	17	0.868	0.955	0.1050
202389_s_at	180.7	199.6	0.14	1.11	AAP-PAP	0.10	17	0.868	0.955	0.5499
224036_s_at	480.0	530.3	0.14	1.11	PPP-PPP	0.10	17	0.868	0.955	0.5441
205932_s_at	419.0	463.1	0.14	1.11	PPP-PPP	0.10	17	0.868	0.955	0.5355
225377_at	1113.5	1230.5	0.14	1.11	PPP-PPP	0.10	17	0.868	0.955	0.5043
228638_at	724.7	800.9	0.14	1.11	PPP-PPP	0.10	17	0.868	0.955	0.4588

201314_at	744.0	822.1	0.14	1.11	PPP-PPP	0.10	17	0.868	0.955	0.4346
226842_at	424.8	469.5	0.14	1.11	PPP-PPP	0.10	17	0.868	0.955	0.3990
227491_at	714.7	789.5	0.14	1.11	PPP-PPP	0.10	17	0.868	0.955	0.3910
243966_at	214.0	236.6	0.14	1.11	PPP-PPP	0.10	17	0.868	0.955	0.3778
225045_at	358.2	395.8	0.14	1.11	PPP-PPP	0.10	17	0.868	0.955	0.3726
212149_at	7969.6	8809.0	0.14	1.11	PPP-PPP	0.10	17	0.868	0.955	0.3501
222672_at	307.8	340.1	0.14	1.11	MPP-PPP	0.10	17	0.868	0.955	0.3426
229292_at	672.6	743.1	0.14	1.11	PPP-PPP	0.10	17	0.868	0.955	0.3244
226217_at	847.7	936.9	0.14	1.11	PPP-PPP	0.10	17	0.868	0.955	0.3181
203514_at	624.1	689.8	0.14	1.11	PPP-PPP	0.10	17	0.868	0.955	0.2881
225527_at	1168.0	1290.8	0.14	1.11	PPP-PPP	0.10	17	0.868	0.955	0.2842
235217_at	87.6	96.8	0.14	1.11	PAP-PPP	0.10	17	0.868	0.955	0.2777
218689_at	1177.5	1300.7	0.14	1.11	PPP-PPP	0.10	17	0.868	0.955	0.2766
224731_at	13274.2	14666.5	0.14	1.11	PPP-PPP	0.10	17	0.868	0.955	0.2211
210561_s_at	1689.7	1867.4	0.14	1.11	PPP-PPP	0.10	17	0.868	0.955	0.1761
200714_x_at	2098.5	2318.8	0.14	1.11	PPP-PPP	0.10	17	0.868	0.955	0.1460
221123_x_at	829.2	916.2	0.14	1.11	MPM-MAP	0.10	17	0.868	0.955	0.1159
208768_x_at	32514.5	35931.4	0.14	1.11	PPP-PPP	0.10	17	0.868	0.955	0.0689
203291_at	762.3	842.5	0.14	1.11	PPP-PPP	0.10	17	0.868	0.955	0.0316
222067_x_at	220.5	243.5	0.14	1.10	PAP-PPP	0.10	17	0.868	0.955	0.5335
223203_at	597.5	659.8	0.14	1.10	PPP-PPP	0.10	17	0.868	0.955	0.5274
206989_s_at	3156.7	3486.4	0.14	1.10	PPP-PPP	0.10	17	0.868	0.955	0.5187
205760_s_at	218.5	241.3	0.14	1.10	PPP-PPP	0.10	17	0.868	0.955	0.5098
204133_at	669.7	739.6	0.14	1.10	PPP-PPP	0.10	17	0.868	0.955	0.4958
212366_at	223.9	247.2	0.14	1.10	PPP-PPP	0.10	17	0.868	0.955	0.4571
222700_at	2617.0	2890.3	0.14	1.10	PPP-PPP	0.10	17	0.868	0.955	0.4227
230619_at	234.8	259.2	0.14	1.10	PPP-PPP	0.10	17	0.868	0.955	0.4132
219062_s_at	687.6	759.5	0.14	1.11	PPP-PPP	0.10	17	0.868	0.955	0.3394
201964_at	563.1	621.6	0.14	1.10	PPP-PPP	0.10	17	0.868	0.955	0.2674
34225_at	780.1	861.3	0.14	1.10	PPP-PPP	0.10	17	0.868	0.955	0.2600
1554089_s_at	5380.5	5941.8	0.14	1.10	PPP-PPP	0.10	17	0.868	0.955	0.2230
200607_s_at	4830.4	5335.0	0.14	1.10	PPP-PPP	0.10	17	0.868	0.955	0.1958
201002_s_at	5750.8	6350.6	0.14	1.10	PPP-PPP	0.10	17	0.868	0.955	0.1838
224690_at	5698.1	6292.2	0.14	1.10	PPP-PPP	0.10	17	0.868	0.955	0.1382
213005_s_at	3381.3	3734.9	0.14	1.11	PPP-PPP	0.10	17	0.868	0.955	0.1370
224844_at	1177.5	1299.9	0.14	1.10	PPP-PPP	0.10	17	0.868	0.955	0.0296
1861_at	638.0	704.1	0.14	1.10	PPP-PPP	0.10	17	0.868	0.955	0.5442
221879_at	376.6	415.6	0.14	1.10	PAP-AAA	0.10	17	0.868	0.955	0.5139
224820_at	2638.5	2912.4	0.14	1.10	PPP-PPP	0.10	17	0.868	0.955	0.4707
220525_s_at	3350.3	3697.0	0.14	1.10	PPP-PPP	0.10	17	0.868	0.955	0.4646
235174_s_at	694.6	766.6	0.14	1.10	PPP-PPP	0.10	17	0.868	0.955	0.4625
223309_x_at	1577.6	1741.0	0.14	1.10	PPP-PPP	0.10	17	0.868	0.955	0.4405

209398_at	336.4	371.2	0.14	1.10	PPP-PPP	0.10	17	0.868	0.955	0.4196
222702_x_at	1873.5	2067.7	0.14	1.10	PPP-PPP	0.10	17	0.868	0.955	0.4166
202330_s_at	1980.1	2184.6	0.14	1.10	PPP-PPP	0.10	17	0.868	0.955	0.3815
219603_s_at	206.7	228.1	0.14	1.10	PPP-PPP	0.10	17	0.868	0.955	0.3092
202108_at	440.7	486.3	0.14	1.10	PPP-PPP	0.10	17	0.868	0.955	0.3019
200053_at	2359.7	2604.4	0.14	1.10	PPP-PPP	0.10	17	0.868	0.955	0.3005
212404_s_at	130.2	143.7	0.14	1.10	PPP-APP	0.10	17	0.868	0.955	0.2527
203346_s_at	777.8	858.2	0.14	1.10	PPP-PPP	0.10	17	0.868	0.955	0.1956
222538_s_at	1420.0	1566.4	0.14	1.10	PPP-PPP	0.10	17	0.868	0.955	0.1891
218412_s_at	1614.8	1782.0	0.14	1.10	PPP-PPP	0.10	17	0.868	0.955	0.1566
211352_s_at	1223.0	1349.6	0.14	1.10	PPP-PPP	0.10	17	0.868	0.955	0.1078
201857_at	4399.6	4854.3	0.14	1.10	PPP-PPP	0.10	17	0.868	0.955	0.0476
241223_x_at	512.5	565.0	0.14	1.10	PPP-PPP	0.10	17	0.868	0.955	0.4742
221953_s_at	476.0	525.0	0.14	1.10	PAP-PPP	0.10	17	0.868	0.955	0.4580
235625_at	428.6	472.7	0.14	1.10	PPP-PPP	0.10	17	0.868	0.955	0.4557
202080_s_at	1217.3	1341.8	0.14	1.10	PPP-PPP	0.10	17	0.868	0.955	0.4372
225344_at	4767.0	5256.5	0.14	1.10	PPP-PPP	0.10	17	0.868	0.955	0.4367
202405_at	385.1	424.6	0.14	1.10	PPP-PPP	0.10	17	0.868	0.955	0.4318
207181_s_at	567.9	626.4	0.14	1.10	PPP-PPP	0.10	17	0.868	0.955	0.4109
201048_x_at	375.3	413.7	0.14	1.10	PPP-PPP	0.10	17	0.868	0.955	0.4031
220261_s_at	396.7	437.5	0.14	1.10	PPP-PPP	0.10	17	0.868	0.955	0.3965
221269_s_at	3849.0	4244.9	0.14	1.10	PPP-PPP	0.10	17	0.868	0.955	0.3946
1557370_s_at	1489.6	1642.8	0.14	1.10	PPP-PPP	0.10	17	0.868	0.955	0.3607
205353_s_at	5673.6	6257.3	0.14	1.10	PPP-PPP	0.10	17	0.868	0.955	0.3523
202491_s_at	3495.5	3853.7	0.14	1.10	PPP-PPP	0.10	17	0.868	0.955	0.3049
203573_s_at	1632.4	1800.1	0.14	1.10	PPP-PPP	0.10	17	0.868	0.955	0.2387
218548_x_at	740.3	816.1	0.14	1.10	PPP-PPP	0.10	17	0.868	0.955	0.2315
217717_s_at	12109.7	13352.4	0.14	1.10	PPP-PPP	0.10	17	0.868	0.955	0.2163
201299_s_at	892.1	983.5	0.14	1.10	PPP-PPP	0.10	17	0.868	0.955	0.1858
222751_at	3122.6	3444.2	0.14	1.10	PPP-PPP	0.10	17	0.868	0.955	0.1399
223003_at	7871.6	8678.0	0.14	1.10	PPP-PPP	0.10	17	0.868	0.955	0.1199
222217_s_at	1124.8	1240.4	0.14	1.10	PPP-PPP	0.10	17	0.868	0.955	0.0502
225498_at	5285.5	5826.5	0.14	1.10	PPP-PPP	0.10	17	0.868	0.955	0.0448
205071_x_at	506.2	557.6	0.14	1.10	PPP-PPP	0.10	17	0.868	0.955	0.5431
237483_at	79.7	87.9	0.14	1.10	AAP-APP	0.10	17	0.868	0.955	0.5160
229741_at	400.7	441.4	0.14	1.10	PPP-PPP	0.10	17	0.868	0.955	0.5129
218425_at	420.7	463.6	0.14	1.10	PPP-PPP	0.10	17	0.868	0.955	0.5084
219050_s_at	383.3	422.3	0.14	1.10	PPP-APP	0.10	17	0.868	0.955	0.4790
224875_at	937.7	1033.5	0.14	1.10	PPP-PPP	0.10	17	0.868	0.955	0.4685
202257_s_at	816.6	899.6	0.14	1.10	PPP-PPP	0.10	17	0.868	0.955	0.4503
202064_s_at	120.9	133.2	0.14	1.10	MPP-PPP	0.10	17	0.868	0.955	0.4457
231873_at	641.0	706.1	0.14	1.10	PPP-PPP	0.10	17	0.868	0.955	0.4123

212752_at	1142.0	1258.0	0.14	1.10	PPP-PPP	0.10	17	0.868	0.955	0.3569
219981_x_at	1329.7	1465.4	0.14	1.10	PPP-PPP	0.10	17	0.868	0.955	0.3557
209216_at	1086.1	1196.7	0.14	1.10	PPP-PPP	0.10	17	0.868	0.955	0.3380
218306_s_at	726.2	800.2	0.14	1.10	PPP-PPP	0.10	17	0.868	0.955	0.3363
209451_at	1716.3	1891.8	0.14	1.10	PPP-PPP	0.10	17	0.868	0.955	0.3277
205709_s_at	1623.4	1789.3	0.14	1.10	PPP-PPP	0.10	17	0.868	0.955	0.3272
212919_at	875.2	964.2	0.14	1.10	PPP-PPP	0.10	17	0.868	0.955	0.3055
200969_at	3099.6	3415.9	0.14	1.10	PPP-PPP	0.10	17	0.868	0.955	0.2970
212693_at	931.1	1025.7	0.14	1.10	PPP-PPP	0.10	17	0.868	0.955	0.2699
213405_at	1240.3	1366.2	0.14	1.10	PPP-PPP	0.10	17	0.868	0.955	0.2369
204518_s_at	1433.2	1579.5	0.14	1.10	PPP-PPP	0.10	17	0.868	0.955	0.2209
1552921_a_at	351.0	386.8	0.14	1.10	PPP-PPP	0.10	17	0.868	0.955	0.2166
223213_s_at	2341.2	2580.5	0.14	1.10	PPP-PPP	0.10	17	0.868	0.955	0.1922
1553956_at	1544.0	1700.8	0.14	1.10	PPP-PPP	0.10	17	0.868	0.955	0.1857
201021_s_at	22106.0	24361.7	0.14	1.10	PPP-PPP	0.10	17	0.868	0.955	0.1490
201429_s_at	47080.8	51872.7	0.14	1.10	PPP-PPP	0.10	17	0.868	0.955	0.1219
201285_at	3062.4	3374.9	0.14	1.10	PPP-PPP	0.10	17	0.868	0.955	0.0620
223677_at	250.8	276.2	0.14	1.10	PPP-PPP	0.10	17	0.868	0.955	0.5407
202745_at	1066.8	1174.7	0.14	1.10	PPP-PPP	0.10	17	0.868	0.955	0.5377
223249_at	1001.9	1103.1	0.14	1.10	PPP-PPP	0.10	17	0.868	0.955	0.5324
215568_x_at	490.9	540.5	0.14	1.10	PPM-PPP	0.10	17	0.868	0.955	0.5270
211891_s_at	197.7	217.7	0.14	1.10	PPP-PPP	0.10	17	0.868	0.955	0.5221
221556_at	568.6	626.1	0.14	1.10	PPP-PPP	0.10	17	0.868	0.955	0.5170
226924_at	528.8	582.5	0.14	1.10	PPP-PPP	0.10	17	0.868	0.955	0.5084
1567224_at	287.2	316.2	0.14	1.10	PPP-PPP	0.10	17	0.868	0.955	0.4890
202525_at	207.1	227.9	0.14	1.10	PPP-PPP	0.10	17	0.868	0.955	0.4449
203674_at	862.2	949.4	0.14	1.10	PPP-PPP	0.10	17	0.868	0.955	0.4436
213227_at	612.2	674.0	0.14	1.10	PPP-PPP	0.10	17	0.868	0.955	0.4402
219164_s_at	251.3	276.7	0.14	1.10	PPP-PPP	0.10	17	0.868	0.955	0.4199
228398_at	136.5	150.3	0.14	1.10	AAP-AMA	0.10	17	0.868	0.955	0.2227
212056_at	586.2	645.4	0.14	1.10	PPP-PMP	0.10	17	0.868	0.955	0.1983
212724_at	3023.5	3330.0	0.14	1.10	PPP-PPP	0.10	17	0.868	0.955	0.1968
224734_at	2241.1	2467.7	0.14	1.10	PPP-PPP	0.10	17	0.868	0.955	0.1669
1554423_a_at	3016.7	3322.5	0.14	1.10	PPP-PPP	0.10	17	0.868	0.955	0.1423
201259_s_at	8683.6	9559.6	0.14	1.10	PPP-PPP	0.10	17	0.868	0.955	0.1129
226662_at	373.2	410.7	0.14	1.10	PPP-PPP	0.10	17	0.868	0.955	0.5497
201109_s_at	1726.2	1898.9	0.14	1.10	PPP-PPP	0.10	17	0.868	0.955	0.5174
239058_at	940.3	1035.0	0.14	1.10	PPP-PPP	0.10	17	0.868	0.955	0.5128
1555920_at	425.7	468.3	0.14	1.10	PPP-PPP	0.10	17	0.868	0.955	0.4619
205716_at	476.0	523.6	0.14	1.10	PPP-PPP	0.10	17	0.868	0.955	0.4576
242389_at	175.7	193.3	0.14	1.10	PPP-PPP	0.10	17	0.868	0.955	0.3888
222679_s_at	731.1	804.3	0.14	1.10	PPP-PPP	0.10	17	0.868	0.955	0.3670

201460_at	1615.2	1777.4	0.14	1.10	PPP-PPP	0.10	17	0.868	0.955	0.3647
221843_s_at	851.4	936.6	0.14	1.10	PPP-PPP	0.10	17	0.868	0.955	0.3524
201401_s_at	401.9	442.2	0.14	1.10	PPP-PPP	0.10	17	0.868	0.955	0.3451
209865_at	508.0	559.0	0.14	1.10	PPP-PPP	0.10	17	0.868	0.955	0.3424
218587_s_at	699.0	769.1	0.14	1.10	PPP-PPP	0.10	17	0.868	0.955	0.3374
225447_at	2903.2	3195.4	0.14	1.10	PPP-PPP	0.10	17	0.868	0.955	0.3271
218150_at	4564.6	5023.3	0.14	1.10	PPP-PPP	0.10	17	0.868	0.955	0.2418
201681_s_at	5078.9	5589.4	0.14	1.10	PPP-PPP	0.10	17	0.868	0.955	0.1853
225932_s_at	4065.4	4474.0	0.14	1.10	PPP-PPP	0.10	17	0.868	0.955	0.1592
212683_at	531.8	585.1	0.14	1.10	PPP-MAP	0.10	17	0.868	0.955	0.1325
212573_at	1310.6	1442.3	0.14	1.10	PPP-PPP	0.10	17	0.868	0.955	0.1257
201990_s_at	649.7	715.1	0.14	1.10	PPP-PPP	0.10	17	0.868	0.955	0.1109
221492_s_at	5564.4	6121.9	0.14	1.10	PPP-PPP	0.10	17	0.868	0.955	0.1096
200675_at	8516.8	9374.7	0.14	1.10	PPP-PPP	0.10	17	0.868	0.955	0.0962
200007_at	11923.6	13122.0	0.14	1.10	PPP-PPP	0.10	17	0.868	0.955	0.0169
204709_s_at	1350.4	1485.0	0.14	1.10	PPP-PPP	0.10	17	0.868	0.955	0.5062
210286_s_at	158.6	174.4	0.14	1.10	PPP-PPP	0.10	17	0.868	0.955	0.4898
209475_at	318.5	350.1	0.14	1.10	PPP-PPP	0.10	17	0.868	0.955	0.4776
223053_x_at	2384.6	2621.8	0.14	1.10	PPP-PPP	0.10	17	0.868	0.955	0.4773
1568597_at	1231.8	1354.2	0.14	1.10	PPP-PPP	0.10	17	0.868	0.955	0.3606
227538_at	1172.6	1289.4	0.14	1.10	PPP-PPP	0.10	17	0.868	0.955	0.2347
224190_x_at	242.8	267.1	0.14	1.10	PPP-PMM	0.10	17	0.868	0.955	0.1997
226545_at	6018.3	6617.5	0.14	1.10	PPP-PPP	0.10	17	0.868	0.955	0.1366
203432_at	2934.8	3226.2	0.14	1.10	PPP-PPP	0.10	17	0.868	0.955	0.0939
201094_at	36333.7	39945.3	0.14	1.10	PPP-PPP	0.10	17	0.868	0.955	0.0324
218740_s_at	1155.6	1270.2	0.14	1.10	PPP-PPP	0.10	17	0.868	0.955	0.5531
213275_x_at	4391.9	4827.5	0.14	1.10	PPP-PPP	0.10	17	0.868	0.955	0.5346
238700_at	254.2	279.4	0.14	1.10	PPP-PPP	0.10	17	0.868	0.955	0.5109
224829_at	316.7	348.1	0.14	1.10	PPP-PPP	0.10	17	0.868	0.955	0.5071
208246_x_at	1350.0	1483.9	0.14	1.10	PPP-PPP	0.10	17	0.868	0.955	0.5069
204209_at	276.3	303.5	0.14	1.10	PPP-PPP	0.10	17	0.868	0.955	0.4946
208706_s_at	3068.7	3372.5	0.14	1.10	PPP-PPP	0.10	17	0.868	0.955	0.4596
221495_s_at	1999.6	2197.1	0.14	1.10	PPP-PPP	0.10	17	0.868	0.955	0.4392
235472_at	249.2	273.9	0.14	1.10	PPP-PPP	0.10	17	0.868	0.955	0.3906
236535_at	322.9	354.7	0.14	1.10	PPP-PPP	0.10	17	0.868	0.955	0.3030
223336_s_at	2930.2	3220.0	0.14	1.10	PPP-PPP	0.10	17	0.868	0.955	0.2920
226324_s_at	313.5	344.5	0.14	1.10	PPP-PAP	0.10	17	0.868	0.955	0.2893
239748_x_at	1558.5	1712.6	0.14	1.10	PPP-PPP	0.10	17	0.868	0.955	0.2799
217851_s_at	4319.2	4745.5	0.14	1.10	PPP-PPP	0.10	17	0.868	0.955	0.2613
209215_at	1079.5	1186.2	0.14	1.10	PPP-PPP	0.10	17	0.868	0.955	0.2475
223389_s_at	1046.9	1150.1	0.14	1.10	PPP-PPP	0.10	17	0.868	0.955	0.2432
229011_at	1191.9	1309.8	0.14	1.10	PPP-PPP	0.10	17	0.868	0.955	0.2231

209260_at	8042.4	8838.1	0.14	1.10	PPP-PPP	0.10	17	0.868	0.955	0.2142
224813_at	3711.2	4077.9	0.14	1.10	PPP-PPP	0.10	17	0.868	0.955	0.2071
218310_at	1525.3	1675.8	0.14	1.10	PPP-PPP	0.10	17	0.868	0.955	0.1865
223025_s_at	769.9	845.9	0.14	1.10	PPP-PPP	0.10	17	0.868	0.955	0.1655
218706_s_at	885.1	972.4	0.14	1.10	PPP-PPP	0.10	17	0.868	0.955	0.1181
241721_at	625.4	687.0	0.14	1.10	PPP-PPP	0.10	17	0.868	0.955	0.1022
205189_s_at	248.5	272.8	0.14	1.10	PPP-PPP	0.10	17	0.868	0.955	0.5574
201579_at	2449.2	2688.6	0.14	1.10	PPP-PPP	0.10	17	0.868	0.955	0.5524
222118_at	341.0	374.5	0.14	1.10	PPP-PPP	0.10	17	0.868	0.955	0.5152
1562456_at	91.7	100.7	0.14	1.10	APP-MPP	0.10	17	0.868	0.955	0.5068
222554_s_at	428.6	470.6	0.14	1.10	PPP-PPP	0.10	17	0.868	0.955	0.4992
227968_at	771.4	846.9	0.14	1.10	PPP-PPP	0.10	17	0.868	0.955	0.4798
225135_at	418.1	459.1	0.14	1.10	PPP-PPP	0.10	17	0.868	0.955	0.4759
219394_at	472.8	519.1	0.14	1.10	PPP-PPP	0.10	17	0.868	0.955	0.4687
239352_at	457.6	502.5	0.14	1.10	PPP-PPP	0.10	17	0.868	0.955	0.4313
204147_s_at	1337.7	1468.8	0.14	1.10	PPP-PPP	0.10	17	0.868	0.955	0.4064
204402_at	200.3	219.9	0.14	1.10	PPP-MPP	0.10	17	0.868	0.955	0.3896
211114_x_at	755.7	829.6	0.14	1.10	PPP-PPP	0.10	17	0.868	0.955	0.3428
229181_s_at	1530.4	1680.9	0.14	1.10	PPP-PPP	0.10	17	0.868	0.955	0.3339
201260_s_at	7246.6	7958.6	0.14	1.10	PPP-PPP	0.10	17	0.868	0.955	0.3054
222514_at	1661.3	1824.0	0.14	1.10	PPP-PPP	0.10	17	0.868	0.955	0.2925
227952_at	1584.9	1740.5	0.14	1.10	PPP-PPP	0.10	17	0.868	0.955	0.2614
202000_at	858.6	942.6	0.14	1.10	PPP-PPP	0.10	17	0.868	0.955	0.2409
228992_at	1252.3	1374.7	0.14	1.10	PPP-PPP	0.10	17	0.868	0.955	0.2393
201567_s_at	2629.4	2886.4	0.14	1.10	PPP-PPP	0.10	17	0.868	0.955	0.2386
202227_s_at	3708.1	4072.9	0.14	1.10	PPP-PPP	0.10	17	0.868	0.955	0.1850
227376_at	1587.3	1742.5	0.14	1.10	PPP-PPP	0.10	17	0.868	0.955	0.1576
209290_s_at	6289.2	6906.4	0.14	1.10	PPP-PPP	0.10	17	0.868	0.955	0.1517
221712_s_at	2416.8	2654.0	0.14	1.10	PPP-PPP	0.10	17	0.868	0.955	0.1344
218791_s_at	387.3	425.2	0.14	1.10	PPP-PPP	0.10	17	0.868	0.955	0.1096
204206_at	1197.4	1315.3	0.14	1.10	PPP-PPP	0.10	17	0.868	0.955	0.0781
37005_at	3196.7	3509.7	0.14	1.10	PPP-PPP	0.10	17	0.868	0.955	0.0741
217595_at	210.5	231.0	0.13	1.10	PPP-PPP	0.10	17	0.868	0.955	0.4591
209227_at	394.2	432.4	0.13	1.10	PPP-PPP	0.10	17	0.868	0.955	0.4228
218774_at	1656.5	1818.2	0.13	1.10	PPP-PPP	0.10	17	0.868	0.955	0.3846
218640_s_at	1048.2	1150.4	0.13	1.10	PPP-PPP	0.10	17	0.868	0.955	0.3822
214246_x_at	1462.3	1604.5	0.13	1.10	PPP-PPP	0.10	17	0.868	0.955	0.3717
209306_s_at	1630.0	1788.1	0.13	1.10	PPP-PPP	0.10	17	0.868	0.955	0.3666
49878_at	2056.0	2256.2	0.13	1.10	PPP-PPP	0.10	17	0.868	0.955	0.3599
202621_at	1609.8	1766.2	0.13	1.10	PPP-PPP	0.10	17	0.868	0.955	0.3481
220788_s_at	254.1	278.9	0.13	1.10	PPP-PPP	0.10	17	0.868	0.955	0.3459
214895_s_at	603.2	662.2	0.13	1.10	PPP-PPP	0.10	17	0.868	0.955	0.3271

202236_s_at	3761.6	4126.3	0.13	1.10	PPP-PPP	0.10	17	0.868	0.955	0.3127
212631_at	902.5	990.0	0.13	1.10	PPP-PPP	0.10	17	0.868	0.955	0.3037
203012_x_at	41609.0	45663.1	0.13	1.10	PPP-PPP	0.10	17	0.868	0.955	0.2223
202636_at	1045.4	1146.9	0.13	1.10	PPP-PPP	0.10	17	0.868	0.955	0.1469
203522_at	345.0	378.5	0.13	1.10	PPA-PPA	0.10	17	0.868	0.955	0.1424
204496_at	1310.3	1437.7	0.13	1.10	PPP-PPP	0.10	17	0.868	0.955	0.1256
201864_at	1145.2	1256.6	0.13	1.10	PPP-PPP	0.10	17	0.868	0.955	0.0888
217812_at	4517.6	4956.0	0.13	1.10	PPP-PPP	0.10	17	0.868	0.955	0.0807
201903_at	8576.5	9410.8	0.13	1.10	PPP-PPP	0.10	17	0.868	0.955	0.0342
218685_s_at	220.9	242.3	0.13	1.10	PPP-PPP	0.10	17	0.868	0.955	0.5465
201165_s_at	2409.0	2641.1	0.13	1.10	PPP-PPP	0.10	17	0.868	0.955	0.5358
219515_at	684.1	750.4	0.13	1.10	PPP-PPP	0.10	17	0.868	0.955	0.5331
205710_at	99.8	109.4	0.13	1.10	PPP-APP	0.10	17	0.868	0.955	0.5190
230972_at	733.9	804.9	0.13	1.10	PPP-PPP	0.10	17	0.868	0.955	0.5136
202611_s_at	738.4	809.5	0.13	1.10	PPP-PPP	0.10	17	0.868	0.955	0.4601
222789_at	939.8	1030.4	0.13	1.10	PPP-PPP	0.10	17	0.868	0.955	0.4101
227808_at	787.6	863.4	0.13	1.10	PPP-PPP	0.10	17	0.868	0.955	0.3805
204731_at	854.6	936.8	0.13	1.10	PPP-PPP	0.10	17	0.868	0.955	0.3754
204761_at	472.8	518.6	0.13	1.10	PPP-PPP	0.10	17	0.868	0.955	0.3474
223024_at	2081.0	2282.1	0.13	1.10	PPP-PPP	0.10	17	0.868	0.955	0.3346
36936_at	4699.3	5152.8	0.13	1.10	PPP-PPP	0.10	17	0.868	0.955	0.3247
201991_s_at	6253.8	6859.6	0.13	1.10	PPP-PPP	0.10	17	0.868	0.955	0.3036
222825_at	1652.1	1811.0	0.13	1.10	PPP-PPP	0.10	17	0.868	0.955	0.2959
200767_s_at	1154.1	1265.7	0.13	1.10	PPP-PPP	0.10	17	0.868	0.955	0.2710
202596_at	4221.4	4629.1	0.13	1.10	PPP-PPP	0.10	17	0.868	0.955	0.2559
212368_at	536.3	588.3	0.13	1.10	PPP-PPP	0.10	17	0.868	0.955	0.2360
230352_at	2881.3	3159.6	0.13	1.10	PPP-PPP	0.10	17	0.868	0.955	0.2304
201575_at	3920.4	4297.5	0.13	1.10	PPP-PPP	0.10	17	0.868	0.955	0.1515
201326_at	6414.8	7034.3	0.13	1.10	PPP-PPP	0.10	17	0.868	0.955	0.1046
200977_s_at	4253.8	4665.5	0.13	1.10	PPP-PPP	0.10	17	0.868	0.955	0.0956
204855_at	20535.2	22517.9	0.13	1.10	PPP-PPP	0.10	17	0.868	0.955	0.0892
37462_i_at	904.0	991.7	0.13	1.10	PPP-PPP	0.10	17	0.868	0.955	0.0316
244495_x_at	893.9	979.5	0.13	1.10	PPP-PPP	0.10	17	0.868	0.955	0.5211
231855_at	441.5	483.6	0.13	1.10	PPP-PPP	0.10	17	0.868	0.955	0.5154
240834_at	100.5	110.2	0.13	1.10	AAP-PAP	0.10	17	0.868	0.955	0.4957
223419_at	607.8	665.9	0.13	1.10	PPP-PPP	0.10	17	0.868	0.955	0.4940
1554006_a_at	492.5	539.8	0.13	1.10	PPP-PPP	0.10	17	0.868	0.955	0.4818
212858_at	403.2	441.8	0.13	1.10	PPP-PPP	0.10	17	0.868	0.955	0.4758
215004_s_at	432.4	473.8	0.13	1.10	PPP-PPP	0.10	17	0.868	0.955	0.4671
223199_at	1582.9	1735.1	0.13	1.10	PPP-PPP	0.10	17	0.868	0.955	0.4515
208051_s_at	1537.0	1684.3	0.13	1.10	PPP-PPP	0.10	17	0.868	0.955	0.4308
217810_x_at	4125.4	4520.3	0.13	1.10	PPP-PPP	0.10	17	0.868	0.955	0.4215

201674_s_at	2372.7	2599.3	0.13	1.10	PPP-PPP	0.10	17	0.868	0.955	0.3225
225127_at	1970.7	2158.9	0.13	1.10	PPP-PPP	0.10	17	0.868	0.955	0.3004
239794_at	125.1	137.1	0.13	1.10	PMP-PPP	0.10	17	0.868	0.955	0.2960
221763_at	1469.2	1610.3	0.13	1.10	PPP-PPP	0.10	17	0.868	0.955	0.2796
226888_at	645.8	707.7	0.13	1.10	PPP-PPP	0.10	17	0.868	0.955	0.2693
204314_s_at	1139.3	1248.7	0.13	1.10	PPP-PPP	0.10	17	0.868	0.955	0.2374
227159_at	233.0	255.3	0.13	1.10	PPP-PPP	0.10	17	0.868	0.955	0.1727
212973_at	1199.8	1314.5	0.13	1.10	PPP-PPP	0.10	17	0.868	0.955	0.1461
210211_s_at	25987.4	28470.7	0.13	1.10	PPP-PPP	0.10	17	0.868	0.955	0.1125
201855_s_at	1155.0	1265.7	0.13	1.10	PPP-PPP	0.10	17	0.868	0.955	0.1020
226690_at	87.5	95.8	0.13	1.10	PPP-PPA	0.10	17	0.868	0.955	0.0658
201568_at	16599.1	18194.7	0.13	1.10	PPP-PPP	0.10	17	0.868	0.955	0.0633
202911_at	5355.4	5867.5	0.13	1.10	PPP-PPP	0.10	17	0.868	0.955	0.0569
211697_x_at	1546.9	1694.8	0.13	1.10	PPP-PPP	0.10	17	0.868	0.955	0.0511
219181_at	815.8	893.4	0.13	1.10	PPP-PPP	0.10	17	0.868	0.955	0.4399
232215_x_at	3061.7	3353.7	0.13	1.10	PPP-PPP	0.10	17	0.868	0.955	0.4377
209861_s_at	9710.8	10635.7	0.13	1.10	PPP-PPP	0.10	17	0.868	0.955	0.4011
1557985_s_at	438.4	480.1	0.13	1.10	PPP-PPP	0.10	17	0.868	0.955	0.3737
218982_s_at	5104.1	5588.6	0.13	1.10	PPP-PPP	0.10	17	0.868	0.955	0.3554
209873_s_at	3170.4	3471.6	0.13	1.10	PPP-PPP	0.10	17	0.868	0.955	0.3493
205283_at	490.3	536.8	0.13	1.10	PPP-PPP	0.10	17	0.868	0.955	0.2387
224934_at	4577.5	5012.8	0.13	1.10	PPP-PPP	0.10	17	0.868	0.955	0.2296
207507_s_at	19593.7	21459.4	0.13	1.10	PPP-PPP	0.10	17	0.868	0.955	0.2002
210775_x_at	330.0	361.5	0.13	1.10	PMP-PPP	0.10	17	0.868	0.955	0.1961
218053_at	7302.8	7994.8	0.13	1.10	PPP-PPP	0.10	17	0.868	0.955	0.1528
200094_s_at	38870.5	42568.5	0.13	1.10	PPP-PPP	0.10	17	0.868	0.955	0.1345
202317_s_at	1132.6	1240.5	0.13	1.10	PPP-PPP	0.10	17	0.868	0.955	0.0533
207941_s_at	8951.4	9800.6	0.13	1.10	PPP-PPP	0.10	17	0.868	0.955	0.0330
205324_s_at	3074.6	3366.9	0.13	1.10	PPP-PPP	0.10	17	0.868	0.955	0.0089
205436_s_at	3188.0	3488.1	0.13	1.09	PPP-PPP	0.10	17	0.868	0.955	0.5471
203386_at	1482.8	1623.1	0.13	1.10	PPP-PPP	0.10	17	0.868	0.955	0.5316
221987_s_at	1366.6	1495.9	0.13	1.10	PPP-PPP	0.10	17	0.868	0.955	0.5157
202766_s_at	127.1	139.1	0.13	1.09	PPP-PPP	0.10	17	0.868	0.955	0.5039
212032_s_at	1193.1	1305.5	0.13	1.09	PPP-PPP	0.10	17	0.868	0.955	0.5011
1559449_a_at	106.7	116.7	0.13	1.09	PAP-AAP	0.10	17	0.868	0.955	0.4536
205394_at	2185.6	2391.3	0.13	1.09	PPP-PPP	0.10	17	0.868	0.955	0.4127
212431_at	566.9	620.1	0.13	1.09	PPP-PPP	0.10	17	0.868	0.955	0.4096
226353_at	2607.0	2853.8	0.13	1.10	PPP-PPP	0.10	17	0.868	0.955	0.3791
203730_s_at	392.2	429.1	0.13	1.09	PPP-PPP	0.10	17	0.868	0.955	0.3377
226916_x_at	614.4	672.2	0.13	1.09	PPP-PPP	0.10	17	0.868	0.955	0.3319
230958_s_at	984.4	1077.0	0.13	1.09	PPP-PPP	0.10	17	0.868	0.955	0.3260
217822_at	4683.3	5124.0	0.13	1.09	PPP-PPP	0.10	17	0.868	0.955	0.3010

226713_at	565.4	618.9	0.13	1.10	PPP-PPP	0.10	17	0.868	0.955	0.2906
222599_s_at	851.6	931.9	0.13	1.09	PPP-PPP	0.10	17	0.868	0.955	0.2724
213199_at	690.7	756.0	0.13	1.09	PPP-PPP	0.10	17	0.868	0.955	0.2722
209837_at	485.7	531.4	0.13	1.09	PPP-PPP	0.10	17	0.868	0.955	0.2656
209112_at	3309.7	3621.4	0.13	1.09	PPP-PPP	0.10	17	0.868	0.955	0.2550
218679_s_at	3733.0	4084.7	0.13	1.09	PPP-PPP	0.10	17	0.868	0.955	0.2381
202407_s_at	1152.5	1260.9	0.13	1.09	PPP-PPP	0.10	17	0.868	0.955	0.2028
1554806_a_at	806.2	881.9	0.13	1.09	PPP-PPP	0.10	17	0.868	0.955	0.1836
212347_x_at	573.8	627.2	0.13	1.09	MMP-PPP	0.10	17	0.868	0.955	0.5102
227592_at	542.1	592.9	0.13	1.09	PPP-PPP	0.10	17	0.868	0.955	0.4955
201684_s_at	1067.3	1166.8	0.13	1.09	PPP-PPP	0.10	17	0.868	0.955	0.4421
211778_s_at	526.1	575.4	0.13	1.09	PPP-PPP	0.10	17	0.868	0.955	0.3963
202298_at	10032.8	10970.2	0.13	1.09	PPP-PPP	0.10	17	0.868	0.955	0.3581
204128_s_at	2097.4	2293.2	0.13	1.09	PPP-PPP	0.10	17	0.868	0.955	0.3549
210048_at	262.2	286.8	0.13	1.09	PPP-PPP	0.10	17	0.868	0.955	0.3195
226041_at	349.4	382.2	0.13	1.09	PPP-PPP	0.10	17	0.868	0.955	0.2942
212566_at	2339.9	2558.7	0.13	1.09	PPP-PPP	0.10	17	0.868	0.955	0.2904
206858_s_at	1658.0	1813.4	0.13	1.09	PPP-PPP	0.10	17	0.868	0.955	0.2454
224856_at	7005.2	7659.7	0.13	1.09	PPP-PPP	0.10	17	0.868	0.955	0.1835
200075_s_at	9710.3	10619.4	0.13	1.09	PPP-PPP	0.10	17	0.868	0.955	0.1748
201760_s_at	5127.9	5607.4	0.13	1.09	PPP-PPP	0.10	17	0.868	0.955	0.1467
202789_at	604.3	660.9	0.13	1.09	PPP-PPP	0.10	17	0.868	0.955	0.0353
208668_x_at	25487.9	27868.9	0.13	1.09	PPP-PPP	0.10	17	0.868	0.955	0.0104
201073_s_at	864.0	943.9	0.13	1.09	PPP-PPP	0.10	17	0.868	0.955	0.5481
236798_at	437.7	478.3	0.13	1.09	PPP-PPP	0.10	17	0.868	0.955	0.5478
231847_at	616.3	673.4	0.13	1.09	PPP-PPP	0.10	17	0.868	0.955	0.5411
217040_x_at	433.3	473.3	0.13	1.09	PPP-APP	0.10	17	0.868	0.955	0.5314
228061_at	226.1	247.2	0.13	1.09	PPP-PPP	0.10	17	0.868	0.955	0.5203
207153_s_at	1124.2	1228.6	0.13	1.09	PPP-PPP	0.10	17	0.868	0.955	0.4689
215586_at	59.2	64.6	0.13	1.09	PMP-PPP	0.10	17	0.868	0.955	0.4170
1557965_at	568.4	621.3	0.13	1.09	PPP-PPP	0.10	17	0.868	0.955	0.4030
203358_s_at	1720.9	1880.7	0.13	1.09	PPP-PPP	0.10	17	0.868	0.955	0.3532
223886_s_at	1153.4	1260.4	0.13	1.09	PPP-PPP	0.10	17	0.868	0.955	0.3118
226405_s_at	906.9	990.9	0.13	1.09	PPP-PPP	0.10	17	0.868	0.955	0.2574
239719_at	425.2	464.6	0.13	1.09	PPP-PPP	0.10	17	0.868	0.955	0.2471
226740_x_at	9826.0	10734.7	0.13	1.09	PPP-PPP	0.10	17	0.868	0.955	0.2363
222104_x_at	1741.5	1903.4	0.13	1.09	PPP-PPP	0.10	17	0.868	0.955	0.2298
203643_at	561.8	614.1	0.13	1.09	PPP-PPP	0.10	17	0.868	0.955	0.2272
204630_s_at	1901.4	2077.8	0.13	1.09	PPP-PPP	0.10	17	0.868	0.955	0.1606
217766_s_at	1616.0	1766.4	0.13	1.09	PPP-PPP	0.10	17	0.868	0.955	0.1597
226481_at	1119.0	1222.6	0.13	1.09	PPP-PPP	0.10	17	0.868	0.955	0.1086
211070_x_at	11635.0	12718.8	0.13	1.09	PPP-PPP	0.10	17	0.868	0.955	0.0636

239084_at	304.5	332.7	0.13	1.09	PPP-PPP	0.10	17	0.868	0.955	0.0596
202333_s_at	4985.3	5448.9	0.13	1.09	PPP-PPP	0.10	17	0.868	0.955	0.0583
225364_at	956.6	1045.2	0.13	1.09	PPP-PPP	0.10	17	0.868	0.955	0.0511
222699_s_at	465.5	508.8	0.13	1.09	PPP-PPP	0.10	17	0.868	0.955	0.0121
220926_s_at	783.0	854.8	0.13	1.09	PPP-PPP	0.10	17	0.868	0.955	0.5295
233544_at	59.3	64.8	0.13	1.09	APA-AAM	0.10	17	0.868	0.955	0.5198
226171_at	235.4	257.1	0.13	1.09	PPP-PPP	0.10	17	0.868	0.955	0.5187
38703_at	1747.7	1908.6	0.13	1.09	PPP-PPP	0.10	17	0.868	0.955	0.4460
202053_s_at	5493.9	6000.8	0.13	1.09	PPP-PPP	0.10	17	0.868	0.955	0.4270
226396_at	443.2	484.1	0.13	1.09	PPP-PPP	0.10	17	0.868	0.955	0.4047
213062_at	1625.4	1775.0	0.13	1.09	PPP-PPP	0.10	17	0.868	0.955	0.4003
219526_at	1305.5	1425.7	0.13	1.09	PPP-PPP	0.10	17	0.868	0.955	0.3987
212055_at	1004.5	1096.7	0.13	1.09	PPP-PPP	0.10	17	0.868	0.955	0.3640
209150_s_at	1864.2	2035.3	0.13	1.09	PPP-PPP	0.10	17	0.868	0.955	0.3551
225558_at	585.3	639.0	0.13	1.09	PPP-PPP	0.10	17	0.868	0.955	0.3318
227508_at	386.9	422.6	0.13	1.09	PPP-PPP	0.10	17	0.868	0.955	0.3265
212305_s_at	872.3	952.5	0.13	1.09	PPP-PPP	0.10	17	0.868	0.955	0.3061
224593_at	5920.8	6464.1	0.13	1.09	PPP-PPP	0.10	17	0.868	0.955	0.2817
201175_at	6672.3	7287.5	0.13	1.09	PPP-PPP	0.10	17	0.868	0.955	0.2730
213477_x_at	40876.2	44644.8	0.13	1.09	PPP-PPP	0.10	17	0.868	0.955	0.2480
209608_s_at	5691.5	6216.8	0.13	1.09	PPP-PPP	0.10	17	0.868	0.955	0.2383
225336_at	2279.5	2488.4	0.13	1.09	PPP-PPP	0.10	17	0.868	0.955	0.2232
214179_s_at	1537.2	1678.4	0.13	1.09	PPP-PPP	0.10	17	0.868	0.955	0.2024
200656_s_at	5624.0	6142.2	0.13	1.09	PPP-PPP	0.10	17	0.868	0.955	0.1519
200034_s_at	31850.6	34792.7	0.13	1.09	PPP-PPP	0.10	17	0.868	0.955	0.1113
214696_at	662.1	723.3	0.13	1.09	PPP-PPP	0.10	17	0.868	0.955	0.0993
208867_s_at	1176.6	1285.3	0.13	1.09	PPP-PPP	0.10	17	0.868	0.955	0.0835
225092_at	1755.5	1917.4	0.13	1.09	PPP-PPP	0.10	17	0.868	0.955	0.0412
212834_at	706.0	770.3	0.13	1.09	PPP-PPP	0.10	17	0.868	0.955	0.5469
225683_x_at	493.4	538.3	0.13	1.09	PPP-PPP	0.10	17	0.868	0.955	0.5274
1566509_s_at	1363.1	1487.8	0.13	1.09	PPP-PPP	0.10	17	0.868	0.955	0.5246
218502_s_at	710.5	775.2	0.13	1.09	PPP-PPP	0.10	17	0.868	0.955	0.5215
209349_at	819.7	894.6	0.13	1.09	PPP-PPP	0.10	17	0.868	0.955	0.4816
212100_s_at	1461.3	1594.5	0.13	1.09	PPP-PPP	0.10	17	0.868	0.955	0.4745
234969_s_at	819.6	894.7	0.13	1.09	PPP-PPP	0.10	17	0.868	0.955	0.4648
219060_at	3187.3	3477.4	0.13	1.09	PPP-PPP	0.10	17	0.868	0.955	0.4504
222681_at	591.6	645.6	0.13	1.09	PPP-PPP	0.10	17	0.868	0.955	0.4428
217125_at	174.3	190.2	0.13	1.09	APP-PAP	0.10	17	0.868	0.955	0.4162
1558511_s_at	3547.0	3870.7	0.13	1.09	PPP-PPP	0.10	17	0.868	0.955	0.4134
201624_at	2277.0	2484.1	0.13	1.09	PPP-PPP	0.10	17	0.868	0.955	0.3877
203970_s_at	1439.1	1570.1	0.13	1.09	PPP-PPP	0.10	17	0.868	0.955	0.3591
203173_s_at	785.2	857.0	0.13	1.09	PPP-PPP	0.10	17	0.868	0.955	0.3164

32836_at	1181.2	1289.2	0.13	1.09	PPP-PPP	0.10	17	0.868	0.955	0.2967
206714_at	365.2	398.5	0.13	1.09	PPP-PPP	0.10	17	0.868	0.955	0.2890
222629_at	254.8	278.1	0.13	1.09	PPP-PPP	0.10	17	0.868	0.955	0.2300
224372_at	49138.2	53617.0	0.13	1.09	PPP-PPP	0.10	17	0.868	0.955	0.1710
1553510_s_at	615.2	671.4	0.13	1.09	PPP-PPP	0.10	17	0.868	0.955	0.1706
244008_at	136.3	148.8	0.13	1.09	PPP-PMM	0.10	17	0.868	0.955	0.1515
202562_s_at	2224.8	2427.6	0.13	1.09	PPP-PPP	0.10	17	0.868	0.955	0.1232
223210_at	1475.3	1609.7	0.13	1.09	PPP-PPP	0.10	17	0.868	0.955	0.0070
203176_s_at	1611.0	1756.6	0.13	1.09	PPP-PPP	0.10	17	0.868	0.955	0.5146
211194_s_at	281.4	306.8	0.13	1.09	PPP-PPP	0.10	17	0.868	0.955	0.4876
229573_at	285.4	311.3	0.13	1.09	PPP-PPP	0.10	17	0.868	0.955	0.4834
227882_at	307.5	335.4	0.13	1.09	APA-AAP	0.10	17	0.868	0.955	0.4566
36829_at	712.9	777.4	0.13	1.09	PPP-PPP	0.10	17	0.868	0.955	0.4117
201617_x_at	3461.2	3774.0	0.13	1.09	PPP-PPP	0.10	17	0.868	0.955	0.4054
1555427_s_at	1031.6	1124.9	0.13	1.09	PPP-PPP	0.10	17	0.868	0.955	0.3314
228310_at	661.8	721.8	0.13	1.09	PPP-PPP	0.10	17	0.868	0.955	0.3219
1569053_at	222.6	242.7	0.13	1.09	PPP-PPP	0.10	17	0.868	0.955	0.3071
209430_at	808.2	881.5	0.13	1.09	PPP-PPP	0.10	17	0.868	0.955	0.2744
204372_s_at	4841.0	5278.7	0.13	1.09	PPP-PPP	0.10	17	0.868	0.955	0.2383
203688_at	689.4	752.0	0.13	1.09	PPP-PPP	0.10	17	0.868	0.955	0.2369
212412_at	5407.9	5899.0	0.13	1.09	PPP-PPP	0.10	17	0.868	0.955	0.2138
224600_at	1676.6	1828.3	0.13	1.09	PPP-PPP	0.10	17	0.868	0.955	0.1849
221604_s_at	579.5	631.6	0.12	1.09	PPP-PPP	0.10	17	0.868	0.955	0.5454
227815_at	297.5	324.2	0.12	1.09	PPP-PMP	0.10	17	0.868	0.955	0.5172
228092_at	427.0	465.3	0.12	1.09	PPP-PPP	0.10	17	0.868	0.955	0.4951
202172_at	1081.8	1179.0	0.12	1.09	PPP-PPP	0.10	17	0.868	0.955	0.4699
226262_at	1097.9	1196.6	0.12	1.09	PPP-PPP	0.10	17	0.868	0.955	0.4625
227961_at	3106.9	3384.9	0.12	1.09	PPP-PPP	0.10	17	0.868	0.955	0.4482
222569_at	656.5	715.5	0.12	1.09	PPP-PPP	0.10	17	0.868	0.955	0.3636
214545_s_at	730.2	795.6	0.12	1.09	PPP-PPP	0.10	17	0.868	0.955	0.3584
201360_at	5788.8	6307.5	0.12	1.09	PPP-PPP	0.10	17	0.868	0.955	0.3575
220305_at	1232.5	1343.6	0.12	1.09	PPP-PPP	0.10	17	0.868	0.955	0.3494
202841_x_at	677.9	739.0	0.12	1.09	PPP-PPP	0.10	17	0.868	0.955	0.3151
208070_s_at	1147.3	1250.5	0.12	1.09	PPP-PPP	0.10	17	0.868	0.955	0.3134
212780_at	767.6	836.5	0.12	1.09	PPP-PPP	0.10	17	0.868	0.955	0.3088
222994_at	7215.3	7861.0	0.12	1.09	PPP-PPP	0.10	17	0.868	0.955	0.2977
212178_s_at	1605.4	1749.8	0.12	1.09	PPP-PPP	0.10	17	0.868	0.955	0.2857
222728_s_at	8854.4	9651.5	0.12	1.09	PPP-PPP	0.10	17	0.868	0.955	0.2777
235587_at	553.1	602.6	0.12	1.09	PPP-PPP	0.10	17	0.868	0.955	0.2704
201656_at	9013.1	9821.3	0.12	1.09	PPP-PPP	0.10	17	0.868	0.955	0.2690
235791_x_at	1685.8	1837.1	0.12	1.09	PPP-PPP	0.10	17	0.868	0.955	0.1712
226148_at	2366.2	2579.1	0.12	1.09	PPP-PPP	0.10	17	0.868	0.955	0.1487

212461_at	5806.4	6327.9	0.12	1.09	PPP-PPP	0.10	17	0.868	0.955	0.0186
200638_s_at	19120.2	20834.5	0.12	1.09	PPP-PPP	0.10	17	0.868	0.955	0.0075
201204_s_at	2015.5	2194.6	0.12	1.09	PPP-PPP	0.10	17	0.868	0.955	0.5435
202582_s_at	3309.0	3604.7	0.12	1.09	PPP-PPP	0.10	17	0.868	0.955	0.5310
203943_at	1061.8	1156.0	0.12	1.09	PPP-PPP	0.10	17	0.868	0.955	0.5195
204568_at	528.3	575.2	0.12	1.09	PPP-PPP	0.10	17	0.868	0.955	0.4929
1555202_a_at	217.0	236.3	0.12	1.09	PPP-AAP	0.10	17	0.868	0.955	0.4885
228026_at	775.1	844.2	0.12	1.09	PPP-PPP	0.10	17	0.868	0.955	0.4517
216684_s_at	318.0	346.4	0.12	1.09	PPP-PPP	0.10	17	0.868	0.955	0.4491
1553101_a_at	927.5	1009.8	0.12	1.09	PPP-PPP	0.10	17	0.868	0.955	0.3065
202209_at	5421.0	5902.3	0.12	1.09	PPP-PPP	0.10	17	0.868	0.955	0.2927
212923_s_at	660.4	719.3	0.12	1.09	PPP-PPP	0.10	17	0.868	0.955	0.2863
217972_at	4028.9	4388.1	0.12	1.09	PPP-PPP	0.10	17	0.868	0.955	0.2380
216843_x_at	1033.2	1125.1	0.12	1.09	PPP-PPP	0.10	17	0.868	0.955	0.2312
200645_at	7203.2	7843.0	0.12	1.09	PPP-PPP	0.10	17	0.868	0.955	0.1200
201078_at	9655.5	10517.5	0.12	1.09	PPP-PPP	0.10	17	0.868	0.955	0.1195
214473_x_at	1008.2	1098.1	0.12	1.09	PPP-PPP	0.10	17	0.868	0.955	0.0776
200097_s_at	11303.8	12305.6	0.12	1.09	PPP-PPP	0.10	17	0.868	0.955	0.0649
227540_at	408.1	444.0	0.12	1.09	PPP-PPP	0.10	17	0.868	0.955	0.5366
229064_s_at	272.3	296.2	0.12	1.09	PPP-PPP	0.10	17	0.868	0.955	0.5349
218459_at	1330.3	1448.0	0.12	1.09	PPP-PPP	0.10	17	0.868	0.955	0.5045
209390_at	516.9	562.6	0.12	1.09	PPP-PPP	0.10	17	0.868	0.955	0.5032
242918_at	187.6	204.1	0.12	1.09	PPP-PPP	0.10	17	0.868	0.955	0.4967
205740_s_at	1689.8	1838.4	0.12	1.09	PPP-PPP	0.10	17	0.868	0.955	0.4709
213943_at	1276.4	1389.2	0.12	1.09	PPP-PPP	0.10	17	0.868	0.955	0.4070
214042_s_at	11297.7	12294.4	0.12	1.09	PPP-PPP	0.10	17	0.868	0.955	0.3805
226915_s_at	2389.2	2600.1	0.12	1.09	PPP-PPP	0.10	17	0.868	0.955	0.3643
203642_s_at	1348.5	1467.3	0.12	1.09	PPP-PPP	0.10	17	0.868	0.955	0.3384
201918_at	2298.3	2502.0	0.12	1.09	PPP-PPP	0.10	17	0.868	0.955	0.3309
224188_s_at	238.9	260.0	0.12	1.09	PPP-PPP	0.10	17	0.868	0.955	0.3168
221425_s_at	1403.4	1527.0	0.12	1.09	PPP-PPP	0.10	17	0.868	0.955	0.2902
223384_s_at	1439.8	1566.9	0.12	1.09	PPP-PPP	0.10	17	0.868	0.955	0.2706
200012_x_at	37199.8	40473.9	0.12	1.09	PPP-PPP	0.10	17	0.868	0.955	0.2587
200872_at	36063.4	39239.0	0.12	1.09	PPP-PPP	0.10	17	0.868	0.955	0.2581
208939_at	2232.7	2430.4	0.12	1.09	PPP-PPP	0.10	17	0.868	0.955	0.2215
205526_s_at	1116.9	1215.4	0.12	1.09	PPP-PPP	0.10	17	0.868	0.955	0.1882
218309_at	4234.0	4608.1	0.12	1.09	PPP-PPP	0.10	17	0.868	0.955	0.1643
1564494_s_at	2516.1	2737.6	0.12	1.09	PPP-PPP	0.10	17	0.868	0.955	0.1356
1555961_a_at	26103.8	28409.2	0.12	1.09	PPP-PPP	0.10	17	0.868	0.955	0.0680
222393_s_at	2551.6	2775.4	0.12	1.09	PPP-PPP	0.10	17	0.868	0.955	0.5243
212747_at	982.2	1068.2	0.12	1.09	PPP-PPP	0.10	17	0.868	0.955	0.4700
227214_at	222.3	241.7	0.12	1.09	PPP-PPP	0.10	17	0.868	0.955	0.4681

212450_at	401.2	436.3	0.12	1.09	PPP-PPP	0.10	17	0.868	0.955	0.4677
201862_s_at	5039.8	5478.9	0.12	1.09	PPP-PPP	0.10	17	0.868	0.955	0.4399
208928_at	668.4	727.1	0.12	1.09	PPP-PPP	0.10	17	0.868	0.955	0.4392
1555844_s_at	8632.1	9387.2	0.12	1.09	PPP-PPP	0.10	17	0.868	0.955	0.4332
218697_at	540.1	587.4	0.12	1.09	PPP-PPP	0.10	17	0.868	0.955	0.4231
214578_s_at	931.7	1013.1	0.12	1.09	PPP-PPP	0.10	17	0.868	0.955	0.3753
215836_s_at	515.9	561.2	0.12	1.09	PPP-MPM	0.10	17	0.868	0.955	0.3562
203460_s_at	1778.8	1934.9	0.12	1.09	PPP-PPP	0.10	17	0.868	0.955	0.3237
225144_at	1700.9	1850.2	0.12	1.09	PPP-PPP	0.10	17	0.868	0.955	0.2862
219024_at	1304.4	1418.4	0.12	1.09	PPP-PPP	0.10	17	0.868	0.955	0.2845
227687_at	2099.6	2284.0	0.12	1.09	PPP-PPP	0.10	17	0.868	0.955	0.2707
202551_s_at	2576.1	2802.1	0.12	1.09	PPP-PPP	0.10	17	0.868	0.955	0.2449
224373_s_at	46221.5	50248.3	0.12	1.09	PPP-PPP	0.10	17	0.868	0.955	0.1637
208030_s_at	1975.5	2147.9	0.12	1.09	PPP-PPP	0.10	17	0.868	0.955	0.1395
213347_x_at	38839.6	42236.7	0.12	1.09	PPP-PPP	0.10	17	0.868	0.955	0.0917
223144_s_at	598.4	650.9	0.12	1.09	PPP-PPP	0.10	17	0.868	0.955	0.0891
223032_x_at	12885.0	14008.4	0.12	1.09	PPP-PPP	0.10	17	0.868	0.955	0.0403
221494_x_at	16207.2	17629.2	0.12	1.09	PPP-PPP	0.10	17	0.868	0.955	0.0414
224068_x_at	2746.1	2987.1	0.12	1.09	PPP-PPP	0.10	17	0.868	0.955	0.0139
203502_at	544.6	592.0	0.12	1.09	PPP-PPP	0.10	17	0.868	0.955	0.5302
218539_at	1488.2	1617.4	0.12	1.09	PPP-PPP	0.10	17	0.868	0.955	0.5299
203350_at	564.1	612.9	0.12	1.09	PPP-PPP	0.10	17	0.868	0.955	0.5236
203959_s_at	366.1	397.9	0.12	1.09	PPP-PPP	0.10	17	0.868	0.955	0.5169
225560_at	283.2	307.8	0.12	1.09	PPP-PPP	0.10	17	0.868	0.955	0.5101
221706_s_at	565.1	614.2	0.12	1.09	PPP-PPP	0.10	17	0.868	0.955	0.4822
222559_s_at	2350.7	2554.6	0.12	1.09	PPP-PPP	0.10	17	0.868	0.955	0.4582
201951_at	3164.0	3438.1	0.12	1.09	PPP-PPP	0.10	17	0.868	0.955	0.4415
227637_at	731.2	794.8	0.12	1.09	PPP-PPP	0.10	17	0.868	0.955	0.4364
225969_at	541.8	588.6	0.12	1.09	PPP-PPP	0.10	17	0.868	0.955	0.4143
223099_s_at	1837.3	1997.3	0.12	1.09	PPP-PPP	0.10	17	0.868	0.955	0.3997
213024_at	807.7	877.9	0.12	1.09	PPP-PPP	0.10	17	0.868	0.955	0.3865
202228_s_at	9118.1	9911.2	0.12	1.09	PPP-PPP	0.10	17	0.868	0.955	0.3177
222553_x_at	2259.1	2455.8	0.12	1.09	PPP-PPP	0.10	17	0.868	0.955	0.2932
242352_at	332.1	360.9	0.12	1.09	PPP-PAP	0.10	17	0.868	0.955	0.2397
235141_at	1403.0	1525.2	0.12	1.09	PPP-PPP	0.10	17	0.868	0.955	0.1777
216960_s_at	545.8	593.1	0.12	1.09	PPP-PPP	0.10	17	0.868	0.955	0.1715
201795_at	7516.7	8170.3	0.12	1.09	PPP-PPP	0.10	17	0.868	0.955	0.1680
201146_at	7536.4	8187.3	0.12	1.09	PPP-PPP	0.10	17	0.868	0.955	0.1327
223118_s_at	554.7	602.4	0.12	1.09	PPP-PPP	0.10	17	0.868	0.955	0.4600
202010_s_at	1320.8	1434.5	0.12	1.09	PPP-PPP	0.10	17	0.868	0.955	0.3937
213090_s_at	1423.3	1545.8	0.12	1.09	PPP-PPP	0.10	17	0.868	0.955	0.3706
224870_at	5044.7	5478.3	0.12	1.09	PPP-PPP	0.10	17	0.868	0.955	0.3631

224753_at	2044.1	2219.7	0.12	1.09	PPP-PPP	0.10	17	0.868	0.955	0.3405
211764_s_at	1866.9	2028.0	0.12	1.09	PPP-PPP	0.10	17	0.868	0.955	0.2658
224439_x_at	3444.5	3741.6	0.12	1.09	PPP-PPP	0.10	17	0.868	0.955	0.2110
201987_at	3768.6	4093.9	0.12	1.09	PPP-PPP	0.10	17	0.868	0.955	0.2026
213399_x_at	12030.1	13060.2	0.12	1.09	PPP-PPP	0.10	17	0.868	0.955	0.1870
244487_at	230.9	250.7	0.12	1.09	PPP-PPP	0.10	17	0.868	0.955	0.1453
203834_s_at	341.6	370.9	0.12	1.09	APP-PMP	0.10	17	0.868	0.955	0.1385
200869_at	48939.7	53163.1	0.12	1.09	PPP-PPP	0.10	17	0.868	0.955	0.1136
210886_x_at	1051.0	1141.1	0.12	1.09	PPP-PPP	0.10	17	0.868	0.955	0.1003
224885_s_at	9376.0	10185.6	0.12	1.09	PPP-PPP	0.10	17	0.868	0.955	0.0440
218243_at	2248.5	2441.0	0.12	1.09	PPP-PPP	0.10	17	0.868	0.955	0.5301
202254_at	214.2	232.4	0.12	1.09	PPP-PPP	0.10	17	0.868	0.955	0.5140
206500_s_at	469.7	509.7	0.12	1.09	PAP-PPP	0.10	17	0.868	0.955	0.5139
226832_at	1262.2	1369.6	0.12	1.09	PPP-PPP	0.10	17	0.868	0.955	0.5071
223470_at	760.7	825.6	0.12	1.09	PPP-PPP	0.10	17	0.868	0.955	0.4784
240616_at	497.6	540.2	0.12	1.09	PPP-PPP	0.10	17	0.868	0.955	0.4625
201281_at	2877.9	3123.4	0.12	1.09	PPP-PPP	0.10	17	0.868	0.955	0.4258
33494_at	595.7	646.5	0.12	1.09	PPP-PPP	0.10	17	0.868	0.955	0.4170
213612_x_at	9301.0	10094.7	0.12	1.09	PPP-PPP	0.10	17	0.868	0.955	0.3561
224751_at	4134.5	4487.1	0.12	1.09	PPP-PPP	0.10	17	0.868	0.955	0.3132
232591_s_at	985.4	1069.4	0.12	1.09	PPP-PPP	0.10	17	0.868	0.955	0.3100
202125_s_at	539.1	585.1	0.12	1.09	PPP-PPP	0.10	17	0.868	0.955	0.2874
200038_s_at	39971.9	43391.2	0.12	1.09	PPP-PPP	0.10	17	0.868	0.955	0.2133
212519_at	7330.1	7953.4	0.12	1.09	PPP-PPP	0.10	17	0.868	0.955	0.1787
212988_x_at	32622.7	35407.9	0.12	1.09	PPP-PPP	0.10	17	0.868	0.955	0.1626
200003_s_at	46694.2	50680.6	0.12	1.09	PPP-PPP	0.10	17	0.868	0.955	0.1362
217753_s_at	26371.4	28618.5	0.12	1.09	PPP-PPP	0.10	17	0.868	0.955	0.0455
203497_at	1547.1	1677.8	0.12	1.08	PPP-PPP	0.10	17	0.868	0.955	0.5277
221849_s_at	265.1	287.6	0.12	1.09	PAP-PPP	0.10	17	0.868	0.955	0.5264
210417_s_at	803.4	871.3	0.12	1.09	PPP-PPP	0.10	17	0.868	0.955	0.4709
225154_at	2731.5	2962.2	0.12	1.08	PPP-PPP	0.10	17	0.868	0.955	0.4680
201988_s_at	430.6	466.8	0.12	1.08	PPP-PPP	0.10	17	0.868	0.955	0.4528
219112_at	658.8	714.6	0.12	1.09	PPP-PPP	0.10	17	0.868	0.955	0.4265
218386_x_at	2921.5	3168.8	0.12	1.09	PPP-PPP	0.10	17	0.868	0.955	0.4149
222606_at	2695.4	2923.0	0.12	1.08	PPP-PPP	0.10	17	0.868	0.955	0.3937
212198_s_at	1171.1	1270.4	0.12	1.09	PPP-PPP	0.10	17	0.868	0.955	0.3871
211240_x_at	2383.3	2584.6	0.12	1.08	PPP-PPP	0.10	17	0.868	0.955	0.3628
212237_at	1201.8	1303.1	0.12	1.08	PPP-PPP	0.10	17	0.868	0.955	0.3521
AFFX-CreX-5_at	20897.2	22669.1	0.12	1.09	PPP-PPP	0.10	17	0.868	0.955	0.3456
231075_x_at	487.2	528.4	0.12	1.08	PPP-PPP	0.10	17	0.868	0.955	0.2973
1555831_s_at	820.3	889.8	0.12	1.09	PPP-PPP	0.10	17	0.868	0.955	0.2957
210453_x_at	26430.1	28657.9	0.12	1.08	PPP-PPP	0.10	17	0.868	0.955	0.2871

208979_at	1935.0	2098.2	0.12	1.08	PPP-PPP	0.10	17	0.868	0.955	0.2232
228615_at	329.8	357.6	0.12	1.08	PPP-PMP	0.10	17	0.868	0.955	0.2150
208808_s_at	16605.1	18011.4	0.12	1.09	PPP-PPP	0.10	17	0.868	0.955	0.1171
212279_at	2949.7	3199.6	0.12	1.09	PPP-PPP	0.10	17	0.868	0.955	0.0629
226477_at	397.1	430.3	0.12	1.08	APP-APP	0.10	17	0.868	0.955	0.5267
202323_s_at	1069.5	1159.4	0.12	1.08	PPP-PPP	0.10	17	0.868	0.955	0.5253
212383_at	677.2	733.8	0.12	1.08	PPP-PPP	0.10	17	0.868	0.955	0.5231
229384_at	406.8	440.9	0.12	1.08	PPP-PPP	0.10	17	0.868	0.955	0.4924
64488_at	525.2	569.0	0.12	1.08	PPP-PPP	0.10	17	0.868	0.955	0.4766
203967_at	740.5	802.4	0.12	1.08	PPP-PPP	0.10	17	0.868	0.955	0.4546
224862_at	1003.0	1087.2	0.12	1.08	PPP-PPP	0.10	17	0.868	0.955	0.4276
227693_at	438.0	474.8	0.12	1.08	PPP-PPP	0.10	17	0.868	0.955	0.4197
227852_at	1218.0	1320.3	0.12	1.08	PPP-PPP	0.10	17	0.868	0.955	0.4002
218777_at	1700.5	1843.2	0.12	1.08	PPP-PPP	0.10	17	0.868	0.955	0.3905
218212_s_at	941.1	1019.8	0.12	1.08	PPP-PPP	0.10	17	0.868	0.955	0.3621
209580_s_at	1644.9	1782.7	0.12	1.08	PPP-PPP	0.10	17	0.868	0.955	0.3411
215548_s_at	1793.2	1943.5	0.12	1.08	PPP-PPP	0.10	17	0.868	0.955	0.3319
202770_s_at	1419.9	1538.9	0.12	1.08	PPP-PPP	0.10	17	0.868	0.955	0.3314
60471_at	283.7	307.4	0.12	1.08	PPP-PPP	0.10	17	0.868	0.955	0.3199
217043_s_at	1142.7	1238.1	0.12	1.08	PPP-PPP	0.10	17	0.868	0.955	0.3188
201252_at	2462.9	2668.2	0.12	1.08	PPP-PPP	0.10	17	0.868	0.955	0.3114
213454_at	1055.4	1143.6	0.12	1.08	PPP-PPP	0.10	17	0.868	0.955	0.2756
203744_at	2402.1	2602.4	0.12	1.08	PPP-PPP	0.10	17	0.868	0.955	0.2545
224946_s_at	744.7	806.9	0.12	1.08	PPP-PPP	0.10	17	0.868	0.955	0.2493
202231_at	20989.7	22752.4	0.12	1.08	PPP-PPP	0.10	17	0.868	0.955	0.1643
201059_at	7861.1	8521.6	0.12	1.08	PPP-PPP	0.10	17	0.868	0.955	0.1265
200837_at	4759.4	5158.7	0.12	1.08	PPP-PPP	0.10	17	0.868	0.955	0.0936
224840_at	21215.7	22987.5	0.12	1.08	PPP-PPP	0.10	17	0.868	0.955	0.0439
209256_s_at	1018.8	1103.9	0.12	1.08	PPP-PPP	0.10	17	0.868	0.955	0.0274
224597_at	11838.9	12830.0	0.12	1.08	PPP-PPP	0.10	17	0.868	0.955	0.0108
201929_s_at	844.1	914.8	0.12	1.08	PPP-PPP	0.10	17	0.868	0.955	0.0065
201901_s_at	4081.7	4421.6	0.12	1.08	PPP-PPP	0.10	17	0.868	0.955	0.5273
228567_at	239.7	259.6	0.12	1.08	PPP-PPP	0.10	17	0.868	0.955	0.5201
222036_s_at	3192.6	3458.4	0.12	1.08	PPP-PPP	0.10	17	0.868	0.955	0.5115
236887_at	57.1	61.8	0.12	1.08	AMA-MMP	0.10	17	0.868	0.955	0.4933
223078_s_at	776.0	840.2	0.12	1.08	PPP-PPP	0.10	17	0.868	0.955	0.4690
232676_x_at	150.0	162.4	0.12	1.08	AAP-APP	0.10	17	0.868	0.955	0.4631
223305_at	505.6	547.5	0.12	1.08	PPP-PPP	0.10	17	0.868	0.955	0.4521
225893_at	1002.6	1085.7	0.12	1.08	PPP-PPP	0.10	17	0.868	0.955	0.4500
1552680_a_at	387.3	419.6	0.12	1.08	PPP-PPP	0.10	17	0.868	0.955	0.4318
201531_at	2082.2	2255.5	0.12	1.08	PPP-PPP	0.10	17	0.868	0.955	0.4187
227931_at	810.9	878.0	0.12	1.08	PPP-PPP	0.10	17	0.868	0.955	0.4123

209488_s_at	1317.4	1426.5	0.12	1.08	PPP-PPP	0.10	17	0.868	0.955	0.3787
209806_at	1173.7	1271.0	0.12	1.08	PPP-PPP	0.10	17	0.868	0.955	0.3715
208766_s_at	12844.5	13908.4	0.12	1.08	PPP-PPP	0.10	17	0.868	0.955	0.3622
219052_at	800.2	866.7	0.12	1.08	PPP-PPP	0.10	17	0.868	0.955	0.3483
219939_s_at	6521.0	7060.4	0.12	1.08	PPP-PPP	0.10	17	0.868	0.955	0.3249
200716_x_at	49777.7	53903.3	0.12	1.08	PPP-PPP	0.10	17	0.868	0.955	0.3187
209759_s_at	2274.7	2463.3	0.12	1.08	PPP-PPP	0.10	17	0.868	0.955	0.3177
221883_at	535.2	579.7	0.12	1.08	PPP-PPP	0.10	17	0.868	0.955	0.3158
227678_at	468.7	507.7	0.12	1.08	PPP-PPP	0.10	17	0.868	0.955	0.3138
221891_x_at	38317.4	41493.2	0.12	1.08	PPP-PPP	0.10	17	0.868	0.955	0.2820
212077_at	14817.2	16042.1	0.12	1.08	PPP-PPP	0.10	17	0.868	0.955	0.2229
222212_s_at	6419.3	6952.5	0.12	1.08	PPP-PPP	0.10	17	0.868	0.955	0.2057
211061_s_at	848.4	918.9	0.12	1.08	PPP-PPP	0.10	17	0.868	0.955	0.1141
224560_at	1608.0	1741.2	0.12	1.08	PPP-PPP	0.10	17	0.868	0.955	0.0735
212239_at	1790.7	1937.4	0.11	1.08	PPP-PPP	0.10	17	0.868	0.955	0.5123
213019_at	902.0	976.0	0.11	1.08	PPP-PPP	0.10	17	0.868	0.955	0.4809
212041_at	3207.5	3472.1	0.11	1.08	PPP-PPP	0.10	17	0.868	0.955	0.4683
235801_at	345.8	374.3	0.11	1.08	PPP-PPP	0.10	17	0.868	0.955	0.4588
216064_s_at	311.3	337.0	0.11	1.08	PPP-PPP	0.10	17	0.868	0.955	0.4469
200864_s_at	1511.5	1636.2	0.11	1.08	PPP-PPP	0.10	17	0.868	0.955	0.4273
223681_s_at	1581.1	1711.0	0.11	1.08	PPP-PPP	0.10	17	0.868	0.955	0.3940
209092_s_at	1770.9	1916.4	0.11	1.08	PPP-PPP	0.10	17	0.868	0.955	0.3182
1554767_s_at	288.7	312.6	0.11	1.08	PPP-PPP	0.10	17	0.868	0.955	0.2905
204711_at	593.6	642.3	0.11	1.08	PMP-APP	0.10	17	0.868	0.955	0.2525
215374_at	99.6	107.9	0.11	1.08	APP-PPP	0.10	17	0.868	0.955	0.2279
209224_s_at	6910.0	7478.0	0.11	1.08	PPP-PPP	0.10	17	0.868	0.955	0.2073
212155_at	3586.1	3880.8	0.11	1.08	PPP-PPP	0.10	17	0.868	0.955	0.2012
200018_at	45813.1	49573.5	0.11	1.08	PPP-PPP	0.10	17	0.868	0.955	0.1874
203459_s_at	1179.6	1277.0	0.11	1.08	PPP-PPP	0.10	17	0.868	0.955	0.1841
228628_at	1934.6	2094.1	0.11	1.08	PPP-PPP	0.10	17	0.868	0.955	0.1804
213794_s_at	1680.2	1818.8	0.11	1.08	PPP-PPP	0.10	17	0.868	0.955	0.1699
204243_at	610.7	660.8	0.11	1.08	PPP-PPP	0.10	17	0.868	0.955	0.1574
201652_at	9172.7	9924.8	0.11	1.08	PPP-PPP	0.10	17	0.868	0.955	0.0423
212057_at	787.8	852.2	0.11	1.08	PPP-PPP	0.10	17	0.868	0.955	0.5030
218036_x_at	1470.4	1590.4	0.11	1.08	PPP-PPP	0.10	17	0.868	0.955	0.4493
225396_at	553.0	597.9	0.11	1.08	PPP-PPP	0.10	17	0.868	0.955	0.4352
224564_s_at	2806.4	3035.3	0.11	1.08	PPP-PPP	0.10	17	0.868	0.955	0.4314
212638_s_at	3352.6	3626.3	0.11	1.08	PPP-PPP	0.10	17	0.868	0.955	0.4001
204062_s_at	458.5	495.8	0.11	1.08	PPP-PPP	0.10	17	0.868	0.955	0.3957
201584_s_at	9522.9	10295.3	0.11	1.08	PPP-PPP	0.10	17	0.868	0.955	0.3613
219215_s_at	2542.3	2748.9	0.11	1.08	PPP-PPP	0.10	17	0.868	0.955	0.3507
214543_x_at	675.5	730.5	0.11	1.08	PPP-APP	0.10	17	0.868	0.955	0.3338

225324_at	1449.9	1568.0	0.11	1.08	PPP-PPP	0.10	17	0.868	0.955	0.2918
1553569_at	54400.5	58826.2	0.11	1.08	PPP-PPP	0.10	17	0.868	0.955	0.2903
212490_at	192.1	207.8	0.11	1.08	PPP-PPP	0.10	17	0.868	0.955	0.2857
222421_at	3019.5	3264.7	0.11	1.08	PPP-PPP	0.10	17	0.868	0.955	0.2602
218383_at	1765.7	1909.0	0.11	1.08	PPP-PPP	0.10	17	0.868	0.955	0.2477
1553588_at	49722.5	53784.2	0.11	1.08	PPP-PPP	0.10	17	0.868	0.955	0.2333
201017_at	2509.4	2713.7	0.11	1.08	PPP-PPP	0.10	17	0.868	0.955	0.2264
202809_s_at	989.6	1070.3	0.11	1.08	PPP-PPP	0.10	17	0.868	0.955	0.2081
213581_at	2076.6	2245.8	0.11	1.08	PPP-PPP	0.10	17	0.868	0.955	0.2029
226131_s_at	50618.5	54728.8	0.11	1.08	PPP-PPP	0.10	17	0.868	0.955	0.1889
200095_x_at	46360.1	50135.4	0.11	1.08	PPP-PPP	0.10	17	0.868	0.955	0.1681
221821_s_at	971.4	1050.8	0.11	1.08	PPP-PPP	0.10	17	0.868	0.955	0.1663
211366_x_at	2077.4	2247.1	0.11	1.08	PPP-PPP	0.10	17	0.868	0.955	0.1617
217720_at	26235.8	28374.8	0.11	1.08	PPP-PPP	0.10	17	0.868	0.955	0.0788
212958_x_at	2989.1	3232.2	0.11	1.08	PPP-PPP	0.10	17	0.868	0.955	0.0659
223219_s_at	832.8	900.0	0.11	1.08	PPP-PPP	0.10	17	0.868	0.955	0.4470
218466_at	233.0	251.9	0.11	1.08	PPP-PPP	0.10	17	0.868	0.955	0.4449
212814_at	739.1	798.8	0.11	1.08	PPP-PPP	0.10	17	0.868	0.955	0.3935
224397_s_at	1291.2	1395.2	0.11	1.08	PPP-PPP	0.10	17	0.868	0.955	0.3607
233878_s_at	2426.1	2621.2	0.11	1.08	PPP-PPP	0.10	17	0.868	0.955	0.3440
227786_at	1672.9	1808.3	0.11	1.08	PPP-PPP	0.10	17	0.868	0.955	0.3124
208856_x_at	41569.3	44920.5	0.11	1.08	PPP-PPP	0.10	17	0.868	0.955	0.2842
221700_s_at	27791.8	30036.4	0.11	1.08	PPP-PPP	0.10	17	0.868	0.955	0.2522
226749_at	2044.2	2209.3	0.11	1.08	PPP-PPP	0.10	17	0.868	0.955	0.1860
230264_s_at	1869.5	2020.3	0.11	1.08	PPP-PPP	0.10	17	0.868	0.955	0.1540
204102_s_at	38794.7	41937.8	0.11	1.08	PPP-PPP	0.10	17	0.868	0.955	0.1555
39891_at	540.3	583.9	0.11	1.08	PPP-PPP	0.10	17	0.868	0.955	0.1482
211943_x_at	41390.5	44718.2	0.11	1.08	PPP-PPP	0.10	17	0.868	0.955	0.1193
209386_at	3007.8	3250.9	0.11	1.08	PPP-PPP	0.10	17	0.868	0.955	0.0774
226715_at	1583.1	1709.4	0.11	1.08	PPP-PPP	0.10	17	0.868	0.955	0.4976
202995_s_at	1151.1	1243.0	0.11	1.08	PPP-PPP	0.10	17	0.868	0.955	0.4962
218024_at	3134.6	3385.3	0.11	1.08	PPP-PPP	0.10	17	0.868	0.955	0.4590
230078_at	1816.9	1962.8	0.11	1.08	PPP-PPP	0.10	17	0.868	0.955	0.4237
1556009_at	100.0	108.0	0.11	1.08	PPP-PPP	0.10	17	0.868	0.955	0.4096
218803_at	917.1	990.7	0.11	1.08	PPP-PPP	0.10	17	0.868	0.955	0.3769
225485_at	580.3	626.5	0.11	1.08	PPP-PPP	0.10	17	0.868	0.955	0.3705
208358_s_at	540.8	584.0	0.11	1.08	PPP-PPP	0.10	17	0.868	0.955	0.3677
221691_x_at	32039.1	34593.3	0.11	1.08	PPP-PPP	0.10	17	0.868	0.955	0.3663
230434_at	395.7	427.4	0.11	1.08	PPP-PPP	0.10	17	0.868	0.955	0.3630
202109_at	1562.4	1687.5	0.11	1.08	PPP-PPP	0.10	17	0.868	0.955	0.3561
AFFX-r2-Ec-bioB-3_at	716.4	773.7	0.11	1.08	PPP-PPP	0.10	17	0.868	0.955	0.3142
40225_at	1817.4	1962.3	0.11	1.08	PPP-PPP	0.10	17	0.868	0.955	0.3124

201361_at	2240.6	2419.3	0.11	1.08	PPP-PPP	0.10	17	0.868	0.955	0.2986
226128_at	6144.4	6635.0	0.11	1.08	PPP-PPP	0.10	17	0.868	0.955	0.2771
209758_s_at	1436.4	1551.5	0.11	1.08	PPP-PPP	0.10	17	0.868	0.955	0.2723
200950_at	7631.6	8240.6	0.11	1.08	PPP-PPP	0.10	17	0.868	0.955	0.2042
212420_at	1489.1	1607.9	0.11	1.08	PPP-PPP	0.10	17	0.868	0.955	0.0807
219098_at	949.0	1024.4	0.11	1.08	PPP-PPP	0.10	17	0.868	0.955	0.5170
242857_at	452.1	487.9	0.11	1.08	PPP-PPP	0.10	17	0.868	0.955	0.4976
228478_at	1045.9	1129.1	0.11	1.08	PPP-PPP	0.10	17	0.868	0.955	0.4975
224950_at	1034.1	1116.3	0.11	1.08	PPP-PPP	0.10	17	0.868	0.955	0.4903
212068_s_at	1191.0	1285.2	0.11	1.08	PPP-PPP	0.10	17	0.868	0.955	0.4697
217943_s_at	1497.9	1616.1	0.11	1.08	PPP-PPP	0.10	17	0.868	0.955	0.4685
223341_s_at	1739.2	1877.7	0.11	1.08	PPP-PPP	0.10	17	0.868	0.955	0.4608
224320_s_at	846.5	913.7	0.11	1.08	PPP-PPP	0.10	17	0.868	0.955	0.4597
224301_x_at	1844.8	1990.3	0.11	1.08	PPP-PPP	0.10	17	0.868	0.955	0.4470
201019_s_at	11940.0	12886.4	0.11	1.08	PPP-PPP	0.10	17	0.868	0.955	0.4473
212184_s_at	1316.7	1421.0	0.11	1.08	PPP-PPP	0.10	17	0.868	0.955	0.4366
238460_at	1316.3	1420.4	0.11	1.08	PPP-PPP	0.10	17	0.868	0.955	0.4237
214527_s_at	2170.6	2343.2	0.11	1.08	PPP-PPP	0.10	17	0.868	0.955	0.3505
216508_x_at	7424.0	8014.7	0.11	1.08	PPP-PPP	0.10	17	0.868	0.955	0.3408
202423_at	2639.0	2847.4	0.11	1.08	PPP-PPP	0.10	17	0.868	0.955	0.2976
209059_s_at	3464.0	3738.4	0.11	1.08	PPP-PPP	0.10	17	0.868	0.955	0.2395
213983_s_at	609.8	658.0	0.11	1.08	PPP-PPP	0.10	17	0.868	0.955	0.1575
223250_at	1706.9	1841.6	0.11	1.08	PPP-PPP	0.10	17	0.868	0.955	0.1379
222626_at	660.5	712.8	0.11	1.08	PPP-PPP	0.10	17	0.868	0.955	0.1277
218138_at	1488.6	1606.9	0.11	1.08	PPP-PPP	0.10	17	0.868	0.955	0.1148
211744_s_at	1389.7	1499.8	0.11	1.08	PPP-PPP	0.10	17	0.868	0.955	0.0821
221311_x_at	1143.1	1232.7	0.11	1.08	PPP-PPP	0.10	17	0.868	0.955	0.4985
213720_s_at	2789.1	3008.4	0.11	1.08	PPP-PPP	0.10	17	0.868	0.955	0.4623
206866_at	631.9	681.5	0.11	1.08	PPP-PPP	0.10	17	0.868	0.955	0.4533
218082_s_at	3471.1	3742.7	0.11	1.08	PPP-PPP	0.10	17	0.868	0.955	0.4028
227769_at	506.3	546.2	0.11	1.08	PPP-PPP	0.10	17	0.868	0.955	0.3898
211038_s_at	753.4	812.5	0.11	1.08	PPP-PPP	0.10	17	0.868	0.955	0.3001
226865_at	703.1	758.1	0.11	1.08	PPP-PPP	0.10	17	0.868	0.955	0.2475
226976_at	1516.3	1635.2	0.11	1.08	PPP-PPP	0.10	17	0.868	0.955	0.1560
208952_s_at	1126.4	1214.8	0.11	1.08	PPP-PPP	0.10	17	0.868	0.955	0.1385
224699_s_at	4255.0	4589.0	0.11	1.08	PPP-PPP	0.10	17	0.868	0.955	0.0282
243296_at	228.1	245.8	0.11	1.08	PMP-PMP	0.10	17	0.868	0.955	0.5089
227473_at	2831.2	3050.6	0.11	1.08	PPP-PPP	0.10	17	0.868	0.955	0.5059
225477_s_at	1686.8	1817.9	0.11	1.08	PPP-PPP	0.10	17	0.868	0.955	0.4833
217936_at	590.7	636.8	0.11	1.08	PPP-PPP	0.10	17	0.868	0.955	0.4673
219235_s_at	789.4	850.7	0.11	1.08	PPP-PPP	0.10	17	0.868	0.955	0.4129
214959_s_at	1014.1	1092.9	0.11	1.08	PPP-PPP	0.10	17	0.868	0.955	0.3833

211025_x_at	7866.3	8476.2	0.11	1.08	PPP-PPP	0.10	17	0.868	0.955	0.3510
212726_at	927.3	999.6	0.11	1.08	PPP-PPP	0.10	17	0.868	0.955	0.3498
208692_at	41728.4	44967.2	0.11	1.08	PPP-PPP	0.10	17	0.868	0.955	0.2256
221808_at	4273.2	4604.1	0.11	1.08	PPP-PPP	0.10	17	0.868	0.955	0.1554
37152_at	764.6	824.2	0.11	1.08	PPP-PPP	0.10	17	0.868	0.955	0.1541
224893_at	4839.4	5214.9	0.11	1.08	PPP-PPP	0.10	17	0.868	0.955	0.0623
201984_s_at	3676.9	3962.3	0.11	1.08	PPP-PPP	0.10	17	0.868	0.955	0.0380
222598_s_at	429.7	463.0	0.11	1.08	PPP-PPP	0.10	17	0.868	0.955	0.5010
218883_s_at	2704.8	2914.0	0.11	1.08	PPP-PPP	0.10	17	0.868	0.955	0.4974
224902_at	942.2	1014.5	0.11	1.08	PPP-PPP	0.10	17	0.868	0.955	0.4915
222576_s_at	279.5	301.0	0.11	1.08	PMP-PPP	0.10	17	0.868	0.955	0.4878
1552419_s_at	107.9	116.2	0.11	1.08	PAA-AAA	0.10	17	0.868	0.955	0.4650
AFFX-r2-Ec-bioC-5_at	2042.3	2199.5	0.11	1.08	PPP-PPP	0.10	17	0.868	0.955	0.4587
208916_at	3247.4	3498.2	0.11	1.08	PPP-PPP	0.10	17	0.868	0.955	0.4055
201277_s_at	18024.9	19411.6	0.11	1.08	PPP-PPP	0.10	17	0.868	0.955	0.3930
202073_at	949.0	1021.8	0.11	1.08	PPP-PPP	0.10	17	0.868	0.955	0.3775
203035_s_at	740.9	797.8	0.11	1.08	PPP-PPP	0.10	17	0.868	0.955	0.3709
1553132_a_at	318.7	343.3	0.11	1.08	PPP-PPP	0.10	17	0.868	0.955	0.3632
215706_x_at	1790.7	1928.4	0.11	1.08	PPP-PPP	0.10	17	0.868	0.955	0.3598
221826_at	755.6	813.9	0.11	1.08	PPP-PPP	0.10	17	0.868	0.955	0.3587
201587_s_at	5574.6	6002.5	0.11	1.08	PPP-PPP	0.10	17	0.868	0.955	0.3412
221922_at	3545.2	3817.5	0.11	1.08	PPP-PPP	0.10	17	0.868	0.955	0.3359
224802_at	6196.1	6671.8	0.11	1.08	PPP-PPP	0.10	17	0.868	0.955	0.3310
201076_at	2010.1	2164.5	0.11	1.08	PPP-PPP	0.10	17	0.868	0.955	0.2994
208734_x_at	4832.9	5204.7	0.11	1.08	PPP-PPP	0.10	17	0.868	0.955	0.2912
222665_at	610.7	657.7	0.11	1.08	PPP-PPP	0.10	17	0.868	0.955	0.2403
226189_at	5240.0	5643.5	0.11	1.08	PPP-PPP	0.10	17	0.868	0.955	0.2073
232296_s_at	1072.9	1155.8	0.11	1.08	PPP-PPP	0.10	17	0.868	0.955	0.1859
211983_x_at	34292.1	36927.7	0.11	1.08	PPP-PPP	0.10	17	0.868	0.955	0.1733
209289_at	5495.4	5917.8	0.11	1.08	PPP-PPP	0.10	17	0.868	0.955	0.1650
221802_s_at	1210.9	1304.0	0.11	1.08	PPP-PPP	0.10	17	0.868	0.955	0.1234
227404_s_at	7512.5	8093.2	0.11	1.08	PPP-PPP	0.10	17	0.868	0.955	0.1017
202117_at	5348.5	5759.6	0.11	1.08	PPP-PPP	0.10	17	0.868	0.955	0.0777
32137_at	1754.5	1889.8	0.11	1.08	PPP-PPP	0.10	17	0.868	0.955	0.0361
213649_at	1176.7	1266.1	0.11	1.08	PPP-PPP	0.10	17	0.868	0.955	0.5019
229512_at	393.6	423.6	0.11	1.08	PPP-PPP	0.10	17	0.868	0.955	0.4915
1554553_s_at	665.1	715.7	0.11	1.08	PPP-PPP	0.10	17	0.868	0.955	0.4765
236458_at	208.3	224.2	0.11	1.08	PPP-PPP	0.10	17	0.868	0.955	0.4567
200594_x_at	11292.9	12157.7	0.11	1.08	PPP-PPP	0.10	17	0.868	0.955	0.4432
215158_s_at	2449.6	2637.2	0.11	1.08	PPP-PPP	0.10	17	0.868	0.955	0.4275
202433_at	1577.5	1698.3	0.11	1.08	PPP-PPP	0.10	17	0.868	0.955	0.4186
222848_at	1535.1	1652.0	0.11	1.08	PPP-PPP	0.10	17	0.868	0.955	0.4107

225133_at	1414.5	1522.2	0.11	1.08	PPP-PPP	0.10	17	0.868	0.955	0.3778
208688_x_at	9832.5	10585.3	0.11	1.08	PPP-PPP	0.10	17	0.868	0.955	0.3777
223338_s_at	2497.2	2688.1	0.11	1.08	PPP-PPP	0.10	17	0.868	0.955	0.3680
226126_at	893.3	961.5	0.11	1.08	PPP-PPP	0.10	17	0.868	0.955	0.3224
204146_at	1335.1	1436.4	0.11	1.08	PPP-PPP	0.10	17	0.868	0.955	0.3004
210241_s_at	449.6	483.9	0.11	1.08	PPP-PPP	0.10	17	0.868	0.955	0.2938
201621_at	1336.8	1438.6	0.11	1.08	PPP-PPP	0.10	17	0.868	0.955	0.2929
218766_s_at	629.5	677.3	0.11	1.08	PPP-PPP	0.10	17	0.868	0.955	0.2138
227452_at	650.6	700.2	0.11	1.08	PPP-PPP	0.10	17	0.868	0.955	0.2066
212204_at	2301.8	2477.0	0.11	1.08	PPP-PPP	0.10	17	0.868	0.955	0.2043
203215_s_at	1652.5	1778.2	0.11	1.08	PPP-PPP	0.10	17	0.868	0.955	0.2027
211569_s_at	2134.6	2296.7	0.11	1.08	PPP-PPP	0.10	17	0.868	0.955	0.1926
225537_at	685.9	738.4	0.11	1.08	PPP-PPP	0.10	17	0.868	0.955	0.1928
201240_s_at	11169.4	12021.1	0.11	1.08	PPP-PPP	0.10	17	0.868	0.955	0.1318
225183_at	1589.4	1709.6	0.11	1.08	PPP-PPP	0.10	17	0.868	0.955	0.5056
211704_s_at	222.6	239.3	0.11	1.08	AAP-AMP	0.10	17	0.868	0.955	0.4890
235709_at	564.1	606.7	0.11	1.08	APA-AAP	0.10	17	0.868	0.955	0.4139
203883_s_at	818.0	879.9	0.11	1.08	PPP-PPP	0.10	17	0.868	0.955	0.3913
223492_s_at	579.5	623.1	0.11	1.08	PPP-PPP	0.10	17	0.868	0.955	0.2881
218051_s_at	1159.7	1246.9	0.11	1.08	PPP-PPP	0.10	17	0.868	0.955	0.2614
203228_at	1674.5	1801.2	0.11	1.08	PPP-PPP	0.10	17	0.868	0.955	0.2185
218068_s_at	1141.9	1228.4	0.11	1.08	PPP-PPP	0.10	17	0.868	0.955	0.1967
204466_s_at	1362.7	1465.2	0.11	1.08	PPP-PPP	0.10	17	0.868	0.955	0.1649
64486_at	1639.9	1764.0	0.11	1.08	PPP-PPP	0.10	17	0.868	0.955	0.1276
1555790_a_at	2441.0	2624.7	0.11	1.08	PPP-PPP	0.10	17	0.868	0.955	0.1180
200089_s_at	34440.2	37036.8	0.11	1.08	PPP-PPP	0.10	17	0.868	0.955	0.1162
229419_at	397.2	426.8	0.10	1.08	PPP-PPP	0.10	17	0.868	0.955	0.5116
210987_x_at	3103.6	3334.5	0.10	1.07	PPP-PPP	0.10	17	0.868	0.955	0.5091
223692_at	178.6	192.0	0.10	1.08	PPP-PPP	0.10	17	0.868	0.955	0.4897
226437_at	852.6	916.1	0.10	1.07	PPP-PPP	0.10	17	0.868	0.955	0.4866
1552978_a_at	769.7	827.3	0.10	1.08	PPP-PPP	0.10	17	0.868	0.955	0.4655
208743_s_at	9147.7	9829.8	0.10	1.08	PPP-PPP	0.10	17	0.868	0.955	0.4635
223984_s_at	900.2	967.7	0.10	1.08	PPP-PPP	0.10	17	0.868	0.955	0.4549
45288_at	143.9	154.7	0.10	1.08	PPP-PPP	0.10	17	0.868	0.955	0.4369
200900_s_at	1241.6	1334.1	0.10	1.08	PPP-PPP	0.10	17	0.868	0.955	0.4052
203906_at	286.5	308.0	0.10	1.08	MAA-APM	0.10	17	0.868	0.955	0.3926
201110_s_at	3038.7	3266.0	0.10	1.08	PPP-PPP	0.10	17	0.868	0.955	0.3802
201343_at	4961.6	5332.3	0.10	1.08	PPP-PPP	0.10	17	0.868	0.955	0.2953
209091_s_at	3889.2	4179.9	0.10	1.08	PPP-PPP	0.10	17	0.868	0.955	0.2542
213646_x_at	36520.3	39248.9	0.10	1.08	PPP-PPP	0.10	17	0.868	0.955	0.1962
220973_s_at	731.0	785.7	0.10	1.08	PPP-PPP	0.10	17	0.868	0.955	0.1952
234302_s_at	1918.6	2061.8	0.10	1.08	PPP-PPP	0.10	17	0.868	0.955	0.1792

213860_x_at	13226.6	14213.8	0.10	1.08	PPP-PPP	0.10	17	0.868	0.955	0.1630
224585_x_at	37320.6	40105.1	0.10	1.08	PPP-PPP	0.10	17	0.868	0.955	0.1063
212097_at	31912.8	34306.3	0.10	1.08	PPP-PPP	0.10	17	0.868	0.955	0.0593
212961_x_at	1242.1	1334.7	0.10	1.08	PPP-PPP	0.10	17	0.868	0.955	0.0265
226472_at	534.3	574.0	0.10	1.07	PPP-PPP	0.10	17	0.868	0.955	0.4984
215111_s_at	10756.3	11556.0	0.10	1.07	PPP-PPP	0.10	17	0.868	0.955	0.4864
208313_s_at	3100.7	3331.1	0.10	1.07	PPP-PPP	0.10	17	0.868	0.955	0.4643
218717_s_at	2652.9	2849.8	0.10	1.07	PPP-PPP	0.10	17	0.868	0.955	0.4494
212411_at	3530.2	3791.6	0.10	1.07	PPP-PPP	0.10	17	0.868	0.955	0.4140
225492_at	3083.0	3310.5	0.10	1.07	PPP-PPP	0.10	17	0.868	0.955	0.3790
202954_at	7927.2	8515.1	0.10	1.07	PPP-PPP	0.10	17	0.868	0.955	0.3272
224580_at	1586.3	1703.4	0.10	1.07	PPP-PPP	0.10	17	0.868	0.955	0.3197
214946_x_at	1385.5	1488.0	0.10	1.07	PPP-PPP	0.10	17	0.868	0.955	0.3011
203002_at	1579.8	1696.7	0.10	1.07	PPP-PPP	0.10	17	0.868	0.955	0.3004
226547_at	1963.5	2109.2	0.10	1.07	PPP-PPP	0.10	17	0.868	0.955	0.2425
200088_x_at	43039.9	46228.1	0.10	1.07	PPP-PPP	0.10	17	0.868	0.955	0.2190
221893_s_at	1289.8	1385.3	0.10	1.07	PPP-PPP	0.10	17	0.868	0.955	0.2118
223077_at	4377.0	4699.9	0.10	1.07	PPP-PPP	0.10	17	0.868	0.955	0.1495
203986_at	336.3	361.2	0.10	1.07	PPP-PPP	0.10	17	0.868	0.955	0.1322
1554480_a_at	1536.3	1650.2	0.10	1.07	PPP-PPP	0.10	17	0.868	0.955	0.1184
202932_at	3218.3	3455.5	0.10	1.07	PPP-PPP	0.10	17	0.868	0.955	0.0426
202090_s_at	18776.4	20153.7	0.10	1.07	PPP-PPP	0.10	17	0.868	0.955	0.4397
232087_at	196.1	210.6	0.10	1.07	PPA-PPP	0.10	17	0.868	0.955	0.4319
209385_s_at	2543.1	2729.7	0.10	1.07	PPP-PPP	0.10	17	0.868	0.955	0.4005
201653_at	13380.4	14357.2	0.10	1.07	PPP-PPP	0.10	17	0.868	0.955	0.3938
213044_at	4288.9	4603.7	0.10	1.07	PPP-PPP	0.10	17	0.868	0.955	0.3887
59644_at	349.9	375.5	0.10	1.07	PPP-PPP	0.10	17	0.868	0.955	0.3873
1553570_x_at	54416.6	58417.3	0.10	1.07	PPP-PPP	0.10	17	0.868	0.955	0.3365
209917_s_at	885.2	949.7	0.10	1.07	PPP-PPP	0.10	17	0.868	0.955	0.3056
217801_at	20646.7	22164.1	0.10	1.07	PPP-PPP	0.10	17	0.868	0.955	0.2726
211963_s_at	12195.4	13086.2	0.10	1.07	PPP-PPP	0.10	17	0.868	0.955	0.2410
201417_at	3105.0	3333.5	0.10	1.07	PPP-PPP	0.10	17	0.868	0.955	0.1879
219378_at	1232.4	1322.4	0.10	1.07	PPP-PPP	0.10	17	0.868	0.955	0.1448
209143_s_at	8605.9	9235.5	0.10	1.07	PPP-PPP	0.10	17	0.868	0.955	0.1370
217492_s_at	2870.6	3081.0	0.10	1.07	PPP-PPP	0.10	17	0.868	0.955	0.1329
218257_s_at	637.5	684.1	0.10	1.07	PPP-PPP	0.10	17	0.868	0.955	0.0717
224465_s_at	462.2	495.8	0.10	1.07	PPP-PPP	0.10	17	0.868	0.955	0.4902
226420_at	806.0	864.6	0.10	1.07	PPP-PPP	0.10	17	0.868	0.955	0.4660
235159_at	381.1	408.8	0.10	1.07	PPP-PPP	0.10	17	0.868	0.955	0.4409
226004_at	559.0	599.3	0.10	1.07	PPP-PPP	0.10	17	0.868	0.955	0.4188
210817_s_at	1779.4	1909.1	0.10	1.07	PPP-PPP	0.10	17	0.868	0.955	0.3218
202845_s_at	1969.4	2112.3	0.10	1.07	PPP-PPP	0.10	17	0.868	0.955	0.2982

218197_s_at	1888.0	2024.3	0.10	1.07	PPP-PPP	0.10	17	0.868	0.955	0.2915
209944_at	3050.4	3271.3	0.10	1.07	PPP-PPP	0.10	17	0.868	0.955	0.2793
203262_s_at	797.5	855.2	0.10	1.07	PPP-PPP	0.10	17	0.868	0.955	0.2595
202078_at	3325.5	3567.5	0.10	1.07	PPP-PPP	0.10	17	0.868	0.955	0.2306
31846_at	5034.0	5399.7	0.10	1.07	PPP-PPP	0.10	17	0.868	0.955	0.2054
226165_at	9005.2	9661.2	0.10	1.07	PPP-PPP	0.10	17	0.868	0.955	0.1923
212826_s_at	30127.5	32317.6	0.10	1.07	PPP-PPP	0.10	17	0.868	0.955	0.1349
211954_s_at	8851.6	9493.1	0.10	1.07	PPP-PPP	0.10	17	0.868	0.955	0.1022
201895_at	932.9	1000.7	0.10	1.07	PPP-PPP	0.10	17	0.868	0.955	0.0944
201979_s_at	1037.9	1113.4	0.10	1.07	PPP-PPP	0.10	17	0.868	0.955	0.0546
218464_s_at	595.8	638.5	0.10	1.07	PPP-PAP	0.10	17	0.868	0.955	0.4995
218997_at	1191.1	1277.0	0.10	1.07	PPP-PPP	0.10	17	0.868	0.955	0.4989
218770_s_at	444.3	476.2	0.10	1.07	PPP-PPP	0.10	17	0.868	0.955	0.4569
226599_at	500.8	536.8	0.10	1.07	PPP-PPP	0.10	17	0.868	0.955	0.4405
224562_at	2522.7	2704.6	0.10	1.07	PPP-PPP	0.10	17	0.868	0.955	0.4274
224784_at	1014.3	1087.1	0.10	1.07	PPP-PPP	0.10	17	0.868	0.955	0.4067
225095_at	487.8	522.8	0.10	1.07	PPP-PPP	0.10	17	0.868	0.955	0.2915
228429_x_at	754.1	808.4	0.10	1.07	PPP-PAP	0.10	17	0.868	0.955	0.2922
210854_x_at	1224.4	1312.3	0.10	1.07	PPP-PPP	0.10	17	0.868	0.955	0.2749
235570_at	694.9	744.6	0.10	1.07	PPP-PPP	0.10	17	0.868	0.955	0.2529
217912_at	1357.1	1454.2	0.10	1.07	PPP-PPP	0.10	17	0.868	0.955	0.2305
225261_x_at	5119.5	5485.0	0.10	1.07	PPP-PPP	0.10	17	0.868	0.955	0.1094
200960_x_at	12458.9	13353.3	0.10	1.07	PPP-PPP	0.10	17	0.868	0.955	0.0812
222499_at	1238.1	1325.7	0.10	1.07	PPP-PPP	0.10	17	0.868	0.955	0.4667
203987_at	5336.1	5713.3	0.10	1.07	PPP-PPP	0.10	17	0.868	0.955	0.4551
1560023_x_at	168.0	179.9	0.10	1.07	PPP-PMP	0.10	17	0.868	0.955	0.4470
214118_x_at	1167.0	1249.8	0.10	1.07	PPP-PPP	0.10	17	0.868	0.955	0.4472
204127_at	3272.8	3505.3	0.10	1.07	PPP-PPP	0.10	17	0.868	0.955	0.4344
209625_at	529.3	566.8	0.10	1.07	PPP-PPP	0.10	17	0.868	0.955	0.4327
218654_s_at	5984.2	6411.2	0.10	1.07	PPP-PPP	0.10	17	0.868	0.955	0.4337
242888_at	412.6	442.0	0.10	1.07	PPP-PPP	0.10	17	0.868	0.955	0.4062
202243_s_at	11561.6	12385.1	0.10	1.07	PPP-PPP	0.10	17	0.868	0.955	0.3809
201899_s_at	3570.7	3824.3	0.10	1.07	PPP-PPP	0.10	17	0.868	0.955	0.3458
223331_s_at	1394.4	1493.8	0.10	1.07	PPP-PPP	0.10	17	0.868	0.955	0.3340
221726_at	7778.8	8332.6	0.10	1.07	PPP-PPP	0.10	17	0.868	0.955	0.3275
222671_s_at	383.9	411.1	0.10	1.07	AAP-AAA	0.10	17	0.868	0.955	0.3066
214141_x_at	10479.6	11220.9	0.10	1.07	PPP-PPP	0.10	17	0.868	0.955	0.2242
210145_at	960.1	1028.5	0.10	1.07	PPP-PPP	0.10	17	0.868	0.955	0.2257
225935_at	1435.2	1537.7	0.10	1.07	PPP-PPP	0.10	17	0.868	0.955	0.2240
224685_at	2128.9	2280.3	0.10	1.07	PPP-PPP	0.10	17	0.868	0.955	0.1915
212398_at	2559.2	2740.5	0.10	1.07	PPP-PPP	0.10	17	0.868	0.955	0.0909
203777_s_at	594.2	635.9	0.10	1.07	PPP-PPP	0.10	17	0.868	0.955	0.4885

213918_s_at	1821.6	1949.9	0.10	1.07	PPP-PPP	0.10	17	0.868	0.955	0.4213
227369_at	1614.6	1728.4	0.10	1.07	PPP-PPP	0.10	17	0.868	0.955	0.4031
208838_at	1563.8	1674.1	0.10	1.07	PPP-PPP	0.10	17	0.868	0.955	0.3951
240145_at	295.5	316.3	0.10	1.07	PPP-PPP	0.10	17	0.868	0.955	0.3855
228408_s_at	1910.7	2044.6	0.10	1.07	PPP-PPP	0.10	17	0.868	0.955	0.3365
201665_x_at	34964.8	37409.7	0.10	1.07	PPP-PPP	0.10	17	0.868	0.955	0.3183
201119_s_at	18733.5	20051.7	0.10	1.07	PPP-PPP	0.10	17	0.868	0.955	0.3146
217196_s_at	3536.5	3785.8	0.10	1.07	PPP-PPP	0.10	17	0.868	0.955	0.3151
210010_s_at	1234.1	1320.8	0.10	1.07	PPP-PPP	0.10	17	0.868	0.955	0.3035
226366_at	630.9	675.1	0.10	1.07	PPP-PPP	0.10	17	0.868	0.955	0.3005
219297_at	450.4	482.1	0.10	1.07	PPP-PPP	0.10	17	0.868	0.955	0.2894
222394_at	742.3	794.5	0.10	1.07	PPP-PPP	0.10	17	0.868	0.955	0.2285
218494_s_at	1472.2	1575.9	0.10	1.07	PPP-PPP	0.10	17	0.868	0.955	0.2226
202858_at	7027.1	7519.3	0.10	1.07	PPP-PPP	0.10	17	0.868	0.955	0.2022
212650_at	784.2	839.3	0.10	1.07	PPP-PPP	0.10	17	0.868	0.955	0.1769
217466_x_at	23448.9	25098.2	0.10	1.07	PPP-PPP	0.10	17	0.868	0.955	0.0665
201115_at	3062.8	3275.6	0.10	1.07	PPP-PPP	0.10	17	0.868	0.955	0.4534
221938_x_at	478.1	511.3	0.10	1.07	PPP-PPP	0.10	17	0.868	0.955	0.4490
204000_at	908.5	971.9	0.10	1.07	PPP-PPP	0.10	17	0.868	0.955	0.4169
224930_x_at	38743.8	41436.2	0.10	1.07	PPP-PPP	0.10	17	0.868	0.955	0.3057
223134_at	1531.8	1638.3	0.10	1.07	PPP-PPP	0.10	17	0.868	0.955	0.3011
202451_at	2149.1	2298.2	0.10	1.07	PPP-PPP	0.10	17	0.868	0.955	0.2926
204170_s_at	13822.7	14789.0	0.10	1.07	PPP-PPP	0.10	17	0.868	0.955	0.2384
200603_at	9250.3	9890.9	0.10	1.07	PPP-PPP	0.10	17	0.868	0.955	0.2276
208908_s_at	2984.4	3192.6	0.10	1.07	PPP-PPP	0.10	17	0.868	0.955	0.2197
200823_x_at	29104.9	31120.0	0.10	1.07	PPP-PPP	0.10	17	0.868	0.955	0.1854
212680_x_at	5647.8	6040.0	0.10	1.07	PPP-PPP	0.10	17	0.868	0.955	0.1569
218185_s_at	3619.5	3872.1	0.10	1.07	PPP-PPP	0.10	17	0.868	0.955	0.1341
226287_at	1631.1	1744.1	0.10	1.07	PPP-PPP	0.10	17	0.868	0.955	0.1237
212449_s_at	15610.5	16699.9	0.10	1.07	PPP-PPP	0.10	17	0.868	0.955	0.1133
222661_at	865.2	925.3	0.10	1.07	PPP-PPP	0.10	17	0.868	0.955	0.1067
224410_s_at	955.3	1021.9	0.10	1.07	PPP-PPP	0.10	17	0.868	0.955	0.0750
207988_s_at	10894.9	11654.2	0.10	1.07	PPP-PPP	0.10	17	0.868	0.955	0.0595
212039_x_at	48934.4	52311.5	0.10	1.07	PPP-PPP	0.10	17	0.868	0.955	0.4529
1554067_at	685.9	733.3	0.10	1.07	PPP-PPP	0.10	17	0.868	0.955	0.4475
204962_s_at	2796.6	2988.1	0.10	1.07	PPP-PPP	0.10	17	0.868	0.955	0.3451
221432_s_at	573.6	612.8	0.10	1.07	APP-APM	0.10	17	0.868	0.955	0.3153
1555037_a_at	4559.0	4872.1	0.10	1.07	PPP-PPP	0.10	17	0.868	0.955	0.3062
231896_s_at	6224.6	6651.4	0.10	1.07	PPP-PPP	0.10	17	0.868	0.955	0.2360
212042_x_at	39659.2	42395.9	0.10	1.07	PPP-PPP	0.10	17	0.868	0.955	0.2274
213911_s_at	23079.0	24667.1	0.10	1.07	PPP-PPP	0.10	17	0.868	0.955	0.1284
211787_s_at	28164.4	30093.5	0.10	1.07	PPP-PPP	0.10	17	0.868	0.955	0.1088

201441_at	14426.1	15420.6	0.10	1.07	PPP-PPP	0.10	17	0.868	0.955	0.0418
212721_at	2543.4	2717.8	0.10	1.07	PPP-PPP	0.10	17	0.868	0.955	0.0057
203356_at	1198.1	1280.1	0.10	1.07	PPP-PPP	0.10	17	0.868	0.955	0.4818
224976_at	670.1	715.8	0.10	1.07	PPP-PPP	0.10	17	0.868	0.955	0.4540
227682_at	1444.1	1542.4	0.10	1.07	PPP-PPP	0.10	17	0.868	0.955	0.4256
212926_at	868.8	927.8	0.10	1.07	PPP-PPP	0.10	17	0.868	0.955	0.3480
222279_at	1105.2	1180.6	0.10	1.07	PPP-PPP	0.10	17	0.868	0.955	0.3462
37577_at	473.5	505.8	0.10	1.07	PPP-PPP	0.10	17	0.868	0.955	0.3390
201971_s_at	1215.3	1298.3	0.10	1.07	PPP-PPP	0.10	17	0.868	0.955	0.2982
217735_s_at	342.1	365.3	0.10	1.07	PPP-PPP	0.10	17	0.868	0.955	0.2896
233974_s_at	210.7	225.0	0.10	1.07	PAA-APA	0.10	17	0.868	0.955	0.2663
211976_at	2096.8	2239.1	0.10	1.07	PPP-PPP	0.10	17	0.868	0.955	0.2511
217917_s_at	6773.1	7231.8	0.10	1.07	PPP-PPP	0.10	17	0.868	0.955	0.0885
226168_at	347.6	370.9	0.09	1.07	APP-PPP	0.10	17	0.868	0.955	0.4457
201230_s_at	1226.2	1309.0	0.09	1.07	PPP-PPP	0.10	17	0.868	0.955	0.4089
200715_x_at	35499.3	37889.4	0.09	1.07	PPP-PPP	0.10	17	0.868	0.955	0.3991
235181_at	298.5	318.7	0.09	1.07	PPP-PPP	0.10	17	0.868	0.955	0.3617
33132_at	1616.0	1724.3	0.09	1.07	PPP-PPP	0.10	17	0.868	0.955	0.3366
224945_at	1357.2	1448.6	0.09	1.07	PPP-PPP	0.10	17	0.868	0.955	0.3310
208698_s_at	3494.8	3730.0	0.09	1.07	PPP-PPP	0.10	17	0.868	0.955	0.2503
213890_x_at	42923.1	45810.5	0.09	1.07	PPP-PPP	0.10	17	0.868	0.955	0.2480
213619_at	21258.0	22695.3	0.09	1.07	PPP-PPP	0.10	17	0.868	0.955	0.2413
223631_s_at	8143.7	8691.2	0.09	1.07	PPP-PPP	0.10	17	0.868	0.955	0.1785
221607_x_at	37189.4	39692.7	0.09	1.07	PPP-PPP	0.10	17	0.868	0.955	0.1532
218450_at	2063.8	2200.6	0.09	1.07	PPP-PPP	0.10	17	0.868	0.955	0.4595
222998_at	2925.7	3120.0	0.09	1.07	PPP-PPP	0.10	17	0.868	0.955	0.4553
225863_s_at	1082.0	1153.8	0.09	1.07	PPP-PPP	0.10	17	0.868	0.955	0.4489
218511_s_at	1359.3	1449.7	0.09	1.07	PPP-PPP	0.10	17	0.868	0.955	0.4382
212502_at	1587.7	1693.9	0.09	1.07	PPP-PPP	0.10	17	0.868	0.955	0.4348
238010_at	311.6	332.4	0.09	1.07	PPP-PPP	0.10	17	0.868	0.955	0.4178
208660_at	9504.2	10139.4	0.09	1.07	PPP-PPP	0.10	17	0.868	0.955	0.4109
228955_at	857.3	914.7	0.09	1.07	PPP-PPP	0.10	17	0.868	0.955	0.3929
226100_at	906.7	967.0	0.09	1.07	PPP-PPP	0.10	17	0.868	0.955	0.3217
223310_x_at	3059.9	3263.3	0.09	1.07	PPP-PPP	0.10	17	0.868	0.955	0.3133
223236_at	1713.9	1828.2	0.09	1.07	PPP-PPP	0.10	17	0.868	0.955	0.2058
217773_s_at	18855.3	20108.0	0.09	1.07	PPP-PPP	0.10	17	0.868	0.955	0.1815
208899_x_at	3019.1	3219.4	0.09	1.07	PPP-PPP	0.10	17	0.868	0.955	0.0935
201275_at	15498.6	16525.8	0.09	1.07	PPP-PPP	0.10	17	0.868	0.955	0.0628
212492_s_at	708.1	754.5	0.09	1.07	PPP-PPP	0.10	17	0.868	0.955	0.4670
212229_s_at	2113.9	2253.4	0.09	1.07	PPP-PPP	0.10	17	0.868	0.955	0.4667
227277_at	1859.8	1982.9	0.09	1.07	PPP-PPP	0.10	17	0.868	0.955	0.4468
203848_at	1197.3	1276.2	0.09	1.07	PPP-PPP	0.10	17	0.868	0.955	0.3968

211368_s_at	1647.7	1756.7	0.09	1.07	PPP-PPP	0.10	17	0.868	0.955	0.3977
200070_at	953.0	1015.4	0.09	1.07	PPP-PPP	0.10	17	0.868	0.955	0.3889
220990_s_at	4312.7	4595.1	0.09	1.07	PPP-PPP	0.10	17	0.868	0.955	0.3694
213867_x_at	43637.7	46516.9	0.09	1.07	PPP-PPP	0.10	17	0.868	0.955	0.2853
209363_s_at	1598.8	1703.6	0.09	1.07	PPP-PPP	0.10	17	0.868	0.955	0.2816
203614_at	1141.5	1216.2	0.09	1.07	PPP-PPP	0.10	17	0.868	0.955	0.2744
214327_x_at	32884.1	35055.8	0.09	1.07	PPP-PPP	0.10	17	0.868	0.955	0.2510
223039_at	1467.3	1564.1	0.09	1.07	PPP-PPP	0.10	17	0.868	0.955	0.2440
235072_s_at	1178.6	1256.5	0.09	1.07	PPP-PPP	0.10	17	0.868	0.955	0.2361
235216_at	203.2	216.6	0.09	1.07	PPP-PPP	0.10	17	0.868	0.955	0.1464
217795_s_at	3195.9	3406.5	0.09	1.07	PPP-PPP	0.10	17	0.868	0.955	0.1378
217934_x_at	3765.6	4013.5	0.09	1.07	PPP-PPP	0.10	17	0.868	0.955	0.1197
209520_s_at	1177.1	1254.4	0.09	1.07	PPP-PPP	0.10	17	0.868	0.955	0.1084
200021_at	33574.5	35788.5	0.09	1.07	PPP-PPP	0.10	17	0.868	0.955	0.0648
209849_s_at	1184.5	1262.0	0.09	1.07	PPP-PPP	0.10	17	0.868	0.955	0.4815
228841_at	2418.7	2576.5	0.09	1.07	PPP-PPP	0.10	17	0.868	0.955	0.4799
206558_at	404.2	430.5	0.09	1.07	PPP-PPP	0.10	17	0.868	0.955	0.4664
225994_at	1579.5	1682.2	0.09	1.07	PPP-PPP	0.10	17	0.868	0.955	0.4465
201573_s_at	4601.3	4902.0	0.09	1.07	PPP-PPP	0.10	17	0.868	0.955	0.4054
210849_s_at	1344.2	1431.6	0.09	1.07	PPP-PPP	0.10	17	0.868	0.955	0.3893
201091_s_at	8430.9	8978.6	0.09	1.07	PPP-PPP	0.10	17	0.868	0.955	0.3802
225455_at	1816.0	1934.6	0.09	1.07	PPP-PPP	0.10	17	0.868	0.955	0.3488
208904_s_at	35458.4	37768.8	0.09	1.07	PPP-PPP	0.10	17	0.868	0.955	0.3468
208826_x_at	32767.1	34901.7	0.09	1.07	PPP-PPP	0.10	17	0.868	0.955	0.3457
213735_s_at	12778.7	13612.7	0.09	1.07	PPP-PPP	0.10	17	0.868	0.955	0.1545
208953_at	1050.2	1118.1	0.09	1.07	PPP-PPP	0.10	17	0.868	0.955	0.4773
224162_s_at	429.9	457.6	0.09	1.06	PPP-PPP	0.10	17	0.868	0.955	0.4595
216942_s_at	1301.1	1385.3	0.09	1.07	PPP-PPP	0.10	17	0.868	0.955	0.4532
229119_s_at	1056.1	1123.8	0.09	1.06	PPP-PPP	0.10	17	0.868	0.955	0.4379
207761_s_at	5649.0	6011.4	0.09	1.06	PPP-PPP	0.10	17	0.868	0.955	0.4221
212959_s_at	1513.6	1610.9	0.09	1.06	PPP-PPP	0.10	17	0.868	0.955	0.3103
200806_s_at	23646.4	25172.8	0.09	1.07	PPP-PPP	0.10	17	0.868	0.955	0.2920
223106_at	9951.4	10595.1	0.09	1.07	PPP-PPP	0.10	17	0.868	0.955	0.2582
222472_at	1044.2	1111.8	0.09	1.07	PPP-PPP	0.10	17	0.868	0.955	0.1909
207801_s_at	2903.7	3090.6	0.09	1.06	PPP-PPP	0.10	17	0.868	0.955	0.1581
45687_at	1035.2	1102.0	0.09	1.07	PPP-PPP	0.10	17	0.868	0.955	0.1359
228123_s_at	1438.6	1531.3	0.09	1.06	PPP-PPP	0.10	17	0.868	0.955	0.1330
1565162_s_at	17623.4	18763.6	0.09	1.07	PPP-PPP	0.10	17	0.868	0.955	0.1018
217485_x_at	897.8	954.7	0.09	1.06	PPP-PPP	0.10	17	0.868	0.955	0.4534
208852_s_at	4822.2	5130.4	0.09	1.06	PPP-PPP	0.10	17	0.868	0.955	0.3884
224376_s_at	7742.6	8233.5	0.09	1.06	PPP-PPP	0.10	17	0.868	0.955	0.3067
AFFX-hum_alu_at	52089.2	55402.1	0.09	1.06	PPP-PPP	0.10	17	0.868	0.955	0.2916

220948_s_at	7074.1	7526.8	0.09	1.06	PPP-PPP	0.10	17	0.868	0.955	0.1760
203190_at	6851.9	7288.8	0.09	1.06	PPP-PPP	0.10	17	0.868	0.955	0.1732
222437_s_at	4644.3	4940.0	0.09	1.06	PPP-PPP	0.10	17	0.868	0.955	0.1304
224778_s_at	1794.8	1908.1	0.09	1.06	PPP-PPP	0.10	17	0.868	0.955	0.4643
224957_at	1433.1	1523.2	0.09	1.06	PPP-PPP	0.10	17	0.868	0.955	0.4207
212435_at	1107.0	1176.9	0.09	1.06	PPP-PPP	0.10	17	0.868	0.955	0.3955
222785_x_at	1218.6	1295.0	0.09	1.06	PPP-PPP	0.10	17	0.868	0.955	0.3929
212790_x_at	53145.0	56482.9	0.09	1.06	PPP-PPP	0.10	17	0.868	0.955	0.3851
217797_at	9744.5	10359.0	0.09	1.06	PPP-PPP	0.10	17	0.868	0.955	0.3411
200651_at	40661.5	43232.2	0.09	1.06	PPP-PPP	0.10	17	0.868	0.955	0.3261
213315_x_at	1304.6	1386.9	0.09	1.06	PPP-PPP	0.10	17	0.868	0.955	0.2953
AFFX-HUMGAPDH/M3	42243.7	44886.1	0.09	1.06	PPP-PPP	0.10	17	0.868	0.955	0.1912
1552628_a_at	2895.5	3078.0	0.09	1.06	PPP-PPP	0.10	17	0.868	0.955	0.1368
201258_at	38808.0	41260.5	0.09	1.06	PPP-PPP	0.10	17	0.868	0.955	0.1256
AFFX-HSAC07/X0035	42977.9	45667.5	0.09	1.06	PPP-PPP	0.10	17	0.868	0.955	0.1002
212597_s_at	1617.6	1718.5	0.09	1.06	PPP-PPP	0.10	17	0.868	0.955	0.4655
219206_x_at	5243.9	5569.1	0.09	1.06	PPP-PPP	0.10	17	0.868	0.955	0.4587
211716_x_at	2135.5	2267.7	0.09	1.06	PPP-PPP	0.10	17	0.868	0.955	0.4569
217964_at	1995.6	2119.3	0.09	1.06	PPP-PPP	0.10	17	0.868	0.955	0.4505
209034_at	2399.2	2548.3	0.09	1.06	PPP-PPP	0.10	17	0.868	0.955	0.4094
202142_at	4906.0	5211.3	0.09	1.06	PPP-PPP	0.10	17	0.868	0.955	0.4044
212370_x_at	1889.6	2006.7	0.09	1.06	PPP-PPP	0.10	17	0.868	0.955	0.3981
227792_at	2416.0	2565.5	0.09	1.06	PPP-PPP	0.10	17	0.868	0.955	0.3937
203455_s_at	11655.9	12382.2	0.09	1.06	PPP-PPP	0.10	17	0.868	0.955	0.3652
200905_x_at	4587.9	4872.6	0.09	1.06	PPP-PPP	0.10	17	0.868	0.955	0.3064
200076_s_at	2530.8	2688.4	0.09	1.06	PPP-PPP	0.10	17	0.868	0.955	0.2652
201480_s_at	842.5	894.6	0.09	1.06	PPP-PPP	0.10	17	0.868	0.955	0.2357
200798_x_at	9214.4	9785.4	0.09	1.06	PPP-PPP	0.10	17	0.868	0.955	0.2133
224810_s_at	2515.1	2671.7	0.09	1.06	PPP-PPP	0.10	17	0.868	0.955	0.1964
231530_s_at	1097.0	1165.1	0.09	1.06	PPP-PPP	0.10	17	0.868	0.955	0.1617
217969_at	4766.1	5062.8	0.09	1.06	PPP-PPP	0.10	17	0.868	0.955	0.1296
202824_s_at	13007.2	13816.2	0.09	1.06	PPP-PPP	0.10	17	0.868	0.955	0.0800
202309_at	7097.6	7539.3	0.09	1.06	PPP-PPP	0.10	17	0.868	0.955	0.0720
200641_s_at	11050.4	11735.2	0.09	1.06	PPP-PPP	0.10	17	0.868	0.955	0.0632
209675_s_at	1711.8	1817.5	0.09	1.06	PPP-PPP	0.10	17	0.868	0.955	0.4519
202358_s_at	625.5	664.0	0.09	1.06	PPP-PPP	0.10	17	0.868	0.955	0.4447
225640_at	1457.0	1546.6	0.09	1.06	PPP-PPP	0.10	17	0.868	0.955	0.3878
214585_s_at	1687.7	1790.9	0.09	1.06	PPP-PPP	0.10	17	0.868	0.955	0.3633
209510_at	2442.0	2591.4	0.09	1.06	PPP-PPP	0.10	17	0.868	0.955	0.3366
218330_s_at	7460.3	7917.4	0.09	1.06	PPP-PPP	0.10	17	0.868	0.955	0.3242
224248_x_at	1081.1	1147.1	0.09	1.06	PPP-PPP	0.10	17	0.868	0.955	0.2354
210835_s_at	7423.4	7878.2	0.09	1.06	PPP-PPP	0.10	17	0.868	0.955	0.1487

34406_at	460.1	488.2	0.09	1.06	PPP-PPP	0.10	17	0.868	0.955	0.1369
213050_at	1279.8	1358.1	0.09	1.06	PPP-PPP	0.10	17	0.868	0.955	0.1200
229235_at	1380.4	1465.5	0.09	1.06	PPP-PPP	0.10	17	0.868	0.955	0.1155
208029_s_at	9376.8	9952.6	0.09	1.06	PPP-PPP	0.10	17	0.868	0.955	0.0435
204868_at	1815.5	1926.3	0.09	1.06	PPP-PPP	0.10	17	0.868	0.955	0.4280
225927_at	808.9	857.9	0.09	1.06	PPP-PPP	0.10	17	0.868	0.955	0.3903
201963_at	2862.1	3035.8	0.09	1.06	PPP-PPP	0.10	17	0.868	0.955	0.3663
209134_s_at	39313.7	41710.4	0.09	1.06	PPP-PPP	0.10	17	0.868	0.955	0.3666
212273_x_at	24773.5	26270.9	0.09	1.06	PPP-PPP	0.10	17	0.868	0.955	0.3503
203422_at	1192.9	1265.3	0.09	1.06	PPP-PPP	0.10	17	0.868	0.955	0.3464
208755_x_at	39915.2	42327.7	0.09	1.06	PPP-PPP	0.10	17	0.868	0.955	0.2078
220199_s_at	5673.8	6019.5	0.09	1.06	PPP-PPP	0.10	17	0.868	0.955	0.2046
208864_s_at	21656.8	22963.7	0.09	1.06	PPP-PPP	0.10	17	0.868	0.955	0.1865
200092_s_at	41654.4	44189.5	0.09	1.06	PPP-PPP	0.10	17	0.868	0.955	0.1791
203316_s_at	23247.0	24652.1	0.09	1.06	PPP-PPP	0.10	17	0.868	0.955	0.0975
226666_at	3394.8	3600.9	0.09	1.06	PPP-PPP	0.10	17	0.868	0.955	0.0967
200074_s_at	27957.7	29627.7	0.08	1.06	PPP-PPP	0.10	17	0.868	0.955	0.4447
213969_x_at	33752.7	35788.3	0.08	1.06	PPP-PPP	0.10	17	0.868	0.955	0.4370
238431_at	840.6	891.1	0.08	1.06	PPP-PPP	0.10	17	0.868	0.955	0.4025
225356_at	2953.6	3129.9	0.08	1.06	PPP-PPP	0.10	17	0.868	0.955	0.3765
201894_s_at	1830.5	1939.8	0.08	1.06	PPP-PPP	0.10	17	0.868	0.955	0.3741
223026_s_at	5899.5	6254.0	0.08	1.06	PPP-PPP	0.10	17	0.868	0.955	0.3643
213687_s_at	25743.6	27292.9	0.08	1.06	PPP-PPP	0.10	17	0.868	0.955	0.3295
230528_s_at	1665.4	1765.6	0.08	1.06	PPP-PPP	0.10	17	0.868	0.955	0.2712
201956_s_at	5388.2	5711.9	0.08	1.06	PPP-PPP	0.10	17	0.868	0.955	0.2339
226031_at	1216.4	1289.6	0.08	1.06	PPP-PPP	0.10	17	0.868	0.955	0.2285
200718_s_at	28636.6	30343.6	0.08	1.06	PPP-PPP	0.10	17	0.868	0.955	0.1485
201626_at	2860.9	3030.9	0.08	1.06	PPP-PPP	0.10	17	0.868	0.955	0.4225
223637_s_at	583.7	618.4	0.08	1.06	PPP-PPP	0.10	17	0.868	0.955	0.4173
222742_s_at	1751.4	1854.7	0.08	1.06	PPP-PPP	0.10	17	0.868	0.955	0.4102
1553299_at	252.6	267.5	0.08	1.06	PPP-PPP	0.10	17	0.868	0.955	0.4001
208781_x_at	5928.4	6280.5	0.08	1.06	PPP-PPP	0.10	17	0.868	0.955	0.3935
203510_at	9369.3	9922.1	0.08	1.06	PPP-PPP	0.10	17	0.868	0.955	0.3852
211666_x_at	32985.5	34928.8	0.08	1.06	PPP-PPP	0.10	17	0.868	0.955	0.3617
221472_at	1758.6	1862.3	0.08	1.06	PPP-PPP	0.10	17	0.868	0.955	0.3450
202346_at	2047.4	2168.7	0.08	1.06	PPP-PPP	0.10	17	0.868	0.955	0.3452
211542_x_at	49830.7	52783.6	0.08	1.06	PPP-PPP	0.10	17	0.868	0.955	0.3449
208120_x_at	840.2	889.8	0.08	1.06	PPP-PPP	0.10	17	0.868	0.955	0.3171
213032_at	965.9	1023.4	0.08	1.06	PPP-PPP	0.10	17	0.868	0.955	0.3042
1568629_s_at	2263.9	2397.7	0.08	1.06	PPP-PPP	0.10	17	0.868	0.955	0.2333
208695_s_at	45206.9	47896.9	0.08	1.06	PPP-PPP	0.10	17	0.868	0.955	0.2217
208641_s_at	17912.8	18973.7	0.08	1.06	PPP-PPP	0.10	17	0.868	0.955	0.1329

211730_s_at	20397.2	21609.7	0.08	1.06	PPP-PPP	0.10	17	0.868	0.955	0.0401
213218_at	159.7	169.1	0.08	1.06	PPM-APP	0.10	17	0.868	0.955	0.3957
219801_at	161.7	171.1	0.08	1.06	PPP-PPP	0.10	17	0.868	0.955	0.3853
225535_s_at	7513.4	7953.6	0.08	1.06	PPP-PPP	0.10	17	0.868	0.955	0.3868
202060_at	4254.8	4502.8	0.08	1.06	PPP-PPP	0.10	17	0.868	0.955	0.3397
209076_s_at	2097.9	2220.2	0.08	1.06	PPP-PPP	0.10	17	0.868	0.955	0.3113
202343_x_at	9809.8	10381.5	0.08	1.06	PPP-PPP	0.10	17	0.868	0.955	0.3084
212112_s_at	1467.3	1552.8	0.08	1.06	PPP-PPP	0.10	17	0.868	0.955	0.3075
201511_at	1622.6	1717.1	0.08	1.06	PPP-PPP	0.10	17	0.868	0.955	0.2994
200061_s_at	36824.4	38985.0	0.08	1.06	PPP-PPP	0.10	17	0.868	0.955	0.2856
213129_s_at	8901.6	9424.3	0.08	1.06	PPP-PPP	0.10	17	0.868	0.955	0.1667
200763_s_at	43040.6	45570.9	0.08	1.06	PPP-PPP	0.10	17	0.868	0.955	0.1437
211681_s_at	646.0	683.4	0.08	1.06	PPP-PPP	0.10	17	0.868	0.955	0.4295
221830_at	4023.6	4254.9	0.08	1.06	PPP-PPP	0.10	17	0.868	0.955	0.4085
218728_s_at	8715.2	9218.3	0.08	1.06	PPP-PPP	0.10	17	0.868	0.955	0.2737
200953_s_at	6020.1	6365.8	0.08	1.06	PPP-PPP	0.10	17	0.868	0.955	0.2636
224876_at	5224.2	5525.3	0.08	1.06	PPP-PPP	0.10	17	0.868	0.955	0.0639
215177_s_at	4656.4	4925.6	0.08	1.06	PPP-PPP	0.10	17	0.868	0.955	0.0342
235470_at	425.7	450.1	0.08	1.06	PPP-PPP	0.10	17	0.868	0.955	0.3842
221434_s_at	8469.7	8954.6	0.08	1.06	PPP-PPP	0.10	17	0.868	0.955	0.3758
226042_at	400.4	423.3	0.08	1.06	PPP-PPP	0.10	17	0.868	0.955	0.3393
200826_at	23095.5	24406.4	0.08	1.06	PPP-PPP	0.10	17	0.868	0.955	0.3182
214351_x_at	42292.6	44712.0	0.08	1.06	PPP-PPP	0.10	17	0.868	0.955	0.3047
222992_s_at	23849.2	25208.4	0.08	1.06	PPP-PPP	0.10	17	0.868	0.955	0.2449
200807_s_at	39379.8	41613.8	0.08	1.06	PPP-PPP	0.10	17	0.868	0.955	0.2034
218011_at	8944.6	9456.2	0.08	1.06	PPP-PPP	0.10	17	0.868	0.955	0.1688
200086_s_at	17639.0	18641.9	0.08	1.06	PPP-PPP	0.10	17	0.868	0.955	0.1596
200941_at	3447.2	3641.9	0.08	1.06	PPP-PPP	0.10	17	0.868	0.955	0.4364
209471_s_at	5544.6	5858.3	0.08	1.06	PPP-PPP	0.10	17	0.868	0.955	0.3875
223010_s_at	4741.8	5010.4	0.08	1.06	PPP-PPP	0.10	17	0.868	0.955	0.3778
204832_s_at	915.8	967.2	0.08	1.06	PPP-PPP	0.10	17	0.868	0.955	0.3588
201406_at	38419.8	40586.0	0.08	1.06	PPP-PPP	0.10	17	0.868	0.955	0.3067
200992_at	10666.4	11269.5	0.08	1.06	PPP-PPP	0.10	17	0.868	0.955	0.2932
200689_x_at	33290.4	35162.5	0.08	1.06	PPP-PPP	0.10	17	0.868	0.955	0.2878
211970_x_at	34506.2	36449.8	0.08	1.06	PPP-PPP	0.10	17	0.868	0.955	0.2671
200673_at	10971.7	11592.2	0.08	1.06	PPP-PPP	0.10	17	0.868	0.955	0.2610
224642_at	1182.6	1248.8	0.08	1.06	PPP-PPP	0.10	17	0.868	0.955	0.2507
200834_s_at	46856.3	49498.1	0.08	1.06	PPP-PPP	0.10	17	0.868	0.955	0.2331
201550_x_at	37120.2	39198.4	0.08	1.06	PPP-PPP	0.10	17	0.868	0.955	0.2129
212074_at	6661.2	7038.1	0.08	1.06	PPP-PPP	0.10	17	0.868	0.955	0.1528
209036_s_at	22410.8	23678.6	0.08	1.06	PPP-PPP	0.10	17	0.868	0.955	0.0970
203387_s_at	1652.1	1744.2	0.08	1.06	PPP-PPP	0.10	17	0.868	0.955	0.4124

202899_s_at	11860.7	12516.6	0.08	1.06	PPP-PPP	0.10	17	0.868	0.955	0.4089
221770_at	1362.0	1438.1	0.08	1.06	PPP-PPP	0.10	17	0.868	0.955	0.3991
202413_s_at	5774.8	6094.3	0.08	1.06	PPP-PPP	0.10	17	0.868	0.955	0.2962
213414_s_at	48815.2	51530.9	0.08	1.06	PPP-PPP	0.10	17	0.868	0.955	0.2901
217819_at	4900.9	5172.8	0.08	1.06	PPP-PPP	0.10	17	0.868	0.955	0.2164
203194_s_at	1232.8	1301.1	0.08	1.06	PPP-PPP	0.10	17	0.868	0.955	0.1856
218495_at	4651.4	4909.2	0.08	1.06	PPP-PPP	0.10	17	0.868	0.955	0.0353
223140_s_at	1762.7	1861.2	0.08	1.06	PPP-PPP	0.10	17	0.868	0.955	0.0354
221775_x_at	35187.7	37128.6	0.08	1.06	PPP-PPP	0.10	17	0.868	0.955	0.4050
203107_x_at	42473.5	44816.2	0.08	1.06	PPP-PPP	0.10	17	0.868	0.955	0.3902
227432_s_at	1432.6	1511.5	0.08	1.06	PPP-PPP	0.10	17	0.868	0.955	0.3807
213828_x_at	38851.2	40979.3	0.08	1.06	PPP-PPP	0.10	17	0.868	0.955	0.3715
212762_s_at	693.9	732.1	0.08	1.06	PPP-PPP	0.10	17	0.868	0.955	0.3728
204064_at	1126.5	1188.5	0.08	1.06	PPP-PPP	0.10	17	0.868	0.955	0.3696
225096_at	3689.9	3891.3	0.08	1.06	PPP-PPP	0.10	17	0.868	0.955	0.3496
229073_at	138.4	146.0	0.08	1.06	PPP-PPP	0.10	17	0.868	0.955	0.3379
223225_s_at	1260.6	1330.1	0.08	1.06	PPP-PPP	0.10	17	0.868	0.955	0.2452
1554575_a_at	798.2	841.7	0.08	1.06	PPP-PPP	0.10	17	0.868	0.955	0.2056
217491_x_at	21432.8	22614.7	0.08	1.06	PPP-PPP	0.10	17	0.868	0.955	0.2014
207243_s_at	30594.5	32277.3	0.08	1.06	PPP-PPP	0.10	17	0.868	0.955	0.1885
200936_at	51379.3	54164.6	0.08	1.05	PPP-PPP	0.10	17	0.868	0.955	0.4076
233665_x_at	1657.5	1747.6	0.08	1.05	PPP-PPP	0.10	17	0.868	0.955	0.3884
223804_s_at	1304.8	1375.3	0.08	1.05	PPP-PPP	0.10	17	0.868	0.955	0.3392
242429_at	238.0	250.9	0.08	1.05	PPP-PPP	0.10	17	0.868	0.955	0.3303
209251_x_at	30827.1	32500.0	0.08	1.05	PPP-PPP	0.10	17	0.868	0.955	0.3152
213503_x_at	36930.0	38940.0	0.08	1.05	PPP-PPP	0.10	17	0.868	0.955	0.3094
222512_at	1025.8	1081.5	0.08	1.05	PPP-PPP	0.10	17	0.868	0.955	0.3067
201395_at	1955.4	2061.6	0.08	1.05	PPP-PPP	0.10	17	0.868	0.955	0.3025
200768_s_at	7491.7	7896.8	0.08	1.05	PPP-PPP	0.10	17	0.868	0.955	0.2518
201924_at	3713.8	3914.5	0.08	1.05	PPP-PPP	0.10	17	0.868	0.955	0.2282
201590_x_at	36080.3	38009.5	0.08	1.05	PPP-PPP	0.10	17	0.868	0.955	0.3814
207508_at	18061.1	19029.3	0.08	1.05	PPP-PPP	0.10	17	0.868	0.955	0.3524
212785_s_at	2255.2	2375.3	0.08	1.05	PPP-PPP	0.10	17	0.868	0.955	0.3264
232524_x_at	1403.9	1478.9	0.08	1.05	PPP-PPP	0.10	17	0.868	0.955	0.2976
213583_x_at	36849.2	38817.9	0.08	1.05	PPP-PPP	0.10	17	0.868	0.955	0.2705
31861_at	499.9	526.6	0.08	1.05	MAP-APP	0.10	17	0.868	0.955	0.2709
201273_s_at	21467.9	22611.0	0.08	1.05	PPP-PPP	0.10	17	0.868	0.955	0.2665
201827_at	1017.4	1071.6	0.08	1.05	PPP-PPP	0.10	17	0.868	0.955	0.1695
213390_at	1756.9	1850.5	0.08	1.05	PPP-PPP	0.10	17	0.868	0.955	0.1193
237563_s_at	6392.8	6732.0	0.08	1.05	PPP-PPP	0.10	17	0.868	0.955	0.0681
208761_s_at	4683.1	4929.9	0.07	1.05	PPP-PPP	0.10	17	0.868	0.955	0.4089
212363_x_at	33314.9	35066.0	0.07	1.05	PPP-PPP	0.10	17	0.868	0.955	0.4067

200001_at	11332.8	11930.0	0.07	1.05	PPP-PPP	0.10	17	0.868	0.955	0.4071
213243_at	1310.5	1379.1	0.07	1.05	PPP-PPP	0.10	17	0.868	0.955	0.4007
212556_at	3040.0	3199.4	0.07	1.05	PPP-PPP	0.10	17	0.868	0.955	0.3904
211972_x_at	41722.9	43928.1	0.07	1.05	PPP-PPP	0.10	17	0.868	0.955	0.3797
213702_x_at	5765.8	6069.2	0.07	1.05	PPP-PPP	0.10	17	0.868	0.955	0.3728
203364_s_at	833.8	877.4	0.07	1.05	PPP-PPP	0.10	17	0.868	0.955	0.3608
229194_at	863.0	908.3	0.07	1.05	PPP-PPP	0.10	17	0.868	0.955	0.3541
201398_s_at	10405.3	10953.5	0.07	1.05	PPP-PPP	0.10	17	0.868	0.955	0.3068
201738_at	4194.8	4414.7	0.07	1.05	PPP-PPP	0.10	17	0.868	0.955	0.2831
200773_x_at	20276.0	21338.2	0.07	1.05	PPP-PPP	0.10	17	0.868	0.955	0.2569
213513_x_at	11421.1	12023.6	0.07	1.05	PPP-PPP	0.10	17	0.868	0.955	0.2541
201022_s_at	28260.3	29743.9	0.07	1.05	PPP-PPP	0.10	17	0.868	0.955	0.2275
203403_s_at	6528.4	6872.8	0.07	1.05	PPP-PPP	0.10	17	0.868	0.955	0.2139
223982_s_at	2300.9	2421.1	0.07	1.05	PPP-PPP	0.10	17	0.868	0.955	0.3809
207585_s_at	15862.6	16689.1	0.07	1.05	PPP-PPP	0.10	17	0.868	0.955	0.3640
225607_at	1174.9	1236.3	0.07	1.05	PPP-PPP	0.10	17	0.868	0.955	0.3395
207700_s_at	3206.3	3373.4	0.07	1.05	PPP-PPP	0.10	17	0.868	0.955	0.2879
201607_at	1122.8	1180.9	0.07	1.05	PPP-PPP	0.10	17	0.868	0.955	0.2489
204366_s_at	2695.5	2835.0	0.07	1.05	PPP-PPP	0.10	17	0.868	0.955	0.2369
202230_s_at	4225.7	4445.9	0.07	1.05	PPP-PPP	0.10	17	0.868	0.955	0.2030
218088_s_at	1792.0	1884.9	0.07	1.05	PPP-PPP	0.10	17	0.868	0.955	0.1334
221952_x_at	18582.9	19552.9	0.07	1.05	PPP-PPP	0.10	17	0.868	0.955	0.0712
203453_at	9437.5	9930.7	0.07	1.05	PPP-PPP	0.10	17	0.868	0.955	0.0515
200062_s_at	48686.8	51173.7	0.07	1.05	PPP-PPP	0.10	17	0.868	0.955	0.3929
214924_s_at	740.7	778.7	0.07	1.05	PPP-PPP	0.10	17	0.868	0.955	0.3694
203972_s_at	1837.4	1931.0	0.07	1.05	PPP-PPP	0.10	17	0.868	0.955	0.3230
217760_at	2268.0	2383.6	0.07	1.05	PPP-PPP	0.10	17	0.868	0.955	0.2937
226779_at	928.7	976.0	0.07	1.05	PPP-PPP	0.10	17	0.868	0.955	0.2519
223002_s_at	3356.7	3527.8	0.07	1.05	PPP-PPP	0.10	17	0.868	0.955	0.0624
217826_s_at	1614.2	1695.3	0.07	1.05	PPP-PPP	0.10	17	0.868	0.955	0.3551
1552477_a_at	3665.0	3849.7	0.07	1.05	PPP-PPP	0.10	17	0.868	0.955	0.3553
225405_at	3825.2	4018.4	0.07	1.05	PPP-PPP	0.10	17	0.868	0.955	0.3493
213923_at	2460.0	2584.7	0.07	1.05	PPP-PPP	0.10	17	0.868	0.955	0.3474
226020_s_at	824.5	866.1	0.07	1.05	PPP-PPP	0.10	17	0.868	0.955	0.3406
212716_s_at	16792.4	17642.6	0.07	1.05	PPP-PPP	0.10	17	0.868	0.955	0.2927
211058_x_at	38481.8	40413.7	0.07	1.05	PPP-PPP	0.10	17	0.868	0.955	0.2311
202029_x_at	30264.6	31800.6	0.07	1.05	PPP-PPP	0.10	17	0.868	0.955	0.2283
33322_i_at	37440.8	39338.8	0.07	1.05	PPP-PPP	0.10	17	0.868	0.955	0.2215
214550_s_at	1778.2	1868.1	0.07	1.05	PPP-PPP	0.10	17	0.868	0.955	0.2042
208628_s_at	33585.5	35281.1	0.07	1.05	PPP-PPP	0.10	17	0.868	0.955	0.1634
235677_at	324.1	340.3	0.07	1.05	PPP-PPP	0.10	17	0.868	0.955	0.0352
218101_s_at	13909.7	14616.3	0.07	1.05	PPP-PPP	0.10	17	0.868	0.955	0.0297

226282_at	6564.3	6894.4	0.07	1.05	PPP-PPP	0.10	17	0.868	0.955	0.0161
208689_s_at	9134.9	9590.2	0.07	1.05	PPP-PPP	0.10	17	0.868	0.955	0.3822
208687_x_at	44474.4	46691.4	0.07	1.05	PPP-PPP	0.10	17	0.868	0.955	0.2764
213699_s_at	18408.9	19318.0	0.07	1.05	PPP-PPP	0.10	17	0.868	0.955	0.2577
218258_at	14091.7	14784.0	0.07	1.05	PPP-PPP	0.10	17	0.868	0.955	0.2872
1553995_a_at	2114.9	2218.5	0.07	1.05	PPP-PPP	0.10	17	0.868	0.955	0.2414
202096_s_at	9104.4	9550.0	0.07	1.05	PPP-PPP	0.10	17	0.868	0.955	0.1377
208909_at	20725.3	21746.7	0.07	1.05	PPP-PPP	0.10	17	0.868	0.955	0.1173
216977_x_at	1877.8	1970.1	0.07	1.05	PPP-PPP	0.10	17	0.868	0.955	0.1117
222572_at	3216.8	3373.7	0.07	1.05	PPP-PPP	0.10	17	0.868	0.955	0.1062
200780_x_at	26580.4	27857.5	0.07	1.05	PPP-PPP	0.10	17	0.868	0.955	0.3596
212330_at	8914.0	9341.9	0.07	1.05	PPP-PPP	0.10	17	0.868	0.955	0.3285
223156_at	2689.1	2818.2	0.07	1.05	PPP-PPP	0.10	17	0.868	0.955	0.2755
218027_at	10659.2	11171.8	0.07	1.05	PPP-PPP	0.10	17	0.868	0.955	0.2472
212639_x_at	41774.3	43796.4	0.07	1.05	PPP-PPP	0.10	17	0.868	0.955	0.2253
209146_at	11353.0	11900.4	0.07	1.05	PPP-PPP	0.10	17	0.868	0.955	0.2216
206656_s_at	3768.0	3950.7	0.07	1.05	PPP-PPP	0.10	17	0.868	0.955	0.0576
224986_s_at	1440.9	1509.3	0.07	1.05	PPP-PPP	0.10	17	0.868	0.955	0.3629
224302_s_at	3257.1	3412.1	0.07	1.05	PPP-PPP	0.10	17	0.868	0.955	0.2921
219960_s_at	4049.4	4241.0	0.07	1.05	PPP-PPP	0.10	17	0.868	0.955	0.2813
225499_at	873.1	914.8	0.07	1.05	PPP-PPP	0.10	17	0.868	0.955	0.1329
214214_s_at	16195.5	16963.3	0.07	1.05	PPP-PPP	0.10	17	0.868	0.955	0.0679
201794_s_at	1210.6	1266.9	0.07	1.05	PPP-PPP	0.10	17	0.868	0.955	0.3446
216899_s_at	741.5	776.1	0.07	1.05	PPP-PPP	0.10	17	0.868	0.955	0.2762
201989_s_at	1136.4	1189.4	0.07	1.05	PPP-PPP	0.10	17	0.868	0.955	0.2246
223591_at	1722.0	1802.4	0.07	1.05	PPP-PPP	0.10	17	0.868	0.955	0.1910
230679_at	247.0	258.6	0.07	1.05	PPP-PPP	0.10	17	0.868	0.955	0.0947
210949_s_at	19268.6	20168.9	0.07	1.05	PPP-PPP	0.10	17	0.868	0.955	0.0305
213377_x_at	43502.1	45504.8	0.07	1.05	PPP-PPP	0.10	17	0.868	0.955	0.3376
212131_at	4195.5	4388.0	0.07	1.05	PPP-PPP	0.10	17	0.868	0.955	0.2448
200957_s_at	5963.4	6238.3	0.07	1.05	PPP-PPP	0.10	17	0.868	0.955	0.2379
34031_i_at	1719.1	1796.9	0.06	1.05	PPP-PPP	0.10	17	0.868	0.955	0.3330
218320_s_at	5763.9	6023.5	0.06	1.05	PPP-PPP	0.10	17	0.868	0.955	0.2566
33323_r_at	40928.4	42781.7	0.06	1.05	PPP-PPP	0.10	17	0.868	0.955	0.2391
218482_at	9089.7	9492.4	0.06	1.04	PPP-PPP	0.10	17	0.868	0.955	0.0668
213588_x_at	25610.8	26743.0	0.06	1.04	PPP-PPP	0.10	17	0.868	0.955	0.2616
218200_s_at	14986.8	15640.4	0.06	1.04	PPP-PPP	0.10	17	0.868	0.955	0.2184
1553764_a_at	3488.9	3643.3	0.06	1.04	PPP-PPP	0.10	17	0.868	0.955	0.1967
222561_at	497.1	519.0	0.06	1.04	PPP-PPP	0.10	17	0.868	0.955	0.1476
211995_x_at	36776.0	38362.8	0.06	1.04	PPP-PPP	0.10	17	0.868	0.955	0.2600
212782_x_at	8252.4	8610.2	0.06	1.04	PPP-PPP	0.10	17	0.868	0.955	0.2612
226230_at	843.8	880.0	0.06	1.04	PPP-PPP	0.10	17	0.868	0.955	0.2018

200804_at	10356.2	10807.0	0.06	1.04	PPP-PPP	0.10	17	0.868	0.955	0.1259
224752_at	4783.6	4988.2	0.06	1.04	PPP-PPP	0.10	17	0.868	0.955	0.2694
218104_at	1592.0	1659.3	0.06	1.04	PPP-PPP	0.10	17	0.868	0.955	0.2579
40189_at	14419.3	15029.6	0.06	1.04	PPP-PPP	0.10	17	0.868	0.955	0.2405
201493_s_at	2923.8	3048.4	0.06	1.04	PPP-PPP	0.10	17	0.868	0.955	0.2123
211662_s_at	18915.5	19723.4	0.06	1.04	PPP-PPP	0.10	17	0.868	0.955	0.0944
200037_s_at	13532.1	14096.9	0.06	1.04	PPP-PPP	0.10	17	0.868	0.955	0.2872
214938_x_at	25164.1	26211.0	0.06	1.04	PPP-PPP	0.10	17	0.868	0.955	0.2596
200619_at	3745.3	3902.9	0.06	1.04	PPP-PPP	0.10	17	0.868	0.955	0.1606
204283_at	1447.1	1506.2	0.06	1.04	PPP-PPP	0.10	17	0.868	0.955	0.2333
205849_s_at	16941.4	17629.0	0.06	1.04	PPP-PPP	0.10	17	0.868	0.955	0.0874
217870_s_at	7213.1	7505.2	0.06	1.04	PPP-PPP	0.10	17	0.868	0.955	0.0822
234107_s_at	6449.4	6701.2	0.06	1.04	PPP-PPP	0.10	17	0.868	0.955	0.1711
217942_at	4854.6	5041.5	0.06	1.04	PPP-PPP	0.10	17	0.868	0.955	0.1437
202475_at	8797.2	9138.2	0.06	1.04	PPP-PPP	0.10	17	0.868	0.955	0.0665
200754_x_at	12383.1	12856.4	0.05	1.04	PPP-PPP	0.10	17	0.868	0.955	0.1811
228605_at	884.9	918.5	0.05	1.04	PPP-PPP	0.10	17	0.868	0.955	0.1606
210624_s_at	1655.4	1717.3	0.05	1.04	PPP-PPP	0.10	17	0.868	0.955	0.1651
1555780_a_at	5360.0	5561.5	0.05	1.04	PPP-PPP	0.10	17	0.868	0.955	0.1323
200873_s_at	16914.4	17544.6	0.05	1.04	PPP-PPP	0.10	17	0.868	0.955	0.1088
200019_s_at	31317.0	32393.6	0.05	1.03	PPP-PPP	0.10	17	0.868	0.955	0.0391
210501_x_at	14720.5	15218.5	0.05	1.03	PPP-PPP	0.10	17	0.868	0.955	0.1211
220655_at	113.7	5.9	-4.27	-19.33	AAA-AAA	0.00	999	1.000	1.000	0.0002
1556655_s_at	2.3	40.5	4.12	17.37	AAA-AAA	0.00	999	1.000	1.000	0.0014
1569354_at	35.4	2.0	-4.11	-17.31	AAA-AAA	0.00	999	1.000	1.000	0.0001
235881_at	2.5	42.9	4.08	16.93	AAA-AAM	0.00	999	1.000	1.000	0.0080
234702_x_at	1.6	26.0	3.99	15.92	AAA-AAA	0.00	999	1.000	1.000	0.0069
216406_at	65.1	4.2	-3.95	-15.46	APA-AAA	0.00	999	1.000	1.000	0.0822
229471_s_at	32.4	2.2	-3.88	-14.75	AAA-AAA	0.00	999	1.000	1.000	0.0030
1554759_at	89.2	6.2	-3.84	-14.34	AAA-AAA	0.00	999	1.000	1.000	0.0002
1561775_at	14.9	1.1	-3.81	-14.00	AAA-AAA	0.00	999	1.000	1.000	0.0088
204913_s_at	2.1	27.3	3.71	13.07	AAA-AAA	0.00	999	1.000	1.000	0.0003
1560997_at	19.9	1.6	-3.68	-12.84	MAA-AAA	0.00	999	1.000	1.000	0.0034
242036_x_at	25.5	2.0	-3.66	-12.62	AAA-AAA	0.00	999	1.000	1.000	0.0100
1553405_a_at	6.3	78.6	3.65	12.56	AAA-AAA	0.00	999	1.000	1.000	0.0037
1569039_s_at	3.9	48.4	3.63	12.41	AAA-AAA	0.00	999	1.000	1.000	0.0039
237900_at	76.0	6.2	-3.62	-12.26	AAA-AAA	0.00	999	1.000	1.000	0.0007
222322_at	36.9	3.0	-3.61	-12.21	AAA-AAA	0.00	999	1.000	1.000	0.0020
242769_at	4.4	53.9	3.60	12.15	AAA-AAA	0.00	999	1.000	1.000	0.0027
207333_at	55.6	4.6	-3.59	-12.04	AAA-AAA	0.00	999	1.000	1.000	0.0015
223822_at	8.4	97.9	3.55	11.68	AAA-AAA	0.00	999	1.000	1.000	0.0175
215767_at	4.7	54.2	3.51	11.43	AAA-AAA	0.00	999	1.000	1.000	0.0289

214593_at	30.0	2.6	-3.51	-11.37	AAA-AAA	0.00	999	1.000	1.000	0.0066
239589_at	3.8	42.9	3.50	11.29	AAA-AAA	0.00	999	1.000	1.000	0.0058
1562582_at	37.0	3.3	-3.49	-11.21	AAA-AAA	0.00	999	1.000	1.000	0.0043
1555391_a_at	2.7	29.9	3.46	11.03	AAA-AAA	0.00	999	1.000	1.000	0.0144
210292_s_at	42.3	3.9	-3.46	-11.00	AAA-AAA	0.00	999	1.000	1.000	0.0037
216463_at	69.8	6.4	-3.46	-10.98	APA-AAA	0.00	999	1.000	1.000	0.0888
224231_at	33.8	3.1	-3.45	-10.92	AAA-AAA	0.00	999	1.000	1.000	0.0026
1559494_at	8.7	94.4	3.44	10.87	AAA-AAA	0.00	999	1.000	1.000	0.0307
1560237_at	5.7	61.5	3.44	10.84	AAA-AAM	0.00	999	1.000	1.000	0.0333
224367_at	4.9	53.6	3.44	10.84	AAA-AAA	0.00	999	1.000	1.000	0.0038
1554460_at	16.3	1.5	-3.43	-10.75	AAA-AAA	0.00	999	1.000	1.000	0.0096
229962_at	13.1	140.3	3.43	10.75	AAA-AAM	0.00	999	1.000	1.000	0.0000
233504_at	5.5	59.4	3.42	10.73	AAA-AAA	0.00	999	1.000	1.000	0.0029
1557434_at	2.1	22.2	3.41	10.66	AAA-AAA	0.00	999	1.000	1.000	0.0253
1556344_at	70.2	6.6	-3.41	-10.63	AAA-AAA	0.00	999	1.000	1.000	0.0009
1562750_at	24.3	2.3	-3.39	-10.47	AAA-AAA	0.00	999	1.000	1.000	0.0117
1557644_at	1.4	14.5	3.38	10.44	AAA-AAA	0.00	999	1.000	1.000	0.0600
239543_s_at	42.1	4.0	-3.38	-10.43	AAA-AAA	0.00	999	1.000	1.000	0.0054
243211_at	1.9	19.9	3.37	10.35	AAA-AAA	0.00	999	1.000	1.000	0.0032
242328_at	4.2	43.0	3.36	10.29	AAA-AAA	0.00	999	1.000	1.000	0.0223
240959_at	3.0	31.0	3.35	10.22	AAA-AAA	0.00	999	1.000	1.000	0.0038
233002_at	13.8	1.4	-3.34	-10.13	AAA-AAA	0.00	999	1.000	1.000	0.0346
211449_at	44.7	4.4	-3.34	-10.13	AAA-AAA	0.00	999	1.000	1.000	0.0006
243522_at	3.1	31.0	3.34	10.12	AAA-AAA	0.00	999	1.000	1.000	0.0012
229973_at	3.0	29.7	3.33	10.06	AAA-AAA	0.00	999	1.000	1.000	0.0555
1564499_at	17.3	1.7	-3.31	-9.92	AAA-AAA	0.00	999	1.000	1.000	0.0102
1556488_s_at	2.9	28.2	3.28	9.73	AAA-AAA	0.00	999	1.000	1.000	0.0051
230001_at	90.2	9.3	-3.28	-9.69	AAA-AAA	0.00	999	1.000	1.000	0.0038
217198_x_at	9.2	89.0	3.27	9.63	AAA-AAA	0.00	999	1.000	1.000	0.0036
1559542_a_at	26.6	2.8	-3.27	-9.62	AAA-AAA	0.00	999	1.000	1.000	0.0031
1564078_at	44.9	4.7	-3.27	-9.61	AAP-AAA	0.00	999	1.000	1.000	0.0433
235616_at	6.8	64.8	3.26	9.58	AAA-AAA	0.00	999	1.000	1.000	0.0186
207194_s_at	7.3	69.9	3.26	9.55	AAA-AAA	0.00	999	1.000	1.000	0.0176
1569764_at	24.6	2.6	-3.26	-9.55	AAA-AAA	0.00	999	1.000	1.000	0.0218
1569891_at	73.4	7.8	-3.24	-9.47	AAA-AAA	0.00	999	1.000	1.000	0.0020
243573_at	26.0	2.8	-3.23	-9.40	AAP-AAA	0.00	999	1.000	1.000	0.0395
1555235_s_at	37.9	4.0	-3.23	-9.40	AAA-AAA	0.00	999	1.000	1.000	0.0633
233309_at	6.3	59.2	3.23	9.38	AAA-AAA	0.00	999	1.000	1.000	0.0009
218625_at	5.0	46.4	3.23	9.35	AAA-AAA	0.00	999	1.000	1.000	0.0001
1562949_at	23.9	2.6	-3.21	-9.26	AAA-AAA	0.00	999	1.000	1.000	0.0037
1558020_at	7.7	71.2	3.21	9.24	AAA-AAA	0.00	999	1.000	1.000	0.0006
237496_at	2.3	21.4	3.20	9.19	AAA-AAA	0.00	999	1.000	1.000	0.0041

229827_at	58.3	6.4	-3.19	-9.12	AAA-AAA	0.00	999	1.000	1.000	0.0005
235773_at	59.7	6.6	-3.18	-9.04	AMA-AAA	0.00	999	1.000	1.000	0.0042
1556267_at	20.8	2.3	-3.18	-9.03	AAA-AAA	0.00	999	1.000	1.000	0.0033
205916_at	12.6	113.7	3.17	8.99	AAA-AAA	0.00	999	1.000	1.000	0.0034
1569377_at	3.8	34.3	3.17	8.97	AAA-AAA	0.00	999	1.000	1.000	0.0014
1557599_a_at	3.0	27.0	3.16	8.92	AAA-AAM	0.00	999	1.000	1.000	0.0323
228343_at	27.5	3.1	-3.15	-8.89	AAA-AAA	0.00	999	1.000	1.000	0.0348
211021_s_at	53.3	6.0	-3.15	-8.88	AAA-AAA	0.00	999	1.000	1.000	0.0003
215572_at	39.2	4.4	-3.15	-8.85	AAA-AAA	0.00	999	1.000	1.000	0.0084
234800_at	3.0	26.8	3.14	8.83	AAA-AAA	0.00	999	1.000	1.000	0.0190
1569450_at	40.2	4.6	-3.14	-8.83	AAA-AAA	0.00	999	1.000	1.000	0.0657
234283_at	5.0	44.2	3.14	8.81	AAA-AAA	0.00	999	1.000	1.000	0.0009
1569855_at	6.3	55.3	3.14	8.80	AAA-AMA	0.00	999	1.000	1.000	0.0582
244386_at	2.8	24.8	3.14	8.80	AAA-AAA	0.00	999	1.000	1.000	0.0027
206025_s_at	66.5	7.6	-3.13	-8.77	AAA-AAA	0.00	999	1.000	1.000	0.0013
1559722_at	16.4	1.9	-3.13	-8.73	AAA-AAA	0.00	999	1.000	1.000	0.0057
242018_at	3.3	28.4	3.12	8.71	AAA-AAA	0.00	999	1.000	1.000	0.0431
231170_at	6.2	53.6	3.12	8.71	AAA-AAA	0.00	999	1.000	1.000	0.0021
1559458_at	4.1	35.3	3.12	8.70	AAA-AAA	0.00	999	1.000	1.000	0.0032
239491_at	3.7	32.3	3.12	8.70	AAA-AAA	0.00	999	1.000	1.000	0.0010
237517_at	28.0	3.3	-3.10	-8.59	AAA-AAA	0.00	999	1.000	1.000	0.0209
239546_at	2.2	18.8	3.09	8.54	AAA-AAA	0.00	999	1.000	1.000	0.0020
224139_at	5.8	49.3	3.09	8.52	AAA-AAA	0.00	999	1.000	1.000	0.0272
240026_x_at	23.6	2.8	-3.09	-8.51	AAA-AAA	0.00	999	1.000	1.000	0.0813
217132_at	3.4	29.2	3.09	8.49	AAA-AAA	0.00	999	1.000	1.000	0.0363
1564595_at	42.1	5.0	-3.09	-8.48	AAA-AAA	0.00	999	1.000	1.000	0.0707
1563070_at	5.8	49.1	3.08	8.48	AAA-AAA	0.00	999	1.000	1.000	0.0309
214770_at	2.9	24.9	3.08	8.47	AAA-AAA	0.00	999	1.000	1.000	0.0133
216408_at	52.1	6.2	-3.08	-8.44	APA-AAA	0.00	999	1.000	1.000	0.1523
1566324_a_at	47.0	5.6	-3.07	-8.39	APA-AAA	0.00	999	1.000	1.000	0.1309
220424_at	51.6	6.1	-3.07	-8.39	AAA-AAA	0.00	999	1.000	1.000	0.0150
238634_x_at	19.1	2.3	-3.07	-8.39	AAA-AAA	0.00	999	1.000	1.000	0.0747
1564459_at	34.5	4.1	-3.06	-8.34	AAA-AAA	0.00	999	1.000	1.000	0.0140
1567059_at	3.2	26.6	3.05	8.31	AAA-AAA	0.00	999	1.000	1.000	0.0338
220218_at	5.5	45.6	3.05	8.28	AAA-AAA	0.00	999	1.000	1.000	0.0003
1562329_at	24.7	3.0	-3.05	-8.27	AAA-AAA	0.00	999	1.000	1.000	0.1439
215973_at	8.7	72.1	3.05	8.26	AAA-AAA	0.00	999	1.000	1.000	0.0017
1569995_at	3.7	30.9	3.05	8.25	AAA-AAA	0.00	999	1.000	1.000	0.0427
1554252_a_at	3.2	26.5	3.04	8.24	AAA-AAA	0.00	999	1.000	1.000	0.0104
1554413_s_at	2.3	18.8	3.04	8.22	AAA-AAA	0.00	999	1.000	1.000	0.0637
1560522_at	45.7	5.6	-3.04	-8.22	AAA-AAA	0.00	999	1.000	1.000	0.0025
236532_at	1.8	15.1	3.03	8.18	AAA-AAA	0.00	999	1.000	1.000	0.1190

1554996_at	3.1	25.4	3.03	8.17	AAA-AAA	0.00	999	1.000	1.000	0.0013
1558900_at	20.5	2.5	-3.03	-8.16	AAA-AAA	0.00	999	1.000	1.000	0.0129
1559597_at	5.3	43.1	3.03	8.16	AAA-AAA	0.00	999	1.000	1.000	0.0103
1566665_at	32.1	4.0	-3.03	-8.14	AAA-AAA	0.00	999	1.000	1.000	0.0666
1569861_at	2.9	23.9	3.03	8.14	AAA-AAA	0.00	999	1.000	1.000	0.0232
1557845_at	25.6	3.2	-3.02	-8.13	AAA-AAA	0.00	999	1.000	1.000	0.0126
233426_at	5.3	43.1	3.02	8.08	AAA-AAA	0.00	999	1.000	1.000	0.0110
214899_at	3.0	24.5	3.01	8.06	AAA-AAA	0.00	999	1.000	1.000	0.1275
243307_at	2.7	22.0	3.01	8.06	AAA-AAA	0.00	999	1.000	1.000	0.0064
215420_at	12.5	100.0	3.01	8.03	AAA-MAA	0.00	999	1.000	1.000	0.0012
205044_at	3.3	26.7	3.00	8.01	AAA-AAA	0.00	999	1.000	1.000	0.0521
216244_at	2.2	17.3	3.00	8.00	AAA-AAA	0.00	999	1.000	1.000	0.0204
221185_s_at	61.1	7.6	-3.00	-7.99	AAA-AAA	0.00	999	1.000	1.000	0.0627
232523_at	3.6	28.4	3.00	7.97	AAA-AAA	0.00	999	1.000	1.000	0.0140
234556_at	20.2	2.5	-2.99	-7.96	AAA-AAA	0.00	999	1.000	1.000	0.0947
1556927_at	15.6	2.0	-2.99	-7.95	AAA-AAA	0.00	999	1.000	1.000	0.0962
211119_at	26.8	3.4	-2.99	-7.95	AAA-AAA	0.00	999	1.000	1.000	0.0127
232345_at	106.0	13.3	-2.99	-7.95	AAA-AAA	0.00	999	1.000	1.000	0.0004
207748_at	14.7	116.0	2.98	7.91	AAA-AAA	0.00	999	1.000	1.000	0.0059
1556500_a_at	4.5	35.2	2.98	7.87	AAA-AAA	0.00	999	1.000	1.000	0.0016
220537_at	6.0	47.5	2.97	7.86	AAA-AAA	0.00	999	1.000	1.000	0.0064
229937_x_at	18.7	2.4	-2.97	-7.85	AAA-AAA	0.00	999	1.000	1.000	0.1436
243102_at	7.6	59.5	2.97	7.83	AAA-AAA	0.00	999	1.000	1.000	0.0736
237945_at	4.4	34.6	2.96	7.80	AAA-AAA	0.00	999	1.000	1.000	0.0001
237101_at	28.5	3.7	-2.96	-7.78	AAA-AAA	0.00	999	1.000	1.000	0.0517
220891_at	23.0	3.0	-2.96	-7.77	AAA-AAA	0.00	999	1.000	1.000	0.0801
216717_at	3.1	23.7	2.96	7.76	AAA-AAA	0.00	999	1.000	1.000	0.0494
232035_at	34.5	4.4	-2.96	-7.76	AAA-AAA	0.00	999	1.000	1.000	0.0153
1552458_at	67.3	8.7	-2.96	-7.75	PAA-AAA	0.00	999	1.000	1.000	0.0714
241974_at	7.1	54.7	2.95	7.75	AAA-AAA	0.00	999	1.000	1.000	0.0023
1570474_s_at	52.5	6.8	-2.95	-7.75	AAA-AAA	0.00	999	1.000	1.000	0.0094
215441_at	2.3	18.0	2.95	7.74	AAA-AAA	0.00	999	1.000	1.000	0.0110
237022_at	3.1	23.8	2.95	7.73	AAA-AAA	0.00	999	1.000	1.000	0.0455
243242_at	5.1	39.4	2.95	7.73	AAA-AAA	0.00	999	1.000	1.000	0.0130
1557542_at	43.9	5.7	-2.95	-7.71	AAA-AAA	0.00	999	1.000	1.000	0.0949
215847_at	16.7	128.4	2.94	7.70	AAA-AAA	0.00	999	1.000	1.000	0.0511
1563357_at	29.3	3.8	-2.94	-7.68	AMA-AAA	0.00	999	1.000	1.000	0.0915
229346_at	74.5	9.7	-2.94	-7.67	AAA-AAA	0.00	999	1.000	1.000	0.0269
216151_at	17.5	2.3	-2.94	-7.66	AAA-AAA	0.00	999	1.000	1.000	0.0522
1560337_at	41.1	5.4	-2.94	-7.65	AAA-AAA	0.00	999	1.000	1.000	0.0038
205206_at	5.7	43.6	2.94	7.65	AAA-AAA	0.00	999	1.000	1.000	0.0132
1563221_at	21.7	2.9	-2.93	-7.63	AAA-AAA	0.00	999	1.000	1.000	0.0510

237995_at	63.0	8.3	-2.93	-7.62	AAA-AAA	0.00	999	1.000	1.000	0.0812
220784_s_at	2.7	20.3	2.93	7.60	AAA-AAA	0.00	999	1.000	1.000	0.0074
1552604_at	58.4	7.7	-2.93	-7.60	AMA-AAA	0.00	999	1.000	1.000	0.0021
241493_at	7.7	57.9	2.92	7.57	AAA-AAA	0.00	999	1.000	1.000	0.0037
215803_at	3.5	26.7	2.92	7.56	AAA-AAA	0.00	999	1.000	1.000	0.0006
243744_at	5.1	38.2	2.92	7.55	AAA-AAA	0.00	999	1.000	1.000	0.0113
216896_at	2.1	16.0	2.92	7.55	AAA-AAA	0.00	999	1.000	1.000	0.0712
219106_s_at	4.9	36.7	2.91	7.52	AAA-AAA	0.00	999	1.000	1.000	0.0807
205113_at	4.5	33.8	2.90	7.48	AAA-AAA	0.00	999	1.000	1.000	0.0199
242955_x_at	1.6	11.8	2.90	7.47	AAA-AAA	0.00	999	1.000	1.000	0.0388
1564368_a_at	1.9	14.4	2.90	7.46	AAA-AAA	0.00	999	1.000	1.000	0.1382
1556329_a_at	22.1	3.0	-2.90	-7.46	AAA-AAA	0.00	999	1.000	1.000	0.0297
231510_at	6.9	51.6	2.90	7.46	AAA-AAA	0.00	999	1.000	1.000	0.0090
1569981_at	4.2	31.0	2.90	7.44	AAA-AAA	0.00	999	1.000	1.000	0.1254
219196_at	10.3	1.4	-2.89	-7.42	AAA-AAA	0.00	999	1.000	1.000	0.0249
229349_at	28.4	3.8	-2.89	-7.42	AAA-AAA	0.00	999	1.000	1.000	0.0058
209460_at	8.3	61.2	2.89	7.39	AAA-AAA	0.00	999	1.000	1.000	0.0037
1552276_a_at	127.8	17.3	-2.89	-7.39	AAA-AAA	0.00	999	1.000	1.000	0.0302
237986_at	7.7	56.8	2.89	7.39	AAA-AAA	0.00	999	1.000	1.000	0.0157
231336_at	4.6	33.9	2.89	7.39	AAA-AAA	0.00	999	1.000	1.000	0.0017
242319_at	4.4	32.4	2.88	7.34	AAA-AAA	0.00	999	1.000	1.000	0.0257
1563503_at	27.7	3.8	-2.88	-7.34	AAA-AAA	0.00	999	1.000	1.000	0.0293
231589_at	11.0	80.7	2.87	7.33	AAA-AAA	0.00	999	1.000	1.000	0.0000
1561139_at	4.0	28.9	2.87	7.32	AAA-AAA	0.00	999	1.000	1.000	0.1447
207912_s_at	21.0	2.9	-2.87	-7.31	APA-AAA	0.00	999	1.000	1.000	0.1135
241006_at	8.9	64.9	2.87	7.30	AAA-AAA	0.00	999	1.000	1.000	0.0016
241291_at	1.8	13.0	2.87	7.30	AAA-AAA	0.00	999	1.000	1.000	0.0115
208475_at	4.6	33.8	2.87	7.29	AAA-AAA	0.00	999	1.000	1.000	0.0107
208561_at	19.6	2.7	-2.86	-7.28	AMA-AAA	0.00	999	1.000	1.000	0.0938
204052_s_at	39.9	5.5	-2.86	-7.26	AAA-AAA	0.00	999	1.000	1.000	0.0629
234277_at	38.3	5.3	-2.86	-7.25	AAA-AAA	0.00	999	1.000	1.000	0.0450
1556779_s_at	6.3	45.6	2.86	7.24	AAA-AAA	0.00	999	1.000	1.000	0.0013
219619_at	2.7	19.8	2.85	7.23	AAA-AAA	0.00	999	1.000	1.000	0.0537
209982_s_at	51.1	7.1	-2.85	-7.22	MAA-AAA	0.00	999	1.000	1.000	0.1388
243274_x_at	1.2	8.4	2.85	7.21	AAA-AAA	0.00	999	1.000	1.000	0.1060
205249_at	36.4	5.1	-2.85	-7.20	AAA-AAA	0.00	999	1.000	1.000	0.0097
243518_at	5.0	36.0	2.85	7.19	AAA-AAA	0.00	999	1.000	1.000	0.0490
235875_at	8.2	58.6	2.85	7.18	AAA-AAA	0.00	999	1.000	1.000	0.0014
240334_at	51.0	7.1	-2.84	-7.18	AAA-AAA	0.00	999	1.000	1.000	0.0394
203009_at	12.6	90.3	2.84	7.18	AAA-AAA	0.00	999	1.000	1.000	0.0101
210893_at	3.0	21.6	2.84	7.18	AAA-AAA	0.00	999	1.000	1.000	0.0886
1562613_at	1.3	9.2	2.84	7.17	AAA-AAA	0.00	999	1.000	1.000	0.1199

213400_s_at	11.8	84.7	2.84	7.16	AAA-AAA	0.00	999	1.000	1.000	0.0010
230456_at	89.5	12.5	-2.84	-7.14	AAA-AAA	0.00	999	1.000	1.000	0.0374
215901_at	6.5	46.3	2.84	7.14	AAA-AAA	0.00	999	1.000	1.000	0.1008
235642_at	4.5	32.2	2.83	7.13	AAA-AAA	0.00	999	1.000	1.000	0.0751
237149_at	22.7	3.2	-2.83	-7.13	AAA-AAA	0.00	999	1.000	1.000	0.0045
220427_at	138.9	19.6	-2.83	-7.10	AAA-AAA	0.00	999	1.000	1.000	0.0003
224213_at	2.3	16.0	2.83	7.10	AAA-AAA	0.00	999	1.000	1.000	0.0260
231669_at	3.2	22.4	2.83	7.10	AAA-AAA	0.00	999	1.000	1.000	0.0101
240309_at	9.8	69.7	2.83	7.09	AAA-AAA	0.00	999	1.000	1.000	0.0006
1556437_at	5.2	36.6	2.83	7.09	AAA-AAA	0.00	999	1.000	1.000	0.0454
235111_at	7.5	53.3	2.83	7.09	AAA-AAA	0.00	999	1.000	1.000	0.0011
233127_at	53.0	7.5	-2.83	-7.09	AAA-AAA	0.00	999	1.000	1.000	0.0024
212478_at	6.8	48.2	2.82	7.08	AAA-AAA	0.00	999	1.000	1.000	0.0013
208552_at	5.5	38.7	2.82	7.08	AAA-AAA	0.00	999	1.000	1.000	0.0011
1562886_at	2.4	16.7	2.82	7.07	AAA-AAA	0.00	999	1.000	1.000	0.0003
234313_at	15.1	2.2	-2.82	-7.05	AAA-AAA	0.00	999	1.000	1.000	0.0389
206391_at	12.4	87.2	2.82	7.05	AAA-AAA	0.00	999	1.000	1.000	0.1474
1566101_at	4.5	31.7	2.81	7.03	AAA-AAA	0.00	999	1.000	1.000	0.0436
1556187_at	4.3	30.2	2.81	7.02	AAA-AAA	0.00	999	1.000	1.000	0.0314
1562754_at	9.1	64.0	2.81	7.01	AAA-AAA	0.00	999	1.000	1.000	0.0075
240093_x_at	12.9	1.8	-2.81	-7.01	AAA-AAA	0.00	999	1.000	1.000	0.1223
232883_at	6.7	47.0	2.81	7.01	AAA-AAA	0.00	999	1.000	1.000	0.0056
1559584_a_at	20.2	2.9	-2.81	-7.00	AAA-AAA	0.00	999	1.000	1.000	0.1103
237758_at	7.4	51.9	2.80	6.98	AAA-AAA	0.00	999	1.000	1.000	0.0302
1556859_a_at	4.3	29.7	2.80	6.95	AAA-AAA	0.00	999	1.000	1.000	0.0692
1564965_at	14.9	2.2	-2.80	-6.95	APA-AAA	0.00	999	1.000	1.000	0.2428
1554920_at	2.9	19.8	2.80	6.94	AAA-AAA	0.00	999	1.000	1.000	0.1522
238957_at	3.7	25.4	2.79	6.94	AAA-AAA	0.00	999	1.000	1.000	0.0056
1560733_at	70.2	10.1	-2.79	-6.93	APP-AAA	0.00	999	1.000	1.000	0.2009
227210_at	4.5	30.8	2.79	6.93	AAA-AAA	0.00	999	1.000	1.000	0.0222
220785_at	4.2	29.2	2.79	6.92	AAA-AAA	0.00	999	1.000	1.000	0.0020
1552657_a_at	11.0	76.2	2.79	6.90	AAA-AAA	0.00	999	1.000	1.000	0.0805
229209_at	6.0	41.0	2.79	6.90	AAA-AAA	0.00	999	1.000	1.000	0.0392
1566047_at	4.6	31.8	2.78	6.88	AAA-AAA	0.00	999	1.000	1.000	0.0009
217400_at	54.5	7.9	-2.78	-6.87	AAA-AAA	0.00	999	1.000	1.000	0.0398
204417_at	60.1	8.8	-2.78	-6.87	AMP-AAA	0.00	999	1.000	1.000	0.1537
241912_at	9.3	63.9	2.78	6.85	AAA-AAA	0.00	999	1.000	1.000	0.0623
225871_at	119.5	17.4	-2.78	-6.85	AAA-AAA	0.00	999	1.000	1.000	0.0324
220405_at	8.7	59.3	2.77	6.83	MAA-AAA	0.00	999	1.000	1.000	0.0816
1566950_at	25.4	3.7	-2.77	-6.83	AAA-AAA	0.00	999	1.000	1.000	0.0041
241677_x_at	5.3	36.4	2.77	6.82	AAA-AAA	0.00	999	1.000	1.000	0.0117
1567182_at	1.7	11.7	2.77	6.82	AAA-AAA	0.00	999	1.000	1.000	0.0959

237104_at	26.0	3.8	-2.77	-6.82	AAA-AAA	0.00	999	1.000	1.000	0.0042
1554355_a_at	7.7	52.7	2.77	6.81	AAA-AAA	0.00	999	1.000	1.000	0.0045
233317_at	14.9	101.1	2.77	6.81	AAA-AAA	0.00	999	1.000	1.000	0.0076
242899_at	4.1	27.5	2.76	6.79	AAA-AAA	0.00	999	1.000	1.000	0.0999
215808_at	19.5	131.8	2.76	6.78	AAA-AAA	0.00	999	1.000	1.000	0.0534
237054_at	6.9	46.7	2.76	6.77	AAA-AAA	0.00	999	1.000	1.000	0.1084
204933_s_at	3.7	24.7	2.76	6.76	AAA-AAA	0.00	999	1.000	1.000	0.0767
207664_at	3.3	22.4	2.75	6.74	AAA-AAA	0.00	999	1.000	1.000	0.0303
1553317_s_at	24.5	3.6	-2.75	-6.74	AAM-AAA	0.00	999	1.000	1.000	0.0071
240138_at	8.1	54.2	2.75	6.73	AAA-AAA	0.00	999	1.000	1.000	0.1184
1562351_at	5.2	34.8	2.75	6.73	AAA-AAA	0.00	999	1.000	1.000	0.0572
1566033_at	8.1	54.3	2.75	6.72	AAA-AAA	0.00	999	1.000	1.000	0.0087
204597_x_at	108.9	16.2	-2.75	-6.71	APA-AAA	0.00	999	1.000	1.000	0.0593
232871_at	9.8	65.4	2.75	6.71	AAA-AAA	0.00	999	1.000	1.000	0.0719
233821_at	1.2	8.0	2.75	6.71	AAA-AAA	0.00	999	1.000	1.000	0.1060
1561761_x_at	40.1	6.0	-2.74	-6.70	AAA-AAA	0.00	999	1.000	1.000	0.0096
205971_s_at	39.2	5.9	-2.74	-6.70	AAA-AAA	0.00	999	1.000	1.000	0.0094
226279_at	46.4	7.0	-2.74	-6.67	AAA-AAA	0.00	999	1.000	1.000	0.0243
231522_at	69.6	10.4	-2.74	-6.66	AAA-AAA	0.00	999	1.000	1.000	0.0009
237445_at	9.3	61.4	2.73	6.63	AAA-AAA	0.00	999	1.000	1.000	0.0179
1552721_a_at	4.7	31.1	2.73	6.62	AAA-AAA	0.00	999	1.000	1.000	0.0010
210375_at	29.3	4.4	-2.73	-6.61	AAA-AAA	0.00	999	1.000	1.000	0.0021
1552553_a_at	28.9	4.4	-2.72	-6.58	AAA-AAA	0.00	999	1.000	1.000	0.0757
240236_at	30.3	4.6	-2.72	-6.57	AAA-AAA	0.00	999	1.000	1.000	0.1179
1555394_at	4.6	30.2	2.72	6.57	AAA-AAA	0.00	999	1.000	1.000	0.1440
1562321_at	14.9	97.5	2.71	6.56	AAA-AAA	0.00	999	1.000	1.000	0.0411
1557742_a_at	29.2	4.5	-2.71	-6.55	AAA-AAA	0.00	999	1.000	1.000	0.2349
1558786_at	21.3	3.3	-2.71	-6.54	AAA-AAA	0.00	999	1.000	1.000	0.0573
244283_x_at	30.2	4.6	-2.71	-6.55	AAA-AAA	0.00	999	1.000	1.000	0.0133
224083_s_at	7.1	1.1	-2.71	-6.54	AAA-AAA	0.00	999	1.000	1.000	0.1429
1555000_at	7.3	47.9	2.71	6.54	AAA-AAM	0.00	999	1.000	1.000	0.0034
220374_at	12.9	83.8	2.70	6.50	AAA-AAA	0.00	999	1.000	1.000	0.0016
1563299_at	5.8	37.5	2.70	6.49	AAA-AAA	0.00	999	1.000	1.000	0.0723
214111_at	4.5	29.0	2.70	6.49	AAA-AAA	0.00	999	1.000	1.000	0.0323
241273_at	2.0	13.1	2.70	6.49	AAA-AAA	0.00	999	1.000	1.000	0.0039
215851_at	7.1	46.3	2.70	6.49	AAA-AAA	0.00	999	1.000	1.000	0.0597
1566096_x_at	6.9	44.6	2.70	6.48	AAA-AAM	0.00	999	1.000	1.000	0.0622
232499_at	7.0	45.4	2.70	6.48	AAA-AAA	0.00	999	1.000	1.000	0.0320
1561959_x_at	1.5	9.9	2.69	6.47	AAA-AAA	0.00	999	1.000	1.000	0.0411
1569385_s_at	7.2	46.3	2.69	6.46	AAA-AAA	0.00	999	1.000	1.000	0.1373
217402_at	11.5	1.8	-2.69	-6.46	AAA-AAA	0.00	999	1.000	1.000	0.0806
231040_at	21.6	3.4	-2.69	-6.44	AAA-AAA	0.00	999	1.000	1.000	0.1610

237467_at	69.3	10.8	-2.69	-6.44	AAA-AAA	0.00	999	1.000	1.000	0.0016
238364_x_at	4.4	28.5	2.69	6.43	AAA-AAA	0.00	999	1.000	1.000	0.1265
232723_at	8.2	52.4	2.68	6.43	AAA-AAA	0.00	999	1.000	1.000	0.0587
230822_at	58.7	9.2	-2.68	-6.42	AAA-AAA	0.00	999	1.000	1.000	0.0146
209200_at	18.0	2.8	-2.68	-6.41	AAA-AAA	0.00	999	1.000	1.000	0.0047
230360_at	61.2	9.5	-2.68	-6.41	AAA-AAA	0.00	999	1.000	1.000	0.0719
1564909_at	3.9	25.1	2.68	6.40	AAA-AAA	0.00	999	1.000	1.000	0.2118
1554732_at	4.5	28.7	2.68	6.40	AAA-AAA	0.00	999	1.000	1.000	0.0869
1560169_at	37.9	5.9	-2.68	-6.39	AAA-AAA	0.00	999	1.000	1.000	0.0050
224071_at	83.9	13.1	-2.68	-6.39	AAA-AAA	0.00	999	1.000	1.000	0.0686
1566202_at	2.2	14.0	2.67	6.37	AAA-AAA	0.00	999	1.000	1.000	0.0647
1559213_at	1.9	11.8	2.67	6.35	AAA-AAA	0.00	999	1.000	1.000	0.0193
1569841_x_at	9.9	63.0	2.66	6.34	AAA-AAA	0.00	999	1.000	1.000	0.0010
1561644_x_at	2.6	16.4	2.66	6.34	AAA-AAA	0.00	999	1.000	1.000	0.1086
205888_s_at	11.8	74.5	2.66	6.33	AAA-AAA	0.00	999	1.000	1.000	0.0718
223756_at	36.2	5.7	-2.66	-6.33	AAA-AAA	0.00	999	1.000	1.000	0.0531
1561550_at	41.9	6.6	-2.66	-6.32	AAA-AAA	0.00	999	1.000	1.000	0.0055
215655_at	19.8	3.1	-2.66	-6.33	AAA-AAA	0.00	999	1.000	1.000	0.0437
236139_at	57.3	9.1	-2.66	-6.32	AAA-AAA	0.00	999	1.000	1.000	0.0829
1565806_at	29.1	4.6	-2.66	-6.30	AAA-AAA	0.00	999	1.000	1.000	0.0977
1562420_at	20.5	3.3	-2.65	-6.27	AAA-AAA	0.00	999	1.000	1.000	0.0916
222910_s_at	52.8	8.4	-2.65	-6.27	AAA-AAA	0.00	999	1.000	1.000	0.0258
243080_at	3.8	24.0	2.65	6.26	AAA-AAA	0.00	999	1.000	1.000	0.0401
234161_at	5.2	32.7	2.65	6.26	AAA-AAA	0.00	999	1.000	1.000	0.1127
211573_x_at	56.0	8.9	-2.65	-6.26	AAA-AAA	0.00	999	1.000	1.000	0.0072
1558809_s_at	5.4	33.6	2.64	6.25	AAA-AAA	0.00	999	1.000	1.000	0.0640
233897_at	6.7	41.5	2.64	6.24	AAA-AAA	0.00	999	1.000	1.000	0.0177
244226_s_at	6.5	40.8	2.64	6.23	AAA-AAA	0.00	999	1.000	1.000	0.0822
223815_at	6.6	40.9	2.64	6.23	AAA-AAA	0.00	999	1.000	1.000	0.0018
1552580_at	30.5	4.9	-2.64	-6.22	AAA-AAA	0.00	999	1.000	1.000	0.0190
1561738_at	7.2	45.0	2.64	6.22	AAA-AAA	0.00	999	1.000	1.000	0.0581
235385_at	1.5	9.1	2.64	6.21	AAA-AAA	0.00	999	1.000	1.000	0.1054
244325_at	16.4	2.7	-2.63	-6.21	AAA-AAA	0.00	999	1.000	1.000	0.0613
240662_at	10.7	1.7	-2.63	-6.20	AAA-AAA	0.00	999	1.000	1.000	0.0118
1556711_at	2.9	18.1	2.63	6.20	AAA-AAA	0.00	999	1.000	1.000	0.0559
233875_at	1.5	9.5	2.63	6.20	AAA-AAA	0.00	999	1.000	1.000	0.0536
232214_x_at	132.4	21.4	-2.63	-6.20	AAA-AAA	0.00	999	1.000	1.000	0.0054
1559735_at	69.6	11.2	-2.63	-6.19	PMP-AAA	0.00	999	1.000	1.000	0.1488
243771_at	35.4	5.7	-2.63	-6.19	AAA-AAA	0.00	999	1.000	1.000	0.0639
223844_at	5.8	35.8	2.63	6.19	AAA-AAA	0.00	999	1.000	1.000	0.1035
243549_at	4.5	28.0	2.63	6.19	AAA-AAA	0.00	999	1.000	1.000	0.1251
242901_at	8.7	53.8	2.63	6.18	AAA-AAA	0.00	999	1.000	1.000	0.0150

244276_at	8.7	53.6	2.63	6.18	AAA-AAA	0.00	999	1.000	1.000	0.1164
1560850_at	5.0	31.0	2.63	6.18	AAA-AAA	0.00	999	1.000	1.000	0.0007
208498_s_at	6.4	39.7	2.63	6.17	AAA-AAA	0.00	999	1.000	1.000	0.0534
238617_at	39.2	6.4	-2.63	-6.17	AAA-AAA	0.00	999	1.000	1.000	0.0008
238149_at	1.6	9.5	2.63	6.17	AAA-AAA	0.00	999	1.000	1.000	0.0675
211239_s_at	33.1	5.4	-2.62	-6.16	AAA-AAA	0.00	999	1.000	1.000	0.0244
239650_at	1.3	8.2	2.62	6.15	AAA-AAA	0.00	999	1.000	1.000	0.1666
1555513_at	19.6	3.2	-2.62	-6.15	AAA-AAA	0.00	999	1.000	1.000	0.0017
1561733_at	4.9	30.2	2.62	6.14	AAA-AAA	0.00	999	1.000	1.000	0.0549
1562086_at	10.6	1.7	-2.61	-6.11	AAA-AAA	0.00	999	1.000	1.000	0.1403
226751_at	68.9	11.3	-2.61	-6.11	AAA-AAA	0.00	999	1.000	1.000	0.0010
229308_at	35.2	5.8	-2.61	-6.10	AAA-AAA	0.00	999	1.000	1.000	0.0498
1570537_a_at	31.7	5.2	-2.61	-6.09	AAA-AAA	0.00	999	1.000	1.000	0.0641
242541_at	7.0	42.6	2.61	6.09	AAA-AAA	0.00	999	1.000	1.000	0.0048
231618_s_at	19.4	3.2	-2.61	-6.08	AAA-AAA	0.00	999	1.000	1.000	0.0395
220393_at	1.5	9.1	2.60	6.08	AAA-AAA	0.00	999	1.000	1.000	0.0484
237247_at	8.0	48.6	2.60	6.08	AAA-AAA	0.00	999	1.000	1.000	0.0576
210823_s_at	13.7	82.9	2.60	6.07	AAA-AAA	0.00	999	1.000	1.000	0.0049
230394_at	10.7	64.8	2.60	6.07	AAA-AAA	0.00	999	1.000	1.000	0.0111
244435_at	1.6	9.6	2.60	6.07	AAA-AAA	0.00	999	1.000	1.000	0.0330
244108_at	81.6	13.5	-2.60	-6.07	AAA-AAA	0.00	999	1.000	1.000	0.0184
205731_s_at	12.4	75.2	2.60	6.07	AAA-AAA	0.00	999	1.000	1.000	0.0075
244309_at	87.7	14.5	-2.60	-6.06	AAA-AAA	0.00	999	1.000	1.000	0.0132
1559988_at	39.1	6.5	-2.60	-6.06	AAA-AAA	0.00	999	1.000	1.000	0.0386
1563068_at	16.3	98.6	2.60	6.06	AAA-AAA	0.00	999	1.000	1.000	0.0192
220585_at	15.9	96.4	2.60	6.06	AAA-AAA	0.00	999	1.000	1.000	0.0555
1569482_at	5.0	30.5	2.60	6.05	AAA-AAA	0.00	999	1.000	1.000	0.1443
206070_s_at	11.3	1.9	-2.60	-6.05	AAA-AAA	0.00	999	1.000	1.000	0.2236
234028_at	4.9	29.3	2.60	6.05	AAA-AAA	0.00	999	1.000	1.000	0.1185
213259_s_at	97.9	16.2	-2.60	-6.05	AAA-AAA	0.00	999	1.000	1.000	0.0206
236107_at	95.0	15.7	-2.60	-6.05	AAA-AAA	0.00	999	1.000	1.000	0.0002
1555397_at	4.0	24.1	2.59	6.04	AAA-AAA	0.00	999	1.000	1.000	0.0606
231287_s_at	10.0	60.3	2.59	6.04	AAA-AAA	0.00	999	1.000	1.000	0.0584
240610_at	7.2	43.4	2.59	6.03	AAA-AAA	0.00	999	1.000	1.000	0.0069
1570039_at	4.6	27.9	2.59	6.03	AAA-AAA	0.00	999	1.000	1.000	0.0575
215932_at	9.2	55.3	2.59	6.03	AAA-AAA	0.00	999	1.000	1.000	0.0296
208564_at	8.7	52.2	2.59	6.03	AAA-AAA	0.00	999	1.000	1.000	0.0316
234057_at	51.7	8.6	-2.59	-6.03	AAA-AAA	0.00	999	1.000	1.000	0.0265
1563225_a_at	68.0	11.3	-2.59	-6.03	AAA-AAA	0.00	999	1.000	1.000	0.0099
240337_at	5.3	31.8	2.59	6.03	AAA-AAA	0.00	999	1.000	1.000	0.0730
206721_at	34.5	5.7	-2.59	-6.02	APA-AAA	0.00	999	1.000	1.000	0.0369
1563681_at	6.5	39.1	2.59	6.02	AAA-AAA	0.00	999	1.000	1.000	0.0001

236864_at	52.2	8.7	-2.59	-6.02	AAA-AAA	0.00	999	1.000	1.000	0.0017
1556757_a_at	35.4	5.9	-2.59	-6.02	AAA-AAA	0.00	999	1.000	1.000	0.0193
222363_at	77.8	12.9	-2.59	-6.01	AAA-AAA	0.00	999	1.000	1.000	0.0356
1562235_s_at	4.9	29.3	2.59	6.01	AAA-AAA	0.00	999	1.000	1.000	0.0964
204301_at	58.7	9.8	-2.59	-6.00	AAA-AAA	0.00	999	1.000	1.000	0.0062
234348_at	2.8	16.7	2.59	6.00	AAA-AAA	0.00	999	1.000	1.000	0.1638
238752_at	35.2	5.9	-2.59	-6.00	AAA-AAA	0.00	999	1.000	1.000	0.0039
1562276_at	3.0	17.8	2.58	5.99	AAA-AAA	0.00	999	1.000	1.000	0.0251
1562738_a_at	21.0	3.5	-2.58	-5.99	AAA-AAA	0.00	999	1.000	1.000	0.0203
214488_at	3.8	23.0	2.58	5.99	AAA-AAA	0.00	999	1.000	1.000	0.1390
211319_at	8.8	52.7	2.58	5.97	AAA-AAA	0.00	999	1.000	1.000	0.0490
227198_at	14.7	2.5	-2.58	-5.97	AAA-AAA	0.00	999	1.000	1.000	0.0731
1563188_at	16.6	2.8	-2.58	-5.96	AAA-AAA	0.00	999	1.000	1.000	0.0544
205560_at	14.3	85.4	2.57	5.96	AAA-AAA	0.00	999	1.000	1.000	0.0504
1556369_a_at	47.1	7.9	-2.57	-5.95	AAA-AAA	0.00	999	1.000	1.000	0.0075
1559575_a_at	11.5	68.5	2.57	5.95	AAA-AAA	0.00	999	1.000	1.000	0.1142
1560034_a_at	14.7	2.5	-2.57	-5.95	AAA-AAA	0.00	999	1.000	1.000	0.0118
233180_at	19.6	116.3	2.57	5.95	AAA-AAA	0.00	999	1.000	1.000	0.0185
236584_at	28.8	4.9	-2.57	-5.95	AAA-AAA	0.00	999	1.000	1.000	0.0402
235622_at	5.9	34.8	2.57	5.94	AAA-AAA	0.00	999	1.000	1.000	0.1737
230496_at	29.4	5.0	-2.57	-5.93	AAA-AAA	0.00	999	1.000	1.000	0.2026
214059_at	21.6	3.7	-2.57	-5.92	AAA-AAA	0.00	999	1.000	1.000	0.0115
234919_s_at	11.2	66.3	2.57	5.92	AAA-AAA	0.00	999	1.000	1.000	0.0084
1557617_at	4.8	28.6	2.57	5.92	AAA-AAA	0.00	999	1.000	1.000	0.1147
206626_x_at	62.8	10.6	-2.56	-5.91	AMA-AAA	0.00	999	1.000	1.000	0.0962
1553365_at	7.7	45.4	2.56	5.91	AAA-AAA	0.00	999	1.000	1.000	0.0330
210196_s_at	50.8	8.6	-2.56	-5.90	AAA-AAA	0.00	999	1.000	1.000	0.0780
239856_at	56.2	9.5	-2.56	-5.90	AAA-AAA	0.00	999	1.000	1.000	0.0001
1556366_s_at	11.1	1.9	-2.56	-5.90	AAA-AAA	0.00	999	1.000	1.000	0.1832
237018_at	9.7	56.9	2.56	5.90	AAA-AAA	0.00	999	1.000	1.000	0.0094
1570396_at	3.7	21.6	2.56	5.88	AAA-AAA	0.00	999	1.000	1.000	0.0721
236996_at	10.6	62.4	2.56	5.88	AAA-AAA	0.00	999	1.000	1.000	0.1234
230244_at	11.5	67.3	2.56	5.88	AAA-AAA	0.00	999	1.000	1.000	0.0523
220064_at	36.7	6.3	-2.55	-5.85	AAA-AAA	0.00	999	1.000	1.000	0.0366
237933_at	34.3	5.9	-2.55	-5.85	AAA-AAA	0.00	999	1.000	1.000	0.0136
237066_at	45.1	7.7	-2.55	-5.85	AAA-AAA	0.00	999	1.000	1.000	0.1663
236473_at	19.4	113.2	2.55	5.85	AAA-AAA	0.00	999	1.000	1.000	0.0062
1562916_at	19.9	3.4	-2.55	-5.84	PAA-AAA	0.00	999	1.000	1.000	0.0927
1570180_at	3.1	17.9	2.55	5.84	AAA-AAA	0.00	999	1.000	1.000	0.0025
224313_at	7.4	43.1	2.54	5.82	AAA-AAA	0.00	999	1.000	1.000	0.0243
215686_x_at	40.9	7.0	-2.54	-5.81	AAA-AAA	0.00	999	1.000	1.000	0.0754
216135_at	3.5	20.1	2.54	5.81	AAA-AAA	0.00	999	1.000	1.000	0.0869

242524_at	31.5	5.4	-2.54	-5.80	AAA-AAA	0.00	999	1.000	1.000	0.0456
242529_x_at	6.5	37.7	2.54	5.80	AAA-AAA	0.00	999	1.000	1.000	0.0074
1562850_at	6.2	36.2	2.54	5.80	AAA-AAA	0.00	999	1.000	1.000	0.0080
213401_s_at	4.0	23.2	2.54	5.79	AAA-AAA	0.00	999	1.000	1.000	0.0970
221241_s_at	8.3	48.2	2.53	5.79	AAA-AAA	0.00	999	1.000	1.000	0.0129
210671_x_at	50.8	8.8	-2.53	-5.79	AAA-AAA	0.00	999	1.000	1.000	0.0745
223863_at	5.1	29.4	2.53	5.79	AAA-AAA	0.00	999	1.000	1.000	0.0301
242536_at	2.4	13.7	2.53	5.78	AAA-AAA	0.00	999	1.000	1.000	0.0714
1566948_at	3.0	17.6	2.53	5.78	AAA-AAA	0.00	999	1.000	1.000	0.0487
217392_at	13.6	2.4	-2.53	-5.78	AAA-AAA	0.00	999	1.000	1.000	0.1774
1564310_a_at	6.7	38.6	2.53	5.78	AAA-AAA	0.00	999	1.000	1.000	0.0265
216089_at	24.1	4.2	-2.53	-5.77	AAA-AAA	0.00	999	1.000	1.000	0.1391
232110_at	50.4	8.7	-2.53	-5.77	AAA-AAA	0.00	999	1.000	1.000	0.0191
238347_at	40.5	7.0	-2.53	-5.77	AAA-AAA	0.00	999	1.000	1.000	0.0482
1552754_a_at	1.8	10.2	2.53	5.76	AAA-AAA	0.00	999	1.000	1.000	0.0965
1564514_at	8.2	47.0	2.53	5.76	AAA-AAA	0.00	999	1.000	1.000	0.0960
1553383_at	13.5	2.3	-2.53	-5.76	AAA-AAA	0.00	999	1.000	1.000	0.1503
214905_at	7.9	45.6	2.53	5.76	AAA-AAA	0.00	999	1.000	1.000	0.0944
1562870_at	45.3	7.9	-2.53	-5.76	AAA-AAA	0.00	999	1.000	1.000	0.0149
1563509_at	63.4	11.0	-2.52	-5.75	AAA-AAA	0.00	999	1.000	1.000	0.1014
205509_at	11.4	65.5	2.52	5.75	AAA-AAA	0.00	999	1.000	1.000	0.1176
204940_at	19.1	3.3	-2.52	-5.75	AAA-AAA	0.00	999	1.000	1.000	0.0182
232253_at	114.2	19.9	-2.52	-5.75	AAA-AAA	0.00	999	1.000	1.000	0.0239
232993_at	4.2	24.0	2.52	5.73	AAA-AAA	0.00	999	1.000	1.000	0.0238
1568908_at	12.1	69.5	2.52	5.73	AAA-AAA	0.00	999	1.000	1.000	0.0173
239756_at	5.7	32.5	2.52	5.72	AAA-AAA	0.00	999	1.000	1.000	0.0431
1554503_a_at	63.5	11.1	-2.52	-5.72	AAA-AAA	0.00	999	1.000	1.000	0.0390
227851_s_at	12.5	71.4	2.52	5.72	AAA-AAA	0.00	999	1.000	1.000	0.0030
217574_at	34.8	6.1	-2.51	-5.70	AAA-PAA	0.00	999	1.000	1.000	0.1311
232217_at	9.7	55.4	2.51	5.70	AAA-AAA	0.00	999	1.000	1.000	0.0010
1562019_at	3.9	22.1	2.51	5.68	AAA-AAA	0.00	999	1.000	1.000	0.0635
237879_at	9.1	51.4	2.51	5.68	AAA-AAA	0.00	999	1.000	1.000	0.1781
243545_at	46.7	8.2	-2.50	-5.67	AAA-AAA	0.00	999	1.000	1.000	0.0436
1558602_a_at	8.8	49.9	2.50	5.67	AAA-AAA	0.00	999	1.000	1.000	0.0068
220384_at	33.5	5.9	-2.50	-5.66	AAA-AAA	0.00	999	1.000	1.000	0.1909
238157_at	14.6	82.4	2.50	5.65	AAA-AAA	0.00	999	1.000	1.000	0.0097
221144_at	31.2	5.5	-2.50	-5.65	AAA-AAA	0.00	999	1.000	1.000	0.1039
1561104_at	4.4	24.9	2.50	5.64	AAA-AAA	0.00	999	1.000	1.000	0.0691
221084_at	23.7	4.2	-2.50	-5.65	AAA-AAA	0.00	999	1.000	1.000	0.0173
224109_at	3.8	21.3	2.50	5.64	AAA-AAA	0.00	999	1.000	1.000	0.0137
229527_s_at	42.8	7.6	-2.50	-5.64	AAA-AAA	0.00	999	1.000	1.000	0.0034
1552673_at	5.2	29.3	2.49	5.64	AAA-AAA	0.00	999	1.000	1.000	0.0680

241264_at	2.4	13.5	2.49	5.63	AAA-AAA	0.00	999	1.000	1.000	0.0650
1561651_s_at	59.2	10.5	-2.49	-5.63	AAA-AAA	0.00	999	1.000	1.000	0.0566
241568_at	41.9	7.5	-2.49	-5.63	AAA-AAA	0.00	999	1.000	1.000	0.0080
1570621_at	4.2	23.5	2.49	5.63	AAA-AAA	0.00	999	1.000	1.000	0.0910
1560416_at	40.7	7.2	-2.49	-5.62	APA-PAA	0.00	999	1.000	1.000	0.1815
1569599_at	4.3	24.2	2.49	5.62	AAA-AAA	0.00	999	1.000	1.000	0.0557
231598_x_at	2.3	12.9	2.49	5.62	AAA-AAA	0.00	999	1.000	1.000	0.0748
1557448_a_at	4.7	26.7	2.49	5.62	AAA-AAA	0.00	999	1.000	1.000	0.0382
1561463_at	28.0	5.0	-2.49	-5.61	AAA-AAA	0.00	999	1.000	1.000	0.0772
220324_at	11.9	2.1	-2.49	-5.61	AAA-AAA	0.00	999	1.000	1.000	0.0158
1563610_at	7.9	44.0	2.49	5.61	AAA-AAA	0.00	999	1.000	1.000	0.0489
1557762_at	1.0	5.6	2.49	5.60	AAA-AAA	0.00	999	1.000	1.000	0.1018
239569_at	11.3	63.1	2.49	5.60	AAA-AAA	0.00	999	1.000	1.000	0.0006
241652_x_at	1.9	10.5	2.48	5.60	AAA-AAA	0.00	999	1.000	1.000	0.1608
1553952_at	32.9	184.1	2.48	5.59	AAA-AAA	0.00	999	1.000	1.000	0.0146
240990_at	6.8	37.7	2.48	5.59	AAA-AAA	0.00	999	1.000	1.000	0.1277
1556921_at	3.2	18.0	2.48	5.59	AAA-AAA	0.00	999	1.000	1.000	0.0343
1556912_at	1.6	8.8	2.48	5.59	AAA-AAA	0.00	999	1.000	1.000	0.0182
212865_s_at	20.2	3.6	-2.48	-5.59	AAA-AAA	0.00	999	1.000	1.000	0.0771
1554686_at	33.8	6.1	-2.48	-5.58	AAA-AAA	0.00	999	1.000	1.000	0.0571
240691_at	5.8	32.2	2.48	5.58	AAA-AAA	0.00	999	1.000	1.000	0.0190
1561232_at	41.1	7.4	-2.48	-5.58	AAA-AAA	0.00	999	1.000	1.000	0.0136
213458_at	2.6	14.6	2.48	5.58	AAA-AAA	0.00	999	1.000	1.000	0.0471
228481_at	45.7	8.2	-2.48	-5.58	AAA-AAA	0.00	999	1.000	1.000	0.0316
223458_at	89.7	16.1	-2.48	-5.58	AAA-AAA	0.00	999	1.000	1.000	0.0089
207744_at	5.9	32.7	2.48	5.57	AAA-AAA	0.00	999	1.000	1.000	0.0289
237009_at	7.0	39.1	2.48	5.57	AAA-AAA	0.00	999	1.000	1.000	0.0237
1555675_at	43.6	7.8	-2.48	-5.57	AAA-AAA	0.00	999	1.000	1.000	0.0792
1556873_at	11.1	2.0	-2.48	-5.56	AAA-AAA	0.00	999	1.000	1.000	0.1467
243332_at	2.7	15.1	2.48	5.57	AAA-AAA	0.00	999	1.000	1.000	0.1024
224219_s_at	2.6	14.5	2.48	5.56	AAA-AAA	0.00	999	1.000	1.000	0.0747
1570186_at	43.9	7.9	-2.47	-5.56	AAA-AAA	0.00	999	1.000	1.000	0.0090
219039_at	36.1	200.7	2.47	5.56	AAA-AAA	0.00	999	1.000	1.000	0.0589
227097_at	17.3	96.0	2.47	5.55	AAA-AAA	0.00	999	1.000	1.000	0.1478
1555020_a_at	73.3	13.2	-2.47	-5.55	AAA-AAA	0.00	999	1.000	1.000	0.0442
1560185_at	3.2	17.5	2.47	5.55	AAA-AAA	0.00	999	1.000	1.000	0.0729
237891_at	7.8	43.5	2.47	5.55	AAA-AAA	0.00	999	1.000	1.000	0.1393
231426_at	6.0	33.4	2.47	5.55	AAA-AAA	0.00	999	1.000	1.000	0.0465
213170_at	10.4	57.6	2.47	5.54	AAA-AAA	0.00	999	1.000	1.000	0.1319
227194_at	23.2	4.2	-2.47	-5.55	AAA-AAA	0.00	999	1.000	1.000	0.0927
1562934_at	8.1	45.1	2.47	5.54	AAA-AAA	0.00	999	1.000	1.000	0.0772
243230_at	8.1	45.1	2.47	5.54	AAA-AAA	0.00	999	1.000	1.000	0.0017

213880_at	11.9	2.2	-2.47	-5.54	AAA-AAA	0.00	999	1.000	1.000	0.0439
233505_at	3.3	18.2	2.47	5.54	AAA-AAA	0.00	999	1.000	1.000	0.1012
1556856_at	3.5	19.3	2.47	5.53	AAA-AAA	0.00	999	1.000	1.000	0.0423
214712_at	6.7	36.8	2.47	5.53	AAA-AAA	0.00	999	1.000	1.000	0.1017
230776_at	7.7	42.4	2.47	5.53	AAA-AAA	0.00	999	1.000	1.000	0.1327
243257_at	2.8	15.4	2.47	5.53	AAA-AAA	0.00	999	1.000	1.000	0.1100
1560705_at	33.0	6.0	-2.47	-5.53	AAA-AAA	0.00	999	1.000	1.000	0.0501
214768_x_at	13.0	72.0	2.47	5.52	AAA-AAA	0.00	999	1.000	1.000	0.0471
1556881_at	18.6	3.4	-2.46	-5.51	AAA-AAA	0.00	999	1.000	1.000	0.0925
1553134_s_at	3.1	16.9	2.46	5.51	AAA-AAA	0.00	999	1.000	1.000	0.0813
210917_at	5.6	30.6	2.46	5.51	AAA-AAA	0.00	999	1.000	1.000	0.0256
232318_s_at	6.1	33.3	2.46	5.51	AAA-AAA	0.00	999	1.000	1.000	0.2356
241585_at	3.4	18.8	2.46	5.50	AAA-AAA	0.00	999	1.000	1.000	0.1373
240848_at	46.3	8.4	-2.46	-5.50	AAA-AAA	0.00	999	1.000	1.000	0.0211
1566947_at	26.2	4.8	-2.46	-5.49	AAA-AAA	0.00	999	1.000	1.000	0.0357
240715_at	4.9	26.7	2.46	5.49	AAA-AAA	0.00	999	1.000	1.000	0.0394
239975_at	53.4	9.7	-2.46	-5.48	AAA-AAA	0.00	999	1.000	1.000	0.0939
244094_at	7.3	40.3	2.46	5.49	AAA-AAA	0.00	999	1.000	1.000	0.0907
213277_at	10.9	59.6	2.45	5.48	AAA-AAA	0.00	999	1.000	1.000	0.0847
230554_at	2.8	15.5	2.45	5.48	AAA-AAA	0.00	999	1.000	1.000	0.0554
1569127_at	88.4	16.2	-2.45	-5.47	AAA-AAA	0.00	999	1.000	1.000	0.1175
211384_s_at	53.7	9.8	-2.45	-5.47	AAA-AAA	0.00	999	1.000	1.000	0.0080
221137_at	70.4	12.9	-2.45	-5.47	PPA-AAA	0.00	999	1.000	1.000	0.1015
241157_at	48.2	8.8	-2.45	-5.47	AAA-AAA	0.00	999	1.000	1.000	0.0004
236313_at	3.1	16.8	2.45	5.47	AAA-AAA	0.00	999	1.000	1.000	0.0536
1557410_at	66.7	12.2	-2.45	-5.46	AAA-AAA	0.00	999	1.000	1.000	0.0529
233780_at	7.8	42.7	2.45	5.46	AAA-AAA	0.00	999	1.000	1.000	0.1013
207932_at	9.0	1.7	-2.45	-5.46	AAA-AAA	0.00	999	1.000	1.000	0.0147
208283_at	24.0	4.4	-2.45	-5.46	AAA-AAA	0.00	999	1.000	1.000	0.0396
1557339_at	3.3	18.1	2.45	5.45	AAA-AAA	0.00	999	1.000	1.000	0.0551
208565_at	50.3	9.2	-2.45	-5.45	AAA-AAA	0.00	999	1.000	1.000	0.1891
1562122_at	10.4	56.8	2.45	5.45	AAA-MAA	0.00	999	1.000	1.000	0.0598
205168_at	52.1	9.6	-2.45	-5.45	AAA-AAA	0.00	999	1.000	1.000	0.0350
234163_at	26.9	4.9	-2.45	-5.45	AAA-AAA	0.00	999	1.000	1.000	0.0925
234077_at	17.7	96.3	2.45	5.45	AAA-AAA	0.00	999	1.000	1.000	0.0442
1553266_at	4.3	23.2	2.45	5.45	AAA-AAA	0.00	999	1.000	1.000	0.2357
217105_at	2.0	10.8	2.45	5.45	AAA-AAA	0.00	999	1.000	1.000	0.1161
201720_s_at	106.3	19.6	-2.44	-5.44	AAA-AAA	0.00	999	1.000	1.000	0.0002
244327_at	37.0	6.8	-2.44	-5.43	AAA-AAA	0.00	999	1.000	1.000	0.0243
244213_at	4.4	24.0	2.44	5.43	AAA-AAM	0.00	999	1.000	1.000	0.0770
229597_s_at	76.6	14.1	-2.44	-5.44	AAA-AAA	0.00	999	1.000	1.000	0.0047
1556682_s_at	5.5	29.9	2.44	5.43	AAA-MAA	0.00	999	1.000	1.000	0.1252

1557733_a_at	4.1	22.0	2.44	5.43	AAA-AAA	0.00	999	1.000	1.000	0.0003
234603_at	7.2	39.0	2.44	5.43	AAA-AAA	0.00	999	1.000	1.000	0.0797
227966_s_at	7.8	42.5	2.44	5.43	AAA-AAA	0.00	999	1.000	1.000	0.0619
229620_at	29.6	5.5	-2.44	-5.43	AAA-AAA	0.00	999	1.000	1.000	0.0146
1568920_at	3.2	17.2	2.44	5.42	AAA-MAA	0.00	999	1.000	1.000	0.0639
1556909_at	16.4	89.0	2.44	5.42	AAA-AAA	0.00	999	1.000	1.000	0.0067
230577_at	2.5	13.6	2.44	5.42	AAA-AAA	0.00	999	1.000	1.000	0.0809
241224_x_at	49.6	9.2	-2.44	-5.42	AAA-AAA	0.00	999	1.000	1.000	0.0407
1556920_s_at	8.9	47.9	2.44	5.42	AAA-AAA	0.00	999	1.000	1.000	0.1285
234378_at	10.7	57.9	2.44	5.42	AAA-AAA	0.00	999	1.000	1.000	0.0140
241330_x_at	12.5	2.3	-2.44	-5.41	AAA-AAA	0.00	999	1.000	1.000	0.0166
206938_at	14.1	76.4	2.44	5.41	AAA-AAA	0.00	999	1.000	1.000	0.0012
215692_s_at	2.6	14.3	2.43	5.40	AAA-AAA	0.00	999	1.000	1.000	0.0379
233667_at	5.3	28.7	2.43	5.40	AAA-AAA	0.00	999	1.000	1.000	0.0077
227362_at	8.2	44.3	2.43	5.40	AAA-AAA	0.00	999	1.000	1.000	0.0889
207302_at	27.5	5.1	-2.43	-5.39	AAA-AAA	0.00	999	1.000	1.000	0.0370
223813_at	8.8	47.6	2.43	5.39	AAA-AAA	0.00	999	1.000	1.000	0.1120
214551_s_at	10.4	55.8	2.43	5.39	AAA-AAA	0.00	999	1.000	1.000	0.0575
1570357_at	48.7	9.1	-2.43	-5.37	AAA-AAA	0.00	999	1.000	1.000	0.0698
240646_at	27.4	5.1	-2.43	-5.37	AAA-AAA	0.00	999	1.000	1.000	0.0314
235987_at	7.7	41.3	2.43	5.37	AAA-AAA	0.00	999	1.000	1.000	0.1457
235671_at	86.4	16.1	-2.42	-5.37	AAA-AAA	0.00	999	1.000	1.000	0.0170
244015_at	11.9	63.9	2.42	5.36	AAA-AAA	0.00	999	1.000	1.000	0.1513
216533_at	3.6	19.2	2.42	5.36	AAA-AAM	0.00	999	1.000	1.000	0.3499
227844_at	5.1	27.4	2.42	5.35	AAA-AAA	0.00	999	1.000	1.000	0.0971
231746_at	8.1	43.5	2.42	5.36	AAA-AAA	0.00	999	1.000	1.000	0.0203
234616_at	20.5	3.8	-2.42	-5.35	AAA-AAA	0.00	999	1.000	1.000	0.0798
235666_at	6.8	36.4	2.42	5.35	AAA-AAA	0.00	999	1.000	1.000	0.0174
1554298_a_at	23.7	4.4	-2.42	-5.34	AAA-AAA	0.00	999	1.000	1.000	0.1296
215146_s_at	31.4	5.9	-2.42	-5.34	AAA-AAA	0.00	999	1.000	1.000	0.0055
219714_s_at	15.7	83.8	2.42	5.34	AAA-AAA	0.00	999	1.000	1.000	0.0564
203876_s_at	7.0	37.3	2.42	5.35	AAA-AAA	0.00	999	1.000	1.000	0.0232
1555144_at	12.0	63.9	2.42	5.34	AAA-AAA	0.00	999	1.000	1.000	0.0873
217245_at	3.6	19.2	2.42	5.34	AAA-AAA	0.00	999	1.000	1.000	0.0995
236516_at	100.6	18.9	-2.42	-5.34	AAA-AAA	0.00	999	1.000	1.000	0.0649
206984_s_at	5.2	27.9	2.42	5.33	AAA-AAA	0.00	999	1.000	1.000	0.1726
239126_at	8.1	43.2	2.41	5.33	AAA-AMA	0.00	999	1.000	1.000	0.0578
227429_at	63.6	11.9	-2.41	-5.33	AAA-AAA	0.00	999	1.000	1.000	0.0005
230483_at	44.3	236.4	2.41	5.33	AAA-AAA	0.00	999	1.000	1.000	0.0450
240433_x_at	25.7	4.8	-2.41	-5.32	AAA-AAA	0.00	999	1.000	1.000	0.0504
221092_at	20.1	3.8	-2.41	-5.32	AAA-AAA	0.00	999	1.000	1.000	0.1731
217262_s_at	12.0	63.7	2.41	5.32	AAA-AAA	0.00	999	1.000	1.000	0.0590

216807_at	85.6	16.1	-2.41	-5.32	AAA-AAA	0.00	999	1.000	1.000	0.0268
239663_x_at	8.8	46.8	2.41	5.31	AAA-AAA	0.00	999	1.000	1.000	0.0248
239371_at	6.5	34.4	2.41	5.31	AAA-AMA	0.00	999	1.000	1.000	0.0071
210264_at	21.6	114.6	2.41	5.31	AAA-AAA	0.00	999	1.000	1.000	0.0010
238641_at	13.6	72.3	2.41	5.31	AAA-AAA	0.00	999	1.000	1.000	0.0368
240855_at	5.3	28.0	2.41	5.30	AAA-AAA	0.00	999	1.000	1.000	0.0372
220270_at	34.7	6.5	-2.41	-5.30	AAA-AAA	0.00	999	1.000	1.000	0.1124
1569728_at	11.9	63.3	2.41	5.30	AAA-AAA	0.00	999	1.000	1.000	0.1697
1553391_at	33.6	6.4	-2.41	-5.30	AAA-AAA	0.00	999	1.000	1.000	0.2343
232774_x_at	2.9	15.1	2.41	5.30	AAA-AAA	0.00	999	1.000	1.000	0.0326
220485_s_at	29.0	5.5	-2.40	-5.28	AAA-AAA	0.00	999	1.000	1.000	0.0393
209821_at	18.6	3.5	-2.40	-5.28	AAA-AAA	0.00	999	1.000	1.000	0.0587
239806_at	21.7	4.1	-2.40	-5.28	AAA-AAA	0.00	999	1.000	1.000	0.2037
1555504_at	16.1	3.0	-2.40	-5.28	AAA-AAA	0.00	999	1.000	1.000	0.1024
237492_at	11.4	2.2	-2.40	-5.27	AAA-AAA	0.00	999	1.000	1.000	0.1157
244121_at	40.6	7.7	-2.40	-5.27	PPA-AAA	0.00	999	1.000	1.000	0.0972
1567361_at	28.1	5.3	-2.40	-5.27	AAA-AAA	0.00	999	1.000	1.000	0.1604
1553915_at	4.8	25.5	2.40	5.27	AAA-AAA	0.00	999	1.000	1.000	0.1446
243679_at	7.6	40.0	2.40	5.27	AAA-AAA	0.00	999	1.000	1.000	0.0493
236737_at	10.7	56.4	2.40	5.27	AAA-AAA	0.00	999	1.000	1.000	0.0141
216219_at	105.0	20.0	-2.40	-5.26	PAA-AAA	0.00	999	1.000	1.000	0.0480
1552898_a_at	36.5	6.9	-2.39	-5.25	AAA-AAA	0.00	999	1.000	1.000	0.1081
244853_at	10.8	56.6	2.39	5.25	AAA-AAA	0.00	999	1.000	1.000	0.0880
201867_s_at	12.2	63.8	2.39	5.24	AAA-AAA	0.00	999	1.000	1.000	0.0881
1556492_a_at	6.8	35.4	2.39	5.23	AAA-AAA	0.00	999	1.000	1.000	0.1119
240059_at	3.3	17.4	2.39	5.23	AAA-AAA	0.00	999	1.000	1.000	0.1791
220484_at	4.5	23.7	2.39	5.23	AAA-AAA	0.00	999	1.000	1.000	0.0342
232941_at	29.5	5.6	-2.39	-5.23	AAA-AAA	0.00	999	1.000	1.000	0.0420
243126_x_at	18.3	3.5	-2.39	-5.22	AAA-AAA	0.00	999	1.000	1.000	0.2745
206620_at	14.5	75.7	2.39	5.22	AAA-AAA	0.00	999	1.000	1.000	0.0656
235548_at	30.4	5.8	-2.39	-5.22	AAA-AAA	0.00	999	1.000	1.000	0.0289
1561064_a_at	5.7	29.7	2.38	5.22	AAA-AAA	0.00	999	1.000	1.000	0.0777
213866_at	8.3	43.1	2.38	5.22	AAA-AAA	0.00	999	1.000	1.000	0.0090
211057_at	58.6	11.2	-2.38	-5.22	AAA-AAA	0.00	999	1.000	1.000	0.1376
214642_x_at	47.6	9.1	-2.38	-5.22	AAA-AAA	0.00	999	1.000	1.000	0.0010
1569235_a_at	6.3	32.9	2.38	5.21	AAA-AAA	0.00	999	1.000	1.000	0.0375
243379_at	3.7	19.2	2.38	5.21	AAA-AAA	0.00	999	1.000	1.000	0.0097
234856_at	3.6	18.8	2.38	5.20	AAA-AAA	0.00	999	1.000	1.000	0.0009
242441_at	7.4	38.3	2.38	5.20	AAA-AAA	0.00	999	1.000	1.000	0.1260
241394_at	4.7	24.6	2.38	5.19	AAA-AAA	0.00	999	1.000	1.000	0.0003
1561657_at	6.2	31.9	2.38	5.19	AAA-AAA	0.00	999	1.000	1.000	0.0006
234150_at	2.9	15.2	2.38	5.19	AAA-AAA	0.00	999	1.000	1.000	0.0903

1553311_at	101.1	19.5	-2.37	-5.18	APP-APA	0.00	999	1.000	1.000	0.0932
1565595_at	9.8	51.0	2.37	5.18	AAA-AAA	0.00	999	1.000	1.000	0.0138
243831_at	15.7	81.5	2.37	5.18	AAA-AAA	0.00	999	1.000	1.000	0.0845
243884_at	8.5	43.8	2.37	5.17	AAA-AAA	0.00	999	1.000	1.000	0.0044
220652_at	4.1	21.3	2.37	5.17	AAA-AAA	0.00	999	1.000	1.000	0.2049
206998_x_at	20.4	105.7	2.37	5.17	AAA-AAA	0.00	999	1.000	1.000	0.0069
229896_at	43.0	8.3	-2.37	-5.17	AAA-AAA	0.00	999	1.000	1.000	0.1697
240965_at	17.7	3.4	-2.37	-5.17	AAA-AAA	0.00	999	1.000	1.000	0.0999
1561212_at	6.5	33.7	2.37	5.17	AAA-AAA	0.00	999	1.000	1.000	0.0851
242623_x_at	4.1	21.0	2.37	5.17	AAA-AAA	0.00	999	1.000	1.000	0.2374
238830_at	77.6	15.0	-2.37	-5.17	AAA-AAA	0.00	999	1.000	1.000	0.0001
228678_at	50.2	9.8	-2.36	-5.15	AAA-AAA	0.00	999	1.000	1.000	0.1760
1563498_s_at	1.9	9.5	2.36	5.14	AAA-AAA	0.00	999	1.000	1.000	0.2375
1568711_a_at	8.1	1.6	-2.36	-5.14	AAA-AAA	0.00	999	1.000	1.000	0.1054
1556272_a_at	7.0	35.9	2.36	5.15	AAA-AAA	0.00	999	1.000	1.000	0.1370
213819_s_at	97.6	19.0	-2.36	-5.14	AAA-AAA	0.00	999	1.000	1.000	0.0077
205430_at	17.0	3.3	-2.36	-5.14	AAA-AAA	0.00	999	1.000	1.000	0.0475
1563787_a_at	12.2	2.4	-2.36	-5.13	AAA-AAA	0.00	999	1.000	1.000	0.1994
213779_at	29.2	150.0	2.36	5.13	AAA-AAA	0.00	999	1.000	1.000	0.0093
234413_at	41.4	8.1	-2.36	-5.13	AAA-AAA	0.00	999	1.000	1.000	0.0460
237997_s_at	44.3	8.6	-2.36	-5.13	AAA-AAA	0.00	999	1.000	1.000	0.0858
241909_at	3.2	16.4	2.36	5.12	AAA-AAA	0.00	999	1.000	1.000	0.0402
1569954_at	5.8	29.5	2.35	5.11	AAA-AAA	0.00	999	1.000	1.000	0.1091
204959_at	3.1	15.9	2.35	5.11	AAA-AAA	0.00	999	1.000	1.000	0.0116
226256_at	12.2	62.4	2.35	5.11	AAA-AAA	0.00	999	1.000	1.000	0.0504
231490_at	5.4	27.7	2.35	5.11	AAA-AAA	0.00	999	1.000	1.000	0.0304
242296_x_at	2.6	13.4	2.35	5.10	AAA-AAA	0.00	999	1.000	1.000	0.0583
239706_x_at	21.0	107.0	2.35	5.10	AAA-AAA	0.00	999	1.000	1.000	0.0188
1569787_at	10.1	51.3	2.35	5.10	AAA-AAA	0.00	999	1.000	1.000	0.0976
211161_s_at	2.3	11.6	2.35	5.10	AAA-AAA	0.00	999	1.000	1.000	0.0958
233184_at	4.0	20.4	2.35	5.10	AAA-AAA	0.00	999	1.000	1.000	0.1732
239696_at	69.1	13.6	-2.35	-5.09	AAA-AAA	0.00	999	1.000	1.000	0.0756
228589_at	8.4	42.9	2.35	5.08	AAA-AAA	0.00	999	1.000	1.000	0.0275
208028_s_at	47.7	9.4	-2.35	-5.08	AAA-AAA	0.00	999	1.000	1.000	0.0423
1560523_at	3.0	15.0	2.34	5.07	AAA-AAA	0.00	999	1.000	1.000	0.0701
1561511_at	8.6	1.7	-2.34	-5.06	AAA-AAA	0.00	999	1.000	1.000	0.0643
206227_at	13.2	66.9	2.34	5.06	AAA-AAA	0.00	999	1.000	1.000	0.0058
234203_at	9.3	47.1	2.34	5.06	AAA-AAA	0.00	999	1.000	1.000	0.1215
1555628_a_at	26.1	132.1	2.34	5.06	AAA-AAA	0.00	999	1.000	1.000	0.0438
1555580_at	7.6	38.4	2.34	5.06	AAA-AAA	0.00	999	1.000	1.000	0.1454
243977_at	79.0	15.6	-2.34	-5.06	AAA-AAA	0.00	999	1.000	1.000	0.0221
212883_at	47.7	9.4	-2.34	-5.06	AAA-AAA	0.00	999	1.000	1.000	0.0776

235328_at	14.7	74.1	2.34	5.06	AAA-AAA	0.00	999	1.000	1.000	0.0105
231582_at	13.0	66.0	2.34	5.06	AAA-AAA	0.00	999	1.000	1.000	0.1056
1568601_at	11.8	59.5	2.34	5.05	AAA-AAA	0.00	999	1.000	1.000	0.0142
230891_at	25.6	5.1	-2.34	-5.05	PPA-AAA	0.00	999	1.000	1.000	0.1652
1558592_at	1.8	9.3	2.34	5.05	AAA-AAA	0.00	999	1.000	1.000	0.0863
230540_at	3.4	17.0	2.34	5.05	AAA-AAA	0.00	999	1.000	1.000	0.1582
241320_at	20.7	4.1	-2.33	-5.04	AAA-AAA	0.00	999	1.000	1.000	0.1014
240144_at	3.3	16.7	2.33	5.04	AAA-AAA	0.00	999	1.000	1.000	0.0751
1552973_at	4.4	22.2	2.33	5.04	AAA-AAA	0.00	999	1.000	1.000	0.0526
243608_at	6.8	34.3	2.33	5.04	AAA-AAA	0.00	999	1.000	1.000	0.0081
238751_at	13.3	67.0	2.33	5.04	AAA-AAA	0.00	999	1.000	1.000	0.2170
229901_at	57.5	11.4	-2.33	-5.04	AAA-AAA	0.00	999	1.000	1.000	0.1774
239178_at	9.7	1.9	-2.33	-5.04	AAA-AAA	0.00	999	1.000	1.000	0.0812
1566805_at	9.8	1.9	-2.33	-5.04	AAA-AAA	0.00	999	1.000	1.000	0.1139
1563263_at	3.2	16.1	2.33	5.03	AAA-AAA	0.00	999	1.000	1.000	0.0444
1568871_at	4.7	23.5	2.33	5.03	AAA-AAA	0.00	999	1.000	1.000	0.2092
217572_at	5.3	26.8	2.33	5.03	AAA-AAA	0.00	999	1.000	1.000	0.0157
237913_at	9.8	49.4	2.33	5.03	AAA-AAA	0.00	999	1.000	1.000	0.1002
243625_at	2.8	14.1	2.33	5.02	AAA-AAA	0.00	999	1.000	1.000	0.0411
204160_s_at	43.3	8.6	-2.33	-5.02	AAA-AAA	0.00	999	1.000	1.000	0.0112
234455_at	9.1	45.6	2.33	5.02	AAA-AAA	0.00	999	1.000	1.000	0.0869
233223_at	19.7	98.8	2.33	5.02	AAA-AAM	0.00	999	1.000	1.000	0.0274
236586_at	34.4	6.9	-2.33	-5.02	AAA-AAA	0.00	999	1.000	1.000	0.2052
241971_at	3.3	16.3	2.33	5.02	AAA-AAA	0.00	999	1.000	1.000	0.1198
1561401_at	6.3	31.4	2.33	5.02	AAA-AAA	0.00	999	1.000	1.000	0.0512
226766_at	3.7	18.5	2.33	5.01	AAA-AAA	0.00	999	1.000	1.000	0.0629
1558945_s_at	18.3	3.7	-2.33	-5.01	AAP-AAA	0.00	999	1.000	1.000	0.1699
220930_s_at	24.1	4.8	-2.32	-5.00	AAA-AAA	0.00	999	1.000	1.000	0.0341
219768_at	4.5	22.3	2.32	5.01	AAA-AAA	0.00	999	1.000	1.000	0.0664
234138_at	10.6	53.0	2.32	5.00	AAA-AAA	0.00	999	1.000	1.000	0.0240
237591_at	16.5	82.5	2.32	5.00	AAA-AAA	0.00	999	1.000	1.000	0.1086
213903_s_at	12.8	64.1	2.32	5.00	AAA-AAA	0.00	999	1.000	1.000	0.0001
237598_at	10.2	50.9	2.32	5.00	AAA-AAA	0.00	999	1.000	1.000	0.0474
1554121_at	4.0	19.9	2.32	4.99	AAA-AAA	0.00	999	1.000	1.000	0.1732
206658_at	8.7	43.5	2.32	4.99	AAA-AAA	0.00	999	1.000	1.000	0.1289
1558595_at	30.6	6.1	-2.32	-4.98	PAA-AAA	0.00	999	1.000	1.000	0.0647
234913_at	41.0	8.2	-2.32	-4.98	AAA-AAA	0.00	999	1.000	1.000	0.0137
1556410_a_at	5.2	25.8	2.32	4.98	AAA-AAA	0.00	999	1.000	1.000	0.0416
206841_at	1.9	9.4	2.32	4.98	AAA-AAA	0.00	999	1.000	1.000	0.0739
1556653_at	8.5	42.0	2.31	4.97	AAA-AAA	0.00	999	1.000	1.000	0.1577
221910_at	36.9	7.4	-2.31	-4.97	AAA-AAA	0.00	999	1.000	1.000	0.0064
1558697_a_at	53.1	10.7	-2.31	-4.97	AAA-AAA	0.00	999	1.000	1.000	0.0035

1562030_at	41.1	204.4	2.31	4.97	AAA-AAA	0.00	999	1.000	1.000	0.0297
211821_x_at	4.6	22.9	2.31	4.97	AAA-AAA	0.00	999	1.000	1.000	0.0511
222326_at	4.1	20.2	2.31	4.97	AAA-AAA	0.00	999	1.000	1.000	0.0108
240408_at	4.7	23.1	2.31	4.96	AAA-AAA	0.00	999	1.000	1.000	0.0438
220849_at	6.7	33.1	2.31	4.96	AAA-AAA	0.00	999	1.000	1.000	0.0840
234747_at	24.8	5.0	-2.31	-4.96	AAA-AAA	0.00	999	1.000	1.000	0.0117
1552269_at	11.6	57.4	2.31	4.95	AAA-AAA	0.00	999	1.000	1.000	0.0754
210133_at	12.7	62.8	2.31	4.95	AAA-AAA	0.00	999	1.000	1.000	0.0560
241010_x_at	12.0	59.5	2.31	4.95	AAA-AAA	0.00	999	1.000	1.000	0.0596
220309_at	7.7	37.9	2.31	4.95	AAA-AAA	0.00	999	1.000	1.000	0.0079
210516_at	16.9	3.4	-2.31	-4.94	AAA-AAA	0.00	999	1.000	1.000	0.0512
237901_at	9.6	47.5	2.31	4.94	AAA-AMA	0.00	999	1.000	1.000	0.1667
1558336_at	35.5	7.2	-2.30	-4.94	AAA-AAA	0.00	999	1.000	1.000	0.1660
231091_x_at	8.8	43.5	2.30	4.94	AAA-AAA	0.00	999	1.000	1.000	0.0575
224237_at	36.6	7.4	-2.30	-4.94	PAA-AAA	0.00	999	1.000	1.000	0.0687
204803_s_at	66.0	13.4	-2.30	-4.93	AAA-AAA	0.00	999	1.000	1.000	0.0437
233434_at	5.6	27.7	2.30	4.93	AAA-AAA	0.00	999	1.000	1.000	0.0847
229373_at	11.9	58.7	2.30	4.94	AAA-AAA	0.00	999	1.000	1.000	0.0007
207442_at	869.0	176.2	-2.30	-4.93	PPP-PAP	0.00	999	1.000	1.000	0.0497
1569832_at	50.0	10.1	-2.30	-4.93	AAA-AAA	0.00	999	1.000	1.000	0.2038
219607_s_at	9.6	2.0	-2.30	-4.93	AAA-AAA	0.00	999	1.000	1.000	0.1199
242933_at	117.3	23.8	-2.30	-4.93	AAA-AAA	0.00	999	1.000	1.000	0.0001
1557876_at	10.5	2.1	-2.30	-4.92	AAA-AAA	0.00	999	1.000	1.000	0.0104
207600_at	53.7	10.9	-2.30	-4.92	AAA-AAA	0.00	999	1.000	1.000	0.0600
1559653_at	24.5	5.0	-2.30	-4.92	AAA-AAA	0.00	999	1.000	1.000	0.1051
207846_at	4.2	20.5	2.30	4.92	AAA-AAA	0.00	999	1.000	1.000	0.0039
1552368_at	17.3	3.5	-2.30	-4.91	AAA-AAA	0.00	999	1.000	1.000	0.0786
220781_at	6.6	32.5	2.30	4.92	AAA-AAA	0.00	999	1.000	1.000	0.0184
1569231_x_at	5.5	27.2	2.30	4.91	AAA-AAA	0.00	999	1.000	1.000	0.1198
1557481_a_at	20.4	4.2	-2.30	-4.91	AAA-AAA	0.00	999	1.000	1.000	0.0339
206664_at	3.8	18.8	2.30	4.91	AAA-AAA	0.00	999	1.000	1.000	0.1953
240336_at	43.1	8.8	-2.29	-4.91	AAA-AAA	0.00	999	1.000	1.000	0.0633
1559507_at	42.6	8.7	-2.29	-4.90	AAA-AAA	0.00	999	1.000	1.000	0.0497
207523_at	7.7	37.8	2.29	4.90	AAA-AAA	0.00	999	1.000	1.000	0.0530
237697_at	12.2	2.5	-2.29	-4.90	AAA-AAA	0.00	999	1.000	1.000	0.0431
235758_at	10.2	49.9	2.29	4.91	AAA-AAA	0.00	999	1.000	1.000	0.0315
236748_at	29.4	6.0	-2.29	-4.91	AAA-AAA	0.00	999	1.000	1.000	0.0098
235892_at	4.5	22.1	2.29	4.90	AAA-AAA	0.00	999	1.000	1.000	0.0029
210294_at	15.5	76.0	2.29	4.89	AAA-AAA	0.00	999	1.000	1.000	0.0004
232618_at	21.4	4.4	-2.29	-4.89	AAA-AAA	0.00	999	1.000	1.000	0.2880
217151_at	17.1	3.5	-2.29	-4.89	AAA-AAA	0.00	999	1.000	1.000	0.0726
231663_s_at	7.2	35.3	2.29	4.88	AAA-AAA	0.00	999	1.000	1.000	0.0052

1561956_at	18.7	3.8	-2.29	-4.88	AAA-AAA	0.00	999	1.000	1.000	0.1096
243763_x_at	9.4	45.7	2.29	4.88	AAA-AAA	0.00	999	1.000	1.000	0.0522
1569188_s_at	94.6	19.4	-2.29	-4.88	AAA-AAA	0.00	999	1.000	1.000	0.1570
1569656_at	6.0	29.1	2.29	4.88	AAA-AAA	0.00	999	1.000	1.000	0.0949
215303_at	31.6	6.5	-2.29	-4.88	AAA-AAA	0.00	999	1.000	1.000	0.1597
1553739_at	6.9	33.4	2.29	4.87	AAA-AAA	0.00	999	1.000	1.000	0.0483
1553544_at	75.2	15.4	-2.29	-4.87	AAA-AAA	0.00	999	1.000	1.000	0.0213
1553247_a_at	3.7	17.8	2.29	4.88	AAA-AAA	0.00	999	1.000	1.000	0.1149
238936_at	7.3	35.6	2.28	4.87	AAA-AAA	0.00	999	1.000	1.000	0.0951
235983_at	60.0	12.3	-2.28	-4.86	AAA-AAA	0.00	999	1.000	1.000	0.1395
236391_at	4.9	23.9	2.28	4.86	AAA-AAA	0.00	999	1.000	1.000	0.0736
205569_at	131.3	27.0	-2.28	-4.86	PPM-AAA	0.00	999	1.000	1.000	0.1133
1562223_at	10.3	2.1	-2.28	-4.86	AAA-AAA	0.00	999	1.000	1.000	0.1877
212701_at	58.0	11.9	-2.28	-4.86	AAA-AAA	0.00	999	1.000	1.000	0.0028
1569332_at	3.0	14.5	2.28	4.85	AAA-AAA	0.00	999	1.000	1.000	0.0520
231717_s_at	3.4	16.6	2.28	4.85	AAA-AAA	0.00	999	1.000	1.000	0.0094
240275_at	4.0	19.2	2.28	4.85	AAA-AAA	0.00	999	1.000	1.000	0.2704
1557840_at	15.7	75.8	2.28	4.85	AAA-AAA	0.00	999	1.000	1.000	0.0446
1554730_at	110.9	22.9	-2.28	-4.84	AAM-AAP	0.00	999	1.000	1.000	0.1397
1562997_a_at	11.4	2.4	-2.28	-4.84	AAA-AAA	0.00	999	1.000	1.000	0.1262
234410_at	19.1	3.9	-2.28	-4.84	AAA-AAA	0.00	999	1.000	1.000	0.1335
210577_at	7.9	38.0	2.27	4.84	AAA-AAA	0.00	999	1.000	1.000	0.0083
215625_at	54.3	11.2	-2.27	-4.83	APA-AAA	0.00	999	1.000	1.000	0.1178
1558862_at	43.9	9.1	-2.27	-4.83	AAA-AAA	0.00	999	1.000	1.000	0.0194
215763_at	2.9	13.9	2.27	4.83	AAA-AAA	0.00	999	1.000	1.000	0.0332
233868_x_at	361.0	74.8	-2.27	-4.83	AAA-AAA	0.00	999	1.000	1.000	0.1209
1560395_at	35.4	7.3	-2.27	-4.82	PPA-AAA	0.00	999	1.000	1.000	0.1447
239674_at	6.8	32.7	2.27	4.82	AAA-AAA	0.00	999	1.000	1.000	0.0029
241061_at	13.2	2.7	-2.27	-4.83	AAA-AAA	0.00	999	1.000	1.000	0.0792
228198_s_at	81.8	17.0	-2.27	-4.82	AAA-AAA	0.00	999	1.000	1.000	0.1227
237978_at	3.0	14.6	2.27	4.82	AAA-AAA	0.00	999	1.000	1.000	0.0779
219872_at	68.0	14.1	-2.27	-4.82	PPA-AAA	0.00	999	1.000	1.000	0.0564
215098_at	28.5	137.2	2.27	4.82	AAA-AAA	0.00	999	1.000	1.000	0.0525
217057_s_at	12.5	60.3	2.27	4.82	AAA-AAA	0.00	999	1.000	1.000	0.0578
1553071_a_at	38.6	8.0	-2.27	-4.81	AAA-AAA	0.00	999	1.000	1.000	0.0358
1561132_at	6.0	28.8	2.27	4.81	AAA-AAA	0.00	999	1.000	1.000	0.0633
221684_s_at	12.2	58.8	2.27	4.81	AAA-AAA	0.00	999	1.000	1.000	0.0872
211178_s_at	17.8	85.8	2.27	4.81	AAA-AAA	0.00	999	1.000	1.000	0.0041
1556451_at	18.3	88.1	2.27	4.81	AAA-AAA	0.00	999	1.000	1.000	0.0212
243564_at	4.3	20.5	2.27	4.81	AAA-AAA	0.00	999	1.000	1.000	0.0460
211592_s_at	11.0	2.3	-2.27	-4.81	AAA-AAA	0.00	999	1.000	1.000	0.0384
217013_at	43.2	9.0	-2.26	-4.80	AAA-AAA	0.00	999	1.000	1.000	0.1598

221724_s_at	5.8	27.8	2.26	4.80	AAA-AAA	0.00	999	1.000	1.000	0.0606
209201_x_at	37.6	180.3	2.26	4.80	AAA-PPP	0.00	999	1.000	1.000	0.0293
232334_at	30.0	6.3	-2.26	-4.80	PAA-AAA	0.00	999	1.000	1.000	0.0886
241160_at	4.1	19.6	2.26	4.79	AAA-AAA	0.00	999	1.000	1.000	0.0639
222961_at	8.3	39.9	2.26	4.80	AAA-AAA	0.00	999	1.000	1.000	0.1026
241987_x_at	13.3	2.8	-2.26	-4.79	AAA-AAA	0.00	999	1.000	1.000	0.1031
216948_at	9.7	46.4	2.26	4.79	AAA-AAA	0.00	999	1.000	1.000	0.1006
221885_at	5.4	25.7	2.26	4.79	AAA-AAA	0.00	999	1.000	1.000	0.0093
1563057_at	34.8	7.3	-2.26	-4.78	AAA-AAA	0.00	999	1.000	1.000	0.0152
241106_at	24.1	115.1	2.26	4.78	AAA-AAA	0.00	999	1.000	1.000	0.0187
236522_at	8.6	41.0	2.26	4.78	AAA-AAA	0.00	999	1.000	1.000	0.1521
219725_at	70.6	14.8	-2.26	-4.78	AAA-AAA	0.00	999	1.000	1.000	0.0185
211305_x_at	11.2	53.3	2.26	4.78	AAA-AAA	0.00	999	1.000	1.000	0.0119
208346_at	49.3	10.3	-2.26	-4.78	AAA-AAA	0.00	999	1.000	1.000	0.0852
224270_at	12.1	57.8	2.26	4.78	AAA-AAA	0.00	999	1.000	1.000	0.0686
205962_at	67.7	14.2	-2.25	-4.77	AAA-AAA	0.00	999	1.000	1.000	0.1798
237582_at	26.1	5.5	-2.25	-4.77	AAA-AAA	0.00	999	1.000	1.000	0.1304
229686_at	9.1	43.2	2.25	4.77	AAA-AAA	0.00	999	1.000	1.000	0.0305
1557642_at	4.5	21.4	2.25	4.77	AAA-AAA	0.00	999	1.000	1.000	0.0146
224142_s_at	4.6	22.0	2.25	4.77	AAA-AAA	0.00	999	1.000	1.000	0.0874
1564367_at	64.1	13.5	-2.25	-4.76	AAA-AAA	0.00	999	1.000	1.000	0.0971
214199_at	8.7	41.6	2.25	4.76	AAA-AAA	0.00	999	1.000	1.000	0.0661
1559443_s_at	97.7	20.5	-2.25	-4.76	AAA-AAA	0.00	999	1.000	1.000	0.0058
211736_at	33.3	7.0	-2.25	-4.75	AAA-AAA	0.00	999	1.000	1.000	0.1481
208352_x_at	10.6	50.2	2.25	4.75	AAA-AAA	0.00	999	1.000	1.000	0.0788
1569609_at	3.4	16.1	2.25	4.75	AAA-AAA	0.00	999	1.000	1.000	0.2220
239730_at	23.9	113.4	2.25	4.75	AAA-AAA	0.00	999	1.000	1.000	0.1847
1554816_at	2.7	12.7	2.25	4.75	AAA-AAA	0.00	999	1.000	1.000	0.2686
243150_at	4.9	23.1	2.25	4.75	AAA-AAA	0.00	999	1.000	1.000	0.0441
56748_at	49.3	10.4	-2.25	-4.75	AAA-AAA	0.00	999	1.000	1.000	0.0384
211499_s_at	36.8	174.5	2.25	4.75	AAA-AAA	0.00	999	1.000	1.000	0.0554
224257_s_at	7.4	35.0	2.25	4.74	AAA-AAA	0.00	999	1.000	1.000	0.0536
237007_at	1.7	8.3	2.25	4.74	AAA-AAA	0.00	999	1.000	1.000	0.1158
235256_s_at	31.1	6.6	-2.24	-4.74	AAA-AAA	0.00	999	1.000	1.000	0.0013
222101_s_at	86.2	18.2	-2.24	-4.74	AAA-AAA	0.00	999	1.000	1.000	0.0023
224165_s_at	8.7	41.3	2.24	4.74	AAA-AAA	0.00	999	1.000	1.000	0.2099
1569486_at	23.5	5.0	-2.24	-4.73	AAA-AAA	0.00	999	1.000	1.000	0.0520
1561230_at	2.2	10.4	2.24	4.73	AAA-AAA	0.00	999	1.000	1.000	0.1214
207546_at	6.6	31.2	2.24	4.73	AAA-AAA	0.00	999	1.000	1.000	0.0421
244501_at	8.3	39.4	2.24	4.73	AAA-AAA	0.00	999	1.000	1.000	0.0180
1569810_at	1.3	5.9	2.24	4.72	AAA-AAA	0.00	999	1.000	1.000	0.0073
208287_at	19.8	93.5	2.24	4.72	AAA-AAA	0.00	999	1.000	1.000	0.0226

235683_at	8.0	37.9	2.24	4.72	AAA-AAA	0.00	999	1.000	1.000	0.1982
1566163_at	9.8	46.3	2.24	4.72	AAA-AAA	0.00	999	1.000	1.000	0.0396
1561519_at	25.4	5.4	-2.24	-4.72	AAA-AAA	0.00	999	1.000	1.000	0.1675
238463_at	21.9	103.1	2.24	4.72	AAA-AAA	0.00	999	1.000	1.000	0.1852
1559559_at	3.6	16.9	2.24	4.71	AAA-AAA	0.00	999	1.000	1.000	0.0910
206460_at	9.6	45.1	2.24	4.71	AAA-AAA	0.00	999	1.000	1.000	0.0081
239092_at	4.0	18.6	2.23	4.70	AAA-AAA	0.00	999	1.000	1.000	0.1545
231711_at	19.8	4.2	-2.23	-4.71	AAA-AAA	0.00	999	1.000	1.000	0.1346
236299_at	59.3	12.6	-2.23	-4.70	AAA-AAA	0.00	999	1.000	1.000	0.1392
1556847_s_at	3.5	16.6	2.23	4.69	AAA-AAA	0.00	999	1.000	1.000	0.1136
244499_at	53.6	11.4	-2.23	-4.69	AAA-AAA	0.00	999	1.000	1.000	0.0641
222135_at	4.4	20.6	2.23	4.69	AAA-AAA	0.00	999	1.000	1.000	0.1002
238133_at	51.8	11.1	-2.23	-4.68	AAA-AAA	0.00	999	1.000	1.000	0.1071
1557037_a_at	40.7	8.7	-2.23	-4.68	AAA-AAA	0.00	999	1.000	1.000	0.2012
238687_x_at	8.5	39.8	2.23	4.68	AAA-AAA	0.00	999	1.000	1.000	0.0066
1559676_a_at	10.5	48.9	2.23	4.68	AAA-AAA	0.00	999	1.000	1.000	0.0390
232279_at	13.5	63.1	2.23	4.68	AAA-AAA	0.00	999	1.000	1.000	0.0401
242768_at	16.5	3.5	-2.23	-4.68	AAA-AAA	0.00	999	1.000	1.000	0.1301
210505_at	16.3	75.9	2.22	4.67	AAA-AAA	0.00	999	1.000	1.000	0.1473
236811_at	37.4	8.0	-2.22	-4.67	AAA-AAA	0.00	999	1.000	1.000	0.1235
240772_at	7.0	32.9	2.22	4.67	AAA-AAA	0.00	999	1.000	1.000	0.0360
243190_at	35.5	7.6	-2.22	-4.67	AAA-AAA	0.00	999	1.000	1.000	0.0300
1559514_at	40.1	8.6	-2.22	-4.67	AAA-AAA	0.00	999	1.000	1.000	0.0387
1568879_a_at	38.9	8.3	-2.22	-4.67	AAA-AAA	0.00	999	1.000	1.000	0.0113
233355_at	4.8	22.2	2.22	4.67	AAA-AAA	0.00	999	1.000	1.000	0.1005
1559433_at	7.5	34.9	2.22	4.66	AAA-AAA	0.00	999	1.000	1.000	0.0729
208518_s_at	16.8	78.5	2.22	4.66	AAA-AAA	0.00	999	1.000	1.000	0.0278
1559282_at	42.7	9.2	-2.22	-4.66	AAA-AAA	0.00	999	1.000	1.000	0.1800
211868_x_at	19.3	89.8	2.22	4.66	AAA-AAA	0.00	999	1.000	1.000	0.0024
236875_at	3.7	17.2	2.22	4.66	AAA-AAA	0.00	999	1.000	1.000	0.0224
1566093_at	55.2	11.9	-2.22	-4.65	AAA-AAA	0.00	999	1.000	1.000	0.1275
1558170_at	5.4	24.9	2.22	4.65	AAA-AAA	0.00	999	1.000	1.000	0.1363
235243_at	48.9	10.5	-2.22	-4.65	AAA-AAA	0.00	999	1.000	1.000	0.0100
232713_at	4.2	19.5	2.22	4.65	AAA-AAA	0.00	999	1.000	1.000	0.2045
242087_x_at	17.0	79.0	2.22	4.65	AAA-AAA	0.00	999	1.000	1.000	0.0237
242189_at	2.9	13.6	2.22	4.65	AAA-AAA	0.00	999	1.000	1.000	0.1033
1562303_at	9.3	43.0	2.22	4.65	AAA-AAA	0.00	999	1.000	1.000	0.1018
217333_at	41.2	8.9	-2.22	-4.65	AAA-AAA	0.00	999	1.000	1.000	0.0140
1554694_at	6.2	28.6	2.22	4.64	AAA-AAA	0.00	999	1.000	1.000	0.1366
237142_at	6.9	32.2	2.22	4.64	AAA-AAA	0.00	999	1.000	1.000	0.0629
1559469_s_at	12.5	57.9	2.21	4.64	AAA-AAA	0.00	999	1.000	1.000	0.0305
233293_at	7.1	33.0	2.21	4.64	AAA-AAA	0.00	999	1.000	1.000	0.1073

235814_at	7.2	33.5	2.21	4.64	AAA-AAA	0.00	999	1.000	1.000	0.0766
228502_at	1.2	5.5	2.21	4.64	AAA-AAA	0.00	999	1.000	1.000	0.0017
230631_s_at	3.8	17.7	2.21	4.64	AAA-AAA	0.00	999	1.000	1.000	0.2087
1560833_at	23.6	5.1	-2.21	-4.63	AAA-AAA	0.00	999	1.000	1.000	0.1402
1553007_a_at	15.6	3.4	-2.21	-4.63	AAA-AAA	0.00	999	1.000	1.000	0.1480
242491_at	8.3	38.5	2.21	4.63	AAA-AAA	0.00	999	1.000	1.000	0.1175
242286_at	29.5	6.4	-2.21	-4.62	AAA-AAA	0.00	999	1.000	1.000	0.1762
243297_at	8.9	41.2	2.21	4.62	AAA-AAA	0.00	999	1.000	1.000	0.0048
1554022_at	38.1	8.2	-2.21	-4.62	AAA-AAA	0.00	999	1.000	1.000	0.0749
223750_s_at	5.2	24.2	2.21	4.62	AAA-AAA	0.00	999	1.000	1.000	0.1197
217452_s_at	6.3	1.4	-2.21	-4.62	AAA-AAA	0.00	999	1.000	1.000	0.1526
241441_at	11.3	2.4	-2.21	-4.61	AAA-AAA	0.00	999	1.000	1.000	0.0185
1559711_at	78.8	17.1	-2.21	-4.62	AAA-AAA	0.00	999	1.000	1.000	0.0650
1561192_at	28.9	6.3	-2.21	-4.61	AAA-AAA	0.00	999	1.000	1.000	0.0202
1566630_at	16.8	77.6	2.20	4.61	AAA-AAA	0.00	999	1.000	1.000	0.0024
1557907_x_at	32.8	7.1	-2.20	-4.61	AAA-AAA	0.00	999	1.000	1.000	0.0286
240090_at	4.2	19.1	2.20	4.60	AAA-AAA	0.00	999	1.000	1.000	0.1038
1562319_at	49.6	10.8	-2.20	-4.60	AAA-AAA	0.00	999	1.000	1.000	0.2045
1554642_at	11.4	52.2	2.20	4.60	AAA-AAA	0.00	999	1.000	1.000	0.1468
1563808_at	19.4	89.3	2.20	4.60	AAA-AAA	0.00	999	1.000	1.000	0.0001
240509_s_at	12.3	2.7	-2.20	-4.60	AAA-AAA	0.00	999	1.000	1.000	0.1004
1559732_at	35.5	7.7	-2.20	-4.60	AAM-AAA	0.00	999	1.000	1.000	0.0227
238317_x_at	27.0	5.9	-2.20	-4.60	AAA-AAA	0.00	999	1.000	1.000	0.1062
237278_x_at	8.8	40.5	2.20	4.60	AAA-AAA	0.00	999	1.000	1.000	0.0782
1563371_at	13.0	2.8	-2.20	-4.59	AAA-AAA	0.00	999	1.000	1.000	0.1347
240226_at	7.5	34.6	2.20	4.59	AAA-AAA	0.00	999	1.000	1.000	0.0105
1553442_a_at	11.1	51.0	2.20	4.59	AAA-AAA	0.00	999	1.000	1.000	0.1330
233659_at	37.3	8.1	-2.20	-4.59	APA-AAA	0.00	999	1.000	1.000	0.0410
1563207_at	5.7	26.2	2.20	4.59	AAA-AAA	0.00	999	1.000	1.000	0.2106
1556627_at	9.3	42.7	2.20	4.59	AAA-AAA	0.00	999	1.000	1.000	0.0538
215047_at	4.1	18.9	2.20	4.59	AAA-AAA	0.00	999	1.000	1.000	0.2067
1559497_at	13.3	2.9	-2.20	-4.58	AAA-AAA	0.00	999	1.000	1.000	0.2774
242238_at	24.3	111.3	2.19	4.58	AAA-AAA	0.00	999	1.000	1.000	0.1075
243269_s_at	65.4	14.3	-2.19	-4.58	AAA-AAA	0.00	999	1.000	1.000	0.1036
237126_at	45.1	9.9	-2.19	-4.58	AAA-AAA	0.00	999	1.000	1.000	0.0566
1566428_at	4.3	19.9	2.19	4.57	AAA-AAA	0.00	999	1.000	1.000	0.1214
229950_s_at	3.0	13.7	2.19	4.57	AAA-AAA	0.00	999	1.000	1.000	0.1464
1563032_at	16.4	3.6	-2.19	-4.57	AAA-AAA	0.00	999	1.000	1.000	0.0721
1554393_a_at	70.0	15.3	-2.19	-4.57	AAA-AAA	0.00	999	1.000	1.000	0.1116
237848_at	12.5	56.9	2.19	4.56	AAA-AAA	0.00	999	1.000	1.000	0.0792
233598_at	32.8	7.2	-2.19	-4.56	AAA-AAA	0.00	999	1.000	1.000	0.0700
1556417_a_at	5.5	25.0	2.19	4.56	AAA-AAA	0.00	999	1.000	1.000	0.1086

233714_at	21.4	4.7	-2.19	-4.55	AAA-AAA	0.00	999	1.000	1.000	0.3337
1564828_at	17.9	81.3	2.19	4.55	AAA-AAA	0.00	999	1.000	1.000	0.0121
228057_at	27.4	6.0	-2.19	-4.55	AAA-AAA	0.00	999	1.000	1.000	0.0346
1561004_at	63.1	13.9	-2.19	-4.55	APA-AAP	0.00	999	1.000	1.000	0.1171
216965_x_at	90.6	19.9	-2.19	-4.55	AAA-AAA	0.00	999	1.000	1.000	0.1146
228784_at	36.6	8.1	-2.19	-4.55	AAA-AAA	0.00	999	1.000	1.000	0.0260
244608_at	20.5	4.5	-2.18	-4.55	AAA-AAA	0.00	999	1.000	1.000	0.1497
1559083_x_at	32.0	7.0	-2.18	-4.54	AAA-AAA	0.00	999	1.000	1.000	0.0095
1559152_at	7.5	34.1	2.18	4.55	AAA-AAA	0.00	999	1.000	1.000	0.0331
1566693_at	10.0	45.3	2.18	4.54	AAA-AAA	0.00	999	1.000	1.000	0.0792
230163_at	12.8	57.9	2.18	4.54	AAA-AAA	0.00	999	1.000	1.000	0.1713
237580_at	3.7	17.0	2.18	4.53	AAA-AAA	0.00	999	1.000	1.000	0.0773
1553191_at	5.2	23.5	2.18	4.53	AAA-AAA	0.00	999	1.000	1.000	0.2312
219385_at	128.3	28.3	-2.18	-4.53	AMA-AAA	0.00	999	1.000	1.000	0.0807
1569817_at	6.6	29.9	2.18	4.53	AAA-MAA	0.00	999	1.000	1.000	0.0904
229406_at	12.5	2.8	-2.18	-4.53	AAA-AAA	0.00	999	1.000	1.000	0.0003
1565684_at	61.7	13.6	-2.18	-4.53	AAA-AAA	0.00	999	1.000	1.000	0.0409
1554958_at	15.9	71.7	2.18	4.52	AAA-AAA	0.00	999	1.000	1.000	0.0555
205148_s_at	19.4	87.9	2.18	4.52	AAA-AAA	0.00	999	1.000	1.000	0.0185
209960_at	2.0	9.1	2.18	4.52	AAA-AAA	0.00	999	1.000	1.000	0.1533
1552993_at	11.8	2.6	-2.18	-4.52	AAA-AAA	0.00	999	1.000	1.000	0.0695
208010_s_at	16.9	3.7	-2.18	-4.52	AAA-AAA	0.00	999	1.000	1.000	0.1751
234124_at	11.6	52.5	2.18	4.52	AAA-AAA	0.00	999	1.000	1.000	0.1038
219396_s_at	25.7	116.2	2.18	4.52	AAA-AAA	0.00	999	1.000	1.000	0.0450
233057_at	72.9	16.1	-2.18	-4.52	AAA-AAA	0.00	999	1.000	1.000	0.0793
236627_at	9.9	44.8	2.18	4.52	AAA-AAA	0.00	999	1.000	1.000	0.1687
225668_at	10.8	48.9	2.18	4.52	AAA-AAA	0.00	999	1.000	1.000	0.0387
230789_at	26.7	5.9	-2.18	-4.52	AAA-AAA	0.00	999	1.000	1.000	0.0998
241288_at	6.2	27.9	2.17	4.51	AAA-AAA	0.00	999	1.000	1.000	0.0549
239488_at	16.0	3.6	-2.17	-4.51	AAA-AAA	0.00	999	1.000	1.000	0.0928
242948_x_at	21.7	4.8	-2.17	-4.51	AAA-AAA	0.00	999	1.000	1.000	0.0070
237232_at	7.3	33.1	2.17	4.51	AAA-AAM	0.00	999	1.000	1.000	0.2094
1562301_at	6.1	1.4	-2.17	-4.50	AAA-AAA	0.00	999	1.000	1.000	0.1560
215673_at	6.0	27.0	2.17	4.50	AAA-AAA	0.00	999	1.000	1.000	0.0274
212671_s_at	32.4	7.2	-2.17	-4.50	AAA-AAA	0.00	999	1.000	1.000	0.2164
242644_at	10.7	48.3	2.17	4.50	AAA-AAA	0.00	999	1.000	1.000	0.0611
242736_at	5.6	25.2	2.17	4.50	AAA-AAA	0.00	999	1.000	1.000	0.0137
1560051_at	10.3	46.2	2.17	4.50	AAA-AAA	0.00	999	1.000	1.000	0.1840
207579_at	36.0	8.0	-2.17	-4.50	AAA-AAA	0.00	999	1.000	1.000	0.1140
229006_at	31.7	7.1	-2.17	-4.50	AAA-AAA	0.00	999	1.000	1.000	0.0544
222259_s_at	33.9	7.5	-2.17	-4.49	AAP-AAA	0.00	999	1.000	1.000	0.2636
217218_at	54.1	12.0	-2.17	-4.50	AAA-AAA	0.00	999	1.000	1.000	0.0212

233954_at	6.8	30.5	2.17	4.50	AAA-AAA	0.00	999	1.000	1.000	0.0358
223582_at	40.4	9.0	-2.17	-4.50	AAA-AAA	0.00	999	1.000	1.000	0.0537
1560990_a_at	5.8	26.2	2.17	4.49	AAA-AAA	0.00	999	1.000	1.000	0.1813
1557576_at	65.2	14.5	-2.17	-4.49	AAA-AAA	0.00	999	1.000	1.000	0.1090
206948_at	12.2	54.7	2.17	4.49	AAA-AAA	0.00	999	1.000	1.000	0.1319
1564841_at	3.3	14.9	2.17	4.49	AAA-AAA	0.00	999	1.000	1.000	0.0062
1558827_a_at	40.0	8.9	-2.16	-4.48	AAA-AAA	0.00	999	1.000	1.000	0.0695
219274_at	36.5	8.2	-2.16	-4.48	AAA-AAA	0.00	999	1.000	1.000	0.2441
208098_at	8.2	36.6	2.16	4.48	AAA-AAA	0.00	999	1.000	1.000	0.1629
222886_at	11.0	49.2	2.16	4.48	AAA-AAA	0.00	999	1.000	1.000	0.1242
216889_s_at	14.4	64.4	2.16	4.47	AAA-AAA	0.00	999	1.000	1.000	0.1182
207516_at	57.3	12.8	-2.16	-4.48	AAA-AAA	0.00	999	1.000	1.000	0.1980
230827_at	4.7	20.9	2.16	4.47	AAA-AAA	0.00	999	1.000	1.000	0.0573
1563157_at	8.8	39.3	2.16	4.47	AAA-AAA	0.00	999	1.000	1.000	0.1170
1552745_at	51.9	11.6	-2.16	-4.47	AMA-AAA	0.00	999	1.000	1.000	0.0785
1564075_a_at	6.0	26.8	2.16	4.47	AAA-AAA	0.00	999	1.000	1.000	0.0154
210611_s_at	20.3	4.6	-2.16	-4.47	AAA-AAA	0.00	999	1.000	1.000	0.1001
1569391_at	3.3	14.9	2.16	4.47	AAA-AAA	0.00	999	1.000	1.000	0.1274
1558373_s_at	10.1	2.3	-2.16	-4.47	AAA-AAA	0.00	999	1.000	1.000	0.1765
1562520_at	28.5	6.4	-2.16	-4.47	AAA-AAA	0.00	999	1.000	1.000	0.0510
207050_at	60.5	13.6	-2.16	-4.46	AAA-AAA	0.00	999	1.000	1.000	0.1061
239776_at	9.9	2.2	-2.16	-4.46	AAA-AAA	0.00	999	1.000	1.000	0.0155
232820_s_at	57.5	12.9	-2.16	-4.46	AAA-AAA	0.00	999	1.000	1.000	0.0110
231161_x_at	17.2	76.8	2.16	4.46	AAA-AAA	0.00	999	1.000	1.000	0.0401
1566086_at	9.1	40.4	2.16	4.45	AAA-AAA	0.00	999	1.000	1.000	0.0932
1570031_at	4.3	19.1	2.15	4.45	AAA-AAA	0.00	999	1.000	1.000	0.1013
1568662_at	10.2	2.3	-2.15	-4.45	AAA-AAA	0.00	999	1.000	1.000	0.1026
223821_s_at	12.1	53.9	2.15	4.45	AAA-AAA	0.00	999	1.000	1.000	0.0008
238385_at	11.8	52.4	2.15	4.44	AAA-AAA	0.00	999	1.000	1.000	0.0128
238062_at	35.0	7.9	-2.15	-4.44	AAA-AAA	0.00	999	1.000	1.000	0.0325
237229_at	13.7	3.1	-2.15	-4.44	AAA-AAA	0.00	999	1.000	1.000	0.0943
228448_at	86.3	19.4	-2.15	-4.44	AMA-AAA	0.00	999	1.000	1.000	0.0723
222941_at	73.6	16.6	-2.15	-4.44	AAA-AAA	0.00	999	1.000	1.000	0.1237
1553494_at	1.9	8.3	2.15	4.44	AAA-AAA	0.00	999	1.000	1.000	0.2524
230140_at	9.9	43.8	2.15	4.44	AAA-AAA	0.00	999	1.000	1.000	0.0107
213931_at	21.4	94.8	2.15	4.43	AAA-AAA	0.00	999	1.000	1.000	0.0287
232929_at	20.3	90.2	2.15	4.44	AAA-AAA	0.00	999	1.000	1.000	0.0777
244260_at	4.8	21.1	2.15	4.43	AAA-AAA	0.00	999	1.000	1.000	0.0759
215244_at	104.8	23.7	-2.15	-4.43	AAA-AAA	0.00	999	1.000	1.000	0.0573
229098_s_at	160.3	36.2	-2.15	-4.43	AAA-AAA	0.00	999	1.000	1.000	0.0351
241030_at	13.3	3.0	-2.15	-4.43	AAA-AAA	0.00	999	1.000	1.000	0.1050
221368_at	6.7	29.6	2.15	4.43	AAA-AAA	0.00	999	1.000	1.000	0.0440

218410_s_at	113.4	25.6	-2.15	-4.43	AAA-AAA	0.00	999	1.000	1.000	0.0765
1565107_x_at	7.8	34.3	2.15	4.42	AAA-AAA	0.00	999	1.000	1.000	0.0849
1564469_at	7.7	1.7	-2.14	-4.42	AAA-AAA	0.00	999	1.000	1.000	0.1352
221345_at	13.7	60.4	2.14	4.42	AAA-AAA	0.00	999	1.000	1.000	0.1418
237486_at	27.1	6.1	-2.14	-4.42	AAA-AAA	0.00	999	1.000	1.000	0.0263
1570574_at	29.3	6.6	-2.14	-4.42	AAA-AAA	0.00	999	1.000	1.000	0.0397
1561345_at	9.1	40.2	2.14	4.42	AAA-AAA	0.00	999	1.000	1.000	0.0280
216414_at	21.4	4.8	-2.14	-4.42	AAA-AAA	0.00	999	1.000	1.000	0.0986
234640_x_at	22.0	5.0	-2.14	-4.42	AAA-AAA	0.00	999	1.000	1.000	0.1081
215168_at	29.4	6.7	-2.14	-4.42	PAA-AAA	0.00	999	1.000	1.000	0.1171
232747_at	3.1	13.5	2.14	4.41	AAA-AAA	0.00	999	1.000	1.000	0.0583
241151_at	3.2	14.2	2.14	4.41	AAA-AAA	0.00	999	1.000	1.000	0.1867
234211_at	59.7	13.5	-2.14	-4.41	AAA-AAA	0.00	999	1.000	1.000	0.0636
234104_at	11.6	51.0	2.14	4.41	AAA-AAA	0.00	999	1.000	1.000	0.0745
237086_at	32.3	7.3	-2.14	-4.41	AAA-AAA	0.00	999	1.000	1.000	0.1187
1568760_at	93.4	21.2	-2.14	-4.41	AAA-AAA	0.00	999	1.000	1.000	0.0762
1558990_at	8.0	35.1	2.14	4.41	AAA-AAA	0.00	999	1.000	1.000	0.1798
208183_at	44.2	10.0	-2.14	-4.41	AAA-AAA	0.00	999	1.000	1.000	0.0260
215185_at	13.2	58.1	2.14	4.41	AAA-AAA	0.00	999	1.000	1.000	0.1729
233047_at	4.8	21.0	2.14	4.41	AAA-AAA	0.00	999	1.000	1.000	0.0811
223929_s_at	10.8	47.6	2.14	4.40	AAA-AAA	0.00	999	1.000	1.000	0.0274
1565565_at	2.8	12.4	2.14	4.40	AAA-AAA	0.00	999	1.000	1.000	0.0211
1565924_a_at	10.6	2.4	-2.14	-4.40	AAA-AAA	0.00	999	1.000	1.000	0.1711
1552757_s_at	17.5	4.0	-2.14	-4.40	AAA-AAA	0.00	999	1.000	1.000	0.1907
238166_s_at	11.3	49.5	2.14	4.40	AAA-AAA	0.00	999	1.000	1.000	0.1947
242130_at	7.6	33.3	2.14	4.40	AAA-AAA	0.00	999	1.000	1.000	0.1986
234157_at	15.1	66.2	2.14	4.40	AAA-AAA	0.00	999	1.000	1.000	0.1032
238072_at	10.0	43.8	2.14	4.40	AAA-AAA	0.00	999	1.000	1.000	0.1393
216039_at	25.2	5.7	-2.14	-4.39	PAA-AAA	0.00	999	1.000	1.000	0.1153
243087_at	19.4	4.4	-2.14	-4.39	AAA-AAA	0.00	999	1.000	1.000	0.0251
235818_at	3.5	15.3	2.14	4.39	AAA-AAA	0.00	999	1.000	1.000	0.2188
1553409_at	23.0	5.2	-2.13	-4.39	AAA-AAA	0.00	999	1.000	1.000	0.0077
204575_s_at	11.2	49.2	2.13	4.39	AAA-AAA	0.00	999	1.000	1.000	0.0599
210055_at	3.6	15.8	2.13	4.39	AAA-AAA	0.00	999	1.000	1.000	0.1143
1554982_a_at	9.1	2.1	-2.13	-4.39	AAA-AAA	0.00	999	1.000	1.000	0.1240
206715_at	3.9	16.9	2.13	4.38	AAA-AAA	0.00	999	1.000	1.000	0.1223
234218_at	28.8	6.6	-2.13	-4.38	AAM-AAA	0.00	999	1.000	1.000	0.0166
227189_at	38.5	8.8	-2.13	-4.38	AAA-AAA	0.00	999	1.000	1.000	0.0817
222289_at	15.8	69.3	2.13	4.38	AAM-AMA	0.00	999	1.000	1.000	0.0583
244232_at	10.1	2.3	-2.13	-4.38	AAA-AAA	0.00	999	1.000	1.000	0.1673
203979_at	77.0	17.6	-2.13	-4.38	AAA-AAA	0.00	999	1.000	1.000	0.0425
224272_at	23.8	5.4	-2.13	-4.38	AAA-AAA	0.00	999	1.000	1.000	0.0391

215192_at	22.3	5.1	-2.13	-4.37	AAA-AAA	0.00	999	1.000	1.000	0.2437
220921_at	8.6	37.7	2.13	4.38	AAA-AAA	0.00	999	1.000	1.000	0.0284
202952_s_at	46.0	10.5	-2.13	-4.37	AAA-AAA	0.00	999	1.000	1.000	0.1350
240863_at	1.9	8.4	2.13	4.37	AAA-AAA	0.00	999	1.000	1.000	0.2109
1555722_at	56.9	13.0	-2.13	-4.37	AAA-AAA	0.00	999	1.000	1.000	0.1323
1562490_at	20.9	4.8	-2.13	-4.37	AAA-AAA	0.00	999	1.000	1.000	0.1318
224515_at	23.8	5.5	-2.13	-4.37	AAA-AAA	0.00	999	1.000	1.000	0.1584
1561702_at	75.6	17.3	-2.13	-4.37	MAA-AAA	0.00	999	1.000	1.000	0.1016
216165_at	22.4	5.1	-2.13	-4.37	AAA-AAA	0.00	999	1.000	1.000	0.0539
1553483_at	15.1	65.7	2.13	4.37	AAA-AAA	0.00	999	1.000	1.000	0.1151
1561741_at	61.9	14.2	-2.13	-4.37	AAA-AAA	0.00	999	1.000	1.000	0.1181
235560_at	38.3	8.8	-2.13	-4.37	AAA-AAA	0.00	999	1.000	1.000	0.0124
240514_at	6.1	26.7	2.13	4.36	AAA-AAA	0.00	999	1.000	1.000	0.0385
210484_s_at	37.6	8.6	-2.13	-4.36	AAA-AAA	0.00	999	1.000	1.000	0.0434
238382_x_at	7.7	33.5	2.13	4.36	AAA-AAA	0.00	999	1.000	1.000	0.0031
244142_at	5.7	24.8	2.12	4.36	AAA-AMA	0.00	999	1.000	1.000	0.2766
228339_at	47.1	10.8	-2.12	-4.36	AAA-AAA	0.00	999	1.000	1.000	0.1110
239822_at	3.0	13.1	2.12	4.35	AAA-AAA	0.00	999	1.000	1.000	0.1409
1569886_a_at	1.7	7.3	2.12	4.35	AAA-AAA	0.00	999	1.000	1.000	0.1669
236959_s_at	5.0	21.6	2.12	4.35	AAA-AAA	0.00	999	1.000	1.000	0.0191
1553433_at	2.7	11.8	2.12	4.35	AAA-AAA	0.00	999	1.000	1.000	0.2249
1559270_at	2.7	11.9	2.12	4.35	AAA-AAA	0.00	999	1.000	1.000	0.0867
207463_x_at	40.5	9.3	-2.12	-4.35	AAA-AAA	0.00	999	1.000	1.000	0.0009
208148_at	6.8	29.6	2.12	4.35	AAA-AAA	0.00	999	1.000	1.000	0.2058
233026_s_at	12.6	54.9	2.12	4.34	AAA-AAA	0.00	999	1.000	1.000	0.0224
202311_s_at	25.7	5.9	-2.12	-4.34	AAA-APA	0.00	999	1.000	1.000	0.2038
241894_at	10.3	44.6	2.12	4.34	AAA-AAA	0.00	999	1.000	1.000	0.0644
228934_x_at	4.8	21.0	2.12	4.34	AAA-AAA	0.00	999	1.000	1.000	0.0149
241963_at	18.5	80.1	2.12	4.34	AAA-AAA	0.00	999	1.000	1.000	0.1866
205906_at	79.1	18.2	-2.12	-4.34	AAA-AAA	0.00	999	1.000	1.000	0.0353
223920_s_at	18.1	78.6	2.12	4.34	AAA-AAA	0.00	999	1.000	1.000	0.0247
240215_at	39.1	9.0	-2.12	-4.33	AAA-AAA	0.00	999	1.000	1.000	0.0122
1555071_at	6.3	27.3	2.12	4.34	AAA-AAA	0.00	999	1.000	1.000	0.1451
221470_s_at	19.5	4.5	-2.12	-4.34	AAA-AAA	0.00	999	1.000	1.000	0.1167
206611_at	38.5	166.7	2.12	4.34	AAA-AAA	0.00	999	1.000	1.000	0.0276
224116_at	33.8	7.8	-2.12	-4.33	AAA-AAA	0.00	999	1.000	1.000	0.0344
1564401_at	5.7	24.7	2.12	4.33	AAA-AAA	0.00	999	1.000	1.000	0.0908
220543_at	67.5	15.6	-2.12	-4.33	AAA-AAA	0.00	999	1.000	1.000	0.0978
222935_x_at	4.0	17.4	2.12	4.33	AAA-AAA	0.00	999	1.000	1.000	0.0066
1555490_s_at	37.2	8.6	-2.11	-4.33	AAA-AAA	0.00	999	1.000	1.000	0.0115
244684_at	14.3	62.0	2.11	4.33	AAA-AAA	0.00	999	1.000	1.000	0.0987
216539_at	31.4	7.3	-2.11	-4.33	AAA-AAA	0.00	999	1.000	1.000	0.1705

236495_at	8.4	36.2	2.11	4.33	AAA-AAA	0.00	999	1.000	1.000	0.0190
1564292_at	15.3	3.5	-2.11	-4.33	MAA-AAA	0.00	999	1.000	1.000	0.1353
222444_at	34.8	8.1	-2.11	-4.33	AAA-AAA	0.00	999	1.000	1.000	0.0872
231797_at	44.4	10.3	-2.11	-4.32	AAP-AAA	0.00	999	1.000	1.000	0.1128
1554773_at	3.8	16.4	2.11	4.32	AAA-AAA	0.00	999	1.000	1.000	0.0129
239977_at	12.0	52.0	2.11	4.32	AAA-AAA	0.00	999	1.000	1.000	0.1297
220376_at	6.5	28.1	2.11	4.32	AAA-AAA	0.00	999	1.000	1.000	0.2070
1559419_at	4.5	19.3	2.11	4.32	AAA-AAM	0.00	999	1.000	1.000	0.1815
220273_at	7.4	31.7	2.11	4.31	AAA-AAA	0.00	999	1.000	1.000	0.0274
233654_at	17.7	76.4	2.11	4.31	AAA-AAA	0.00	999	1.000	1.000	0.1301
1561462_at	24.2	5.6	-2.11	-4.31	AAA-AAA	0.00	999	1.000	1.000	0.0607
215259_s_at	9.7	41.7	2.11	4.31	AAA-AAA	0.00	999	1.000	1.000	0.0248
237987_x_at	3.5	15.2	2.11	4.31	AAA-AAA	0.00	999	1.000	1.000	0.1976
233663_s_at	55.7	12.9	-2.11	-4.30	AAA-AAA	0.00	999	1.000	1.000	0.2367
214776_x_at	7.1	30.6	2.11	4.30	AAA-AAA	0.00	999	1.000	1.000	0.1982
220425_x_at	9.9	42.7	2.11	4.30	AAA-AAA	0.00	999	1.000	1.000	0.2044
234822_at	8.9	38.4	2.11	4.30	AAA-AAA	0.00	999	1.000	1.000	0.1428
228206_at	42.4	9.9	-2.11	-4.30	AAA-AAA	0.00	999	1.000	1.000	0.0374
241426_at	65.1	15.2	-2.10	-4.30	AAA-AAA	0.00	999	1.000	1.000	0.0172
1558887_at	5.6	23.9	2.10	4.30	AAA-AAA	0.00	999	1.000	1.000	0.1561
231619_at	16.7	71.9	2.10	4.30	AAA-AAA	0.00	999	1.000	1.000	0.1128
1565002_at	10.1	43.4	2.10	4.29	AAA-AAA	0.00	999	1.000	1.000	0.1953
240973_s_at	34.0	7.9	-2.10	-4.29	AAA-AAA	0.00	999	1.000	1.000	0.1091
236950_s_at	2.3	9.9	2.10	4.29	AAA-AAA	0.00	999	1.000	1.000	0.1148
243577_at	12.0	51.5	2.10	4.29	AAA-MAA	0.00	999	1.000	1.000	0.0378
223326_s_at	35.2	151.1	2.10	4.29	AAA-AAA	0.00	999	1.000	1.000	0.0130
1560111_at	2.9	12.3	2.10	4.28	AAA-AAA	0.00	999	1.000	1.000	0.2076
211771_s_at	5.7	24.5	2.10	4.28	AAA-AAA	0.00	999	1.000	1.000	0.2014
209400_at	30.4	7.1	-2.10	-4.28	AAA-AAA	0.00	999	1.000	1.000	0.1388
241479_at	2.0	8.6	2.10	4.28	AAA-AAA	0.00	999	1.000	1.000	0.1117
217483_at	22.9	97.9	2.10	4.28	AAA-AAA	0.00	999	1.000	1.000	0.0136
234123_at	11.1	47.3	2.10	4.28	AAA-AAM	0.00	999	1.000	1.000	0.0903
240265_at	3.8	16.3	2.10	4.28	AAA-AAA	0.00	999	1.000	1.000	0.2314
221300_at	15.6	66.6	2.10	4.27	AAA-AAA	0.00	999	1.000	1.000	0.0095
232755_at	5.4	23.2	2.10	4.28	AAA-AAA	0.00	999	1.000	1.000	0.0082
213122_at	22.9	5.4	-2.09	-4.27	AAM-AAA	0.00	999	1.000	1.000	0.1777
235307_at	7.8	33.4	2.09	4.27	AAA-AAA	0.00	999	1.000	1.000	0.0339
1556371_at	5.1	21.8	2.09	4.27	AAA-AAA	0.00	999	1.000	1.000	0.0960
216625_at	18.1	4.2	-2.09	-4.27	AAA-AAA	0.00	999	1.000	1.000	0.0956
234377_at	10.9	46.6	2.09	4.27	AAA-AAA	0.00	999	1.000	1.000	0.0997
241740_at	34.1	8.0	-2.09	-4.26	AAA-AAA	0.00	999	1.000	1.000	0.1097
239537_at	8.8	37.4	2.09	4.26	AAA-AAA	0.00	999	1.000	1.000	0.0074

240787_at	9.5	40.3	2.09	4.26	AAA-AAA	0.00	999	1.000	1.000	0.0350
204443_at	34.7	147.9	2.09	4.26	AAA-AAA	0.00	999	1.000	1.000	0.1363
208501_at	49.8	11.7	-2.09	-4.26	AAA-AAA	0.00	999	1.000	1.000	0.0894
1555939_at	5.7	1.3	-2.09	-4.26	AAA-AAA	0.00	999	1.000	1.000	0.1166
211242_x_at	56.1	13.2	-2.09	-4.26	AAA-AAA	0.00	999	1.000	1.000	0.1878
238659_at	4.5	19.3	2.09	4.26	AAA-AAA	0.00	999	1.000	1.000	0.0736
1558107_at	24.1	5.7	-2.09	-4.26	AAP-AAA	0.00	999	1.000	1.000	0.0884
242244_at	5.5	23.5	2.09	4.25	AAA-AAA	0.00	999	1.000	1.000	0.1459
244837_at	28.2	6.6	-2.09	-4.25	AAA-AAA	0.00	999	1.000	1.000	0.0498
234529_at	17.9	4.2	-2.09	-4.25	AAA-AAA	0.00	999	1.000	1.000	0.2107
232722_at	16.4	69.5	2.09	4.25	AAA-AAA	0.00	999	1.000	1.000	0.1811
243622_at	22.0	93.3	2.09	4.25	AAA-AAA	0.00	999	1.000	1.000	0.0198
1562336_at	8.0	34.1	2.09	4.25	AAA-AAA	0.00	999	1.000	1.000	0.0291
213920_at	60.0	14.1	-2.09	-4.25	AAA-AAA	0.00	999	1.000	1.000	0.1222
205145_s_at	125.9	29.6	-2.09	-4.25	PPP-APA	0.00	999	1.000	1.000	0.1366
240913_at	29.0	6.8	-2.09	-4.25	AAA-AAA	0.00	999	1.000	1.000	0.0902
220450_at	15.4	65.2	2.09	4.25	AAA-AAA	0.00	999	1.000	1.000	0.0881
208554_at	21.6	5.1	-2.09	-4.25	AAA-AAA	0.00	999	1.000	1.000	0.0199
1559370_at	57.0	13.4	-2.09	-4.24	AAA-PAA	0.00	999	1.000	1.000	0.3102
212681_at	14.6	3.5	-2.09	-4.24	AAA-AAA	0.00	999	1.000	1.000	0.1465
236824_at	8.0	1.9	-2.09	-4.24	AAA-AAA	0.00	999	1.000	1.000	0.1655
1569956_at	4.4	18.7	2.08	4.24	AAA-AAA	0.00	999	1.000	1.000	0.2743
243212_at	45.9	10.8	-2.08	-4.24	AAA-AAA	0.00	999	1.000	1.000	0.0222
223939_at	25.0	5.9	-2.08	-4.24	AAA-AAA	0.00	999	1.000	1.000	0.0494
1569783_at	3.8	15.9	2.08	4.23	AAA-AAA	0.00	999	1.000	1.000	0.0467
206372_at	79.7	18.9	-2.08	-4.22	AAA-AAA	0.00	999	1.000	1.000	0.0008
207280_at	9.5	40.2	2.08	4.22	AAA-AAA	0.00	999	1.000	1.000	0.0273
236763_at	10.8	45.5	2.08	4.23	AAA-AAA	0.00	999	1.000	1.000	0.0856
1562778_at	58.4	13.8	-2.08	-4.22	AAA-AAA	0.00	999	1.000	1.000	0.0804
240133_x_at	28.1	6.7	-2.08	-4.22	AAA-AAA	0.00	999	1.000	1.000	0.0691
217230_at	8.0	33.7	2.08	4.22	AAA-AAA	0.00	999	1.000	1.000	0.2017
204850_s_at	5.8	24.7	2.08	4.22	AAA-AAA	0.00	999	1.000	1.000	0.0749
213938_at	45.3	10.7	-2.08	-4.22	AAA-AAA	0.00	999	1.000	1.000	0.1941
213192_at	84.8	20.1	-2.08	-4.22	AAA-AAA	0.00	999	1.000	1.000	0.1484
234370_at	35.2	8.4	-2.08	-4.22	AAA-AAA	0.00	999	1.000	1.000	0.0496
242732_at	49.7	11.8	-2.08	-4.21	AAA-AAA	0.00	999	1.000	1.000	0.0787
207437_at	4.1	17.2	2.08	4.21	AAA-AAA	0.00	999	1.000	1.000	0.2884
241822_at	19.8	83.4	2.07	4.21	AAA-AAA	0.00	999	1.000	1.000	0.0747
236871_s_at	3.8	16.2	2.07	4.21	AAA-AAA	0.00	999	1.000	1.000	0.0646
230286_at	61.1	14.5	-2.07	-4.21	AAA-AAA	0.00	999	1.000	1.000	0.1081
1566798_at	22.2	5.3	-2.07	-4.21	AAA-AAA	0.00	999	1.000	1.000	0.1746
234917_at	8.2	34.5	2.07	4.21	AAA-AAA	0.00	999	1.000	1.000	0.1422

1552658_a_at	73.5	17.5	-2.07	-4.20	MAP-AAM	0.00	999	1.000	1.000	0.0615
204284_at	13.5	56.6	2.07	4.20	AAA-AAA	0.00	999	1.000	1.000	0.0686
1555025_at	9.6	40.2	2.07	4.20	AAA-AAA	0.00	999	1.000	1.000	0.0821
226695_at	71.8	17.1	-2.07	-4.20	AAA-AAA	0.00	999	1.000	1.000	0.0930
241904_at	67.5	16.1	-2.07	-4.20	AAA-AAA	0.00	999	1.000	1.000	0.0844
233492_s_at	110.7	26.4	-2.07	-4.20	AAA-AAA	0.00	999	1.000	1.000	0.0891
240950_s_at	18.4	77.1	2.07	4.19	AAA-AAA	0.00	999	1.000	1.000	0.0500
1554322_a_at	17.4	72.8	2.07	4.19	AAA-AAA	0.00	999	1.000	1.000	0.0844
242603_x_at	21.2	5.1	-2.07	-4.19	AAA-AAA	0.00	999	1.000	1.000	0.1079
206883_x_at	23.4	98.2	2.07	4.19	AAA-AAA	0.00	999	1.000	1.000	0.0544
232329_at	9.0	37.7	2.07	4.19	AAA-AAA	0.00	999	1.000	1.000	0.0051
1569102_at	7.7	32.3	2.07	4.19	AAA-AAA	0.00	999	1.000	1.000	0.1109
1566459_at	36.4	8.7	-2.07	-4.19	AAA-AAA	0.00	999	1.000	1.000	0.0372
1556898_at	6.9	29.0	2.07	4.19	AAA-AAA	0.00	999	1.000	1.000	0.1316
215768_at	31.0	7.4	-2.07	-4.19	AAA-AAA	0.00	999	1.000	1.000	0.1770
221033_s_at	13.6	57.0	2.07	4.19	AAA-AAA	0.00	999	1.000	1.000	0.0212
1554927_at	54.4	13.0	-2.07	-4.19	AAA-AAA	0.00	999	1.000	1.000	0.0503
213921_at	12.1	50.8	2.07	4.19	AAA-AAA	0.00	999	1.000	1.000	0.1833
228593_at	13.0	54.6	2.07	4.19	AAA-AAA	0.00	999	1.000	1.000	0.1150
230876_at	2.6	10.8	2.07	4.18	AAA-AAA	0.00	999	1.000	1.000	0.2400
241032_at	8.5	35.5	2.06	4.18	AAA-AAA	0.00	999	1.000	1.000	0.1341
1561140_at	4.4	18.4	2.06	4.18	AAA-AAA	0.00	999	1.000	1.000	0.0786
228843_at	39.8	9.5	-2.06	-4.18	AAP-AAA	0.00	999	1.000	1.000	0.1167
1565858_at	17.5	72.9	2.06	4.18	AAA-AAA	0.00	999	1.000	1.000	0.0944
1561754_at	3.2	13.3	2.06	4.18	AAA-AAA	0.00	999	1.000	1.000	0.2576
1560317_s_at	4.2	17.3	2.06	4.18	AAA-AAA	0.00	999	1.000	1.000	0.1787
214509_at	28.5	6.8	-2.06	-4.17	AAA-AAA	0.00	999	1.000	1.000	0.0274
234183_at	41.3	9.9	-2.06	-4.17	AAA-AAA	0.00	999	1.000	1.000	0.0148
233335_at	2.7	11.1	2.06	4.17	AAA-AAA	0.00	999	1.000	1.000	0.1671
1552412_a_at	30.8	7.4	-2.06	-4.17	AAA-AAA	0.00	999	1.000	1.000	0.0595
1554394_at	23.5	5.6	-2.06	-4.17	AAA-AAA	0.00	999	1.000	1.000	0.0056
1553546_at	11.4	2.7	-2.06	-4.17	AAA-AAA	0.00	999	1.000	1.000	0.2606
1555073_at	33.4	8.0	-2.06	-4.17	AAA-AAA	0.00	999	1.000	1.000	0.0314
215350_at	3.9	16.1	2.06	4.17	AAA-AAA	0.00	999	1.000	1.000	0.0642
217343_at	56.7	13.6	-2.06	-4.17	APA-AAA	0.00	999	1.000	1.000	0.0586
241853_at	2.8	11.5	2.06	4.17	AAA-AAA	0.00	999	1.000	1.000	0.2866
237356_at	22.1	5.3	-2.06	-4.17	AAA-AAA	0.00	999	1.000	1.000	0.1301
236592_at	27.2	113.5	2.06	4.17	AAA-AAA	0.00	999	1.000	1.000	0.0269
239732_x_at	19.4	4.7	-2.06	-4.16	AAA-AAA	0.00	999	1.000	1.000	0.1318
1557658_at	7.1	29.6	2.06	4.17	AAA-AAA	0.00	999	1.000	1.000	0.0603
1553374_at	3.4	14.3	2.06	4.16	AAA-AAA	0.00	999	1.000	1.000	0.1123
233831_at	45.9	11.0	-2.06	-4.17	AAA-AAA	0.00	999	1.000	1.000	0.0025

1555839_a_at	4.5	18.5	2.06	4.16	AAA-AAA	0.00	999	1.000	1.000	0.1760
1560260_at	7.1	1.7	-2.06	-4.16	AAA-AAA	0.00	999	1.000	1.000	0.0774
214158_s_at	17.1	71.2	2.06	4.16	AAA-AAA	0.00	999	1.000	1.000	0.0757
234893_s_at	23.3	5.6	-2.06	-4.16	AAA-AAA	0.00	999	1.000	1.000	0.0957
1565795_at	38.2	9.2	-2.06	-4.16	AAA-AAA	0.00	999	1.000	1.000	0.1345
1558725_at	25.8	6.2	-2.06	-4.16	AAA-AAA	0.00	999	1.000	1.000	0.2577
1559377_at	37.9	9.1	-2.06	-4.16	AAA-AAA	0.00	999	1.000	1.000	0.1093
1558780_a_at	48.7	11.7	-2.06	-4.16	AAA-AAA	0.00	999	1.000	1.000	0.0014
1553340_s_at	52.2	12.6	-2.06	-4.16	AAA-AAA	0.00	999	1.000	1.000	0.0185
231128_at	37.7	9.1	-2.06	-4.16	AAA-AAA	0.00	999	1.000	1.000	0.1479
1565633_at	2.2	9.1	2.05	4.15	AAA-AAA	0.00	999	1.000	1.000	0.1393
234704_at	63.3	15.3	-2.05	-4.15	MAA-AAA	0.00	999	1.000	1.000	0.1380
230043_at	64.7	15.6	-2.05	-4.15	PAA-AAA	0.00	999	1.000	1.000	0.1358
236354_at	61.4	14.8	-2.05	-4.15	AAA-AAA	0.00	999	1.000	1.000	0.2290
224012_at	2.0	8.3	2.05	4.15	AAA-AAA	0.00	999	1.000	1.000	0.1570
216068_at	27.0	6.5	-2.05	-4.15	PPM-AAA	0.00	999	1.000	1.000	0.1191
228086_at	8.1	33.6	2.05	4.15	AAA-AAA	0.00	999	1.000	1.000	0.1639
229150_at	13.8	57.1	2.05	4.15	AAA-AAA	0.00	999	1.000	1.000	0.0430
241181_x_at	98.3	23.7	-2.05	-4.15	AAA-AAA	0.00	999	1.000	1.000	0.2302
206962_x_at	35.8	8.7	-2.05	-4.14	AAA-AAA	0.00	999	1.000	1.000	0.0944
239519_at	48.5	11.7	-2.05	-4.14	AAP-AAA	0.00	999	1.000	1.000	0.1751
1566162_x_at	21.0	5.1	-2.05	-4.14	AAA-AAA	0.00	999	1.000	1.000	0.1292
1562439_at	2.9	12.1	2.05	4.14	AAA-AAA	0.00	999	1.000	1.000	0.1560
243914_at	8.3	34.3	2.05	4.14	AAA-AAA	0.00	999	1.000	1.000	0.1055
228692_at	14.8	3.6	-2.05	-4.14	AAA-AAA	0.00	999	1.000	1.000	0.1495
1552736_a_at	8.8	36.6	2.05	4.14	AAA-AAA	0.00	999	1.000	1.000	0.1839
1560063_a_at	4.0	16.7	2.05	4.14	AAA-AAA	0.00	999	1.000	1.000	0.0093
212158_at	5.9	24.5	2.05	4.13	AAA-AAA	0.00	999	1.000	1.000	0.0985
217073_x_at	18.9	78.1	2.05	4.13	AAA-AAA	0.00	999	1.000	1.000	0.0304
234101_at	11.6	47.8	2.05	4.13	AAA-AAA	0.00	999	1.000	1.000	0.0947
222639_s_at	45.7	11.1	-2.05	-4.13	AAA-AAA	0.00	999	1.000	1.000	0.1102
1570625_at	46.0	11.1	-2.05	-4.13	AAA-AAA	0.00	999	1.000	1.000	0.1398
1561272_at	8.2	34.0	2.05	4.13	AAA-AAA	0.00	999	1.000	1.000	0.0521
235821_at	11.3	46.4	2.05	4.13	AAA-AAA	0.00	999	1.000	1.000	0.0977
241131_at	62.3	15.1	-2.04	-4.12	AAA-AAA	0.00	999	1.000	1.000	0.1231
209854_s_at	11.6	47.8	2.04	4.13	AAA-AAA	0.00	999	1.000	1.000	0.1307
1560118_at	6.3	25.9	2.04	4.12	AAA-AAA	0.00	999	1.000	1.000	0.1502
214297_at	11.1	45.6	2.04	4.12	AAA-AAA	0.00	999	1.000	1.000	0.1291
206863_x_at	38.1	9.2	-2.04	-4.12	AAA-AAA	0.00	999	1.000	1.000	0.1245
207815_at	3.1	12.6	2.04	4.12	AAA-AAA	0.00	999	1.000	1.000	0.3014
207552_at	6.5	26.9	2.04	4.12	AAA-AAA	0.00	999	1.000	1.000	0.0344
1555197_a_at	4.8	19.9	2.04	4.12	AAA-AAA	0.00	999	1.000	1.000	0.0946

242206_at	10.0	2.4	-2.04	-4.12	AAA-AAA	0.00	999	1.000	1.000	0.1136
1552878_at	8.0	1.9	-2.04	-4.12	AAA-AAA	0.00	999	1.000	1.000	0.1834
244867_at	23.8	5.8	-2.04	-4.12	AAA-AAA	0.00	999	1.000	1.000	0.1549
220037_s_at	21.3	5.2	-2.04	-4.12	AAA-AAA	0.00	999	1.000	1.000	0.0480
206442_at	10.6	2.6	-2.04	-4.12	AAA-AAA	0.00	999	1.000	1.000	0.1733
220859_at	14.5	59.8	2.04	4.11	AAA-AAA	0.00	999	1.000	1.000	0.0306
210838_s_at	69.5	16.9	-2.04	-4.11	AAA-AAA	0.00	999	1.000	1.000	0.0301
211831_s_at	6.6	27.3	2.04	4.11	AAA-AAA	0.00	999	1.000	1.000	0.0925
226436_at	20.2	4.9	-2.04	-4.11	AAA-AAA	0.00	999	1.000	1.000	0.0369
231351_at	10.0	41.1	2.04	4.11	AAA-AAA	0.00	999	1.000	1.000	0.0712
224112_at	5.7	23.4	2.04	4.11	AAA-AAA	0.00	999	1.000	1.000	0.2154
217381_s_at	16.4	67.4	2.04	4.11	AAA-AAA	0.00	999	1.000	1.000	0.2390
205715_at	24.3	5.9	-2.04	-4.11	AAA-AAA	0.00	999	1.000	1.000	0.0530
211843_x_at	5.3	21.7	2.04	4.11	AAA-AAA	0.00	999	1.000	1.000	0.0235
1554777_at	8.5	34.9	2.04	4.10	AAA-AAA	0.00	999	1.000	1.000	0.1838
217138_x_at	35.2	8.6	-2.04	-4.10	AAA-AAA	0.00	999	1.000	1.000	0.0418
237374_at	22.0	5.4	-2.04	-4.10	AAA-AAA	0.00	999	1.000	1.000	0.2177
226182_s_at	52.0	12.7	-2.04	-4.10	AAA-AAA	0.00	999	1.000	1.000	0.0076
224194_at	47.2	11.5	-2.04	-4.10	AAA-AAA	0.00	999	1.000	1.000	0.1980
244342_at	15.8	64.7	2.04	4.10	AAA-MAA	0.00	999	1.000	1.000	0.0013
1563241_at	9.2	37.5	2.03	4.09	AAA-AAA	0.00	999	1.000	1.000	0.1116
240599_x_at	2.9	11.7	2.03	4.09	AAA-AAA	0.00	999	1.000	1.000	0.1976
1566938_at	36.8	9.0	-2.03	-4.09	APA-AAA	0.00	999	1.000	1.000	0.1529
244415_at	9.7	39.5	2.03	4.09	AAA-AAA	0.00	999	1.000	1.000	0.0528
205183_at	3.7	15.1	2.03	4.09	AAA-AAA	0.00	999	1.000	1.000	0.0916
222756_s_at	61.7	15.1	-2.03	-4.09	AAA-AAA	0.00	999	1.000	1.000	0.1899
239166_at	7.9	32.3	2.03	4.09	AAA-AAA	0.00	999	1.000	1.000	0.0437
AFFX-ThrX-3_at	21.5	5.3	-2.03	-4.09	AAA-AAA	0.00	999	1.000	1.000	0.0585
236339_at	2.9	11.7	2.03	4.09	AAA-AAA	0.00	999	1.000	1.000	0.1957
1564789_at	3.5	14.5	2.03	4.08	AAA-AAA	0.00	999	1.000	1.000	0.1354
1556599_s_at	2.4	10.0	2.03	4.09	AAA-AAA	0.00	999	1.000	1.000	0.3017
235746_s_at	7.2	29.3	2.03	4.08	AAA-AAA	0.00	999	1.000	1.000	0.1373
223667_at	3.0	12.0	2.03	4.08	AAA-AAA	0.00	999	1.000	1.000	0.1466
229774_at	10.4	42.6	2.03	4.08	AAA-AAA	0.00	999	1.000	1.000	0.0659
1569500_at	154.2	37.8	-2.03	-4.08	AAA-AAA	0.00	999	1.000	1.000	0.0783
1557523_at	66.7	16.4	-2.03	-4.08	AAA-AAA	0.00	999	1.000	1.000	0.0123
243014_at	11.4	46.5	2.03	4.08	AAA-AAA	0.00	999	1.000	1.000	0.2101
212736_at	82.1	20.1	-2.03	-4.08	AAA-AAA	0.00	999	1.000	1.000	0.0857
1553549_at	25.5	6.3	-2.03	-4.07	AAA-AAA	0.00	999	1.000	1.000	0.1500
207038_at	37.3	9.2	-2.03	-4.08	AAA-AAA	0.00	999	1.000	1.000	0.0760
234293_x_at	20.0	81.4	2.03	4.07	AAA-AAA	0.00	999	1.000	1.000	0.0936
233771_at	67.3	16.5	-2.03	-4.08	AAA-AAA	0.00	999	1.000	1.000	0.1417

243105_at	64.0	15.7	-2.03	-4.07	AAA-AAA	0.00	999	1.000	1.000	0.1321
206408_at	52.7	12.9	-2.03	-4.07	AAA-AAA	0.00	999	1.000	1.000	0.0516
209498_at	93.2	22.9	-2.03	-4.07	AAA-AAA	0.00	999	1.000	1.000	0.0986
237382_at	13.4	54.5	2.03	4.07	AAA-AAA	0.00	999	1.000	1.000	0.0760
223704_s_at	34.5	8.5	-2.03	-4.07	AAA-AAA	0.00	999	1.000	1.000	0.2162
241858_at	53.6	13.2	-2.03	-4.07	AMP-AAA	0.00	999	1.000	1.000	0.1867
241578_x_at	1.6	6.4	2.03	4.07	AAA-AAA	0.00	999	1.000	1.000	0.1685
208353_x_at	10.3	41.8	2.03	4.07	AAA-AAA	0.00	999	1.000	1.000	0.1067
1565338_x_at	5.1	20.8	2.02	4.07	AAA-AAA	0.00	999	1.000	1.000	0.1406
207142_at	16.4	4.0	-2.02	-4.07	AAA-AAA	0.00	999	1.000	1.000	0.1642
238854_at	25.8	104.7	2.02	4.06	AAA-AAA	0.00	999	1.000	1.000	0.0861
203998_s_at	3.9	15.9	2.02	4.06	AAA-AAA	0.00	999	1.000	1.000	0.1935
236056_s_at	29.8	7.3	-2.02	-4.06	AAA-AAA	0.00	999	1.000	1.000	0.2883
210875_s_at	47.7	11.8	-2.02	-4.06	AAA-AAA	0.00	999	1.000	1.000	0.0082
238368_at	39.2	9.7	-2.02	-4.06	AAA-AAA	0.00	999	1.000	1.000	0.2569
1560153_at	89.9	22.2	-2.02	-4.06	APP-AAA	0.00	999	1.000	1.000	0.1840
243605_at	1.7	7.0	2.02	4.06	AAA-AAA	0.00	999	1.000	1.000	0.0422
220552_at	4.9	19.8	2.02	4.06	AAA-AAA	0.00	999	1.000	1.000	0.2965
1561125_at	58.0	14.3	-2.02	-4.05	AAP-AAA	0.00	999	1.000	1.000	0.2015
1566046_at	9.2	37.2	2.02	4.05	AAA-AAA	0.00	999	1.000	1.000	0.1270
241232_x_at	25.8	6.4	-2.02	-4.05	AAA-AAA	0.00	999	1.000	1.000	0.1243
1554633_a_at	15.7	3.9	-2.02	-4.05	AAA-AAA	0.00	999	1.000	1.000	0.0932
214903_at	11.1	44.8	2.02	4.05	AAA-AAA	0.00	999	1.000	1.000	0.1071
238137_at	9.6	38.9	2.02	4.05	AAA-AAA	0.00	999	1.000	1.000	0.1443
232392_at	40.9	165.2	2.02	4.04	AAA-AAA	0.00	999	1.000	1.000	0.0967
230694_at	13.5	54.7	2.02	4.05	AAA-AAA	0.00	999	1.000	1.000	0.0166
243351_at	64.2	15.9	-2.02	-4.04	AAA-AAA	0.00	999	1.000	1.000	0.2105
216198_at	11.6	46.9	2.02	4.04	AAA-AAA	0.00	999	1.000	1.000	0.1065
216369_at	28.6	7.1	-2.02	-4.04	AAA-AAA	0.00	999	1.000	1.000	0.0661
234994_at	12.7	3.1	-2.02	-4.04	AAA-AAA	0.00	999	1.000	1.000	0.2300
224329_s_at	36.5	147.6	2.02	4.04	AAA-AAA	0.00	999	1.000	1.000	0.0254
242980_at	40.0	9.9	-2.01	-4.04	AAA-AAA	0.00	999	1.000	1.000	0.0099
235876_at	29.5	7.3	-2.01	-4.04	AAA-AAA	0.00	999	1.000	1.000	0.0291
232298_at	32.8	8.1	-2.01	-4.04	AAA-AAA	0.00	999	1.000	1.000	0.0894
221261_x_at	24.7	6.1	-2.01	-4.04	AAA-AAA	0.00	999	1.000	1.000	0.1507
217229_at	6.0	1.5	-2.01	-4.04	AAA-AAA	0.00	999	1.000	1.000	0.0993
208102_s_at	8.2	33.2	2.01	4.04	AAA-AAA	0.00	999	1.000	1.000	0.0580
1552913_at	1.5	5.9	2.01	4.03	AAA-AAA	0.00	999	1.000	1.000	0.0378
1552825_at	2.5	10.0	2.01	4.03	AAA-AAA	0.00	999	1.000	1.000	0.1142
237645_at	14.0	56.5	2.01	4.03	AAA-AAA	0.00	999	1.000	1.000	0.1267
1561577_at	8.1	32.8	2.01	4.03	AAA-AAA	0.00	999	1.000	1.000	0.1441
221644_s_at	39.9	9.9	-2.01	-4.03	AAA-AAA	0.00	999	1.000	1.000	0.0189

1554864_a_at	79.8	19.8	-2.01	-4.03	AAA-AAA	0.00	999	1.000	1.000	0.0723
219779_at	25.0	100.6	2.01	4.03	AAA-AAA	0.00	999	1.000	1.000	0.1527
232538_at	39.2	9.7	-2.01	-4.03	AAA-AAA	0.00	999	1.000	1.000	0.0457
220614_s_at	4.8	19.2	2.01	4.03	AAA-AAA	0.00	999	1.000	1.000	0.0747
217583_at	28.6	7.1	-2.01	-4.03	AAA-AAA	0.00	999	1.000	1.000	0.0197
211700_s_at	12.4	49.7	2.01	4.02	AAA-AAA	0.00	999	1.000	1.000	0.1184
233126_s_at	9.9	39.7	2.01	4.02	AAA-AAA	0.00	999	1.000	1.000	0.0044
1556798_a_at	17.6	4.4	-2.01	-4.02	AAA-AAA	0.00	999	1.000	1.000	0.2859
207607_at	6.0	1.5	-2.01	-4.02	AAA-AAA	0.00	999	1.000	1.000	0.0891
1569207_s_at	53.6	13.3	-2.01	-4.02	AAA-AAA	0.00	999	1.000	1.000	0.0519
1557821_at	8.3	33.3	2.01	4.02	AAA-AAA	0.00	999	1.000	1.000	0.1543
220415_at	46.1	11.5	-2.01	-4.02	AAA-AAA	0.00	999	1.000	1.000	0.1256
222276_at	50.5	202.9	2.01	4.02	AAA-MAM	0.00	999	1.000	1.000	0.1667
1556994_at	93.1	23.2	-2.01	-4.02	AAA-AAA	0.00	999	1.000	1.000	0.0169
1554504_at	17.7	4.4	-2.01	-4.02	AAA-AAA	0.00	999	1.000	1.000	0.1717
210551_s_at	24.6	6.1	-2.01	-4.02	AAA-AAA	0.00	999	1.000	1.000	0.0845
1563147_at	10.0	40.1	2.01	4.01	AAA-AAA	0.00	999	1.000	1.000	0.1203
241120_s_at	4.1	16.6	2.01	4.01	AAA-AAA	0.00	999	1.000	1.000	0.0897
235358_at	60.6	15.1	-2.01	-4.01	AAA-AAA	0.00	999	1.000	1.000	0.1074
233212_at	18.3	73.5	2.01	4.01	AAA-AAA	0.00	999	1.000	1.000	0.0816
239401_at	27.9	7.0	-2.00	-4.01	AAA-AAA	0.00	999	1.000	1.000	0.0996
1566171_at	26.1	104.6	2.00	4.01	AAA-AAA	0.00	999	1.000	1.000	0.0372
224208_at	11.4	45.5	2.00	4.01	AAA-AAA	0.00	999	1.000	1.000	0.1441
234615_at	46.6	11.6	-2.00	-4.01	PAM-AAA	0.00	999	1.000	1.000	0.2872
1570189_at	8.0	31.9	2.00	4.01	AAA-AAA	0.00	999	1.000	1.000	0.0009
213248_at	71.5	17.8	-2.00	-4.01	AAA-AAA	0.00	999	1.000	1.000	0.1148
235665_at	5.2	20.7	2.00	4.00	AAA-AAA	0.00	999	1.000	1.000	0.0877
243511_at	53.3	13.3	-2.00	-4.00	AAA-AAP	0.00	999	1.000	1.000	0.2311
1557775_a_at	35.3	8.8	-2.00	-4.00	AAA-AAA	0.00	999	1.000	1.000	0.1052
1560418_at	2.3	9.0	2.00	4.00	AAA-AAA	0.00	999	1.000	1.000	0.1327
207887_s_at	11.1	44.4	2.00	4.00	AAA-AAA	0.00	999	1.000	1.000	0.0519
1564051_at	18.2	72.8	2.00	4.00	AAA-AAA	0.00	999	1.000	1.000	0.0467
1561358_at	10.8	43.4	2.00	4.00	AAA-AAA	0.00	999	1.000	1.000	0.1005
220832_at	5.3	21.3	2.00	4.00	AAA-AAA	0.00	999	1.000	1.000	0.2162
1562914_a_at	31.7	7.9	-2.00	-4.00	AAA-AAA	0.00	999	1.000	1.000	0.1564
243574_at	5.5	22.0	2.00	4.00	AAA-AAA	0.00	999	1.000	1.000	0.1985
208195_at	10.9	43.5	2.00	4.00	AAA-AAA	0.00	999	1.000	1.000	0.1024
205040_at	21.8	5.5	-2.00	-3.99	AAA-AAA	0.00	999	1.000	1.000	0.1365
234457_at	2.4	9.6	2.00	4.00	AAA-AAA	0.00	999	1.000	1.000	0.3208
1566473_a_at	18.4	73.2	2.00	3.99	AAA-AAA	0.00	999	1.000	1.000	0.0474
217288_at	3.9	15.5	2.00	3.99	AAA-AAA	0.00	999	1.000	1.000	0.1356
219782_s_at	3.5	13.9	2.00	3.99	AAA-AAA	0.00	999	1.000	1.000	0.2040

1566766_a_at	16.9	67.5	2.00	3.99	AAA-AAA	0.00	999	1.000	1.000	0.2317
1560887_a_at	3.0	12.0	2.00	3.99	AAA-AAA	0.00	999	1.000	1.000	0.0754
204802_at	4.4	17.7	2.00	3.99	AAA-AAA	0.00	999	1.000	1.000	0.2079
211674_x_at	23.8	6.0	-2.00	-3.99	AAA-AAA	0.00	999	1.000	1.000	0.0579
235128_at	32.0	127.6	1.99	3.98	AAA-AAA	0.00	999	1.000	1.000	0.0985
237874_at	11.2	2.8	-1.99	-3.98	AAA-AAA	0.00	999	1.000	1.000	0.3201
228377_at	5.8	23.1	1.99	3.98	AAA-AAA	0.00	999	1.000	1.000	0.0738
228083_at	7.0	27.7	1.99	3.98	AAA-AAA	0.00	999	1.000	1.000	0.0777
1568722_at	5.1	20.3	1.99	3.98	AAA-AAA	0.00	999	1.000	1.000	0.0181
240537_s_at	18.6	73.8	1.99	3.98	AAA-AAA	0.00	999	1.000	1.000	0.0084
210872_x_at	39.7	10.0	-1.99	-3.98	AAA-AAA	0.00	999	1.000	1.000	0.0729
223957_at	16.3	64.7	1.99	3.98	AAA-AAA	0.00	999	1.000	1.000	0.0526
241958_at	26.2	104.3	1.99	3.98	AAA-AAA	0.00	999	1.000	1.000	0.1151
1555605_x_at	9.1	36.1	1.99	3.98	AAA-AAA	0.00	999	1.000	1.000	0.2894
244621_x_at	6.7	26.5	1.99	3.98	AAA-AAA	0.00	999	1.000	1.000	0.2582
232880_at	23.3	92.5	1.99	3.98	AAA-AAA	0.00	999	1.000	1.000	0.2106
236694_at	4.6	18.3	1.99	3.97	AAA-AAA	0.00	999	1.000	1.000	0.1924
243815_at	93.2	23.5	-1.99	-3.97	PAA-AAA	0.00	999	1.000	1.000	0.1282
242516_x_at	2.0	8.1	1.99	3.97	AAA-AAA	0.00	999	1.000	1.000	0.1862
1567174_at	9.1	2.3	-1.99	-3.97	AAA-AAA	0.00	999	1.000	1.000	0.0710
210636_at	8.3	33.1	1.99	3.97	AAA-AAA	0.00	999	1.000	1.000	0.1049
238478_at	82.7	20.8	-1.99	-3.97	MAA-AAA	0.00	999	1.000	1.000	0.2095
210204_s_at	97.1	24.5	-1.99	-3.97	AAP-PAA	0.00	999	1.000	1.000	0.1679
213544_at	6.8	26.9	1.99	3.97	AAA-AAA	0.00	999	1.000	1.000	0.2124
229459_at	23.7	6.0	-1.99	-3.97	AAA-AAA	0.00	999	1.000	1.000	0.0032
224357_s_at	10.4	41.4	1.99	3.97	AAA-AAA	0.00	999	1.000	1.000	0.0466
243390_at	2.5	9.9	1.99	3.97	AAA-AAA	0.00	999	1.000	1.000	0.2967
207072_at	16.3	64.6	1.99	3.96	AAA-AAA	0.00	999	1.000	1.000	0.0483
209554_at	7.9	31.3	1.99	3.96	AAA-AAA	0.00	999	1.000	1.000	0.1572
1561506_at	44.3	11.2	-1.99	-3.96	AAA-AAA	0.00	999	1.000	1.000	0.1119
220624_s_at	15.3	3.9	-1.99	-3.96	AAA-AAA	0.00	999	1.000	1.000	0.1042
240743_at	10.5	41.5	1.99	3.96	AAA-AAA	0.00	999	1.000	1.000	0.0520
235963_at	8.4	33.3	1.99	3.96	AAA-AAA	0.00	999	1.000	1.000	0.0802
222300_at	15.6	61.9	1.99	3.96	AAA-AAA	0.00	999	1.000	1.000	0.1566
224081_at	18.2	4.6	-1.98	-3.96	APA-AAA	0.00	999	1.000	1.000	0.2949
200784_s_at	32.5	128.4	1.98	3.96	AAA-AAA	0.00	999	1.000	1.000	0.0192
1556331_a_at	4.2	16.6	1.98	3.95	AAA-AAA	0.00	999	1.000	1.000	0.0873
1561301_at	36.1	9.1	-1.98	-3.95	AAA-AAA	0.00	999	1.000	1.000	0.1147
221126_at	7.6	29.9	1.98	3.95	AAA-AAA	0.00	999	1.000	1.000	0.0586
207647_at	14.1	3.6	-1.98	-3.95	AAA-AAA	0.00	999	1.000	1.000	0.1200
228821_at	6.5	25.6	1.98	3.95	AAA-AAA	0.00	999	1.000	1.000	0.1001
231754_at	19.6	5.0	-1.98	-3.95	AAA-AAA	0.00	999	1.000	1.000	0.0344

239500_at	34.5	8.7	-1.98	-3.95	AAA-AAA	0.00	999	1.000	1.000	0.1434
208434_at	3.0	11.7	1.98	3.95	AAA-AAA	0.00	999	1.000	1.000	0.2092
232869_at	30.6	121.0	1.98	3.95	AAA-AAA	0.00	999	1.000	1.000	0.0122
1570384_at	12.2	48.0	1.98	3.95	AAA-AAA	0.00	999	1.000	1.000	0.1085
1570035_at	31.7	8.0	-1.98	-3.95	AAA-AAA	0.00	999	1.000	1.000	0.0639
240050_s_at	11.1	43.8	1.98	3.95	AAA-AAA	0.00	999	1.000	1.000	0.2488
231702_at	4.1	16.3	1.98	3.95	AAA-AAA	0.00	999	1.000	1.000	0.1859
1568906_at	13.4	52.9	1.98	3.95	AAA-AAA	0.00	999	1.000	1.000	0.1051
1566576_at	10.7	42.2	1.98	3.94	AAA-AAA	0.00	999	1.000	1.000	0.1185
1561236_at	37.5	9.5	-1.98	-3.95	AAA-AAA	0.00	999	1.000	1.000	0.2665
1560174_at	17.0	4.3	-1.98	-3.94	AAA-AAA	0.00	999	1.000	1.000	0.3709
216425_at	19.3	76.1	1.98	3.94	AAA-AAA	0.00	999	1.000	1.000	0.1391
225817_at	3.3	13.1	1.98	3.94	AAA-AAA	0.00	999	1.000	1.000	0.1437
223074_s_at	12.4	48.8	1.98	3.94	AAA-AAA	0.00	999	1.000	1.000	0.1172
240645_at	39.2	10.0	-1.98	-3.94	AAA-AAA	0.00	999	1.000	1.000	0.1243
1561096_at	8.5	33.6	1.98	3.94	AAA-AAA	0.00	999	1.000	1.000	0.0719
1555085_at	14.0	55.0	1.98	3.94	AAA-AAA	0.00	999	1.000	1.000	0.0535
1568872_at	21.3	5.4	-1.98	-3.93	AAA-AAA	0.00	999	1.000	1.000	0.0783
201280_s_at	26.8	6.8	-1.98	-3.93	AAA-AAA	0.00	999	1.000	1.000	0.2241
210450_at	4.6	18.1	1.98	3.93	AAA-AAA	0.00	999	1.000	1.000	0.1289
210741_at	4.8	18.8	1.98	3.93	AAA-AAA	0.00	999	1.000	1.000	0.0614
238105_x_at	17.7	69.6	1.98	3.93	AAA-AAA	0.00	999	1.000	1.000	0.0194
1563073_at	5.1	20.2	1.97	3.93	AAA-AAA	0.00	999	1.000	1.000	0.2212
1556266_a_at	11.4	44.6	1.97	3.93	AAA-AAA	0.00	999	1.000	1.000	0.0365
228202_at	12.2	3.1	-1.97	-3.93	AAA-AAA	0.00	999	1.000	1.000	0.1811
1570273_at	3.6	14.2	1.97	3.93	AAA-AAA	0.00	999	1.000	1.000	0.2755
1561882_at	8.8	34.5	1.97	3.93	AAA-AAA	0.00	999	1.000	1.000	0.0717
230323_s_at	17.6	4.5	-1.97	-3.93	AAA-AAA	0.00	999	1.000	1.000	0.0974
1562747_at	6.7	26.3	1.97	3.92	AAA-AAA	0.00	999	1.000	1.000	0.2714
244169_x_at	11.2	2.8	-1.97	-3.92	AAA-AAA	0.00	999	1.000	1.000	0.3250
211685_s_at	28.1	7.2	-1.97	-3.92	AAA-AAA	0.00	999	1.000	1.000	0.1083
241177_at	2.0	7.7	1.97	3.92	AAA-AAA	0.00	999	1.000	1.000	0.1651
1565702_at	5.6	21.9	1.97	3.92	AAA-AAA	0.00	999	1.000	1.000	0.1879
214282_at	7.6	29.7	1.97	3.92	AAA-AAA	0.00	999	1.000	1.000	0.0144
220518_at	14.9	3.8	-1.97	-3.92	AAA-AAA	0.00	999	1.000	1.000	0.1289
220830_at	35.8	9.1	-1.97	-3.92	AAA-AAA	0.00	999	1.000	1.000	0.2569
226303_at	19.2	4.9	-1.97	-3.92	AAA-AAA	0.00	999	1.000	1.000	0.0657
1555332_at	4.5	17.7	1.97	3.92	AAA-AAA	0.00	999	1.000	1.000	0.1742
204096_s_at	26.6	104.1	1.97	3.92	AAA-AAA	0.00	999	1.000	1.000	0.1245
207715_at	58.0	14.8	-1.97	-3.92	AAA-AAA	0.00	999	1.000	1.000	0.0556
233040_at	6.4	25.2	1.97	3.92	AAA-AAA	0.00	999	1.000	1.000	0.1577
1554937_x_at	60.0	15.3	-1.97	-3.91	AAA-AAA	0.00	999	1.000	1.000	0.0550

214405_at	3.8	15.0	1.97	3.91	AAA-AAA	0.00	999	1.000	1.000	0.2099
233900_at	9.7	37.8	1.97	3.91	AAA-AAA	0.00	999	1.000	1.000	0.0524
230900_at	8.1	2.1	-1.97	-3.91	AAA-AAA	0.00	999	1.000	1.000	0.0893
1555335_at	19.0	4.9	-1.97	-3.91	AAA-AAA	0.00	999	1.000	1.000	0.0511
205028_at	4.1	16.1	1.97	3.91	AAA-AAA	0.00	999	1.000	1.000	0.1051
209638_x_at	11.0	42.8	1.97	3.91	AMA-AAA	0.00	999	1.000	1.000	0.1460
1555963_x_at	7.2	28.2	1.97	3.91	AAA-AAA	0.00	999	1.000	1.000	0.1046
208271_at	12.4	48.4	1.97	3.91	AAA-AAA	0.00	999	1.000	1.000	0.0110
1557836_at	69.7	17.9	-1.97	-3.90	AAA-AAA	0.00	999	1.000	1.000	0.0231
1556531_at	7.4	28.8	1.96	3.90	AAA-AAA	0.00	999	1.000	1.000	0.1303
1553037_a_at	15.4	4.0	-1.96	-3.90	AAA-AAA	0.00	999	1.000	1.000	0.1654
217589_at	40.5	10.4	-1.96	-3.90	AAA-AAA	0.00	999	1.000	1.000	0.1141
1562818_at	13.4	52.0	1.96	3.90	AAA-AAA	0.00	999	1.000	1.000	0.1787
242893_at	6.9	26.9	1.96	3.90	AAA-AAA	0.00	999	1.000	1.000	0.2571
232712_at	12.0	3.1	-1.96	-3.90	AAA-AAA	0.00	999	1.000	1.000	0.2266
237750_at	5.1	20.0	1.96	3.90	AAA-AAA	0.00	999	1.000	1.000	0.1753
1564444_at	89.0	22.8	-1.96	-3.90	PAA-MAA	0.00	999	1.000	1.000	0.2970
241969_at	32.9	8.4	-1.96	-3.90	AAA-AAA	0.00	999	1.000	1.000	0.1971
216633_s_at	18.8	73.1	1.96	3.90	AAA-AAA	0.00	999	1.000	1.000	0.0895
205940_at	15.6	4.0	-1.96	-3.90	AAA-AAA	0.00	999	1.000	1.000	0.2466
230709_x_at	23.3	6.0	-1.96	-3.90	AAA-AAA	0.00	999	1.000	1.000	0.1174
1555135_at	6.4	24.9	1.96	3.89	AAA-AAA	0.00	999	1.000	1.000	0.0936
231389_at	23.7	6.1	-1.96	-3.89	AAA-AAA	0.00	999	1.000	1.000	0.0039
231429_at	51.4	13.2	-1.96	-3.89	AAA-AAA	0.00	999	1.000	1.000	0.1868
1560098_at	17.4	4.5	-1.96	-3.89	AAA-AAA	0.00	999	1.000	1.000	0.1676
217431_x_at	26.8	6.9	-1.96	-3.89	AAA-AAA	0.00	999	1.000	1.000	0.0780
242478_at	81.1	20.9	-1.96	-3.89	AAA-AAA	0.00	999	1.000	1.000	0.2640
1567379_at	10.4	2.7	-1.96	-3.89	AAA-AAA	0.00	999	1.000	1.000	0.1625
1562431_x_at	2.3	9.0	1.96	3.88	AAA-AAA	0.00	999	1.000	1.000	0.2641
215614_at	11.9	46.2	1.96	3.88	AAA-AAA	0.00	999	1.000	1.000	0.0386
220188_at	19.5	75.9	1.96	3.88	AAA-AAA	0.00	999	1.000	1.000	0.2072
205841_at	12.9	50.1	1.96	3.88	AAA-AAA	0.00	999	1.000	1.000	0.1836
234145_at	8.7	33.8	1.96	3.88	AAA-AAA	0.00	999	1.000	1.000	0.0561
227240_at	39.2	10.1	-1.96	-3.88	AAA-AAA	0.00	999	1.000	1.000	0.0547
231308_at	43.6	11.2	-1.96	-3.88	AAA-AAA	0.00	999	1.000	1.000	0.0537
239018_at	43.4	11.2	-1.96	-3.88	AAP-AAA	0.00	999	1.000	1.000	0.0755
240553_at	20.8	5.4	-1.96	-3.88	AAA-AAA	0.00	999	1.000	1.000	0.2290
243544_at	5.5	21.1	1.96	3.88	AAA-AAA	0.00	999	1.000	1.000	0.1537
243932_at	9.6	37.2	1.96	3.88	AAA-AAA	0.00	999	1.000	1.000	0.2093
205866_at	46.1	11.9	-1.96	-3.88	AAA-AAA	0.00	999	1.000	1.000	0.0066
206705_at	20.9	81.1	1.96	3.88	AAA-AAA	0.00	999	1.000	1.000	0.1959
204931_at	5.5	21.2	1.96	3.88	AAA-AAA	0.00	999	1.000	1.000	0.1479

209242_at	17.4	4.5	-1.96	-3.88	AAA-AAA	0.00	999	1.000	1.000	0.2318
231491_at	6.8	26.3	1.96	3.88	AAA-AAA	0.00	999	1.000	1.000	0.2074
243929_at	12.7	3.3	-1.96	-3.88	AAA-AAA	0.00	999	1.000	1.000	0.0734
220416_at	9.0	35.0	1.96	3.88	AAA-AAA	0.00	999	1.000	1.000	0.1000
207228_at	8.6	33.2	1.95	3.88	AAA-AAA	0.00	999	1.000	1.000	0.0957
244548_at	9.2	35.8	1.95	3.87	AAA-AAA	0.00	999	1.000	1.000	0.2420
219331_s_at	33.7	8.7	-1.95	-3.87	AAA-AAA	0.00	999	1.000	1.000	0.0071
203632_s_at	20.7	5.4	-1.95	-3.87	AAA-AAA	0.00	999	1.000	1.000	0.1277
229053_at	55.6	14.4	-1.95	-3.87	AAA-AAA	0.00	999	1.000	1.000	0.0239
1566935_at	10.6	41.1	1.95	3.87	AAA-AAA	0.00	999	1.000	1.000	0.1455
217008_s_at	88.5	22.9	-1.95	-3.87	APA-AAA	0.00	999	1.000	1.000	0.1749
203060_s_at	110.4	28.6	-1.95	-3.87	AAA-PAA	0.00	999	1.000	1.000	0.2283
242519_at	21.1	5.5	-1.95	-3.87	AAA-AAA	0.00	999	1.000	1.000	0.2258
237912_at	39.8	10.3	-1.95	-3.87	AAA-AAA	0.00	999	1.000	1.000	0.2542
238928_at	4.0	15.4	1.95	3.87	AAA-AAA	0.00	999	1.000	1.000	0.0580
209483_s_at	34.6	8.9	-1.95	-3.86	AAP-AAA	0.00	999	1.000	1.000	0.0729
221054_s_at	2.2	8.5	1.95	3.86	AAA-AAA	0.00	999	1.000	1.000	0.0117
243541_at	110.7	28.7	-1.95	-3.86	APA-PAA	0.00	999	1.000	1.000	0.1836
243935_at	23.0	6.0	-1.95	-3.86	AAA-AAA	0.00	999	1.000	1.000	0.1708
220715_at	59.7	15.5	-1.95	-3.86	AAA-AAA	0.00	999	1.000	1.000	0.2583
205153_s_at	128.2	33.2	-1.95	-3.86	AAA-AAA	0.00	999	1.000	1.000	0.0666
234872_at	3.6	13.9	1.95	3.86	AAA-AAA	0.00	999	1.000	1.000	0.2269
1563469_at	42.0	10.9	-1.95	-3.86	AAA-AAA	0.00	999	1.000	1.000	0.0924
212732_at	14.6	56.2	1.95	3.86	AAA-AAA	0.00	999	1.000	1.000	0.0136
238671_at	20.6	5.4	-1.95	-3.86	AAA-AAA	0.00	999	1.000	1.000	0.2217
1563174_at	13.8	53.1	1.95	3.85	AAA-AAA	0.00	999	1.000	1.000	0.0503
223854_at	13.1	50.5	1.95	3.85	AAA-AAA	0.00	999	1.000	1.000	0.1129
1558636_s_at	4.5	17.2	1.95	3.85	AAA-AAA	0.00	999	1.000	1.000	0.1900
239685_at	64.3	16.7	-1.94	-3.85	AAA-AAA	0.00	999	1.000	1.000	0.1252
1554141_s_at	4.3	16.4	1.94	3.85	AAA-AAA	0.00	999	1.000	1.000	0.1018
1560087_a_at	11.8	45.4	1.94	3.85	AAA-AAA	0.00	999	1.000	1.000	0.1208
242682_at	8.5	32.8	1.94	3.84	AAA-AAA	0.00	999	1.000	1.000	0.2469
1556394_a_at	6.7	25.9	1.94	3.84	AAA-AAA	0.00	999	1.000	1.000	0.1312
208040_s_at	36.3	139.5	1.94	3.84	AAA-AAA	0.00	999	1.000	1.000	0.0590
1570064_at	68.3	17.8	-1.94	-3.84	AAA-AAA	0.00	999	1.000	1.000	0.0659
215478_at	55.5	14.5	-1.94	-3.84	APP-AAA	0.00	999	1.000	1.000	0.1763
210506_at	42.8	11.2	-1.94	-3.84	AAA-AAA	0.00	999	1.000	1.000	0.0923
233067_at	23.9	6.2	-1.94	-3.84	AAA-AAA	0.00	999	1.000	1.000	0.0702
232280_at	46.7	12.2	-1.94	-3.84	AAA-AAA	0.00	999	1.000	1.000	0.1011
1563620_at	22.4	5.9	-1.94	-3.83	AAA-AAA	0.00	999	1.000	1.000	0.2473
1563904_at	1.7	6.7	1.94	3.83	AAA-AAA	0.00	999	1.000	1.000	0.2010
244348_at	4.9	18.6	1.94	3.83	AAA-AAA	0.00	999	1.000	1.000	0.0773

215423_at	16.0	61.2	1.94	3.83	AAA-AAA	0.00	999	1.000	1.000	0.2796
232643_at	7.5	28.8	1.94	3.83	AAA-AAA	0.00	999	1.000	1.000	0.0771
1562607_at	7.6	28.9	1.94	3.83	AAA-AAA	0.00	999	1.000	1.000	0.1211
1566475_at	21.2	81.3	1.94	3.83	AAA-AAA	0.00	999	1.000	1.000	0.0192
220356_at	2.0	7.5	1.94	3.83	AAA-AAA	0.00	999	1.000	1.000	0.0590
1554778_at	7.8	2.0	-1.94	-3.83	AAA-AAA	0.00	999	1.000	1.000	0.1435
208323_s_at	23.2	88.6	1.94	3.83	AAA-AAA	0.00	999	1.000	1.000	0.2779
235900_at	48.0	12.5	-1.94	-3.83	AAA-AAA	0.00	999	1.000	1.000	0.1142
242216_at	3.2	12.1	1.94	3.82	AAA-AAA	0.00	999	1.000	1.000	0.1928
216365_x_at	5.4	20.8	1.94	3.82	AAA-AAA	0.00	999	1.000	1.000	0.0012
241060_x_at	4.6	17.7	1.93	3.82	AAA-AAA	0.00	999	1.000	1.000	0.0392
215697_at	62.3	16.3	-1.93	-3.82	AAA-AAA	0.00	999	1.000	1.000	0.1431
1558160_at	5.5	20.8	1.93	3.82	AAA-AAA	0.00	999	1.000	1.000	0.0215
1554908_at	4.8	18.4	1.93	3.82	AAA-AAA	0.00	999	1.000	1.000	0.0621
1556259_at	15.1	57.6	1.93	3.82	AAA-AAA	0.00	999	1.000	1.000	0.0996
241085_at	16.1	61.3	1.93	3.82	AAA-MAA	0.00	999	1.000	1.000	0.0793
1557066_at	36.8	9.6	-1.93	-3.82	AAA-AAA	0.00	999	1.000	1.000	0.2708
1554071_at	3.5	13.4	1.93	3.81	AAA-AAA	0.00	999	1.000	1.000	0.2393
240426_at	25.6	6.7	-1.93	-3.81	AAA-AAA	0.00	999	1.000	1.000	0.3280
1561249_a_at	23.5	6.2	-1.93	-3.81	AAA-AAA	0.00	999	1.000	1.000	0.1566
238080_at	44.0	11.5	-1.93	-3.81	AAA-AAA	0.00	999	1.000	1.000	0.0952
241038_at	6.9	26.2	1.93	3.81	AAA-AAA	0.00	999	1.000	1.000	0.0654
208401_s_at	15.4	58.5	1.93	3.81	AAA-AAA	0.00	999	1.000	1.000	0.0403
1563573_at	58.4	15.4	-1.93	-3.81	APA-AAA	0.00	999	1.000	1.000	0.0877
1568755_a_at	8.8	33.5	1.93	3.81	AAA-AAA	0.00	999	1.000	1.000	0.1535
1566041_at	29.5	7.8	-1.93	-3.80	AAA-AAA	0.00	999	1.000	1.000	0.2953
1554506_x_at	36.6	9.6	-1.93	-3.80	AAA-AAA	0.00	999	1.000	1.000	0.1714
243283_at	53.5	14.1	-1.93	-3.80	AAA-AAA	0.00	999	1.000	1.000	0.1036
243822_at	16.1	4.2	-1.93	-3.81	AAA-AAA	0.00	999	1.000	1.000	0.1522
236747_at	35.3	9.3	-1.93	-3.81	AAA-AAA	0.00	999	1.000	1.000	0.0608
239011_at	13.7	3.6	-1.93	-3.80	AAA-AAA	0.00	999	1.000	1.000	0.0769
232801_at	8.9	33.8	1.93	3.80	AAA-AAA	0.00	999	1.000	1.000	0.1768
1556997_a_at	5.1	19.4	1.93	3.80	AAA-AAA	0.00	999	1.000	1.000	0.2321
213719_s_at	11.6	44.2	1.93	3.80	AAA-AAA	0.00	999	1.000	1.000	0.0922
220828_s_at	4.0	15.3	1.93	3.80	AAA-AAA	0.00	999	1.000	1.000	0.0556
206726_at	21.8	5.7	-1.93	-3.80	AAA-AAA	0.00	999	1.000	1.000	0.0687
229889_at	15.7	4.1	-1.93	-3.80	AAA-AAA	0.00	999	1.000	1.000	0.0682
241189_at	6.6	25.1	1.93	3.80	AAA-AAA	0.00	999	1.000	1.000	0.0770
241881_at	8.5	32.4	1.93	3.80	AAA-AAA	0.00	999	1.000	1.000	0.2168
1557765_at	3.0	11.5	1.93	3.80	AAA-AAA	0.00	999	1.000	1.000	0.0521
216632_at	11.8	3.1	-1.93	-3.80	AAA-AAA	0.00	999	1.000	1.000	0.2841
226988_s_at	13.9	52.9	1.93	3.80	AAA-AAA	0.00	999	1.000	1.000	0.0456

219989_s_at	32.1	8.5	-1.92	-3.79	AAA-AAA	0.00	999	1.000	1.000	0.1638
234795_at	39.8	10.5	-1.92	-3.80	AAA-AAA	0.00	999	1.000	1.000	0.1765
238429_at	105.5	27.8	-1.92	-3.79	AAA-AAA	0.00	999	1.000	1.000	0.0987
242910_x_at	12.6	3.3	-1.92	-3.79	AAA-AAA	0.00	999	1.000	1.000	0.1561
1566899_at	15.9	4.2	-1.92	-3.79	AAP-AAA	0.00	999	1.000	1.000	0.1882
1563055_at	2.5	9.4	1.92	3.79	AAA-AAA	0.00	999	1.000	1.000	0.3932
221183_at	5.3	20.1	1.92	3.79	AAA-AAA	0.00	999	1.000	1.000	0.0932
206985_at	75.0	19.8	-1.92	-3.79	AAA-AAA	0.00	999	1.000	1.000	0.0090
229363_at	19.9	75.3	1.92	3.79	AAA-AAA	0.00	999	1.000	1.000	0.0869
240195_at	39.4	10.4	-1.92	-3.79	AAA-AAA	0.00	999	1.000	1.000	0.1744
1570196_at	9.3	35.3	1.92	3.78	AAA-AAA	0.00	999	1.000	1.000	0.1803
234207_at	6.3	24.0	1.92	3.79	AAA-AAA	0.00	999	1.000	1.000	0.0933
224354_at	27.5	7.3	-1.92	-3.78	AAA-AAA	0.00	999	1.000	1.000	0.1064
219845_at	13.2	49.9	1.92	3.78	AAA-AAA	0.00	999	1.000	1.000	0.1298
1553635_s_at	13.5	3.6	-1.92	-3.78	AAA-AAA	0.00	999	1.000	1.000	0.1801
220294_at	2.3	8.6	1.92	3.78	AAA-AAA	0.00	999	1.000	1.000	0.1821
207989_at	46.7	12.4	-1.92	-3.78	AAA-AAA	0.00	999	1.000	1.000	0.1461
240799_at	8.1	30.5	1.92	3.78	AAA-AAA	0.00	999	1.000	1.000	0.2097
234951_s_at	120.6	31.9	-1.92	-3.78	AAA-AAA	0.00	999	1.000	1.000	0.2794
235863_at	7.6	28.7	1.92	3.78	AAA-AAA	0.00	999	1.000	1.000	0.1615
237193_s_at	11.0	41.5	1.92	3.78	AAA-AAA	0.00	999	1.000	1.000	0.1496
228470_at	5.9	22.2	1.92	3.78	AAA-AAA	0.00	999	1.000	1.000	0.1204
1567575_at	24.6	6.5	-1.92	-3.77	AAA-AAA	0.00	999	1.000	1.000	0.1830
230670_at	8.7	33.0	1.92	3.77	AAA-AAA	0.00	999	1.000	1.000	0.0277
1557430_at	3.9	14.5	1.92	3.77	AAA-AAA	0.00	999	1.000	1.000	0.1894
231379_at	18.2	4.8	-1.92	-3.77	AAA-AAA	0.00	999	1.000	1.000	0.1459
1568735_at	44.5	11.8	-1.91	-3.77	AAA-AAA	0.00	999	1.000	1.000	0.2219
207427_at	67.6	17.9	-1.91	-3.77	AAA-AAA	0.00	999	1.000	1.000	0.0026
238046_x_at	7.5	28.3	1.91	3.77	AAA-AAA	0.00	999	1.000	1.000	0.0641
236272_at	23.4	6.2	-1.91	-3.77	AAA-AAA	0.00	999	1.000	1.000	0.2668
236575_at	12.3	3.3	-1.91	-3.77	AAA-AAA	0.00	999	1.000	1.000	0.2242
230377_s_at	20.4	5.4	-1.91	-3.77	AAA-AAA	0.00	999	1.000	1.000	0.0405
239359_at	59.2	15.7	-1.91	-3.77	AAA-AAA	0.00	999	1.000	1.000	0.1761
1553456_at	35.5	9.4	-1.91	-3.77	AAA-AAA	0.00	999	1.000	1.000	0.1120
222344_at	10.1	37.9	1.91	3.77	AAA-AAA	0.00	999	1.000	1.000	0.3178
243551_at	9.2	34.8	1.91	3.76	AAA-AAA	0.00	999	1.000	1.000	0.0481
215508_at	7.1	26.9	1.91	3.77	AAA-AAA	0.00	999	1.000	1.000	0.3363
217250_s_at	12.3	3.3	-1.91	-3.76	AAA-AAA	0.00	999	1.000	1.000	0.0555
1556421_at	6.9	1.8	-1.91	-3.76	AAA-AAA	0.00	999	1.000	1.000	0.0903
1553171_x_at	72.0	19.1	-1.91	-3.76	AAA-AAA	0.00	999	1.000	1.000	0.1498
231046_at	6.6	24.8	1.91	3.76	AAA-AAA	0.00	999	1.000	1.000	0.1075
1570515_a_at	4.2	15.7	1.91	3.76	AAA-AAA	0.00	999	1.000	1.000	0.1434

217210_at	90.8	24.2	-1.91	-3.76	AAA-AAA	0.00	999	1.000	1.000	0.2369
207371_at	3.5	13.1	1.91	3.76	AAA-AAA	0.00	999	1.000	1.000	0.0773
230145_at	70.5	18.8	-1.91	-3.76	AAA-AAA	0.00	999	1.000	1.000	0.1617
1552705_at	6.5	24.4	1.91	3.76	AAA-AAA	0.00	999	1.000	1.000	0.2475
1561767_at	12.7	3.4	-1.91	-3.75	AAA-AAA	0.00	999	1.000	1.000	0.1522
243937_x_at	61.2	16.3	-1.91	-3.75	AAA-AAA	0.00	999	1.000	1.000	0.0508
217305_s_at	17.5	65.5	1.91	3.75	AAA-AAA	0.00	999	1.000	1.000	0.0138
234890_at	16.1	60.3	1.91	3.75	AAA-AAA	0.00	999	1.000	1.000	0.0321
243186_at	40.7	10.9	-1.91	-3.75	PAA-AAA	0.00	999	1.000	1.000	0.0720
1562998_at	2.5	9.5	1.91	3.75	AAA-MAA	0.00	999	1.000	1.000	0.3551
1556192_x_at	22.3	6.0	-1.91	-3.75	AAA-AAA	0.00	999	1.000	1.000	0.1557
242742_at	9.2	34.3	1.91	3.75	AAA-AAA	0.00	999	1.000	1.000	0.0718
230717_at	42.4	11.3	-1.91	-3.75	PAA-AAA	0.00	999	1.000	1.000	0.3473
1566271_x_at	16.2	4.3	-1.91	-3.75	AAA-AAA	0.00	999	1.000	1.000	0.0531
1561737_at	24.8	6.6	-1.91	-3.75	AAA-AAA	0.00	999	1.000	1.000	0.3394
228264_at	122.6	32.7	-1.91	-3.75	AAA-AAA	0.00	999	1.000	1.000	0.1255
1554147_s_at	7.7	28.9	1.90	3.74	AAA-AAA	0.00	999	1.000	1.000	0.0152
214944_at	19.0	71.1	1.90	3.74	AAA-AAA	0.00	999	1.000	1.000	0.1324
228507_at	5.9	22.2	1.90	3.74	AAA-AAA	0.00	999	1.000	1.000	0.0647
1560517_s_at	5.6	21.0	1.90	3.74	AAA-AAA	0.00	999	1.000	1.000	0.1420
206396_at	31.9	8.5	-1.90	-3.74	AAA-AAA	0.00	999	1.000	1.000	0.2198
205826_at	7.6	28.3	1.90	3.74	AAA-AAA	0.00	999	1.000	1.000	0.3004
210321_at	24.5	6.6	-1.90	-3.74	AAA-AAA	0.00	999	1.000	1.000	0.0436
1564787_at	19.0	71.1	1.90	3.74	AAA-AAA	0.00	999	1.000	1.000	0.1783
1569421_at	53.6	14.3	-1.90	-3.74	AAA-AAA	0.00	999	1.000	1.000	0.0629
1552566_at	9.9	2.7	-1.90	-3.74	AAA-AAA	0.00	999	1.000	1.000	0.0808
1555353_at	9.2	34.4	1.90	3.74	AAA-AAA	0.00	999	1.000	1.000	0.1528
211923_s_at	3.2	11.9	1.90	3.74	AAA-AAA	0.00	999	1.000	1.000	0.0736
208208_at	15.4	57.7	1.90	3.74	AAA-AAA	0.00	999	1.000	1.000	0.0499
33197_at	86.8	23.2	-1.90	-3.74	APM-AAA	0.00	999	1.000	1.000	0.2364
243032_at	6.6	24.7	1.90	3.74	AAA-AAA	0.00	999	1.000	1.000	0.2117
221266_s_at	42.8	11.5	-1.90	-3.74	AAA-AAA	0.00	999	1.000	1.000	0.1746
205614_x_at	32.8	122.5	1.90	3.74	AAA-AAA	0.00	999	1.000	1.000	0.0035
207759_s_at	62.9	16.8	-1.90	-3.73	AAA-AAA	0.00	999	1.000	1.000	0.0274
237653_at	20.5	76.4	1.90	3.74	AAA-AAA	0.00	999	1.000	1.000	0.1109
40687_at	63.7	17.1	-1.90	-3.73	AAP-AAA	0.00	999	1.000	1.000	0.1307
227629_at	52.9	14.2	-1.90	-3.73	PPA-APA	0.00	999	1.000	1.000	0.3658
1556899_at	20.4	76.0	1.90	3.73	AAA-AAA	0.00	999	1.000	1.000	0.1622
210861_s_at	5.8	21.7	1.90	3.73	AAA-AAA	0.00	999	1.000	1.000	0.3711
235779_at	18.8	5.0	-1.90	-3.73	AAA-AAA	0.00	999	1.000	1.000	0.2001
1563696_at	8.3	2.2	-1.90	-3.73	AAA-AAA	0.00	999	1.000	1.000	0.2493
241133_at	14.2	53.0	1.90	3.73	AAA-AAA	0.00	999	1.000	1.000	0.0572

1552846_s_at	112.2	30.1	-1.90	-3.73	AAA-AAA	0.00	999	1.000	1.000	0.1519
244112_x_at	2.6	9.7	1.90	3.73	AAA-AAA	0.00	999	1.000	1.000	0.3141
206144_at	41.0	11.0	-1.90	-3.73	AAA-AAA	0.00	999	1.000	1.000	0.1288
236877_at	15.6	58.1	1.90	3.73	AAA-AAA	0.00	999	1.000	1.000	0.2388
1558672_at	15.6	4.2	-1.90	-3.73	AAA-AAA	0.00	999	1.000	1.000	0.0422
1561294_a_at	36.9	9.9	-1.90	-3.73	AAA-AAA	0.00	999	1.000	1.000	0.2343
219738_s_at	15.1	56.3	1.90	3.73	AAA-AAA	0.00	999	1.000	1.000	0.0752
223612_s_at	14.4	53.5	1.90	3.73	AAA-AAA	0.00	999	1.000	1.000	0.0272
241128_at	5.3	19.7	1.90	3.72	AAA-AAA	0.00	999	1.000	1.000	0.1966
206237_s_at	18.9	70.2	1.90	3.73	AAA-AAA	0.00	999	1.000	1.000	0.1061
233396_s_at	11.3	41.9	1.90	3.73	AAA-AAA	0.00	999	1.000	1.000	0.2362
224405_at	10.2	2.7	-1.90	-3.73	AAA-AAA	0.00	999	1.000	1.000	0.2063
231741_at	80.0	21.5	-1.90	-3.72	AAA-AAA	0.00	999	1.000	1.000	0.1042
1564709_at	89.5	24.1	-1.90	-3.72	MPP-AAA	0.00	999	1.000	1.000	0.0469
1568939_at	4.0	15.0	1.90	3.72	AAA-AAA	0.00	999	1.000	1.000	0.1717
1570048_at	16.0	59.7	1.90	3.72	AAA-AAA	0.00	999	1.000	1.000	0.0990
215432_at	3.9	14.5	1.90	3.72	AAA-AAA	0.00	999	1.000	1.000	0.1715
220767_at	41.0	11.0	-1.90	-3.72	AAA-AAA	0.00	999	1.000	1.000	0.0656
208747_s_at	26.7	7.2	-1.90	-3.72	AAA-AAA	0.00	999	1.000	1.000	0.1139
227845_s_at	14.4	53.6	1.90	3.72	AAA-AAA	0.00	999	1.000	1.000	0.1262
212706_at	46.9	12.6	-1.89	-3.72	PAA-AAP	0.00	999	1.000	1.000	0.3462
1553296_at	26.6	7.1	-1.89	-3.72	AAA-AAA	0.00	999	1.000	1.000	0.3009
201354_s_at	257.3	69.3	-1.89	-3.72	AAA-AAA	0.00	999	1.000	1.000	0.3146
205306_x_at	9.7	36.0	1.89	3.72	AAA-AAA	0.00	999	1.000	1.000	0.1545
1554847_at	70.7	19.0	-1.89	-3.72	AAA-AAA	0.00	999	1.000	1.000	0.2708
244253_at	6.9	25.4	1.89	3.71	AAA-AAA	0.00	999	1.000	1.000	0.0725
231520_at	15.8	58.6	1.89	3.71	AAA-AAA	0.00	999	1.000	1.000	0.0095
1561003_at	6.6	24.6	1.89	3.71	AAA-AAA	0.00	999	1.000	1.000	0.1104
204943_at	17.2	64.0	1.89	3.71	AAA-AAA	0.00	999	1.000	1.000	0.0405
209671_x_at	71.1	19.2	-1.89	-3.71	AAA-AAA	0.00	999	1.000	1.000	0.1561
1566528_at	14.5	53.9	1.89	3.71	AAA-AAA	0.00	999	1.000	1.000	0.0276
210108_at	26.3	7.1	-1.89	-3.71	AAA-AAA	0.00	999	1.000	1.000	0.1893
233160_at	11.1	40.9	1.89	3.71	AAA-AAA	0.00	999	1.000	1.000	0.0365
233412_x_at	14.6	54.1	1.89	3.71	AAA-AAA	0.00	999	1.000	1.000	0.1104
1561474_at	8.2	30.3	1.89	3.70	AAA-AAA	0.00	999	1.000	1.000	0.2020
234721_s_at	9.5	35.1	1.89	3.71	AAA-AAA	0.00	999	1.000	1.000	0.1321
235858_at	26.1	7.1	-1.89	-3.71	AAA-AAA	0.00	999	1.000	1.000	0.1433
231802_at	9.1	33.7	1.89	3.71	AAA-AAA	0.00	999	1.000	1.000	0.1314
230550_at	8.3	30.8	1.89	3.70	AAA-AAA	0.00	999	1.000	1.000	0.2096
242771_at	15.5	57.3	1.89	3.70	AAA-AAA	0.00	999	1.000	1.000	0.1088
220711_at	63.5	17.2	-1.89	-3.70	AAA-AAA	0.00	999	1.000	1.000	0.0003
1558957_s_at	12.1	3.3	-1.89	-3.70	AAA-AAA	0.00	999	1.000	1.000	0.4143

238839_at	10.0	37.0	1.89	3.70	AAA-AAA	0.00	999	1.000	1.000	0.0877
1559928_at	29.6	8.0	-1.89	-3.70	PAA-AAA	0.00	999	1.000	1.000	0.1460
206274_s_at	9.4	34.9	1.89	3.70	AAA-AAA	0.00	999	1.000	1.000	0.1577
227613_at	46.7	12.6	-1.89	-3.70	AMA-AAA	0.00	999	1.000	1.000	0.0085
220574_at	18.3	67.7	1.89	3.69	AAA-AAA	0.00	999	1.000	1.000	0.2098
220896_at	26.9	99.2	1.89	3.69	AAA-AAA	0.00	999	1.000	1.000	0.0072
1555912_at	64.4	17.4	-1.88	-3.69	AAA-AAA	0.00	999	1.000	1.000	0.0575
234097_s_at	13.3	3.6	-1.88	-3.69	AAA-AAA	0.00	999	1.000	1.000	0.2624
229910_at	13.2	48.8	1.88	3.69	AAA-AAA	0.00	999	1.000	1.000	0.1354
1561347_a_at	11.7	3.2	-1.88	-3.69	AAA-AAA	0.00	999	1.000	1.000	0.3675
1567054_at	16.5	60.8	1.88	3.69	AAA-AAA	0.00	999	1.000	1.000	0.1035
1556383_at	68.5	18.6	-1.88	-3.69	AMA-AAA	0.00	999	1.000	1.000	0.1427
215868_x_at	31.6	8.6	-1.88	-3.69	AAA-AAA	0.00	999	1.000	1.000	0.1874
212977_at	14.5	53.3	1.88	3.69	AAA-AAA	0.00	999	1.000	1.000	0.0271
244267_at	15.4	4.2	-1.88	-3.68	AAA-AAA	0.00	999	1.000	1.000	0.1312
216473_x_at	37.6	10.2	-1.88	-3.68	AAA-AAA	0.00	999	1.000	1.000	0.1658
211579_at	7.9	29.1	1.88	3.68	AAA-AAA	0.00	999	1.000	1.000	0.0227
241496_at	27.6	101.5	1.88	3.68	AAA-AAA	0.00	999	1.000	1.000	0.0783
241411_at	117.1	31.8	-1.88	-3.68	AAA-AAA	0.00	999	1.000	1.000	0.0236
1561422_at	5.2	19.2	1.88	3.68	AAA-AAA	0.00	999	1.000	1.000	0.0373
243430_at	21.0	77.3	1.88	3.68	AAA-AAA	0.00	999	1.000	1.000	0.0620
1559925_s_at	7.9	29.2	1.88	3.68	AAA-AAA	0.00	999	1.000	1.000	0.2423
213042_s_at	6.6	24.2	1.88	3.68	AAA-AAA	0.00	999	1.000	1.000	0.1309
241837_at	10.1	37.0	1.88	3.68	AAA-AAA	0.00	999	1.000	1.000	0.1055
1570015_at	16.4	4.5	-1.88	-3.68	AAA-AAA	0.00	999	1.000	1.000	0.1504
1567986_at	28.7	7.8	-1.88	-3.68	AAM-AAA	0.00	999	1.000	1.000	0.0964
1562392_at	36.0	9.8	-1.88	-3.68	AAA-AAA	0.00	999	1.000	1.000	0.0948
233721_x_at	36.0	9.8	-1.88	-3.68	AAA-AAA	0.00	999	1.000	1.000	0.1494
1563560_at	35.6	9.7	-1.88	-3.68	AAA-AAA	0.00	999	1.000	1.000	0.0530
236914_at	43.7	11.9	-1.88	-3.68	AAA-AAA	0.00	999	1.000	1.000	0.0643
241296_at	2.8	10.2	1.88	3.67	AAA-AAA	0.00	999	1.000	1.000	0.1946
243632_at	17.2	4.7	-1.88	-3.67	AAA-AAA	0.00	999	1.000	1.000	0.0828
1565918_a_at	71.3	19.4	-1.88	-3.67	PAA-AMA	0.00	999	1.000	1.000	0.1354
1552523_a_at	32.1	8.7	-1.88	-3.67	AAA-AAA	0.00	999	1.000	1.000	0.0472
206231_at	200.0	54.5	-1.88	-3.67	AAA-AAA	0.00	999	1.000	1.000	0.0461
241251_at	6.5	24.0	1.88	3.67	AAA-AAA	0.00	999	1.000	1.000	0.0311
1552760_at	10.5	2.9	-1.88	-3.67	AAA-AAA	0.00	999	1.000	1.000	0.0376
244179_x_at	40.1	10.9	-1.88	-3.67	AAA-AAA	0.00	999	1.000	1.000	0.0989
240023_at	30.5	111.7	1.87	3.67	AAA-AAA	0.00	999	1.000	1.000	0.2938
240960_at	11.6	42.4	1.87	3.67	AAA-AAA	0.00	999	1.000	1.000	0.0033
1553385_at	36.6	10.0	-1.87	-3.67	AAA-AAA	0.00	999	1.000	1.000	0.0489
1553949_at	9.3	34.0	1.87	3.67	AAA-AAA	0.00	999	1.000	1.000	0.0605

1561278_at	4.5	16.6	1.87	3.67	AAA-AAA	0.00	999	1.000	1.000	0.0041
243689_s_at	8.2	30.1	1.87	3.66	AAA-AAA	0.00	999	1.000	1.000	0.1162
243483_at	2.7	9.7	1.87	3.66	AAA-AAA	0.00	999	1.000	1.000	0.1939
213580_at	5.1	18.8	1.87	3.66	AAA-AAA	0.00	999	1.000	1.000	0.1033
227734_s_at	21.6	5.9	-1.87	-3.66	AAA-AAA	0.00	999	1.000	1.000	0.3028
228137_s_at	31.1	8.5	-1.87	-3.66	AAA-AAA	0.00	999	1.000	1.000	0.1236
238862_at	28.2	7.7	-1.87	-3.66	AAA-AAA	0.00	999	1.000	1.000	0.0411
240905_at	5.8	21.3	1.87	3.65	AAA-AAA	0.00	999	1.000	1.000	0.0918
241929_at	51.4	14.1	-1.87	-3.65	MAP-AAA	0.00	999	1.000	1.000	0.1279
240022_at	5.9	21.6	1.87	3.65	AAA-AAA	0.00	999	1.000	1.000	0.0436
209582_s_at	16.3	59.6	1.87	3.65	AAA-AAA	0.00	999	1.000	1.000	0.0554
237359_at	16.6	60.6	1.87	3.65	AAA-AAA	0.00	999	1.000	1.000	0.0714
236403_at	9.5	34.8	1.87	3.65	AAA-AAA	0.00	999	1.000	1.000	0.1477
207938_at	71.8	19.7	-1.87	-3.65	PAA-AAA	0.00	999	1.000	1.000	0.2106
1554702_at	13.1	47.7	1.87	3.65	AAA-AAA	0.00	999	1.000	1.000	0.1534
1559810_at	11.7	42.6	1.87	3.65	AAA-AAA	0.00	999	1.000	1.000	0.0969
1559195_at	12.8	3.5	-1.87	-3.65	AAA-AAA	0.00	999	1.000	1.000	0.0177
215212_at	9.9	36.2	1.87	3.65	AAA-AAM	0.00	999	1.000	1.000	0.1289
220339_s_at	114.8	31.5	-1.87	-3.65	AAA-AAA	0.00	999	1.000	1.000	0.0600
234808_x_at	7.7	28.2	1.87	3.65	AAA-AAA	0.00	999	1.000	1.000	0.1014
242049_s_at	9.8	35.6	1.87	3.65	AAA-AAA	0.00	999	1.000	1.000	0.0972
213339_at	55.8	15.3	-1.87	-3.65	AAA-AAA	0.00	999	1.000	1.000	0.1758
1562696_at	23.3	6.4	-1.87	-3.64	AAA-AAA	0.00	999	1.000	1.000	0.0256
1553886_at	4.4	1.2	-1.87	-3.64	AAA-AAA	0.00	999	1.000	1.000	0.2673
243127_x_at	8.1	29.7	1.87	3.64	AAA-AAA	0.00	999	1.000	1.000	0.2080
206941_x_at	16.6	60.3	1.87	3.64	AAA-AAA	0.00	999	1.000	1.000	0.1765
207236_at	5.7	20.7	1.87	3.64	AAA-AAA	0.00	999	1.000	1.000	0.1623
229601_at	8.3	30.1	1.87	3.64	AAA-AAA	0.00	999	1.000	1.000	0.0445
1552875_a_at	4.4	15.9	1.86	3.64	AAA-AAA	0.00	999	1.000	1.000	0.0128
205798_at	82.8	22.8	-1.86	-3.64	AAA-AAA	0.00	999	1.000	1.000	0.0055
215798_at	111.6	30.7	-1.86	-3.64	PAA-AAA	0.00	999	1.000	1.000	0.1558
1557633_at	55.1	15.2	-1.86	-3.64	AAA-AAA	0.00	999	1.000	1.000	0.0064
1560483_at	3.5	12.8	1.86	3.64	AAA-AAA	0.00	999	1.000	1.000	0.2794
237094_at	30.0	8.3	-1.86	-3.64	AAA-AAA	0.00	999	1.000	1.000	0.0188
244755_at	82.7	22.8	-1.86	-3.64	AAA-AAA	0.00	999	1.000	1.000	0.0925
232634_at	36.4	10.0	-1.86	-3.64	AAA-AAA	0.00	999	1.000	1.000	0.0981
232426_at	12.5	45.3	1.86	3.64	AAA-AAA	0.00	999	1.000	1.000	0.0978
240187_at	7.9	28.7	1.86	3.63	AAA-AAA	0.00	999	1.000	1.000	0.1932
206045_s_at	14.1	51.3	1.86	3.63	AAA-AAA	0.00	999	1.000	1.000	0.1641
234861_at	8.4	30.5	1.86	3.63	AAA-AAA	0.00	999	1.000	1.000	0.2415
242067_at	17.7	64.1	1.86	3.63	AAA-AAA	0.00	999	1.000	1.000	0.2311
221228_s_at	8.5	30.8	1.86	3.63	AAA-AAA	0.00	999	1.000	1.000	0.0843

208586_s_at	15.4	4.2	-1.86	-3.63	AAA-AAA	0.00	999	1.000	1.000	0.1324
229090_at	129.2	35.6	-1.86	-3.63	PPP-AAM	0.00	999	1.000	1.000	0.1078
240972_at	6.1	22.1	1.86	3.63	AAA-AAA	0.00	999	1.000	1.000	0.1731
240440_at	12.5	45.4	1.86	3.63	AAA-AAA	0.00	999	1.000	1.000	0.1920
217575_s_at	8.0	2.2	-1.86	-3.63	AAA-AAA	0.00	999	1.000	1.000	0.1210
219955_at	43.5	12.0	-1.86	-3.63	AAA-AAA	0.00	999	1.000	1.000	0.0216
1557026_at	86.0	23.7	-1.86	-3.63	APP-AAA	0.00	999	1.000	1.000	0.0395
1561899_at	12.3	44.7	1.86	3.62	AAA-AAA	0.00	999	1.000	1.000	0.0942
213560_at	32.2	116.9	1.86	3.63	AAA-AAA	0.00	999	1.000	1.000	0.1979
207873_x_at	46.2	12.7	-1.86	-3.63	AAA-AAA	0.00	999	1.000	1.000	0.1359
234458_at	19.5	5.4	-1.86	-3.62	AAA-AAA	0.00	999	1.000	1.000	0.2347
227544_at	8.7	31.5	1.86	3.62	AAA-AAA	0.00	999	1.000	1.000	0.0175
240099_at	15.3	55.3	1.86	3.62	AAA-AAA	0.00	999	1.000	1.000	0.0214
223561_at	11.7	42.2	1.86	3.62	AAA-AAA	0.00	999	1.000	1.000	0.2159
216030_s_at	6.7	24.3	1.86	3.62	AAA-AAA	0.00	999	1.000	1.000	0.2649
224136_at	17.3	62.6	1.86	3.62	AAA-AAA	0.00	999	1.000	1.000	0.2985
1557780_at	48.9	13.5	-1.85	-3.61	AAA-AAA	0.00	999	1.000	1.000	0.2383
1562080_at	25.5	7.0	-1.85	-3.62	AAA-AAA	0.00	999	1.000	1.000	0.2363
1560201_at	16.9	4.7	-1.85	-3.62	AAA-AAA	0.00	999	1.000	1.000	0.0333
236964_at	9.7	35.2	1.85	3.62	AAA-AAA	0.00	999	1.000	1.000	0.2408
1565776_at	2.2	7.8	1.85	3.61	AAA-AAA	0.00	999	1.000	1.000	0.0141
1554436_a_at	13.5	48.6	1.85	3.61	AAA-AAA	0.00	999	1.000	1.000	0.0645
206423_at	31.5	8.7	-1.85	-3.61	AAA-AAA	0.00	999	1.000	1.000	0.0772
222296_at	20.1	5.6	-1.85	-3.61	AAA-AAA	0.00	999	1.000	1.000	0.0157
1562491_at	3.2	11.4	1.85	3.61	AAA-AAA	0.00	999	1.000	1.000	0.3133
215961_at	5.0	18.1	1.85	3.61	AAA-AAA	0.00	999	1.000	1.000	0.1069
220539_at	14.3	51.5	1.85	3.61	AAA-AAA	0.00	999	1.000	1.000	0.1491
1564299_at	11.8	3.3	-1.85	-3.61	APA-AAA	0.00	999	1.000	1.000	0.2674
242128_at	6.0	21.6	1.85	3.61	AAA-AAA	0.00	999	1.000	1.000	0.3244
240564_x_at	3.5	12.8	1.85	3.61	AAA-AAA	0.00	999	1.000	1.000	0.2884
237125_at	61.5	17.1	-1.85	-3.61	AAA-AAA	0.00	999	1.000	1.000	0.0477
1553531_at	6.0	21.8	1.85	3.60	AAA-AAA	0.00	999	1.000	1.000	0.1078
217287_s_at	8.0	28.9	1.85	3.61	AAA-AAA	0.00	999	1.000	1.000	0.2665
233042_at	59.4	16.5	-1.85	-3.61	AAA-AAA	0.00	999	1.000	1.000	0.0728
235303_at	26.9	7.5	-1.85	-3.60	AAP-AAA	0.00	999	1.000	1.000	0.1634
1560542_at	17.9	5.0	-1.85	-3.60	AAA-AAA	0.00	999	1.000	1.000	0.0394
211829_s_at	10.2	36.7	1.85	3.60	AAA-AAA	0.00	999	1.000	1.000	0.3552
234284_at	5.0	18.1	1.85	3.60	AAA-AAA	0.00	999	1.000	1.000	0.1367
1568832_a_at	29.4	8.2	-1.85	-3.60	AAM-AAA	0.00	999	1.000	1.000	0.2155
1565900_at	47.4	13.2	-1.85	-3.60	AAA-AAA	0.00	999	1.000	1.000	0.1527
233491_at	3.8	13.6	1.85	3.60	AAA-AAA	0.00	999	1.000	1.000	0.0079
240498_at	13.7	49.3	1.85	3.60	AAA-AAA	0.00	999	1.000	1.000	0.1421

237984_x_at	5.3	19.1	1.85	3.60	AAA-AAA	0.00	999	1.000	1.000	0.2848
224032_x_at	9.5	34.1	1.85	3.60	AAA-AAA	0.00	999	1.000	1.000	0.0657
1562669_at	14.3	4.0	-1.85	-3.59	AAA-AAA	0.00	999	1.000	1.000	0.1629
233394_at	23.0	82.6	1.85	3.60	AAA-AAA	0.00	999	1.000	1.000	0.0955
236221_at	40.3	11.2	-1.85	-3.59	AAA-AAA	0.00	999	1.000	1.000	0.2425
232570_s_at	5.5	19.6	1.85	3.59	AAA-AAA	0.00	999	1.000	1.000	0.0574
237453_at	85.8	23.9	-1.85	-3.59	AAA-AAA	0.00	999	1.000	1.000	0.1291
204539_s_at	6.7	24.1	1.84	3.59	AAA-AAA	0.00	999	1.000	1.000	0.1648
233249_at	42.7	11.9	-1.84	-3.59	AAA-AAA	0.00	999	1.000	1.000	0.0570
228731_at	13.9	3.9	-1.84	-3.59	AAA-AAA	0.00	999	1.000	1.000	0.2516
239454_at	15.4	55.3	1.84	3.59	AAA-AAA	0.00	999	1.000	1.000	0.1248
244424_at	13.7	49.0	1.84	3.59	AAA-AAA	0.00	999	1.000	1.000	0.0704
233956_at	12.6	45.1	1.84	3.59	AAA-AAA	0.00	999	1.000	1.000	0.1283
233610_at	20.0	5.6	-1.84	-3.59	AAA-AAA	0.00	999	1.000	1.000	0.1661
1567540_at	11.8	42.3	1.84	3.59	AAA-AAA	0.00	999	1.000	1.000	0.1412
221186_at	14.7	52.6	1.84	3.58	AAA-AAA	0.00	999	1.000	1.000	0.1315
228215_at	7.3	26.3	1.84	3.59	AAA-AAA	0.00	999	1.000	1.000	0.1491
204114_at	22.2	6.2	-1.84	-3.58	AAA-AAA	0.00	999	1.000	1.000	0.0539
233805_at	25.5	7.1	-1.84	-3.58	AAA-AAA	0.00	999	1.000	1.000	0.0258
237902_at	4.0	14.4	1.84	3.58	AAA-AAA	0.00	999	1.000	1.000	0.1789
228679_at	34.5	9.6	-1.84	-3.58	AAA-AAA	0.00	999	1.000	1.000	0.2981
1553087_at	8.4	29.9	1.84	3.58	AAA-AAA	0.00	999	1.000	1.000	0.0593
217684_at	23.9	85.7	1.84	3.58	AAA-AAA	0.00	999	1.000	1.000	0.1195
234409_at	4.0	14.4	1.84	3.58	AAA-AAA	0.00	999	1.000	1.000	0.1203
237739_at	13.3	47.4	1.84	3.58	AAA-AAA	0.00	999	1.000	1.000	0.1675
229357_at	20.5	5.7	-1.84	-3.58	AAA-AAA	0.00	999	1.000	1.000	0.1604
213125_at	50.4	14.1	-1.84	-3.58	APA-AAA	0.00	999	1.000	1.000	0.1668
241533_at	44.6	12.5	-1.84	-3.57	AAA-AAA	0.00	999	1.000	1.000	0.0109
1558423_at	13.1	46.9	1.84	3.58	AAA-AAA	0.00	999	1.000	1.000	0.0543
211533_at	3.6	12.9	1.84	3.58	AAA-AAA	0.00	999	1.000	1.000	0.3305
236861_at	56.9	15.9	-1.84	-3.57	PAA-AAA	0.00	999	1.000	1.000	0.1112
1560566_at	16.5	4.6	-1.84	-3.57	AAA-AAA	0.00	999	1.000	1.000	0.1296
215619_at	3.2	11.4	1.84	3.57	AAA-AAA	0.00	999	1.000	1.000	0.2782
206637_at	5.3	18.8	1.84	3.57	AAA-AAA	0.00	999	1.000	1.000	0.2130
236660_at	34.2	9.6	-1.84	-3.57	AAA-AAA	0.00	999	1.000	1.000	0.2298
228199_at	5.8	20.8	1.84	3.57	AAA-AAA	0.00	999	1.000	1.000	0.0722
211925_s_at	11.6	3.2	-1.84	-3.57	AAA-AAA	0.00	999	1.000	1.000	0.2682
241834_at	33.8	9.5	-1.83	-3.56	AAA-AAA	0.00	999	1.000	1.000	0.1488
1557809_a_at	13.5	48.1	1.83	3.57	AAA-AAA	0.00	999	1.000	1.000	0.0021
237634_at	13.9	49.6	1.83	3.57	AAA-AAM	0.00	999	1.000	1.000	0.1936
1566984_at	8.4	30.0	1.83	3.56	AAA-AAA	0.00	999	1.000	1.000	0.2570
1556592_at	6.0	1.7	-1.83	-3.56	AAA-AAA	0.00	999	1.000	1.000	0.3375

1553462_at	9.4	33.3	1.83	3.56	AAA-AAA	0.00	999	1.000	1.000	0.2244
212466_at	20.3	72.5	1.83	3.56	AAA-AAA	0.00	999	1.000	1.000	0.0009
205471_s_at	11.1	39.4	1.83	3.56	AAA-AAA	0.00	999	1.000	1.000	0.1543
1553328_a_at	8.1	28.9	1.83	3.56	AAA-AAA	0.00	999	1.000	1.000	0.1043
1560940_at	8.9	31.5	1.83	3.56	AAA-AAA	0.00	999	1.000	1.000	0.1329
233848_x_at	5.2	18.4	1.83	3.56	AAA-AAA	0.00	999	1.000	1.000	0.0404
1562946_at	21.9	77.9	1.83	3.56	AAA-AAA	0.00	999	1.000	1.000	0.0490
240285_at	30.4	8.6	-1.83	-3.56	AAA-AAA	0.00	999	1.000	1.000	0.1998
207583_at	10.8	3.0	-1.83	-3.56	AAA-AAA	0.00	999	1.000	1.000	0.1256
229309_at	5.0	17.8	1.83	3.56	AAA-AAA	0.00	999	1.000	1.000	0.1907
241765_at	36.4	10.3	-1.83	-3.55	AAP-AAA	0.00	999	1.000	1.000	0.2188
1552511_a_at	4.9	17.3	1.83	3.56	AAA-AAA	0.00	999	1.000	1.000	0.4467
216851_at	50.9	14.3	-1.83	-3.56	AAA-AAA	0.00	999	1.000	1.000	0.1566
203853_s_at	37.4	133.0	1.83	3.56	AAA-AAA	0.00	999	1.000	1.000	0.0230
241077_at	4.4	15.7	1.83	3.55	AAA-AAA	0.00	999	1.000	1.000	0.2136
1553228_at	34.2	9.6	-1.83	-3.55	AAA-AAA	0.00	999	1.000	1.000	0.0176
210436_at	10.2	36.2	1.83	3.55	AAA-AAA	0.00	999	1.000	1.000	0.1968
208400_at	58.0	16.3	-1.83	-3.55	AAA-AAA	0.00	999	1.000	1.000	0.1587
242767_at	17.4	61.6	1.83	3.55	AAA-AMA	0.00	999	1.000	1.000	0.0671
215332_s_at	9.7	34.5	1.83	3.55	AAA-AAA	0.00	999	1.000	1.000	0.1421
217366_at	6.0	21.4	1.83	3.55	AAA-AAA	0.00	999	1.000	1.000	0.1096
232701_at	16.9	59.9	1.83	3.55	AAA-AAA	0.00	999	1.000	1.000	0.1894
231187_at	22.9	6.5	-1.83	-3.55	AAA-AAA	0.00	999	1.000	1.000	0.1233
236421_at	32.2	9.1	-1.83	-3.55	APA-AAA	0.00	999	1.000	1.000	0.1968
1563704_at	23.5	6.6	-1.83	-3.55	AAA-AAA	0.00	999	1.000	1.000	0.0143
1569259_at	11.6	41.3	1.83	3.55	AAA-AAA	0.00	999	1.000	1.000	0.0405
1557292_a_at	10.2	36.2	1.83	3.55	AAA-AAA	0.00	999	1.000	1.000	0.2158
238399_x_at	4.9	17.2	1.83	3.55	AAA-AAA	0.00	999	1.000	1.000	0.0659
227130_s_at	11.6	41.2	1.83	3.55	AAA-AAA	0.00	999	1.000	1.000	0.1890
240453_at	24.8	7.0	-1.83	-3.55	AAA-AAA	0.00	999	1.000	1.000	0.2646
1561689_at	37.9	10.7	-1.83	-3.55	AAA-AAA	0.00	999	1.000	1.000	0.1196
214708_at	28.2	8.0	-1.83	-3.55	AAA-AAA	0.00	999	1.000	1.000	0.1157
233561_at	14.8	52.4	1.83	3.55	AAA-AAA	0.00	999	1.000	1.000	0.1825
1569610_at	8.6	2.4	-1.83	-3.54	AAA-AAA	0.00	999	1.000	1.000	0.1801
1561130_at	6.0	21.2	1.83	3.54	AAA-AAA	0.00	999	1.000	1.000	0.0688
207482_at	16.6	58.8	1.83	3.54	AAA-AAA	0.00	999	1.000	1.000	0.0514
1570578_at	3.9	13.8	1.82	3.54	AAA-AAA	0.00	999	1.000	1.000	0.2679
1569192_at	16.6	4.7	-1.82	-3.54	AAA-AAA	0.00	999	1.000	1.000	0.3422
202988_s_at	27.6	7.8	-1.82	-3.54	AAA-AAA	0.00	999	1.000	1.000	0.3164
233439_at	28.7	101.7	1.82	3.54	AAA-AMA	0.00	999	1.000	1.000	0.2450
1570308_at	9.4	2.7	-1.82	-3.54	AAA-AAA	0.00	999	1.000	1.000	0.1548
1555898_at	17.5	61.8	1.82	3.54	AAA-AAM	0.00	999	1.000	1.000	0.1783

243885_x_at	4.3	15.0	1.82	3.54	AAA-AAA	0.00	999	1.000	1.000	0.2648
215666_at	3.3	11.5	1.82	3.54	AAA-AAA	0.00	999	1.000	1.000	0.3108
217335_at	38.1	10.8	-1.82	-3.54	AAA-AAA	0.00	999	1.000	1.000	0.1460
206269_at	52.9	15.0	-1.82	-3.54	AAA-AAA	0.00	999	1.000	1.000	0.1748
243250_at	39.9	11.3	-1.82	-3.54	AAA-AAA	0.00	999	1.000	1.000	0.0722
229916_at	12.5	3.5	-1.82	-3.54	AAA-AAA	0.00	999	1.000	1.000	0.2697
239222_at	26.4	7.5	-1.82	-3.53	AAA-AAA	0.00	999	1.000	1.000	0.0293
243254_at	4.9	17.2	1.82	3.53	AAA-AAA	0.00	999	1.000	1.000	0.2549
205038_at	14.3	50.7	1.82	3.53	AAA-AAA	0.00	999	1.000	1.000	0.0557
223483_at	10.6	3.0	-1.82	-3.53	AAA-AAA	0.00	999	1.000	1.000	0.0198
1556629_a_at	7.2	25.4	1.82	3.53	AAA-AAA	0.00	999	1.000	1.000	0.2395
229749_at	11.6	41.1	1.82	3.53	AAA-AAA	0.00	999	1.000	1.000	0.0249
230552_at	38.3	10.9	-1.82	-3.53	AAA-AAA	0.00	999	1.000	1.000	0.0054
1566043_at	7.2	25.3	1.82	3.53	AAA-AAA	0.00	999	1.000	1.000	0.2260
1562244_at	4.8	17.0	1.82	3.53	AAA-AAA	0.00	999	1.000	1.000	0.0949
1561983_at	4.5	16.0	1.82	3.53	AAA-AAA	0.00	999	1.000	1.000	0.3160
215077_at	7.0	24.7	1.82	3.53	AAA-AAA	0.00	999	1.000	1.000	0.0877
211111_at	30.3	8.6	-1.82	-3.53	AAA-AAA	0.00	999	1.000	1.000	0.1002
228837_at	22.0	6.2	-1.82	-3.53	AAA-AAA	0.00	999	1.000	1.000	0.1186
1559131_a_at	8.2	28.8	1.82	3.53	AAA-AAA	0.00	999	1.000	1.000	0.2760
204627_s_at	28.3	8.0	-1.82	-3.53	AAA-AAA	0.00	999	1.000	1.000	0.3637
233379_at	18.7	5.3	-1.82	-3.52	AAA-AAA	0.00	999	1.000	1.000	0.1310
236433_at	14.8	4.2	-1.82	-3.52	AAA-AAA	0.00	999	1.000	1.000	0.2793
241247_at	7.0	24.5	1.82	3.52	AAA-AAA	0.00	999	1.000	1.000	0.2547
1562810_at	30.9	108.7	1.82	3.52	AAA-AAA	0.00	999	1.000	1.000	0.2044
1555252_a_at	7.9	27.9	1.82	3.52	AAA-AAA	0.00	999	1.000	1.000	0.1930
208126_s_at	30.7	8.7	-1.82	-3.52	AAA-AAA	0.00	999	1.000	1.000	0.0359
236493_at	23.7	6.7	-1.82	-3.52	AAA-AAA	0.00	999	1.000	1.000	0.1613
1556086_at	62.1	17.6	-1.82	-3.52	PPA-AAA	0.00	999	1.000	1.000	0.1259
241476_at	23.4	6.6	-1.81	-3.52	AAA-AAA	0.00	999	1.000	1.000	0.0918
1567853_at	8.3	29.2	1.81	3.52	AAA-AAA	0.00	999	1.000	1.000	0.0380
1553147_at	13.3	3.8	-1.81	-3.52	AAA-AAA	0.00	999	1.000	1.000	0.0502
1559689_a_at	8.6	30.0	1.81	3.52	AAA-AAA	0.00	999	1.000	1.000	0.0812
219671_at	21.6	76.0	1.81	3.52	AAA-AAA	0.00	999	1.000	1.000	0.2429
236068_s_at	28.5	8.1	-1.81	-3.52	AAA-AAA	0.00	999	1.000	1.000	0.1075
242545_at	116.6	33.2	-1.81	-3.52	AAP-AAA	0.00	999	1.000	1.000	0.1777
240506_at	41.6	11.9	-1.81	-3.51	AAA-AAA	0.00	999	1.000	1.000	0.1171
1553319_at	3.0	10.5	1.81	3.51	AAA-AAA	0.00	999	1.000	1.000	0.0639
241395_at	46.4	13.2	-1.81	-3.51	AAA-AAA	0.00	999	1.000	1.000	0.3000
232249_at	41.7	11.9	-1.81	-3.51	AAA-AAA	0.00	999	1.000	1.000	0.2195
1561414_at	6.6	1.9	-1.81	-3.51	AAA-AAA	0.00	999	1.000	1.000	0.1804
1559571_a_at	24.9	7.1	-1.81	-3.51	AAA-AAA	0.00	999	1.000	1.000	0.2373

233455_at	35.1	10.0	-1.81	-3.51	AAA-AAA	0.00	999	1.000	1.000	0.0087
241847_at	69.2	19.8	-1.81	-3.51	AAA-AAA	0.00	999	1.000	1.000	0.0146
1560751_at	11.3	39.6	1.81	3.51	AAA-AAA	0.00	999	1.000	1.000	0.2490
1562426_a_at	102.4	29.3	-1.81	-3.50	APA-AAA	0.00	999	1.000	1.000	0.1842
1569445_at	10.1	35.5	1.81	3.50	AAA-AAA	0.00	999	1.000	1.000	0.2078
1565912_at	26.3	7.5	-1.81	-3.50	AAA-AAA	0.00	999	1.000	1.000	0.1045
243238_at	13.2	46.2	1.81	3.50	AAA-AAA	0.00	999	1.000	1.000	0.2347
204425_at	9.3	32.5	1.81	3.50	AAA-AAA	0.00	999	1.000	1.000	0.0062
234415_x_at	61.9	17.7	-1.81	-3.50	AAA-AAA	0.00	999	1.000	1.000	0.0580
238689_at	52.8	184.7	1.81	3.50	AAA-AAA	0.00	999	1.000	1.000	0.0698
229643_at	21.6	75.7	1.81	3.50	AAA-AAA	0.00	999	1.000	1.000	0.0356
204439_at	49.7	14.2	-1.81	-3.50	PAP-AAA	0.00	999	1.000	1.000	0.0336
1566884_at	13.9	48.5	1.81	3.50	AAA-AAA	0.00	999	1.000	1.000	0.3293
1559861_at	8.0	27.9	1.81	3.50	AAA-AAA	0.00	999	1.000	1.000	0.0901
1553052_at	6.3	22.0	1.81	3.50	AAA-AAA	0.00	999	1.000	1.000	0.3750
219566_at	126.1	36.1	-1.81	-3.50	AAA-AAA	0.00	999	1.000	1.000	0.0095
206107_at	25.7	7.4	-1.81	-3.50	AAA-AAA	0.00	999	1.000	1.000	0.0667
207278_s_at	17.1	59.9	1.81	3.50	AAA-AAA	0.00	999	1.000	1.000	0.0710
233392_at	1.7	5.8	1.81	3.50	AAA-AAA	0.00	999	1.000	1.000	0.1336
236908_at	29.0	101.5	1.81	3.50	AAA-MAA	0.00	999	1.000	1.000	0.0540
216189_at	39.4	11.3	-1.81	-3.50	PPA-AAA	0.00	999	1.000	1.000	0.1009
1555383_a_at	6.7	23.5	1.81	3.50	AAA-AAA	0.00	999	1.000	1.000	0.0673
206615_s_at	3.1	11.0	1.81	3.50	AAA-AAA	0.00	999	1.000	1.000	0.2786
1555097_a_at	33.2	9.5	-1.80	-3.49	AAA-AAA	0.00	999	1.000	1.000	0.3407
204701_s_at	114.5	32.8	-1.80	-3.49	AAA-AAA	0.00	999	1.000	1.000	0.1639
241753_at	40.4	11.6	-1.80	-3.49	MPA-AAA	0.00	999	1.000	1.000	0.1550
1564672_at	6.3	1.8	-1.80	-3.49	AAA-AAA	0.00	999	1.000	1.000	0.1573
1562965_at	11.7	40.7	1.80	3.49	AAA-AAA	0.00	999	1.000	1.000	0.0274
215687_x_at	45.2	12.9	-1.80	-3.49	AAA-AAA	0.00	999	1.000	1.000	0.2242
234508_at	80.8	23.2	-1.80	-3.49	AAA-AAA	0.00	999	1.000	1.000	0.1835
220340_at	24.9	86.8	1.80	3.49	AAA-AAA	0.00	999	1.000	1.000	0.0071
233728_at	15.8	55.1	1.80	3.49	AAA-AAA	0.00	999	1.000	1.000	0.1030
243192_at	9.9	34.5	1.80	3.48	AAA-AAA	0.00	999	1.000	1.000	0.3191
215196_at	14.9	51.8	1.80	3.48	AAA-AAA	0.00	999	1.000	1.000	0.1481
203717_at	40.5	11.6	-1.80	-3.48	AAA-AAA	0.00	999	1.000	1.000	0.3309
208062_s_at	29.6	8.5	-1.80	-3.48	AAA-AAA	0.00	999	1.000	1.000	0.2834
239591_at	5.4	18.9	1.80	3.48	AAA-AAA	0.00	999	1.000	1.000	0.3393
216767_at	66.8	19.2	-1.80	-3.48	AAA-AAA	0.00	999	1.000	1.000	0.1320
229986_at	53.4	15.3	-1.80	-3.48	AAA-AAA	0.00	999	1.000	1.000	0.1062
240816_at	13.6	3.9	-1.80	-3.48	AAA-AAA	0.00	999	1.000	1.000	0.0223
206152_at	101.2	29.1	-1.80	-3.48	AAA-AAA	0.00	999	1.000	1.000	0.1164
207013_s_at	30.8	106.9	1.80	3.48	AAA-AAA	0.00	999	1.000	1.000	0.1462

231971_at	67.2	19.3	-1.80	-3.48	AMA-AAA	0.00	999	1.000	1.000	0.0250
244854_at	31.1	9.0	-1.80	-3.48	AAA-AAA	0.00	999	1.000	1.000	0.2799
209722_s_at	17.2	59.7	1.80	3.48	AAA-AAA	0.00	999	1.000	1.000	0.1759
231421_at	18.0	5.2	-1.80	-3.48	AAA-AAA	0.00	999	1.000	1.000	0.0494
1559813_at	13.1	45.3	1.80	3.47	AAA-AAA	0.00	999	1.000	1.000	0.0440
244549_at	9.5	33.0	1.80	3.47	AAA-AAA	0.00	999	1.000	1.000	0.0539
1559244_at	5.8	20.1	1.80	3.47	AAA-AAA	0.00	999	1.000	1.000	0.2375
1560182_at	8.0	27.6	1.80	3.47	AAA-AAA	0.00	999	1.000	1.000	0.1498
215360_at	6.7	23.1	1.80	3.47	AAA-AAA	0.00	999	1.000	1.000	0.1384
229169_at	13.6	3.9	-1.80	-3.47	AAA-AAA	0.00	999	1.000	1.000	0.1793
239306_at	10.9	38.0	1.80	3.47	AAA-AAA	0.00	999	1.000	1.000	0.2619
244690_at	6.4	22.2	1.80	3.47	AAA-AAA	0.00	999	1.000	1.000	0.0527
1564584_at	31.3	9.0	-1.79	-3.47	AAA-AAA	0.00	999	1.000	1.000	0.0900
239255_at	25.4	88.2	1.79	3.47	AAA-AAA	0.00	999	1.000	1.000	0.1603
240518_at	87.1	25.1	-1.79	-3.47	AAA-AAA	0.00	999	1.000	1.000	0.0487
216623_x_at	1.8	6.2	1.79	3.47	AAA-MAA	0.00	999	1.000	1.000	0.3482
1570222_at	14.0	4.0	-1.79	-3.47	APA-AAA	0.00	999	1.000	1.000	0.3104
1563143_at	29.0	8.4	-1.79	-3.46	AAA-AAA	0.00	999	1.000	1.000	0.2678
244528_at	9.0	31.2	1.79	3.46	AAA-AAA	0.00	999	1.000	1.000	0.2828
213661_at	15.9	54.9	1.79	3.46	AAA-AAA	0.00	999	1.000	1.000	0.0132
215115_x_at	14.8	51.2	1.79	3.46	AAA-AAA	0.00	999	1.000	1.000	0.2002
220116_at	8.9	30.7	1.79	3.46	AAA-AAA	0.00	999	1.000	1.000	0.2946
211371_at	10.6	36.6	1.79	3.46	AAA-AAA	0.00	999	1.000	1.000	0.0407
230199_at	16.8	4.8	-1.79	-3.46	AAA-AAA	0.00	999	1.000	1.000	0.2693
1569794_at	13.1	45.5	1.79	3.46	AAA-AAA	0.00	999	1.000	1.000	0.2961
1552564_at	27.1	7.8	-1.79	-3.46	AAA-AAA	0.00	999	1.000	1.000	0.2998
244137_at	15.7	4.5	-1.79	-3.46	AAA-AAA	0.00	999	1.000	1.000	0.3125
234129_at	4.5	15.5	1.79	3.46	AAA-AAA	0.00	999	1.000	1.000	0.0342
227449_at	8.7	30.1	1.79	3.46	AAA-AAA	0.00	999	1.000	1.000	0.3525
231685_at	46.0	13.3	-1.79	-3.46	AAA-AAA	0.00	999	1.000	1.000	0.1651
1553226_at	32.1	9.3	-1.79	-3.46	AAA-AAA	0.00	999	1.000	1.000	0.4103
225815_at	10.0	34.7	1.79	3.46	AAA-AAA	0.00	999	1.000	1.000	0.1368
231036_at	30.3	104.7	1.79	3.46	AAA-AAA	0.00	999	1.000	1.000	0.0310
1554126_at	711.1	205.7	-1.79	-3.46	PPP-PAP	0.00	999	1.000	1.000	0.2628
1554222_at	22.8	78.6	1.79	3.46	AAA-AAA	0.00	999	1.000	1.000	0.0187
244541_x_at	21.2	6.1	-1.79	-3.46	AAA-AAA	0.00	999	1.000	1.000	0.1866
211902_x_at	36.3	10.5	-1.79	-3.46	AAA-AAA	0.00	999	1.000	1.000	0.0699
240209_at	79.1	22.9	-1.79	-3.45	AAA-AAA	0.00	999	1.000	1.000	0.0026
242852_at	20.1	5.8	-1.79	-3.45	AAA-AAA	0.00	999	1.000	1.000	0.1206
220622_at	13.1	3.8	-1.79	-3.45	AAA-AAA	0.00	999	1.000	1.000	0.1659
234461_at	21.4	74.1	1.79	3.45	AAA-AAA	0.00	999	1.000	1.000	0.1789
240286_at	4.7	16.3	1.79	3.45	AAA-AAA	0.00	999	1.000	1.000	0.2165

1567681_at	14.6	4.2	-1.79	-3.45	AAA-AAA	0.00	999	1.000	1.000	0.1880
210031_at	72.2	20.9	-1.79	-3.45	AAA-AAA	0.00	999	1.000	1.000	0.0387
223877_at	35.6	10.3	-1.79	-3.45	AAA-AAA	0.00	999	1.000	1.000	0.2125
231595_at	125.6	36.4	-1.79	-3.45	AAA-AAA	0.00	999	1.000	1.000	0.2521
1564400_at	11.7	40.4	1.79	3.45	AAA-AAA	0.00	999	1.000	1.000	0.2706
1563452_at	14.6	50.4	1.79	3.45	AAA-AAA	0.00	999	1.000	1.000	0.0630
1566424_at	49.6	14.4	-1.79	-3.45	AAA-AAA	0.00	999	1.000	1.000	0.0841
210600_s_at	1.8	6.1	1.79	3.45	AAA-AAA	0.00	999	1.000	1.000	0.0881
227834_at	7.6	2.2	-1.79	-3.45	AAA-AAA	0.00	999	1.000	1.000	0.1191
210115_at	133.4	38.7	-1.79	-3.45	PAP-PAA	0.00	999	1.000	1.000	0.2805
236731_at	19.8	5.8	-1.79	-3.45	AAA-AAA	0.00	999	1.000	1.000	0.1896
239406_at	42.8	147.2	1.78	3.44	AAA-AAA	0.00	999	1.000	1.000	0.1132
220117_at	6.3	21.6	1.78	3.44	AAA-AAA	0.00	999	1.000	1.000	0.1645
238466_at	5.5	19.0	1.78	3.44	AAA-AAA	0.00	999	1.000	1.000	0.3390
237132_at	13.3	45.9	1.78	3.44	AAA-AAA	0.00	999	1.000	1.000	0.0230
230902_at	29.7	8.6	-1.78	-3.44	AAA-AAA	0.00	999	1.000	1.000	0.1890
1569947_at	16.1	4.7	-1.78	-3.44	AAA-AAA	0.00	999	1.000	1.000	0.2951
244286_at	18.8	64.6	1.78	3.44	AAA-AAA	0.00	999	1.000	1.000	0.0734
232855_at	7.0	23.9	1.78	3.44	AAA-AAA	0.00	999	1.000	1.000	0.1481
232302_at	25.4	7.4	-1.78	-3.44	AAA-AAA	0.00	999	1.000	1.000	0.1406
229124_at	48.5	14.1	-1.78	-3.44	AAA-AAA	0.00	999	1.000	1.000	0.0946
1558383_at	34.0	9.9	-1.78	-3.44	APP-AMA	0.00	999	1.000	1.000	0.2675
241868_at	13.7	4.0	-1.78	-3.44	AAA-AAA	0.00	999	1.000	1.000	0.4029
238152_at	21.3	6.2	-1.78	-3.44	AAA-AAA	0.00	999	1.000	1.000	0.2554
220845_at	25.3	7.4	-1.78	-3.44	AAA-AAA	0.00	999	1.000	1.000	0.2265
237863_at	25.0	7.3	-1.78	-3.44	AAA-AAA	0.00	999	1.000	1.000	0.1807
204963_at	13.7	47.0	1.78	3.44	AAA-AAA	0.00	999	1.000	1.000	0.3291
243132_at	11.4	39.3	1.78	3.43	AAA-AAA	0.00	999	1.000	1.000	0.1269
243516_at	5.1	17.7	1.78	3.43	AAA-AAA	0.00	999	1.000	1.000	0.0200
232942_at	52.1	15.2	-1.78	-3.43	AAA-AAA	0.00	999	1.000	1.000	0.1581
237738_at	20.0	5.8	-1.78	-3.43	AAA-AAA	0.00	999	1.000	1.000	0.2130
241892_at	88.4	25.8	-1.78	-3.43	APP-PAA	0.00	999	1.000	1.000	0.1552
240987_at	10.1	34.6	1.78	3.43	AAA-AAA	0.00	999	1.000	1.000	0.0700
240574_at	20.6	70.4	1.78	3.43	AAA-AAA	0.00	999	1.000	1.000	0.1820
244817_at	14.1	4.1	-1.78	-3.43	AAA-AAA	0.00	999	1.000	1.000	0.2198
243025_at	24.5	83.9	1.78	3.43	AAA-AAA	0.00	999	1.000	1.000	0.2025
226210_s_at	19.2	5.6	-1.78	-3.43	AAA-AAA	0.00	999	1.000	1.000	0.0571
1557721_at	16.7	57.1	1.78	3.42	AAA-AAA	0.00	999	1.000	1.000	0.0073
1561567_at	11.6	3.4	-1.78	-3.43	AAA-AAA	0.00	999	1.000	1.000	0.0879
1559264_at	3.7	12.7	1.78	3.43	AAA-AAA	0.00	999	1.000	1.000	0.0941
233729_at	25.3	7.4	-1.78	-3.42	AAA-AAA	0.00	999	1.000	1.000	0.1197
238527_at	31.2	106.8	1.78	3.42	AAA-AAA	0.00	999	1.000	1.000	0.0889

224341_x_at	2.9	9.8	1.78	3.42	AAA-AAA	0.00	999	1.000	1.000	0.0980
239636_at	31.5	9.2	-1.77	-3.42	AAA-AAA	0.00	999	1.000	1.000	0.0036
234342_at	57.5	16.8	-1.77	-3.42	AAA-AAA	0.00	999	1.000	1.000	0.2620
236925_at	39.7	11.6	-1.77	-3.42	AAA-AAA	0.00	999	1.000	1.000	0.2037
235843_at	38.5	11.3	-1.77	-3.42	AAP-AAA	0.00	999	1.000	1.000	0.2489
1557793_at	8.4	28.8	1.77	3.42	AAA-AAA	0.00	999	1.000	1.000	0.2767
219950_s_at	83.0	24.3	-1.77	-3.42	AAA-AAA	0.00	999	1.000	1.000	0.3065
235777_at	15.4	4.5	-1.77	-3.42	AAA-AAA	0.00	999	1.000	1.000	0.3490
1557136_at	38.9	11.4	-1.77	-3.42	AAA-AAA	0.00	999	1.000	1.000	0.0388
233769_at	7.9	26.8	1.77	3.42	AAA-AAA	0.00	999	1.000	1.000	0.1915
233813_at	5.2	17.8	1.77	3.42	AAA-AAA	0.00	999	1.000	1.000	0.1353
237620_at	82.9	24.3	-1.77	-3.42	AAA-AAA	0.00	999	1.000	1.000	0.0397
214584_x_at	35.2	10.3	-1.77	-3.41	AAA-AAA	0.00	999	1.000	1.000	0.0683
211413_s_at	6.6	22.6	1.77	3.41	AAA-AAA	0.00	999	1.000	1.000	0.2085
237682_at	12.4	3.6	-1.77	-3.41	AAA-AAA	0.00	999	1.000	1.000	0.0341
224174_at	24.5	83.6	1.77	3.41	AAA-AAA	0.00	999	1.000	1.000	0.0273
241976_at	47.8	14.0	-1.77	-3.41	AAA-AAA	0.00	999	1.000	1.000	0.0199
1559333_at	17.6	5.2	-1.77	-3.41	AAA-AAA	0.00	999	1.000	1.000	0.1679
210127_at	18.2	62.0	1.77	3.41	AAA-AAA	0.00	999	1.000	1.000	0.2641
208412_s_at	9.6	32.6	1.77	3.41	AAA-AAA	0.00	999	1.000	1.000	0.1034
228974_at	15.8	4.6	-1.77	-3.41	AAA-AAA	0.00	999	1.000	1.000	0.2537
1560576_at	23.5	80.1	1.77	3.41	AAA-AAA	0.00	999	1.000	1.000	0.1107
208471_at	15.5	52.7	1.77	3.41	AAA-AAA	0.00	999	1.000	1.000	0.0691
228399_at	3.2	10.8	1.77	3.41	AAA-AAA	0.00	999	1.000	1.000	0.0262
1552583_s_at	11.6	39.3	1.77	3.41	AAA-AAA	0.00	999	1.000	1.000	0.2292
233074_at	32.9	9.7	-1.77	-3.41	AAA-AAA	0.00	999	1.000	1.000	0.0854
239477_at	3.8	13.0	1.77	3.40	AAA-AAA	0.00	999	1.000	1.000	0.2196
212415_at	60.9	17.9	-1.77	-3.40	AAA-AAA	0.00	999	1.000	1.000	0.0355
201907_x_at	28.0	95.1	1.77	3.40	AAA-AAA	0.00	999	1.000	1.000	0.2423
237959_at	8.6	29.3	1.77	3.40	AAA-AAA	0.00	999	1.000	1.000	0.0597
235891_at	11.2	3.3	-1.77	-3.40	AAA-AAA	0.00	999	1.000	1.000	0.1812
236645_at	7.9	27.0	1.77	3.40	AAA-AAA	0.00	999	1.000	1.000	0.0570
1555269_a_at	60.3	17.7	-1.77	-3.40	AAA-AAA	0.00	999	1.000	1.000	0.1132
220619_at	11.0	37.4	1.77	3.40	AAA-AAA	0.00	999	1.000	1.000	0.0988
222379_at	35.9	10.6	-1.77	-3.40	AAA-AAA	0.00	999	1.000	1.000	0.2059
211824_x_at	38.8	11.4	-1.77	-3.40	AAA-AAA	0.00	999	1.000	1.000	0.1438
233311_at	32.9	9.7	-1.77	-3.40	AAA-AAA	0.00	999	1.000	1.000	0.0410
227939_s_at	41.7	12.3	-1.77	-3.40	AAA-AAA	0.00	999	1.000	1.000	0.1747
240100_at	44.5	13.1	-1.76	-3.40	AAA-AAA	0.00	999	1.000	1.000	0.0400
1566874_at	37.4	11.0	-1.76	-3.40	AAA-AAA	0.00	999	1.000	1.000	0.0388
244445_at	9.2	31.2	1.76	3.40	AAA-AAA	0.00	999	1.000	1.000	0.1303
1559840_s_at	4.9	16.5	1.76	3.40	AAA-AAA	0.00	999	1.000	1.000	0.1283

1558984_at	36.9	10.9	-1.76	-3.40	AAA-AAA	0.00	999	1.000	1.000	0.1027
210997_at	12.9	3.8	-1.76	-3.40	AAA-AAA	0.00	999	1.000	1.000	0.1756
234444_at	18.4	62.4	1.76	3.40	AAA-AAA	0.00	999	1.000	1.000	0.1594
223565_at	3.8	12.8	1.76	3.40	AAA-AAA	0.00	999	1.000	1.000	0.1600
229116_at	8.9	2.6	-1.76	-3.40	AAA-AAA	0.00	999	1.000	1.000	0.1417
1552402_at	34.0	10.0	-1.76	-3.39	AAA-AAA	0.00	999	1.000	1.000	0.2167
1555749_at	8.7	2.6	-1.76	-3.39	AAA-AAA	0.00	999	1.000	1.000	0.1659
242788_at	14.6	49.4	1.76	3.39	AAA-AAA	0.00	999	1.000	1.000	0.1316
229565_x_at	2.5	8.6	1.76	3.39	AAA-AAA	0.00	999	1.000	1.000	0.0037
217038_at	40.7	12.0	-1.76	-3.39	AAA-AAA	0.00	999	1.000	1.000	0.0552
207580_at	2.5	8.5	1.76	3.39	AAA-AAA	0.00	999	1.000	1.000	0.2252
233420_at	37.6	11.1	-1.76	-3.39	AAA-AAA	0.00	999	1.000	1.000	0.0619
1556282_at	3.7	12.5	1.76	3.39	AAA-AAA	0.00	999	1.000	1.000	0.3719
1559341_at	2.2	7.5	1.76	3.39	AAA-AAA	0.00	999	1.000	1.000	0.2181
234636_at	16.0	54.2	1.76	3.39	AAA-AAA	0.00	999	1.000	1.000	0.1275
232267_at	8.0	2.4	-1.76	-3.39	AAA-AAA	0.00	999	1.000	1.000	0.0134
230422_at	4.4	15.1	1.76	3.39	AAA-AAA	0.00	999	1.000	1.000	0.2798
241419_at	28.9	97.9	1.76	3.39	AAA-AAA	0.00	999	1.000	1.000	0.0874
1562787_at	14.0	4.1	-1.76	-3.39	AAA-AAA	0.00	999	1.000	1.000	0.0332
1554819_a_at	12.3	41.6	1.76	3.39	AAA-AAA	0.00	999	1.000	1.000	0.1053
207069_s_at	7.8	26.6	1.76	3.39	AAA-AAA	0.00	999	1.000	1.000	0.0640
205278_at	26.2	7.8	-1.76	-3.39	AAA-AAA	0.00	999	1.000	1.000	0.2922
224074_at	34.3	10.1	-1.76	-3.39	AAA-AAA	0.00	999	1.000	1.000	0.2945
1565809_x_at	2.9	9.8	1.76	3.38	AAA-AAA	0.00	999	1.000	1.000	0.1996
232651_at	10.5	35.7	1.76	3.39	AAA-AAA	0.00	999	1.000	1.000	0.0785
232664_at	9.6	2.8	-1.76	-3.38	AAA-AAA	0.00	999	1.000	1.000	0.0606
233628_at	6.0	20.2	1.76	3.38	AAA-AAA	0.00	999	1.000	1.000	0.2034
243957_at	28.8	8.5	-1.76	-3.38	AAA-AAA	0.00	999	1.000	1.000	0.1912
213769_at	44.0	13.0	-1.76	-3.38	AAA-AAA	0.00	999	1.000	1.000	0.0060
214554_at	54.2	16.0	-1.76	-3.38	AAA-AAA	0.00	999	1.000	1.000	0.2283
217385_at	7.3	24.7	1.76	3.38	AAA-AAA	0.00	999	1.000	1.000	0.1328
217012_at	34.7	10.3	-1.76	-3.38	APA-AAA	0.00	999	1.000	1.000	0.3574
240313_at	14.7	4.4	-1.76	-3.38	AAA-AAA	0.00	999	1.000	1.000	0.0956
206409_at	6.3	21.3	1.76	3.38	AAA-AAA	0.00	999	1.000	1.000	0.0579
237108_x_at	43.5	146.8	1.76	3.38	AAA-AAA	0.00	999	1.000	1.000	0.0524
228558_at	14.0	47.3	1.76	3.38	AAA-AAA	0.00	999	1.000	1.000	0.2457
1552379_at	15.4	4.6	-1.75	-3.37	AAP-AAA	0.00	999	1.000	1.000	0.1748
1569835_at	13.4	4.0	-1.75	-3.37	AAA-AAA	0.00	999	1.000	1.000	0.1481
239791_at	20.7	6.2	-1.75	-3.37	AAA-AAA	0.00	999	1.000	1.000	0.1905
1566896_at	3.2	10.9	1.75	3.37	AAA-AAA	0.00	999	1.000	1.000	0.1166
243809_at	8.2	27.8	1.75	3.37	AAA-AAA	0.00	999	1.000	1.000	0.3262
216415_at	26.1	7.8	-1.75	-3.37	AAA-AAA	0.00	999	1.000	1.000	0.2500

213752_at	19.6	66.1	1.75	3.37	AAA-AAA	0.00	999	1.000	1.000	0.1110
234823_at	29.8	8.8	-1.75	-3.37	AAA-AAA	0.00	999	1.000	1.000	0.1408
240866_at	16.1	54.2	1.75	3.37	AAA-AAA	0.00	999	1.000	1.000	0.2729
1566865_at	7.6	25.7	1.75	3.37	AAA-AAA	0.00	999	1.000	1.000	0.1807
1561378_at	15.4	4.6	-1.75	-3.37	AAA-AAA	0.00	999	1.000	1.000	0.1401
243009_at	25.8	86.9	1.75	3.37	AAA-AAA	0.00	999	1.000	1.000	0.1329
205663_at	65.3	19.4	-1.75	-3.37	AAA-AAA	0.00	999	1.000	1.000	0.3050
239301_at	6.7	2.0	-1.75	-3.37	AAA-AAA	0.00	999	1.000	1.000	0.3290
1570182_at	11.0	37.2	1.75	3.37	AAA-AAA	0.00	999	1.000	1.000	0.2722
1554045_at	75.7	22.5	-1.75	-3.37	AAA-AAA	0.00	999	1.000	1.000	0.1250
1553808_a_at	18.6	5.5	-1.75	-3.37	AAA-AAA	0.00	999	1.000	1.000	0.2435
243300_at	20.1	67.6	1.75	3.37	AAA-AAA	0.00	999	1.000	1.000	0.0792
205884_at	40.2	11.9	-1.75	-3.37	AAA-AAA	0.00	999	1.000	1.000	0.3394
208512_s_at	24.5	82.7	1.75	3.37	AAA-AAA	0.00	999	1.000	1.000	0.0507
235710_at	20.6	69.2	1.75	3.37	AAA-AAA	0.00	999	1.000	1.000	0.1275
237201_at	7.2	24.1	1.75	3.37	AAA-AAA	0.00	999	1.000	1.000	0.1246
230715_at	10.1	33.9	1.75	3.37	AAA-AAA	0.00	999	1.000	1.000	0.1931
1558813_at	19.4	5.8	-1.75	-3.37	AAA-AAA	0.00	999	1.000	1.000	0.0585
1555313_a_at	4.5	15.2	1.75	3.37	AAA-AAA	0.00	999	1.000	1.000	0.4055
244203_at	5.1	17.0	1.75	3.37	AAA-AAA	0.00	999	1.000	1.000	0.0954
1556877_at	26.1	7.8	-1.75	-3.36	AMA-APA	0.00	999	1.000	1.000	0.3153
231310_at	46.8	13.9	-1.75	-3.36	PAP-PAA	0.00	999	1.000	1.000	0.3308
223858_at	10.4	35.0	1.75	3.36	AAA-AAA	0.00	999	1.000	1.000	0.0532
238571_at	79.3	23.6	-1.75	-3.36	APA-AAA	0.00	999	1.000	1.000	0.0653
220866_at	57.5	17.1	-1.75	-3.36	APA-AAA	0.00	999	1.000	1.000	0.0817
232400_at	36.3	10.8	-1.75	-3.36	AAA-AAA	0.00	999	1.000	1.000	0.2647
238009_at	87.3	26.0	-1.75	-3.36	AAA-AAP	0.00	999	1.000	1.000	0.1261
241605_at	13.4	45.2	1.75	3.36	AAA-AAA	0.00	999	1.000	1.000	0.0163
1555369_at	7.0	23.4	1.75	3.36	AAA-AAA	0.00	999	1.000	1.000	0.0858
243850_at	9.3	31.3	1.75	3.36	AAA-AAA	0.00	999	1.000	1.000	0.0934
1559987_at	25.6	85.9	1.75	3.36	AAA-AAA	0.00	999	1.000	1.000	0.0339
211402_x_at	107.2	31.9	-1.75	-3.36	AAA-AAA	0.00	999	1.000	1.000	0.1374
208263_at	11.8	39.7	1.75	3.36	AAA-AAA	0.00	999	1.000	1.000	0.0488
241471_at	88.8	26.5	-1.75	-3.36	AAM-AAP	0.00	999	1.000	1.000	0.0942
239313_at	101.4	30.2	-1.75	-3.36	AAA-AAA	0.00	999	1.000	1.000	0.2471
1559437_at	20.6	69.2	1.75	3.36	AAA-AAA	0.00	999	1.000	1.000	0.0726
219414_at	33.4	10.0	-1.75	-3.36	AAA-AAA	0.00	999	1.000	1.000	0.3330
207372_s_at	37.5	11.2	-1.75	-3.36	AAA-AAA	0.00	999	1.000	1.000	0.0321
222377_at	65.5	19.5	-1.75	-3.36	AAA-AAA	0.00	999	1.000	1.000	0.0873
205885_s_at	33.5	10.0	-1.75	-3.35	AAA-PAA	0.00	999	1.000	1.000	0.1892
239446_x_at	51.6	15.4	-1.75	-3.35	AAA-AAA	0.00	999	1.000	1.000	0.2583
237885_at	16.5	4.9	-1.75	-3.35	AAA-AAA	0.00	999	1.000	1.000	0.3329

228657_at	35.7	10.7	-1.75	-3.35	AAA-AAA	0.00	999	1.000	1.000	0.2215
237284_at	73.3	21.9	-1.75	-3.35	AAP-APA	0.00	999	1.000	1.000	0.0759
226218_at	64.7	19.3	-1.75	-3.35	AAP-AAA	0.00	999	1.000	1.000	0.1948
206306_at	44.5	13.3	-1.75	-3.35	APA-PAA	0.00	999	1.000	1.000	0.2325
215440_s_at	48.4	14.4	-1.75	-3.35	AAA-AAA	0.00	999	1.000	1.000	0.0864
236112_at	15.2	50.8	1.75	3.35	AAA-AAA	0.00	999	1.000	1.000	0.2270
235873_at	6.2	20.7	1.74	3.35	AAA-AAA	0.00	999	1.000	1.000	0.0892
231148_at	28.7	96.0	1.74	3.35	AAA-AAA	0.00	999	1.000	1.000	0.1548
1557455_s_at	305.7	91.3	-1.74	-3.35	AAP-AAP	0.00	999	1.000	1.000	0.1945
1562955_at	39.2	11.7	-1.74	-3.35	AAA-AAA	0.00	999	1.000	1.000	0.2115
1558010_s_at	10.5	35.1	1.74	3.35	AAA-AAA	0.00	999	1.000	1.000	0.1632
1560469_at	11.9	3.6	-1.74	-3.35	AAA-AAA	0.00	999	1.000	1.000	0.1153
219666_at	7.0	23.5	1.74	3.35	AAA-AAA	0.00	999	1.000	1.000	0.0944
221913_at	39.2	131.1	1.74	3.35	AAA-AAA	0.00	999	1.000	1.000	0.0880
1552882_a_at	7.2	24.0	1.74	3.35	AAA-AAA	0.00	999	1.000	1.000	0.0953
1557704_a_at	28.0	8.4	-1.74	-3.34	AAA-AAA	0.00	999	1.000	1.000	0.1223
1553761_at	11.9	39.9	1.74	3.34	AAA-AAA	0.00	999	1.000	1.000	0.1933
214481_at	9.2	30.7	1.74	3.34	AAA-AAA	0.00	999	1.000	1.000	0.2002
233501_at	34.8	116.5	1.74	3.35	AAA-AAA	0.00	999	1.000	1.000	0.0457
1560264_at	39.5	11.8	-1.74	-3.34	AAP-AAA	0.00	999	1.000	1.000	0.1518
1570360_s_at	15.0	4.5	-1.74	-3.34	AAA-AAA	0.00	999	1.000	1.000	0.0126
209963_s_at	11.5	38.4	1.74	3.34	AAA-AAA	0.00	999	1.000	1.000	0.1199
237530_at	2.6	8.7	1.74	3.34	AAA-AAA	0.00	999	1.000	1.000	0.3473
226228_at	2.1	7.1	1.74	3.34	AAA-AAA	0.00	999	1.000	1.000	0.2201
233343_at	25.4	7.6	-1.74	-3.34	AAA-PAA	0.00	999	1.000	1.000	0.3721
1560263_at	15.6	52.1	1.74	3.34	AAA-AAA	0.00	999	1.000	1.000	0.1361
214796_at	12.5	41.7	1.74	3.34	AAA-AAA	0.00	999	1.000	1.000	0.1410
209277_at	47.7	14.3	-1.74	-3.34	AAA-AAA	0.00	999	1.000	1.000	0.1303
235284_s_at	16.6	5.0	-1.74	-3.34	AAA-AAA	0.00	999	1.000	1.000	0.1259
1555365_x_at	5.1	1.5	-1.74	-3.34	AAA-AAA	0.00	999	1.000	1.000	0.1874
216358_at	56.0	16.8	-1.74	-3.34	AAA-AAA	0.00	999	1.000	1.000	0.0778
205334_at	81.8	24.5	-1.74	-3.34	AAA-AAA	0.00	999	1.000	1.000	0.0275
239241_at	5.4	17.9	1.74	3.34	AAA-AAA	0.00	999	1.000	1.000	0.0898
1569652_at	34.8	10.4	-1.74	-3.34	AAA-AAA	0.00	999	1.000	1.000	0.0808
1556065_at	34.5	10.3	-1.74	-3.34	MAA-AAA	0.00	999	1.000	1.000	0.2091
202434_s_at	8.7	29.1	1.74	3.34	AAA-AAA	0.00	999	1.000	1.000	0.1048
234918_at	10.5	34.9	1.74	3.34	AAA-AAA	0.00	999	1.000	1.000	0.0350
1561597_x_at	8.5	28.2	1.74	3.33	AAA-AAA	0.00	999	1.000	1.000	0.0545
203498_at	38.9	11.7	-1.74	-3.33	AAA-AAA	0.00	999	1.000	1.000	0.2230
1557533_at	6.6	22.1	1.74	3.33	AAA-AAA	0.00	999	1.000	1.000	0.2065
1562229_at	7.1	23.6	1.74	3.33	AAA-AAA	0.00	999	1.000	1.000	0.3559
244578_at	20.0	6.0	-1.74	-3.33	AAA-AAA	0.00	999	1.000	1.000	0.2565

212924_s_at	70.4	21.1	-1.74	-3.33	AAA-AAA	0.00	999	1.000	1.000	0.0193
203828_s_at	131.3	39.4	-1.74	-3.33	AAA-AAA	0.00	999	1.000	1.000	0.0976
230395_at	32.3	9.7	-1.74	-3.33	AAA-AAA	0.00	999	1.000	1.000	0.1894
233778_at	20.9	6.3	-1.74	-3.33	PAA-AAA	0.00	999	1.000	1.000	0.2568
211842_s_at	49.6	14.9	-1.74	-3.33	AAP-AAA	0.00	999	1.000	1.000	0.2738
244883_at	7.2	23.8	1.74	3.33	AAA-AAA	0.00	999	1.000	1.000	0.2062
1556257_at	10.0	3.0	-1.74	-3.33	AAA-AAA	0.00	999	1.000	1.000	0.3762
236025_at	5.5	18.2	1.74	3.33	AAA-AAA	0.00	999	1.000	1.000	0.1402
224426_s_at	13.4	4.0	-1.74	-3.33	AAA-AAA	0.00	999	1.000	1.000	0.1841
1560559_at	5.6	18.6	1.73	3.33	AAA-AAA	0.00	999	1.000	1.000	0.1894
230698_at	6.6	21.9	1.73	3.33	AAA-AAA	0.00	999	1.000	1.000	0.2080
244467_at	57.5	17.3	-1.73	-3.32	PAA-APA	0.00	999	1.000	1.000	0.2087
1565000_a_at	27.7	92.0	1.73	3.32	AAA-AAA	0.00	999	1.000	1.000	0.3424
1570362_at	207.4	62.4	-1.73	-3.33	AAA-AAA	0.00	999	1.000	1.000	0.0748
1568817_at	10.1	33.7	1.73	3.32	AAA-AAA	0.00	999	1.000	1.000	0.2215
1570134_at	18.1	60.1	1.73	3.33	AAA-AAA	0.00	999	1.000	1.000	0.1172
1554302_s_at	12.1	40.1	1.73	3.32	AAA-AAA	0.00	999	1.000	1.000	0.1247
243440_at	57.3	17.2	-1.73	-3.33	AAA-AAA	0.00	999	1.000	1.000	0.1324
215525_at	47.2	14.2	-1.73	-3.33	AAA-AAA	0.00	999	1.000	1.000	0.1993
209539_at	27.8	92.3	1.73	3.32	AAA-AAA	0.00	999	1.000	1.000	0.0509
230497_at	18.4	61.1	1.73	3.32	AAA-AAA	0.00	999	1.000	1.000	0.2473
241439_at	9.2	30.6	1.73	3.32	AAA-AAA	0.00	999	1.000	1.000	0.3260
1563087_at	2.2	7.3	1.73	3.32	AAA-AAA	0.00	999	1.000	1.000	0.0837
241625_at	4.7	15.7	1.73	3.32	AAA-AAA	0.00	999	1.000	1.000	0.1180
1568612_at	5.7	19.0	1.73	3.32	AAA-AAA	0.00	999	1.000	1.000	0.0381
1558451_at	5.3	17.7	1.73	3.32	AAA-AAA	0.00	999	1.000	1.000	0.1212
1562163_at	3.5	11.7	1.73	3.32	AAA-AAA	0.00	999	1.000	1.000	0.2274
216708_x_at	15.1	4.5	-1.73	-3.32	AAA-AAA	0.00	999	1.000	1.000	0.0923
207773_x_at	26.3	7.9	-1.73	-3.32	AAA-AAA	0.00	999	1.000	1.000	0.3040
232824_at	29.1	8.8	-1.73	-3.32	AAA-AAA	0.00	999	1.000	1.000	0.1233
216276_s_at	35.9	10.8	-1.73	-3.32	AAA-AAA	0.00	999	1.000	1.000	0.0822
235657_at	4.5	14.9	1.73	3.32	AAA-AAA	0.00	999	1.000	1.000	0.1181
234833_at	2.3	7.7	1.73	3.32	AAA-AAA	0.00	999	1.000	1.000	0.3581
227714_s_at	34.9	10.5	-1.73	-3.32	AAA-AAA	0.00	999	1.000	1.000	0.2538
232265_at	5.6	18.4	1.73	3.32	AAA-AAA	0.00	999	1.000	1.000	0.0425
215609_at	11.9	39.5	1.73	3.32	AAA-AAA	0.00	999	1.000	1.000	0.2199
217267_s_at	13.7	4.1	-1.73	-3.32	AAA-AAA	0.00	999	1.000	1.000	0.1995
208462_s_at	33.4	10.1	-1.73	-3.32	AAA-AAA	0.00	999	1.000	1.000	0.1636
1566511_at	22.3	6.7	-1.73	-3.32	AAA-AAA	0.00	999	1.000	1.000	0.2807
1556541_s_at	7.1	23.4	1.73	3.32	AAA-AAA	0.00	999	1.000	1.000	0.1564
1559044_at	12.7	42.1	1.73	3.31	AAA-AAA	0.00	999	1.000	1.000	0.3031
1560763_at	15.9	4.8	-1.73	-3.31	AAA-AAA	0.00	999	1.000	1.000	0.1961

215809_at	60.8	18.4	-1.73	-3.32	AAA-AAA	0.00	999	1.000	1.000	0.0040
212813_at	77.1	23.3	-1.73	-3.32	AAA-AAA	0.00	999	1.000	1.000	0.3452
205033_s_at	21.1	69.9	1.73	3.31	AAA-AAA	0.00	999	1.000	1.000	0.2174
210065_s_at	9.1	30.1	1.73	3.32	AAA-AAA	0.00	999	1.000	1.000	0.2326
207641_at	116.0	35.0	-1.73	-3.32	AAA-AAA	0.00	999	1.000	1.000	0.0859
234656_x_at	7.7	25.5	1.73	3.32	AAA-AAA	0.00	999	1.000	1.000	0.1573
237073_at	6.2	20.4	1.73	3.32	AAA-AAA	0.00	999	1.000	1.000	0.0676
229936_at	21.0	69.5	1.73	3.31	AAA-AAA	0.00	999	1.000	1.000	0.0418
232786_at	35.1	10.6	-1.73	-3.31	PPA-AAA	0.00	999	1.000	1.000	0.2184
239414_at	79.3	23.9	-1.73	-3.31	AAA-AAA	0.00	999	1.000	1.000	0.1168
1565888_at	3.5	11.7	1.73	3.31	AAA-AAA	0.00	999	1.000	1.000	0.0385
1560856_at	45.0	13.6	-1.73	-3.31	AAA-AAA	0.00	999	1.000	1.000	0.1916
220496_at	89.1	26.9	-1.73	-3.31	AAA-AAA	0.00	999	1.000	1.000	0.1479
238809_at	12.2	40.4	1.73	3.31	AAA-AAA	0.00	999	1.000	1.000	0.2832
231487_at	6.7	2.0	-1.73	-3.31	AAA-AAA	0.00	999	1.000	1.000	0.0710
232421_at	87.3	26.4	-1.73	-3.31	AAA-AAA	0.00	999	1.000	1.000	0.2756
1569881_at	20.8	6.3	-1.73	-3.31	AAA-AAA	0.00	999	1.000	1.000	0.2829
244626_at	19.6	64.8	1.73	3.31	AAA-AAA	0.00	999	1.000	1.000	0.1088
206219_s_at	22.0	72.9	1.73	3.31	AAA-AAA	0.00	999	1.000	1.000	0.0750
1553131_a_at	16.0	52.9	1.73	3.31	AAA-AAA	0.00	999	1.000	1.000	0.1851
236651_at	7.4	24.6	1.73	3.31	AAA-AAA	0.00	999	1.000	1.000	0.0736
229929_at	33.1	10.0	-1.73	-3.31	AAA-AAA	0.00	999	1.000	1.000	0.0976
211890_x_at	6.6	21.7	1.73	3.31	AAA-AAA	0.00	999	1.000	1.000	0.0873
233789_at	2.2	7.3	1.73	3.31	AAA-AAA	0.00	999	1.000	1.000	0.1494
208104_s_at	257.0	77.8	-1.72	-3.30	AAP-AAA	0.00	999	1.000	1.000	0.2767
1564777_at	14.9	49.3	1.72	3.30	AAA-AAA	0.00	999	1.000	1.000	0.3230
241301_at	14.4	47.7	1.72	3.30	AAA-AAA	0.00	999	1.000	1.000	0.1930
239182_at	25.9	7.8	-1.72	-3.30	AAA-AAA	0.00	999	1.000	1.000	0.3882
1556972_at	19.5	64.3	1.72	3.30	AAA-AAA	0.00	999	1.000	1.000	0.1292
207087_x_at	8.6	28.2	1.72	3.30	AAA-AAA	0.00	999	1.000	1.000	0.0268
208211_s_at	27.0	89.0	1.72	3.30	AAA-AAA	0.00	999	1.000	1.000	0.2431
238325_s_at	6.3	20.7	1.72	3.30	AAA-AAA	0.00	999	1.000	1.000	0.1364
231294_at	28.2	8.5	-1.72	-3.30	AAA-AAA	0.00	999	1.000	1.000	0.0662
1561890_at	79.8	24.2	-1.72	-3.30	AAA-AAA	0.00	999	1.000	1.000	0.1345
229596_at	5.2	17.1	1.72	3.30	AAA-AAA	0.00	999	1.000	1.000	0.1776
217268_at	2.5	8.4	1.72	3.30	AAA-AAA	0.00	999	1.000	1.000	0.3105
206128_at	77.9	23.6	-1.72	-3.30	AAA-AAA	0.00	999	1.000	1.000	0.1176
204086_at	45.9	13.9	-1.72	-3.30	AAA-AAA	0.00	999	1.000	1.000	0.2625
1560184_at	19.0	5.8	-1.72	-3.30	AAA-PAA	0.00	999	1.000	1.000	0.3195
1563351_at	7.5	2.3	-1.72	-3.30	AAA-AAA	0.00	999	1.000	1.000	0.2585
1565848_at	24.3	80.2	1.72	3.30	AAA-AAA	0.00	999	1.000	1.000	0.0516
1556062_at	36.8	11.2	-1.72	-3.30	MAA-AAA	0.00	999	1.000	1.000	0.3516

244138_at	22.1	72.9	1.72	3.30	AAA-AAA	0.00	999	1.000	1.000	0.2910
219908_at	2.9	9.4	1.72	3.30	AAA-AAA	0.00	999	1.000	1.000	0.0927
206322_at	18.2	60.1	1.72	3.30	AAA-AAA	0.00	999	1.000	1.000	0.2691
207901_at	12.8	42.1	1.72	3.30	AAA-AAA	0.00	999	1.000	1.000	0.0202
1552482_at	9.8	32.1	1.72	3.30	AAA-AAA	0.00	999	1.000	1.000	0.1637
1553443_at	17.4	57.2	1.72	3.30	AAA-AAA	0.00	999	1.000	1.000	0.0821
229151_at	18.1	5.5	-1.72	-3.29	PAA-AAA	0.00	999	1.000	1.000	0.2427
234352_x_at	14.1	4.3	-1.72	-3.29	AAA-AAA	0.00	999	1.000	1.000	0.2837
1569772_x_at	19.0	5.8	-1.72	-3.29	PAA-AAA	0.00	999	1.000	1.000	0.0727
243535_at	26.1	7.9	-1.72	-3.29	AAA-AAA	0.00	999	1.000	1.000	0.2417
219440_at	17.8	5.4	-1.72	-3.29	AAA-AAA	0.00	999	1.000	1.000	0.1009
228957_at	23.7	7.2	-1.72	-3.29	AAA-AAA	0.00	999	1.000	1.000	0.1251
1553193_at	38.1	11.6	-1.72	-3.29	AAP-AAA	0.00	999	1.000	1.000	0.2647
241539_at	15.2	4.6	-1.72	-3.29	AAA-AAA	0.00	999	1.000	1.000	0.1109
243812_at	58.6	17.8	-1.72	-3.29	AAA-AAA	0.00	999	1.000	1.000	0.1336
239295_at	54.2	16.5	-1.72	-3.29	AAA-AAA	0.00	999	1.000	1.000	0.0360
220479_at	5.2	17.0	1.72	3.28	AAA-AAA	0.00	999	1.000	1.000	0.0677
220153_at	37.1	121.8	1.72	3.29	AAA-AAA	0.00	999	1.000	1.000	0.2442
219562_at	35.4	116.2	1.72	3.28	AAA-AAA	0.00	999	1.000	1.000	0.2625
206439_at	15.4	4.7	-1.72	-3.29	AAA-AAA	0.00	999	1.000	1.000	0.4456
206997_s_at	72.3	237.6	1.72	3.28	AAA-AAA	0.00	999	1.000	1.000	0.0845
210194_at	3.4	11.2	1.72	3.29	AAA-AAA	0.00	999	1.000	1.000	0.1137
1557906_at	19.6	64.3	1.72	3.28	AAA-AAA	0.00	999	1.000	1.000	0.0407
219465_at	12.0	39.3	1.72	3.28	AAA-AAA	0.00	999	1.000	1.000	0.0940
202517_at	58.8	17.9	-1.72	-3.28	AAA-AAA	0.00	999	1.000	1.000	0.1983
238926_at	22.4	6.8	-1.72	-3.28	AAA-AAA	0.00	999	1.000	1.000	0.0573
1566964_at	9.7	31.7	1.71	3.28	AAA-AAA	0.00	999	1.000	1.000	0.2294
243610_at	9.8	32.1	1.71	3.28	AAA-AAA	0.00	999	1.000	1.000	0.2372
1561055_at	7.0	23.0	1.71	3.28	AAA-AAA	0.00	999	1.000	1.000	0.0849
244263_at	7.4	24.2	1.71	3.28	AAA-AAA	0.00	999	1.000	1.000	0.0484
217410_at	24.8	81.3	1.71	3.28	AAA-AAA	0.00	999	1.000	1.000	0.0648
238184_at	5.7	18.8	1.71	3.28	AAA-AAA	0.00	999	1.000	1.000	0.0860
1564872_at	14.0	45.9	1.71	3.28	AAA-AAA	0.00	999	1.000	1.000	0.1454
1570188_at	5.5	17.9	1.71	3.28	AAA-AAA	0.00	999	1.000	1.000	0.2130
1558931_at	6.5	21.2	1.71	3.28	AAA-AAA	0.00	999	1.000	1.000	0.1455
1558875_at	66.7	20.3	-1.71	-3.28	AAA-AAA	0.00	999	1.000	1.000	0.0935
215197_at	8.1	26.7	1.71	3.28	AAA-AAA	0.00	999	1.000	1.000	0.1108
236226_at	25.6	7.8	-1.71	-3.28	AAA-AAA	0.00	999	1.000	1.000	0.2504
237268_at	11.4	37.3	1.71	3.28	AAA-AAA	0.00	999	1.000	1.000	0.1874
229427_at	31.8	104.2	1.71	3.28	AAA-AAA	0.00	999	1.000	1.000	0.1033
211214_s_at	11.3	37.0	1.71	3.28	AAA-AAA	0.00	999	1.000	1.000	0.0993
234867_at	14.2	46.6	1.71	3.28	AAA-AAA	0.00	999	1.000	1.000	0.0002

231690_at	30.6	9.4	-1.71	-3.28	AAA-AAA	0.00	999	1.000	1.000	0.0175
1564294_at	5.7	18.5	1.71	3.27	AAA-AAA	0.00	999	1.000	1.000	0.0424
1565576_at	67.0	20.5	-1.71	-3.27	AAA-AAA	0.00	999	1.000	1.000	0.1519
242095_at	22.7	6.9	-1.71	-3.27	AAA-AAA	0.00	999	1.000	1.000	0.2676
1557027_at	46.0	14.1	-1.71	-3.27	AAA-AAA	0.00	999	1.000	1.000	0.0664
214340_at	31.4	102.8	1.71	3.27	AAA-AAA	0.00	999	1.000	1.000	0.3534
220899_at	50.5	15.4	-1.71	-3.27	AAA-AAA	0.00	999	1.000	1.000	0.1402
210738_s_at	3.7	12.1	1.71	3.27	AAA-AAA	0.00	999	1.000	1.000	0.2651
1563136_at	2.5	8.1	1.71	3.27	AAA-AAA	0.00	999	1.000	1.000	0.1662
1570517_at	3.4	11.3	1.71	3.27	AAA-AAA	0.00	999	1.000	1.000	0.2029
215602_at	11.3	36.8	1.71	3.27	AAA-AAA	0.00	999	1.000	1.000	0.3036
206677_at	16.2	53.1	1.71	3.27	AAA-AAA	0.00	999	1.000	1.000	0.1432
205275_at	67.9	20.8	-1.71	-3.27	AAA-AAA	0.00	999	1.000	1.000	0.0361
208164_s_at	9.2	30.0	1.71	3.27	AAA-AAA	0.00	999	1.000	1.000	0.2269
235438_at	2.3	7.6	1.71	3.27	AAA-AAA	0.00	999	1.000	1.000	0.1763
1566152_a_at	3.7	12.0	1.71	3.27	AAA-AAA	0.00	999	1.000	1.000	0.2347
219152_at	25.0	81.6	1.71	3.27	AAA-AAA	0.00	999	1.000	1.000	0.2033
233837_at	32.7	106.8	1.71	3.27	AAA-AAA	0.00	999	1.000	1.000	0.2627
237673_at	3.7	12.2	1.71	3.27	AAA-AAA	0.00	999	1.000	1.000	0.1921
229222_at	2.8	9.0	1.71	3.27	AAA-AAA	0.00	999	1.000	1.000	0.0357
1565065_at	8.7	28.3	1.71	3.27	AAA-AAA	0.00	999	1.000	1.000	0.0773
206388_at	63.2	19.4	-1.71	-3.27	AAA-AAA	0.00	999	1.000	1.000	0.1902
216373_at	12.9	42.0	1.71	3.27	AAA-AAA	0.00	999	1.000	1.000	0.1996
219243_at	4.2	13.6	1.71	3.27	AAA-AAA	0.00	999	1.000	1.000	0.1963
1559605_a_at	31.0	9.5	-1.71	-3.26	AAA-AAA	0.00	999	1.000	1.000	0.1507
1560369_at	40.4	12.4	-1.71	-3.26	AAA-AAA	0.00	999	1.000	1.000	0.3310
238330_s_at	72.9	22.4	-1.71	-3.26	AAA-AAA	0.00	999	1.000	1.000	0.1871
1570098_at	25.8	7.9	-1.71	-3.26	AAA-AAA	0.00	999	1.000	1.000	0.2605
240585_at	3.1	10.2	1.71	3.26	AAA-AAA	0.00	999	1.000	1.000	0.2300
214517_at	67.1	20.6	-1.71	-3.26	AAA-AAA	0.00	999	1.000	1.000	0.1636
204754_at	11.4	37.1	1.71	3.26	AAA-AAA	0.00	999	1.000	1.000	0.1940
229288_at	36.2	11.1	-1.71	-3.26	AAA-AAA	0.00	999	1.000	1.000	0.2207
1564545_a_at	22.2	6.8	-1.70	-3.26	AAA-AAA	0.00	999	1.000	1.000	0.0564
243889_at	32.0	9.8	-1.70	-3.26	AAA-AAA	0.00	999	1.000	1.000	0.0284
206696_at	50.4	15.5	-1.70	-3.26	AAA-AAA	0.00	999	1.000	1.000	0.0714
204938_s_at	6.2	1.9	-1.70	-3.26	AAA-AAA	0.00	999	1.000	1.000	0.0618
239950_at	2.9	9.6	1.70	3.26	AAA-AAA	0.00	999	1.000	1.000	0.2991
203305_at	14.4	47.0	1.70	3.26	AAA-AAA	0.00	999	1.000	1.000	0.1552
208298_at	24.4	7.5	-1.70	-3.26	AAA-AAA	0.00	999	1.000	1.000	0.1273
236637_at	20.3	6.3	-1.70	-3.26	AAA-AAA	0.00	999	1.000	1.000	0.2734
243569_at	148.0	45.6	-1.70	-3.25	AMA-AAA	0.00	999	1.000	1.000	0.1669
1559506_x_at	1.6	5.3	1.70	3.25	AAA-AAA	0.00	999	1.000	1.000	0.2110

204708_at	28.0	8.6	-1.70	-3.25	AAA-AAA	0.00	999	1.000	1.000	0.0022
209447_at	29.3	95.3	1.70	3.25	AAA-AAA	0.00	999	1.000	1.000	0.0635
208294_x_at	5.0	16.3	1.70	3.25	AAA-AAA	0.00	999	1.000	1.000	0.1565
1558021_at	66.2	20.4	-1.70	-3.25	APA-AAA	0.00	999	1.000	1.000	0.1084
1556699_at	70.1	21.6	-1.70	-3.25	AAA-AAA	0.00	999	1.000	1.000	0.2073
210079_x_at	6.1	19.9	1.70	3.25	AAA-AAA	0.00	999	1.000	1.000	0.0709
233670_at	8.7	2.7	-1.70	-3.25	AAA-AAA	0.00	999	1.000	1.000	0.2395
238381_x_at	4.8	15.7	1.70	3.25	AAA-AAA	0.00	999	1.000	1.000	0.1035
232443_at	7.7	25.1	1.70	3.25	AAA-AAA	0.00	999	1.000	1.000	0.1410
210599_at	80.4	24.8	-1.70	-3.24	AAA-AAA	0.00	999	1.000	1.000	0.2113
211565_at	21.8	6.7	-1.70	-3.25	AAA-AAA	0.00	999	1.000	1.000	0.2261
233267_at	9.3	30.1	1.70	3.24	AAA-AAA	0.00	999	1.000	1.000	0.1748
1565714_at	25.7	7.9	-1.70	-3.24	AAA-AAA	0.00	999	1.000	1.000	0.1365
1566900_at	19.3	62.5	1.70	3.24	AAA-AAA	0.00	999	1.000	1.000	0.2848
1561311_at	66.1	20.4	-1.70	-3.24	AAA-AAA	0.00	999	1.000	1.000	0.2368
242630_at	23.1	74.8	1.70	3.24	AAA-AAA	0.00	999	1.000	1.000	0.1076
1561886_a_at	35.1	10.8	-1.70	-3.24	AAA-AAA	0.00	999	1.000	1.000	0.1358
210726_at	67.9	21.0	-1.70	-3.24	AAA-AAA	0.00	999	1.000	1.000	0.0720
210657_s_at	29.5	9.1	-1.70	-3.24	AAA-AAA	0.00	999	1.000	1.000	0.1408
234172_at	21.6	6.7	-1.70	-3.24	AAA-AAA	0.00	999	1.000	1.000	0.2818
231323_at	7.6	24.6	1.70	3.24	AAA-AAA	0.00	999	1.000	1.000	0.2090
242466_at	82.3	25.4	-1.70	-3.24	AAP-AAA	0.00	999	1.000	1.000	0.1600
244736_at	11.2	36.4	1.70	3.24	AAA-AAA	0.00	999	1.000	1.000	0.0151
1554952_s_at	94.9	29.3	-1.70	-3.24	AAA-AAA	0.00	999	1.000	1.000	0.0679
234235_at	8.9	2.7	-1.70	-3.24	AAA-AAA	0.00	999	1.000	1.000	0.3007
1563496_at	22.7	7.0	-1.70	-3.24	AAA-AAA	0.00	999	1.000	1.000	0.1761
1557869_at	27.1	8.4	-1.70	-3.24	AAA-AAA	0.00	999	1.000	1.000	0.3320
213131_at	10.6	34.3	1.70	3.24	AAA-AAA	0.00	999	1.000	1.000	0.2152
214447_at	44.3	143.5	1.70	3.24	AAA-AAA	0.00	999	1.000	1.000	0.1175
1565903_at	45.7	14.1	-1.69	-3.24	AAA-AAA	0.00	999	1.000	1.000	0.0575
1560714_at	2.9	9.5	1.69	3.24	AAA-AAA	0.00	999	1.000	1.000	0.2633
216047_x_at	30.6	98.9	1.69	3.24	AAA-AAA	0.00	999	1.000	1.000	0.0902
201744_s_at	5.9	1.8	-1.69	-3.24	AAA-AAA	0.00	999	1.000	1.000	0.1872
210467_x_at	36.2	11.2	-1.69	-3.24	AAA-AAA	0.00	999	1.000	1.000	0.1448
1562611_at	2.4	7.7	1.69	3.23	AAA-AAA	0.00	999	1.000	1.000	0.1313
1557861_at	15.0	4.6	-1.69	-3.23	AAA-AAA	0.00	999	1.000	1.000	0.0656
1561606_at	3.0	9.8	1.69	3.23	AAA-AAA	0.00	999	1.000	1.000	0.2245
216468_s_at	10.0	3.1	-1.69	-3.23	AAA-AAA	0.00	999	1.000	1.000	0.2149
236300_at	39.3	12.1	-1.69	-3.23	MAA-AAA	0.00	999	1.000	1.000	0.2333
240422_at	18.1	58.5	1.69	3.23	AAA-AAA	0.00	999	1.000	1.000	0.1983
1558262_at	8.3	27.0	1.69	3.23	AAA-AAA	0.00	999	1.000	1.000	0.1906
220449_at	60.5	18.7	-1.69	-3.23	AAA-AAA	0.00	999	1.000	1.000	0.1744

204409_s_at	44.2	13.7	-1.69	-3.23	AAA-AAA	0.00	999	1.000	1.000	0.1315
234016_at	9.9	31.9	1.69	3.23	AAA-AAA	0.00	999	1.000	1.000	0.1427
1562907_at	43.8	13.6	-1.69	-3.23	AAA-AAA	0.00	999	1.000	1.000	0.2230
1568919_at	20.9	6.5	-1.69	-3.23	AAA-AAA	0.00	999	1.000	1.000	0.0155
1562309_s_at	3.0	9.6	1.69	3.23	AAA-AAA	0.00	999	1.000	1.000	0.0305
215120_s_at	19.0	5.9	-1.69	-3.23	AAA-AAA	0.00	999	1.000	1.000	0.0479
207039_at	20.7	6.4	-1.69	-3.23	AAA-AAA	0.00	999	1.000	1.000	0.2948
234225_at	8.4	27.3	1.69	3.23	AAA-AAA	0.00	999	1.000	1.000	0.2087
236987_at	57.4	17.8	-1.69	-3.23	AAA-AAA	0.00	999	1.000	1.000	0.1419
1568751_at	7.9	2.5	-1.69	-3.23	AAA-AAA	0.00	999	1.000	1.000	0.3820
219134_at	3.3	10.7	1.69	3.23	AAA-AAA	0.00	999	1.000	1.000	0.0438
207542_s_at	26.7	86.3	1.69	3.23	AAA-AAA	0.00	999	1.000	1.000	0.3077
236067_at	36.5	117.7	1.69	3.23	AAA-AAA	0.00	999	1.000	1.000	0.0673
1562630_at	4.8	15.4	1.69	3.22	AAA-AAA	0.00	999	1.000	1.000	0.0335
239924_at	20.5	66.2	1.69	3.22	AAA-AAA	0.00	999	1.000	1.000	0.1344
1559622_at	10.2	32.9	1.69	3.22	AAA-AAA	0.00	999	1.000	1.000	0.0700
229080_at	46.6	14.5	-1.69	-3.23	AAA-AAA	0.00	999	1.000	1.000	0.1563
1563466_at	55.3	17.2	-1.69	-3.22	PAA-AAA	0.00	999	1.000	1.000	0.2576
AFFX-r2-Bs-thr-5_s_at	64.5	20.0	-1.69	-3.22	PMA-MAA	0.00	999	1.000	1.000	0.2997
1565558_at	45.1	14.0	-1.69	-3.22	AAA-AAA	0.00	999	1.000	1.000	0.1179
239874_at	6.1	19.8	1.69	3.22	AAA-AAA	0.00	999	1.000	1.000	0.2778
240605_at	24.4	7.6	-1.69	-3.22	AAA-AAA	0.00	999	1.000	1.000	0.1440
1554745_at	53.3	16.6	-1.69	-3.22	AAA-AAA	0.00	999	1.000	1.000	0.1747
1560679_at	10.0	3.1	-1.69	-3.22	AAA-AAA	0.00	999	1.000	1.000	0.2004
213862_at	15.2	4.7	-1.69	-3.22	AAA-AAA	0.00	999	1.000	1.000	0.2437
237148_at	6.3	20.3	1.69	3.22	AAA-AAA	0.00	999	1.000	1.000	0.1269
1564610_at	28.1	8.7	-1.69	-3.22	AAA-AAA	0.00	999	1.000	1.000	0.1957
1554368_at	5.3	1.6	-1.69	-3.22	AAA-AAA	0.00	999	1.000	1.000	0.2842
214013_s_at	18.4	5.7	-1.69	-3.22	AAA-AAA	0.00	999	1.000	1.000	0.2342
206947_at	23.5	7.3	-1.69	-3.22	AAA-AAA	0.00	999	1.000	1.000	0.0209
237802_at	15.4	4.8	-1.69	-3.22	AAA-AAA	0.00	999	1.000	1.000	0.1646
224017_at	2.1	6.6	1.69	3.22	AAA-AAA	0.00	999	1.000	1.000	0.0673
232389_at	10.1	32.7	1.69	3.22	AAA-AAA	0.00	999	1.000	1.000	0.1133
1566337_x_at	38.7	12.0	-1.69	-3.22	AAA-AAA	0.00	999	1.000	1.000	0.3721
216634_at	10.7	34.4	1.69	3.22	AAA-AAA	0.00	999	1.000	1.000	0.1018
213814_s_at	67.4	21.0	-1.69	-3.22	AAA-AAA	0.00	999	1.000	1.000	0.1861
205124_at	7.1	22.9	1.69	3.22	AAA-AAA	0.00	999	1.000	1.000	0.0411
239527_at	21.1	68.0	1.69	3.22	AAA-AAA	0.00	999	1.000	1.000	0.1560
1566136_at	67.7	21.1	-1.69	-3.22	AAA-AAA	0.00	999	1.000	1.000	0.0109
231341_at	7.2	23.1	1.69	3.22	AAA-AAA	0.00	999	1.000	1.000	0.0871
1559203_s_at	29.5	9.2	-1.68	-3.21	APA-AAA	0.00	999	1.000	1.000	0.1618
1562923_at	29.3	9.1	-1.68	-3.21	AAA-AAA	0.00	999	1.000	1.000	0.3525

243397_at	59.4	18.5	-1.68	-3.21	AAA-AAA	0.00	999	1.000	1.000	0.3384
217397_at	87.2	27.1	-1.68	-3.21	AAA-AAA	0.00	999	1.000	1.000	0.1196
219932_at	57.3	17.8	-1.68	-3.21	AAA-AAA	0.00	999	1.000	1.000	0.0539
231398_at	13.7	4.3	-1.68	-3.21	AAA-AAA	0.00	999	1.000	1.000	0.0223
233998_x_at	20.7	6.4	-1.68	-3.21	AAA-AAA	0.00	999	1.000	1.000	0.2383
238123_at	82.3	25.6	-1.68	-3.21	AAA-AAA	0.00	999	1.000	1.000	0.2120
235761_at	14.0	44.9	1.68	3.21	AAA-AAA	0.00	999	1.000	1.000	0.0034
239974_at	97.6	30.4	-1.68	-3.21	PAA-AAA	0.00	999	1.000	1.000	0.1673
1569755_at	2.2	7.0	1.68	3.21	AAA-AAA	0.00	999	1.000	1.000	0.2580
220829_s_at	3.2	10.3	1.68	3.21	AAA-AAA	0.00	999	1.000	1.000	0.2372
207529_at	69.2	21.6	-1.68	-3.21	AAA-AAA	0.00	999	1.000	1.000	0.1305
233584_at	6.0	19.3	1.68	3.21	AAA-AMA	0.00	999	1.000	1.000	0.2873
237417_at	9.8	3.1	-1.68	-3.21	AAA-AAA	0.00	999	1.000	1.000	0.0162
230299_s_at	11.5	37.0	1.68	3.21	AAA-AAA	0.00	999	1.000	1.000	0.1611
206826_at	50.0	15.6	-1.68	-3.20	PPA-AAA	0.00	999	1.000	1.000	0.1166
215144_at	32.1	10.0	-1.68	-3.21	AAA-AAA	0.00	999	1.000	1.000	0.1252
232397_at	5.7	1.8	-1.68	-3.20	AAA-AAA	0.00	999	1.000	1.000	0.2650
209700_x_at	45.1	14.1	-1.68	-3.20	AAA-AAP	0.00	999	1.000	1.000	0.4329
1563529_at	11.8	37.7	1.68	3.20	AAA-AAA	0.00	999	1.000	1.000	0.2031
1568943_at	67.8	21.2	-1.68	-3.20	AAA-AAA	0.00	999	1.000	1.000	0.0088
222287_at	7.1	2.2	-1.68	-3.20	AAA-AAA	0.00	999	1.000	1.000	0.3750
1555342_a_at	17.1	5.3	-1.68	-3.20	AAA-AAA	0.00	999	1.000	1.000	0.1717
241679_at	57.4	17.9	-1.68	-3.20	APA-AAA	0.00	999	1.000	1.000	0.0559
1566673_at	25.4	7.9	-1.68	-3.20	APA-AAA	0.00	999	1.000	1.000	0.1003
1565329_at	8.4	26.9	1.68	3.20	AAA-AAA	0.00	999	1.000	1.000	0.1122
201045_s_at	12.0	38.2	1.68	3.20	AAA-AAA	0.00	999	1.000	1.000	0.0503
209541_at	5.9	19.0	1.68	3.20	AAA-AAA	0.00	999	1.000	1.000	0.2290
237656_at	5.4	17.3	1.68	3.20	AAA-AAA	0.00	999	1.000	1.000	0.0928
236769_at	36.2	11.3	-1.68	-3.20	AAA-AAA	0.00	999	1.000	1.000	0.1620
230931_at	38.8	12.2	-1.68	-3.20	AMA-AAA	0.00	999	1.000	1.000	0.2442
1563114_at	13.7	43.9	1.68	3.20	AAA-AAA	0.00	999	1.000	1.000	0.0285
239800_at	58.0	18.1	-1.68	-3.20	AAA-AAA	0.00	999	1.000	1.000	0.2674
1560154_a_at	8.1	25.8	1.68	3.20	AAA-AAA	0.00	999	1.000	1.000	0.1388
216050_at	6.0	19.1	1.68	3.20	AAA-AAA	0.00	999	1.000	1.000	0.2878
238126_at	17.3	55.2	1.68	3.20	AAA-AAA	0.00	999	1.000	1.000	0.1990
1561509_at	49.0	15.4	-1.68	-3.19	AAA-AAA	0.00	999	1.000	1.000	0.3039
209687_at	5.9	18.8	1.68	3.19	AAA-AAA	0.00	999	1.000	1.000	0.0308
1569525_s_at	27.4	8.6	-1.67	-3.19	AAA-AAA	0.00	999	1.000	1.000	0.2103
216636_at	13.8	4.3	-1.67	-3.19	AAA-AAA	0.00	999	1.000	1.000	0.1776
216695_s_at	18.5	59.2	1.67	3.19	AAA-AAA	0.00	999	1.000	1.000	0.3480
213946_s_at	39.7	12.4	-1.67	-3.19	AAA-AAA	0.00	999	1.000	1.000	0.2823
204648_at	11.8	37.5	1.67	3.19	AAA-AAA	0.00	999	1.000	1.000	0.0199

225960_at	10.3	32.9	1.67	3.19	AAA-AAA	0.00	999	1.000	1.000	0.0845
1565810_at	30.4	9.5	-1.67	-3.19	PAA-AAA	0.00	999	1.000	1.000	0.2939
242186_x_at	2.7	8.7	1.67	3.19	AAA-AAA	0.00	999	1.000	1.000	0.2824
1556459_at	16.2	5.1	-1.67	-3.19	AAA-AAA	0.00	999	1.000	1.000	0.1881
1553887_at	49.7	15.6	-1.67	-3.19	AMA-AAA	0.00	999	1.000	1.000	0.0890
214546_s_at	30.2	9.5	-1.67	-3.19	AAA-AAA	0.00	999	1.000	1.000	0.2700
1566285_at	13.9	44.3	1.67	3.19	AAA-AAA	0.00	999	1.000	1.000	0.1966
238309_x_at	20.0	6.3	-1.67	-3.19	AAA-AAA	0.00	999	1.000	1.000	0.3016
221698_s_at	45.0	14.1	-1.67	-3.18	AMA-AAA	0.00	999	1.000	1.000	0.1338
234548_at	13.8	4.3	-1.67	-3.18	AAA-AAA	0.00	999	1.000	1.000	0.4349
237017_s_at	20.7	6.5	-1.67	-3.18	AAA-AAA	0.00	999	1.000	1.000	0.3728
223915_at	10.1	32.3	1.67	3.19	AAA-AAA	0.00	999	1.000	1.000	0.1755
228945_s_at	64.2	20.2	-1.67	-3.18	AAA-AAA	0.00	999	1.000	1.000	0.0717
208176_at	33.5	106.7	1.67	3.18	AAA-AAA	0.00	999	1.000	1.000	0.1430
240397_x_at	23.4	7.4	-1.67	-3.18	AAA-AAA	0.00	999	1.000	1.000	0.2853
240418_at	21.2	6.7	-1.67	-3.18	AAA-AAA	0.00	999	1.000	1.000	0.2900
214520_at	31.7	100.7	1.67	3.18	AAA-AAA	0.00	999	1.000	1.000	0.0625
219537_x_at	55.5	17.4	-1.67	-3.18	AAA-AAA	0.00	999	1.000	1.000	0.2895
206925_at	7.4	23.4	1.67	3.18	AAA-AAA	0.00	999	1.000	1.000	0.3540
234420_at	37.3	11.7	-1.67	-3.18	AAA-AAA	0.00	999	1.000	1.000	0.2773
233972_s_at	4.6	14.6	1.67	3.18	AAA-AAA	0.00	999	1.000	1.000	0.0070
221377_s_at	60.8	19.1	-1.67	-3.18	PAP-APA	0.00	999	1.000	1.000	0.2156
241575_at	18.8	5.9	-1.67	-3.18	AAA-AAA	0.00	999	1.000	1.000	0.1216
1552337_s_at	9.5	30.0	1.67	3.18	AAA-AAA	0.00	999	1.000	1.000	0.2652
216978_x_at	10.9	34.6	1.67	3.18	AAA-AAA	0.00	999	1.000	1.000	0.2415
238384_x_at	3.6	1.1	-1.67	-3.18	AAA-AAA	0.00	999	1.000	1.000	0.1999
238755_at	33.5	10.5	-1.67	-3.18	AAA-AAA	0.00	999	1.000	1.000	0.2040
236767_at	23.0	72.9	1.67	3.18	AAA-AAA	0.00	999	1.000	1.000	0.1716
236938_at	83.5	26.3	-1.67	-3.18	AAA-AAA	0.00	999	1.000	1.000	0.2042
215890_at	52.9	16.7	-1.67	-3.18	AAA-AAA	0.00	999	1.000	1.000	0.1996
211188_at	18.4	58.4	1.67	3.18	AAA-AAA	0.00	999	1.000	1.000	0.3043
1560851_at	5.3	1.7	-1.67	-3.17	AAA-AAA	0.00	999	1.000	1.000	0.0897
205876_at	14.8	4.7	-1.67	-3.17	AAA-AAA	0.00	999	1.000	1.000	0.2781
1560614_at	24.5	7.7	-1.67	-3.17	AAA-AAA	0.00	999	1.000	1.000	0.1139
207663_x_at	6.7	21.3	1.67	3.17	AAA-AAA	0.00	999	1.000	1.000	0.2647
1553055_a_at	108.1	34.1	-1.66	-3.17	PAA-AAA	0.00	999	1.000	1.000	0.0361
1569002_x_at	30.5	96.6	1.66	3.17	AAA-AAA	0.00	999	1.000	1.000	0.1234
239979_at	28.9	9.1	-1.66	-3.17	AAA-AAA	0.00	999	1.000	1.000	0.2802
1552436_a_at	6.9	22.0	1.66	3.17	AAA-AAA	0.00	999	1.000	1.000	0.3091
1553078_at	90.3	28.5	-1.66	-3.17	AAA-AAA	0.00	999	1.000	1.000	0.1541
1556262_at	4.2	13.2	1.66	3.17	AAA-AAA	0.00	999	1.000	1.000	0.0342
243832_at	41.5	13.1	-1.66	-3.17	AAA-PAA	0.00	999	1.000	1.000	0.2164

242432_at	4.4	14.0	1.66	3.17	AAA-AAA	0.00	999	1.000	1.000	0.3134
1569812_at	11.6	36.7	1.66	3.17	AAA-AAA	0.00	999	1.000	1.000	0.1248
1554912_at	9.3	2.9	-1.66	-3.17	AAA-AAA	0.00	999	1.000	1.000	0.1090
1560915_at	13.7	4.3	-1.66	-3.17	AAA-AAA	0.00	999	1.000	1.000	0.2214
1560164_at	19.6	6.2	-1.66	-3.17	AAA-AAA	0.00	999	1.000	1.000	0.3454
1560059_at	100.7	31.8	-1.66	-3.17	AAA-AAA	0.00	999	1.000	1.000	0.0982
206768_at	12.0	37.9	1.66	3.17	AAA-AAA	0.00	999	1.000	1.000	0.0070
235326_at	27.4	8.7	-1.66	-3.17	AAA-AAA	0.00	999	1.000	1.000	0.3160
236376_at	19.3	61.2	1.66	3.17	AAA-AAA	0.00	999	1.000	1.000	0.0225
228728_at	48.8	15.4	-1.66	-3.17	AAA-AAA	0.00	999	1.000	1.000	0.1349
206134_at	40.5	12.8	-1.66	-3.17	PAA-APA	0.00	999	1.000	1.000	0.2187
1564635_a_at	6.5	20.5	1.66	3.17	AAA-AAA	0.00	999	1.000	1.000	0.2565
1556713_at	3.7	11.8	1.66	3.17	AAA-AAA	0.00	999	1.000	1.000	0.2286
214858_at	17.1	5.4	-1.66	-3.17	AAA-AAA	0.00	999	1.000	1.000	0.2073
234925_at	33.7	10.7	-1.66	-3.16	AAA-AAA	0.00	999	1.000	1.000	0.1877
1570272_at	7.7	24.3	1.66	3.16	AAA-AAA	0.00	999	1.000	1.000	0.2690
1560409_at	25.8	8.2	-1.66	-3.16	AAA-AAA	0.00	999	1.000	1.000	0.1380
216093_at	50.6	16.0	-1.66	-3.16	PPP-AAM	0.00	999	1.000	1.000	0.3295
242332_at	27.9	88.1	1.66	3.16	AAA-AAA	0.00	999	1.000	1.000	0.1289
1559295_at	15.1	47.7	1.66	3.16	AAA-AAA	0.00	999	1.000	1.000	0.0644
214971_s_at	27.2	86.0	1.66	3.16	AAA-AAA	0.00	999	1.000	1.000	0.0496
220107_s_at	33.3	10.5	-1.66	-3.16	AAA-AAA	0.00	999	1.000	1.000	0.2605
217858_s_at	9.0	28.4	1.66	3.16	AAA-AAA	0.00	999	1.000	1.000	0.1664
204415_at	49.8	15.8	-1.66	-3.16	AAA-AAA	0.00	999	1.000	1.000	0.1674
231044_at	16.5	52.0	1.66	3.16	AAA-AAA	0.00	999	1.000	1.000	0.2026
243200_at	16.3	5.2	-1.66	-3.16	AAA-AAA	0.00	999	1.000	1.000	0.1656
220808_at	49.9	15.8	-1.66	-3.16	AAA-AAA	0.00	999	1.000	1.000	0.0563
219761_at	11.1	35.1	1.66	3.16	AAA-AAA	0.00	999	1.000	1.000	0.0033
237469_at	11.0	34.7	1.66	3.16	AAA-AAA	0.00	999	1.000	1.000	0.0227
233927_at	37.8	12.0	-1.66	-3.16	APA-AAP	0.00	999	1.000	1.000	0.4404
1556786_at	20.1	6.4	-1.66	-3.16	AAA-AAA	0.00	999	1.000	1.000	0.2676
237797_at	3.2	10.2	1.66	3.16	AAA-AAA	0.00	999	1.000	1.000	0.2391
221416_at	40.6	12.9	-1.66	-3.16	AAA-AAA	0.00	999	1.000	1.000	0.1137
211331_x_at	55.6	17.6	-1.66	-3.15	AAA-AAA	0.00	999	1.000	1.000	0.1668
232857_at	81.3	25.8	-1.66	-3.15	AAA-AAA	0.00	999	1.000	1.000	0.1208
225518_x_at	18.6	5.9	-1.66	-3.15	AAA-AAA	0.00	999	1.000	1.000	0.0800
1554409_at	9.6	30.1	1.66	3.15	AAA-AAA	0.00	999	1.000	1.000	0.2890
235568_at	109.5	34.8	-1.66	-3.15	PPP-AAA	0.00	999	1.000	1.000	0.0914
1560025_at	36.2	11.5	-1.66	-3.15	PPM-AAA	0.00	999	1.000	1.000	0.3038
1559169_at	18.7	58.9	1.66	3.15	AAA-AAA	0.00	999	1.000	1.000	0.0830
204915_s_at	12.3	38.6	1.66	3.15	AAA-AAA	0.00	999	1.000	1.000	0.2657
232377_at	5.1	16.0	1.66	3.15	AAA-AAA	0.00	999	1.000	1.000	0.2142

229484_at	103.4	32.8	-1.66	-3.15	AAA-AAM	0.00	999	1.000	1.000	0.1552
207183_at	111.2	35.3	-1.65	-3.15	AAA-AAA	0.00	999	1.000	1.000	0.0649
234895_at	32.5	10.3	-1.65	-3.15	AAA-AAA	0.00	999	1.000	1.000	0.0645
232677_at	30.3	9.6	-1.65	-3.15	AAA-AAA	0.00	999	1.000	1.000	0.2075
208166_at	33.3	10.6	-1.65	-3.15	PAA-AAA	0.00	999	1.000	1.000	0.3649
212999_x_at	23.6	7.5	-1.65	-3.15	AAA-AAA	0.00	999	1.000	1.000	0.2434
238676_at	34.7	109.0	1.65	3.15	AAA-AAA	0.00	999	1.000	1.000	0.0443
238255_at	11.9	37.3	1.65	3.15	AAA-AAA	0.00	999	1.000	1.000	0.1174
231980_at	1.8	5.6	1.65	3.15	AAA-AAA	0.00	999	1.000	1.000	0.3499
1569446_x_at	16.9	5.4	-1.65	-3.14	AAA-AAA	0.00	999	1.000	1.000	0.1346
1566749_at	2.5	7.8	1.65	3.14	AAA-AAA	0.00	999	1.000	1.000	0.4285
217609_at	20.9	65.8	1.65	3.14	AAA-AAA	0.00	999	1.000	1.000	0.0678
230650_at	34.0	10.8	-1.65	-3.14	PAA-AAA	0.00	999	1.000	1.000	0.2149
215378_at	26.6	8.5	-1.65	-3.14	PAA-AAA	0.00	999	1.000	1.000	0.2907
242964_at	23.7	7.5	-1.65	-3.14	AAP-AAA	0.00	999	1.000	1.000	0.3771
240328_at	77.2	24.6	-1.65	-3.14	AAA-AAA	0.00	999	1.000	1.000	0.0206
205878_at	59.4	18.9	-1.65	-3.14	AAA-AAA	0.00	999	1.000	1.000	0.0945
234927_s_at	43.2	13.8	-1.65	-3.14	AAA-AAA	0.00	999	1.000	1.000	0.1354
232898_at	9.5	29.7	1.65	3.14	AAA-AAA	0.00	999	1.000	1.000	0.2754
228290_at	20.5	64.4	1.65	3.14	AAA-AAA	0.00	999	1.000	1.000	0.2567
230760_at	8.4	2.7	-1.65	-3.14	AAA-AAA	0.00	999	1.000	1.000	0.4436
230214_at	92.1	29.4	-1.65	-3.14	PAA-AAA	0.00	999	1.000	1.000	0.0697
241234_at	47.6	149.3	1.65	3.14	AAA-AAA	0.00	999	1.000	1.000	0.0917
240617_at	15.7	49.2	1.65	3.14	AAA-AAA	0.00	999	1.000	1.000	0.1806
1558793_at	8.9	2.8	-1.65	-3.14	AAA-AAA	0.00	999	1.000	1.000	0.3080
202283_at	74.4	23.7	-1.65	-3.14	AAA-AAA	0.00	999	1.000	1.000	0.1593
222172_at	15.4	4.9	-1.65	-3.14	AAA-AAA	0.00	999	1.000	1.000	0.1382
231474_at	9.9	31.2	1.65	3.14	AAA-AAA	0.00	999	1.000	1.000	0.2676
231642_at	15.6	49.0	1.65	3.14	AAA-AAA	0.00	999	1.000	1.000	0.2395
221342_at	10.9	34.1	1.65	3.14	AAA-AAA	0.00	999	1.000	1.000	0.0540
217757_at	5.5	17.2	1.65	3.14	AAA-AAA	0.00	999	1.000	1.000	0.1983
211731_x_at	15.5	48.5	1.65	3.14	AAA-AAA	0.00	999	1.000	1.000	0.1580
231475_at	85.6	27.3	-1.65	-3.14	AAA-AAA	0.00	999	1.000	1.000	0.2744
1563881_at	4.5	14.1	1.65	3.13	AAA-AAA	0.00	999	1.000	1.000	0.2180
238514_at	41.7	13.3	-1.65	-3.13	AAA-AAA	0.00	999	1.000	1.000	0.0207
227709_at	60.8	19.4	-1.65	-3.13	AAA-AAA	0.00	999	1.000	1.000	0.3070
240544_at	34.8	11.1	-1.65	-3.13	MAA-AAA	0.00	999	1.000	1.000	0.1495
1553634_a_at	16.7	5.3	-1.65	-3.13	AAA-AAA	0.00	999	1.000	1.000	0.2284
242977_at	11.4	35.6	1.65	3.13	AAA-AAA	0.00	999	1.000	1.000	0.0891
237162_at	15.1	4.8	-1.65	-3.13	AAA-AAA	0.00	999	1.000	1.000	0.0965
231220_at	13.2	41.3	1.65	3.13	AAA-AAA	0.00	999	1.000	1.000	0.2264
232255_at	21.6	67.5	1.65	3.13	AAA-AAA	0.00	999	1.000	1.000	0.0469

206784_at	23.2	7.4	-1.65	-3.13	AAA-AAA	0.00	999	1.000	1.000	0.1361
234865_at	6.2	2.0	-1.65	-3.13	AAA-AAA	0.00	999	1.000	1.000	0.2382
244202_at	49.5	15.8	-1.65	-3.13	AAA-AAA	0.00	999	1.000	1.000	0.0558
221151_at	21.1	6.7	-1.65	-3.13	AAA-AAA	0.00	999	1.000	1.000	0.0962
241080_at	31.7	98.9	1.64	3.13	AAA-AAA	0.00	999	1.000	1.000	0.0500
217184_s_at	68.5	21.9	-1.64	-3.13	AAA-AAA	0.00	999	1.000	1.000	0.1722
217070_at	49.6	15.9	-1.64	-3.13	AAA-AAA	0.00	999	1.000	1.000	0.1589
233786_at	9.6	29.9	1.64	3.13	AAA-AAA	0.00	999	1.000	1.000	0.2798
236134_at	26.5	82.9	1.64	3.13	AAA-AAA	0.00	999	1.000	1.000	0.0868
1570261_at	9.4	3.0	-1.64	-3.12	AAA-AAA	0.00	999	1.000	1.000	0.0869
240525_at	57.9	18.6	-1.64	-3.12	AAA-AAA	0.00	999	1.000	1.000	0.3174
1554946_at	8.8	27.5	1.64	3.12	AAA-AAA	0.00	999	1.000	1.000	0.3239
206893_at	9.0	28.0	1.64	3.12	AAA-MAA	0.00	999	1.000	1.000	0.1034
1554225_a_at	17.2	5.5	-1.64	-3.12	AAA-AAA	0.00	999	1.000	1.000	0.0773
243520_x_at	6.9	2.2	-1.64	-3.12	AAA-AAA	0.00	999	1.000	1.000	0.2413
233326_at	8.2	25.4	1.64	3.12	AAA-AAA	0.00	999	1.000	1.000	0.0550
233602_at	7.2	2.3	-1.64	-3.12	AAA-AAA	0.00	999	1.000	1.000	0.2460
1558469_at	54.8	17.6	-1.64	-3.12	AAA-AAP	0.00	999	1.000	1.000	0.2763
240968_at	38.7	12.4	-1.64	-3.12	AAA-AAA	0.00	999	1.000	1.000	0.1056
1565699_at	26.6	8.5	-1.64	-3.12	AAA-AAA	0.00	999	1.000	1.000	0.1755
1553534_at	37.2	11.9	-1.64	-3.12	AAA-AAA	0.00	999	1.000	1.000	0.1193
1555264_a_at	76.4	24.5	-1.64	-3.12	AAA-AAA	0.00	999	1.000	1.000	0.2313
213652_at	20.4	6.5	-1.64	-3.12	AAA-AAA	0.00	999	1.000	1.000	0.1415
206817_x_at	12.3	38.2	1.64	3.12	AAA-AAA	0.00	999	1.000	1.000	0.1103
238019_at	15.9	49.5	1.64	3.12	AAA-AAA	0.00	999	1.000	1.000	0.1921
1563462_at	1.9	5.9	1.64	3.12	AAA-AAA	0.00	999	1.000	1.000	0.0033
1552818_a_at	12.3	3.9	-1.64	-3.12	AAA-AAA	0.00	999	1.000	1.000	0.2597
1562558_at	10.8	3.5	-1.64	-3.12	AAA-AAA	0.00	999	1.000	1.000	0.3186
208005_at	10.7	33.4	1.64	3.12	AAA-AAA	0.00	999	1.000	1.000	0.2278
237077_at	15.2	47.4	1.64	3.12	AAA-AAA	0.00	999	1.000	1.000	0.0641
236727_at	40.6	13.0	-1.64	-3.12	AAA-AAA	0.00	999	1.000	1.000	0.1274
226632_at	67.2	21.6	-1.64	-3.12	AAA-AAA	0.00	999	1.000	1.000	0.0719
230560_at	24.7	7.9	-1.64	-3.12	AAA-AAA	0.00	999	1.000	1.000	0.1370
230807_at	39.5	123.1	1.64	3.12	AAA-AAA	0.00	999	1.000	1.000	0.2362
1564407_a_at	11.8	36.8	1.64	3.11	AAA-AAA	0.00	999	1.000	1.000	0.2525
1570078_a_at	20.9	65.2	1.64	3.12	AAA-AAA	0.00	999	1.000	1.000	0.2927
220854_at	9.3	28.9	1.64	3.12	AAA-MAA	0.00	999	1.000	1.000	0.2733
217121_at	61.1	19.6	-1.64	-3.11	AAA-AAA	0.00	999	1.000	1.000	0.2623
235004_at	13.1	40.7	1.64	3.11	AAA-AAA	0.00	999	1.000	1.000	0.1888
231014_at	23.8	7.7	-1.64	-3.12	AAA-AAA	0.00	999	1.000	1.000	0.1496
242945_at	18.3	57.1	1.64	3.11	AAA-AAA	0.00	999	1.000	1.000	0.3024
233586_s_at	38.9	12.5	-1.64	-3.11	AAA-AAA	0.00	999	1.000	1.000	0.1386

228335_at	4.9	15.3	1.64	3.11	AAA-AAA	0.00	999	1.000	1.000	0.1821
224358_s_at	3.2	9.9	1.64	3.11	AAA-AAA	0.00	999	1.000	1.000	0.3419
241019_at	45.5	14.6	-1.64	-3.11	AAA-AAA	0.00	999	1.000	1.000	0.0257
1557022_at	28.1	87.4	1.64	3.11	AAA-AAA	0.00	999	1.000	1.000	0.0693
1556814_a_at	6.0	18.7	1.64	3.11	AAA-AAA	0.00	999	1.000	1.000	0.3002
206585_at	57.5	18.5	-1.64	-3.11	AAA-AAA	0.00	999	1.000	1.000	0.0922
209996_x_at	65.5	21.1	-1.64	-3.11	AAA-AAA	0.00	999	1.000	1.000	0.2080
208108_s_at	8.6	26.8	1.64	3.11	AAA-AAA	0.00	999	1.000	1.000	0.0156
234957_at	17.0	52.7	1.64	3.11	AAA-AAA	0.00	999	1.000	1.000	0.2126
227336_at	39.2	12.6	-1.64	-3.11	AAA-AAA	0.00	999	1.000	1.000	0.1505
64064_at	56.8	176.7	1.64	3.11	AAA-AAA	0.00	999	1.000	1.000	0.1186
229728_at	29.9	92.9	1.64	3.11	AAA-AAA	0.00	999	1.000	1.000	0.0522
1568852_x_at	5.8	17.9	1.64	3.11	AAA-AAA	0.00	999	1.000	1.000	0.4031
1556760_a_at	10.2	31.6	1.64	3.11	AAA-AAA	0.00	999	1.000	1.000	0.0252
1553356_at	14.6	4.7	-1.64	-3.11	AAA-AAA	0.00	999	1.000	1.000	0.2992
1555546_a_at	31.8	10.2	-1.64	-3.11	AAA-AAA	0.00	999	1.000	1.000	0.0502
221429_x_at	21.9	68.2	1.64	3.11	AAA-AAA	0.00	999	1.000	1.000	0.2148
201766_at	93.7	30.1	-1.64	-3.11	AAA-AAA	0.00	999	1.000	1.000	0.1595
1556148_s_at	26.9	8.7	-1.64	-3.11	AAP-AAA	0.00	999	1.000	1.000	0.1472
236023_at	70.3	22.6	-1.64	-3.11	PAP-AAA	0.00	999	1.000	1.000	0.1686
238054_at	58.6	18.9	-1.64	-3.11	AAA-AAA	0.00	999	1.000	1.000	0.1718
236501_at	12.7	39.4	1.64	3.11	AAA-AAA	0.00	999	1.000	1.000	0.1421
336_at	25.0	77.7	1.64	3.11	AAA-AAA	0.00	999	1.000	1.000	0.0510
237415_at	64.2	20.7	-1.63	-3.10	APA-PAA	0.00	999	1.000	1.000	0.2824
1562688_at	5.9	18.3	1.63	3.10	AAA-AAA	0.00	999	1.000	1.000	0.1958
220029_at	93.3	30.1	-1.63	-3.10	AAA-AAA	0.00	999	1.000	1.000	0.0491
234996_at	3.2	9.8	1.63	3.11	AAA-AAA	0.00	999	1.000	1.000	0.2416
229142_s_at	114.0	36.7	-1.63	-3.11	AAA-AAA	0.00	999	1.000	1.000	0.0568
241086_at	5.1	15.7	1.63	3.10	AAA-AAA	0.00	999	1.000	1.000	0.1825
1561692_at	4.4	13.6	1.63	3.10	AAA-AAM	0.00	999	1.000	1.000	0.4384
213412_at	9.4	29.1	1.63	3.10	AAA-AAA	0.00	999	1.000	1.000	0.3402
209082_s_at	22.8	70.8	1.63	3.10	AAA-AAA	0.00	999	1.000	1.000	0.3324
232361_s_at	31.7	98.4	1.63	3.10	AAA-AAA	0.00	999	1.000	1.000	0.1533
217690_at	41.9	130.0	1.63	3.10	AAA-AAA	0.00	999	1.000	1.000	0.1589
205151_s_at	4.7	14.7	1.63	3.10	AAA-AAA	0.00	999	1.000	1.000	0.1811
204758_s_at	11.9	36.9	1.63	3.10	AAA-AAA	0.00	999	1.000	1.000	0.2864
230325_at	10.2	31.5	1.63	3.10	AAA-AAA	0.00	999	1.000	1.000	0.1352
242459_at	24.3	7.9	-1.63	-3.10	AAA-AAA	0.00	999	1.000	1.000	0.2296
1561025_at	9.1	3.0	-1.63	-3.10	AAA-AAA	0.00	999	1.000	1.000	0.2475
216195_at	55.2	17.8	-1.63	-3.10	AAA-AAA	0.00	999	1.000	1.000	0.0819
221304_at	41.2	13.3	-1.63	-3.10	AAA-AAA	0.00	999	1.000	1.000	0.1556
217085_at	2.2	6.9	1.63	3.10	AAA-AAA	0.00	999	1.000	1.000	0.1827

219760_at	19.1	59.1	1.63	3.10	AAA-AAA	0.00	999	1.000	1.000	0.2803
208395_s_at	13.7	42.3	1.63	3.10	AAA-AAA	0.00	999	1.000	1.000	0.0816
232468_at	8.2	25.5	1.63	3.10	AAA-AAA	0.00	999	1.000	1.000	0.1373
241398_at	25.3	78.4	1.63	3.10	AAA-AMA	0.00	999	1.000	1.000	0.0296
239183_at	6.1	18.9	1.63	3.09	AAA-AAA	0.00	999	1.000	1.000	0.3628
1560202_at	8.5	2.7	-1.63	-3.10	AAA-AAA	0.00	999	1.000	1.000	0.1670
207363_at	9.7	29.9	1.63	3.10	AAA-AAA	0.00	999	1.000	1.000	0.2141
232599_at	6.5	20.0	1.63	3.10	AAA-AAA	0.00	999	1.000	1.000	0.1230
229913_at	23.5	72.8	1.63	3.09	AAA-AAA	0.00	999	1.000	1.000	0.2370
229271_x_at	3.1	9.5	1.63	3.10	AAA-AAA	0.00	999	1.000	1.000	0.4444
220381_at	51.5	16.6	-1.63	-3.09	AAA-AAA	0.00	999	1.000	1.000	0.1122
207101_at	30.9	10.0	-1.63	-3.09	AAA-AAA	0.00	999	1.000	1.000	0.2597
204309_at	16.8	5.4	-1.63	-3.09	AAA-AAA	0.00	999	1.000	1.000	0.2856
207349_s_at	6.4	19.8	1.63	3.09	AAA-AAA	0.00	999	1.000	1.000	0.1703
235401_s_at	7.6	23.4	1.63	3.09	AAA-AAA	0.00	999	1.000	1.000	0.1120
233323_at	38.4	118.8	1.63	3.09	AAA-AAA	0.00	999	1.000	1.000	0.1056
236131_at	18.8	58.1	1.63	3.09	AAA-AAA	0.00	999	1.000	1.000	0.3351
228288_at	18.3	56.6	1.63	3.09	AAA-AAA	0.00	999	1.000	1.000	0.1950
1569879_a_at	95.3	30.9	-1.63	-3.09	AAA-AAA	0.00	999	1.000	1.000	0.1775
1561525_at	26.9	8.7	-1.63	-3.09	AAA-AAA	0.00	999	1.000	1.000	0.2434
216448_at	11.1	34.2	1.63	3.09	AAA-AAA	0.00	999	1.000	1.000	0.1449
208239_at	17.9	5.8	-1.63	-3.09	AAA-AAA	0.00	999	1.000	1.000	0.1559
230823_at	5.4	16.8	1.63	3.09	AAA-AAA	0.00	999	1.000	1.000	0.1225
221461_at	23.7	7.7	-1.63	-3.09	APA-AAA	0.00	999	1.000	1.000	0.1018
1556494_at	48.7	15.8	-1.63	-3.09	AAA-AAA	0.00	999	1.000	1.000	0.1092
1558247_s_at	88.3	28.6	-1.63	-3.09	AAA-AAA	0.00	999	1.000	1.000	0.2376
243533_x_at	11.0	33.9	1.63	3.09	AAA-AAA	0.00	999	1.000	1.000	0.0146
214914_at	5.5	1.8	-1.63	-3.09	AAA-AAA	0.00	999	1.000	1.000	0.0519
221010_s_at	29.9	92.4	1.63	3.09	AAA-AMA	0.00	999	1.000	1.000	0.0754
234314_at	6.3	19.6	1.63	3.09	AAA-AAA	0.00	999	1.000	1.000	0.2948
1562690_at	28.2	9.1	-1.63	-3.09	AAA-AAA	0.00	999	1.000	1.000	0.1760
242686_at	34.4	11.2	-1.63	-3.09	AAA-AAA	0.00	999	1.000	1.000	0.3230
1561658_at	24.3	7.9	-1.63	-3.09	AAM-AAA	0.00	999	1.000	1.000	0.1351
216720_at	17.0	5.5	-1.63	-3.09	AAA-AAA	0.00	999	1.000	1.000	0.1581
208334_at	28.0	9.1	-1.63	-3.09	AAA-AAA	0.00	999	1.000	1.000	0.2554
230436_s_at	76.1	24.6	-1.63	-3.09	AAA-AAA	0.00	999	1.000	1.000	0.0024
1555139_a_at	48.9	15.9	-1.63	-3.09	AAA-AAA	0.00	999	1.000	1.000	0.2697
1561218_s_at	46.3	15.0	-1.63	-3.08	AAA-AAA	0.00	999	1.000	1.000	0.3075
216498_at	19.8	61.2	1.63	3.09	AAA-AAA	0.00	999	1.000	1.000	0.2657
232947_at	3.5	10.8	1.63	3.08	AAA-AAA	0.00	999	1.000	1.000	0.1916
237304_at	8.3	25.5	1.63	3.08	AAA-AAA	0.00	999	1.000	1.000	0.0600
226709_at	10.0	30.8	1.63	3.08	AAA-AAA	0.00	999	1.000	1.000	0.1757

240201_at	36.9	12.0	-1.62	-3.08	AAA-APA	0.00	999	1.000	1.000	0.3702
1564202_at	8.2	25.2	1.62	3.08	AAA-AAA	0.00	999	1.000	1.000	0.2846
1564158_a_at	4.0	12.2	1.62	3.08	AAA-AAA	0.00	999	1.000	1.000	0.2245
1556924_at	44.1	14.3	-1.62	-3.08	AAA-AAA	0.00	999	1.000	1.000	0.2485
207848_at	8.5	26.0	1.62	3.08	AAA-AAA	0.00	999	1.000	1.000	0.1528
224226_at	16.1	5.2	-1.62	-3.08	AAA-AAA	0.00	999	1.000	1.000	0.1378
229372_at	21.0	6.8	-1.62	-3.08	AAA-AAA	0.00	999	1.000	1.000	0.0921
241307_at	20.4	6.6	-1.62	-3.08	AAA-AAA	0.00	999	1.000	1.000	0.0768
1569410_at	2.4	7.3	1.62	3.08	AAA-AAA	0.00	999	1.000	1.000	0.2539
1562541_at	4.2	13.0	1.62	3.08	AAA-AAA	0.00	999	1.000	1.000	0.0975
244698_at	73.1	23.7	-1.62	-3.08	AAA-AAA	0.00	999	1.000	1.000	0.3640
213723_s_at	22.8	7.4	-1.62	-3.08	AAA-AAA	0.00	999	1.000	1.000	0.1039
220106_at	86.2	28.0	-1.62	-3.08	AAA-AAA	0.00	999	1.000	1.000	0.0440
205694_at	9.9	30.5	1.62	3.08	AMA-AAA	0.00	999	1.000	1.000	0.3903
210444_at	43.8	14.2	-1.62	-3.08	AAA-AAA	0.00	999	1.000	1.000	0.1495
233781_s_at	17.3	53.2	1.62	3.08	AAA-AAA	0.00	999	1.000	1.000	0.0925
231666_at	9.2	28.4	1.62	3.08	AAA-AAA	0.00	999	1.000	1.000	0.0917
242906_at	23.7	7.7	-1.62	-3.08	AAP-AAA	0.00	999	1.000	1.000	0.2511
240439_at	43.1	14.0	-1.62	-3.08	AAA-AAA	0.00	999	1.000	1.000	0.0732
239939_at	17.0	52.5	1.62	3.08	AAA-AAA	0.00	999	1.000	1.000	0.2445
1557380_at	63.6	20.7	-1.62	-3.08	AAA-AAA	0.00	999	1.000	1.000	0.1562
1556624_at	4.2	12.8	1.62	3.08	AAA-AAA	0.00	999	1.000	1.000	0.4479
1562220_at	41.1	126.6	1.62	3.08	AAA-AAA	0.00	999	1.000	1.000	0.0038
1561713_at	6.5	20.1	1.62	3.08	AAA-AAA	0.00	999	1.000	1.000	0.2597
1561530_at	30.9	95.2	1.62	3.08	AAA-AAA	0.00	999	1.000	1.000	0.0772
207262_at	30.0	9.8	-1.62	-3.08	AAA-AAA	0.00	999	1.000	1.000	0.4221
209081_s_at	35.0	11.4	-1.62	-3.08	AAA-AAA	0.00	999	1.000	1.000	0.1253
208488_s_at	87.8	28.6	-1.62	-3.08	AAA-AAA	0.00	999	1.000	1.000	0.2833
1567254_at	3.9	12.0	1.62	3.08	AAA-AAA	0.00	999	1.000	1.000	0.0645
243177_at	13.4	4.4	-1.62	-3.08	AAA-AAA	0.00	999	1.000	1.000	0.1784
242591_at	16.0	5.2	-1.62	-3.08	AAA-AAA	0.00	999	1.000	1.000	0.2603
244106_at	11.2	34.5	1.62	3.08	AAA-AAA	0.00	999	1.000	1.000	0.1302
234453_s_at	13.6	4.4	-1.62	-3.08	AAA-AAA	0.00	999	1.000	1.000	0.1137
228132_at	20.9	6.8	-1.62	-3.08	AAA-AAA	0.00	999	1.000	1.000	0.2251
231805_at	17.6	5.7	-1.62	-3.08	AAA-AAA	0.00	999	1.000	1.000	0.2002
1556207_a_at	4.8	1.6	-1.62	-3.08	AAA-AAA	0.00	999	1.000	1.000	0.3723
220569_at	16.0	49.1	1.62	3.07	AAA-AAA	0.00	999	1.000	1.000	0.0900
217330_at	20.0	61.4	1.62	3.07	AAA-AAA	0.00	999	1.000	1.000	0.0057
207193_at	23.7	72.9	1.62	3.07	AAA-AAA	0.00	999	1.000	1.000	0.3451
202751_at	36.7	113.0	1.62	3.08	AAA-AAA	0.00	999	1.000	1.000	0.0147
235503_at	16.0	5.2	-1.62	-3.07	AAA-AAA	0.00	999	1.000	1.000	0.3307
238224_at	1.6	4.8	1.62	3.07	AAA-AAA	0.00	999	1.000	1.000	0.2589

241856_at	4.4	13.6	1.62	3.07	AAA-AAA	0.00	999	1.000	1.000	0.3461
1555431_a_at	51.2	16.7	-1.62	-3.07	AAA-AAA	0.00	999	1.000	1.000	0.1030
206100_at	18.9	58.2	1.62	3.07	AAA-AAA	0.00	999	1.000	1.000	0.3084
223720_at	68.3	22.3	-1.62	-3.07	AAA-AAA	0.00	999	1.000	1.000	0.1662
1561247_at	64.3	21.0	-1.62	-3.07	PAA-AAA	0.00	999	1.000	1.000	0.1254
239218_at	6.5	19.8	1.62	3.07	AAA-AAA	0.00	999	1.000	1.000	0.3330
206761_at	9.1	28.0	1.62	3.07	AAA-AAA	0.00	999	1.000	1.000	0.3249
234715_at	6.8	20.9	1.62	3.07	AAA-AAA	0.00	999	1.000	1.000	0.2468
237862_at	24.9	8.1	-1.62	-3.07	AAA-AAA	0.00	999	1.000	1.000	0.1588
230627_at	30.1	9.8	-1.62	-3.07	AAA-AAA	0.00	999	1.000	1.000	0.2308
1563559_at	10.9	33.6	1.62	3.07	AAA-AAA	0.00	999	1.000	1.000	0.3156
1569124_at	14.4	4.7	-1.62	-3.07	AAA-AAA	0.00	999	1.000	1.000	0.2053
240775_at	12.1	37.2	1.62	3.07	AAA-AAA	0.00	999	1.000	1.000	0.1421
1561765_at	1.8	5.6	1.62	3.07	AAA-AAA	0.00	999	1.000	1.000	0.2868
231138_at	5.8	17.7	1.62	3.07	AAA-AAA	0.00	999	1.000	1.000	0.1599
1560798_at	8.5	2.8	-1.62	-3.07	AAA-AAA	0.00	999	1.000	1.000	0.2443
215957_at	2.9	8.9	1.62	3.07	AAA-AAA	0.00	999	1.000	1.000	0.3215
215311_at	36.3	11.9	-1.62	-3.07	AAA-AAA	0.00	999	1.000	1.000	0.2952
52255_s_at	15.9	48.6	1.62	3.07	AAA-AAA	0.00	999	1.000	1.000	0.0481
230635_at	8.2	25.1	1.62	3.07	AAA-AAA	0.00	999	1.000	1.000	0.0953
244630_at	4.8	14.6	1.62	3.06	AAA-AAA	0.00	999	1.000	1.000	0.1511
212695_at	20.0	61.2	1.62	3.06	AAA-AAA	0.00	999	1.000	1.000	0.2138
234744_x_at	105.3	34.4	-1.62	-3.06	AAA-AAA	0.00	999	1.000	1.000	0.0165
221941_at	43.1	14.1	-1.62	-3.06	AAA-AAA	0.00	999	1.000	1.000	0.1385
1558334_a_at	12.8	39.2	1.61	3.06	AAA-AAA	0.00	999	1.000	1.000	0.1243
244173_at	4.1	12.6	1.61	3.06	AAA-AAA	0.00	999	1.000	1.000	0.1970
207276_at	6.2	2.0	-1.61	-3.06	AAA-AAA	0.00	999	1.000	1.000	0.2927
234619_at	33.4	10.9	-1.61	-3.06	AAA-AAA	0.00	999	1.000	1.000	0.3121
235981_at	15.5	47.6	1.61	3.06	AAA-AAA	0.00	999	1.000	1.000	0.2098
1558007_s_at	34.5	11.3	-1.61	-3.06	AAA-AAA	0.00	999	1.000	1.000	0.0517
220190_s_at	3.3	10.0	1.61	3.06	AAA-AAA	0.00	999	1.000	1.000	0.1249
203698_s_at	6.0	18.3	1.61	3.06	AAA-AAA	0.00	999	1.000	1.000	0.2778
224290_at	25.7	8.4	-1.61	-3.06	AAA-AAA	0.00	999	1.000	1.000	0.3124
1568951_at	81.4	26.6	-1.61	-3.06	APA-AAA	0.00	999	1.000	1.000	0.1054
240437_at	14.1	43.0	1.61	3.06	AAA-AAA	0.00	999	1.000	1.000	0.2717
220010_at	31.6	10.4	-1.61	-3.06	AAA-AAA	0.00	999	1.000	1.000	0.1620
234664_at	19.5	6.4	-1.61	-3.06	AAA-AAA	0.00	999	1.000	1.000	0.2100
235938_at	15.9	48.7	1.61	3.06	AAA-AAA	0.00	999	1.000	1.000	0.2174
230510_at	9.5	28.9	1.61	3.06	AAA-AAA	0.00	999	1.000	1.000	0.0860
1556210_at	33.5	11.0	-1.61	-3.06	PAA-AAA	0.00	999	1.000	1.000	0.2062
1564887_at	37.0	112.9	1.61	3.05	AAA-AAA	0.00	999	1.000	1.000	0.1293
1556497_a_at	19.1	6.2	-1.61	-3.05	AAA-AAA	0.00	999	1.000	1.000	0.2017

1555116_s_at	3.4	10.4	1.61	3.06	AAA-AAA	0.00	999	1.000	1.000	0.3446
1554988_at	2.4	7.4	1.61	3.05	AAA-AAA	0.00	999	1.000	1.000	0.2816
1561493_at	16.9	5.5	-1.61	-3.05	AAA-AAA	0.00	999	1.000	1.000	0.5170
1562367_at	16.7	5.5	-1.61	-3.05	AAA-AAA	0.00	999	1.000	1.000	0.3294
234507_at	19.2	58.7	1.61	3.06	AAA-AAA	0.00	999	1.000	1.000	0.1411
244599_at	61.4	20.1	-1.61	-3.05	AAA-AAA	0.00	999	1.000	1.000	0.2798
216660_at	3.4	10.4	1.61	3.05	AAA-AAA	0.00	999	1.000	1.000	0.0790
208252_s_at	18.9	6.2	-1.61	-3.05	AAA-AAA	0.00	999	1.000	1.000	0.0822
236539_at	5.4	16.3	1.61	3.05	AAA-AAA	0.00	999	1.000	1.000	0.2109
221412_at	39.3	12.9	-1.61	-3.05	PAA-AAA	0.00	999	1.000	1.000	0.3251
244862_at	31.5	10.3	-1.61	-3.05	AAA-AAA	0.00	999	1.000	1.000	0.3281
220004_at	8.7	26.4	1.61	3.05	AAA-AAA	0.00	999	1.000	1.000	0.0230
208145_at	6.2	18.8	1.61	3.05	AAA-AAA	0.00	999	1.000	1.000	0.0621
235087_at	34.6	11.3	-1.61	-3.05	AAA-AAA	0.00	999	1.000	1.000	0.2377
233508_at	2.3	7.1	1.61	3.05	AAA-AAA	0.00	999	1.000	1.000	0.0241
237896_at	11.5	35.1	1.61	3.05	AAA-AAA	0.00	999	1.000	1.000	0.2798
223706_at	99.7	32.7	-1.61	-3.05	AAA-AAA	0.00	999	1.000	1.000	0.1050
239087_at	11.4	3.7	-1.61	-3.05	AAA-AAA	0.00	999	1.000	1.000	0.0568
216998_s_at	8.1	24.7	1.61	3.05	AAA-AAA	0.00	999	1.000	1.000	0.2284
209315_at	35.0	106.6	1.61	3.05	AAA-AAA	0.00	999	1.000	1.000	0.1682
233046_at	5.0	15.3	1.61	3.05	AAA-AAA	0.00	999	1.000	1.000	0.0980
237422_at	8.1	2.6	-1.61	-3.05	AAA-AAA	0.00	999	1.000	1.000	0.3878
235975_at	57.6	18.9	-1.61	-3.05	AAA-AAA	0.00	999	1.000	1.000	0.0165
237258_at	27.6	9.0	-1.61	-3.05	AAA-AAA	0.00	999	1.000	1.000	0.1534
1562528_at	18.3	6.0	-1.61	-3.05	PAA-AAA	0.00	999	1.000	1.000	0.3272
240341_at	16.8	5.5	-1.61	-3.05	AAA-AAA	0.00	999	1.000	1.000	0.3129
1554547_at	24.7	8.1	-1.61	-3.05	AAA-AAA	0.00	999	1.000	1.000	0.2111
206869_at	7.6	23.1	1.61	3.05	AAA-AAA	0.00	999	1.000	1.000	0.1750
204583_x_at	54.7	18.0	-1.61	-3.05	AAA-AAA	0.00	999	1.000	1.000	0.0212
210274_at	7.5	22.9	1.61	3.05	AAA-AAA	0.00	999	1.000	1.000	0.0559
240030_at	33.6	102.2	1.61	3.05	AAA-AAA	0.00	999	1.000	1.000	0.0595
244582_at	3.0	9.0	1.61	3.04	AAA-AAA	0.00	999	1.000	1.000	0.3746
217273_at	57.1	18.8	-1.61	-3.04	AAA-AAA	0.00	999	1.000	1.000	0.1458
240218_at	24.4	8.0	-1.61	-3.04	AAA-AAA	0.00	999	1.000	1.000	0.3847
1553380_at	11.8	3.9	-1.61	-3.04	AAA-AAA	0.00	999	1.000	1.000	0.3115
223903_at	35.6	11.7	-1.61	-3.04	AAA-AAA	0.00	999	1.000	1.000	0.0975
231156_at	17.9	54.4	1.61	3.04	AMA-AAA	0.00	999	1.000	1.000	0.2645
1568287_at	7.2	2.4	-1.60	-3.04	AAA-AAA	0.00	999	1.000	1.000	0.1448
1562481_at	4.1	12.5	1.60	3.04	AAA-AAA	0.00	999	1.000	1.000	0.2388
1561860_at	12.6	38.3	1.60	3.04	AAA-AAA	0.00	999	1.000	1.000	0.2873
214636_at	9.6	29.2	1.60	3.04	AAA-AAA	0.00	999	1.000	1.000	0.2735
1563572_at	21.9	7.2	-1.60	-3.04	AAA-PAA	0.00	999	1.000	1.000	0.3706

240092_at	27.6	83.8	1.60	3.04	AAA-AAA	0.00	999	1.000	1.000	0.1813
1558611_at	5.3	16.0	1.60	3.04	AAA-AAA	0.00	999	1.000	1.000	0.2070
243175_at	26.0	8.6	-1.60	-3.04	AAA-AAA	0.00	999	1.000	1.000	0.1834
1561083_at	11.1	33.6	1.60	3.04	AAA-AAA	0.00	999	1.000	1.000	0.0762
206597_at	18.0	54.8	1.60	3.04	AAA-AAA	0.00	999	1.000	1.000	0.2669
203765_at	33.2	10.9	-1.60	-3.04	AAA-AAA	0.00	999	1.000	1.000	0.1601
1564178_at	27.2	82.7	1.60	3.04	AAA-AAA	0.00	999	1.000	1.000	0.0121
1555646_at	21.3	7.0	-1.60	-3.04	AAA-AAA	0.00	999	1.000	1.000	0.1095
1560137_at	12.4	37.6	1.60	3.04	AAA-AAA	0.00	999	1.000	1.000	0.1707
211613_s_at	121.5	40.0	-1.60	-3.04	MAA-AAA	0.00	999	1.000	1.000	0.3400
235672_at	8.5	25.7	1.60	3.04	AAA-AAA	0.00	999	1.000	1.000	0.3148
232827_at	62.2	20.5	-1.60	-3.04	AAA-AAA	0.00	999	1.000	1.000	0.1671
241576_at	21.6	7.1	-1.60	-3.03	AAA-AAA	0.00	999	1.000	1.000	0.2370
1557009_a_at	1.5	4.6	1.60	3.03	AAA-AAA	0.00	999	1.000	1.000	0.3621
243183_at	1.7	5.3	1.60	3.03	AAA-AAA	0.00	999	1.000	1.000	0.3109
220205_at	27.5	9.1	-1.60	-3.03	AAA-AAA	0.00	999	1.000	1.000	0.1639
233683_at	17.0	5.6	-1.60	-3.04	AAA-AAA	0.00	999	1.000	1.000	0.2278
228979_at	1.8	5.5	1.60	3.03	AAA-AAA	0.00	999	1.000	1.000	0.3815
230014_at	16.4	49.8	1.60	3.03	AAA-AAA	0.00	999	1.000	1.000	0.0857
1564474_at	4.1	12.3	1.60	3.03	AAA-AAA	0.00	999	1.000	1.000	0.2797
1563160_at	17.8	5.9	-1.60	-3.03	AAA-AAA	0.00	999	1.000	1.000	0.1244
241089_at	6.1	18.6	1.60	3.03	AAA-AAA	0.00	999	1.000	1.000	0.3707
1553084_at	8.4	25.5	1.60	3.03	AAA-AAA	0.00	999	1.000	1.000	0.1770
213338_at	29.3	9.7	-1.60	-3.03	AAA-AAA	0.00	999	1.000	1.000	0.2100
206434_at	9.1	27.6	1.60	3.03	AAA-AAA	0.00	999	1.000	1.000	0.1419
234230_at	10.9	33.1	1.60	3.03	AAA-AAM	0.00	999	1.000	1.000	0.1276
239671_at	54.2	17.9	-1.60	-3.03	AAA-AAA	0.00	999	1.000	1.000	0.2066
241022_at	8.7	26.3	1.60	3.03	AAA-AAA	0.00	999	1.000	1.000	0.2251
1554496_at	25.1	75.9	1.60	3.03	AAA-AAA	0.00	999	1.000	1.000	0.1957
232924_at	4.2	12.7	1.60	3.03	AAA-AAA	0.00	999	1.000	1.000	0.1059
237974_at	28.9	9.6	-1.60	-3.03	AAA-AAA	0.00	999	1.000	1.000	0.2820
236728_at	37.4	12.3	-1.60	-3.03	AAA-AAA	0.00	999	1.000	1.000	0.0953
223841_s_at	5.0	15.3	1.60	3.03	AAA-AAA	0.00	999	1.000	1.000	0.1783
1558768_at	12.4	37.5	1.60	3.03	AAA-AAA	0.00	999	1.000	1.000	0.2402
216418_at	9.9	30.0	1.60	3.03	AAA-AAA	0.00	999	1.000	1.000	0.0740
214120_at	3.4	10.3	1.60	3.03	AAA-AAA	0.00	999	1.000	1.000	0.3282
201547_at	13.5	40.8	1.60	3.03	AAA-AAA	0.00	999	1.000	1.000	0.1130
238051_x_at	11.2	33.8	1.60	3.03	AAA-AAA	0.00	999	1.000	1.000	0.0104
231549_at	28.9	9.5	-1.60	-3.03	AAA-AAA	0.00	999	1.000	1.000	0.2731
239339_at	22.9	69.3	1.60	3.03	AAA-MAA	0.00	999	1.000	1.000	0.0933
1566469_at	4.4	13.3	1.60	3.03	AAA-AAA	0.00	999	1.000	1.000	0.2179
1566748_at	24.2	8.0	-1.60	-3.03	AAA-AAA	0.00	999	1.000	1.000	0.0893

215806_x_at	10.6	32.0	1.60	3.03	AAA-AAA	0.00	999	1.000	1.000	0.1434
206955_at	50.4	152.5	1.60	3.02	AAA-AAA	0.00	999	1.000	1.000	0.1053
238905_at	17.2	52.1	1.60	3.03	AAA-AAA	0.00	999	1.000	1.000	0.1201
241645_at	9.1	27.4	1.60	3.02	AAA-AAA	0.00	999	1.000	1.000	0.1980
1569955_at	2.4	7.4	1.60	3.02	AAA-AAA	0.00	999	1.000	1.000	0.1017
1566485_at	23.2	7.7	-1.60	-3.02	AAA-AAA	0.00	999	1.000	1.000	0.1840
1553285_s_at	6.4	19.4	1.60	3.02	AAA-AAA	0.00	999	1.000	1.000	0.3070
214675_at	50.0	16.6	-1.60	-3.02	AAA-AAA	0.00	999	1.000	1.000	0.0742
232507_at	4.9	14.9	1.60	3.02	AAA-AAA	0.00	999	1.000	1.000	0.1746
1570126_at	29.9	9.9	-1.60	-3.02	AAA-AAA	0.00	999	1.000	1.000	0.0880
1553229_at	34.7	11.5	-1.60	-3.02	AAA-AAA	0.00	999	1.000	1.000	0.1791
244204_at	5.7	17.1	1.60	3.02	AAA-AAA	0.00	999	1.000	1.000	0.2718
216164_at	30.3	91.5	1.60	3.02	AAA-AAA	0.00	999	1.000	1.000	0.2751
213316_at	7.1	21.4	1.60	3.02	AAA-AAA	0.00	999	1.000	1.000	0.2594
218186_at	29.5	9.8	-1.59	-3.02	AAA-AAA	0.00	999	1.000	1.000	0.3138
228772_at	7.6	23.0	1.59	3.02	AAA-AAA	0.00	999	1.000	1.000	0.1725
1566135_at	25.1	8.3	-1.59	-3.02	AAA-AAA	0.00	999	1.000	1.000	0.2759
1557336_at	37.5	12.4	-1.59	-3.02	AAA-AAA	0.00	999	1.000	1.000	0.1319
205862_at	21.8	7.2	-1.59	-3.02	AAA-AAA	0.00	999	1.000	1.000	0.3155
237138_at	4.1	12.4	1.59	3.02	AAA-AAA	0.00	999	1.000	1.000	0.2787
1553740_a_at	61.7	20.5	-1.59	-3.01	AAA-AAA	0.00	999	1.000	1.000	0.1051
223812_at	16.0	48.3	1.59	3.02	AAA-AAA	0.00	999	1.000	1.000	0.2643
1563519_at	23.7	7.9	-1.59	-3.01	AAA-AAA	0.00	999	1.000	1.000	0.1732
1566861_at	10.0	30.0	1.59	3.01	AAA-AAA	0.00	999	1.000	1.000	0.0010
216658_at	90.5	30.1	-1.59	-3.01	AAA-AAA	0.00	999	1.000	1.000	0.2045
218863_s_at	8.5	2.8	-1.59	-3.01	AAA-AAA	0.00	999	1.000	1.000	0.3332
234600_at	13.7	4.5	-1.59	-3.01	AAA-AAA	0.00	999	1.000	1.000	0.3317
1555540_at	31.3	10.4	-1.59	-3.01	AAA-AAA	0.00	999	1.000	1.000	0.3301
214469_at	33.1	11.0	-1.59	-3.01	AAA-AAA	0.00	999	1.000	1.000	0.2907
206605_at	8.5	25.7	1.59	3.01	AAA-AAA	0.00	999	1.000	1.000	0.0161
211484_s_at	89.1	29.6	-1.59	-3.01	AAA-AAA	0.00	999	1.000	1.000	0.0309
222256_s_at	125.8	41.8	-1.59	-3.01	MAA-AAA	0.00	999	1.000	1.000	0.2075
1553536_at	50.0	16.6	-1.59	-3.01	AAA-AAA	0.00	999	1.000	1.000	0.0212
234227_at	21.2	7.1	-1.59	-3.01	AAA-AAA	0.00	999	1.000	1.000	0.2312
1552669_at	59.3	19.7	-1.59	-3.01	AAA-AAA	0.00	999	1.000	1.000	0.1075
1553636_at	2.7	8.1	1.59	3.01	AAA-AAA	0.00	999	1.000	1.000	0.1052
220827_at	19.2	57.8	1.59	3.01	AAA-AAA	0.00	999	1.000	1.000	0.1700
216957_at	12.2	36.6	1.59	3.01	AAA-AAA	0.00	999	1.000	1.000	0.2267
206001_at	25.3	76.0	1.59	3.01	AAA-AAA	0.00	999	1.000	1.000	0.1738
236792_at	16.6	5.5	-1.59	-3.01	AAA-AAA	0.00	999	1.000	1.000	0.1064
1552639_at	49.5	16.5	-1.59	-3.01	APA-AAA	0.00	999	1.000	1.000	0.2776
236190_at	74.9	24.9	-1.59	-3.01	APA-AAA	0.00	999	1.000	1.000	0.3081

241802_x_at	29.6	9.9	-1.59	-3.00	AAA-AAA	0.00	999	1.000	1.000	0.0826
206512_at	17.2	5.7	-1.59	-3.00	AAA-AAA	0.00	999	1.000	1.000	0.0481
222225_at	22.4	67.4	1.59	3.01	AAA-AAA	0.00	999	1.000	1.000	0.2249
209779_at	58.2	19.4	-1.59	-3.00	PAA-AAA	0.00	999	1.000	1.000	0.2753
240182_at	29.2	9.7	-1.59	-3.00	AAP-AAA	0.00	999	1.000	1.000	0.3051
1561175_at	25.5	76.4	1.59	3.00	AAA-AAA	0.00	999	1.000	1.000	0.0521
215674_at	16.5	5.5	-1.59	-3.00	AAA-AAA	0.00	999	1.000	1.000	0.3718
207080_s_at	6.1	18.2	1.59	3.00	AAA-AAA	0.00	999	1.000	1.000	0.1956
224028_at	22.3	7.4	-1.59	-3.00	AAA-AAA	0.00	999	1.000	1.000	0.2574
230463_at	38.6	12.9	-1.59	-3.00	AAA-AAA	0.00	999	1.000	1.000	0.2537
1564227_at	39.9	119.7	1.59	3.00	AAA-AAA	0.00	999	1.000	1.000	0.1614
1564567_at	5.8	17.2	1.59	3.00	AAA-AAA	0.00	999	1.000	1.000	0.1069
1555244_at	20.1	6.7	-1.59	-3.00	AAA-AAA	0.00	999	1.000	1.000	0.2906
1559630_at	4.0	12.0	1.59	3.00	AAA-AAA	0.00	999	1.000	1.000	0.1149
215514_at	4.5	13.4	1.59	3.00	AAA-AAA	0.00	999	1.000	1.000	0.4099
216420_at	34.9	11.6	-1.59	-3.00	AAA-AAA	0.00	999	1.000	1.000	0.1800
217674_at	23.6	7.9	-1.59	-3.00	AAM-AAA	0.00	999	1.000	1.000	0.0778
233112_at	15.3	45.8	1.59	3.00	AAA-AAA	0.00	999	1.000	1.000	0.2319
228863_at	26.8	8.9	-1.59	-3.00	AAA-AAA	0.00	999	1.000	1.000	0.1493
230390_at	7.7	23.0	1.59	3.00	AAA-AAA	0.00	999	1.000	1.000	0.0615
1561102_at	6.9	20.6	1.58	3.00	AAA-AAA	0.00	999	1.000	1.000	0.2402
1564209_at	10.8	32.4	1.58	3.00	AAA-AAA	0.00	999	1.000	1.000	0.0219
1565337_at	5.5	16.5	1.58	3.00	AAA-AAA	0.00	999	1.000	1.000	0.2727
1555853_at	21.9	65.6	1.58	3.00	AAA-AAA	0.00	999	1.000	1.000	0.2270
1405_i_at	22.9	7.6	-1.58	-3.00	AAA-AAA	0.00	999	1.000	1.000	0.3465
205363_at	25.3	75.7	1.58	3.00	AAA-AMA	0.00	999	1.000	1.000	0.1653
1563107_at	16.0	5.3	-1.58	-3.00	AAA-AAA	0.00	999	1.000	1.000	0.1052
1556447_at	3.1	9.3	1.58	2.99	AAA-AAA	0.00	999	1.000	1.000	0.1150
1553747_at	5.7	17.1	1.58	2.99	AAA-AAA	0.00	999	1.000	1.000	0.2591
244076_at	11.6	34.6	1.58	2.99	AAA-AAA	0.00	999	1.000	1.000	0.2370
218353_at	37.5	12.5	-1.58	-2.99	AAA-AAA	0.00	999	1.000	1.000	0.1011
205952_at	10.2	30.6	1.58	2.99	AAA-AAA	0.00	999	1.000	1.000	0.0997
228872_at	23.3	69.9	1.58	2.99	AAA-AAA	0.00	999	1.000	1.000	0.2131
1553931_at	49.2	16.4	-1.58	-2.99	AAP-MAA	0.00	999	1.000	1.000	0.4883
1566986_at	28.6	9.5	-1.58	-2.99	AAA-AAA	0.00	999	1.000	1.000	0.2273
243603_at	77.0	25.7	-1.58	-2.99	AAA-AAA	0.00	999	1.000	1.000	0.0426
243694_at	16.8	50.3	1.58	2.99	AAA-AAM	0.00	999	1.000	1.000	0.0683
216638_s_at	13.1	39.1	1.58	2.99	AAA-AAA	0.00	999	1.000	1.000	0.0483
204152_s_at	62.3	20.8	-1.58	-2.99	AAA-AAA	0.00	999	1.000	1.000	0.2202
235150_at	5.9	17.7	1.58	2.99	AAA-AAA	0.00	999	1.000	1.000	0.1945
203854_at	89.8	30.0	-1.58	-2.99	PMP-AAM	0.00	999	1.000	1.000	0.0527
235471_at	50.2	16.8	-1.58	-2.99	AAP-AAA	0.00	999	1.000	1.000	0.1000

1554813_at	16.9	50.4	1.58	2.99	AAA-AAA	0.00	999	1.000	1.000	0.1605
1559108_at	4.4	13.1	1.58	2.99	AAA-AAA	0.00	999	1.000	1.000	0.2163
219727_at	51.8	17.3	-1.58	-2.99	AAA-AAA	0.00	999	1.000	1.000	0.3237
210865_at	28.3	84.7	1.58	2.99	AAA-AAA	0.00	999	1.000	1.000	0.0061
227664_at	17.2	5.8	-1.58	-2.99	AAA-AAA	0.00	999	1.000	1.000	0.3447
214284_s_at	19.4	6.5	-1.58	-2.99	APA-AAA	0.00	999	1.000	1.000	0.2277
1563943_at	33.7	100.6	1.58	2.99	AAA-AAA	0.00	999	1.000	1.000	0.2090
240325_x_at	29.3	87.5	1.58	2.99	AAA-AAA	0.00	999	1.000	1.000	0.1019
1558356_at	7.9	23.7	1.58	2.99	AAA-AAA	0.00	999	1.000	1.000	0.2261
1554751_at	19.5	6.5	-1.58	-2.99	AAA-AAA	0.00	999	1.000	1.000	0.4120
204683_at	10.4	31.0	1.58	2.99	AAA-AAA	0.00	999	1.000	1.000	0.3161
229170_s_at	6.3	18.7	1.58	2.99	AAA-AAA	0.00	999	1.000	1.000	0.4783
204007_at	29.7	10.0	-1.58	-2.98	AAP-AAA	0.00	999	1.000	1.000	0.1901
1561361_at	87.0	29.2	-1.58	-2.98	AAA-AAA	0.00	999	1.000	1.000	0.3261
219075_at	35.3	105.3	1.58	2.98	AAA-AAA	0.00	999	1.000	1.000	0.3333
239458_at	12.5	37.3	1.58	2.98	AAA-AAA	0.00	999	1.000	1.000	0.2980
240598_at	71.9	24.1	-1.58	-2.98	AAA-AAA	0.00	999	1.000	1.000	0.0284
244234_at	54.3	161.9	1.58	2.98	AAA-AAA	0.00	999	1.000	1.000	0.0944
215534_at	38.0	12.8	-1.58	-2.98	AAA-AAA	0.00	999	1.000	1.000	0.0703
221376_at	18.3	54.5	1.58	2.98	AAA-AAA	0.00	999	1.000	1.000	0.1698
204414_at	3.5	10.5	1.58	2.98	AAA-AAA	0.00	999	1.000	1.000	0.2064
208307_at	18.8	6.3	-1.58	-2.98	AAA-AAA	0.00	999	1.000	1.000	0.1428
234102_at	10.4	31.1	1.58	2.98	AAA-AAA	0.00	999	1.000	1.000	0.0610
228392_at	25.4	75.9	1.58	2.98	AAA-AAA	0.00	999	1.000	1.000	0.0138
228218_at	16.1	48.1	1.58	2.98	AAA-AAA	0.00	999	1.000	1.000	0.0535
1563169_at	16.0	5.4	-1.58	-2.98	AAA-AAA	0.00	999	1.000	1.000	0.2200
1555717_at	18.9	6.4	-1.58	-2.98	AAA-AAA	0.00	999	1.000	1.000	0.3537
215787_at	37.5	12.6	-1.58	-2.98	AAA-AAA	0.00	999	1.000	1.000	0.0209
206013_s_at	3.3	9.9	1.58	2.98	AAA-AAA	0.00	999	1.000	1.000	0.1243
209888_s_at	43.3	14.5	-1.58	-2.98	AAA-AAA	0.00	999	1.000	1.000	0.2031
210957_s_at	3.4	10.1	1.58	2.98	AAA-AAA	0.00	999	1.000	1.000	0.0445
207888_at	6.0	2.0	-1.58	-2.98	AAA-AAA	0.00	999	1.000	1.000	0.0294
229935_s_at	4.0	12.0	1.58	2.98	AAA-AAA	0.00	999	1.000	1.000	0.1136
243497_at	43.8	14.7	-1.57	-2.98	AAA-AAA	0.00	999	1.000	1.000	0.2065
1561584_at	4.6	13.7	1.57	2.98	AAA-AAA	0.00	999	1.000	1.000	0.2635
1560038_at	13.7	40.8	1.57	2.98	AAA-AAA	0.00	999	1.000	1.000	0.1671
235596_at	20.6	61.2	1.57	2.98	AAA-AAA	0.00	999	1.000	1.000	0.0170
228028_at	34.1	101.5	1.57	2.98	AAA-AAA	0.00	999	1.000	1.000	0.0022
240364_at	17.2	5.8	-1.57	-2.98	AAA-AAA	0.00	999	1.000	1.000	0.1251
240539_at	3.8	11.3	1.57	2.98	AAA-AAA	0.00	999	1.000	1.000	0.2638
214702_at	8.9	3.0	-1.57	-2.98	AAA-AAA	0.00	999	1.000	1.000	0.1723
234824_at	8.4	25.0	1.57	2.98	AAA-AAA	0.00	999	1.000	1.000	0.3983

240163_at	33.3	11.2	-1.57	-2.97	AAA-AAA	0.00	999	1.000	1.000	0.2810
241015_at	31.4	10.6	-1.57	-2.97	AAA-AAA	0.00	999	1.000	1.000	0.3909
214382_at	6.9	20.4	1.57	2.97	AAA-AAA	0.00	999	1.000	1.000	0.0961
217361_at	17.9	6.0	-1.57	-2.97	AAA-AAA	0.00	999	1.000	1.000	0.2907
219759_at	16.1	5.4	-1.57	-2.97	AAA-AAA	0.00	999	1.000	1.000	0.1906
204746_s_at	78.8	234.3	1.57	2.97	AAA-AAA	0.00	999	1.000	1.000	0.0740
242222_at	37.9	12.8	-1.57	-2.97	AAA-AAA	0.00	999	1.000	1.000	0.1825
242276_at	35.2	11.8	-1.57	-2.97	AAA-AAA	0.00	999	1.000	1.000	0.1102
207250_at	20.6	6.9	-1.57	-2.97	AAA-AAA	0.00	999	1.000	1.000	0.0900
204823_at	70.0	23.6	-1.57	-2.97	AAA-AAA	0.00	999	1.000	1.000	0.2723
234850_at	13.4	39.8	1.57	2.97	AAA-AAA	0.00	999	1.000	1.000	0.1121
235077_at	5.6	16.7	1.57	2.97	AAA-AAA	0.00	999	1.000	1.000	0.1001
234079_at	79.3	26.7	-1.57	-2.97	APA-AAA	0.00	999	1.000	1.000	0.1297
243330_at	6.2	18.5	1.57	2.97	AAA-AAA	0.00	999	1.000	1.000	0.0529
243953_at	8.7	25.8	1.57	2.97	AAA-AAA	0.00	999	1.000	1.000	0.2422
206806_at	5.9	17.4	1.57	2.97	AAA-AAA	0.00	999	1.000	1.000	0.2548
234853_s_at	3.1	9.3	1.57	2.97	AAA-AAA	0.00	999	1.000	1.000	0.2514
228591_at	108.1	36.4	-1.57	-2.97	AAA-AAA	0.00	999	1.000	1.000	0.0811
222092_at	11.7	34.8	1.57	2.97	AAA-AAA	0.00	999	1.000	1.000	0.1616
1569748_at	7.7	22.8	1.57	2.97	AAA-AAA	0.00	999	1.000	1.000	0.1400
240676_at	11.0	3.7	-1.57	-2.97	AAA-AAA	0.00	999	1.000	1.000	0.1191
1554700_at	3.7	11.0	1.57	2.97	AAA-AAA	0.00	999	1.000	1.000	0.2952
242833_at	24.9	8.4	-1.57	-2.97	AAA-AAA	0.00	999	1.000	1.000	0.4305
1560042_at	9.9	29.3	1.57	2.97	AAA-AAA	0.00	999	1.000	1.000	0.2569
231555_at	16.9	50.1	1.57	2.96	AAA-AAA	0.00	999	1.000	1.000	0.0545
1568900_a_at	16.5	5.6	-1.57	-2.96	AAA-AAA	0.00	999	1.000	1.000	0.4017
1555454_at	4.6	13.5	1.57	2.96	AAA-AAA	0.00	999	1.000	1.000	0.2619
215028_at	6.6	19.5	1.57	2.96	AAA-AAA	0.00	999	1.000	1.000	0.3288
219780_at	18.4	6.2	-1.57	-2.96	AAA-AAA	0.00	999	1.000	1.000	0.1405
232989_s_at	55.6	18.8	-1.57	-2.96	AAA-AAA	0.00	999	1.000	1.000	0.1561
232496_at	5.8	17.2	1.57	2.96	AAA-AAA	0.00	999	1.000	1.000	0.1682
1570044_at	79.7	26.9	-1.57	-2.96	AAP-AAA	0.00	999	1.000	1.000	0.0639
1570032_at	85.5	28.9	-1.57	-2.96	AAA-APA	0.00	999	1.000	1.000	0.1330
241621_at	6.0	17.9	1.57	2.96	AAA-AMA	0.00	999	1.000	1.000	0.1885
1553802_a_at	7.2	21.4	1.57	2.96	AAA-AAA	0.00	999	1.000	1.000	0.4246
1561703_at	5.0	14.7	1.57	2.96	AAA-AAA	0.00	999	1.000	1.000	0.3247
220824_at	20.7	61.4	1.57	2.96	AAA-AAA	0.00	999	1.000	1.000	0.1102
220325_at	38.3	12.9	-1.57	-2.96	AAA-AAA	0.00	999	1.000	1.000	0.3385
222964_at	25.5	8.6	-1.57	-2.96	AAA-AAA	0.00	999	1.000	1.000	0.1750
231469_at	58.2	19.7	-1.57	-2.96	AAA-AAA	0.00	999	1.000	1.000	0.1498
1563906_at	12.2	36.0	1.57	2.96	AAA-AAA	0.00	999	1.000	1.000	0.0034
1557778_at	3.0	9.0	1.57	2.96	AAA-AAA	0.00	999	1.000	1.000	0.2151

243021_at	21.6	7.3	-1.57	-2.96	AAA-AAA	0.00	999	1.000	1.000	0.1227
226622_at	8.7	25.6	1.57	2.96	AAA-AAA	0.00	999	1.000	1.000	0.2036
222797_at	13.2	39.2	1.57	2.96	AAA-AAA	0.00	999	1.000	1.000	0.0998
229893_at	12.6	37.3	1.57	2.96	AAA-AAA	0.00	999	1.000	1.000	0.1845
230546_at	32.3	95.5	1.57	2.96	AAA-AAA	0.00	999	1.000	1.000	0.2171
241446_at	53.2	18.0	-1.56	-2.96	AAA-AAA	0.00	999	1.000	1.000	0.2962
241378_at	45.1	15.3	-1.56	-2.96	MAA-AAA	0.00	999	1.000	1.000	0.2673
239906_at	9.6	28.4	1.56	2.96	AAA-AAA	0.00	999	1.000	1.000	0.4053
1556456_at	71.1	24.1	-1.56	-2.96	AAA-AAA	0.00	999	1.000	1.000	0.0060
1555158_at	31.6	10.7	-1.56	-2.96	AAA-AAA	0.00	999	1.000	1.000	0.2172
204722_at	26.4	8.9	-1.56	-2.96	AAA-AAA	0.00	999	1.000	1.000	0.1047
243073_at	18.1	6.1	-1.56	-2.96	AAA-AAA	0.00	999	1.000	1.000	0.0927
220062_s_at	21.3	63.0	1.56	2.95	AAA-AAA	0.00	999	1.000	1.000	0.1232
204345_at	146.3	49.5	-1.56	-2.95	AAA-AAA	0.00	999	1.000	1.000	0.0518
234052_at	11.7	34.4	1.56	2.95	AAA-AAA	0.00	999	1.000	1.000	0.0672
229042_s_at	62.1	21.0	-1.56	-2.95	AAA-AAA	0.00	999	1.000	1.000	0.3987
1570650_at	11.2	33.1	1.56	2.95	AAA-AAA	0.00	999	1.000	1.000	0.0753
1570651_at	48.9	16.6	-1.56	-2.95	AAA-AAA	0.00	999	1.000	1.000	0.0400
1556231_a_at	39.7	13.4	-1.56	-2.95	AAA-AAA	0.00	999	1.000	1.000	0.2122
215863_at	23.2	68.5	1.56	2.95	AAA-AAA	0.00	999	1.000	1.000	0.2302
230349_at	17.3	5.8	-1.56	-2.95	AAA-AAA	0.00	999	1.000	1.000	0.0748
228128_x_at	106.6	36.1	-1.56	-2.95	PPP-PAA	0.00	999	1.000	1.000	0.0369
1569021_at	43.8	14.8	-1.56	-2.95	PPA-AAA	0.00	999	1.000	1.000	0.2242
241830_at	26.0	76.6	1.56	2.95	AAA-AAA	0.00	999	1.000	1.000	0.1309
214867_at	30.3	89.5	1.56	2.95	AAA-AAA	0.00	999	1.000	1.000	0.0356
229802_at	22.9	7.8	-1.56	-2.95	AAA-AAA	0.00	999	1.000	1.000	0.3470
1570038_at	15.1	44.6	1.56	2.95	AAA-AAA	0.00	999	1.000	1.000	0.0622
239262_at	10.3	3.5	-1.56	-2.95	AAA-AAA	0.00	999	1.000	1.000	0.2539
240811_at	14.2	4.8	-1.56	-2.95	AAA-AAA	0.00	999	1.000	1.000	0.4316
1561062_a_at	21.6	63.8	1.56	2.95	AAA-AAA	0.00	999	1.000	1.000	0.2869
243480_at	10.8	31.8	1.56	2.95	AAA-AAA	0.00	999	1.000	1.000	0.0615
211817_s_at	51.8	17.6	-1.56	-2.95	AAA-AAA	0.00	999	1.000	1.000	0.1865
234003_at	57.5	19.5	-1.56	-2.95	AMA-AAA	0.00	999	1.000	1.000	0.2248
226086_at	67.0	22.7	-1.56	-2.95	AAA-AAA	0.00	999	1.000	1.000	0.2141
232078_at	33.1	97.6	1.56	2.95	AAA-AAA	0.00	999	1.000	1.000	0.2066
1562730_a_at	31.9	10.8	-1.56	-2.95	AAA-AAA	0.00	999	1.000	1.000	0.2865
1562661_at	48.2	16.3	-1.56	-2.95	AAA-AAA	0.00	999	1.000	1.000	0.2567
1562562_at	45.1	15.3	-1.56	-2.95	AAA-AAA	0.00	999	1.000	1.000	0.1244
217698_at	7.6	22.5	1.56	2.95	AAA-AAA	0.00	999	1.000	1.000	0.1237
238625_at	15.8	5.4	-1.56	-2.95	AAA-AAA	0.00	999	1.000	1.000	0.2726
216467_s_at	91.3	31.0	-1.56	-2.94	PAP-AAA	0.00	999	1.000	1.000	0.1250
1558645_at	6.5	19.1	1.56	2.94	AAA-AAA	0.00	999	1.000	1.000	0.4268

217093_at	8.4	24.7	1.56	2.94	AAA-AMA	0.00	999	1.000	1.000	0.1197
207366_at	93.6	31.8	-1.56	-2.94	AAA-AAA	0.00	999	1.000	1.000	0.0677
227831_at	35.0	11.9	-1.56	-2.94	AAA-AAA	0.00	999	1.000	1.000	0.1390
232243_at	23.3	68.7	1.56	2.94	AAA-AAA	0.00	999	1.000	1.000	0.2527
204289_at	17.3	5.9	-1.56	-2.94	AAA-AAA	0.00	999	1.000	1.000	0.2381
237776_at	55.1	18.7	-1.56	-2.94	AAA-AAA	0.00	999	1.000	1.000	0.0045
239397_at	17.5	51.3	1.56	2.94	AAA-AAA	0.00	999	1.000	1.000	0.2785
207210_at	9.9	3.4	-1.56	-2.94	AAA-AAA	0.00	999	1.000	1.000	0.2226
210629_x_at	15.3	45.0	1.56	2.94	AAA-AAA	0.00	999	1.000	1.000	0.1691
237775_x_at	23.2	7.9	-1.56	-2.94	AAA-AAA	0.00	999	1.000	1.000	0.2026
1559939_at	17.1	50.1	1.56	2.94	AAA-AAA	0.00	999	1.000	1.000	0.1703
244560_at	10.5	30.7	1.56	2.94	AAA-AAA	0.00	999	1.000	1.000	0.1365
214532_x_at	33.1	97.3	1.56	2.94	AAA-AAA	0.00	999	1.000	1.000	0.2063
220814_at	23.1	7.9	-1.56	-2.94	AAA-AAA	0.00	999	1.000	1.000	0.3278
221661_at	42.3	14.4	-1.56	-2.94	AAA-AAA	0.00	999	1.000	1.000	0.0172
234481_at	64.8	22.1	-1.56	-2.94	AAA-AAA	0.00	999	1.000	1.000	0.0963
238379_x_at	8.7	25.5	1.56	2.94	AAA-AAM	0.00	999	1.000	1.000	0.4463
237057_at	55.7	19.0	-1.56	-2.94	AAA-AAA	0.00	999	1.000	1.000	0.1575
1568899_at	16.7	48.9	1.55	2.94	AAA-AMA	0.00	999	1.000	1.000	0.1361
1559948_at	12.3	36.2	1.55	2.94	AAA-AAA	0.00	999	1.000	1.000	0.1111
1560325_at	5.0	1.7	-1.55	-2.94	AAA-AAA	0.00	999	1.000	1.000	0.0641
207075_at	55.4	18.9	-1.55	-2.94	AAA-AAA	0.00	999	1.000	1.000	0.0543
202498_s_at	13.8	40.6	1.55	2.94	AAA-AAA	0.00	999	1.000	1.000	0.0333
211142_x_at	40.2	117.9	1.55	2.94	AAA-AAA	0.00	999	1.000	1.000	0.0054
237224_at	13.4	4.6	-1.55	-2.94	AAA-AAA	0.00	999	1.000	1.000	0.0909
229754_at	63.1	21.5	-1.55	-2.93	APP-AAA	0.00	999	1.000	1.000	0.2164
1568826_at	12.9	37.9	1.55	2.94	AAA-AAA	0.00	999	1.000	1.000	0.2009
243500_at	6.8	20.0	1.55	2.93	AAA-AAA	0.00	999	1.000	1.000	0.2700
244572_at	60.6	20.7	-1.55	-2.94	AAA-AAA	0.00	999	1.000	1.000	0.0489
213602_s_at	66.6	22.7	-1.55	-2.94	AAA-AAA	0.00	999	1.000	1.000	0.1400
204834_at	40.7	13.9	-1.55	-2.93	AAA-AAA	0.00	999	1.000	1.000	0.1185
1561997_at	4.8	14.0	1.55	2.93	AAA-AAA	0.00	999	1.000	1.000	0.4654
1559022_at	10.2	30.0	1.55	2.93	AAA-AAA	0.00	999	1.000	1.000	0.1978
244460_at	23.9	8.1	-1.55	-2.93	AAA-AAA	0.00	999	1.000	1.000	0.2057
216065_at	31.7	10.8	-1.55	-2.93	AAA-AAA	0.00	999	1.000	1.000	0.2467
205727_at	39.6	116.0	1.55	2.93	AAA-AAA	0.00	999	1.000	1.000	0.0924
232544_at	19.4	6.6	-1.55	-2.93	AAA-AAA	0.00	999	1.000	1.000	0.1165
228600_x_at	7.6	22.3	1.55	2.93	AAA-AAA	0.00	999	1.000	1.000	0.2206
230617_at	31.1	91.0	1.55	2.93	AAA-AAA	0.00	999	1.000	1.000	0.1126
1562942_at	36.1	12.3	-1.55	-2.93	AAA-AAA	0.00	999	1.000	1.000	0.2650
217502_at	25.8	8.8	-1.55	-2.93	AAA-AAA	0.00	999	1.000	1.000	0.2228
229639_s_at	7.4	21.6	1.55	2.93	AAA-AAA	0.00	999	1.000	1.000	0.3198

230489_at	9.5	27.7	1.55	2.93	AAA-AAA	0.00	999	1.000	1.000	0.2172
207145_at	12.7	4.3	-1.55	-2.93	APA-AAA	0.00	999	1.000	1.000	0.2989
1556606_at	21.9	64.1	1.55	2.93	AAA-AAA	0.00	999	1.000	1.000	0.0725
217332_at	60.6	20.7	-1.55	-2.93	AAA-AAA	0.00	999	1.000	1.000	0.1101
206485_at	39.9	116.7	1.55	2.93	AAA-AAA	0.00	999	1.000	1.000	0.0801
207312_at	10.4	30.4	1.55	2.93	AAA-AAA	0.00	999	1.000	1.000	0.2962
225511_at	3.9	11.5	1.55	2.93	AAA-AAA	0.00	999	1.000	1.000	0.1546
241169_at	14.6	42.7	1.55	2.93	AAA-AAA	0.00	999	1.000	1.000	0.1567
240167_at	5.7	16.6	1.55	2.93	AAA-AAA	0.00	999	1.000	1.000	0.1163
1568851_at	16.7	48.9	1.55	2.93	AAA-AAA	0.00	999	1.000	1.000	0.2194
244833_at	9.7	28.5	1.55	2.93	AAA-AAA	0.00	999	1.000	1.000	0.0267
1553604_at	10.9	3.7	-1.55	-2.93	AAA-AAA	0.00	999	1.000	1.000	0.4008
202746_at	4.3	12.7	1.55	2.93	AAA-AAA	0.00	999	1.000	1.000	0.3484
231177_at	62.3	21.3	-1.55	-2.93	AAA-AAA	0.00	999	1.000	1.000	0.3343
231849_at	61.3	20.9	-1.55	-2.93	AAA-AAA	0.00	999	1.000	1.000	0.2137
235236_at	117.6	40.2	-1.55	-2.93	PPP-AAP	0.00	999	1.000	1.000	0.0460
239065_at	17.1	49.9	1.55	2.92	AAA-AAA	0.00	999	1.000	1.000	0.1222
1558193_at	5.5	16.1	1.55	2.92	AAA-AAA	0.00	999	1.000	1.000	0.0732
1553789_a_at	43.7	15.0	-1.55	-2.93	AAA-AAA	0.00	999	1.000	1.000	0.2789
1561217_at	8.2	24.0	1.55	2.92	AAA-AAA	0.00	999	1.000	1.000	0.2336
207133_x_at	24.5	71.6	1.55	2.93	AAA-AAA	0.00	999	1.000	1.000	0.0266
204713_s_at	44.0	15.0	-1.55	-2.93	AAA-AAA	0.00	999	1.000	1.000	0.3564
211079_s_at	19.8	57.9	1.55	2.93	AAA-AAA	0.00	999	1.000	1.000	0.1263
234364_at	23.4	68.3	1.55	2.92	AAA-AAA	0.00	999	1.000	1.000	0.0451
233022_at	28.2	82.6	1.55	2.93	AAA-AAA	0.00	999	1.000	1.000	0.0434
243952_at	34.8	101.7	1.55	2.92	AAA-AAA	0.00	999	1.000	1.000	0.4228
207955_at	16.2	47.2	1.55	2.92	AAA-AAA	0.00	999	1.000	1.000	0.1244
208411_x_at	12.1	4.2	-1.55	-2.92	AAA-AAA	0.00	999	1.000	1.000	0.0138
1559839_at	11.2	3.9	-1.55	-2.92	PAA-AAA	0.00	999	1.000	1.000	0.4761
242446_at	5.9	17.2	1.55	2.92	AAA-AAA	0.00	999	1.000	1.000	0.0730
240034_at	4.5	13.2	1.55	2.92	AAA-AAA	0.00	999	1.000	1.000	0.1399
240264_at	32.3	94.2	1.55	2.92	AAA-MAA	0.00	999	1.000	1.000	0.0169
1561237_at	16.4	5.6	-1.55	-2.92	AAA-AAA	0.00	999	1.000	1.000	0.2297
213210_at	38.8	113.2	1.55	2.92	AAA-AAA	0.00	999	1.000	1.000	0.1920
236489_at	10.4	30.4	1.55	2.92	AAA-AAA	0.00	999	1.000	1.000	0.3486
227192_at	17.6	51.4	1.55	2.92	AAA-AAA	0.00	999	1.000	1.000	0.1800
230925_at	18.0	52.6	1.55	2.92	AAA-AAA	0.00	999	1.000	1.000	0.3145
229052_at	41.0	14.1	-1.55	-2.92	AAA-AAA	0.00	999	1.000	1.000	0.0694
239573_at	3.4	9.8	1.55	2.92	AAA-AAA	0.00	999	1.000	1.000	0.1048
1553963_at	2.4	7.1	1.55	2.92	AAA-AAA	0.00	999	1.000	1.000	0.2653
243298_at	44.2	15.2	-1.55	-2.92	AAA-AAA	0.00	999	1.000	1.000	0.1774
243637_at	5.3	15.4	1.55	2.92	AAA-AAA	0.00	999	1.000	1.000	0.2845

215391_at	10.4	30.3	1.55	2.92	AAA-AAA	0.00	999	1.000	1.000	0.0165
216832_at	28.8	84.0	1.55	2.92	AAA-AAA	0.00	999	1.000	1.000	0.2634
211552_s_at	110.1	37.7	-1.55	-2.92	AAA-AAA	0.00	999	1.000	1.000	0.0506
207925_at	20.0	6.8	-1.55	-2.92	AAA-AAA	0.00	999	1.000	1.000	0.2502
237859_at	4.5	13.1	1.55	2.92	AAA-AAA	0.00	999	1.000	1.000	0.0482
232077_s_at	11.0	32.1	1.55	2.92	AAA-AAA	0.00	999	1.000	1.000	0.2237
229579_s_at	82.0	28.1	-1.55	-2.92	AAA-AAA	0.00	999	1.000	1.000	0.3137
230238_at	53.6	18.4	-1.55	-2.92	AAA-AAA	0.00	999	1.000	1.000	0.2597
238198_at	82.5	28.3	-1.54	-2.92	AAM-AAP	0.00	999	1.000	1.000	0.0539
1559651_at	3.7	10.8	1.54	2.92	AAA-AAA	0.00	999	1.000	1.000	0.1653
221138_s_at	5.1	14.8	1.54	2.92	AAA-AAA	0.00	999	1.000	1.000	0.2059
207544_s_at	12.2	35.4	1.54	2.92	AAA-AAA	0.00	999	1.000	1.000	0.3078
232495_x_at	53.7	156.6	1.54	2.92	AAA-AAA	0.00	999	1.000	1.000	0.0612
241551_at	33.2	11.4	-1.54	-2.91	AAA-AAA	0.00	999	1.000	1.000	0.3303
1563426_a_at	25.3	73.8	1.54	2.91	AAA-AAA	0.00	999	1.000	1.000	0.3147
1566608_at	4.0	11.5	1.54	2.91	AAA-AAA	0.00	999	1.000	1.000	0.3470
210068_s_at	30.8	10.6	-1.54	-2.91	AAA-AAA	0.00	999	1.000	1.000	0.3578
210883_x_at	53.9	157.2	1.54	2.92	AAA-AAM	0.00	999	1.000	1.000	0.0001
234570_at	12.8	37.4	1.54	2.92	AAA-AAA	0.00	999	1.000	1.000	0.2671
232731_x_at	13.1	38.3	1.54	2.91	AAA-AAA	0.00	999	1.000	1.000	0.2951
237376_at	11.8	34.2	1.54	2.91	AAA-AAA	0.00	999	1.000	1.000	0.3405
241305_at	15.4	5.3	-1.54	-2.91	AAA-AAA	0.00	999	1.000	1.000	0.2806
240812_at	3.5	10.2	1.54	2.91	AAA-AAA	0.00	999	1.000	1.000	0.3887
1557515_at	13.6	4.7	-1.54	-2.91	AAA-AAA	0.00	999	1.000	1.000	0.2120
1562597_at	27.1	78.9	1.54	2.91	AAA-AAA	0.00	999	1.000	1.000	0.1610
221331_x_at	13.0	38.0	1.54	2.91	AAA-AAA	0.00	999	1.000	1.000	0.2609
221310_at	27.1	9.3	-1.54	-2.91	AAA-AAA	0.00	999	1.000	1.000	0.1512
235979_at	17.3	50.4	1.54	2.91	AAA-AAA	0.00	999	1.000	1.000	0.0406
1563099_at	2.3	6.6	1.54	2.91	AAA-AAA	0.00	999	1.000	1.000	0.3712
1563379_at	17.5	6.0	-1.54	-2.91	AAA-AAA	0.00	999	1.000	1.000	0.2668
1560010_a_at	5.2	15.0	1.54	2.91	AAA-AAA	0.00	999	1.000	1.000	0.0663
216432_at	36.0	12.4	-1.54	-2.91	AAA-AAA	0.00	999	1.000	1.000	0.2548
202273_at	37.5	12.9	-1.54	-2.91	AAA-AAA	0.00	999	1.000	1.000	0.0656
240849_at	73.6	25.3	-1.54	-2.91	AAA-AAA	0.00	999	1.000	1.000	0.2293
1554384_at	45.6	15.7	-1.54	-2.91	AAA-AAA	0.00	999	1.000	1.000	0.0336
1553943_at	28.5	82.8	1.54	2.91	AAA-AAA	0.00	999	1.000	1.000	0.0712
1555403_a_at	25.3	8.7	-1.54	-2.91	AAA-AAA	0.00	999	1.000	1.000	0.1527
1555286_at	14.9	5.1	-1.54	-2.91	AAA-AAA	0.00	999	1.000	1.000	0.1569
243776_at	3.5	10.3	1.54	2.91	AAA-AAA	0.00	999	1.000	1.000	0.1202
244012_x_at	21.3	61.8	1.54	2.91	AAA-AAA	0.00	999	1.000	1.000	0.0754
216801_at	57.0	165.7	1.54	2.91	AAA-AAA	0.00	999	1.000	1.000	0.1415
220256_s_at	19.3	56.1	1.54	2.91	AAA-AAA	0.00	999	1.000	1.000	0.0464

237756_at	14.0	4.8	-1.54	-2.91	AAA-AAA	0.00	999	1.000	1.000	0.2722
236736_at	58.0	19.9	-1.54	-2.91	AAA-AAA	0.00	999	1.000	1.000	0.1849
231701_s_at	93.4	32.1	-1.54	-2.91	AAA-AAA	0.00	999	1.000	1.000	0.2966
1565026_a_at	2.1	6.0	1.54	2.91	AAA-AAA	0.00	999	1.000	1.000	0.2233
1569378_at	3.9	11.3	1.54	2.91	AAA-AAA	0.00	999	1.000	1.000	0.1624
1567238_at	10.4	3.6	-1.54	-2.91	AAA-AAA	0.00	999	1.000	1.000	0.2743
223794_at	14.6	5.0	-1.54	-2.91	AAA-AAA	0.00	999	1.000	1.000	0.1881
1559127_x_at	79.6	27.4	-1.54	-2.90	AAA-PAA	0.00	999	1.000	1.000	0.2785
1564504_at	10.2	29.5	1.54	2.90	AAA-MAA	0.00	999	1.000	1.000	0.1559
241047_at	14.2	41.2	1.54	2.90	AAA-AAA	0.00	999	1.000	1.000	0.0543
1555647_a_at	6.0	17.3	1.54	2.91	AAA-AAA	0.00	999	1.000	1.000	0.3161
221338_at	23.6	8.1	-1.54	-2.90	AAA-AAA	0.00	999	1.000	1.000	0.3931
216972_at	5.8	16.8	1.54	2.91	AAA-AAA	0.00	999	1.000	1.000	0.3323
237433_at	4.4	12.8	1.54	2.90	AAA-AAA	0.00	999	1.000	1.000	0.4363
232247_at	14.1	41.0	1.54	2.90	AAA-AAA	0.00	999	1.000	1.000	0.2237
231652_at	7.8	22.7	1.54	2.91	AAA-AAA	0.00	999	1.000	1.000	0.1263
1553114_a_at	172.0	59.3	-1.54	-2.90	AAA-AAA	0.00	999	1.000	1.000	0.1511
244149_at	52.6	18.1	-1.54	-2.90	AAA-AAA	0.00	999	1.000	1.000	0.1288
234959_at	7.0	20.4	1.54	2.90	AAA-AAA	0.00	999	1.000	1.000	0.4807
228060_at	66.8	193.9	1.54	2.90	AAA-AAA	0.00	999	1.000	1.000	0.0815
214774_x_at	4.0	1.4	-1.54	-2.90	AAA-AAA	0.00	999	1.000	1.000	0.2618
205691_at	45.0	15.5	-1.54	-2.90	AAA-AAA	0.00	999	1.000	1.000	0.0836
205405_at	12.2	35.3	1.54	2.90	AAA-AAA	0.00	999	1.000	1.000	0.3017
210477_x_at	12.9	37.4	1.54	2.90	AAA-AAA	0.00	999	1.000	1.000	0.1759
234934_at	22.0	63.7	1.54	2.90	AAA-AAA	0.00	999	1.000	1.000	0.2324
236635_at	18.4	53.4	1.54	2.90	AAA-AAA	0.00	999	1.000	1.000	0.1643
210271_at	52.3	18.1	-1.54	-2.90	APA-PAA	0.00	999	1.000	1.000	0.3639
239302_s_at	81.2	28.0	-1.53	-2.90	AAA-AAA	0.00	999	1.000	1.000	0.2480
1557862_at	44.1	15.2	-1.53	-2.90	AAA-AAA	0.00	999	1.000	1.000	0.2680
243277_x_at	5.9	17.0	1.53	2.90	AAA-AAA	0.00	999	1.000	1.000	0.3026
1561129_at	10.5	3.6	-1.53	-2.90	AAA-AAA	0.00	999	1.000	1.000	0.4515
213953_at	10.0	29.0	1.53	2.90	AAA-AAA	0.00	999	1.000	1.000	0.2589
214138_at	35.7	103.3	1.53	2.90	AAA-AAA	0.00	999	1.000	1.000	0.0610
236452_at	12.4	36.0	1.53	2.90	AAA-AAA	0.00	999	1.000	1.000	0.0746
236519_at	6.3	18.1	1.53	2.90	AAA-AAA	0.00	999	1.000	1.000	0.3284
241590_at	14.7	5.1	-1.53	-2.89	AAA-AAA	0.00	999	1.000	1.000	0.2067
240924_x_at	19.8	6.8	-1.53	-2.89	AAA-AAA	0.00	999	1.000	1.000	0.1775
211619_s_at	29.9	86.6	1.53	2.89	AAA-AAA	0.00	999	1.000	1.000	0.1909
240476_at	25.4	8.8	-1.53	-2.89	AAA-AAA	0.00	999	1.000	1.000	0.2833
1553856_s_at	37.2	12.9	-1.53	-2.89	AAA-AAA	0.00	999	1.000	1.000	0.1925
1562112_at	5.1	14.7	1.53	2.89	AAA-AAA	0.00	999	1.000	1.000	0.2887
1561962_at	2.7	7.8	1.53	2.89	AAA-AAA	0.00	999	1.000	1.000	0.1092

219820_at	10.4	3.6	-1.53	-2.89	AAA-AAA	0.00	999	1.000	1.000	0.0860
238162_at	20.2	7.0	-1.53	-2.89	AAA-AAA	0.00	999	1.000	1.000	0.0652
238343_x_at	11.1	3.9	-1.53	-2.89	AAA-AAA	0.00	999	1.000	1.000	0.4656
236965_at	59.2	20.5	-1.53	-2.89	AAA-AAA	0.00	999	1.000	1.000	0.3143
215879_at	11.3	3.9	-1.53	-2.89	AAA-AAA	0.00	999	1.000	1.000	0.1746
215122_at	25.4	8.8	-1.53	-2.89	AAA-AAA	0.00	999	1.000	1.000	0.4057
220269_at	23.1	8.0	-1.53	-2.89	AAA-AAA	0.00	999	1.000	1.000	0.2404
232739_at	20.5	59.3	1.53	2.89	AAA-AAA	0.00	999	1.000	1.000	0.0867
237447_at	29.0	10.0	-1.53	-2.89	AAA-AAA	0.00	999	1.000	1.000	0.1450
228969_at	5.5	15.8	1.53	2.89	AAA-AAA	0.00	999	1.000	1.000	0.0575
209776_s_at	152.5	52.8	-1.53	-2.89	MPP-AAM	0.00	999	1.000	1.000	0.1309
240346_at	6.2	17.9	1.53	2.89	AAA-AAA	0.00	999	1.000	1.000	0.1236
1569998_at	48.4	16.8	-1.53	-2.89	AAA-AAA	0.00	999	1.000	1.000	0.2965
241056_at	22.5	7.8	-1.53	-2.89	AAA-AAA	0.00	999	1.000	1.000	0.1706
1558828_s_at	10.5	3.6	-1.53	-2.89	AAA-AAA	0.00	999	1.000	1.000	0.0874
210429_at	18.5	6.4	-1.53	-2.89	AAA-AAA	0.00	999	1.000	1.000	0.3342
228549_at	72.6	25.2	-1.53	-2.89	PAA-AAA	0.00	999	1.000	1.000	0.0413
1562621_at	10.7	30.8	1.53	2.89	AAA-AAA	0.00	999	1.000	1.000	0.0744
1566500_at	9.5	27.4	1.53	2.89	AAA-AAA	0.00	999	1.000	1.000	0.3525
239188_at	79.2	27.4	-1.53	-2.89	MAA-AAA	0.00	999	1.000	1.000	0.1758
1555763_x_at	26.1	75.2	1.53	2.89	AAA-AAA	0.00	999	1.000	1.000	0.0246
218541_s_at	1.4	4.0	1.53	2.89	AAA-AAA	0.00	999	1.000	1.000	0.3399
211617_at	5.9	17.0	1.53	2.89	AAA-AAA	0.00	999	1.000	1.000	0.2700
234606_at	16.9	48.9	1.53	2.89	AAA-AAM	0.00	999	1.000	1.000	0.3836
238636_at	98.4	34.1	-1.53	-2.89	AAA-AAA	0.00	999	1.000	1.000	0.0853
241194_at	22.4	7.8	-1.53	-2.89	AAA-AAA	0.00	999	1.000	1.000	0.2153
241253_at	46.8	16.2	-1.53	-2.88	AAA-AAA	0.00	999	1.000	1.000	0.1566
210953_at	23.0	8.0	-1.53	-2.89	AAA-AAA	0.00	999	1.000	1.000	0.2367
1570579_at	15.7	5.5	-1.53	-2.88	AAA-AAA	0.00	999	1.000	1.000	0.0983
239384_at	36.9	106.5	1.53	2.88	AAA-AAA	0.00	999	1.000	1.000	0.1238
209038_s_at	81.7	28.3	-1.53	-2.88	AAA-AAA	0.00	999	1.000	1.000	0.2351
226900_at	4.9	14.0	1.53	2.88	AAA-AAA	0.00	999	1.000	1.000	0.3362
222223_s_at	12.7	36.7	1.53	2.88	AAA-AAA	0.00	999	1.000	1.000	0.1232
231801_at	14.2	40.8	1.53	2.88	AAA-AAA	0.00	999	1.000	1.000	0.2424
228938_at	5.4	15.6	1.53	2.88	AAA-AAA	0.00	999	1.000	1.000	0.2807
1554950_at	28.3	81.4	1.53	2.88	AAA-AAA	0.00	999	1.000	1.000	0.2586
216388_s_at	29.4	84.6	1.53	2.88	AAA-AAA	0.00	999	1.000	1.000	0.1710
223859_at	24.2	8.4	-1.53	-2.88	AAA-AAA	0.00	999	1.000	1.000	0.0487
231912_s_at	54.1	155.8	1.53	2.88	AAA-AAA	0.00	999	1.000	1.000	0.0016
1564790_at	9.1	26.3	1.53	2.88	AAA-AAA	0.00	999	1.000	1.000	0.2013
1561158_at	21.1	7.3	-1.53	-2.88	AAA-AAA	0.00	999	1.000	1.000	0.3009
209197_at	56.8	19.8	-1.53	-2.88	AAA-MAA	0.00	999	1.000	1.000	0.3066

234376_at	16.0	5.6	-1.53	-2.88	AAA-AAA	0.00	999	1.000	1.000	0.3012
236396_at	35.5	12.3	-1.53	-2.88	AAA-AAA	0.00	999	1.000	1.000	0.2257
35974_at	4.6	13.2	1.53	2.88	AAA-AAA	0.00	999	1.000	1.000	0.1080
231442_at	12.2	35.0	1.53	2.88	AAA-AAA	0.00	999	1.000	1.000	0.3016
229306_at	16.3	46.9	1.53	2.88	AAA-AAA	0.00	999	1.000	1.000	0.0164
237322_at	69.3	24.1	-1.52	-2.88	APA-AAA	0.00	999	1.000	1.000	0.1102
227317_at	50.8	17.7	-1.52	-2.88	AAP-AAA	0.00	999	1.000	1.000	0.3166
1563327_a_at	21.2	7.4	-1.52	-2.88	AAA-AAA	0.00	999	1.000	1.000	0.1217
239530_at	77.2	26.9	-1.52	-2.88	AAA-AAA	0.00	999	1.000	1.000	0.0924
1554725_at	12.7	4.4	-1.52	-2.88	AAA-AAA	0.00	999	1.000	1.000	0.2723
1560385_x_at	7.3	20.9	1.52	2.88	AAA-AAA	0.00	999	1.000	1.000	0.2112
208600_s_at	106.8	37.1	-1.52	-2.88	AAA-AAA	0.00	999	1.000	1.000	0.1687
37201_at	17.4	50.1	1.52	2.88	AAA-AAA	0.00	999	1.000	1.000	0.1984
217072_at	10.9	31.2	1.52	2.87	AAA-AAA	0.00	999	1.000	1.000	0.2876
219456_s_at	24.7	71.1	1.52	2.88	AAA-AAA	0.00	999	1.000	1.000	0.1805
206601_s_at	11.6	33.4	1.52	2.87	AAA-AAA	0.00	999	1.000	1.000	0.2164
211732_x_at	10.8	31.0	1.52	2.87	AAA-AAA	0.00	999	1.000	1.000	0.1706
235752_at	9.5	27.4	1.52	2.87	AAA-AAA	0.00	999	1.000	1.000	0.0272
1570350_at	102.1	35.5	-1.52	-2.87	MAA-AAA	0.00	999	1.000	1.000	0.1046
216245_at	11.6	4.0	-1.52	-2.87	AAA-AAA	0.00	999	1.000	1.000	0.1874
231331_at	10.3	29.6	1.52	2.87	AAA-AAA	0.00	999	1.000	1.000	0.1053
237831_x_at	31.9	11.1	-1.52	-2.87	APP-AAA	0.00	999	1.000	1.000	0.1682
1562428_at	71.2	24.8	-1.52	-2.87	APA-PAA	0.00	999	1.000	1.000	0.3153
1566862_at	12.6	4.4	-1.52	-2.87	AAA-AAA	0.00	999	1.000	1.000	0.4116
219994_at	16.0	46.0	1.52	2.87	AAA-AAA	0.00	999	1.000	1.000	0.1479
206021_at	21.2	7.4	-1.52	-2.87	AAA-AAA	0.00	999	1.000	1.000	0.3675
206999_at	35.5	12.4	-1.52	-2.87	AAA-AAA	0.00	999	1.000	1.000	0.3606
211266_s_at	47.9	16.7	-1.52	-2.87	AAA-AAA	0.00	999	1.000	1.000	0.2381
233246_at	69.0	24.1	-1.52	-2.87	PPP-AAP	0.00	999	1.000	1.000	0.1716
1555932_at	11.4	4.0	-1.52	-2.87	AAA-AAA	0.00	999	1.000	1.000	0.3095
237225_at	15.9	5.6	-1.52	-2.87	AAA-AAA	0.00	999	1.000	1.000	0.1249
1570233_at	9.4	3.3	-1.52	-2.87	AAA-AAA	0.00	999	1.000	1.000	0.3878
1557797_a_at	7.0	20.1	1.52	2.87	AAA-AAM	0.00	999	1.000	1.000	0.3750
243352_at	18.3	52.4	1.52	2.87	AAA-AAA	0.00	999	1.000	1.000	0.3206
1561846_s_at	14.2	40.8	1.52	2.87	AAA-AAA	0.00	999	1.000	1.000	0.2778
234039_at	36.2	103.8	1.52	2.87	AAA-AAA	0.00	999	1.000	1.000	0.2492
237253_at	10.7	3.7	-1.52	-2.87	AAA-AAA	0.00	999	1.000	1.000	0.3333
229172_at	34.0	97.4	1.52	2.87	AAA-AAA	0.00	999	1.000	1.000	0.1076
1554724_at	9.8	28.1	1.52	2.86	AAA-AAA	0.00	999	1.000	1.000	0.3387
214799_at	8.0	22.9	1.52	2.87	AAA-AAA	0.00	999	1.000	1.000	0.0754
220448_at	12.7	4.4	-1.52	-2.86	AAA-AAA	0.00	999	1.000	1.000	0.2898
229203_at	32.5	11.3	-1.52	-2.87	AAA-AAA	0.00	999	1.000	1.000	0.3121

1561951_at	15.3	5.3	-1.52	-2.86	APA-AAA	0.00	999	1.000	1.000	0.3061
1569206_at	32.9	94.1	1.52	2.86	AAA-AAA	0.00	999	1.000	1.000	0.1118
1558607_at	53.8	18.8	-1.52	-2.86	AAA-AAA	0.00	999	1.000	1.000	0.1501
1559030_a_at	24.2	8.5	-1.52	-2.86	AAA-AAA	0.00	999	1.000	1.000	0.2007
215642_at	44.7	15.6	-1.52	-2.86	AAA-AAA	0.00	999	1.000	1.000	0.0167
204051_s_at	74.8	26.2	-1.52	-2.86	PAA-AAA	0.00	999	1.000	1.000	0.0742
1569923_s_at	46.0	16.1	-1.52	-2.86	AAP-AAA	0.00	999	1.000	1.000	0.2216
238442_at	68.7	24.0	-1.52	-2.86	AAA-AAP	0.00	999	1.000	1.000	0.2304
1564772_at	3.6	10.4	1.52	2.86	AAA-AAA	0.00	999	1.000	1.000	0.2611
241889_at	8.2	23.5	1.52	2.86	AAA-AAA	0.00	999	1.000	1.000	0.1170
236579_at	35.5	12.4	-1.52	-2.86	AAA-AAA	0.00	999	1.000	1.000	0.2386
230702_at	8.7	24.9	1.52	2.86	AAA-AAA	0.00	999	1.000	1.000	0.2998
1570219_at	21.4	7.5	-1.52	-2.86	AAA-AAA	0.00	999	1.000	1.000	0.2320
1558119_at	80.1	28.0	-1.52	-2.86	AAA-AAA	0.00	999	1.000	1.000	0.0061
220538_at	150.2	52.5	-1.52	-2.86	AAA-AAA	0.00	999	1.000	1.000	0.0825
219612_s_at	25.4	8.9	-1.52	-2.86	AAA-AAA	0.00	999	1.000	1.000	0.2528
219621_at	21.8	62.3	1.52	2.86	AAA-AAA	0.00	999	1.000	1.000	0.2004
231712_at	16.4	5.8	-1.52	-2.86	AAA-AAA	0.00	999	1.000	1.000	0.1062
1552457_a_at	9.1	3.2	-1.51	-2.86	AAA-AAA	0.00	999	1.000	1.000	0.1584
220290_at	123.7	43.3	-1.51	-2.86	AAA-AAA	0.00	999	1.000	1.000	0.1205
221149_at	11.7	4.1	-1.51	-2.86	AAA-AAA	0.00	999	1.000	1.000	0.3004
207401_at	59.7	20.9	-1.51	-2.86	AAM-AAA	0.00	999	1.000	1.000	0.1637
1561545_at	6.7	19.1	1.51	2.85	AAA-AAA	0.00	999	1.000	1.000	0.1348
216423_at	11.8	33.6	1.51	2.85	AAA-AAA	0.00	999	1.000	1.000	0.1458
221152_at	11.1	31.5	1.51	2.85	AAA-AAA	0.00	999	1.000	1.000	0.3750
206757_at	15.7	5.5	-1.51	-2.85	AAA-AAA	0.00	999	1.000	1.000	0.2889
233336_at	13.4	38.2	1.51	2.86	AAA-AAA	0.00	999	1.000	1.000	0.2664
237338_at	58.3	20.4	-1.51	-2.85	AAA-AAA	0.00	999	1.000	1.000	0.0127
231506_at	23.2	8.1	-1.51	-2.85	AAA-AAA	0.00	999	1.000	1.000	0.3331
240350_at	18.6	52.9	1.51	2.85	AAA-AAA	0.00	999	1.000	1.000	0.1862
233373_at	13.3	37.9	1.51	2.85	AAA-AAA	0.00	999	1.000	1.000	0.4146
224499_s_at	19.9	7.0	-1.51	-2.85	AAA-AAA	0.00	999	1.000	1.000	0.0805
231898_x_at	6.9	2.4	-1.51	-2.85	AAA-AAA	0.00	999	1.000	1.000	0.3933
220169_at	57.6	20.2	-1.51	-2.85	PPA-AAA	0.00	999	1.000	1.000	0.3071
220377_at	19.9	7.0	-1.51	-2.85	AAA-AAA	0.00	999	1.000	1.000	0.0070
220978_at	8.3	23.6	1.51	2.85	AAA-AAA	0.00	999	1.000	1.000	0.2024
207451_at	15.2	43.2	1.51	2.85	AAA-AAA	0.00	999	1.000	1.000	0.1279
227149_at	10.1	28.9	1.51	2.85	AAA-AAA	0.00	999	1.000	1.000	0.4968
1565554_at	49.6	17.4	-1.51	-2.85	AAA-AAA	0.00	999	1.000	1.000	0.2497
1562643_at	51.7	18.1	-1.51	-2.85	AAA-AAA	0.00	999	1.000	1.000	0.2400
239938_x_at	1.3	3.8	1.51	2.85	AAA-AAA	0.00	999	1.000	1.000	0.1206
1557261_at	7.6	21.6	1.51	2.85	AAA-AAA	0.00	999	1.000	1.000	0.1312

1559884_at	6.7	2.4	-1.51	-2.85	AAA-AAA	0.00	999	1.000	1.000	0.3078
244620_at	14.8	5.2	-1.51	-2.85	AAA-AAA	0.00	999	1.000	1.000	0.1563
210536_s_at	57.0	20.0	-1.51	-2.85	AAA-AAA	0.00	999	1.000	1.000	0.2207
236765_at	10.2	29.1	1.51	2.85	AAA-AAA	0.00	999	1.000	1.000	0.2899
233945_at	48.8	17.1	-1.51	-2.85	AAA-PAA	0.00	999	1.000	1.000	0.2047
240622_at	36.8	12.9	-1.51	-2.85	AAP-AAA	0.00	999	1.000	1.000	0.4290
1565701_at	62.7	22.0	-1.51	-2.85	AAA-AAA	0.00	999	1.000	1.000	0.1134
1561881_at	6.5	18.6	1.51	2.85	AAA-AAA	0.00	999	1.000	1.000	0.3064
221375_at	17.2	48.8	1.51	2.85	AAA-AAA	0.00	999	1.000	1.000	0.1639
217394_at	10.1	28.8	1.51	2.85	AAA-AAA	0.00	999	1.000	1.000	0.2798
208172_s_at	8.6	24.4	1.51	2.85	AAA-AAA	0.00	999	1.000	1.000	0.1864
234518_at	38.5	13.5	-1.51	-2.85	AAA-AAA	0.00	999	1.000	1.000	0.2967
227282_at	12.1	34.5	1.51	2.85	AAA-AAA	0.00	999	1.000	1.000	0.1138
222725_s_at	5.5	15.7	1.51	2.85	AAA-AAA	0.00	999	1.000	1.000	0.1706
1552543_a_at	112.2	39.5	-1.51	-2.85	PPP-AAA	0.00	999	1.000	1.000	0.0968
241519_at	16.3	5.7	-1.51	-2.84	AAA-AAA	0.00	999	1.000	1.000	0.2129
216624_s_at	39.5	112.4	1.51	2.84	AAA-AAA	0.00	999	1.000	1.000	0.2228
220855_at	7.4	21.1	1.51	2.84	AAA-AAM	0.00	999	1.000	1.000	0.3311
201137_s_at	16.5	47.0	1.51	2.84	AAA-AAA	0.00	999	1.000	1.000	0.1742
238855_at	40.6	115.5	1.51	2.84	AAA-AAA	0.00	999	1.000	1.000	0.2102
231507_at	7.7	22.0	1.51	2.84	AAA-AAA	0.00	999	1.000	1.000	0.3764
240058_at	4.0	11.2	1.51	2.84	AAA-AAA	0.00	999	1.000	1.000	0.3170
1569944_at	22.6	64.3	1.51	2.84	AAA-AAA	0.00	999	1.000	1.000	0.1537
1555164_at	58.9	20.7	-1.51	-2.84	AAA-AAA	0.00	999	1.000	1.000	0.1945
216108_at	15.3	43.5	1.51	2.84	AAA-AAA	0.00	999	1.000	1.000	0.1764
239944_at	32.8	93.1	1.51	2.84	AAA-AAA	0.00	999	1.000	1.000	0.2519
1560752_at	8.2	23.2	1.51	2.84	AAA-AAA	0.00	999	1.000	1.000	0.2689
232766_at	17.6	50.0	1.51	2.84	AAA-AAA	0.00	999	1.000	1.000	0.1090
229047_at	67.7	23.9	-1.51	-2.84	AAA-AAA	0.00	999	1.000	1.000	0.2799
1552396_at	13.0	36.9	1.51	2.84	AAA-AAA	0.00	999	1.000	1.000	0.0450
1554716_s_at	39.0	13.8	-1.51	-2.84	AAA-AAA	0.00	999	1.000	1.000	0.2216
1560832_at	14.3	40.6	1.51	2.84	AAA-AAA	0.00	999	1.000	1.000	0.2688
213749_at	12.5	35.4	1.51	2.84	AAA-AAA	0.00	999	1.000	1.000	0.1306
214024_s_at	12.8	36.3	1.51	2.84	AAA-AAA	0.00	999	1.000	1.000	0.1218
211786_at	23.4	8.2	-1.51	-2.84	AAA-AAA	0.00	999	1.000	1.000	0.3799
211633_x_at	18.4	52.3	1.51	2.84	AAA-AAA	0.00	999	1.000	1.000	0.2062
238629_x_at	19.2	54.5	1.51	2.84	AAA-AAA	0.00	999	1.000	1.000	0.1651
227631_at	81.7	28.8	-1.51	-2.84	AAA-AAA	0.00	999	1.000	1.000	0.0983
229485_x_at	7.4	20.9	1.51	2.84	AAA-AAA	0.00	999	1.000	1.000	0.0898
241220_at	11.1	3.9	-1.50	-2.84	AAA-AAA	0.00	999	1.000	1.000	0.2120
242090_x_at	8.8	3.1	-1.50	-2.84	AAA-AAA	0.00	999	1.000	1.000	0.2660
1561966_at	13.5	38.3	1.50	2.84	AAA-AAA	0.00	999	1.000	1.000	0.3065

219604_s_at	35.4	12.5	-1.50	-2.84	AAA-AAA	0.00	999	1.000	1.000	0.1174
211469_s_at	53.3	18.8	-1.50	-2.84	AAA-AAA	0.00	999	1.000	1.000	0.2367
233997_at	21.4	60.6	1.50	2.83	AAA-AAA	0.00	999	1.000	1.000	0.2245
237113_at	5.2	14.8	1.50	2.84	AAA-AAA	0.00	999	1.000	1.000	0.2337
222885_at	6.9	19.6	1.50	2.83	AAA-AAA	0.00	999	1.000	1.000	0.1928
223732_at	16.8	47.7	1.50	2.84	AAA-AAA	0.00	999	1.000	1.000	0.0787
242166_at	58.5	20.6	-1.50	-2.83	AAA-AAA	0.00	999	1.000	1.000	0.2444
1568606_at	16.2	45.9	1.50	2.83	AMA-AAA	0.00	999	1.000	1.000	0.3939
240832_at	1.9	5.3	1.50	2.83	AAA-AAA	0.00	999	1.000	1.000	0.1093
1560806_at	8.4	23.8	1.50	2.83	AAA-AAA	0.00	999	1.000	1.000	0.2930
220806_x_at	4.9	13.8	1.50	2.83	AAA-AAA	0.00	999	1.000	1.000	0.2828
217162_at	8.4	23.8	1.50	2.83	AAA-AAA	0.00	999	1.000	1.000	0.0197
209327_s_at	30.2	10.7	-1.50	-2.83	AAA-AAA	0.00	999	1.000	1.000	0.2995
238763_at	20.7	58.6	1.50	2.83	AAA-AAA	0.00	999	1.000	1.000	0.4204
222222_s_at	239.3	84.5	-1.50	-2.83	AAA-AAA	0.00	999	1.000	1.000	0.2394
232260_at	15.1	5.3	-1.50	-2.83	AAA-AAA	0.00	999	1.000	1.000	0.2562
1562673_at	36.8	13.0	-1.50	-2.83	AAA-AAA	0.00	999	1.000	1.000	0.0143
1563868_a_at	4.0	11.4	1.50	2.83	AAA-AAA	0.00	999	1.000	1.000	0.0473
244081_at	8.8	25.0	1.50	2.83	AAA-AAA	0.00	999	1.000	1.000	0.3261
202554_s_at	26.2	74.0	1.50	2.83	AAA-AAA	0.00	999	1.000	1.000	0.1481
207984_s_at	132.1	46.7	-1.50	-2.83	AAA-AAA	0.00	999	1.000	1.000	0.2174
233360_at	17.2	48.8	1.50	2.83	AAA-AAA	0.00	999	1.000	1.000	0.1615
1562727_at	43.0	15.2	-1.50	-2.83	PMA-AAP	0.00	999	1.000	1.000	0.3098
1566694_at	6.3	17.7	1.50	2.83	AAA-AAA	0.00	999	1.000	1.000	0.3721
240540_at	30.3	85.7	1.50	2.83	AAA-AAA	0.00	999	1.000	1.000	0.1039
244747_at	32.6	11.5	-1.50	-2.83	AAA-AAA	0.00	999	1.000	1.000	0.3063
1558101_at	11.8	33.4	1.50	2.83	AAA-AAA	0.00	999	1.000	1.000	0.1901
1555430_at	5.6	15.9	1.50	2.83	AAA-AAA	0.00	999	1.000	1.000	0.0908
243659_at	33.7	95.2	1.50	2.83	AAA-AAA	0.00	999	1.000	1.000	0.1733
237416_at	3.9	11.0	1.50	2.83	AAA-AAA	0.00	999	1.000	1.000	0.3285
237546_at	20.8	7.4	-1.50	-2.83	AAA-AAA	0.00	999	1.000	1.000	0.3590
235824_at	9.2	26.1	1.50	2.83	AAA-AAA	0.00	999	1.000	1.000	0.0741
1565111_x_at	3.2	9.1	1.50	2.83	AAA-AAA	0.00	999	1.000	1.000	0.3404
1570226_at	3.2	9.2	1.50	2.83	AAA-AAA	0.00	999	1.000	1.000	0.3184
1553036_at	8.1	22.9	1.50	2.83	AAA-AAA	0.00	999	1.000	1.000	0.0258
210737_at	34.2	96.7	1.50	2.83	AAA-AAA	0.00	999	1.000	1.000	0.2096
227390_at	43.6	15.4	-1.50	-2.83	AAA-AAA	0.00	999	1.000	1.000	0.3620
219655_at	249.4	88.3	-1.50	-2.83	PPP-PAP	0.00	999	1.000	1.000	0.2019
1554929_at	38.2	13.5	-1.50	-2.82	AAA-AAA	0.00	999	1.000	1.000	0.0532
1559737_at	4.3	12.2	1.50	2.83	AAA-AAA	0.00	999	1.000	1.000	0.2203
219173_at	62.5	22.1	-1.50	-2.82	AAA-AAA	0.00	999	1.000	1.000	0.1968
1569543_at	15.2	42.9	1.50	2.82	AAA-AAA	0.00	999	1.000	1.000	0.2194

219712_s_at	34.4	12.2	-1.50	-2.82	AAA-AAA	0.00	999	1.000	1.000	0.1193
206476_s_at	23.7	8.4	-1.50	-2.82	AAA-AAA	0.00	999	1.000	1.000	0.2841
203111_s_at	55.3	156.2	1.50	2.82	AAA-AAA	0.00	999	1.000	1.000	0.1710
238246_at	24.2	8.6	-1.50	-2.82	AAA-AAA	0.00	999	1.000	1.000	0.3119
1556138_a_at	7.8	22.1	1.50	2.82	AAA-AAA	0.00	999	1.000	1.000	0.3503
220562_at	10.9	30.7	1.50	2.82	AAA-AAA	0.00	999	1.000	1.000	0.1252
237841_at	8.9	25.2	1.50	2.82	AAA-AAA	0.00	999	1.000	1.000	0.3301
237562_at	26.4	9.3	-1.50	-2.82	AAA-AAA	0.00	999	1.000	1.000	0.3595
227697_at	38.3	13.6	-1.50	-2.82	AAA-AAA	0.00	999	1.000	1.000	0.3268
231742_at	7.0	19.8	1.50	2.82	AAA-AAA	0.00	999	1.000	1.000	0.0990
211913_s_at	72.6	25.7	-1.50	-2.82	APP-AAA	0.00	999	1.000	1.000	0.1469
1569932_at	32.5	91.6	1.50	2.82	AAA-MAA	0.00	999	1.000	1.000	0.1119
1552849_at	4.3	12.2	1.50	2.82	AAA-AAA	0.00	999	1.000	1.000	0.1804
235656_s_at	29.3	10.4	-1.50	-2.82	AAA-AAA	0.00	999	1.000	1.000	0.2608
235825_at	18.0	6.4	-1.50	-2.82	AAA-AAA	0.00	999	1.000	1.000	0.3012
216222_s_at	116.3	41.3	-1.49	-2.82	AAP-AAA	0.00	999	1.000	1.000	0.3326
1562908_at	28.7	10.2	-1.49	-2.82	AAA-AAA	0.00	999	1.000	1.000	0.2709
244635_s_at	6.2	17.6	1.49	2.82	AAA-AAA	0.00	999	1.000	1.000	0.3505
215386_at	6.9	19.5	1.49	2.82	AAA-AAA	0.00	999	1.000	1.000	0.2067
221161_at	31.5	11.2	-1.49	-2.82	AAA-AAA	0.00	999	1.000	1.000	0.2763
234015_at	27.9	9.9	-1.49	-2.82	AAA-AAA	0.00	999	1.000	1.000	0.1598
1570424_at	40.3	14.3	-1.49	-2.82	APA-AAA	0.00	999	1.000	1.000	0.2236
1563907_at	5.5	15.5	1.49	2.82	AAA-AAA	0.00	999	1.000	1.000	0.2976
242000_at	42.7	15.2	-1.49	-2.82	AAA-AAA	0.00	999	1.000	1.000	0.1863
212154_at	21.6	60.8	1.49	2.82	AAA-AAA	0.00	999	1.000	1.000	0.0651
210244_at	19.6	55.1	1.49	2.81	AAA-AAA	0.00	999	1.000	1.000	0.1750
211556_at	18.5	6.6	-1.49	-2.82	AAA-AAA	0.00	999	1.000	1.000	0.2662
233629_at	3.6	10.1	1.49	2.82	AAA-AAA	0.00	999	1.000	1.000	0.1963
229324_x_at	15.0	42.3	1.49	2.82	AAA-AAA	0.00	999	1.000	1.000	0.2798
1553526_at	72.9	25.9	-1.49	-2.81	AAA-AAA	0.00	999	1.000	1.000	0.2267
243399_at	10.2	3.6	-1.49	-2.81	AAA-AAA	0.00	999	1.000	1.000	0.3209
242600_at	19.2	54.0	1.49	2.81	AAA-AAA	0.00	999	1.000	1.000	0.1375
212489_at	15.0	5.3	-1.49	-2.81	AAA-AAA	0.00	999	1.000	1.000	0.1422
207472_at	4.0	11.2	1.49	2.81	AAA-AAA	0.00	999	1.000	1.000	0.1175
234601_x_at	5.5	15.3	1.49	2.81	AAA-AAA	0.00	999	1.000	1.000	0.2915
228368_at	9.0	25.4	1.49	2.81	AAA-AAA	0.00	999	1.000	1.000	0.1542
230250_at	50.4	18.0	-1.49	-2.81	PAA-AAA	0.00	999	1.000	1.000	0.2186
1569677_a_at	28.3	10.1	-1.49	-2.81	APA-AAA	0.00	999	1.000	1.000	0.3465
242792_at	14.0	5.0	-1.49	-2.81	AAA-AAA	0.00	999	1.000	1.000	0.2646
214725_at	17.8	6.4	-1.49	-2.81	AAA-AAA	0.00	999	1.000	1.000	0.1249
220347_at	47.6	16.9	-1.49	-2.81	AAA-AAA	0.00	999	1.000	1.000	0.1213
206198_s_at	4.1	11.6	1.49	2.81	AAA-AAA	0.00	999	1.000	1.000	0.3748

202997_s_at	20.6	7.3	-1.49	-2.81	AAA-AAA	0.00	999	1.000	1.000	0.2660
232982_at	15.8	44.3	1.49	2.81	AAA-AAA	0.00	999	1.000	1.000	0.1445
235942_at	28.6	10.2	-1.49	-2.81	AAA-AAA	0.00	999	1.000	1.000	0.2437
231047_at	55.3	19.7	-1.49	-2.81	AAA-AAA	0.00	999	1.000	1.000	0.2321
230217_at	5.4	15.1	1.49	2.81	AAA-AAA	0.00	999	1.000	1.000	0.2410
242183_at	13.0	36.6	1.49	2.81	AAA-AAA	0.00	999	1.000	1.000	0.2149
1566490_at	27.1	9.7	-1.49	-2.81	AAA-AAA	0.00	999	1.000	1.000	0.0107
1556983_a_at	8.8	24.8	1.49	2.81	AAA-AAA	0.00	999	1.000	1.000	0.2155
244707_at	17.5	6.2	-1.49	-2.81	AAA-AAA	0.00	999	1.000	1.000	0.1913
1552515_at	15.9	44.7	1.49	2.81	AAA-AAA	0.00	999	1.000	1.000	0.2097
219695_at	74.2	26.4	-1.49	-2.81	AAA-AAA	0.00	999	1.000	1.000	0.1126
1565613_at	11.4	4.1	-1.49	-2.81	AAA-AAA	0.00	999	1.000	1.000	0.2882
1555793_a_at	20.6	7.3	-1.49	-2.81	AAA-AAA	0.00	999	1.000	1.000	0.2089
206831_s_at	49.7	17.7	-1.49	-2.81	AAA-AAA	0.00	999	1.000	1.000	0.1237
1570332_at	6.8	19.2	1.49	2.81	AAA-AAA	0.00	999	1.000	1.000	0.1800
1556445_at	144.6	51.6	-1.49	-2.81	AAA-AAA	0.00	999	1.000	1.000	0.2431
1555726_at	4.1	11.5	1.49	2.81	AAA-AAA	0.00	999	1.000	1.000	0.4750
1559624_at	47.8	17.0	-1.49	-2.80	AAA-AAA	0.00	999	1.000	1.000	0.1540
217396_at	38.4	13.7	-1.49	-2.81	AAA-AAA	0.00	999	1.000	1.000	0.1164
207929_at	113.0	40.3	-1.49	-2.81	AAA-AAA	0.00	999	1.000	1.000	0.3267
238408_at	8.2	22.9	1.49	2.81	AAA-AAA	0.00	999	1.000	1.000	0.3911
37892_at	4.8	13.4	1.49	2.81	AAA-AAA	0.00	999	1.000	1.000	0.0891
230247_at	12.9	36.1	1.49	2.81	AAA-AAA	0.00	999	1.000	1.000	0.3228
1553970_s_at	12.4	34.9	1.49	2.80	AAA-AAA	0.00	999	1.000	1.000	0.1844
1561290_at	3.1	8.8	1.49	2.80	AAA-AAA	0.00	999	1.000	1.000	0.2126
1559021_at	30.3	85.0	1.49	2.80	AAA-AAA	0.00	999	1.000	1.000	0.1563
211380_s_at	10.3	28.9	1.49	2.80	AAA-AAA	0.00	999	1.000	1.000	0.2310
237098_at	11.1	31.2	1.49	2.80	AAA-AAA	0.00	999	1.000	1.000	0.1710
232290_at	50.2	17.9	-1.49	-2.80	AAA-AAA	0.00	999	1.000	1.000	0.2983
1569353_at	10.4	29.0	1.49	2.80	AAA-AAA	0.00	999	1.000	1.000	0.2764
1565925_at	4.1	11.5	1.49	2.80	AAA-AAA	0.00	999	1.000	1.000	0.3559
1557717_at	10.0	27.9	1.49	2.80	AAA-AAA	0.00	999	1.000	1.000	0.1480
204546_at	89.7	32.0	-1.49	-2.80	AAA-AAA	0.00	999	1.000	1.000	0.0405
232606_at	3.2	8.9	1.49	2.80	AAA-AAA	0.00	999	1.000	1.000	0.3786
241053_at	105.5	37.7	-1.49	-2.80	PAP-PAP	0.00	999	1.000	1.000	0.2269
1566295_at	9.6	26.8	1.49	2.80	AAA-AAA	0.00	999	1.000	1.000	0.1924
1554865_at	16.1	5.8	-1.49	-2.80	AAA-AAA	0.00	999	1.000	1.000	0.3895
242567_at	31.1	11.1	-1.49	-2.80	AAA-AAA	0.00	999	1.000	1.000	0.1350
1559600_at	18.9	52.9	1.49	2.80	AAA-AAA	0.00	999	1.000	1.000	0.0961
1560782_at	17.9	50.0	1.49	2.80	AAA-AAA	0.00	999	1.000	1.000	0.0911
219888_at	75.3	26.9	-1.49	-2.80	AAA-AAA	0.00	999	1.000	1.000	0.3194
208376_at	13.5	37.9	1.49	2.80	AAA-AAA	0.00	999	1.000	1.000	0.2801

243081_at	14.0	39.3	1.48	2.80	AAA-AAA	0.00	999	1.000	1.000	0.1970
214392_at	28.7	80.2	1.48	2.80	AAA-AAA	0.00	999	1.000	1.000	0.0882
220481_at	49.5	17.7	-1.48	-2.80	AAA-AAA	0.00	999	1.000	1.000	0.0755
236331_at	60.9	21.8	-1.48	-2.80	AAA-AAA	0.00	999	1.000	1.000	0.2094
230984_s_at	9.7	27.2	1.48	2.80	AAA-AAA	0.00	999	1.000	1.000	0.1494
1556935_at	43.9	15.7	-1.48	-2.80	AAA-PAA	0.00	999	1.000	1.000	0.1420
242084_at	54.9	19.6	-1.48	-2.80	AAA-AAA	0.00	999	1.000	1.000	0.1145
1570546_a_at	10.5	3.8	-1.48	-2.79	AAA-AAA	0.00	999	1.000	1.000	0.0321
1558077_s_at	52.7	18.8	-1.48	-2.80	AAA-AAA	0.00	999	1.000	1.000	0.1009
1557850_at	6.4	18.0	1.48	2.80	AAA-AAA	0.00	999	1.000	1.000	0.2395
216213_at	10.7	29.9	1.48	2.80	AAA-AAA	0.00	999	1.000	1.000	0.2959
227749_at	18.7	52.3	1.48	2.80	AAA-AAA	0.00	999	1.000	1.000	0.2877
229048_at	9.5	26.5	1.48	2.79	AAA-AAA	0.00	999	1.000	1.000	0.3457
239860_at	69.1	24.7	-1.48	-2.79	PAA-AAA	0.00	999	1.000	1.000	0.3249
214886_s_at	10.5	3.8	-1.48	-2.79	AAA-AAA	0.00	999	1.000	1.000	0.1907
207294_at	9.1	3.2	-1.48	-2.79	AAA-AAA	0.00	999	1.000	1.000	0.2197
230628_at	24.5	68.5	1.48	2.79	AAA-AAA	0.00	999	1.000	1.000	0.0952
1558857_at	18.7	52.1	1.48	2.79	AAA-AAA	0.00	999	1.000	1.000	0.0907
221115_s_at	94.6	33.9	-1.48	-2.79	AAA-AAA	0.00	999	1.000	1.000	0.2428
1566917_at	24.7	68.9	1.48	2.79	AAA-AAA	0.00	999	1.000	1.000	0.2720
240678_at	18.9	6.8	-1.48	-2.79	AAA-AAA	0.00	999	1.000	1.000	0.1525
1557024_at	10.1	3.6	-1.48	-2.79	AAA-AAA	0.00	999	1.000	1.000	0.2585
216541_x_at	4.2	11.7	1.48	2.79	AAA-AAA	0.00	999	1.000	1.000	0.2930
205983_at	24.4	8.8	-1.48	-2.79	AAA-AAA	0.00	999	1.000	1.000	0.0711
237371_at	23.0	8.2	-1.48	-2.79	AAA-AAA	0.00	999	1.000	1.000	0.3016
1570314_at	39.7	14.2	-1.48	-2.79	AAA-AAA	0.00	999	1.000	1.000	0.0238
1563679_at	7.8	21.7	1.48	2.79	AAA-AAA	0.00	999	1.000	1.000	0.2998
1563209_a_at	12.5	4.5	-1.48	-2.79	AAA-AAA	0.00	999	1.000	1.000	0.1644
1552661_at	2.1	5.8	1.48	2.79	AAA-AAA	0.00	999	1.000	1.000	0.2300
1561198_at	3.1	8.7	1.48	2.79	AAA-AAA	0.00	999	1.000	1.000	0.2106
1561575_at	5.5	15.2	1.48	2.79	AAA-AAA	0.00	999	1.000	1.000	0.2823
1560791_at	18.8	6.7	-1.48	-2.79	AAA-AAA	0.00	999	1.000	1.000	0.2836
211087_x_at	68.1	189.7	1.48	2.79	AAA-AAA	0.00	999	1.000	1.000	0.1943
234475_x_at	68.5	24.6	-1.48	-2.79	AAA-AAA	0.00	999	1.000	1.000	0.1866
234115_s_at	6.3	2.3	-1.48	-2.79	AAA-AAA	0.00	999	1.000	1.000	0.2934
238258_at	8.8	3.2	-1.48	-2.79	AAA-AAA	0.00	999	1.000	1.000	0.2188
236426_at	48.6	17.4	-1.48	-2.79	AAA-AAA	0.00	999	1.000	1.000	0.1626
225423_x_at	11.3	31.5	1.48	2.79	AAA-AAA	0.00	999	1.000	1.000	0.0170
239813_at	23.6	65.7	1.48	2.79	AAA-AAA	0.00	999	1.000	1.000	0.3763
1557122_s_at	19.2	53.5	1.48	2.79	AAA-AAA	0.00	999	1.000	1.000	0.1203
217037_at	2.7	7.5	1.48	2.79	AAA-AAA	0.00	999	1.000	1.000	0.0728
237348_at	18.2	50.7	1.48	2.79	AAA-AAA	0.00	999	1.000	1.000	0.2825

238837_at	8.2	22.7	1.48	2.79	AAA-AAA	0.00	999	1.000	1.000	0.2891
1564139_at	12.8	4.6	-1.48	-2.78	AAA-AAA	0.00	999	1.000	1.000	0.3678
1563340_at	19.1	53.0	1.48	2.78	AAA-AAA	0.00	999	1.000	1.000	0.3954
240886_at	7.9	21.9	1.48	2.78	AAA-AAA	0.00	999	1.000	1.000	0.2394
1553209_at	32.0	89.0	1.48	2.78	MAA-MAA	0.00	999	1.000	1.000	0.2619
244466_at	13.8	38.4	1.48	2.79	AAA-AAA	0.00	999	1.000	1.000	0.0309
214825_at	13.0	36.3	1.48	2.78	AAA-AAA	0.00	999	1.000	1.000	0.3581
234908_s_at	26.8	9.6	-1.48	-2.78	AAA-AAA	0.00	999	1.000	1.000	0.2504
224144_at	25.5	70.9	1.48	2.79	AAA-AAA	0.00	999	1.000	1.000	0.1610
232039_at	22.8	8.2	-1.48	-2.78	AAA-AAA	0.00	999	1.000	1.000	0.1974
214536_at	80.4	28.9	-1.48	-2.78	MAA-PAA	0.00	999	1.000	1.000	0.1959
1565705_x_at	7.4	20.5	1.48	2.78	AAA-AAA	0.00	999	1.000	1.000	0.0926
1552484_at	25.9	72.0	1.48	2.78	AAA-AAA	0.00	999	1.000	1.000	0.1826
216736_at	26.1	72.5	1.48	2.78	AAA-AAA	0.00	999	1.000	1.000	0.1561
220929_at	9.4	26.2	1.48	2.78	AAA-AAA	0.00	999	1.000	1.000	0.2349
205875_s_at	29.4	81.9	1.48	2.78	AAA-AAA	0.00	999	1.000	1.000	0.1226
204472_at	65.9	23.7	-1.48	-2.78	AAA-AAA	0.00	999	1.000	1.000	0.3596
235795_at	2.0	5.5	1.48	2.78	AAA-AAA	0.00	999	1.000	1.000	0.3182
226504_at	20.3	56.6	1.48	2.78	AAA-AAA	0.00	999	1.000	1.000	0.0197
1570204_at	9.0	3.2	-1.48	-2.78	AAA-AAA	0.00	999	1.000	1.000	0.3532
1566126_at	9.6	3.5	-1.48	-2.78	AAA-AAA	0.00	999	1.000	1.000	0.1627
1556355_x_at	11.1	30.8	1.48	2.78	AAA-AAA	0.00	999	1.000	1.000	0.1123
220152_at	34.4	12.4	-1.48	-2.78	AAA-AAA	0.00	999	1.000	1.000	0.2373
221174_at	7.9	21.9	1.48	2.78	AAA-AAA	0.00	999	1.000	1.000	0.3243
210697_at	7.7	21.3	1.48	2.78	AAA-AAA	0.00	999	1.000	1.000	0.2432
207576_x_at	13.7	38.0	1.48	2.78	AAA-AAA	0.00	999	1.000	1.000	0.1259
1570307_s_at	7.6	21.2	1.47	2.78	AAA-AAA	0.00	999	1.000	1.000	0.2365
1552675_at	6.6	2.4	-1.47	-2.78	AAA-AAA	0.00	999	1.000	1.000	0.1833
1556467_at	27.1	75.3	1.47	2.78	AAA-AAA	0.00	999	1.000	1.000	0.2747
244420_at	71.4	25.7	-1.47	-2.78	AAA-AAA	0.00	999	1.000	1.000	0.0408
1558971_at	4.6	12.9	1.47	2.78	AAA-AAA	0.00	999	1.000	1.000	0.3630
211879_x_at	6.6	18.4	1.47	2.78	AAA-AAA	0.00	999	1.000	1.000	0.1857
204851_s_at	15.7	5.7	-1.47	-2.78	AAA-AAA	0.00	999	1.000	1.000	0.2522
238177_at	13.3	36.8	1.47	2.78	AAA-AAA	0.00	999	1.000	1.000	0.3078
226856_at	9.6	26.8	1.47	2.78	AAA-AAA	0.00	999	1.000	1.000	0.4775
227695_at	70.6	25.4	-1.47	-2.78	AAA-AAA	0.00	999	1.000	1.000	0.0254
241192_at	6.1	16.8	1.47	2.78	AAA-AAA	0.00	999	1.000	1.000	0.0173
1561776_at	4.6	12.6	1.47	2.78	AAA-AAA	0.00	999	1.000	1.000	0.3268
221365_at	24.0	8.7	-1.47	-2.78	AAA-AAA	0.00	999	1.000	1.000	0.3007
203760_s_at	31.3	11.3	-1.47	-2.78	AAA-AAA	0.00	999	1.000	1.000	0.2061
208474_at	27.5	76.4	1.47	2.78	AAA-AAA	0.00	999	1.000	1.000	0.0720
239380_at	65.1	23.5	-1.47	-2.77	PAP-AAA	0.00	999	1.000	1.000	0.2713

244884_at	30.1	10.9	-1.47	-2.77	AAA-AAA	0.00	999	1.000	1.000	0.0644
1559585_at	15.9	44.0	1.47	2.77	AAA-AAA	0.00	999	1.000	1.000	0.1229
213767_at	3.8	10.5	1.47	2.77	AAA-AAA	0.00	999	1.000	1.000	0.0689
217102_at	75.3	27.1	-1.47	-2.77	AAA-AAA	0.00	999	1.000	1.000	0.2757
228317_at	15.4	42.6	1.47	2.78	AAA-AAA	0.00	999	1.000	1.000	0.2247
223757_at	25.4	9.1	-1.47	-2.77	AAA-AAA	0.00	999	1.000	1.000	0.3305
229714_at	20.2	7.3	-1.47	-2.77	AAA-AAA	0.00	999	1.000	1.000	0.2098
1552424_at	16.9	6.1	-1.47	-2.77	AAA-AAA	0.00	999	1.000	1.000	0.4370
217557_s_at	99.8	36.0	-1.47	-2.77	AAA-AAA	0.00	999	1.000	1.000	0.1908
206496_at	10.9	30.3	1.47	2.77	AAA-AAA	0.00	999	1.000	1.000	0.0816
230476_at	11.7	32.3	1.47	2.77	AAA-AAA	0.00	999	1.000	1.000	0.0821
227929_at	36.8	13.3	-1.47	-2.77	MPA-AAA	0.00	999	1.000	1.000	0.1408
216216_at	8.8	24.4	1.47	2.77	AAA-AAA	0.00	999	1.000	1.000	0.3023
221070_s_at	13.0	4.7	-1.47	-2.77	AAA-AAA	0.00	999	1.000	1.000	0.1726
216953_s_at	19.0	6.9	-1.47	-2.77	AAA-AAA	0.00	999	1.000	1.000	0.3091
233016_at	27.1	9.8	-1.47	-2.77	AAA-AAA	0.00	999	1.000	1.000	0.2998
231467_at	70.8	25.5	-1.47	-2.77	AAA-AAA	0.00	999	1.000	1.000	0.0142
244512_at	79.6	28.8	-1.47	-2.77	APA-AAA	0.00	999	1.000	1.000	0.1221
240402_at	7.0	19.4	1.47	2.77	AAA-AAA	0.00	999	1.000	1.000	0.2477
1555816_at	6.5	17.9	1.47	2.77	AAA-AAA	0.00	999	1.000	1.000	0.3365
242972_at	48.9	135.5	1.47	2.77	AAA-AAA	0.00	999	1.000	1.000	0.1742
233097_x_at	13.5	37.3	1.47	2.77	AAA-AAA	0.00	999	1.000	1.000	0.1152
236341_at	4.7	13.1	1.47	2.77	AAA-AAA	0.00	999	1.000	1.000	0.3351
230943_at	34.0	12.3	-1.47	-2.77	AAA-AAA	0.00	999	1.000	1.000	0.2797
231668_x_at	11.3	31.3	1.47	2.77	AAA-AAA	0.00	999	1.000	1.000	0.2789
1562879_at	24.7	8.9	-1.47	-2.77	AAA-AAA	0.00	999	1.000	1.000	0.3035
210464_at	6.4	17.8	1.47	2.77	AAA-AAA	0.00	999	1.000	1.000	0.3633
233741_at	66.4	183.8	1.47	2.77	AAA-AAA	0.00	999	1.000	1.000	0.0550
229079_at	24.4	67.4	1.47	2.77	AAA-AAA	0.00	999	1.000	1.000	0.1070
243111_at	45.8	16.6	-1.47	-2.77	AAA-AAA	0.00	999	1.000	1.000	0.1124
1561371_at	13.3	36.7	1.47	2.76	AAA-AAA	0.00	999	1.000	1.000	0.3534
1558899_s_at	56.2	20.3	-1.47	-2.77	AAA-AAA	0.00	999	1.000	1.000	0.1637
215908_at	57.7	20.9	-1.47	-2.77	AAA-AAA	0.00	999	1.000	1.000	0.2500
210382_at	8.8	24.2	1.47	2.76	AAA-AAA	0.00	999	1.000	1.000	0.1701
240132_at	11.8	4.3	-1.47	-2.76	AAA-AAA	0.00	999	1.000	1.000	0.3632
1570050_at	10.6	29.2	1.47	2.76	AAA-AAA	0.00	999	1.000	1.000	0.2727
1556701_at	21.4	59.1	1.47	2.76	AAA-AAA	0.00	999	1.000	1.000	0.2714
243363_at	73.6	26.7	-1.47	-2.76	AAA-AAA	0.00	999	1.000	1.000	0.2058
1561685_a_at	14.1	39.1	1.47	2.76	AAA-AAA	0.00	999	1.000	1.000	0.0072
221114_at	119.7	43.3	-1.47	-2.76	AAA-AAA	0.00	999	1.000	1.000	0.0560
217556_at	20.6	56.9	1.47	2.76	AAA-AAA	0.00	999	1.000	1.000	0.2512
205506_at	8.9	24.5	1.47	2.76	AAA-AAA	0.00	999	1.000	1.000	0.1844

237635_at	11.5	31.8	1.47	2.76	AAA-AAA	0.00	999	1.000	1.000	0.0790
242082_at	15.7	43.3	1.47	2.76	AAA-AAA	0.00	999	1.000	1.000	0.2323
243115_at	14.5	5.3	-1.47	-2.76	AAA-AAA	0.00	999	1.000	1.000	0.3458
213740_s_at	8.0	22.2	1.47	2.76	AAA-AAA	0.00	999	1.000	1.000	0.2279
221541_at	21.6	59.6	1.47	2.76	AAA-AAA	0.00	999	1.000	1.000	0.1262
217244_at	13.5	37.4	1.47	2.76	AAA-AAA	0.00	999	1.000	1.000	0.1947
206366_x_at	34.6	12.5	-1.47	-2.76	AAA-AAA	0.00	999	1.000	1.000	0.1026
204973_at	100.2	36.3	-1.47	-2.76	AAA-AAA	0.00	999	1.000	1.000	0.1718
208221_s_at	26.6	73.5	1.47	2.76	AAA-AAA	0.00	999	1.000	1.000	0.1055
233050_at	6.2	2.3	-1.47	-2.76	AAA-AAA	0.00	999	1.000	1.000	0.3389
233356_at	50.4	18.3	-1.47	-2.76	AAA-AAA	0.00	999	1.000	1.000	0.1839
237185_at	15.7	43.3	1.47	2.76	AAA-AAA	0.00	999	1.000	1.000	0.1179
225122_at	37.0	13.4	-1.47	-2.76	AAA-AAA	0.00	999	1.000	1.000	0.2345
229328_at	22.7	8.2	-1.47	-2.76	AAA-AAA	0.00	999	1.000	1.000	0.3856
1566739_at	29.7	81.9	1.46	2.76	AAA-AAA	0.00	999	1.000	1.000	0.2462
1552261_at	25.7	71.0	1.46	2.76	AAA-AMA	0.00	999	1.000	1.000	0.1710
220148_at	33.0	12.0	-1.46	-2.76	PAA-AAA	0.00	999	1.000	1.000	0.3498
1552607_at	73.7	26.7	-1.46	-2.76	AAA-AAA	0.00	999	1.000	1.000	0.0003
1554781_at	8.3	3.0	-1.46	-2.76	AAA-AAA	0.00	999	1.000	1.000	0.3274
1560439_at	94.2	259.8	1.46	2.76	AAA-AAA	0.00	999	1.000	1.000	0.0388
215846_at	11.3	31.3	1.46	2.76	AAA-AAA	0.00	999	1.000	1.000	0.2655
213894_at	4.7	13.0	1.46	2.76	AAA-AAA	0.00	999	1.000	1.000	0.2982
209914_s_at	30.7	84.7	1.46	2.76	AAA-AAA	0.00	999	1.000	1.000	0.1261
238079_at	17.2	47.5	1.46	2.76	AAA-AAA	0.00	999	1.000	1.000	0.1880
227230_s_at	53.8	19.5	-1.46	-2.76	AAA-PAM	0.00	999	1.000	1.000	0.2941
205111_s_at	40.5	14.7	-1.46	-2.76	AAA-AAA	0.00	999	1.000	1.000	0.1656
237248_at	13.3	36.7	1.46	2.76	MAA-AAA	0.00	999	1.000	1.000	0.2790
223385_at	87.8	31.9	-1.46	-2.75	APP-AAA	0.00	999	1.000	1.000	0.1096
242165_at	8.5	23.3	1.46	2.75	AAA-AAA	0.00	999	1.000	1.000	0.1934
240671_at	15.6	5.7	-1.46	-2.75	AAA-AAA	0.00	999	1.000	1.000	0.3295
1556697_at	1.7	4.7	1.46	2.75	AAA-AAA	0.00	999	1.000	1.000	0.3780
216478_at	31.9	11.6	-1.46	-2.75	AAA-AAA	0.00	999	1.000	1.000	0.3809
237589_at	19.9	54.6	1.46	2.75	AAA-AAA	0.00	999	1.000	1.000	0.1016
1565820_x_at	6.3	17.5	1.46	2.75	AAA-AAA	0.00	999	1.000	1.000	0.3748
240814_at	14.8	40.8	1.46	2.75	AAA-AAA	0.00	999	1.000	1.000	0.3529
221795_at	7.6	20.9	1.46	2.75	AAA-AAA	0.00	999	1.000	1.000	0.4815
206889_at	12.9	35.5	1.46	2.75	AAA-AAA	0.00	999	1.000	1.000	0.1082
234196_at	28.8	10.5	-1.46	-2.75	AAA-AAA	0.00	999	1.000	1.000	0.1711
229122_x_at	13.3	36.6	1.46	2.75	AAA-AAA	0.00	999	1.000	1.000	0.1881
229159_at	11.6	4.2	-1.46	-2.75	AAA-AAA	0.00	999	1.000	1.000	0.3662
243687_at	14.7	40.5	1.46	2.75	AAA-AAA	0.00	999	1.000	1.000	0.1878
216329_at	8.0	21.9	1.46	2.75	AAA-AAA	0.00	999	1.000	1.000	0.2063

217433_at	16.5	45.4	1.46	2.75	AAA-AAA	0.00	999	1.000	1.000	0.2913
205721_at	20.7	56.8	1.46	2.75	AAA-AAA	0.00	999	1.000	1.000	0.0531
234478_at	38.3	13.9	-1.46	-2.75	AAA-AAA	0.00	999	1.000	1.000	0.1323
236296_x_at	68.3	24.8	-1.46	-2.75	AAA-AAA	0.00	999	1.000	1.000	0.1570
236162_at	68.4	24.9	-1.46	-2.75	AAA-AAA	0.00	999	1.000	1.000	0.2813
229508_at	7.1	19.5	1.46	2.75	AAA-AAA	0.00	999	1.000	1.000	0.1292
210920_x_at	46.6	17.0	-1.46	-2.75	AAA-PAA	0.00	999	1.000	1.000	0.3626
1565927_s_at	54.5	19.9	-1.46	-2.75	AAA-AAA	0.00	999	1.000	1.000	0.1259
1570136_at	3.9	10.7	1.46	2.75	AAA-AAA	0.00	999	1.000	1.000	0.4118
206290_s_at	10.1	27.7	1.46	2.75	AAA-AAA	0.00	999	1.000	1.000	0.0079
207051_at	109.9	40.0	-1.46	-2.75	AAA-AAA	0.00	999	1.000	1.000	0.2176
205674_x_at	31.5	86.6	1.46	2.75	AAA-MAA	0.00	999	1.000	1.000	0.3792
235700_at	4.8	13.2	1.46	2.75	AAA-AAA	0.00	999	1.000	1.000	0.4202
231758_at	5.1	14.1	1.46	2.75	AAA-AAA	0.00	999	1.000	1.000	0.0670
216740_at	50.5	18.4	-1.46	-2.75	AAP-AAA	0.00	999	1.000	1.000	0.1814
1564377_at	31.9	11.6	-1.46	-2.75	AAA-AAA	0.00	999	1.000	1.000	0.1310
239103_at	27.7	10.1	-1.46	-2.75	AAA-AAA	0.00	999	1.000	1.000	0.0888
1563187_at	11.4	4.1	-1.46	-2.75	AAA-AAA	0.00	999	1.000	1.000	0.2195
1563557_at	10.4	28.5	1.46	2.75	AAA-AAA	0.00	999	1.000	1.000	0.3902
1569873_at	9.0	3.3	-1.46	-2.75	AAA-AAA	0.00	999	1.000	1.000	0.3405
1560637_at	9.6	26.4	1.46	2.75	AAA-AAA	0.00	999	1.000	1.000	0.3278
207447_s_at	14.1	5.1	-1.46	-2.75	AAA-AAA	0.00	999	1.000	1.000	0.3071
232649_at	37.1	101.7	1.46	2.75	AAA-AAA	0.00	999	1.000	1.000	0.3010
242226_at	28.8	78.8	1.46	2.74	AAA-AAA	0.00	999	1.000	1.000	0.2709
1561973_at	14.7	40.4	1.46	2.74	AAA-AAM	0.00	999	1.000	1.000	0.2969
232760_at	5.4	2.0	-1.46	-2.74	AAA-AAA	0.00	999	1.000	1.000	0.3850
231364_at	8.9	24.5	1.46	2.74	AAA-AAA	0.00	999	1.000	1.000	0.2917
231268_at	27.5	75.4	1.46	2.74	AAA-AAA	0.00	999	1.000	1.000	0.0164
239624_at	18.9	6.9	-1.45	-2.74	AAA-AAA	0.00	999	1.000	1.000	0.3020
1553938_a_at	12.3	33.7	1.45	2.74	AAA-AAA	0.00	999	1.000	1.000	0.3021
1555569_a_at	30.3	82.9	1.45	2.74	AAA-AAA	0.00	999	1.000	1.000	0.0622
242942_at	88.4	32.3	-1.45	-2.74	AAA-AAA	0.00	999	1.000	1.000	0.1155
208304_at	16.7	6.1	-1.45	-2.74	AAA-AAA	0.00	999	1.000	1.000	0.2132
231127_at	18.8	6.9	-1.45	-2.74	AAA-AAA	0.00	999	1.000	1.000	0.3773
231628_s_at	16.3	6.0	-1.45	-2.74	AAA-AAA	0.00	999	1.000	1.000	0.2572
1566709_at	55.6	20.3	-1.45	-2.74	AAP-AAA	0.00	999	1.000	1.000	0.2658
1557616_at	28.0	76.7	1.45	2.74	AAA-AAA	0.00	999	1.000	1.000	0.3244
1555444_a_at	29.1	10.6	-1.45	-2.74	AAA-AAA	0.00	999	1.000	1.000	0.4214
231340_at	6.1	16.6	1.45	2.74	AAA-AAA	0.00	999	1.000	1.000	0.2566
241033_at	51.9	19.0	-1.45	-2.74	PAP-MAA	0.00	999	1.000	1.000	0.3559
215872_at	7.6	20.8	1.45	2.74	AAA-AAA	0.00	999	1.000	1.000	0.3259
238339_x_at	70.5	25.8	-1.45	-2.74	AAA-AAA	0.00	999	1.000	1.000	0.1600

236442_at	53.6	19.6	-1.45	-2.74	AAA-AAA	0.00	999	1.000	1.000	0.1848
221880_s_at	14.7	40.2	1.45	2.74	AAA-AAA	0.00	999	1.000	1.000	0.2339
221862_at	29.2	79.8	1.45	2.74	AAA-AAA	0.00	999	1.000	1.000	0.3214
1561228_at	19.6	7.2	-1.45	-2.73	AAA-AAA	0.00	999	1.000	1.000	0.3617
244099_at	34.2	93.6	1.45	2.73	AAA-AAA	0.00	999	1.000	1.000	0.1348
215705_at	44.1	120.6	1.45	2.73	AAA-AAA	0.00	999	1.000	1.000	0.1746
216566_at	6.2	16.9	1.45	2.73	AAA-AAA	0.00	999	1.000	1.000	0.3731
216685_s_at	47.1	17.2	-1.45	-2.73	AAA-AAA	0.00	999	1.000	1.000	0.2859
206584_at	5.2	14.2	1.45	2.73	AAA-AAA	0.00	999	1.000	1.000	0.4030
203850_s_at	17.2	6.3	-1.45	-2.74	AAA-AAA	0.00	999	1.000	1.000	0.3928
207393_at	22.2	8.1	-1.45	-2.74	AAA-AAA	0.00	999	1.000	1.000	0.1676
237983_at	7.6	20.9	1.45	2.73	AAA-AAA	0.00	999	1.000	1.000	0.1556
236395_at	3.1	8.4	1.45	2.73	AAA-AAA	0.00	999	1.000	1.000	0.2296
223509_at	44.0	120.2	1.45	2.73	AAA-AAA	0.00	999	1.000	1.000	0.1056
231930_at	12.8	35.0	1.45	2.73	AAA-AAA	0.00	999	1.000	1.000	0.2352
1566032_at	81.3	29.7	-1.45	-2.73	APA-PAM	0.00	999	1.000	1.000	0.3980
1557565_a_at	24.3	8.9	-1.45	-2.73	AAA-AAA	0.00	999	1.000	1.000	0.2951
207497_s_at	19.2	52.6	1.45	2.73	AAA-AAA	0.00	999	1.000	1.000	0.1925
209557_s_at	32.6	89.1	1.45	2.73	AAA-AAA	0.00	999	1.000	1.000	0.0675
231692_at	7.2	19.6	1.45	2.73	AAA-AAA	0.00	999	1.000	1.000	0.3030
239351_at	5.5	15.0	1.45	2.73	AAA-AAA	0.00	999	1.000	1.000	0.2350
240639_at	19.5	7.1	-1.45	-2.73	AAA-AAA	0.00	999	1.000	1.000	0.0766
244392_at	4.7	12.8	1.45	2.73	AAA-AAA	0.00	999	1.000	1.000	0.0270
244667_at	34.4	93.9	1.45	2.73	AAA-AAA	0.00	999	1.000	1.000	0.0939
221609_s_at	47.7	17.5	-1.45	-2.73	AAA-AAA	0.00	999	1.000	1.000	0.2485
207500_at	62.7	23.0	-1.45	-2.73	AAA-AAA	0.00	999	1.000	1.000	0.0632
233711_at	4.0	10.9	1.45	2.73	AAA-AAA	0.00	999	1.000	1.000	0.3951
228342_s_at	12.2	33.3	1.45	2.73	AAA-AAA	0.00	999	1.000	1.000	0.1559
241299_at	4.5	12.2	1.45	2.73	AAA-AAA	0.00	999	1.000	1.000	0.3205
1569551_at	10.2	27.7	1.45	2.73	AAA-AAA	0.00	999	1.000	1.000	0.2493
1566447_at	48.9	17.9	-1.45	-2.73	AAA-AAA	0.00	999	1.000	1.000	0.0696
243107_at	55.5	20.3	-1.45	-2.73	AAA-AAA	0.00	999	1.000	1.000	0.0981
1562165_at	8.4	23.0	1.45	2.73	AAA-AAA	0.00	999	1.000	1.000	0.3397
1559812_at	15.5	42.2	1.45	2.73	AAA-AAA	0.00	999	1.000	1.000	0.2521
243785_at	35.7	13.1	-1.45	-2.73	AAA-AAA	0.00	999	1.000	1.000	0.2965
213559_s_at	12.1	32.9	1.45	2.73	AAA-AAA	0.00	999	1.000	1.000	0.3423
236179_at	10.5	3.8	-1.45	-2.73	AAA-AAA	0.00	999	1.000	1.000	0.4122
1554772_at	30.4	11.2	-1.45	-2.72	APA-AAA	0.00	999	1.000	1.000	0.4172
241427_x_at	30.7	83.7	1.45	2.72	AAA-AAA	0.00	999	1.000	1.000	0.1342
241087_at	28.8	78.4	1.45	2.73	AAA-AAA	0.00	999	1.000	1.000	0.3900
1566772_at	32.0	11.8	-1.45	-2.73	AAA-AAA	0.00	999	1.000	1.000	0.2844
244048_x_at	3.1	8.4	1.45	2.73	AAA-AAA	0.00	999	1.000	1.000	0.5220

215105_at	28.8	78.4	1.45	2.73	AAA-AAA	0.00	999	1.000	1.000	0.1975
208493_at	12.3	4.5	-1.45	-2.73	AAA-AAA	0.00	999	1.000	1.000	0.1561
233863_at	10.8	29.5	1.45	2.73	AAA-AAA	0.00	999	1.000	1.000	0.2246
229479_at	12.0	4.4	-1.45	-2.72	AAA-AAA	0.00	999	1.000	1.000	0.2861
1562785_at	12.6	34.4	1.45	2.72	AAA-AAA	0.00	999	1.000	1.000	0.2180
239156_at	22.9	62.4	1.45	2.72	AAA-AAA	0.00	999	1.000	1.000	0.3838
1567064_at	8.1	3.0	-1.45	-2.72	AAA-AAA	0.00	999	1.000	1.000	0.3522
1567173_at	3.9	10.6	1.45	2.72	AAA-AAA	0.00	999	1.000	1.000	0.4379
1554158_at	67.2	24.7	-1.45	-2.72	AAA-AAA	0.00	999	1.000	1.000	0.1184
220540_at	3.0	8.2	1.45	2.72	AAA-AAA	0.00	999	1.000	1.000	0.3877
219379_x_at	39.0	106.1	1.45	2.72	AAA-AAA	0.00	999	1.000	1.000	0.2050
235631_at	40.7	15.0	-1.45	-2.72	AAA-AAA	0.00	999	1.000	1.000	0.3056
226446_at	30.8	84.0	1.45	2.72	AAA-AAA	0.00	999	1.000	1.000	0.1254
241108_at	11.4	31.1	1.44	2.72	AAA-AAA	0.00	999	1.000	1.000	0.3363
214418_at	15.0	5.5	-1.44	-2.72	AAA-AAA	0.00	999	1.000	1.000	0.0773
221444_at	13.6	36.9	1.44	2.72	AAA-AAA	0.00	999	1.000	1.000	0.2040
206395_at	12.6	34.3	1.44	2.72	AAA-AAA	0.00	999	1.000	1.000	0.3801
205852_at	58.2	21.4	-1.44	-2.72	AAA-AAA	0.00	999	1.000	1.000	0.3069
205582_s_at	17.1	6.3	-1.44	-2.72	AAA-AAA	0.00	999	1.000	1.000	0.0052
224532_at	16.5	45.0	1.44	2.72	AAA-AAA	0.00	999	1.000	1.000	0.3806
242548_x_at	18.4	50.1	1.44	2.72	AAA-AAA	0.00	999	1.000	1.000	0.1417
1552971_at	2.8	7.6	1.44	2.72	AAA-AAA	0.00	999	1.000	1.000	0.1821
1553857_at	29.7	80.8	1.44	2.72	AAA-AAA	0.00	999	1.000	1.000	0.1340
206430_at	45.4	16.7	-1.44	-2.72	AAA-AAA	0.00	999	1.000	1.000	0.1150
AFFX-TrpnX-M_at	4.7	12.9	1.44	2.72	AAA-AAA	0.00	999	1.000	1.000	0.0709
231236_at	8.3	22.5	1.44	2.72	AAA-AAA	0.00	999	1.000	1.000	0.1799
239113_at	17.0	46.1	1.44	2.72	AAA-AAA	0.00	999	1.000	1.000	0.2617
1557825_at	11.7	31.9	1.44	2.72	AAA-AAA	0.00	999	1.000	1.000	0.3936
1555363_s_at	10.0	27.3	1.44	2.72	AAA-AAA	0.00	999	1.000	1.000	0.1342
1559879_a_at	11.0	29.9	1.44	2.72	AAA-AAA	0.00	999	1.000	1.000	0.3117
205182_s_at	36.2	98.4	1.44	2.72	AAA-AAA	0.00	999	1.000	1.000	0.2049
234279_at	5.5	14.9	1.44	2.72	AAA-AAA	0.00	999	1.000	1.000	0.0659
232919_at	30.7	83.5	1.44	2.72	AAA-AAA	0.00	999	1.000	1.000	0.1900
235877_at	11.1	30.2	1.44	2.72	AAA-AAA	0.00	999	1.000	1.000	0.0980
232250_at	13.0	35.4	1.44	2.72	AAA-AAA	0.00	999	1.000	1.000	0.3238
230467_at	76.2	28.1	-1.44	-2.72	AAA-AAA	0.00	999	1.000	1.000	0.2718
239257_at	11.2	30.4	1.44	2.72	AAA-AAA	0.00	999	1.000	1.000	0.2893
240530_at	63.1	23.2	-1.44	-2.72	AAA-AAA	0.00	999	1.000	1.000	0.3526
1558397_at	4.6	12.5	1.44	2.72	AAA-AAA	0.00	999	1.000	1.000	0.2730
1557900_at	81.8	30.1	-1.44	-2.71	AAA-AAA	0.00	999	1.000	1.000	0.0120
1561216_at	4.4	11.9	1.44	2.72	AAA-AAA	0.00	999	1.000	1.000	0.3534
1562308_at	30.2	11.1	-1.44	-2.72	AAA-AAA	0.00	999	1.000	1.000	0.1375

243901_at	12.3	4.5	-1.44	-2.72	AAA-AAA	0.00	999	1.000	1.000	0.3828
1560238_at	17.6	6.5	-1.44	-2.71	AAA-AAA	0.00	999	1.000	1.000	0.2101
228489_at	43.2	15.9	-1.44	-2.72	AAA-AAA	0.00	999	1.000	1.000	0.3120
1565846_at	18.0	6.6	-1.44	-2.71	AAA-AAA	0.00	999	1.000	1.000	0.2529
1557560_at	14.8	5.4	-1.44	-2.71	AAA-AAA	0.00	999	1.000	1.000	0.3794
236939_at	25.3	9.3	-1.44	-2.71	AAA-AAA	0.00	999	1.000	1.000	0.4947
1565601_at	10.3	27.9	1.44	2.71	AAA-AAA	0.00	999	1.000	1.000	0.1105
241337_at	26.6	9.8	-1.44	-2.71	AAA-AAA	0.00	999	1.000	1.000	0.3171
239673_at	25.6	9.4	-1.44	-2.71	AAA-AAA	0.00	999	1.000	1.000	0.2281
1557147_a_at	37.8	13.9	-1.44	-2.71	AAA-AAA	0.00	999	1.000	1.000	0.0882
1561985_at	5.3	14.3	1.44	2.71	AAA-AAA	0.00	999	1.000	1.000	0.4679
237474_at	9.5	25.8	1.44	2.71	AAA-AAA	0.00	999	1.000	1.000	0.2122
231560_at	7.7	20.9	1.44	2.71	AAA-AAA	0.00	999	1.000	1.000	0.3884
231206_at	10.2	27.6	1.44	2.71	AAA-AAA	0.00	999	1.000	1.000	0.3628
242320_at	81.6	30.1	-1.44	-2.71	AAA-AAA	0.00	999	1.000	1.000	0.0510
1567252_at	17.8	48.3	1.44	2.71	AAA-AAA	0.00	999	1.000	1.000	0.3095
1555314_at	19.1	7.1	-1.44	-2.71	AAA-AAA	0.00	999	1.000	1.000	0.3032
243144_at	9.3	25.1	1.44	2.71	AAA-AAA	0.00	999	1.000	1.000	0.1507
1561538_at	49.7	134.6	1.44	2.71	AAA-AAA	0.00	999	1.000	1.000	0.1528
1561564_at	1.6	4.3	1.44	2.71	AAA-AAA	0.00	999	1.000	1.000	0.3159
243691_at	13.1	35.4	1.44	2.71	AAA-AAA	0.00	999	1.000	1.000	0.0899
215831_at	3.1	8.3	1.44	2.71	AAA-AAA	0.00	999	1.000	1.000	0.1681
220168_at	12.1	32.7	1.44	2.71	AAA-AAM	0.00	999	1.000	1.000	0.3392
234083_at	39.7	14.7	-1.44	-2.71	AAA-AAA	0.00	999	1.000	1.000	0.2195
232494_at	18.5	50.2	1.44	2.71	AAA-AAA	0.00	999	1.000	1.000	0.2202
1561467_at	46.0	17.0	-1.44	-2.71	PAA-AAA	0.00	999	1.000	1.000	0.3830
1566158_at	5.5	14.9	1.44	2.71	AAA-AAA	0.00	999	1.000	1.000	0.2686
1557353_at	33.3	90.3	1.44	2.71	MAA-AAA	0.00	999	1.000	1.000	0.2179
1561320_at	55.6	20.5	-1.44	-2.71	MAA-AAA	0.00	999	1.000	1.000	0.0202
1558888_x_at	26.0	9.6	-1.44	-2.71	AAA-AAA	0.00	999	1.000	1.000	0.2348
1560590_s_at	41.8	15.4	-1.44	-2.71	AAA-AAA	0.00	999	1.000	1.000	0.1880
214586_at	7.5	20.3	1.44	2.71	AAA-AAA	0.00	999	1.000	1.000	0.2259
207047_s_at	39.7	14.7	-1.44	-2.71	AAA-AAA	0.00	999	1.000	1.000	0.2271
209721_s_at	30.7	83.2	1.44	2.71	AAA-AAA	0.00	999	1.000	1.000	0.1389
239368_at	54.3	147.1	1.44	2.71	AAA-AAA	0.00	999	1.000	1.000	0.1674
213250_at	27.1	73.3	1.44	2.71	AAA-AAA	0.00	999	1.000	1.000	0.2207
209775_x_at	13.3	36.1	1.44	2.71	AAA-AAA	0.00	999	1.000	1.000	0.1388
208068_x_at	103.9	38.4	-1.44	-2.71	AAA-AAA	0.00	999	1.000	1.000	0.1200
234452_at	85.8	31.7	-1.44	-2.71	AAA-AAA	0.00	999	1.000	1.000	0.1725
224115_at	33.1	12.2	-1.44	-2.71	AAA-AAA	0.00	999	1.000	1.000	0.3389
231227_at	13.8	5.1	-1.44	-2.71	AAA-AAA	0.00	999	1.000	1.000	0.4215
1569824_at	24.2	8.9	-1.44	-2.70	AAA-AAA	0.00	999	1.000	1.000	0.2864

235916_at	30.5	11.3	-1.44	-2.70	AAA-AAA	0.00	999	1.000	1.000	0.3193
231307_at	4.9	13.4	1.44	2.70	AAA-AAA	0.00	999	1.000	1.000	0.2610
1560405_at	94.0	34.8	-1.43	-2.70	AAP-PAA	0.00	999	1.000	1.000	0.1924
207220_at	46.7	17.3	-1.43	-2.70	AAP-AAA	0.00	999	1.000	1.000	0.2249
205502_at	51.0	18.9	-1.43	-2.70	AAA-AAA	0.00	999	1.000	1.000	0.2220
1563026_at	6.4	2.4	-1.43	-2.70	AAA-AAA	0.00	999	1.000	1.000	0.3329
1562189_at	19.9	7.4	-1.43	-2.70	AAA-AAA	0.00	999	1.000	1.000	0.3840
220422_at	55.6	20.6	-1.43	-2.70	AAA-AAA	0.00	999	1.000	1.000	0.0852
207026_s_at	16.4	44.2	1.43	2.70	AAA-AAA	0.00	999	1.000	1.000	0.0629
221128_at	114.9	42.6	-1.43	-2.70	PPA-AAP	0.00	999	1.000	1.000	0.1091
219898_at	39.1	14.5	-1.43	-2.70	AAA-PAA	0.00	999	1.000	1.000	0.3546
239051_at	21.4	57.8	1.43	2.70	AAA-AAA	0.00	999	1.000	1.000	0.0267
1556004_at	4.8	12.9	1.43	2.70	AAA-AAA	0.00	999	1.000	1.000	0.0577
1560683_at	15.7	5.8	-1.43	-2.70	AAA-AAA	0.00	999	1.000	1.000	0.3040
210226_at	140.5	52.1	-1.43	-2.70	AAA-AAA	0.00	999	1.000	1.000	0.1578
224379_at	15.0	5.6	-1.43	-2.70	AAA-AAA	0.00	999	1.000	1.000	0.3448
237829_at	60.8	22.6	-1.43	-2.70	APA-AAA	0.00	999	1.000	1.000	0.0454
239039_at	12.3	33.2	1.43	2.70	AAA-AAA	0.00	999	1.000	1.000	0.0779
1569528_at	14.1	5.2	-1.43	-2.70	AAA-AAA	0.00	999	1.000	1.000	0.4632
1566030_at	6.0	16.2	1.43	2.70	AAA-AAA	0.00	999	1.000	1.000	0.1431
1559763_at	35.2	13.1	-1.43	-2.70	AAA-AAA	0.00	999	1.000	1.000	0.4305
217571_at	8.1	21.8	1.43	2.70	AAA-AAA	0.00	999	1.000	1.000	0.3904
234408_at	7.0	18.9	1.43	2.70	AAA-AAA	0.00	999	1.000	1.000	0.2667
228058_at	82.7	30.7	-1.43	-2.69	APA-AAA	0.00	999	1.000	1.000	0.2579
1562924_at	29.5	11.0	-1.43	-2.69	AAA-AAA	0.00	999	1.000	1.000	0.2455
1559148_at	91.7	34.1	-1.43	-2.69	AAA-AAA	0.00	999	1.000	1.000	0.1984
215940_at	13.9	5.2	-1.43	-2.69	AAA-AAA	0.00	999	1.000	1.000	0.3924
210940_s_at	4.9	13.3	1.43	2.70	AAA-AAA	0.00	999	1.000	1.000	0.1672
235884_at	25.4	68.5	1.43	2.70	AAA-AAA	0.00	999	1.000	1.000	0.0911
227028_s_at	87.8	32.6	-1.43	-2.69	AAA-AAA	0.00	999	1.000	1.000	0.0932
223558_at	301.7	112.1	-1.43	-2.69	AAA-AAP	0.00	999	1.000	1.000	0.2458
1569858_at	30.6	11.4	-1.43	-2.69	AAA-AAA	0.00	999	1.000	1.000	0.3229
239833_at	20.5	55.1	1.43	2.69	AAA-AAA	0.00	999	1.000	1.000	0.3531
1552807_a_at	16.9	45.6	1.43	2.69	AAA-AAA	0.00	999	1.000	1.000	0.1809
243145_at	11.1	29.9	1.43	2.69	AAA-AAA	0.00	999	1.000	1.000	0.2437
243869_at	64.6	24.0	-1.43	-2.69	AAA-AAA	0.00	999	1.000	1.000	0.1948
214981_at	2.7	7.3	1.43	2.69	AAA-AAA	0.00	999	1.000	1.000	0.4519
220314_at	35.7	13.3	-1.43	-2.69	AAA-AAA	0.00	999	1.000	1.000	0.3247
206655_s_at	4.3	11.6	1.43	2.69	AAA-AAA	0.00	999	1.000	1.000	0.0636
234456_at	53.9	145.0	1.43	2.69	AAA-AAA	0.00	999	1.000	1.000	0.0361
236988_x_at	10.4	28.1	1.43	2.69	AAA-AAA	0.00	999	1.000	1.000	0.2405
1562310_at	3.1	8.4	1.43	2.69	AAA-AAA	0.00	999	1.000	1.000	0.5440

221111_at	5.2	14.0	1.43	2.69	AAA-AAA	0.00	999	1.000	1.000	0.4162
207412_x_at	6.1	16.5	1.43	2.69	AAA-AAA	0.00	999	1.000	1.000	0.0078
209047_at	19.1	7.1	-1.43	-2.69	AAA-AAA	0.00	999	1.000	1.000	0.1524
241622_at	3.2	8.6	1.43	2.69	AAA-AAA	0.00	999	1.000	1.000	0.2309
1566433_at	10.4	28.1	1.43	2.69	AAA-AAA	0.00	999	1.000	1.000	0.1874
1558777_at	12.5	33.6	1.43	2.69	AAA-AAA	0.00	999	1.000	1.000	0.2425
1553394_a_at	58.3	21.7	-1.43	-2.69	MAA-AAA	0.00	999	1.000	1.000	0.1377
1553851_at	13.3	5.0	-1.43	-2.69	AAA-AAA	0.00	999	1.000	1.000	0.3280
1562245_a_at	7.7	2.9	-1.43	-2.69	AAA-AAA	0.00	999	1.000	1.000	0.4483
1559533_at	47.2	17.6	-1.43	-2.69	AAA-AAA	0.00	999	1.000	1.000	0.1011
244200_at	112.7	41.9	-1.43	-2.69	AAA-AAA	0.00	999	1.000	1.000	0.1941
221076_at	23.0	8.6	-1.43	-2.69	AAA-AAA	0.00	999	1.000	1.000	0.2066
205599_at	19.6	52.8	1.43	2.69	AAA-AAA	0.00	999	1.000	1.000	0.2634
1566440_at	17.0	45.7	1.43	2.69	AAA-AAA	0.00	999	1.000	1.000	0.3564
243836_at	15.5	5.8	-1.43	-2.69	AAA-AAA	0.00	999	1.000	1.000	0.1854
244061_at	6.4	17.2	1.43	2.69	AAA-AAA	0.00	999	1.000	1.000	0.2751
215902_at	13.6	36.4	1.43	2.69	AAA-AAA	0.00	999	1.000	1.000	0.3997
234130_at	25.5	9.5	-1.43	-2.69	AAA-AAA	0.00	999	1.000	1.000	0.1887
231315_at	17.8	6.6	-1.43	-2.69	AAA-AAA	0.00	999	1.000	1.000	0.1353
229195_at	36.5	98.0	1.43	2.69	AAA-AAA	0.00	999	1.000	1.000	0.1929
1566461_at	4.4	11.7	1.43	2.69	AAA-AAA	0.00	999	1.000	1.000	0.2674
214287_s_at	20.8	55.9	1.43	2.69	AAA-AAA	0.00	999	1.000	1.000	0.2840
206502_s_at	4.6	12.2	1.43	2.69	AAA-AAA	0.00	999	1.000	1.000	0.4331
205753_at	4.8	12.9	1.43	2.69	AAA-AAA	0.00	999	1.000	1.000	0.1879
233944_at	5.2	14.0	1.43	2.68	AAA-AAA	0.00	999	1.000	1.000	0.3127
223901_at	28.9	10.8	-1.43	-2.69	AAA-AAA	0.00	999	1.000	1.000	0.0943
229600_s_at	27.7	10.3	-1.43	-2.69	AAA-AAA	0.00	999	1.000	1.000	0.2757
229761_at	12.3	32.9	1.43	2.69	AAA-AAA	0.00	999	1.000	1.000	0.1641
240693_at	4.5	12.0	1.42	2.68	AAA-AAA	0.00	999	1.000	1.000	0.2816
214608_s_at	12.1	32.5	1.42	2.68	AAA-AAA	0.00	999	1.000	1.000	0.2638
207820_at	22.5	8.4	-1.42	-2.68	AAA-AAA	0.00	999	1.000	1.000	0.3257
207787_at	16.3	6.1	-1.42	-2.68	AAA-AAA	0.00	999	1.000	1.000	0.1416
241325_at	66.1	24.7	-1.42	-2.68	AAA-AAA	0.00	999	1.000	1.000	0.2205
1563015_at	9.2	24.8	1.42	2.68	AAA-AAA	0.00	999	1.000	1.000	0.0723
239325_at	32.8	12.2	-1.42	-2.68	AAA-AAA	0.00	999	1.000	1.000	0.3519
1552718_at	42.9	16.0	-1.42	-2.68	AAA-AAA	0.00	999	1.000	1.000	0.2950
1560750_at	10.3	27.6	1.42	2.68	AAA-AAA	0.00	999	1.000	1.000	0.0982
215232_at	46.0	123.2	1.42	2.68	MAA-AAA	0.00	999	1.000	1.000	0.2161
216819_at	10.3	3.8	-1.42	-2.68	AAA-AAA	0.00	999	1.000	1.000	0.2509
211915_s_at	20.2	7.5	-1.42	-2.68	AAA-AAA	0.00	999	1.000	1.000	0.1192
217562_at	5.9	15.8	1.42	2.68	AAA-AAA	0.00	999	1.000	1.000	0.3947
205589_at	30.8	82.6	1.42	2.68	AAA-AAA	0.00	999	1.000	1.000	0.3832

229780_at	49.9	133.8	1.42	2.68	AAA-AAA	0.00	999	1.000	1.000	0.1007
1553894_at	45.6	17.0	-1.42	-2.68	AAA-AAP	0.00	999	1.000	1.000	0.3747
241970_at	16.1	43.1	1.42	2.68	MAA-AAA	0.00	999	1.000	1.000	0.1946
239037_at	30.0	11.2	-1.42	-2.68	AAA-AAA	0.00	999	1.000	1.000	0.2143
1554383_a_at	12.3	33.1	1.42	2.68	AAA-AAA	0.00	999	1.000	1.000	0.2725
1561012_at	8.7	23.3	1.42	2.68	AAA-AAA	0.00	999	1.000	1.000	0.2873
217417_at	6.4	17.0	1.42	2.68	AAA-AAA	0.00	999	1.000	1.000	0.0815
219902_at	21.1	56.6	1.42	2.68	AAA-AAA	0.00	999	1.000	1.000	0.0677
207589_at	11.6	31.1	1.42	2.68	AAA-AAA	0.00	999	1.000	1.000	0.1407
234245_at	15.1	40.5	1.42	2.68	AAA-AAA	0.00	999	1.000	1.000	0.2188
243580_at	11.3	30.2	1.42	2.68	AAA-AAA	0.00	999	1.000	1.000	0.2460
1562022_s_at	84.2	225.5	1.42	2.68	AAA-AAA	0.00	999	1.000	1.000	0.1202
1561897_at	29.9	80.1	1.42	2.68	AAA-AAA	0.00	999	1.000	1.000	0.1303
1560013_at	35.9	96.1	1.42	2.68	AAA-AAA	0.00	999	1.000	1.000	0.2678
206475_x_at	12.5	33.4	1.42	2.68	AAA-AAA	0.00	999	1.000	1.000	0.2871
211462_s_at	16.4	44.0	1.42	2.68	AAA-AAA	0.00	999	1.000	1.000	0.2648
234559_at	26.0	9.7	-1.42	-2.68	AAA-AAA	0.00	999	1.000	1.000	0.3705
237878_at	54.0	144.5	1.42	2.68	AAA-AAA	0.00	999	1.000	1.000	0.0672
231963_at	230.9	86.2	-1.42	-2.68	AAA-AAA	0.00	999	1.000	1.000	0.4053
230418_s_at	8.7	23.2	1.42	2.68	AAA-AAA	0.00	999	1.000	1.000	0.2871
218925_s_at	36.2	13.5	-1.42	-2.68	APP-AAA	0.00	999	1.000	1.000	0.2079
1561099_at	21.3	8.0	-1.42	-2.68	AAA-AAA	0.00	999	1.000	1.000	0.3754
205751_at	5.4	2.0	-1.42	-2.68	AAA-AAA	0.00	999	1.000	1.000	0.0520
233094_at	7.4	19.7	1.42	2.68	AAA-AAA	0.00	999	1.000	1.000	0.4609
233259_at	19.7	7.3	-1.42	-2.68	AAA-AAA	0.00	999	1.000	1.000	0.2796
233682_at	12.7	4.8	-1.42	-2.68	AAA-AAA	0.00	999	1.000	1.000	0.4016
227343_at	30.9	11.6	-1.42	-2.68	AAA-AAA	0.00	999	1.000	1.000	0.1501
1561121_at	37.7	14.1	-1.42	-2.68	AAA-AAA	0.00	999	1.000	1.000	0.3515
214604_at	44.1	16.5	-1.42	-2.67	AAA-AAA	0.00	999	1.000	1.000	0.2403
214704_at	22.3	8.4	-1.42	-2.68	AAA-AAA	0.00	999	1.000	1.000	0.1257
207139_at	18.1	48.3	1.42	2.68	AAA-AAA	0.00	999	1.000	1.000	0.3349
207935_s_at	38.1	101.7	1.42	2.67	AAA-AAA	0.00	999	1.000	1.000	0.3052
233994_at	11.7	31.2	1.42	2.68	AAA-AAA	0.00	999	1.000	1.000	0.2417
222888_at	24.5	9.2	-1.42	-2.68	AAA-AAA	0.00	999	1.000	1.000	0.1416
230093_at	39.5	14.8	-1.42	-2.67	AAA-AAA	0.00	999	1.000	1.000	0.3764
241216_at	41.6	15.6	-1.42	-2.67	AAA-AAP	0.00	999	1.000	1.000	0.2354
241311_at	8.0	21.3	1.42	2.67	AAA-AAA	0.00	999	1.000	1.000	0.3175
241841_at	36.4	97.2	1.42	2.67	AAA-AAA	0.00	999	1.000	1.000	0.0659
240998_at	19.8	52.9	1.42	2.67	AAA-AAA	0.00	999	1.000	1.000	0.2559
1557402_at	14.8	5.6	-1.42	-2.67	AAA-AAA	0.00	999	1.000	1.000	0.2819
1553920_at	6.8	18.1	1.42	2.67	AAA-AAA	0.00	999	1.000	1.000	0.4100
243674_at	49.9	18.7	-1.42	-2.67	AAA-AAA	0.00	999	1.000	1.000	0.1172

216702_x_at	23.3	62.3	1.42	2.67	AAA-AAA	0.00	999	1.000	1.000	0.1399
217259_at	13.9	37.2	1.42	2.67	AAA-AAA	0.00	999	1.000	1.000	0.3249
204945_at	26.2	69.9	1.42	2.67	AAA-AAA	0.00	999	1.000	1.000	0.2812
228644_s_at	64.8	24.3	-1.42	-2.67	AAA-AAA	0.00	999	1.000	1.000	0.2068
220565_at	30.9	11.6	-1.42	-2.67	AAA-AAA	0.00	999	1.000	1.000	0.2985
236454_at	14.4	38.5	1.42	2.67	AAA-AAA	0.00	999	1.000	1.000	0.0862
228682_at	35.8	13.4	-1.42	-2.67	AAA-AAA	0.00	999	1.000	1.000	0.1501
231174_s_at	28.1	10.5	-1.42	-2.67	APA-AAA	0.00	999	1.000	1.000	0.3112
1554634_at	30.5	11.4	-1.42	-2.67	AAP-AAA	0.00	999	1.000	1.000	0.4174
241854_at	81.5	30.6	-1.42	-2.67	AAA-AAA	0.00	999	1.000	1.000	0.0564
1568717_a_at	71.6	26.8	-1.42	-2.67	AAA-AAA	0.00	999	1.000	1.000	0.1739
217422_s_at	4.6	12.2	1.42	2.67	AAA-AAA	0.00	999	1.000	1.000	0.0032
217023_x_at	60.7	22.8	-1.42	-2.67	AAA-AAA	0.00	999	1.000	1.000	0.2093
1562079_at	12.9	4.9	-1.42	-2.67	APA-AAA	0.00	999	1.000	1.000	0.4623
1563052_at	12.3	4.6	-1.42	-2.67	AAA-AAA	0.00	999	1.000	1.000	0.5574
238954_at	8.1	21.6	1.42	2.67	AAA-AAA	0.00	999	1.000	1.000	0.2639
1552319_a_at	25.5	67.9	1.42	2.67	AAA-AAA	0.00	999	1.000	1.000	0.2608
1554697_at	7.5	20.1	1.42	2.67	AAA-AAA	0.00	999	1.000	1.000	0.1609
1555800_at	48.9	18.3	-1.42	-2.67	AAA-AAA	0.00	999	1.000	1.000	0.1755
1555655_at	6.7	18.0	1.42	2.67	AAA-AAA	0.00	999	1.000	1.000	0.3361
217153_at	81.8	30.7	-1.42	-2.67	AAA-AAA	0.00	999	1.000	1.000	0.1434
210546_x_at	16.7	6.3	-1.42	-2.67	AAA-AAA	0.00	999	1.000	1.000	0.1901
1562249_at	14.5	5.4	-1.41	-2.67	AAA-APA	0.00	999	1.000	1.000	0.5555
239136_at	8.2	21.8	1.41	2.66	AAA-AAA	0.00	999	1.000	1.000	0.0608
1555102_at	11.0	4.1	-1.41	-2.67	AAA-AAA	0.00	999	1.000	1.000	0.5116
243979_at	2.0	5.3	1.41	2.67	AAA-AAA	0.00	999	1.000	1.000	0.0916
214354_x_at	169.0	63.4	-1.41	-2.67	AAA-AAA	0.00	999	1.000	1.000	0.1508
206517_at	5.6	14.8	1.41	2.66	AAA-AAA	0.00	999	1.000	1.000	0.1100
234411_x_at	66.0	24.8	-1.41	-2.66	AAA-AAA	0.00	999	1.000	1.000	0.0545
236364_at	64.9	24.4	-1.41	-2.67	AAA-AAA	0.00	999	1.000	1.000	0.1447
235998_at	81.1	30.4	-1.41	-2.66	AAA-AAA	0.00	999	1.000	1.000	0.0608
226811_at	17.2	45.9	1.41	2.66	AAA-AAA	0.00	999	1.000	1.000	0.1804
230410_at	15.6	41.5	1.41	2.66	AAA-AAA	0.00	999	1.000	1.000	0.1201
1564314_at	11.7	31.2	1.41	2.66	AAA-AAA	0.00	999	1.000	1.000	0.3529
1569942_at	20.5	7.7	-1.41	-2.66	AAA-AAA	0.00	999	1.000	1.000	0.3324
1555585_a_at	7.0	18.7	1.41	2.66	AAA-AAA	0.00	999	1.000	1.000	0.0382
206154_at	58.9	22.1	-1.41	-2.66	AAA-AAA	0.00	999	1.000	1.000	0.3688
204424_s_at	15.2	40.6	1.41	2.66	AAA-AAA	0.00	999	1.000	1.000	0.3855
235859_at	15.9	42.3	1.41	2.66	AAA-AAA	0.00	999	1.000	1.000	0.2413
229972_at	11.8	4.4	-1.41	-2.66	AAA-AAA	0.00	999	1.000	1.000	0.1182
1558653_at	11.9	31.8	1.41	2.66	AAA-AAA	0.00	999	1.000	1.000	0.2615
216667_at	16.0	42.5	1.41	2.66	AAA-AAA	0.00	999	1.000	1.000	0.0654

220434_at	10.2	27.2	1.41	2.66	AAA-AAA	0.00	999	1.000	1.000	0.2890
238441_at	7.2	19.0	1.41	2.66	AAA-AAA	0.00	999	1.000	1.000	0.3800
229302_at	31.2	11.7	-1.41	-2.66	AAA-AAA	0.00	999	1.000	1.000	0.3850
1564463_at	27.6	73.5	1.41	2.66	AAA-AAA	0.00	999	1.000	1.000	0.1942
243503_at	38.9	14.6	-1.41	-2.66	AAA-AAA	0.00	999	1.000	1.000	0.1812
211914_x_at	33.6	89.4	1.41	2.66	AAA-AAA	0.00	999	1.000	1.000	0.0305
221394_at	42.7	16.1	-1.41	-2.66	AAA-AAA	0.00	999	1.000	1.000	0.1810
207923_x_at	61.9	23.3	-1.41	-2.66	AAA-AAA	0.00	999	1.000	1.000	0.1985
236691_at	20.4	7.7	-1.41	-2.66	AAA-AAA	0.00	999	1.000	1.000	0.2086
229918_at	16.8	44.7	1.41	2.66	AAA-AAA	0.00	999	1.000	1.000	0.1231
216094_at	23.4	62.1	1.41	2.66	AAA-AAA	0.00	999	1.000	1.000	0.3878
207161_at	30.7	11.5	-1.41	-2.66	AMA-AAA	0.00	999	1.000	1.000	0.2099
234041_at	41.4	15.6	-1.41	-2.66	AAA-AAA	0.00	999	1.000	1.000	0.3212
229848_at	25.9	9.8	-1.41	-2.66	AAA-AAA	0.00	999	1.000	1.000	0.1136
1553936_a_at	7.3	19.3	1.41	2.66	AAA-AAA	0.00	999	1.000	1.000	0.2324
1564291_at	6.0	15.9	1.41	2.66	AAA-AAA	0.00	999	1.000	1.000	0.0996
1570302_at	28.6	10.8	-1.41	-2.65	AAA-AAA	0.00	999	1.000	1.000	0.4170
1563776_at	9.6	3.6	-1.41	-2.65	AAA-AAA	0.00	999	1.000	1.000	0.0501
240102_at	19.5	51.9	1.41	2.65	AAA-AAA	0.00	999	1.000	1.000	0.1805
1556671_s_at	37.1	14.0	-1.41	-2.65	AAA-AAA	0.00	999	1.000	1.000	0.0818
1555705_a_at	112.4	42.4	-1.41	-2.65	AAA-AAA	0.00	999	1.000	1.000	0.0578
219310_at	13.0	34.5	1.41	2.65	AAA-AAA	0.00	999	1.000	1.000	0.2349
206849_at	30.2	11.4	-1.41	-2.65	AAA-AAA	0.00	999	1.000	1.000	0.5241
224146_s_at	5.0	13.2	1.41	2.65	AAA-AAA	0.00	999	1.000	1.000	0.2315
241161_at	13.3	35.4	1.41	2.65	AAA-AAA	0.00	999	1.000	1.000	0.2367
241758_at	22.4	59.3	1.41	2.65	AAA-AAA	0.00	999	1.000	1.000	0.1043
240485_at	26.0	9.8	-1.41	-2.65	AAA-AAA	0.00	999	1.000	1.000	0.2910
1557623_at	30.8	11.6	-1.41	-2.65	AAA-AAA	0.00	999	1.000	1.000	0.3902
213257_at	44.3	16.7	-1.41	-2.65	AAA-AAA	0.00	999	1.000	1.000	0.3645
220471_s_at	46.2	17.4	-1.41	-2.65	AAA-AAA	0.00	999	1.000	1.000	0.2696
227657_at	64.0	24.1	-1.41	-2.65	AAA-AAA	0.00	999	1.000	1.000	0.1076
1570291_at	9.1	24.2	1.41	2.65	AAA-AAA	0.00	999	1.000	1.000	0.2965
244784_at	23.7	62.9	1.41	2.65	AAA-AAA	0.00	999	1.000	1.000	0.0560
215039_at	29.4	78.0	1.41	2.65	AAA-AAA	0.00	999	1.000	1.000	0.3540
214223_at	30.2	80.0	1.41	2.65	AAA-AAA	0.00	999	1.000	1.000	0.3443
220878_at	3.0	8.1	1.41	2.65	AAA-AAA	0.00	999	1.000	1.000	0.3726
217701_x_at	77.4	29.2	-1.41	-2.65	AAA-AAA	0.00	999	1.000	1.000	0.1577
236739_at	6.4	16.8	1.41	2.65	AAA-AAA	0.00	999	1.000	1.000	0.4338
231689_at	11.4	30.1	1.41	2.65	AAA-AAA	0.00	999	1.000	1.000	0.1618
232015_at	33.8	12.8	-1.41	-2.65	AAA-AAA	0.00	999	1.000	1.000	0.1892
1569474_at	25.7	68.1	1.41	2.65	AAA-AAA	0.00	999	1.000	1.000	0.3553
1554533_at	21.2	8.0	-1.41	-2.65	AAA-AAA	0.00	999	1.000	1.000	0.0577

1555031_at	4.9	12.9	1.41	2.65	AAA-AAA	0.00	999	1.000	1.000	0.1761
220093_at	14.8	39.1	1.41	2.65	AAA-AAA	0.00	999	1.000	1.000	0.1690
AFFX-ThrX-5_at	25.3	9.5	-1.41	-2.65	AAA-AAA	0.00	999	1.000	1.000	0.2606
241891_at	19.9	7.5	-1.40	-2.65	AAA-AAA	0.00	999	1.000	1.000	0.3646
1566844_at	84.2	31.8	-1.40	-2.65	AAA-AAA	0.00	999	1.000	1.000	0.1879
1562142_at	8.3	22.0	1.40	2.65	AAA-AAA	0.00	999	1.000	1.000	0.2121
213854_at	36.1	95.5	1.40	2.65	AAA-AAA	0.00	999	1.000	1.000	0.0690
209552_at	62.9	23.8	-1.40	-2.65	AAA-AAA	0.00	999	1.000	1.000	0.3126
237742_at	18.8	49.7	1.40	2.65	AAA-AAA	0.00	999	1.000	1.000	0.2126
230987_at	37.0	98.0	1.40	2.65	AAA-AAA	0.00	999	1.000	1.000	0.1379
204199_at	48.1	18.2	-1.40	-2.65	AAA-AAA	0.00	999	1.000	1.000	0.0389
226992_at	39.6	15.0	-1.40	-2.65	AAA-AAA	0.00	999	1.000	1.000	0.1363
1569587_at	5.9	15.5	1.40	2.64	AAA-AAA	0.00	999	1.000	1.000	0.3640
216140_at	29.0	11.0	-1.40	-2.64	AAA-AAA	0.00	999	1.000	1.000	0.4733
215112_x_at	10.7	28.2	1.40	2.64	AAA-AAA	0.00	999	1.000	1.000	0.0796
217290_at	28.0	10.6	-1.40	-2.64	AAA-AAA	0.00	999	1.000	1.000	0.0611
206995_x_at	17.4	6.6	-1.40	-2.64	AAA-AAA	0.00	999	1.000	1.000	0.2512
231213_at	21.0	8.0	-1.40	-2.64	AAA-AAA	0.00	999	1.000	1.000	0.2991
1564192_at	10.7	4.1	-1.40	-2.64	AAA-AAA	0.00	999	1.000	1.000	0.1390
1563833_at	35.0	13.3	-1.40	-2.64	AAA-AAA	0.00	999	1.000	1.000	0.2653
241895_at	7.2	19.0	1.40	2.64	AAA-AAA	0.00	999	1.000	1.000	0.1785
214054_at	76.4	28.9	-1.40	-2.64	AAA-AAA	0.00	999	1.000	1.000	0.1217
215110_at	20.1	7.6	-1.40	-2.64	AAA-AAA	0.00	999	1.000	1.000	0.2703
220025_at	15.1	5.7	-1.40	-2.64	AAA-AAA	0.00	999	1.000	1.000	0.2008
207854_at	8.0	21.1	1.40	2.64	AAA-AAA	0.00	999	1.000	1.000	0.1616
208155_x_at	36.9	14.0	-1.40	-2.64	AAA-AAA	0.00	999	1.000	1.000	0.3801
237055_at	32.4	85.6	1.40	2.64	AAA-AAA	0.00	999	1.000	1.000	0.2110
1570145_at	59.1	22.4	-1.40	-2.64	AAA-AAA	0.00	999	1.000	1.000	0.2393
1555387_at	20.8	54.9	1.40	2.64	AAA-AAA	0.00	999	1.000	1.000	0.1144
243775_at	24.7	9.4	-1.40	-2.64	AAA-AAA	0.00	999	1.000	1.000	0.2166
210606_x_at	32.2	12.2	-1.40	-2.64	AAA-AAA	0.00	999	1.000	1.000	0.2113
230227_at	9.9	3.7	-1.40	-2.64	AAA-AAA	0.00	999	1.000	1.000	0.2214
240250_at	57.3	21.7	-1.40	-2.64	APA-PAA	0.00	999	1.000	1.000	0.2460
239501_at	24.0	9.1	-1.40	-2.64	AAA-AAA	0.00	999	1.000	1.000	0.3919
216782_at	17.3	6.6	-1.40	-2.64	AAA-AAA	0.00	999	1.000	1.000	0.2051
236130_at	49.1	18.6	-1.40	-2.64	AAA-AAA	0.00	999	1.000	1.000	0.3553
242762_s_at	32.2	12.2	-1.40	-2.64	MAA-AAA	0.00	999	1.000	1.000	0.3769
220678_at	11.6	30.6	1.40	2.64	AAA-AAA	0.00	999	1.000	1.000	0.0854
205790_at	24.0	63.2	1.40	2.64	AAA-AAA	0.00	999	1.000	1.000	0.2099
234522_at	17.2	45.3	1.40	2.64	AAA-AAA	0.00	999	1.000	1.000	0.1719
237579_at	5.7	14.9	1.40	2.64	AAA-AAA	0.00	999	1.000	1.000	0.0747
237196_at	12.9	4.9	-1.40	-2.64	AAA-AAA	0.00	999	1.000	1.000	0.2212

231409_at	12.8	33.8	1.40	2.64	AAA-AAA	0.00	999	1.000	1.000	0.4245
1570454_at	17.9	6.8	-1.40	-2.63	AAA-AAA	0.00	999	1.000	1.000	0.2707
239365_at	5.0	13.3	1.40	2.63	AAA-AAA	0.00	999	1.000	1.000	0.1428
1560007_at	18.8	49.4	1.40	2.63	AAA-AAA	0.00	999	1.000	1.000	0.2492
210437_at	34.9	13.2	-1.40	-2.63	AAA-AAA	0.00	999	1.000	1.000	0.1523
1556823_s_at	23.0	60.4	1.40	2.63	AAA-AAA	0.00	999	1.000	1.000	0.1669
1557495_at	28.6	10.9	-1.40	-2.63	AAA-AAA	0.00	999	1.000	1.000	0.0528
1562167_a_at	6.1	16.0	1.40	2.63	AAA-AAA	0.00	999	1.000	1.000	0.1528
219113_x_at	14.3	37.7	1.40	2.63	AAA-AAA	0.00	999	1.000	1.000	0.0745
210523_at	19.9	52.2	1.40	2.63	AAA-AAA	0.00	999	1.000	1.000	0.1201
1557673_at	83.7	31.8	-1.40	-2.63	AAA-AAA	0.00	999	1.000	1.000	0.1443
210565_at	15.7	6.0	-1.40	-2.63	AAA-AAA	0.00	999	1.000	1.000	0.2495
233768_at	43.9	16.7	-1.40	-2.63	AAA-AAA	0.00	999	1.000	1.000	0.0357
233661_at	33.2	12.6	-1.40	-2.63	AAA-AAA	0.00	999	1.000	1.000	0.2316
229977_at	100.9	38.4	-1.40	-2.63	AAA-AAA	0.00	999	1.000	1.000	0.1103
241207_at	9.8	25.6	1.39	2.63	AAA-AAA	0.00	999	1.000	1.000	0.3199
242267_x_at	63.8	24.3	-1.39	-2.63	AAA-AAA	0.00	999	1.000	1.000	0.2647
1561653_at	1.9	5.0	1.39	2.63	AAA-AAA	0.00	999	1.000	1.000	0.4572
230661_at	40.9	15.6	-1.39	-2.63	AAA-AAA	0.00	999	1.000	1.000	0.1765
1562873_at	9.7	25.5	1.39	2.63	AAA-AAA	0.00	999	1.000	1.000	0.4613
1568728_s_at	74.9	28.5	-1.39	-2.63	AAA-AAA	0.00	999	1.000	1.000	0.0458
244824_at	41.0	15.6	-1.39	-2.63	AAA-AAA	0.00	999	1.000	1.000	0.3932
1556224_a_at	63.9	24.3	-1.39	-2.63	AAA-AAA	0.00	999	1.000	1.000	0.1162
216307_at	5.3	13.9	1.39	2.63	AAA-AAA	0.00	999	1.000	1.000	0.3261
214425_at	101.5	38.6	-1.39	-2.63	AAA-AAA	0.00	999	1.000	1.000	0.1321
205285_s_at	17.9	47.1	1.39	2.63	AAA-AAA	0.00	999	1.000	1.000	0.1955
207745_at	48.4	18.4	-1.39	-2.63	AAA-AAA	0.00	999	1.000	1.000	0.0646
208162_s_at	10.8	28.4	1.39	2.63	AAA-AAA	0.00	999	1.000	1.000	0.3626
233207_at	17.6	46.3	1.39	2.63	AAA-AAA	0.00	999	1.000	1.000	0.2745
233517_s_at	20.5	7.8	-1.39	-2.63	AAA-AAA	0.00	999	1.000	1.000	0.0077
236276_at	16.0	6.1	-1.39	-2.63	AAA-AAA	0.00	999	1.000	1.000	0.1466
238869_at	87.5	33.4	-1.39	-2.62	AAP-AAP	0.00	999	1.000	1.000	0.3495
1564327_at	10.1	26.5	1.39	2.62	AAA-AAA	0.00	999	1.000	1.000	0.4016
242076_at	25.5	67.0	1.39	2.63	AAA-AAA	0.00	999	1.000	1.000	0.0856
213450_s_at	68.0	178.4	1.39	2.62	AAA-AAA	0.00	999	1.000	1.000	0.0314
219829_at	31.3	11.9	-1.39	-2.62	AAA-AAA	0.00	999	1.000	1.000	0.2255
241044_x_at	2.1	5.4	1.39	2.62	AAA-AAA	0.00	999	1.000	1.000	0.3435
1561988_at	44.7	17.1	-1.39	-2.62	AAA-AAA	0.00	999	1.000	1.000	0.1481
1559687_at	66.6	25.4	-1.39	-2.62	MAA-AAA	0.00	999	1.000	1.000	0.0981
217686_at	84.2	32.1	-1.39	-2.62	AAA-AAA	0.00	999	1.000	1.000	0.2587
202196_s_at	5.6	14.6	1.39	2.62	AAA-AAA	0.00	999	1.000	1.000	0.4193
211163_s_at	25.9	67.8	1.39	2.62	AAA-AAA	0.00	999	1.000	1.000	0.1164

237250_at	6.2	2.3	-1.39	-2.62	AAA-AAA	0.00	999	1.000	1.000	0.1477
1555725_a_at	55.2	21.1	-1.39	-2.62	AAA-APA	0.00	999	1.000	1.000	0.2360
219948_x_at	36.0	13.7	-1.39	-2.62	AAP-AAA	0.00	999	1.000	1.000	0.2668
240192_at	5.6	14.6	1.39	2.62	AAA-AAA	0.00	999	1.000	1.000	0.1519
217242_at	35.7	13.6	-1.39	-2.62	AAA-AAA	0.00	999	1.000	1.000	0.3851
202075_s_at	12.6	33.1	1.39	2.62	AAA-AAA	0.00	999	1.000	1.000	0.3713
231367_s_at	10.1	26.5	1.39	2.62	AAA-AAA	0.00	999	1.000	1.000	0.4251
231190_at	17.5	45.7	1.39	2.62	AAA-AAA	0.00	999	1.000	1.000	0.3403
231066_s_at	25.1	9.6	-1.39	-2.62	AAA-AAA	0.00	999	1.000	1.000	0.2884
1569673_at	41.9	16.0	-1.39	-2.62	AAP-PAA	0.00	999	1.000	1.000	0.3954
1552923_a_at	30.1	78.8	1.39	2.62	AAA-AAA	0.00	999	1.000	1.000	0.2309
1553435_at	8.1	21.2	1.39	2.62	AAA-AAA	0.00	999	1.000	1.000	0.2252
235576_at	30.7	80.5	1.39	2.62	AAA-AAM	0.00	999	1.000	1.000	0.2520
230218_at	4.5	11.7	1.39	2.62	AAA-AAA	0.00	999	1.000	1.000	0.2596
1570282_at	8.5	22.3	1.39	2.62	AAA-AAA	0.00	999	1.000	1.000	0.0480
1553093_a_at	6.1	16.0	1.39	2.62	AAA-AAA	0.00	999	1.000	1.000	0.2121
1556156_at	5.3	14.0	1.39	2.62	AAA-AAA	0.00	999	1.000	1.000	0.3358
242795_at	3.8	9.9	1.39	2.62	AAA-AAA	0.00	999	1.000	1.000	0.1890
1562010_x_at	70.2	26.8	-1.39	-2.62	AAA-AAA	0.00	999	1.000	1.000	0.2952
220818_s_at	4.6	12.0	1.39	2.62	AAA-AAA	0.00	999	1.000	1.000	0.0235
206067_s_at	24.9	9.5	-1.39	-2.62	AAA-AAA	0.00	999	1.000	1.000	0.0193
204714_s_at	109.1	41.7	-1.39	-2.62	AAA-AAA	0.00	999	1.000	1.000	0.1679
235468_at	19.1	7.3	-1.39	-2.62	AAA-AAA	0.00	999	1.000	1.000	0.1513
232791_at	23.9	9.1	-1.39	-2.62	AAA-AAA	0.00	999	1.000	1.000	0.3457
223540_at	36.8	96.2	1.39	2.62	AAA-AAA	0.00	999	1.000	1.000	0.2149
231242_at	11.1	29.1	1.39	2.62	AAA-AAA	0.00	999	1.000	1.000	0.2383
230111_at	8.7	22.8	1.39	2.62	AAA-AAA	0.00	999	1.000	1.000	0.3309
240423_at	31.8	12.2	-1.39	-2.62	AAA-AAA	0.00	999	1.000	1.000	0.3984
213160_at	14.5	5.6	-1.39	-2.62	AAA-AAA	0.00	999	1.000	1.000	0.2159
1555019_at	30.7	11.7	-1.39	-2.61	AAA-AAA	0.00	999	1.000	1.000	0.3091
243651_at	50.8	19.4	-1.39	-2.61	AAA-AAA	0.00	999	1.000	1.000	0.1883
214635_at	94.5	36.2	-1.39	-2.61	AAA-AAA	0.00	999	1.000	1.000	0.0398
217325_at	56.7	21.7	-1.39	-2.61	MAA-AAA	0.00	999	1.000	1.000	0.0332
218805_at	39.5	103.2	1.39	2.61	AAA-AAA	0.00	999	1.000	1.000	0.0766
244760_at	21.6	8.3	-1.39	-2.61	AAA-PAA	0.00	999	1.000	1.000	0.4026
1569721_at	21.8	8.3	-1.39	-2.61	AAA-AAA	0.00	999	1.000	1.000	0.3260
1557359_at	14.1	5.4	-1.39	-2.61	AAA-AAA	0.00	999	1.000	1.000	0.2609
1557639_at	12.9	33.7	1.39	2.61	AAA-AAA	0.00	999	1.000	1.000	0.3802
214438_at	14.7	38.4	1.39	2.61	AAA-AAA	0.00	999	1.000	1.000	0.4129
221108_at	29.1	11.1	-1.39	-2.61	AAA-AAA	0.00	999	1.000	1.000	0.0095
236558_at	10.4	27.1	1.39	2.61	AAA-AAA	0.00	999	1.000	1.000	0.3420
240338_at	47.1	18.1	-1.38	-2.61	PAA-AAP	0.00	999	1.000	1.000	0.2551

241107_at	40.4	15.5	-1.38	-2.61	AAA-AAA	0.00	999	1.000	1.000	0.3782
1560142_at	7.3	2.8	-1.38	-2.61	AAA-AAA	0.00	999	1.000	1.000	0.3184
214856_at	6.5	17.0	1.38	2.61	AAA-AAA	0.00	999	1.000	1.000	0.2331
220410_s_at	8.7	22.8	1.38	2.61	AAA-AAA	0.00	999	1.000	1.000	0.2028
206212_at	44.9	117.1	1.38	2.61	AAA-AAA	0.00	999	1.000	1.000	0.2803
204994_at	80.7	30.9	-1.38	-2.61	AAA-AAA	0.00	999	1.000	1.000	0.3550
1569434_at	75.6	29.0	-1.38	-2.61	AAP-AAA	0.00	999	1.000	1.000	0.3602
239340_at	13.9	36.2	1.38	2.61	AAA-AAA	0.00	999	1.000	1.000	0.2936
1562009_a_at	16.9	44.1	1.38	2.61	AAA-AAA	0.00	999	1.000	1.000	0.0935
216340_s_at	94.3	36.1	-1.38	-2.61	AAA-AAA	0.00	999	1.000	1.000	0.1156
214176_s_at	25.5	66.4	1.38	2.61	AAA-AAA	0.00	999	1.000	1.000	0.1498
234275_at	19.4	50.7	1.38	2.61	AAA-AAA	0.00	999	1.000	1.000	0.2590
234765_at	36.8	14.1	-1.38	-2.61	AAA-AAA	0.00	999	1.000	1.000	0.3828
239980_at	39.5	15.2	-1.38	-2.61	AAA-AAA	0.00	999	1.000	1.000	0.3361
241037_at	32.6	12.5	-1.38	-2.61	AAA-AAA	0.00	999	1.000	1.000	0.1947
1554825_at	68.7	26.4	-1.38	-2.61	AAA-AAA	0.00	999	1.000	1.000	0.2866
221075_s_at	6.1	15.8	1.38	2.61	AAA-AAA	0.00	999	1.000	1.000	0.1300
210184_at	10.0	26.0	1.38	2.61	AAA-AAA	0.00	999	1.000	1.000	0.2004
232691_at	15.7	6.0	-1.38	-2.61	AAA-AAA	0.00	999	1.000	1.000	0.3649
233416_at	27.6	10.6	-1.38	-2.61	AAA-AAA	0.00	999	1.000	1.000	0.1977
236039_at	13.4	35.0	1.38	2.61	AAA-AAA	0.00	999	1.000	1.000	0.2118
231508_s_at	97.4	37.4	-1.38	-2.61	AAA-AAA	0.00	999	1.000	1.000	0.1586
229629_at	14.8	38.7	1.38	2.61	AAA-AAA	0.00	999	1.000	1.000	0.3124
207402_at	50.9	19.6	-1.38	-2.60	PAA-AAA	0.00	999	1.000	1.000	0.0622
221176_x_at	157.1	60.4	-1.38	-2.60	APA-AAA	0.00	999	1.000	1.000	0.1174
1569064_at	14.5	5.6	-1.38	-2.61	AAA-AAA	0.00	999	1.000	1.000	0.2560
1568746_a_at	15.4	5.9	-1.38	-2.60	AAA-AAA	0.00	999	1.000	1.000	0.5091
1566181_at	15.2	39.7	1.38	2.61	AAA-AAA	0.00	999	1.000	1.000	0.2297
240864_at	27.8	10.7	-1.38	-2.61	AAA-AAA	0.00	999	1.000	1.000	0.2552
1560725_at	4.7	12.2	1.38	2.60	AAA-AAA	0.00	999	1.000	1.000	0.1921
234190_at	25.3	9.7	-1.38	-2.60	AAA-AAA	0.00	999	1.000	1.000	0.2065
224295_at	10.2	3.9	-1.38	-2.61	AAA-AAA	0.00	999	1.000	1.000	0.1412
206690_at	23.0	59.9	1.38	2.60	AAA-AAA	0.00	999	1.000	1.000	0.3666
231509_at	38.8	14.9	-1.38	-2.60	AAA-AAA	0.00	999	1.000	1.000	0.1234
232261_at	10.0	26.1	1.38	2.60	AAA-AAA	0.00	999	1.000	1.000	0.2530
242109_at	61.6	23.7	-1.38	-2.60	AAP-AAA	0.00	999	1.000	1.000	0.1174
1569193_at	20.5	7.9	-1.38	-2.60	AAA-AAA	0.00	999	1.000	1.000	0.0389
240966_at	6.8	17.8	1.38	2.60	AAA-AAA	0.00	999	1.000	1.000	0.1452
219831_at	104.5	40.2	-1.38	-2.60	AAA-AAA	0.00	999	1.000	1.000	0.0561
233244_at	20.4	53.1	1.38	2.60	AAA-AAA	0.00	999	1.000	1.000	0.1386
236447_at	54.6	21.0	-1.38	-2.60	AAA-AAA	0.00	999	1.000	1.000	0.2949
216217_at	57.4	22.1	-1.38	-2.60	AAP-AAA	0.00	999	1.000	1.000	0.1209

221030_s_at	57.0	21.9	-1.38	-2.60	AAP-AAA	0.00	999	1.000	1.000	0.2061
219054_at	44.8	17.2	-1.38	-2.60	AAP-PAA	0.00	999	1.000	1.000	0.2431
1563346_at	14.2	36.9	1.38	2.60	AAA-AAA	0.00	999	1.000	1.000	0.1745
229604_at	8.4	21.9	1.38	2.60	AAA-AAA	0.00	999	1.000	1.000	0.2962
243915_at	15.3	5.9	-1.38	-2.60	PAA-AAA	0.00	999	1.000	1.000	0.3447
242454_at	85.1	32.8	-1.38	-2.60	AAA-AAA	0.00	999	1.000	1.000	0.1927
1558062_at	34.6	13.3	-1.38	-2.60	AAA-AAA	0.00	999	1.000	1.000	0.2911
1562455_at	59.4	22.9	-1.38	-2.60	AAA-AAA	0.00	999	1.000	1.000	0.3161
237770_at	5.2	13.5	1.38	2.60	AAA-AAA	0.00	999	1.000	1.000	0.0883
236193_at	25.4	66.1	1.38	2.60	AAA-AAA	0.00	999	1.000	1.000	0.1358
237178_at	31.7	12.2	-1.38	-2.60	AAA-AAA	0.00	999	1.000	1.000	0.1143
239484_at	58.3	151.3	1.38	2.60	AAA-AAA	0.00	999	1.000	1.000	0.0395
239210_at	28.5	11.0	-1.38	-2.60	AAA-AAA	0.00	999	1.000	1.000	0.2246
1555385_at	16.7	43.4	1.38	2.60	AAA-AAA	0.00	999	1.000	1.000	0.4022
215801_at	40.1	15.5	-1.38	-2.60	AAA-AAA	0.00	999	1.000	1.000	0.2891
219708_at	73.7	28.4	-1.38	-2.60	AAA-AAA	0.00	999	1.000	1.000	0.2771
234350_at	12.8	33.3	1.38	2.60	AAA-AAA	0.00	999	1.000	1.000	0.4367
1569168_at	4.5	11.7	1.38	2.59	AAA-AAA	0.00	999	1.000	1.000	0.4950
1570246_at	3.9	10.2	1.38	2.59	AAA-AAA	0.00	999	1.000	1.000	0.3735
1552713_a_at	27.3	70.7	1.38	2.59	AAA-AAA	0.00	999	1.000	1.000	0.1243
1559634_at	11.4	29.5	1.38	2.59	AAA-AAA	0.00	999	1.000	1.000	0.4193
215695_s_at	22.0	8.5	-1.38	-2.59	MAA-AAA	0.00	999	1.000	1.000	0.2823
221003_s_at	81.3	31.3	-1.38	-2.59	AAA-AAA	0.00	999	1.000	1.000	0.2827
234238_at	24.1	9.3	-1.38	-2.59	AAA-AAA	0.00	999	1.000	1.000	0.1389
233157_x_at	13.9	5.4	-1.38	-2.59	AAA-AAA	0.00	999	1.000	1.000	0.3327
235951_s_at	9.2	23.9	1.38	2.59	AAA-AAA	0.00	999	1.000	1.000	0.0895
222022_at	86.1	33.2	-1.38	-2.59	AAA-AAA	0.00	999	1.000	1.000	0.1046
230511_at	92.3	35.6	-1.37	-2.59	PAA-AAA	0.00	999	1.000	1.000	0.1262
1563119_at	20.9	54.1	1.37	2.59	AAA-AAA	0.00	999	1.000	1.000	0.3435
239584_at	58.4	22.5	-1.37	-2.59	AAA-AAA	0.00	999	1.000	1.000	0.2035
1569811_at	48.5	18.7	-1.37	-2.59	AAM-AAA	0.00	999	1.000	1.000	0.1890
240652_at	10.3	4.0	-1.37	-2.59	AAA-AAA	0.00	999	1.000	1.000	0.2057
204468_s_at	6.8	2.6	-1.37	-2.59	AAA-AAA	0.00	999	1.000	1.000	0.0560
208231_at	14.0	36.2	1.37	2.59	AAA-AAA	0.00	999	1.000	1.000	0.1166
237651_x_at	10.0	3.8	-1.37	-2.59	AAA-AAA	0.00	999	1.000	1.000	0.1404
231218_at	20.4	52.8	1.37	2.59	AAA-AAA	0.00	999	1.000	1.000	0.1794
232388_at	68.1	26.3	-1.37	-2.59	AAA-AAA	0.00	999	1.000	1.000	0.3362
1562969_at	28.3	10.9	-1.37	-2.59	AAA-AAA	0.00	999	1.000	1.000	0.1731
1568921_at	9.6	24.9	1.37	2.59	AAA-AAA	0.00	999	1.000	1.000	0.0044
1562260_at	8.5	21.9	1.37	2.59	AAA-AAA	0.00	999	1.000	1.000	0.3209
243732_at	10.3	26.7	1.37	2.59	AAA-AAA	0.00	999	1.000	1.000	0.2653
1560109_s_at	4.1	10.5	1.37	2.59	AAA-AAA	0.00	999	1.000	1.000	0.1292

215013_s_at	56.9	22.0	-1.37	-2.59	AAA-AAA	0.00	999	1.000	1.000	0.2070
233791_at	45.7	17.6	-1.37	-2.59	AAA-AAA	0.00	999	1.000	1.000	0.0776
228585_at	6.3	16.3	1.37	2.59	AAA-AAA	0.00	999	1.000	1.000	0.3700
1555659_a_at	28.1	72.8	1.37	2.59	AAA-AAA	0.00	999	1.000	1.000	0.3113
1554923_at	12.2	4.7	-1.37	-2.59	AAA-AAA	0.00	999	1.000	1.000	0.2087
243239_at	7.7	19.9	1.37	2.59	AAA-AAA	0.00	999	1.000	1.000	0.0211
206221_at	12.0	4.7	-1.37	-2.59	AAA-AAA	0.00	999	1.000	1.000	0.1067
238515_at	98.0	37.9	-1.37	-2.59	PPP-AAA	0.00	999	1.000	1.000	0.0673
242493_at	3.1	7.9	1.37	2.59	AAA-AAA	0.00	999	1.000	1.000	0.3364
1558448_a_at	33.3	12.9	-1.37	-2.59	AAA-AAA	0.00	999	1.000	1.000	0.3429
214819_at	34.8	89.9	1.37	2.59	AAA-AAA	0.00	999	1.000	1.000	0.1732
201062_at	15.5	6.0	-1.37	-2.59	AAA-AAA	0.00	999	1.000	1.000	0.4254
209977_at	12.6	32.6	1.37	2.59	AAA-AAA	0.00	999	1.000	1.000	0.1925
241040_at	54.1	20.9	-1.37	-2.59	AAP-AAA	0.00	999	1.000	1.000	0.1562
1570001_at	45.4	17.6	-1.37	-2.58	AAP-AAA	0.00	999	1.000	1.000	0.1772
237788_at	29.9	11.6	-1.37	-2.59	APA-AAA	0.00	999	1.000	1.000	0.3248
1563335_at	17.4	6.7	-1.37	-2.59	AAA-AAA	0.00	999	1.000	1.000	0.2324
240943_at	26.4	68.3	1.37	2.58	AAA-AAA	0.00	999	1.000	1.000	0.2444
1552922_at	2.3	6.0	1.37	2.59	AAA-AAA	0.00	999	1.000	1.000	0.4021
1561240_at	14.4	5.6	-1.37	-2.59	AAA-AAA	0.00	999	1.000	1.000	0.3629
220283_at	12.5	32.3	1.37	2.59	AAA-AAA	0.00	999	1.000	1.000	0.0725
219568_x_at	11.1	28.7	1.37	2.58	AAA-AAA	0.00	999	1.000	1.000	0.1520
219781_s_at	84.9	32.8	-1.37	-2.58	AAA-AAA	0.00	999	1.000	1.000	0.1481
234780_at	20.3	7.9	-1.37	-2.58	AAA-AAA	0.00	999	1.000	1.000	0.1914
233138_at	36.7	94.8	1.37	2.59	AAA-AAA	0.00	999	1.000	1.000	0.2337
238159_at	14.3	37.0	1.37	2.59	AAA-AAA	0.00	999	1.000	1.000	0.0563
227344_at	57.3	22.2	-1.37	-2.59	AAA-AAA	0.00	999	1.000	1.000	0.0892
231581_at	6.2	15.9	1.37	2.59	AAA-AAA	0.00	999	1.000	1.000	0.4360
229756_at	26.4	68.2	1.37	2.59	AAA-AAA	0.00	999	1.000	1.000	0.2806
229290_at	39.9	15.4	-1.37	-2.59	AAA-AAA	0.00	999	1.000	1.000	0.4403
1557436_at	11.2	29.0	1.37	2.58	AAA-AAA	0.00	999	1.000	1.000	0.2282
215355_at	5.7	14.7	1.37	2.58	AAA-AAA	0.00	999	1.000	1.000	0.5454
221051_s_at	64.0	24.8	-1.37	-2.58	AAA-AAA	0.00	999	1.000	1.000	0.2403
206309_at	47.9	18.5	-1.37	-2.58	AAA-AAA	0.00	999	1.000	1.000	0.3105
204926_at	18.1	46.8	1.37	2.58	AAA-AAA	0.00	999	1.000	1.000	0.3499
238371_s_at	24.4	9.4	-1.37	-2.58	AAA-AAA	0.00	999	1.000	1.000	0.1167
236548_at	7.8	3.0	-1.37	-2.58	AAA-AAA	0.00	999	1.000	1.000	0.4403
216975_x_at	8.4	21.7	1.37	2.58	AAA-AAA	0.00	999	1.000	1.000	0.1723
204953_at	8.3	21.4	1.37	2.58	AAA-AAA	0.00	999	1.000	1.000	0.2582
210724_at	15.7	40.5	1.37	2.58	AAA-AAA	0.00	999	1.000	1.000	0.3442
233288_at	13.2	34.1	1.37	2.58	AAA-AAA	0.00	999	1.000	1.000	0.1837
1562625_at	34.1	13.2	-1.37	-2.58	AAA-AAA	0.00	999	1.000	1.000	0.1398

1556131_s_at	29.6	11.5	-1.37	-2.58	AAA-AAA	0.00	999	1.000	1.000	0.1924
1554142_at	2.2	5.5	1.37	2.58	AAA-AAA	0.00	999	1.000	1.000	0.4044
1555711_x_at	51.7	20.0	-1.37	-2.58	AAA-AAA	0.00	999	1.000	1.000	0.2147
1561492_at	8.5	22.0	1.37	2.58	AAA-AAA	0.00	999	1.000	1.000	0.1364
243723_at	2.6	6.6	1.37	2.58	AAA-AAA	0.00	999	1.000	1.000	0.3647
238878_at	22.2	8.6	-1.37	-2.58	AAA-AAA	0.00	999	1.000	1.000	0.1027
240379_at	65.6	25.5	-1.37	-2.58	AAA-AAA	0.00	999	1.000	1.000	0.0836
1569700_s_at	25.7	66.1	1.37	2.58	AAA-AAA	0.00	999	1.000	1.000	0.2122
1557483_at	8.7	22.5	1.37	2.58	AAA-AAA	0.00	999	1.000	1.000	0.0573
244717_x_at	33.3	12.9	-1.37	-2.58	AAA-AAA	0.00	999	1.000	1.000	0.3935
244383_at	30.9	12.0	-1.37	-2.58	AAA-AAA	0.00	999	1.000	1.000	0.1000
214339_s_at	78.8	30.6	-1.37	-2.58	AAA-AAA	0.00	999	1.000	1.000	0.2776
220810_at	6.7	2.6	-1.37	-2.58	AAA-AAA	0.00	999	1.000	1.000	0.4755
206547_s_at	16.1	41.4	1.37	2.58	AAA-AAA	0.00	999	1.000	1.000	0.4608
209631_s_at	10.5	27.2	1.37	2.58	AAA-AAA	0.00	999	1.000	1.000	0.3351
238592_at	10.3	4.0	-1.37	-2.58	AAA-AAA	0.00	999	1.000	1.000	0.1839
1564713_a_at	90.0	34.9	-1.37	-2.58	AAA-AAA	0.00	999	1.000	1.000	0.2841
241667_x_at	50.4	19.6	-1.37	-2.58	AAA-AAA	0.00	999	1.000	1.000	0.2794
1557921_s_at	8.2	21.0	1.37	2.58	AAA-AAA	0.00	999	1.000	1.000	0.2922
1557832_at	12.5	4.9	-1.37	-2.58	AAA-AAA	0.00	999	1.000	1.000	0.4565
218468_s_at	4.8	12.3	1.37	2.58	AAA-AAA	0.00	999	1.000	1.000	0.4278
207224_s_at	53.4	20.7	-1.37	-2.58	AAA-AAA	0.00	999	1.000	1.000	0.1268
234328_at	23.6	60.7	1.37	2.58	AAA-AAA	0.00	999	1.000	1.000	0.2088
237015_at	3.9	10.0	1.37	2.58	AAA-AAA	0.00	999	1.000	1.000	0.4002
232423_at	29.8	76.6	1.37	2.58	AAA-AAA	0.00	999	1.000	1.000	0.2770
233115_at	40.3	15.6	-1.36	-2.57	AAA-APA	0.00	999	1.000	1.000	0.4416
1564403_at	27.9	71.8	1.36	2.57	AAA-AAA	0.00	999	1.000	1.000	0.1520
1562459_at	19.0	7.4	-1.36	-2.57	AAA-AAA	0.00	999	1.000	1.000	0.3104
243741_at	16.8	43.3	1.36	2.57	AAA-AAA	0.00	999	1.000	1.000	0.3078
231241_at	20.7	53.2	1.36	2.57	AAA-AAA	0.00	999	1.000	1.000	0.3372
207732_s_at	102.3	39.8	-1.36	-2.57	PAA-AAA	0.00	999	1.000	1.000	0.3462
1570644_at	36.7	14.3	-1.36	-2.57	AAA-AAA	0.00	999	1.000	1.000	0.2308
1566182_at	43.3	111.5	1.36	2.57	AAA-AAA	0.00	999	1.000	1.000	0.0052
207710_at	22.8	58.7	1.36	2.57	AAA-AAA	0.00	999	1.000	1.000	0.2162
208496_x_at	7.5	19.2	1.36	2.57	AAA-AAA	0.00	999	1.000	1.000	0.2388
234072_at	29.0	74.6	1.36	2.57	AAA-AAA	0.00	999	1.000	1.000	0.0853
234253_at	7.3	18.8	1.36	2.57	AAA-AAA	0.00	999	1.000	1.000	0.2514
238541_at	30.5	78.5	1.36	2.57	AAA-AAA	0.00	999	1.000	1.000	0.4340
1560800_at	4.8	12.3	1.36	2.57	AAA-AAA	0.00	999	1.000	1.000	0.4162
1560435_at	63.5	24.7	-1.36	-2.57	AAA-AAA	0.00	999	1.000	1.000	0.2269
216645_at	29.6	76.2	1.36	2.57	AAA-AAA	0.00	999	1.000	1.000	0.1510
209933_s_at	11.8	30.4	1.36	2.57	AAA-AAA	0.00	999	1.000	1.000	0.1883

211307_s_at	4.2	10.8	1.36	2.57	AAA-AAA	0.00	999	1.000	1.000	0.0207
207899_at	29.3	11.4	-1.36	-2.57	AAA-AAA	0.00	999	1.000	1.000	0.0545
242544_x_at	2.2	5.6	1.36	2.57	AAA-AAA	0.00	999	1.000	1.000	0.3495
1566762_at	6.0	15.4	1.36	2.57	AAA-AAA	0.00	999	1.000	1.000	0.5225
244400_at	9.5	24.5	1.36	2.57	AAA-AAA	0.00	999	1.000	1.000	0.1779
210815_s_at	5.0	12.8	1.36	2.57	AAA-AAA	0.00	999	1.000	1.000	0.0894
209693_at	5.7	14.7	1.36	2.57	AAA-AAA	0.00	999	1.000	1.000	0.4041
237944_at	16.9	43.3	1.36	2.57	AAA-AAA	0.00	999	1.000	1.000	0.2418
237998_at	4.1	10.5	1.36	2.57	AAA-AAA	0.00	999	1.000	1.000	0.2142
227472_at	102.3	39.9	-1.36	-2.57	APA-AAA	0.00	999	1.000	1.000	0.0906
1561238_at	37.2	14.5	-1.36	-2.57	AAA-AAA	0.00	999	1.000	1.000	0.2304
243153_at	45.5	17.7	-1.36	-2.57	AAA-AAA	0.00	999	1.000	1.000	0.0878
219701_at	7.1	18.1	1.36	2.57	AAA-AAA	0.00	999	1.000	1.000	0.3754
219223_at	76.0	29.6	-1.36	-2.57	AAA-AAA	0.00	999	1.000	1.000	0.3018
234621_at	4.0	1.6	-1.36	-2.57	AAA-AAA	0.00	999	1.000	1.000	0.2099
234307_s_at	11.3	29.1	1.36	2.57	AAA-AAA	0.00	999	1.000	1.000	0.1715
233606_at	6.4	16.5	1.36	2.57	AAA-AAA	0.00	999	1.000	1.000	0.2502
230324_at	20.9	53.7	1.36	2.57	AAA-AAA	0.00	999	1.000	1.000	0.1961
240051_at	4.9	12.7	1.36	2.57	AAA-AAA	0.00	999	1.000	1.000	0.2296
1553797_a_at	10.9	27.8	1.36	2.57	AAA-AAA	0.00	999	1.000	1.000	0.2615
243532_at	28.5	11.1	-1.36	-2.57	AAA-AAA	0.00	999	1.000	1.000	0.3887
243381_at	5.8	14.8	1.36	2.57	AAA-AAA	0.00	999	1.000	1.000	0.4084
1560346_at	32.6	12.7	-1.36	-2.57	AAM-AAA	0.00	999	1.000	1.000	0.2019
214165_s_at	3.7	9.5	1.36	2.57	AAA-AAA	0.00	999	1.000	1.000	0.1774
202885_s_at	17.5	45.0	1.36	2.57	AAA-AAA	0.00	999	1.000	1.000	0.1661
241712_at	9.4	24.2	1.36	2.56	AAA-AAA	0.00	999	1.000	1.000	0.4480
1554231_a_at	9.9	25.3	1.36	2.56	AAA-AAA	0.00	999	1.000	1.000	0.3162
1555703_at	47.2	18.4	-1.36	-2.56	AAA-AAA	0.00	999	1.000	1.000	0.1796
206525_at	23.6	9.2	-1.36	-2.56	AAA-AAA	0.00	999	1.000	1.000	0.3053
208291_s_at	22.9	8.9	-1.36	-2.56	AAA-AAA	0.00	999	1.000	1.000	0.3184
238334_at	9.3	23.8	1.36	2.56	AAA-AAA	0.00	999	1.000	1.000	0.0795
222803_at	2.6	6.7	1.36	2.56	AAA-AAA	0.00	999	1.000	1.000	0.3639
224362_at	7.2	18.4	1.36	2.56	AAA-AAA	0.00	999	1.000	1.000	0.2600
231489_x_at	6.6	16.9	1.36	2.56	AAA-AAA	0.00	999	1.000	1.000	0.3903
206553_at	63.7	24.9	-1.36	-2.56	APA-AAP	0.00	999	1.000	1.000	0.2588
240512_x_at	51.3	20.0	-1.36	-2.56	PPA-PAM	0.00	999	1.000	1.000	0.3039
1566098_at	25.1	64.4	1.36	2.56	AAA-AAA	0.00	999	1.000	1.000	0.3844
1554749_s_at	86.7	33.8	-1.36	-2.56	AAA-AAA	0.00	999	1.000	1.000	0.1864
216303_s_at	64.1	25.0	-1.36	-2.56	AAA-AAA	0.00	999	1.000	1.000	0.0984
221134_at	112.6	44.0	-1.36	-2.56	AAA-AAA	0.00	999	1.000	1.000	0.2146
217477_at	25.6	65.7	1.36	2.56	AAA-AAA	0.00	999	1.000	1.000	0.0231
233232_at	9.9	25.4	1.36	2.56	AAA-AAA	0.00	999	1.000	1.000	0.2788

224072_s_at	29.2	11.4	-1.36	-2.56	AAA-AAA	0.00	999	1.000	1.000	0.2501
1563830_a_at	12.7	5.0	-1.36	-2.56	AAA-AAA	0.00	999	1.000	1.000	0.0032
240707_at	26.1	10.2	-1.36	-2.56	AAA-AAA	0.00	999	1.000	1.000	0.3867
1553620_at	4.3	11.1	1.36	2.56	AAA-AAA	0.00	999	1.000	1.000	0.2776
1553601_a_at	8.6	21.9	1.36	2.56	AAA-AAA	0.00	999	1.000	1.000	0.1388
243027_at	9.7	24.9	1.36	2.56	AAA-AAA	0.00	999	1.000	1.000	0.4031
1560595_at	10.2	4.0	-1.36	-2.56	AAA-AAA	0.00	999	1.000	1.000	0.2739
219890_at	20.5	8.0	-1.36	-2.56	AAA-AAA	0.00	999	1.000	1.000	0.1165
208432_s_at	31.5	12.3	-1.36	-2.56	AAA-AAA	0.00	999	1.000	1.000	0.4502
237315_at	18.3	46.9	1.36	2.56	AAA-AAA	0.00	999	1.000	1.000	0.0576
230605_at	55.4	141.8	1.36	2.56	AAA-AAA	0.00	999	1.000	1.000	0.0185
1556533_at	127.6	49.9	-1.36	-2.56	AAP-AAA	0.00	999	1.000	1.000	0.4215
1552685_a_at	99.5	38.9	-1.36	-2.56	AAA-AAA	0.00	999	1.000	1.000	0.2020
1556453_at	29.1	11.4	-1.36	-2.56	AAA-AAA	0.00	999	1.000	1.000	0.1793
1557998_at	8.3	3.2	-1.36	-2.56	AAA-AAA	0.00	999	1.000	1.000	0.3111
243103_at	11.0	28.1	1.36	2.56	AAA-AAA	0.00	999	1.000	1.000	0.2813
221320_at	76.9	30.1	-1.36	-2.56	AAA-AAA	0.00	999	1.000	1.000	0.0735
220869_at	17.4	44.5	1.36	2.56	AAA-AAA	0.00	999	1.000	1.000	0.2679
207197_at	15.6	6.1	-1.36	-2.56	AAA-AAA	0.00	999	1.000	1.000	0.4089
207639_at	6.0	15.3	1.36	2.56	AAA-AAA	0.00	999	1.000	1.000	0.3284
230750_at	105.7	41.3	-1.36	-2.56	AAA-AAA	0.00	999	1.000	1.000	0.1950
242415_at	22.5	8.8	-1.35	-2.56	AAA-AAA	0.00	999	1.000	1.000	0.3666
216469_at	8.6	21.9	1.35	2.56	AAA-AAA	0.00	999	1.000	1.000	0.2776
217423_at	79.4	31.1	-1.35	-2.56	AMA-AAA	0.00	999	1.000	1.000	0.1621
228888_at	81.6	31.9	-1.35	-2.56	AAA-AAA	0.00	999	1.000	1.000	0.2510
241153_at	32.8	12.9	-1.35	-2.56	AAA-AAA	0.00	999	1.000	1.000	0.3649
1568656_at	29.9	76.4	1.35	2.56	AAA-MAA	0.00	999	1.000	1.000	0.1543
1554655_a_at	9.0	23.1	1.35	2.56	AAA-AAA	0.00	999	1.000	1.000	0.2329
214453_s_at	53.7	21.0	-1.35	-2.56	AAA-AAA	0.00	999	1.000	1.000	0.0816
206463_s_at	29.5	11.6	-1.35	-2.55	AAA-AAA	0.00	999	1.000	1.000	0.2773
207102_at	22.6	8.8	-1.35	-2.55	AAA-AAA	0.00	999	1.000	1.000	0.3666
206755_at	28.4	11.1	-1.35	-2.55	AAA-AAA	0.00	999	1.000	1.000	0.3749
210134_x_at	27.7	10.9	-1.35	-2.56	AAA-AAA	0.00	999	1.000	1.000	0.2134
211741_x_at	15.1	38.6	1.35	2.56	AAA-AAA	0.00	999	1.000	1.000	0.3703
237551_at	5.9	15.0	1.35	2.56	AAA-AAA	0.00	999	1.000	1.000	0.4293
237570_x_at	8.3	21.2	1.35	2.55	AAA-AAA	0.00	999	1.000	1.000	0.3901
238489_at	41.0	16.1	-1.35	-2.55	AAA-AAA	0.00	999	1.000	1.000	0.2580
231641_at	26.3	10.3	-1.35	-2.55	AAA-AAA	0.00	999	1.000	1.000	0.3201
224101_x_at	29.2	11.4	-1.35	-2.55	AAP-AAP	0.00	999	1.000	1.000	0.2655
239052_at	4.2	10.8	1.35	2.55	AAA-AAA	0.00	999	1.000	1.000	0.3299
1559707_at	5.9	2.3	-1.35	-2.55	AAA-AAA	0.00	999	1.000	1.000	0.0538
215659_at	5.7	14.6	1.35	2.55	AAA-AAA	0.00	999	1.000	1.000	0.2142

213638_at	25.3	9.9	-1.35	-2.55	AAA-AAA	0.00	999	1.000	1.000	0.4311
234571_at	30.3	11.9	-1.35	-2.55	AAA-AAA	0.00	999	1.000	1.000	0.2013
227269_s_at	16.2	41.4	1.35	2.55	AAA-AAA	0.00	999	1.000	1.000	0.3325
1570639_at	13.5	34.4	1.35	2.55	AAA-AAA	0.00	999	1.000	1.000	0.3106
1569203_at	21.1	8.3	-1.35	-2.55	AAA-AAA	0.00	999	1.000	1.000	0.3864
1556625_a_at	19.4	7.6	-1.35	-2.55	AAA-AAA	0.00	999	1.000	1.000	0.4638
1554418_s_at	4.2	10.8	1.35	2.55	AAA-AAA	0.00	999	1.000	1.000	0.3763
1553464_at	200.9	78.8	-1.35	-2.55	AAA-AAA	0.00	999	1.000	1.000	0.1683
216647_at	53.4	136.3	1.35	2.55	AAA-AAA	0.00	999	1.000	1.000	0.1089
216580_at	29.5	11.6	-1.35	-2.55	AAA-AAA	0.00	999	1.000	1.000	0.4668
220302_at	9.7	3.8	-1.35	-2.55	AAA-AAA	0.00	999	1.000	1.000	0.3133
219279_at	15.9	6.2	-1.35	-2.55	AAA-AAA	0.00	999	1.000	1.000	0.2512
206579_at	25.3	64.5	1.35	2.55	AAA-AAA	0.00	999	1.000	1.000	0.2283
210326_at	29.7	11.7	-1.35	-2.55	AAA-AAA	0.00	999	1.000	1.000	0.1145
222285_at	8.6	21.9	1.35	2.55	AAA-AAA	0.00	999	1.000	1.000	0.2011
1569073_x_at	174.4	68.4	-1.35	-2.55	APP-AAP	0.00	999	1.000	1.000	0.2600
208139_s_at	72.8	28.6	-1.35	-2.55	APA-AAA	0.00	999	1.000	1.000	0.3869
1562909_at	3.3	8.5	1.35	2.55	AAA-AAA	0.00	999	1.000	1.000	0.1231
1570212_a_at	17.3	6.8	-1.35	-2.55	AAA-AAA	0.00	999	1.000	1.000	0.1892
217314_at	10.5	26.8	1.35	2.55	AAA-AAA	0.00	999	1.000	1.000	0.2267
217075_x_at	12.3	4.8	-1.35	-2.55	AAA-AAA	0.00	999	1.000	1.000	0.1883
207240_s_at	3.7	9.5	1.35	2.55	AAA-AAA	0.00	999	1.000	1.000	0.3921
235252_at	62.8	160.1	1.35	2.55	AAA-MAA	0.00	999	1.000	1.000	0.0641
235115_at	45.1	17.7	-1.35	-2.55	AAA-AAA	0.00	999	1.000	1.000	0.0302
228276_at	12.9	32.9	1.35	2.55	AAA-AAA	0.00	999	1.000	1.000	0.2785
1554831_x_at	14.4	5.7	-1.35	-2.55	PAA-AAA	0.00	999	1.000	1.000	0.3209
1562722_at	43.2	17.0	-1.35	-2.55	AAA-AAA	0.00	999	1.000	1.000	0.2615
241618_at	26.9	10.6	-1.35	-2.55	AAA-AAA	0.00	999	1.000	1.000	0.1367
1561611_at	38.3	97.5	1.35	2.55	AAA-AAA	0.00	999	1.000	1.000	0.1653
244403_at	6.8	2.7	-1.35	-2.55	AAA-AAA	0.00	999	1.000	1.000	0.0715
215367_at	13.7	34.9	1.35	2.55	AAA-AAA	0.00	999	1.000	1.000	0.2358
207012_at	22.1	8.7	-1.35	-2.55	MAA-AAA	0.00	999	1.000	1.000	0.5107
211298_s_at	49.1	19.3	-1.35	-2.55	AAA-AAA	0.00	999	1.000	1.000	0.1105
229703_at	32.1	81.8	1.35	2.55	AAA-AAA	0.00	999	1.000	1.000	0.4039
205789_at	112.3	44.1	-1.35	-2.55	PPP-AAP	0.00	999	1.000	1.000	0.0574
1570318_at	12.7	5.0	-1.35	-2.55	AAA-AAA	0.00	999	1.000	1.000	0.1980
1569900_at	10.6	27.0	1.35	2.55	AAA-AAA	0.00	999	1.000	1.000	0.0700
242902_at	43.4	17.1	-1.35	-2.55	AAA-AAA	0.00	999	1.000	1.000	0.1826
1559274_at	25.8	10.1	-1.35	-2.55	AAM-AAA	0.00	999	1.000	1.000	0.2566
210834_s_at	6.7	17.0	1.35	2.55	AAA-MAA	0.00	999	1.000	1.000	0.3041
233591_at	4.2	10.6	1.35	2.55	AAA-AAA	0.00	999	1.000	1.000	0.5353
229275_at	14.2	36.0	1.35	2.55	AAA-AAA	0.00	999	1.000	1.000	0.2414

238458_at	7.1	2.8	-1.35	-2.54	APA-AAA	0.00	999	1.000	1.000	0.5552
1564631_at	5.4	13.7	1.35	2.54	AAA-AAA	0.00	999	1.000	1.000	0.5558
240134_at	31.9	81.2	1.35	2.54	AAA-AAA	0.00	999	1.000	1.000	0.3312
1566767_at	19.6	7.7	-1.35	-2.54	AAA-AAA	0.00	999	1.000	1.000	0.0656
1554914_at	55.9	22.0	-1.35	-2.54	AAA-AAA	0.00	999	1.000	1.000	0.1585
216352_x_at	26.7	68.0	1.35	2.54	AAA-AAA	0.00	999	1.000	1.000	0.1033
214889_at	8.7	22.1	1.35	2.54	AAA-AAA	0.00	999	1.000	1.000	0.1923
209371_s_at	30.4	77.4	1.35	2.54	AAA-AAA	0.00	999	1.000	1.000	0.1568
234933_at	5.4	13.8	1.35	2.54	AAA-AAA	0.00	999	1.000	1.000	0.3888
224085_at	21.3	8.4	-1.35	-2.54	AAA-AAA	0.00	999	1.000	1.000	0.2955
231353_at	16.8	42.7	1.35	2.54	AAA-AAA	0.00	999	1.000	1.000	0.2422
231551_at	4.5	11.5	1.35	2.54	AAA-AAA	0.00	999	1.000	1.000	0.3203
232404_at	17.3	6.8	-1.35	-2.54	AAA-AAA	0.00	999	1.000	1.000	0.1605
230073_at	13.1	33.2	1.35	2.54	AAA-AAA	0.00	999	1.000	1.000	0.3655
236942_at	123.4	48.6	-1.35	-2.54	PAA-AAA	0.00	999	1.000	1.000	0.4364
241536_at	21.8	8.6	-1.35	-2.54	AAA-AAA	0.00	999	1.000	1.000	0.4634
239396_at	119.2	46.9	-1.35	-2.54	AAA-AAA	0.00	999	1.000	1.000	0.2260
1553879_a_at	12.5	31.8	1.35	2.54	AAA-AAA	0.00	999	1.000	1.000	0.2837
205693_at	30.6	77.7	1.35	2.54	AAA-AAA	0.00	999	1.000	1.000	0.0854
207110_at	18.4	7.2	-1.35	-2.54	AAA-AAA	0.00	999	1.000	1.000	0.2506
208147_s_at	8.8	22.5	1.35	2.54	AAA-AAA	0.00	999	1.000	1.000	0.0518
235913_at	48.8	19.2	-1.35	-2.54	AAA-AAA	0.00	999	1.000	1.000	0.2943
228891_at	39.7	15.6	-1.35	-2.54	AAA-AAA	0.00	999	1.000	1.000	0.3712
222177_s_at	51.4	20.2	-1.35	-2.54	AAA-AAA	0.00	999	1.000	1.000	0.2078
232236_at	8.5	21.6	1.35	2.54	AAA-AAA	0.00	999	1.000	1.000	0.3201
1561642_at	45.1	17.8	-1.35	-2.54	AAA-APA	0.00	999	1.000	1.000	0.1874
239203_at	30.9	78.5	1.35	2.54	AAA-AAA	0.00	999	1.000	1.000	0.1354
1554299_at	7.3	18.5	1.35	2.54	AAA-AAA	0.00	999	1.000	1.000	0.2427
243030_at	84.8	33.4	-1.35	-2.54	AAA-AAA	0.00	999	1.000	1.000	0.4004
237177_at	7.7	19.6	1.35	2.54	AAA-AAA	0.00	999	1.000	1.000	0.2519
227148_at	2.9	7.4	1.35	2.54	AAA-AAA	0.00	999	1.000	1.000	0.2661
240249_at	16.4	41.5	1.34	2.54	AAA-AAA	0.00	999	1.000	1.000	0.0995
1560506_at	22.0	8.7	-1.34	-2.54	AAA-AAA	0.00	999	1.000	1.000	0.1394
244017_at	30.4	12.0	-1.34	-2.54	AAA-AAA	0.00	999	1.000	1.000	0.1549
215571_at	5.2	13.2	1.34	2.54	AAA-AAA	0.00	999	1.000	1.000	0.4758
206914_at	9.6	3.8	-1.34	-2.54	AAA-AAA	0.00	999	1.000	1.000	0.0828
234558_at	42.2	16.6	-1.34	-2.54	AAA-AAA	0.00	999	1.000	1.000	0.2769
234964_at	4.5	11.5	1.34	2.54	AAA-AAA	0.00	999	1.000	1.000	0.3745
1560228_at	15.2	38.7	1.34	2.54	AAA-AAA	0.00	999	1.000	1.000	0.2159
215795_at	13.3	33.8	1.34	2.54	AAA-AAA	0.00	999	1.000	1.000	0.3506
202157_s_at	49.3	19.4	-1.34	-2.54	AMA-AAA	0.00	999	1.000	1.000	0.1162
226286_at	19.7	50.0	1.34	2.54	AAA-AAA	0.00	999	1.000	1.000	0.0574

1565769_at	10.7	27.1	1.34	2.54	AAA-AAA	0.00	999	1.000	1.000	0.2961
1554602_at	17.0	43.2	1.34	2.54	AAA-AAA	0.00	999	1.000	1.000	0.4154
1553865_a_at	93.0	36.7	-1.34	-2.54	AAA-AAA	0.00	999	1.000	1.000	0.0679
244622_at	19.8	50.1	1.34	2.53	AAA-AAA	0.00	999	1.000	1.000	0.2526
207900_at	35.6	14.1	-1.34	-2.54	AAA-AAA	0.00	999	1.000	1.000	0.1365
232717_at	7.2	18.2	1.34	2.54	AAA-AAA	0.00	999	1.000	1.000	0.3379
233577_at	17.3	6.8	-1.34	-2.54	AAA-AAA	0.00	999	1.000	1.000	0.2247
228235_at	35.3	13.9	-1.34	-2.54	AAA-AAA	0.00	999	1.000	1.000	0.4551
1563367_at	4.3	10.9	1.34	2.53	AAA-AAA	0.00	999	1.000	1.000	0.0500
1562152_at	3.8	9.7	1.34	2.53	AAA-AAA	0.00	999	1.000	1.000	0.1692
243040_at	4.9	12.4	1.34	2.53	AAA-AAA	0.00	999	1.000	1.000	0.1650
1559880_at	7.7	19.6	1.34	2.53	AAA-AAA	0.00	999	1.000	1.000	0.1699
211182_x_at	10.6	26.8	1.34	2.53	AAA-AAA	0.00	999	1.000	1.000	0.3160
235626_at	18.6	47.2	1.34	2.53	AAA-AAA	0.00	999	1.000	1.000	0.2286
238101_at	5.0	12.5	1.34	2.53	AAA-AAA	0.00	999	1.000	1.000	0.2927
237175_at	9.8	24.8	1.34	2.53	AAA-AMA	0.00	999	1.000	1.000	0.2639
237202_at	64.2	162.6	1.34	2.53	AAA-AAA	0.00	999	1.000	1.000	0.1110
229847_at	28.2	11.1	-1.34	-2.53	AAA-AAA	0.00	999	1.000	1.000	0.1495
229254_at	12.5	31.6	1.34	2.53	MAA-AAA	0.00	999	1.000	1.000	0.3403
1563106_at	11.8	30.0	1.34	2.53	AAA-AAA	0.00	999	1.000	1.000	0.0443
1553364_at	36.1	14.3	-1.34	-2.53	AAA-AAA	0.00	999	1.000	1.000	0.1626
221330_at	4.3	10.9	1.34	2.53	AAA-AAA	0.00	999	1.000	1.000	0.1243
217017_at	18.8	47.6	1.34	2.53	AAA-AAA	0.00	999	1.000	1.000	0.2871
232985_s_at	25.7	65.0	1.34	2.53	AAA-AAA	0.00	999	1.000	1.000	0.1916
236280_at	3.6	9.0	1.34	2.53	AAA-AAA	0.00	999	1.000	1.000	0.3401
229963_at	9.3	23.5	1.34	2.53	AAA-AAA	0.00	999	1.000	1.000	0.3819
239951_at	31.2	12.3	-1.34	-2.53	PAA-AAA	0.00	999	1.000	1.000	0.3827
1565265_at	30.0	11.9	-1.34	-2.53	AAA-AAA	0.00	999	1.000	1.000	0.3880
1555527_at	5.1	12.9	1.34	2.53	AAA-AAA	0.00	999	1.000	1.000	0.0738
242849_at	23.9	60.5	1.34	2.53	AAA-AAA	0.00	999	1.000	1.000	0.1797
220510_at	9.9	25.0	1.34	2.53	AAA-AAA	0.00	999	1.000	1.000	0.1878
237266_at	27.9	70.6	1.34	2.53	AAA-AAA	0.00	999	1.000	1.000	0.1021
231682_at	19.5	7.7	-1.34	-2.53	AAA-AAA	0.00	999	1.000	1.000	0.2682
1556583_a_at	17.2	6.8	-1.34	-2.53	AAA-AAA	0.00	999	1.000	1.000	0.4992
1556670_at	18.0	45.6	1.34	2.53	AAA-AAA	0.00	999	1.000	1.000	0.3626
216974_at	4.6	11.7	1.34	2.53	AAA-AAA	0.00	999	1.000	1.000	0.3921
236982_at	48.2	19.1	-1.34	-2.53	AAA-AAA	0.00	999	1.000	1.000	0.0568
231092_s_at	10.5	26.5	1.34	2.53	AAA-AAA	0.00	999	1.000	1.000	0.2486
206085_s_at	76.1	30.1	-1.34	-2.53	PPA-AAP	0.00	999	1.000	1.000	0.3418
1564306_at	4.3	10.8	1.34	2.53	AAA-AAA	0.00	999	1.000	1.000	0.3734
241221_at	20.1	7.9	-1.34	-2.53	AAA-AAA	0.00	999	1.000	1.000	0.0993
1563118_at	23.1	9.2	-1.34	-2.53	AAA-AAA	0.00	999	1.000	1.000	0.3461

1562082_at	6.3	15.8	1.34	2.53	AAA-AAA	0.00	999	1.000	1.000	0.3786
206942_s_at	95.5	37.8	-1.34	-2.53	AAA-AAA	0.00	999	1.000	1.000	0.0213
208069_x_at	62.8	24.9	-1.34	-2.53	AAA-AAA	0.00	999	1.000	1.000	0.2745
230584_at	49.5	19.6	-1.34	-2.53	AAA-AAA	0.00	999	1.000	1.000	0.2616
1563753_at	29.3	73.9	1.34	2.52	AAA-AAA	0.00	999	1.000	1.000	0.0067
221546_at	15.7	39.5	1.34	2.52	AAA-AAA	0.00	999	1.000	1.000	0.1737
219852_s_at	63.9	25.3	-1.34	-2.52	AAA-AAA	0.00	999	1.000	1.000	0.0241
210302_s_at	29.4	11.7	-1.34	-2.52	AAA-AAA	0.00	999	1.000	1.000	0.1908
208111_at	15.5	6.2	-1.34	-2.52	AAA-AAA	0.00	999	1.000	1.000	0.2555
234288_at	6.8	17.1	1.34	2.53	AAA-AAA	0.00	999	1.000	1.000	0.3765
236681_at	78.7	31.2	-1.34	-2.52	AAA-AAA	0.00	999	1.000	1.000	0.1398
235999_at	82.0	32.5	-1.33	-2.52	APP-AAA	0.00	999	1.000	1.000	0.3271
240143_at	16.0	6.3	-1.33	-2.52	AAA-PAA	0.00	999	1.000	1.000	0.4728
239608_at	18.5	46.7	1.33	2.52	AAA-AAA	0.00	999	1.000	1.000	0.2609
1553295_at	28.7	11.4	-1.33	-2.52	AAA-AAA	0.00	999	1.000	1.000	0.1800
216398_at	58.6	23.2	-1.33	-2.52	AAA-AAA	0.00	999	1.000	1.000	0.1214
204588_s_at	68.4	27.1	-1.33	-2.52	AAA-AAA	0.00	999	1.000	1.000	0.1921
237590_at	14.0	5.6	-1.33	-2.52	AAA-AAA	0.00	999	1.000	1.000	0.1289
235944_at	7.3	2.9	-1.33	-2.52	AAA-AAA	0.00	999	1.000	1.000	0.3010
237182_at	21.4	8.5	-1.33	-2.52	APA-AAA	0.00	999	1.000	1.000	0.1618
1557419_a_at	82.4	32.7	-1.33	-2.52	PAA-AAA	0.00	999	1.000	1.000	0.2365
219870_at	94.5	37.5	-1.33	-2.52	AAP-AAA	0.00	999	1.000	1.000	0.2837
240977_at	40.7	16.1	-1.33	-2.52	AAA-AAA	0.00	999	1.000	1.000	0.3667
1556584_at	27.8	70.1	1.33	2.52	AAA-AAA	0.00	999	1.000	1.000	0.1824
1560988_a_at	12.0	30.3	1.33	2.52	AAA-AAA	0.00	999	1.000	1.000	0.1153
209880_s_at	10.4	26.1	1.33	2.52	AAA-AAA	0.00	999	1.000	1.000	0.3402
207895_at	7.2	18.3	1.33	2.52	AAA-AAA	0.00	999	1.000	1.000	0.3653
234623_x_at	5.3	13.3	1.33	2.52	AAA-AAA	0.00	999	1.000	1.000	0.3760
234309_at	4.6	11.7	1.33	2.52	AAA-AAA	0.00	999	1.000	1.000	0.1459
237119_at	9.4	23.8	1.33	2.52	AAA-AAA	0.00	999	1.000	1.000	0.5663
237010_at	109.6	43.5	-1.33	-2.52	AAA-AAA	0.00	999	1.000	1.000	0.0467
228406_at	21.7	8.6	-1.33	-2.52	AAA-AAA	0.00	999	1.000	1.000	0.3382
228143_at	44.4	17.6	-1.33	-2.52	MAA-AAA	0.00	999	1.000	1.000	0.1549
230704_s_at	62.8	24.9	-1.33	-2.52	AAA-AAA	0.00	999	1.000	1.000	0.1173
220186_s_at	141.2	56.1	-1.33	-2.52	APA-AAA	0.00	999	1.000	1.000	0.2041
1562752_at	8.4	3.3	-1.33	-2.52	AAA-AAA	0.00	999	1.000	1.000	0.1926
1569925_at	24.7	9.8	-1.33	-2.52	AAA-AAA	0.00	999	1.000	1.000	0.1177
1565799_at	16.1	6.4	-1.33	-2.52	AAA-AAA	0.00	999	1.000	1.000	0.4088
1565804_at	6.8	17.0	1.33	2.52	AAA-AAA	0.00	999	1.000	1.000	0.3605
240670_at	41.3	16.4	-1.33	-2.52	AAA-AAA	0.00	999	1.000	1.000	0.3015
243214_at	11.4	28.7	1.33	2.52	AAA-AAA	0.00	999	1.000	1.000	0.2520
244584_at	32.0	12.7	-1.33	-2.52	AAA-AAA	0.00	999	1.000	1.000	0.0457

216845_x_at	29.1	73.3	1.33	2.52	AAA-AAA	0.00	999	1.000	1.000	0.0763
217692_at	71.8	28.5	-1.33	-2.52	AAA-AAA	0.00	999	1.000	1.000	0.3416
210479_s_at	12.7	31.9	1.33	2.52	AAA-AAA	0.00	999	1.000	1.000	0.1467
210537_s_at	25.9	65.2	1.33	2.52	AAA-AAA	0.00	999	1.000	1.000	0.2195
234552_at	21.8	54.8	1.33	2.52	AAA-AAA	0.00	999	1.000	1.000	0.2931
233792_at	35.7	14.2	-1.33	-2.52	AAA-AAA	0.00	999	1.000	1.000	0.4871
228150_at	8.7	3.5	-1.33	-2.52	AAA-AAA	0.00	999	1.000	1.000	0.3688
1555303_at	87.5	34.8	-1.33	-2.52	PAA-AAA	0.00	999	1.000	1.000	0.0337
1552925_at	12.8	5.1	-1.33	-2.52	AAA-AAA	0.00	999	1.000	1.000	0.1859
1556742_at	7.8	19.7	1.33	2.52	AAA-AAA	0.00	999	1.000	1.000	0.1829
1558600_a_at	11.8	29.6	1.33	2.52	AAA-AAA	0.00	999	1.000	1.000	0.3496
215634_at	18.2	7.2	-1.33	-2.52	AAA-AAA	0.00	999	1.000	1.000	0.1989
220983_s_at	8.4	21.1	1.33	2.52	AAA-AAA	0.00	999	1.000	1.000	0.3323
207924_x_at	12.2	30.8	1.33	2.52	AAA-AAA	0.00	999	1.000	1.000	0.0970
239628_at	39.9	15.9	-1.33	-2.51	AAA-AAA	0.00	999	1.000	1.000	0.3163
239427_at	30.4	76.3	1.33	2.51	AAA-AAA	0.00	999	1.000	1.000	0.2936
1553769_at	14.4	36.1	1.33	2.51	AAA-AAA	0.00	999	1.000	1.000	0.0986
244606_at	5.0	12.6	1.33	2.51	AAA-AAA	0.00	999	1.000	1.000	0.2405
243994_at	5.3	13.2	1.33	2.52	AAA-AAA	0.00	999	1.000	1.000	0.2179
208163_s_at	23.1	58.1	1.33	2.51	AAA-AAA	0.00	999	1.000	1.000	0.2336
236245_at	15.8	39.8	1.33	2.51	AAA-AAA	0.00	999	1.000	1.000	0.1400
239386_at	54.5	21.7	-1.33	-2.51	AAA-AAA	0.00	999	1.000	1.000	0.2679
1552381_at	16.0	40.1	1.33	2.51	AAA-AAA	0.00	999	1.000	1.000	0.2262
215789_s_at	46.3	18.4	-1.33	-2.51	AAA-AAA	0.00	999	1.000	1.000	0.3168
234059_at	13.0	32.7	1.33	2.51	AAA-AAA	0.00	999	1.000	1.000	0.1196
236897_at	21.3	53.5	1.33	2.51	AAA-AAM	0.00	999	1.000	1.000	0.4595
228849_at	28.5	71.5	1.33	2.51	AAA-AAA	0.00	999	1.000	1.000	0.2953
232415_at	21.3	8.5	-1.33	-2.51	AAA-AAA	0.00	999	1.000	1.000	0.1627
241245_at	18.1	7.2	-1.33	-2.51	AAA-AAA	0.00	999	1.000	1.000	0.4165
1566969_at	10.8	4.3	-1.33	-2.51	AAA-AAA	0.00	999	1.000	1.000	0.4710
1552311_a_at	120.7	48.1	-1.33	-2.51	AAA-AAA	0.00	999	1.000	1.000	0.2317
1560099_at	3.8	9.5	1.33	2.51	AAA-AAA	0.00	999	1.000	1.000	0.2564
216148_at	4.8	12.1	1.33	2.51	AAA-AAA	0.00	999	1.000	1.000	0.3645
221017_s_at	10.5	26.4	1.33	2.51	AAA-AAA	0.00	999	1.000	1.000	0.2452
207063_at	9.5	3.8	-1.33	-2.51	AAA-AAA	0.00	999	1.000	1.000	0.0774
207996_s_at	3.4	8.6	1.33	2.51	AAA-AAA	0.00	999	1.000	1.000	0.3813
222337_at	22.7	9.1	-1.33	-2.51	AAA-AAA	0.00	999	1.000	1.000	0.2398
224191_x_at	33.1	13.2	-1.33	-2.51	AMA-AAA	0.00	999	1.000	1.000	0.3492
231248_at	9.4	23.5	1.33	2.51	AAA-AAA	0.00	999	1.000	1.000	0.2460
231131_at	7.4	3.0	-1.33	-2.51	AAA-AAA	0.00	999	1.000	1.000	0.4131
205508_at	108.0	43.1	-1.33	-2.51	APA-AAA	0.00	999	1.000	1.000	0.3640
244016_at	25.1	10.0	-1.33	-2.51	AAA-APA	0.00	999	1.000	1.000	0.4576

241582_at	18.8	47.1	1.33	2.51	AAA-AAA	0.00	999	1.000	1.000	0.1293
213563_s_at	40.4	16.1	-1.33	-2.51	AAA-AAA	0.00	999	1.000	1.000	0.1017
214809_at	9.8	24.6	1.33	2.51	AAA-AAA	0.00	999	1.000	1.000	0.3879
220713_at	57.3	22.9	-1.33	-2.51	AAA-AAA	0.00	999	1.000	1.000	0.3604
207611_at	28.6	11.4	-1.33	-2.51	AAA-AAA	0.00	999	1.000	1.000	0.1052
238091_at	7.5	3.0	-1.33	-2.51	AAA-AAA	0.00	999	1.000	1.000	0.0799
241873_at	12.8	5.1	-1.33	-2.51	AAA-AAA	0.00	999	1.000	1.000	0.3436
1563465_at	5.2	13.1	1.33	2.51	AAA-AAA	0.00	999	1.000	1.000	0.3976
1556465_at	19.0	7.6	-1.33	-2.51	AAA-AAA	0.00	999	1.000	1.000	0.1954
1555044_a_at	13.7	5.4	-1.33	-2.51	AAA-AAA	0.00	999	1.000	1.000	0.2898
1555550_at	17.5	43.8	1.33	2.51	AAA-AAA	0.00	999	1.000	1.000	0.1621
1562216_at	12.0	4.8	-1.33	-2.51	AAA-AAA	0.00	999	1.000	1.000	0.0935
235377_at	15.8	39.6	1.33	2.51	AAA-AAA	0.00	999	1.000	1.000	0.1262
233641_s_at	17.9	44.9	1.33	2.51	AAA-AAA	0.00	999	1.000	1.000	0.1646
228471_at	20.7	51.8	1.33	2.51	AAA-AAA	0.00	999	1.000	1.000	0.3199
224111_x_at	47.6	119.4	1.33	2.51	AAA-AAA	0.00	999	1.000	1.000	0.2872
233438_at	18.2	7.3	-1.33	-2.51	AAP-AAA	0.00	999	1.000	1.000	0.4736
1564387_at	44.3	17.7	-1.33	-2.51	AAA-AAA	0.00	999	1.000	1.000	0.2115
1566544_at	8.5	3.4	-1.33	-2.51	AAA-AAA	0.00	999	1.000	1.000	0.4976
1566848_x_at	17.3	43.3	1.33	2.51	AAA-AAA	0.00	999	1.000	1.000	0.1593
1558972_s_at	4.0	9.9	1.33	2.51	AAA-AAA	0.00	999	1.000	1.000	0.4819
214312_at	16.7	41.9	1.33	2.51	AAA-AAA	0.00	999	1.000	1.000	0.2885
228513_at	29.1	72.9	1.33	2.51	AAA-MAM	0.00	999	1.000	1.000	0.2383
241115_at	31.4	12.6	-1.32	-2.50	APA-AAA	0.00	999	1.000	1.000	0.3952
1566991_at	12.3	4.9	-1.32	-2.50	AAA-AAA	0.00	999	1.000	1.000	0.1625
1557848_at	42.2	16.9	-1.32	-2.50	AAA-AAA	0.00	999	1.000	1.000	0.3512
1555082_a_at	33.3	83.4	1.32	2.50	AAA-AAA	0.00	999	1.000	1.000	0.0489
242695_at	38.3	95.8	1.32	2.50	AAA-AAA	0.00	999	1.000	1.000	0.2470
216688_at	48.3	19.3	-1.32	-2.50	AAA-AAA	0.00	999	1.000	1.000	0.2379
213817_at	3.6	1.4	-1.32	-2.50	AAA-AAA	0.00	999	1.000	1.000	0.2419
207804_s_at	9.8	24.5	1.32	2.50	AAA-AAA	0.00	999	1.000	1.000	0.1516
237672_at	6.5	16.2	1.32	2.50	AAA-AAA	0.00	999	1.000	1.000	0.2044
236999_at	14.1	35.3	1.32	2.50	AAA-AMA	0.00	999	1.000	1.000	0.1236
223725_at	23.5	58.9	1.32	2.50	AAA-AAA	0.00	999	1.000	1.000	0.0954
240396_at	7.3	18.3	1.32	2.50	AAA-AAA	0.00	999	1.000	1.000	0.2471
1566099_at	49.8	19.9	-1.32	-2.50	AAA-AAA	0.00	999	1.000	1.000	0.2340
1557877_s_at	19.6	7.8	-1.32	-2.50	AAA-AAA	0.00	999	1.000	1.000	0.3517
1553492_a_at	14.8	37.1	1.32	2.50	AAA-AAA	0.00	999	1.000	1.000	0.2963
243416_at	19.7	49.2	1.32	2.50	AAA-AAA	0.00	999	1.000	1.000	0.4026
244072_at	35.6	14.2	-1.32	-2.50	AAA-AAA	0.00	999	1.000	1.000	0.3356
216990_at	7.6	3.1	-1.32	-2.50	AAA-AAA	0.00	999	1.000	1.000	0.2274
206234_s_at	28.7	71.7	1.32	2.50	AAA-AAA	0.00	999	1.000	1.000	0.2826

237662_at	55.6	22.2	-1.32	-2.50	AAA-AAA	0.00	999	1.000	1.000	0.2827
236909_at	5.5	13.7	1.32	2.50	AAA-AAA	0.00	999	1.000	1.000	0.0877
233195_at	127.1	50.8	-1.32	-2.50	PAP-PAA	0.00	999	1.000	1.000	0.1954
241901_at	45.5	18.2	-1.32	-2.50	AAA-AAA	0.00	999	1.000	1.000	0.1834
1557057_a_at	3.0	7.6	1.32	2.50	AAA-AAA	0.00	999	1.000	1.000	0.0634
1557225_at	8.8	22.0	1.32	2.50	AAA-AAA	0.00	999	1.000	1.000	0.4377
243787_at	19.4	7.8	-1.32	-2.50	AAA-AAA	0.00	999	1.000	1.000	0.1049
217581_at	14.9	6.0	-1.32	-2.50	AAA-AAA	0.00	999	1.000	1.000	0.0333
236625_at	12.2	30.5	1.32	2.50	AAA-AAA	0.00	999	1.000	1.000	0.1568
236587_at	40.2	16.1	-1.32	-2.50	AAA-AAA	0.00	999	1.000	1.000	0.1695
236474_at	75.2	30.1	-1.32	-2.50	AAA-AAA	0.00	999	1.000	1.000	0.3283
237267_at	7.2	18.1	1.32	2.50	AAA-AAA	0.00	999	1.000	1.000	0.2567
232227_at	29.5	11.8	-1.32	-2.50	AAA-AAA	0.00	999	1.000	1.000	0.2370
242164_s_at	44.9	18.0	-1.32	-2.50	AAA-AAA	0.00	999	1.000	1.000	0.1116
1563856_at	9.2	23.1	1.32	2.50	AAA-AAA	0.00	999	1.000	1.000	0.3809
1570100_at	27.2	10.9	-1.32	-2.50	AAA-AAA	0.00	999	1.000	1.000	0.3510
1556777_a_at	15.3	38.2	1.32	2.50	AAA-AAA	0.00	999	1.000	1.000	0.3698
220126_at	14.8	37.0	1.32	2.50	AAA-AAA	0.00	999	1.000	1.000	0.1431
206852_at	9.4	3.7	-1.32	-2.50	AAA-AAA	0.00	999	1.000	1.000	0.1416
225163_at	24.3	9.7	-1.32	-2.50	AAA-AAA	0.00	999	1.000	1.000	0.3791
226417_at	28.7	11.5	-1.32	-2.50	AAA-AAA	0.00	999	1.000	1.000	0.1470
228533_at	74.5	29.8	-1.32	-2.50	AAA-AAA	0.00	999	1.000	1.000	0.3164
1563453_at	43.4	17.4	-1.32	-2.50	AAA-AAA	0.00	999	1.000	1.000	0.4437
244720_at	7.3	18.3	1.32	2.50	AAA-AAA	0.00	999	1.000	1.000	0.2013
243428_at	18.5	46.2	1.32	2.50	AAA-AAA	0.00	999	1.000	1.000	0.2673
213217_at	12.1	30.1	1.32	2.50	AAA-AAA	0.00	999	1.000	1.000	0.2035
221018_s_at	9.5	23.6	1.32	2.50	AAA-AAA	0.00	999	1.000	1.000	0.2674
205445_at	28.1	70.0	1.32	2.50	AAA-AAA	0.00	999	1.000	1.000	0.3364
228044_at	3.3	8.2	1.32	2.50	AAA-AAA	0.00	999	1.000	1.000	0.1174
231833_at	33.0	82.3	1.32	2.50	AAA-AAA	0.00	999	1.000	1.000	0.0269
230515_at	7.4	18.5	1.32	2.50	AAA-AAA	0.00	999	1.000	1.000	0.2133
242867_x_at	52.6	21.1	-1.32	-2.50	PAA-AAA	0.00	999	1.000	1.000	0.1650
1563606_a_at	9.2	22.9	1.32	2.49	AAA-AAA	0.00	999	1.000	1.000	0.4538
240856_at	26.9	10.8	-1.32	-2.50	AAA-AAA	0.00	999	1.000	1.000	0.3630
1566168_at	9.7	24.2	1.32	2.49	AAA-AAA	0.00	999	1.000	1.000	0.2567
1557692_a_at	2.7	6.6	1.32	2.49	AAA-AAA	0.00	999	1.000	1.000	0.4440
206455_s_at	26.1	65.1	1.32	2.50	AAA-AAA	0.00	999	1.000	1.000	0.1103
233497_at	28.1	11.3	-1.32	-2.49	AAA-AAA	0.00	999	1.000	1.000	0.2947
237412_at	135.4	54.3	-1.32	-2.50	AAA-AAA	0.00	999	1.000	1.000	0.1089
1569076_a_at	78.8	31.6	-1.32	-2.49	MAA-AAA	0.00	999	1.000	1.000	0.3244
239497_at	35.6	88.6	1.32	2.49	AAA-AAA	0.00	999	1.000	1.000	0.2275
1566124_a_at	4.4	10.9	1.32	2.49	AAA-AAA	0.00	999	1.000	1.000	0.4044

1558803_at	6.6	16.3	1.32	2.49	AAA-AAA	0.00	999	1.000	1.000	0.3099
1555229_a_at	14.7	5.9	-1.32	-2.49	AAA-AAA	0.00	999	1.000	1.000	0.4438
213888_s_at	52.9	21.2	-1.32	-2.49	AAA-AAA	0.00	999	1.000	1.000	0.1970
233745_at	8.9	22.2	1.32	2.49	AAA-AAA	0.00	999	1.000	1.000	0.2651
237926_s_at	16.1	6.5	-1.32	-2.49	AAA-AAA	0.00	999	1.000	1.000	0.2556
236180_at	6.1	15.2	1.32	2.49	AAA-AAA	0.00	999	1.000	1.000	0.1960
223913_s_at	12.5	31.1	1.32	2.49	AAA-AAA	0.00	999	1.000	1.000	0.4150
231322_at	57.1	22.9	-1.32	-2.49	AAA-AAA	0.00	999	1.000	1.000	0.1664
1562716_at	44.0	17.7	-1.32	-2.49	AAA-PAA	0.00	999	1.000	1.000	0.3398
1553504_at	68.7	27.6	-1.32	-2.49	AAP-AAP	0.00	999	1.000	1.000	0.3553
241579_at	9.9	24.7	1.32	2.49	AAA-AAA	0.00	999	1.000	1.000	0.4944
217260_x_at	31.7	12.7	-1.32	-2.49	AAA-AAA	0.00	999	1.000	1.000	0.4473
207457_s_at	37.9	15.2	-1.32	-2.49	AAA-AAA	0.00	999	1.000	1.000	0.3174
205320_at	81.1	32.6	-1.32	-2.49	MAA-PAA	0.00	999	1.000	1.000	0.4169
244421_at	28.3	70.6	1.32	2.49	AAA-AAA	0.00	999	1.000	1.000	0.1528
244273_at	8.2	20.5	1.32	2.49	AAA-AAA	0.00	999	1.000	1.000	0.1362
216462_at	25.9	10.4	-1.32	-2.49	AAA-AAA	0.00	999	1.000	1.000	0.2243
216464_x_at	8.8	22.0	1.32	2.49	AAA-AAA	0.00	999	1.000	1.000	0.3909
220035_at	21.5	53.6	1.32	2.49	AAA-AAA	0.00	999	1.000	1.000	0.3015
205665_at	38.8	96.6	1.32	2.49	MAA-AAA	0.00	999	1.000	1.000	0.2292
236119_s_at	86.4	34.7	-1.32	-2.49	AAA-AAA	0.00	999	1.000	1.000	0.1586
243108_at	48.9	19.7	-1.32	-2.49	PAA-APA	0.00	999	1.000	1.000	0.3738
242070_at	15.3	6.2	-1.32	-2.49	AAA-AAA	0.00	999	1.000	1.000	0.0957
1558387_at	6.5	2.6	-1.32	-2.49	AAA-AAA	0.00	999	1.000	1.000	0.4649
1561134_at	13.3	33.0	1.32	2.49	AAA-AAA	0.00	999	1.000	1.000	0.4631
215187_at	11.9	29.6	1.32	2.49	AAA-AAA	0.00	999	1.000	1.000	0.1880
214811_at	13.9	34.6	1.32	2.49	AAA-AAA	0.00	999	1.000	1.000	0.2240
234231_at	18.8	46.9	1.32	2.49	AAA-AAA	0.00	999	1.000	1.000	0.1045
227403_at	37.0	92.1	1.32	2.49	AAA-AAA	0.00	999	1.000	1.000	0.3502
231386_at	11.6	28.8	1.32	2.49	AAA-AAA	0.00	999	1.000	1.000	0.0861
231525_at	15.1	37.6	1.32	2.49	AAA-AAA	0.00	999	1.000	1.000	0.0134
232660_at	79.5	32.0	-1.31	-2.49	PPA-AAA	0.00	999	1.000	1.000	0.1380
1562835_at	15.8	6.3	-1.31	-2.49	AAA-AAA	0.00	999	1.000	1.000	0.4095
240714_at	15.4	6.2	-1.31	-2.49	AAA-AAA	0.00	999	1.000	1.000	0.5346
1555257_a_at	10.0	24.9	1.31	2.49	AAA-AAA	0.00	999	1.000	1.000	0.4987
215914_at	80.7	32.5	-1.31	-2.49	AAA-AAA	0.00	999	1.000	1.000	0.2523
220567_at	24.2	60.0	1.31	2.49	AAA-AAA	0.00	999	1.000	1.000	0.3085
209504_s_at	20.3	50.5	1.31	2.49	AAA-AAA	0.00	999	1.000	1.000	0.0037
228886_at	36.0	89.4	1.31	2.49	AAA-AAA	0.00	999	1.000	1.000	0.1494
1569838_at	7.2	17.8	1.31	2.48	AAA-AAA	0.00	999	1.000	1.000	0.3573
1569459_a_at	17.6	7.1	-1.31	-2.49	AAA-AAA	0.00	999	1.000	1.000	0.5704
244870_at	37.2	92.5	1.31	2.49	AAA-AAA	0.00	999	1.000	1.000	0.0176

1553194_at	9.9	24.7	1.31	2.49	AAA-AAA	0.00	999	1.000	1.000	0.2860
1561668_at	17.5	7.1	-1.31	-2.48	AAA-AAA	0.00	999	1.000	1.000	0.2741
216884_at	14.1	5.7	-1.31	-2.49	AAA-AAA	0.00	999	1.000	1.000	0.2235
207119_at	3.5	8.6	1.31	2.48	AAA-AAA	0.00	999	1.000	1.000	0.2368
204400_at	23.0	9.3	-1.31	-2.48	AAA-AAA	0.00	999	1.000	1.000	0.2051
203084_at	15.3	37.9	1.31	2.49	AAA-AAA	0.00	999	1.000	1.000	0.2507
210618_at	49.0	19.7	-1.31	-2.49	AAA-AAA	0.00	999	1.000	1.000	0.2346
233250_x_at	42.3	17.0	-1.31	-2.48	AAA-AAA	0.00	999	1.000	1.000	0.3426
241210_at	23.6	58.6	1.31	2.48	AAA-AAA	0.00	999	1.000	1.000	0.3006
1561255_at	36.8	14.8	-1.31	-2.48	AAA-AAA	0.00	999	1.000	1.000	0.3088
206482_at	66.1	26.6	-1.31	-2.48	AMA-AAA	0.00	999	1.000	1.000	0.1637
232967_at	29.0	11.7	-1.31	-2.48	AAA-AAA	0.00	999	1.000	1.000	0.4073
226471_at	12.8	31.8	1.31	2.48	AAA-AAA	0.00	999	1.000	1.000	0.1216
230686_s_at	55.0	22.1	-1.31	-2.48	AAA-AAA	0.00	999	1.000	1.000	0.1895
230081_at	26.8	10.8	-1.31	-2.48	AAA-AAA	0.00	999	1.000	1.000	0.1828
1564996_at	2.8	7.1	1.31	2.48	AAA-AAA	0.00	999	1.000	1.000	0.1894
1563771_a_at	4.3	10.7	1.31	2.48	AAA-AAA	0.00	999	1.000	1.000	0.4118
1569020_at	52.2	129.5	1.31	2.48	AAA-AAA	0.00	999	1.000	1.000	0.1612
1552626_a_at	16.9	41.8	1.31	2.48	AAA-AAA	0.00	999	1.000	1.000	0.2868
1552501_a_at	10.2	25.4	1.31	2.48	AAA-AAA	0.00	999	1.000	1.000	0.3370
1560721_at	28.2	11.4	-1.31	-2.48	AAA-AAA	0.00	999	1.000	1.000	0.2230
215658_at	26.2	10.6	-1.31	-2.48	AAA-AAA	0.00	999	1.000	1.000	0.2745
214128_at	50.1	20.2	-1.31	-2.48	AAA-AAA	0.00	999	1.000	1.000	0.2825
209573_s_at	32.6	13.1	-1.31	-2.48	AAA-AAA	0.00	999	1.000	1.000	0.2731
232647_at	34.6	85.8	1.31	2.48	AAA-AAA	0.00	999	1.000	1.000	0.1415
238595_at	54.0	21.8	-1.31	-2.48	AAA-AAA	0.00	999	1.000	1.000	0.3095
238319_at	78.1	31.5	-1.31	-2.48	AAA-AAA	0.00	999	1.000	1.000	0.1461
235831_at	23.8	9.6	-1.31	-2.48	AAA-APA	0.00	999	1.000	1.000	0.4428
240278_at	20.8	8.4	-1.31	-2.48	AAA-AAA	0.00	999	1.000	1.000	0.2878
244367_at	17.2	6.9	-1.31	-2.48	AAA-AAA	0.00	999	1.000	1.000	0.0815
221383_at	14.3	35.5	1.31	2.48	AAA-AAA	0.00	999	1.000	1.000	0.1908
211067_s_at	27.3	11.0	-1.31	-2.48	AAA-AAA	0.00	999	1.000	1.000	0.4149
226613_at	86.2	34.8	-1.31	-2.48	AAA-AAA	0.00	999	1.000	1.000	0.1192
227389_x_at	10.3	25.5	1.31	2.48	AAA-AAA	0.00	999	1.000	1.000	0.1102
226248_s_at	55.7	22.5	-1.31	-2.48	AAA-AAA	0.00	999	1.000	1.000	0.2225
201859_at	375.3	151.5	-1.31	-2.48	PPP-APP	0.00	999	1.000	1.000	0.0500
1561362_at	2.4	5.9	1.31	2.48	AAA-AAA	0.00	999	1.000	1.000	0.4205
243611_at	32.9	13.3	-1.31	-2.48	AAA-AAA	0.00	999	1.000	1.000	0.2750
1558999_x_at	10.9	27.0	1.31	2.48	AMA-AAA	0.00	999	1.000	1.000	0.3418
215429_s_at	84.4	209.0	1.31	2.48	AAA-AAA	0.00	999	1.000	1.000	0.2310
206208_at	40.1	16.2	-1.31	-2.48	AAA-AAA	0.00	999	1.000	1.000	0.4309
208265_at	22.0	8.9	-1.31	-2.48	AAA-AAA	0.00	999	1.000	1.000	0.2687

231084_at	4.1	10.0	1.31	2.48	AAA-AAA	0.00	999	1.000	1.000	0.3572
240098_at	74.8	30.2	-1.31	-2.48	AAA-AAA	0.00	999	1.000	1.000	0.1102
240289_at	87.3	35.3	-1.31	-2.48	AAA-AAA	0.00	999	1.000	1.000	0.3212
216706_x_at	28.3	11.4	-1.31	-2.48	AAA-AAA	0.00	999	1.000	1.000	0.3230
230688_at	21.2	8.6	-1.31	-2.48	AAA-AAA	0.00	999	1.000	1.000	0.2204
1569345_at	6.1	15.0	1.31	2.47	AAA-AAA	0.00	999	1.000	1.000	0.2006
1556964_s_at	27.5	11.1	-1.31	-2.47	AAA-AAA	0.00	999	1.000	1.000	0.3443
244612_at	7.2	17.7	1.31	2.47	AAA-AAA	0.00	999	1.000	1.000	0.4071
220697_at	3.2	8.0	1.31	2.47	AAA-AAA	0.00	999	1.000	1.000	0.3377
205993_s_at	83.6	33.8	-1.31	-2.47	AMP-AAA	0.00	999	1.000	1.000	0.3715
1566462_at	9.3	23.1	1.31	2.47	AAA-AAA	0.00	999	1.000	1.000	0.2570
243362_s_at	24.5	60.7	1.31	2.47	AAA-AAA	0.00	999	1.000	1.000	0.3057
1559275_x_at	7.6	3.1	-1.31	-2.47	AAA-AAA	0.00	999	1.000	1.000	0.3167
213219_at	19.3	7.8	-1.31	-2.47	AAA-AAA	0.00	999	1.000	1.000	0.2765
220504_at	8.1	3.3	-1.31	-2.47	AAA-AAA	0.00	999	1.000	1.000	0.3920
217239_x_at	20.8	51.5	1.31	2.47	AAA-AAA	0.00	999	1.000	1.000	0.0007
235597_s_at	4.8	11.9	1.31	2.47	AAA-AAA	0.00	999	1.000	1.000	0.2572
228166_at	80.2	32.4	-1.31	-2.47	AAA-AAA	0.00	999	1.000	1.000	0.1069
228300_at	12.4	30.7	1.31	2.47	AAA-MAA	0.00	999	1.000	1.000	0.2946
222823_at	125.8	50.9	-1.31	-2.47	AAA-AAA	0.00	999	1.000	1.000	0.2232
1564165_at	77.7	31.5	-1.31	-2.47	AAA-AAP	0.00	999	1.000	1.000	0.3228
1570371_a_at	53.1	21.5	-1.31	-2.47	AAA-AAA	0.00	999	1.000	1.000	0.3572
239036_at	26.3	10.6	-1.31	-2.47	AAA-AAA	0.00	999	1.000	1.000	0.1989
1555132_at	38.3	15.5	-1.31	-2.47	AAA-AAA	0.00	999	1.000	1.000	0.4137
1561680_at	8.0	19.7	1.31	2.47	AAA-AAA	0.00	999	1.000	1.000	0.3775
244101_at	12.0	29.5	1.31	2.47	AAA-AAA	0.00	999	1.000	1.000	0.2690
1559992_a_at	14.9	6.0	-1.31	-2.47	AAA-AAA	0.00	999	1.000	1.000	0.3635
213725_x_at	44.4	18.0	-1.31	-2.47	AAA-AAA	0.00	999	1.000	1.000	0.3534
231511_at	39.0	15.8	-1.31	-2.47	AAA-AAA	0.00	999	1.000	1.000	0.1306
1561222_at	15.9	6.4	-1.30	-2.47	APA-AAA	0.00	999	1.000	1.000	0.4795
239911_at	23.6	9.6	-1.30	-2.47	AAA-AAA	0.00	999	1.000	1.000	0.4049
1565876_x_at	22.2	9.0	-1.30	-2.47	AAA-AAA	0.00	999	1.000	1.000	0.4556
1560552_a_at	36.5	14.8	-1.30	-2.47	AAA-AAA	0.00	999	1.000	1.000	0.3312
218537_at	66.2	26.8	-1.30	-2.47	AAA-AAA	0.00	999	1.000	1.000	0.0727
232639_at	17.0	6.9	-1.30	-2.47	AAA-AAA	0.00	999	1.000	1.000	0.2078
221605_s_at	76.2	30.9	-1.30	-2.47	PAP-AAA	0.00	999	1.000	1.000	0.0976
1569272_at	2.1	5.2	1.30	2.47	AAA-AAA	0.00	999	1.000	1.000	0.1248
240358_at	49.6	20.1	-1.30	-2.47	AAA-AAA	0.00	999	1.000	1.000	0.2954
220554_at	72.9	29.6	-1.30	-2.47	AAA-AAA	0.00	999	1.000	1.000	0.1405
209993_at	12.3	30.3	1.30	2.47	AAA-AAA	0.00	999	1.000	1.000	0.1942
238256_at	31.3	12.7	-1.30	-2.47	AAA-AAA	0.00	999	1.000	1.000	0.2648
236084_at	19.3	47.6	1.30	2.47	AAA-AAA	0.00	999	1.000	1.000	0.0967

224192_at	46.1	18.7	-1.30	-2.47	AAA-AAA	0.00	999	1.000	1.000	0.1051
231737_at	21.2	52.3	1.30	2.47	AAA-AAA	0.00	999	1.000	1.000	0.1681
229661_at	15.9	39.2	1.30	2.47	AAA-AAA	0.00	999	1.000	1.000	0.2993
1555990_at	80.7	32.7	-1.30	-2.47	AAA-AAA	0.00	999	1.000	1.000	0.3099
220707_s_at	127.0	51.5	-1.30	-2.47	AAA-AAA	0.00	999	1.000	1.000	0.2942
217683_at	19.7	48.7	1.30	2.47	AAA-AAA	0.00	999	1.000	1.000	0.3731
206029_at	43.0	17.4	-1.30	-2.47	AAA-AAA	0.00	999	1.000	1.000	0.2669
207085_x_at	65.9	26.8	-1.30	-2.47	AAA-AAA	0.00	999	1.000	1.000	0.1998
210081_at	42.1	17.1	-1.30	-2.47	AAA-AAA	0.00	999	1.000	1.000	0.3907
232828_at	27.5	11.1	-1.30	-2.47	AAA-AAA	0.00	999	1.000	1.000	0.2092
228371_s_at	15.0	37.0	1.30	2.47	AAA-AAA	0.00	999	1.000	1.000	0.1556
1559595_at	14.2	5.8	-1.30	-2.47	AAA-AAA	0.00	999	1.000	1.000	0.2289
219270_at	71.8	29.1	-1.30	-2.46	AAA-AAA	0.00	999	1.000	1.000	0.1247
227076_at	71.9	29.2	-1.30	-2.46	AAA-AAA	0.00	999	1.000	1.000	0.1810
1563044_at	4.3	10.6	1.30	2.46	AAA-AAA	0.00	999	1.000	1.000	0.2229
240381_at	40.7	100.3	1.30	2.46	AAA-AAA	0.00	999	1.000	1.000	0.2446
1567060_at	37.1	15.1	-1.30	-2.46	AAA-AAA	0.00	999	1.000	1.000	0.2363
1553367_a_at	126.4	51.3	-1.30	-2.46	AAA-AAA	0.00	999	1.000	1.000	0.1783
1553898_a_at	2.2	5.4	1.30	2.46	AAA-AAA	0.00	999	1.000	1.000	0.0592
1555169_at	57.4	23.3	-1.30	-2.46	AAA-AAA	0.00	999	1.000	1.000	0.2928
206310_at	10.3	25.4	1.30	2.46	AAA-AAA	0.00	999	1.000	1.000	0.1947
204410_at	8.3	20.5	1.30	2.46	AAA-AAA	0.00	999	1.000	1.000	0.2087
208568_at	47.4	116.8	1.30	2.46	AAA-AAA	0.00	999	1.000	1.000	0.2995
232142_at	9.8	24.1	1.30	2.46	AAA-AAA	0.00	999	1.000	1.000	0.2472
233419_at	45.4	18.5	-1.30	-2.46	APP-APA	0.00	999	1.000	1.000	0.2735
244449_at	78.8	32.0	-1.30	-2.46	AAP-AAA	0.00	999	1.000	1.000	0.3572
240758_at	10.6	26.1	1.30	2.46	AAA-AAA	0.00	999	1.000	1.000	0.2852
1552536_at	26.5	65.1	1.30	2.46	AAA-AAA	0.00	999	1.000	1.000	0.1118
1561106_at	6.5	16.0	1.30	2.46	AAA-AAA	0.00	999	1.000	1.000	0.3563
243991_at	2.1	5.2	1.30	2.46	AAA-AAA	0.00	999	1.000	1.000	0.1564
220722_s_at	16.6	6.8	-1.30	-2.46	AAA-AAA	0.00	999	1.000	1.000	0.4351
205387_s_at	18.8	46.3	1.30	2.46	AAA-AAA	0.00	999	1.000	1.000	0.2190
238212_at	25.2	10.2	-1.30	-2.46	AAA-AAA	0.00	999	1.000	1.000	0.4825
230818_at	11.6	28.6	1.30	2.46	AAA-AAA	0.00	999	1.000	1.000	0.2834
240559_at	6.9	16.9	1.30	2.46	AAA-AAA	0.00	999	1.000	1.000	0.3570
1553972_a_at	72.8	29.6	-1.30	-2.46	AAA-AAA	0.00	999	1.000	1.000	0.0777
1560097_at	92.0	37.4	-1.30	-2.46	AAA-AAA	0.00	999	1.000	1.000	0.3584
215020_at	5.5	13.4	1.30	2.46	AAA-AAA	0.00	999	1.000	1.000	0.0653
202063_s_at	33.1	81.4	1.30	2.46	AAA-AAA	0.00	999	1.000	1.000	0.2837
235454_at	34.7	14.1	-1.30	-2.46	AAA-AAA	0.00	999	1.000	1.000	0.2018
236351_at	7.2	17.7	1.30	2.46	AAA-AAA	0.00	999	1.000	1.000	0.1204
1565840_at	22.4	9.1	-1.30	-2.46	AAA-AAA	0.00	999	1.000	1.000	0.3758

1556709_a_at	18.3	44.9	1.30	2.46	AAA-AAA	0.00	999	1.000	1.000	0.0810
1554663_a_at	14.2	5.8	-1.30	-2.46	AAA-AAA	0.00	999	1.000	1.000	0.3056
1556256_a_at	4.1	10.0	1.30	2.46	AAA-AAA	0.00	999	1.000	1.000	0.1167
243437_at	24.2	59.4	1.30	2.46	AAA-AAA	0.00	999	1.000	1.000	0.2589
215531_s_at	8.0	19.8	1.30	2.46	AAA-AAA	0.00	999	1.000	1.000	0.2120
212797_at	41.6	102.3	1.30	2.46	AAA-AAA	0.00	999	1.000	1.000	0.2542
205935_at	8.2	20.1	1.30	2.46	AAA-AAA	0.00	999	1.000	1.000	0.0485
208538_at	11.4	4.6	-1.30	-2.46	AAA-AAA	0.00	999	1.000	1.000	0.2292
234374_at	9.8	4.0	-1.30	-2.46	AAA-AAA	0.00	999	1.000	1.000	0.0858
226299_at	57.9	142.2	1.30	2.46	AAA-AAA	0.00	999	1.000	1.000	0.1189
228794_at	21.4	8.7	-1.30	-2.46	AAA-AAA	0.00	999	1.000	1.000	0.5143
223496_s_at	15.1	6.2	-1.30	-2.46	AAA-AAA	0.00	999	1.000	1.000	0.3418
229386_at	28.0	11.4	-1.30	-2.46	AAA-AAA	0.00	999	1.000	1.000	0.3882
241684_at	67.0	27.3	-1.30	-2.46	AAP-AAA	0.00	999	1.000	1.000	0.1754
242802_x_at	18.2	7.4	-1.30	-2.46	AAA-PAA	0.00	999	1.000	1.000	0.4609
215861_at	72.4	29.5	-1.30	-2.46	AAP-PAP	0.00	999	1.000	1.000	0.4886
1552903_at	43.3	17.6	-1.30	-2.46	AAA-AAA	0.00	999	1.000	1.000	0.1831
1552986_at	23.1	56.8	1.30	2.46	AAA-AAA	0.00	999	1.000	1.000	0.0727
1560697_at	6.6	16.3	1.30	2.46	AAA-AAA	0.00	999	1.000	1.000	0.2290
206787_at	24.4	9.9	-1.30	-2.46	AAA-AAA	0.00	999	1.000	1.000	0.3802
240335_at	45.1	18.4	-1.30	-2.46	PPA-AAA	0.00	999	1.000	1.000	0.2696
1561277_at	51.5	21.0	-1.30	-2.45	MAA-APA	0.00	999	1.000	1.000	0.5146
1569596_at	19.7	8.0	-1.30	-2.45	AAA-AAA	0.00	999	1.000	1.000	0.4255
1565978_a_at	15.4	6.3	-1.30	-2.45	AAA-AAA	0.00	999	1.000	1.000	0.4994
240729_at	26.6	65.2	1.30	2.45	AAA-AAA	0.00	999	1.000	1.000	0.1971
1558372_at	6.3	15.5	1.30	2.45	AAA-AAA	0.00	999	1.000	1.000	0.4610
1553474_at	2.8	6.8	1.30	2.45	AAA-AAA	0.00	999	1.000	1.000	0.3758
242572_at	7.1	17.4	1.30	2.45	AAA-AAA	0.00	999	1.000	1.000	0.4088
206466_at	21.0	51.5	1.30	2.45	AAA-AAA	0.00	999	1.000	1.000	0.2677
209889_at	12.1	29.8	1.30	2.45	AAA-AAA	0.00	999	1.000	1.000	0.2203
228874_at	7.5	18.5	1.30	2.45	AAA-AAA	0.00	999	1.000	1.000	0.3283
224054_at	57.2	23.3	-1.30	-2.45	AAA-AAA	0.00	999	1.000	1.000	0.2928
228792_at	16.6	6.8	-1.29	-2.45	APA-PAA	0.00	999	1.000	1.000	0.4413
239495_at	14.5	35.5	1.29	2.45	AAA-AAA	0.00	999	1.000	1.000	0.3335
215226_at	9.7	23.8	1.29	2.45	AAA-AMA	0.00	999	1.000	1.000	0.2939
213797_at	16.3	6.6	-1.29	-2.45	AAA-AAA	0.00	999	1.000	1.000	0.1946
221321_s_at	13.6	33.4	1.29	2.45	AAA-AAA	0.00	999	1.000	1.000	0.3023
232434_at	36.8	15.0	-1.29	-2.45	AAA-AAA	0.00	999	1.000	1.000	0.3154
231761_at	69.7	28.4	-1.29	-2.45	AAA-AAA	0.00	999	1.000	1.000	0.1512
1563297_s_at	8.4	20.6	1.29	2.45	AAA-AAA	0.00	999	1.000	1.000	0.3214
244829_at	20.6	8.4	-1.29	-2.45	AAA-AAA	0.00	999	1.000	1.000	0.4212
1561558_at	3.4	8.3	1.29	2.45	AAA-AAA	0.00	999	1.000	1.000	0.2022

211588_s_at	50.7	20.7	-1.29	-2.45	AAA-AAA	0.00	999	1.000	1.000	0.1959
233071_at	20.8	8.5	-1.29	-2.45	AAA-AAA	0.00	999	1.000	1.000	0.2018
223629_at	23.5	9.6	-1.29	-2.45	AAA-AAA	0.00	999	1.000	1.000	0.5446
231228_at	13.8	33.7	1.29	2.45	AAA-AAA	0.00	999	1.000	1.000	0.0700
207184_at	168.8	68.9	-1.29	-2.45	APA-AAA	0.00	999	1.000	1.000	0.0830
215494_at	36.0	14.7	-1.29	-2.45	PAA-AAM	0.00	999	1.000	1.000	0.2817
238318_at	44.3	18.1	-1.29	-2.45	PAA-AAM	0.00	999	1.000	1.000	0.3375
1556685_at	16.8	41.2	1.29	2.45	AAA-AAA	0.00	999	1.000	1.000	0.4049
1553593_a_at	5.5	13.4	1.29	2.45	AAA-AAA	0.00	999	1.000	1.000	0.0031
214064_at	34.6	14.1	-1.29	-2.45	AAA-AAA	0.00	999	1.000	1.000	0.2442
207496_at	23.3	9.5	-1.29	-2.45	AAA-AAA	0.00	999	1.000	1.000	0.2694
234222_at	12.7	31.2	1.29	2.45	AAA-AAA	0.00	999	1.000	1.000	0.3839
1562023_at	38.0	15.5	-1.29	-2.45	APP-AAA	0.00	999	1.000	1.000	0.3225
241092_at	32.7	80.0	1.29	2.45	AAA-AAA	0.00	999	1.000	1.000	0.1830
1557373_at	18.9	46.2	1.29	2.45	AAA-AAA	0.00	999	1.000	1.000	0.2658
1558651_at	31.0	12.7	-1.29	-2.45	AAA-AAA	0.00	999	1.000	1.000	0.5330
1554333_at	9.7	4.0	-1.29	-2.45	AAA-AAA	0.00	999	1.000	1.000	0.5549
1560413_at	11.4	4.7	-1.29	-2.45	AAA-AAA	0.00	999	1.000	1.000	0.3519
216778_s_at	13.0	5.3	-1.29	-2.45	AAA-AAA	0.00	999	1.000	1.000	0.4664
216113_at	5.9	14.3	1.29	2.45	AAA-AAA	0.00	999	1.000	1.000	0.3155
206494_s_at	24.2	9.9	-1.29	-2.45	AAA-AAA	0.00	999	1.000	1.000	0.3771
234535_at	5.2	12.7	1.29	2.45	AAA-AAA	0.00	999	1.000	1.000	0.3499
238359_at	30.7	12.5	-1.29	-2.45	AAA-AAA	0.00	999	1.000	1.000	0.3466
223737_x_at	15.3	6.3	-1.29	-2.45	AAA-AAA	0.00	999	1.000	1.000	0.1863
231362_at	4.7	11.4	1.29	2.45	AAA-AAA	0.00	999	1.000	1.000	0.0561
231747_at	11.3	27.6	1.29	2.45	AAA-AAA	0.00	999	1.000	1.000	0.2297
1570398_at	3.0	7.4	1.29	2.45	AAA-AAA	0.00	999	1.000	1.000	0.4762
244453_at	104.8	42.8	-1.29	-2.45	AAA-AAA	0.00	999	1.000	1.000	0.2614
244206_at	11.5	28.0	1.29	2.45	AAA-AAA	0.00	999	1.000	1.000	0.2496
216909_at	73.3	30.0	-1.29	-2.45	AAA-AAA	0.00	999	1.000	1.000	0.0580
219436_s_at	5.5	13.4	1.29	2.45	AAA-AAA	0.00	999	1.000	1.000	0.1889
219554_at	56.8	23.2	-1.29	-2.45	AAA-AAA	0.00	999	1.000	1.000	0.4159
206415_at	13.5	33.1	1.29	2.45	AMA-AAA	0.00	999	1.000	1.000	0.3377
222780_s_at	19.2	46.8	1.29	2.44	AAA-AAA	0.00	999	1.000	1.000	0.3455
232390_at	3.9	1.6	-1.29	-2.45	AAA-AAA	0.00	999	1.000	1.000	0.1492
1566803_at	19.5	47.6	1.29	2.44	AAA-AAA	0.00	999	1.000	1.000	0.2713
1562381_at	8.0	3.3	-1.29	-2.44	AAA-AAA	0.00	999	1.000	1.000	0.3778
1559771_at	12.3	30.1	1.29	2.44	AAA-AAA	0.00	999	1.000	1.000	0.2217
1559760_at	5.6	2.3	-1.29	-2.44	AAA-AAA	0.00	999	1.000	1.000	0.4242
243960_x_at	8.5	20.8	1.29	2.44	AAA-AAA	0.00	999	1.000	1.000	0.1163
214019_at	73.5	30.1	-1.29	-2.44	AMA-AAA	0.00	999	1.000	1.000	0.3048
206359_at	4.8	11.6	1.29	2.44	AAA-AAA	0.00	999	1.000	1.000	0.0937

210190_at	14.7	6.0	-1.29	-2.44	AAA-AAA	0.00	999	1.000	1.000	0.0997
237854_at	40.7	99.4	1.29	2.44	AAA-AAA	0.00	999	1.000	1.000	0.2252
224370_s_at	24.4	10.0	-1.29	-2.44	AAA-AAA	0.00	999	1.000	1.000	0.2468
231743_at	27.2	11.1	-1.29	-2.44	AAA-AAA	0.00	999	1.000	1.000	0.2401
240803_at	85.1	34.9	-1.29	-2.44	APP-AAP	0.00	999	1.000	1.000	0.1953
1569156_at	37.4	15.3	-1.29	-2.44	AAA-AAA	0.00	999	1.000	1.000	0.2704
242649_x_at	23.3	9.6	-1.29	-2.44	AAA-AAA	0.00	999	1.000	1.000	0.2190
1559073_at	37.2	15.3	-1.29	-2.44	AAA-AAA	0.00	999	1.000	1.000	0.3982
244482_at	3.6	8.7	1.29	2.44	AAA-AAA	0.00	999	1.000	1.000	0.4655
216204_at	43.2	17.7	-1.29	-2.44	AAA-AAA	0.00	999	1.000	1.000	0.2065
238329_at	15.1	36.8	1.29	2.44	AAA-AAA	0.00	999	1.000	1.000	0.1172
220191_at	6.0	14.7	1.29	2.44	AAA-AAA	0.00	999	1.000	1.000	0.0568
207968_s_at	19.9	48.5	1.29	2.44	AAA-AAA	0.00	999	1.000	1.000	0.2689
207397_s_at	4.1	1.7	-1.29	-2.44	AAA-AAA	0.00	999	1.000	1.000	0.4125
233327_at	8.1	19.7	1.29	2.44	AAA-AAA	0.00	999	1.000	1.000	0.2269
233374_at	18.0	43.8	1.29	2.44	AAA-AAA	0.00	999	1.000	1.000	0.2358
233593_at	25.0	10.3	-1.29	-2.44	AAA-AAA	0.00	999	1.000	1.000	0.0865
236345_at	16.6	40.4	1.29	2.44	AAA-AAA	0.00	999	1.000	1.000	0.2935
230770_at	78.3	32.1	-1.29	-2.44	AAA-AAA	0.00	999	1.000	1.000	0.2125
1561461_at	3.9	9.5	1.29	2.44	AAA-AAA	0.00	999	1.000	1.000	0.4246
1560187_at	25.1	10.3	-1.29	-2.44	AAA-AAA	0.00	999	1.000	1.000	0.3687
220506_at	28.6	11.7	-1.29	-2.44	AAA-AAA	0.00	999	1.000	1.000	0.4511
239739_at	14.8	36.0	1.29	2.44	AAA-AAA	0.00	999	1.000	1.000	0.3674
243091_at	17.5	42.7	1.29	2.44	AAA-AAA	0.00	999	1.000	1.000	0.2651
1561924_at	6.0	14.7	1.29	2.44	AAA-AAA	0.00	999	1.000	1.000	0.1950
220361_at	10.0	24.5	1.29	2.44	AAA-AAA	0.00	999	1.000	1.000	0.3946
220000_at	52.7	21.6	-1.29	-2.44	AAA-AAA	0.00	999	1.000	1.000	0.1944
234388_at	29.8	12.3	-1.29	-2.44	AAA-AAA	0.00	999	1.000	1.000	0.3101
236246_x_at	63.5	26.1	-1.29	-2.44	AAA-AAA	0.00	999	1.000	1.000	0.1575
241155_at	13.9	5.7	-1.28	-2.44	AAA-AAA	0.00	999	1.000	1.000	0.3515
241956_at	32.1	78.1	1.28	2.44	AAA-AAA	0.00	999	1.000	1.000	0.2101
1570252_at	14.7	35.9	1.28	2.43	AAA-AAA	0.00	999	1.000	1.000	0.2062
1557720_s_at	20.6	50.1	1.28	2.44	AAA-AAA	0.00	999	1.000	1.000	0.2858
1554528_at	37.9	15.6	-1.28	-2.44	AAA-AAA	0.00	999	1.000	1.000	0.4513
219958_at	51.3	125.0	1.28	2.44	AAA-AAA	0.00	999	1.000	1.000	0.0763
218746_at	48.0	116.9	1.28	2.44	AAA-AAA	0.00	999	1.000	1.000	0.2825
205119_s_at	99.4	40.8	-1.28	-2.44	AAA-AAA	0.00	999	1.000	1.000	0.2813
210583_at	50.7	20.8	-1.28	-2.44	AAA-AAA	0.00	999	1.000	1.000	0.2012
235605_at	38.4	93.5	1.28	2.44	AAA-AAA	0.00	999	1.000	1.000	0.3784
235044_at	18.4	7.5	-1.28	-2.44	AAA-AAA	0.00	999	1.000	1.000	0.4144
238370_x_at	5.2	12.7	1.28	2.44	AAA-AAA	0.00	999	1.000	1.000	0.4631
229117_s_at	42.9	17.6	-1.28	-2.44	AAA-AAA	0.00	999	1.000	1.000	0.4191

242299_at	39.2	16.1	-1.28	-2.43	PAA-AAA	0.00	999	1.000	1.000	0.2440
239737_at	20.3	49.3	1.28	2.43	AAA-AAA	0.00	999	1.000	1.000	0.3623
1557233_at	12.8	31.1	1.28	2.43	AAA-AAA	0.00	999	1.000	1.000	0.1598
1555155_at	54.1	131.7	1.28	2.43	AAA-AAA	0.00	999	1.000	1.000	0.2436
243602_at	16.3	6.7	-1.28	-2.43	AAA-AAA	0.00	999	1.000	1.000	0.0877
220259_at	4.3	10.5	1.28	2.43	AAA-AAA	0.00	999	1.000	1.000	0.1797
211618_s_at	6.0	14.6	1.28	2.43	AAA-AAA	0.00	999	1.000	1.000	0.4129
207772_s_at	13.1	32.0	1.28	2.43	AAA-AAA	0.00	999	1.000	1.000	0.3014
230801_at	19.4	47.2	1.28	2.43	AAA-AAA	0.00	999	1.000	1.000	0.3229
1558963_at	30.9	12.7	-1.28	-2.43	APA-AAA	0.00	999	1.000	1.000	0.4008
242571_at	10.4	4.3	-1.28	-2.43	AAA-AAA	0.00	999	1.000	1.000	0.5391
1561527_at	3.3	8.1	1.28	2.43	AAA-AAA	0.00	999	1.000	1.000	0.3914
215106_at	68.7	28.2	-1.28	-2.43	AAA-AAA	0.00	999	1.000	1.000	0.0881
234290_x_at	30.0	73.1	1.28	2.43	AAA-AAA	0.00	999	1.000	1.000	0.2372
229759_s_at	39.5	16.2	-1.28	-2.43	AAA-AAA	0.00	999	1.000	1.000	0.2999
228921_at	27.6	67.2	1.28	2.43	AAA-AAA	0.00	999	1.000	1.000	0.3010
230593_at	32.4	13.3	-1.28	-2.43	AAA-AAA	0.00	999	1.000	1.000	0.1740
239670_at	20.4	49.6	1.28	2.43	AAA-AAA	0.00	999	1.000	1.000	0.4017
214514_at	6.2	15.1	1.28	2.43	AAA-AAA	0.00	999	1.000	1.000	0.2601
220568_at	10.7	4.4	-1.28	-2.43	AAA-AAA	0.00	999	1.000	1.000	0.2561
237762_at	3.5	8.5	1.28	2.43	AAA-AAA	0.00	999	1.000	1.000	0.2938
237180_at	80.3	33.1	-1.28	-2.43	AAA-AAA	0.00	999	1.000	1.000	0.4150
228804_at	49.1	119.3	1.28	2.43	AAA-AAA	0.00	999	1.000	1.000	0.1220
228762_at	60.5	146.9	1.28	2.43	AAA-AAM	0.00	999	1.000	1.000	0.1271
229555_at	10.7	25.9	1.28	2.43	AAA-AAA	0.00	999	1.000	1.000	0.0556
224014_at	61.8	25.4	-1.28	-2.43	PPA-AAA	0.00	999	1.000	1.000	0.2216
1555339_at	10.0	24.2	1.28	2.43	AAA-AAA	0.00	999	1.000	1.000	0.4189
244004_at	14.1	5.8	-1.28	-2.43	AAA-AAA	0.00	999	1.000	1.000	0.3968
219401_at	217.3	89.5	-1.28	-2.43	MAA-AAA	0.00	999	1.000	1.000	0.0277
204343_at	75.5	31.1	-1.28	-2.43	AAA-AAA	0.00	999	1.000	1.000	0.1072
241324_at	10.0	24.3	1.28	2.43	AAA-AAA	0.00	999	1.000	1.000	0.1371
1557235_at	12.1	29.5	1.28	2.43	AAA-AAA	0.00	999	1.000	1.000	0.3524
243342_at	9.5	23.1	1.28	2.43	AAA-AAA	0.00	999	1.000	1.000	0.3242
1561806_at	68.2	28.1	-1.28	-2.43	AAA-AAA	0.00	999	1.000	1.000	0.1119
211273_s_at	5.2	12.6	1.28	2.43	AAA-AAA	0.00	999	1.000	1.000	0.0832
234100_at	8.4	3.5	-1.28	-2.43	AAA-AAA	0.00	999	1.000	1.000	0.1997
234281_at	7.5	3.1	-1.28	-2.43	AAA-AAA	0.00	999	1.000	1.000	0.0530
236141_at	6.4	15.6	1.28	2.43	AAA-AAA	0.00	999	1.000	1.000	0.3397
223678_s_at	8.6	3.5	-1.28	-2.43	AAA-AAA	0.00	999	1.000	1.000	0.0462
231372_at	8.3	20.1	1.28	2.43	AAA-AAA	0.00	999	1.000	1.000	0.3139
230716_at	21.2	8.7	-1.28	-2.43	AAA-AAA	0.00	999	1.000	1.000	0.2272
1563120_at	9.2	22.2	1.28	2.43	AAA-AAA	0.00	999	1.000	1.000	0.3689

1570528_at	7.3	17.7	1.28	2.43	AAA-AAA	0.00	999	1.000	1.000	0.2552
240164_at	12.4	30.1	1.28	2.43	AAA-AAA	0.00	999	1.000	1.000	0.2049
1569630_a_at	5.8	14.0	1.28	2.43	AAA-AAA	0.00	999	1.000	1.000	0.4298
1555377_at	19.0	46.0	1.28	2.43	AAA-AAA	0.00	999	1.000	1.000	0.0468
219674_s_at	79.7	32.9	-1.28	-2.43	AAA-AAM	0.00	999	1.000	1.000	0.2613
219471_at	8.6	20.8	1.28	2.43	AAA-AAA	0.00	999	1.000	1.000	0.3548
224498_x_at	31.0	12.8	-1.28	-2.43	AAA-AAA	0.00	999	1.000	1.000	0.2238
228936_at	15.0	36.3	1.28	2.43	AAA-AAA	0.00	999	1.000	1.000	0.0292
232605_s_at	113.7	46.9	-1.28	-2.42	APP-AAA	0.00	999	1.000	1.000	0.0361
218694_at	48.0	19.8	-1.28	-2.42	PAA-AAA	0.00	999	1.000	1.000	0.3657
1561589_a_at	19.4	47.0	1.28	2.42	AAA-AAA	0.00	999	1.000	1.000	0.3140
205786_s_at	17.8	43.1	1.28	2.42	AAA-AAA	0.00	999	1.000	1.000	0.0347
AFFX-r2-Bs-dap-M_at	3.5	8.5	1.28	2.42	AAA-AAA	0.00	999	1.000	1.000	0.2817
233953_at	18.0	7.4	-1.28	-2.42	AAA-AAA	0.00	999	1.000	1.000	0.5301
236601_at	6.1	14.7	1.28	2.42	AAA-AAA	0.00	999	1.000	1.000	0.1072
222960_at	43.6	105.7	1.28	2.42	AAA-AAA	0.00	999	1.000	1.000	0.2520
229608_at	38.6	93.6	1.28	2.42	AAA-AAA	0.00	999	1.000	1.000	0.2075
231201_at	112.6	46.5	-1.28	-2.42	PAA-AAA	0.00	999	1.000	1.000	0.0785
1556323_at	20.5	8.5	-1.28	-2.42	APA-AAA	0.00	999	1.000	1.000	0.2326
241749_at	5.8	14.0	1.28	2.42	AAA-AAA	0.00	999	1.000	1.000	0.3693
1563295_at	48.5	117.5	1.28	2.42	AAA-AAA	0.00	999	1.000	1.000	0.1023
240404_at	24.0	58.0	1.28	2.42	AAA-AAA	0.00	999	1.000	1.000	0.1374
1568191_at	81.3	33.6	-1.28	-2.42	AAA-AAA	0.00	999	1.000	1.000	0.1984
1557615_a_at	12.9	31.3	1.28	2.42	AAA-AAA	0.00	999	1.000	1.000	0.1984
216496_s_at	12.8	5.3	-1.28	-2.42	AAA-AAA	0.00	999	1.000	1.000	0.1338
219425_at	19.0	46.0	1.28	2.42	AAA-AAA	0.00	999	1.000	1.000	0.3209
211190_x_at	28.9	11.9	-1.28	-2.42	AAA-AAA	0.00	999	1.000	1.000	0.1250
208222_at	30.9	74.8	1.28	2.42	AAA-AAA	0.00	999	1.000	1.000	0.2026
234659_at	6.7	16.2	1.28	2.42	AAA-AAA	0.00	999	1.000	1.000	0.4659
237657_at	10.4	4.3	-1.28	-2.42	AAA-AAA	0.00	999	1.000	1.000	0.1993
236464_at	25.0	10.3	-1.28	-2.42	AAA-AAA	0.00	999	1.000	1.000	0.4179
228705_at	17.1	41.4	1.28	2.42	AAA-AAA	0.00	999	1.000	1.000	0.3105
231231_at	34.6	14.3	-1.28	-2.42	AAA-AAA	0.00	999	1.000	1.000	0.0314
217481_x_at	64.8	26.8	-1.28	-2.42	PPP-AMA	0.00	999	1.000	1.000	0.2145
232851_at	63.2	26.1	-1.28	-2.42	AAA-AAP	0.00	999	1.000	1.000	0.2894
242410_s_at	13.8	5.7	-1.28	-2.42	AAA-AAA	0.00	999	1.000	1.000	0.5947
1570065_at	85.9	35.5	-1.28	-2.42	AAA-AAA	0.00	999	1.000	1.000	0.3591
237423_at	17.9	7.4	-1.28	-2.42	AAA-AAA	0.00	999	1.000	1.000	0.1493
236669_at	17.4	42.1	1.28	2.42	AAA-AAA	0.00	999	1.000	1.000	0.0392
236894_at	15.3	6.3	-1.28	-2.42	AAA-AAA	0.00	999	1.000	1.000	0.4693
211032_at	53.6	22.2	-1.27	-2.42	APP-APA	0.00	999	1.000	1.000	0.0781
1561433_at	76.8	31.8	-1.27	-2.42	AAP-AAA	0.00	999	1.000	1.000	0.2854

240091_at	48.6	20.1	-1.27	-2.42	APA-AAA	0.00	999	1.000	1.000	0.2920
1570587_at	19.6	8.1	-1.27	-2.42	AAA-AAA	0.00	999	1.000	1.000	0.3812
1569487_at	11.5	27.8	1.27	2.42	AAA-AAA	0.00	999	1.000	1.000	0.2089
1557777_at	32.3	13.4	-1.27	-2.42	AAA-AAA	0.00	999	1.000	1.000	0.2691
1553196_a_at	5.9	14.3	1.27	2.42	AAA-AAA	0.00	999	1.000	1.000	0.1547
243879_at	31.0	74.9	1.27	2.42	AAA-AAA	0.00	999	1.000	1.000	0.1528
205821_at	34.8	84.2	1.27	2.42	AAA-AAA	0.00	999	1.000	1.000	0.1556
204626_s_at	65.0	26.9	-1.27	-2.42	AAA-AAA	0.00	999	1.000	1.000	0.1092
237892_at	51.9	21.5	-1.27	-2.42	AAA-AAA	0.00	999	1.000	1.000	0.2128
237139_at	13.0	31.4	1.27	2.42	AAA-AAA	0.00	999	1.000	1.000	0.3050
230899_at	22.7	54.8	1.27	2.42	AAA-AAA	0.00	999	1.000	1.000	0.1933
1559003_a_at	128.4	53.1	-1.27	-2.42	APP-AMA	0.00	999	1.000	1.000	0.2121
1562776_at	18.1	43.7	1.27	2.42	AAA-AAA	0.00	999	1.000	1.000	0.5144
1557621_at	19.6	47.2	1.27	2.42	AAA-AAA	0.00	999	1.000	1.000	0.2474
1560510_at	6.4	15.5	1.27	2.42	AAA-AAA	0.00	999	1.000	1.000	0.2666
220556_at	6.5	2.7	-1.27	-2.42	AAA-AAA	0.00	999	1.000	1.000	0.0314
221157_s_at	18.0	43.4	1.27	2.42	AAA-AAA	0.00	999	1.000	1.000	0.1507
206259_at	18.3	7.6	-1.27	-2.42	AAA-AAA	0.00	999	1.000	1.000	0.0948
206140_at	70.9	29.3	-1.27	-2.42	AAA-AAA	0.00	999	1.000	1.000	0.2698
237552_at	10.3	4.3	-1.27	-2.42	AAA-AAA	0.00	999	1.000	1.000	0.3279
222847_s_at	24.6	10.2	-1.27	-2.42	AAA-AAA	0.00	999	1.000	1.000	0.2831
233383_at	37.3	15.5	-1.27	-2.42	APA-AAP	0.00	999	1.000	1.000	0.3766
1557534_at	1.3	3.2	1.27	2.42	AAA-AAA	0.00	999	1.000	1.000	0.1539
243208_x_at	48.8	20.2	-1.27	-2.42	AAA-AAA	0.00	999	1.000	1.000	0.1063
1560706_at	19.0	45.8	1.27	2.42	AAA-AAA	0.00	999	1.000	1.000	0.2845
216584_at	9.1	21.9	1.27	2.42	AAA-AAA	0.00	999	1.000	1.000	0.1543
220111_s_at	6.0	14.6	1.27	2.41	AAA-AAA	0.00	999	1.000	1.000	0.4153
220850_at	11.4	4.7	-1.27	-2.41	AAA-AAA	0.00	999	1.000	1.000	0.1840
219823_at	61.5	158.6	1.27	2.42	MMP-PPP	0.00	999	1.000	1.000	0.0243
234063_at	54.2	22.5	-1.27	-2.42	AAA-AAA	0.00	999	1.000	1.000	0.1860
233034_at	8.7	3.6	-1.27	-2.42	AAA-AAA	0.00	999	1.000	1.000	0.3776
227302_s_at	43.2	17.9	-1.27	-2.42	AAA-AAA	0.00	999	1.000	1.000	0.1568
234565_x_at	95.1	39.4	-1.27	-2.41	APA-AAA	0.00	999	1.000	1.000	0.2580
237988_at	19.2	8.0	-1.27	-2.41	PAA-AAA	0.00	999	1.000	1.000	0.3150
240657_at	9.8	23.6	1.27	2.41	AAA-AAA	0.00	999	1.000	1.000	0.3593
1556128_a_at	28.7	69.1	1.27	2.41	AAA-AAA	0.00	999	1.000	1.000	0.4828
1561146_at	10.1	24.3	1.27	2.41	AAA-AAA	0.00	999	1.000	1.000	0.3544
216424_at	6.3	2.6	-1.27	-2.41	AAA-AAA	0.00	999	1.000	1.000	0.3104
217160_at	7.3	17.6	1.27	2.41	AAA-AAA	0.00	999	1.000	1.000	0.2629
211324_s_at	4.6	11.1	1.27	2.41	AAA-AAA	0.00	999	1.000	1.000	0.3102
237518_at	156.0	64.7	-1.27	-2.41	AAA-AAA	0.00	999	1.000	1.000	0.0704
238298_at	7.9	19.0	1.27	2.41	AAA-AAA	0.00	999	1.000	1.000	0.1686

228790_at	35.5	14.7	-1.27	-2.41	AAA-AAA	0.00	999	1.000	1.000	0.4484
1553666_at	35.9	14.9	-1.27	-2.41	AAP-AAA	0.00	999	1.000	1.000	0.4066
1559033_at	25.0	60.3	1.27	2.41	AAA-AAA	0.00	999	1.000	1.000	0.2723
233031_at	13.1	5.4	-1.27	-2.41	AAA-AAA	0.00	999	1.000	1.000	0.3268
237880_at	11.3	27.2	1.27	2.41	AAA-AAA	0.00	999	1.000	1.000	0.3448
238850_at	21.8	9.0	-1.27	-2.41	AAA-AAA	0.00	999	1.000	1.000	0.3487
222949_at	52.1	21.6	-1.27	-2.41	AAA-AAA	0.00	999	1.000	1.000	0.1127
243571_at	35.3	14.7	-1.27	-2.41	AAA-AAA	0.00	999	1.000	1.000	0.0793
1562038_at	9.8	23.6	1.27	2.41	AAA-AAA	0.00	999	1.000	1.000	0.3086
207120_at	5.8	14.0	1.27	2.41	AAA-AAA	0.00	999	1.000	1.000	0.1392
210147_at	16.4	39.6	1.27	2.41	MAA-AAA	0.00	999	1.000	1.000	0.3355
237993_at	33.2	13.8	-1.27	-2.41	AAA-AAA	0.00	999	1.000	1.000	0.1340
1562972_at	12.0	5.0	-1.27	-2.41	AAA-AAA	0.00	999	1.000	1.000	0.4514
242455_at	11.7	28.2	1.27	2.41	AAA-AAA	0.00	999	1.000	1.000	0.4462
240194_at	41.5	17.2	-1.27	-2.41	AAA-AAA	0.00	999	1.000	1.000	0.2564
1569777_a_at	4.4	10.6	1.27	2.41	AAA-AAA	0.00	999	1.000	1.000	0.3962
1556127_at	57.6	23.9	-1.27	-2.41	AAA-AAA	0.00	999	1.000	1.000	0.5799
216795_at	27.1	11.3	-1.27	-2.41	AAA-AAA	0.00	999	1.000	1.000	0.4885
212618_at	121.4	50.4	-1.27	-2.41	AAA-AAA	0.00	999	1.000	1.000	0.3806
221204_s_at	34.1	82.2	1.27	2.41	AAA-AAA	0.00	999	1.000	1.000	0.3236
228756_at	109.5	45.5	-1.27	-2.41	AAA-AAA	0.00	999	1.000	1.000	0.1452
240566_at	30.8	12.8	-1.27	-2.41	AAA-AAA	0.00	999	1.000	1.000	0.2916
240519_at	95.1	39.5	-1.27	-2.41	AAA-AAA	0.00	999	1.000	1.000	0.1838
1554335_at	13.3	5.5	-1.27	-2.41	AAA-AAA	0.00	999	1.000	1.000	0.1471
1562278_at	40.9	17.0	-1.27	-2.41	AAA-AAA	0.00	999	1.000	1.000	0.3297
220799_at	6.5	15.6	1.27	2.41	AAA-AAA	0.00	999	1.000	1.000	0.2513
207006_s_at	22.9	9.5	-1.27	-2.41	AAA-AAA	0.00	999	1.000	1.000	0.2209
236319_at	24.0	10.0	-1.27	-2.41	AAA-AAA	0.00	999	1.000	1.000	0.2804
236499_at	41.0	98.6	1.27	2.41	AAA-AAA	0.00	999	1.000	1.000	0.1780
1552751_a_at	43.6	105.0	1.27	2.41	AAA-AAA	0.00	999	1.000	1.000	0.0109
1553592_x_at	39.5	94.9	1.27	2.41	AAA-AAA	0.00	999	1.000	1.000	0.1105
221544_s_at	62.2	149.7	1.27	2.41	AAA-AAA	0.00	999	1.000	1.000	0.0410
207033_at	12.5	30.2	1.27	2.41	AAA-AAA	0.00	999	1.000	1.000	0.3350
236543_at	9.7	23.3	1.27	2.41	AAA-AAA	0.00	999	1.000	1.000	0.4213
223676_at	30.6	12.7	-1.27	-2.41	AAA-AAA	0.00	999	1.000	1.000	0.1640
1569277_at	6.3	15.0	1.27	2.40	AAM-AAA	0.00	999	1.000	1.000	0.4334
233545_at	32.5	13.5	-1.27	-2.40	AAA-AAA	0.00	999	1.000	1.000	0.2684
237261_at	30.8	12.8	-1.27	-2.40	AAA-AAA	0.00	999	1.000	1.000	0.3688
228601_at	10.4	25.0	1.27	2.40	AAA-AAA	0.00	999	1.000	1.000	0.2018
1566183_at	23.2	55.8	1.26	2.40	AAA-AAA	0.00	999	1.000	1.000	0.0767
1556804_s_at	22.7	9.5	-1.26	-2.40	AAA-AAA	0.00	999	1.000	1.000	0.3728
221402_at	98.5	41.0	-1.26	-2.40	AAA-AAA	0.00	999	1.000	1.000	0.3666

201638_s_at	138.3	57.6	-1.26	-2.40	AAM-AAA	0.00	999	1.000	1.000	0.4837
209443_at	11.8	28.3	1.26	2.40	AAA-AAA	0.00	999	1.000	1.000	0.3532
237593_at	4.4	10.5	1.26	2.40	AAA-AAA	0.00	999	1.000	1.000	0.2995
236060_at	41.2	98.9	1.26	2.40	AAA-AAA	0.00	999	1.000	1.000	0.2456
231422_x_at	91.9	38.3	-1.26	-2.40	AAA-AAA	0.00	999	1.000	1.000	0.2799
241025_at	23.8	9.9	-1.26	-2.40	AAA-AAA	0.00	999	1.000	1.000	0.4321
1562002_at	118.8	49.5	-1.26	-2.40	AAA-AAA	0.00	999	1.000	1.000	0.2151
215300_s_at	8.7	21.0	1.26	2.40	AAA-AAA	0.00	999	1.000	1.000	0.2429
233377_at	6.5	15.6	1.26	2.40	AAA-AAA	0.00	999	1.000	1.000	0.3998
238194_at	11.5	27.6	1.26	2.40	AAA-AAA	0.00	999	1.000	1.000	0.3030
228827_at	22.0	9.2	-1.26	-2.40	AAA-AAA	0.00	999	1.000	1.000	0.3374
222102_at	8.9	21.4	1.26	2.40	AAA-AAA	0.00	999	1.000	1.000	0.0817
223753_s_at	54.8	22.9	-1.26	-2.40	AAA-AAP	0.00	999	1.000	1.000	0.1548
1557896_at	3.2	7.8	1.26	2.40	AAA-AAA	0.00	999	1.000	1.000	0.3058
1554891_at	13.9	5.8	-1.26	-2.40	AAA-AAA	0.00	999	1.000	1.000	0.0902
1560943_s_at	71.7	29.9	-1.26	-2.40	AAA-AAA	0.00	999	1.000	1.000	0.3084
1562240_at	4.8	11.6	1.26	2.40	AAA-AAA	0.00	999	1.000	1.000	0.2657
242666_at	47.7	19.9	-1.26	-2.40	AAA-AAA	0.00	999	1.000	1.000	0.0554
243038_at	8.8	21.2	1.26	2.40	AAA-AAA	0.00	999	1.000	1.000	0.2684
216665_s_at	5.4	13.0	1.26	2.40	AAA-AAA	0.00	999	1.000	1.000	0.4705
217095_x_at	26.0	62.3	1.26	2.40	AAA-AAA	0.00	999	1.000	1.000	0.2710
201496_x_at	36.1	86.6	1.26	2.40	AAA-AAA	0.00	999	1.000	1.000	0.1532
238697_at	10.0	24.0	1.26	2.40	AAA-AAA	0.00	999	1.000	1.000	0.4437
237308_at	31.5	13.1	-1.26	-2.40	PAA-AAA	0.00	999	1.000	1.000	0.2176
217246_s_at	72.4	30.2	-1.26	-2.40	AAP-APA	0.00	999	1.000	1.000	0.2936
1570281_at	93.4	39.0	-1.26	-2.40	AAA-AAA	0.00	999	1.000	1.000	0.1946
241415_at	11.6	27.7	1.26	2.40	AAA-AAA	0.00	999	1.000	1.000	0.0981
1565826_at	6.5	15.6	1.26	2.40	AAA-AAA	0.00	999	1.000	1.000	0.2245
1560955_at	37.3	15.6	-1.26	-2.40	AAA-AAA	0.00	999	1.000	1.000	0.2539
214388_at	36.4	15.2	-1.26	-2.40	AAA-AAA	0.00	999	1.000	1.000	0.3432
232745_x_at	29.5	12.3	-1.26	-2.40	AAA-AAA	0.00	999	1.000	1.000	0.3945
1564547_x_at	21.3	51.0	1.26	2.40	AAA-AMA	0.00	999	1.000	1.000	0.0845
240838_s_at	23.2	9.7	-1.26	-2.40	AAA-AAA	0.00	999	1.000	1.000	0.2854
1558745_at	30.0	12.5	-1.26	-2.39	AAA-AAA	0.00	999	1.000	1.000	0.1868
1560958_s_at	26.7	64.0	1.26	2.40	AAA-AAA	0.00	999	1.000	1.000	0.2175
1559277_at	16.5	6.9	-1.26	-2.40	AAA-AAA	0.00	999	1.000	1.000	0.3453
227468_at	8.3	19.9	1.26	2.40	AAA-AAA	0.00	999	1.000	1.000	0.0280
230940_at	4.1	9.9	1.26	2.40	AAA-AAA	0.00	999	1.000	1.000	0.0010
AFFX-DapX-5_at	39.9	16.7	-1.26	-2.39	APA-AAA	0.00	999	1.000	1.000	0.3096
243962_at	55.3	23.1	-1.26	-2.39	PAA-AAA	0.00	999	1.000	1.000	0.3317
241442_at	13.6	32.6	1.26	2.39	AAA-AAA	0.00	999	1.000	1.000	0.4106
1562732_at	41.6	17.4	-1.26	-2.39	AAA-AAA	0.00	999	1.000	1.000	0.3697

239353_at	16.1	38.6	1.26	2.39	AMA-AAA	0.00	999	1.000	1.000	0.2946
1553253_at	39.4	94.2	1.26	2.39	AAA-AAA	0.00	999	1.000	1.000	0.1633
1561834_a_at	17.1	7.1	-1.26	-2.39	AAA-AAA	0.00	999	1.000	1.000	0.1464
215305_at	25.3	10.6	-1.26	-2.39	AAA-AAA	0.00	999	1.000	1.000	0.0723
213456_at	9.4	22.4	1.26	2.39	AAA-AAA	0.00	999	1.000	1.000	0.3258
214371_at	28.6	11.9	-1.26	-2.39	AAA-AAA	0.00	999	1.000	1.000	0.2640
221384_at	12.4	5.2	-1.26	-2.39	AAA-AAA	0.00	999	1.000	1.000	0.0184
211122_s_at	21.8	9.1	-1.26	-2.39	AAA-AAA	0.00	999	1.000	1.000	0.2024
223045_at	15.1	36.2	1.26	2.39	AAA-AAA	0.00	999	1.000	1.000	0.3441
220275_at	124.8	52.2	-1.26	-2.39	PAP-PAA	0.00	999	1.000	1.000	0.3167
1570541_s_at	9.4	22.6	1.26	2.39	AAA-AAA	0.00	999	1.000	1.000	0.1738
243834_at	45.3	108.3	1.26	2.39	AAA-AAA	0.00	999	1.000	1.000	0.0255
212278_x_at	21.9	9.2	-1.26	-2.39	AAA-AAA	0.00	999	1.000	1.000	0.1221
217700_at	26.4	63.1	1.26	2.39	AAA-AAA	0.00	999	1.000	1.000	0.2139
211001_at	21.0	50.2	1.26	2.39	AAA-AAA	0.00	999	1.000	1.000	0.1830
236289_at	8.7	20.9	1.26	2.39	AAA-AAA	0.00	999	1.000	1.000	0.2530
241855_s_at	20.9	50.0	1.26	2.39	AAA-AAA	0.00	999	1.000	1.000	0.2339
1569462_x_at	24.4	10.2	-1.26	-2.39	AAA-AAA	0.00	999	1.000	1.000	0.3596
1558765_a_at	70.6	29.5	-1.26	-2.39	AAA-AAA	0.00	999	1.000	1.000	0.1391
1554798_at	50.5	21.1	-1.26	-2.39	AAA-AAA	0.00	999	1.000	1.000	0.3733
215565_at	4.1	9.8	1.26	2.39	AAA-AAA	0.00	999	1.000	1.000	0.1260
216812_at	10.5	25.1	1.26	2.39	AAA-AAA	0.00	999	1.000	1.000	0.4374
213533_at	138.4	57.9	-1.26	-2.39	AAA-AAA	0.00	999	1.000	1.000	0.2790
213981_at	36.5	87.1	1.26	2.39	AAA-AAA	0.00	999	1.000	1.000	0.0709
220505_at	12.1	28.9	1.26	2.39	AAA-AAA	0.00	999	1.000	1.000	0.0495
207352_s_at	63.9	26.7	-1.26	-2.39	AAA-AAA	0.00	999	1.000	1.000	0.2340
232624_at	11.6	27.6	1.26	2.39	AAA-AAA	0.00	999	1.000	1.000	0.2405
216466_at	34.5	14.5	-1.26	-2.39	AMP-AAA	0.00	999	1.000	1.000	0.0752
1564077_at	9.7	4.1	-1.26	-2.39	AAA-AAA	0.00	999	1.000	1.000	0.2707
1557139_at	8.3	19.9	1.26	2.39	AAA-AAA	0.00	999	1.000	1.000	0.3191
1560100_at	30.4	12.7	-1.26	-2.39	AAA-AAA	0.00	999	1.000	1.000	0.0232
220059_at	5.4	12.8	1.26	2.39	AAA-AAA	0.00	999	1.000	1.000	0.2836
203191_at	4.7	11.3	1.26	2.39	AAA-AAA	0.00	999	1.000	1.000	0.3556
211645_x_at	5.3	12.7	1.26	2.39	AAA-AAA	0.00	999	1.000	1.000	0.0578
208213_s_at	6.7	15.9	1.26	2.39	AAA-AAA	0.00	999	1.000	1.000	0.0483
208359_s_at	11.2	26.8	1.26	2.39	AAA-AAA	0.00	999	1.000	1.000	0.2979
228175_at	70.1	29.4	-1.26	-2.39	MAA-AAA	0.00	999	1.000	1.000	0.1738
229931_at	9.7	23.1	1.26	2.39	AAA-AAA	0.00	999	1.000	1.000	0.2885
206157_at	153.5	64.3	-1.26	-2.39	PPP-PPA	0.00	999	1.000	1.000	0.0913
1559627_at	54.4	22.8	-1.26	-2.39	PAA-AAA	0.00	999	1.000	1.000	0.2783
215669_at	42.5	17.8	-1.26	-2.39	PAA-AAA	0.00	999	1.000	1.000	0.3121
1562982_at	19.4	8.1	-1.26	-2.39	AAA-AAA	0.00	999	1.000	1.000	0.4017

241043_at	41.1	17.2	-1.26	-2.39	AAA-AAA	0.00	999	1.000	1.000	0.0830
219140_s_at	14.3	34.1	1.26	2.39	AAA-AAA	0.00	999	1.000	1.000	0.4479
205810_s_at	70.8	29.7	-1.26	-2.39	AAA-AAA	0.00	999	1.000	1.000	0.2428
236390_at	113.5	47.5	-1.26	-2.39	AAM-AAA	0.00	999	1.000	1.000	0.3688
222226_at	8.8	21.0	1.26	2.39	AAA-AAA	0.00	999	1.000	1.000	0.0473
223985_at	15.3	6.4	-1.26	-2.39	AAA-AAA	0.00	999	1.000	1.000	0.1954
231516_at	17.3	41.3	1.26	2.39	AAA-AAA	0.00	999	1.000	1.000	0.4819
231533_at	73.8	30.9	-1.26	-2.39	AAA-AAA	0.00	999	1.000	1.000	0.4078
230373_at	27.9	11.7	-1.26	-2.39	AAA-AAA	0.00	999	1.000	1.000	0.3032
239214_at	6.2	14.7	1.25	2.39	AAA-AAA	0.00	999	1.000	1.000	0.2652
220114_s_at	37.8	90.1	1.25	2.39	AAA-AAA	0.00	999	1.000	1.000	0.0565
219777_at	31.0	13.0	-1.25	-2.39	AAA-AAA	0.00	999	1.000	1.000	0.3791
202977_s_at	62.7	26.3	-1.25	-2.39	AAA-AAA	0.00	999	1.000	1.000	0.2542
204987_at	31.8	13.4	-1.25	-2.39	AAA-AAA	0.00	999	1.000	1.000	0.1297
62987_r_at	180.8	431.0	1.25	2.39	AAA-AAA	0.00	999	1.000	1.000	0.1368
219984_s_at	31.3	13.1	-1.25	-2.38	PAA-AAA	0.00	999	1.000	1.000	0.1299
243702_at	89.5	37.6	-1.25	-2.38	PPP-PPA	0.00	999	1.000	1.000	0.3416
241101_at	8.8	3.7	-1.25	-2.38	AAA-AAA	0.00	999	1.000	1.000	0.4955
1552716_at	36.6	87.3	1.25	2.38	AAA-AAA	0.00	999	1.000	1.000	0.2285
1555626_a_at	15.2	36.3	1.25	2.38	AAA-AAA	0.00	999	1.000	1.000	0.2111
243620_at	6.7	16.0	1.25	2.38	AAA-AAA	0.00	999	1.000	1.000	0.2558
1560431_at	5.2	12.4	1.25	2.38	AAA-AAA	0.00	999	1.000	1.000	0.3793
219455_at	8.3	19.9	1.25	2.38	AAA-AAA	0.00	999	1.000	1.000	0.2840
209813_x_at	25.1	10.5	-1.25	-2.38	AAA-AAA	0.00	999	1.000	1.000	0.3677
211419_s_at	26.0	62.0	1.25	2.38	AAA-AAA	0.00	999	1.000	1.000	0.0602
211836_s_at	12.0	28.7	1.25	2.38	AAA-AAA	0.00	999	1.000	1.000	0.2486
222918_at	39.0	93.0	1.25	2.38	AAA-AAA	0.00	999	1.000	1.000	0.1735
242231_at	49.1	20.6	-1.25	-2.38	AAA-AAA	0.00	999	1.000	1.000	0.2985
239055_at	18.1	7.6	-1.25	-2.38	AAA-AAA	0.00	999	1.000	1.000	0.1375
239048_at	15.9	37.9	1.25	2.38	AAA-AAA	0.00	999	1.000	1.000	0.2218
240548_at	20.6	8.6	-1.25	-2.38	AAA-AAA	0.00	999	1.000	1.000	0.3982
1555935_s_at	11.0	26.1	1.25	2.38	AAA-AAA	0.00	999	1.000	1.000	0.4160
1561161_at	15.2	36.1	1.25	2.38	AAA-AAA	0.00	999	1.000	1.000	0.3109
216853_x_at	49.0	20.6	-1.25	-2.38	AAA-AAA	0.00	999	1.000	1.000	0.2920
215060_at	52.6	22.1	-1.25	-2.38	AAA-AAA	0.00	999	1.000	1.000	0.0353
207066_at	25.5	10.7	-1.25	-2.38	AAA-AAA	0.00	999	1.000	1.000	0.1275
236775_s_at	11.1	26.5	1.25	2.38	AAA-AAA	0.00	999	1.000	1.000	0.1567
241888_at	29.4	12.4	-1.25	-2.38	APA-AAA	0.00	999	1.000	1.000	0.1323
1560189_at	17.4	41.3	1.25	2.38	AAA-AAA	0.00	999	1.000	1.000	0.1512
217546_at	31.4	13.2	-1.25	-2.38	AAA-AAA	0.00	999	1.000	1.000	0.3858
204755_x_at	22.7	9.5	-1.25	-2.38	AAA-AAA	0.00	999	1.000	1.000	0.5515
211044_at	14.0	33.4	1.25	2.38	AAA-AAA	0.00	999	1.000	1.000	0.2529

207518_at	57.5	24.2	-1.25	-2.38	AAM-AAA	0.00	999	1.000	1.000	0.1371
236534_at	37.1	15.6	-1.25	-2.38	AAA-AAA	0.00	999	1.000	1.000	0.1635
229777_at	14.1	5.9	-1.25	-2.38	AAA-AAA	0.00	999	1.000	1.000	0.0511
216302_at	225.1	94.7	-1.25	-2.38	PPA-AAP	0.00	999	1.000	1.000	0.2288
1557776_at	9.4	22.3	1.25	2.38	AAA-AAA	0.00	999	1.000	1.000	0.5385
1554199_at	65.4	27.5	-1.25	-2.38	AAA-AAA	0.00	999	1.000	1.000	0.4505
1556107_at	32.1	76.4	1.25	2.38	AAA-AAA	0.00	999	1.000	1.000	0.1432
1555318_at	9.7	23.0	1.25	2.38	AAA-AAA	0.00	999	1.000	1.000	0.1115
213432_at	20.6	8.7	-1.25	-2.38	AAA-AAA	0.00	999	1.000	1.000	0.1205
219853_at	6.4	15.3	1.25	2.38	AAA-AAA	0.00	999	1.000	1.000	0.0650
206111_at	100.3	42.2	-1.25	-2.38	AAM-AAA	0.00	999	1.000	1.000	0.0089
204200_s_at	7.8	18.6	1.25	2.38	AAA-AAA	0.00	999	1.000	1.000	0.0019
224067_at	15.9	6.7	-1.25	-2.38	AAA-AAA	0.00	999	1.000	1.000	0.4639
224292_at	7.0	16.7	1.25	2.38	AAA-AAA	0.00	999	1.000	1.000	0.2699
231100_at	7.9	18.8	1.25	2.38	AAA-AAA	0.00	999	1.000	1.000	0.2050
241410_at	36.8	87.5	1.25	2.38	AAA-AAA	0.00	999	1.000	1.000	0.2045
1561000_at	18.5	43.9	1.25	2.38	AAA-AAA	0.00	999	1.000	1.000	0.4607
1562600_at	9.5	22.6	1.25	2.38	AAA-AAA	0.00	999	1.000	1.000	0.3769
233615_at	13.2	5.5	-1.25	-2.38	AAA-AAA	0.00	999	1.000	1.000	0.5186
237256_at	11.7	27.9	1.25	2.38	AAA-AAA	0.00	999	1.000	1.000	0.3060
228407_at	44.6	106.0	1.25	2.38	AAA-AAA	0.00	999	1.000	1.000	0.1468
239704_at	58.0	137.8	1.25	2.38	AAA-AAA	0.00	999	1.000	1.000	0.1830
1565874_at	11.7	27.7	1.25	2.38	AAA-AAA	0.00	999	1.000	1.000	0.3964
1554615_at	35.1	14.8	-1.25	-2.38	AAA-AAA	0.00	999	1.000	1.000	0.3890
207056_s_at	10.8	4.6	-1.25	-2.37	AAA-AAA	0.00	999	1.000	1.000	0.3884
210504_at	21.7	9.1	-1.25	-2.38	AAA-AAA	0.00	999	1.000	1.000	0.3512
234842_at	22.0	9.2	-1.25	-2.38	AAA-AAA	0.00	999	1.000	1.000	0.1705
235836_at	20.3	8.6	-1.25	-2.38	AAA-AAA	0.00	999	1.000	1.000	0.1333
228681_x_at	3.0	7.2	1.25	2.37	AAA-AAA	0.00	999	1.000	1.000	0.0150
222323_at	56.4	23.7	-1.25	-2.38	AAA-AAA	0.00	999	1.000	1.000	0.4233
1557710_at	37.7	15.9	-1.25	-2.37	AAA-AAA	0.00	999	1.000	1.000	0.3746
1555526_a_at	25.1	59.7	1.25	2.37	AAA-AAA	0.00	999	1.000	1.000	0.2329
1559311_at	32.1	13.5	-1.25	-2.37	AAA-AAA	0.00	999	1.000	1.000	0.0373
1560764_at	26.9	11.3	-1.25	-2.37	AAA-AAA	0.00	999	1.000	1.000	0.0653
216659_at	6.9	16.4	1.25	2.37	AAA-AAA	0.00	999	1.000	1.000	0.3195
215043_s_at	14.8	35.2	1.25	2.37	AAA-AAA	0.00	999	1.000	1.000	0.2726
222253_s_at	14.3	33.9	1.25	2.37	AAA-AAA	0.00	999	1.000	1.000	0.2681
229239_x_at	59.4	25.0	-1.25	-2.37	AAA-AAA	0.00	999	1.000	1.000	0.1624
1553013_at	45.9	19.4	-1.25	-2.37	AAP-AAA	0.00	999	1.000	1.000	0.3519
240933_at	60.4	25.5	-1.25	-2.37	AAA-AAA	0.00	999	1.000	1.000	0.0703
1556956_at	104.7	44.1	-1.25	-2.37	AAA-AAA	0.00	999	1.000	1.000	0.1558
244794_at	5.8	13.7	1.25	2.37	AAA-AAA	0.00	999	1.000	1.000	0.0835

1553265_at	20.4	8.6	-1.25	-2.37	AAA-AAA	0.00	999	1.000	1.000	0.3662
1559111_a_at	10.9	25.8	1.25	2.37	AAA-AAA	0.00	999	1.000	1.000	0.4225
1560209_at	15.3	36.2	1.25	2.37	AAA-AAA	0.00	999	1.000	1.000	0.1039
219589_s_at	31.3	74.2	1.25	2.37	AAA-AAA	0.00	999	1.000	1.000	0.4296
211003_x_at	50.8	21.4	-1.25	-2.37	AAA-AAA	0.00	999	1.000	1.000	0.3686
208337_s_at	3.1	7.3	1.25	2.37	AAA-AAA	0.00	999	1.000	1.000	0.1409
238395_at	77.0	32.5	-1.25	-2.37	AAA-AAA	0.00	999	1.000	1.000	0.3420
236228_at	11.5	27.4	1.25	2.37	AAA-AAA	0.00	999	1.000	1.000	0.4006
236230_at	28.0	11.8	-1.25	-2.37	AAA-AAA	0.00	999	1.000	1.000	0.3129
229161_at	39.2	92.9	1.25	2.37	AAA-AAA	0.00	999	1.000	1.000	0.1562
1552569_a_at	9.3	21.9	1.25	2.37	AAA-AAA	0.00	999	1.000	1.000	0.2056
1553351_at	8.0	19.0	1.25	2.37	AAA-AAA	0.00	999	1.000	1.000	0.1703
243669_s_at	49.5	20.9	-1.25	-2.37	AAA-AAA	0.00	999	1.000	1.000	0.1067
242998_at	21.8	51.6	1.25	2.37	AAA-AAA	0.00	999	1.000	1.000	0.2334
206489_s_at	10.9	25.8	1.25	2.37	AAA-AAA	0.00	999	1.000	1.000	0.1820
233353_at	50.4	21.2	-1.25	-2.37	AAA-AAA	0.00	999	1.000	1.000	0.3749
237796_at	4.0	9.4	1.25	2.37	AAA-AAA	0.00	999	1.000	1.000	0.0407
235929_s_at	54.6	23.1	-1.25	-2.37	AAA-AAA	0.00	999	1.000	1.000	0.1992
236874_at	14.2	33.8	1.25	2.37	AAA-AAA	0.00	999	1.000	1.000	0.3104
1569750_at	16.1	38.1	1.24	2.37	AAA-AAA	0.00	999	1.000	1.000	0.1037
1569938_at	36.3	15.3	-1.24	-2.37	AAA-AAA	0.00	999	1.000	1.000	0.1710
1555631_at	8.3	19.8	1.24	2.37	AAA-AAA	0.00	999	1.000	1.000	0.0668
244670_at	4.9	11.7	1.24	2.37	AAA-AAA	0.00	999	1.000	1.000	0.2347
236302_at	7.1	16.7	1.24	2.37	AAA-AAA	0.00	999	1.000	1.000	0.4768
231150_at	33.8	14.3	-1.24	-2.37	AAA-AAA	0.00	999	1.000	1.000	0.1970
230555_s_at	34.8	14.7	-1.24	-2.37	AAA-AAA	0.00	999	1.000	1.000	0.4444
1569201_a_at	45.7	19.3	-1.24	-2.37	PAP-AAA	0.00	999	1.000	1.000	0.0795
240631_at	44.9	19.0	-1.24	-2.37	AAA-AAA	0.00	999	1.000	1.000	0.0788
220511_s_at	45.1	19.1	-1.24	-2.37	AAA-AAA	0.00	999	1.000	1.000	0.3265
207113_s_at	42.1	17.8	-1.24	-2.37	AAA-AAA	0.00	999	1.000	1.000	0.1593
210300_at	51.3	121.5	1.24	2.37	AAA-AAA	0.00	999	1.000	1.000	0.0464
234544_at	3.8	9.0	1.24	2.37	AAA-AAA	0.00	999	1.000	1.000	0.2799
232753_at	40.1	95.0	1.24	2.37	AAA-AAA	0.00	999	1.000	1.000	0.2240
236177_s_at	5.3	12.7	1.24	2.37	AAA-AAA	0.00	999	1.000	1.000	0.1213
221886_at	12.7	30.1	1.24	2.37	AAA-AAA	0.00	999	1.000	1.000	0.2187
222334_at	84.1	35.5	-1.24	-2.37	AAA-AAA	0.00	999	1.000	1.000	0.2737
209883_at	75.6	31.9	-1.24	-2.37	AAP-APA	0.00	999	1.000	1.000	0.1797
230626_at	85.1	36.0	-1.24	-2.37	APA-AAP	0.00	999	1.000	1.000	0.3218
1557274_at	25.6	10.8	-1.24	-2.37	APA-AAA	0.00	999	1.000	1.000	0.4549
1563427_at	25.5	10.8	-1.24	-2.37	AAA-AAA	0.00	999	1.000	1.000	0.3645
1568814_at	25.0	59.1	1.24	2.37	AAA-AAA	0.00	999	1.000	1.000	0.4605
1566863_at	19.9	47.2	1.24	2.37	AAA-AAA	0.00	999	1.000	1.000	0.3376

1561136_at	23.2	9.8	-1.24	-2.37	AAA-AAA	0.00	999	1.000	1.000	0.0742
215788_at	52.2	123.5	1.24	2.37	AAA-AAA	0.00	999	1.000	1.000	0.2964
212914_at	54.6	129.2	1.24	2.37	AAA-AAA	0.00	999	1.000	1.000	0.1512
214419_s_at	16.3	38.5	1.24	2.37	AAA-AAA	0.00	999	1.000	1.000	0.2413
205287_s_at	93.0	39.3	-1.24	-2.37	AAA-AAA	0.00	999	1.000	1.000	0.4173
207736_s_at	6.6	2.8	-1.24	-2.37	AAA-AAA	0.00	999	1.000	1.000	0.1015
235261_at	11.6	27.4	1.24	2.37	AAA-AAA	0.00	999	1.000	1.000	0.0487
237857_at	16.5	39.1	1.24	2.37	AAA-AAA	0.00	999	1.000	1.000	0.3070
237259_at	3.4	8.0	1.24	2.37	AAA-AAA	0.00	999	1.000	1.000	0.2705
223044_at	19.4	8.2	-1.24	-2.37	AAA-MAA	0.00	999	1.000	1.000	0.4865
232275_s_at	2.2	5.3	1.24	2.37	AAA-AAA	0.00	999	1.000	1.000	0.0587
1566442_at	39.7	16.8	-1.24	-2.36	AAA-AAA	0.00	999	1.000	1.000	0.3274
1566703_at	15.8	6.7	-1.24	-2.36	AAA-AAA	0.00	999	1.000	1.000	0.2316
1553590_at	20.7	49.0	1.24	2.36	AAA-AAA	0.00	999	1.000	1.000	0.1434
215613_at	10.3	24.3	1.24	2.36	AAA-AAA	0.00	999	1.000	1.000	0.5092
219319_at	70.0	29.6	-1.24	-2.36	AAA-AAA	0.00	999	1.000	1.000	0.0853
207612_at	8.0	18.9	1.24	2.36	AAA-AAA	0.00	999	1.000	1.000	0.1665
231492_at	28.3	12.0	-1.24	-2.36	AAA-AAA	0.00	999	1.000	1.000	0.4753
231058_at	6.3	14.8	1.24	2.36	AAA-AAA	0.00	999	1.000	1.000	0.2113
1556852_a_at	26.6	62.7	1.24	2.36	AAA-AAA	0.00	999	1.000	1.000	0.1998
1554593_s_at	7.5	3.2	-1.24	-2.36	AAA-AAA	0.00	999	1.000	1.000	0.3776
1561591_at	19.3	45.7	1.24	2.36	AAA-AAA	0.00	999	1.000	1.000	0.2958
1559800_a_at	21.4	9.0	-1.24	-2.36	AAA-AAA	0.00	999	1.000	1.000	0.3393
207819_s_at	20.5	8.7	-1.24	-2.36	AAA-AAA	0.00	999	1.000	1.000	0.2727
207726_at	10.8	25.5	1.24	2.36	AAA-AAA	0.00	999	1.000	1.000	0.3631
227868_at	15.3	36.1	1.24	2.36	AAA-AAA	0.00	999	1.000	1.000	0.1699
1555153_s_at	48.0	20.3	-1.24	-2.36	PAA-AAA	0.00	999	1.000	1.000	0.2484
1555610_at	31.1	13.2	-1.24	-2.36	AMP-AAA	0.00	999	1.000	1.000	0.3531
240333_at	39.3	92.7	1.24	2.36	AAA-AAA	0.00	999	1.000	1.000	0.2703
1569911_at	152.7	64.7	-1.24	-2.36	AAA-AAA	0.00	999	1.000	1.000	0.1284
1565768_at	6.4	15.0	1.24	2.36	AAA-AAA	0.00	999	1.000	1.000	0.1394
1553004_at	55.9	23.7	-1.24	-2.36	AAA-AAA	0.00	999	1.000	1.000	0.3665
1562250_at	11.0	26.0	1.24	2.36	AAA-AAA	0.00	999	1.000	1.000	0.3700
220670_at	8.9	3.8	-1.24	-2.36	AAA-AAA	0.00	999	1.000	1.000	0.3553
217592_at	50.9	120.2	1.24	2.36	AAA-AAA	0.00	999	1.000	1.000	0.2071
219516_at	68.4	29.0	-1.24	-2.36	AAA-AAA	0.00	999	1.000	1.000	0.1568
204749_at	6.4	2.7	-1.24	-2.36	AAA-AAA	0.00	999	1.000	1.000	0.4964
230119_at	98.0	41.5	-1.24	-2.36	AMA-AAA	0.00	999	1.000	1.000	0.4813
1566720_at	100.0	42.4	-1.24	-2.36	PPA-PAA	0.00	999	1.000	1.000	0.1801
242274_x_at	17.8	7.6	-1.24	-2.36	AAA-AAA	0.00	999	1.000	1.000	0.3471
239933_x_at	7.2	16.9	1.24	2.36	AAA-AAA	0.00	999	1.000	1.000	0.1863
1557117_at	23.4	55.2	1.24	2.36	AAA-AAA	0.00	999	1.000	1.000	0.4711

1552939_at	4.1	9.8	1.24	2.36	AAA-AAA	0.00	999	1.000	1.000	0.5692
1558987_at	12.5	29.4	1.24	2.36	AAA-AAA	0.00	999	1.000	1.000	0.3110
220108_at	13.6	31.9	1.24	2.36	AAA-AAA	0.00	999	1.000	1.000	0.4427
206246_at	29.0	12.3	-1.24	-2.36	AAA-AAA	0.00	999	1.000	1.000	0.1000
211646_at	20.8	8.8	-1.24	-2.36	AAA-AAA	0.00	999	1.000	1.000	0.4202
234633_at	8.7	20.6	1.24	2.36	AAA-AAA	0.00	999	1.000	1.000	0.1419
223691_at	13.4	31.5	1.24	2.36	AAA-AAA	0.00	999	1.000	1.000	0.3384
1563178_at	11.0	26.0	1.24	2.36	AAA-AAA	0.00	999	1.000	1.000	0.1531
1570167_at	23.7	10.1	-1.24	-2.36	AAA-AAA	0.00	999	1.000	1.000	0.2477
1567377_at	5.5	12.9	1.24	2.36	AAA-AAA	0.00	999	1.000	1.000	0.3489
1568660_a_at	29.4	12.5	-1.24	-2.36	AAA-AAA	0.00	999	1.000	1.000	0.1846
1561386_at	69.3	29.4	-1.24	-2.36	AAA-AAA	0.00	999	1.000	1.000	0.0895
1561635_at	17.9	7.6	-1.24	-2.36	AAA-AAA	0.00	999	1.000	1.000	0.3143
220390_at	15.3	36.0	1.24	2.36	AAA-AAA	0.00	999	1.000	1.000	0.2190
210931_at	10.1	4.3	-1.24	-2.36	AAA-AAA	0.00	999	1.000	1.000	0.3640
235851_s_at	36.6	15.5	-1.24	-2.36	AAA-AAA	0.00	999	1.000	1.000	0.3289
222076_at	14.4	6.1	-1.24	-2.36	AAA-AAA	0.00	999	1.000	1.000	0.3629
232226_at	45.7	19.4	-1.24	-2.36	AAA-AAA	0.00	999	1.000	1.000	0.3087
1559598_at	50.1	21.3	-1.24	-2.36	AAP-AAP	0.00	999	1.000	1.000	0.3774
1564561_at	19.9	8.4	-1.24	-2.36	AAA-AAA	0.00	999	1.000	1.000	0.2688
241637_at	22.0	51.8	1.24	2.36	AAA-AAA	0.00	999	1.000	1.000	0.2895
239485_at	19.2	8.2	-1.24	-2.36	AAA-AAA	0.00	999	1.000	1.000	0.1851
1560617_at	5.4	12.8	1.24	2.36	AAA-AAA	0.00	999	1.000	1.000	0.1781
244215_at	64.2	27.3	-1.24	-2.36	AAA-AAA	0.00	999	1.000	1.000	0.1985
217222_at	12.4	29.2	1.24	2.36	AAA-AAA	0.00	999	1.000	1.000	0.4222
233710_at	13.4	31.6	1.24	2.36	AAA-AAA	0.00	999	1.000	1.000	0.2725
223644_s_at	41.8	17.7	-1.24	-2.36	AAA-AAA	0.00	999	1.000	1.000	0.0912
229137_at	51.9	22.0	-1.24	-2.36	AAA-AAA	0.00	999	1.000	1.000	0.1985
240266_at	50.3	21.4	-1.24	-2.35	APP-AAP	0.00	999	1.000	1.000	0.4138
1569758_at	28.0	65.8	1.24	2.35	AAA-AAA	0.00	999	1.000	1.000	0.3074
1567272_at	9.6	22.5	1.24	2.35	AAA-AAA	0.00	999	1.000	1.000	0.3206
1558995_at	24.7	58.1	1.24	2.35	AAA-AAA	0.00	999	1.000	1.000	0.4629
232829_at	11.3	4.8	-1.24	-2.35	AAA-AAA	0.00	999	1.000	1.000	0.0738
231721_at	10.3	4.4	-1.24	-2.35	AAA-AAA	0.00	999	1.000	1.000	0.1025
223564_s_at	228.0	96.9	-1.23	-2.35	AAA-AAP	0.00	999	1.000	1.000	0.3411
1563078_at	26.2	61.7	1.23	2.35	AAA-AAA	0.00	999	1.000	1.000	0.2216
1569054_at	5.3	12.5	1.23	2.35	AAA-AAA	0.00	999	1.000	1.000	0.2391
1570084_at	6.5	15.4	1.23	2.35	AAA-AAA	0.00	999	1.000	1.000	0.2500
1561488_at	18.9	8.0	-1.23	-2.35	AAA-AAA	0.00	999	1.000	1.000	0.3548
244614_at	26.9	63.3	1.23	2.35	AAA-AAA	0.00	999	1.000	1.000	0.0429
244124_at	25.1	59.1	1.23	2.35	AAA-AAA	0.00	999	1.000	1.000	0.1297
208387_s_at	50.7	21.6	-1.23	-2.35	AAA-AAA	0.00	999	1.000	1.000	0.4527

236385_at	13.3	5.7	-1.23	-2.35	AAA-AAA	0.00	999	1.000	1.000	0.3214
227154_at	20.6	8.8	-1.23	-2.35	AAA-AAA	0.00	999	1.000	1.000	0.2958
1562983_at	56.8	24.1	-1.23	-2.35	AAP-APA	0.00	999	1.000	1.000	0.4067
242107_x_at	2.9	1.2	-1.23	-2.35	AAA-AAA	0.00	999	1.000	1.000	0.3607
239783_at	74.9	176.2	1.23	2.35	AAA-AAA	0.00	999	1.000	1.000	0.2189
241029_at	2.6	6.1	1.23	2.35	AAA-AAA	0.00	999	1.000	1.000	0.0995
240756_at	17.9	7.6	-1.23	-2.35	AAA-AAA	0.00	999	1.000	1.000	0.1972
1562066_at	9.6	22.5	1.23	2.35	AAA-AAA	0.00	999	1.000	1.000	0.1611
244319_at	20.6	48.4	1.23	2.35	AAA-AAA	0.00	999	1.000	1.000	0.3271
206493_at	42.8	18.2	-1.23	-2.35	AAA-AAA	0.00	999	1.000	1.000	0.0970
210354_at	13.1	30.8	1.23	2.35	AAA-AAA	0.00	999	1.000	1.000	0.2907
211109_at	30.4	12.9	-1.23	-2.35	AAA-AAA	0.00	999	1.000	1.000	0.2763
235171_at	18.8	44.2	1.23	2.35	AAA-AAA	0.00	999	1.000	1.000	0.3434
237840_at	34.5	14.7	-1.23	-2.35	AAA-AAA	0.00	999	1.000	1.000	0.2741
220392_at	48.6	20.7	-1.23	-2.35	APA-AMA	0.00	999	1.000	1.000	0.2954
244866_at	13.5	5.8	-1.23	-2.35	AAA-AAA	0.00	999	1.000	1.000	0.2841
1554706_at	4.1	1.7	-1.23	-2.35	AAA-AAA	0.00	999	1.000	1.000	0.4697
1553499_s_at	40.0	93.9	1.23	2.35	AAA-AAA	0.00	999	1.000	1.000	0.0246
210579_s_at	34.6	14.7	-1.23	-2.35	AAA-AAA	0.00	999	1.000	1.000	0.3883
207308_at	38.8	16.5	-1.23	-2.35	AMA-AAA	0.00	999	1.000	1.000	0.3156
236915_at	14.0	32.8	1.23	2.35	AAA-AAA	0.00	999	1.000	1.000	0.4294
222325_at	11.4	4.9	-1.23	-2.35	AAA-AAA	0.00	999	1.000	1.000	0.3020
1554648_a_at	81.5	34.7	-1.23	-2.35	AMA-APP	0.00	999	1.000	1.000	0.4082
241544_at	14.3	33.6	1.23	2.35	AAA-AAA	0.00	999	1.000	1.000	0.4089
1556695_a_at	10.9	25.7	1.23	2.35	AAA-AAA	0.00	999	1.000	1.000	0.2693
1562300_at	17.8	41.8	1.23	2.35	AAA-AAA	0.00	999	1.000	1.000	0.2285
243036_at	2.2	5.2	1.23	2.35	AAA-AAA	0.00	999	1.000	1.000	0.4021
215900_at	28.2	66.1	1.23	2.35	AAA-AAA	0.00	999	1.000	1.000	0.1759
232846_s_at	4.7	11.1	1.23	2.35	AAA-AAA	0.00	999	1.000	1.000	0.3533
237539_at	4.6	10.8	1.23	2.35	AAA-AAA	0.00	999	1.000	1.000	0.2266
231482_at	32.6	76.5	1.23	2.35	AAA-AAA	0.00	999	1.000	1.000	0.1845
1554516_at	96.4	226.0	1.23	2.35	AAA-AAA	0.00	999	1.000	1.000	0.1720
1561403_at	137.4	58.6	-1.23	-2.35	AAA-AAA	0.00	999	1.000	1.000	0.1378
1562412_at	56.7	24.2	-1.23	-2.35	AAA-AAA	0.00	999	1.000	1.000	0.3008
1560309_s_at	21.6	9.2	-1.23	-2.35	AAA-AAA	0.00	999	1.000	1.000	0.2093
1560253_at	20.4	47.9	1.23	2.35	AAA-AAA	0.00	999	1.000	1.000	0.1614
214452_at	16.1	37.8	1.23	2.35	AAA-AAA	0.00	999	1.000	1.000	0.2845
217440_at	5.2	12.3	1.23	2.35	AAA-AAA	0.00	999	1.000	1.000	0.5222
201235_s_at	54.5	127.8	1.23	2.35	AAA-MAA	0.00	999	1.000	1.000	0.0392
208470_s_at	13.5	5.8	-1.23	-2.35	AAA-AAA	0.00	999	1.000	1.000	0.2759
227976_at	18.3	7.8	-1.23	-2.35	AAA-AAA	0.00	999	1.000	1.000	0.4268
1570283_at	25.6	60.0	1.23	2.35	AAA-AAA	0.00	999	1.000	1.000	0.1717

239765_at	5.0	11.8	1.23	2.34	AAA-AAA	0.00	999	1.000	1.000	0.2514
1552842_at	25.5	10.9	-1.23	-2.34	AAA-AAA	0.00	999	1.000	1.000	0.2591
1556084_at	27.8	65.1	1.23	2.34	AAA-AAA	0.00	999	1.000	1.000	0.1274
221525_at	47.0	20.0	-1.23	-2.34	AAA-AAA	0.00	999	1.000	1.000	0.4210
233987_at	9.3	21.7	1.23	2.34	AAA-AAA	0.00	999	1.000	1.000	0.1735
238348_x_at	32.4	13.8	-1.23	-2.35	AAA-AAA	0.00	999	1.000	1.000	0.3066
227231_at	3.2	7.5	1.23	2.34	AAA-AAA	0.00	999	1.000	1.000	0.3737
229496_at	68.2	29.1	-1.23	-2.34	AAA-AAA	0.00	999	1.000	1.000	0.3636
242066_at	38.7	90.7	1.23	2.34	AAA-AAA	0.00	999	1.000	1.000	0.2280
1569823_at	4.7	11.1	1.23	2.34	AAA-AAA	0.00	999	1.000	1.000	0.3195
1565998_at	45.5	19.4	-1.23	-2.34	AAA-AAA	0.00	999	1.000	1.000	0.3318
1560257_at	8.6	20.0	1.23	2.34	AAA-AAA	0.00	999	1.000	1.000	0.4528
215779_s_at	12.0	28.1	1.23	2.34	AAA-AAA	0.00	999	1.000	1.000	0.3817
214207_s_at	12.8	30.0	1.23	2.34	AAA-AAA	0.00	999	1.000	1.000	0.3859
217206_at	35.2	15.0	-1.23	-2.34	AAA-AAA	0.00	999	1.000	1.000	0.4089
208468_at	9.7	22.7	1.23	2.34	AAA-AAA	0.00	999	1.000	1.000	0.2601
234901_at	6.4	14.9	1.23	2.34	AAA-AAA	0.00	999	1.000	1.000	0.2188
223926_at	28.8	12.3	-1.23	-2.34	AAA-AAA	0.00	999	1.000	1.000	0.3185
232294_at	28.2	12.1	-1.23	-2.34	AAA-AAA	0.00	999	1.000	1.000	0.2777
1553486_a_at	31.9	13.6	-1.23	-2.34	AAA-AAA	0.00	999	1.000	1.000	0.4293
1559229_at	5.9	13.9	1.23	2.34	AAA-AAA	0.00	999	1.000	1.000	0.0726
244063_at	15.3	35.9	1.23	2.34	AAA-AMA	0.00	999	1.000	1.000	0.0875
215491_at	24.5	57.4	1.23	2.34	AAA-AAA	0.00	999	1.000	1.000	0.0336
216779_at	20.3	47.6	1.23	2.34	AAA-AAA	0.00	999	1.000	1.000	0.3070
211705_s_at	7.6	17.7	1.23	2.34	AAA-AAA	0.00	999	1.000	1.000	0.1406
211262_at	58.3	24.9	-1.23	-2.34	AAA-AAA	0.00	999	1.000	1.000	0.3155
233882_s_at	12.5	29.2	1.23	2.34	AAA-AAA	0.00	999	1.000	1.000	0.4650
237800_at	13.8	32.3	1.23	2.34	AAA-AAA	0.00	999	1.000	1.000	0.3503
241591_at	22.5	52.5	1.23	2.34	AAA-AAA	0.00	999	1.000	1.000	0.2218
242444_at	17.0	39.9	1.23	2.34	AAA-AAA	0.00	999	1.000	1.000	0.0811
240009_at	15.0	6.4	-1.23	-2.34	AAA-AAA	0.00	999	1.000	1.000	0.3547
1556937_at	23.4	54.7	1.23	2.34	AAA-AAA	0.00	999	1.000	1.000	0.3042
1556038_at	13.0	5.6	-1.23	-2.34	AAA-AAA	0.00	999	1.000	1.000	0.2612
242601_at	4.4	10.4	1.23	2.34	AAA-AAA	0.00	999	1.000	1.000	0.4145
1559756_at	21.6	50.5	1.23	2.34	AAA-AAA	0.00	999	1.000	1.000	0.2867
215176_x_at	5.4	12.7	1.23	2.34	AAA-AAA	0.00	999	1.000	1.000	0.3388
214068_at	62.6	26.8	-1.23	-2.34	AAA-AAA	0.00	999	1.000	1.000	0.2078
219018_s_at	281.4	120.3	-1.23	-2.34	AAA-AAA	0.00	999	1.000	1.000	0.2112
203902_at	10.7	4.6	-1.23	-2.34	AAA-AAA	0.00	999	1.000	1.000	0.3255
207491_at	80.9	34.6	-1.23	-2.34	AAA-AAA	0.00	999	1.000	1.000	0.3002
208570_at	23.4	10.0	-1.23	-2.34	AAA-AAA	0.00	999	1.000	1.000	0.2207
227463_at	24.4	10.4	-1.23	-2.34	AAA-AAA	0.00	999	1.000	1.000	0.4334

222181_at	15.1	6.5	-1.23	-2.34	AAA-AAA	0.00	999	1.000	1.000	0.4685
230612_at	8.0	18.6	1.23	2.34	AAA-AAA	0.00	999	1.000	1.000	0.2870
222224_at	61.8	26.5	-1.23	-2.34	AAP-AMA	0.00	999	1.000	1.000	0.4142
241199_x_at	4.1	9.6	1.23	2.34	AAA-AAA	0.00	999	1.000	1.000	0.2543
1570433_at	19.2	44.9	1.23	2.34	AAA-AAA	0.00	999	1.000	1.000	0.4520
1561699_a_at	11.4	4.9	-1.23	-2.34	AAA-AAA	0.00	999	1.000	1.000	0.4610
1559186_at	26.7	11.4	-1.23	-2.34	AAA-AAA	0.00	999	1.000	1.000	0.3631
1560543_at	5.5	12.8	1.23	2.34	AAA-AAA	0.00	999	1.000	1.000	0.2074
216257_at	15.4	6.6	-1.23	-2.34	AAA-AAA	0.00	999	1.000	1.000	0.1445
221086_s_at	15.5	6.6	-1.23	-2.34	AAA-AAA	0.00	999	1.000	1.000	0.3794
205117_at	9.6	22.4	1.23	2.34	AAA-AAA	0.00	999	1.000	1.000	0.1552
228695_at	4.8	11.3	1.23	2.34	AAA-AAA	0.00	999	1.000	1.000	0.4546
224065_at	30.6	71.6	1.23	2.34	AAA-AAA	0.00	999	1.000	1.000	0.0874
224082_at	55.6	129.9	1.23	2.34	AAA-AAA	0.00	999	1.000	1.000	0.1827
1569765_at	32.2	75.1	1.22	2.34	AAA-AAA	0.00	999	1.000	1.000	0.4094
1554108_at	13.2	30.9	1.22	2.34	AAA-AAA	0.00	999	1.000	1.000	0.1036
243883_at	12.7	29.6	1.22	2.34	AAA-AAA	0.00	999	1.000	1.000	0.2599
220204_s_at	13.8	32.2	1.22	2.34	AAA-AAA	0.00	999	1.000	1.000	0.2575
220851_at	9.3	21.7	1.22	2.34	AAA-AAA	0.00	999	1.000	1.000	0.3250
202902_s_at	33.3	14.3	-1.22	-2.34	AAA-AAA	0.00	999	1.000	1.000	0.2779
233947_s_at	9.6	4.1	-1.22	-2.34	AAA-AAA	0.00	999	1.000	1.000	0.1796
224003_at	22.9	53.5	1.22	2.34	AAA-AAA	0.00	999	1.000	1.000	0.1168
230019_s_at	81.0	34.7	-1.22	-2.34	AAA-AAA	0.00	999	1.000	1.000	0.3594
1561200_at	35.7	15.3	-1.22	-2.33	AAP-AAA	0.00	999	1.000	1.000	0.1401
202458_at	124.0	53.1	-1.22	-2.33	AAP-APA	0.00	999	1.000	1.000	0.1479
240343_at	30.8	13.2	-1.22	-2.33	AAA-AAA	0.00	999	1.000	1.000	0.4642
244602_at	15.8	36.8	1.22	2.33	AAA-AAA	0.00	999	1.000	1.000	0.1723
210441_at	5.4	12.5	1.22	2.33	AAA-AAA	0.00	999	1.000	1.000	0.3054
209867_s_at	5.4	12.5	1.22	2.34	AAA-AAA	0.00	999	1.000	1.000	0.2179
211411_at	6.7	15.6	1.22	2.33	AAA-AAA	0.00	999	1.000	1.000	0.2317
210921_at	4.3	9.9	1.22	2.34	AAA-AAA	0.00	999	1.000	1.000	0.3497
232521_at	89.2	208.2	1.22	2.33	AAA-AAA	0.00	999	1.000	1.000	0.0073
1557099_at	54.9	23.5	-1.22	-2.33	APP-AAP	0.00	999	1.000	1.000	0.3538
1556505_at	128.8	55.2	-1.22	-2.33	AAA-AAA	0.00	999	1.000	1.000	0.3137
1561667_at	113.5	48.7	-1.22	-2.33	AAA-AAA	0.00	999	1.000	1.000	0.1629
1561565_at	8.5	19.9	1.22	2.33	AAA-AAA	0.00	999	1.000	1.000	0.3519
220341_s_at	75.7	176.5	1.22	2.33	AAA-AAA	0.00	999	1.000	1.000	0.2559
235494_at	11.3	4.8	-1.22	-2.33	AAA-AAA	0.00	999	1.000	1.000	0.4040
234847_at	3.9	9.1	1.22	2.33	AAA-AAA	0.00	999	1.000	1.000	0.2793
236851_x_at	22.7	53.0	1.22	2.33	AAA-AAA	0.00	999	1.000	1.000	0.1472
227922_x_at	42.6	99.3	1.22	2.33	AAA-AAA	0.00	999	1.000	1.000	0.3686
222112_at	14.0	32.7	1.22	2.33	AAA-AAA	0.00	999	1.000	1.000	0.2470

229706_at	25.5	59.5	1.22	2.33	AAA-AAA	0.00	999	1.000	1.000	0.1635
204550_x_at	68.0	29.2	-1.22	-2.33	AAA-APA	0.00	999	1.000	1.000	0.3651
241362_at	49.3	114.9	1.22	2.33	AAA-AAA	0.00	999	1.000	1.000	0.1263
240003_at	57.7	24.7	-1.22	-2.33	AAA-AAA	0.00	999	1.000	1.000	0.3925
1566505_at	27.7	11.9	-1.22	-2.33	AAA-AAA	0.00	999	1.000	1.000	0.3706
240921_at	13.9	32.5	1.22	2.33	AAA-AAA	0.00	999	1.000	1.000	0.2246
1568689_at	22.8	9.8	-1.22	-2.33	AAA-AAA	0.00	999	1.000	1.000	0.4791
243143_at	5.5	12.8	1.22	2.33	AAA-AAA	0.00	999	1.000	1.000	0.4047
243053_x_at	19.6	45.6	1.22	2.33	AAA-AAA	0.00	999	1.000	1.000	0.3314
221441_at	9.2	4.0	-1.22	-2.33	AAA-AAA	0.00	999	1.000	1.000	0.3937
236713_at	45.8	19.6	-1.22	-2.33	AAA-AAA	0.00	999	1.000	1.000	0.4717
231783_at	25.2	10.8	-1.22	-2.33	AAA-AAA	0.00	999	1.000	1.000	0.4247
1565706_at	7.7	17.9	1.22	2.33	AAA-AAA	0.00	999	1.000	1.000	0.3606
1556718_s_at	41.9	18.0	-1.22	-2.33	AAA-AAA	0.00	999	1.000	1.000	0.3086
1558322_a_at	11.7	27.4	1.22	2.33	AAA-AAA	0.00	999	1.000	1.000	0.4205
1560404_a_at	25.2	10.8	-1.22	-2.33	AAA-AAA	0.00	999	1.000	1.000	0.3474
206159_at	26.1	11.2	-1.22	-2.33	AAA-AAA	0.00	999	1.000	1.000	0.4783
233423_at	21.7	50.7	1.22	2.33	AAA-AAA	0.00	999	1.000	1.000	0.2491
228133_s_at	13.3	5.7	-1.22	-2.33	AAA-AAA	0.00	999	1.000	1.000	0.0031
224262_at	41.4	17.8	-1.22	-2.33	AAA-AAA	0.00	999	1.000	1.000	0.3705
1570215_at	44.5	19.1	-1.22	-2.33	AAA-AAA	0.00	999	1.000	1.000	0.3476
1555601_at	3.0	7.0	1.22	2.33	AAA-AAA	0.00	999	1.000	1.000	0.5243
220498_at	36.7	15.8	-1.22	-2.33	AAA-AAA	0.00	999	1.000	1.000	0.2561
217376_at	11.1	25.8	1.22	2.33	AAA-AAA	0.00	999	1.000	1.000	0.3206
206487_at	156.5	364.2	1.22	2.33	AAA-AAA	0.00	999	1.000	1.000	0.0878
206895_at	5.7	2.4	-1.22	-2.33	AAA-AAA	0.00	999	1.000	1.000	0.0392
AFFX-ThrX-M_at	10.1	23.4	1.22	2.33	AAA-AAA	0.00	999	1.000	1.000	0.1916
210118_s_at	3042.1	1307.5	-1.22	-2.33	PPP-PPP	0.00	999	1.000	1.000	0.0248
1555581_a_at	14.2	33.0	1.22	2.33	AAA-AAA	0.00	999	1.000	1.000	0.1447
1560642_at	7.4	3.2	-1.22	-2.33	AAA-AAA	0.00	999	1.000	1.000	0.3368
206319_s_at	23.1	53.8	1.22	2.33	AAA-AAA	0.00	999	1.000	1.000	0.3535
205911_at	134.7	57.9	-1.22	-2.33	AAA-MAA	0.00	999	1.000	1.000	0.4704
233213_at	9.1	21.1	1.22	2.33	AAA-AAA	0.00	999	1.000	1.000	0.2590
1556659_at	36.8	15.8	-1.22	-2.33	AAP-AAA	0.00	999	1.000	1.000	0.2605
1553094_at	4.2	9.6	1.22	2.32	AAA-AAA	0.00	999	1.000	1.000	0.3377
1558915_a_at	12.6	29.3	1.22	2.33	AAA-AAA	0.00	999	1.000	1.000	0.1020
220421_at	10.5	24.5	1.22	2.32	AAA-AAA	0.00	999	1.000	1.000	0.3410
206427_s_at	10.8	25.2	1.22	2.33	AAA-AAA	0.00	999	1.000	1.000	0.3625
211132_at	10.3	24.0	1.22	2.32	AAA-AAA	0.00	999	1.000	1.000	0.3608
237767_at	4.7	10.9	1.22	2.32	AAA-AAA	0.00	999	1.000	1.000	0.1745
234921_at	23.9	10.3	-1.22	-2.32	PAA-AAA	0.00	999	1.000	1.000	0.5099
1565728_at	6.7	15.5	1.22	2.32	AAA-AAA	0.00	999	1.000	1.000	0.2028

1556963_at	24.0	55.7	1.22	2.32	AAA-AAA	0.00	999	1.000	1.000	0.3261
1555985_at	9.4	21.8	1.22	2.32	AAA-AAA	0.00	999	1.000	1.000	0.2282
243168_at	5.9	13.8	1.22	2.32	AAA-AAA	0.00	999	1.000	1.000	0.1208
1559723_s_at	16.0	37.2	1.22	2.32	AAA-AAA	0.00	999	1.000	1.000	0.4021
211226_at	51.1	22.0	-1.22	-2.32	AAA-AAA	0.00	999	1.000	1.000	0.2822
227789_at	52.3	22.5	-1.22	-2.32	AAA-AAA	0.00	999	1.000	1.000	0.3010
1564413_at	35.3	82.0	1.22	2.32	AAA-AAA	0.00	999	1.000	1.000	0.2742
1570359_at	13.0	30.2	1.22	2.32	AAA-AAA	0.00	999	1.000	1.000	0.3398
1556364_at	28.6	66.4	1.22	2.32	AAA-AAA	0.00	999	1.000	1.000	0.2555
1555727_at	10.1	23.5	1.22	2.32	AAA-AAA	0.00	999	1.000	1.000	0.4329
243328_at	12.7	5.5	-1.22	-2.32	AAA-AAA	0.00	999	1.000	1.000	0.5522
243477_at	31.5	73.2	1.22	2.32	AAA-AAA	0.00	999	1.000	1.000	0.0390
215906_at	17.6	40.7	1.22	2.32	AAA-AAA	0.00	999	1.000	1.000	0.4215
216278_at	5.8	13.5	1.22	2.32	AAA-AAA	0.00	999	1.000	1.000	0.4682
213524_s_at	28.6	12.3	-1.22	-2.32	AAA-AAA	0.00	999	1.000	1.000	0.0168
206386_at	12.1	28.1	1.22	2.32	AAA-AAA	0.00	999	1.000	1.000	0.3546
210895_s_at	19.5	8.4	-1.22	-2.32	AAA-AAA	0.00	999	1.000	1.000	0.0797
208065_at	4.3	10.0	1.22	2.32	AAA-AAA	0.00	999	1.000	1.000	0.3559
237520_x_at	2.3	5.3	1.22	2.32	AAA-AAA	0.00	999	1.000	1.000	0.4918
237587_at	22.0	9.5	-1.22	-2.32	AAA-AAA	0.00	999	1.000	1.000	0.2266
228165_at	3.5	8.1	1.22	2.32	AAA-AAA	0.00	999	1.000	1.000	0.0152
222485_at	49.4	21.3	-1.22	-2.32	AAA-AAA	0.00	999	1.000	1.000	0.3772
224038_at	13.5	31.4	1.22	2.32	AAA-AAA	0.00	999	1.000	1.000	0.3124
230459_s_at	12.1	28.0	1.22	2.32	AAA-AAA	0.00	999	1.000	1.000	0.0867
239097_at	31.4	72.8	1.21	2.32	AAA-AAA	0.00	999	1.000	1.000	0.1689
1562885_at	86.6	37.3	-1.21	-2.32	AAA-AAA	0.00	999	1.000	1.000	0.2513
1568574_x_at	17.2	7.4	-1.21	-2.32	AAA-AAA	0.00	999	1.000	1.000	0.2704
1562532_at	28.6	66.3	1.21	2.32	AAA-AAA	0.00	999	1.000	1.000	0.2068
244096_at	9.1	21.1	1.21	2.32	AAA-AAA	0.00	999	1.000	1.000	0.4180
233365_at	19.7	45.8	1.21	2.32	AAA-AAA	0.00	999	1.000	1.000	0.3126
1558784_at	33.7	78.1	1.21	2.32	AAA-AAA	0.00	999	1.000	1.000	0.1304
1558589_at	42.6	18.4	-1.21	-2.32	AAA-AAA	0.00	999	1.000	1.000	0.2742
205712_at	56.4	24.3	-1.21	-2.32	AAA-AAA	0.00	999	1.000	1.000	0.3190
206004_at	86.5	37.3	-1.21	-2.32	AAA-AAA	0.00	999	1.000	1.000	0.1359
210446_at	22.7	52.6	1.21	2.32	AAA-AAA	0.00	999	1.000	1.000	0.1769
233724_at	22.1	51.2	1.21	2.32	AAA-AAA	0.00	999	1.000	1.000	0.2518
224533_s_at	10.4	24.1	1.21	2.32	AAA-AAA	0.00	999	1.000	1.000	0.3436
231096_at	14.4	33.4	1.21	2.32	AAA-AAA	0.00	999	1.000	1.000	0.4687
1559257_a_at	59.9	25.9	-1.21	-2.32	PAA-AAA	0.00	999	1.000	1.000	0.1360
1557275_a_at	12.6	29.1	1.21	2.32	AAA-AAA	0.00	999	1.000	1.000	0.4635
1555536_at	115.0	49.6	-1.21	-2.32	AAA-AAA	0.00	999	1.000	1.000	0.1850
1561215_at	7.0	16.3	1.21	2.32	AAA-AAA	0.00	999	1.000	1.000	0.2709

1559692_at	28.1	12.1	-1.21	-2.32	AAA-AAA	0.00	999	1.000	1.000	0.2077
234334_s_at	18.0	7.8	-1.21	-2.32	AAA-AAA	0.00	999	1.000	1.000	0.4436
234287_at	88.1	38.0	-1.21	-2.32	AAA-AAA	0.00	999	1.000	1.000	0.2614
238257_at	5.6	13.1	1.21	2.32	AAA-AAA	0.00	999	1.000	1.000	0.4188
224538_s_at	46.5	107.6	1.21	2.32	AAA-MAA	0.00	999	1.000	1.000	0.2333
231602_at	93.3	40.3	-1.21	-2.32	AAA-PAA	0.00	999	1.000	1.000	0.4431
239006_at	2.3	5.3	1.21	2.32	AAA-AAA	0.00	999	1.000	1.000	0.2581
1566814_at	17.7	40.8	1.21	2.31	AAA-AAA	0.00	999	1.000	1.000	0.3442
1554779_s_at	30.6	13.2	-1.21	-2.31	AAA-AAA	0.00	999	1.000	1.000	0.2140
1554954_at	20.1	46.5	1.21	2.32	AAA-AAA	0.00	999	1.000	1.000	0.4040
244326_at	33.7	14.5	-1.21	-2.32	AAA-AAA	0.00	999	1.000	1.000	0.2458
215356_at	9.8	22.7	1.21	2.32	AAA-AAA	0.00	999	1.000	1.000	0.1695
233535_at	24.4	56.5	1.21	2.32	AAA-AAA	0.00	999	1.000	1.000	0.2345
236676_at	34.9	80.7	1.21	2.32	AAA-AAA	0.00	999	1.000	1.000	0.1701
230315_at	36.2	15.7	-1.21	-2.32	AAA-AAA	0.00	999	1.000	1.000	0.0866
234099_at	80.5	34.8	-1.21	-2.31	AAA-APA	0.00	999	1.000	1.000	0.3334
1557583_at	31.4	13.6	-1.21	-2.31	PAA-APA	0.00	999	1.000	1.000	0.5035
241162_at	4.9	11.3	1.21	2.31	AAA-AAA	0.00	999	1.000	1.000	0.4082
1562847_at	8.0	18.6	1.21	2.31	AAA-AAA	0.00	999	1.000	1.000	0.2992
1552687_a_at	12.1	28.1	1.21	2.31	AAA-AAA	0.00	999	1.000	1.000	0.4395
206064_s_at	42.3	97.8	1.21	2.31	AAA-AAA	0.00	999	1.000	1.000	0.0532
207008_at	18.6	42.9	1.21	2.31	AAA-AAA	0.00	999	1.000	1.000	0.2104
228884_at	53.6	23.2	-1.21	-2.31	AAA-AAA	0.00	999	1.000	1.000	0.2427
231140_at	12.8	5.5	-1.21	-2.31	AAA-AAA	0.00	999	1.000	1.000	0.2720
1569494_at	3.8	8.9	1.21	2.31	AAA-AAA	0.00	999	1.000	1.000	0.4209
1565734_at	36.5	84.4	1.21	2.31	AAA-AAA	0.00	999	1.000	1.000	0.3301
240586_at	16.2	7.0	-1.21	-2.31	AAA-AAA	0.00	999	1.000	1.000	0.4694
1555018_at	10.8	4.7	-1.21	-2.31	AAA-AAA	0.00	999	1.000	1.000	0.6091
215035_at	4.6	10.7	1.21	2.31	AAA-AAA	0.00	999	1.000	1.000	0.1656
220490_at	68.1	29.5	-1.21	-2.31	AAA-AAA	0.00	999	1.000	1.000	0.4378
203472_s_at	47.9	20.7	-1.21	-2.31	AAA-AAA	0.00	999	1.000	1.000	0.0577
234769_at	15.3	35.4	1.21	2.31	AAA-AAA	0.00	999	1.000	1.000	0.3132
237871_x_at	4.5	10.4	1.21	2.31	AAA-AAA	0.00	999	1.000	1.000	0.4167
237219_at	6.0	13.9	1.21	2.31	AAA-AAA	0.00	999	1.000	1.000	0.4680
222378_at	8.4	3.6	-1.21	-2.31	AAA-AAA	0.00	999	1.000	1.000	0.3104
1559404_a_at	35.6	15.4	-1.21	-2.31	PAA-AAA	0.00	999	1.000	1.000	0.2876
241926_s_at	13.2	30.5	1.21	2.31	AAA-AAA	0.00	999	1.000	1.000	0.4112
1560573_at	50.5	21.9	-1.21	-2.31	AAA-AAA	0.00	999	1.000	1.000	0.3208
216441_at	5.5	12.6	1.21	2.31	AAA-AAA	0.00	999	1.000	1.000	0.3641
212707_s_at	24.8	10.8	-1.21	-2.31	AAA-AAA	0.00	999	1.000	1.000	0.2653
220677_s_at	63.9	27.6	-1.21	-2.31	AAA-AAA	0.00	999	1.000	1.000	0.1756
206964_at	14.6	33.8	1.21	2.31	AAA-AAA	0.00	999	1.000	1.000	0.4355

227823_at	41.7	18.0	-1.21	-2.31	AAA-AAA	0.00	999	1.000	1.000	0.1110
229628_s_at	16.8	38.7	1.21	2.31	AAA-AAA	0.00	999	1.000	1.000	0.3865
1552835_at	37.6	16.3	-1.21	-2.31	APM-AAA	0.00	999	1.000	1.000	0.4612
1566468_at	6.5	14.9	1.21	2.31	AAA-AAA	0.00	999	1.000	1.000	0.4714
1553630_at	5.0	11.5	1.21	2.31	AAA-AAA	0.00	999	1.000	1.000	0.2977
1555892_s_at	21.4	49.5	1.21	2.31	AAA-AAA	0.00	999	1.000	1.000	0.2550
243467_at	11.9	27.5	1.21	2.31	AAA-AAA	0.00	999	1.000	1.000	0.1489
1562029_at	10.7	4.6	-1.21	-2.31	AAA-AAA	0.00	999	1.000	1.000	0.1041
1560695_at	9.9	22.8	1.21	2.31	AAA-AAA	0.00	999	1.000	1.000	0.5305
221747_at	71.8	31.1	-1.21	-2.31	AAA-AAA	0.00	999	1.000	1.000	0.3248
216922_x_at	44.9	19.5	-1.21	-2.31	AAA-AAA	0.00	999	1.000	1.000	0.4998
208529_at	3.9	9.1	1.21	2.31	AAA-AAA	0.00	999	1.000	1.000	0.3204
235246_at	17.3	39.8	1.21	2.31	AAA-AAA	0.00	999	1.000	1.000	0.2325
236870_at	6.2	14.4	1.21	2.31	AAA-AAA	0.00	999	1.000	1.000	0.4683
223997_at	23.1	53.4	1.21	2.31	AAA-AAM	0.00	999	1.000	1.000	0.2736
229999_at	4.2	9.6	1.21	2.31	AAA-AAA	0.00	999	1.000	1.000	0.3618
230365_at	33.4	14.5	-1.21	-2.31	AAA-AAA	0.00	999	1.000	1.000	0.4085
240288_at	47.5	20.6	-1.21	-2.31	PAA-AAA	0.00	999	1.000	1.000	0.2226
241514_at	19.5	8.5	-1.21	-2.31	AAA-AAA	0.00	999	1.000	1.000	0.1146
240327_at	25.0	10.8	-1.21	-2.31	AAA-AAA	0.00	999	1.000	1.000	0.4097
1555076_at	86.0	37.3	-1.21	-2.31	AAA-AAA	0.00	999	1.000	1.000	0.3808
215141_at	7.5	17.2	1.21	2.31	AAA-AAA	0.00	999	1.000	1.000	0.3332
221668_s_at	14.3	32.9	1.21	2.31	AAA-AAA	0.00	999	1.000	1.000	0.3127
220928_s_at	26.9	11.7	-1.21	-2.31	AAA-AAA	0.00	999	1.000	1.000	0.3064
210814_at	10.1	23.2	1.21	2.31	AAA-AAA	0.00	999	1.000	1.000	0.4377
237934_at	17.8	41.0	1.21	2.31	AAA-AAA	0.00	999	1.000	1.000	0.2058
1566109_at	10.7	4.6	-1.21	-2.31	AAA-AAA	0.00	999	1.000	1.000	0.3711
240748_at	86.1	37.3	-1.21	-2.31	AAA-AAA	0.00	999	1.000	1.000	0.2692
244857_at	13.9	32.0	1.21	2.31	AAA-AAA	0.00	999	1.000	1.000	0.2196
1562458_at	40.9	17.7	-1.21	-2.31	AAA-AAA	0.00	999	1.000	1.000	0.5213
1561650_s_at	17.2	39.7	1.21	2.31	AAA-AAM	0.00	999	1.000	1.000	0.3812
221198_at	9.8	4.3	-1.21	-2.31	AAA-AAA	0.00	999	1.000	1.000	0.3588
220003_at	43.1	18.7	-1.21	-2.31	AAA-AAA	0.00	999	1.000	1.000	0.2031
204339_s_at	11.0	25.4	1.21	2.31	AAA-AAA	0.00	999	1.000	1.000	0.2244
205605_at	22.5	51.9	1.21	2.31	AAA-AAA	0.00	999	1.000	1.000	0.1626
204515_at	43.1	18.7	-1.21	-2.31	AAA-AAA	0.00	999	1.000	1.000	0.0257
234269_at	25.2	10.9	-1.21	-2.31	AAA-AAA	0.00	999	1.000	1.000	0.3862
232725_s_at	19.4	8.4	-1.21	-2.31	AAA-AAA	0.00	999	1.000	1.000	0.4004
237353_at	16.0	6.9	-1.21	-2.31	AAA-AAA	0.00	999	1.000	1.000	0.4349
223875_s_at	28.6	66.0	1.21	2.31	AAA-AAA	0.00	999	1.000	1.000	0.1731
230881_at	10.5	24.2	1.21	2.31	AAA-AAA	0.00	999	1.000	1.000	0.2234
1552506_at	4.5	10.4	1.20	2.30	AAA-AAA	0.00	999	1.000	1.000	0.0452

1562440_at	10.6	24.4	1.20	2.30	AAA-AAA	0.00	999	1.000	1.000	0.4532
1561678_at	7.6	17.5	1.20	2.30	AAA-AAA	0.00	999	1.000	1.000	0.2090
1561540_at	33.3	14.5	-1.20	-2.30	AAA-AAA	0.00	999	1.000	1.000	0.2353
214162_at	21.5	9.3	-1.20	-2.30	AAA-AAA	0.00	999	1.000	1.000	0.2120
220910_at	33.6	77.3	1.20	2.30	AAA-AAA	0.00	999	1.000	1.000	0.2700
221226_s_at	14.6	33.6	1.20	2.30	AAA-AAA	0.00	999	1.000	1.000	0.1960
216994_s_at	42.5	18.5	-1.20	-2.31	AAA-AAA	0.00	999	1.000	1.000	0.2249
207074_s_at	4.6	10.5	1.20	2.30	AAA-AAA	0.00	999	1.000	1.000	0.2445
210682_at	14.8	34.1	1.20	2.30	AAA-AAA	0.00	999	1.000	1.000	0.1643
233430_at	38.0	16.5	-1.20	-2.31	AAA-AAA	0.00	999	1.000	1.000	0.2196
237774_at	16.1	7.0	-1.20	-2.30	AAA-AAA	0.00	999	1.000	1.000	0.1184
236823_at	18.2	7.9	-1.20	-2.30	AAA-AAA	0.00	999	1.000	1.000	0.2097
231935_at	12.5	5.4	-1.20	-2.30	AAA-AAA	0.00	999	1.000	1.000	0.1403
1561627_at	44.0	19.1	-1.20	-2.30	APA-AAA	0.00	999	1.000	1.000	0.1473
214522_x_at	85.3	37.1	-1.20	-2.30	AAA-AAP	0.00	999	1.000	1.000	0.3816
241205_at	7.2	16.6	1.20	2.30	AAA-AAA	0.00	999	1.000	1.000	0.5223
1565544_at	7.0	16.2	1.20	2.30	AAA-AAA	0.00	999	1.000	1.000	0.4125
1567101_at	12.6	29.0	1.20	2.30	AAA-MAA	0.00	999	1.000	1.000	0.2488
244195_at	11.5	5.0	-1.20	-2.30	AAA-AAA	0.00	999	1.000	1.000	0.4954
206256_at	31.4	72.3	1.20	2.30	AAA-AAA	0.00	999	1.000	1.000	0.2406
230896_at	4.7	2.1	-1.20	-2.30	AAA-AAA	0.00	999	1.000	1.000	0.3218
208425_s_at	143.2	62.2	-1.20	-2.30	PAA-AAA	0.00	999	1.000	1.000	0.2229
229707_at	89.5	38.9	-1.20	-2.30	AAP-AAA	0.00	999	1.000	1.000	0.3460
1568856_at	37.6	16.4	-1.20	-2.30	AAM-AAA	0.00	999	1.000	1.000	0.1895
1562004_x_at	52.0	119.6	1.20	2.30	AAA-AAA	0.00	999	1.000	1.000	0.0715
1560391_at	50.1	21.8	-1.20	-2.30	AAA-AAA	0.00	999	1.000	1.000	0.2586
215703_at	8.0	18.4	1.20	2.30	AAA-AAA	0.00	999	1.000	1.000	0.3904
215965_at	9.2	4.0	-1.20	-2.30	AAA-AAA	0.00	999	1.000	1.000	0.2213
217101_at	4.5	10.3	1.20	2.30	AAA-AAA	0.00	999	1.000	1.000	0.1655
208526_at	30.4	69.8	1.20	2.30	AAA-AAA	0.00	999	1.000	1.000	0.1943
235504_at	49.9	21.7	-1.20	-2.30	AAA-AAA	0.00	999	1.000	1.000	0.1896
233935_at	33.8	77.7	1.20	2.30	AAA-AAA	0.00	999	1.000	1.000	0.1107
233604_at	10.4	4.5	-1.20	-2.30	AAA-AAA	0.00	999	1.000	1.000	0.4157
238703_at	135.6	58.9	-1.20	-2.30	AAA-AAA	0.00	999	1.000	1.000	0.2279
225952_at	87.1	37.9	-1.20	-2.30	AAA-AAA	0.00	999	1.000	1.000	0.2394
230814_at	19.6	45.0	1.20	2.30	AAA-AAA	0.00	999	1.000	1.000	0.2294
204793_at	46.9	20.4	-1.20	-2.30	AAA-PAA	0.00	999	1.000	1.000	0.3774
207428_x_at	396.8	172.6	-1.20	-2.30	PPA-PAP	0.00	999	1.000	1.000	0.4852
1569818_at	16.4	37.6	1.20	2.30	AAA-AAA	0.00	999	1.000	1.000	0.4698
216117_at	20.6	47.3	1.20	2.30	AAA-AAA	0.00	999	1.000	1.000	0.2048
217435_x_at	18.7	43.0	1.20	2.30	AAA-AAA	0.00	999	1.000	1.000	0.1886
232673_at	35.1	15.3	-1.20	-2.30	AAA-AAA	0.00	999	1.000	1.000	0.0326

227706_at	65.9	151.5	1.20	2.30	AAA-AAA	0.00	999	1.000	1.000	0.0779
1560029_a_at	45.8	20.0	-1.20	-2.30	PAA-MAA	0.00	999	1.000	1.000	0.3299
240985_at	44.4	19.3	-1.20	-2.30	AMA-MPA	0.00	999	1.000	1.000	0.3879
242223_at	37.1	85.3	1.20	2.30	AAA-AAA	0.00	999	1.000	1.000	0.3707
1563728_at	18.3	42.0	1.20	2.30	AAA-AAA	0.00	999	1.000	1.000	0.3370
240021_at	29.4	67.5	1.20	2.30	AAA-AAA	0.00	999	1.000	1.000	0.2716
239326_at	16.2	7.0	-1.20	-2.30	AAA-AAA	0.00	999	1.000	1.000	0.2241
1567056_at	17.2	7.5	-1.20	-2.30	AAA-AAA	0.00	999	1.000	1.000	0.2227
1557690_x_at	52.8	121.4	1.20	2.30	AAA-AAA	0.00	999	1.000	1.000	0.3063
1554301_at	21.2	9.2	-1.20	-2.30	AAA-AAA	0.00	999	1.000	1.000	0.2566
219755_at	48.5	111.3	1.20	2.30	AAA-AAA	0.00	999	1.000	1.000	0.2413
207743_at	13.6	31.3	1.20	2.30	AAA-AAA	0.00	999	1.000	1.000	0.1943
209737_at	13.2	30.3	1.20	2.30	AAA-AAM	0.00	999	1.000	1.000	0.3698
235655_at	12.0	27.6	1.20	2.30	AAA-AAA	0.00	999	1.000	1.000	0.1430
236746_at	15.6	35.7	1.20	2.30	AAA-AAA	0.00	999	1.000	1.000	0.3242
243185_at	83.8	36.5	-1.20	-2.30	APA-AAA	0.00	999	1.000	1.000	0.0552
222367_at	44.8	19.5	-1.20	-2.30	APA-AAA	0.00	999	1.000	1.000	0.4200
241542_at	30.0	13.1	-1.20	-2.30	AAA-AAA	0.00	999	1.000	1.000	0.4351
1568896_at	3.9	9.1	1.20	2.30	AAA-AAA	0.00	999	1.000	1.000	0.5013
1566515_at	4.9	11.1	1.20	2.30	AAA-AAA	0.00	999	1.000	1.000	0.3600
1567273_at	8.2	18.9	1.20	2.30	AAA-AAA	0.00	999	1.000	1.000	0.2812
240455_at	56.6	24.6	-1.20	-2.30	AAA-AAA	0.00	999	1.000	1.000	0.3266
240768_x_at	2.7	6.1	1.20	2.30	AAA-AAA	0.00	999	1.000	1.000	0.2785
243133_at	24.9	57.2	1.20	2.30	AAA-AAA	0.00	999	1.000	1.000	0.0356
207758_at	20.8	9.1	-1.20	-2.30	AAA-AAA	0.00	999	1.000	1.000	0.2056
236171_at	16.2	37.1	1.20	2.30	AAA-AAA	0.00	999	1.000	1.000	0.4126
240762_at	13.9	32.0	1.20	2.30	AAA-AAA	0.00	999	1.000	1.000	0.1362
1552389_at	17.9	41.1	1.20	2.29	AAA-AAA	0.00	999	1.000	1.000	0.2385
244590_at	16.3	7.1	-1.20	-2.29	AAA-AAA	0.00	999	1.000	1.000	0.2919
216517_at	22.7	9.9	-1.20	-2.29	AAA-AAA	0.00	999	1.000	1.000	0.3133
209828_s_at	11.8	27.1	1.20	2.29	AAA-AAA	0.00	999	1.000	1.000	0.4302
208064_s_at	24.1	55.3	1.20	2.29	AAA-AAA	0.00	999	1.000	1.000	0.1211
1566204_at	10.7	4.7	-1.20	-2.29	AAA-AAA	0.00	999	1.000	1.000	0.3739
1553449_at	7.3	3.2	-1.20	-2.29	AAA-AAA	0.00	999	1.000	1.000	0.1322
1553518_at	10.9	24.9	1.20	2.29	AAA-AAA	0.00	999	1.000	1.000	0.4882
214837_at	14.3	32.8	1.20	2.29	AAA-AAA	0.00	999	1.000	1.000	0.3423
220002_at	20.9	48.0	1.20	2.29	AAA-AAA	0.00	999	1.000	1.000	0.2242
211389_x_at	9.3	21.4	1.20	2.29	AAA-AAA	0.00	999	1.000	1.000	0.2738
208227_x_at	13.1	30.0	1.20	2.29	AAA-AAA	0.00	999	1.000	1.000	0.2371
236260_at	7.0	16.0	1.20	2.29	AAA-AAA	0.00	999	1.000	1.000	0.1487
232340_at	16.5	37.7	1.20	2.29	AAA-AAA	0.00	999	1.000	1.000	0.3209
229524_at	7.7	17.7	1.20	2.29	AAA-AAA	0.00	999	1.000	1.000	0.4059

219400_at	120.4	52.6	-1.20	-2.29	APA-AAA	0.00	999	1.000	1.000	0.0927
1570284_x_at	10.8	4.7	-1.20	-2.29	AAA-AAA	0.00	999	1.000	1.000	0.4779
241403_at	63.3	27.6	-1.20	-2.29	AAA-AAA	0.00	999	1.000	1.000	0.4635
242257_at	6.3	14.4	1.20	2.29	AAA-AAA	0.00	999	1.000	1.000	0.4539
239884_at	10.8	24.7	1.20	2.29	AAA-AAA	0.00	999	1.000	1.000	0.3678
1569431_at	61.3	26.7	-1.20	-2.29	AAA-AAA	0.00	999	1.000	1.000	0.2699
240444_x_at	40.0	17.5	-1.20	-2.29	AAA-AAA	0.00	999	1.000	1.000	0.4403
1552504_a_at	62.3	142.6	1.20	2.29	AAA-AAA	0.00	999	1.000	1.000	0.2858
1559353_at	2.3	5.4	1.20	2.29	AAA-AAA	0.00	999	1.000	1.000	0.3591
219643_at	3.6	1.6	-1.20	-2.29	AAA-AAA	0.00	999	1.000	1.000	0.1721
210015_s_at	21.2	48.5	1.20	2.29	AAA-AAA	0.00	999	1.000	1.000	0.1265
209173_at	9.3	21.4	1.20	2.29	AAA-AAA	0.00	999	1.000	1.000	0.0980
237124_at	21.5	9.4	-1.20	-2.29	AAA-AAA	0.00	999	1.000	1.000	0.4817
231601_at	16.8	38.4	1.20	2.29	AAA-AAA	0.00	999	1.000	1.000	0.4567
230852_at	16.4	37.6	1.20	2.29	AAA-AAA	0.00	999	1.000	1.000	0.2548
1565650_at	9.3	21.3	1.20	2.29	AAA-AAA	0.00	999	1.000	1.000	0.4742
239017_at	32.7	74.9	1.20	2.29	AAA-AAA	0.00	999	1.000	1.000	0.2213
239918_at	45.8	20.0	-1.20	-2.29	AAA-AAA	0.00	999	1.000	1.000	0.4240
1555185_x_at	169.2	73.9	-1.20	-2.29	AAA-AAA	0.00	999	1.000	1.000	0.1297
243585_at	35.0	15.3	-1.20	-2.29	AAA-AAA	0.00	999	1.000	1.000	0.2841
210078_s_at	25.3	58.0	1.20	2.29	AAA-AAA	0.00	999	1.000	1.000	0.3665
228847_at	14.5	33.2	1.20	2.29	AAA-AAA	0.00	999	1.000	1.000	0.3360
241341_at	24.0	54.8	1.19	2.29	AAA-AAA	0.00	999	1.000	1.000	0.4082
238961_s_at	14.3	6.3	-1.19	-2.29	AAA-AAA	0.00	999	1.000	1.000	0.4474
1567440_at	64.6	28.2	-1.19	-2.29	AAA-AAA	0.00	999	1.000	1.000	0.1594
1560760_s_at	5.6	12.9	1.19	2.29	AAA-AAA	0.00	999	1.000	1.000	0.5674
221715_at	91.1	39.8	-1.19	-2.29	AAA-AAA	0.00	999	1.000	1.000	0.2523
210684_s_at	11.0	25.1	1.19	2.29	AAA-AAA	0.00	999	1.000	1.000	0.1628
209840_s_at	6.5	14.9	1.19	2.29	AAA-AAA	0.00	999	1.000	1.000	0.3468
238338_at	18.3	41.9	1.19	2.29	AAA-AAA	0.00	999	1.000	1.000	0.1745
225619_at	2.6	5.8	1.19	2.29	AAA-AAA	0.00	999	1.000	1.000	0.4343
227662_at	13.7	6.0	-1.19	-2.29	AAA-AAA	0.00	999	1.000	1.000	0.5891
214455_at	39.2	17.2	-1.19	-2.29	AAA-AAP	0.00	999	1.000	1.000	0.4675
241142_at	5.0	11.5	1.19	2.29	AAA-AAA	0.00	999	1.000	1.000	0.2275
1562100_at	45.5	19.9	-1.19	-2.29	AAA-AAA	0.00	999	1.000	1.000	0.4922
242777_at	4.3	9.8	1.19	2.29	AAA-AAA	0.00	999	1.000	1.000	0.3291
216355_at	28.8	65.9	1.19	2.29	AAA-AAA	0.00	999	1.000	1.000	0.0249
207221_at	5.5	12.6	1.19	2.29	AAA-AAA	0.00	999	1.000	1.000	0.2588
207195_at	11.1	25.3	1.19	2.29	AAA-AAA	0.00	999	1.000	1.000	0.2683
210332_at	55.1	24.1	-1.19	-2.29	AAA-AAA	0.00	999	1.000	1.000	0.2150
237644_at	18.5	42.2	1.19	2.29	AAA-AAA	0.00	999	1.000	1.000	0.1295
224134_at	31.8	13.9	-1.19	-2.29	AAA-AAA	0.00	999	1.000	1.000	0.2481

1560797_s_at	10.8	24.7	1.19	2.29	AAA-AAA	0.00	999	1.000	1.000	0.4315
214217_at	36.3	15.9	-1.19	-2.29	AAA-AAA	0.00	999	1.000	1.000	0.3276
233457_at	29.1	12.8	-1.19	-2.28	AAA-AAA	0.00	999	1.000	1.000	0.2691
231496_at	12.8	29.3	1.19	2.29	AAA-AAA	0.00	999	1.000	1.000	0.5064
231705_at	19.9	8.7	-1.19	-2.29	AAA-AAA	0.00	999	1.000	1.000	0.1693
242739_at	77.2	33.8	-1.19	-2.28	AAA-AAP	0.00	999	1.000	1.000	0.3498
205714_s_at	19.2	43.8	1.19	2.28	AAA-AAA	0.00	999	1.000	1.000	0.3905
205344_at	8.4	19.1	1.19	2.28	AAA-AAA	0.00	999	1.000	1.000	0.0619
205253_at	19.1	43.6	1.19	2.28	AAA-AAA	0.00	999	1.000	1.000	0.1874
237233_at	7.6	3.3	-1.19	-2.28	AAA-AAA	0.00	999	1.000	1.000	0.5206
221912_s_at	29.5	12.9	-1.19	-2.28	AAA-AAA	0.00	999	1.000	1.000	0.0501
201981_at	95.3	41.8	-1.19	-2.28	PAP-AAA	0.00	999	1.000	1.000	0.1558
208544_at	89.0	39.0	-1.19	-2.28	AAA-APA	0.00	999	1.000	1.000	0.3655
242039_at	19.2	43.9	1.19	2.28	AAA-AAA	0.00	999	1.000	1.000	0.4288
244744_at	41.7	18.3	-1.19	-2.28	AAA-AAA	0.00	999	1.000	1.000	0.2599
1560138_at	49.9	21.9	-1.19	-2.28	AAM-AAA	0.00	999	1.000	1.000	0.3088
214146_s_at	5.4	12.4	1.19	2.28	AAA-AAA	0.00	999	1.000	1.000	0.1981
206765_at	12.8	29.1	1.19	2.28	AAA-AAA	0.00	999	1.000	1.000	0.2753
228493_at	31.2	13.7	-1.19	-2.28	AAA-AAA	0.00	999	1.000	1.000	0.3107
1562970_at	25.7	11.3	-1.19	-2.28	AAA-AAA	0.00	999	1.000	1.000	0.2590
1566278_at	8.9	20.3	1.19	2.28	AAA-AAA	0.00	999	1.000	1.000	0.3798
1555103_s_at	6.7	15.3	1.19	2.28	AAA-AAA	0.00	999	1.000	1.000	0.2905
216169_at	7.3	16.6	1.19	2.28	AAA-AAA	0.00	999	1.000	1.000	0.2172
203756_at	56.7	129.2	1.19	2.28	AAA-AAA	0.00	999	1.000	1.000	0.0542
234812_at	11.4	5.0	-1.19	-2.28	AAA-AAA	0.00	999	1.000	1.000	0.1285
237812_at	11.6	5.1	-1.19	-2.28	AAA-AAA	0.00	999	1.000	1.000	0.4374
231470_at	23.2	10.2	-1.19	-2.28	AAA-AAA	0.00	999	1.000	1.000	0.4310
232335_at	103.0	45.2	-1.19	-2.28	AAA-AAA	0.00	999	1.000	1.000	0.0650
229179_at	7.0	15.9	1.19	2.28	AAA-AAA	0.00	999	1.000	1.000	0.3099
229291_at	55.4	24.3	-1.19	-2.28	AAA-AAA	0.00	999	1.000	1.000	0.1571
234634_at	48.8	21.4	-1.19	-2.28	PPA-AAA	0.00	999	1.000	1.000	0.0857
224444_s_at	269.9	118.5	-1.19	-2.28	PAM-AAA	0.00	999	1.000	1.000	0.1068
240281_at	9.0	20.6	1.19	2.28	AAA-AAA	0.00	999	1.000	1.000	0.2831
239913_at	3.8	1.7	-1.19	-2.28	AAA-AAA	0.00	999	1.000	1.000	0.4662
1559593_a_at	8.7	19.8	1.19	2.28	AAA-AAA	0.00	999	1.000	1.000	0.2222
1558975_at	29.2	66.6	1.19	2.28	AAA-AAA	0.00	999	1.000	1.000	0.0602
243728_at	7.7	17.6	1.19	2.28	AAA-AAA	0.00	999	1.000	1.000	0.3439
237380_at	13.3	30.3	1.19	2.28	AAA-AAA	0.00	999	1.000	1.000	0.0958
1564688_a_at	5.1	11.5	1.19	2.28	AAA-AAA	0.00	999	1.000	1.000	0.4998
1568666_at	11.9	27.1	1.19	2.28	AAA-AAA	0.00	999	1.000	1.000	0.0384
1554842_at	13.2	30.0	1.19	2.28	AAA-AAA	0.00	999	1.000	1.000	0.0933
1554969_x_at	17.9	40.6	1.19	2.28	AAA-AAA	0.00	999	1.000	1.000	0.2951

205972_at	15.8	36.0	1.19	2.28	AAA-AAA	0.00	999	1.000	1.000	0.3431
205307_s_at	10.0	22.9	1.19	2.28	AAA-AAA	0.00	999	1.000	1.000	0.3272
233024_at	53.3	23.4	-1.19	-2.28	AAA-AAA	0.00	999	1.000	1.000	0.2637
238566_at	29.4	12.9	-1.19	-2.28	AAA-AAA	0.00	999	1.000	1.000	0.1303
236753_at	14.4	32.8	1.19	2.28	AAA-AAA	0.00	999	1.000	1.000	0.4049
232192_at	41.7	18.3	-1.19	-2.28	AAA-AAA	0.00	999	1.000	1.000	0.1789
1562645_at	12.1	5.3	-1.19	-2.28	AAA-AAA	0.00	999	1.000	1.000	0.3130
1556158_at	1.7	3.9	1.19	2.28	AAA-AAA	0.00	999	1.000	1.000	0.0600
243199_at	13.0	29.7	1.19	2.28	AAA-AAA	0.00	999	1.000	1.000	0.2920
237712_at	20.5	46.6	1.19	2.28	AAA-AAA	0.00	999	1.000	1.000	0.3259
237894_at	29.0	12.8	-1.19	-2.28	AAA-AAA	0.00	999	1.000	1.000	0.4603
228056_s_at	2.8	6.4	1.19	2.28	AAA-AAA	0.00	999	1.000	1.000	0.1723
1554203_at	58.0	25.5	-1.19	-2.27	PAA-AAA	0.00	999	1.000	1.000	0.0576
1562110_at	48.7	21.4	-1.19	-2.27	PPA-PAP	0.00	999	1.000	1.000	0.5697
1570290_at	27.1	11.9	-1.19	-2.27	AAA-AAA	0.00	999	1.000	1.000	0.1979
240384_at	66.2	29.1	-1.19	-2.27	AAA-AAA	0.00	999	1.000	1.000	0.1700
239123_at	30.2	68.8	1.19	2.27	AAA-AAA	0.00	999	1.000	1.000	0.3870
1556439_at	3.9	8.9	1.19	2.27	AAA-AAA	0.00	999	1.000	1.000	0.3152
1560844_at	50.7	22.3	-1.19	-2.27	AAA-AAA	0.00	999	1.000	1.000	0.3302
211434_s_at	13.9	31.5	1.19	2.27	AAA-AAA	0.00	999	1.000	1.000	0.5097
234530_s_at	19.0	43.3	1.19	2.27	AAA-AAA	0.00	999	1.000	1.000	0.4416
232595_at	22.6	51.3	1.19	2.27	AAA-AAA	0.00	999	1.000	1.000	0.2303
237403_at	9.0	20.5	1.19	2.27	AAA-AAA	0.00	999	1.000	1.000	0.2131
227258_at	39.0	17.1	-1.19	-2.27	AAA-AAA	0.00	999	1.000	1.000	0.2797
228740_at	5.4	12.3	1.19	2.27	AAA-AAA	0.00	999	1.000	1.000	0.4304
231454_at	29.9	67.9	1.19	2.27	AAA-AAA	0.00	999	1.000	1.000	0.1557
231194_at	15.8	36.0	1.19	2.27	AAA-AAA	0.00	999	1.000	1.000	0.1442
209774_x_at	1235.0	543.6	-1.18	-2.27	PPP-PPP	0.00	999	1.000	1.000	0.0332
1570191_at	107.8	47.5	-1.18	-2.27	AAA-AAA	0.00	999	1.000	1.000	0.4350
240484_at	68.9	30.3	-1.18	-2.27	AAA-AAA	0.00	999	1.000	1.000	0.3033
1559567_at	7.9	3.5	-1.18	-2.27	AAA-AAA	0.00	999	1.000	1.000	0.1063
214567_s_at	2.5	5.6	1.18	2.27	AAA-AAA	0.00	999	1.000	1.000	0.0106
229164_s_at	86.8	197.2	1.18	2.27	AAA-AAA	0.00	999	1.000	1.000	0.4929
230755_at	18.6	42.3	1.18	2.27	AAA-AAA	0.00	999	1.000	1.000	0.4531
237163_x_at	138.2	60.8	-1.18	-2.27	PPA-AAA	0.00	999	1.000	1.000	0.0446
241404_at	63.7	28.1	-1.18	-2.27	PAA-AAA	0.00	999	1.000	1.000	0.2769
241777_x_at	9.6	4.2	-1.18	-2.27	AAA-AAA	0.00	999	1.000	1.000	0.2857
1566499_at	53.3	23.5	-1.18	-2.27	AAA-AAA	0.00	999	1.000	1.000	0.1685
240538_at	82.5	36.3	-1.18	-2.27	AAA-AAA	0.00	999	1.000	1.000	0.2303
1556351_at	2.7	6.2	1.18	2.27	AAA-AAA	0.00	999	1.000	1.000	0.4747
1559578_at	42.2	95.9	1.18	2.27	AAA-AAA	0.00	999	1.000	1.000	0.2670
244558_at	34.2	77.6	1.18	2.27	AAA-AAA	0.00	999	1.000	1.000	0.0554

220069_at	51.1	22.5	-1.18	-2.27	AAA-AAA	0.00	999	1.000	1.000	0.2760
220109_at	5.3	12.1	1.18	2.27	AAA-AAA	0.00	999	1.000	1.000	0.3980
217395_at	35.6	80.9	1.18	2.27	AAA-AAA	0.00	999	1.000	1.000	0.0453
219323_s_at	16.1	36.6	1.18	2.27	AAA-AAA	0.00	999	1.000	1.000	0.3595
237154_at	71.6	31.5	-1.18	-2.27	AMA-AAA	0.00	999	1.000	1.000	0.3272
237313_at	19.7	44.7	1.18	2.27	AAA-AAA	0.00	999	1.000	1.000	0.1819
223503_at	14.9	33.9	1.18	2.27	AAA-MAA	0.00	999	1.000	1.000	0.3899
1554748_at	20.8	47.1	1.18	2.27	AAA-AAA	0.00	999	1.000	1.000	0.2224
1556350_a_at	31.1	13.7	-1.18	-2.27	AAA-AAA	0.00	999	1.000	1.000	0.1318
1561152_a_at	44.9	19.8	-1.18	-2.27	AAA-AAA	0.00	999	1.000	1.000	0.1211
244237_at	40.1	17.7	-1.18	-2.27	AAA-AAA	0.00	999	1.000	1.000	0.4031
1559910_at	63.4	143.8	1.18	2.27	AAA-AAA	0.00	999	1.000	1.000	0.1109
237876_at	11.3	5.0	-1.18	-2.27	AAA-AAA	0.00	999	1.000	1.000	0.5399
224555_x_at	68.6	155.7	1.18	2.27	AAA-AAA	0.00	999	1.000	1.000	0.1676
1564338_at	45.0	19.8	-1.18	-2.27	AAA-AAA	0.00	999	1.000	1.000	0.0926
1565775_at	20.1	45.6	1.18	2.27	AAA-AAA	0.00	999	1.000	1.000	0.2439
1567281_at	23.0	10.1	-1.18	-2.27	AAA-AAA	0.00	999	1.000	1.000	0.4849
242813_at	127.2	56.1	-1.18	-2.27	AAA-AAA	0.00	999	1.000	1.000	0.4564
243920_x_at	29.7	13.1	-1.18	-2.27	AAA-AAA	0.00	999	1.000	1.000	0.2345
214477_at	8.1	18.4	1.18	2.27	AAA-AAA	0.00	999	1.000	1.000	0.2454
233847_x_at	5.9	2.6	-1.18	-2.27	AAA-AAA	0.00	999	1.000	1.000	0.4885
238278_at	11.6	26.3	1.18	2.27	AAA-AAA	0.00	999	1.000	1.000	0.2241
236203_at	1.7	3.9	1.18	2.27	AAA-AAA	0.00	999	1.000	1.000	0.4338
236048_at	16.9	7.5	-1.18	-2.27	AAA-AAA	0.00	999	1.000	1.000	0.3304
227704_at	109.1	48.1	-1.18	-2.27	AAA-AAA	0.00	999	1.000	1.000	0.4920
229387_at	5.0	11.2	1.18	2.27	AAA-AAA	0.00	999	1.000	1.000	0.4264
230084_at	104.4	46.0	-1.18	-2.27	AAA-AAA	0.00	999	1.000	1.000	0.2734
227771_at	100.7	44.4	-1.18	-2.27	PPA-AAA	0.00	999	1.000	1.000	0.0555
1564473_at	44.4	19.6	-1.18	-2.27	APM-AAA	0.00	999	1.000	1.000	0.2911
238391_at	15.1	6.7	-1.18	-2.27	AAA-AAP	0.00	999	1.000	1.000	0.3705
239931_at	9.9	22.4	1.18	2.27	AAA-AAA	0.00	999	1.000	1.000	0.2543
240845_at	7.1	3.1	-1.18	-2.27	AAA-AAA	0.00	999	1.000	1.000	0.4220
1556796_at	36.0	81.5	1.18	2.27	AAA-AAA	0.00	999	1.000	1.000	0.2361
1562549_at	14.9	33.7	1.18	2.27	AAA-AAA	0.00	999	1.000	1.000	0.3727
211904_x_at	30.7	69.5	1.18	2.27	AAA-AAA	0.00	999	1.000	1.000	0.4048
206454_s_at	31.2	70.6	1.18	2.27	AAA-AAA	0.00	999	1.000	1.000	0.2449
206049_at	66.1	149.8	1.18	2.27	AAA-AAA	0.00	999	1.000	1.000	0.3864
206780_at	14.3	32.3	1.18	2.27	AAA-AAA	0.00	999	1.000	1.000	0.0880
AFFX-LysX-M_at	20.8	9.2	-1.18	-2.27	AAA-AAA	0.00	999	1.000	1.000	0.1241
224120_at	25.2	57.1	1.18	2.27	AAA-AAA	0.00	999	1.000	1.000	0.1304
224310_s_at	7.3	16.6	1.18	2.27	AAA-AAA	0.00	999	1.000	1.000	0.0556
232234_at	24.3	55.1	1.18	2.27	AAA-AAA	0.00	999	1.000	1.000	0.2666

202191_s_at	34.0	15.0	-1.18	-2.26	PMA-AAA	0.00	999	1.000	1.000	0.3742
241141_at	16.3	36.8	1.18	2.26	AAA-AAA	0.00	999	1.000	1.000	0.4515
1563591_at	3.9	8.7	1.18	2.26	AAA-AAA	0.00	999	1.000	1.000	0.1626
1570445_a_at	3.9	8.9	1.18	2.26	AAA-AAA	0.00	999	1.000	1.000	0.2572
1560288_at	5.1	11.6	1.18	2.26	AAA-AAA	0.00	999	1.000	1.000	0.2233
213003_s_at	49.9	113.0	1.18	2.27	AAA-AMA	0.00	999	1.000	1.000	0.3967
217454_at	3.6	8.1	1.18	2.27	AAA-AAA	0.00	999	1.000	1.000	0.5120
219841_at	10.7	4.7	-1.18	-2.27	AAA-AAA	0.00	999	1.000	1.000	0.4233
202150_s_at	21.2	47.9	1.18	2.26	AAA-AAA	0.00	999	1.000	1.000	0.4440
207593_at	130.0	57.5	-1.18	-2.26	AAA-AAA	0.00	999	1.000	1.000	0.1797
233693_at	115.3	50.9	-1.18	-2.26	AAA-AAA	0.00	999	1.000	1.000	0.0181
1566581_at	72.3	32.0	-1.18	-2.26	MAP-AAA	0.00	999	1.000	1.000	0.1717
233371_at	34.6	15.3	-1.18	-2.26	PAA-AAA	0.00	999	1.000	1.000	0.4050
210848_at	26.2	11.6	-1.18	-2.26	APA-AAA	0.00	999	1.000	1.000	0.4831
1552828_at	11.0	24.8	1.18	2.26	AAA-AAA	0.00	999	1.000	1.000	0.3912
1557849_at	10.3	4.5	-1.18	-2.26	AAA-AAA	0.00	999	1.000	1.000	0.4026
1561777_at	18.6	8.2	-1.18	-2.26	AAA-AAA	0.00	999	1.000	1.000	0.3851
223745_at	28.4	64.3	1.18	2.26	AAA-AAA	0.00	999	1.000	1.000	0.3448
220297_at	34.3	15.2	-1.18	-2.26	APA-AAA	0.00	999	1.000	1.000	0.3560
237117_at	43.8	19.4	-1.18	-2.26	AAA-PAA	0.00	999	1.000	1.000	0.3800
218279_s_at	45.6	20.2	-1.18	-2.26	APA-AAA	0.00	999	1.000	1.000	0.4812
1569663_at	12.1	5.4	-1.18	-2.26	AAA-AAA	0.00	999	1.000	1.000	0.5510
1560772_a_at	5.5	12.4	1.18	2.26	AAA-AAA	0.00	999	1.000	1.000	0.1484
213611_at	8.2	18.5	1.18	2.26	AAA-AAA	0.00	999	1.000	1.000	0.0580
206068_s_at	22.7	10.0	-1.18	-2.26	AAA-AAA	0.00	999	1.000	1.000	0.4577
206823_at	32.0	14.2	-1.18	-2.26	AAA-AAA	0.00	999	1.000	1.000	0.3202
203670_at	11.6	26.2	1.18	2.26	AAA-AAA	0.00	999	1.000	1.000	0.1914
211791_s_at	16.2	36.7	1.18	2.26	AAA-AAA	0.00	999	1.000	1.000	0.1048
233234_at	12.9	5.7	-1.18	-2.26	AAA-AAA	0.00	999	1.000	1.000	0.4803
233907_s_at	23.2	52.6	1.18	2.26	AAA-AAA	0.00	999	1.000	1.000	0.1967
236508_at	26.2	59.3	1.18	2.26	AAA-AAA	0.00	999	1.000	1.000	0.2219
235972_at	5.8	13.2	1.18	2.26	AAA-AAA	0.00	999	1.000	1.000	0.0465
237204_at	27.6	12.2	-1.18	-2.26	AAA-AAA	0.00	999	1.000	1.000	0.2800
231696_x_at	108.1	47.8	-1.18	-2.26	PAA-AAA	0.00	999	1.000	1.000	0.0862
230744_at	71.4	31.6	-1.18	-2.26	APA-AAA	0.00	999	1.000	1.000	0.4226
1564590_a_at	21.0	47.5	1.18	2.26	AAA-AAA	0.00	999	1.000	1.000	0.3226
1563097_at	19.8	8.8	-1.18	-2.26	AAA-AAA	0.00	999	1.000	1.000	0.4686
1556651_at	45.1	20.0	-1.18	-2.26	AAA-AAA	0.00	999	1.000	1.000	0.4290
214958_s_at	84.7	37.5	-1.18	-2.26	AAA-AAA	0.00	999	1.000	1.000	0.0767
204196_x_at	30.0	67.9	1.18	2.26	AAA-AAA	0.00	999	1.000	1.000	0.3518
1554030_at	93.3	41.3	-1.18	-2.26	AMP-AAA	0.00	999	1.000	1.000	0.0807
1561984_at	36.6	82.7	1.18	2.26	AAA-AAA	0.00	999	1.000	1.000	0.0839

206280_at	14.3	6.3	-1.18	-2.26	AAA-AAA	0.00	999	1.000	1.000	0.0569
229591_at	33.1	74.7	1.18	2.26	AAA-AAA	0.00	999	1.000	1.000	0.0287
1567237_at	24.7	10.9	-1.17	-2.26	AAA-AAA	0.00	999	1.000	1.000	0.4509
1560935_s_at	25.6	57.8	1.17	2.26	AAA-AAA	0.00	999	1.000	1.000	0.0595
243498_at	12.6	28.3	1.17	2.26	AAA-AAA	0.00	999	1.000	1.000	0.1388
242667_at	4.6	10.3	1.17	2.26	AAA-AAA	0.00	999	1.000	1.000	0.3684
242654_at	21.1	47.6	1.17	2.26	AAA-AAA	0.00	999	1.000	1.000	0.1708
220077_at	27.9	63.0	1.17	2.26	AAA-AAA	0.00	999	1.000	1.000	0.2131
206334_at	22.0	49.6	1.17	2.26	AAA-AAA	0.00	999	1.000	1.000	0.3775
237748_at	11.6	26.1	1.17	2.26	AAA-AAA	0.00	999	1.000	1.000	0.0581
238310_at	4.0	8.9	1.17	2.26	AAA-AAA	0.00	999	1.000	1.000	0.1860
237044_s_at	89.6	39.7	-1.17	-2.26	AAA-AAA	0.00	999	1.000	1.000	0.1223
1563744_a_at	14.0	31.5	1.17	2.25	AAA-AAA	0.00	999	1.000	1.000	0.2876
1556325_at	3.7	8.4	1.17	2.25	AAA-AAA	0.00	999	1.000	1.000	0.2420
1553213_a_at	2.5	5.6	1.17	2.26	AAA-AAA	0.00	999	1.000	1.000	0.0463
1562372_at	10.4	23.4	1.17	2.25	AAA-AAA	0.00	999	1.000	1.000	0.4496
234429_at	5.9	13.2	1.17	2.25	AAA-AAA	0.00	999	1.000	1.000	0.0420
228873_at	17.0	38.3	1.17	2.25	AAA-AAA	0.00	999	1.000	1.000	0.3391
231620_at	13.7	6.1	-1.17	-2.25	AAA-AAA	0.00	999	1.000	1.000	0.3486
229702_at	41.8	18.6	-1.17	-2.26	AAA-AAA	0.00	999	1.000	1.000	0.2396
235830_at	142.5	63.3	-1.17	-2.25	PAP-PAP	0.00	999	1.000	1.000	0.3923
242347_at	30.3	68.3	1.17	2.25	AAA-AAA	0.00	999	1.000	1.000	0.0192
239547_at	25.0	11.1	-1.17	-2.25	AAA-AAA	0.00	999	1.000	1.000	0.2223
1554728_at	10.3	23.2	1.17	2.25	AAA-AAA	0.00	999	1.000	1.000	0.5194
1559827_at	3.7	8.3	1.17	2.25	AAA-AAA	0.00	999	1.000	1.000	0.4774
202588_at	103.2	45.8	-1.17	-2.25	MAA-AAA	0.00	999	1.000	1.000	0.2519
208233_at	12.3	5.4	-1.17	-2.25	AAA-AAA	0.00	999	1.000	1.000	0.1467
236618_at	9.0	4.0	-1.17	-2.25	AAA-AAA	0.00	999	1.000	1.000	0.4243
237305_at	17.6	7.8	-1.17	-2.25	AAA-AAA	0.00	999	1.000	1.000	0.4397
227287_at	8.6	19.4	1.17	2.25	AAA-AAA	0.00	999	1.000	1.000	0.0468
1565771_at	7.0	15.7	1.17	2.25	AAA-AAA	0.00	999	1.000	1.000	0.0863
1562295_at	31.0	13.7	-1.17	-2.25	AAA-AAA	0.00	999	1.000	1.000	0.0880
1560012_at	25.0	11.1	-1.17	-2.25	AAA-AAA	0.00	999	1.000	1.000	0.4140
215782_at	23.4	52.7	1.17	2.25	AAA-AAA	0.00	999	1.000	1.000	0.5060
202828_s_at	43.5	97.9	1.17	2.25	AAA-AAA	0.00	999	1.000	1.000	0.2360
233237_at	17.0	38.2	1.17	2.25	AAA-AAA	0.00	999	1.000	1.000	0.3378
214091_s_at	86.0	38.2	-1.17	-2.25	PPA-AAA	0.00	999	1.000	1.000	0.3075
1568813_at	131.8	58.5	-1.17	-2.25	AAA-AAA	0.00	999	1.000	1.000	0.2907
1564414_a_at	70.7	31.4	-1.17	-2.25	AAA-AAA	0.00	999	1.000	1.000	0.1887
1553502_a_at	19.0	8.4	-1.17	-2.25	AAA-AAA	0.00	999	1.000	1.000	0.1405
1553234_at	45.1	101.5	1.17	2.25	AAA-AAA	0.00	999	1.000	1.000	0.2899
206758_at	12.3	27.7	1.17	2.25	AAA-AAA	0.00	999	1.000	1.000	0.2609

233772_at	2.6	5.9	1.17	2.25	AAA-AAA	0.00	999	1.000	1.000	0.5284
236740_at	7.8	3.5	-1.17	-2.25	AAA-AAA	0.00	999	1.000	1.000	0.2432
241074_at	75.4	33.6	-1.17	-2.25	PPA-AAA	0.00	999	1.000	1.000	0.1585
241586_at	9.5	21.4	1.17	2.25	AAA-AAA	0.00	999	1.000	1.000	0.4357
242291_at	5.3	11.9	1.17	2.25	AAA-AAA	0.00	999	1.000	1.000	0.2689
242344_at	3.6	8.2	1.17	2.25	AAA-AAA	0.00	999	1.000	1.000	0.5009
1566037_at	33.5	14.9	-1.17	-2.25	AAA-AAA	0.00	999	1.000	1.000	0.4078
1555991_s_at	31.1	13.9	-1.17	-2.25	AAA-AAA	0.00	999	1.000	1.000	0.3273
1560971_a_at	7.7	17.2	1.17	2.25	AAA-AAA	0.00	999	1.000	1.000	0.2864
207174_at	4.3	9.7	1.17	2.25	AAA-AAA	0.00	999	1.000	1.000	0.5112
235276_at	57.2	25.4	-1.17	-2.25	AAA-AAA	0.00	999	1.000	1.000	0.2320
232750_at	61.9	27.5	-1.17	-2.25	AAA-AAA	0.00	999	1.000	1.000	0.4370
1568905_at	5.4	12.1	1.17	2.25	AAA-AAA	0.00	999	1.000	1.000	0.2778
239472_at	15.1	6.7	-1.17	-2.25	AAA-AAA	0.00	999	1.000	1.000	0.5353
1553350_at	23.9	10.7	-1.17	-2.25	AAA-AAA	0.00	999	1.000	1.000	0.0898
216839_at	13.8	6.2	-1.17	-2.25	AAA-AAA	0.00	999	1.000	1.000	0.3845
217525_at	18.6	8.3	-1.17	-2.25	AAA-AAA	0.00	999	1.000	1.000	0.4506
211809_x_at	46.8	105.2	1.17	2.25	AAA-AAA	0.00	999	1.000	1.000	0.4352
224351_at	6.1	13.7	1.17	2.25	AAA-AAA	0.00	999	1.000	1.000	0.4331
1557571_at	3.2	7.1	1.17	2.25	AAA-AAA	0.00	999	1.000	1.000	0.3744
1557897_at	60.5	135.9	1.17	2.25	AAA-AAA	0.00	999	1.000	1.000	0.4016
1554566_at	23.0	51.7	1.17	2.25	AAA-AAA	0.00	999	1.000	1.000	0.4026
214265_at	7.1	16.0	1.17	2.25	AAA-AAA	0.00	999	1.000	1.000	0.5180
210880_s_at	87.6	39.0	-1.17	-2.25	AAA-AAA	0.00	999	1.000	1.000	0.2802
234070_at	51.9	116.5	1.17	2.25	MAA-AAA	0.00	999	1.000	1.000	0.1140
224107_at	38.2	17.0	-1.17	-2.25	AAA-AAA	0.00	999	1.000	1.000	0.1282
223669_at	4.6	2.1	-1.17	-2.25	AAA-AAA	0.00	999	1.000	1.000	0.0957
232109_at	20.0	8.9	-1.17	-2.25	AAA-AAA	0.00	999	1.000	1.000	0.1730
229130_at	43.9	19.6	-1.17	-2.25	AAA-AAA	0.00	999	1.000	1.000	0.3797
1562919_at	4.7	10.5	1.17	2.24	AAA-AAA	0.00	999	1.000	1.000	0.3017
239336_at	19.1	8.5	-1.17	-2.24	AAA-AAA	0.00	999	1.000	1.000	0.3406
1554242_a_at	60.0	26.7	-1.17	-2.24	AAA-AAA	0.00	999	1.000	1.000	0.3706
1562586_at	25.0	11.1	-1.17	-2.24	AAA-AAA	0.00	999	1.000	1.000	0.1443
1559478_at	22.5	10.0	-1.17	-2.24	AAA-AAA	0.00	999	1.000	1.000	0.0704
214069_at	51.7	23.1	-1.17	-2.24	AAA-AAA	0.00	999	1.000	1.000	0.3578
217411_s_at	8.2	18.5	1.17	2.24	AAA-AAA	0.00	999	1.000	1.000	0.3447
217530_at	8.4	18.9	1.17	2.25	AAA-AAA	0.00	999	1.000	1.000	0.3609
203750_s_at	91.1	40.6	-1.17	-2.24	AAA-AAA	0.00	999	1.000	1.000	0.2755
210012_s_at	47.7	21.3	-1.17	-2.24	AAA-AAA	0.00	999	1.000	1.000	0.3830
234246_at	15.5	34.7	1.17	2.24	AAA-AAA	0.00	999	1.000	1.000	0.2547
232588_at	22.7	50.9	1.17	2.24	AAA-AAA	0.00	999	1.000	1.000	0.2408
232737_s_at	15.4	6.9	-1.17	-2.24	AAA-AAA	0.00	999	1.000	1.000	0.2379

228048_at	32.2	14.4	-1.17	-2.24	AAA-AAA	0.00	999	1.000	1.000	0.5035
203709_at	63.1	141.6	1.17	2.24	AAA-AAA	0.00	999	1.000	1.000	0.2311
234688_x_at	16.1	36.0	1.17	2.24	AAA-AAA	0.00	999	1.000	1.000	0.5245
237721_s_at	8.9	19.9	1.17	2.24	AAA-AAA	0.00	999	1.000	1.000	0.2215
1553859_at	54.6	24.4	-1.16	-2.24	AMA-APA	0.00	999	1.000	1.000	0.2916
1564138_at	15.1	33.8	1.16	2.24	AAA-AAA	0.00	999	1.000	1.000	0.5206
1567912_s_at	3.6	8.1	1.16	2.24	AAA-AAA	0.00	999	1.000	1.000	0.3124
1567183_s_at	4.2	9.4	1.16	2.24	AAA-AAA	0.00	999	1.000	1.000	0.2843
1558481_s_at	19.5	43.8	1.16	2.24	AAA-AAA	0.00	999	1.000	1.000	0.2018
1557874_at	27.6	12.3	-1.16	-2.24	AAA-AAA	0.00	999	1.000	1.000	0.4694
1554475_a_at	64.7	144.9	1.16	2.24	AAA-AAM	0.00	999	1.000	1.000	0.2492
1560794_at	12.3	27.6	1.16	2.24	AAA-AAA	0.00	999	1.000	1.000	0.3369
215055_at	38.5	86.2	1.16	2.24	AAA-AAA	0.00	999	1.000	1.000	0.0888
219309_at	62.4	27.9	-1.16	-2.24	AAA-AAA	0.00	999	1.000	1.000	0.2874
205267_at	12.3	27.6	1.16	2.24	AAA-AAA	0.00	999	1.000	1.000	0.4244
205043_at	4.0	9.1	1.16	2.24	AAA-AAA	0.00	999	1.000	1.000	0.1122
211315_s_at	51.1	22.8	-1.16	-2.24	AAA-AAA	0.00	999	1.000	1.000	0.3006
207561_s_at	60.8	27.1	-1.16	-2.24	AAA-AAA	0.00	999	1.000	1.000	0.0525
208476_s_at	7.7	17.3	1.16	2.24	AAA-AAA	0.00	999	1.000	1.000	0.3534
234482_at	13.6	6.1	-1.16	-2.24	AAA-AAA	0.00	999	1.000	1.000	0.2018
233664_at	42.3	18.9	-1.16	-2.24	AAA-AAA	0.00	999	1.000	1.000	0.3195
238630_at	116.2	51.9	-1.16	-2.24	AAA-AAA	0.00	999	1.000	1.000	0.3067
236120_at	7.9	17.6	1.16	2.24	AAA-AAA	0.00	999	1.000	1.000	0.4128
227690_at	33.2	14.8	-1.16	-2.24	AAA-AAA	0.00	999	1.000	1.000	0.4164
232068_s_at	1.7	3.9	1.16	2.24	AAA-AAA	0.00	999	1.000	1.000	0.4160
241357_at	29.6	66.2	1.16	2.24	AAA-AAA	0.00	999	1.000	1.000	0.1376
238944_at	27.0	60.5	1.16	2.24	AAA-AAA	0.00	999	1.000	1.000	0.3765
1562653_at	20.1	44.9	1.16	2.24	AAA-AAA	0.00	999	1.000	1.000	0.2458
240047_at	8.6	19.3	1.16	2.24	AAA-AAA	0.00	999	1.000	1.000	0.1076
1569756_at	10.3	4.6	-1.16	-2.24	AAA-AAA	0.00	999	1.000	1.000	0.3933
241121_at	24.2	10.8	-1.16	-2.24	AAA-AAA	0.00	999	1.000	1.000	0.4887
1553268_at	20.0	44.7	1.16	2.24	AAA-AAA	0.00	999	1.000	1.000	0.4044
242926_at	23.5	10.5	-1.16	-2.24	AAA-AAA	0.00	999	1.000	1.000	0.5319
217568_at	40.3	18.0	-1.16	-2.24	AAA-AAA	0.00	999	1.000	1.000	0.5715
206370_at	25.7	11.5	-1.16	-2.24	AAA-AAA	0.00	999	1.000	1.000	0.2719
205611_at	19.0	42.5	1.16	2.24	AAA-AAA	0.00	999	1.000	1.000	0.3734
237273_at	13.9	6.2	-1.16	-2.24	AAA-AAA	0.00	999	1.000	1.000	0.5072
215331_at	65.1	29.1	-1.16	-2.24	PPP-AAA	0.00	999	1.000	1.000	0.1942
1555034_at	9.9	4.4	-1.16	-2.24	APA-AAA	0.00	999	1.000	1.000	0.5912
1562961_at	3.5	7.9	1.16	2.24	AAA-AAA	0.00	999	1.000	1.000	0.2870
241049_at	51.3	22.9	-1.16	-2.24	AAA-AAA	0.00	999	1.000	1.000	0.3436
1555627_s_at	10.0	22.3	1.16	2.24	AAA-AAA	0.00	999	1.000	1.000	0.3451

232934_at	25.0	55.8	1.16	2.24	AAA-AAA	0.00	999	1.000	1.000	0.1222
233552_at	6.4	2.8	-1.16	-2.24	AAA-AAA	0.00	999	1.000	1.000	0.3966
226884_at	13.2	5.9	-1.16	-2.24	AAA-AAA	0.00	999	1.000	1.000	0.3627
224909_s_at	45.8	20.5	-1.16	-2.24	AAA-AAA	0.00	999	1.000	1.000	0.2462
231176_at	33.2	14.8	-1.16	-2.24	AAA-AAA	0.00	999	1.000	1.000	0.2492
232444_at	16.5	7.4	-1.16	-2.24	AAA-AAA	0.00	999	1.000	1.000	0.3581
241796_x_at	2.0	4.4	1.16	2.24	AAA-AAA	0.00	999	1.000	1.000	0.4450
1570420_at	53.8	24.1	-1.16	-2.24	AAA-AAA	0.00	999	1.000	1.000	0.4680
240377_at	7.6	17.0	1.16	2.24	AAA-AAA	0.00	999	1.000	1.000	0.0689
242951_at	28.5	63.7	1.16	2.24	AAA-AAA	0.00	999	1.000	1.000	0.2585
1559656_a_at	26.9	12.0	-1.16	-2.24	AAA-AAA	0.00	999	1.000	1.000	0.4961
244316_at	24.2	10.8	-1.16	-2.24	AAA-AAA	0.00	999	1.000	1.000	0.4023
1559297_at	20.9	46.7	1.16	2.24	AAA-AAA	0.00	999	1.000	1.000	0.0288
220187_at	15.8	7.1	-1.16	-2.24	AAA-AAA	0.00	999	1.000	1.000	0.4996
238910_at	10.7	23.8	1.16	2.24	AAA-AAA	0.00	999	1.000	1.000	0.3947
240213_at	10.7	24.0	1.16	2.24	AAA-AAA	0.00	999	1.000	1.000	0.2043
243099_at	35.3	78.8	1.16	2.23	AAA-AMA	0.00	999	1.000	1.000	0.1549
1558976_x_at	19.3	43.2	1.16	2.24	AAA-AAA	0.00	999	1.000	1.000	0.5053
221372_s_at	21.0	47.0	1.16	2.23	AAA-AAA	0.00	999	1.000	1.000	0.2895
206963_s_at	19.4	8.7	-1.16	-2.24	AAA-AAA	0.00	999	1.000	1.000	0.2516
205473_at	58.7	131.2	1.16	2.23	AAA-AAA	0.00	999	1.000	1.000	0.1040
211196_at	31.7	14.2	-1.16	-2.24	AAA-AAA	0.00	999	1.000	1.000	0.3412
233620_at	3.7	8.3	1.16	2.23	AAA-AAA	0.00	999	1.000	1.000	0.5461
236057_at	42.0	94.0	1.16	2.24	AAA-AAA	0.00	999	1.000	1.000	0.3610
244378_at	69.0	30.9	-1.16	-2.23	APA-PAA	0.00	999	1.000	1.000	0.1992
244769_at	19.9	44.4	1.16	2.23	AAA-AAA	0.00	999	1.000	1.000	0.1245
1554943_at	33.8	75.5	1.16	2.23	AAA-AAA	0.00	999	1.000	1.000	0.3014
1562510_at	31.7	14.2	-1.16	-2.23	AAA-AAA	0.00	999	1.000	1.000	0.3352
243913_at	49.1	109.6	1.16	2.23	AAA-AAA	0.00	999	1.000	1.000	0.2211
216777_at	7.2	16.0	1.16	2.23	AAA-AAA	0.00	999	1.000	1.000	0.5114
203950_s_at	53.4	119.3	1.16	2.23	AAA-AAA	0.00	999	1.000	1.000	0.1888
238780_s_at	65.7	29.4	-1.16	-2.23	AAA-AAA	0.00	999	1.000	1.000	0.3509
225753_at	132.5	59.4	-1.16	-2.23	PAP-PAA	0.00	999	1.000	1.000	0.3655
236672_at	68.5	30.7	-1.16	-2.23	PPA-AAA	0.00	999	1.000	1.000	0.4993
1562729_at	71.8	32.2	-1.16	-2.23	AAA-AAA	0.00	999	1.000	1.000	0.1759
241703_at	26.9	12.0	-1.16	-2.23	AAA-AAA	0.00	999	1.000	1.000	0.3977
1560562_a_at	7.7	3.4	-1.16	-2.23	AAA-AAA	0.00	999	1.000	1.000	0.1058
213484_at	100.6	45.1	-1.16	-2.23	AAA-AAA	0.00	999	1.000	1.000	0.2631
220923_s_at	36.2	16.2	-1.16	-2.23	AAA-AAA	0.00	999	1.000	1.000	0.2866
202508_s_at	9.7	4.3	-1.16	-2.23	AAA-AAA	0.00	999	1.000	1.000	0.4211
236422_at	54.4	24.4	-1.16	-2.23	AAA-AAA	0.00	999	1.000	1.000	0.1662
224048_at	12.3	27.4	1.16	2.23	AAA-AAA	0.00	999	1.000	1.000	0.2913

242937_at	86.0	38.6	-1.16	-2.23	PPM-AAP	0.00	999	1.000	1.000	0.0323
237210_at	61.1	27.4	-1.16	-2.23	AAP-APA	0.00	999	1.000	1.000	0.3151
241547_at	16.8	37.4	1.16	2.23	AAA-AAA	0.00	999	1.000	1.000	0.3180
1569909_at	31.4	70.0	1.16	2.23	AAA-AAA	0.00	999	1.000	1.000	0.3952
244095_at	57.8	25.9	-1.16	-2.23	AAA-AAA	0.00	999	1.000	1.000	0.3449
220184_at	70.8	172.4	1.16	2.23	PMP-PPP	0.00	999	1.000	1.000	0.0112
221662_s_at	192.9	86.5	-1.16	-2.23	AAA-AAA	0.00	999	1.000	1.000	0.2390
218960_at	10.2	22.6	1.16	2.23	AAA-AAA	0.00	999	1.000	1.000	0.3999
211223_at	48.5	108.3	1.16	2.23	AAA-AAA	0.00	999	1.000	1.000	0.2764
237296_at	8.7	19.3	1.16	2.23	AAA-AAA	0.00	999	1.000	1.000	0.2583
1556413_a_at	57.7	25.9	-1.16	-2.23	PPA-PAP	0.00	999	1.000	1.000	0.4895
241285_at	60.6	135.1	1.16	2.23	AAA-AMA	0.00	999	1.000	1.000	0.2168
1568870_at	6.4	14.3	1.16	2.23	AAA-AAA	0.00	999	1.000	1.000	0.3998
1568898_at	8.9	4.0	-1.16	-2.23	AAA-AAA	0.00	999	1.000	1.000	0.5292
239837_at	10.1	22.6	1.16	2.23	AAA-AAA	0.00	999	1.000	1.000	0.4087
1555662_s_at	39.1	17.6	-1.16	-2.23	AAA-AAA	0.00	999	1.000	1.000	0.3180
1558649_at	17.4	38.9	1.16	2.23	AAA-AAA	0.00	999	1.000	1.000	0.3409
220727_at	34.5	76.8	1.16	2.23	AAA-AAA	0.00	999	1.000	1.000	0.0987
220224_at	8.4	3.8	-1.16	-2.23	AAA-AAA	0.00	999	1.000	1.000	0.1356
221608_at	6.0	13.4	1.16	2.23	AAA-AAA	0.00	999	1.000	1.000	0.3447
204663_at	34.9	77.7	1.16	2.23	AAA-AAA	0.00	999	1.000	1.000	0.2832
238873_at	21.7	9.7	-1.16	-2.23	AAA-AAA	0.00	999	1.000	1.000	0.3159
1570593_at	3.5	7.7	1.16	2.23	AAA-AAA	0.00	999	1.000	1.000	0.3709
220544_at	7.1	15.8	1.16	2.23	AAA-AAA	0.00	999	1.000	1.000	0.3253
220692_at	53.7	24.1	-1.16	-2.23	AAA-AAA	0.00	999	1.000	1.000	0.3791
220211_at	4.7	10.4	1.16	2.23	AAA-AAA	0.00	999	1.000	1.000	0.4074
206751_s_at	16.8	37.3	1.16	2.23	AAA-AAA	0.00	999	1.000	1.000	0.1606
234652_at	11.7	26.1	1.16	2.23	AAA-AAA	0.00	999	1.000	1.000	0.2373
232886_at	21.7	48.4	1.16	2.23	AAA-AAA	0.00	999	1.000	1.000	0.1274
233452_at	15.9	7.1	-1.16	-2.23	AAA-AAA	0.00	999	1.000	1.000	0.0770
236175_at	13.2	29.4	1.16	2.23	AAA-AAA	0.00	999	1.000	1.000	0.4286
230311_s_at	90.2	200.9	1.16	2.23	AAA-AAA	0.00	999	1.000	1.000	0.1687
216992_s_at	95.7	43.0	-1.15	-2.23	APA-AAA	0.00	999	1.000	1.000	0.0647
241312_at	48.9	22.0	-1.15	-2.23	AAA-AAA	0.00	999	1.000	1.000	0.4355
240122_at	9.4	4.2	-1.15	-2.23	AAA-AAA	0.00	999	1.000	1.000	0.3562
1565760_at	10.0	4.5	-1.15	-2.23	AAA-AAA	0.00	999	1.000	1.000	0.2710
1553873_at	8.4	3.8	-1.15	-2.23	AAA-AAA	0.00	999	1.000	1.000	0.0804
214204_at	33.2	14.9	-1.15	-2.23	AAA-AAA	0.00	999	1.000	1.000	0.3621
206736_x_at	10.6	4.8	-1.15	-2.23	AAA-AAA	0.00	999	1.000	1.000	0.2707
234647_at	55.7	25.0	-1.15	-2.23	AAA-AAA	0.00	999	1.000	1.000	0.1432
237357_at	3.4	7.6	1.15	2.23	AAA-AAA	0.00	999	1.000	1.000	0.3135
238679_at	20.7	46.0	1.15	2.23	AAA-AAA	0.00	999	1.000	1.000	0.4215

231558_at	6.6	14.8	1.15	2.23	AAA-AAA	0.00	999	1.000	1.000	0.3516
232173_at	13.6	30.4	1.15	2.23	AAA-AAA	0.00	999	1.000	1.000	0.3531
233400_at	46.2	20.8	-1.15	-2.22	APP-APA	0.00	999	1.000	1.000	0.3772
1555309_a_at	9.7	21.7	1.15	2.22	AAA-AAA	0.00	999	1.000	1.000	0.1849
215312_at	14.3	6.4	-1.15	-2.22	AAA-AAA	0.00	999	1.000	1.000	0.6052
237821_at	14.9	6.7	-1.15	-2.22	AAA-AAA	0.00	999	1.000	1.000	0.4299
236040_at	20.6	45.7	1.15	2.22	AAA-AAA	0.00	999	1.000	1.000	0.1850
227799_at	16.5	36.7	1.15	2.22	AAA-AAA	0.00	999	1.000	1.000	0.3291
228274_at	30.5	13.7	-1.15	-2.22	AAA-AAA	0.00	999	1.000	1.000	0.0732
231986_at	23.1	10.4	-1.15	-2.22	AAA-AAA	0.00	999	1.000	1.000	0.1660
230273_at	27.1	12.2	-1.15	-2.22	AAA-AAA	0.00	999	1.000	1.000	0.4303
1563721_at	8.1	3.6	-1.15	-2.22	AAA-AAA	0.00	999	1.000	1.000	0.4216
241705_at	8.4	18.7	1.15	2.22	AAA-AAA	0.00	999	1.000	1.000	0.4376
240819_at	4.5	10.0	1.15	2.22	AAA-AAA	0.00	999	1.000	1.000	0.5578
220331_at	8.9	19.9	1.15	2.22	AAA-AAA	0.00	999	1.000	1.000	0.1820
219422_at	32.4	72.0	1.15	2.22	AAA-AAA	0.00	999	1.000	1.000	0.2245
219167_at	63.6	28.6	-1.15	-2.22	AAA-AAA	0.00	999	1.000	1.000	0.0171
224176_s_at	37.8	84.0	1.15	2.22	AAA-AAA	0.00	999	1.000	1.000	0.0203
224396_s_at	5.9	13.2	1.15	2.22	AAA-AAA	0.00	999	1.000	1.000	0.4530
1569104_a_at	38.7	17.4	-1.15	-2.22	AAA-AAA	0.00	999	1.000	1.000	0.2448
214413_at	3.9	8.7	1.15	2.22	AAA-AAA	0.00	999	1.000	1.000	0.0019
220699_s_at	28.8	13.0	-1.15	-2.22	AAA-AAA	0.00	999	1.000	1.000	0.4137
220042_x_at	118.8	53.5	-1.15	-2.22	AAA-AAA	0.00	999	1.000	1.000	0.3692
220397_at	26.5	58.9	1.15	2.22	AAA-AAA	0.00	999	1.000	1.000	0.1867
217689_at	41.4	91.9	1.15	2.22	AAA-AAA	0.00	999	1.000	1.000	0.0329
204292_x_at	119.3	53.7	-1.15	-2.22	AAA-AAA	0.00	999	1.000	1.000	0.2628
211604_x_at	10.3	23.0	1.15	2.22	AAA-AAA	0.00	999	1.000	1.000	0.2407
235367_at	38.7	17.4	-1.15	-2.22	AAA-AAA	0.00	999	1.000	1.000	0.3025
233962_at	17.5	7.9	-1.15	-2.22	AAA-AAA	0.00	999	1.000	1.000	0.3858
237363_at	4.9	10.9	1.15	2.22	AAA-AAA	0.00	999	1.000	1.000	0.3158
236733_at	28.2	12.7	-1.15	-2.22	AAA-AAA	0.00	999	1.000	1.000	0.2486
236684_at	10.5	23.2	1.15	2.22	AAA-AAA	0.00	999	1.000	1.000	0.2347
221930_at	12.8	28.4	1.15	2.22	AAA-AAA	0.00	999	1.000	1.000	0.1626
230995_at	22.9	10.3	-1.15	-2.22	AAA-AAA	0.00	999	1.000	1.000	0.4996
230487_at	10.4	23.0	1.15	2.22	AAA-AAA	0.00	999	1.000	1.000	0.3022
1553765_a_at	1.9	4.2	1.15	2.22	AAA-AAA	0.00	999	1.000	1.000	0.3740
206532_at	10.9	24.2	1.15	2.22	AAA-AAA	0.00	999	1.000	1.000	0.3475
206759_at	11.1	24.7	1.15	2.22	AAA-AAA	0.00	999	1.000	1.000	0.2150
224020_at	21.2	47.1	1.15	2.22	AAA-AAA	0.00	999	1.000	1.000	0.2208
220764_at	102.7	46.3	-1.15	-2.22	PAA-PAP	0.00	999	1.000	1.000	0.3032
1563090_at	183.6	82.8	-1.15	-2.22	AAA-AAM	0.00	999	1.000	1.000	0.1624
240840_s_at	18.3	8.3	-1.15	-2.22	AAA-AAA	0.00	999	1.000	1.000	0.3968

1552365_at	12.6	5.7	-1.15	-2.22	AAA-AAA	0.00	999	1.000	1.000	0.2718
242594_at	70.4	31.8	-1.15	-2.22	AAA-AAA	0.00	999	1.000	1.000	0.3368
1561912_at	12.8	5.8	-1.15	-2.22	AAA-AAA	0.00	999	1.000	1.000	0.5082
210833_at	10.3	4.7	-1.15	-2.22	AAA-AAA	0.00	999	1.000	1.000	0.3479
222176_at	23.2	10.5	-1.15	-2.22	AAA-AAA	0.00	999	1.000	1.000	0.3988
231568_at	20.9	9.4	-1.15	-2.22	AAA-AAA	0.00	999	1.000	1.000	0.4787
231757_at	5.6	12.5	1.15	2.22	AAA-AAA	0.00	999	1.000	1.000	0.2608
228985_at	38.7	17.4	-1.15	-2.22	AAA-AAA	0.00	999	1.000	1.000	0.2403
230830_at	11.5	5.2	-1.15	-2.22	AAA-AAA	0.00	999	1.000	1.000	0.0897
1570482_at	36.6	16.5	-1.15	-2.22	AMP-AAA	0.00	999	1.000	1.000	0.3648
1568675_at	6.7	14.8	1.15	2.22	AAA-AAA	0.00	999	1.000	1.000	0.1122
1552758_at	24.0	10.8	-1.15	-2.22	AAA-AAA	0.00	999	1.000	1.000	0.3099
1554999_at	7.3	16.3	1.15	2.22	AAA-AAA	0.00	999	1.000	1.000	0.0792
213663_s_at	10.9	24.2	1.15	2.22	AAA-AAA	0.00	999	1.000	1.000	0.3528
220616_at	45.1	99.8	1.15	2.22	AAA-AAA	0.00	999	1.000	1.000	0.1305
217434_at	24.3	11.0	-1.15	-2.22	AAA-AAA	0.00	999	1.000	1.000	0.4952
210000_s_at	29.2	13.2	-1.15	-2.22	AAA-AAA	0.00	999	1.000	1.000	0.4063
211425_x_at	35.2	15.9	-1.15	-2.22	AAA-AAA	0.00	999	1.000	1.000	0.3018
207398_at	13.3	29.5	1.15	2.22	AAA-AAA	0.00	999	1.000	1.000	0.2861
226772_s_at	26.0	57.7	1.15	2.22	AAA-AAA	0.00	999	1.000	1.000	0.2503
222013_x_at	115.5	52.1	-1.15	-2.22	AAA-AAA	0.00	999	1.000	1.000	0.1258
222298_at	22.3	10.0	-1.15	-2.22	AAA-AAA	0.00	999	1.000	1.000	0.4067
224001_at	39.0	86.4	1.15	2.22	AAA-AAA	0.00	999	1.000	1.000	0.2184
231300_at	48.3	107.1	1.15	2.22	AAA-AAA	0.00	999	1.000	1.000	0.1876
1561333_at	45.4	20.5	-1.15	-2.21	APA-AAA	0.00	999	1.000	1.000	0.2691
1566513_a_at	71.4	32.3	-1.15	-2.21	APA-AAP	0.00	999	1.000	1.000	0.3183
239945_at	10.8	24.0	1.15	2.22	AAA-MAA	0.00	999	1.000	1.000	0.5226
1553372_at	11.0	24.3	1.15	2.21	AAA-AAA	0.00	999	1.000	1.000	0.1279
1561942_x_at	20.0	44.4	1.15	2.22	AAA-AAA	0.00	999	1.000	1.000	0.0803
243870_at	8.6	19.0	1.15	2.21	AAA-AAA	0.00	999	1.000	1.000	0.3017
215237_at	44.1	97.6	1.15	2.21	AAA-AAA	0.00	999	1.000	1.000	0.1673
217382_at	35.3	78.1	1.15	2.21	AAA-AAA	0.00	999	1.000	1.000	0.2076
210819_x_at	21.2	46.9	1.15	2.21	AAA-AAA	0.00	999	1.000	1.000	0.4320
235318_at	39.6	17.9	-1.15	-2.21	AAA-APA	0.00	999	1.000	1.000	0.2084
240993_at	7.0	15.4	1.15	2.21	AAA-AAA	0.00	999	1.000	1.000	0.3436
1561590_a_at	12.7	5.8	-1.15	-2.21	AAA-AAA	0.00	999	1.000	1.000	0.4802
1553199_at	2.2	5.0	1.15	2.21	AAA-AAA	0.00	999	1.000	1.000	0.0511
1561455_at	25.1	11.4	-1.15	-2.21	AAA-AAA	0.00	999	1.000	1.000	0.3960
1561335_at	58.2	26.3	-1.15	-2.21	AAA-AAA	0.00	999	1.000	1.000	0.4081
1562254_at	17.6	38.9	1.15	2.21	AAA-AAA	0.00	999	1.000	1.000	0.3484
1559579_at	66.5	30.1	-1.15	-2.21	AAA-AAA	0.00	999	1.000	1.000	0.2896
205838_at	24.1	53.4	1.15	2.21	AAA-AAA	0.00	999	1.000	1.000	0.1877

235129_at	12.3	27.1	1.15	2.21	AAA-AAA	0.00	999	1.000	1.000	0.4909
237504_at	46.7	103.3	1.15	2.21	AAA-AAA	0.00	999	1.000	1.000	0.1751
237049_at	2.7	5.9	1.15	2.21	AAA-AAA	0.00	999	1.000	1.000	0.3340
224117_at	40.5	89.7	1.15	2.21	AAA-AAA	0.00	999	1.000	1.000	0.2704
223861_at	3.8	8.4	1.15	2.21	AAA-AAA	0.00	999	1.000	1.000	0.3429
225375_at	479.2	216.7	-1.15	-2.21	PPP-PAP	0.00	999	1.000	1.000	0.3513
241405_at	46.4	21.0	-1.15	-2.21	AAA-AAA	0.00	999	1.000	1.000	0.4092
1563860_at	10.5	4.7	-1.15	-2.21	AAA-AAA	0.00	999	1.000	1.000	0.2329
240936_at	12.6	27.8	1.15	2.21	AAA-AAA	0.00	999	1.000	1.000	0.4245
1566215_at	4.5	9.9	1.15	2.21	AAA-AAA	0.00	999	1.000	1.000	0.2534
1553640_at	58.0	26.2	-1.15	-2.21	AAA-AAA	0.00	999	1.000	1.000	0.2593
244188_at	8.9	19.6	1.15	2.21	AAA-AAA	0.00	999	1.000	1.000	0.5082
216932_at	26.1	57.8	1.15	2.21	AAA-AAA	0.00	999	1.000	1.000	0.0664
205514_at	6.3	14.0	1.15	2.21	AAA-AAA	0.00	999	1.000	1.000	0.5582
232900_at	16.8	37.1	1.15	2.21	AAA-AAA	0.00	999	1.000	1.000	0.4067
230941_at	11.9	5.4	-1.15	-2.21	AAA-AAA	0.00	999	1.000	1.000	0.3104
229392_s_at	23.2	51.2	1.15	2.21	AAA-AAA	0.00	999	1.000	1.000	0.0752
231925_at	37.7	17.1	-1.14	-2.21	PAP-AAP	0.00	999	1.000	1.000	0.2619
1565689_at	46.5	102.8	1.14	2.21	AAA-AAA	0.00	999	1.000	1.000	0.2248
244346_at	5.9	13.1	1.14	2.21	AAA-AAM	0.00	999	1.000	1.000	0.4885
243761_at	38.3	17.3	-1.14	-2.21	AAA-AAA	0.00	999	1.000	1.000	0.1950
244027_at	11.1	24.6	1.14	2.21	AAA-AAA	0.00	999	1.000	1.000	0.4953
220817_at	15.1	6.8	-1.14	-2.21	AAA-AAA	0.00	999	1.000	1.000	0.2886
233818_at	20.4	9.2	-1.14	-2.21	AAA-AAA	0.00	999	1.000	1.000	0.4275
237437_s_at	12.0	26.4	1.14	2.21	AAA-AAA	0.00	999	1.000	1.000	0.1006
237819_at	62.0	28.1	-1.14	-2.21	AAP-AAA	0.00	999	1.000	1.000	0.1010
242014_at	54.0	24.4	-1.14	-2.21	APP-AAA	0.00	999	1.000	1.000	0.1491
216873_s_at	152.7	69.1	-1.14	-2.21	MAP-AAA	0.00	999	1.000	1.000	0.1874
1562713_a_at	13.7	30.2	1.14	2.21	AAA-AAA	0.00	999	1.000	1.000	0.3437
242461_at	28.5	62.9	1.14	2.21	AAA-AAA	0.00	999	1.000	1.000	0.0622
239668_at	9.1	20.1	1.14	2.21	AAA-AAA	0.00	999	1.000	1.000	0.5726
214255_at	55.9	123.5	1.14	2.21	AAA-AAA	0.00	999	1.000	1.000	0.2153
220984_s_at	7.8	3.5	-1.14	-2.21	AAA-AAA	0.00	999	1.000	1.000	0.1428
205221_at	6.0	13.2	1.14	2.21	AAA-AAA	0.00	999	1.000	1.000	0.2621
233990_at	16.6	36.6	1.14	2.21	AAA-AAA	0.00	999	1.000	1.000	0.4501
231282_at	9.4	20.8	1.14	2.21	AAA-AAA	0.00	999	1.000	1.000	0.4810
1556328_at	2.8	6.3	1.14	2.21	AAA-AAA	0.00	999	1.000	1.000	0.2178
1561155_at	40.7	89.8	1.14	2.21	AAA-AAA	0.00	999	1.000	1.000	0.0120
244442_at	25.9	57.1	1.14	2.21	AAA-AAA	0.00	999	1.000	1.000	0.2558
1560419_at	9.6	4.4	-1.14	-2.21	AAA-AAA	0.00	999	1.000	1.000	0.5288
216571_at	21.1	9.6	-1.14	-2.21	AAA-AAA	0.00	999	1.000	1.000	0.2147
216984_x_at	30.4	13.8	-1.14	-2.21	AAA-AAA	0.00	999	1.000	1.000	0.2191

203861_s_at	32.7	14.8	-1.14	-2.21	AAA-AAA	0.00	999	1.000	1.000	0.0574
238715_at	47.9	105.6	1.14	2.21	AAA-AAA	0.00	999	1.000	1.000	0.2000
223751_x_at	45.8	20.8	-1.14	-2.21	AAA-AAA	0.00	999	1.000	1.000	0.4296
229725_at	3.8	8.5	1.14	2.21	AAA-AAA	0.00	999	1.000	1.000	0.4270
229376_at	10.3	22.6	1.14	2.21	AAA-AAA	0.00	999	1.000	1.000	0.3659
239542_at	38.2	17.3	-1.14	-2.21	APA-AAA	0.00	999	1.000	1.000	0.3391
230664_at	65.5	29.7	-1.14	-2.21	PAA-AAA	0.00	999	1.000	1.000	0.4938
215592_at	101.8	46.2	-1.14	-2.21	AAA-AAA	0.00	999	1.000	1.000	0.0570
237602_at	21.6	9.8	-1.14	-2.21	AAA-AAA	0.00	999	1.000	1.000	0.4538
227232_at	24.0	53.0	1.14	2.21	AAA-AAA	0.00	999	1.000	1.000	0.2693
1564264_at	34.0	74.9	1.14	2.20	AAA-AAA	0.00	999	1.000	1.000	0.2184
1563082_at	11.5	25.3	1.14	2.20	AAA-AAA	0.00	999	1.000	1.000	0.3169
239905_at	10.3	4.7	-1.14	-2.20	AAA-AAA	0.00	999	1.000	1.000	0.4707
243391_x_at	7.4	16.3	1.14	2.20	AAA-AAA	0.00	999	1.000	1.000	0.1978
213781_at	20.8	9.4	-1.14	-2.20	AAA-AAA	0.00	999	1.000	1.000	0.2010
213162_at	43.7	19.8	-1.14	-2.20	AAA-AAA	0.00	999	1.000	1.000	0.4853
214368_at	22.1	10.0	-1.14	-2.20	AAA-AAA	0.00	999	1.000	1.000	0.1270
217219_at	8.8	19.3	1.14	2.20	AAA-AAA	0.00	999	1.000	1.000	0.0405
206440_at	3.6	8.0	1.14	2.20	AAA-AAA	0.00	999	1.000	1.000	0.1810
206811_at	22.4	10.2	-1.14	-2.20	AAA-AAA	0.00	999	1.000	1.000	0.4561
209886_s_at	19.5	43.1	1.14	2.20	AAA-AAA	0.00	999	1.000	1.000	0.3528
232943_at	15.5	34.2	1.14	2.20	AAA-AAA	0.00	999	1.000	1.000	0.3162
238200_at	21.2	46.6	1.14	2.20	AAA-AAA	0.00	999	1.000	1.000	0.2480
228163_at	34.0	15.4	-1.14	-2.20	AAA-AAA	0.00	999	1.000	1.000	0.4441
205518_s_at	50.7	23.0	-1.14	-2.20	PAP-AAA	0.00	999	1.000	1.000	0.2444
241271_at	3.8	8.3	1.14	2.20	AAA-AAA	0.00	999	1.000	1.000	0.4612
1569637_at	46.5	21.1	-1.14	-2.20	AMA-AAA	0.00	999	1.000	1.000	0.2954
1569405_at	13.4	29.4	1.14	2.20	AAA-MAA	0.00	999	1.000	1.000	0.3284
239917_at	44.1	97.1	1.14	2.20	AAA-MAA	0.00	999	1.000	1.000	0.3614
1561473_at	4.0	8.8	1.14	2.20	AAA-AAA	0.00	999	1.000	1.000	0.4644
215689_s_at	23.0	10.5	-1.14	-2.20	AAA-AAA	0.00	999	1.000	1.000	0.3798
205892_s_at	3.9	8.6	1.14	2.20	AAA-AAA	0.00	999	1.000	1.000	0.3042
206741_at	8.3	18.2	1.14	2.20	AAA-AAA	0.00	999	1.000	1.000	0.1253
218978_s_at	109.7	49.8	-1.14	-2.20	AAP-AAA	0.00	999	1.000	1.000	0.2201
240041_at	7.6	16.7	1.14	2.20	AAA-AAA	0.00	999	1.000	1.000	0.0234
240532_at	14.5	31.9	1.14	2.20	AAA-AAA	0.00	999	1.000	1.000	0.2739
1556645_s_at	28.3	62.4	1.14	2.20	AAA-AAA	0.00	999	1.000	1.000	0.0924
243079_x_at	35.9	79.0	1.14	2.20	AAA-AAA	0.00	999	1.000	1.000	0.1626
215685_s_at	78.2	35.5	-1.14	-2.20	AAA-AAA	0.00	999	1.000	1.000	0.0370
221153_s_at	17.0	37.4	1.14	2.20	AAA-MAA	0.00	999	1.000	1.000	0.2737
210368_at	20.9	45.9	1.14	2.20	AAA-AAA	0.00	999	1.000	1.000	0.3269
233796_at	30.0	13.7	-1.14	-2.20	AAA-AAA	0.00	999	1.000	1.000	0.0427

236502_at	30.6	13.9	-1.14	-2.20	AAA-AAA	0.00	999	1.000	1.000	0.4694
226141_at	18.7	41.2	1.14	2.20	AAA-AAA	0.00	999	1.000	1.000	0.3022
230054_at	75.3	34.2	-1.14	-2.20	AAA-AAA	0.00	999	1.000	1.000	0.4585
212599_at	56.6	25.7	-1.14	-2.20	AAP-AAA	0.00	999	1.000	1.000	0.3834
222356_at	39.3	17.9	-1.14	-2.20	PAA-APA	0.00	999	1.000	1.000	0.4525
1559568_at	52.2	23.7	-1.14	-2.20	AAA-AAA	0.00	999	1.000	1.000	0.1041
243697_at	15.6	7.1	-1.14	-2.20	AAA-AAA	0.00	999	1.000	1.000	0.2889
216761_at	8.6	18.9	1.14	2.20	AAA-AAA	0.00	999	1.000	1.000	0.1608
206646_at	32.6	71.7	1.14	2.20	AAA-AAA	0.00	999	1.000	1.000	0.4041
210476_s_at	28.7	13.0	-1.14	-2.20	AAA-AAA	0.00	999	1.000	1.000	0.2699
210652_s_at	13.8	30.4	1.14	2.20	AAA-AAA	0.00	999	1.000	1.000	0.3070
234198_at	13.3	6.1	-1.14	-2.20	AAA-AAA	0.00	999	1.000	1.000	0.3199
226827_at	31.6	69.4	1.14	2.20	AAA-AAA	0.00	999	1.000	1.000	0.1086
244544_at	119.0	54.2	-1.14	-2.20	AAA-AAP	0.00	999	1.000	1.000	0.1863
243627_at	32.5	14.8	-1.14	-2.20	APA-AAP	0.00	999	1.000	1.000	0.5082
1563277_at	16.9	7.7	-1.14	-2.20	AAA-AAA	0.00	999	1.000	1.000	0.4131
240197_at	42.9	19.5	-1.14	-2.20	AAA-AAA	0.00	999	1.000	1.000	0.3772
240067_at	8.2	3.7	-1.14	-2.20	AAA-AAA	0.00	999	1.000	1.000	0.1879
1553166_at	76.2	167.6	1.14	2.20	AAA-MAA	0.00	999	1.000	1.000	0.2790
216642_at	31.8	14.5	-1.14	-2.20	AAA-AAA	0.00	999	1.000	1.000	0.1804
220591_s_at	12.7	27.8	1.14	2.20	AAA-AAA	0.00	999	1.000	1.000	0.1672
221233_s_at	8.4	18.4	1.14	2.20	AAA-AAA	0.00	999	1.000	1.000	0.4021
217263_x_at	25.7	56.4	1.14	2.20	AAA-AAA	0.00	999	1.000	1.000	0.4589
235601_at	44.3	97.3	1.14	2.20	AAA-AAA	0.00	999	1.000	1.000	0.1367
236098_at	44.0	20.0	-1.14	-2.20	AAA-AAA	0.00	999	1.000	1.000	0.0777
231996_at	60.8	133.6	1.14	2.20	AAA-AAA	0.00	999	1.000	1.000	0.3586
229641_at	600.3	273.4	-1.14	-2.20	PPP-PPP	0.00	999	1.000	1.000	0.0358
1555545_at	82.5	37.6	-1.14	-2.20	PPA-AAA	0.00	999	1.000	1.000	0.1521
1565614_at	26.5	12.1	-1.14	-2.20	APA-AAA	0.00	999	1.000	1.000	0.1598
239550_at	62.7	28.6	-1.14	-2.20	MAA-AAP	0.00	999	1.000	1.000	0.3652
239308_at	101.7	46.3	-1.14	-2.20	AAA-AAA	0.00	999	1.000	1.000	0.1170
1569696_at	41.2	18.8	-1.14	-2.20	AAA-AAA	0.00	999	1.000	1.000	0.4775
240745_at	7.4	16.3	1.14	2.20	AAA-AAA	0.00	999	1.000	1.000	0.3188
1554346_at	69.1	151.8	1.14	2.20	AAA-AAA	0.00	999	1.000	1.000	0.0207
1554140_at	8.8	19.4	1.14	2.20	AAA-AAA	0.00	999	1.000	1.000	0.3651
1559252_a_at	16.1	35.4	1.14	2.20	AAA-AAA	0.00	999	1.000	1.000	0.2272
217415_at	21.8	47.9	1.14	2.20	AAA-AAA	0.00	999	1.000	1.000	0.3530
208394_x_at	17.6	38.6	1.14	2.20	AAA-AAA	0.00	999	1.000	1.000	0.3937
1555544_a_at	45.9	20.9	-1.13	-2.20	AAP-AAA	0.00	999	1.000	1.000	0.4201
242288_s_at	23.1	50.6	1.13	2.19	AAA-AAA	0.00	999	1.000	1.000	0.2387
1562670_at	20.8	45.5	1.13	2.20	AAA-AAA	0.00	999	1.000	1.000	0.3509
1554699_at	5.1	11.2	1.13	2.19	AAA-AAA	0.00	999	1.000	1.000	0.2419

1555567_s_at	12.7	5.8	-1.13	-2.20	AAA-AAA	0.00	999	1.000	1.000	0.2908
244434_at	2.0	4.3	1.13	2.20	AAA-AAA	0.00	999	1.000	1.000	0.4438
220902_at	27.7	12.6	-1.13	-2.19	AAA-AAA	0.00	999	1.000	1.000	0.1659
211191_at	6.6	3.0	-1.13	-2.19	AAA-AAA	0.00	999	1.000	1.000	0.0762
229739_s_at	54.0	24.6	-1.13	-2.19	AAA-AAA	0.00	999	1.000	1.000	0.1080
230033_at	52.6	115.5	1.13	2.20	AAA-AAA	0.00	999	1.000	1.000	0.2500
242065_x_at	91.3	200.2	1.13	2.19	AAA-AAA	0.00	999	1.000	1.000	0.3550
1566854_at	68.3	31.1	-1.13	-2.19	AAA-AAA	0.00	999	1.000	1.000	0.3068
1566841_at	107.6	49.1	-1.13	-2.19	AAA-AAA	0.00	999	1.000	1.000	0.3760
244313_at	13.9	6.3	-1.13	-2.19	AAA-AAA	0.00	999	1.000	1.000	0.5377
1560277_a_at	54.5	24.9	-1.13	-2.19	AAA-AAA	0.00	999	1.000	1.000	0.4233
205613_at	24.1	11.0	-1.13	-2.19	AAA-AAA	0.00	999	1.000	1.000	0.1769
210755_at	43.5	19.9	-1.13	-2.19	AAA-AAA	0.00	999	1.000	1.000	0.2645
211203_s_at	25.9	56.9	1.13	2.19	AAA-AAA	0.00	999	1.000	1.000	0.5592
236638_at	17.0	7.8	-1.13	-2.19	AAA-AAA	0.00	999	1.000	1.000	0.1395
228616_at	26.4	57.9	1.13	2.19	AAA-AAA	0.00	999	1.000	1.000	0.2455
230425_at	13.8	6.3	-1.13	-2.19	AAA-AAA	0.00	999	1.000	1.000	0.3746
1570014_at	15.3	7.0	-1.13	-2.19	AAA-AAA	0.00	999	1.000	1.000	0.0034
240938_at	3.1	6.8	1.13	2.19	AAA-AAA	0.00	999	1.000	1.000	0.2856
1565896_at	16.5	7.5	-1.13	-2.19	AAA-AAA	0.00	999	1.000	1.000	0.6122
240741_x_at	9.2	4.2	-1.13	-2.19	AAA-AAA	0.00	999	1.000	1.000	0.1201
210535_at	105.5	48.2	-1.13	-2.19	AAA-AAA	0.00	999	1.000	1.000	0.2424
208210_at	60.0	27.4	-1.13	-2.19	AAA-AAA	0.00	999	1.000	1.000	0.4201
235336_at	17.1	7.8	-1.13	-2.19	AAA-AAA	0.00	999	1.000	1.000	0.4485
233918_at	90.5	41.3	-1.13	-2.19	AAA-AAA	0.00	999	1.000	1.000	0.2725
237243_at	5.0	11.0	1.13	2.19	AAA-AAA	0.00	999	1.000	1.000	0.3478
226933_s_at	3.6	7.9	1.13	2.19	AAA-AAA	0.00	999	1.000	1.000	0.5030
222878_s_at	37.7	17.2	-1.13	-2.19	AAA-AAA	0.00	999	1.000	1.000	0.1583
229041_s_at	114.7	52.4	-1.13	-2.19	AAA-AAA	0.00	999	1.000	1.000	0.1389
230254_at	39.3	86.1	1.13	2.19	AAA-AAA	0.00	999	1.000	1.000	0.0576
1563310_a_at	11.2	24.4	1.13	2.19	AAA-AAA	0.00	999	1.000	1.000	0.3775
241137_at	31.9	69.9	1.13	2.19	AAA-AAA	0.00	999	1.000	1.000	0.2242
1559714_at	65.4	29.8	-1.13	-2.19	AAA-AAA	0.00	999	1.000	1.000	0.3317
207672_at	4.7	10.2	1.13	2.19	AAA-AAA	0.00	999	1.000	1.000	0.3037
234707_x_at	7.3	15.9	1.13	2.19	AAA-AAA	0.00	999	1.000	1.000	0.0066
234878_at	50.8	23.2	-1.13	-2.19	AAA-AAA	0.00	999	1.000	1.000	0.2638
237529_at	10.2	4.7	-1.13	-2.19	AAA-AAA	0.00	999	1.000	1.000	0.0461
238215_at	27.1	59.3	1.13	2.19	AAA-AAA	0.00	999	1.000	1.000	0.2593
237171_at	54.4	24.8	-1.13	-2.19	AAA-AAA	0.00	999	1.000	1.000	0.5291
237078_at	9.7	21.2	1.13	2.19	AAA-AAA	0.00	999	1.000	1.000	0.2880
228241_at	12.3	5.6	-1.13	-2.19	AAA-AAA	0.00	999	1.000	1.000	0.2460
224300_x_at	91.4	41.7	-1.13	-2.19	AAA-AAA	0.00	999	1.000	1.000	0.0809

216835_s_at	107.5	49.1	-1.13	-2.19	AAA-AAP	0.00	999	1.000	1.000	0.1873
207916_at	38.4	17.5	-1.13	-2.19	AAA-PAA	0.00	999	1.000	1.000	0.3868
1568736_s_at	28.6	13.1	-1.13	-2.19	AAA-MAA	0.00	999	1.000	1.000	0.4855
1558804_at	68.6	31.3	-1.13	-2.19	AAA-AAA	0.00	999	1.000	1.000	0.2838
221129_at	41.9	19.1	-1.13	-2.19	AAA-AAA	0.00	999	1.000	1.000	0.3791
233306_at	29.8	13.6	-1.13	-2.19	AAA-AAA	0.00	999	1.000	1.000	0.3949
223469_at	59.8	27.3	-1.13	-2.19	AAA-AAA	0.00	999	1.000	1.000	0.2055
232479_at	17.5	38.3	1.13	2.19	AAA-AAA	0.00	999	1.000	1.000	0.2864
1566867_at	6.8	14.8	1.13	2.19	AAA-AAA	0.00	999	1.000	1.000	0.3131
1557501_a_at	46.1	21.1	-1.13	-2.19	AAA-AAA	0.00	999	1.000	1.000	0.4017
1555157_at	16.4	36.0	1.13	2.19	AAA-AAA	0.00	999	1.000	1.000	0.1818
211446_at	12.4	27.1	1.13	2.19	AAA-AAA	0.00	999	1.000	1.000	0.4738
234742_at	15.9	34.8	1.13	2.19	AAA-AAA	0.00	999	1.000	1.000	0.4114
238749_at	16.1	35.1	1.13	2.19	AAA-AAA	0.00	999	1.000	1.000	0.2130
238367_s_at	12.9	28.2	1.13	2.19	AAA-AAA	0.00	999	1.000	1.000	0.4459
231363_at	65.3	29.9	-1.13	-2.19	AAA-AAA	0.00	999	1.000	1.000	0.2997
229941_at	8.4	18.4	1.13	2.19	AAA-AAA	0.00	999	1.000	1.000	0.2714
1563088_a_at	26.5	57.9	1.13	2.19	AAA-AAA	0.00	999	1.000	1.000	0.4552
1562678_at	29.7	64.9	1.13	2.19	AAA-AAA	0.00	999	1.000	1.000	0.4193
243441_at	18.5	40.5	1.13	2.19	AAA-AAA	0.00	999	1.000	1.000	0.2847
1559679_a_at	7.9	17.2	1.13	2.19	AAA-AAA	0.00	999	1.000	1.000	0.0062
214384_s_at	11.2	24.5	1.13	2.19	AAA-AAA	0.00	999	1.000	1.000	0.2849
220318_at	55.5	25.4	-1.13	-2.19	AAA-AAA	0.00	999	1.000	1.000	0.4612
220950_s_at	20.5	44.9	1.13	2.19	AAA-AAA	0.00	999	1.000	1.000	0.1573
221191_at	59.6	130.2	1.13	2.19	AAA-AAA	0.00	999	1.000	1.000	0.2190
210698_at	7.9	17.2	1.13	2.19	AAA-AAA	0.00	999	1.000	1.000	0.4996
210032_s_at	37.6	17.2	-1.13	-2.19	AAA-AAA	0.00	999	1.000	1.000	0.1729
207841_at	11.7	25.6	1.13	2.19	AAA-AAA	0.00	999	1.000	1.000	0.2423
234042_at	22.5	49.2	1.13	2.19	AAA-AAA	0.00	999	1.000	1.000	0.3613
222827_s_at	64.5	29.6	-1.13	-2.18	MPA-AAA	0.00	999	1.000	1.000	0.4980
239861_at	26.5	57.8	1.13	2.18	AAA-AAA	0.00	999	1.000	1.000	0.3036
239173_at	56.4	123.1	1.13	2.19	AAA-AAA	0.00	999	1.000	1.000	0.3647
1567179_at	7.3	3.3	-1.13	-2.18	AAA-AAA	0.00	999	1.000	1.000	0.3634
1558525_at	18.4	40.1	1.13	2.18	AAA-AAA	0.00	999	1.000	1.000	0.4452
213721_at	29.5	64.4	1.13	2.19	AAA-AAA	0.00	999	1.000	1.000	0.2588
206478_at	31.9	69.7	1.13	2.19	AAA-AAA	0.00	999	1.000	1.000	0.1899
205713_s_at	8.7	19.1	1.13	2.18	AAA-AAA	0.00	999	1.000	1.000	0.4673
203697_at	13.1	28.5	1.13	2.18	AAA-AAA	0.00	999	1.000	1.000	0.4066
233091_at	21.3	9.7	-1.13	-2.19	AAA-AAA	0.00	999	1.000	1.000	0.3691
237759_at	16.8	36.6	1.13	2.18	AAA-AAA	0.00	999	1.000	1.000	0.2937
238927_at	8.6	18.8	1.13	2.19	AAA-AAA	0.00	999	1.000	1.000	0.1957
238316_at	35.5	77.6	1.13	2.19	AAA-AAA	0.00	999	1.000	1.000	0.3257

238265_x_at	87.7	40.2	-1.13	-2.18	AAA-AMA	0.00	999	1.000	1.000	0.1841
232428_at	28.0	12.8	-1.13	-2.18	AAA-AAA	0.00	999	1.000	1.000	0.2851
239349_at	11.1	24.2	1.13	2.18	AAA-AAA	0.00	999	1.000	1.000	0.5381
1558000_at	4.6	10.0	1.13	2.18	AAA-AAA	0.00	999	1.000	1.000	0.4110
1561499_at	11.4	5.2	-1.13	-2.18	AAA-AAA	0.00	999	1.000	1.000	0.3765
212575_at	47.7	104.0	1.13	2.18	AAA-AAA	0.00	999	1.000	1.000	0.2894
219884_at	27.0	58.8	1.13	2.18	AAA-AAA	0.00	999	1.000	1.000	0.2615
236218_at	26.1	11.9	-1.13	-2.18	AAA-AAA	0.00	999	1.000	1.000	0.1602
232070_at	44.2	96.4	1.13	2.18	AAA-AAA	0.00	999	1.000	1.000	0.2980
229218_at	29.6	13.5	-1.13	-2.18	AAA-AAA	0.00	999	1.000	1.000	0.4412
212901_s_at	213.8	98.0	-1.13	-2.18	PPP-AAP	0.00	999	1.000	1.000	0.1484
1562905_at	13.8	6.3	-1.13	-2.18	AAA-AAA	0.00	999	1.000	1.000	0.3363
1557107_at	7.9	3.6	-1.13	-2.18	AAA-AAA	0.00	999	1.000	1.000	0.4758
1557242_at	49.4	107.8	1.13	2.18	AAA-AAA	0.00	999	1.000	1.000	0.1723
1556476_at	22.4	10.3	-1.13	-2.18	AAA-AAA	0.00	999	1.000	1.000	0.2504
242936_at	16.3	35.6	1.13	2.18	AAA-AAA	0.00	999	1.000	1.000	0.1182
243765_at	22.6	49.4	1.13	2.18	AAA-AAA	0.00	999	1.000	1.000	0.1935
204816_s_at	27.7	12.7	-1.13	-2.18	AAA-AAA	0.00	999	1.000	1.000	0.2870
207964_x_at	32.2	70.3	1.13	2.18	AAA-AAA	0.00	999	1.000	1.000	0.2328
229825_at	41.5	90.5	1.13	2.18	AAA-AAA	0.00	999	1.000	1.000	0.0407
230677_at	7.4	16.1	1.13	2.18	AAA-AAA	0.00	999	1.000	1.000	0.3321
1560212_a_at	13.2	28.8	1.12	2.18	AAA-AAA	0.00	999	1.000	1.000	0.3760
221859_at	104.7	48.0	-1.12	-2.18	AAA-AAA	0.00	999	1.000	1.000	0.1764
206814_at	9.4	20.4	1.12	2.18	AAA-AAA	0.00	999	1.000	1.000	0.1543
235175_at	12.2	26.6	1.12	2.18	AAA-AAA	0.00	999	1.000	1.000	0.5132
233751_at	6.3	2.9	-1.12	-2.18	AAA-AAA	0.00	999	1.000	1.000	0.3514
237227_at	35.7	77.8	1.12	2.18	AAA-AAA	0.00	999	1.000	1.000	0.2381
228887_x_at	12.3	26.8	1.12	2.18	AAA-AAA	0.00	999	1.000	1.000	0.2514
1563978_at	21.0	9.6	-1.12	-2.18	AAA-AAA	0.00	999	1.000	1.000	0.1898
240888_at	10.9	23.8	1.12	2.18	AAA-AAA	0.00	999	1.000	1.000	0.4557
221062_at	35.5	16.3	-1.12	-2.18	AAA-AAA	0.00	999	1.000	1.000	0.5427
235221_at	46.9	102.1	1.12	2.18	AAA-AAA	0.00	999	1.000	1.000	0.4089
235480_at	7.4	16.2	1.12	2.18	AAA-AAA	0.00	999	1.000	1.000	0.4420
235592_at	53.0	24.3	-1.12	-2.18	AAA-AAM	0.00	999	1.000	1.000	0.3980
1557296_at	21.5	9.9	-1.12	-2.18	AAA-AAA	0.00	999	1.000	1.000	0.2461
243348_at	13.0	6.0	-1.12	-2.18	AAA-AAA	0.00	999	1.000	1.000	0.4099
1562173_a_at	27.9	12.8	-1.12	-2.18	AAA-AAA	0.00	999	1.000	1.000	0.2867
217570_x_at	44.9	20.6	-1.12	-2.18	AAA-AAA	0.00	999	1.000	1.000	0.2544
206267_s_at	51.2	23.5	-1.12	-2.18	AAA-AAA	0.00	999	1.000	1.000	0.3105
209930_s_at	20.4	9.4	-1.12	-2.18	AAA-AAA	0.00	999	1.000	1.000	0.4032
234152_at	51.1	23.5	-1.12	-2.18	AAA-AAA	0.00	999	1.000	1.000	0.3946
234783_at	61.2	28.1	-1.12	-2.18	AAA-AAA	0.00	999	1.000	1.000	0.3688

223722_at	22.7	10.4	-1.12	-2.18	AAA-AAA	0.00	999	1.000	1.000	0.4443
229313_at	27.1	12.4	-1.12	-2.18	AAA-AAA	0.00	999	1.000	1.000	0.4785
230846_at	12.3	5.7	-1.12	-2.18	AAA-AAA	0.00	999	1.000	1.000	0.3350
1553418_a_at	38.5	17.7	-1.12	-2.18	AAP-AAA	0.00	999	1.000	1.000	0.4720
241566_at	11.3	24.6	1.12	2.18	AAA-AAA	0.00	999	1.000	1.000	0.0886
239637_at	28.1	61.0	1.12	2.18	AAA-AAA	0.00	999	1.000	1.000	0.3443
1569603_at	26.5	12.2	-1.12	-2.18	AAA-AAA	0.00	999	1.000	1.000	0.4844
1557551_at	6.9	15.1	1.12	2.18	AAA-AAA	0.00	999	1.000	1.000	0.1669
1555199_at	2.7	5.9	1.12	2.18	AAA-AAA	0.00	999	1.000	1.000	0.3124
214396_s_at	60.8	28.0	-1.12	-2.18	AAA-AAA	0.00	999	1.000	1.000	0.5124
217514_at	14.5	31.6	1.12	2.17	AAA-AAA	0.00	999	1.000	1.000	0.3152
203570_at	119.1	54.8	-1.12	-2.18	AAA-AAA	0.00	999	1.000	1.000	0.1301
211621_at	24.9	54.2	1.12	2.18	AAA-AAA	0.00	999	1.000	1.000	0.1727
233986_s_at	25.4	55.2	1.12	2.18	AAA-AAA	0.00	999	1.000	1.000	0.1804
238432_at	27.7	60.3	1.12	2.18	AAA-AAA	0.00	999	1.000	1.000	0.2317
242027_at	25.3	55.0	1.12	2.17	AAA-AAA	0.00	999	1.000	1.000	0.2772
240040_at	56.4	26.0	-1.12	-2.17	AAA-AAA	0.00	999	1.000	1.000	0.0749
1566502_at	58.8	127.7	1.12	2.17	AAA-AAA	0.00	999	1.000	1.000	0.0824
1557453_at	38.5	83.7	1.12	2.17	AAA-AAA	0.00	999	1.000	1.000	0.2303
1557535_at	18.6	40.4	1.12	2.17	AAA-AAA	0.00	999	1.000	1.000	0.3486
216552_x_at	13.0	28.3	1.12	2.17	AAA-AAA	0.00	999	1.000	1.000	0.1478
220821_at	3.2	7.0	1.12	2.17	AAA-AAA	0.00	999	1.000	1.000	0.0329
217615_at	31.8	14.6	-1.12	-2.17	AAA-AAA	0.00	999	1.000	1.000	0.1403
236890_at	12.3	26.6	1.12	2.17	AAA-AAA	0.00	999	1.000	1.000	0.3969
1570364_at	19.8	43.1	1.12	2.17	AAA-AAA	0.00	999	1.000	1.000	0.3953
240946_at	14.2	30.8	1.12	2.17	AAA-AAA	0.00	999	1.000	1.000	0.4421
1552542_s_at	20.7	9.5	-1.12	-2.17	AAA-AAA	0.00	999	1.000	1.000	0.5349
1556095_at	2.2	4.7	1.12	2.17	AAA-AAA	0.00	999	1.000	1.000	0.3749
243432_at	17.7	38.4	1.12	2.17	AAA-AAA	0.00	999	1.000	1.000	0.2424
1559909_a_at	24.6	11.3	-1.12	-2.17	AAA-AAA	0.00	999	1.000	1.000	0.3455
214646_at	16.4	35.6	1.12	2.17	AAA-AAA	0.00	999	1.000	1.000	0.3364
221370_at	17.4	37.7	1.12	2.17	AAA-AAA	0.00	999	1.000	1.000	0.3235
219605_at	34.6	16.0	-1.12	-2.17	AAA-AAA	0.00	999	1.000	1.000	0.3693
234134_at	8.6	3.9	-1.12	-2.17	AAA-AAA	0.00	999	1.000	1.000	0.3979
236407_at	19.9	9.2	-1.12	-2.17	AAA-AAA	0.00	999	1.000	1.000	0.2826
231076_at	9.0	19.6	1.12	2.17	AAA-AAA	0.00	999	1.000	1.000	0.3058
241932_at	118.2	54.4	-1.12	-2.17	AAA-AAA	0.00	999	1.000	1.000	0.2019
1563425_at	51.1	23.5	-1.12	-2.17	AAA-AAA	0.00	999	1.000	1.000	0.2397
244802_at	7.3	15.9	1.12	2.17	AAA-AAA	0.00	999	1.000	1.000	0.4630
1557707_at	17.4	37.7	1.12	2.17	AAA-AAA	0.00	999	1.000	1.000	0.3681
243460_at	17.7	8.1	-1.12	-2.17	AAA-AAA	0.00	999	1.000	1.000	0.4402
1560581_at	4.9	10.7	1.12	2.17	AAA-AAA	0.00	999	1.000	1.000	0.5038

1559986_at	2.3	4.9	1.12	2.17	AAA-AAA	0.00	999	1.000	1.000	0.3759
205270_s_at	15.5	7.1	-1.12	-2.17	AAA-AAA	0.00	999	1.000	1.000	0.1781
204869_at	28.4	13.1	-1.12	-2.17	AAA-AAA	0.00	999	1.000	1.000	0.1703
207881_at	47.5	21.9	-1.12	-2.17	AAA-AAA	0.00	999	1.000	1.000	0.3607
228409_at	23.7	10.9	-1.12	-2.17	AAA-AAA	0.00	999	1.000	1.000	0.2251
231359_at	6.5	14.0	1.12	2.17	AAA-AAA	0.00	999	1.000	1.000	0.3448
231098_at	13.6	6.3	-1.12	-2.17	AAA-AAA	0.00	999	1.000	1.000	0.3698
231977_at	8.7	19.0	1.12	2.17	AAA-AAA	0.00	999	1.000	1.000	0.4278
229611_at	20.2	9.3	-1.12	-2.17	AAA-AAA	0.00	999	1.000	1.000	0.2302
1568787_at	12.2	5.6	-1.12	-2.17	AAA-AAA	0.00	999	1.000	1.000	0.3045
239424_at	26.7	58.0	1.12	2.17	AAA-AAA	0.00	999	1.000	1.000	0.4233
1557562_at	36.1	16.7	-1.12	-2.17	AAA-AAA	0.00	999	1.000	1.000	0.4583
216561_x_at	29.9	13.8	-1.12	-2.17	AAA-AAA	0.00	999	1.000	1.000	0.5066
227360_at	78.2	36.0	-1.12	-2.17	AAA-AAA	0.00	999	1.000	1.000	0.2730
231318_at	23.4	10.8	-1.12	-2.17	AAA-AAA	0.00	999	1.000	1.000	0.3184
1570285_at	20.3	9.4	-1.12	-2.17	AAA-AAA	0.00	999	1.000	1.000	0.4618
1562853_x_at	104.5	48.2	-1.12	-2.17	AAA-AAA	0.00	999	1.000	1.000	0.2745
239420_at	32.5	15.0	-1.12	-2.17	AAA-AAA	0.00	999	1.000	1.000	0.0915
1555267_at	50.6	109.7	1.12	2.17	AAA-AAA	0.00	999	1.000	1.000	0.2572
213265_at	31.4	68.1	1.12	2.17	AAA-AAA	0.00	999	1.000	1.000	0.1148
217217_at	11.5	5.3	-1.12	-2.17	AAA-AAA	0.00	999	1.000	1.000	0.4033
206080_at	29.5	63.9	1.12	2.17	AAA-AAA	0.00	999	1.000	1.000	0.4978
236653_at	30.1	65.2	1.12	2.17	AAA-AAA	0.00	999	1.000	1.000	0.2356
223800_s_at	19.7	9.1	-1.12	-2.17	AAA-AAA	0.00	999	1.000	1.000	0.6078
230895_at	7.9	17.2	1.12	2.17	AAA-AAA	0.00	999	1.000	1.000	0.3931
232132_at	43.9	95.1	1.12	2.17	AAA-AAM	0.00	999	1.000	1.000	0.0123
231615_at	42.9	19.8	-1.12	-2.17	AAA-AAA	0.00	999	1.000	1.000	0.3604
1568732_at	9.1	4.2	-1.12	-2.17	AAA-AAA	0.00	999	1.000	1.000	0.4409
239689_at	15.2	32.9	1.12	2.17	AAA-AAA	0.00	999	1.000	1.000	0.4093
1556490_a_at	5.5	11.8	1.12	2.17	AAA-AAA	0.00	999	1.000	1.000	0.4033
217418_x_at	27.4	12.7	-1.12	-2.17	AAA-AAA	0.00	999	1.000	1.000	0.3724
209841_s_at	28.7	62.2	1.12	2.17	AAA-AAA	0.00	999	1.000	1.000	0.3997
211205_x_at	112.0	51.7	-1.12	-2.17	AAA-AAA	0.00	999	1.000	1.000	0.2827
207534_at	11.6	25.2	1.12	2.17	AAA-AAA	0.00	999	1.000	1.000	0.2970
230865_at	10.6	4.9	-1.12	-2.17	AAA-AAA	0.00	999	1.000	1.000	0.4785
231110_at	64.8	29.9	-1.12	-2.17	AAA-AAA	0.00	999	1.000	1.000	0.2250
1570414_x_at	53.2	24.6	-1.11	-2.16	AAA-AAA	0.00	999	1.000	1.000	0.3969
1557052_at	21.7	10.0	-1.11	-2.17	AAA-AAA	0.00	999	1.000	1.000	0.4600
1558820_a_at	25.2	54.7	1.11	2.17	AAA-AAA	0.00	999	1.000	1.000	0.3430
1560973_a_at	1.9	4.1	1.11	2.17	AAA-AAA	0.00	999	1.000	1.000	0.1875
1561785_at	24.6	11.4	-1.11	-2.17	AAA-AAA	0.00	999	1.000	1.000	0.3346
242958_x_at	15.6	7.2	-1.11	-2.16	AAA-AAA	0.00	999	1.000	1.000	0.6182

209730_at	43.3	93.8	1.11	2.16	AAA-AAA	0.00	999	1.000	1.000	0.0722
234446_at	45.3	98.0	1.11	2.17	AAA-AAA	0.00	999	1.000	1.000	0.0476
238354_x_at	5.3	11.4	1.11	2.16	AAA-AAA	0.00	999	1.000	1.000	0.0909
237188_x_at	51.7	111.9	1.11	2.17	AAA-AAA	0.00	999	1.000	1.000	0.2624
231395_at	26.6	12.3	-1.11	-2.17	AAA-AAA	0.00	999	1.000	1.000	0.1552
230242_at	50.4	23.3	-1.11	-2.16	AAA-AAA	0.00	999	1.000	1.000	0.1018
241152_at	12.2	26.4	1.11	2.16	AAA-AAA	0.00	999	1.000	1.000	0.2205
1557248_at	27.0	12.5	-1.11	-2.16	AAA-AAA	0.00	999	1.000	1.000	0.3314
1555129_at	66.7	30.9	-1.11	-2.16	AAA-AAA	0.00	999	1.000	1.000	0.2398
221165_s_at	35.5	76.8	1.11	2.16	AAA-AAA	0.00	999	1.000	1.000	0.1191
211649_x_at	11.2	24.3	1.11	2.16	AAA-AAA	0.00	999	1.000	1.000	0.2394
235491_at	53.8	24.9	-1.11	-2.16	AAA-AAA	0.00	999	1.000	1.000	0.4653
233790_at	9.4	4.3	-1.11	-2.16	AAA-AAA	0.00	999	1.000	1.000	0.0841
236374_at	21.8	47.2	1.11	2.16	AAA-AAA	0.00	999	1.000	1.000	0.4285
236270_at	60.1	27.8	-1.11	-2.16	AAA-AAA	0.00	999	1.000	1.000	0.3603
236701_at	36.4	78.7	1.11	2.16	AAA-AAA	0.00	999	1.000	1.000	0.2506
222930_s_at	82.7	38.2	-1.11	-2.16	AAA-AAA	0.00	999	1.000	1.000	0.4203
231687_at	20.8	9.6	-1.11	-2.16	AAA-AAA	0.00	999	1.000	1.000	0.2474
230187_s_at	35.2	16.3	-1.11	-2.16	AAA-AAA	0.00	999	1.000	1.000	0.4102
239969_at	12.3	26.5	1.11	2.16	AAA-AAA	0.00	999	1.000	1.000	0.2787
1556312_at	7.2	15.5	1.11	2.16	AAA-AAA	0.00	999	1.000	1.000	0.3318
1561417_x_at	4.4	9.5	1.11	2.16	AAA-AAA	0.00	999	1.000	1.000	0.6039
243798_at	8.6	18.6	1.11	2.16	AAA-AAA	0.00	999	1.000	1.000	0.0198
216196_at	51.1	23.6	-1.11	-2.16	AAA-AAA	0.00	999	1.000	1.000	0.3594
213759_at	15.2	7.0	-1.11	-2.16	AAA-AAA	0.00	999	1.000	1.000	0.3731
213512_at	22.6	48.9	1.11	2.16	AAA-AAA	0.00	999	1.000	1.000	0.2419
206418_at	6.9	14.8	1.11	2.16	AAA-AAA	0.00	999	1.000	1.000	0.1651
208344_x_at	28.8	62.3	1.11	2.16	AAA-AAA	0.00	999	1.000	1.000	0.3491
228658_at	29.8	64.3	1.11	2.16	AAA-AAA	0.00	999	1.000	1.000	0.2304
229462_at	15.4	7.1	-1.11	-2.16	AAA-AAA	0.00	999	1.000	1.000	0.2799
230639_at	14.6	31.5	1.11	2.16	AAA-AAA	0.00	999	1.000	1.000	0.3270
244230_at	32.3	15.0	-1.11	-2.16	APA-PAA	0.00	999	1.000	1.000	0.3742
1567341_at	23.7	51.1	1.11	2.16	AAA-AAA	0.00	999	1.000	1.000	0.5041
1561323_at	9.5	20.6	1.11	2.16	AAA-AAA	0.00	999	1.000	1.000	0.0962
1562190_at	2.9	6.2	1.11	2.16	AAA-AAA	0.00	999	1.000	1.000	0.1403
1559061_at	10.6	22.8	1.11	2.16	AAA-AAA	0.00	999	1.000	1.000	0.5094
1560871_a_at	21.1	45.6	1.11	2.16	AAA-AAA	0.00	999	1.000	1.000	0.3452
203930_s_at	11.6	5.4	-1.11	-2.16	AAA-AAA	0.00	999	1.000	1.000	0.2512
209072_at	70.7	32.7	-1.11	-2.16	AAA-AAA	0.00	999	1.000	1.000	0.2377
237763_at	15.4	7.1	-1.11	-2.16	AAA-AAA	0.00	999	1.000	1.000	0.2558
224540_at	11.7	25.2	1.11	2.16	AAA-AAA	0.00	999	1.000	1.000	0.3165
231256_at	35.5	16.4	-1.11	-2.16	AAA-AAA	0.00	999	1.000	1.000	0.2427

229337_at	30.3	65.5	1.11	2.16	AAA-AAA	0.00	999	1.000	1.000	0.4758
230319_at	2.9	6.2	1.11	2.16	AAA-AAA	0.00	999	1.000	1.000	0.4419
238313_at	74.8	34.6	-1.11	-2.16	AAP-MAA	0.00	999	1.000	1.000	0.2561
222051_s_at	55.5	25.7	-1.11	-2.16	APA-PAA	0.00	999	1.000	1.000	0.3854
1563638_at	8.3	18.0	1.11	2.16	AAA-AAA	0.00	999	1.000	1.000	0.4250
215953_at	21.0	45.4	1.11	2.16	AAA-AAA	0.00	999	1.000	1.000	0.4247
217464_at	28.2	13.0	-1.11	-2.16	AAA-AAA	0.00	999	1.000	1.000	0.2810
217228_s_at	19.0	41.0	1.11	2.16	AAA-AAA	0.00	999	1.000	1.000	0.4309
205683_x_at	14.6	31.6	1.11	2.16	AAA-AAA	0.00	999	1.000	1.000	0.2199
207082_at	9.5	20.5	1.11	2.16	AAA-AAA	0.00	999	1.000	1.000	0.3358
206539_s_at	34.0	15.7	-1.11	-2.16	AAA-AAA	0.00	999	1.000	1.000	0.1531
207849_at	8.0	3.7	-1.11	-2.16	AAA-AAA	0.00	999	1.000	1.000	0.5812
233765_at	18.1	8.4	-1.11	-2.16	AAA-AAA	0.00	999	1.000	1.000	0.2272
238734_at	15.3	7.1	-1.11	-2.16	AAA-AAA	0.00	999	1.000	1.000	0.5127
238100_at	25.6	11.9	-1.11	-2.16	AAA-AAA	0.00	999	1.000	1.000	0.3651
224495_at	51.5	23.8	-1.11	-2.16	AAA-AAA	0.00	999	1.000	1.000	0.2628
1562737_at	6.9	3.2	-1.11	-2.16	AAA-AAA	0.00	999	1.000	1.000	0.6364
1563839_at	11.5	5.3	-1.11	-2.16	AAA-AAA	0.00	999	1.000	1.000	0.4461
240352_at	8.7	18.7	1.11	2.16	AAA-AAA	0.00	999	1.000	1.000	0.2984
1561251_at	77.6	36.0	-1.11	-2.16	AAA-AAA	0.00	999	1.000	1.000	0.1095
1562007_at	13.7	29.6	1.11	2.16	AAA-AAA	0.00	999	1.000	1.000	0.1924
216097_at	10.0	21.5	1.11	2.16	AAA-AAA	0.00	999	1.000	1.000	0.4813
201750_s_at	151.4	70.2	-1.11	-2.16	AAA-AAA	0.00	999	1.000	1.000	0.0315
205378_s_at	4.7	10.2	1.11	2.16	AAA-AAA	0.00	999	1.000	1.000	0.1377
222922_at	11.2	5.2	-1.11	-2.16	AAA-AAA	0.00	999	1.000	1.000	0.1550
241634_at	52.1	24.2	-1.11	-2.16	APA-PAA	0.00	999	1.000	1.000	0.4475
1556887_at	104.0	48.2	-1.11	-2.16	AAA-AAA	0.00	999	1.000	1.000	0.3723
1556783_a_at	32.4	15.0	-1.11	-2.16	AAA-AAA	0.00	999	1.000	1.000	0.0137
243244_at	24.9	11.6	-1.11	-2.16	AAA-AAA	0.00	999	1.000	1.000	0.4430
216083_s_at	14.4	31.0	1.11	2.16	AAA-AAA	0.00	999	1.000	1.000	0.1074
212937_s_at	16.0	7.4	-1.11	-2.16	AAA-AAA	0.00	999	1.000	1.000	0.3169
220935_s_at	101.5	218.9	1.11	2.16	AAA-AAA	0.00	999	1.000	1.000	0.4506
203547_at	28.8	62.0	1.11	2.16	AAA-AAA	0.00	999	1.000	1.000	0.2327
211165_x_at	45.5	21.1	-1.11	-2.16	AAA-AAA	0.00	999	1.000	1.000	0.2652
233187_s_at	12.7	5.9	-1.11	-2.16	AAA-AAA	0.00	999	1.000	1.000	0.2022
236258_at	9.8	21.2	1.11	2.16	AAA-AAA	0.00	999	1.000	1.000	0.2145
1563089_at	31.4	14.6	-1.11	-2.15	AAA-AAA	0.00	999	1.000	1.000	0.3612
1567656_at	3.7	1.7	-1.11	-2.16	AAA-AAA	0.00	999	1.000	1.000	0.4850
244762_at	20.9	45.1	1.11	2.16	AAA-AAA	0.00	999	1.000	1.000	0.2443
243289_at	19.4	41.7	1.11	2.15	AAA-AAA	0.00	999	1.000	1.000	0.4506
243137_at	21.5	46.4	1.11	2.15	AAA-AAA	0.00	999	1.000	1.000	0.2730
220551_at	13.5	29.2	1.11	2.15	AAA-AAA	0.00	999	1.000	1.000	0.3921

206840_at	15.5	33.3	1.11	2.15	AAA-AAA	0.00	999	1.000	1.000	0.2549
235553_at	39.3	84.6	1.11	2.15	AAA-AAA	0.00	999	1.000	1.000	0.4131
238588_at	31.9	68.7	1.11	2.15	AAA-AAA	0.00	999	1.000	1.000	0.0560
237996_at	42.6	19.8	-1.11	-2.15	AAA-PPA	0.00	999	1.000	1.000	0.5235
242386_x_at	146.6	68.1	-1.11	-2.15	AAA-AAA	0.00	999	1.000	1.000	0.1819
1567378_x_at	21.0	9.8	-1.11	-2.15	AAA-AAA	0.00	999	1.000	1.000	0.3734
1559953_at	12.4	26.6	1.11	2.15	AAA-AMA	0.00	999	1.000	1.000	0.3476
212962_at	21.9	10.2	-1.11	-2.15	AAA-AAA	0.00	999	1.000	1.000	0.1577
206545_at	5.7	12.3	1.11	2.15	AAA-AAA	0.00	999	1.000	1.000	0.5880
221936_x_at	22.7	48.9	1.11	2.15	AAA-AAA	0.00	999	1.000	1.000	0.2359
229837_s_at	117.0	54.4	-1.11	-2.15	AAA-AAA	0.00	999	1.000	1.000	0.3375
229096_at	36.4	16.9	-1.11	-2.15	AAA-AAA	0.00	999	1.000	1.000	0.1793
1562973_at	11.8	5.5	-1.11	-2.15	AAA-AAA	0.00	999	1.000	1.000	0.3804
1558574_at	13.9	6.4	-1.11	-2.15	AAA-AAA	0.00	999	1.000	1.000	0.2751
243227_at	10.9	23.4	1.11	2.15	AAA-AAA	0.00	999	1.000	1.000	0.0188
243725_at	48.0	22.3	-1.11	-2.15	AAA-AMA	0.00	999	1.000	1.000	0.4373
216273_at	93.6	43.5	-1.11	-2.15	AAA-AAA	0.00	999	1.000	1.000	0.2518
211887_x_at	12.4	26.8	1.11	2.15	AAA-AAA	0.00	999	1.000	1.000	0.3126
213406_at	44.9	20.9	-1.11	-2.15	AAA-AAA	0.00	999	1.000	1.000	0.0714
221121_at	29.1	62.6	1.11	2.15	AAA-AAA	0.00	999	1.000	1.000	0.0393
216955_at	35.9	77.2	1.11	2.15	AAA-AAA	0.00	999	1.000	1.000	0.3582
211455_at	103.4	48.1	-1.11	-2.15	AAA-AAA	0.00	999	1.000	1.000	0.4363
211215_x_at	24.2	51.9	1.11	2.15	AAA-AAA	0.00	999	1.000	1.000	0.1720
235133_at	72.5	33.7	-1.11	-2.15	AAA-AAA	0.00	999	1.000	1.000	0.2759
237855_at	8.3	17.8	1.11	2.15	AAA-AAA	0.00	999	1.000	1.000	0.1380
238192_at	9.0	19.3	1.11	2.15	AAA-AAA	0.00	999	1.000	1.000	0.3629
227311_at	21.0	45.1	1.11	2.15	AAA-AAA	0.00	999	1.000	1.000	0.3897
231683_at	18.9	40.6	1.11	2.15	AAA-AAA	0.00	999	1.000	1.000	0.1599
229915_at	6.5	14.0	1.11	2.15	AAA-AAA	0.00	999	1.000	1.000	0.1649
217607_x_at	63.9	29.7	-1.10	-2.15	APA-PAP	0.00	999	1.000	1.000	0.3933
1554309_at	21.5	10.0	-1.10	-2.15	AAA-APA	0.00	999	1.000	1.000	0.4942
241317_at	4.9	10.6	1.10	2.15	AAA-AAA	0.00	999	1.000	1.000	0.0263
240069_at	43.8	20.4	-1.10	-2.15	AAA-AAA	0.00	999	1.000	1.000	0.4289
1555148_a_at	13.8	6.4	-1.10	-2.15	AAA-AAA	0.00	999	1.000	1.000	0.4492
243272_at	15.3	32.8	1.10	2.15	AAA-AAA	0.00	999	1.000	1.000	0.2042
1561408_at	18.9	40.5	1.10	2.15	AAA-AAA	0.00	999	1.000	1.000	0.3903
1561670_at	13.1	6.1	-1.10	-2.15	AAA-AAA	0.00	999	1.000	1.000	0.2197
216395_at	17.7	38.0	1.10	2.15	AAA-AAA	0.00	999	1.000	1.000	0.3585
216300_x_at	47.3	101.7	1.10	2.15	AAA-AAA	0.00	999	1.000	1.000	0.4747
216293_at	55.3	118.9	1.10	2.15	AAA-AAA	0.00	999	1.000	1.000	0.2299
236776_at	28.1	60.5	1.10	2.15	AAA-AAA	0.00	999	1.000	1.000	0.3061
228862_at	44.8	20.9	-1.10	-2.15	AAA-AAA	0.00	999	1.000	1.000	0.3422

224104_at	8.4	18.1	1.10	2.15	AAA-AAA	0.00	999	1.000	1.000	0.2260
223895_s_at	59.5	27.7	-1.10	-2.15	AAA-AAA	0.00	999	1.000	1.000	0.0311
230720_at	23.1	10.8	-1.10	-2.15	AAA-AAA	0.00	999	1.000	1.000	0.2996
225166_at	206.7	96.2	-1.10	-2.15	PPP-PPA	0.00	999	1.000	1.000	0.0318
214022_s_at	451.2	210.0	-1.10	-2.15	PPP-PPP	0.00	999	1.000	1.000	0.1055
234168_at	78.4	36.5	-1.10	-2.15	APP-PAM	0.00	999	1.000	1.000	0.1926
1554261_at	31.7	14.7	-1.10	-2.15	PAA-AAA	0.00	999	1.000	1.000	0.4774
240136_at	6.5	13.9	1.10	2.15	AAA-AAA	0.00	999	1.000	1.000	0.4372
239652_at	24.6	11.4	-1.10	-2.15	AAA-MAA	0.00	999	1.000	1.000	0.5643
244865_at	51.7	24.1	-1.10	-2.15	AAA-AAA	0.00	999	1.000	1.000	0.1542
1557833_at	16.6	7.7	-1.10	-2.15	AAA-AAA	0.00	999	1.000	1.000	0.2775
1553498_at	37.7	17.5	-1.10	-2.15	AAA-AAA	0.00	999	1.000	1.000	0.4625
1561407_at	29.3	62.9	1.10	2.15	AAA-AAA	0.00	999	1.000	1.000	0.1882
242793_at	8.9	19.0	1.10	2.15	AAA-AAA	0.00	999	1.000	1.000	0.0793
238238_at	16.8	36.0	1.10	2.15	AAA-AAA	0.00	999	1.000	1.000	0.4202
227774_s_at	11.5	24.8	1.10	2.15	AAA-AAA	0.00	999	1.000	1.000	0.5046
227396_at	88.8	41.4	-1.10	-2.15	AAP-AAA	0.00	999	1.000	1.000	0.1141
1570412_at	5.5	11.8	1.10	2.15	AAA-AAA	0.00	999	1.000	1.000	0.0985
240533_at	20.4	43.7	1.10	2.15	AAA-AAA	0.00	999	1.000	1.000	0.2520
208322_s_at	159.7	74.4	-1.10	-2.15	AAA-AAA	0.00	999	1.000	1.000	0.3510
237752_at	5.5	11.8	1.10	2.15	AAA-AAA	0.00	999	1.000	1.000	0.0458
237555_at	47.9	102.8	1.10	2.15	AAA-AAA	0.00	999	1.000	1.000	0.4551
230246_at	15.7	33.7	1.10	2.15	AAA-AAA	0.00	999	1.000	1.000	0.0763
239362_at	13.7	6.4	-1.10	-2.15	AAA-AAA	0.00	999	1.000	1.000	0.3253
1566432_at	28.9	13.5	-1.10	-2.15	AAA-AAA	0.00	999	1.000	1.000	0.1192
240802_at	3.0	6.5	1.10	2.15	AAA-AAA	0.00	999	1.000	1.000	0.3103
1561211_at	37.7	17.6	-1.10	-2.15	AAA-AAA	0.00	999	1.000	1.000	0.3642
214528_s_at	12.6	5.9	-1.10	-2.15	AAA-AAA	0.00	999	1.000	1.000	0.1162
214498_at	65.6	30.6	-1.10	-2.15	AAA-AAA	0.00	999	1.000	1.000	0.1881
203859_s_at	74.1	34.5	-1.10	-2.15	AAA-AAA	0.00	999	1.000	1.000	0.0856
210586_x_at	19.6	42.1	1.10	2.14	AAA-AAA	0.00	999	1.000	1.000	0.2724
238556_at	18.2	39.0	1.10	2.14	AAA-AAA	0.00	999	1.000	1.000	0.4043
238817_at	9.0	19.3	1.10	2.15	AAA-AAA	0.00	999	1.000	1.000	0.2587
221921_s_at	35.0	16.3	-1.10	-2.15	AAA-AAA	0.00	999	1.000	1.000	0.3880
222205_x_at	17.8	8.3	-1.10	-2.15	AAA-AAA	0.00	999	1.000	1.000	0.2342
236277_at	184.8	86.2	-1.10	-2.14	PPP-PPP	0.00	999	1.000	1.000	0.0471
242526_at	17.7	37.9	1.10	2.14	AAA-AAA	0.00	999	1.000	1.000	0.3237
1569406_at	4.0	8.5	1.10	2.14	AAA-AAA	0.00	999	1.000	1.000	0.5787
1567861_at	12.7	5.9	-1.10	-2.14	AAA-AAA	0.00	999	1.000	1.000	0.5473
1554354_at	4.5	9.6	1.10	2.14	AAA-AAA	0.00	999	1.000	1.000	0.2345
1553523_at	35.9	16.7	-1.10	-2.14	AAA-AAA	0.00	999	1.000	1.000	0.4428
1553258_at	22.9	10.7	-1.10	-2.14	AAA-AAA	0.00	999	1.000	1.000	0.4509

221181_at	8.1	17.3	1.10	2.14	AAA-AAA	0.00	999	1.000	1.000	0.2369
219194_at	44.9	96.3	1.10	2.14	AAA-AAA	0.00	999	1.000	1.000	0.3614
206886_x_at	36.1	16.8	-1.10	-2.14	AAA-AAA	0.00	999	1.000	1.000	0.3351
202158_s_at	4.4	9.4	1.10	2.14	AAA-AAA	0.00	999	1.000	1.000	0.4359
208192_at	4.4	2.0	-1.10	-2.14	AAA-AAA	0.00	999	1.000	1.000	0.1844
235379_at	16.2	7.5	-1.10	-2.14	AAA-AAA	0.00	999	1.000	1.000	0.4394
226435_at	31.0	14.5	-1.10	-2.14	AAA-AAA	0.00	999	1.000	1.000	0.3215
224201_s_at	83.9	39.1	-1.10	-2.14	AAA-AAA	0.00	999	1.000	1.000	0.1540
231378_at	78.6	168.6	1.10	2.14	AAA-AAA	0.00	999	1.000	1.000	0.1246
231915_at	8.0	3.7	-1.10	-2.14	AAA-AAA	0.00	999	1.000	1.000	0.0724
230260_s_at	6.5	13.9	1.10	2.14	AAA-AAA	0.00	999	1.000	1.000	0.3761
209261_s_at	261.6	122.2	-1.10	-2.14	PAP-PAP	0.00	999	1.000	1.000	0.3498
1570653_at	41.9	19.6	-1.10	-2.14	AAA-AAA	0.00	999	1.000	1.000	0.4239
240357_at	115.0	53.7	-1.10	-2.14	AAA-AAA	0.00	999	1.000	1.000	0.0475
1566449_at	3.3	7.0	1.10	2.14	AAA-AAA	0.00	999	1.000	1.000	0.4602
1553871_at	140.1	65.4	-1.10	-2.14	AAA-AAA	0.00	999	1.000	1.000	0.1699
1559587_at	22.5	48.1	1.10	2.14	AAA-AAA	0.00	999	1.000	1.000	0.2419
207572_at	31.5	67.4	1.10	2.14	AAA-AAA	0.00	999	1.000	1.000	0.4088
233594_at	3.0	6.5	1.10	2.14	AAA-AAA	0.00	999	1.000	1.000	0.4007
230460_at	111.7	52.2	-1.10	-2.14	AAA-AAA	0.00	999	1.000	1.000	0.2752
210306_at	38.0	17.8	-1.10	-2.14	AAA-AAP	0.00	999	1.000	1.000	0.4398
240810_at	21.2	9.9	-1.10	-2.14	AAA-AAA	0.00	999	1.000	1.000	0.0757
1559405_a_at	5.5	11.8	1.10	2.14	AAA-AAA	0.00	999	1.000	1.000	0.1996
1560455_at	16.3	7.6	-1.10	-2.14	AAA-AAA	0.00	999	1.000	1.000	0.3334
209908_s_at	20.5	43.8	1.10	2.14	AAA-AMA	0.00	999	1.000	1.000	0.2097
233650_at	53.9	25.2	-1.10	-2.14	AAM-AAA	0.00	999	1.000	1.000	0.3592
228449_at	50.4	23.5	-1.10	-2.14	AAA-AAA	0.00	999	1.000	1.000	0.2221
223499_at	35.5	16.6	-1.10	-2.14	AAA-AAA	0.00	999	1.000	1.000	0.5004
211106_at	87.6	41.0	-1.10	-2.14	PMA-AAA	0.00	999	1.000	1.000	0.2077
1563597_at	14.3	6.7	-1.10	-2.14	AAA-AAA	0.00	999	1.000	1.000	0.2335
1568805_at	13.2	28.2	1.10	2.14	AAA-AAA	0.00	999	1.000	1.000	0.1531
239499_at	10.0	21.5	1.10	2.14	AAA-AAA	0.00	999	1.000	1.000	0.3943
212951_at	17.4	8.1	-1.10	-2.14	AAA-AAA	0.00	999	1.000	1.000	0.1842
214652_at	3.1	6.5	1.10	2.14	AAA-AAA	0.00	999	1.000	1.000	0.1108
211805_s_at	57.9	27.0	-1.10	-2.14	AAA-AAA	0.00	999	1.000	1.000	0.2548
207994_s_at	4.8	10.2	1.10	2.14	AAA-AAA	0.00	999	1.000	1.000	0.3044
234598_at	11.9	5.5	-1.10	-2.14	AAA-AAA	0.00	999	1.000	1.000	0.1961
222971_at	35.1	75.0	1.10	2.14	AAA-AAA	0.00	999	1.000	1.000	0.2095
231413_at	10.9	5.1	-1.10	-2.14	AAA-AAA	0.00	999	1.000	1.000	0.2891
208849_at	50.0	23.4	-1.10	-2.14	PPA-AAP	0.00	999	1.000	1.000	0.3798
1565192_at	17.8	38.0	1.10	2.14	AAA-AAA	0.00	999	1.000	1.000	0.0955
241333_x_at	52.5	112.1	1.10	2.14	AAA-AAA	0.00	999	1.000	1.000	0.0943

1552924_a_at	125.9	58.9	-1.10	-2.14	AAA-AAA	0.00	999	1.000	1.000	0.0509
1562265_at	38.0	81.3	1.10	2.14	AAA-AAA	0.00	999	1.000	1.000	0.2130
215612_at	10.3	21.9	1.10	2.14	AAA-AAA	0.00	999	1.000	1.000	0.2348
221032_s_at	22.8	48.8	1.10	2.14	AAA-AAA	0.00	999	1.000	1.000	0.1727
233981_at	21.0	9.8	-1.10	-2.14	AAA-AAA	0.00	999	1.000	1.000	0.4279
234007_at	7.0	14.9	1.10	2.14	AAA-AAA	0.00	999	1.000	1.000	0.4022
222213_x_at	14.0	29.8	1.10	2.14	MAA-AAM	0.00	999	1.000	1.000	0.3648
238499_at	161.6	75.7	-1.10	-2.14	PPA-PAP	0.00	999	1.000	1.000	0.2865
240555_at	13.5	6.3	-1.10	-2.14	AAA-AAA	0.00	999	1.000	1.000	0.3919
1567248_at	3.3	7.0	1.10	2.14	AAA-AAA	0.00	999	1.000	1.000	0.3343
1553792_at	2.9	1.3	-1.10	-2.14	AAA-AAA	0.00	999	1.000	1.000	0.0963
244576_at	10.9	23.3	1.10	2.14	AAA-AAA	0.00	999	1.000	1.000	0.3178
215459_at	35.8	16.8	-1.10	-2.14	AAA-AAA	0.00	999	1.000	1.000	0.4503
220782_x_at	165.2	77.4	-1.10	-2.14	AAA-AAA	0.00	999	1.000	1.000	0.0567
217131_at	9.9	21.2	1.10	2.14	AAA-AAA	0.00	999	1.000	1.000	0.4456
204704_s_at	6.7	3.1	-1.10	-2.14	AAA-AAA	0.00	999	1.000	1.000	0.5932
234504_at	4.0	8.6	1.10	2.14	AAA-AAA	0.00	999	1.000	1.000	0.3255
234777_at	37.8	80.9	1.10	2.14	AAA-AAA	0.00	999	1.000	1.000	0.1750
224043_s_at	11.2	5.2	-1.10	-2.14	AAA-AAA	0.00	999	1.000	1.000	0.4300
231309_at	78.4	36.7	-1.10	-2.14	AAA-AAA	0.00	999	1.000	1.000	0.2082
229165_at	43.8	20.5	-1.10	-2.14	AAA-AAA	0.00	999	1.000	1.000	0.2329
1563074_at	16.0	7.5	-1.09	-2.13	AAA-AAA	0.00	999	1.000	1.000	0.1609
241973_x_at	54.8	25.7	-1.09	-2.13	AAA-AAA	0.00	999	1.000	1.000	0.4141
240027_at	4.1	8.9	1.09	2.14	AAA-AAA	0.00	999	1.000	1.000	0.5243
241005_at	27.1	12.7	-1.09	-2.14	AAA-AAA	0.00	999	1.000	1.000	0.3658
239189_at	30.8	65.7	1.09	2.13	AAA-AAA	0.00	999	1.000	1.000	0.3514
1555111_at	2.6	1.2	-1.09	-2.13	AAA-AAA	0.00	999	1.000	1.000	0.0036
1555171_at	5.2	11.0	1.09	2.14	AAA-AAA	0.00	999	1.000	1.000	0.3579
216353_s_at	7.7	16.4	1.09	2.13	AAM-AAA	0.00	999	1.000	1.000	0.5329
213950_s_at	17.2	36.8	1.09	2.13	AAA-AAA	0.00	999	1.000	1.000	0.3900
220900_at	11.0	5.1	-1.09	-2.14	AAA-AAA	0.00	999	1.000	1.000	0.5028
216963_s_at	23.1	49.3	1.09	2.14	AAA-AAA	0.00	999	1.000	1.000	0.5137
203558_at	23.1	10.8	-1.09	-2.14	AAA-AAA	0.00	999	1.000	1.000	0.2302
209723_at	19.4	9.1	-1.09	-2.14	AAA-AAA	0.00	999	1.000	1.000	0.3526
234881_at	38.2	17.9	-1.09	-2.14	AAA-AAA	0.00	999	1.000	1.000	0.1292
228262_at	6.3	13.4	1.09	2.13	AAA-AAA	0.00	999	1.000	1.000	0.2176
222057_at	74.3	34.8	-1.09	-2.14	AAA-AAA	0.00	999	1.000	1.000	0.3120
230197_s_at	25.4	11.9	-1.09	-2.13	AAA-AAA	0.00	999	1.000	1.000	0.5208
239088_at	33.0	15.5	-1.09	-2.13	AAA-AAA	0.00	999	1.000	1.000	0.3910
242360_at	76.1	35.7	-1.09	-2.13	AAA-AAA	0.00	999	1.000	1.000	0.0897
1565861_at	7.1	15.2	1.09	2.13	AAA-AAA	0.00	999	1.000	1.000	0.3461
207737_at	9.0	4.2	-1.09	-2.13	AAA-AAA	0.00	999	1.000	1.000	0.4348

237714_at	8.1	17.3	1.09	2.13	AAA-AAA	0.00	999	1.000	1.000	0.1756
237490_at	3.7	7.9	1.09	2.13	AAA-AAA	0.00	999	1.000	1.000	0.4877
1570505_at	5.6	12.0	1.09	2.13	AAA-AAA	0.00	999	1.000	1.000	0.5551
1568770_at	21.4	10.0	-1.09	-2.13	AAA-AAA	0.00	999	1.000	1.000	0.0160
1566580_at	43.5	92.7	1.09	2.13	AAA-AAA	0.00	999	1.000	1.000	0.0368
1567682_x_at	4.6	9.8	1.09	2.13	AAA-AAA	0.00	999	1.000	1.000	0.5135
243701_at	7.8	3.7	-1.09	-2.13	AAA-AAA	0.00	999	1.000	1.000	0.4017
212664_at	108.1	50.7	-1.09	-2.13	AAA-AAA	0.00	999	1.000	1.000	0.1577
216968_at	37.2	17.5	-1.09	-2.13	AAA-AAA	0.00	999	1.000	1.000	0.3396
233871_at	64.1	30.1	-1.09	-2.13	AMA-AAA	0.00	999	1.000	1.000	0.0980
237686_at	18.5	39.4	1.09	2.13	AAA-AAA	0.00	999	1.000	1.000	0.3480
238125_at	5.4	11.4	1.09	2.13	AAA-AAA	0.00	999	1.000	1.000	0.4406
237269_at	10.1	21.4	1.09	2.13	AAA-AAA	0.00	999	1.000	1.000	0.0832
222722_at	19.4	41.3	1.09	2.13	AAA-AAA	0.00	999	1.000	1.000	0.2169
222956_at	115.1	54.0	-1.09	-2.13	PMP-AAP	0.00	999	1.000	1.000	0.5235
238942_at	11.5	24.5	1.09	2.13	AAA-AAA	0.00	999	1.000	1.000	0.1059
1568603_at	6.2	2.9	-1.09	-2.13	AAA-AAA	0.00	999	1.000	1.000	0.6270
1555708_a_at	5.4	11.5	1.09	2.13	AAA-AAA	0.00	999	1.000	1.000	0.5323
243139_at	12.5	26.7	1.09	2.13	AAA-AAA	0.00	999	1.000	1.000	0.3174
1561116_at	18.1	38.6	1.09	2.13	AAA-AAA	0.00	999	1.000	1.000	0.4705
1565228_s_at	3.9	1.8	-1.09	-2.13	AAA-AAA	0.00	999	1.000	1.000	0.1485
1560399_a_at	6.0	12.8	1.09	2.13	AAA-AAA	0.00	999	1.000	1.000	0.4775
212659_s_at	46.4	98.7	1.09	2.13	AAA-AAA	0.00	999	1.000	1.000	0.3322
220456_at	7.6	16.2	1.09	2.13	AAA-AAA	0.00	999	1.000	1.000	0.5991
208548_at	18.4	39.2	1.09	2.13	AAA-AAA	0.00	999	1.000	1.000	0.1966
237463_at	30.1	64.1	1.09	2.13	AAA-AAA	0.00	999	1.000	1.000	0.2407
238600_at	20.1	9.5	-1.09	-2.13	AAA-AAA	0.00	999	1.000	1.000	0.1713
237328_at	15.9	7.5	-1.09	-2.13	AAA-AAA	0.00	999	1.000	1.000	0.1558
230585_at	17.5	37.3	1.09	2.13	AAA-AAA	0.00	999	1.000	1.000	0.5284
221657_s_at	265.9	124.9	-1.09	-2.13	PAP-AAP	0.00	999	1.000	1.000	0.1672
239832_at	5.2	11.2	1.09	2.13	AAA-AAA	0.00	999	1.000	1.000	0.1678
244685_at	13.9	6.5	-1.09	-2.13	AAA-AAA	0.00	999	1.000	1.000	0.3004
244082_at	48.6	103.5	1.09	2.13	AAA-AAA	0.00	999	1.000	1.000	0.0147
215407_s_at	21.9	10.3	-1.09	-2.13	AAA-AAA	0.00	999	1.000	1.000	0.4081
220970_s_at	7.0	15.0	1.09	2.13	AAA-AAA	0.00	999	1.000	1.000	0.1952
210750_s_at	119.6	56.2	-1.09	-2.13	AAA-AAA	0.00	999	1.000	1.000	0.2970
207750_at	21.1	9.9	-1.09	-2.13	AAA-AAA	0.00	999	1.000	1.000	0.3619
237890_at	32.0	68.2	1.09	2.13	AAA-AAA	0.00	999	1.000	1.000	0.3079
230393_at	110.9	52.1	-1.09	-2.13	AAA-AAA	0.00	999	1.000	1.000	0.2261
1557374_at	54.4	25.6	-1.09	-2.13	PPP-PAA	0.00	999	1.000	1.000	0.2077
240412_s_at	14.2	6.7	-1.09	-2.13	AAA-AAA	0.00	999	1.000	1.000	0.4763
1555395_at	18.4	8.7	-1.09	-2.13	AAA-AAA	0.00	999	1.000	1.000	0.3107

1561354_at	8.8	18.7	1.09	2.13	AAA-AAA	0.00	999	1.000	1.000	0.3757
216277_at	32.0	15.1	-1.09	-2.13	AAA-AAA	0.00	999	1.000	1.000	0.4007
210189_at	42.8	20.1	-1.09	-2.13	AAA-AAA	0.00	999	1.000	1.000	0.2228
237244_at	16.2	7.6	-1.09	-2.13	AAA-AAA	0.00	999	1.000	1.000	0.5198
222828_at	7.8	16.5	1.09	2.13	AAA-AAA	0.00	999	1.000	1.000	0.0965
230819_at	17.8	37.9	1.09	2.13	AAA-AAA	0.00	999	1.000	1.000	0.4851
230107_at	83.3	39.2	-1.09	-2.13	AAA-AAM	0.00	999	1.000	1.000	0.2648
222088_s_at	76.6	36.0	-1.09	-2.13	PPA-AAM	0.00	999	1.000	1.000	0.1859
1569386_at	20.1	9.4	-1.09	-2.13	AAA-AAA	0.00	999	1.000	1.000	0.2567
240754_at	27.6	13.0	-1.09	-2.13	AAA-AAA	0.00	999	1.000	1.000	0.4166
1556778_at	1.9	4.0	1.09	2.13	AAA-AAA	0.00	999	1.000	1.000	0.1253
1561561_x_at	23.3	49.4	1.09	2.13	AAA-AAA	0.00	999	1.000	1.000	0.1939
213496_at	19.2	9.0	-1.09	-2.13	AAA-AAA	0.00	999	1.000	1.000	0.5258
219005_at	12.8	27.3	1.09	2.13	AAA-AAA	0.00	999	1.000	1.000	0.1418
206009_at	28.9	61.4	1.09	2.13	AAA-AAA	0.00	999	1.000	1.000	0.1902
207292_s_at	77.8	36.6	-1.09	-2.13	AAA-AAA	0.00	999	1.000	1.000	0.1493
232557_at	11.4	24.2	1.09	2.13	AAA-AAA	0.00	999	1.000	1.000	0.3050
1563005_at	33.7	71.6	1.09	2.13	AAA-AAA	0.00	999	1.000	1.000	0.2664
239881_at	48.2	22.7	-1.09	-2.12	AAA-AAA	0.00	999	1.000	1.000	0.5367
240683_at	47.5	100.9	1.09	2.12	AAA-AAA	0.00	999	1.000	1.000	0.1355
1557386_at	20.9	44.3	1.09	2.12	AAA-AAA	0.00	999	1.000	1.000	0.3616
1554524_a_at	4.2	8.9	1.09	2.12	AAA-AAA	0.00	999	1.000	1.000	0.1573
1553882_at	4.6	9.8	1.09	2.12	AAA-AAA	0.00	999	1.000	1.000	0.2749
210881_s_at	16.5	7.8	-1.09	-2.13	AAA-AAA	0.00	999	1.000	1.000	0.3707
211422_at	51.0	24.0	-1.09	-2.12	AAP-AAM	0.00	999	1.000	1.000	0.4536
1554349_at	6.8	14.3	1.09	2.12	AAA-AAA	0.00	999	1.000	1.000	0.4842
1561760_s_at	38.0	17.9	-1.09	-2.12	AAA-AAA	0.00	999	1.000	1.000	0.3783
215671_at	15.9	7.5	-1.09	-2.12	AAA-AAA	0.00	999	1.000	1.000	0.4491
214285_at	19.1	40.6	1.09	2.12	AAA-AAA	0.00	999	1.000	1.000	0.3936
220639_at	16.4	7.7	-1.09	-2.12	AAA-AAA	0.00	999	1.000	1.000	0.4477
221296_at	34.1	16.1	-1.09	-2.12	AAA-AAA	0.00	999	1.000	1.000	0.3361
205818_at	10.6	22.5	1.09	2.12	AAA-AAA	0.00	999	1.000	1.000	0.4475
234766_at	35.9	16.9	-1.09	-2.12	AAA-AAA	0.00	999	1.000	1.000	0.4150
224488_s_at	33.2	15.6	-1.09	-2.12	AAA-AAA	0.00	999	1.000	1.000	0.4786
224412_s_at	11.2	23.7	1.09	2.12	AAA-AAA	0.00	999	1.000	1.000	0.4103
239738_at	22.3	10.5	-1.09	-2.12	APA-AAA	0.00	999	1.000	1.000	0.3575
1562638_at	2.8	6.0	1.09	2.12	AAA-AAA	0.00	999	1.000	1.000	0.4509
1569617_at	35.8	16.9	-1.09	-2.12	AAA-AAA	0.00	999	1.000	1.000	0.4400
1566979_at	13.1	6.2	-1.09	-2.12	AAA-AAA	0.00	999	1.000	1.000	0.4959
1554129_a_at	18.1	38.4	1.09	2.12	AAA-AAA	0.00	999	1.000	1.000	0.5327
1555273_at	9.3	4.4	-1.09	-2.12	AAA-AAA	0.00	999	1.000	1.000	0.3294
1560758_at	13.3	28.2	1.09	2.12	AAA-AAA	0.00	999	1.000	1.000	0.4222

214308_s_at	20.8	44.1	1.09	2.12	AAA-AAA	0.00	999	1.000	1.000	0.5133
206424_at	12.7	27.0	1.09	2.12	AAA-AAA	0.00	999	1.000	1.000	0.2041
224123_at	52.0	24.5	-1.09	-2.12	AAA-AAA	0.00	999	1.000	1.000	0.2828
231693_at	9.0	19.1	1.09	2.12	AAA-AAA	0.00	999	1.000	1.000	0.1022
239305_at	65.8	31.1	-1.08	-2.12	AAP-AAA	0.00	999	1.000	1.000	0.3338
1552877_s_at	41.8	19.7	-1.08	-2.12	AAA-AAA	0.00	999	1.000	1.000	0.0079
244861_at	153.9	72.6	-1.08	-2.12	AAA-AAA	0.00	999	1.000	1.000	0.1505
1557641_at	6.2	13.1	1.08	2.12	AAA-AAA	0.00	999	1.000	1.000	0.4862
1556261_a_at	7.1	15.0	1.08	2.12	AAA-AAA	0.00	999	1.000	1.000	0.5582
1562215_at	13.8	6.5	-1.08	-2.12	AAA-AAA	0.00	999	1.000	1.000	0.5125
220641_at	82.5	38.9	-1.08	-2.12	AAA-AAA	0.00	999	1.000	1.000	0.3421
220061_at	13.3	28.3	1.08	2.12	AAA-AAA	0.00	999	1.000	1.000	0.3206
217146_at	20.2	9.5	-1.08	-2.12	AAA-AAA	0.00	999	1.000	1.000	0.4258
219090_at	13.6	6.4	-1.08	-2.12	AAA-AAA	0.00	999	1.000	1.000	0.0171
205831_at	42.5	90.0	1.08	2.12	AMA-MMA	0.00	999	1.000	1.000	0.2504
208596_s_at	14.1	29.9	1.08	2.12	AAA-AAA	0.00	999	1.000	1.000	0.1274
236919_at	28.1	59.6	1.08	2.12	AAA-AAA	0.00	999	1.000	1.000	0.0036
225987_at	41.6	88.1	1.08	2.12	AAA-AAA	0.00	999	1.000	1.000	0.1505
231420_at	19.8	42.1	1.08	2.12	AAA-AAA	0.00	999	1.000	1.000	0.2173
229286_at	16.0	33.9	1.08	2.12	AAA-AAA	0.00	999	1.000	1.000	0.0381
1563477_at	50.4	23.8	-1.08	-2.12	AAA-AAA	0.00	999	1.000	1.000	0.3830
239467_at	21.2	10.0	-1.08	-2.12	AAA-AAA	0.00	999	1.000	1.000	0.4915
1561669_at	16.7	35.4	1.08	2.12	AAA-MAA	0.00	999	1.000	1.000	0.3311
216529_at	2.1	4.4	1.08	2.12	AAA-AAA	0.00	999	1.000	1.000	0.1406
220528_at	6.8	14.4	1.08	2.12	AAA-AAA	0.00	999	1.000	1.000	0.3793
219153_s_at	73.1	34.5	-1.08	-2.12	AAA-AAA	0.00	999	1.000	1.000	0.2147
205025_at	55.5	117.5	1.08	2.12	AAA-AAA	0.00	999	1.000	1.000	0.2102
207797_s_at	17.8	37.7	1.08	2.12	AAA-AAA	0.00	999	1.000	1.000	0.4390
232956_at	10.1	21.4	1.08	2.12	AAA-AAA	0.00	999	1.000	1.000	0.3652
232735_at	54.5	25.7	-1.08	-2.12	AAA-AAA	0.00	999	1.000	1.000	0.2316
237660_at	9.4	4.5	-1.08	-2.12	AAA-AAA	0.00	999	1.000	1.000	0.5519
225840_at	49.4	23.3	-1.08	-2.12	AAA-AAA	0.00	999	1.000	1.000	0.2962
231684_at	4.4	9.3	1.08	2.12	AAA-AAA	0.00	999	1.000	1.000	0.1863
230182_at	47.2	99.9	1.08	2.12	AAA-AAA	0.00	999	1.000	1.000	0.3183
1560533_at	60.3	28.5	-1.08	-2.12	APP-AAA	0.00	999	1.000	1.000	0.1846
242303_at	38.9	82.3	1.08	2.12	AAA-AAA	0.00	999	1.000	1.000	0.3957
1568804_at	10.1	21.5	1.08	2.12	AAA-AAA	0.00	999	1.000	1.000	0.1035
240541_at	13.1	27.7	1.08	2.12	AAA-AAA	0.00	999	1.000	1.000	0.3402
1553461_at	32.9	15.5	-1.08	-2.12	AAA-AAA	0.00	999	1.000	1.000	0.2551
1562453_at	5.1	10.8	1.08	2.12	AAA-AAA	0.00	999	1.000	1.000	0.1123
1560265_at	12.3	5.8	-1.08	-2.12	AAA-AAA	0.00	999	1.000	1.000	0.5622
213100_at	69.6	147.2	1.08	2.12	AAA-AAA	0.00	999	1.000	1.000	0.1875

218573_at	14.0	29.7	1.08	2.12	AAA-AAA	0.00	999	1.000	1.000	0.0993
206730_at	10.4	4.9	-1.08	-2.12	AAA-AAA	0.00	999	1.000	1.000	0.4936
236893_at	3.8	8.0	1.08	2.12	AAA-AAA	0.00	999	1.000	1.000	0.1362
231375_at	10.8	22.9	1.08	2.12	AAA-AAA	0.00	999	1.000	1.000	0.1360
232474_at	55.5	26.2	-1.08	-2.12	AAA-AAA	0.00	999	1.000	1.000	0.5539
229118_at	30.1	63.7	1.08	2.12	AAA-AAA	0.00	999	1.000	1.000	0.2764
240238_at	42.8	20.2	-1.08	-2.12	AAA-AAA	0.00	999	1.000	1.000	0.3080
1566658_at	15.1	7.1	-1.08	-2.12	AAA-AAA	0.00	999	1.000	1.000	0.3206
243033_at	3.9	8.3	1.08	2.12	AAA-AAA	0.00	999	1.000	1.000	0.4677
214307_at	16.8	7.9	-1.08	-2.12	AAA-AAA	0.00	999	1.000	1.000	0.1237
234889_at	15.0	31.8	1.08	2.12	AAA-AAA	0.00	999	1.000	1.000	0.2521
231678_s_at	13.0	6.1	-1.08	-2.12	AAA-AAA	0.00	999	1.000	1.000	0.2171
230431_at	19.8	9.3	-1.08	-2.12	AAA-AAA	0.00	999	1.000	1.000	0.1408
1552954_at	45.6	21.6	-1.08	-2.11	PAP-AAA	0.00	999	1.000	1.000	0.1541
239099_at	24.4	11.5	-1.08	-2.11	AAA-AAA	0.00	999	1.000	1.000	0.4336
239263_at	46.4	98.1	1.08	2.11	AAA-AAA	0.00	999	1.000	1.000	0.0808
1567622_at	75.9	160.4	1.08	2.11	AAA-AAA	0.00	999	1.000	1.000	0.0831
1567270_at	14.4	6.8	-1.08	-2.11	AAA-AAA	0.00	999	1.000	1.000	0.3049
1561854_at	4.2	2.0	-1.08	-2.11	AAA-AAA	0.00	999	1.000	1.000	0.5023
244279_at	6.7	14.2	1.08	2.11	AAA-AAA	0.00	999	1.000	1.000	0.1431
215370_at	29.4	62.2	1.08	2.11	AAA-AAA	0.00	999	1.000	1.000	0.4688
205749_at	12.0	25.3	1.08	2.11	AAA-AAA	0.00	999	1.000	1.000	0.4809
1564949_at	27.8	13.2	-1.08	-2.11	PAM-AAA	0.00	999	1.000	1.000	0.3792
1569731_at	9.5	4.5	-1.08	-2.11	AAA-AAA	0.00	999	1.000	1.000	0.1515
244380_at	9.0	18.9	1.08	2.11	AAA-AAA	0.00	999	1.000	1.000	0.3016
210587_at	11.6	24.6	1.08	2.11	AAA-AAA	0.00	999	1.000	1.000	0.4740
231053_at	10.2	21.6	1.08	2.11	AAA-AAA	0.00	999	1.000	1.000	0.3346
229816_at	13.7	29.0	1.08	2.11	AAA-AAA	0.00	999	1.000	1.000	0.3031
242060_x_at	23.4	11.1	-1.08	-2.11	AAA-AAA	0.00	999	1.000	1.000	0.4006
218975_at	12.9	27.1	1.08	2.11	AAA-AAA	0.00	999	1.000	1.000	0.2654
210297_s_at	16.9	35.8	1.08	2.11	AAA-AAA	0.00	999	1.000	1.000	0.1961
234676_s_at	13.7	29.0	1.08	2.11	AAA-AAA	0.00	999	1.000	1.000	0.3741
235669_at	38.6	81.6	1.08	2.11	AAA-AAA	0.00	999	1.000	1.000	0.2737
236252_at	9.7	20.4	1.08	2.11	AAA-AAA	0.00	999	1.000	1.000	0.2316
231708_at	50.1	105.8	1.08	2.11	AAA-AAA	0.00	999	1.000	1.000	0.2892
1565073_at	19.3	40.8	1.08	2.11	AAA-AAA	0.00	999	1.000	1.000	0.3362
1563692_at	12.2	25.7	1.08	2.11	AAA-AAA	0.00	999	1.000	1.000	0.4158
1563235_at	20.6	9.8	-1.08	-2.11	AAA-AAA	0.00	999	1.000	1.000	0.3085
240804_at	15.6	32.8	1.08	2.11	AAA-AAA	0.00	999	1.000	1.000	0.2182
1557908_at	31.5	66.5	1.08	2.11	AAA-AAA	0.00	999	1.000	1.000	0.1492
1553874_a_at	9.6	4.6	-1.08	-2.11	AAA-AAA	0.00	999	1.000	1.000	0.2398
1561721_a_at	5.2	11.0	1.08	2.11	AMA-AAA	0.00	999	1.000	1.000	0.4419

216234_s_at	47.8	100.9	1.08	2.11	AAA-AAA	0.00	999	1.000	1.000	0.2038
212531_at	31.0	14.7	-1.08	-2.11	AAA-AAA	0.00	999	1.000	1.000	0.4007
222709_at	169.4	80.3	-1.08	-2.11	AAA-AAA	0.00	999	1.000	1.000	0.2474
232307_at	41.5	87.5	1.08	2.11	AAA-AAA	0.00	999	1.000	1.000	0.4381
241171_at	14.1	29.7	1.08	2.11	AAA-AAA	0.00	999	1.000	1.000	0.0640
242494_at	30.7	64.7	1.08	2.11	AAA-AAA	0.00	999	1.000	1.000	0.0086
239805_at	32.5	15.4	-1.08	-2.11	AAA-AAA	0.00	999	1.000	1.000	0.3955
1552667_a_at	7.3	15.5	1.08	2.11	AAA-AAA	0.00	999	1.000	1.000	0.1031
1553817_at	5.3	11.1	1.08	2.11	AAA-AAA	0.00	999	1.000	1.000	0.4113
1553232_at	25.1	11.9	-1.08	-2.11	AAA-AAA	0.00	999	1.000	1.000	0.4535
1562217_at	11.4	24.0	1.08	2.11	AAA-AAA	0.00	999	1.000	1.000	0.4900
215152_at	17.6	37.2	1.08	2.11	AAA-AAA	0.00	999	1.000	1.000	0.0943
212258_s_at	20.9	9.9	-1.08	-2.11	AAA-AAA	0.00	999	1.000	1.000	0.4056
211990_at	13.1	27.7	1.08	2.11	AAA-AAA	0.00	999	1.000	1.000	0.4076
215008_at	3.9	8.2	1.08	2.11	AAA-AAA	0.00	999	1.000	1.000	0.4655
214557_at	38.9	81.9	1.08	2.11	AAA-AAA	0.00	999	1.000	1.000	0.2019
220887_at	43.2	20.5	-1.08	-2.11	AAA-AAA	0.00	999	1.000	1.000	0.4501
217634_at	20.5	43.2	1.08	2.11	AAA-AAA	0.00	999	1.000	1.000	0.4078
219797_at	25.8	12.3	-1.08	-2.11	AAA-AAA	0.00	999	1.000	1.000	0.3895
207756_at	9.5	20.0	1.08	2.11	AAA-AAA	0.00	999	1.000	1.000	0.4430
234860_at	7.1	15.0	1.08	2.11	AAA-AAA	0.00	999	1.000	1.000	0.2846
231179_at	67.8	32.2	-1.08	-2.11	AAA-AAA	0.00	999	1.000	1.000	0.3906
227206_at	52.4	24.9	-1.08	-2.11	PAA-AAP	0.00	999	1.000	1.000	0.3832
1570337_at	16.3	34.3	1.08	2.11	AAA-AAA	0.00	999	1.000	1.000	0.1776
239795_at	51.1	24.2	-1.08	-2.11	AAA-AAA	0.00	999	1.000	1.000	0.2788
241119_at	4.4	9.3	1.08	2.11	AAA-AAA	0.00	999	1.000	1.000	0.1485
1552468_a_at	89.4	42.4	-1.08	-2.11	AAA-AAA	0.00	999	1.000	1.000	0.2701
242817_at	99.2	47.1	-1.08	-2.11	AAA-AAA	0.00	999	1.000	1.000	0.0313
244228_at	21.2	10.1	-1.08	-2.11	AAA-AAA	0.00	999	1.000	1.000	0.4093
219943_s_at	16.6	34.9	1.08	2.11	AAA-AAA	0.00	999	1.000	1.000	0.4712
219114_at	45.3	21.5	-1.08	-2.11	AAA-AAA	0.00	999	1.000	1.000	0.1553
203841_x_at	19.8	9.4	-1.08	-2.11	AAA-AAA	0.00	999	1.000	1.000	0.3633
238845_at	14.9	7.1	-1.08	-2.11	AAA-AAA	0.00	999	1.000	1.000	0.3138
232225_at	29.0	61.1	1.08	2.11	AAA-AAA	0.00	999	1.000	1.000	0.4581
204174_at	341.0	162.0	-1.07	-2.11	PPP-APA	0.00	999	1.000	1.000	0.0245
239211_at	73.5	34.9	-1.07	-2.11	PPA-AAA	0.00	999	1.000	1.000	0.0963
235464_at	55.4	26.3	-1.07	-2.11	AAA-AAP	0.00	999	1.000	1.000	0.4249
1564438_at	51.6	24.5	-1.07	-2.11	AAA-AAA	0.00	999	1.000	1.000	0.2810
239836_at	14.5	6.9	-1.07	-2.11	AAA-AAA	0.00	999	1.000	1.000	0.2090
1552521_a_at	22.9	10.9	-1.07	-2.11	AAA-AAA	0.00	999	1.000	1.000	0.3412
1560318_at	33.7	71.0	1.07	2.11	AAA-AAA	0.00	999	1.000	1.000	0.0748
220057_at	43.7	20.7	-1.07	-2.11	AAA-AAA	0.00	999	1.000	1.000	0.3060

234008_s_at	47.1	99.1	1.07	2.11	AAA-AAA	0.00	999	1.000	1.000	0.1223
224296_x_at	8.7	18.3	1.07	2.11	AAA-AAA	0.00	999	1.000	1.000	0.2978
214606_at	43.7	20.8	-1.07	-2.10	MPP-AAA	0.00	999	1.000	1.000	0.0900
242414_at	60.9	128.2	1.07	2.10	AAA-AAA	0.00	999	1.000	1.000	0.0231
240779_at	26.6	12.7	-1.07	-2.10	AAA-AAA	0.00	999	1.000	1.000	0.2251
1562559_at	11.9	5.7	-1.07	-2.10	AAA-AAA	0.00	999	1.000	1.000	0.0256
1562563_at	23.3	48.9	1.07	2.10	AAA-AAA	0.00	999	1.000	1.000	0.0014
1560565_at	12.0	5.7	-1.07	-2.10	AAA-AAA	0.00	999	1.000	1.000	0.3351
216427_at	24.4	11.6	-1.07	-2.10	AAA-AAA	0.00	999	1.000	1.000	0.5224
218851_s_at	12.6	26.5	1.07	2.10	AAA-AAA	0.00	999	1.000	1.000	0.5931
205572_at	15.1	7.2	-1.07	-2.10	AAA-AAA	0.00	999	1.000	1.000	0.3414
234071_at	10.7	22.6	1.07	2.10	AAA-AAA	0.00	999	1.000	1.000	0.4060
238266_at	36.1	17.2	-1.07	-2.10	AAA-AAA	0.00	999	1.000	1.000	0.4193
223865_at	9.9	20.7	1.07	2.10	AAA-AAA	0.00	999	1.000	1.000	0.1064
230674_at	9.6	20.2	1.07	2.10	AAA-AAA	0.00	999	1.000	1.000	0.0522
229381_at	93.6	44.5	-1.07	-2.10	AAP-AMA	0.00	999	1.000	1.000	0.3533
241886_x_at	13.1	6.2	-1.07	-2.10	AAA-AAA	0.00	999	1.000	1.000	0.4425
240531_at	10.9	5.2	-1.07	-2.10	AAA-AAA	0.00	999	1.000	1.000	0.0465
1555256_at	43.9	20.9	-1.07	-2.10	AAA-MAA	0.00	999	1.000	1.000	0.4801
215998_at	7.7	16.2	1.07	2.10	AAA-AAA	0.00	999	1.000	1.000	0.4715
205685_at	49.5	23.5	-1.07	-2.10	AAA-AAA	0.00	999	1.000	1.000	0.3164
237023_at	86.1	40.9	-1.07	-2.10	AAA-AAA	0.00	999	1.000	1.000	0.4042
231237_x_at	8.6	18.1	1.07	2.10	AAA-AAA	0.00	999	1.000	1.000	0.4596
230353_at	49.6	104.2	1.07	2.10	AAA-AAA	0.00	999	1.000	1.000	0.4200
239287_at	58.8	28.0	-1.07	-2.10	APM-AAA	0.00	999	1.000	1.000	0.2446
1562623_at	6.0	12.6	1.07	2.10	AAA-AAA	0.00	999	1.000	1.000	0.3837
241046_at	5.7	12.1	1.07	2.10	AAA-AAA	0.00	999	1.000	1.000	0.5334
1552281_at	51.6	108.4	1.07	2.10	AAA-AAA	0.00	999	1.000	1.000	0.2784
1554607_at	9.5	20.0	1.07	2.10	AAA-AAA	0.00	999	1.000	1.000	0.5941
1556075_at	12.4	26.1	1.07	2.10	AAA-AAA	0.00	999	1.000	1.000	0.6228
214648_at	22.7	47.6	1.07	2.10	AAA-AAA	0.00	999	1.000	1.000	0.3176
220805_at	64.7	30.8	-1.07	-2.10	AAA-AAA	0.00	999	1.000	1.000	0.2128
205578_at	3.7	7.8	1.07	2.10	AAA-AAA	0.00	999	1.000	1.000	0.1413
232741_at	23.2	11.1	-1.07	-2.10	AAA-AAA	0.00	999	1.000	1.000	0.5140
233905_at	10.9	22.8	1.07	2.10	AAA-AAA	0.00	999	1.000	1.000	0.2389
238623_at	45.2	21.5	-1.07	-2.10	AAA-AAA	0.00	999	1.000	1.000	0.4775
222087_at	98.8	207.4	1.07	2.10	AAA-AAA	0.00	999	1.000	1.000	0.2390
231883_at	26.1	54.8	1.07	2.10	AAA-AAA	0.00	999	1.000	1.000	0.3282
237301_at	91.7	43.7	-1.07	-2.10	PAM-AAA	0.00	999	1.000	1.000	0.1466
1554343_a_at	31.9	15.2	-1.07	-2.10	APA-AAA	0.00	999	1.000	1.000	0.4318
1564004_at	11.4	23.8	1.07	2.10	AAA-AAA	0.00	999	1.000	1.000	0.0507
1556715_at	33.5	70.2	1.07	2.10	AAA-AAA	0.00	999	1.000	1.000	0.1832

243285_at	54.8	26.1	-1.07	-2.10	AAA-AAA	0.00	999	1.000	1.000	0.0390
243336_at	33.1	69.6	1.07	2.10	AAA-AAA	0.00	999	1.000	1.000	0.4201
1559461_at	84.0	40.0	-1.07	-2.10	AAA-AAA	0.00	999	1.000	1.000	0.4578
215107_s_at	3.7	7.8	1.07	2.10	AAA-AAA	0.00	999	1.000	1.000	0.3470
206743_s_at	5.7	12.0	1.07	2.10	AAA-AAA	0.00	999	1.000	1.000	0.2737
203271_s_at	67.0	140.8	1.07	2.10	AAA-AAA	0.00	999	1.000	1.000	0.0107
232933_at	34.8	16.6	-1.07	-2.10	AAA-AAA	0.00	999	1.000	1.000	0.3613
1559648_at	42.0	20.0	-1.07	-2.10	APA-AAA	0.00	999	1.000	1.000	0.3647
211336_x_at	56.6	27.0	-1.07	-2.10	AAA-AAA	0.00	999	1.000	1.000	0.3029
224289_s_at	22.0	10.5	-1.07	-2.10	AAA-AAA	0.00	999	1.000	1.000	0.3165
231377_at	63.1	30.1	-1.07	-2.10	AAA-AAA	0.00	999	1.000	1.000	0.1326
231799_at	7.9	16.6	1.07	2.10	AAA-AAA	0.00	999	1.000	1.000	0.1609
226777_at	63.5	30.3	-1.07	-2.10	PPA-AAM	0.00	999	1.000	1.000	0.3461
241619_at	17.6	8.4	-1.07	-2.10	AAA-AAA	0.00	999	1.000	1.000	0.4244
239310_at	21.0	44.0	1.07	2.10	AAA-AAA	0.00	999	1.000	1.000	0.3471
1568934_at	54.7	26.1	-1.07	-2.10	AAA-AAA	0.00	999	1.000	1.000	0.4014
1570264_at	4.8	10.1	1.07	2.10	AAA-AAA	0.00	999	1.000	1.000	0.1799
243685_at	5.4	11.3	1.07	2.10	AAA-AAA	0.00	999	1.000	1.000	0.4013
235238_at	13.0	27.3	1.07	2.10	AAA-AAA	0.00	999	1.000	1.000	0.1387
233403_x_at	39.8	83.4	1.07	2.10	AAA-AAA	0.00	999	1.000	1.000	0.4238
236509_at	25.6	53.7	1.07	2.10	AAA-AAA	0.00	999	1.000	1.000	0.3592
236063_at	8.4	4.0	-1.07	-2.10	AAA-AAA	0.00	999	1.000	1.000	0.3569
209616_s_at	230.5	110.0	-1.07	-2.10	PPP-PAP	0.00	999	1.000	1.000	0.0953
1562999_x_at	8.9	18.7	1.07	2.10	AAA-AAA	0.00	999	1.000	1.000	0.3727
241760_x_at	6.3	3.0	-1.07	-2.10	AAA-AAA	0.00	999	1.000	1.000	0.5683
239762_at	9.5	19.8	1.07	2.10	AAA-AAA	0.00	999	1.000	1.000	0.0746
1566195_at	78.5	37.5	-1.07	-2.09	AAA-AAA	0.00	999	1.000	1.000	0.1523
1562316_at	11.8	24.8	1.07	2.09	AAA-AAA	0.00	999	1.000	1.000	0.3642
243085_at	35.8	17.1	-1.07	-2.10	AAA-AAA	0.00	999	1.000	1.000	0.4252
1561555_at	24.0	11.5	-1.07	-2.10	AAA-AAA	0.00	999	1.000	1.000	0.4588
1559086_at	11.9	24.8	1.07	2.10	AAA-AAA	0.00	999	1.000	1.000	0.4666
212488_at	33.2	15.8	-1.07	-2.10	AAA-AAA	0.00	999	1.000	1.000	0.5464
222178_s_at	11.7	5.6	-1.07	-2.10	AAA-AAA	0.00	999	1.000	1.000	0.2879
232051_at	39.7	83.2	1.07	2.10	AAA-AAA	0.00	999	1.000	1.000	0.2485
1566880_at	7.6	15.9	1.07	2.09	AAA-AAA	0.00	999	1.000	1.000	0.2590
1555661_at	75.9	36.2	-1.07	-2.09	AAA-AAA	0.00	999	1.000	1.000	0.2928
243255_at	37.9	18.1	-1.07	-2.09	AAA-AAA	0.00	999	1.000	1.000	0.3446
215502_at	14.8	7.1	-1.07	-2.09	AAA-AAA	0.00	999	1.000	1.000	0.0081
215454_x_at	18.4	8.8	-1.07	-2.09	AAA-AAA	0.00	999	1.000	1.000	0.3870
216439_at	25.8	12.3	-1.07	-2.09	AAA-AAA	0.00	999	1.000	1.000	0.2305
220151_at	7.8	16.3	1.07	2.09	AAA-AAA	0.00	999	1.000	1.000	0.0476
206119_at	15.8	33.1	1.07	2.09	AAA-AAA	0.00	999	1.000	1.000	0.2868

210911_at	7.9	16.4	1.07	2.09	AAA-AAA	0.00	999	1.000	1.000	0.3458
237106_at	72.6	34.7	-1.07	-2.09	APA-AAA	0.00	999	1.000	1.000	0.3216
1555289_at	83.8	40.1	-1.07	-2.09	APA-AAA	0.00	999	1.000	1.000	0.3235
1569254_s_at	25.6	12.2	-1.07	-2.09	AAA-AAA	0.00	999	1.000	1.000	0.3163
1567380_at	3.0	6.2	1.07	2.09	AAA-AAA	0.00	999	1.000	1.000	0.4771
1553889_at	27.4	13.1	-1.07	-2.09	AAA-AAA	0.00	999	1.000	1.000	0.0558
244601_at	24.0	50.2	1.07	2.09	AAA-AAA	0.00	999	1.000	1.000	0.1078
215949_x_at	20.3	42.4	1.07	2.09	AAA-AAA	0.00	999	1.000	1.000	0.1951
220249_at	5.2	10.8	1.07	2.09	AAA-AAA	0.00	999	1.000	1.000	0.0380
205057_s_at	11.6	24.3	1.07	2.09	AAA-AAA	0.00	999	1.000	1.000	0.2075
210173_at	12.3	5.9	-1.07	-2.09	AAA-AAA	0.00	999	1.000	1.000	0.2077
211428_at	12.3	25.8	1.07	2.09	AAA-AAA	0.00	999	1.000	1.000	0.3460
1568706_s_at	14.6	30.5	1.06	2.09	AAA-AAA	0.00	999	1.000	1.000	0.4181
1556913_a_at	14.6	7.0	-1.06	-2.09	AAA-AAA	0.00	999	1.000	1.000	0.4385
1557176_a_at	15.5	32.4	1.06	2.09	AAA-AAA	0.00	999	1.000	1.000	0.4335
220310_at	81.6	39.0	-1.06	-2.09	AAA-AAA	0.00	999	1.000	1.000	0.4360
221442_at	15.8	33.0	1.06	2.09	AAA-AAA	0.00	999	1.000	1.000	0.5004
221122_at	44.9	93.9	1.06	2.09	AAA-AAA	0.00	999	1.000	1.000	0.3105
206019_at	97.9	204.6	1.06	2.09	AAA-AAA	0.00	999	1.000	1.000	0.2735
233579_at	11.1	23.2	1.06	2.09	AAA-AAA	0.00	999	1.000	1.000	0.4232
231263_at	28.7	60.1	1.06	2.09	AAA-AAA	0.00	999	1.000	1.000	0.4595
232460_at	61.3	29.3	-1.06	-2.09	AAA-AAA	0.00	999	1.000	1.000	0.3252
229951_x_at	11.9	24.9	1.06	2.09	AAA-AAA	0.00	999	1.000	1.000	0.4599
1561080_at	21.9	10.5	-1.06	-2.09	AAP-APA	0.00	999	1.000	1.000	0.6333
1562601_at	15.5	32.4	1.06	2.09	AAA-AAA	0.00	999	1.000	1.000	0.2657
1569770_x_at	2.5	5.2	1.06	2.09	AAA-AAA	0.00	999	1.000	1.000	0.4833
1555083_at	115.2	55.1	-1.06	-2.09	AAA-AAA	0.00	999	1.000	1.000	0.4555
216586_at	17.3	36.1	1.06	2.09	AAA-AAA	0.00	999	1.000	1.000	0.4151
214248_s_at	15.0	7.2	-1.06	-2.09	AAA-AAA	0.00	999	1.000	1.000	0.2533
207251_at	10.6	22.1	1.06	2.09	AAA-AAA	0.00	999	1.000	1.000	0.1017
232840_at	17.1	8.2	-1.06	-2.09	AAA-AAA	0.00	999	1.000	1.000	0.3318
237631_at	17.0	35.6	1.06	2.09	AAA-AAA	0.00	999	1.000	1.000	0.3316
227201_at	46.5	97.3	1.06	2.09	AAA-AAA	0.00	999	1.000	1.000	0.2309
222098_s_at	10.0	20.9	1.06	2.09	AAA-AAA	0.00	999	1.000	1.000	0.5291
229709_at	21.4	44.8	1.06	2.09	AAA-AAA	0.00	999	1.000	1.000	0.2343
1558573_at	73.2	35.1	-1.06	-2.09	AAP-AAA	0.00	999	1.000	1.000	0.1806
240507_at	87.7	42.0	-1.06	-2.09	AAP-AAA	0.00	999	1.000	1.000	0.1903
242093_at	10.5	21.8	1.06	2.09	AAA-AAA	0.00	999	1.000	1.000	0.3814
1562698_x_at	31.6	66.0	1.06	2.09	AAA-AAA	0.00	999	1.000	1.000	0.3520
240503_at	12.8	26.7	1.06	2.09	AAA-AAA	0.00	999	1.000	1.000	0.4349
243235_at	6.5	3.1	-1.06	-2.09	AAA-AAA	0.00	999	1.000	1.000	0.1764
1559790_at	8.0	16.6	1.06	2.09	AAA-AAA	0.00	999	1.000	1.000	0.1585

208372_s_at	40.3	84.2	1.06	2.09	AAA-AAA	0.00	999	1.000	1.000	0.0665
234244_at	6.9	14.4	1.06	2.09	AAA-AAA	0.00	999	1.000	1.000	0.5434
237766_at	51.6	24.7	-1.06	-2.09	AAA-AAA	0.00	999	1.000	1.000	0.4949
236100_at	19.9	9.6	-1.06	-2.09	AAA-AAA	0.00	999	1.000	1.000	0.3315
227409_at	48.4	23.2	-1.06	-2.09	AAA-AAA	0.00	999	1.000	1.000	0.4775
1562728_at	28.3	59.1	1.06	2.09	AAA-AAA	0.00	999	1.000	1.000	0.2212
240152_at	3.1	6.5	1.06	2.09	AAA-AAA	0.00	999	1.000	1.000	0.1512
239341_at	9.0	18.7	1.06	2.09	AAA-AAA	0.00	999	1.000	1.000	0.4296
239345_at	20.0	9.6	-1.06	-2.09	AAA-AAA	0.00	999	1.000	1.000	0.3859
240719_at	6.9	3.3	-1.06	-2.09	AAA-AAA	0.00	999	1.000	1.000	0.1770
244789_at	14.2	6.8	-1.06	-2.09	AAA-AAA	0.00	999	1.000	1.000	0.2818
1569481_s_at	85.4	40.9	-1.06	-2.09	AAA-AAA	0.00	999	1.000	1.000	0.3732
1562272_at	18.1	37.8	1.06	2.09	AAA-AAA	0.00	999	1.000	1.000	0.4924
1561719_at	20.2	9.7	-1.06	-2.09	AAA-AAA	0.00	999	1.000	1.000	0.4568
215209_at	11.9	24.8	1.06	2.09	AAA-AAA	0.00	999	1.000	1.000	0.3553
215563_s_at	11.8	24.7	1.06	2.09	AAA-AAA	0.00	999	1.000	1.000	0.3411
211909_x_at	18.9	9.1	-1.06	-2.09	AAA-AAA	0.00	999	1.000	1.000	0.5137
217056_at	5.3	11.0	1.06	2.09	AAA-AAA	0.00	999	1.000	1.000	0.4464
219851_at	12.7	26.4	1.06	2.09	AAA-AAA	0.00	999	1.000	1.000	0.4542
205979_at	8.6	4.1	-1.06	-2.09	AAA-AAA	0.00	999	1.000	1.000	0.3746
204960_at	13.8	6.6	-1.06	-2.09	AAA-AAA	0.00	999	1.000	1.000	0.3453
233951_at	26.5	55.2	1.06	2.09	AAA-AAA	0.00	999	1.000	1.000	0.3648
223123_s_at	62.6	30.0	-1.06	-2.09	AAA-AAA	0.00	999	1.000	1.000	0.3433
231029_at	5.3	11.1	1.06	2.09	AAA-AAA	0.00	999	1.000	1.000	0.4935
217520_x_at	109.8	52.7	-1.06	-2.09	PAP-AAA	0.00	999	1.000	1.000	0.0397
1564134_at	11.6	24.1	1.06	2.09	AAA-AAA	0.00	999	1.000	1.000	0.4363
1565242_at	8.4	4.0	-1.06	-2.09	AAA-AAA	0.00	999	1.000	1.000	0.5311
238966_at	12.5	26.1	1.06	2.09	AAA-AAA	0.00	999	1.000	1.000	0.3147
1567878_at	4.9	10.1	1.06	2.09	AAA-AAA	0.00	999	1.000	1.000	0.6330
1557314_at	30.4	14.6	-1.06	-2.09	AAA-AAA	0.00	999	1.000	1.000	0.4150
1561477_at	3.5	7.3	1.06	2.09	AAA-AAA	0.00	999	1.000	1.000	0.5405
1561384_a_at	17.1	8.2	-1.06	-2.09	AAA-AAA	0.00	999	1.000	1.000	0.1287
213784_at	51.2	106.7	1.06	2.09	AAA-AAA	0.00	999	1.000	1.000	0.3123
219669_at	29.7	61.9	1.06	2.09	AAA-AAA	0.00	999	1.000	1.000	0.0361
207456_at	15.3	7.3	-1.06	-2.09	AAA-AAA	0.00	999	1.000	1.000	0.4660
235315_at	146.8	70.4	-1.06	-2.09	AAA-AAA	0.00	999	1.000	1.000	0.1527
232046_at	21.8	10.5	-1.06	-2.09	AAA-AAA	0.00	999	1.000	1.000	0.4014
1564358_at	62.2	29.9	-1.06	-2.08	AAA-PAA	0.00	999	1.000	1.000	0.2242
1558769_s_at	34.8	16.7	-1.06	-2.08	AAA-AAA	0.00	999	1.000	1.000	0.5091
1554219_at	53.8	25.8	-1.06	-2.08	AAA-AAA	0.00	999	1.000	1.000	0.1503
243458_at	22.9	11.0	-1.06	-2.08	AAA-AAA	0.00	999	1.000	1.000	0.3896
1560383_at	8.0	3.8	-1.06	-2.08	AAA-AAA	0.00	999	1.000	1.000	0.4768

216772_at	23.0	11.1	-1.06	-2.08	AAA-AAA	0.00	999	1.000	1.000	0.5060
217617_at	6.3	13.2	1.06	2.08	AAA-AAA	0.00	999	1.000	1.000	0.1744
219370_at	27.7	13.3	-1.06	-2.08	AAA-AAA	0.00	999	1.000	1.000	0.5223
207091_at	13.8	28.8	1.06	2.08	AAA-AAA	0.00	999	1.000	1.000	0.3090
210550_s_at	21.4	10.3	-1.06	-2.08	AAA-AAA	0.00	999	1.000	1.000	0.2726
207979_s_at	37.1	17.8	-1.06	-2.08	AAA-AAA	0.00	999	1.000	1.000	0.2446
209269_s_at	20.0	41.6	1.06	2.08	AAA-AAA	0.00	999	1.000	1.000	0.1928
238187_at	20.2	9.7	-1.06	-2.08	AAA-AAA	0.00	999	1.000	1.000	0.0789
238253_at	40.2	19.3	-1.06	-2.08	AAA-AAA	0.00	999	1.000	1.000	0.2633
231222_at	16.3	34.0	1.06	2.08	AAA-AAA	0.00	999	1.000	1.000	0.2225
1569680_at	70.3	33.8	-1.06	-2.08	PPA-AAA	0.00	999	1.000	1.000	0.1248
1553205_at	175.2	84.1	-1.06	-2.08	PPA-APA	0.00	999	1.000	1.000	0.1597
233054_at	33.9	16.3	-1.06	-2.08	APP-PAA	0.00	999	1.000	1.000	0.6309
1563137_at	5.1	10.6	1.06	2.08	AAA-AAA	0.00	999	1.000	1.000	0.1801
240305_at	7.1	14.8	1.06	2.08	AAA-AAA	0.00	999	1.000	1.000	0.0916
239871_at	4.2	8.7	1.06	2.08	AAA-AAA	0.00	999	1.000	1.000	0.5277
1553927_at	15.8	7.6	-1.06	-2.08	AAA-AAA	0.00	999	1.000	1.000	0.3192
1554960_at	24.1	11.6	-1.06	-2.08	AAA-AAA	0.00	999	1.000	1.000	0.6107
1561828_at	4.8	10.0	1.06	2.08	AAA-AAA	0.00	999	1.000	1.000	0.4972
1559354_a_at	101.0	48.5	-1.06	-2.08	AAA-AAA	0.00	999	1.000	1.000	0.2399
216128_at	44.1	21.2	-1.06	-2.08	AAA-AAA	0.00	999	1.000	1.000	0.2567
213731_s_at	28.1	13.5	-1.06	-2.08	AAA-AAA	0.00	999	1.000	1.000	0.5043
213997_at	6.8	14.1	1.06	2.08	AAA-AAA	0.00	999	1.000	1.000	0.2865
217649_at	29.9	62.3	1.06	2.08	AMA-AAA	0.00	999	1.000	1.000	0.1562
217663_at	23.1	48.0	1.06	2.08	AAA-AAA	0.00	999	1.000	1.000	0.4500
228509_at	6.1	12.6	1.06	2.08	AAA-AAA	0.00	999	1.000	1.000	0.2391
228788_at	6.5	13.6	1.06	2.08	AAA-AAA	0.00	999	1.000	1.000	0.4809
229870_at	80.1	166.7	1.06	2.08	AAA-AAA	0.00	999	1.000	1.000	0.2898
234574_at	45.0	21.6	-1.06	-2.08	APA-AAA	0.00	999	1.000	1.000	0.5028
1563344_at	53.0	110.3	1.06	2.08	AAA-AAA	0.00	999	1.000	1.000	0.3263
1568869_at	114.1	54.8	-1.06	-2.08	AAA-AAA	0.00	999	1.000	1.000	0.2106
240875_at	14.6	7.0	-1.06	-2.08	AAA-AAA	0.00	999	1.000	1.000	0.0125
1557585_at	19.4	40.3	1.06	2.08	AMA-AAA	0.00	999	1.000	1.000	0.2643
1561557_at	12.5	25.9	1.06	2.08	AAA-AAA	0.00	999	1.000	1.000	0.3990
1560527_at	14.2	6.8	-1.06	-2.08	AAA-AAA	0.00	999	1.000	1.000	0.1355
208915_s_at	342.6	164.7	-1.06	-2.08	AAA-MAA	0.00	999	1.000	1.000	0.0403
229938_at	8.8	18.4	1.06	2.08	AAA-AAA	0.00	999	1.000	1.000	0.2212
242253_at	75.8	36.5	-1.06	-2.08	PPP-AAP	0.00	999	1.000	1.000	0.0441
241140_at	29.4	61.1	1.06	2.08	AAA-AAA	0.00	999	1.000	1.000	0.2556
1564067_x_at	82.0	39.4	-1.06	-2.08	AAA-AAA	0.00	999	1.000	1.000	0.3050
1568915_at	13.0	27.0	1.06	2.08	AAA-AAA	0.00	999	1.000	1.000	0.3576
1553051_s_at	21.6	10.4	-1.06	-2.08	AAA-AAA	0.00	999	1.000	1.000	0.2018

216002_at	18.2	37.8	1.06	2.08	AAA-AAA	0.00	999	1.000	1.000	0.4217
206470_at	5.2	10.9	1.06	2.08	AAA-AAA	0.00	999	1.000	1.000	0.3103
206934_at	13.0	6.3	-1.06	-2.08	AAA-AAA	0.00	999	1.000	1.000	0.2995
235445_at	34.9	16.8	-1.06	-2.08	AAA-AAA	0.00	999	1.000	1.000	0.6009
233449_at	13.9	28.9	1.06	2.08	AAA-AAA	0.00	999	1.000	1.000	0.2734
243301_at	45.1	21.7	-1.06	-2.08	AAP-AAA	0.00	999	1.000	1.000	0.0667
1564472_at	23.6	49.0	1.06	2.08	AAA-AAA	0.00	999	1.000	1.000	0.1949
1569929_at	11.5	23.8	1.06	2.08	AAA-AAA	0.00	999	1.000	1.000	0.4557
240641_at	4.1	8.5	1.06	2.08	AAA-AAA	0.00	999	1.000	1.000	0.3184
1558661_at	4.9	10.2	1.06	2.08	AAA-AAA	0.00	999	1.000	1.000	0.1611
243934_at	50.6	24.4	-1.06	-2.08	AAA-AAA	0.00	999	1.000	1.000	0.4389
206341_at	45.8	22.0	-1.06	-2.08	AAA-AAA	0.00	999	1.000	1.000	0.2424
236901_at	7.1	14.7	1.06	2.08	AAA-AAA	0.00	999	1.000	1.000	0.5133
226578_s_at	119.0	247.2	1.06	2.08	AAA-AAA	0.00	999	1.000	1.000	0.0279
231512_at	2.6	5.5	1.06	2.08	AAA-AAA	0.00	999	1.000	1.000	0.3862
1564315_at	18.5	38.4	1.05	2.08	AAA-AAA	0.00	999	1.000	1.000	0.1976
240045_at	27.2	13.1	-1.05	-2.08	AAA-AAA	0.00	999	1.000	1.000	0.4013
240340_at	59.5	28.6	-1.05	-2.08	AAA-AAA	0.00	999	1.000	1.000	0.3000
243779_at	4.0	8.3	1.05	2.08	AAA-AAA	0.00	999	1.000	1.000	0.3898
215340_at	8.0	16.6	1.05	2.08	AAA-AAA	0.00	999	1.000	1.000	0.3857
214621_at	9.8	20.4	1.05	2.08	AAA-AAA	0.00	999	1.000	1.000	0.2467
205459_s_at	54.2	112.6	1.05	2.08	AAM-AAM	0.00	999	1.000	1.000	0.4488
233961_at	16.6	34.4	1.05	2.08	AAA-AAA	0.00	999	1.000	1.000	0.1288
233904_at	8.8	18.3	1.05	2.08	AAA-AAA	0.00	999	1.000	1.000	0.4758
237312_at	31.0	14.9	-1.05	-2.08	AAA-AAA	0.00	999	1.000	1.000	0.3098
231054_at	36.5	75.9	1.05	2.08	AAA-AAA	0.00	999	1.000	1.000	0.2449
243818_at	225.0	108.4	-1.05	-2.08	PPP-AAA	0.00	999	1.000	1.000	0.1162
202510_s_at	300.9	145.0	-1.05	-2.08	PPP-MAA	0.00	999	1.000	1.000	0.1701
219730_at	127.6	61.5	-1.05	-2.08	MAP-APP	0.00	999	1.000	1.000	0.2771
1566690_at	30.2	62.6	1.05	2.08	AAA-AAA	0.00	999	1.000	1.000	0.2971
241103_at	12.9	6.2	-1.05	-2.07	AAA-AAA	0.00	999	1.000	1.000	0.4955
1556473_at	11.3	23.4	1.05	2.08	AAA-AAA	0.00	999	1.000	1.000	0.4555
1553314_a_at	10.3	21.4	1.05	2.08	AAA-AAA	0.00	999	1.000	1.000	0.4267
1555215_a_at	55.8	26.9	-1.05	-2.08	AAA-AAA	0.00	999	1.000	1.000	0.1984
243505_at	3.4	7.1	1.05	2.08	AAA-AAA	0.00	999	1.000	1.000	0.0641
1561100_at	15.2	31.5	1.05	2.08	AAA-AAA	0.00	999	1.000	1.000	0.6005
216691_at	9.4	4.6	-1.05	-2.08	AAA-AAA	0.00	999	1.000	1.000	0.2340
214434_at	50.3	104.4	1.05	2.08	AAA-AAA	0.00	999	1.000	1.000	0.1618
218232_at	8.3	17.3	1.05	2.07	AAA-AAA	0.00	999	1.000	1.000	0.2457
210533_at	5.7	11.9	1.05	2.08	AAA-AAA	0.00	999	1.000	1.000	0.5040
210035_s_at	12.4	25.7	1.05	2.08	AAA-AAA	0.00	999	1.000	1.000	0.3885
207360_s_at	18.1	37.6	1.05	2.07	AAA-AAA	0.00	999	1.000	1.000	0.0690

235018_at	17.9	37.1	1.05	2.07	AAA-AAA	0.00	999	1.000	1.000	0.4095
237927_at	9.3	19.3	1.05	2.08	AAA-AAA	0.00	999	1.000	1.000	0.2818
236455_at	22.7	47.0	1.05	2.08	AAA-AAA	0.00	999	1.000	1.000	0.2195
237048_at	29.5	61.2	1.05	2.07	AAA-AAA	0.00	999	1.000	1.000	0.4592
226427_s_at	10.4	5.0	-1.05	-2.08	AAA-AAA	0.00	999	1.000	1.000	0.2774
229280_s_at	44.9	21.7	-1.05	-2.08	AAA-AAA	0.00	999	1.000	1.000	0.3647
242041_at	72.9	35.1	-1.05	-2.07	PPP-AAP	0.00	999	1.000	1.000	0.3198
233304_at	44.6	21.5	-1.05	-2.07	AAA-AAP	0.00	999	1.000	1.000	0.4824
1566514_at	52.5	25.3	-1.05	-2.07	AAA-MAA	0.00	999	1.000	1.000	0.3658
1557745_at	8.9	18.5	1.05	2.07	AAA-AAA	0.00	999	1.000	1.000	0.3555
1556249_a_at	63.5	131.7	1.05	2.07	AAA-AAA	0.00	999	1.000	1.000	0.2139
215419_at	52.4	25.2	-1.05	-2.07	AAA-AAA	0.00	999	1.000	1.000	0.3519
214174_s_at	147.9	71.4	-1.05	-2.07	AAA-AAA	0.00	999	1.000	1.000	0.0173
218406_x_at	25.1	12.1	-1.05	-2.07	AAA-AAA	0.00	999	1.000	1.000	0.1966
201495_x_at	12.9	6.2	-1.05	-2.07	AAA-AAA	0.00	999	1.000	1.000	0.1362
209866_s_at	13.4	6.5	-1.05	-2.07	AAA-AAA	0.00	999	1.000	1.000	0.2683
211606_at	30.3	14.6	-1.05	-2.07	AAA-AAA	0.00	999	1.000	1.000	0.4453
237723_at	21.8	10.5	-1.05	-2.07	AAA-AAA	0.00	999	1.000	1.000	0.5175
231028_at	5.3	2.5	-1.05	-2.07	AAA-AAA	0.00	999	1.000	1.000	0.2653
241414_at	114.4	55.2	-1.05	-2.07	AAA-AAA	0.00	999	1.000	1.000	0.4791
239744_at	16.4	7.9	-1.05	-2.07	AAA-AAA	0.00	999	1.000	1.000	0.0931
244851_at	8.6	4.1	-1.05	-2.07	AAA-AAA	0.00	999	1.000	1.000	0.1179
1561088_at	13.1	6.3	-1.05	-2.07	AAA-AAA	0.00	999	1.000	1.000	0.4505
1559488_at	26.5	12.8	-1.05	-2.07	AAA-AAA	0.00	999	1.000	1.000	0.6224
217350_at	21.1	10.2	-1.05	-2.07	AAA-AAA	0.00	999	1.000	1.000	0.4183
217174_s_at	12.7	26.4	1.05	2.07	AAA-AAA	0.00	999	1.000	1.000	0.4176
206365_at	92.1	44.5	-1.05	-2.07	AAA-AAA	0.00	999	1.000	1.000	0.2948
211436_at	10.2	21.1	1.05	2.07	AAA-AAA	0.00	999	1.000	1.000	0.4516
238189_at	4.3	9.0	1.05	2.07	AAA-AAA	0.00	999	1.000	1.000	0.3004
228321_s_at	30.0	14.5	-1.05	-2.07	AAA-AAA	0.00	999	1.000	1.000	0.4486
222257_s_at	27.5	13.3	-1.05	-2.07	AAA-AAA	0.00	999	1.000	1.000	0.2158
224273_at	21.3	44.1	1.05	2.07	AAA-AAA	0.00	999	1.000	1.000	0.2878
231333_at	47.1	97.6	1.05	2.07	AAA-AAA	0.00	999	1.000	1.000	0.3142
241746_at	53.0	25.6	-1.05	-2.07	APA-AAA	0.00	999	1.000	1.000	0.0677
1565585_at	3.0	6.2	1.05	2.07	AAA-AAA	0.00	999	1.000	1.000	0.2476
1553657_at	61.8	29.9	-1.05	-2.07	AAA-AAA	0.00	999	1.000	1.000	0.1444
1560908_at	15.3	31.7	1.05	2.07	AAA-AAA	0.00	999	1.000	1.000	0.1684
217265_at	4.6	9.5	1.05	2.07	AAA-AAA	0.00	999	1.000	1.000	0.1353
206043_s_at	20.3	42.1	1.05	2.07	AAA-AAA	0.00	999	1.000	1.000	0.3313
209392_at	27.6	13.3	-1.05	-2.07	AAA-AAA	0.00	999	1.000	1.000	0.3883
236460_at	44.6	92.3	1.05	2.07	AAA-AAA	0.00	999	1.000	1.000	0.0814
1559092_at	39.3	19.0	-1.05	-2.07	AAA-AAP	0.00	999	1.000	1.000	0.3443

1560720_at	33.7	16.3	-1.05	-2.07	APP-AAA	0.00	999	1.000	1.000	0.4935
242502_at	20.8	43.0	1.05	2.07	AAA-AAA	0.00	999	1.000	1.000	0.3299
1561314_at	37.0	17.9	-1.05	-2.07	AAA-AAA	0.00	999	1.000	1.000	0.1617
220389_at	67.8	32.8	-1.05	-2.07	AAA-AAA	0.00	999	1.000	1.000	0.2836
204570_at	10.0	4.8	-1.05	-2.07	AAA-AAA	0.00	999	1.000	1.000	0.0265
234380_x_at	24.8	51.4	1.05	2.07	AAA-AAA	0.00	999	1.000	1.000	0.1817
227178_at	45.7	94.6	1.05	2.07	AAA-AAA	0.00	999	1.000	1.000	0.3286
232308_at	35.9	17.4	-1.05	-2.07	AAA-AAA	0.00	999	1.000	1.000	0.2340
244282_at	72.1	34.9	-1.05	-2.07	APP-AAA	0.00	999	1.000	1.000	0.2342
1563273_at	49.5	102.3	1.05	2.07	AAA-AAA	0.00	999	1.000	1.000	0.3527
1569940_at	23.5	11.3	-1.05	-2.07	AAA-AAA	0.00	999	1.000	1.000	0.5399
1569847_at	66.7	137.9	1.05	2.07	AAA-AAA	0.00	999	1.000	1.000	0.0350
239921_at	16.6	34.3	1.05	2.07	AAA-AAA	0.00	999	1.000	1.000	0.3702
1569375_at	26.0	12.6	-1.05	-2.07	AAA-AAA	0.00	999	1.000	1.000	0.4068
1553880_at	15.8	7.7	-1.05	-2.07	AAA-AAA	0.00	999	1.000	1.000	0.4461
213768_s_at	18.6	38.4	1.05	2.07	AAA-AAA	0.00	999	1.000	1.000	0.2035
216869_at	56.7	27.4	-1.05	-2.07	AAA-AAA	0.00	999	1.000	1.000	0.2618
219046_s_at	18.7	38.7	1.05	2.07	AAA-AAA	0.00	999	1.000	1.000	0.3900
203784_s_at	28.2	58.4	1.05	2.07	AAA-AAA	0.00	999	1.000	1.000	0.2084
205529_s_at	22.0	10.6	-1.05	-2.07	AAA-AAA	0.00	999	1.000	1.000	0.5044
236693_at	11.4	5.5	-1.05	-2.07	AAA-AAA	0.00	999	1.000	1.000	0.2993
227225_at	23.5	11.4	-1.05	-2.07	AAA-AAA	0.00	999	1.000	1.000	0.4536
222324_at	24.8	51.3	1.05	2.07	AAA-AAA	0.00	999	1.000	1.000	0.3195
223567_at	13.5	27.9	1.05	2.07	AAA-AAA	0.00	999	1.000	1.000	0.4061
231457_at	6.5	13.5	1.05	2.07	AAA-AAA	0.00	999	1.000	1.000	0.1213
230602_at	16.2	33.5	1.05	2.07	AAA-AAA	0.00	999	1.000	1.000	0.4373
242105_at	5.8	12.1	1.05	2.07	AAA-AAA	0.00	999	1.000	1.000	0.0380
1563300_at	65.5	31.7	-1.05	-2.07	AAA-AAA	0.00	999	1.000	1.000	0.2355
240493_at	69.6	33.7	-1.05	-2.07	AAA-AAA	0.00	999	1.000	1.000	0.2033
1556333_at	18.7	9.1	-1.05	-2.07	AAA-AAA	0.00	999	1.000	1.000	0.4312
216666_at	56.6	27.4	-1.05	-2.07	AAA-AAA	0.00	999	1.000	1.000	0.4192
206363_at	7.3	3.5	-1.05	-2.07	AAA-AAA	0.00	999	1.000	1.000	0.0554
204707_s_at	15.0	31.0	1.05	2.07	AAA-AAA	0.00	999	1.000	1.000	0.3592
210673_x_at	54.5	26.4	-1.05	-2.07	AAA-AAA	0.00	999	1.000	1.000	0.1446
238366_at	34.0	70.3	1.05	2.07	AAA-AAA	0.00	999	1.000	1.000	0.2745
236051_at	4.2	8.7	1.05	2.07	AAA-AAA	0.00	999	1.000	1.000	0.1552
237222_at	62.3	30.2	-1.05	-2.07	AAA-AAA	0.00	999	1.000	1.000	0.4176
222004_s_at	5.8	12.0	1.05	2.07	AAA-AAA	0.00	999	1.000	1.000	0.2353
204205_at	153.6	74.3	-1.05	-2.07	PPP-AAA	0.00	999	1.000	1.000	0.2898
244329_at	19.3	39.9	1.05	2.07	AAA-AAA	0.00	999	1.000	1.000	0.5063
216603_at	6.0	12.4	1.05	2.07	AAA-AAA	0.00	999	1.000	1.000	0.1451
220480_at	5.3	10.9	1.05	2.07	AAA-AAA	0.00	999	1.000	1.000	0.2403

208562_s_at	14.9	7.2	-1.05	-2.06	AAA-AAA	0.00	999	1.000	1.000	0.4677
237249_at	30.6	63.2	1.05	2.07	AAA-AAA	0.00	999	1.000	1.000	0.3027
232211_at	26.5	54.8	1.05	2.07	AAA-AAA	0.00	999	1.000	1.000	0.5013
232258_at	6.6	13.6	1.05	2.07	AAA-AAA	0.00	999	1.000	1.000	0.4098
1569415_at	166.4	80.6	-1.05	-2.06	AAA-PAA	0.00	999	1.000	1.000	0.5185
240795_at	65.1	31.5	-1.05	-2.06	PAM-MAP	0.00	999	1.000	1.000	0.5602
242239_at	43.6	21.1	-1.05	-2.06	AAA-AAA	0.00	999	1.000	1.000	0.3220
1563797_at	3.2	6.6	1.05	2.06	AAA-AAA	0.00	999	1.000	1.000	0.2522
239993_at	22.7	46.8	1.05	2.06	AAA-AAA	0.00	999	1.000	1.000	0.4593
1569337_at	12.6	6.1	-1.05	-2.06	AAA-AAA	0.00	999	1.000	1.000	0.1062
1561728_a_at	15.1	31.1	1.05	2.06	AAA-AAA	0.00	999	1.000	1.000	0.3184
243647_at	9.9	4.8	-1.05	-2.06	AAA-AAA	0.00	999	1.000	1.000	0.5083
215635_at	26.7	55.1	1.05	2.06	AAA-AAA	0.00	999	1.000	1.000	0.3995
220065_at	3.7	7.6	1.05	2.06	AAA-AAA	0.00	999	1.000	1.000	0.0949
207865_s_at	29.3	14.2	-1.05	-2.06	AAA-AAA	0.00	999	1.000	1.000	0.3594
237731_at	6.7	13.8	1.05	2.06	AAA-AAA	0.00	999	1.000	1.000	0.3129
227573_s_at	40.0	19.4	-1.05	-2.06	AAA-AAA	0.00	999	1.000	1.000	0.2926
229040_at	53.1	25.8	-1.05	-2.06	AAA-AAA	0.00	999	1.000	1.000	0.3088
239844_x_at	78.8	38.2	-1.04	-2.06	AAA-AAA	0.00	999	1.000	1.000	0.1491
1558378_a_at	61.8	127.5	1.04	2.06	AAA-AAA	0.00	999	1.000	1.000	0.2660
1558337_at	7.5	3.7	-1.04	-2.06	AAA-AAA	0.00	999	1.000	1.000	0.0639
1557541_at	10.5	21.7	1.04	2.06	AAA-AAA	0.00	999	1.000	1.000	0.4283
1554340_a_at	7.0	14.4	1.04	2.06	AAA-AAA	0.00	999	1.000	1.000	0.2905
1559412_at	43.5	21.1	-1.04	-2.06	AAA-AAA	0.00	999	1.000	1.000	0.3664
1560540_x_at	8.8	4.3	-1.04	-2.06	AAA-AAA	0.00	999	1.000	1.000	0.6326
1560881_a_at	128.6	62.4	-1.04	-2.06	AAA-AAA	0.00	999	1.000	1.000	0.1589
215975_x_at	36.8	75.9	1.04	2.06	AAA-AAA	0.00	999	1.000	1.000	0.2977
206909_at	14.7	7.1	-1.04	-2.06	AAA-AAA	0.00	999	1.000	1.000	0.2318
236348_at	17.3	8.4	-1.04	-2.06	AAA-AAA	0.00	999	1.000	1.000	0.3949
224546_at	14.4	29.6	1.04	2.06	AAA-AAA	0.00	999	1.000	1.000	0.3549
231159_at	45.2	93.3	1.04	2.06	AAA-AAA	0.00	999	1.000	1.000	0.2376
240441_at	18.6	9.0	-1.04	-2.06	AAA-AAA	0.00	999	1.000	1.000	0.4479
1557399_at	73.1	35.5	-1.04	-2.06	AAA-AAA	0.00	999	1.000	1.000	0.2728
1556504_at	4.2	8.7	1.04	2.06	AAA-AAA	0.00	999	1.000	1.000	0.3478
1558565_at	14.1	6.8	-1.04	-2.06	AAA-AAA	0.00	999	1.000	1.000	0.1901
1553137_s_at	32.2	66.3	1.04	2.06	AAA-AAA	0.00	999	1.000	1.000	0.2020
1556253_s_at	21.8	10.6	-1.04	-2.06	AAA-AAA	0.00	999	1.000	1.000	0.4946
1561197_at	6.0	12.4	1.04	2.06	AAA-AAA	0.00	999	1.000	1.000	0.4912
234228_at	12.8	26.3	1.04	2.06	AAA-AAA	0.00	999	1.000	1.000	0.3791
235121_at	16.2	33.3	1.04	2.06	AAA-AAA	0.00	999	1.000	1.000	0.3004
232819_s_at	57.4	118.2	1.04	2.06	AAA-AAA	0.00	999	1.000	1.000	0.2316
233408_at	7.5	15.4	1.04	2.06	AAA-AAA	0.00	999	1.000	1.000	0.2751

223505_s_at	35.7	73.5	1.04	2.06	AAA-AAA	0.00	999	1.000	1.000	0.1615
231744_at	55.0	26.7	-1.04	-2.06	AAA-AAP	0.00	999	1.000	1.000	0.2254
233425_at	46.5	22.6	-1.04	-2.06	AAA-AAP	0.00	999	1.000	1.000	0.2319
1563002_at	4.3	8.8	1.04	2.06	AAA-AAA	0.00	999	1.000	1.000	0.4847
1560322_at	48.9	23.8	-1.04	-2.06	AAA-AAA	0.00	999	1.000	1.000	0.1810
216286_at	14.9	30.6	1.04	2.06	AAA-AAA	0.00	999	1.000	1.000	0.4970
213589_s_at	22.7	11.0	-1.04	-2.06	AAA-AAA	0.00	999	1.000	1.000	0.4059
215102_at	10.0	20.5	1.04	2.06	AAA-AAA	0.00	999	1.000	1.000	0.5654
219593_at	8.3	17.0	1.04	2.06	AAA-AAA	0.00	999	1.000	1.000	0.0156
235525_at	63.0	129.8	1.04	2.06	AAA-AAA	0.00	999	1.000	1.000	0.0395
233674_at	63.5	130.7	1.04	2.06	AAA-AAA	0.00	999	1.000	1.000	0.2493
237065_s_at	51.1	24.8	-1.04	-2.06	AAA-AAA	0.00	999	1.000	1.000	0.3033
237335_at	50.9	24.7	-1.04	-2.06	AAA-AAA	0.00	999	1.000	1.000	0.3130
224131_at	7.4	15.3	1.04	2.06	AAA-AAA	0.00	999	1.000	1.000	0.2668
1558293_at	64.9	31.5	-1.04	-2.06	PPP-PAA	0.00	999	1.000	1.000	0.1962
240961_at	3.3	6.8	1.04	2.06	AAA-AAA	0.00	999	1.000	1.000	0.5077
1566463_at	3.0	6.1	1.04	2.06	AAA-AAA	0.00	999	1.000	1.000	0.3507
1556564_at	89.9	43.7	-1.04	-2.06	AAA-AAA	0.00	999	1.000	1.000	0.1891
1562005_at	25.3	52.0	1.04	2.06	AAA-AAA	0.00	999	1.000	1.000	0.4465
244444_at	23.0	11.2	-1.04	-2.06	AAA-AAA	0.00	999	1.000	1.000	0.3372
217442_at	16.8	8.2	-1.04	-2.06	AAA-AAA	0.00	999	1.000	1.000	0.3261
206318_at	5.8	11.9	1.04	2.06	AAA-AAA	0.00	999	1.000	1.000	0.4550
206624_at	5.6	2.7	-1.04	-2.06	AAA-AAA	0.00	999	1.000	1.000	0.3455
210185_at	56.3	115.8	1.04	2.06	AAA-AAA	0.00	999	1.000	1.000	0.4616
233066_at	37.3	18.1	-1.04	-2.06	AAA-AAA	0.00	999	1.000	1.000	0.3399
237393_at	58.4	28.4	-1.04	-2.06	AAA-AAA	0.00	999	1.000	1.000	0.1749
236749_at	82.3	40.0	-1.04	-2.06	PMA-APP	0.00	999	1.000	1.000	0.3039
1561663_at	36.8	17.9	-1.04	-2.06	APA-PAA	0.00	999	1.000	1.000	0.4861
214561_at	20.6	10.0	-1.04	-2.06	AAP-AAA	0.00	999	1.000	1.000	0.5615
1567859_at	4.8	9.9	1.04	2.06	AAA-AAA	0.00	999	1.000	1.000	0.3259
244208_at	10.0	20.6	1.04	2.06	AAA-AAA	0.00	999	1.000	1.000	0.2714
205590_at	4.6	9.4	1.04	2.06	AAA-AAA	0.00	999	1.000	1.000	0.4394
207816_at	13.1	6.4	-1.04	-2.06	AAA-AAA	0.00	999	1.000	1.000	0.2629
235421_at	13.5	27.8	1.04	2.06	AAA-AAA	0.00	999	1.000	1.000	0.0879
227323_at	5.5	11.3	1.04	2.06	AAA-AAA	0.00	999	1.000	1.000	0.4559
228474_s_at	49.6	24.1	-1.04	-2.06	AAA-AAA	0.00	999	1.000	1.000	0.3403
229688_at	45.3	22.0	-1.04	-2.06	AAA-AAA	0.00	999	1.000	1.000	0.1117
221349_at	26.0	12.6	-1.04	-2.05	AAP-AAA	0.00	999	1.000	1.000	0.4824
241487_at	31.3	15.2	-1.04	-2.06	AAP-AAP	0.00	999	1.000	1.000	0.4894
1562686_at	10.7	22.1	1.04	2.06	AAA-AAA	0.00	999	1.000	1.000	0.5981
1553798_a_at	18.2	37.3	1.04	2.05	AAA-AAA	0.00	999	1.000	1.000	0.2979
1561710_at	14.9	7.3	-1.04	-2.06	MAA-AAA	0.00	999	1.000	1.000	0.6263

213770_at	121.2	59.0	-1.04	-2.06	AMA-AAA	0.00	999	1.000	1.000	0.1078
214977_at	11.1	5.4	-1.04	-2.05	AAA-AAA	0.00	999	1.000	1.000	0.2797
208247_at	65.8	32.0	-1.04	-2.06	AAA-AAA	0.00	999	1.000	1.000	0.2505
235522_at	33.6	69.1	1.04	2.06	AAA-AAA	0.00	999	1.000	1.000	0.2089
231633_at	9.5	19.6	1.04	2.05	AAA-AAA	0.00	999	1.000	1.000	0.2555
1557177_at	12.0	5.9	-1.04	-2.05	AAP-AAA	0.00	999	1.000	1.000	0.6270
1568791_s_at	15.1	30.9	1.04	2.05	AAA-AAA	0.00	999	1.000	1.000	0.5031
1555040_at	15.4	7.5	-1.04	-2.05	AAA-AAA	0.00	999	1.000	1.000	0.4506
1555558_at	6.4	13.1	1.04	2.05	AAA-AAA	0.00	999	1.000	1.000	0.4864
209670_at	44.1	21.5	-1.04	-2.05	AAA-AAA	0.00	999	1.000	1.000	0.3112
234877_x_at	140.3	68.3	-1.04	-2.05	AAA-AAA	0.00	999	1.000	1.000	0.2655
231120_x_at	26.0	53.4	1.04	2.05	AAA-AAA	0.00	999	1.000	1.000	0.0888
1564232_at	38.9	79.8	1.04	2.05	AAA-AAA	0.00	999	1.000	1.000	0.3327
1557089_at	18.4	9.0	-1.04	-2.05	AAA-AAA	0.00	999	1.000	1.000	0.5636
1552771_a_at	8.9	4.3	-1.04	-2.05	AAA-AAA	0.00	999	1.000	1.000	0.1828
1558628_at	6.2	12.7	1.04	2.05	AAA-AAA	0.00	999	1.000	1.000	0.4196
205744_at	53.0	25.8	-1.04	-2.05	AAA-AAA	0.00	999	1.000	1.000	0.2939
203915_at	56.8	27.7	-1.04	-2.05	AAA-AAA	0.00	999	1.000	1.000	0.2882
234536_at	23.1	11.2	-1.04	-2.05	AAA-AAA	0.00	999	1.000	1.000	0.2288
230908_at	76.4	37.3	-1.04	-2.05	AAA-AAA	0.00	999	1.000	1.000	0.1664
231080_at	13.2	27.0	1.04	2.05	AAA-AAA	0.00	999	1.000	1.000	0.5207
232069_at	11.6	23.7	1.04	2.05	AAA-AAA	0.00	999	1.000	1.000	0.4099
1565880_at	59.2	28.9	-1.04	-2.05	PPA-AAA	0.00	999	1.000	1.000	0.2139
1552579_a_at	71.7	35.0	-1.04	-2.05	PAP-AAA	0.00	999	1.000	1.000	0.2508
1564426_x_at	32.5	66.6	1.04	2.05	AAA-AAA	0.00	999	1.000	1.000	0.1778
1566425_at	55.9	27.3	-1.04	-2.05	AAA-AAA	0.00	999	1.000	1.000	0.4822
216576_x_at	7.4	15.1	1.04	2.05	AAA-AAA	0.00	999	1.000	1.000	0.0232
216707_at	13.2	6.4	-1.04	-2.05	AAA-AAA	0.00	999	1.000	1.000	0.2735
221308_at	40.7	83.5	1.04	2.05	AAA-AAA	0.00	999	1.000	1.000	0.4428
206876_at	25.1	12.2	-1.04	-2.05	AAA-AAA	0.00	999	1.000	1.000	0.3790
234560_at	68.8	141.1	1.04	2.05	AAA-AAA	0.00	999	1.000	1.000	0.4743
233569_at	7.9	3.9	-1.04	-2.05	AAA-AAA	0.00	999	1.000	1.000	0.2235
236773_at	16.4	8.0	-1.04	-2.05	AAA-AAA	0.00	999	1.000	1.000	0.4077
224022_x_at	14.8	30.4	1.04	2.05	AAA-AMA	0.00	999	1.000	1.000	0.0230
231339_at	6.8	13.9	1.04	2.05	AAA-AAA	0.00	999	1.000	1.000	0.2854
231480_at	6.3	12.8	1.04	2.05	AAA-AAA	0.00	999	1.000	1.000	0.0822
1553794_at	58.6	28.6	-1.04	-2.05	PAA-AAA	0.00	999	1.000	1.000	0.1396
1562231_at	28.7	14.0	-1.04	-2.05	AAA-AAA	0.00	999	1.000	1.000	0.4051
244270_at	35.7	17.4	-1.04	-2.05	AAA-AAA	0.00	999	1.000	1.000	0.2797
217444_at	10.9	22.3	1.04	2.05	AAA-AAA	0.00	999	1.000	1.000	0.5351
200939_s_at	35.1	17.1	-1.04	-2.05	AAA-AAA	0.00	999	1.000	1.000	0.3283
209355_s_at	13.8	6.7	-1.04	-2.05	AAA-AAA	0.00	999	1.000	1.000	0.1108

234076_at	9.6	19.7	1.04	2.05	AAA-AAA	0.00	999	1.000	1.000	0.5570
233331_at	13.7	28.1	1.04	2.05	AAA-AAA	0.00	999	1.000	1.000	0.5232
237340_at	15.9	7.8	-1.04	-2.05	AAA-AAA	0.00	999	1.000	1.000	0.2898
231122_x_at	6.5	13.4	1.04	2.05	AAA-AAA	0.00	999	1.000	1.000	0.2259
1563302_at	63.0	30.7	-1.03	-2.05	AAM-AAA	0.00	999	1.000	1.000	0.2081
1569122_at	6.8	3.3	-1.03	-2.05	AAA-AAA	0.00	999	1.000	1.000	0.4794
1568796_at	71.5	146.4	1.03	2.05	AAA-AAA	0.00	999	1.000	1.000	0.1899
240300_at	16.2	33.2	1.03	2.05	AAA-AAA	0.00	999	1.000	1.000	0.3173
240927_at	3.1	6.4	1.03	2.05	AAA-AAA	0.00	999	1.000	1.000	0.5680
1570181_a_at	33.6	16.4	-1.03	-2.05	AAA-AAA	0.00	999	1.000	1.000	0.5027
1556676_a_at	11.8	24.2	1.03	2.05	AAA-AAA	0.00	999	1.000	1.000	0.3078
242651_at	29.2	59.7	1.03	2.05	AAA-AAA	0.00	999	1.000	1.000	0.3207
210307_s_at	38.9	19.0	-1.03	-2.05	AAA-AAA	0.00	999	1.000	1.000	0.3462
228422_at	48.4	23.7	-1.03	-2.05	AAA-AAA	0.00	999	1.000	1.000	0.3289
231597_x_at	8.2	16.9	1.03	2.05	AAA-AAA	0.00	999	1.000	1.000	0.5468
241650_x_at	123.3	60.3	-1.03	-2.05	AAA-AAA	0.00	999	1.000	1.000	0.3112
1563480_at	46.2	22.6	-1.03	-2.05	AAA-AAA	0.00	999	1.000	1.000	0.2196
1567527_at	11.2	23.0	1.03	2.05	AAA-AAA	0.00	999	1.000	1.000	0.3907
205988_at	46.3	94.8	1.03	2.05	AAA-AAA	0.00	999	1.000	1.000	0.2452
237985_at	16.2	7.9	-1.03	-2.05	AAA-AAA	0.00	999	1.000	1.000	0.3464
223467_at	43.6	21.3	-1.03	-2.05	AAA-AAA	0.00	999	1.000	1.000	0.0836
231498_at	12.1	24.8	1.03	2.05	AAA-AAA	0.00	999	1.000	1.000	0.3910
1564937_at	15.1	7.4	-1.03	-2.04	AAA-AAA	0.00	999	1.000	1.000	0.5338
1563513_at	61.4	30.0	-1.03	-2.05	AAA-AAA	0.00	999	1.000	1.000	0.4154
240298_at	27.4	13.4	-1.03	-2.04	AAA-AAA	0.00	999	1.000	1.000	0.2972
240578_at	6.3	12.8	1.03	2.05	AAA-AAA	0.00	999	1.000	1.000	0.5630
1556889_s_at	6.6	3.2	-1.03	-2.05	AAA-AAA	0.00	999	1.000	1.000	0.3953
1555664_at	6.6	13.4	1.03	2.05	AAA-AAA	0.00	999	1.000	1.000	0.2914
1560863_a_at	5.3	10.9	1.03	2.04	AAA-AAA	0.00	999	1.000	1.000	0.5725
206447_at	8.2	16.7	1.03	2.04	AAA-AAA	0.00	999	1.000	1.000	0.5172
1557223_at	48.2	23.6	-1.03	-2.04	PAP-PAA	0.00	999	1.000	1.000	0.5305
241567_at	3.5	1.7	-1.03	-2.04	AAA-AAA	0.00	999	1.000	1.000	0.3544
1562854_at	25.2	12.3	-1.03	-2.04	AAA-AAA	0.00	999	1.000	1.000	0.5565
241669_x_at	37.3	76.1	1.03	2.04	AAA-AAA	0.00	999	1.000	1.000	0.1486
1569086_at	42.3	86.5	1.03	2.04	AAA-AAM	0.00	999	1.000	1.000	0.0178
239803_at	9.9	20.2	1.03	2.04	AAA-AAA	0.00	999	1.000	1.000	0.1967
215247_at	53.1	108.4	1.03	2.04	AAA-AAA	0.00	999	1.000	1.000	0.0725
217119_s_at	90.5	44.3	-1.03	-2.04	AAA-AAA	0.00	999	1.000	1.000	0.2602
206798_x_at	15.9	32.5	1.03	2.04	AAA-AAA	0.00	999	1.000	1.000	0.1665
203398_s_at	22.5	45.9	1.03	2.04	AAA-AAA	0.00	999	1.000	1.000	0.1880
203220_s_at	168.3	82.4	-1.03	-2.04	AAA-AAA	0.00	999	1.000	1.000	0.0511
211570_s_at	10.9	22.3	1.03	2.04	AAA-AAA	0.00	999	1.000	1.000	0.3800

207527_at	39.1	79.8	1.03	2.04	AAA-AAA	0.00	999	1.000	1.000	0.3098
1565894_at	38.2	78.1	1.03	2.04	AAA-AAA	0.00	999	1.000	1.000	0.1121
1554583_a_at	12.2	6.0	-1.03	-2.04	AAA-AAA	0.00	999	1.000	1.000	0.1328
1561691_at	24.8	50.7	1.03	2.04	AAA-AAA	0.00	999	1.000	1.000	0.2343
1561856_at	9.9	4.9	-1.03	-2.04	AAA-AAA	0.00	999	1.000	1.000	0.5323
1559590_at	28.4	13.9	-1.03	-2.04	AAA-AAA	0.00	999	1.000	1.000	0.6059
243774_at	40.0	19.6	-1.03	-2.04	AAA-AAA	0.00	999	1.000	1.000	0.1909
215870_s_at	5.7	2.8	-1.03	-2.04	AAA-AAA	0.00	999	1.000	1.000	0.4062
203835_at	21.8	10.7	-1.03	-2.04	AAA-AAA	0.00	999	1.000	1.000	0.1926
205056_s_at	18.5	9.1	-1.03	-2.04	AAA-AAA	0.00	999	1.000	1.000	0.3616
210998_s_at	19.9	9.7	-1.03	-2.04	AAA-AAA	0.00	999	1.000	1.000	0.3639
237480_at	6.8	13.9	1.03	2.04	AAA-AAA	0.00	999	1.000	1.000	0.5475
221916_at	31.5	64.4	1.03	2.04	AAA-AAA	0.00	999	1.000	1.000	0.0900
231015_at	147.0	300.2	1.03	2.04	AAA-AAA	0.00	999	1.000	1.000	0.1785
229570_at	68.3	139.3	1.03	2.04	AAA-AAA	0.00	999	1.000	1.000	0.2032
242582_at	30.4	14.9	-1.03	-2.04	PAA-AAA	0.00	999	1.000	1.000	0.3282
1564718_at	7.1	14.5	1.03	2.04	AAA-AAA	0.00	999	1.000	1.000	0.4911
1570295_at	37.7	76.8	1.03	2.04	AAA-AAA	0.00	999	1.000	1.000	0.3010
1556923_at	36.7	18.0	-1.03	-2.04	AAA-AAA	0.00	999	1.000	1.000	0.3431
243666_at	3.1	6.2	1.03	2.04	AAA-AAA	0.00	999	1.000	1.000	0.6061
216016_at	7.3	3.6	-1.03	-2.04	AAA-AAA	0.00	999	1.000	1.000	0.1108
217177_s_at	28.6	14.0	-1.03	-2.04	AAA-AAA	0.00	999	1.000	1.000	0.5370
210972_x_at	43.8	21.5	-1.03	-2.04	AAA-AAA	0.00	999	1.000	1.000	0.3490
209574_s_at	33.4	68.1	1.03	2.04	AAA-AAA	0.00	999	1.000	1.000	0.1127
234653_at	17.2	8.5	-1.03	-2.04	AAA-AAA	0.00	999	1.000	1.000	0.2982
234185_at	14.1	6.9	-1.03	-2.04	AAA-AAA	0.00	999	1.000	1.000	0.4853
235270_at	33.8	16.6	-1.03	-2.04	AAA-AAA	0.00	999	1.000	1.000	0.4018
238036_at	15.2	7.5	-1.03	-2.04	AAA-AAA	0.00	999	1.000	1.000	0.2304
1562981_at	80.3	39.4	-1.03	-2.04	AAA-AAA	0.00	999	1.000	1.000	0.3851
1563450_at	3.0	1.5	-1.03	-2.04	AAA-AAA	0.00	999	1.000	1.000	0.1708
243240_at	51.4	25.2	-1.03	-2.04	AAA-AAA	0.00	999	1.000	1.000	0.2168
207242_s_at	6.4	13.0	1.03	2.04	AAA-AAA	0.00	999	1.000	1.000	0.0837
203325_s_at	29.3	14.4	-1.03	-2.04	AAA-AAA	0.00	999	1.000	1.000	0.3914
234234_at	58.7	28.8	-1.03	-2.04	AAA-AAA	0.00	999	1.000	1.000	0.2117
229895_s_at	51.7	25.4	-1.03	-2.04	AAA-AAA	0.00	999	1.000	1.000	0.5814
240088_at	27.4	55.8	1.03	2.04	AAA-AAA	0.00	999	1.000	1.000	0.3625
1556552_a_at	69.1	33.9	-1.03	-2.04	AAA-AAA	0.00	999	1.000	1.000	0.2529
1559695_a_at	7.7	15.8	1.03	2.04	AAA-AAA	0.00	999	1.000	1.000	0.4412
243998_at	11.5	23.4	1.03	2.04	AAA-AAA	0.00	999	1.000	1.000	0.3940
219857_at	2.5	5.0	1.03	2.04	AAA-AAA	0.00	999	1.000	1.000	0.0995
206957_at	60.7	29.8	-1.03	-2.04	AAA-AAA	0.00	999	1.000	1.000	0.1770
206616_s_at	7.3	15.0	1.03	2.04	AAA-AAA	0.00	999	1.000	1.000	0.2627

211301_at	14.6	7.2	-1.03	-2.04	AAA-AAA	0.00	999	1.000	1.000	0.2396
207902_at	25.9	52.7	1.03	2.04	AAA-AAA	0.00	999	1.000	1.000	0.4420
236015_at	7.0	3.4	-1.03	-2.04	AAA-AAA	0.00	999	1.000	1.000	0.2114
231167_at	117.9	57.9	-1.03	-2.04	MAP-AAA	0.00	999	1.000	1.000	0.0717
222727_s_at	157.6	77.4	-1.03	-2.04	PAA-AAP	0.00	999	1.000	1.000	0.1705
213541_s_at	35.1	17.2	-1.03	-2.04	AAA-AAA	0.00	999	1.000	1.000	0.2485
207140_at	4.2	8.6	1.03	2.04	AAA-AAA	0.00	999	1.000	1.000	0.1017
205680_at	24.1	11.9	-1.03	-2.04	AAA-AAA	0.00	999	1.000	1.000	0.4790
231514_at	26.0	52.9	1.03	2.04	AAA-AAA	0.00	999	1.000	1.000	0.4100
232026_at	24.5	50.0	1.03	2.04	AAA-AMA	0.00	999	1.000	1.000	0.4162
229771_at	19.4	39.5	1.03	2.04	AAA-AAA	0.00	999	1.000	1.000	0.4864
1554947_at	33.8	16.6	-1.03	-2.03	PAA-AAA	0.00	999	1.000	1.000	0.2992
241518_at	19.2	9.4	-1.03	-2.04	AAA-AAA	0.00	999	1.000	1.000	0.2558
1565859_at	13.4	6.6	-1.03	-2.04	AAA-AAA	0.00	999	1.000	1.000	0.2533
1570235_at	12.1	24.6	1.03	2.04	AAA-AAA	0.00	999	1.000	1.000	0.1981
1570168_at	21.3	10.5	-1.03	-2.04	AAA-AAA	0.00	999	1.000	1.000	0.4257
1557476_at	18.7	38.1	1.03	2.04	AAA-AAA	0.00	999	1.000	1.000	0.4866
242677_at	28.6	14.1	-1.03	-2.03	AAA-AAA	0.00	999	1.000	1.000	0.4368
244437_at	39.5	19.4	-1.03	-2.04	AAA-AAA	0.00	999	1.000	1.000	0.2278
221205_at	20.3	10.0	-1.03	-2.04	AAA-AAA	0.00	999	1.000	1.000	0.4866
211442_x_at	40.8	83.0	1.03	2.04	AAA-AAA	0.00	999	1.000	1.000	0.2391
237726_at	58.0	28.5	-1.03	-2.03	AAA-AAA	0.00	999	1.000	1.000	0.2597
237406_at	11.6	5.7	-1.03	-2.04	AAA-AAA	0.00	999	1.000	1.000	0.4026
218874_s_at	133.6	65.7	-1.02	-2.03	AAA-AAP	0.00	999	1.000	1.000	0.4200
1563660_at	1.6	3.2	1.02	2.03	AAA-AAA	0.00	999	1.000	1.000	0.5388
241623_at	54.5	26.8	-1.02	-2.03	AAA-AAA	0.00	999	1.000	1.000	0.1130
1559785_at	7.0	14.2	1.02	2.03	AAA-AAA	0.00	999	1.000	1.000	0.5363
244676_s_at	26.3	53.4	1.02	2.03	AAA-AAA	0.00	999	1.000	1.000	0.1741
213438_at	11.7	23.7	1.02	2.03	AAA-AAA	0.00	999	1.000	1.000	0.5334
220343_at	12.9	26.2	1.02	2.03	AAA-AAA	0.00	999	1.000	1.000	0.1756
221289_at	6.8	13.8	1.02	2.03	AAA-AAA	0.00	999	1.000	1.000	0.3456
217163_at	10.5	5.1	-1.02	-2.03	AAA-AAA	0.00	999	1.000	1.000	0.4471
210305_at	46.3	22.8	-1.02	-2.03	AAA-AAA	0.00	999	1.000	1.000	0.3752
211690_at	16.0	7.9	-1.02	-2.03	AAA-AAA	0.00	999	1.000	1.000	0.2857
234549_at	45.5	92.5	1.02	2.03	AAA-AAA	0.00	999	1.000	1.000	0.0947
234212_at	8.7	4.3	-1.02	-2.03	AAA-AAA	0.00	999	1.000	1.000	0.5070
234785_at	16.8	34.1	1.02	2.03	AAA-AAA	0.00	999	1.000	1.000	0.3190
232785_at	24.4	49.7	1.02	2.03	AAA-AAA	0.00	999	1.000	1.000	0.3288
232805_at	11.8	24.1	1.02	2.03	AAA-AAA	0.00	999	1.000	1.000	0.4383
238220_at	9.9	20.1	1.02	2.03	AAA-AAA	0.00	999	1.000	1.000	0.5655
224805_s_at	42.0	20.6	-1.02	-2.03	AAA-AAA	0.00	999	1.000	1.000	0.2723
226266_at	10.8	21.9	1.02	2.03	AAA-AAA	0.00	999	1.000	1.000	0.3423

227911_at	7.3	14.8	1.02	2.03	AAA-AAA	0.00	999	1.000	1.000	0.4638
51158_at	25.8	52.4	1.02	2.03	AAA-AAA	0.00	999	1.000	1.000	0.2382
216979_at	62.6	30.8	-1.02	-2.03	APA-AAA	0.00	999	1.000	1.000	0.2956
232875_at	48.3	23.8	-1.02	-2.03	PAA-AAA	0.00	999	1.000	1.000	0.3565
230024_at	146.2	71.9	-1.02	-2.03	AAP-AAA	0.00	999	1.000	1.000	0.4272
242504_at	13.5	27.4	1.02	2.03	AAA-AAA	0.00	999	1.000	1.000	0.4298
241952_at	6.5	13.2	1.02	2.03	AAA-AAA	0.00	999	1.000	1.000	0.4339
244795_at	37.3	75.8	1.02	2.03	AAA-AAA	0.00	999	1.000	1.000	0.3046
244388_at	25.9	52.5	1.02	2.03	AAA-AAA	0.00	999	1.000	1.000	0.1966
214063_s_at	30.8	15.2	-1.02	-2.03	AAA-AAA	0.00	999	1.000	1.000	0.2171
217030_at	78.6	38.7	-1.02	-2.03	AAA-AAA	0.00	999	1.000	1.000	0.2883
206338_at	14.5	29.5	1.02	2.03	AAA-AAA	0.00	999	1.000	1.000	0.4775
207410_s_at	11.4	5.6	-1.02	-2.03	AAA-AAA	0.00	999	1.000	1.000	0.3439
233141_s_at	30.2	14.9	-1.02	-2.03	AAA-AAA	0.00	999	1.000	1.000	0.2160
233733_at	36.4	17.9	-1.02	-2.03	AAA-AAA	0.00	999	1.000	1.000	0.5629
237720_at	8.6	4.2	-1.02	-2.03	AAA-AAA	0.00	999	1.000	1.000	0.4184
237858_s_at	36.1	17.8	-1.02	-2.03	AAA-AAA	0.00	999	1.000	1.000	0.1675
223715_at	35.1	17.3	-1.02	-2.03	AAA-AAA	0.00	999	1.000	1.000	0.0980
1564685_a_at	3.1	6.3	1.02	2.03	AAA-AAA	0.00	999	1.000	1.000	0.4426
242192_at	9.1	18.4	1.02	2.03	AAA-AAA	0.00	999	1.000	1.000	0.3619
240065_at	19.0	38.7	1.02	2.03	AAA-AAA	0.00	999	1.000	1.000	0.4293
1552557_a_at	40.5	19.9	-1.02	-2.03	AAA-AAA	0.00	999	1.000	1.000	0.4102
1562251_a_at	31.3	63.6	1.02	2.03	AAA-AAA	0.00	999	1.000	1.000	0.3415
1562514_at	3.9	7.9	1.02	2.03	AAA-AAA	0.00	999	1.000	1.000	0.5615
215664_s_at	5.0	10.1	1.02	2.03	AAA-AAA	0.00	999	1.000	1.000	0.0508
221169_s_at	36.4	17.9	-1.02	-2.03	AAA-AAA	0.00	999	1.000	1.000	0.1330
206367_at	9.0	18.3	1.02	2.03	AAA-AAA	0.00	999	1.000	1.000	0.0216
201372_s_at	63.9	31.5	-1.02	-2.03	MAA-AMA	0.00	999	1.000	1.000	0.4192
205368_at	19.1	38.7	1.02	2.03	AAM-AAA	0.00	999	1.000	1.000	0.3531
204540_at	89.8	44.2	-1.02	-2.03	AAA-AAA	0.00	999	1.000	1.000	0.3092
211421_s_at	8.5	17.3	1.02	2.03	AAA-AAA	0.00	999	1.000	1.000	0.4917
224550_s_at	8.7	17.8	1.02	2.03	AAA-AAA	0.00	999	1.000	1.000	0.2989
222342_at	4.4	9.0	1.02	2.03	AAA-AAA	0.00	999	1.000	1.000	0.2119
224297_s_at	63.1	128.1	1.02	2.03	AAA-AAA	0.00	999	1.000	1.000	0.2595
231781_s_at	11.1	22.5	1.02	2.03	AAA-AAA	0.00	999	1.000	1.000	0.4012
241828_x_at	40.4	82.0	1.02	2.03	AAA-AAA	0.00	999	1.000	1.000	0.3059
1569960_at	6.6	13.4	1.02	2.03	AAA-AAA	0.00	999	1.000	1.000	0.5824
1555253_at	2.7	5.5	1.02	2.03	AAA-AAA	0.00	999	1.000	1.000	0.3241
1559807_at	31.6	15.6	-1.02	-2.03	AAA-AAA	0.00	999	1.000	1.000	0.3361
1558906_a_at	42.1	85.4	1.02	2.03	AAA-AAM	0.00	999	1.000	1.000	0.1347
221197_s_at	104.6	51.5	-1.02	-2.03	AAA-AAA	0.00	999	1.000	1.000	0.2984
221061_at	47.3	95.9	1.02	2.03	AAA-AAA	0.00	999	1.000	1.000	0.2287

204368_at	44.9	91.2	1.02	2.03	AAA-AAA	0.00	999	1.000	1.000	0.3816
235706_at	63.5	31.3	-1.02	-2.03	AAA-AAA	0.00	999	1.000	1.000	0.3077
232729_at	31.5	64.0	1.02	2.03	AAA-AAA	0.00	999	1.000	1.000	0.4200
228538_at	23.2	11.4	-1.02	-2.03	AAA-AAA	0.00	999	1.000	1.000	0.4884
232420_x_at	392.9	193.8	-1.02	-2.03	APA-APA	0.00	999	1.000	1.000	0.1091
239155_at	47.9	97.0	1.02	2.03	AAA-AAA	0.00	999	1.000	1.000	0.0172
1566995_at	8.1	4.0	-1.02	-2.03	AAA-AAA	0.00	999	1.000	1.000	0.4599
1557214_at	7.6	15.4	1.02	2.03	AAA-AAA	0.00	999	1.000	1.000	0.4267
1554481_a_at	52.3	25.8	-1.02	-2.03	AAA-AAA	0.00	999	1.000	1.000	0.2671
1555713_at	36.1	73.3	1.02	2.03	AAA-AAA	0.00	999	1.000	1.000	0.3794
242745_at	90.2	44.5	-1.02	-2.03	AAA-AAA	0.00	999	1.000	1.000	0.3735
1562093_at	2.7	5.6	1.02	2.03	AAA-AAA	0.00	999	1.000	1.000	0.5372
1562476_at	48.8	24.1	-1.02	-2.03	AAA-AAA	0.00	999	1.000	1.000	0.3685
1559623_at	19.4	39.3	1.02	2.03	AAA-AAA	0.00	999	1.000	1.000	0.3520
1559618_at	34.9	70.8	1.02	2.03	AAA-AAA	0.00	999	1.000	1.000	0.1566
244609_at	11.6	5.7	-1.02	-2.03	AAA-AAA	0.00	999	1.000	1.000	0.5264
215279_at	33.7	68.3	1.02	2.03	AAA-AAA	0.00	999	1.000	1.000	0.3643
221413_at	45.1	22.2	-1.02	-2.03	AAA-AAA	0.00	999	1.000	1.000	0.4299
217194_at	21.6	10.6	-1.02	-2.03	AAA-AAA	0.00	999	1.000	1.000	0.4030
205941_s_at	38.0	77.1	1.02	2.03	AAA-AAA	0.00	999	1.000	1.000	0.5085
233474_at	13.2	26.8	1.02	2.03	AAA-AAA	0.00	999	1.000	1.000	0.2696
230568_x_at	47.1	23.2	-1.02	-2.03	AAA-AAA	0.00	999	1.000	1.000	0.2294
1563589_at	18.1	8.9	-1.02	-2.03	APA-AAA	0.00	999	1.000	1.000	0.6245
1570103_at	14.4	7.1	-1.02	-2.03	AAA-AAA	0.00	999	1.000	1.000	0.4082
240359_at	29.0	58.7	1.02	2.03	AAA-AAA	0.00	999	1.000	1.000	0.2918
239793_at	60.9	30.0	-1.02	-2.03	AAA-AAA	0.00	999	1.000	1.000	0.3963
1566688_at	16.4	8.1	-1.02	-2.03	AAA-AAA	0.00	999	1.000	1.000	0.4454
1556308_at	45.4	91.9	1.02	2.03	AAA-AAA	0.00	999	1.000	1.000	0.3703
1562247_at	34.5	17.0	-1.02	-2.03	AAA-AAA	0.00	999	1.000	1.000	0.4753
243780_at	8.4	17.1	1.02	2.03	AAA-AAA	0.00	999	1.000	1.000	0.3767
215349_at	7.7	15.7	1.02	2.03	AAA-AAA	0.00	999	1.000	1.000	0.3271
215542_at	6.0	12.1	1.02	2.03	AAA-AAA	0.00	999	1.000	1.000	0.2969
219954_s_at	56.5	27.9	-1.02	-2.03	AAA-AAA	0.00	999	1.000	1.000	0.0995
234588_at	11.2	22.6	1.02	2.03	AAA-AAA	0.00	999	1.000	1.000	0.2889
237751_x_at	7.3	14.8	1.02	2.03	AAA-AAA	0.00	999	1.000	1.000	0.4706
237625_s_at	9.0	18.3	1.02	2.03	AAA-AAA	0.00	999	1.000	1.000	0.6175
230859_at	15.1	30.6	1.02	2.03	AAA-AAA	0.00	999	1.000	1.000	0.4559
232459_at	42.7	86.6	1.02	2.03	AAA-AAM	0.00	999	1.000	1.000	0.0728
231729_s_at	7.4	15.0	1.02	2.03	AAA-AAA	0.00	999	1.000	1.000	0.2277
230025_at	83.2	41.1	-1.02	-2.03	AAA-AAA	0.00	999	1.000	1.000	0.3385
1558944_at	12.5	6.1	-1.02	-2.03	AAP-AAA	0.00	999	1.000	1.000	0.4415
241461_at	9.2	18.7	1.02	2.03	AAA-AAA	0.00	999	1.000	1.000	0.3261

1557605_a_at	85.9	42.4	-1.02	-2.03	AAA-AAA	0.00	999	1.000	1.000	0.2030
1553403_at	8.7	4.3	-1.02	-2.03	AAA-AAA	0.00	999	1.000	1.000	0.1050
215104_at	31.2	15.4	-1.02	-2.03	AAA-AAA	0.00	999	1.000	1.000	0.4801
236554_x_at	8.1	16.4	1.02	2.03	AAA-AAA	0.00	999	1.000	1.000	0.3869
228101_at	44.8	22.1	-1.02	-2.03	AAA-AAA	0.00	999	1.000	1.000	0.3764
231347_at	9.9	4.9	-1.02	-2.03	AAA-AAA	0.00	999	1.000	1.000	0.2897
213474_at	87.4	43.2	-1.02	-2.02	PAA-AAA	0.00	999	1.000	1.000	0.3807
1564117_at	152.5	75.4	-1.02	-2.02	APA-AAA	0.00	999	1.000	1.000	0.4771
241182_at	12.3	6.1	-1.02	-2.02	AAA-AAA	0.00	999	1.000	1.000	0.3420
1566456_at	79.0	39.1	-1.02	-2.02	AAA-AAA	0.00	999	1.000	1.000	0.4104
242808_at	28.0	56.7	1.02	2.02	AAM-AAA	0.00	999	1.000	1.000	0.4349
220736_at	10.5	5.2	-1.02	-2.02	AAA-AAA	0.00	999	1.000	1.000	0.6415
220759_at	3.9	7.8	1.02	2.02	AAA-AAA	0.00	999	1.000	1.000	0.2626
238628_s_at	83.5	41.3	-1.02	-2.02	AAA-AAA	0.00	999	1.000	1.000	0.3643
223747_x_at	10.1	20.4	1.02	2.02	AAA-AAA	0.00	999	1.000	1.000	0.3277
1556265_at	226.7	112.1	-1.02	-2.02	APP-AAA	0.00	999	1.000	1.000	0.1811
1564236_at	5.9	2.9	-1.02	-2.02	AAA-AAA	0.00	999	1.000	1.000	0.2762
1562683_a_at	16.4	8.1	-1.02	-2.02	AAA-AAA	0.00	999	1.000	1.000	0.4637
1566771_at	19.6	9.7	-1.02	-2.02	AAA-AAA	0.00	999	1.000	1.000	0.5168
244681_at	66.0	133.5	1.02	2.02	AAA-AAA	0.00	999	1.000	1.000	0.0533
205563_at	21.8	10.8	-1.02	-2.02	AAA-AAA	0.00	999	1.000	1.000	0.3493
210710_at	77.9	38.5	-1.02	-2.02	AAA-AAA	0.00	999	1.000	1.000	0.0513
208182_x_at	31.6	15.6	-1.02	-2.02	AAA-AAA	0.00	999	1.000	1.000	0.5773
231889_at	18.4	37.1	1.02	2.02	AAA-AAA	0.00	999	1.000	1.000	0.5551
223371_s_at	114.8	56.8	-1.02	-2.02	APA-AAA	0.00	999	1.000	1.000	0.2203
233783_at	100.4	49.7	-1.02	-2.02	AAA-AAP	0.00	999	1.000	1.000	0.4053
239116_at	9.4	4.7	-1.02	-2.02	AAA-AAA	0.00	999	1.000	1.000	0.3426
1562793_at	37.3	18.5	-1.02	-2.02	AAA-AAA	0.00	999	1.000	1.000	0.3265
242409_at	22.9	11.3	-1.02	-2.02	AAA-AAA	0.00	999	1.000	1.000	0.4300
1558889_at	11.2	22.6	1.02	2.02	AAA-AAA	0.00	999	1.000	1.000	0.2595
212831_at	27.9	56.4	1.02	2.02	AAA-AAA	0.00	999	1.000	1.000	0.5253
204918_s_at	29.0	14.4	-1.02	-2.02	AAA-AAA	0.00	999	1.000	1.000	0.4373
210393_at	10.9	22.0	1.02	2.02	AAA-AAA	0.00	999	1.000	1.000	0.5030
207652_s_at	42.8	21.2	-1.02	-2.02	AAA-AAA	0.00	999	1.000	1.000	0.4642
234187_at	45.4	91.8	1.02	2.02	AAA-AAA	0.00	999	1.000	1.000	0.3457
234264_at	12.5	25.3	1.02	2.02	AAA-AAA	0.00	999	1.000	1.000	0.4903
232892_at	60.9	123.0	1.02	2.02	AAA-AAA	0.00	999	1.000	1.000	0.1057
236419_at	39.5	79.8	1.02	2.02	AAA-AAA	0.00	999	1.000	1.000	0.4979
229824_at	35.0	17.3	-1.02	-2.02	AAA-AAA	0.00	999	1.000	1.000	0.3307
230341_x_at	43.1	87.1	1.02	2.02	AAA-AAA	0.00	999	1.000	1.000	0.4495
1555100_at	31.6	15.7	-1.01	-2.02	AAA-AAP	0.00	999	1.000	1.000	0.4303
1569089_a_at	17.1	8.4	-1.01	-2.02	AAA-AAA	0.00	999	1.000	1.000	0.5763

244773_at	6.3	12.7	1.01	2.02	AAA-AAA	0.00	999	1.000	1.000	0.5508
1558109_x_at	27.9	56.2	1.01	2.02	AAA-AAA	0.00	999	1.000	1.000	0.3229
220378_at	27.9	13.8	-1.01	-2.02	AAA-AAA	0.00	999	1.000	1.000	0.4636
217264_s_at	28.3	57.1	1.01	2.02	AAA-AAA	0.00	999	1.000	1.000	0.4151
204885_s_at	91.0	45.0	-1.01	-2.02	AAA-AAA	0.00	999	1.000	1.000	0.0799
211313_s_at	12.7	25.7	1.01	2.02	AAA-AAA	0.00	999	1.000	1.000	0.5357
233718_at	15.0	7.4	-1.01	-2.02	AAA-AAA	0.00	999	1.000	1.000	0.5453
237549_at	8.5	17.2	1.01	2.02	AAA-AAA	0.00	999	1.000	1.000	0.3921
222838_at	6.0	12.1	1.01	2.02	AAA-AAA	0.00	999	1.000	1.000	0.5478
1552509_a_at	53.1	26.3	-1.01	-2.02	APA-AAA	0.00	999	1.000	1.000	0.0409
227307_at	343.5	170.2	-1.01	-2.02	PAA-AAA	0.00	999	1.000	1.000	0.0982
219066_at	190.4	94.3	-1.01	-2.02	AAP-AAP	0.00	999	1.000	1.000	0.3817
1563887_at	9.4	19.1	1.01	2.02	AAA-AAA	0.00	999	1.000	1.000	0.5260
1554962_a_at	35.9	72.5	1.01	2.02	AAA-AAA	0.00	999	1.000	1.000	0.2645
213780_at	5.6	11.3	1.01	2.02	AAA-AAA	0.00	999	1.000	1.000	0.1605
234967_at	9.2	18.5	1.01	2.02	AAA-AAA	0.00	999	1.000	1.000	0.5443
236643_s_at	34.6	17.2	-1.01	-2.02	AAA-AAA	0.00	999	1.000	1.000	0.4232
228836_at	53.5	107.9	1.01	2.02	AAA-AAA	0.00	999	1.000	1.000	0.3346
231151_at	28.1	13.9	-1.01	-2.02	AAA-AAA	0.00	999	1.000	1.000	0.3278
235541_at	169.6	84.1	-1.01	-2.02	PAA-AAA	0.00	999	1.000	1.000	0.0383
1568827_at	36.3	73.1	1.01	2.02	AAA-AAA	0.00	999	1.000	1.000	0.0099
240984_at	43.2	21.4	-1.01	-2.02	AAA-AAA	0.00	999	1.000	1.000	0.1932
1553043_a_at	17.2	34.6	1.01	2.02	AAA-AAA	0.00	999	1.000	1.000	0.2367
243128_at	37.0	18.4	-1.01	-2.02	AAA-AAA	0.00	999	1.000	1.000	0.5016
243060_at	18.6	9.2	-1.01	-2.02	AAA-AAA	0.00	999	1.000	1.000	0.4299
1560380_at	1.5	3.1	1.01	2.02	AAA-AAA	0.00	999	1.000	1.000	0.0551
215637_at	31.4	63.4	1.01	2.02	AAA-AAA	0.00	999	1.000	1.000	0.2708
216618_at	12.5	25.1	1.01	2.02	AAA-MAA	0.00	999	1.000	1.000	0.5175
206264_at	50.5	25.0	-1.01	-2.02	AAA-AAA	0.00	999	1.000	1.000	0.1882
211586_s_at	3.6	7.2	1.01	2.02	AAA-AAA	0.00	999	1.000	1.000	0.1523
234434_at	24.2	48.9	1.01	2.02	AAA-AAA	0.00	999	1.000	1.000	0.2183
234595_at	7.1	14.4	1.01	2.02	AAA-AAA	0.00	999	1.000	1.000	0.2070
236963_at	11.8	23.8	1.01	2.02	AAA-AAA	0.00	999	1.000	1.000	0.5491
215495_s_at	149.5	74.2	-1.01	-2.02	PPP-AAP	0.00	999	1.000	1.000	0.0650
217380_s_at	28.2	14.0	-1.01	-2.02	PAA-AAA	0.00	999	1.000	1.000	0.5367
239073_at	34.3	17.0	-1.01	-2.02	AAA-AAA	0.00	999	1.000	1.000	0.1968
1557665_at	25.8	12.8	-1.01	-2.02	AAA-AAA	0.00	999	1.000	1.000	0.5093
1553736_at	50.2	24.9	-1.01	-2.02	AAA-AAA	0.00	999	1.000	1.000	0.1661
1553465_a_at	13.9	6.9	-1.01	-2.02	AAA-AAA	0.00	999	1.000	1.000	0.5718
244500_s_at	96.3	47.8	-1.01	-2.02	AAA-AAA	0.00	999	1.000	1.000	0.2597
212587_s_at	9.2	18.6	1.01	2.02	AAA-AAA	0.00	999	1.000	1.000	0.5627
216874_at	12.4	25.0	1.01	2.02	AAA-AAA	0.00	999	1.000	1.000	0.6017

203886_s_at	7.2	14.5	1.01	2.02	AAA-AAA	0.00	999	1.000	1.000	0.0747
234658_at	28.6	14.2	-1.01	-2.02	AAA-AAA	0.00	999	1.000	1.000	0.4253
235342_at	16.6	8.3	-1.01	-2.02	AAA-AAA	0.00	999	1.000	1.000	0.3525
234871_at	6.2	12.5	1.01	2.02	AAA-AAA	0.00	999	1.000	1.000	0.4801
233828_at	13.7	27.6	1.01	2.02	AAA-AAA	0.00	999	1.000	1.000	0.4937
228918_at	58.2	117.3	1.01	2.02	AAA-AAA	0.00	999	1.000	1.000	0.1088
1562720_at	13.7	27.6	1.01	2.01	AAA-AAA	0.00	999	1.000	1.000	0.4654
1570471_at	17.0	34.3	1.01	2.01	AAA-AAA	0.00	999	1.000	1.000	0.2736
240731_at	3.0	6.1	1.01	2.01	AAA-AAA	0.00	999	1.000	1.000	0.4132
1554976_a_at	7.7	3.8	-1.01	-2.01	AAA-AAA	0.00	999	1.000	1.000	0.2413
1559045_at	15.0	30.2	1.01	2.01	AAA-AAA	0.00	999	1.000	1.000	0.3380
215733_x_at	12.4	25.0	1.01	2.01	AAA-AAA	0.00	999	1.000	1.000	0.2892
206937_at	37.0	18.4	-1.01	-2.01	AAA-AAA	0.00	999	1.000	1.000	0.1673
211589_at	31.4	15.6	-1.01	-2.01	AAA-AAA	0.00	999	1.000	1.000	0.4745
208422_at	10.6	5.3	-1.01	-2.01	AAA-AAA	0.00	999	1.000	1.000	0.3976
232787_at	39.6	79.7	1.01	2.02	AAA-AAA	0.00	999	1.000	1.000	0.4028
236291_at	84.4	41.9	-1.01	-2.01	AAA-AAA	0.00	999	1.000	1.000	0.5692
227570_at	32.2	64.8	1.01	2.01	AAA-AAA	0.00	999	1.000	1.000	0.3875
222966_at	3.3	6.7	1.01	2.01	AAA-AAA	0.00	999	1.000	1.000	0.5698
223887_at	39.2	19.5	-1.01	-2.01	AAA-AAA	0.00	999	1.000	1.000	0.5330
1565653_at	20.8	41.8	1.01	2.01	AAA-AAA	0.00	999	1.000	1.000	0.3701
241744_x_at	2.8	5.7	1.01	2.01	AAA-AAA	0.00	999	1.000	1.000	0.4867
240055_at	12.3	6.1	-1.01	-2.01	AAA-AAA	0.00	999	1.000	1.000	0.3321
243151_at	27.0	54.3	1.01	2.01	AAA-AAA	0.00	999	1.000	1.000	0.4111
242749_at	82.8	41.1	-1.01	-2.01	AAA-AAA	0.00	999	1.000	1.000	0.2352
1562157_at	12.7	6.3	-1.01	-2.01	AAA-AAA	0.00	999	1.000	1.000	0.3939
243778_at	6.8	13.7	1.01	2.01	AAA-AAA	0.00	999	1.000	1.000	0.4177
215830_at	9.5	4.7	-1.01	-2.01	AAA-AAA	0.00	999	1.000	1.000	0.0805
203559_s_at	50.4	101.5	1.01	2.01	AAA-AAM	0.00	999	1.000	1.000	0.2224
205507_at	65.8	132.5	1.01	2.01	AAA-AAA	0.00	999	1.000	1.000	0.0635
211820_x_at	21.2	10.5	-1.01	-2.01	AAA-AAA	0.00	999	1.000	1.000	0.3590
240135_x_at	19.8	39.7	1.01	2.01	AAA-AAA	0.00	999	1.000	1.000	0.4061
1552456_a_at	9.1	18.3	1.01	2.01	AAA-AAA	0.00	999	1.000	1.000	0.2131
1555417_a_at	35.2	70.8	1.01	2.01	AAA-AAA	0.00	999	1.000	1.000	0.0365
214292_at	8.2	16.4	1.01	2.01	AAA-AAA	0.00	999	1.000	1.000	0.2426
219339_s_at	55.1	110.8	1.01	2.01	AAA-AAA	0.00	999	1.000	1.000	0.4222
234366_x_at	108.0	53.7	-1.01	-2.01	AAA-AAA	0.00	999	1.000	1.000	0.3247
235157_at	54.2	27.0	-1.01	-2.01	MAP-AAA	0.00	999	1.000	1.000	0.1315
239062_at	8.6	17.3	1.01	2.01	AAA-AAA	0.00	999	1.000	1.000	0.3900
1569032_at	20.4	41.1	1.01	2.01	AAA-AAA	0.00	999	1.000	1.000	0.4608
240522_at	8.1	16.3	1.01	2.01	AAA-AAA	0.00	999	1.000	1.000	0.2785
1556392_a_at	7.0	14.0	1.01	2.01	AAA-AAA	0.00	999	1.000	1.000	0.4660

1558842_at	20.7	10.3	-1.01	-2.01	AAA-AAA	0.00	999	1.000	1.000	0.2066
1553376_a_at	19.4	9.6	-1.01	-2.01	AAA-AAA	0.00	999	1.000	1.000	0.5693
1554776_at	7.5	15.0	1.01	2.01	AAA-AAA	0.00	999	1.000	1.000	0.4601
244053_at	33.0	66.4	1.01	2.01	AAA-AAA	0.00	999	1.000	1.000	0.2194
217489_s_at	13.7	27.5	1.01	2.01	AAA-AAA	0.00	999	1.000	1.000	0.2339
204536_s_at	59.2	29.5	-1.01	-2.01	AAA-AAA	0.00	999	1.000	1.000	0.4082
234776_at	2.6	5.2	1.01	2.01	AAA-AAA	0.00	999	1.000	1.000	0.4988
226304_at	36.4	18.1	-1.01	-2.01	AAA-AAA	0.00	999	1.000	1.000	0.4664
228798_x_at	75.3	151.3	1.01	2.01	AAA-AAA	0.00	999	1.000	1.000	0.1088
244448_at	53.7	26.7	-1.01	-2.01	PPA-AAA	0.00	999	1.000	1.000	0.1372
204762_s_at	71.2	35.4	-1.01	-2.01	PAA-MAA	0.00	999	1.000	1.000	0.1933
205150_s_at	34.7	17.3	-1.01	-2.01	AAA-PAA	0.00	999	1.000	1.000	0.5397
241574_s_at	40.1	20.0	-1.01	-2.01	AAA-AAA	0.00	999	1.000	1.000	0.3086
1570405_at	40.6	81.5	1.01	2.01	AAA-AAA	0.00	999	1.000	1.000	0.2189
1564022_at	10.0	5.0	-1.01	-2.01	AAA-AAA	0.00	999	1.000	1.000	0.3617
1556554_at	6.3	12.6	1.01	2.01	AAA-AAA	0.00	999	1.000	1.000	0.4990
1553641_a_at	23.3	11.6	-1.01	-2.01	AAA-AAA	0.00	999	1.000	1.000	0.3892
214249_at	11.6	23.2	1.01	2.01	AAA-AAA	0.00	999	1.000	1.000	0.4991
220646_s_at	68.7	34.2	-1.01	-2.01	AAA-AAA	0.00	999	1.000	1.000	0.3015
234830_at	5.0	10.1	1.01	2.01	AAA-AAA	0.00	999	1.000	1.000	0.4784
233088_at	29.8	14.8	-1.01	-2.01	AAA-AAA	0.00	999	1.000	1.000	0.4792
237953_at	9.6	19.4	1.01	2.01	AAA-AAA	0.00	999	1.000	1.000	0.3158
237325_at	29.4	14.7	-1.01	-2.01	AAA-AAA	0.00	999	1.000	1.000	0.2971
224476_s_at	40.1	80.4	1.01	2.01	AAA-AAA	0.00	999	1.000	1.000	0.3197
223944_at	33.0	66.3	1.01	2.01	AAA-AAA	0.00	999	1.000	1.000	0.2358
224005_at	55.0	110.5	1.01	2.01	AAA-AAA	0.00	999	1.000	1.000	0.1920
214256_at	60.9	30.3	-1.01	-2.01	APA-AAA	0.00	999	1.000	1.000	0.2652
1561030_at	9.7	4.8	-1.01	-2.01	AAA-AAA	0.00	999	1.000	1.000	0.5960
213620_s_at	24.6	49.5	1.01	2.01	AAA-AAA	0.00	999	1.000	1.000	0.2555
231738_at	17.7	35.5	1.01	2.01	AAA-AAA	0.00	999	1.000	1.000	0.4502
230539_at	18.4	37.0	1.01	2.01	AAA-AAA	0.00	999	1.000	1.000	0.5484
240751_at	8.7	4.3	-1.00	-2.01	AAA-AAA	0.00	999	1.000	1.000	0.1517
1556616_a_at	25.7	51.5	1.00	2.01	AAA-AAA	0.00	999	1.000	1.000	0.3577
1555011_at	7.5	15.1	1.00	2.01	AAA-AAA	0.00	999	1.000	1.000	0.1536
1560842_a_at	33.3	16.6	-1.00	-2.01	AAA-AAA	0.00	999	1.000	1.000	0.0922
244157_x_at	44.2	88.8	1.00	2.01	AAA-AAA	0.00	999	1.000	1.000	0.3356
221420_at	11.2	22.4	1.00	2.01	AAA-AAM	0.00	999	1.000	1.000	0.5387
205908_s_at	22.7	11.3	-1.00	-2.01	AAA-AAA	0.00	999	1.000	1.000	0.3400
206954_at	71.6	35.7	-1.00	-2.01	AAA-AAA	0.00	999	1.000	1.000	0.4621
207103_at	13.5	6.7	-1.00	-2.01	AAA-AAA	0.00	999	1.000	1.000	0.2772
232821_at	43.6	21.7	-1.00	-2.01	AAA-AAA	0.00	999	1.000	1.000	0.1274
237487_at	28.2	56.5	1.00	2.01	AAA-AAA	0.00	999	1.000	1.000	0.3388

236936_at	12.0	24.1	1.00	2.01	AAA-AAA	0.00	999	1.000	1.000	0.2173
230989_s_at	15.4	31.0	1.00	2.01	AAA-AAA	0.00	999	1.000	1.000	0.5999
230939_at	30.6	61.4	1.00	2.01	AAA-AAA	0.00	999	1.000	1.000	0.4336
219928_s_at	162.9	81.3	-1.00	-2.00	PPP-AAP	0.00	999	1.000	1.000	0.1542
222936_s_at	77.9	38.9	-1.00	-2.00	PAM-APA	0.00	999	1.000	1.000	0.4259
240612_at	46.5	93.2	1.00	2.00	AAA-AAA	0.00	999	1.000	1.000	0.2306
1556663_s_at	12.1	6.0	-1.00	-2.00	AAA-AAA	0.00	999	1.000	1.000	0.3587
1555648_at	8.8	4.4	-1.00	-2.00	AAA-AAA	0.00	999	1.000	1.000	0.2977
244588_at	20.7	10.3	-1.00	-2.01	AAA-AAA	0.00	999	1.000	1.000	0.5233
244463_at	4.7	2.4	-1.00	-2.01	AAA-AAA	0.00	999	1.000	1.000	0.3107
206076_at	19.7	9.9	-1.00	-2.00	AAA-AAA	0.00	999	1.000	1.000	0.2008
201279_s_at	36.8	73.7	1.00	2.00	AAA-AAA	0.00	999	1.000	1.000	0.3354
210397_at	12.2	24.5	1.00	2.00	AAA-AAA	0.00	999	1.000	1.000	0.4107
210073_at	3.5	7.0	1.00	2.00	AAA-AAA	0.00	999	1.000	1.000	0.3501
233708_at	17.7	35.5	1.00	2.00	AAA-AAA	0.00	999	1.000	1.000	0.4877
233624_at	28.4	14.2	-1.00	-2.00	AAA-AAA	0.00	999	1.000	1.000	0.4441
237277_at	13.2	6.6	-1.00	-2.00	AAA-AAA	0.00	999	1.000	1.000	0.5496
223646_s_at	33.3	16.6	-1.00	-2.00	AAA-AAA	0.00	999	1.000	1.000	0.4941
229260_at	17.4	34.9	1.00	2.00	AAA-AAA	0.00	999	1.000	1.000	0.3807
230553_at	17.9	35.9	1.00	2.00	AAA-AAA	0.00	999	1.000	1.000	0.4303
1569885_at	25.6	12.8	-1.00	-2.00	AAA-AAA	0.00	999	1.000	1.000	0.4619
1557042_at	47.2	23.6	-1.00	-2.00	AAA-AAA	0.00	999	1.000	1.000	0.2703
1562341_at	45.6	22.8	-1.00	-2.00	AAA-AAA	0.00	999	1.000	1.000	0.1136
216138_at	22.5	45.1	1.00	2.00	AAA-AAA	0.00	999	1.000	1.000	0.2695
217281_x_at	5.3	10.7	1.00	2.00	AAA-AAA	0.00	999	1.000	1.000	0.1089
208000_at	47.8	23.9	-1.00	-2.00	AAA-AAA	0.00	999	1.000	1.000	0.4505
234306_s_at	15.0	7.5	-1.00	-2.00	AAA-AAA	0.00	999	1.000	1.000	0.1562
236797_at	15.2	30.4	1.00	2.00	AAA-AAA	0.00	999	1.000	1.000	0.4038
226459_at	30.8	15.4	-1.00	-2.00	AAA-AAA	0.00	999	1.000	1.000	0.3050
230962_at	2.6	5.1	1.00	2.00	AAA-AAA	0.00	999	1.000	1.000	0.3627
230161_at	100.9	202.1	1.00	2.00	AAA-AAA	0.00	999	1.000	1.000	0.2654
233481_at	75.2	37.6	-1.00	-2.00	APA-AAP	0.00	999	1.000	1.000	0.3823
1570108_at	48.0	96.1	1.00	2.00	AAA-AAA	0.00	999	1.000	1.000	0.1476
1570229_at	88.2	44.1	-1.00	-2.00	AAA-AAA	0.00	999	1.000	1.000	0.4237
239187_at	60.3	30.1	-1.00	-2.00	AAA-AAA	0.00	999	1.000	1.000	0.4561
1556239_a_at	17.5	35.1	1.00	2.00	AAA-AAM	0.00	999	1.000	1.000	0.4109
1561415_at	2.9	1.5	-1.00	-2.00	AAA-AAA	0.00	999	1.000	1.000	0.4540
206155_at	15.8	31.7	1.00	2.00	AAA-AAA	0.00	999	1.000	1.000	0.4654
238819_at	13.1	6.6	-1.00	-2.00	AAA-AAA	0.00	999	1.000	1.000	0.6132
222197_s_at	16.2	32.4	1.00	2.00	AAA-AAA	0.00	999	1.000	1.000	0.2716
239502_at	54.1	27.1	-1.00	-2.00	APA-AAA	0.00	999	1.000	1.000	0.0999
1570628_at	7.8	3.9	-1.00	-2.00	AAA-AAA	0.00	999	1.000	1.000	0.3823

1552461_at	18.2	9.1	-1.00	-2.00	AAA-AAA	0.00	999	1.000	1.000	0.4555
1557888_at	7.5	15.0	1.00	2.00	AAA-AAA	0.00	999	1.000	1.000	0.3961
244522_at	3.8	7.6	1.00	2.00	AAA-AAA	0.00	999	1.000	1.000	0.3436
1560490_at	12.3	24.6	1.00	2.00	AAA-AAA	0.00	999	1.000	1.000	0.5775
211878_at	5.2	10.4	1.00	2.00	AAA-AAA	0.00	999	1.000	1.000	0.4664
210894_s_at	34.9	69.7	1.00	2.00	AAM-AAA	0.00	999	1.000	1.000	0.3857
233387_s_at	54.9	109.9	1.00	2.00	AAA-AAA	0.00	999	1.000	1.000	0.2999
227711_at	68.1	34.1	-1.00	-2.00	AAA-AAA	0.00	999	1.000	1.000	0.0704
232131_at	20.2	10.1	-1.00	-2.00	AAA-AAA	0.00	999	1.000	1.000	0.4684
229619_at	17.7	35.4	1.00	2.00	AAA-AAA	0.00	999	1.000	1.000	0.5359
242334_at	3.9	7.9	1.00	2.00	AAA-AAA	0.00	999	1.000	1.000	0.2026
240395_at	28.5	14.3	-1.00	-2.00	AAA-AAA	0.00	999	1.000	1.000	0.4287
1566701_at	33.1	66.0	1.00	2.00	AAA-AAA	0.00	999	1.000	1.000	0.2777
1560937_at	10.6	5.3	-1.00	-2.00	AAA-AAA	0.00	999	1.000	1.000	0.5266
215477_at	27.5	13.8	-1.00	-2.00	AAA-AAA	0.00	999	1.000	1.000	0.2455
216385_at	46.7	93.3	1.00	2.00	AAA-AAA	0.00	999	1.000	1.000	0.1473
216917_s_at	48.4	24.2	-1.00	-2.00	AAA-AAA	0.00	999	1.000	1.000	0.0515
211393_at	37.9	75.7	1.00	2.00	AAA-AAA	0.00	999	1.000	1.000	0.3860
235537_at	61.6	30.8	-1.00	-2.00	AAA-AAA	0.00	999	1.000	1.000	0.3155
232792_at	21.9	11.0	-1.00	-2.00	AAA-AAA	0.00	999	1.000	1.000	0.5656
235940_at	7.1	14.2	1.00	2.00	AAA-AAA	0.00	999	1.000	1.000	0.5684
237203_at	82.0	41.0	-1.00	-2.00	AAA-AAA	0.00	999	1.000	1.000	0.2884
241323_at	19.6	9.8	-1.00	-2.00	PAA-AAA	0.00	999	1.000	1.000	0.5996
241388_at	56.1	112.0	1.00	2.00	AAA-AAM	0.00	999	1.000	1.000	0.1385
1557898_at	5.6	11.2	1.00	2.00	AAA-AAA	0.00	999	1.000	1.000	0.3875
216672_s_at	9.3	18.5	1.00	2.00	AAA-AAA	0.00	999	1.000	1.000	0.3870
215117_at	4.7	9.4	1.00	2.00	AAA-AAA	0.00	999	1.000	1.000	0.5563
207299_s_at	7.7	3.8	-1.00	-2.00	AAA-AAA	0.00	999	1.000	1.000	0.1678
235373_at	41.3	20.7	-1.00	-2.00	AAA-AAA	0.00	999	1.000	1.000	0.4962
232539_at	44.3	22.2	-1.00	-2.00	AAA-AAA	0.00	999	1.000	1.000	0.4983
238178_at	7.6	3.8	-1.00	-2.00	AAA-AAA	0.00	999	1.000	1.000	0.6406
238351_x_at	17.6	8.8	-1.00	-2.00	AAA-AAA	0.00	999	1.000	1.000	0.3794
228484_s_at	16.1	32.2	1.00	2.00	AAA-AAA	0.00	999	1.000	1.000	0.5151
229019_at	134.1	67.1	-1.00	-2.00	MAA-AMA	0.00	999	1.000	1.000	0.3533
230117_at	15.8	7.9	-1.00	-2.00	AAA-AAA	0.00	999	1.000	1.000	0.4495
202628_s_at	1869.4	936.5	-1.00	-2.00	PPP-PPP	0.00	999	1.000	1.000	0.0372
216719_s_at	44.3	22.2	-1.00	-2.00	AMA-APA	0.00	999	1.000	1.000	0.3643
1570584_at	8.1	16.1	1.00	2.00	AAA-AAA	0.00	999	1.000	1.000	0.3691
1563899_at	12.6	6.3	-1.00	-2.00	AAA-AAA	0.00	999	1.000	1.000	0.5261
240970_x_at	15.9	31.7	1.00	2.00	AAA-AAA	0.00	999	1.000	1.000	0.4473
242715_at	11.9	6.0	-1.00	-2.00	AAA-AAA	0.00	999	1.000	1.000	0.1044
216061_x_at	67.2	134.1	1.00	2.00	AAA-AAA	0.00	999	1.000	1.000	0.0097

216410_at	10.7	5.4	-1.00	-2.00	AAA-AAA	0.00	999	1.000	1.000	0.5513
216390_at	16.8	8.4	-1.00	-2.00	AAA-AAA	0.00	999	1.000	1.000	0.0106
220512_at	36.3	18.2	-1.00	-2.00	AAA-AAA	0.00	999	1.000	1.000	0.5270
207706_at	10.2	5.1	-1.00	-2.00	AAA-AAA	0.00	999	1.000	1.000	0.5921
235487_at	21.1	42.1	1.00	2.00	AAA-AAA	0.00	999	1.000	1.000	0.4017
233644_at	24.2	12.1	-1.00	-2.00	AAA-AAA	0.00	999	1.000	1.000	0.3208
1563635_at	23.2	11.7	-1.00	-1.99	AAA-AAA	0.00	999	1.000	1.000	0.3006
1569732_at	85.6	42.9	-1.00	-2.00	AAA-AAA	0.00	999	1.000	1.000	0.2451
1553900_s_at	50.4	25.2	-1.00	-2.00	AAA-AAA	0.00	999	1.000	1.000	0.2606
1560963_a_at	5.3	10.7	1.00	1.99	AAA-AAA	0.00	999	1.000	1.000	0.3148
1562121_at	8.8	4.4	-1.00	-2.00	AAA-AAA	0.00	999	1.000	1.000	0.2920
1561516_at	7.0	13.9	1.00	2.00	AAA-AAA	0.00	999	1.000	1.000	0.4678
213543_at	15.3	7.7	-1.00	-2.00	AAA-AAA	0.00	999	1.000	1.000	0.4860
207838_x_at	13.5	26.8	1.00	2.00	AAA-AAA	0.00	999	1.000	1.000	0.4245
232990_at	46.7	93.2	1.00	2.00	AAA-AAA	0.00	999	1.000	1.000	0.0139
232754_at	16.6	33.1	1.00	2.00	AAA-AAA	0.00	999	1.000	1.000	0.3442
237280_at	6.2	12.3	1.00	1.99	AAA-AAA	0.00	999	1.000	1.000	0.0433
227644_at	10.5	20.9	1.00	1.99	AAA-AAA	0.00	999	1.000	1.000	0.2673
1565923_at	4.3	8.6	1.00	1.99	AAA-AAA	0.00	999	1.000	1.000	0.5499
243504_at	38.4	19.3	-1.00	-1.99	AAA-AAA	0.00	999	1.000	1.000	0.2867
216007_at	21.8	11.0	-1.00	-1.99	AAA-AAA	0.00	999	1.000	1.000	0.3723
213241_at	29.7	14.9	-1.00	-1.99	AAA-AAA	0.00	999	1.000	1.000	0.3269
207259_at	6.1	12.1	1.00	1.99	AAA-AAA	0.00	999	1.000	1.000	0.1657
206651_s_at	6.1	12.1	1.00	1.99	AAA-AAA	0.00	999	1.000	1.000	0.2446
235386_at	22.8	11.5	-1.00	-1.99	AAA-AAA	0.00	999	1.000	1.000	0.2928
237087_at	17.0	8.5	-1.00	-1.99	AAA-AAA	0.00	999	1.000	1.000	0.3125
232373_at	64.9	129.4	1.00	1.99	AAA-AAA	0.00	999	1.000	1.000	0.5352
231175_at	47.7	23.9	-0.99	-1.99	AMM-PAA	0.00	999	1.000	1.000	0.4230
219268_at	118.5	59.5	-0.99	-1.99	PAA-PMA	0.00	999	1.000	1.000	0.4886
1552590_a_at	22.4	44.6	0.99	1.99	AAA-AAA	0.00	999	1.000	1.000	0.3572
1555056_at	35.1	70.0	0.99	1.99	AAA-AAA	0.00	999	1.000	1.000	0.5957
214883_at	33.4	66.4	0.99	1.99	AAA-AAA	0.00	999	1.000	1.000	0.3325
211476_at	5.1	2.6	-0.99	-1.99	AAA-AAA	0.00	999	1.000	1.000	0.2504
234298_at	33.8	67.4	0.99	1.99	AAA-AAA	0.00	999	1.000	1.000	0.0075
233920_at	39.0	19.6	-0.99	-1.99	AAA-AAA	0.00	999	1.000	1.000	0.3795
238698_at	46.7	23.4	-0.99	-1.99	AAA-AAA	0.00	999	1.000	1.000	0.4288
229188_s_at	15.7	31.2	0.99	1.99	AAA-AAA	0.00	999	1.000	1.000	0.4983
233488_at	121.3	61.0	-0.99	-1.99	PPP-APA	0.00	999	1.000	1.000	0.0667
217128_s_at	115.6	58.1	-0.99	-1.99	AAP-AMA	0.00	999	1.000	1.000	0.2127
1564451_at	15.8	31.4	0.99	1.99	AAA-AAA	0.00	999	1.000	1.000	0.5690
241292_at	21.9	43.6	0.99	1.99	AAA-AAA	0.00	999	1.000	1.000	0.2992
1565748_at	19.6	39.0	0.99	1.99	AAA-AAA	0.00	999	1.000	1.000	0.1423

240710_at	13.1	6.6	-0.99	-1.99	AAA-AAA	0.00	999	1.000	1.000	0.2822
1558189_a_at	10.7	5.4	-0.99	-1.99	AAA-AAA	0.00	999	1.000	1.000	0.4680
212810_s_at	30.8	61.4	0.99	1.99	AAA-AAA	0.00	999	1.000	1.000	0.5423
220476_s_at	73.2	36.8	-0.99	-1.99	AAA-AAA	0.00	999	1.000	1.000	0.2675
207043_s_at	23.4	46.6	0.99	1.99	AAA-AAA	0.00	999	1.000	1.000	0.2717
206541_at	37.5	18.9	-0.99	-1.99	AAA-AAA	0.00	999	1.000	1.000	0.3234
237428_at	29.3	14.7	-0.99	-1.99	AAA-AAA	0.00	999	1.000	1.000	0.3176
232391_at	9.1	18.1	0.99	1.99	AAA-AAA	0.00	999	1.000	1.000	0.3144
1556730_at	32.4	16.3	-0.99	-1.99	PAA-AAP	0.00	999	1.000	1.000	0.5771
240517_at	29.0	14.6	-0.99	-1.99	AAA-AAA	0.00	999	1.000	1.000	0.4172
1556411_s_at	12.4	6.2	-0.99	-1.99	AAA-AAA	0.00	999	1.000	1.000	0.2125
243191_at	11.6	23.0	0.99	1.99	AAA-AAA	0.00	999	1.000	1.000	0.5142
243838_at	26.9	53.5	0.99	1.99	AAA-AAA	0.00	999	1.000	1.000	0.3701
1560347_at	8.5	17.0	0.99	1.99	AAA-AAA	0.00	999	1.000	1.000	0.4493
206719_at	21.2	42.2	0.99	1.99	AAA-AAA	0.00	999	1.000	1.000	0.2680
204787_at	23.4	11.8	-0.99	-1.99	AAA-AAA	0.00	999	1.000	1.000	0.2322
210121_at	8.0	4.0	-0.99	-1.99	AAA-AAA	0.00	999	1.000	1.000	0.5551
234421_s_at	19.7	39.1	0.99	1.99	AAA-AAA	0.00	999	1.000	1.000	0.3421
234569_at	5.9	11.6	0.99	1.99	AAA-AAA	0.00	999	1.000	1.000	0.2867
224505_s_at	44.8	22.5	-0.99	-1.99	AAA-AAA	0.00	999	1.000	1.000	0.3882
228269_x_at	135.4	68.1	-0.99	-1.99	AAA-AAA	0.00	999	1.000	1.000	0.1037
228055_at	17.3	34.5	0.99	1.99	AAA-AAA	0.00	999	1.000	1.000	0.4309
231653_at	21.9	43.5	0.99	1.99	AAA-AAA	0.00	999	1.000	1.000	0.4101
229499_at	30.2	15.2	-0.99	-1.99	AAA-AAA	0.00	999	1.000	1.000	0.1469
230586_s_at	10.4	5.2	-0.99	-1.99	AAA-AAA	0.00	999	1.000	1.000	0.4532
211506_s_at	232.3	116.9	-0.99	-1.99	PPP-PAP	0.00	999	1.000	1.000	0.2386
233722_at	27.2	13.7	-0.99	-1.99	PAA-AAA	0.00	999	1.000	1.000	0.4198
244775_at	17.5	8.8	-0.99	-1.99	PAA-AAA	0.00	999	1.000	1.000	0.4638
241513_at	7.1	14.2	0.99	1.99	AAA-AAA	0.00	999	1.000	1.000	0.2473
239319_at	11.6	5.9	-0.99	-1.99	AAA-AAA	0.00	999	1.000	1.000	0.4662
1560091_a_at	7.7	15.3	0.99	1.99	AAA-AAA	0.00	999	1.000	1.000	0.4885
1561084_at	4.9	9.8	0.99	1.99	AAA-AAA	0.00	999	1.000	1.000	0.3902
215913_s_at	11.1	22.0	0.99	1.99	AAA-AAA	0.00	999	1.000	1.000	0.5580
238505_at	33.5	66.5	0.99	1.99	AAA-AAA	0.00	999	1.000	1.000	0.3735
224384_s_at	22.4	44.4	0.99	1.99	AAA-AAA	0.00	999	1.000	1.000	0.3658
1567304_at	10.3	5.2	-0.99	-1.99	AAA-AAA	0.00	999	1.000	1.000	0.1464
244887_at	3.5	7.0	0.99	1.99	AAA-AAA	0.00	999	1.000	1.000	0.2948
1554131_at	5.9	3.0	-0.99	-1.99	AAA-AAA	0.00	999	1.000	1.000	0.6672
1555120_at	14.4	28.5	0.99	1.99	AAA-AAA	0.00	999	1.000	1.000	0.4959
220469_at	49.6	25.0	-0.99	-1.99	AAA-AAA	0.00	999	1.000	1.000	0.4384
210690_at	8.4	16.7	0.99	1.99	AAA-AAA	0.00	999	1.000	1.000	0.5962
207959_s_at	9.4	18.7	0.99	1.99	AAA-AAA	0.00	999	1.000	1.000	0.4144

234337_at	13.8	27.4	0.99	1.99	AAA-AAA	0.00	999	1.000	1.000	0.4057
235225_at	12.2	24.3	0.99	1.99	AAA-AAA	0.00	999	1.000	1.000	0.5091
222743_s_at	127.6	64.3	-0.99	-1.99	AAA-AAA	0.00	999	1.000	1.000	0.1232
224275_at	3.8	7.5	0.99	1.99	AAA-AAA	0.00	999	1.000	1.000	0.4204
239344_at	22.8	45.3	0.99	1.99	AAA-AAA	0.00	999	1.000	1.000	0.4019
240883_at	5.2	10.3	0.99	1.99	AAA-AAA	0.00	999	1.000	1.000	0.2034
219533_at	3.6	7.2	0.99	1.99	AAA-AAA	0.00	999	1.000	1.000	0.4983
234773_x_at	46.6	23.5	-0.99	-1.99	AAA-AAA	0.00	999	1.000	1.000	0.4744
237214_at	15.0	29.8	0.99	1.99	AAA-AAA	0.00	999	1.000	1.000	0.2616
227781_x_at	26.8	13.5	-0.99	-1.99	AAA-AAA	0.00	999	1.000	1.000	0.5103
1563963_at	50.0	25.2	-0.99	-1.98	AAA-AAA	0.00	999	1.000	1.000	0.0580
1566906_at	11.2	22.2	0.99	1.98	AAA-AAA	0.00	999	1.000	1.000	0.2638
1557289_s_at	10.4	20.6	0.99	1.98	AAA-AAA	0.00	999	1.000	1.000	0.3122
1556769_a_at	41.3	82.0	0.99	1.98	AAA-AAA	0.00	999	1.000	1.000	0.3786
215962_at	6.1	3.1	-0.99	-1.98	AAA-AAA	0.00	999	1.000	1.000	0.5869
220260_at	165.6	83.5	-0.99	-1.98	AAM-AAA	0.00	999	1.000	1.000	0.0813
211363_s_at	47.6	24.0	-0.99	-1.98	AAA-AAA	0.00	999	1.000	1.000	0.2305
208115_x_at	101.4	51.1	-0.99	-1.98	AAA-AAA	0.00	999	1.000	1.000	0.1559
236806_at	59.2	117.4	0.99	1.98	AAA-AAA	0.00	999	1.000	1.000	0.0852
226804_at	6.3	12.5	0.99	1.98	AAA-AAA	0.00	999	1.000	1.000	0.1483
228617_at	26.6	52.7	0.99	1.98	AAA-AAA	0.00	999	1.000	1.000	0.4133
231739_at	5.8	11.5	0.99	1.98	AAA-AAA	0.00	999	1.000	1.000	0.1957
229623_at	24.1	12.1	-0.99	-1.98	AAA-AAA	0.00	999	1.000	1.000	0.4749
229136_s_at	75.4	38.0	-0.99	-1.98	AAA-AAA	0.00	999	1.000	1.000	0.2970
243116_at	55.3	27.9	-0.99	-1.98	AAA-AAP	0.00	999	1.000	1.000	0.4199
242262_x_at	4.9	9.6	0.99	1.98	AAA-AAA	0.00	999	1.000	1.000	0.4614
1570030_at	12.0	23.7	0.99	1.98	AAA-AAA	0.00	999	1.000	1.000	0.5949
240897_at	22.5	11.4	-0.99	-1.98	AAA-AAA	0.00	999	1.000	1.000	0.2984
1554062_at	22.6	11.4	-0.99	-1.98	AAA-AAA	0.00	999	1.000	1.000	0.4407
242780_at	56.9	112.8	0.99	1.98	AAA-AAA	0.00	999	1.000	1.000	0.1026
1560773_at	14.3	28.2	0.99	1.98	AAA-AAA	0.00	999	1.000	1.000	0.4478
215172_at	8.0	15.9	0.99	1.98	AAA-AAA	0.00	999	1.000	1.000	0.5307
211876_x_at	65.5	33.0	-0.99	-1.98	AAA-AAA	0.00	999	1.000	1.000	0.0320
220911_s_at	8.0	15.8	0.99	1.98	AAA-AAA	0.00	999	1.000	1.000	0.3398
201792_at	36.0	18.2	-0.99	-1.98	AAA-AAA	0.00	999	1.000	1.000	0.3390
202291_s_at	6.5	12.8	0.99	1.98	AAA-AAA	0.00	999	1.000	1.000	0.0441
207640_x_at	28.6	14.4	-0.99	-1.98	AAA-AAA	0.00	999	1.000	1.000	0.5096
233949_s_at	18.2	36.1	0.99	1.98	AAA-AAA	0.00	999	1.000	1.000	0.3855
222863_at	106.5	53.8	-0.99	-1.98	AAA-AAA	0.00	999	1.000	1.000	0.0095
223280_x_at	12.2	24.1	0.99	1.98	AAA-AAA	0.00	999	1.000	1.000	0.1690
223820_at	18.2	36.1	0.99	1.98	AAA-AAA	0.00	999	1.000	1.000	0.2058
231811_at	50.6	25.5	-0.99	-1.98	AAA-AAA	0.00	999	1.000	1.000	0.3912

233453_at	51.5	26.0	-0.99	-1.98	AAA-APA	0.00	999	1.000	1.000	0.5631
1570200_at	19.0	37.7	0.99	1.98	AAA-AAA	0.00	999	1.000	1.000	0.3847
1562306_at	33.6	16.9	-0.99	-1.98	AAA-AAA	0.00	999	1.000	1.000	0.4667
234751_s_at	12.1	24.0	0.99	1.98	AAA-AAA	0.00	999	1.000	1.000	0.3428
236484_at	47.5	24.0	-0.99	-1.98	AAA-AAA	0.00	999	1.000	1.000	0.2579
1559000_at	77.2	39.0	-0.99	-1.98	AAP-AAP	0.00	999	1.000	1.000	0.1963
228940_at	47.8	24.2	-0.99	-1.98	PAA-PAA	0.00	999	1.000	1.000	0.4056
1565825_at	17.9	35.4	0.99	1.98	AAA-AAA	0.00	999	1.000	1.000	0.3804
1558210_at	16.7	8.4	-0.99	-1.98	AAA-AAA	0.00	999	1.000	1.000	0.4068
243131_x_at	69.3	137.1	0.99	1.98	AAA-AAA	0.00	999	1.000	1.000	0.4204
244566_at	20.6	40.8	0.99	1.98	AAA-AAA	0.00	999	1.000	1.000	0.4794
214597_at	9.8	19.3	0.99	1.98	AAA-AAA	0.00	999	1.000	1.000	0.4909
207930_at	26.1	51.6	0.99	1.98	AAA-AAA	0.00	999	1.000	1.000	0.3844
228586_at	14.3	7.2	-0.99	-1.98	AAA-AAA	0.00	999	1.000	1.000	0.3127
232208_at	29.5	58.5	0.99	1.98	AAA-AAA	0.00	999	1.000	1.000	0.4136
230438_at	72.9	36.8	-0.99	-1.98	AAA-AAA	0.00	999	1.000	1.000	0.2437
200982_s_at	75.3	38.1	-0.98	-1.98	MPA-AAA	0.00	999	1.000	1.000	0.4205
1564881_at	6.4	12.7	0.98	1.98	AAA-AAA	0.00	999	1.000	1.000	0.2036
1552910_at	18.9	37.3	0.98	1.98	AAA-AAA	0.00	999	1.000	1.000	0.3811
1552280_at	7.4	14.6	0.98	1.98	AAA-AAA	0.00	999	1.000	1.000	0.5340
1561256_at	13.8	7.0	-0.98	-1.98	AAA-AAA	0.00	999	1.000	1.000	0.2517
243575_at	14.0	27.7	0.98	1.98	AAA-AAA	0.00	999	1.000	1.000	0.3599
221093_at	16.7	8.5	-0.98	-1.98	AAA-AAA	0.00	999	1.000	1.000	0.4158
206083_at	13.0	25.7	0.98	1.98	AAA-AAA	0.00	999	1.000	1.000	0.1559
202409_at	25.7	50.8	0.98	1.98	AAA-AAA	0.00	999	1.000	1.000	0.0870
204487_s_at	13.7	27.1	0.98	1.98	AAA-AAA	0.00	999	1.000	1.000	0.4415
209769_s_at	20.1	39.7	0.98	1.98	AAA-AAA	0.00	999	1.000	1.000	0.3617
234811_at	24.2	47.9	0.98	1.98	AAA-AAA	0.00	999	1.000	1.000	0.0578
237067_at	46.7	23.6	-0.98	-1.98	AAA-AAA	0.00	999	1.000	1.000	0.2054
222278_at	12.9	25.6	0.98	1.98	AAA-AAA	0.00	999	1.000	1.000	0.1848
242827_x_at	80.2	40.6	-0.98	-1.98	AAA-AAP	0.00	999	1.000	1.000	0.4502
219788_at	47.1	23.9	-0.98	-1.98	PAA-PAA	0.00	999	1.000	1.000	0.4951
1568914_at	36.6	18.5	-0.98	-1.98	AAA-AAA	0.00	999	1.000	1.000	0.3517
1566222_at	16.4	8.3	-0.98	-1.98	AAA-AAA	0.00	999	1.000	1.000	0.5555
1567036_at	10.2	20.2	0.98	1.98	AAA-AAA	0.00	999	1.000	1.000	0.4564
1557079_at	3.5	1.8	-0.98	-1.98	AAA-AAA	0.00	999	1.000	1.000	0.4700
244735_at	9.0	17.7	0.98	1.98	AAA-AAA	0.00	999	1.000	1.000	0.5681
1554942_a_at	66.5	131.4	0.98	1.98	AAA-AAA	0.00	999	1.000	1.000	0.0924
1561593_at	4.1	8.1	0.98	1.98	AAA-AAA	0.00	999	1.000	1.000	0.3593
1559134_a_at	6.4	12.6	0.98	1.98	AAA-AAA	0.00	999	1.000	1.000	0.5307
216747_at	34.7	17.6	-0.98	-1.98	AAA-AAA	0.00	999	1.000	1.000	0.4266
220903_at	26.1	13.2	-0.98	-1.98	AAA-AAA	0.00	999	1.000	1.000	0.2274

219647_at	18.9	9.6	-0.98	-1.98	AAA-AAA	0.00	999	1.000	1.000	0.4915
211712_s_at	21.9	43.3	0.98	1.98	AAA-AAA	0.00	999	1.000	1.000	0.5008
234361_at	24.7	12.5	-0.98	-1.98	AAA-AAA	0.00	999	1.000	1.000	0.3674
235239_at	49.4	25.0	-0.98	-1.98	AAA-AAA	0.00	999	1.000	1.000	0.1362
225979_at	122.6	62.1	-0.98	-1.98	MPP-AAA	0.00	999	1.000	1.000	0.1240
216791_at	132.8	67.2	-0.98	-1.98	APP-AAP	0.00	999	1.000	1.000	0.2208
207678_s_at	17.2	8.7	-0.98	-1.98	AAA-AAP	0.00	999	1.000	1.000	0.5541
1557366_at	21.6	10.9	-0.98	-1.98	AAA-AAA	0.00	999	1.000	1.000	0.1288
244721_at	14.3	28.1	0.98	1.98	AAA-AAA	0.00	999	1.000	1.000	0.1962
1555338_s_at	28.9	14.6	-0.98	-1.98	AAA-AAA	0.00	999	1.000	1.000	0.5592
1555173_at	9.8	19.4	0.98	1.98	AAA-AAA	0.00	999	1.000	1.000	0.4435
1555002_at	7.0	3.5	-0.98	-1.98	AAA-AAA	0.00	999	1.000	1.000	0.4940
244266_at	8.9	4.5	-0.98	-1.98	AAA-AAA	0.00	999	1.000	1.000	0.2149
220118_at	13.0	25.7	0.98	1.98	AAA-AAA	0.00	999	1.000	1.000	0.1896
219867_at	22.9	45.2	0.98	1.98	AAA-AAA	0.00	999	1.000	1.000	0.4340
210626_at	14.2	28.0	0.98	1.98	AAA-AAA	0.00	999	1.000	1.000	0.3385
210954_s_at	20.7	10.5	-0.98	-1.98	AAA-AAA	0.00	999	1.000	1.000	0.3568
232738_at	6.1	3.1	-0.98	-1.98	AAA-AAA	0.00	999	1.000	1.000	0.3777
227339_at	29.4	58.0	0.98	1.98	AAA-AAA	0.00	999	1.000	1.000	0.5280
224236_s_at	10.8	5.5	-0.98	-1.98	AAA-AAA	0.00	999	1.000	1.000	0.2680
230675_at	9.8	19.4	0.98	1.98	AAA-AAA	0.00	999	1.000	1.000	0.2289
229985_at	25.2	12.8	-0.98	-1.98	AAA-AAA	0.00	999	1.000	1.000	0.4806
1557948_at	141.5	71.7	-0.98	-1.97	MMP-APA	0.00	999	1.000	1.000	0.2375
241664_x_at	37.7	19.1	-0.98	-1.97	AAA-APA	0.00	999	1.000	1.000	0.3382
244505_at	16.1	8.2	-0.98	-1.97	APA-AAA	0.00	999	1.000	1.000	0.6379
240825_at	49.2	25.0	-0.98	-1.97	AAA-AAA	0.00	999	1.000	1.000	0.2338
240692_at	25.5	50.3	0.98	1.97	AAA-AAA	0.00	999	1.000	1.000	0.3466
1557021_s_at	11.1	5.6	-0.98	-1.97	AAA-AAA	0.00	999	1.000	1.000	0.5435
244432_at	10.4	20.6	0.98	1.97	AAA-AAA	0.00	999	1.000	1.000	0.4845
1559154_at	16.1	31.8	0.98	1.97	AAA-AAA	0.00	999	1.000	1.000	0.4704
243794_at	26.0	51.3	0.98	1.97	AMA-AAA	0.00	999	1.000	1.000	0.0240
216328_at	21.1	10.7	-0.98	-1.97	AAA-AAA	0.00	999	1.000	1.000	0.4931
207202_s_at	13.0	6.6	-0.98	-1.97	AAA-AAA	0.00	999	1.000	1.000	0.1650
210712_at	10.9	21.6	0.98	1.97	AAA-AAA	0.00	999	1.000	1.000	0.4337
207539_s_at	12.7	6.5	-0.98	-1.97	AAA-AAA	0.00	999	1.000	1.000	0.1685
209291_at	27.6	14.0	-0.98	-1.97	AAA-AAA	0.00	999	1.000	1.000	0.1472
234058_at	30.6	15.5	-0.98	-1.97	AAA-AAA	0.00	999	1.000	1.000	0.3990
223702_x_at	21.7	42.9	0.98	1.97	AAA-AAA	0.00	999	1.000	1.000	0.3908
232257_s_at	3.4	6.6	0.98	1.97	AAA-AAA	0.00	999	1.000	1.000	0.4346
1557518_a_at	24.1	47.6	0.98	1.97	AAA-AAA	0.00	999	1.000	1.000	0.4411
1556175_at	10.2	5.2	-0.98	-1.97	AAA-AAA	0.00	999	1.000	1.000	0.4005
243122_at	30.8	15.6	-0.98	-1.97	AAA-AAA	0.00	999	1.000	1.000	0.4751

244089_at	18.7	9.5	-0.98	-1.97	AAA-AAA	0.00	999	1.000	1.000	0.3763
221262_s_at	43.8	22.2	-0.98	-1.97	AAA-AAA	0.00	999	1.000	1.000	0.3954
218475_at	129.0	65.4	-0.98	-1.97	AAA-AAA	0.00	999	1.000	1.000	0.4820
210789_x_at	74.5	147.0	0.98	1.97	AAA-AAA	0.00	999	1.000	1.000	0.0282
234319_at	10.1	20.0	0.98	1.97	AAA-AAA	0.00	999	1.000	1.000	0.4566
237461_at	21.7	11.0	-0.98	-1.97	AAA-AAA	0.00	999	1.000	1.000	0.5883
1570238_at	87.5	44.4	-0.98	-1.97	APA-AAA	0.00	999	1.000	1.000	0.2865
231661_at	41.1	20.8	-0.98	-1.97	AAA-PAA	0.00	999	1.000	1.000	0.4933
240414_at	33.6	17.1	-0.98	-1.97	AAA-AAA	0.00	999	1.000	1.000	0.3078
1556571_at	19.9	39.1	0.98	1.97	AAA-AAA	0.00	999	1.000	1.000	0.2710
1556145_a_at	10.5	5.3	-0.98	-1.97	AAA-AAA	0.00	999	1.000	1.000	0.4059
243034_at	10.6	20.8	0.98	1.97	AAA-AAA	0.00	999	1.000	1.000	0.4466
242856_at	9.5	18.7	0.98	1.97	AAA-AAA	0.00	999	1.000	1.000	0.2984
219601_s_at	28.7	14.6	-0.98	-1.97	AAA-AAA	0.00	999	1.000	1.000	0.4313
206143_at	7.9	4.0	-0.98	-1.97	AAA-AAA	0.00	999	1.000	1.000	0.3388
205703_at	26.1	13.2	-0.98	-1.97	AAA-AAA	0.00	999	1.000	1.000	0.6200
204475_at	14.8	7.5	-0.98	-1.97	AAA-AAA	0.00	999	1.000	1.000	0.5949
208224_at	38.1	19.3	-0.98	-1.97	AAA-AAA	0.00	999	1.000	1.000	0.3478
235734_at	18.8	37.1	0.98	1.97	AAA-AAA	0.00	999	1.000	1.000	0.3538
233367_at	18.6	9.4	-0.98	-1.97	AAA-AAA	0.00	999	1.000	1.000	0.4896
225911_at	11.5	22.7	0.98	1.97	AAA-AAA	0.00	999	1.000	1.000	0.4785
222952_s_at	45.1	22.9	-0.98	-1.97	AAA-AAA	0.00	999	1.000	1.000	0.4167
223933_at	39.5	77.8	0.98	1.97	AAA-AAA	0.00	999	1.000	1.000	0.3135
1569067_at	36.4	71.7	0.98	1.97	AAA-AAA	0.00	999	1.000	1.000	0.3172
1554324_s_at	55.5	28.1	-0.98	-1.97	AAA-AAA	0.00	999	1.000	1.000	0.2782
1555746_at	50.9	25.8	-0.98	-1.97	AAA-AAA	0.00	999	1.000	1.000	0.2791
1553064_at	4.9	9.6	0.98	1.97	AAA-AAA	0.00	999	1.000	1.000	0.2242
220101_x_at	28.8	14.6	-0.98	-1.97	AAA-AAA	0.00	999	1.000	1.000	0.5747
206398_s_at	24.0	12.2	-0.98	-1.97	AAA-AAA	0.00	999	1.000	1.000	0.4437
206528_at	10.3	5.3	-0.98	-1.97	AAA-AAA	0.00	999	1.000	1.000	0.2656
210016_at	10.1	20.0	0.98	1.97	AAA-AAA	0.00	999	1.000	1.000	0.4699
234285_at	18.2	35.8	0.98	1.97	AAA-AAA	0.00	999	1.000	1.000	0.1105
224214_at	10.2	5.2	-0.98	-1.97	AAA-AAA	0.00	999	1.000	1.000	0.2241
210662_at	77.9	39.6	-0.98	-1.97	PPP-APA	0.00	999	1.000	1.000	0.0657
241377_s_at	19.4	9.9	-0.98	-1.97	AAA-AAA	0.00	999	1.000	1.000	0.4503
1553066_at	17.7	9.0	-0.98	-1.97	AAA-AAA	0.00	999	1.000	1.000	0.2883
213673_x_at	16.9	33.2	0.98	1.97	AAA-AAA	0.00	999	1.000	1.000	0.4030
207910_at	10.4	5.3	-0.98	-1.97	AAA-AAA	0.00	999	1.000	1.000	0.6093
208373_s_at	28.3	14.4	-0.98	-1.97	AAA-AAA	0.00	999	1.000	1.000	0.4967
235135_at	116.7	59.3	-0.98	-1.97	AAA-AAA	0.00	999	1.000	1.000	0.3593
234724_x_at	15.7	8.0	-0.98	-1.97	AAA-AAA	0.00	999	1.000	1.000	0.1024
233282_at	23.7	12.0	-0.98	-1.97	AAA-AAA	0.00	999	1.000	1.000	0.5333

237677_at	10.3	20.3	0.98	1.97	AAA-AAA	0.00	999	1.000	1.000	0.2466
222097_at	82.6	42.0	-0.98	-1.97	AAA-MAA	0.00	999	1.000	1.000	0.4490
230966_at	32.6	16.5	-0.98	-1.97	AAA-AAA	0.00	999	1.000	1.000	0.0108
242219_at	37.6	19.1	-0.98	-1.97	AAA-AAA	0.00	999	1.000	1.000	0.1838
1562817_at	14.0	27.6	0.98	1.97	AAA-AAA	0.00	999	1.000	1.000	0.4135
239959_x_at	180.3	91.6	-0.98	-1.97	MAA-AAA	0.00	999	1.000	1.000	0.3176
220920_at	32.1	16.3	-0.98	-1.97	AAA-AAA	0.00	999	1.000	1.000	0.5520
218825_at	90.8	46.2	-0.98	-1.97	AAA-AAA	0.00	999	1.000	1.000	0.3281
217710_x_at	61.4	31.2	-0.98	-1.97	AAA-AAA	0.00	999	1.000	1.000	0.3860
206007_at	7.2	14.1	0.98	1.97	AAA-AAA	0.00	999	1.000	1.000	0.4648
235932_x_at	3.7	7.2	0.98	1.97	AAA-AAA	0.00	999	1.000	1.000	0.0905
230731_x_at	31.0	60.9	0.98	1.97	AAA-AAA	0.00	999	1.000	1.000	0.2438
240072_at	53.5	27.2	-0.98	-1.97	PAA-AAA	0.00	999	1.000	1.000	0.0375
220951_s_at	85.9	43.7	-0.98	-1.97	AAA-PAA	0.00	999	1.000	1.000	0.4978
242520_s_at	5.4	10.5	0.98	1.97	AAA-AAA	0.00	999	1.000	1.000	0.3939
1569716_at	12.0	23.5	0.98	1.97	AAA-AAA	0.00	999	1.000	1.000	0.4684
1569849_at	19.4	38.1	0.98	1.97	AAA-AAA	0.00	999	1.000	1.000	0.3997
239199_at	18.7	36.7	0.98	1.97	AAA-AAA	0.00	999	1.000	1.000	0.4134
1554872_a_at	19.4	9.9	-0.98	-1.97	AAA-AAA	0.00	999	1.000	1.000	0.2670
214537_at	113.2	57.6	-0.98	-1.97	AAA-AAA	0.00	999	1.000	1.000	0.1967
220664_at	7.4	14.5	0.98	1.97	AAA-AAA	0.00	999	1.000	1.000	0.5224
208385_at	5.8	11.4	0.98	1.97	AAA-AAA	0.00	999	1.000	1.000	0.2022
234013_at	16.6	32.7	0.98	1.97	AAA-AAA	0.00	999	1.000	1.000	0.2638
238360_s_at	15.1	29.7	0.98	1.97	AAA-AAA	0.00	999	1.000	1.000	0.4129
222677_x_at	7.9	15.5	0.98	1.97	AAA-AAA	0.00	999	1.000	1.000	0.0572
224088_at	135.4	68.9	-0.98	-1.97	AAA-AAA	0.00	999	1.000	1.000	0.1324
231244_at	11.2	22.0	0.98	1.97	AAA-AAA	0.00	999	1.000	1.000	0.2634
1569741_at	54.6	27.8	-0.97	-1.96	PAA-AAA	0.00	999	1.000	1.000	0.2102
241629_at	31.0	15.8	-0.97	-1.97	APA-AAA	0.00	999	1.000	1.000	0.5249
235915_at	13.4	6.8	-0.97	-1.97	APA-AAA	0.00	999	1.000	1.000	0.5428
1569325_at	59.0	30.0	-0.97	-1.96	AAA-AAA	0.00	999	1.000	1.000	0.3509
1556484_at	52.2	102.4	0.97	1.96	AAA-AAA	0.00	999	1.000	1.000	0.3724
1558414_at	61.6	31.4	-0.97	-1.97	AAA-AAA	0.00	999	1.000	1.000	0.3474
242757_at	11.3	22.2	0.97	1.97	AAA-AAA	0.00	999	1.000	1.000	0.1710
1561619_at	46.1	90.5	0.97	1.97	AAA-AAA	0.00	999	1.000	1.000	0.4149
220628_s_at	13.4	6.8	-0.97	-1.97	AAA-AAA	0.00	999	1.000	1.000	0.0471
209793_at	6.3	12.3	0.97	1.97	AAA-AAA	0.00	999	1.000	1.000	0.4756
207733_x_at	87.5	44.5	-0.97	-1.97	AAA-AAA	0.00	999	1.000	1.000	0.3901
207673_at	11.5	22.5	0.97	1.97	AAA-AAA	0.00	999	1.000	1.000	0.2038
228384_s_at	8.5	16.8	0.97	1.97	AAA-AAA	0.00	999	1.000	1.000	0.2798
221959_at	69.6	35.4	-0.97	-1.97	AAA-AAA	0.00	999	1.000	1.000	0.2470
1555687_a_at	23.7	12.1	-0.97	-1.96	AAP-AAA	0.00	999	1.000	1.000	0.4285

1566873_at	14.3	7.3	-0.97	-1.96	AAA-AAA	0.00	999	1.000	1.000	0.3304
215328_at	26.5	13.5	-0.97	-1.96	AAA-AAA	0.00	999	1.000	1.000	0.3810
216320_x_at	108.5	213.1	0.97	1.96	AAA-AAA	0.00	999	1.000	1.000	0.0294
213075_at	32.3	16.5	-0.97	-1.96	AAA-AAA	0.00	999	1.000	1.000	0.3823
219873_at	24.8	12.6	-0.97	-1.96	AAA-AAA	0.00	999	1.000	1.000	0.5163
208364_at	9.5	18.6	0.97	1.96	AAA-AAA	0.00	999	1.000	1.000	0.3174
234550_at	13.4	6.8	-0.97	-1.96	AAA-AAA	0.00	999	1.000	1.000	0.6959
238690_at	18.9	37.1	0.97	1.96	AAA-AAA	0.00	999	1.000	1.000	0.2072
222904_s_at	52.5	26.8	-0.97	-1.96	APP-AAA	0.00	999	1.000	1.000	0.0400
241512_at	68.7	134.7	0.97	1.96	AAA-AAA	0.00	999	1.000	1.000	0.4123
240077_at	22.0	43.1	0.97	1.96	AAA-AAA	0.00	999	1.000	1.000	0.5093
1552592_at	7.5	14.7	0.97	1.96	AAA-AAA	0.00	999	1.000	1.000	0.3294
1558666_at	31.6	62.0	0.97	1.96	AAA-AAA	0.00	999	1.000	1.000	0.2831
1554790_at	65.2	33.3	-0.97	-1.96	AAA-MAA	0.00	999	1.000	1.000	0.3446
1555410_at	37.5	73.5	0.97	1.96	MAA-AAA	0.00	999	1.000	1.000	0.2881
1561330_at	6.3	12.4	0.97	1.96	AAA-AAA	0.00	999	1.000	1.000	0.2870
243438_at	14.9	29.3	0.97	1.96	AAA-AAA	0.00	999	1.000	1.000	0.4186
214043_at	12.6	6.5	-0.97	-1.96	AAA-AAA	0.00	999	1.000	1.000	0.2510
220578_at	26.6	52.2	0.97	1.96	AAA-AAA	0.00	999	1.000	1.000	0.4435
210545_at	39.9	20.4	-0.97	-1.96	AAA-AAA	0.00	999	1.000	1.000	0.0686
234935_at	24.6	12.6	-0.97	-1.96	AAA-AAA	0.00	999	1.000	1.000	0.2988
238770_at	6.8	13.4	0.97	1.96	AAA-AAA	0.00	999	1.000	1.000	0.2576
236061_at	26.1	51.3	0.97	1.96	AAA-AAA	0.00	999	1.000	1.000	0.2595
224169_at	14.1	7.2	-0.97	-1.96	AAA-AAA	0.00	999	1.000	1.000	0.2919
238626_at	42.4	21.6	-0.97	-1.96	PPA-AAA	0.00	999	1.000	1.000	0.3519
244298_at	51.3	26.2	-0.97	-1.96	AAA-AAP	0.00	999	1.000	1.000	0.3950
1552303_a_at	59.6	30.4	-0.97	-1.96	AAA-AAA	0.00	999	1.000	1.000	0.2739
242782_x_at	31.1	61.0	0.97	1.96	AAA-AAA	0.00	999	1.000	1.000	0.1988
211869_at	10.9	5.6	-0.97	-1.96	AAA-AAA	0.00	999	1.000	1.000	0.0041
207433_at	11.1	21.7	0.97	1.96	AAA-AAA	0.00	999	1.000	1.000	0.4393
234300_s_at	17.1	8.7	-0.97	-1.96	AAA-AAA	0.00	999	1.000	1.000	0.4295
238727_at	15.0	29.4	0.97	1.96	AAA-AAA	0.00	999	1.000	1.000	0.0797
238721_at	19.7	10.0	-0.97	-1.96	AAA-AAA	0.00	999	1.000	1.000	0.5412
236415_at	14.5	7.4	-0.97	-1.96	AAA-AAA	0.00	999	1.000	1.000	0.3058
222374_at	6.9	13.6	0.97	1.96	AAA-AAA	0.00	999	1.000	1.000	0.3048
1566715_at	39.6	20.2	-0.97	-1.96	APA-AAA	0.00	999	1.000	1.000	0.3033
1564190_x_at	67.0	131.3	0.97	1.96	AAA-AAA	0.00	999	1.000	1.000	0.0356
239286_at	12.8	25.2	0.97	1.96	AAA-AAA	0.00	999	1.000	1.000	0.5315
1554541_a_at	13.1	25.7	0.97	1.96	AAA-AAA	0.00	999	1.000	1.000	0.4815
1556125_at	27.2	53.2	0.97	1.96	AAM-AMA	0.00	999	1.000	1.000	0.0665
1561566_at	11.2	21.9	0.97	1.96	AAA-AAA	0.00	999	1.000	1.000	0.5563
1559459_at	13.4	6.9	-0.97	-1.96	AAA-AAA	0.00	999	1.000	1.000	0.2206

1559842_at	2.2	4.3	0.97	1.96	AAA-AAA	0.00	999	1.000	1.000	0.3972
244694_at	11.2	5.7	-0.97	-1.96	AAA-AAA	0.00	999	1.000	1.000	0.0708
202497_x_at	18.5	36.2	0.97	1.96	AAA-AAA	0.00	999	1.000	1.000	0.4471
209962_at	84.8	43.3	-0.97	-1.96	AAA-AAA	0.00	999	1.000	1.000	0.0146
237835_at	51.7	26.4	-0.97	-1.96	AAA-AAA	0.00	999	1.000	1.000	0.2856
236929_at	6.8	13.2	0.97	1.96	AAA-AAA	0.00	999	1.000	1.000	0.0768
229125_at	23.4	45.8	0.97	1.96	AAA-AAA	0.00	999	1.000	1.000	0.2892
230632_at	7.0	13.7	0.97	1.96	AAA-AAA	0.00	999	1.000	1.000	0.4148
230428_at	51.5	100.8	0.97	1.96	AAA-AAA	0.00	999	1.000	1.000	0.3228
236679_x_at	163.9	83.8	-0.97	-1.96	PAA-APA	0.00	999	1.000	1.000	0.1491
1566605_at	22.1	11.3	-0.97	-1.96	PAA-AMA	0.00	999	1.000	1.000	0.5235
223655_at	58.9	30.1	-0.97	-1.96	APA-AAA	0.00	999	1.000	1.000	0.5817
1562893_at	16.6	8.5	-0.97	-1.96	AAA-AAA	0.00	999	1.000	1.000	0.5731
1569234_at	17.1	8.8	-0.97	-1.96	AAA-AAA	0.00	999	1.000	1.000	0.4685
240944_at	21.6	11.1	-0.97	-1.96	AAA-AAA	0.00	999	1.000	1.000	0.2889
1567359_at	5.1	2.6	-0.97	-1.96	AAA-AAA	0.00	999	1.000	1.000	0.3856
1557517_a_at	28.4	14.5	-0.97	-1.96	AAA-AAA	0.00	999	1.000	1.000	0.0593
1561928_s_at	120.6	61.6	-0.97	-1.96	AAA-AAA	0.00	999	1.000	1.000	0.1859
213537_at	7.7	15.0	0.97	1.96	AAA-AAA	0.00	999	1.000	1.000	0.4887
227958_s_at	6.5	12.6	0.97	1.96	AAA-AAA	0.00	999	1.000	1.000	0.5051
231542_at	20.3	39.7	0.97	1.96	AAA-AAA	0.00	999	1.000	1.000	0.3398
229102_at	30.5	59.7	0.97	1.96	AAA-AAA	0.00	999	1.000	1.000	0.1768
228929_at	52.9	103.6	0.97	1.96	AAA-AAA	0.00	999	1.000	1.000	0.4305
209051_s_at	198.7	101.6	-0.97	-1.96	AAP-AAP	0.00	999	1.000	1.000	0.5427
241013_at	8.2	16.1	0.97	1.96	AAA-AAA	0.00	999	1.000	1.000	0.1116
243232_at	6.9	3.5	-0.97	-1.96	AAA-AAA	0.00	999	1.000	1.000	0.4415
1559492_at	1.6	3.0	0.97	1.96	AAA-AAA	0.00	999	1.000	1.000	0.5655
244540_at	27.4	14.0	-0.97	-1.96	AAA-AAA	0.00	999	1.000	1.000	0.4396
203413_at	24.9	48.7	0.97	1.96	AAA-AAA	0.00	999	1.000	1.000	0.4331
230392_at	21.1	41.3	0.97	1.96	AAA-AAA	0.00	999	1.000	1.000	0.4327
230746_s_at	83.6	42.7	-0.97	-1.96	PPP-MAM	0.00	999	1.000	1.000	0.0714
239109_at	44.2	22.6	-0.97	-1.96	AAP-AAA	0.00	999	1.000	1.000	0.4240
218183_at	76.4	39.1	-0.97	-1.96	PAA-AAA	0.00	999	1.000	1.000	0.4681
241270_at	50.0	25.6	-0.97	-1.96	AAA-AAA	0.00	999	1.000	1.000	0.5240
1562628_at	4.7	2.4	-0.97	-1.96	AAA-AAA	0.00	999	1.000	1.000	0.6717
1568248_x_at	11.6	5.9	-0.97	-1.96	AAA-AAA	0.00	999	1.000	1.000	0.4336
1554300_a_at	13.0	6.7	-0.97	-1.95	AAA-AAA	0.00	999	1.000	1.000	0.5144
1553586_at	33.0	64.6	0.97	1.95	AAA-AAA	0.00	999	1.000	1.000	0.2492
220704_at	9.7	18.9	0.97	1.96	AAM-AAA	0.00	999	1.000	1.000	0.5046
232508_at	64.0	32.7	-0.97	-1.95	AAA-AAA	0.00	999	1.000	1.000	0.2657
233494_at	7.0	13.7	0.97	1.96	AAA-AAA	0.00	999	1.000	1.000	0.3921
225233_at	26.4	13.5	-0.97	-1.95	AAA-AAA	0.00	999	1.000	1.000	0.3216

227915_at	11.8	6.0	-0.97	-1.96	AAA-AAA	0.00	999	1.000	1.000	0.2807
230137_at	15.8	30.9	0.97	1.95	AAA-AAA	0.00	999	1.000	1.000	0.3111
1563654_at	23.0	45.0	0.97	1.95	AAA-AAA	0.00	999	1.000	1.000	0.4913
240980_at	17.1	8.8	-0.97	-1.95	AAA-AAA	0.00	999	1.000	1.000	0.6138
240873_x_at	9.5	18.5	0.97	1.95	AAA-AAA	0.00	999	1.000	1.000	0.3131
1558368_s_at	34.8	68.0	0.97	1.95	AAA-AAA	0.00	999	1.000	1.000	0.1876
1561252_at	23.2	11.9	-0.97	-1.95	AAA-AAA	0.00	999	1.000	1.000	0.3787
1561304_a_at	11.3	22.1	0.97	1.95	AAA-AAA	0.00	999	1.000	1.000	0.4656
244521_at	22.5	11.5	-0.97	-1.95	AAA-AAA	0.00	999	1.000	1.000	0.4282
1560748_at	13.8	26.9	0.97	1.95	AAA-AAA	0.00	999	1.000	1.000	0.6213
203591_s_at	13.8	26.9	0.97	1.95	AAA-AAA	0.00	999	1.000	1.000	0.4381
234427_at	3.1	6.1	0.97	1.95	AAA-AAA	0.00	999	1.000	1.000	0.1601
232519_at	22.0	43.0	0.97	1.95	AAA-AAA	0.00	999	1.000	1.000	0.3560
226940_at	9.5	4.8	-0.97	-1.95	AAA-AAA	0.00	999	1.000	1.000	0.2337
227879_at	27.3	53.4	0.97	1.95	AAA-AAA	0.00	999	1.000	1.000	0.2369
242306_at	35.4	69.1	0.97	1.95	AAA-AAA	0.00	999	1.000	1.000	0.3653
1556401_a_at	5.3	10.3	0.97	1.95	AAA-AAA	0.00	999	1.000	1.000	0.0848
244787_at	8.0	15.6	0.97	1.95	AAA-AAA	0.00	999	1.000	1.000	0.3635
1553864_at	3.7	7.2	0.97	1.95	AAA-AAA	0.00	999	1.000	1.000	0.4908
1561521_at	3.2	6.3	0.97	1.95	AAA-AAA	0.00	999	1.000	1.000	0.4058
1558938_at	20.5	40.0	0.97	1.95	AAA-AMA	0.00	999	1.000	1.000	0.3044
220679_s_at	6.4	12.5	0.97	1.95	AAA-AAA	0.00	999	1.000	1.000	0.0244
206301_at	17.5	9.0	-0.97	-1.95	AAA-AAA	0.00	999	1.000	1.000	0.2971
205475_at	74.1	38.0	-0.97	-1.95	AAA-AAA	0.00	999	1.000	1.000	0.2752
211053_at	14.7	7.5	-0.97	-1.95	AAA-AAA	0.00	999	1.000	1.000	0.1385
208349_at	43.9	22.5	-0.97	-1.95	AAA-AAA	0.00	999	1.000	1.000	0.1224
235663_at	45.1	23.1	-0.97	-1.95	AAA-AAA	0.00	999	1.000	1.000	0.2665
233752_s_at	4.9	9.5	0.97	1.95	AAA-AAA	0.00	999	1.000	1.000	0.4319
233916_at	21.6	11.1	-0.97	-1.95	AAA-AAA	0.00	999	1.000	1.000	0.3948
238956_at	7.8	15.3	0.96	1.95	AAA-AAA	0.00	999	1.000	1.000	0.4572
240017_at	28.2	54.9	0.96	1.95	AAA-AAA	0.00	999	1.000	1.000	0.4339
1558826_at	11.0	5.6	-0.96	-1.95	AAA-AAA	0.00	999	1.000	1.000	0.4273
1552398_a_at	11.1	21.6	0.96	1.95	AAA-AAA	0.00	999	1.000	1.000	0.5048
1556155_at	94.5	48.4	-0.96	-1.95	AAA-AAA	0.00	999	1.000	1.000	0.3196
1561605_at	14.9	29.1	0.96	1.95	AAA-AAA	0.00	999	1.000	1.000	0.1841
243813_at	8.9	17.4	0.96	1.95	AAA-AAA	0.00	999	1.000	1.000	0.5654
1560715_at	5.4	10.5	0.96	1.95	AAA-AAA	0.00	999	1.000	1.000	0.2140
217578_at	9.1	17.8	0.96	1.95	AAA-AAA	0.00	999	1.000	1.000	0.4021
217460_at	13.6	7.0	-0.96	-1.95	AAA-AAA	0.00	999	1.000	1.000	0.2961
207237_at	10.4	20.2	0.96	1.95	AAA-AAA	0.00	999	1.000	1.000	0.2238
207148_x_at	9.0	4.6	-0.96	-1.95	AAA-AAA	0.00	999	1.000	1.000	0.4657
210330_at	12.2	23.8	0.96	1.95	AAA-AAA	0.00	999	1.000	1.000	0.4348

207708_at	64.0	32.8	-0.96	-1.95	AAA-AAA	0.00	999	1.000	1.000	0.4502
207550_at	15.2	29.7	0.96	1.95	AAA-AAA	0.00	999	1.000	1.000	0.4086
233967_at	40.7	20.9	-0.96	-1.95	AAA-AAA	0.00	999	1.000	1.000	0.4903
233448_s_at	11.8	22.9	0.96	1.95	AAA-AAA	0.00	999	1.000	1.000	0.3716
49306_at	12.3	6.3	-0.96	-1.95	AAA-AAA	0.00	999	1.000	1.000	0.2392
230877_at	16.8	8.6	-0.96	-1.95	AAA-AAA	0.00	999	1.000	1.000	0.4582
1555671_at	57.6	29.5	-0.96	-1.95	APP-AAA	0.00	999	1.000	1.000	0.0699
215971_at	56.1	28.8	-0.96	-1.95	APA-AAA	0.00	999	1.000	1.000	0.3025
214955_at	171.1	87.8	-0.96	-1.95	AAA-PAA	0.00	999	1.000	1.000	0.3030
223924_at	43.7	22.4	-0.96	-1.95	APA-AAA	0.00	999	1.000	1.000	0.4686
224202_at	19.2	9.8	-0.96	-1.95	APA-AAA	0.00	999	1.000	1.000	0.5787
1569763_at	46.7	91.1	0.96	1.95	AAA-AAA	0.00	999	1.000	1.000	0.3690
241042_at	8.3	16.3	0.96	1.95	AAA-AAA	0.00	999	1.000	1.000	0.4983
1562226_at	10.3	5.3	-0.96	-1.95	AAA-AAA	0.00	999	1.000	1.000	0.5891
1561528_at	46.8	24.0	-0.96	-1.95	AAA-AAA	0.00	999	1.000	1.000	0.2963
244428_at	116.2	59.6	-0.96	-1.95	AAA-AAA	0.00	999	1.000	1.000	0.3449
243756_at	11.2	5.7	-0.96	-1.95	AAA-AAA	0.00	999	1.000	1.000	0.5616
219949_at	10.4	20.2	0.96	1.95	AAA-AAA	0.00	999	1.000	1.000	0.4606
203483_at	21.0	10.8	-0.96	-1.95	AAA-AAA	0.00	999	1.000	1.000	0.4102
222831_at	55.8	108.8	0.96	1.95	AAA-AAA	0.00	999	1.000	1.000	0.1097
1565581_at	22.1	43.1	0.96	1.95	AAA-AAA	0.00	999	1.000	1.000	0.2851
1562751_at	55.9	28.7	-0.96	-1.95	AAA-AAA	0.00	999	1.000	1.000	0.3793
1554629_at	2.8	5.4	0.96	1.95	AAA-AAA	0.00	999	1.000	1.000	0.3370
243722_at	57.3	29.4	-0.96	-1.95	AAA-AAA	0.00	999	1.000	1.000	0.4117
244140_at	15.7	30.5	0.96	1.95	AAA-AAA	0.00	999	1.000	1.000	0.5337
215369_at	14.4	7.4	-0.96	-1.95	AAA-AAA	0.00	999	1.000	1.000	0.5285
215363_x_at	5.8	11.3	0.96	1.95	AAA-AAA	0.00	999	1.000	1.000	0.1652
213479_at	6.2	12.2	0.96	1.95	AAA-AAA	0.00	999	1.000	1.000	0.3564
213013_at	55.2	28.3	-0.96	-1.95	AAA-AAA	0.00	999	1.000	1.000	0.1616
219640_at	141.4	275.6	0.96	1.95	AAA-AAA	0.00	999	1.000	1.000	0.3365
210922_at	10.6	5.4	-0.96	-1.95	AAA-AAA	0.00	999	1.000	1.000	0.3563
208888_s_at	16.7	8.6	-0.96	-1.95	AAA-AAA	0.00	999	1.000	1.000	0.2788
234038_at	4.2	2.2	-0.96	-1.95	AAA-AAA	0.00	999	1.000	1.000	0.2320
233546_at	12.8	6.6	-0.96	-1.95	AAA-AAA	0.00	999	1.000	1.000	0.6733
238088_at	8.6	16.8	0.96	1.95	AAA-AAA	0.00	999	1.000	1.000	0.5494
222921_s_at	17.2	8.8	-0.96	-1.95	AAA-AAA	0.00	999	1.000	1.000	0.3053
230452_at	41.8	81.5	0.96	1.95	AAA-AAA	0.00	999	1.000	1.000	0.2084
218608_at	248.1	127.4	-0.96	-1.95	APA-AAA	0.00	999	1.000	1.000	0.1045
1564070_s_at	43.9	22.6	-0.96	-1.95	AAA-AAA	0.00	999	1.000	1.000	0.3047
241611_s_at	6.8	13.3	0.96	1.95	AAA-AAA	0.00	999	1.000	1.000	0.5216
240031_at	35.2	68.5	0.96	1.95	AAA-AAA	0.00	999	1.000	1.000	0.3715
1553607_at	4.8	9.4	0.96	1.95	AAA-AAA	0.00	999	1.000	1.000	0.5446

1553829_at	7.4	3.8	-0.96	-1.95	AAA-AAA	0.00	999	1.000	1.000	0.4020
243270_at	16.5	32.0	0.96	1.95	AAA-AAA	0.00	999	1.000	1.000	0.3374
243364_at	7.4	14.5	0.96	1.95	AAA-AAA	0.00	999	1.000	1.000	0.4163
208130_s_at	35.4	68.9	0.96	1.95	AAA-AAA	0.00	999	1.000	1.000	0.4779
237112_at	23.1	11.9	-0.96	-1.95	AAA-AAA	0.00	999	1.000	1.000	0.4352
227483_s_at	15.3	7.9	-0.96	-1.95	AAA-AAA	0.00	999	1.000	1.000	0.2839
227848_at	38.0	19.5	-0.96	-1.95	AAA-AAA	0.00	999	1.000	1.000	0.4839
222723_at	20.2	39.4	0.96	1.95	AAA-AAA	0.00	999	1.000	1.000	0.3504
222813_at	13.6	26.5	0.96	1.95	AAA-AAA	0.00	999	1.000	1.000	0.4846
230911_at	13.9	7.1	-0.96	-1.95	AAA-AAA	0.00	999	1.000	1.000	0.4670
231672_at	12.2	23.8	0.96	1.95	AAA-AAA	0.00	999	1.000	1.000	0.5215
215150_at	99.0	50.9	-0.96	-1.95	PPM-AAP	0.00	999	1.000	1.000	0.0553
1562411_at	134.1	68.9	-0.96	-1.95	PAP-AAA	0.00	999	1.000	1.000	0.0813
1563000_at	27.6	14.2	-0.96	-1.95	AAA-PAA	0.00	999	1.000	1.000	0.4993
1553413_at	14.2	27.5	0.96	1.95	AAA-AAA	0.00	999	1.000	1.000	0.2876
1555060_a_at	23.0	11.8	-0.96	-1.95	AAA-AAA	0.00	999	1.000	1.000	0.2906
1558920_at	4.7	2.4	-0.96	-1.95	AAA-AAA	0.00	999	1.000	1.000	0.4863
1560753_at	32.4	63.0	0.96	1.95	AAA-AAA	0.00	999	1.000	1.000	0.5318
235467_s_at	96.0	49.3	-0.96	-1.95	AMA-AAA	0.00	999	1.000	1.000	0.0543
233856_at	54.6	28.1	-0.96	-1.95	AMA-MAA	0.00	999	1.000	1.000	0.3362
237898_at	9.2	17.9	0.96	1.95	AAA-AAA	0.00	999	1.000	1.000	0.6397
238661_at	13.4	6.9	-0.96	-1.95	AAA-AAA	0.00	999	1.000	1.000	0.5287
232327_at	6.1	3.1	-0.96	-1.95	AAA-AAA	0.00	999	1.000	1.000	0.2620
208035_at	114.9	59.1	-0.96	-1.94	PPA-AAA	0.00	999	1.000	1.000	0.0901
243828_at	78.0	40.1	-0.96	-1.94	AAP-AAA	0.00	999	1.000	1.000	0.3267
240919_at	93.3	48.0	-0.96	-1.94	PAP-MPA	0.00	999	1.000	1.000	0.3743
1569033_at	55.2	107.3	0.96	1.94	AAA-AAA	0.00	999	1.000	1.000	0.4040
239786_at	31.0	60.3	0.96	1.94	AAA-AAA	0.00	999	1.000	1.000	0.3426
1552296_at	13.9	7.2	-0.96	-1.94	AAA-AAA	0.00	999	1.000	1.000	0.4233
1559828_at	20.2	10.4	-0.96	-1.94	AAA-AAA	0.00	999	1.000	1.000	0.3729
244564_at	17.4	33.8	0.96	1.94	AAA-AAA	0.00	999	1.000	1.000	0.1616
221136_at	27.3	53.0	0.96	1.94	AAA-AAA	0.00	999	1.000	1.000	0.4658
217531_at	12.3	23.9	0.96	1.94	AAA-AAA	0.00	999	1.000	1.000	0.3983
217699_at	34.3	17.6	-0.96	-1.94	AAA-AAA	0.00	999	1.000	1.000	0.3704
201785_at	66.3	128.8	0.96	1.95	AAA-AAA	0.00	999	1.000	1.000	0.1217
235627_at	64.9	33.4	-0.96	-1.94	AAA-AAA	0.00	999	1.000	1.000	0.1817
227985_at	47.9	24.6	-0.96	-1.94	AAA-AAA	0.00	999	1.000	1.000	0.5648
221933_at	7.3	14.2	0.96	1.94	AAA-AAA	0.00	999	1.000	1.000	0.7249
229566_at	400.1	777.5	0.96	1.94	AAA-AAM	0.00	999	1.000	1.000	0.0457
1557882_at	39.6	20.4	-0.96	-1.94	PPA-AMA	0.00	999	1.000	1.000	0.4128
207977_s_at	24.5	12.6	-0.96	-1.94	AAA-APA	0.00	999	1.000	1.000	0.5616
1557330_at	114.2	58.8	-0.96	-1.94	AAA-AAA	0.00	999	1.000	1.000	0.3609

1560898_at	18.2	9.4	-0.96	-1.94	AAA-AAA	0.00	999	1.000	1.000	0.4586
204265_s_at	57.2	111.0	0.96	1.94	AAA-AAM	0.00	999	1.000	1.000	0.1102
211250_s_at	54.8	106.5	0.96	1.94	AAA-AAA	0.00	999	1.000	1.000	0.1693
234943_at	32.1	62.4	0.96	1.94	AAA-AAA	0.00	999	1.000	1.000	0.1332
229133_s_at	11.4	22.1	0.96	1.94	AAA-AAA	0.00	999	1.000	1.000	0.4034
208324_at	70.6	36.4	-0.96	-1.94	PAA-AAA	0.00	999	1.000	1.000	0.3610
232446_at	50.9	26.2	-0.96	-1.94	AAP-AAA	0.00	999	1.000	1.000	0.4683
224490_s_at	68.7	35.4	-0.96	-1.94	PAP-PAP	0.00	999	1.000	1.000	0.5334
231093_at	40.7	21.0	-0.96	-1.94	PPA-MPA	0.00	999	1.000	1.000	0.5687
1562641_at	36.5	70.9	0.96	1.94	AAA-AAA	0.00	999	1.000	1.000	0.3565
244664_at	81.9	42.2	-0.96	-1.94	AAA-AAA	0.00	999	1.000	1.000	0.3733
215804_at	16.3	8.4	-0.96	-1.94	AAA-AAA	0.00	999	1.000	1.000	0.0504
215866_at	54.4	105.7	0.96	1.94	AAA-AAA	0.00	999	1.000	1.000	0.1424
215552_s_at	20.6	10.6	-0.96	-1.94	AAA-AAA	0.00	999	1.000	1.000	0.4912
216330_s_at	74.2	38.2	-0.96	-1.94	AAA-AAA	0.00	999	1.000	1.000	0.3290
219536_s_at	213.8	110.1	-0.96	-1.94	AAA-AAA	0.00	999	1.000	1.000	0.0809
207190_at	20.2	10.4	-0.96	-1.94	AAA-AAA	0.00	999	1.000	1.000	0.1917
208495_at	33.4	17.2	-0.96	-1.94	AAA-AAA	0.00	999	1.000	1.000	0.2923
233574_at	16.4	31.8	0.96	1.94	AAA-AAA	0.00	999	1.000	1.000	0.2552
238790_at	24.1	46.8	0.96	1.94	AAA-AAA	0.00	999	1.000	1.000	0.3108
236962_at	22.1	11.4	-0.96	-1.94	AAA-AAA	0.00	999	1.000	1.000	0.2960
225168_at	28.0	54.3	0.96	1.94	AAA-AAA	0.00	999	1.000	1.000	0.3938
231361_at	8.6	16.7	0.96	1.94	AAA-MAA	0.00	999	1.000	1.000	0.5469
231320_at	79.8	41.1	-0.96	-1.94	AAA-AAA	0.00	999	1.000	1.000	0.3615
230932_at	60.5	31.2	-0.96	-1.94	AAP-APA	0.00	999	1.000	1.000	0.1451
1563565_at	14.3	7.4	-0.96	-1.94	AAA-AAA	0.00	999	1.000	1.000	0.3660
240101_at	9.0	4.6	-0.96	-1.94	AAA-AAA	0.00	999	1.000	1.000	0.1811
239964_at	24.0	12.4	-0.96	-1.94	AAA-AAA	0.00	999	1.000	1.000	0.2330
1558544_at	15.4	30.0	0.96	1.94	AAA-AAA	0.00	999	1.000	1.000	0.3109
1553757_at	27.7	53.8	0.96	1.94	AAA-AAA	0.00	999	1.000	1.000	0.4672
1560104_at	42.0	21.6	-0.96	-1.94	AAA-AAA	0.00	999	1.000	1.000	0.4186
215307_at	32.2	16.6	-0.96	-1.94	AAA-AAA	0.00	999	1.000	1.000	0.4444
216371_at	30.3	58.9	0.96	1.94	AAA-AAA	0.00	999	1.000	1.000	0.2773
214936_at	20.7	40.1	0.96	1.94	AAA-AAA	0.00	999	1.000	1.000	0.3579
221072_at	18.1	35.1	0.96	1.94	AAA-AAA	0.00	999	1.000	1.000	0.2314
233701_at	45.6	88.5	0.96	1.94	AAA-AAA	0.00	999	1.000	1.000	0.2338
236448_at	10.6	5.4	-0.96	-1.94	AAA-AAA	0.00	999	1.000	1.000	0.3211
1569729_a_at	7.2	3.7	-0.96	-1.94	AAP-AAA	0.00	999	1.000	1.000	0.5417
243758_at	24.9	12.9	-0.96	-1.94	APA-AAA	0.00	999	1.000	1.000	0.5727
1562687_x_at	33.4	17.2	-0.96	-1.94	AAA-AAA	0.00	999	1.000	1.000	0.6679
242002_at	11.5	5.9	-0.96	-1.94	AAA-AAA	0.00	999	1.000	1.000	0.5544
1561148_at	34.4	17.8	-0.96	-1.94	AAA-AAA	0.00	999	1.000	1.000	0.6081

1562573_at	9.2	17.9	0.96	1.94	AAA-AAA	0.00	999	1.000	1.000	0.5283
244436_at	12.8	6.6	-0.96	-1.94	AAA-AAA	0.00	999	1.000	1.000	0.5874
243854_at	15.6	8.0	-0.96	-1.94	AAA-MAA	0.00	999	1.000	1.000	0.5636
211173_at	40.8	21.0	-0.96	-1.94	AAA-AAA	0.00	999	1.000	1.000	0.1669
207330_at	77.7	40.1	-0.96	-1.94	AAA-AAA	0.00	999	1.000	1.000	0.2686
234855_at	33.8	17.4	-0.96	-1.94	AAA-AAA	0.00	999	1.000	1.000	0.0340
232663_s_at	16.7	32.4	0.96	1.94	AAA-AAA	0.00	999	1.000	1.000	0.3568
230983_at	23.4	45.3	0.96	1.94	AAA-AAA	0.00	999	1.000	1.000	0.4771
229592_at	43.7	22.6	-0.96	-1.94	AAA-AAA	0.00	999	1.000	1.000	0.5194
1553826_a_at	26.7	51.8	0.95	1.94	AAA-AAA	0.00	999	1.000	1.000	0.1886
1561113_at	12.1	23.4	0.95	1.94	AAA-AAA	0.00	999	1.000	1.000	0.4854
1560062_at	9.6	18.7	0.95	1.94	AAA-AAA	0.00	999	1.000	1.000	0.2927
216651_s_at	11.2	21.8	0.95	1.94	AAA-AAA	0.00	999	1.000	1.000	0.5274
205309_at	48.2	24.9	-0.95	-1.94	AAA-AAA	0.00	999	1.000	1.000	0.5249
237895_at	78.6	152.2	0.95	1.94	AAA-AAA	0.00	999	1.000	1.000	0.1391
205180_s_at	239.9	123.9	-0.95	-1.94	PAA-PAP	0.00	999	1.000	1.000	0.1652
226029_at	69.8	36.1	-0.95	-1.94	PAA-AAA	0.00	999	1.000	1.000	0.2473
240004_at	25.3	13.1	-0.95	-1.94	AAA-AAA	0.00	999	1.000	1.000	0.3859
240342_at	32.1	62.1	0.95	1.94	AAA-AAA	0.00	999	1.000	1.000	0.3912
1567374_at	16.2	31.3	0.95	1.94	AAA-AAA	0.00	999	1.000	1.000	0.5416
243152_at	6.5	12.6	0.95	1.94	AAA-AAA	0.00	999	1.000	1.000	0.4316
207149_at	4.3	8.2	0.95	1.94	AAA-AAA	0.00	999	1.000	1.000	0.3479
211798_x_at	111.7	57.7	-0.95	-1.94	AAA-AAA	0.00	999	1.000	1.000	0.2954
233009_at	22.1	42.8	0.95	1.94	AAA-AAA	0.00	999	1.000	1.000	0.4611
232711_at	15.1	7.8	-0.95	-1.94	AAA-AAA	0.00	999	1.000	1.000	0.3778
228018_at	10.8	5.6	-0.95	-1.94	AAA-AAA	0.00	999	1.000	1.000	0.4072
232143_at	20.9	40.4	0.95	1.94	AAA-AAA	0.00	999	1.000	1.000	0.5564
241231_at	41.3	79.9	0.95	1.94	AAA-AAA	0.00	999	1.000	1.000	0.3810
1563109_at	36.8	19.0	-0.95	-1.94	AAA-AAA	0.00	999	1.000	1.000	0.4233
1557812_a_at	21.9	42.4	0.95	1.94	AAA-AAA	0.00	999	1.000	1.000	0.4035
1554060_s_at	23.3	45.0	0.95	1.93	AAA-AAA	0.00	999	1.000	1.000	0.1757
243873_at	7.8	4.1	-0.95	-1.93	AAA-AAA	0.00	999	1.000	1.000	0.1537
221178_at	6.8	13.2	0.95	1.93	AAA-AAA	0.00	999	1.000	1.000	0.3505
216937_s_at	25.5	49.3	0.95	1.93	AAA-AAA	0.00	999	1.000	1.000	0.4434
211523_at	12.7	24.6	0.95	1.94	AAA-AAA	0.00	999	1.000	1.000	0.5532
234451_at	44.2	85.5	0.95	1.93	AAA-AAA	0.00	999	1.000	1.000	0.1764
233826_at	5.7	11.0	0.95	1.93	AAA-AAA	0.00	999	1.000	1.000	0.2451
237845_at	15.6	8.1	-0.95	-1.94	AAA-AAA	0.00	999	1.000	1.000	0.5884
237638_at	52.0	100.6	0.95	1.94	AAA-AAA	0.00	999	1.000	1.000	0.3051
224552_s_at	11.9	6.1	-0.95	-1.94	AAA-AAA	0.00	999	1.000	1.000	0.1296
224030_s_at	38.6	74.7	0.95	1.94	AAA-AAA	0.00	999	1.000	1.000	0.0429
231575_at	21.1	40.8	0.95	1.94	AAA-AAA	0.00	999	1.000	1.000	0.4980

229449_at	15.0	28.9	0.95	1.93	AAA-AAA	0.00	999	1.000	1.000	0.3649
211343_s_at	51.3	26.5	-0.95	-1.93	PPA-AAA	0.00	999	1.000	1.000	0.1058
237051_at	62.7	32.5	-0.95	-1.93	AAP-PAA	0.00	999	1.000	1.000	0.4033
241278_at	16.1	8.3	-0.95	-1.93	AAA-AAA	0.00	999	1.000	1.000	0.5603
239360_at	59.7	30.9	-0.95	-1.93	AAA-AAA	0.00	999	1.000	1.000	0.2164
1562415_a_at	17.2	8.9	-0.95	-1.93	AAA-AAA	0.00	999	1.000	1.000	0.3078
1561164_at	11.7	22.5	0.95	1.93	AAA-AAA	0.00	999	1.000	1.000	0.1973
216742_at	7.0	13.4	0.95	1.93	AAA-AAA	0.00	999	1.000	1.000	0.2586
207257_at	10.8	5.6	-0.95	-1.93	AAA-AAA	0.00	999	1.000	1.000	0.5646
232514_at	18.1	35.0	0.95	1.93	AAA-AAA	0.00	999	1.000	1.000	0.2852
235947_at	11.2	5.8	-0.95	-1.93	AAA-AAA	0.00	999	1.000	1.000	0.4552
230991_at	21.8	42.1	0.95	1.93	AAA-AAA	0.00	999	1.000	1.000	0.2913
232368_at	28.2	14.6	-0.95	-1.93	AAP-AMA	0.00	999	1.000	1.000	0.2002
242971_at	90.8	47.0	-0.95	-1.93	PAP-APA	0.00	999	1.000	1.000	0.3843
230828_at	60.0	31.1	-0.95	-1.93	APP-AAA	0.00	999	1.000	1.000	0.4020
216045_at	33.4	17.3	-0.95	-1.93	AAA-AAP	0.00	999	1.000	1.000	0.4566
1557403_s_at	49.1	25.4	-0.95	-1.93	AAA-AAP	0.00	999	1.000	1.000	0.4790
1564753_at	33.9	65.4	0.95	1.93	AAA-AAA	0.00	999	1.000	1.000	0.1408
239015_at	73.7	38.1	-0.95	-1.93	AAA-AAA	0.00	999	1.000	1.000	0.1945
1569291_at	73.7	142.4	0.95	1.93	AAA-AAA	0.00	999	1.000	1.000	0.1770
1555079_at	6.7	3.5	-0.95	-1.93	AAA-AAA	0.00	999	1.000	1.000	0.4048
1559715_at	38.7	74.8	0.95	1.93	AAA-AAA	0.00	999	1.000	1.000	0.5080
216166_at	44.5	23.0	-0.95	-1.93	AAA-AAA	0.00	999	1.000	1.000	0.4158
220373_at	13.9	7.2	-0.95	-1.93	AAA-AAA	0.00	999	1.000	1.000	0.2199
221584_s_at	48.0	24.9	-0.95	-1.93	AAA-AAA	0.00	999	1.000	1.000	0.3923
232906_at	16.6	32.1	0.95	1.93	AAA-AAA	0.00	999	1.000	1.000	0.5732
233784_at	20.5	39.5	0.95	1.93	AAA-AAA	0.00	999	1.000	1.000	0.5419
225259_at	48.6	25.2	-0.95	-1.93	AAA-AAM	0.00	999	1.000	1.000	0.2462
226139_at	38.8	74.9	0.95	1.93	AAA-AAA	0.00	999	1.000	1.000	0.0546
230126_s_at	57.2	29.6	-0.95	-1.93	AAA-AAA	0.00	999	1.000	1.000	0.3214
230303_at	11.1	21.4	0.95	1.93	AAA-AAA	0.00	999	1.000	1.000	0.5421
236571_at	97.0	50.3	-0.95	-1.93	AAM-PAA	0.00	999	1.000	1.000	0.1882
1564359_a_at	21.7	11.2	-0.95	-1.93	MAA-AAA	0.00	999	1.000	1.000	0.3929
1564357_at	34.8	18.0	-0.95	-1.93	AAA-AAA	0.00	999	1.000	1.000	0.3283
1570194_x_at	10.0	5.2	-0.95	-1.93	AAA-AAA	0.00	999	1.000	1.000	0.2117
239206_at	8.0	15.5	0.95	1.93	AAA-AAA	0.00	999	1.000	1.000	0.5655
1554984_a_at	64.6	33.5	-0.95	-1.93	AAA-AAA	0.00	999	1.000	1.000	0.5544
1560910_at	30.9	59.6	0.95	1.93	AAA-AAA	0.00	999	1.000	1.000	0.3785
1561556_at	23.7	45.7	0.95	1.93	AAA-AAA	0.00	999	1.000	1.000	0.2136
243800_at	7.6	4.0	-0.95	-1.93	AAA-AAA	0.00	999	1.000	1.000	0.4725
243954_at	13.0	25.1	0.95	1.93	AAA-AAA	0.00	999	1.000	1.000	0.4588
212224_at	10.9	5.7	-0.95	-1.93	AAA-AAA	0.00	999	1.000	1.000	0.0636

219670_at	8.7	16.8	0.95	1.93	AAA-AAA	0.00	999	1.000	1.000	0.4727
238797_at	109.4	56.7	-0.95	-1.93	AAA-AAA	0.00	999	1.000	1.000	0.3279
222974_at	16.0	8.3	-0.95	-1.93	AAA-AAA	0.00	999	1.000	1.000	0.4976
1569560_at	16.3	31.5	0.95	1.93	AAA-AAA	0.00	999	1.000	1.000	0.4288
240656_at	42.2	81.5	0.95	1.93	AAA-AAA	0.00	999	1.000	1.000	0.0756
1552377_s_at	165.3	85.7	-0.95	-1.93	AAA-AAA	0.00	999	1.000	1.000	0.2161
1561137_s_at	2.7	5.2	0.95	1.93	AAA-AAA	0.00	999	1.000	1.000	0.3748
1562591_a_at	13.1	25.2	0.95	1.93	AAA-AAA	0.00	999	1.000	1.000	0.6556
214099_s_at	66.8	128.8	0.95	1.93	AAA-AAA	0.00	999	1.000	1.000	0.1429
217624_at	68.0	35.3	-0.95	-1.93	AAA-AAA	0.00	999	1.000	1.000	0.0375
219144_at	32.8	63.2	0.95	1.93	AAA-AAA	0.00	999	1.000	1.000	0.1345
204486_at	6.4	12.3	0.95	1.93	AAA-AAA	0.00	999	1.000	1.000	0.5406
235097_at	28.7	55.4	0.95	1.93	AAA-AAA	0.00	999	1.000	1.000	0.3942
237707_at	69.1	35.8	-0.95	-1.93	AAA-AAA	0.00	999	1.000	1.000	0.2356
236835_at	30.6	15.9	-0.95	-1.93	AAA-AAA	0.00	999	1.000	1.000	0.5925
228878_s_at	6.2	11.9	0.95	1.93	AAA-AAA	0.00	999	1.000	1.000	0.2120
229445_at	26.1	13.6	-0.95	-1.93	AAA-AAA	0.00	999	1.000	1.000	0.5234
1564008_at	26.2	50.5	0.95	1.93	AAA-AAA	0.00	999	1.000	1.000	0.1932
241882_at	44.9	86.5	0.95	1.93	AAA-AAA	0.00	999	1.000	1.000	0.1661
240267_at	11.1	21.3	0.95	1.93	AAA-MAA	0.00	999	1.000	1.000	0.5347
1556536_at	6.1	11.8	0.95	1.93	AAA-AAA	0.00	999	1.000	1.000	0.1578
1558603_at	6.9	13.3	0.95	1.93	AAA-AAA	0.00	999	1.000	1.000	0.5074
244281_at	15.9	8.3	-0.95	-1.93	AAA-AAA	0.00	999	1.000	1.000	0.4566
1560526_at	4.0	2.1	-0.95	-1.93	AAA-AAA	0.00	999	1.000	1.000	0.4263
213975_s_at	8.4	4.4	-0.95	-1.93	AAA-AAA	0.00	999	1.000	1.000	0.4630
220478_at	8.0	15.4	0.95	1.93	AAA-AAA	0.00	999	1.000	1.000	0.2627
220938_s_at	58.2	112.1	0.95	1.93	AAA-AAA	0.00	999	1.000	1.000	0.2748
234386_s_at	26.9	13.9	-0.95	-1.93	AAA-AAA	0.00	999	1.000	1.000	0.3869
233761_at	85.5	164.7	0.95	1.93	AAA-AAA	0.00	999	1.000	1.000	0.0348
231591_at	9.4	18.0	0.95	1.93	AAA-AAA	0.00	999	1.000	1.000	0.4327
213662_at	50.2	26.1	-0.95	-1.93	PPA-AAA	0.00	999	1.000	1.000	0.1759
230204_at	46.0	23.9	-0.95	-1.93	APA-AAA	0.00	999	1.000	1.000	0.4076
241351_at	28.6	55.1	0.95	1.93	AAA-AAA	0.00	999	1.000	1.000	0.4670
1570385_at	22.6	43.6	0.95	1.93	AAA-AAA	0.00	999	1.000	1.000	0.1576
1565898_at	5.7	11.0	0.95	1.93	AAA-AAA	0.00	999	1.000	1.000	0.3952
1567612_at	14.0	27.0	0.95	1.93	AAA-AAA	0.00	999	1.000	1.000	0.4460
1567663_at	1.6	3.0	0.95	1.93	AAA-AAA	0.00	999	1.000	1.000	0.3275
1556694_a_at	35.6	18.5	-0.95	-1.93	AAA-AAA	0.00	999	1.000	1.000	0.2142
1562193_at	5.1	2.7	-0.95	-1.93	AAA-AAA	0.00	999	1.000	1.000	0.3340
1562418_at	11.3	21.7	0.95	1.93	AAA-AAA	0.00	999	1.000	1.000	0.4459
244168_s_at	35.1	67.6	0.95	1.93	AAA-AAA	0.00	999	1.000	1.000	0.3335
219824_at	33.0	63.5	0.95	1.93	AAA-AAA	0.00	999	1.000	1.000	0.3303

210660_at	44.5	23.1	-0.95	-1.93	AAA-AAA	0.00	999	1.000	1.000	0.1942
207666_x_at	25.6	13.3	-0.95	-1.93	AAA-AAA	0.00	999	1.000	1.000	0.4740
208134_x_at	12.5	24.1	0.95	1.93	AAA-AAA	0.00	999	1.000	1.000	0.2984
236463_at	35.2	67.7	0.95	1.93	AAA-AAA	0.00	999	1.000	1.000	0.2934
226226_at	77.2	40.1	-0.95	-1.93	AAA-AAA	0.00	999	1.000	1.000	0.1508
231264_at	59.1	30.7	-0.95	-1.93	AAA-AAA	0.00	999	1.000	1.000	0.3800
231485_at	40.6	21.1	-0.95	-1.93	AAA-AAA	0.00	999	1.000	1.000	0.5385
1568365_at	28.3	14.7	-0.95	-1.93	PPA-AAA	0.00	999	1.000	1.000	0.5505
1568698_at	28.2	54.3	0.95	1.93	AAA-AAA	0.00	999	1.000	1.000	0.5174
1554867_a_at	18.5	35.5	0.95	1.93	AAA-AAA	0.00	999	1.000	1.000	0.3708
1554012_at	8.2	15.7	0.95	1.93	AAA-AAA	0.00	999	1.000	1.000	0.5471
1555678_at	16.4	31.6	0.95	1.93	AAA-AAA	0.00	999	1.000	1.000	0.2854
1559849_at	4.5	8.6	0.95	1.93	AAA-AAA	0.00	999	1.000	1.000	0.6047
213914_s_at	14.8	28.4	0.95	1.93	AAA-AAA	0.00	999	1.000	1.000	0.5559
221389_at	20.8	10.8	-0.95	-1.93	AAA-AAA	0.00	999	1.000	1.000	0.4459
205837_s_at	10.2	5.3	-0.95	-1.93	AAM-AAA	0.00	999	1.000	1.000	0.5560
206103_at	9.4	18.1	0.95	1.93	AAA-AAA	0.00	999	1.000	1.000	0.2748
207138_at	12.2	6.4	-0.95	-1.93	AAA-AAA	0.00	999	1.000	1.000	0.4718
210472_at	40.1	20.8	-0.95	-1.93	AAA-AAA	0.00	999	1.000	1.000	0.0608
233797_s_at	30.5	15.8	-0.95	-1.93	AAA-AAA	0.00	999	1.000	1.000	0.6027
238376_at	22.0	42.4	0.95	1.93	AAA-AAA	0.00	999	1.000	1.000	0.2966
231636_s_at	14.6	28.0	0.95	1.93	AAA-AAA	0.00	999	1.000	1.000	0.5931
231749_at	14.7	7.6	-0.95	-1.93	AAA-AAA	0.00	999	1.000	1.000	0.3591
1565780_at	19.1	36.8	0.94	1.92	AAA-AAA	0.00	999	1.000	1.000	0.5715
1557012_a_at	23.8	45.7	0.94	1.92	AAA-AAA	0.00	999	1.000	1.000	0.2374
243076_x_at	65.4	34.0	-0.94	-1.92	AAA-AAA	0.00	999	1.000	1.000	0.5369
214996_at	28.2	14.6	-0.94	-1.92	AAA-AAA	0.00	999	1.000	1.000	0.0828
207226_at	20.9	10.9	-0.94	-1.92	AAA-AAA	0.00	999	1.000	1.000	0.4086
205023_at	63.3	32.9	-0.94	-1.92	AAA-AAA	0.00	999	1.000	1.000	0.5485
209683_at	35.0	67.4	0.94	1.92	AAA-MAM	0.00	999	1.000	1.000	0.1992
238302_at	17.4	9.1	-0.94	-1.92	AAA-AAA	0.00	999	1.000	1.000	0.2669
236070_at	93.6	48.7	-0.94	-1.92	AAA-AAA	0.00	999	1.000	1.000	0.4110
236812_at	75.0	39.0	-0.94	-1.92	AAA-AAA	0.00	999	1.000	1.000	0.2228
231826_at	42.5	22.1	-0.94	-1.92	AAA-AAA	0.00	999	1.000	1.000	0.3288
236845_at	70.2	36.5	-0.94	-1.92	APP-AAA	0.00	999	1.000	1.000	0.3494
217145_at	15.7	8.2	-0.94	-1.92	AAP-AAA	0.00	999	1.000	1.000	0.6810
241492_at	21.9	11.4	-0.94	-1.92	AAA-AAA	0.00	999	1.000	1.000	0.3281
1564568_at	38.0	73.0	0.94	1.92	AAA-AAA	0.00	999	1.000	1.000	0.2991
240185_at	43.3	22.5	-0.94	-1.92	AAA-AAA	0.00	999	1.000	1.000	0.3300
1566963_at	9.0	17.4	0.94	1.92	AAA-AAA	0.00	999	1.000	1.000	0.4878
1558053_s_at	16.3	8.5	-0.94	-1.92	AAA-AAA	0.00	999	1.000	1.000	0.3491
1562373_at	6.4	12.2	0.94	1.92	AAA-AAA	0.00	999	1.000	1.000	0.4695

243941_at	23.7	45.5	0.94	1.92	AAA-AAA	0.00	999	1.000	1.000	0.1734
220798_x_at	15.6	29.9	0.94	1.92	AAA-AAA	0.00	999	1.000	1.000	0.3376
219255_x_at	20.8	10.8	-0.94	-1.92	AAA-AAA	0.00	999	1.000	1.000	0.3115
206709_x_at	54.9	105.5	0.94	1.92	AAA-AAA	0.00	999	1.000	1.000	0.0542
203470_s_at	65.8	126.5	0.94	1.92	AAA-AAA	0.00	999	1.000	1.000	0.1356
209975_at	18.1	34.8	0.94	1.92	AAA-AAA	0.00	999	1.000	1.000	0.5475
238141_s_at	16.3	8.5	-0.94	-1.92	AAA-AAA	0.00	999	1.000	1.000	0.5268
210584_s_at	58.3	30.3	-0.94	-1.92	MAA-AAP	0.00	999	1.000	1.000	0.2150
1563373_at	4.1	7.8	0.94	1.92	AAA-AAA	0.00	999	1.000	1.000	0.3605
1568843_at	26.6	51.0	0.94	1.92	AAA-AAA	0.00	999	1.000	1.000	0.4404
1569666_s_at	42.8	82.3	0.94	1.92	AAA-AAA	0.00	999	1.000	1.000	0.1177
1566927_at	46.7	89.6	0.94	1.92	AAA-AAA	0.00	999	1.000	1.000	0.3369
240667_at	10.7	20.5	0.94	1.92	AAA-AAA	0.00	999	1.000	1.000	0.6291
244850_at	11.8	22.6	0.94	1.92	AAA-AAA	0.00	999	1.000	1.000	0.2908
1554378_a_at	3.5	6.8	0.94	1.92	AAA-AAA	0.00	999	1.000	1.000	0.2357
242670_at	9.8	18.9	0.94	1.92	AAA-AAA	0.00	999	1.000	1.000	0.2105
205311_at	21.7	41.7	0.94	1.92	AAA-AAA	0.00	999	1.000	1.000	0.3244
238426_at	10.9	5.7	-0.94	-1.92	AAA-AAA	0.00	999	1.000	1.000	0.2675
238747_at	30.8	16.0	-0.94	-1.92	AAA-AAA	0.00	999	1.000	1.000	0.4650
227168_at	12.8	24.7	0.94	1.92	AAA-AAA	0.00	999	1.000	1.000	0.3648
228895_s_at	27.9	53.7	0.94	1.92	AAA-AAA	0.00	999	1.000	1.000	0.3576
222281_s_at	21.3	40.9	0.94	1.92	AAA-AAA	0.00	999	1.000	1.000	0.2583
208084_at	147.0	76.6	-0.94	-1.92	APA-APA	0.00	999	1.000	1.000	0.3540
1568828_at	13.4	25.6	0.94	1.92	AAA-AAA	0.00	999	1.000	1.000	0.3932
239260_at	6.6	12.7	0.94	1.92	AAA-AAA	0.00	999	1.000	1.000	0.4822
239970_at	35.3	67.7	0.94	1.92	AAA-AAA	0.00	999	1.000	1.000	0.0210
1553425_at	28.0	14.6	-0.94	-1.92	AAA-AAA	0.00	999	1.000	1.000	0.3733
216003_at	3.8	7.3	0.94	1.92	AAA-AAA	0.00	999	1.000	1.000	0.0698
216005_at	36.8	19.2	-0.94	-1.92	AAA-AAA	0.00	999	1.000	1.000	0.1769
216744_at	11.0	5.7	-0.94	-1.92	AAA-AAA	0.00	999	1.000	1.000	0.4728
214640_at	6.4	12.3	0.94	1.92	AAA-AAA	0.00	999	1.000	1.000	0.5926
203609_s_at	16.2	31.2	0.94	1.92	AAA-AAA	0.00	999	1.000	1.000	0.4203
207327_at	35.2	18.3	-0.94	-1.92	AAA-AAA	0.00	999	1.000	1.000	0.1625
208573_s_at	85.7	44.6	-0.94	-1.92	AAA-AAA	0.00	999	1.000	1.000	0.3107
234422_at	7.3	3.8	-0.94	-1.92	AAA-AAA	0.00	999	1.000	1.000	0.1427
232656_at	16.0	8.3	-0.94	-1.92	AAA-AAA	0.00	999	1.000	1.000	0.3554
236392_at	6.4	12.4	0.94	1.92	AAA-AAA	0.00	999	1.000	1.000	0.2044
225996_at	4.0	2.1	-0.94	-1.92	AAA-AAA	0.00	999	1.000	1.000	0.5141
222332_at	9.9	18.9	0.94	1.92	AAA-AAA	0.00	999	1.000	1.000	0.5787
223951_at	69.0	35.9	-0.94	-1.92	AAA-AAA	0.00	999	1.000	1.000	0.0221
230545_at	55.7	29.0	-0.94	-1.92	AAA-AAA	0.00	999	1.000	1.000	0.4342
233467_s_at	82.8	43.2	-0.94	-1.92	PPA-AAA	0.00	999	1.000	1.000	0.2215

1563886_at	8.9	17.0	0.94	1.92	AAA-AAA	0.00	999	1.000	1.000	0.2195
241995_at	50.0	26.1	-0.94	-1.92	AAA-AAA	0.00	999	1.000	1.000	0.4773
1569775_at	13.9	26.6	0.94	1.92	AAA-AAA	0.00	999	1.000	1.000	0.3264
1556916_a_at	15.1	7.9	-0.94	-1.92	AAA-AAA	0.00	999	1.000	1.000	0.3241
216898_s_at	33.8	17.6	-0.94	-1.92	AAA-AAA	0.00	999	1.000	1.000	0.4437
207027_at	14.4	7.5	-0.94	-1.92	AAA-AAA	0.00	999	1.000	1.000	0.2189
203400_s_at	13.3	25.6	0.94	1.92	AAA-AAA	0.00	999	1.000	1.000	0.4669
209752_at	22.5	43.2	0.94	1.92	AAA-AAA	0.00	999	1.000	1.000	0.5798
208563_x_at	11.0	21.1	0.94	1.92	AAA-AAA	0.00	999	1.000	1.000	0.4209
208180_s_at	17.0	32.6	0.94	1.92	AAA-AAA	0.00	999	1.000	1.000	0.3283
233428_at	62.7	120.3	0.94	1.92	AAA-AAA	0.00	999	1.000	1.000	0.0943
236091_at	8.4	16.1	0.94	1.92	AAA-AAA	0.00	999	1.000	1.000	0.2994
231557_at	22.6	43.4	0.94	1.92	AAA-AAA	0.00	999	1.000	1.000	0.2375
214775_at	61.9	32.3	-0.94	-1.92	AAA-APA	0.00	999	1.000	1.000	0.4799
1553846_at	9.5	18.2	0.94	1.92	AAA-AAA	0.00	999	1.000	1.000	0.2973
244615_x_at	28.6	14.9	-0.94	-1.92	AAA-AAA	0.00	999	1.000	1.000	0.4935
244509_at	60.9	116.8	0.94	1.92	AAA-AAA	0.00	999	1.000	1.000	0.0714
215258_at	27.1	14.1	-0.94	-1.92	AAA-AAA	0.00	999	1.000	1.000	0.3726
215384_s_at	23.6	12.3	-0.94	-1.92	AAA-AAA	0.00	999	1.000	1.000	0.5564
214232_at	172.2	89.9	-0.94	-1.92	MAA-AAM	0.00	999	1.000	1.000	0.4299
220766_at	32.0	16.7	-0.94	-1.92	AAA-AAA	0.00	999	1.000	1.000	0.5532
220649_at	5.2	10.0	0.94	1.92	AAA-AAA	0.00	999	1.000	1.000	0.5295
210619_s_at	42.1	21.9	-0.94	-1.92	AAA-AAA	0.00	999	1.000	1.000	0.1654
211351_at	11.9	6.2	-0.94	-1.92	AAA-AAA	0.00	999	1.000	1.000	0.5884
233921_s_at	75.8	39.6	-0.94	-1.92	AAA-AAA	0.00	999	1.000	1.000	0.1872
236293_at	7.2	13.9	0.94	1.92	AAA-AAA	0.00	999	1.000	1.000	0.3922
51226_at	40.1	20.9	-0.94	-1.92	AAA-AAA	0.00	999	1.000	1.000	0.2046
231005_at	60.1	115.2	0.94	1.92	AAA-AAA	0.00	999	1.000	1.000	0.2355
231082_at	78.6	41.0	-0.94	-1.92	AAA-AAA	0.00	999	1.000	1.000	0.3754
231865_at	139.2	72.6	-0.94	-1.92	AAA-AAA	0.00	999	1.000	1.000	0.2908
230166_at	104.6	54.6	-0.94	-1.92	AAP-APA	0.00	999	1.000	1.000	0.1510
1566208_at	7.2	13.9	0.94	1.92	AAA-AAA	0.00	999	1.000	1.000	0.4076
1568685_at	13.9	26.7	0.94	1.92	AAA-AAA	0.00	999	1.000	1.000	0.6126
1555499_a_at	60.5	31.6	-0.94	-1.92	AAA-AAA	0.00	999	1.000	1.000	0.2642
243154_at	38.5	20.1	-0.94	-1.92	AAA-AAA	0.00	999	1.000	1.000	0.4040
1559263_s_at	10.6	5.5	-0.94	-1.92	AAA-AAA	0.00	999	1.000	1.000	0.5629
205692_s_at	11.3	21.6	0.94	1.92	AAA-AAA	0.00	999	1.000	1.000	0.3366
206742_at	20.3	39.0	0.94	1.92	AAA-AAA	0.00	999	1.000	1.000	0.5448
207562_at	23.7	12.4	-0.94	-1.92	AAA-AAA	0.00	999	1.000	1.000	0.4720
236088_at	37.6	19.6	-0.94	-1.92	AAA-AAA	0.00	999	1.000	1.000	0.0025
228224_at	10.9	20.8	0.94	1.92	AAA-AAA	0.00	999	1.000	1.000	0.2519
231373_at	12.2	6.4	-0.94	-1.92	AAA-AAA	0.00	999	1.000	1.000	0.1602

231261_at	6.9	13.2	0.94	1.92	AAA-AAA	0.00	999	1.000	1.000	0.1830
1568743_at	29.3	15.3	-0.94	-1.91	AAA-AAA	0.00	999	1.000	1.000	0.3529
1552327_at	4.7	9.1	0.94	1.92	AAA-AAA	0.00	999	1.000	1.000	0.4593
1553815_a_at	46.7	24.4	-0.94	-1.91	AAA-AAA	0.00	999	1.000	1.000	0.3911
243359_at	5.5	10.5	0.94	1.91	AAA-AAA	0.00	999	1.000	1.000	0.1880
1562358_at	7.8	4.1	-0.94	-1.92	AAA-AAA	0.00	999	1.000	1.000	0.1898
1561534_at	15.3	8.0	-0.94	-1.92	AAA-AAA	0.00	999	1.000	1.000	0.2175
244594_x_at	39.3	20.5	-0.94	-1.92	AAA-AAA	0.00	999	1.000	1.000	0.5143
220997_s_at	15.2	8.0	-0.94	-1.91	AAA-AAA	0.00	999	1.000	1.000	0.5853
206890_at	111.8	58.4	-0.94	-1.91	AAA-AAA	0.00	999	1.000	1.000	0.0481
210702_s_at	3.8	7.3	0.94	1.92	AAA-AAA	0.00	999	1.000	1.000	0.4988
210929_s_at	66.9	34.9	-0.94	-1.92	AAA-MMA	0.00	999	1.000	1.000	0.4369
233444_at	8.0	15.2	0.94	1.91	AAA-AAA	0.00	999	1.000	1.000	0.3582
227084_at	45.3	23.7	-0.94	-1.91	AAA-AAA	0.00	999	1.000	1.000	0.6005
202627_s_at	1803.5	942.7	-0.94	-1.91	PPP-PPP	0.00	999	1.000	1.000	0.0336
240369_at	52.8	27.6	-0.94	-1.91	AAA-AAA	0.00	999	1.000	1.000	0.4652
1558825_s_at	18.6	35.6	0.94	1.91	AAA-AAA	0.00	999	1.000	1.000	0.1210
1558608_a_at	15.6	29.9	0.94	1.91	AAA-AAA	0.00	999	1.000	1.000	0.2129
1558041_a_at	111.1	58.1	-0.94	-1.91	AAA-AAA	0.00	999	1.000	1.000	0.1143
1561479_at	22.9	12.0	-0.94	-1.91	AAA-AAA	0.00	999	1.000	1.000	0.4764
208440_at	10.3	19.6	0.94	1.91	AAA-AAA	0.00	999	1.000	1.000	0.3921
232458_at	6.9	13.2	0.94	1.91	AAA-AAA	0.00	999	1.000	1.000	0.3971
231610_at	43.1	22.5	-0.94	-1.91	AAA-AAA	0.00	999	1.000	1.000	0.3916
229806_at	39.3	20.6	-0.94	-1.91	AAA-AAA	0.00	999	1.000	1.000	0.3661
215143_at	68.6	35.9	-0.94	-1.91	APP-AAA	0.00	999	1.000	1.000	0.1058
241213_x_at	35.3	18.4	-0.94	-1.91	AAA-AAA	0.00	999	1.000	1.000	0.1460
1556377_s_at	28.3	14.8	-0.94	-1.91	AAA-AAA	0.00	999	1.000	1.000	0.3871
1554327_a_at	142.7	273.0	0.94	1.91	AAA-AAA	0.00	999	1.000	1.000	0.2219
1555811_at	62.2	118.9	0.94	1.91	AAA-AAA	0.00	999	1.000	1.000	0.4195
1559326_at	41.9	21.9	-0.94	-1.91	AAA-AAA	0.00	999	1.000	1.000	0.4490
1560030_at	8.1	15.5	0.94	1.91	AAA-AAA	0.00	999	1.000	1.000	0.4158
216416_at	40.5	21.2	-0.94	-1.91	AAA-AAA	0.00	999	1.000	1.000	0.5854
221275_s_at	6.9	13.3	0.94	1.91	AAA-AAA	0.00	999	1.000	1.000	0.0961
219740_at	17.6	9.2	-0.94	-1.91	AAA-AAA	0.00	999	1.000	1.000	0.3956
235312_s_at	55.0	28.8	-0.94	-1.91	AAA-AAA	0.00	999	1.000	1.000	0.1958
235194_at	190.2	99.5	-0.94	-1.91	AAA-AAM	0.00	999	1.000	1.000	0.2969
223816_at	6.7	12.9	0.94	1.91	AAA-AAA	0.00	999	1.000	1.000	0.5266
229808_at	23.2	44.4	0.94	1.91	AAA-AAA	0.00	999	1.000	1.000	0.3934
228997_at	118.8	227.0	0.94	1.91	AAA-AAA	0.00	999	1.000	1.000	0.0845
1570131_at	9.3	17.8	0.93	1.91	AAA-AAA	0.00	999	1.000	1.000	0.3221
1561392_at	24.9	13.0	-0.93	-1.91	AAA-AAA	0.00	999	1.000	1.000	0.4655
1560909_x_at	11.0	5.7	-0.93	-1.91	AAA-AAA	0.00	999	1.000	1.000	0.1885

1560168_at	4.1	7.9	0.93	1.91	AAA-AAA	0.00	999	1.000	1.000	0.3114
208426_x_at	58.6	30.7	-0.93	-1.91	AAA-AAA	0.00	999	1.000	1.000	0.1625
234639_x_at	7.6	14.6	0.93	1.91	AAA-AAA	0.00	999	1.000	1.000	0.3448
236264_at	12.2	23.4	0.93	1.91	AAA-AAA	0.00	999	1.000	1.000	0.6170
237318_at	8.2	4.3	-0.93	-1.91	AAA-AAA	0.00	999	1.000	1.000	0.0207
230806_s_at	72.1	37.7	-0.93	-1.91	AAA-AAA	0.00	999	1.000	1.000	0.5716
1569642_at	94.2	49.3	-0.93	-1.91	AAA-AAA	0.00	999	1.000	1.000	0.4619
1552808_at	13.7	7.2	-0.93	-1.91	AAA-AAA	0.00	999	1.000	1.000	0.2187
244816_at	11.1	21.2	0.93	1.91	AAA-AAA	0.00	999	1.000	1.000	0.3487
1558631_at	91.1	47.7	-0.93	-1.91	AAA-AAA	0.00	999	1.000	1.000	0.2729
1557630_s_at	20.1	38.3	0.93	1.91	AAA-AAA	0.00	999	1.000	1.000	0.3622
1560276_at	15.2	8.0	-0.93	-1.91	AAA-AAA	0.00	999	1.000	1.000	0.3803
215451_s_at	46.0	87.9	0.93	1.91	AAA-AAA	0.00	999	1.000	1.000	0.3526
214122_at	27.9	53.3	0.93	1.91	AAA-AAA	0.00	999	1.000	1.000	0.3728
214412_at	10.7	5.6	-0.93	-1.91	AAA-AAA	0.00	999	1.000	1.000	0.0067
211872_s_at	23.5	44.9	0.93	1.91	AAA-AAA	0.00	999	1.000	1.000	0.2431
236216_at	19.6	10.3	-0.93	-1.91	AAA-AAA	0.00	999	1.000	1.000	0.2595
222871_at	6.7	12.7	0.93	1.91	AAA-AAA	0.00	999	1.000	1.000	0.2078
223872_at	9.8	5.1	-0.93	-1.91	AAA-AAA	0.00	999	1.000	1.000	0.4560
239165_at	142.7	74.8	-0.93	-1.91	PAP-AAA	0.00	999	1.000	1.000	0.3050
1554680_s_at	40.4	21.2	-0.93	-1.91	AAA-AAA	0.00	999	1.000	1.000	0.4472
1555396_s_at	7.2	13.7	0.93	1.91	AAA-AAA	0.00	999	1.000	1.000	0.1326
243138_at	39.6	75.6	0.93	1.91	AAA-AAA	0.00	999	1.000	1.000	0.4802
243531_at	125.3	65.6	-0.93	-1.91	AAA-AAM	0.00	999	1.000	1.000	0.4327
213055_at	122.8	64.4	-0.93	-1.91	AAA-AAA	0.00	999	1.000	1.000	0.3609
221112_at	81.2	42.6	-0.93	-1.91	AAA-AAA	0.00	999	1.000	1.000	0.3286
206988_at	39.0	20.4	-0.93	-1.91	AAA-AAA	0.00	999	1.000	1.000	0.5017
203477_at	7.1	13.5	0.93	1.91	AAA-AAA	0.00	999	1.000	1.000	0.3327
203029_s_at	15.5	29.7	0.93	1.91	AAA-AAA	0.00	999	1.000	1.000	0.4751
237851_at	17.0	8.9	-0.93	-1.91	AAA-AAA	0.00	999	1.000	1.000	0.1601
236418_at	5.4	10.3	0.93	1.91	AAA-AAA	0.00	999	1.000	1.000	0.1376
205398_s_at	436.8	229.1	-0.93	-1.91	PPP-PAP	0.00	999	1.000	1.000	0.0917
242217_s_at	86.9	45.6	-0.93	-1.91	AAA-AAA	0.00	999	1.000	1.000	0.3857
242022_at	47.7	91.0	0.93	1.91	AAA-AAA	0.00	999	1.000	1.000	0.2692
1552691_at	18.0	9.4	-0.93	-1.91	AAA-AAA	0.00	999	1.000	1.000	0.5664
1554517_x_at	124.3	237.0	0.93	1.91	AMA-AMA	0.00	999	1.000	1.000	0.4095
1556213_a_at	13.0	24.7	0.93	1.91	AAA-AAA	0.00	999	1.000	1.000	0.5630
1560957_at	20.5	10.8	-0.93	-1.91	AAA-AAA	0.00	999	1.000	1.000	0.4729
211827_s_at	14.0	26.7	0.93	1.91	AAA-AAA	0.00	999	1.000	1.000	0.1697
211642_at	6.0	3.2	-0.93	-1.91	AAA-AAA	0.00	999	1.000	1.000	0.5228
208046_at	28.4	14.9	-0.93	-1.91	AAA-AAA	0.00	999	1.000	1.000	0.4730
207465_at	9.0	4.7	-0.93	-1.91	AAA-AAA	0.00	999	1.000	1.000	0.5494

208558_at	34.8	18.3	-0.93	-1.91	AAA-AAA	0.00	999	1.000	1.000	0.4767
237852_at	74.2	38.9	-0.93	-1.91	AMA-AAA	0.00	999	1.000	1.000	0.5013
238355_at	3.4	6.4	0.93	1.91	AAA-AAA	0.00	999	1.000	1.000	0.4025
222361_at	16.1	30.7	0.93	1.91	AAA-AAA	0.00	999	1.000	1.000	0.4632
221594_at	60.3	31.7	-0.93	-1.91	AAP-AAA	0.00	999	1.000	1.000	0.1280
239309_at	15.0	7.9	-0.93	-1.91	AAA-AAA	0.00	999	1.000	1.000	0.3013
239304_at	39.9	20.9	-0.93	-1.91	AAA-AAA	0.00	999	1.000	1.000	0.4367
1553702_at	39.9	21.0	-0.93	-1.91	AAA-AAA	0.00	999	1.000	1.000	0.2892
1555995_a_at	30.5	58.1	0.93	1.91	AAA-AAA	0.00	999	1.000	1.000	0.5410
243566_at	20.9	11.0	-0.93	-1.91	AAA-AAA	0.00	999	1.000	1.000	0.5272
243075_at	12.2	23.3	0.93	1.91	AAA-AAA	0.00	999	1.000	1.000	0.3701
215155_at	14.6	27.8	0.93	1.91	AAA-AAA	0.00	999	1.000	1.000	0.3023
213745_at	4.9	2.6	-0.93	-1.91	AAA-AAA	0.00	999	1.000	1.000	0.2113
204273_at	2.2	4.2	0.93	1.91	AAA-AAA	0.00	999	1.000	1.000	0.5251
AFFX-TrpnX-5_at	26.0	13.6	-0.93	-1.91	AAA-AAA	0.00	999	1.000	1.000	0.4293
205200_at	27.0	14.2	-0.93	-1.91	AAA-AAA	0.00	999	1.000	1.000	0.3277
234220_at	12.3	6.5	-0.93	-1.91	AAA-AAA	0.00	999	1.000	1.000	0.3024
234874_at	70.8	37.2	-0.93	-1.91	AAA-AAA	0.00	999	1.000	1.000	0.2734
237811_at	13.6	26.0	0.93	1.91	AAA-AAA	0.00	999	1.000	1.000	0.0740
236838_at	18.2	34.6	0.93	1.91	AAA-AAA	0.00	999	1.000	1.000	0.2096
223754_at	23.4	44.5	0.93	1.91	AAA-AAA	0.00	999	1.000	1.000	0.5827
223663_at	97.0	50.9	-0.93	-1.91	AAA-AAA	0.00	999	1.000	1.000	0.2728
216180_s_at	85.4	44.9	-0.93	-1.91	AAA-AAP	0.00	999	1.000	1.000	0.2095
1566178_x_at	20.5	39.1	0.93	1.90	AAA-AAA	0.00	999	1.000	1.000	0.0279
1565814_at	38.7	20.3	-0.93	-1.90	AAA-AAA	0.00	999	1.000	1.000	0.3294
1558124_at	15.5	29.6	0.93	1.91	AAA-AAA	0.00	999	1.000	1.000	0.3332
1560980_a_at	1.8	3.5	0.93	1.91	AAA-AAA	0.00	999	1.000	1.000	0.6040
1561926_at	19.9	10.5	-0.93	-1.90	AAA-AAA	0.00	999	1.000	1.000	0.6433
1560684_x_at	17.8	9.3	-0.93	-1.90	AAA-AAA	0.00	999	1.000	1.000	0.4817
215408_at	36.5	19.2	-0.93	-1.90	AAA-AAA	0.00	999	1.000	1.000	0.5265
232724_at	37.3	19.6	-0.93	-1.90	AAA-AAA	0.00	999	1.000	1.000	0.5489
237809_at	19.0	10.0	-0.93	-1.90	AAA-AAA	0.00	999	1.000	1.000	0.4904
236323_at	5.5	10.4	0.93	1.90	AAA-AAA	0.00	999	1.000	1.000	0.3172
236768_at	2.7	5.1	0.93	1.90	AAA-AAA	0.00	999	1.000	1.000	0.4363
225895_at	36.7	19.3	-0.93	-1.90	AAA-AAA	0.00	999	1.000	1.000	0.4368
232144_at	13.0	24.7	0.93	1.90	AAA-AAA	0.00	999	1.000	1.000	0.3897
231665_at	30.5	16.0	-0.93	-1.90	AAA-AAA	0.00	999	1.000	1.000	0.5669
228973_at	25.6	13.4	-0.93	-1.90	AAA-AAA	0.00	999	1.000	1.000	0.3982
1557569_at	47.0	24.7	-0.93	-1.90	AAA-AAA	0.00	999	1.000	1.000	0.5703
1554279_a_at	102.9	54.1	-0.93	-1.90	AAA-AAA	0.00	999	1.000	1.000	0.3632
1554049_s_at	87.3	166.1	0.93	1.90	AAA-AAM	0.00	999	1.000	1.000	0.2470
1561261_at	7.2	13.7	0.93	1.90	AAA-AAA	0.00	999	1.000	1.000	0.5945

1562290_at	10.9	5.7	-0.93	-1.90	AAA-AAA	0.00	999	1.000	1.000	0.6085
214090_at	35.1	18.4	-0.93	-1.90	AAA-AAA	0.00	999	1.000	1.000	0.5452
204692_at	196.4	103.2	-0.93	-1.90	AAA-AAA	0.00	999	1.000	1.000	0.1203
204553_x_at	151.3	79.5	-0.93	-1.90	AAA-AAA	0.00	999	1.000	1.000	0.4619
207603_at	20.0	10.5	-0.93	-1.90	AAA-AAA	0.00	999	1.000	1.000	0.2038
209070_s_at	36.2	19.0	-0.93	-1.90	AAA-AAA	0.00	999	1.000	1.000	0.4920
237281_at	10.6	20.1	0.93	1.90	AAA-AAA	0.00	999	1.000	1.000	0.6017
57588_at	11.2	21.3	0.93	1.90	AAA-AAA	0.00	999	1.000	1.000	0.3784
231154_x_at	20.9	39.8	0.93	1.90	AAA-AAA	0.00	999	1.000	1.000	0.4619
229612_at	53.1	27.9	-0.93	-1.90	AAA-AAA	0.00	999	1.000	1.000	0.1511
219434_at	81.9	43.1	-0.93	-1.90	APA-AAA	0.00	999	1.000	1.000	0.1394
1552732_at	23.0	12.1	-0.93	-1.90	AAA-AAP	0.00	999	1.000	1.000	0.4544
233630_at	46.0	24.2	-0.93	-1.90	APA-AAP	0.00	999	1.000	1.000	0.5075
241631_at	22.7	12.0	-0.93	-1.90	AAA-AAA	0.00	999	1.000	1.000	0.3151
1563279_at	7.5	14.2	0.93	1.90	AAA-AAA	0.00	999	1.000	1.000	0.2895
1568883_at	13.9	26.3	0.93	1.90	AAA-AAA	0.00	999	1.000	1.000	0.5226
1552900_a_at	7.4	14.1	0.93	1.90	AAA-AAA	0.00	999	1.000	1.000	0.3229
242642_at	31.5	16.6	-0.93	-1.90	AAA-AAA	0.00	999	1.000	1.000	0.4705
1559930_at	19.9	10.5	-0.93	-1.90	AAA-AAA	0.00	999	1.000	1.000	0.2557
205325_at	5.9	11.3	0.93	1.90	AAA-AAA	0.00	999	1.000	1.000	0.2809
207567_at	15.0	28.5	0.93	1.90	AAA-AAA	0.00	999	1.000	1.000	0.2799
236541_at	40.0	21.0	-0.93	-1.90	AAA-AAA	0.00	999	1.000	1.000	0.2904
222942_s_at	42.4	80.7	0.93	1.90	AAA-AAA	0.00	999	1.000	1.000	0.2096
225474_at	171.3	90.2	-0.93	-1.90	PPA-MPP	0.00	999	1.000	1.000	0.0473
238402_s_at	223.0	117.4	-0.93	-1.90	MPA-AAA	0.00	999	1.000	1.000	0.1177
240018_at	28.0	14.7	-0.93	-1.90	PPA-APA	0.00	999	1.000	1.000	0.4433
1564603_at	36.2	68.7	0.93	1.90	AAA-AAA	0.00	999	1.000	1.000	0.2042
1562723_at	20.0	38.0	0.93	1.90	AAA-AAA	0.00	999	1.000	1.000	0.5820
241890_at	11.4	21.8	0.93	1.90	AAA-AAA	0.00	999	1.000	1.000	0.4975
1564621_a_at	5.1	9.6	0.93	1.90	AAA-AAA	0.00	999	1.000	1.000	0.0239
1554855_at	5.7	3.0	-0.93	-1.90	AAA-AAA	0.00	999	1.000	1.000	0.0600
1555471_a_at	11.4	21.6	0.93	1.90	AAA-AAA	0.00	999	1.000	1.000	0.4713
1555375_at	3.3	6.2	0.93	1.90	AAA-AAA	0.00	999	1.000	1.000	0.5194
1559667_at	138.4	72.8	-0.93	-1.90	AAA-AAA	0.00	999	1.000	1.000	0.1559
205724_at	25.1	47.7	0.93	1.90	AAA-AAA	0.00	999	1.000	1.000	0.1826
226997_at	24.3	46.3	0.93	1.90	AAA-AAA	0.00	999	1.000	1.000	0.5508
223630_at	132.3	69.6	-0.93	-1.90	AAA-AAA	0.00	999	1.000	1.000	0.0549
1559042_at	21.5	11.4	-0.93	-1.90	APA-AAP	0.00	999	1.000	1.000	0.5685
241742_at	7.0	13.3	0.93	1.90	AAA-AAA	0.00	999	1.000	1.000	0.4076
239244_at	9.1	17.2	0.93	1.90	AAA-AAA	0.00	999	1.000	1.000	0.5757
1554371_at	76.0	40.0	-0.93	-1.90	AAA-AAA	0.00	999	1.000	1.000	0.0374
243172_at	15.2	8.0	-0.93	-1.90	AAA-AAA	0.00	999	1.000	1.000	0.5171

1561387_a_at	8.2	15.5	0.93	1.90	AAA-AAA	0.00	999	1.000	1.000	0.4832
221334_s_at	40.4	76.6	0.93	1.90	AAA-AAM	0.00	999	1.000	1.000	0.3587
221624_at	14.3	27.1	0.93	1.90	AAA-AAA	0.00	999	1.000	1.000	0.4566
206556_at	69.3	36.5	-0.93	-1.90	AAA-AAA	0.00	999	1.000	1.000	0.3396
232727_at	43.4	82.4	0.93	1.90	AAA-AAA	0.00	999	1.000	1.000	0.3983
237916_at	37.8	19.9	-0.93	-1.90	AAA-AAA	0.00	999	1.000	1.000	0.1322
223235_s_at	13.9	7.3	-0.93	-1.90	AAA-AAA	0.00	999	1.000	1.000	0.6491
231432_at	7.8	14.9	0.93	1.90	AAA-AAA	0.00	999	1.000	1.000	0.5411
239962_at	6.1	3.2	-0.92	-1.90	AAA-AAA	0.00	999	1.000	1.000	0.3304
1569355_at	10.5	19.9	0.92	1.90	AAA-AAA	0.00	999	1.000	1.000	0.2938
1561085_at	96.7	51.0	-0.92	-1.90	AAM-AAA	0.00	999	1.000	1.000	0.0585
1559789_a_at	19.2	10.1	-0.92	-1.90	AAA-AAA	0.00	999	1.000	1.000	0.4828
215724_at	5.6	10.7	0.92	1.90	AAA-AAA	0.00	999	1.000	1.000	0.4059
215839_at	9.8	18.6	0.92	1.90	AAA-AAA	0.00	999	1.000	1.000	0.3618
216543_at	8.0	4.2	-0.92	-1.90	AAA-AAA	0.00	999	1.000	1.000	0.4160
221639_x_at	60.5	31.9	-0.92	-1.90	AAA-AAA	0.00	999	1.000	1.000	0.2772
203471_s_at	24.7	46.9	0.92	1.90	AAA-AAA	0.00	999	1.000	1.000	0.2421
210390_s_at	13.8	7.2	-0.92	-1.90	AAA-AAA	0.00	999	1.000	1.000	0.5850
207981_s_at	26.5	14.0	-0.92	-1.90	AAM-AAA	0.00	999	1.000	1.000	0.4378
234862_at	78.7	41.5	-0.92	-1.90	AAA-AAA	0.00	999	1.000	1.000	0.2617
224095_at	4.5	8.5	0.92	1.90	AAA-AAA	0.00	999	1.000	1.000	0.2848
241589_at	8.8	16.6	0.92	1.90	AAA-AAA	0.00	999	1.000	1.000	0.3504
241148_at	6.5	12.3	0.92	1.90	AAA-AAA	0.00	999	1.000	1.000	0.0907
241598_at	37.6	19.8	-0.92	-1.90	AAA-AAA	0.00	999	1.000	1.000	0.5913
1552552_s_at	22.1	11.7	-0.92	-1.90	AAA-AAA	0.00	999	1.000	1.000	0.5997
242791_at	26.8	14.2	-0.92	-1.90	AAA-AAA	0.00	999	1.000	1.000	0.5619
203131_at	16.0	8.4	-0.92	-1.90	AAA-AAA	0.00	999	1.000	1.000	0.5698
211500_at	15.0	28.4	0.92	1.90	AAA-AAA	0.00	999	1.000	1.000	0.4245
208196_x_at	33.0	17.4	-0.92	-1.90	AAA-AAA	0.00	999	1.000	1.000	0.4907
238228_at	117.5	62.0	-0.92	-1.90	AAA-AAA	0.00	999	1.000	1.000	0.2653
232314_at	11.7	22.1	0.92	1.90	AAA-AAA	0.00	999	1.000	1.000	0.4746
229476_s_at	17.5	9.2	-0.92	-1.90	AAA-AAA	0.00	999	1.000	1.000	0.4809
1562791_at	13.5	7.1	-0.92	-1.90	AAA-AAA	0.00	999	1.000	1.000	0.3814
1563643_at	13.6	25.7	0.92	1.89	MAA-AAA	0.00	999	1.000	1.000	0.4944
1552722_at	9.7	5.1	-0.92	-1.90	AAA-AAA	0.00	999	1.000	1.000	0.6343
244751_at	58.1	30.7	-0.92	-1.89	AAA-AAA	0.00	999	1.000	1.000	0.2465
243726_at	7.1	3.7	-0.92	-1.90	AAA-AAA	0.00	999	1.000	1.000	0.5547
215974_at	22.7	43.0	0.92	1.90	AAA-AAA	0.00	999	1.000	1.000	0.5275
202831_at	84.8	44.8	-0.92	-1.89	AAA-AAA	0.00	999	1.000	1.000	0.4342
207342_at	4.8	9.1	0.92	1.90	AAA-AAA	0.00	999	1.000	1.000	0.2417
208560_at	11.0	20.8	0.92	1.90	AAA-AAA	0.00	999	1.000	1.000	0.4588
209199_s_at	17.0	32.1	0.92	1.90	AAA-AAA	0.00	999	1.000	1.000	0.5429

234642_at	13.0	6.8	-0.92	-1.89	AAA-AAA	0.00	999	1.000	1.000	0.1510
238868_at	21.7	11.5	-0.92	-1.89	AAA-AAA	0.00	999	1.000	1.000	0.5589
241276_at	37.5	71.0	0.92	1.89	AAA-AAA	0.00	999	1.000	1.000	0.1813
242197_x_at	9.2	17.4	0.92	1.89	AAA-AAA	0.00	999	1.000	1.000	0.4684
1563370_at	4.3	8.2	0.92	1.89	AAA-AAA	0.00	999	1.000	1.000	0.2522
1570146_at	3.9	7.4	0.92	1.89	AAA-AAA	0.00	999	1.000	1.000	0.2803
1552409_a_at	50.9	26.9	-0.92	-1.89	AAA-AAA	0.00	999	1.000	1.000	0.4568
243581_at	5.8	3.1	-0.92	-1.89	AAA-AAA	0.00	999	1.000	1.000	0.0960
213068_at	34.7	18.3	-0.92	-1.89	AAA-AAA	0.00	999	1.000	1.000	0.3289
221583_s_at	19.5	10.3	-0.92	-1.89	AAA-AAA	0.00	999	1.000	1.000	0.5370
203980_at	12.8	6.8	-0.92	-1.89	AAA-AAA	0.00	999	1.000	1.000	0.5892
205302_at	17.0	32.1	0.92	1.89	AAA-AAA	0.00	999	1.000	1.000	0.2735
204548_at	20.3	10.7	-0.92	-1.89	AAA-AAA	0.00	999	1.000	1.000	0.4757
234256_at	19.0	36.0	0.92	1.89	AAA-AAA	0.00	999	1.000	1.000	0.3161
233285_at	12.8	24.3	0.92	1.89	AMA-AAA	0.00	999	1.000	1.000	0.5636
233422_at	10.6	5.6	-0.92	-1.89	AAA-AAA	0.00	999	1.000	1.000	0.2007
236287_at	6.5	3.4	-0.92	-1.89	AAA-AAA	0.00	999	1.000	1.000	0.1735
227782_at	24.5	13.0	-0.92	-1.89	AAA-AAA	0.00	999	1.000	1.000	0.1288
224179_s_at	36.2	19.1	-0.92	-1.89	AAA-AAA	0.00	999	1.000	1.000	0.4666
206456_at	29.0	15.3	-0.92	-1.89	AAP-AAA	0.00	999	1.000	1.000	0.3378
242309_at	38.9	20.6	-0.92	-1.89	APA-AAA	0.00	999	1.000	1.000	0.4101
230665_at	53.4	28.2	-0.92	-1.89	PAA-PAA	0.00	999	1.000	1.000	0.5008
1563103_at	67.4	35.6	-0.92	-1.89	AAA-AAA	0.00	999	1.000	1.000	0.1296
1563584_at	46.3	24.5	-0.92	-1.89	AAA-AAA	0.00	999	1.000	1.000	0.1336
1566288_at	12.6	6.7	-0.92	-1.89	AAA-AAA	0.00	999	1.000	1.000	0.5932
1567387_at	6.3	11.9	0.92	1.89	AAA-AAA	0.00	999	1.000	1.000	0.3811
1558795_at	12.1	6.4	-0.92	-1.89	AAA-AAA	0.00	999	1.000	1.000	0.6580
1560520_at	39.1	74.0	0.92	1.89	AAA-AAA	0.00	999	1.000	1.000	0.2614
214645_at	14.7	27.7	0.92	1.89	AAA-AAA	0.00	999	1.000	1.000	0.4617
214254_at	22.5	42.6	0.92	1.89	AAA-AAA	0.00	999	1.000	1.000	0.4059
210757_x_at	121.2	64.0	-0.92	-1.89	AAA-AAA	0.00	999	1.000	1.000	0.2460
234320_at	10.6	5.6	-0.92	-1.89	AAA-AAA	0.00	999	1.000	1.000	0.0452
238119_at	79.5	150.4	0.92	1.89	AAA-MMA	0.00	999	1.000	1.000	0.1029
235945_at	13.4	25.3	0.92	1.89	AAA-MAA	0.00	999	1.000	1.000	0.4475
1559601_at	13.0	6.9	-0.92	-1.89	APA-AAA	0.00	999	1.000	1.000	0.5377
1569917_at	20.7	39.1	0.92	1.89	AAA-AAA	0.00	999	1.000	1.000	0.3830
240463_at	14.4	7.6	-0.92	-1.89	AAA-AAA	0.00	999	1.000	1.000	0.5690
1552355_s_at	21.7	41.1	0.92	1.89	AAA-AAA	0.00	999	1.000	1.000	0.2034
244100_at	21.4	40.4	0.92	1.89	AAA-AAA	0.00	999	1.000	1.000	0.2777
216043_x_at	57.8	109.3	0.92	1.89	AAA-AAA	0.00	999	1.000	1.000	0.0911
214633_at	12.0	22.6	0.92	1.89	AAA-AAA	0.00	999	1.000	1.000	0.4526
234296_s_at	2.0	3.7	0.92	1.89	AAA-AAA	0.00	999	1.000	1.000	0.0537

235460_at	13.4	7.1	-0.92	-1.89	AAA-AAA	0.00	999	1.000	1.000	0.3171
237918_at	24.6	13.0	-0.92	-1.89	AAA-AAA	0.00	999	1.000	1.000	0.2930
38521_at	35.1	66.4	0.92	1.89	AAA-AAA	0.00	999	1.000	1.000	0.2313
230067_at	28.7	54.2	0.92	1.89	AAA-AAA	0.00	999	1.000	1.000	0.4341
1553713_a_at	164.3	87.0	-0.92	-1.89	PPA-AAA	0.00	999	1.000	1.000	0.1013
206508_at	1002.8	530.8	-0.92	-1.89	PAP-PPP	0.00	999	1.000	1.000	0.1030
1564198_a_at	23.9	12.7	-0.92	-1.89	AAA-AAA	0.00	999	1.000	1.000	0.4744
1569250_at	34.7	65.6	0.92	1.89	AAA-MAA	0.00	999	1.000	1.000	0.5154
1569681_at	8.7	4.6	-0.92	-1.89	AAA-AAA	0.00	999	1.000	1.000	0.4205
1553362_at	4.1	7.8	0.92	1.89	AAA-AAA	0.00	999	1.000	1.000	0.4498
242957_at	39.9	75.4	0.92	1.89	AAA-AAA	0.00	999	1.000	1.000	0.2458
1560767_at	41.3	78.0	0.92	1.89	AAA-AAA	0.00	999	1.000	1.000	0.1944
215734_at	63.1	119.2	0.92	1.89	AAA-AAA	0.00	999	1.000	1.000	0.2375
213195_at	83.3	44.1	-0.92	-1.89	AAA-AAA	0.00	999	1.000	1.000	0.3237
206712_at	11.8	22.2	0.92	1.89	AAA-AAA	0.00	999	1.000	1.000	0.6397
211525_s_at	3.5	6.6	0.92	1.89	AAA-AAA	0.00	999	1.000	1.000	0.5382
211796_s_at	24.7	13.1	-0.92	-1.89	AAA-AAA	0.00	999	1.000	1.000	0.5577
233029_at	27.0	14.3	-0.92	-1.89	AAA-AAA	0.00	999	1.000	1.000	0.4367
232970_at	58.1	30.7	-0.92	-1.89	AAA-AAA	0.00	999	1.000	1.000	0.4279
238038_at	34.1	64.4	0.92	1.89	AAA-AAA	0.00	999	1.000	1.000	0.3389
236286_at	11.3	21.3	0.92	1.89	AAA-AAA	0.00	999	1.000	1.000	0.0537
231397_at	13.4	25.4	0.92	1.89	AAA-AAA	0.00	999	1.000	1.000	0.3931
231273_x_at	13.2	24.9	0.92	1.89	AAA-AAA	0.00	999	1.000	1.000	0.3971
220776_at	94.8	50.2	-0.92	-1.89	PPM-MAP	0.00	999	1.000	1.000	0.0336
234387_at	46.1	24.4	-0.92	-1.89	AAP-PPA	0.00	999	1.000	1.000	0.6085
1564533_at	8.9	16.7	0.92	1.89	AAA-AAA	0.00	999	1.000	1.000	0.4942
241791_at	15.6	8.3	-0.92	-1.89	AAA-AAA	0.00	999	1.000	1.000	0.1293
1566538_at	68.8	36.5	-0.92	-1.89	AAA-AAA	0.00	999	1.000	1.000	0.1656
241083_at	3.1	5.9	0.92	1.89	AAA-AAA	0.00	999	1.000	1.000	0.5554
241113_at	16.4	31.0	0.92	1.89	AAA-AAA	0.00	999	1.000	1.000	0.4202
1552827_s_at	36.8	19.5	-0.92	-1.89	AAA-AAA	0.00	999	1.000	1.000	0.4939
1553605_a_at	7.0	3.7	-0.92	-1.89	MAA-AAA	0.00	999	1.000	1.000	0.6866
1559616_x_at	32.0	60.3	0.92	1.89	AAA-AAA	0.00	999	1.000	1.000	0.5423
216265_x_at	14.1	26.6	0.92	1.89	AAA-AAA	0.00	999	1.000	1.000	0.1488
216289_at	21.2	40.1	0.92	1.89	AAA-AAA	0.00	999	1.000	1.000	0.3739
214000_s_at	98.3	52.1	-0.92	-1.89	AMA-AAA	0.00	999	1.000	1.000	0.1876
220142_at	41.3	77.9	0.92	1.89	AAA-AAA	0.00	999	1.000	1.000	0.2412
207118_s_at	5.2	9.9	0.92	1.89	AAA-AAA	0.00	999	1.000	1.000	0.0382
205299_s_at	14.9	28.2	0.92	1.89	AAA-AAA	0.00	999	1.000	1.000	0.5629
205632_s_at	26.9	50.8	0.92	1.89	AAA-AAA	0.00	999	1.000	1.000	0.2877
207886_s_at	6.7	12.6	0.92	1.89	AAA-AAA	0.00	999	1.000	1.000	0.1878
233836_at	49.3	26.1	-0.92	-1.89	AAA-AAA	0.00	999	1.000	1.000	0.4913

233930_at	13.2	25.0	0.92	1.89	AAA-AAA	0.00	999	1.000	1.000	0.3189
227739_at	87.8	165.7	0.92	1.89	AAA-AAA	0.00	999	1.000	1.000	0.1000
231958_at	32.4	61.3	0.92	1.89	AAA-AAA	0.00	999	1.000	1.000	0.2974
228922_at	18.7	35.3	0.92	1.89	AAA-AAA	0.00	999	1.000	1.000	0.5286
1568868_at	28.4	15.1	-0.92	-1.89	AAA-AAP	0.00	999	1.000	1.000	0.4469
241986_at	2.1	3.9	0.92	1.89	AAA-AAA	0.00	999	1.000	1.000	0.2508
1569270_at	28.8	15.3	-0.92	-1.89	AAA-AAA	0.00	999	1.000	1.000	0.2763
239429_at	18.8	10.0	-0.92	-1.89	AAA-AAA	0.00	999	1.000	1.000	0.3360
1556872_s_at	12.6	6.7	-0.92	-1.89	AAA-AAA	0.00	999	1.000	1.000	0.1996
1555191_a_at	26.3	13.9	-0.92	-1.89	AAA-AAA	0.00	999	1.000	1.000	0.5466
1555230_a_at	2.1	4.0	0.92	1.89	AAA-AAA	0.00	999	1.000	1.000	0.0938
1559208_at	6.3	12.0	0.92	1.89	AAA-AAA	0.00	999	1.000	1.000	0.6587
213945_s_at	36.5	19.3	-0.92	-1.89	AAA-AAA	0.00	999	1.000	1.000	0.4317
211008_s_at	68.7	36.4	-0.92	-1.89	AAA-AAA	0.00	999	1.000	1.000	0.1461
235448_at	60.3	32.0	-0.92	-1.89	AAA-AAA	0.00	999	1.000	1.000	0.4753
234828_at	21.5	11.4	-0.92	-1.89	AAA-AAA	0.00	999	1.000	1.000	0.0951
232736_s_at	79.3	42.0	-0.92	-1.89	AAA-AAA	0.00	999	1.000	1.000	0.2341
226172_at	56.3	29.8	-0.92	-1.89	AMA-AAA	0.00	999	1.000	1.000	0.1301
228579_at	65.4	123.5	0.92	1.89	AAM-MAA	0.00	999	1.000	1.000	0.1635
218954_s_at	141.6	75.1	-0.92	-1.89	AAP-AAA	0.00	999	1.000	1.000	0.2719
226487_at	94.3	50.0	-0.92	-1.89	APM-APA	0.00	999	1.000	1.000	0.3985
230748_at	53.3	28.2	-0.92	-1.89	APP-PAP	0.00	999	1.000	1.000	0.5510
1562684_at	17.3	32.7	0.92	1.89	AAA-AAA	0.00	999	1.000	1.000	0.5035
240996_at	50.7	26.9	-0.92	-1.89	AAA-AAA	0.00	999	1.000	1.000	0.4782
240653_at	21.4	40.4	0.92	1.89	AAA-AAA	0.00	999	1.000	1.000	0.5331
1554886_a_at	58.0	30.8	-0.92	-1.89	AAA-AAA	0.00	999	1.000	1.000	0.3070
1555245_s_at	16.7	8.9	-0.92	-1.89	AAA-AAA	0.00	999	1.000	1.000	0.6495
216139_s_at	77.1	40.9	-0.92	-1.89	AAA-AAA	0.00	999	1.000	1.000	0.2687
214480_at	5.4	10.2	0.92	1.89	AAA-AAA	0.00	999	1.000	1.000	0.0936
234209_at	10.7	20.2	0.92	1.89	AAA-AAA	0.00	999	1.000	1.000	0.5492
238293_at	27.9	52.7	0.92	1.89	AAA-AAA	0.00	999	1.000	1.000	0.2662
236652_at	32.7	17.3	-0.92	-1.89	AAA-AAA	0.00	999	1.000	1.000	0.4543
226388_at	22.9	12.2	-0.92	-1.89	AAA-AAA	0.00	999	1.000	1.000	0.6445
229008_at	4.1	7.7	0.92	1.89	AAA-AAA	0.00	999	1.000	1.000	0.0591
238971_at	11.8	6.2	-0.91	-1.88	AAA-AAA	0.00	999	1.000	1.000	0.0083
241624_at	16.0	30.1	0.91	1.88	AAA-AAA	0.00	999	1.000	1.000	0.4300
1553512_at	24.8	46.8	0.91	1.89	AAA-AAA	0.00	999	1.000	1.000	0.2705
243204_at	6.6	12.5	0.91	1.88	AAA-AAA	0.00	999	1.000	1.000	0.5602
1562054_at	4.3	2.3	-0.91	-1.88	AAA-AAA	0.00	999	1.000	1.000	0.2289
1561573_at	32.8	61.8	0.91	1.89	AAA-AAA	0.00	999	1.000	1.000	0.0431
1559945_at	54.6	102.8	0.91	1.88	AAA-AAA	0.00	999	1.000	1.000	0.6343
215270_at	17.6	33.2	0.91	1.89	AAA-AAA	0.00	999	1.000	1.000	0.2539

214602_at	6.0	11.2	0.91	1.89	AAA-AAA	0.00	999	1.000	1.000	0.4549
205244_s_at	12.3	6.5	-0.91	-1.88	AAA-AAA	0.00	999	1.000	1.000	0.3854
207533_at	26.1	13.8	-0.91	-1.89	AAA-AAA	0.00	999	1.000	1.000	0.3792
236734_at	7.3	13.8	0.91	1.89	AAA-AAA	0.00	999	1.000	1.000	0.4143
236990_at	12.0	22.6	0.91	1.88	AAA-AAA	0.00	999	1.000	1.000	0.4491
213934_s_at	195.9	104.1	-0.91	-1.88	PPP-PPA	0.00	999	1.000	1.000	0.1227
214121_x_at	160.3	85.1	-0.91	-1.88	PAA-AAA	0.00	999	1.000	1.000	0.3254
226439_s_at	40.3	21.4	-0.91	-1.88	AAP-AAA	0.00	999	1.000	1.000	0.4143
241597_at	30.8	16.3	-0.91	-1.88	AAA-AAA	0.00	999	1.000	1.000	0.1556
1566339_at	12.6	6.7	-0.91	-1.88	AAA-AAA	0.00	999	1.000	1.000	0.5953
1561443_at	46.6	24.8	-0.91	-1.88	AAA-AAA	0.00	999	1.000	1.000	0.4124
215860_at	26.1	49.2	0.91	1.88	AAA-AAA	0.00	999	1.000	1.000	0.3290
220203_at	27.1	14.4	-0.91	-1.88	AAA-AAA	0.00	999	1.000	1.000	0.3986
205422_s_at	3.6	6.7	0.91	1.88	AAA-AAA	0.00	999	1.000	1.000	0.3380
208242_at	18.8	10.0	-0.91	-1.88	AAA-AAA	0.00	999	1.000	1.000	0.3436
233454_at	40.8	21.7	-0.91	-1.88	AAA-AAA	0.00	999	1.000	1.000	0.2720
222050_at	40.5	21.5	-0.91	-1.88	AAA-AAA	0.00	999	1.000	1.000	0.1844
232106_s_at	93.1	175.2	0.91	1.88	AAA-AAA	0.00	999	1.000	1.000	0.3815
241150_at	12.5	23.5	0.91	1.88	AAA-AAA	0.00	999	1.000	1.000	0.3636
241381_at	19.2	36.2	0.91	1.88	AAA-AAA	0.00	999	1.000	1.000	0.4171
242430_at	5.8	11.0	0.91	1.88	AAA-AAA	0.00	999	1.000	1.000	0.1739
1552950_at	23.7	12.6	-0.91	-1.88	AAA-AAA	0.00	999	1.000	1.000	0.3775
243429_at	38.6	72.5	0.91	1.88	AAA-AAA	0.00	999	1.000	1.000	0.5038
1559438_at	17.0	32.0	0.91	1.88	AAA-AAA	0.00	999	1.000	1.000	0.4243
216815_at	13.5	25.5	0.91	1.88	AAA-AAA	0.00	999	1.000	1.000	0.4500
210414_at	50.8	95.5	0.91	1.88	AAA-AAA	0.00	999	1.000	1.000	0.3498
234883_x_at	25.5	48.0	0.91	1.88	AAA-AAA	0.00	999	1.000	1.000	0.4963
232756_at	19.8	37.2	0.91	1.88	AAA-AAA	0.00	999	1.000	1.000	0.3151
235832_at	10.3	19.4	0.91	1.88	AAA-AAA	0.00	999	1.000	1.000	0.4502
237069_s_at	4.9	9.3	0.91	1.88	AAA-AAA	0.00	999	1.000	1.000	0.4424
240922_at	14.6	27.4	0.91	1.88	AAA-AAA	0.00	999	1.000	1.000	0.6012
1566609_at	21.8	41.0	0.91	1.88	AAA-AAA	0.00	999	1.000	1.000	0.0784
1556929_at	18.8	35.4	0.91	1.88	AAA-AAA	0.00	999	1.000	1.000	0.5444
1552632_a_at	16.8	31.5	0.91	1.88	AAA-AAA	0.00	999	1.000	1.000	0.4334
1553326_at	13.4	7.2	-0.91	-1.88	AAA-AAA	0.00	999	1.000	1.000	0.5561
216530_at	58.7	110.4	0.91	1.88	AAA-AAA	0.00	999	1.000	1.000	0.1385
214523_at	81.9	43.5	-0.91	-1.88	AAA-AAA	0.00	999	1.000	1.000	0.3235
207107_at	13.3	25.0	0.91	1.88	AAA-AAA	0.00	999	1.000	1.000	0.5094
234113_at	34.0	64.0	0.91	1.88	AAA-AAA	0.00	999	1.000	1.000	0.3855
235723_at	11.4	21.4	0.91	1.88	AAA-AAA	0.00	999	1.000	1.000	0.4156
233946_at	142.0	75.5	-0.91	-1.88	AAM-AAM	0.00	999	1.000	1.000	0.0846
238877_at	19.7	10.5	-0.91	-1.88	AAA-AAA	0.00	999	1.000	1.000	0.5997

224147_at	59.5	31.7	-0.91	-1.88	AAA-AAA	0.00	999	1.000	1.000	0.3975
232300_at	145.5	77.4	-0.91	-1.88	PPP-PPA	0.00	999	1.000	1.000	0.2810
233716_at	18.6	9.9	-0.91	-1.88	PAA-AAA	0.00	999	1.000	1.000	0.3959
1569602_at	20.3	10.8	-0.91	-1.88	AAA-AAA	0.00	999	1.000	1.000	0.5267
1558411_at	7.7	14.4	0.91	1.88	AAA-AAA	0.00	999	1.000	1.000	0.6267
1561857_at	19.1	35.9	0.91	1.88	AAA-AAA	0.00	999	1.000	1.000	0.3821
210487_at	15.2	8.1	-0.91	-1.88	AAA-AAA	0.00	999	1.000	1.000	0.4589
210832_x_at	79.2	42.1	-0.91	-1.88	AAA-AAA	0.00	999	1.000	1.000	0.2699
238730_at	9.2	17.2	0.91	1.88	AAA-AAA	0.00	999	1.000	1.000	0.3734
241676_x_at	17.1	9.1	-0.91	-1.88	AAA-AAA	0.00	999	1.000	1.000	0.5900
1567044_s_at	17.9	33.6	0.91	1.88	AAA-AAA	0.00	999	1.000	1.000	0.0610
1552826_at	15.4	28.8	0.91	1.88	AAA-AAM	0.00	999	1.000	1.000	0.2292
1554646_at	106.4	56.7	-0.91	-1.88	AAA-AAA	0.00	999	1.000	1.000	0.2288
242716_at	8.7	4.6	-0.91	-1.88	AAA-AAA	0.00	999	1.000	1.000	0.3567
242804_at	26.5	49.7	0.91	1.88	AAA-AAA	0.00	999	1.000	1.000	0.3284
215676_at	40.0	75.0	0.91	1.88	AAA-AAA	0.00	999	1.000	1.000	0.4280
215951_at	134.7	71.8	-0.91	-1.88	AAA-AAA	0.00	999	1.000	1.000	0.0620
214026_s_at	9.7	5.2	-0.91	-1.88	AAA-AAA	0.00	999	1.000	1.000	0.2290
216876_s_at	10.6	5.6	-0.91	-1.88	AAA-AAA	0.00	999	1.000	1.000	0.5812
203813_s_at	7.3	13.8	0.91	1.88	AAA-AAA	0.00	999	1.000	1.000	0.2442
207597_at	40.4	21.5	-0.91	-1.88	MAA-AAA	0.00	999	1.000	1.000	0.2847
237505_at	10.2	19.2	0.91	1.88	AAA-AAA	0.00	999	1.000	1.000	0.6026
235845_at	12.6	23.6	0.91	1.88	AAA-AAA	0.00	999	1.000	1.000	0.4676
236044_at	15.6	29.3	0.91	1.88	AAA-AAA	0.00	999	1.000	1.000	0.5299
227024_s_at	26.8	14.3	-0.91	-1.88	AAA-AAA	0.00	999	1.000	1.000	0.4407
224547_at	62.9	33.5	-0.91	-1.88	AMA-AAA	0.00	999	1.000	1.000	0.5338
227795_at	18.3	34.4	0.91	1.88	AAA-AAA	0.00	999	1.000	1.000	0.0800
223557_s_at	16.1	30.1	0.91	1.88	AAA-AAA	0.00	999	1.000	1.000	0.4981
233291_at	36.6	19.5	-0.91	-1.88	PAA-AAA	0.00	999	1.000	1.000	0.3085
236178_at	55.8	29.7	-0.91	-1.88	AAP-AAP	0.00	999	1.000	1.000	0.3946
214681_at	84.1	44.8	-0.91	-1.88	AAP-APA	0.00	999	1.000	1.000	0.4714
217697_at	15.0	8.0	-0.91	-1.88	PAA-AAA	0.00	999	1.000	1.000	0.6677
240353_s_at	22.8	12.2	-0.91	-1.88	AAA-AAA	0.00	999	1.000	1.000	0.0510
241112_at	38.7	20.6	-0.91	-1.88	AAA-AAA	0.00	999	1.000	1.000	0.3696
1568655_a_at	14.4	27.1	0.91	1.88	AAA-AAA	0.00	999	1.000	1.000	0.5697
1557679_at	39.0	20.8	-0.91	-1.88	AAA-AAA	0.00	999	1.000	1.000	0.1218
216163_at	14.3	26.8	0.91	1.88	AAA-AAA	0.00	999	1.000	1.000	0.1437
211892_s_at	70.1	37.3	-0.91	-1.88	AAA-AAA	0.00	999	1.000	1.000	0.4179
213442_x_at	91.8	48.9	-0.91	-1.88	AAA-AAA	0.00	999	1.000	1.000	0.3728
216871_at	41.8	78.5	0.91	1.88	AAA-MAA	0.00	999	1.000	1.000	0.1426
233235_x_at	23.8	12.7	-0.91	-1.88	AAA-AAA	0.00	999	1.000	1.000	0.0344
236430_at	34.3	18.3	-0.91	-1.88	AAA-AAA	0.00	999	1.000	1.000	0.5699

236972_at	12.3	6.6	-0.91	-1.88	AAA-AAA	0.00	999	1.000	1.000	0.4833
222384_at	11.6	6.2	-0.91	-1.88	AAA-AAA	0.00	999	1.000	1.000	0.5811
223789_s_at	64.9	121.7	0.91	1.88	AAA-AAA	0.00	999	1.000	1.000	0.1514
223755_at	6.0	11.2	0.91	1.88	AAA-AAA	0.00	999	1.000	1.000	0.0541
230340_s_at	53.9	101.1	0.91	1.88	AAA-AAA	0.00	999	1.000	1.000	0.0625
231255_at	365.7	195.0	-0.91	-1.88	PPA-PAP	0.00	999	1.000	1.000	0.3744
242037_at	90.3	169.4	0.91	1.88	AAA-AAA	0.00	999	1.000	1.000	0.0220
1570276_a_at	13.1	7.0	-0.91	-1.88	AAA-AAA	0.00	999	1.000	1.000	0.4576
1555765_a_at	305.7	163.1	-0.91	-1.88	MAA-AAA	0.00	999	1.000	1.000	0.0117
1556167_at	28.1	15.0	-0.91	-1.88	AAA-AAA	0.00	999	1.000	1.000	0.4615
243218_at	41.4	77.5	0.91	1.87	AAA-AAA	0.00	999	1.000	1.000	0.4739
242660_at	6.7	3.6	-0.91	-1.88	AAA-AAA	0.00	999	1.000	1.000	0.6202
1559674_at	57.0	107.0	0.91	1.88	AAA-AAA	0.00	999	1.000	1.000	0.3077
216528_at	23.8	44.6	0.91	1.88	AAA-AAA	0.00	999	1.000	1.000	0.4163
206432_at	34.1	64.0	0.91	1.88	AAA-AAA	0.00	999	1.000	1.000	0.0112
206516_at	15.1	28.3	0.91	1.88	AAA-AAA	0.00	999	1.000	1.000	0.5406
211494_s_at	16.6	31.1	0.91	1.88	AAA-AAA	0.00	999	1.000	1.000	0.4507
207906_at	4.9	9.1	0.91	1.88	AAA-AAA	0.00	999	1.000	1.000	0.3095
233116_at	73.9	138.6	0.91	1.88	AAA-AMA	0.00	999	1.000	1.000	0.2418
232817_at	8.3	4.4	-0.91	-1.88	AAA-AAA	0.00	999	1.000	1.000	0.5675
237414_at	29.1	15.5	-0.91	-1.88	AAA-AAA	0.00	999	1.000	1.000	0.0890
223823_at	14.7	27.6	0.91	1.88	AAA-AAA	0.00	999	1.000	1.000	0.2185
230883_at	14.1	7.5	-0.91	-1.88	AAA-AAA	0.00	999	1.000	1.000	0.6144
1566840_at	7.7	14.4	0.91	1.87	AAA-AAA	0.00	999	1.000	1.000	0.5708
243392_at	10.5	19.6	0.91	1.87	AAA-AAA	0.00	999	1.000	1.000	0.0165
216023_at	39.8	21.2	-0.91	-1.87	AAA-AAA	0.00	999	1.000	1.000	0.1302
217084_at	2.5	4.7	0.91	1.87	AAA-AAA	0.00	999	1.000	1.000	0.0025
217130_at	89.7	47.9	-0.91	-1.87	AAA-AAA	0.00	999	1.000	1.000	0.1693
205444_at	14.3	26.8	0.91	1.87	AAA-AAA	0.00	999	1.000	1.000	0.6344
233876_at	10.0	18.8	0.91	1.87	AAA-AAA	0.00	999	1.000	1.000	0.4661
238245_at	20.3	38.1	0.91	1.87	AAA-AAA	0.00	999	1.000	1.000	0.4319
223889_at	19.3	36.2	0.91	1.87	AAA-AAA	0.00	999	1.000	1.000	0.4881
230838_s_at	49.5	26.4	-0.91	-1.87	AAA-AAA	0.00	999	1.000	1.000	0.3544
230049_at	46.9	87.9	0.91	1.87	AAA-AAA	0.00	999	1.000	1.000	0.2932
1563512_at	110.5	59.0	-0.91	-1.87	APM-AAA	0.00	999	1.000	1.000	0.2873
239829_at	31.7	59.4	0.91	1.87	AAA-AAA	0.00	999	1.000	1.000	0.3570
1558619_at	15.8	29.6	0.91	1.87	AAA-AAA	0.00	999	1.000	1.000	0.1254
1553077_at	8.7	16.3	0.91	1.87	AAA-AAA	0.00	999	1.000	1.000	0.4752
1552990_at	5.7	3.1	-0.91	-1.87	AAA-AAA	0.00	999	1.000	1.000	0.1804
1556170_at	7.1	13.3	0.91	1.87	AAA-AAA	0.00	999	1.000	1.000	0.6433
1561219_x_at	13.5	25.2	0.91	1.87	AAA-AAA	0.00	999	1.000	1.000	0.5400
244221_at	34.4	18.4	-0.91	-1.87	AAA-AAA	0.00	999	1.000	1.000	0.2862

1560424_at	7.2	13.4	0.91	1.87	AAA-AAA	0.00	999	1.000	1.000	0.6544
221861_at	26.7	50.0	0.91	1.87	AAA-AAA	0.00	999	1.000	1.000	0.1511
234543_at	17.8	9.5	-0.91	-1.87	AAA-AAA	0.00	999	1.000	1.000	0.5409
235102_x_at	28.1	52.5	0.91	1.87	AAA-AAA	0.00	999	1.000	1.000	0.5783
234031_at	6.2	11.6	0.91	1.87	AAA-AAA	0.00	999	1.000	1.000	0.1137
238852_at	14.6	7.8	-0.91	-1.87	AAA-AAA	0.00	999	1.000	1.000	0.5344
1560862_at	41.5	22.2	-0.90	-1.87	APA-AAA	0.00	999	1.000	1.000	0.5724
239098_at	99.0	52.9	-0.90	-1.87	AAA-AAA	0.00	999	1.000	1.000	0.3363
1562832_at	69.6	37.2	-0.90	-1.87	AAA-AAA	0.00	999	1.000	1.000	0.0313
240560_at	16.9	9.1	-0.90	-1.87	AAA-AAA	0.00	999	1.000	1.000	0.2930
240700_at	132.1	247.2	0.90	1.87	AAA-AAM	0.00	999	1.000	1.000	0.1389
1567140_at	28.2	15.1	-0.90	-1.87	AAA-AAA	0.00	999	1.000	1.000	0.2112
240718_at	4.9	9.1	0.90	1.87	AAA-AAA	0.00	999	1.000	1.000	0.4776
1557478_at	117.9	220.7	0.90	1.87	AAA-AAA	0.00	999	1.000	1.000	0.0784
1556643_at	23.2	43.4	0.90	1.87	AAA-AAA	0.00	999	1.000	1.000	0.4301
1553446_at	16.8	9.0	-0.90	-1.87	AAA-AAA	0.00	999	1.000	1.000	0.6257
243482_at	74.8	40.0	-0.90	-1.87	AAA-AAA	0.00	999	1.000	1.000	0.3753
216275_at	76.9	41.1	-0.90	-1.87	AAA-AAA	0.00	999	1.000	1.000	0.0891
217405_x_at	109.4	58.5	-0.90	-1.87	AAA-AAA	0.00	999	1.000	1.000	0.3743
203444_s_at	18.6	10.0	-0.90	-1.87	AAA-AAA	0.00	999	1.000	1.000	0.3789
210340_s_at	9.0	4.8	-0.90	-1.87	AAA-AAA	0.00	999	1.000	1.000	0.3234
235720_at	51.9	27.8	-0.90	-1.87	AAA-AAA	0.00	999	1.000	1.000	0.3821
232838_at	28.7	15.3	-0.90	-1.87	AAA-AAA	0.00	999	1.000	1.000	0.4820
231368_at	81.3	43.4	-0.90	-1.87	AAA-AAA	0.00	999	1.000	1.000	0.0485
207096_at	123.8	66.2	-0.90	-1.87	PAP-APA	0.00	999	1.000	1.000	0.3347
243317_at	38.2	20.4	-0.90	-1.87	AAA-APA	0.00	999	1.000	1.000	0.4982
1563532_at	21.2	39.5	0.90	1.87	AAA-AAA	0.00	999	1.000	1.000	0.5322
1553320_s_at	11.2	6.0	-0.90	-1.87	AAA-AAA	0.00	999	1.000	1.000	0.6197
1562432_at	20.6	38.5	0.90	1.87	AAA-AAA	0.00	999	1.000	1.000	0.4670
215829_at	54.7	29.2	-0.90	-1.87	AAA-AAA	0.00	999	1.000	1.000	0.2697
221306_at	88.1	164.8	0.90	1.87	AAA-AMA	0.00	999	1.000	1.000	0.0058
221455_s_at	38.3	71.6	0.90	1.87	AAA-AAA	0.00	999	1.000	1.000	0.3144
221179_at	7.3	13.6	0.90	1.87	AAA-AAA	0.00	999	1.000	1.000	0.1764
237448_at	5.2	9.7	0.90	1.87	AAA-AAA	0.00	999	1.000	1.000	0.4776
224183_at	14.8	27.8	0.90	1.87	AAA-AAA	0.00	999	1.000	1.000	0.4537
231245_s_at	7.5	13.9	0.90	1.87	AAA-AAA	0.00	999	1.000	1.000	0.2611
230920_at	5.3	9.9	0.90	1.87	AAA-AAA	0.00	999	1.000	1.000	0.3245
231211_s_at	10.3	19.2	0.90	1.87	AAA-AAA	0.00	999	1.000	1.000	0.4056
239281_at	51.5	96.2	0.90	1.87	AAA-AAA	0.00	999	1.000	1.000	0.3703
1569661_at	7.7	14.3	0.90	1.87	AAA-AAA	0.00	999	1.000	1.000	0.5940
1560814_a_at	49.9	26.7	-0.90	-1.87	AAA-AAA	0.00	999	1.000	1.000	0.5258
243657_at	11.1	6.0	-0.90	-1.87	AAA-AAA	0.00	999	1.000	1.000	0.3254

1559421_at	55.8	29.9	-0.90	-1.87	AAA-AAA	0.00	999	1.000	1.000	0.4294
219464_at	124.4	66.6	-0.90	-1.87	AAA-AAA	0.00	999	1.000	1.000	0.1122
205166_at	25.9	48.5	0.90	1.87	AAA-AAA	0.00	999	1.000	1.000	0.3885
207519_at	49.7	26.6	-0.90	-1.87	AAA-AAA	0.00	999	1.000	1.000	0.2919
233699_at	17.4	32.5	0.90	1.87	AAA-AAA	0.00	999	1.000	1.000	0.4151
238288_at	16.8	9.0	-0.90	-1.87	AAA-AAA	0.00	999	1.000	1.000	0.4811
1558002_at	78.8	42.2	-0.90	-1.87	AAA-AAP	0.00	999	1.000	1.000	0.5033
1554332_a_at	87.5	46.8	-0.90	-1.87	AAA-AAA	0.00	999	1.000	1.000	0.3365
1561639_at	31.5	16.9	-0.90	-1.87	AAA-AAA	0.00	999	1.000	1.000	0.5583
1559029_at	5.7	10.7	0.90	1.87	AAA-AAA	0.00	999	1.000	1.000	0.4934
1560422_at	11.6	21.7	0.90	1.87	AAA-AAA	0.00	999	1.000	1.000	0.5831
216648_s_at	56.3	30.1	-0.90	-1.87	AAA-AAA	0.00	999	1.000	1.000	0.1579
214296_x_at	85.1	45.6	-0.90	-1.87	AAA-AAA	0.00	999	1.000	1.000	0.4875
217584_at	38.2	71.3	0.90	1.87	AAA-AAA	0.00	999	1.000	1.000	0.2377
217006_x_at	17.0	31.7	0.90	1.87	AAA-AAA	0.00	999	1.000	1.000	0.4727
219179_at	14.6	27.2	0.90	1.87	AAA-AAA	0.00	999	1.000	1.000	0.3800
206800_at	66.7	35.7	-0.90	-1.87	AAA-AAA	0.00	999	1.000	1.000	0.3689
203618_at	74.5	39.9	-0.90	-1.87	AAA-AAA	0.00	999	1.000	1.000	0.3614
201858_s_at	28.1	15.0	-0.90	-1.87	AAA-AAA	0.00	999	1.000	1.000	0.4645
207864_at	8.2	15.4	0.90	1.87	AAA-AAA	0.00	999	1.000	1.000	0.3554
207466_at	21.2	39.6	0.90	1.87	AAA-AAA	0.00	999	1.000	1.000	0.3629
227552_at	30.0	56.0	0.90	1.87	AAA-AAA	0.00	999	1.000	1.000	0.4257
224371_at	43.9	23.5	-0.90	-1.87	AAA-AAA	0.00	999	1.000	1.000	0.4161
230366_at	10.2	19.1	0.90	1.87	AAA-AAA	0.00	999	1.000	1.000	0.5752
1554604_at	88.3	47.3	-0.90	-1.87	PAA-AAA	0.00	999	1.000	1.000	0.0443
224733_at	155.2	83.1	-0.90	-1.87	PPP-PAP	0.00	999	1.000	1.000	0.4064
1567698_x_at	16.2	8.7	-0.90	-1.87	APA-AAA	0.00	999	1.000	1.000	0.4335
242897_at	43.7	23.4	-0.90	-1.87	PPA-AAP	0.00	999	1.000	1.000	0.5544
1563186_at	5.7	10.6	0.90	1.87	AAA-AAA	0.00	999	1.000	1.000	0.6128
241861_at	5.7	10.6	0.90	1.87	AAA-AAA	0.00	999	1.000	1.000	0.5511
1555441_at	43.2	23.2	-0.90	-1.87	AAA-AAA	0.00	999	1.000	1.000	0.6034
243341_at	9.9	18.4	0.90	1.87	AAA-AAA	0.00	999	1.000	1.000	0.5527
206437_at	28.6	53.3	0.90	1.87	AAA-AAA	0.00	999	1.000	1.000	0.3424
205737_at	30.8	16.5	-0.90	-1.87	AAA-AAA	0.00	999	1.000	1.000	0.3397
205924_at	15.5	8.3	-0.90	-1.87	AAA-AAA	0.00	999	1.000	1.000	0.3509
206815_at	6.6	12.3	0.90	1.87	AAA-AAA	0.00	999	1.000	1.000	0.3296
206720_at	25.3	47.2	0.90	1.87	AAA-AAA	0.00	999	1.000	1.000	0.2864
211640_x_at	5.6	10.4	0.90	1.87	AAA-AAA	0.00	999	1.000	1.000	0.0918
234144_at	14.0	26.1	0.90	1.87	AAA-AAA	0.00	999	1.000	1.000	0.4318
236307_at	20.0	10.7	-0.90	-1.87	AAA-AAA	0.00	999	1.000	1.000	0.0546
224239_at	16.4	30.6	0.90	1.87	AAA-AAA	0.00	999	1.000	1.000	0.5735
224058_s_at	36.6	19.6	-0.90	-1.87	AAP-AAA	0.00	999	1.000	1.000	0.5468

240638_at	19.6	36.5	0.90	1.87	PAA-APA	0.00	999	1.000	1.000	0.6648
239111_at	18.6	34.8	0.90	1.87	AAA-AAA	0.00	999	1.000	1.000	0.4035
1569048_s_at	43.1	80.4	0.90	1.87	AAA-AAA	0.00	999	1.000	1.000	0.2631
240178_at	14.8	27.6	0.90	1.87	AAA-AAA	0.00	999	1.000	1.000	0.4019
1570102_at	4.8	2.6	-0.90	-1.87	AAA-AAA	0.00	999	1.000	1.000	0.3460
1566441_at	4.1	7.6	0.90	1.87	AAA-AAA	0.00	999	1.000	1.000	0.0897
1556941_a_at	15.2	8.1	-0.90	-1.87	AAA-AAA	0.00	999	1.000	1.000	0.4785
242840_at	13.5	25.2	0.90	1.87	AAA-AAA	0.00	999	1.000	1.000	0.1012
1560226_at	39.2	21.0	-0.90	-1.86	AAA-AAA	0.00	999	1.000	1.000	0.4479
215205_x_at	20.5	11.0	-0.90	-1.87	AAA-AAA	0.00	999	1.000	1.000	0.4027
216428_x_at	30.2	56.3	0.90	1.87	AAA-AAA	0.00	999	1.000	1.000	0.1379
217412_at	12.1	22.5	0.90	1.87	AAA-AAA	0.00	999	1.000	1.000	0.3415
219894_at	9.4	17.5	0.90	1.87	AAA-AAA	0.00	999	1.000	1.000	0.0908
201438_at	32.2	60.0	0.90	1.87	AAA-AAA	0.00	999	1.000	1.000	0.2545
203130_s_at	14.6	27.1	0.90	1.87	AAA-AAA	0.00	999	1.000	1.000	0.3681
210553_x_at	26.7	14.3	-0.90	-1.86	AAA-AAA	0.00	999	1.000	1.000	0.3984
210341_at	11.3	21.0	0.90	1.87	AAA-AAA	0.00	999	1.000	1.000	0.4248
227812_at	7.3	13.6	0.90	1.87	AAA-AAA	0.00	999	1.000	1.000	0.4133
222911_s_at	29.6	15.8	-0.90	-1.87	AAA-AAA	0.00	999	1.000	1.000	0.4020
209094_at	1165.2	625.1	-0.90	-1.86	PPP-PPP	0.00	999	1.000	1.000	0.1056
1565616_at	18.1	9.7	-0.90	-1.86	AAA-AAA	0.00	999	1.000	1.000	0.4606
1562866_at	13.0	24.1	0.90	1.86	AAA-AAA	0.00	999	1.000	1.000	0.4549
1566888_at	6.6	3.5	-0.90	-1.86	AAA-AAA	0.00	999	1.000	1.000	0.4038
244793_at	11.1	6.0	-0.90	-1.86	AAA-AAA	0.00	999	1.000	1.000	0.5234
1557914_s_at	4.4	8.1	0.90	1.86	AAA-AAA	0.00	999	1.000	1.000	0.4642
1552293_at	26.5	49.4	0.90	1.86	AAA-AAA	0.00	999	1.000	1.000	0.5691
1558882_at	2.8	1.5	-0.90	-1.86	AAA-AAA	0.00	999	1.000	1.000	0.2403
216152_at	102.7	55.1	-0.90	-1.86	AAA-AAA	0.00	999	1.000	1.000	0.5350
216131_at	20.3	37.8	0.90	1.86	AAA-AAA	0.00	999	1.000	1.000	0.2893
220894_x_at	40.4	75.3	0.90	1.86	AAA-AAA	0.00	999	1.000	1.000	0.4657
205785_at	48.8	91.0	0.90	1.86	AAA-AAA	0.00	999	1.000	1.000	0.0320
207771_at	54.2	101.1	0.90	1.86	AAA-AAA	0.00	999	1.000	1.000	0.3881
234519_at	21.6	40.2	0.90	1.86	AAA-AAA	0.00	999	1.000	1.000	0.5558
238358_x_at	4.6	8.6	0.90	1.86	AAA-AAA	0.00	999	1.000	1.000	0.5478
236867_at	18.5	34.6	0.90	1.86	AAA-AAA	0.00	999	1.000	1.000	0.2239
223953_s_at	6.7	12.6	0.90	1.86	AAA-AAA	0.00	999	1.000	1.000	0.3284
1556700_a_at	31.6	17.0	-0.90	-1.86	AAP-AAA	0.00	999	1.000	1.000	0.4919
1564855_at	22.6	12.1	-0.90	-1.86	AAA-AAA	0.00	999	1.000	1.000	0.5822
1563725_at	12.0	22.3	0.90	1.86	AAA-AAA	0.00	999	1.000	1.000	0.5654
1569644_at	11.2	20.9	0.90	1.86	AAA-AAA	0.00	999	1.000	1.000	0.2375
239468_at	4.6	8.6	0.90	1.86	AAA-AAA	0.00	999	1.000	1.000	0.6953
242765_at	17.9	33.4	0.90	1.86	AAA-AAA	0.00	999	1.000	1.000	0.5382

243797_at	10.1	18.9	0.90	1.86	AAA-AAA	0.00	999	1.000	1.000	0.4182
214978_s_at	10.6	5.7	-0.90	-1.86	AAA-AAA	0.00	999	1.000	1.000	0.3600
220623_s_at	21.2	39.4	0.90	1.86	AAA-AAA	0.00	999	1.000	1.000	0.2406
234846_at	16.2	30.2	0.90	1.86	AAA-AAA	0.00	999	1.000	1.000	0.3963
241656_at	26.0	14.0	-0.90	-1.86	AAA-APA	0.00	999	1.000	1.000	0.4989
235343_at	35.1	18.9	-0.90	-1.86	PAA-PAA	0.00	999	1.000	1.000	0.6178
239715_at	54.7	29.4	-0.90	-1.86	AAA-MAA	0.00	999	1.000	1.000	0.6233
239675_at	11.8	6.4	-0.90	-1.86	AAA-AAA	0.00	999	1.000	1.000	0.2913
240575_at	4.3	2.3	-0.90	-1.86	AAA-AAA	0.00	999	1.000	1.000	0.3277
1558832_at	35.1	18.9	-0.90	-1.86	AAA-AAA	0.00	999	1.000	1.000	0.4262
1554165_at	6.7	12.4	0.90	1.86	AAA-AAA	0.00	999	1.000	1.000	0.4726
1555090_x_at	41.5	77.3	0.90	1.86	AAA-AAA	0.00	999	1.000	1.000	0.3431
1561512_at	11.9	22.1	0.90	1.86	AAA-AAA	0.00	999	1.000	1.000	0.5102
243155_at	16.3	8.8	-0.90	-1.86	AAA-AAA	0.00	999	1.000	1.000	0.2931
244034_at	51.0	94.8	0.90	1.86	AAA-AAA	0.00	999	1.000	1.000	0.0040
213200_at	8.0	14.9	0.90	1.86	AAA-AAA	0.00	999	1.000	1.000	0.3943
214400_at	15.0	8.0	-0.90	-1.86	AAA-AAA	0.00	999	1.000	1.000	0.3251
220100_at	7.0	13.1	0.90	1.86	AAA-AAA	0.00	999	1.000	1.000	0.5809
211841_s_at	68.8	128.0	0.90	1.86	AAA-AAA	0.00	999	1.000	1.000	0.1588
235066_at	12.8	6.9	-0.90	-1.86	AAA-AAA	0.00	999	1.000	1.000	0.6175
237824_at	19.1	35.4	0.90	1.86	AAA-AAA	0.00	999	1.000	1.000	0.3775
226959_at	25.4	47.3	0.90	1.86	AAA-AAA	0.00	999	1.000	1.000	0.5490
235288_at	84.2	45.3	-0.90	-1.86	PAP-AAA	0.00	999	1.000	1.000	0.2587
1564449_at	38.3	71.3	0.90	1.86	AAA-AAA	0.00	999	1.000	1.000	0.4635
240480_at	2.0	3.7	0.90	1.86	AAA-AAA	0.00	999	1.000	1.000	0.4454
243896_at	8.2	15.2	0.90	1.86	AAA-AAA	0.00	999	1.000	1.000	0.4120
207093_s_at	33.5	62.4	0.90	1.86	AAA-AAA	0.00	999	1.000	1.000	0.4606
233134_at	9.5	17.6	0.90	1.86	AAA-AAA	0.00	999	1.000	1.000	0.3694
237951_at	7.4	4.0	-0.90	-1.86	AAA-AAA	0.00	999	1.000	1.000	0.4494
238312_s_at	7.5	13.9	0.90	1.86	AAA-AAA	0.00	999	1.000	1.000	0.5353
227743_at	13.7	25.5	0.90	1.86	AAA-AAA	0.00	999	1.000	1.000	0.2416
1556754_at	58.9	109.4	0.89	1.86	AAA-AAA	0.00	999	1.000	1.000	0.2397
1561060_at	20.1	37.4	0.89	1.86	AAA-AAA	0.00	999	1.000	1.000	0.5157
1562383_at	4.5	8.4	0.89	1.86	AAA-AAA	0.00	999	1.000	1.000	0.1921
1560859_at	2.9	5.4	0.89	1.86	AAA-AAA	0.00	999	1.000	1.000	0.3591
216820_at	11.7	6.3	-0.89	-1.86	AAA-AAA	0.00	999	1.000	1.000	0.3688
216748_at	32.4	60.3	0.89	1.86	AAA-AAM	0.00	999	1.000	1.000	0.3727
237965_at	32.1	17.3	-0.89	-1.86	AAA-AAA	0.00	999	1.000	1.000	0.4061
236335_at	11.6	21.5	0.89	1.86	AAA-AAA	0.00	999	1.000	1.000	0.4284
236783_at	60.0	32.3	-0.89	-1.86	AAA-AAA	0.00	999	1.000	1.000	0.3897
228713_s_at	79.3	42.6	-0.89	-1.86	AAA-AAA	0.00	999	1.000	1.000	0.3679
229691_at	9.6	17.9	0.89	1.86	AAA-AAA	0.00	999	1.000	1.000	0.5434

229207_x_at	24.2	13.0	-0.89	-1.86	AAA-AAA	0.00	999	1.000	1.000	0.5183
229947_at	15.1	8.2	-0.89	-1.86	AAA-AAA	0.00	999	1.000	1.000	0.3893
1563587_at	73.0	39.3	-0.89	-1.86	AAA-APA	0.00	999	1.000	1.000	0.4535
243717_at	64.5	34.7	-0.89	-1.86	AAA-AAA	0.00	999	1.000	1.000	0.3106
216183_at	19.3	10.4	-0.89	-1.86	AAA-AAA	0.00	999	1.000	1.000	0.4525
205799_s_at	20.7	38.5	0.89	1.86	AAA-MAM	0.00	999	1.000	1.000	0.5575
211140_s_at	15.1	28.0	0.89	1.86	AAA-AAA	0.00	999	1.000	1.000	0.2401
208416_s_at	40.5	75.1	0.89	1.86	AAA-AAA	0.00	999	1.000	1.000	0.4198
234055_s_at	32.4	60.2	0.89	1.86	AAA-AAA	0.00	999	1.000	1.000	0.4734
235524_at	11.9	6.4	-0.89	-1.86	AAA-AAA	0.00	999	1.000	1.000	0.2489
233130_at	18.8	34.8	0.89	1.86	AAA-AAA	0.00	999	1.000	1.000	0.4941
237477_at	2.2	4.2	0.89	1.86	AAA-AAA	0.00	999	1.000	1.000	0.6325
238297_at	7.3	13.5	0.89	1.86	AAA-AAA	0.00	999	1.000	1.000	0.4304
236926_at	60.4	32.5	-0.89	-1.86	AAA-AAA	0.00	999	1.000	1.000	0.4957
227480_at	216.1	116.5	-0.89	-1.86	PPP-PPA	0.00	999	1.000	1.000	0.1465
205099_s_at	31.9	17.2	-0.89	-1.86	APA-AAA	0.00	999	1.000	1.000	0.1712
206121_at	58.8	31.7	-0.89	-1.86	AAP-AAA	0.00	999	1.000	1.000	0.4434
240776_at	11.9	6.4	-0.89	-1.86	AAA-AAA	0.00	999	1.000	1.000	0.3859
216017_s_at	113.3	210.2	0.89	1.86	AAA-AAA	0.00	999	1.000	1.000	0.3210
213197_at	9.7	5.2	-0.89	-1.86	AAA-AAA	0.00	999	1.000	1.000	0.5975
219812_at	52.7	97.7	0.89	1.86	AAA-AAA	0.00	999	1.000	1.000	0.1367
232832_at	4.3	8.0	0.89	1.86	AAA-AAA	0.00	999	1.000	1.000	0.1579
236786_at	34.7	64.4	0.89	1.86	AAA-MAA	0.00	999	1.000	1.000	0.5412
224090_s_at	8.8	16.4	0.89	1.86	AAA-AAA	0.00	999	1.000	1.000	0.4111
231052_at	37.0	68.6	0.89	1.86	AAA-AAA	0.00	999	1.000	1.000	0.3885
232465_at	42.6	79.0	0.89	1.86	AAA-AAA	0.00	999	1.000	1.000	0.1687
229879_at	104.4	56.3	-0.89	-1.86	AAA-AAA	0.00	999	1.000	1.000	0.3623
1563569_at	12.7	23.6	0.89	1.86	AAA-AAA	0.00	999	1.000	1.000	0.4696
1568884_at	6.3	11.8	0.89	1.85	AAA-AAA	0.00	999	1.000	1.000	0.2727
239971_at	17.3	9.3	-0.89	-1.86	AAA-AAA	0.00	999	1.000	1.000	0.3543
1552733_at	15.8	29.2	0.89	1.86	AAA-AAA	0.00	999	1.000	1.000	0.5018
1556806_at	44.4	82.4	0.89	1.85	AAA-AAA	0.00	999	1.000	1.000	0.0758
1558034_s_at	18.7	10.1	-0.89	-1.86	AAA-AAA	0.00	999	1.000	1.000	0.3625
1555282_a_at	105.0	56.6	-0.89	-1.86	AAA-AAA	0.00	999	1.000	1.000	0.2563
1561204_at	18.7	10.1	-0.89	-1.86	AAA-AAA	0.00	999	1.000	1.000	0.6452
1561587_at	13.2	24.4	0.89	1.85	AAA-AAA	0.00	999	1.000	1.000	0.5135
1559342_a_at	8.7	4.7	-0.89	-1.86	AAA-AAA	0.00	999	1.000	1.000	0.5338
216446_at	21.2	39.3	0.89	1.85	AAA-AAA	0.00	999	1.000	1.000	0.1872
212275_s_at	72.6	134.7	0.89	1.86	AAA-AAA	0.00	999	1.000	1.000	0.3723
204260_at	27.6	14.9	-0.89	-1.86	AAA-AAA	0.00	999	1.000	1.000	0.5571
210113_s_at	17.3	9.3	-0.89	-1.85	AAA-AAA	0.00	999	1.000	1.000	0.2164
211694_at	2.9	5.3	0.89	1.86	AAA-AAA	0.00	999	1.000	1.000	0.3320

235583_at	25.3	13.7	-0.89	-1.85	AAA-AAA	0.00	999	1.000	1.000	0.4702
233653_at	4.3	7.9	0.89	1.86	AAA-AAA	0.00	999	1.000	1.000	0.5399
238645_at	29.3	15.8	-0.89	-1.85	AAA-AAA	0.00	999	1.000	1.000	0.5528
226646_at	25.5	47.4	0.89	1.85	AAA-AAA	0.00	999	1.000	1.000	0.4747
224406_s_at	40.0	74.2	0.89	1.86	AAA-AAA	0.00	999	1.000	1.000	0.5740
1562745_at	32.6	17.6	-0.89	-1.85	AAA-AAA	0.00	999	1.000	1.000	0.4191
1566081_at	12.6	6.8	-0.89	-1.85	AAA-AAA	0.00	999	1.000	1.000	0.4874
1556650_at	24.7	45.7	0.89	1.85	AAA-AAA	0.00	999	1.000	1.000	0.3349
221148_at	66.0	35.6	-0.89	-1.85	AAA-AAA	0.00	999	1.000	1.000	0.3528
217296_at	31.2	57.9	0.89	1.85	AAA-AAA	0.00	999	1.000	1.000	0.4938
211348_s_at	40.5	21.9	-0.89	-1.85	AAA-AAA	0.00	999	1.000	1.000	0.4807
208515_at	48.7	26.3	-0.89	-1.85	AAA-AAA	0.00	999	1.000	1.000	0.0349
234462_at	14.4	7.8	-0.89	-1.85	AAA-AAA	0.00	999	1.000	1.000	0.1154
237499_at	5.7	3.1	-0.89	-1.85	AAA-AAA	0.00	999	1.000	1.000	0.1296
238487_at	40.0	74.2	0.89	1.85	AAA-AAA	0.00	999	1.000	1.000	0.3523
236729_at	20.4	37.9	0.89	1.85	AAA-AAA	0.00	999	1.000	1.000	0.2464
224045_x_at	25.9	14.0	-0.89	-1.85	AAA-AAA	0.00	999	1.000	1.000	0.2137
230965_at	41.3	22.3	-0.89	-1.85	AAA-AAA	0.00	999	1.000	1.000	0.4738
230914_at	10.2	5.5	-0.89	-1.85	AAA-AAA	0.00	999	1.000	1.000	0.0538
1564319_at	50.9	27.5	-0.89	-1.85	AAA-AAA	0.00	999	1.000	1.000	0.2105
1566903_at	19.1	35.3	0.89	1.85	AAA-AAA	0.00	999	1.000	1.000	0.3619
1560901_at	45.5	84.3	0.89	1.85	AAA-AAA	0.00	999	1.000	1.000	0.2338
215817_at	47.2	25.5	-0.89	-1.85	AAA-AAA	0.00	999	1.000	1.000	0.3118
213783_at	31.3	16.9	-0.89	-1.85	AAA-AAA	0.00	999	1.000	1.000	0.0737
215137_at	26.6	49.2	0.89	1.85	AAA-AAA	0.00	999	1.000	1.000	0.5610
214451_at	13.9	25.7	0.89	1.85	AAA-AAA	0.00	999	1.000	1.000	0.5657
205886_at	38.9	21.0	-0.89	-1.85	AAA-AAA	0.00	999	1.000	1.000	0.4633
237521_x_at	28.0	15.1	-0.89	-1.85	AMA-AAA	0.00	999	1.000	1.000	0.4530
236152_at	5.0	9.3	0.89	1.85	AAA-AAA	0.00	999	1.000	1.000	0.1671
236849_at	133.8	72.2	-0.89	-1.85	AAA-AAA	0.00	999	1.000	1.000	0.4470
221928_at	36.1	66.8	0.89	1.85	AAA-AAA	0.00	999	1.000	1.000	0.4597
204702_s_at	185.7	100.4	-0.89	-1.85	PPP-PPP	0.00	999	1.000	1.000	0.0300
210786_s_at	441.4	238.5	-0.89	-1.85	PPP-PPP	0.00	999	1.000	1.000	0.0576
1561615_s_at	21.9	40.5	0.89	1.85	AAA-AAA	0.00	999	1.000	1.000	0.3935
1562822_at	8.9	16.5	0.89	1.85	AAA-AAA	0.00	999	1.000	1.000	0.5903
1568488_at	16.7	9.0	-0.89	-1.85	AAA-AAA	0.00	999	1.000	1.000	0.5117
1556759_at	4.4	2.4	-0.89	-1.85	AAA-AAA	0.00	999	1.000	1.000	0.5589
244251_at	11.6	21.4	0.89	1.85	AAA-AAA	0.00	999	1.000	1.000	0.5629
216590_at	7.0	13.0	0.89	1.85	AAA-AAA	0.00	999	1.000	1.000	0.3576
221395_at	11.1	6.0	-0.89	-1.85	AAA-AAA	0.00	999	1.000	1.000	0.3086
210143_at	9.3	17.1	0.89	1.85	AAA-AAA	0.00	999	1.000	1.000	0.3355
208288_at	10.4	19.3	0.89	1.85	AAA-AAA	0.00	999	1.000	1.000	0.4996

234382_x_at	18.6	34.3	0.89	1.85	AAA-AMA	0.00	999	1.000	1.000	0.5998
233170_at	17.9	33.1	0.89	1.85	AAA-AAA	0.00	999	1.000	1.000	0.1750
233554_at	42.3	78.3	0.89	1.85	AAA-AAA	0.00	999	1.000	1.000	0.4420
236807_at	17.8	32.8	0.89	1.85	AAA-AAA	0.00	999	1.000	1.000	0.5473
227281_at	27.1	14.6	-0.89	-1.85	AAA-AAA	0.00	999	1.000	1.000	0.2741
214995_s_at	47.5	25.7	-0.89	-1.85	PAA-AAA	0.00	999	1.000	1.000	0.5686
241601_at	56.2	30.4	-0.89	-1.85	AAA-AAA	0.00	999	1.000	1.000	0.4944
1563420_at	5.3	9.7	0.89	1.85	AAA-AAA	0.00	999	1.000	1.000	0.4799
239473_x_at	34.9	64.6	0.89	1.85	AAA-AAA	0.00	999	1.000	1.000	0.4506
240999_at	7.2	13.2	0.89	1.85	AAA-AAA	0.00	999	1.000	1.000	0.4223
1561037_a_at	132.3	71.6	-0.89	-1.85	MAA-AAA	0.00	999	1.000	1.000	0.4272
244080_at	6.9	12.8	0.89	1.85	AAA-AAA	0.00	999	1.000	1.000	0.2492
215865_at	31.5	58.2	0.89	1.85	AAA-AAA	0.00	999	1.000	1.000	0.5096
216459_x_at	79.1	42.8	-0.89	-1.85	AAA-AAA	0.00	999	1.000	1.000	0.3521
220045_at	50.0	27.0	-0.89	-1.85	AAA-AAA	0.00	999	1.000	1.000	0.5971
217179_x_at	10.9	20.1	0.89	1.85	AAA-AAA	0.00	999	1.000	1.000	0.4716
206397_x_at	14.2	26.3	0.89	1.85	AAA-AAA	0.00	999	1.000	1.000	0.2354
234075_at	6.2	11.5	0.89	1.85	AAA-AAA	0.00	999	1.000	1.000	0.6213
235813_at	16.3	30.1	0.89	1.85	AAA-AAA	0.00	999	1.000	1.000	0.5476
222592_s_at	10.1	18.6	0.89	1.85	AAA-AAA	0.00	999	1.000	1.000	0.5373
230978_at	18.2	9.9	-0.89	-1.85	AAA-AAA	0.00	999	1.000	1.000	0.4123
228935_at	11.3	6.1	-0.89	-1.85	AAA-AAA	0.00	999	1.000	1.000	0.6112
1564238_a_at	60.2	32.6	-0.89	-1.85	PPP-AAA	0.00	999	1.000	1.000	0.0973
232809_s_at	155.6	84.2	-0.89	-1.85	PMP-AAP	0.00	999	1.000	1.000	0.1603
216259_at	32.2	17.4	-0.89	-1.85	AAA-PAA	0.00	999	1.000	1.000	0.4860
220170_at	12.8	7.0	-0.89	-1.85	APA-AAA	0.00	999	1.000	1.000	0.6878
241671_x_at	20.3	11.0	-0.89	-1.85	AAA-AAA	0.00	999	1.000	1.000	0.4352
244742_at	4.4	2.4	-0.89	-1.85	AAA-AAA	0.00	999	1.000	1.000	0.3544
1552450_a_at	24.6	13.3	-0.89	-1.85	AAA-AAA	0.00	999	1.000	1.000	0.2874
1557637_at	20.2	37.4	0.89	1.85	AAA-AAA	0.00	999	1.000	1.000	0.2918
243195_s_at	14.5	7.8	-0.89	-1.85	AAA-AAA	0.00	999	1.000	1.000	0.1797
1561940_at	35.5	19.2	-0.89	-1.85	AAA-AAA	0.00	999	1.000	1.000	0.4159
206834_at	33.5	18.1	-0.89	-1.85	AAA-AAA	0.00	999	1.000	1.000	0.4515
205577_at	12.0	22.3	0.89	1.85	AAA-AAA	0.00	999	1.000	1.000	0.5562
210408_s_at	21.4	11.6	-0.89	-1.85	AAA-AAA	0.00	999	1.000	1.000	0.3310
233247_x_at	10.7	19.7	0.89	1.85	AAA-AAA	0.00	999	1.000	1.000	0.4814
233236_at	33.3	61.6	0.89	1.85	AAA-AAA	0.00	999	1.000	1.000	0.3127
237749_at	6.4	11.8	0.89	1.85	AAA-AAA	0.00	999	1.000	1.000	0.5202
238136_at	13.2	24.4	0.89	1.85	AAA-AAA	0.00	999	1.000	1.000	0.0222
222897_s_at	155.1	83.9	-0.89	-1.85	AAA-AAA	0.00	999	1.000	1.000	0.0030
232223_at	14.8	8.0	-0.89	-1.85	AAA-AAA	0.00	999	1.000	1.000	0.4422
224166_at	90.5	49.0	-0.89	-1.85	AAP-AAA	0.00	999	1.000	1.000	0.1011

1570270_at	32.0	17.4	-0.89	-1.85	AAP-AAA	0.00	999	1.000	1.000	0.4217
241304_at	7.8	4.3	-0.89	-1.85	AAA-AAA	0.00	999	1.000	1.000	0.3908
1565758_at	12.8	6.9	-0.89	-1.85	AAA-AAA	0.00	999	1.000	1.000	0.0769
1556919_at	32.9	60.7	0.89	1.85	AAA-AAA	0.00	999	1.000	1.000	0.2852
1556468_at	26.5	48.9	0.89	1.85	AAA-AAA	0.00	999	1.000	1.000	0.4418
1555214_a_at	2.4	4.5	0.89	1.85	AAA-AAA	0.00	999	1.000	1.000	0.1254
1553839_at	46.1	25.0	-0.89	-1.85	AAA-AAA	0.00	999	1.000	1.000	0.4604
1561532_at	46.0	24.9	-0.89	-1.85	AAA-AAA	0.00	999	1.000	1.000	0.3621
244671_at	8.2	15.1	0.89	1.85	AAA-AAA	0.00	999	1.000	1.000	0.3770
213245_at	79.1	42.8	-0.89	-1.85	AAA-AAA	0.00	999	1.000	1.000	0.2072
220246_at	5.5	10.1	0.89	1.85	AAA-AAA	0.00	999	1.000	1.000	0.3516
220409_at	7.2	13.3	0.89	1.85	AAA-AAA	0.00	999	1.000	1.000	0.5569
217359_s_at	19.9	10.8	-0.89	-1.85	AAA-AAA	0.00	999	1.000	1.000	0.6023
210129_s_at	64.3	34.8	-0.89	-1.85	AAA-AAA	0.00	999	1.000	1.000	0.5161
211470_s_at	22.0	11.9	-0.89	-1.85	AAA-AAA	0.00	999	1.000	1.000	0.5178
233885_at	19.7	10.7	-0.89	-1.85	AAA-AAA	0.00	999	1.000	1.000	0.1990
224506_s_at	7.9	14.6	0.89	1.85	AAA-AAA	0.00	999	1.000	1.000	0.3503
228267_at	13.4	24.7	0.89	1.85	AAA-AAA	0.00	999	1.000	1.000	0.0925
204279_at	194.6	105.5	-0.88	-1.85	PAP-AAA	0.00	999	1.000	1.000	0.0741
1568970_at	5.2	9.7	0.88	1.85	AAA-AAA	0.00	999	1.000	1.000	0.4837
1552646_at	74.6	137.7	0.88	1.85	AAA-AAA	0.00	999	1.000	1.000	0.1695
244306_at	28.1	51.8	0.88	1.85	AAA-AAA	0.00	999	1.000	1.000	0.0317
1560846_at	5.6	10.3	0.88	1.85	AAA-AAA	0.00	999	1.000	1.000	0.5781
215072_x_at	40.4	74.5	0.88	1.85	AAA-AAA	0.00	999	1.000	1.000	0.4824
204551_s_at	30.0	55.4	0.88	1.85	AAA-AAA	0.00	999	1.000	1.000	0.2858
209347_s_at	4.2	7.8	0.88	1.85	AAA-AAA	0.00	999	1.000	1.000	0.5082
232998_at	20.5	11.1	-0.88	-1.85	AAA-AAA	0.00	999	1.000	1.000	0.4529
232718_at	24.0	13.0	-0.88	-1.85	AAA-AAA	0.00	999	1.000	1.000	0.2619
237079_at	14.6	7.9	-0.88	-1.85	AAA-AAA	0.00	999	1.000	1.000	0.1808
226743_at	5.5	10.1	0.88	1.85	AAA-AAA	0.00	999	1.000	1.000	0.3790
229607_at	46.1	25.0	-0.88	-1.85	AAA-AAA	0.00	999	1.000	1.000	0.2740
230797_s_at	28.2	15.3	-0.88	-1.85	AAA-AAA	0.00	999	1.000	1.000	0.4852
237242_at	83.4	45.2	-0.88	-1.84	PAP-PAA	0.00	999	1.000	1.000	0.2294
235372_at	107.8	58.5	-0.88	-1.84	PPP-AAA	0.00	999	1.000	1.000	0.3826
1566607_at	25.7	13.9	-0.88	-1.85	AAA-AAA	0.00	999	1.000	1.000	0.6319
1565837_at	6.2	11.3	0.88	1.84	AAA-AAA	0.00	999	1.000	1.000	0.6702
239191_at	15.6	8.5	-0.88	-1.84	AAA-AAA	0.00	999	1.000	1.000	0.2637
1552868_at	28.3	52.2	0.88	1.85	AAA-AAA	0.00	999	1.000	1.000	0.4359
1553389_at	47.4	25.7	-0.88	-1.84	AAA-AAA	0.00	999	1.000	1.000	0.2719
1555265_at	27.2	50.1	0.88	1.84	AAA-AMA	0.00	999	1.000	1.000	0.2499
220532_s_at	52.4	28.4	-0.88	-1.85	AAA-AAA	0.00	999	1.000	1.000	0.4098
219882_at	13.9	25.6	0.88	1.85	AAA-AAA	0.00	999	1.000	1.000	0.5637

210699_at	7.7	14.2	0.88	1.84	AAA-AAA	0.00	999	1.000	1.000	0.4901
231528_at	7.4	13.6	0.88	1.84	AAA-AAA	0.00	999	1.000	1.000	0.3210
243862_at	30.8	16.7	-0.88	-1.84	AAP-AAA	0.00	999	1.000	1.000	0.1081
230322_at	63.2	34.3	-0.88	-1.84	APA-AAA	0.00	999	1.000	1.000	0.5107
1564964_at	5.2	9.6	0.88	1.84	AAA-AAA	0.00	999	1.000	1.000	0.6491
1556960_a_at	12.7	6.9	-0.88	-1.84	AAA-AAA	0.00	999	1.000	1.000	0.3331
1558310_s_at	14.4	26.6	0.88	1.84	AAA-AAA	0.00	999	1.000	1.000	0.3386
214818_at	45.3	24.6	-0.88	-1.84	AAA-AAA	0.00	999	1.000	1.000	0.3094
214495_at	12.6	23.2	0.88	1.84	AAA-AAA	0.00	999	1.000	1.000	0.2421
217001_x_at	106.8	58.0	-0.88	-1.84	AAA-AAA	0.00	999	1.000	1.000	0.4426
205114_s_at	8.0	14.8	0.88	1.84	AAA-AAA	0.00	999	1.000	1.000	0.4360
210270_at	17.2	31.7	0.88	1.84	AAA-AAA	0.00	999	1.000	1.000	0.2069
210991_s_at	53.3	28.9	-0.88	-1.84	AAA-AAA	0.00	999	1.000	1.000	0.5068
235574_at	5.4	10.0	0.88	1.84	AAA-AAA	0.00	999	1.000	1.000	0.5435
233278_at	6.5	11.9	0.88	1.84	AAA-AAA	0.00	999	1.000	1.000	0.2788
236504_x_at	50.8	27.5	-0.88	-1.84	AAA-AAA	0.00	999	1.000	1.000	0.5359
228892_at	52.5	96.8	0.88	1.84	AAA-AAA	0.00	999	1.000	1.000	0.2789
229669_at	35.5	19.3	-0.88	-1.84	AAA-AAA	0.00	999	1.000	1.000	0.3583
1556474_a_at	32.8	17.8	-0.88	-1.84	MAA-AAA	0.00	999	1.000	1.000	0.5001
1558480_at	4.0	7.4	0.88	1.84	AAA-AAA	0.00	999	1.000	1.000	0.5553
1555099_at	5.9	3.2	-0.88	-1.84	AAA-AAA	0.00	999	1.000	1.000	0.4089
242789_at	41.3	22.4	-0.88	-1.84	AAA-AAA	0.00	999	1.000	1.000	0.5475
244163_at	30.0	16.3	-0.88	-1.84	AAA-AAA	0.00	999	1.000	1.000	0.5762
215129_at	46.5	85.6	0.88	1.84	AAA-AAA	0.00	999	1.000	1.000	0.4626
217358_at	28.5	52.4	0.88	1.84	AAA-AAA	0.00	999	1.000	1.000	0.4354
211687_x_at	11.3	20.8	0.88	1.84	AAA-AAA	0.00	999	1.000	1.000	0.5550
235586_at	19.1	35.2	0.88	1.84	AAA-AAA	0.00	999	1.000	1.000	0.4143
233471_at	8.9	4.9	-0.88	-1.84	AAA-AAA	0.00	999	1.000	1.000	0.4061
237618_at	49.0	90.2	0.88	1.84	AAA-AAA	0.00	999	1.000	1.000	0.3879
236055_at	45.9	84.6	0.88	1.84	AAA-MMA	0.00	999	1.000	1.000	0.2856
236960_at	19.7	10.7	-0.88	-1.84	AAA-AAA	0.00	999	1.000	1.000	0.0204
224092_at	33.4	61.5	0.88	1.84	AAA-AAA	0.00	999	1.000	1.000	0.3375
224385_s_at	9.2	17.0	0.88	1.84	AAA-AAA	0.00	999	1.000	1.000	0.3400
229252_at	54.0	29.3	-0.88	-1.84	AAA-AAA	0.00	999	1.000	1.000	0.4129
209469_at	66.1	35.9	-0.88	-1.84	PAA-AAA	0.00	999	1.000	1.000	0.0663
242778_at	64.5	35.0	-0.88	-1.84	PMA-PAM	0.00	999	1.000	1.000	0.4471
211508_s_at	13.6	7.4	-0.88	-1.84	AAA-AAP	0.00	999	1.000	1.000	0.6587
1563222_at	45.2	83.1	0.88	1.84	AAA-AAA	0.00	999	1.000	1.000	0.3787
240901_at	9.9	5.4	-0.88	-1.84	AAA-AAA	0.00	999	1.000	1.000	0.3616
1556876_s_at	7.8	14.3	0.88	1.84	AAA-AAA	0.00	999	1.000	1.000	0.4882
1552798_a_at	13.2	7.2	-0.88	-1.84	AAA-AAA	0.00	999	1.000	1.000	0.5724
1553437_at	81.9	44.5	-0.88	-1.84	AAA-AAA	0.00	999	1.000	1.000	0.4797

1554188_at	41.0	75.5	0.88	1.84	AAA-AAA	0.00	999	1.000	1.000	0.2859
1561786_at	29.3	15.9	-0.88	-1.84	AAA-AAA	0.00	999	1.000	1.000	0.3841
215618_at	16.8	30.8	0.88	1.84	AAA-AAA	0.00	999	1.000	1.000	0.5430
205834_s_at	96.0	52.2	-0.88	-1.84	AAA-AAA	0.00	999	1.000	1.000	0.1830
207151_at	51.4	27.9	-0.88	-1.84	AAA-AAA	0.00	999	1.000	1.000	0.1736
211680_at	6.6	3.6	-0.88	-1.84	AAA-AAA	0.00	999	1.000	1.000	0.2271
211143_x_at	46.2	85.0	0.88	1.84	AAA-AAA	0.00	999	1.000	1.000	0.5574
234384_at	33.1	60.8	0.88	1.84	AAA-AAA	0.00	999	1.000	1.000	0.3603
235950_at	7.7	4.2	-0.88	-1.84	AAA-AAA	0.00	999	1.000	1.000	0.1687
234557_at	61.3	33.3	-0.88	-1.84	APP-AAA	0.00	999	1.000	1.000	0.5037
1563219_at	10.3	18.9	0.88	1.84	AAA-AAA	0.00	999	1.000	1.000	0.1158
239799_at	20.8	38.3	0.88	1.84	AAA-AAA	0.00	999	1.000	1.000	0.4788
1552588_a_at	3.2	5.9	0.88	1.84	AAA-AAA	0.00	999	1.000	1.000	0.1094
1556775_at	6.9	12.6	0.88	1.84	AAA-AAA	0.00	999	1.000	1.000	0.4906
1553002_at	15.8	8.6	-0.88	-1.84	AAA-AAA	0.00	999	1.000	1.000	0.6415
1561609_at	55.5	30.2	-0.88	-1.84	AAA-AAA	0.00	999	1.000	1.000	0.5139
1563542_a_at	54.6	29.7	-0.88	-1.84	AAA-AAA	0.00	999	1.000	1.000	0.2385
219308_s_at	17.9	32.8	0.88	1.84	AAA-AAA	0.00	999	1.000	1.000	0.4695
205969_at	7.4	13.5	0.88	1.84	AAA-AAA	0.00	999	1.000	1.000	0.2535
211832_s_at	25.9	14.1	-0.88	-1.84	AAA-AAA	0.00	999	1.000	1.000	0.5686
208121_s_at	20.7	38.1	0.88	1.84	AAA-AAA	0.00	999	1.000	1.000	0.2945
238209_at	59.9	32.6	-0.88	-1.84	AAA-AAA	0.00	999	1.000	1.000	0.3925
237061_at	20.2	11.0	-0.88	-1.84	AAA-AAA	0.00	999	1.000	1.000	0.4852
226548_at	54.8	29.8	-0.88	-1.84	AAA-AAA	0.00	999	1.000	1.000	0.0724
221996_s_at	52.5	96.5	0.88	1.84	AAA-MAA	0.00	999	1.000	1.000	0.1819
223628_at	48.9	90.0	0.88	1.84	AAA-AAA	0.00	999	1.000	1.000	0.3881
1554808_at	76.6	41.7	-0.88	-1.84	PAA-AAM	0.00	999	1.000	1.000	0.1274
238955_at	94.2	51.3	-0.88	-1.84	PAA-AAA	0.00	999	1.000	1.000	0.2455
240374_at	44.1	81.0	0.88	1.84	AAA-AAA	0.00	999	1.000	1.000	0.1935
1569537_at	26.6	48.8	0.88	1.84	AAA-AAA	0.00	999	1.000	1.000	0.3261
1556925_at	48.7	26.5	-0.88	-1.84	AAA-AAA	0.00	999	1.000	1.000	0.2841
1552830_at	49.9	27.2	-0.88	-1.84	AAA-AAA	0.00	999	1.000	1.000	0.4371
1558121_at	3.5	1.9	-0.88	-1.84	AAA-AAA	0.00	999	1.000	1.000	0.0948
1554379_a_at	32.6	17.8	-0.88	-1.84	AAA-AAA	0.00	999	1.000	1.000	0.6005
220927_s_at	7.8	14.3	0.88	1.84	AAA-AAA	0.00	999	1.000	1.000	0.3555
210432_s_at	2.8	5.1	0.88	1.84	AAA-AAA	0.00	999	1.000	1.000	0.4344
210116_at	7.2	13.2	0.88	1.84	AAA-AAA	0.00	999	1.000	1.000	0.4424
234495_at	22.3	40.9	0.88	1.84	AAA-AAA	0.00	999	1.000	1.000	0.3088
238544_at	30.1	16.4	-0.88	-1.84	AAA-AAA	0.00	999	1.000	1.000	0.5680
226971_at	23.9	13.0	-0.88	-1.84	AAA-AAA	0.00	999	1.000	1.000	0.6701
228632_at	34.2	18.6	-0.88	-1.84	AAA-AAA	0.00	999	1.000	1.000	0.1329
231021_at	75.1	138.0	0.88	1.84	AAA-AAA	0.00	999	1.000	1.000	0.2188

213319_s_at	85.5	46.5	-0.88	-1.84	PAA-AAA	0.00	999	1.000	1.000	0.1370
1570239_a_at	38.7	71.1	0.88	1.84	AAA-AAA	0.00	999	1.000	1.000	0.4532
1558181_at	13.8	25.3	0.88	1.84	AAA-AAA	0.00	999	1.000	1.000	0.3263
1558300_at	12.8	6.9	-0.88	-1.84	AAA-AAA	0.00	999	1.000	1.000	0.0521
1557566_at	6.9	3.8	-0.88	-1.84	AAA-AAA	0.00	999	1.000	1.000	0.5643
1555151_s_at	9.0	16.6	0.88	1.84	AAA-AAA	0.00	999	1.000	1.000	0.4198
216746_at	19.6	10.7	-0.88	-1.84	AAA-AAA	0.00	999	1.000	1.000	0.5059
216413_at	3.6	6.6	0.88	1.84	AAA-AAA	0.00	999	1.000	1.000	0.2374
220102_at	26.0	47.7	0.88	1.84	AAA-AAA	0.00	999	1.000	1.000	0.1920
219993_at	18.9	34.7	0.88	1.84	AAA-AAA	0.00	999	1.000	1.000	0.1947
237540_at	9.6	5.2	-0.88	-1.84	AAA-AAA	0.00	999	1.000	1.000	0.6042
229680_at	18.2	9.9	-0.88	-1.84	AAA-AAA	0.00	999	1.000	1.000	0.1100
229438_at	152.5	83.0	-0.88	-1.84	AAA-AAA	0.00	999	1.000	1.000	0.0091
229976_at	33.1	60.8	0.88	1.84	AAA-AAA	0.00	999	1.000	1.000	0.2928
216989_at	93.8	51.1	-0.88	-1.84	MPA-AAA	0.00	999	1.000	1.000	0.1592
1561016_at	12.0	6.6	-0.88	-1.84	AAA-PAA	0.00	999	1.000	1.000	0.6350
241483_at	4.4	8.1	0.88	1.84	AAA-AAA	0.00	999	1.000	1.000	0.4004
1564773_x_at	19.6	36.0	0.88	1.84	AAA-AAA	0.00	999	1.000	1.000	0.4635
1570085_at	12.6	23.2	0.88	1.84	AAA-AAA	0.00	999	1.000	1.000	0.2445
240317_at	7.6	14.0	0.88	1.84	AAA-AAA	0.00	999	1.000	1.000	0.3337
1558075_at	62.3	33.9	-0.88	-1.84	AAM-AAA	0.00	999	1.000	1.000	0.4535
1553264_a_at	84.4	46.0	-0.88	-1.84	AAA-AAA	0.00	999	1.000	1.000	0.1723
216332_at	24.7	13.5	-0.88	-1.84	AAA-AAA	0.00	999	1.000	1.000	0.3548
216911_s_at	6.3	11.6	0.88	1.84	AAA-AAA	0.00	999	1.000	1.000	0.1945
238314_x_at	5.6	3.0	-0.88	-1.84	AAA-AAA	0.00	999	1.000	1.000	0.6510
237155_at	31.0	16.9	-0.88	-1.84	AAA-AAA	0.00	999	1.000	1.000	0.3901
223583_at	38.5	70.6	0.88	1.84	AAA-AAA	0.00	999	1.000	1.000	0.0411
229866_at	11.7	21.6	0.88	1.84	AAA-AAA	0.00	999	1.000	1.000	0.5831
214987_at	67.0	36.5	-0.88	-1.83	AAA-APA	0.00	999	1.000	1.000	0.4052
239639_at	13.2	24.2	0.88	1.83	AAA-AAA	0.00	999	1.000	1.000	0.6041
1561424_at	9.7	5.3	-0.88	-1.83	AAA-AAA	0.00	999	1.000	1.000	0.4932
1561662_at	6.8	12.5	0.88	1.83	AAA-AAA	0.00	999	1.000	1.000	0.4219
216155_at	23.3	12.7	-0.88	-1.83	AAA-AAA	0.00	999	1.000	1.000	0.5898
217298_at	5.8	10.6	0.88	1.83	AAA-AAA	0.00	999	1.000	1.000	0.2461
203937_s_at	98.4	53.7	-0.88	-1.83	AAA-AAA	0.00	999	1.000	1.000	0.1498
211154_at	20.6	37.9	0.88	1.83	AAA-AAA	0.00	999	1.000	1.000	0.4341
235278_at	37.6	69.0	0.88	1.83	AAA-AAA	0.00	999	1.000	1.000	0.2815
224942_at	56.0	30.5	-0.88	-1.83	AAA-AAA	0.00	999	1.000	1.000	0.4654
225379_at	26.9	49.4	0.88	1.83	AAA-AAA	0.00	999	1.000	1.000	0.4238
232379_at	13.3	7.3	-0.88	-1.83	AAA-AAA	0.00	999	1.000	1.000	0.5604
212667_at	216.9	118.4	-0.87	-1.83	PPP-PPA	0.00	999	1.000	1.000	0.0947
231888_at	94.4	51.5	-0.87	-1.83	PPP-APA	0.00	999	1.000	1.000	0.2620

1564097_at	7.0	12.8	0.87	1.83	AAA-AAA	0.00	999	1.000	1.000	0.6295
1555096_at	47.7	87.4	0.87	1.83	AAA-AAA	0.00	999	1.000	1.000	0.2389
1555209_at	18.5	10.1	-0.87	-1.83	AAA-AAA	0.00	999	1.000	1.000	0.2388
234817_at	40.6	22.1	-0.87	-1.83	AAA-AAA	0.00	999	1.000	1.000	0.5370
236029_at	21.4	11.7	-0.87	-1.83	AAA-AAA	0.00	999	1.000	1.000	0.3863
224527_at	4.3	7.8	0.87	1.83	AAA-AAA	0.00	999	1.000	1.000	0.0766
224126_at	3.0	5.6	0.87	1.83	AAA-AAA	0.00	999	1.000	1.000	0.5814
224388_s_at	27.9	51.1	0.87	1.83	AAA-AAA	0.00	999	1.000	1.000	0.5517
230548_at	61.3	33.4	-0.87	-1.83	AAA-AAA	0.00	999	1.000	1.000	0.3905
234265_at	41.1	22.5	-0.87	-1.83	AAP-AAA	0.00	999	1.000	1.000	0.4677
1564143_at	20.4	37.4	0.87	1.83	AAA-AAA	0.00	999	1.000	1.000	0.4432
1565282_at	14.9	27.3	0.87	1.83	AAA-AAA	0.00	999	1.000	1.000	0.4860
1570213_at	1.5	2.8	0.87	1.83	AAA-AAA	0.00	999	1.000	1.000	0.5303
240909_at	20.1	36.9	0.87	1.83	AAA-AAA	0.00	999	1.000	1.000	0.5213
244638_at	20.1	36.8	0.87	1.83	AAA-AAA	0.00	999	1.000	1.000	0.2169
205269_at	10.4	19.1	0.87	1.83	AAA-AAA	0.00	999	1.000	1.000	0.4748
209842_at	63.6	34.7	-0.87	-1.83	AAA-AAA	0.00	999	1.000	1.000	0.3871
232995_at	35.4	19.3	-0.87	-1.83	AAA-AAA	0.00	999	1.000	1.000	0.5239
237676_at	12.3	6.7	-0.87	-1.83	AAA-AAA	0.00	999	1.000	1.000	0.5988
222089_s_at	37.2	20.3	-0.87	-1.83	AAA-AAA	0.00	999	1.000	1.000	0.1877
229990_at	15.5	28.4	0.87	1.83	AAA-AAA	0.00	999	1.000	1.000	0.1479
1556395_at	13.6	7.5	-0.87	-1.83	AAA-AAA	0.00	999	1.000	1.000	0.5998
1559249_at	42.0	76.9	0.87	1.83	AAA-AAA	0.00	999	1.000	1.000	0.3462
214624_at	21.8	11.9	-0.87	-1.83	AAA-AAA	0.00	999	1.000	1.000	0.2583
215138_s_at	129.2	70.6	-0.87	-1.83	AAA-AAA	0.00	999	1.000	1.000	0.3099
221672_s_at	62.9	115.1	0.87	1.83	AAA-AAA	0.00	999	1.000	1.000	0.2980
221457_s_at	16.0	8.8	-0.87	-1.83	AAA-AAA	0.00	999	1.000	1.000	0.4179
207156_at	30.5	55.8	0.87	1.83	AAA-AAA	0.00	999	1.000	1.000	0.3491
209887_at	28.5	15.6	-0.87	-1.83	AAA-AAA	0.00	999	1.000	1.000	0.6011
211198_s_at	19.4	10.6	-0.87	-1.83	AAA-AAA	0.00	999	1.000	1.000	0.3243
207596_at	27.3	50.0	0.87	1.83	AAA-AAA	0.00	999	1.000	1.000	0.0154
238061_at	18.3	10.0	-0.87	-1.83	AAA-AAA	0.00	999	1.000	1.000	0.0179
235785_at	131.5	71.8	-0.87	-1.83	AAA-AAA	0.00	999	1.000	1.000	0.2493
228344_s_at	15.9	29.0	0.87	1.83	AAA-AAA	0.00	999	1.000	1.000	0.3005
227733_at	89.2	163.3	0.87	1.83	AAA-AAA	0.00	999	1.000	1.000	0.1871
230831_at	66.7	36.4	-0.87	-1.83	AAA-AAA	0.00	999	1.000	1.000	0.0978
230671_at	67.5	36.9	-0.87	-1.83	APA-AAP	0.00	999	1.000	1.000	0.3874
239886_at	108.6	59.4	-0.87	-1.83	AAP-AAP	0.00	999	1.000	1.000	0.4129
239552_at	63.2	34.5	-0.87	-1.83	AAP-AAA	0.00	999	1.000	1.000	0.4702
240109_at	7.5	13.7	0.87	1.83	AAA-AAA	0.00	999	1.000	1.000	0.4965
1567390_at	42.8	23.4	-0.87	-1.83	AAA-AAA	0.00	999	1.000	1.000	0.5831
240464_at	13.6	24.9	0.87	1.83	AAA-AAA	0.00	999	1.000	1.000	0.2320

1553746_a_at	10.7	19.5	0.87	1.83	AAA-AAA	0.00	999	1.000	1.000	0.5332
244596_at	29.7	16.3	-0.87	-1.83	AAA-AAA	0.00	999	1.000	1.000	0.4708
1560615_a_at	32.8	60.1	0.87	1.83	AAM-AAA	0.00	999	1.000	1.000	0.4176
243830_at	20.9	11.4	-0.87	-1.83	AAA-AAA	0.00	999	1.000	1.000	0.6305
219693_at	73.9	40.4	-0.87	-1.83	AAA-AAA	0.00	999	1.000	1.000	0.3014
202327_s_at	41.3	22.6	-0.87	-1.83	AAA-AAA	0.00	999	1.000	1.000	0.0571
211135_x_at	48.8	26.7	-0.87	-1.83	AAA-AAA	0.00	999	1.000	1.000	0.2703
233706_at	12.3	6.7	-0.87	-1.83	AAA-AAA	0.00	999	1.000	1.000	0.3397
233478_at	27.5	50.3	0.87	1.83	AAA-AAA	0.00	999	1.000	1.000	0.4775
237604_at	13.7	25.0	0.87	1.83	AAA-AAA	0.00	999	1.000	1.000	0.5290
236161_at	10.8	19.8	0.87	1.83	AAA-AAA	0.00	999	1.000	1.000	0.4653
236658_at	8.3	15.2	0.87	1.83	AAA-AAA	0.00	999	1.000	1.000	0.3623
236880_at	18.4	33.7	0.87	1.83	AAA-AAA	0.00	999	1.000	1.000	0.3211
223601_at	38.8	70.9	0.87	1.83	AAA-AAA	0.00	999	1.000	1.000	0.3615
232450_at	10.3	18.8	0.87	1.83	AAA-AAA	0.00	999	1.000	1.000	0.6752
230128_at	16.3	29.8	0.87	1.83	AAA-AAA	0.00	999	1.000	1.000	0.5188
210871_x_at	269.3	147.4	-0.87	-1.83	PAP-PAP	0.00	999	1.000	1.000	0.0383
236529_at	85.8	47.0	-0.87	-1.83	APA-AAA	0.00	999	1.000	1.000	0.3321
232981_s_at	157.8	86.4	-0.87	-1.83	AAP-AAA	0.00	999	1.000	1.000	0.3823
1562932_at	14.4	7.9	-0.87	-1.83	AAA-AAA	0.00	999	1.000	1.000	0.5636
242120_at	38.0	69.5	0.87	1.83	AAA-AAA	0.00	999	1.000	1.000	0.2634
1563081_at	60.5	110.5	0.87	1.83	AAA-AAA	0.00	999	1.000	1.000	0.1543
1569321_at	89.7	49.1	-0.87	-1.83	AAA-AAA	0.00	999	1.000	1.000	0.4195
1569753_at	15.1	27.6	0.87	1.83	AAA-AAA	0.00	999	1.000	1.000	0.3235
239592_at	82.9	45.4	-0.87	-1.83	AAA-AAA	0.00	999	1.000	1.000	0.4486
1570225_at	7.3	13.4	0.87	1.83	AAA-AAA	0.00	999	1.000	1.000	0.3622
1554224_at	8.6	4.7	-0.87	-1.83	AAA-AAA	0.00	999	1.000	1.000	0.4027
1561346_at	190.4	348.0	0.87	1.83	AAA-AAA	0.00	999	1.000	1.000	0.3022
210324_at	4.7	8.6	0.87	1.83	AAA-AAA	0.00	999	1.000	1.000	0.2882
207293_s_at	18.2	33.2	0.87	1.83	AAA-AAA	0.00	999	1.000	1.000	0.4362
230271_at	22.7	41.5	0.87	1.83	AAA-AAA	0.00	999	1.000	1.000	0.3717
201243_s_at	1577.7	863.6	-0.87	-1.83	PPP-PPP	0.00	999	1.000	1.000	0.0443
233518_at	71.2	39.0	-0.87	-1.83	APA-AAP	0.00	999	1.000	1.000	0.5297
241211_at	15.7	28.7	0.87	1.83	MAA-AAA	0.00	999	1.000	1.000	0.6114
1567010_at	10.8	5.9	-0.87	-1.83	AAA-AAA	0.00	999	1.000	1.000	0.0297
1553721_at	12.6	23.1	0.87	1.83	AAA-AAA	0.00	999	1.000	1.000	0.6224
1555794_at	3.3	6.1	0.87	1.83	AAA-AAA	0.00	999	1.000	1.000	0.2650
1564591_a_at	12.9	7.1	-0.87	-1.83	AAA-AAA	0.00	999	1.000	1.000	0.4505
1561421_a_at	10.3	18.9	0.87	1.83	AAA-AAA	0.00	999	1.000	1.000	0.1739
215893_x_at	23.7	43.3	0.87	1.83	AAA-AAA	0.00	999	1.000	1.000	0.3249
217626_at	30.2	55.1	0.87	1.83	AAA-AAA	0.00	999	1.000	1.000	0.3385
217517_x_at	19.5	35.6	0.87	1.83	AAA-AAA	0.00	999	1.000	1.000	0.5301

206131_at	42.2	77.1	0.87	1.83	AAA-AAA	0.00	999	1.000	1.000	0.1871
211221_at	10.7	19.5	0.87	1.83	AAA-AAA	0.00	999	1.000	1.000	0.4587
222042_x_at	26.3	14.4	-0.87	-1.83	AAA-AAA	0.00	999	1.000	1.000	0.5858
223699_at	32.7	17.9	-0.87	-1.83	AAA-AAA	0.00	999	1.000	1.000	0.4268
231027_at	9.9	18.1	0.87	1.83	AAA-AAA	0.00	999	1.000	1.000	0.2470
229039_at	36.9	20.2	-0.87	-1.83	AAA-AAA	0.00	999	1.000	1.000	0.2693
230601_s_at	55.8	30.6	-0.87	-1.83	AAA-AAA	0.00	999	1.000	1.000	0.4160
234270_at	70.8	38.8	-0.87	-1.83	AAP-AAA	0.00	999	1.000	1.000	0.2658
1561453_at	60.5	33.1	-0.87	-1.83	AAP-MAA	0.00	999	1.000	1.000	0.4967
1564251_at	22.2	12.1	-0.87	-1.83	AAA-AAA	0.00	999	1.000	1.000	0.4889
1552549_a_at	17.1	31.2	0.87	1.83	AAA-AAA	0.00	999	1.000	1.000	0.4112
1552946_at	17.4	31.7	0.87	1.83	AAA-AAA	0.00	999	1.000	1.000	0.5356
1556598_at	2.5	4.6	0.87	1.83	AAA-AAA	0.00	999	1.000	1.000	0.4582
214913_at	33.2	18.2	-0.87	-1.83	AAA-AAA	0.00	999	1.000	1.000	0.5484
214243_s_at	6.0	11.0	0.87	1.83	AAA-AAA	0.00	999	1.000	1.000	0.1298
218864_at	15.1	27.5	0.87	1.83	AAA-AAA	0.00	999	1.000	1.000	0.0534
211107_s_at	32.0	17.5	-0.87	-1.83	AAA-AAA	0.00	999	1.000	1.000	0.3391
232601_at	89.1	162.6	0.87	1.83	AAA-AAA	0.00	999	1.000	1.000	0.3377
233521_at	9.0	4.9	-0.87	-1.83	AAA-AAA	0.00	999	1.000	1.000	0.1306
236003_x_at	7.3	4.0	-0.87	-1.83	AAA-AAA	0.00	999	1.000	1.000	0.1720
229752_at	25.8	14.1	-0.87	-1.83	AAA-AAA	0.00	999	1.000	1.000	0.4316
1562267_s_at	42.7	23.4	-0.87	-1.82	AAA-PAM	0.00	999	1.000	1.000	0.6239
1563312_at	19.0	10.4	-0.87	-1.82	AAA-AAA	0.00	999	1.000	1.000	0.4074
240184_at	42.3	77.2	0.87	1.82	AAA-AAA	0.00	999	1.000	1.000	0.2089
1566232_at	53.9	29.5	-0.87	-1.82	AAA-AAA	0.00	999	1.000	1.000	0.0345
1552845_at	24.6	44.8	0.87	1.82	AAA-AAA	0.00	999	1.000	1.000	0.2457
1561094_a_at	12.9	7.1	-0.87	-1.82	AAA-AAA	0.00	999	1.000	1.000	0.6418
243447_at	24.0	13.2	-0.87	-1.82	AAA-AAA	0.00	999	1.000	1.000	0.4089
1559697_a_at	18.8	10.3	-0.87	-1.82	AAA-AAA	0.00	999	1.000	1.000	0.6022
212545_s_at	43.5	79.3	0.87	1.82	AAA-AAA	0.00	999	1.000	1.000	0.4564
207216_at	63.1	34.6	-0.87	-1.82	AAA-AAA	0.00	999	1.000	1.000	0.5395
201203_s_at	109.0	198.8	0.87	1.82	AAA-AAA	0.00	999	1.000	1.000	0.2069
210394_x_at	25.5	46.4	0.87	1.82	AAA-AAA	0.00	999	1.000	1.000	0.2730
210126_at	26.6	14.6	-0.87	-1.82	AAA-AAA	0.00	999	1.000	1.000	0.4628
235250_at	39.9	21.9	-0.87	-1.82	AAA-AAA	0.00	999	1.000	1.000	0.3973
237957_at	3.4	6.2	0.87	1.82	AAA-AAA	0.00	999	1.000	1.000	0.4449
226722_at	1834.8	1007.0	-0.87	-1.82	PPP-PPP	0.00	999	1.000	1.000	0.0418
1562990_at	7.4	13.5	0.87	1.82	AAA-AAA	0.00	999	1.000	1.000	0.5990
1570439_at	24.4	44.5	0.87	1.82	AAA-AAA	0.00	999	1.000	1.000	0.2603
1552503_at	28.7	52.3	0.87	1.82	AAA-AAA	0.00	999	1.000	1.000	0.4961
1561489_at	5.0	9.1	0.87	1.82	AAA-AAA	0.00	999	1.000	1.000	0.4922
1560742_at	22.4	12.3	-0.87	-1.82	AAA-AAA	0.00	999	1.000	1.000	0.4335

221401_at	16.3	9.0	-0.87	-1.82	AAA-AAA	0.00	999	1.000	1.000	0.2948
216982_x_at	8.5	15.5	0.87	1.82	AAA-AAA	0.00	999	1.000	1.000	0.5877
207362_at	8.8	16.1	0.87	1.82	AAA-AAA	0.00	999	1.000	1.000	0.4791
207547_s_at	31.7	57.7	0.87	1.82	AAA-AAA	0.00	999	1.000	1.000	0.4607
236136_at	28.2	51.5	0.87	1.82	AAA-AAA	0.00	999	1.000	1.000	0.5329
228080_at	7.8	14.2	0.87	1.82	AAA-AAA	0.00	999	1.000	1.000	0.1643
1562443_at	56.0	30.8	-0.87	-1.82	PAA-AAA	0.00	999	1.000	1.000	0.5142
1570330_at	32.5	17.9	-0.87	-1.82	AAA-AAA	0.00	999	1.000	1.000	0.4109
240981_at	20.0	11.0	-0.87	-1.82	AAA-AAA	0.00	999	1.000	1.000	0.3901
1557328_at	21.8	12.0	-0.87	-1.82	AAA-AAA	0.00	999	1.000	1.000	0.4496
1555429_at	91.6	50.3	-0.87	-1.82	AAA-AAA	0.00	999	1.000	1.000	0.1613
243275_at	15.2	27.7	0.87	1.82	AAA-AAA	0.00	999	1.000	1.000	0.3356
1562275_at	6.8	12.4	0.87	1.82	AAA-AAA	0.00	999	1.000	1.000	0.5274
1562477_at	17.4	31.7	0.87	1.82	AAA-AAA	0.00	999	1.000	1.000	0.3926
1562468_at	12.0	21.8	0.87	1.82	AAA-AAA	0.00	999	1.000	1.000	0.5166
216022_at	61.9	112.8	0.87	1.82	AAA-AAM	0.00	999	1.000	1.000	0.0733
215199_at	26.6	14.6	-0.87	-1.82	AAA-AAA	0.00	999	1.000	1.000	0.6294
213685_at	151.5	83.2	-0.87	-1.82	AAA-AAA	0.00	999	1.000	1.000	0.0169
205907_s_at	9.2	16.8	0.87	1.82	AAA-AAA	0.00	999	1.000	1.000	0.6361
205259_at	32.6	59.4	0.87	1.82	AAA-AAA	0.00	999	1.000	1.000	0.0239
210483_at	33.5	18.4	-0.87	-1.82	AAA-AAA	0.00	999	1.000	1.000	0.3209
210906_x_at	5.7	10.4	0.87	1.82	AAA-AAA	0.00	999	1.000	1.000	0.4848
234903_at	8.4	15.2	0.87	1.82	AAA-AAA	0.00	999	1.000	1.000	0.5254
234006_s_at	36.3	20.0	-0.87	-1.82	AAA-AAA	0.00	999	1.000	1.000	0.3804
236156_at	7.4	4.1	-0.87	-1.82	AAA-AAA	0.00	999	1.000	1.000	0.3608
212067_s_at	265.2	145.7	-0.86	-1.82	PPP-PPP	0.00	999	1.000	1.000	0.1135
204035_at	36.4	20.0	-0.86	-1.82	APA-AAA	0.00	999	1.000	1.000	0.3214
1558340_at	92.4	50.8	-0.86	-1.82	AAA-AAP	0.00	999	1.000	1.000	0.3727
1559034_at	39.1	71.2	0.86	1.82	AAA-AAA	0.00	999	1.000	1.000	0.2580
243742_at	62.1	34.1	-0.86	-1.82	AAA-AAA	0.00	999	1.000	1.000	0.3445
215406_at	6.9	12.5	0.86	1.82	AAA-AAA	0.00	999	1.000	1.000	0.3782
221852_at	11.7	6.5	-0.86	-1.82	AAA-AAA	0.00	999	1.000	1.000	0.1697
205944_s_at	51.9	94.5	0.86	1.82	AAA-AAA	0.00	999	1.000	1.000	0.2607
234939_s_at	94.0	51.6	-0.86	-1.82	AAA-AAA	0.00	999	1.000	1.000	0.4818
228421_s_at	15.2	27.6	0.86	1.82	AAA-AAA	0.00	999	1.000	1.000	0.3267
231113_at	3.3	6.0	0.86	1.82	AAA-AAA	0.00	999	1.000	1.000	0.5131
1561624_at	102.0	56.1	-0.86	-1.82	PAA-AAA	0.00	999	1.000	1.000	0.0832
1566147_a_at	60.4	109.8	0.86	1.82	AAA-AAA	0.00	999	1.000	1.000	0.1487
1558463_s_at	66.9	121.7	0.86	1.82	AAA-AAA	0.00	999	1.000	1.000	0.1413
211877_s_at	4.9	8.9	0.86	1.82	AAA-AAA	0.00	999	1.000	1.000	0.3319
220620_at	42.5	77.3	0.86	1.82	AAA-AAA	0.00	999	1.000	1.000	0.1463
220231_at	39.9	72.5	0.86	1.82	AAA-AAA	0.00	999	1.000	1.000	0.3731

206589_at	5.7	10.4	0.86	1.82	AAA-AAA	0.00	999	1.000	1.000	0.4886
205268_s_at	125.0	68.7	-0.86	-1.82	AAA-AAA	0.00	999	1.000	1.000	0.0211
210132_at	213.2	117.2	-0.86	-1.82	AAA-AAA	0.00	999	1.000	1.000	0.3106
233196_at	57.1	103.8	0.86	1.82	AAA-AAA	0.00	999	1.000	1.000	0.3641
232833_at	59.5	32.8	-0.86	-1.82	AAA-APP	0.00	999	1.000	1.000	0.2632
1564241_at	44.6	24.5	-0.86	-1.82	AMA-AAA	0.00	999	1.000	1.000	0.4065
1563053_at	9.3	5.1	-0.86	-1.82	AAA-AAA	0.00	999	1.000	1.000	0.5415
1556449_at	12.2	22.2	0.86	1.82	AAA-AAA	0.00	999	1.000	1.000	0.4359
1554268_at	41.0	74.4	0.86	1.82	AAA-AAA	0.00	999	1.000	1.000	0.1560
1553574_at	24.8	13.7	-0.86	-1.82	MAA-AAA	0.00	999	1.000	1.000	0.6228
221237_s_at	54.0	98.1	0.86	1.82	AAA-AAA	0.00	999	1.000	1.000	0.5139
207015_s_at	16.9	30.8	0.86	1.82	AAA-AAA	0.00	999	1.000	1.000	0.5644
206700_s_at	42.2	23.2	-0.86	-1.82	AAA-AAA	0.00	999	1.000	1.000	0.5377
206535_at	2.8	5.0	0.86	1.82	AAA-AAA	0.00	999	1.000	1.000	0.3420
202878_s_at	27.7	15.2	-0.86	-1.82	AAA-AAA	0.00	999	1.000	1.000	0.4466
207885_at	9.4	5.2	-0.86	-1.82	AAA-AAA	0.00	999	1.000	1.000	0.5503
208590_x_at	8.2	14.9	0.86	1.82	AAA-AAA	0.00	999	1.000	1.000	0.3683
237786_at	14.8	27.0	0.86	1.82	AAA-AAA	0.00	999	1.000	1.000	0.5193
238217_at	9.6	17.4	0.86	1.82	AAA-AAA	0.00	999	1.000	1.000	0.3399
236958_at	68.7	124.9	0.86	1.82	AAA-AAA	0.00	999	1.000	1.000	0.1650
231755_at	6.7	12.2	0.86	1.82	AAA-AAA	0.00	999	1.000	1.000	0.4486
231699_at	58.8	32.4	-0.86	-1.82	APA-AAA	0.00	999	1.000	1.000	0.0797
1567519_at	38.9	70.7	0.86	1.82	AAA-AAA	0.00	999	1.000	1.000	0.0932
1556590_s_at	6.4	11.7	0.86	1.82	AAA-AAA	0.00	999	1.000	1.000	0.5599
1553009_s_at	3.9	7.1	0.86	1.82	AAA-AAA	0.00	999	1.000	1.000	0.0531
1562057_at	6.7	3.7	-0.86	-1.82	AAA-AAA	0.00	999	1.000	1.000	0.1004
1559079_at	15.7	8.6	-0.86	-1.82	AAA-AAA	0.00	999	1.000	1.000	0.2177
213993_at	3.8	6.9	0.86	1.82	AAA-AAA	0.00	999	1.000	1.000	0.4716
211237_s_at	6.6	12.0	0.86	1.82	AAA-AAA	0.00	999	1.000	1.000	0.0256
237533_at	34.9	19.2	-0.86	-1.82	AAA-AAA	0.00	999	1.000	1.000	0.3847
236500_at	22.6	41.1	0.86	1.82	AAA-AAA	0.00	999	1.000	1.000	0.5228
1564356_at	5.1	9.2	0.86	1.81	AAA-AAA	0.00	999	1.000	1.000	0.4207
241350_at	2.7	5.0	0.86	1.82	AAA-AAA	0.00	999	1.000	1.000	0.0428
241600_at	14.8	26.8	0.86	1.82	AAA-AAA	0.00	999	1.000	1.000	0.3488
239553_at	7.2	13.1	0.86	1.82	AAA-AAA	0.00	999	1.000	1.000	0.2770
1565889_at	18.5	33.6	0.86	1.82	AAA-AAA	0.00	999	1.000	1.000	0.5708
215702_s_at	4.2	7.7	0.86	1.82	AAA-AAA	0.00	999	1.000	1.000	0.6294
214598_at	15.0	27.3	0.86	1.81	AAA-AAA	0.00	999	1.000	1.000	0.3267
221259_s_at	8.1	14.6	0.86	1.82	AAA-AAA	0.00	999	1.000	1.000	0.3020
217513_at	12.5	22.7	0.86	1.82	AAA-AAA	0.00	999	1.000	1.000	0.5304
206534_at	18.4	33.4	0.86	1.82	AAA-AAA	0.00	999	1.000	1.000	0.4874
233649_at	20.3	36.9	0.86	1.82	AAA-AAA	0.00	999	1.000	1.000	0.5517

233512_at	6.6	11.9	0.86	1.82	AAA-AAA	0.00	999	1.000	1.000	0.5630
237684_at	15.5	28.2	0.86	1.82	AAA-AAA	0.00	999	1.000	1.000	0.3088
236306_at	16.7	9.2	-0.86	-1.82	AAA-AAA	0.00	999	1.000	1.000	0.6770
228016_s_at	8.4	15.3	0.86	1.82	AAA-AAA	0.00	999	1.000	1.000	0.4458
231008_at	28.5	51.8	0.86	1.82	AAA-AAA	0.00	999	1.000	1.000	0.2537
229382_at	18.1	32.8	0.86	1.82	AAA-AAA	0.00	999	1.000	1.000	0.4261
238739_at	118.2	65.1	-0.86	-1.81	PAP-PAA	0.00	999	1.000	1.000	0.0416
1570080_at	5.6	10.1	0.86	1.81	AAA-AAA	0.00	999	1.000	1.000	0.7215
241018_at	11.7	6.5	-0.86	-1.81	AAA-AAA	0.00	999	1.000	1.000	0.5599
1556687_a_at	15.5	8.6	-0.86	-1.81	AAA-AAA	0.00	999	1.000	1.000	0.5505
216579_at	17.0	9.4	-0.86	-1.81	AAA-AAA	0.00	999	1.000	1.000	0.0319
214871_x_at	32.5	58.9	0.86	1.81	AAA-AAA	0.00	999	1.000	1.000	0.3972
217183_at	17.6	31.9	0.86	1.81	AAA-AAA	0.00	999	1.000	1.000	0.2517
210040_at	42.3	76.6	0.86	1.81	AAA-AAA	0.00	999	1.000	1.000	0.2748
237691_x_at	9.3	16.8	0.86	1.81	AAA-AAA	0.00	999	1.000	1.000	0.4074
228366_at	56.9	103.2	0.86	1.81	AAA-AAA	0.00	999	1.000	1.000	0.1659
224402_s_at	10.0	18.1	0.86	1.81	AAA-AAA	0.00	999	1.000	1.000	0.5004
224253_at	27.9	15.4	-0.86	-1.81	AAA-AAA	0.00	999	1.000	1.000	0.3084
229530_at	17.4	31.5	0.86	1.81	AAA-AAA	0.00	999	1.000	1.000	0.4053
230442_at	80.0	145.1	0.86	1.81	AAA-AAA	0.00	999	1.000	1.000	0.1026
224924_at	158.4	87.4	-0.86	-1.81	PPP-PAP	0.00	999	1.000	1.000	0.2967
242144_at	67.9	123.1	0.86	1.81	AAA-AAA	0.00	999	1.000	1.000	0.1745
1563524_a_at	23.6	13.0	-0.86	-1.81	AAA-AAA	0.00	999	1.000	1.000	0.5268
240208_at	3.5	6.3	0.86	1.81	AAA-AAA	0.00	999	1.000	1.000	0.5213
1569616_at	35.0	19.3	-0.86	-1.81	AAA-AAA	0.00	999	1.000	1.000	0.4094
1566868_at	52.4	28.9	-0.86	-1.81	AAA-AAA	0.00	999	1.000	1.000	0.1205
207111_at	34.0	61.7	0.86	1.81	AAA-AAA	0.00	999	1.000	1.000	0.4779
207752_x_at	75.3	41.5	-0.86	-1.81	AAA-AAA	0.00	999	1.000	1.000	0.3407
208317_at	9.1	5.0	-0.86	-1.81	AAA-AAA	0.00	999	1.000	1.000	0.0199
227241_at	6.9	3.8	-0.86	-1.81	AAA-AAA	0.00	999	1.000	1.000	0.4744
227971_at	14.8	26.9	0.86	1.81	AAA-AAA	0.00	999	1.000	1.000	0.5845
241724_x_at	244.6	135.1	-0.86	-1.81	PPP-APM	0.00	999	1.000	1.000	0.1601
1552649_a_at	71.2	39.3	-0.86	-1.81	PAA-PAA	0.00	999	1.000	1.000	0.5783
1557802_at	18.7	10.4	-0.86	-1.81	AAP-AAA	0.00	999	1.000	1.000	0.6737
240368_at	27.4	49.6	0.86	1.81	AAA-AAA	0.00	999	1.000	1.000	0.3258
1552879_a_at	11.8	6.5	-0.86	-1.81	AAA-AAA	0.00	999	1.000	1.000	0.5455
1560548_at	4.0	7.2	0.86	1.81	AAA-AAA	0.00	999	1.000	1.000	0.1751
1560175_at	28.6	51.8	0.86	1.81	AAA-AAA	0.00	999	1.000	1.000	0.2139
214534_at	25.7	46.6	0.86	1.81	AAA-AAA	0.00	999	1.000	1.000	0.3438
207328_at	8.0	14.5	0.86	1.81	AAA-AAA	0.00	999	1.000	1.000	0.6462
207681_at	30.7	16.9	-0.86	-1.81	AAA-AAA	0.00	999	1.000	1.000	0.3336
234404_at	14.4	7.9	-0.86	-1.81	AAA-AAA	0.00	999	1.000	1.000	0.2124

234184_at	17.3	31.3	0.86	1.81	AAA-AAA	0.00	999	1.000	1.000	0.5536
236308_at	11.4	20.6	0.86	1.81	AAA-MAA	0.00	999	1.000	1.000	0.5560
231446_at	7.1	12.9	0.86	1.81	AAA-AAA	0.00	999	1.000	1.000	0.3775
232472_at	22.4	40.6	0.86	1.81	AAA-AAA	0.00	999	1.000	1.000	0.5878
239503_at	69.9	38.6	-0.86	-1.81	PAP-PAA	0.00	999	1.000	1.000	0.3175
240186_at	28.1	15.5	-0.86	-1.81	APA-AAA	0.00	999	1.000	1.000	0.5901
244390_at	29.9	16.5	-0.86	-1.81	AAA-AAP	0.00	999	1.000	1.000	0.6267
238953_at	59.2	32.7	-0.86	-1.81	AAA-AAA	0.00	999	1.000	1.000	0.5095
1563287_at	24.2	13.4	-0.86	-1.81	AAA-AAA	0.00	999	1.000	1.000	0.4288
241951_at	37.9	21.0	-0.86	-1.81	MAA-AAA	0.00	999	1.000	1.000	0.2952
1569983_at	16.9	9.4	-0.86	-1.81	AAA-AAA	0.00	999	1.000	1.000	0.6378
240735_at	8.7	15.7	0.86	1.81	AAA-AAA	0.00	999	1.000	1.000	0.3079
1558176_at	17.0	9.4	-0.86	-1.81	AAA-AAA	0.00	999	1.000	1.000	0.3568
1561289_at	16.2	9.0	-0.86	-1.81	AAA-AAA	0.00	999	1.000	1.000	0.5547
1561059_a_at	38.8	21.4	-0.86	-1.81	AAA-AAA	0.00	999	1.000	1.000	0.5504
244212_at	16.7	9.2	-0.86	-1.81	AAA-AAA	0.00	999	1.000	1.000	0.3631
214846_s_at	25.7	14.2	-0.86	-1.81	AAA-AAA	0.00	999	1.000	1.000	0.3796
220507_s_at	12.0	21.7	0.86	1.81	AAA-AAA	0.00	999	1.000	1.000	0.5480
217032_at	8.0	14.6	0.86	1.81	AAA-AAA	0.00	999	1.000	1.000	0.2269
202456_s_at	16.8	30.4	0.86	1.81	AAA-AAA	0.00	999	1.000	1.000	0.3744
209547_s_at	166.2	91.8	-0.86	-1.81	AAA-AAA	0.00	999	1.000	1.000	0.4123
236158_at	15.3	8.5	-0.86	-1.81	AAA-AAA	0.00	999	1.000	1.000	0.4604
223782_s_at	10.5	18.9	0.86	1.81	AAA-AAA	0.00	999	1.000	1.000	0.5343
232036_at	38.8	70.2	0.86	1.81	AAA-AAA	0.00	999	1.000	1.000	0.2879
230437_s_at	37.8	68.4	0.86	1.81	AAA-AAA	0.00	999	1.000	1.000	0.1219
242496_at	5.6	3.1	-0.86	-1.81	AAA-AAA	0.00	999	1.000	1.000	0.3252
242009_at	12.5	6.9	-0.86	-1.81	AAA-AAA	0.00	999	1.000	1.000	0.3062
1557181_s_at	6.7	3.7	-0.86	-1.81	AAA-AAA	0.00	999	1.000	1.000	0.3325
1555976_s_at	69.7	126.1	0.86	1.81	AAA-AAA	0.00	999	1.000	1.000	0.0020
1559551_at	30.2	54.5	0.86	1.81	AAA-AAA	0.00	999	1.000	1.000	0.1289
215589_at	21.5	38.9	0.86	1.81	AAA-AAA	0.00	999	1.000	1.000	0.3368
220233_at	92.3	51.0	-0.86	-1.81	AAA-AAA	0.00	999	1.000	1.000	0.2079
234589_at	6.9	12.4	0.86	1.81	AAA-AAA	0.00	999	1.000	1.000	0.3424
233846_at	35.8	64.8	0.86	1.81	AAA-AAA	0.00	999	1.000	1.000	0.2877
222915_s_at	11.3	20.4	0.86	1.81	AAA-AAA	0.00	999	1.000	1.000	0.4119
231686_at	9.0	16.3	0.86	1.81	AAA-AAA	0.00	999	1.000	1.000	0.5304
229024_at	16.8	30.3	0.86	1.81	AAA-AAA	0.00	999	1.000	1.000	0.4357
206277_at	294.0	162.6	-0.85	-1.81	PPA-APP	0.00	999	1.000	1.000	0.0365
210822_at	144.9	80.2	-0.85	-1.81	AAA-APA	0.00	999	1.000	1.000	0.0951
1566448_at	15.7	28.3	0.85	1.81	AAA-AAA	0.00	999	1.000	1.000	0.2797
242724_x_at	31.6	17.5	-0.85	-1.81	AAA-AAA	0.00	999	1.000	1.000	0.3679
214865_at	9.9	5.5	-0.85	-1.81	AAA-AAA	0.00	999	1.000	1.000	0.3129

214591_at	16.2	29.3	0.85	1.81	AAA-AAA	0.00	999	1.000	1.000	0.3437
208606_s_at	5.1	9.3	0.85	1.81	AAA-AAA	0.00	999	1.000	1.000	0.0402
234223_at	8.1	14.6	0.85	1.81	AAA-AAA	0.00	999	1.000	1.000	0.4618
236176_at	12.1	21.8	0.85	1.81	AAA-AAA	0.00	999	1.000	1.000	0.3439
228383_at	11.5	20.9	0.85	1.81	AAA-AAA	0.00	999	1.000	1.000	0.1452
223828_s_at	7.5	13.5	0.85	1.81	AAA-AAA	0.00	999	1.000	1.000	0.1835
230732_s_at	8.0	4.4	-0.85	-1.81	AAA-AAA	0.00	999	1.000	1.000	0.1500
230225_x_at	2.9	5.2	0.85	1.81	AAA-AAA	0.00	999	1.000	1.000	0.5631
220402_at	51.3	28.4	-0.85	-1.81	PAA-AAA	0.00	999	1.000	1.000	0.2505
235737_at	74.3	41.2	-0.85	-1.81	PAA-AAA	0.00	999	1.000	1.000	0.3917
204972_at	51.2	28.4	-0.85	-1.81	APA-AAA	0.00	999	1.000	1.000	0.4941
239115_at	62.9	113.7	0.85	1.81	AAA-AAA	0.00	999	1.000	1.000	0.4339
239991_at	7.2	13.0	0.85	1.81	AAA-AAA	0.00	999	1.000	1.000	0.2957
1553241_at	24.8	44.8	0.85	1.81	AAA-AAA	0.00	999	1.000	1.000	0.6390
1555165_a_at	27.0	48.8	0.85	1.81	AAA-AAA	0.00	999	1.000	1.000	0.4607
1562274_at	95.4	52.8	-0.85	-1.81	AAA-AAA	0.00	999	1.000	1.000	0.1403
243703_x_at	8.9	16.0	0.85	1.81	AAA-AAA	0.00	999	1.000	1.000	0.4188
205804_s_at	48.7	27.0	-0.85	-1.81	AAA-AAA	0.00	999	1.000	1.000	0.4747
206859_s_at	9.0	16.2	0.85	1.81	AAA-AAA	0.00	999	1.000	1.000	0.3583
208435_s_at	21.4	38.7	0.85	1.81	AAA-AAA	0.00	999	1.000	1.000	0.3682
236187_s_at	49.2	27.2	-0.85	-1.81	AAA-AAA	0.00	999	1.000	1.000	0.5322
230950_at	24.8	13.7	-0.85	-1.81	AAA-AAA	0.00	999	1.000	1.000	0.6584
1569566_at	61.2	33.9	-0.85	-1.81	AAA-AAA	0.00	999	1.000	1.000	0.2356
1566094_at	5.1	9.2	0.85	1.81	AAA-AAA	0.00	999	1.000	1.000	0.5038
244805_at	3.6	6.4	0.85	1.81	AAA-AAA	0.00	999	1.000	1.000	0.5256
1553318_at	25.0	45.2	0.85	1.81	AAA-AAA	0.00	999	1.000	1.000	0.4850
1554722_at	5.7	3.2	-0.85	-1.81	AAA-AAA	0.00	999	1.000	1.000	0.3095
243578_at	26.7	14.8	-0.85	-1.81	AAA-AAA	0.00	999	1.000	1.000	0.2918
242991_at	32.7	18.1	-0.85	-1.81	AAA-AAA	0.00	999	1.000	1.000	0.4724
216027_at	2.9	5.3	0.85	1.81	AAA-AAA	0.00	999	1.000	1.000	0.4553
213561_at	159.7	88.5	-0.85	-1.81	AAA-AAA	0.00	999	1.000	1.000	0.1321
235822_at	73.7	133.1	0.85	1.81	AAA-AAA	0.00	999	1.000	1.000	0.2862
222134_at	13.6	7.5	-0.85	-1.81	AAA-AAA	0.00	999	1.000	1.000	0.4923
223873_s_at	11.2	20.2	0.85	1.81	AAA-AAA	0.00	999	1.000	1.000	0.4703
232321_at	39.5	71.2	0.85	1.81	AAA-AAA	0.00	999	1.000	1.000	0.0766
231544_s_at	41.9	23.2	-0.85	-1.80	APP-APA	0.00	999	1.000	1.000	0.5355
233830_at	18.8	10.4	-0.85	-1.80	AAA-PAA	0.00	999	1.000	1.000	0.6929
1564274_at	9.5	17.0	0.85	1.80	AAA-AAA	0.00	999	1.000	1.000	0.4333
240792_at	12.9	23.3	0.85	1.80	AAA-AAA	0.00	999	1.000	1.000	0.5218
1559180_at	8.7	15.7	0.85	1.80	AAA-AAA	0.00	999	1.000	1.000	0.4791
1559982_s_at	28.3	51.0	0.85	1.80	AAA-AAA	0.00	999	1.000	1.000	0.2927
216797_at	6.0	10.9	0.85	1.80	AAA-AAA	0.00	999	1.000	1.000	0.2978

216405_at	26.1	14.5	-0.85	-1.80	AAA-AAA	0.00	999	1.000	1.000	0.5336
217321_x_at	34.9	19.3	-0.85	-1.80	AAA-AAA	0.00	999	1.000	1.000	0.0364
205697_at	17.0	9.4	-0.85	-1.80	AAA-AAA	0.00	999	1.000	1.000	0.4343
207004_at	59.7	107.8	0.85	1.80	AAA-AAA	0.00	999	1.000	1.000	0.0613
207792_at	23.6	42.6	0.85	1.80	AAA-AAA	0.00	999	1.000	1.000	0.3374
234355_s_at	46.9	84.7	0.85	1.80	AAA-AAA	0.00	999	1.000	1.000	0.2615
233978_at	29.3	52.8	0.85	1.80	AAA-AAA	0.00	999	1.000	1.000	0.4841
238378_at	9.6	5.3	-0.85	-1.80	AAA-AAA	0.00	999	1.000	1.000	0.5911
238459_x_at	52.4	29.1	-0.85	-1.80	AAA-AAA	0.00	999	1.000	1.000	0.0227
222571_at	84.4	152.3	0.85	1.80	AAA-AAA	0.00	999	1.000	1.000	0.0744
231041_at	91.5	165.0	0.85	1.80	AAA-AAA	0.00	999	1.000	1.000	0.1209
241172_at	20.3	11.2	-0.85	-1.80	AAA-AAA	0.00	999	1.000	1.000	0.5392
1562386_s_at	11.5	20.7	0.85	1.80	AAA-AAA	0.00	999	1.000	1.000	0.3927
1561419_at	20.1	36.2	0.85	1.80	AAA-AAA	0.00	999	1.000	1.000	0.3687
1559745_at	34.7	62.5	0.85	1.80	AAA-AAM	0.00	999	1.000	1.000	0.4256
216704_at	22.8	41.0	0.85	1.80	AAA-AAA	0.00	999	1.000	1.000	0.5126
217285_at	13.6	24.6	0.85	1.80	AAA-AAA	0.00	999	1.000	1.000	0.4646
211265_at	10.4	5.8	-0.85	-1.80	AAA-AAA	0.00	999	1.000	1.000	0.5562
237558_at	23.6	13.1	-0.85	-1.80	AAA-AAA	0.00	999	1.000	1.000	0.6021
231677_at	55.8	31.0	-0.85	-1.80	AAA-AAA	0.00	999	1.000	1.000	0.1244
229375_at	83.7	150.8	0.85	1.80	AAA-AAA	0.00	999	1.000	1.000	0.3886
210538_s_at	2033.8	1129.1	-0.85	-1.80	PPP-PPP	0.00	999	1.000	1.000	0.0583
1567679_at	9.1	5.0	-0.85	-1.80	AAA-AAA	0.00	999	1.000	1.000	0.6146
240828_at	5.5	9.8	0.85	1.80	AAA-AAA	0.00	999	1.000	1.000	0.4035
1554235_at	3.4	6.1	0.85	1.80	AAA-AAA	0.00	999	1.000	1.000	0.6249
1555322_at	67.9	37.7	-0.85	-1.80	AAA-AAA	0.00	999	1.000	1.000	0.3976
1562482_at	10.7	5.9	-0.85	-1.80	AAA-AAA	0.00	999	1.000	1.000	0.2159
216436_at	29.4	16.3	-0.85	-1.80	AAA-AAA	0.00	999	1.000	1.000	0.3020
216447_at	13.7	7.6	-0.85	-1.80	AAA-AAA	0.00	999	1.000	1.000	0.3842
213597_s_at	51.4	92.5	0.85	1.80	AAA-AAA	0.00	999	1.000	1.000	0.4645
219668_at	40.3	72.6	0.85	1.80	AAA-AAA	0.00	999	1.000	1.000	0.2838
207540_s_at	82.9	149.4	0.85	1.80	AAA-AAA	0.00	999	1.000	1.000	0.4272
209690_s_at	17.4	31.3	0.85	1.80	AAA-AAA	0.00	999	1.000	1.000	0.4038
208305_at	8.2	14.8	0.85	1.80	AAA-AAA	0.00	999	1.000	1.000	0.4296
234669_x_at	61.2	34.0	-0.85	-1.80	AAA-AAA	0.00	999	1.000	1.000	0.3004
234580_at	10.8	19.4	0.85	1.80	AAA-AAA	0.00	999	1.000	1.000	0.2140
238362_at	15.5	8.6	-0.85	-1.80	AAM-AAA	0.00	999	1.000	1.000	0.5530
227265_at	2.8	5.0	0.85	1.80	AAA-AAA	0.00	999	1.000	1.000	0.0684
222929_at	8.4	15.1	0.85	1.80	AAA-AAA	0.00	999	1.000	1.000	0.0420
230441_at	41.5	74.8	0.85	1.80	AAA-AAA	0.00	999	1.000	1.000	0.2452
214379_at	61.9	34.4	-0.85	-1.80	APA-AAA	0.00	999	1.000	1.000	0.1504
235893_at	90.7	50.4	-0.85	-1.80	PAM-AMP	0.00	999	1.000	1.000	0.5223

241185_at	24.1	43.3	0.85	1.80	AAA-AAA	0.00	999	1.000	1.000	0.4662
240052_at	36.0	20.0	-0.85	-1.80	AAA-AAA	0.00	999	1.000	1.000	0.3727
1568840_at	11.1	6.1	-0.85	-1.80	AAA-AAA	0.00	999	1.000	1.000	0.5931
1557395_at	10.2	5.7	-0.85	-1.80	AAA-AAA	0.00	999	1.000	1.000	0.6543
1558670_at	70.5	39.2	-0.85	-1.80	AAA-AAA	0.00	999	1.000	1.000	0.1859
213268_at	21.1	38.0	0.85	1.80	AAA-AAA	0.00	999	1.000	1.000	0.5083
220194_at	12.6	7.0	-0.85	-1.80	AAA-AAA	0.00	999	1.000	1.000	0.4409
207021_at	24.4	13.5	-0.85	-1.80	AAA-AAA	0.00	999	1.000	1.000	0.3942
210451_at	7.4	13.3	0.85	1.80	AAA-AAA	0.00	999	1.000	1.000	0.1968
208038_at	10.4	18.6	0.85	1.80	AAA-AAA	0.00	999	1.000	1.000	0.5710
235582_at	54.0	97.1	0.85	1.80	AAA-AAA	0.00	999	1.000	1.000	0.4036
232526_at	69.1	38.4	-0.85	-1.80	AAM-AAA	0.00	999	1.000	1.000	0.4481
236644_at	57.3	31.8	-0.85	-1.80	AAA-AAA	0.00	999	1.000	1.000	0.0688
228819_at	311.6	173.1	-0.85	-1.80	AAA-AAA	0.00	999	1.000	1.000	0.0889
228752_at	34.3	61.7	0.85	1.80	AAA-AAA	0.00	999	1.000	1.000	0.1744
230864_at	50.7	28.2	-0.85	-1.80	AAA-AAA	0.00	999	1.000	1.000	0.5458
232177_at	15.6	8.7	-0.85	-1.80	AAA-AAA	0.00	999	1.000	1.000	0.3133
230318_at	62.1	111.8	0.85	1.80	AAA-AAA	0.00	999	1.000	1.000	0.1663
202638_s_at	170.0	94.5	-0.85	-1.80	PAA-AAA	0.00	999	1.000	1.000	0.2279
226836_at	101.7	56.6	-0.85	-1.80	APP-AAA	0.00	999	1.000	1.000	0.3269
1556434_at	60.2	33.5	-0.85	-1.80	PPA-PAP	0.00	999	1.000	1.000	0.5400
241747_s_at	45.7	82.2	0.85	1.80	AAA-AAA	0.00	999	1.000	1.000	0.0617
239640_at	13.9	25.0	0.85	1.80	AAA-AAA	0.00	999	1.000	1.000	0.4078
240893_at	11.3	6.3	-0.85	-1.80	AAA-AAA	0.00	999	1.000	1.000	0.4542
1556954_at	12.1	6.7	-0.85	-1.80	AAA-AAA	0.00	999	1.000	1.000	0.5735
1558762_a_at	118.7	65.9	-0.85	-1.80	AAA-AAA	0.00	999	1.000	1.000	0.3209
244797_at	23.3	12.9	-0.85	-1.80	AAA-AAA	0.00	999	1.000	1.000	0.5398
1553842_at	36.4	20.2	-0.85	-1.80	AAA-MAA	0.00	999	1.000	1.000	0.5318
1561683_at	66.6	119.8	0.85	1.80	AAA-AMA	0.00	999	1.000	1.000	0.2575
1559901_s_at	14.1	25.4	0.85	1.80	AAA-AAA	0.00	999	1.000	1.000	0.4001
214644_at	6.8	12.2	0.85	1.80	AAA-AAA	0.00	999	1.000	1.000	0.4097
219772_s_at	4.3	7.7	0.85	1.80	AAA-AAA	0.00	999	1.000	1.000	0.3920
209823_x_at	10.2	18.4	0.85	1.80	AAA-AAA	0.00	999	1.000	1.000	0.0314
236400_at	40.9	22.8	-0.85	-1.80	AAA-AAA	0.00	999	1.000	1.000	0.0287
236266_at	13.1	7.3	-0.85	-1.80	AAA-AAA	0.00	999	1.000	1.000	0.0422
228136_s_at	48.6	87.5	0.85	1.80	AAA-AAA	0.00	999	1.000	1.000	0.2589
228995_at	9.4	16.9	0.85	1.80	AAA-AAA	0.00	999	1.000	1.000	0.6353
219199_at	138.7	77.2	-0.85	-1.80	PAP-MAA	0.00	999	1.000	1.000	0.1265
236105_at	113.6	63.2	-0.85	-1.80	PPP-PPA	0.00	999	1.000	1.000	0.1619
206731_at	49.0	27.3	-0.85	-1.80	PAP-MAA	0.00	999	1.000	1.000	0.3670
242252_at	40.5	22.5	-0.85	-1.80	APP-PAA	0.00	999	1.000	1.000	0.4630
242246_x_at	31.7	56.9	0.85	1.80	MAA-AAA	0.00	999	1.000	1.000	0.4747

240362_at	29.1	16.2	-0.85	-1.80	AAA-AAA	0.00	999	1.000	1.000	0.4945
241138_at	59.3	33.0	-0.85	-1.80	AAA-AAA	0.00	999	1.000	1.000	0.4395
239162_at	59.9	33.3	-0.85	-1.80	AAA-AAA	0.00	999	1.000	1.000	0.2029
1560895_at	3.6	6.4	0.85	1.80	AAA-AAA	0.00	999	1.000	1.000	0.1696
243478_at	10.8	6.0	-0.85	-1.80	AAA-AAA	0.00	999	1.000	1.000	0.7090
216939_s_at	15.8	8.8	-0.85	-1.80	AAA-AAA	0.00	999	1.000	1.000	0.2253
211011_at	15.2	27.4	0.85	1.80	AAA-AAA	0.00	999	1.000	1.000	0.6001
223768_at	14.6	8.1	-0.85	-1.80	AAA-AAA	0.00	999	1.000	1.000	0.0319
223634_at	44.6	24.8	-0.85	-1.80	AAA-AAA	0.00	999	1.000	1.000	0.5778
1564175_at	12.4	6.9	-0.85	-1.80	AAA-AAA	0.00	999	1.000	1.000	0.4672
242295_at	6.8	12.3	0.85	1.80	AAA-AAA	0.00	999	1.000	1.000	0.4020
1569402_at	5.8	10.5	0.85	1.80	AAA-AAA	0.00	999	1.000	1.000	0.1775
1566967_at	25.5	14.2	-0.85	-1.80	AAA-AAA	0.00	999	1.000	1.000	0.6629
1559194_a_at	46.7	83.9	0.85	1.80	AAA-AAA	0.00	999	1.000	1.000	0.3064
1560636_a_at	12.0	21.6	0.85	1.80	AAA-AAA	0.00	999	1.000	1.000	0.4836
233238_s_at	3.8	6.9	0.85	1.80	AAA-AAA	0.00	999	1.000	1.000	0.5841
231586_at	16.8	30.2	0.85	1.80	AAA-AAA	0.00	999	1.000	1.000	0.3539
229440_at	7.5	13.6	0.85	1.80	AAA-AAA	0.00	999	1.000	1.000	0.2251
233162_at	21.1	11.7	-0.84	-1.80	APA-AAA	0.00	999	1.000	1.000	0.5436
240294_at	83.9	150.7	0.84	1.80	AAA-AAA	0.00	999	1.000	1.000	0.3785
239764_at	30.2	16.8	-0.84	-1.80	AAA-AAA	0.00	999	1.000	1.000	0.4748
240822_at	21.6	38.8	0.84	1.80	AAA-AAA	0.00	999	1.000	1.000	0.4932
1552391_at	16.8	9.4	-0.84	-1.80	AAA-AAA	0.00	999	1.000	1.000	0.6816
1555435_at	15.4	27.6	0.84	1.80	AAA-AAA	0.00	999	1.000	1.000	0.5534
243097_x_at	28.2	50.6	0.84	1.80	AAA-AAA	0.00	999	1.000	1.000	0.5992
244069_at	11.8	21.2	0.84	1.80	AAA-AAA	0.00	999	1.000	1.000	0.5338
214330_at	59.2	106.3	0.84	1.80	AAA-AAA	0.00	999	1.000	1.000	0.2670
205848_at	15.3	27.5	0.84	1.80	AAA-AAA	0.00	999	1.000	1.000	0.3854
203561_at	31.8	17.7	-0.84	-1.80	AAA-AAA	0.00	999	1.000	1.000	0.0778
210280_at	33.6	18.7	-0.84	-1.80	AAA-AAA	0.00	999	1.000	1.000	0.5500
208366_at	15.4	8.6	-0.84	-1.80	AAA-AAA	0.00	999	1.000	1.000	0.5881
235690_at	18.6	33.4	0.84	1.79	AAA-AAA	0.00	999	1.000	1.000	0.4457
237886_at	19.7	35.3	0.84	1.80	AAA-AAA	0.00	999	1.000	1.000	0.4249
236438_at	27.5	15.3	-0.84	-1.80	AAA-AAA	0.00	999	1.000	1.000	0.4937
230833_at	9.9	5.5	-0.84	-1.80	AAA-AAA	0.00	999	1.000	1.000	0.1051
230255_at	6.6	11.9	0.84	1.80	AAA-AAA	0.00	999	1.000	1.000	0.3376
213141_at	261.4	145.7	-0.84	-1.79	MMP-AAP	0.00	999	1.000	1.000	0.0273
239083_at	253.8	141.6	-0.84	-1.79	PPP-PPP	0.00	999	1.000	1.000	0.3148
1564049_at	12.8	7.1	-0.84	-1.79	AAA-AAA	0.00	999	1.000	1.000	0.5020
241673_x_at	25.0	14.0	-0.84	-1.79	AAA-AAA	0.00	999	1.000	1.000	0.2607
1556679_at	30.8	17.2	-0.84	-1.79	AAA-AAA	0.00	999	1.000	1.000	0.2507
1558299_at	25.8	46.3	0.84	1.79	AAA-AAA	0.00	999	1.000	1.000	0.5398

1554683_a_at	27.3	15.2	-0.84	-1.79	AAA-AAA	0.00	999	1.000	1.000	0.4125
1558874_a_at	13.2	23.7	0.84	1.79	AAA-AAA	0.00	999	1.000	1.000	0.5660
216075_at	67.8	37.8	-0.84	-1.79	AAA-AAA	0.00	999	1.000	1.000	0.3562
213636_at	52.1	29.1	-0.84	-1.79	AAA-AAA	0.00	999	1.000	1.000	0.5007
214229_at	16.2	9.0	-0.84	-1.79	AAA-AAA	0.00	999	1.000	1.000	0.4065
206898_at	13.3	23.9	0.84	1.79	AAA-AAA	0.00	999	1.000	1.000	0.3418
1255_g_at	11.5	20.7	0.84	1.79	AAA-AMA	0.00	999	1.000	1.000	0.5808
210557_x_at	96.1	53.6	-0.84	-1.79	AAA-AAA	0.00	999	1.000	1.000	0.1744
237941_at	51.4	28.7	-0.84	-1.79	AAA-AMA	0.00	999	1.000	1.000	0.4237
237585_at	16.0	8.9	-0.84	-1.79	AAA-AAA	0.00	999	1.000	1.000	0.3808
222899_at	37.1	20.7	-0.84	-1.79	AAM-AAA	0.00	999	1.000	1.000	0.5106
231505_s_at	97.5	174.9	0.84	1.79	AAA-AAA	0.00	999	1.000	1.000	0.3404
206746_at	70.8	39.5	-0.84	-1.79	AAA-APP	0.00	999	1.000	1.000	0.2887
235317_at	259.1	144.6	-0.84	-1.79	AAP-AAA	0.00	999	1.000	1.000	0.3656
242308_at	33.2	59.6	0.84	1.79	AAA-AAA	0.00	999	1.000	1.000	0.4284
1569969_a_at	6.8	3.8	-0.84	-1.79	AAA-AAA	0.00	999	1.000	1.000	0.6415
1555463_a_at	8.5	15.2	0.84	1.79	AAA-AAA	0.00	999	1.000	1.000	0.5046
244570_at	6.8	3.8	-0.84	-1.79	AAA-AAA	0.00	999	1.000	1.000	0.0250
243714_at	16.5	29.5	0.84	1.79	AAA-AAA	0.00	999	1.000	1.000	0.5290
214947_at	23.6	42.2	0.84	1.79	AAA-AAA	0.00	999	1.000	1.000	0.5016
214311_at	45.7	81.9	0.84	1.79	AAA-AAA	0.00	999	1.000	1.000	0.2214
219524_s_at	29.7	53.2	0.84	1.79	AAA-AAA	0.00	999	1.000	1.000	0.2311
206060_s_at	7.3	13.1	0.84	1.79	AAA-AAA	0.00	999	1.000	1.000	0.4592
207329_at	8.6	15.4	0.84	1.79	AAA-AAA	0.00	999	1.000	1.000	0.6266
238777_x_at	62.4	111.8	0.84	1.79	AAA-AAA	0.00	999	1.000	1.000	0.0292
238132_at	10.2	5.7	-0.84	-1.79	AAA-AAA	0.00	999	1.000	1.000	0.3068
235922_at	18.7	10.5	-0.84	-1.79	AAA-AAA	0.00	999	1.000	1.000	0.4390
230405_at	57.6	32.1	-0.84	-1.79	AAA-AAA	0.00	999	1.000	1.000	0.3655
1552701_a_at	321.2	179.3	-0.84	-1.79	PPP-PPP	0.00	999	1.000	1.000	0.0468
236304_at	33.5	18.7	-0.84	-1.79	PAA-PAA	0.00	999	1.000	1.000	0.4199
242468_at	42.5	23.7	-0.84	-1.79	MAA-APA	0.00	999	1.000	1.000	0.4382
1565621_at	36.2	20.2	-0.84	-1.79	AAA-AAA	0.00	999	1.000	1.000	0.4999
241244_at	55.4	30.9	-0.84	-1.79	AAA-MAA	0.00	999	1.000	1.000	0.2017
1563659_at	7.8	14.0	0.84	1.79	AAA-AAA	0.00	999	1.000	1.000	0.1156
1566217_at	14.1	7.9	-0.84	-1.79	AAA-AAA	0.00	999	1.000	1.000	0.5290
1559313_at	16.1	9.0	-0.84	-1.79	AAA-AAA	0.00	999	1.000	1.000	0.2252
215752_at	6.2	11.2	0.84	1.79	AAA-AAA	0.00	999	1.000	1.000	0.5084
215887_at	68.1	121.9	0.84	1.79	AAA-AAA	0.00	999	1.000	1.000	0.1638
215290_at	21.9	39.3	0.84	1.79	AAA-AAA	0.00	999	1.000	1.000	0.5166
206421_s_at	85.1	47.5	-0.84	-1.79	AAA-AAA	0.00	999	1.000	1.000	0.3162
209708_at	29.1	52.1	0.84	1.79	AAA-AAA	0.00	999	1.000	1.000	0.5685
234681_s_at	62.6	35.0	-0.84	-1.79	AAA-AAA	0.00	999	1.000	1.000	0.4802

237560_at	27.1	15.1	-0.84	-1.79	AAA-AAA	0.00	999	1.000	1.000	0.4951
238167_at	27.8	49.8	0.84	1.79	AAA-AAA	0.00	999	1.000	1.000	0.2132
238003_at	19.9	35.7	0.84	1.79	AAA-AAA	0.00	999	1.000	1.000	0.5674
228294_s_at	16.2	9.0	-0.84	-1.79	AAA-AAA	0.00	999	1.000	1.000	0.3226
203172_at	292.0	163.1	-0.84	-1.79	APA-AAP	0.00	999	1.000	1.000	0.2776
244506_at	119.1	66.6	-0.84	-1.79	AAA-AAP	0.00	999	1.000	1.000	0.3745
206378_at	15.0	8.4	-0.84	-1.79	AAP-AAA	0.00	999	1.000	1.000	0.6838
1569532_a_at	69.0	123.5	0.84	1.79	AAA-AAA	0.00	999	1.000	1.000	0.5727
1556901_s_at	5.0	9.0	0.84	1.79	AAA-AAA	0.00	999	1.000	1.000	0.0582
1553566_at	14.8	26.6	0.84	1.79	AAA-AAA	0.00	999	1.000	1.000	0.1710
1553888_at	8.2	4.6	-0.84	-1.79	AAA-AAA	0.00	999	1.000	1.000	0.2292
243698_at	16.0	8.9	-0.84	-1.79	AAA-AAA	0.00	999	1.000	1.000	0.0078
236102_at	3.8	6.8	0.84	1.79	AAA-AAA	0.00	999	1.000	1.000	0.2634
222864_s_at	31.3	17.5	-0.84	-1.79	AAA-AAA	0.00	999	1.000	1.000	0.4349
47550_at	11.3	20.2	0.84	1.79	AAA-AAA	0.00	999	1.000	1.000	0.5033
231626_at	15.9	8.9	-0.84	-1.79	AAA-AAA	0.00	999	1.000	1.000	0.0577
229205_at	18.8	10.5	-0.84	-1.79	AAA-AAA	0.00	999	1.000	1.000	0.5129
204288_s_at	102.8	57.5	-0.84	-1.79	AAP-AAA	0.00	999	1.000	1.000	0.4813
241660_at	12.1	6.8	-0.84	-1.79	AAA-AAA	0.00	999	1.000	1.000	0.6310
1553527_at	42.2	23.6	-0.84	-1.79	AAA-AAA	0.00	999	1.000	1.000	0.6332
242595_at	19.3	34.4	0.84	1.79	AAA-AAA	0.00	999	1.000	1.000	0.4648
1559144_x_at	14.4	25.7	0.84	1.79	AAA-AAA	0.00	999	1.000	1.000	0.6947
204562_at	47.4	84.8	0.84	1.79	AAA-AAA	0.00	999	1.000	1.000	0.4971
210677_at	9.7	17.4	0.84	1.79	AAA-AAA	0.00	999	1.000	1.000	0.0704
233128_at	18.9	33.8	0.84	1.79	AAA-AAA	0.00	999	1.000	1.000	0.1325
237969_at	22.9	12.8	-0.84	-1.79	AAA-AAA	0.00	999	1.000	1.000	0.6255
236666_s_at	65.7	36.7	-0.84	-1.79	AAA-AAA	0.00	999	1.000	1.000	0.4484
228677_s_at	89.4	50.0	-0.84	-1.79	AAA-AAA	0.00	999	1.000	1.000	0.5033
231623_at	14.1	25.2	0.84	1.79	AAA-AAA	0.00	999	1.000	1.000	0.4604
1570117_at	8.0	4.5	-0.84	-1.79	AAA-AAA	0.00	999	1.000	1.000	0.5930
1568673_s_at	5.5	3.1	-0.84	-1.79	AAA-AAA	0.00	999	1.000	1.000	0.6155
1567022_at	37.4	20.9	-0.84	-1.79	AAA-AAA	0.00	999	1.000	1.000	0.4377
1555088_x_at	72.9	130.3	0.84	1.79	AAA-AAA	0.00	999	1.000	1.000	0.0531
244423_at	15.5	8.7	-0.84	-1.79	AAA-AAA	0.00	999	1.000	1.000	0.5253
1559296_at	4.3	7.6	0.84	1.79	AAA-AAA	0.00	999	1.000	1.000	0.6685
215372_x_at	73.9	132.1	0.84	1.79	AAA-AAA	0.00	999	1.000	1.000	0.5328
218599_at	90.0	160.9	0.84	1.79	AAA-AAA	0.00	999	1.000	1.000	0.4352
205719_s_at	28.7	16.0	-0.84	-1.79	AAA-AAA	0.00	999	1.000	1.000	0.4135
206926_s_at	58.4	104.3	0.84	1.79	AAA-AAA	0.00	999	1.000	1.000	0.1861
206650_at	56.4	31.5	-0.84	-1.79	AAA-AAA	0.00	999	1.000	1.000	0.1939
206822_s_at	22.1	12.4	-0.84	-1.79	AAA-AAA	0.00	999	1.000	1.000	0.4809
235118_at	1.9	3.4	0.84	1.79	AAA-AAA	0.00	999	1.000	1.000	0.4888

237345_at	41.2	23.1	-0.84	-1.79	AAA-AAA	0.00	999	1.000	1.000	0.3507
222219_s_at	38.5	21.5	-0.84	-1.79	AAA-AAA	0.00	999	1.000	1.000	0.4944
224153_s_at	8.6	15.5	0.84	1.79	AAA-AAA	0.00	999	1.000	1.000	0.2849
231384_at	6.9	3.8	-0.84	-1.79	AAA-AAA	0.00	999	1.000	1.000	0.6409
243688_at	95.8	53.6	-0.84	-1.79	PAP-AAA	0.00	999	1.000	1.000	0.2908
220027_s_at	97.4	54.5	-0.84	-1.79	AAP-AAA	0.00	999	1.000	1.000	0.3909
240043_at	8.1	14.4	0.84	1.79	AAA-AAA	0.00	999	1.000	1.000	0.4528
1558301_a_at	9.8	5.5	-0.84	-1.79	AAA-AAA	0.00	999	1.000	1.000	0.6277
244731_at	3.4	1.9	-0.84	-1.79	AAA-AAA	0.00	999	1.000	1.000	0.3986
243824_at	15.2	27.2	0.84	1.79	AAA-AAA	0.00	999	1.000	1.000	0.4599
243987_at	21.3	11.9	-0.84	-1.79	AAA-AAA	0.00	999	1.000	1.000	0.6101
213565_s_at	23.5	13.1	-0.84	-1.79	AAA-AAA	0.00	999	1.000	1.000	0.4911
221366_at	6.7	11.9	0.84	1.79	AAA-AAA	0.00	999	1.000	1.000	0.2563
217284_x_at	36.3	64.9	0.84	1.79	AAA-AAA	0.00	999	1.000	1.000	0.4762
205666_at	13.5	24.2	0.84	1.79	AAA-AAA	0.00	999	1.000	1.000	0.5269
209948_at	111.9	62.7	-0.84	-1.79	AAA-AAA	0.00	999	1.000	1.000	0.1351
209474_s_at	7.3	13.1	0.84	1.79	AAA-AAA	0.00	999	1.000	1.000	0.4850
237053_at	16.8	30.0	0.84	1.79	AAA-AAA	0.00	999	1.000	1.000	0.4669
1561763_at	33.4	18.7	-0.84	-1.79	AAA-PAA	0.00	999	1.000	1.000	0.5074
1562926_at	57.6	32.3	-0.84	-1.79	AAA-AAA	0.00	999	1.000	1.000	0.4486
243106_at	4.7	8.4	0.84	1.79	AAA-AAA	0.00	999	1.000	1.000	0.4482
1559147_at	3.2	1.8	-0.84	-1.79	AAA-AAA	0.00	999	1.000	1.000	0.0869
1560633_a_at	34.9	19.6	-0.84	-1.79	AAA-AAA	0.00	999	1.000	1.000	0.5374
221393_at	9.1	16.2	0.84	1.79	AAA-AAA	0.00	999	1.000	1.000	0.4521
207677_s_at	8.8	4.9	-0.84	-1.79	AAA-AAA	0.00	999	1.000	1.000	0.5634
225491_at	4.5	8.0	0.84	1.79	AAA-AAA	0.00	999	1.000	1.000	0.4989
222053_at	9.0	16.1	0.84	1.79	AAA-AAA	0.00	999	1.000	1.000	0.5272
221914_at	12.6	7.1	-0.84	-1.79	AAA-AAA	0.00	999	1.000	1.000	0.6285
229925_at	50.5	28.3	-0.84	-1.79	AAA-AAA	0.00	999	1.000	1.000	0.5141
229820_at	39.7	22.2	-0.84	-1.79	AAA-AAA	0.00	999	1.000	1.000	0.4900
230307_at	36.5	20.4	-0.84	-1.79	AAA-AAA	0.00	999	1.000	1.000	0.4461
242205_at	105.5	59.2	-0.84	-1.78	PAA-AAA	0.00	999	1.000	1.000	0.0936
207464_at	58.9	33.0	-0.84	-1.78	PAP-AAP	0.00	999	1.000	1.000	0.1610
205100_at	193.3	108.3	-0.84	-1.78	PAP-AAP	0.00	999	1.000	1.000	0.2400
215445_x_at	67.6	37.9	-0.84	-1.78	PAP-MAA	0.00	999	1.000	1.000	0.2704
241438_at	12.4	7.0	-0.84	-1.78	AAA-AAA	0.00	999	1.000	1.000	0.4097
239968_at	33.8	19.0	-0.84	-1.78	MAA-AAA	0.00	999	1.000	1.000	0.6617
1553906_s_at	301.2	168.9	-0.84	-1.78	AAA-AAA	0.00	999	1.000	1.000	0.4495
220441_at	97.5	54.7	-0.84	-1.78	AAA-AAA	0.00	999	1.000	1.000	0.0353
217003_s_at	40.6	22.8	-0.84	-1.78	AAA-AAA	0.00	999	1.000	1.000	0.5912
206329_at	16.8	9.4	-0.84	-1.78	AAA-AAA	0.00	999	1.000	1.000	0.4069
211492_s_at	22.1	12.4	-0.84	-1.78	AAA-AAA	0.00	999	1.000	1.000	0.5580

234301_s_at	23.0	12.9	-0.84	-1.78	AAA-AAA	0.00	999	1.000	1.000	0.5364
224380_s_at	18.5	10.4	-0.84	-1.78	AAA-AAA	0.00	999	1.000	1.000	0.4976
231313_at	3.6	6.4	0.84	1.78	AAA-AAA	0.00	999	1.000	1.000	0.0600
238767_at	98.1	55.1	-0.83	-1.78	AAP-PAA	0.00	999	1.000	1.000	0.4644
241225_at	35.0	19.6	-0.83	-1.78	AAP-AAA	0.00	999	1.000	1.000	0.5970
1564056_at	6.3	11.3	0.83	1.78	AAA-AAA	0.00	999	1.000	1.000	0.5056
1570190_at	5.8	3.2	-0.83	-1.78	AAA-AAA	0.00	999	1.000	1.000	0.2745
1554032_at	6.7	11.9	0.83	1.78	AAA-AAA	0.00	999	1.000	1.000	0.6153
1555645_at	15.1	8.4	-0.83	-1.78	AAA-AAA	0.00	999	1.000	1.000	0.2222
1561978_at	24.4	13.7	-0.83	-1.78	AAA-AAA	0.00	999	1.000	1.000	0.4289
244363_at	49.6	88.5	0.83	1.78	AAA-AAA	0.00	999	1.000	1.000	0.2377
217110_s_at	19.5	10.9	-0.83	-1.78	AAA-AAA	0.00	999	1.000	1.000	0.6503
206282_at	12.5	7.0	-0.83	-1.78	AAA-AAA	0.00	999	1.000	1.000	0.5910
202986_at	20.1	35.9	0.83	1.78	AAA-AAA	0.00	999	1.000	1.000	0.4925
211562_s_at	32.9	18.4	-0.83	-1.78	AAA-AAA	0.00	999	1.000	1.000	0.4619
209437_s_at	18.3	10.2	-0.83	-1.78	AAA-AAA	0.00	999	1.000	1.000	0.5237
236572_at	17.1	9.6	-0.83	-1.78	AAA-AAA	0.00	999	1.000	1.000	0.0943
236567_at	27.9	15.6	-0.83	-1.78	AAA-AAA	0.00	999	1.000	1.000	0.3257
236810_at	16.2	28.8	0.83	1.78	AAA-AAA	0.00	999	1.000	1.000	0.4560
221901_at	14.8	26.4	0.83	1.78	AAA-AAA	0.00	999	1.000	1.000	0.0154
223856_at	39.9	22.4	-0.83	-1.78	AAA-AAA	0.00	999	1.000	1.000	0.2398
1570347_at	122.5	68.8	-0.83	-1.78	AAA-AAP	0.00	999	1.000	1.000	0.3867
241581_at	46.5	26.1	-0.83	-1.78	PAA-AAA	0.00	999	1.000	1.000	0.3993
232483_at	95.0	53.3	-0.83	-1.78	PPA-APP	0.00	999	1.000	1.000	0.4329
231584_s_at	91.7	51.5	-0.83	-1.78	AAA-PAA	0.00	999	1.000	1.000	0.5234
1566518_at	57.5	32.3	-0.83	-1.78	AAA-AAA	0.00	999	1.000	1.000	0.5351
1560241_at	32.3	18.1	-0.83	-1.78	AAA-AAA	0.00	999	1.000	1.000	0.4881
216726_at	10.6	6.0	-0.83	-1.78	AAA-AAA	0.00	999	1.000	1.000	0.0895
221409_at	70.9	39.8	-0.83	-1.78	AAA-AAA	0.00	999	1.000	1.000	0.4764
207060_at	22.6	40.3	0.83	1.78	AAA-AAA	0.00	999	1.000	1.000	0.3111
206881_s_at	6.1	10.8	0.83	1.78	AAA-AAA	0.00	999	1.000	1.000	0.1370
205557_at	35.9	64.0	0.83	1.78	AAA-AAA	0.00	999	1.000	1.000	0.3165
208268_at	26.9	15.1	-0.83	-1.78	AAA-AAA	0.00	999	1.000	1.000	0.5938
242752_at	114.7	64.4	-0.83	-1.78	PPP-PPP	0.00	999	1.000	1.000	0.0586
1556639_at	10.3	5.8	-0.83	-1.78	AAA-AAA	0.00	999	1.000	1.000	0.4314
1559109_a_at	45.1	25.3	-0.83	-1.78	AAA-AAA	0.00	999	1.000	1.000	0.3789
244207_at	43.6	77.7	0.83	1.78	AAA-AAA	0.00	999	1.000	1.000	0.3695
244001_at	9.6	5.4	-0.83	-1.78	AAA-AAA	0.00	999	1.000	1.000	0.5226
216724_at	20.8	37.0	0.83	1.78	AAA-AAA	0.00	999	1.000	1.000	0.6356
211468_s_at	42.2	75.1	0.83	1.78	AAA-AAA	0.00	999	1.000	1.000	0.3561
235591_at	7.3	13.0	0.83	1.78	AAA-AAA	0.00	999	1.000	1.000	0.6620
233581_at	20.0	11.2	-0.83	-1.78	AAA-AAA	0.00	999	1.000	1.000	0.5390

232449_at	14.8	26.3	0.83	1.78	AAA-AAA	0.00	999	1.000	1.000	0.4045
236719_at	113.6	63.9	-0.83	-1.78	MAP-AAA	0.00	999	1.000	1.000	0.2630
216372_at	79.9	44.9	-0.83	-1.78	AAP-PAA	0.00	999	1.000	1.000	0.3966
1564699_at	4.4	2.5	-0.83	-1.78	AAA-AAA	0.00	999	1.000	1.000	0.5523
241259_at	40.0	22.5	-0.83	-1.78	AAA-AAA	0.00	999	1.000	1.000	0.3467
1570208_at	6.4	11.4	0.83	1.78	AAA-AAA	0.00	999	1.000	1.000	0.5349
240820_at	19.0	10.7	-0.83	-1.78	AAA-AAA	0.00	999	1.000	1.000	0.5577
1553696_s_at	52.1	29.3	-0.83	-1.78	AAA-AAA	0.00	999	1.000	1.000	0.3505
1556159_at	3.8	6.7	0.83	1.78	AAA-AAA	0.00	999	1.000	1.000	0.2147
243210_at	59.8	106.4	0.83	1.78	AAA-AAA	0.00	999	1.000	1.000	0.0673
1559057_at	37.2	20.9	-0.83	-1.78	AAA-AAA	0.00	999	1.000	1.000	0.4235
212592_at	2.0	3.6	0.83	1.78	AAA-AAA	0.00	999	1.000	1.000	0.5135
214847_s_at	35.2	19.8	-0.83	-1.78	AAA-AAA	0.00	999	1.000	1.000	0.5067
220431_at	7.7	13.6	0.83	1.78	AAA-AAA	0.00	999	1.000	1.000	0.3045
207289_at	88.2	49.6	-0.83	-1.78	AAA-AAA	0.00	999	1.000	1.000	0.4322
208157_at	7.4	13.2	0.83	1.78	AAA-AAA	0.00	999	1.000	1.000	0.3915
234494_x_at	54.1	30.4	-0.83	-1.78	AAA-AAA	0.00	999	1.000	1.000	0.4503
222372_at	35.9	20.2	-0.83	-1.78	AAA-AAA	0.00	999	1.000	1.000	0.2977
231316_at	26.8	47.7	0.83	1.78	AAA-AAA	0.00	999	1.000	1.000	0.3676
229626_at	14.1	25.1	0.83	1.78	AAA-AAA	0.00	999	1.000	1.000	0.5422
229227_at	30.0	53.3	0.83	1.78	AAA-AAA	0.00	999	1.000	1.000	0.1017
230041_at	11.0	19.6	0.83	1.78	AAA-AAA	0.00	999	1.000	1.000	0.5477
1564262_at	54.6	30.7	-0.83	-1.78	AAA-APA	0.00	999	1.000	1.000	0.2670
229335_at	65.5	36.8	-0.83	-1.78	MPA-AAP	0.00	999	1.000	1.000	0.4087
1563801_at	18.0	32.0	0.83	1.78	AAA-AAA	0.00	999	1.000	1.000	0.5202
1568866_at	21.3	12.0	-0.83	-1.78	AAA-AAA	0.00	999	1.000	1.000	0.4218
1557682_a_at	28.4	50.5	0.83	1.78	AAA-AAA	0.00	999	1.000	1.000	0.2769
1555568_at	20.7	36.9	0.83	1.78	AAA-AAA	0.00	999	1.000	1.000	0.4111
242825_at	15.3	8.6	-0.83	-1.78	AAA-AAA	0.00	999	1.000	1.000	0.5196
214353_at	86.6	48.7	-0.83	-1.78	AAA-AAA	0.00	999	1.000	1.000	0.2586
217472_at	88.0	49.5	-0.83	-1.78	AAA-AAA	0.00	999	1.000	1.000	0.2836
206252_s_at	13.3	23.7	0.83	1.78	AAA-AAA	0.00	999	1.000	1.000	0.5171
205627_at	49.9	28.1	-0.83	-1.78	AAA-AAA	0.00	999	1.000	1.000	0.5754
234173_s_at	42.6	23.9	-0.83	-1.78	AAA-AAA	0.00	999	1.000	1.000	0.3576
235664_at	14.2	25.3	0.83	1.78	AAA-AAA	0.00	999	1.000	1.000	0.2157
232804_at	10.9	19.5	0.83	1.78	AAA-AAA	0.00	999	1.000	1.000	0.5604
227677_at	44.7	25.1	-0.83	-1.78	AAA-AAA	0.00	999	1.000	1.000	0.5363
89977_at	21.9	39.0	0.83	1.78	AAA-AAA	0.00	999	1.000	1.000	0.4361
221141_x_at	107.0	60.2	-0.83	-1.78	PAA-AAA	0.00	999	1.000	1.000	0.3577
213667_at	189.6	106.8	-0.83	-1.78	PAA-AAA	0.00	999	1.000	1.000	0.4683
220278_at	30.5	17.2	-0.83	-1.78	PAA-AAA	0.00	999	1.000	1.000	0.5119
1561291_at	5.8	3.3	-0.83	-1.78	AAA-AAA	0.00	999	1.000	1.000	0.1372

1561126_at	23.3	41.4	0.83	1.78	AAA-AAA	0.00	999	1.000	1.000	0.4810
244083_at	5.9	3.3	-0.83	-1.78	AAA-AAA	0.00	999	1.000	1.000	0.6670
213488_at	49.3	27.7	-0.83	-1.78	AAA-AAA	0.00	999	1.000	1.000	0.2169
221177_at	25.2	44.7	0.83	1.78	AAA-AAA	0.00	999	1.000	1.000	0.0375
217280_x_at	26.3	14.8	-0.83	-1.78	AAA-AAA	0.00	999	1.000	1.000	0.5870
216866_s_at	14.9	8.4	-0.83	-1.78	AAA-AAA	0.00	999	1.000	1.000	0.4272
218792_s_at	34.0	60.4	0.83	1.78	AAA-AAA	0.00	999	1.000	1.000	0.3048
206348_s_at	78.0	138.5	0.83	1.78	AAA-AAA	0.00	999	1.000	1.000	0.1061
204124_at	22.7	40.4	0.83	1.78	AAA-AAA	0.00	999	1.000	1.000	0.2991
209237_s_at	69.2	123.0	0.83	1.78	AAA-AAA	0.00	999	1.000	1.000	0.0122
208333_at	7.0	12.4	0.83	1.78	AAA-AAA	0.00	999	1.000	1.000	0.0329
227838_at	25.2	14.2	-0.83	-1.78	AAA-AAA	0.00	999	1.000	1.000	0.5892
224094_at	15.0	26.6	0.83	1.78	AAA-AAA	0.00	999	1.000	1.000	0.3846
231993_at	18.8	33.5	0.83	1.78	AAA-AAA	0.00	999	1.000	1.000	0.6962
1553772_at	48.1	27.1	-0.83	-1.78	APA-MAP	0.00	999	1.000	1.000	0.4020
242543_at	29.9	53.0	0.83	1.78	AAA-AAA	0.00	999	1.000	1.000	0.3199
1563867_at	29.6	52.6	0.83	1.78	AAA-AAA	0.00	999	1.000	1.000	0.4183
239984_at	10.6	18.8	0.83	1.78	AAA-AAA	0.00	999	1.000	1.000	0.0350
1556608_a_at	10.0	5.6	-0.83	-1.78	AAA-AAA	0.00	999	1.000	1.000	0.1338
1555227_a_at	75.2	42.4	-0.83	-1.78	AAA-AAA	0.00	999	1.000	1.000	0.2654
244555_at	35.0	19.7	-0.83	-1.78	AAA-AAA	0.00	999	1.000	1.000	0.4432
213712_at	4.4	7.8	0.83	1.78	AAA-AAA	0.00	999	1.000	1.000	0.3279
220462_at	25.2	44.7	0.83	1.78	AAA-AAA	0.00	999	1.000	1.000	0.3359
220786_s_at	19.3	10.9	-0.83	-1.78	AAA-AAA	0.00	999	1.000	1.000	0.5684
217669_s_at	9.8	5.5	-0.83	-1.78	AAA-AAA	0.00	999	1.000	1.000	0.0788
206642_at	23.6	13.3	-0.83	-1.78	AAA-AAA	0.00	999	1.000	1.000	0.4554
207729_at	5.6	10.0	0.83	1.78	AAA-AAA	0.00	999	1.000	1.000	0.4549
232571_at	68.6	38.6	-0.83	-1.78	AAA-AAA	0.00	999	1.000	1.000	0.3180
233883_at	10.2	18.1	0.83	1.78	AAA-AAA	0.00	999	1.000	1.000	0.4601
237799_at	32.7	58.1	0.83	1.78	AAA-AAA	0.00	999	1.000	1.000	0.3627
238423_at	79.6	44.9	-0.83	-1.78	AAA-AAA	0.00	999	1.000	1.000	0.3850
227427_at	65.7	116.7	0.83	1.78	AAA-AAA	0.00	999	1.000	1.000	0.2898
32128_at	15.4	27.4	0.83	1.78	AAA-AAA	0.00	999	1.000	1.000	0.4695
232042_at	6.4	11.4	0.83	1.78	AAA-AAA	0.00	999	1.000	1.000	0.1096
229177_at	18.3	10.3	-0.83	-1.78	AAA-AAA	0.00	999	1.000	1.000	0.2619
1558604_a_at	97.2	54.8	-0.83	-1.77	AAP-MAP	0.00	999	1.000	1.000	0.4499
1553561_at	40.0	22.5	-0.83	-1.77	PAA-AAP	0.00	999	1.000	1.000	0.5260
1570328_s_at	46.7	26.3	-0.83	-1.77	AAA-AAA	0.00	999	1.000	1.000	0.3810
239531_at	94.4	167.6	0.83	1.77	AAA-MAA	0.00	999	1.000	1.000	0.0522
1555904_at	30.0	16.9	-0.83	-1.77	AAA-AAA	0.00	999	1.000	1.000	0.3585
243180_at	12.4	7.0	-0.83	-1.77	AAA-AAA	0.00	999	1.000	1.000	0.3163
242729_at	35.6	20.0	-0.83	-1.77	AAA-AAA	0.00	999	1.000	1.000	0.4779

1561714_a_at	31.0	17.5	-0.83	-1.77	AAM-AAA	0.00	999	1.000	1.000	0.4615
1559633_a_at	24.6	13.9	-0.83	-1.77	AAA-AAA	0.00	999	1.000	1.000	0.4766
1559862_at	6.3	11.2	0.83	1.77	AAA-AAA	0.00	999	1.000	1.000	0.4793
244148_at	20.5	11.6	-0.83	-1.77	AAA-AAA	0.00	999	1.000	1.000	0.3098
217345_at	7.2	4.1	-0.83	-1.77	AAA-AAA	0.00	999	1.000	1.000	0.4014
218638_s_at	64.6	36.4	-0.83	-1.77	AAA-AAA	0.00	999	1.000	1.000	0.5354
207272_at	16.1	9.1	-0.83	-1.77	AAA-AAA	0.00	999	1.000	1.000	0.4040
211577_s_at	84.1	47.4	-0.83	-1.77	AAA-AAA	0.00	999	1.000	1.000	0.4347
207356_at	15.7	8.9	-0.83	-1.77	AAA-AAA	0.00	999	1.000	1.000	0.2737
207494_s_at	22.1	39.2	0.83	1.78	AAA-AAA	0.00	999	1.000	1.000	0.0891
208251_at	49.8	88.4	0.83	1.77	AAA-AAA	0.00	999	1.000	1.000	0.1935
233514_x_at	20.8	37.0	0.83	1.77	AAA-AAA	0.00	999	1.000	1.000	0.5472
236189_at	9.8	5.5	-0.83	-1.77	AAA-AAA	0.00	999	1.000	1.000	0.2542
231219_at	27.6	15.5	-0.83	-1.77	AAA-AAA	0.00	999	1.000	1.000	0.1814
229867_at	69.2	122.7	0.83	1.77	AAA-AAA	0.00	999	1.000	1.000	0.1432
230764_at	57.6	102.1	0.83	1.77	AAA-AAA	0.00	999	1.000	1.000	0.1408
1569138_a_at	134.1	75.7	-0.83	-1.77	PAA-AAA	0.00	999	1.000	1.000	0.1032
1564208_x_at	32.3	57.2	0.83	1.77	AAA-AAA	0.00	999	1.000	1.000	0.3212
1565637_at	8.4	14.8	0.83	1.77	AAA-AAA	0.00	999	1.000	1.000	0.6063
1570480_s_at	23.8	42.2	0.83	1.77	AAA-AAA	0.00	999	1.000	1.000	0.4629
239337_at	7.1	4.0	-0.83	-1.77	AAA-AAA	0.00	999	1.000	1.000	0.5086
1569387_at	30.6	54.2	0.83	1.77	AAA-AAA	0.00	999	1.000	1.000	0.5452
1569383_s_at	28.2	49.9	0.83	1.77	AAA-AAA	0.00	999	1.000	1.000	0.2464
240962_at	96.8	54.6	-0.83	-1.77	AAA-AAA	0.00	999	1.000	1.000	0.4405
241134_at	7.3	12.9	0.83	1.77	AAA-AAA	0.00	999	1.000	1.000	0.0207
1556601_a_at	5.5	9.7	0.83	1.77	AAA-AAA	0.00	999	1.000	1.000	0.4089
1553573_s_at	23.7	42.0	0.83	1.77	AAA-AAA	0.00	999	1.000	1.000	0.3583
1553675_at	14.5	25.7	0.83	1.77	AAA-AAA	0.00	999	1.000	1.000	0.5266
1553046_s_at	10.3	18.2	0.83	1.77	AAA-AAA	0.00	999	1.000	1.000	0.4365
1561961_at	38.6	21.8	-0.83	-1.77	AAA-AAA	0.00	999	1.000	1.000	0.3876
1559766_at	33.6	59.6	0.83	1.77	AAA-AAA	0.00	999	1.000	1.000	0.2811
1560744_at	11.1	19.6	0.83	1.77	AAA-AAA	0.00	999	1.000	1.000	0.5097
206313_at	15.5	27.5	0.83	1.77	AAA-AAA	0.00	999	1.000	1.000	0.5724
205860_x_at	6.6	3.7	-0.83	-1.77	AAA-AAA	0.00	999	1.000	1.000	0.3844
203993_x_at	8.3	14.8	0.83	1.77	AAA-AAA	0.00	999	1.000	1.000	0.1909
205544_s_at	15.3	8.7	-0.83	-1.77	AAA-AAA	0.00	999	1.000	1.000	0.5633
210783_x_at	60.6	107.4	0.83	1.77	AAA-AAA	0.00	999	1.000	1.000	0.3532
233601_at	56.7	32.0	-0.83	-1.77	AAA-AAA	0.00	999	1.000	1.000	0.4055
238175_at	4.6	8.2	0.83	1.77	AAA-AAA	0.00	999	1.000	1.000	0.6410
237270_at	113.1	63.8	-0.83	-1.77	AAA-AAA	0.00	999	1.000	1.000	0.2847
231288_at	30.7	54.4	0.83	1.77	AAA-AAA	0.00	999	1.000	1.000	0.3414
232017_at	40.5	71.8	0.83	1.77	AAA-AAA	0.00	999	1.000	1.000	0.6001

238646_at	60.8	34.3	-0.83	-1.77	PAA-AAA	0.00	999	1.000	1.000	0.0822
1565563_at	3.9	2.2	-0.83	-1.77	MAA-AAA	0.00	999	1.000	1.000	0.6507
1564402_at	13.6	7.7	-0.83	-1.77	AAA-AAA	0.00	999	1.000	1.000	0.5483
1569580_a_at	8.3	14.6	0.83	1.77	AAA-AAA	0.00	999	1.000	1.000	0.5406
240958_at	63.3	35.7	-0.83	-1.77	AAA-AAA	0.00	999	1.000	1.000	0.3076
1565843_s_at	124.3	70.2	-0.83	-1.77	AAA-AAA	0.00	999	1.000	1.000	0.2841
1567030_at	7.6	13.4	0.83	1.77	AAA-AAA	0.00	999	1.000	1.000	0.4124
1556770_a_at	13.0	23.0	0.83	1.77	AAA-AAA	0.00	999	1.000	1.000	0.5426
1554692_at	46.5	26.2	-0.83	-1.77	AAA-AAM	0.00	999	1.000	1.000	0.3892
1556244_s_at	5.6	9.9	0.83	1.77	AAA-AAA	0.00	999	1.000	1.000	0.6078
216018_at	38.7	68.5	0.83	1.77	AAA-AAA	0.00	999	1.000	1.000	0.4272
221369_at	26.4	14.9	-0.83	-1.77	AAA-AAA	0.00	999	1.000	1.000	0.5865
206701_x_at	7.3	4.1	-0.83	-1.77	AAA-AAA	0.00	999	1.000	1.000	0.3746
207676_at	55.7	31.5	-0.83	-1.77	AAA-AAA	0.00	999	1.000	1.000	0.5643
233240_at	15.9	28.1	0.83	1.77	AAA-AAA	0.00	999	1.000	1.000	0.5084
237532_at	31.4	55.5	0.83	1.77	AAA-AAA	0.00	999	1.000	1.000	0.5505
238779_at	5.8	10.3	0.83	1.77	AAA-AAA	0.00	999	1.000	1.000	0.6920
235883_at	109.9	62.0	-0.83	-1.77	AAA-AAA	0.00	999	1.000	1.000	0.1704
222908_at	68.5	38.7	-0.83	-1.77	AAA-AAA	0.00	999	1.000	1.000	0.1268
232315_at	30.7	17.3	-0.83	-1.77	AAA-AAA	0.00	999	1.000	1.000	0.2245
212552_at	5327.6	3008.6	-0.82	-1.77	PPP-PPP	0.00	999	1.000	1.000	0.0959
1562682_at	12.9	7.3	-0.82	-1.77	AAA-AAA	0.00	999	1.000	1.000	0.5309
1569074_at	9.0	16.0	0.82	1.77	AAA-AAA	0.00	999	1.000	1.000	0.4128
240438_at	58.0	32.8	-0.82	-1.77	AAA-AAA	0.00	999	1.000	1.000	0.2896
1557674_s_at	12.7	22.4	0.82	1.77	AAA-AAA	0.00	999	1.000	1.000	0.4650
243479_at	2.8	1.6	-0.82	-1.77	AAA-AAA	0.00	999	1.000	1.000	0.4918
216808_at	15.8	28.0	0.82	1.77	AAA-AAA	0.00	999	1.000	1.000	0.4907
216458_at	26.9	47.6	0.82	1.77	AAA-AAA	0.00	999	1.000	1.000	0.4136
214890_s_at	3.2	5.6	0.82	1.77	AAA-AAA	0.00	999	1.000	1.000	0.3405
218870_at	4.3	7.6	0.82	1.77	AAA-AAA	0.00	999	1.000	1.000	0.4375
206807_s_at	177.2	100.1	-0.82	-1.77	AAA-AAA	0.00	999	1.000	1.000	0.4935
207454_at	5.1	9.1	0.82	1.77	AAA-AAA	0.00	999	1.000	1.000	0.2020
209396_s_at	36.3	64.2	0.82	1.77	AAA-AAA	0.00	999	1.000	1.000	0.2155
208481_at	18.4	10.4	-0.82	-1.77	AAA-AAA	0.00	999	1.000	1.000	0.6790
234282_at	35.1	19.8	-0.82	-1.77	AAA-AAA	0.00	999	1.000	1.000	0.4527
237481_at	16.5	29.2	0.82	1.77	AAA-AAA	0.00	999	1.000	1.000	0.4293
236199_at	20.7	11.7	-0.82	-1.77	AAA-AAA	0.00	999	1.000	1.000	0.3951
222795_s_at	109.2	61.7	-0.82	-1.77	AAA-AAA	0.00	999	1.000	1.000	0.4256
232111_at	11.0	6.2	-0.82	-1.77	AAA-AAA	0.00	999	1.000	1.000	0.2770
229416_at	14.4	25.5	0.82	1.77	AAA-AAA	0.00	999	1.000	1.000	0.5334
228933_at	176.6	99.9	-0.82	-1.77	PPA-PAP	0.00	999	1.000	1.000	0.2282
241780_at	4.8	2.7	-0.82	-1.77	AAA-AAA	0.00	999	1.000	1.000	0.4301

1570415_at	10.5	18.5	0.82	1.77	AAA-AAA	0.00	999	1.000	1.000	0.4000
239894_at	5.5	9.7	0.82	1.77	AAA-AAA	0.00	999	1.000	1.000	0.2297
241048_at	60.5	34.2	-0.82	-1.77	AAA-AAA	0.00	999	1.000	1.000	0.0669
1568598_at	21.9	12.4	-0.82	-1.77	AAA-AAA	0.00	999	1.000	1.000	0.5412
1561504_s_at	11.2	6.4	-0.82	-1.77	AAA-AAA	0.00	999	1.000	1.000	0.5257
243468_at	30.6	17.3	-0.82	-1.77	AAA-AAA	0.00	999	1.000	1.000	0.5395
220455_at	29.9	16.9	-0.82	-1.77	AAA-AAA	0.00	999	1.000	1.000	0.2318
207155_at	18.0	31.8	0.82	1.77	AAA-AAA	0.00	999	1.000	1.000	0.3929
203768_s_at	33.2	58.6	0.82	1.77	AAA-AAA	0.00	999	1.000	1.000	0.6725
238930_at	5.9	10.4	0.82	1.77	AAA-AAA	0.00	999	1.000	1.000	0.1183
238275_at	9.9	17.4	0.82	1.77	AAA-AAA	0.00	999	1.000	1.000	0.3447
236191_at	6.5	11.5	0.82	1.77	AAA-AAA	0.00	999	1.000	1.000	0.5139
237221_at	15.3	27.0	0.82	1.77	AAA-AAA	0.00	999	1.000	1.000	0.0697
225867_at	64.5	114.2	0.82	1.77	AAA-AAA	0.00	999	1.000	1.000	0.2997
224271_x_at	80.2	45.3	-0.82	-1.77	AAA-AAA	0.00	999	1.000	1.000	0.3262
230971_x_at	20.0	11.3	-0.82	-1.77	AAA-AAA	0.00	999	1.000	1.000	0.5644
229004_at	37.5	66.3	0.82	1.77	AAA-AAA	0.00	999	1.000	1.000	0.1030
242766_at	99.2	56.1	-0.82	-1.77	PMA-APM	0.00	999	1.000	1.000	0.1066
220988_s_at	77.3	43.7	-0.82	-1.77	AAP-MAA	0.00	999	1.000	1.000	0.3241
1557826_at	14.1	8.0	-0.82	-1.77	PAA-AAA	0.00	999	1.000	1.000	0.6398
1564679_at	17.9	31.6	0.82	1.77	AAA-AAA	0.00	999	1.000	1.000	0.6656
241945_at	52.7	93.1	0.82	1.77	AAA-MAA	0.00	999	1.000	1.000	0.5091
241949_at	11.1	6.3	-0.82	-1.77	AAA-AAA	0.00	999	1.000	1.000	0.1557
1568764_x_at	106.4	60.2	-0.82	-1.77	AAA-AAA	0.00	999	1.000	1.000	0.2148
1569953_at	30.8	17.4	-0.82	-1.77	AAA-AAA	0.00	999	1.000	1.000	0.5616
1555022_at	35.1	19.9	-0.82	-1.77	AAA-AAA	0.00	999	1.000	1.000	0.4611
243506_at	14.1	24.9	0.82	1.77	AAA-AAA	0.00	999	1.000	1.000	0.5391
242643_x_at	8.4	14.8	0.82	1.77	AAA-AAA	0.00	999	1.000	1.000	0.5002
216255_s_at	10.7	6.0	-0.82	-1.77	AAA-AAA	0.00	999	1.000	1.000	0.5787
234546_at	10.2	18.1	0.82	1.77	AAA-AAA	0.00	999	1.000	1.000	0.5151
234802_at	43.2	24.5	-0.82	-1.77	AAA-AAA	0.00	999	1.000	1.000	0.1731
237413_at	8.9	15.8	0.82	1.77	AAA-AAA	0.00	999	1.000	1.000	0.6427
237583_at	38.6	68.3	0.82	1.77	AAA-AAA	0.00	999	1.000	1.000	0.4569
228413_s_at	12.9	22.8	0.82	1.77	AAA-AAA	0.00	999	1.000	1.000	0.5677
231675_s_at	11.2	19.9	0.82	1.77	AAA-AAA	0.00	999	1.000	1.000	0.1978
230391_at	33.1	58.5	0.82	1.77	AAA-AAA	0.00	999	1.000	1.000	0.4594
209473_at	61.4	34.8	-0.82	-1.77	APA-AAA	0.00	999	1.000	1.000	0.4430
242970_at	69.1	39.1	-0.82	-1.77	PAA-AAA	0.00	999	1.000	1.000	0.4518
1562989_at	4.9	8.7	0.82	1.77	AAA-AAA	0.00	999	1.000	1.000	0.1391
1562933_at	9.7	17.2	0.82	1.77	AAA-AAA	0.00	999	1.000	1.000	0.5001
1566080_at	5.0	8.9	0.82	1.77	AAA-AAA	0.00	999	1.000	1.000	0.3595
240500_at	32.2	18.3	-0.82	-1.77	AAA-AAA	0.00	999	1.000	1.000	0.5325

1556917_a_at	11.1	19.7	0.82	1.77	AAA-AAA	0.00	999	1.000	1.000	0.6953
1552858_at	8.5	4.8	-0.82	-1.77	AAA-AAA	0.00	999	1.000	1.000	0.6327
1554033_at	7.3	12.8	0.82	1.77	AAA-AAA	0.00	999	1.000	1.000	0.5685
1554945_x_at	21.3	37.7	0.82	1.77	AAA-AAA	0.00	999	1.000	1.000	0.4230
1561122_a_at	24.9	14.1	-0.82	-1.77	AAA-AAA	0.00	999	1.000	1.000	0.0864
1562073_at	50.8	28.7	-0.82	-1.77	AAA-AAA	0.00	999	1.000	1.000	0.1424
1560769_at	9.3	16.5	0.82	1.77	AAA-AAA	0.00	999	1.000	1.000	0.6184
216512_s_at	45.1	79.6	0.82	1.77	AAA-AAA	0.00	999	1.000	1.000	0.3578
210258_at	6.2	10.9	0.82	1.77	AAA-AAA	0.00	999	1.000	1.000	0.4850
232930_at	52.0	29.4	-0.82	-1.77	AAA-AAA	0.00	999	1.000	1.000	0.3203
233502_at	7.1	4.0	-0.82	-1.77	AAA-AAA	0.00	999	1.000	1.000	0.6438
238016_s_at	40.6	23.0	-0.82	-1.77	AAA-AAA	0.00	999	1.000	1.000	0.4579
235770_at	5.9	3.3	-0.82	-1.77	AAA-AAA	0.00	999	1.000	1.000	0.3191
236213_at	10.5	18.5	0.82	1.77	AAA-AAA	0.00	999	1.000	1.000	0.2035
227346_at	4.9	8.7	0.82	1.77	AAA-AAA	0.00	999	1.000	1.000	0.4671
224482_s_at	24.0	42.3	0.82	1.77	AAA-AAA	0.00	999	1.000	1.000	0.5962
226079_at	86.7	49.1	-0.82	-1.77	AAA-AAA	0.00	999	1.000	1.000	0.1677
1564194_a_at	44.8	25.4	-0.82	-1.77	APA-AAA	0.00	999	1.000	1.000	0.4654
241572_at	10.4	18.4	0.82	1.77	AAA-AAA	0.00	999	1.000	1.000	0.5124
1563360_at	55.1	31.2	-0.82	-1.77	AAA-AAA	0.00	999	1.000	1.000	0.4085
1563185_at	21.5	12.2	-0.82	-1.77	AAA-AAA	0.00	999	1.000	1.000	0.6131
1569872_a_at	73.9	41.9	-0.82	-1.77	AAA-AAA	0.00	999	1.000	1.000	0.1096
1569785_at	3.4	6.0	0.82	1.77	AAA-AAA	0.00	999	1.000	1.000	0.3849
1552768_at	11.7	6.7	-0.82	-1.77	AAA-AAA	0.00	999	1.000	1.000	0.2528
1557807_a_at	38.9	22.0	-0.82	-1.77	AAA-AAA	0.00	999	1.000	1.000	0.6388
1554406_a_at	19.9	35.1	0.82	1.77	AAA-AAA	0.00	999	1.000	1.000	0.4675
243082_at	15.8	9.0	-0.82	-1.77	AAA-AAA	0.00	999	1.000	1.000	0.4599
1561592_at	5.7	10.1	0.82	1.77	AAA-AAA	0.00	999	1.000	1.000	0.6255
244136_at	33.6	59.4	0.82	1.77	AMA-AAA	0.00	999	1.000	1.000	0.5326
216549_s_at	12.8	7.3	-0.82	-1.77	AAA-AAA	0.00	999	1.000	1.000	0.5753
216287_at	30.6	54.0	0.82	1.77	AAA-AAA	0.00	999	1.000	1.000	0.0471
220438_at	100.3	56.8	-0.82	-1.77	AAA-AAA	0.00	999	1.000	1.000	0.4509
234709_at	5.6	9.8	0.82	1.77	AAA-AAA	0.00	999	1.000	1.000	0.6387
238664_s_at	20.3	35.8	0.82	1.77	AAA-AAA	0.00	999	1.000	1.000	0.4364
225990_at	50.6	89.4	0.82	1.77	AAA-AAA	0.00	999	1.000	1.000	0.0132
231745_at	33.7	19.1	-0.82	-1.77	AAA-AAA	0.00	999	1.000	1.000	0.3240
229383_at	25.7	14.6	-0.82	-1.77	AAA-AAA	0.00	999	1.000	1.000	0.4095
230269_at	7.5	13.2	0.82	1.77	AAA-AAA	0.00	999	1.000	1.000	0.2887
213492_at	65.6	37.2	-0.82	-1.77	APA-AAA	0.00	999	1.000	1.000	0.4194
234459_at	96.6	54.8	-0.82	-1.76	AAA-AAP	0.00	999	1.000	1.000	0.4367
243959_at	36.4	20.7	-0.82	-1.76	PAP-AAP	0.00	999	1.000	1.000	0.5266
241511_at	3.2	5.6	0.82	1.77	AAA-AAA	0.00	999	1.000	1.000	0.0086

241204_at	8.4	4.7	-0.82	-1.77	AAA-AAA	0.00	999	1.000	1.000	0.1832
239298_at	9.4	5.3	-0.82	-1.77	AAA-AAA	0.00	999	1.000	1.000	0.5589
241136_at	9.8	5.5	-0.82	-1.76	AAA-AAA	0.00	999	1.000	1.000	0.6487
240665_at	15.5	8.8	-0.82	-1.76	AAA-AAA	0.00	999	1.000	1.000	0.6232
1556822_s_at	7.6	13.3	0.82	1.76	AAA-AAA	0.00	999	1.000	1.000	0.1748
1561618_at	15.2	26.7	0.82	1.76	AAA-AAA	0.00	999	1.000	1.000	0.6289
220350_at	12.4	22.0	0.82	1.77	AAA-AAA	0.00	999	1.000	1.000	0.4088
210402_at	28.6	16.2	-0.82	-1.76	AAA-AAA	0.00	999	1.000	1.000	0.5115
210100_s_at	122.5	69.4	-0.82	-1.76	AAA-AAA	0.00	999	1.000	1.000	0.4085
235717_at	2.6	4.5	0.82	1.76	AAA-AAA	0.00	999	1.000	1.000	0.4773
236378_at	23.9	42.2	0.82	1.76	AAA-AAA	0.00	999	1.000	1.000	0.5487
236771_at	19.7	34.7	0.82	1.76	AAA-AAA	0.00	999	1.000	1.000	0.3644
236646_at	20.1	35.4	0.82	1.76	AAA-AAA	0.00	999	1.000	1.000	0.4161
228424_at	99.3	56.3	-0.82	-1.76	AAA-AAA	0.00	999	1.000	1.000	0.4108
241389_at	32.8	18.6	-0.82	-1.76	AAA-AAA	0.00	999	1.000	1.000	0.3468
1569297_at	9.6	5.5	-0.82	-1.76	AAA-AAA	0.00	999	1.000	1.000	0.5861
1569479_at	11.0	19.3	0.82	1.76	AAA-AAA	0.00	999	1.000	1.000	0.3871
1566645_at	16.9	9.6	-0.82	-1.76	AAA-AAA	0.00	999	1.000	1.000	0.6233
243394_at	13.2	23.2	0.82	1.76	AAA-AAA	0.00	999	1.000	1.000	0.2778
244330_at	52.7	29.9	-0.82	-1.76	AAA-AAA	0.00	999	1.000	1.000	0.5053
243903_at	54.1	30.7	-0.82	-1.76	AAA-AAA	0.00	999	1.000	1.000	0.3044
220811_at	42.6	24.2	-0.82	-1.76	AAA-AAA	0.00	999	1.000	1.000	0.3957
220718_at	12.3	7.0	-0.82	-1.76	AAA-AAA	0.00	999	1.000	1.000	0.4348
218380_at	18.2	32.0	0.82	1.76	AAA-AAA	0.00	999	1.000	1.000	0.6646
237615_at	6.0	3.4	-0.82	-1.76	AAA-AAA	0.00	999	1.000	1.000	0.4082
237029_at	16.7	29.4	0.82	1.76	AAA-AAA	0.00	999	1.000	1.000	0.4973
226878_at	38.8	68.4	0.82	1.76	AAA-AAA	0.00	999	1.000	1.000	0.2322
224487_at	34.9	61.5	0.82	1.76	AAA-AAA	0.00	999	1.000	1.000	0.2564
230869_at	8.4	4.8	-0.82	-1.76	AAA-AAA	0.00	999	1.000	1.000	0.5924
232438_at	79.5	140.1	0.82	1.76	AAA-AAM	0.00	999	1.000	1.000	0.1735
241550_at	6.1	10.8	0.82	1.76	AAA-AAA	0.00	999	1.000	1.000	0.5527
1565811_at	30.9	17.5	-0.82	-1.76	AAA-AAA	0.00	999	1.000	1.000	0.5221
244839_at	5.3	9.3	0.82	1.76	AAA-AAA	0.00	999	1.000	1.000	0.6768
1553458_at	58.1	33.0	-0.82	-1.76	AAA-AAA	0.00	999	1.000	1.000	0.3336
1553556_at	10.3	18.2	0.82	1.76	AAA-AAA	0.00	999	1.000	1.000	0.6677
1554160_a_at	90.9	160.2	0.82	1.76	AAA-AAA	0.00	999	1.000	1.000	0.4153
1559665_at	3.5	6.2	0.82	1.76	AAA-AAA	0.00	999	1.000	1.000	0.4815
216754_at	10.8	18.9	0.82	1.76	AAA-AAA	0.00	999	1.000	1.000	0.4818
234844_at	6.0	10.5	0.82	1.76	AAA-AAA	0.00	999	1.000	1.000	0.6820
228096_at	114.2	64.8	-0.82	-1.76	AAA-AAA	0.00	999	1.000	1.000	0.0309
224100_s_at	37.1	21.1	-0.82	-1.76	AAA-AAA	0.00	999	1.000	1.000	0.5864
241876_at	148.2	84.2	-0.82	-1.76	MPP-PAA	0.00	999	1.000	1.000	0.1403

205934_at	42.5	24.1	-0.82	-1.76	PAP-AAP	0.00	999	1.000	1.000	0.1639
242032_at	103.7	58.9	-0.82	-1.76	MPP-MAP	0.00	999	1.000	1.000	0.2060
36742_at	82.7	47.0	-0.82	-1.76	PAA-APA	0.00	999	1.000	1.000	0.3155
217315_s_at	47.5	27.0	-0.82	-1.76	AAA-PAA	0.00	999	1.000	1.000	0.3412
1560222_at	35.9	20.4	-0.82	-1.76	AAP-MAA	0.00	999	1.000	1.000	0.5021
1554557_at	100.4	57.0	-0.82	-1.76	PPP-APP	0.00	999	1.000	1.000	0.5465
1552386_at	36.1	20.5	-0.82	-1.76	AMA-PPA	0.00	999	1.000	1.000	0.5496
1566680_at	7.7	4.4	-0.82	-1.76	AAA-AAA	0.00	999	1.000	1.000	0.1963
1555509_a_at	10.8	18.9	0.82	1.76	AAA-AAA	0.00	999	1.000	1.000	0.6166
213108_at	7.9	13.8	0.82	1.76	AAA-AAA	0.00	999	1.000	1.000	0.3302
221231_s_at	14.9	26.2	0.82	1.76	AAA-AAA	0.00	999	1.000	1.000	0.4029
216924_s_at	49.4	28.1	-0.82	-1.76	AAA-AAA	0.00	999	1.000	1.000	0.4477
235290_at	36.6	64.5	0.82	1.76	AAA-AAA	0.00	999	1.000	1.000	0.4931
233499_at	28.6	16.3	-0.82	-1.76	AAA-AAA	0.00	999	1.000	1.000	0.2632
237508_at	12.3	7.0	-0.82	-1.76	AAA-AAA	0.00	999	1.000	1.000	0.6410
231979_at	32.0	18.2	-0.82	-1.76	AAA-AAA	0.00	999	1.000	1.000	0.6581
230468_s_at	5.1	9.0	0.82	1.76	AAA-AAA	0.00	999	1.000	1.000	0.4439
203844_at	51.6	29.3	-0.82	-1.76	PAP-AAP	0.00	999	1.000	1.000	0.0693
226756_at	538.2	306.0	-0.82	-1.76	PPP-PPP	0.00	999	1.000	1.000	0.2069
242860_at	61.1	34.8	-0.82	-1.76	APA-AAP	0.00	999	1.000	1.000	0.6509
242177_at	48.0	84.4	0.82	1.76	AAA-AAA	0.00	999	1.000	1.000	0.1971
239868_at	31.3	55.0	0.82	1.76	AAA-AAA	0.00	999	1.000	1.000	0.2773
1556514_at	26.4	15.0	-0.82	-1.76	AAA-AAA	0.00	999	1.000	1.000	0.4975
243366_s_at	38.8	22.1	-0.82	-1.76	AAA-AAA	0.00	999	1.000	1.000	0.5812
1560869_a_at	7.9	14.0	0.82	1.76	AAA-AAA	0.00	999	1.000	1.000	0.5204
214390_s_at	15.3	26.9	0.82	1.76	AAA-AAA	0.00	999	1.000	1.000	0.1959
217251_x_at	8.9	15.6	0.82	1.76	AAA-AAA	0.00	999	1.000	1.000	0.2611
206927_s_at	61.8	35.1	-0.82	-1.76	AAA-AAA	0.00	999	1.000	1.000	0.5913
211597_s_at	11.8	6.7	-0.82	-1.76	AAA-AAA	0.00	999	1.000	1.000	0.5068
211005_at	13.3	23.4	0.82	1.76	AAA-AAA	0.00	999	1.000	1.000	0.4743
207766_at	55.0	96.8	0.82	1.76	AAA-AAA	0.00	999	1.000	1.000	0.4605
209010_s_at	45.9	26.1	-0.82	-1.76	AAA-AAA	0.00	999	1.000	1.000	0.4528
234117_at	37.7	21.5	-0.82	-1.76	AAA-AAA	0.00	999	1.000	1.000	0.3417
235914_at	16.0	9.1	-0.82	-1.76	AAA-AAA	0.00	999	1.000	1.000	0.1909
60084_at	26.0	45.7	0.82	1.76	AAA-AAA	0.00	999	1.000	1.000	0.3967
232041_at	22.5	12.8	-0.82	-1.76	AAA-AAA	0.00	999	1.000	1.000	0.5271
234219_at	63.2	35.9	-0.81	-1.76	APP-APA	0.00	999	1.000	1.000	0.0390
232456_at	61.9	35.2	-0.81	-1.76	APA-APA	0.00	999	1.000	1.000	0.5073
1559276_at	18.3	10.4	-0.81	-1.76	APA-AAA	0.00	999	1.000	1.000	0.5656
1565657_at	7.6	4.3	-0.81	-1.76	AAA-AAA	0.00	999	1.000	1.000	0.6741
240096_at	5.4	9.5	0.81	1.76	AAA-AAA	0.00	999	1.000	1.000	0.4478
1556762_a_at	24.5	43.0	0.81	1.76	AAA-AAA	0.00	999	1.000	1.000	0.6070

1557763_at	26.2	14.9	-0.81	-1.76	AAA-AAA	0.00	999	1.000	1.000	0.3824
1553844_a_at	55.8	98.1	0.81	1.76	AAA-AAA	0.00	999	1.000	1.000	0.4939
1553025_at	17.4	9.9	-0.81	-1.76	AAA-AAA	0.00	999	1.000	1.000	0.5095
1555277_a_at	30.0	52.7	0.81	1.76	MAA-AAA	0.00	999	1.000	1.000	0.3869
1561938_at	12.5	7.1	-0.81	-1.76	AAA-AAA	0.00	999	1.000	1.000	0.4933
1559645_at	80.7	45.9	-0.81	-1.76	AAA-AAA	0.00	999	1.000	1.000	0.3116
220396_at	13.5	23.7	0.81	1.76	AAA-AAA	0.00	999	1.000	1.000	0.6432
217449_at	36.5	64.3	0.81	1.76	AAA-AAA	0.00	999	1.000	1.000	0.1836
204223_at	32.1	18.3	-0.81	-1.76	AAA-AAA	0.00	999	1.000	1.000	0.4882
209597_s_at	12.6	22.1	0.81	1.76	AAA-AAA	0.00	999	1.000	1.000	0.6116
208383_s_at	11.6	6.6	-0.81	-1.76	AAA-AAA	0.00	999	1.000	1.000	0.5074
237454_at	38.2	21.7	-0.81	-1.76	AAA-AAA	0.00	999	1.000	1.000	0.6244
228246_s_at	13.4	23.5	0.81	1.76	AAA-AAA	0.00	999	1.000	1.000	0.4139
231768_at	10.4	18.4	0.81	1.76	AAA-AAA	0.00	999	1.000	1.000	0.1340
237516_at	20.7	11.8	-0.81	-1.76	AAA-AAP	0.00	999	1.000	1.000	0.6052
241230_at	58.7	103.2	0.81	1.76	AAA-AAA	0.00	999	1.000	1.000	0.0035
1556618_at	60.8	34.6	-0.81	-1.76	AAA-AAA	0.00	999	1.000	1.000	0.5221
244847_at	81.5	46.4	-0.81	-1.76	AAA-AAA	0.00	999	1.000	1.000	0.1968
244739_at	47.3	26.9	-0.81	-1.76	AAA-AAA	0.00	999	1.000	1.000	0.1923
1558556_at	17.0	29.9	0.81	1.76	AAA-AAA	0.00	999	1.000	1.000	0.2705
244776_at	14.0	8.0	-0.81	-1.76	AAA-AAA	0.00	999	1.000	1.000	0.4511
1556166_x_at	29.7	16.9	-0.81	-1.76	AAA-AAA	0.00	999	1.000	1.000	0.5808
1561596_at	4.4	7.8	0.81	1.76	AAA-AAA	0.00	999	1.000	1.000	0.4851
244355_at	12.1	21.2	0.81	1.76	AAA-AAA	0.00	999	1.000	1.000	0.3824
1558959_at	51.3	29.2	-0.81	-1.76	AAA-AAA	0.00	999	1.000	1.000	0.6237
243976_at	107.3	61.1	-0.81	-1.76	AAA-AAA	0.00	999	1.000	1.000	0.4289
204428_s_at	18.9	33.2	0.81	1.76	AAA-AAA	0.00	999	1.000	1.000	0.5553
209847_at	16.5	29.0	0.81	1.76	AAA-AAA	0.00	999	1.000	1.000	0.6203
207897_at	65.0	37.0	-0.81	-1.76	AAA-AAA	0.00	999	1.000	1.000	0.4064
234781_at	7.0	12.3	0.81	1.76	AAA-AAA	0.00	999	1.000	1.000	0.3073
237440_at	50.1	28.5	-0.81	-1.76	AAA-AAA	0.00	999	1.000	1.000	0.2003
237571_at	26.6	15.1	-0.81	-1.76	AAA-AAA	0.00	999	1.000	1.000	0.5266
228431_at	19.3	33.9	0.81	1.76	AAA-AAA	0.00	999	1.000	1.000	0.4554
223563_at	38.8	22.1	-0.81	-1.76	AAA-AAA	0.00	999	1.000	1.000	0.6082
230909_at	9.1	5.2	-0.81	-1.76	AAA-AAA	0.00	999	1.000	1.000	0.1564
231707_at	21.0	11.9	-0.81	-1.76	AAA-AAA	0.00	999	1.000	1.000	0.4642
239350_at	96.5	169.3	0.81	1.76	AAA-AAA	0.00	999	1.000	1.000	0.0973
1553321_a_at	21.8	38.3	0.81	1.76	AAA-AAA	0.00	999	1.000	1.000	0.4879
243418_at	34.3	19.6	-0.81	-1.76	AAA-AAA	0.00	999	1.000	1.000	0.6253
216107_at	54.8	31.2	-0.81	-1.76	AAA-AAA	0.00	999	1.000	1.000	0.4072
220958_at	63.9	112.1	0.81	1.76	AAA-AAA	0.00	999	1.000	1.000	0.0259
219893_at	56.6	99.4	0.81	1.76	AAA-AAA	0.00	999	1.000	1.000	0.3855

206933_s_at	8.0	14.1	0.81	1.76	AAA-AAA	0.00	999	1.000	1.000	0.1156
204192_at	13.7	24.0	0.81	1.76	AAA-AAA	0.00	999	1.000	1.000	0.2521
209686_at	11.1	6.3	-0.81	-1.76	AAA-AAA	0.00	999	1.000	1.000	0.4450
224256_at	21.3	37.3	0.81	1.76	AAA-AAA	0.00	999	1.000	1.000	0.4751
223610_at	35.0	61.5	0.81	1.76	AAA-AAA	0.00	999	1.000	1.000	0.4035
238523_at	598.3	341.1	-0.81	-1.75	PPP-APP	0.00	999	1.000	1.000	0.0633
1563224_at	41.2	23.5	-0.81	-1.75	AAA-AAA	0.00	999	1.000	1.000	0.5624
1566898_at	6.6	11.5	0.81	1.75	AAA-AAA	0.00	999	1.000	1.000	0.5228
1557669_at	13.1	7.4	-0.81	-1.75	AAA-AAA	0.00	999	1.000	1.000	0.3427
1559394_a_at	15.6	8.9	-0.81	-1.76	AAA-AAA	0.00	999	1.000	1.000	0.3353
220700_at	13.2	23.1	0.81	1.75	AAA-AAA	0.00	999	1.000	1.000	0.4503
206640_x_at	8.0	14.0	0.81	1.76	AAA-AAA	0.00	999	1.000	1.000	0.2686
205185_at	46.5	81.5	0.81	1.75	AAA-AAA	0.00	999	1.000	1.000	0.2906
211626_x_at	28.6	16.3	-0.81	-1.76	AAA-AAA	0.00	999	1.000	1.000	0.2425
208200_at	33.7	59.1	0.81	1.76	AAA-AAA	0.00	999	1.000	1.000	0.1949
208354_s_at	6.4	11.3	0.81	1.76	AAA-AAA	0.00	999	1.000	1.000	0.0504
235748_s_at	21.5	37.6	0.81	1.75	AAA-AAA	0.00	999	1.000	1.000	0.5938
232593_at	22.9	40.2	0.81	1.75	AAA-AAA	0.00	999	1.000	1.000	0.5819
233742_at	14.3	25.1	0.81	1.75	AAA-AAA	0.00	999	1.000	1.000	0.1758
227055_at	54.6	95.7	0.81	1.75	AAA-AAA	0.00	999	1.000	1.000	0.1702
230036_at	18.0	31.6	0.81	1.76	AAA-AAA	0.00	999	1.000	1.000	0.5517
1566487_at	3.7	6.5	0.81	1.75	AAA-AAA	0.00	999	1.000	1.000	0.4827
239215_at	27.9	48.9	0.81	1.75	AAA-AAA	0.00	999	1.000	1.000	0.3844
1570177_at	23.3	40.8	0.81	1.75	AAA-AAA	0.00	999	1.000	1.000	0.4928
243587_x_at	12.1	6.9	-0.81	-1.75	AAA-AAA	0.00	999	1.000	1.000	0.3978
216546_s_at	15.1	26.4	0.81	1.75	AAA-AAA	0.00	999	1.000	1.000	0.1251
215124_at	12.1	21.1	0.81	1.75	AAA-AAA	0.00	999	1.000	1.000	0.3325
220693_at	68.6	39.1	-0.81	-1.75	AAA-AMA	0.00	999	1.000	1.000	0.0679
206513_at	63.8	36.4	-0.81	-1.75	AAA-AAA	0.00	999	1.000	1.000	0.2186
205163_at	47.3	27.0	-0.81	-1.75	AAA-AAA	0.00	999	1.000	1.000	0.5869
211207_s_at	12.0	21.1	0.81	1.75	AAA-AAA	0.00	999	1.000	1.000	0.5189
232611_at	52.6	92.2	0.81	1.75	AAA-AAA	0.00	999	1.000	1.000	0.3324
232490_s_at	146.8	83.7	-0.81	-1.75	AAA-AAA	0.00	999	1.000	1.000	0.4565
233386_at	7.2	12.6	0.81	1.75	AAA-AAA	0.00	999	1.000	1.000	0.5078
237368_at	5.1	8.9	0.81	1.75	AAA-AAA	0.00	999	1.000	1.000	0.0089
224551_s_at	33.9	59.4	0.81	1.75	AAA-AAA	0.00	999	1.000	1.000	0.2171
223344_s_at	20.6	36.1	0.81	1.75	AAA-AAA	0.00	999	1.000	1.000	0.5136
202052_s_at	2266.3	1293.3	-0.81	-1.75	PPP-PPP	0.00	999	1.000	1.000	0.0293
240557_at	210.4	120.1	-0.81	-1.75	PPP-PPP	0.00	999	1.000	1.000	0.0763
242094_at	75.9	43.3	-0.81	-1.75	PAA-AAA	0.00	999	1.000	1.000	0.1925
219892_at	35.2	20.1	-0.81	-1.75	PAA-AAA	0.00	999	1.000	1.000	0.5533
1568888_at	1.9	3.4	0.81	1.75	AAA-AAA	0.00	999	1.000	1.000	0.3149

217420_s_at	20.9	36.6	0.81	1.75	AAA-AAA	0.00	999	1.000	1.000	0.5421
203994_s_at	21.6	37.8	0.81	1.75	AAA-AAA	0.00	999	1.000	1.000	0.4213
235986_at	7.1	12.4	0.81	1.75	AAA-AAA	0.00	999	1.000	1.000	0.3478
231937_at	33.5	19.1	-0.81	-1.75	AAA-AAA	0.00	999	1.000	1.000	0.5173
221407_at	152.2	86.9	-0.81	-1.75	PAA-AAA	0.00	999	1.000	1.000	0.1203
38149_at	78.5	44.8	-0.81	-1.75	AAP-AAA	0.00	999	1.000	1.000	0.5528
1563607_x_at	105.6	60.3	-0.81	-1.75	AAA-AAA	0.00	999	1.000	1.000	0.0881
1557645_at	25.2	44.1	0.81	1.75	AAA-AAA	0.00	999	1.000	1.000	0.5118
1554259_at	33.5	58.6	0.81	1.75	AAA-AAA	0.00	999	1.000	1.000	0.2205
1553282_at	10.3	18.0	0.81	1.75	AAA-AAA	0.00	999	1.000	1.000	0.5061
1553447_at	25.3	14.5	-0.81	-1.75	AAA-AAA	0.00	999	1.000	1.000	0.5411
1561452_at	12.2	21.3	0.81	1.75	AAA-AAA	0.00	999	1.000	1.000	0.6076
212821_at	63.7	36.4	-0.81	-1.75	AAA-AAA	0.00	999	1.000	1.000	0.5604
214361_s_at	29.6	51.8	0.81	1.75	AAA-AAA	0.00	999	1.000	1.000	0.4907
221382_at	22.5	39.4	0.81	1.75	AAA-AAA	0.00	999	1.000	1.000	0.3942
217326_x_at	15.1	8.6	-0.81	-1.75	AAA-AAA	0.00	999	1.000	1.000	0.3151
216927_at	13.1	23.0	0.81	1.75	AAA-AAA	0.00	999	1.000	1.000	0.5049
209978_s_at	16.4	28.8	0.81	1.75	AAA-AAA	0.00	999	1.000	1.000	0.5326
211103_at	28.9	16.5	-0.81	-1.75	AAA-AAA	0.00	999	1.000	1.000	0.5355
232674_at	37.0	64.8	0.81	1.75	AAA-AAA	0.00	999	1.000	1.000	0.4960
238409_x_at	11.9	20.9	0.81	1.75	AAA-AAA	0.00	999	1.000	1.000	0.5107
224508_at	24.1	42.1	0.81	1.75	AAA-AAA	0.00	999	1.000	1.000	0.1022
202376_at	596.9	341.2	-0.81	-1.75	PPP-PPP	0.00	999	1.000	1.000	0.0406
241433_at	171.2	97.9	-0.81	-1.75	PPP-PPP	0.00	999	1.000	1.000	0.1419
1562637_at	10.5	18.3	0.81	1.75	AAA-AAA	0.00	999	1.000	1.000	0.4930
1568782_at	55.0	31.4	-0.81	-1.75	AAA-AAA	0.00	999	1.000	1.000	0.4871
239620_at	6.5	11.5	0.81	1.75	AAA-AAA	0.00	999	1.000	1.000	0.5923
1557321_a_at	7.8	13.7	0.81	1.75	AAA-AAA	0.00	999	1.000	1.000	0.5383
1556511_a_at	10.2	5.8	-0.81	-1.75	AAA-AAA	0.00	999	1.000	1.000	0.1764
244855_at	3.6	6.2	0.81	1.75	AAA-AAA	0.00	999	1.000	1.000	0.3004
1560162_at	28.7	50.2	0.81	1.75	AAA-AAA	0.00	999	1.000	1.000	0.3660
1560219_at	31.1	54.3	0.81	1.75	AAA-AAA	0.00	999	1.000	1.000	0.4419
219716_at	108.3	61.9	-0.81	-1.75	AAA-AAA	0.00	999	1.000	1.000	0.0931
204390_at	8.7	15.2	0.81	1.75	AAA-AAA	0.00	999	1.000	1.000	0.1006
204712_at	7.5	4.3	-0.81	-1.75	AAA-AAA	0.00	999	1.000	1.000	0.6076
208017_s_at	75.0	42.9	-0.81	-1.75	AAA-AAA	0.00	999	1.000	1.000	0.4562
234741_at	3.7	6.4	0.81	1.75	AAA-AAA	0.00	999	1.000	1.000	0.5245
229921_at	36.8	21.0	-0.81	-1.75	AAA-AAA	0.00	999	1.000	1.000	0.2976
230849_at	30.6	53.5	0.81	1.75	AAA-AAA	0.00	999	1.000	1.000	0.4371
241977_s_at	88.0	50.3	-0.81	-1.75	PPA-AAP	0.00	999	1.000	1.000	0.3265
1553837_at	15.1	8.7	-0.81	-1.75	AAA-AAA	0.00	999	1.000	1.000	0.0276
1553811_at	31.5	55.1	0.81	1.75	AAA-AAA	0.00	999	1.000	1.000	0.5534

1559063_at	46.1	80.5	0.81	1.75	AAA-AAA	0.00	999	1.000	1.000	0.3426
244567_at	19.0	33.2	0.81	1.75	AAA-AAA	0.00	999	1.000	1.000	0.2923
221236_s_at	26.9	47.0	0.81	1.75	AAA-AAA	0.00	999	1.000	1.000	0.4201
219765_at	11.9	20.7	0.81	1.75	AAA-AAA	0.00	999	1.000	1.000	0.3889
205850_s_at	13.0	7.5	-0.81	-1.75	AAA-AAA	0.00	999	1.000	1.000	0.5768
207913_at	16.8	9.6	-0.81	-1.75	AAA-AAA	0.00	999	1.000	1.000	0.0371
209031_at	17.7	10.1	-0.81	-1.75	AAA-AAA	0.00	999	1.000	1.000	0.6399
235794_at	10.5	18.3	0.81	1.75	AAA-AAA	0.00	999	1.000	1.000	0.6186
227646_at	34.1	19.5	-0.81	-1.75	AAA-AAA	0.00	999	1.000	1.000	0.4387
231562_at	19.8	11.3	-0.81	-1.75	AAA-AAA	0.00	999	1.000	1.000	0.0120
230458_at	33.3	58.3	0.81	1.75	AAA-AAA	0.00	999	1.000	1.000	0.3493
224940_s_at	114.2	65.4	-0.81	-1.75	PPP-PPP	0.00	999	1.000	1.000	0.1108
1553945_at	70.4	40.3	-0.81	-1.75	APA-AAA	0.00	999	1.000	1.000	0.2194
235332_at	129.2	73.9	-0.81	-1.75	APP-PAA	0.00	999	1.000	1.000	0.2222
229927_at	30.3	17.4	-0.81	-1.75	PAA-AAA	0.00	999	1.000	1.000	0.6104
1564803_at	16.4	28.6	0.81	1.75	AAA-AAA	0.00	999	1.000	1.000	0.2678
1564972_x_at	37.0	21.2	-0.81	-1.75	AAA-AAA	0.00	999	1.000	1.000	0.5836
1561539_at	25.7	44.9	0.81	1.75	AAA-AAA	0.00	999	1.000	1.000	0.5111
1562046_at	9.2	16.1	0.81	1.75	AAA-AAA	0.00	999	1.000	1.000	0.3951
244480_at	14.7	25.7	0.81	1.75	AAA-AAA	0.00	999	1.000	1.000	0.4292
211772_x_at	11.8	20.6	0.81	1.75	AAA-AAA	0.00	999	1.000	1.000	0.1242
208218_s_at	30.7	53.6	0.81	1.75	AAA-AAA	0.00	999	1.000	1.000	0.5998
208204_s_at	20.8	36.4	0.81	1.75	AAA-AAA	0.00	999	1.000	1.000	0.2648
233484_at	52.4	30.0	-0.81	-1.75	AAA-AAA	0.00	999	1.000	1.000	0.5116
231283_at	4.7	8.3	0.81	1.75	AAA-AAA	0.00	999	1.000	1.000	0.5535
230765_at	10.1	17.7	0.81	1.75	AAA-AAA	0.00	999	1.000	1.000	0.3646
238867_at	88.8	50.9	-0.80	-1.75	PAP-AAA	0.00	999	1.000	1.000	0.2918
1566097_at	29.5	51.5	0.80	1.75	AAA-AAA	0.00	999	1.000	1.000	0.4812
1557290_at	11.9	6.8	-0.80	-1.75	AAA-AAA	0.00	999	1.000	1.000	0.6372
1558476_at	13.5	7.7	-0.80	-1.75	AAA-AAA	0.00	999	1.000	1.000	0.3283
1557604_at	4.1	7.2	0.80	1.75	AAA-AAA	0.00	999	1.000	1.000	0.4351
1552315_at	21.6	37.7	0.80	1.75	AAA-AAA	0.00	999	1.000	1.000	0.5688
1554897_s_at	37.4	65.3	0.80	1.75	AAA-AAA	0.00	999	1.000	1.000	0.4404
1553044_at	20.5	35.8	0.80	1.75	AAA-AAA	0.00	999	1.000	1.000	0.4898
1559621_at	5.4	3.1	-0.80	-1.75	AAA-AAA	0.00	999	1.000	1.000	0.2338
1560885_x_at	1.9	3.3	0.80	1.75	AAA-AAA	0.00	999	1.000	1.000	0.4765
216157_at	121.7	69.7	-0.80	-1.75	AAA-AAA	0.00	999	1.000	1.000	0.0763
216357_at	19.4	11.1	-0.80	-1.75	AAA-AAA	0.00	999	1.000	1.000	0.5157
206774_at	29.9	17.2	-0.80	-1.75	AAA-AAA	0.00	999	1.000	1.000	0.6186
202637_s_at	188.5	107.9	-0.80	-1.75	AAA-AAA	0.00	999	1.000	1.000	0.4887
204724_s_at	8.1	4.7	-0.80	-1.75	AAA-AAA	0.00	999	1.000	1.000	0.0270
210781_x_at	9.3	16.3	0.80	1.75	AAA-AAA	0.00	999	1.000	1.000	0.0965

210161_at	50.0	87.3	0.80	1.75	AAA-AAA	0.00	999	1.000	1.000	0.5540
234483_at	13.4	7.7	-0.80	-1.75	AAA-AAA	0.00	999	1.000	1.000	0.3993
233258_at	6.9	12.0	0.80	1.75	AAA-AAA	0.00	999	1.000	1.000	0.1438
232577_at	68.0	118.7	0.80	1.75	AAA-AAA	0.00	999	1.000	1.000	0.3647
238731_at	31.3	18.0	-0.80	-1.75	AAA-AAA	0.00	999	1.000	1.000	0.5320
237992_at	11.6	6.6	-0.80	-1.75	AAA-AAA	0.00	999	1.000	1.000	0.6080
237056_at	271.0	155.3	-0.80	-1.75	PPP-MPP	0.00	999	1.000	1.000	0.0193
239101_at	231.9	132.9	-0.80	-1.75	PPP-PPP	0.00	999	1.000	1.000	0.0547
229402_at	103.4	59.3	-0.80	-1.74	PAP-AAA	0.00	999	1.000	1.000	0.1050
1569760_at	27.4	47.8	0.80	1.75	AAA-AAA	0.00	999	1.000	1.000	0.4925
241039_at	10.2	5.8	-0.80	-1.74	AAA-AAA	0.00	999	1.000	1.000	0.3944
240607_at	72.7	126.8	0.80	1.74	AAA-AAA	0.00	999	1.000	1.000	0.4422
1557206_at	6.9	12.0	0.80	1.75	AAA-AAA	0.00	999	1.000	1.000	0.7044
1552320_a_at	27.8	15.9	-0.80	-1.75	MAA-AAA	0.00	999	1.000	1.000	0.5635
1555914_a_at	4.0	7.0	0.80	1.74	AAA-AAA	0.00	999	1.000	1.000	0.1949
1553027_a_at	25.1	14.4	-0.80	-1.75	AAA-AAA	0.00	999	1.000	1.000	0.1025
1560896_a_at	18.9	33.0	0.80	1.75	AAA-AAA	0.00	999	1.000	1.000	0.5600
217134_at	28.6	16.4	-0.80	-1.74	AAA-AAA	0.00	999	1.000	1.000	0.4039
211235_s_at	28.4	16.3	-0.80	-1.75	AAA-AAA	0.00	999	1.000	1.000	0.5670
237378_at	26.3	15.1	-0.80	-1.75	AAA-AAA	0.00	999	1.000	1.000	0.4783
237070_at	7.1	12.4	0.80	1.75	AAA-AAA	0.00	999	1.000	1.000	0.5261
230996_at	38.0	66.4	0.80	1.74	AAA-AAA	0.00	999	1.000	1.000	0.4729
229821_at	156.6	89.8	-0.80	-1.74	AAA-AAA	0.00	999	1.000	1.000	0.3317
229578_at	396.8	227.6	-0.80	-1.74	PPP-PPP	0.00	999	1.000	1.000	0.1004
227380_x_at	142.0	81.4	-0.80	-1.74	PPA-PAA	0.00	999	1.000	1.000	0.2176
1563040_s_at	3.9	2.2	-0.80	-1.74	AAA-AAA	0.00	999	1.000	1.000	0.6118
241732_at	11.8	20.6	0.80	1.74	AAA-AAA	0.00	999	1.000	1.000	0.3264
240760_at	23.3	40.6	0.80	1.74	AAA-AAA	0.00	999	1.000	1.000	0.3504
1552340_at	19.5	11.2	-0.80	-1.74	AAA-AAA	0.00	999	1.000	1.000	0.4454
1564697_a_at	13.8	24.1	0.80	1.74	AAA-AAA	0.00	999	1.000	1.000	0.2904
1566229_a_at	16.9	29.4	0.80	1.74	AAA-AAA	0.00	999	1.000	1.000	0.5335
1560496_at	50.5	29.0	-0.80	-1.74	AAA-AAA	0.00	999	1.000	1.000	0.5568
215748_at	42.4	73.9	0.80	1.74	AAA-AAA	0.00	999	1.000	1.000	0.3971
216788_at	16.0	28.0	0.80	1.74	AAA-AAA	0.00	999	1.000	1.000	0.4751
214089_at	15.0	26.2	0.80	1.74	AAA-AAA	0.00	999	1.000	1.000	0.4553
206480_at	14.5	8.3	-0.80	-1.74	AAA-AAA	0.00	999	1.000	1.000	0.1384
209763_at	45.6	26.1	-0.80	-1.74	AAA-AAA	0.00	999	1.000	1.000	0.5673
211010_s_at	47.0	26.9	-0.80	-1.74	AAA-AAA	0.00	999	1.000	1.000	0.5137
208605_s_at	21.9	12.6	-0.80	-1.74	AAA-AAA	0.00	999	1.000	1.000	0.5522
234607_at	23.3	40.5	0.80	1.74	AAA-AAA	0.00	999	1.000	1.000	0.3319
234930_at	6.0	3.4	-0.80	-1.74	AAA-AAA	0.00	999	1.000	1.000	0.3579
235104_at	54.0	31.0	-0.80	-1.74	AAA-AAA	0.00	999	1.000	1.000	0.4186

238099_at	58.1	33.3	-0.80	-1.74	AAA-AAA	0.00	999	1.000	1.000	0.3919
236370_at	64.3	112.1	0.80	1.74	AAA-AAA	0.00	999	1.000	1.000	0.1946
236095_at	28.6	16.4	-0.80	-1.74	AAA-AAA	0.00	999	1.000	1.000	0.3385
229934_at	6.3	3.6	-0.80	-1.74	AAA-AAA	0.00	999	1.000	1.000	0.0566
230603_at	31.0	17.8	-0.80	-1.74	AAA-AAA	0.00	999	1.000	1.000	0.5340
206662_at	667.9	383.2	-0.80	-1.74	PPP-PPP	0.00	999	1.000	1.000	0.1831
230695_s_at	45.9	26.3	-0.80	-1.74	AAA-PAA	0.00	999	1.000	1.000	0.6266
239242_at	98.9	56.8	-0.80	-1.74	AAA-AAA	0.00	999	1.000	1.000	0.0656
1565763_at	22.8	39.8	0.80	1.74	AAA-AAA	0.00	999	1.000	1.000	0.4128
1558711_at	19.4	33.8	0.80	1.74	AAA-AAA	0.00	999	1.000	1.000	0.4865
1560906_at	27.9	16.0	-0.80	-1.74	AAA-AAA	0.00	999	1.000	1.000	0.1359
243667_at	57.8	33.2	-0.80	-1.74	AAA-AAA	0.00	999	1.000	1.000	0.3881
216063_at	39.0	67.9	0.80	1.74	AAA-AAM	0.00	999	1.000	1.000	0.1402
217320_at	6.0	3.5	-0.80	-1.74	AAA-AAA	0.00	999	1.000	1.000	0.5668
220022_at	4.9	8.4	0.80	1.74	AAA-AAA	0.00	999	1.000	1.000	0.5004
204150_at	61.2	35.1	-0.80	-1.74	AAA-AAA	0.00	999	1.000	1.000	0.2108
207852_at	16.8	9.6	-0.80	-1.74	AAA-AAA	0.00	999	1.000	1.000	0.6539
234175_at	26.3	45.9	0.80	1.74	AAA-AAA	0.00	999	1.000	1.000	0.2969
237910_x_at	35.9	20.6	-0.80	-1.74	MAA-AAA	0.00	999	1.000	1.000	0.5738
236538_at	5.8	3.3	-0.80	-1.74	AAA-AAA	0.00	999	1.000	1.000	0.7036
210279_at	24.7	14.2	-0.80	-1.74	AAA-AAP	0.00	999	1.000	1.000	0.5828
240103_at	4.1	7.2	0.80	1.74	AAA-AAA	0.00	999	1.000	1.000	0.5207
1566726_at	13.4	23.3	0.80	1.74	AAA-AAA	0.00	999	1.000	1.000	0.4843
240619_at	4.6	8.0	0.80	1.74	AAA-AAA	0.00	999	1.000	1.000	0.3836
1560286_s_at	25.6	44.6	0.80	1.74	AAA-AAA	0.00	999	1.000	1.000	0.2528
203863_at	4.9	8.5	0.80	1.74	AAA-AAA	0.00	999	1.000	1.000	0.5119
209830_s_at	7.0	12.3	0.80	1.74	AAA-AAA	0.00	999	1.000	1.000	0.3901
232863_at	45.2	78.8	0.80	1.74	AAA-AAA	0.00	999	1.000	1.000	0.3360
232364_at	29.8	17.1	-0.80	-1.74	AAA-AAA	0.00	999	1.000	1.000	0.4743
231910_at	18.0	10.4	-0.80	-1.74	AAA-AAA	0.00	999	1.000	1.000	0.4597
232006_at	18.7	10.8	-0.80	-1.74	AAA-AAA	0.00	999	1.000	1.000	0.5030
208144_s_at	99.7	57.3	-0.80	-1.74	PAP-PPA	0.00	999	1.000	1.000	0.1297
216661_x_at	101.8	58.5	-0.80	-1.74	PAA-AAA	0.00	999	1.000	1.000	0.1889
1570391_at	2.4	4.2	0.80	1.74	AAA-AAA	0.00	999	1.000	1.000	0.5664
1553070_a_at	36.1	62.8	0.80	1.74	AAA-AAA	0.00	999	1.000	1.000	0.3501
242622_x_at	38.2	21.9	-0.80	-1.74	AAA-AAA	0.00	999	1.000	1.000	0.4466
214610_at	19.1	33.1	0.80	1.74	AAA-AAA	0.00	999	1.000	1.000	0.3432
215042_at	13.6	23.6	0.80	1.74	AAA-AAA	0.00	999	1.000	1.000	0.5177
220579_at	5.8	10.1	0.80	1.74	AAA-AAA	0.00	999	1.000	1.000	0.5356
205638_at	9.6	5.5	-0.80	-1.74	AAA-AAA	0.00	999	1.000	1.000	0.7132
207554_x_at	62.0	107.8	0.80	1.74	AAA-AAA	0.00	999	1.000	1.000	0.1133
208889_s_at	97.5	56.0	-0.80	-1.74	AAA-AAA	0.00	999	1.000	1.000	0.1869

223313_s_at	4.0	7.0	0.80	1.74	AAA-AAA	0.00	999	1.000	1.000	0.1627
218704_at	186.9	107.5	-0.80	-1.74	PPP-APA	0.00	999	1.000	1.000	0.2484
1557063_at	17.8	10.2	-0.80	-1.74	PAA-AAA	0.00	999	1.000	1.000	0.5670
1564212_at	32.0	18.4	-0.80	-1.74	AAA-AAA	0.00	999	1.000	1.000	0.4129
1555640_at	5.2	9.0	0.80	1.74	AAA-AAA	0.00	999	1.000	1.000	0.5952
1561270_at	46.9	81.5	0.80	1.74	AAA-MAA	0.00	999	1.000	1.000	0.3138
242638_at	22.4	38.9	0.80	1.74	AAA-AAA	0.00	999	1.000	1.000	0.4976
243757_at	18.5	32.1	0.80	1.74	AAA-AAA	0.00	999	1.000	1.000	0.4829
218657_at	239.7	417.0	0.80	1.74	AAA-AAA	0.00	999	1.000	1.000	0.0885
202229_s_at	85.9	149.4	0.80	1.74	AAA-AAA	0.00	999	1.000	1.000	0.0946
207876_s_at	104.7	60.2	-0.80	-1.74	AMA-AAM	0.00	999	1.000	1.000	0.3794
209601_at	9.5	16.5	0.80	1.74	AAA-AAA	0.00	999	1.000	1.000	0.2827
234525_at	26.3	15.1	-0.80	-1.74	AAA-AAA	0.00	999	1.000	1.000	0.2799
234538_at	3.2	5.5	0.80	1.74	AAA-AAA	0.00	999	1.000	1.000	0.6973
234576_at	7.0	12.1	0.80	1.74	AAA-AAA	0.00	999	1.000	1.000	0.2040
234805_at	21.0	12.1	-0.80	-1.74	AAA-AAA	0.00	999	1.000	1.000	0.6065
238206_at	20.5	11.8	-0.80	-1.74	AAA-AAA	0.00	999	1.000	1.000	0.5481
236410_x_at	81.3	141.3	0.80	1.74	AAA-AAA	0.00	999	1.000	1.000	0.5905
228219_s_at	16.0	27.9	0.80	1.74	AAA-AAA	0.00	999	1.000	1.000	0.5756
224006_at	8.7	15.1	0.80	1.74	AAA-AAA	0.00	999	1.000	1.000	0.4935
220596_at	106.6	61.3	-0.80	-1.74	APP-AAP	0.00	999	1.000	1.000	0.1175
229677_at	145.5	83.7	-0.80	-1.74	PAP-PAP	0.00	999	1.000	1.000	0.4169
1570575_at	5.8	10.1	0.80	1.74	AAA-AAA	0.00	999	1.000	1.000	0.2600
242342_at	18.8	32.6	0.80	1.74	AAA-AAA	0.00	999	1.000	1.000	0.5098
1566169_at	5.0	2.9	-0.80	-1.74	AAA-AAA	0.00	999	1.000	1.000	0.3752
240709_at	4.3	7.4	0.80	1.74	AAA-AAA	0.00	999	1.000	1.000	0.5496
1555192_at	14.9	25.9	0.80	1.74	AAA-AAA	0.00	999	1.000	1.000	0.5066
213519_s_at	30.1	52.2	0.80	1.74	AAA-AAA	0.00	999	1.000	1.000	0.2467
214309_s_at	24.9	14.3	-0.80	-1.74	AAA-AAA	0.00	999	1.000	1.000	0.4872
221557_s_at	24.7	14.2	-0.80	-1.74	AAA-AAA	0.00	999	1.000	1.000	0.3664
221023_s_at	25.9	45.1	0.80	1.74	AAA-AAA	0.00	999	1.000	1.000	0.4456
217439_at	23.6	41.0	0.80	1.74	AAA-AAA	0.00	999	1.000	1.000	0.6464
203382_s_at	10.5	18.3	0.80	1.74	AAA-AAA	0.00	999	1.000	1.000	0.3163
204674_at	19.3	33.5	0.80	1.74	AAA-AAA	0.00	999	1.000	1.000	0.5828
233872_x_at	5.9	10.3	0.80	1.74	AAA-AAA	0.00	999	1.000	1.000	0.4706
236687_at	29.2	50.7	0.80	1.74	AAA-AAA	0.00	999	1.000	1.000	0.2417
1553298_at	17.6	10.1	-0.80	-1.74	APA-AAA	0.00	999	1.000	1.000	0.3033
231914_at	188.4	108.5	-0.80	-1.74	PAA-AAA	0.00	999	1.000	1.000	0.3768
208410_x_at	49.3	28.4	-0.80	-1.74	AAP-APA	0.00	999	1.000	1.000	0.3818
1566952_at	27.8	16.0	-0.80	-1.74	AAA-AAP	0.00	999	1.000	1.000	0.4773
210109_at	57.3	33.0	-0.80	-1.74	AAA-AAP	0.00	999	1.000	1.000	0.6070
1564220_a_at	9.1	15.8	0.80	1.74	AAA-AAA	0.00	999	1.000	1.000	0.5830

240674_at	6.7	11.7	0.80	1.74	AAA-AAA	0.00	999	1.000	1.000	0.5587
1556586_x_at	50.6	87.9	0.80	1.74	AAA-AAA	0.00	999	1.000	1.000	0.4312
1552948_at	15.5	26.9	0.80	1.74	AAA-AAA	0.00	999	1.000	1.000	0.4392
244438_at	78.9	45.4	-0.80	-1.74	AAA-AAA	0.00	999	1.000	1.000	0.0689
243711_at	4.8	8.3	0.80	1.74	AAA-AAA	0.00	999	1.000	1.000	0.4452
215132_at	21.2	36.8	0.80	1.74	AAA-AAA	0.00	999	1.000	1.000	0.4679
217536_x_at	4.9	8.4	0.80	1.74	AAA-AAA	0.00	999	1.000	1.000	0.5689
205489_at	23.7	41.2	0.80	1.74	AAA-AAA	0.00	999	1.000	1.000	0.5061
207778_at	32.7	18.8	-0.80	-1.74	AAA-AAA	0.00	999	1.000	1.000	0.1002
234691_at	4.7	8.2	0.80	1.74	AAA-AAA	0.00	999	1.000	1.000	0.4978
236017_at	17.5	30.4	0.80	1.74	AAA-AAA	0.00	999	1.000	1.000	0.5813
227933_at	23.4	40.6	0.80	1.74	AAA-AAA	0.00	999	1.000	1.000	0.5832
229953_x_at	50.0	28.8	-0.80	-1.74	PAP-AAM	0.00	999	1.000	1.000	0.0889
233131_at	34.4	19.8	-0.80	-1.74	AAP-AAA	0.00	999	1.000	1.000	0.5118
233839_at	16.9	9.7	-0.80	-1.74	APA-AAA	0.00	999	1.000	1.000	0.5804
224363_at	29.4	16.9	-0.80	-1.74	MAA-APA	0.00	999	1.000	1.000	0.5880
241557_x_at	7.6	4.4	-0.80	-1.74	AAA-AAA	0.00	999	1.000	1.000	0.6672
1564160_at	2.8	1.6	-0.80	-1.74	AAA-AAA	0.00	999	1.000	1.000	0.0809
1558816_at	43.7	75.9	0.80	1.74	AAA-AAA	0.00	999	1.000	1.000	0.1509
1553428_at	31.2	54.1	0.80	1.74	AAA-AAA	0.00	999	1.000	1.000	0.1057
1559097_at	65.9	114.4	0.80	1.74	AMA-AAA	0.00	999	1.000	1.000	0.0105
216181_at	13.9	24.1	0.80	1.74	AAA-AAA	0.00	999	1.000	1.000	0.0690
212562_s_at	40.2	69.7	0.80	1.74	AAA-AAA	0.00	999	1.000	1.000	0.4280
213335_s_at	9.7	5.6	-0.80	-1.74	AAA-AAA	0.00	999	1.000	1.000	0.4108
219907_at	132.2	76.2	-0.80	-1.74	AAA-AAA	0.00	999	1.000	1.000	0.0381
228828_at	46.5	26.8	-0.80	-1.74	AAA-AAA	0.00	999	1.000	1.000	0.1233
231325_at	8.8	15.3	0.80	1.74	AAA-AAA	0.00	999	1.000	1.000	0.4715
232239_at	68.7	39.6	-0.79	-1.73	PAA-APA	0.00	999	1.000	1.000	0.3478
1566551_at	12.4	21.4	0.79	1.73	AAA-PAA	0.00	999	1.000	1.000	0.7111
241521_at	20.0	34.7	0.79	1.73	AAA-AAA	0.00	999	1.000	1.000	0.3919
239431_at	81.6	47.0	-0.79	-1.73	AAA-AAA	0.00	999	1.000	1.000	0.3181
239389_at	89.3	51.5	-0.79	-1.73	AAA-AAA	0.00	999	1.000	1.000	0.2865
240447_at	28.7	16.6	-0.79	-1.73	AAA-AAA	0.00	999	1.000	1.000	0.5817
1553497_at	11.3	6.5	-0.79	-1.73	AAA-AAA	0.00	999	1.000	1.000	0.4373
1555127_at	8.2	14.2	0.79	1.73	AAA-AAM	0.00	999	1.000	1.000	0.5078
1561676_at	8.4	14.5	0.79	1.73	AAA-AAA	0.00	999	1.000	1.000	0.5545
214467_at	15.4	8.9	-0.79	-1.73	AAA-AAA	0.00	999	1.000	1.000	0.7246
217688_at	15.0	26.1	0.79	1.73	AAA-AAA	0.00	999	1.000	1.000	0.3947
202509_s_at	51.0	29.4	-0.79	-1.73	AAA-AAA	0.00	999	1.000	1.000	0.2566
233857_s_at	40.0	69.3	0.79	1.73	AAM-AAA	0.00	999	1.000	1.000	0.3848
233928_at	17.1	9.8	-0.79	-1.73	AAA-AAA	0.00	999	1.000	1.000	0.3646
236585_at	62.4	36.0	-0.79	-1.73	AAA-AAA	0.00	999	1.000	1.000	0.4264

235901_at	91.2	158.2	0.79	1.73	AAA-AAA	0.00	999	1.000	1.000	0.0796
235965_at	38.1	22.0	-0.79	-1.73	AAA-AAA	0.00	999	1.000	1.000	0.2656
232289_at	41.7	72.4	0.79	1.73	AAA-AAA	0.00	999	1.000	1.000	0.1131
204854_at	136.8	79.0	-0.79	-1.73	APA-AAA	0.00	999	1.000	1.000	0.3034
231763_at	308.6	178.1	-0.79	-1.73	PPP-MAP	0.00	999	1.000	1.000	0.4714
241238_at	31.7	18.3	-0.79	-1.73	AAA-AAA	0.00	999	1.000	1.000	0.4017
240308_at	3.6	6.2	0.79	1.73	AAA-AAA	0.00	999	1.000	1.000	0.6415
1566269_at	6.4	11.1	0.79	1.73	AAA-AAA	0.00	999	1.000	1.000	0.1451
239177_at	34.7	60.1	0.79	1.73	AAA-AAA	0.00	999	1.000	1.000	0.4325
1558248_at	9.3	16.1	0.79	1.73	AAA-AAA	0.00	999	1.000	1.000	0.3521
1552455_at	32.1	18.6	-0.79	-1.73	AAA-AAA	0.00	999	1.000	1.000	0.5238
1556067_a_at	32.4	18.7	-0.79	-1.73	AAA-AAA	0.00	999	1.000	1.000	0.6144
1555262_a_at	9.5	16.5	0.79	1.73	AAA-AAA	0.00	999	1.000	1.000	0.4169
1561497_at	48.0	27.7	-0.79	-1.73	AAA-AAA	0.00	999	1.000	1.000	0.3632
242914_at	45.1	26.0	-0.79	-1.73	AAA-AAA	0.00	999	1.000	1.000	0.6111
214420_s_at	8.0	13.8	0.79	1.73	AAA-AAA	0.00	999	1.000	1.000	0.2113
221337_s_at	24.0	13.9	-0.79	-1.73	AAA-AAA	0.00	999	1.000	1.000	0.3244
217090_at	26.8	15.5	-0.79	-1.73	AAA-AAA	0.00	999	1.000	1.000	0.5487
208193_at	5.3	9.2	0.79	1.73	AAA-AAA	0.00	999	1.000	1.000	0.4284
234803_at	11.8	6.8	-0.79	-1.73	AAA-AAA	0.00	999	1.000	1.000	0.5516
237727_at	67.2	38.7	-0.79	-1.73	AAA-AAA	0.00	999	1.000	1.000	0.2190
237399_at	9.0	15.5	0.79	1.73	AAA-AAA	0.00	999	1.000	1.000	0.5714
238326_at	15.4	8.9	-0.79	-1.73	AAA-AAA	0.00	999	1.000	1.000	0.2718
229869_at	74.4	128.8	0.79	1.73	AAA-AAA	0.00	999	1.000	1.000	0.0303
238476_at	109.9	63.5	-0.79	-1.73	PPA-APP	0.00	999	1.000	1.000	0.2154
219230_at	41.0	23.7	-0.79	-1.73	PAA-AAA	0.00	999	1.000	1.000	0.6514
1556382_a_at	50.0	28.9	-0.79	-1.73	AAA-AAA	0.00	999	1.000	1.000	0.3228
1554491_a_at	21.6	37.4	0.79	1.73	AAM-AAA	0.00	999	1.000	1.000	0.4805
216350_s_at	46.8	81.0	0.79	1.73	AAA-AAA	0.00	999	1.000	1.000	0.0441
206602_s_at	25.7	14.9	-0.79	-1.73	AAA-AAA	0.00	999	1.000	1.000	0.6313
209987_s_at	6.2	10.7	0.79	1.73	AAA-AAA	0.00	999	1.000	1.000	0.5571
211816_x_at	16.1	27.9	0.79	1.73	AAA-AAA	0.00	999	1.000	1.000	0.5499
234695_x_at	25.6	44.3	0.79	1.73	AAA-AAA	0.00	999	1.000	1.000	0.5270
234750_at	24.5	42.3	0.79	1.73	AAA-AAA	0.00	999	1.000	1.000	0.5015
236461_at	29.9	51.8	0.79	1.73	AAA-AAA	0.00	999	1.000	1.000	0.3962
225292_at	133.6	77.2	-0.79	-1.73	AAA-AAA	0.00	999	1.000	1.000	0.2665
229284_at	104.9	60.6	-0.79	-1.73	AAA-AAA	0.00	999	1.000	1.000	0.0879
238882_at	37.5	21.7	-0.79	-1.73	APA-AAA	0.00	999	1.000	1.000	0.6432
1563071_at	26.8	15.5	-0.79	-1.73	AAA-AAA	0.00	999	1.000	1.000	0.5171
240054_at	64.3	111.3	0.79	1.73	AAA-AAA	0.00	999	1.000	1.000	0.2496
240253_at	21.7	12.5	-0.79	-1.73	AAA-AAA	0.00	999	1.000	1.000	0.5286
240868_at	13.9	24.0	0.79	1.73	AAA-MAA	0.00	999	1.000	1.000	0.6631

1556884_a_at	21.0	12.2	-0.79	-1.73	AAA-AAA	0.00	999	1.000	1.000	0.4646
244757_at	16.3	28.2	0.79	1.73	AAA-AAA	0.00	999	1.000	1.000	0.3661
1552411_at	23.4	40.5	0.79	1.73	AAA-AAA	0.00	999	1.000	1.000	0.2964
215661_at	31.3	18.1	-0.79	-1.73	AAA-AAA	0.00	999	1.000	1.000	0.3918
215306_at	13.4	23.3	0.79	1.73	AAA-AAA	0.00	999	1.000	1.000	0.6748
215002_at	5.1	8.9	0.79	1.73	AAA-AAA	0.00	999	1.000	1.000	0.5766
218832_x_at	22.5	13.0	-0.79	-1.73	AAA-AAA	0.00	999	1.000	1.000	0.5382
206570_s_at	67.6	39.1	-0.79	-1.73	AAA-AAA	0.00	999	1.000	1.000	0.5051
206799_at	19.1	11.0	-0.79	-1.73	AAA-AAA	0.00	999	1.000	1.000	0.6213
205001_s_at	21.6	12.5	-0.79	-1.73	AAA-AAA	0.00	999	1.000	1.000	0.4177
207944_at	26.1	15.1	-0.79	-1.73	AAA-AAA	0.00	999	1.000	1.000	0.2847
234128_at	60.3	104.4	0.79	1.73	AAA-AAA	0.00	999	1.000	1.000	0.4988
236562_at	13.1	7.6	-0.79	-1.73	AAA-AAA	0.00	999	1.000	1.000	0.5551
1555564_a_at	63.7	36.9	-0.79	-1.73	AAA-PAA	0.00	999	1.000	1.000	0.0840
234440_at	68.4	39.5	-0.79	-1.73	PPP-AAP	0.00	999	1.000	1.000	0.1418
1563023_at	25.4	14.7	-0.79	-1.73	AAA-AAA	0.00	999	1.000	1.000	0.5358
1567105_at	23.8	13.8	-0.79	-1.73	AAA-AAA	0.00	999	1.000	1.000	0.1232
1554584_at	23.4	13.5	-0.79	-1.73	AAA-AAA	0.00	999	1.000	1.000	0.4143
1553799_at	3.4	5.9	0.79	1.73	AAA-AAA	0.00	999	1.000	1.000	0.2952
1555862_s_at	37.1	21.5	-0.79	-1.73	AAA-AAA	0.00	999	1.000	1.000	0.4961
214373_at	47.9	82.9	0.79	1.73	AAA-AAA	0.00	999	1.000	1.000	0.3908
220192_x_at	157.3	91.0	-0.79	-1.73	AAA-AAA	0.00	999	1.000	1.000	0.5106
206266_at	24.3	14.0	-0.79	-1.73	AAA-AAA	0.00	999	1.000	1.000	0.5934
205551_at	17.8	10.3	-0.79	-1.73	AAA-AAA	0.00	999	1.000	1.000	0.4017
204543_at	8.2	14.2	0.79	1.73	AAA-AAA	0.00	999	1.000	1.000	0.3801
234966_at	9.3	16.1	0.79	1.73	AAA-AAA	0.00	999	1.000	1.000	0.4950
238113_at	34.8	60.2	0.79	1.73	AAA-AAA	0.00	999	1.000	1.000	0.0583
222072_at	40.9	23.7	-0.79	-1.73	AAA-AAA	0.00	999	1.000	1.000	0.4084
224411_at	16.1	27.8	0.79	1.73	AAA-AAA	0.00	999	1.000	1.000	0.3760
231099_at	12.3	7.1	-0.79	-1.73	AAA-AAA	0.00	999	1.000	1.000	0.6233
220497_at	54.4	31.5	-0.79	-1.73	APA-PAA	0.00	999	1.000	1.000	0.4858
221626_at	74.5	43.1	-0.79	-1.73	AAP-PAP	0.00	999	1.000	1.000	0.5777
241502_x_at	2.8	4.8	0.79	1.73	AAA-AAA	0.00	999	1.000	1.000	0.5951
1570516_s_at	15.5	9.0	-0.79	-1.73	AAA-AAA	0.00	999	1.000	1.000	0.4867
1566672_at	31.4	54.3	0.79	1.73	AAA-AAA	0.00	999	1.000	1.000	0.1798
1556471_at	18.8	32.4	0.79	1.73	AAA-AAA	0.00	999	1.000	1.000	0.6810
1558537_x_at	9.0	15.5	0.79	1.73	AAA-AAA	0.00	999	1.000	1.000	0.3118
1553069_at	21.5	37.1	0.79	1.73	AAA-AAA	0.00	999	1.000	1.000	0.2221
243599_at	7.0	4.0	-0.79	-1.73	AAA-AAA	0.00	999	1.000	1.000	0.4016
244366_at	42.9	74.1	0.79	1.73	AAA-AAA	0.00	999	1.000	1.000	0.2686
1560181_at	39.5	22.9	-0.79	-1.73	AAA-AAA	0.00	999	1.000	1.000	0.5277
215738_at	61.4	35.5	-0.79	-1.73	AAA-AAA	0.00	999	1.000	1.000	0.1129

204197_s_at	43.1	74.4	0.79	1.73	AAA-AAA	0.00	999	1.000	1.000	0.2928
232783_at	22.8	39.4	0.79	1.73	AAA-AAA	0.00	999	1.000	1.000	0.3059
229534_at	29.6	17.1	-0.79	-1.73	AAA-AAA	0.00	999	1.000	1.000	0.3587
229152_at	20.4	35.2	0.79	1.73	AAA-AAA	0.00	999	1.000	1.000	0.2829
230804_at	3.8	6.6	0.79	1.73	AAA-AAA	0.00	999	1.000	1.000	0.6770
229883_at	96.7	56.0	-0.79	-1.73	PPM-AAA	0.00	999	1.000	1.000	0.1783
1554119_at	85.5	49.5	-0.79	-1.73	PPP-APP	0.00	999	1.000	1.000	0.3110
216179_x_at	91.4	52.9	-0.79	-1.73	PAP-PAA	0.00	999	1.000	1.000	0.5623
1570352_at	5.5	9.5	0.79	1.73	AAA-AAA	0.00	999	1.000	1.000	0.6541
239343_at	15.9	27.4	0.79	1.73	AAA-AAA	0.00	999	1.000	1.000	0.5913
1569874_s_at	7.0	12.1	0.79	1.73	AAA-AAA	0.00	999	1.000	1.000	0.6066
239777_at	9.1	5.3	-0.79	-1.73	AAA-AAA	0.00	999	1.000	1.000	0.5821
240949_x_at	14.0	8.1	-0.79	-1.73	AAA-AAA	0.00	999	1.000	1.000	0.6459
1568286_at	3.0	5.2	0.79	1.73	AAA-AAA	0.00	999	1.000	1.000	0.6210
1558821_s_at	107.2	62.1	-0.79	-1.73	AAA-AAA	0.00	999	1.000	1.000	0.3322
1555587_at	30.4	17.6	-0.79	-1.73	AAA-AAA	0.00	999	1.000	1.000	0.4242
1559914_at	25.8	44.6	0.79	1.73	AAA-AMA	0.00	999	1.000	1.000	0.2028
216333_x_at	21.4	36.9	0.79	1.73	AAA-AAA	0.00	999	1.000	1.000	0.1919
203601_s_at	51.8	89.4	0.79	1.73	AAA-AAA	0.00	999	1.000	1.000	0.4622
205564_at	62.0	35.9	-0.79	-1.73	AAA-AAA	0.00	999	1.000	1.000	0.4945
211083_s_at	3.8	6.6	0.79	1.73	AAA-AAA	0.00	999	1.000	1.000	0.0523
207742_s_at	10.6	18.3	0.79	1.73	AAA-AAA	0.00	999	1.000	1.000	0.2899
207653_at	39.1	22.6	-0.79	-1.73	AAA-AAA	0.00	999	1.000	1.000	0.3919
233763_at	68.9	119.0	0.79	1.73	AAA-AAA	0.00	999	1.000	1.000	0.1033
238388_x_at	19.9	11.5	-0.79	-1.73	AAA-AAA	0.00	999	1.000	1.000	0.1289
238491_at	35.1	60.6	0.79	1.73	AAA-AAA	0.00	999	1.000	1.000	0.1579
225333_at	154.1	89.2	-0.79	-1.73	AAA-AAA	0.00	999	1.000	1.000	0.4822
216062_at	50.1	29.0	-0.79	-1.73	PAA-AAA	0.00	999	1.000	1.000	0.5581
241505_at	17.9	10.4	-0.79	-1.73	AAA-AAA	0.00	999	1.000	1.000	0.4880
241265_x_at	29.0	50.0	0.79	1.73	AMA-AAA	0.00	999	1.000	1.000	0.6484
1563884_at	44.6	25.9	-0.79	-1.73	AAA-AAA	0.00	999	1.000	1.000	0.0981
1569264_at	91.5	53.0	-0.79	-1.73	AMA-AAA	0.00	999	1.000	1.000	0.0129
1568987_at	305.9	177.2	-0.79	-1.73	AAA-AAA	0.00	999	1.000	1.000	0.0525
1566301_at	9.0	15.4	0.79	1.73	AAA-AAA	0.00	999	1.000	1.000	0.5783
1556513_at	20.9	36.0	0.79	1.73	AAA-AAA	0.00	999	1.000	1.000	0.5374
1559820_at	46.3	80.0	0.79	1.73	AAA-AAA	0.00	999	1.000	1.000	0.0734
243891_at	27.6	16.0	-0.79	-1.73	AAA-AAA	0.00	999	1.000	1.000	0.3588
220836_at	8.0	13.8	0.79	1.73	AAA-AAA	0.00	999	1.000	1.000	0.6072
208357_x_at	18.9	32.6	0.79	1.73	AAA-AAA	0.00	999	1.000	1.000	0.0994
234336_s_at	21.0	12.2	-0.79	-1.73	AAA-AAA	0.00	999	1.000	1.000	0.4083
221990_at	18.5	10.7	-0.79	-1.73	AAA-AAA	0.00	999	1.000	1.000	0.2369
230970_at	58.1	100.3	0.79	1.73	AAA-AAA	0.00	999	1.000	1.000	0.2260

230905_at	44.2	25.6	-0.79	-1.73	AAA-AAA	0.00	999	1.000	1.000	0.3096
231759_at	25.9	15.0	-0.79	-1.73	AAA-AAA	0.00	999	1.000	1.000	0.4411
229154_at	6.5	11.2	0.79	1.73	AAA-AAA	0.00	999	1.000	1.000	0.5642
1565910_at	2.3	4.0	0.79	1.72	AAA-AAA	0.00	999	1.000	1.000	0.2372
1552999_a_at	16.1	9.3	-0.79	-1.72	AAA-AAA	0.00	999	1.000	1.000	0.6171
216058_s_at	65.2	37.8	-0.79	-1.73	AAA-AAA	0.00	999	1.000	1.000	0.4955
216134_at	51.4	29.8	-0.79	-1.72	AAA-AAA	0.00	999	1.000	1.000	0.3199
221611_s_at	63.8	37.0	-0.79	-1.73	AAA-AAA	0.00	999	1.000	1.000	0.5108
205939_at	33.4	57.7	0.79	1.72	AAA-AAA	0.00	999	1.000	1.000	0.0817
205989_s_at	4.4	7.5	0.79	1.72	AAA-AAA	0.00	999	1.000	1.000	0.6563
211218_at	30.1	17.5	-0.79	-1.72	AAA-AAA	0.00	999	1.000	1.000	0.5082
207909_x_at	4.1	2.4	-0.79	-1.72	AAA-AAA	0.00	999	1.000	1.000	0.4798
234575_at	40.1	23.3	-0.79	-1.73	AAA-AAA	0.00	999	1.000	1.000	0.3655
238415_at	10.3	6.0	-0.79	-1.72	AAA-AAA	0.00	999	1.000	1.000	0.2559
225015_s_at	65.3	112.7	0.79	1.73	AAA-AAA	0.00	999	1.000	1.000	0.5532
230596_at	21.2	12.3	-0.79	-1.73	AAA-AAA	0.00	999	1.000	1.000	0.6307
224099_at	73.5	42.6	-0.79	-1.72	PPP-PAP	0.00	999	1.000	1.000	0.1924
220968_s_at	255.3	148.1	-0.79	-1.72	PAA-AAA	0.00	999	1.000	1.000	0.2876
205943_at	38.8	22.5	-0.79	-1.72	AMA-PAA	0.00	999	1.000	1.000	0.3811
1562895_at	16.3	9.5	-0.79	-1.72	AAA-AAA	0.00	999	1.000	1.000	0.3195
241811_x_at	4.1	2.4	-0.79	-1.72	AAA-AAA	0.00	999	1.000	1.000	0.4704
240920_at	17.7	10.3	-0.79	-1.72	AAA-AAA	0.00	999	1.000	1.000	0.0018
1552555_at	35.8	61.7	0.79	1.72	AAA-AAA	0.00	999	1.000	1.000	0.6162
215885_at	10.5	6.1	-0.79	-1.72	AAA-AAA	0.00	999	1.000	1.000	0.5356
214324_at	22.5	13.0	-0.79	-1.72	AAA-AAA	0.00	999	1.000	1.000	0.5237
207166_at	5.1	8.7	0.79	1.72	AAA-AAA	0.00	999	1.000	1.000	0.4925
209812_x_at	30.5	17.7	-0.79	-1.72	AAA-AAA	0.00	999	1.000	1.000	0.6003
208282_x_at	10.1	5.9	-0.79	-1.72	AAA-AAA	0.00	999	1.000	1.000	0.5783
234524_at	10.2	17.5	0.79	1.72	AAA-AAA	0.00	999	1.000	1.000	0.6476
231114_at	32.1	18.6	-0.79	-1.72	AAA-AAA	0.00	999	1.000	1.000	0.1838
1557120_at	249.6	145.0	-0.78	-1.72	PPP-PPP	0.00	999	1.000	1.000	0.0321
1564933_at	8.9	15.4	0.78	1.72	AAA-AAA	0.00	999	1.000	1.000	0.5186
1569792_a_at	28.0	16.3	-0.78	-1.72	AAA-AAA	0.00	999	1.000	1.000	0.4760
1557438_at	20.2	11.8	-0.78	-1.72	AAA-AAA	0.00	999	1.000	1.000	0.5775
244370_at	10.4	6.0	-0.78	-1.72	AAA-AAA	0.00	999	1.000	1.000	0.6157
1559889_at	31.3	18.2	-0.78	-1.72	AAA-AAA	0.00	999	1.000	1.000	0.4263
1560295_at	20.9	36.0	0.78	1.72	AAA-AAA	0.00	999	1.000	1.000	0.4182
219718_at	119.2	69.2	-0.78	-1.72	AAA-AAA	0.00	999	1.000	1.000	0.4498
208318_s_at	12.4	21.4	0.78	1.72	AAA-AAA	0.00	999	1.000	1.000	0.3904
237367_x_at	63.5	109.4	0.78	1.72	AAA-AAA	0.00	999	1.000	1.000	0.6290
224049_at	75.9	130.7	0.78	1.72	AAA-AAA	0.00	999	1.000	1.000	0.0916
231250_at	14.2	8.2	-0.78	-1.72	AAA-AAA	0.00	999	1.000	1.000	0.3833

226614_s_at	1602.5	931.5	-0.78	-1.72	PPP-PPP	0.00	999	1.000	1.000	0.0341
241530_at	6.5	3.8	-0.78	-1.72	AAA-AAA	0.00	999	1.000	1.000	0.4825
241165_at	29.5	17.2	-0.78	-1.72	AAA-AAA	0.00	999	1.000	1.000	0.2794
241368_at	86.6	149.0	0.78	1.72	AAA-AAM	0.00	999	1.000	1.000	0.1995
241957_x_at	72.0	41.8	-0.78	-1.72	AAA-AAA	0.00	999	1.000	1.000	0.5005
1556427_s_at	53.7	31.2	-0.78	-1.72	AAA-AAA	0.00	999	1.000	1.000	0.5335
1562273_at	43.3	25.2	-0.78	-1.72	AAA-AAA	0.00	999	1.000	1.000	0.5391
213840_s_at	100.4	58.4	-0.78	-1.72	AAA-AAA	0.00	999	1.000	1.000	0.4494
215131_at	103.3	177.6	0.78	1.72	AAA-AMA	0.00	999	1.000	1.000	0.0388
214461_at	6.1	3.6	-0.78	-1.72	AAA-AAA	0.00	999	1.000	1.000	0.1711
220819_at	45.4	26.4	-0.78	-1.72	AAA-AAA	0.00	999	1.000	1.000	0.4702
217021_at	20.1	34.6	0.78	1.72	AAA-AAA	0.00	999	1.000	1.000	0.3874
205013_s_at	61.4	105.6	0.78	1.72	AAA-AAA	0.00	999	1.000	1.000	0.2396
211369_at	13.2	7.7	-0.78	-1.72	AAA-AAA	0.00	999	1.000	1.000	0.5055
208584_at	6.4	11.1	0.78	1.72	AAA-AAA	0.00	999	1.000	1.000	0.3417
234329_at	11.4	6.6	-0.78	-1.72	AAA-AAA	0.00	999	1.000	1.000	0.3388
234905_at	36.6	63.0	0.78	1.72	AAA-AAA	0.00	999	1.000	1.000	0.4394
233281_at	12.3	7.1	-0.78	-1.72	AAA-AAA	0.00	999	1.000	1.000	0.3578
233993_at	16.7	28.7	0.78	1.72	AAA-AAA	0.00	999	1.000	1.000	0.2074
233611_at	9.9	5.8	-0.78	-1.72	AAA-AAA	0.00	999	1.000	1.000	0.6181
223627_at	19.8	34.1	0.78	1.72	AAA-AAA	0.00	999	1.000	1.000	0.6081
236281_x_at	210.7	122.5	-0.78	-1.72	PPP-PPP	0.00	999	1.000	1.000	0.0611
207575_at	41.5	24.1	-0.78	-1.72	APA-APA	0.00	999	1.000	1.000	0.5776
240417_at	21.6	37.2	0.78	1.72	AAA-AAA	0.00	999	1.000	1.000	0.6448
1566737_at	12.9	7.5	-0.78	-1.72	AAA-AAA	0.00	999	1.000	1.000	0.6396
240755_at	22.9	39.4	0.78	1.72	AAA-AAA	0.00	999	1.000	1.000	0.4998
1557835_at	4.2	7.2	0.78	1.72	AAA-AAA	0.00	999	1.000	1.000	0.2594
1561440_at	39.4	67.8	0.78	1.72	AAA-AAA	0.00	999	1.000	1.000	0.3803
1561436_at	66.0	38.4	-0.78	-1.72	AAA-AAA	0.00	999	1.000	1.000	0.4169
1562384_at	21.2	36.5	0.78	1.72	AAA-AAA	0.00	999	1.000	1.000	0.3490
244244_at	32.5	18.9	-0.78	-1.72	AAA-AAA	0.00	999	1.000	1.000	0.6102
214062_x_at	216.5	125.9	-0.78	-1.72	AAA-AAA	0.00	999	1.000	1.000	0.4188
220196_at	10.0	17.2	0.78	1.72	AAA-AAA	0.00	999	1.000	1.000	0.4842
220245_at	101.6	59.1	-0.78	-1.72	AAA-AAA	0.00	999	1.000	1.000	0.5681
217061_s_at	48.2	82.8	0.78	1.72	AAA-AAA	0.00	999	1.000	1.000	0.4959
204419_x_at	34.9	20.3	-0.78	-1.72	AAA-AAA	0.00	999	1.000	1.000	0.5753
207475_at	52.6	30.6	-0.78	-1.72	AAA-AAA	0.00	999	1.000	1.000	0.5672
234053_at	20.3	11.8	-0.78	-1.72	AAA-AAA	0.00	999	1.000	1.000	0.3284
235267_at	13.1	22.5	0.78	1.72	AAA-AAA	0.00	999	1.000	1.000	0.6553
235098_at	34.4	59.1	0.78	1.72	AAA-AAA	0.00	999	1.000	1.000	0.1489
233086_at	8.3	14.2	0.78	1.72	AAA-AAA	0.00	999	1.000	1.000	0.5405
237743_at	27.0	15.7	-0.78	-1.72	MAA-AAA	0.00	999	1.000	1.000	0.6154

238033_at	11.6	20.0	0.78	1.72	AAA-AAA	0.00	999	1.000	1.000	0.3531
227553_at	29.4	50.5	0.78	1.72	AAA-AAM	0.00	999	1.000	1.000	0.4858
229497_at	11.7	6.8	-0.78	-1.72	AAA-AAA	0.00	999	1.000	1.000	0.4175
1563069_at	5.6	9.6	0.78	1.72	AAA-AAA	0.00	999	1.000	1.000	0.2978
240425_x_at	4.8	8.2	0.78	1.72	AAA-AAA	0.00	999	1.000	1.000	0.1133
240782_at	9.7	16.7	0.78	1.72	AAA-AAA	0.00	999	1.000	1.000	0.4580
1552755_at	6.7	3.9	-0.78	-1.72	AAA-AAA	0.00	999	1.000	1.000	0.2219
1552804_a_at	104.8	180.1	0.78	1.72	AAA-AAA	0.00	999	1.000	1.000	0.2075
1557656_at	30.7	52.8	0.78	1.72	AAA-AAA	0.00	999	1.000	1.000	0.6111
1555488_at	58.4	34.0	-0.78	-1.72	AAA-AAA	0.00	999	1.000	1.000	0.2491
216605_s_at	12.3	7.2	-0.78	-1.72	AAA-AAA	0.00	999	1.000	1.000	0.6382
205337_at	17.3	10.1	-0.78	-1.72	AAA-AAA	0.00	999	1.000	1.000	0.5436
204891_s_at	13.6	23.4	0.78	1.72	AAA-AAA	0.00	999	1.000	1.000	0.5651
210431_at	68.2	39.7	-0.78	-1.72	AAA-AAA	0.00	999	1.000	1.000	0.5812
207493_x_at	19.6	33.7	0.78	1.72	AAA-AAA	0.00	999	1.000	1.000	0.2906
208225_at	12.2	21.0	0.78	1.72	AAA-AAA	0.00	999	1.000	1.000	0.6026
233870_at	35.1	60.2	0.78	1.72	AAA-AAA	0.00	999	1.000	1.000	0.6022
226475_at	192.4	112.0	-0.78	-1.72	AAA-AAA	0.00	999	1.000	1.000	0.3290
224157_at	20.8	35.7	0.78	1.72	AAA-AAA	0.00	999	1.000	1.000	0.4560
240131_at	78.9	45.9	-0.78	-1.72	MPP-PAA	0.00	999	1.000	1.000	0.3205
1563671_at	32.4	18.8	-0.78	-1.72	AAA-AAA	0.00	999	1.000	1.000	0.6350
240858_at	7.6	4.4	-0.78	-1.72	AAA-AAA	0.00	999	1.000	1.000	0.2107
1554752_a_at	18.2	10.6	-0.78	-1.72	AAA-AAA	0.00	999	1.000	1.000	0.2619
243938_x_at	25.2	14.7	-0.78	-1.72	AAA-AAA	0.00	999	1.000	1.000	0.6322
214038_at	40.3	23.5	-0.78	-1.72	AAA-AAA	0.00	999	1.000	1.000	0.5195
220745_at	23.3	40.0	0.78	1.72	AAA-AAA	0.00	999	1.000	1.000	0.4090
218591_s_at	57.2	98.1	0.78	1.72	AAA-AAA	0.00	999	1.000	1.000	0.2249
211093_at	1.8	3.1	0.78	1.72	AAA-AAA	0.00	999	1.000	1.000	0.6064
207674_at	27.3	15.9	-0.78	-1.72	AAA-AAA	0.00	999	1.000	1.000	0.3341
240587_x_at	75.5	44.0	-0.78	-1.72	PAA-AAA	0.00	999	1.000	1.000	0.3481
208034_s_at	62.7	36.5	-0.78	-1.72	AAA-AAP	0.00	999	1.000	1.000	0.3815
230261_at	24.9	14.5	-0.78	-1.72	PAA-AAA	0.00	999	1.000	1.000	0.4529
238356_at	50.8	29.6	-0.78	-1.72	PPA-AAA	0.00	999	1.000	1.000	0.4685
1561167_at	30.3	17.7	-0.78	-1.72	MAA-AAP	0.00	999	1.000	1.000	0.6676
1568752_s_at	3.6	2.1	-0.78	-1.72	AAA-AAA	0.00	999	1.000	1.000	0.5103
1568663_a_at	26.7	45.8	0.78	1.72	AAA-AAA	0.00	999	1.000	1.000	0.3552
1568190_at	4.3	7.3	0.78	1.72	AAA-AAA	0.00	999	1.000	1.000	0.7015
1552979_at	20.6	12.0	-0.78	-1.72	AAA-AAA	0.00	999	1.000	1.000	0.5945
1558128_at	47.1	27.5	-0.78	-1.72	AAA-AAA	0.00	999	1.000	1.000	0.4036
244479_at	36.0	61.7	0.78	1.72	AAA-AAA	0.00	999	1.000	1.000	0.2836
205385_at	33.2	19.3	-0.78	-1.72	AAA-AAA	0.00	999	1.000	1.000	0.3682
207833_s_at	87.3	149.8	0.78	1.72	AAA-AAA	0.00	999	1.000	1.000	0.3627

237351_at	42.5	24.8	-0.78	-1.72	AAA-AAA	0.00	999	1.000	1.000	0.4075
228808_s_at	13.4	22.9	0.78	1.72	AAA-AAA	0.00	999	1.000	1.000	0.4693
223836_at	35.0	20.4	-0.78	-1.72	AAA-AAA	0.00	999	1.000	1.000	0.2313
231788_at	65.4	38.2	-0.78	-1.72	AAA-AAA	0.00	999	1.000	1.000	0.4106
1565580_s_at	49.7	29.0	-0.78	-1.72	AAA-AAA	0.00	999	1.000	1.000	0.4924
1562943_at	23.8	40.7	0.78	1.72	AAA-AAA	0.00	999	1.000	1.000	0.4870
1563854_s_at	66.6	38.8	-0.78	-1.72	AAA-AAA	0.00	999	1.000	1.000	0.0155
240461_at	26.0	15.2	-0.78	-1.72	AAA-AAA	0.00	999	1.000	1.000	0.5014
1567241_at	52.0	30.4	-0.78	-1.72	AAA-AAA	0.00	999	1.000	1.000	0.4602
1557208_at	15.3	26.2	0.78	1.72	AMA-AMA	0.00	999	1.000	1.000	0.6703
1558477_at	5.9	10.2	0.78	1.72	AAA-AAA	0.00	999	1.000	1.000	0.6605
243665_s_at	4.2	7.2	0.78	1.71	AAA-AAA	0.00	999	1.000	1.000	0.6749
1559582_at	50.7	86.9	0.78	1.72	AAA-AAA	0.00	999	1.000	1.000	0.2980
220680_at	14.0	24.0	0.78	1.72	AAA-AAA	0.00	999	1.000	1.000	0.4701
210847_x_at	97.2	166.7	0.78	1.72	AAA-AAA	0.00	999	1.000	1.000	0.4488
207578_s_at	90.4	155.0	0.78	1.72	AAA-AAA	0.00	999	1.000	1.000	0.6295
232492_at	24.8	14.5	-0.78	-1.71	AAA-AAA	0.00	999	1.000	1.000	0.5569
233737_s_at	37.9	64.9	0.78	1.71	AAA-MAA	0.00	999	1.000	1.000	0.5148
229636_at	20.2	11.8	-0.78	-1.72	AAA-AAA	0.00	999	1.000	1.000	0.3965
242421_at	87.5	51.1	-0.78	-1.71	APP-AAP	0.00	999	1.000	1.000	0.1586
211431_s_at	136.8	79.9	-0.78	-1.71	APA-AAA	0.00	999	1.000	1.000	0.2306
234156_at	148.6	86.7	-0.78	-1.71	PPP-PAP	0.00	999	1.000	1.000	0.3231
244859_at	32.3	18.9	-0.78	-1.71	AAA-AAP	0.00	999	1.000	1.000	0.6279
239932_at	10.9	6.4	-0.78	-1.71	AAA-AAA	0.00	999	1.000	1.000	0.3975
1554308_s_at	6.7	11.4	0.78	1.71	AAA-AAA	0.00	999	1.000	1.000	0.4835
1561341_at	11.0	6.4	-0.78	-1.71	AAA-AAA	0.00	999	1.000	1.000	0.3246
215923_s_at	52.6	30.7	-0.78	-1.71	AAA-AAA	0.00	999	1.000	1.000	0.5549
216802_at	19.2	11.2	-0.78	-1.71	AAA-AAA	0.00	999	1.000	1.000	0.4967
213925_at	30.4	52.2	0.78	1.71	AAA-AAA	0.00	999	1.000	1.000	0.2942
221631_at	109.1	63.7	-0.78	-1.71	AAA-AAA	0.00	999	1.000	1.000	0.5381
219847_at	37.5	64.3	0.78	1.71	AAA-AAA	0.00	999	1.000	1.000	0.4998
206986_at	13.5	7.9	-0.78	-1.71	AAA-AAA	0.00	999	1.000	1.000	0.4191
207907_at	19.6	11.4	-0.78	-1.71	AAA-AAA	0.00	999	1.000	1.000	0.5466
232707_at	34.7	59.4	0.78	1.71	AAA-AAA	0.00	999	1.000	1.000	0.0957
232491_at	26.7	45.8	0.78	1.71	AAA-AAA	0.00	999	1.000	1.000	0.3618
233681_at	9.5	16.2	0.78	1.71	AAA-AAA	0.00	999	1.000	1.000	0.6162
233915_at	6.5	11.2	0.78	1.71	AAA-AAA	0.00	999	1.000	1.000	0.5351
224979_s_at	81.6	139.9	0.78	1.71	AAA-AAA	0.00	999	1.000	1.000	0.1829
223579_s_at	10.1	5.9	-0.78	-1.71	AAA-AAA	0.00	999	1.000	1.000	0.5538
229542_at	8.0	13.7	0.78	1.71	AAA-AAA	0.00	999	1.000	1.000	0.4140
229738_at	21.4	36.6	0.78	1.71	AAA-AAA	0.00	999	1.000	1.000	0.4017
229320_at	9.8	16.8	0.78	1.71	AAA-AAA	0.00	999	1.000	1.000	0.1544

235402_at	23.2	39.6	0.78	1.71	AAP-APA	0.00	999	1.000	1.000	0.6702
1563226_at	8.4	4.9	-0.78	-1.71	AAA-AAA	0.00	999	1.000	1.000	0.4017
1563523_at	38.1	22.3	-0.78	-1.71	AAA-AAA	0.00	999	1.000	1.000	0.4334
1569712_at	8.4	14.4	0.78	1.71	AAA-AAA	0.00	999	1.000	1.000	0.4893
1570171_at	46.9	27.4	-0.78	-1.71	AAA-AAA	0.00	999	1.000	1.000	0.5670
1552787_at	57.5	33.6	-0.78	-1.71	AAA-AAA	0.00	999	1.000	1.000	0.0785
243596_at	101.0	59.0	-0.78	-1.71	AAA-AAA	0.00	999	1.000	1.000	0.2016
243334_at	4.9	8.3	0.78	1.71	AAA-AAA	0.00	999	1.000	1.000	0.4683
244210_at	11.0	18.8	0.78	1.71	AAA-AAA	0.00	999	1.000	1.000	0.5665
244075_at	44.1	75.5	0.78	1.71	MAA-AAA	0.00	999	1.000	1.000	0.2240
220023_at	16.0	27.5	0.78	1.71	AAA-AAA	0.00	999	1.000	1.000	0.2864
206361_at	94.3	55.1	-0.78	-1.71	AAA-AAA	0.00	999	1.000	1.000	0.4625
210303_at	13.2	7.7	-0.78	-1.71	AAA-AAA	0.00	999	1.000	1.000	0.5758
208448_x_at	34.1	19.9	-0.78	-1.71	AAA-AAA	0.00	999	1.000	1.000	0.2340
234098_at	11.0	6.4	-0.78	-1.71	AAA-AAA	0.00	999	1.000	1.000	0.2173
232920_at	37.5	21.9	-0.78	-1.71	AAA-AAA	0.00	999	1.000	1.000	0.5341
233697_at	6.0	10.3	0.78	1.71	AAA-AAA	0.00	999	1.000	1.000	0.2502
224305_s_at	22.2	13.0	-0.78	-1.71	AAA-AAA	0.00	999	1.000	1.000	0.5253
204774_at	330.5	193.2	-0.78	-1.71	PPP-PPP	0.00	999	1.000	1.000	0.0638
202833_s_at	198.9	116.2	-0.78	-1.71	PAP-AAP	0.00	999	1.000	1.000	0.0652
240160_x_at	1.8	3.1	0.78	1.71	AAA-AAA	0.00	999	1.000	1.000	0.3000
240427_at	18.8	11.0	-0.78	-1.71	AAA-AAA	0.00	999	1.000	1.000	0.5849
239444_at	55.1	32.2	-0.78	-1.71	AAA-AAA	0.00	999	1.000	1.000	0.0616
1570262_at	11.2	19.1	0.78	1.71	AAA-AAA	0.00	999	1.000	1.000	0.6512
1558782_a_at	58.6	34.3	-0.78	-1.71	AAA-AAM	0.00	999	1.000	1.000	0.5330
1557857_a_at	34.0	58.1	0.78	1.71	AAA-AAA	0.00	999	1.000	1.000	0.2216
1553529_at	16.0	27.5	0.78	1.71	AAA-AAA	0.00	999	1.000	1.000	0.6122
1561187_at	8.0	13.6	0.78	1.71	AAA-AAA	0.00	999	1.000	1.000	0.4967
1562026_at	207.3	121.1	-0.78	-1.71	AAA-AAA	0.00	999	1.000	1.000	0.2539
244039_x_at	7.0	4.1	-0.78	-1.71	AAA-AAA	0.00	999	1.000	1.000	0.2500
206163_at	11.3	19.3	0.78	1.71	AAA-AAA	0.00	999	1.000	1.000	0.5654
211437_at	10.9	18.7	0.78	1.71	AAA-AAA	0.00	999	1.000	1.000	0.5900
208142_at	20.9	12.2	-0.78	-1.71	AAA-AAA	0.00	999	1.000	1.000	0.2727
241361_at	130.1	76.1	-0.77	-1.71	AAA-AAA	0.00	999	1.000	1.000	0.0539
1563677_at	35.1	20.5	-0.77	-1.71	AAA-AAA	0.00	999	1.000	1.000	0.1543
1552304_at	21.8	12.8	-0.77	-1.71	AAA-AAA	0.00	999	1.000	1.000	0.6766
1557796_at	4.9	8.3	0.77	1.71	AAA-AAA	0.00	999	1.000	1.000	0.1596
1555250_a_at	26.6	45.5	0.77	1.71	AAA-AAA	0.00	999	1.000	1.000	0.2070
1559159_at	56.7	33.2	-0.77	-1.71	AAA-AAA	0.00	999	1.000	1.000	0.5070
216619_at	3.6	6.2	0.77	1.71	AAA-AAA	0.00	999	1.000	1.000	0.5920
217091_at	5.7	3.3	-0.77	-1.71	AAA-AAA	0.00	999	1.000	1.000	0.5337
205859_at	159.6	93.4	-0.77	-1.71	AAA-AAA	0.00	999	1.000	1.000	0.0144

204220_at	5.2	9.0	0.77	1.71	AAA-AAA	0.00	999	1.000	1.000	0.2841
207617_at	27.2	46.4	0.77	1.71	AAA-AAA	0.00	999	1.000	1.000	0.3906
227742_at	16.2	9.4	-0.77	-1.71	AAA-AAA	0.00	999	1.000	1.000	0.6216
1556982_at	135.1	79.0	-0.77	-1.71	PAA-AAA	0.00	999	1.000	1.000	0.3511
243028_x_at	10.3	6.0	-0.77	-1.71	AAP-AAA	0.00	999	1.000	1.000	0.5975
241144_at	19.4	11.4	-0.77	-1.71	AAA-AAA	0.00	999	1.000	1.000	0.3217
1552763_at	8.8	15.0	0.77	1.71	AAA-AAA	0.00	999	1.000	1.000	0.4226
1556190_s_at	41.4	24.2	-0.77	-1.71	AAA-AAA	0.00	999	1.000	1.000	0.4673
1556154_a_at	54.8	93.6	0.77	1.71	AAA-AAA	0.00	999	1.000	1.000	0.2145
1555557_a_at	5.9	10.1	0.77	1.71	AAA-AAA	0.00	999	1.000	1.000	0.5161
243621_at	8.0	13.7	0.77	1.71	AAA-AAA	0.00	999	1.000	1.000	0.4275
215194_at	27.8	16.3	-0.77	-1.71	AAA-AAA	0.00	999	1.000	1.000	0.5898
214380_at	11.6	6.8	-0.77	-1.71	AAA-AAA	0.00	999	1.000	1.000	0.0509
233510_s_at	27.5	16.1	-0.77	-1.71	AAA-AAA	0.00	999	1.000	1.000	0.2086
231335_at	26.1	44.5	0.77	1.71	AAA-AAA	0.00	999	1.000	1.000	0.4474
231181_at	16.0	9.4	-0.77	-1.71	AAA-AAA	0.00	999	1.000	1.000	0.4709
235959_at	124.0	72.6	-0.77	-1.71	PPP-AMP	0.00	999	1.000	1.000	0.3614
240367_at	71.3	121.7	0.77	1.71	AAA-AAA	0.00	999	1.000	1.000	0.1193
1569688_at	27.4	16.1	-0.77	-1.71	AAA-AAA	0.00	999	1.000	1.000	0.7081
1570140_at	22.8	13.3	-0.77	-1.71	AAA-AAA	0.00	999	1.000	1.000	0.5078
1552997_a_at	43.0	73.4	0.77	1.71	AAA-AAA	0.00	999	1.000	1.000	0.3489
1559151_at	36.4	21.3	-0.77	-1.71	AAM-AAA	0.00	999	1.000	1.000	0.4466
206791_s_at	150.8	88.3	-0.77	-1.71	AAA-AAA	0.00	999	1.000	1.000	0.0976
208509_s_at	48.9	28.6	-0.77	-1.71	AAA-AAA	0.00	999	1.000	1.000	0.5630
233854_x_at	30.0	17.6	-0.77	-1.71	AAA-AAA	0.00	999	1.000	1.000	0.3309
238886_at	24.3	41.4	0.77	1.71	AAA-AAA	0.00	999	1.000	1.000	0.5580
237168_at	12.5	7.3	-0.77	-1.71	AAA-AAA	0.00	999	1.000	1.000	0.6948
228463_at	13.3	22.7	0.77	1.71	AAA-AAA	0.00	999	1.000	1.000	0.5556
222773_s_at	52.0	30.5	-0.77	-1.71	AAA-AAA	0.00	999	1.000	1.000	0.4550
223830_s_at	12.7	7.5	-0.77	-1.71	AAA-AAA	0.00	999	1.000	1.000	0.3658
223883_s_at	9.1	5.3	-0.77	-1.71	AAA-AAA	0.00	999	1.000	1.000	0.2161
232408_at	19.7	33.7	0.77	1.71	AAA-AAA	0.00	999	1.000	1.000	0.6028
242636_at	66.9	39.2	-0.77	-1.71	PPP-PAA	0.00	999	1.000	1.000	0.2449
239239_at	31.3	53.4	0.77	1.71	AAA-AAA	0.00	999	1.000	1.000	0.5530
1552532_a_at	23.2	13.6	-0.77	-1.71	AAA-AAA	0.00	999	1.000	1.000	0.5016
1553582_a_at	26.8	45.8	0.77	1.71	AAA-AAA	0.00	999	1.000	1.000	0.3354
1560905_at	32.3	19.0	-0.77	-1.71	AAA-AAA	0.00	999	1.000	1.000	0.4762
243475_at	57.1	97.5	0.77	1.71	AAA-AAA	0.00	999	1.000	1.000	0.5198
1558919_a_at	55.7	32.6	-0.77	-1.71	AAA-AAA	0.00	999	1.000	1.000	0.4692
215379_x_at	40.3	23.6	-0.77	-1.71	AAA-AAA	0.00	999	1.000	1.000	0.5030
215396_at	7.8	4.6	-0.77	-1.71	AAA-AAA	0.00	999	1.000	1.000	0.5187
222898_s_at	12.4	21.1	0.77	1.71	AAA-AAA	0.00	999	1.000	1.000	0.2386

224056_at	12.2	7.1	-0.77	-1.71	AAA-AAA	0.00	999	1.000	1.000	0.5438
239745_at	4.6	7.8	0.77	1.71	AAA-AAA	0.00	999	1.000	1.000	0.4816
1566824_at	31.5	18.5	-0.77	-1.71	AAA-AAA	0.00	999	1.000	1.000	0.3563
1552974_at	4.2	7.1	0.77	1.71	AAA-AAA	0.00	999	1.000	1.000	0.5660
1554554_at	9.9	16.9	0.77	1.71	AAA-AAA	0.00	999	1.000	1.000	0.0516
1554787_at	3.7	6.3	0.77	1.71	AAA-AAA	0.00	999	1.000	1.000	0.1713
1556309_s_at	27.5	46.9	0.77	1.71	AAA-AAA	0.00	999	1.000	1.000	0.4681
1561234_at	1.7	2.9	0.77	1.71	AAA-AAA	0.00	999	1.000	1.000	0.2223
1562166_at	24.7	42.2	0.77	1.71	AAA-AAA	0.00	999	1.000	1.000	0.5478
1559631_at	14.9	25.4	0.77	1.71	AAA-AAA	0.00	999	1.000	1.000	0.5495
214415_at	30.0	17.6	-0.77	-1.71	AAA-AAA	0.00	999	1.000	1.000	0.3306
206732_at	12.9	7.6	-0.77	-1.71	AAA-AAA	0.00	999	1.000	1.000	0.1462
204406_at	13.7	23.3	0.77	1.71	AAA-AAA	0.00	999	1.000	1.000	0.4887
234516_at	45.5	26.7	-0.77	-1.71	AAA-AAA	0.00	999	1.000	1.000	0.4018
238808_at	12.3	21.0	0.77	1.71	AAA-AAA	0.00	999	1.000	1.000	0.4476
237027_at	29.6	50.5	0.77	1.71	AAA-AAA	0.00	999	1.000	1.000	0.4636
230521_at	29.8	50.9	0.77	1.71	AAA-AAA	0.00	999	1.000	1.000	0.2850
1562259_at	67.2	39.4	-0.77	-1.70	APA-MAA	0.00	999	1.000	1.000	0.0235
1562111_at	30.6	17.9	-0.77	-1.71	AAA-APA	0.00	999	1.000	1.000	0.4038
1558606_s_at	35.8	21.0	-0.77	-1.70	AAP-AAA	0.00	999	1.000	1.000	0.5567
1558179_at	23.1	13.5	-0.77	-1.70	AAA-AAA	0.00	999	1.000	1.000	0.3769
1553793_a_at	69.2	40.6	-0.77	-1.70	AAM-AAA	0.00	999	1.000	1.000	0.2081
1556181_at	88.3	51.8	-0.77	-1.70	AAA-AAA	0.00	999	1.000	1.000	0.5262
216798_at	26.9	45.9	0.77	1.70	AAA-AAA	0.00	999	1.000	1.000	0.4973
216149_at	30.7	52.3	0.77	1.70	AAA-AAA	0.00	999	1.000	1.000	0.4041
216367_at	5.5	9.4	0.77	1.70	AAA-AAA	0.00	999	1.000	1.000	0.1072
219722_s_at	66.4	39.0	-0.77	-1.70	AAA-AAA	0.00	999	1.000	1.000	0.4440
206660_at	21.7	37.0	0.77	1.70	AAA-AAA	0.00	999	1.000	1.000	0.5419
204782_at	16.8	28.6	0.77	1.70	AAA-AAA	0.00	999	1.000	1.000	0.5854
233404_at	13.0	7.7	-0.77	-1.70	AAA-AAA	0.00	999	1.000	1.000	0.6231
233580_at	31.3	53.3	0.77	1.70	AAA-AAA	0.00	999	1.000	1.000	0.4646
236599_at	85.6	50.2	-0.77	-1.71	AAA-AAA	0.00	999	1.000	1.000	0.2990
237329_at	24.3	41.4	0.77	1.70	AMA-AAA	0.00	999	1.000	1.000	0.1193
228475_at	34.9	59.5	0.77	1.70	AAA-AAA	0.00	999	1.000	1.000	0.3188
37986_at	109.3	64.2	-0.77	-1.70	AAA-AAA	0.00	999	1.000	1.000	0.0544
230856_at	26.0	44.4	0.77	1.70	AAA-AAA	0.00	999	1.000	1.000	0.4535
232299_at	13.3	22.6	0.77	1.70	AAA-AAA	0.00	999	1.000	1.000	0.3933
231573_at	83.8	49.2	-0.77	-1.70	AAA-AAA	0.00	999	1.000	1.000	0.3750
220453_at	75.9	44.6	-0.77	-1.70	APA-AAM	0.00	999	1.000	1.000	0.0519
220701_at	74.5	43.7	-0.77	-1.70	AAA-AAP	0.00	999	1.000	1.000	0.1204
242385_at	13.9	8.1	-0.77	-1.70	AAA-AAA	0.00	999	1.000	1.000	0.6377
1569095_at	112.3	66.0	-0.77	-1.70	AAA-AAA	0.00	999	1.000	1.000	0.4134

1561478_at	8.2	14.0	0.77	1.70	AAA-AAA	0.00	999	1.000	1.000	0.6158
1561355_at	52.4	30.8	-0.77	-1.70	AAA-AAA	0.00	999	1.000	1.000	0.5997
215573_at	15.2	8.9	-0.77	-1.70	AAA-AAA	0.00	999	1.000	1.000	0.4735
219521_at	20.3	11.9	-0.77	-1.70	AAA-AAA	0.00	999	1.000	1.000	0.3298
206671_at	47.2	80.4	0.77	1.70	AAA-AAA	0.00	999	1.000	1.000	0.3804
211808_s_at	57.1	33.5	-0.77	-1.70	AAA-AAA	0.00	999	1.000	1.000	0.2967
208531_at	21.5	12.6	-0.77	-1.70	AAA-AAA	0.00	999	1.000	1.000	0.3265
234109_x_at	67.7	115.2	0.77	1.70	AAA-AAA	0.00	999	1.000	1.000	0.2566
236375_at	4.9	8.3	0.77	1.70	AAA-AAA	0.00	999	1.000	1.000	0.4584
236801_at	19.5	11.5	-0.77	-1.70	AAA-AAA	0.00	999	1.000	1.000	0.5824
226857_at	54.0	31.7	-0.77	-1.70	AAA-AAA	0.00	999	1.000	1.000	0.3810
225720_at	8.9	5.2	-0.77	-1.70	AAA-AAA	0.00	999	1.000	1.000	0.3575
223801_s_at	25.1	14.7	-0.77	-1.70	AAA-AAA	0.00	999	1.000	1.000	0.0843
231010_at	24.8	14.5	-0.77	-1.70	AMA-AAA	0.00	999	1.000	1.000	0.6162
229569_at	5.6	9.6	0.77	1.70	AAA-AAA	0.00	999	1.000	1.000	0.5168
1566656_a_at	88.3	51.9	-0.77	-1.70	AAP-PAP	0.00	999	1.000	1.000	0.0593
233277_at	76.4	44.9	-0.77	-1.70	AAP-AAA	0.00	999	1.000	1.000	0.1461
1570013_at	10.0	5.9	-0.77	-1.70	AAA-AAA	0.00	999	1.000	1.000	0.0926
1570169_at	22.7	38.6	0.77	1.70	AAA-AAA	0.00	999	1.000	1.000	0.3424
1554377_a_at	17.8	10.5	-0.77	-1.70	AAA-AAA	0.00	999	1.000	1.000	0.4724
214904_at	36.6	62.2	0.77	1.70	AAA-AMA	0.00	999	1.000	1.000	0.1536
221182_at	7.1	4.2	-0.77	-1.70	AAA-AAA	0.00	999	1.000	1.000	0.6124
234755_x_at	1.6	2.8	0.77	1.70	AAA-AAA	0.00	999	1.000	1.000	0.5042
235811_at	97.7	57.4	-0.77	-1.70	AAA-AAA	0.00	999	1.000	1.000	0.4952
228109_at	15.3	26.1	0.77	1.70	AAA-AAA	0.00	999	1.000	1.000	0.5310
236977_at	56.0	33.0	-0.77	-1.70	PAA-AAA	0.00	999	1.000	1.000	0.4861
1569745_at	26.5	45.0	0.77	1.70	AAA-AAA	0.00	999	1.000	1.000	0.2367
239521_at	16.2	9.5	-0.77	-1.70	AAA-AAA	0.00	999	1.000	1.000	0.5101
1565836_at	12.4	21.1	0.77	1.70	AAA-AAA	0.00	999	1.000	1.000	0.5899
240642_at	6.6	11.3	0.77	1.70	AAA-AAA	0.00	999	1.000	1.000	0.2210
214110_s_at	312.9	184.0	-0.77	-1.70	MMA-AAA	0.00	999	1.000	1.000	0.0162
212913_at	17.2	29.2	0.77	1.70	AAA-AAA	0.00	999	1.000	1.000	0.5633
220709_at	5.0	8.5	0.77	1.70	AAA-AAA	0.00	999	1.000	1.000	0.2179
210227_at	29.9	17.6	-0.77	-1.70	AAA-AAA	0.00	999	1.000	1.000	0.5665
211567_at	24.0	40.8	0.77	1.70	AAA-AAA	0.00	999	1.000	1.000	0.1832
211260_at	30.8	52.3	0.77	1.70	AAA-AAA	0.00	999	1.000	1.000	0.4464
207940_x_at	11.3	19.1	0.77	1.70	AAA-AAA	0.00	999	1.000	1.000	0.4920
208489_at	35.5	20.9	-0.77	-1.70	AAA-AAA	0.00	999	1.000	1.000	0.2888
234673_at	6.3	3.7	-0.77	-1.70	AAA-AAA	0.00	999	1.000	1.000	0.5286
236896_at	20.1	11.8	-0.77	-1.70	AAA-AAA	0.00	999	1.000	1.000	0.2858
236667_at	225.0	132.4	-0.77	-1.70	PPP-AAP	0.00	999	1.000	1.000	0.1221
214012_at	66.7	39.3	-0.77	-1.70	PPP-PPP	0.00	999	1.000	1.000	0.1880

1562281_at	38.9	22.9	-0.77	-1.70	PPA-PAA	0.00	999	1.000	1.000	0.6061
243408_at	26.5	45.0	0.77	1.70	AAA-AAA	0.00	999	1.000	1.000	0.5231
244318_at	5.8	9.8	0.77	1.70	AAA-AAA	0.00	999	1.000	1.000	0.7132
215741_x_at	90.0	53.0	-0.77	-1.70	AAA-AAA	0.00	999	1.000	1.000	0.6106
215365_at	12.0	7.1	-0.77	-1.70	AAA-AAA	0.00	999	1.000	1.000	0.2726
216452_at	45.4	26.7	-0.77	-1.70	AAA-AAA	0.00	999	1.000	1.000	0.3478
216925_s_at	16.9	28.7	0.77	1.70	AAA-AAA	0.00	999	1.000	1.000	0.4360
217114_at	52.3	88.9	0.77	1.70	AAA-AAA	0.00	999	1.000	1.000	0.3122
206943_at	92.7	54.5	-0.77	-1.70	AAA-AAA	0.00	999	1.000	1.000	0.2203
207872_s_at	19.8	33.6	0.77	1.70	AAA-AAA	0.00	999	1.000	1.000	0.2440
207557_s_at	15.6	26.6	0.77	1.70	AAA-AAA	0.00	999	1.000	1.000	0.7026
208206_s_at	44.9	26.4	-0.77	-1.70	AAA-AAA	0.00	999	1.000	1.000	0.3768
233233_at	23.0	13.5	-0.77	-1.70	AAA-AAA	0.00	999	1.000	1.000	0.5248
237573_at	7.2	12.2	0.77	1.70	AAA-AAA	0.00	999	1.000	1.000	0.6943
227419_x_at	19.3	11.4	-0.77	-1.70	AAA-AAA	0.00	999	1.000	1.000	0.5517
223973_at	16.7	9.8	-0.77	-1.70	AAA-AAA	0.00	999	1.000	1.000	0.5492
229536_at	12.7	7.5	-0.77	-1.70	AAA-AAA	0.00	999	1.000	1.000	0.3669
230799_at	105.6	62.2	-0.76	-1.70	AAP-AAA	0.00	999	1.000	1.000	0.0349
202644_s_at	839.7	494.3	-0.76	-1.70	PPP-PPP	0.00	999	1.000	1.000	0.0663
1564882_at	30.0	17.7	-0.76	-1.70	AAA-AAA	0.00	999	1.000	1.000	0.5115
1562632_at	41.9	24.7	-0.76	-1.70	AAA-AAA	0.00	999	1.000	1.000	0.3576
241007_at	24.4	41.4	0.76	1.70	AAA-AAA	0.00	999	1.000	1.000	0.5133
1559352_a_at	79.6	46.9	-0.76	-1.70	AAA-AAA	0.00	999	1.000	1.000	0.2212
243050_at	36.0	21.2	-0.76	-1.70	AAA-AAA	0.00	999	1.000	1.000	0.5921
1559429_a_at	15.0	25.5	0.76	1.70	AAA-AAA	0.00	999	1.000	1.000	0.4818
244058_at	20.3	11.9	-0.76	-1.70	AAA-AAA	0.00	999	1.000	1.000	0.4936
216656_at	9.5	16.1	0.76	1.70	AAA-AAA	0.00	999	1.000	1.000	0.4228
216781_at	3.7	6.2	0.76	1.70	AAA-AAA	0.00	999	1.000	1.000	0.1744
216099_at	4.5	7.6	0.76	1.70	AAA-AAA	0.00	999	1.000	1.000	0.5461
217018_at	7.5	12.8	0.76	1.70	AAA-AAA	0.00	999	1.000	1.000	0.4821
233154_at	13.5	7.9	-0.76	-1.70	AAA-AAA	0.00	999	1.000	1.000	0.6755
232657_at	7.5	4.4	-0.76	-1.70	AAA-AAA	0.00	999	1.000	1.000	0.3051
233509_at	44.4	75.4	0.76	1.70	AAA-AAA	0.00	999	1.000	1.000	0.2846
231278_at	23.8	40.4	0.76	1.70	AAA-AAA	0.00	999	1.000	1.000	0.4985
231444_at	9.6	16.3	0.76	1.70	AAA-AAA	0.00	999	1.000	1.000	0.5960
222812_s_at	135.6	79.9	-0.76	-1.70	PPA-PAA	0.00	999	1.000	1.000	0.2128
241300_at	237.6	140.1	-0.76	-1.70	APP-AAA	0.00	999	1.000	1.000	0.3228
241392_at	87.3	51.5	-0.76	-1.70	AAA-AAA	0.00	999	1.000	1.000	0.1135
1569401_at	11.9	20.3	0.76	1.70	AAA-AAA	0.00	999	1.000	1.000	0.5724
241125_at	21.1	12.5	-0.76	-1.70	AAA-AAA	0.00	999	1.000	1.000	0.2661
1556195_a_at	38.1	64.6	0.76	1.70	AAA-AAA	0.00	999	1.000	1.000	0.5952
1556072_at	4.8	8.2	0.76	1.70	AAA-AAA	0.00	999	1.000	1.000	0.1439

216317_x_at	50.9	30.0	-0.76	-1.70	AAA-AAA	0.00	999	1.000	1.000	0.0942
216914_at	26.6	45.1	0.76	1.70	AAA-AAA	0.00	999	1.000	1.000	0.1222
219900_s_at	109.5	185.8	0.76	1.70	AAA-AAA	0.00	999	1.000	1.000	0.0313
203548_s_at	6.6	3.9	-0.76	-1.70	AAA-AAA	0.00	999	1.000	1.000	0.4681
205212_s_at	17.3	29.3	0.76	1.70	AAA-AAA	0.00	999	1.000	1.000	0.6121
204845_s_at	5.1	8.6	0.76	1.70	AAA-AAA	0.00	999	1.000	1.000	0.6471
208244_at	20.7	12.2	-0.76	-1.70	AAA-AAA	0.00	999	1.000	1.000	0.1430
232631_at	44.2	75.0	0.76	1.70	AAA-AAA	0.00	999	1.000	1.000	0.2343
236583_at	24.2	14.3	-0.76	-1.70	AAA-AAA	0.00	999	1.000	1.000	0.5473
236108_at	16.4	9.7	-0.76	-1.70	AAA-AAA	0.00	999	1.000	1.000	0.0492
228796_at	21.5	36.4	0.76	1.70	AAA-AAA	0.00	999	1.000	1.000	0.3712
231532_at	6.4	10.9	0.76	1.70	AAA-AAA	0.00	999	1.000	1.000	0.2633
242237_at	53.6	31.6	-0.76	-1.70	PAA-AAA	0.00	999	1.000	1.000	0.0399
236332_at	108.5	64.0	-0.76	-1.70	MPA-AAA	0.00	999	1.000	1.000	0.1933
220550_at	44.8	26.4	-0.76	-1.70	PPA-AAP	0.00	999	1.000	1.000	0.2636
231857_s_at	227.2	134.0	-0.76	-1.70	PPP-PAP	0.00	999	1.000	1.000	0.3143
1564253_at	49.5	29.2	-0.76	-1.70	AAA-AAA	0.00	999	1.000	1.000	0.4373
1562783_at	24.6	41.7	0.76	1.70	AAA-AAA	0.00	999	1.000	1.000	0.4302
1554771_at	10.5	17.8	0.76	1.70	AAA-AAA	0.00	999	1.000	1.000	0.4209
1555613_a_at	14.6	8.6	-0.76	-1.70	AAA-AAA	0.00	999	1.000	1.000	0.2066
243223_at	47.7	28.2	-0.76	-1.70	AAA-AAA	0.00	999	1.000	1.000	0.3852
244127_at	21.0	35.6	0.76	1.70	AAA-AAA	0.00	999	1.000	1.000	0.3869
215601_at	3.3	5.6	0.76	1.70	AAA-AAA	0.00	999	1.000	1.000	0.2778
215593_at	6.1	10.3	0.76	1.70	AAA-AAA	0.00	999	1.000	1.000	0.2856
210642_at	24.9	14.7	-0.76	-1.70	AAA-AAA	0.00	999	1.000	1.000	0.6246
209810_at	17.6	10.4	-0.76	-1.70	AAA-AAA	0.00	999	1.000	1.000	0.3375
228599_at	24.3	41.3	0.76	1.70	AAA-AAA	0.00	999	1.000	1.000	0.1760
224128_at	73.9	43.6	-0.76	-1.70	AAA-AAA	0.00	999	1.000	1.000	0.3880
237664_at	83.1	49.0	-0.76	-1.70	AAP-AMA	0.00	999	1.000	1.000	0.2515
238985_at	24.7	41.9	0.76	1.69	AAA-AAA	0.00	999	1.000	1.000	0.4490
1566115_at	8.7	14.8	0.76	1.70	AAA-AAA	0.00	999	1.000	1.000	0.5729
1555895_at	55.3	93.7	0.76	1.70	AAA-AAA	0.00	999	1.000	1.000	0.4967
1554977_at	41.6	24.6	-0.76	-1.69	AAA-AAA	0.00	999	1.000	1.000	0.6079
1555311_at	78.0	132.1	0.76	1.70	AAA-AAA	0.00	999	1.000	1.000	0.0882
244649_at	29.9	17.7	-0.76	-1.70	AAA-AAA	0.00	999	1.000	1.000	0.5638
217054_at	21.4	36.3	0.76	1.70	AAA-AAA	0.00	999	1.000	1.000	0.3563
208312_s_at	9.8	16.7	0.76	1.69	AAA-AAA	0.00	999	1.000	1.000	0.0656
234080_at	6.8	11.6	0.76	1.70	AAA-AAA	0.00	999	1.000	1.000	0.6451
235137_at	32.1	19.0	-0.76	-1.69	AAA-AAA	0.00	999	1.000	1.000	0.6262
237554_at	25.3	14.9	-0.76	-1.69	AAA-AAA	0.00	999	1.000	1.000	0.3339
238555_at	45.4	26.8	-0.76	-1.70	AAA-AAA	0.00	999	1.000	1.000	0.5256
228426_at	5.8	9.8	0.76	1.69	AAA-AAA	0.00	999	1.000	1.000	0.6350

231603_at	3.7	6.2	0.76	1.70	AAA-AAA	0.00	999	1.000	1.000	0.5182
1554509_a_at	233.9	138.2	-0.76	-1.69	APP-APA	0.00	999	1.000	1.000	0.1040
234581_at	46.0	27.1	-0.76	-1.69	PAA-APA	0.00	999	1.000	1.000	0.2352
1569316_at	66.2	39.1	-0.76	-1.69	PPA-AAA	0.00	999	1.000	1.000	0.4973
215622_x_at	76.6	45.3	-0.76	-1.69	AAA-AAP	0.00	999	1.000	1.000	0.5280
216612_x_at	27.5	16.2	-0.76	-1.69	AAP-AAA	0.00	999	1.000	1.000	0.6056
1564510_at	23.7	14.0	-0.76	-1.69	AAA-AAA	0.00	999	1.000	1.000	0.5988
241809_at	198.2	117.1	-0.76	-1.69	AAA-AAA	0.00	999	1.000	1.000	0.3260
239703_at	44.7	75.8	0.76	1.69	AAA-AAA	0.00	999	1.000	1.000	0.3343
1566606_a_at	9.5	16.1	0.76	1.69	AAA-AAA	0.00	999	1.000	1.000	0.5761
1552932_at	19.1	11.3	-0.76	-1.69	AAA-AAA	0.00	999	1.000	1.000	0.4487
219541_at	53.5	31.6	-0.76	-1.69	AAA-AAA	0.00	999	1.000	1.000	0.2932
219057_at	58.5	34.6	-0.76	-1.69	AAA-AAA	0.00	999	1.000	1.000	0.5072
205772_s_at	25.5	15.0	-0.76	-1.69	AAA-AAA	0.00	999	1.000	1.000	0.5109
211811_s_at	12.9	21.9	0.76	1.69	AAA-AAA	0.00	999	1.000	1.000	0.4530
234010_at	19.9	11.8	-0.76	-1.69	AAA-AAA	0.00	999	1.000	1.000	0.5093
238196_at	7.7	4.6	-0.76	-1.69	AAA-AAA	0.00	999	1.000	1.000	0.5239
236093_at	52.9	31.2	-0.76	-1.69	AAA-AAA	0.00	999	1.000	1.000	0.3412
237298_at	10.5	17.8	0.76	1.69	AAA-AAA	0.00	999	1.000	1.000	0.3399
222857_s_at	52.4	31.0	-0.76	-1.69	AAA-AAA	0.00	999	1.000	1.000	0.2462
231664_at	11.3	19.2	0.76	1.69	AAA-AAA	0.00	999	1.000	1.000	0.1042
229660_at	51.8	87.7	0.76	1.69	AAA-AAA	0.00	999	1.000	1.000	0.3920
230272_at	5.0	3.0	-0.76	-1.69	AAA-AAA	0.00	999	1.000	1.000	0.2215
206504_at	55.0	32.5	-0.76	-1.69	PAA-AMA	0.00	999	1.000	1.000	0.0290
241367_at	77.7	45.9	-0.76	-1.69	AAA-AAA	0.00	999	1.000	1.000	0.4290
1564074_at	6.8	11.4	0.76	1.69	AAA-AAA	0.00	999	1.000	1.000	0.4396
241003_at	25.4	15.0	-0.76	-1.69	AAA-AAA	0.00	999	1.000	1.000	0.4583
1565722_at	9.5	16.0	0.76	1.69	AAA-AAA	0.00	999	1.000	1.000	0.4590
1568667_s_at	107.1	181.2	0.76	1.69	AAA-AAA	0.00	999	1.000	1.000	0.1309
1554175_at	26.2	44.3	0.76	1.69	AAA-AAA	0.00	999	1.000	1.000	0.4952
1561451_a_at	35.1	59.4	0.76	1.69	AAA-AAA	0.00	999	1.000	1.000	0.4944
1561098_at	3.6	6.1	0.76	1.69	AAA-AAA	0.00	999	1.000	1.000	0.2321
1562311_at	14.4	24.4	0.76	1.69	AAA-AAA	0.00	999	1.000	1.000	0.4198
234618_at	41.1	24.3	-0.76	-1.69	AAA-AAA	0.00	999	1.000	1.000	0.5985
232602_at	12.7	21.4	0.76	1.69	AAA-AAA	0.00	999	1.000	1.000	0.4979
237395_at	31.8	18.8	-0.76	-1.69	AAA-AAA	0.00	999	1.000	1.000	0.4091
238481_at	3.6	6.1	0.76	1.69	AAA-AAA	0.00	999	1.000	1.000	0.6985
228160_at	19.3	32.6	0.76	1.69	AAA-AAA	0.00	999	1.000	1.000	0.5711
231563_at	15.7	9.3	-0.76	-1.69	AAA-AAA	0.00	999	1.000	1.000	0.3360
238788_at	127.4	75.3	-0.76	-1.69	PPP-PPP	0.00	999	1.000	1.000	0.0354
238524_at	31.6	18.7	-0.76	-1.69	AAP-AAA	0.00	999	1.000	1.000	0.5132
240852_at	13.4	22.7	0.76	1.69	AAA-AAA	0.00	999	1.000	1.000	0.5469

1570121_at	7.2	4.3	-0.76	-1.69	AAA-AAA	0.00	999	1.000	1.000	0.5015
1553572_a_at	12.7	21.4	0.76	1.69	AAA-AAA	0.00	999	1.000	1.000	0.4646
1559586_at	39.2	66.3	0.76	1.69	AAA-AAA	0.00	999	1.000	1.000	0.1729
1559526_at	43.3	25.6	-0.76	-1.69	AAA-AAA	0.00	999	1.000	1.000	0.2542
1559920_a_at	20.5	12.2	-0.76	-1.69	AAA-AAA	0.00	999	1.000	1.000	0.5630
243955_at	23.6	14.0	-0.76	-1.69	AAA-AAA	0.00	999	1.000	1.000	0.1762
216227_at	25.2	14.9	-0.76	-1.69	AAA-AAA	0.00	999	1.000	1.000	0.3706
220723_s_at	18.8	11.1	-0.76	-1.69	AAA-AAA	0.00	999	1.000	1.000	0.5849
220976_s_at	27.5	46.4	0.76	1.69	AAA-AAA	0.00	999	1.000	1.000	0.5465
207229_at	8.7	14.7	0.76	1.69	AAA-AAA	0.00	999	1.000	1.000	0.4547
205008_s_at	33.9	20.0	-0.76	-1.69	AAA-AAA	0.00	999	1.000	1.000	0.5430
208285_at	19.0	11.2	-0.76	-1.69	AAA-AAA	0.00	999	1.000	1.000	0.5432
238060_s_at	23.6	39.9	0.76	1.69	AAA-AAA	0.00	999	1.000	1.000	0.5386
230953_at	15.6	9.2	-0.76	-1.69	AAA-AAA	0.00	999	1.000	1.000	0.4280
210195_s_at	79.7	47.2	-0.76	-1.69	PPP-PAP	0.00	999	1.000	1.000	0.0889
240355_at	27.0	45.6	0.76	1.69	AAA-AAA	0.00	999	1.000	1.000	0.4362
239464_at	48.5	82.0	0.76	1.69	AAA-AAA	0.00	999	1.000	1.000	0.3764
239549_at	83.2	140.6	0.76	1.69	AAA-AAA	0.00	999	1.000	1.000	0.1793
1553822_at	3.8	2.2	-0.76	-1.69	AAA-AAA	0.00	999	1.000	1.000	0.5568
1553239_at	48.8	82.5	0.76	1.69	AAA-AAA	0.00	999	1.000	1.000	0.3402
1561280_at	2.5	1.5	-0.76	-1.69	AAA-AAA	0.00	999	1.000	1.000	0.2297
1562083_at	13.3	7.8	-0.76	-1.69	AAA-AAA	0.00	999	1.000	1.000	0.4235
242611_at	22.9	38.6	0.76	1.69	AAA-AAA	0.00	999	1.000	1.000	0.5366
244344_at	41.1	24.3	-0.76	-1.69	AAA-AAA	0.00	999	1.000	1.000	0.4745
244571_s_at	56.8	33.6	-0.76	-1.69	AAA-AAA	0.00	999	1.000	1.000	0.4457
1560877_a_at	21.4	12.7	-0.76	-1.69	AAA-AAA	0.00	999	1.000	1.000	0.6189
215915_at	7.9	13.3	0.76	1.69	AAA-AAA	0.00	999	1.000	1.000	0.4128
215904_at	7.9	13.3	0.76	1.69	AAA-AAA	0.00	999	1.000	1.000	0.6208
214261_s_at	10.9	18.4	0.76	1.69	AAA-AAA	0.00	999	1.000	1.000	0.5216
206133_at	37.6	63.6	0.76	1.69	AAA-AAA	0.00	999	1.000	1.000	0.0325
206577_at	14.7	8.7	-0.76	-1.69	AAA-AAA	0.00	999	1.000	1.000	0.5546
201610_at	15.3	9.1	-0.76	-1.69	AAA-AAA	0.00	999	1.000	1.000	0.4096
201982_s_at	94.8	56.1	-0.76	-1.69	AAA-AAA	0.00	999	1.000	1.000	0.3007
234487_at	23.7	40.1	0.76	1.69	AAA-AAA	0.00	999	1.000	1.000	0.5575
234582_at	3.7	6.3	0.76	1.69	AAA-AAA	0.00	999	1.000	1.000	0.6002
238872_at	9.2	15.5	0.76	1.69	AAA-AAA	0.00	999	1.000	1.000	0.1329
241063_at	16.4	27.6	0.76	1.69	AAA-APA	0.00	999	1.000	1.000	0.6774
1565556_at	43.4	25.7	-0.76	-1.69	AAA-AAA	0.00	999	1.000	1.000	0.6725
1567686_at	41.1	69.5	0.76	1.69	AAA-AAA	0.00	999	1.000	1.000	0.3529
1556944_at	4.1	6.9	0.76	1.69	AAA-AAA	0.00	999	1.000	1.000	0.6732
1552852_a_at	9.5	16.0	0.76	1.69	AAA-AAA	0.00	999	1.000	1.000	0.5513
1559141_s_at	6.6	11.1	0.76	1.69	AAA-AAA	0.00	999	1.000	1.000	0.6244

211020_at	8.2	13.8	0.76	1.69	AAA-AAA	0.00	999	1.000	1.000	0.5998
234563_at	10.3	17.5	0.76	1.69	AAA-AAA	0.00	999	1.000	1.000	0.6199
230616_at	93.2	55.2	-0.76	-1.69	PAP-AAA	0.00	999	1.000	1.000	0.0309
225567_at	712.5	422.2	-0.76	-1.69	PPP-PPP	0.00	999	1.000	1.000	0.0543
224276_at	59.9	35.5	-0.76	-1.69	PPP-PAA	0.00	999	1.000	1.000	0.2623
1563621_at	48.0	28.4	-0.76	-1.69	PAA-AAA	0.00	999	1.000	1.000	0.5398
241525_at	3.9	6.5	0.76	1.69	AAA-AAA	0.00	999	1.000	1.000	0.1172
1564786_at	12.2	20.5	0.76	1.69	AAA-AAA	0.00	999	1.000	1.000	0.5100
1570115_at	19.9	11.8	-0.76	-1.69	MAA-AAA	0.00	999	1.000	1.000	0.5206
240615_at	18.4	31.0	0.76	1.69	AAA-AAA	0.00	999	1.000	1.000	0.5601
1556238_at	13.4	7.9	-0.76	-1.69	AAA-AAA	0.00	999	1.000	1.000	0.6898
1570355_a_at	13.4	22.7	0.76	1.69	AAA-AAA	0.00	999	1.000	1.000	0.3041
213824_at	16.2	9.6	-0.76	-1.69	AAA-AAA	0.00	999	1.000	1.000	0.4944
221651_x_at	35.4	21.0	-0.76	-1.69	AAA-AAA	0.00	999	1.000	1.000	0.1822
218272_at	78.0	131.6	0.76	1.69	AAA-AAA	0.00	999	1.000	1.000	0.3914
235726_at	15.0	25.4	0.76	1.69	AAA-AAA	0.00	999	1.000	1.000	0.5226
231728_at	8.3	14.1	0.76	1.69	AAA-AAA	0.00	999	1.000	1.000	0.5724
211981_at	466.2	276.4	-0.75	-1.69	PMP-AAP	0.00	999	1.000	1.000	0.1131
1570339_x_at	15.2	25.6	0.75	1.69	AAA-AAA	0.00	999	1.000	1.000	0.6735
1563993_at	27.9	47.1	0.75	1.69	AAA-AAA	0.00	999	1.000	1.000	0.4743
215598_at	44.1	74.4	0.75	1.69	AAA-AAA	0.00	999	1.000	1.000	0.1275
220705_s_at	101.4	60.1	-0.75	-1.69	AAA-AAA	0.00	999	1.000	1.000	0.3034
203296_s_at	11.0	6.5	-0.75	-1.69	AAA-AAA	0.00	999	1.000	1.000	0.5236
208128_x_at	14.4	24.2	0.75	1.69	AAA-AAA	0.00	999	1.000	1.000	0.4769
233308_at	72.9	43.2	-0.75	-1.69	AAA-AAA	0.00	999	1.000	1.000	0.5911
233405_at	16.4	9.7	-0.75	-1.69	AAA-AAA	0.00	999	1.000	1.000	0.5127
237932_at	10.8	6.4	-0.75	-1.69	AAA-AAA	0.00	999	1.000	1.000	0.5245
238720_at	8.7	14.7	0.75	1.69	AAA-AAA	0.00	999	1.000	1.000	0.6743
235778_s_at	6.8	4.0	-0.75	-1.69	AAA-AAA	0.00	999	1.000	1.000	0.5766
228233_at	27.4	16.3	-0.75	-1.69	AAA-AAA	0.00	999	1.000	1.000	0.6542
230889_at	18.2	30.6	0.75	1.69	AAA-AAA	0.00	999	1.000	1.000	0.5917
230371_at	12.9	21.8	0.75	1.69	AAA-AAA	0.00	999	1.000	1.000	0.2249
200665_s_at	605.8	359.4	-0.75	-1.69	PPP-PPP	0.00	999	1.000	1.000	0.0217
216150_at	78.1	46.3	-0.75	-1.69	PAM-AAA	0.00	999	1.000	1.000	0.2837
238564_at	55.9	33.2	-0.75	-1.69	PAP-AAA	0.00	999	1.000	1.000	0.3799
242187_s_at	5.9	9.9	0.75	1.69	AAA-AAA	0.00	999	1.000	1.000	0.4831
1563902_at	7.3	12.3	0.75	1.69	AAA-AAA	0.00	999	1.000	1.000	0.1077
1558540_s_at	30.9	18.3	-0.75	-1.69	AAA-AAA	0.00	999	1.000	1.000	0.4994
1559289_at	14.9	8.9	-0.75	-1.69	AAA-AAA	0.00	999	1.000	1.000	0.6150
221095_s_at	25.9	15.4	-0.75	-1.69	AAA-AAA	0.00	999	1.000	1.000	0.2943
219815_at	53.4	90.0	0.75	1.69	AAA-AAA	0.00	999	1.000	1.000	0.4738
219059_s_at	23.4	39.4	0.75	1.69	AAA-AAA	0.00	999	1.000	1.000	0.4882

206135_at	5.3	9.0	0.75	1.69	AAA-AAA	0.00	999	1.000	1.000	0.6779
203389_at	171.7	101.9	-0.75	-1.69	AMA-AAA	0.00	999	1.000	1.000	0.1036
204002_s_at	77.3	130.3	0.75	1.69	AAA-AAA	0.00	999	1.000	1.000	0.2638
233446_at	22.0	13.0	-0.75	-1.69	AAA-AAA	0.00	999	1.000	1.000	0.6361
224079_at	92.4	155.6	0.75	1.69	MAM-AAM	0.00	999	1.000	1.000	0.2303
232358_at	76.7	129.3	0.75	1.69	AAA-AAA	0.00	999	1.000	1.000	0.2902
1561310_at	80.4	47.7	-0.75	-1.68	APP-PAP	0.00	999	1.000	1.000	0.5434
242348_at	10.2	6.0	-0.75	-1.68	AAA-AAA	0.00	999	1.000	1.000	0.6537
1563332_at	13.5	22.8	0.75	1.68	AAA-AAA	0.00	999	1.000	1.000	0.5112
1565784_at	28.8	48.6	0.75	1.69	AAA-AAA	0.00	999	1.000	1.000	0.4965
1554707_at	36.3	21.5	-0.75	-1.68	AAA-AAA	0.00	999	1.000	1.000	0.1480
1559652_at	6.4	3.8	-0.75	-1.69	AAA-AAA	0.00	999	1.000	1.000	0.5440
1560495_at	22.8	13.5	-0.75	-1.68	AAA-AAA	0.00	999	1.000	1.000	0.5182
215486_at	22.5	13.3	-0.75	-1.68	AAA-AAA	0.00	999	1.000	1.000	0.7153
214166_at	27.1	45.7	0.75	1.68	AAA-AAA	0.00	999	1.000	1.000	0.4607
220098_at	15.8	26.7	0.75	1.68	AAA-AAA	0.00	999	1.000	1.000	0.3998
202796_at	78.4	46.5	-0.75	-1.68	AAA-AAA	0.00	999	1.000	1.000	0.4898
204482_at	20.1	11.9	-0.75	-1.69	AAA-AAA	0.00	999	1.000	1.000	0.0495
207837_at	18.6	31.3	0.75	1.69	AAA-AAM	0.00	999	1.000	1.000	0.6132
209243_s_at	9.2	5.5	-0.75	-1.68	AAA-AAA	0.00	999	1.000	1.000	0.5669
237606_at	11.3	19.1	0.75	1.68	AAA-AAA	0.00	999	1.000	1.000	0.5457
229481_at	49.4	29.3	-0.75	-1.68	AAA-AAA	0.00	999	1.000	1.000	0.5016
1570301_at	44.9	26.7	-0.75	-1.68	AAP-AAA	0.00	999	1.000	1.000	0.2194
1553775_at	87.4	51.9	-0.75	-1.68	PAP-PAA	0.00	999	1.000	1.000	0.2222
1564658_at	88.5	52.6	-0.75	-1.68	PMA-AMA	0.00	999	1.000	1.000	0.5996
240256_at	11.1	18.6	0.75	1.68	AAA-AAA	0.00	999	1.000	1.000	0.5161
240860_at	79.0	47.0	-0.75	-1.68	AAA-AAA	0.00	999	1.000	1.000	0.2770
1557816_a_at	13.7	23.1	0.75	1.68	AAA-AAA	0.00	999	1.000	1.000	0.5162
1556199_a_at	4.3	7.2	0.75	1.68	AAA-AAA	0.00	999	1.000	1.000	0.5615
1555357_at	15.9	9.4	-0.75	-1.68	AAA-AAA	0.00	999	1.000	1.000	0.4537
1562585_at	14.4	8.5	-0.75	-1.68	AAA-AAA	0.00	999	1.000	1.000	0.2770
1561671_at	37.1	22.0	-0.75	-1.68	AAA-AAA	0.00	999	1.000	1.000	0.5101
244166_at	50.2	29.9	-0.75	-1.68	AAA-AAA	0.00	999	1.000	1.000	0.6567
216687_x_at	12.8	21.5	0.75	1.68	AAA-AAA	0.00	999	1.000	1.000	0.5797
213874_at	87.2	51.8	-0.75	-1.68	AAA-AAA	0.00	999	1.000	1.000	0.4752
221810_at	36.6	21.7	-0.75	-1.68	AAA-AAA	0.00	999	1.000	1.000	0.6646
221603_at	128.7	76.5	-0.75	-1.68	AAA-AAA	0.00	999	1.000	1.000	0.0821
219659_at	35.8	60.3	0.75	1.68	AAA-AAA	0.00	999	1.000	1.000	0.4933
205274_at	12.3	20.6	0.75	1.68	AAA-AAA	0.00	999	1.000	1.000	0.1785
205556_at	10.3	6.1	-0.75	-1.68	AAA-AAA	0.00	999	1.000	1.000	0.6385
210215_at	27.1	16.1	-0.75	-1.68	AAA-AAA	0.00	999	1.000	1.000	0.4834
210311_at	52.1	31.0	-0.75	-1.68	AAA-AAA	0.00	999	1.000	1.000	0.3062

211451_s_at	8.7	14.7	0.75	1.68	AAA-AAA	0.00	999	1.000	1.000	0.5427
234879_at	8.1	13.6	0.75	1.68	AAA-AAA	0.00	999	1.000	1.000	0.5694
233738_at	14.8	8.8	-0.75	-1.68	AAA-AAA	0.00	999	1.000	1.000	0.6553
233424_at	17.5	29.4	0.75	1.68	AAA-AAA	0.00	999	1.000	1.000	0.3915
237925_at	20.6	34.7	0.75	1.68	AAA-AAA	0.00	999	1.000	1.000	0.5727
224170_s_at	27.0	16.0	-0.75	-1.68	AAA-AAA	0.00	999	1.000	1.000	0.5727
224000_at	96.0	57.1	-0.75	-1.68	AAA-AAA	0.00	999	1.000	1.000	0.6179
231476_at	24.7	41.6	0.75	1.68	AAA-AAA	0.00	999	1.000	1.000	0.4973
232204_at	3.2	5.4	0.75	1.68	AAA-AAA	0.00	999	1.000	1.000	0.4452
213954_at	353.1	209.9	-0.75	-1.68	PPP-PPP	0.00	999	1.000	1.000	0.1130
231067_s_at	378.8	225.3	-0.75	-1.68	PPP-PPP	0.00	999	1.000	1.000	0.1135
220460_at	51.3	30.5	-0.75	-1.68	APM-APA	0.00	999	1.000	1.000	0.1952
1569334_at	4.2	7.1	0.75	1.68	AAA-AAA	0.00	999	1.000	1.000	0.7000
1570024_at	17.9	30.2	0.75	1.68	AAA-AAA	0.00	999	1.000	1.000	0.6343
244823_at	15.9	9.5	-0.75	-1.68	AAA-AAA	0.00	999	1.000	1.000	0.1129
1557588_at	24.5	14.6	-0.75	-1.68	AAA-AAA	0.00	999	1.000	1.000	0.2329
243203_at	20.3	34.2	0.75	1.68	AAA-AAA	0.00	999	1.000	1.000	0.3408
1559138_a_at	9.9	16.7	0.75	1.68	AAA-AAA	0.00	999	1.000	1.000	0.3599
215821_x_at	139.4	82.9	-0.75	-1.68	AAA-AAA	0.00	999	1.000	1.000	0.2400
217311_at	6.3	10.5	0.75	1.68	AAA-AAA	0.00	999	1.000	1.000	0.3782
205779_at	21.5	12.8	-0.75	-1.68	AAA-AAA	0.00	999	1.000	1.000	0.5212
201868_s_at	5.4	9.1	0.75	1.68	AAA-AAA	0.00	999	1.000	1.000	0.5410
234136_at	7.8	13.1	0.75	1.68	AAA-AAA	0.00	999	1.000	1.000	0.5304
235517_at	63.8	107.3	0.75	1.68	AAA-AAA	0.00	999	1.000	1.000	0.5019
233707_at	47.4	79.8	0.75	1.68	AMA-AAA	0.00	999	1.000	1.000	0.0796
238233_at	8.1	13.6	0.75	1.68	AAA-AAA	0.00	999	1.000	1.000	0.4351
236117_at	50.8	85.5	0.75	1.68	AAA-AAA	0.00	999	1.000	1.000	0.5371
231342_at	49.3	83.0	0.75	1.68	AAA-AAA	0.00	999	1.000	1.000	0.2325
231276_at	70.4	118.4	0.75	1.68	AAA-AAA	0.00	999	1.000	1.000	0.1908
220440_at	48.6	28.9	-0.75	-1.68	AAP-AAA	0.00	999	1.000	1.000	0.3439
1565812_at	15.6	9.3	-0.75	-1.68	AAA-AAA	0.00	999	1.000	1.000	0.1612
1556781_at	19.0	11.3	-0.75	-1.68	AAA-AAA	0.00	999	1.000	1.000	0.5750
1553776_at	13.7	8.2	-0.75	-1.68	AAA-AAA	0.00	999	1.000	1.000	0.6254
243567_at	11.8	19.8	0.75	1.68	AAA-AAA	0.00	999	1.000	1.000	0.6012
1562208_a_at	56.6	95.1	0.75	1.68	AAA-AAA	0.00	999	1.000	1.000	0.2729
243680_at	7.6	4.5	-0.75	-1.68	AAA-AAA	0.00	999	1.000	1.000	0.3988
1561367_a_at	23.1	38.9	0.75	1.68	AAA-AAA	0.00	999	1.000	1.000	0.4537
215715_at	11.4	19.2	0.75	1.68	AAA-AAA	0.00	999	1.000	1.000	0.0140
215709_at	23.3	39.1	0.75	1.68	AAA-AAA	0.00	999	1.000	1.000	0.5680
216728_at	9.8	16.5	0.75	1.68	AAA-AAA	0.00	999	1.000	1.000	0.3596
213201_s_at	143.3	85.3	-0.75	-1.68	AAA-AAA	0.00	999	1.000	1.000	0.0685
221660_at	61.8	103.8	0.75	1.68	AAA-AAA	0.00	999	1.000	1.000	0.4918

217115_at	23.7	39.8	0.75	1.68	AAA-AAA	0.00	999	1.000	1.000	0.5775
219564_at	34.2	20.4	-0.75	-1.68	AAA-AAA	0.00	999	1.000	1.000	0.5784
211782_at	25.5	15.2	-0.75	-1.68	AAA-AAA	0.00	999	1.000	1.000	0.3490
234197_at	11.6	6.9	-0.75	-1.68	AAA-AAA	0.00	999	1.000	1.000	0.5967
234205_at	10.4	17.4	0.75	1.68	AAA-AAA	0.00	999	1.000	1.000	0.5926
238584_at	9.3	15.6	0.75	1.68	AAA-AAA	0.00	999	1.000	1.000	0.7057
228451_at	33.0	19.6	-0.75	-1.68	AAA-AAA	0.00	999	1.000	1.000	0.5260
228689_at	62.3	37.1	-0.75	-1.68	AAA-AAA	0.00	999	1.000	1.000	0.5420
227821_at	96.4	57.3	-0.75	-1.68	AAA-AAA	0.00	999	1.000	1.000	0.5194
222963_s_at	15.3	9.1	-0.75	-1.68	AAA-AAA	0.00	999	1.000	1.000	0.2379
230381_at	113.9	67.7	-0.75	-1.68	AAA-AAA	0.00	999	1.000	1.000	0.1493
224197_s_at	121.9	72.6	-0.75	-1.68	PAP-PPA	0.00	999	1.000	1.000	0.1227
1563318_s_at	176.2	104.9	-0.75	-1.68	MAP-AAA	0.00	999	1.000	1.000	0.2388
43511_s_at	76.6	45.7	-0.75	-1.68	AAA-PAA	0.00	999	1.000	1.000	0.4591
241642_x_at	77.4	46.1	-0.75	-1.68	AAA-AAA	0.00	999	1.000	1.000	0.6115
239279_at	26.5	15.8	-0.75	-1.68	AAA-AAA	0.00	999	1.000	1.000	0.4050
239321_at	6.9	4.1	-0.75	-1.68	AAA-AAA	0.00	999	1.000	1.000	0.5076
1552662_a_at	29.2	17.4	-0.75	-1.68	AAA-AAA	0.00	999	1.000	1.000	0.5547
1557068_at	4.7	7.8	0.75	1.68	AAA-AAA	0.00	999	1.000	1.000	0.4330
1559174_at	36.1	21.5	-0.75	-1.68	AAA-AAA	0.00	999	1.000	1.000	0.4306
214968_at	122.1	72.7	-0.75	-1.68	AAA-AAA	0.00	999	1.000	1.000	0.3678
214275_at	19.9	33.4	0.75	1.68	AAA-AAA	0.00	999	1.000	1.000	0.4898
221035_s_at	20.7	12.3	-0.75	-1.68	AAA-AAA	0.00	999	1.000	1.000	0.5981
206215_at	20.3	12.1	-0.75	-1.68	AAA-AAA	0.00	999	1.000	1.000	0.4216
207295_at	51.0	85.6	0.75	1.68	AAA-MAA	0.00	999	1.000	1.000	0.1653
233800_at	16.9	28.3	0.75	1.68	AAA-AAA	0.00	999	1.000	1.000	0.6566
235380_at	39.3	23.4	-0.75	-1.68	AAA-PAA	0.00	999	1.000	1.000	0.4952
241269_at	6.0	10.0	0.75	1.68	AAA-AAA	0.00	999	1.000	1.000	0.4960
239323_at	22.5	13.4	-0.75	-1.68	AAA-AAA	0.00	999	1.000	1.000	0.4381
1556623_at	11.2	18.8	0.75	1.68	AAA-AAA	0.00	999	1.000	1.000	0.5966
244577_at	14.2	8.4	-0.75	-1.68	AAA-AAA	0.00	999	1.000	1.000	0.1247
221367_at	28.0	47.0	0.75	1.68	AAA-AAA	0.00	999	1.000	1.000	0.5334
205007_s_at	25.8	15.4	-0.75	-1.68	AAA-AAA	0.00	999	1.000	1.000	0.6073
209992_at	29.6	49.7	0.75	1.68	AAA-AAA	0.00	999	1.000	1.000	0.4819
209785_s_at	20.8	12.4	-0.75	-1.68	AAA-AAA	0.00	999	1.000	1.000	0.5022
233129_at	42.0	25.0	-0.75	-1.68	AAA-AAA	0.00	999	1.000	1.000	0.4375
237761_at	46.7	78.5	0.75	1.68	AAA-AAA	0.00	999	1.000	1.000	0.1965
236315_at	18.9	11.3	-0.75	-1.68	AAA-AAA	0.00	999	1.000	1.000	0.5039
237080_at	18.2	10.8	-0.75	-1.68	AAA-AAA	0.00	999	1.000	1.000	0.1477
222746_s_at	137.1	81.7	-0.75	-1.68	MPP-AAP	0.00	999	1.000	1.000	0.0217
241766_at	22.9	38.3	0.75	1.68	AAA-AAA	0.00	999	1.000	1.000	0.6231
239972_at	38.4	22.9	-0.75	-1.68	AAA-AAA	0.00	999	1.000	1.000	0.3191

1569361_a_at	27.7	46.4	0.75	1.68	AAA-AAA	0.00	999	1.000	1.000	0.5590
240561_at	26.2	15.6	-0.75	-1.68	AAA-AAA	0.00	999	1.000	1.000	0.4803
243340_at	14.1	8.4	-0.75	-1.68	AAA-AAA	0.00	999	1.000	1.000	0.2967
242927_at	11.8	19.8	0.75	1.68	AAA-AAA	0.00	999	1.000	1.000	0.6043
220499_at	17.6	10.5	-0.75	-1.68	AAA-AAA	0.00	999	1.000	1.000	0.5761
220319_s_at	51.1	85.6	0.75	1.68	AAA-AAA	0.00	999	1.000	1.000	0.4436
204582_s_at	19.2	32.2	0.75	1.68	AAA-AAA	0.00	999	1.000	1.000	0.3690
235742_at	55.2	32.9	-0.75	-1.68	AAA-AAA	0.00	999	1.000	1.000	0.3199
234024_at	25.0	42.0	0.75	1.68	AAA-AAA	0.00	999	1.000	1.000	0.2069
227217_at	15.9	26.6	0.75	1.68	AAA-AAA	0.00	999	1.000	1.000	0.4705
223323_x_at	113.0	189.5	0.75	1.68	AAA-AAA	0.00	999	1.000	1.000	0.5102
241222_at	68.5	40.9	-0.75	-1.68	AAA-PAA	0.00	999	1.000	1.000	0.2816
215917_at	5.4	9.0	0.75	1.68	AAA-PAA	0.00	999	1.000	1.000	0.6981
242307_at	60.1	100.7	0.75	1.68	AAA-AAM	0.00	999	1.000	1.000	0.3396
1563825_at	11.9	7.1	-0.75	-1.68	AAA-AAA	0.00	999	1.000	1.000	0.6048
239328_at	43.3	72.5	0.75	1.68	AAA-AAA	0.00	999	1.000	1.000	0.3241
240975_x_at	21.5	36.0	0.75	1.68	AAA-AAA	0.00	999	1.000	1.000	0.5134
1556737_at	33.2	55.7	0.75	1.68	AAA-AAA	0.00	999	1.000	1.000	0.0883
1562169_at	5.5	9.2	0.75	1.68	AAA-AAA	0.00	999	1.000	1.000	0.6933
215594_at	53.0	31.6	-0.75	-1.68	AAA-AAA	0.00	999	1.000	1.000	0.4257
216700_at	13.7	23.0	0.75	1.68	AAA-AAA	0.00	999	1.000	1.000	0.2997
213536_s_at	37.4	22.3	-0.75	-1.68	AAA-AAA	0.00	999	1.000	1.000	0.3329
206801_at	41.7	69.8	0.75	1.68	AAA-AAA	0.00	999	1.000	1.000	0.6302
207874_s_at	33.1	19.8	-0.75	-1.68	AAA-AAA	0.00	999	1.000	1.000	0.3249
234149_at	22.0	36.9	0.75	1.68	AAA-AAA	0.00	999	1.000	1.000	0.4791
224113_at	9.9	16.5	0.75	1.68	AAA-AAA	0.00	999	1.000	1.000	0.2349
231445_at	10.8	6.4	-0.75	-1.68	AAA-AAA	0.00	999	1.000	1.000	0.1883
226512_at	180.3	107.7	-0.74	-1.67	PPP-PPP	0.00	999	1.000	1.000	0.0287
237515_at	152.6	91.2	-0.74	-1.67	PPP-PPP	0.00	999	1.000	1.000	0.0936
214769_at	72.0	43.0	-0.74	-1.68	AAA-AAP	0.00	999	1.000	1.000	0.3264
221915_s_at	83.0	49.6	-0.74	-1.68	MMP-APA	0.00	999	1.000	1.000	0.3817
241146_at	26.4	15.8	-0.74	-1.67	AAA-AAA	0.00	999	1.000	1.000	0.4761
241168_at	24.3	14.5	-0.74	-1.68	AAA-AAA	0.00	999	1.000	1.000	0.4829
1569776_at	18.5	11.1	-0.74	-1.67	AAA-AAA	0.00	999	1.000	1.000	0.6251
243389_at	6.1	10.2	0.74	1.68	AAA-AAA	0.00	999	1.000	1.000	0.4087
1559523_at	31.1	18.6	-0.74	-1.68	AAA-AAA	0.00	999	1.000	1.000	0.6388
215633_x_at	7.8	13.1	0.74	1.68	AAA-AAA	0.00	999	1.000	1.000	0.1363
207077_at	21.1	35.3	0.74	1.67	AAA-AAA	0.00	999	1.000	1.000	0.4958
210772_at	34.6	58.0	0.74	1.68	AAA-AMA	0.00	999	1.000	1.000	0.4207
237385_at	9.6	5.7	-0.74	-1.67	AAA-AAA	0.00	999	1.000	1.000	0.0045
224096_at	56.0	33.5	-0.74	-1.67	AAA-AAA	0.00	999	1.000	1.000	0.5831
1570070_at	285.8	170.8	-0.74	-1.67	PPP-PAP	0.00	999	1.000	1.000	0.0545

242894_at	17.3	10.3	-0.74	-1.67	PAA-AAA	0.00	999	1.000	1.000	0.6683
241190_at	17.5	29.3	0.74	1.67	AAA-AAA	0.00	999	1.000	1.000	0.5694
240200_at	11.4	6.8	-0.74	-1.67	AAA-AAA	0.00	999	1.000	1.000	0.0222
240330_at	8.9	15.0	0.74	1.67	AAA-AAA	0.00	999	1.000	1.000	0.6719
1556420_s_at	15.9	9.5	-0.74	-1.67	AAA-AAA	0.00	999	1.000	1.000	0.2081
244846_at	25.7	43.1	0.74	1.67	AAA-AAA	0.00	999	1.000	1.000	0.4793
244198_at	12.6	21.1	0.74	1.67	AAA-AAA	0.00	999	1.000	1.000	0.6275
217323_at	10.5	17.6	0.74	1.67	AAA-AAA	0.00	999	1.000	1.000	0.6722
205612_at	2.4	4.1	0.74	1.67	AAA-AAA	0.00	999	1.000	1.000	0.5388
207810_at	9.8	5.9	-0.74	-1.67	AAA-AAA	0.00	999	1.000	1.000	0.5487
227583_at	90.7	54.2	-0.74	-1.67	AAA-AAM	0.00	999	1.000	1.000	0.4962
223866_at	16.2	9.7	-0.74	-1.67	AAA-AAA	0.00	999	1.000	1.000	0.4753
232430_at	65.1	38.9	-0.74	-1.67	AAA-AAA	0.00	999	1.000	1.000	0.5186
215730_at	58.9	35.2	-0.74	-1.67	APA-AAP	0.00	999	1.000	1.000	0.4469
232381_s_at	78.8	47.1	-0.74	-1.67	AAA-AAP	0.00	999	1.000	1.000	0.5341
241508_at	51.4	30.7	-0.74	-1.67	AAA-AAA	0.00	999	1.000	1.000	0.5825
240064_at	6.7	11.1	0.74	1.67	AAA-AAA	0.00	999	1.000	1.000	0.2922
1566185_at	51.8	31.0	-0.74	-1.67	AAA-AAA	0.00	999	1.000	1.000	0.5507
1565821_at	38.1	63.7	0.74	1.67	AAA-AAA	0.00	999	1.000	1.000	0.5316
1565915_at	17.0	28.4	0.74	1.67	AAA-AMA	0.00	999	1.000	1.000	0.5305
1566833_x_at	5.3	8.8	0.74	1.67	AAA-AAA	0.00	999	1.000	1.000	0.2994
1556970_at	35.5	21.2	-0.74	-1.67	AAA-AAA	0.00	999	1.000	1.000	0.6284
1552605_s_at	18.7	31.3	0.74	1.67	AAA-AAA	0.00	999	1.000	1.000	0.3056
1556683_x_at	33.9	20.3	-0.74	-1.67	AAA-AAA	0.00	999	1.000	1.000	0.5992
1555175_a_at	38.4	23.0	-0.74	-1.67	AAA-AAA	0.00	999	1.000	1.000	0.5624
1560630_at	94.4	56.4	-0.74	-1.67	AAA-AAA	0.00	999	1.000	1.000	0.3732
214839_at	21.3	35.6	0.74	1.67	AAA-AAA	0.00	999	1.000	1.000	0.2382
220687_at	18.0	10.8	-0.74	-1.67	AAA-AAA	0.00	999	1.000	1.000	0.6006
216966_at	19.5	11.7	-0.74	-1.67	AAA-AAA	0.00	999	1.000	1.000	0.5400
218293_x_at	52.0	31.1	-0.74	-1.67	AAA-AAA	0.00	999	1.000	1.000	0.0775
210964_s_at	16.0	26.7	0.74	1.67	AAA-AAA	0.00	999	1.000	1.000	0.5429
208437_at	41.7	25.0	-0.74	-1.67	AAA-AAA	0.00	999	1.000	1.000	0.5656
233135_at	100.0	59.8	-0.74	-1.67	AMA-AAA	0.00	999	1.000	1.000	0.2386
232813_s_at	13.9	8.3	-0.74	-1.67	AAA-AAA	0.00	999	1.000	1.000	0.1813
49111_at	59.9	35.8	-0.74	-1.67	AAA-AAA	0.00	999	1.000	1.000	0.2166
222959_at	11.8	7.0	-0.74	-1.67	AAA-AAA	0.00	999	1.000	1.000	0.7364
222345_at	16.3	9.8	-0.74	-1.67	AAA-AAA	0.00	999	1.000	1.000	0.5820
230916_at	26.8	16.0	-0.74	-1.67	AAA-AAA	0.00	999	1.000	1.000	0.1499
212343_at	154.0	92.1	-0.74	-1.67	PPP-PPP	0.00	999	1.000	1.000	0.0166
243326_at	126.3	75.5	-0.74	-1.67	PPA-PAA	0.00	999	1.000	1.000	0.2679
203115_at	134.2	80.3	-0.74	-1.67	PMP-PAP	0.00	999	1.000	1.000	0.3000
241554_at	17.8	10.6	-0.74	-1.67	AAA-AAA	0.00	999	1.000	1.000	0.6257

240323_at	10.8	18.1	0.74	1.67	AAA-AAA	0.00	999	1.000	1.000	0.5671
1569738_at	89.1	53.3	-0.74	-1.67	AAA-AAA	0.00	999	1.000	1.000	0.0345
241088_at	6.9	11.6	0.74	1.67	AAA-AAA	0.00	999	1.000	1.000	0.5150
1556940_at	10.7	6.4	-0.74	-1.67	AAA-AAA	0.00	999	1.000	1.000	0.7057
1553560_at	10.5	6.3	-0.74	-1.67	AAA-AAA	0.00	999	1.000	1.000	0.5818
243311_at	6.6	11.1	0.74	1.67	AAA-AAA	0.00	999	1.000	1.000	0.5674
1560604_at	14.3	23.9	0.74	1.67	AAA-AAA	0.00	999	1.000	1.000	0.3077
244156_at	22.7	13.6	-0.74	-1.67	AAA-AAA	0.00	999	1.000	1.000	0.6318
215319_at	10.3	17.2	0.74	1.67	AAA-AAA	0.00	999	1.000	1.000	0.2248
221351_at	28.9	48.3	0.74	1.67	AAA-AAA	0.00	999	1.000	1.000	0.6119
206944_at	96.4	57.7	-0.74	-1.67	AAA-AAA	0.00	999	1.000	1.000	0.4320
206692_at	10.1	16.9	0.74	1.67	AAA-AAA	0.00	999	1.000	1.000	0.4620
202999_s_at	7.8	13.1	0.74	1.67	AAA-AAA	0.00	999	1.000	1.000	0.5477
205272_s_at	6.9	11.6	0.74	1.67	AAA-AAA	0.00	999	1.000	1.000	0.1557
211728_s_at	38.9	23.3	-0.74	-1.67	AAA-AAA	0.00	999	1.000	1.000	0.6303
207972_at	7.1	11.8	0.74	1.67	AAA-AAA	0.00	999	1.000	1.000	0.5544
236593_at	57.5	96.2	0.74	1.67	AAA-AAA	0.00	999	1.000	1.000	0.4647
224529_s_at	78.6	47.0	-0.74	-1.67	AAA-AAA	0.00	999	1.000	1.000	0.5172
224466_s_at	19.0	31.7	0.74	1.67	AAA-AAA	0.00	999	1.000	1.000	0.2501
221980_at	12.8	21.4	0.74	1.67	AAA-AAA	0.00	999	1.000	1.000	0.4058
230749_s_at	26.7	16.0	-0.74	-1.67	AAA-AAA	0.00	999	1.000	1.000	0.3945
207510_at	221.9	132.8	-0.74	-1.67	PPP-APM	0.00	999	1.000	1.000	0.0337
243163_at	24.8	14.8	-0.74	-1.67	APA-AAA	0.00	999	1.000	1.000	0.3306
1560080_at	29.5	17.7	-0.74	-1.67	AMA-PAA	0.00	999	1.000	1.000	0.5719
230401_at	102.9	61.6	-0.74	-1.67	APA-AAM	0.00	999	1.000	1.000	0.6445
242211_x_at	23.2	13.9	-0.74	-1.67	AAA-AAA	0.00	999	1.000	1.000	0.6187
1563190_at	5.8	9.7	0.74	1.67	AAA-AAA	0.00	999	1.000	1.000	0.3158
1570046_at	34.6	20.7	-0.74	-1.67	AAA-AAA	0.00	999	1.000	1.000	0.0969
239909_at	7.3	12.1	0.74	1.67	AAA-AAA	0.00	999	1.000	1.000	0.1244
1557451_at	5.6	9.3	0.74	1.67	AAA-AAA	0.00	999	1.000	1.000	0.6046
1553965_x_at	3.0	4.9	0.74	1.67	AAA-AAA	0.00	999	1.000	1.000	0.3950
1555340_x_at	11.1	6.7	-0.74	-1.67	AAA-AAA	0.00	999	1.000	1.000	0.1752
244118_at	6.2	3.7	-0.74	-1.67	MAA-AAA	0.00	999	1.000	1.000	0.7440
216013_at	14.3	24.0	0.74	1.67	AAA-AAA	0.00	999	1.000	1.000	0.6001
214270_s_at	72.8	43.6	-0.74	-1.67	AAA-AAA	0.00	999	1.000	1.000	0.4253
220133_at	43.6	26.1	-0.74	-1.67	AAA-AAA	0.00	999	1.000	1.000	0.4851
217277_at	50.7	30.4	-0.74	-1.67	AAA-AAA	0.00	999	1.000	1.000	0.5366
208332_at	58.6	97.8	0.74	1.67	AAA-AAA	0.00	999	1.000	1.000	0.6023
[truncated: 13,392,339 more chars]
