# Supplementary material for: KLF8: so different in ovarian and breast cancer
Source: Oncoscience. 2014 Apr 30;1(4):248–9. doi: 10.18632/oncoscience.34 (PMC4256715; doi:10.18632/oncoscience.34)
Supplement: Supplementary file 2 [file oncoscience-01-0248-s002.pdf]

Transcript	Control Group Signal	Exp Group Signal	Est Log Ratio	Raw Est Fold Change	Affy PA Calls Cntl Grp-Exp Grp		Delta (PADE)	Set (PADE)	FDR for Accumulated Sets (PADE)	FDR for Specific Set (PADE)		Individual Transcript p-value
**Group Size**	**3.0**	**3.0**										
1559656_a_at	48.2	4.4	-3.44	-10.85	MAP-AAA		2.00	1	0.000	0.000		0.0000
224022_x_at	1.8	17.7	3.33	10.04	AAA-PAA		2.00	1	0.000	0.000		0.0001
214930_at	14.0	98.4	2.81	7.01	AAA-MPP		2.00	1	0.000	0.000		0.0001
1569023_a_at	35.1	3.1	-3.50	-11.29	PAA-AAA		1.60	2	0.250	1.000		0.0003
1569941_at	34.0	1.6	-4.40	-21.05	PAA-AAA		0.90	3	0.333	0.348		0.0043
1559882_at	45.1	3.1	-3.87	-14.67	APA-AAA		0.90	3	0.333	0.348		0.0029
237764_at	64.1	4.5	-3.85	-14.39	PAA-AAA		0.90	3	0.333	0.348		0.0052
216097_at	33.0	2.5	-3.73	-13.28	APA-AAA		0.90	3	0.333	0.348		0.0028
1566115_at	31.4	2.6	-3.60	-12.11	AAP-AAA		0.90	3	0.333	0.348		0.0015
232567_at	67.3	7.2	-3.22	-9.34	AAP-AAA		0.90	3	0.333	0.348		0.0049
216638_s_at	47.5	6.1	-2.96	-7.78	PAA-AAA		0.90	3	0.333	0.348		0.0043
241244_at	60.4	9.6	-2.66	-6.32	PAA-AAA		0.90	3	0.333	0.348		0.0017
1564887_at	91.2	14.7	-2.64	-6.23	PAA-AAA		0.90	3	0.333	0.348		0.0033
231187_at	8.5	46.2	2.44	5.44	AAA-APA		0.90	3	0.333	0.348		0.0005
239701_at	67.9	15.3	-2.15	-4.43	APA-AAA		0.90	3	0.333	0.348		0.0013
210150_s_at	304.3	105.5	-1.53	-2.88	PAA-AAA		0.90	3	0.333	0.348		0.0011
234685_x_at	113.0	40.8	-1.47	-2.77	APA-APA		0.90	3	0.333	0.348		0.0019
1555775_a_at	101.2	38.9	-1.38	-2.60	PPP-AAA		0.90	3	0.333	0.348		0.0011
1567101_at	67.6	27.0	-1.32	-2.50	APA-APA		0.90	3	0.333	0.348		0.0009
241890_at	68.1	27.5	-1.31	-2.48	PPP-AAA		0.90	3	0.333	0.348		0.0013
240939_x_at	75.6	30.7	-1.30	-2.46	PPP-PPP		0.90	3	0.333	0.348		0.0014
216639_at	50.7	21.1	-1.27	-2.41	PPA-AAA		0.90	3	0.333	0.348		0.0013
208467_at	177.5	86.3	-1.04	-2.06	AAP-AAA		0.90	3	0.333	0.348		0.0004
235110_at	135.5	68.7	-0.98	-1.97	PPP-APP		0.90	3	0.333	0.348		0.0001
234670_at	78.1	41.3	-0.92	-1.89	PPP-PPP		0.90	3	0.333	0.348		0.0004
243099_at	96.4	60.8	-0.67	-1.59	AAP-APA		0.90	3	0.333	0.348		0.0000
1558017_s_at	225.5	144.4	-0.64	-1.56	PPP-PPP		0.90	3	0.333	0.348		0.0001
235491_at	111.8	11.3	-3.31	-9.89	PPA-AAA		0.80	4	0.343	0.375		0.0052
1554947_at	47.7	9.9	-2.27	-4.82	APA-AAA		0.80	4	0.343	0.375		0.0050
1555289_at	100.5	24.0	-2.07	-4.19	PAA-AAA		0.80	4	0.343	0.375		0.0048
203949_at	88.5	28.3	-1.65	-3.13	PAA-AAA		0.80	4	0.343	0.375		0.0036
243100_at	85.2	30.3	-1.49	-2.81	APA-APA		0.80	4	0.343	0.375		0.0031
232605_s_at	92.6	33.4	-1.47	-2.77	PAA-AMA		0.80	4	0.343	0.375		0.0031
1555702_a_at	139.8	56.8	-1.30	-2.46	APA-PPA		0.80	4	0.343	0.375		0.0019
227609_at	31.3	114.1	1.88	3.68	PPP-PPP		0.08	4	0.343	0.375		0.0062
1564816_at	177.1	75.7	-1.23	-2.34	PAA-APP		0.80	4	0.343	0.375		0.0020
240751_at	49.1	3.0	-4.05	-16.55	APA-AAA		0.70	5	0.354	0.367		0.0095
207872_s_at	60.2	5.2	-3.54	-11.65	APA-AAA		0.70	5	0.354	0.367		0.0111

242378_at	79.0	8.3	-3.25	-9.49	AAP-AAA	0.70	5	0.354	0.367	0.0114
244672_at	106.5	13.6	-2.97	-7.83	PAA-AAA	0.70	5	0.354	0.367	0.0113
237265_at	32.7	4.5	-2.87	-7.32	APA-AAA	0.70	5	0.354	0.367	0.0085
1554108_at	39.4	7.0	-2.49	-5.60	APA-AAA	0.70	5	0.354	0.367	0.0062
1556413_a_at	59.4	11.7	-2.34	-5.06	APP-AAA	0.70	5	0.354	0.367	0.0096
203922_s_at	74.3	19.4	-1.94	-3.83	PAA-AAA	0.70	5	0.354	0.367	0.0069
216828_at	53.6	14.5	-1.89	-3.70	AMP-AAA	0.70	5	0.354	0.367	0.0056
213721_at	82.9	23.1	-1.84	-3.59	APP-AAA	0.70	5	0.354	0.367	0.0049
234731_at	195.1	62.3	-1.65	-3.13	APP-APP	0.70	5	0.354	0.367	0.0072
223395_at	573.9	196.6	-1.55	-2.92	PPP-PPP	0.70	5	0.354	0.367	0.0063
1552497_a_at	100.1	34.5	-1.54	-2.90	APM-AAA	0.70	5	0.354	0.367	0.0058
1556896_at	55.6	19.8	-1.49	-2.81	AAP-AAA	0.70	5	0.354	0.367	0.0057
240409_at	68.1	24.9	-1.45	-2.74	PAP-AAA	0.70	5	0.354	0.367	0.0064
1569499_at	73.6	27.2	-1.44	-2.71	APP-APA	0.70	5	0.354	0.367	0.0052
1556082_a_at	128.7	50.2	-1.36	-2.56	PPP-APP	0.70	5	0.354	0.367	0.0032
233485_at	49.4	19.5	-1.34	-2.53	PAA-AMA	0.70	5	0.354	0.367	0.0034
233585_at	157.3	69.6	-1.18	-2.26	APA-AAA	0.70	5	0.354	0.367	0.0026
1570269_at	60.3	26.7	-1.17	-2.26	AAP-AAA	0.70	5	0.354	0.367	0.0040
219232_s_at	82.8	36.9	-1.17	-2.25	APM-MAP	0.70	5	0.354	0.367	0.0027
214860_at	119.0	55.4	-1.10	-2.15	AAM-AAP	0.70	5	0.354	0.367	0.0025
227725_at	28.8	58.2	1.01	2.02	AAA-APP	0.07	5	0.354	0.367	0.0091
222905_s_at	302.3	153.2	-0.98	-1.97	PPP-PPP	0.70	5	0.354	0.367	0.0033
241093_at	83.8	43.8	-0.93	-1.91	APA-PAM	0.70	5	0.354	0.367	0.0022
208257_x_at	598.9	339.0	-0.82	-1.77	PPP-PPP	0.70	5	0.354	0.367	0.0010
222303_at	234.9	136.2	-0.79	-1.73	PAP-PPP	0.70	5	0.354	0.367	0.0016
232773_at	92.8	53.8	-0.79	-1.72	PPP-PPP	0.70	5	0.354	0.367	0.0009
1552671_a_at	190.1	117.4	-0.70	-1.62	PAP-PPP	0.70	5	0.354	0.367	0.0006
208191_x_at	1229.1	763.0	-0.69	-1.61	PPP-PPP	0.70	5	0.354	0.367	0.0005
220007_at	435.2	295.1	-0.56	-1.48	PPP-PPP	0.70	5	0.354	0.367	0.0003
241581_at	2.9	27.2	3.21	9.27	AAA-AAP	0.60	6	0.380	0.414	0.0036
220534_at	6.6	58.1	3.15	8.86	AAA-MPA	0.60	6	0.380	0.414	0.0025
206457_s_at	5.8	43.3	2.91	7.50	AAA-APA	0.60	6	0.380	0.414	0.0039
220481_at	10.1	72.3	2.84	7.15	AAA-APA	0.60	6	0.380	0.414	0.0061
1565684_at	4.4	31.1	2.83	7.11	AAA-PPA	0.60	6	0.380	0.414	0.0039
207010_at	67.1	11.6	-2.53	-5.79	MPM-AAA	0.60	6	0.380	0.414	0.0155
233366_at	40.1	8.3	-2.28	-4.86	APA-AAA	0.60	6	0.380	0.414	0.0114
236147_at	72.3	17.4	-2.05	-4.15	APA-AAA	0.60	6	0.380	0.414	0.0107
216856_s_at	9.2	37.3	2.02	4.05	AAA-AAP	0.60	6	0.380	0.414	0.0027
1564083_at	23.9	6.0	-2.00	-3.99	PAA-AAA	0.60	6	0.380	0.414	0.0119
217028_at	6.1	20.0	1.68	3.25	APA-PPP	0.60	6	0.380	0.414	0.0067
243438_at	139.2	44.5	-1.65	-3.13	APA-AAP	0.60	6	0.380	0.414	0.0102
237086_at	14.6	44.5	1.61	3.05	AAA-MPP	0.60	6	0.380	0.414	0.0025

208711_s_at	1243.3	3646.4	1.58	3.01	PPP-PPP	0.60	6	0.370	0.413	0.0009
216522_at	77.8	25.5	-1.61	-3.05	AAP-AAA	0.60	6	0.380	0.414	0.0079
206376_at	100.5	303.1	1.59	3.02	PAA-PPP	0.60	6	0.380	0.414	0.0009
202563_at	57.7	19.5	-1.57	-2.97	AAP-AAA	0.60	6	0.380	0.414	0.0081
229816_at	61.1	20.6	-1.57	-2.96	PAA-AAA	0.60	6	0.380	0.414	0.0092
1566284_at	84.6	29.1	-1.54	-2.90	AAA-AAP	0.60	6	0.380	0.414	0.0082
230795_at	194.0	537.5	1.47	2.77	APP-PPP	0.60	6	0.380	0.414	0.0018
205841_at	93.2	34.4	-1.44	-2.71	PPP-AAA	0.60	6	0.380	0.414	0.0074
216248_s_at	38.0	14.1	-1.43	-2.70	AAP-AAA	0.60	6	0.380	0.414	0.0086
235275_at	23.4	61.9	1.41	2.65	AAA-APA	0.60	6	0.380	0.414	0.0018
1553031_at	91.0	34.6	-1.40	-2.63	PPP-AAA	0.60	6	0.380	0.414	0.0073
215882_at	81.4	31.8	-1.36	-2.56	AAP-AAA	0.60	6	0.380	0.414	0.0084
227578_at	67.2	171.4	1.35	2.55	AAP-APP	0.60	6	0.380	0.414	0.0020
208712_at	1921.6	4861.6	1.34	2.53	PPP-PPP	0.60	6	0.380	0.414	0.0036
1555812_a_at	143.5	359.7	1.33	2.51	APP-PPP	0.60	6	0.380	0.414	0.0025
227597_at	179.6	71.8	-1.32	-2.50	APP-AAA	0.60	6	0.380	0.414	0.0062
217204_at	17.2	43.0	1.32	2.50	AAA-APP	0.60	6	0.380	0.414	0.0006
227203_at	102.9	251.8	1.29	2.45	PAP-PPP	0.60	6	0.380	0.414	0.0013
220145_at	126.0	300.3	1.25	2.39	APA-PPP	0.60	6	0.380	0.414	0.0018
218807_at	112.7	48.1	-1.23	-2.35	PAA-AAA	0.60	6	0.380	0.414	0.0069
216902_s_at	242.7	532.9	1.14	2.20	PPP-PPP	0.60	6	0.380	0.414	0.0006
229883_at	116.2	53.1	-1.13	-2.19	AAA-PAA	0.60	6	0.380	0.414	0.0074
1569815_x_at	65.9	30.2	-1.13	-2.18	PPA-AAA	0.60	6	0.380	0.414	0.0064
202202_s_at	819.3	1765.8	1.11	2.16	PPP-PPP	0.60	6	0.380	0.414	0.0018
228857_at	72.3	148.7	1.04	2.06	APP-PPP	0.60	6	0.380	0.414	0.0002
228223_at	103.5	206.6	1.00	2.00	AAA-PPP	0.60	6	0.380	0.414	0.0014
1553881_at	60.6	120.8	1.00	1.99	AAP-PPP	0.60	6	0.380	0.414	0.0010
236486_at	150.7	76.6	-0.98	-1.97	APP-PPP	0.60	6	0.380	0.414	0.0049
201106_at	9352.8	4766.3	-0.97	-1.96	PPP-PPP	0.60	6	0.380	0.414	0.0036
210967_x_at	157.4	81.0	-0.96	-1.94	AAA-PAP	0.60	6	0.380	0.414	0.0048
211540_s_at	87.1	164.7	0.92	1.89	PAA-PAP	0.60	6	0.380	0.414	0.0003
214454_at	226.3	125.9	-0.85	-1.80	APP-AAM	0.60	6	0.380	0.414	0.0026
216885_s_at	288.3	160.4	-0.85	-1.80	PPP-PPP	0.60	6	0.380	0.414	0.0034
214337_at	106.5	60.4	-0.82	-1.76	PAP-PAP	0.60	6	0.380	0.414	0.0033
220033_at	249.0	142.8	-0.80	-1.74	PPP-PPP	0.60	6	0.380	0.414	0.0022
244294_at	110.0	68.8	-0.68	-1.60	AAP-AAP	0.60	6	0.380	0.414	0.0020
207164_s_at	124.5	78.0	-0.68	-1.60	PPP-PMP	0.60	6	0.380	0.414	0.0021
1558430_at	100.1	63.9	-0.65	-1.57	AAP-AAA	0.60	6	0.380	0.414	0.0014
219590_x_at	2934.6	1987.9	-0.56	-1.48	PPP-PPP	0.60	6	0.380	0.414	0.0004
204298_s_at	12093.3	8433.6	-0.52	-1.43	PPP-PPP	0.60	6	0.380	0.414	0.0003
200917_s_at	1049.6	734.6	-0.52	-1.43	PPP-PPP	0.60	6	0.380	0.414	0.0003
240915_at	33.8	2.6	-3.69	-12.92	PAA-AAA	0.50	7	0.420	0.469	0.0234

1552819_at	39.8	4.4	-3.20	-9.16	APA-AAA	0.50	7	0.420	0.469	0.0244
224034_at	3.9	31.3	3.02	8.08	AAA-PAA	0.50	7	0.420	0.469	0.0070
210126_at	100.3	12.8	-2.97	-7.83	AAP-AAA	0.50	7	0.420	0.469	0.0266
1563637_at	32.8	4.4	-2.91	-7.54	PAP-AAA	0.50	7	0.420	0.469	0.0287
235306_at	37.3	5.2	-2.85	-7.21	PAA-AAA	0.50	7	0.420	0.469	0.0187
210579_s_at	75.9	10.7	-2.83	-7.11	PAA-AAA	0.50	7	0.420	0.469	0.0230
211149_at	44.7	6.8	-2.73	-6.62	PAA-AAA	0.50	7	0.420	0.469	0.0272
229215_at	66.1	10.4	-2.67	-6.38	AAP-AAA	0.50	7	0.420	0.469	0.0276
232690_at	137.8	22.6	-2.61	-6.11	PAA-AAA	0.50	7	0.420	0.469	0.0215
211171_s_at	51.4	10.3	-2.32	-4.98	PAA-AAA	0.50	7	0.420	0.469	0.0252
1553204_at	107.5	22.5	-2.26	-4.78	PAA-AAA	0.50	7	0.420	0.469	0.0197
215569_at	105.1	23.9	-2.14	-4.40	APA-AAP	0.50	7	0.420	0.469	0.0225
1561719_at	58.0	14.8	-1.97	-3.93	APP-AAA	0.50	7	0.420	0.469	0.0220
1560051_at	36.4	9.3	-1.97	-3.91	APA-AAA	0.50	7	0.420	0.469	0.0213
223798_at	28.4	107.4	1.92	3.79	AAA-APA	0.50	7	0.420	0.469	0.0047
235310_at	71.4	19.9	-1.84	-3.58	PAA-AAA	0.50	7	0.420	0.469	0.0158
1562888_at	49.9	14.1	-1.82	-3.54	PAA-AAA	0.50	7	0.420	0.469	0.0181
230616_at	91.6	27.2	-1.75	-3.37	APA-AAA	0.50	7	0.420	0.469	0.0195
1560480_at	112.8	33.5	-1.75	-3.37	PMA-AAA	0.50	7	0.420	0.469	0.0225
231090_s_at	121.8	408.0	1.74	3.35	PPP-PPP	0.50	7	0.420	0.469	0.0048
1552794_a_at	72.0	23.7	-1.60	-3.04	PPA-AAA	0.50	7	0.420	0.469	0.0129
241240_at	62.7	20.7	-1.60	-3.03	AMP-AAA	0.50	7	0.420	0.469	0.0177
229781_at	45.2	15.1	-1.59	-3.01	PAA-AAA	0.50	7	0.420	0.469	0.0171
244393_x_at	33.5	11.3	-1.57	-2.97	PAP-AAA	0.50	7	0.420	0.469	0.0206
1558797_at	33.3	11.3	-1.56	-2.95	APA-AAA	0.50	7	0.420	0.469	0.0187
1555320_a_at	58.9	20.0	-1.56	-2.94	PAA-AAA	0.50	7	0.420	0.469	0.0128
223640_at	146.1	50.2	-1.54	-2.91	PPP-AAP	0.50	7	0.420	0.469	0.0160
1559350_at	64.8	22.6	-1.52	-2.86	PAP-AAA	0.50	7	0.420	0.469	0.0178
216882_s_at	117.8	42.2	-1.48	-2.79	APA-PAA	0.50	7	0.420	0.469	0.0193
214557_at	81.2	29.7	-1.45	-2.73	PAA-AAA	0.50	7	0.420	0.469	0.0179
233583_at	72.6	26.8	-1.44	-2.71	PPA-AAA	0.50	7	0.420	0.469	0.0126
232324_x_at	71.0	26.6	-1.42	-2.68	APA-AAP	0.50	7	0.420	0.469	0.0119
238096_at	188.8	71.3	-1.41	-2.65	APA-AAP	0.50	7	0.420	0.469	0.0135
227672_at	196.6	74.4	-1.40	-2.64	AAA-PAA	0.50	7	0.420	0.469	0.0143
238319_at	105.5	40.1	-1.40	-2.63	APA-AAA	0.50	7	0.420	0.469	0.0113
234079_at	128.3	49.2	-1.38	-2.61	APA-AAA	0.50	7	0.420	0.469	0.0139
208417_at	127.6	49.4	-1.37	-2.58	AAP-AAA	0.50	7	0.420	0.469	0.0181
238586_at	62.3	24.7	-1.33	-2.52	PMP-AAA	0.50	7	0.420	0.469	0.0184
217435_x_at	77.1	30.7	-1.33	-2.51	AAP-AAA	0.50	7	0.420	0.469	0.0170
207135_at	76.0	30.4	-1.33	-2.51	PPP-AAA	0.50	7	0.420	0.469	0.0127
241634_at	97.4	39.5	-1.30	-2.47	PAP-AAA	0.50	7	0.420	0.469	0.0099
232999_at	58.8	23.9	-1.30	-2.46	MPA-AAA	0.50	7	0.420	0.469	0.0170

209267_s_at	86.0	211.0	1.30	2.45	PMP-PPP	0.50	7	0.420	0.469	0.0033
1552455_at	114.1	47.8	-1.26	-2.39	PAP-AAA	0.50	7	0.420	0.469	0.0095
220357_s_at	105.2	44.2	-1.25	-2.38	AAP-AAM	0.50	7	0.420	0.469	0.0162
1569867_at	121.6	51.2	-1.25	-2.38	PPA-AAA	0.50	7	0.420	0.469	0.0100
204584_at	420.7	178.6	-1.24	-2.36	PPP-PPP	0.50	7	0.420	0.469	0.0126
243055_at	150.6	64.5	-1.22	-2.33	PPP-PPP	0.50	7	0.420	0.469	0.0174
216467_s_at	86.2	37.1	-1.22	-2.33	PPA-AAA	0.50	7	0.420	0.469	0.0164
220375_s_at	258.7	116.7	-1.15	-2.22	PPP-PPM	0.50	7	0.420	0.469	0.0110
205708_s_at	183.4	82.8	-1.15	-2.22	PPM-PAP	0.50	7	0.420	0.469	0.0089
1555176_at	89.0	40.2	-1.15	-2.21	PPM-AAM	0.50	7	0.420	0.469	0.0099
1554027_a_at	95.8	44.2	-1.12	-2.17	PPP-APP	0.50	7	0.420	0.469	0.0122
215838_at	64.3	29.8	-1.11	-2.16	PPA-AAP	0.50	7	0.420	0.469	0.0130
237051_at	97.3	45.1	-1.11	-2.16	PPP-PAP	0.50	7	0.420	0.469	0.0112
242290_at	110.8	52.3	-1.08	-2.12	PPP-APA	0.50	7	0.420	0.469	0.0103
234631_at	175.8	83.2	-1.08	-2.11	PPP-MPP	0.50	7	0.420	0.469	0.0117
228602_at	64.8	30.9	-1.07	-2.10	APP-AMA	0.50	7	0.420	0.469	0.0075
222213_x_at	63.7	30.8	-1.05	-2.07	PPA-AMA	0.50	7	0.420	0.469	0.0107
222219_s_at	98.8	48.1	-1.04	-2.05	APP-PPA	0.50	7	0.420	0.469	0.0124
236599_at	277.6	135.9	-1.03	-2.04	PPP-PAA	0.50	7	0.420	0.469	0.0079
222370_x_at	95.3	47.9	-0.99	-1.99	PPP-PAP	0.50	7	0.420	0.469	0.0075
226931_at	230.4	117.0	-0.98	-1.97	PPP-PPP	0.50	7	0.420	0.469	0.0116
224058_s_at	78.6	40.0	-0.97	-1.96	MAP-AAP	0.50	7	0.420	0.469	0.0087
220672_at	76.6	39.0	-0.97	-1.96	PPA-PPP	0.50	7	0.420	0.469	0.0070
1567069_at	67.1	34.6	-0.96	-1.94	PAA-AAA	0.50	7	0.420	0.469	0.0098
235101_at	82.7	43.5	-0.93	-1.90	AMA-APA	0.50	7	0.420	0.469	0.0082
217706_at	46.4	24.4	-0.92	-1.90	AAP-AAA	0.50	7	0.420	0.469	0.0072
1570566_at	54.0	28.7	-0.91	-1.88	PPA-APP	0.50	7	0.420	0.469	0.0120
239140_at	64.3	34.5	-0.90	-1.86	PPP-APP	0.50	7	0.420	0.469	0.0090
1567013_at	216.0	116.2	-0.89	-1.86	PPP-PPP	0.50	7	0.420	0.469	0.0081
202571_s_at	237.3	130.3	-0.86	-1.82	AAP-AAM	0.50	7	0.420	0.469	0.0088
211340_s_at	292.6	161.8	-0.86	-1.81	PAP-PPP	0.50	7	0.420	0.469	0.0100
242760_x_at	158.2	285.3	0.85	1.80	PPP-PPP	0.50	7	0.420	0.469	0.0010
1559048_at	262.4	146.4	-0.84	-1.79	PPP-PPP	0.50	7	0.420	0.469	0.0046
235856_at	85.6	47.9	-0.84	-1.79	AAA-AAP	0.50	7	0.420	0.469	0.0088
241474_at	285.2	162.0	-0.82	-1.76	PPP-PMP	0.50	7	0.420	0.469	0.0100
1562830_at	120.7	70.0	-0.79	-1.72	PPP-APP	0.50	7	0.420	0.469	0.0048
236662_at	127.3	74.0	-0.78	-1.72	PPP-PMP	0.50	7	0.420	0.469	0.0057
205456_at	93.3	55.4	-0.75	-1.69	AAA-APA	0.50	7	0.420	0.469	0.0072
243253_at	93.8	55.9	-0.75	-1.68	APP-PPP	0.50	7	0.420	0.469	0.0047
1569053_at	273.0	162.6	-0.75	-1.68	PPP-AMP	0.50	7	0.420	0.469	0.0036
208035_at	92.4	55.1	-0.75	-1.68	PAP-PPP	0.50	7	0.420	0.469	0.0050
238843_at	491.4	309.2	-0.67	-1.59	PPP-PPP	0.50	7	0.420	0.469	0.0053

1316_at	151.8	96.3	-0.66	-1.58	PPP-PPP	0.50	7	0.420	0.469	0.0037
235954_at	138.1	87.9	-0.65	-1.57	PPP-PPP	0.50	7	0.420	0.469	0.0034
212425_at	144.2	93.9	-0.62	-1.54	PPP-PPP	0.50	7	0.420	0.469	0.0036
241800_x_at	91.5	59.6	-0.62	-1.54	PAA-AAA	0.50	7	0.420	0.469	0.0045
1561638_at	48.2	31.5	-0.61	-1.53	PPP-APA	0.50	7	0.420	0.469	0.0031
220580_at	187.1	123.1	-0.60	-1.52	PPP-PPP	0.50	7	0.420	0.469	0.0036
202182_at	1090.2	728.5	-0.58	-1.50	PPP-PPP	0.50	7	0.420	0.469	0.0019
228797_at	312.4	218.9	-0.51	-1.43	PPP-PPP	0.50	7	0.420	0.469	0.0021
1569013_s_at	174.3	122.1	-0.51	-1.43	PPP-PPP	0.50	7	0.420	0.469	0.0009
226080_at	469.5	342.3	-0.46	-1.37	PPP-PPP	0.50	7	0.420	0.469	0.0013
242710_at	117.1	92.5	-0.34	-1.27	PPP-PPP	0.50	7	0.420	0.469	0.0000
1557745_at	48.4	4.1	-3.57	-11.88	AAP-AAA	0.40	8	0.500	0.565	0.0363
237123_x_at	38.3	3.3	-3.52	-11.48	AAP-AAA	0.40	8	0.500	0.565	0.0511
228732_at	44.3	4.4	-3.33	-10.05	APA-AAA	0.40	8	0.500	0.565	0.0519
1557374_at	74.9	7.5	-3.32	-10.01	PAM-AAA	0.40	8	0.500	0.565	0.0427
1565635_at	61.5	6.4	-3.26	-9.57	PAM-AAA	0.40	8	0.500	0.565	0.0554
1566295_at	43.4	4.8	-3.19	-9.11	PPA-AAA	0.40	8	0.500	0.565	0.0545
1561339_at	54.1	6.0	-3.19	-9.10	APA-AAA	0.40	8	0.500	0.565	0.0424
236035_at	23.4	2.7	-3.12	-8.69	PAA-AAA	0.40	8	0.500	0.565	0.0582
233002_at	8.1	66.2	3.04	8.21	AAA-APP	0.40	8	0.500	0.565	0.0104
233301_at	49.8	6.2	-3.00	-7.97	PAA-AAA	0.40	8	0.500	0.565	0.0333
1564765_at	49.6	6.4	-2.95	-7.71	APA-AAA	0.40	8	0.500	0.565	0.0433
207207_at	44.7	5.9	-2.93	-7.63	PAA-AAA	0.40	8	0.500	0.565	0.0582
233671_at	59.3	7.9	-2.90	-7.47	APA-AAA	0.40	8	0.500	0.565	0.0569
1562313_at	30.9	4.2	-2.89	-7.41	AAP-AAA	0.40	8	0.500	0.565	0.0312
237831_x_at	20.6	2.8	-2.87	-7.30	APA-AAA	0.40	8	0.500	0.565	0.0320
240413_at	29.5	4.1	-2.84	-7.17	AAP-AAA	0.40	8	0.500	0.565	0.0406
242464_at	43.6	6.2	-2.83	-7.09	PAA-AAA	0.40	8	0.500	0.565	0.0591
239967_at	52.4	7.5	-2.80	-6.95	PAA-AAA	0.40	8	0.500	0.565	0.0451
234692_at	13.6	2.0	-2.78	-6.86	AAP-AAA	0.40	8	0.500	0.565	0.0393
1559914_at	40.0	6.4	-2.66	-6.30	AAP-AAA	0.40	8	0.500	0.565	0.0581
244767_at	26.7	4.3	-2.62	-6.15	PAA-AAA	0.40	8	0.500	0.565	0.0454
221319_at	52.4	9.1	-2.52	-5.73	APP-AAA	0.40	8	0.500	0.565	0.0507
1560995_s_at	50.7	8.9	-2.51	-5.69	APP-AAA	0.40	8	0.500	0.565	0.0387
209956_s_at	38.5	6.9	-2.48	-5.58	PAA-AAA	0.40	8	0.500	0.565	0.0517
222300_at	45.1	8.2	-2.47	-5.54	PAA-AAA	0.40	8	0.500	0.565	0.0385
207840_at	84.0	15.4	-2.45	-5.44	PAA-AAM	0.40	8	0.500	0.565	0.0395
232729_at	97.2	18.0	-2.43	-5.40	PAA-AAA	0.40	8	0.500	0.565	0.0566
240431_at	48.8	9.2	-2.41	-5.32	PAA-AAA	0.40	8	0.500	0.565	0.0502
1561523_at	29.8	5.8	-2.36	-5.13	AAP-AAA	0.40	8	0.500	0.565	0.0332
243346_at	39.3	7.8	-2.34	-5.06	PAA-AAA	0.40	8	0.500	0.565	0.0352
205291_at	98.6	19.6	-2.33	-5.02	APA-AAA	0.40	8	0.500	0.565	0.0399

205225_at	66.4	13.6	-2.29	-4.88	APA-AAA	0.40	8	0.500	0.565	0.0566
237479_at	31.6	6.7	-2.23	-4.70	AAP-AAA	0.40	8	0.500	0.565	0.0293
214392_at	54.2	11.6	-2.23	-4.69	AAP-AAA	0.40	8	0.500	0.565	0.0379
206938_at	41.8	9.0	-2.22	-4.65	APA-AAA	0.40	8	0.500	0.565	0.0532
216259_at	87.6	19.1	-2.20	-4.60	PPA-MAA	0.40	8	0.500	0.565	0.0280
1559746_a_at	69.6	15.2	-2.19	-4.57	PAA-AAP	0.40	8	0.500	0.565	0.0461
238235_at	147.8	32.6	-2.18	-4.54	PPA-AAA	0.40	8	0.500	0.565	0.0406
220805_at	82.8	18.8	-2.14	-4.42	APA-AAA	0.40	8	0.500	0.565	0.0312
222957_at	119.1	28.4	-2.07	-4.19	PAA-AAA	0.40	8	0.500	0.565	0.0275
240517_at	65.9	15.8	-2.06	-4.16	APA-AAA	0.40	8	0.500	0.565	0.0520
237117_at	37.1	9.1	-2.03	-4.09	APA-AAA	0.40	8	0.500	0.565	0.0516
238850_at	26.5	6.5	-2.03	-4.09	APA-AAA	0.40	8	0.500	0.565	0.0541
231349_at	89.2	22.1	-2.01	-4.04	AAA-AAP	0.40	8	0.500	0.565	0.0299
222058_at	82.9	20.7	-2.00	-4.01	APP-AAA	0.40	8	0.500	0.565	0.0517
240933_at	61.8	15.9	-1.96	-3.88	APP-AAA	0.40	8	0.500	0.565	0.0319
236397_at	42.0	11.2	-1.90	-3.74	PAA-AAA	0.40	8	0.500	0.565	0.0408
212587_s_at	39.7	10.9	-1.87	-3.64	APA-AAA	0.40	8	0.500	0.565	0.0343
1559835_at	66.1	18.5	-1.84	-3.57	PPA-AAA	0.40	8	0.500	0.565	0.0494
1555000_at	45.4	13.0	-1.80	-3.48	APA-AAA	0.40	8	0.500	0.565	0.0447
1566889_at	55.4	16.7	-1.73	-3.31	APP-AAA	0.40	8	0.500	0.565	0.0348
244075_at	87.1	26.3	-1.73	-3.31	APA-AAA	0.40	8	0.500	0.565	0.0442
1564338_at	67.7	20.6	-1.72	-3.28	PAA-AAA	0.40	8	0.500	0.565	0.0405
1555034_at	28.8	8.9	-1.70	-3.25	AAP-AAA	0.40	8	0.500	0.565	0.0356
1554801_at	98.2	30.7	-1.68	-3.20	MAP-AAA	0.40	8	0.500	0.565	0.0319
239723_at	47.6	15.0	-1.67	-3.17	APA-AAA	0.40	8	0.500	0.565	0.0297
1559522_at	143.6	45.4	-1.66	-3.16	AAP-AAA	0.40	8	0.500	0.565	0.0371
238621_at	80.3	25.7	-1.64	-3.12	AAP-AAA	0.40	8	0.500	0.565	0.0453
1564052_at	60.2	19.6	-1.62	-3.06	PPA-AAA	0.40	8	0.500	0.565	0.0498
232913_at	94.1	30.9	-1.61	-3.05	PAA-AAM	0.40	8	0.500	0.565	0.0293
236521_at	60.1	20.2	-1.57	-2.98	PMP-AAA	0.40	8	0.500	0.565	0.0288
220360_at	61.9	20.8	-1.57	-2.97	APA-AAA	0.40	8	0.500	0.565	0.0349
243108_at	54.1	18.3	-1.57	-2.96	MPP-AAA	0.40	8	0.500	0.565	0.0419
215583_at	81.2	27.7	-1.55	-2.93	AAP-AAA	0.40	8	0.500	0.565	0.0347
1554710_at	30.8	10.6	-1.55	-2.92	PAA-AAA	0.40	8	0.500	0.565	0.0360
244352_at	79.3	27.3	-1.54	-2.91	AMA-AAP	0.40	8	0.500	0.565	0.0468
211334_at	84.2	29.2	-1.53	-2.89	PPA-AAA	0.40	8	0.500	0.565	0.0462
214159_at	64.7	22.4	-1.53	-2.88	AAP-AAA	0.40	8	0.500	0.565	0.0337
1563038_at	59.9	21.0	-1.51	-2.86	PAP-AAA	0.40	8	0.500	0.565	0.0327
1554686_at	47.3	16.8	-1.49	-2.82	APP-AAA	0.40	8	0.500	0.565	0.0448
1554443_s_at	51.7	18.6	-1.48	-2.78	AAP-AAA	0.40	8	0.500	0.565	0.0439
216615_s_at	93.4	33.8	-1.47	-2.76	PAA-AAA	0.40	8	0.500	0.565	0.0242
217197_x_at	69.8	25.8	-1.44	-2.71	APA-AAA	0.40	8	0.500	0.565	0.0222

240372_at	91.1	33.9	-1.43	-2.69	PAP-AAA	0.40	8	0.500	0.565	0.0431
219823_at	62.6	167.8	1.42	2.68	APP-PPP	0.04	8	0.500	0.565	0.0320
240660_at	57.4	21.4	-1.42	-2.68	APA-AAA	0.40	8	0.500	0.565	0.0272
210873_x_at	88.9	33.4	-1.41	-2.66	PPA-AAA	0.40	8	0.500	0.565	0.0230
210493_s_at	66.1	24.9	-1.41	-2.66	PAA-AAA	0.40	8	0.500	0.565	0.0424
231937_at	81.9	31.0	-1.40	-2.64	PAA-AAA	0.40	8	0.500	0.565	0.0256
202597_at	102.4	38.9	-1.40	-2.63	AAP-AAA	0.40	8	0.500	0.565	0.0270
1559928_at	62.7	24.2	-1.37	-2.59	APA-AAA	0.40	8	0.500	0.565	0.0341
1560493_a_at	86.8	33.7	-1.37	-2.58	PAP-PAA	0.40	8	0.500	0.565	0.0275
244297_at	53.6	21.1	-1.35	-2.54	PAA-AAA	0.40	8	0.500	0.565	0.0305
232494_at	67.5	26.6	-1.34	-2.54	PAA-AAA	0.40	8	0.500	0.565	0.0279
208360_s_at	83.0	32.8	-1.34	-2.53	MMA-PAA	0.40	8	0.500	0.565	0.0371
1564878_at	75.3	29.9	-1.33	-2.52	PPP-PAA	0.40	8	0.500	0.565	0.0248
242176_at	140.6	56.0	-1.33	-2.51	PPP-PPP	0.40	8	0.500	0.565	0.0276
52651_at	110.6	44.2	-1.33	-2.51	APP-PPA	0.40	8	0.500	0.565	0.0356
238413_at	62.9	25.1	-1.32	-2.50	AAA-APA	0.40	8	0.500	0.565	0.0289
210939_s_at	100.7	40.3	-1.32	-2.50	APP-AAA	0.40	8	0.500	0.565	0.0400
1562797_at	105.0	42.1	-1.32	-2.50	AAP-AAA	0.40	8	0.500	0.565	0.0216
1557203_at	185.0	74.7	-1.31	-2.48	PPA-AAA	0.40	8	0.500	0.565	0.0220
208423_s_at	72.8	29.4	-1.31	-2.48	PAA-AAA	0.40	8	0.500	0.565	0.0232
244420_at	90.6	36.7	-1.31	-2.47	APP-APA	0.40	8	0.500	0.565	0.0443
1560222_at	66.8	27.3	-1.29	-2.45	AAP-APA	0.40	8	0.500	0.565	0.0264
236484_at	92.1	37.6	-1.29	-2.45	AAP-AAP	0.40	8	0.500	0.565	0.0438
227524_at	94.7	38.7	-1.29	-2.45	AAP-AAA	0.40	8	0.500	0.565	0.0258
236149_at	131.3	53.7	-1.29	-2.44	PPP-AMP	0.40	8	0.500	0.565	0.0310
224048_at	130.4	53.7	-1.28	-2.43	PPP-AAP	0.40	8	0.500	0.565	0.0271
232833_at	55.9	23.1	-1.27	-2.42	APA-AAA	0.40	8	0.500	0.565	0.0237
237081_at	112.5	46.7	-1.27	-2.41	AMP-APP	0.40	8	0.500	0.565	0.0196
243403_x_at	73.9	30.7	-1.27	-2.41	PPA-AAA	0.40	8	0.500	0.565	0.0319
242130_at	31.8	13.2	-1.26	-2.40	APA-AAA	0.40	8	0.500	0.565	0.0233
231006_at	123.6	51.7	-1.26	-2.39	PAA-AAA	0.40	8	0.500	0.565	0.0189
1558624_at	84.3	35.3	-1.26	-2.39	AAP-AAA	0.40	8	0.500	0.565	0.0334
1554786_at	51.4	21.6	-1.25	-2.38	AAP-AAA	0.40	8	0.500	0.565	0.0301
236010_at	22.9	54.5	1.25	2.38	AAA-PPP	0.40	8	0.500	0.565	0.0038
207568_at	101.1	42.6	-1.25	-2.37	PPP-AAA	0.40	8	0.500	0.565	0.0233
230539_at	50.7	21.5	-1.24	-2.36	APA-AAA	0.40	8	0.500	0.565	0.0402
1569039_s_at	55.7	23.7	-1.23	-2.35	PAP-AAA	0.40	8	0.500	0.565	0.0210
240712_s_at	49.7	21.2	-1.23	-2.35	PPP-AAM	0.40	8	0.500	0.565	0.0252
215062_at	149.3	64.6	-1.21	-2.31	PPP-APP	0.40	8	0.500	0.565	0.0320
1561202_at	88.7	38.4	-1.21	-2.31	APP-AAA	0.40	8	0.500	0.565	0.0258
236851_x_at	73.6	31.9	-1.21	-2.31	AMP-PAA	0.40	8	0.500	0.565	0.0281
242719_at	86.9	37.9	-1.20	-2.29	APP-AAP	0.40	8	0.500	0.565	0.0275

216540_at	63.6	27.9	-1.19	-2.28	AAP-AAA	0.40	8	0.500	0.565	0.0321
243443_at	96.4	42.3	-1.19	-2.28	AAA-APA	0.40	8	0.500	0.565	0.0389
233053_at	74.3	32.7	-1.19	-2.28	PPP-AAA	0.40	8	0.500	0.565	0.0339
216440_at	42.3	18.6	-1.18	-2.27	AMP-AAA	0.40	8	0.500	0.565	0.0321
230837_at	109.2	48.1	-1.18	-2.27	APP-PAP	0.40	8	0.500	0.565	0.0349
239946_at	85.1	37.5	-1.18	-2.27	APP-AAP	0.40	8	0.500	0.565	0.0375
217077_s_at	94.0	41.5	-1.18	-2.26	APP-AAP	0.40	8	0.500	0.565	0.0370
233807_at	96.5	43.5	-1.15	-2.22	AAP-AAM	0.40	8	0.500	0.565	0.0289
208134_x_at	172.7	78.1	-1.15	-2.21	PAP-AMA	0.40	8	0.500	0.565	0.0288
1552477_a_at	53.3	24.2	-1.14	-2.20	AAP-AAA	0.40	8	0.500	0.565	0.0383
244003_at	73.1	33.3	-1.14	-2.20	PAA-PPP	0.40	8	0.500	0.565	0.0184
1555206_at	102.3	46.7	-1.13	-2.19	PAP-AAA	0.40	8	0.500	0.565	0.0205
236488_s_at	192.0	419.3	1.13	2.18	PPP-PPP	0.40	8	0.500	0.565	0.0038
242280_x_at	74.7	34.3	-1.12	-2.18	PPP-APP	0.40	8	0.500	0.565	0.0277
203021_at	140.0	64.8	-1.11	-2.16	AAA-PAA	0.40	8	0.500	0.565	0.0269
217699_at	74.6	34.6	-1.11	-2.15	AAP-AAP	0.40	8	0.500	0.565	0.0218
222375_at	74.6	35.2	-1.09	-2.12	APP-APP	0.40	8	0.500	0.565	0.0349
1570447_at	158.0	74.8	-1.08	-2.11	APP-AAA	0.40	8	0.500	0.565	0.0302
211362_s_at	69.0	32.7	-1.08	-2.11	PAA-AAA	0.40	8	0.500	0.565	0.0280
1561332_at	90.8	43.2	-1.07	-2.10	AAA-MAP	0.40	8	0.500	0.565	0.0245
1561909_at	90.0	43.8	-1.04	-2.05	PAA-AAA	0.40	8	0.500	0.565	0.0298
1570237_at	132.4	64.5	-1.04	-2.05	PMA-PPA	0.40	8	0.500	0.565	0.0194
232632_at	137.7	67.1	-1.04	-2.05	PPA-PAP	0.40	8	0.500	0.565	0.0217
1569738_at	76.4	37.6	-1.02	-2.03	PAA-AAA	0.40	8	0.500	0.565	0.0219
210730_s_at	80.0	39.4	-1.02	-2.03	AAP-AAA	0.40	8	0.500	0.565	0.0233
1566953_x_at	52.3	25.9	-1.02	-2.02	AAP-AAA	0.40	8	0.500	0.565	0.0366
210704_at	70.4	34.9	-1.01	-2.01	PAA-AAA	0.40	8	0.500	0.565	0.0180
213536_s_at	105.5	52.4	-1.01	-2.01	AAA-APA	0.40	8	0.500	0.565	0.0200
233127_at	79.0	39.3	-1.01	-2.01	MPA-AAA	0.40	8	0.500	0.565	0.0306
241875_at	56.8	28.3	-1.00	-2.00	APP-AAA	0.40	8	0.500	0.565	0.0188
217355_at	75.6	37.8	-1.00	-2.00	PAA-AAP	0.40	8	0.500	0.565	0.0277
233198_at	80.7	40.4	-1.00	-1.99	PAM-AAA	0.40	8	0.500	0.565	0.0332
1557406_s_at	80.7	40.8	-0.98	-1.98	PAA-AAA	0.40	8	0.500	0.565	0.0162
225407_at	213.9	108.9	-0.97	-1.96	PAA-AAA	0.40	8	0.500	0.565	0.0186
1564308_a_at	100.7	51.5	-0.97	-1.95	PAP-PAP	0.40	8	0.500	0.565	0.0232
209598_at	638.3	329.3	-0.96	-1.94	PPP-PPP	0.40	8	0.500	0.565	0.0323
236270_at	117.5	60.8	-0.95	-1.93	PPP-PPP	0.40	8	0.500	0.565	0.0289
1562388_at	91.7	47.5	-0.95	-1.93	AAA-AAP	0.40	8	0.500	0.565	0.0328
231437_at	174.0	90.7	-0.94	-1.92	PPP-APP	0.40	8	0.500	0.565	0.0302
239614_x_at	93.9	49.3	-0.93	-1.91	APP-AAP	0.40	8	0.500	0.565	0.0196
211460_at	74.3	39.0	-0.93	-1.91	AAP-AAA	0.40	8	0.500	0.565	0.0275
1555624_a_at	110.8	58.3	-0.93	-1.90	MPA-AAP	0.40	8	0.500	0.565	0.0285

232809_s_at	162.8	85.8	-0.92	-1.90	PAA-AMP	0.40	8	0.500	0.565	0.0210
1558381_a_at	165.1	87.3	-0.92	-1.89	PPM-AAM	0.40	8	0.500	0.565	0.0279
237330_at	96.9	51.3	-0.92	-1.89	PPP-PAP	0.40	8	0.500	0.565	0.0297
1553133_at	68.2	36.2	-0.92	-1.89	PPP-APA	0.40	8	0.500	0.565	0.0216
219674_s_at	89.3	47.4	-0.91	-1.88	PAA-PAA	0.40	8	0.500	0.565	0.0333
235394_at	198.5	372.6	0.91	1.88	PPP-PPP	0.40	8	0.500	0.565	0.0023
1557207_s_at	117.3	62.6	-0.91	-1.87	AAP-AMA	0.40	8	0.500	0.565	0.0152
232495_x_at	128.2	68.8	-0.90	-1.86	MAM-APA	0.40	8	0.500	0.565	0.0155
239031_at	122.7	65.9	-0.90	-1.86	APP-PAP	0.40	8	0.500	0.565	0.0134
242687_at	234.5	126.3	-0.89	-1.86	PPP-APP	0.40	8	0.500	0.565	0.0273
230701_x_at	674.9	363.8	-0.89	-1.86	PPP-PPP	0.40	8	0.500	0.565	0.0254
233614_at	104.4	56.7	-0.88	-1.84	PPP-APP	0.40	8	0.500	0.565	0.0305
1558606_s_at	59.3	32.3	-0.88	-1.84	APP-APM	0.40	8	0.500	0.565	0.0226
210717_at	98.3	53.7	-0.87	-1.83	APP-APP	0.40	8	0.500	0.565	0.0211
226791_at	256.4	140.6	-0.87	-1.82	APA-AAA	0.40	8	0.500	0.565	0.0294
240557_at	213.2	117.3	-0.86	-1.82	PPP-PPP	0.40	8	0.500	0.565	0.0206
242668_x_at	316.9	174.5	-0.86	-1.82	PPP-APA	0.40	8	0.500	0.565	0.0182
228034_x_at	142.2	78.6	-0.86	-1.81	PAA-AAP	0.40	8	0.500	0.565	0.0119
215852_x_at	95.8	53.0	-0.85	-1.81	APA-AAA	0.40	8	0.500	0.565	0.0229
214091_s_at	95.3	52.9	-0.85	-1.80	PAP-PAA	0.40	8	0.500	0.565	0.0290
1560296_at	287.7	159.9	-0.85	-1.80	PPP-APP	0.40	8	0.500	0.565	0.0280
216036_x_at	281.2	156.4	-0.85	-1.80	PMP-APM	0.40	8	0.500	0.565	0.0289
1569114_at	145.0	81.2	-0.84	-1.79	PMP-PPP	0.40	8	0.500	0.565	0.0251
1559132_at	300.2	169.4	-0.83	-1.77	PPP-PPP	0.40	8	0.500	0.565	0.0239
216568_x_at	173.7	98.3	-0.82	-1.77	PPP-APP	0.40	8	0.500	0.565	0.0159
239771_at	155.0	87.9	-0.82	-1.76	PPP-PPP	0.40	8	0.500	0.565	0.0114
217487_x_at	79.1	44.9	-0.82	-1.76	PPA-PAA	0.40	8	0.500	0.565	0.0124
206099_at	122.4	69.6	-0.81	-1.76	AAP-AAA	0.40	8	0.500	0.565	0.0180
1561915_at	108.8	62.1	-0.81	-1.75	MPA-MPP	0.40	8	0.500	0.565	0.0150
243981_at	73.4	41.9	-0.81	-1.75	PPP-PPP	0.40	8	0.500	0.565	0.0109
230346_x_at	148.2	85.0	-0.80	-1.74	PAP-PPP	0.40	8	0.500	0.565	0.0249
243213_at	177.7	102.2	-0.80	-1.74	PPP-PPP	0.40	8	0.500	0.565	0.0205
1555108_at	86.5	50.1	-0.79	-1.73	PMP-APA	0.40	8	0.500	0.565	0.0278
202507_s_at	230.0	133.4	-0.79	-1.73	PAP-PPP	0.40	8	0.500	0.565	0.0147
217107_at	112.0	65.2	-0.78	-1.72	APP-PPA	0.40	8	0.500	0.565	0.0187
233476_at	239.7	139.8	-0.78	-1.72	PAA-AAA	0.40	8	0.500	0.565	0.0265
216678_at	274.6	160.6	-0.77	-1.71	PPP-APP	0.40	8	0.500	0.565	0.0107
1570166_a_at	157.2	91.9	-0.77	-1.71	PAA-AAP	0.40	8	0.500	0.565	0.0267
205957_at	92.7	54.3	-0.77	-1.71	MAP-AAA	0.40	8	0.500	0.565	0.0147
216769_x_at	86.5	50.9	-0.77	-1.70	APP-MAA	0.40	8	0.500	0.565	0.0207
201690_s_at	90.4	53.3	-0.76	-1.70	PAP-PAP	0.40	8	0.500	0.565	0.0199
1559598_at	49.9	29.5	-0.76	-1.69	APA-AAA	0.40	8	0.500	0.565	0.0228

71933_at	182.2	108.4	-0.75	-1.68	APP-AAP	0.40	8	0.500	0.565	0.0222
213685_at	244.8	146.2	-0.74	-1.68	PAA-AAP	0.40	8	0.500	0.565	0.0149
1566402_at	206.4	123.3	-0.74	-1.67	PPP-PPP	0.40	8	0.500	0.565	0.0114
227407_at	355.6	590.3	0.73	1.66	PPP-PPP	0.40	8	0.500	0.565	0.0011
202437_s_at	119.6	72.1	-0.73	-1.66	PPP-PPM	0.40	8	0.500	0.565	0.0182
1570425_s_at	103.1	62.2	-0.73	-1.66	PAP-APA	0.40	8	0.500	0.565	0.0177
218574_s_at	598.7	363.2	-0.72	-1.65	PPP-PPP	0.40	8	0.500	0.565	0.0241
37004_at	88.3	53.8	-0.72	-1.64	PAA-AAP	0.40	8	0.500	0.565	0.0187
236780_at	136.1	83.0	-0.71	-1.64	PPP-MAP	0.40	8	0.500	0.565	0.0089
219591_at	149.4	91.1	-0.71	-1.64	PAA-AAA	0.40	8	0.500	0.565	0.0215
1557659_a_at	65.8	40.3	-0.71	-1.64	PAP-AAP	0.40	8	0.500	0.565	0.0114
208315_x_at	311.3	190.6	-0.71	-1.63	PPP-PPP	0.40	8	0.500	0.565	0.0195
211252_x_at	319.4	198.1	-0.69	-1.61	MAA-PPA	0.40	8	0.500	0.565	0.0174
216294_s_at	166.0	104.0	-0.67	-1.60	PPP-PAM	0.40	8	0.500	0.565	0.0083
215376_at	55.0	34.5	-0.67	-1.59	AAA-AAP	0.40	8	0.500	0.565	0.0179
238508_at	490.5	308.3	-0.67	-1.59	PPP-PPP	0.40	8	0.500	0.565	0.0091
1553015_a_at	937.2	590.9	-0.67	-1.59	PPP-PPP	0.40	8	0.500	0.565	0.0078
234308_at	109.6	69.5	-0.66	-1.58	APP-AAA	0.40	8	0.500	0.565	0.0086
221860_at	316.1	200.8	-0.66	-1.57	MPP-PPP	0.40	8	0.500	0.565	0.0147
208917_x_at	335.2	213.2	-0.65	-1.57	APP-PPP	0.40	8	0.500	0.565	0.0095
65521_at	496.0	315.7	-0.65	-1.57	PPP-PPP	0.40	8	0.500	0.565	0.0096
243343_at	136.3	86.8	-0.65	-1.57	PPP-AAP	0.40	8	0.500	0.565	0.0185
206965_at	56.1	35.9	-0.65	-1.56	PAP-AAP	0.40	8	0.500	0.565	0.0169
238185_at	124.7	80.4	-0.63	-1.55	PPP-PPP	0.40	8	0.500	0.565	0.0130
225499_at	221.9	143.2	-0.63	-1.55	AAP-AMP	0.40	8	0.500	0.565	0.0151
239633_at	125.5	81.1	-0.63	-1.55	AAA-APP	0.40	8	0.500	0.565	0.0093
232221_x_at	271.8	175.7	-0.63	-1.55	PPP-PPP	0.40	8	0.500	0.565	0.0092
210984_x_at	367.5	240.2	-0.61	-1.53	PPP-PPP	0.40	8	0.500	0.565	0.0162
224673_at	352.4	231.1	-0.61	-1.53	PPP-PPP	0.40	8	0.500	0.565	0.0186
200752_s_at	601.4	394.9	-0.61	-1.52	PPP-PPP	0.40	8	0.500	0.565	0.0057
213935_at	214.9	142.1	-0.60	-1.51	PPP-PPP	0.40	8	0.500	0.565	0.0091
1556555_at	169.5	112.8	-0.59	-1.50	PPP-PPP	0.40	8	0.500	0.565	0.0073
211285_s_at	1948.4	2915.2	0.58	1.50	PPP-PPP	0.40	8	0.500	0.565	0.0003
241375_at	91.4	61.2	-0.58	-1.49	PAP-PPP	0.40	8	0.500	0.565	0.0092
210305_at	764.7	512.5	-0.58	-1.49	PPP-PPP	0.40	8	0.500	0.565	0.0147
210058_at	144.2	96.7	-0.58	-1.49	PPP-PPP	0.40	8	0.500	0.565	0.0155
231023_at	250.0	169.4	-0.56	-1.48	PPP-PPP	0.40	8	0.500	0.565	0.0142
238644_at	105.2	71.3	-0.56	-1.48	PPP-AMP	0.40	8	0.500	0.565	0.0046
229354_at	588.7	401.5	-0.55	-1.47	PPP-PPP	0.40	8	0.500	0.565	0.0089
1561908_a_at	114.5	78.2	-0.55	-1.46	PPA-PPP	0.40	8	0.500	0.565	0.0134
242871_at	457.3	315.7	-0.54	-1.45	AAA-APA	0.40	8	0.500	0.565	0.0061
230611_at	191.2	132.3	-0.53	-1.45	PMP-APP	0.40	8	0.500	0.565	0.0137

235974_at	115.4	80.0	-0.53	-1.44	APM-AAA	0.40	8	0.500	0.565	0.0103
203139_at	708.2	493.4	-0.52	-1.44	PPP-PPP	0.40	8	0.500	0.565	0.0051
238895_at	100.5	70.3	-0.52	-1.43	PPP-PPP	0.40	8	0.500	0.565	0.0086
230196_x_at	74.5	52.5	-0.50	-1.42	AAP-AAA	0.40	8	0.500	0.565	0.0104
215628_x_at	556.4	395.9	-0.49	-1.41	PPP-PPP	0.40	8	0.500	0.565	0.0101
203769_s_at	81.4	58.1	-0.49	-1.40	APP-PPM	0.40	8	0.500	0.565	0.0024
217916_s_at	3279.7	2350.1	-0.48	-1.40	PPP-PPP	0.40	8	0.500	0.565	0.0054
225789_at	1161.1	833.8	-0.48	-1.39	PPP-PPP	0.40	8	0.500	0.565	0.0066
224105_x_at	102.4	73.5	-0.48	-1.39	AAP-AAA	0.40	8	0.500	0.565	0.0048
242473_at	198.1	142.9	-0.47	-1.39	PPP-PPP	0.40	8	0.500	0.565	0.0031
219202_at	378.0	276.2	-0.45	-1.37	PPP-PPP	0.40	8	0.500	0.565	0.0051
213234_at	387.0	283.0	-0.45	-1.37	APA-PPP	0.40	8	0.500	0.565	0.0017
231764_at	929.8	709.3	-0.39	-1.31	PPP-PPP	0.40	8	0.500	0.565	0.0005
210138_at	1601.7	1229.7	-0.38	-1.30	PPP-PPP	0.40	8	0.500	0.565	0.0009
225303_at	927.3	713.2	-0.38	-1.30	PPP-PPP	0.40	8	0.500	0.565	0.0029
221951_at	388.4	299.5	-0.38	-1.30	PPP-PPP	0.40	8	0.500	0.565	0.0010
204267_x_at	1430.7	1103.6	-0.38	-1.30	PPP-PPP	0.40	8	0.500	0.565	0.0021
1557415_s_at	167.2	129.0	-0.37	-1.30	PPP-PPP	0.40	8	0.500	0.565	0.0025
238896_at	328.0	253.3	-0.37	-1.30	AMP-PPP	0.40	8	0.500	0.565	0.0018
233919_s_at	558.0	430.9	-0.37	-1.30	PPP-PPP	0.40	8	0.500	0.565	0.0022
209735_at	490.6	380.1	-0.37	-1.29	PPP-PPP	0.40	8	0.500	0.565	0.0004
205370_x_at	3152.9	2537.2	-0.31	-1.24	PPP-PPP	0.40	8	0.500	0.565	0.0006
221923_s_at	22857.7	18811.9	-0.28	-1.22	PPP-PPP	0.40	8	0.500	0.565	0.0000
217157_x_at	80.3	7.1	-3.50	-11.31	AAP-AAA	0.30	9	0.571	0.631	0.0856
1556357_s_at	34.5	3.5	-3.32	-9.97	PAA-APA	0.30	9	0.571	0.631	0.1090
238717_at	53.0	5.8	-3.19	-9.15	AAP-AAA	0.30	9	0.571	0.631	0.0638
230718_at	34.5	3.9	-3.15	-8.85	APA-AAA	0.30	9	0.571	0.631	0.1061
1553920_at	34.6	4.3	-3.02	-8.14	APA-AAA	0.30	9	0.571	0.631	0.0677
233149_at	140.7	18.4	-2.94	-7.65	PAA-AAP	0.30	9	0.571	0.631	0.1097
1556185_a_at	27.6	3.7	-2.92	-7.56	AAP-AAA	0.30	9	0.571	0.631	0.0748
220552_at	40.9	5.5	-2.90	-7.48	APA-AAA	0.30	9	0.571	0.631	0.1117
221174_at	27.0	3.7	-2.89	-7.40	PAA-AAA	0.30	9	0.571	0.631	0.0943
216947_at	32.9	4.6	-2.83	-7.13	AAP-AAA	0.30	9	0.571	0.631	0.0614
209829_at	39.3	5.7	-2.78	-6.89	PAP-AAA	0.30	9	0.571	0.631	0.0615
1559575_a_at	72.6	10.6	-2.78	-6.85	AAA-APA	0.30	9	0.571	0.631	0.0896
1563997_at	94.0	14.3	-2.71	-6.56	PAM-AAA	0.30	9	0.571	0.631	0.1050
1565638_at	44.4	6.9	-2.70	-6.48	APP-AAA	0.30	9	0.571	0.631	0.0851
239868_at	52.6	8.4	-2.65	-6.27	PAA-AAP	0.30	9	0.571	0.631	0.1024
235699_at	102.1	16.6	-2.62	-6.16	AAP-AAA	0.30	9	0.571	0.631	0.0718
227702_at	23.5	3.8	-2.62	-6.15	PAA-AAA	0.30	9	0.571	0.631	0.0952
215878_at	61.4	10.0	-2.62	-6.15	PPA-AAA	0.30	9	0.571	0.631	0.0665
240647_at	26.3	4.3	-2.62	-6.13	PAA-AAA	0.30	9	0.571	0.631	0.0723

228219_s_at	57.1	9.3	-2.62	-6.13	PAM-AAA	0.30	9	0.571	0.631	0.0684
205385_at	58.6	9.6	-2.61	-6.12	PAP-AAA	0.30	9	0.571	0.631	0.0733
1560153_at	68.4	11.6	-2.56	-5.91	APA-AAA	0.30	9	0.571	0.631	0.1056
207561_s_at	81.8	14.0	-2.55	-5.85	PMP-AAA	0.30	9	0.571	0.631	0.1043
222860_s_at	103.5	18.0	-2.52	-5.75	MAM-AAP	0.30	9	0.571	0.631	0.0731
237147_at	34.1	6.0	-2.50	-5.67	MPP-AAA	0.30	9	0.571	0.631	0.0776
239196_at	118.2	20.9	-2.50	-5.66	PAA-AAA	0.30	9	0.571	0.631	0.1073
231191_at	74.9	13.4	-2.48	-5.58	PAA-AAA	0.30	9	0.571	0.631	0.0790
222145_at	113.0	20.3	-2.47	-5.55	APA-AAA	0.30	9	0.571	0.631	0.0867
244101_at	38.1	7.0	-2.45	-5.47	PAA-AAA	0.30	9	0.571	0.631	0.0936
244314_at	10.9	2.0	-2.44	-5.42	AAP-AAA	0.30	9	0.571	0.631	0.0926
207051_at	76.6	14.3	-2.42	-5.36	AAP-AAA	0.30	9	0.571	0.631	0.1053
1552544_at	70.7	13.2	-2.42	-5.34	PPA-AAA	0.30	9	0.571	0.631	0.0703
226828_s_at	67.8	12.9	-2.39	-5.25	PAP-AAP	0.30	9	0.571	0.631	0.1111
238098_at	64.4	12.3	-2.39	-5.24	APA-AAA	0.30	9	0.571	0.631	0.0979
220623_s_at	50.1	10.0	-2.33	-5.01	PAA-AAA	0.30	9	0.571	0.631	0.1017
213430_at	64.6	13.0	-2.32	-4.98	PAA-AAP	0.30	9	0.571	0.631	0.0716
229309_at	26.7	5.4	-2.31	-4.95	APA-AAA	0.30	9	0.571	0.631	0.1036
1553177_at	41.3	8.6	-2.27	-4.81	AAP-AAA	0.30	9	0.571	0.631	0.0786
216816_at	99.5	21.0	-2.24	-4.74	AAP-AAA	0.30	9	0.571	0.631	0.0755
211523_at	41.9	8.9	-2.24	-4.72	APA-AAA	0.30	9	0.571	0.631	0.1021
215463_at	43.4	9.3	-2.23	-4.68	APA-AAA	0.30	9	0.571	0.631	0.0575
234634_at	37.8	8.2	-2.21	-4.62	AAP-AAA	0.30	9	0.571	0.631	0.0977
215850_s_at	41.9	9.1	-2.21	-4.62	AAA-AAP	0.30	9	0.571	0.631	0.0607
207596_at	47.3	10.3	-2.20	-4.59	AAP-AAA	0.30	9	0.571	0.631	0.0899
214397_at	32.5	7.2	-2.18	-4.54	APM-AAA	0.30	9	0.571	0.631	0.0883
220891_at	43.8	9.7	-2.17	-4.51	AAA-APA	0.30	9	0.571	0.631	0.0622
1560550_at	68.0	15.2	-2.16	-4.47	PAA-AAA	0.30	9	0.571	0.631	0.1030
203808_at	90.6	20.3	-2.16	-4.46	PPP-AAA	0.30	9	0.571	0.631	0.0624
1569904_x_at	49.6	11.2	-2.15	-4.43	PAA-AAA	0.30	9	0.571	0.631	0.0582
220158_at	71.8	16.4	-2.13	-4.37	PAA-AAA	0.30	9	0.571	0.631	0.0595
1569808_at	33.4	7.7	-2.11	-4.31	PAA-AAA	0.30	9	0.571	0.631	0.1008
242227_at	54.8	12.9	-2.09	-4.26	PAA-AAA	0.30	9	0.571	0.631	0.0882
205815_at	53.8	12.8	-2.08	-4.21	PAA-AAA	0.30	9	0.571	0.631	0.0832
1559731_x_at	68.7	16.6	-2.05	-4.14	APM-AAA	0.30	9	0.571	0.631	0.0997
209242_at	26.0	6.3	-2.04	-4.12	APA-AAA	0.30	9	0.571	0.631	0.0881
215741_x_at	108.9	27.2	-2.00	-4.01	AAP-AAA	0.30	9	0.571	0.631	0.0614
240978_at	61.9	15.5	-2.00	-4.00	APA-AAA	0.30	9	0.571	0.631	0.0957
209348_s_at	41.5	10.4	-2.00	-3.99	AAP-AAA	0.30	9	0.571	0.631	0.1018
224240_s_at	79.6	20.4	-1.97	-3.91	PAA-AAA	0.30	9	0.571	0.631	0.1075
1557878_at	72.8	18.7	-1.96	-3.90	APA-AAP	0.30	9	0.571	0.631	0.0748
206214_at	48.7	12.5	-1.96	-3.89	PAA-AAA	0.30	9	0.571	0.631	0.0860

1569607_s_at	130.4	33.6	-1.96	-3.88	AAA-APA	0.30	9	0.571	0.631	0.0609
1558645_at	27.0	7.0	-1.95	-3.86	PAA-AAA	0.30	9	0.571	0.631	0.0586
1559712_at	73.0	19.1	-1.94	-3.83	AAP-AAA	0.30	9	0.571	0.631	0.0722
220149_at	72.4	19.0	-1.93	-3.81	AMA-PAA	0.30	9	0.571	0.631	0.0657
1566267_at	33.8	8.9	-1.93	-3.80	PAA-AAA	0.30	9	0.571	0.631	0.0670
243541_at	100.3	26.6	-1.91	-3.77	AAP-PAA	0.30	9	0.571	0.631	0.0928
230415_at	108.2	28.8	-1.91	-3.75	APA-AAA	0.30	9	0.571	0.631	0.0564
242252_at	41.9	11.2	-1.90	-3.73	APA-AAA	0.30	9	0.571	0.631	0.0895
242253_at	76.2	20.6	-1.89	-3.71	PMP-AAP	0.30	9	0.571	0.631	0.0733
1566868_at	70.7	19.4	-1.87	-3.66	APA-AAA	0.30	9	0.571	0.631	0.0655
1561740_at	20.6	5.7	-1.86	-3.64	PAA-AAA	0.30	9	0.571	0.631	0.0888
237958_at	82.0	22.6	-1.86	-3.63	PAA-MAA	0.30	9	0.571	0.631	0.0780
237542_at	58.4	16.3	-1.84	-3.58	AAP-AAA	0.30	9	0.571	0.631	0.0747
232725_s_at	44.2	12.5	-1.82	-3.52	AAP-AAA	0.30	9	0.571	0.631	0.0613
240456_at	77.9	22.2	-1.81	-3.52	APP-AAA	0.30	9	0.571	0.631	0.0596
243745_at	82.2	23.4	-1.81	-3.51	AAP-AAA	0.30	9	0.571	0.631	0.0980
1555505_a_at	53.4	15.5	-1.79	-3.45	AAP-AAA	0.30	9	0.571	0.631	0.0957
242817_at	68.2	19.8	-1.79	-3.45	APA-AAA	0.30	9	0.571	0.631	0.0580
216444_at	36.0	10.5	-1.78	-3.43	PAM-AAA	0.30	9	0.571	0.631	0.0570
227705_at	61.0	208.5	1.77	3.42	PPP-PPP	0.30	9	0.571	0.631	0.0078
242274_x_at	41.2	12.1	-1.77	-3.40	APP-AAA	0.30	9	0.571	0.631	0.0915
1570315_at	95.1	28.1	-1.76	-3.38	PPA-PAA	0.30	9	0.571	0.631	0.0863
244837_at	63.1	19.2	-1.72	-3.29	APA-AAA	0.30	9	0.571	0.631	0.1023
213733_at	88.1	27.0	-1.71	-3.27	AAP-AAA	0.30	9	0.571	0.631	0.0879
234430_at	82.2	25.3	-1.70	-3.25	AAP-AAA	0.30	9	0.571	0.631	0.0984
242973_at	53.3	16.6	-1.69	-3.22	APA-AAA	0.30	9	0.571	0.631	0.0747
1557620_a_at	63.2	19.9	-1.67	-3.18	APA-AAA	0.30	9	0.571	0.631	0.0971
228780_at	39.0	12.4	-1.66	-3.15	AAP-AAA	0.30	9	0.571	0.631	0.0514
241383_at	53.3	17.4	-1.62	-3.06	PAA-AAA	0.30	9	0.571	0.631	0.0574
242364_x_at	253.1	82.6	-1.62	-3.06	PPP-AAA	0.30	9	0.571	0.631	0.1002
1568981_at	72.7	24.0	-1.60	-3.02	APA-AAP	0.30	9	0.571	0.631	0.0960
243650_at	69.1	22.9	-1.60	-3.02	PMM-AAA	0.30	9	0.571	0.631	0.0804
215013_s_at	90.0	30.0	-1.59	-3.00	APA-AAP	0.30	9	0.571	0.631	0.0964
1563840_at	65.0	21.9	-1.57	-2.97	PAA-AAA	0.30	9	0.571	0.631	0.0662
236954_at	97.7	33.1	-1.56	-2.96	PPP-AAP	0.30	9	0.571	0.631	0.0957
227371_at	113.4	38.8	-1.55	-2.92	AAP-AAP	0.30	9	0.571	0.631	0.0811
240874_at	48.8	17.0	-1.53	-2.88	APA-AAA	0.30	9	0.571	0.631	0.0633
216773_at	59.0	20.5	-1.52	-2.88	MAP-AAA	0.30	9	0.571	0.631	0.0707
207919_at	97.5	33.9	-1.52	-2.88	APA-AAA	0.30	9	0.571	0.631	0.0829
238086_at	119.3	41.7	-1.52	-2.86	APP-AAA	0.30	9	0.571	0.631	0.0507
1562009_a_at	42.6	14.9	-1.52	-2.86	PPA-APA	0.30	9	0.571	0.631	0.0922
1566222_at	24.2	8.5	-1.51	-2.85	PAA-AAA	0.30	9	0.571	0.631	0.0979

217390_x_at	135.7	47.7	-1.51	-2.84	APP-APA	0.30	9	0.571	0.631	0.0664
1569176_at	45.7	16.1	-1.51	-2.84	AAP-AAA	0.30	9	0.571	0.631	0.0536
237232_at	36.4	12.9	-1.49	-2.82	AAP-AAA	0.30	9	0.571	0.631	0.0835
1556153_s_at	61.0	21.7	-1.49	-2.81	PAA-AAA	0.30	9	0.571	0.631	0.0503
229335_at	37.9	13.5	-1.49	-2.81	AAP-AAA	0.30	9	0.571	0.631	0.0577
234610_at	84.0	30.1	-1.48	-2.79	AAP-AAA	0.30	9	0.571	0.631	0.0872
1559827_at	128.8	355.9	1.47	2.76	PPP-PPP	0.30	9	0.571	0.631	0.0072
214421_x_at	109.6	39.8	-1.46	-2.76	PPP-APA	0.30	9	0.571	0.631	0.0501
223611_s_at	57.8	21.3	-1.44	-2.72	AAP-APA	0.30	9	0.571	0.631	0.0765
1562619_at	51.6	19.2	-1.43	-2.69	APA-AAA	0.30	9	0.571	0.631	0.0934
237013_at	87.7	32.8	-1.42	-2.67	AAP-AAA	0.30	9	0.571	0.631	0.0659
1563970_at	45.1	17.0	-1.41	-2.65	PAA-AAP	0.30	9	0.571	0.631	0.0831
215342_s_at	37.3	14.1	-1.41	-2.65	PAA-AAA	0.30	9	0.571	0.631	0.0691
239878_at	54.4	20.6	-1.40	-2.64	AAA-AAP	0.30	9	0.571	0.631	0.0624
1553726_s_at	51.1	19.4	-1.40	-2.63	PMA-AAP	0.30	9	0.571	0.631	0.0837
216733_s_at	43.3	16.5	-1.39	-2.63	PAA-AAA	0.30	9	0.571	0.631	0.0849
1557003_at	64.2	24.5	-1.39	-2.62	APA-AAP	0.30	9	0.571	0.631	0.0942
244467_at	69.1	26.6	-1.38	-2.60	APA-AAA	0.30	9	0.571	0.631	0.0764
244230_at	40.4	15.7	-1.36	-2.57	PAA-AAA	0.30	9	0.571	0.631	0.0691
230280_at	43.7	17.0	-1.36	-2.57	AAP-AAA	0.30	9	0.571	0.631	0.0781
1555014_x_at	96.6	38.8	-1.32	-2.49	APP-PAP	0.30	9	0.571	0.631	0.0684
234167_at	41.1	16.5	-1.32	-2.49	PAA-AAA	0.30	9	0.571	0.631	0.0915
228534_s_at	58.9	23.9	-1.30	-2.46	PPA-AAA	0.30	9	0.571	0.631	0.0531
238556_at	85.1	34.9	-1.29	-2.44	PAA-AAA	0.30	9	0.571	0.631	0.0511
241899_at	95.8	39.5	-1.28	-2.43	APA-AAA	0.30	9	0.571	0.631	0.0723
244093_at	67.8	28.0	-1.28	-2.42	PPA-MAA	0.30	9	0.571	0.631	0.0578
215397_x_at	99.0	41.1	-1.27	-2.41	PPP-APP	0.30	9	0.571	0.631	0.0765
237605_at	47.3	19.7	-1.26	-2.40	PAA-AAA	0.30	9	0.571	0.631	0.0896
231202_at	36.6	15.3	-1.26	-2.40	AAA-AAP	0.30	9	0.571	0.631	0.0855
1560133_at	50.7	21.2	-1.26	-2.39	AMP-APA	0.30	9	0.571	0.631	0.0528
1569496_s_at	104.4	43.7	-1.26	-2.39	APA-AAA	0.30	9	0.571	0.631	0.0578
230500_at	70.1	29.4	-1.25	-2.39	PPA-AAP	0.30	9	0.571	0.631	0.0881
234760_at	100.4	42.2	-1.25	-2.38	PPP-PAA	0.30	9	0.571	0.631	0.0450
1566760_at	77.1	32.4	-1.25	-2.38	AAP-AAA	0.30	9	0.571	0.631	0.0496
238886_at	80.7	34.4	-1.23	-2.35	AAP-AAA	0.30	9	0.571	0.631	0.0672
231244_at	58.1	24.8	-1.23	-2.34	AAA-APA	0.30	9	0.571	0.631	0.0752
233307_x_at	260.0	111.5	-1.22	-2.33	AMA-APA	0.30	9	0.571	0.631	0.0435
239481_at	46.6	20.1	-1.22	-2.32	APA-AAA	0.30	9	0.571	0.631	0.0751
239585_at	68.9	30.0	-1.20	-2.29	PAP-AAA	0.30	9	0.571	0.631	0.0898
227266_s_at	81.0	35.4	-1.20	-2.29	APP-AAA	0.30	9	0.571	0.631	0.0500
244317_at	73.9	32.3	-1.19	-2.29	PAA-AAA	0.30	9	0.571	0.631	0.0470
205991_s_at	95.7	41.9	-1.19	-2.28	PPP-PAP	0.30	9	0.571	0.631	0.0556

233358_at	145.3	63.9	-1.19	-2.28	MAA-APP	0.30	9	0.571	0.631	0.0913
222882_s_at	100.9	44.5	-1.18	-2.27	PAA-AAA	0.30	9	0.571	0.631	0.0589
231723_at	221.3	99.4	-1.16	-2.23	PPP-APP	0.30	9	0.571	0.631	0.0507
1565880_at	96.0	43.2	-1.15	-2.22	PPP-PPA	0.30	9	0.571	0.631	0.0628
1559765_a_at	169.6	76.5	-1.15	-2.22	PPP-AMA	0.30	9	0.571	0.631	0.0443
1558450_at	85.1	38.4	-1.15	-2.22	APM-AAA	0.30	9	0.571	0.631	0.0497
1564798_at	154.3	70.0	-1.14	-2.20	PPP-PPM	0.30	9	0.571	0.631	0.0436
208281_x_at	58.1	26.4	-1.14	-2.20	APP-MAP	0.30	9	0.571	0.631	0.0502
236187_s_at	48.7	22.2	-1.13	-2.19	AAP-AAA	0.30	9	0.571	0.631	0.0576
220184_at	13.3	29.0	1.13	2.18	AAP-APP	0.30	9	0.571	0.631	0.0421
1564921_at	97.3	44.9	-1.12	-2.17	APA-AAA	0.30	9	0.571	0.631	0.0479
201893_x_at	278.8	129.0	-1.11	-2.16	MAP-AAP	0.30	9	0.571	0.631	0.0575
241992_at	47.5	22.1	-1.10	-2.15	AAP-AAA	0.30	9	0.571	0.631	0.0444
206766_at	123.4	57.6	-1.10	-2.14	PPP-PPP	0.30	9	0.571	0.631	0.0748
1569741_at	76.4	35.7	-1.10	-2.14	AAA-APA	0.30	9	0.571	0.631	0.0696
1563715_at	96.8	45.2	-1.10	-2.14	PPP-PAP	0.30	9	0.571	0.631	0.0689
242244_at	72.3	33.8	-1.10	-2.14	APA-AAM	0.30	9	0.571	0.631	0.0511
217630_at	97.4	45.7	-1.09	-2.13	AAA-AAP	0.30	9	0.571	0.631	0.0543
237058_x_at	68.5	32.2	-1.09	-2.13	PAA-AAA	0.30	9	0.571	0.631	0.0564
243461_at	90.9	42.8	-1.09	-2.13	APP-PAA	0.30	9	0.571	0.631	0.0828
1562598_at	63.1	29.9	-1.08	-2.11	MPP-AAP	0.30	9	0.571	0.631	0.0465
242604_at	49.5	23.5	-1.07	-2.10	APA-AAA	0.30	9	0.571	0.631	0.0582
1560625_s_at	80.3	38.1	-1.07	-2.10	PPP-AAA	0.30	9	0.571	0.631	0.0582
238809_at	46.8	22.3	-1.07	-2.10	APA-AAA	0.30	9	0.571	0.631	0.0799
1562222_at	64.8	30.9	-1.07	-2.10	APP-AAA	0.30	9	0.571	0.631	0.0687
1555671_at	46.1	22.0	-1.07	-2.10	AAP-AAA	0.30	9	0.571	0.631	0.0835
1552520_at	49.2	23.4	-1.07	-2.10	AAM-AAP	0.30	9	0.571	0.631	0.0754
233456_at	70.4	33.7	-1.07	-2.09	PAA-PAP	0.30	9	0.571	0.631	0.0442
1562514_at	28.9	13.9	-1.06	-2.08	AAP-AAA	0.30	9	0.571	0.631	0.0621
237367_x_at	150.1	72.0	-1.06	-2.08	PAP-APA	0.30	9	0.571	0.631	0.0719
239949_at	102.2	49.4	-1.05	-2.07	PAA-AAA	0.30	9	0.571	0.631	0.0583
236564_at	54.3	26.3	-1.05	-2.07	APA-AAA	0.30	9	0.571	0.631	0.0563
232986_at	68.6	33.3	-1.04	-2.06	APA-AAA	0.30	9	0.571	0.631	0.0394
243435_at	161.1	78.2	-1.04	-2.06	AAP-AAM	0.30	9	0.571	0.631	0.0424
224016_at	86.3	41.9	-1.04	-2.06	MAA-APA	0.30	9	0.571	0.631	0.0599
238578_at	98.2	47.8	-1.04	-2.05	PPA-AMP	0.30	9	0.571	0.631	0.0706
210057_at	293.6	601.3	1.03	2.05	PPP-PPP	0.30	9	0.571	0.631	0.0045
1559977_a_at	166.5	81.5	-1.03	-2.04	PPP-APA	0.30	9	0.571	0.631	0.0630
232427_at	54.9	26.9	-1.03	-2.04	PAA-AAA	0.30	9	0.571	0.631	0.0696
234182_at	107.7	53.1	-1.02	-2.03	AAP-APM	0.30	9	0.571	0.631	0.0670
210943_s_at	254.9	126.5	-1.01	-2.02	PPP-APP	0.30	9	0.571	0.631	0.0519
1570087_at	118.0	58.6	-1.01	-2.01	PAP-APA	0.30	9	0.571	0.631	0.0435

33323_r_at	182.4	90.6	-1.01	-2.01	PPP-PPA	0.30	9	0.571	0.631	0.0497
243095_at	99.1	49.4	-1.00	-2.01	AAP-AAA	0.30	9	0.571	0.631	0.0646
230834_at	49.7	24.8	-1.00	-2.00	APA-AAA	0.30	9	0.571	0.631	0.0420
219086_at	181.6	90.7	-1.00	-2.00	PPP-PPP	0.30	9	0.571	0.631	0.0445
225721_at	156.4	78.2	-1.00	-2.00	APP-AAP	0.30	9	0.571	0.631	0.0391
1555529_at	153.8	77.0	-1.00	-2.00	PPP-PPA	0.30	9	0.571	0.631	0.0765
237721_s_at	32.8	16.5	-0.99	-1.99	APA-AAA	0.30	9	0.571	0.631	0.0649
243974_at	122.1	61.4	-0.99	-1.99	APP-AMP	0.30	9	0.571	0.631	0.0723
236352_at	110.4	55.7	-0.99	-1.98	APA-AAA	0.30	9	0.571	0.631	0.0427
236817_at	89.9	45.5	-0.98	-1.98	PPP-APP	0.30	9	0.571	0.631	0.0429
1552301_a_at	102.1	51.7	-0.98	-1.98	PMP-APA	0.30	9	0.571	0.631	0.0669
230113_at	50.8	25.7	-0.98	-1.97	PAA-AAA	0.30	9	0.571	0.631	0.0791
244618_at	65.9	33.5	-0.98	-1.97	APA-AAA	0.30	9	0.571	0.631	0.0587
1559256_at	57.7	29.4	-0.97	-1.96	AAP-AAA	0.30	9	0.571	0.631	0.0650
1555078_at	132.9	67.8	-0.97	-1.96	APA-AAP	0.30	9	0.571	0.631	0.0377
1569405_at	39.4	20.1	-0.97	-1.96	AAP-AAA	0.30	9	0.571	0.631	0.0549
234594_at	103.9	53.1	-0.97	-1.96	PPP-AAA	0.30	9	0.571	0.631	0.0823
230157_at	122.5	62.7	-0.97	-1.96	AAA-PAA	0.30	9	0.571	0.631	0.0401
215597_x_at	51.7	26.5	-0.97	-1.95	APA-AAA	0.30	9	0.571	0.631	0.0702
233657_at	128.7	65.9	-0.97	-1.95	PPP-PAA	0.30	9	0.571	0.631	0.0791
220372_at	115.6	59.4	-0.96	-1.95	APP-PPP	0.30	9	0.571	0.631	0.0450
228975_at	81.6	42.0	-0.96	-1.94	PPA-AAA	0.30	9	0.571	0.631	0.0597
233290_at	43.1	22.3	-0.95	-1.93	AAA-AAP	0.30	9	0.571	0.631	0.0591
226086_at	52.6	27.3	-0.95	-1.93	AAP-AAA	0.30	9	0.571	0.631	0.0686
1558905_at	39.9	20.7	-0.95	-1.93	PAA-AAA	0.30	9	0.571	0.631	0.0439
214582_at	57.9	30.1	-0.94	-1.92	AMP-APP	0.30	9	0.571	0.631	0.0358
232571_at	80.1	41.6	-0.94	-1.92	AAM-AAP	0.30	9	0.571	0.631	0.0644
230248_x_at	32.6	17.0	-0.94	-1.91	AAP-AAA	0.30	9	0.571	0.631	0.0428
1562681_at	69.5	36.3	-0.94	-1.92	PPA-APA	0.30	9	0.571	0.631	0.0514
228154_at	132.9	69.5	-0.94	-1.91	PAP-PPP	0.30	9	0.571	0.631	0.0749
219315_s_at	74.7	39.1	-0.93	-1.91	AAP-AAP	0.30	9	0.571	0.631	0.0352
223658_at	133.0	69.7	-0.93	-1.91	APA-AAA	0.30	9	0.571	0.631	0.0462
233089_at	145.2	76.1	-0.93	-1.91	PAA-AAA	0.30	9	0.571	0.631	0.0409
241478_at	115.7	60.7	-0.93	-1.91	PPA-AAA	0.30	9	0.571	0.631	0.0554
234266_at	68.8	36.2	-0.93	-1.90	AAP-AAA	0.30	9	0.571	0.631	0.0640
1561896_at	97.7	51.9	-0.91	-1.88	APM-AAA	0.30	9	0.571	0.631	0.0715
1560112_at	93.5	49.8	-0.91	-1.88	APP-APA	0.30	9	0.571	0.631	0.0346
208140_s_at	124.7	66.5	-0.91	-1.88	PAA-AAA	0.30	9	0.571	0.631	0.0523
231212_x_at	276.4	147.4	-0.91	-1.88	PMP-PPP	0.30	9	0.571	0.631	0.0358
243705_at	71.3	38.0	-0.91	-1.87	APA-AAP	0.30	9	0.571	0.631	0.0497
235538_at	79.2	42.5	-0.90	-1.87	PPP-APP	0.30	9	0.571	0.631	0.0363
243730_at	97.5	52.3	-0.90	-1.87	PAA-AAA	0.30	9	0.571	0.631	0.0599

215584_at	183.4	98.4	-0.90	-1.86	PMP-AMA	0.30	9	0.571	0.631	0.0528
209594_x_at	584.8	313.9	-0.90	-1.86	PPP-PPP	0.30	9	0.571	0.631	0.0472
1561618_at	74.2	39.9	-0.90	-1.86	AAA-PAA	0.30	9	0.571	0.631	0.0452
1560449_at	95.0	51.0	-0.90	-1.86	AAP-PAA	0.30	9	0.571	0.631	0.0488
223930_at	85.3	46.1	-0.89	-1.85	PMP-PPP	0.30	9	0.571	0.631	0.0539
240149_at	81.1	43.9	-0.89	-1.85	PPA-AAP	0.30	9	0.571	0.631	0.0624
240229_at	102.3	55.5	-0.88	-1.84	APA-AAA	0.30	9	0.571	0.631	0.0678
1553983_at	656.4	356.2	-0.88	-1.84	PPP-MPP	0.30	9	0.571	0.631	0.0706
223497_at	122.4	225.6	0.88	1.84	PPP-PPP	0.30	9	0.571	0.631	0.0029
241943_at	97.0	52.8	-0.88	-1.84	APA-APP	0.30	9	0.571	0.631	0.0504
242144_at	103.0	56.0	-0.88	-1.84	PMA-AAA	0.30	9	0.571	0.631	0.0606
235447_at	89.9	165.1	0.88	1.84	PPP-PPP	0.30	9	0.571	0.631	0.0023
232395_x_at	68.9	37.5	-0.88	-1.84	APP-AAP	0.30	9	0.571	0.631	0.0329
236707_at	92.5	50.6	-0.87	-1.83	AAP-AAA	0.30	9	0.571	0.631	0.0745
229329_s_at	128.8	70.4	-0.87	-1.83	AAA-APA	0.30	9	0.571	0.631	0.0346
202154_x_at	8260.2	15103.2	0.87	1.83	PPP-PPP	0.30	9	0.571	0.631	0.0028
216344_at	65.0	35.6	-0.87	-1.83	PAA-AAP	0.30	9	0.571	0.631	0.0593
236422_at	66.4	36.5	-0.87	-1.82	PAA-AAA	0.30	9	0.571	0.631	0.0710
239451_at	141.5	77.8	-0.86	-1.82	PPA-APP	0.30	9	0.571	0.631	0.0343
222179_at	69.8	38.4	-0.86	-1.82	PAA-AAA	0.30	9	0.571	0.631	0.0526
242986_at	77.0	42.4	-0.86	-1.82	AAP-AAP	0.30	9	0.571	0.631	0.0729
208588_at	66.7	36.7	-0.86	-1.82	PPM-PAA	0.30	9	0.571	0.631	0.0515
219252_s_at	125.3	69.1	-0.86	-1.81	PMP-APP	0.30	9	0.571	0.631	0.0548
1553407_at	89.9	49.6	-0.86	-1.81	PPP-AAP	0.30	9	0.571	0.631	0.0736
213072_at	197.3	109.0	-0.86	-1.81	AAP-PAP	0.30	9	0.571	0.631	0.0463
230148_at	66.5	36.8	-0.86	-1.81	APA-AAA	0.30	9	0.571	0.631	0.0596
201422_at	219.7	121.9	-0.85	-1.80	PAA-MPP	0.30	9	0.571	0.631	0.0325
214955_at	109.6	60.8	-0.85	-1.80	AAA-AAP	0.30	9	0.571	0.631	0.0394
1559126_at	137.8	76.4	-0.85	-1.80	PAM-MPP	0.30	9	0.571	0.631	0.0735
225408_at	112.7	62.7	-0.85	-1.80	APP-APA	0.30	9	0.571	0.631	0.0576
1564424_at	112.9	63.0	-0.84	-1.79	PPP-MPP	0.30	9	0.571	0.631	0.0342
1560443_at	141.2	78.8	-0.84	-1.79	AAP-APA	0.30	9	0.571	0.631	0.0647
241719_at	114.6	64.1	-0.84	-1.79	APP-AAP	0.30	9	0.571	0.631	0.0437
1565150_at	64.0	35.8	-0.84	-1.79	PPP-APP	0.30	9	0.571	0.631	0.0513
235750_at	280.5	157.5	-0.83	-1.78	PAP-AAP	0.30	9	0.571	0.631	0.0433
212316_at	89.5	50.3	-0.83	-1.78	PAP-APA	0.30	9	0.571	0.631	0.0306
217559_at	203.2	114.4	-0.83	-1.78	PPP-PPP	0.30	9	0.571	0.631	0.0512
229921_at	207.4	116.8	-0.83	-1.78	AAA-AAP	0.30	9	0.571	0.631	0.0633
1555894_s_at	220.8	124.4	-0.83	-1.77	PAP-AAA	0.30	9	0.571	0.631	0.0538
224175_s_at	113.9	64.3	-0.82	-1.77	APP-PPA	0.30	9	0.571	0.631	0.0460
221294_at	78.6	44.4	-0.82	-1.77	AAP-AAA	0.30	9	0.571	0.631	0.0646
1559682_at	230.7	130.5	-0.82	-1.77	PPP-PPA	0.30	9	0.571	0.631	0.0460

230672_at	155.0	87.7	-0.82	-1.77	AAP-PAP	0.30	9	0.571	0.631	0.0357
219618_at	329.3	186.8	-0.82	-1.76	PPP-PPP	0.30	9	0.571	0.631	0.0588
226448_at	132.7	75.5	-0.82	-1.76	PPP-MPA	0.30	9	0.571	0.631	0.0566
244580_at	150.8	85.7	-0.81	-1.76	APP-PAA	0.30	9	0.571	0.631	0.0345
1557114_a_at	136.3	77.6	-0.81	-1.76	PPA-APP	0.30	9	0.571	0.631	0.0537
243302_at	68.5	39.1	-0.81	-1.75	PAA-AMP	0.30	9	0.571	0.631	0.0349
1564207_at	347.9	198.8	-0.81	-1.75	PAP-PPP	0.30	9	0.571	0.631	0.0328
239734_at	85.1	48.7	-0.81	-1.75	PPP-AAA	0.30	9	0.571	0.631	0.0523
206088_at	100.2	57.3	-0.81	-1.75	AAP-PPP	0.30	9	0.571	0.631	0.0320
229455_at	77.5	44.3	-0.81	-1.75	PPA-PPP	0.30	9	0.571	0.631	0.0685
1566231_at	108.3	62.0	-0.80	-1.75	AMP-AAP	0.30	9	0.571	0.631	0.0294
211203_s_at	107.2	61.4	-0.80	-1.75	APM-AAP	0.30	9	0.571	0.631	0.0393
225449_at	199.4	114.2	-0.80	-1.75	AAP-AAA	0.30	9	0.571	0.631	0.0646
218940_at	385.9	673.8	0.80	1.75	PPP-PPP	0.30	9	0.571	0.631	0.0019
233218_at	91.9	52.7	-0.80	-1.74	PMA-APA	0.30	9	0.571	0.631	0.0383
238564_at	40.8	23.4	-0.80	-1.74	APA-APA	0.30	9	0.571	0.631	0.0539
240015_at	95.8	55.1	-0.80	-1.74	PPA-APA	0.30	9	0.571	0.631	0.0442
243261_at	201.4	116.3	-0.79	-1.73	PAA-AAA	0.30	9	0.571	0.631	0.0401
238089_at	136.5	79.1	-0.79	-1.73	PPP-PPP	0.30	9	0.571	0.631	0.0503
211092_s_at	351.1	203.4	-0.79	-1.73	APP-MPP	0.30	9	0.571	0.631	0.0552
201149_s_at	141.1	81.9	-0.79	-1.72	APP-APP	0.30	9	0.571	0.631	0.0499
239036_at	86.9	50.5	-0.78	-1.72	AAP-AAP	0.30	9	0.571	0.631	0.0364
213478_at	441.1	256.8	-0.78	-1.72	PPP-PPP	0.30	9	0.571	0.631	0.0350
240417_at	68.6	39.9	-0.78	-1.72	AAP-AAA	0.30	9	0.571	0.631	0.0558
236580_at	132.3	77.2	-0.78	-1.71	AAA-AAP	0.30	9	0.571	0.631	0.0407
242743_at	126.1	73.7	-0.78	-1.71	APP-APP	0.30	9	0.571	0.631	0.0307
211551_at	152.4	89.6	-0.77	-1.70	PPP-APP	0.30	9	0.571	0.631	0.0587
241366_at	97.0	165.1	0.77	1.70	APP-PPP	0.30	9	0.571	0.631	0.0020
1552899_at	80.9	47.7	-0.76	-1.69	PPP-AAP	0.30	9	0.571	0.631	0.0467
239741_at	146.5	86.5	-0.76	-1.69	PAP-PPP	0.30	9	0.571	0.631	0.0273
241613_at	217.6	129.1	-0.75	-1.69	PPA-PPP	0.30	9	0.571	0.631	0.0394
207252_at	118.9	70.5	-0.75	-1.69	PPA-AAA	0.30	9	0.571	0.631	0.0485
232797_at	158.9	94.3	-0.75	-1.69	PPP-PPP	0.30	9	0.571	0.631	0.0632
203416_at	133.7	79.4	-0.75	-1.69	PPP-PPP	0.30	9	0.571	0.631	0.0684
238696_at	113.2	67.5	-0.75	-1.68	PPP-MPP	0.30	9	0.571	0.631	0.0575
242380_at	69.0	41.2	-0.75	-1.68	PPP-AAP	0.30	9	0.571	0.631	0.0658
229562_at	100.8	60.2	-0.74	-1.68	AAA-AAP	0.30	9	0.571	0.631	0.0486
1559576_at	92.1	55.0	-0.74	-1.68	APA-AMA	0.30	9	0.571	0.631	0.0625
209703_x_at	95.0	56.8	-0.74	-1.67	AAP-AAA	0.30	9	0.571	0.631	0.0624
1561141_at	72.5	43.4	-0.74	-1.67	AAA-AAP	0.30	9	0.571	0.631	0.0457
239437_at	156.7	93.9	-0.74	-1.67	MAA-AAP	0.30	9	0.571	0.631	0.0657
232772_at	94.8	56.8	-0.74	-1.67	APA-AAA	0.30	9	0.571	0.631	0.0361

238114_at	82.8	49.8	-0.73	-1.66	MPP-AAA	0.30	9	0.571	0.631	0.0362
214175_x_at	3041.9	1834.5	-0.73	-1.66	PPP-PPP	0.30	9	0.571	0.631	0.0549
241984_at	198.8	120.2	-0.73	-1.65	PPP-PPP	0.30	9	0.571	0.631	0.0665
237372_at	135.6	82.2	-0.72	-1.65	PPA-APA	0.30	9	0.571	0.631	0.0616
1559711_at	75.1	45.5	-0.72	-1.65	PAA-AAA	0.30	9	0.571	0.631	0.0461
205602_x_at	526.4	319.4	-0.72	-1.65	PPP-PPP	0.30	9	0.571	0.631	0.0370
217539_at	64.1	38.9	-0.72	-1.65	PPP-AMM	0.30	9	0.571	0.631	0.0428
242043_s_at	116.8	70.9	-0.72	-1.65	AAP-AAP	0.30	9	0.571	0.631	0.0518
1558327_at	119.0	72.3	-0.72	-1.65	AAP-AMP	0.30	9	0.571	0.631	0.0522
235303_at	55.4	33.7	-0.72	-1.64	APP-AAA	0.30	9	0.571	0.631	0.0618
1570133_at	91.5	55.8	-0.71	-1.64	PPP-PAP	0.30	9	0.571	0.631	0.0491
215821_x_at	692.8	422.8	-0.71	-1.64	PPP-PPP	0.30	9	0.571	0.631	0.0641
231727_s_at	244.0	149.0	-0.71	-1.64	PAP-PPP	0.30	9	0.571	0.631	0.0599
238988_at	186.5	114.3	-0.71	-1.63	AAP-AAP	0.30	9	0.571	0.631	0.0574
237327_at	81.5	50.0	-0.71	-1.63	AAP-AAP	0.30	9	0.571	0.631	0.0630
1556597_a_at	143.9	88.2	-0.71	-1.63	APP-PPP	0.30	9	0.571	0.631	0.0650
215087_at	131.3	80.6	-0.71	-1.63	APP-AAP	0.30	9	0.571	0.631	0.0385
230897_at	77.4	47.5	-0.70	-1.63	PPP-APA	0.30	9	0.571	0.631	0.0457
241928_at	87.5	53.8	-0.70	-1.63	PPA-APA	0.30	9	0.571	0.631	0.0615
214185_at	166.8	102.8	-0.70	-1.62	PPP-PPP	0.30	9	0.571	0.631	0.0641
236742_at	96.4	59.5	-0.70	-1.62	AMA-APA	0.30	9	0.571	0.631	0.0418
209271_at	1072.5	662.6	-0.70	-1.62	PPP-PPP	0.30	9	0.571	0.631	0.0589
223882_at	162.4	100.4	-0.69	-1.62	PPP-PPP	0.30	9	0.571	0.631	0.0419
1558540_s_at	139.0	86.0	-0.69	-1.62	PPM-PAM	0.30	9	0.571	0.631	0.0242
241065_x_at	128.1	79.2	-0.69	-1.62	APP-MAM	0.30	9	0.571	0.631	0.0377
1560974_s_at	58.5	36.3	-0.69	-1.61	APA-AAA	0.30	9	0.571	0.631	0.0357
1558604_a_at	99.0	61.5	-0.69	-1.61	PAP-AAA	0.30	9	0.571	0.631	0.0410
242780_at	111.3	69.2	-0.69	-1.61	AAP-AAP	0.30	9	0.571	0.631	0.0537
239023_at	102.2	63.8	-0.68	-1.60	PPP-PPP	0.30	9	0.571	0.631	0.0466
1568734_a_at	136.3	85.2	-0.68	-1.60	PMA-AAP	0.30	9	0.571	0.631	0.0279
1564911_at	153.5	95.9	-0.68	-1.60	PPP-PPP	0.30	9	0.571	0.631	0.0331
215435_at	100.8	63.0	-0.68	-1.60	PPP-MPP	0.30	9	0.571	0.631	0.0279
221590_s_at	92.1	57.6	-0.68	-1.60	PPP-PPP	0.30	9	0.571	0.631	0.0614
210623_at	75.5	47.3	-0.67	-1.60	PAM-AAA	0.30	9	0.571	0.631	0.0484
1556385_at	609.5	383.3	-0.67	-1.59	PPP-PPP	0.30	9	0.571	0.631	0.0456
1556542_a_at	128.0	80.6	-0.67	-1.59	APP-PPP	0.30	9	0.571	0.631	0.0252
242819_at	74.6	47.1	-0.67	-1.59	PAP-AAP	0.30	9	0.571	0.631	0.0340
1566602_at	112.2	70.8	-0.67	-1.59	APA-APA	0.30	9	0.571	0.631	0.0397
1566512_at	180.3	114.0	-0.66	-1.58	APP-PPP	0.30	9	0.571	0.631	0.0358
241725_at	183.4	116.3	-0.66	-1.58	PPP-PPM	0.30	9	0.571	0.631	0.0336
221466_at	75.3	47.8	-0.66	-1.58	PAA-APA	0.30	9	0.571	0.631	0.0537
240393_at	118.6	75.3	-0.66	-1.58	AAA-PAA	0.30	9	0.571	0.631	0.0584

243004_at	138.0	87.7	-0.65	-1.57	APP-AAP	0.30	9	0.571	0.631	0.0559
1557418_at	178.5	113.7	-0.65	-1.57	PPP-PPP	0.30	9	0.571	0.631	0.0311
204684_at	553.3	353.5	-0.65	-1.57	PPP-PPP	0.30	9	0.571	0.631	0.0354
226260_x_at	213.2	136.4	-0.64	-1.56	PPP-PPP	0.30	9	0.571	0.631	0.0374
213202_at	215.2	137.8	-0.64	-1.56	PPP-AMM	0.30	9	0.571	0.631	0.0222
240165_at	238.5	152.8	-0.64	-1.56	PPP-PPP	0.30	9	0.571	0.631	0.0214
204924_at	109.4	70.2	-0.64	-1.56	PPA-AAA	0.30	9	0.571	0.631	0.0267
216814_at	67.2	43.1	-0.64	-1.56	MPP-AAP	0.30	9	0.571	0.631	0.0267
206825_at	4698.3	3021.6	-0.64	-1.56	PPP-PPP	0.30	9	0.571	0.631	0.0301
1555888_at	80.4	51.8	-0.63	-1.55	APP-APA	0.30	9	0.571	0.631	0.0214
215069_at	312.6	201.4	-0.63	-1.55	PPP-PPP	0.30	9	0.571	0.631	0.0449
244792_at	83.7	54.0	-0.63	-1.55	MPP-APP	0.30	9	0.571	0.631	0.0414
225630_at	137.2	88.6	-0.63	-1.55	PPP-PPP	0.30	9	0.571	0.631	0.0521
241898_at	34.0	22.0	-0.63	-1.55	AAA-AAP	0.30	9	0.571	0.631	0.0565
233041_x_at	667.7	431.7	-0.63	-1.55	PPP-PPP	0.30	9	0.571	0.631	0.0308
210926_at	1339.3	868.2	-0.63	-1.54	PPP-PPP	0.30	9	0.571	0.631	0.0303
243719_at	74.7	48.5	-0.62	-1.54	AAP-APA	0.30	9	0.571	0.631	0.0232
1554972_at	68.5	44.5	-0.62	-1.54	AAA-AAP	0.30	9	0.571	0.631	0.0332
239841_at	249.8	162.6	-0.62	-1.54	PPP-PPP	0.30	9	0.571	0.631	0.0487
215645_at	170.1	110.7	-0.62	-1.54	AAP-APA	0.30	9	0.571	0.631	0.0475
241432_at	52.2	34.0	-0.62	-1.54	PPA-AAA	0.30	9	0.571	0.631	0.0521
204447_at	336.5	219.4	-0.62	-1.53	PPP-PPP	0.30	9	0.571	0.631	0.0225
218326_s_at	629.2	410.6	-0.62	-1.53	PPP-PPP	0.30	9	0.571	0.631	0.0280
242134_at	139.3	91.1	-0.61	-1.53	PPP-MPP	0.30	9	0.571	0.631	0.0276
205846_at	409.9	268.1	-0.61	-1.53	PPP-PPP	0.30	9	0.571	0.631	0.0347
1565620_at	183.3	119.8	-0.61	-1.53	PPP-PPP	0.30	9	0.571	0.631	0.0410
235800_at	81.3	53.3	-0.61	-1.53	APP-APP	0.30	9	0.571	0.631	0.0222
220777_at	74.1	48.5	-0.61	-1.53	PAA-AAA	0.30	9	0.571	0.631	0.0351
241223_x_at	689.5	452.8	-0.61	-1.52	PPP-PPP	0.30	9	0.571	0.631	0.0214
201613_s_at	519.6	341.4	-0.61	-1.52	PPP-PPP	0.30	9	0.571	0.631	0.0494
242325_at	112.9	74.4	-0.60	-1.52	PAP-APA	0.30	9	0.571	0.631	0.0239
243780_at	71.1	46.9	-0.60	-1.52	PAP-AAP	0.30	9	0.571	0.631	0.0448
1569555_at	85.7	56.5	-0.60	-1.52	PPA-AAA	0.30	9	0.571	0.631	0.0457
235996_at	104.8	69.3	-0.60	-1.51	PMP-AAP	0.30	9	0.571	0.631	0.0448
216187_x_at	3246.0	2154.2	-0.59	-1.51	PPP-PPP	0.30	9	0.571	0.631	0.0504
219977_at	95.2	63.2	-0.59	-1.51	MPM-APP	0.30	9	0.571	0.631	0.0280
225875_s_at	600.6	399.0	-0.59	-1.51	APP-PPP	0.30	9	0.571	0.631	0.0332
214060_at	180.9	120.3	-0.59	-1.50	PPP-MPP	0.30	9	0.571	0.631	0.0201
1559780_at	115.5	76.8	-0.59	-1.50	PPP-MMP	0.30	9	0.571	0.631	0.0273
216554_s_at	357.7	237.8	-0.59	-1.51	PPP-PPP	0.30	9	0.571	0.631	0.0310
232912_at	105.5	70.2	-0.59	-1.50	PPP-PPP	0.30	9	0.571	0.631	0.0359
223036_at	85.4	56.8	-0.59	-1.50	PPP-PPP	0.30	9	0.571	0.631	0.0505

234956_at	174.0	115.8	-0.59	-1.50	PPA-PPP	0.30	9	0.571	0.631	0.0215
204635_at	202.8	135.1	-0.59	-1.50	PPP-PPP	0.30	9	0.571	0.631	0.0417
233122_at	110.9	73.9	-0.59	-1.50	PPP-PPP	0.30	9	0.571	0.631	0.0247
240076_at	83.2	55.4	-0.59	-1.50	PAA-AAA	0.30	9	0.571	0.631	0.0259
213763_at	545.7	364.3	-0.58	-1.50	PAP-PPP	0.30	9	0.571	0.631	0.0269
229492_at	390.9	261.4	-0.58	-1.50	PAA-PPP	0.30	9	0.571	0.631	0.0232
216347_s_at	171.7	114.9	-0.58	-1.49	PAP-AAM	0.30	9	0.571	0.631	0.0370
235826_at	196.7	131.6	-0.58	-1.49	PPP-PPP	0.30	9	0.571	0.631	0.0474
242594_at	122.7	82.1	-0.58	-1.50	AAA-APA	0.30	9	0.571	0.631	0.0509
243353_at	67.3	45.0	-0.58	-1.49	APA-PAA	0.30	9	0.571	0.631	0.0416
203391_at	1526.3	1022.2	-0.58	-1.49	PPP-PPP	0.30	9	0.571	0.631	0.0331
AFFX-r2-Bs-thr-5_s_at	63.6	42.6	-0.58	-1.49	APA-PPP	0.30	9	0.571	0.631	0.0195
1553974_at	483.4	324.6	-0.58	-1.49	PPP-PPP	0.30	9	0.571	0.631	0.0231
211387_x_at	294.6	197.9	-0.57	-1.49	PPP-PPP	0.30	9	0.571	0.631	0.0244
1559425_at	73.9	49.6	-0.57	-1.49	APM-AAA	0.30	9	0.571	0.631	0.0463
235322_at	242.5	163.2	-0.57	-1.49	AAP-APA	0.30	9	0.571	0.631	0.0268
209602_s_at	120.0	80.7	-0.57	-1.49	AAP-APM	0.30	9	0.571	0.631	0.0434
204501_at	254.6	171.4	-0.57	-1.49	PPP-PPA	0.30	9	0.571	0.631	0.0298
224940_s_at	677.1	456.0	-0.57	-1.49	PPP-PPP	0.30	9	0.571	0.631	0.0354
240240_at	168.1	113.8	-0.56	-1.48	APP-PMP	0.30	9	0.571	0.631	0.0310
201250_s_at	1883.2	1274.4	-0.56	-1.48	PPP-PPP	0.30	9	0.571	0.631	0.0368
227209_at	82.8	56.1	-0.56	-1.48	PAP-PMP	0.30	9	0.571	0.631	0.0478
1569180_at	71.4	48.4	-0.56	-1.48	MPP-APP	0.30	9	0.571	0.631	0.0329
1560503_a_at	131.4	89.2	-0.56	-1.47	PPP-PAP	0.30	9	0.571	0.631	0.0460
228983_at	172.4	117.1	-0.56	-1.47	PPA-APP	0.30	9	0.571	0.631	0.0466
202039_at	581.1	395.9	-0.55	-1.47	PPP-PPP	0.30	9	0.571	0.631	0.0300
1556180_at	175.5	119.7	-0.55	-1.47	PPP-PPP	0.30	9	0.571	0.631	0.0468
212093_s_at	159.8	109.2	-0.55	-1.46	MPP-APP	0.30	9	0.571	0.631	0.0199
237652_at	98.0	67.0	-0.55	-1.46	MAP-PPP	0.30	9	0.571	0.631	0.0280
223510_at	240.4	164.6	-0.55	-1.46	PAP-APP	0.30	9	0.571	0.631	0.0352
232810_at	104.0	71.3	-0.55	-1.46	AAP-AAP	0.30	9	0.571	0.631	0.0406
233779_x_at	232.4	159.3	-0.55	-1.46	PPP-PPP	0.30	9	0.571	0.631	0.0311
243570_at	147.5	101.2	-0.54	-1.46	PPP-PPP	0.30	9	0.571	0.631	0.0169
217344_at	82.9	56.9	-0.54	-1.46	PAA-AAP	0.30	9	0.571	0.631	0.0241
204073_s_at	687.1	472.0	-0.54	-1.46	PPP-PPP	0.30	9	0.571	0.631	0.0251
39313_at	81.9	56.3	-0.54	-1.45	PPP-PPP	0.30	9	0.571	0.631	0.0276
220014_at	594.4	408.9	-0.54	-1.45	PPP-PPP	0.30	9	0.571	0.631	0.0285
1568627_at	159.5	109.8	-0.54	-1.45	PPP-PPP	0.30	9	0.571	0.631	0.0252
238889_at	162.2	111.6	-0.54	-1.45	PPP-PPP	0.30	9	0.571	0.631	0.0322
219445_at	323.8	222.9	-0.54	-1.45	PMP-PPP	0.30	9	0.571	0.631	0.0340
240355_at	125.3	86.3	-0.54	-1.45	APA-AAP	0.30	9	0.571	0.631	0.0319
59433_at	120.3	82.9	-0.54	-1.45	PPP-APA	0.30	9	0.571	0.631	0.0317

1568704_a_at	367.0	253.3	-0.54	-1.45	AAA-APA	0.30	9	0.571	0.631	0.0196
206169_x_at	369.5	255.1	-0.54	-1.45	AMP-MAA	0.30	9	0.571	0.631	0.0437
235529_x_at	2502.5	1729.9	-0.53	-1.45	PPP-PPP	0.30	9	0.571	0.631	0.0283
215130_s_at	195.8	135.5	-0.53	-1.45	PPP-PPP	0.30	9	0.571	0.631	0.0330
217610_at	187.9	130.1	-0.53	-1.45	APP-MAP	0.30	9	0.571	0.631	0.0336
208339_at	30.1	20.8	-0.53	-1.44	AAA-APA	0.30	9	0.571	0.631	0.0403
230777_s_at	492.2	342.3	-0.52	-1.44	PAP-PMP	0.30	9	0.571	0.631	0.0367
1560596_at	104.0	72.8	-0.51	-1.43	PPP-PPP	0.30	9	0.571	0.631	0.0309
1569668_at	75.4	52.9	-0.51	-1.43	AAA-APA	0.30	9	0.571	0.631	0.0196
211089_s_at	145.4	102.3	-0.51	-1.42	PPP-PPP	0.30	9	0.571	0.631	0.0250
242228_at	236.6	166.6	-0.51	-1.42	PPP-PPP	0.30	9	0.571	0.631	0.0305
207112_s_at	101.4	71.5	-0.50	-1.42	PPA-AMP	0.30	9	0.571	0.631	0.0170
210735_s_at	440.7	311.0	-0.50	-1.42	PPP-PPP	0.30	9	0.571	0.631	0.0135
244498_x_at	119.8	84.6	-0.50	-1.42	PAP-PPP	0.30	9	0.571	0.631	0.0214
230761_at	177.6	125.4	-0.50	-1.42	PPP-PPP	0.30	9	0.571	0.631	0.0400
224746_at	862.0	609.5	-0.50	-1.41	AAP-PPP	0.30	9	0.571	0.631	0.0144
205140_at	414.6	293.4	-0.50	-1.41	PPP-PPP	0.30	9	0.571	0.631	0.0287
244597_at	152.5	107.9	-0.50	-1.41	PAP-AMA	0.30	9	0.571	0.631	0.0328
232096_x_at	120.9	85.5	-0.50	-1.41	PPP-PPP	0.30	9	0.571	0.631	0.0342
237444_at	162.1	114.7	-0.50	-1.41	PPP-PPP	0.30	9	0.571	0.631	0.0252
1554701_a_at	383.5	271.9	-0.50	-1.41	PPP-PPP	0.30	9	0.571	0.631	0.0253
240382_at	232.1	164.5	-0.50	-1.41	PPP-PPP	0.30	9	0.571	0.631	0.0323
244481_at	95.0	67.5	-0.49	-1.41	AAP-APA	0.30	9	0.571	0.631	0.0135
237746_at	89.9	63.9	-0.49	-1.41	PPA-APP	0.30	9	0.571	0.631	0.0148
221002_s_at	870.1	618.5	-0.49	-1.41	PPP-PPP	0.30	9	0.571	0.631	0.0234
224392_s_at	320.3	227.8	-0.49	-1.41	PPP-PPP	0.30	9	0.571	0.631	0.0364
224447_s_at	3299.1	2349.4	-0.49	-1.40	PPP-PPP	0.30	9	0.571	0.631	0.0363
243794_at	59.5	42.4	-0.49	-1.40	APP-PAA	0.30	9	0.571	0.631	0.0333
214699_x_at	179.2	127.8	-0.49	-1.40	PPP-PPP	0.30	9	0.571	0.631	0.0324
215291_at	134.6	96.2	-0.49	-1.40	APP-MPP	0.30	9	0.571	0.631	0.0126
235107_at	269.5	192.9	-0.48	-1.40	PAP-APP	0.30	9	0.571	0.631	0.0129
212969_x_at	413.1	296.4	-0.48	-1.39	PPP-PPP	0.30	9	0.571	0.631	0.0194
222297_x_at	1267.9	909.7	-0.48	-1.39	PPP-PPP	0.30	9	0.571	0.631	0.0223
209238_at	1424.1	1027.2	-0.47	-1.39	PPP-PPP	0.30	9	0.571	0.631	0.0342
243052_at	143.4	103.5	-0.47	-1.39	APA-PAA	0.30	9	0.571	0.631	0.0170
227347_x_at	337.7	244.4	-0.47	-1.38	APA-PPP	0.30	9	0.571	0.631	0.0140
205367_at	270.0	195.4	-0.47	-1.38	PAA-PPP	0.30	9	0.571	0.631	0.0232
205160_at	275.6	199.8	-0.46	-1.38	PPP-PPP	0.30	9	0.571	0.631	0.0234
1556695_a_at	105.5	76.5	-0.46	-1.38	PPP-PPP	0.30	9	0.571	0.631	0.0294
225803_at	570.4	414.0	-0.46	-1.38	PPP-PPP	0.30	9	0.571	0.631	0.0279
225446_at	318.8	231.7	-0.46	-1.38	APP-PPP	0.30	9	0.571	0.631	0.0252
222479_s_at	2596.2	1888.2	-0.46	-1.38	PPP-PPP	0.30	9	0.571	0.631	0.0104

236254_at	243.3	177.1	-0.46	-1.37	PAP-PPP	0.30	9	0.571	0.631	0.0243
229200_at	549.5	400.1	-0.46	-1.37	PPP-PPP	0.30	9	0.571	0.631	0.0353
222931_s_at	219.1	159.6	-0.46	-1.37	PPP-PPP	0.30	9	0.571	0.631	0.0178
231106_at	301.3	219.9	-0.45	-1.37	PPP-PPP	0.30	9	0.571	0.631	0.0299
213203_at	1322.5	1811.4	0.45	1.37	PPP-PPP	0.30	9	0.571	0.631	0.0001
230404_at	84.0	61.4	-0.45	-1.37	PPP-PPP	0.30	9	0.571	0.631	0.0213
234111_at	243.6	178.2	-0.45	-1.37	PPP-PPP	0.30	9	0.571	0.631	0.0340
212351_at	1067.9	784.4	-0.45	-1.36	PPP-PPP	0.30	9	0.571	0.631	0.0226
230250_at	839.3	617.9	-0.44	-1.36	PPP-PPP	0.30	9	0.571	0.631	0.0124
1554873_at	142.1	104.9	-0.44	-1.36	PPP-PPP	0.30	9	0.571	0.631	0.0116
242201_at	164.2	121.2	-0.44	-1.35	PPP-PPP	0.30	9	0.571	0.631	0.0089
241968_at	271.8	201.0	-0.44	-1.35	APP-PPP	0.30	9	0.571	0.631	0.0231
1554004_a_at	793.6	587.4	-0.43	-1.35	PPP-PPP	0.30	9	0.571	0.631	0.0174
1554523_a_at	303.6	225.0	-0.43	-1.35	APP-APP	0.30	9	0.571	0.631	0.0163
213615_at	182.0	135.6	-0.43	-1.34	AAP-AAP	0.30	9	0.571	0.631	0.0249
238714_at	399.5	299.6	-0.42	-1.33	PPP-PPP	0.30	9	0.571	0.631	0.0185
202162_s_at	1025.0	770.0	-0.41	-1.33	PPP-PPP	0.30	9	0.571	0.631	0.0277
219490_s_at	589.2	443.1	-0.41	-1.33	PPP-PPP	0.30	9	0.571	0.631	0.0142
220973_s_at	332.9	251.0	-0.41	-1.33	PPP-PPP	0.30	9	0.571	0.631	0.0271
219639_x_at	1275.3	963.4	-0.41	-1.32	PPP-PPP	0.30	9	0.571	0.631	0.0202
228023_x_at	383.0	291.2	-0.40	-1.32	PPP-PPP	0.30	9	0.571	0.631	0.0062
228464_at	217.3	165.4	-0.39	-1.31	PPP-PPP	0.30	9	0.571	0.631	0.0113
211097_s_at	131.5	100.4	-0.39	-1.31	PAM-PAP	0.30	9	0.571	0.631	0.0195
228714_at	176.8	135.0	-0.39	-1.31	APP-PPA	0.30	9	0.571	0.631	0.0243
216442_x_at	38027.8	29035.4	-0.39	-1.31	PPP-PPP	0.30	9	0.571	0.631	0.0244
203790_s_at	2336.1	1786.5	-0.39	-1.31	PPP-PPP	0.30	9	0.571	0.631	0.0096
208980_s_at	30467.3	23297.8	-0.39	-1.31	PPP-PPP	0.30	9	0.571	0.631	0.0188
216178_x_at	152.1	116.4	-0.39	-1.31	PPP-PPP	0.30	9	0.571	0.631	0.0071
234981_x_at	2726.0	2086.9	-0.39	-1.31	PPP-PPP	0.30	9	0.571	0.631	0.0070
201982_s_at	244.7	187.3	-0.39	-1.31	MPP-PPP	0.30	9	0.571	0.631	0.0080
200651_at	40383.9	30947.9	-0.38	-1.31	PPP-PPP	0.30	9	0.571	0.631	0.0143
205282_at	800.0	614.5	-0.38	-1.30	PPP-PPP	0.30	9	0.571	0.631	0.0050
222327_x_at	264.6	203.2	-0.38	-1.30	PPP-PPP	0.30	9	0.571	0.631	0.0063
201968_s_at	2068.5	1588.5	-0.38	-1.30	PPP-PPP	0.30	9	0.571	0.631	0.0110
1553567_s_at	40417.4	31052.0	-0.38	-1.30	PPP-PPP	0.30	9	0.571	0.631	0.0150
210235_s_at	682.7	524.9	-0.38	-1.30	PPP-PPP	0.30	9	0.571	0.631	0.0173
1554248_at	194.8	150.4	-0.37	-1.30	PPP-PPP	0.30	9	0.571	0.631	0.0083
207018_s_at	93.7	72.7	-0.37	-1.29	PPP-PPP	0.30	9	0.571	0.631	0.0130
1007_s_at	1454.4	1128.3	-0.37	-1.29	PPP-PPP	0.30	9	0.571	0.631	0.0139
227298_at	332.7	258.3	-0.37	-1.29	PPP-PPP	0.30	9	0.571	0.631	0.0061
212859_x_at	19460.6	15115.0	-0.37	-1.29	PPP-PPP	0.30	9	0.571	0.631	0.0185
203309_s_at	139.4	108.7	-0.36	-1.28	PPP-PPP	0.30	9	0.571	0.631	0.0052

219001_s_at	604.3	471.2	-0.36	-1.28	PPP-PPP	0.30	9	0.571	0.631	0.0050
213583_x_at	37861.0	29566.3	-0.36	-1.28	PPP-PPP	0.30	9	0.571	0.631	0.0183
217905_at	1711.9	1339.1	-0.35	-1.28	PPP-PPP	0.30	9	0.571	0.631	0.0027
215541_s_at	615.3	481.4	-0.35	-1.28	PPP-PPP	0.30	9	0.571	0.631	0.0041
205130_at	4053.9	3175.9	-0.35	-1.28	PPP-PPP	0.30	9	0.571	0.631	0.0028
219418_at	601.0	471.4	-0.35	-1.28	PPP-PPP	0.30	9	0.571	0.631	0.0113
218332_at	22217.0	17452.2	-0.35	-1.27	PPP-PPP	0.30	9	0.571	0.631	0.0170
232371_at	101.8	80.1	-0.35	-1.27	AAP-PPP	0.30	9	0.571	0.631	0.0032
1553263_at	228.3	179.5	-0.35	-1.27	AAM-AAP	0.30	9	0.571	0.631	0.0032
224707_at	2177.3	1712.1	-0.35	-1.27	PPP-PPP	0.30	9	0.571	0.631	0.0067
227534_at	1877.5	1478.0	-0.35	-1.27	PPP-PPP	0.30	9	0.571	0.631	0.0084
212376_s_at	952.8	751.7	-0.34	-1.27	PPP-PPP	0.30	9	0.571	0.631	0.0104
230937_at	151.2	119.7	-0.34	-1.26	APP-APP	0.30	9	0.571	0.631	0.0126
203113_s_at	17135.8	13599.0	-0.33	-1.26	PPP-PPP	0.30	9	0.571	0.631	0.0088
226898_s_at	146.4	116.3	-0.33	-1.26	PPP-PPP	0.30	9	0.571	0.631	0.0059
206405_x_at	419.6	333.6	-0.33	-1.26	PPP-PPP	0.30	9	0.571	0.631	0.0049
215383_x_at	763.3	607.0	-0.33	-1.26	PPP-PPP	0.30	9	0.571	0.631	0.0104
214394_x_at	22848.4	18180.5	-0.33	-1.26	PPP-PPP	0.30	9	0.571	0.631	0.0105
226739_at	968.3	775.8	-0.32	-1.25	PPP-PPP	0.30	9	0.571	0.631	0.0080
201596_x_at	32499.9	26044.7	-0.32	-1.25	PPP-PPP	0.30	9	0.571	0.631	0.0076
230600_at	95.3	76.5	-0.32	-1.25	PAP-PAP	0.30	9	0.571	0.631	0.0020
200936_at	42970.8	34512.7	-0.32	-1.25	PPP-PPP	0.30	9	0.571	0.631	0.0031
218640_s_at	1236.5	997.3	-0.31	-1.24	PPP-PPP	0.30	9	0.571	0.631	0.0064
212277_at	1339.8	1082.3	-0.31	-1.24	PPP-PPP	0.30	9	0.571	0.631	0.0055
39817_s_at	1026.1	831.1	-0.30	-1.24	PPP-PPP	0.30	9	0.571	0.631	0.0016
217466_x_at	23435.1	18990.0	-0.30	-1.23	PPP-PPP	0.30	9	0.571	0.631	0.0034
225947_at	1670.7	1356.4	-0.30	-1.23	PPP-PPP	0.30	9	0.571	0.631	0.0026
212801_at	1432.4	1173.3	-0.29	-1.22	PPP-PPP	0.30	9	0.571	0.631	0.0033
201323_at	8680.2	7112.4	-0.29	-1.22	PPP-PPP	0.30	9	0.571	0.631	0.0061
220174_at	425.8	349.0	-0.29	-1.22	PPP-PPP	0.30	9	0.571	0.631	0.0069
203065_s_at	16142.6	13508.4	-0.26	-1.20	PPP-PPP	0.30	9	0.571	0.631	0.0003
215321_at	28.3	3.8	-2.92	-7.54	PAA-AAA	0.20	10	0.678	0.723	0.1386
1558579_at	18.7	2.6	-2.86	-7.26	APA-AAA	0.20	10	0.678	0.723	0.1370
209687_at	7.7	55.9	2.85	7.23	AAA-AAP	0.20	10	0.678	0.723	0.0234
1561516_at	21.0	3.2	-2.73	-6.63	APA-AAA	0.20	10	0.678	0.723	0.2159
1566860_at	6.4	41.7	2.71	6.53	AAA-PAA	0.20	10	0.678	0.723	0.0173
1561328_at	16.6	2.6	-2.69	-6.46	APA-AAA	0.20	10	0.678	0.723	0.1900
243601_at	19.9	3.2	-2.66	-6.33	APA-AAA	0.20	10	0.678	0.723	0.1313
206603_at	22.9	3.6	-2.66	-6.33	AAP-AAA	0.20	10	0.678	0.723	0.1579
227806_at	12.1	76.2	2.66	6.31	AAA-APA	0.20	10	0.678	0.723	0.0154
237762_at	22.8	3.7	-2.63	-6.20	APA-AAA	0.20	10	0.678	0.723	0.1485
237586_at	38.9	6.3	-2.62	-6.16	APA-AAA	0.20	10	0.678	0.723	0.1238

217400_at	48.6	7.9	-2.62	-6.13	PPA-AAA	0.20	10	0.678	0.723	0.1347
239962_at	16.6	2.8	-2.55	-5.86	PAA-AAA	0.20	10	0.678	0.723	0.1818
231303_at	26.0	4.5	-2.53	-5.79	APA-AAA	0.20	10	0.678	0.723	0.1331
221453_at	102.9	17.9	-2.53	-5.76	PPA-AAA	0.20	10	0.678	0.723	0.1592
205267_at	16.6	2.9	-2.52	-5.75	PAA-AAA	0.20	10	0.678	0.723	0.1416
1564544_x_at	80.7	14.1	-2.52	-5.72	APA-AAA	0.20	10	0.678	0.723	0.1213
1570622_at	22.8	4.0	-2.51	-5.70	APA-AAA	0.20	10	0.678	0.723	0.1869
1554778_at	11.5	2.0	-2.51	-5.68	AAP-AAA	0.20	10	0.678	0.723	0.2059
1570126_at	57.5	10.3	-2.49	-5.60	APA-AAA	0.20	10	0.678	0.723	0.1389
235335_at	11.0	2.0	-2.48	-5.58	PAA-AAA	0.20	10	0.678	0.723	0.1651
237001_at	42.4	7.7	-2.46	-5.48	APA-AAA	0.20	10	0.678	0.723	0.1203
233241_at	52.1	9.5	-2.45	-5.47	APM-AAA	0.20	10	0.678	0.723	0.1814
206201_s_at	6.4	1.2	-2.44	-5.44	APA-AAA	0.20	10	0.678	0.723	0.1504
1558217_at	37.0	7.0	-2.40	-5.27	PAP-AAA	0.20	10	0.678	0.723	0.1411
1569969_a_at	10.6	2.0	-2.40	-5.27	PAA-AAA	0.20	10	0.678	0.723	0.2048
1568900_a_at	6.3	32.5	2.37	5.18	AAA-AAP	0.20	10	0.678	0.723	0.0144
1557593_at	13.7	2.7	-2.37	-5.17	PAA-AAA	0.20	10	0.678	0.723	0.1781
242250_at	35.5	6.9	-2.36	-5.15	AAA-PAA	0.20	10	0.678	0.723	0.2344
1560306_at	45.0	8.8	-2.36	-5.13	PMP-AAA	0.20	10	0.678	0.723	0.1875
236944_at	43.9	8.6	-2.36	-5.11	AAP-AAA	0.20	10	0.678	0.723	0.1337
241397_at	61.3	12.1	-2.34	-5.08	AMM-PAA	0.20	10	0.678	0.723	0.1535
1561229_at	22.2	4.5	-2.30	-4.91	APA-AAA	0.20	10	0.678	0.723	0.1810
234832_at	43.0	8.8	-2.28	-4.86	APA-AAA	0.20	10	0.678	0.723	0.1559
223645_s_at	54.4	11.3	-2.27	-4.83	PAA-AAA	0.20	10	0.678	0.723	0.1380
237282_s_at	9.8	2.0	-2.27	-4.81	AAP-AAA	0.20	10	0.678	0.723	0.1410
206420_at	18.9	3.9	-2.26	-4.80	PAA-AAA	0.20	10	0.678	0.723	0.1877
224324_at	39.5	8.2	-2.26	-4.79	PAA-AAA	0.20	10	0.678	0.723	0.1564
1560080_at	38.0	8.0	-2.26	-4.77	AAP-AAA	0.20	10	0.678	0.723	0.1920
241444_at	60.2	12.8	-2.24	-4.71	PAA-AAA	0.20	10	0.678	0.723	0.1306
1555728_a_at	50.4	10.9	-2.21	-4.63	AAA-APA	0.20	10	0.678	0.723	0.1193
242513_x_at	37.5	8.1	-2.21	-4.62	APA-AAA	0.20	10	0.678	0.723	0.2316
1566437_at	41.4	9.0	-2.21	-4.61	AAA-PAA	0.20	10	0.678	0.723	0.1261
240620_at	105.0	23.0	-2.19	-4.57	AAA-AAP	0.20	10	0.678	0.723	0.1384
243378_at	52.8	11.6	-2.19	-4.56	AAP-AAP	0.20	10	0.678	0.723	0.1807
205984_at	59.3	13.1	-2.18	-4.52	AAA-AAP	0.20	10	0.678	0.723	0.1925
244257_at	73.3	16.2	-2.18	-4.52	PAA-AAA	0.20	10	0.678	0.723	0.2373
231114_at	43.8	9.7	-2.17	-4.51	AAP-AAA	0.20	10	0.678	0.723	0.1697
1569433_at	17.4	3.9	-2.16	-4.48	APA-AAA	0.20	10	0.678	0.723	0.1900
213006_at	26.9	6.0	-2.15	-4.45	AAM-AAP	0.20	10	0.678	0.723	0.2436
233046_at	22.5	5.1	-2.15	-4.42	AAA-AAP	0.20	10	0.678	0.723	0.1458
241804_at	37.0	8.4	-2.14	-4.40	AAP-AAA	0.20	10	0.678	0.723	0.2423
214369_s_at	52.6	12.0	-2.14	-4.40	APA-AAA	0.20	10	0.678	0.723	0.1903

1561198_at	28.9	6.6	-2.13	-4.37	PAA-PAA	0.20	10	0.678	0.723	0.1192
214598_at	48.7	11.2	-2.12	-4.34	AAP-AAA	0.20	10	0.678	0.723	0.1298
1563656_at	59.1	13.6	-2.12	-4.34	PAP-AAP	0.20	10	0.678	0.723	0.2012
216408_at	52.6	12.2	-2.11	-4.32	PPA-AAA	0.20	10	0.678	0.723	0.1184
239814_at	73.9	17.1	-2.11	-4.32	PAA-AAA	0.20	10	0.678	0.723	0.1868
1556491_at	31.4	7.3	-2.11	-4.31	APA-AAM	0.20	10	0.678	0.723	0.2203
243163_at	36.4	8.5	-2.10	-4.29	APA-AAA	0.20	10	0.678	0.723	0.2223
208587_s_at	74.1	17.4	-2.09	-4.25	MAA-PAA	0.20	10	0.678	0.723	0.2072
236802_at	70.8	16.7	-2.08	-4.24	AAP-AAA	0.20	10	0.678	0.723	0.1362
232251_at	37.4	8.9	-2.08	-4.22	PAA-AAA	0.20	10	0.678	0.723	0.1275
1563649_at	41.9	9.9	-2.08	-4.21	APA-AAP	0.20	10	0.678	0.723	0.1142
206239_s_at	61.8	14.7	-2.07	-4.21	PAA-AAA	0.20	10	0.678	0.723	0.1809
1566764_at	28.5	6.9	-2.05	-4.13	AAP-AAA	0.20	10	0.678	0.723	0.1576
237193_s_at	57.7	14.0	-2.04	-4.12	AAA-APA	0.20	10	0.678	0.723	0.2102
1560489_at	85.2	20.8	-2.04	-4.10	PAM-AAA	0.20	10	0.678	0.723	0.1223
242897_at	31.3	7.7	-2.03	-4.09	PAA-AAA	0.20	10	0.678	0.723	0.1273
206610_s_at	71.4	17.6	-2.02	-4.06	PMA-AAA	0.20	10	0.678	0.723	0.1435
220626_at	61.9	15.3	-2.02	-4.05	APA-AAA	0.20	10	0.678	0.723	0.1398
233136_at	6.8	1.7	-2.02	-4.05	PAA-AAA	0.20	10	0.678	0.723	0.2723
242765_at	59.5	14.8	-2.01	-4.02	AAP-AAA	0.20	10	0.678	0.723	0.2609
208235_x_at	26.3	6.6	-2.01	-4.02	APA-AAA	0.20	10	0.678	0.723	0.2335
239467_at	20.9	5.2	-2.01	-4.02	AAP-AAA	0.20	10	0.678	0.723	0.2426
207958_at	40.1	10.1	-1.99	-3.97	APA-AAA	0.20	10	0.678	0.723	0.2121
243316_x_at	54.8	13.8	-1.99	-3.96	PAA-AAA	0.20	10	0.678	0.723	0.1293
243015_at	36.4	9.2	-1.98	-3.96	APA-AAA	0.20	10	0.678	0.723	0.1132
214630_at	30.2	7.7	-1.98	-3.93	PAA-AAA	0.20	10	0.678	0.723	0.2102
241193_at	74.5	18.9	-1.98	-3.93	APA-PAA	0.20	10	0.678	0.723	0.2668
238702_at	53.4	13.6	-1.98	-3.93	AAA-APA	0.20	10	0.678	0.723	0.1317
242329_at	113.5	29.0	-1.97	-3.92	APA-APM	0.20	10	0.678	0.723	0.2495
214233_at	42.7	10.9	-1.97	-3.92	PPA-AAA	0.20	10	0.678	0.723	0.2012
204800_s_at	70.3	18.1	-1.96	-3.89	AAA-AAP	0.20	10	0.678	0.723	0.1358
233130_at	21.1	5.4	-1.96	-3.88	PAA-AAA	0.20	10	0.678	0.723	0.2186
207699_at	144.7	37.5	-1.95	-3.86	PAA-AAA	0.20	10	0.678	0.723	0.1564
233771_at	173.4	44.9	-1.95	-3.86	PAA-AMP	0.20	10	0.678	0.723	0.1558
205414_s_at	73.6	19.2	-1.94	-3.83	PAA-AAA	0.20	10	0.678	0.723	0.1620
1553396_a_at	119.6	31.3	-1.93	-3.82	APA-AAA	0.20	10	0.678	0.723	0.2334
1557540_at	92.9	24.5	-1.92	-3.80	PPA-AAA	0.20	10	0.678	0.723	0.1898
232461_at	51.2	13.5	-1.92	-3.79	AAP-AAA	0.20	10	0.678	0.723	0.1185
220595_at	37.4	9.9	-1.92	-3.77	AMP-AAA	0.20	10	0.678	0.723	0.2376
223764_x_at	41.8	11.2	-1.91	-3.75	MPA-AAA	0.20	10	0.678	0.723	0.1829
233721_x_at	56.0	15.0	-1.91	-3.75	PPP-APA	0.20	10	0.678	0.723	0.2266
230942_at	79.4	21.4	-1.89	-3.71	AMA-AAP	0.20	10	0.678	0.723	0.2240

204580_at	22.9	6.2	-1.88	-3.69	AAP-AAA	0.20	10	0.678	0.723	0.2201
1564336_at	80.5	21.9	-1.88	-3.68	APA-AAP	0.20	10	0.678	0.723	0.1375
232164_s_at	61.9	16.9	-1.87	-3.67	AAA-AAP	0.20	10	0.678	0.723	0.2425
214186_s_at	54.4	14.9	-1.87	-3.66	PAA-AAA	0.20	10	0.678	0.723	0.2028
1557206_at	20.7	5.7	-1.87	-3.66	PAA-AAA	0.20	10	0.678	0.723	0.2713
242175_at	43.5	11.9	-1.87	-3.65	AMP-AAP	0.20	10	0.678	0.723	0.2592
1570326_at	61.4	16.8	-1.87	-3.65	APA-AAA	0.20	10	0.678	0.723	0.1359
1562470_at	22.9	6.3	-1.87	-3.65	APA-AAA	0.20	10	0.678	0.723	0.2395
235982_at	24.2	6.6	-1.87	-3.64	PAA-AAA	0.20	10	0.678	0.723	0.1977
242412_at	53.6	14.7	-1.86	-3.64	PAA-AAA	0.20	10	0.678	0.723	0.1099
238229_at	69.5	19.1	-1.86	-3.64	APA-AAA	0.20	10	0.678	0.723	0.2133
206290_s_at	114.0	31.3	-1.86	-3.64	MMP-AMP	0.20	10	0.678	0.723	0.1930
237088_at	79.5	21.9	-1.86	-3.63	PAM-AAA	0.20	10	0.678	0.723	0.1270
1555718_x_at	18.5	5.1	-1.86	-3.63	PAA-AAA	0.20	10	0.678	0.723	0.1734
234447_at	91.6	25.3	-1.86	-3.63	PAP-AAA	0.20	10	0.678	0.723	0.1891
227526_at	59.1	16.3	-1.85	-3.62	APA-AAA	0.20	10	0.678	0.723	0.1303
1552463_at	51.1	14.2	-1.85	-3.61	PAA-AAA	0.20	10	0.678	0.723	0.1481
236423_at	34.9	9.7	-1.85	-3.60	AAA-AAP	0.20	10	0.678	0.723	0.2024
235905_at	53.1	14.8	-1.85	-3.59	AAP-AAA	0.20	10	0.678	0.723	0.2472
228988_at	17.0	4.7	-1.84	-3.59	PAA-AAA	0.20	10	0.678	0.723	0.2183
217217_at	37.7	10.5	-1.84	-3.58	PAA-AAA	0.20	10	0.678	0.723	0.1322
1552412_a_at	33.0	9.2	-1.84	-3.57	PAA-AAA	0.20	10	0.678	0.723	0.2194
242599_at	31.1	8.7	-1.84	-3.57	PAA-AAA	0.20	10	0.678	0.723	0.2392
216553_x_at	44.7	12.5	-1.84	-3.57	AAA-APA	0.20	10	0.678	0.723	0.2757
235909_at	55.0	15.4	-1.83	-3.57	PAA-PAA	0.20	10	0.678	0.723	0.2516
1557363_a_at	38.0	10.7	-1.83	-3.56	APA-AAA	0.20	10	0.678	0.723	0.2290
221409_at	91.6	25.8	-1.83	-3.55	MAA-AAP	0.20	10	0.678	0.723	0.1446
220931_at	31.1	8.8	-1.83	-3.55	AAA-AAP	0.20	10	0.678	0.723	0.2720
1562527_at	42.9	12.1	-1.83	-3.54	AMA-AAP	0.20	10	0.678	0.723	0.2463
227874_at	39.4	11.2	-1.82	-3.54	PAA-AAA	0.20	10	0.678	0.723	0.1730
242713_at	82.0	23.2	-1.82	-3.53	PAA-AAM	0.20	10	0.678	0.723	0.1831
228897_at	106.2	30.0	-1.82	-3.53	PPP-PAA	0.20	10	0.678	0.723	0.2499
234276_at	70.8	20.0	-1.82	-3.53	APP-APP	0.20	10	0.678	0.723	0.2509
1553601_a_at	34.5	9.8	-1.82	-3.52	PAA-AAA	0.20	10	0.678	0.723	0.1749
234204_at	45.0	12.8	-1.81	-3.52	PAA-PAA	0.20	10	0.678	0.723	0.2660
1561210_at	38.8	11.0	-1.81	-3.51	PPA-AAA	0.20	10	0.678	0.723	0.1422
1557758_at	30.0	8.6	-1.81	-3.51	PAA-AAA	0.20	10	0.678	0.723	0.1791
220585_at	76.9	21.9	-1.81	-3.51	APA-AAA	0.20	10	0.678	0.723	0.2351
1554307_at	45.8	13.1	-1.81	-3.50	APA-AAA	0.20	10	0.678	0.723	0.1664
243983_at	129.3	37.0	-1.81	-3.50	APP-MAA	0.20	10	0.678	0.723	0.1830
233556_at	69.3	19.9	-1.80	-3.49	AAP-AAA	0.20	10	0.678	0.723	0.1472
233465_at	21.4	6.1	-1.80	-3.49	AAP-AAA	0.20	10	0.678	0.723	0.2058

244290_at	44.2	12.7	-1.80	-3.49	PAA-AAA	0.20	10	0.678	0.723	0.1152
243488_at	48.5	13.9	-1.80	-3.48	PAP-AAA	0.20	10	0.678	0.723	0.1303
1563120_at	34.1	9.8	-1.80	-3.48	AAP-AAA	0.20	10	0.678	0.723	0.1596
207404_s_at	43.8	12.7	-1.79	-3.46	PAP-AAM	0.20	10	0.678	0.723	0.2319
233675_s_at	8.0	2.3	-1.79	-3.46	AAP-AAA	0.20	10	0.678	0.723	0.2388
240290_at	106.0	30.7	-1.79	-3.46	PAP-MAA	0.20	10	0.678	0.723	0.1764
233187_s_at	37.7	11.0	-1.78	-3.44	AAM-APA	0.20	10	0.678	0.723	0.1764
222071_s_at	54.4	15.8	-1.78	-3.44	APM-AAA	0.20	10	0.678	0.723	0.1413
231690_at	22.9	6.7	-1.78	-3.43	PAA-AAA	0.20	10	0.678	0.723	0.2245
234455_at	30.5	8.9	-1.78	-3.43	MAA-AAP	0.20	10	0.678	0.723	0.2725
228715_at	55.6	16.3	-1.77	-3.42	APP-PAA	0.20	10	0.678	0.723	0.2297
239664_at	24.2	7.1	-1.77	-3.41	APA-AAA	0.20	10	0.678	0.723	0.2618
243424_at	58.5	17.2	-1.77	-3.41	APA-AAA	0.20	10	0.678	0.723	0.1488
1556595_at	44.3	13.0	-1.77	-3.40	AAP-AAA	0.20	10	0.678	0.723	0.1723
232795_at	28.8	8.5	-1.76	-3.39	PAA-AAA	0.20	10	0.678	0.723	0.1433
237712_at	38.4	11.3	-1.76	-3.39	PAA-AAA	0.20	10	0.678	0.723	0.1980
1568830_at	63.5	18.8	-1.76	-3.39	APA-AAP	0.20	10	0.678	0.723	0.2405
1552767_a_at	57.5	17.0	-1.76	-3.38	AAP-AAA	0.20	10	0.678	0.723	0.1266
1560318_at	47.5	14.1	-1.76	-3.38	AAM-AAP	0.20	10	0.678	0.723	0.2706
232891_at	116.8	34.6	-1.76	-3.38	PAP-AAA	0.20	10	0.678	0.723	0.1138
216588_at	33.7	10.1	-1.75	-3.36	PAA-AAA	0.20	10	0.678	0.723	0.1135
216217_at	53.0	15.8	-1.75	-3.36	AAA-APA	0.20	10	0.678	0.723	0.2242
243708_at	32.8	9.8	-1.75	-3.35	PAA-AAA	0.20	10	0.678	0.723	0.2406
238016_s_at	75.2	22.5	-1.74	-3.35	AAP-AAA	0.20	10	0.678	0.723	0.2175
235900_at	67.0	20.0	-1.74	-3.35	PAA-AAA	0.20	10	0.678	0.723	0.1516
243401_at	18.8	5.6	-1.74	-3.34	AAA-AAP	0.20	10	0.678	0.723	0.1308
1556656_at	169.8	51.0	-1.74	-3.33	AAA-AAP	0.20	10	0.678	0.723	0.2425
215639_at	51.0	15.3	-1.73	-3.33	PAA-AAM	0.20	10	0.678	0.723	0.2349
1556898_at	41.2	12.4	-1.73	-3.33	APA-AAA	0.20	10	0.678	0.723	0.1271
1567559_s_at	56.7	17.1	-1.73	-3.32	APA-AAA	0.20	10	0.678	0.723	0.1821
209697_at	65.6	19.8	-1.73	-3.32	AAA-PAA	0.20	10	0.678	0.723	0.1372
240744_at	48.0	14.5	-1.72	-3.30	PAA-AAA	0.20	10	0.678	0.723	0.1499
1556730_at	33.5	10.1	-1.72	-3.30	APA-AAA	0.20	10	0.678	0.723	0.2484
226829_at	53.5	16.2	-1.72	-3.30	APA-AAA	0.20	10	0.678	0.723	0.2444
238760_at	167.0	50.6	-1.72	-3.30	APP-APA	0.20	10	0.678	0.723	0.2505
1566882_at	24.3	7.4	-1.72	-3.29	PAA-AAA	0.20	10	0.678	0.723	0.2533
222381_at	34.5	10.5	-1.72	-3.29	AAP-AAA	0.20	10	0.678	0.723	0.2698
235944_at	85.0	25.9	-1.72	-3.29	PPA-AAA	0.20	10	0.678	0.723	0.1329
216162_at	74.1	22.6	-1.72	-3.28	AAA-AAP	0.20	10	0.678	0.723	0.2344
240769_at	43.7	13.4	-1.71	-3.27	AAP-AAA	0.20	10	0.678	0.723	0.1629
1564438_at	66.7	20.4	-1.71	-3.27	APP-APP	0.20	10	0.678	0.723	0.1729
205777_at	108.3	33.2	-1.71	-3.26	MAA-APA	0.20	10	0.678	0.723	0.1304

236962_at	55.5	17.0	-1.71	-3.26	PPA-AAA	0.20	10	0.678	0.723	0.1389
1566725_at	28.6	8.8	-1.70	-3.25	APA-AAA	0.20	10	0.678	0.723	0.2714
227393_at	57.5	17.7	-1.70	-3.25	AAP-AAA	0.20	10	0.678	0.723	0.2640
231273_x_at	26.6	8.2	-1.69	-3.23	PAA-AAA	0.20	10	0.678	0.723	0.1111
1554519_at	36.0	11.2	-1.69	-3.23	AAP-AAA	0.20	10	0.678	0.723	0.1512
1562550_at	75.6	23.5	-1.69	-3.22	AAP-APA	0.20	10	0.678	0.723	0.1043
1570022_at	30.8	9.5	-1.69	-3.22	PAA-AAP	0.20	10	0.678	0.723	0.2346
1555168_a_at	65.2	20.3	-1.68	-3.21	AAA-APA	0.20	10	0.678	0.723	0.2487
1570200_at	18.5	59.4	1.68	3.21	AAA-AAP	0.20	10	0.678	0.723	0.0141
211189_x_at	78.0	24.4	-1.68	-3.20	PMA-AAA	0.20	10	0.678	0.723	0.1291
204271_s_at	28.1	89.9	1.68	3.19	AAA-PMP	0.20	10	0.678	0.723	0.0153
1552572_a_at	51.8	16.2	-1.68	-3.19	AAA-PAA	0.20	10	0.678	0.723	0.1406
1562950_at	55.0	17.2	-1.68	-3.19	AAP-AAA	0.20	10	0.678	0.723	0.1887
236919_at	72.2	22.6	-1.68	-3.19	APA-APA	0.20	10	0.678	0.723	0.2317
205727_at	107.5	33.7	-1.67	-3.19	AAP-AAA	0.20	10	0.678	0.723	0.2307
217212_s_at	86.6	27.2	-1.67	-3.19	APA-AAM	0.20	10	0.678	0.723	0.2062
244723_at	39.2	12.3	-1.67	-3.19	PAA-AAA	0.20	10	0.678	0.723	0.2349
236803_at	77.4	24.4	-1.67	-3.18	AAP-AAA	0.20	10	0.678	0.723	0.1993
244785_at	34.5	10.9	-1.67	-3.17	APA-AAA	0.20	10	0.678	0.723	0.1915
205533_s_at	32.2	10.2	-1.66	-3.17	PAA-AAA	0.20	10	0.678	0.723	0.1734
1555516_at	56.9	18.0	-1.66	-3.16	AAA-PAA	0.20	10	0.678	0.723	0.1910
204365_s_at	50.3	16.0	-1.66	-3.15	PAA-AAA	0.20	10	0.678	0.723	0.1864
236672_at	69.4	22.1	-1.65	-3.15	PAA-AAP	0.20	10	0.678	0.723	0.1582
1563884_at	43.8	13.9	-1.65	-3.15	APP-AAP	0.20	10	0.678	0.723	0.1544
1562955_at	95.7	30.5	-1.65	-3.14	MPP-AAA	0.20	10	0.678	0.723	0.1465
207256_at	6.4	2.0	-1.65	-3.14	APA-AAA	0.20	10	0.678	0.723	0.2366
1556920_s_at	40.5	12.9	-1.65	-3.13	PAA-AAA	0.20	10	0.678	0.723	0.1885
217659_at	69.9	22.5	-1.64	-3.11	APA-AAP	0.20	10	0.678	0.723	0.2353
233121_at	68.4	22.0	-1.64	-3.11	MPA-APA	0.20	10	0.678	0.723	0.2458
236767_at	80.2	25.8	-1.63	-3.11	AAP-AAA	0.20	10	0.678	0.723	0.1580
232000_at	28.1	9.1	-1.63	-3.10	AAA-PAA	0.20	10	0.678	0.723	0.2024
241850_at	31.6	10.2	-1.63	-3.10	PAA-AAA	0.20	10	0.678	0.723	0.1649
1565535_x_at	86.0	27.7	-1.63	-3.10	AAP-AAA	0.20	10	0.678	0.723	0.1886
238502_at	36.7	11.9	-1.63	-3.09	APA-AAA	0.20	10	0.678	0.723	0.1030
229690_at	170.6	55.4	-1.62	-3.08	AAP-AAP	0.20	10	0.678	0.723	0.1820
223357_s_at	46.5	15.1	-1.62	-3.07	AAA-APA	0.20	10	0.678	0.723	0.1347
206168_at	50.1	16.3	-1.62	-3.07	APA-AAA	0.20	10	0.678	0.723	0.1417
239646_at	81.3	26.5	-1.62	-3.07	PMP-APP	0.20	10	0.678	0.723	0.1885
240964_at	59.1	19.3	-1.61	-3.06	APP-AAA	0.20	10	0.678	0.723	0.1462
1561266_at	52.9	17.3	-1.61	-3.06	AAA-AAP	0.20	10	0.678	0.723	0.1521
229243_at	97.8	32.2	-1.60	-3.04	APP-AAP	0.20	10	0.678	0.723	0.2608
1565839_a_at	43.5	14.3	-1.60	-3.03	PAA-AAA	0.20	10	0.678	0.723	0.1891

239381_at	85.0	28.1	-1.60	-3.03	AAP-AAA	0.20	10	0.678	0.723	0.2001
219743_at	22.5	7.4	-1.60	-3.03	APA-AAA	0.20	10	0.678	0.723	0.2307
1562885_at	59.9	19.8	-1.60	-3.02	APA-AAA	0.20	10	0.678	0.723	0.2263
235462_at	50.1	16.6	-1.60	-3.02	PAA-AAA	0.20	10	0.678	0.723	0.1165
1565876_x_at	37.6	12.5	-1.60	-3.02	PPA-AAA	0.20	10	0.678	0.723	0.2035
1566165_at	40.0	13.3	-1.59	-3.02	AAA-APA	0.20	10	0.678	0.723	0.1808
234813_at	31.4	10.4	-1.59	-3.02	PAA-AAA	0.20	10	0.678	0.723	0.2483
1553945_at	73.9	24.6	-1.59	-3.01	APA-AAA	0.20	10	0.678	0.723	0.1116
235547_at	52.1	17.3	-1.59	-3.01	PMP-APA	0.20	10	0.678	0.723	0.1289
233890_at	57.6	19.2	-1.59	-3.00	PAA-AAA	0.20	10	0.678	0.723	0.1085
40687_at	53.9	17.9	-1.59	-3.01	AAP-PAA	0.20	10	0.678	0.723	0.1236
233040_at	28.6	9.6	-1.59	-3.00	AAP-AAA	0.20	10	0.678	0.723	0.1209
241398_at	42.6	14.2	-1.59	-3.00	APA-AAA	0.20	10	0.678	0.723	0.1447
242702_at	46.8	15.6	-1.58	-3.00	PPM-AAA	0.20	10	0.678	0.723	0.1067
238531_x_at	102.3	34.1	-1.58	-3.00	MAA-APA	0.20	10	0.678	0.723	0.1566
200865_at	35.3	11.8	-1.58	-2.99	APA-AAA	0.20	10	0.678	0.723	0.2108
215173_at	55.5	18.6	-1.58	-2.98	AAP-AAA	0.20	10	0.678	0.723	0.1662
1569882_at	30.7	10.3	-1.57	-2.98	AAA-APA	0.20	10	0.678	0.723	0.2700
237103_at	63.7	21.5	-1.57	-2.97	APA-AAA	0.20	10	0.678	0.723	0.1058
1565974_at	72.9	24.7	-1.57	-2.96	APP-AAA	0.20	10	0.678	0.723	0.1586
227296_at	95.8	32.4	-1.56	-2.96	AAP-AAA	0.20	10	0.678	0.723	0.1053
1564932_at	46.7	15.8	-1.56	-2.96	APA-AAA	0.20	10	0.678	0.723	0.2004
230341_x_at	133.7	45.3	-1.56	-2.95	PAA-AAA	0.20	10	0.678	0.723	0.2599
242488_at	28.8	9.8	-1.56	-2.95	PAM-AAA	0.20	10	0.678	0.723	0.2449
223876_at	78.6	26.7	-1.56	-2.95	PPP-AAP	0.20	10	0.678	0.723	0.1502
227885_at	37.7	12.9	-1.55	-2.93	AAP-AAA	0.20	10	0.678	0.723	0.1751
244485_at	33.9	11.6	-1.55	-2.94	APA-AAA	0.20	10	0.678	0.723	0.2125
211718_at	33.8	11.5	-1.55	-2.94	AAA-PAA	0.20	10	0.678	0.723	0.2264
242709_s_at	69.8	23.8	-1.55	-2.93	PAA-APA	0.20	10	0.678	0.723	0.2711
1570009_at	24.5	8.4	-1.55	-2.93	PAA-AAA	0.20	10	0.678	0.723	0.1252
244490_at	59.2	20.3	-1.55	-2.92	AAP-AAA	0.20	10	0.678	0.723	0.2312
240625_at	34.3	11.8	-1.55	-2.92	PAA-AAA	0.20	10	0.678	0.723	0.2722
240110_at	74.9	25.7	-1.54	-2.92	AAP-PAA	0.20	10	0.678	0.723	0.1934
228195_at	62.6	21.5	-1.54	-2.91	PAP-AAP	0.20	10	0.678	0.723	0.1395
229722_at	76.2	26.4	-1.53	-2.89	PAA-AAA	0.20	10	0.678	0.723	0.2269
1566735_at	63.5	22.0	-1.53	-2.89	PAA-AAA	0.20	10	0.678	0.723	0.1008
220918_at	162.9	56.5	-1.53	-2.88	PAP-AAP	0.20	10	0.678	0.723	0.2633
206279_at	46.6	16.2	-1.53	-2.88	AAP-PAA	0.20	10	0.678	0.723	0.2391
217464_at	29.9	10.4	-1.53	-2.88	APA-AAA	0.20	10	0.678	0.723	0.1185
1561813_at	28.7	10.0	-1.53	-2.88	PAA-AAA	0.20	10	0.678	0.723	0.1620
236846_at	51.6	18.0	-1.52	-2.87	AAA-PAA	0.20	10	0.678	0.723	0.1455
1558636_s_at	35.5	12.4	-1.52	-2.86	AAA-AAP	0.20	10	0.678	0.723	0.1367

217637_at	123.8	43.4	-1.51	-2.85	PAP-AAA	0.20	10	0.678	0.723	0.2183
217570_x_at	48.1	16.9	-1.51	-2.85	APA-AAA	0.20	10	0.678	0.723	0.2613
238213_at	62.9	22.1	-1.51	-2.85	AAP-AAP	0.20	10	0.678	0.723	0.1717
227058_at	172.0	60.5	-1.51	-2.84	APP-AAA	0.20	10	0.678	0.723	0.1299
210373_at	88.1	31.2	-1.50	-2.83	AAA-AAP	0.20	10	0.678	0.723	0.1265
1558428_at	9.8	3.5	-1.50	-2.83	AAP-AAA	0.20	10	0.678	0.723	0.2276
1566965_at	56.3	20.0	-1.50	-2.82	PAM-AAA	0.20	10	0.678	0.723	0.2468
211493_x_at	169.8	60.3	-1.49	-2.82	PAA-AMP	0.20	10	0.678	0.723	0.1749
205680_at	64.9	23.1	-1.49	-2.81	PAA-AAA	0.20	10	0.678	0.723	0.1010
232761_at	187.6	66.8	-1.49	-2.81	PPP-AAA	0.20	10	0.678	0.723	0.1510
1554951_at	63.2	22.5	-1.49	-2.81	APP-AAA	0.20	10	0.678	0.723	0.2042
211393_at	73.6	26.2	-1.49	-2.81	AAA-PAA	0.20	10	0.678	0.723	0.2378
1552722_at	22.1	7.9	-1.49	-2.80	AAA-AAP	0.20	10	0.678	0.723	0.2505
223977_s_at	28.1	10.0	-1.49	-2.80	MAP-AAA	0.20	10	0.678	0.723	0.1959
209168_at	38.4	13.8	-1.48	-2.80	AAA-PAA	0.20	10	0.678	0.723	0.1399
1553432_s_at	96.0	34.4	-1.48	-2.79	AAP-AAA	0.20	10	0.678	0.723	0.1298
1560069_at	83.5	29.9	-1.48	-2.79	AAA-APA	0.20	10	0.678	0.723	0.2300
215860_at	72.9	26.1	-1.48	-2.79	AAP-AAA	0.20	10	0.678	0.723	0.1618
234556_at	39.7	14.2	-1.48	-2.79	APA-APA	0.20	10	0.678	0.723	0.1515
1570479_at	77.6	27.8	-1.48	-2.79	AMP-AAA	0.20	10	0.678	0.723	0.1870
1569588_x_at	74.0	26.6	-1.48	-2.79	AAP-APA	0.20	10	0.678	0.723	0.2328
1556682_s_at	56.9	20.4	-1.48	-2.79	PAP-APP	0.20	10	0.678	0.723	0.2507
234668_at	21.5	7.7	-1.48	-2.79	AAA-AAP	0.20	10	0.678	0.723	0.2618
242014_at	32.8	11.8	-1.48	-2.78	AAA-AAP	0.20	10	0.678	0.723	0.1313
213426_s_at	98.6	35.5	-1.47	-2.78	APP-APM	0.20	10	0.678	0.723	0.1778
1569696_at	48.4	17.5	-1.47	-2.77	PAA-AAA	0.20	10	0.678	0.723	0.2187
235185_s_at	42.5	15.4	-1.47	-2.76	PAA-AAA	0.20	10	0.678	0.723	0.1017
1560942_at	104.5	37.9	-1.46	-2.76	APA-PAA	0.20	10	0.678	0.723	0.1845
241981_at	38.9	14.1	-1.46	-2.76	AAA-APA	0.20	10	0.678	0.723	0.2135
210360_s_at	48.8	17.8	-1.46	-2.75	AAA-AAP	0.20	10	0.678	0.723	0.1960
231929_at	46.7	17.0	-1.46	-2.75	AAA-AAP	0.20	10	0.678	0.723	0.2248
201147_s_at	162.2	59.0	-1.46	-2.75	AAP-AAA	0.20	10	0.678	0.723	0.2487
207728_at	81.6	29.7	-1.46	-2.75	APP-APP	0.20	10	0.678	0.723	0.2577
233664_at	87.8	32.0	-1.46	-2.74	PAP-AAM	0.20	10	0.678	0.723	0.1076
206353_at	111.2	40.6	-1.46	-2.74	APA-PAA	0.20	10	0.678	0.723	0.2452
1554203_at	54.1	19.8	-1.45	-2.73	PPA-AAA	0.20	10	0.678	0.723	0.2410
206462_s_at	66.6	24.5	-1.45	-2.72	AAP-MPA	0.20	10	0.678	0.723	0.2383
236325_at	41.8	15.4	-1.44	-2.72	PPM-AAA	0.20	10	0.678	0.723	0.1616
233213_at	31.8	11.7	-1.44	-2.72	APA-AAA	0.20	10	0.678	0.723	0.1505
240908_at	44.0	16.3	-1.44	-2.71	AAA-AAP	0.20	10	0.678	0.723	0.1588
1558691_a_at	23.4	8.6	-1.44	-2.71	AAP-AAA	0.20	10	0.678	0.723	0.1958
215132_at	45.1	16.7	-1.43	-2.69	PMA-AMA	0.20	10	0.678	0.723	0.1614

219381_at	76.5	28.5	-1.43	-2.69	AAA-AAP	0.20	10	0.678	0.723	0.1321
212560_at	47.0	17.5	-1.43	-2.69	AAP-APA	0.20	10	0.678	0.723	0.2625
244831_at	70.8	26.3	-1.43	-2.69	PPA-APA	0.20	10	0.678	0.723	0.2396
1557394_at	119.4	44.5	-1.42	-2.68	AAP-AAP	0.20	10	0.678	0.723	0.2216
1562238_at	36.2	13.5	-1.42	-2.68	APA-AAA	0.20	10	0.678	0.723	0.1692
234143_at	120.0	44.8	-1.42	-2.68	MMP-AAA	0.20	10	0.678	0.723	0.1443
240968_at	47.3	17.7	-1.42	-2.67	PAA-AAA	0.20	10	0.678	0.723	0.1314
216001_at	76.8	28.8	-1.42	-2.67	PAA-AAA	0.20	10	0.678	0.723	0.1319
1559735_at	50.0	18.8	-1.42	-2.67	APA-AAA	0.20	10	0.678	0.723	0.1510
238134_at	55.8	20.9	-1.42	-2.67	AAP-AAA	0.20	10	0.678	0.723	0.1765
242638_at	40.0	15.0	-1.42	-2.67	AAP-AAA	0.20	10	0.678	0.723	0.2180
239492_at	25.2	9.5	-1.41	-2.66	AAP-AAA	0.20	10	0.678	0.723	0.2573
231696_x_at	79.5	30.0	-1.41	-2.65	APA-PAA	0.20	10	0.678	0.723	0.2579
214861_at	72.7	27.4	-1.41	-2.65	AAA-APA	0.20	10	0.678	0.723	0.1643
1561270_at	75.5	28.6	-1.40	-2.64	AAP-PAA	0.20	10	0.678	0.723	0.1460
240629_at	41.2	15.6	-1.40	-2.64	AAA-APA	0.20	10	0.678	0.723	0.2443
208395_s_at	40.6	15.4	-1.40	-2.64	AAP-AAA	0.20	10	0.678	0.723	0.1557
236904_x_at	59.9	22.7	-1.40	-2.64	AAA-AAP	0.20	10	0.678	0.723	0.2451
1553599_a_at	59.0	22.5	-1.39	-2.62	AAA-PAA	0.20	10	0.678	0.723	0.2455
1556933_at	39.6	15.1	-1.39	-2.62	PPA-AAA	0.20	10	0.678	0.723	0.2419
206479_at	42.2	16.1	-1.39	-2.61	PAA-AAA	0.20	10	0.678	0.723	0.1817
1558649_at	67.9	26.1	-1.38	-2.60	PPA-AAA	0.20	10	0.678	0.723	0.1119
240542_at	81.1	31.2	-1.38	-2.60	PMM-AAP	0.20	10	0.678	0.723	0.1064
210414_at	71.1	27.3	-1.38	-2.60	APP-AAA	0.20	10	0.678	0.723	0.2124
1564318_at	79.2	30.5	-1.38	-2.60	AAA-AAP	0.20	10	0.678	0.723	0.1445
216865_at	58.1	22.4	-1.38	-2.60	APA-AAA	0.20	10	0.678	0.723	0.2141
208292_at	51.8	20.0	-1.37	-2.59	MPA-AAA	0.20	10	0.678	0.723	0.2102
242741_x_at	40.4	15.6	-1.37	-2.59	AAP-APA	0.20	10	0.678	0.723	0.2137
1561529_at	33.9	13.1	-1.37	-2.59	AAA-AAP	0.20	10	0.678	0.723	0.2155
231683_at	38.7	15.0	-1.37	-2.59	PAA-AAA	0.20	10	0.678	0.723	0.2243
238884_at	105.9	41.0	-1.37	-2.58	APP-AMA	0.20	10	0.678	0.723	0.2429
240022_at	36.7	14.3	-1.37	-2.58	APA-AAA	0.20	10	0.678	0.723	0.2499
243604_at	112.3	43.7	-1.36	-2.57	AAP-AMA	0.20	10	0.678	0.723	0.1677
223415_at	112.9	44.0	-1.36	-2.57	AMP-AAA	0.20	10	0.678	0.723	0.1223
218353_at	27.0	10.5	-1.36	-2.57	APA-AAA	0.20	10	0.678	0.723	0.2490
236379_at	98.3	38.3	-1.36	-2.56	APP-APP	0.20	10	0.678	0.723	0.1700
232336_at	83.1	32.5	-1.35	-2.55	APA-AAA	0.20	10	0.678	0.723	0.1163
211106_at	68.9	27.0	-1.35	-2.56	PMA-AAA	0.20	10	0.678	0.723	0.2673
1554604_at	57.1	22.4	-1.35	-2.55	AAA-AAP	0.20	10	0.678	0.723	0.2059
1569898_a_at	83.7	32.9	-1.35	-2.54	AAA-APA	0.20	10	0.678	0.723	0.2485
235503_at	43.0	16.9	-1.35	-2.54	PAP-APA	0.20	10	0.678	0.723	0.2163
1555021_a_at	81.3	32.0	-1.34	-2.54	APA-PAA	0.20	10	0.678	0.723	0.1852

241334_at	67.5	26.6	-1.34	-2.54	AAP-AAA	0.20	10	0.678	0.723	0.2542
1561602_at	38.2	15.1	-1.34	-2.54	AAA-APA	0.20	10	0.678	0.723	0.2260
226504_at	196.9	77.7	-1.34	-2.53	PPP-APA	0.20	10	0.678	0.723	0.2467
204536_s_at	56.9	22.5	-1.34	-2.53	AAP-AAA	0.20	10	0.678	0.723	0.1173
1570588_at	138.1	54.6	-1.34	-2.53	MAA-AAP	0.20	10	0.678	0.723	0.1256
220491_at	122.0	48.2	-1.34	-2.53	PMA-AAP	0.20	10	0.678	0.723	0.1325
243686_at	50.4	19.9	-1.34	-2.53	AAA-APA	0.20	10	0.678	0.723	0.2004
213703_at	89.4	35.4	-1.34	-2.53	MPP-APP	0.20	10	0.678	0.723	0.1907
242016_at	33.6	13.3	-1.34	-2.52	APA-AAA	0.20	10	0.678	0.723	0.1670
220321_s_at	51.7	20.5	-1.33	-2.52	AMA-AAP	0.20	10	0.678	0.723	0.2193
1558356_at	69.5	27.6	-1.33	-2.52	APP-APA	0.20	10	0.678	0.723	0.1216
241765_at	30.5	12.1	-1.33	-2.51	PAA-AAA	0.20	10	0.678	0.723	0.1595
240359_at	80.1	31.9	-1.33	-2.51	PAA-AAA	0.20	10	0.678	0.723	0.2080
1554812_at	44.1	17.6	-1.33	-2.51	AAP-AAA	0.20	10	0.678	0.723	0.1550
234131_at	49.8	19.9	-1.32	-2.50	APP-AAA	0.20	10	0.678	0.723	0.2197
220767_at	62.0	24.8	-1.32	-2.50	AAA-PAA	0.20	10	0.678	0.723	0.2261
215875_at	81.1	32.4	-1.32	-2.50	AAA-APA	0.20	10	0.678	0.723	0.2568
214200_s_at	81.0	32.4	-1.32	-2.50	AAA-APP	0.20	10	0.678	0.723	0.1448
221051_s_at	86.3	34.6	-1.32	-2.49	AAP-AAP	0.20	10	0.678	0.723	0.1925
241646_s_at	55.7	22.3	-1.32	-2.49	APP-APA	0.20	10	0.678	0.723	0.2553
218692_at	121.7	48.9	-1.32	-2.49	PPP-PAA	0.20	10	0.678	0.723	0.1187
1560834_a_at	13.3	5.3	-1.32	-2.49	PAA-AAA	0.20	10	0.678	0.723	0.1633
210015_s_at	75.0	30.1	-1.32	-2.49	AAP-AAP	0.20	10	0.678	0.723	0.1673
1558230_at	115.8	46.5	-1.32	-2.49	PPA-AAA	0.20	10	0.678	0.723	0.2146
238145_at	62.2	25.0	-1.31	-2.48	APA-AAA	0.20	10	0.678	0.723	0.2224
243796_at	195.2	78.7	-1.31	-2.48	PAP-APP	0.20	10	0.678	0.723	0.1253
207263_x_at	76.2	30.9	-1.30	-2.47	MPA-PAA	0.20	10	0.678	0.723	0.2100
1552393_at	39.8	16.2	-1.30	-2.47	AAA-AAP	0.20	10	0.678	0.723	0.2632
242811_x_at	50.2	20.5	-1.30	-2.45	AAP-AAA	0.20	10	0.678	0.723	0.2591
241174_at	70.7	28.8	-1.29	-2.45	PAP-AAA	0.20	10	0.678	0.723	0.1433
216961_s_at	95.7	39.1	-1.29	-2.45	PAP-AAP	0.20	10	0.678	0.723	0.2167
229659_s_at	59.3	24.2	-1.29	-2.45	PAA-AAA	0.20	10	0.678	0.723	0.1486
232592_at	122.4	50.1	-1.29	-2.44	MPA-AAP	0.20	10	0.678	0.723	0.2483
1564241_at	31.0	12.7	-1.29	-2.44	AAA-APA	0.20	10	0.678	0.723	0.2645
1563800_at	87.0	35.7	-1.29	-2.44	AAP-PAA	0.20	10	0.678	0.723	0.1677
217561_at	75.9	31.1	-1.29	-2.44	PAA-AMP	0.20	10	0.678	0.723	0.1546
1562034_at	109.2	44.8	-1.29	-2.44	APA-AMA	0.20	10	0.678	0.723	0.1573
244022_at	76.4	31.3	-1.29	-2.44	APP-APP	0.20	10	0.678	0.723	0.2579
1561642_at	48.7	20.0	-1.28	-2.43	PPP-AAA	0.20	10	0.678	0.723	0.2537
215561_s_at	66.1	27.3	-1.28	-2.42	PMP-APP	0.20	10	0.678	0.723	0.1343
216123_x_at	65.1	26.9	-1.27	-2.42	APA-APP	0.20	10	0.678	0.723	0.2176
214634_at	106.5	44.1	-1.27	-2.42	PAA-AAA	0.20	10	0.678	0.723	0.2397

241490_s_at	70.0	29.3	-1.26	-2.39	AAP-AAM	0.20	10	0.678	0.723	0.1593
217655_at	126.5	53.0	-1.26	-2.39	APP-APP	0.20	10	0.678	0.723	0.2434
217560_at	130.9	55.0	-1.25	-2.38	AAP-AAA	0.20	10	0.678	0.723	0.1345
206179_s_at	40.3	16.9	-1.25	-2.38	AAP-AAA	0.20	10	0.678	0.723	0.1652
244298_at	39.1	16.4	-1.25	-2.38	APA-AAA	0.20	10	0.678	0.723	0.1712
211813_x_at	117.7	49.6	-1.25	-2.38	MAA-AAP	0.20	10	0.678	0.723	0.1614
208182_x_at	48.7	20.5	-1.25	-2.37	AAA-APA	0.20	10	0.678	0.723	0.1557
1563414_at	71.7	30.2	-1.25	-2.37	APM-AAA	0.20	10	0.678	0.723	0.2179
1554266_at	67.1	28.3	-1.25	-2.37	PAP-AAP	0.20	10	0.678	0.723	0.2317
229788_s_at	50.5	21.3	-1.24	-2.37	APA-AAM	0.20	10	0.678	0.723	0.1774
1562282_at	56.7	24.0	-1.24	-2.36	AAA-PAP	0.20	10	0.678	0.723	0.2335
233066_at	42.1	17.8	-1.24	-2.36	PAA-AAA	0.20	10	0.678	0.723	0.1438
1557505_a_at	100.4	42.5	-1.24	-2.36	AAP-APP	0.20	10	0.678	0.723	0.2568
235023_at	41.2	97.2	1.24	2.36	AAA-PPP	0.20	10	0.678	0.723	0.0076
232985_s_at	54.3	23.1	-1.24	-2.36	PAA-APA	0.20	10	0.678	0.723	0.1658
231653_at	82.8	35.2	-1.24	-2.35	PAP-AAA	0.20	10	0.678	0.723	0.1037
241076_at	75.9	32.3	-1.23	-2.35	APA-AAA	0.20	10	0.678	0.723	0.0968
237124_at	50.6	21.6	-1.23	-2.35	APM-AAP	0.20	10	0.678	0.723	0.1125
231592_at	89.8	38.3	-1.23	-2.34	APA-APA	0.20	10	0.678	0.723	0.1826
242843_at	86.0	36.7	-1.23	-2.34	AAP-AAA	0.20	10	0.678	0.723	0.1656
205489_at	104.9	44.8	-1.23	-2.34	PAA-AAA	0.20	10	0.678	0.723	0.2591
242545_at	96.5	41.2	-1.23	-2.34	APA-AAA	0.20	10	0.678	0.723	0.2018
1570156_s_at	98.6	42.3	-1.22	-2.33	PPP-AAP	0.20	10	0.678	0.723	0.1015
1562741_at	78.6	33.8	-1.22	-2.33	APP-PAA	0.20	10	0.678	0.723	0.2446
1570251_at	60.8	26.2	-1.22	-2.33	AAP-AAP	0.20	10	0.678	0.723	0.1856
234775_at	130.9	56.4	-1.21	-2.32	AAA-AAP	0.20	10	0.678	0.723	0.1248
204687_at	107.1	46.2	-1.21	-2.32	AAA-PAA	0.20	10	0.678	0.723	0.1429
237456_at	134.2	57.9	-1.21	-2.32	PAP-AAA	0.20	10	0.678	0.723	0.2243
208486_at	99.4	42.9	-1.21	-2.32	APP-PAA	0.20	10	0.678	0.723	0.1959
1556520_at	50.6	21.9	-1.21	-2.31	PMA-AAA	0.20	10	0.678	0.723	0.1806
238869_at	170.0	73.8	-1.21	-2.31	PPA-AAP	0.20	10	0.678	0.723	0.1817
223408_s_at	177.0	76.8	-1.20	-2.30	APM-AMP	0.20	10	0.678	0.723	0.1144
1558053_s_at	65.2	28.3	-1.20	-2.30	APA-AAP	0.20	10	0.678	0.723	0.2418
233623_at	92.6	40.3	-1.20	-2.30	AAP-AAA	0.20	10	0.678	0.723	0.1279
243319_at	85.4	37.2	-1.20	-2.30	PAP-APP	0.20	10	0.678	0.723	0.1857
223253_at	33.2	14.5	-1.20	-2.30	AAA-APA	0.20	10	0.678	0.723	0.2459
230386_at	93.9	40.9	-1.20	-2.30	AMA-APA	0.20	10	0.678	0.723	0.1047
1557292_a_at	76.0	33.2	-1.20	-2.29	APA-APA	0.20	10	0.678	0.723	0.1569
241266_at	42.7	18.7	-1.19	-2.29	AAP-AAA	0.20	10	0.678	0.723	0.1587
232706_s_at	245.1	107.4	-1.19	-2.28	APM-AAA	0.20	10	0.678	0.723	0.1692
1557000_at	176.6	77.5	-1.19	-2.28	APA-AAA	0.20	10	0.678	0.723	0.0975
242959_at	72.1	31.7	-1.19	-2.28	AAP-AAA	0.20	10	0.678	0.723	0.2216

215854_at	83.5	36.7	-1.19	-2.27	AAP-APP	0.20	10	0.678	0.723	0.1146
239936_at	48.9	21.5	-1.18	-2.27	APA-AAP	0.20	10	0.678	0.723	0.1399
216871_at	44.6	19.6	-1.18	-2.27	PAA-AAA	0.20	10	0.678	0.723	0.2282
224199_at	47.8	21.1	-1.18	-2.27	PAP-AAA	0.20	10	0.678	0.723	0.1153
206573_at	62.3	27.6	-1.18	-2.26	APA-AAA	0.20	10	0.678	0.723	0.2304
1564784_at	48.5	21.4	-1.18	-2.26	PAA-AAA	0.20	10	0.678	0.723	0.2502
236529_at	90.7	40.2	-1.18	-2.26	PAA-AAA	0.20	10	0.678	0.723	0.2461
218934_s_at	50.2	22.3	-1.17	-2.26	APM-AAA	0.20	10	0.678	0.723	0.1025
234615_at	64.3	28.5	-1.17	-2.25	MPA-MAA	0.20	10	0.678	0.723	0.1031
239438_at	43.3	19.2	-1.17	-2.25	PAA-AAA	0.20	10	0.678	0.723	0.1946
233429_at	67.7	30.1	-1.17	-2.25	PAA-AAA	0.20	10	0.678	0.723	0.2212
235680_at	98.0	43.7	-1.17	-2.25	APP-AAP	0.20	10	0.678	0.723	0.1891
231166_at	72.5	32.4	-1.16	-2.24	PAA-APA	0.20	10	0.678	0.723	0.2061
232161_x_at	39.9	17.9	-1.16	-2.23	APA-APA	0.20	10	0.678	0.723	0.1569
241184_x_at	37.8	16.9	-1.16	-2.23	APA-PAP	0.20	10	0.678	0.723	0.2023
1569335_a_at	130.4	58.4	-1.16	-2.23	AAP-MAA	0.20	10	0.678	0.723	0.1061
217120_s_at	81.5	36.5	-1.16	-2.23	PAA-AAA	0.20	10	0.678	0.723	0.1399
1566642_at	71.5	32.0	-1.16	-2.23	AAA-APA	0.20	10	0.678	0.723	0.1901
1561611_at	86.8	38.9	-1.16	-2.23	PAA-AAA	0.20	10	0.678	0.723	0.2167
242975_s_at	95.8	43.0	-1.16	-2.23	PPP-APP	0.20	10	0.678	0.723	0.2283
239426_at	58.7	26.4	-1.16	-2.23	AAP-APP	0.20	10	0.678	0.723	0.1250
244716_x_at	175.5	78.8	-1.16	-2.23	AMP-AAA	0.20	10	0.678	0.723	0.0971
234786_at	52.0	23.5	-1.15	-2.22	PAA-AAA	0.20	10	0.678	0.723	0.2010
1559640_at	33.1	14.9	-1.15	-2.22	APA-AAA	0.20	10	0.678	0.723	0.2225
227769_at	33.6	15.2	-1.15	-2.22	PAA-AAP	0.20	10	0.678	0.723	0.2454
215797_at	57.2	25.8	-1.15	-2.22	AAP-AAA	0.20	10	0.678	0.723	0.2365
223835_x_at	47.1	21.3	-1.15	-2.21	APA-AAA	0.20	10	0.678	0.723	0.1896
230223_at	155.9	70.6	-1.14	-2.21	PAA-APP	0.20	10	0.678	0.723	0.1035
215063_x_at	251.8	114.2	-1.14	-2.20	APP-APA	0.20	10	0.678	0.723	0.2118
221841_s_at	355.9	161.8	-1.14	-2.20	PPP-PPP	0.20	10	0.678	0.723	0.0054
229009_at	163.6	360.6	1.14	2.20	AAM-MPP	0.20	10	0.678	0.723	0.0069
232610_at	93.3	42.4	-1.14	-2.20	PAA-AAP	0.20	10	0.678	0.723	0.1121
230926_s_at	61.3	135.0	1.14	2.20	AAP-PPP	0.20	10	0.678	0.723	0.0102
238076_at	49.7	22.6	-1.14	-2.20	AAP-AAA	0.20	10	0.678	0.723	0.2359
224062_x_at	47.9	21.8	-1.14	-2.20	PAA-AAA	0.20	10	0.678	0.723	0.2523
234350_at	45.8	20.8	-1.14	-2.20	AAA-AAP	0.20	10	0.678	0.723	0.2342
222904_s_at	49.1	22.4	-1.13	-2.20	PPA-PAA	0.20	10	0.678	0.723	0.1629
1569940_at	71.7	32.7	-1.13	-2.19	PMA-AAA	0.20	10	0.678	0.723	0.2046
243379_at	34.7	15.8	-1.13	-2.19	AAA-AAP	0.20	10	0.678	0.723	0.1104
237424_at	65.8	30.0	-1.13	-2.19	APA-APA	0.20	10	0.678	0.723	0.1174
240778_at	98.6	44.9	-1.13	-2.19	APP-AAA	0.20	10	0.678	0.723	0.1574
215378_at	42.2	19.3	-1.13	-2.19	PAA-AAA	0.20	10	0.678	0.723	0.2376

219314_s_at	173.9	79.5	-1.13	-2.19	PAA-APA	0.20	10	0.678	0.723	0.1776
215868_x_at	77.4	35.3	-1.13	-2.19	PPP-AAA	0.20	10	0.678	0.723	0.1985
211374_x_at	103.8	47.5	-1.13	-2.19	APP-AAA	0.20	10	0.678	0.723	0.2280
208306_x_at	65.8	30.1	-1.13	-2.19	AAA-PAA	0.20	10	0.678	0.723	0.2482
1557782_s_at	93.5	42.8	-1.13	-2.18	PAA-AAM	0.20	10	0.678	0.723	0.1296
220306_at	92.8	42.6	-1.13	-2.18	PPA-AAA	0.20	10	0.678	0.723	0.1370
207717_s_at	305.5	140.3	-1.12	-2.18	PPP-PPP	0.20	10	0.678	0.723	0.2041
244520_at	91.2	41.9	-1.12	-2.18	AAA-PAA	0.20	10	0.678	0.723	0.1815
239082_at	40.8	18.8	-1.12	-2.17	PAA-AAA	0.20	10	0.678	0.723	0.2384
244658_at	70.8	32.6	-1.12	-2.17	APA-APP	0.20	10	0.678	0.723	0.1611
1556768_at	138.1	63.8	-1.12	-2.17	AAA-APA	0.20	10	0.678	0.723	0.1126
1570375_at	69.7	32.3	-1.11	-2.16	MAA-APA	0.20	10	0.678	0.723	0.1980
1561442_at	31.9	14.8	-1.11	-2.16	AAA-PAA	0.20	10	0.678	0.723	0.2094
214403_x_at	133.7	62.0	-1.11	-2.15	AAA-PAA	0.20	10	0.678	0.723	0.1396
231180_at	63.1	29.3	-1.11	-2.15	AAP-AAA	0.20	10	0.678	0.723	0.1308
207936_x_at	144.9	67.4	-1.11	-2.15	APP-PAP	0.20	10	0.678	0.723	0.1727
208048_at	62.0	28.8	-1.10	-2.15	PAA-AAA	0.20	10	0.678	0.723	0.1925
219574_at	43.8	20.4	-1.10	-2.15	AAP-AAA	0.20	10	0.678	0.723	0.1940
1565975_at	42.8	20.0	-1.10	-2.14	AAP-AAA	0.20	10	0.678	0.723	0.2101
1569138_a_at	132.1	61.7	-1.10	-2.14	APA-AAA	0.20	10	0.678	0.723	0.2470
216792_at	65.4	30.6	-1.10	-2.14	PPA-AAA	0.20	10	0.678	0.723	0.2431
220833_at	71.4	33.5	-1.09	-2.13	APA-APA	0.20	10	0.678	0.723	0.1381
1556464_a_at	195.8	91.9	-1.09	-2.13	PAP-PPA	0.20	10	0.678	0.723	0.2185
1553024_at	17.6	8.3	-1.09	-2.13	APA-AAA	0.20	10	0.678	0.723	0.1950
234030_at	56.0	26.4	-1.08	-2.12	PPA-AAA	0.20	10	0.678	0.723	0.1010
231888_at	98.5	46.5	-1.08	-2.12	PPP-MAA	0.20	10	0.678	0.723	0.1880
214775_at	86.4	40.8	-1.08	-2.12	PMA-AAP	0.20	10	0.678	0.723	0.1255
220210_at	80.7	38.2	-1.08	-2.11	AAA-PAA	0.20	10	0.678	0.723	0.2026
240621_at	93.3	44.2	-1.08	-2.11	PAA-AAP	0.20	10	0.678	0.723	0.0989
218413_s_at	67.5	32.1	-1.07	-2.10	PAM-APA	0.20	10	0.678	0.723	0.1733
1563495_at	56.8	27.0	-1.07	-2.10	PAA-PAA	0.20	10	0.678	0.723	0.2423
1561187_at	55.9	26.6	-1.07	-2.10	APA-PAA	0.20	10	0.678	0.723	0.2006
239504_at	88.8	42.3	-1.07	-2.10	PPM-APP	0.20	10	0.678	0.723	0.1591
236257_at	59.2	28.3	-1.06	-2.09	AAP-AAP	0.20	10	0.678	0.723	0.2303
230828_at	70.2	33.6	-1.06	-2.09	PAP-APA	0.20	10	0.678	0.723	0.1730
1569444_at	92.5	44.3	-1.06	-2.09	AMP-AAA	0.20	10	0.678	0.723	0.0937
1569652_at	64.4	30.9	-1.06	-2.09	AAA-APA	0.20	10	0.678	0.723	0.0970
239122_at	102.1	48.9	-1.06	-2.09	APA-PAA	0.20	10	0.678	0.723	0.1732
242889_x_at	276.3	132.6	-1.06	-2.09	MAP-AAP	0.20	10	0.678	0.723	0.0962
49111_at	93.5	45.0	-1.06	-2.08	PPA-APP	0.20	10	0.678	0.723	0.1129
1567375_at	91.0	43.8	-1.06	-2.08	PAP-AAP	0.20	10	0.678	0.723	0.0898
240370_at	99.0	47.7	-1.05	-2.08	PPP-APP	0.20	10	0.678	0.723	0.2029

222301_at	113.8	54.9	-1.05	-2.07	PPM-APA	0.20	10	0.678	0.723	0.2085
1564215_at	43.7	21.1	-1.05	-2.07	PAP-AAA	0.20	10	0.678	0.723	0.1318
205856_at	29.9	14.4	-1.05	-2.07	PAA-AAA	0.20	10	0.678	0.723	0.0988
1563949_at	89.9	43.4	-1.05	-2.07	AAA-PAA	0.20	10	0.678	0.723	0.1530
209966_x_at	69.3	33.5	-1.05	-2.07	AAP-AAA	0.20	10	0.678	0.723	0.1885
211696_x_at	104.8	50.7	-1.05	-2.07	PPM-APA	0.20	10	0.678	0.723	0.2001
232546_at	35.3	17.1	-1.05	-2.07	APA-AAA	0.20	10	0.678	0.723	0.2287
207575_at	38.1	18.5	-1.05	-2.07	AAA-APP	0.20	10	0.678	0.723	0.2380
241466_at	93.7	45.4	-1.05	-2.06	APA-AAA	0.20	10	0.678	0.723	0.1254
1565132_at	54.4	26.4	-1.04	-2.06	APA-APA	0.20	10	0.678	0.723	0.2028
237484_at	49.4	24.0	-1.04	-2.06	APA-APA	0.20	10	0.678	0.723	0.2204
244050_at	176.6	364.0	1.04	2.06	PPP-PPP	0.20	10	0.678	0.723	0.0103
1564444_at	73.6	35.8	-1.04	-2.06	PPA-AAP	0.20	10	0.678	0.723	0.1731
1569415_at	237.4	115.5	-1.04	-2.06	AAP-AAA	0.20	10	0.678	0.723	0.2442
215368_at	44.5	21.7	-1.04	-2.05	PAA-AAA	0.20	10	0.678	0.723	0.2092
1565806_at	24.4	11.9	-1.04	-2.05	AAP-PAA	0.20	10	0.678	0.723	0.2384
241461_at	43.7	21.3	-1.04	-2.05	AAP-AAA	0.20	10	0.678	0.723	0.1628
239471_at	67.2	32.8	-1.03	-2.05	PPP-AAP	0.20	10	0.678	0.723	0.1254
1554800_at	143.9	70.3	-1.03	-2.05	AAP-AAA	0.20	10	0.678	0.723	0.0977
219183_s_at	181.8	88.8	-1.03	-2.05	APA-AAA	0.20	10	0.678	0.723	0.1744
234208_at	68.6	33.5	-1.03	-2.05	AAP-AAA	0.20	10	0.678	0.723	0.1812
1570090_at	93.7	45.8	-1.03	-2.05	AAA-APA	0.20	10	0.678	0.723	0.2078
243808_at	46.4	94.8	1.03	2.04	AAA-PPP	0.20	10	0.678	0.723	0.0090
242794_at	55.0	27.0	-1.03	-2.04	APA-PPA	0.20	10	0.678	0.723	0.0938
1566452_at	82.9	40.6	-1.03	-2.04	APA-AAA	0.20	10	0.678	0.723	0.1485
219732_at	77.3	37.9	-1.03	-2.04	APA-AAA	0.20	10	0.678	0.723	0.1425
222771_s_at	213.6	434.3	1.02	2.03	PPP-PPP	0.20	10	0.678	0.723	0.0100
231265_at	83.0	40.9	-1.02	-2.03	AAP-AAA	0.20	10	0.678	0.723	0.1489
220898_at	103.4	51.0	-1.02	-2.03	APA-AAA	0.20	10	0.678	0.723	0.0896
224547_at	91.8	45.3	-1.02	-2.03	APP-AAA	0.20	10	0.678	0.723	0.1200
1553990_at	122.9	60.7	-1.02	-2.03	APA-PAA	0.20	10	0.678	0.723	0.1298
215041_s_at	74.8	36.9	-1.02	-2.03	AAP-AAA	0.20	10	0.678	0.723	0.1841
1566665_at	38.2	18.9	-1.02	-2.02	APA-AAA	0.20	10	0.678	0.723	0.2190
232002_at	116.2	57.5	-1.02	-2.02	APM-APA	0.20	10	0.678	0.723	0.0986
208161_s_at	88.2	43.7	-1.02	-2.02	PPA-AMA	0.20	10	0.678	0.723	0.2395
231020_at	46.7	23.1	-1.01	-2.02	PAA-AAA	0.20	10	0.678	0.723	0.2193
224424_x_at	78.9	39.1	-1.01	-2.02	APM-AAA	0.20	10	0.678	0.723	0.2450
229063_s_at	288.0	143.0	-1.01	-2.02	PPP-PPP	0.20	10	0.678	0.723	0.0910
1555387_at	113.8	56.6	-1.01	-2.01	AAP-AAM	0.20	10	0.678	0.723	0.1176
220366_at	76.6	38.1	-1.01	-2.01	PAA-PAA	0.20	10	0.678	0.723	0.2055
228439_at	57.3	28.5	-1.01	-2.01	PAA-AAA	0.20	10	0.678	0.723	0.1608
1561390_at	104.3	52.0	-1.01	-2.01	AAP-AAA	0.20	10	0.678	0.723	0.1771

239784_at	479.3	238.9	-1.00	-2.01	PPP-PPP	0.20	10	0.678	0.723	0.0923
1561363_a_at	33.8	16.9	-1.00	-2.01	APA-AAA	0.20	10	0.678	0.723	0.2326
237866_at	41.3	20.6	-1.00	-2.00	PAA-AAA	0.20	10	0.678	0.723	0.1095
213204_at	134.6	67.2	-1.00	-2.00	AAP-AAP	0.20	10	0.678	0.723	0.2000
243627_at	50.6	25.2	-1.00	-2.00	APA-AAP	0.20	10	0.678	0.723	0.2319
206352_s_at	522.8	261.0	-1.00	-2.00	PPP-PAP	0.20	10	0.678	0.723	0.2298
1567686_at	55.7	27.8	-1.00	-2.00	AAP-AAA	0.20	10	0.678	0.723	0.2559
243764_at	52.5	26.2	-1.00	-2.00	PAP-AAA	0.20	10	0.678	0.723	0.1395
217020_at	84.2	42.1	-1.00	-2.00	APA-AAA	0.20	10	0.678	0.723	0.2492
214745_at	53.3	26.7	-1.00	-2.00	PPP-AAA	0.20	10	0.678	0.723	0.2106
241074_at	87.1	43.7	-1.00	-1.99	AAP-AAA	0.20	10	0.678	0.723	0.1113
218835_at	66.2	33.2	-1.00	-1.99	APA-AAA	0.20	10	0.678	0.723	0.1432
1557875_at	79.4	39.8	-1.00	-1.99	APP-AAA	0.20	10	0.678	0.723	0.2019
207969_x_at	87.7	44.1	-0.99	-1.99	PMA-AMA	0.20	10	0.678	0.723	0.2276
213953_at	37.9	19.0	-0.99	-1.99	AAA-APP	0.20	10	0.678	0.723	0.2541
239929_at	74.4	37.4	-0.99	-1.99	PAA-APA	0.20	10	0.678	0.723	0.1059
1559600_at	83.5	41.9	-0.99	-1.99	AAA-AAP	0.20	10	0.678	0.723	0.1243
204981_at	172.4	86.7	-0.99	-1.99	PPP-PPP	0.20	10	0.678	0.723	0.1483
231733_at	36.5	18.3	-0.99	-1.99	AAP-AAA	0.20	10	0.678	0.723	0.1533
205247_at	151.1	76.1	-0.99	-1.99	MAP-PPA	0.20	10	0.678	0.723	0.1023
1563488_at	51.1	25.8	-0.99	-1.98	PAA-AAA	0.20	10	0.678	0.723	0.1841
236943_at	53.2	26.9	-0.99	-1.98	APA-AAA	0.20	10	0.678	0.723	0.1320
216556_x_at	138.6	70.0	-0.99	-1.98	APA-AAP	0.20	10	0.678	0.723	0.1384
214279_s_at	118.0	59.6	-0.99	-1.98	PAA-APP	0.20	10	0.678	0.723	0.2214
238445_x_at	213.2	107.8	-0.98	-1.98	PAP-MPP	0.20	10	0.678	0.723	0.0858
1569305_a_at	180.7	91.4	-0.98	-1.98	AAP-AMP	0.20	10	0.678	0.723	0.1304
1565911_at	64.1	32.4	-0.98	-1.98	PAA-AAA	0.20	10	0.678	0.723	0.2165
240410_at	108.5	54.9	-0.98	-1.98	APP-APM	0.20	10	0.678	0.723	0.1679
214058_at	128.9	65.3	-0.98	-1.97	PPP-AAA	0.20	10	0.678	0.723	0.1009
209280_at	165.9	84.1	-0.98	-1.97	PPM-PAP	0.20	10	0.678	0.723	0.1474
210584_s_at	75.3	38.2	-0.98	-1.97	PAA-AAA	0.20	10	0.678	0.723	0.1814
215331_at	62.4	31.7	-0.98	-1.97	AMP-AAA	0.20	10	0.678	0.723	0.1830
223437_at	140.1	71.2	-0.98	-1.97	AAA-PAA	0.20	10	0.678	0.723	0.1488
220995_at	60.6	30.9	-0.97	-1.96	AAP-PAA	0.20	10	0.678	0.723	0.2134
1566809_a_at	84.4	43.0	-0.97	-1.96	APA-PAA	0.20	10	0.678	0.723	0.1543
234009_at	35.8	18.3	-0.97	-1.96	AAA-AAP	0.20	10	0.678	0.723	0.1896
234202_at	43.8	22.4	-0.97	-1.96	AAA-PAA	0.20	10	0.678	0.723	0.2472
232247_at	203.9	104.0	-0.97	-1.96	AAA-PPP	0.20	10	0.678	0.723	0.2488
235991_at	101.2	51.7	-0.97	-1.96	AAP-AAA	0.20	10	0.678	0.723	0.1368
232526_at	82.2	42.0	-0.97	-1.96	AMP-AAA	0.20	10	0.678	0.723	0.1346
1555042_at	79.1	40.5	-0.97	-1.96	AAP-AAA	0.20	10	0.678	0.723	0.1443
1561526_at	75.0	38.4	-0.97	-1.95	PMA-AAM	0.20	10	0.678	0.723	0.2306

238259_at	154.3	79.1	-0.96	-1.95	APA-AAA	0.20	10	0.678	0.723	0.1009
235108_at	97.1	49.8	-0.96	-1.95	PAM-AAA	0.20	10	0.678	0.723	0.1235
221234_s_at	42.1	21.6	-0.96	-1.95	AAP-AAP	0.20	10	0.678	0.723	0.0956
230707_at	77.7	39.9	-0.96	-1.95	APP-AAA	0.20	10	0.678	0.723	0.1903
217793_at	108.8	55.9	-0.96	-1.95	AAP-PAA	0.20	10	0.678	0.723	0.1911
202903_at	221.0	429.7	0.96	1.95	PPP-PPP	0.20	10	0.678	0.723	0.0061
206583_at	330.2	642.0	0.96	1.94	PPP-PPP	0.20	10	0.678	0.723	0.0076
1554837_a_at	98.7	50.9	-0.96	-1.94	APP-AAA	0.20	10	0.678	0.723	0.1927
239149_at	79.8	41.1	-0.96	-1.94	AAP-AAM	0.20	10	0.678	0.723	0.2170
1562960_at	82.6	42.6	-0.95	-1.94	PPA-AAP	0.20	10	0.678	0.723	0.1530
236955_at	40.2	20.8	-0.95	-1.94	APA-AAA	0.20	10	0.678	0.723	0.1833
231428_at	131.5	67.9	-0.95	-1.94	PPA-MAA	0.20	10	0.678	0.723	0.2197
229523_at	65.6	33.9	-0.95	-1.93	PAA-AAP	0.20	10	0.678	0.723	0.0913
206704_at	120.1	62.1	-0.95	-1.94	PPP-PPP	0.20	10	0.678	0.723	0.1523
205245_at	85.8	44.4	-0.95	-1.93	PAA-AAA	0.20	10	0.678	0.723	0.2509
230665_at	94.6	49.0	-0.95	-1.93	PPP-AAA	0.20	10	0.678	0.723	0.1630
227793_at	327.2	170.2	-0.94	-1.92	APP-MAP	0.20	10	0.678	0.723	0.1428
1563075_s_at	46.3	24.1	-0.94	-1.92	PAA-AAA	0.20	10	0.678	0.723	0.1609
234128_at	131.4	68.5	-0.94	-1.92	PAA-AAA	0.20	10	0.678	0.723	0.1835
1553453_at	69.3	36.2	-0.94	-1.92	APA-AAA	0.20	10	0.678	0.723	0.1050
233844_at	107.9	56.4	-0.94	-1.91	APA-AAA	0.20	10	0.678	0.723	0.1243
204550_x_at	193.0	100.9	-0.94	-1.91	AAP-APA	0.20	10	0.678	0.723	0.1075
238410_x_at	68.2	35.7	-0.93	-1.91	APP-AAA	0.20	10	0.678	0.723	0.1210
228786_at	604.8	316.7	-0.93	-1.91	APP-PAP	0.20	10	0.678	0.723	0.1883
238113_at	39.6	20.7	-0.93	-1.91	PAA-AAA	0.20	10	0.678	0.723	0.2088
210315_at	44.2	23.2	-0.93	-1.91	PAA-AAA	0.20	10	0.678	0.723	0.1478
1561258_at	71.6	37.6	-0.93	-1.91	PAA-AAA	0.20	10	0.678	0.723	0.1431
237914_s_at	85.6	44.9	-0.93	-1.91	PAM-APA	0.20	10	0.678	0.723	0.2086
1565562_at	73.9	38.7	-0.93	-1.91	APP-AAA	0.20	10	0.678	0.723	0.2460
1567703_at	31.8	16.7	-0.93	-1.91	AAA-AAP	0.20	10	0.678	0.723	0.1338
242830_at	55.8	29.3	-0.93	-1.90	AAP-AAM	0.20	10	0.678	0.723	0.1922
242425_at	85.6	45.0	-0.93	-1.90	PAP-AAA	0.20	10	0.678	0.723	0.1961
239714_at	89.0	46.8	-0.93	-1.90	PAA-PAP	0.20	10	0.678	0.723	0.1120
203185_at	74.9	39.5	-0.92	-1.90	MPP-AAP	0.20	10	0.678	0.723	0.1260
232108_at	49.3	26.0	-0.92	-1.90	PAM-AAP	0.20	10	0.678	0.723	0.1667
216002_at	55.9	29.5	-0.92	-1.90	PPA-APA	0.20	10	0.678	0.723	0.1767
1553658_at	93.6	49.5	-0.92	-1.89	AAP-PPA	0.20	10	0.678	0.723	0.0983
235194_at	130.6	69.1	-0.92	-1.89	AMA-AAP	0.20	10	0.678	0.723	0.1246
242173_at	49.8	26.4	-0.92	-1.89	AAA-AAP	0.20	10	0.678	0.723	0.1577
229992_at	76.1	40.3	-0.92	-1.89	APP-AAA	0.20	10	0.678	0.723	0.1635
1569912_at	49.6	26.3	-0.92	-1.89	PAA-AAA	0.20	10	0.678	0.723	0.1093
228632_at	80.9	42.9	-0.92	-1.89	PPA-PAA	0.20	10	0.678	0.723	0.0833

237901_at	63.3	33.6	-0.91	-1.88	AAP-AAA	0.20	10	0.678	0.723	0.2492
205106_at	104.0	55.2	-0.91	-1.88	APA-AAA	0.20	10	0.678	0.723	0.1094
241917_at	40.5	21.5	-0.91	-1.88	AAP-AAA	0.20	10	0.678	0.723	0.1165
207591_s_at	137.5	73.1	-0.91	-1.88	PAA-PAP	0.20	10	0.678	0.723	0.1578
212404_s_at	77.0	144.7	0.91	1.88	APP-PPP	0.20	10	0.678	0.723	0.0063
232425_at	103.0	54.8	-0.91	-1.88	PAA-AMP	0.20	10	0.678	0.723	0.1743
1552887_at	104.6	55.7	-0.91	-1.88	AAA-PAA	0.20	10	0.678	0.723	0.0847
241788_x_at	146.2	77.9	-0.91	-1.88	AMM-AAP	0.20	10	0.678	0.723	0.1497
220963_s_at	110.5	59.0	-0.91	-1.87	PPP-PAP	0.20	10	0.678	0.723	0.0810
225906_at	418.3	223.6	-0.90	-1.87	AAP-MAA	0.20	10	0.678	0.723	0.1152
215576_at	65.7	35.1	-0.90	-1.87	MAA-AAP	0.20	10	0.678	0.723	0.2185
241075_at	49.9	26.7	-0.90	-1.87	MPA-PAA	0.20	10	0.678	0.723	0.1686
1552609_s_at	50.9	27.3	-0.90	-1.86	PPA-AAA	0.20	10	0.678	0.723	0.1353
228831_s_at	67.2	36.0	-0.90	-1.86	APA-AAP	0.20	10	0.678	0.723	0.2230
218144_s_at	276.0	148.1	-0.90	-1.86	AAA-APA	0.20	10	0.678	0.723	0.2492
243174_at	119.7	64.3	-0.90	-1.86	PPP-PPP	0.20	10	0.678	0.723	0.1180
1569887_a_at	61.2	32.9	-0.90	-1.86	AAA-APA	0.20	10	0.678	0.723	0.1800
236294_at	66.9	124.5	0.90	1.86	AAP-AAA	0.20	10	0.678	0.723	0.0066
233374_at	64.8	34.9	-0.89	-1.86	APA-AAA	0.20	10	0.678	0.723	0.1602
240158_at	53.4	28.9	-0.89	-1.85	PPA-AAA	0.20	10	0.678	0.723	0.1169
205328_at	63.2	34.1	-0.89	-1.85	PPA-AAA	0.20	10	0.678	0.723	0.1558
217647_at	93.6	50.6	-0.89	-1.85	PAA-AAA	0.20	10	0.678	0.723	0.0979
214019_at	69.8	37.8	-0.89	-1.85	PAA-AAA	0.20	10	0.678	0.723	0.1512
1566201_at	35.1	19.0	-0.88	-1.85	AAA-APA	0.20	10	0.678	0.723	0.1891
219309_at	138.1	74.8	-0.88	-1.85	AAA-AAP	0.20	10	0.678	0.723	0.2332
239842_x_at	191.2	103.7	-0.88	-1.84	PPP-APP	0.20	10	0.678	0.723	0.1903
229760_at	131.1	71.1	-0.88	-1.84	PMP-PAP	0.20	10	0.678	0.723	0.1729
1566695_at	45.6	24.7	-0.88	-1.84	APA-AAA	0.20	10	0.678	0.723	0.2283
1557309_at	53.7	29.2	-0.88	-1.84	AAP-APA	0.20	10	0.678	0.723	0.0909
242315_at	124.9	68.0	-0.88	-1.84	PAP-AAP	0.20	10	0.678	0.723	0.1516
243453_at	164.7	89.6	-0.88	-1.84	PAP-APA	0.20	10	0.678	0.723	0.1296
240113_at	73.9	40.2	-0.88	-1.84	APA-AAA	0.20	10	0.678	0.723	0.1656
232685_at	202.3	110.1	-0.88	-1.84	AAP-APP	0.20	10	0.678	0.723	0.2189
238829_at	227.3	417.6	0.88	1.84	PPP-PPP	0.20	10	0.678	0.723	0.0059
210247_at	84.3	45.9	-0.88	-1.84	PPP-AAA	0.20	10	0.678	0.723	0.1164
223179_at	83.3	152.9	0.88	1.84	AAM-PPA	0.20	10	0.678	0.723	0.0077
242462_at	91.3	49.8	-0.87	-1.83	AAP-AAA	0.20	10	0.678	0.723	0.1566
214933_at	82.0	44.7	-0.87	-1.83	PPP-AAA	0.20	10	0.678	0.723	0.2351
237632_at	64.8	35.3	-0.87	-1.83	AAP-APA	0.20	10	0.678	0.723	0.2403
1563077_at	126.5	69.1	-0.87	-1.83	MPA-AAA	0.20	10	0.678	0.723	0.0946
211416_x_at	109.0	59.5	-0.87	-1.83	APP-APA	0.20	10	0.678	0.723	0.2251
207987_s_at	42.0	22.9	-0.87	-1.83	APA-AAA	0.20	10	0.678	0.723	0.2402

230925_at	76.3	41.8	-0.87	-1.83	PPA-AAA	0.20	10	0.678	0.723	0.1089
205554_s_at	81.7	44.7	-0.87	-1.83	PAA-AAA	0.20	10	0.678	0.723	0.1280
1553387_at	105.4	57.7	-0.87	-1.83	PAA-AAP	0.20	10	0.678	0.723	0.2046
236807_at	58.4	31.9	-0.87	-1.83	PAA-AAA	0.20	10	0.678	0.723	0.2128
230849_at	55.1	30.2	-0.87	-1.83	PAA-AAM	0.20	10	0.678	0.723	0.0839
228528_at	84.7	46.5	-0.87	-1.82	APA-AAP	0.20	10	0.678	0.723	0.0903
233443_at	99.0	54.3	-0.87	-1.82	PAA-AAP	0.20	10	0.678	0.723	0.0784
1561166_a_at	44.8	24.6	-0.87	-1.82	AAP-AAA	0.20	10	0.678	0.723	0.1520
235921_at	238.1	130.9	-0.86	-1.82	PPP-MPA	0.20	10	0.678	0.723	0.0894
215163_at	87.9	48.3	-0.86	-1.82	APA-PPP	0.20	10	0.678	0.723	0.1052
240765_at	201.7	110.8	-0.86	-1.82	PPP-PPP	0.20	10	0.678	0.723	0.1162
239452_at	109.2	60.1	-0.86	-1.82	PPA-MAA	0.20	10	0.678	0.723	0.2110
211339_s_at	80.7	44.5	-0.86	-1.81	AMP-AAP	0.20	10	0.678	0.723	0.2394
1559672_a_at	61.9	34.2	-0.86	-1.81	PPA-APA	0.20	10	0.678	0.723	0.0852
216331_at	269.8	148.9	-0.86	-1.81	AAA-AAP	0.20	10	0.678	0.723	0.1427
227115_at	207.6	114.8	-0.86	-1.81	APP-PAA	0.20	10	0.678	0.723	0.0854
236354_at	74.2	41.0	-0.86	-1.81	PAA-AAA	0.20	10	0.678	0.723	0.1067
1556835_s_at	134.4	74.3	-0.86	-1.81	PPP-PPA	0.20	10	0.678	0.723	0.1462
1561056_a_at	134.3	74.3	-0.85	-1.81	PPM-APP	0.20	10	0.678	0.723	0.1798
242882_at	92.2	51.0	-0.85	-1.81	APA-AAA	0.20	10	0.678	0.723	0.2185
1557488_at	34.4	19.0	-0.85	-1.81	AAP-PAA	0.20	10	0.678	0.723	0.0843
211722_s_at	71.9	39.8	-0.85	-1.81	AAP-AAA	0.20	10	0.678	0.723	0.2443
226023_at	163.2	90.4	-0.85	-1.81	APP-AAA	0.20	10	0.678	0.723	0.1071
236001_at	123.2	68.3	-0.85	-1.81	PPP-PAA	0.20	10	0.678	0.723	0.1719
210894_s_at	121.6	67.4	-0.85	-1.81	APA-AAP	0.20	10	0.678	0.723	0.1886
231868_at	64.2	35.6	-0.85	-1.80	AAP-PAA	0.20	10	0.678	0.723	0.0999
231399_at	87.5	48.5	-0.85	-1.80	AAA-APA	0.20	10	0.678	0.723	0.1744
231368_at	113.2	62.8	-0.85	-1.80	AAA-PAA	0.20	10	0.678	0.723	0.0961
239396_at	134.1	74.5	-0.85	-1.80	PAA-APA	0.20	10	0.678	0.723	0.2109
1557666_s_at	165.6	92.0	-0.85	-1.80	PPP-APP	0.20	10	0.678	0.723	0.0949
236619_at	88.4	49.3	-0.84	-1.80	PPP-PAM	0.20	10	0.678	0.723	0.1045
244749_at	74.3	41.4	-0.84	-1.79	APP-APA	0.20	10	0.678	0.723	0.0982
228235_at	283.0	157.8	-0.84	-1.79	PAP-PPP	0.20	10	0.678	0.723	0.1189
214906_x_at	116.6	65.1	-0.84	-1.79	AAA-APA	0.20	10	0.678	0.723	0.0788
1555526_a_at	64.1	115.0	0.84	1.79	MPP-PPP	0.20	10	0.678	0.723	0.0072
239840_at	77.5	43.3	-0.84	-1.79	AAP-AAA	0.20	10	0.678	0.723	0.1085
1559667_at	134.8	75.3	-0.84	-1.79	PAA-APA	0.20	10	0.678	0.723	0.2269
219470_x_at	165.8	297.0	0.84	1.79	PPP-PPP	0.20	10	0.678	0.723	0.0045
241150_at	128.9	72.0	-0.84	-1.79	MAP-APP	0.20	10	0.678	0.723	0.0818
240727_s_at	87.7	49.0	-0.84	-1.79	AAA-PPA	0.20	10	0.678	0.723	0.1623
219999_at	80.4	44.9	-0.84	-1.79	APP-PAA	0.20	10	0.678	0.723	0.2111
1569573_at	67.8	38.0	-0.84	-1.79	AAA-APA	0.20	10	0.678	0.723	0.1756

238648_at	59.9	33.6	-0.84	-1.79	PAA-AAP	0.20	10	0.678	0.723	0.2254
232701_at	158.8	88.9	-0.84	-1.79	PPP-AAP	0.20	10	0.678	0.723	0.1003
227629_at	44.9	25.1	-0.84	-1.79	PPA-AAM	0.20	10	0.678	0.723	0.1589
1557541_at	34.6	19.4	-0.84	-1.79	AAP-AAA	0.20	10	0.678	0.723	0.2272
235140_at	93.3	52.3	-0.84	-1.78	APA-AAA	0.20	10	0.678	0.723	0.1672
225626_at	81.7	45.8	-0.84	-1.78	APA-PPA	0.20	10	0.678	0.723	0.2045
241750_x_at	39.9	22.4	-0.83	-1.78	PAA-AAA	0.20	10	0.678	0.723	0.1285
1554538_at	104.4	58.5	-0.83	-1.78	APA-PAA	0.20	10	0.678	0.723	0.1911
208849_at	46.5	26.1	-0.83	-1.78	APA-APA	0.20	10	0.678	0.723	0.2185
1552690_a_at	89.0	50.0	-0.83	-1.78	APA-APA	0.20	10	0.678	0.723	0.0797
209911_x_at	395.1	703.8	0.83	1.78	PPP-PPP	0.20	10	0.678	0.723	0.0055
239615_at	124.3	69.9	-0.83	-1.78	APA-AAA	0.20	10	0.678	0.723	0.2382
235768_at	177.3	99.8	-0.83	-1.78	PMP-AAP	0.20	10	0.678	0.723	0.1770
225301_s_at	196.4	110.5	-0.83	-1.78	PPP-AAP	0.20	10	0.678	0.723	0.1899
214558_at	81.2	45.7	-0.83	-1.78	AAA-PAA	0.20	10	0.678	0.723	0.2116
216938_x_at	190.0	107.1	-0.83	-1.77	APA-AAA	0.20	10	0.678	0.723	0.1691
224650_at	128.2	72.3	-0.83	-1.77	PPP-PPP	0.20	10	0.678	0.723	0.1763
1552848_a_at	52.8	29.8	-0.83	-1.77	PAA-AAA	0.20	10	0.678	0.723	0.2387
219491_at	423.2	238.9	-0.83	-1.77	PPP-PPP	0.20	10	0.678	0.723	0.0840
240835_at	56.9	32.1	-0.83	-1.77	PPA-AAP	0.20	10	0.678	0.723	0.2031
225587_at	135.9	76.8	-0.82	-1.77	APP-AAP	0.20	10	0.678	0.723	0.0852
236671_at	242.3	137.1	-0.82	-1.77	PPP-PPP	0.20	10	0.678	0.723	0.0743
1554344_s_at	474.8	268.5	-0.82	-1.77	PAP-PPP	0.20	10	0.678	0.723	0.1562
1562894_at	40.3	22.8	-0.82	-1.77	PAA-AAA	0.20	10	0.678	0.723	0.2392
239847_at	192.2	108.8	-0.82	-1.77	PPP-PPP	0.20	10	0.678	0.723	0.0978
242737_at	66.4	37.6	-0.82	-1.77	APP-PAP	0.20	10	0.678	0.723	0.1702
208039_at	147.8	83.7	-0.82	-1.77	PPP-MPP	0.20	10	0.678	0.723	0.0849
1555306_a_at	246.0	139.4	-0.82	-1.77	PPP-PPP	0.20	10	0.678	0.723	0.0997
221306_at	119.1	67.6	-0.82	-1.76	APA-AAP	0.20	10	0.678	0.723	0.1317
240539_at	65.6	37.2	-0.82	-1.76	PPA-PAP	0.20	10	0.678	0.723	0.1549
210387_at	64.7	114.2	0.82	1.76	AAP-PPP	0.20	10	0.678	0.723	0.0045
215375_x_at	96.4	54.7	-0.82	-1.76	AMA-PAP	0.20	10	0.678	0.723	0.0878
1561631_at	88.8	50.4	-0.82	-1.76	PPP-APP	0.20	10	0.678	0.723	0.0901
1557744_at	76.8	43.6	-0.82	-1.76	AAP-AAA	0.20	10	0.678	0.723	0.0912
234611_at	115.0	65.5	-0.81	-1.76	PMP-PAA	0.20	10	0.678	0.723	0.2269
241489_at	90.6	51.6	-0.81	-1.76	APP-APP	0.20	10	0.678	0.723	0.1025
219782_s_at	66.1	37.7	-0.81	-1.76	AAP-APA	0.20	10	0.678	0.723	0.1551
229483_at	157.7	89.8	-0.81	-1.76	PPP-PPP	0.20	10	0.678	0.723	0.2339
207095_at	89.4	50.9	-0.81	-1.76	PMM-PPA	0.20	10	0.678	0.723	0.1928
1560070_at	84.3	48.1	-0.81	-1.75	APM-AAA	0.20	10	0.678	0.723	0.1174
1555272_at	224.4	128.1	-0.81	-1.75	PAP-APP	0.20	10	0.678	0.723	0.1234
244014_x_at	38.9	22.2	-0.81	-1.75	PAA-AAP	0.20	10	0.678	0.723	0.2195

239159_at	171.7	98.1	-0.81	-1.75	AAA-AAP	0.20	10	0.678	0.723	0.0900
238218_at	91.8	52.5	-0.81	-1.75	AAA-APA	0.20	10	0.678	0.723	0.0748
237515_at	35.8	20.5	-0.81	-1.75	AAA-PAA	0.20	10	0.678	0.723	0.1145
239371_at	52.2	29.9	-0.81	-1.75	PAP-AAA	0.20	10	0.678	0.723	0.1455
201650_at	3400.1	1946.2	-0.81	-1.75	PPP-PPP	0.20	10	0.678	0.723	0.1925
236079_at	152.5	87.6	-0.80	-1.74	PPP-MPP	0.20	10	0.678	0.723	0.1469
207490_at	202.3	116.3	-0.80	-1.74	AAA-PAA	0.20	10	0.678	0.723	0.1607
242678_at	42.1	24.2	-0.80	-1.74	AAP-APA	0.20	10	0.678	0.723	0.2144
1561097_at	76.7	44.2	-0.80	-1.74	PPM-AAA	0.20	10	0.678	0.723	0.2055
1560405_at	73.8	42.5	-0.80	-1.74	PAA-AAA	0.20	10	0.678	0.723	0.2100
240443_at	174.0	100.2	-0.80	-1.74	APP-AAA	0.20	10	0.678	0.723	0.1567
244248_at	119.9	69.1	-0.80	-1.74	AAA-PAM	0.20	10	0.678	0.723	0.1098
242298_x_at	95.5	55.1	-0.80	-1.74	AMP-AAA	0.20	10	0.678	0.723	0.1701
1555437_at	109.5	63.2	-0.79	-1.73	AAP-PPP	0.20	10	0.678	0.723	0.1016
1570108_at	83.6	48.2	-0.79	-1.73	APP-AAA	0.20	10	0.678	0.723	0.1728
227475_at	112.3	64.8	-0.79	-1.73	AAP-PAA	0.20	10	0.678	0.723	0.1837
1554202_x_at	135.7	78.4	-0.79	-1.73	APP-PAA	0.20	10	0.678	0.723	0.0854
1557813_at	167.8	97.0	-0.79	-1.73	PPP-PAP	0.20	10	0.678	0.723	0.0791
1561135_at	75.8	43.8	-0.79	-1.73	APP-APP	0.20	10	0.678	0.723	0.1479
234900_at	115.7	66.8	-0.79	-1.73	APP-PPA	0.20	10	0.678	0.723	0.1628
235796_at	132.7	76.6	-0.79	-1.73	APP-AAP	0.20	10	0.678	0.723	0.1937
236332_at	52.7	30.5	-0.79	-1.73	PAA-AMA	0.20	10	0.678	0.723	0.2078
1569448_at	180.1	104.2	-0.79	-1.73	AAP-AAA	0.20	10	0.678	0.723	0.0893
230417_at	126.4	73.2	-0.79	-1.73	AAM-PAM	0.20	10	0.678	0.723	0.2086
209871_s_at	77.1	133.1	0.79	1.73	AAA-PPP	0.20	10	0.678	0.723	0.0057
227394_at	96.3	55.8	-0.79	-1.72	PAA-PAA	0.20	10	0.678	0.723	0.2053
1569701_at	50.9	29.5	-0.79	-1.72	MAP-AAA	0.20	10	0.678	0.723	0.2160
219883_at	133.0	77.2	-0.79	-1.72	AAM-APA	0.20	10	0.678	0.723	0.1712
234971_x_at	289.5	168.1	-0.78	-1.72	PAA-PAA	0.20	10	0.678	0.723	0.1718
1558903_at	41.3	24.0	-0.78	-1.72	AAP-APA	0.20	10	0.678	0.723	0.1676
1557645_at	60.3	35.1	-0.78	-1.72	APA-AAM	0.20	10	0.678	0.723	0.1677
206311_s_at	56.5	32.9	-0.78	-1.72	PAA-AAA	0.20	10	0.678	0.723	0.1258
224417_at	52.9	30.9	-0.78	-1.72	AAA-AAP	0.20	10	0.678	0.723	0.2152
217446_x_at	182.8	106.6	-0.78	-1.72	PPP-PPP	0.20	10	0.678	0.723	0.1584
204927_at	398.7	232.7	-0.78	-1.71	AAP-AAM	0.20	10	0.678	0.723	0.0728
1562576_at	55.8	32.6	-0.78	-1.71	PAA-APA	0.20	10	0.678	0.723	0.0906
223674_s_at	75.7	44.2	-0.78	-1.71	MPP-MPP	0.20	10	0.678	0.723	0.2349
1564378_a_at	452.4	264.5	-0.77	-1.71	PPP-PPP	0.20	10	0.678	0.723	0.1663
1558504_at	141.8	82.9	-0.77	-1.71	AAP-AAA	0.20	10	0.678	0.723	0.2271
244225_x_at	64.3	37.6	-0.77	-1.71	APA-APA	0.20	10	0.678	0.723	0.2368
236223_s_at	159.3	272.5	0.77	1.71	PPP-PPP	0.20	10	0.678	0.723	0.0049
208223_s_at	83.0	48.6	-0.77	-1.71	AAP-AMP	0.20	10	0.678	0.723	0.1411

1569730_at	110.9	65.0	-0.77	-1.71	AAA-PAA	0.20	10	0.678	0.723	0.2336
1569501_at	144.8	84.9	-0.77	-1.71	APA-AAM	0.20	10	0.678	0.723	0.0946
242125_at	147.0	86.2	-0.77	-1.71	PPP-PAP	0.20	10	0.678	0.723	0.1628
1552839_at	57.8	33.9	-0.77	-1.71	PAA-AMA	0.20	10	0.678	0.723	0.0720
1561360_at	127.7	75.1	-0.77	-1.70	PPP-PMP	0.20	10	0.678	0.723	0.1547
232710_at	111.0	65.3	-0.77	-1.70	PPP-MAA	0.20	10	0.678	0.723	0.2089
243880_at	77.5	45.6	-0.76	-1.70	PAP-PAP	0.20	10	0.678	0.723	0.1179
216279_at	63.6	37.5	-0.76	-1.70	PAA-APA	0.20	10	0.678	0.723	0.2247
1556309_s_at	126.1	74.4	-0.76	-1.70	MAP-AAA	0.20	10	0.678	0.723	0.0874
219168_s_at	231.4	136.5	-0.76	-1.70	PPA-AMP	0.20	10	0.678	0.723	0.1625
229602_at	119.0	70.2	-0.76	-1.69	PPP-MPA	0.20	10	0.678	0.723	0.1214
230696_at	372.5	219.9	-0.76	-1.69	AAP-AMP	0.20	10	0.678	0.723	0.2223
220760_x_at	655.9	388.3	-0.76	-1.69	PPP-PPP	0.20	10	0.678	0.723	0.1267
241948_at	56.7	33.6	-0.76	-1.69	APA-APA	0.20	10	0.678	0.723	0.1335
242726_at	110.0	65.2	-0.76	-1.69	AAP-AAP	0.20	10	0.678	0.723	0.2202
227468_at	324.3	192.3	-0.76	-1.69	PPP-PPP	0.20	10	0.678	0.723	0.1013
243450_at	100.3	59.5	-0.75	-1.69	AAA-AAP	0.20	10	0.678	0.723	0.2234
208389_s_at	71.7	42.5	-0.75	-1.69	APA-AAA	0.20	10	0.678	0.723	0.2308
239709_at	101.5	60.2	-0.75	-1.69	AAA-APA	0.20	10	0.678	0.723	0.2349
205511_at	68.7	40.7	-0.75	-1.69	PPP-APP	0.20	10	0.678	0.723	0.1902
228022_at	79.8	47.3	-0.75	-1.69	PPP-AAA	0.20	10	0.678	0.723	0.2145
235511_at	85.3	50.7	-0.75	-1.68	PPA-APA	0.20	10	0.678	0.723	0.1326
208573_s_at	74.4	44.2	-0.75	-1.68	AAA-AAP	0.20	10	0.678	0.723	0.0854
216409_at	88.3	52.5	-0.75	-1.68	APA-PAA	0.20	10	0.678	0.723	0.0875
241945_at	92.8	55.2	-0.75	-1.68	AMA-PAA	0.20	10	0.678	0.723	0.1716
208216_at	94.0	56.0	-0.75	-1.68	PMP-APP	0.20	10	0.678	0.723	0.2249
204269_at	173.2	103.2	-0.75	-1.68	MPM-MAA	0.20	10	0.678	0.723	0.1290
223432_at	166.0	98.9	-0.75	-1.68	AAA-PAA	0.20	10	0.678	0.723	0.1942
229867_at	88.5	52.8	-0.75	-1.68	AAP-AAP	0.20	10	0.678	0.723	0.1365
232257_s_at	72.6	43.3	-0.75	-1.68	PPA-AAP	0.20	10	0.678	0.723	0.1727
242730_at	83.7	49.9	-0.75	-1.68	PPA-APP	0.20	10	0.678	0.723	0.1842
242287_at	117.5	70.2	-0.74	-1.68	PMP-PPP	0.20	10	0.678	0.723	0.0931
1559436_x_at	918.8	549.0	-0.74	-1.67	APP-PMP	0.20	10	0.678	0.723	0.0897
239910_at	92.1	55.0	-0.74	-1.67	APP-APA	0.20	10	0.678	0.723	0.1350
232152_at	51.7	30.9	-0.74	-1.67	AMA-AAP	0.20	10	0.678	0.723	0.0894
223459_s_at	228.1	136.4	-0.74	-1.67	PPP-PPP	0.20	10	0.678	0.723	0.1461
1561085_at	103.8	62.2	-0.74	-1.67	AAA-APA	0.20	10	0.678	0.723	0.0799
233114_at	78.8	47.2	-0.74	-1.67	AMA-AAP	0.20	10	0.678	0.723	0.1507
230949_at	126.9	76.1	-0.74	-1.67	AAA-AAP	0.20	10	0.678	0.723	0.0987
221426_s_at	105.6	63.3	-0.74	-1.67	AAP-AAA	0.20	10	0.678	0.723	0.1019
240269_at	64.1	38.5	-0.74	-1.67	AAP-AAA	0.20	10	0.678	0.723	0.1428
1554717_a_at	80.7	48.4	-0.74	-1.67	APP-PPP	0.20	10	0.678	0.723	0.1580

218677_at	84.1	50.5	-0.74	-1.67	PPA-AAA	0.20	10	0.678	0.723	0.2105
204993_at	129.2	77.6	-0.74	-1.66	AAP-APA	0.20	10	0.678	0.723	0.0718
232179_at	88.7	53.3	-0.74	-1.66	APP-AAA	0.20	10	0.678	0.723	0.1124
231061_at	123.5	74.2	-0.73	-1.66	PPP-PPP	0.20	10	0.678	0.723	0.0779
1552970_s_at	65.8	39.6	-0.73	-1.66	AMP-AAA	0.20	10	0.678	0.723	0.0838
229457_at	108.5	65.3	-0.73	-1.66	PPP-PPA	0.20	10	0.678	0.723	0.1078
1559535_s_at	69.4	41.8	-0.73	-1.66	PMA-APP	0.20	10	0.678	0.723	0.0993
1565844_at	68.3	41.2	-0.73	-1.66	AAA-PAP	0.20	10	0.678	0.723	0.1088
1562033_at	121.6	73.4	-0.73	-1.66	MAM-APP	0.20	10	0.678	0.723	0.1382
203765_at	85.2	51.4	-0.73	-1.66	APP-PPP	0.20	10	0.678	0.723	0.1466
221616_s_at	92.5	55.9	-0.73	-1.66	MAA-APP	0.20	10	0.678	0.723	0.0702
231097_at	222.8	134.6	-0.73	-1.66	PPP-APP	0.20	10	0.678	0.723	0.0992
1557729_at	142.1	85.9	-0.73	-1.66	PAM-APP	0.20	10	0.678	0.723	0.1264
1561976_at	153.3	92.6	-0.73	-1.66	PMA-APA	0.20	10	0.678	0.723	0.1669
216119_s_at	120.9	73.1	-0.73	-1.65	PPA-AAP	0.20	10	0.678	0.723	0.1109
227505_at	144.4	87.3	-0.73	-1.65	PAA-AAP	0.20	10	0.678	0.723	0.1143
205899_at	136.7	82.7	-0.73	-1.65	PAP-PPP	0.20	10	0.678	0.723	0.1566
244154_at	106.3	64.3	-0.73	-1.65	PPP-AAP	0.20	10	0.678	0.723	0.1770
226069_at	141.3	85.7	-0.72	-1.65	PPA-PMP	0.20	10	0.678	0.723	0.1303
243063_at	58.3	35.3	-0.72	-1.65	PPA-PAA	0.20	10	0.678	0.723	0.1389
202626_s_at	732.2	443.8	-0.72	-1.65	PPP-PPP	0.20	10	0.678	0.723	0.1434
230973_at	845.3	512.9	-0.72	-1.65	PPP-PPP	0.20	10	0.678	0.723	0.0727
1558534_at	64.5	39.2	-0.72	-1.65	PAA-PAA	0.20	10	0.678	0.723	0.1850
236123_at	81.8	49.7	-0.72	-1.65	AAP-AAA	0.20	10	0.678	0.723	0.1174
244082_at	72.5	44.0	-0.72	-1.65	APA-AAA	0.20	10	0.678	0.723	0.1230
203389_at	292.0	177.3	-0.72	-1.65	MPP-PPP	0.20	10	0.678	0.723	0.0889
242399_at	97.9	59.5	-0.72	-1.65	APM-PAP	0.20	10	0.678	0.723	0.0945
230033_at	103.6	63.0	-0.72	-1.65	AAP-AAA	0.20	10	0.678	0.723	0.2243
227869_at	461.1	280.6	-0.72	-1.64	PPP-PPP	0.20	10	0.678	0.723	0.1068
217114_at	97.0	59.0	-0.72	-1.64	PPA-AAP	0.20	10	0.678	0.723	0.2163
229817_at	151.8	92.4	-0.72	-1.64	PPA-AAP	0.20	10	0.678	0.723	0.0873
221173_at	48.6	29.6	-0.72	-1.64	APA-AAA	0.20	10	0.678	0.723	0.2304
205585_at	90.8	149.0	0.71	1.64	AAA-PPP	0.20	10	0.678	0.723	0.0045
230507_at	53.6	32.7	-0.71	-1.64	APA-APA	0.20	10	0.678	0.723	0.2243
239409_at	109.3	66.7	-0.71	-1.64	AAP-APP	0.20	10	0.678	0.723	0.2015
235155_at	54.3	33.2	-0.71	-1.64	AAP-AAA	0.20	10	0.678	0.723	0.1312
204590_x_at	304.9	186.4	-0.71	-1.64	PPP-APP	0.20	10	0.678	0.723	0.1157
1560733_at	72.5	44.4	-0.71	-1.64	APP-APA	0.20	10	0.678	0.723	0.0739
241907_at	90.2	55.2	-0.71	-1.63	AAP-APM	0.20	10	0.678	0.723	0.0751
235634_at	67.2	41.1	-0.71	-1.63	APP-AAA	0.20	10	0.678	0.723	0.0958
1561871_at	257.6	157.7	-0.71	-1.63	PAA-AAP	0.20	10	0.678	0.723	0.1128
240013_at	89.6	146.4	0.71	1.63	APP-PPP	0.20	10	0.678	0.723	0.0025

1564547_x_at	56.2	34.4	-0.71	-1.63	APA-PMP	0.20	10	0.678	0.723	0.1176
1558529_s_at	137.1	84.0	-0.71	-1.63	PMA-AMP	0.20	10	0.678	0.723	0.1470
242960_at	43.8	26.8	-0.71	-1.63	PAA-AAA	0.20	10	0.678	0.723	0.1882
224974_at	171.0	278.9	0.71	1.63	PPP-PPP	0.20	10	0.678	0.723	0.0034
33322_i_at	504.8	309.7	-0.71	-1.63	PPP-PPP	0.20	10	0.678	0.723	0.0734
219287_at	159.6	97.9	-0.71	-1.63	PPP-AAP	0.20	10	0.678	0.723	0.0949
1556148_s_at	83.0	50.9	-0.71	-1.63	APP-AAA	0.20	10	0.678	0.723	0.1694
222350_at	35.9	22.0	-0.71	-1.63	APA-APA	0.20	10	0.678	0.723	0.1707
1556250_at	80.2	49.3	-0.70	-1.63	AAP-AAA	0.20	10	0.678	0.723	0.0704
237750_at	56.2	34.5	-0.70	-1.63	PAA-AAP	0.20	10	0.678	0.723	0.0981
244150_at	65.7	40.3	-0.70	-1.63	AAA-APA	0.20	10	0.678	0.723	0.1126
1553828_at	84.0	51.6	-0.70	-1.63	PAA-AAP	0.20	10	0.678	0.723	0.0865
220694_at	200.9	123.4	-0.70	-1.63	PPP-APP	0.20	10	0.678	0.723	0.1238
1554205_s_at	105.1	64.6	-0.70	-1.63	PPP-PPA	0.20	10	0.678	0.723	0.1057
235340_at	72.9	44.8	-0.70	-1.63	PAA-AAA	0.20	10	0.678	0.723	0.1295
241433_at	189.9	116.8	-0.70	-1.63	PPP-APP	0.20	10	0.678	0.723	0.0929
217331_at	141.2	86.9	-0.70	-1.63	AAA-AAP	0.20	10	0.678	0.723	0.1135
234168_at	50.8	31.3	-0.70	-1.63	AAM-AMP	0.20	10	0.678	0.723	0.2265
232319_at	54.1	33.3	-0.70	-1.63	AAP-AAP	0.20	10	0.678	0.723	0.2281
216374_at	60.1	37.0	-0.70	-1.63	APP-AAP	0.20	10	0.678	0.723	0.0689
205405_at	429.6	264.4	-0.70	-1.63	PPM-AMA	0.20	10	0.678	0.723	0.1063
213141_at	215.9	132.9	-0.70	-1.62	PPP-PPA	0.20	10	0.678	0.723	0.2240
243491_at	62.2	38.3	-0.70	-1.62	MAA-PPM	0.20	10	0.678	0.723	0.1381
234509_at	49.6	30.6	-0.70	-1.62	APM-AAP	0.20	10	0.678	0.723	0.1735
215422_at	52.4	32.3	-0.70	-1.62	PAA-AAA	0.20	10	0.678	0.723	0.1897
242365_at	149.4	92.1	-0.70	-1.62	APP-AAP	0.20	10	0.678	0.723	0.2190
1560474_at	91.0	56.1	-0.70	-1.62	PPP-PPP	0.20	10	0.678	0.723	0.2102
227492_at	712.9	440.0	-0.70	-1.62	PPP-PPP	0.20	10	0.678	0.723	0.0693
215855_s_at	152.6	94.2	-0.70	-1.62	PPP-PAP	0.20	10	0.678	0.723	0.1572
206263_at	101.9	62.9	-0.70	-1.62	APA-AAP	0.20	10	0.678	0.723	0.2024
1554448_at	73.8	45.5	-0.70	-1.62	PPP-PAP	0.20	10	0.678	0.723	0.2129
233914_s_at	141.5	87.5	-0.70	-1.62	PPP-PPP	0.20	10	0.678	0.723	0.1527
1566700_at	127.3	78.6	-0.70	-1.62	PPP-AAA	0.20	10	0.678	0.723	0.1956
214321_at	341.7	211.2	-0.69	-1.62	PPP-PPP	0.20	10	0.678	0.723	0.1281
1556111_s_at	203.6	125.9	-0.69	-1.62	PPP-PPP	0.20	10	0.678	0.723	0.1406
242732_at	58.4	36.2	-0.69	-1.62	AAA-AAP	0.20	10	0.678	0.723	0.1053
231985_at	87.7	54.2	-0.69	-1.62	APP-PPA	0.20	10	0.678	0.723	0.2312
214376_at	59.6	36.9	-0.69	-1.62	PAA-AAA	0.20	10	0.678	0.723	0.1944
207037_at	94.8	58.8	-0.69	-1.61	PAM-AAP	0.20	10	0.678	0.723	0.1507
227694_at	225.3	139.6	-0.69	-1.61	PPP-PPP	0.20	10	0.678	0.723	0.1863
1560198_at	176.4	109.4	-0.69	-1.61	APA-AAM	0.20	10	0.678	0.723	0.0937
242763_at	129.8	80.4	-0.69	-1.61	MPP-PPM	0.20	10	0.678	0.723	0.1105

239284_at	110.9	68.7	-0.69	-1.61	MPP-AAP	0.20	10	0.678	0.723	0.1283
207986_x_at	285.1	176.7	-0.69	-1.61	PPP-PPP	0.20	10	0.678	0.723	0.1320
240803_at	101.4	62.9	-0.69	-1.61	APP-AAP	0.20	10	0.678	0.723	0.0886
230571_at	141.3	87.7	-0.69	-1.61	MPM-APP	0.20	10	0.678	0.723	0.2039
227532_at	66.4	41.2	-0.69	-1.61	PPA-AAA	0.20	10	0.678	0.723	0.1681
221240_s_at	137.4	85.3	-0.69	-1.61	AAA-APA	0.20	10	0.678	0.723	0.2226
1559706_at	92.2	57.3	-0.69	-1.61	PAP-APA	0.20	10	0.678	0.723	0.1000
228136_s_at	143.7	89.3	-0.69	-1.61	PAP-AAP	0.20	10	0.678	0.723	0.1435
236597_at	39.6	24.6	-0.69	-1.61	PAA-AAA	0.20	10	0.678	0.723	0.2178
216272_x_at	644.0	401.0	-0.68	-1.61	PPP-PPP	0.20	10	0.678	0.723	0.0786
243001_at	148.4	92.5	-0.68	-1.61	PPP-PPP	0.20	10	0.678	0.723	0.1860
207678_s_at	59.4	37.1	-0.68	-1.60	PMA-AAA	0.20	10	0.678	0.723	0.0713
205844_at	84.6	52.8	-0.68	-1.60	AAP-APA	0.20	10	0.678	0.723	0.1887
228866_at	259.2	161.9	-0.68	-1.60	APP-PPP	0.20	10	0.678	0.723	0.1661
216203_at	92.7	57.9	-0.68	-1.60	PPP-APP	0.20	10	0.678	0.723	0.1819
1554835_a_at	129.0	80.6	-0.68	-1.60	PAM-APA	0.20	10	0.678	0.723	0.0813
214664_at	95.3	59.5	-0.68	-1.60	PPP-APP	0.20	10	0.678	0.723	0.0844
232242_at	64.8	40.5	-0.68	-1.60	PAP-AAP	0.20	10	0.678	0.723	0.0848
231776_at	48.3	30.2	-0.68	-1.60	APA-AAA	0.20	10	0.678	0.723	0.1630
216155_at	66.7	41.7	-0.68	-1.60	APP-AAP	0.20	10	0.678	0.723	0.1852
227086_at	546.4	341.7	-0.68	-1.60	PPP-PPP	0.20	10	0.678	0.723	0.2019
232239_at	109.3	68.4	-0.68	-1.60	PPP-PAP	0.20	10	0.678	0.723	0.1987
211896_s_at	134.5	84.2	-0.68	-1.60	PPP-APP	0.20	10	0.678	0.723	0.2205
242861_at	106.6	66.8	-0.68	-1.60	PPP-AAP	0.20	10	0.678	0.723	0.0910
240824_at	49.4	31.0	-0.67	-1.60	AAP-AAP	0.20	10	0.678	0.723	0.2062
233528_s_at	147.1	92.3	-0.67	-1.59	AAP-AAP	0.20	10	0.678	0.723	0.2001
233881_s_at	190.4	119.4	-0.67	-1.60	PAA-PPP	0.20	10	0.678	0.723	0.2216
210195_s_at	106.2	66.7	-0.67	-1.59	PPP-PPM	0.20	10	0.678	0.723	0.0723
238909_at	153.0	96.0	-0.67	-1.59	AAP-PPP	0.20	10	0.678	0.723	0.1293
1556905_at	69.1	43.4	-0.67	-1.59	PAP-AAP	0.20	10	0.678	0.723	0.1331
215986_at	93.1	58.4	-0.67	-1.59	AAA-APP	0.20	10	0.678	0.723	0.1486
242841_at	95.3	59.9	-0.67	-1.59	PPP-PPP	0.20	10	0.678	0.723	0.1855
1558759_s_at	72.7	45.7	-0.67	-1.59	APA-AAA	0.20	10	0.678	0.723	0.0776
1568892_at	58.8	37.0	-0.67	-1.59	PAP-AAP	0.20	10	0.678	0.723	0.1128
217778_at	377.2	237.4	-0.67	-1.59	PPP-PPP	0.20	10	0.678	0.723	0.0818
242994_at	180.1	113.4	-0.67	-1.59	PPP-PPP	0.20	10	0.678	0.723	0.1662
235592_at	216.9	136.6	-0.67	-1.59	PPP-PPP	0.20	10	0.678	0.723	0.1249
242699_at	84.8	53.4	-0.67	-1.59	PPM-PPP	0.20	10	0.678	0.723	0.1590
204898_at	87.6	55.2	-0.67	-1.59	APA-APA	0.20	10	0.678	0.723	0.2279
220717_at	33.9	21.4	-0.67	-1.59	APP-AAP	0.20	10	0.678	0.723	0.1530
221019_s_at	62.9	39.6	-0.67	-1.59	AAA-PAA	0.20	10	0.678	0.723	0.2067
243003_at	136.0	85.7	-0.67	-1.59	APP-APP	0.20	10	0.678	0.723	0.2213

212400_at	353.7	223.2	-0.67	-1.59	APP-PPA	0.20	10	0.678	0.723	0.0724
233315_at	177.7	112.1	-0.67	-1.59	PPP-PPP	0.20	10	0.678	0.723	0.0827
202436_s_at	177.5	112.0	-0.66	-1.59	PAP-MAP	0.20	10	0.678	0.723	0.1348
243051_at	88.3	55.8	-0.66	-1.58	PMA-PAA	0.20	10	0.678	0.723	0.2057
232925_at	140.9	89.1	-0.66	-1.58	APP-APA	0.20	10	0.678	0.723	0.1994
1564709_at	61.0	38.6	-0.66	-1.58	AAA-APA	0.20	10	0.678	0.723	0.2241
207520_at	97.1	61.4	-0.66	-1.58	PPP-APP	0.20	10	0.678	0.723	0.1684
227179_at	231.5	146.5	-0.66	-1.58	PPP-PPP	0.20	10	0.678	0.723	0.0699
242485_at	161.3	102.1	-0.66	-1.58	AAP-AAP	0.20	10	0.678	0.723	0.1543
233226_at	89.0	56.4	-0.66	-1.58	AAP-AAA	0.20	10	0.678	0.723	0.0886
238422_at	164.0	103.9	-0.66	-1.58	APP-APA	0.20	10	0.678	0.723	0.1318
239607_at	185.6	117.5	-0.66	-1.58	PPA-AAA	0.20	10	0.678	0.723	0.1479
211825_s_at	97.0	61.5	-0.66	-1.58	MPP-PPP	0.20	10	0.678	0.723	0.0885
234036_x_at	112.7	71.4	-0.66	-1.58	PAP-APP	0.20	10	0.678	0.723	0.1334
242111_at	220.1	139.5	-0.66	-1.58	PPP-PPP	0.20	10	0.678	0.723	0.1676
236725_at	147.7	93.8	-0.66	-1.58	APP-AAA	0.20	10	0.678	0.723	0.1407
236034_at	69.7	44.3	-0.66	-1.57	PAA-AAM	0.20	10	0.678	0.723	0.0814
244859_at	111.6	70.9	-0.66	-1.57	PPP-AMP	0.20	10	0.678	0.723	0.0887
238356_at	83.3	52.9	-0.66	-1.57	PAP-PAA	0.20	10	0.678	0.723	0.1775
230593_at	74.5	47.3	-0.65	-1.57	AAP-AAA	0.20	10	0.678	0.723	0.1999
237183_at	52.9	33.6	-0.65	-1.57	APA-AMP	0.20	10	0.678	0.723	0.1933
242920_at	75.3	47.9	-0.65	-1.57	PPA-AAP	0.20	10	0.678	0.723	0.1483
1557581_x_at	57.2	36.5	-0.65	-1.57	APA-AAA	0.20	10	0.678	0.723	0.1454
241595_at	135.0	86.0	-0.65	-1.57	AAP-AAP	0.20	10	0.678	0.723	0.1814
244032_at	179.9	282.4	0.65	1.57	PPP-PPP	0.20	10	0.678	0.723	0.0039
1554933_at	61.6	39.3	-0.65	-1.57	APA-APA	0.20	10	0.678	0.723	0.1227
222511_x_at	264.1	168.4	-0.65	-1.57	AAA-AAP	0.20	10	0.678	0.723	0.1764
205199_at	237.3	151.4	-0.65	-1.57	PPP-PPP	0.20	10	0.678	0.723	0.0791
226778_at	250.6	159.9	-0.65	-1.57	PPP-PPP	0.20	10	0.678	0.723	0.1102
241053_at	84.9	54.2	-0.65	-1.57	APA-MAP	0.20	10	0.678	0.723	0.2225
205482_x_at	1000.9	639.5	-0.65	-1.57	PPP-PPP	0.20	10	0.678	0.723	0.0652
1569105_at	325.7	208.4	-0.65	-1.56	PAA-AAA	0.20	10	0.678	0.723	0.0710
232446_at	212.9	136.1	-0.65	-1.56	PPP-PPP	0.20	10	0.678	0.723	0.1602
231034_s_at	97.5	152.5	0.65	1.56	PAP-PPP	0.20	10	0.678	0.723	0.0027
226078_at	458.2	293.1	-0.64	-1.56	PPP-PPP	0.20	10	0.678	0.723	0.0892
223409_at	108.1	69.2	-0.64	-1.56	PPP-AAP	0.20	10	0.678	0.723	0.0984
44040_at	183.8	117.7	-0.64	-1.56	MAP-AAA	0.20	10	0.678	0.723	0.1024
223438_s_at	157.2	100.7	-0.64	-1.56	MPM-AAP	0.20	10	0.678	0.723	0.0817
205966_at	121.6	77.9	-0.64	-1.56	MPP-APP	0.20	10	0.678	0.723	0.1594
238619_at	343.7	220.2	-0.64	-1.56	PPP-PPP	0.20	10	0.678	0.723	0.1388
215218_s_at	311.5	199.9	-0.64	-1.56	PPP-PPP	0.20	10	0.678	0.723	0.1010
227079_at	533.8	342.5	-0.64	-1.56	PPP-PPP	0.20	10	0.678	0.723	0.1457

226786_at	877.8	563.8	-0.64	-1.56	PPP-PPP	0.20	10	0.678	0.723	0.1255
238951_at	207.0	133.0	-0.64	-1.56	PPP-PPP	0.20	10	0.678	0.723	0.1320
217518_at	135.9	87.3	-0.64	-1.56	PAP-APA	0.20	10	0.678	0.723	0.1873
242535_at	94.6	60.8	-0.64	-1.56	MPA-AAM	0.20	10	0.678	0.723	0.0658
1558996_at	157.9	101.5	-0.64	-1.56	PPP-APP	0.20	10	0.678	0.723	0.0986
1566166_at	65.9	42.4	-0.64	-1.56	MPP-APP	0.20	10	0.678	0.723	0.0629
222187_x_at	241.5	155.3	-0.64	-1.56	PPP-PPP	0.20	10	0.678	0.723	0.1051
242413_at	68.6	44.1	-0.64	-1.56	AAP-PPP	0.20	10	0.678	0.723	0.1289
236852_at	121.3	78.0	-0.64	-1.56	PAA-AAA	0.20	10	0.678	0.723	0.1301
227954_at	509.6	792.7	0.64	1.56	PPP-PPP	0.20	10	0.678	0.723	0.0030
1566550_at	69.5	44.7	-0.64	-1.56	AAP-AAA	0.20	10	0.678	0.723	0.1059
215604_x_at	658.0	423.5	-0.64	-1.55	PPP-PPP	0.20	10	0.678	0.723	0.1400
240148_at	162.4	104.6	-0.64	-1.55	PAP-APA	0.20	10	0.678	0.723	0.0723
229047_at	61.8	39.8	-0.64	-1.55	PAA-AAP	0.20	10	0.678	0.723	0.1107
230767_at	96.2	61.9	-0.64	-1.55	AAA-APP	0.20	10	0.678	0.723	0.1855
1552947_x_at	54.0	34.8	-0.64	-1.55	AAA-AAP	0.20	10	0.678	0.723	0.2023
1554465_s_at	148.2	230.1	0.64	1.55	PPP-PPP	0.20	10	0.678	0.723	0.0034
209925_at	128.6	82.9	-0.63	-1.55	PPP-PPP	0.20	10	0.678	0.723	0.0607
1560477_a_at	145.8	94.0	-0.63	-1.55	PAP-AAP	0.20	10	0.678	0.723	0.1023
228577_x_at	223.8	144.1	-0.63	-1.55	PPP-PPP	0.20	10	0.678	0.723	0.1182
216735_x_at	100.5	64.7	-0.63	-1.55	MAA-AAP	0.20	10	0.678	0.723	0.1331
241976_at	103.5	66.7	-0.63	-1.55	PAA-AAA	0.20	10	0.678	0.723	0.1182
217703_x_at	824.0	531.5	-0.63	-1.55	PPP-PPP	0.20	10	0.678	0.723	0.1202
204642_at	548.8	354.0	-0.63	-1.55	PPP-PPP	0.20	10	0.678	0.723	0.2069
1560883_s_at	103.9	67.1	-0.63	-1.55	AAA-APA	0.20	10	0.678	0.723	0.1131
239796_x_at	75.8	48.9	-0.63	-1.55	APP-PAA	0.20	10	0.678	0.723	0.2193
217042_at	143.5	92.7	-0.63	-1.55	APP-APP	0.20	10	0.678	0.723	0.1602
AFFX-r2-Bs-phe-3_at	71.7	46.3	-0.63	-1.55	PAA-AAP	0.20	10	0.678	0.723	0.0585
1559426_at	72.8	47.0	-0.63	-1.55	AAA-PAA	0.20	10	0.678	0.723	0.0988
1568748_at	65.3	42.2	-0.63	-1.55	PPA-MAA	0.20	10	0.678	0.723	0.1959
238193_at	186.2	120.4	-0.63	-1.55	PPP-APP	0.20	10	0.678	0.723	0.0880
233824_at	164.1	106.1	-0.63	-1.55	PPP-PPP	0.20	10	0.678	0.723	0.1115
230537_at	100.6	65.1	-0.63	-1.55	PMA-AAA	0.20	10	0.678	0.723	0.1542
207089_at	47.3	30.6	-0.63	-1.55	PAM-AAA	0.20	10	0.678	0.723	0.2136
219949_at	215.3	139.4	-0.63	-1.55	PPP-APP	0.20	10	0.678	0.723	0.1952
217045_x_at	181.3	117.5	-0.63	-1.54	APA-PAA	0.20	10	0.678	0.723	0.1031
1561194_at	85.5	55.4	-0.63	-1.54	PAA-APA	0.20	10	0.678	0.723	0.1740
204691_x_at	219.6	142.3	-0.63	-1.54	PPP-PPP	0.20	10	0.678	0.723	0.1849
226796_at	181.1	117.3	-0.63	-1.54	PMP-PPP	0.20	10	0.678	0.723	0.2090
1554703_at	143.6	93.1	-0.63	-1.54	APP-AAP	0.20	10	0.678	0.723	0.2238
219707_at	280.1	181.7	-0.62	-1.54	APA-AAP	0.20	10	0.678	0.723	0.0673
221967_at	235.6	152.8	-0.62	-1.54	PPA-PPP	0.20	10	0.678	0.723	0.1125

210253_at	140.6	91.3	-0.62	-1.54	PPP-PPP	0.20	10	0.678	0.723	0.1308
243642_x_at	134.1	87.2	-0.62	-1.54	PAP-APP	0.20	10	0.678	0.723	0.0803
235823_at	67.6	44.0	-0.62	-1.54	AAP-APA	0.20	10	0.678	0.723	0.1001
227809_at	116.2	75.5	-0.62	-1.54	AAA-AAP	0.20	10	0.678	0.723	0.1664
201709_s_at	1461.0	950.0	-0.62	-1.54	PPP-PPP	0.20	10	0.678	0.723	0.0787
232922_s_at	110.2	71.7	-0.62	-1.54	PAP-AAP	0.20	10	0.678	0.723	0.0800
216067_at	78.1	50.9	-0.62	-1.54	PPP-AAP	0.20	10	0.678	0.723	0.1199
238464_at	91.0	59.3	-0.62	-1.53	APP-APP	0.20	10	0.678	0.723	0.1561
243350_at	179.8	117.2	-0.62	-1.53	PPP-APP	0.20	10	0.678	0.723	0.1589
1554314_at	246.8	161.0	-0.62	-1.53	PPP-PPP	0.20	10	0.678	0.723	0.0778
240474_x_at	69.2	45.2	-0.62	-1.53	MAA-AAP	0.20	10	0.678	0.723	0.1189
228685_at	190.4	124.2	-0.62	-1.53	APP-AAP	0.20	10	0.678	0.723	0.1368
238650_x_at	485.8	317.1	-0.62	-1.53	PPP-PPP	0.20	10	0.678	0.723	0.1468
1559101_at	84.4	55.1	-0.62	-1.53	AMA-APP	0.20	10	0.678	0.723	0.1912
239201_at	328.1	214.0	-0.62	-1.53	PAP-PAP	0.20	10	0.678	0.723	0.1913
202663_at	132.0	86.2	-0.62	-1.53	PPP-PPP	0.20	10	0.678	0.723	0.0783
240363_at	130.5	85.3	-0.61	-1.53	PPP-PAP	0.20	10	0.678	0.723	0.1921
239335_at	146.1	95.4	-0.61	-1.53	APA-APA	0.20	10	0.678	0.723	0.2074
218921_at	240.2	157.0	-0.61	-1.53	PPP-PPA	0.20	10	0.678	0.723	0.0831
1568857_a_at	56.0	36.6	-0.61	-1.53	APA-AAA	0.20	10	0.678	0.723	0.1021
1568796_at	185.9	121.6	-0.61	-1.53	PAA-AAA	0.20	10	0.678	0.723	0.1314
240689_at	130.7	85.4	-0.61	-1.53	PAP-AAA	0.20	10	0.678	0.723	0.1438
220482_s_at	112.1	73.3	-0.61	-1.53	PPP-PPP	0.20	10	0.678	0.723	0.1438
213348_at	172.7	113.0	-0.61	-1.53	PPP-AAP	0.20	10	0.678	0.723	0.0617
244756_at	191.0	125.0	-0.61	-1.53	PAP-AAM	0.20	10	0.678	0.723	0.0832
1556928_at	127.2	83.2	-0.61	-1.53	AAP-AAA	0.20	10	0.678	0.723	0.0882
1556629_a_at	60.5	39.6	-0.61	-1.53	APP-AAP	0.20	10	0.678	0.723	0.1339
40020_at	77.9	51.0	-0.61	-1.53	APP-APA	0.20	10	0.678	0.723	0.1424
1556829_at	37.7	24.7	-0.61	-1.53	APA-AAM	0.20	10	0.678	0.723	0.1847
202914_s_at	160.2	104.9	-0.61	-1.53	PPA-MPP	0.20	10	0.678	0.723	0.0903
223698_at	64.8	42.5	-0.61	-1.53	AAP-AAP	0.20	10	0.678	0.723	0.2058
229882_at	152.8	100.2	-0.61	-1.53	PAM-AAP	0.20	10	0.678	0.723	0.0576
224288_x_at	468.1	307.0	-0.61	-1.53	APP-AAP	0.20	10	0.678	0.723	0.0656
224877_s_at	454.8	298.1	-0.61	-1.53	PPP-PPA	0.20	10	0.678	0.723	0.0886
239258_at	121.7	79.8	-0.61	-1.53	PPP-PPP	0.20	10	0.678	0.723	0.1104
210695_s_at	186.1	122.2	-0.61	-1.52	AAA-APP	0.20	10	0.678	0.723	0.0575
218299_at	873.8	573.3	-0.61	-1.52	AMM-PPM	0.20	10	0.678	0.723	0.1215
1553670_at	117.1	76.9	-0.61	-1.52	PAM-AAA	0.20	10	0.678	0.723	0.0955
225972_at	351.3	230.6	-0.61	-1.52	PPP-PPP	0.20	10	0.678	0.723	0.1782
233017_x_at	208.1	136.7	-0.61	-1.52	PPP-PPP	0.20	10	0.678	0.723	0.0613
210876_at	234.5	154.0	-0.61	-1.52	PPP-PPP	0.20	10	0.678	0.723	0.1139
204139_x_at	270.3	177.8	-0.60	-1.52	PPP-PPP	0.20	10	0.678	0.723	0.0573

227948_at	250.5	164.7	-0.60	-1.52	PPP-PPP	0.20	10	0.678	0.723	0.0750
241391_at	187.4	123.3	-0.60	-1.52	PPP-PPP	0.20	10	0.678	0.723	0.1600
229055_at	98.7	65.0	-0.60	-1.52	APA-AAA	0.20	10	0.678	0.723	0.1567
1565935_at	270.9	178.4	-0.60	-1.52	PAA-PPP	0.20	10	0.678	0.723	0.2123
220232_at	115.6	76.1	-0.60	-1.52	PPP-AAP	0.20	10	0.678	0.723	0.1226
222313_at	114.3	75.3	-0.60	-1.52	PPP-APP	0.20	10	0.678	0.723	0.1565
209763_at	1125.4	741.9	-0.60	-1.52	PPP-PPP	0.20	10	0.678	0.723	0.0920
210991_s_at	99.7	65.7	-0.60	-1.52	AAA-PAM	0.20	10	0.678	0.723	0.1782
230054_at	108.4	71.5	-0.60	-1.52	AAP-APM	0.20	10	0.678	0.723	0.0768
243973_at	82.4	54.4	-0.60	-1.52	AMA-PAP	0.20	10	0.678	0.723	0.1535
238419_at	255.1	168.2	-0.60	-1.52	PPP-PPP	0.20	10	0.678	0.723	0.2201
228228_at	139.4	92.1	-0.60	-1.51	AAA-PPA	0.20	10	0.678	0.723	0.0582
233977_at	142.0	93.8	-0.60	-1.51	MPP-PPP	0.20	10	0.678	0.723	0.1105
237673_at	36.6	24.2	-0.60	-1.51	APA-AAP	0.20	10	0.678	0.723	0.2020
222082_at	72.4	47.9	-0.60	-1.51	AAA-APP	0.20	10	0.678	0.723	0.0998
207747_s_at	145.1	95.9	-0.60	-1.51	PAP-PAP	0.20	10	0.678	0.723	0.1225
210647_x_at	148.4	98.1	-0.60	-1.51	PPP-PAP	0.20	10	0.678	0.723	0.1856
216051_x_at	148.1	97.9	-0.60	-1.51	PPA-APA	0.20	10	0.678	0.723	0.1892
229119_s_at	674.0	1019.4	0.60	1.51	PPP-PPP	0.20	10	0.678	0.723	0.0024
215083_at	201.7	133.5	-0.60	-1.51	PPP-PPP	0.20	10	0.678	0.723	0.0730
223052_x_at	156.8	103.8	-0.60	-1.51	PPP-AMP	0.20	10	0.678	0.723	0.0794
220124_at	96.8	64.1	-0.60	-1.51	AAP-MPA	0.20	10	0.678	0.723	0.0933
1563474_at	26.8	17.8	-0.60	-1.51	AAA-AAP	0.20	10	0.678	0.723	0.1044
236109_at	180.0	119.2	-0.60	-1.51	PPP-PPP	0.20	10	0.678	0.723	0.1850
211768_at	118.1	78.3	-0.59	-1.51	APA-PAA	0.20	10	0.678	0.723	0.1036
202928_s_at	192.0	127.3	-0.59	-1.51	PMA-APA	0.20	10	0.678	0.723	0.1155
226546_at	1233.1	817.0	-0.59	-1.51	PPP-PPP	0.20	10	0.678	0.723	0.2028
228790_at	178.0	117.9	-0.59	-1.51	PPP-APP	0.20	10	0.678	0.723	0.2080
238812_at	178.2	118.1	-0.59	-1.51	AAP-APP	0.20	10	0.678	0.723	0.1728
1553594_a_at	294.4	195.4	-0.59	-1.51	PPA-PPP	0.20	10	0.678	0.723	0.0597
232768_at	124.1	82.3	-0.59	-1.51	AAP-MPA	0.20	10	0.678	0.723	0.1159
233533_at	470.1	311.8	-0.59	-1.51	PPP-PPA	0.20	10	0.678	0.723	0.1406
209438_at	161.9	107.6	-0.59	-1.51	PPP-PPP	0.20	10	0.678	0.723	0.1106
232410_at	85.5	56.9	-0.59	-1.50	AAP-PAM	0.20	10	0.678	0.723	0.0813
229642_at	155.2	103.2	-0.59	-1.50	AAP-MAP	0.20	10	0.678	0.723	0.1253
240025_x_at	76.6	51.0	-0.59	-1.50	APP-AAA	0.20	10	0.678	0.723	0.1262
239188_at	136.6	90.8	-0.59	-1.50	AAP-MAP	0.20	10	0.678	0.723	0.1936
214244_s_at	161.4	107.3	-0.59	-1.51	PPP-PPP	0.20	10	0.678	0.723	0.2068
1554468_s_at	239.8	159.6	-0.59	-1.50	PAA-MMA	0.20	10	0.678	0.723	0.0618
229998_x_at	200.2	133.2	-0.59	-1.50	MAP-PPA	0.20	10	0.678	0.723	0.1418
235959_at	82.6	55.0	-0.59	-1.50	PAP-PPP	0.20	10	0.678	0.723	0.1958
207638_at	28.9	19.3	-0.59	-1.50	AAA-APA	0.20	10	0.678	0.723	0.1556

217177_s_at	73.9	49.2	-0.59	-1.50	APP-PMP	0.20	10	0.678	0.723	0.1945
238456_at	119.5	79.6	-0.59	-1.50	AAA-APP	0.20	10	0.678	0.723	0.2065
212465_at	2344.8	1563.5	-0.59	-1.50	PPP-PPP	0.20	10	0.678	0.723	0.0734
233037_at	51.0	34.0	-0.59	-1.50	APA-AAP	0.20	10	0.678	0.723	0.1286
1561673_at	51.7	34.5	-0.58	-1.50	AAP-APP	0.20	10	0.678	0.723	0.1667
209708_at	510.7	340.9	-0.58	-1.50	PPP-PPP	0.20	10	0.678	0.723	0.0947
219308_s_at	246.2	164.4	-0.58	-1.50	PAP-PPP	0.20	10	0.678	0.723	0.1509
239264_at	60.3	40.3	-0.58	-1.50	APA-APP	0.20	10	0.678	0.723	0.1711
242665_at	267.7	178.9	-0.58	-1.50	PPP-PPP	0.20	10	0.678	0.723	0.1123
206079_at	344.6	230.2	-0.58	-1.50	APA-PPP	0.20	10	0.678	0.723	0.1611
1552719_at	120.6	80.6	-0.58	-1.50	PAP-AAM	0.20	10	0.678	0.723	0.1827
242829_x_at	163.2	109.1	-0.58	-1.50	PAP-AAA	0.20	10	0.678	0.723	0.1753
1561310_at	77.8	52.1	-0.58	-1.49	AAA-AAP	0.20	10	0.678	0.723	0.1007
226455_at	232.3	155.5	-0.58	-1.49	PPP-PPP	0.20	10	0.678	0.723	0.1491
238392_at	37.3	25.0	-0.58	-1.49	AAA-AAP	0.20	10	0.678	0.723	0.1927
204738_s_at	318.1	213.1	-0.58	-1.49	AAA-AAP	0.20	10	0.678	0.723	0.1088
241387_at	205.6	137.7	-0.58	-1.49	PPP-PPP	0.20	10	0.678	0.723	0.1132
211607_x_at	328.0	219.8	-0.58	-1.49	PPP-PPP	0.20	10	0.678	0.723	0.1443
204893_s_at	293.1	196.5	-0.58	-1.49	AAP-AAP	0.20	10	0.678	0.723	0.0974
204065_at	579.1	388.3	-0.58	-1.49	PPP-PPP	0.20	10	0.678	0.723	0.1237
234047_at	71.1	47.7	-0.58	-1.49	PPA-APP	0.20	10	0.678	0.723	0.0951
237513_at	89.3	59.9	-0.58	-1.49	APA-AAP	0.20	10	0.678	0.723	0.1198
231412_at	133.7	89.8	-0.58	-1.49	PPP-PPP	0.20	10	0.678	0.723	0.0590
213739_at	180.9	121.5	-0.57	-1.49	PAA-AAP	0.20	10	0.678	0.723	0.1396
230992_at	73.8	49.6	-0.57	-1.49	AAA-APA	0.20	10	0.678	0.723	0.1798
239540_at	74.7	50.2	-0.57	-1.49	AAA-APP	0.20	10	0.678	0.723	0.2108
203793_x_at	412.6	277.3	-0.57	-1.49	PPP-PPP	0.20	10	0.678	0.723	0.2042
232800_at	134.9	90.8	-0.57	-1.49	PPP-PPP	0.20	10	0.678	0.723	0.1137
1565566_a_at	68.6	46.2	-0.57	-1.49	PPP-PPP	0.20	10	0.678	0.723	0.1254
204830_x_at	273.3	183.8	-0.57	-1.49	PPP-PPP	0.20	10	0.678	0.723	0.1513
236038_at	60.3	40.5	-0.57	-1.49	AAP-PAA	0.20	10	0.678	0.723	0.2138
243112_at	128.0	86.2	-0.57	-1.49	PPP-PPP	0.20	10	0.678	0.723	0.0598
231502_at	62.7	42.2	-0.57	-1.49	PPP-AAP	0.20	10	0.678	0.723	0.0984
243626_at	94.6	63.6	-0.57	-1.49	MPM-AAP	0.20	10	0.678	0.723	0.1082
243718_at	122.1	82.3	-0.57	-1.49	AAA-AAP	0.20	10	0.678	0.723	0.1916
220089_at	237.0	159.6	-0.57	-1.49	PPP-PPP	0.20	10	0.678	0.723	0.0912
221071_at	68.3	46.0	-0.57	-1.49	PAA-AAA	0.20	10	0.678	0.723	0.1471
241100_at	119.4	80.5	-0.57	-1.48	APP-PAP	0.20	10	0.678	0.723	0.0785
208159_x_at	929.4	626.7	-0.57	-1.48	PPP-PAP	0.20	10	0.678	0.723	0.1068
205305_at	112.5	75.9	-0.57	-1.48	PMP-PPP	0.20	10	0.678	0.723	0.1185
204029_at	219.5	148.1	-0.57	-1.48	PAP-AAP	0.20	10	0.678	0.723	0.1449
215978_x_at	2027.8	1368.9	-0.57	-1.48	PPP-PPP	0.20	10	0.678	0.723	0.0907

239372_at	81.7	55.1	-0.57	-1.48	PPP-PPP	0.20	10	0.678	0.723	0.0918
220566_at	151.4	102.2	-0.57	-1.48	PPP-PPA	0.20	10	0.678	0.723	0.1522
216006_at	62.5	42.2	-0.57	-1.48	PPA-APP	0.20	10	0.678	0.723	0.2028
224132_at	210.4	142.1	-0.57	-1.48	PPP-PAP	0.20	10	0.678	0.723	0.0991
1560014_s_at	86.8	58.7	-0.57	-1.48	PMA-APP	0.20	10	0.678	0.723	0.1243
208276_at	80.2	54.2	-0.57	-1.48	AAP-APA	0.20	10	0.678	0.723	0.0684
235367_at	457.2	309.0	-0.57	-1.48	MPP-PPP	0.20	10	0.678	0.723	0.0838
230062_at	175.0	118.3	-0.57	-1.48	PPP-PAP	0.20	10	0.678	0.723	0.1058
236282_at	210.2	142.0	-0.57	-1.48	PPP-PPP	0.20	10	0.678	0.723	0.1175
237768_x_at	464.8	314.3	-0.57	-1.48	PPP-PPP	0.20	10	0.678	0.723	0.1766
215643_at	91.0	61.6	-0.56	-1.48	PPP-PAP	0.20	10	0.678	0.723	0.1173
216450_x_at	477.9	323.3	-0.56	-1.48	PPP-PPP	0.20	10	0.678	0.723	0.1302
206027_at	181.7	122.8	-0.56	-1.48	MAM-PPA	0.20	10	0.678	0.723	0.1400
233231_at	59.7	40.4	-0.56	-1.48	AAP-AAA	0.20	10	0.678	0.723	0.0710
232386_at	95.0	64.3	-0.56	-1.48	PPP-PMP	0.20	10	0.678	0.723	0.0945
222857_s_at	236.6	160.1	-0.56	-1.48	PAA-APP	0.20	10	0.678	0.723	0.1509
234545_at	87.7	59.4	-0.56	-1.48	AAA-PAA	0.20	10	0.678	0.723	0.1625
1562289_at	111.5	75.5	-0.56	-1.48	PAM-MAA	0.20	10	0.678	0.723	0.1705
232392_at	203.5	137.7	-0.56	-1.48	MPP-APP	0.20	10	0.678	0.723	0.1875
224852_at	703.6	476.2	-0.56	-1.48	PPP-PPP	0.20	10	0.678	0.723	0.1910
214291_at	313.6	212.4	-0.56	-1.48	PPP-PPP	0.20	10	0.678	0.723	0.1124
226365_at	111.7	75.7	-0.56	-1.48	AAA-PAP	0.20	10	0.678	0.723	0.0791
210232_at	183.6	124.5	-0.56	-1.48	PAA-APM	0.20	10	0.678	0.723	0.1018
204035_at	1165.6	790.4	-0.56	-1.48	PPP-PPP	0.20	10	0.678	0.723	0.1829
227745_at	98.3	66.7	-0.56	-1.47	APP-PPP	0.20	10	0.678	0.723	0.1970
208018_s_at	66.6	45.3	-0.56	-1.47	AAA-AAP	0.20	10	0.678	0.723	0.0643
1569905_at	172.4	117.1	-0.56	-1.47	PPP-PPP	0.20	10	0.678	0.723	0.1020
239211_at	53.0	36.0	-0.56	-1.47	PAA-AAA	0.20	10	0.678	0.723	0.1911
219537_x_at	169.6	115.2	-0.56	-1.47	PPP-PPP	0.20	10	0.678	0.723	0.1153
1554784_at	45.4	30.9	-0.56	-1.47	AAP-AAA	0.20	10	0.678	0.723	0.1227
229511_at	103.7	70.6	-0.56	-1.47	PPP-PPP	0.20	10	0.678	0.723	0.0798
211866_x_at	234.7	159.6	-0.56	-1.47	PPP-PPP	0.20	10	0.678	0.723	0.0864
228247_at	294.8	200.6	-0.56	-1.47	PPP-PPP	0.20	10	0.678	0.723	0.0686
242635_s_at	124.8	85.0	-0.56	-1.47	PPP-PMP	0.20	10	0.678	0.723	0.0886
241464_s_at	161.7	110.1	-0.56	-1.47	AAP-AAP	0.20	10	0.678	0.723	0.1503
230215_at	95.2	64.8	-0.56	-1.47	AAP-AMA	0.20	10	0.678	0.723	0.2035
203623_at	162.6	110.8	-0.55	-1.47	AAP-AAP	0.20	10	0.678	0.723	0.1105
236901_at	236.0	160.9	-0.55	-1.47	PPP-PPP	0.20	10	0.678	0.723	0.1839
244031_at	82.0	55.9	-0.55	-1.47	APP-APP	0.20	10	0.678	0.723	0.1225
208511_at	768.8	524.3	-0.55	-1.47	PPP-PPP	0.20	10	0.678	0.723	0.2094
236722_at	156.5	106.8	-0.55	-1.47	AAA-PAA	0.20	10	0.678	0.723	0.2113
242094_at	113.1	77.2	-0.55	-1.47	AMM-AAP	0.20	10	0.678	0.723	0.0810

205580_s_at	431.9	295.0	-0.55	-1.46	PPP-PPP	0.20	10	0.678	0.723	0.0536
217363_x_at	492.3	336.2	-0.55	-1.46	PPP-PPP	0.20	10	0.678	0.723	0.1817
229987_at	89.6	61.3	-0.55	-1.46	APP-PMM	0.20	10	0.678	0.723	0.1198
207234_at	62.0	42.4	-0.55	-1.46	AAA-PPA	0.20	10	0.678	0.723	0.1692
201369_s_at	131.4	89.9	-0.55	-1.46	PAA-AAP	0.20	10	0.678	0.723	0.1987
214481_at	75.4	51.6	-0.55	-1.46	AAA-PAA	0.20	10	0.678	0.723	0.1833
228801_at	286.3	196.1	-0.55	-1.46	PPP-PPP	0.20	10	0.678	0.723	0.1165
226473_at	424.1	290.6	-0.55	-1.46	PPP-APP	0.20	10	0.678	0.723	0.1460
242080_at	220.1	150.8	-0.55	-1.46	AAP-AAP	0.20	10	0.678	0.723	0.1077
242645_at	98.8	67.7	-0.55	-1.46	APP-PAP	0.20	10	0.678	0.723	0.1543
243262_at	57.2	39.2	-0.55	-1.46	AAP-PPP	0.20	10	0.678	0.723	0.1846
221189_s_at	523.7	359.3	-0.54	-1.46	APP-PPP	0.20	10	0.678	0.723	0.0648
206543_at	68.2	46.8	-0.54	-1.46	PPP-AAP	0.20	10	0.678	0.723	0.0922
236923_x_at	240.4	165.0	-0.54	-1.46	MPP-PPP	0.20	10	0.678	0.723	0.1208
215298_at	67.5	46.4	-0.54	-1.46	AAA-AAP	0.20	10	0.678	0.723	0.1382
208075_s_at	86.7	59.5	-0.54	-1.46	AAP-AAA	0.20	10	0.678	0.723	0.1425
227078_at	220.1	151.1	-0.54	-1.46	PPP-PAP	0.20	10	0.678	0.723	0.1599
228354_at	465.7	319.8	-0.54	-1.46	PPP-PPP	0.20	10	0.678	0.723	0.0562
212095_s_at	312.2	214.5	-0.54	-1.46	PPP-PPP	0.20	10	0.678	0.723	0.0968
1556058_s_at	159.2	109.5	-0.54	-1.46	APA-PPA	0.20	10	0.678	0.723	0.1525
231988_x_at	151.2	103.9	-0.54	-1.46	AAP-MAA	0.20	10	0.678	0.723	0.1603
225191_at	170.5	117.2	-0.54	-1.45	PPP-APA	0.20	10	0.678	0.723	0.0486
234759_at	84.6	58.2	-0.54	-1.45	APP-APP	0.20	10	0.678	0.723	0.0667
243582_at	501.1	344.7	-0.54	-1.45	PPP-PPP	0.20	10	0.678	0.723	0.1083
1564129_a_at	291.2	200.5	-0.54	-1.45	PPP-PPP	0.20	10	0.678	0.723	0.1191
209952_s_at	113.9	78.5	-0.54	-1.45	AAP-MAA	0.20	10	0.678	0.723	0.1835
238108_at	127.3	87.8	-0.54	-1.45	APP-PPP	0.20	10	0.678	0.723	0.0981
210524_x_at	633.0	436.9	-0.54	-1.45	PPP-PPP	0.20	10	0.678	0.723	0.0673
205891_at	4074.6	2812.9	-0.54	-1.45	PPP-PPP	0.20	10	0.678	0.723	0.1108
242550_at	320.0	220.9	-0.54	-1.45	PPP-PPP	0.20	10	0.678	0.723	0.1751
223185_s_at	97.9	67.6	-0.53	-1.45	PPP-PAP	0.20	10	0.678	0.723	0.0559
233713_at	118.0	81.5	-0.53	-1.45	PPP-PPP	0.20	10	0.678	0.723	0.1030
234573_at	73.5	50.8	-0.53	-1.45	AMA-AAP	0.20	10	0.678	0.723	0.1351
230579_at	234.9	162.3	-0.53	-1.45	PPM-APA	0.20	10	0.678	0.723	0.2060
209947_at	252.2	174.4	-0.53	-1.45	PPP-PPP	0.20	10	0.678	0.723	0.0899
209532_at	131.9	190.8	0.53	1.45	PPP-PPP	0.20	10	0.678	0.723	0.0010
1560698_a_at	146.4	101.3	-0.53	-1.45	PPM-APP	0.20	10	0.678	0.723	0.0968
231157_at	133.1	92.1	-0.53	-1.45	AAA-AAP	0.20	10	0.678	0.723	0.1290
240971_x_at	132.3	91.5	-0.53	-1.45	MPP-APP	0.20	10	0.678	0.723	0.1315
1563497_at	100.9	69.7	-0.53	-1.45	PPP-PPP	0.20	10	0.678	0.723	0.1477
38892_at	332.2	230.0	-0.53	-1.45	PPP-PPP	0.20	10	0.678	0.723	0.1112
1558882_at	70.7	102.2	0.53	1.45	PPM-PPP	0.20	10	0.678	0.723	0.0007

1568605_at	240.9	166.8	-0.53	-1.44	PPA-APP	0.20	10	0.678	0.723	0.0789
213882_at	75.5	52.3	-0.53	-1.44	PPP-APA	0.20	10	0.678	0.723	0.1064
1564776_at	53.9	37.3	-0.53	-1.44	PAA-AAA	0.20	10	0.678	0.723	0.1180
216750_at	133.5	92.5	-0.53	-1.44	PPP-PPA	0.20	10	0.678	0.723	0.1582
243977_at	158.1	109.6	-0.53	-1.44	APP-PPP	0.20	10	0.678	0.723	0.1859
238006_at	151.4	105.0	-0.53	-1.44	PPP-PPA	0.20	10	0.678	0.723	0.0999
209826_at	186.3	129.1	-0.53	-1.44	PPA-APP	0.20	10	0.678	0.723	0.1495
1554076_s_at	304.7	211.5	-0.53	-1.44	PPP-PPP	0.20	10	0.678	0.723	0.1074
207571_x_at	95.6	66.4	-0.53	-1.44	PAA-AAA	0.20	10	0.678	0.723	0.0864
242531_at	63.1	43.8	-0.53	-1.44	AAP-APP	0.20	10	0.678	0.723	0.0947
218815_s_at	510.3	354.4	-0.53	-1.44	PPP-PPP	0.20	10	0.678	0.723	0.0990
1561453_at	84.0	58.3	-0.53	-1.44	AAA-AAP	0.20	10	0.678	0.723	0.1752
1561989_at	82.0	56.9	-0.53	-1.44	APM-AAA	0.20	10	0.678	0.723	0.1930
242258_at	55.1	38.3	-0.53	-1.44	PPP-MPP	0.20	10	0.678	0.723	0.2029
1569342_at	154.2	107.1	-0.53	-1.44	APP-PPP	0.20	10	0.678	0.723	0.0568
1557283_a_at	77.2	53.6	-0.53	-1.44	PMP-AAA	0.20	10	0.678	0.723	0.1064
1555843_at	208.4	144.8	-0.53	-1.44	PPP-PPP	0.20	10	0.678	0.723	0.1347
396_f_at	532.0	369.7	-0.53	-1.44	PAA-AAA	0.20	10	0.678	0.723	0.1381
240020_at	59.0	41.0	-0.53	-1.44	APP-PPA	0.20	10	0.678	0.723	0.1632
219602_s_at	317.6	220.9	-0.52	-1.44	PPP-PPP	0.20	10	0.678	0.723	0.0481
237310_at	311.2	216.4	-0.52	-1.44	PPP-PPP	0.20	10	0.678	0.723	0.1061
236129_at	65.3	45.4	-0.52	-1.44	APA-APP	0.20	10	0.678	0.723	0.1377
214225_at	113.0	78.6	-0.52	-1.44	PPP-PPP	0.20	10	0.678	0.723	0.1531
1564028_s_at	60.0	41.8	-0.52	-1.44	PPA-APP	0.20	10	0.678	0.723	0.1856
238167_at	79.6	55.4	-0.52	-1.44	PAA-AAA	0.20	10	0.678	0.723	0.1944
242091_at	51.7	36.0	-0.52	-1.44	AAP-PAP	0.20	10	0.678	0.723	0.1956
215492_x_at	210.6	146.6	-0.52	-1.44	AMP-AAA	0.20	10	0.678	0.723	0.1764
213125_at	74.5	51.9	-0.52	-1.44	APP-AMP	0.20	10	0.678	0.723	0.1812
211027_s_at	156.1	108.8	-0.52	-1.44	PPM-PPP	0.20	10	0.678	0.723	0.0679
207749_s_at	874.0	609.1	-0.52	-1.44	PPP-PPP	0.20	10	0.678	0.723	0.0847
214707_x_at	389.3	271.3	-0.52	-1.44	PPP-PPP	0.20	10	0.678	0.723	0.0958
230210_at	163.0	113.7	-0.52	-1.43	PAP-MPP	0.20	10	0.678	0.723	0.1563
224766_at	269.4	187.8	-0.52	-1.43	PPP-PPP	0.20	10	0.678	0.723	0.1739
215067_x_at	406.9	284.1	-0.52	-1.43	PPP-PMP	0.20	10	0.678	0.723	0.0494
204537_s_at	181.2	126.5	-0.52	-1.43	PPP-PPP	0.20	10	0.678	0.723	0.0968
229798_s_at	232.5	162.4	-0.52	-1.43	AMA-APP	0.20	10	0.678	0.723	0.1303
230769_at	43.8	30.6	-0.52	-1.43	AAP-AAP	0.20	10	0.678	0.723	0.1748
219361_s_at	1006.5	702.8	-0.52	-1.43	PPP-PPP	0.20	10	0.678	0.723	0.1912
208415_x_at	1048.9	732.7	-0.52	-1.43	PPP-PPP	0.20	10	0.678	0.723	0.2012
36829_at	350.2	244.7	-0.52	-1.43	PPP-PPP	0.20	10	0.678	0.723	0.0994
239375_at	251.5	175.7	-0.52	-1.43	PPP-PPP	0.20	10	0.678	0.723	0.1061
1559059_s_at	65.5	45.8	-0.52	-1.43	AAP-PAP	0.20	10	0.678	0.723	0.1772

203074_at	2647.4	1851.7	-0.52	-1.43	PPP-PPP	0.20	10	0.678	0.723	0.0977
220215_at	241.6	168.9	-0.52	-1.43	PPP-PPP	0.20	10	0.678	0.723	0.1301
235334_at	78.3	54.8	-0.52	-1.43	PPA-PPM	0.20	10	0.678	0.723	0.1624
244047_at	81.2	56.8	-0.52	-1.43	AAP-PAP	0.20	10	0.678	0.723	0.1634
205214_at	408.1	285.5	-0.52	-1.43	PPP-PPP	0.20	10	0.678	0.723	0.0594
208510_s_at	314.7	220.3	-0.52	-1.43	PPP-PPP	0.20	10	0.678	0.723	0.1197
212086_x_at	3408.3	2386.1	-0.51	-1.43	PPP-PPP	0.20	10	0.678	0.723	0.1570
232527_at	156.2	109.4	-0.51	-1.43	PPA-AAP	0.20	10	0.678	0.723	0.1784
1570569_at	213.7	149.8	-0.51	-1.43	PAA-AAA	0.20	10	0.678	0.723	0.0519
222207_x_at	923.8	647.5	-0.51	-1.43	PPP-PPP	0.20	10	0.678	0.723	0.1094
203932_at	141.4	99.1	-0.51	-1.43	AAP-PAP	0.20	10	0.678	0.723	0.1153
240576_at	146.6	102.7	-0.51	-1.43	PMP-AAP	0.20	10	0.678	0.723	0.1203
58900_at	706.9	495.6	-0.51	-1.43	PPP-PPP	0.20	10	0.678	0.723	0.1295
236023_at	65.3	45.7	-0.51	-1.43	PAA-AAP	0.20	10	0.678	0.723	0.1397
1569091_at	190.9	133.8	-0.51	-1.43	PPP-PPP	0.20	10	0.678	0.723	0.1410
239164_at	134.1	94.1	-0.51	-1.43	PPP-PPP	0.20	10	0.678	0.723	0.0632
202335_s_at	110.7	77.7	-0.51	-1.43	PPP-PPP	0.20	10	0.678	0.723	0.1031
218330_s_at	1492.8	1047.4	-0.51	-1.43	PPP-PPP	0.20	10	0.678	0.723	0.1190
209766_at	147.0	103.2	-0.51	-1.43	PPP-PPP	0.20	10	0.678	0.723	0.1342
1556110_at	136.7	95.9	-0.51	-1.43	APA-MPP	0.20	10	0.678	0.723	0.1561
228958_at	159.6	112.0	-0.51	-1.42	PMA-PAP	0.20	10	0.678	0.723	0.0453
1556228_a_at	143.9	101.1	-0.51	-1.42	PPP-PPP	0.20	10	0.678	0.723	0.0884
204202_at	183.7	129.0	-0.51	-1.42	PPP-PPP	0.20	10	0.678	0.723	0.0965
235467_s_at	161.2	113.2	-0.51	-1.42	MPP-PMP	0.20	10	0.678	0.723	0.0977
1562861_at	138.1	96.9	-0.51	-1.43	PPP-PPP	0.20	10	0.678	0.723	0.1037
231599_x_at	200.5	141.0	-0.51	-1.42	PPP-PPP	0.20	10	0.678	0.723	0.1869
207303_at	360.3	253.6	-0.51	-1.42	PPP-PPP	0.20	10	0.678	0.723	0.0590
205920_at	120.3	84.7	-0.51	-1.42	APP-APA	0.20	10	0.678	0.723	0.1658
208099_x_at	239.5	168.5	-0.51	-1.42	PPP-PPP	0.20	10	0.678	0.723	0.1775
1557751_at	138.5	97.5	-0.51	-1.42	PPP-PPP	0.20	10	0.678	0.723	0.0742
1554018_at	84.4	59.4	-0.51	-1.42	PPP-MPP	0.20	10	0.678	0.723	0.1483
219028_at	247.9	174.5	-0.51	-1.42	PPP-PAP	0.20	10	0.678	0.723	0.1750
234485_at	302.0	212.9	-0.51	-1.42	PPP-PPP	0.20	10	0.678	0.723	0.0860
206966_s_at	206.7	145.7	-0.51	-1.42	MPP-PPP	0.20	10	0.678	0.723	0.1359
222183_x_at	408.3	287.7	-0.51	-1.42	PPP-PPP	0.20	10	0.678	0.723	0.1394
214806_at	234.0	164.9	-0.51	-1.42	PAP-PPP	0.20	10	0.678	0.723	0.1792
226840_at	359.4	253.5	-0.50	-1.42	PPA-APP	0.20	10	0.678	0.723	0.0620
1570210_x_at	403.3	284.4	-0.50	-1.42	PPP-PPP	0.20	10	0.678	0.723	0.0765
229735_s_at	225.4	159.0	-0.50	-1.42	PPP-PPP	0.20	10	0.678	0.723	0.0903
238549_at	290.1	204.6	-0.50	-1.42	PPP-PPP	0.20	10	0.678	0.723	0.1178
230408_at	145.2	102.4	-0.50	-1.42	PPP-APP	0.20	10	0.678	0.723	0.0861
237475_x_at	1645.6	1161.0	-0.50	-1.42	PPP-PPP	0.20	10	0.678	0.723	0.1487

235298_at	178.6	126.0	-0.50	-1.42	PPP-PMP	0.20	10	0.678	0.723	0.1499
231880_at	688.1	485.5	-0.50	-1.42	PPP-PPP	0.20	10	0.678	0.723	0.1931
242610_x_at	108.9	76.9	-0.50	-1.42	PAA-PAA	0.20	10	0.678	0.723	0.0918
211014_s_at	239.0	168.7	-0.50	-1.42	PPP-PPP	0.20	10	0.678	0.723	0.1346
214031_s_at	76.8	54.3	-0.50	-1.42	AAA-PAA	0.20	10	0.678	0.723	0.1413
242724_x_at	84.2	59.4	-0.50	-1.42	APA-APP	0.20	10	0.678	0.723	0.1990
204817_at	1491.3	1054.8	-0.50	-1.41	PPP-PPP	0.20	10	0.678	0.723	0.0499
226484_at	235.0	166.2	-0.50	-1.41	PPP-APP	0.20	10	0.678	0.723	0.0531
222349_x_at	192.0	135.7	-0.50	-1.41	PPP-PMP	0.20	10	0.678	0.723	0.1173
236470_at	58.0	41.1	-0.50	-1.41	APP-PPP	0.20	10	0.678	0.723	0.1086
241438_at	52.3	37.0	-0.50	-1.41	AAP-AMP	0.20	10	0.678	0.723	0.1239
239288_at	353.1	249.8	-0.50	-1.41	PPP-PPP	0.20	10	0.678	0.723	0.1320
1552822_at	83.2	58.9	-0.50	-1.41	PAP-APP	0.20	10	0.678	0.723	0.0514
1554312_at	151.2	107.0	-0.50	-1.41	APM-PPP	0.20	10	0.678	0.723	0.0693
233300_at	264.8	187.6	-0.50	-1.41	MPP-APP	0.20	10	0.678	0.723	0.1307
203128_at	531.0	376.4	-0.50	-1.41	PPP-PPP	0.20	10	0.678	0.723	0.0938
226071_at	93.9	66.5	-0.50	-1.41	AAP-AAP	0.20	10	0.678	0.723	0.1457
215421_at	58.4	41.4	-0.50	-1.41	AAP-AAP	0.20	10	0.678	0.723	0.1881
203379_at	370.6	262.8	-0.50	-1.41	PPP-PPP	0.20	10	0.678	0.723	0.0870
242888_at	105.3	74.7	-0.50	-1.41	PPA-PPP	0.20	10	0.678	0.723	0.1247
243624_at	141.9	100.7	-0.50	-1.41	PPP-PPP	0.20	10	0.678	0.723	0.1443
236969_at	130.5	92.6	-0.50	-1.41	AAP-MPP	0.20	10	0.678	0.723	0.0452
207076_s_at	126.5	89.8	-0.50	-1.41	PAP-PPP	0.20	10	0.678	0.723	0.0475
210969_at	50.0	35.5	-0.50	-1.41	AAA-APA	0.20	10	0.678	0.723	0.0708
205210_at	492.1	349.1	-0.50	-1.41	PPP-PPP	0.20	10	0.678	0.723	0.1771
242431_at	173.4	123.1	-0.49	-1.41	PPP-PPP	0.20	10	0.678	0.723	0.0992
242614_at	164.4	116.7	-0.49	-1.41	PPP-PPP	0.20	10	0.678	0.723	0.1299
222168_at	332.7	236.2	-0.49	-1.41	PPP-PPP	0.20	10	0.678	0.723	0.1850
1560433_at	282.3	200.6	-0.49	-1.41	APP-PPP	0.20	10	0.678	0.723	0.1219
226140_s_at	971.0	689.9	-0.49	-1.41	PPP-PPP	0.20	10	0.678	0.723	0.1367
217548_at	141.3	100.4	-0.49	-1.41	APP-APP	0.20	10	0.678	0.723	0.1640
1570243_at	101.2	71.9	-0.49	-1.41	MPM-PAP	0.20	10	0.678	0.723	0.1796
204999_s_at	233.6	166.1	-0.49	-1.41	PPA-PPP	0.20	10	0.678	0.723	0.1588
220398_at	85.0	60.5	-0.49	-1.41	PAA-AAA	0.20	10	0.678	0.723	0.0532
217667_at	151.1	107.6	-0.49	-1.40	PPP-PPP	0.20	10	0.678	0.723	0.0717
227320_at	640.7	456.1	-0.49	-1.41	PPP-PPP	0.20	10	0.678	0.723	0.0748
235677_at	384.6	274.0	-0.49	-1.40	PPP-PPP	0.20	10	0.678	0.723	0.0527
229218_at	1247.1	888.6	-0.49	-1.40	PPP-PPP	0.20	10	0.678	0.723	0.1122
241164_at	79.2	56.5	-0.49	-1.40	PPP-PPP	0.20	10	0.678	0.723	0.1463
219779_at	1430.5	1019.7	-0.49	-1.40	PPP-PPP	0.20	10	0.678	0.723	0.0511
203641_s_at	223.1	159.0	-0.49	-1.40	AAA-MPA	0.20	10	0.678	0.723	0.0821
221205_at	133.8	95.5	-0.49	-1.40	PPP-PMP	0.20	10	0.678	0.723	0.0698

1565661_x_at	133.0	94.9	-0.49	-1.40	AMP-AAP	0.20	10	0.678	0.723	0.1022
1552278_a_at	103.2	73.7	-0.49	-1.40	APA-AAA	0.20	10	0.678	0.723	0.1293
236320_at	104.9	74.8	-0.49	-1.40	AAA-AAP	0.20	10	0.678	0.723	0.1306
226121_at	361.9	258.7	-0.49	-1.40	PPP-PPP	0.20	10	0.678	0.723	0.0976
236329_at	159.9	114.3	-0.49	-1.40	PPP-PPP	0.20	10	0.678	0.723	0.1401
221681_s_at	78.4	56.1	-0.49	-1.40	PAP-PAP	0.20	10	0.678	0.723	0.1977
202488_s_at	242.6	173.5	-0.48	-1.40	PPP-PPP	0.20	10	0.678	0.723	0.0527
235031_at	101.6	72.6	-0.48	-1.40	PPP-AAP	0.20	10	0.678	0.723	0.1832
209130_at	2809.8	3928.5	0.48	1.40	PPP-PPP	0.20	10	0.678	0.723	0.0009
224563_at	201.9	144.4	-0.48	-1.40	APA-APP	0.20	10	0.678	0.723	0.0407
219337_at	265.8	190.2	-0.48	-1.40	PPP-APP	0.20	10	0.678	0.723	0.0809
223740_at	95.9	68.6	-0.48	-1.40	AAA-PPP	0.20	10	0.678	0.723	0.0842
243909_x_at	304.2	217.6	-0.48	-1.40	PPP-PPP	0.20	10	0.678	0.723	0.1724
242932_at	159.8	114.4	-0.48	-1.40	PPP-PPP	0.20	10	0.678	0.723	0.0728
230235_at	144.8	103.7	-0.48	-1.40	APA-APP	0.20	10	0.678	0.723	0.1917
215237_at	113.4	81.2	-0.48	-1.40	PAA-AAA	0.20	10	0.678	0.723	0.1120
204665_at	60.8	43.6	-0.48	-1.40	MAA-AAP	0.20	10	0.678	0.723	0.1672
209440_at	7049.7	5056.1	-0.48	-1.39	PPP-PPP	0.20	10	0.678	0.723	0.0491
1565265_at	135.6	97.2	-0.48	-1.40	APP-AAP	0.20	10	0.678	0.723	0.0814
2028_s_at	550.8	395.1	-0.48	-1.39	PPP-PPP	0.20	10	0.678	0.723	0.0645
239930_at	99.2	71.2	-0.48	-1.39	AAA-PAA	0.20	10	0.678	0.723	0.0941
243895_x_at	92.9	66.7	-0.48	-1.39	APP-PAA	0.20	10	0.678	0.723	0.0971
225616_at	496.6	356.4	-0.48	-1.39	PPP-PPP	0.20	10	0.678	0.723	0.1106
222571_at	289.1	207.7	-0.48	-1.39	PAP-PPM	0.20	10	0.678	0.723	0.1354
227106_at	162.8	116.9	-0.48	-1.39	AAP-PAA	0.20	10	0.678	0.723	0.1807
243376_at	68.8	49.4	-0.48	-1.39	PAA-AAA	0.20	10	0.678	0.723	0.1835
233775_x_at	710.5	510.5	-0.48	-1.39	PPP-PPP	0.20	10	0.678	0.723	0.0622
215249_at	44.7	32.1	-0.48	-1.39	AAA-AAP	0.20	10	0.678	0.723	0.0787
1557582_at	219.9	158.0	-0.48	-1.39	PAA-AAA	0.20	10	0.678	0.723	0.0804
223123_s_at	81.7	58.7	-0.48	-1.39	PAP-APP	0.20	10	0.678	0.723	0.1071
208241_at	131.5	94.5	-0.48	-1.39	PPP-PPP	0.20	10	0.678	0.723	0.1795
235478_at	128.9	92.7	-0.48	-1.39	PPP-PPP	0.20	10	0.678	0.723	0.0639
239866_at	117.3	84.3	-0.48	-1.39	MAA-PPP	0.20	10	0.678	0.723	0.0970
240190_at	100.2	72.1	-0.48	-1.39	AAP-APP	0.20	10	0.678	0.723	0.1224
242152_at	39.1	28.1	-0.48	-1.39	AAP-AAA	0.20	10	0.678	0.723	0.1415
1556081_at	57.2	41.1	-0.48	-1.39	PAP-APP	0.20	10	0.678	0.723	0.1775
232516_x_at	135.1	97.3	-0.47	-1.39	PPP-PPP	0.20	10	0.678	0.723	0.0555
212701_at	163.3	117.5	-0.47	-1.39	AAA-AAP	0.20	10	0.678	0.723	0.1030
236749_at	78.6	56.6	-0.47	-1.39	PPP-AAA	0.20	10	0.678	0.723	0.1591
210152_at	64.2	46.2	-0.47	-1.39	AMP-PMP	0.20	10	0.678	0.723	0.1695
1563014_at	91.9	66.2	-0.47	-1.39	PPP-PMP	0.20	10	0.678	0.723	0.1917
218653_at	719.2	518.2	-0.47	-1.39	PPP-PPP	0.20	10	0.678	0.723	0.0804

203749_s_at	465.5	335.5	-0.47	-1.39	PPP-PPP	0.20	10	0.678	0.723	0.0995
1557064_s_at	128.3	92.5	-0.47	-1.39	PPA-PPP	0.20	10	0.678	0.723	0.0810
239035_at	113.1	81.5	-0.47	-1.39	AAP-PPA	0.20	10	0.678	0.723	0.0949
225533_at	1050.6	757.6	-0.47	-1.39	PPP-PPP	0.20	10	0.678	0.723	0.1301
237365_at	77.1	55.6	-0.47	-1.39	PPP-AAM	0.20	10	0.678	0.723	0.1483
241202_at	129.2	93.1	-0.47	-1.39	MPP-PPP	0.20	10	0.678	0.723	0.1490
220707_s_at	315.9	228.0	-0.47	-1.39	AAA-APA	0.20	10	0.678	0.723	0.0473
228333_at	346.7	250.2	-0.47	-1.39	PPP-PPP	0.20	10	0.678	0.723	0.0601
1564069_at	291.2	210.0	-0.47	-1.39	PPP-PPP	0.20	10	0.678	0.723	0.1122
209378_s_at	671.0	484.6	-0.47	-1.39	PPP-PPP	0.20	10	0.678	0.723	0.0514
205201_at	670.1	483.9	-0.47	-1.39	PPP-PPP	0.20	10	0.678	0.723	0.0542
217508_s_at	300.9	217.2	-0.47	-1.39	PPP-PPP	0.20	10	0.678	0.723	0.1148
206648_at	81.7	59.0	-0.47	-1.39	APP-APP	0.20	10	0.678	0.723	0.1156
221307_at	55.5	40.1	-0.47	-1.39	AAA-AAP	0.20	10	0.678	0.723	0.1230
219576_at	548.4	396.0	-0.47	-1.39	PPP-PPP	0.20	10	0.678	0.723	0.1446
242847_at	195.1	140.9	-0.47	-1.39	PPP-PPP	0.20	10	0.678	0.723	0.1603
1569139_s_at	127.8	92.3	-0.47	-1.38	PPP-PPP	0.20	10	0.678	0.723	0.0780
213250_at	166.0	120.0	-0.47	-1.38	AAA-PAA	0.20	10	0.678	0.723	0.1103
228200_at	266.6	192.7	-0.47	-1.38	PPP-PPP	0.20	10	0.678	0.723	0.1489
232331_at	303.0	219.0	-0.47	-1.38	PPP-PPP	0.20	10	0.678	0.723	0.1590
205822_s_at	328.7	237.6	-0.47	-1.38	PPP-PPP	0.20	10	0.678	0.723	0.1656
216806_at	653.8	472.8	-0.47	-1.38	MPP-PPP	0.20	10	0.678	0.723	0.0466
235530_at	228.6	165.4	-0.47	-1.38	PAP-APP	0.20	10	0.678	0.723	0.1226
218265_at	434.4	314.2	-0.47	-1.38	PPP-PPP	0.20	10	0.678	0.723	0.1762
234156_at	119.8	86.7	-0.47	-1.38	PPP-APP	0.20	10	0.678	0.723	0.0460
231101_at	502.8	364.0	-0.47	-1.38	PPP-PPP	0.20	10	0.678	0.723	0.0697
225756_at	742.7	537.8	-0.47	-1.38	PPP-PPP	0.20	10	0.678	0.723	0.0741
1553192_at	67.7	49.0	-0.47	-1.38	APA-APP	0.20	10	0.678	0.723	0.1117
1555565_s_at	254.2	184.0	-0.47	-1.38	PPP-APP	0.20	10	0.678	0.723	0.1173
231629_x_at	284.3	205.9	-0.47	-1.38	AAP-PPA	0.20	10	0.678	0.723	0.1577
204663_at	139.2	100.8	-0.47	-1.38	AAP-PPP	0.20	10	0.678	0.723	0.1685
221874_at	58.6	42.4	-0.47	-1.38	APA-APA	0.20	10	0.678	0.723	0.1891
243236_at	69.4	50.3	-0.47	-1.38	PPA-PPP	0.20	10	0.678	0.723	0.0427
205794_s_at	139.6	101.2	-0.47	-1.38	PMA-PMP	0.20	10	0.678	0.723	0.0972
222302_at	613.4	444.3	-0.47	-1.38	PPP-PPP	0.20	10	0.678	0.723	0.1360
201838_s_at	150.0	108.6	-0.47	-1.38	PMP-PMP	0.20	10	0.678	0.723	0.1753
209524_at	2444.7	3374.8	0.47	1.38	PPP-PPP	0.20	10	0.678	0.723	0.0004
239948_at	101.4	73.5	-0.46	-1.38	PPP-PPP	0.20	10	0.678	0.723	0.1434
215191_at	90.1	65.3	-0.46	-1.38	PPP-APP	0.20	10	0.678	0.723	0.1551
228944_at	218.1	158.2	-0.46	-1.38	PPP-PPP	0.20	10	0.678	0.723	0.0657
209426_s_at	165.2	119.9	-0.46	-1.38	PPP-PPP	0.20	10	0.678	0.723	0.1012
1570571_at	115.8	84.0	-0.46	-1.38	PPP-APP	0.20	10	0.678	0.723	0.1132

206983_at	35.9	26.1	-0.46	-1.38	AAA-APA	0.20	10	0.678	0.723	0.1770
238166_s_at	148.7	108.0	-0.46	-1.38	PPP-PPP	0.20	10	0.678	0.723	0.1414
232436_at	196.4	142.7	-0.46	-1.38	PPP-PPP	0.20	10	0.678	0.723	0.0803
214298_x_at	147.4	107.0	-0.46	-1.38	PPA-PPP	0.20	10	0.678	0.723	0.1277
230424_at	692.3	502.9	-0.46	-1.38	PPP-PPP	0.20	10	0.678	0.723	0.1883
204916_at	337.5	245.3	-0.46	-1.38	PPP-PPP	0.20	10	0.678	0.723	0.0694
214277_at	140.7	102.3	-0.46	-1.38	PPP-PPP	0.20	10	0.678	0.723	0.0938
235679_at	211.8	154.0	-0.46	-1.38	PPP-PPP	0.20	10	0.678	0.723	0.0952
220154_at	195.5	142.2	-0.46	-1.38	PPP-PPP	0.20	10	0.678	0.723	0.1207
206912_at	65.2	47.4	-0.46	-1.38	APP-AAP	0.20	10	0.678	0.723	0.0515
1555515_a_at	112.7	82.0	-0.46	-1.37	AAA-AAP	0.20	10	0.678	0.723	0.1570
1555205_at	73.1	53.1	-0.46	-1.38	PAA-AAA	0.20	10	0.678	0.723	0.1629
225478_at	1067.1	776.7	-0.46	-1.37	PPP-PPP	0.20	10	0.678	0.723	0.0366
203920_at	149.5	108.8	-0.46	-1.37	PPP-PPP	0.20	10	0.678	0.723	0.0854
205020_s_at	1757.5	1279.3	-0.46	-1.37	PPP-PPP	0.20	10	0.678	0.723	0.1341
203125_x_at	490.8	357.3	-0.46	-1.37	PPP-PPP	0.20	10	0.678	0.723	0.1365
225791_at	505.8	368.4	-0.46	-1.37	APP-PAP	0.20	10	0.678	0.723	0.1491
1555238_at	216.4	157.7	-0.46	-1.37	PAA-AAA	0.20	10	0.678	0.723	0.1694
1563364_at	70.0	51.1	-0.46	-1.37	PPP-PPP	0.20	10	0.678	0.723	0.1364
227148_at	88.2	64.3	-0.46	-1.37	PAP-PPP	0.20	10	0.678	0.723	0.1402
209700_x_at	256.2	186.8	-0.46	-1.37	PPP-PPP	0.20	10	0.678	0.723	0.1592
226382_at	124.6	90.9	-0.46	-1.37	PPP-PPP	0.20	10	0.678	0.723	0.0853
238516_at	163.3	119.1	-0.46	-1.37	PPP-PPP	0.20	10	0.678	0.723	0.1442
1562733_at	241.6	176.2	-0.46	-1.37	PPP-PPP	0.20	10	0.678	0.723	0.1629
1568600_at	79.5	58.0	-0.45	-1.37	PPP-PPP	0.20	10	0.678	0.723	0.1149
205986_at	115.4	84.3	-0.45	-1.37	PAA-AAA	0.20	10	0.678	0.723	0.1550
213670_x_at	533.9	389.9	-0.45	-1.37	PPP-PPP	0.20	10	0.678	0.723	0.0359
208238_x_at	790.7	577.6	-0.45	-1.37	PPP-PPP	0.20	10	0.678	0.723	0.0718
224548_at	117.2	85.6	-0.45	-1.37	AMA-APA	0.20	10	0.678	0.723	0.0373
212992_at	368.2	269.3	-0.45	-1.37	APP-MPM	0.20	10	0.678	0.723	0.0433
224210_s_at	82.8	60.5	-0.45	-1.37	APP-AAP	0.20	10	0.678	0.723	0.0777
205642_at	163.6	119.6	-0.45	-1.37	PAP-PPP	0.20	10	0.678	0.723	0.1222
210680_s_at	76.9	56.3	-0.45	-1.37	AMA-PAA	0.20	10	0.678	0.723	0.1621
213431_x_at	227.1	166.3	-0.45	-1.37	AMA-AAP	0.20	10	0.678	0.723	0.0423
221222_s_at	100.4	73.5	-0.45	-1.37	APP-PPP	0.20	10	0.678	0.723	0.1001
202091_at	79.2	57.9	-0.45	-1.37	PAP-PMP	0.20	10	0.678	0.723	0.1574
1555606_a_at	69.5	51.0	-0.45	-1.37	PPP-PPP	0.20	10	0.678	0.723	0.0529
1554245_x_at	470.0	344.3	-0.45	-1.37	AAA-AAP	0.20	10	0.678	0.723	0.1543
228429_x_at	522.3	383.0	-0.45	-1.36	MAP-AAA	0.20	10	0.678	0.723	0.0801
1570249_x_at	207.3	152.0	-0.45	-1.37	PPP-PPP	0.20	10	0.678	0.723	0.0857
228498_at	3148.2	2309.4	-0.45	-1.36	PPP-PPP	0.20	10	0.678	0.723	0.0602
243370_at	149.4	109.6	-0.45	-1.36	PPP-PPP	0.20	10	0.678	0.723	0.0704

242143_at	136.0	99.8	-0.45	-1.36	PAP-PPP	0.20	10	0.678	0.723	0.1539
243484_x_at	173.0	126.9	-0.45	-1.36	MAP-PPP	0.20	10	0.678	0.723	0.1587
1554474_a_at	768.7	564.3	-0.45	-1.36	PPP-PPP	0.20	10	0.678	0.723	0.1030
231448_at	87.4	64.2	-0.45	-1.36	MAA-APA	0.20	10	0.678	0.723	0.1086
234464_s_at	1138.2	835.8	-0.45	-1.36	PPP-PPP	0.20	10	0.678	0.723	0.1884
213344_s_at	214.0	157.2	-0.45	-1.36	PPP-PPP	0.20	10	0.678	0.723	0.0945
238888_at	108.5	79.7	-0.45	-1.36	PAP-PPP	0.20	10	0.678	0.723	0.0993
230780_at	87.3	64.2	-0.44	-1.36	PPP-PAA	0.20	10	0.678	0.723	0.0454
1555350_at	126.2	92.8	-0.44	-1.36	PPP-PPP	0.20	10	0.678	0.723	0.0498
211487_x_at	40388.5	29698.9	-0.44	-1.36	PPP-PPP	0.20	10	0.678	0.723	0.0535
206943_at	142.2	104.6	-0.44	-1.36	PPA-PPP	0.20	10	0.678	0.723	0.1432
238130_at	175.6	129.2	-0.44	-1.36	APP-APA	0.20	10	0.678	0.723	0.1835
242969_at	80.2	59.0	-0.44	-1.36	MPP-PAP	0.20	10	0.678	0.723	0.1765
232180_at	89.2	65.7	-0.44	-1.36	APP-MPP	0.20	10	0.678	0.723	0.1773
215833_s_at	170.9	125.8	-0.44	-1.36	AAP-AAA	0.20	10	0.678	0.723	0.1834
1560476_at	155.4	114.5	-0.44	-1.36	AAP-MMM	0.20	10	0.678	0.723	0.0678
225254_at	222.9	164.3	-0.44	-1.36	AAA-APP	0.20	10	0.678	0.723	0.0891
243452_at	66.5	49.0	-0.44	-1.36	AAA-APA	0.20	10	0.678	0.723	0.1109
1556255_a_at	67.1	49.4	-0.44	-1.36	AAA-PAM	0.20	10	0.678	0.723	0.1340
226929_at	190.3	140.2	-0.44	-1.36	PPP-APP	0.20	10	0.678	0.723	0.1572
1558652_at	90.9	67.0	-0.44	-1.36	APP-APP	0.20	10	0.678	0.723	0.1649
215537_x_at	642.8	473.9	-0.44	-1.36	PPP-PPP	0.20	10	0.678	0.723	0.0979
1560817_at	96.0	70.7	-0.44	-1.36	AMA-PPP	0.20	10	0.678	0.723	0.1312
237419_at	128.9	95.0	-0.44	-1.36	PPP-PPP	0.20	10	0.678	0.723	0.1792
242195_x_at	1887.9	1392.1	-0.44	-1.36	PPP-PPP	0.20	10	0.678	0.723	0.0907
208149_x_at	978.6	722.0	-0.44	-1.36	PPP-PPP	0.20	10	0.678	0.723	0.1804
217347_at	538.5	397.5	-0.44	-1.36	PPP-PPP	0.20	10	0.678	0.723	0.0344
1567079_at	135.4	99.9	-0.44	-1.36	PPP-APA	0.20	10	0.678	0.723	0.0954
241840_at	129.1	95.3	-0.44	-1.35	AAA-AAP	0.20	10	0.678	0.723	0.1059
1567527_at	79.0	58.3	-0.44	-1.35	AAA-PPP	0.20	10	0.678	0.723	0.1809
1556105_at	96.6	71.3	-0.44	-1.35	PPP-PPP	0.20	10	0.678	0.723	0.1809
215994_x_at	1538.0	1136.3	-0.44	-1.35	PPP-PPP	0.20	10	0.678	0.723	0.0457
220050_at	136.7	101.0	-0.44	-1.35	PPP-PPP	0.20	10	0.678	0.723	0.0948
218681_s_at	2005.4	1481.1	-0.44	-1.35	PPP-PPP	0.20	10	0.678	0.723	0.0995
207159_x_at	222.1	164.1	-0.44	-1.35	PPP-PPP	0.20	10	0.678	0.723	0.1099
226728_at	257.9	190.5	-0.44	-1.35	PPP-MPP	0.20	10	0.678	0.723	0.1479
1569604_at	141.4	104.4	-0.44	-1.35	PPP-APP	0.20	10	0.678	0.723	0.1757
230759_at	191.3	141.4	-0.44	-1.35	PPP-PPP	0.20	10	0.678	0.723	0.0336
241986_at	235.0	173.7	-0.44	-1.35	PPP-PPP	0.20	10	0.678	0.723	0.1033
233061_at	210.1	155.3	-0.44	-1.35	PPP-PPP	0.20	10	0.678	0.723	0.1096
224818_at	1130.8	836.1	-0.44	-1.35	PPP-PPP	0.20	10	0.678	0.723	0.1108
212912_at	243.4	180.0	-0.44	-1.35	PPP-PPP	0.20	10	0.678	0.723	0.1584

203412_at	621.7	459.7	-0.44	-1.35	PPP-PPP	0.20	10	0.678	0.723	0.0404
209357_at	6998.1	5175.7	-0.44	-1.35	PPP-PPP	0.20	10	0.678	0.723	0.1094
229852_at	224.8	166.4	-0.43	-1.35	APP-PPP	0.20	10	0.678	0.723	0.0771
202350_s_at	3107.6	2299.9	-0.43	-1.35	PPP-PPP	0.20	10	0.678	0.723	0.1121
236545_at	236.1	174.7	-0.43	-1.35	PPP-PPP	0.20	10	0.678	0.723	0.1127
238532_at	318.5	235.8	-0.43	-1.35	PPP-PPP	0.20	10	0.678	0.723	0.1570
238331_at	148.2	109.7	-0.43	-1.35	AAA-APA	0.20	10	0.678	0.723	0.0425
1554536_at	94.7	70.2	-0.43	-1.35	APP-AAP	0.20	10	0.678	0.723	0.1222
239404_at	140.4	104.1	-0.43	-1.35	PPP-PPP	0.20	10	0.678	0.723	0.1247
206662_at	5934.6	4402.3	-0.43	-1.35	PPP-PPP	0.20	10	0.678	0.723	0.0498
211720_x_at	46732.2	34658.4	-0.43	-1.35	PPP-PPP	0.20	10	0.678	0.723	0.0574
222130_s_at	1266.1	939.3	-0.43	-1.35	PPP-PPP	0.20	10	0.678	0.723	0.0919
221797_at	311.5	231.0	-0.43	-1.35	PMP-PPP	0.20	10	0.678	0.723	0.1470
229351_at	293.0	217.5	-0.43	-1.35	PPP-PPP	0.20	10	0.678	0.723	0.0377
235635_at	140.4	104.3	-0.43	-1.35	PPP-PPP	0.20	10	0.678	0.723	0.0774
209984_at	275.6	204.6	-0.43	-1.35	PPP-PAP	0.20	10	0.678	0.723	0.0961
218750_at	964.9	716.1	-0.43	-1.35	PPP-PPP	0.20	10	0.678	0.723	0.1244
210743_s_at	64.4	47.8	-0.43	-1.35	AAP-APA	0.20	10	0.678	0.723	0.0944
241520_x_at	176.8	131.4	-0.43	-1.35	PMM-AMA	0.20	10	0.678	0.723	0.1131
209738_x_at	397.5	295.2	-0.43	-1.35	PPP-PPP	0.20	10	0.678	0.723	0.1337
228257_at	818.8	608.4	-0.43	-1.35	PPP-PPP	0.20	10	0.678	0.723	0.1355
219189_at	283.5	210.6	-0.43	-1.35	PPP-PPP	0.20	10	0.678	0.723	0.1581
226727_at	1136.9	845.2	-0.43	-1.35	PPP-PPP	0.20	10	0.678	0.723	0.0497
228664_at	422.2	313.8	-0.43	-1.35	PPP-PPP	0.20	10	0.678	0.723	0.1820
227770_at	442.4	329.1	-0.43	-1.34	PPP-PPP	0.20	10	0.678	0.723	0.0878
1566956_at	603.3	448.6	-0.43	-1.35	APP-MAP	0.20	10	0.678	0.723	0.1412
244544_at	129.8	96.5	-0.43	-1.35	PPA-MAP	0.20	10	0.678	0.723	0.1722
227446_s_at	240.4	178.8	-0.43	-1.35	PPP-PPP	0.20	10	0.678	0.723	0.1723
232781_at	78.0	58.0	-0.43	-1.34	PPA-MAP	0.20	10	0.678	0.723	0.1803
208856_x_at	44692.1	33257.1	-0.43	-1.34	PPP-PPP	0.20	10	0.678	0.723	0.0816
208834_x_at	60295.3	44912.9	-0.43	-1.34	PPP-PPP	0.20	10	0.678	0.723	0.0456
1568594_s_at	193.7	144.3	-0.43	-1.34	PPP-PPP	0.20	10	0.678	0.723	0.0996
201329_s_at	838.8	624.6	-0.43	-1.34	PPP-PPP	0.20	10	0.678	0.723	0.1208
233319_x_at	278.4	207.4	-0.43	-1.34	PPP-PPP	0.20	10	0.678	0.723	0.1518
202720_at	2658.7	1980.5	-0.43	-1.34	PPP-PPP	0.20	10	0.678	0.723	0.1650
242225_at	168.5	125.5	-0.42	-1.34	PPP-PPP	0.20	10	0.678	0.723	0.0977
224808_s_at	692.8	516.4	-0.42	-1.34	PPP-PPP	0.20	10	0.678	0.723	0.0996
1562294_x_at	60.2	44.8	-0.42	-1.34	PAP-PPP	0.20	10	0.678	0.723	0.1190
239730_at	179.6	134.0	-0.42	-1.34	PPP-MPP	0.20	10	0.678	0.723	0.0519
201136_at	10402.0	7759.4	-0.42	-1.34	PPP-PPP	0.20	10	0.678	0.723	0.0594
227233_at	51.1	38.1	-0.42	-1.34	AAM-PAM	0.20	10	0.678	0.723	0.0845
240217_s_at	101.1	75.4	-0.42	-1.34	PPP-APP	0.20	10	0.678	0.723	0.1415

1566144_at	210.5	157.2	-0.42	-1.34	PPP-PPP	0.20	10	0.678	0.723	0.0744
242569_at	139.4	104.0	-0.42	-1.34	PMP-PPP	0.20	10	0.678	0.723	0.1448
230376_at	200.1	149.4	-0.42	-1.34	PPP-PPP	0.20	10	0.678	0.723	0.1458
238034_at	824.7	616.1	-0.42	-1.34	PPP-PPP	0.20	10	0.678	0.723	0.1824
225840_at	233.7	174.6	-0.42	-1.34	PPP-PPP	0.20	10	0.678	0.723	0.0602
244320_at	84.7	63.4	-0.42	-1.34	PMP-APP	0.20	10	0.678	0.723	0.1815
239586_at	149.8	112.1	-0.42	-1.34	APM-APA	0.20	10	0.678	0.723	0.0926
235765_at	434.7	325.2	-0.42	-1.34	PPP-PPP	0.20	10	0.678	0.723	0.1775
206343_s_at	2052.8	1536.4	-0.42	-1.34	PPP-PPP	0.20	10	0.678	0.723	0.1533
40446_at	2005.9	1501.4	-0.42	-1.34	PPP-PPP	0.20	10	0.678	0.723	0.1700
1557091_at	242.4	181.5	-0.42	-1.34	PPP-PPP	0.20	10	0.678	0.723	0.0498
225548_at	468.7	351.1	-0.42	-1.34	PMP-PPP	0.20	10	0.678	0.723	0.0582
1559517_a_at	341.0	255.4	-0.42	-1.34	PPP-PPP	0.20	10	0.678	0.723	0.1317
238417_at	273.4	204.8	-0.42	-1.34	PPP-PPP	0.20	10	0.678	0.723	0.1788
203107_x_at	48707.0	36513.1	-0.42	-1.33	PPP-PPP	0.20	10	0.678	0.723	0.0349
227372_s_at	289.3	216.8	-0.42	-1.33	PPP-PPP	0.20	10	0.678	0.723	0.0762
210139_s_at	3339.1	2502.3	-0.42	-1.33	PPP-PPP	0.20	10	0.678	0.723	0.0912
222060_at	188.0	141.0	-0.42	-1.33	PAA-AMP	0.20	10	0.678	0.723	0.0941
212736_at	696.9	522.2	-0.42	-1.34	PPP-PPP	0.20	10	0.678	0.723	0.1188
207624_s_at	212.6	159.3	-0.42	-1.34	PPP-PPP	0.20	10	0.678	0.723	0.1584
209276_s_at	1745.1	1308.8	-0.42	-1.33	PPP-PPP	0.20	10	0.678	0.723	0.1065
1554659_at	120.5	90.4	-0.42	-1.33	AAM-APA	0.20	10	0.678	0.723	0.1419
206063_x_at	286.2	214.7	-0.41	-1.33	PPP-APP	0.20	10	0.678	0.723	0.0821
33132_at	1138.3	854.4	-0.41	-1.33	PPP-PPP	0.20	10	0.678	0.723	0.0840
216531_at	149.9	112.5	-0.41	-1.33	PPP-PPP	0.20	10	0.678	0.723	0.1675
214753_at	518.0	389.0	-0.41	-1.33	APP-PAP	0.20	10	0.678	0.723	0.0619
235190_at	1213.5	911.2	-0.41	-1.33	PPP-PPP	0.20	10	0.678	0.723	0.0862
231022_at	180.0	135.2	-0.41	-1.33	PAA-APM	0.20	10	0.678	0.723	0.1719
220613_s_at	87.8	66.0	-0.41	-1.33	AAP-MMP	0.20	10	0.678	0.723	0.0299
208016_s_at	134.4	101.1	-0.41	-1.33	PPP-PPP	0.20	10	0.678	0.723	0.1713
222753_s_at	2712.6	2039.8	-0.41	-1.33	PPP-PPP	0.20	10	0.678	0.723	0.0636
222903_s_at	84.8	63.8	-0.41	-1.33	PPP-PAP	0.20	10	0.678	0.723	0.1715
1552423_at	127.8	96.2	-0.41	-1.33	PPP-PMP	0.20	10	0.678	0.723	0.0429
242103_at	100.0	75.3	-0.41	-1.33	APP-AAA	0.20	10	0.678	0.723	0.0630
218901_at	54.9	41.3	-0.41	-1.33	APP-PAP	0.20	10	0.678	0.723	0.0718
227334_at	614.1	462.4	-0.41	-1.33	PPP-PPP	0.20	10	0.678	0.723	0.0526
1557611_at	157.0	118.2	-0.41	-1.33	PAA-APA	0.20	10	0.678	0.723	0.1223
235803_at	198.5	149.6	-0.41	-1.33	PPP-PPP	0.20	10	0.678	0.723	0.1478
223569_at	297.9	224.3	-0.41	-1.33	PPP-PPP	0.20	10	0.678	0.723	0.1651
1552836_at	178.6	134.6	-0.41	-1.33	PPP-PPA	0.20	10	0.678	0.723	0.0377
229193_at	138.2	104.2	-0.41	-1.33	PPP-PPP	0.20	10	0.678	0.723	0.0404
217398_x_at	45091.6	33984.7	-0.41	-1.33	PPP-PPP	0.20	10	0.678	0.723	0.0606

235268_at	38.2	28.8	-0.41	-1.33	AAA-APA	0.20	10	0.678	0.723	0.0776
201033_x_at	44144.6	33280.9	-0.41	-1.33	PPP-PPP	0.20	10	0.678	0.723	0.0910
201426_s_at	47887.1	36082.9	-0.41	-1.33	PPP-PPP	0.20	10	0.678	0.723	0.0937
216230_x_at	229.0	172.6	-0.41	-1.33	PPP-PPP	0.20	10	0.678	0.723	0.1199
231136_at	102.4	77.2	-0.41	-1.33	MPA-PPP	0.20	10	0.678	0.723	0.1218
1559876_at	97.7	73.6	-0.41	-1.33	MAA-AAP	0.20	10	0.678	0.723	0.1622
238303_at	72.1	54.3	-0.41	-1.33	PPP-APP	0.20	10	0.678	0.723	0.1639
205896_at	652.8	492.4	-0.41	-1.33	PPP-PPP	0.20	10	0.678	0.723	0.1238
219138_at	190.4	143.6	-0.41	-1.33	AAA-APP	0.20	10	0.678	0.723	0.1310
217967_s_at	2560.2	1931.5	-0.41	-1.33	PPP-PPP	0.20	10	0.678	0.723	0.1310
226355_at	977.4	737.6	-0.41	-1.33	PPP-PPP	0.20	10	0.678	0.723	0.0296
201310_s_at	10468.7	7899.0	-0.41	-1.33	PPP-PPP	0.20	10	0.678	0.723	0.1495
205667_at	789.4	595.8	-0.41	-1.33	PPP-PPP	0.20	10	0.678	0.723	0.1756
632_at	638.9	482.6	-0.41	-1.32	PPP-PPP	0.20	10	0.678	0.723	0.0915
202812_at	322.1	243.3	-0.41	-1.32	PAP-PPP	0.20	10	0.678	0.723	0.0924
203180_at	17976.8	13586.2	-0.40	-1.32	PPP-PPP	0.20	10	0.678	0.723	0.0393
211250_s_at	126.3	95.5	-0.40	-1.32	APP-APP	0.20	10	0.678	0.723	0.0480
210809_s_at	39757.3	30052.2	-0.40	-1.32	PPP-PPP	0.20	10	0.678	0.723	0.0489
201665_x_at	42701.1	32264.8	-0.40	-1.32	PPP-PPP	0.20	10	0.678	0.723	0.0714
217168_s_at	5573.4	4214.1	-0.40	-1.32	PPP-PPP	0.20	10	0.678	0.723	0.0526
216407_at	177.2	134.0	-0.40	-1.32	APP-PAP	0.20	10	0.678	0.723	0.0539
223053_x_at	2997.5	2267.7	-0.40	-1.32	PPP-PPP	0.20	10	0.678	0.723	0.0544
200021_at	39588.4	29949.6	-0.40	-1.32	PPP-PPP	0.20	10	0.678	0.723	0.0659
226889_at	556.3	420.8	-0.40	-1.32	PPP-PPP	0.20	10	0.678	0.723	0.1694
214785_at	511.8	387.2	-0.40	-1.32	PPP-PPP	0.20	10	0.678	0.723	0.1707
201481_s_at	1821.0	1378.4	-0.40	-1.32	PPP-PPP	0.20	10	0.678	0.723	0.0349
48106_at	279.4	211.4	-0.40	-1.32	APP-PPP	0.20	10	0.678	0.723	0.0498
223031_s_at	862.6	652.8	-0.40	-1.32	PPP-PPP	0.20	10	0.678	0.723	0.0765
206908_s_at	1009.1	763.5	-0.40	-1.32	PPP-PPP	0.20	10	0.678	0.723	0.1214
236344_at	651.8	493.4	-0.40	-1.32	PPP-PPP	0.20	10	0.678	0.723	0.1398
218278_at	2278.3	1727.0	-0.40	-1.32	PPP-PPP	0.20	10	0.678	0.723	0.0516
237878_at	217.3	164.8	-0.40	-1.32	MAP-APP	0.20	10	0.678	0.723	0.1124
207917_at	243.5	184.6	-0.40	-1.32	PMP-PPP	0.20	10	0.678	0.723	0.1161
1558094_s_at	232.3	176.1	-0.40	-1.32	PPP-AMA	0.20	10	0.678	0.723	0.1210
240111_at	165.0	125.0	-0.40	-1.32	PPP-PPP	0.20	10	0.678	0.723	0.1343
222810_s_at	1152.7	874.3	-0.40	-1.32	PPP-PPP	0.20	10	0.678	0.723	0.0597
202079_s_at	275.8	209.2	-0.40	-1.32	PPP-PPP	0.20	10	0.678	0.723	0.1247
207536_s_at	134.1	101.7	-0.40	-1.32	PPP-PPP	0.20	10	0.678	0.723	0.1355
218262_at	462.0	350.6	-0.40	-1.32	PPP-PPP	0.20	10	0.678	0.723	0.0488
1552641_s_at	1065.0	808.0	-0.40	-1.32	PPP-PPP	0.20	10	0.678	0.723	0.0872
239342_at	158.1	119.9	-0.40	-1.32	PPP-PPP	0.20	10	0.678	0.723	0.1176
1558330_x_at	313.2	237.6	-0.40	-1.32	AAP-PAA	0.20	10	0.678	0.723	0.1688

215156_at	178.4	135.4	-0.40	-1.32	PMA-PAP	0.20	10	0.678	0.723	0.1450
244502_at	83.7	63.6	-0.40	-1.32	PPA-APA	0.20	10	0.678	0.723	0.1472
212699_at	174.6	132.7	-0.40	-1.32	APP-PPP	0.20	10	0.678	0.723	0.0609
240554_at	123.0	93.5	-0.40	-1.32	AMM-PAP	0.20	10	0.678	0.723	0.0831
205872_x_at	1443.8	1097.3	-0.40	-1.32	PPP-PPP	0.20	10	0.678	0.723	0.0874
239978_at	209.2	159.0	-0.40	-1.32	PPP-PPP	0.20	10	0.678	0.723	0.1380
43511_s_at	111.6	84.8	-0.40	-1.32	PMP-MAP	0.20	10	0.678	0.723	0.1431
222786_at	482.3	366.6	-0.40	-1.32	AMA-MAP	0.20	10	0.678	0.723	0.1577
224002_s_at	778.7	592.1	-0.40	-1.32	PPP-PPP	0.20	10	0.678	0.723	0.0271
205248_at	540.9	411.2	-0.40	-1.32	PPP-PPP	0.20	10	0.678	0.723	0.0392
1553570_x_at	55190.8	41985.1	-0.40	-1.32	PPP-PPP	0.20	10	0.678	0.723	0.0551
221798_x_at	53520.4	40709.0	-0.40	-1.32	PPP-PPP	0.20	10	0.678	0.723	0.0725
215223_s_at	1486.3	1130.7	-0.40	-1.32	PPP-PPP	0.20	10	0.678	0.723	0.0757
215718_s_at	404.9	307.9	-0.40	-1.32	PPP-PPP	0.20	10	0.678	0.723	0.0881
221895_at	522.3	397.3	-0.40	-1.32	PPP-PPP	0.20	10	0.678	0.723	0.0910
241722_x_at	248.9	189.4	-0.40	-1.32	PPP-PPP	0.20	10	0.678	0.723	0.0911
1554428_s_at	301.9	229.5	-0.40	-1.32	MAA-PAA	0.20	10	0.678	0.723	0.1293
223109_at	1278.3	973.0	-0.39	-1.31	PPP-PPP	0.20	10	0.678	0.723	0.0357
226467_at	586.3	446.1	-0.39	-1.31	PPP-APP	0.20	10	0.678	0.723	0.1181
214892_x_at	423.1	322.1	-0.39	-1.31	APA-PAA	0.20	10	0.678	0.723	0.1225
205013_s_at	227.1	172.9	-0.39	-1.31	PPP-PPP	0.20	10	0.678	0.723	0.1266
AFFX-hum_alu_at	57785.8	44002.8	-0.39	-1.31	PPP-PPP	0.20	10	0.678	0.723	0.0452
214922_at	105.3	80.2	-0.39	-1.31	AAA-PAP	0.20	10	0.678	0.723	0.0496
221867_at	188.1	143.4	-0.39	-1.31	PAP-AAA	0.20	10	0.678	0.723	0.0398
200781_s_at	40514.2	30883.8	-0.39	-1.31	PPP-PPP	0.20	10	0.678	0.723	0.0407
222166_at	53.5	40.8	-0.39	-1.31	APA-AMA	0.20	10	0.678	0.723	0.0789
213867_x_at	50305.5	38337.4	-0.39	-1.31	PPP-PPP	0.20	10	0.678	0.723	0.0799
228312_at	92.7	70.6	-0.39	-1.31	MAA-PAA	0.20	10	0.678	0.723	0.1442
211123_at	137.6	105.0	-0.39	-1.31	PPA-APA	0.20	10	0.678	0.723	0.0470
1554213_at	158.6	121.0	-0.39	-1.31	PPP-PPP	0.20	10	0.678	0.723	0.0761
212788_x_at	47339.3	36094.2	-0.39	-1.31	PPP-PPP	0.20	10	0.678	0.723	0.0988
228008_at	196.8	150.1	-0.39	-1.31	APP-APM	0.20	10	0.678	0.723	0.1260
225753_at	233.9	178.4	-0.39	-1.31	APP-PPP	0.20	10	0.678	0.723	0.1660
238826_x_at	159.4	121.7	-0.39	-1.31	PPP-PPP	0.20	10	0.678	0.723	0.0257
208860_s_at	592.3	452.0	-0.39	-1.31	PPP-PPP	0.20	10	0.678	0.723	0.0713
1557432_at	97.9	74.7	-0.39	-1.31	APP-PPP	0.20	10	0.678	0.723	0.0919
1560371_at	85.1	64.9	-0.39	-1.31	AAA-AAP	0.20	10	0.678	0.723	0.1362
213367_at	155.5	118.7	-0.39	-1.31	PPA-APP	0.20	10	0.678	0.723	0.1615
212537_x_at	39441.1	30120.1	-0.39	-1.31	PPP-PPP	0.20	10	0.678	0.723	0.0378
204854_at	412.4	314.9	-0.39	-1.31	PPP-PPP	0.20	10	0.678	0.723	0.0399
223316_at	780.1	595.6	-0.39	-1.31	PPP-PPP	0.20	10	0.678	0.723	0.0710
206382_s_at	4765.1	3637.9	-0.39	-1.31	PPP-PPP	0.20	10	0.678	0.723	0.0756

1559109_a_at	103.8	79.2	-0.39	-1.31	MPP-APP	0.20	10	0.678	0.723	0.1594
241596_at	120.1	91.8	-0.39	-1.31	PPA-APM	0.20	10	0.678	0.723	0.0578
200817_x_at	54225.5	41444.1	-0.39	-1.31	PPP-PPP	0.20	10	0.678	0.723	0.0630
243318_at	272.5	208.2	-0.39	-1.31	AAA-AAP	0.20	10	0.678	0.723	0.0734
217966_s_at	904.3	691.2	-0.39	-1.31	PPP-PPP	0.20	10	0.678	0.723	0.1080
203518_at	448.2	342.4	-0.39	-1.31	PPP-PPP	0.20	10	0.678	0.723	0.1670
1554520_at	58.7	44.9	-0.39	-1.31	AAP-AAA	0.20	10	0.678	0.723	0.1074
217456_x_at	1710.3	1307.7	-0.39	-1.31	PPP-PPP	0.20	10	0.678	0.723	0.1080
228850_s_at	1125.4	860.7	-0.39	-1.31	PPP-PPP	0.20	10	0.678	0.723	0.1173
233445_at	203.5	155.6	-0.39	-1.31	PPP-PPP	0.20	10	0.678	0.723	0.1354
223684_s_at	609.6	466.6	-0.39	-1.31	APP-APP	0.20	10	0.678	0.723	0.0466
243005_at	78.8	60.3	-0.39	-1.31	APP-PPP	0.20	10	0.678	0.723	0.0869
212581_x_at	43210.2	33085.5	-0.39	-1.31	PPP-PPP	0.20	10	0.678	0.723	0.0557
209656_s_at	3299.4	2527.3	-0.39	-1.31	PPP-PPP	0.20	10	0.678	0.723	0.1003
214605_x_at	274.1	210.1	-0.38	-1.31	PPP-PPP	0.20	10	0.678	0.723	0.0628
226146_at	788.2	604.1	-0.38	-1.31	PPP-PPP	0.20	10	0.678	0.723	0.1080
218782_s_at	4312.4	3304.9	-0.38	-1.31	PPP-PPP	0.20	10	0.678	0.723	0.1339
241517_at	121.8	93.3	-0.38	-1.31	PPP-PPP	0.20	10	0.678	0.723	0.1455
AFFX-r2-P1-cre-3_at	46232.9	35449.3	-0.38	-1.30	PPP-PPP	0.20	10	0.678	0.723	0.0365
214020_x_at	493.9	378.8	-0.38	-1.30	MPP-PPP	0.20	10	0.678	0.723	0.0373
212391_x_at	53905.1	41347.0	-0.38	-1.30	PPP-PPP	0.20	10	0.678	0.723	0.0391
223425_at	726.3	556.8	-0.38	-1.30	PPP-PPP	0.20	10	0.678	0.723	0.0704
229558_at	730.0	559.9	-0.38	-1.30	PPP-PPP	0.20	10	0.678	0.723	0.1200
219026_s_at	211.7	162.3	-0.38	-1.30	PPP-PPP	0.20	10	0.678	0.723	0.1534
212578_x_at	37177.6	28529.4	-0.38	-1.30	PPP-PPP	0.20	10	0.678	0.723	0.0348
216993_s_at	158.1	121.3	-0.38	-1.30	AAP-AAM	0.20	10	0.678	0.723	0.1218
1559249_at	411.2	315.6	-0.38	-1.30	PPP-PPP	0.20	10	0.678	0.723	0.1685
1562794_at	99.2	76.2	-0.38	-1.30	APP-MPP	0.20	10	0.678	0.723	0.0680
201429_s_at	53224.3	40880.2	-0.38	-1.30	PPP-PPP	0.20	10	0.678	0.723	0.0739
218770_s_at	826.1	634.2	-0.38	-1.30	PPP-PPP	0.20	10	0.678	0.723	0.1090
244091_at	209.3	160.8	-0.38	-1.30	PPP-PPP	0.20	10	0.678	0.723	0.1126
32094_at	641.3	493.2	-0.38	-1.30	PPP-PPP	0.20	10	0.678	0.723	0.0415
218440_at	621.5	477.8	-0.38	-1.30	PPP-PPP	0.20	10	0.678	0.723	0.0999
204099_at	460.7	354.3	-0.38	-1.30	PPP-PPP	0.20	10	0.678	0.723	0.1684
201257_x_at	54359.9	41840.1	-0.38	-1.30	PPP-PPP	0.20	10	0.678	0.723	0.0635
221016_s_at	263.4	202.7	-0.38	-1.30	PPP-PPP	0.20	10	0.678	0.723	0.1323
203034_s_at	50393.4	38804.5	-0.38	-1.30	PPP-PPP	0.20	10	0.678	0.723	0.0352
1553301_a_at	88.6	68.3	-0.38	-1.30	PPA-APP	0.20	10	0.678	0.723	0.0930
222164_at	248.6	191.5	-0.38	-1.30	PPP-PPP	0.20	10	0.678	0.723	0.0451
213084_x_at	57013.7	43936.8	-0.38	-1.30	PPP-PPP	0.20	10	0.678	0.723	0.0496
224248_x_at	1077.1	829.9	-0.38	-1.30	PPP-PPP	0.20	10	0.678	0.723	0.1431
211152_s_at	945.1	728.9	-0.38	-1.30	PPP-PPP	0.20	10	0.678	0.723	0.0249

220631_at	562.4	433.8	-0.38	-1.30	MMP-PAP	0.20	10	0.678	0.723	0.0358
211542_x_at	54612.8	42139.9	-0.37	-1.30	PPP-PPP	0.20	10	0.678	0.723	0.0692
243329_at	113.1	87.3	-0.37	-1.30	PPP-PPP	0.20	10	0.678	0.723	0.0820
202113_s_at	4356.0	3364.3	-0.37	-1.30	PPP-PPP	0.20	10	0.678	0.723	0.0277
1560275_at	241.3	186.3	-0.37	-1.30	PPP-PPP	0.20	10	0.678	0.723	0.0446
212039_x_at	42495.9	32811.3	-0.37	-1.30	PPP-PPP	0.20	10	0.678	0.723	0.0455
224594_x_at	49014.8	37838.9	-0.37	-1.30	PPP-PPP	0.20	10	0.678	0.723	0.0765
227546_x_at	1185.0	915.2	-0.37	-1.30	PPP-PPP	0.20	10	0.678	0.723	0.1119
200807_s_at	42278.8	32658.6	-0.37	-1.30	PPP-PPP	0.20	10	0.678	0.723	0.0231
206092_x_at	263.1	203.3	-0.37	-1.29	PPP-PPP	0.20	10	0.678	0.723	0.0426
223485_at	260.4	201.3	-0.37	-1.29	PPP-PPP	0.20	10	0.678	0.723	0.0488
243747_at	86.0	66.5	-0.37	-1.30	APP-MPP	0.20	10	0.678	0.723	0.1219
215157_x_at	31277.8	24188.0	-0.37	-1.29	PPP-PPP	0.20	10	0.678	0.723	0.0421
230708_at	216.5	167.4	-0.37	-1.29	PPP-PPP	0.20	10	0.678	0.723	0.0780
1557094_at	523.3	404.7	-0.37	-1.29	PPP-PPP	0.20	10	0.678	0.723	0.1232
207223_s_at	312.7	241.8	-0.37	-1.29	PPP-PPP	0.20	10	0.678	0.723	0.1269
210286_s_at	163.9	126.8	-0.37	-1.29	PPP-PPP	0.20	10	0.678	0.723	0.1654
222651_s_at	326.0	252.3	-0.37	-1.29	PPP-PPP	0.20	10	0.678	0.723	0.1142
231274_s_at	937.6	726.0	-0.37	-1.29	PPP-PPP	0.20	10	0.678	0.723	0.0345
200025_s_at	40940.3	31706.2	-0.37	-1.29	PPP-PPP	0.20	10	0.678	0.723	0.0437
214882_s_at	12472.4	9657.4	-0.37	-1.29	PPP-PPP	0.20	10	0.678	0.723	0.0816
AFFX-M27830_5_at	3000.3	2323.1	-0.37	-1.29	PPP-PPP	0.20	10	0.678	0.723	0.1054
216505_x_at	6654.9	5152.4	-0.37	-1.29	PPP-PPP	0.20	10	0.678	0.723	0.1473
240793_at	106.6	82.5	-0.37	-1.29	PPP-PPM	0.20	10	0.678	0.723	0.1521
200094_s_at	33622.2	26052.8	-0.37	-1.29	PPP-PPP	0.20	10	0.678	0.723	0.0258
229175_at	436.7	338.3	-0.37	-1.29	PPP-PPP	0.20	10	0.678	0.723	0.0512
215387_x_at	165.1	127.9	-0.37	-1.29	AAP-PPP	0.20	10	0.678	0.723	0.1033
202148_s_at	1894.7	1468.2	-0.37	-1.29	PPP-PPP	0.20	10	0.678	0.723	0.1067
234074_at	191.5	148.5	-0.37	-1.29	APP-APP	0.20	10	0.678	0.723	0.0462
218255_s_at	355.5	275.7	-0.37	-1.29	PPP-PPP	0.20	10	0.678	0.723	0.0728
208743_s_at	10474.5	8121.1	-0.37	-1.29	PPP-PPP	0.20	10	0.678	0.723	0.0855
202656_s_at	2233.3	1732.2	-0.37	-1.29	PPP-PPP	0.20	10	0.678	0.723	0.1485
219020_at	666.1	516.5	-0.37	-1.29	PPP-PPP	0.20	10	0.678	0.723	0.1638
AFFX-HSAC07/X0035	47853.6	37125.2	-0.37	-1.29	PPP-PPP	0.20	10	0.678	0.723	0.0435
229962_at	144.6	112.2	-0.37	-1.29	AAA-APP	0.20	10	0.678	0.723	0.1262
232183_at	602.4	467.4	-0.37	-1.29	PPP-PPP	0.20	10	0.678	0.723	0.1513
211799_x_at	790.2	613.5	-0.37	-1.29	PPP-PPP	0.20	10	0.678	0.723	0.0606
230031_at	895.6	695.3	-0.37	-1.29	PPP-PPP	0.20	10	0.678	0.723	0.0704
220178_at	463.0	359.5	-0.37	-1.29	APP-PPP	0.20	10	0.678	0.723	0.0860
202715_at	2265.7	1758.9	-0.37	-1.29	PPP-PPP	0.20	10	0.678	0.723	0.0866
217092_x_at	6413.5	4978.8	-0.37	-1.29	PPP-PPP	0.20	10	0.678	0.723	0.0996
203301_s_at	1307.8	1015.9	-0.36	-1.29	PPP-PPP	0.20	10	0.678	0.723	0.0433

1554599_x_at	583.5	453.5	-0.36	-1.29	PPP-PPP	0.20	10	0.678	0.723	0.0692
200095_x_at	50666.8	39356.7	-0.36	-1.29	PPP-PPP	0.20	10	0.678	0.723	0.0794
228453_at	941.5	732.0	-0.36	-1.29	PPP-PPP	0.20	10	0.678	0.723	0.0222
214838_at	319.4	248.3	-0.36	-1.29	PAM-AAP	0.20	10	0.678	0.723	0.0904
241812_at	138.9	108.0	-0.36	-1.29	APP-PPP	0.20	10	0.678	0.723	0.1493
1560588_at	98.1	76.3	-0.36	-1.29	PAA-AAA	0.20	10	0.678	0.723	0.1552
1558969_a_at	166.7	129.7	-0.36	-1.29	PPP-AAP	0.20	10	0.678	0.723	0.0310
213646_x_at	44547.4	34669.1	-0.36	-1.29	PPP-PPP	0.20	10	0.678	0.723	0.0933
208972_s_at	2737.6	2129.4	-0.36	-1.29	PPP-PPP	0.20	10	0.678	0.723	0.1196
200949_x_at	32691.3	25448.0	-0.36	-1.29	PPP-PPP	0.20	10	0.678	0.723	0.0385
200801_x_at	57036.3	44438.3	-0.36	-1.28	PPP-PPP	0.20	10	0.678	0.723	0.0574
200081_s_at	41040.2	31980.6	-0.36	-1.28	PPP-PPP	0.20	10	0.678	0.723	0.0602
214003_x_at	53067.7	41352.6	-0.36	-1.28	PPP-PPP	0.20	10	0.678	0.723	0.0839
211378_x_at	47939.3	37369.6	-0.36	-1.28	PPP-PPP	0.20	10	0.678	0.723	0.0772
201090_x_at	41195.8	32116.5	-0.36	-1.28	PPP-PPP	0.20	10	0.678	0.723	0.0872
216945_x_at	223.0	173.8	-0.36	-1.28	PPP-PPP	0.20	10	0.678	0.723	0.1102
1566696_at	69.5	54.2	-0.36	-1.28	AAA-MAP	0.20	10	0.678	0.723	0.1142
203017_s_at	1057.3	824.7	-0.36	-1.28	PPP-PPP	0.20	10	0.678	0.723	0.0678
213999_at	81.1	63.2	-0.36	-1.28	AAP-AAA	0.20	10	0.678	0.723	0.1282
201925_s_at	1912.4	1491.8	-0.36	-1.28	PPP-PPP	0.20	10	0.678	0.723	0.1332
227047_x_at	941.7	734.7	-0.36	-1.28	PPP-PPP	0.20	10	0.678	0.723	0.1378
219838_at	472.4	369.2	-0.36	-1.28	PPP-PPP	0.20	10	0.678	0.723	0.1049
221029_s_at	3543.1	2770.9	-0.36	-1.28	PPP-PPP	0.20	10	0.678	0.723	0.0189
244197_x_at	1054.6	824.7	-0.36	-1.28	PPP-PPP	0.20	10	0.678	0.723	0.0205
206559_x_at	47511.7	37136.3	-0.36	-1.28	PPP-PPP	0.20	10	0.678	0.723	0.0438
211936_at	30902.2	24183.9	-0.35	-1.28	PPP-PPP	0.20	10	0.678	0.723	0.0252
226527_at	638.9	500.0	-0.35	-1.28	PPP-PPP	0.20	10	0.678	0.723	0.0326
211073_x_at	41347.7	32343.1	-0.35	-1.28	PPP-PPP	0.20	10	0.678	0.723	0.0368
212433_x_at	44875.5	35110.9	-0.35	-1.28	PPP-PPP	0.20	10	0.678	0.723	0.0475
213801_x_at	43588.1	34097.9	-0.35	-1.28	PPP-PPP	0.20	10	0.678	0.723	0.0506
222343_at	66.5	52.0	-0.35	-1.28	APP-APP	0.20	10	0.678	0.723	0.1423
227114_at	315.8	247.3	-0.35	-1.28	PPP-PPP	0.20	10	0.678	0.723	0.0442
222672_at	365.3	286.0	-0.35	-1.28	PPP-PAP	0.20	10	0.678	0.723	0.0452
209251_x_at	38460.6	30111.4	-0.35	-1.28	PPP-PPP	0.20	10	0.678	0.723	0.0586
201550_x_at	44523.2	34866.0	-0.35	-1.28	PPP-PPP	0.20	10	0.678	0.723	0.0796
234749_s_at	342.7	268.2	-0.35	-1.28	PPP-PPP	0.20	10	0.678	0.723	0.0892
1559025_at	104.1	81.5	-0.35	-1.28	APM-PMP	0.20	10	0.678	0.723	0.1090
228336_at	723.9	566.9	-0.35	-1.28	PPP-PPP	0.20	10	0.678	0.723	0.1416
236985_at	229.2	179.4	-0.35	-1.28	PPP-PPP	0.20	10	0.678	0.723	0.1536
1569906_s_at	340.2	266.5	-0.35	-1.28	PPP-PPP	0.20	10	0.678	0.723	0.0387
219133_at	1019.9	799.0	-0.35	-1.28	PPP-PPP	0.20	10	0.678	0.723	0.0388
211972_x_at	40054.7	31391.9	-0.35	-1.28	PPP-PPP	0.20	10	0.678	0.723	0.0657

205873_at	379.7	297.6	-0.35	-1.28	PPP-PPP	0.20	10	0.678	0.723	0.1022
235032_at	1395.3	1093.1	-0.35	-1.28	PPP-PPP	0.20	10	0.678	0.723	0.1444
1552618_at	678.3	531.7	-0.35	-1.28	PPP-PPP	0.20	10	0.678	0.723	0.0312
206113_s_at	1370.5	1074.7	-0.35	-1.28	PPP-PPP	0.20	10	0.678	0.723	0.0616
200834_s_at	42492.7	33307.9	-0.35	-1.28	PPP-PPP	0.20	10	0.678	0.723	0.0647
47069_at	458.7	359.6	-0.35	-1.28	PPP-PPP	0.20	10	0.678	0.723	0.1549
213453_x_at	42677.3	33493.6	-0.35	-1.27	PPP-PPP	0.20	10	0.678	0.723	0.0490
209835_x_at	8902.0	6983.3	-0.35	-1.28	PPP-PPP	0.20	10	0.678	0.723	0.0936
1555848_at	265.3	208.2	-0.35	-1.27	PPP-PPP	0.20	10	0.678	0.723	0.1273
224519_at	296.4	232.5	-0.35	-1.28	PPP-PPP	0.20	10	0.678	0.723	0.1574
212270_x_at	38298.4	30061.5	-0.35	-1.27	PPP-PPP	0.20	10	0.678	0.723	0.0239
226712_at	464.0	364.4	-0.35	-1.27	PPP-PPP	0.20	10	0.678	0.723	0.0365
224930_x_at	41486.5	32582.9	-0.35	-1.27	PPP-PPP	0.20	10	0.678	0.723	0.0877
213377_x_at	43430.1	34119.2	-0.35	-1.27	PPP-PPP	0.20	10	0.678	0.723	0.0302
221643_s_at	210.2	165.2	-0.35	-1.27	PPP-PPP	0.20	10	0.678	0.723	0.0469
219419_at	701.3	550.9	-0.35	-1.27	PPP-PPP	0.20	10	0.678	0.723	0.0632
AFFX-HSAC07/X0035	45109.3	35443.3	-0.35	-1.27	PPP-PPP	0.20	10	0.678	0.723	0.0732
202515_at	3063.8	2406.4	-0.35	-1.27	PPP-PPP	0.20	10	0.678	0.723	0.0751
213486_at	118.2	92.8	-0.35	-1.27	MPM-APA	0.20	10	0.678	0.723	0.1428
208692_at	42073.2	33077.4	-0.35	-1.27	PPP-PPP	0.20	10	0.678	0.723	0.0202
AFFX-HSAC07/X0035	39449.5	31009.6	-0.35	-1.27	PPP-PPP	0.20	10	0.678	0.723	0.0480
201492_s_at	48325.9	37989.3	-0.35	-1.27	PPP-PPP	0.20	10	0.678	0.723	0.0488
221850_x_at	647.5	509.0	-0.35	-1.27	PPP-PPP	0.20	10	0.678	0.723	0.0650
228011_at	1601.4	1259.2	-0.35	-1.27	PPP-PPP	0.20	10	0.678	0.723	0.0661
230892_at	65.6	51.6	-0.35	-1.27	AAP-AAP	0.20	10	0.678	0.723	0.1214
1557300_s_at	161.5	127.0	-0.35	-1.27	MAP-PPP	0.20	10	0.678	0.723	0.1481
AFFX-HUMGAPDH/M3	46021.3	36208.5	-0.35	-1.27	PPP-PPP	0.20	10	0.678	0.723	0.0323
200717_x_at	49303.2	38777.6	-0.35	-1.27	PPP-PPP	0.20	10	0.678	0.723	0.0387
200725_x_at	28542.4	22449.1	-0.35	-1.27	PPP-PPP	0.20	10	0.678	0.723	0.0652
208825_x_at	58792.8	46247.0	-0.35	-1.27	PPP-PPP	0.20	10	0.678	0.723	0.0983
215446_s_at	12059.8	9496.3	-0.35	-1.27	PPP-PPP	0.20	10	0.678	0.723	0.0326
215195_at	927.1	729.9	-0.35	-1.27	PPP-PPP	0.20	10	0.678	0.723	0.0334
211978_x_at	48946.8	38525.9	-0.35	-1.27	PPP-PPP	0.20	10	0.678	0.723	0.0646
211995_x_at	41395.1	32591.8	-0.35	-1.27	PPP-PPP	0.20	10	0.678	0.723	0.0798
202501_at	2934.2	2309.6	-0.35	-1.27	PPP-PPP	0.20	10	0.678	0.723	0.0822
212869_x_at	47714.9	37568.8	-0.35	-1.27	PPP-PPP	0.20	10	0.678	0.723	0.1114
215497_s_at	217.3	171.1	-0.35	-1.27	AAP-AAA	0.20	10	0.678	0.723	0.1340
227443_at	455.9	359.2	-0.34	-1.27	PPP-PPP	0.20	10	0.678	0.723	0.0239
200092_s_at	41649.9	32814.6	-0.34	-1.27	PPP-PPP	0.20	10	0.678	0.723	0.0544
201105_at	38274.8	30147.8	-0.34	-1.27	PPP-PPP	0.20	10	0.678	0.723	0.0575
235815_at	105.8	83.4	-0.34	-1.27	APA-PPP	0.20	10	0.678	0.723	0.1100
48612_at	1005.3	792.8	-0.34	-1.27	PPP-PPP	0.20	10	0.678	0.723	0.1027

219626_at	526.2	414.8	-0.34	-1.27	PPP-PPP	0.20	10	0.678	0.723	0.1051
231204_at	57.2	45.1	-0.34	-1.27	PPP-APP	0.20	10	0.678	0.723	0.1477
235041_at	490.6	386.9	-0.34	-1.27	PPP-PPP	0.20	10	0.678	0.723	0.0413
AFFX-HUMGAPDH/M3	42947.9	33885.8	-0.34	-1.27	PPP-PPP	0.20	10	0.678	0.723	0.0484
203012_x_at	43185.1	34076.4	-0.34	-1.27	PPP-PPP	0.20	10	0.678	0.723	0.0887
208646_at	22226.5	17535.2	-0.34	-1.27	PPP-PPP	0.20	10	0.678	0.723	0.1116
216336_x_at	14129.8	11159.0	-0.34	-1.27	PPP-PPP	0.20	10	0.678	0.723	0.0374
201511_at	2014.5	1590.8	-0.34	-1.27	PPP-PPP	0.20	10	0.678	0.723	0.0695
204044_at	285.0	225.2	-0.34	-1.27	PPP-PPP	0.20	10	0.678	0.723	0.0214
212790_x_at	50293.5	39742.0	-0.34	-1.27	PPP-PPP	0.20	10	0.678	0.723	0.0245
219238_at	467.9	369.6	-0.34	-1.27	PPP-APP	0.20	10	0.678	0.723	0.0290
202403_s_at	29525.3	23323.0	-0.34	-1.27	PPP-PPP	0.20	10	0.678	0.723	0.0425
211058_x_at	43225.4	34140.7	-0.34	-1.27	PPP-PPP	0.20	10	0.678	0.723	0.0773
212212_s_at	798.6	630.9	-0.34	-1.27	PPP-PPP	0.20	10	0.678	0.723	0.0788
1555091_at	224.7	177.6	-0.34	-1.27	PPP-PPP	0.20	10	0.678	0.723	0.1252
221691_x_at	34142.1	26984.6	-0.34	-1.27	PPP-PPP	0.20	10	0.678	0.723	0.0239
212661_x_at	51329.5	40594.0	-0.34	-1.26	PPP-PPP	0.20	10	0.678	0.723	0.0784
1552257_a_at	2672.6	2112.5	-0.34	-1.27	PPP-PPP	0.20	10	0.678	0.723	0.0864
238547_at	204.0	161.4	-0.34	-1.26	AAP-PPP	0.20	10	0.678	0.723	0.0276
214271_x_at	33449.1	26465.5	-0.34	-1.26	PPP-PPP	0.20	10	0.678	0.723	0.0489
1553538_s_at	54782.8	43342.0	-0.34	-1.26	PPP-PPP	0.20	10	0.678	0.723	0.0577
201293_x_at	49078.1	38816.4	-0.34	-1.26	PPP-PPP	0.20	10	0.678	0.723	0.0842
1558292_s_at	942.1	745.5	-0.34	-1.26	PPP-PPP	0.20	10	0.678	0.723	0.0986
1558685_a_at	664.0	525.4	-0.34	-1.26	PPP-PPP	0.20	10	0.678	0.723	0.1412
211927_x_at	37733.4	29899.2	-0.34	-1.26	PPP-PPP	0.20	10	0.678	0.723	0.0312
239577_at	119.2	94.4	-0.34	-1.26	PPP-PPP	0.20	10	0.678	0.723	0.0470
206688_s_at	2565.8	2032.6	-0.34	-1.26	PPP-PPP	0.20	10	0.678	0.723	0.0496
212639_x_at	52127.7	41298.5	-0.34	-1.26	PPP-PPP	0.20	10	0.678	0.723	0.0683
1555860_x_at	209.7	166.2	-0.34	-1.26	PPP-PPP	0.20	10	0.678	0.723	0.0701
215883_at	122.0	96.7	-0.34	-1.26	PPP-MPP	0.20	10	0.678	0.723	0.1431
209121_x_at	6647.1	5268.2	-0.34	-1.26	PPP-PPP	0.20	10	0.678	0.723	0.0435
AFFX-r2-Ec-bioB-3_at	847.0	671.4	-0.34	-1.26	PPP-PPP	0.20	10	0.678	0.723	0.0458
220960_x_at	34794.9	27586.8	-0.34	-1.26	PPP-PPP	0.20	10	0.678	0.723	0.0461
200809_x_at	37031.7	29363.9	-0.34	-1.26	PPP-PPP	0.20	10	0.678	0.723	0.0617
204892_x_at	47165.4	37383.3	-0.34	-1.26	PPP-PPP	0.20	10	0.678	0.723	0.0653
200741_s_at	41792.2	33141.6	-0.34	-1.26	PPP-PPP	0.20	10	0.678	0.723	0.0669
212363_x_at	39164.0	31041.9	-0.34	-1.26	PPP-PPP	0.20	10	0.678	0.723	0.0767
239748_x_at	1624.3	1287.8	-0.34	-1.26	PPP-PPP	0.20	10	0.678	0.723	0.1234
1555778_a_at	29080.4	23071.4	-0.33	-1.26	PPP-PPP	0.20	10	0.678	0.723	0.0207
215823_x_at	25795.4	20462.7	-0.33	-1.26	PPP-PPP	0.20	10	0.678	0.723	0.0210
200819_s_at	41157.4	32655.7	-0.33	-1.26	PPP-PPP	0.20	10	0.678	0.723	0.0545
214221_at	514.9	408.6	-0.33	-1.26	PPP-PPP	0.20	10	0.678	0.723	0.1365

200088_x_at	40859.7	32440.4	-0.33	-1.26	PPP-PPP	0.20	10	0.678	0.723	0.0429
200704_at	5881.5	4669.7	-0.33	-1.26	PPP-PPP	0.20	10	0.678	0.723	0.0734
221607_x_at	43432.7	34491.9	-0.33	-1.26	PPP-PPP	0.20	10	0.678	0.723	0.0751
211970_x_at	40723.1	32329.4	-0.33	-1.26	PPP-PPP	0.20	10	0.678	0.723	0.1088
219809_at	878.6	697.8	-0.33	-1.26	PPP-PPP	0.20	10	0.678	0.723	0.1164
206323_x_at	2417.8	1920.1	-0.33	-1.26	PPP-PPP	0.20	10	0.678	0.723	0.0371
200063_s_at	41067.6	32623.0	-0.33	-1.26	PPP-PPP	0.20	10	0.678	0.723	0.0805
242981_at	343.3	272.7	-0.33	-1.26	PPP-PPP	0.20	10	0.678	0.723	0.0951
201286_at	732.7	582.0	-0.33	-1.26	PPP-PPP	0.20	10	0.678	0.723	0.1209
229532_at	163.8	130.3	-0.33	-1.26	PPA-PPP	0.20	10	0.678	0.723	0.1477
211345_x_at	32726.5	26032.5	-0.33	-1.26	PPP-PPP	0.20	10	0.678	0.723	0.0239
1553569_at	53566.7	42613.9	-0.33	-1.26	PPP-PPP	0.20	10	0.678	0.723	0.0567
223466_x_at	1355.9	1078.7	-0.33	-1.26	PPP-PPP	0.20	10	0.678	0.723	0.0589
213803_at	4787.6	3808.4	-0.33	-1.26	PPP-PPP	0.20	10	0.678	0.723	0.1440
214328_s_at	44866.3	35708.6	-0.33	-1.26	PPP-PPP	0.20	10	0.678	0.723	0.0419
210646_x_at	52134.9	41494.5	-0.33	-1.26	PPP-PPP	0.20	10	0.678	0.723	0.0570
244788_at	146.0	116.3	-0.33	-1.26	PPP-PPM	0.20	10	0.678	0.723	0.1289
208645_s_at	45529.5	36282.8	-0.33	-1.26	PPP-PPP	0.20	10	0.678	0.723	0.0569
221276_s_at	1762.5	1404.2	-0.33	-1.26	PPP-PPP	0.20	10	0.678	0.723	0.0628
225654_at	125.4	99.9	-0.33	-1.26	PAA-AAP	0.20	10	0.678	0.723	0.0986
226885_at	386.5	308.0	-0.33	-1.26	PPP-PPP	0.20	10	0.678	0.723	0.1215
1553588_at	44249.2	35275.7	-0.33	-1.25	PPP-PPP	0.20	10	0.678	0.723	0.0494
201279_s_at	3219.3	2565.9	-0.33	-1.26	PPP-PPP	0.20	10	0.678	0.723	0.0513
200062_s_at	41536.1	33122.9	-0.33	-1.25	PPP-PPP	0.20	10	0.678	0.723	0.0531
212605_s_at	736.3	587.1	-0.33	-1.25	PPP-PPP	0.20	10	0.678	0.723	0.0605
236609_at	238.9	190.5	-0.33	-1.25	PPP-PPP	0.20	10	0.678	0.723	0.0820
1554220_a_at	78.4	62.5	-0.33	-1.26	APA-AAA	0.20	10	0.678	0.723	0.1168
222125_s_at	773.9	616.8	-0.33	-1.26	MPP-PPP	0.20	10	0.678	0.723	0.1170
221775_x_at	36262.7	28920.0	-0.33	-1.25	PPP-PPP	0.20	10	0.678	0.723	0.0366
201406_at	41044.5	32737.0	-0.33	-1.25	PPP-PPP	0.20	10	0.678	0.723	0.0688
213445_at	410.1	327.3	-0.33	-1.25	PPP-PPP	0.20	10	0.678	0.723	0.0878
233564_s_at	263.2	210.0	-0.33	-1.25	PAP-APP	0.20	10	0.678	0.723	0.1053
239718_at	142.8	114.1	-0.32	-1.25	PPP-PPP	0.20	10	0.678	0.723	0.0532
221629_x_at	673.7	538.3	-0.32	-1.25	PPP-PPP	0.20	10	0.678	0.723	0.1247
203304_at	1019.4	814.2	-0.32	-1.25	PPP-PPP	0.20	10	0.678	0.723	0.1389
227224_at	1289.2	1031.2	-0.32	-1.25	PPP-PPP	0.20	10	0.678	0.723	0.0277
213169_at	387.0	309.5	-0.32	-1.25	PPP-PPP	0.20	10	0.678	0.723	0.1221
232467_at	127.7	102.2	-0.32	-1.25	PPP-PPP	0.20	10	0.678	0.723	0.1305
232052_at	267.9	214.4	-0.32	-1.25	PPP-PPP	0.20	10	0.678	0.723	0.0427
219933_at	7214.1	5773.2	-0.32	-1.25	PPP-PPP	0.20	10	0.678	0.723	0.0777
211296_x_at	41371.6	33114.7	-0.32	-1.25	PPP-PPP	0.20	10	0.678	0.723	0.0783
219269_at	488.4	391.0	-0.32	-1.25	PPP-PPP	0.20	10	0.678	0.723	0.0801

211719_x_at	38523.7	30841.4	-0.32	-1.25	PPP-PPP	0.20	10	0.678	0.723	0.0902
230186_at	281.3	225.1	-0.32	-1.25	PPP-PPP	0.20	10	0.678	0.723	0.0943
209964_s_at	180.7	144.6	-0.32	-1.25	APP-PPP	0.20	10	0.678	0.723	0.1371
217740_x_at	47413.8	37974.1	-0.32	-1.25	PPP-PPP	0.20	10	0.678	0.723	0.0825
220288_at	46.7	37.4	-0.32	-1.25	APA-AAA	0.20	10	0.678	0.723	0.1075
227968_at	762.3	610.8	-0.32	-1.25	PPP-PPP	0.20	10	0.678	0.723	0.1286
218267_at	556.6	445.9	-0.32	-1.25	PPP-PPP	0.20	10	0.678	0.723	0.1344
211983_x_at	41658.1	33396.9	-0.32	-1.25	PPP-PPP	0.20	10	0.678	0.723	0.0463
212185_x_at	35446.9	28406.0	-0.32	-1.25	PPP-PPP	0.20	10	0.678	0.723	0.0822
204253_s_at	242.9	194.9	-0.32	-1.25	PPP-PPP	0.20	10	0.678	0.723	0.0485
201049_s_at	42886.5	34393.8	-0.32	-1.25	PPP-PPP	0.20	10	0.678	0.723	0.0624
1556393_at	70.4	56.5	-0.32	-1.25	MPP-APP	0.20	10	0.678	0.723	0.0753
227507_at	473.2	379.6	-0.32	-1.25	PPP-PPP	0.20	10	0.678	0.723	0.1018
211666_x_at	26686.6	21423.7	-0.32	-1.25	PPP-PPP	0.20	10	0.678	0.723	0.0378
209329_x_at	4578.3	3674.4	-0.32	-1.25	PPP-PPP	0.20	10	0.678	0.723	0.0463
202649_x_at	43170.4	34647.3	-0.32	-1.25	PPP-PPP	0.20	10	0.678	0.723	0.0553
209725_at	1214.3	974.5	-0.32	-1.25	PPP-PPP	0.20	10	0.678	0.723	0.0825
211765_x_at	48929.1	39273.6	-0.32	-1.25	PPP-PPP	0.20	10	0.678	0.723	0.1042
200680_x_at	33423.3	26840.0	-0.32	-1.25	PPP-PPP	0.20	10	0.678	0.723	0.0130
211943_x_at	35914.4	28845.1	-0.32	-1.25	PPP-PPP	0.20	10	0.678	0.723	0.0262
200823_x_at	29368.6	23597.1	-0.32	-1.25	PPP-PPP	0.20	10	0.678	0.723	0.0278
201134_x_at	22865.7	18361.9	-0.32	-1.25	PPP-PPP	0.20	10	0.678	0.723	0.0324
208502_s_at	820.9	659.3	-0.32	-1.25	PPP-PPP	0.20	10	0.678	0.723	0.0506
213890_x_at	39440.8	31680.8	-0.32	-1.25	PPP-PPP	0.20	10	0.678	0.723	0.0624
200909_s_at	35217.4	28311.3	-0.32	-1.24	PPP-PPP	0.20	10	0.678	0.723	0.0133
213829_x_at	268.1	215.6	-0.32	-1.24	PPP-PPP	0.20	10	0.678	0.723	0.0934
1562474_at	199.7	160.5	-0.32	-1.24	PPP-PPP	0.20	10	0.678	0.723	0.1043
212933_x_at	32117.5	25840.7	-0.31	-1.24	PPP-PPP	0.20	10	0.678	0.723	0.0551
1555542_at	189.1	152.2	-0.31	-1.24	PPP-PPP	0.20	10	0.678	0.723	0.0576
213187_x_at	35333.3	28418.3	-0.31	-1.24	PPP-PPP	0.20	10	0.678	0.723	0.0996
201944_at	11100.6	8928.5	-0.31	-1.24	PPP-PPP	0.20	10	0.678	0.723	0.1010
217643_x_at	94.1	75.7	-0.31	-1.24	PAP-MPA	0.20	10	0.678	0.723	0.1118
208815_x_at	9433.0	7586.4	-0.31	-1.24	PPP-PPP	0.20	10	0.678	0.723	0.1167
209899_s_at	6054.6	4873.5	-0.31	-1.24	PPP-PPP	0.20	10	0.678	0.723	0.0594
212734_x_at	41533.9	33443.0	-0.31	-1.24	PPP-PPP	0.20	10	0.678	0.723	0.0768
200674_s_at	36281.2	29214.6	-0.31	-1.24	PPP-PPP	0.20	10	0.678	0.723	0.1021
202775_s_at	695.3	559.5	-0.31	-1.24	PPP-PPP	0.20	10	0.678	0.723	0.1175
40359_at	1066.5	858.9	-0.31	-1.24	PPP-PPP	0.20	10	0.678	0.723	0.0747
201984_s_at	1808.2	1456.8	-0.31	-1.24	PPP-PPP	0.20	10	0.678	0.723	0.0956
209608_s_at	4759.5	3836.1	-0.31	-1.24	PPP-PPP	0.20	10	0.678	0.723	0.0247
203367_at	3871.7	3122.0	-0.31	-1.24	PPP-PPP	0.20	10	0.678	0.723	0.0638
211750_x_at	38237.4	30832.2	-0.31	-1.24	PPP-PPP	0.20	10	0.678	0.723	0.0810

217733_s_at	50920.7	41057.4	-0.31	-1.24	PPP-PPP	0.20	10	0.678	0.723	0.0823
1557961_s_at	449.7	362.5	-0.31	-1.24	PPP-PPP	0.20	10	0.678	0.723	0.0925
211072_x_at	41763.4	33670.9	-0.31	-1.24	PPP-PPP	0.20	10	0.678	0.723	0.0955
212284_x_at	46985.4	37875.1	-0.31	-1.24	PPP-PPP	0.20	10	0.678	0.723	0.1079
223086_x_at	8814.1	7110.9	-0.31	-1.24	PPP-PPP	0.20	10	0.678	0.723	0.0203
209134_s_at	40252.5	32463.7	-0.31	-1.24	PPP-PPP	0.20	10	0.678	0.723	0.0348
211755_s_at	26984.6	21760.3	-0.31	-1.24	PPP-PPP	0.20	10	0.678	0.723	0.0405
204473_s_at	459.6	370.8	-0.31	-1.24	PPP-PPP	0.20	10	0.678	0.723	0.0729
211034_s_at	463.7	373.9	-0.31	-1.24	PPP-PPP	0.20	10	0.678	0.723	0.0779
221927_s_at	791.3	638.6	-0.31	-1.24	PPP-PPP	0.20	10	0.678	0.723	0.0412
200926_at	55159.6	44517.0	-0.31	-1.24	PPP-PPP	0.20	10	0.678	0.723	0.0789
212464_s_at	35089.0	28336.2	-0.31	-1.24	PPP-PPP	0.20	10	0.678	0.723	0.0266
208718_at	14131.3	11411.9	-0.31	-1.24	PPP-PPP	0.20	10	0.678	0.723	0.0340
212816_s_at	564.4	455.8	-0.31	-1.24	PPP-PPP	0.20	10	0.678	0.723	0.0359
211982_x_at	3941.2	3183.3	-0.31	-1.24	PPP-PPP	0.20	10	0.678	0.723	0.0878
201543_s_at	3249.1	2624.3	-0.31	-1.24	PPP-PPP	0.20	10	0.678	0.723	0.0919
213614_x_at	48563.6	39234.1	-0.31	-1.24	PPP-PPP	0.20	10	0.678	0.723	0.1028
34063_at	134.6	108.7	-0.31	-1.24	AMP-APP	0.20	10	0.678	0.723	0.1122
225958_at	507.3	409.9	-0.31	-1.24	PPP-PPP	0.20	10	0.678	0.723	0.1317
200016_x_at	41173.1	33273.7	-0.31	-1.24	PPP-PPP	0.20	10	0.678	0.723	0.0217
211787_s_at	35107.0	28383.8	-0.31	-1.24	PPP-PPP	0.20	10	0.678	0.723	0.0356
224060_s_at	1118.3	903.7	-0.31	-1.24	PPP-PPP	0.20	10	0.678	0.723	0.0376
213477_x_at	43080.1	34830.5	-0.31	-1.24	PPP-PPP	0.20	10	0.678	0.723	0.0600
227042_at	1266.4	1023.9	-0.31	-1.24	PPP-PPP	0.20	10	0.678	0.723	0.0635
210427_x_at	36298.1	29339.1	-0.31	-1.24	PPP-PPP	0.20	10	0.678	0.723	0.0657
235534_at	183.0	147.9	-0.31	-1.24	APP-PAP	0.20	10	0.678	0.723	0.0832
230380_at	291.9	236.0	-0.31	-1.24	PPP-PPP	0.20	10	0.678	0.723	0.0856
210338_s_at	41456.4	33524.7	-0.31	-1.24	PPP-PPP	0.20	10	0.678	0.723	0.0464
212049_at	672.4	544.3	-0.31	-1.24	PPP-PPP	0.20	10	0.678	0.723	0.0220
226819_at	202.7	164.1	-0.31	-1.24	APP-APP	0.20	10	0.678	0.723	0.0246
235917_at	166.0	134.4	-0.31	-1.24	MPP-PPP	0.20	10	0.678	0.723	0.0296
1552309_a_at	3883.1	3143.2	-0.31	-1.24	PPP-PPP	0.20	10	0.678	0.723	0.0307
217884_at	1699.0	1375.4	-0.31	-1.24	PPP-PPP	0.20	10	0.678	0.723	0.0537
215253_s_at	975.1	789.1	-0.31	-1.24	PPP-PPP	0.20	10	0.678	0.723	0.0961
208858_s_at	2294.4	1856.9	-0.31	-1.24	PPP-PPP	0.20	10	0.678	0.723	0.0999
213107_at	841.4	681.5	-0.30	-1.24	PPP-PPP	0.20	10	0.678	0.723	0.0205
222804_x_at	1151.5	932.5	-0.30	-1.24	PPP-PPP	0.20	10	0.678	0.723	0.0688
211121_s_at	268.9	217.7	-0.30	-1.24	PPP-PPP	0.20	10	0.678	0.723	0.1176
240254_at	87.6	71.0	-0.30	-1.23	APA-AAP	0.20	10	0.678	0.723	0.0135
214167_s_at	31940.9	25890.3	-0.30	-1.23	PPP-PPP	0.20	10	0.678	0.723	0.0203
223671_x_at	2585.4	2095.1	-0.30	-1.23	PPP-PPP	0.20	10	0.678	0.723	0.1048
208904_s_at	31509.6	25565.5	-0.30	-1.23	PPP-PPP	0.20	10	0.678	0.723	0.0601

226945_at	378.9	307.3	-0.30	-1.23	PPP-PPP	0.20	10	0.678	0.723	0.0680
224585_x_at	43197.7	35037.3	-0.30	-1.23	PPP-PPP	0.20	10	0.678	0.723	0.0815
235833_at	122.8	99.6	-0.30	-1.23	PPP-PPP	0.20	10	0.678	0.723	0.0913
223433_at	1512.5	1227.1	-0.30	-1.23	PPP-PPP	0.20	10	0.678	0.723	0.1274
37586_at	218.2	176.9	-0.30	-1.23	PPP-PAP	0.20	10	0.678	0.723	0.1316
212251_at	7168.0	5820.1	-0.30	-1.23	PPP-PPP	0.20	10	0.678	0.723	0.0213
225624_at	202.5	164.4	-0.30	-1.23	AAM-APM	0.20	10	0.678	0.723	0.0795
41220_at	7223.8	5862.8	-0.30	-1.23	PPP-PPP	0.20	10	0.678	0.723	0.0892
200024_at	30531.9	24801.4	-0.30	-1.23	PPP-PPP	0.20	10	0.678	0.723	0.0164
225504_at	403.7	327.9	-0.30	-1.23	PPP-MPP	0.20	10	0.678	0.723	0.0514
213588_x_at	23641.4	19197.8	-0.30	-1.23	PPP-PPP	0.20	10	0.678	0.723	0.0686
213460_x_at	755.9	614.1	-0.30	-1.23	PPP-PPP	0.20	10	0.678	0.723	0.1120
212703_at	308.6	250.7	-0.30	-1.23	PPP-PPP	0.20	10	0.678	0.723	0.1289
212314_at	446.2	362.5	-0.30	-1.23	PPP-PPP	0.20	10	0.678	0.723	0.1317
244842_x_at	323.5	263.0	-0.30	-1.23	PPP-PPP	0.20	10	0.678	0.723	0.0236
224057_s_at	1568.7	1274.8	-0.30	-1.23	PPP-PPP	0.20	10	0.678	0.723	0.0434
201217_x_at	47985.7	39006.4	-0.30	-1.23	PPP-PPP	0.20	10	0.678	0.723	0.0906
1555255_a_at	260.3	211.5	-0.30	-1.23	PPP-PPP	0.20	10	0.678	0.723	0.1233
242218_at	781.1	635.3	-0.30	-1.23	PPP-PPP	0.20	10	0.678	0.723	0.0239
200032_s_at	45181.8	36772.1	-0.30	-1.23	PPP-PPP	0.20	10	0.678	0.723	0.0410
223068_at	2597.5	2114.6	-0.30	-1.23	PPP-PPP	0.20	10	0.678	0.723	0.0931
201590_x_at	35812.9	29155.7	-0.30	-1.23	PPP-PPP	0.20	10	0.678	0.723	0.1183
202024_at	3454.5	2812.8	-0.30	-1.23	PPP-PPP	0.20	10	0.678	0.723	0.0318
200689_x_at	31966.8	26030.2	-0.30	-1.23	PPP-PPP	0.20	10	0.678	0.723	0.0323
213414_s_at	45057.4	36708.6	-0.30	-1.23	PPP-PPP	0.20	10	0.678	0.723	0.0599
211762_s_at	27289.1	22223.1	-0.30	-1.23	PPP-PPP	0.20	10	0.678	0.723	0.0828
238940_at	146.2	119.1	-0.30	-1.23	AAA-PAA	0.20	10	0.678	0.723	0.1024
211452_x_at	8272.6	6740.7	-0.30	-1.23	PPP-PPP	0.20	10	0.678	0.723	0.0094
242056_at	203.6	165.9	-0.30	-1.23	PPP-PPP	0.20	10	0.678	0.723	0.0290
213175_s_at	24694.2	20128.3	-0.30	-1.23	PPP-PPP	0.20	10	0.678	0.723	0.0594
203832_at	10143.0	8268.6	-0.30	-1.23	PPP-PPP	0.20	10	0.678	0.723	0.0843
204015_s_at	705.9	575.4	-0.30	-1.23	MPP-PPP	0.20	10	0.678	0.723	0.1107
219819_s_at	3247.9	2647.7	-0.30	-1.23	PPP-PPP	0.20	10	0.678	0.723	0.1192
210534_s_at	624.4	509.2	-0.29	-1.23	PPP-PPP	0.20	10	0.678	0.723	0.0198
208696_at	22328.2	18214.9	-0.29	-1.23	PPP-PPP	0.20	10	0.678	0.723	0.0527
218077_s_at	818.7	667.5	-0.29	-1.23	PPP-PPP	0.20	10	0.678	0.723	0.1282
201231_s_at	36779.1	30028.9	-0.29	-1.23	PPP-PPP	0.20	10	0.678	0.723	0.0175
213564_x_at	31674.5	25849.6	-0.29	-1.23	PPP-PPP	0.20	10	0.678	0.723	0.0231
226350_at	4090.3	3341.9	-0.29	-1.22	PPP-PPP	0.20	10	0.678	0.723	0.0274
229563_s_at	42526.5	34723.3	-0.29	-1.23	PPP-PPP	0.20	10	0.678	0.723	0.0398
239053_at	543.8	444.2	-0.29	-1.22	PPP-PPP	0.20	10	0.678	0.723	0.0865
203171_s_at	946.4	773.0	-0.29	-1.22	PPP-PPP	0.20	10	0.678	0.723	0.1051

213969_x_at	32930.8	26910.3	-0.29	-1.22	PPP-PPP	0.20	10	0.678	0.723	0.0547
230100_x_at	340.3	278.1	-0.29	-1.22	PPP-PPP	0.20	10	0.678	0.723	0.0774
202206_at	1634.7	1335.8	-0.29	-1.22	PPP-PPP	0.20	10	0.678	0.723	0.0813
212988_x_at	37919.5	30991.0	-0.29	-1.22	PPP-PPP	0.20	10	0.678	0.723	0.1038
213511_s_at	2720.5	2223.3	-0.29	-1.22	PPP-PPP	0.20	10	0.678	0.723	0.1169
200650_s_at	38340.8	31368.7	-0.29	-1.22	PPP-PPP	0.20	10	0.678	0.723	0.0257
201947_s_at	27380.0	22398.6	-0.29	-1.22	PPP-PPP	0.20	10	0.678	0.723	0.0371
200029_at	40415.6	33057.8	-0.29	-1.22	PPP-PPP	0.20	10	0.678	0.723	0.0546
243790_at	70.8	57.9	-0.29	-1.22	PAA-MPP	0.20	10	0.678	0.723	0.0572
36907_at	764.3	625.1	-0.29	-1.22	PPP-PPP	0.20	10	0.678	0.723	0.0690
216520_s_at	43746.3	35773.1	-0.29	-1.22	PPP-PPP	0.20	10	0.678	0.723	0.0712
210386_s_at	3657.6	2991.5	-0.29	-1.22	PPP-PPP	0.20	10	0.678	0.723	0.0814
240616_at	375.0	306.7	-0.29	-1.22	PPP-PPP	0.20	10	0.678	0.723	0.0935
235004_at	2389.0	1954.4	-0.29	-1.22	PPP-PPP	0.20	10	0.678	0.723	0.1004
213214_x_at	40390.8	33077.4	-0.29	-1.22	PPP-PPP	0.20	10	0.678	0.723	0.0469
205094_at	466.7	382.2	-0.29	-1.22	PPP-PPP	0.20	10	0.678	0.723	0.0743
201592_at	12073.7	9889.5	-0.29	-1.22	PPP-PPP	0.20	10	0.678	0.723	0.0772
212369_at	1161.6	952.2	-0.29	-1.22	PPP-PPP	0.20	10	0.678	0.723	0.0209
208029_s_at	8856.1	7257.4	-0.29	-1.22	PPP-PPP	0.20	10	0.678	0.723	0.0231
224372_at	48522.6	39775.8	-0.29	-1.22	PPP-PPP	0.20	10	0.678	0.723	0.0356
210495_x_at	36241.9	29694.6	-0.29	-1.22	PPP-PPP	0.20	10	0.678	0.723	0.0444
225794_s_at	2903.0	2378.5	-0.29	-1.22	PPP-PPP	0.20	10	0.678	0.723	0.0756
200030_s_at	26581.8	21785.4	-0.29	-1.22	PPP-PPP	0.20	10	0.678	0.723	0.0770
224796_at	5811.9	4767.9	-0.29	-1.22	PPP-PPP	0.20	10	0.678	0.723	0.0100
210018_x_at	1040.1	853.2	-0.29	-1.22	PPP-PPP	0.20	10	0.678	0.723	0.0162
201254_x_at	47923.5	39294.9	-0.29	-1.22	PPP-PPP	0.20	10	0.678	0.723	0.0551
213347_x_at	38993.8	31971.1	-0.29	-1.22	PPP-PPP	0.20	10	0.678	0.723	0.0617
201303_at	8676.5	7114.9	-0.29	-1.22	PPP-PPP	0.20	10	0.678	0.723	0.0941
211576_s_at	1886.8	1547.4	-0.29	-1.22	PPP-PPP	0.20	10	0.678	0.723	0.1077
200010_at	34883.1	28651.6	-0.28	-1.22	PPP-PPP	0.20	10	0.678	0.723	0.0197
228970_at	2798.7	2298.1	-0.28	-1.22	PPP-PPP	0.20	10	0.678	0.723	0.1196
208627_s_at	25997.2	21363.3	-0.28	-1.22	PPP-PPP	0.20	10	0.678	0.723	0.0297
214684_at	961.7	790.3	-0.28	-1.22	PPP-PPP	0.20	10	0.678	0.723	0.0446
202951_at	2621.4	2153.9	-0.28	-1.22	PPP-PPP	0.20	10	0.678	0.723	0.0837
211456_x_at	20524.4	16864.3	-0.28	-1.22	PPP-PPP	0.20	10	0.678	0.723	0.0924
200002_at	32273.1	26537.9	-0.28	-1.22	PPP-PPP	0.20	10	0.678	0.723	0.0528
232274_at	142.8	117.4	-0.28	-1.22	AAA-AAP	0.20	10	0.678	0.723	0.1201
221039_s_at	3454.6	2842.6	-0.28	-1.22	PPP-PPP	0.20	10	0.678	0.723	0.0097
200038_s_at	41630.0	34261.3	-0.28	-1.22	PPP-PPP	0.20	10	0.678	0.723	0.0175
231857_s_at	359.6	296.0	-0.28	-1.22	PPP-PPP	0.20	10	0.678	0.723	0.0694
202029_x_at	30688.2	25257.1	-0.28	-1.22	PPP-PPP	0.20	10	0.678	0.723	0.0704
41386_i_at	540.3	444.6	-0.28	-1.22	PPP-PPP	0.20	10	0.678	0.723	0.1100

213048_s_at	25167.4	20728.5	-0.28	-1.21	PPP-PPP	0.20	10	0.678	0.723	0.0294
223198_x_at	1161.1	956.0	-0.28	-1.22	PPP-PPP	0.20	10	0.678	0.723	0.0743
200031_s_at	43963.7	36226.1	-0.28	-1.21	PPP-PPP	0.20	10	0.678	0.723	0.0292
200017_at	44151.2	36394.0	-0.28	-1.21	PPP-PPP	0.20	10	0.678	0.723	0.0378
211956_s_at	41670.5	34339.1	-0.28	-1.21	PPP-PPP	0.20	10	0.678	0.723	0.0642
215963_x_at	34614.5	28532.2	-0.28	-1.21	PPP-PPP	0.20	10	0.678	0.723	0.0683
207153_s_at	1385.8	1141.8	-0.28	-1.21	PPP-PPP	0.20	10	0.678	0.723	0.0694
219071_x_at	1602.9	1320.8	-0.28	-1.21	PPP-PPP	0.20	10	0.678	0.723	0.0873
226131_s_at	50239.0	41416.1	-0.28	-1.21	PPP-PPP	0.20	10	0.678	0.723	0.0910
212135_s_at	1395.8	1150.5	-0.28	-1.21	PPP-PPP	0.20	10	0.678	0.723	0.0940
221575_at	503.8	415.2	-0.28	-1.21	PPP-PPP	0.20	10	0.678	0.723	0.1013
213645_at	520.9	429.3	-0.28	-1.21	AAP-APP	0.20	10	0.678	0.723	0.1171
212042_x_at	36374.7	29990.7	-0.28	-1.21	PPP-PPP	0.20	10	0.678	0.723	0.0089
228990_at	630.6	520.3	-0.28	-1.21	PPP-PPP	0.20	10	0.678	0.723	0.0255
226215_s_at	2330.8	1922.6	-0.28	-1.21	PPP-PPP	0.20	10	0.678	0.723	0.0264
214041_x_at	1190.3	981.5	-0.28	-1.21	PPP-PPP	0.20	10	0.678	0.723	0.0389
200018_at	43833.4	36162.8	-0.28	-1.21	PPP-PPP	0.20	10	0.678	0.723	0.0554
204905_s_at	5451.4	4500.5	-0.28	-1.21	PPP-PPP	0.20	10	0.678	0.723	0.0737
1555009_a_at	338.5	279.6	-0.28	-1.21	PPP-PPP	0.20	10	0.678	0.723	0.0389
221700_s_at	25330.3	20933.3	-0.28	-1.21	PPP-PPP	0.20	10	0.678	0.723	0.0194
200082_s_at	34246.9	28298.6	-0.28	-1.21	PPP-PPP	0.20	10	0.678	0.723	0.0218
244517_x_at	74.5	61.6	-0.28	-1.21	PPP-PPP	0.20	10	0.678	0.723	0.0559
226511_at	434.3	358.8	-0.28	-1.21	PPP-PPP	0.20	10	0.678	0.723	0.0703
205167_s_at	1218.9	1007.5	-0.28	-1.21	PPP-PPP	0.20	10	0.678	0.723	0.0878
212923_s_at	2988.0	2470.4	-0.27	-1.21	PPP-PPP	0.20	10	0.678	0.723	0.0854
213738_s_at	26887.2	22244.2	-0.27	-1.21	PPP-PPP	0.20	10	0.678	0.723	0.0260
217150_s_at	934.6	773.7	-0.27	-1.21	PPP-PPP	0.20	10	0.678	0.723	0.0357
216457_s_at	5747.9	4758.3	-0.27	-1.21	PPP-PPP	0.20	10	0.678	0.723	0.0619
200869_at	41571.2	34428.1	-0.27	-1.21	PPP-PPP	0.20	10	0.678	0.723	0.0199
1558641_at	1211.1	1003.0	-0.27	-1.21	PPP-PPP	0.20	10	0.678	0.723	0.0330
200014_s_at	14286.7	11832.9	-0.27	-1.21	PPP-PPP	0.20	10	0.678	0.723	0.0544
212954_at	706.8	585.4	-0.27	-1.21	PPP-PPP	0.20	10	0.678	0.723	0.0635
214316_x_at	519.2	429.9	-0.27	-1.21	PPP-PPP	0.20	10	0.678	0.723	0.0715
213941_x_at	35222.8	29163.1	-0.27	-1.21	PPP-PPP	0.20	10	0.678	0.723	0.0779
200818_at	16064.8	13318.0	-0.27	-1.21	PPP-PPP	0.20	10	0.678	0.723	0.0130
210754_s_at	1035.4	858.0	-0.27	-1.21	PPP-PPP	0.20	10	0.678	0.723	0.0285
200933_x_at	43327.8	35915.5	-0.27	-1.21	PPP-PPP	0.20	10	0.678	0.723	0.0475
200064_at	30914.6	25627.1	-0.27	-1.21	PPP-PPP	0.20	10	0.678	0.723	0.0732
217353_at	228.3	189.2	-0.27	-1.21	PPP-PPP	0.20	10	0.678	0.723	0.0880
202931_x_at	1196.0	991.4	-0.27	-1.21	PPP-PPP	0.20	10	0.678	0.723	0.0983
218366_x_at	2720.8	2256.2	-0.27	-1.21	PPP-PPP	0.20	10	0.678	0.723	0.0196
211714_x_at	40109.0	33254.2	-0.27	-1.21	PPP-PPP	0.20	10	0.678	0.723	0.0793

209384_at	1925.5	1598.1	-0.27	-1.21	PPP-PPP	0.20	10	0.678	0.723	0.0195
214351_x_at	40196.0	33356.7	-0.27	-1.21	PPP-PPP	0.20	10	0.678	0.723	0.0237
209510_at	2378.0	1973.4	-0.27	-1.21	PPP-PPP	0.20	10	0.678	0.723	0.0309
225646_at	10514.0	8723.4	-0.27	-1.21	PPP-PPP	0.20	10	0.678	0.723	0.0528
230257_s_at	2104.0	1745.8	-0.27	-1.21	PPP-PPP	0.20	10	0.678	0.723	0.0641
208767_s_at	10721.1	8896.4	-0.27	-1.21	PPP-PPP	0.20	10	0.678	0.723	0.0950
242515_x_at	1789.1	1486.1	-0.27	-1.20	PPP-PPP	0.20	10	0.678	0.723	0.0140
225676_s_at	8338.7	6923.3	-0.27	-1.20	PPP-PPP	0.20	10	0.678	0.723	0.0431
206992_s_at	645.8	536.2	-0.27	-1.20	PPP-PPP	0.20	10	0.678	0.723	0.0483
1555961_a_at	17564.6	14591.1	-0.27	-1.20	PPP-PPP	0.20	10	0.678	0.723	0.0657
51192_at	237.9	197.6	-0.27	-1.20	PPP-PPP	0.20	10	0.678	0.723	0.0790
212724_at	22905.8	19017.2	-0.27	-1.20	PPP-PPP	0.20	10	0.678	0.723	0.0815
200743_s_at	2784.6	2312.7	-0.27	-1.20	PPP-PPP	0.20	10	0.678	0.723	0.0939
208635_x_at	38758.5	32202.8	-0.27	-1.20	PPP-PPP	0.20	10	0.678	0.723	0.0847
242324_x_at	980.7	815.8	-0.27	-1.20	PPP-PPP	0.20	10	0.678	0.723	0.0060
208309_s_at	1180.1	981.4	-0.27	-1.20	PPP-PPP	0.20	10	0.678	0.723	0.0247
AFFX-HUMGAPDH/M3	44257.5	36815.2	-0.27	-1.20	PPP-PPP	0.20	10	0.678	0.723	0.0361
206687_s_at	105.8	88.0	-0.27	-1.20	AAM-APA	0.20	10	0.678	0.723	0.0779
201643_x_at	1252.1	1041.8	-0.27	-1.20	PPP-PPP	0.20	10	0.678	0.723	0.0145
35179_at	175.0	145.7	-0.27	-1.20	AMP-PPP	0.20	10	0.678	0.723	0.0163
225467_s_at	400.0	332.9	-0.27	-1.20	PPP-PPP	0.20	10	0.678	0.723	0.0475
218632_at	833.4	693.3	-0.27	-1.20	PPP-PPP	0.20	10	0.678	0.723	0.0481
212595_s_at	3014.7	2508.6	-0.27	-1.20	PPP-PPP	0.20	10	0.678	0.723	0.0515
223512_at	745.7	620.8	-0.27	-1.20	PPP-PPP	0.20	10	0.678	0.723	0.0910
226206_at	1508.4	1256.2	-0.26	-1.20	PPP-PPP	0.20	10	0.678	0.723	0.0701
227094_at	818.6	682.0	-0.26	-1.20	PPP-PPP	0.20	10	0.678	0.723	0.0119
200022_at	39840.0	33205.0	-0.26	-1.20	PPP-PPP	0.20	10	0.678	0.723	0.0211
202233_s_at	16648.5	13877.1	-0.26	-1.20	PPP-PPP	0.20	10	0.678	0.723	0.0361
200750_s_at	38360.2	31968.0	-0.26	-1.20	PPP-PPP	0.20	10	0.678	0.723	0.0374
1553575_at	1959.1	1632.8	-0.26	-1.20	PPP-PPP	0.20	10	0.678	0.723	0.0585
201769_at	4909.4	4093.0	-0.26	-1.20	PPP-PPP	0.20	10	0.678	0.723	0.0304
217491_x_at	18506.6	15429.7	-0.26	-1.20	PPP-PPP	0.20	10	0.678	0.723	0.0308
231292_at	134.5	112.2	-0.26	-1.20	PPP-PPP	0.20	10	0.678	0.723	0.1064
208695_s_at	43770.0	36536.8	-0.26	-1.20	PPP-PPP	0.20	10	0.678	0.723	0.0221
231234_at	516.7	431.4	-0.26	-1.20	PPP-PPP	0.20	10	0.678	0.723	0.0312
202820_at	1077.2	898.6	-0.26	-1.20	PPP-PPP	0.20	10	0.678	0.723	0.0366
225189_s_at	1694.6	1414.0	-0.26	-1.20	PPP-PPP	0.20	10	0.678	0.723	0.0372
214214_s_at	18537.9	15471.1	-0.26	-1.20	PPP-PPP	0.20	10	0.678	0.723	0.0710
200716_x_at	47128.2	39338.7	-0.26	-1.20	PPP-PPP	0.20	10	0.678	0.723	0.0914
235072_s_at	2866.1	2392.9	-0.26	-1.20	PPP-PPP	0.20	10	0.678	0.723	0.0110
223728_at	415.6	347.0	-0.26	-1.20	PPP-PPP	0.20	10	0.678	0.723	0.0660
210142_x_at	2971.0	2481.0	-0.26	-1.20	PPP-PPP	0.20	10	0.678	0.723	0.0756

1557372_at	98.9	82.6	-0.26	-1.20	PPP-PPP	0.20	10	0.678	0.723	0.0341
202584_at	458.8	383.4	-0.26	-1.20	PPP-PPP	0.20	10	0.678	0.723	0.0848
1565611_at	171.5	143.4	-0.26	-1.20	PPP-PPP	0.20	10	0.678	0.723	0.0149
207169_x_at	693.4	579.8	-0.26	-1.20	PPP-PPP	0.20	10	0.678	0.723	0.0206
205206_at	494.7	413.6	-0.26	-1.20	PPP-PPP	0.20	10	0.678	0.723	0.0751
213113_s_at	2511.2	2100.3	-0.26	-1.20	PPP-PPP	0.20	10	0.678	0.723	0.0837
213700_s_at	314.2	262.8	-0.26	-1.20	MPP-PPP	0.20	10	0.678	0.723	0.0863
208768_x_at	33355.5	27912.4	-0.26	-1.20	PPP-PPP	0.20	10	0.678	0.723	0.0425
200099_s_at	43691.4	36572.1	-0.26	-1.20	PPP-PPP	0.20	10	0.678	0.723	0.0473
213659_at	163.4	136.7	-0.26	-1.20	PPP-PPP	0.20	10	0.678	0.723	0.0593
204102_s_at	35722.8	29894.3	-0.26	-1.20	PPP-PPP	0.20	10	0.678	0.723	0.0767
218407_x_at	2179.0	1823.2	-0.26	-1.20	PPP-PPP	0.20	10	0.678	0.723	0.0849
222821_s_at	1427.4	1195.4	-0.26	-1.19	PPP-PPP	0.20	10	0.678	0.723	0.0082
218888_s_at	5848.0	4897.4	-0.26	-1.19	PPP-PPP	0.20	10	0.678	0.723	0.0656
208697_s_at	19216.5	16088.2	-0.26	-1.19	PPP-PPP	0.20	10	0.678	0.723	0.0700
200963_x_at	43689.8	36592.3	-0.26	-1.19	PPP-PPP	0.20	10	0.678	0.723	0.0861
218572_at	989.3	828.6	-0.26	-1.19	PPP-PPP	0.20	10	0.678	0.723	0.0929
210220_at	4784.2	4005.4	-0.26	-1.19	PPP-PPP	0.20	10	0.678	0.723	0.0974
202870_s_at	8057.7	6747.7	-0.26	-1.19	PPP-PPP	0.20	10	0.678	0.723	0.1015
211623_s_at	14908.2	12493.4	-0.26	-1.19	PPP-PPP	0.20	10	0.678	0.723	0.0342
208668_x_at	30050.3	25176.5	-0.26	-1.19	PPP-PPP	0.20	10	0.678	0.723	0.0365
234687_x_at	231.1	193.7	-0.26	-1.19	PPP-PPP	0.20	10	0.678	0.723	0.0629
201066_at	10555.4	8848.2	-0.26	-1.19	PPP-PPP	0.20	10	0.678	0.723	0.0656
224187_x_at	44441.1	37244.6	-0.26	-1.19	PPP-PPP	0.20	10	0.678	0.723	0.0772
208794_s_at	5543.8	4649.6	-0.25	-1.19	PPP-PPP	0.20	10	0.678	0.723	0.0201
207721_x_at	19598.2	16432.6	-0.25	-1.19	PPP-PPP	0.20	10	0.678	0.723	0.0729
217994_x_at	1657.5	1391.1	-0.25	-1.19	PPP-PPP	0.20	10	0.678	0.723	0.0033
212742_at	1688.7	1416.9	-0.25	-1.19	PPP-PPP	0.20	10	0.678	0.723	0.0071
207243_s_at	41448.7	34783.3	-0.25	-1.19	PPP-PPP	0.20	10	0.678	0.723	0.0391
213356_x_at	36099.0	30290.5	-0.25	-1.19	PPP-PPP	0.20	10	0.678	0.723	0.0437
212563_at	2989.5	2508.0	-0.25	-1.19	PPP-PPP	0.20	10	0.678	0.723	0.0578
214143_x_at	39408.9	33075.0	-0.25	-1.19	PPP-PPP	0.20	10	0.678	0.723	0.0786
212320_at	18383.0	15420.9	-0.25	-1.19	PPP-PPP	0.20	10	0.678	0.723	0.0984
215073_s_at	3629.7	3046.9	-0.25	-1.19	PPP-PPP	0.20	10	0.678	0.723	0.0877
218058_at	896.8	753.5	-0.25	-1.19	PPP-PPP	0.20	10	0.678	0.723	0.0033
200003_s_at	42198.5	35468.2	-0.25	-1.19	PPP-PPP	0.20	10	0.678	0.723	0.0312
228324_at	1202.6	1010.7	-0.25	-1.19	PPP-PPP	0.20	10	0.678	0.723	0.0423
209864_at	392.0	329.7	-0.25	-1.19	PPP-PPP	0.20	10	0.678	0.723	0.0498
212233_at	5590.4	4700.8	-0.25	-1.19	PPP-PPP	0.20	10	0.678	0.723	0.0578
212137_at	14127.8	11883.8	-0.25	-1.19	PPP-PPP	0.20	10	0.678	0.723	0.0814
201364_s_at	1963.5	1650.8	-0.25	-1.19	PPP-PPP	0.20	10	0.678	0.723	0.0842
209577_at	358.3	301.5	-0.25	-1.19	MPP-PPP	0.20	10	0.678	0.723	0.0637

202740_at	906.7	763.4	-0.25	-1.19	PPP-PPP	0.20	10	0.678	0.723	0.0131
209297_at	1244.2	1047.5	-0.25	-1.19	PPP-PPP	0.20	10	0.678	0.723	0.0412
222982_x_at	18088.6	15244.9	-0.25	-1.19	PPP-PPP	0.20	10	0.678	0.723	0.0073
213080_x_at	38445.4	32428.9	-0.25	-1.19	PPP-PPP	0.20	10	0.678	0.723	0.0481
203384_s_at	308.6	260.3	-0.25	-1.19	PPP-PPP	0.20	10	0.678	0.723	0.0504
208874_x_at	1380.6	1165.1	-0.25	-1.19	PPP-PPP	0.20	10	0.678	0.723	0.0021
200937_s_at	34994.6	29521.6	-0.25	-1.19	PPP-PPP	0.20	10	0.678	0.723	0.0207
202519_at	1282.2	1082.3	-0.25	-1.19	PPP-PPP	0.20	10	0.678	0.723	0.0508
203277_at	2070.3	1746.7	-0.25	-1.19	PPP-PPP	0.20	10	0.678	0.723	0.0719
224995_at	2455.0	2072.9	-0.24	-1.18	PPP-PPP	0.20	10	0.678	0.723	0.0140
201258_at	32868.3	27760.0	-0.24	-1.18	PPP-PPP	0.20	10	0.678	0.723	0.0168
205126_at	1891.6	1596.7	-0.24	-1.19	PPP-PPP	0.20	10	0.678	0.723	0.0256
212472_at	3641.3	3074.0	-0.24	-1.19	PPP-PPP	0.20	10	0.678	0.723	0.0461
201530_x_at	30513.3	25772.2	-0.24	-1.18	PPP-PPP	0.20	10	0.678	0.723	0.0726
228810_at	463.5	391.7	-0.24	-1.18	PPP-PPP	0.20	10	0.678	0.723	0.0029
203261_at	3273.0	2765.0	-0.24	-1.18	PPP-PPP	0.20	10	0.678	0.723	0.0506
AFFX-r2-Ec-bioC-5_at	2219.0	1875.5	-0.24	-1.18	PPP-PPP	0.20	10	0.678	0.723	0.0921
201983_s_at	4855.9	4105.5	-0.24	-1.18	PPP-PPP	0.20	10	0.678	0.723	0.0550
227796_at	889.2	751.6	-0.24	-1.18	PPP-PPP	0.20	10	0.678	0.723	0.0573
200853_at	34547.9	29222.8	-0.24	-1.18	PPP-PPP	0.20	10	0.678	0.723	0.0769
212063_at	20996.2	17753.5	-0.24	-1.18	PPP-PPP	0.20	10	0.678	0.723	0.0830
221893_s_at	355.2	300.6	-0.24	-1.18	PPP-PPP	0.20	10	0.678	0.723	0.0122
208641_s_at	19642.8	16619.9	-0.24	-1.18	PPP-PPP	0.20	10	0.678	0.723	0.0216
218358_at	5737.4	4854.5	-0.24	-1.18	PPP-PPP	0.20	10	0.678	0.723	0.0317
212191_x_at	33489.9	28342.6	-0.24	-1.18	PPP-PPP	0.20	10	0.678	0.723	0.0682
218350_s_at	8167.8	6918.1	-0.24	-1.18	PPP-PPP	0.20	10	0.678	0.723	0.0230
1552516_a_at	309.7	262.5	-0.24	-1.18	PPP-PPP	0.20	10	0.678	0.723	0.0161
201322_at	22131.7	18752.9	-0.24	-1.18	PPP-PPP	0.20	10	0.678	0.723	0.0293
206332_s_at	3988.5	3382.0	-0.24	-1.18	PPP-PPP	0.20	10	0.678	0.723	0.0231
220924_s_at	15491.6	13135.0	-0.24	-1.18	PPP-PPP	0.20	10	0.678	0.723	0.0301
226507_at	1122.8	951.7	-0.24	-1.18	PPP-PPP	0.20	10	0.678	0.723	0.0462
205981_s_at	1461.9	1240.4	-0.24	-1.18	PPP-PPP	0.20	10	0.678	0.723	0.0172
221952_x_at	23344.1	19807.8	-0.24	-1.18	PPP-PPP	0.20	10	0.678	0.723	0.0264
203585_at	180.6	153.2	-0.24	-1.18	APP-PPP	0.20	10	0.678	0.723	0.0447
1553551_s_at	32191.2	27309.4	-0.24	-1.18	PPP-PPP	0.20	10	0.678	0.723	0.0642
237563_s_at	634.1	538.2	-0.24	-1.18	PPP-PPP	0.20	10	0.678	0.723	0.0817
200956_s_at	6315.6	5360.7	-0.24	-1.18	PPP-PPP	0.20	10	0.678	0.723	0.0475
225755_at	520.4	441.8	-0.24	-1.18	PPP-PPP	0.20	10	0.678	0.723	0.0864
217492_s_at	3874.8	3292.5	-0.24	-1.18	PPP-PPP	0.20	10	0.678	0.723	0.0026
202432_at	3992.2	3392.1	-0.24	-1.18	PPP-PPP	0.20	10	0.678	0.723	0.0436
211572_s_at	190.4	161.8	-0.24	-1.18	PPP-PPP	0.20	10	0.678	0.723	0.0758
201350_at	877.2	746.1	-0.23	-1.18	PPP-PPP	0.20	10	0.678	0.723	0.0155

201770_at	4880.5	4149.7	-0.23	-1.18	PPP-PPP	0.20	10	0.678	0.723	0.0206
218754_at	2577.9	2192.4	-0.23	-1.18	PPP-PPP	0.20	10	0.678	0.723	0.0539
208905_at	26037.9	22143.3	-0.23	-1.18	PPP-PPP	0.20	10	0.678	0.723	0.0635
1564521_x_at	3860.6	3283.7	-0.23	-1.18	PPP-PPP	0.20	10	0.678	0.723	0.0066
209478_at	3439.7	2926.9	-0.23	-1.18	PPP-PPP	0.20	10	0.678	0.723	0.0249
205053_at	2226.0	1893.7	-0.23	-1.18	PPP-PPP	0.20	10	0.678	0.723	0.0633
201094_at	36980.5	31457.0	-0.23	-1.18	PPP-PPP	0.20	10	0.678	0.723	0.0675
224160_s_at	1192.9	1016.0	-0.23	-1.17	PPP-PPP	0.20	10	0.678	0.723	0.0320
200735_x_at	36257.4	30869.1	-0.23	-1.18	PPP-PPP	0.20	10	0.678	0.723	0.0411
203667_at	20415.0	17396.1	-0.23	-1.17	PPP-PPP	0.20	10	0.678	0.723	0.0314
200705_s_at	28391.7	24186.1	-0.23	-1.17	PPP-PPP	0.20	10	0.678	0.723	0.0800
225844_at	3227.0	2751.4	-0.23	-1.17	PPP-PPP	0.20	10	0.678	0.723	0.0272
217719_at	27187.8	23204.0	-0.23	-1.17	PPP-PPP	0.20	10	0.678	0.723	0.0263
215773_x_at	3658.3	3121.6	-0.23	-1.17	PPP-PPP	0.20	10	0.678	0.723	0.0321
211327_x_at	292.9	249.9	-0.23	-1.17	PPP-PPP	0.20	10	0.678	0.723	0.0412
203383_s_at	719.7	614.1	-0.23	-1.17	PPP-PPP	0.20	10	0.678	0.723	0.0432
225943_at	1118.7	954.7	-0.23	-1.17	PPP-PPP	0.20	10	0.678	0.723	0.0518
200077_s_at	29796.2	25429.0	-0.23	-1.17	PPP-PPP	0.20	10	0.678	0.723	0.0570
229447_x_at	9403.3	8022.4	-0.23	-1.17	PPP-PPP	0.20	10	0.678	0.723	0.0645
203401_at	4242.9	3622.7	-0.23	-1.17	PPP-PPP	0.20	10	0.678	0.723	0.0145
218482_at	6796.8	5803.2	-0.23	-1.17	PPP-PPP	0.20	10	0.678	0.723	0.0155
207265_s_at	2818.8	2406.6	-0.23	-1.17	PPP-PPP	0.20	10	0.678	0.723	0.0513
214804_at	1459.2	1246.8	-0.23	-1.17	PPP-PPP	0.20	10	0.678	0.723	0.0332
217746_s_at	6878.0	5877.4	-0.23	-1.17	PPP-PPP	0.20	10	0.678	0.723	0.0737
201853_s_at	3307.4	2827.7	-0.23	-1.17	PPP-PPP	0.20	10	0.678	0.723	0.0031
201900_s_at	1968.6	1682.9	-0.23	-1.17	PPP-PPP	0.20	10	0.678	0.723	0.0640
232215_x_at	4088.8	3496.9	-0.23	-1.17	PPP-PPP	0.20	10	0.678	0.723	0.0722
213881_x_at	26764.1	22892.1	-0.23	-1.17	PPP-PPP	0.20	10	0.678	0.723	0.0416
208864_s_at	22220.6	19012.4	-0.23	-1.17	PPP-PPP	0.20	10	0.678	0.723	0.0621
201020_at	3605.2	3086.7	-0.22	-1.17	PPP-PPP	0.20	10	0.678	0.723	0.0623
212079_s_at	194.9	167.1	-0.22	-1.17	PPP-PPP	0.20	10	0.678	0.723	0.0120
201846_s_at	2167.8	1859.2	-0.22	-1.17	PPP-PPP	0.20	10	0.678	0.723	0.0585
215832_x_at	1175.7	1009.0	-0.22	-1.17	PPP-PPP	0.20	10	0.678	0.723	0.0146
214941_s_at	3216.2	2758.7	-0.22	-1.17	PPP-PPP	0.20	10	0.678	0.723	0.0424
209953_s_at	5743.8	4928.5	-0.22	-1.17	PPP-PPP	0.20	10	0.678	0.723	0.0425
224822_at	6091.5	5227.2	-0.22	-1.17	PPP-PPP	0.20	10	0.678	0.723	0.0515
218237_s_at	5749.4	4932.4	-0.22	-1.17	PPP-PPP	0.20	10	0.678	0.723	0.0562
225464_at	16243.9	13948.2	-0.22	-1.17	PPP-PPP	0.20	10	0.678	0.723	0.0246
217871_s_at	24742.6	21236.3	-0.22	-1.17	PPP-PPP	0.20	10	0.678	0.723	0.0339
217725_x_at	14325.3	12301.6	-0.22	-1.17	PPP-PPP	0.20	10	0.678	0.723	0.0551
200886_s_at	23548.4	20213.6	-0.22	-1.17	PPP-PPP	0.20	10	0.678	0.723	0.0654
204521_at	4317.5	3710.6	-0.22	-1.16	PPP-PPP	0.20	10	0.678	0.723	0.0215

222064_s_at	729.4	627.3	-0.22	-1.16	PPP-PPP	0.20	10	0.678	0.723	0.0146
208628_s_at	33413.4	28723.5	-0.22	-1.16	PPP-PPP	0.20	10	0.678	0.723	0.0584
209665_at	832.7	716.1	-0.22	-1.16	PPP-PPP	0.20	10	0.678	0.723	0.0602
210087_s_at	1298.3	1116.5	-0.22	-1.16	PPP-PPP	0.20	10	0.678	0.723	0.0664
223032_x_at	14669.6	12622.2	-0.22	-1.16	PPP-PPP	0.20	10	0.678	0.723	0.0322
219032_x_at	1311.0	1127.6	-0.22	-1.16	PPP-PPP	0.20	10	0.678	0.723	0.0642
201632_at	4827.0	4154.6	-0.22	-1.16	PPP-PPP	0.20	10	0.678	0.723	0.0311
200647_x_at	16286.8	14022.2	-0.22	-1.16	PPP-PPP	0.20	10	0.678	0.723	0.0363
233665_x_at	2024.7	1743.2	-0.22	-1.16	PPP-PPP	0.20	10	0.678	0.723	0.0393
208884_s_at	1699.3	1463.0	-0.22	-1.16	PPP-PPP	0.20	10	0.678	0.723	0.0405
217294_s_at	25969.3	22357.5	-0.22	-1.16	PPP-PPP	0.20	10	0.678	0.723	0.0428
223171_at	1473.0	1268.2	-0.22	-1.16	PPP-PPP	0.20	10	0.678	0.723	0.0454
201400_at	12001.3	10333.5	-0.22	-1.16	PPP-PPP	0.20	10	0.678	0.723	0.0530
1553088_a_at	190.0	163.5	-0.22	-1.16	APP-APP	0.20	10	0.678	0.723	0.0538
204602_at	16990.7	14632.0	-0.22	-1.16	PPP-PPP	0.20	10	0.678	0.723	0.0611
201919_at	1723.9	1483.9	-0.22	-1.16	PPP-PPP	0.20	10	0.678	0.723	0.0633
227060_at	415.3	357.7	-0.22	-1.16	AAA-APP	0.20	10	0.678	0.723	0.0304
200888_s_at	26731.5	23048.3	-0.21	-1.16	PPP-PPP	0.20	10	0.678	0.723	0.0170
226528_at	1176.5	1014.7	-0.21	-1.16	PPP-PPP	0.20	10	0.678	0.723	0.0494
224479_s_at	1632.5	1407.1	-0.21	-1.16	PPP-PPP	0.20	10	0.678	0.723	0.0648
217816_s_at	6419.2	5532.9	-0.21	-1.16	PPP-PPP	0.20	10	0.678	0.723	0.0649
223062_s_at	10235.7	8831.5	-0.21	-1.16	PPP-PPP	0.20	10	0.678	0.723	0.0427
209218_at	4054.4	3498.5	-0.21	-1.16	PPP-PPP	0.20	10	0.678	0.723	0.0450
205300_s_at	917.1	791.0	-0.21	-1.16	PPP-PPP	0.20	10	0.678	0.723	0.0553
201812_s_at	13327.1	11502.6	-0.21	-1.16	PPP-PPP	0.20	10	0.678	0.723	0.0270
222992_s_at	19951.9	17224.4	-0.21	-1.16	PPP-PPP	0.20	10	0.678	0.723	0.0446
211530_x_at	1288.9	1112.7	-0.21	-1.16	PPP-PPP	0.20	10	0.678	0.723	0.0482
201990_s_at	573.7	495.5	-0.21	-1.16	PPP-PPP	0.20	10	0.678	0.723	0.0128
203342_at	820.0	708.5	-0.21	-1.16	PPP-PPP	0.20	10	0.678	0.723	0.0227
35160_at	568.8	491.5	-0.21	-1.16	PPP-PPP	0.20	10	0.678	0.723	0.0581
218557_at	2020.2	1746.1	-0.21	-1.16	PPP-PPP	0.20	10	0.678	0.723	0.0008
211997_x_at	28609.6	24729.2	-0.21	-1.16	PPP-PPP	0.20	10	0.678	0.723	0.0402
225823_at	3036.0	2624.6	-0.21	-1.16	PPP-PPP	0.20	10	0.678	0.723	0.0496
229325_at	762.0	658.8	-0.21	-1.16	PPP-PPP	0.20	10	0.678	0.723	0.0562
200068_s_at	18154.2	15698.1	-0.21	-1.16	PPP-PPP	0.20	10	0.678	0.723	0.0580
202830_s_at	437.4	378.6	-0.21	-1.16	AAP-MPP	0.20	10	0.678	0.723	0.0578
216342_x_at	23279.4	20162.7	-0.21	-1.16	PPP-PPP	0.20	10	0.678	0.723	0.0280
216971_s_at	1375.8	1191.5	-0.21	-1.16	PPP-PPP	0.20	10	0.678	0.723	0.0386
218041_x_at	17193.8	14910.6	-0.21	-1.15	PPP-PPP	0.20	10	0.678	0.723	0.0113
200638_s_at	13447.6	11657.7	-0.21	-1.15	PPP-PPP	0.20	10	0.678	0.723	0.0415
202073_at	1033.3	895.9	-0.21	-1.15	PPP-PPP	0.20	10	0.678	0.723	0.0532
226434_at	666.4	578.3	-0.21	-1.15	PPM-PPP	0.20	10	0.678	0.723	0.0181

200792_at	20266.9	17583.7	-0.21	-1.15	PPP-PPP	0.20	10	0.678	0.723	0.0251
55705_at	1998.9	1734.2	-0.21	-1.15	PPP-PPP	0.20	10	0.678	0.723	0.0275
215792_s_at	1767.9	1533.7	-0.21	-1.15	PPP-PPP	0.20	10	0.678	0.723	0.0300
206929_s_at	1298.3	1126.7	-0.21	-1.15	PPP-PPP	0.20	10	0.678	0.723	0.0424
202399_s_at	2121.6	1840.9	-0.21	-1.15	PPP-PPP	0.20	10	0.678	0.723	0.0517
204180_s_at	405.8	352.2	-0.20	-1.15	PPP-PPP	0.20	10	0.678	0.723	0.0145
213858_at	220.5	191.6	-0.20	-1.15	AAP-AAA	0.20	10	0.678	0.723	0.0207
226715_at	890.8	774.6	-0.20	-1.15	PPP-PPP	0.20	10	0.678	0.723	0.0429
203714_s_at	3561.4	3098.6	-0.20	-1.15	PPP-PPP	0.20	10	0.678	0.723	0.0359
200074_s_at	24421.3	21248.6	-0.20	-1.15	PPP-PPP	0.20	10	0.678	0.723	0.0460
223337_at	1438.5	1252.9	-0.20	-1.15	PPP-PPP	0.20	10	0.678	0.723	0.0168
225948_at	2112.0	1839.4	-0.20	-1.15	PPP-PPP	0.20	10	0.678	0.723	0.0187
212658_at	2485.8	2164.8	-0.20	-1.15	PPP-PPP	0.20	10	0.678	0.723	0.0239
224999_at	3307.9	2881.9	-0.20	-1.15	PPP-PPP	0.20	10	0.678	0.723	0.0238
226231_at	1131.2	985.7	-0.20	-1.15	PPP-PPP	0.20	10	0.678	0.723	0.0308
213129_s_at	8815.7	7678.7	-0.20	-1.15	PPP-PPP	0.20	10	0.678	0.723	0.0494
218672_at	2050.1	1785.4	-0.20	-1.15	PPP-PPP	0.20	10	0.678	0.723	0.0517
212159_x_at	2313.8	2016.8	-0.20	-1.15	PPP-PPP	0.20	10	0.678	0.723	0.0162
203040_s_at	1272.4	1109.3	-0.20	-1.15	PPP-PPP	0.20	10	0.678	0.723	0.0203
202299_s_at	7919.2	6909.1	-0.20	-1.15	PPP-PPP	0.20	10	0.678	0.723	0.0104
207705_s_at	233.5	203.8	-0.20	-1.15	PPP-PPP	0.20	10	0.678	0.723	0.0269
201391_at	4121.5	3600.3	-0.20	-1.15	PPP-PPP	0.20	10	0.678	0.723	0.0350
212967_x_at	17890.6	15638.6	-0.19	-1.14	PPP-PPP	0.20	10	0.678	0.723	0.0240
216326_s_at	2279.9	1994.0	-0.19	-1.14	PPP-PPP	0.20	10	0.678	0.723	0.0229
208756_at	13065.1	11434.3	-0.19	-1.14	PPP-PPP	0.20	10	0.678	0.723	0.0326
217768_at	19445.2	17016.7	-0.19	-1.14	PPP-PPP	0.20	10	0.678	0.723	0.0352
201946_s_at	16849.8	14762.2	-0.19	-1.14	PPP-PPP	0.20	10	0.678	0.723	0.0085
200957_s_at	6147.5	5393.5	-0.19	-1.14	PPP-PPP	0.20	10	0.678	0.723	0.0209
213059_at	3146.9	2760.5	-0.19	-1.14	PPP-PPP	0.20	10	0.678	0.723	0.0252
200023_s_at	22173.6	19458.9	-0.19	-1.14	PPP-PPP	0.20	10	0.678	0.723	0.0172
214100_x_at	789.9	693.5	-0.19	-1.14	PPP-PPP	0.20	10	0.678	0.723	0.0184
202115_s_at	1346.7	1182.4	-0.19	-1.14	PPP-PPP	0.20	10	0.678	0.723	0.0368
212426_s_at	21406.9	18799.3	-0.19	-1.14	PPP-PPP	0.20	10	0.678	0.723	0.0183
225093_at	1189.9	1046.2	-0.19	-1.14	PPP-PPP	0.20	10	0.678	0.723	0.0191
231890_at	243.0	213.8	-0.19	-1.14	PPP-PPP	0.20	10	0.678	0.723	0.0280
209916_at	1100.5	971.7	-0.18	-1.13	PPP-PPP	0.20	10	0.678	0.723	0.0122
212097_at	25652.6	22665.2	-0.18	-1.13	PPP-PPP	0.20	10	0.678	0.723	0.0219
201487_at	6396.4	5652.7	-0.18	-1.13	PPP-PPP	0.20	10	0.678	0.723	0.0302
207039_at	4425.4	3915.7	-0.18	-1.13	PPP-PPP	0.20	10	0.678	0.723	0.0190
206238_s_at	681.1	602.7	-0.18	-1.13	PPP-PPP	0.20	10	0.678	0.723	0.0032
221190_s_at	1129.3	999.4	-0.18	-1.13	PPP-PPP	0.20	10	0.678	0.723	0.0219
213709_at	328.0	290.4	-0.18	-1.13	PPP-PPP	0.20	10	0.678	0.723	0.0251

201292_at	7545.6	6681.6	-0.18	-1.13	PPP-PPP	0.20	10	0.678	0.723	0.0030
225340_s_at	10622.5	9416.0	-0.17	-1.13	PPP-PPP	0.20	10	0.678	0.723	0.0044
208682_s_at	1676.6	1486.1	-0.17	-1.13	PPP-PPP	0.20	10	0.678	0.723	0.0121
226020_s_at	715.9	634.8	-0.17	-1.13	PPP-PPP	0.20	10	0.678	0.723	0.0140
211609_x_at	6538.7	5802.8	-0.17	-1.13	PPP-PPP	0.20	10	0.678	0.723	0.0018
200631_s_at	16383.9	14553.6	-0.17	-1.13	PPP-PPP	0.20	10	0.678	0.723	0.0045
214039_s_at	17379.3	15514.8	-0.16	-1.12	PPP-PPP	0.20	10	0.678	0.723	0.0160
202418_at	3722.1	3338.4	-0.16	-1.12	PPP-PPP	0.20	10	0.678	0.723	0.0072
208117_s_at	1583.1	1419.4	-0.16	-1.12	PPP-PPP	0.20	10	0.678	0.723	0.0079
205292_s_at	23151.1	20804.3	-0.15	-1.11	PPP-PPP	0.20	10	0.678	0.723	0.0052
209340_at	10690.7	9700.2	-0.14	-1.10	PPP-PPP	0.20	10	0.678	0.723	0.0030
219567_s_at	6.7	91.3	3.78	13.69	AAA-PPP	0.10	11	0.861	0.984	0.0345
210258_at	3.2	29.4	3.21	9.23	AAA-APA	0.10	11	0.861	0.984	0.0574
242049_s_at	9.5	60.3	2.67	6.38	AAA-PAA	0.10	11	0.861	0.984	0.0733
1552765_x_at	4.8	29.6	2.63	6.18	AAA-AAP	0.10	11	0.861	0.984	0.0285
243573_at	7.4	43.9	2.56	5.91	AAA-AAP	0.10	11	0.861	0.984	0.0390
239895_at	4.6	26.2	2.52	5.75	AAA-APP	0.10	11	0.861	0.984	0.0274
222307_at	11.9	67.6	2.51	5.71	AAA-APP	0.10	11	0.861	0.984	0.0576
216062_at	9.9	45.0	2.18	4.54	AAA-MMP	0.10	11	0.861	0.984	0.0434
210941_at	8.1	36.4	2.16	4.48	AAA-MAP	0.10	11	0.861	0.984	0.0644
203561_at	19.6	4.4	-2.14	-4.42	AAA-PAA	0.10	11	0.861	0.984	0.3117
220064_at	22.1	5.0	-2.14	-4.42	PAA-AAA	0.10	11	0.861	0.984	0.3383
219740_at	15.5	67.0	2.11	4.32	AAA-APA	0.10	11	0.861	0.984	0.0491
214740_at	18.1	77.5	2.10	4.28	AAA-AAP	0.10	11	0.861	0.984	0.0272
229799_s_at	9.6	40.8	2.09	4.26	AAA-PAA	0.10	11	0.861	0.984	0.0237
240273_at	45.6	11.1	-2.03	-4.10	AAA-PAA	0.10	11	0.861	0.984	0.2820
215653_at	8.3	2.1	-2.03	-4.07	AAP-AAA	0.10	11	0.861	0.984	0.2860
235821_at	50.3	12.5	-2.01	-4.02	APA-AAA	0.10	11	0.861	0.984	0.2849
206355_at	8.9	35.2	1.98	3.94	AAA-APP	0.10	11	0.861	0.984	0.0383
230309_at	16.8	4.3	-1.96	-3.89	PAP-AAA	0.10	11	0.861	0.984	0.3106
214893_x_at	20.6	79.1	1.94	3.84	AAA-PAA	0.10	11	0.861	0.984	0.0626
205208_at	98.0	25.9	-1.92	-3.78	AAP-AAA	0.10	11	0.861	0.984	0.3699
1562352_at	23.6	6.3	-1.91	-3.76	AAA-APA	0.10	11	0.861	0.984	0.2874
215086_at	39.3	10.4	-1.91	-3.76	APA-APP	0.10	11	0.861	0.984	0.3374
240251_at	49.4	13.2	-1.91	-3.75	AAM-APP	0.10	11	0.861	0.984	0.2816
217440_at	12.3	3.3	-1.91	-3.75	APM-AAA	0.10	11	0.861	0.984	0.3027
1566605_at	46.3	12.4	-1.91	-3.75	APP-AAA	0.10	11	0.861	0.984	0.3293
244714_at	9.5	35.5	1.90	3.73	AAA-PAA	0.10	11	0.861	0.984	0.0508
1566137_s_at	39.7	10.7	-1.89	-3.71	AAP-AAA	0.10	11	0.861	0.984	0.3002
220578_at	76.1	20.5	-1.89	-3.70	PAA-AAA	0.10	11	0.861	0.984	0.3178
232399_at	42.9	11.6	-1.88	-3.69	AAA-APA	0.10	11	0.861	0.984	0.3298
1570135_at	57.4	16.0	-1.84	-3.59	PMM-AAP	0.10	11	0.861	0.984	0.2978

213919_at	110.1	30.8	-1.84	-3.57	PAP-PAP	0.10	11	0.861	0.984	0.2890
243878_at	14.6	4.1	-1.83	-3.56	PAA-AAA	0.10	11	0.861	0.984	0.4008
230523_at	12.6	44.7	1.83	3.56	AAA-PPA	0.10	11	0.861	0.984	0.0483
1555373_at	42.9	12.2	-1.82	-3.54	AAP-AAP	0.10	11	0.861	0.984	0.3030
237355_at	17.5	61.7	1.82	3.53	AAA-PPM	0.10	11	0.861	0.984	0.0537
234472_at	30.0	8.5	-1.82	-3.53	AAA-AAP	0.10	11	0.861	0.984	0.3260
210611_s_at	15.6	4.4	-1.81	-3.51	PAA-AAA	0.10	11	0.861	0.984	0.2911
238919_at	17.8	62.2	1.81	3.50	AAA-PPP	0.10	11	0.861	0.984	0.0607
1562094_at	12.7	3.7	-1.79	-3.46	AAP-AAA	0.10	11	0.861	0.984	0.2990
229591_at	92.3	26.8	-1.79	-3.45	PAA-AAA	0.10	11	0.861	0.984	0.3397
1552747_a_at	24.3	7.0	-1.79	-3.45	APA-AAA	0.10	11	0.861	0.984	0.3562
233164_x_at	94.4	27.6	-1.77	-3.42	PMA-APA	0.10	11	0.861	0.984	0.3193
236285_at	72.0	21.2	-1.77	-3.41	PPM-APA	0.10	11	0.861	0.984	0.2839
1556232_at	48.0	14.1	-1.77	-3.40	AAA-PAA	0.10	11	0.861	0.984	0.3267
1563881_at	8.9	2.6	-1.76	-3.38	AAP-AAA	0.10	11	0.861	0.984	0.3781
233327_at	35.2	10.4	-1.75	-3.37	AAA-AAP	0.10	11	0.861	0.984	0.3421
231636_s_at	22.5	6.8	-1.72	-3.29	PAA-AAA	0.10	11	0.861	0.984	0.3109
1552939_at	24.2	7.4	-1.72	-3.29	AAP-APA	0.10	11	0.861	0.984	0.3144
208154_at	103.6	31.7	-1.71	-3.26	APP-APP	0.10	11	0.861	0.984	0.2783
214676_x_at	48.2	14.9	-1.69	-3.23	APA-AAA	0.10	11	0.861	0.984	0.3120
238049_at	61.7	19.2	-1.69	-3.22	APA-AAP	0.10	11	0.861	0.984	0.2958
243485_at	58.9	18.4	-1.68	-3.20	APP-PAP	0.10	11	0.861	0.984	0.3166
236793_at	13.2	4.2	-1.67	-3.18	AAP-AAA	0.10	11	0.861	0.984	0.3347
218591_s_at	26.2	83.1	1.67	3.18	AAA-APP	0.10	11	0.861	0.984	0.0547
1569124_at	21.1	6.7	-1.66	-3.15	AAP-AAA	0.10	11	0.861	0.984	0.3105
207008_at	26.2	8.3	-1.65	-3.15	AAP-AAA	0.10	11	0.861	0.984	0.3357
1565702_at	11.5	3.7	-1.65	-3.14	PAA-AAA	0.10	11	0.861	0.984	0.3402
211508_s_at	16.7	5.3	-1.65	-3.14	MPA-AAA	0.10	11	0.861	0.984	0.4181
1563876_at	35.5	11.3	-1.65	-3.14	PPA-APA	0.10	11	0.861	0.984	0.4259
215457_at	38.9	12.4	-1.65	-3.13	AAA-APM	0.10	11	0.861	0.984	0.4288
1569403_at	35.9	11.6	-1.64	-3.11	PAA-AAP	0.10	11	0.861	0.984	0.3197
211295_x_at	82.6	26.7	-1.63	-3.09	PPA-APA	0.10	11	0.861	0.984	0.3610
238317_x_at	54.0	17.5	-1.63	-3.08	MPP-APP	0.10	11	0.861	0.984	0.3621
233502_at	43.4	14.2	-1.62	-3.06	AAP-AAA	0.10	11	0.861	0.984	0.2854
220909_at	57.5	18.8	-1.61	-3.05	AAA-APA	0.10	11	0.861	0.984	0.3193
207394_at	70.5	23.1	-1.61	-3.05	APP-APA	0.10	11	0.861	0.984	0.2956
242500_at	46.2	15.1	-1.61	-3.05	AAP-AAA	0.10	11	0.861	0.984	0.3034
1554907_a_at	80.8	26.5	-1.61	-3.05	PMA-AMA	0.10	11	0.861	0.984	0.3291
201261_x_at	58.4	19.2	-1.61	-3.04	AAP-AAA	0.10	11	0.861	0.984	0.3397
219975_x_at	42.9	14.2	-1.60	-3.02	PAA-PAA	0.10	11	0.861	0.984	0.2776
1563519_at	23.9	7.9	-1.59	-3.02	PAA-AAA	0.10	11	0.861	0.984	0.4157
214417_s_at	30.6	10.2	-1.59	-3.01	AAP-AMA	0.10	11	0.861	0.984	0.3409

1555313_a_at	14.0	4.7	-1.59	-3.00	AAP-AAA	0.10	11	0.861	0.984	0.4787
244594_x_at	57.6	19.2	-1.58	-3.00	AAA-APP	0.10	11	0.861	0.984	0.3166
243746_at	87.9	29.4	-1.58	-3.00	AAP-APP	0.10	11	0.861	0.984	0.2800
243465_at	62.6	21.1	-1.57	-2.97	AMA-MAP	0.10	11	0.861	0.984	0.2803
1562736_at	17.3	5.9	-1.57	-2.97	AAA-PAA	0.10	11	0.861	0.984	0.4462
1562473_at	17.5	5.9	-1.57	-2.96	PAA-AAA	0.10	11	0.861	0.984	0.3977
237377_at	70.0	23.8	-1.56	-2.95	PPA-AAP	0.10	11	0.861	0.984	0.3589
212611_at	70.3	24.0	-1.55	-2.93	APA-AAA	0.10	11	0.861	0.984	0.2992
1558893_a_at	11.8	4.0	-1.55	-2.92	AAP-AAA	0.10	11	0.861	0.984	0.3258
1554958_at	76.0	26.1	-1.54	-2.91	AAP-AAA	0.10	11	0.861	0.984	0.3408
1564836_at	32.3	11.1	-1.54	-2.90	AAA-PAA	0.10	11	0.861	0.984	0.4019
235763_at	22.7	7.8	-1.53	-2.89	PAA-AAA	0.10	11	0.861	0.984	0.3804
240442_at	54.4	18.9	-1.53	-2.89	MAA-APA	0.10	11	0.861	0.984	0.3629
234534_at	63.9	22.3	-1.52	-2.86	PAM-AAA	0.10	11	0.861	0.984	0.3320
215252_at	45.7	130.8	1.52	2.86	AAA-APP	0.10	11	0.861	0.984	0.0595
216753_at	62.8	22.0	-1.51	-2.86	PAA-AAA	0.10	11	0.861	0.984	0.2920
228108_at	17.8	50.6	1.51	2.85	AAA-PPP	0.10	11	0.861	0.984	0.0244
226636_at	124.3	43.7	-1.51	-2.85	AAP-AAM	0.10	11	0.861	0.984	0.3282
242965_at	53.0	18.7	-1.51	-2.84	AAA-APA	0.10	11	0.861	0.984	0.4368
1556849_at	28.1	9.9	-1.50	-2.83	AAM-AAP	0.10	11	0.861	0.984	0.3981
206111_at	57.8	20.4	-1.50	-2.83	APP-AAA	0.10	11	0.861	0.984	0.2975
211298_s_at	34.8	12.3	-1.50	-2.83	PAA-AAA	0.10	11	0.861	0.984	0.4290
1562591_a_at	51.4	18.2	-1.50	-2.83	AAM-AAP	0.10	11	0.861	0.984	0.3814
1565810_at	41.5	14.7	-1.50	-2.82	APP-AAA	0.10	11	0.861	0.984	0.3702
1563348_at	41.3	14.7	-1.49	-2.82	AAP-AAP	0.10	11	0.861	0.984	0.3214
1560696_x_at	79.0	28.1	-1.49	-2.82	PAP-AAP	0.10	11	0.861	0.984	0.3474
1561368_at	35.3	12.5	-1.49	-2.81	PAA-AAA	0.10	11	0.861	0.984	0.3362
234209_at	30.1	10.7	-1.49	-2.81	AAA-AAP	0.10	11	0.861	0.984	0.3300
239262_at	21.9	7.8	-1.49	-2.80	PAA-AAA	0.10	11	0.861	0.984	0.4442
242622_x_at	55.4	19.8	-1.49	-2.80	AAA-AAP	0.10	11	0.861	0.984	0.3127
213960_at	38.1	13.6	-1.49	-2.80	APA-AAA	0.10	11	0.861	0.984	0.3144
227425_at	75.6	27.1	-1.48	-2.79	PPA-AAA	0.10	11	0.861	0.984	0.2929
1570424_at	13.8	5.0	-1.48	-2.79	APA-AAA	0.10	11	0.861	0.984	0.4012
207527_at	50.0	18.0	-1.47	-2.78	PAA-AAA	0.10	11	0.861	0.984	0.3120
208212_s_at	45.2	16.3	-1.47	-2.77	AAP-AAA	0.10	11	0.861	0.984	0.2739
220405_at	15.6	5.6	-1.47	-2.77	APA-AAA	0.10	11	0.861	0.984	0.3731
1564097_at	9.7	3.5	-1.47	-2.76	PAA-AAA	0.10	11	0.861	0.984	0.3102
211592_s_at	38.2	13.8	-1.47	-2.76	AAA-AAP	0.10	11	0.861	0.984	0.4108
244368_x_at	90.2	32.7	-1.46	-2.76	AAP-PAP	0.10	11	0.861	0.984	0.3758
223153_x_at	93.3	256.6	1.46	2.75	AAA-APA	0.10	11	0.861	0.984	0.0225
1570376_at	45.9	16.7	-1.46	-2.74	AAP-AAA	0.10	11	0.861	0.984	0.3162
1558494_at	25.1	9.2	-1.46	-2.74	PAA-AAA	0.10	11	0.861	0.984	0.4701

238205_at	9.5	25.9	1.45	2.73	AAA-AAP	0.10	11	0.861	0.984	0.0346
241329_s_at	64.4	23.6	-1.45	-2.73	AAA-PAA	0.10	11	0.861	0.984	0.3620
231029_at	11.9	4.4	-1.45	-2.72	APA-AAA	0.10	11	0.861	0.984	0.4831
231983_at	149.7	55.0	-1.44	-2.72	PPA-APA	0.10	11	0.861	0.984	0.3007
221648_s_at	39.2	14.4	-1.44	-2.72	AAA-AAP	0.10	11	0.861	0.984	0.3458
1556486_at	45.4	16.7	-1.44	-2.71	AAP-AAA	0.10	11	0.861	0.984	0.3489
241823_at	92.3	34.0	-1.44	-2.71	PPP-APP	0.10	11	0.861	0.984	0.3646
242540_at	37.3	13.7	-1.44	-2.71	MAM-APP	0.10	11	0.861	0.984	0.4692
226375_at	162.2	440.0	1.44	2.71	MPA-PPP	0.10	11	0.861	0.984	0.0312
1561389_at	72.9	26.9	-1.44	-2.71	APA-MAA	0.10	11	0.861	0.984	0.3328
209802_at	60.5	22.4	-1.44	-2.71	AAA-PMA	0.10	11	0.861	0.984	0.3602
205397_x_at	117.1	315.3	1.43	2.69	AAA-APP	0.10	11	0.861	0.984	0.0558
241249_at	34.3	12.7	-1.43	-2.69	PAA-AAA	0.10	11	0.861	0.984	0.4143
1559441_s_at	35.9	13.3	-1.43	-2.69	APA-PAA	0.10	11	0.861	0.984	0.4150
221201_s_at	34.9	93.9	1.43	2.69	AAA-PPM	0.10	11	0.861	0.984	0.0217
234450_at	89.7	33.4	-1.42	-2.68	APA-AAA	0.10	11	0.861	0.984	0.3460
207437_at	54.4	20.3	-1.42	-2.67	AAA-PAP	0.10	11	0.861	0.984	0.3099
216415_at	27.2	10.2	-1.42	-2.67	AAA-PAA	0.10	11	0.861	0.984	0.3234
238121_at	63.8	170.4	1.42	2.67	PPP-PPP	0.10	11	0.861	0.984	0.0280
231689_at	62.1	23.5	-1.40	-2.64	AAA-MPA	0.10	11	0.861	0.984	0.4020
205030_at	35.1	13.3	-1.40	-2.64	PAA-AAA	0.10	11	0.861	0.984	0.4510
1558595_at	11.2	29.6	1.40	2.64	AAA-AMP	0.10	11	0.861	0.984	0.0425
219764_at	22.7	8.6	-1.40	-2.63	PAA-AAA	0.10	11	0.861	0.984	0.2995
231541_s_at	44.1	16.8	-1.39	-2.63	APA-AAA	0.10	11	0.861	0.984	0.3782
240895_at	22.0	8.4	-1.39	-2.62	PAA-AAA	0.10	11	0.861	0.984	0.4385
1554672_at	65.3	24.9	-1.39	-2.62	PPA-AAP	0.10	11	0.861	0.984	0.3431
220897_at	27.7	10.6	-1.39	-2.62	PAA-APA	0.10	11	0.861	0.984	0.3514
243961_at	59.7	22.9	-1.38	-2.61	AAP-APA	0.10	11	0.861	0.984	0.4159
1568934_at	21.3	8.2	-1.38	-2.61	PAA-AAA	0.10	11	0.861	0.984	0.4174
240228_at	39.8	15.3	-1.38	-2.61	AAA-PAA	0.10	11	0.861	0.984	0.3493
240931_s_at	43.2	16.6	-1.38	-2.60	AAA-APA	0.10	11	0.861	0.984	0.2757
207346_at	229.7	88.3	-1.38	-2.60	PPM-APA	0.10	11	0.861	0.984	0.3058
242894_at	25.5	9.8	-1.38	-2.60	AAA-AAP	0.10	11	0.861	0.984	0.4774
220518_at	16.3	6.3	-1.37	-2.59	APA-AAA	0.10	11	0.861	0.984	0.3847
222856_at	78.1	30.2	-1.37	-2.58	APA-AAP	0.10	11	0.861	0.984	0.3033
227868_at	77.0	29.9	-1.37	-2.58	AAA-APA	0.10	11	0.861	0.984	0.3570
237727_at	71.9	27.9	-1.37	-2.58	PAA-AAA	0.10	11	0.861	0.984	0.4148
1561468_at	31.0	12.0	-1.37	-2.58	AAA-APA	0.10	11	0.861	0.984	0.4005
231644_at	42.0	16.3	-1.37	-2.58	AAA-PAA	0.10	11	0.861	0.984	0.4250
217606_at	68.8	177.6	1.37	2.58	AAA-APP	0.10	11	0.861	0.984	0.0369
231926_at	140.8	54.6	-1.37	-2.58	PAP-APP	0.10	11	0.861	0.984	0.3853
1560712_at	19.5	7.6	-1.36	-2.57	AAA-AAP	0.10	11	0.861	0.984	0.4355

222932_at	31.2	12.2	-1.36	-2.56	PAA-AAA	0.10	11	0.861	0.984	0.3458
205320_at	61.1	23.9	-1.35	-2.56	MAA-AAP	0.10	11	0.861	0.984	0.3036
235920_at	32.9	12.9	-1.35	-2.56	AMP-AAA	0.10	11	0.861	0.984	0.3158
217524_x_at	12.2	4.8	-1.35	-2.55	PAA-AAA	0.10	11	0.861	0.984	0.2826
233797_s_at	33.0	12.9	-1.35	-2.55	AAP-AAA	0.10	11	0.861	0.984	0.5038
244534_at	42.9	109.4	1.35	2.55	AAA-APA	0.10	11	0.861	0.984	0.0161
1560284_at	26.8	10.6	-1.35	-2.54	AAA-PAA	0.10	11	0.861	0.984	0.3264
1564760_at	50.3	19.8	-1.34	-2.54	AAA-PAA	0.10	11	0.861	0.984	0.2988
238363_at	35.0	13.8	-1.34	-2.54	APA-PAA	0.10	11	0.861	0.984	0.3400
243347_at	9.4	3.7	-1.34	-2.54	PAA-AAA	0.10	11	0.861	0.984	0.4898
214256_at	29.5	11.6	-1.34	-2.53	PAA-AAA	0.10	11	0.861	0.984	0.4052
1561300_at	28.9	11.4	-1.34	-2.53	PAA-AAA	0.10	11	0.861	0.984	0.3942
1554034_a_at	47.3	18.8	-1.33	-2.52	AAA-APA	0.10	11	0.861	0.984	0.3587
1557286_at	66.7	26.5	-1.33	-2.52	APP-PAP	0.10	11	0.861	0.984	0.3287
234475_x_at	50.9	20.3	-1.33	-2.51	AAP-AAA	0.10	11	0.861	0.984	0.2988
235499_at	53.7	21.3	-1.33	-2.51	PAA-APA	0.10	11	0.861	0.984	0.3756
215526_at	42.7	17.0	-1.33	-2.51	AAA-APA	0.10	11	0.861	0.984	0.4488
236986_at	119.6	47.6	-1.33	-2.51	AAA-PAA	0.10	11	0.861	0.984	0.3167
222953_at	26.8	10.7	-1.33	-2.51	PAA-AAA	0.10	11	0.861	0.984	0.3265
214837_at	33.2	13.2	-1.33	-2.51	APA-AAA	0.10	11	0.861	0.984	0.4399
241916_at	42.0	16.8	-1.33	-2.51	PAA-AAA	0.10	11	0.861	0.984	0.3688
203474_at	32.8	13.1	-1.32	-2.50	AAA-APA	0.10	11	0.861	0.984	0.4020
222367_at	49.6	19.9	-1.32	-2.50	AAA-APA	0.10	11	0.861	0.984	0.3383
233705_at	41.8	16.7	-1.32	-2.50	MAA-PAA	0.10	11	0.861	0.984	0.2702
222676_at	61.9	24.8	-1.32	-2.50	APM-AAM	0.10	11	0.861	0.984	0.2907
1563104_at	93.3	37.4	-1.32	-2.49	AAP-AAM	0.10	11	0.861	0.984	0.3312
216580_at	41.5	16.7	-1.32	-2.49	APP-AAA	0.10	11	0.861	0.984	0.3356
1566585_at	17.8	7.1	-1.32	-2.49	PAA-AAA	0.10	11	0.861	0.984	0.3937
219014_at	61.2	24.6	-1.31	-2.49	AAP-AAA	0.10	11	0.861	0.984	0.3087
240807_at	59.8	24.1	-1.31	-2.48	AAA-AAP	0.10	11	0.861	0.984	0.2889
242248_at	28.2	11.4	-1.31	-2.48	APA-AAP	0.10	11	0.861	0.984	0.4239
237573_at	29.6	11.9	-1.31	-2.48	AAA-APP	0.10	11	0.861	0.984	0.4586
1555191_a_at	20.5	8.3	-1.31	-2.48	PAA-AAA	0.10	11	0.861	0.984	0.4293
1560021_at	22.7	55.9	1.30	2.47	AAA-PAA	0.10	11	0.861	0.984	0.0449
1555988_a_at	46.1	18.7	-1.30	-2.46	PAA-AAP	0.10	11	0.861	0.984	0.2905
237565_at	34.5	14.0	-1.30	-2.46	AAP-AAA	0.10	11	0.861	0.984	0.3716
207174_at	14.6	5.9	-1.30	-2.46	PAA-AAA	0.10	11	0.861	0.984	0.3822
217111_at	55.2	22.5	-1.30	-2.46	PAA-AAA	0.10	11	0.861	0.984	0.4172
210078_s_at	96.6	237.4	1.30	2.46	APP-PPP	0.10	11	0.861	0.984	0.0418
1565689_at	69.7	28.4	-1.30	-2.46	AAM-AAP	0.10	11	0.861	0.984	0.3015
1562759_at	36.5	14.9	-1.29	-2.45	AAA-APA	0.10	11	0.861	0.984	0.3860
227176_at	49.5	20.3	-1.28	-2.44	APA-AAA	0.10	11	0.861	0.984	0.3449

1561089_at	47.6	19.6	-1.28	-2.43	MAA-AAP	0.10	11	0.861	0.984	0.4245
244504_x_at	80.0	33.0	-1.28	-2.43	PMA-AAP	0.10	11	0.861	0.984	0.4048
1561691_at	53.0	21.9	-1.28	-2.43	APA-AAA	0.10	11	0.861	0.984	0.2779
239605_x_at	27.7	11.5	-1.28	-2.42	PAA-PAA	0.10	11	0.861	0.984	0.3891
234059_at	48.6	20.1	-1.28	-2.42	PAA-AMP	0.10	11	0.861	0.984	0.3515
1552386_at	19.0	7.9	-1.28	-2.42	APA-AAA	0.10	11	0.861	0.984	0.4536
231174_s_at	40.8	16.9	-1.27	-2.42	PAP-PAA	0.10	11	0.861	0.984	0.3500
231487_at	12.8	5.3	-1.27	-2.41	AAA-PAA	0.10	11	0.861	0.984	0.5668
1560634_a_at	25.7	10.7	-1.27	-2.41	PAA-AAA	0.10	11	0.861	0.984	0.4987
234907_x_at	27.8	11.5	-1.27	-2.41	APA-AAA	0.10	11	0.861	0.984	0.3509
214533_at	23.8	9.9	-1.27	-2.41	PAA-AAA	0.10	11	0.861	0.984	0.3847
237874_at	20.6	8.6	-1.27	-2.40	AAA-PAA	0.10	11	0.861	0.984	0.4061
1560823_at	7.7	3.2	-1.26	-2.40	APA-AAA	0.10	11	0.861	0.984	0.4875
244625_at	46.2	19.3	-1.26	-2.40	AAA-AAP	0.10	11	0.861	0.984	0.3007
1560320_a_at	72.4	30.2	-1.26	-2.39	PAA-AAA	0.10	11	0.861	0.984	0.4571
1559400_s_at	68.7	28.7	-1.26	-2.40	AMP-APA	0.10	11	0.861	0.984	0.4850
234931_at	99.2	41.5	-1.26	-2.39	AAP-PAA	0.10	11	0.861	0.984	0.2971
1561405_s_at	32.0	13.4	-1.26	-2.39	PAA-AAA	0.10	11	0.861	0.984	0.4484
221104_s_at	42.1	17.6	-1.25	-2.39	APA-APA	0.10	11	0.861	0.984	0.2913
213336_at	125.8	52.8	-1.25	-2.39	AMA-AAP	0.10	11	0.861	0.984	0.4335
233569_at	17.7	7.4	-1.25	-2.38	AAP-AAA	0.10	11	0.861	0.984	0.4287
1562715_at	15.8	6.6	-1.25	-2.38	APA-AAA	0.10	11	0.861	0.984	0.3524
207642_at	51.2	21.5	-1.25	-2.38	APA-APA	0.10	11	0.861	0.984	0.3995
228243_at	39.6	94.2	1.25	2.38	AAA-PPP	0.10	11	0.861	0.984	0.0552
240380_at	77.6	32.7	-1.25	-2.38	APM-PAP	0.10	11	0.861	0.984	0.3832
215154_at	54.2	22.9	-1.25	-2.37	PAA-AAA	0.10	11	0.861	0.984	0.4181
1562107_at	24.4	10.3	-1.24	-2.37	AAA-AAP	0.10	11	0.861	0.984	0.3832
220054_at	41.6	17.6	-1.24	-2.37	PAA-AAA	0.10	11	0.861	0.984	0.4146
203723_at	63.4	150.1	1.24	2.37	AAA-APA	0.10	11	0.861	0.984	0.0381
218610_s_at	70.5	29.8	-1.24	-2.37	PPA-PAP	0.10	11	0.861	0.984	0.3165
211425_x_at	27.6	11.7	-1.24	-2.36	PAA-AAA	0.10	11	0.861	0.984	0.4996
211990_at	51.7	21.9	-1.24	-2.36	AAA-PPA	0.10	11	0.861	0.984	0.4336
1558791_at	43.8	18.6	-1.24	-2.36	APA-AAA	0.10	11	0.861	0.984	0.5180
220231_at	43.4	18.4	-1.24	-2.36	APA-AAA	0.10	11	0.861	0.984	0.2675
243473_at	30.1	12.8	-1.24	-2.36	AAP-AAP	0.10	11	0.861	0.984	0.2690
1563391_at	71.0	30.1	-1.24	-2.36	AAA-APA	0.10	11	0.861	0.984	0.2741
1559624_at	41.8	17.8	-1.24	-2.36	PAA-AAP	0.10	11	0.861	0.984	0.2856
1562308_at	34.9	14.8	-1.24	-2.36	PAA-AAA	0.10	11	0.861	0.984	0.3371
213499_at	90.4	38.4	-1.24	-2.36	APM-AAA	0.10	11	0.861	0.984	0.3649
215219_at	61.0	25.9	-1.24	-2.35	AAA-APA	0.10	11	0.861	0.984	0.3031
210500_at	42.4	18.1	-1.23	-2.35	AAA-AAP	0.10	11	0.861	0.984	0.2942
233389_at	28.9	12.3	-1.23	-2.35	APA-PAA	0.10	11	0.861	0.984	0.5697

243351_at	55.0	23.5	-1.23	-2.35	PAA-MAA	0.10	11	0.861	0.984	0.3746
1556410_a_at	31.1	13.3	-1.23	-2.34	PAA-AAA	0.10	11	0.861	0.984	0.3053
1567859_at	45.6	19.5	-1.23	-2.34	AAA-PAA	0.10	11	0.861	0.984	0.4459
222317_at	10.6	4.5	-1.23	-2.34	AAP-AAA	0.10	11	0.861	0.984	0.5408
1569998_at	42.2	18.0	-1.23	-2.34	APA-AAP	0.10	11	0.861	0.984	0.5754
237692_at	19.3	8.3	-1.22	-2.33	AAP-AAA	0.10	11	0.861	0.984	0.5505
1566093_at	40.0	17.3	-1.21	-2.32	APA-AAA	0.10	11	0.861	0.984	0.3949
244112_x_at	18.1	7.8	-1.21	-2.31	AAP-AAA	0.10	11	0.861	0.984	0.3351
1555489_at	46.2	20.0	-1.21	-2.31	APA-APA	0.10	11	0.861	0.984	0.3498
223873_s_at	17.7	7.6	-1.21	-2.31	AAA-PAA	0.10	11	0.861	0.984	0.4843
235347_at	176.9	409.3	1.21	2.31	PPP-PPP	0.10	11	0.861	0.984	0.0153
236262_at	39.6	17.2	-1.21	-2.31	AAA-APA	0.10	11	0.861	0.984	0.3462
243324_x_at	15.5	6.7	-1.20	-2.30	AAA-APA	0.10	11	0.861	0.984	0.3578
215777_at	44.5	19.3	-1.20	-2.30	APA-AAA	0.10	11	0.861	0.984	0.2793
1561255_at	21.4	9.3	-1.20	-2.30	APA-AAA	0.10	11	0.861	0.984	0.4314
227509_x_at	134.9	309.9	1.20	2.30	PPP-PPP	0.10	11	0.861	0.984	0.0368
231560_at	29.4	12.8	-1.20	-2.30	AAA-PPA	0.10	11	0.861	0.984	0.3758
1560144_at	20.5	8.9	-1.20	-2.29	AAA-AAP	0.10	11	0.861	0.984	0.3690
206729_at	92.4	40.4	-1.19	-2.29	MPM-APA	0.10	11	0.861	0.984	0.3569
232737_s_at	13.9	6.1	-1.19	-2.29	APA-AAA	0.10	11	0.861	0.984	0.4994
1560385_x_at	24.9	10.9	-1.19	-2.29	APA-AAA	0.10	11	0.861	0.984	0.3501
243610_at	34.3	15.1	-1.19	-2.28	APA-AAA	0.10	11	0.861	0.984	0.3218
223326_s_at	145.3	63.9	-1.19	-2.28	AAA-PAA	0.10	11	0.861	0.984	0.4547
215478_at	35.3	15.5	-1.19	-2.27	APP-APA	0.10	11	0.861	0.984	0.3471
213974_at	34.3	15.1	-1.19	-2.27	PMA-PAA	0.10	11	0.861	0.984	0.4169
209606_at	23.3	10.3	-1.18	-2.27	AAP-AAA	0.10	11	0.861	0.984	0.3377
205236_x_at	56.4	24.8	-1.18	-2.27	PAA-AAA	0.10	11	0.861	0.984	0.3390
1555303_at	84.8	37.4	-1.18	-2.27	APM-AAP	0.10	11	0.861	0.984	0.2890
242532_at	25.2	11.1	-1.18	-2.27	AAA-PAA	0.10	11	0.861	0.984	0.3682
1556202_at	85.8	37.8	-1.18	-2.27	PPA-AAP	0.10	11	0.861	0.984	0.4161
204086_at	34.5	15.2	-1.18	-2.27	PAA-AAA	0.10	11	0.861	0.984	0.5729
1560755_at	42.7	18.9	-1.18	-2.27	AAP-AAA	0.10	11	0.861	0.984	0.4291
239086_at	65.2	28.8	-1.18	-2.26	AAA-PPA	0.10	11	0.861	0.984	0.3113
207912_s_at	10.4	4.6	-1.18	-2.26	AAA-APA	0.10	11	0.861	0.984	0.5148
236596_at	29.3	13.0	-1.18	-2.26	PAA-AAA	0.10	11	0.861	0.984	0.3662
243646_at	68.9	30.6	-1.17	-2.26	APA-AAP	0.10	11	0.861	0.984	0.3195
1569580_a_at	18.0	8.0	-1.17	-2.26	AAP-AAA	0.10	11	0.861	0.984	0.4082
236424_at	91.8	40.7	-1.17	-2.26	AAP-PPA	0.10	11	0.861	0.984	0.4905
219040_at	108.3	48.1	-1.17	-2.25	PAA-AAA	0.10	11	0.861	0.984	0.2825
1556936_at	22.9	10.2	-1.17	-2.25	PPA-AAA	0.10	11	0.861	0.984	0.3872
1559140_at	56.5	25.1	-1.17	-2.25	AAA-PAA	0.10	11	0.861	0.984	0.3875
1570020_at	26.9	12.0	-1.17	-2.25	AAA-AAP	0.10	11	0.861	0.984	0.4306

217455_s_at	85.0	37.9	-1.17	-2.25	APA-PAA	0.10	11	0.861	0.984	0.3118
244528_at	22.1	9.9	-1.17	-2.24	APA-AAA	0.10	11	0.861	0.984	0.4758
243139_at	27.4	12.2	-1.17	-2.24	PAA-AAA	0.10	11	0.861	0.984	0.5034
234604_at	63.3	28.3	-1.17	-2.24	APP-APP	0.10	11	0.861	0.984	0.3279
1555123_at	45.8	20.5	-1.16	-2.24	APA-AAP	0.10	11	0.861	0.984	0.5054
236806_at	89.9	40.3	-1.16	-2.23	AAA-APA	0.10	11	0.861	0.984	0.3165
244408_at	29.2	13.1	-1.15	-2.22	AAP-AAA	0.10	11	0.861	0.984	0.2809
203891_s_at	137.3	305.2	1.15	2.22	PPP-PPP	0.10	11	0.861	0.984	0.0222
234559_at	22.8	10.3	-1.15	-2.22	PAA-AAA	0.10	11	0.861	0.984	0.5288
1555433_at	37.9	17.1	-1.15	-2.22	AAP-AAA	0.10	11	0.861	0.984	0.3718
217692_at	71.4	32.3	-1.15	-2.21	PAA-AAA	0.10	11	0.861	0.984	0.3315
215659_at	45.2	20.4	-1.15	-2.21	PAA-APP	0.10	11	0.861	0.984	0.3832
236239_at	80.1	36.3	-1.14	-2.21	APA-AAA	0.10	11	0.861	0.984	0.3424
1558469_at	44.2	20.1	-1.14	-2.21	APA-AAA	0.10	11	0.861	0.984	0.3817
244313_at	20.4	9.2	-1.14	-2.21	PAA-AMA	0.10	11	0.861	0.984	0.3826
1560095_s_at	46.9	21.3	-1.14	-2.20	PAA-AAP	0.10	11	0.861	0.984	0.3690
214974_x_at	117.4	258.8	1.14	2.21	PPP-PPP	0.10	11	0.861	0.984	0.0229
242079_at	49.0	22.2	-1.14	-2.20	APA-AAA	0.10	11	0.861	0.984	0.4911
241418_at	65.3	29.7	-1.14	-2.20	AAA-PAA	0.10	11	0.861	0.984	0.4007
209335_at	63.3	28.8	-1.14	-2.20	APA-AAA	0.10	11	0.861	0.984	0.2843
221309_at	34.0	15.5	-1.14	-2.20	AAA-PAA	0.10	11	0.861	0.984	0.3955
226961_at	24.9	11.4	-1.13	-2.19	AAP-AAA	0.10	11	0.861	0.984	0.4023
1553325_at	39.5	18.0	-1.13	-2.20	PAA-AAA	0.10	11	0.861	0.984	0.5332
1570076_at	28.5	13.0	-1.13	-2.19	AAP-AAP	0.10	11	0.861	0.984	0.4891
1554293_at	31.0	14.1	-1.13	-2.19	APA-AAP	0.10	11	0.861	0.984	0.4093
239903_at	68.0	31.1	-1.13	-2.19	AAA-AAP	0.10	11	0.861	0.984	0.3105
214986_x_at	408.0	186.7	-1.13	-2.19	PMA-APP	0.10	11	0.861	0.984	0.3112
214046_at	6.1	2.8	-1.13	-2.18	APA-AAA	0.10	11	0.861	0.984	0.4539
205578_at	244.4	112.1	-1.12	-2.18	PAP-AAP	0.10	11	0.861	0.984	0.3291
243957_at	43.7	20.1	-1.12	-2.18	AAA-AAP	0.10	11	0.861	0.984	0.4257
1569673_at	39.8	18.3	-1.12	-2.18	PMA-AAA	0.10	11	0.861	0.984	0.4694
1562311_at	40.1	18.4	-1.12	-2.18	AAA-PMA	0.10	11	0.861	0.984	0.2728
207064_s_at	82.3	37.8	-1.12	-2.18	PPP-AAP	0.10	11	0.861	0.984	0.2905
1563489_at	106.3	48.9	-1.12	-2.18	AAP-PAA	0.10	11	0.861	0.984	0.3060
223217_s_at	211.8	460.5	1.12	2.17	PPP-PPP	0.10	11	0.861	0.984	0.0386
235348_at	109.2	237.3	1.12	2.17	PPP-PPP	0.10	11	0.861	0.984	0.0264
242977_at	19.1	8.8	-1.12	-2.17	PPA-AAA	0.10	11	0.861	0.984	0.4153
1559952_x_at	60.9	28.2	-1.11	-2.16	APA-PAA	0.10	11	0.861	0.984	0.3949
230610_at	59.8	27.7	-1.11	-2.16	PPA-AAA	0.10	11	0.861	0.984	0.3839
238029_s_at	45.9	21.2	-1.11	-2.16	PAP-AAP	0.10	11	0.861	0.984	0.4262
241149_at	46.3	21.4	-1.11	-2.16	PPA-PAP	0.10	11	0.861	0.984	0.5433
231356_at	23.0	10.7	-1.11	-2.16	APA-AAA	0.10	11	0.861	0.984	0.4138

207653_at	24.2	11.3	-1.11	-2.16	AAA-AAP	0.10	11	0.861	0.984	0.5114
236139_at	65.7	30.5	-1.11	-2.15	PAA-AAM	0.10	11	0.861	0.984	0.5478
1553153_at	90.3	42.0	-1.11	-2.15	PPP-PPA	0.10	11	0.861	0.984	0.5415
230080_at	81.7	175.7	1.11	2.15	MAA-PAP	0.10	11	0.861	0.984	0.0499
216711_s_at	155.5	72.4	-1.10	-2.15	PMP-AAP	0.10	11	0.861	0.984	0.3208
230402_at	35.5	16.6	-1.10	-2.14	AAA-AAP	0.10	11	0.861	0.984	0.5042
205830_at	171.2	366.7	1.10	2.14	PPM-PPP	0.10	11	0.861	0.984	0.0423
220893_at	39.9	18.7	-1.10	-2.14	AAP-AAA	0.10	11	0.861	0.984	0.3189
232009_at	38.6	18.0	-1.10	-2.14	AAP-APA	0.10	11	0.861	0.984	0.3693
1560770_at	36.0	16.8	-1.10	-2.14	AAP-AAA	0.10	11	0.861	0.984	0.4829
221152_at	23.2	10.9	-1.09	-2.13	AAA-APA	0.10	11	0.861	0.984	0.5806
1560209_at	32.7	69.6	1.09	2.13	AAA-AAP	0.10	11	0.861	0.984	0.0425
201694_s_at	1013.5	2157.8	1.09	2.13	PPP-PPP	0.10	11	0.861	0.984	0.0167
239833_at	83.0	39.1	-1.09	-2.13	AAA-APM	0.10	11	0.861	0.984	0.5121
1570035_at	20.2	42.9	1.09	2.13	AAA-AAP	0.10	11	0.861	0.984	0.0466
206891_at	112.1	52.8	-1.09	-2.12	PPA-AMA	0.10	11	0.861	0.984	0.3394
240511_at	51.1	24.1	-1.08	-2.12	AAP-AAA	0.10	11	0.861	0.984	0.2778
206548_at	76.6	36.1	-1.08	-2.12	PPP-PPA	0.10	11	0.861	0.984	0.2615
234063_at	43.6	20.6	-1.08	-2.12	AAP-AAA	0.10	11	0.861	0.984	0.3938
1569201_a_at	38.1	18.0	-1.08	-2.12	APA-AAP	0.10	11	0.861	0.984	0.4005
238344_at	39.9	18.9	-1.08	-2.12	PAA-AAA	0.10	11	0.861	0.984	0.3536
233415_at	34.4	16.3	-1.08	-2.11	PPA-AAP	0.10	11	0.861	0.984	0.5104
232263_at	232.5	491.5	1.08	2.11	PPP-PPP	0.10	11	0.861	0.984	0.0227
220073_s_at	73.1	34.6	-1.08	-2.11	AAP-PAA	0.10	11	0.861	0.984	0.3655
203631_s_at	54.3	114.5	1.08	2.11	AAA-PAP	0.10	11	0.861	0.984	0.0398
1559589_a_at	33.8	16.1	-1.07	-2.10	APA-AAA	0.10	11	0.861	0.984	0.4498
207369_at	43.5	20.8	-1.07	-2.10	APP-AAP	0.10	11	0.861	0.984	0.2628
232689_at	47.3	22.6	-1.07	-2.10	AAA-PAA	0.10	11	0.861	0.984	0.3257
238127_at	25.8	12.3	-1.07	-2.10	AAA-AAP	0.10	11	0.861	0.984	0.3947
237918_at	29.6	14.1	-1.07	-2.10	APP-AAA	0.10	11	0.861	0.984	0.4777
1564360_a_at	37.5	18.0	-1.06	-2.08	APA-AAP	0.10	11	0.861	0.984	0.3950
215938_s_at	83.5	40.1	-1.06	-2.08	APA-PPA	0.10	11	0.861	0.984	0.4330
242057_at	39.4	18.9	-1.06	-2.08	APA-PAP	0.10	11	0.861	0.984	0.5371
244149_at	87.4	181.4	1.05	2.08	AAA-PPP	0.10	11	0.861	0.984	0.0338
238560_at	79.6	38.4	-1.05	-2.07	AAA-AAP	0.10	11	0.861	0.984	0.3254
243807_at	138.7	66.9	-1.05	-2.07	AAP-AAP	0.10	11	0.861	0.984	0.2780
233548_at	18.1	8.7	-1.05	-2.07	AAA-APA	0.10	11	0.861	0.984	0.3411
240078_at	24.3	11.8	-1.05	-2.07	AAA-AAP	0.10	11	0.861	0.984	0.4575
1570270_at	53.2	25.7	-1.05	-2.07	MAA-AAP	0.10	11	0.861	0.984	0.4989
1561417_x_at	20.3	9.8	-1.05	-2.07	PAA-AAA	0.10	11	0.861	0.984	0.5582
208410_x_at	53.6	25.9	-1.05	-2.07	PAA-PAA	0.10	11	0.861	0.984	0.2775
238814_at	91.8	44.3	-1.05	-2.07	PAA-AAA	0.10	11	0.861	0.984	0.2831

224004_at	52.3	25.3	-1.05	-2.07	PAM-AAP	0.10	11	0.861	0.984	0.3693
241380_at	64.5	31.2	-1.05	-2.07	AAP-AAA	0.10	11	0.861	0.984	0.4225
217695_x_at	65.4	31.7	-1.05	-2.07	AAP-PAA	0.10	11	0.861	0.984	0.4923
221680_s_at	75.7	36.6	-1.05	-2.07	PPP-APP	0.10	11	0.861	0.984	0.2911
212886_at	38.7	18.8	-1.05	-2.07	AAP-AAA	0.10	11	0.861	0.984	0.4106
208481_at	34.6	16.8	-1.05	-2.06	PAA-AAA	0.10	11	0.861	0.984	0.4766
205932_s_at	174.0	358.8	1.04	2.06	PPP-PPP	0.10	11	0.861	0.984	0.0161
221974_at	113.4	233.5	1.04	2.06	PPP-PPP	0.10	11	0.861	0.984	0.0175
217701_x_at	97.7	47.5	-1.04	-2.06	AAP-AAA	0.10	11	0.861	0.984	0.2757
235710_at	82.4	40.1	-1.04	-2.06	PAA-AAA	0.10	11	0.861	0.984	0.3143
223374_s_at	49.6	102.0	1.04	2.06	PAP-PPP	0.10	11	0.861	0.984	0.0295
227647_at	49.3	24.0	-1.04	-2.05	AAP-AAA	0.10	11	0.861	0.984	0.2924
203473_at	58.8	28.6	-1.04	-2.06	AAP-AAA	0.10	11	0.861	0.984	0.4159
1569930_at	55.2	26.9	-1.04	-2.05	PPA-APA	0.10	11	0.861	0.984	0.3580
242907_at	39.3	19.2	-1.03	-2.05	APA-APP	0.10	11	0.861	0.984	0.3957
240279_at	35.4	17.3	-1.03	-2.05	AAA-AAP	0.10	11	0.861	0.984	0.3416
227899_at	101.5	49.6	-1.03	-2.05	APA-AAP	0.10	11	0.861	0.984	0.3476
210269_s_at	138.6	283.3	1.03	2.05	AAA-PPP	0.10	11	0.861	0.984	0.0228
221670_s_at	88.1	43.1	-1.03	-2.04	APA-AAA	0.10	11	0.861	0.984	0.3981
1555688_s_at	38.4	18.8	-1.03	-2.04	APA-AAA	0.10	11	0.861	0.984	0.4171
244633_at	48.7	99.3	1.03	2.04	APA-PPP	0.10	11	0.861	0.984	0.0215
242416_at	75.3	37.0	-1.03	-2.04	PMA-AAP	0.10	11	0.861	0.984	0.2729
204545_at	78.3	38.5	-1.02	-2.03	AAP-AAA	0.10	11	0.861	0.984	0.3225
236590_at	70.9	34.9	-1.02	-2.03	APA-AAA	0.10	11	0.861	0.984	0.2605
215856_at	115.1	56.7	-1.02	-2.03	PPA-MPA	0.10	11	0.861	0.984	0.2708
220988_s_at	54.2	26.7	-1.02	-2.03	AAP-AAA	0.10	11	0.861	0.984	0.3733
241608_at	35.0	17.3	-1.02	-2.03	PAA-AMA	0.10	11	0.861	0.984	0.3530
1563724_at	33.5	68.0	1.02	2.03	AAA-APP	0.10	11	0.861	0.984	0.0354
242421_at	60.4	29.8	-1.02	-2.03	APA-APA	0.10	11	0.861	0.984	0.3829
239408_at	41.9	20.7	-1.02	-2.03	PAA-AAA	0.10	11	0.861	0.984	0.5797
1552519_at	51.5	25.4	-1.02	-2.03	PAP-AAA	0.10	11	0.861	0.984	0.2822
236481_at	93.4	46.1	-1.02	-2.03	AAA-PAA	0.10	11	0.861	0.984	0.3528
217666_at	41.1	83.1	1.02	2.03	AAP-PPM	0.10	11	0.861	0.984	0.0434
228564_at	285.8	578.0	1.02	2.02	PPP-PPP	0.10	11	0.861	0.984	0.0485
207413_s_at	78.8	39.0	-1.02	-2.02	PAA-AAA	0.10	11	0.861	0.984	0.3145
1555155_at	97.5	48.2	-1.02	-2.02	APA-AAA	0.10	11	0.861	0.984	0.3757
240717_at	10.4	5.2	-1.02	-2.02	AAA-AAP	0.10	11	0.861	0.984	0.5170
1570231_at	25.9	12.8	-1.02	-2.02	AAP-AAA	0.10	11	0.861	0.984	0.5564
216564_at	66.5	32.9	-1.01	-2.02	PAA-AAA	0.10	11	0.861	0.984	0.2913
230064_at	53.3	26.4	-1.01	-2.02	AAP-APA	0.10	11	0.861	0.984	0.3437
216662_at	40.5	20.1	-1.01	-2.02	APA-AAA	0.10	11	0.861	0.984	0.4210
231380_at	41.1	20.4	-1.01	-2.02	AAA-PAA	0.10	11	0.861	0.984	0.2985

232779_at	33.6	16.7	-1.01	-2.02	AAA-AAP	0.10	11	0.861	0.984	0.4641
229621_x_at	47.3	23.5	-1.01	-2.02	AAP-AAA	0.10	11	0.861	0.984	0.2879
1554295_x_at	43.7	21.7	-1.01	-2.02	AAA-AAP	0.10	11	0.861	0.984	0.4593
231961_at	46.9	23.3	-1.01	-2.01	AAP-MAM	0.10	11	0.861	0.984	0.4325
222706_at	161.0	323.7	1.01	2.01	AAP-PPP	0.10	11	0.861	0.984	0.0175
223765_s_at	149.7	300.5	1.01	2.01	PPP-PPP	0.10	11	0.861	0.984	0.0448
1559374_at	54.7	27.3	-1.01	-2.01	APP-PAP	0.10	11	0.861	0.984	0.3020
216478_at	29.3	14.6	-1.01	-2.01	AAA-APA	0.10	11	0.861	0.984	0.4118
233209_at	87.0	43.4	-1.00	-2.01	AAA-PPA	0.10	11	0.861	0.984	0.4263
237383_at	45.8	22.9	-1.00	-2.00	AAP-AAP	0.10	11	0.861	0.984	0.5145
239352_at	43.0	86.0	1.00	2.00	AAA-PPP	0.10	11	0.861	0.984	0.0114
239631_at	41.6	20.8	-1.00	-2.00	MPA-APA	0.10	11	0.861	0.984	0.5073
223634_at	142.5	71.3	-1.00	-2.00	APP-PAP	0.10	11	0.861	0.984	0.2862
1561152_a_at	41.6	20.8	-1.00	-2.00	AAA-AAP	0.10	11	0.861	0.984	0.3109
220591_s_at	44.7	22.4	-1.00	-1.99	MPA-APM	0.10	11	0.861	0.984	0.3058
201964_at	330.0	658.0	1.00	1.99	PPP-PPP	0.10	11	0.861	0.984	0.0250
220169_at	51.3	25.7	-1.00	-1.99	AAP-PAA	0.10	11	0.861	0.984	0.4110
219312_s_at	24.6	49.1	1.00	1.99	AAA-PPP	0.10	11	0.861	0.984	0.0371
221663_x_at	74.2	37.3	-0.99	-1.99	AAP-AAA	0.10	11	0.861	0.984	0.2809
210029_at	51.0	25.7	-0.99	-1.99	PPA-AAA	0.10	11	0.861	0.984	0.5341
220545_s_at	19.2	9.7	-0.99	-1.98	APA-AAA	0.10	11	0.861	0.984	0.5069
211786_at	73.6	37.1	-0.99	-1.98	AAA-APA	0.10	11	0.861	0.984	0.4620
1569961_at	82.6	41.7	-0.99	-1.98	APA-AAP	0.10	11	0.861	0.984	0.4662
1564468_at	20.4	10.3	-0.98	-1.98	AAA-AAP	0.10	11	0.861	0.984	0.5433
227318_at	51.1	25.9	-0.98	-1.98	APA-APA	0.10	11	0.861	0.984	0.4045
210393_at	24.6	12.5	-0.98	-1.98	APA-AAA	0.10	11	0.861	0.984	0.5596
219585_at	72.9	37.0	-0.98	-1.97	APA-AAA	0.10	11	0.861	0.984	0.4381
227525_at	42.2	83.3	0.98	1.97	AAA-AMP	0.10	11	0.861	0.984	0.0169
238634_x_at	16.1	8.2	-0.98	-1.97	AAA-AAP	0.10	11	0.861	0.984	0.4570
221195_at	83.5	42.4	-0.98	-1.97	APP-AAP	0.10	11	0.861	0.984	0.3470
240552_at	73.8	37.5	-0.98	-1.97	AMP-AAP	0.10	11	0.861	0.984	0.5043
229201_at	29.1	14.8	-0.97	-1.96	PAA-AAA	0.10	11	0.861	0.984	0.3614
243988_at	50.5	25.7	-0.97	-1.96	AAA-AAP	0.10	11	0.861	0.984	0.3708
223995_at	80.4	40.9	-0.97	-1.96	PPP-AMP	0.10	11	0.861	0.984	0.2918
1560066_at	30.0	15.3	-0.97	-1.96	PAA-AAA	0.10	11	0.861	0.984	0.3109
233285_at	36.0	18.3	-0.97	-1.96	AAP-MAA	0.10	11	0.861	0.984	0.4652
243159_x_at	41.5	21.2	-0.97	-1.96	APA-AAA	0.10	11	0.861	0.984	0.3236
238769_at	114.9	58.6	-0.97	-1.96	AAM-APA	0.10	11	0.861	0.984	0.3987
1552727_s_at	28.9	14.7	-0.97	-1.96	PAP-PAA	0.10	11	0.861	0.984	0.4672
241532_at	9.3	4.7	-0.97	-1.96	AAA-PAA	0.10	11	0.861	0.984	0.5718
226747_at	178.7	350.1	0.97	1.96	PPP-PPP	0.10	11	0.861	0.984	0.0192
222073_at	37.5	19.2	-0.97	-1.96	APA-PAA	0.10	11	0.861	0.984	0.4992

207526_s_at	51.2	26.2	-0.97	-1.96	APA-AAA	0.10	11	0.861	0.984	0.4172
209569_x_at	50.1	25.7	-0.97	-1.95	PAA-APA	0.10	11	0.861	0.984	0.3475
233701_at	93.1	47.7	-0.96	-1.95	AAA-APA	0.10	11	0.861	0.984	0.3251
237263_at	79.5	40.8	-0.96	-1.95	APA-PAA	0.10	11	0.861	0.984	0.2969
1563512_at	65.9	33.8	-0.96	-1.95	APP-APA	0.10	11	0.861	0.984	0.3220
224433_s_at	193.8	99.5	-0.96	-1.95	AAA-APP	0.10	11	0.861	0.984	0.3136
232670_at	78.6	40.3	-0.96	-1.95	AAP-AAP	0.10	11	0.861	0.984	0.5344
216621_at	63.7	32.7	-0.96	-1.95	AAA-APA	0.10	11	0.861	0.984	0.3645
220908_at	50.0	25.7	-0.96	-1.95	APA-AAA	0.10	11	0.861	0.984	0.3855
215861_at	50.1	25.7	-0.96	-1.95	APA-AAA	0.10	11	0.861	0.984	0.3986
225971_at	492.3	958.0	0.96	1.95	PPP-PPP	0.10	11	0.861	0.984	0.0237
1554388_at	130.7	67.2	-0.96	-1.95	AAP-APP	0.10	11	0.861	0.984	0.4877
221155_x_at	144.7	74.4	-0.96	-1.94	PPA-PPP	0.10	11	0.861	0.984	0.3688
207349_s_at	17.2	8.8	-0.96	-1.94	AAA-APA	0.10	11	0.861	0.984	0.5558
1566952_at	33.0	17.0	-0.96	-1.94	AAP-AAA	0.10	11	0.861	0.984	0.2562
1557513_a_at	89.8	46.3	-0.96	-1.94	AAP-AAA	0.10	11	0.861	0.984	0.3897
242307_at	181.1	93.4	-0.96	-1.94	MAA-PAP	0.10	11	0.861	0.984	0.2999
1555774_at	49.4	25.5	-0.95	-1.94	PAA-AAA	0.10	11	0.861	0.984	0.3510
241091_at	76.9	39.7	-0.95	-1.94	APP-APP	0.10	11	0.861	0.984	0.4381
1560888_x_at	37.6	19.4	-0.95	-1.94	APP-AAA	0.10	11	0.861	0.984	0.4579
232841_at	144.6	74.8	-0.95	-1.93	PAA-PAA	0.10	11	0.861	0.984	0.2691
239412_at	50.0	25.9	-0.95	-1.93	APA-AAA	0.10	11	0.861	0.984	0.3568
238615_at	35.8	18.5	-0.95	-1.93	AAA-PAA	0.10	11	0.861	0.984	0.3874
201288_at	394.4	762.3	0.95	1.93	PPP-PPP	0.10	11	0.861	0.984	0.0151
237819_at	55.6	28.8	-0.95	-1.93	AAP-AAA	0.10	11	0.861	0.984	0.2748
227643_at	50.5	26.2	-0.95	-1.93	PAA-APA	0.10	11	0.861	0.984	0.3690
244770_at	30.4	15.8	-0.95	-1.93	PAA-APA	0.10	11	0.861	0.984	0.4999
244293_at	42.6	22.1	-0.95	-1.93	PAA-AAA	0.10	11	0.861	0.984	0.4433
222075_s_at	48.3	93.2	0.95	1.93	AAA-APP	0.10	11	0.861	0.984	0.0299
216084_at	26.9	14.0	-0.95	-1.93	APA-PAA	0.10	11	0.861	0.984	0.3706
1556114_a_at	65.2	33.8	-0.95	-1.93	PAA-AAA	0.10	11	0.861	0.984	0.4428
217190_x_at	86.7	45.0	-0.95	-1.93	AAA-MPA	0.10	11	0.861	0.984	0.5113
1562764_at	64.5	33.5	-0.95	-1.93	PAA-AAA	0.10	11	0.861	0.984	0.3179
234326_at	63.8	33.2	-0.94	-1.92	AAA-AAP	0.10	11	0.861	0.984	0.3050
37547_at	53.9	28.0	-0.94	-1.92	APP-APA	0.10	11	0.861	0.984	0.3810
241946_at	80.7	155.3	0.94	1.92	PPP-PPP	0.10	11	0.861	0.984	0.0425
243770_at	49.6	25.8	-0.94	-1.92	APA-AAP	0.10	11	0.861	0.984	0.2701
242498_x_at	68.6	35.7	-0.94	-1.92	APA-PAP	0.10	11	0.861	0.984	0.4402
231535_x_at	41.9	21.9	-0.94	-1.92	PAA-AAA	0.10	11	0.861	0.984	0.4773
1555634_a_at	44.7	23.3	-0.94	-1.92	PAA-AAA	0.10	11	0.861	0.984	0.2553
1553315_at	60.9	31.7	-0.94	-1.92	PPP-APP	0.10	11	0.861	0.984	0.2809
201884_at	27.3	14.2	-0.94	-1.92	AAA-AAP	0.10	11	0.861	0.984	0.3523

204296_at	68.5	35.8	-0.94	-1.92	AAA-APA	0.10	11	0.861	0.984	0.3518
237491_at	32.5	17.0	-0.94	-1.92	AAA-APA	0.10	11	0.861	0.984	0.4743
237891_at	16.0	8.3	-0.94	-1.92	AAA-AAP	0.10	11	0.861	0.984	0.5681
219468_s_at	88.8	46.4	-0.94	-1.92	AAP-PAA	0.10	11	0.861	0.984	0.4113
230297_x_at	83.3	43.5	-0.94	-1.91	APA-AAA	0.10	11	0.861	0.984	0.4999
226485_at	203.3	389.4	0.94	1.92	PPP-PPP	0.10	11	0.861	0.984	0.0103
233365_at	62.0	32.4	-0.93	-1.91	PAA-AAP	0.10	11	0.861	0.984	0.3593
1559754_at	85.1	44.5	-0.93	-1.91	MPA-APP	0.10	11	0.861	0.984	0.4523
204381_at	115.1	220.0	0.93	1.91	AAA-PPP	0.10	11	0.861	0.984	0.0198
220719_at	80.0	41.9	-0.93	-1.91	PPA-PAP	0.10	11	0.861	0.984	0.2724
220439_at	45.5	23.8	-0.93	-1.91	APA-AAA	0.10	11	0.861	0.984	0.2919
215474_at	66.6	34.9	-0.93	-1.91	AAA-AAP	0.10	11	0.861	0.984	0.2686
239544_at	43.1	22.6	-0.93	-1.91	PAA-AAA	0.10	11	0.861	0.984	0.4391
243748_at	40.1	21.1	-0.93	-1.91	AAA-AAP	0.10	11	0.861	0.984	0.2930
244436_at	17.6	9.3	-0.93	-1.90	AAP-AAA	0.10	11	0.861	0.984	0.4935
1552841_s_at	30.0	15.8	-0.93	-1.90	AAA-AAP	0.10	11	0.861	0.984	0.3389
1563975_at	56.6	29.8	-0.92	-1.90	AAA-PAA	0.10	11	0.861	0.984	0.5238
244104_at	39.9	21.0	-0.92	-1.89	PAA-AAA	0.10	11	0.861	0.984	0.5189
1565668_at	24.0	12.7	-0.92	-1.89	PAA-AAA	0.10	11	0.861	0.984	0.3264
230905_at	43.0	22.7	-0.92	-1.89	PAA-PAA	0.10	11	0.861	0.984	0.3621
207850_at	79.5	150.3	0.92	1.89	AAP-AMP	0.10	11	0.861	0.984	0.0427
1563461_at	36.8	19.5	-0.92	-1.89	APA-APA	0.10	11	0.861	0.984	0.3279
228155_at	164.0	309.8	0.92	1.89	PPP-PPP	0.10	11	0.861	0.984	0.0214
231008_at	65.6	34.8	-0.92	-1.89	PPA-APA	0.10	11	0.861	0.984	0.3783
215456_at	25.1	13.3	-0.92	-1.89	AAP-AAA	0.10	11	0.861	0.984	0.4079
228542_at	136.8	258.0	0.92	1.89	PPP-PPP	0.10	11	0.861	0.984	0.0172
206005_s_at	132.9	250.5	0.92	1.89	PPP-PPP	0.10	11	0.861	0.984	0.0101
230295_at	162.7	86.3	-0.91	-1.88	AMP-PAP	0.10	11	0.861	0.984	0.2667
1566577_at	51.7	27.4	-0.91	-1.89	PAA-AAP	0.10	11	0.861	0.984	0.3091
210368_at	82.6	155.5	0.91	1.88	AAA-AAP	0.10	11	0.861	0.984	0.0283
1569780_at	23.2	12.3	-0.91	-1.88	APA-AAA	0.10	11	0.861	0.984	0.5286
201117_s_at	139.8	263.1	0.91	1.88	PPP-PPP	0.10	11	0.861	0.984	0.0312
239004_at	270.0	143.6	-0.91	-1.88	APP-AMP	0.10	11	0.861	0.984	0.3106
241772_at	30.9	16.4	-0.91	-1.88	PAA-AAA	0.10	11	0.861	0.984	0.4442
219795_at	19.8	10.5	-0.91	-1.88	APA-AAA	0.10	11	0.861	0.984	0.5720
1554514_at	30.2	16.1	-0.91	-1.88	PAA-AAA	0.10	11	0.861	0.984	0.5743
224091_at	27.4	14.6	-0.91	-1.88	AAA-AAP	0.10	11	0.861	0.984	0.5277
233538_s_at	52.5	28.0	-0.91	-1.87	APA-PAP	0.10	11	0.861	0.984	0.3783
210380_s_at	46.0	24.5	-0.91	-1.87	AAA-APA	0.10	11	0.861	0.984	0.3545
232272_at	51.1	95.8	0.91	1.87	APP-PPP	0.10	11	0.861	0.984	0.0313
214329_x_at	42.3	22.6	-0.90	-1.87	APM-AAA	0.10	11	0.861	0.984	0.4008
232675_s_at	183.1	98.0	-0.90	-1.87	APA-AAP	0.10	11	0.861	0.984	0.2961

231577_s_at	1020.1	1904.2	0.90	1.87	PPP-PPP	0.10	11	0.861	0.984	0.0327
238638_at	82.1	44.0	-0.90	-1.86	AMM-PMA	0.10	11	0.861	0.984	0.2730
1554941_at	31.4	16.8	-0.90	-1.87	PAA-AAA	0.10	11	0.861	0.984	0.5394
228063_s_at	375.5	699.5	0.90	1.86	PMP-PPP	0.10	11	0.861	0.984	0.0438
221172_at	112.4	60.4	-0.90	-1.86	MAA-AAP	0.10	11	0.861	0.984	0.2753
240461_at	29.8	16.0	-0.90	-1.86	AAA-AAP	0.10	11	0.861	0.984	0.4407
220650_s_at	32.6	17.5	-0.90	-1.86	APA-AAA	0.10	11	0.861	0.984	0.4958
205131_x_at	122.7	228.3	0.90	1.86	AAA-PPP	0.10	11	0.861	0.984	0.0396
230798_at	63.6	34.2	-0.89	-1.86	APA-AAA	0.10	11	0.861	0.984	0.3674
243557_at	51.9	28.0	-0.89	-1.86	APA-AAP	0.10	11	0.861	0.984	0.2827
1558293_at	42.5	22.9	-0.89	-1.85	PAP-AAP	0.10	11	0.861	0.984	0.2859
239627_at	66.5	35.9	-0.89	-1.85	PPA-AMA	0.10	11	0.861	0.984	0.4472
215116_s_at	186.3	345.3	0.89	1.85	AAA-AAP	0.10	11	0.861	0.984	0.0229
229568_at	122.3	226.7	0.89	1.85	PMP-PPP	0.10	11	0.861	0.984	0.0164
215771_x_at	136.5	73.7	-0.89	-1.85	APP-PAA	0.10	11	0.861	0.984	0.2745
228623_at	67.0	36.2	-0.89	-1.85	AAP-AAP	0.10	11	0.861	0.984	0.4968
217202_s_at	147.3	272.8	0.89	1.85	AAA-MPP	0.10	11	0.861	0.984	0.0149
241695_s_at	44.4	24.0	-0.89	-1.85	AAP-AAP	0.10	11	0.861	0.984	0.3341
243066_at	48.2	26.1	-0.89	-1.85	AAP-AAA	0.10	11	0.861	0.984	0.4126
232502_at	35.1	19.0	-0.89	-1.85	PAP-AAM	0.10	11	0.861	0.984	0.4303
237215_s_at	509.7	942.8	0.89	1.85	PPP-PPP	0.10	11	0.861	0.984	0.0365
1552991_at	40.8	22.1	-0.89	-1.85	AAA-PAA	0.10	11	0.861	0.984	0.5091
235898_at	72.2	39.1	-0.89	-1.85	AAA-PAA	0.10	11	0.861	0.984	0.2607
232100_at	37.3	20.2	-0.89	-1.85	PAA-PAM	0.10	11	0.861	0.984	0.4944
223218_s_at	681.9	1259.0	0.89	1.85	PPP-PPP	0.10	11	0.861	0.984	0.0169
207757_at	38.6	20.9	-0.88	-1.84	AAA-AAP	0.10	11	0.861	0.984	0.4439
235069_at	85.2	46.3	-0.88	-1.84	PAA-APP	0.10	11	0.861	0.984	0.5143
1561906_at	34.0	18.4	-0.88	-1.84	AAP-AAA	0.10	11	0.861	0.984	0.5686
210409_at	32.1	17.5	-0.88	-1.84	AAP-AAP	0.10	11	0.861	0.984	0.5378
202966_at	30.7	16.7	-0.88	-1.84	APA-AAA	0.10	11	0.861	0.984	0.5626
234127_at	55.8	30.4	-0.88	-1.84	APA-AAP	0.10	11	0.861	0.984	0.3796
241978_at	110.5	60.1	-0.88	-1.84	APA-PMP	0.10	11	0.861	0.984	0.2568
211792_s_at	241.6	444.0	0.88	1.84	PMP-PPP	0.10	11	0.861	0.984	0.0145
215723_s_at	30.5	16.6	-0.88	-1.84	APP-AAA	0.10	11	0.861	0.984	0.4716
239127_at	94.5	51.6	-0.87	-1.83	PAA-AAA	0.10	11	0.861	0.984	0.3797
237645_at	41.2	22.5	-0.87	-1.83	AAA-PAA	0.10	11	0.861	0.984	0.5659
206336_at	1739.7	3188.4	0.87	1.83	PPP-PPP	0.10	11	0.861	0.984	0.0339
229759_s_at	494.3	904.6	0.87	1.83	PPP-PPP	0.10	11	0.861	0.984	0.0232
234192_s_at	87.9	160.6	0.87	1.83	PPP-PPP	0.10	11	0.861	0.984	0.0092
1553269_at	45.8	25.1	-0.87	-1.83	AAA-APA	0.10	11	0.861	0.984	0.3587
229831_at	18.3	10.0	-0.87	-1.83	AAA-PAA	0.10	11	0.861	0.984	0.3829
241446_at	30.0	16.4	-0.87	-1.83	PAA-AAA	0.10	11	0.861	0.984	0.4357

239263_at	76.2	41.7	-0.87	-1.83	APP-AAM	0.10	11	0.861	0.984	0.5240
242530_at	79.9	43.8	-0.87	-1.83	PAA-AAP	0.10	11	0.861	0.984	0.2863
237408_at	65.4	35.8	-0.87	-1.82	PPA-PPA	0.10	11	0.861	0.984	0.2506
229293_at	171.7	94.1	-0.87	-1.82	PPA-AAA	0.10	11	0.861	0.984	0.2574
1553686_at	114.2	62.6	-0.87	-1.82	PPP-APA	0.10	11	0.861	0.984	0.3092
243938_x_at	94.0	51.6	-0.87	-1.82	PPP-APP	0.10	11	0.861	0.984	0.3408
240419_at	96.7	176.2	0.87	1.82	PAA-PPP	0.10	11	0.861	0.984	0.0405
243920_x_at	38.0	20.8	-0.87	-1.82	PPA-APA	0.10	11	0.861	0.984	0.2764
1564960_at	54.8	30.1	-0.87	-1.82	PPA-APA	0.10	11	0.861	0.984	0.4527
1556373_a_at	81.4	44.7	-0.86	-1.82	PAA-AMM	0.10	11	0.861	0.984	0.2564
241805_at	27.2	14.9	-0.86	-1.82	AAA-PAA	0.10	11	0.861	0.984	0.5573
239385_at	52.2	28.7	-0.86	-1.82	PPA-APP	0.10	11	0.861	0.984	0.5530
237814_at	23.7	13.1	-0.86	-1.82	AAA-AAP	0.10	11	0.861	0.984	0.5617
1560257_at	29.6	16.3	-0.86	-1.82	AAP-AAA	0.10	11	0.861	0.984	0.2531
229786_at	179.7	99.0	-0.86	-1.82	MPA-PAP	0.10	11	0.861	0.984	0.2704
1560647_at	37.8	20.8	-0.86	-1.82	PAA-AAP	0.10	11	0.861	0.984	0.4606
206576_s_at	66.4	36.6	-0.86	-1.81	PAA-AAA	0.10	11	0.861	0.984	0.5024
1564854_at	33.2	18.3	-0.86	-1.81	AAA-APA	0.10	11	0.861	0.984	0.5286
221594_at	37.1	20.5	-0.86	-1.81	AAP-AAP	0.10	11	0.861	0.984	0.4225
206459_s_at	64.7	35.7	-0.86	-1.81	APP-AAA	0.10	11	0.861	0.984	0.4283
221571_at	649.4	1175.9	0.86	1.81	PPP-PPP	0.10	11	0.861	0.984	0.0396
215185_at	83.5	46.1	-0.86	-1.81	AAA-PAA	0.10	11	0.861	0.984	0.2593
214153_at	149.4	82.6	-0.86	-1.81	PPA-PPP	0.10	11	0.861	0.984	0.3024
1556834_at	36.7	20.4	-0.85	-1.81	AMA-APA	0.10	11	0.861	0.984	0.2715
230928_at	34.6	19.1	-0.85	-1.81	APA-APA	0.10	11	0.861	0.984	0.5522
210783_x_at	82.5	149.0	0.85	1.81	AAA-PPA	0.10	11	0.861	0.984	0.0132
227189_at	45.3	25.1	-0.85	-1.81	APA-AAA	0.10	11	0.861	0.984	0.5028
238281_at	52.7	29.2	-0.85	-1.80	AAP-AMP	0.10	11	0.861	0.984	0.2805
228029_at	463.3	835.9	0.85	1.80	PPP-PPP	0.10	11	0.861	0.984	0.0245
213264_at	429.8	775.1	0.85	1.80	PPP-PPP	0.10	11	0.861	0.984	0.0244
220863_at	55.9	31.0	-0.85	-1.80	AAP-AAA	0.10	11	0.861	0.984	0.3960
1557155_a_at	27.6	49.7	0.85	1.80	PAA-AAP	0.10	11	0.861	0.984	0.0373
1558052_at	93.1	51.7	-0.85	-1.80	AAA-PPP	0.10	11	0.861	0.984	0.2609
224323_s_at	72.5	40.3	-0.85	-1.80	AMP-AMM	0.10	11	0.861	0.984	0.3902
228260_at	39.8	22.1	-0.85	-1.80	AAA-APA	0.10	11	0.861	0.984	0.3156
220463_at	77.0	42.8	-0.85	-1.80	AAA-APA	0.10	11	0.861	0.984	0.3194
239744_at	18.4	10.2	-0.85	-1.80	PAA-AAA	0.10	11	0.861	0.984	0.4640
1568866_at	26.2	14.5	-0.85	-1.80	AAA-APA	0.10	11	0.861	0.984	0.2742
41469_at	42.8	23.8	-0.85	-1.80	PAA-AAA	0.10	11	0.861	0.984	0.4309
244168_s_at	51.3	28.5	-0.85	-1.80	AAP-AAA	0.10	11	0.861	0.984	0.5055
203868_s_at	77.6	139.6	0.85	1.80	APP-PPP	0.10	11	0.861	0.984	0.0389
209060_x_at	332.8	597.8	0.85	1.80	PPP-PPP	0.10	11	0.861	0.984	0.0261

240831_at	124.5	69.5	-0.84	-1.79	PAA-PAA	0.10	11	0.861	0.984	0.2799
220452_x_at	300.9	167.8	-0.84	-1.79	APA-APA	0.10	11	0.861	0.984	0.3280
242756_at	52.3	29.2	-0.84	-1.79	APP-APP	0.10	11	0.861	0.984	0.5425
232789_at	71.9	40.1	-0.84	-1.79	APA-APA	0.10	11	0.861	0.984	0.4656
240532_at	19.7	11.0	-0.84	-1.79	APA-AAA	0.10	11	0.861	0.984	0.4840
243527_at	85.5	47.8	-0.84	-1.79	PPP-AAP	0.10	11	0.861	0.984	0.4437
229586_at	383.6	686.7	0.84	1.79	PPP-PPP	0.10	11	0.861	0.984	0.0148
1556493_a_at	46.4	25.9	-0.84	-1.79	AAM-APA	0.10	11	0.861	0.984	0.4650
237861_at	93.4	52.4	-0.84	-1.78	MAA-PMA	0.10	11	0.861	0.984	0.2516
1568817_at	30.9	17.3	-0.84	-1.79	AAP-AAA	0.10	11	0.861	0.984	0.5651
209522_s_at	520.6	928.9	0.84	1.79	PPP-PPP	0.10	11	0.861	0.984	0.0223
1553859_at	41.8	23.4	-0.84	-1.78	AAA-AAP	0.10	11	0.861	0.984	0.3408
1560874_at	45.0	25.2	-0.83	-1.78	AAA-AAP	0.10	11	0.861	0.984	0.3258
230671_at	83.9	47.0	-0.83	-1.78	APA-APP	0.10	11	0.861	0.984	0.3392
1556543_at	55.5	31.2	-0.83	-1.78	PPP-AAP	0.10	11	0.861	0.984	0.4483
226926_at	24.4	13.7	-0.83	-1.78	AAP-AAA	0.10	11	0.861	0.984	0.5493
225738_at	322.6	574.1	0.83	1.78	PPP-PPP	0.10	11	0.861	0.984	0.0186
242754_at	71.2	40.0	-0.83	-1.78	AAA-PAA	0.10	11	0.861	0.984	0.4901
233457_at	35.3	19.9	-0.83	-1.78	APA-AAA	0.10	11	0.861	0.984	0.2812
219250_s_at	52.2	29.4	-0.83	-1.78	AAP-APP	0.10	11	0.861	0.984	0.3750
1562777_at	46.8	26.3	-0.83	-1.78	AAP-AAP	0.10	11	0.861	0.984	0.5622
215589_at	29.1	16.4	-0.83	-1.78	AAA-AAP	0.10	11	0.861	0.984	0.5725
1562091_at	40.4	22.8	-0.83	-1.77	APA-AAA	0.10	11	0.861	0.984	0.4361
1566999_at	38.3	21.6	-0.83	-1.77	APP-AAA	0.10	11	0.861	0.984	0.5652
203699_s_at	104.1	58.8	-0.83	-1.77	AAA-AAP	0.10	11	0.861	0.984	0.4015
242479_s_at	37.8	21.4	-0.83	-1.77	AAA-APA	0.10	11	0.861	0.984	0.4360
1554549_a_at	103.5	183.2	0.83	1.77	MMA-PPP	0.10	11	0.861	0.984	0.0308
241947_at	29.7	16.8	-0.82	-1.77	PAA-AAA	0.10	11	0.861	0.984	0.4087
205435_s_at	109.3	61.8	-0.82	-1.77	AAA-AAP	0.10	11	0.861	0.984	0.4133
1561286_a_at	119.7	67.8	-0.82	-1.77	APP-APP	0.10	11	0.861	0.984	0.4217
222950_at	49.3	28.0	-0.82	-1.76	APA-AAA	0.10	11	0.861	0.984	0.3762
208920_at	284.6	502.0	0.82	1.76	PPP-PPP	0.10	11	0.861	0.984	0.0196
228921_at	49.6	28.1	-0.82	-1.76	AAP-AAA	0.10	11	0.861	0.984	0.4218
228119_at	72.5	41.1	-0.82	-1.76	AAP-AAA	0.10	11	0.861	0.984	0.4267
1558331_at	84.3	47.8	-0.82	-1.76	APP-APP	0.10	11	0.861	0.984	0.4608
219456_s_at	71.4	125.7	0.82	1.76	AAA-APA	0.10	11	0.861	0.984	0.0345
244471_x_at	70.0	39.8	-0.82	-1.76	APA-PAA	0.10	11	0.861	0.984	0.3238
244121_at	34.0	19.3	-0.81	-1.76	AAA-PAA	0.10	11	0.861	0.984	0.4100
1558120_at	61.3	34.9	-0.81	-1.76	PAP-AAP	0.10	11	0.861	0.984	0.4496
221605_s_at	40.5	23.0	-0.81	-1.76	AAP-AAA	0.10	11	0.861	0.984	0.5422
1564820_at	39.4	22.4	-0.81	-1.76	AAP-AAA	0.10	11	0.861	0.984	0.4521
233830_at	44.6	25.4	-0.81	-1.76	AAP-AMA	0.10	11	0.861	0.984	0.5104

214878_at	73.9	129.6	0.81	1.76	APP-PPP	0.10	11	0.861	0.984	0.0297
229127_at	51.3	29.3	-0.81	-1.75	AAA-AAP	0.10	11	0.861	0.984	0.4512
224014_at	31.0	17.7	-0.81	-1.75	AAA-APA	0.10	11	0.861	0.984	0.5352
1553212_at	56.5	32.3	-0.81	-1.75	PAA-APA	0.10	11	0.861	0.984	0.3649
238623_at	306.1	535.3	0.81	1.75	PPP-PPP	0.10	11	0.861	0.984	0.0385
242137_at	84.9	48.6	-0.81	-1.75	AAA-AAP	0.10	11	0.861	0.984	0.3092
236776_at	54.2	31.1	-0.80	-1.74	AAA-PPA	0.10	11	0.861	0.984	0.2911
224909_s_at	147.8	84.7	-0.80	-1.75	PPP-PPA	0.10	11	0.861	0.984	0.4891
1552734_at	67.7	38.8	-0.80	-1.74	APA-MAP	0.10	11	0.861	0.984	0.3575
205349_at	67.7	38.8	-0.80	-1.74	PAA-PAA	0.10	11	0.861	0.984	0.4799
222800_at	44.9	78.2	0.80	1.74	AAA-APP	0.10	11	0.861	0.984	0.0313
215919_s_at	73.6	128.2	0.80	1.74	MAP-PPP	0.10	11	0.861	0.984	0.0154
233083_at	28.1	48.9	0.80	1.74	AAA-APM	0.10	11	0.861	0.984	0.0340
1565811_at	94.6	54.3	-0.80	-1.74	AAA-AAP	0.10	11	0.861	0.984	0.3542
236875_at	31.1	17.9	-0.80	-1.74	APA-AAA	0.10	11	0.861	0.984	0.5157
220776_at	87.2	50.2	-0.80	-1.74	PPP-PAP	0.10	11	0.861	0.984	0.3648
214339_s_at	53.8	93.3	0.80	1.74	AAA-AAP	0.10	11	0.861	0.984	0.0193
220040_x_at	123.2	213.7	0.80	1.74	AAA-APP	0.10	11	0.861	0.984	0.0157
215055_at	95.0	54.8	-0.79	-1.73	PPA-AAA	0.10	11	0.861	0.984	0.4774
228954_at	97.9	169.8	0.79	1.73	APA-PPP	0.10	11	0.861	0.984	0.0284
200646_s_at	299.5	519.3	0.79	1.73	PPP-PPP	0.10	11	0.861	0.984	0.0106
220516_at	54.0	31.2	-0.79	-1.73	PAA-AAA	0.10	11	0.861	0.984	0.5577
215199_at	71.5	41.4	-0.79	-1.73	APA-APP	0.10	11	0.861	0.984	0.5010
215238_s_at	84.9	49.2	-0.79	-1.73	APA-APA	0.10	11	0.861	0.984	0.5687
223170_at	320.4	553.2	0.79	1.73	PPP-PPP	0.10	11	0.861	0.984	0.0183
1554901_at	62.8	36.4	-0.79	-1.73	AAA-PAA	0.10	11	0.861	0.984	0.2730
243915_at	39.7	23.0	-0.79	-1.73	PAA-AAA	0.10	11	0.861	0.984	0.4133
239848_at	84.0	48.6	-0.79	-1.73	PAP-APP	0.10	11	0.861	0.984	0.4298
235875_at	51.7	30.0	-0.79	-1.73	AAA-APP	0.10	11	0.861	0.984	0.5561
227739_at	307.5	178.3	-0.79	-1.73	AAA-APA	0.10	11	0.861	0.984	0.2731
234965_at	60.3	34.9	-0.79	-1.73	PAA-AAA	0.10	11	0.861	0.984	0.3746
231585_at	26.6	15.4	-0.79	-1.72	APA-AAA	0.10	11	0.861	0.984	0.5310
235363_at	125.0	215.6	0.79	1.73	PPP-PPP	0.10	11	0.861	0.984	0.0241
218908_at	252.0	434.6	0.79	1.73	APP-PPP	0.10	11	0.861	0.984	0.0237
1559980_at	50.7	29.4	-0.79	-1.72	APA-AAP	0.10	11	0.861	0.984	0.4120
1560553_at	95.8	55.6	-0.79	-1.72	AMP-AAP	0.10	11	0.861	0.984	0.5642
242676_at	77.8	45.2	-0.78	-1.72	APM-PPM	0.10	11	0.861	0.984	0.2436
216809_at	53.3	31.0	-0.78	-1.72	PPP-APA	0.10	11	0.861	0.984	0.4304
1557305_at	28.7	16.7	-0.78	-1.72	APA-AAA	0.10	11	0.861	0.984	0.5333
226192_at	125.3	72.8	-0.78	-1.72	PAA-PAA	0.10	11	0.861	0.984	0.2579
215019_x_at	104.0	60.5	-0.78	-1.72	AAP-AMA	0.10	11	0.861	0.984	0.2868
205669_at	71.2	41.4	-0.78	-1.72	PPP-AAP	0.10	11	0.861	0.984	0.3029

242373_at	81.2	47.2	-0.78	-1.72	AAA-PAA	0.10	11	0.861	0.984	0.3855
1562122_at	18.1	10.5	-0.78	-1.72	AAP-AAA	0.10	11	0.861	0.984	0.5740
1560446_at	55.7	32.4	-0.78	-1.72	PAP-PMA	0.10	11	0.861	0.984	0.2646
227969_at	41.1	23.9	-0.78	-1.72	AAA-AAP	0.10	11	0.861	0.984	0.3884
232014_at	184.0	315.9	0.78	1.72	MPP-PPP	0.10	11	0.861	0.984	0.0118
241784_x_at	68.2	39.8	-0.78	-1.72	AAA-PAA	0.10	11	0.861	0.984	0.2775
1558688_at	1471.9	859.1	-0.78	-1.71	PPP-PPP	0.10	11	0.861	0.984	0.3022
1554980_a_at	148.8	86.9	-0.78	-1.71	PPA-PAP	0.10	11	0.861	0.984	0.3030
207973_x_at	43.0	25.1	-0.78	-1.71	PAA-AAA	0.10	11	0.861	0.984	0.4225
208561_at	47.5	27.8	-0.78	-1.71	APA-APA	0.10	11	0.861	0.984	0.3765
241688_at	55.7	32.5	-0.78	-1.71	PPA-APA	0.10	11	0.861	0.984	0.3883
201564_s_at	1026.8	1755.5	0.77	1.71	PPP-PPP	0.10	11	0.861	0.984	0.0116
243559_at	34.7	20.3	-0.77	-1.71	PAA-AAP	0.10	11	0.861	0.984	0.5570
224305_s_at	32.5	19.1	-0.77	-1.70	PAA-AAA	0.10	11	0.861	0.984	0.4145
244575_at	99.6	58.5	-0.77	-1.70	PAA-PAA	0.10	11	0.861	0.984	0.3909
241331_at	48.7	28.6	-0.77	-1.70	AAA-APP	0.10	11	0.861	0.984	0.5519
1554229_at	111.2	65.3	-0.77	-1.70	PPA-PPA	0.10	11	0.861	0.984	0.3021
1559895_x_at	55.2	32.5	-0.77	-1.70	APA-PPA	0.10	11	0.861	0.984	0.3202
223821_s_at	31.3	18.4	-0.77	-1.70	AAA-AAP	0.10	11	0.861	0.984	0.4239
228424_at	135.4	79.6	-0.77	-1.70	APP-MPA	0.10	11	0.861	0.984	0.4373
206807_s_at	81.6	48.0	-0.77	-1.70	APA-AAA	0.10	11	0.861	0.984	0.5094
1570089_at	53.5	31.5	-0.77	-1.70	AAP-AAA	0.10	11	0.861	0.984	0.5312
242255_at	97.4	165.5	0.77	1.70	MAP-PPP	0.10	11	0.861	0.984	0.0183
218428_s_at	402.2	683.5	0.77	1.70	PPP-PPP	0.10	11	0.861	0.984	0.0321
212466_at	56.1	95.2	0.77	1.70	AAA-AAP	0.10	11	0.861	0.984	0.0078
242949_x_at	132.1	77.8	-0.76	-1.70	PAA-MAA	0.10	11	0.861	0.984	0.2471
211368_s_at	116.7	198.2	0.76	1.70	APP-PPP	0.10	11	0.861	0.984	0.0168
242199_at	71.0	41.8	-0.76	-1.70	PAP-PAA	0.10	11	0.861	0.984	0.4038
228047_at	86.1	50.8	-0.76	-1.70	PPP-AMP	0.10	11	0.861	0.984	0.4088
1565722_at	37.2	21.9	-0.76	-1.69	PAA-AAA	0.10	11	0.861	0.984	0.3765
232705_at	76.4	45.1	-0.76	-1.70	AAM-APP	0.10	11	0.861	0.984	0.4440
203417_at	412.6	699.2	0.76	1.70	PPP-PPP	0.10	11	0.861	0.984	0.0231
232295_at	74.1	43.7	-0.76	-1.69	PPP-APP	0.10	11	0.861	0.984	0.3344
226910_at	632.4	1071.0	0.76	1.69	PPP-PPP	0.10	11	0.861	0.984	0.0160
221756_at	107.4	63.5	-0.76	-1.69	PAA-AAA	0.10	11	0.861	0.984	0.3885
1561532_at	87.1	51.5	-0.76	-1.69	PAA-PAA	0.10	11	0.861	0.984	0.2792
1553644_at	103.0	61.0	-0.76	-1.69	PPP-PPP	0.10	11	0.861	0.984	0.3464
206524_at	78.7	46.6	-0.76	-1.69	PAA-AAA	0.10	11	0.861	0.984	0.5258
221953_s_at	413.2	698.2	0.76	1.69	MPP-PPP	0.10	11	0.861	0.984	0.0354
238946_at	64.3	38.1	-0.76	-1.69	AMP-APP	0.10	11	0.861	0.984	0.3589
227450_at	61.6	36.5	-0.76	-1.69	PPA-AAP	0.10	11	0.861	0.984	0.4775
233539_at	76.9	45.6	-0.76	-1.69	PPA-APP	0.10	11	0.861	0.984	0.4025

234937_x_at	94.6	56.2	-0.75	-1.69	AAP-PAP	0.10	11	0.861	0.984	0.3804
241382_at	64.1	38.1	-0.75	-1.68	PPA-MMA	0.10	11	0.861	0.984	0.3702
227888_at	64.2	38.1	-0.75	-1.68	AAA-PAA	0.10	11	0.861	0.984	0.3745
210226_at	29.1	17.3	-0.75	-1.69	APA-AAA	0.10	11	0.861	0.984	0.5215
221646_s_at	68.6	40.7	-0.75	-1.69	PAA-APP	0.10	11	0.861	0.984	0.5527
206112_at	39.0	23.2	-0.75	-1.68	PAA-AAA	0.10	11	0.861	0.984	0.3441
215620_at	84.6	50.3	-0.75	-1.68	PPA-APP	0.10	11	0.861	0.984	0.3985
229187_at	34.0	57.2	0.75	1.68	APP-PAP	0.10	11	0.861	0.984	0.0200
220954_s_at	169.4	285.1	0.75	1.68	PPP-PPP	0.10	11	0.861	0.984	0.0078
229956_at	37.0	62.2	0.75	1.68	AAA-PPP	0.10	11	0.861	0.984	0.0172
209695_at	98.2	58.4	-0.75	-1.68	PAA-PPA	0.10	11	0.861	0.984	0.4146
239093_at	39.9	23.8	-0.75	-1.68	PAM-AAM	0.10	11	0.861	0.984	0.3950
204129_at	202.4	120.5	-0.75	-1.68	APP-PAP	0.10	11	0.861	0.984	0.3978
231901_at	286.4	480.9	0.75	1.68	APA-PPP	0.10	11	0.861	0.984	0.0346
1555561_a_at	214.9	360.8	0.75	1.68	PPP-PPP	0.10	11	0.861	0.984	0.0246
241430_at	88.3	52.6	-0.75	-1.68	APA-AAA	0.10	11	0.861	0.984	0.3192
210089_s_at	165.3	277.2	0.75	1.68	APA-PPP	0.10	11	0.861	0.984	0.0191
230775_s_at	79.9	47.7	-0.75	-1.68	AAP-AAA	0.10	11	0.861	0.984	0.4388
33148_at	307.0	514.6	0.75	1.68	PPP-PPP	0.10	11	0.861	0.984	0.0202
238965_at	56.8	33.9	-0.74	-1.68	AAA-AAP	0.10	11	0.861	0.984	0.5611
238620_at	50.6	30.2	-0.74	-1.67	AAA-AAP	0.10	11	0.861	0.984	0.2397
227268_at	268.5	449.4	0.74	1.67	PPP-PPP	0.10	11	0.861	0.984	0.0187
1570470_at	38.8	23.2	-0.74	-1.67	AAA-PAP	0.10	11	0.861	0.984	0.2428
1562499_at	31.4	18.8	-0.74	-1.67	AAP-AAA	0.10	11	0.861	0.984	0.4864
228381_at	72.3	43.3	-0.74	-1.67	AAP-AAA	0.10	11	0.861	0.984	0.4056
1559340_at	44.4	26.6	-0.74	-1.67	PAA-AAA	0.10	11	0.861	0.984	0.5443
217520_x_at	167.2	279.3	0.74	1.67	APP-PPP	0.10	11	0.861	0.984	0.0066
223593_at	141.4	236.0	0.74	1.67	APP-PPP	0.10	11	0.861	0.984	0.0067
239660_at	48.3	29.0	-0.74	-1.67	MPA-AAM	0.10	11	0.861	0.984	0.3042
217133_x_at	53.7	32.2	-0.74	-1.67	APA-PAP	0.10	11	0.861	0.984	0.5296
216950_s_at	70.6	42.5	-0.73	-1.66	APA-AAA	0.10	11	0.861	0.984	0.4375
1558885_at	69.5	41.8	-0.73	-1.66	PPP-PAP	0.10	11	0.861	0.984	0.2592
229224_x_at	76.2	45.9	-0.73	-1.66	AAA-APP	0.10	11	0.861	0.984	0.2685
227673_at	48.9	29.4	-0.73	-1.66	AAA-APA	0.10	11	0.861	0.984	0.5399
224458_at	199.7	331.9	0.73	1.66	PPP-PPP	0.10	11	0.861	0.984	0.0280
212414_s_at	243.3	403.9	0.73	1.66	PPP-MPP	0.10	11	0.861	0.984	0.0340
221103_s_at	48.2	29.1	-0.73	-1.66	PAA-APP	0.10	11	0.861	0.984	0.4006
1554334_a_at	48.6	29.3	-0.73	-1.66	AAP-AAA	0.10	11	0.861	0.984	0.5146
243407_at	65.1	39.3	-0.73	-1.66	PAA-AAP	0.10	11	0.861	0.984	0.2416
206345_s_at	72.3	43.6	-0.73	-1.66	PAA-AMA	0.10	11	0.861	0.984	0.2627
213000_at	850.5	1409.1	0.73	1.66	PPP-PPP	0.10	11	0.861	0.984	0.0104
217014_s_at	57.0	34.4	-0.73	-1.66	APM-AAA	0.10	11	0.861	0.984	0.2556

240319_at	54.0	32.6	-0.73	-1.66	APA-PPA	0.10	11	0.861	0.984	0.5695
244369_at	36.4	22.0	-0.73	-1.66	APA-PAP	0.10	11	0.861	0.984	0.5711
215400_x_at	40.7	24.6	-0.73	-1.65	APA-AAA	0.10	11	0.861	0.984	0.4164
1562161_at	30.8	18.6	-0.73	-1.65	AAA-AAP	0.10	11	0.861	0.984	0.2514
205500_at	39.8	24.1	-0.72	-1.65	PAA-AAA	0.10	11	0.861	0.984	0.5429
204160_s_at	162.0	267.6	0.72	1.65	PPP-PPP	0.10	11	0.861	0.984	0.0160
212683_at	417.9	690.0	0.72	1.65	APP-PPP	0.10	11	0.861	0.984	0.0142
228531_at	570.1	941.8	0.72	1.65	PPP-PPP	0.10	11	0.861	0.984	0.0063
214984_at	33.9	20.5	-0.72	-1.65	AAP-APA	0.10	11	0.861	0.984	0.4494
1568805_at	28.2	17.1	-0.72	-1.65	AAP-AAA	0.10	11	0.861	0.984	0.4186
203003_at	87.8	53.2	-0.72	-1.65	APP-APP	0.10	11	0.861	0.984	0.4877
238630_at	102.5	168.8	0.72	1.65	AAA-APP	0.10	11	0.861	0.984	0.0115
1553823_a_at	38.3	23.3	-0.72	-1.64	APA-AAA	0.10	11	0.861	0.984	0.2632
232315_at	104.3	171.4	0.72	1.64	PPP-PPP	0.10	11	0.861	0.984	0.0119
238087_at	296.4	180.4	-0.72	-1.64	APP-PPP	0.10	11	0.861	0.984	0.3107
224327_s_at	143.3	87.3	-0.72	-1.64	PAA-AAA	0.10	11	0.861	0.984	0.4534
1553207_at	120.9	73.7	-0.72	-1.64	AAA-PAA	0.10	11	0.861	0.984	0.3123
233731_at	64.1	39.1	-0.72	-1.64	AAP-APA	0.10	11	0.861	0.984	0.3512
231631_at	75.6	46.1	-0.72	-1.64	AAA-PAA	0.10	11	0.861	0.984	0.3974
204523_at	247.8	406.8	0.72	1.64	PPP-PPP	0.10	11	0.861	0.984	0.0094
209521_s_at	83.0	50.6	-0.71	-1.64	AAA-APA	0.10	11	0.861	0.984	0.3068
1564209_at	39.0	23.8	-0.71	-1.64	APM-AAA	0.10	11	0.861	0.984	0.2414
228694_at	240.8	394.6	0.71	1.64	PPP-PPP	0.10	11	0.861	0.984	0.0282
205359_at	52.9	32.4	-0.71	-1.64	MPA-AAA	0.10	11	0.861	0.984	0.2778
212558_at	107.2	65.7	-0.71	-1.63	PMP-PMP	0.10	11	0.861	0.984	0.2397
1556272_a_at	41.4	25.3	-0.71	-1.63	APA-AAM	0.10	11	0.861	0.984	0.2685
239561_at	115.5	70.8	-0.71	-1.63	APA-AAP	0.10	11	0.861	0.984	0.2664
201744_s_at	1218.3	746.7	-0.71	-1.63	PPP-PPP	0.10	11	0.861	0.984	0.3187
1567539_at	99.4	61.0	-0.71	-1.63	APA-PAA	0.10	11	0.861	0.984	0.3957
242778_at	53.5	32.8	-0.71	-1.63	AAA-APA	0.10	11	0.861	0.984	0.4519
202157_s_at	58.1	35.7	-0.70	-1.63	PAP-PAP	0.10	11	0.861	0.984	0.3077
239983_at	66.9	41.1	-0.70	-1.63	PPP-APP	0.10	11	0.861	0.984	0.3441
206672_at	91.7	56.3	-0.70	-1.63	PAA-AAA	0.10	11	0.861	0.984	0.4434
231239_at	34.3	21.0	-0.70	-1.63	AAA-AMP	0.10	11	0.861	0.984	0.5426
1566195_at	116.3	71.4	-0.70	-1.63	AAA-PPA	0.10	11	0.861	0.984	0.2940
224425_x_at	58.5	35.9	-0.70	-1.63	AMP-AAA	0.10	11	0.861	0.984	0.3040
218227_at	529.0	861.2	0.70	1.63	PPP-PPP	0.10	11	0.861	0.984	0.0243
1565852_at	107.2	65.9	-0.70	-1.63	AAP-PAA	0.10	11	0.861	0.984	0.3314
235436_at	154.3	250.8	0.70	1.63	PPP-PPP	0.10	11	0.861	0.984	0.0165
1563513_at	44.6	27.4	-0.70	-1.62	AAA-APA	0.10	11	0.861	0.984	0.4950
207229_at	30.9	19.0	-0.70	-1.63	MAP-AAA	0.10	11	0.861	0.984	0.5405
1560214_at	143.3	88.3	-0.70	-1.62	PAA-AAA	0.10	11	0.861	0.984	0.3514

233449_at	25.5	15.7	-0.70	-1.62	AAP-AAA	0.10	11	0.861	0.984	0.5154
230480_at	71.0	43.8	-0.70	-1.62	AAA-APA	0.10	11	0.861	0.984	0.2450
224259_at	48.5	29.9	-0.70	-1.62	AAA-PAA	0.10	11	0.861	0.984	0.4885
240690_at	166.2	102.6	-0.70	-1.62	PPP-PPP	0.10	11	0.861	0.984	0.2349
1554591_at	106.1	65.5	-0.70	-1.62	PAA-MAM	0.10	11	0.861	0.984	0.3715
234783_at	42.6	26.4	-0.70	-1.62	AAP-MAM	0.10	11	0.861	0.984	0.2879
201741_x_at	164.9	101.9	-0.70	-1.62	PAP-AAP	0.10	11	0.861	0.984	0.3003
1561430_s_at	55.7	34.5	-0.70	-1.62	APA-PAA	0.10	11	0.861	0.984	0.4642
225933_at	574.8	930.6	0.70	1.62	PPP-PPP	0.10	11	0.861	0.984	0.0296
243294_at	27.5	17.0	-0.69	-1.62	AAP-AAP	0.10	11	0.861	0.984	0.4737
234977_at	111.1	179.8	0.69	1.62	PPP-PPP	0.10	11	0.861	0.984	0.0211
237939_at	119.6	74.0	-0.69	-1.62	PPP-PPP	0.10	11	0.861	0.984	0.2400
233112_at	91.3	56.5	-0.69	-1.62	AAP-PAM	0.10	11	0.861	0.984	0.4489
219570_at	70.0	113.2	0.69	1.62	PPP-PPP	0.10	11	0.861	0.984	0.0288
1560141_at	75.0	46.5	-0.69	-1.62	APP-AAP	0.10	11	0.861	0.984	0.2881
240107_at	40.4	25.0	-0.69	-1.62	PPA-AAP	0.10	11	0.861	0.984	0.4123
222456_s_at	69.8	43.2	-0.69	-1.61	PPA-APA	0.10	11	0.861	0.984	0.3361
1555159_at	76.5	47.4	-0.69	-1.62	APP-PPA	0.10	11	0.861	0.984	0.3880
231838_at	72.4	44.9	-0.69	-1.61	APA-PAA	0.10	11	0.861	0.984	0.3197
224036_s_at	198.8	320.7	0.69	1.61	PPP-PPP	0.10	11	0.861	0.984	0.0158
1567045_at	81.9	50.8	-0.69	-1.61	AAA-AAP	0.10	11	0.861	0.984	0.3045
240801_at	48.0	29.8	-0.69	-1.61	PAA-PAA	0.10	11	0.861	0.984	0.3551
242517_at	58.6	36.4	-0.69	-1.61	PAA-AAA	0.10	11	0.861	0.984	0.3870
210219_at	113.8	183.4	0.69	1.61	PPM-PMP	0.10	11	0.861	0.984	0.0132
236881_at	49.8	30.9	-0.69	-1.61	AAA-AAP	0.10	11	0.861	0.984	0.2625
240529_at	56.5	35.1	-0.69	-1.61	AAP-AAA	0.10	11	0.861	0.984	0.5477
1556328_at	31.3	19.5	-0.69	-1.61	AAA-AAP	0.10	11	0.861	0.984	0.3870
224442_at	44.5	27.6	-0.69	-1.61	AAA-AAP	0.10	11	0.861	0.984	0.4962
243612_at	164.7	102.4	-0.69	-1.61	PPP-APP	0.10	11	0.861	0.984	0.3582
1562686_at	36.6	22.8	-0.68	-1.61	PAA-AAA	0.10	11	0.861	0.984	0.4118
229754_at	112.3	70.0	-0.68	-1.61	APM-PPA	0.10	11	0.861	0.984	0.2522
1556743_at	61.4	38.3	-0.68	-1.60	APP-MPP	0.10	11	0.861	0.984	0.2959
235659_at	88.7	55.3	-0.68	-1.60	AAA-APA	0.10	11	0.861	0.984	0.3899
237946_at	48.7	30.4	-0.68	-1.60	AAA-PAA	0.10	11	0.861	0.984	0.2843
222169_x_at	106.6	66.6	-0.68	-1.60	PAA-AAP	0.10	11	0.861	0.984	0.3894
1561133_at	68.9	43.0	-0.68	-1.60	AAA-AAP	0.10	11	0.861	0.984	0.5140
237239_at	194.3	121.4	-0.68	-1.60	PAP-AMP	0.10	11	0.861	0.984	0.3678
1553479_at	86.7	54.2	-0.68	-1.60	AAA-APP	0.10	11	0.861	0.984	0.4040
230569_at	96.7	154.6	0.68	1.60	PPP-PPP	0.10	11	0.861	0.984	0.0226
232396_at	87.1	54.5	-0.68	-1.60	APP-AAP	0.10	11	0.861	0.984	0.2335
213418_at	87.5	54.8	-0.68	-1.60	AAA-PPA	0.10	11	0.861	0.984	0.4303
1557823_s_at	28.4	17.8	-0.68	-1.60	AAA-AAP	0.10	11	0.861	0.984	0.4923

227102_at	141.4	226.0	0.68	1.60	PPP-PPP	0.10	11	0.861	0.984	0.0222
232928_at	52.1	32.6	-0.68	-1.60	AAP-AAA	0.10	11	0.861	0.984	0.2898
236561_at	98.4	61.7	-0.67	-1.60	AAP-AAP	0.10	11	0.861	0.984	0.4009
222031_at	58.5	36.7	-0.67	-1.59	PPP-AAP	0.10	11	0.861	0.984	0.3277
1553780_at	154.4	96.9	-0.67	-1.59	PPP-APA	0.10	11	0.861	0.984	0.3773
216161_at	94.2	59.1	-0.67	-1.60	APA-AAP	0.10	11	0.861	0.984	0.3928
1565346_a_at	51.0	32.0	-0.67	-1.59	APA-APA	0.10	11	0.861	0.984	0.2396
221448_s_at	60.7	38.1	-0.67	-1.59	AAA-PAA	0.10	11	0.861	0.984	0.3140
206209_s_at	82.1	51.6	-0.67	-1.59	PPP-APA	0.10	11	0.861	0.984	0.3528
237628_at	27.9	17.5	-0.67	-1.59	AAA-APA	0.10	11	0.861	0.984	0.5079
237008_at	55.7	35.0	-0.67	-1.59	PMP-AAA	0.10	11	0.861	0.984	0.5539
228400_at	163.2	259.7	0.67	1.59	PPP-PPP	0.10	11	0.861	0.984	0.0134
1562612_at	63.9	40.2	-0.67	-1.59	PPP-AMP	0.10	11	0.861	0.984	0.3537
210617_at	69.1	43.5	-0.67	-1.59	AAP-AAA	0.10	11	0.861	0.984	0.4722
1552499_a_at	68.8	43.3	-0.67	-1.59	PAP-AAP	0.10	11	0.861	0.984	0.3121
219551_at	128.8	204.4	0.67	1.59	APP-PPP	0.10	11	0.861	0.984	0.0075
203498_at	36.6	23.1	-0.67	-1.59	PAA-APA	0.10	11	0.861	0.984	0.4053
1557400_at	118.2	74.6	-0.67	-1.59	PPP-PPP	0.10	11	0.861	0.984	0.2592
1563770_at	57.5	36.3	-0.66	-1.58	PAA-AAA	0.10	11	0.861	0.984	0.2321
238977_at	82.4	52.0	-0.66	-1.58	PAP-PPP	0.10	11	0.861	0.984	0.3426
233375_at	95.5	60.3	-0.66	-1.59	PPP-APP	0.10	11	0.861	0.984	0.3821
222237_s_at	195.1	309.2	0.66	1.58	PPP-PPP	0.10	11	0.861	0.984	0.0100
244231_at	77.3	48.8	-0.66	-1.58	AAA-APA	0.10	11	0.861	0.984	0.2537
1559655_at	32.3	20.4	-0.66	-1.58	PAA-AAA	0.10	11	0.861	0.984	0.4835
227555_s_at	158.3	100.2	-0.66	-1.58	MPA-AAA	0.10	11	0.861	0.984	0.2885
244440_at	74.3	47.0	-0.66	-1.58	APP-APA	0.10	11	0.861	0.984	0.3579
1564151_at	60.6	38.3	-0.66	-1.58	PAA-AAP	0.10	11	0.861	0.984	0.3597
1557518_a_at	37.8	23.9	-0.66	-1.58	PAA-AAA	0.10	11	0.861	0.984	0.4281
233469_at	85.8	54.3	-0.66	-1.58	APA-AAA	0.10	11	0.861	0.984	0.3284
1563063_at	76.8	48.6	-0.66	-1.58	AAA-APA	0.10	11	0.861	0.984	0.4840
238073_at	63.4	40.1	-0.66	-1.58	PAA-AAA	0.10	11	0.861	0.984	0.4798
241687_at	151.4	238.9	0.66	1.58	APP-PPP	0.10	11	0.861	0.984	0.0293
233599_at	88.8	56.3	-0.66	-1.58	APA-AAP	0.10	11	0.861	0.984	0.4410
1560369_at	81.9	52.0	-0.66	-1.58	PPM-PAA	0.10	11	0.861	0.984	0.2539
233630_at	41.4	26.3	-0.65	-1.57	AAP-AAA	0.10	11	0.861	0.984	0.2504
222962_s_at	897.6	1411.9	0.65	1.57	PPP-PPP	0.10	11	0.861	0.984	0.0235
235038_at	76.3	120.1	0.65	1.57	APP-PAA	0.10	11	0.861	0.984	0.0108
234541_s_at	35.7	22.7	-0.65	-1.57	AAP-AAA	0.10	11	0.861	0.984	0.2317
204532_x_at	142.6	90.8	-0.65	-1.57	AMP-APA	0.10	11	0.861	0.984	0.2365
78330_at	60.2	38.3	-0.65	-1.57	AAP-PAP	0.10	11	0.861	0.984	0.3738
244459_at	52.6	33.5	-0.65	-1.57	PAA-AAP	0.10	11	0.861	0.984	0.5214
230092_at	92.5	58.9	-0.65	-1.57	PMA-APA	0.10	11	0.861	0.984	0.5256

1554367_at	91.7	58.4	-0.65	-1.57	PPA-PMA	0.10	11	0.861	0.984	0.2659
243109_at	44.8	28.6	-0.65	-1.57	APP-MAA	0.10	11	0.861	0.984	0.4370
204055_s_at	68.6	43.8	-0.65	-1.57	APP-APP	0.10	11	0.861	0.984	0.2377
243037_at	177.7	113.4	-0.65	-1.57	PPP-PMP	0.10	11	0.861	0.984	0.2755
235376_at	69.6	44.4	-0.65	-1.57	AAA-APP	0.10	11	0.861	0.984	0.4053
1553141_at	75.2	48.0	-0.65	-1.57	PAA-APA	0.10	11	0.861	0.984	0.2738
1555248_a_at	53.3	34.0	-0.65	-1.57	PPA-APA	0.10	11	0.861	0.984	0.4120
228329_at	38.9	24.8	-0.65	-1.57	AAA-AAP	0.10	11	0.861	0.984	0.5078
221627_at	124.0	79.2	-0.65	-1.57	AAP-AAA	0.10	11	0.861	0.984	0.2438
215694_at	73.8	47.2	-0.65	-1.57	PPP-APP	0.10	11	0.861	0.984	0.2568
203457_at	267.4	170.9	-0.65	-1.56	PPP-PPP	0.10	11	0.861	0.984	0.2715
213475_s_at	72.2	46.1	-0.65	-1.56	PPP-PAP	0.10	11	0.861	0.984	0.3522
203692_s_at	314.8	492.7	0.65	1.57	PPP-PPP	0.10	11	0.861	0.984	0.0208
233770_at	75.8	48.5	-0.65	-1.56	PPP-APP	0.10	11	0.861	0.984	0.4740
228501_at	76.9	49.2	-0.65	-1.56	APA-PPA	0.10	11	0.861	0.984	0.5684
236401_at	35.5	22.7	-0.64	-1.56	PAA-AAA	0.10	11	0.861	0.984	0.5475
205768_s_at	72.8	46.7	-0.64	-1.56	AAP-AAP	0.10	11	0.861	0.984	0.4948
213927_at	191.4	298.9	0.64	1.56	PPP-PPP	0.10	11	0.861	0.984	0.0172
1568931_at	46.0	29.5	-0.64	-1.56	APA-AAA	0.10	11	0.861	0.984	0.4429
226136_at	1281.3	1999.6	0.64	1.56	PPP-PPP	0.10	11	0.861	0.984	0.0145
225845_at	448.0	699.3	0.64	1.56	PPP-PPP	0.10	11	0.861	0.984	0.0135
228481_at	755.1	484.2	-0.64	-1.56	PPP-PPP	0.10	11	0.861	0.984	0.2440
206133_at	75.0	48.1	-0.64	-1.56	MAA-PPA	0.10	11	0.861	0.984	0.4918
244439_at	98.9	154.2	0.64	1.56	PPA-PPP	0.10	11	0.861	0.984	0.0130
232422_at	78.1	50.1	-0.64	-1.56	AAP-AAA	0.10	11	0.861	0.984	0.4330
235055_x_at	61.7	39.6	-0.64	-1.56	APA-AAA	0.10	11	0.861	0.984	0.4997
1562106_at	55.6	35.7	-0.64	-1.56	APM-PPA	0.10	11	0.861	0.984	0.5292
235345_at	90.3	58.0	-0.64	-1.56	PPP-PPA	0.10	11	0.861	0.984	0.3336
214424_s_at	47.1	30.3	-0.64	-1.56	APP-PAA	0.10	11	0.861	0.984	0.4452
239673_at	59.4	38.2	-0.64	-1.56	APA-AAA	0.10	11	0.861	0.984	0.2270
217365_at	142.0	91.3	-0.64	-1.56	PPP-PAP	0.10	11	0.861	0.984	0.2899
1569408_at	47.0	30.2	-0.64	-1.56	AAP-APP	0.10	11	0.861	0.984	0.4313
239611_at	35.9	23.1	-0.64	-1.55	APA-AAA	0.10	11	0.861	0.984	0.2554
234486_at	77.2	49.7	-0.64	-1.55	AAP-MMM	0.10	11	0.861	0.984	0.2533
243874_at	83.2	53.6	-0.64	-1.55	PPA-AAP	0.10	11	0.861	0.984	0.2675
202745_at	958.4	1487.9	0.64	1.55	PPP-PPP	0.10	11	0.861	0.984	0.0211
236198_at	30.0	19.4	-0.63	-1.55	PAA-AAA	0.10	11	0.861	0.984	0.3435
233597_at	45.0	29.0	-0.63	-1.55	AAP-AAA	0.10	11	0.861	0.984	0.4971
237287_at	111.3	71.7	-0.63	-1.55	PAA-AAM	0.10	11	0.861	0.984	0.3324
1555845_at	291.5	452.0	0.63	1.55	PPP-PPP	0.10	11	0.861	0.984	0.0194
206579_at	51.0	32.9	-0.63	-1.55	APP-AAA	0.10	11	0.861	0.984	0.3458
206801_at	559.9	361.2	-0.63	-1.55	PPP-PPP	0.10	11	0.861	0.984	0.3514

233461_x_at	214.8	332.9	0.63	1.55	PPP-PPP	0.10	11	0.861	0.984	0.0243
233650_at	48.3	31.2	-0.63	-1.55	APA-AAP	0.10	11	0.861	0.984	0.2471
1568974_at	74.2	47.9	-0.63	-1.55	PPP-AAA	0.10	11	0.861	0.984	0.2861
210095_s_at	10193.8	6583.1	-0.63	-1.55	PPP-PPP	0.10	11	0.861	0.984	0.3528
215287_at	141.7	91.5	-0.63	-1.55	MAP-PPP	0.10	11	0.861	0.984	0.4539
222662_at	145.3	225.0	0.63	1.55	PPP-PPP	0.10	11	0.861	0.984	0.0196
237465_at	92.8	143.8	0.63	1.55	PPM-PPP	0.10	11	0.861	0.984	0.0056
1566513_a_at	78.6	50.8	-0.63	-1.55	AAA-PAA	0.10	11	0.861	0.984	0.2266
205943_at	58.4	37.7	-0.63	-1.55	PPA-PAP	0.10	11	0.861	0.984	0.2854
243267_x_at	83.4	53.9	-0.63	-1.55	PAM-AAA	0.10	11	0.861	0.984	0.4936
215139_at	59.9	38.7	-0.63	-1.55	APA-APP	0.10	11	0.861	0.984	0.5618
1563841_at	155.0	100.2	-0.63	-1.55	PAA-AAA	0.10	11	0.861	0.984	0.2814
242030_at	89.4	57.8	-0.63	-1.55	APA-APA	0.10	11	0.861	0.984	0.3654
243428_at	60.6	39.2	-0.63	-1.55	APA-AAA	0.10	11	0.861	0.984	0.4092
212143_s_at	3491.4	2259.8	-0.63	-1.55	PPP-PPP	0.10	11	0.861	0.984	0.2728
242008_at	63.8	41.3	-0.63	-1.55	PAA-APA	0.10	11	0.861	0.984	0.2733
210102_at	43.8	28.3	-0.63	-1.55	PAA-AAA	0.10	11	0.861	0.984	0.3572
216783_at	52.9	34.3	-0.63	-1.54	APP-PPA	0.10	11	0.861	0.984	0.2651
230174_at	141.3	218.2	0.63	1.54	PPP-PPP	0.10	11	0.861	0.984	0.0193
233264_at	67.8	44.0	-0.63	-1.54	APA-PAP	0.10	11	0.861	0.984	0.3232
244605_at	70.2	45.5	-0.63	-1.54	PAA-PAA	0.10	11	0.861	0.984	0.4123
238396_at	66.1	42.9	-0.63	-1.54	AAA-APA	0.10	11	0.861	0.984	0.5247
220326_s_at	342.1	221.9	-0.63	-1.54	PPA-AAP	0.10	11	0.861	0.984	0.3674
221890_at	354.3	229.9	-0.62	-1.54	AAA-PAA	0.10	11	0.861	0.984	0.2293
212349_at	377.3	581.6	0.62	1.54	PPP-PPP	0.10	11	0.861	0.984	0.0276
221224_s_at	82.4	53.5	-0.62	-1.54	PAA-APA	0.10	11	0.861	0.984	0.2480
1563376_at	34.1	22.2	-0.62	-1.54	PAP-AMA	0.10	11	0.861	0.984	0.5604
231763_at	400.0	615.9	0.62	1.54	PPP-PPP	0.10	11	0.861	0.984	0.0281
243875_at	27.8	18.1	-0.62	-1.54	AAA-PAA	0.10	11	0.861	0.984	0.5278
1562300_at	27.6	17.9	-0.62	-1.54	AAA-PAA	0.10	11	0.861	0.984	0.4179
234848_at	26.3	17.1	-0.62	-1.54	AAA-APA	0.10	11	0.861	0.984	0.4928
209305_s_at	463.7	713.0	0.62	1.54	PPP-PPP	0.10	11	0.861	0.984	0.0123
239716_at	64.8	42.2	-0.62	-1.54	PPP-PAP	0.10	11	0.861	0.984	0.3303
236365_at	33.4	21.8	-0.62	-1.54	APA-AAA	0.10	11	0.861	0.984	0.3336
244679_at	76.9	50.1	-0.62	-1.54	APP-PAP	0.10	11	0.861	0.984	0.3611
1568889_at	42.0	27.4	-0.62	-1.53	PAA-AAP	0.10	11	0.861	0.984	0.5268
205939_at	46.7	30.4	-0.62	-1.54	PAA-AAA	0.10	11	0.861	0.984	0.5456
1561129_at	37.8	24.6	-0.62	-1.53	APA-AAA	0.10	11	0.861	0.984	0.2515
233239_at	78.4	51.1	-0.62	-1.53	PPP-APP	0.10	11	0.861	0.984	0.2825
217406_at	162.1	105.7	-0.62	-1.53	PMA-APA	0.10	11	0.861	0.984	0.2979
1566363_at	113.0	73.8	-0.62	-1.53	PAA-APA	0.10	11	0.861	0.984	0.2678
239247_at	43.2	28.2	-0.62	-1.53	APA-APM	0.10	11	0.861	0.984	0.2782

1554906_a_at	69.1	45.1	-0.62	-1.53	PPP-APP	0.10	11	0.861	0.984	0.2961
240354_at	46.0	30.0	-0.62	-1.53	AAA-APP	0.10	11	0.861	0.984	0.3086
241888_at	24.3	15.8	-0.62	-1.53	AAA-AAP	0.10	11	0.861	0.984	0.3975
204316_at	262.9	171.6	-0.62	-1.53	PPP-PPP	0.10	11	0.861	0.984	0.2522
209631_s_at	184.8	120.7	-0.62	-1.53	PPP-PPP	0.10	11	0.861	0.984	0.3585
216021_s_at	53.2	34.7	-0.62	-1.53	PAA-AAA	0.10	11	0.861	0.984	0.3662
1557749_at	63.2	41.3	-0.61	-1.53	AAP-AAA	0.10	11	0.861	0.984	0.3925
210367_s_at	91.8	140.6	0.61	1.53	PAP-PPA	0.10	11	0.861	0.984	0.0057
236396_at	109.1	71.4	-0.61	-1.53	APA-PAP	0.10	11	0.861	0.984	0.2318
1556951_at	86.7	56.7	-0.61	-1.53	APP-AAP	0.10	11	0.861	0.984	0.3217
209851_at	86.2	56.4	-0.61	-1.53	PAA-PAP	0.10	11	0.861	0.984	0.3662
242514_at	85.4	55.8	-0.61	-1.53	APP-AAA	0.10	11	0.861	0.984	0.4116
236769_at	71.9	47.0	-0.61	-1.53	PAA-MPP	0.10	11	0.861	0.984	0.2304
233030_at	249.5	163.2	-0.61	-1.53	APP-APP	0.10	11	0.861	0.984	0.2553
236409_at	48.1	31.5	-0.61	-1.53	PPP-AAP	0.10	11	0.861	0.984	0.3172
1562918_at	43.4	28.4	-0.61	-1.53	AAA-PAA	0.10	11	0.861	0.984	0.3977
1567031_at	37.1	24.3	-0.61	-1.53	AAP-AAA	0.10	11	0.861	0.984	0.4487
227471_at	270.1	412.7	0.61	1.53	PPP-PPP	0.10	11	0.861	0.984	0.0236
233427_x_at	288.9	189.1	-0.61	-1.53	MPP-AAA	0.10	11	0.861	0.984	0.3555
215496_at	70.3	46.0	-0.61	-1.53	AAA-PAA	0.10	11	0.861	0.984	0.3607
236479_at	68.3	44.7	-0.61	-1.53	PAA-PAA	0.10	11	0.861	0.984	0.4605
1559388_a_at	183.4	120.1	-0.61	-1.53	PAA-PAA	0.10	11	0.861	0.984	0.3706
207158_at	46.0	30.1	-0.61	-1.53	PAA-AAA	0.10	11	0.861	0.984	0.5262
242331_x_at	59.6	39.1	-0.61	-1.53	PAA-PPP	0.10	11	0.861	0.984	0.2636
237290_at	178.3	117.0	-0.61	-1.52	PPP-PPP	0.10	11	0.861	0.984	0.2468
224990_at	265.1	404.0	0.61	1.52	PPP-PPP	0.10	11	0.861	0.984	0.0094
1554625_at	150.8	99.0	-0.61	-1.52	PAA-AAA	0.10	11	0.861	0.984	0.2445
243490_at	127.0	83.4	-0.61	-1.52	PPP-APP	0.10	11	0.861	0.984	0.3492
1569782_at	24.4	16.1	-0.61	-1.52	AAA-PAA	0.10	11	0.861	0.984	0.5454
242069_at	215.1	141.4	-0.61	-1.52	AAP-PPA	0.10	11	0.861	0.984	0.2590
209818_s_at	283.2	186.1	-0.61	-1.52	PPP-PPP	0.10	11	0.861	0.984	0.2623
237496_at	53.3	35.1	-0.61	-1.52	AAP-MAP	0.10	11	0.861	0.984	0.3044
238499_at	96.5	63.4	-0.61	-1.52	PPP-PPP	0.10	11	0.861	0.984	0.4890
216101_at	90.2	59.3	-0.61	-1.52	PAP-APA	0.10	11	0.861	0.984	0.4876
205783_at	87.9	57.9	-0.60	-1.52	AAA-PAA	0.10	11	0.861	0.984	0.3333
211751_at	45.1	29.7	-0.60	-1.52	PAA-APA	0.10	11	0.861	0.984	0.4320
211254_x_at	143.0	94.1	-0.60	-1.52	APP-APA	0.10	11	0.861	0.984	0.2413
1553326_at	35.5	23.4	-0.60	-1.52	AAA-APA	0.10	11	0.861	0.984	0.2868
237187_at	44.7	29.4	-0.60	-1.52	AAA-AAP	0.10	11	0.861	0.984	0.5442
227131_at	546.3	829.4	0.60	1.52	PPP-PPP	0.10	11	0.861	0.984	0.0108
213401_s_at	72.9	48.0	-0.60	-1.52	AAA-APA	0.10	11	0.861	0.984	0.3189
223916_s_at	73.5	48.4	-0.60	-1.52	AAP-MPA	0.10	11	0.861	0.984	0.2816

212301_at	1548.9	2349.8	0.60	1.52	PPP-PPP	0.10	11	0.861	0.984	0.0218
244783_at	98.6	65.0	-0.60	-1.52	PPP-MPP	0.10	11	0.861	0.984	0.2550
211488_s_at	54.4	35.9	-0.60	-1.52	AAP-AAA	0.10	11	0.861	0.984	0.3464
1564067_x_at	72.1	47.6	-0.60	-1.52	APA-AAA	0.10	11	0.861	0.984	0.4821
208316_s_at	347.5	229.5	-0.60	-1.52	PPA-PPA	0.10	11	0.861	0.984	0.2774
243019_at	60.7	40.1	-0.60	-1.52	AAP-PAA	0.10	11	0.861	0.984	0.3758
230038_at	75.7	50.0	-0.60	-1.52	PAA-PAM	0.10	11	0.861	0.984	0.4636
238668_at	60.7	40.1	-0.60	-1.51	AAP-APA	0.10	11	0.861	0.984	0.2552
1560282_at	32.3	21.3	-0.60	-1.51	APA-AAA	0.10	11	0.861	0.984	0.4767
212606_at	383.0	579.6	0.60	1.51	PPP-PPP	0.10	11	0.861	0.984	0.0224
214057_at	134.2	203.1	0.60	1.51	PPP-PPP	0.10	11	0.861	0.984	0.0152
216499_at	103.6	68.5	-0.60	-1.51	PPA-APA	0.10	11	0.861	0.984	0.3249
207389_at	158.1	104.5	-0.60	-1.51	PPM-PAA	0.10	11	0.861	0.984	0.3922
216043_x_at	74.6	49.4	-0.60	-1.51	AAA-PAA	0.10	11	0.861	0.984	0.5464
218950_at	389.0	588.6	0.60	1.51	PPP-PPP	0.10	11	0.861	0.984	0.0114
220572_at	70.3	46.5	-0.60	-1.51	APA-MPP	0.10	11	0.861	0.984	0.3359
216112_at	155.9	103.2	-0.60	-1.51	MMA-APA	0.10	11	0.861	0.984	0.2474
215415_s_at	71.5	47.3	-0.60	-1.51	AAP-AAA	0.10	11	0.861	0.984	0.3648
236796_at	41.3	27.3	-0.60	-1.51	AAA-AAP	0.10	11	0.861	0.984	0.4747
215874_at	85.3	56.5	-0.59	-1.51	AAA-AMP	0.10	11	0.861	0.984	0.2319
1558322_a_at	47.6	31.6	-0.59	-1.51	PPA-AAA	0.10	11	0.861	0.984	0.4419
1562080_at	39.2	26.0	-0.59	-1.51	PAA-AAA	0.10	11	0.861	0.984	0.2461
216658_at	99.6	66.0	-0.59	-1.51	AAA-PAA	0.10	11	0.861	0.984	0.2573
1557586_s_at	94.5	62.7	-0.59	-1.51	PPP-MPP	0.10	11	0.861	0.984	0.3110
232483_at	149.4	99.1	-0.59	-1.51	PMP-PPP	0.10	11	0.861	0.984	0.3470
231854_at	69.6	46.2	-0.59	-1.51	PPA-APA	0.10	11	0.861	0.984	0.4287
201888_s_at	572.6	863.7	0.59	1.51	PPP-PPP	0.10	11	0.861	0.984	0.0232
228427_at	64.5	42.8	-0.59	-1.51	APA-APP	0.10	11	0.861	0.984	0.5080
227448_at	171.7	114.0	-0.59	-1.51	PPP-PPP	0.10	11	0.861	0.984	0.2907
229648_at	66.9	44.4	-0.59	-1.51	PPM-AAP	0.10	11	0.861	0.984	0.3124
209783_at	114.5	76.0	-0.59	-1.51	AAA-APP	0.10	11	0.861	0.984	0.3917
209806_at	2704.6	4073.3	0.59	1.51	PPP-PPP	0.10	11	0.861	0.984	0.0064
214682_at	256.7	170.6	-0.59	-1.51	APP-APP	0.10	11	0.861	0.984	0.2279
239245_at	71.8	47.7	-0.59	-1.51	PPA-APA	0.10	11	0.861	0.984	0.3756
221916_at	166.7	110.7	-0.59	-1.51	PPP-PPP	0.10	11	0.861	0.984	0.4569
217651_at	68.8	45.8	-0.59	-1.50	AAM-APA	0.10	11	0.861	0.984	0.2569
1552976_at	113.3	75.3	-0.59	-1.51	APP-AAA	0.10	11	0.861	0.984	0.3252
1559127_x_at	61.6	41.0	-0.59	-1.50	AAA-AAP	0.10	11	0.861	0.984	0.5194
235766_x_at	184.4	277.4	0.59	1.51	PPP-PPP	0.10	11	0.861	0.984	0.0212
231449_at	74.6	49.6	-0.59	-1.50	PPP-PAP	0.10	11	0.861	0.984	0.2331
214826_at	93.8	62.4	-0.59	-1.50	AAA-APA	0.10	11	0.861	0.984	0.2864
227975_at	200.7	133.6	-0.59	-1.50	APP-PAP	0.10	11	0.861	0.984	0.3644

237561_x_at	176.9	117.7	-0.59	-1.50	PMA-AAP	0.10	11	0.861	0.984	0.2565
206028_s_at	76.4	50.9	-0.59	-1.50	PMA-AAA	0.10	11	0.861	0.984	0.3394
238784_at	132.6	199.2	0.59	1.50	PPP-PPP	0.10	11	0.861	0.984	0.0166
210525_x_at	52.5	35.0	-0.59	-1.50	APA-APM	0.10	11	0.861	0.984	0.4097
228886_at	67.4	44.9	-0.59	-1.50	MAA-APA	0.10	11	0.861	0.984	0.4497
212478_at	103.3	68.9	-0.59	-1.50	AAM-AAP	0.10	11	0.861	0.984	0.2921
1564078_at	34.0	22.7	-0.59	-1.50	AAA-PAA	0.10	11	0.861	0.984	0.4261
238204_at	41.0	27.3	-0.59	-1.50	AAA-PAA	0.10	11	0.861	0.984	0.5397
233195_at	84.1	56.1	-0.58	-1.50	APA-AAP	0.10	11	0.861	0.984	0.5099
1562742_at	31.4	20.9	-0.58	-1.50	PAA-AAA	0.10	11	0.861	0.984	0.5274
231873_at	531.4	796.5	0.58	1.50	PPP-PPP	0.10	11	0.861	0.984	0.0192
230414_s_at	71.3	47.6	-0.58	-1.50	AAP-AAA	0.10	11	0.861	0.984	0.3332
1560716_at	50.2	33.6	-0.58	-1.50	PAA-AAA	0.10	11	0.861	0.984	0.2631
242992_at	74.9	50.0	-0.58	-1.50	PPA-APA	0.10	11	0.861	0.984	0.2920
1566340_at	77.8	52.0	-0.58	-1.50	APA-AAP	0.10	11	0.861	0.984	0.2208
1565559_at	42.1	28.2	-0.58	-1.50	APA-AAA	0.10	11	0.861	0.984	0.4073
219638_at	84.7	56.6	-0.58	-1.50	APA-APP	0.10	11	0.861	0.984	0.4158
213194_at	555.1	830.2	0.58	1.50	PPP-PPP	0.10	11	0.861	0.984	0.0141
1565696_at	72.5	48.5	-0.58	-1.49	PAP-AAA	0.10	11	0.861	0.984	0.3424
226226_at	89.5	60.0	-0.58	-1.49	AAA-PAA	0.10	11	0.861	0.984	0.4048
227921_at	664.1	991.9	0.58	1.49	PPP-PPP	0.10	11	0.861	0.984	0.0129
206839_at	105.2	70.5	-0.58	-1.49	PAA-AAA	0.10	11	0.861	0.984	0.3106
240538_at	124.6	83.5	-0.58	-1.49	AMP-AAP	0.10	11	0.861	0.984	0.2275
210812_at	96.4	64.6	-0.58	-1.49	AAA-APA	0.10	11	0.861	0.984	0.2291
229765_at	288.6	193.5	-0.58	-1.49	PPP-PPP	0.10	11	0.861	0.984	0.4706
217230_at	95.4	142.3	0.58	1.49	MPA-AAA	0.10	11	0.861	0.984	0.0188
232636_at	39.1	58.4	0.58	1.49	PAA-APP	0.10	11	0.861	0.984	0.0149
213447_at	178.0	265.6	0.58	1.49	PPP-PPP	0.10	11	0.861	0.984	0.0089
215505_s_at	101.6	151.5	0.58	1.49	AAA-APP	0.10	11	0.861	0.984	0.0235
1554623_x_at	56.3	37.8	-0.58	-1.49	AAP-AAA	0.10	11	0.861	0.984	0.2959
212800_at	202.7	301.9	0.58	1.49	PPP-PPP	0.10	11	0.861	0.984	0.0209
238311_at	73.0	49.0	-0.57	-1.49	AAP-APP	0.10	11	0.861	0.984	0.2275
208037_s_at	109.5	73.6	-0.57	-1.49	PMA-AAA	0.10	11	0.861	0.984	0.2972
242774_at	87.2	58.6	-0.57	-1.49	AAP-AAP	0.10	11	0.861	0.984	0.3278
1554237_at	126.6	85.0	-0.57	-1.49	AMA-APA	0.10	11	0.861	0.984	0.4602
244389_at	76.5	51.4	-0.57	-1.49	PAP-AAP	0.10	11	0.861	0.984	0.5218
224929_at	268.4	399.5	0.57	1.49	MPP-MPP	0.10	11	0.861	0.984	0.0198
1561705_at	98.5	66.2	-0.57	-1.49	PAP-PPP	0.10	11	0.861	0.984	0.3325
205362_s_at	228.6	340.1	0.57	1.49	PPP-PPP	0.10	11	0.861	0.984	0.0168
222128_at	85.2	57.4	-0.57	-1.49	APA-APP	0.10	11	0.861	0.984	0.2582
224514_x_at	110.5	74.3	-0.57	-1.49	AAP-PPA	0.10	11	0.861	0.984	0.3180
239535_at	90.0	60.5	-0.57	-1.49	PAP-AMA	0.10	11	0.861	0.984	0.4870

1569791_at	95.5	64.3	-0.57	-1.49	PPP-PPP	0.10	11	0.861	0.984	0.2581
218551_at	298.0	200.5	-0.57	-1.49	AAP-AMP	0.10	11	0.861	0.984	0.2758
202987_at	61.5	41.4	-0.57	-1.49	PPP-MPM	0.10	11	0.861	0.984	0.3642
215575_at	37.9	25.5	-0.57	-1.49	AAP-APA	0.10	11	0.861	0.984	0.4301
236449_at	74.7	110.9	0.57	1.49	APP-PPP	0.10	11	0.861	0.984	0.0148
223507_at	358.8	532.9	0.57	1.49	PPP-PPP	0.10	11	0.861	0.984	0.0097
1555448_at	98.9	66.6	-0.57	-1.49	PPP-PPP	0.10	11	0.861	0.984	0.2471
217663_at	102.9	69.3	-0.57	-1.48	PMP-MPP	0.10	11	0.861	0.984	0.2512
235556_at	105.5	71.1	-0.57	-1.49	PPP-PPA	0.10	11	0.861	0.984	0.2649
1564010_at	72.6	48.9	-0.57	-1.49	PPA-AAA	0.10	11	0.861	0.984	0.2867
209567_at	1736.4	2577.9	0.57	1.49	PPP-PPP	0.10	11	0.861	0.984	0.0112
1560872_at	47.2	31.8	-0.57	-1.48	APA-AAA	0.10	11	0.861	0.984	0.5464
224923_at	185.7	125.3	-0.57	-1.48	PPP-PPA	0.10	11	0.861	0.984	0.2164
219774_at	564.0	380.5	-0.57	-1.48	PPP-PPP	0.10	11	0.861	0.984	0.3641
1556950_s_at	78.0	52.7	-0.57	-1.48	APA-APA	0.10	11	0.861	0.984	0.3576
1557375_at	89.8	60.6	-0.57	-1.48	PAP-PPM	0.10	11	0.861	0.984	0.4083
241022_at	57.6	38.9	-0.57	-1.48	AAA-AAP	0.10	11	0.861	0.984	0.2803
238767_at	118.1	79.8	-0.57	-1.48	PPA-APA	0.10	11	0.861	0.984	0.3315
223756_at	54.7	37.0	-0.57	-1.48	PAA-AAP	0.10	11	0.861	0.984	0.5233
231584_s_at	92.5	62.5	-0.57	-1.48	APP-PAP	0.10	11	0.861	0.984	0.2158
201689_s_at	53.1	35.9	-0.57	-1.48	AAA-APP	0.10	11	0.861	0.984	0.2957
240282_at	274.9	185.8	-0.57	-1.48	PPP-PPP	0.10	11	0.861	0.984	0.3288
205092_x_at	87.6	59.2	-0.57	-1.48	PPP-PPM	0.10	11	0.861	0.984	0.3974
215766_at	132.0	89.3	-0.56	-1.48	APP-AAP	0.10	11	0.861	0.984	0.2636
239578_at	125.6	85.0	-0.56	-1.48	AAP-AAP	0.10	11	0.861	0.984	0.2649
217124_at	138.0	93.4	-0.56	-1.48	PPP-PPM	0.10	11	0.861	0.984	0.2854
1554740_a_at	267.2	394.6	0.56	1.48	PPP-PPP	0.10	11	0.861	0.984	0.0135
242784_at	84.8	57.4	-0.56	-1.48	APP-PAA	0.10	11	0.861	0.984	0.2674
206298_at	302.4	204.9	-0.56	-1.48	PMP-PPP	0.10	11	0.861	0.984	0.2784
215377_at	64.1	43.4	-0.56	-1.48	MAP-AAP	0.10	11	0.861	0.984	0.4194
227818_at	548.8	810.2	0.56	1.48	PPP-PPP	0.10	11	0.861	0.984	0.0144
211388_s_at	73.0	49.4	-0.56	-1.48	PAA-AAA	0.10	11	0.861	0.984	0.3407
235401_s_at	38.3	25.9	-0.56	-1.48	APA-APA	0.10	11	0.861	0.984	0.4226
237143_at	57.3	38.9	-0.56	-1.47	PAA-AAA	0.10	11	0.861	0.984	0.2325
232094_at	109.5	74.2	-0.56	-1.47	PPP-PPP	0.10	11	0.861	0.984	0.2359
223583_at	85.8	58.2	-0.56	-1.48	PAA-AAA	0.10	11	0.861	0.984	0.3101
231132_at	77.7	52.7	-0.56	-1.48	AAP-AAA	0.10	11	0.861	0.984	0.3680
203965_at	200.6	136.3	-0.56	-1.47	PPA-PMA	0.10	11	0.861	0.984	0.4437
218965_s_at	176.7	120.1	-0.56	-1.47	PAA-AAP	0.10	11	0.861	0.984	0.2271
1558702_at	87.7	59.6	-0.56	-1.47	PAP-AMP	0.10	11	0.861	0.984	0.2433
241913_at	66.5	45.2	-0.56	-1.47	PPA-PAP	0.10	11	0.861	0.984	0.3310
219358_s_at	108.0	73.4	-0.56	-1.47	AAA-APA	0.10	11	0.861	0.984	0.4378

235409_at	175.7	258.5	0.56	1.47	PPP-PPP	0.10	11	0.861	0.984	0.0054
239199_at	97.5	66.3	-0.56	-1.47	APP-AMP	0.10	11	0.861	0.984	0.2533
222720_x_at	110.4	75.1	-0.56	-1.47	PPP-APP	0.10	11	0.861	0.984	0.4298
1561155_at	59.5	40.5	-0.56	-1.47	APA-AAP	0.10	11	0.861	0.984	0.4547
1562719_at	94.4	64.3	-0.56	-1.47	PMP-PPA	0.10	11	0.861	0.984	0.2643
1557996_at	439.7	299.2	-0.56	-1.47	PPP-PPP	0.10	11	0.861	0.984	0.2935
215907_at	142.8	97.3	-0.55	-1.47	AAP-AMP	0.10	11	0.861	0.984	0.2313
242835_s_at	68.9	47.0	-0.55	-1.47	APP-AMA	0.10	11	0.861	0.984	0.3705
232696_at	45.0	30.7	-0.55	-1.47	PAA-AAA	0.10	11	0.861	0.984	0.2659
244700_at	60.8	41.4	-0.55	-1.47	MAP-AAP	0.10	11	0.861	0.984	0.3806
242642_at	35.7	24.3	-0.55	-1.47	AAP-AAP	0.10	11	0.861	0.984	0.4882
214666_x_at	198.4	290.9	0.55	1.47	APP-PAP	0.10	11	0.861	0.984	0.0114
237725_x_at	65.2	44.5	-0.55	-1.47	APP-APP	0.10	11	0.861	0.984	0.3939
1558601_at	52.7	36.0	-0.55	-1.46	AAP-AAA	0.10	11	0.861	0.984	0.3279
215353_at	81.5	55.7	-0.55	-1.46	APA-APA	0.10	11	0.861	0.984	0.5500
1567286_at	80.2	54.8	-0.55	-1.46	AAA-APA	0.10	11	0.861	0.984	0.2900
213481_at	100.5	68.7	-0.55	-1.46	PAP-PAP	0.10	11	0.861	0.984	0.3486
221872_at	108.1	73.9	-0.55	-1.46	PAP-AAP	0.10	11	0.861	0.984	0.4819
1553280_at	129.2	88.5	-0.55	-1.46	PPP-PPA	0.10	11	0.861	0.984	0.3096
230365_at	39.8	27.2	-0.55	-1.46	AAA-APA	0.10	11	0.861	0.984	0.3921
1569647_at	56.5	38.7	-0.55	-1.46	AMA-APA	0.10	11	0.861	0.984	0.4129
1557818_x_at	73.9	50.6	-0.55	-1.46	APA-AAP	0.10	11	0.861	0.984	0.4412
1561624_at	54.1	37.1	-0.55	-1.46	MAP-AAA	0.10	11	0.861	0.984	0.2281
213681_at	198.7	136.1	-0.55	-1.46	PAP-PMP	0.10	11	0.861	0.984	0.2366
1560071_a_at	77.9	53.4	-0.55	-1.46	AAA-MAP	0.10	11	0.861	0.984	0.2470
225380_at	312.4	214.0	-0.55	-1.46	AAP-PPP	0.10	11	0.861	0.984	0.2513
238231_at	47.5	32.6	-0.55	-1.46	APP-AAA	0.10	11	0.861	0.984	0.4248
230791_at	79.7	54.7	-0.54	-1.46	PPP-APA	0.10	11	0.861	0.984	0.2232
1556831_at	119.3	81.8	-0.54	-1.46	PMP-PMP	0.10	11	0.861	0.984	0.3093
220855_at	94.6	64.9	-0.54	-1.46	PPP-APP	0.10	11	0.861	0.984	0.3140
238534_at	77.0	52.8	-0.54	-1.46	AAA-AAP	0.10	11	0.861	0.984	0.3697
242460_at	185.9	127.5	-0.54	-1.46	AAA-AAP	0.10	11	0.861	0.984	0.3783
223796_at	48.4	33.2	-0.54	-1.46	AAA-PAA	0.10	11	0.861	0.984	0.4351
207871_s_at	334.6	487.9	0.54	1.46	PPP-PPP	0.10	11	0.861	0.984	0.0125
239560_at	95.5	65.5	-0.54	-1.46	PAA-AAA	0.10	11	0.861	0.984	0.2305
241268_x_at	245.3	168.3	-0.54	-1.46	PPM-PPP	0.10	11	0.861	0.984	0.4494
242822_at	106.1	72.9	-0.54	-1.46	PAP-MAP	0.10	11	0.861	0.984	0.2216
239679_at	93.0	63.9	-0.54	-1.46	APP-PPP	0.10	11	0.861	0.984	0.2690
235479_at	153.3	105.3	-0.54	-1.46	PPP-PPP	0.10	11	0.861	0.984	0.3068
1557257_at	111.3	76.4	-0.54	-1.46	PPP-PPP	0.10	11	0.861	0.984	0.3183
242212_at	37.3	25.6	-0.54	-1.46	PPA-AAA	0.10	11	0.861	0.984	0.4759
210829_s_at	345.5	237.4	-0.54	-1.46	PPP-PPP	0.10	11	0.861	0.984	0.2585

1565557_at	46.0	31.6	-0.54	-1.46	AAA-AAP	0.10	11	0.861	0.984	0.4010
232978_at	53.7	36.9	-0.54	-1.46	APP-PAP	0.10	11	0.861	0.984	0.4886
227867_at	143.7	98.8	-0.54	-1.45	AAP-AAA	0.10	11	0.861	0.984	0.2219
242868_at	60.0	41.3	-0.54	-1.45	PAA-AAP	0.10	11	0.861	0.984	0.3539
224724_at	98.6	67.8	-0.54	-1.45	MPM-APA	0.10	11	0.861	0.984	0.3634
1563090_at	167.3	115.1	-0.54	-1.45	PPP-PAP	0.10	11	0.861	0.984	0.3752
1560486_at	50.9	35.0	-0.54	-1.45	MPP-APP	0.10	11	0.861	0.984	0.4760
240868_at	74.7	51.4	-0.54	-1.45	APP-APP	0.10	11	0.861	0.984	0.3020
1559077_at	35.2	24.2	-0.54	-1.45	AAA-PAA	0.10	11	0.861	0.984	0.3254
224920_x_at	413.3	600.4	0.54	1.45	PPP-PPP	0.10	11	0.861	0.984	0.0162
210471_s_at	146.1	212.3	0.54	1.45	PPP-PPP	0.10	11	0.861	0.984	0.0105
1562625_at	60.7	41.8	-0.54	-1.45	AMA-PPA	0.10	11	0.861	0.984	0.2741
243593_s_at	129.2	89.0	-0.54	-1.45	PPA-APP	0.10	11	0.861	0.984	0.3117
220887_at	46.5	32.1	-0.54	-1.45	PAA-AAA	0.10	11	0.861	0.984	0.3133
1566597_at	40.0	27.5	-0.54	-1.45	PAP-APA	0.10	11	0.861	0.984	0.4748
1552856_at	70.4	48.4	-0.54	-1.45	AAP-PAA	0.10	11	0.861	0.984	0.4760
213325_at	932.1	1353.7	0.54	1.45	PPP-PPP	0.10	11	0.861	0.984	0.0055
1569491_at	38.7	26.7	-0.54	-1.45	AAM-APA	0.10	11	0.861	0.984	0.2173
1559966_a_at	164.1	113.1	-0.54	-1.45	PPP-PMP	0.10	11	0.861	0.984	0.2640
1553145_at	98.4	67.8	-0.54	-1.45	APP-APP	0.10	11	0.861	0.984	0.2889
230066_at	57.6	39.7	-0.54	-1.45	AAP-AAA	0.10	11	0.861	0.984	0.3065
1567166_at	124.2	85.6	-0.54	-1.45	PPA-AAA	0.10	11	0.861	0.984	0.3386
218509_at	281.9	194.5	-0.54	-1.45	PAA-APP	0.10	11	0.861	0.984	0.2456
232474_at	72.9	50.3	-0.54	-1.45	AAA-PAA	0.10	11	0.861	0.984	0.3656
1566190_at	52.3	36.1	-0.54	-1.45	AAP-AAA	0.10	11	0.861	0.984	0.4627
243576_at	75.6	52.2	-0.54	-1.45	APP-AAA	0.10	11	0.861	0.984	0.2745
1556931_at	115.0	79.4	-0.54	-1.45	APA-AAP	0.10	11	0.861	0.984	0.3186
206557_at	72.3	49.9	-0.54	-1.45	APA-PPA	0.10	11	0.861	0.984	0.4704
232525_at	151.2	104.4	-0.53	-1.45	AAA-APP	0.10	11	0.861	0.984	0.2529
206001_at	328.4	226.8	-0.53	-1.45	PPP-PPP	0.10	11	0.861	0.984	0.3482
241602_at	203.2	140.3	-0.53	-1.45	AAM-PAP	0.10	11	0.861	0.984	0.3717
231121_at	58.4	84.6	0.53	1.45	PPP-PPP	0.10	11	0.861	0.984	0.0198
224346_at	111.2	76.9	-0.53	-1.45	PAM-AAA	0.10	11	0.861	0.984	0.2310
1555487_a_at	384.2	265.4	-0.53	-1.45	PPP-PPP	0.10	11	0.861	0.984	0.2506
220254_at	250.3	362.2	0.53	1.45	PPP-PPP	0.10	11	0.861	0.984	0.0183
235788_at	85.4	59.1	-0.53	-1.45	APP-PAA	0.10	11	0.861	0.984	0.4066
227300_at	35.7	24.7	-0.53	-1.45	PAA-APA	0.10	11	0.861	0.984	0.4902
232437_at	198.0	137.1	-0.53	-1.44	APP-AAA	0.10	11	0.861	0.984	0.2335
202821_s_at	238.8	165.3	-0.53	-1.44	PPP-PPP	0.10	11	0.861	0.984	0.2734
204166_at	485.4	336.1	-0.53	-1.44	PPA-PPA	0.10	11	0.861	0.984	0.2126
244660_at	61.0	42.3	-0.53	-1.44	APA-APP	0.10	11	0.861	0.984	0.3334
227671_at	1766.1	2550.7	0.53	1.44	PPP-PPP	0.10	11	0.861	0.984	0.0105

241381_at	70.3	48.7	-0.53	-1.44	PAA-PAA	0.10	11	0.861	0.984	0.3336
210486_at	101.0	70.0	-0.53	-1.44	AAP-PAA	0.10	11	0.861	0.984	0.3556
208124_s_at	163.9	113.6	-0.53	-1.44	AMP-PAP	0.10	11	0.861	0.984	0.3827
226718_at	91.7	63.6	-0.53	-1.44	AAP-PPP	0.10	11	0.861	0.984	0.2304
204179_at	59.2	41.0	-0.53	-1.44	AAP-AMA	0.10	11	0.861	0.984	0.5591
212956_at	636.7	917.7	0.53	1.44	PPP-PPP	0.10	11	0.861	0.984	0.0097
220596_at	60.7	42.1	-0.53	-1.44	APA-AAA	0.10	11	0.861	0.984	0.4352
1555501_s_at	977.4	1408.2	0.53	1.44	PPP-PPP	0.10	11	0.861	0.984	0.0023
1553178_a_at	172.9	120.1	-0.53	-1.44	AAP-AAM	0.10	11	0.861	0.984	0.3896
1569521_s_at	163.2	113.4	-0.53	-1.44	MAP-PPA	0.10	11	0.861	0.984	0.2343
209596_at	351.3	244.1	-0.53	-1.44	PPP-PPP	0.10	11	0.861	0.984	0.2995
222866_s_at	132.6	92.1	-0.53	-1.44	PPP-PMA	0.10	11	0.861	0.984	0.3469
220453_at	100.0	69.5	-0.53	-1.44	AAP-PMA	0.10	11	0.861	0.984	0.4045
218518_at	804.0	1156.5	0.53	1.44	PPP-PPP	0.10	11	0.861	0.984	0.0047
237076_at	42.3	29.4	-0.52	-1.44	AAA-PAP	0.10	11	0.861	0.984	0.3722
1561560_at	58.3	40.5	-0.52	-1.44	PMA-APA	0.10	11	0.861	0.984	0.2085
229064_s_at	150.1	104.5	-0.52	-1.44	PPP-APP	0.10	11	0.861	0.984	0.3011
204884_s_at	91.0	63.3	-0.52	-1.44	PPP-PPA	0.10	11	0.861	0.984	0.3102
1564746_at	119.8	83.4	-0.52	-1.44	AAA-PAA	0.10	11	0.861	0.984	0.2128
237664_at	118.2	82.3	-0.52	-1.44	PPP-PPP	0.10	11	0.861	0.984	0.2265
236046_at	163.5	113.8	-0.52	-1.44	PPP-PPP	0.10	11	0.861	0.984	0.2273
1562948_at	109.0	75.9	-0.52	-1.44	PPP-PPP	0.10	11	0.861	0.984	0.2370
210801_at	69.4	48.3	-0.52	-1.44	PPA-AAP	0.10	11	0.861	0.984	0.3791
204479_at	353.3	246.0	-0.52	-1.44	AMA-PPP	0.10	11	0.861	0.984	0.4217
1570596_at	41.0	28.6	-0.52	-1.44	AAP-AAP	0.10	11	0.861	0.984	0.2824
216867_s_at	52.8	36.9	-0.52	-1.43	AAA-AAP	0.10	11	0.861	0.984	0.2138
232918_at	213.2	148.7	-0.52	-1.43	PPP-PPP	0.10	11	0.861	0.984	0.2906
242346_x_at	111.6	77.8	-0.52	-1.43	APA-PPP	0.10	11	0.861	0.984	0.3629
1568755_a_at	30.2	21.1	-0.52	-1.43	AAA-APA	0.10	11	0.861	0.984	0.5134
215304_at	148.7	103.8	-0.52	-1.43	PPP-APP	0.10	11	0.861	0.984	0.2363
205016_at	51.2	35.7	-0.52	-1.43	AAA-APA	0.10	11	0.861	0.984	0.4548
232991_at	122.9	85.8	-0.52	-1.43	APP-PPP	0.10	11	0.861	0.984	0.4777
234797_at	128.6	89.7	-0.52	-1.43	PAA-AAA	0.10	11	0.861	0.984	0.5536
230060_at	117.4	82.0	-0.52	-1.43	PPP-PPP	0.10	11	0.861	0.984	0.2140
1558938_at	58.2	40.7	-0.52	-1.43	AAP-AAA	0.10	11	0.861	0.984	0.5493
1553167_a_at	104.9	150.3	0.52	1.43	PPP-PPP	0.10	11	0.861	0.984	0.0148
1554534_at	161.4	112.8	-0.52	-1.43	PPP-PPP	0.10	11	0.861	0.984	0.2642
217484_at	53.6	37.5	-0.52	-1.43	PPA-AAA	0.10	11	0.861	0.984	0.3089
232362_at	203.8	142.4	-0.52	-1.43	PPP-PPP	0.10	11	0.861	0.984	0.3716
224106_at	94.0	65.7	-0.52	-1.43	AAP-APA	0.10	11	0.861	0.984	0.2144
239657_x_at	100.3	70.2	-0.52	-1.43	PPA-AAA	0.10	11	0.861	0.984	0.2619
205710_at	60.3	42.2	-0.52	-1.43	APP-AAP	0.10	11	0.861	0.984	0.2952

211795_s_at	57.0	39.9	-0.52	-1.43	APP-PAA	0.10	11	0.861	0.984	0.4341
214174_s_at	107.8	75.4	-0.52	-1.43	AAP-AAA	0.10	11	0.861	0.984	0.4878
235062_at	55.6	38.9	-0.52	-1.43	PPP-PMP	0.10	11	0.861	0.984	0.3522
233726_at	121.4	84.9	-0.52	-1.43	PPA-PPA	0.10	11	0.861	0.984	0.4409
242993_at	64.2	44.9	-0.52	-1.43	PPA-APP	0.10	11	0.861	0.984	0.4584
221991_at	156.3	109.5	-0.51	-1.43	AAA-PPP	0.10	11	0.861	0.984	0.2210
235786_at	162.9	114.1	-0.51	-1.43	PPP-PPP	0.10	11	0.861	0.984	0.2790
227622_at	876.5	613.8	-0.51	-1.43	PPP-PPP	0.10	11	0.861	0.984	0.2961
236304_at	111.9	78.4	-0.51	-1.43	PPP-PPP	0.10	11	0.861	0.984	0.3121
233333_x_at	180.4	126.3	-0.51	-1.43	PAP-APA	0.10	11	0.861	0.984	0.4167
213295_at	289.6	413.4	0.51	1.43	PPP-PPP	0.10	11	0.861	0.984	0.0078
238412_at	246.9	173.0	-0.51	-1.43	PPP-PPP	0.10	11	0.861	0.984	0.3319
1559201_a_at	79.0	55.4	-0.51	-1.43	APP-APA	0.10	11	0.861	0.984	0.3707
238409_x_at	45.7	32.0	-0.51	-1.43	PAA-AAP	0.10	11	0.861	0.984	0.3852
219078_at	342.3	488.4	0.51	1.43	PPP-PPP	0.10	11	0.861	0.984	0.0028
212915_at	292.0	204.7	-0.51	-1.43	PPP-PPP	0.10	11	0.861	0.984	0.2194
1558942_at	837.5	587.3	-0.51	-1.43	PPP-PPP	0.10	11	0.861	0.984	0.2887
206590_x_at	192.1	134.6	-0.51	-1.43	PPP-PPA	0.10	11	0.861	0.984	0.3425
227703_s_at	143.4	204.5	0.51	1.43	PPP-PPP	0.10	11	0.861	0.984	0.0040
37022_at	118.0	82.8	-0.51	-1.43	AMM-APP	0.10	11	0.861	0.984	0.2380
243540_at	103.1	72.3	-0.51	-1.43	PAA-PAP	0.10	11	0.861	0.984	0.3250
1555788_a_at	312.1	218.9	-0.51	-1.43	PPP-PPP	0.10	11	0.861	0.984	0.3562
238307_at	69.5	48.8	-0.51	-1.43	MAP-PAA	0.10	11	0.861	0.984	0.3826
217607_x_at	80.8	56.7	-0.51	-1.43	PPP-PPP	0.10	11	0.861	0.984	0.4428
236524_at	99.9	70.1	-0.51	-1.43	PPP-AAP	0.10	11	0.861	0.984	0.4439
224099_at	42.0	29.4	-0.51	-1.43	MPA-PMA	0.10	11	0.861	0.984	0.5041
244340_x_at	48.2	33.9	-0.51	-1.43	APA-PPA	0.10	11	0.861	0.984	0.2124
215123_at	517.6	363.4	-0.51	-1.42	PPP-PPP	0.10	11	0.861	0.984	0.2899
224823_at	1963.0	2795.2	0.51	1.42	PPP-PPP	0.10	11	0.861	0.984	0.0175
1559691_at	71.7	50.4	-0.51	-1.42	PPP-APP	0.10	11	0.861	0.984	0.2907
1557036_at	57.7	40.5	-0.51	-1.42	APP-APP	0.10	11	0.861	0.984	0.3299
225656_at	274.7	193.0	-0.51	-1.42	PPP-PPP	0.10	11	0.861	0.984	0.4197
215470_at	356.0	250.4	-0.51	-1.42	PPP-PPP	0.10	11	0.861	0.984	0.2586
225375_at	521.5	741.5	0.51	1.42	PPP-PPP	0.10	11	0.861	0.984	0.0182
1553947_at	235.6	335.0	0.51	1.42	PPP-PPP	0.10	11	0.861	0.984	0.0055
215302_at	62.0	43.6	-0.51	-1.42	PAA-AAP	0.10	11	0.861	0.984	0.3054
1557685_at	153.1	107.7	-0.51	-1.42	PPA-PPP	0.10	11	0.861	0.984	0.3251
216847_at	74.6	52.5	-0.51	-1.42	AMA-APA	0.10	11	0.861	0.984	0.3892
213832_at	44.6	31.4	-0.51	-1.42	PAA-AAA	0.10	11	0.861	0.984	0.4458
1569368_at	85.2	60.0	-0.51	-1.42	PPP-AAP	0.10	11	0.861	0.984	0.3112
207041_at	97.7	68.8	-0.51	-1.42	PMA-PAP	0.10	11	0.861	0.984	0.3897
232658_at	131.8	92.9	-0.51	-1.42	MAA-PMP	0.10	11	0.861	0.984	0.2446

203982_s_at	58.7	41.3	-0.51	-1.42	AAA-APA	0.10	11	0.861	0.984	0.2688
237498_at	103.9	73.2	-0.51	-1.42	PPP-MPP	0.10	11	0.861	0.984	0.2944
244026_at	180.5	127.1	-0.51	-1.42	PPP-PPP	0.10	11	0.861	0.984	0.4328
233399_x_at	215.5	151.9	-0.51	-1.42	APP-PPP	0.10	11	0.861	0.984	0.4750
237713_at	77.4	54.6	-0.51	-1.42	APA-AAA	0.10	11	0.861	0.984	0.5366
225934_at	115.7	164.2	0.51	1.42	AAA-APP	0.10	11	0.861	0.984	0.0178
203996_s_at	79.3	55.9	-0.50	-1.42	AAP-AAP	0.10	11	0.861	0.984	0.4310
230789_at	241.0	341.9	0.50	1.42	PPP-PPP	0.10	11	0.861	0.984	0.0067
242092_at	105.4	74.4	-0.50	-1.42	AAP-AMA	0.10	11	0.861	0.984	0.2671
222827_s_at	100.8	71.1	-0.50	-1.42	AMP-MPP	0.10	11	0.861	0.984	0.2787
232989_s_at	85.1	60.1	-0.50	-1.42	APA-AAA	0.10	11	0.861	0.984	0.3855
219694_at	186.6	264.6	0.50	1.42	PPP-PPP	0.10	11	0.861	0.984	0.0157
242739_at	143.4	101.3	-0.50	-1.42	AMP-APP	0.10	11	0.861	0.984	0.3600
1556015_a_at	208.5	147.5	-0.50	-1.41	PAM-APA	0.10	11	0.861	0.984	0.2792
206417_at	72.4	51.2	-0.50	-1.41	PPA-AAA	0.10	11	0.861	0.984	0.3090
238068_at	400.6	283.3	-0.50	-1.41	PPP-PPP	0.10	11	0.861	0.984	0.3397
238593_at	104.9	74.2	-0.50	-1.42	PPP-PMP	0.10	11	0.861	0.984	0.3437
230888_at	60.7	42.9	-0.50	-1.42	PPP-APA	0.10	11	0.861	0.984	0.3447
238406_x_at	202.4	143.1	-0.50	-1.41	AAP-APA	0.10	11	0.861	0.984	0.3640
212806_at	106.3	75.2	-0.50	-1.41	PMP-PPP	0.10	11	0.861	0.984	0.3949
225919_s_at	166.5	235.6	0.50	1.42	PPP-PPP	0.10	11	0.861	0.984	0.0116
216833_x_at	104.4	73.9	-0.50	-1.41	APP-AAA	0.10	11	0.861	0.984	0.2168
228076_s_at	221.5	156.8	-0.50	-1.41	PPP-PPP	0.10	11	0.861	0.984	0.2910
203936_s_at	148.3	104.9	-0.50	-1.41	PPP-PPP	0.10	11	0.861	0.984	0.3512
236210_at	115.0	81.4	-0.50	-1.41	AAA-AAP	0.10	11	0.861	0.984	0.4548
222577_at	87.4	61.8	-0.50	-1.41	AAP-AAP	0.10	11	0.861	0.984	0.4553
235576_at	81.7	57.8	-0.50	-1.41	AAA-APA	0.10	11	0.861	0.984	0.4597
237388_at	134.7	95.4	-0.50	-1.41	PPP-APP	0.10	11	0.861	0.984	0.2760
215393_s_at	85.3	60.4	-0.50	-1.41	AAA-AAP	0.10	11	0.861	0.984	0.3095
235670_at	69.1	49.0	-0.50	-1.41	PPA-AAP	0.10	11	0.861	0.984	0.3096
242332_at	115.9	82.0	-0.50	-1.41	PAA-PAM	0.10	11	0.861	0.984	0.3343
236150_at	144.1	102.0	-0.50	-1.41	MPP-PPA	0.10	11	0.861	0.984	0.5066
1557279_at	99.1	70.2	-0.50	-1.41	AAA-PAA	0.10	11	0.861	0.984	0.3365
228278_at	211.4	149.8	-0.50	-1.41	PPP-PPP	0.10	11	0.861	0.984	0.3568
229808_at	66.4	47.1	-0.50	-1.41	AAP-AAP	0.10	11	0.861	0.984	0.3628
220175_s_at	2661.9	1886.0	-0.50	-1.41	PPP-PPP	0.10	11	0.861	0.984	0.3802
214915_at	52.1	36.9	-0.50	-1.41	PAP-AAP	0.10	11	0.861	0.984	0.4562
208205_at	114.4	81.1	-0.50	-1.41	MPP-PAA	0.10	11	0.861	0.984	0.5073
1569312_at	440.4	312.2	-0.50	-1.41	PPP-PPP	0.10	11	0.861	0.984	0.3170
1557080_s_at	62.7	44.5	-0.50	-1.41	PAP-PPA	0.10	11	0.861	0.984	0.4159
206969_at	865.5	613.7	-0.50	-1.41	PPP-PPP	0.10	11	0.861	0.984	0.5101
206536_s_at	127.5	90.4	-0.50	-1.41	APP-PPP	0.10	11	0.861	0.984	0.2440

204923_at	104.3	74.0	-0.50	-1.41	PPP-AAA	0.10	11	0.861	0.984	0.2559
233109_at	96.3	68.3	-0.50	-1.41	PPP-APP	0.10	11	0.861	0.984	0.2790
226379_s_at	162.7	115.5	-0.50	-1.41	PPP-PPA	0.10	11	0.861	0.984	0.2830
241950_at	116.3	82.5	-0.50	-1.41	AAP-PAP	0.10	11	0.861	0.984	0.3411
222886_at	66.8	47.4	-0.50	-1.41	APA-AAP	0.10	11	0.861	0.984	0.5506
209040_s_at	558.1	786.7	0.50	1.41	PPP-PPP	0.10	11	0.861	0.984	0.0124
1555993_at	74.2	52.7	-0.49	-1.41	PPM-PPA	0.10	11	0.861	0.984	0.3611
225631_at	89.4	63.5	-0.49	-1.41	APA-AAA	0.10	11	0.861	0.984	0.4501
208670_s_at	2687.0	3784.9	0.49	1.41	PPP-PPP	0.10	11	0.861	0.984	0.0174
1554671_a_at	232.5	165.2	-0.49	-1.41	MMP-AAA	0.10	11	0.861	0.984	0.2186
208300_at	90.6	64.4	-0.49	-1.41	AAM-APA	0.10	11	0.861	0.984	0.3023
244674_at	139.5	99.1	-0.49	-1.41	PPP-MPP	0.10	11	0.861	0.984	0.3319
239788_at	92.8	66.0	-0.49	-1.41	PPP-MPP	0.10	11	0.861	0.984	0.2735
238248_at	54.3	38.6	-0.49	-1.41	MAA-APP	0.10	11	0.861	0.984	0.3901
221270_s_at	339.4	241.3	-0.49	-1.41	AAA-AMP	0.10	11	0.861	0.984	0.4128
1568620_at	58.7	41.7	-0.49	-1.41	AAA-PAA	0.10	11	0.861	0.984	0.4520
220603_s_at	36.2	50.9	0.49	1.41	AAP-APA	0.10	11	0.861	0.984	0.0064
234394_at	171.9	122.4	-0.49	-1.41	PPP-PPP	0.10	11	0.861	0.984	0.2036
1560723_at	51.1	36.3	-0.49	-1.41	PAA-PAA	0.10	11	0.861	0.984	0.2926
239242_at	99.1	70.5	-0.49	-1.41	PAP-MAP	0.10	11	0.861	0.984	0.4667
1555145_at	119.2	84.8	-0.49	-1.41	PAA-APP	0.10	11	0.861	0.984	0.3166
1570382_at	60.3	42.9	-0.49	-1.40	AAP-AAM	0.10	11	0.861	0.984	0.3169
232193_at	76.4	54.4	-0.49	-1.40	APP-PAP	0.10	11	0.861	0.984	0.5175
225798_at	602.9	846.6	0.49	1.40	PPP-PPP	0.10	11	0.861	0.984	0.0016
1556049_at	240.4	171.3	-0.49	-1.40	PPP-APP	0.10	11	0.861	0.984	0.2281
227943_at	101.5	72.4	-0.49	-1.40	MAP-APP	0.10	11	0.861	0.984	0.2626
205011_at	94.9	67.7	-0.49	-1.40	PAA-AAA	0.10	11	0.861	0.984	0.3276
222780_s_at	107.4	76.6	-0.49	-1.40	PPP-APP	0.10	11	0.861	0.984	0.3573
217475_s_at	127.5	91.0	-0.49	-1.40	PPP-PPP	0.10	11	0.861	0.984	0.2667
234081_at	30.3	21.6	-0.49	-1.40	PAA-AAA	0.10	11	0.861	0.984	0.2509
210954_s_at	65.7	46.9	-0.49	-1.40	AAM-APP	0.10	11	0.861	0.984	0.4242
228347_at	461.1	645.8	0.49	1.40	PPP-PPP	0.10	11	0.861	0.984	0.0154
221802_s_at	367.9	262.9	-0.49	-1.40	PPP-PPP	0.10	11	0.861	0.984	0.2117
1557527_at	515.2	368.0	-0.49	-1.40	PPP-PPP	0.10	11	0.861	0.984	0.3252
230944_at	64.1	45.8	-0.49	-1.40	PAA-APA	0.10	11	0.861	0.984	0.5269
202996_at	493.9	353.0	-0.48	-1.40	PPP-PPP	0.10	11	0.861	0.984	0.2451
226271_at	317.1	226.7	-0.48	-1.40	APP-PPP	0.10	11	0.861	0.984	0.2700
204462_s_at	1285.7	919.4	-0.48	-1.40	PPP-PPP	0.10	11	0.861	0.984	0.3929
235497_at	139.2	194.7	0.48	1.40	PPP-PPP	0.10	11	0.861	0.984	0.0071
1557616_at	132.1	94.6	-0.48	-1.40	PAP-APP	0.10	11	0.861	0.984	0.2607
238846_at	162.0	115.9	-0.48	-1.40	PPP-PPP	0.10	11	0.861	0.984	0.2610
234322_at	102.0	73.0	-0.48	-1.40	APM-PPP	0.10	11	0.861	0.984	0.2625

41553_at	108.7	77.7	-0.48	-1.40	PPA-APA	0.10	11	0.861	0.984	0.2834
1566885_at	200.6	143.6	-0.48	-1.40	PPP-PPP	0.10	11	0.861	0.984	0.3173
214747_at	70.8	50.7	-0.48	-1.40	AAA-AAP	0.10	11	0.861	0.984	0.3356
235730_at	101.2	72.4	-0.48	-1.40	PPP-APP	0.10	11	0.861	0.984	0.4405
224202_at	76.2	54.5	-0.48	-1.40	APP-APP	0.10	11	0.861	0.984	0.2297
225828_at	230.3	164.9	-0.48	-1.40	APP-AAA	0.10	11	0.861	0.984	0.2549
1560485_at	130.9	93.7	-0.48	-1.40	PPP-PPP	0.10	11	0.861	0.984	0.2644
235452_at	116.2	83.2	-0.48	-1.40	PPA-PPP	0.10	11	0.861	0.984	0.3836
233294_at	94.7	67.8	-0.48	-1.40	APA-AAP	0.10	11	0.861	0.984	0.4053
1553585_a_at	60.4	43.2	-0.48	-1.40	PAA-AAA	0.10	11	0.861	0.984	0.4708
206631_at	361.7	259.1	-0.48	-1.40	PPP-PPP	0.10	11	0.861	0.984	0.2067
226717_at	134.7	96.5	-0.48	-1.40	AAM-AAP	0.10	11	0.861	0.984	0.3687
209003_at	836.3	1167.4	0.48	1.40	PPP-PPP	0.10	11	0.861	0.984	0.0042
205222_at	87.5	62.7	-0.48	-1.40	PPP-PAP	0.10	11	0.861	0.984	0.2374
219956_at	316.3	226.7	-0.48	-1.40	PPP-PPP	0.10	11	0.861	0.984	0.2773
206084_at	371.2	266.0	-0.48	-1.40	PPP-PPP	0.10	11	0.861	0.984	0.3042
1552930_at	217.5	155.9	-0.48	-1.40	PPP-PPP	0.10	11	0.861	0.984	0.3112
239401_at	78.4	56.2	-0.48	-1.40	AAA-AAP	0.10	11	0.861	0.984	0.4830
1567361_at	129.6	93.0	-0.48	-1.40	PPP-AAP	0.10	11	0.861	0.984	0.5252
1560609_at	41.4	29.7	-0.48	-1.39	APA-AAP	0.10	11	0.861	0.984	0.2811
244215_at	78.1	56.0	-0.48	-1.39	PAA-AAA	0.10	11	0.861	0.984	0.4851
230736_at	112.6	80.9	-0.48	-1.39	AAA-PPP	0.10	11	0.861	0.984	0.2241
1563468_at	113.0	81.2	-0.48	-1.39	MPP-APP	0.10	11	0.861	0.984	0.3703
1552670_a_at	92.3	66.3	-0.48	-1.39	AAP-APP	0.10	11	0.861	0.984	0.4516
243462_s_at	206.8	148.5	-0.48	-1.39	PPP-PPP	0.10	11	0.861	0.984	0.2213
1555100_at	67.9	48.8	-0.48	-1.39	PPP-MAA	0.10	11	0.861	0.984	0.4314
239179_at	104.9	75.4	-0.48	-1.39	AAP-AAM	0.10	11	0.861	0.984	0.2578
1569539_at	57.3	41.2	-0.48	-1.39	APP-AAP	0.10	11	0.861	0.984	0.2623
232172_at	129.6	93.2	-0.48	-1.39	PAP-AMP	0.10	11	0.861	0.984	0.2810
1565843_s_at	150.0	107.8	-0.48	-1.39	AAP-AMA	0.10	11	0.861	0.984	0.3295
210004_at	214.9	154.5	-0.48	-1.39	PPP-PPP	0.10	11	0.861	0.984	0.3460
1553533_at	63.7	45.8	-0.48	-1.39	APA-MAA	0.10	11	0.861	0.984	0.4724
1561050_a_at	43.8	31.5	-0.48	-1.39	PAA-AAA	0.10	11	0.861	0.984	0.5057
224952_at	1190.2	856.1	-0.48	-1.39	PPP-PPP	0.10	11	0.861	0.984	0.2246
232455_x_at	224.8	161.7	-0.48	-1.39	PPP-PPP	0.10	11	0.861	0.984	0.2662
1566851_at	89.2	64.2	-0.48	-1.39	PPA-PPP	0.10	11	0.861	0.984	0.3905
229346_at	47.7	34.3	-0.48	-1.39	PAA-AAA	0.10	11	0.861	0.984	0.5247
219215_s_at	394.3	283.9	-0.47	-1.39	PPP-PPP	0.10	11	0.861	0.984	0.2452
239049_at	122.3	88.0	-0.47	-1.39	PPP-PPP	0.10	11	0.861	0.984	0.3174
225314_at	1499.4	2083.0	0.47	1.39	PPP-PPP	0.10	11	0.861	0.984	0.0053
1556088_at	104.0	74.9	-0.47	-1.39	PPP-APP	0.10	11	0.861	0.984	0.2393
236911_at	39.0	28.1	-0.47	-1.39	AAP-AAP	0.10	11	0.861	0.984	0.2889

1557584_at	115.5	83.2	-0.47	-1.39	PPA-PPP	0.10	11	0.861	0.984	0.2822
221030_s_at	120.4	86.8	-0.47	-1.39	PPP-PPP	0.10	11	0.861	0.984	0.3359
232668_at	53.0	38.2	-0.47	-1.39	AAP-AAP	0.10	11	0.861	0.984	0.3493
1558758_s_at	85.2	61.5	-0.47	-1.39	AAP-AAP	0.10	11	0.861	0.984	0.4667
243682_at	87.8	63.3	-0.47	-1.39	AAA-AAP	0.10	11	0.861	0.984	0.5281
230879_at	157.0	113.3	-0.47	-1.39	PPP-PPP	0.10	11	0.861	0.984	0.2040
232093_at	96.3	69.5	-0.47	-1.39	APP-AAM	0.10	11	0.861	0.984	0.2304
242508_at	87.4	63.1	-0.47	-1.39	PPA-AAP	0.10	11	0.861	0.984	0.3430
206721_at	27.0	19.5	-0.47	-1.39	APM-AAA	0.10	11	0.861	0.984	0.5539
235318_at	184.9	256.3	0.47	1.39	PPP-PPP	0.10	11	0.861	0.984	0.0015
1562546_at	44.8	32.4	-0.47	-1.39	PMP-AAP	0.10	11	0.861	0.984	0.3212
227501_at	119.6	86.3	-0.47	-1.39	PAP-APP	0.10	11	0.861	0.984	0.3659
1552536_at	84.0	60.7	-0.47	-1.39	PAA-PAP	0.10	11	0.861	0.984	0.3938
215284_at	33.8	24.4	-0.47	-1.39	AAA-APA	0.10	11	0.861	0.984	0.4171
244646_at	91.9	66.4	-0.47	-1.38	AAA-APA	0.10	11	0.861	0.984	0.2872
213029_at	601.4	434.4	-0.47	-1.38	PPP-PPP	0.10	11	0.861	0.984	0.3152
220283_at	69.3	50.1	-0.47	-1.39	AAP-AAA	0.10	11	0.861	0.984	0.4574
215001_s_at	1101.5	1524.9	0.47	1.38	PPP-PPP	0.10	11	0.861	0.984	0.0119
228423_at	429.9	595.0	0.47	1.38	PPP-PPP	0.10	11	0.861	0.984	0.0070
1566146_x_at	127.1	91.9	-0.47	-1.38	PPA-MPP	0.10	11	0.861	0.984	0.2479
235412_at	65.8	47.6	-0.47	-1.38	AAA-APA	0.10	11	0.861	0.984	0.3506
224835_at	118.4	85.7	-0.47	-1.38	PAA-PPP	0.10	11	0.861	0.984	0.4011
213231_at	113.1	81.9	-0.47	-1.38	AAP-AAP	0.10	11	0.861	0.984	0.3546
240206_at	77.9	56.4	-0.47	-1.38	PAP-PMP	0.10	11	0.861	0.984	0.4983
232264_at	171.8	124.4	-0.47	-1.38	PPP-PPP	0.10	11	0.861	0.984	0.2186
232458_at	266.1	192.7	-0.47	-1.38	PPP-PPP	0.10	11	0.861	0.984	0.3278
233155_at	69.2	50.2	-0.47	-1.38	PAA-PAP	0.10	11	0.861	0.984	0.4244
221238_at	49.1	35.5	-0.47	-1.38	MPA-MAA	0.10	11	0.861	0.984	0.4357
1570511_at	82.1	59.5	-0.47	-1.38	APP-APP	0.10	11	0.861	0.984	0.4457
237271_at	33.8	24.5	-0.47	-1.38	AAP-AAA	0.10	11	0.861	0.984	0.5097
222826_at	1553.5	2144.4	0.47	1.38	PPP-PPP	0.10	11	0.861	0.984	0.0016
240534_at	95.8	69.5	-0.46	-1.38	AAA-PAA	0.10	11	0.861	0.984	0.4474
1556940_at	79.4	57.6	-0.46	-1.38	PAP-PPM	0.10	11	0.861	0.984	0.5120
236568_at	101.0	73.2	-0.46	-1.38	AAA-PAP	0.10	11	0.861	0.984	0.2342
1554101_a_at	106.0	76.9	-0.46	-1.38	APP-APP	0.10	11	0.861	0.984	0.2415
236953_s_at	83.2	60.4	-0.46	-1.38	MPP-PPP	0.10	11	0.861	0.984	0.2645
205467_at	71.4	51.8	-0.46	-1.38	AAA-AAP	0.10	11	0.861	0.984	0.2951
237864_at	74.1	53.8	-0.46	-1.38	AAA-MAP	0.10	11	0.861	0.984	0.3158
1561571_at	171.8	124.7	-0.46	-1.38	PPA-PPP	0.10	11	0.861	0.984	0.3254
224218_s_at	122.5	88.9	-0.46	-1.38	APP-MAP	0.10	11	0.861	0.984	0.2068
207818_s_at	58.7	42.6	-0.46	-1.38	AAP-AAA	0.10	11	0.861	0.984	0.2839
241027_at	68.5	49.7	-0.46	-1.38	PAP-PPP	0.10	11	0.861	0.984	0.2917

223390_at	221.2	160.6	-0.46	-1.38	PPP-PAM	0.10	11	0.861	0.984	0.2943
216197_at	94.9	68.9	-0.46	-1.38	APP-APP	0.10	11	0.861	0.984	0.3289
230742_at	158.1	114.8	-0.46	-1.38	PPP-PPP	0.10	11	0.861	0.984	0.4118
243786_at	175.5	127.5	-0.46	-1.38	PPP-PPP	0.10	11	0.861	0.984	0.2281
1560049_at	123.0	89.4	-0.46	-1.38	PPA-PPP	0.10	11	0.861	0.984	0.3896
219742_at	105.9	77.0	-0.46	-1.38	MPP-APP	0.10	11	0.861	0.984	0.4260
215088_s_at	1933.1	2660.5	0.46	1.38	PPP-PPP	0.10	11	0.861	0.984	0.0016
242628_at	59.7	43.4	-0.46	-1.38	APP-PPA	0.10	11	0.861	0.984	0.2475
226679_at	554.0	402.9	-0.46	-1.38	PPP-PPP	0.10	11	0.861	0.984	0.3204
230173_at	61.2	44.5	-0.46	-1.38	PAP-AAA	0.10	11	0.861	0.984	0.4407
229638_at	62.0	45.1	-0.46	-1.38	AAA-PAA	0.10	11	0.861	0.984	0.4683
218154_at	366.5	266.4	-0.46	-1.38	PPP-PPP	0.10	11	0.861	0.984	0.4787
1554648_a_at	48.3	35.1	-0.46	-1.38	AAA-AAP	0.10	11	0.861	0.984	0.4812
213781_at	56.5	41.1	-0.46	-1.38	AAA-APA	0.10	11	0.861	0.984	0.4998
212978_at	175.1	240.8	0.46	1.38	PPP-PPP	0.10	11	0.861	0.984	0.0145
1559916_a_at	63.5	46.2	-0.46	-1.37	APA-PAA	0.10	11	0.861	0.984	0.2375
222245_s_at	147.0	107.0	-0.46	-1.37	AAP-APA	0.10	11	0.861	0.984	0.2995
226872_at	167.8	122.1	-0.46	-1.38	MPP-APP	0.10	11	0.861	0.984	0.4358
230832_at	138.5	100.8	-0.46	-1.37	PPP-MPP	0.10	11	0.861	0.984	0.2223
239223_s_at	127.0	92.4	-0.46	-1.37	AMA-PPP	0.10	11	0.861	0.984	0.2481
234503_at	40.1	29.2	-0.46	-1.37	AAA-PMP	0.10	11	0.861	0.984	0.2889
219241_x_at	265.1	192.9	-0.46	-1.37	APP-PAP	0.10	11	0.861	0.984	0.2991
211222_s_at	109.8	80.0	-0.46	-1.37	PAA-AAA	0.10	11	0.861	0.984	0.2070
227213_at	249.2	181.6	-0.46	-1.37	PPP-PPP	0.10	11	0.861	0.984	0.3407
243179_at	142.8	104.1	-0.46	-1.37	PPP-PPP	0.10	11	0.861	0.984	0.3662
210155_at	77.3	56.3	-0.46	-1.37	AAA-APA	0.10	11	0.861	0.984	0.3681
221415_s_at	85.7	62.4	-0.46	-1.37	APA-AAP	0.10	11	0.861	0.984	0.5008
211610_at	121.3	88.4	-0.46	-1.37	PPP-PPP	0.10	11	0.861	0.984	0.2552
1553805_at	73.1	53.3	-0.46	-1.37	PPA-PAP	0.10	11	0.861	0.984	0.3870
224276_at	60.9	44.4	-0.46	-1.37	AMP-PPA	0.10	11	0.861	0.984	0.4506
211653_x_at	233.0	169.9	-0.46	-1.37	APA-MPA	0.10	11	0.861	0.984	0.5164
204330_s_at	204.6	280.6	0.46	1.37	PPP-PPP	0.10	11	0.861	0.984	0.0068
201981_at	412.1	300.6	-0.46	-1.37	PPP-PPP	0.10	11	0.861	0.984	0.2776
230090_at	277.3	202.3	-0.46	-1.37	PPP-PPP	0.10	11	0.861	0.984	0.2787
1563745_a_at	71.5	52.1	-0.46	-1.37	APA-AAA	0.10	11	0.861	0.984	0.3230
236971_at	86.8	63.3	-0.46	-1.37	AAA-AMP	0.10	11	0.861	0.984	0.3850
240789_at	48.4	35.3	-0.46	-1.37	AAP-APP	0.10	11	0.861	0.984	0.4868
228279_s_at	96.7	70.5	-0.46	-1.37	AAA-PAA	0.10	11	0.861	0.984	0.4880
1559170_at	135.0	98.5	-0.45	-1.37	PPP-PPP	0.10	11	0.861	0.984	0.2444
1559069_at	68.2	49.8	-0.45	-1.37	APP-AAA	0.10	11	0.861	0.984	0.2858
240847_at	44.8	32.7	-0.45	-1.37	PAA-PAA	0.10	11	0.861	0.984	0.5414
202886_s_at	556.1	762.1	0.45	1.37	PPP-PPP	0.10	11	0.861	0.984	0.0097

222678_s_at	380.5	521.1	0.45	1.37	PPP-PPP	0.10	11	0.861	0.984	0.0094
1553637_s_at	62.9	45.9	-0.45	-1.37	PPA-PAA	0.10	11	0.861	0.984	0.2689
235733_at	681.9	498.2	-0.45	-1.37	PPP-PPP	0.10	11	0.861	0.984	0.2841
221053_s_at	71.8	52.4	-0.45	-1.37	PAA-AAA	0.10	11	0.861	0.984	0.2640
216206_x_at	103.5	75.6	-0.45	-1.37	APP-APM	0.10	11	0.861	0.984	0.3265
236913_at	152.5	111.4	-0.45	-1.37	AAA-APP	0.10	11	0.861	0.984	0.3335
201759_at	183.7	134.2	-0.45	-1.37	PMA-APP	0.10	11	0.861	0.984	0.3699
1559409_a_at	68.5	50.1	-0.45	-1.37	PAA-AAM	0.10	11	0.861	0.984	0.4297
205536_at	163.2	119.4	-0.45	-1.37	PPP-PPP	0.10	11	0.861	0.984	0.1998
209151_x_at	202.0	147.7	-0.45	-1.37	PAA-AAP	0.10	11	0.861	0.984	0.2107
1554030_at	124.3	90.9	-0.45	-1.37	APP-PPM	0.10	11	0.861	0.984	0.2384
232135_at	125.1	91.6	-0.45	-1.37	APA-AAA	0.10	11	0.861	0.984	0.2702
233010_at	81.8	59.8	-0.45	-1.37	PPP-AAP	0.10	11	0.861	0.984	0.4111
1555632_at	81.1	59.3	-0.45	-1.37	PPA-AMA	0.10	11	0.861	0.984	0.4453
241523_at	46.3	33.9	-0.45	-1.37	AAA-PAA	0.10	11	0.861	0.984	0.2854
206990_at	63.5	46.5	-0.45	-1.37	AAA-APA	0.10	11	0.861	0.984	0.3646
1556336_at	79.1	57.9	-0.45	-1.37	PPA-PPA	0.10	11	0.861	0.984	0.3781
244523_at	175.8	128.7	-0.45	-1.37	PPP-PPP	0.10	11	0.861	0.984	0.3979
242371_x_at	231.4	169.4	-0.45	-1.37	APP-PPP	0.10	11	0.861	0.984	0.4039
1566043_at	53.0	38.8	-0.45	-1.37	PAA-AAA	0.10	11	0.861	0.984	0.2209
201822_at	132.0	96.7	-0.45	-1.37	PAP-APA	0.10	11	0.861	0.984	0.2980
231116_at	125.3	91.8	-0.45	-1.37	PPA-PPA	0.10	11	0.861	0.984	0.3390
222208_s_at	111.2	81.5	-0.45	-1.37	PPP-PAP	0.10	11	0.861	0.984	0.3695
241905_at	105.7	77.5	-0.45	-1.36	APP-PPP	0.10	11	0.861	0.984	0.1913
222647_at	373.4	273.7	-0.45	-1.36	PPP-PPP	0.10	11	0.861	0.984	0.2345
237917_at	40.5	29.7	-0.45	-1.36	MPP-APA	0.10	11	0.861	0.984	0.3474
222862_s_at	1331.7	976.2	-0.45	-1.36	PPP-PPP	0.10	11	0.861	0.984	0.3826
215430_at	70.3	51.6	-0.45	-1.36	AAP-AAA	0.10	11	0.861	0.984	0.3924
238921_at	72.6	53.2	-0.45	-1.36	PAA-AAA	0.10	11	0.861	0.984	0.4398
1554432_x_at	344.3	252.6	-0.45	-1.36	APA-PMA	0.10	11	0.861	0.984	0.2401
235938_at	174.2	127.8	-0.45	-1.36	APP-PPP	0.10	11	0.861	0.984	0.2416
226704_at	171.9	126.1	-0.45	-1.36	PPP-PPP	0.10	11	0.861	0.984	0.2626
217052_x_at	173.0	126.9	-0.45	-1.36	PPM-APP	0.10	11	0.861	0.984	0.3799
224924_at	65.1	47.7	-0.45	-1.36	AAP-AAA	0.10	11	0.861	0.984	0.4552
1554685_a_at	114.7	84.2	-0.45	-1.36	AAP-AAP	0.10	11	0.861	0.984	0.3356
239826_at	66.6	48.9	-0.45	-1.36	APA-AAA	0.10	11	0.861	0.984	0.3362
241555_at	80.1	58.8	-0.45	-1.36	APA-AAP	0.10	11	0.861	0.984	0.3591
237163_x_at	120.4	88.4	-0.45	-1.36	PPP-PPP	0.10	11	0.861	0.984	0.4316
223196_s_at	378.5	277.9	-0.45	-1.36	MPA-PPP	0.10	11	0.861	0.984	0.5392
204557_s_at	327.9	446.6	0.45	1.36	PPP-PPP	0.10	11	0.861	0.984	0.0096
229317_at	211.4	155.3	-0.45	-1.36	PPP-PPP	0.10	11	0.861	0.984	0.2716
237470_at	90.1	66.2	-0.45	-1.36	AAP-APM	0.10	11	0.861	0.984	0.3396

207022_s_at	141.5	103.9	-0.45	-1.36	PAA-AAA	0.10	11	0.861	0.984	0.3883
1552863_a_at	343.4	252.4	-0.44	-1.36	PPP-PPP	0.10	11	0.861	0.984	0.1972
209951_s_at	116.1	85.4	-0.44	-1.36	PPP-PPP	0.10	11	0.861	0.984	0.2250
244631_at	56.7	41.7	-0.44	-1.36	APA-APP	0.10	11	0.861	0.984	0.2709
243579_at	298.7	219.7	-0.44	-1.36	PPP-PPP	0.10	11	0.861	0.984	0.1972
241847_at	93.3	68.6	-0.44	-1.36	PAM-PPP	0.10	11	0.861	0.984	0.2119
241988_x_at	63.6	46.8	-0.44	-1.36	APP-AAP	0.10	11	0.861	0.984	0.2246
228827_at	304.6	224.0	-0.44	-1.36	APP-PPP	0.10	11	0.861	0.984	0.3067
210822_at	265.9	195.6	-0.44	-1.36	PPP-APP	0.10	11	0.861	0.984	0.3073
1570503_at	54.4	40.0	-0.44	-1.36	AAP-AAA	0.10	11	0.861	0.984	0.4048
228387_at	136.4	100.3	-0.44	-1.36	PMP-APP	0.10	11	0.861	0.984	0.4189
237548_at	54.3	39.9	-0.44	-1.36	AAP-AAP	0.10	11	0.861	0.984	0.4648
234446_at	106.4	78.3	-0.44	-1.36	PAA-AAA	0.10	11	0.861	0.984	0.5131
229885_at	167.3	227.5	0.44	1.36	PPP-PPP	0.10	11	0.861	0.984	0.0128
227467_at	1037.6	763.8	-0.44	-1.36	PPP-PPP	0.10	11	0.861	0.984	0.2094
219722_s_at	139.7	102.8	-0.44	-1.36	AAA-PAA	0.10	11	0.861	0.984	0.2261
229559_at	67.8	49.9	-0.44	-1.36	AAA-AAP	0.10	11	0.861	0.984	0.4184
210182_at	107.0	78.7	-0.44	-1.36	PAA-PAA	0.10	11	0.861	0.984	0.4314
1554161_at	37.8	27.8	-0.44	-1.36	AAP-APA	0.10	11	0.861	0.984	0.5156
238353_at	106.9	78.7	-0.44	-1.36	PAP-PAA	0.10	11	0.861	0.984	0.2535
1562406_at	74.5	54.9	-0.44	-1.36	PAP-APP	0.10	11	0.861	0.984	0.3844
209963_s_at	106.0	78.1	-0.44	-1.36	PAA-AAP	0.10	11	0.861	0.984	0.4389
220223_at	288.7	212.7	-0.44	-1.36	PPP-PMP	0.10	11	0.861	0.984	0.4581
1554079_at	152.9	112.7	-0.44	-1.36	PAP-APP	0.10	11	0.861	0.984	0.4154
237690_at	46.0	33.9	-0.44	-1.36	AAA-PAA	0.10	11	0.861	0.984	0.4603
235829_at	74.9	55.2	-0.44	-1.36	MPP-PAP	0.10	11	0.861	0.984	0.5173
235330_at	159.0	215.7	0.44	1.36	AAA-PPP	0.10	11	0.861	0.984	0.0053
207689_at	76.2	56.2	-0.44	-1.36	APA-PAP	0.10	11	0.861	0.984	0.3715
239922_at	83.7	61.8	-0.44	-1.35	APP-PPP	0.10	11	0.861	0.984	0.1900
232759_at	145.1	107.1	-0.44	-1.35	PAP-APP	0.10	11	0.861	0.984	0.2062
1553225_s_at	116.3	85.9	-0.44	-1.36	AAP-PAA	0.10	11	0.861	0.984	0.2367
217601_at	166.1	122.6	-0.44	-1.36	AAP-APM	0.10	11	0.861	0.984	0.2662
1569107_s_at	192.7	142.3	-0.44	-1.35	PPP-PPP	0.10	11	0.861	0.984	0.2443
240499_at	118.7	87.6	-0.44	-1.35	PPP-PPP	0.10	11	0.861	0.984	0.2963
231400_s_at	87.0	64.3	-0.44	-1.35	PAA-AAA	0.10	11	0.861	0.984	0.4373
204813_at	124.6	92.0	-0.44	-1.35	PPP-PMP	0.10	11	0.861	0.984	0.5133
229246_at	173.0	127.9	-0.44	-1.35	APA-APP	0.10	11	0.861	0.984	0.1936
1563458_at	119.0	88.0	-0.44	-1.35	PPA-APP	0.10	11	0.861	0.984	0.1997
215221_at	175.0	129.4	-0.44	-1.35	MPM-APP	0.10	11	0.861	0.984	0.2424
232477_at	41.0	30.3	-0.44	-1.35	AAM-APA	0.10	11	0.861	0.984	0.2548
232016_at	70.1	51.8	-0.44	-1.35	APA-AAA	0.10	11	0.861	0.984	0.2796
207379_at	151.6	112.1	-0.44	-1.35	PPP-APP	0.10	11	0.861	0.984	0.2838

1562263_at	96.2	71.1	-0.44	-1.35	APA-AAA	0.10	11	0.861	0.984	0.3586
213561_at	120.7	89.2	-0.44	-1.35	AAP-MAA	0.10	11	0.861	0.984	0.3619
220823_at	82.1	60.7	-0.44	-1.35	APP-AAM	0.10	11	0.861	0.984	0.3994
215525_at	75.8	56.0	-0.44	-1.35	PMP-APP	0.10	11	0.861	0.984	0.4282
1558199_at	329.1	243.2	-0.44	-1.35	PPP-PPP	0.10	11	0.861	0.984	0.4999
224627_at	169.3	125.1	-0.44	-1.35	APP-PAP	0.10	11	0.861	0.984	0.5364
1562283_at	79.4	58.7	-0.44	-1.35	APP-PAP	0.10	11	0.861	0.984	0.5407
1555348_at	113.5	84.0	-0.44	-1.35	PPP-AAA	0.10	11	0.861	0.984	0.2460
243423_at	89.2	66.0	-0.44	-1.35	MAP-PPA	0.10	11	0.861	0.984	0.3112
204494_s_at	255.7	189.2	-0.44	-1.35	PPA-PPP	0.10	11	0.861	0.984	0.3827
228380_at	109.3	80.9	-0.44	-1.35	AMP-PMM	0.10	11	0.861	0.984	0.3893
242270_at	83.4	61.8	-0.43	-1.35	PPP-PAP	0.10	11	0.861	0.984	0.2450
209031_at	410.5	303.9	-0.43	-1.35	PPP-PPP	0.10	11	0.861	0.984	0.2570
236367_at	139.9	103.6	-0.43	-1.35	APP-AMP	0.10	11	0.861	0.984	0.3388
232601_at	189.2	140.2	-0.43	-1.35	APP-APP	0.10	11	0.861	0.984	0.4638
242133_s_at	455.3	614.8	0.43	1.35	PPP-PPP	0.10	11	0.861	0.984	0.0046
1563612_at	45.6	33.8	-0.43	-1.35	APA-AAA	0.10	11	0.861	0.984	0.2867
216164_at	152.1	112.8	-0.43	-1.35	AAP-MPA	0.10	11	0.861	0.984	0.3705
242361_at	105.7	78.4	-0.43	-1.35	PPP-APP	0.10	11	0.861	0.984	0.4274
236569_at	128.6	95.3	-0.43	-1.35	PPA-APP	0.10	11	0.861	0.984	0.4534
240450_at	69.0	51.1	-0.43	-1.35	MPP-APP	0.10	11	0.861	0.984	0.4549
241815_at	61.6	45.6	-0.43	-1.35	PPA-PAP	0.10	11	0.861	0.984	0.4740
205855_at	141.7	105.2	-0.43	-1.35	APP-PAP	0.10	11	0.861	0.984	0.2914
244515_at	173.3	128.5	-0.43	-1.35	PPP-PPP	0.10	11	0.861	0.984	0.3191
243388_at	186.4	138.3	-0.43	-1.35	PPP-PPP	0.10	11	0.861	0.984	0.3693
1558648_at	130.9	97.1	-0.43	-1.35	PPP-MPP	0.10	11	0.861	0.984	0.3898
236605_at	73.2	54.3	-0.43	-1.35	APP-APP	0.10	11	0.861	0.984	0.5267
232720_at	88.4	65.7	-0.43	-1.35	PAP-PPP	0.10	11	0.861	0.984	0.2406
202970_at	195.6	145.1	-0.43	-1.35	PPP-PPP	0.10	11	0.861	0.984	0.2976
207300_s_at	73.4	54.4	-0.43	-1.35	AAA-APP	0.10	11	0.861	0.984	0.3263
241679_at	109.7	81.4	-0.43	-1.35	PPP-APP	0.10	11	0.861	0.984	0.4197
244387_at	111.8	83.0	-0.43	-1.35	PPP-PPP	0.10	11	0.861	0.984	0.4433
228876_at	163.7	121.6	-0.43	-1.35	PPA-APP	0.10	11	0.861	0.984	0.2370
236910_at	41.2	30.6	-0.43	-1.35	APP-AAA	0.10	11	0.861	0.984	0.3462
218913_s_at	179.8	133.5	-0.43	-1.35	APM-APA	0.10	11	0.861	0.984	0.4315
236697_at	36.5	27.1	-0.43	-1.35	APA-AAA	0.10	11	0.861	0.984	0.4984
207484_s_at	171.0	127.0	-0.43	-1.35	APM-AAP	0.10	11	0.861	0.984	0.5011
206586_at	141.4	105.2	-0.43	-1.35	PAA-APA	0.10	11	0.861	0.984	0.1886
1559156_at	120.2	89.4	-0.43	-1.35	APP-APP	0.10	11	0.861	0.984	0.1940
225650_at	408.5	303.7	-0.43	-1.35	PPP-PPP	0.10	11	0.861	0.984	0.1950
205399_at	106.1	78.8	-0.43	-1.35	APM-AAP	0.10	11	0.861	0.984	0.2286
239798_at	89.6	66.6	-0.43	-1.35	APP-APP	0.10	11	0.861	0.984	0.2354

239759_at	51.5	38.3	-0.43	-1.35	PAA-PPP	0.10	11	0.861	0.984	0.2799
234255_at	61.2	45.5	-0.43	-1.35	APA-AMA	0.10	11	0.861	0.984	0.4683
1554867_a_at	148.6	110.5	-0.43	-1.35	PAP-PPP	0.10	11	0.861	0.984	0.2569
1570185_at	336.9	250.6	-0.43	-1.34	APP-APP	0.10	11	0.861	0.984	0.2695
239668_at	90.7	67.5	-0.43	-1.34	AAA-PAA	0.10	11	0.861	0.984	0.4118
1565716_at	126.5	94.1	-0.43	-1.35	MPP-PPP	0.10	11	0.861	0.984	0.4233
242131_at	411.0	305.9	-0.43	-1.34	PPP-PPP	0.10	11	0.861	0.984	0.2123
207130_at	132.3	98.5	-0.43	-1.34	PPP-APP	0.10	11	0.861	0.984	0.2265
216387_x_at	770.6	573.8	-0.43	-1.34	PPP-APA	0.10	11	0.861	0.984	0.3580
234923_at	115.2	85.8	-0.43	-1.34	PPP-PPP	0.10	11	0.861	0.984	0.3333
1565920_at	66.6	49.6	-0.43	-1.34	PAA-APP	0.10	11	0.861	0.984	0.3370
227331_at	455.7	339.7	-0.42	-1.34	PPP-PPP	0.10	11	0.861	0.984	0.2211
239872_at	24.6	18.4	-0.42	-1.34	APA-AAA	0.10	11	0.861	0.984	0.3236
213178_s_at	288.4	215.0	-0.42	-1.34	PPP-PMP	0.10	11	0.861	0.984	0.3427
1564974_at	126.2	94.0	-0.42	-1.34	PPP-PPP	0.10	11	0.861	0.984	0.3441
242182_x_at	47.1	35.1	-0.42	-1.34	APP-PPP	0.10	11	0.861	0.984	0.2518
225957_at	198.8	148.3	-0.42	-1.34	PPP-PPP	0.10	11	0.861	0.984	0.3073
239063_at	63.0	47.0	-0.42	-1.34	AAM-APA	0.10	11	0.861	0.984	0.4030
203837_at	162.6	121.3	-0.42	-1.34	PPP-PPP	0.10	11	0.861	0.984	0.5228
227260_at	780.2	582.3	-0.42	-1.34	PPP-PPP	0.10	11	0.861	0.984	0.2356
228253_at	262.3	195.9	-0.42	-1.34	PPP-PPP	0.10	11	0.861	0.984	0.2142
211124_s_at	200.0	149.3	-0.42	-1.34	PPP-PPP	0.10	11	0.861	0.984	0.2516
239233_at	59.6	44.5	-0.42	-1.34	PPA-APP	0.10	11	0.861	0.984	0.2676
222746_s_at	74.9	56.0	-0.42	-1.34	PAA-APA	0.10	11	0.861	0.984	0.3587
220287_at	102.5	76.6	-0.42	-1.34	PPP-MPP	0.10	11	0.861	0.984	0.3596
225839_at	183.5	137.0	-0.42	-1.34	PPP-PAP	0.10	11	0.861	0.984	0.3713
1557590_at	89.6	67.0	-0.42	-1.34	MPP-PAA	0.10	11	0.861	0.984	0.3734
232604_at	61.7	46.1	-0.42	-1.34	AAA-APA	0.10	11	0.861	0.984	0.3929
211778_s_at	101.2	75.6	-0.42	-1.34	MPP-PPA	0.10	11	0.861	0.984	0.5387
235308_at	626.3	468.2	-0.42	-1.34	PPP-PPP	0.10	11	0.861	0.984	0.2058
201309_x_at	3819.7	2855.1	-0.42	-1.34	PPP-PPP	0.10	11	0.861	0.984	0.2360
226672_s_at	161.1	120.4	-0.42	-1.34	APP-AAM	0.10	11	0.861	0.984	0.3121
238913_at	178.5	133.5	-0.42	-1.34	PPP-PPP	0.10	11	0.861	0.984	0.2349
215233_at	176.6	132.1	-0.42	-1.34	MPP-PPP	0.10	11	0.861	0.984	0.2917
240307_at	217.9	163.0	-0.42	-1.34	APP-PPP	0.10	11	0.861	0.984	0.3096
242285_at	66.7	49.9	-0.42	-1.34	AAA-APA	0.10	11	0.861	0.984	0.3234
228882_at	345.4	258.3	-0.42	-1.34	PPP-PPP	0.10	11	0.861	0.984	0.4502
202072_at	1159.8	867.2	-0.42	-1.34	PAP-PPP	0.10	11	0.861	0.984	0.4517
206706_at	335.3	250.9	-0.42	-1.34	PAP-MMP	0.10	11	0.861	0.984	0.2044
239318_at	59.7	44.7	-0.42	-1.34	MAP-PPA	0.10	11	0.861	0.984	0.4075
236799_at	38.9	29.1	-0.42	-1.34	PAA-AAP	0.10	11	0.861	0.984	0.4328
220353_at	158.2	118.5	-0.42	-1.34	AAP-PPP	0.10	11	0.861	0.984	0.2037

215521_at	112.9	84.6	-0.42	-1.34	PPP-PPP	0.10	11	0.861	0.984	0.2148
228506_at	137.3	102.9	-0.42	-1.34	APP-AAP	0.10	11	0.861	0.984	0.2236
239655_at	53.1	39.7	-0.42	-1.34	PAP-MMP	0.10	11	0.861	0.984	0.2377
239799_at	75.1	56.2	-0.42	-1.34	APA-AAA	0.10	11	0.861	0.984	0.2975
230524_at	189.8	142.2	-0.42	-1.34	PPP-PPP	0.10	11	0.861	0.984	0.3053
203469_s_at	283.5	212.3	-0.42	-1.34	PPP-APP	0.10	11	0.861	0.984	0.3124
1564773_x_at	49.0	36.7	-0.42	-1.34	AAA-AAP	0.10	11	0.861	0.984	0.3459
243434_at	154.1	115.4	-0.42	-1.34	AMP-APA	0.10	11	0.861	0.984	0.3621
234366_x_at	88.1	66.0	-0.42	-1.34	AAA-AAP	0.10	11	0.861	0.984	0.4380
228906_at	119.9	89.8	-0.42	-1.34	PPP-PPP	0.10	11	0.861	0.984	0.4453
241801_at	72.8	54.5	-0.42	-1.34	PPP-PPP	0.10	11	0.861	0.984	0.4537
230375_at	83.2	62.3	-0.42	-1.34	AAA-APA	0.10	11	0.861	0.984	0.4734
1558831_x_at	93.6	70.1	-0.42	-1.34	AAP-AAM	0.10	11	0.861	0.984	0.4920
220096_at	93.0	69.7	-0.42	-1.33	APP-PPP	0.10	11	0.861	0.984	0.3213
226132_s_at	102.2	76.6	-0.42	-1.33	APA-AAA	0.10	11	0.861	0.984	0.3460
1565889_at	70.9	53.1	-0.42	-1.33	APA-AAA	0.10	11	0.861	0.984	0.4027
240759_at	34.1	25.6	-0.42	-1.34	AAA-AAP	0.10	11	0.861	0.984	0.5399
1554646_at	222.8	167.1	-0.42	-1.33	PPP-PPP	0.10	11	0.861	0.984	0.2045
212691_at	1738.8	1304.2	-0.42	-1.33	PPP-PPP	0.10	11	0.861	0.984	0.2135
230788_at	99.6	74.7	-0.42	-1.33	PAA-AAA	0.10	11	0.861	0.984	0.2979
201722_s_at	1670.4	2227.8	0.42	1.33	PPP-PPP	0.10	11	0.861	0.984	0.0101
1559054_a_at	98.3	73.8	-0.41	-1.33	PPP-APP	0.10	11	0.861	0.984	0.1858
1558705_at	128.6	96.5	-0.41	-1.33	PPA-AAA	0.10	11	0.861	0.984	0.2294
228587_at	333.5	250.3	-0.41	-1.33	APP-PPP	0.10	11	0.861	0.984	0.4635
239462_at	183.8	138.0	-0.41	-1.33	PPP-PPP	0.10	11	0.861	0.984	0.2343
206186_at	104.3	78.3	-0.41	-1.33	APM-PAA	0.10	11	0.861	0.984	0.2585
1562012_at	78.7	59.1	-0.41	-1.33	PPP-PAP	0.10	11	0.861	0.984	0.3444
239235_at	113.8	85.5	-0.41	-1.33	AAA-PPP	0.10	11	0.861	0.984	0.3937
233815_at	48.8	36.6	-0.41	-1.33	APP-AAP	0.10	11	0.861	0.984	0.3952
1553681_a_at	119.1	89.4	-0.41	-1.33	PAA-AAA	0.10	11	0.861	0.984	0.4933
1556279_at	87.0	65.4	-0.41	-1.33	PAP-APA	0.10	11	0.861	0.984	0.2491
207176_s_at	129.5	97.3	-0.41	-1.33	PPA-APP	0.10	11	0.861	0.984	0.3322
212374_at	203.3	152.8	-0.41	-1.33	MPA-APP	0.10	11	0.861	0.984	0.3815
201056_at	350.8	263.9	-0.41	-1.33	PPP-PPP	0.10	11	0.861	0.984	0.2739
240421_x_at	159.6	120.0	-0.41	-1.33	PPP-PPP	0.10	11	0.861	0.984	0.2847
203787_at	483.2	363.5	-0.41	-1.33	PPP-PPP	0.10	11	0.861	0.984	0.3427
235808_at	148.7	112.0	-0.41	-1.33	PPP-APP	0.10	11	0.861	0.984	0.2607
240128_at	152.8	115.0	-0.41	-1.33	AAP-APA	0.10	11	0.861	0.984	0.2608
1570070_at	179.9	135.5	-0.41	-1.33	APA-PMP	0.10	11	0.861	0.984	0.3658
242512_at	51.4	38.7	-0.41	-1.33	AAA-AAP	0.10	11	0.861	0.984	0.4502
214918_at	99.7	75.1	-0.41	-1.33	APP-APP	0.10	11	0.861	0.984	0.5073
240929_at	157.7	118.8	-0.41	-1.33	AAA-APA	0.10	11	0.861	0.984	0.1811

224728_at	494.9	372.8	-0.41	-1.33	PPP-PPP	0.10	11	0.861	0.984	0.1909
1563318_s_at	113.3	85.3	-0.41	-1.33	AMP-APA	0.10	11	0.861	0.984	0.3658
225825_at	271.4	204.4	-0.41	-1.33	PPP-PPP	0.10	11	0.861	0.984	0.3678
222382_x_at	219.4	165.2	-0.41	-1.33	PAP-AAP	0.10	11	0.861	0.984	0.4115
237577_at	52.2	39.3	-0.41	-1.33	APP-AAA	0.10	11	0.861	0.984	0.5000
222865_x_at	126.5	95.3	-0.41	-1.33	APA-AAA	0.10	11	0.861	0.984	0.2003
210701_at	235.9	177.7	-0.41	-1.33	PPP-PPP	0.10	11	0.861	0.984	0.2184
1562524_at	52.1	39.2	-0.41	-1.33	PAP-AAA	0.10	11	0.861	0.984	0.4916
228832_at	177.1	133.6	-0.41	-1.33	AAP-AAP	0.10	11	0.861	0.984	0.1895
227009_at	200.2	151.0	-0.41	-1.33	AAM-APA	0.10	11	0.861	0.984	0.2371
223460_at	302.1	227.8	-0.41	-1.33	PMP-PPP	0.10	11	0.861	0.984	0.2569
233090_at	200.2	151.0	-0.41	-1.33	APM-APM	0.10	11	0.861	0.984	0.2887
239503_at	53.9	40.7	-0.41	-1.33	APP-PMP	0.10	11	0.861	0.984	0.4954
230119_at	119.9	90.5	-0.41	-1.33	MAP-PAA	0.10	11	0.861	0.984	0.5013
214535_s_at	254.1	191.7	-0.41	-1.33	APP-APM	0.10	11	0.861	0.984	0.2161
236122_at	159.5	120.3	-0.41	-1.33	PPP-PPP	0.10	11	0.861	0.984	0.2229
226074_at	138.3	104.4	-0.41	-1.33	PPP-PPP	0.10	11	0.861	0.984	0.2398
228175_at	120.7	91.1	-0.41	-1.33	APP-PPP	0.10	11	0.861	0.984	0.3829
216697_at	107.9	81.5	-0.41	-1.33	PPP-APP	0.10	11	0.861	0.984	0.4934
242282_at	413.2	312.0	-0.41	-1.32	AAP-MAA	0.10	11	0.861	0.984	0.2729
244086_at	119.0	89.9	-0.41	-1.32	APP-AAA	0.10	11	0.861	0.984	0.2942
220737_at	49.8	37.6	-0.41	-1.33	AAA-MPA	0.10	11	0.861	0.984	0.3119
219664_s_at	441.1	333.2	-0.41	-1.32	PPP-PPP	0.10	11	0.861	0.984	0.3130
1566549_at	155.5	117.5	-0.41	-1.32	APP-PAP	0.10	11	0.861	0.984	0.5087
203502_at	637.8	844.8	0.41	1.32	PPP-PPP	0.10	11	0.861	0.984	0.0033
226655_at	99.3	75.0	-0.40	-1.32	AAP-AAA	0.10	11	0.861	0.984	0.1796
243908_at	70.4	53.2	-0.40	-1.32	PPP-PPP	0.10	11	0.861	0.984	0.1850
216292_at	120.6	91.1	-0.40	-1.32	PAA-APA	0.10	11	0.861	0.984	0.1992
217671_at	62.8	47.4	-0.40	-1.32	AAP-APP	0.10	11	0.861	0.984	0.3112
226848_at	328.7	248.5	-0.40	-1.32	PPP-PPP	0.10	11	0.861	0.984	0.3437
222796_at	88.9	67.2	-0.40	-1.32	PMP-PAP	0.10	11	0.861	0.984	0.5318
1556163_a_at	102.9	77.8	-0.40	-1.32	APA-PAA	0.10	11	0.861	0.984	0.5470
1563030_at	195.7	148.0	-0.40	-1.32	PAM-APP	0.10	11	0.861	0.984	0.2067
221185_s_at	94.8	71.7	-0.40	-1.32	APP-PAA	0.10	11	0.861	0.984	0.3589
242696_at	212.6	160.8	-0.40	-1.32	PPP-PPP	0.10	11	0.861	0.984	0.4571
235686_at	119.2	90.1	-0.40	-1.32	PAP-APP	0.10	11	0.861	0.984	0.5149
227113_at	100.8	76.3	-0.40	-1.32	APA-AAA	0.10	11	0.861	0.984	0.1898
222996_s_at	770.7	583.3	-0.40	-1.32	PPP-PPP	0.10	11	0.861	0.984	0.2115
236560_at	168.5	127.5	-0.40	-1.32	MPP-PAA	0.10	11	0.861	0.984	0.2142
1557801_x_at	141.4	107.0	-0.40	-1.32	PPP-PPP	0.10	11	0.861	0.984	0.2237
216109_at	132.0	99.9	-0.40	-1.32	PPP-PPP	0.10	11	0.861	0.984	0.3155
228441_s_at	73.6	55.7	-0.40	-1.32	PAP-APA	0.10	11	0.861	0.984	0.3434

201283_s_at	80.8	61.1	-0.40	-1.32	AAA-AAP	0.10	11	0.861	0.984	0.3463
220488_s_at	140.1	106.0	-0.40	-1.32	PMP-APP	0.10	11	0.861	0.984	0.4159
1561486_at	42.3	32.0	-0.40	-1.32	AMP-APA	0.10	11	0.861	0.984	0.4772
232533_at	55.5	42.1	-0.40	-1.32	APA-AAM	0.10	11	0.861	0.984	0.2080
231005_at	174.2	131.9	-0.40	-1.32	AAA-AAP	0.10	11	0.861	0.984	0.2874
205319_at	68.9	52.2	-0.40	-1.32	AAA-PAA	0.10	11	0.861	0.984	0.4884
232473_at	176.2	133.4	-0.40	-1.32	AAP-APP	0.10	11	0.861	0.984	0.5393
210765_at	170.0	128.8	-0.40	-1.32	MAP-MPA	0.10	11	0.861	0.984	0.2088
225999_at	94.1	71.3	-0.40	-1.32	PPP-APP	0.10	11	0.861	0.984	0.2167
228769_at	233.3	176.8	-0.40	-1.32	PPP-PPP	0.10	11	0.861	0.984	0.2248
232921_at	114.3	86.6	-0.40	-1.32	PPP-PPA	0.10	11	0.861	0.984	0.2770
230766_at	368.4	279.1	-0.40	-1.32	PPP-PPP	0.10	11	0.861	0.984	0.2779
235221_at	126.8	96.1	-0.40	-1.32	PAA-APA	0.10	11	0.861	0.984	0.3323
242753_x_at	85.7	65.0	-0.40	-1.32	APP-PPP	0.10	11	0.861	0.984	0.3526
236172_at	91.3	69.2	-0.40	-1.32	AAP-PAA	0.10	11	0.861	0.984	0.4632
229589_x_at	55.4	42.0	-0.40	-1.32	AAA-APP	0.10	11	0.861	0.984	0.4938
205695_at	96.0	72.7	-0.40	-1.32	PAA-AAA	0.10	11	0.861	0.984	0.5421
232442_at	65.2	86.0	0.40	1.32	AAA-AMP	0.10	11	0.861	0.984	0.0055
222674_at	356.6	270.5	-0.40	-1.32	PPP-PMP	0.10	11	0.861	0.984	0.2430
210654_at	337.7	256.1	-0.40	-1.32	PPP-PPP	0.10	11	0.861	0.984	0.2691
38157_at	402.2	305.0	-0.40	-1.32	PPP-PPP	0.10	11	0.861	0.984	0.3376
215532_x_at	31.3	23.7	-0.40	-1.32	PPA-PAA	0.10	11	0.861	0.984	0.4348
225495_x_at	80.3	60.9	-0.40	-1.32	AAA-PAA	0.10	11	0.861	0.984	0.4592
1559514_at	34.0	25.8	-0.40	-1.32	AAA-AAP	0.10	11	0.861	0.984	0.5272
208472_at	75.7	57.4	-0.40	-1.32	APM-PPP	0.10	11	0.861	0.984	0.5396
1555858_at	163.7	124.2	-0.40	-1.32	PPP-PPP	0.10	11	0.861	0.984	0.1919
1557008_at	93.8	71.2	-0.40	-1.32	PAA-APA	0.10	11	0.861	0.984	0.2774
219800_s_at	37.5	28.5	-0.40	-1.32	PPA-AAP	0.10	11	0.861	0.984	0.3477
244372_at	32.2	24.4	-0.40	-1.32	PAA-AAA	0.10	11	0.861	0.984	0.4181
239198_at	78.7	59.8	-0.40	-1.32	PAP-PPA	0.10	11	0.861	0.984	0.4462
214015_at	134.5	102.1	-0.40	-1.32	APA-PPM	0.10	11	0.861	0.984	0.1937
226724_s_at	103.0	78.2	-0.40	-1.32	AAA-AAP	0.10	11	0.861	0.984	0.3467
209359_x_at	209.0	158.7	-0.40	-1.32	APP-PPP	0.10	11	0.861	0.984	0.3955
242320_at	197.0	149.6	-0.40	-1.32	MPP-APP	0.10	11	0.861	0.984	0.4439
1555673_at	5975.7	4539.0	-0.40	-1.32	PPP-PPP	0.10	11	0.861	0.984	0.4707
1559108_at	149.8	113.8	-0.40	-1.32	PAP-APP	0.10	11	0.861	0.984	0.5205
244312_at	62.3	47.4	-0.40	-1.32	PPP-PMP	0.10	11	0.861	0.984	0.2541
1553992_s_at	203.2	154.5	-0.40	-1.32	PPP-PPP	0.10	11	0.861	0.984	0.2548
217152_at	110.5	83.9	-0.40	-1.32	APA-APP	0.10	11	0.861	0.984	0.3471
206974_at	56.1	42.6	-0.40	-1.32	AAA-PAA	0.10	11	0.861	0.984	0.3709
244655_at	64.8	49.2	-0.40	-1.32	PPA-APP	0.10	11	0.861	0.984	0.3861
205832_at	741.9	563.7	-0.40	-1.32	PPP-PPP	0.10	11	0.861	0.984	0.4010

218911_at	606.1	797.4	0.40	1.32	PPP-PPP	0.10	11	0.861	0.984	0.0032
213884_s_at	122.9	93.4	-0.40	-1.32	AAP-PAA	0.10	11	0.861	0.984	0.1889
235968_at	153.7	116.9	-0.40	-1.32	AAM-PAA	0.10	11	0.861	0.984	0.1941
239937_at	299.4	227.7	-0.40	-1.32	PPP-PPP	0.10	11	0.861	0.984	0.1987
229733_s_at	2781.1	2115.3	-0.40	-1.32	PPP-PPP	0.10	11	0.861	0.984	0.2606
1560982_at	111.5	84.8	-0.40	-1.32	AAA-AAP	0.10	11	0.861	0.984	0.2734
1561749_at	110.9	84.3	-0.40	-1.32	AAA-APP	0.10	11	0.861	0.984	0.2894
238787_at	69.5	52.8	-0.40	-1.32	AAA-PAA	0.10	11	0.861	0.984	0.2967
1558075_at	59.5	45.2	-0.40	-1.32	PAA-AAA	0.10	11	0.861	0.984	0.4010
229216_s_at	61.0	46.4	-0.40	-1.32	AMA-PAP	0.10	11	0.861	0.984	0.4243
218337_at	426.7	324.7	-0.39	-1.31	PPP-PPP	0.10	11	0.861	0.984	0.1878
37802_r_at	70.5	53.6	-0.39	-1.31	PPP-PPP	0.10	11	0.861	0.984	0.2445
217340_at	79.5	60.5	-0.39	-1.31	PAP-MAA	0.10	11	0.861	0.984	0.2526
216821_at	116.1	88.3	-0.39	-1.31	PAA-AAA	0.10	11	0.861	0.984	0.3185
222344_at	227.0	172.7	-0.39	-1.31	PPP-PPP	0.10	11	0.861	0.984	0.3289
1557804_at	140.9	107.2	-0.39	-1.31	PPP-APP	0.10	11	0.861	0.984	0.3552
206950_at	684.2	520.9	-0.39	-1.31	PPP-PPP	0.10	11	0.861	0.984	0.1765
204455_at	137.5	104.7	-0.39	-1.31	PPP-PPP	0.10	11	0.861	0.984	0.2062
227814_at	248.1	189.0	-0.39	-1.31	PPP-PPP	0.10	11	0.861	0.984	0.2550
232885_at	74.3	56.5	-0.39	-1.31	MPP-APA	0.10	11	0.861	0.984	0.3403
56829_at	286.8	218.4	-0.39	-1.31	PPP-PPP	0.10	11	0.861	0.984	0.3512
230667_at	157.2	119.6	-0.39	-1.31	APP-AAP	0.10	11	0.861	0.984	0.3571
240627_x_at	36.2	27.6	-0.39	-1.31	PAP-APA	0.10	11	0.861	0.984	0.4359
229570_at	192.0	146.2	-0.39	-1.31	APP-APA	0.10	11	0.861	0.984	0.4437
233607_at	90.4	68.9	-0.39	-1.31	APP-PAP	0.10	11	0.861	0.984	0.4540
235095_at	89.0	67.8	-0.39	-1.31	AMA-AAP	0.10	11	0.861	0.984	0.4773
217127_at	497.1	378.9	-0.39	-1.31	PPP-PPP	0.10	11	0.861	0.984	0.1771
213520_at	413.4	314.9	-0.39	-1.31	PPP-PPP	0.10	11	0.861	0.984	0.2183
241827_at	236.0	179.9	-0.39	-1.31	APP-PPP	0.10	11	0.861	0.984	0.2501
223871_x_at	360.9	275.0	-0.39	-1.31	PPP-PPP	0.10	11	0.861	0.984	0.2901
1557649_at	135.7	103.4	-0.39	-1.31	PPA-MAP	0.10	11	0.861	0.984	0.3767
239536_at	78.6	59.9	-0.39	-1.31	PPP-APP	0.10	11	0.861	0.984	0.4125
35150_at	307.3	234.1	-0.39	-1.31	PPP-PPP	0.10	11	0.861	0.984	0.4186
212786_at	288.5	219.8	-0.39	-1.31	PPP-PPP	0.10	11	0.861	0.984	0.4614
207447_s_at	31.8	24.2	-0.39	-1.31	AAA-APP	0.10	11	0.861	0.984	0.5392
240263_at	238.6	182.0	-0.39	-1.31	PPP-PPP	0.10	11	0.861	0.984	0.1928
1562074_a_at	72.2	55.1	-0.39	-1.31	AAP-MAA	0.10	11	0.861	0.984	0.3418
1569713_at	86.8	66.2	-0.39	-1.31	MAP-PPP	0.10	11	0.861	0.984	0.4876
206481_s_at	173.6	132.4	-0.39	-1.31	PAP-PPP	0.10	11	0.861	0.984	0.5324
203894_at	276.2	210.7	-0.39	-1.31	PPP-PPP	0.10	11	0.861	0.984	0.3191
241785_at	89.9	68.6	-0.39	-1.31	PPA-APP	0.10	11	0.861	0.984	0.3353
223999_at	69.1	52.7	-0.39	-1.31	PAM-PPA	0.10	11	0.861	0.984	0.3452

212942_s_at	2711.2	2068.4	-0.39	-1.31	PPP-PPP	0.10	11	0.861	0.984	0.3537
1557170_at	205.7	156.9	-0.39	-1.31	APP-PPA	0.10	11	0.861	0.984	0.3975
243118_at	122.9	93.8	-0.39	-1.31	APA-PPP	0.10	11	0.861	0.984	0.4052
218706_s_at	827.3	631.7	-0.39	-1.31	PPP-PPP	0.10	11	0.861	0.984	0.1800
211809_x_at	303.2	231.5	-0.39	-1.31	PPP-PPP	0.10	11	0.861	0.984	0.4120
244642_at	87.5	66.8	-0.39	-1.31	APA-AAA	0.10	11	0.861	0.984	0.4760
215445_x_at	72.0	55.0	-0.39	-1.31	APP-AAP	0.10	11	0.861	0.984	0.5346
233955_x_at	1263.8	965.8	-0.39	-1.31	PPP-PPP	0.10	11	0.861	0.984	0.2304
1569362_at	100.6	76.9	-0.39	-1.31	PAA-APP	0.10	11	0.861	0.984	0.2710
204205_at	151.8	116.1	-0.39	-1.31	PAA-PPP	0.10	11	0.861	0.984	0.3273
232879_at	123.5	94.4	-0.39	-1.31	PPP-PPP	0.10	11	0.861	0.984	0.3406
213635_s_at	104.7	80.0	-0.39	-1.31	PPM-PPP	0.10	11	0.861	0.984	0.3460
1559039_at	106.5	81.4	-0.39	-1.31	AAP-AAP	0.10	11	0.861	0.984	0.4507
1554852_a_at	69.4	53.1	-0.39	-1.31	APA-AAA	0.10	11	0.861	0.984	0.4886
238402_s_at	289.9	221.6	-0.39	-1.31	PPP-PPP	0.10	11	0.861	0.984	0.2029
1562505_at	82.5	63.1	-0.39	-1.31	PPP-PPP	0.10	11	0.861	0.984	0.2253
1566990_x_at	333.0	254.6	-0.39	-1.31	APP-APA	0.10	11	0.861	0.984	0.2612
244129_at	133.7	102.2	-0.39	-1.31	APA-AAA	0.10	11	0.861	0.984	0.2900
220319_s_at	165.1	126.3	-0.39	-1.31	AAA-APP	0.10	11	0.861	0.984	0.3212
235000_at	206.5	270.1	0.39	1.31	PPP-PPP	0.10	11	0.861	0.984	0.0070
220078_at	185.5	141.9	-0.39	-1.31	PPP-PPP	0.10	11	0.861	0.984	0.1884
219550_at	432.8	331.1	-0.39	-1.31	PPP-PPA	0.10	11	0.861	0.984	0.2159
210198_s_at	75.4	57.7	-0.39	-1.31	PAA-PAP	0.10	11	0.861	0.984	0.2978
241866_at	112.4	86.0	-0.39	-1.31	PPP-APP	0.10	11	0.861	0.984	0.3016
242750_at	79.9	61.2	-0.39	-1.31	APP-PPA	0.10	11	0.861	0.984	0.3679
211880_x_at	79.3	60.7	-0.39	-1.31	AAA-AAP	0.10	11	0.861	0.984	0.2635
237813_at	123.5	94.6	-0.39	-1.31	PPP-PPP	0.10	11	0.861	0.984	0.3123
1557196_a_at	73.6	56.4	-0.39	-1.31	AAP-APA	0.10	11	0.861	0.984	0.3835
32137_at	125.7	96.2	-0.39	-1.31	APP-PPA	0.10	11	0.861	0.984	0.5201
221045_s_at	620.2	475.3	-0.38	-1.31	PPP-PPP	0.10	11	0.861	0.984	0.2536
235939_at	143.8	110.2	-0.38	-1.31	PPP-PPP	0.10	11	0.861	0.984	0.3906
217149_x_at	177.2	135.8	-0.38	-1.31	PPP-PPP	0.10	11	0.861	0.984	0.5020
235337_at	85.5	65.6	-0.38	-1.30	PMA-PPP	0.10	11	0.861	0.984	0.2295
226639_at	325.5	249.6	-0.38	-1.30	PPP-PPP	0.10	11	0.861	0.984	0.2386
229210_at	108.2	83.0	-0.38	-1.30	PAA-APP	0.10	11	0.861	0.984	0.2870
220180_at	200.7	153.9	-0.38	-1.30	PPP-PPP	0.10	11	0.861	0.984	0.3284
228455_at	152.0	116.6	-0.38	-1.30	PPP-PPP	0.10	11	0.861	0.984	0.3767
1564277_a_at	174.2	133.5	-0.38	-1.30	APA-PPA	0.10	11	0.861	0.984	0.4235
206763_at	67.9	52.1	-0.38	-1.30	AAP-AAA	0.10	11	0.861	0.984	0.4972
226912_at	70.0	53.7	-0.38	-1.30	PAP-AAA	0.10	11	0.861	0.984	0.5334
204783_at	359.4	468.7	0.38	1.30	PPP-PPP	0.10	11	0.861	0.984	0.0049
207953_at	132.5	101.7	-0.38	-1.30	PPP-APP	0.10	11	0.861	0.984	0.3026

221801_x_at	46.2	35.4	-0.38	-1.30	PAA-APA	0.10	11	0.861	0.984	0.3866
206921_at	63.2	48.5	-0.38	-1.30	AAA-APA	0.10	11	0.861	0.984	0.3921
228307_at	107.2	82.3	-0.38	-1.30	APP-AAP	0.10	11	0.861	0.984	0.5122
203700_s_at	75.7	58.1	-0.38	-1.30	PAA-AAM	0.10	11	0.861	0.984	0.5256
241627_x_at	100.8	77.4	-0.38	-1.30	APP-PPP	0.10	11	0.861	0.984	0.5392
1563166_at	43.7	33.5	-0.38	-1.30	AAA-AAP	0.10	11	0.861	0.984	0.1867
239538_at	191.6	147.2	-0.38	-1.30	PPP-MPP	0.10	11	0.861	0.984	0.2167
218296_x_at	757.8	581.8	-0.38	-1.30	PPP-PPP	0.10	11	0.861	0.984	0.2228
242052_at	300.9	231.1	-0.38	-1.30	PPP-PPP	0.10	11	0.861	0.984	0.2722
227810_at	110.3	84.7	-0.38	-1.30	PPP-PPA	0.10	11	0.861	0.984	0.4457
232958_at	89.7	68.9	-0.38	-1.30	PPP-PPP	0.10	11	0.861	0.984	0.2023
209883_at	122.5	94.2	-0.38	-1.30	PPA-PPP	0.10	11	0.861	0.984	0.2693
242671_at	164.1	126.1	-0.38	-1.30	PPP-PPP	0.10	11	0.861	0.984	0.4109
204155_s_at	170.1	130.7	-0.38	-1.30	PPP-PPM	0.10	11	0.861	0.984	0.1949
1555374_at	175.5	134.9	-0.38	-1.30	PPP-PMM	0.10	11	0.861	0.984	0.2902
209991_x_at	62.5	48.0	-0.38	-1.30	APA-AAA	0.10	11	0.861	0.984	0.3237
229567_at	109.1	83.9	-0.38	-1.30	APA-PAA	0.10	11	0.861	0.984	0.3379
232489_at	144.0	110.7	-0.38	-1.30	APP-PPP	0.10	11	0.861	0.984	0.3860
206183_s_at	85.5	65.7	-0.38	-1.30	APP-APP	0.10	11	0.861	0.984	0.4361
232522_at	104.2	80.1	-0.38	-1.30	APP-AAP	0.10	11	0.861	0.984	0.2251
221940_at	395.6	304.5	-0.38	-1.30	PPP-PMP	0.10	11	0.861	0.984	0.2316
239801_at	179.7	138.3	-0.38	-1.30	AAP-PPP	0.10	11	0.861	0.984	0.3114
244763_at	90.4	69.6	-0.38	-1.30	AMP-PPP	0.10	11	0.861	0.984	0.3929
226777_at	801.9	616.9	-0.38	-1.30	PPP-PPP	0.10	11	0.861	0.984	0.4882
213826_s_at	165.3	127.2	-0.38	-1.30	APP-PAA	0.10	11	0.861	0.984	0.5133
211150_s_at	835.0	1084.9	0.38	1.30	PPP-PPP	0.10	11	0.861	0.984	0.0060
240314_at	96.3	74.2	-0.38	-1.30	PPM-PMP	0.10	11	0.861	0.984	0.3239
220015_at	234.5	180.6	-0.38	-1.30	PMM-PPP	0.10	11	0.861	0.984	0.3345
218246_at	480.5	370.1	-0.38	-1.30	AAA-PAA	0.10	11	0.861	0.984	0.3488
231057_at	139.3	107.3	-0.38	-1.30	PPP-APP	0.10	11	0.861	0.984	0.5025
1569318_at	57.1	44.0	-0.38	-1.30	AAP-AAP	0.10	11	0.861	0.984	0.1756
205255_x_at	529.1	407.8	-0.38	-1.30	PPP-PPP	0.10	11	0.861	0.984	0.2574
222038_s_at	85.7	66.1	-0.38	-1.30	PPP-PAP	0.10	11	0.861	0.984	0.3033
213022_s_at	300.8	231.8	-0.38	-1.30	PPM-PPP	0.10	11	0.861	0.984	0.3893
231517_at	330.9	255.1	-0.38	-1.30	PPP-APP	0.10	11	0.861	0.984	0.1692
222632_s_at	488.6	376.6	-0.38	-1.30	PPP-PPP	0.10	11	0.861	0.984	0.1773
226197_at	154.4	119.1	-0.38	-1.30	MAP-APP	0.10	11	0.861	0.984	0.1798
210250_x_at	8991.5	6933.3	-0.38	-1.30	PPP-PPP	0.10	11	0.861	0.984	0.1959
203778_at	384.2	296.4	-0.38	-1.30	PPP-PPP	0.10	11	0.861	0.984	0.2113
232540_x_at	148.6	114.6	-0.38	-1.30	PPA-PPP	0.10	11	0.861	0.984	0.3644
238898_at	100.1	77.1	-0.38	-1.30	AAP-AAA	0.10	11	0.861	0.984	0.4306
217200_x_at	256.3	197.9	-0.37	-1.30	MPP-PPP	0.10	11	0.861	0.984	0.1831

236114_at	202.2	155.9	-0.37	-1.30	PPP-PPP	0.10	11	0.861	0.984	0.2367
215324_at	70.3	54.2	-0.37	-1.30	AAA-AAP	0.10	11	0.861	0.984	0.3707
244018_at	85.3	65.9	-0.37	-1.30	AMA-AAP	0.10	11	0.861	0.984	0.4326
238568_s_at	120.1	92.7	-0.37	-1.30	AAP-APP	0.10	11	0.861	0.984	0.4517
227675_at	113.7	87.8	-0.37	-1.30	PAP-AAP	0.10	11	0.861	0.984	0.2030
227515_at	239.4	184.9	-0.37	-1.30	PAP-PAP	0.10	11	0.861	0.984	0.2106
222622_at	1987.6	1534.4	-0.37	-1.30	PPP-PPP	0.10	11	0.861	0.984	0.2114
201638_s_at	139.7	107.8	-0.37	-1.30	AAP-AAP	0.10	11	0.861	0.984	0.2602
230229_at	207.9	160.6	-0.37	-1.30	PPP-PPP	0.10	11	0.861	0.984	0.2988
238851_at	192.2	148.4	-0.37	-1.30	PPP-MPP	0.10	11	0.861	0.984	0.3214
236668_at	68.6	53.0	-0.37	-1.30	PAA-MAP	0.10	11	0.861	0.984	0.4685
244418_at	80.5	62.2	-0.37	-1.30	PAP-APP	0.10	11	0.861	0.984	0.4767
239760_at	150.9	116.6	-0.37	-1.30	AAM-APM	0.10	11	0.861	0.984	0.4833
241970_at	97.9	75.6	-0.37	-1.30	APA-MPP	0.10	11	0.861	0.984	0.5042
238032_at	216.5	167.2	-0.37	-1.30	AAP-AAP	0.10	11	0.861	0.984	0.5172
230570_at	83.3	64.3	-0.37	-1.30	PPP-PPP	0.10	11	0.861	0.984	0.5203
227605_at	279.0	361.2	0.37	1.30	PPP-PPP	0.10	11	0.861	0.984	0.0020
214715_x_at	1735.0	1340.6	-0.37	-1.29	PPP-PPP	0.10	11	0.861	0.984	0.1890
208949_s_at	5243.7	4053.1	-0.37	-1.29	PPP-PPP	0.10	11	0.861	0.984	0.2173
218837_s_at	685.2	529.3	-0.37	-1.29	PPP-PPP	0.10	11	0.861	0.984	0.2567
60528_at	360.4	278.6	-0.37	-1.29	PPP-MPP	0.10	11	0.861	0.984	0.2754
207524_at	431.3	333.2	-0.37	-1.29	PPP-PPP	0.10	11	0.861	0.984	0.2861
242602_x_at	213.6	165.0	-0.37	-1.29	PPP-PPP	0.10	11	0.861	0.984	0.3653
1566142_at	83.7	64.6	-0.37	-1.29	MPA-PAP	0.10	11	0.861	0.984	0.3660
205357_s_at	447.8	345.9	-0.37	-1.29	PPP-PPP	0.10	11	0.861	0.984	0.3822
216628_at	79.3	61.3	-0.37	-1.30	PMA-APP	0.10	11	0.861	0.984	0.3936
207608_x_at	204.0	157.7	-0.37	-1.29	PPA-APP	0.10	11	0.861	0.984	0.5299
228917_at	186.4	144.2	-0.37	-1.29	PPP-PPP	0.10	11	0.861	0.984	0.2458
1557810_at	152.0	117.6	-0.37	-1.29	PPP-PPP	0.10	11	0.861	0.984	0.2556
240815_at	157.4	121.7	-0.37	-1.29	PPP-APP	0.10	11	0.861	0.984	0.2559
209777_s_at	415.0	320.8	-0.37	-1.29	AAA-MAP	0.10	11	0.861	0.984	0.3138
216516_at	38.6	29.9	-0.37	-1.29	AAP-APA	0.10	11	0.861	0.984	0.3574
241922_at	161.2	124.7	-0.37	-1.29	PPP-PPP	0.10	11	0.861	0.984	0.3717
232849_at	105.5	81.6	-0.37	-1.29	PPA-PPP	0.10	11	0.861	0.984	0.4541
239516_at	127.1	98.4	-0.37	-1.29	PPP-PPP	0.10	11	0.861	0.984	0.3267
1552729_at	294.5	227.9	-0.37	-1.29	APP-AAP	0.10	11	0.861	0.984	0.3367
239163_at	78.7	60.8	-0.37	-1.29	PPA-AAP	0.10	11	0.861	0.984	0.3459
232667_at	292.3	226.3	-0.37	-1.29	PPP-PPP	0.10	11	0.861	0.984	0.3925
236540_at	88.7	68.7	-0.37	-1.29	APA-AMP	0.10	11	0.861	0.984	0.2155
205923_at	257.2	199.2	-0.37	-1.29	PPP-PPP	0.10	11	0.861	0.984	0.2231
221686_s_at	201.6	156.1	-0.37	-1.29	MMA-APP	0.10	11	0.861	0.984	0.3009
233480_at	140.5	108.8	-0.37	-1.29	MPP-PPP	0.10	11	0.861	0.984	0.4614

238933_at	111.6	86.4	-0.37	-1.29	PPA-PAP	0.10	11	0.861	0.984	0.4949
239926_at	86.7	67.1	-0.37	-1.29	AAP-APA	0.10	11	0.861	0.984	0.5240
234154_at	34.3	26.6	-0.37	-1.29	AAA-PAA	0.10	11	0.861	0.984	0.5253
220942_x_at	3947.6	3057.9	-0.37	-1.29	PPP-PPP	0.10	11	0.861	0.984	0.2226
206900_x_at	119.9	92.9	-0.37	-1.29	PPP-PPP	0.10	11	0.861	0.984	0.2231
1562723_at	86.2	66.8	-0.37	-1.29	PAM-AAM	0.10	11	0.861	0.984	0.2346
209088_s_at	1018.7	789.4	-0.37	-1.29	PPP-PPP	0.10	11	0.861	0.984	0.2479
225252_at	2773.5	2148.8	-0.37	-1.29	PPP-PPP	0.10	11	0.861	0.984	0.2603
222283_at	120.0	93.0	-0.37	-1.29	PPP-PPP	0.10	11	0.861	0.984	0.3247
1562484_at	89.3	69.2	-0.37	-1.29	AAP-AAP	0.10	11	0.861	0.984	0.3816
238282_at	37.3	28.9	-0.37	-1.29	AAP-AAA	0.10	11	0.861	0.984	0.3961
203754_s_at	164.2	127.3	-0.37	-1.29	AAM-APM	0.10	11	0.861	0.984	0.4209
233690_at	85.2	66.1	-0.37	-1.29	PAP-AAP	0.10	11	0.861	0.984	0.4785
201727_s_at	1286.0	1659.2	0.37	1.29	PPP-PPP	0.10	11	0.861	0.984	0.0029
238580_at	77.8	60.4	-0.37	-1.29	AAA-APA	0.10	11	0.861	0.984	0.1732
209018_s_at	981.2	760.6	-0.37	-1.29	PPP-PPP	0.10	11	0.861	0.984	0.1865
215011_at	450.0	349.0	-0.37	-1.29	PPP-PPP	0.10	11	0.861	0.984	0.2300
213361_at	653.6	506.8	-0.37	-1.29	PPP-PPP	0.10	11	0.861	0.984	0.2359
1567081_x_at	232.0	179.9	-0.37	-1.29	PPP-PPP	0.10	11	0.861	0.984	0.2379
1563315_s_at	482.2	373.8	-0.37	-1.29	PPP-PPP	0.10	11	0.861	0.984	0.2922
204653_at	3803.3	2949.6	-0.37	-1.29	PPP-PPP	0.10	11	0.861	0.984	0.3032
239757_at	98.6	76.4	-0.37	-1.29	MPP-APP	0.10	11	0.861	0.984	0.3964
240919_at	86.5	67.1	-0.37	-1.29	PPP-PPP	0.10	11	0.861	0.984	0.4434
242246_x_at	92.5	71.7	-0.37	-1.29	PMA-AMP	0.10	11	0.861	0.984	0.4763
208958_at	63.1	49.0	-0.37	-1.29	AAA-AAP	0.10	11	0.861	0.984	0.5141
209295_at	2175.0	2805.9	0.37	1.29	PPP-PPP	0.10	11	0.861	0.984	0.0029
210527_x_at	1141.2	885.7	-0.37	-1.29	PPP-PPP	0.10	11	0.861	0.984	0.1722
1553698_a_at	76.4	59.3	-0.37	-1.29	MPA-AAA	0.10	11	0.861	0.984	0.2191
232674_at	140.6	109.0	-0.37	-1.29	AAA-MPP	0.10	11	0.861	0.984	0.2539
224037_at	88.4	68.6	-0.37	-1.29	APM-AAP	0.10	11	0.861	0.984	0.3498
224454_at	211.7	164.3	-0.37	-1.29	PPP-PPP	0.10	11	0.861	0.984	0.3788
228296_at	85.1	66.0	-0.37	-1.29	PPA-AAA	0.10	11	0.861	0.984	0.4132
222279_at	424.6	329.5	-0.37	-1.29	PPP-PPP	0.10	11	0.861	0.984	0.4938
214688_at	113.0	87.7	-0.37	-1.29	PPP-PPP	0.10	11	0.861	0.984	0.5066
230035_at	68.4	53.1	-0.37	-1.29	AAA-APA	0.10	11	0.861	0.984	0.5367
208595_s_at	368.5	474.8	0.37	1.29	AAA-PAP	0.10	11	0.861	0.984	0.0024
209030_s_at	496.0	385.1	-0.37	-1.29	PPP-PPP	0.10	11	0.861	0.984	0.2635
204187_at	187.0	145.3	-0.37	-1.29	AAP-PPP	0.10	11	0.861	0.984	0.3077
1554869_at	114.1	88.6	-0.37	-1.29	APP-PPA	0.10	11	0.861	0.984	0.3362
210656_at	80.6	62.6	-0.37	-1.29	AAP-APP	0.10	11	0.861	0.984	0.4028
233511_at	151.8	117.9	-0.37	-1.29	PPM-PPP	0.10	11	0.861	0.984	0.4378
210598_at	90.5	70.3	-0.36	-1.29	MPP-PPP	0.10	11	0.861	0.984	0.2327

226439_s_at	99.7	77.5	-0.36	-1.29	PPP-PPP	0.10	11	0.861	0.984	0.3193
214617_at	106.0	82.4	-0.36	-1.29	AAA-APA	0.10	11	0.861	0.984	0.4210
1553386_at	88.6	68.8	-0.36	-1.29	AMA-PPA	0.10	11	0.861	0.984	0.5157
222988_s_at	2122.3	2730.6	0.36	1.29	PPP-PPP	0.10	11	0.861	0.984	0.0049
235144_at	65.9	51.2	-0.36	-1.29	PPP-APP	0.10	11	0.861	0.984	0.1965
237619_at	78.3	60.9	-0.36	-1.29	PPA-APP	0.10	11	0.861	0.984	0.2384
1564344_at	55.8	43.4	-0.36	-1.29	AAP-AAM	0.10	11	0.861	0.984	0.3053
1554795_a_at	804.1	625.2	-0.36	-1.29	PPP-PPP	0.10	11	0.861	0.984	0.3730
225067_at	465.5	362.0	-0.36	-1.29	PPP-PPP	0.10	11	0.861	0.984	0.3816
212805_at	261.4	203.4	-0.36	-1.29	PPP-PPP	0.10	11	0.861	0.984	0.3966
213971_s_at	1351.7	1051.3	-0.36	-1.29	PPP-PPP	0.10	11	0.861	0.984	0.4188
1566739_at	87.2	67.8	-0.36	-1.29	PPA-PAP	0.10	11	0.861	0.984	0.5226
203685_at	122.3	95.2	-0.36	-1.29	PPP-PPP	0.10	11	0.861	0.984	0.2329
227700_x_at	1494.9	1163.2	-0.36	-1.29	PPP-PPP	0.10	11	0.861	0.984	0.2786
236607_at	92.1	71.7	-0.36	-1.29	PPP-PPP	0.10	11	0.861	0.984	0.2905
213490_s_at	416.8	324.2	-0.36	-1.29	PPP-PPP	0.10	11	0.861	0.984	0.2922
1555336_a_at	77.1	60.0	-0.36	-1.29	AAA-AAP	0.10	11	0.861	0.984	0.3070
1556201_at	70.4	54.8	-0.36	-1.29	MPP-APP	0.10	11	0.861	0.984	0.3559
235559_at	161.1	125.3	-0.36	-1.29	PPP-PPP	0.10	11	0.861	0.984	0.3803
229694_at	210.1	163.5	-0.36	-1.29	PPA-PPP	0.10	11	0.861	0.984	0.3929
224522_s_at	213.1	165.8	-0.36	-1.29	PAA-AAA	0.10	11	0.861	0.984	0.4810
231969_at	39.9	31.1	-0.36	-1.29	PMA-AAP	0.10	11	0.861	0.984	0.4919
236059_at	83.6	65.0	-0.36	-1.29	PPP-MPP	0.10	11	0.861	0.984	0.5061
37079_at	303.4	390.0	0.36	1.29	PPP-PPP	0.10	11	0.861	0.984	0.0040
1557630_s_at	97.3	75.8	-0.36	-1.28	AAP-APA	0.10	11	0.861	0.984	0.2022
228020_at	258.7	201.4	-0.36	-1.29	PPP-PPP	0.10	11	0.861	0.984	0.2315
200939_s_at	167.3	130.3	-0.36	-1.28	PPP-PPP	0.10	11	0.861	0.984	0.2326
206689_x_at	581.5	452.6	-0.36	-1.29	PPP-PPP	0.10	11	0.861	0.984	0.2715
219849_at	106.6	83.0	-0.36	-1.28	AAA-MAP	0.10	11	0.861	0.984	0.3296
207375_s_at	182.3	141.9	-0.36	-1.28	AAA-PAA	0.10	11	0.861	0.984	0.4089
215006_at	127.5	99.3	-0.36	-1.29	AMP-PAP	0.10	11	0.861	0.984	0.4333
229144_at	182.0	141.7	-0.36	-1.28	PPM-PAP	0.10	11	0.861	0.984	0.4521
202709_at	92.8	72.3	-0.36	-1.28	PPA-APP	0.10	11	0.861	0.984	0.5093
229029_at	283.0	220.6	-0.36	-1.28	PPP-PPP	0.10	11	0.861	0.984	0.1899
214216_s_at	170.4	132.8	-0.36	-1.28	APP-APP	0.10	11	0.861	0.984	0.2057
213098_at	627.7	489.2	-0.36	-1.28	APP-PPP	0.10	11	0.861	0.984	0.2128
229238_at	351.6	274.1	-0.36	-1.28	APP-PAA	0.10	11	0.861	0.984	0.2579
222366_at	232.3	181.0	-0.36	-1.28	PPP-PPP	0.10	11	0.861	0.984	0.2942
237264_at	52.2	40.7	-0.36	-1.28	APA-AAA	0.10	11	0.861	0.984	0.4265
1563874_at	49.2	38.3	-0.36	-1.28	AAA-AAP	0.10	11	0.861	0.984	0.4597
237023_at	108.9	84.9	-0.36	-1.28	AAA-APA	0.10	11	0.861	0.984	0.4830
231233_at	106.4	82.9	-0.36	-1.28	AAA-PAP	0.10	11	0.861	0.984	0.5273

36920_at	160.3	205.7	0.36	1.28	PPP-PPP	0.10	11	0.861	0.984	0.0030
219403_s_at	122.0	95.1	-0.36	-1.28	PAP-AMA	0.10	11	0.861	0.984	0.1784
242362_at	199.7	155.7	-0.36	-1.28	PPP-PPP	0.10	11	0.861	0.984	0.5229
223884_at	108.4	84.6	-0.36	-1.28	AAM-PPP	0.10	11	0.861	0.984	0.1674
1556582_at	925.8	722.1	-0.36	-1.28	PPP-PPP	0.10	11	0.861	0.984	0.1940
219855_at	533.0	415.7	-0.36	-1.28	PPP-PPP	0.10	11	0.861	0.984	0.2401
233575_s_at	612.9	478.4	-0.36	-1.28	PPP-PPP	0.10	11	0.861	0.984	0.2600
228482_at	160.2	125.0	-0.36	-1.28	PPP-PPP	0.10	11	0.861	0.984	0.3046
244699_at	140.6	109.7	-0.36	-1.28	AAP-AAA	0.10	11	0.861	0.984	0.3295
241467_at	170.1	132.7	-0.36	-1.28	APM-PPA	0.10	11	0.861	0.984	0.3446
238295_at	106.3	82.9	-0.36	-1.28	AMP-PPA	0.10	11	0.861	0.984	0.3727
216698_x_at	186.4	145.4	-0.36	-1.28	APP-APP	0.10	11	0.861	0.984	0.3885
220957_at	39.5	30.8	-0.36	-1.28	APA-APA	0.10	11	0.861	0.984	0.5205
218892_at	99.7	77.8	-0.36	-1.28	AAP-AAM	0.10	11	0.861	0.984	0.5318
233757_x_at	658.2	514.0	-0.36	-1.28	AMP-PPA	0.10	11	0.861	0.984	0.1905
207160_at	117.7	91.9	-0.36	-1.28	PPP-PPP	0.10	11	0.861	0.984	0.2564
41856_at	163.8	127.9	-0.36	-1.28	PPP-PPP	0.10	11	0.861	0.984	0.3291
236111_at	32.3	25.2	-0.36	-1.28	AAA-APA	0.10	11	0.861	0.984	0.4438
222612_at	157.8	123.2	-0.36	-1.28	PPP-PPP	0.10	11	0.861	0.984	0.4614
236976_at	181.5	141.7	-0.36	-1.28	AMP-MAA	0.10	11	0.861	0.984	0.1722
34846_at	213.7	166.9	-0.36	-1.28	PMP-PAP	0.10	11	0.861	0.984	0.1853
236247_at	256.6	200.5	-0.36	-1.28	PPP-PPP	0.10	11	0.861	0.984	0.2793
234135_x_at	199.2	155.7	-0.36	-1.28	PPP-APP	0.10	11	0.861	0.984	0.3006
237034_at	404.7	316.2	-0.36	-1.28	PPP-PPP	0.10	11	0.861	0.984	0.3020
216056_at	132.4	103.4	-0.36	-1.28	APP-MMA	0.10	11	0.861	0.984	0.3083
205215_at	145.5	113.7	-0.36	-1.28	PAA-PPM	0.10	11	0.861	0.984	0.3660
233891_at	93.0	72.7	-0.36	-1.28	APA-MAP	0.10	11	0.861	0.984	0.4606
226647_at	136.6	106.7	-0.36	-1.28	PPA-AAA	0.10	11	0.861	0.984	0.4787
215207_x_at	544.2	696.3	0.36	1.28	PPP-PPP	0.10	11	0.861	0.984	0.0018
233866_at	161.1	126.0	-0.36	-1.28	PPP-PPP	0.10	11	0.861	0.984	0.1645
210435_at	49.9	39.0	-0.36	-1.28	AAA-AAP	0.10	11	0.861	0.984	0.2146
224513_s_at	1359.7	1063.1	-0.36	-1.28	PPP-PPP	0.10	11	0.861	0.984	0.2172
1552303_a_at	165.9	129.7	-0.36	-1.28	APA-APP	0.10	11	0.861	0.984	0.2374
215588_x_at	708.3	553.7	-0.36	-1.28	PPP-PPP	0.10	11	0.861	0.984	0.3339
241935_at	170.4	133.2	-0.36	-1.28	PPP-PPP	0.10	11	0.861	0.984	0.4770
206184_at	219.1	171.5	-0.35	-1.28	PPP-PPP	0.10	11	0.861	0.984	0.2314
227535_at	109.9	86.0	-0.35	-1.28	PPP-PPP	0.10	11	0.861	0.984	0.2777
213981_at	291.8	228.3	-0.35	-1.28	APP-PPP	0.10	11	0.861	0.984	0.2845
239071_at	219.7	171.9	-0.35	-1.28	AAP-AAA	0.10	11	0.861	0.984	0.3564
223537_s_at	786.6	615.4	-0.35	-1.28	PPP-PPP	0.10	11	0.861	0.984	0.3838
243868_at	96.5	75.5	-0.35	-1.28	AAP-PPP	0.10	11	0.861	0.984	0.5101
1553155_x_at	85.9	67.3	-0.35	-1.28	APP-PPP	0.10	11	0.861	0.984	0.1660

1559401_a_at	99.8	78.1	-0.35	-1.28	AAP-AAP	0.10	11	0.861	0.984	0.1795
212730_at	343.1	268.7	-0.35	-1.28	PPP-PPP	0.10	11	0.861	0.984	0.2265
212146_at	828.8	648.9	-0.35	-1.28	PPP-PPP	0.10	11	0.861	0.984	0.2329
227470_at	427.1	334.3	-0.35	-1.28	PPP-PAP	0.10	11	0.861	0.984	0.3461
1556984_at	124.8	97.7	-0.35	-1.28	AAM-AAP	0.10	11	0.861	0.984	0.3828
232702_at	94.4	73.9	-0.35	-1.28	APA-AAP	0.10	11	0.861	0.984	0.4586
210733_at	472.2	369.8	-0.35	-1.28	PPP-PPP	0.10	11	0.861	0.984	0.4657
234440_at	57.6	45.1	-0.35	-1.28	APA-APA	0.10	11	0.861	0.984	0.4897
1553181_at	87.0	68.1	-0.35	-1.28	PPP-PPP	0.10	11	0.861	0.984	0.5269
81811_at	196.5	153.9	-0.35	-1.28	PPP-PPP	0.10	11	0.861	0.984	0.1856
221964_at	461.5	361.6	-0.35	-1.28	PPP-PPP	0.10	11	0.861	0.984	0.3778
1554558_at	53.0	41.6	-0.35	-1.28	PAP-PPA	0.10	11	0.861	0.984	0.3812
236404_at	97.2	76.2	-0.35	-1.28	PPA-APP	0.10	11	0.861	0.984	0.4403
1554621_at	44.1	34.6	-0.35	-1.28	APA-APA	0.10	11	0.861	0.984	0.1692
226783_at	148.2	116.2	-0.35	-1.28	APP-PPP	0.10	11	0.861	0.984	0.2013
213138_at	212.0	166.3	-0.35	-1.28	PAA-PPA	0.10	11	0.861	0.984	0.2208
214355_x_at	248.1	194.5	-0.35	-1.28	AAA-APM	0.10	11	0.861	0.984	0.2689
236386_at	105.2	82.5	-0.35	-1.28	PAP-PPP	0.10	11	0.861	0.984	0.2893
242673_at	223.0	174.8	-0.35	-1.28	PPP-PPP	0.10	11	0.861	0.984	0.3021
205231_s_at	213.1	167.1	-0.35	-1.28	PPP-PPP	0.10	11	0.861	0.984	0.3269
229914_at	146.6	114.9	-0.35	-1.28	AAA-APP	0.10	11	0.861	0.984	0.3291
212858_at	347.7	272.7	-0.35	-1.28	PPP-PPP	0.10	11	0.861	0.984	0.3613
236797_at	53.8	42.2	-0.35	-1.28	PMA-AAA	0.10	11	0.861	0.984	0.4185
240482_at	131.0	102.8	-0.35	-1.28	PPA-PPP	0.10	11	0.861	0.984	0.4567
233943_x_at	176.4	138.3	-0.35	-1.28	PPP-PPP	0.10	11	0.861	0.984	0.4690
226055_at	174.8	137.1	-0.35	-1.28	APP-PAP	0.10	11	0.861	0.984	0.5089
229427_at	371.3	291.4	-0.35	-1.27	PPP-PPP	0.10	11	0.861	0.984	0.1589
220856_x_at	4452.7	3494.0	-0.35	-1.27	PPP-PPP	0.10	11	0.861	0.984	0.1941
220352_x_at	462.5	362.8	-0.35	-1.28	PPP-PPP	0.10	11	0.861	0.984	0.2519
212289_at	281.0	358.2	0.35	1.28	PPP-PPP	0.10	11	0.861	0.984	0.0040
212052_s_at	1644.9	1291.2	-0.35	-1.27	PPP-PPP	0.10	11	0.861	0.984	0.1588
207730_x_at	1321.2	1037.3	-0.35	-1.27	PPP-PPP	0.10	11	0.861	0.984	0.1796
221800_s_at	596.1	468.0	-0.35	-1.27	PPP-PPP	0.10	11	0.861	0.984	0.2331
201937_s_at	767.6	602.8	-0.35	-1.27	PPP-PPP	0.10	11	0.861	0.984	0.2838
223416_at	15484.5	12154.3	-0.35	-1.27	PPP-PPP	0.10	11	0.861	0.984	0.3060
208779_x_at	652.4	512.3	-0.35	-1.27	PPP-PPP	0.10	11	0.861	0.984	0.3204
243492_at	271.4	213.0	-0.35	-1.27	PPP-PPP	0.10	11	0.861	0.984	0.4239
1554444_s_at	234.3	183.9	-0.35	-1.27	AAA-APA	0.10	11	0.861	0.984	0.5231
210261_at	186.6	146.6	-0.35	-1.27	PPP-PPP	0.10	11	0.861	0.984	0.1611
233065_at	217.3	170.8	-0.35	-1.27	PPP-PPP	0.10	11	0.861	0.984	0.1986
1558682_at	170.3	133.8	-0.35	-1.27	PPP-PPP	0.10	11	0.861	0.984	0.4676
214001_x_at	291.1	228.9	-0.35	-1.27	PPP-PPP	0.10	11	0.861	0.984	0.2155

38918_at	166.0	130.5	-0.35	-1.27	PPP-PPP	0.10	11	0.861	0.984	0.2394
205730_s_at	321.3	252.7	-0.35	-1.27	PPP-PPP	0.10	11	0.861	0.984	0.3121
230565_at	49.2	38.7	-0.35	-1.27	AAP-AAA	0.10	11	0.861	0.984	0.3503
242480_at	65.8	51.7	-0.35	-1.27	PPA-PPP	0.10	11	0.861	0.984	0.3921
1556706_at	58.8	46.2	-0.35	-1.27	AAP-PAP	0.10	11	0.861	0.984	0.4410
236458_at	149.7	117.8	-0.35	-1.27	AAA-AAP	0.10	11	0.861	0.984	0.2500
219082_at	323.9	254.9	-0.35	-1.27	AAP-AAP	0.10	11	0.861	0.984	0.2775
228000_at	275.3	216.6	-0.35	-1.27	PPM-MPP	0.10	11	0.861	0.984	0.2784
239232_at	98.8	77.7	-0.35	-1.27	PAP-APP	0.10	11	0.861	0.984	0.3620
227884_at	352.0	276.9	-0.35	-1.27	PPP-PPP	0.10	11	0.861	0.984	0.4498
1569077_x_at	139.9	110.1	-0.35	-1.27	PPA-AAA	0.10	11	0.861	0.984	0.4521
212172_at	182.7	143.9	-0.35	-1.27	PPP-PPP	0.10	11	0.861	0.984	0.1598
218554_s_at	183.6	144.5	-0.35	-1.27	PPP-PPP	0.10	11	0.861	0.984	0.3177
209083_at	154.5	121.6	-0.35	-1.27	AAP-AAP	0.10	11	0.861	0.984	0.3958
238322_s_at	158.1	124.5	-0.35	-1.27	PPP-PPP	0.10	11	0.861	0.984	0.5247
1557945_at	140.9	111.1	-0.34	-1.27	MPP-PPP	0.10	11	0.861	0.984	0.2231
242007_at	48.0	37.8	-0.34	-1.27	PAA-APP	0.10	11	0.861	0.984	0.2331
203297_s_at	1056.0	832.1	-0.34	-1.27	PPP-PPP	0.10	11	0.861	0.984	0.2620
1566110_at	119.9	94.5	-0.34	-1.27	PAA-PPA	0.10	11	0.861	0.984	0.3958
215029_at	171.4	135.0	-0.34	-1.27	PPP-PPP	0.10	11	0.861	0.984	0.4147
238142_at	168.1	132.5	-0.34	-1.27	PPP-PPP	0.10	11	0.861	0.984	0.4782
230481_at	63.8	50.3	-0.34	-1.27	AAP-AAP	0.10	11	0.861	0.984	0.1679
242304_at	245.0	193.2	-0.34	-1.27	PPP-PPP	0.10	11	0.861	0.984	0.2240
1558148_x_at	74.6	58.8	-0.34	-1.27	AAA-AAP	0.10	11	0.861	0.984	0.2698
1555198_x_at	77.6	61.1	-0.34	-1.27	PAA-AAA	0.10	11	0.861	0.984	0.2994
204720_s_at	480.9	379.1	-0.34	-1.27	PPP-PPP	0.10	11	0.861	0.984	0.3945
1556442_x_at	970.8	765.1	-0.34	-1.27	PPP-PPP	0.10	11	0.861	0.984	0.5106
229240_at	100.5	79.2	-0.34	-1.27	PPA-PPP	0.10	11	0.861	0.984	0.5140
229951_x_at	154.7	122.0	-0.34	-1.27	PPP-PPP	0.10	11	0.861	0.984	0.5315
220841_s_at	463.5	365.6	-0.34	-1.27	PPP-PPP	0.10	11	0.861	0.984	0.3050
233182_x_at	89.8	70.8	-0.34	-1.27	PPP-PPP	0.10	11	0.861	0.984	0.3710
1554274_a_at	146.4	115.5	-0.34	-1.27	APP-PPP	0.10	11	0.861	0.984	0.3745
210290_at	267.8	211.3	-0.34	-1.27	PPP-PPP	0.10	11	0.861	0.984	0.4311
209640_at	69.8	55.1	-0.34	-1.27	AAA-APP	0.10	11	0.861	0.984	0.4357
241756_at	172.6	136.1	-0.34	-1.27	PAP-MPP	0.10	11	0.861	0.984	0.4376
211256_x_at	655.6	517.3	-0.34	-1.27	PPP-PPP	0.10	11	0.861	0.984	0.4406
203892_at	138.8	109.5	-0.34	-1.27	APP-PPP	0.10	11	0.861	0.984	0.4808
203617_x_at	929.3	733.7	-0.34	-1.27	PPP-PPP	0.10	11	0.861	0.984	0.1747
215198_s_at	181.7	143.4	-0.34	-1.27	PPP-APP	0.10	11	0.861	0.984	0.2506
219018_s_at	395.1	311.9	-0.34	-1.27	AMA-APM	0.10	11	0.861	0.984	0.2695
242450_at	501.4	395.8	-0.34	-1.27	PPP-PPP	0.10	11	0.861	0.984	0.3126
242573_at	77.6	61.3	-0.34	-1.27	APP-PPP	0.10	11	0.861	0.984	0.3375

224907_s_at	179.2	141.5	-0.34	-1.27	PAP-MAP	0.10	11	0.861	0.984	0.4127
215281_x_at	99.4	78.5	-0.34	-1.27	PPP-PPP	0.10	11	0.861	0.984	0.4841
216278_at	34.6	27.3	-0.34	-1.27	AAP-AAA	0.10	11	0.861	0.984	0.5058
206129_s_at	222.6	175.8	-0.34	-1.27	PPP-PPP	0.10	11	0.861	0.984	0.1643
223241_at	1214.8	959.8	-0.34	-1.27	PPP-PPP	0.10	11	0.861	0.984	0.1721
244752_at	97.3	76.8	-0.34	-1.27	AAA-APA	0.10	11	0.861	0.984	0.1821
206996_x_at	122.7	96.9	-0.34	-1.27	APP-AAA	0.10	11	0.861	0.984	0.1977
1562378_s_at	373.0	294.6	-0.34	-1.27	PPP-PPA	0.10	11	0.861	0.984	0.2132
244552_at	176.2	139.3	-0.34	-1.27	PPM-PPP	0.10	11	0.861	0.984	0.2146
218613_at	602.2	475.9	-0.34	-1.27	PPP-PPP	0.10	11	0.861	0.984	0.2194
201905_s_at	332.6	262.8	-0.34	-1.27	PMP-PPP	0.10	11	0.861	0.984	0.2343
203035_s_at	601.2	474.9	-0.34	-1.27	PPP-PPP	0.10	11	0.861	0.984	0.2486
215070_x_at	85.0	67.1	-0.34	-1.27	APA-PAA	0.10	11	0.861	0.984	0.2703
237747_at	150.4	118.8	-0.34	-1.27	APP-PPP	0.10	11	0.861	0.984	0.2937
1557238_s_at	89.2	70.5	-0.34	-1.27	PPP-PPP	0.10	11	0.861	0.984	0.2940
219268_at	117.5	92.8	-0.34	-1.27	PPP-APP	0.10	11	0.861	0.984	0.3874
1566403_at	70.0	55.3	-0.34	-1.27	APP-MPP	0.10	11	0.861	0.984	0.4312
233419_at	63.5	50.2	-0.34	-1.27	AAP-PPP	0.10	11	0.861	0.984	0.4518
239603_x_at	132.6	104.7	-0.34	-1.27	PAP-PPP	0.10	11	0.861	0.984	0.4982
235525_at	83.0	65.5	-0.34	-1.27	APA-AAA	0.10	11	0.861	0.984	0.5326
217191_x_at	254.5	201.2	-0.34	-1.27	PPP-PPP	0.10	11	0.861	0.984	0.1613
204014_at	298.1	235.6	-0.34	-1.27	PPP-PPP	0.10	11	0.861	0.984	0.2410
217189_s_at	239.7	189.5	-0.34	-1.26	PPP-PPP	0.10	11	0.861	0.984	0.2427
235926_at	234.1	185.0	-0.34	-1.27	PPP-PPP	0.10	11	0.861	0.984	0.2507
241797_at	87.9	69.5	-0.34	-1.27	AMA-APA	0.10	11	0.861	0.984	0.3292
219874_at	296.7	234.6	-0.34	-1.27	PPP-PPP	0.10	11	0.861	0.984	0.3302
213596_at	137.8	108.9	-0.34	-1.27	APP-PAP	0.10	11	0.861	0.984	0.3450
242024_at	158.8	125.5	-0.34	-1.27	AAP-AAP	0.10	11	0.861	0.984	0.4082
204745_x_at	5221.8	4131.2	-0.34	-1.26	PPP-PPP	0.10	11	0.861	0.984	0.1711
211026_s_at	2573.3	2035.3	-0.34	-1.26	PPP-PPP	0.10	11	0.861	0.984	0.1750
232775_at	119.9	94.8	-0.34	-1.26	PAA-AAM	0.10	11	0.861	0.984	0.3330
203426_s_at	180.0	142.4	-0.34	-1.26	APP-PPP	0.10	11	0.861	0.984	0.3435
214587_at	80.4	63.6	-0.34	-1.26	PPP-PPP	0.10	11	0.861	0.984	0.3988
205741_s_at	69.9	55.3	-0.34	-1.26	PAP-PAP	0.10	11	0.861	0.984	0.4365
241630_at	80.9	64.0	-0.34	-1.26	APA-APA	0.10	11	0.861	0.984	0.4423
1558706_a_at	93.4	73.9	-0.34	-1.26	PPP-PAP	0.10	11	0.861	0.984	0.4636
230208_at	93.3	73.8	-0.34	-1.26	AAA-PAA	0.10	11	0.861	0.984	0.4866
206053_at	243.9	193.1	-0.34	-1.26	PPP-PPP	0.10	11	0.861	0.984	0.1703
208447_s_at	3778.8	2990.8	-0.34	-1.26	PPP-PPP	0.10	11	0.861	0.984	0.1715
237469_at	122.3	96.8	-0.34	-1.26	PPP-PPP	0.10	11	0.861	0.984	0.2756
242474_s_at	55.3	43.7	-0.34	-1.26	AAA-APP	0.10	11	0.861	0.984	0.3588
211433_x_at	605.7	479.4	-0.34	-1.26	PPP-PPP	0.10	11	0.861	0.984	0.3709

1553205_at	242.3	191.8	-0.34	-1.26	PPP-PPM	0.10	11	0.861	0.984	0.3841
229001_at	151.7	120.0	-0.34	-1.26	APP-PPP	0.10	11	0.861	0.984	0.4278
222941_at	106.0	83.9	-0.34	-1.26	AAP-APP	0.10	11	0.861	0.984	0.4594
219794_at	53.7	42.5	-0.34	-1.26	AAA-AAP	0.10	11	0.861	0.984	0.4980
225864_at	393.0	311.0	-0.34	-1.26	PPP-PPP	0.10	11	0.861	0.984	0.5253
225255_at	227.2	180.0	-0.34	-1.26	PPP-PPP	0.10	11	0.861	0.984	0.2680
226410_at	691.7	548.2	-0.34	-1.26	PPP-PPP	0.10	11	0.861	0.984	0.3070
226171_at	151.7	120.2	-0.34	-1.26	APP-PPP	0.10	11	0.861	0.984	0.3216
204475_at	34288.9	27179.2	-0.34	-1.26	PPP-PPP	0.10	11	0.861	0.984	0.3537
218833_at	191.5	151.8	-0.34	-1.26	PPP-PPP	0.10	11	0.861	0.984	0.4802
234560_at	81.8	64.9	-0.34	-1.26	AAA-AAP	0.10	11	0.861	0.984	0.5268
233467_s_at	79.0	62.6	-0.33	-1.26	AAP-APA	0.10	11	0.861	0.984	0.1797
239218_at	739.8	587.0	-0.33	-1.26	PPP-PPP	0.10	11	0.861	0.984	0.2239
208106_x_at	361.2	286.5	-0.33	-1.26	APP-AAP	0.10	11	0.861	0.984	0.2973
203848_at	841.4	667.3	-0.33	-1.26	PPP-PPP	0.10	11	0.861	0.984	0.3154
235450_at	108.6	86.1	-0.33	-1.26	PMP-PPP	0.10	11	0.861	0.984	0.4443
221978_at	53.4	42.4	-0.33	-1.26	AAA-AAP	0.10	11	0.861	0.984	0.1662
228843_at	237.4	188.5	-0.33	-1.26	PPP-PPP	0.10	11	0.861	0.984	0.1723
201926_s_at	1406.7	1117.1	-0.33	-1.26	PPP-PPP	0.10	11	0.861	0.984	0.2015
1560680_at	60.1	47.7	-0.33	-1.26	APA-AMP	0.10	11	0.861	0.984	0.2585
204948_s_at	2836.5	2251.2	-0.33	-1.26	PPP-PPP	0.10	11	0.861	0.984	0.2990
229784_at	269.9	214.4	-0.33	-1.26	APP-AAP	0.10	11	0.861	0.984	0.3135
1552457_a_at	111.1	88.2	-0.33	-1.26	APP-PPP	0.10	11	0.861	0.984	0.3173
203083_at	5824.9	4625.6	-0.33	-1.26	PPP-PPP	0.10	11	0.861	0.984	0.3284
1553186_x_at	629.1	499.3	-0.33	-1.26	PPP-PPP	0.10	11	0.861	0.984	0.3537
232055_at	187.2	148.7	-0.33	-1.26	PPP-PPP	0.10	11	0.861	0.984	0.3590
235349_at	159.3	126.5	-0.33	-1.26	PPP-PPP	0.10	11	0.861	0.984	0.4174
236862_at	84.4	67.0	-0.33	-1.26	PAA-APP	0.10	11	0.861	0.984	0.4325
212549_at	719.5	571.3	-0.33	-1.26	PPP-PPP	0.10	11	0.861	0.984	0.4596
243805_at	850.3	675.6	-0.33	-1.26	PPP-PPP	0.10	11	0.861	0.984	0.1626
1566785_x_at	226.3	179.8	-0.33	-1.26	PPP-PPP	0.10	11	0.861	0.984	0.2003
207365_x_at	1092.2	867.9	-0.33	-1.26	PPP-PPP	0.10	11	0.861	0.984	0.3058
221326_s_at	205.0	162.8	-0.33	-1.26	PPP-PPP	0.10	11	0.861	0.984	0.3094
221211_s_at	1463.4	1162.4	-0.33	-1.26	PPP-PPP	0.10	11	0.861	0.984	0.4121
238506_at	289.6	364.6	0.33	1.26	PPP-PPP	0.10	11	0.861	0.984	0.0001
227037_at	725.8	577.1	-0.33	-1.26	PPP-PPP	0.10	11	0.861	0.984	0.1761
229014_at	1140.1	906.4	-0.33	-1.26	PPP-PPP	0.10	11	0.861	0.984	0.2127
211417_x_at	190.8	151.7	-0.33	-1.26	AAM-PPA	0.10	11	0.861	0.984	0.2168
242511_at	118.0	93.8	-0.33	-1.26	PMP-PPP	0.10	11	0.861	0.984	0.2185
221011_s_at	380.1	302.3	-0.33	-1.26	PPP-PPP	0.10	11	0.861	0.984	0.3006
214967_at	141.4	112.4	-0.33	-1.26	PPP-PPP	0.10	11	0.861	0.984	0.3077
225078_at	216.0	171.8	-0.33	-1.26	PPP-PPP	0.10	11	0.861	0.984	0.3330

217665_at	120.7	96.0	-0.33	-1.26	PPP-PPP	0.10	11	0.861	0.984	0.3332
228147_at	58.6	46.6	-0.33	-1.26	PAP-AMP	0.10	11	0.861	0.984	0.4236
214474_at	128.1	101.9	-0.33	-1.26	PPM-PPP	0.10	11	0.861	0.984	0.4516
206438_x_at	477.4	379.4	-0.33	-1.26	AMP-PMP	0.10	11	0.861	0.984	0.4543
232881_at	76.0	60.4	-0.33	-1.26	PAP-APP	0.10	11	0.861	0.984	0.5189
220048_at	132.1	105.0	-0.33	-1.26	AAP-AAA	0.10	11	0.861	0.984	0.5196
226599_at	240.0	190.9	-0.33	-1.26	PPP-PPP	0.10	11	0.861	0.984	0.1537
1552712_a_at	431.2	343.1	-0.33	-1.26	PPP-PPP	0.10	11	0.861	0.984	0.1608
235623_at	209.6	166.7	-0.33	-1.26	APP-PPA	0.10	11	0.861	0.984	0.3325
233005_at	120.6	96.0	-0.33	-1.26	PPP-PPP	0.10	11	0.861	0.984	0.3515
209006_s_at	486.2	386.8	-0.33	-1.26	PPP-PPP	0.10	11	0.861	0.984	0.3870
234032_at	223.2	177.6	-0.33	-1.26	PPP-PPP	0.10	11	0.861	0.984	0.3890
1553684_at	66.7	53.1	-0.33	-1.26	APM-PPA	0.10	11	0.861	0.984	0.4214
229514_at	147.0	117.0	-0.33	-1.26	PPA-APP	0.10	11	0.861	0.984	0.4588
240798_at	133.5	106.2	-0.33	-1.26	PPP-PPP	0.10	11	0.861	0.984	0.4900
225605_at	144.4	114.8	-0.33	-1.26	PPP-PPP	0.10	11	0.861	0.984	0.4989
1569578_at	64.6	51.4	-0.33	-1.26	AAA-AAP	0.10	11	0.861	0.984	0.5042
215206_at	312.7	248.9	-0.33	-1.26	MPP-MPP	0.10	11	0.861	0.984	0.1615
233380_s_at	637.9	507.8	-0.33	-1.26	PPP-PPP	0.10	11	0.861	0.984	0.2090
219879_s_at	303.1	241.4	-0.33	-1.26	PPP-PPP	0.10	11	0.861	0.984	0.2442
224980_at	345.5	275.0	-0.33	-1.26	PPP-PPP	0.10	11	0.861	0.984	0.2831
234315_at	140.7	112.0	-0.33	-1.26	PPA-PPP	0.10	11	0.861	0.984	0.2910
210942_s_at	282.0	224.5	-0.33	-1.26	PPP-PPP	0.10	11	0.861	0.984	0.3601
207416_s_at	320.4	255.1	-0.33	-1.26	PPM-PPP	0.10	11	0.861	0.984	0.3769
237681_at	104.1	82.9	-0.33	-1.26	PAA-AAA	0.10	11	0.861	0.984	0.4292
237502_at	70.2	55.9	-0.33	-1.26	PMA-APP	0.10	11	0.861	0.984	0.4434
227748_at	672.4	535.4	-0.33	-1.26	PPP-PPP	0.10	11	0.861	0.984	0.5131
239498_at	107.4	85.5	-0.33	-1.26	APP-AAP	0.10	11	0.861	0.984	0.5169
213254_at	383.0	481.0	0.33	1.26	PPP-PPP	0.10	11	0.861	0.984	0.0010
240277_at	789.2	628.7	-0.33	-1.26	PPP-PPP	0.10	11	0.861	0.984	0.1596
213642_at	611.3	486.9	-0.33	-1.26	PPP-PPP	0.10	11	0.861	0.984	0.2056
214164_x_at	1010.0	804.9	-0.33	-1.26	PPP-PPP	0.10	11	0.861	0.984	0.2219
207564_x_at	582.0	463.8	-0.33	-1.26	PPP-PPP	0.10	11	0.861	0.984	0.2369
230906_at	345.3	275.1	-0.33	-1.26	PPM-MPP	0.10	11	0.861	0.984	0.2723
225056_at	130.6	104.0	-0.33	-1.26	PAP-AAM	0.10	11	0.861	0.984	0.2761
226093_at	659.6	525.5	-0.33	-1.26	PPP-PPP	0.10	11	0.861	0.984	0.2913
239528_at	84.9	67.7	-0.33	-1.26	PAA-MAA	0.10	11	0.861	0.984	0.3947
232022_at	87.3	69.5	-0.33	-1.26	PPP-APP	0.10	11	0.861	0.984	0.4237
220446_s_at	72.3	57.6	-0.33	-1.26	AAP-PPM	0.10	11	0.861	0.984	0.4250
1552258_at	130.8	104.2	-0.33	-1.26	APM-APA	0.10	11	0.861	0.984	0.5252
232266_x_at	3086.4	2461.1	-0.33	-1.25	PPP-PPP	0.10	11	0.861	0.984	0.1468
205996_s_at	1894.5	1510.0	-0.33	-1.26	PPP-PPP	0.10	11	0.861	0.984	0.1774

216242_x_at	763.6	608.6	-0.33	-1.26	PPP-PPP	0.10	11	0.861	0.984	0.1948
1555883_s_at	204.8	163.2	-0.33	-1.25	PPA-APP	0.10	11	0.861	0.984	0.2626
213046_at	488.2	389.1	-0.33	-1.26	PPP-PPP	0.10	11	0.861	0.984	0.2942
218711_s_at	147.1	117.2	-0.33	-1.25	PPP-PPP	0.10	11	0.861	0.984	0.3213
209147_s_at	782.0	623.5	-0.33	-1.25	PPP-PPP	0.10	11	0.861	0.984	0.3282
215545_at	140.3	111.8	-0.33	-1.26	PPP-PPP	0.10	11	0.861	0.984	0.3399
202016_at	15139.5	12067.0	-0.33	-1.26	PPP-PPP	0.10	11	0.861	0.984	0.4117
219766_at	207.7	165.6	-0.33	-1.25	PPP-PAM	0.10	11	0.861	0.984	0.4398
219348_at	348.2	277.5	-0.33	-1.25	PPP-PPP	0.10	11	0.861	0.984	0.4471
203131_at	1199.1	955.9	-0.33	-1.25	PPP-PPP	0.10	11	0.861	0.984	0.4602
1563272_at	77.9	62.1	-0.33	-1.25	PPP-AAM	0.10	11	0.861	0.984	0.4664
219563_at	120.7	96.2	-0.33	-1.25	APP-PPP	0.10	11	0.861	0.984	0.4958
1558695_at	219.3	174.9	-0.33	-1.25	PPP-PPP	0.10	11	0.861	0.984	0.1584
203323_at	2432.6	1940.6	-0.33	-1.25	PPP-PPP	0.10	11	0.861	0.984	0.1671
235234_at	598.5	477.3	-0.33	-1.25	PPP-PPP	0.10	11	0.861	0.984	0.1702
1555913_at	363.8	290.3	-0.33	-1.25	APP-PPP	0.10	11	0.861	0.984	0.2198
212839_s_at	146.4	116.8	-0.33	-1.25	PPP-PPP	0.10	11	0.861	0.984	0.2926
1559331_x_at	67.8	54.1	-0.33	-1.25	MAA-APA	0.10	11	0.861	0.984	0.2931
226461_at	202.5	161.5	-0.33	-1.25	PPP-PPP	0.10	11	0.861	0.984	0.3698
1567359_at	92.7	74.0	-0.33	-1.25	PPP-PPP	0.10	11	0.861	0.984	0.4326
219304_s_at	278.9	222.6	-0.33	-1.25	PPP-PPP	0.10	11	0.861	0.984	0.4422
235989_at	101.6	81.1	-0.33	-1.25	AAA-AAP	0.10	11	0.861	0.984	0.4871
223022_s_at	2783.4	2222.8	-0.33	-1.25	PPP-PPP	0.10	11	0.861	0.984	0.1804
214919_s_at	546.0	435.8	-0.33	-1.25	PPP-PPP	0.10	11	0.861	0.984	0.2875
243707_at	167.2	133.4	-0.33	-1.25	PPP-PPP	0.10	11	0.861	0.984	0.3058
229310_at	256.0	204.4	-0.33	-1.25	PPP-PPP	0.10	11	0.861	0.984	0.3379
234300_s_at	87.3	69.7	-0.33	-1.25	APP-APA	0.10	11	0.861	0.984	0.3546
205919_at	274.5	219.2	-0.33	-1.25	PPP-PPP	0.10	11	0.861	0.984	0.3802
224049_at	88.4	70.6	-0.33	-1.25	AAA-PAA	0.10	11	0.861	0.984	0.4545
1555194_at	46.1	36.8	-0.33	-1.25	PAM-PAA	0.10	11	0.861	0.984	0.5109
238666_at	107.3	85.7	-0.32	-1.25	PPA-PAP	0.10	11	0.861	0.984	0.2580
219501_at	347.8	277.9	-0.32	-1.25	PPP-PPP	0.10	11	0.861	0.984	0.2885
242818_x_at	112.5	89.8	-0.32	-1.25	PPP-PPP	0.10	11	0.861	0.984	0.2992
203705_s_at	1385.4	1106.6	-0.32	-1.25	PPP-PPP	0.10	11	0.861	0.984	0.3610
216650_at	246.1	196.6	-0.32	-1.25	PPP-PPP	0.10	11	0.861	0.984	0.3827
229141_at	97.1	77.6	-0.32	-1.25	PPP-PAP	0.10	11	0.861	0.984	0.4056
239379_at	133.4	106.6	-0.32	-1.25	APP-APP	0.10	11	0.861	0.984	0.5104
219709_x_at	562.8	449.6	-0.32	-1.25	APP-PAP	0.10	11	0.861	0.984	0.5182
208741_at	283.5	226.4	-0.32	-1.25	PPP-PPP	0.10	11	0.861	0.984	0.5225
1556678_a_at	69.0	55.2	-0.32	-1.25	PAA-AAA	0.10	11	0.861	0.984	0.2539
203162_s_at	1984.0	1586.2	-0.32	-1.25	PPP-PPP	0.10	11	0.861	0.984	0.2675
212390_at	803.5	642.4	-0.32	-1.25	PPP-PPP	0.10	11	0.861	0.984	0.2996

220730_at	269.3	215.2	-0.32	-1.25	AAP-PMP	0.10	11	0.861	0.984	0.3072
231243_s_at	153.6	122.8	-0.32	-1.25	PPP-PPP	0.10	11	0.861	0.984	0.3482
231540_at	65.1	52.1	-0.32	-1.25	PAA-PPP	0.10	11	0.861	0.984	0.3782
230452_at	120.9	96.7	-0.32	-1.25	PPP-PPP	0.10	11	0.861	0.984	0.3784
213650_at	563.4	450.4	-0.32	-1.25	PPP-PPP	0.10	11	0.861	0.984	0.4017
221750_at	667.6	533.8	-0.32	-1.25	PPP-PPP	0.10	11	0.861	0.984	0.4089
234939_s_at	136.9	109.4	-0.32	-1.25	APA-PAA	0.10	11	0.861	0.984	0.4268
216249_at	70.0	56.0	-0.32	-1.25	AAA-APA	0.10	11	0.861	0.984	0.4530
219383_at	194.0	155.2	-0.32	-1.25	PPP-PPP	0.10	11	0.861	0.984	0.2018
238011_at	187.9	150.3	-0.32	-1.25	PPP-PPP	0.10	11	0.861	0.984	0.2426
241620_at	87.3	69.8	-0.32	-1.25	PPP-AMP	0.10	11	0.861	0.984	0.2932
1560922_s_at	149.1	119.3	-0.32	-1.25	PPP-PPP	0.10	11	0.861	0.984	0.3483
234929_s_at	208.2	166.6	-0.32	-1.25	AAA-PPP	0.10	11	0.861	0.984	0.3919
202145_at	930.7	745.3	-0.32	-1.25	PPP-PPP	0.10	11	0.861	0.984	0.1883
210791_s_at	185.8	148.7	-0.32	-1.25	PPM-APP	0.10	11	0.861	0.984	0.1984
221882_s_at	826.3	661.4	-0.32	-1.25	PPP-PPP	0.10	11	0.861	0.984	0.2144
52078_at	1211.0	969.5	-0.32	-1.25	PPP-PPP	0.10	11	0.861	0.984	0.2174
1555358_a_at	192.2	153.8	-0.32	-1.25	PPP-PPP	0.10	11	0.861	0.984	0.2811
243136_at	85.1	68.1	-0.32	-1.25	PPA-PAP	0.10	11	0.861	0.984	0.2899
211815_s_at	141.6	113.4	-0.32	-1.25	APP-PPP	0.10	11	0.861	0.984	0.2907
238452_at	385.5	308.7	-0.32	-1.25	PPP-PPP	0.10	11	0.861	0.984	0.2974
1554860_at	79.5	63.6	-0.32	-1.25	PAA-APP	0.10	11	0.861	0.984	0.4440
233592_at	87.0	69.7	-0.32	-1.25	AAP-AAA	0.10	11	0.861	0.984	0.4645
207951_at	47.0	37.6	-0.32	-1.25	AMA-APA	0.10	11	0.861	0.984	0.4934
212158_at	666.0	533.6	-0.32	-1.25	PPP-PPP	0.10	11	0.861	0.984	0.2056
242151_at	155.0	124.1	-0.32	-1.25	PPP-APP	0.10	11	0.861	0.984	0.2152
230930_at	608.0	487.1	-0.32	-1.25	PPP-PPP	0.10	11	0.861	0.984	0.2188
211842_s_at	59.5	47.7	-0.32	-1.25	APM-PPP	0.10	11	0.861	0.984	0.2281
242828_at	487.8	390.7	-0.32	-1.25	PPP-PPP	0.10	11	0.861	0.984	0.2922
228839_s_at	1108.6	887.8	-0.32	-1.25	PPP-PPP	0.10	11	0.861	0.984	0.3169
236267_at	279.9	224.3	-0.32	-1.25	PPP-PAP	0.10	11	0.861	0.984	0.3547
213562_s_at	549.6	440.3	-0.32	-1.25	PPP-PPP	0.10	11	0.861	0.984	0.3592
243031_at	198.2	158.7	-0.32	-1.25	PPP-PPP	0.10	11	0.861	0.984	0.4681
244791_at	45.6	36.5	-0.32	-1.25	AAP-APA	0.10	11	0.861	0.984	0.4688
237783_at	110.0	88.2	-0.32	-1.25	PPA-MPP	0.10	11	0.861	0.984	0.1694
228388_at	125.5	100.6	-0.32	-1.25	AAA-PAA	0.10	11	0.861	0.984	0.1969
238571_at	83.3	66.8	-0.32	-1.25	APP-APA	0.10	11	0.861	0.984	0.2929
241360_at	107.4	86.1	-0.32	-1.25	PPP-PPP	0.10	11	0.861	0.984	0.3497
207604_s_at	353.9	283.6	-0.32	-1.25	PPP-PPP	0.10	11	0.861	0.984	0.4205
209010_s_at	322.5	258.5	-0.32	-1.25	PPP-PPP	0.10	11	0.861	0.984	0.4310
236700_at	143.2	114.8	-0.32	-1.25	PPP-APP	0.10	11	0.861	0.984	0.4976
225852_at	2542.5	2037.7	-0.32	-1.25	PPP-PPP	0.10	11	0.861	0.984	0.5189

208826_x_at	20956.6	16807.8	-0.32	-1.25	PPP-PPP	0.10	11	0.861	0.984	0.1762
217576_x_at	165.0	132.3	-0.32	-1.25	PPP-PAP	0.10	11	0.861	0.984	0.1995
226310_at	507.3	406.9	-0.32	-1.25	PPP-PPP	0.10	11	0.861	0.984	0.3960
231756_at	87.7	70.4	-0.32	-1.25	AMA-APA	0.10	11	0.861	0.984	0.4626
1562831_a_at	74.0	59.4	-0.32	-1.25	APA-PAP	0.10	11	0.861	0.984	0.4657
225374_at	924.3	741.9	-0.32	-1.25	PPP-PPP	0.10	11	0.861	0.984	0.1421
234989_at	2455.8	1971.2	-0.32	-1.25	PPP-PPP	0.10	11	0.861	0.984	0.2736
1554348_s_at	130.6	104.8	-0.32	-1.25	MPP-PPP	0.10	11	0.861	0.984	0.4008
233142_at	101.9	81.8	-0.32	-1.25	AAA-PPA	0.10	11	0.861	0.984	0.4312
231277_x_at	247.8	198.9	-0.32	-1.25	APP-APA	0.10	11	0.861	0.984	0.4698
1555892_s_at	68.0	54.6	-0.32	-1.25	AAA-AAP	0.10	11	0.861	0.984	0.5045
220979_s_at	918.5	737.4	-0.32	-1.25	PPP-PPP	0.10	11	0.861	0.984	0.5135
211078_s_at	78.6	63.1	-0.32	-1.25	PPP-MPP	0.10	11	0.861	0.984	0.5189
215022_x_at	256.7	206.2	-0.32	-1.25	PPP-PPP	0.10	11	0.861	0.984	0.1572
201927_s_at	584.5	469.4	-0.32	-1.25	PPP-PPP	0.10	11	0.861	0.984	0.2240
229155_at	59.7	48.0	-0.32	-1.25	AAA-PPA	0.10	11	0.861	0.984	0.3870
1556886_a_at	96.9	77.8	-0.32	-1.25	PPP-PPP	0.10	11	0.861	0.984	0.4764
232748_at	66.7	53.6	-0.32	-1.25	PPP-MAP	0.10	11	0.861	0.984	0.4861
223350_x_at	2904.7	2334.7	-0.32	-1.24	PPP-PPP	0.10	11	0.861	0.984	0.1410
42361_g_at	379.7	305.2	-0.32	-1.24	PPP-PPP	0.10	11	0.861	0.984	0.1640
218114_at	504.7	405.8	-0.32	-1.24	PPP-AMP	0.10	11	0.861	0.984	0.1731
214594_x_at	1463.7	1176.9	-0.32	-1.24	PPP-PPP	0.10	11	0.861	0.984	0.3364
221096_s_at	310.0	249.2	-0.32	-1.24	PPP-PPP	0.10	11	0.861	0.984	0.3447
1555598_a_at	96.8	77.8	-0.32	-1.24	AAA-APP	0.10	11	0.861	0.984	0.4258
230815_at	76.7	61.6	-0.32	-1.24	PMA-AMA	0.10	11	0.861	0.984	0.4676
1564498_at	137.4	110.5	-0.32	-1.24	APA-AAA	0.10	11	0.861	0.984	0.4746
235264_at	185.1	148.9	-0.31	-1.24	PPP-PPP	0.10	11	0.861	0.984	0.1618
1566761_a_at	80.3	64.6	-0.31	-1.24	PAA-PAA	0.10	11	0.861	0.984	0.2038
214395_x_at	155.8	125.3	-0.31	-1.24	PPP-PMP	0.10	11	0.861	0.984	0.2906
1553967_at	156.9	126.3	-0.31	-1.24	PAP-PMP	0.10	11	0.861	0.984	0.3117
242487_at	130.7	105.2	-0.31	-1.24	AAP-PPP	0.10	11	0.861	0.984	0.3182
243847_at	72.9	58.7	-0.31	-1.24	AAP-APP	0.10	11	0.861	0.984	0.3471
1554919_s_at	103.2	83.0	-0.31	-1.24	AAP-AAP	0.10	11	0.861	0.984	0.4125
211621_at	70.1	56.4	-0.31	-1.24	AAA-APP	0.10	11	0.861	0.984	0.4399
242403_at	296.3	238.4	-0.31	-1.24	PPP-PPP	0.10	11	0.861	0.984	0.4696
230581_at	76.6	61.6	-0.31	-1.24	PAP-PPP	0.10	11	0.861	0.984	0.4841
230884_s_at	318.4	396.0	0.31	1.24	PPP-PPP	0.10	11	0.861	0.984	0.0016
229073_at	95.5	76.9	-0.31	-1.24	PPP-APP	0.10	11	0.861	0.984	0.1487
205661_s_at	735.6	592.1	-0.31	-1.24	PPP-PPP	0.10	11	0.861	0.984	0.1945
243043_at	87.6	70.6	-0.31	-1.24	AAP-AMM	0.10	11	0.861	0.984	0.2576
1560754_at	160.5	129.2	-0.31	-1.24	PPP-PPP	0.10	11	0.861	0.984	0.2624
218788_s_at	3919.4	3155.2	-0.31	-1.24	PPP-PPP	0.10	11	0.861	0.984	0.2802

236696_at	92.7	74.7	-0.31	-1.24	PPP-PPP	0.10	11	0.861	0.984	0.2870
217166_at	151.7	122.1	-0.31	-1.24	PPP-PAP	0.10	11	0.861	0.984	0.3230
1564203_at	80.9	65.1	-0.31	-1.24	PPP-PPP	0.10	11	0.861	0.984	0.3374
242506_at	113.2	91.2	-0.31	-1.24	PPP-PAP	0.10	11	0.861	0.984	0.3634
229357_at	128.9	103.7	-0.31	-1.24	PPP-PPP	0.10	11	0.861	0.984	0.3872
213517_at	362.4	291.8	-0.31	-1.24	PPP-PPP	0.10	11	0.861	0.984	0.4446
231695_at	86.1	69.3	-0.31	-1.24	AAP-AAP	0.10	11	0.861	0.984	0.4688
240574_at	68.3	55.0	-0.31	-1.24	APA-APP	0.10	11	0.861	0.984	0.4738
219834_at	50.6	40.7	-0.31	-1.24	PPP-APP	0.10	11	0.861	0.984	0.4896
229434_at	805.0	648.4	-0.31	-1.24	PPP-PPP	0.10	11	0.861	0.984	0.1526
223964_x_at	342.6	276.1	-0.31	-1.24	PPP-PPP	0.10	11	0.861	0.984	0.1657
203886_s_at	527.5	425.0	-0.31	-1.24	PPP-PPP	0.10	11	0.861	0.984	0.2251
235640_at	265.4	213.8	-0.31	-1.24	AMP-APP	0.10	11	0.861	0.984	0.2705
230385_at	151.0	121.6	-0.31	-1.24	AAA-AAP	0.10	11	0.861	0.984	0.3693
236116_at	67.8	54.6	-0.31	-1.24	AAP-AAP	0.10	11	0.861	0.984	0.3990
240125_at	85.7	69.0	-0.31	-1.24	PPM-PAA	0.10	11	0.861	0.984	0.4204
229002_at	107.1	86.3	-0.31	-1.24	PAP-PAA	0.10	11	0.861	0.984	0.2381
1556821_x_at	461.6	372.2	-0.31	-1.24	PPP-PPP	0.10	11	0.861	0.984	0.2398
226781_at	537.5	433.1	-0.31	-1.24	PPP-PPP	0.10	11	0.861	0.984	0.2886
243451_at	80.1	64.6	-0.31	-1.24	PAP-PAA	0.10	11	0.861	0.984	0.2955
222286_at	115.3	92.9	-0.31	-1.24	PPP-PPP	0.10	11	0.861	0.984	0.3115
219472_at	281.2	226.6	-0.31	-1.24	PPP-PPP	0.10	11	0.861	0.984	0.3420
214297_at	111.2	89.7	-0.31	-1.24	AAP-MPP	0.10	11	0.861	0.984	0.3826
228999_at	254.5	205.2	-0.31	-1.24	PPP-PPP	0.10	11	0.861	0.984	0.4449
239100_x_at	103.0	83.0	-0.31	-1.24	PMP-AMP	0.10	11	0.861	0.984	0.4992
202732_at	1970.1	1589.4	-0.31	-1.24	PPP-PPP	0.10	11	0.861	0.984	0.1454
230720_at	1260.8	1017.2	-0.31	-1.24	PPP-PPP	0.10	11	0.861	0.984	0.1489
201037_at	12608.1	10171.7	-0.31	-1.24	PPP-PPP	0.10	11	0.861	0.984	0.1688
224182_x_at	200.3	161.6	-0.31	-1.24	APP-PMP	0.10	11	0.861	0.984	0.2300
210349_at	211.2	170.3	-0.31	-1.24	PAP-MPP	0.10	11	0.861	0.984	0.2370
231225_at	130.7	105.4	-0.31	-1.24	PPP-PPP	0.10	11	0.861	0.984	0.2472
1556062_at	69.7	56.2	-0.31	-1.24	PPP-PPP	0.10	11	0.861	0.984	0.4249
230109_at	546.5	440.9	-0.31	-1.24	PAP-PPP	0.10	11	0.861	0.984	0.4438
204514_at	547.6	442.0	-0.31	-1.24	PPP-PPP	0.10	11	0.861	0.984	0.3624
215534_at	116.8	94.3	-0.31	-1.24	PPA-PPP	0.10	11	0.861	0.984	0.4521
1565881_at	55.0	44.4	-0.31	-1.24	APP-PPP	0.10	11	0.861	0.984	0.5162
236291_at	109.0	88.0	-0.31	-1.24	APA-APP	0.10	11	0.861	0.984	0.5171
208623_s_at	8202.7	6627.7	-0.31	-1.24	PPP-PPP	0.10	11	0.861	0.984	0.1837
235179_at	112.7	91.0	-0.31	-1.24	AMP-APP	0.10	11	0.861	0.984	0.1997
204434_at	399.7	322.8	-0.31	-1.24	PPP-PPP	0.10	11	0.861	0.984	0.2252
204377_s_at	96.7	78.1	-0.31	-1.24	PAP-APA	0.10	11	0.861	0.984	0.3877
206047_at	137.8	111.3	-0.31	-1.24	AAA-PAP	0.10	11	0.861	0.984	0.4352

222769_at	115.6	93.4	-0.31	-1.24	PPP-PPP	0.10	11	0.861	0.984	0.4464
236492_at	248.9	201.1	-0.31	-1.24	PPP-APP	0.10	11	0.861	0.984	0.4817
238868_at	150.8	121.8	-0.31	-1.24	PPP-PPP	0.10	11	0.861	0.984	0.4830
1560370_x_at	70.3	56.8	-0.31	-1.24	PPA-PMA	0.10	11	0.861	0.984	0.4933
221698_s_at	47.6	38.4	-0.31	-1.24	AAM-PAP	0.10	11	0.861	0.984	0.5022
241910_x_at	200.8	162.2	-0.31	-1.24	PAP-MPP	0.10	11	0.861	0.984	0.5062
227048_at	842.6	681.3	-0.31	-1.24	PPP-PPP	0.10	11	0.861	0.984	0.3074
226438_at	100.0	80.8	-0.31	-1.24	PPP-PPP	0.10	11	0.861	0.984	0.3803
235208_at	231.6	187.2	-0.31	-1.24	PPP-PPP	0.10	11	0.861	0.984	0.3993
230048_at	257.4	208.0	-0.31	-1.24	MPP-PPP	0.10	11	0.861	0.984	0.4296
211356_x_at	125.4	101.3	-0.31	-1.24	MPA-PPP	0.10	11	0.861	0.984	0.4442
239804_at	124.9	101.0	-0.31	-1.24	PPP-PAP	0.10	11	0.861	0.984	0.4487
230904_at	102.1	82.5	-0.31	-1.24	PPP-PPP	0.10	11	0.861	0.984	0.4625
214924_s_at	874.0	707.2	-0.31	-1.24	PPP-PPP	0.10	11	0.861	0.984	0.1362
AFFX-r2-Ec-bioD-5_at	12179.9	9852.1	-0.31	-1.24	PPP-PPP	0.10	11	0.861	0.984	0.1773
211180_x_at	249.6	201.8	-0.31	-1.24	PPP-PPP	0.10	11	0.861	0.984	0.1902
236760_at	98.9	80.0	-0.31	-1.24	PPP-PPP	0.10	11	0.861	0.984	0.2248
216997_x_at	649.7	525.4	-0.31	-1.24	PPP-PPP	0.10	11	0.861	0.984	0.2552
1557455_s_at	1123.5	909.0	-0.31	-1.24	PPP-PPP	0.10	11	0.861	0.984	0.2574
225598_at	209.7	169.6	-0.31	-1.24	PPP-PPP	0.10	11	0.861	0.984	0.3235
243514_at	208.8	168.9	-0.31	-1.24	PPP-PPP	0.10	11	0.861	0.984	0.3430
220137_at	227.7	184.2	-0.31	-1.24	PPP-PPP	0.10	11	0.861	0.984	0.3677
210918_at	50.2	40.6	-0.31	-1.24	AMP-AAA	0.10	11	0.861	0.984	0.4874
209381_x_at	549.0	444.4	-0.31	-1.24	PPP-PPP	0.10	11	0.861	0.984	0.1759
228367_at	1058.3	856.9	-0.31	-1.24	PPP-PPP	0.10	11	0.861	0.984	0.1786
1558329_at	244.8	198.2	-0.31	-1.24	AAP-PPA	0.10	11	0.861	0.984	0.1825
1555847_a_at	507.7	411.1	-0.31	-1.24	PPP-PPP	0.10	11	0.861	0.984	0.2175
209163_at	291.6	236.0	-0.31	-1.24	PPP-PPP	0.10	11	0.861	0.984	0.2180
201171_at	146.7	118.8	-0.31	-1.24	APA-PPP	0.10	11	0.861	0.984	0.2671
229903_x_at	459.8	372.2	-0.31	-1.24	PPP-PPP	0.10	11	0.861	0.984	0.2996
244398_x_at	98.7	79.9	-0.31	-1.24	PPP-PPP	0.10	11	0.861	0.984	0.3229
208180_s_at	45.7	37.0	-0.31	-1.24	AAA-AAP	0.10	11	0.861	0.984	0.4280
208960_s_at	1197.1	968.7	-0.31	-1.24	PPP-PPP	0.10	11	0.861	0.984	0.4317
230604_at	105.6	85.5	-0.30	-1.24	PPP-PPP	0.10	11	0.861	0.984	0.1460
201216_at	6299.9	5102.5	-0.30	-1.24	PPP-PPP	0.10	11	0.861	0.984	0.2088
237311_at	209.2	169.5	-0.30	-1.23	PPP-PPP	0.10	11	0.861	0.984	0.2374
218094_s_at	418.4	339.0	-0.30	-1.23	PPP-PPP	0.10	11	0.861	0.984	0.2557
212092_at	590.8	478.7	-0.30	-1.23	PPP-PPP	0.10	11	0.861	0.984	0.3149
213789_at	664.9	538.5	-0.30	-1.24	PPP-PPP	0.10	11	0.861	0.984	0.3263
216466_at	126.7	102.6	-0.30	-1.23	PPP-PPP	0.10	11	0.861	0.984	0.3729
241768_at	63.4	51.4	-0.30	-1.24	AAM-PAA	0.10	11	0.861	0.984	0.4571
241359_at	146.9	119.0	-0.30	-1.24	PPP-PPP	0.10	11	0.861	0.984	0.5195

232946_s_at	292.6	237.2	-0.30	-1.23	PPP-PPP	0.10	11	0.861	0.984	0.1523
207783_x_at	45314.8	36718.2	-0.30	-1.23	PPP-PPP	0.10	11	0.861	0.984	0.1570
214338_at	354.2	287.2	-0.30	-1.23	MPA-PPP	0.10	11	0.861	0.984	0.1686
203912_s_at	491.6	398.3	-0.30	-1.23	PPP-PPP	0.10	11	0.861	0.984	0.1968
242370_at	70.1	56.8	-0.30	-1.23	MPA-PAP	0.10	11	0.861	0.984	0.2082
37953_s_at	125.6	101.8	-0.30	-1.23	PPA-PPP	0.10	11	0.861	0.984	0.2872
203666_at	72.0	58.4	-0.30	-1.23	AAA-APA	0.10	11	0.861	0.984	0.3029
1555786_s_at	524.0	424.8	-0.30	-1.23	PPP-PPP	0.10	11	0.861	0.984	0.3080
49077_at	4300.7	3485.3	-0.30	-1.23	PPP-PPP	0.10	11	0.861	0.984	0.3344
1561687_a_at	77.7	62.9	-0.30	-1.23	PPP-APP	0.10	11	0.861	0.984	0.3571
1553438_at	154.6	125.3	-0.30	-1.23	APA-PAP	0.10	11	0.861	0.984	0.3639
212070_at	278.8	225.9	-0.30	-1.23	PPP-PPP	0.10	11	0.861	0.984	0.3844
230533_at	304.2	246.6	-0.30	-1.23	PPP-PPP	0.10	11	0.861	0.984	0.3984
228647_at	68.9	55.9	-0.30	-1.23	MAP-APA	0.10	11	0.861	0.984	0.4068
232333_at	199.8	162.0	-0.30	-1.23	PPP-PPP	0.10	11	0.861	0.984	0.4811
232587_at	130.0	105.4	-0.30	-1.23	PAP-PAP	0.10	11	0.861	0.984	0.4912
205045_at	152.5	123.6	-0.30	-1.23	PPA-APP	0.10	11	0.861	0.984	0.5096
226519_s_at	210.1	170.4	-0.30	-1.23	PPP-PPP	0.10	11	0.861	0.984	0.1427
204158_s_at	400.4	324.7	-0.30	-1.23	APP-PPA	0.10	11	0.861	0.984	0.2202
201012_at	17401.9	14116.0	-0.30	-1.23	PPP-PPP	0.10	11	0.861	0.984	0.2390
235819_at	165.8	134.5	-0.30	-1.23	PPP-PPP	0.10	11	0.861	0.984	0.3043
222524_s_at	359.8	291.9	-0.30	-1.23	PAP-PPP	0.10	11	0.861	0.984	0.3302
227912_s_at	182.6	148.1	-0.30	-1.23	PMA-PPP	0.10	11	0.861	0.984	0.3313
204268_at	545.0	442.5	-0.30	-1.23	PPP-PPP	0.10	11	0.861	0.984	0.1517
210034_s_at	159.7	129.6	-0.30	-1.23	PPP-PPP	0.10	11	0.861	0.984	0.1575
230509_at	168.8	137.0	-0.30	-1.23	AAA-PAM	0.10	11	0.861	0.984	0.2212
1554317_s_at	553.9	449.7	-0.30	-1.23	PPP-PPP	0.10	11	0.861	0.984	0.2286
1562281_at	77.8	63.2	-0.30	-1.23	MPP-APP	0.10	11	0.861	0.984	0.2562
220632_s_at	255.6	207.5	-0.30	-1.23	APP-APP	0.10	11	0.861	0.984	0.3187
203802_x_at	1503.7	1220.7	-0.30	-1.23	PPP-PPP	0.10	11	0.861	0.984	0.3220
239046_at	110.3	89.6	-0.30	-1.23	PPP-MPP	0.10	11	0.861	0.984	0.4067
229251_s_at	200.8	163.0	-0.30	-1.23	MAP-APP	0.10	11	0.861	0.984	0.4792
211343_s_at	1637.6	1328.9	-0.30	-1.23	PPP-PPP	0.10	11	0.861	0.984	0.5040
214674_at	159.4	129.4	-0.30	-1.23	PPM-APP	0.10	11	0.861	0.984	0.5232
222115_x_at	212.1	172.4	-0.30	-1.23	PPP-PPP	0.10	11	0.861	0.984	0.2369
232994_s_at	937.7	761.8	-0.30	-1.23	PPP-PPP	0.10	11	0.861	0.984	0.2677
221566_s_at	127.0	103.2	-0.30	-1.23	AAA-AAP	0.10	11	0.861	0.984	0.3027
230727_at	173.3	140.7	-0.30	-1.23	PPP-PPP	0.10	11	0.861	0.984	0.3043
216126_at	116.4	94.6	-0.30	-1.23	AMA-AAP	0.10	11	0.861	0.984	0.3305
219142_at	102.3	83.1	-0.30	-1.23	AAP-PPP	0.10	11	0.861	0.984	0.3594
226021_at	1802.9	1464.8	-0.30	-1.23	PPP-PPP	0.10	11	0.861	0.984	0.3677
1559139_at	192.6	156.4	-0.30	-1.23	APA-PPP	0.10	11	0.861	0.984	0.4018

238660_at	122.4	99.4	-0.30	-1.23	MPP-PPP	0.10	11	0.861	0.984	0.4057
219147_s_at	133.4	108.4	-0.30	-1.23	PPP-PPP	0.10	11	0.861	0.984	0.4670
1563283_at	164.6	133.8	-0.30	-1.23	APP-PAP	0.10	11	0.861	0.984	0.5103
220275_at	86.4	70.2	-0.30	-1.23	AAA-AAP	0.10	11	0.861	0.984	0.1582
202367_at	250.1	203.2	-0.30	-1.23	AAP-PPA	0.10	11	0.861	0.984	0.1846
221561_at	2515.7	2044.4	-0.30	-1.23	PPP-PPP	0.10	11	0.861	0.984	0.2283
226559_at	286.3	232.7	-0.30	-1.23	MMP-PAA	0.10	11	0.861	0.984	0.2620
224516_s_at	1227.8	998.3	-0.30	-1.23	PPP-PPP	0.10	11	0.861	0.984	0.3401
242555_at	132.4	107.7	-0.30	-1.23	PPP-PPP	0.10	11	0.861	0.984	0.3648
1554830_a_at	341.0	277.2	-0.30	-1.23	PPP-PPP	0.10	11	0.861	0.984	0.3842
1560402_at	108.8	88.4	-0.30	-1.23	MPP-PPP	0.10	11	0.861	0.984	0.3876
241041_at	97.8	79.5	-0.30	-1.23	PPP-PAP	0.10	11	0.861	0.984	0.4602
239282_at	68.9	56.0	-0.30	-1.23	AAP-AAP	0.10	11	0.861	0.984	0.4826
212505_s_at	559.1	454.9	-0.30	-1.23	PPP-PPP	0.10	11	0.861	0.984	0.1350
225071_at	2398.2	1951.0	-0.30	-1.23	PPP-PPP	0.10	11	0.861	0.984	0.1364
222147_s_at	292.1	237.5	-0.30	-1.23	APP-PPP	0.10	11	0.861	0.984	0.1625
218133_s_at	2783.4	2263.7	-0.30	-1.23	PPP-PPP	0.10	11	0.861	0.984	0.1687
215504_x_at	756.2	615.1	-0.30	-1.23	PPP-AAP	0.10	11	0.861	0.984	0.1944
221187_s_at	183.1	148.9	-0.30	-1.23	AAP-PPP	0.10	11	0.861	0.984	0.2084
1570248_at	121.8	99.0	-0.30	-1.23	PPP-PPP	0.10	11	0.861	0.984	0.2244
214656_x_at	2309.5	1878.4	-0.30	-1.23	PPP-PPP	0.10	11	0.861	0.984	0.2636
222110_at	104.2	84.8	-0.30	-1.23	AAP-AAA	0.10	11	0.861	0.984	0.2918
242167_at	125.3	101.9	-0.30	-1.23	APP-APP	0.10	11	0.861	0.984	0.3785
239917_at	117.0	95.2	-0.30	-1.23	AAA-PPP	0.10	11	0.861	0.984	0.3865
207061_at	163.9	133.3	-0.30	-1.23	PPP-APP	0.10	11	0.861	0.984	0.4540
210818_s_at	139.3	113.4	-0.30	-1.23	PPP-PPP	0.10	11	0.861	0.984	0.5124
225709_at	116.1	94.5	-0.30	-1.23	MAP-MPA	0.10	11	0.861	0.984	0.1409
223988_x_at	3322.3	2703.7	-0.30	-1.23	PPP-PPP	0.10	11	0.861	0.984	0.1542
1559347_at	96.6	78.6	-0.30	-1.23	PPP-PPP	0.10	11	0.861	0.984	0.1559
231198_at	71.6	58.3	-0.30	-1.23	PAA-AAA	0.10	11	0.861	0.984	0.2445
208370_s_at	7584.0	6172.9	-0.30	-1.23	PPP-PPP	0.10	11	0.861	0.984	0.2772
228699_at	60.6	49.4	-0.30	-1.23	PMM-APA	0.10	11	0.861	0.984	0.3499
243614_s_at	152.8	124.4	-0.30	-1.23	APA-AAA	0.10	11	0.861	0.984	0.3856
207920_x_at	259.1	210.8	-0.30	-1.23	PPP-PPP	0.10	11	0.861	0.984	0.3895
232018_at	154.5	125.8	-0.30	-1.23	PMP-PPP	0.10	11	0.861	0.984	0.4161
240770_at	444.1	361.4	-0.30	-1.23	PPP-PPP	0.10	11	0.861	0.984	0.4286
223808_s_at	1631.5	1327.7	-0.30	-1.23	PPP-PPP	0.10	11	0.861	0.984	0.4526
235325_at	147.9	120.4	-0.30	-1.23	APA-PAP	0.10	11	0.861	0.984	0.5011
1565868_at	180.1	146.6	-0.30	-1.23	PPP-PPP	0.10	11	0.861	0.984	0.5052
241941_at	129.3	105.3	-0.30	-1.23	PPP-PPP	0.10	11	0.861	0.984	0.1872
209410_s_at	396.8	323.1	-0.30	-1.23	PPP-PPP	0.10	11	0.861	0.984	0.3672
226341_at	379.0	308.7	-0.30	-1.23	PPP-PPP	0.10	11	0.861	0.984	0.3679

208118_x_at	512.5	417.5	-0.30	-1.23	PPP-PPP	0.10	11	0.861	0.984	0.4026
227992_s_at	165.3	134.6	-0.30	-1.23	MPA-AAP	0.10	11	0.861	0.984	0.4150
233003_at	235.1	191.5	-0.30	-1.23	PPP-PPP	0.10	11	0.861	0.984	0.4272
1552942_at	125.0	101.8	-0.30	-1.23	PPP-PPP	0.10	11	0.861	0.984	0.4456
206091_at	185.1	150.8	-0.30	-1.23	PPP-PPP	0.10	11	0.861	0.984	0.4942
222165_x_at	320.1	260.7	-0.30	-1.23	PPP-PPP	0.10	11	0.861	0.984	0.5021
203056_s_at	178.6	145.6	-0.30	-1.23	AAP-APP	0.10	11	0.861	0.984	0.1747
226404_at	133.4	108.8	-0.30	-1.23	PPP-PPP	0.10	11	0.861	0.984	0.1950
202030_at	1195.7	974.3	-0.30	-1.23	PPP-PPP	0.10	11	0.861	0.984	0.2119
220734_s_at	552.3	450.0	-0.30	-1.23	PPP-PPP	0.10	11	0.861	0.984	0.2344
220112_at	78.7	64.1	-0.30	-1.23	AAP-PAP	0.10	11	0.861	0.984	0.2510
231806_s_at	389.1	317.2	-0.30	-1.23	PPP-PPP	0.10	11	0.861	0.984	0.3479
209240_at	3684.6	3002.5	-0.30	-1.23	PPP-PPP	0.10	11	0.861	0.984	0.3615
232454_at	83.7	68.2	-0.30	-1.23	PPP-PPP	0.10	11	0.861	0.984	0.3668
1568849_at	250.9	204.6	-0.30	-1.23	PPP-PPP	0.10	11	0.861	0.984	0.3860
239121_at	131.1	106.8	-0.30	-1.23	AAA-APP	0.10	11	0.861	0.984	0.3956
220465_at	162.7	132.6	-0.30	-1.23	APP-AAP	0.10	11	0.861	0.984	0.4546
234562_x_at	95.8	78.1	-0.30	-1.23	PPP-PPP	0.10	11	0.861	0.984	0.4608
240435_at	86.2	70.3	-0.29	-1.23	APA-PAP	0.10	11	0.861	0.984	0.2011
204458_at	510.2	416.2	-0.29	-1.23	PPP-PPP	0.10	11	0.861	0.984	0.2027
201201_at	9303.3	7589.0	-0.29	-1.23	PPP-PPP	0.10	11	0.861	0.984	0.2323
1557718_at	84.1	68.6	-0.29	-1.23	AAP-APP	0.10	11	0.861	0.984	0.2854
208384_s_at	207.4	169.1	-0.29	-1.23	PPP-PPP	0.10	11	0.861	0.984	0.4738
244414_at	265.6	216.6	-0.29	-1.23	PPP-PPP	0.10	11	0.861	0.984	0.5196
235296_at	1180.8	963.5	-0.29	-1.23	PPP-PPP	0.10	11	0.861	0.984	0.1769
223907_s_at	670.4	547.3	-0.29	-1.23	PPP-PPP	0.10	11	0.861	0.984	0.2029
227517_s_at	4872.8	3976.4	-0.29	-1.23	PPP-PPP	0.10	11	0.861	0.984	0.2260
226920_at	128.2	104.6	-0.29	-1.23	AAA-APP	0.10	11	0.861	0.984	0.2727
227062_at	873.8	712.9	-0.29	-1.23	PPP-PPP	0.10	11	0.861	0.984	0.2902
227025_at	338.4	276.1	-0.29	-1.23	PPP-PPP	0.10	11	0.861	0.984	0.3037
218261_at	1131.5	923.3	-0.29	-1.23	PPP-MMP	0.10	11	0.861	0.984	0.3489
228555_at	378.3	308.8	-0.29	-1.23	PPP-PPP	0.10	11	0.861	0.984	0.4178
237190_at	90.7	74.0	-0.29	-1.23	PPA-APA	0.10	11	0.861	0.984	0.4484
218485_s_at	277.1	226.1	-0.29	-1.23	APP-PPP	0.10	11	0.861	0.984	0.4587
201347_x_at	3182.6	2600.1	-0.29	-1.22	PPP-PPP	0.10	11	0.861	0.984	0.1738
1553108_at	311.5	254.4	-0.29	-1.22	PPP-PPP	0.10	11	0.861	0.984	0.1918
227516_at	1021.4	834.1	-0.29	-1.23	PPP-PPP	0.10	11	0.861	0.984	0.1977
222540_s_at	560.5	457.8	-0.29	-1.23	PPP-PPP	0.10	11	0.861	0.984	0.2160
204275_at	741.2	605.6	-0.29	-1.22	PPP-PPP	0.10	11	0.861	0.984	0.2198
223100_s_at	6250.5	5104.9	-0.29	-1.22	PPP-PPP	0.10	11	0.861	0.984	0.2299
1556387_at	55.7	45.5	-0.29	-1.23	AAA-APP	0.10	11	0.861	0.984	0.2384
203042_at	2195.6	1792.8	-0.29	-1.23	PPP-PPP	0.10	11	0.861	0.984	0.2689

1569041_at	98.7	80.6	-0.29	-1.22	PPP-PPP	0.10	11	0.861	0.984	0.3268
226665_at	203.2	166.0	-0.29	-1.22	PPP-PPP	0.10	11	0.861	0.984	0.4067
242849_at	41.9	34.2	-0.29	-1.23	APP-AAP	0.10	11	0.861	0.984	0.4196
238800_s_at	102.0	83.3	-0.29	-1.22	APP-PAP	0.10	11	0.861	0.984	0.4432
207991_x_at	66.4	54.3	-0.29	-1.22	AAA-AAP	0.10	11	0.861	0.984	0.4728
226795_at	432.3	353.0	-0.29	-1.22	PPP-PAP	0.10	11	0.861	0.984	0.4978
209413_at	468.7	383.1	-0.29	-1.22	PPP-PPP	0.10	11	0.861	0.984	0.1400
218848_at	300.6	245.7	-0.29	-1.22	APP-PPP	0.10	11	0.861	0.984	0.2044
225577_at	866.5	708.1	-0.29	-1.22	PPP-PPP	0.10	11	0.861	0.984	0.2173
209180_at	4619.1	3774.1	-0.29	-1.22	PPP-PPP	0.10	11	0.861	0.984	0.3028
1558256_at	133.8	109.4	-0.29	-1.22	PPP-PPP	0.10	11	0.861	0.984	0.3106
224994_at	824.6	673.9	-0.29	-1.22	PPP-PPP	0.10	11	0.861	0.984	0.3110
230028_at	235.6	192.5	-0.29	-1.22	PPP-MPP	0.10	11	0.861	0.984	0.4509
242448_at	306.9	250.9	-0.29	-1.22	PPP-PPP	0.10	11	0.861	0.984	0.4900
200061_s_at	37957.5	31041.2	-0.29	-1.22	PPP-PPP	0.10	11	0.861	0.984	0.1284
229426_at	352.5	288.4	-0.29	-1.22	PPP-PPP	0.10	11	0.861	0.984	0.1454
201266_at	20153.0	16485.7	-0.29	-1.22	PPP-PPP	0.10	11	0.861	0.984	0.1494
202155_s_at	467.9	382.6	-0.29	-1.22	PPP-APP	0.10	11	0.861	0.984	0.2487
203615_x_at	339.0	277.4	-0.29	-1.22	PPP-PPP	0.10	11	0.861	0.984	0.3908
231087_at	94.2	77.1	-0.29	-1.22	PPP-PPP	0.10	11	0.861	0.984	0.4496
214733_s_at	767.1	627.7	-0.29	-1.22	PPP-PPP	0.10	11	0.861	0.984	0.1801
228369_at	271.4	222.1	-0.29	-1.22	PPP-PPP	0.10	11	0.861	0.984	0.2724
214972_at	137.9	112.9	-0.29	-1.22	PPP-PPP	0.10	11	0.861	0.984	0.3639
244425_at	84.1	68.9	-0.29	-1.22	PAP-AAP	0.10	11	0.861	0.984	0.3682
205299_s_at	82.0	67.1	-0.29	-1.22	PPP-PPP	0.10	11	0.861	0.984	0.3785
217984_at	546.8	447.7	-0.29	-1.22	PPP-PPP	0.10	11	0.861	0.984	0.3831
232486_at	93.7	76.7	-0.29	-1.22	APP-PPP	0.10	11	0.861	0.984	0.4011
228821_at	109.8	89.9	-0.29	-1.22	PPP-PPP	0.10	11	0.861	0.984	0.4188
239909_at	312.4	255.6	-0.29	-1.22	PPP-PPP	0.10	11	0.861	0.984	0.4322
212991_at	97.8	80.0	-0.29	-1.22	APP-APP	0.10	11	0.861	0.984	0.4495
223797_at	150.3	123.1	-0.29	-1.22	PPP-PPP	0.10	11	0.861	0.984	0.4597
1556602_at	45.1	36.9	-0.29	-1.22	AAA-APP	0.10	11	0.861	0.984	0.5030
207020_at	240.7	197.1	-0.29	-1.22	PPP-PPP	0.10	11	0.861	0.984	0.1327
215412_x_at	243.7	199.7	-0.29	-1.22	PPP-PPP	0.10	11	0.861	0.984	0.2089
217156_at	88.6	72.6	-0.29	-1.22	APP-PAP	0.10	11	0.861	0.984	0.2835
242356_at	328.9	269.4	-0.29	-1.22	PPP-PPP	0.10	11	0.861	0.984	0.2858
242424_at	247.6	202.7	-0.29	-1.22	PPP-PPP	0.10	11	0.861	0.984	0.2925
219354_at	297.3	243.5	-0.29	-1.22	PPP-PPP	0.10	11	0.861	0.984	0.3700
1555741_at	97.4	79.8	-0.29	-1.22	AAA-PAA	0.10	11	0.861	0.984	0.4152
1556657_at	107.2	87.8	-0.29	-1.22	AAP-APP	0.10	11	0.861	0.984	0.4195
231055_at	80.7	66.1	-0.29	-1.22	PPP-PPP	0.10	11	0.861	0.984	0.4965
220703_at	105.4	86.4	-0.29	-1.22	AAA-PPP	0.10	11	0.861	0.984	0.1313

AFFX-r2-P1-cre-5_at	33699.5	27623.2	-0.29	-1.22	PPP-PPP	0.10	11	0.861	0.984	0.1613
226420_at	477.7	391.4	-0.29	-1.22	PPP-PPP	0.10	11	0.861	0.984	0.1720
AFFX-r2-Ec-bioC-3_at	2645.2	2167.9	-0.29	-1.22	PPP-PPP	0.10	11	0.861	0.984	0.2143
208721_s_at	1479.8	1213.2	-0.29	-1.22	PPP-PPP	0.10	11	0.861	0.984	0.2773
223187_s_at	3832.5	3140.4	-0.29	-1.22	PPP-PPP	0.10	11	0.861	0.984	0.2889
205088_at	419.6	344.0	-0.29	-1.22	PPP-PPP	0.10	11	0.861	0.984	0.3086
207013_s_at	120.1	98.5	-0.29	-1.22	AAP-AAA	0.10	11	0.861	0.984	0.3196
1569251_a_at	94.5	77.5	-0.29	-1.22	AAA-APA	0.10	11	0.861	0.984	0.3514
214976_at	163.8	134.3	-0.29	-1.22	PPP-APP	0.10	11	0.861	0.984	0.3762
238207_at	98.6	80.8	-0.29	-1.22	PAA-APP	0.10	11	0.861	0.984	0.4011
216402_at	50.3	41.3	-0.29	-1.22	PAA-AMA	0.10	11	0.861	0.984	0.4449
235152_at	127.9	104.9	-0.29	-1.22	PPP-APP	0.10	11	0.861	0.984	0.4768
225168_at	3898.7	3196.6	-0.29	-1.22	PPP-PPP	0.10	11	0.861	0.984	0.1407
235990_at	229.7	188.4	-0.29	-1.22	PMP-PPP	0.10	11	0.861	0.984	0.1513
226049_at	889.6	729.8	-0.29	-1.22	PPP-PPP	0.10	11	0.861	0.984	0.1694
1567224_at	679.4	557.3	-0.29	-1.22	PPP-PPP	0.10	11	0.861	0.984	0.2200
235386_at	45.3	37.1	-0.29	-1.22	AAP-AAA	0.10	11	0.861	0.984	0.3642
238000_at	60.9	49.9	-0.29	-1.22	PPP-APP	0.10	11	0.861	0.984	0.3860
1570567_at	96.3	79.0	-0.29	-1.22	APP-APP	0.10	11	0.861	0.984	0.4307
225051_at	387.6	317.8	-0.29	-1.22	PPP-PPP	0.10	11	0.861	0.984	0.4567
238570_at	95.3	78.2	-0.29	-1.22	AAA-APA	0.10	11	0.861	0.984	0.4584
230000_at	182.8	150.0	-0.29	-1.22	PPP-PPP	0.10	11	0.861	0.984	0.4690
237179_at	78.0	64.0	-0.29	-1.22	APA-PAP	0.10	11	0.861	0.984	0.5087
230077_at	285.2	233.9	-0.29	-1.22	PPP-PPP	0.10	11	0.861	0.984	0.5169
201214_s_at	1800.2	1477.6	-0.29	-1.22	PPP-PPP	0.10	11	0.861	0.984	0.1266
227674_at	378.1	310.3	-0.29	-1.22	PPP-PPP	0.10	11	0.861	0.984	0.1339
200790_at	30101.5	24703.8	-0.29	-1.22	PPP-PPP	0.10	11	0.861	0.984	0.1546
215169_at	48.7	39.9	-0.29	-1.22	PAA-AAA	0.10	11	0.861	0.984	0.2125
216383_at	1329.4	1091.0	-0.29	-1.22	PPP-PPP	0.10	11	0.861	0.984	0.2939
222120_at	166.4	136.6	-0.29	-1.22	PPP-PPP	0.10	11	0.861	0.984	0.3060
236075_s_at	740.6	607.9	-0.29	-1.22	PPP-PPP	0.10	11	0.861	0.984	0.3440
242042_s_at	75.1	61.7	-0.29	-1.22	AAP-AAA	0.10	11	0.861	0.984	0.4141
244822_at	123.8	101.6	-0.29	-1.22	PPP-PPP	0.10	11	0.861	0.984	0.4229
212777_at	369.2	303.1	-0.29	-1.22	PPP-PPP	0.10	11	0.861	0.984	0.4331
1558847_at	151.3	124.2	-0.29	-1.22	PPP-PPP	0.10	11	0.861	0.984	0.4532
228170_at	39.1	32.1	-0.29	-1.22	APA-AAP	0.10	11	0.861	0.984	0.4785
241747_s_at	137.9	113.2	-0.29	-1.22	PPP-PPP	0.10	11	0.861	0.984	0.4800
243591_at	286.6	235.1	-0.29	-1.22	PPP-PPP	0.10	11	0.861	0.984	0.5074
1552631_a_at	117.8	96.7	-0.29	-1.22	PAA-PAA	0.10	11	0.861	0.984	0.5095
218588_s_at	438.8	360.5	-0.28	-1.22	PPP-PPP	0.10	11	0.861	0.984	0.1457
212192_at	1311.4	1077.1	-0.28	-1.22	PPP-PPP	0.10	11	0.861	0.984	0.1752
212142_at	1014.3	833.2	-0.28	-1.22	PPP-PPP	0.10	11	0.861	0.984	0.2220

203678_at	520.1	427.0	-0.28	-1.22	PPP-PPP	0.10	11	0.861	0.984	0.2283
221599_at	759.8	624.0	-0.28	-1.22	PPP-PPP	0.10	11	0.861	0.984	0.2497
242751_at	311.9	256.2	-0.28	-1.22	PPP-PPP	0.10	11	0.861	0.984	0.3049
238299_at	280.3	230.3	-0.28	-1.22	PPP-PPP	0.10	11	0.861	0.984	0.3135
239469_at	115.9	95.2	-0.28	-1.22	PPA-PPP	0.10	11	0.861	0.984	0.3639
233595_at	373.1	306.4	-0.28	-1.22	PPP-PPP	0.10	11	0.861	0.984	0.4520
219609_at	124.1	101.9	-0.28	-1.22	AAP-APP	0.10	11	0.861	0.984	0.4576
208640_at	21003.9	17261.1	-0.28	-1.22	PPP-PPP	0.10	11	0.861	0.984	0.1541
226168_at	655.3	538.7	-0.28	-1.22	PPP-PPP	0.10	11	0.861	0.984	0.2573
242387_at	118.2	97.2	-0.28	-1.22	APA-PPA	0.10	11	0.861	0.984	0.3269
240873_x_at	191.9	157.7	-0.28	-1.22	PPP-PPP	0.10	11	0.861	0.984	0.3333
217550_at	255.3	209.7	-0.28	-1.22	PPP-PPP	0.10	11	0.861	0.984	0.3459
204437_s_at	138.5	113.9	-0.28	-1.22	PAA-AAA	0.10	11	0.861	0.984	0.3751
202750_s_at	1777.1	1460.9	-0.28	-1.22	PPP-PPP	0.10	11	0.861	0.984	0.3754
236274_at	129.5	106.4	-0.28	-1.22	PPA-APA	0.10	11	0.861	0.984	0.3947
214716_at	99.5	81.8	-0.28	-1.22	APP-PPP	0.10	11	0.861	0.984	0.4452
205166_at	174.7	143.6	-0.28	-1.22	PPP-PPP	0.10	11	0.861	0.984	0.5157
219522_at	1900.2	1562.5	-0.28	-1.22	PPP-PPP	0.10	11	0.861	0.984	0.1891
202295_s_at	177.4	145.9	-0.28	-1.22	PPP-PPP	0.10	11	0.861	0.984	0.1894
244547_at	139.4	114.7	-0.28	-1.22	PPP-PPP	0.10	11	0.861	0.984	0.2888
235705_at	545.8	449.0	-0.28	-1.22	PPP-PPP	0.10	11	0.861	0.984	0.2905
225733_at	562.7	463.0	-0.28	-1.22	PPP-PPP	0.10	11	0.861	0.984	0.2934
228608_at	303.9	249.9	-0.28	-1.22	PPP-PPP	0.10	11	0.861	0.984	0.3363
1570143_at	150.1	123.4	-0.28	-1.22	PPP-PPP	0.10	11	0.861	0.984	0.3811
229474_at	143.1	117.7	-0.28	-1.22	PPP-APP	0.10	11	0.861	0.984	0.5061
211574_s_at	761.9	627.2	-0.28	-1.22	PPP-PPP	0.10	11	0.861	0.984	0.1365
228003_at	234.3	192.8	-0.28	-1.22	PPP-PPP	0.10	11	0.861	0.984	0.1392
209185_s_at	1863.4	1533.9	-0.28	-1.22	PPP-PPP	0.10	11	0.861	0.984	0.1713
227285_at	201.8	166.1	-0.28	-1.22	PAA-APP	0.10	11	0.861	0.984	0.1782
226081_at	531.8	437.5	-0.28	-1.22	PPP-PPP	0.10	11	0.861	0.984	0.2027
234944_s_at	682.8	562.0	-0.28	-1.22	PPP-PPP	0.10	11	0.861	0.984	0.2268
241626_at	61.5	50.6	-0.28	-1.22	APP-AAA	0.10	11	0.861	0.984	0.3292
1554378_a_at	146.4	120.5	-0.28	-1.22	PPP-PPP	0.10	11	0.861	0.984	0.3301
233841_s_at	229.3	188.7	-0.28	-1.22	PPP-PPP	0.10	11	0.861	0.984	0.3719
1552575_a_at	306.3	252.1	-0.28	-1.22	APP-AAP	0.10	11	0.861	0.984	0.4046
223349_s_at	1280.0	1053.7	-0.28	-1.22	PPP-PPP	0.10	11	0.861	0.984	0.4369
217478_s_at	79.5	65.4	-0.28	-1.22	PPP-APA	0.10	11	0.861	0.984	0.4645
213816_s_at	243.4	200.3	-0.28	-1.22	PPP-PPP	0.10	11	0.861	0.984	0.4740
203324_s_at	7724.6	6362.2	-0.28	-1.21	PPP-PPP	0.10	11	0.861	0.984	0.1325
229672_at	297.3	244.9	-0.28	-1.21	PPP-PPP	0.10	11	0.861	0.984	0.1648
210208_x_at	5006.8	4124.0	-0.28	-1.21	PPP-PPP	0.10	11	0.861	0.984	0.2070
229054_at	126.0	103.8	-0.28	-1.21	APP-PPP	0.10	11	0.861	0.984	0.2596

225239_at	2796.4	2302.9	-0.28	-1.21	PPP-PPP	0.10	11	0.861	0.984	0.2742
222879_s_at	307.8	253.5	-0.28	-1.21	APA-PAP	0.10	11	0.861	0.984	0.3394
223226_x_at	578.9	476.9	-0.28	-1.21	PPP-PPP	0.10	11	0.861	0.984	0.3622
226433_at	97.9	80.6	-0.28	-1.21	APP-AAA	0.10	11	0.861	0.984	0.4712
224338_s_at	255.2	210.3	-0.28	-1.21	PPP-PAP	0.10	11	0.861	0.984	0.1868
218626_at	1093.7	901.3	-0.28	-1.21	PPP-PPP	0.10	11	0.861	0.984	0.2217
212860_at	585.4	482.5	-0.28	-1.21	PPP-PPP	0.10	11	0.861	0.984	0.2282
201896_s_at	1757.5	1448.5	-0.28	-1.21	PPP-PPP	0.10	11	0.861	0.984	0.2297
228696_at	377.5	311.1	-0.28	-1.21	PPP-PPP	0.10	11	0.861	0.984	0.2590
212948_at	780.7	643.6	-0.28	-1.21	PPP-PPP	0.10	11	0.861	0.984	0.3298
207687_at	74.6	61.5	-0.28	-1.21	PAP-PAP	0.10	11	0.861	0.984	0.3915
1561207_at	58.5	48.2	-0.28	-1.21	AAP-AMA	0.10	11	0.861	0.984	0.4929
232899_at	586.2	483.5	-0.28	-1.21	PPP-PPP	0.10	11	0.861	0.984	0.1312
226509_at	363.9	300.2	-0.28	-1.21	PPP-PPP	0.10	11	0.861	0.984	0.1372
242126_at	214.3	176.8	-0.28	-1.21	PPP-PPP	0.10	11	0.861	0.984	0.1394
221435_x_at	1270.3	1047.6	-0.28	-1.21	PPP-PPP	0.10	11	0.861	0.984	0.1444
235501_at	236.6	195.1	-0.28	-1.21	PPP-PPP	0.10	11	0.861	0.984	0.1473
208452_x_at	268.8	221.7	-0.28	-1.21	PPP-PPP	0.10	11	0.861	0.984	0.1510
202109_at	1838.2	1516.0	-0.28	-1.21	PPP-PPP	0.10	11	0.861	0.984	0.1796
200688_at	450.7	371.6	-0.28	-1.21	PPP-PPP	0.10	11	0.861	0.984	0.2146
213534_s_at	203.0	167.4	-0.28	-1.21	PPP-PPP	0.10	11	0.861	0.984	0.3174
214136_at	189.9	156.6	-0.28	-1.21	PPP-PPP	0.10	11	0.861	0.984	0.3753
203706_s_at	1779.6	1467.5	-0.28	-1.21	PPP-PPP	0.10	11	0.861	0.984	0.3979
1561167_at	52.9	43.7	-0.28	-1.21	PAP-APP	0.10	11	0.861	0.984	0.4138
1558922_at	114.9	94.7	-0.28	-1.21	PPP-PPP	0.10	11	0.861	0.984	0.4607
203117_s_at	202.3	166.8	-0.28	-1.21	PPP-PPP	0.10	11	0.861	0.984	0.4632
229398_at	97.8	80.7	-0.28	-1.21	PPP-PPP	0.10	11	0.861	0.984	0.4870
213726_x_at	30962.0	25550.2	-0.28	-1.21	PPP-PPP	0.10	11	0.861	0.984	0.1293
212876_at	462.7	382.0	-0.28	-1.21	PPP-PPP	0.10	11	0.861	0.984	0.1585
229198_at	225.3	185.9	-0.28	-1.21	APP-PPM	0.10	11	0.861	0.984	0.1613
208581_x_at	23669.8	19533.9	-0.28	-1.21	PPP-PPP	0.10	11	0.861	0.984	0.1780
204214_s_at	4840.2	3995.7	-0.28	-1.21	PPP-PPP	0.10	11	0.861	0.984	0.3148
225266_at	308.8	254.7	-0.28	-1.21	PPP-PPP	0.10	11	0.861	0.984	0.3174
217367_s_at	273.0	225.3	-0.28	-1.21	PPP-PPP	0.10	11	0.861	0.984	0.3259
241938_at	117.7	97.1	-0.28	-1.21	PPA-MPP	0.10	11	0.861	0.984	0.3729
1558761_a_at	171.7	141.7	-0.28	-1.21	PPM-PMP	0.10	11	0.861	0.984	0.4748
218463_s_at	992.0	819.2	-0.28	-1.21	PPP-PPP	0.10	11	0.861	0.984	0.1284
212419_at	727.0	600.3	-0.28	-1.21	PPP-PPP	0.10	11	0.861	0.984	0.1293
236703_at	140.9	116.3	-0.28	-1.21	PAP-PAP	0.10	11	0.861	0.984	0.2119
1570198_x_at	295.6	244.0	-0.28	-1.21	PAP-PPP	0.10	11	0.861	0.984	0.2363
223161_at	227.7	188.1	-0.28	-1.21	PPA-PPP	0.10	11	0.861	0.984	0.2511
222721_at	109.5	90.4	-0.28	-1.21	AAP-AAP	0.10	11	0.861	0.984	0.2710

212543_at	560.1	462.4	-0.28	-1.21	PPP-PPP	0.10	11	0.861	0.984	0.2715
209364_at	703.2	580.9	-0.28	-1.21	PPP-PPP	0.10	11	0.861	0.984	0.2817
223894_s_at	790.8	653.0	-0.28	-1.21	PPP-PPP	0.10	11	0.861	0.984	0.3335
210395_x_at	199.5	164.8	-0.28	-1.21	PPP-PPP	0.10	11	0.861	0.984	0.3463
205609_at	174.8	144.3	-0.28	-1.21	PPP-PPP	0.10	11	0.861	0.984	0.3525
220608_s_at	73.0	60.3	-0.28	-1.21	PAA-PPP	0.10	11	0.861	0.984	0.3911
202839_s_at	1945.8	1606.5	-0.28	-1.21	PPP-PPP	0.10	11	0.861	0.984	0.5121
242812_at	197.5	163.3	-0.28	-1.21	PPP-PPP	0.10	11	0.861	0.984	0.1265
204360_s_at	226.5	187.1	-0.28	-1.21	PPP-PPP	0.10	11	0.861	0.984	0.1361
221799_at	548.3	453.2	-0.28	-1.21	AAP-PPP	0.10	11	0.861	0.984	0.1842
208319_s_at	6455.1	5334.7	-0.28	-1.21	PPP-PPP	0.10	11	0.861	0.984	0.2014
235094_at	906.0	748.8	-0.28	-1.21	PPP-PPP	0.10	11	0.861	0.984	0.2050
235198_at	886.4	732.8	-0.28	-1.21	PPP-PPP	0.10	11	0.861	0.984	0.2324
206972_s_at	182.9	151.1	-0.28	-1.21	APP-PPP	0.10	11	0.861	0.984	0.2709
222282_at	158.9	131.3	-0.28	-1.21	PMP-PAP	0.10	11	0.861	0.984	0.3313
234710_s_at	472.2	390.3	-0.28	-1.21	PPP-PPP	0.10	11	0.861	0.984	0.3799
239830_at	87.2	72.1	-0.28	-1.21	PPP-APP	0.10	11	0.861	0.984	0.4020
1566243_at	82.3	68.1	-0.28	-1.21	PPP-MPP	0.10	11	0.861	0.984	0.4898
222040_at	247.5	204.6	-0.28	-1.21	PPP-PPP	0.10	11	0.861	0.984	0.4988
227999_at	146.3	121.0	-0.27	-1.21	APP-PPP	0.10	11	0.861	0.984	0.1378
231319_x_at	1227.9	1015.5	-0.27	-1.21	PPP-PPP	0.10	11	0.861	0.984	0.1587
219650_at	1615.7	1336.3	-0.27	-1.21	PPP-PPP	0.10	11	0.861	0.984	0.1929
212303_x_at	833.2	689.2	-0.27	-1.21	PPP-PPP	0.10	11	0.861	0.984	0.2295
229374_at	259.7	214.8	-0.27	-1.21	PPP-PPP	0.10	11	0.861	0.984	0.3887
212054_x_at	1072.9	887.3	-0.27	-1.21	PPP-PPP	0.10	11	0.861	0.984	0.4494
219844_at	117.2	97.0	-0.27	-1.21	AAM-APP	0.10	11	0.861	0.984	0.1391
243067_at	86.1	71.3	-0.27	-1.21	AAP-AAA	0.10	11	0.861	0.984	0.1810
223465_at	659.0	545.3	-0.27	-1.21	PPP-PPP	0.10	11	0.861	0.984	0.2634
220956_s_at	948.8	785.4	-0.27	-1.21	PPP-PPP	0.10	11	0.861	0.984	0.3106
238154_at	94.9	78.5	-0.27	-1.21	PAP-PPP	0.10	11	0.861	0.984	0.4657
1553349_at	101.7	84.1	-0.27	-1.21	PPP-APP	0.10	11	0.861	0.984	0.4690
240181_at	110.0	91.0	-0.27	-1.21	PPA-PPP	0.10	11	0.861	0.984	0.4772
241737_x_at	118.7	98.3	-0.27	-1.21	PPA-AAP	0.10	11	0.861	0.984	0.1474
223619_x_at	504.1	417.4	-0.27	-1.21	PPP-PPP	0.10	11	0.861	0.984	0.2445
208194_s_at	165.6	137.2	-0.27	-1.21	PPP-PPP	0.10	11	0.861	0.984	0.3995
235652_at	203.2	168.4	-0.27	-1.21	PPP-MPP	0.10	11	0.861	0.984	0.1623
210927_x_at	13177.4	10918.5	-0.27	-1.21	PPP-PPP	0.10	11	0.861	0.984	0.1963
214844_s_at	179.0	148.4	-0.27	-1.21	PPP-PPP	0.10	11	0.861	0.984	0.2362
219986_s_at	91.4	75.7	-0.27	-1.21	APA-AAA	0.10	11	0.861	0.984	0.2401
241425_at	354.8	294.1	-0.27	-1.21	PPP-PPP	0.10	11	0.861	0.984	0.2404
223847_s_at	655.7	543.5	-0.27	-1.21	PPP-PPP	0.10	11	0.861	0.984	0.2429
212953_x_at	6811.8	5644.1	-0.27	-1.21	PPP-PPP	0.10	11	0.861	0.984	0.2792

203018_s_at	572.5	474.6	-0.27	-1.21	PPP-PPP	0.10	11	0.861	0.984	0.3070
226329_s_at	2253.5	1868.1	-0.27	-1.21	PPP-PPP	0.10	11	0.861	0.984	0.3348
210947_s_at	944.5	782.5	-0.27	-1.21	PPP-PPP	0.10	11	0.861	0.984	0.3482
204795_at	403.1	334.0	-0.27	-1.21	AAP-MMP	0.10	11	0.861	0.984	0.3527
233197_at	99.7	82.6	-0.27	-1.21	APP-PPP	0.10	11	0.861	0.984	0.3952
216682_s_at	99.4	82.3	-0.27	-1.21	PPP-APP	0.10	11	0.861	0.984	0.4110
227205_at	866.1	717.7	-0.27	-1.21	PPP-PPP	0.10	11	0.861	0.984	0.4372
236685_at	145.7	120.7	-0.27	-1.21	PPP-PPP	0.10	11	0.861	0.984	0.5029
203262_s_at	2432.2	2017.0	-0.27	-1.21	PPP-PPP	0.10	11	0.861	0.984	0.1347
1570155_at	479.2	397.4	-0.27	-1.21	PPP-PPP	0.10	11	0.861	0.984	0.2004
232231_at	1546.8	1282.8	-0.27	-1.21	PPP-PPP	0.10	11	0.861	0.984	0.2156
221989_at	1200.4	995.6	-0.27	-1.21	PPP-PPP	0.10	11	0.861	0.984	0.2496
1555351_s_at	328.3	272.2	-0.27	-1.21	PPP-PPP	0.10	11	0.861	0.984	0.2554
207172_s_at	365.1	302.7	-0.27	-1.21	PPP-PPP	0.10	11	0.861	0.984	0.2737
207992_s_at	463.3	384.3	-0.27	-1.21	PPP-PPP	0.10	11	0.861	0.984	0.4074
1560031_at	606.4	503.3	-0.27	-1.21	PPP-PPP	0.10	11	0.861	0.984	0.1144
210841_s_at	345.1	286.4	-0.27	-1.21	PPP-PPP	0.10	11	0.861	0.984	0.1799
201338_x_at	8950.8	7427.1	-0.27	-1.21	PPP-PPP	0.10	11	0.861	0.984	0.2394
1558393_at	249.2	206.8	-0.27	-1.21	AAA-AAP	0.10	11	0.861	0.984	0.2762
223617_x_at	1191.4	988.7	-0.27	-1.21	PPP-PPP	0.10	11	0.861	0.984	0.2907
212090_at	983.3	816.1	-0.27	-1.21	PPP-PPP	0.10	11	0.861	0.984	0.3393
218679_s_at	1867.0	1549.8	-0.27	-1.21	PPP-PPP	0.10	11	0.861	0.984	0.3776
227854_at	243.8	202.4	-0.27	-1.21	APP-APP	0.10	11	0.861	0.984	0.3781
206100_at	98.7	81.9	-0.27	-1.21	PPP-PAP	0.10	11	0.861	0.984	0.4503
1562022_s_at	249.2	206.8	-0.27	-1.21	AAP-AAA	0.10	11	0.861	0.984	0.4749
1565329_at	49.6	41.2	-0.27	-1.21	APA-PAP	0.10	11	0.861	0.984	0.4784
218744_s_at	194.8	161.6	-0.27	-1.21	APP-PPA	0.10	11	0.861	0.984	0.4836
222376_at	205.0	170.2	-0.27	-1.20	PPP-PPP	0.10	11	0.861	0.984	0.2104
201829_at	7057.7	5861.0	-0.27	-1.20	PPP-PPP	0.10	11	0.861	0.984	0.2319
241975_at	52.9	43.9	-0.27	-1.20	PPP-AAP	0.10	11	0.861	0.984	0.2879
1556347_at	63.3	52.6	-0.27	-1.20	APP-PMP	0.10	11	0.861	0.984	0.3005
241887_at	70.7	58.7	-0.27	-1.20	PPP-PPP	0.10	11	0.861	0.984	0.3902
237136_at	77.1	64.0	-0.27	-1.21	AAP-AMA	0.10	11	0.861	0.984	0.4714
242034_at	60.9	50.6	-0.27	-1.21	APP-MPP	0.10	11	0.861	0.984	0.5004
226875_at	662.0	550.3	-0.27	-1.20	PPP-PPP	0.10	11	0.861	0.984	0.1122
244008_at	83.9	69.7	-0.27	-1.20	PAA-APA	0.10	11	0.861	0.984	0.1956
242044_at	79.5	66.1	-0.27	-1.20	PPA-AAA	0.10	11	0.861	0.984	0.3254
213849_s_at	74.1	61.6	-0.27	-1.20	PAP-PAP	0.10	11	0.861	0.984	0.3774
233572_s_at	194.1	161.4	-0.27	-1.20	AAA-AAP	0.10	11	0.861	0.984	0.3818
201621_at	533.2	443.2	-0.27	-1.20	AAA-PPA	0.10	11	0.861	0.984	0.5070
243615_at	101.0	84.0	-0.27	-1.20	PAA-PMP	0.10	11	0.861	0.984	0.1382
203712_at	3241.9	2696.7	-0.27	-1.20	PPP-PPP	0.10	11	0.861	0.984	0.2080

210178_x_at	3312.0	2754.9	-0.27	-1.20	PPP-PPP	0.10	11	0.861	0.984	0.2243
216381_x_at	374.5	311.5	-0.27	-1.20	PPP-PPP	0.10	11	0.861	0.984	0.2719
232141_at	486.2	404.4	-0.27	-1.20	PPP-PPP	0.10	11	0.861	0.984	0.3837
228259_s_at	138.5	115.2	-0.27	-1.20	MPP-PPP	0.10	11	0.861	0.984	0.4173
1566079_at	195.6	162.6	-0.27	-1.20	PPP-PPP	0.10	11	0.861	0.984	0.4388
219295_s_at	73.1	60.8	-0.27	-1.20	PPP-PPA	0.10	11	0.861	0.984	0.4719
215177_s_at	535.5	445.2	-0.27	-1.20	PPP-PPP	0.10	11	0.861	0.984	0.4751
212680_x_at	6139.3	5110.3	-0.27	-1.20	PPP-PPP	0.10	11	0.861	0.984	0.1161
203060_s_at	10736.0	8932.3	-0.27	-1.20	PPP-PPP	0.10	11	0.861	0.984	0.1407
218069_at	5812.6	4836.2	-0.27	-1.20	PPP-PPP	0.10	11	0.861	0.984	0.1560
200012_x_at	34284.2	28536.0	-0.27	-1.20	PPP-PPP	0.10	11	0.861	0.984	0.1566
238609_at	408.4	340.0	-0.27	-1.20	PPP-PPP	0.10	11	0.861	0.984	0.1774
226324_s_at	439.0	365.3	-0.27	-1.20	APP-PPP	0.10	11	0.861	0.984	0.2102
201331_s_at	2324.1	1934.4	-0.27	-1.20	PPP-PPP	0.10	11	0.861	0.984	0.2655
221821_s_at	1465.0	1219.1	-0.27	-1.20	PPP-PPP	0.10	11	0.861	0.984	0.2719
215947_s_at	3767.5	3136.1	-0.27	-1.20	PPP-PPP	0.10	11	0.861	0.984	0.2792
217602_at	101.8	84.7	-0.27	-1.20	PAP-APP	0.10	11	0.861	0.984	0.3319
209558_s_at	173.5	144.4	-0.27	-1.20	AAA-APP	0.10	11	0.861	0.984	0.4418
205276_s_at	124.5	103.6	-0.27	-1.20	AMP-PAA	0.10	11	0.861	0.984	0.4807
226274_at	297.7	247.7	-0.27	-1.20	PPP-PPP	0.10	11	0.861	0.984	0.4929
222029_x_at	1750.9	1458.0	-0.26	-1.20	PPP-PPP	0.10	11	0.861	0.984	0.1322
241407_at	100.7	83.9	-0.26	-1.20	APP-PPP	0.10	11	0.861	0.984	0.1343
225465_at	566.8	471.9	-0.26	-1.20	PPP-PPP	0.10	11	0.861	0.984	0.1368
209971_x_at	6427.5	5352.4	-0.26	-1.20	PPP-PPP	0.10	11	0.861	0.984	0.1601
208273_at	43.2	36.0	-0.26	-1.20	APA-APA	0.10	11	0.861	0.984	0.2700
1554537_at	160.9	134.0	-0.26	-1.20	PPP-PPP	0.10	11	0.861	0.984	0.2804
1569685_at	91.6	76.3	-0.26	-1.20	PAM-AAP	0.10	11	0.861	0.984	0.3792
203725_at	5086.9	4236.3	-0.26	-1.20	PPP-PPP	0.10	11	0.861	0.984	0.3828
238970_at	84.7	70.6	-0.26	-1.20	PPP-PAP	0.10	11	0.861	0.984	0.4111
228709_at	144.0	119.9	-0.26	-1.20	PPP-PPP	0.10	11	0.861	0.984	0.4382
238104_at	98.2	81.8	-0.26	-1.20	AAP-AAA	0.10	11	0.861	0.984	0.4687
229966_at	226.8	189.0	-0.26	-1.20	PPP-PPP	0.10	11	0.861	0.984	0.1467
222713_s_at	269.7	224.7	-0.26	-1.20	PPP-PPP	0.10	11	0.861	0.984	0.1815
1560509_at	172.4	143.7	-0.26	-1.20	APP-MPP	0.10	11	0.861	0.984	0.2630
214734_at	259.7	216.4	-0.26	-1.20	PPP-PPP	0.10	11	0.861	0.984	0.2950
1558486_at	90.2	75.2	-0.26	-1.20	APM-APM	0.10	11	0.861	0.984	0.3251
203627_at	1682.2	1402.3	-0.26	-1.20	PPP-PPP	0.10	11	0.861	0.984	0.3293
203482_at	947.2	789.5	-0.26	-1.20	PPP-PPP	0.10	11	0.861	0.984	0.3496
1566507_a_at	61.9	51.6	-0.26	-1.20	PAP-AMP	0.10	11	0.861	0.984	0.3512
239600_at	91.0	75.8	-0.26	-1.20	PPP-MPP	0.10	11	0.861	0.984	0.4028
235894_at	116.1	96.8	-0.26	-1.20	PPP-AAP	0.10	11	0.861	0.984	0.4338
1569409_x_at	244.9	204.0	-0.26	-1.20	PPP-PPP	0.10	11	0.861	0.984	0.4554

212772_s_at	124.2	103.5	-0.26	-1.20	MPP-APP	0.10	11	0.861	0.984	0.4553
207152_at	93.2	77.6	-0.26	-1.20	PAP-PPP	0.10	11	0.861	0.984	0.4669
226273_at	155.7	129.8	-0.26	-1.20	PPP-PPP	0.10	11	0.861	0.984	0.4730
230850_at	69.9	58.2	-0.26	-1.20	PPP-AAP	0.10	11	0.861	0.984	0.4786
224286_at	193.6	161.3	-0.26	-1.20	PAA-AAA	0.10	11	0.861	0.984	0.4999
201713_s_at	4659.3	3884.6	-0.26	-1.20	PPP-PPP	0.10	11	0.861	0.984	0.1611
207165_at	8175.3	6816.2	-0.26	-1.20	PPP-PPP	0.10	11	0.861	0.984	0.1875
202369_s_at	6349.8	5293.9	-0.26	-1.20	PPP-PPP	0.10	11	0.861	0.984	0.1903
221227_x_at	1125.4	938.6	-0.26	-1.20	PPP-PPP	0.10	11	0.861	0.984	0.1982
214437_s_at	3437.5	2866.9	-0.26	-1.20	PPP-PPP	0.10	11	0.861	0.984	0.2050
214902_x_at	645.9	538.6	-0.26	-1.20	PPP-PPP	0.10	11	0.861	0.984	0.2103
244458_at	118.8	99.1	-0.26	-1.20	PPA-APA	0.10	11	0.861	0.984	0.2771
225810_at	643.6	536.7	-0.26	-1.20	PPP-PPP	0.10	11	0.861	0.984	0.3336
224495_at	126.2	105.3	-0.26	-1.20	APA-AAA	0.10	11	0.861	0.984	0.3828
1559902_at	211.4	176.3	-0.26	-1.20	PPP-PPP	0.10	11	0.861	0.984	0.4411
1559222_at	106.0	88.4	-0.26	-1.20	PPP-PAP	0.10	11	0.861	0.984	0.4464
209322_s_at	254.5	212.3	-0.26	-1.20	PPP-PPP	0.10	11	0.861	0.984	0.4714
233589_x_at	678.2	565.6	-0.26	-1.20	PPP-PPP	0.10	11	0.861	0.984	0.5128
200633_at	44732.7	37318.4	-0.26	-1.20	PPP-PPP	0.10	11	0.861	0.984	0.1137
213503_x_at	35011.4	29224.0	-0.26	-1.20	PPP-PPP	0.10	11	0.861	0.984	0.1351
233563_s_at	832.5	694.9	-0.26	-1.20	PPP-PPP	0.10	11	0.861	0.984	0.2081
205107_s_at	201.1	167.8	-0.26	-1.20	AAP-PPP	0.10	11	0.861	0.984	0.2204
214948_s_at	3029.5	2527.6	-0.26	-1.20	PPP-PPP	0.10	11	0.861	0.984	0.2979
220661_s_at	443.3	369.9	-0.26	-1.20	PPP-PPP	0.10	11	0.861	0.984	0.3076
232365_at	74.2	61.9	-0.26	-1.20	PPA-PPP	0.10	11	0.861	0.984	0.4196
218022_at	461.8	385.3	-0.26	-1.20	MAP-PPP	0.10	11	0.861	0.984	0.4211
230291_s_at	430.5	359.2	-0.26	-1.20	PPP-PPP	0.10	11	0.861	0.984	0.4229
204062_s_at	195.2	162.9	-0.26	-1.20	PPP-PPP	0.10	11	0.861	0.984	0.4390
215731_s_at	421.0	351.4	-0.26	-1.20	PPP-PPP	0.10	11	0.861	0.984	0.4773
226088_at	556.0	464.4	-0.26	-1.20	APP-PPP	0.10	11	0.861	0.984	0.1095
1557049_at	223.1	186.3	-0.26	-1.20	PPP-PPP	0.10	11	0.861	0.984	0.1475
213093_at	2923.0	2440.3	-0.26	-1.20	PPP-PPP	0.10	11	0.861	0.984	0.1799
1554329_x_at	79.4	66.3	-0.26	-1.20	PAP-APP	0.10	11	0.861	0.984	0.2993
231943_at	84.4	70.4	-0.26	-1.20	PAP-PPP	0.10	11	0.861	0.984	0.3109
230180_at	705.6	589.2	-0.26	-1.20	PPP-PPP	0.10	11	0.861	0.984	0.3114
202575_at	138.2	115.4	-0.26	-1.20	AAP-AAP	0.10	11	0.861	0.984	0.3923
220642_x_at	1216.5	1016.0	-0.26	-1.20	PPP-PPP	0.10	11	0.861	0.984	0.4075
243229_at	149.4	124.7	-0.26	-1.20	PPP-MPP	0.10	11	0.861	0.984	0.4576
1553466_at	87.4	73.0	-0.26	-1.20	APA-AAA	0.10	11	0.861	0.984	0.4668
227655_at	551.3	460.5	-0.26	-1.20	PPP-PPP	0.10	11	0.861	0.984	0.4899
227399_at	1599.4	1336.2	-0.26	-1.20	PPP-PPP	0.10	11	0.861	0.984	0.1329
243280_at	259.0	216.5	-0.26	-1.20	AAA-AAP	0.10	11	0.861	0.984	0.1706

1554310_a_at	138.8	116.0	-0.26	-1.20	PPA-APA	0.10	11	0.861	0.984	0.1928
219573_at	205.6	171.8	-0.26	-1.20	PPM-AAP	0.10	11	0.861	0.984	0.2151
213274_s_at	1023.0	855.0	-0.26	-1.20	PPP-PPP	0.10	11	0.861	0.984	0.2272
238465_at	1096.3	916.2	-0.26	-1.20	PPP-PPP	0.10	11	0.861	0.984	0.2290
225804_at	259.5	216.9	-0.26	-1.20	PPP-PPP	0.10	11	0.861	0.984	0.2546
226035_at	1001.4	836.9	-0.26	-1.20	PPP-PPP	0.10	11	0.861	0.984	0.2615
203354_s_at	288.4	241.0	-0.26	-1.20	PPP-APP	0.10	11	0.861	0.984	0.3415
204727_at	215.9	180.4	-0.26	-1.20	PPP-PPP	0.10	11	0.861	0.984	0.4147
242313_at	81.5	68.1	-0.26	-1.20	PPP-PPP	0.10	11	0.861	0.984	0.4569
220473_s_at	130.8	109.3	-0.26	-1.20	PPP-PPP	0.10	11	0.861	0.984	0.4772
210679_x_at	428.5	358.1	-0.26	-1.20	PPP-PPP	0.10	11	0.861	0.984	0.4876
219146_at	1886.3	1577.4	-0.26	-1.20	PPP-PPP	0.10	11	0.861	0.984	0.1524
209000_s_at	669.2	559.6	-0.26	-1.20	PPP-PPP	0.10	11	0.861	0.984	0.1793
202499_s_at	551.8	461.5	-0.26	-1.20	PPP-PPP	0.10	11	0.861	0.984	0.2169
216977_x_at	4328.3	3619.1	-0.26	-1.20	PPP-PPP	0.10	11	0.861	0.984	0.2528
201431_s_at	5097.5	4263.1	-0.26	-1.20	PPP-PPP	0.10	11	0.861	0.984	0.2813
228245_s_at	159.7	133.5	-0.26	-1.20	MPP-PPP	0.10	11	0.861	0.984	0.3104
229315_at	86.3	72.2	-0.26	-1.20	MPP-PPP	0.10	11	0.861	0.984	0.3687
236975_at	59.5	49.8	-0.26	-1.20	PAP-PMP	0.10	11	0.861	0.984	0.3721
210870_s_at	131.3	109.8	-0.26	-1.20	MPP-PPP	0.10	11	0.861	0.984	0.3981
243411_at	80.4	67.3	-0.26	-1.20	PAA-AAA	0.10	11	0.861	0.984	0.4591
236808_at	138.5	115.9	-0.26	-1.20	PPP-PPP	0.10	11	0.861	0.984	0.4693
1558785_a_at	113.3	94.7	-0.26	-1.20	MAA-PPP	0.10	11	0.861	0.984	0.5007
221501_x_at	5008.1	4190.6	-0.26	-1.20	PPP-PPP	0.10	11	0.861	0.984	0.2119
208823_s_at	921.7	771.2	-0.26	-1.20	PPP-PPP	0.10	11	0.861	0.984	0.2221
240271_at	92.7	77.5	-0.26	-1.20	APP-PPP	0.10	11	0.861	0.984	0.2602
230213_at	141.6	118.5	-0.26	-1.20	MPA-PPP	0.10	11	0.861	0.984	0.2645
215436_at	73.1	61.2	-0.26	-1.20	PAP-PPP	0.10	11	0.861	0.984	0.3687
1555271_a_at	152.4	127.5	-0.26	-1.20	PPP-PPP	0.10	11	0.861	0.984	0.3962
221475_s_at	37198.2	31144.0	-0.26	-1.19	PPP-PPP	0.10	11	0.861	0.984	0.1082
200763_s_at	42517.1	35605.3	-0.26	-1.19	PPP-PPP	0.10	11	0.861	0.984	0.1505
236772_s_at	109.9	92.1	-0.26	-1.19	PPP-PPP	0.10	11	0.861	0.984	0.1635
225993_at	463.0	387.8	-0.26	-1.19	PPP-PPP	0.10	11	0.861	0.984	0.1906
224558_s_at	771.1	645.7	-0.26	-1.19	PPP-PPP	0.10	11	0.861	0.984	0.2095
209453_at	502.8	421.0	-0.26	-1.19	PPP-PPP	0.10	11	0.861	0.984	0.2168
203173_s_at	1022.8	856.4	-0.26	-1.19	PPP-PPP	0.10	11	0.861	0.984	0.2834
227540_at	401.3	336.1	-0.26	-1.19	PPP-PPP	0.10	11	0.861	0.984	0.2953
201050_at	1184.4	992.1	-0.26	-1.19	PPP-PPP	0.10	11	0.861	0.984	0.2981
241156_at	59.9	50.2	-0.26	-1.19	APM-MPA	0.10	11	0.861	0.984	0.3326
204024_at	217.0	181.7	-0.26	-1.19	PPP-PPP	0.10	11	0.861	0.984	0.3471
228013_at	562.9	471.6	-0.26	-1.19	PPP-PPP	0.10	11	0.861	0.984	0.3601
204922_at	166.2	139.2	-0.26	-1.19	MAP-PAP	0.10	11	0.861	0.984	0.3963

230240_at	352.1	294.9	-0.26	-1.19	PAA-AAP	0.10	11	0.861	0.984	0.4230
214748_at	166.1	139.1	-0.26	-1.19	PPP-PPP	0.10	11	0.861	0.984	0.4902
208966_x_at	4413.1	3698.2	-0.26	-1.19	PPP-PPP	0.10	11	0.861	0.984	0.1099
201433_s_at	7491.8	6277.3	-0.26	-1.19	PPP-PPP	0.10	11	0.861	0.984	0.1342
204741_at	208.0	174.4	-0.26	-1.19	PAA-APP	0.10	11	0.861	0.984	0.1559
218882_s_at	3038.5	2547.0	-0.26	-1.19	PPP-PPP	0.10	11	0.861	0.984	0.2071
218460_at	3146.5	2636.7	-0.26	-1.19	PPP-PPP	0.10	11	0.861	0.984	0.2119
1552481_s_at	124.7	104.5	-0.26	-1.19	PPP-PPP	0.10	11	0.861	0.984	0.2507
227206_at	76.3	63.9	-0.26	-1.19	APA-PMP	0.10	11	0.861	0.984	0.3484
227223_at	543.7	455.6	-0.26	-1.19	PPP-PPP	0.10	11	0.861	0.984	0.4652
214048_at	196.4	164.6	-0.26	-1.19	PPP-PPP	0.10	11	0.861	0.984	0.4881
205836_s_at	273.8	229.5	-0.25	-1.19	PPP-PPP	0.10	11	0.861	0.984	0.2678
1556755_s_at	136.8	114.8	-0.25	-1.19	APP-PAA	0.10	11	0.861	0.984	0.3136
241762_at	68.9	57.8	-0.25	-1.19	APP-APA	0.10	11	0.861	0.984	0.3154
1553120_at	123.3	103.4	-0.25	-1.19	PPP-PPP	0.10	11	0.861	0.984	0.3307
1554414_a_at	670.2	561.8	-0.25	-1.19	PPP-PPP	0.10	11	0.861	0.984	0.3615
202288_at	435.2	365.0	-0.25	-1.19	PPP-PPP	0.10	11	0.861	0.984	0.4842
208604_s_at	87.5	73.4	-0.25	-1.19	AAA-PPM	0.10	11	0.861	0.984	0.4914
205427_at	784.5	658.4	-0.25	-1.19	PPP-PPP	0.10	11	0.861	0.984	0.1057
210211_s_at	33979.1	28522.2	-0.25	-1.19	PPP-PPP	0.10	11	0.861	0.984	0.1099
224849_at	1495.5	1254.6	-0.25	-1.19	PPP-PPP	0.10	11	0.861	0.984	0.1126
211094_s_at	260.0	218.1	-0.25	-1.19	PPP-PPP	0.10	11	0.861	0.984	0.1969
226084_at	4366.6	3665.4	-0.25	-1.19	PPP-PPP	0.10	11	0.861	0.984	0.1978
226579_at	1015.5	852.0	-0.25	-1.19	PPP-PPP	0.10	11	0.861	0.984	0.2925
213992_at	242.0	203.1	-0.25	-1.19	PPP-PPP	0.10	11	0.861	0.984	0.2984
222835_at	931.3	781.6	-0.25	-1.19	PPP-PPP	0.10	11	0.861	0.984	0.3070
242191_at	832.1	698.5	-0.25	-1.19	PPP-PPP	0.10	11	0.861	0.984	0.3144
240928_at	458.8	385.0	-0.25	-1.19	PPP-PPP	0.10	11	0.861	0.984	0.3225
1568923_at	99.6	83.6	-0.25	-1.19	APM-PPP	0.10	11	0.861	0.984	0.3379
230605_at	150.6	126.4	-0.25	-1.19	AAA-AAP	0.10	11	0.861	0.984	0.3650
205233_s_at	231.4	194.2	-0.25	-1.19	AAP-PPM	0.10	11	0.861	0.984	0.4008
204367_at	1428.8	1199.4	-0.25	-1.19	PPP-PPP	0.10	11	0.861	0.984	0.4214
1567222_x_at	130.7	109.7	-0.25	-1.19	AAP-PPP	0.10	11	0.861	0.984	0.4290
209017_s_at	2814.4	2363.3	-0.25	-1.19	PPP-PPP	0.10	11	0.861	0.984	0.1434
225898_at	4262.5	3578.5	-0.25	-1.19	PPP-PPP	0.10	11	0.861	0.984	0.2335
206920_s_at	619.8	520.5	-0.25	-1.19	PPP-PPP	0.10	11	0.861	0.984	0.2365
227121_at	262.1	220.0	-0.25	-1.19	PPP-PPP	0.10	11	0.861	0.984	0.2397
227997_at	141.7	119.0	-0.25	-1.19	PPP-PPP	0.10	11	0.861	0.984	0.2569
228686_at	140.0	117.6	-0.25	-1.19	PPP-PPP	0.10	11	0.861	0.984	0.3253
225622_at	110.5	92.8	-0.25	-1.19	PPP-PPP	0.10	11	0.861	0.984	0.3639
217618_x_at	331.2	278.3	-0.25	-1.19	PPP-PPP	0.10	11	0.861	0.984	0.1136
206431_x_at	993.6	835.1	-0.25	-1.19	PPP-PPP	0.10	11	0.861	0.984	0.1582

1552275_s_at	406.2	341.5	-0.25	-1.19	PPP-PPP	0.10	11	0.861	0.984	0.1748
238472_at	163.9	137.7	-0.25	-1.19	MPP-APP	0.10	11	0.861	0.984	0.2175
235429_at	389.7	327.5	-0.25	-1.19	PPP-PPP	0.10	11	0.861	0.984	0.2771
241824_at	209.1	175.7	-0.25	-1.19	PPP-PPP	0.10	11	0.861	0.984	0.3141
228570_at	145.4	122.2	-0.25	-1.19	APP-PPP	0.10	11	0.861	0.984	0.3191
226201_at	266.2	223.6	-0.25	-1.19	PPP-PPP	0.10	11	0.861	0.984	0.4128
224566_at	2209.1	1856.6	-0.25	-1.19	PPP-PPP	0.10	11	0.861	0.984	0.4262
226454_at	202.1	169.8	-0.25	-1.19	PPP-PPP	0.10	11	0.861	0.984	0.4269
207798_s_at	171.5	144.1	-0.25	-1.19	PPA-APP	0.10	11	0.861	0.984	0.4329
202321_at	192.9	162.1	-0.25	-1.19	PPP-PPP	0.10	11	0.861	0.984	0.4366
202207_at	2809.8	2363.2	-0.25	-1.19	PPP-PPP	0.10	11	0.861	0.984	0.1187
205412_at	6005.8	5049.6	-0.25	-1.19	PPP-PPP	0.10	11	0.861	0.984	0.1201
221216_s_at	658.1	553.5	-0.25	-1.19	PPP-PPP	0.10	11	0.861	0.984	0.1882
218873_at	578.0	486.1	-0.25	-1.19	PPP-PPP	0.10	11	0.861	0.984	0.2497
201278_at	3186.2	2679.7	-0.25	-1.19	PPP-PPP	0.10	11	0.861	0.984	0.3033
230886_at	194.4	163.5	-0.25	-1.19	PPP-PPP	0.10	11	0.861	0.984	0.3141
232037_at	157.4	132.4	-0.25	-1.19	PPM-AAP	0.10	11	0.861	0.984	0.3398
240451_at	178.0	149.7	-0.25	-1.19	PPP-PPP	0.10	11	0.861	0.984	0.4298
212738_at	557.7	468.9	-0.25	-1.19	PAP-PPP	0.10	11	0.861	0.984	0.4632
1565886_at	119.0	100.1	-0.25	-1.19	PPP-PPP	0.10	11	0.861	0.984	0.4643
225945_at	2226.4	1873.7	-0.25	-1.19	PPP-PPP	0.10	11	0.861	0.984	0.1067
217310_s_at	249.7	210.1	-0.25	-1.19	PPP-PPP	0.10	11	0.861	0.984	0.1709
235356_at	94.0	79.1	-0.25	-1.19	PPP-PPP	0.10	11	0.861	0.984	0.1773
211454_x_at	853.3	717.8	-0.25	-1.19	PPP-PPP	0.10	11	0.861	0.984	0.2116
218739_at	952.9	801.8	-0.25	-1.19	PPP-PPP	0.10	11	0.861	0.984	0.2507
225177_at	312.7	263.2	-0.25	-1.19	PPP-PPP	0.10	11	0.861	0.984	0.3519
236305_at	94.9	79.8	-0.25	-1.19	AAA-APP	0.10	11	0.861	0.984	0.3543
222744_s_at	231.7	195.1	-0.25	-1.19	MPP-PPP	0.10	11	0.861	0.984	0.3751
233638_s_at	413.7	348.2	-0.25	-1.19	PPP-PPP	0.10	11	0.861	0.984	0.4029
229099_at	813.6	684.4	-0.25	-1.19	PPP-PPP	0.10	11	0.861	0.984	0.4460
1557112_a_at	423.1	356.0	-0.25	-1.19	PPP-PPP	0.10	11	0.861	0.984	0.4494
203906_at	200.6	168.8	-0.25	-1.19	AAA-AAP	0.10	11	0.861	0.984	0.4686
226536_at	2232.3	1879.4	-0.25	-1.19	PPP-PPP	0.10	11	0.861	0.984	0.1009
1562228_s_at	216.8	182.5	-0.25	-1.19	APP-PPP	0.10	11	0.861	0.984	0.1260
219654_at	5541.1	4665.7	-0.25	-1.19	PPP-PPP	0.10	11	0.861	0.984	0.1526
209488_s_at	2552.6	2149.5	-0.25	-1.19	PPP-PPP	0.10	11	0.861	0.984	0.2297
210434_x_at	14740.9	12416.1	-0.25	-1.19	PPP-PPP	0.10	11	0.861	0.984	0.2554
1554475_a_at	168.7	142.0	-0.25	-1.19	AAA-PAA	0.10	11	0.861	0.984	0.2689
210112_at	714.0	601.3	-0.25	-1.19	PPP-PPP	0.10	11	0.861	0.984	0.2748
209910_at	149.0	125.5	-0.25	-1.19	PPP-APA	0.10	11	0.861	0.984	0.3081
212733_at	857.4	722.1	-0.25	-1.19	PPP-PPP	0.10	11	0.861	0.984	0.3224
215242_at	80.1	67.5	-0.25	-1.19	APP-PPP	0.10	11	0.861	0.984	0.3514

226033_at	614.4	517.4	-0.25	-1.19	PPP-PPP	0.10	11	0.861	0.984	0.4061
1555906_s_at	892.2	751.4	-0.25	-1.19	PPP-PPP	0.10	11	0.861	0.984	0.4191
1553750_a_at	114.0	96.0	-0.25	-1.19	AMP-PPP	0.10	11	0.861	0.984	0.4331
222162_s_at	3974.3	3346.0	-0.25	-1.19	PPP-PPP	0.10	11	0.861	0.984	0.4731
235164_at	179.8	151.4	-0.25	-1.19	PAP-APP	0.10	11	0.861	0.984	0.4932
218580_x_at	8891.6	7491.7	-0.25	-1.19	PPP-PPP	0.10	11	0.861	0.984	0.1948
1560348_at	240.7	202.8	-0.25	-1.19	AAP-PPP	0.10	11	0.861	0.984	0.1950
205226_at	218.6	184.1	-0.25	-1.19	PPP-PMP	0.10	11	0.861	0.984	0.1977
224345_x_at	3772.9	3178.9	-0.25	-1.19	PPP-PPP	0.10	11	0.861	0.984	0.2206
224334_s_at	1488.9	1254.3	-0.25	-1.19	PPP-PPP	0.10	11	0.861	0.984	0.3123
211812_s_at	51.4	43.3	-0.25	-1.19	AAA-APA	0.10	11	0.861	0.984	0.4020
242207_at	148.4	125.0	-0.25	-1.19	APP-PPP	0.10	11	0.861	0.984	0.4076
206208_at	70.7	59.6	-0.25	-1.19	PAA-AAP	0.10	11	0.861	0.984	0.4478
226144_at	206.0	173.6	-0.25	-1.19	PPP-PPP	0.10	11	0.861	0.984	0.4584
200924_s_at	3439.0	2897.0	-0.25	-1.19	PPP-PPP	0.10	11	0.861	0.984	0.4910
230108_at	198.0	166.9	-0.25	-1.19	PPP-PPP	0.10	11	0.861	0.984	0.4954
209622_at	415.7	350.6	-0.25	-1.19	PPP-PPP	0.10	11	0.861	0.984	0.1226
202220_at	2600.7	2193.3	-0.25	-1.19	PPP-PPP	0.10	11	0.861	0.984	0.1489
229983_at	802.2	676.5	-0.25	-1.19	PPP-PPP	0.10	11	0.861	0.984	0.2159
213136_at	1805.3	1522.1	-0.25	-1.19	PPP-PPP	0.10	11	0.861	0.984	0.2670
230503_at	250.1	210.9	-0.25	-1.19	PPP-PPP	0.10	11	0.861	0.984	0.3281
200606_at	8391.5	7077.9	-0.25	-1.19	PPP-PPP	0.10	11	0.861	0.984	0.3849
212723_at	4125.9	3480.1	-0.25	-1.19	PPP-PPP	0.10	11	0.861	0.984	0.4138
203960_s_at	1797.7	1515.8	-0.25	-1.19	PPP-PPP	0.10	11	0.861	0.984	0.4161
220272_at	497.4	419.5	-0.25	-1.19	PPP-PPP	0.10	11	0.861	0.984	0.4243
232541_at	671.4	566.2	-0.25	-1.19	PPP-PPP	0.10	11	0.861	0.984	0.4640
227665_at	1113.9	939.2	-0.25	-1.19	PPP-PPP	0.10	11	0.861	0.984	0.4945
204657_s_at	491.5	414.8	-0.25	-1.19	PPP-PPP	0.10	11	0.861	0.984	0.0948
211502_s_at	396.6	334.7	-0.25	-1.19	PPP-PPP	0.10	11	0.861	0.984	0.1349
224580_at	735.2	620.4	-0.25	-1.19	PPP-PPP	0.10	11	0.861	0.984	0.2937
232506_s_at	143.4	121.0	-0.25	-1.19	PMP-PPA	0.10	11	0.861	0.984	0.3500
200962_at	248.9	210.1	-0.25	-1.19	APP-MPP	0.10	11	0.861	0.984	0.3691
242337_at	191.5	161.6	-0.25	-1.19	PPP-PAP	0.10	11	0.861	0.984	0.3900
238803_at	98.2	82.9	-0.25	-1.19	PPP-PMP	0.10	11	0.861	0.984	0.4288
54037_at	276.7	233.5	-0.25	-1.19	PPP-PPP	0.10	11	0.861	0.984	0.4635
225545_at	1665.3	1406.1	-0.24	-1.18	PPP-PPP	0.10	11	0.861	0.984	0.1308
207943_x_at	785.6	663.2	-0.24	-1.19	PPP-PPP	0.10	11	0.861	0.984	0.1554
1557409_at	666.9	563.0	-0.24	-1.18	PPP-PPP	0.10	11	0.861	0.984	0.1918
203526_s_at	433.1	365.8	-0.24	-1.18	PPP-PPP	0.10	11	0.861	0.984	0.2312
220094_s_at	5090.7	4298.1	-0.24	-1.18	PPP-PPP	0.10	11	0.861	0.984	0.2772
237172_at	96.5	81.5	-0.24	-1.19	AAA-PPP	0.10	11	0.861	0.984	0.3223
223149_s_at	97.3	82.2	-0.24	-1.19	PPA-PPP	0.10	11	0.861	0.984	0.4579

210128_s_at	124.6	105.2	-0.24	-1.18	AMP-PPP	0.10	11	0.861	0.984	0.4621
208987_s_at	578.3	488.4	-0.24	-1.18	APP-PPP	0.10	11	0.861	0.984	0.4646
211969_at	40857.4	34524.7	-0.24	-1.18	PPP-PPP	0.10	11	0.861	0.984	0.1345
225366_at	879.0	742.8	-0.24	-1.18	PPP-PPP	0.10	11	0.861	0.984	0.1577
231017_at	499.7	422.2	-0.24	-1.18	PPP-PPP	0.10	11	0.861	0.984	0.1649
221196_x_at	1951.6	1648.9	-0.24	-1.18	PPP-PPP	0.10	11	0.861	0.984	0.2005
244511_at	135.6	114.6	-0.24	-1.18	PPP-PPP	0.10	11	0.861	0.984	0.2388
205083_at	895.3	756.4	-0.24	-1.18	PPP-PPP	0.10	11	0.861	0.984	0.4002
222849_s_at	658.4	556.2	-0.24	-1.18	PPP-PPP	0.10	11	0.861	0.984	0.4363
229579_s_at	267.3	225.9	-0.24	-1.18	PPP-PPP	0.10	11	0.861	0.984	0.4758
218698_at	2492.1	2107.7	-0.24	-1.18	PPP-PPP	0.10	11	0.861	0.984	0.0998
224232_s_at	2766.9	2339.3	-0.24	-1.18	PPP-PPP	0.10	11	0.861	0.984	0.1457
208705_s_at	24064.4	20350.5	-0.24	-1.18	PPP-PPP	0.10	11	0.861	0.984	0.1523
217825_s_at	1216.8	1028.7	-0.24	-1.18	PPP-PPP	0.10	11	0.861	0.984	0.1643
227554_at	1520.2	1285.7	-0.24	-1.18	PPP-PPP	0.10	11	0.861	0.984	0.2136
200036_s_at	26530.8	22439.7	-0.24	-1.18	PPP-PPP	0.10	11	0.861	0.984	0.2158
223230_at	1303.9	1102.4	-0.24	-1.18	PPP-PPP	0.10	11	0.861	0.984	0.2582
227763_at	69.4	58.7	-0.24	-1.18	AAP-PAA	0.10	11	0.861	0.984	0.2905
206096_at	359.3	303.9	-0.24	-1.18	PPP-PPP	0.10	11	0.861	0.984	0.2952
217586_x_at	521.3	440.7	-0.24	-1.18	PPP-PPP	0.10	11	0.861	0.984	0.3611
1553139_s_at	130.8	110.6	-0.24	-1.18	AMA-PAP	0.10	11	0.861	0.984	0.4359
227082_at	177.3	149.9	-0.24	-1.18	PPP-PPP	0.10	11	0.861	0.984	0.4360
34260_at	472.5	399.5	-0.24	-1.18	PPP-PPP	0.10	11	0.861	0.984	0.4505
206033_s_at	155.6	131.6	-0.24	-1.18	PPP-PPP	0.10	11	0.861	0.984	0.4731
223649_s_at	1990.0	1684.4	-0.24	-1.18	PPP-PPP	0.10	11	0.861	0.984	0.0910
206473_at	380.7	322.0	-0.24	-1.18	PPP-PPP	0.10	11	0.861	0.984	0.1039
223334_at	9251.0	7829.5	-0.24	-1.18	PPP-PPP	0.10	11	0.861	0.984	0.1587
226935_s_at	2510.4	2124.4	-0.24	-1.18	PPP-PPP	0.10	11	0.861	0.984	0.1589
1557047_at	332.6	281.6	-0.24	-1.18	APP-PPP	0.10	11	0.861	0.984	0.1623
218005_at	2900.2	2453.7	-0.24	-1.18	PPP-PPP	0.10	11	0.861	0.984	0.2031
244659_at	164.3	139.1	-0.24	-1.18	PPP-MPP	0.10	11	0.861	0.984	0.2495
212262_at	2082.8	1762.9	-0.24	-1.18	PPP-PPP	0.10	11	0.861	0.984	0.2540
238478_at	406.5	344.0	-0.24	-1.18	PPP-PPP	0.10	11	0.861	0.984	0.2961
211926_s_at	9267.7	7840.5	-0.24	-1.18	PPP-PPP	0.10	11	0.861	0.984	0.3431
1556409_a_at	122.4	103.6	-0.24	-1.18	APP-PPP	0.10	11	0.861	0.984	0.3941
216226_at	116.5	98.6	-0.24	-1.18	PPA-AMA	0.10	11	0.861	0.984	0.4021
232269_x_at	469.2	397.1	-0.24	-1.18	PPP-PPP	0.10	11	0.861	0.984	0.4370
208929_x_at	34845.6	29502.0	-0.24	-1.18	PPP-PPP	0.10	11	0.861	0.984	0.0982
201122_x_at	6048.5	5120.2	-0.24	-1.18	PPP-PPP	0.10	11	0.861	0.984	0.1558
218335_x_at	1207.5	1022.2	-0.24	-1.18	PPP-PPP	0.10	11	0.861	0.984	0.1926
209026_x_at	33232.0	28137.9	-0.24	-1.18	PPP-PPP	0.10	11	0.861	0.984	0.2062
221876_at	245.9	208.2	-0.24	-1.18	PPA-PAM	0.10	11	0.861	0.984	0.2380

47773_at	434.1	367.5	-0.24	-1.18	PPP-PPP	0.10	11	0.861	0.984	0.2767
244474_at	137.5	116.5	-0.24	-1.18	PPP-PPP	0.10	11	0.861	0.984	0.2937
222452_s_at	1306.1	1105.6	-0.24	-1.18	PPP-PPP	0.10	11	0.861	0.984	0.2959
213429_at	1379.9	1168.3	-0.24	-1.18	PPP-PPP	0.10	11	0.861	0.984	0.3067
210767_at	102.3	86.6	-0.24	-1.18	PAP-MPM	0.10	11	0.861	0.984	0.3120
233146_at	92.3	78.1	-0.24	-1.18	PPA-AAA	0.10	11	0.861	0.984	0.3309
221912_s_at	237.3	201.0	-0.24	-1.18	PPP-PPP	0.10	11	0.861	0.984	0.3439
242117_at	52.9	44.8	-0.24	-1.18	APA-APA	0.10	11	0.861	0.984	0.4284
209604_s_at	553.0	468.3	-0.24	-1.18	PPP-PPP	0.10	11	0.861	0.984	0.4470
1562271_x_at	103.7	87.8	-0.24	-1.18	PPA-PPP	0.10	11	0.861	0.984	0.4620
1558586_at	52.9	44.8	-0.24	-1.18	PAA-AAA	0.10	11	0.861	0.984	0.4642
242162_at	84.5	71.5	-0.24	-1.18	PPM-PPP	0.10	11	0.861	0.984	0.5022
225463_x_at	1359.7	1152.0	-0.24	-1.18	PPP-PPP	0.10	11	0.861	0.984	0.1674
201421_s_at	1860.4	1576.2	-0.24	-1.18	PPP-PPP	0.10	11	0.861	0.984	0.1745
209969_s_at	574.6	486.7	-0.24	-1.18	PPP-PPP	0.10	11	0.861	0.984	0.2036
222133_s_at	238.0	201.7	-0.24	-1.18	PPP-PPP	0.10	11	0.861	0.984	0.2141
244038_at	404.4	342.6	-0.24	-1.18	PPP-PPP	0.10	11	0.861	0.984	0.2393
233625_x_at	1744.8	1477.9	-0.24	-1.18	PPP-PPP	0.10	11	0.861	0.984	0.2617
235475_at	103.7	87.8	-0.24	-1.18	AAP-APP	0.10	11	0.861	0.984	0.3540
225526_at	838.1	710.1	-0.24	-1.18	PPP-PPP	0.10	11	0.861	0.984	0.3762
232508_at	92.7	78.6	-0.24	-1.18	PAA-APM	0.10	11	0.861	0.984	0.4541
1557189_at	148.8	126.0	-0.24	-1.18	MAP-PPP	0.10	11	0.861	0.984	0.4662
232293_at	117.0	99.1	-0.24	-1.18	PAA-PPM	0.10	11	0.861	0.984	0.4812
200943_at	23761.1	20152.9	-0.24	-1.18	PPP-PPP	0.10	11	0.861	0.984	0.0982
200059_s_at	24017.0	20364.1	-0.24	-1.18	PPP-PPP	0.10	11	0.861	0.984	0.1007
214692_s_at	402.9	341.6	-0.24	-1.18	PPP-PPP	0.10	11	0.861	0.984	0.1386
226040_at	372.1	315.5	-0.24	-1.18	PPP-PPP	0.10	11	0.861	0.984	0.1499
201093_x_at	4328.2	3669.2	-0.24	-1.18	PPP-PPP	0.10	11	0.861	0.984	0.1572
224988_at	810.0	687.0	-0.24	-1.18	PPP-PPP	0.10	11	0.861	0.984	0.1568
239742_at	965.0	818.3	-0.24	-1.18	PPP-PPP	0.10	11	0.861	0.984	0.1584
225365_at	3650.1	3095.7	-0.24	-1.18	PPP-PPP	0.10	11	0.861	0.984	0.1752
206237_s_at	181.7	154.0	-0.24	-1.18	PPP-PPP	0.10	11	0.861	0.984	0.2313
205457_at	477.4	404.8	-0.24	-1.18	PPP-PPP	0.10	11	0.861	0.984	0.2772
235866_at	128.6	109.1	-0.24	-1.18	PPP-APP	0.10	11	0.861	0.984	0.3370
235204_at	830.5	704.0	-0.24	-1.18	PPP-PPP	0.10	11	0.861	0.984	0.3604
227563_at	132.3	112.2	-0.24	-1.18	PPP-PPP	0.10	11	0.861	0.984	0.3923
204947_at	530.6	449.8	-0.24	-1.18	APP-APP	0.10	11	0.861	0.984	0.4633
1552927_at	58.4	49.5	-0.24	-1.18	AAM-PPP	0.10	11	0.861	0.984	0.4841
201786_s_at	5981.9	5076.8	-0.24	-1.18	PPP-PPP	0.10	11	0.861	0.984	0.1130
211710_x_at	33365.2	28318.2	-0.24	-1.18	PPP-PPP	0.10	11	0.861	0.984	0.1288
201382_at	163.9	139.1	-0.24	-1.18	PAP-PPP	0.10	11	0.861	0.984	0.1438
218529_at	1401.4	1189.0	-0.24	-1.18	PPP-PPP	0.10	11	0.861	0.984	0.2111

222093_s_at	339.2	287.8	-0.24	-1.18	MPA-PAP	0.10	11	0.861	0.984	0.2185
202862_at	1183.0	1003.9	-0.24	-1.18	PPP-PPP	0.10	11	0.861	0.984	0.2703
240781_x_at	55.8	47.4	-0.24	-1.18	AMA-PAA	0.10	11	0.861	0.984	0.3433
1554102_a_at	118.6	100.6	-0.24	-1.18	PPP-PPP	0.10	11	0.861	0.984	0.3708
219891_at	177.6	150.7	-0.24	-1.18	PAP-PPA	0.10	11	0.861	0.984	0.3793
209746_s_at	447.5	379.8	-0.24	-1.18	PPP-PPP	0.10	11	0.861	0.984	0.4015
202692_s_at	526.6	446.8	-0.24	-1.18	PPP-PPP	0.10	11	0.861	0.984	0.4722
222030_at	323.9	275.0	-0.24	-1.18	PPP-PPP	0.10	11	0.861	0.984	0.0928
239897_at	242.4	205.8	-0.24	-1.18	APP-PPP	0.10	11	0.861	0.984	0.1687
229548_at	135.4	115.0	-0.24	-1.18	PMP-PAP	0.10	11	0.861	0.984	0.3839
230779_at	367.5	312.1	-0.24	-1.18	PPP-PPP	0.10	11	0.861	0.984	0.3895
226418_at	168.3	142.9	-0.24	-1.18	PPP-PPP	0.10	11	0.861	0.984	0.4282
220671_at	127.1	107.9	-0.24	-1.18	APP-PAP	0.10	11	0.861	0.984	0.4533
235702_at	155.6	132.1	-0.24	-1.18	PPP-PPP	0.10	11	0.861	0.984	0.4611
230320_at	144.3	122.5	-0.24	-1.18	PAA-AAP	0.10	11	0.861	0.984	0.4780
226482_s_at	495.6	420.9	-0.24	-1.18	PPP-PPP	0.10	11	0.861	0.984	0.4793
232778_at	148.2	125.9	-0.24	-1.18	PPP-PPP	0.10	11	0.861	0.984	0.4958
228742_at	92.8	78.8	-0.24	-1.18	PAA-AAP	0.10	11	0.861	0.984	0.4993
226611_s_at	4079.2	3464.9	-0.24	-1.18	PPP-PPP	0.10	11	0.861	0.984	0.0974
232559_at	69.0	58.6	-0.24	-1.18	PPP-AAP	0.10	11	0.861	0.984	0.1412
223342_at	434.7	369.4	-0.24	-1.18	PPP-PPP	0.10	11	0.861	0.984	0.1557
225853_at	3830.8	3255.7	-0.24	-1.18	PPP-PPP	0.10	11	0.861	0.984	0.1639
242073_at	282.5	240.1	-0.24	-1.18	PPP-PPP	0.10	11	0.861	0.984	0.2022
221038_at	170.6	145.0	-0.24	-1.18	PPP-PPP	0.10	11	0.861	0.984	0.2248
204183_s_at	196.4	166.8	-0.24	-1.18	APA-AAA	0.10	11	0.861	0.984	0.3466
228722_at	269.3	228.8	-0.24	-1.18	PPP-PPP	0.10	11	0.861	0.984	0.3945
220866_at	116.1	98.6	-0.24	-1.18	APP-APP	0.10	11	0.861	0.984	0.3980
218530_at	515.5	438.0	-0.24	-1.18	APP-PPP	0.10	11	0.861	0.984	0.4097
204627_s_at	162.8	138.3	-0.24	-1.18	APP-APP	0.10	11	0.861	0.984	0.4189
226054_at	890.0	756.4	-0.24	-1.18	PPP-PPP	0.10	11	0.861	0.984	0.4244
1553672_at	198.3	168.5	-0.24	-1.18	PPP-PPP	0.10	11	0.861	0.984	0.4290
218666_s_at	228.6	194.3	-0.24	-1.18	PMP-PPP	0.10	11	0.861	0.984	0.4336
224186_s_at	456.9	388.2	-0.24	-1.18	PPP-PPP	0.10	11	0.861	0.984	0.4479
201387_s_at	12528.6	10653.3	-0.23	-1.18	PPP-PPP	0.10	11	0.861	0.984	0.1120
203494_s_at	2150.9	1829.3	-0.23	-1.18	PPP-PPP	0.10	11	0.861	0.984	0.1217
216547_at	786.3	668.3	-0.23	-1.18	PPP-PPP	0.10	11	0.861	0.984	0.1306
219079_at	1066.8	907.0	-0.23	-1.18	PPP-PPP	0.10	11	0.861	0.984	0.1630
218477_at	4145.1	3525.6	-0.23	-1.18	PPP-PPP	0.10	11	0.861	0.984	0.1694
203515_s_at	1006.2	855.8	-0.23	-1.18	PPP-PPP	0.10	11	0.861	0.984	0.1722
212057_at	694.2	590.4	-0.23	-1.18	PPP-PPP	0.10	11	0.861	0.984	0.1879
212166_at	2170.0	1844.6	-0.23	-1.18	PPP-PPP	0.10	11	0.861	0.984	0.2221
221632_s_at	417.3	355.0	-0.23	-1.18	PMP-PPP	0.10	11	0.861	0.984	0.2276

213944_x_at	1397.5	1188.3	-0.23	-1.18	PPP-PPP	0.10	11	0.861	0.984	0.2739
203656_at	562.4	478.3	-0.23	-1.18	PPP-PPP	0.10	11	0.861	0.984	0.3000
1555950_a_at	1850.1	1572.7	-0.23	-1.18	PPP-PPP	0.10	11	0.861	0.984	0.3045
201165_s_at	2211.2	1880.0	-0.23	-1.18	PPP-PPP	0.10	11	0.861	0.984	0.3466
241303_x_at	321.6	273.4	-0.23	-1.18	PPA-PPP	0.10	11	0.861	0.984	0.3803
1569477_at	83.1	70.7	-0.23	-1.18	APP-PPP	0.10	11	0.861	0.984	0.4699
208915_s_at	487.0	414.0	-0.23	-1.18	MPP-PPP	0.10	11	0.861	0.984	0.4719
208714_at	4424.9	3764.9	-0.23	-1.18	PPP-PPP	0.10	11	0.861	0.984	0.0856
203404_at	1774.5	1510.3	-0.23	-1.18	PPP-PPP	0.10	11	0.861	0.984	0.0987
201577_at	25054.0	21323.4	-0.23	-1.18	PPP-PPP	0.10	11	0.861	0.984	0.1104
208073_x_at	6851.7	5831.5	-0.23	-1.18	PPP-PPP	0.10	11	0.861	0.984	0.1251
239888_at	218.3	185.8	-0.23	-1.18	PPP-PPP	0.10	11	0.861	0.984	0.1565
221954_at	443.0	376.9	-0.23	-1.18	PPP-PPP	0.10	11	0.861	0.984	0.1896
232730_at	94.0	80.0	-0.23	-1.18	PAP-PAM	0.10	11	0.861	0.984	0.2072
220160_s_at	539.3	458.8	-0.23	-1.18	PPP-PPP	0.10	11	0.861	0.984	0.3282
229829_at	178.5	151.8	-0.23	-1.18	AAP-APP	0.10	11	0.861	0.984	0.3637
214436_at	339.8	289.2	-0.23	-1.18	PPP-PPP	0.10	11	0.861	0.984	0.3705
239532_at	123.8	105.4	-0.23	-1.18	AAA-APA	0.10	11	0.861	0.984	0.3891
232230_at	68.0	57.9	-0.23	-1.18	APM-PAP	0.10	11	0.861	0.984	0.4033
231927_at	270.7	230.3	-0.23	-1.18	PPP-PPP	0.10	11	0.861	0.984	0.4244
203140_at	1712.7	1458.6	-0.23	-1.17	PPP-PPP	0.10	11	0.861	0.984	0.0890
207618_s_at	1428.0	1216.2	-0.23	-1.17	PPP-PPP	0.10	11	0.861	0.984	0.1018
1567032_s_at	92.3	78.5	-0.23	-1.18	PPP-PPP	0.10	11	0.861	0.984	0.1199
202279_at	4085.7	3477.7	-0.23	-1.18	PPP-PPP	0.10	11	0.861	0.984	0.1224
223780_s_at	271.3	230.9	-0.23	-1.18	PPP-PPP	0.10	11	0.861	0.984	0.1293
209286_at	3843.8	3272.6	-0.23	-1.18	PPP-PPP	0.10	11	0.861	0.984	0.1305
224815_at	5313.6	4525.1	-0.23	-1.17	PPP-PPP	0.10	11	0.861	0.984	0.1347
227561_at	1380.4	1175.6	-0.23	-1.17	PPP-PPP	0.10	11	0.861	0.984	0.1665
238637_at	354.9	302.2	-0.23	-1.17	PPA-APP	0.10	11	0.861	0.984	0.1780
1569392_at	96.3	82.0	-0.23	-1.18	AAP-PAP	0.10	11	0.861	0.984	0.1815
208977_x_at	30492.2	25961.1	-0.23	-1.18	PPP-PPP	0.10	11	0.861	0.984	0.2480
230747_s_at	229.9	195.8	-0.23	-1.17	PPP-PPP	0.10	11	0.861	0.984	0.2570
1558116_x_at	220.0	187.3	-0.23	-1.17	MAA-APP	0.10	11	0.861	0.984	0.3381
209530_at	351.5	299.3	-0.23	-1.18	PPP-PPP	0.10	11	0.861	0.984	0.3483
206448_at	216.5	184.3	-0.23	-1.18	PPP-PPP	0.10	11	0.861	0.984	0.3561
222566_at	139.2	118.5	-0.23	-1.17	AAP-PPP	0.10	11	0.861	0.984	0.3777
210277_at	548.0	466.6	-0.23	-1.17	PPP-PPP	0.10	11	0.861	0.984	0.4157
219263_at	166.1	141.4	-0.23	-1.18	PPP-PPP	0.10	11	0.861	0.984	0.4321
1569464_at	142.7	121.5	-0.23	-1.18	APP-PPP	0.10	11	0.861	0.984	0.4534
218856_at	1037.8	883.6	-0.23	-1.18	PPP-PPP	0.10	11	0.861	0.984	0.4575
204933_s_at	568.5	483.9	-0.23	-1.18	PPP-PPP	0.10	11	0.861	0.984	0.4607
228445_at	193.9	165.1	-0.23	-1.17	APA-PPP	0.10	11	0.861	0.984	0.4734

229417_at	164.3	139.9	-0.23	-1.18	PPP-PPP	0.10	11	0.861	0.984	0.4914
202082_s_at	1625.9	1385.5	-0.23	-1.17	PPP-PPP	0.10	11	0.861	0.984	0.0942
200976_s_at	6007.2	5118.9	-0.23	-1.17	PPP-PPP	0.10	11	0.861	0.984	0.1300
218874_s_at	187.0	159.3	-0.23	-1.17	APP-AAP	0.10	11	0.861	0.984	0.1503
222841_s_at	871.9	743.2	-0.23	-1.17	PPP-PPP	0.10	11	0.861	0.984	0.1651
208807_s_at	685.2	583.9	-0.23	-1.17	PPM-AAP	0.10	11	0.861	0.984	0.1665
212539_at	2896.1	2467.2	-0.23	-1.17	PPP-PPP	0.10	11	0.861	0.984	0.2204
227455_at	990.7	844.3	-0.23	-1.17	PPP-PPP	0.10	11	0.861	0.984	0.2272
1555679_a_at	881.8	751.1	-0.23	-1.17	PPP-PPP	0.10	11	0.861	0.984	0.2931
239775_at	101.1	86.2	-0.23	-1.17	APP-AMP	0.10	11	0.861	0.984	0.3247
230206_at	728.4	620.6	-0.23	-1.17	PPP-PPP	0.10	11	0.861	0.984	0.3683
202676_x_at	617.5	526.3	-0.23	-1.17	APP-PPP	0.10	11	0.861	0.984	0.4691
201112_s_at	20875.6	17798.3	-0.23	-1.17	PPP-PPP	0.10	11	0.861	0.984	0.0903
214427_at	3231.2	2755.9	-0.23	-1.17	PPP-PPP	0.10	11	0.861	0.984	0.1093
208908_s_at	3361.7	2867.2	-0.23	-1.17	PPP-PPP	0.10	11	0.861	0.984	0.1299
215617_at	803.7	685.5	-0.23	-1.17	PPP-PPP	0.10	11	0.861	0.984	0.1565
209467_s_at	807.9	688.9	-0.23	-1.17	PPP-PPP	0.10	11	0.861	0.984	0.1699
1555779_a_at	162.5	138.5	-0.23	-1.17	PAA-AAA	0.10	11	0.861	0.984	0.3524
214459_x_at	5274.8	4498.0	-0.23	-1.17	PPP-PPP	0.10	11	0.861	0.984	0.3875
217720_at	26335.3	22477.4	-0.23	-1.17	PPP-PPP	0.10	11	0.861	0.984	0.0831
204254_s_at	989.7	844.5	-0.23	-1.17	PPP-PPP	0.10	11	0.861	0.984	0.1039
205835_s_at	129.0	110.1	-0.23	-1.17	PPP-PPP	0.10	11	0.861	0.984	0.2188
224473_x_at	725.7	619.0	-0.23	-1.17	PAM-APM	0.10	11	0.861	0.984	0.2388
227607_at	1702.5	1452.5	-0.23	-1.17	PPP-PPP	0.10	11	0.861	0.984	0.2414
238902_at	100.6	85.8	-0.23	-1.17	PPP-APP	0.10	11	0.861	0.984	0.2491
232106_s_at	206.0	175.8	-0.23	-1.17	AAA-PPA	0.10	11	0.861	0.984	0.2771
237568_at	124.9	106.5	-0.23	-1.17	APP-PAP	0.10	11	0.861	0.984	0.2897
224624_at	3678.6	3139.5	-0.23	-1.17	PPP-PPP	0.10	11	0.861	0.984	0.2941
1555370_a_at	94.6	80.7	-0.23	-1.17	PPP-PPP	0.10	11	0.861	0.984	0.3469
236941_at	102.8	87.7	-0.23	-1.17	PPP-PPP	0.10	11	0.861	0.984	0.4655
203899_s_at	364.7	311.1	-0.23	-1.17	PPP-PPP	0.10	11	0.861	0.984	0.4703
234936_s_at	362.6	309.4	-0.23	-1.17	PPP-PPP	0.10	11	0.861	0.984	0.4931
200966_x_at	23457.5	20022.3	-0.23	-1.17	PPP-PPP	0.10	11	0.861	0.984	0.0997
226229_s_at	472.8	403.8	-0.23	-1.17	AAA-AAP	0.10	11	0.861	0.984	0.1015
227656_at	417.1	356.2	-0.23	-1.17	PPP-APP	0.10	11	0.861	0.984	0.1377
212737_at	1481.9	1265.1	-0.23	-1.17	PPP-PPP	0.10	11	0.861	0.984	0.1661
1555910_at	942.6	804.8	-0.23	-1.17	PPP-PPP	0.10	11	0.861	0.984	0.1664
AFFX-HUMISGF3A/M9	2114.0	1805.3	-0.23	-1.17	PPP-PPP	0.10	11	0.861	0.984	0.1668
208805_at	21386.7	18258.4	-0.23	-1.17	PPP-PPP	0.10	11	0.861	0.984	0.1683
1569470_a_at	282.6	241.3	-0.23	-1.17	AAA-AAP	0.10	11	0.861	0.984	0.2076
200877_at	24281.9	20730.3	-0.23	-1.17	PPP-PPP	0.10	11	0.861	0.984	0.2262
210529_s_at	810.1	691.8	-0.23	-1.17	PPP-PPP	0.10	11	0.861	0.984	0.2774

206621_s_at	13381.3	11426.0	-0.23	-1.17	PPP-PPP	0.10	11	0.861	0.984	0.2819
226876_at	7998.3	6831.3	-0.23	-1.17	PPP-PPP	0.10	11	0.861	0.984	0.2974
242293_at	265.6	226.8	-0.23	-1.17	PPP-PPP	0.10	11	0.861	0.984	0.3100
215037_s_at	312.9	267.2	-0.23	-1.17	PPP-PPP	0.10	11	0.861	0.984	0.3825
232130_at	437.5	373.6	-0.23	-1.17	PPP-PPP	0.10	11	0.861	0.984	0.4181
203438_at	2488.2	2124.8	-0.23	-1.17	PPP-PPP	0.10	11	0.861	0.984	0.4693
204228_at	3623.3	3095.8	-0.23	-1.17	PPP-PPP	0.10	11	0.861	0.984	0.1263
217645_at	470.5	401.9	-0.23	-1.17	PPP-PPP	0.10	11	0.861	0.984	0.1326
201544_x_at	10695.4	9135.1	-0.23	-1.17	PPP-PPP	0.10	11	0.861	0.984	0.1515
212364_at	2805.9	2397.5	-0.23	-1.17	PPP-PPP	0.10	11	0.861	0.984	0.1577
228075_x_at	1295.2	1106.9	-0.23	-1.17	PPP-PPP	0.10	11	0.861	0.984	0.1727
224720_at	1579.9	1350.3	-0.23	-1.17	PPP-PPP	0.10	11	0.861	0.984	0.2863
216308_x_at	2439.5	2083.9	-0.23	-1.17	PPP-PPP	0.10	11	0.861	0.984	0.3532
223963_s_at	249.7	213.3	-0.23	-1.17	PPP-PPP	0.10	11	0.861	0.984	0.3665
1569709_at	74.6	63.7	-0.23	-1.17	AAA-AAP	0.10	11	0.861	0.984	0.4052
241937_s_at	2577.1	2201.2	-0.23	-1.17	PPP-PPP	0.10	11	0.861	0.984	0.4075
204107_at	291.0	248.7	-0.23	-1.17	AAM-PPP	0.10	11	0.861	0.984	0.4214
201357_s_at	1204.5	1028.8	-0.23	-1.17	PPP-PPP	0.10	11	0.861	0.984	0.4417
218451_at	2421.9	2069.2	-0.23	-1.17	PPP-PPP	0.10	11	0.861	0.984	0.4697
210757_x_at	3048.9	2606.5	-0.23	-1.17	PPP-PPP	0.10	11	0.861	0.984	0.0952
201697_s_at	6914.5	5912.1	-0.23	-1.17	PPP-PPP	0.10	11	0.861	0.984	0.1052
205111_s_at	124.9	106.8	-0.23	-1.17	PPP-PPP	0.10	11	0.861	0.984	0.1072
40850_at	329.3	281.6	-0.23	-1.17	MPM-PPP	0.10	11	0.861	0.984	0.2178
226711_at	1497.5	1280.7	-0.23	-1.17	PPP-PPP	0.10	11	0.861	0.984	0.2291
228012_at	243.5	208.2	-0.23	-1.17	PPP-PPP	0.10	11	0.861	0.984	0.2615
233571_x_at	1606.8	1374.0	-0.23	-1.17	PPP-PPP	0.10	11	0.861	0.984	0.3084
223347_at	550.7	471.0	-0.23	-1.17	PPP-PPP	0.10	11	0.861	0.984	0.3343
203361_s_at	363.7	311.0	-0.23	-1.17	PPP-PPP	0.10	11	0.861	0.984	0.3494
218783_at	295.9	253.0	-0.23	-1.17	PPP-PPP	0.10	11	0.861	0.984	0.3559
223079_s_at	1412.6	1207.6	-0.23	-1.17	PPP-PPP	0.10	11	0.861	0.984	0.3644
203510_at	5018.4	4290.0	-0.23	-1.17	PPP-PPP	0.10	11	0.861	0.984	0.4910
201622_at	2466.9	2110.8	-0.23	-1.17	PPP-PPP	0.10	11	0.861	0.984	0.0802
202990_at	3548.0	3036.0	-0.23	-1.17	PPP-PPP	0.10	11	0.861	0.984	0.0921
213476_x_at	20748.2	17748.5	-0.23	-1.17	PPP-PPP	0.10	11	0.861	0.984	0.1292
220688_s_at	4765.4	4076.7	-0.23	-1.17	PPP-PPP	0.10	11	0.861	0.984	0.1504
AFFX-r2-Ec-bioD-3_at	11755.1	10057.3	-0.23	-1.17	PPP-PPP	0.10	11	0.861	0.984	0.1529
225398_at	937.5	802.0	-0.23	-1.17	PPP-PPP	0.10	11	0.861	0.984	0.1542
226610_at	3625.1	3101.0	-0.23	-1.17	PPP-PPP	0.10	11	0.861	0.984	0.1982
1555784_s_at	678.7	580.5	-0.23	-1.17	PPP-PPP	0.10	11	0.861	0.984	0.2062
202628_s_at	25555.2	21860.0	-0.23	-1.17	PPP-PPP	0.10	11	0.861	0.984	0.2341
239602_at	79.0	67.6	-0.23	-1.17	APA-APP	0.10	11	0.861	0.984	0.2562
226105_at	369.9	316.5	-0.23	-1.17	PPP-PPP	0.10	11	0.861	0.984	0.2575

1554256_a_at	123.7	105.8	-0.23	-1.17	APP-AAA	0.10	11	0.861	0.984	0.2687
235368_at	207.2	177.3	-0.23	-1.17	PPP-PPP	0.10	11	0.861	0.984	0.3400
215705_at	134.0	114.6	-0.23	-1.17	PAA-PPP	0.10	11	0.861	0.984	0.3517
244202_at	69.1	59.2	-0.23	-1.17	AAP-AAA	0.10	11	0.861	0.984	0.4664
239794_at	183.2	156.8	-0.23	-1.17	PPP-PPP	0.10	11	0.861	0.984	0.4768
239068_at	69.7	59.6	-0.23	-1.17	PAP-PPA	0.10	11	0.861	0.984	0.4813
222000_at	2920.7	2500.3	-0.22	-1.17	PPP-PPP	0.10	11	0.861	0.984	0.0839
202767_at	890.1	762.0	-0.22	-1.17	PPP-PPP	0.10	11	0.861	0.984	0.0964
215566_x_at	876.7	750.8	-0.22	-1.17	PPP-PPP	0.10	11	0.861	0.984	0.1259
213146_at	153.2	131.2	-0.22	-1.17	PPP-PPP	0.10	11	0.861	0.984	0.1309
200625_s_at	19231.3	16462.6	-0.22	-1.17	PPP-PPP	0.10	11	0.861	0.984	0.1403
228289_at	286.3	245.2	-0.22	-1.17	PPP-PPP	0.10	11	0.861	0.984	0.1433
228114_x_at	872.6	747.3	-0.22	-1.17	PPP-PPP	0.10	11	0.861	0.984	0.1913
228019_s_at	4626.7	3960.8	-0.22	-1.17	PPP-PPP	0.10	11	0.861	0.984	0.1937
215515_at	159.8	136.8	-0.22	-1.17	PPP-PPP	0.10	11	0.861	0.984	0.3226
217888_s_at	938.0	803.0	-0.22	-1.17	PPP-PPP	0.10	11	0.861	0.984	0.3230
214231_s_at	235.3	201.4	-0.22	-1.17	PPP-PPP	0.10	11	0.861	0.984	0.3303
235810_at	272.8	233.6	-0.22	-1.17	PPP-PPP	0.10	11	0.861	0.984	0.4877
218700_s_at	696.0	596.5	-0.22	-1.17	PPP-PPP	0.10	11	0.861	0.984	0.0907
219162_s_at	2333.6	1999.1	-0.22	-1.17	PPP-PPP	0.10	11	0.861	0.984	0.1169
204074_s_at	696.4	596.8	-0.22	-1.17	PPP-PPP	0.10	11	0.861	0.984	0.1824
211471_s_at	229.8	196.9	-0.22	-1.17	PPA-PPP	0.10	11	0.861	0.984	0.1929
228688_at	220.2	188.7	-0.22	-1.17	PPP-PPP	0.10	11	0.861	0.984	0.2816
220011_at	709.8	608.3	-0.22	-1.17	PPP-PPP	0.10	11	0.861	0.984	0.3323
212663_at	1486.4	1273.9	-0.22	-1.17	PPP-PPP	0.10	11	0.861	0.984	0.3829
235864_at	142.7	122.2	-0.22	-1.17	PPP-APP	0.10	11	0.861	0.984	0.4438
213054_at	142.2	121.9	-0.22	-1.17	PPP-PPP	0.10	11	0.861	0.984	0.4851
216449_x_at	7948.2	6812.5	-0.22	-1.17	PPP-PPP	0.10	11	0.861	0.984	0.0923
31861_at	513.6	440.2	-0.22	-1.17	PPP-PPP	0.10	11	0.861	0.984	0.0944
200098_s_at	9173.8	7865.5	-0.22	-1.17	PPP-PPP	0.10	11	0.861	0.984	0.1040
200779_at	19442.1	16666.0	-0.22	-1.17	PPP-PPP	0.10	11	0.861	0.984	0.1475
214794_at	4188.4	3590.6	-0.22	-1.17	PPP-PPP	0.10	11	0.861	0.984	0.1789
218970_s_at	1259.0	1079.5	-0.22	-1.17	PPP-PPP	0.10	11	0.861	0.984	0.2128
218849_s_at	651.5	558.4	-0.22	-1.17	PPP-PPP	0.10	11	0.861	0.984	0.2323
1564063_a_at	225.5	193.3	-0.22	-1.17	PPP-PPP	0.10	11	0.861	0.984	0.2566
203053_at	5430.2	4656.8	-0.22	-1.17	PPP-PPP	0.10	11	0.861	0.984	0.2851
1569024_at	128.9	110.5	-0.22	-1.17	PPP-PPP	0.10	11	0.861	0.984	0.2911
209466_x_at	411.6	353.0	-0.22	-1.17	PPP-PPP	0.10	11	0.861	0.984	0.2952
206308_at	341.1	292.3	-0.22	-1.17	PPP-PPP	0.10	11	0.861	0.984	0.3508
37425_g_at	310.6	266.3	-0.22	-1.17	PPP-PPP	0.10	11	0.861	0.984	0.3550
1555614_at	115.5	99.0	-0.22	-1.17	MPP-PPP	0.10	11	0.861	0.984	0.4632
233832_at	138.4	118.6	-0.22	-1.17	APA-PPP	0.10	11	0.861	0.984	0.4632

204991_s_at	453.5	388.8	-0.22	-1.17	PPP-PPP	0.10	11	0.861	0.984	0.4807
230653_at	118.3	101.4	-0.22	-1.17	PMA-PPP	0.10	11	0.861	0.984	0.4906
213812_s_at	2749.6	2359.4	-0.22	-1.17	PPP-PPP	0.10	11	0.861	0.984	0.0761
202043_s_at	10256.4	8802.6	-0.22	-1.17	PPP-PPP	0.10	11	0.861	0.984	0.1133
218014_at	4005.0	3435.7	-0.22	-1.17	PPP-PPP	0.10	11	0.861	0.984	0.1278
223445_at	1029.3	883.1	-0.22	-1.17	PPP-PPP	0.10	11	0.861	0.984	0.1415
242261_at	141.3	121.2	-0.22	-1.17	PPP-PPP	0.10	11	0.861	0.984	0.1456
202615_at	1938.4	1662.6	-0.22	-1.17	PPP-PPP	0.10	11	0.861	0.984	0.1613
209254_at	694.5	595.8	-0.22	-1.17	PPP-PPP	0.10	11	0.861	0.984	0.1870
224621_at	7034.2	6035.2	-0.22	-1.17	PPP-PPP	0.10	11	0.861	0.984	0.2055
226942_at	861.7	739.2	-0.22	-1.17	PPP-PPP	0.10	11	0.861	0.984	0.2146
201562_s_at	531.7	456.2	-0.22	-1.17	PPP-PPP	0.10	11	0.861	0.984	0.2351
202368_s_at	3125.8	2681.1	-0.22	-1.17	PPP-PPP	0.10	11	0.861	0.984	0.2469
221783_at	582.1	499.4	-0.22	-1.17	APA-MPP	0.10	11	0.861	0.984	0.3484
227255_at	271.3	232.8	-0.22	-1.17	PPP-PPP	0.10	11	0.861	0.984	0.3700
206708_at	118.0	101.2	-0.22	-1.17	AAP-PAP	0.10	11	0.861	0.984	0.4438
211921_x_at	28422.9	24398.2	-0.22	-1.17	PPP-PPP	0.10	11	0.861	0.984	0.0839
208476_s_at	1200.1	1030.4	-0.22	-1.17	PPP-PPP	0.10	11	0.861	0.984	0.0865
202938_x_at	243.3	208.9	-0.22	-1.17	PMP-PPP	0.10	11	0.861	0.984	0.1503
205756_s_at	131.0	112.5	-0.22	-1.17	PPP-PPP	0.10	11	0.861	0.984	0.1759
222659_at	1578.2	1354.6	-0.22	-1.17	PPP-PPP	0.10	11	0.861	0.984	0.2132
226734_at	418.3	359.2	-0.22	-1.17	PPP-PPP	0.10	11	0.861	0.984	0.2195
232012_at	204.3	175.3	-0.22	-1.17	APP-MPP	0.10	11	0.861	0.984	0.2472
203800_s_at	3243.7	2785.1	-0.22	-1.17	PPP-PPP	0.10	11	0.861	0.984	0.2694
201182_s_at	948.2	814.4	-0.22	-1.16	PPP-PPP	0.10	11	0.861	0.984	0.3671
229467_at	262.9	225.8	-0.22	-1.16	PPP-PPP	0.10	11	0.861	0.984	0.3963
202618_s_at	254.4	218.5	-0.22	-1.16	AAP-AMP	0.10	11	0.861	0.984	0.4183
215791_at	132.2	113.4	-0.22	-1.17	PPP-PPP	0.10	11	0.861	0.984	0.4216
232060_at	121.1	103.9	-0.22	-1.17	PPP-PPP	0.10	11	0.861	0.984	0.4266
244569_at	175.9	151.0	-0.22	-1.16	PPP-PPP	0.10	11	0.861	0.984	0.4753
218495_at	4601.7	3954.4	-0.22	-1.16	PPP-PPP	0.10	11	0.861	0.984	0.0764
202613_at	4572.0	3928.7	-0.22	-1.16	PPP-PPP	0.10	11	0.861	0.984	0.1350
1555730_a_at	27598.5	23708.7	-0.22	-1.16	PPP-PPP	0.10	11	0.861	0.984	0.1697
1553968_a_at	493.8	424.3	-0.22	-1.16	PPP-PPP	0.10	11	0.861	0.984	0.1736
223826_s_at	208.1	178.8	-0.22	-1.16	AAP-APP	0.10	11	0.861	0.984	0.1886
217942_at	5127.6	4405.7	-0.22	-1.16	PPP-PPP	0.10	11	0.861	0.984	0.2066
232573_at	106.2	91.3	-0.22	-1.16	APP-APP	0.10	11	0.861	0.984	0.2667
1555301_a_at	115.2	99.0	-0.22	-1.16	PPP-PPP	0.10	11	0.861	0.984	0.2994
226892_at	600.4	516.0	-0.22	-1.16	PPP-PPP	0.10	11	0.861	0.984	0.3066
209928_s_at	268.4	230.6	-0.22	-1.16	PPP-PPP	0.10	11	0.861	0.984	0.3525
222986_s_at	2067.8	1777.2	-0.22	-1.16	PPP-PPP	0.10	11	0.861	0.984	0.3588
228708_at	849.6	729.8	-0.22	-1.16	PPP-PPP	0.10	11	0.861	0.984	0.4366

204169_at	829.6	712.7	-0.22	-1.16	PPP-PPP	0.10	11	0.861	0.984	0.4589
227616_at	265.9	228.5	-0.22	-1.16	PPP-PPP	0.10	11	0.861	0.984	0.4707
205529_s_at	272.9	234.4	-0.22	-1.16	PPP-PPP	0.10	11	0.861	0.984	0.4733
221622_s_at	6692.2	5754.2	-0.22	-1.16	PPP-PPP	0.10	11	0.861	0.984	0.0888
208907_s_at	2514.4	2161.7	-0.22	-1.16	PPP-PPP	0.10	11	0.861	0.984	0.1131
200932_s_at	5569.0	4787.0	-0.22	-1.16	PPP-PPP	0.10	11	0.861	0.984	0.1299
203091_at	1528.3	1314.3	-0.22	-1.16	PPP-PPP	0.10	11	0.861	0.984	0.1793
201064_s_at	6916.1	5947.7	-0.22	-1.16	PPP-PPP	0.10	11	0.861	0.984	0.2046
225484_at	1633.6	1404.6	-0.22	-1.16	PPP-PPP	0.10	11	0.861	0.984	0.2260
36552_at	1025.0	881.2	-0.22	-1.16	PPP-PPP	0.10	11	0.861	0.984	0.2480
209411_s_at	521.9	448.8	-0.22	-1.16	PPP-PPP	0.10	11	0.861	0.984	0.2914
1552738_a_at	70.2	60.3	-0.22	-1.16	AAA-APM	0.10	11	0.861	0.984	0.3335
232098_at	404.2	347.6	-0.22	-1.16	PPP-PPP	0.10	11	0.861	0.984	0.3655
200859_x_at	8617.7	7409.3	-0.22	-1.16	PPP-PPP	0.10	11	0.861	0.984	0.3742
215182_x_at	203.2	174.6	-0.22	-1.16	PPP-PPP	0.10	11	0.861	0.984	0.4183
236215_at	66.1	56.8	-0.22	-1.16	PPP-PPP	0.10	11	0.861	0.984	0.4898
201274_at	9194.1	7909.4	-0.22	-1.16	PPP-PPP	0.10	11	0.861	0.984	0.0892
1555823_at	1592.5	1369.9	-0.22	-1.16	PPP-PPP	0.10	11	0.861	0.984	0.1089
216348_at	2257.7	1941.9	-0.22	-1.16	PPP-PPP	0.10	11	0.861	0.984	0.1135
228357_at	1635.1	1406.8	-0.22	-1.16	PPP-PPP	0.10	11	0.861	0.984	0.1189
1553162_x_at	282.7	243.2	-0.22	-1.16	PPP-PPP	0.10	11	0.861	0.984	0.1408
212110_at	7748.2	6668.4	-0.22	-1.16	PPP-PPP	0.10	11	0.861	0.984	0.1485
200663_at	25119.5	21614.0	-0.22	-1.16	PPP-PPP	0.10	11	0.861	0.984	0.1625
218905_at	2048.9	1762.8	-0.22	-1.16	PPP-PPP	0.10	11	0.861	0.984	0.1778
209825_s_at	4694.3	4038.0	-0.22	-1.16	PPP-PPP	0.10	11	0.861	0.984	0.1887
214327_x_at	27982.2	24066.5	-0.22	-1.16	PPP-PPP	0.10	11	0.861	0.984	0.2004
204976_s_at	3020.0	2598.2	-0.22	-1.16	PPP-PPP	0.10	11	0.861	0.984	0.2121
211068_x_at	3906.8	3361.6	-0.22	-1.16	PPP-PPP	0.10	11	0.861	0.984	0.2595
227560_at	478.2	411.3	-0.22	-1.16	PPP-PPP	0.10	11	0.861	0.984	0.3466
244133_at	149.8	128.9	-0.22	-1.16	PPA-PPP	0.10	11	0.861	0.984	0.3719
1560622_at	290.9	250.3	-0.22	-1.16	PPP-PPP	0.10	11	0.861	0.984	0.4151
1555875_at	324.4	279.1	-0.22	-1.16	PPP-PPP	0.10	11	0.861	0.984	0.4165
225454_at	1163.7	1001.3	-0.22	-1.16	PPP-PPP	0.10	11	0.861	0.984	0.4633
226823_at	2206.1	1899.0	-0.22	-1.16	PPP-PPP	0.10	11	0.861	0.984	0.0981
201938_at	17432.5	15010.4	-0.22	-1.16	PPP-PPP	0.10	11	0.861	0.984	0.0989
45572_s_at	2631.8	2265.3	-0.22	-1.16	PPP-PPP	0.10	11	0.861	0.984	0.1029
218942_at	1128.2	971.1	-0.22	-1.16	PPP-PPP	0.10	11	0.861	0.984	0.1042
201154_x_at	31897.2	27457.6	-0.22	-1.16	PPP-PPP	0.10	11	0.861	0.984	0.1660
214334_x_at	4167.6	3587.7	-0.22	-1.16	PPP-PPP	0.10	11	0.861	0.984	0.1993
228280_at	1317.7	1134.8	-0.22	-1.16	PPP-PPP	0.10	11	0.861	0.984	0.2233
210835_s_at	6050.6	5209.1	-0.22	-1.16	PPP-PPP	0.10	11	0.861	0.984	0.2249
37152_at	3086.5	2656.4	-0.22	-1.16	PPP-PPP	0.10	11	0.861	0.984	0.2422

238818_at	160.9	138.5	-0.22	-1.16	AAA-PPP	0.10	11	0.861	0.984	0.2784
243256_at	188.2	162.0	-0.22	-1.16	PPP-PPP	0.10	11	0.861	0.984	0.2840
227100_at	1029.8	886.5	-0.22	-1.16	PPP-PPP	0.10	11	0.861	0.984	0.2854
223188_at	434.5	374.1	-0.22	-1.16	PPP-PPP	0.10	11	0.861	0.984	0.3444
205752_s_at	138.3	119.1	-0.22	-1.16	AAA-APA	0.10	11	0.861	0.984	0.3620
121_at	562.8	484.7	-0.22	-1.16	PPP-PPP	0.10	11	0.861	0.984	0.3843
229948_at	123.1	106.0	-0.22	-1.16	PPP-APP	0.10	11	0.861	0.984	0.4340
214991_s_at	259.1	223.1	-0.22	-1.16	PPP-PPP	0.10	11	0.861	0.984	0.4496
224493_x_at	874.5	753.0	-0.22	-1.16	PPP-PPP	0.10	11	0.861	0.984	0.4730
226949_at	642.7	553.8	-0.22	-1.16	PPP-PPP	0.10	11	0.861	0.984	0.1119
224623_at	6083.8	5240.1	-0.22	-1.16	PPP-PPP	0.10	11	0.861	0.984	0.1259
224807_at	406.6	350.2	-0.22	-1.16	PPP-PPP	0.10	11	0.861	0.984	0.1343
226116_at	3238.1	2789.3	-0.22	-1.16	PPP-PPP	0.10	11	0.861	0.984	0.1371
1556176_at	124.6	107.4	-0.22	-1.16	PPP-PPP	0.10	11	0.861	0.984	0.1898
221619_s_at	19896.6	17141.8	-0.22	-1.16	PPP-PPP	0.10	11	0.861	0.984	0.2340
226496_at	1415.2	1218.9	-0.22	-1.16	PPP-PPP	0.10	11	0.861	0.984	0.2552
226361_at	413.3	356.2	-0.22	-1.16	PPP-PPP	0.10	11	0.861	0.984	0.2551
212383_at	745.5	642.4	-0.22	-1.16	PPP-PPP	0.10	11	0.861	0.984	0.2873
209587_at	210.4	181.3	-0.22	-1.16	AMP-MPA	0.10	11	0.861	0.984	0.3350
226425_at	1249.2	1076.4	-0.22	-1.16	PPP-PPP	0.10	11	0.861	0.984	0.3410
203629_s_at	653.3	562.7	-0.22	-1.16	PPP-PPP	0.10	11	0.861	0.984	0.3729
232560_at	247.0	212.8	-0.22	-1.16	PPP-PPP	0.10	11	0.861	0.984	0.3726
222046_at	134.4	115.9	-0.22	-1.16	APP-MMP	0.10	11	0.861	0.984	0.4344
209599_s_at	151.1	130.1	-0.22	-1.16	AMP-PAP	0.10	11	0.861	0.984	0.4399
222062_at	175.3	151.1	-0.22	-1.16	PAP-PPP	0.10	11	0.861	0.984	0.4431
205104_at	133.4	114.9	-0.22	-1.16	PAA-AAP	0.10	11	0.861	0.984	0.4847
201516_at	5688.6	4905.3	-0.21	-1.16	PPP-PPP	0.10	11	0.861	0.984	0.0996
204909_at	127.4	109.8	-0.21	-1.16	APA-APP	0.10	11	0.861	0.984	0.1623
214894_x_at	1710.0	1474.7	-0.21	-1.16	PPP-PPP	0.10	11	0.861	0.984	0.1993
203834_s_at	970.6	836.6	-0.21	-1.16	PPP-PPP	0.10	11	0.861	0.984	0.2211
224769_at	2968.6	2559.9	-0.21	-1.16	PPP-PPP	0.10	11	0.861	0.984	0.2254
226495_at	1196.3	1031.2	-0.21	-1.16	PPP-PPP	0.10	11	0.861	0.984	0.3309
237329_at	150.5	129.7	-0.21	-1.16	PPP-PPP	0.10	11	0.861	0.984	0.3824
230133_at	372.9	321.4	-0.21	-1.16	PPP-PPP	0.10	11	0.861	0.984	0.4674
200706_s_at	3338.7	2879.7	-0.21	-1.16	PPP-PPP	0.10	11	0.861	0.984	0.0867
224948_at	15703.6	13547.0	-0.21	-1.16	PPP-PPP	0.10	11	0.861	0.984	0.0921
210555_s_at	627.1	541.0	-0.21	-1.16	PPP-PPP	0.10	11	0.861	0.984	0.0939
215136_s_at	5345.3	4611.9	-0.21	-1.16	PPP-PPP	0.10	11	0.861	0.984	0.1231
225126_at	1045.5	901.9	-0.21	-1.16	PPP-PPP	0.10	11	0.861	0.984	0.1300
205379_at	1611.2	1390.2	-0.21	-1.16	PPP-PPP	0.10	11	0.861	0.984	0.1435
208659_at	19819.2	17094.6	-0.21	-1.16	PPP-PPP	0.10	11	0.861	0.984	0.2171
211501_s_at	5277.8	4554.0	-0.21	-1.16	PPP-PPP	0.10	11	0.861	0.984	0.2226

235783_at	1851.3	1596.8	-0.21	-1.16	PPP-PPP	0.10	11	0.861	0.984	0.2425
211724_x_at	892.3	770.1	-0.21	-1.16	PPP-PPP	0.10	11	0.861	0.984	0.2792
1557242_at	169.3	146.0	-0.21	-1.16	PPP-APP	0.10	11	0.861	0.984	0.2894
218083_at	696.2	600.5	-0.21	-1.16	PPP-PPP	0.10	11	0.861	0.984	0.3065
204654_s_at	824.6	711.6	-0.21	-1.16	PPP-PPP	0.10	11	0.861	0.984	0.3305
209380_s_at	671.2	579.0	-0.21	-1.16	PPP-PPP	0.10	11	0.861	0.984	0.3546
200696_s_at	453.2	390.9	-0.21	-1.16	PPP-PPP	0.10	11	0.861	0.984	0.3690
223231_at	4187.0	3615.3	-0.21	-1.16	PPP-PPP	0.10	11	0.861	0.984	0.0783
200013_at	36061.5	31131.0	-0.21	-1.16	PPP-PPP	0.10	11	0.861	0.984	0.0795
1552978_a_at	593.2	512.3	-0.21	-1.16	PPP-PPP	0.10	11	0.861	0.984	0.0796
216680_s_at	172.1	148.6	-0.21	-1.16	APP-APP	0.10	11	0.861	0.984	0.0975
201819_at	906.3	782.4	-0.21	-1.16	PPP-PPP	0.10	11	0.861	0.984	0.1382
213612_x_at	11344.0	9793.8	-0.21	-1.16	PPP-PPP	0.10	11	0.861	0.984	0.1429
221247_s_at	1296.4	1119.0	-0.21	-1.16	PPP-PPP	0.10	11	0.861	0.984	0.1494
1555764_s_at	5572.2	4810.8	-0.21	-1.16	PPP-PPP	0.10	11	0.861	0.984	0.1515
203123_s_at	839.6	725.0	-0.21	-1.16	PPP-PPP	0.10	11	0.861	0.984	0.1945
218080_x_at	2315.2	1998.8	-0.21	-1.16	PPP-PPP	0.10	11	0.861	0.984	0.2056
218088_s_at	3412.5	2945.4	-0.21	-1.16	PPP-PPP	0.10	11	0.861	0.984	0.2145
208636_at	21531.2	18592.0	-0.21	-1.16	PPP-PPP	0.10	11	0.861	0.984	0.2280
219439_at	1466.9	1266.5	-0.21	-1.16	PPP-PPP	0.10	11	0.861	0.984	0.2331
61297_at	228.6	197.4	-0.21	-1.16	PPP-PPP	0.10	11	0.861	0.984	0.2381
229593_at	353.2	304.9	-0.21	-1.16	PPP-PPP	0.10	11	0.861	0.984	0.2443
204251_s_at	187.7	162.0	-0.21	-1.16	PPP-PPP	0.10	11	0.861	0.984	0.2666
213256_at	1507.6	1301.4	-0.21	-1.16	PPP-PPP	0.10	11	0.861	0.984	0.3091
212311_at	223.5	193.0	-0.21	-1.16	PPP-PPP	0.10	11	0.861	0.984	0.3221
210243_s_at	1452.6	1254.0	-0.21	-1.16	PPP-PPP	0.10	11	0.861	0.984	0.3334
212614_at	3393.8	2929.0	-0.21	-1.16	PPP-PPP	0.10	11	0.861	0.984	0.3610
228955_at	379.7	327.9	-0.21	-1.16	PPP-PPP	0.10	11	0.861	0.984	0.4628
219024_at	514.7	444.4	-0.21	-1.16	PPP-PPP	0.10	11	0.861	0.984	0.4722
204054_at	362.3	312.8	-0.21	-1.16	PPP-PPP	0.10	11	0.861	0.984	0.4825
201282_at	554.2	478.7	-0.21	-1.16	PPP-PPP	0.10	11	0.861	0.984	0.1010
202810_at	8751.1	7559.0	-0.21	-1.16	PPP-PPP	0.10	11	0.861	0.984	0.1438
243966_at	152.8	132.0	-0.21	-1.16	PPP-PPP	0.10	11	0.861	0.984	0.1542
229632_s_at	1558.1	1346.1	-0.21	-1.16	PPP-PPP	0.10	11	0.861	0.984	0.1698
202382_s_at	3253.9	2810.2	-0.21	-1.16	PPP-PPP	0.10	11	0.861	0.984	0.1721
231810_at	1868.5	1613.8	-0.21	-1.16	PPP-PPP	0.10	11	0.861	0.984	0.2230
222514_at	3133.3	2706.7	-0.21	-1.16	PPP-PPP	0.10	11	0.861	0.984	0.2554
218850_s_at	245.1	211.9	-0.21	-1.16	PPP-PPP	0.10	11	0.861	0.984	0.2764
1557411_s_at	1341.3	1158.5	-0.21	-1.16	PPP-PPP	0.10	11	0.861	0.984	0.2812
227263_at	497.3	429.7	-0.21	-1.16	PPP-PPP	0.10	11	0.861	0.984	0.2898
202842_s_at	1422.1	1229.0	-0.21	-1.16	PPP-PPP	0.10	11	0.861	0.984	0.2938
210720_s_at	356.8	308.3	-0.21	-1.16	PPP-PPP	0.10	11	0.861	0.984	0.3102

212136_at	1702.5	1471.0	-0.21	-1.16	PPP-PPP	0.10	11	0.861	0.984	0.3338
202937_x_at	1923.7	1661.4	-0.21	-1.16	PPP-PPP	0.10	11	0.861	0.984	0.3636
222757_s_at	761.5	658.0	-0.21	-1.16	PPP-PPP	0.10	11	0.861	0.984	0.3779
1555292_at	330.1	285.1	-0.21	-1.16	PPP-PPP	0.10	11	0.861	0.984	0.3832
238807_at	377.0	325.6	-0.21	-1.16	PPP-AAA	0.10	11	0.861	0.984	0.3986
239719_at	125.9	108.8	-0.21	-1.16	AAP-PPP	0.10	11	0.861	0.984	0.4330
213813_x_at	204.0	176.3	-0.21	-1.16	AAP-PPM	0.10	11	0.861	0.984	0.4747
48031_r_at	79.0	68.3	-0.21	-1.16	APA-AAP	0.10	11	0.861	0.984	0.4773
218305_at	1994.6	1724.6	-0.21	-1.16	PPP-PPP	0.10	11	0.861	0.984	0.0670
226569_s_at	555.1	479.8	-0.21	-1.16	PPP-PPP	0.10	11	0.861	0.984	0.0797
201119_s_at	11648.7	10073.2	-0.21	-1.16	PPP-PPP	0.10	11	0.861	0.984	0.1027
201505_at	4427.9	3827.0	-0.21	-1.16	PPP-PPP	0.10	11	0.861	0.984	0.1282
200009_at	17725.0	15325.9	-0.21	-1.16	PPP-PPP	0.10	11	0.861	0.984	0.1505
218420_s_at	496.1	429.1	-0.21	-1.16	PPP-PPP	0.10	11	0.861	0.984	0.1863
228588_s_at	2348.6	2030.8	-0.21	-1.16	PPP-PPP	0.10	11	0.861	0.984	0.1919
203746_s_at	3729.7	3224.8	-0.21	-1.16	PPP-PPP	0.10	11	0.861	0.984	0.2314
222522_x_at	2094.2	1810.6	-0.21	-1.16	PPP-PPP	0.10	11	0.861	0.984	0.2347
218584_at	509.9	440.8	-0.21	-1.16	PPP-PPP	0.10	11	0.861	0.984	0.2538
221739_at	5901.6	5101.6	-0.21	-1.16	PPP-PPP	0.10	11	0.861	0.984	0.2907
203054_s_at	608.7	526.1	-0.21	-1.16	PPP-PPP	0.10	11	0.861	0.984	0.3140
242538_at	438.8	379.3	-0.21	-1.16	PPP-PPP	0.10	11	0.861	0.984	0.3611
201539_s_at	116.9	101.0	-0.21	-1.16	APP-APA	0.10	11	0.861	0.984	0.3767
212257_s_at	260.7	225.5	-0.21	-1.16	PPP-PPP	0.10	11	0.861	0.984	0.3876
1554321_a_at	371.4	321.1	-0.21	-1.16	PPP-PPP	0.10	11	0.861	0.984	0.4306
211942_x_at	30084.8	26020.5	-0.21	-1.16	PPP-PPP	0.10	11	0.861	0.984	0.0862
210101_x_at	5265.7	4555.4	-0.21	-1.16	PPP-PPP	0.10	11	0.861	0.984	0.1422
200096_s_at	13547.4	11721.5	-0.21	-1.16	PPP-PPP	0.10	11	0.861	0.984	0.1494
209091_s_at	6046.0	5230.4	-0.21	-1.16	PPP-PPP	0.10	11	0.861	0.984	0.1555
203773_x_at	2709.2	2343.9	-0.21	-1.16	PPP-PPP	0.10	11	0.861	0.984	0.2016
235331_x_at	407.5	352.4	-0.21	-1.16	PPP-PPP	0.10	11	0.861	0.984	0.2146
225866_at	6700.1	5798.3	-0.21	-1.16	PPP-PPP	0.10	11	0.861	0.984	0.2547
218032_at	655.7	567.2	-0.21	-1.16	PPP-PPP	0.10	11	0.861	0.984	0.2797
38043_at	390.4	337.7	-0.21	-1.16	AAP-PPP	0.10	11	0.861	0.984	0.3137
214035_x_at	2284.7	1976.3	-0.21	-1.16	PPP-PPP	0.10	11	0.861	0.984	0.3221
226731_at	167.8	145.2	-0.21	-1.16	PPP-PPP	0.10	11	0.861	0.984	0.4704
200000_s_at	6807.2	5891.9	-0.21	-1.16	PPP-PPP	0.10	11	0.861	0.984	0.1323
236141_at	84.3	73.0	-0.21	-1.16	MPP-APP	0.10	11	0.861	0.984	0.1434
215016_x_at	4187.7	3625.4	-0.21	-1.16	PPP-PPP	0.10	11	0.861	0.984	0.1541
220576_at	84.4	73.1	-0.21	-1.16	PPP-PPP	0.10	11	0.861	0.984	0.1914
205698_s_at	74.8	64.8	-0.21	-1.16	PAP-APP	0.10	11	0.861	0.984	0.2092
222039_at	2739.7	2372.7	-0.21	-1.16	PPP-PPP	0.10	11	0.861	0.984	0.2771
47105_at	417.5	361.5	-0.21	-1.16	PPP-PPP	0.10	11	0.861	0.984	0.3309

209284_s_at	1087.3	941.5	-0.21	-1.16	PPP-PPP	0.10	11	0.861	0.984	0.3960
215600_x_at	1030.9	892.2	-0.21	-1.16	PPP-PPP	0.10	11	0.861	0.984	0.4152
219122_s_at	1139.7	986.3	-0.21	-1.16	PPP-PPP	0.10	11	0.861	0.984	0.4172
203230_at	1038.7	899.2	-0.21	-1.16	PPP-PPP	0.10	11	0.861	0.984	0.4753
201277_s_at	21300.1	18450.9	-0.21	-1.15	PPP-PPP	0.10	11	0.861	0.984	0.0833
220948_s_at	4991.0	4322.8	-0.21	-1.16	PPP-PPP	0.10	11	0.861	0.984	0.0866
240891_at	236.0	204.4	-0.21	-1.15	PAP-APP	0.10	11	0.861	0.984	0.0962
202627_s_at	22794.0	19747.3	-0.21	-1.15	PPP-PPP	0.10	11	0.861	0.984	0.2331
203654_s_at	2003.1	1735.5	-0.21	-1.15	PPP-PPP	0.10	11	0.861	0.984	0.2408
218651_s_at	2356.9	2042.4	-0.21	-1.15	PPP-PPP	0.10	11	0.861	0.984	0.2829
206968_s_at	216.1	187.3	-0.21	-1.15	PPP-PPP	0.10	11	0.861	0.984	0.3552
227572_at	298.5	258.7	-0.21	-1.15	PPP-PPP	0.10	11	0.861	0.984	0.3869
222416_at	4206.5	3643.2	-0.21	-1.16	PPP-PPP	0.10	11	0.861	0.984	0.4283
213551_x_at	629.3	545.6	-0.21	-1.15	PPP-PPP	0.10	11	0.861	0.984	0.0797
204978_at	711.9	617.4	-0.21	-1.15	PPP-PPP	0.10	11	0.861	0.984	0.0905
202585_s_at	250.3	217.0	-0.21	-1.15	PPP-PPP	0.10	11	0.861	0.984	0.1498
201987_at	4304.3	3731.4	-0.21	-1.15	PPP-PPP	0.10	11	0.861	0.984	0.1669
209186_at	12246.9	10617.5	-0.21	-1.15	PPP-PPP	0.10	11	0.861	0.984	0.1796
236930_at	76.0	65.9	-0.21	-1.15	AAP-AAM	0.10	11	0.861	0.984	0.1880
1554003_at	365.6	316.8	-0.21	-1.15	PPP-PPP	0.10	11	0.861	0.984	0.2253
217855_x_at	3150.6	2732.2	-0.21	-1.15	PPP-PPP	0.10	11	0.861	0.984	0.2529
221693_s_at	1078.8	935.2	-0.21	-1.15	PPP-PPP	0.10	11	0.861	0.984	0.2584
208679_s_at	27926.4	24218.6	-0.21	-1.15	PPP-PPP	0.10	11	0.861	0.984	0.3006
209082_s_at	413.7	358.8	-0.21	-1.15	PPP-PPP	0.10	11	0.861	0.984	0.3589
230194_at	368.6	319.6	-0.21	-1.15	PPP-PPP	0.10	11	0.861	0.984	0.3932
1570410_at	193.4	167.7	-0.21	-1.15	PPM-PPP	0.10	11	0.861	0.984	0.4087
201557_at	252.3	218.8	-0.21	-1.15	APA-AMA	0.10	11	0.861	0.984	0.4162
218423_x_at	1269.7	1101.0	-0.21	-1.15	PPP-PPP	0.10	11	0.861	0.984	0.4629
1560274_at	53.2	46.1	-0.21	-1.15	AAA-PAP	0.10	11	0.861	0.984	0.4777
211566_x_at	600.7	521.1	-0.21	-1.15	PPP-PPP	0.10	11	0.861	0.984	0.0631
224750_at	1218.3	1056.8	-0.21	-1.15	PPP-PPP	0.10	11	0.861	0.984	0.0653
210821_x_at	1057.4	917.1	-0.21	-1.15	PPP-PPP	0.10	11	0.861	0.984	0.0670
200754_x_at	17149.2	14882.5	-0.21	-1.15	PPP-PPP	0.10	11	0.861	0.984	0.1065
221580_s_at	9115.1	7908.2	-0.21	-1.15	PPP-PPP	0.10	11	0.861	0.984	0.1270
217960_s_at	2152.8	1867.5	-0.21	-1.15	PPP-PPP	0.10	11	0.861	0.984	0.1377
225433_at	3523.4	3056.1	-0.21	-1.15	PPP-PPP	0.10	11	0.861	0.984	0.1717
201306_s_at	19542.6	16950.8	-0.21	-1.15	PPP-PPP	0.10	11	0.861	0.984	0.1815
208709_s_at	3471.2	3010.6	-0.21	-1.15	PPP-PPP	0.10	11	0.861	0.984	0.1949
201536_at	1328.9	1153.0	-0.21	-1.15	PPP-PPP	0.10	11	0.861	0.984	0.2063
238199_x_at	2904.6	2520.7	-0.21	-1.15	PPP-PPP	0.10	11	0.861	0.984	0.2078
231078_at	262.7	227.9	-0.21	-1.15	PPP-PPP	0.10	11	0.861	0.984	0.3218
227630_at	1095.0	949.9	-0.21	-1.15	PPP-PPP	0.10	11	0.861	0.984	0.3521

235717_at	125.9	109.2	-0.21	-1.15	PPP-PPP	0.10	11	0.861	0.984	0.3986
227994_x_at	1480.2	1284.3	-0.21	-1.15	PPP-PPP	0.10	11	0.861	0.984	0.4209
227625_s_at	13444.0	11660.6	-0.21	-1.15	PPP-PPP	0.10	11	0.861	0.984	0.4588
209534_x_at	544.4	472.7	-0.20	-1.15	PPP-PPP	0.10	11	0.861	0.984	0.0898
205550_s_at	1622.1	1408.5	-0.20	-1.15	PPP-PPP	0.10	11	0.861	0.984	0.0952
219335_at	590.6	512.7	-0.20	-1.15	PPP-PPP	0.10	11	0.861	0.984	0.1069
216620_s_at	2067.3	1794.9	-0.20	-1.15	PPP-PPP	0.10	11	0.861	0.984	0.1383
228495_at	825.9	717.0	-0.20	-1.15	PPP-PPP	0.10	11	0.861	0.984	0.2304
236883_at	109.7	95.2	-0.20	-1.15	APM-APP	0.10	11	0.861	0.984	0.2339
223206_s_at	1236.4	1073.1	-0.20	-1.15	PPP-PPP	0.10	11	0.861	0.984	0.2429
200896_x_at	9392.1	8152.3	-0.20	-1.15	PPP-PPP	0.10	11	0.861	0.984	0.2464
203229_s_at	906.0	786.5	-0.20	-1.15	PPP-PPP	0.10	11	0.861	0.984	0.2567
232511_at	143.1	124.2	-0.20	-1.15	PPP-PPP	0.10	11	0.861	0.984	0.2599
234471_s_at	260.8	226.3	-0.20	-1.15	PPP-PPP	0.10	11	0.861	0.984	0.3101
230314_at	107.5	93.3	-0.20	-1.15	PPP-PPP	0.10	11	0.861	0.984	0.3489
1554356_at	178.5	154.9	-0.20	-1.15	AAP-MMP	0.10	11	0.861	0.984	0.3521
233974_s_at	328.9	285.6	-0.20	-1.15	PPP-PPP	0.10	11	0.861	0.984	0.3537
226721_at	1033.3	896.8	-0.20	-1.15	PPP-PPP	0.10	11	0.861	0.984	0.3629
225647_s_at	10154.8	8819.5	-0.20	-1.15	PPP-PPP	0.10	11	0.861	0.984	0.0585
225127_at	2690.0	2337.5	-0.20	-1.15	PPP-PPP	0.10	11	0.861	0.984	0.0854
201030_x_at	28112.1	24427.7	-0.20	-1.15	PPP-PPP	0.10	11	0.861	0.984	0.1050
200020_at	9476.2	8234.3	-0.20	-1.15	PPP-PPP	0.10	11	0.861	0.984	0.1501
225294_s_at	3825.2	3323.8	-0.20	-1.15	PPP-PPP	0.10	11	0.861	0.984	0.1508
210983_s_at	8715.8	7570.8	-0.20	-1.15	PPP-PPP	0.10	11	0.861	0.984	0.1621
222477_s_at	1954.2	1697.9	-0.20	-1.15	PPP-PPP	0.10	11	0.861	0.984	0.1679
203616_at	2257.1	1960.9	-0.20	-1.15	PPP-PPP	0.10	11	0.861	0.984	0.1704
216521_s_at	902.6	784.3	-0.20	-1.15	PPP-PPP	0.10	11	0.861	0.984	0.1813
235591_at	3480.5	3023.8	-0.20	-1.15	PPP-PPP	0.10	11	0.861	0.984	0.2048
201044_x_at	220.4	191.5	-0.20	-1.15	PPP-PPP	0.10	11	0.861	0.984	0.2069
209714_s_at	4608.7	4005.0	-0.20	-1.15	PPP-PPP	0.10	11	0.861	0.984	0.2368
32837_at	1136.0	986.9	-0.20	-1.15	PPP-PPP	0.10	11	0.861	0.984	0.2643
1552621_at	827.2	718.3	-0.20	-1.15	PPP-PPP	0.10	11	0.861	0.984	0.3273
228399_at	126.5	109.9	-0.20	-1.15	AAP-PAA	0.10	11	0.861	0.984	0.3845
223553_s_at	179.3	155.7	-0.20	-1.15	PPP-PPP	0.10	11	0.861	0.984	0.3842
219489_s_at	2281.5	1982.4	-0.20	-1.15	PPP-PPP	0.10	11	0.861	0.984	0.3920
1559102_at	50.2	43.6	-0.20	-1.15	APP-PPP	0.10	11	0.861	0.984	0.4082
227015_at	319.1	277.3	-0.20	-1.15	PPP-PPP	0.10	11	0.861	0.984	0.4185
219182_at	443.5	385.4	-0.20	-1.15	PPP-PPP	0.10	11	0.861	0.984	0.4248
244128_x_at	226.6	196.8	-0.20	-1.15	PPP-PPP	0.10	11	0.861	0.984	0.4338
224523_s_at	4234.0	3681.6	-0.20	-1.15	PPP-PPP	0.10	11	0.861	0.984	0.1209
220865_s_at	1569.8	1364.4	-0.20	-1.15	PPP-PPP	0.10	11	0.861	0.984	0.2709
238828_at	193.9	168.5	-0.20	-1.15	PPP-PPP	0.10	11	0.861	0.984	0.3110

229086_at	195.9	170.3	-0.20	-1.15	PPP-PPP	0.10	11	0.861	0.984	0.3211
212282_at	3800.3	3304.5	-0.20	-1.15	PPP-PPP	0.10	11	0.861	0.984	0.3261
212445_s_at	2874.7	2499.3	-0.20	-1.15	PPP-PPP	0.10	11	0.861	0.984	0.3427
1568629_s_at	1392.8	1210.5	-0.20	-1.15	PPP-PPP	0.10	11	0.861	0.984	0.4025
228306_at	526.2	457.6	-0.20	-1.15	PPP-PPP	0.10	11	0.861	0.984	0.4114
233473_x_at	215.6	187.4	-0.20	-1.15	AAP-MPP	0.10	11	0.861	0.984	0.4298
210589_s_at	997.5	867.4	-0.20	-1.15	PPP-PPP	0.10	11	0.861	0.984	0.4408
213864_s_at	18895.6	16437.1	-0.20	-1.15	PPP-PPP	0.10	11	0.861	0.984	0.1149
202000_at	1296.0	1127.5	-0.20	-1.15	PPP-PPP	0.10	11	0.861	0.984	0.1340
200665_s_at	22027.1	19164.7	-0.20	-1.15	PPP-PPP	0.10	11	0.861	0.984	0.1527
224665_at	2156.8	1876.8	-0.20	-1.15	PPP-PPP	0.10	11	0.861	0.984	0.1899
201126_s_at	1585.1	1379.2	-0.20	-1.15	PPP-PPP	0.10	11	0.861	0.984	0.1910
223252_at	645.0	561.2	-0.20	-1.15	PPP-PPP	0.10	11	0.861	0.984	0.2194
220610_s_at	827.8	720.2	-0.20	-1.15	PPP-PPP	0.10	11	0.861	0.984	0.2708
203781_at	7621.4	6631.9	-0.20	-1.15	PPP-PPP	0.10	11	0.861	0.984	0.2749
201349_at	1070.0	930.6	-0.20	-1.15	PPP-PPP	0.10	11	0.861	0.984	0.2949
219662_at	1038.1	902.9	-0.20	-1.15	PPP-PPP	0.10	11	0.861	0.984	0.3002
230443_at	130.0	113.1	-0.20	-1.15	AAP-PMP	0.10	11	0.861	0.984	0.3649
202924_s_at	197.9	172.1	-0.20	-1.15	APP-PPP	0.10	11	0.861	0.984	0.3771
1554780_a_at	613.4	533.4	-0.20	-1.15	PPP-PPP	0.10	11	0.861	0.984	0.4120
219842_at	130.3	113.5	-0.20	-1.15	PPP-APP	0.10	11	0.861	0.984	0.0560
202655_at	4718.7	4106.7	-0.20	-1.15	PPP-PPP	0.10	11	0.861	0.984	0.0946
223158_s_at	2069.3	1801.5	-0.20	-1.15	PPP-PPP	0.10	11	0.861	0.984	0.1112
227836_at	1057.8	921.0	-0.20	-1.15	PPP-PPP	0.10	11	0.861	0.984	0.1250
215691_x_at	8026.2	6989.6	-0.20	-1.15	PPP-PPP	0.10	11	0.861	0.984	0.1744
206499_s_at	1842.2	1603.5	-0.20	-1.15	PPP-PPP	0.10	11	0.861	0.984	0.2092
203762_s_at	362.6	315.7	-0.20	-1.15	PPP-PPP	0.10	11	0.861	0.984	0.2085
213427_at	2985.8	2599.5	-0.20	-1.15	PPP-PPP	0.10	11	0.861	0.984	0.2384
201851_at	1995.7	1737.7	-0.20	-1.15	PPP-PPP	0.10	11	0.861	0.984	0.2493
226184_at	1896.2	1650.4	-0.20	-1.15	PPP-PPP	0.10	11	0.861	0.984	0.2704
226360_at	579.4	504.4	-0.20	-1.15	PPP-PPP	0.10	11	0.861	0.984	0.2773
227167_s_at	2647.1	2304.4	-0.20	-1.15	PPP-PPP	0.10	11	0.861	0.984	0.3174
201895_at	1121.6	976.3	-0.20	-1.15	PPP-PPP	0.10	11	0.861	0.984	0.3332
202993_at	748.0	651.1	-0.20	-1.15	PPP-PPP	0.10	11	0.861	0.984	0.3477
201639_s_at	1312.2	1142.0	-0.20	-1.15	PPP-PPP	0.10	11	0.861	0.984	0.3564
229319_at	316.8	275.8	-0.20	-1.15	PPP-PPP	0.10	11	0.861	0.984	0.4185
218492_s_at	822.9	716.6	-0.20	-1.15	PPP-PPP	0.10	11	0.861	0.984	0.4695
212216_at	1023.4	891.7	-0.20	-1.15	PPP-PPP	0.10	11	0.861	0.984	0.0647
231894_at	964.5	840.3	-0.20	-1.15	PPP-PPP	0.10	11	0.861	0.984	0.0716
226568_at	352.9	307.5	-0.20	-1.15	PPP-PPP	0.10	11	0.861	0.984	0.0815
219061_s_at	1114.7	970.9	-0.20	-1.15	PPP-PPP	0.10	11	0.861	0.984	0.1244
212833_at	4701.9	4096.8	-0.20	-1.15	PPP-PPP	0.10	11	0.861	0.984	0.1241

216295_s_at	13766.6	11994.5	-0.20	-1.15	PPP-PPP	0.10	11	0.861	0.984	0.1833
209439_s_at	443.2	386.1	-0.20	-1.15	PPP-PPP	0.10	11	0.861	0.984	0.2193
202484_s_at	6355.4	5535.6	-0.20	-1.15	PPP-PPP	0.10	11	0.861	0.984	0.2279
224670_at	909.6	792.5	-0.20	-1.15	PPP-PPP	0.10	11	0.861	0.984	0.2608
232028_at	202.9	176.8	-0.20	-1.15	PPP-PPP	0.10	11	0.861	0.984	0.3047
207122_x_at	339.1	295.4	-0.20	-1.15	PPP-PAP	0.10	11	0.861	0.984	0.3194
241900_at	219.2	191.0	-0.20	-1.15	PPP-PPP	0.10	11	0.861	0.984	0.3263
224162_s_at	546.3	475.9	-0.20	-1.15	PPP-PPP	0.10	11	0.861	0.984	0.3547
200670_at	4018.3	3501.1	-0.20	-1.15	PPP-PPP	0.10	11	0.861	0.984	0.3792
218813_s_at	302.1	263.2	-0.20	-1.15	PPP-PPP	0.10	11	0.861	0.984	0.4016
221766_s_at	1716.5	1495.5	-0.20	-1.15	PPP-PPP	0.10	11	0.861	0.984	0.4618
213052_at	3192.6	2782.3	-0.20	-1.15	PPP-PPP	0.10	11	0.861	0.984	0.0685
216215_s_at	3612.8	3150.4	-0.20	-1.15	PPP-PPP	0.10	11	0.861	0.984	0.0834
212759_s_at	265.3	231.3	-0.20	-1.15	PAP-PPP	0.10	11	0.861	0.984	0.1276
221488_s_at	10577.3	9222.4	-0.20	-1.15	PPP-PPP	0.10	11	0.861	0.984	0.1652
209303_at	5020.3	4375.0	-0.20	-1.15	PPP-PPP	0.10	11	0.861	0.984	0.1878
219207_at	935.4	815.5	-0.20	-1.15	PPP-PPP	0.10	11	0.861	0.984	0.2645
217499_x_at	812.9	708.6	-0.20	-1.15	PPP-PPP	0.10	11	0.861	0.984	0.3123
206316_s_at	984.4	858.4	-0.20	-1.15	PPP-PPP	0.10	11	0.861	0.984	0.3780
213207_s_at	300.1	261.5	-0.20	-1.15	PMA-AAP	0.10	11	0.861	0.984	0.4365
224969_at	628.4	547.8	-0.20	-1.15	PPP-PPP	0.10	11	0.861	0.984	0.4702
34406_at	295.7	257.7	-0.20	-1.15	PPP-PPP	0.10	11	0.861	0.984	0.4732
228908_s_at	563.5	491.7	-0.20	-1.15	PPP-PPP	0.10	11	0.861	0.984	0.1180
204143_s_at	688.2	600.3	-0.20	-1.15	PPP-PPP	0.10	11	0.861	0.984	0.1338
226946_at	752.6	656.3	-0.20	-1.15	PPP-PPP	0.10	11	0.861	0.984	0.1506
200055_at	4257.4	3714.2	-0.20	-1.15	PPP-PPP	0.10	11	0.861	0.984	0.1714
208843_s_at	5006.6	4368.0	-0.20	-1.15	PPP-PPP	0.10	11	0.861	0.984	0.2343
221806_s_at	1887.8	1646.6	-0.20	-1.15	PPP-PPP	0.10	11	0.861	0.984	0.2463
213218_at	335.4	292.6	-0.20	-1.15	PPP-PPP	0.10	11	0.861	0.984	0.2536
218112_at	2086.7	1820.9	-0.20	-1.15	PPP-PPP	0.10	11	0.861	0.984	0.2734
1554008_at	671.5	585.9	-0.20	-1.15	PPP-PPP	0.10	11	0.861	0.984	0.2831
215737_x_at	435.7	380.1	-0.20	-1.15	PPP-PPP	0.10	11	0.861	0.984	0.2979
213406_at	45.1	39.3	-0.20	-1.15	APA-APA	0.10	11	0.861	0.984	0.3021
44065_at	812.3	708.4	-0.20	-1.15	PPP-PPP	0.10	11	0.861	0.984	0.3036
204599_s_at	2086.6	1820.6	-0.20	-1.15	PPP-PPP	0.10	11	0.861	0.984	0.3240
223945_x_at	1128.6	984.5	-0.20	-1.15	PPP-PPP	0.10	11	0.861	0.984	0.3314
213627_at	1106.1	965.8	-0.20	-1.15	PPP-PPP	0.10	11	0.861	0.984	0.0518
211976_at	1140.3	995.4	-0.20	-1.15	PPP-PPP	0.10	11	0.861	0.984	0.0690
211939_x_at	32600.7	28456.5	-0.20	-1.15	PPP-PPP	0.10	11	0.861	0.984	0.1137
211036_x_at	7180.3	6266.2	-0.20	-1.15	PPP-PPP	0.10	11	0.861	0.984	0.1412
224738_x_at	16530.5	14434.4	-0.20	-1.15	PPP-PPP	0.10	11	0.861	0.984	0.1583
216306_x_at	15798.7	13791.4	-0.20	-1.15	PPP-PPP	0.10	11	0.861	0.984	0.1724

200709_at	15089.3	13174.9	-0.20	-1.15	PPP-PPP	0.10	11	0.861	0.984	0.1798
212022_s_at	4634.2	4045.6	-0.20	-1.15	PPP-PPP	0.10	11	0.861	0.984	0.1948
237158_s_at	518.1	452.2	-0.20	-1.15	PPP-PPP	0.10	11	0.861	0.984	0.3390
212473_s_at	11224.9	9796.8	-0.20	-1.15	PPP-PPP	0.10	11	0.861	0.984	0.3680
208112_x_at	594.8	519.2	-0.20	-1.15	PPP-PPP	0.10	11	0.861	0.984	0.4201
1560250_s_at	63.9	55.8	-0.20	-1.15	AAA-APP	0.10	11	0.861	0.984	0.4215
242556_at	127.4	111.2	-0.20	-1.15	AAP-AAP	0.10	11	0.861	0.984	0.4339
222770_s_at	1026.8	896.7	-0.20	-1.15	PPP-PPP	0.10	11	0.861	0.984	0.4618
202036_s_at	7543.4	6586.1	-0.20	-1.15	PPP-PPP	0.10	11	0.861	0.984	0.4660
212931_at	965.0	843.2	-0.20	-1.14	PPP-PPP	0.10	11	0.861	0.984	0.0677
225992_at	1026.3	896.3	-0.20	-1.15	PPP-PPP	0.10	11	0.861	0.984	0.0860
213619_at	21517.7	18791.0	-0.20	-1.15	PPP-PPP	0.10	11	0.861	0.984	0.0874
212779_at	924.3	807.4	-0.20	-1.15	PPP-PPP	0.10	11	0.861	0.984	0.1089
225520_at	3432.1	2998.7	-0.20	-1.15	PPP-PPP	0.10	11	0.861	0.984	0.1890
1569003_at	1000.8	874.3	-0.20	-1.15	PPP-PPP	0.10	11	0.861	0.984	0.2048
227477_at	1525.3	1332.7	-0.20	-1.15	PPP-PPP	0.10	11	0.861	0.984	0.2175
218059_at	4971.5	4344.4	-0.20	-1.14	PPP-PPP	0.10	11	0.861	0.984	0.2715
221589_s_at	387.2	338.3	-0.20	-1.15	PPP-PPP	0.10	11	0.861	0.984	0.3381
221628_s_at	243.4	212.6	-0.20	-1.15	PPP-PPP	0.10	11	0.861	0.984	0.3855
200723_s_at	13177.2	11519.6	-0.19	-1.14	PPP-PPP	0.10	11	0.861	0.984	0.0855
209104_s_at	15380.3	13446.6	-0.19	-1.14	PPP-PPP	0.10	11	0.861	0.984	0.1375
227692_at	1709.5	1494.8	-0.19	-1.14	PPP-PPP	0.10	11	0.861	0.984	0.1413
205702_at	305.4	267.0	-0.19	-1.14	MPM-MPP	0.10	11	0.861	0.984	0.2046
220890_s_at	7679.3	6711.8	-0.19	-1.14	PPP-PPP	0.10	11	0.861	0.984	0.2209
225077_at	547.6	478.9	-0.19	-1.14	PPP-PPP	0.10	11	0.861	0.984	0.2506
228308_at	469.1	410.2	-0.19	-1.14	PPP-PPP	0.10	11	0.861	0.984	0.3348
214728_x_at	3702.9	3238.0	-0.19	-1.14	PPP-PPP	0.10	11	0.861	0.984	0.3351
218441_s_at	292.8	255.9	-0.19	-1.14	MPP-PPP	0.10	11	0.861	0.984	0.3837
236527_at	198.7	173.7	-0.19	-1.14	PPP-MPP	0.10	11	0.861	0.984	0.3864
240066_at	155.0	135.6	-0.19	-1.14	PPP-PPP	0.10	11	0.861	0.984	0.3869
228566_at	389.9	340.9	-0.19	-1.14	PMP-MPP	0.10	11	0.861	0.984	0.4091
213323_s_at	282.6	247.0	-0.19	-1.14	PPP-PPP	0.10	11	0.861	0.984	0.4110
229676_at	1067.8	933.2	-0.19	-1.14	PPP-PPP	0.10	11	0.861	0.984	0.4488
203166_at	2000.9	1748.7	-0.19	-1.14	PPP-PPP	0.10	11	0.861	0.984	0.4578
227313_at	580.9	507.9	-0.19	-1.14	PPP-PPP	0.10	11	0.861	0.984	0.4686
200718_s_at	26231.4	22954.1	-0.19	-1.14	PPP-PPP	0.10	11	0.861	0.984	0.0518
213648_at	367.4	321.4	-0.19	-1.14	PPP-PPP	0.10	11	0.861	0.984	0.0636
217823_s_at	2907.1	2542.6	-0.19	-1.14	PPP-PPP	0.10	11	0.861	0.984	0.0958
222044_at	503.9	440.9	-0.19	-1.14	PPP-PPP	0.10	11	0.861	0.984	0.1474
1558117_s_at	186.4	163.1	-0.19	-1.14	PPP-PPP	0.10	11	0.861	0.984	0.1743
212213_x_at	1493.7	1307.0	-0.19	-1.14	PPP-PPP	0.10	11	0.861	0.984	0.1806
205909_at	2245.8	1965.1	-0.19	-1.14	PPP-PPP	0.10	11	0.861	0.984	0.1818

214074_s_at	444.9	389.0	-0.19	-1.14	PPP-PPP	0.10	11	0.861	0.984	0.2231
232209_x_at	1434.9	1255.1	-0.19	-1.14	PPP-PPP	0.10	11	0.861	0.984	0.2457
209191_at	23902.1	20914.5	-0.19	-1.14	PPP-PPP	0.10	11	0.861	0.984	0.2609
207198_s_at	3311.9	2896.5	-0.19	-1.14	PPP-PPP	0.10	11	0.861	0.984	0.2635
212355_at	1324.4	1158.3	-0.19	-1.14	PPP-PPP	0.10	11	0.861	0.984	0.2654
212808_at	662.0	579.0	-0.19	-1.14	PPP-PPP	0.10	11	0.861	0.984	0.2807
229115_at	1769.0	1547.3	-0.19	-1.14	PPP-PPP	0.10	11	0.861	0.984	0.2813
1556242_a_at	218.8	191.4	-0.19	-1.14	PPP-PPP	0.10	11	0.861	0.984	0.2945
202537_s_at	2478.9	2168.5	-0.19	-1.14	PPP-PPP	0.10	11	0.861	0.984	0.3247
228436_at	109.1	95.5	-0.19	-1.14	PPP-PPP	0.10	11	0.861	0.984	0.3420
202770_s_at	394.6	345.2	-0.19	-1.14	PPP-PPP	0.10	11	0.861	0.984	0.3760
224196_x_at	2468.0	2160.1	-0.19	-1.14	PPP-PPP	0.10	11	0.861	0.984	0.1342
200599_s_at	19604.6	17165.1	-0.19	-1.14	PPP-PPP	0.10	11	0.861	0.984	0.1388
211746_x_at	16987.5	14870.2	-0.19	-1.14	PPP-PPP	0.10	11	0.861	0.984	0.1629
218431_at	788.8	690.5	-0.19	-1.14	MPP-PPP	0.10	11	0.861	0.984	0.1722
202787_s_at	481.4	421.4	-0.19	-1.14	PPP-PPP	0.10	11	0.861	0.984	0.2127
225639_at	1738.0	1521.5	-0.19	-1.14	PPP-PPP	0.10	11	0.861	0.984	0.2447
211711_s_at	1521.9	1332.0	-0.19	-1.14	PPP-PPP	0.10	11	0.861	0.984	0.2775
209655_s_at	896.0	784.6	-0.19	-1.14	PPP-PPP	0.10	11	0.861	0.984	0.3084
203355_s_at	932.5	816.5	-0.19	-1.14	PPP-PPP	0.10	11	0.861	0.984	0.3568
226743_at	1068.1	934.9	-0.19	-1.14	PPP-PPP	0.10	11	0.861	0.984	0.3838
226349_at	1399.4	1224.9	-0.19	-1.14	PPP-PPP	0.10	11	0.861	0.984	0.4638
208021_s_at	2005.2	1757.0	-0.19	-1.14	PPP-PPP	0.10	11	0.861	0.984	0.1913
202542_s_at	5749.6	5035.2	-0.19	-1.14	PPP-PPP	0.10	11	0.861	0.984	0.2088
213747_at	204.1	178.7	-0.19	-1.14	PPP-PPP	0.10	11	0.861	0.984	0.2301
227430_at	178.8	156.6	-0.19	-1.14	PPP-PPP	0.10	11	0.861	0.984	0.2381
207332_s_at	14440.2	12646.1	-0.19	-1.14	PPP-PPP	0.10	11	0.861	0.984	0.2652
222814_s_at	130.0	113.9	-0.19	-1.14	APA-APA	0.10	11	0.861	0.984	0.2687
213005_s_at	791.3	693.1	-0.19	-1.14	PPP-PPP	0.10	11	0.861	0.984	0.2767
215568_x_at	443.6	388.6	-0.19	-1.14	PPP-PPM	0.10	11	0.861	0.984	0.3065
208693_s_at	14459.1	12669.9	-0.19	-1.14	PPP-PPP	0.10	11	0.861	0.984	0.3225
202320_at	1076.5	942.7	-0.19	-1.14	PPP-PPP	0.10	11	0.861	0.984	0.3403
1563919_a_at	180.2	157.9	-0.19	-1.14	PPP-PPP	0.10	11	0.861	0.984	0.3926
216862_s_at	753.6	660.0	-0.19	-1.14	PPP-PPP	0.10	11	0.861	0.984	0.4275
203717_at	190.4	166.8	-0.19	-1.14	AAP-PMP	0.10	11	0.861	0.984	0.4492
222610_s_at	827.2	725.2	-0.19	-1.14	PPP-PPP	0.10	11	0.861	0.984	0.0741
1560116_a_at	4909.6	4302.5	-0.19	-1.14	PPP-PPP	0.10	11	0.861	0.984	0.0956
202009_at	862.0	755.5	-0.19	-1.14	PPP-PPP	0.10	11	0.861	0.984	0.1326
1553718_at	657.8	576.5	-0.19	-1.14	PPP-PPP	0.10	11	0.861	0.984	0.1508
224741_x_at	8593.9	7535.1	-0.19	-1.14	PPP-PPP	0.10	11	0.861	0.984	0.2243
231904_at	348.4	305.4	-0.19	-1.14	PPP-PPP	0.10	11	0.861	0.984	0.2270
242691_at	444.3	389.4	-0.19	-1.14	PPP-PPP	0.10	11	0.861	0.984	0.2475

222231_s_at	16275.7	14268.0	-0.19	-1.14	PPP-PPP	0.10	11	0.861	0.984	0.2587
224610_at	12946.3	11347.9	-0.19	-1.14	PPP-PPP	0.10	11	0.861	0.984	0.2596
243759_at	172.6	151.3	-0.19	-1.14	PPP-PPP	0.10	11	0.861	0.984	0.2720
217930_s_at	584.0	511.9	-0.19	-1.14	PPP-PPP	0.10	11	0.861	0.984	0.3473
223458_at	178.2	156.2	-0.19	-1.14	PPP-PPP	0.10	11	0.861	0.984	0.3568
239753_at	109.7	96.2	-0.19	-1.14	PPP-PPP	0.10	11	0.861	0.984	0.3768
219334_s_at	190.0	166.5	-0.19	-1.14	PPP-PPP	0.10	11	0.861	0.984	0.3820
218855_at	372.1	326.1	-0.19	-1.14	PPP-PPP	0.10	11	0.861	0.984	0.3884
230869_at	1012.3	887.3	-0.19	-1.14	PPP-PPP	0.10	11	0.861	0.984	0.4001
204578_at	150.8	132.2	-0.19	-1.14	PPP-PPP	0.10	11	0.861	0.984	0.4227
203823_at	1640.9	1438.2	-0.19	-1.14	PPP-PPP	0.10	11	0.861	0.984	0.4237
236657_at	411.9	361.2	-0.19	-1.14	PPP-PPP	0.10	11	0.861	0.984	0.4404
221840_at	242.8	212.7	-0.19	-1.14	PPP-PPP	0.10	11	0.861	0.984	0.4617
217736_s_at	3843.3	3371.3	-0.19	-1.14	PPP-PPP	0.10	11	0.861	0.984	0.0754
227416_s_at	4546.0	3988.9	-0.19	-1.14	PPP-PPP	0.10	11	0.861	0.984	0.0769
223413_s_at	2802.4	2458.4	-0.19	-1.14	PPP-PPP	0.10	11	0.861	0.984	0.1285
202189_x_at	12092.7	10605.9	-0.19	-1.14	PPP-PPP	0.10	11	0.861	0.984	0.1582
218919_at	1114.4	977.5	-0.19	-1.14	PPP-PPP	0.10	11	0.861	0.984	0.1661
222411_s_at	16199.5	14210.5	-0.19	-1.14	PPP-PPP	0.10	11	0.861	0.984	0.1896
217745_s_at	11529.1	10110.6	-0.19	-1.14	PPP-PPP	0.10	11	0.861	0.984	0.2550
218512_at	4153.7	3644.8	-0.19	-1.14	PPP-PPP	0.10	11	0.861	0.984	0.2621
220521_s_at	70.3	61.7	-0.19	-1.14	APP-PPP	0.10	11	0.861	0.984	0.2935
202328_s_at	194.8	170.8	-0.19	-1.14	PPP-PPP	0.10	11	0.861	0.984	0.2980
209077_at	2804.0	2459.3	-0.19	-1.14	PPP-PPP	0.10	11	0.861	0.984	0.3009
215385_at	210.0	184.2	-0.19	-1.14	PPP-PAP	0.10	11	0.861	0.984	0.3401
212607_at	2914.5	2556.6	-0.19	-1.14	PPP-PPP	0.10	11	0.861	0.984	0.3554
229940_at	265.5	232.9	-0.19	-1.14	PPP-PPP	0.10	11	0.861	0.984	0.3559
1553169_at	185.8	162.9	-0.19	-1.14	MAM-PPA	0.10	11	0.861	0.984	0.3563
226760_at	2917.6	2558.5	-0.19	-1.14	PPP-PPP	0.10	11	0.861	0.984	0.3663
206095_s_at	4676.2	4100.9	-0.19	-1.14	PPP-PPP	0.10	11	0.861	0.984	0.4173
203045_at	561.1	492.2	-0.19	-1.14	PPP-PPP	0.10	11	0.861	0.984	0.4396
218106_s_at	4036.6	3544.1	-0.19	-1.14	PPP-PPP	0.10	11	0.861	0.984	0.0818
202998_s_at	13521.6	11866.2	-0.19	-1.14	PPP-PPP	0.10	11	0.861	0.984	0.0928
212470_at	2627.8	2307.3	-0.19	-1.14	PPP-PPP	0.10	11	0.861	0.984	0.1308
213446_s_at	4983.1	4374.8	-0.19	-1.14	PPP-PPP	0.10	11	0.861	0.984	0.1661
243673_at	107.9	94.8	-0.19	-1.14	PPP-PPP	0.10	11	0.861	0.984	0.2029
227279_at	3177.3	2788.3	-0.19	-1.14	PPP-PPP	0.10	11	0.861	0.984	0.2385
219420_s_at	1677.0	1472.5	-0.19	-1.14	PPP-PPP	0.10	11	0.861	0.984	0.2738
44702_at	1928.6	1692.8	-0.19	-1.14	PPP-PPP	0.10	11	0.861	0.984	0.2923
225362_at	388.2	340.7	-0.19	-1.14	PPP-PPP	0.10	11	0.861	0.984	0.3274
209875_s_at	444.8	390.4	-0.19	-1.14	PPP-PPP	0.10	11	0.861	0.984	0.3818
200004_at	31254.3	27452.3	-0.19	-1.14	PPP-PPP	0.10	11	0.861	0.984	0.0413

201327_s_at	13315.4	11699.3	-0.19	-1.14	PPP-PPP	0.10	11	0.861	0.984	0.1253
207559_s_at	940.4	826.1	-0.19	-1.14	PPP-PPP	0.10	11	0.861	0.984	0.1348
59644_at	936.8	822.9	-0.19	-1.14	PPP-PPP	0.10	11	0.861	0.984	0.1457
217809_at	13542.7	11895.4	-0.19	-1.14	PPP-PPP	0.10	11	0.861	0.984	0.1749
213011_s_at	19291.0	16945.4	-0.19	-1.14	PPP-PPP	0.10	11	0.861	0.984	0.1755
224987_at	487.6	428.2	-0.19	-1.14	PPP-PPP	0.10	11	0.861	0.984	0.2221
226297_at	1192.9	1048.3	-0.19	-1.14	PPP-PPP	0.10	11	0.861	0.984	0.3027
226967_at	602.0	529.0	-0.19	-1.14	PPP-PPP	0.10	11	0.861	0.984	0.3282
224826_at	356.9	313.4	-0.19	-1.14	PPP-PPP	0.10	11	0.861	0.984	0.3352
220060_s_at	1205.1	1058.8	-0.19	-1.14	PPP-PPP	0.10	11	0.861	0.984	0.4075
205711_x_at	17987.0	15814.6	-0.19	-1.14	PPP-PPP	0.10	11	0.861	0.984	0.0432
202922_at	557.1	489.6	-0.19	-1.14	PPP-PPP	0.10	11	0.861	0.984	0.0498
202671_s_at	2569.3	2257.9	-0.19	-1.14	PPP-PPP	0.10	11	0.861	0.984	0.0582
201318_s_at	22415.6	19705.8	-0.19	-1.14	PPP-PPP	0.10	11	0.861	0.984	0.1192
202824_s_at	11564.9	10166.0	-0.19	-1.14	PPP-PPP	0.10	11	0.861	0.984	0.1500
201918_at	1925.3	1692.6	-0.19	-1.14	PPP-PPP	0.10	11	0.861	0.984	0.2010
223167_s_at	268.6	236.1	-0.19	-1.14	PPP-PPP	0.10	11	0.861	0.984	0.2141
223417_at	1066.0	937.1	-0.19	-1.14	PPP-PPP	0.10	11	0.861	0.984	0.2146
213860_x_at	11330.4	9961.7	-0.19	-1.14	PPP-PPP	0.10	11	0.861	0.984	0.2156
221746_at	759.6	667.6	-0.19	-1.14	PPP-PPP	0.10	11	0.861	0.984	0.2165
238879_at	306.9	269.8	-0.19	-1.14	PPP-PPP	0.10	11	0.861	0.984	0.2290
225891_at	320.9	282.2	-0.19	-1.14	APA-AMM	0.10	11	0.861	0.984	0.4124
238585_at	275.8	242.5	-0.19	-1.14	PPP-PPP	0.10	11	0.861	0.984	0.4273
1558839_at	87.4	76.8	-0.19	-1.14	AAP-AAP	0.10	11	0.861	0.984	0.4461
201453_x_at	16155.1	14210.9	-0.19	-1.14	PPP-PPP	0.10	11	0.861	0.984	0.0591
224913_s_at	4534.6	3988.4	-0.19	-1.14	PPP-PPP	0.10	11	0.861	0.984	0.0719
200806_s_at	23615.2	20771.0	-0.19	-1.14	PPP-PPP	0.10	11	0.861	0.984	0.0916
210283_x_at	3812.7	3354.6	-0.19	-1.14	PPP-PPP	0.10	11	0.861	0.984	0.1514
210213_s_at	9273.6	8156.1	-0.19	-1.14	PPP-PPP	0.10	11	0.861	0.984	0.1969
222427_s_at	4938.9	4344.9	-0.19	-1.14	PPP-PPP	0.10	11	0.861	0.984	0.1993
224841_x_at	8496.4	7476.0	-0.19	-1.14	PPP-PPP	0.10	11	0.861	0.984	0.2360
224856_at	309.7	272.3	-0.19	-1.14	PPP-PPP	0.10	11	0.861	0.984	0.2824
200748_s_at	32165.6	28290.9	-0.19	-1.14	PPP-PPP	0.10	11	0.861	0.984	0.3727
223602_at	512.5	450.7	-0.19	-1.14	PPP-PPP	0.10	11	0.861	0.984	0.3973
238526_at	93.3	82.1	-0.19	-1.14	AAP-APA	0.10	11	0.861	0.984	0.3989
223205_s_at	883.4	777.1	-0.19	-1.14	PPP-PPP	0.10	11	0.861	0.984	0.4141
221050_s_at	797.1	701.3	-0.19	-1.14	PPA-PPP	0.10	11	0.861	0.984	0.4364
201956_s_at	2335.6	2055.1	-0.19	-1.14	PPP-PPP	0.10	11	0.861	0.984	0.4395
238969_at	67.1	59.1	-0.19	-1.14	APP-PPP	0.10	11	0.861	0.984	0.4606
203537_at	1727.9	1520.5	-0.18	-1.14	PPP-PPP	0.10	11	0.861	0.984	0.0654
215230_x_at	14651.5	12897.5	-0.18	-1.14	PPP-PPP	0.10	11	0.861	0.984	0.1032
214687_x_at	25026.6	22023.5	-0.18	-1.14	PPP-PPP	0.10	11	0.861	0.984	0.1890

212341_at	1697.8	1494.9	-0.18	-1.14	PPP-PPP	0.10	11	0.861	0.984	0.1907
222036_s_at	3561.1	3134.5	-0.18	-1.14	PPP-PPP	0.10	11	0.861	0.984	0.2282
201630_s_at	4323.7	3804.7	-0.18	-1.14	PPP-PPP	0.10	11	0.861	0.984	0.2820
205173_x_at	1974.3	1737.7	-0.18	-1.14	PPP-PPP	0.10	11	0.861	0.984	0.2843
221753_at	1216.9	1071.5	-0.18	-1.14	PPP-PPP	0.10	11	0.861	0.984	0.3284
212203_x_at	4172.0	3672.1	-0.18	-1.14	PPP-PPP	0.10	11	0.861	0.984	0.3664
229072_at	161.4	142.1	-0.18	-1.14	PPP-PPP	0.10	11	0.861	0.984	0.3886
208541_x_at	691.5	608.9	-0.18	-1.14	PPP-PPP	0.10	11	0.861	0.984	0.3879
1563531_at	64.6	56.9	-0.18	-1.14	APP-PPP	0.10	11	0.861	0.984	0.4046
202322_s_at	1653.0	1456.4	-0.18	-1.14	PPP-PPP	0.10	11	0.861	0.984	0.0447
203033_x_at	7324.2	6453.3	-0.18	-1.14	PPP-PPP	0.10	11	0.861	0.984	0.0837
201276_at	1047.4	922.4	-0.18	-1.14	PPP-PPP	0.10	11	0.861	0.984	0.2260
221844_x_at	5730.2	5047.7	-0.18	-1.14	PPP-PPP	0.10	11	0.861	0.984	0.2304
205570_at	223.7	197.1	-0.18	-1.14	PPP-PPP	0.10	11	0.861	0.984	0.2609
230050_at	266.4	234.7	-0.18	-1.14	PPP-PPP	0.10	11	0.861	0.984	0.2655
211863_x_at	249.4	219.7	-0.18	-1.14	PPP-PPP	0.10	11	0.861	0.984	0.2669
224869_s_at	1836.9	1617.7	-0.18	-1.14	PPP-PPP	0.10	11	0.861	0.984	0.2681
209406_at	4053.4	3570.6	-0.18	-1.14	PPP-PPP	0.10	11	0.861	0.984	0.2689
220892_s_at	4277.4	3767.6	-0.18	-1.14	PPP-PPP	0.10	11	0.861	0.984	0.2784
229418_at	305.2	268.9	-0.18	-1.14	PPP-PPP	0.10	11	0.861	0.984	0.2985
229097_at	4960.5	4369.8	-0.18	-1.14	PPP-PPP	0.10	11	0.861	0.984	0.3565
211284_s_at	1213.2	1068.6	-0.18	-1.14	PPP-PPP	0.10	11	0.861	0.984	0.3858
201489_at	10438.0	9192.8	-0.18	-1.14	PPP-PPP	0.10	11	0.861	0.984	0.4586
202854_at	8658.3	7634.1	-0.18	-1.13	PPP-PPP	0.10	11	0.861	0.984	0.0861
200786_at	17653.8	15561.6	-0.18	-1.13	PPP-PPP	0.10	11	0.861	0.984	0.0961
217753_s_at	27260.8	24024.5	-0.18	-1.14	PPP-PPP	0.10	11	0.861	0.984	0.1142
212551_at	2831.5	2495.2	-0.18	-1.14	PPP-PPP	0.10	11	0.861	0.984	0.1282
225403_at	1490.3	1314.1	-0.18	-1.13	PPP-PPP	0.10	11	0.861	0.984	0.1302
232551_at	178.0	156.9	-0.18	-1.14	PPP-APP	0.10	11	0.861	0.984	0.1597
206102_at	4678.1	4124.0	-0.18	-1.13	PPP-PPP	0.10	11	0.861	0.984	0.1704
201922_at	20215.7	17817.5	-0.18	-1.14	PPP-PPP	0.10	11	0.861	0.984	0.1734
1554010_at	397.8	350.6	-0.18	-1.14	PPP-PPP	0.10	11	0.861	0.984	0.2144
226281_at	3868.8	3411.1	-0.18	-1.13	PPP-PPP	0.10	11	0.861	0.984	0.2548
203058_s_at	4603.1	4058.6	-0.18	-1.13	PPP-PPP	0.10	11	0.861	0.984	0.2859
222545_s_at	162.6	143.3	-0.18	-1.13	AAA-AAP	0.10	11	0.861	0.984	0.3362
225143_at	1242.1	1095.0	-0.18	-1.13	PPP-PPP	0.10	11	0.861	0.984	0.4058
226207_at	674.0	594.3	-0.18	-1.13	PPP-PPP	0.10	11	0.861	0.984	0.4380
212943_at	2948.5	2601.6	-0.18	-1.13	PPP-PPP	0.10	11	0.861	0.984	0.0664
200640_at	15819.9	13954.1	-0.18	-1.13	PPP-PPP	0.10	11	0.861	0.984	0.0681
223311_s_at	664.9	586.6	-0.18	-1.13	PPP-PPP	0.10	11	0.861	0.984	0.1088
218959_at	138.7	122.3	-0.18	-1.13	AAP-PPM	0.10	11	0.861	0.984	0.1233
202292_x_at	1010.3	891.1	-0.18	-1.13	PPP-PPP	0.10	11	0.861	0.984	0.1324

202657_s_at	2246.0	1981.2	-0.18	-1.13	PPP-PPP	0.10	11	0.861	0.984	0.1370
201600_at	21896.8	19317.4	-0.18	-1.13	PPP-PPP	0.10	11	0.861	0.984	0.1397
203150_at	2098.0	1851.3	-0.18	-1.13	PPP-PPP	0.10	11	0.861	0.984	0.1495
217795_s_at	2992.4	2640.5	-0.18	-1.13	PPP-PPP	0.10	11	0.861	0.984	0.1706
1570151_at	100.9	89.0	-0.18	-1.13	PPP-PPP	0.10	11	0.861	0.984	0.1867
226510_at	1023.1	902.2	-0.18	-1.13	PPP-PPP	0.10	11	0.861	0.984	0.2040
218732_at	3062.6	2701.3	-0.18	-1.13	PPP-PPP	0.10	11	0.861	0.984	0.2109
209372_x_at	2848.9	2513.6	-0.18	-1.13	PPP-PPP	0.10	11	0.861	0.984	0.2344
208290_s_at	7688.6	6779.8	-0.18	-1.13	PPP-PPP	0.10	11	0.861	0.984	0.3378
240385_at	110.7	97.7	-0.18	-1.13	PPP-PPP	0.10	11	0.861	0.984	0.3391
218307_at	909.5	802.1	-0.18	-1.13	PPP-PPP	0.10	11	0.861	0.984	0.3966
243259_at	98.5	86.9	-0.18	-1.13	AMP-APP	0.10	11	0.861	0.984	0.3994
244404_at	122.8	108.4	-0.18	-1.13	APP-PPP	0.10	11	0.861	0.984	0.4024
235532_at	134.6	118.7	-0.18	-1.13	PAM-AAM	0.10	11	0.861	0.984	0.4096
229465_s_at	912.9	805.5	-0.18	-1.13	PPP-PPP	0.10	11	0.861	0.984	0.4411
224958_at	970.5	855.9	-0.18	-1.13	PPP-PPP	0.10	11	0.861	0.984	0.4530
1563130_a_at	85.8	75.7	-0.18	-1.13	PAA-PPP	0.10	11	0.861	0.984	0.0485
201631_s_at	17256.0	15231.5	-0.18	-1.13	PPP-PPP	0.10	11	0.861	0.984	0.1041
203594_at	4775.4	4216.4	-0.18	-1.13	PPP-PPP	0.10	11	0.861	0.984	0.1076
214960_at	776.5	685.2	-0.18	-1.13	PPP-PPP	0.10	11	0.861	0.984	0.1622
212263_at	4583.7	4046.5	-0.18	-1.13	PPP-PPP	0.10	11	0.861	0.984	0.2174
228153_at	372.9	329.1	-0.18	-1.13	PPP-PPP	0.10	11	0.861	0.984	0.2316
217716_s_at	6837.4	6036.7	-0.18	-1.13	PPP-PPP	0.10	11	0.861	0.984	0.2616
214864_s_at	3050.8	2692.5	-0.18	-1.13	PPP-PPP	0.10	11	0.861	0.984	0.2645
208622_s_at	4461.5	3938.7	-0.18	-1.13	PPP-PPP	0.10	11	0.861	0.984	0.2856
227934_at	584.6	515.8	-0.18	-1.13	PPP-PPP	0.10	11	0.861	0.984	0.2990
201353_s_at	640.3	565.1	-0.18	-1.13	PPP-PPP	0.10	11	0.861	0.984	0.3527
224903_at	7334.7	6476.8	-0.18	-1.13	PPP-PPP	0.10	11	0.861	0.984	0.0995
203022_at	3345.7	2955.3	-0.18	-1.13	PPP-PPP	0.10	11	0.861	0.984	0.1317
224749_at	639.4	565.0	-0.18	-1.13	PPP-PPP	0.10	11	0.861	0.984	0.1424
201103_x_at	10842.0	9576.2	-0.18	-1.13	PPP-PPP	0.10	11	0.861	0.984	0.1475
229942_at	261.9	231.3	-0.18	-1.13	PPP-PPP	0.10	11	0.861	0.984	0.1481
215128_at	321.4	283.8	-0.18	-1.13	PPP-PPP	0.10	11	0.861	0.984	0.1519
201249_at	122.7	108.4	-0.18	-1.13	PPA-AAM	0.10	11	0.861	0.984	0.2210
224173_s_at	822.4	726.2	-0.18	-1.13	PPP-PPP	0.10	11	0.861	0.984	0.2606
33814_at	838.2	740.4	-0.18	-1.13	PPP-PPP	0.10	11	0.861	0.984	0.3285
235792_x_at	883.4	780.2	-0.18	-1.13	PPP-PPP	0.10	11	0.861	0.984	0.3564
218131_s_at	2485.8	2196.2	-0.18	-1.13	PPP-PPP	0.10	11	0.861	0.984	0.3644
233276_at	198.9	175.7	-0.18	-1.13	MAP-PPP	0.10	11	0.861	0.984	0.4213
241734_at	1125.9	995.3	-0.18	-1.13	PPP-PPP	0.10	11	0.861	0.984	0.0321
208308_s_at	7958.1	7032.7	-0.18	-1.13	PPP-PPP	0.10	11	0.861	0.984	0.0403
225147_at	1177.2	1040.8	-0.18	-1.13	PPP-PPP	0.10	11	0.861	0.984	0.1043

218867_s_at	876.4	774.9	-0.18	-1.13	PPP-PPP	0.10	11	0.861	0.984	0.1093
203377_s_at	613.4	542.2	-0.18	-1.13	PPP-PPP	0.10	11	0.861	0.984	0.1329
218125_s_at	451.1	398.7	-0.18	-1.13	PPP-PPP	0.10	11	0.861	0.984	0.1448
219502_at	1289.9	1140.4	-0.18	-1.13	PPP-PPP	0.10	11	0.861	0.984	0.1486
225285_at	13912.7	12297.4	-0.18	-1.13	PPP-PPP	0.10	11	0.861	0.984	0.2159
216044_x_at	1153.1	1019.3	-0.18	-1.13	PPP-PPP	0.10	11	0.861	0.984	0.2429
210125_s_at	10517.9	9295.6	-0.18	-1.13	PPP-PPP	0.10	11	0.861	0.984	0.2464
209600_s_at	664.0	587.1	-0.18	-1.13	PPP-PPP	0.10	11	0.861	0.984	0.2716
210985_s_at	107.5	95.0	-0.18	-1.13	PPP-PPP	0.10	11	0.861	0.984	0.3046
41657_at	484.8	428.6	-0.18	-1.13	PMP-PPP	0.10	11	0.861	0.984	0.3140
1552277_a_at	5509.5	4869.8	-0.18	-1.13	PPP-PPP	0.10	11	0.861	0.984	0.3241
212014_x_at	7635.7	6749.9	-0.18	-1.13	PPP-PPP	0.10	11	0.861	0.984	0.3759
211686_s_at	2687.4	2375.4	-0.18	-1.13	PPP-PPP	0.10	11	0.861	0.984	0.4091
209890_at	3033.9	2684.1	-0.18	-1.13	PPP-PPP	0.10	11	0.861	0.984	0.0500
201403_s_at	4879.3	4316.1	-0.18	-1.13	PPP-PPP	0.10	11	0.861	0.984	0.0601
217356_s_at	11895.3	10525.5	-0.18	-1.13	PPP-PPP	0.10	11	0.861	0.984	0.0674
214452_at	2377.1	2103.3	-0.18	-1.13	PPP-PPP	0.10	11	0.861	0.984	0.0980
201844_s_at	1889.6	1671.1	-0.18	-1.13	PPP-PPP	0.10	11	0.861	0.984	0.0996
209545_s_at	2721.6	2406.7	-0.18	-1.13	PPP-PPP	0.10	11	0.861	0.984	0.1036
205136_s_at	335.5	296.6	-0.18	-1.13	PPP-PPP	0.10	11	0.861	0.984	0.1381
225436_at	1929.7	1707.4	-0.18	-1.13	PPP-PPP	0.10	11	0.861	0.984	0.1367
225400_at	1440.9	1274.9	-0.18	-1.13	PPP-PPP	0.10	11	0.861	0.984	0.1526
217893_s_at	1723.5	1524.3	-0.18	-1.13	PPP-PPP	0.10	11	0.861	0.984	0.1661
218049_s_at	3843.4	3398.9	-0.18	-1.13	PPP-PPP	0.10	11	0.861	0.984	0.1946
225095_at	687.3	608.1	-0.18	-1.13	PPP-PPP	0.10	11	0.861	0.984	0.2109
207616_s_at	1455.2	1287.5	-0.18	-1.13	PPP-PPP	0.10	11	0.861	0.984	0.2186
1569527_at	125.0	110.6	-0.18	-1.13	PPP-PPP	0.10	11	0.861	0.984	0.2628
217140_s_at	8001.7	7075.6	-0.18	-1.13	PPP-PPP	0.10	11	0.861	0.984	0.3086
215690_x_at	1904.9	1685.0	-0.18	-1.13	PPP-PPP	0.10	11	0.861	0.984	0.3324
200824_at	6788.6	6006.6	-0.18	-1.13	PPP-PPP	0.10	11	0.861	0.984	0.3431
206468_s_at	796.1	704.1	-0.18	-1.13	PPP-PPP	0.10	11	0.861	0.984	0.3439
218526_s_at	2136.3	1889.1	-0.18	-1.13	PPP-PPP	0.10	11	0.861	0.984	0.3470
225004_at	405.3	358.5	-0.18	-1.13	PPP-PPP	0.10	11	0.861	0.984	0.3784
218507_at	824.7	729.4	-0.18	-1.13	PPP-PPP	0.10	11	0.861	0.984	0.3919
211774_s_at	420.5	371.8	-0.18	-1.13	PPP-PPP	0.10	11	0.861	0.984	0.4050
231959_at	772.6	683.4	-0.18	-1.13	PPP-PPP	0.10	11	0.861	0.984	0.4279
207821_s_at	1322.6	1169.9	-0.18	-1.13	PPP-PPP	0.10	11	0.861	0.984	0.4295
203098_at	1804.8	1596.8	-0.18	-1.13	PPP-PPP	0.10	11	0.861	0.984	0.4556
202916_s_at	1232.3	1090.5	-0.18	-1.13	PPP-PPP	0.10	11	0.861	0.984	0.0319
201219_at	1097.7	971.8	-0.18	-1.13	PPP-PPP	0.10	11	0.861	0.984	0.0335
202144_s_at	11635.2	10298.8	-0.18	-1.13	PPP-PPP	0.10	11	0.861	0.984	0.0624
200703_at	24134.5	21362.3	-0.18	-1.13	PPP-PPP	0.10	11	0.861	0.984	0.0702

217632_at	77.3	68.4	-0.18	-1.13	PPP-PPP	0.10	11	0.861	0.984	0.1259
216508_x_at	7329.5	6488.4	-0.18	-1.13	PPP-PPP	0.10	11	0.861	0.984	0.1730
212379_at	2594.4	2295.7	-0.18	-1.13	PPP-PPP	0.10	11	0.861	0.984	0.1745
209226_s_at	9745.1	8624.4	-0.18	-1.13	PPP-PPP	0.10	11	0.861	0.984	0.2830
218364_at	961.3	850.9	-0.18	-1.13	PPP-PPP	0.10	11	0.861	0.984	0.4051
227195_at	2922.0	2586.7	-0.18	-1.13	PPP-PPP	0.10	11	0.861	0.984	0.4441
222229_x_at	17850.7	15806.9	-0.18	-1.13	PPP-PPP	0.10	11	0.861	0.984	0.0418
211955_at	7096.6	6283.9	-0.18	-1.13	PPP-PPP	0.10	11	0.861	0.984	0.0984
225204_at	1812.3	1604.8	-0.18	-1.13	PPP-PPP	0.10	11	0.861	0.984	0.1657
1564031_a_at	397.2	351.9	-0.18	-1.13	PPP-PPP	0.10	11	0.861	0.984	0.1666
208872_s_at	7143.7	6329.2	-0.18	-1.13	PPP-PPP	0.10	11	0.861	0.984	0.1734
226177_at	4120.1	3648.8	-0.18	-1.13	PPP-PPP	0.10	11	0.861	0.984	0.2113
203016_s_at	1335.5	1182.9	-0.18	-1.13	PPP-PPP	0.10	11	0.861	0.984	0.2169
201977_s_at	395.6	350.4	-0.18	-1.13	PPP-PPP	0.10	11	0.861	0.984	0.2284
220408_x_at	1005.0	890.1	-0.18	-1.13	PPP-PPP	0.10	11	0.861	0.984	0.2875
224844_at	2473.1	2190.5	-0.18	-1.13	PPP-PPP	0.10	11	0.861	0.984	0.3333
1598_g_at	2145.2	1900.6	-0.18	-1.13	PPP-PPP	0.10	11	0.861	0.984	0.3332
214995_s_at	87.6	77.7	-0.18	-1.13	AAA-PPP	0.10	11	0.861	0.984	0.3346
218189_s_at	4895.7	4337.9	-0.18	-1.13	PPP-PPP	0.10	11	0.861	0.984	0.3381
218948_at	353.6	313.2	-0.18	-1.13	APP-PPP	0.10	11	0.861	0.984	0.4052
1559232_a_at	116.5	103.2	-0.18	-1.13	APA-AMA	0.10	11	0.861	0.984	0.4126
205540_s_at	72.5	64.2	-0.18	-1.13	MAA-APP	0.10	11	0.861	0.984	0.4398
223097_at	1706.1	1512.1	-0.17	-1.13	PPP-PPP	0.10	11	0.861	0.984	0.0555
200850_s_at	6809.7	6037.5	-0.17	-1.13	PPP-PPP	0.10	11	0.861	0.984	0.0708
203857_s_at	1281.7	1136.2	-0.17	-1.13	PPP-PPP	0.10	11	0.861	0.984	0.0914
202391_at	14801.7	13123.1	-0.17	-1.13	PPP-PPP	0.10	11	0.861	0.984	0.0962
201506_at	30274.7	26835.1	-0.17	-1.13	PPP-PPP	0.10	11	0.861	0.984	0.1126
214363_s_at	17005.6	15072.2	-0.17	-1.13	PPP-PPP	0.10	11	0.861	0.984	0.1323
209262_s_at	1389.4	1231.9	-0.17	-1.13	PPP-PPP	0.10	11	0.861	0.984	0.1524
201111_at	5057.9	4481.9	-0.17	-1.13	PPP-PPP	0.10	11	0.861	0.984	0.1646
226751_at	4935.6	4374.3	-0.17	-1.13	PPP-PPP	0.10	11	0.861	0.984	0.1779
204175_at	2160.0	1914.9	-0.17	-1.13	PPP-PPP	0.10	11	0.861	0.984	0.2532
222884_at	839.8	744.5	-0.17	-1.13	PPP-PPP	0.10	11	0.861	0.984	0.2738
227531_at	1835.5	1627.2	-0.17	-1.13	PPP-PPP	0.10	11	0.861	0.984	0.3295
209062_x_at	481.7	427.0	-0.17	-1.13	PPP-PPP	0.10	11	0.861	0.984	0.3306
222501_s_at	221.1	195.9	-0.17	-1.13	APP-PPP	0.10	11	0.861	0.984	0.3523
219926_at	2001.0	1774.1	-0.17	-1.13	PPP-PPP	0.10	11	0.861	0.984	0.3529
217830_s_at	908.9	805.6	-0.17	-1.13	PPP-PPP	0.10	11	0.861	0.984	0.3622
1552928_s_at	90.4	80.1	-0.17	-1.13	AAA-PPA	0.10	11	0.861	0.984	0.4257
228041_at	351.5	311.7	-0.17	-1.13	PPP-PPP	0.10	11	0.861	0.984	0.4285
221350_at	297.3	263.5	-0.17	-1.13	PPP-PPP	0.10	11	0.861	0.984	0.4309
210740_s_at	902.0	799.3	-0.17	-1.13	PPP-PPP	0.10	11	0.861	0.984	0.4443

219778_at	283.8	251.5	-0.17	-1.13	PPP-PPP	0.10	11	0.861	0.984	0.4499
200005_at	14069.5	12481.0	-0.17	-1.13	PPP-PPP	0.10	11	0.861	0.984	0.0444
210892_s_at	751.6	666.8	-0.17	-1.13	PPP-PPP	0.10	11	0.861	0.984	0.0467
227013_at	2638.8	2340.8	-0.17	-1.13	PPP-PPP	0.10	11	0.861	0.984	0.1184
212050_at	1092.7	969.0	-0.17	-1.13	PPP-PPP	0.10	11	0.861	0.984	0.1437
224681_at	4381.4	3887.2	-0.17	-1.13	PPP-PPP	0.10	11	0.861	0.984	0.1469
224312_x_at	1589.9	1410.2	-0.17	-1.13	PPP-PPP	0.10	11	0.861	0.984	0.1957
213574_s_at	7438.7	6596.4	-0.17	-1.13	PPP-PPP	0.10	11	0.861	0.984	0.2100
211954_s_at	10190.4	9039.7	-0.17	-1.13	PPP-PPP	0.10	11	0.861	0.984	0.2158
203495_at	249.6	221.4	-0.17	-1.13	PPP-PPP	0.10	11	0.861	0.984	0.2176
238010_at	529.8	470.1	-0.17	-1.13	PPP-PPP	0.10	11	0.861	0.984	0.2178
209265_s_at	3179.8	2820.4	-0.17	-1.13	PPP-PPP	0.10	11	0.861	0.984	0.2365
223251_s_at	902.6	800.4	-0.17	-1.13	PPP-PPP	0.10	11	0.861	0.984	0.2753
31845_at	1930.0	1712.5	-0.17	-1.13	PPP-PPP	0.10	11	0.861	0.984	0.2935
223711_s_at	978.3	867.9	-0.17	-1.13	PPP-PPP	0.10	11	0.861	0.984	0.3230
203579_s_at	657.2	582.9	-0.17	-1.13	PPP-PPP	0.10	11	0.861	0.984	0.3453
206659_at	151.7	134.6	-0.17	-1.13	PPP-PPP	0.10	11	0.861	0.984	0.3939
206661_at	162.3	144.0	-0.17	-1.13	PPP-PPP	0.10	11	0.861	0.984	0.4183
238587_at	800.9	710.3	-0.17	-1.13	PPP-PPP	0.10	11	0.861	0.984	0.4305
227039_at	543.6	482.0	-0.17	-1.13	PPP-PAP	0.10	11	0.861	0.984	0.4311
226621_at	3813.8	3386.0	-0.17	-1.13	PPP-PPP	0.10	11	0.861	0.984	0.0832
226129_at	159.8	141.8	-0.17	-1.13	PPP-PPP	0.10	11	0.861	0.984	0.1083
226740_x_at	12026.2	10673.8	-0.17	-1.13	PPP-PPP	0.10	11	0.861	0.984	0.1175
218464_s_at	641.3	569.1	-0.17	-1.13	PPP-PPP	0.10	11	0.861	0.984	0.1558
219192_at	4402.6	3908.3	-0.17	-1.13	PPP-PPP	0.10	11	0.861	0.984	0.1764
228204_at	534.6	474.7	-0.17	-1.13	PPP-PPP	0.10	11	0.861	0.984	0.1866
226185_at	60.6	53.8	-0.17	-1.13	PPP-AAP	0.10	11	0.861	0.984	0.2217
223234_at	2422.5	2150.5	-0.17	-1.13	PPP-PPP	0.10	11	0.861	0.984	0.2655
218418_s_at	2545.6	2259.8	-0.17	-1.13	PPP-PPP	0.10	11	0.861	0.984	0.2751
209011_at	1371.6	1217.4	-0.17	-1.13	PPP-PPP	0.10	11	0.861	0.984	0.2891
208393_s_at	1463.6	1299.1	-0.17	-1.13	PPP-PPP	0.10	11	0.861	0.984	0.3363
204572_s_at	154.6	137.3	-0.17	-1.13	PPP-PPP	0.10	11	0.861	0.984	0.3523
209008_x_at	8056.5	7152.8	-0.17	-1.13	PPP-PPP	0.10	11	0.861	0.984	0.3880
235577_at	176.1	156.3	-0.17	-1.13	AAM-APP	0.10	11	0.861	0.984	0.4195
203041_s_at	3200.4	2839.9	-0.17	-1.13	PPP-PPP	0.10	11	0.861	0.984	0.4303
204142_at	717.3	636.7	-0.17	-1.13	PPP-PPP	0.10	11	0.861	0.984	0.4402
242584_at	595.1	528.1	-0.17	-1.13	PPP-PPP	0.10	11	0.861	0.984	0.4460
242045_at	90.7	80.5	-0.17	-1.13	PPP-PPP	0.10	11	0.861	0.984	0.4493
200840_at	26033.7	23123.5	-0.17	-1.13	PPP-PPP	0.10	11	0.861	0.984	0.0864
202442_at	12232.9	10862.3	-0.17	-1.13	PPP-PPP	0.10	11	0.861	0.984	0.0995
215529_x_at	726.6	645.3	-0.17	-1.13	PPP-PPP	0.10	11	0.861	0.984	0.1409
212791_at	1348.7	1197.8	-0.17	-1.13	PPP-PPP	0.10	11	0.861	0.984	0.1587

224478_s_at	1279.8	1137.0	-0.17	-1.13	PPP-PPP	0.10	11	0.861	0.984	0.1804
224810_s_at	9601.4	8528.7	-0.17	-1.13	PPP-PPP	0.10	11	0.861	0.984	0.1918
221779_at	1955.1	1737.0	-0.17	-1.13	PPP-PPP	0.10	11	0.861	0.984	0.2218
215000_s_at	6652.0	5908.9	-0.17	-1.13	PPP-PPP	0.10	11	0.861	0.984	0.2299
225030_at	5021.9	4460.8	-0.17	-1.13	PPP-PPP	0.10	11	0.861	0.984	0.3160
221999_at	426.8	379.2	-0.17	-1.13	PPP-PPP	0.10	11	0.861	0.984	0.3567
212123_at	1328.6	1179.8	-0.17	-1.13	PPP-PPP	0.10	11	0.861	0.984	0.3791
209181_s_at	10924.8	9706.5	-0.17	-1.13	PPP-PPP	0.10	11	0.861	0.984	0.3899
236515_at	392.9	349.1	-0.17	-1.13	PPP-PPP	0.10	11	0.861	0.984	0.4144
202475_at	5036.3	4476.6	-0.17	-1.13	PPP-PPP	0.10	11	0.861	0.984	0.0268
239115_at	246.6	219.2	-0.17	-1.13	PPP-PPP	0.10	11	0.861	0.984	0.0364
234725_s_at	372.7	331.2	-0.17	-1.13	PPP-PPP	0.10	11	0.861	0.984	0.0474
223035_s_at	5087.6	4522.4	-0.17	-1.13	PPP-PPP	0.10	11	0.861	0.984	0.0542
202309_at	8143.7	7240.2	-0.17	-1.13	PPP-PPP	0.10	11	0.861	0.984	0.0974
211852_s_at	408.0	362.7	-0.17	-1.13	PPP-PPP	0.10	11	0.861	0.984	0.1162
200873_s_at	22797.0	20264.5	-0.17	-1.13	PPP-PPP	0.10	11	0.861	0.984	0.1388
201455_s_at	2500.9	2222.7	-0.17	-1.13	PPP-PPP	0.10	11	0.861	0.984	0.2169
209252_at	2053.7	1826.0	-0.17	-1.13	PPP-PPP	0.10	11	0.861	0.984	0.2159
208683_at	23689.0	21051.6	-0.17	-1.13	PPP-PPP	0.10	11	0.861	0.984	0.2563
41037_at	1298.1	1154.2	-0.17	-1.13	PPP-PPP	0.10	11	0.861	0.984	0.2798
52005_at	1008.6	896.3	-0.17	-1.13	PPP-PPP	0.10	11	0.861	0.984	0.2908
1560060_s_at	2949.1	2621.8	-0.17	-1.13	PPP-PPP	0.10	11	0.861	0.984	0.3280
202800_at	236.9	210.6	-0.17	-1.13	MPP-PPM	0.10	11	0.861	0.984	0.3430
228610_at	134.5	119.6	-0.17	-1.13	PPP-PPP	0.10	11	0.861	0.984	0.4203
1773_at	401.6	357.0	-0.17	-1.13	PPP-PPP	0.10	11	0.861	0.984	0.4244
221538_s_at	750.2	667.4	-0.17	-1.12	PPP-PPP	0.10	11	0.861	0.984	0.0293
202427_s_at	3626.0	3225.1	-0.17	-1.12	PPP-PPP	0.10	11	0.861	0.984	0.0320
208684_at	5458.6	4856.1	-0.17	-1.12	PPP-PPP	0.10	11	0.861	0.984	0.0468
202491_s_at	3375.6	3003.2	-0.17	-1.12	PPP-PPP	0.10	11	0.861	0.984	0.1081
220261_s_at	1033.9	919.7	-0.17	-1.12	PPP-PPP	0.10	11	0.861	0.984	0.1672
201251_at	21097.0	18759.1	-0.17	-1.13	PPP-PPP	0.10	11	0.861	0.984	0.1685
202591_s_at	15742.1	13998.5	-0.17	-1.13	PPP-PPP	0.10	11	0.861	0.984	0.1691
221685_s_at	6368.4	5664.5	-0.17	-1.12	PPP-PPP	0.10	11	0.861	0.984	0.1820
211986_at	3356.5	2985.5	-0.17	-1.12	PPP-PPP	0.10	11	0.861	0.984	0.1972
204842_x_at	2458.6	2186.7	-0.17	-1.12	PPP-PPP	0.10	11	0.861	0.984	0.2161
212077_at	21815.9	19399.7	-0.17	-1.13	PPP-PPP	0.10	11	0.861	0.984	0.2261
223057_s_at	1114.6	991.7	-0.17	-1.12	PPP-PPP	0.10	11	0.861	0.984	0.2922
200714_x_at	2973.0	2644.5	-0.17	-1.12	PPP-PPP	0.10	11	0.861	0.984	0.3121
219737_s_at	618.3	549.8	-0.17	-1.12	PPP-PPP	0.10	11	0.861	0.984	0.3132
34868_at	743.5	661.4	-0.17	-1.12	PPP-PPP	0.10	11	0.861	0.984	0.3260
225373_at	313.8	279.2	-0.17	-1.12	PPP-PPP	0.10	11	0.861	0.984	0.3339
212627_s_at	1751.3	1557.4	-0.17	-1.12	PPP-PPP	0.10	11	0.861	0.984	0.3356

217939_s_at	1238.4	1101.7	-0.17	-1.12	PPP-PPP	0.10	11	0.861	0.984	0.3407
216071_x_at	428.8	381.4	-0.17	-1.12	PPP-PPP	0.10	11	0.861	0.984	0.3531
228161_at	147.7	131.4	-0.17	-1.12	AAP-AAA	0.10	11	0.861	0.984	0.4232
201077_s_at	15433.2	13740.8	-0.17	-1.12	PPP-PPP	0.10	11	0.861	0.984	0.0328
1554167_a_at	2633.6	2344.1	-0.17	-1.12	PPP-PPP	0.10	11	0.861	0.984	0.1098
201608_s_at	6284.8	5593.4	-0.17	-1.12	PPP-PPP	0.10	11	0.861	0.984	0.1304
202647_s_at	2891.6	2573.1	-0.17	-1.12	PPP-PPP	0.10	11	0.861	0.984	0.1963
221494_x_at	10089.8	8980.7	-0.17	-1.12	PPP-PPP	0.10	11	0.861	0.984	0.2123
210094_s_at	1691.5	1505.3	-0.17	-1.12	PPP-PPP	0.10	11	0.861	0.984	0.2444
212600_s_at	8411.4	7486.2	-0.17	-1.12	PPP-PPP	0.10	11	0.861	0.984	0.3003
200791_s_at	7464.1	6641.8	-0.17	-1.12	PPP-PPP	0.10	11	0.861	0.984	0.3177
210201_x_at	1538.2	1368.8	-0.17	-1.12	PPP-PPP	0.10	11	0.861	0.984	0.3487
208820_at	3379.6	3008.9	-0.17	-1.12	PPP-PPP	0.10	11	0.861	0.984	0.3482
218550_s_at	245.3	218.3	-0.17	-1.12	APP-PPP	0.10	11	0.861	0.984	0.3646
209403_at	361.4	321.6	-0.17	-1.12	PMP-PPP	0.10	11	0.861	0.984	0.3724
1555284_at	45.5	40.5	-0.17	-1.12	AAA-AAP	0.10	11	0.861	0.984	0.4059
232405_at	127.9	113.8	-0.17	-1.12	APP-PPP	0.10	11	0.861	0.984	0.4289
203814_s_at	556.9	495.6	-0.17	-1.12	PPP-PPP	0.10	11	0.861	0.984	0.4332
201626_at	2827.9	2517.1	-0.17	-1.12	PPP-PPP	0.10	11	0.861	0.984	0.4395
200773_x_at	18061.6	16084.6	-0.17	-1.12	PPP-PPP	0.10	11	0.861	0.984	0.0269
213699_s_at	25032.8	22293.7	-0.17	-1.12	PPP-PPP	0.10	11	0.861	0.984	0.0427
200844_s_at	14204.3	12647.4	-0.17	-1.12	PPP-PPP	0.10	11	0.861	0.984	0.1011
224586_x_at	18231.0	16241.2	-0.17	-1.12	PPP-PPP	0.10	11	0.861	0.984	0.1026
237411_at	2352.5	2095.3	-0.17	-1.12	PPP-PPP	0.10	11	0.861	0.984	0.1151
225712_at	2899.3	2581.8	-0.17	-1.12	PPP-PPP	0.10	11	0.861	0.984	0.1377
230656_s_at	6786.5	6045.6	-0.17	-1.12	PPP-PPP	0.10	11	0.861	0.984	0.1486
212250_at	6224.3	5542.8	-0.17	-1.12	PPP-PPP	0.10	11	0.861	0.984	0.1584
233292_s_at	511.1	455.1	-0.17	-1.12	PPP-PPP	0.10	11	0.861	0.984	0.1830
204604_at	1507.8	1342.6	-0.17	-1.12	PPP-PPP	0.10	11	0.861	0.984	0.1976
217934_x_at	3974.2	3540.0	-0.17	-1.12	PPP-PPP	0.10	11	0.861	0.984	0.2595
217407_x_at	515.9	459.5	-0.17	-1.12	MPP-PPP	0.10	11	0.861	0.984	0.2995
203345_s_at	2682.4	2388.7	-0.17	-1.12	PPP-PPP	0.10	11	0.861	0.984	0.3032
217165_x_at	7898.2	7035.8	-0.17	-1.12	PPP-PPP	0.10	11	0.861	0.984	0.3285
221931_s_at	7710.8	6866.8	-0.17	-1.12	PPP-PPP	0.10	11	0.861	0.984	0.3538
219623_at	499.6	445.0	-0.17	-1.12	PPP-PPP	0.10	11	0.861	0.984	0.3870
242062_at	185.7	165.4	-0.17	-1.12	AAP-PPP	0.10	11	0.861	0.984	0.3951
238865_at	86.5	77.0	-0.17	-1.12	AAP-APP	0.10	11	0.861	0.984	0.4228
218027_at	9343.4	8325.7	-0.17	-1.12	PPP-PPP	0.10	11	0.861	0.984	0.0560
201356_at	4176.3	3721.5	-0.17	-1.12	PPP-PPP	0.10	11	0.861	0.984	0.0707
210949_s_at	15504.2	13816.2	-0.17	-1.12	PPP-PPP	0.10	11	0.861	0.984	0.0895
201950_x_at	7901.8	7040.8	-0.17	-1.12	PPP-PPP	0.10	11	0.861	0.984	0.2351
238045_at	197.1	175.7	-0.17	-1.12	PPP-PPP	0.10	11	0.861	0.984	0.2733

218601_at	1057.2	942.4	-0.17	-1.12	PAA-PAP	0.10	11	0.861	0.984	0.3402
213549_at	1351.0	1204.3	-0.17	-1.12	PPP-PPP	0.10	11	0.861	0.984	0.3406
200034_s_at	31979.1	28515.1	-0.17	-1.12	PPP-PPP	0.10	11	0.861	0.984	0.0243
221737_at	985.9	879.3	-0.17	-1.12	PPP-PPP	0.10	11	0.861	0.984	0.0249
201574_at	10518.0	9379.4	-0.17	-1.12	PPP-PPP	0.10	11	0.861	0.984	0.0354
214501_s_at	4560.0	4066.9	-0.17	-1.12	PPP-PPP	0.10	11	0.861	0.984	0.0411
217724_at	17455.7	15564.3	-0.17	-1.12	PPP-PPP	0.10	11	0.861	0.984	0.1321
202698_x_at	20897.1	18635.6	-0.17	-1.12	PPP-PPP	0.10	11	0.861	0.984	0.1959
204314_s_at	1396.2	1245.5	-0.17	-1.12	PPP-PPP	0.10	11	0.861	0.984	0.1985
211779_x_at	2140.3	1908.9	-0.17	-1.12	PPP-PPP	0.10	11	0.861	0.984	0.1995
218569_s_at	572.8	510.8	-0.17	-1.12	PPP-PPP	0.10	11	0.861	0.984	0.2079
201752_s_at	1429.4	1274.5	-0.17	-1.12	PPP-PPP	0.10	11	0.861	0.984	0.2118
226302_at	3121.1	2782.9	-0.17	-1.12	PPP-PPP	0.10	11	0.861	0.984	0.2204
208726_s_at	20348.3	18150.3	-0.17	-1.12	PPP-PPP	0.10	11	0.861	0.984	0.2284
218637_at	496.6	443.0	-0.17	-1.12	PPP-PPP	0.10	11	0.861	0.984	0.2685
222993_at	5020.8	4476.9	-0.17	-1.12	PPP-PPP	0.10	11	0.861	0.984	0.2812
210543_s_at	2285.3	2038.5	-0.17	-1.12	PPP-PPP	0.10	11	0.861	0.984	0.3260
226024_at	3122.1	2785.4	-0.17	-1.12	PPP-PPP	0.10	11	0.861	0.984	0.3326
230903_s_at	246.8	220.1	-0.17	-1.12	PPP-PPP	0.10	11	0.861	0.984	0.4035
233999_s_at	388.9	347.0	-0.17	-1.12	PPP-PPP	0.10	11	0.861	0.984	0.4270
224945_at	1716.4	1530.9	-0.17	-1.12	PPP-PPP	0.10	11	0.861	0.984	0.4295
201754_at	12334.0	11009.0	-0.16	-1.12	PPP-PPP	0.10	11	0.861	0.984	0.0599
227181_at	192.2	171.6	-0.16	-1.12	PPP-PPP	0.10	11	0.861	0.984	0.0971
200843_s_at	4743.2	4232.6	-0.16	-1.12	PPP-PPP	0.10	11	0.861	0.984	0.1284
223441_at	863.6	770.9	-0.16	-1.12	PPP-PPP	0.10	11	0.861	0.984	0.1814
201885_s_at	12272.6	10955.6	-0.16	-1.12	PPP-PPP	0.10	11	0.861	0.984	0.1951
226972_s_at	170.0	151.7	-0.16	-1.12	PAP-PPP	0.10	11	0.861	0.984	0.2154
203316_s_at	16601.4	14812.7	-0.16	-1.12	PPP-PPP	0.10	11	0.861	0.984	0.2162
201821_s_at	7861.4	7016.7	-0.16	-1.12	PPP-PPP	0.10	11	0.861	0.984	0.2377
202872_at	1617.5	1443.3	-0.16	-1.12	PPP-PPP	0.10	11	0.861	0.984	0.2527
219679_s_at	1636.7	1460.7	-0.16	-1.12	PPP-PPP	0.10	11	0.861	0.984	0.2685
214092_x_at	1416.5	1264.5	-0.16	-1.12	PPP-PPP	0.10	11	0.861	0.984	0.2771
205621_at	1240.5	1107.2	-0.16	-1.12	PPP-PPP	0.10	11	0.861	0.984	0.3088
218066_at	215.4	192.2	-0.16	-1.12	PPA-APA	0.10	11	0.861	0.984	0.3170
219921_s_at	221.3	197.6	-0.16	-1.12	MAP-APP	0.10	11	0.861	0.984	0.3267
208782_at	13503.3	12051.6	-0.16	-1.12	PPP-PPP	0.10	11	0.861	0.984	0.3742
224708_at	1401.3	1250.4	-0.16	-1.12	PPP-PPP	0.10	11	0.861	0.984	0.3877
202379_s_at	3031.4	2704.9	-0.16	-1.12	PPP-PPP	0.10	11	0.861	0.984	0.3989
209624_s_at	548.6	489.5	-0.16	-1.12	PPP-PPP	0.10	11	0.861	0.984	0.4344
200782_at	18553.2	16565.5	-0.16	-1.12	PPP-PPP	0.10	11	0.861	0.984	0.0935
222609_s_at	2889.9	2581.1	-0.16	-1.12	PPP-PPP	0.10	11	0.861	0.984	0.0967
205263_at	3446.7	3078.7	-0.16	-1.12	PPP-PPP	0.10	11	0.861	0.984	0.1142

224806_at	2126.6	1900.0	-0.16	-1.12	PPP-PPP	0.10	11	0.861	0.984	0.1196
222122_s_at	2021.9	1805.6	-0.16	-1.12	PPP-PPP	0.10	11	0.861	0.984	0.1369
212893_at	2833.7	2531.0	-0.16	-1.12	PPP-PPP	0.10	11	0.861	0.984	0.1407
208870_x_at	19770.2	17664.0	-0.16	-1.12	PPP-PPP	0.10	11	0.861	0.984	0.1938
240449_at	147.1	131.4	-0.16	-1.12	AAP-PPP	0.10	11	0.861	0.984	0.2047
37966_at	500.9	447.5	-0.16	-1.12	PPP-PPP	0.10	11	0.861	0.984	0.2444
201706_s_at	1110.1	991.8	-0.16	-1.12	PPP-PPP	0.10	11	0.861	0.984	0.2748
209093_s_at	816.0	728.6	-0.16	-1.12	PPP-PPP	0.10	11	0.861	0.984	0.3052
211060_x_at	1768.5	1579.7	-0.16	-1.12	PPP-PPP	0.10	11	0.861	0.984	0.3114
221537_at	424.0	378.7	-0.16	-1.12	PPP-PPP	0.10	11	0.861	0.984	0.3811
228293_at	932.4	832.7	-0.16	-1.12	PPP-PPP	0.10	11	0.861	0.984	0.4276
217122_s_at	2173.3	1942.0	-0.16	-1.12	PPP-PPP	0.10	11	0.861	0.984	0.0314
210826_x_at	967.7	865.1	-0.16	-1.12	PPP-PPP	0.10	11	0.861	0.984	0.0368
227267_at	845.6	755.6	-0.16	-1.12	PPP-PPP	0.10	11	0.861	0.984	0.1003
214280_x_at	4251.0	3800.8	-0.16	-1.12	PPP-PPP	0.10	11	0.861	0.984	0.1183
219083_at	2448.6	2188.0	-0.16	-1.12	PPP-PPP	0.10	11	0.861	0.984	0.1545
203511_s_at	3903.8	3490.2	-0.16	-1.12	PPP-PPP	0.10	11	0.861	0.984	0.1660
204837_at	955.6	854.0	-0.16	-1.12	PPP-PPP	0.10	11	0.861	0.984	0.1818
208616_s_at	23216.9	20754.6	-0.16	-1.12	PPP-PPP	0.10	11	0.861	0.984	0.2260
211325_x_at	652.9	583.6	-0.16	-1.12	APP-PPP	0.10	11	0.861	0.984	0.2295
205733_at	1239.0	1107.6	-0.16	-1.12	PPP-PPP	0.10	11	0.861	0.984	0.2673
226275_at	160.4	143.4	-0.16	-1.12	APP-PPP	0.10	11	0.861	0.984	0.2776
228930_at	608.2	543.5	-0.16	-1.12	PPP-PPP	0.10	11	0.861	0.984	0.2881
209195_s_at	369.1	329.8	-0.16	-1.12	APA-APM	0.10	11	0.861	0.984	0.3104
214938_x_at	25817.1	23068.7	-0.16	-1.12	PPP-PPP	0.10	11	0.861	0.984	0.3122
204305_at	717.2	640.8	-0.16	-1.12	PPP-PPP	0.10	11	0.861	0.984	0.3196
202025_x_at	423.7	378.7	-0.16	-1.12	PPP-PPP	0.10	11	0.861	0.984	0.3426
211949_s_at	1206.8	1078.5	-0.16	-1.12	PPP-PPP	0.10	11	0.861	0.984	0.4270
221934_s_at	812.6	726.3	-0.16	-1.12	APP-PPP	0.10	11	0.861	0.984	0.4346
226759_at	243.5	217.7	-0.16	-1.12	PPP-PPP	0.10	11	0.861	0.984	0.4392
202675_at	4634.1	4144.0	-0.16	-1.12	PPP-PPP	0.10	11	0.861	0.984	0.0392
204490_s_at	7678.1	6866.3	-0.16	-1.12	PPP-PPP	0.10	11	0.861	0.984	0.0511
217747_s_at	23084.5	20641.1	-0.16	-1.12	PPP-PPP	0.10	11	0.861	0.984	0.0562
37012_at	9389.2	8399.4	-0.16	-1.12	PPP-PPP	0.10	11	0.861	0.984	0.1034
212171_x_at	934.0	835.3	-0.16	-1.12	PPP-PPP	0.10	11	0.861	0.984	0.1435
228721_at	316.6	283.2	-0.16	-1.12	PPP-PPP	0.10	11	0.861	0.984	0.1930
210097_s_at	11076.1	9908.0	-0.16	-1.12	PPP-PPP	0.10	11	0.861	0.984	0.2646
218398_at	3822.3	3419.0	-0.16	-1.12	PPP-PPP	0.10	11	0.861	0.984	0.2727
213608_s_at	1970.0	1762.3	-0.16	-1.12	PPP-PPP	0.10	11	0.861	0.984	0.3326
200019_s_at	25995.7	23267.3	-0.16	-1.12	PPP-PPP	0.10	11	0.861	0.984	0.0418
231938_at	422.0	377.6	-0.16	-1.12	PPP-PPP	0.10	11	0.861	0.984	0.0551
218481_at	1603.7	1435.3	-0.16	-1.12	PPP-PPP	0.10	11	0.861	0.984	0.1005

212798_s_at	2824.9	2528.6	-0.16	-1.12	PPP-PPP	0.10	11	0.861	0.984	0.1260
212145_at	3917.5	3507.1	-0.16	-1.12	PPP-PPP	0.10	11	0.861	0.984	0.1272
222673_x_at	2288.7	2048.7	-0.16	-1.12	PPP-PPP	0.10	11	0.861	0.984	0.1587
202846_s_at	998.4	893.6	-0.16	-1.12	PPP-PPP	0.10	11	0.861	0.984	0.1627
208517_x_at	29886.6	26757.0	-0.16	-1.12	PPP-PPP	0.10	11	0.861	0.984	0.1791
203554_x_at	17174.8	15369.9	-0.16	-1.12	PPP-PPP	0.10	11	0.861	0.984	0.2128
218263_s_at	1787.8	1599.7	-0.16	-1.12	PPP-PPP	0.10	11	0.861	0.984	0.2228
213301_x_at	1085.1	971.5	-0.16	-1.12	PPP-PPP	0.10	11	0.861	0.984	0.2210
227639_at	1277.2	1143.0	-0.16	-1.12	PPP-PPP	0.10	11	0.861	0.984	0.2409
213031_s_at	1727.8	1546.9	-0.16	-1.12	PPP-PPP	0.10	11	0.861	0.984	0.2450
219598_s_at	6025.3	5391.0	-0.16	-1.12	PPP-PPP	0.10	11	0.861	0.984	0.2522
201572_x_at	3677.7	3292.4	-0.16	-1.12	PPP-PPP	0.10	11	0.861	0.984	0.2543
200793_s_at	3232.2	2892.4	-0.16	-1.12	PPP-PPP	0.10	11	0.861	0.984	0.2912
223307_at	4329.0	3874.0	-0.16	-1.12	PPP-PPP	0.10	11	0.861	0.984	0.3100
210916_s_at	4770.1	4269.8	-0.16	-1.12	PPP-PPP	0.10	11	0.861	0.984	0.3113
214500_at	1719.5	1538.8	-0.16	-1.12	PPP-PPP	0.10	11	0.861	0.984	0.3223
211761_s_at	6085.6	5447.0	-0.16	-1.12	PPP-PPP	0.10	11	0.861	0.984	0.3716
208873_s_at	2931.3	2622.7	-0.16	-1.12	PPP-PPP	0.10	11	0.861	0.984	0.3981
202779_s_at	14059.2	12584.7	-0.16	-1.12	PPP-PPP	0.10	11	0.861	0.984	0.3997
221751_at	3470.5	3106.6	-0.16	-1.12	PPP-PPP	0.10	11	0.861	0.984	0.4216
205006_s_at	1425.8	1276.1	-0.16	-1.12	PPP-PPP	0.10	11	0.861	0.984	0.4258
208777_s_at	8508.4	7620.2	-0.16	-1.12	PPP-PPP	0.10	11	0.861	0.984	0.0339
211558_s_at	1275.5	1142.6	-0.16	-1.12	PPP-PPP	0.10	11	0.861	0.984	0.0398
200658_s_at	8144.7	7295.2	-0.16	-1.12	PPP-PPP	0.10	11	0.861	0.984	0.0729
230588_s_at	914.4	819.1	-0.16	-1.12	PPP-PPP	0.10	11	0.861	0.984	0.0793
212784_at	495.1	443.3	-0.16	-1.12	PPP-PPP	0.10	11	0.861	0.984	0.1158
221620_s_at	1490.2	1334.9	-0.16	-1.12	PPP-PPP	0.10	11	0.861	0.984	0.1171
213687_s_at	19575.9	17528.8	-0.16	-1.12	PPP-PPP	0.10	11	0.861	0.984	0.1272
201241_at	9544.4	8548.5	-0.16	-1.12	PPP-PPP	0.10	11	0.861	0.984	0.1408
202416_at	2523.8	2260.7	-0.16	-1.12	PPP-PPP	0.10	11	0.861	0.984	0.1693
200645_at	9833.6	8806.8	-0.16	-1.12	PPP-PPP	0.10	11	0.861	0.984	0.1713
208813_at	2980.8	2669.5	-0.16	-1.12	PPP-PPP	0.10	11	0.861	0.984	0.1764
200973_s_at	3229.3	2892.3	-0.16	-1.12	PPP-PPP	0.10	11	0.861	0.984	0.1881
205324_s_at	4744.2	4248.0	-0.16	-1.12	PPP-PPP	0.10	11	0.861	0.984	0.2154
226257_x_at	3052.6	2734.2	-0.16	-1.12	PPP-PPP	0.10	11	0.861	0.984	0.2382
209428_s_at	637.5	571.0	-0.16	-1.12	PPP-PPP	0.10	11	0.861	0.984	0.2471
222652_s_at	445.1	398.6	-0.16	-1.12	PPP-PPP	0.10	11	0.861	0.984	0.2722
200615_s_at	3133.0	2805.9	-0.16	-1.12	PPP-PPP	0.10	11	0.861	0.984	0.2833
212922_s_at	1922.8	1721.8	-0.16	-1.12	PPP-PPP	0.10	11	0.861	0.984	0.3181
217346_at	319.3	286.0	-0.16	-1.12	PPP-PPP	0.10	11	0.861	0.984	0.3201
225468_at	1011.5	906.1	-0.16	-1.12	PPP-PPP	0.10	11	0.861	0.984	0.3588
207143_at	225.0	201.5	-0.16	-1.12	PPP-PPP	0.10	11	0.861	0.984	0.3619

209417_s_at	267.9	239.9	-0.16	-1.12	PPP-PPP	0.10	11	0.861	0.984	0.3868
218631_at	1222.5	1094.6	-0.16	-1.12	PPP-PPP	0.10	11	0.861	0.984	0.3883
206074_s_at	11493.2	10292.6	-0.16	-1.12	PPP-PPP	0.10	11	0.861	0.984	0.4210
205953_at	297.6	266.6	-0.16	-1.12	PPP-PPP	0.10	11	0.861	0.984	0.0563
207081_s_at	835.9	749.4	-0.16	-1.12	PPP-PPP	0.10	11	0.861	0.984	0.0557
209049_s_at	2658.8	2382.5	-0.16	-1.12	PPP-PPP	0.10	11	0.861	0.984	0.0650
202313_at	4936.1	4423.6	-0.16	-1.12	PPP-PPP	0.10	11	0.861	0.984	0.0820
212520_s_at	1472.5	1319.6	-0.16	-1.12	PPP-PPP	0.10	11	0.861	0.984	0.1141
202579_x_at	5325.7	4772.7	-0.16	-1.12	PPP-PPP	0.10	11	0.861	0.984	0.1458
208766_s_at	15284.0	13701.9	-0.16	-1.12	PPP-PPP	0.10	11	0.861	0.984	0.1794
232300_at	170.5	152.9	-0.16	-1.12	PPP-PPP	0.10	11	0.861	0.984	0.1896
217848_s_at	19504.6	17482.6	-0.16	-1.12	PPP-PPP	0.10	11	0.861	0.984	0.2487
1555905_a_at	404.9	363.1	-0.16	-1.12	PPP-PPP	0.10	11	0.861	0.984	0.2519
221508_at	173.8	155.8	-0.16	-1.12	PPP-PPP	0.10	11	0.861	0.984	0.2657
222628_s_at	867.9	777.7	-0.16	-1.12	PPP-PPP	0.10	11	0.861	0.984	0.3023
201714_at	6501.9	5827.9	-0.16	-1.12	PPP-PPP	0.10	11	0.861	0.984	0.3181
209100_at	4275.4	3832.4	-0.16	-1.12	PPP-PPP	0.10	11	0.861	0.984	0.3250
202413_s_at	9266.4	8307.9	-0.16	-1.12	PPP-PPP	0.10	11	0.861	0.984	0.3330
208152_s_at	16870.8	15121.3	-0.16	-1.12	PPP-PPP	0.10	11	0.861	0.984	0.3356
229307_at	1077.6	966.0	-0.16	-1.12	PPP-PPP	0.10	11	0.861	0.984	0.3850
212130_x_at	18785.2	16834.9	-0.16	-1.12	PPP-PPP	0.10	11	0.861	0.984	0.3861
223017_at	6629.3	5941.9	-0.16	-1.12	PPP-PPP	0.10	11	0.861	0.984	0.4148
200944_s_at	18358.5	16460.2	-0.16	-1.12	PPP-PPP	0.10	11	0.861	0.984	0.0280
223085_at	941.4	844.3	-0.16	-1.12	PPP-PPP	0.10	11	0.861	0.984	0.0517
224781_s_at	5480.5	4914.1	-0.16	-1.12	PPP-PPP	0.10	11	0.861	0.984	0.1369
208680_at	24847.3	22286.8	-0.16	-1.12	PPP-PPP	0.10	11	0.861	0.984	0.1389
226169_at	808.2	724.7	-0.16	-1.12	PPP-PPP	0.10	11	0.861	0.984	0.1454
210028_s_at	2252.9	2020.5	-0.16	-1.12	PPP-PPP	0.10	11	0.861	0.984	0.1871
211968_s_at	34770.2	31194.4	-0.16	-1.12	PPP-PPP	0.10	11	0.861	0.984	0.1866
200993_at	9476.3	8500.1	-0.16	-1.12	PPP-PPP	0.10	11	0.861	0.984	0.1914
208690_s_at	9170.9	8226.5	-0.16	-1.12	PPP-PPP	0.10	11	0.861	0.984	0.1954
217833_at	2790.3	2502.8	-0.16	-1.12	PPP-PPP	0.10	11	0.861	0.984	0.1977
211793_s_at	1784.9	1600.4	-0.16	-1.12	PPP-PPP	0.10	11	0.861	0.984	0.2087
218622_at	5863.7	5259.9	-0.16	-1.12	PPP-PPP	0.10	11	0.861	0.984	0.2299
222875_at	3312.9	2971.4	-0.16	-1.12	PPP-PPP	0.10	11	0.861	0.984	0.2372
205516_x_at	1989.5	1784.7	-0.16	-1.12	PPP-PPP	0.10	11	0.861	0.984	0.2831
201584_s_at	9241.3	8286.8	-0.16	-1.12	PPP-PPP	0.10	11	0.861	0.984	0.2859
211940_x_at	21339.2	19136.6	-0.16	-1.12	PPP-PPP	0.10	11	0.861	0.984	0.3040
212796_s_at	1046.9	938.8	-0.16	-1.12	PPP-PPP	0.10	11	0.861	0.984	0.3495
225344_at	2088.3	1872.9	-0.16	-1.12	PPP-PPP	0.10	11	0.861	0.984	0.3604
224950_at	342.9	307.5	-0.16	-1.12	PPP-PPP	0.10	11	0.861	0.984	0.3735
210645_s_at	5369.7	4815.9	-0.16	-1.12	PPP-PPP	0.10	11	0.861	0.984	0.3735

1552684_a_at	127.3	114.2	-0.16	-1.12	AAP-PPP	0.10	11	0.861	0.984	0.4192
1553928_at	361.7	324.4	-0.16	-1.12	PPP-PPP	0.10	11	0.861	0.984	0.4323
208653_s_at	3476.8	3120.6	-0.16	-1.11	PPP-PPP	0.10	11	0.861	0.984	0.1244
201113_at	8863.3	7956.2	-0.16	-1.11	PPP-PPP	0.10	11	0.861	0.984	0.1466
226517_at	9510.8	8535.4	-0.16	-1.11	PPP-PPP	0.10	11	0.861	0.984	0.1709
218103_at	3102.4	2785.1	-0.16	-1.11	PPP-PPP	0.10	11	0.861	0.984	0.1880
200746_s_at	9825.8	8819.2	-0.16	-1.11	PPP-PPP	0.10	11	0.861	0.984	0.2504
50277_at	2558.7	2295.8	-0.16	-1.11	PPP-PPP	0.10	11	0.861	0.984	0.2627
228318_s_at	495.6	444.7	-0.16	-1.11	PPP-PPP	0.10	11	0.861	0.984	0.2639
213062_at	1918.4	1721.6	-0.16	-1.11	PPP-PPP	0.10	11	0.861	0.984	0.2713
212117_at	1798.6	1613.8	-0.16	-1.12	PPP-PPP	0.10	11	0.861	0.984	0.2834
202757_at	2087.4	1873.8	-0.16	-1.11	PPP-PPP	0.10	11	0.861	0.984	0.2994
206284_x_at	2261.8	2030.0	-0.16	-1.11	PPP-PPP	0.10	11	0.861	0.984	0.3177
225878_at	3286.6	2950.0	-0.16	-1.11	PPP-PPP	0.10	11	0.861	0.984	0.3863
1556035_s_at	850.1	762.9	-0.16	-1.11	PPP-PPP	0.10	11	0.861	0.984	0.3948
234095_at	86.3	77.5	-0.16	-1.11	PPP-PPP	0.10	11	0.861	0.984	0.4039
221264_s_at	466.3	418.6	-0.16	-1.11	PPP-PPP	0.10	11	0.861	0.984	0.4229
201923_at	11199.4	10055.9	-0.16	-1.11	PPP-PPP	0.10	11	0.861	0.984	0.0656
1556006_s_at	359.6	323.0	-0.16	-1.11	PPP-PPP	0.10	11	0.861	0.984	0.0893
224879_at	3624.7	3254.7	-0.16	-1.11	PPP-PPP	0.10	11	0.861	0.984	0.1398
229846_s_at	1514.2	1359.9	-0.16	-1.11	PPP-PPP	0.10	11	0.861	0.984	0.1730
200006_at	19930.7	17905.0	-0.16	-1.11	PPP-PPP	0.10	11	0.861	0.984	0.1827
225417_at	959.2	861.2	-0.16	-1.11	PPP-PPP	0.10	11	0.861	0.984	0.1892
201381_x_at	11148.3	10015.7	-0.16	-1.11	PPP-PPP	0.10	11	0.861	0.984	0.1906
1556060_a_at	1772.9	1592.0	-0.16	-1.11	PPP-PPP	0.10	11	0.861	0.984	0.2085
210762_s_at	10186.5	9150.4	-0.16	-1.11	PPP-PPP	0.10	11	0.861	0.984	0.2094
212002_at	1165.5	1046.7	-0.16	-1.11	PPP-PPP	0.10	11	0.861	0.984	0.2427
201777_s_at	565.7	508.1	-0.16	-1.11	PPP-PPP	0.10	11	0.861	0.984	0.2462
218817_at	1274.3	1144.3	-0.16	-1.11	PPP-PPP	0.10	11	0.861	0.984	0.2503
224675_at	5944.5	5339.8	-0.16	-1.11	PPP-PPP	0.10	11	0.861	0.984	0.2684
202808_at	1524.1	1369.1	-0.16	-1.11	PPP-PPP	0.10	11	0.861	0.984	0.3130
223076_s_at	2214.4	1989.4	-0.16	-1.11	PPP-PPP	0.10	11	0.861	0.984	0.3266
203794_at	761.7	684.0	-0.16	-1.11	PPP-PPP	0.10	11	0.861	0.984	0.3349
218034_at	3881.6	3486.2	-0.16	-1.11	PPP-PPP	0.10	11	0.861	0.984	0.3506
203183_s_at	316.7	284.4	-0.16	-1.11	PPP-PPP	0.10	11	0.861	0.984	0.3513
241599_at	367.8	330.4	-0.16	-1.11	PPP-PPP	0.10	11	0.861	0.984	0.3582
208924_at	6618.9	5944.8	-0.16	-1.11	PPP-PPP	0.10	11	0.861	0.984	0.3771
223493_at	229.4	206.0	-0.16	-1.11	PPP-PPP	0.10	11	0.861	0.984	0.3874
202514_at	1822.0	1636.3	-0.16	-1.11	PPP-PPP	0.10	11	0.861	0.984	0.4037
209341_s_at	665.7	598.0	-0.16	-1.11	PPP-PPP	0.10	11	0.861	0.984	0.4196
224721_at	2206.4	1982.8	-0.15	-1.11	PPP-PPP	0.10	11	0.861	0.984	0.0180
216241_s_at	9202.8	8272.0	-0.15	-1.11	PPP-PPP	0.10	11	0.861	0.984	0.0474

210716_s_at	967.0	869.1	-0.15	-1.11	PPP-PPP	0.10	11	0.861	0.984	0.0524
206158_s_at	16730.4	15032.5	-0.15	-1.11	PPP-PPP	0.10	11	0.861	0.984	0.0921
201588_at	12470.9	11209.5	-0.15	-1.11	PPP-PPP	0.10	11	0.861	0.984	0.1715
219939_s_at	8086.8	7267.6	-0.15	-1.11	PPP-PPP	0.10	11	0.861	0.984	0.1915
208758_at	7584.1	6814.8	-0.15	-1.11	PPP-PPP	0.10	11	0.861	0.984	0.2136
201573_s_at	5517.5	4957.7	-0.15	-1.11	PPP-PPP	0.10	11	0.861	0.984	0.2207
200841_s_at	1360.0	1222.2	-0.15	-1.11	PPP-PPP	0.10	11	0.861	0.984	0.2369
200764_s_at	4019.3	3613.4	-0.15	-1.11	PPP-PPP	0.10	11	0.861	0.984	0.2480
204045_at	1348.8	1212.3	-0.15	-1.11	PPP-PPP	0.10	11	0.861	0.984	0.2568
225043_at	966.7	869.0	-0.15	-1.11	PPP-PPP	0.10	11	0.861	0.984	0.2846
200889_s_at	4747.9	4267.3	-0.15	-1.11	PPP-PPP	0.10	11	0.861	0.984	0.2964
204693_at	1553.3	1395.8	-0.15	-1.11	PPP-PPP	0.10	11	0.861	0.984	0.3417
1568658_at	1115.7	1002.5	-0.15	-1.11	PPP-PPP	0.10	11	0.861	0.984	0.3418
1562849_at	174.6	156.9	-0.15	-1.11	PPP-PPP	0.10	11	0.861	0.984	0.3459
227765_at	977.5	878.7	-0.15	-1.11	PPP-PPP	0.10	11	0.861	0.984	0.3531
1555832_s_at	3201.2	2876.8	-0.15	-1.11	PPP-PPP	0.10	11	0.861	0.984	0.3636
219322_s_at	510.5	459.0	-0.15	-1.11	PPP-PPP	0.10	11	0.861	0.984	0.3648
221780_s_at	1839.2	1653.4	-0.15	-1.11	PPP-PPP	0.10	11	0.861	0.984	0.3672
211801_x_at	492.7	442.9	-0.15	-1.11	PPP-PPP	0.10	11	0.861	0.984	0.3893
46167_at	1719.7	1545.9	-0.15	-1.11	PPP-PPP	0.10	11	0.861	0.984	0.4106
201656_at	1474.0	1324.7	-0.15	-1.11	PPP-PPP	0.10	11	0.861	0.984	0.4139
202697_at	4667.0	4198.7	-0.15	-1.11	PPP-PPP	0.10	11	0.861	0.984	0.0253
224992_s_at	456.1	410.1	-0.15	-1.11	PPP-PPP	0.10	11	0.861	0.984	0.1363
212082_s_at	28954.1	26034.4	-0.15	-1.11	PPP-PPP	0.10	11	0.861	0.984	0.1390
222774_s_at	2715.7	2442.9	-0.15	-1.11	PPP-PPP	0.10	11	0.861	0.984	0.1389
208642_s_at	14923.6	13422.1	-0.15	-1.11	PPP-PPP	0.10	11	0.861	0.984	0.1508
202832_at	735.0	661.2	-0.15	-1.11	PPP-PPP	0.10	11	0.861	0.984	0.1521
213798_s_at	19622.2	17650.1	-0.15	-1.11	PPP-PPP	0.10	11	0.861	0.984	0.1527
202932_at	3135.6	2819.5	-0.15	-1.11	PPP-PPP	0.10	11	0.861	0.984	0.1563
200048_s_at	11757.7	10577.0	-0.15	-1.11	PPP-PPP	0.10	11	0.861	0.984	0.1619
1557055_s_at	790.8	711.4	-0.15	-1.11	PPP-PPP	0.10	11	0.861	0.984	0.1682
228089_x_at	1385.2	1246.1	-0.15	-1.11	PPP-PPP	0.10	11	0.861	0.984	0.1934
200740_s_at	15001.1	13495.1	-0.15	-1.11	PPP-PPP	0.10	11	0.861	0.984	0.2221
219297_at	747.6	672.6	-0.15	-1.11	PPP-PPP	0.10	11	0.861	0.984	0.2258
207805_s_at	5722.6	5146.2	-0.15	-1.11	PPP-PPP	0.10	11	0.861	0.984	0.2410
215313_x_at	11516.0	10359.6	-0.15	-1.11	PPP-PPP	0.10	11	0.861	0.984	0.2688
202799_at	2712.4	2438.9	-0.15	-1.11	PPP-PPP	0.10	11	0.861	0.984	0.3020
228987_at	288.4	259.5	-0.15	-1.11	PPP-PPP	0.10	11	0.861	0.984	0.3081
219641_at	516.9	464.7	-0.15	-1.11	PPP-PPP	0.10	11	0.861	0.984	0.3107
1555945_s_at	3818.0	3433.7	-0.15	-1.11	PPP-PPP	0.10	11	0.861	0.984	0.3231
1553535_a_at	2669.1	2401.2	-0.15	-1.11	PPP-PPP	0.10	11	0.861	0.984	0.3234
212693_at	1132.8	1018.9	-0.15	-1.11	PPP-PPP	0.10	11	0.861	0.984	0.3332

225234_at	663.2	596.6	-0.15	-1.11	PPP-PPP	0.10	11	0.861	0.984	0.3355
229664_at	334.9	301.3	-0.15	-1.11	MPP-PPP	0.10	11	0.861	0.984	0.3622
202264_s_at	2341.7	2105.8	-0.15	-1.11	PPP-PPP	0.10	11	0.861	0.984	0.3635
200759_x_at	3225.6	2902.4	-0.15	-1.11	PPP-PPP	0.10	11	0.861	0.984	0.0208
236155_at	365.0	328.6	-0.15	-1.11	PPP-PPP	0.10	11	0.861	0.984	0.0240
225309_at	2514.0	2262.2	-0.15	-1.11	PPP-PPP	0.10	11	0.861	0.984	0.1014
218853_s_at	2694.0	2424.2	-0.15	-1.11	PPP-PPP	0.10	11	0.861	0.984	0.1195
200613_at	10652.8	9588.9	-0.15	-1.11	PPP-PPP	0.10	11	0.861	0.984	0.1511
202529_at	2853.1	2567.1	-0.15	-1.11	PPP-PPP	0.10	11	0.861	0.984	0.1802
219017_at	1567.2	1410.7	-0.15	-1.11	PPP-PPP	0.10	11	0.861	0.984	0.1943
218217_at	886.2	797.6	-0.15	-1.11	PPP-PPP	0.10	11	0.861	0.984	0.2456
201696_at	4262.7	3837.3	-0.15	-1.11	PPP-PPP	0.10	11	0.861	0.984	0.2567
244647_at	187.3	168.6	-0.15	-1.11	PPP-PPP	0.10	11	0.861	0.984	0.2929
212462_at	928.6	835.8	-0.15	-1.11	PPP-PPP	0.10	11	0.861	0.984	0.3345
201778_s_at	1995.2	1795.5	-0.15	-1.11	PPP-PPP	0.10	11	0.861	0.984	0.3589
209575_at	926.5	833.9	-0.15	-1.11	PPP-PPP	0.10	11	0.861	0.984	0.3634
210968_s_at	29266.0	26345.0	-0.15	-1.11	PPP-PPP	0.10	11	0.861	0.984	0.3783
213980_s_at	3310.2	2980.1	-0.15	-1.11	PPP-PPP	0.10	11	0.861	0.984	0.3953
218101_s_at	14107.8	12709.3	-0.15	-1.11	PPP-PPP	0.10	11	0.861	0.984	0.0863
212747_at	569.9	513.2	-0.15	-1.11	PPP-PPP	0.10	11	0.861	0.984	0.1172
1554667_s_at	862.4	776.7	-0.15	-1.11	PPP-PPP	0.10	11	0.861	0.984	0.1194
1555841_at	4521.7	4073.1	-0.15	-1.11	PPP-PPP	0.10	11	0.861	0.984	0.1406
208900_s_at	3712.5	3342.5	-0.15	-1.11	PPP-PPP	0.10	11	0.861	0.984	0.2000
228099_at	269.1	242.4	-0.15	-1.11	PPP-PPP	0.10	11	0.861	0.984	0.2075
1555815_a_at	759.1	683.7	-0.15	-1.11	PPP-PPP	0.10	11	0.861	0.984	0.2092
202431_s_at	4655.6	4191.5	-0.15	-1.11	PPP-PPP	0.10	11	0.861	0.984	0.2476
226094_at	1757.1	1582.7	-0.15	-1.11	PPP-PPP	0.10	11	0.861	0.984	0.2463
227186_s_at	6192.2	5576.9	-0.15	-1.11	PPP-PPP	0.10	11	0.861	0.984	0.2579
227075_at	866.2	780.3	-0.15	-1.11	PPP-PPP	0.10	11	0.861	0.984	0.3382
219193_at	1089.0	980.9	-0.15	-1.11	PPP-PPP	0.10	11	0.861	0.984	0.3476
223419_at	377.0	339.5	-0.15	-1.11	PPP-PPP	0.10	11	0.861	0.984	0.4233
225105_at	11236.6	10126.5	-0.15	-1.11	PPP-PPP	0.10	11	0.861	0.984	0.0493
227436_at	981.3	884.6	-0.15	-1.11	PPP-PPP	0.10	11	0.861	0.984	0.0544
218716_x_at	1938.8	1746.8	-0.15	-1.11	PPP-PPP	0.10	11	0.861	0.984	0.1006
208910_s_at	20274.6	18272.4	-0.15	-1.11	PPP-PPP	0.10	11	0.861	0.984	0.1245
221870_at	3009.1	2711.6	-0.15	-1.11	PPP-PPP	0.10	11	0.861	0.984	0.1371
224955_at	7700.6	6940.4	-0.15	-1.11	PPP-PPP	0.10	11	0.861	0.984	0.1643
208736_at	12319.4	11099.2	-0.15	-1.11	PPP-PPP	0.10	11	0.861	0.984	0.2009
221548_s_at	1942.7	1750.6	-0.15	-1.11	PPP-PPP	0.10	11	0.861	0.984	0.2623
213688_at	222.7	200.7	-0.15	-1.11	PPP-PPP	0.10	11	0.861	0.984	0.3203
206357_at	181.6	163.8	-0.15	-1.11	PPP-PPP	0.10	11	0.861	0.984	0.3277
212223_at	768.1	692.2	-0.15	-1.11	PPP-PPP	0.10	11	0.861	0.984	0.3386

217834_s_at	8293.3	7472.1	-0.15	-1.11	PPP-PPP	0.10	11	0.861	0.984	0.3725
218669_at	1668.3	1503.5	-0.15	-1.11	PPP-PPP	0.10	11	0.861	0.984	0.4090
215533_s_at	358.4	322.9	-0.15	-1.11	MPP-PPP	0.10	11	0.861	0.984	0.4105
201897_s_at	9604.3	8663.0	-0.15	-1.11	PPP-PPP	0.10	11	0.861	0.984	0.0827
200045_at	4079.0	3678.6	-0.15	-1.11	PPP-PPP	0.10	11	0.861	0.984	0.1583
214359_s_at	21985.6	19830.5	-0.15	-1.11	PPP-PPP	0.10	11	0.861	0.984	0.1625
210378_s_at	1056.3	952.6	-0.15	-1.11	PPP-PPP	0.10	11	0.861	0.984	0.1805
207320_x_at	8047.2	7257.0	-0.15	-1.11	PPP-PPP	0.10	11	0.861	0.984	0.2072
222448_s_at	3048.8	2749.9	-0.15	-1.11	PPP-PPP	0.10	11	0.861	0.984	0.2285
212669_at	739.9	667.2	-0.15	-1.11	PPP-PPP	0.10	11	0.861	0.984	0.2497
204405_x_at	6496.8	5861.2	-0.15	-1.11	PPP-PPP	0.10	11	0.861	0.984	0.3528
218270_at	2250.8	2029.2	-0.15	-1.11	PPP-PPP	0.10	11	0.861	0.984	0.3638
216503_s_at	393.8	355.4	-0.15	-1.11	PPP-PMP	0.10	11	0.861	0.984	0.0789
201000_at	4955.2	4473.4	-0.15	-1.11	PPP-PPP	0.10	11	0.861	0.984	0.0923
221676_s_at	14562.3	13142.0	-0.15	-1.11	PPP-PPP	0.10	11	0.861	0.984	0.1170
203688_at	1787.5	1613.7	-0.15	-1.11	PPP-PPP	0.10	11	0.861	0.984	0.1399
211662_s_at	16669.8	15043.7	-0.15	-1.11	PPP-PPP	0.10	11	0.861	0.984	0.1819
212375_at	876.0	790.8	-0.15	-1.11	PPP-PPP	0.10	11	0.861	0.984	0.2051
231896_s_at	11189.2	10098.0	-0.15	-1.11	PPP-PPP	0.10	11	0.861	0.984	0.2244
215867_x_at	904.0	816.2	-0.15	-1.11	PPP-PPP	0.10	11	0.861	0.984	0.2252
201289_at	18234.0	16459.8	-0.15	-1.11	PPP-PPP	0.10	11	0.861	0.984	0.2906
218229_s_at	1575.2	1421.5	-0.15	-1.11	PPP-PPP	0.10	11	0.861	0.984	0.2988
202373_s_at	2068.4	1866.8	-0.15	-1.11	PPP-PPP	0.10	11	0.861	0.984	0.3055
212491_s_at	2721.3	2456.4	-0.15	-1.11	PPP-PPP	0.10	11	0.861	0.984	0.3109
225652_at	1816.1	1639.0	-0.15	-1.11	PPP-PPP	0.10	11	0.861	0.984	0.3582
218403_at	3551.7	3206.2	-0.15	-1.11	PPP-PPP	0.10	11	0.861	0.984	0.3602
201202_at	11738.9	10596.1	-0.15	-1.11	PPP-PPP	0.10	11	0.861	0.984	0.4087
1553218_a_at	749.7	676.4	-0.15	-1.11	PPP-PPP	0.10	11	0.861	0.984	0.4105
218671_s_at	3555.9	3212.5	-0.15	-1.11	PPP-PPP	0.10	11	0.861	0.984	0.0129
226694_at	12367.6	11172.0	-0.15	-1.11	PPP-PPP	0.10	11	0.861	0.984	0.1132
202927_at	1614.9	1458.8	-0.15	-1.11	PPP-PPP	0.10	11	0.861	0.984	0.1196
215980_s_at	238.2	215.1	-0.15	-1.11	PPP-PPP	0.10	11	0.861	0.984	0.1809
202138_x_at	5183.6	4682.1	-0.15	-1.11	PPP-PPP	0.10	11	0.861	0.984	0.2140
212429_s_at	1957.4	1768.1	-0.15	-1.11	PPP-PPP	0.10	11	0.861	0.984	0.3109
235241_at	863.7	780.2	-0.15	-1.11	PPP-PPP	0.10	11	0.861	0.984	0.3169
200090_at	6346.3	5732.9	-0.15	-1.11	PPP-PPP	0.10	11	0.861	0.984	0.3296
202317_s_at	1563.0	1411.3	-0.15	-1.11	PPP-PPP	0.10	11	0.861	0.984	0.3347
201587_s_at	9399.6	8489.6	-0.15	-1.11	PPP-PPP	0.10	11	0.861	0.984	0.3363
201004_at	7282.4	6576.4	-0.15	-1.11	PPP-PPP	0.10	11	0.861	0.984	0.3401
218313_s_at	2252.5	2034.7	-0.15	-1.11	PPP-PPP	0.10	11	0.861	0.984	0.3405
216202_s_at	1191.1	1075.6	-0.15	-1.11	PPP-PPP	0.10	11	0.861	0.984	0.3434
208842_s_at	5008.5	4524.4	-0.15	-1.11	PPP-PPP	0.10	11	0.861	0.984	0.3948

37424_at	463.9	418.9	-0.15	-1.11	PPP-PPP	0.10	11	0.861	0.984	0.4071
205545_x_at	5875.3	5311.3	-0.15	-1.11	PPP-PPP	0.10	11	0.861	0.984	0.0543
201663_s_at	7222.7	6529.6	-0.15	-1.11	PPP-PPP	0.10	11	0.861	0.984	0.1950
222738_at	522.2	472.1	-0.15	-1.11	PPP-PPP	0.10	11	0.861	0.984	0.2000
219972_s_at	1194.5	1079.2	-0.15	-1.11	PPP-PPP	0.10	11	0.861	0.984	0.2038
208764_s_at	11248.1	10169.0	-0.15	-1.11	PPP-PPP	0.10	11	0.861	0.984	0.2048
204594_s_at	1099.7	993.6	-0.15	-1.11	PPP-PPP	0.10	11	0.861	0.984	0.2727
205423_at	721.7	652.0	-0.15	-1.11	PPP-PPP	0.10	11	0.861	0.984	0.2760
209256_s_at	878.7	794.2	-0.15	-1.11	PPP-PPP	0.10	11	0.861	0.984	0.3023
201386_s_at	6965.9	6294.4	-0.15	-1.11	PPP-PPP	0.10	11	0.861	0.984	0.3160
211271_x_at	10396.6	9397.9	-0.15	-1.11	PPP-PPP	0.10	11	0.861	0.984	0.3869
223105_s_at	17375.4	15717.7	-0.15	-1.11	PPP-PPP	0.10	11	0.861	0.984	0.1206
79005_at	859.6	777.2	-0.15	-1.11	PPP-PPP	0.10	11	0.861	0.984	0.1507
226277_at	913.2	825.6	-0.15	-1.11	PPP-PPP	0.10	11	0.861	0.984	0.1745
223207_x_at	5218.7	4720.6	-0.15	-1.11	PPP-PPP	0.10	11	0.861	0.984	0.2075
213101_s_at	24429.1	22089.5	-0.15	-1.11	PPP-PPP	0.10	11	0.861	0.984	0.2203
221891_x_at	36443.9	32964.2	-0.15	-1.11	PPP-PPP	0.10	11	0.861	0.984	0.2418
200641_s_at	6117.4	5531.2	-0.15	-1.11	PPP-PPP	0.10	11	0.861	0.984	0.2465
221903_s_at	908.3	821.5	-0.15	-1.11	APP-PPP	0.10	11	0.861	0.984	0.2935
211228_s_at	892.3	806.8	-0.15	-1.11	PPP-PPP	0.10	11	0.861	0.984	0.3246
1553956_at	1416.5	1280.7	-0.15	-1.11	PPP-PPP	0.10	11	0.861	0.984	0.3668
215781_s_at	317.7	287.3	-0.15	-1.11	PPP-PPP	0.10	11	0.861	0.984	0.4052
201080_at	932.9	843.4	-0.15	-1.11	PPP-PPP	0.10	11	0.861	0.984	0.4078
222428_s_at	4346.9	3932.5	-0.15	-1.11	PPP-PPP	0.10	11	0.861	0.984	0.4101
213842_x_at	1629.6	1474.6	-0.14	-1.11	PPP-PPP	0.10	11	0.861	0.984	0.0120
207668_x_at	11085.6	10032.4	-0.14	-1.11	PPP-PPP	0.10	11	0.861	0.984	0.0586
201239_s_at	6769.5	6124.9	-0.14	-1.11	PPP-PPP	0.10	11	0.861	0.984	0.0908
203133_at	18709.1	16932.6	-0.14	-1.11	PPP-PPP	0.10	11	0.861	0.984	0.1687
211725_s_at	3234.0	2926.9	-0.14	-1.11	PPP-PPP	0.10	11	0.861	0.984	0.1890
1554029_a_at	629.1	569.2	-0.14	-1.11	PPP-PPP	0.10	11	0.861	0.984	0.2809
221593_s_at	1369.1	1238.9	-0.14	-1.11	PPP-PPP	0.10	11	0.861	0.984	0.3244
209633_at	1839.3	1664.8	-0.14	-1.11	PPP-PPP	0.10	11	0.861	0.984	0.3352
210554_s_at	6282.8	5685.3	-0.14	-1.11	PPP-PPP	0.10	11	0.861	0.984	0.3691
208433_s_at	556.5	503.6	-0.14	-1.11	PPP-PPP	0.10	11	0.861	0.984	0.3979
243024_at	216.6	196.2	-0.14	-1.10	PAP-PPP	0.10	11	0.861	0.984	0.0706
218235_s_at	4387.4	3972.9	-0.14	-1.10	PPP-PPP	0.10	11	0.861	0.984	0.1426
230793_at	325.5	294.8	-0.14	-1.10	PPP-PPP	0.10	11	0.861	0.984	0.1794
210428_s_at	2428.8	2199.2	-0.14	-1.10	PPP-PPP	0.10	11	0.861	0.984	0.2103
227257_s_at	1053.7	954.3	-0.14	-1.10	PPP-PPP	0.10	11	0.861	0.984	0.2342
216988_s_at	14586.7	13208.2	-0.14	-1.10	PPP-PPP	0.10	11	0.861	0.984	0.2477
235709_at	962.5	871.6	-0.14	-1.10	PPP-PPP	0.10	11	0.861	0.984	0.3292
201928_at	2015.9	1825.7	-0.14	-1.10	PPP-PPP	0.10	11	0.861	0.984	0.3524

202422_s_at	1654.4	1498.5	-0.14	-1.10	PPP-PPP	0.10	11	0.861	0.984	0.3574
225226_at	863.5	782.1	-0.14	-1.10	PPP-PPP	0.10	11	0.861	0.984	0.3748
202567_at	15935.1	14439.5	-0.14	-1.10	PPP-PPP	0.10	11	0.861	0.984	0.1779
202281_at	714.3	647.3	-0.14	-1.10	PPP-PPP	0.10	11	0.861	0.984	0.2033
202052_s_at	6874.7	6231.0	-0.14	-1.10	PPP-PPP	0.10	11	0.861	0.984	0.2039
209120_at	9555.7	8660.3	-0.14	-1.10	PPP-PPP	0.10	11	0.861	0.984	0.2152
215091_s_at	9021.9	8175.2	-0.14	-1.10	PPP-PPP	0.10	11	0.861	0.984	0.2222
214512_s_at	11133.2	10091.1	-0.14	-1.10	PPP-PPP	0.10	11	0.861	0.984	0.2629
214446_at	928.8	841.7	-0.14	-1.10	PPP-PPP	0.10	11	0.861	0.984	0.2754
203411_s_at	4730.0	4287.4	-0.14	-1.10	PPP-PPP	0.10	11	0.861	0.984	0.2961
207266_x_at	3559.5	3225.5	-0.14	-1.10	PPP-PPP	0.10	11	0.861	0.984	0.3024
219405_at	794.7	720.3	-0.14	-1.10	PPP-PPP	0.10	11	0.861	0.984	0.3015
202696_at	3110.4	2819.3	-0.14	-1.10	PPP-PPP	0.10	11	0.861	0.984	0.3175
201440_at	1897.0	1719.0	-0.14	-1.10	PPP-PPP	0.10	11	0.861	0.984	0.3189
226586_at	327.0	296.5	-0.14	-1.10	PAP-PPP	0.10	11	0.861	0.984	0.3711
215111_s_at	6342.4	5748.9	-0.14	-1.10	PPP-PPP	0.10	11	0.861	0.984	0.3990
1566303_s_at	2105.4	1907.4	-0.14	-1.10	PPP-PPP	0.10	11	0.861	0.984	0.4030
224607_s_at	5531.2	5015.7	-0.14	-1.10	PPP-PPP	0.10	11	0.861	0.984	0.0356
241871_at	372.4	337.7	-0.14	-1.10	PPP-PPP	0.10	11	0.861	0.984	0.1504
200738_s_at	19705.1	17869.3	-0.14	-1.10	PPP-PPP	0.10	11	0.861	0.984	0.1534
209076_s_at	3963.3	3595.0	-0.14	-1.10	PPP-PPP	0.10	11	0.861	0.984	0.1547
201774_s_at	2811.1	2549.2	-0.14	-1.10	PPP-PPP	0.10	11	0.861	0.984	0.1664
243852_at	1282.6	1163.2	-0.14	-1.10	PPP-PPP	0.10	11	0.861	0.984	0.2273
223021_x_at	3203.7	2906.3	-0.14	-1.10	PPP-PPP	0.10	11	0.861	0.984	0.2320
211015_s_at	8669.5	7860.4	-0.14	-1.10	PPP-PPP	0.10	11	0.861	0.984	0.2454
213366_x_at	20265.6	18377.9	-0.14	-1.10	PPP-PPP	0.10	11	0.861	0.984	0.2537
203606_at	10834.8	9828.4	-0.14	-1.10	PPP-PPP	0.10	11	0.861	0.984	0.2803
224347_x_at	370.1	335.6	-0.14	-1.10	PPP-PPP	0.10	11	0.861	0.984	0.3185
202686_s_at	8284.0	7511.6	-0.14	-1.10	PPP-PPP	0.10	11	0.861	0.984	0.3307
203055_s_at	354.4	321.3	-0.14	-1.10	PPP-PPP	0.10	11	0.861	0.984	0.3380
213655_at	20590.5	18677.5	-0.14	-1.10	PPP-PPP	0.10	11	0.861	0.984	0.3533
210396_s_at	4004.7	3631.5	-0.14	-1.10	PPP-PPP	0.10	11	0.861	0.984	0.3708
242862_x_at	218.2	197.9	-0.14	-1.10	PPP-PPP	0.10	11	0.861	0.984	0.3804
200775_s_at	19526.6	17715.8	-0.14	-1.10	PPP-PPP	0.10	11	0.861	0.984	0.0463
202055_at	1165.6	1057.6	-0.14	-1.10	PPP-PPP	0.10	11	0.861	0.984	0.1154
204020_at	3266.1	2963.8	-0.14	-1.10	PPP-PPP	0.10	11	0.861	0.984	0.1263
210563_x_at	1655.0	1502.1	-0.14	-1.10	PPP-PPP	0.10	11	0.861	0.984	0.1769
203343_at	3498.8	3174.9	-0.14	-1.10	PPP-PPP	0.10	11	0.861	0.984	0.1919
225389_at	3400.2	3084.8	-0.14	-1.10	PPP-PPP	0.10	11	0.861	0.984	0.2578
226102_at	703.5	638.5	-0.14	-1.10	PPP-PPP	0.10	11	0.861	0.984	0.2821
202419_at	1939.6	1759.7	-0.14	-1.10	PPP-PPP	0.10	11	0.861	0.984	0.2871
226092_at	3261.7	2960.9	-0.14	-1.10	PPP-PPP	0.10	11	0.861	0.984	0.3211

221474_at	21953.3	19925.3	-0.14	-1.10	PPP-PPP	0.10	11	0.861	0.984	0.3325
224891_at	4052.4	3678.7	-0.14	-1.10	PPP-PPP	0.10	11	0.861	0.984	0.3517
227590_at	316.6	287.4	-0.14	-1.10	MPP-PPP	0.10	11	0.861	0.984	0.3575
201680_x_at	4358.3	3955.7	-0.14	-1.10	PPP-PPP	0.10	11	0.861	0.984	0.3674
225089_at	315.6	286.5	-0.14	-1.10	PPP-PPP	0.10	11	0.861	0.984	0.3721
212015_x_at	8265.9	7499.1	-0.14	-1.10	PPP-PPP	0.10	11	0.861	0.984	0.3828
204502_at	754.8	685.6	-0.14	-1.10	PPP-PPP	0.10	11	0.861	0.984	0.1422
207023_x_at	4842.9	4399.3	-0.14	-1.10	PPP-PPP	0.10	11	0.861	0.984	0.1906
222883_at	1393.1	1265.1	-0.14	-1.10	PPP-PPP	0.10	11	0.861	0.984	0.2193
201995_at	5560.2	5048.1	-0.14	-1.10	PPP-PPP	0.10	11	0.861	0.984	0.2578
200912_s_at	13277.3	12054.5	-0.14	-1.10	PPP-PPP	0.10	11	0.861	0.984	0.2593
210501_x_at	9086.6	8252.9	-0.14	-1.10	PPP-PPP	0.10	11	0.861	0.984	0.2985
200661_at	4255.3	3864.6	-0.14	-1.10	PPP-PPP	0.10	11	0.861	0.984	0.3251
218479_s_at	498.5	452.8	-0.14	-1.10	PPP-PPP	0.10	11	0.861	0.984	0.3602
1557370_s_at	821.1	746.0	-0.14	-1.10	PPP-PPP	0.10	11	0.861	0.984	0.0432
218715_at	4847.2	4404.4	-0.14	-1.10	PPP-PPP	0.10	11	0.861	0.984	0.0503
200858_s_at	26398.9	23986.1	-0.14	-1.10	PPP-PPP	0.10	11	0.861	0.984	0.1166
202609_at	5509.4	5006.3	-0.14	-1.10	PPP-PPP	0.10	11	0.861	0.984	0.1341
221512_at	921.8	837.9	-0.14	-1.10	AAP-PPP	0.10	11	0.861	0.984	0.2005
214800_x_at	13006.9	11824.2	-0.14	-1.10	PPP-PPP	0.10	11	0.861	0.984	0.2310
207467_x_at	5335.1	4847.1	-0.14	-1.10	PPP-PPP	0.10	11	0.861	0.984	0.2364
223133_at	11724.7	10656.4	-0.14	-1.10	PPP-PPP	0.10	11	0.861	0.984	0.2471
209852_x_at	4506.0	4094.7	-0.14	-1.10	PPP-PPP	0.10	11	0.861	0.984	0.2714
217763_s_at	1831.1	1663.9	-0.14	-1.10	PPP-PPP	0.10	11	0.861	0.984	0.3475
213044_at	5832.0	5298.9	-0.14	-1.10	PPP-PPP	0.10	11	0.861	0.984	0.3796
202062_s_at	735.0	667.8	-0.14	-1.10	PPP-PPP	0.10	11	0.861	0.984	0.3871
217941_s_at	6934.2	6299.6	-0.14	-1.10	PPP-PPP	0.10	11	0.861	0.984	0.3955
224373_s_at	41343.3	37593.0	-0.14	-1.10	PPP-PPP	0.10	11	0.861	0.984	0.0056
201305_x_at	11927.2	10843.2	-0.14	-1.10	PPP-PPP	0.10	11	0.861	0.984	0.0698
201097_s_at	20907.7	19015.9	-0.14	-1.10	PPP-PPP	0.10	11	0.861	0.984	0.1416
217883_at	14638.5	13310.1	-0.14	-1.10	PPP-PPP	0.10	11	0.861	0.984	0.1680
209059_s_at	5849.5	5317.9	-0.14	-1.10	PPP-PPP	0.10	11	0.861	0.984	0.1962
219636_s_at	376.4	342.3	-0.14	-1.10	PPP-PPP	0.10	11	0.861	0.984	0.2433
212449_s_at	8203.6	7461.0	-0.14	-1.10	PPP-PPP	0.10	11	0.861	0.984	0.2493
239171_at	66.8	60.7	-0.14	-1.10	AMA-PAA	0.10	11	0.861	0.984	0.2751
218756_s_at	587.9	534.7	-0.14	-1.10	PPP-PPP	0.10	11	0.861	0.984	0.2865
202681_at	973.4	884.9	-0.14	-1.10	PPP-PPP	0.10	11	0.861	0.984	0.3094
208621_s_at	3339.9	3036.8	-0.14	-1.10	PPP-PPP	0.10	11	0.861	0.984	0.3089
218244_at	2611.0	2375.0	-0.14	-1.10	PPP-PPP	0.10	11	0.861	0.984	0.3177
213454_at	1686.0	1533.5	-0.14	-1.10	PPP-PPP	0.10	11	0.861	0.984	0.3475
202412_s_at	4451.3	4046.9	-0.14	-1.10	PPP-PPP	0.10	11	0.861	0.984	0.3517
201644_at	1279.9	1164.1	-0.14	-1.10	PPP-PPP	0.10	11	0.861	0.984	0.3680

200839_s_at	9119.6	8291.3	-0.14	-1.10	PPP-PPP	0.10	11	0.861	0.984	0.3763
208749_x_at	3202.8	2911.9	-0.14	-1.10	PPP-PPP	0.10	11	0.861	0.984	0.3824
211052_s_at	417.5	379.7	-0.14	-1.10	MPP-PPP	0.10	11	0.861	0.984	0.3825
201672_s_at	14263.9	12975.0	-0.14	-1.10	PPP-PPP	0.10	11	0.861	0.984	0.3960
1554499_s_at	175.6	159.6	-0.14	-1.10	AAA-AAP	0.10	11	0.861	0.984	0.3984
210418_s_at	4265.2	3880.1	-0.14	-1.10	PPP-PPP	0.10	11	0.861	0.984	0.0153
201215_at	20166.5	18348.1	-0.14	-1.10	PPP-PPP	0.10	11	0.861	0.984	0.0767
200594_x_at	13814.4	12568.9	-0.14	-1.10	PPP-PPP	0.10	11	0.861	0.984	0.1319
202471_s_at	2627.2	2390.7	-0.14	-1.10	PPP-PPP	0.10	11	0.861	0.984	0.1378
201669_s_at	17226.2	15678.3	-0.14	-1.10	PPP-PPP	0.10	11	0.861	0.984	0.1799
200667_at	6196.1	5637.2	-0.14	-1.10	PPP-PPP	0.10	11	0.861	0.984	0.2309
217225_x_at	10372.7	9438.0	-0.14	-1.10	PPP-PPP	0.10	11	0.861	0.984	0.2666
225885_at	2536.4	2308.6	-0.14	-1.10	PPP-PPP	0.10	11	0.861	0.984	0.2896
226801_s_at	9274.6	8441.5	-0.14	-1.10	PPP-PPP	0.10	11	0.861	0.984	0.2983
212971_at	8290.7	7542.6	-0.14	-1.10	PPP-PPP	0.10	11	0.861	0.984	0.3018
211749_s_at	7279.0	6622.3	-0.14	-1.10	PPP-PPP	0.10	11	0.861	0.984	0.3217
209429_x_at	2248.2	2045.4	-0.14	-1.10	PPP-PPP	0.10	11	0.861	0.984	0.3293
223182_s_at	628.8	572.0	-0.14	-1.10	PPP-PPP	0.10	11	0.861	0.984	0.3372
216438_s_at	34540.1	31443.5	-0.14	-1.10	PPP-PPP	0.10	11	0.861	0.984	0.3522
201817_at	3178.5	2891.9	-0.14	-1.10	PPP-PPP	0.10	11	0.861	0.984	0.3587
201326_at	6891.8	6271.2	-0.14	-1.10	PPP-PPP	0.10	11	0.861	0.984	0.3770
208755_x_at	23800.1	21665.6	-0.14	-1.10	PPP-PPP	0.10	11	0.861	0.984	0.3763
218467_at	11349.2	10328.8	-0.14	-1.10	PPP-PPP	0.10	11	0.861	0.984	0.3810
213266_at	400.2	364.1	-0.14	-1.10	PPP-PPP	0.10	11	0.861	0.984	0.3877
208634_s_at	4615.2	4202.9	-0.14	-1.10	PPP-PPP	0.10	11	0.861	0.984	0.0108
216041_x_at	1747.1	1590.7	-0.14	-1.10	PPP-PPP	0.10	11	0.861	0.984	0.0379
213535_s_at	9989.9	9095.0	-0.14	-1.10	PPP-PPP	0.10	11	0.861	0.984	0.1650
209919_x_at	150.0	136.6	-0.14	-1.10	APA-APA	0.10	11	0.861	0.984	0.1865
204050_s_at	12353.3	11250.8	-0.14	-1.10	PPP-PPP	0.10	11	0.861	0.984	0.3533
218365_s_at	936.9	853.4	-0.14	-1.10	PPP-PPP	0.10	11	0.861	0.984	0.3722
213047_x_at	16179.5	14746.0	-0.13	-1.10	PPP-PPP	0.10	11	0.861	0.984	0.0638
1568954_s_at	1458.2	1329.1	-0.13	-1.10	PPP-PPP	0.10	11	0.861	0.984	0.1288
224880_at	10592.3	9650.2	-0.13	-1.10	PPP-PPP	0.10	11	0.861	0.984	0.1890
202428_x_at	12394.2	11298.1	-0.13	-1.10	PPP-PPP	0.10	11	0.861	0.984	0.2007
202362_at	7851.7	7155.4	-0.13	-1.10	PPP-PPP	0.10	11	0.861	0.984	0.2067
218611_at	4420.7	4029.3	-0.13	-1.10	PPP-PPP	0.10	11	0.861	0.984	0.2131
201022_s_at	27678.3	25229.1	-0.13	-1.10	PPP-PPP	0.10	11	0.861	0.984	0.2150
237571_at	207.4	189.0	-0.13	-1.10	PPP-PPP	0.10	11	0.861	0.984	0.2230
225308_s_at	1541.0	1403.9	-0.13	-1.10	PPP-PPP	0.10	11	0.861	0.984	0.2424
1554696_s_at	4935.6	4499.2	-0.13	-1.10	PPP-PPP	0.10	11	0.861	0.984	0.2550
201618_x_at	1560.6	1422.5	-0.13	-1.10	PPP-PPP	0.10	11	0.861	0.984	0.3295
214731_at	236.8	215.8	-0.13	-1.10	PPP-PPP	0.10	11	0.861	0.984	0.3589

204017_at	5571.7	5076.4	-0.13	-1.10	PPP-PPP	0.10	11	0.861	0.984	0.3640
203893_at	5130.5	4675.2	-0.13	-1.10	PPP-PPP	0.10	11	0.861	0.984	0.3918
209009_at	10503.6	9577.3	-0.13	-1.10	PPP-PPP	0.10	11	0.861	0.984	0.0100
209487_at	4118.6	3754.7	-0.13	-1.10	PPP-PPP	0.10	11	0.861	0.984	0.0571
200657_at	21540.8	19642.6	-0.13	-1.10	PPP-PPP	0.10	11	0.861	0.984	0.0815
222614_at	496.1	452.3	-0.13	-1.10	PPP-PPP	0.10	11	0.861	0.984	0.1909
213399_x_at	16516.8	15063.3	-0.13	-1.10	PPP-PPP	0.10	11	0.861	0.984	0.2197
218997_at	2816.6	2567.9	-0.13	-1.10	PPP-PPP	0.10	11	0.861	0.984	0.2554
215926_x_at	399.3	364.2	-0.13	-1.10	PAA-AAA	0.10	11	0.861	0.984	0.2608
223476_s_at	509.8	464.7	-0.13	-1.10	PPP-PPP	0.10	11	0.861	0.984	0.2694
200845_s_at	12308.8	11224.0	-0.13	-1.10	PPP-PPP	0.10	11	0.861	0.984	0.2735
226296_s_at	13221.4	12058.1	-0.13	-1.10	PPP-PPP	0.10	11	0.861	0.984	0.2780
208818_s_at	6895.1	6287.6	-0.13	-1.10	PPP-PPP	0.10	11	0.861	0.984	0.3056
218494_s_at	1189.5	1084.5	-0.13	-1.10	PPP-PPP	0.10	11	0.861	0.984	0.3120
202110_at	11606.2	10582.4	-0.13	-1.10	PPP-PPP	0.10	11	0.861	0.984	0.3415
228284_at	1244.4	1134.6	-0.13	-1.10	PPP-PPP	0.10	11	0.861	0.984	0.3469
214709_s_at	10260.2	9358.4	-0.13	-1.10	PPP-PPP	0.10	11	0.861	0.984	0.3479
213979_s_at	1442.4	1315.1	-0.13	-1.10	PPP-PPP	0.10	11	0.861	0.984	0.3581
217840_at	1846.7	1683.6	-0.13	-1.10	PPP-PPP	0.10	11	0.861	0.984	0.3835
210978_s_at	7108.4	6487.5	-0.13	-1.10	PPP-PPP	0.10	11	0.861	0.984	0.0154
201874_at	2249.1	2053.0	-0.13	-1.10	PPP-PPP	0.10	11	0.861	0.984	0.0790
222975_s_at	12156.1	11090.9	-0.13	-1.10	PPP-PPP	0.10	11	0.861	0.984	0.1226
213358_at	1325.8	1209.6	-0.13	-1.10	PPP-PPP	0.10	11	0.861	0.984	0.2160
209155_s_at	2685.7	2450.5	-0.13	-1.10	PPP-PPP	0.10	11	0.861	0.984	0.2356
200628_s_at	2605.3	2377.2	-0.13	-1.10	PPP-PPP	0.10	11	0.861	0.984	0.2583
212610_at	12918.4	11787.4	-0.13	-1.10	PPP-PPP	0.10	11	0.861	0.984	0.2652
201717_at	4138.0	3776.0	-0.13	-1.10	PPP-PPP	0.10	11	0.861	0.984	0.2898
203405_at	6886.7	6285.0	-0.13	-1.10	PPP-PPP	0.10	11	0.861	0.984	0.3131
211864_s_at	6637.5	6057.3	-0.13	-1.10	PPP-PPP	0.10	11	0.861	0.984	0.3212
231056_at	265.3	242.0	-0.13	-1.10	AMP-PMP	0.10	11	0.861	0.984	0.3563
226312_at	2196.3	2004.2	-0.13	-1.10	PPP-PPP	0.10	11	0.861	0.984	0.3556
227085_at	2028.5	1851.3	-0.13	-1.10	PPP-PPP	0.10	11	0.861	0.984	0.3666
204700_x_at	1437.1	1311.3	-0.13	-1.10	PPP-PPP	0.10	11	0.861	0.984	0.3711
208914_at	238.6	217.8	-0.13	-1.10	PPP-PPP	0.10	11	0.861	0.984	0.3704
51176_at	2571.4	2348.0	-0.13	-1.10	PPP-PPP	0.10	11	0.861	0.984	0.0081
203105_s_at	2899.2	2647.4	-0.13	-1.10	PPP-PPP	0.10	11	0.861	0.984	0.0561
202261_at	1542.8	1408.7	-0.13	-1.10	PPP-PPP	0.10	11	0.861	0.984	0.0633
213491_x_at	15195.3	13872.2	-0.13	-1.10	PPP-PPP	0.10	11	0.861	0.984	0.1528
202680_at	2027.1	1850.7	-0.13	-1.10	PPP-PPP	0.10	11	0.861	0.984	0.1624
201225_s_at	5643.8	5155.0	-0.13	-1.10	PPP-PPP	0.10	11	0.861	0.984	0.2225
208361_s_at	552.5	504.6	-0.13	-1.10	PPP-PPP	0.10	11	0.861	0.984	0.2280
207034_s_at	430.2	392.9	-0.13	-1.10	PPP-PPP	0.10	11	0.861	0.984	0.3711

225399_at	4061.2	3711.9	-0.13	-1.09	PPP-PPP	0.10	11	0.861	0.984	0.0323
200830_at	16908.9	15452.0	-0.13	-1.09	PPP-PPP	0.10	11	0.861	0.984	0.0378
218200_s_at	10760.0	9832.3	-0.13	-1.09	PPP-PPP	0.10	11	0.861	0.984	0.1719
205474_at	1964.5	1795.5	-0.13	-1.09	PPP-PPP	0.10	11	0.861	0.984	0.1804
221823_at	862.7	788.2	-0.13	-1.10	PPP-PPP	0.10	11	0.861	0.984	0.2342
211945_s_at	28372.5	25934.4	-0.13	-1.09	PPP-PPP	0.10	11	0.861	0.984	0.2675
224870_at	3008.0	2748.0	-0.13	-1.10	PPP-PPP	0.10	11	0.861	0.984	0.2876
202282_at	4391.4	4011.7	-0.13	-1.10	PPP-PPP	0.10	11	0.861	0.984	0.2882
201472_at	9777.9	8937.4	-0.13	-1.09	PPP-PPP	0.10	11	0.861	0.984	0.3057
213887_s_at	6202.6	5669.0	-0.13	-1.09	PPP-PPP	0.10	11	0.861	0.984	0.3156
201476_s_at	4394.0	4014.2	-0.13	-1.10	PPP-PPP	0.10	11	0.861	0.984	0.3299
217938_s_at	8574.7	7837.7	-0.13	-1.09	PPP-PPP	0.10	11	0.861	0.984	0.3365
224984_at	2517.5	2300.5	-0.13	-1.09	PPP-PPP	0.10	11	0.861	0.984	0.3399
217992_s_at	1922.6	1757.0	-0.13	-1.09	PPP-PPP	0.10	11	0.861	0.984	0.3452
209884_s_at	1009.7	923.0	-0.13	-1.09	PPP-PPP	0.10	11	0.861	0.984	0.3526
217982_s_at	20205.0	18459.3	-0.13	-1.10	PPP-PPP	0.10	11	0.861	0.984	0.3570
209898_x_at	977.6	893.3	-0.13	-1.09	PPP-PPP	0.10	11	0.861	0.984	0.3627
215596_s_at	2461.5	2250.3	-0.13	-1.09	PPP-PPP	0.10	11	0.861	0.984	0.0605
210460_s_at	5382.9	4923.6	-0.13	-1.09	PPP-PPP	0.10	11	0.861	0.984	0.0983
211047_x_at	15226.3	13928.0	-0.13	-1.09	PPP-PPP	0.10	11	0.861	0.984	0.1237
219539_at	2592.0	2370.9	-0.13	-1.09	PPP-PPP	0.10	11	0.861	0.984	0.1292
203545_at	4141.9	3788.7	-0.13	-1.09	PPP-PPP	0.10	11	0.861	0.984	0.1640
228299_at	4250.8	3887.6	-0.13	-1.09	PPP-PPP	0.10	11	0.861	0.984	0.2097
200995_at	3596.2	3288.4	-0.13	-1.09	PPP-PPP	0.10	11	0.861	0.984	0.2214
210638_s_at	3403.8	3112.2	-0.13	-1.09	PPP-PPP	0.10	11	0.861	0.984	0.2695
227249_at	1799.1	1644.6	-0.13	-1.09	PPP-PPP	0.10	11	0.861	0.984	0.2937
201518_at	9779.7	8943.7	-0.13	-1.09	PPP-PPP	0.10	11	0.861	0.984	0.2926
204190_at	827.9	757.3	-0.13	-1.09	PPP-PPP	0.10	11	0.861	0.984	0.3105
214845_s_at	5842.3	5342.4	-0.13	-1.09	PPP-PPP	0.10	11	0.861	0.984	0.3325
213823_at	691.6	632.3	-0.13	-1.09	PPP-PPP	0.10	11	0.861	0.984	0.3495
243864_at	270.2	247.1	-0.13	-1.09	PPP-PPP	0.10	11	0.861	0.984	0.3575
209190_s_at	4322.1	3953.5	-0.13	-1.09	PPP-PPP	0.10	11	0.861	0.984	0.3632
AFFX-r2-Ec-bioB-5_at	832.6	762.0	-0.13	-1.09	PPP-PPP	0.10	11	0.861	0.984	0.1407
223831_x_at	1243.6	1138.3	-0.13	-1.09	PPP-PPP	0.10	11	0.861	0.984	0.1558
211270_x_at	11331.2	10367.5	-0.13	-1.09	PPP-PPP	0.10	11	0.861	0.984	0.1912
229803_s_at	975.1	892.4	-0.13	-1.09	PPP-PPP	0.10	11	0.861	0.984	0.2342
212140_at	2185.0	1999.3	-0.13	-1.09	PPP-PPP	0.10	11	0.861	0.984	0.2658
225722_at	563.0	515.1	-0.13	-1.09	PPP-PPP	0.10	11	0.861	0.984	0.2670
201275_at	5182.0	4741.7	-0.13	-1.09	PPP-PPP	0.10	11	0.861	0.984	0.2704
228776_at	3689.1	3375.3	-0.13	-1.09	PPP-PPP	0.10	11	0.861	0.984	0.3303
227386_s_at	1218.9	1115.0	-0.13	-1.09	PPP-PPP	0.10	11	0.861	0.984	0.3500
211212_s_at	1027.4	940.0	-0.13	-1.09	PPP-PPP	0.10	11	0.861	0.984	0.3624

202804_at	1928.7	1766.1	-0.13	-1.09	PPP-PPP	0.10	11	0.861	0.984	0.0752
217969_at	2309.4	2114.4	-0.13	-1.09	PPP-PPP	0.10	11	0.861	0.984	0.0848
228335_at	16855.6	15436.5	-0.13	-1.09	PPP-PPP	0.10	11	0.861	0.984	0.0853
201906_s_at	567.0	519.4	-0.13	-1.09	PPP-PPP	0.10	11	0.861	0.984	0.1132
208672_s_at	16631.1	15231.0	-0.13	-1.09	PPP-PPP	0.10	11	0.861	0.984	0.1535
222423_at	1552.9	1422.0	-0.13	-1.09	PPP-PPP	0.10	11	0.861	0.984	0.1865
222623_s_at	3318.9	3038.2	-0.13	-1.09	PPP-PPP	0.10	11	0.861	0.984	0.2661
212774_at	3461.9	3170.5	-0.13	-1.09	PPP-PPP	0.10	11	0.861	0.984	0.2717
235509_at	1531.9	1402.9	-0.13	-1.09	PPP-PPP	0.10	11	0.861	0.984	0.3038
211928_at	5576.4	5105.9	-0.13	-1.09	PPP-PPP	0.10	11	0.861	0.984	0.3052
223034_s_at	10650.4	9754.0	-0.13	-1.09	PPP-PPP	0.10	11	0.861	0.984	0.3216
222637_at	1882.9	1723.7	-0.13	-1.09	PPP-PPP	0.10	11	0.861	0.984	0.3453
221647_s_at	5550.2	5084.0	-0.13	-1.09	PPP-PPP	0.10	11	0.861	0.984	0.3683
1555495_a_at	3808.8	3489.7	-0.13	-1.09	PPP-PPP	0.10	11	0.861	0.984	0.0119
211938_at	6965.6	6381.7	-0.13	-1.09	PPP-PPP	0.10	11	0.861	0.984	0.0289
209896_s_at	5006.8	4589.0	-0.13	-1.09	PPP-PPP	0.10	11	0.861	0.984	0.0836
212254_s_at	3747.0	3434.6	-0.13	-1.09	PPP-PPP	0.10	11	0.861	0.984	0.0917
208652_at	10720.1	9825.6	-0.13	-1.09	PPP-PPP	0.10	11	0.861	0.984	0.1602
213115_at	551.2	505.1	-0.13	-1.09	PPP-PPP	0.10	11	0.861	0.984	0.2987
41047_at	847.8	777.1	-0.13	-1.09	PPP-PPP	0.10	11	0.861	0.984	0.3056
201609_x_at	4914.6	4503.0	-0.13	-1.09	PPP-PPP	0.10	11	0.861	0.984	0.3284
213312_at	382.8	350.7	-0.13	-1.09	PPP-PPP	0.10	11	0.861	0.984	0.3315
203621_at	5882.0	5391.4	-0.13	-1.09	PPP-PPP	0.10	11	0.861	0.984	0.3459
225492_at	2792.0	2559.1	-0.13	-1.09	PPP-PPP	0.10	11	0.861	0.984	0.3696
216274_s_at	9717.7	8913.4	-0.13	-1.09	PPP-PPP	0.10	11	0.861	0.984	0.0024
204170_s_at	20108.6	18442.3	-0.13	-1.09	PPP-PPP	0.10	11	0.861	0.984	0.0199
200057_s_at	17390.0	15943.2	-0.13	-1.09	PPP-PPP	0.10	11	0.861	0.984	0.0938
218006_s_at	1439.3	1319.7	-0.13	-1.09	PPP-PPP	0.10	11	0.861	0.984	0.1429
201345_s_at	6058.2	5554.3	-0.13	-1.09	PPP-PPP	0.10	11	0.861	0.984	0.1856
204140_at	1287.9	1181.0	-0.13	-1.09	PPP-PPP	0.10	11	0.861	0.984	0.1862
208965_s_at	1825.4	1673.6	-0.13	-1.09	PPP-PPP	0.10	11	0.861	0.984	0.1874
208845_at	15034.0	13786.7	-0.13	-1.09	PPP-PPP	0.10	11	0.861	0.984	0.2078
222754_at	1896.4	1738.7	-0.13	-1.09	PPP-PPP	0.10	11	0.861	0.984	0.2191
218768_at	5333.6	4891.1	-0.13	-1.09	PPP-PPP	0.10	11	0.861	0.984	0.2509
1553117_a_at	905.1	830.0	-0.13	-1.09	PPP-PPP	0.10	11	0.861	0.984	0.2561
211509_s_at	26698.8	24489.9	-0.13	-1.09	PPP-PPP	0.10	11	0.861	0.984	0.2671
203916_at	476.7	437.0	-0.13	-1.09	PPP-PPP	0.10	11	0.861	0.984	0.2800
228450_at	139.3	127.7	-0.13	-1.09	APA-PPP	0.10	11	0.861	0.984	0.2864
219910_at	460.5	422.4	-0.13	-1.09	PPP-PPP	0.10	11	0.861	0.984	0.2947
222649_at	1110.9	1018.5	-0.13	-1.09	PPP-PPP	0.10	11	0.861	0.984	0.3317
224915_x_at	10609.1	9728.5	-0.13	-1.09	PPP-PPP	0.10	11	0.861	0.984	0.3344
219306_at	1016.2	931.9	-0.13	-1.09	PPP-PPP	0.10	11	0.861	0.984	0.3514

230735_at	71.4	65.5	-0.13	-1.09	PAA-AAA	0.10	11	0.861	0.984	0.3603
226099_at	6725.6	6172.6	-0.12	-1.09	PPP-PPP	0.10	11	0.861	0.984	0.0046
226544_x_at	1797.2	1649.2	-0.12	-1.09	PPP-PPP	0.10	11	0.861	0.984	0.1317
201740_at	4028.0	3696.6	-0.12	-1.09	PPP-PPP	0.10	11	0.861	0.984	0.1490
230722_at	573.1	525.9	-0.12	-1.09	PPP-PPP	0.10	11	0.861	0.984	0.1629
218249_at	2358.8	2165.2	-0.12	-1.09	PPP-PPP	0.10	11	0.861	0.984	0.1617
201866_s_at	1463.6	1342.6	-0.12	-1.09	PPP-PPP	0.10	11	0.861	0.984	0.1680
209390_at	769.2	705.7	-0.12	-1.09	PPP-PPP	0.10	11	0.861	0.984	0.1896
1555760_a_at	1410.1	1294.4	-0.12	-1.09	PPP-PPP	0.10	11	0.861	0.984	0.1993
209511_at	5028.4	4615.0	-0.12	-1.09	PPP-PPP	0.10	11	0.861	0.984	0.2576
200978_at	12958.3	11891.3	-0.12	-1.09	PPP-PPP	0.10	11	0.861	0.984	0.2715
200916_at	15233.3	13978.3	-0.12	-1.09	PPP-PPP	0.10	11	0.861	0.984	0.2777
224884_at	268.2	246.0	-0.12	-1.09	PPP-PPP	0.10	11	0.861	0.984	0.2865
203565_s_at	1513.4	1388.3	-0.12	-1.09	PPP-PPP	0.10	11	0.861	0.984	0.2880
218556_at	3233.2	2967.0	-0.12	-1.09	PPP-PPP	0.10	11	0.861	0.984	0.2885
239246_at	141.9	130.2	-0.12	-1.09	PPP-PPP	0.10	11	0.861	0.984	0.2947
224284_x_at	699.5	641.9	-0.12	-1.09	PPP-PPP	0.10	11	0.861	0.984	0.3043
201010_s_at	2657.3	2438.1	-0.12	-1.09	PPP-PPP	0.10	11	0.861	0.984	0.3490
205849_s_at	12905.9	11850.7	-0.12	-1.09	PPP-PPP	0.10	11	0.861	0.984	0.0120
208855_s_at	12435.6	11416.1	-0.12	-1.09	PPP-PPP	0.10	11	0.861	0.984	0.0980
218830_at	9096.4	8353.0	-0.12	-1.09	PPP-PPP	0.10	11	0.861	0.984	0.1339
202047_s_at	4200.1	3856.0	-0.12	-1.09	PPP-PPP	0.10	11	0.861	0.984	0.1659
226505_x_at	1957.5	1797.1	-0.12	-1.09	PPP-PPP	0.10	11	0.861	0.984	0.2409
200870_at	17370.3	15955.3	-0.12	-1.09	PPP-PPP	0.10	11	0.861	0.984	0.2512
225125_at	3360.9	3087.3	-0.12	-1.09	PPP-PPP	0.10	11	0.861	0.984	0.2881
202520_s_at	4132.8	3794.3	-0.12	-1.09	PPP-PPP	0.10	11	0.861	0.984	0.3011
200910_at	14509.8	13321.8	-0.12	-1.09	PPP-PPP	0.10	11	0.861	0.984	0.3049
201629_s_at	3779.4	3471.4	-0.12	-1.09	PPP-PPP	0.10	11	0.861	0.984	0.3049
206059_at	463.7	425.7	-0.12	-1.09	PPP-PPP	0.10	11	0.861	0.984	0.3162
201317_s_at	13863.4	12728.8	-0.12	-1.09	PPP-PPP	0.10	11	0.861	0.984	0.3423
202786_at	2431.9	2232.4	-0.12	-1.09	PPP-PPP	0.10	11	0.861	0.984	0.3440
202753_at	11058.2	10156.4	-0.12	-1.09	PPP-PPP	0.10	11	0.861	0.984	0.3559
219979_s_at	2065.1	1897.3	-0.12	-1.09	PPP-PPP	0.10	11	0.861	0.984	0.0825
225050_at	1320.2	1213.4	-0.12	-1.09	PPP-PPP	0.10	11	0.861	0.984	0.0963
212459_x_at	2091.0	1921.1	-0.12	-1.09	PPP-PPP	0.10	11	0.861	0.984	0.1749
227556_at	1361.2	1250.5	-0.12	-1.09	PPP-PPP	0.10	11	0.861	0.984	0.2216
229092_at	474.8	436.2	-0.12	-1.09	PPP-PPP	0.10	11	0.861	0.984	0.2268
214150_x_at	11363.1	10440.5	-0.12	-1.09	PPP-PPP	0.10	11	0.861	0.984	0.2319
210092_at	3721.8	3420.2	-0.12	-1.09	PPP-PPP	0.10	11	0.861	0.984	0.2422
224629_at	9762.8	8969.4	-0.12	-1.09	PPP-PPP	0.10	11	0.861	0.984	0.2510
200990_at	7148.1	6566.8	-0.12	-1.09	PPP-PPP	0.10	11	0.861	0.984	0.3306
208808_s_at	13984.3	12851.4	-0.12	-1.09	PPP-PPP	0.10	11	0.861	0.984	0.3350

218654_s_at	3491.4	3207.4	-0.12	-1.09	PPP-PPP	0.10	11	0.861	0.984	0.3573
208717_at	2752.5	2530.3	-0.12	-1.09	PPP-PPP	0.10	11	0.861	0.984	0.0427
200093_s_at	14105.8	12968.1	-0.12	-1.09	PPP-PPP	0.10	11	0.861	0.984	0.1493
222426_at	709.5	652.5	-0.12	-1.09	PPP-PPP	0.10	11	0.861	0.984	0.2050
221839_s_at	2456.0	2259.0	-0.12	-1.09	PPP-PPP	0.10	11	0.861	0.984	0.2357
201359_at	10675.9	9819.5	-0.12	-1.09	PPP-PPP	0.10	11	0.861	0.984	0.2496
204361_s_at	688.1	632.6	-0.12	-1.09	PPP-PPP	0.10	11	0.861	0.984	0.2543
202244_at	16236.2	14925.9	-0.12	-1.09	PPP-PPP	0.10	11	0.861	0.984	0.2982
225799_at	7312.7	6726.3	-0.12	-1.09	PPP-PPP	0.10	11	0.861	0.984	0.3298
202589_at	17936.3	16494.4	-0.12	-1.09	PPP-PPP	0.10	11	0.861	0.984	0.3396
201479_at	7406.0	6811.2	-0.12	-1.09	PPP-PPP	0.10	11	0.861	0.984	0.3545
207585_s_at	17815.6	16392.4	-0.12	-1.09	PPP-PPP	0.10	11	0.861	0.984	0.0470
215416_s_at	6876.6	6329.8	-0.12	-1.09	PPP-PPP	0.10	11	0.861	0.984	0.0547
227141_at	2479.7	2282.0	-0.12	-1.09	PPP-PPP	0.10	11	0.861	0.984	0.0853
1555278_a_at	2750.3	2530.8	-0.12	-1.09	PPP-PPP	0.10	11	0.861	0.984	0.1030
223279_s_at	2894.7	2664.4	-0.12	-1.09	PPP-PPP	0.10	11	0.861	0.984	0.1037
225829_at	525.6	483.7	-0.12	-1.09	PPP-PPP	0.10	11	0.861	0.984	0.1105
219913_s_at	808.0	743.5	-0.12	-1.09	PPP-PPP	0.10	11	0.861	0.984	0.1733
227364_at	13012.1	11969.5	-0.12	-1.09	PPP-PPP	0.10	11	0.861	0.984	0.1772
226124_at	609.7	561.1	-0.12	-1.09	PPP-PPP	0.10	11	0.861	0.984	0.1875
209450_at	875.8	805.8	-0.12	-1.09	PPP-PPP	0.10	11	0.861	0.984	0.2312
229984_at	203.1	186.9	-0.12	-1.09	APP-PPP	0.10	11	0.861	0.984	0.2452
224867_at	10265.7	9449.5	-0.12	-1.09	PPP-PPP	0.10	11	0.861	0.984	0.2462
209939_x_at	2348.1	2160.9	-0.12	-1.09	PPP-PPP	0.10	11	0.861	0.984	0.2576
225614_at	1944.1	1788.7	-0.12	-1.09	PPP-PPP	0.10	11	0.861	0.984	0.3088
201535_at	2112.1	1943.1	-0.12	-1.09	PPP-PPP	0.10	11	0.861	0.984	0.3127
228196_s_at	533.0	490.4	-0.12	-1.09	PPP-PPP	0.10	11	0.861	0.984	0.3312
AFFX-CreX-3_at	27101.2	24949.6	-0.12	-1.09	PPP-PPP	0.10	11	0.861	0.984	0.0311
232432_s_at	2210.7	2036.3	-0.12	-1.09	PPP-PPP	0.10	11	0.861	0.984	0.0377
203967_at	1844.2	1698.6	-0.12	-1.09	PPP-PPP	0.10	11	0.861	0.984	0.0508
201847_at	4727.8	4352.6	-0.12	-1.09	PPP-PPP	0.10	11	0.861	0.984	0.1474
201568_at	14243.3	13117.2	-0.12	-1.09	PPP-PPP	0.10	11	0.861	0.984	0.1844
212673_at	4955.9	4564.6	-0.12	-1.09	PPP-PPP	0.10	11	0.861	0.984	0.1882
200708_at	5938.7	5469.9	-0.12	-1.09	PPP-PPP	0.10	11	0.861	0.984	0.2315
203586_s_at	444.9	409.6	-0.12	-1.09	PPP-PPP	0.10	11	0.861	0.984	0.2461
221253_s_at	12116.8	11156.8	-0.12	-1.09	PPP-PPP	0.10	11	0.861	0.984	0.2832
48659_at	1011.0	930.9	-0.12	-1.09	PPP-PPP	0.10	11	0.861	0.984	0.3129
201994_at	23244.8	21417.4	-0.12	-1.09	PPP-PPP	0.10	11	0.861	0.984	0.0354
215952_s_at	28755.7	26505.0	-0.12	-1.09	PPP-PPP	0.10	11	0.861	0.984	0.1138
223027_at	1294.0	1192.1	-0.12	-1.09	PPP-PPP	0.10	11	0.861	0.984	0.2621
214288_s_at	19463.2	17938.8	-0.12	-1.09	PPP-PPP	0.10	11	0.861	0.984	0.2989
218593_at	3167.7	2920.9	-0.12	-1.09	PPP-PPP	0.10	11	0.861	0.984	0.0758

201013_s_at	12722.3	11734.1	-0.12	-1.08	PPP-PPP	0.10	11	0.861	0.984	0.1180
208744_x_at	6342.1	5850.0	-0.12	-1.08	PPP-PPP	0.10	11	0.861	0.984	0.2274
218374_s_at	1141.1	1051.9	-0.12	-1.09	PPP-PPP	0.10	11	0.861	0.984	0.2821
217927_at	14295.1	13180.5	-0.12	-1.09	PPP-PPP	0.10	11	0.861	0.984	0.2980
222199_s_at	1148.9	1059.6	-0.12	-1.08	PPP-PPP	0.10	11	0.861	0.984	0.3276
200726_at	18829.9	17373.0	-0.12	-1.08	PPP-PPP	0.10	11	0.861	0.984	0.0152
209298_s_at	847.8	782.3	-0.12	-1.08	PPP-PPP	0.10	11	0.861	0.984	0.1079
203462_x_at	8092.3	7467.3	-0.12	-1.08	PPP-PPP	0.10	11	0.861	0.984	0.1094
223664_x_at	834.4	770.2	-0.12	-1.08	PPP-PPP	0.10	11	0.861	0.984	0.1721
200060_s_at	9543.7	8805.7	-0.12	-1.08	PPP-PPP	0.10	11	0.861	0.984	0.2030
204053_x_at	3190.1	2944.5	-0.12	-1.08	PPP-PPP	0.10	11	0.861	0.984	0.2528
206558_at	368.0	339.5	-0.12	-1.08	PPP-PPP	0.10	11	0.861	0.984	0.2658
202907_s_at	1894.9	1748.4	-0.12	-1.08	PPP-PPP	0.10	11	0.861	0.984	0.2871
227698_s_at	1041.8	961.3	-0.12	-1.08	PPP-PPP	0.10	11	0.861	0.984	0.3088
208258_s_at	142.7	131.6	-0.12	-1.08	AAA-PAA	0.10	11	0.861	0.984	0.3202
207127_s_at	4230.9	3902.7	-0.12	-1.08	PPP-PPP	0.10	11	0.861	0.984	0.3238
236679_x_at	341.2	315.0	-0.12	-1.08	PPP-PPP	0.10	11	0.861	0.984	0.1659
201144_s_at	12598.5	11635.7	-0.12	-1.08	PPP-PPP	0.10	11	0.861	0.984	0.1834
205273_s_at	3969.4	3666.5	-0.12	-1.08	PPP-PPP	0.10	11	0.861	0.984	0.1949
228690_s_at	11188.7	10334.0	-0.12	-1.08	PPP-PPP	0.10	11	0.861	0.984	0.2061
208752_x_at	19358.6	17874.9	-0.12	-1.08	PPP-PPP	0.10	11	0.861	0.984	0.2307
210453_x_at	12025.4	11101.4	-0.12	-1.08	PPP-PPP	0.10	11	0.861	0.984	0.2460
234302_s_at	2820.8	2605.3	-0.12	-1.08	PPP-PPP	0.10	11	0.861	0.984	0.2864
235545_at	1533.5	1416.0	-0.12	-1.08	PPP-PPP	0.10	11	0.861	0.984	0.3158
224761_at	6144.2	5673.1	-0.12	-1.08	PPP-PPP	0.10	11	0.861	0.984	0.3212
209154_at	19257.2	17779.0	-0.12	-1.08	PPP-PPP	0.10	11	0.861	0.984	0.3267
204616_at	6854.5	6331.2	-0.12	-1.08	PPP-PPP	0.10	11	0.861	0.984	0.3343
200058_s_at	7024.1	6489.8	-0.11	-1.08	PPP-PPP	0.10	11	0.861	0.984	0.0972
218046_s_at	3799.0	3509.9	-0.11	-1.08	PPP-PPP	0.10	11	0.861	0.984	0.1046
209709_s_at	4114.5	3800.6	-0.11	-1.08	PPP-PPP	0.10	11	0.861	0.984	0.1867
202167_s_at	1682.5	1554.5	-0.11	-1.08	PPP-PPP	0.10	11	0.861	0.984	0.2090
218026_at	4092.9	3782.5	-0.11	-1.08	PPP-PPP	0.10	11	0.861	0.984	0.2324
222530_s_at	4137.0	3824.0	-0.11	-1.08	PPP-PPP	0.10	11	0.861	0.984	0.2645
209517_s_at	2117.8	1957.2	-0.11	-1.08	PPP-PPP	0.10	11	0.861	0.984	0.2715
213753_x_at	2476.2	2288.6	-0.11	-1.08	PPP-PPP	0.10	11	0.861	0.984	0.2910
1554691_a_at	2326.7	2149.6	-0.11	-1.08	PPP-PPP	0.10	11	0.861	0.984	0.3075
216262_s_at	382.9	353.8	-0.11	-1.08	AMM-MPP	0.10	11	0.861	0.984	0.3348
201894_s_at	3011.5	2784.1	-0.11	-1.08	PPP-PPP	0.10	11	0.861	0.984	0.0246
208619_at	6446.1	5958.8	-0.11	-1.08	PPP-PPP	0.10	11	0.861	0.984	0.0757
221702_s_at	2239.5	2070.6	-0.11	-1.08	PPP-PPP	0.10	11	0.861	0.984	0.1632
201870_at	2622.3	2424.7	-0.11	-1.08	PPP-PPP	0.10	11	0.861	0.984	0.2496
225903_at	727.7	672.9	-0.11	-1.08	PPP-PPP	0.10	11	0.861	0.984	0.3202

220239_at	2259.6	2090.6	-0.11	-1.08	PPP-PPP	0.10	11	0.861	0.984	0.1097
201240_s_at	11091.8	10263.8	-0.11	-1.08	PPP-PPP	0.10	11	0.861	0.984	0.1170
213911_s_at	27742.6	25676.1	-0.11	-1.08	PPP-PPP	0.10	11	0.861	0.984	0.1265
222976_s_at	18957.2	17545.8	-0.11	-1.08	PPP-PPP	0.10	11	0.861	0.984	0.1446
203076_s_at	2625.9	2429.1	-0.11	-1.08	PPP-PPP	0.10	11	0.861	0.984	0.1791
226042_at	708.8	655.7	-0.11	-1.08	PPP-PPP	0.10	11	0.861	0.984	0.1962
225552_x_at	9602.5	8886.7	-0.11	-1.08	PPP-PPP	0.10	11	0.861	0.984	0.2026
203719_at	1915.8	1772.8	-0.11	-1.08	PPP-PPP	0.10	11	0.861	0.984	0.2138
202123_s_at	2081.9	1925.8	-0.11	-1.08	PPP-PPP	0.10	11	0.861	0.984	0.2164
212048_s_at	7834.7	7247.1	-0.11	-1.08	PPP-PPP	0.10	11	0.861	0.984	0.2348
211037_s_at	409.3	378.8	-0.11	-1.08	PPP-PPP	0.10	11	0.861	0.984	0.2359
221452_s_at	9863.2	9127.6	-0.11	-1.08	PPP-PPP	0.10	11	0.861	0.984	0.2501
225232_at	2832.5	2621.6	-0.11	-1.08	PPP-PPP	0.10	11	0.861	0.984	0.2543
1554501_at	167.6	155.0	-0.11	-1.08	PPP-PPP	0.10	11	0.861	0.984	0.2569
222852_at	543.1	502.4	-0.11	-1.08	PPP-PPP	0.10	11	0.861	0.984	0.2709
202139_at	2232.7	2066.4	-0.11	-1.08	PPP-PPP	0.10	11	0.861	0.984	0.2715
217786_at	6272.2	5804.4	-0.11	-1.08	PPP-PPP	0.10	11	0.861	0.984	0.2917
225888_at	1109.3	1026.4	-0.11	-1.08	PPP-PPP	0.10	11	0.861	0.984	0.3108
207507_s_at	13487.1	12482.7	-0.11	-1.08	PPP-PPP	0.10	11	0.861	0.984	0.3172
200994_at	8591.9	7956.1	-0.11	-1.08	PPP-PPP	0.10	11	0.861	0.984	0.1188
223005_s_at	2176.1	2014.5	-0.11	-1.08	PPP-PPP	0.10	11	0.861	0.984	0.1480
225350_s_at	1904.2	1762.8	-0.11	-1.08	PPP-PPP	0.10	11	0.861	0.984	0.1612
218617_at	1435.8	1329.5	-0.11	-1.08	PPP-PPP	0.10	11	0.861	0.984	0.1842
225000_at	4754.9	4401.3	-0.11	-1.08	PPP-PPP	0.10	11	0.861	0.984	0.2322
226922_at	1045.4	967.8	-0.11	-1.08	PPP-PPP	0.10	11	0.861	0.984	0.2525
210153_s_at	2913.7	2698.3	-0.11	-1.08	PPP-PPP	0.10	11	0.861	0.984	0.2549
233588_x_at	1577.6	1461.2	-0.11	-1.08	PPP-PPP	0.10	11	0.861	0.984	0.2730
201128_s_at	10819.8	10016.2	-0.11	-1.08	PPP-PPP	0.10	11	0.861	0.984	0.2911
214629_x_at	26008.5	24078.4	-0.11	-1.08	PPP-PPP	0.10	11	0.861	0.984	0.2910
212175_s_at	4011.5	3713.9	-0.11	-1.08	PPP-PPP	0.10	11	0.861	0.984	0.3058
218771_at	966.1	895.0	-0.11	-1.08	PPP-PPP	0.10	11	0.861	0.984	0.1336
221791_s_at	23370.7	21648.5	-0.11	-1.08	PPP-PPP	0.10	11	0.861	0.984	0.1522
200614_at	23815.7	22060.7	-0.11	-1.08	PPP-PPP	0.10	11	0.861	0.984	0.1983
1556499_s_at	29861.4	27667.0	-0.11	-1.08	PPP-PPP	0.10	11	0.861	0.984	0.3061
209127_s_at	2183.8	2025.1	-0.11	-1.08	PPP-PPP	0.10	11	0.861	0.984	0.0332
223040_at	5235.7	4854.9	-0.11	-1.08	PPP-PPP	0.10	11	0.861	0.984	0.0945
208661_s_at	4348.5	4030.9	-0.11	-1.08	PPP-PPP	0.10	11	0.861	0.984	0.1347
215380_s_at	9045.6	8384.9	-0.11	-1.08	PPP-PPP	0.10	11	0.861	0.984	0.1745
211759_x_at	4858.6	4504.6	-0.11	-1.08	PPP-PPP	0.10	11	0.861	0.984	0.2204
222747_s_at	492.6	456.7	-0.11	-1.08	PPP-PPP	0.10	11	0.861	0.984	0.2492
201482_at	3357.7	3113.4	-0.11	-1.08	PPP-PPP	0.10	11	0.861	0.984	0.2581
207945_s_at	2724.5	2526.5	-0.11	-1.08	PPP-PPP	0.10	11	0.861	0.984	0.2606

210470_x_at	7164.1	6640.8	-0.11	-1.08	PPP-PPP	0.10	11	0.861	0.984	0.2716
212857_x_at	18583.4	17229.8	-0.11	-1.08	PPP-PPP	0.10	11	0.861	0.984	0.2707
226947_at	476.6	442.0	-0.11	-1.08	PPP-PPP	0.10	11	0.861	0.984	0.2767
213995_at	1235.9	1145.8	-0.11	-1.08	PPP-PPP	0.10	11	0.861	0.984	0.2921
235713_at	573.4	531.8	-0.11	-1.08	PPP-PPP	0.10	11	0.861	0.984	0.2978
200027_at	15010.1	13913.4	-0.11	-1.08	PPP-PPP	0.10	11	0.861	0.984	0.3048
218497_s_at	4226.9	3922.8	-0.11	-1.08	PPP-PPP	0.10	11	0.861	0.984	0.0313
218803_at	1399.6	1298.3	-0.11	-1.08	PPP-PPP	0.10	11	0.861	0.984	0.0696
200681_at	15527.8	14404.1	-0.11	-1.08	PPP-PPP	0.10	11	0.861	0.984	0.2161
200655_s_at	8158.3	7568.7	-0.11	-1.08	PPP-PPP	0.10	11	0.861	0.984	0.2335
203425_s_at	281.8	261.4	-0.11	-1.08	PPP-PPP	0.10	11	0.861	0.984	0.3100
211758_x_at	5572.8	5173.2	-0.11	-1.08	PPP-PPP	0.10	11	0.861	0.984	0.0844
212782_x_at	7883.5	7320.6	-0.11	-1.08	PPP-PPP	0.10	11	0.861	0.984	0.1359
200826_at	20475.4	19016.8	-0.11	-1.08	PPP-PPP	0.10	11	0.861	0.984	0.2095
201061_s_at	2222.8	2063.4	-0.11	-1.08	PPP-PPP	0.10	11	0.861	0.984	0.2406
204744_s_at	14752.0	13702.4	-0.11	-1.08	PPP-PPP	0.10	11	0.861	0.984	0.1438
224837_at	4803.0	4463.7	-0.11	-1.08	PPP-PPP	0.10	11	0.861	0.984	0.2410
225008_at	803.5	746.4	-0.11	-1.08	PPP-PPP	0.10	11	0.861	0.984	0.2536
226705_at	1020.5	948.2	-0.11	-1.08	PPP-PPP	0.10	11	0.861	0.984	0.2565
202336_s_at	6037.3	5609.6	-0.11	-1.08	PPP-PPP	0.10	11	0.861	0.984	0.2569
225213_at	2656.7	2467.9	-0.11	-1.08	PPP-PPP	0.10	11	0.861	0.984	0.2614
201160_s_at	13528.0	12573.7	-0.11	-1.08	PPP-PPP	0.10	11	0.861	0.984	0.2653
200089_s_at	27547.1	25596.6	-0.11	-1.08	PPP-PPP	0.10	11	0.861	0.984	0.2791
212530_at	14256.9	13253.9	-0.11	-1.08	PPP-PPP	0.10	11	0.861	0.984	0.1081
203204_s_at	721.9	671.0	-0.11	-1.08	PPP-PPP	0.10	11	0.861	0.984	0.2771
218233_s_at	14376.3	13363.4	-0.11	-1.08	PPP-PPP	0.10	11	0.861	0.984	0.2796
212908_at	880.3	819.1	-0.10	-1.08	PPP-PPP	0.10	11	0.861	0.984	0.0489
1554365_a_at	2215.1	2060.4	-0.10	-1.08	PPP-PPP	0.10	11	0.861	0.984	0.1087
200822_x_at	18895.6	17583.8	-0.10	-1.08	PPP-PPP	0.10	11	0.861	0.984	0.1339
212139_at	2632.5	2449.4	-0.10	-1.08	PPP-PPP	0.10	11	0.861	0.984	0.1867
222683_at	3325.6	3095.3	-0.10	-1.07	PPP-PPP	0.10	11	0.861	0.984	0.2052
1559946_s_at	10255.1	9542.2	-0.10	-1.08	PPP-PPP	0.10	11	0.861	0.984	0.2108
1555943_at	646.8	601.8	-0.10	-1.08	PPP-PPP	0.10	11	0.861	0.984	0.2482
223071_at	7224.2	6722.5	-0.10	-1.08	PPP-PPP	0.10	11	0.861	0.984	0.2820
203336_s_at	5710.7	5313.6	-0.10	-1.08	PPP-PPP	0.10	11	0.861	0.984	0.2848
203908_at	1079.4	1004.6	-0.10	-1.07	PPP-PPP	0.10	11	0.861	0.984	0.2919
211937_at	6126.2	5705.9	-0.10	-1.07	PPP-PPP	0.10	11	0.861	0.984	0.0890
204647_at	1815.8	1690.1	-0.10	-1.07	PPP-PPP	0.10	11	0.861	0.984	0.1134
202858_at	13284.5	12368.3	-0.10	-1.07	PPP-PPP	0.10	11	0.861	0.984	0.1599
223356_s_at	1136.4	1058.1	-0.10	-1.07	PPP-PPP	0.10	11	0.861	0.984	0.2250
205851_at	833.6	776.8	-0.10	-1.07	PPP-PPP	0.10	11	0.861	0.984	0.1609
225976_at	1827.0	1702.7	-0.10	-1.07	PPP-PPP	0.10	11	0.861	0.984	0.1610

1553694_a_at	784.4	730.8	-0.10	-1.07	PPP-PPP	0.10	11	0.861	0.984	0.2028
202246_s_at	11037.3	10281.5	-0.10	-1.07	PPP-PPP	0.10	11	0.861	0.984	0.2480
203447_at	2394.9	2231.1	-0.10	-1.07	PPP-PPP	0.10	11	0.861	0.984	0.2682
202408_s_at	4181.0	3897.3	-0.10	-1.07	PPP-PPP	0.10	11	0.861	0.984	0.0291
227908_at	654.8	610.7	-0.10	-1.07	PPP-PPP	0.10	11	0.861	0.984	0.0633
200618_at	12845.5	11975.7	-0.10	-1.07	PPP-PPP	0.10	11	0.861	0.984	0.1148
208967_s_at	5576.8	5201.3	-0.10	-1.07	PPP-PPP	0.10	11	0.861	0.984	0.1927
207573_x_at	10029.7	9352.8	-0.10	-1.07	PPP-PPP	0.10	11	0.861	0.984	0.2710
223339_at	1140.3	1063.9	-0.10	-1.07	PPP-PPP	0.10	11	0.861	0.984	0.2215
221726_at	5995.8	5597.9	-0.10	-1.07	PPP-PPP	0.10	11	0.861	0.984	0.0881
207040_s_at	13138.0	12263.6	-0.10	-1.07	PPP-PPP	0.10	11	0.861	0.984	0.0913
217919_s_at	6284.5	5866.8	-0.10	-1.07	PPP-PPP	0.10	11	0.861	0.984	0.2025
209503_s_at	8657.4	8081.2	-0.10	-1.07	PPP-PPP	0.10	11	0.861	0.984	0.2120
211297_s_at	3940.0	3679.2	-0.10	-1.07	PPP-PPP	0.10	11	0.861	0.984	0.2114
235113_at	4063.6	3793.0	-0.10	-1.07	PPP-PPP	0.10	11	0.861	0.984	0.2226
218078_s_at	1354.5	1264.5	-0.10	-1.07	PPP-PPP	0.10	11	0.861	0.984	0.2405
204067_at	519.4	484.9	-0.10	-1.07	PPP-PPP	0.10	11	0.861	0.984	0.2552
204031_s_at	17789.3	16616.5	-0.10	-1.07	PPP-PPP	0.10	11	0.861	0.984	0.1032
200072_s_at	10815.7	10103.4	-0.10	-1.07	PPP-PPP	0.10	11	0.861	0.984	0.1312
218135_at	4808.7	4492.1	-0.10	-1.07	PPP-PPP	0.10	11	0.861	0.984	0.1542
209449_at	2063.7	1929.9	-0.10	-1.07	PPP-PPP	0.10	11	0.861	0.984	0.0820
217802_s_at	9618.2	8994.9	-0.10	-1.07	PPP-PPP	0.10	11	0.861	0.984	0.0859
210027_s_at	10770.1	10069.3	-0.10	-1.07	PPP-PPP	0.10	11	0.861	0.984	0.1188
209123_at	4780.9	4468.4	-0.10	-1.07	PPP-PPP	0.10	11	0.861	0.984	0.2279
201180_s_at	10747.0	10055.6	-0.10	-1.07	PPP-PPP	0.10	11	0.861	0.984	0.0140
212180_at	3195.5	2989.9	-0.10	-1.07	PPP-PPP	0.10	11	0.861	0.984	0.1242
213287_s_at	6368.6	5961.8	-0.10	-1.07	PPP-PPP	0.10	11	0.861	0.984	0.1710
223299_at	1599.8	1497.9	-0.10	-1.07	PPP-PPP	0.10	11	0.861	0.984	0.2129
209853_s_at	4485.8	4199.5	-0.10	-1.07	PPP-PPP	0.10	11	0.861	0.984	0.2190
201220_x_at	6251.0	5850.9	-0.10	-1.07	PPP-PPP	0.10	11	0.861	0.984	0.2309
207071_s_at	3364.6	3153.4	-0.09	-1.07	PPP-PPP	0.10	11	0.861	0.984	0.1336
224885_s_at	5257.2	4926.2	-0.09	-1.07	PPP-PPP	0.10	11	0.861	0.984	0.1829
219510_at	918.6	861.4	-0.09	-1.07	PPP-PPP	0.10	11	0.861	0.984	0.0124
218387_s_at	1391.4	1304.7	-0.09	-1.07	PPP-PPP	0.10	11	0.861	0.984	0.1364
202069_s_at	2530.0	2374.7	-0.09	-1.07	PPP-PPP	0.10	11	0.861	0.984	0.0503
242711_x_at	249.5	234.2	-0.09	-1.07	PPP-PPP	0.10	11	0.861	0.984	0.1954
224908_s_at	1932.2	1813.6	-0.09	-1.07	PPP-PPP	0.10	11	0.861	0.984	0.2168
225793_at	2825.3	2654.9	-0.09	-1.06	PPP-PPP	0.10	11	0.861	0.984	0.0077
221570_s_at	8988.2	8446.9	-0.09	-1.06	PPP-PPP	0.10	11	0.861	0.984	0.0418
38158_at	1982.4	1862.8	-0.09	-1.06	PPP-PPP	0.10	11	0.861	0.984	0.1219
203663_s_at	14814.3	13924.2	-0.09	-1.06	PPP-PPP	0.10	11	0.861	0.984	0.1383
207168_s_at	12424.6	11681.0	-0.09	-1.06	PPP-PPP	0.10	11	0.861	0.984	0.1774

200700_s_at	6695.7	6297.5	-0.09	-1.06	PPP-PPP	0.10	11	0.861	0.984	0.2066
201398_s_at	6386.9	6013.0	-0.09	-1.06	PPP-PPP	0.10	11	0.861	0.984	0.1212
204216_s_at	3008.2	2833.9	-0.09	-1.06	PPP-PPP	0.10	11	0.861	0.984	0.0412
209318_x_at	1866.3	1759.6	-0.09	-1.06	PPP-PPP	0.10	11	0.861	0.984	0.0430
204023_at	4136.9	3901.2	-0.09	-1.06	PPP-PPP	0.10	11	0.861	0.984	0.0663
202486_at	1083.6	1023.3	-0.08	-1.06	PPP-PPP	0.10	11	0.861	0.984	0.0860
206445_s_at	12050.5	11396.3	-0.08	-1.06	PPP-PPP	0.10	11	0.861	0.984	0.0756
208742_s_at	12398.6	11741.8	-0.08	-1.06	PPP-PPP	0.10	11	0.861	0.984	0.0998
223016_x_at	3317.5	3141.0	-0.08	-1.06	PPP-PPP	0.10	11	0.861	0.984	0.1125
217739_s_at	3588.4	3398.9	-0.08	-1.06	PPP-PPP	0.10	11	0.861	0.984	0.1315
210996_s_at	7647.8	7258.3	-0.08	-1.05	PPP-PPP	0.10	11	0.861	0.984	0.0477
1569277_at	32.7	1.2	-4.78	-27.40	MAA-AAA	0.00	999	1.000	1.000	0.0006
208331_at	44.7	2.0	-4.52	-22.89	AAA-AAA	0.00	999	1.000	1.000	0.0007
234244_at	29.9	1.3	-4.52	-22.87	AAA-AAA	0.00	999	1.000	1.000	0.0001
1566951_at	63.2	3.3	-4.26	-19.10	AAA-AAA	0.00	999	1.000	1.000	0.0001
231669_at	32.3	1.7	-4.25	-19.01	AAA-AAA	0.00	999	1.000	1.000	0.0210
234358_at	32.1	1.8	-4.18	-18.09	AAA-AAA	0.00	999	1.000	1.000	0.0023
1556026_at	25.5	1.4	-4.15	-17.77	AAA-AAA	0.00	999	1.000	1.000	0.0011
1553821_at	62.5	3.6	-4.11	-17.29	AAA-AAA	0.00	999	1.000	1.000	0.0028
1566600_at	44.6	2.7	-4.05	-16.57	AAA-AAA	0.00	999	1.000	1.000	0.0103
242625_at	23.5	1.4	-4.03	-16.33	AAA-AAA	0.00	999	1.000	1.000	0.0077
1557233_at	33.8	2.1	-4.00	-15.95	AAA-AAA	0.00	999	1.000	1.000	0.0080
228163_at	41.1	2.6	-3.97	-15.61	AAA-AAA	0.00	999	1.000	1.000	0.0048
1565588_at	55.4	3.6	-3.93	-15.29	AAA-AAA	0.00	999	1.000	1.000	0.0009
1562566_at	19.7	1.3	-3.92	-15.14	AAA-AAA	0.00	999	1.000	1.000	0.0015
240171_at	50.4	3.4	-3.90	-14.97	AMA-AAA	0.00	999	1.000	1.000	0.0029
209795_at	16.9	1.1	-3.88	-14.75	AAA-AAA	0.00	999	1.000	1.000	0.0024
1557836_at	58.6	4.0	-3.88	-14.69	AAA-AAA	0.00	999	1.000	1.000	0.0007
1561132_at	54.0	3.7	-3.87	-14.63	AAA-AAA	0.00	999	1.000	1.000	0.0011
231802_at	51.9	3.6	-3.87	-14.61	AAA-AAA	0.00	999	1.000	1.000	0.0008
234078_at	61.6	4.3	-3.85	-14.43	AAA-AAA	0.00	999	1.000	1.000	0.0044
242221_at	24.5	1.7	-3.83	-14.20	AAA-AAA	0.00	999	1.000	1.000	0.0011
215851_at	56.2	4.0	-3.82	-14.16	AAA-AAA	0.00	999	1.000	1.000	0.0364
1569287_at	29.5	2.1	-3.82	-14.13	MAA-AAA	0.00	999	1.000	1.000	0.0041
1565644_at	47.4	3.4	-3.79	-13.82	AAA-AAA	0.00	999	1.000	1.000	0.0007
231563_at	52.1	3.9	-3.76	-13.53	AAA-AAA	0.00	999	1.000	1.000	0.0095
1558653_at	39.0	2.9	-3.75	-13.49	AAA-AAA	0.00	999	1.000	1.000	0.0002
1562916_at	29.8	2.3	-3.71	-13.08	AAA-AAA	0.00	999	1.000	1.000	0.0059
1570261_at	24.8	1.9	-3.70	-13.01	AAA-AAA	0.00	999	1.000	1.000	0.0525
237281_at	32.4	2.5	-3.70	-12.96	AAA-AAA	0.00	999	1.000	1.000	0.0055
1570082_x_at	53.1	4.2	-3.67	-12.75	AAA-AAA	0.00	999	1.000	1.000	0.0119
232448_at	22.7	1.8	-3.65	-12.56	AAA-AAA	0.00	999	1.000	1.000	0.0019

242906_at	37.9	3.0	-3.64	-12.47	AAA-AAA	0.00	999	1.000	1.000	0.0690
1563916_at	64.8	5.2	-3.64	-12.48	AAA-AAA	0.00	999	1.000	1.000	0.0042
1563089_at	60.2	4.9	-3.63	-12.41	AAA-AAA	0.00	999	1.000	1.000	0.0207
228167_at	29.7	2.4	-3.62	-12.33	AAA-AAA	0.00	999	1.000	1.000	0.0013
224384_s_at	44.7	3.7	-3.60	-12.13	AAA-AAA	0.00	999	1.000	1.000	0.0019
1566839_at	64.1	5.4	-3.58	-11.96	AAA-AAA	0.00	999	1.000	1.000	0.0017
1554012_at	25.0	2.1	-3.57	-11.90	AAA-AAA	0.00	999	1.000	1.000	0.0179
240386_at	25.6	2.2	-3.57	-11.85	AAA-AAA	0.00	999	1.000	1.000	0.0114
1565787_at	36.7	3.1	-3.57	-11.84	AAA-AAA	0.00	999	1.000	1.000	0.0350
223646_s_at	62.0	5.3	-3.56	-11.81	AAA-AAA	0.00	999	1.000	1.000	0.0007
1556069_s_at	48.5	4.1	-3.56	-11.77	AAA-AAA	0.00	999	1.000	1.000	0.0039
216258_s_at	21.0	1.8	-3.55	-11.73	AAA-AAA	0.00	999	1.000	1.000	0.0119
236694_at	2.3	26.6	3.55	11.73	AAA-AAA	0.00	999	1.000	1.000	0.0119
240207_at	32.9	2.8	-3.55	-11.71	AMA-AAA	0.00	999	1.000	1.000	0.0249
1558995_at	64.7	5.5	-3.55	-11.70	AAA-AAA	0.00	999	1.000	1.000	0.0042
235881_at	46.7	4.0	-3.55	-11.67	AAA-AAA	0.00	999	1.000	1.000	0.0362
233184_at	31.3	2.7	-3.54	-11.65	AAA-AAA	0.00	999	1.000	1.000	0.0213
239621_at	27.7	2.4	-3.54	-11.61	AAA-AAA	0.00	999	1.000	1.000	0.0074
1564945_at	51.0	4.4	-3.53	-11.57	AMA-AAA	0.00	999	1.000	1.000	0.0040
1556447_at	16.4	1.4	-3.52	-11.50	AAA-AAA	0.00	999	1.000	1.000	0.0577
1565628_at	58.0	5.1	-3.52	-11.46	AAA-AAA	0.00	999	1.000	1.000	0.0145
216401_x_at	75.3	6.6	-3.52	-11.43	AAA-AAA	0.00	999	1.000	1.000	0.0001
1552870_s_at	23.0	2.0	-3.49	-11.26	AAA-AAA	0.00	999	1.000	1.000	0.0060
1553614_a_at	46.2	4.1	-3.49	-11.22	AAA-AAA	0.00	999	1.000	1.000	0.0022
243317_at	38.6	3.5	-3.48	-11.14	AAA-AAA	0.00	999	1.000	1.000	0.0002
243549_at	16.4	1.5	-3.48	-11.14	AAA-AAA	0.00	999	1.000	1.000	0.0039
1569824_at	42.3	3.8	-3.47	-11.11	AAA-AAA	0.00	999	1.000	1.000	0.0190
240081_at	3.1	33.9	3.47	11.05	AAA-AAA	0.00	999	1.000	1.000	0.0036
1558813_at	27.8	2.5	-3.47	-11.04	AAA-AAA	0.00	999	1.000	1.000	0.0116
1560168_at	12.5	1.1	-3.45	-10.90	AAA-AAA	0.00	999	1.000	1.000	0.0122
231341_at	29.1	2.7	-3.44	-10.88	AAA-AAA	0.00	999	1.000	1.000	0.0006
1556589_at	42.6	3.9	-3.44	-10.85	AAA-AAA	0.00	999	1.000	1.000	0.0168
220627_at	74.3	6.9	-3.43	-10.79	AAA-AAA	0.00	999	1.000	1.000	0.0536
1563507_at	4.3	46.1	3.43	10.76	AAA-AMA	0.00	999	1.000	1.000	0.0790
1559653_at	32.9	3.1	-3.43	-10.75	AAA-AAA	0.00	999	1.000	1.000	0.0124
231626_at	34.4	3.2	-3.43	-10.75	AAA-AAA	0.00	999	1.000	1.000	0.0003
1564351_at	36.4	3.4	-3.42	-10.70	AAA-AAA	0.00	999	1.000	1.000	0.0002
205814_at	60.8	5.7	-3.42	-10.70	AAA-AAA	0.00	999	1.000	1.000	0.0002
239968_at	31.9	3.0	-3.41	-10.64	AAA-AAA	0.00	999	1.000	1.000	0.0078
244537_at	54.3	5.1	-3.41	-10.64	AAA-AAA	0.00	999	1.000	1.000	0.0042
1569234_at	55.9	5.3	-3.41	-10.59	AAA-AAA	0.00	999	1.000	1.000	0.0025
210698_at	67.1	6.4	-3.39	-10.46	AAA-AAA	0.00	999	1.000	1.000	0.0115

1557004_at	42.8	4.1	-3.38	-10.39	AAA-AAA	0.00	999	1.000	1.000	0.0001
1559154_at	5.2	53.6	3.38	10.37	AAA-AAA	0.00	999	1.000	1.000	0.0062
233688_at	59.8	5.8	-3.37	-10.37	AAA-AAA	0.00	999	1.000	1.000	0.0002
230220_at	31.9	3.1	-3.37	-10.36	AAA-AAA	0.00	999	1.000	1.000	0.0738
222247_at	56.7	5.5	-3.37	-10.36	MAM-AAA	0.00	999	1.000	1.000	0.0003
215612_at	46.3	4.5	-3.37	-10.33	AAA-AAA	0.00	999	1.000	1.000	0.0081
214618_at	29.3	2.8	-3.37	-10.32	AAA-AAA	0.00	999	1.000	1.000	0.0065
233681_at	19.4	1.9	-3.37	-10.32	AAA-AAA	0.00	999	1.000	1.000	0.0474
1564309_at	23.8	2.3	-3.36	-10.28	AAA-AAA	0.00	999	1.000	1.000	0.0407
240514_at	54.5	5.3	-3.36	-10.26	AAA-AAA	0.00	999	1.000	1.000	0.0012
207106_s_at	71.6	7.0	-3.36	-10.26	AAA-AAA	0.00	999	1.000	1.000	0.0008
232846_s_at	28.3	2.8	-3.35	-10.18	AAA-AAA	0.00	999	1.000	1.000	0.0019
1570528_at	43.7	4.3	-3.34	-10.14	AAA-AAA	0.00	999	1.000	1.000	0.0173
233370_at	42.1	4.2	-3.34	-10.13	AAA-AAA	0.00	999	1.000	1.000	0.0367
1569354_at	27.6	2.7	-3.34	-10.12	AAA-AAA	0.00	999	1.000	1.000	0.0062
229478_x_at	4.0	39.5	3.32	9.99	AAA-AAA	0.00	999	1.000	1.000	0.0999
237395_at	22.8	2.3	-3.32	-9.97	AAA-AAA	0.00	999	1.000	1.000	0.0125
221608_at	33.7	3.4	-3.31	-9.94	AAA-AAA	0.00	999	1.000	1.000	0.0050
224191_x_at	84.8	8.5	-3.31	-9.94	AAA-AAA	0.00	999	1.000	1.000	0.0651
211122_s_at	3.0	30.1	3.31	9.93	AAA-AAA	0.00	999	1.000	1.000	0.0140
1568190_at	24.7	2.5	-3.31	-9.91	AAA-AAA	0.00	999	1.000	1.000	0.0389
224017_at	11.8	1.2	-3.31	-9.89	AAA-AAA	0.00	999	1.000	1.000	0.0237
229244_at	26.3	2.7	-3.30	-9.87	AAA-AAA	0.00	999	1.000	1.000	0.0225
217037_at	34.7	3.5	-3.30	-9.83	AAA-AAA	0.00	999	1.000	1.000	0.0364
221439_at	89.7	9.2	-3.29	-9.77	AAA-AAA	0.00	999	1.000	1.000	0.0102
1553482_at	19.8	2.0	-3.29	-9.76	AAA-AAA	0.00	999	1.000	1.000	0.0481
219059_s_at	60.0	6.2	-3.29	-9.75	AAA-AAA	0.00	999	1.000	1.000	0.0157
205737_at	75.5	7.8	-3.28	-9.73	AAA-AAA	0.00	999	1.000	1.000	0.0294
237649_at	39.6	4.1	-3.27	-9.66	AAA-AAA	0.00	999	1.000	1.000	0.0256
1569375_at	33.3	3.5	-3.27	-9.61	MAA-AAA	0.00	999	1.000	1.000	0.0032
211766_s_at	48.2	5.1	-3.26	-9.55	AAA-AAA	0.00	999	1.000	1.000	0.0006
235732_at	32.4	3.4	-3.25	-9.54	AAA-AAA	0.00	999	1.000	1.000	0.0314
237806_s_at	20.9	2.2	-3.25	-9.52	AAA-AAA	0.00	999	1.000	1.000	0.0278
216929_x_at	96.5	10.2	-3.24	-9.44	AAA-AAA	0.00	999	1.000	1.000	0.0423
205386_s_at	5.7	54.2	3.24	9.44	AAA-AAA	0.00	999	1.000	1.000	0.0096
203902_at	29.0	3.1	-3.24	-9.42	AAA-AAA	0.00	999	1.000	1.000	0.0033
211906_s_at	34.0	3.6	-3.23	-9.41	AAA-AAA	0.00	999	1.000	1.000	0.0201
206517_at	40.7	4.3	-3.23	-9.39	AAA-AAA	0.00	999	1.000	1.000	0.0077
231131_at	21.1	2.3	-3.23	-9.37	AAA-AAA	0.00	999	1.000	1.000	0.0003
240480_at	13.9	1.5	-3.23	-9.37	AAA-AAA	0.00	999	1.000	1.000	0.0543
233663_s_at	33.7	3.6	-3.23	-9.35	AAA-AAA	0.00	999	1.000	1.000	0.0519
1563115_at	39.6	4.3	-3.22	-9.33	AAA-AAA	0.00	999	1.000	1.000	0.0003

226769_at	81.7	8.8	-3.22	-9.33	AAA-AAA	0.00	999	1.000	1.000	0.0714
1553260_s_at	26.2	2.8	-3.22	-9.32	AAA-AAA	0.00	999	1.000	1.000	0.0132
221129_at	39.9	4.3	-3.22	-9.32	AAA-AAA	0.00	999	1.000	1.000	0.0003
1552592_at	21.7	2.3	-3.22	-9.30	AAA-AAA	0.00	999	1.000	1.000	0.0391
203961_at	38.4	4.1	-3.22	-9.30	AAA-AAA	0.00	999	1.000	1.000	0.0029
241853_at	1.9	17.5	3.22	9.30	AAA-AAA	0.00	999	1.000	1.000	0.0110
1556903_at	39.4	4.3	-3.21	-9.28	AAM-AAA	0.00	999	1.000	1.000	0.0022
217245_at	25.8	2.8	-3.21	-9.27	AAA-AAA	0.00	999	1.000	1.000	0.0262
241110_at	42.9	4.6	-3.21	-9.26	AAA-AAA	0.00	999	1.000	1.000	0.0011
205699_at	51.6	5.6	-3.21	-9.26	AAA-AAA	0.00	999	1.000	1.000	0.0205
1562414_at	47.3	5.1	-3.21	-9.25	AAA-AAA	0.00	999	1.000	1.000	0.0097
1560542_at	19.5	2.1	-3.21	-9.23	AAA-AAA	0.00	999	1.000	1.000	0.0030
1557639_at	31.4	3.4	-3.21	-9.23	AAA-AAA	0.00	999	1.000	1.000	0.0305
209915_s_at	31.3	3.4	-3.20	-9.18	AAA-AAA	0.00	999	1.000	1.000	0.0242
1560483_at	22.4	2.4	-3.20	-9.17	AAA-AAA	0.00	999	1.000	1.000	0.0107
210420_at	52.2	5.7	-3.20	-9.16	AAA-AAA	0.00	999	1.000	1.000	0.0047
237528_at	28.5	3.1	-3.20	-9.16	AMA-AAA	0.00	999	1.000	1.000	0.0786
220435_at	49.7	5.4	-3.19	-9.15	AAA-AAA	0.00	999	1.000	1.000	0.0546
1566439_at	11.7	1.3	-3.19	-9.11	AAA-AAA	0.00	999	1.000	1.000	0.0418
230084_at	83.0	9.1	-3.19	-9.11	AAA-AAA	0.00	999	1.000	1.000	0.0226
241872_at	58.3	6.4	-3.19	-9.09	AAA-AAA	0.00	999	1.000	1.000	0.0012
1566268_at	114.7	12.6	-3.18	-9.09	AAA-AAA	0.00	999	1.000	1.000	0.0126
241995_at	5.7	51.4	3.18	9.06	AAA-AAA	0.00	999	1.000	1.000	0.0010
205617_at	45.0	5.0	-3.18	-9.05	AMA-AAA	0.00	999	1.000	1.000	0.0940
1565554_at	62.1	6.9	-3.18	-9.04	AAA-AAA	0.00	999	1.000	1.000	0.0146
222137_at	95.5	10.6	-3.18	-9.04	AAA-AAA	0.00	999	1.000	1.000	0.0113
1553485_at	51.2	5.7	-3.17	-9.02	MAA-AAA	0.00	999	1.000	1.000	0.0292
232117_at	3.8	34.1	3.17	8.98	AAA-AAA	0.00	999	1.000	1.000	0.0562
207817_at	39.6	4.4	-3.17	-8.97	AAA-AAA	0.00	999	1.000	1.000	0.0213
232663_s_at	55.7	6.2	-3.16	-8.96	AAA-AAA	0.00	999	1.000	1.000	0.0472
231060_at	33.9	3.8	-3.16	-8.96	AAA-AAA	0.00	999	1.000	1.000	0.0391
1570235_at	27.8	3.1	-3.16	-8.95	AAA-AAA	0.00	999	1.000	1.000	0.0044
1558795_at	45.7	5.1	-3.16	-8.95	AAA-AAA	0.00	999	1.000	1.000	0.0488
237781_at	60.6	6.8	-3.16	-8.94	AAA-AAA	0.00	999	1.000	1.000	0.0193
1567247_at	12.4	1.4	-3.16	-8.93	AAA-AAA	0.00	999	1.000	1.000	0.0929
1557366_at	1.3	11.2	3.15	8.90	AAA-AAA	0.00	999	1.000	1.000	0.0362
208279_s_at	45.9	5.2	-3.15	-8.85	AAA-AAA	0.00	999	1.000	1.000	0.0200
232945_at	4.8	42.1	3.15	8.85	AAA-AAA	0.00	999	1.000	1.000	0.0129
240604_at	40.3	4.6	-3.12	-8.71	AAA-AAA	0.00	999	1.000	1.000	0.0009
239650_at	19.1	2.2	-3.12	-8.71	AAA-AAA	0.00	999	1.000	1.000	0.0591
1569126_at	42.4	4.9	-3.12	-8.68	MAA-AAA	0.00	999	1.000	1.000	0.0448
241096_at	52.0	6.0	-3.12	-8.66	AAA-AAA	0.00	999	1.000	1.000	0.0000

207680_x_at	52.4	6.1	-3.12	-8.66	AAA-AAA	0.00	999	1.000	1.000	0.0639
1552552_s_at	19.5	2.3	-3.11	-8.65	AAA-AAA	0.00	999	1.000	1.000	0.0959
1562703_at	67.6	7.8	-3.11	-8.65	AAA-AAA	0.00	999	1.000	1.000	0.0121
211447_s_at	22.1	2.6	-3.10	-8.60	AAA-AAA	0.00	999	1.000	1.000	0.0424
234357_at	30.1	3.5	-3.10	-8.59	AAA-AAA	0.00	999	1.000	1.000	0.0294
239529_at	27.9	3.3	-3.10	-8.59	AAA-AAA	0.00	999	1.000	1.000	0.0036
233045_at	1.6	13.9	3.10	8.58	AAA-AAA	0.00	999	1.000	1.000	0.0016
240864_at	32.8	3.8	-3.10	-8.58	AAA-AAA	0.00	999	1.000	1.000	0.0064
1561414_at	11.7	1.4	-3.10	-8.57	AAA-AAA	0.00	999	1.000	1.000	0.0707
207615_s_at	65.8	7.7	-3.10	-8.57	AAA-AAA	0.00	999	1.000	1.000	0.0013
1558001_s_at	51.2	6.0	-3.10	-8.54	AAA-AAA	0.00	999	1.000	1.000	0.0170
238265_x_at	90.0	10.5	-3.09	-8.53	AAA-AAA	0.00	999	1.000	1.000	0.0325
236714_at	34.0	4.0	-3.09	-8.50	AAA-AAA	0.00	999	1.000	1.000	0.0479
208017_s_at	29.1	3.4	-3.09	-8.50	AAA-AAA	0.00	999	1.000	1.000	0.0063
206219_s_at	76.8	9.1	-3.08	-8.47	AAA-AAA	0.00	999	1.000	1.000	0.0043
243922_at	78.5	9.3	-3.08	-8.46	AAA-AAA	0.00	999	1.000	1.000	0.0735
217583_at	15.9	1.9	-3.08	-8.46	AAA-AAA	0.00	999	1.000	1.000	0.0056
236968_at	44.1	5.2	-3.08	-8.46	AAA-AAA	0.00	999	1.000	1.000	0.0200
203812_at	149.5	17.7	-3.08	-8.43	MAA-AAA	0.00	999	1.000	1.000	0.0032
1569988_at	14.2	1.7	-3.08	-8.43	AAA-AAA	0.00	999	1.000	1.000	0.0123
1559807_at	18.2	2.2	-3.07	-8.39	AAA-AAA	0.00	999	1.000	1.000	0.0462
214420_s_at	23.7	2.8	-3.07	-8.39	AAA-AAA	0.00	999	1.000	1.000	0.0719
230986_at	23.0	2.7	-3.07	-8.39	AAA-AAA	0.00	999	1.000	1.000	0.0703
1563685_at	19.0	2.3	-3.07	-8.37	AAA-AAA	0.00	999	1.000	1.000	0.0492
243150_at	30.4	3.6	-3.06	-8.36	AAA-AAA	0.00	999	1.000	1.000	0.0734
1562815_at	33.0	4.0	-3.06	-8.35	AAA-AAA	0.00	999	1.000	1.000	0.0784
240605_at	23.6	2.8	-3.06	-8.34	AAA-AAA	0.00	999	1.000	1.000	0.0067
216314_at	15.9	1.9	-3.05	-8.31	AAA-AAA	0.00	999	1.000	1.000	0.0784
239858_at	31.6	3.8	-3.05	-8.28	AAA-AAA	0.00	999	1.000	1.000	0.0101
1566673_at	3.2	26.7	3.05	8.28	AAA-AMA	0.00	999	1.000	1.000	0.0519
1561754_at	15.1	1.8	-3.05	-8.28	AAA-AAA	0.00	999	1.000	1.000	0.0312
236074_at	3.6	30.0	3.05	8.27	AAA-AAA	0.00	999	1.000	1.000	0.0093
220997_s_at	3.3	27.6	3.05	8.26	AAA-AAA	0.00	999	1.000	1.000	0.0322
207834_at	50.5	6.1	-3.04	-8.25	AAA-AAA	0.00	999	1.000	1.000	0.0412
236876_at	39.4	4.8	-3.04	-8.24	AAA-AAA	0.00	999	1.000	1.000	0.0288
213952_s_at	72.5	8.8	-3.04	-8.22	AAA-AAA	0.00	999	1.000	1.000	0.0076
1560172_at	27.0	3.3	-3.04	-8.22	MAA-AAA	0.00	999	1.000	1.000	0.0554
1556351_at	10.7	1.3	-3.04	-8.21	AAA-AAA	0.00	999	1.000	1.000	0.0420
216681_at	18.7	2.3	-3.04	-8.19	AAA-AAA	0.00	999	1.000	1.000	0.0321
206045_s_at	22.2	2.7	-3.04	-8.20	AAA-AAA	0.00	999	1.000	1.000	0.0439
221886_at	44.6	5.5	-3.03	-8.17	AAA-AAA	0.00	999	1.000	1.000	0.0030
224351_at	38.5	4.7	-3.03	-8.18	AAA-AAA	0.00	999	1.000	1.000	0.0455

210690_at	26.5	3.2	-3.03	-8.17	AAA-AAA	0.00	999	1.000	1.000	0.0727
238661_at	19.1	2.3	-3.03	-8.16	AAA-AAA	0.00	999	1.000	1.000	0.0119
207392_x_at	80.4	9.9	-3.03	-8.16	AAA-AAA	0.00	999	1.000	1.000	0.0072
216058_s_at	57.9	7.1	-3.03	-8.15	AAA-AAA	0.00	999	1.000	1.000	0.0341
239518_at	67.2	8.3	-3.03	-8.15	AAA-AAA	0.00	999	1.000	1.000	0.0115
230447_at	20.0	2.5	-3.02	-8.13	AAA-AAA	0.00	999	1.000	1.000	0.0893
232056_at	34.9	4.3	-3.02	-8.12	AAM-AAA	0.00	999	1.000	1.000	0.0908
210472_at	37.8	4.7	-3.02	-8.11	AAA-AAA	0.00	999	1.000	1.000	0.0269
1570116_at	55.3	6.8	-3.02	-8.10	AAA-AAA	0.00	999	1.000	1.000	0.0475
205829_at	38.2	4.7	-3.02	-8.10	AAA-AAA	0.00	999	1.000	1.000	0.0419
234702_x_at	10.8	1.3	-3.02	-8.08	AAA-AAA	0.00	999	1.000	1.000	0.1044
1561590_a_at	21.5	2.7	-3.01	-8.08	AAA-AAA	0.00	999	1.000	1.000	0.0162
229916_at	15.5	1.9	-3.01	-8.08	AAA-AAA	0.00	999	1.000	1.000	0.0178
1562829_at	34.2	4.2	-3.01	-8.07	AAA-AAA	0.00	999	1.000	1.000	0.0250
222336_at	5.1	41.2	3.01	8.03	AAA-AAP	0.00	999	1.000	1.000	0.0962
1554820_at	15.4	1.9	-3.01	-8.03	AAA-AAA	0.00	999	1.000	1.000	0.0729
231317_at	151.0	18.8	-3.01	-8.04	AAA-AAA	0.00	999	1.000	1.000	0.0071
208495_at	41.8	5.2	-3.01	-8.03	AAA-AAA	0.00	999	1.000	1.000	0.0318
240922_at	2.1	16.7	3.00	8.02	AAA-AAA	0.00	999	1.000	1.000	0.0137
1564117_at	169.2	21.1	-3.00	-8.01	AAA-AAA	0.00	999	1.000	1.000	0.0314
1568926_x_at	17.7	2.2	-3.00	-8.00	AAA-AAA	0.00	999	1.000	1.000	0.0430
231616_at	51.3	6.4	-3.00	-8.00	AAA-AAA	0.00	999	1.000	1.000	0.0437
1566256_s_at	44.4	5.6	-3.00	-8.00	AAA-AAA	0.00	999	1.000	1.000	0.0793
217709_at	12.7	1.6	-3.00	-8.00	AAA-AAA	0.00	999	1.000	1.000	0.0535
1563814_at	32.4	4.1	-2.99	-7.96	AAA-AAA	0.00	999	1.000	1.000	0.0241
1568764_x_at	16.2	2.0	-2.99	-7.96	AAA-AAA	0.00	999	1.000	1.000	0.0132
234090_at	45.4	5.7	-2.99	-7.95	AAA-AAA	0.00	999	1.000	1.000	0.0096
215655_at	36.1	4.5	-2.99	-7.95	AAA-AAA	0.00	999	1.000	1.000	0.0330
243339_at	19.6	2.5	-2.99	-7.95	AAA-AAA	0.00	999	1.000	1.000	0.1411
206119_at	53.6	6.8	-2.99	-7.94	AAA-AAA	0.00	999	1.000	1.000	0.0243
1560372_at	10.8	1.4	-2.99	-7.94	AAA-AAA	0.00	999	1.000	1.000	0.1268
1570262_at	45.2	5.7	-2.99	-7.92	AAA-AAA	0.00	999	1.000	1.000	0.0106
216529_at	13.0	1.6	-2.99	-7.92	AAA-AAA	0.00	999	1.000	1.000	0.0749
1561461_at	31.6	4.0	-2.98	-7.91	AAA-AAA	0.00	999	1.000	1.000	0.0362
1557874_at	29.2	3.7	-2.98	-7.91	AAA-AAA	0.00	999	1.000	1.000	0.0322
215388_s_at	5.1	40.3	2.98	7.90	AAA-PAM	0.00	999	1.000	1.000	0.0888
1552940_at	56.2	7.1	-2.98	-7.90	AAA-AAA	0.00	999	1.000	1.000	0.0009
244205_at	58.9	7.5	-2.98	-7.87	AAA-AAA	0.00	999	1.000	1.000	0.0812
208222_at	96.7	12.3	-2.98	-7.87	AAA-AAA	0.00	999	1.000	1.000	0.0096
241311_at	16.6	2.1	-2.97	-7.86	AAA-AAA	0.00	999	1.000	1.000	0.0065
221348_at	32.4	4.1	-2.97	-7.86	AAA-AAA	0.00	999	1.000	1.000	0.0354
237436_at	44.1	5.6	-2.97	-7.86	AAA-AAA	0.00	999	1.000	1.000	0.1197

229625_at	45.8	5.8	-2.97	-7.86	AAA-AAA	0.00	999	1.000	1.000	0.0868
234960_at	47.9	6.1	-2.97	-7.85	AAA-AAA	0.00	999	1.000	1.000	0.0014
1558793_at	1.7	13.7	2.97	7.85	AAA-AAA	0.00	999	1.000	1.000	0.0319
222959_at	28.4	3.6	-2.97	-7.85	AAA-AAA	0.00	999	1.000	1.000	0.0634
237020_at	19.5	2.5	-2.97	-7.85	AAA-AAA	0.00	999	1.000	1.000	0.0323
1568711_a_at	9.6	1.2	-2.97	-7.82	AAA-AAA	0.00	999	1.000	1.000	0.0742
1567009_at	14.5	1.9	-2.97	-7.82	AAA-AAA	0.00	999	1.000	1.000	0.0649
208329_at	37.4	4.8	-2.97	-7.81	AAA-AAA	0.00	999	1.000	1.000	0.0213
1560614_at	29.0	3.7	-2.96	-7.79	AAA-AAA	0.00	999	1.000	1.000	0.0041
1565874_at	10.5	1.3	-2.96	-7.79	AAA-AAA	0.00	999	1.000	1.000	0.0267
217231_s_at	60.3	7.7	-2.96	-7.79	AAA-AAA	0.00	999	1.000	1.000	0.0009
239994_at	49.0	6.3	-2.96	-7.77	AAA-AAA	0.00	999	1.000	1.000	0.0052
1565742_at	19.7	2.5	-2.96	-7.76	AAA-AAA	0.00	999	1.000	1.000	0.0699
1561123_at	28.4	3.7	-2.96	-7.76	AAA-AAA	0.00	999	1.000	1.000	0.0015
1558834_s_at	4.3	33.0	2.96	7.76	AAA-AAA	0.00	999	1.000	1.000	0.0018
1568902_at	26.2	3.4	-2.95	-7.75	AAA-AAA	0.00	999	1.000	1.000	0.0777
241902_at	26.0	3.4	-2.95	-7.74	AAA-AAA	0.00	999	1.000	1.000	0.0069
1564220_a_at	29.5	3.8	-2.95	-7.72	AAA-AAA	0.00	999	1.000	1.000	0.0423
207504_at	52.9	6.9	-2.95	-7.70	AAA-AAA	0.00	999	1.000	1.000	0.0101
224275_at	27.9	3.6	-2.95	-7.70	AAA-AAA	0.00	999	1.000	1.000	0.0168
1565923_at	20.2	2.6	-2.94	-7.69	AAA-AAA	0.00	999	1.000	1.000	0.0325
1554865_at	9.9	1.3	-2.94	-7.69	AAA-AAA	0.00	999	1.000	1.000	0.0495
236039_at	40.1	5.2	-2.94	-7.68	AAA-AAA	0.00	999	1.000	1.000	0.0086
207725_at	34.5	4.5	-2.94	-7.68	AAA-AAA	0.00	999	1.000	1.000	0.1134
1561497_at	65.4	8.5	-2.94	-7.67	AAA-AAA	0.00	999	1.000	1.000	0.0088
211712_s_at	24.6	3.2	-2.94	-7.67	AAA-AAA	0.00	999	1.000	1.000	0.0080
238099_at	86.2	11.2	-2.94	-7.68	AAA-AAA	0.00	999	1.000	1.000	0.0019
203649_s_at	39.7	5.2	-2.94	-7.67	AAA-AAA	0.00	999	1.000	1.000	0.0372
217175_at	48.9	6.4	-2.94	-7.65	AAA-AAA	0.00	999	1.000	1.000	0.1371
235931_at	19.7	2.6	-2.94	-7.65	AAA-AAA	0.00	999	1.000	1.000	0.0760
244173_at	22.7	3.0	-2.93	-7.64	AAA-AAA	0.00	999	1.000	1.000	0.1132
241507_x_at	24.1	3.2	-2.93	-7.64	AAA-AAA	0.00	999	1.000	1.000	0.0042
219812_at	72.1	9.5	-2.93	-7.63	AAA-AAA	0.00	999	1.000	1.000	0.0065
241519_at	37.8	5.0	-2.93	-7.62	AAA-AAA	0.00	999	1.000	1.000	0.0044
240965_at	27.1	3.6	-2.93	-7.61	AAA-AAA	0.00	999	1.000	1.000	0.0791
1559419_at	2.5	19.3	2.93	7.61	AAA-AAA	0.00	999	1.000	1.000	0.0894
234800_at	9.5	1.3	-2.93	-7.59	AAA-AAA	0.00	999	1.000	1.000	0.0732
1552801_at	49.8	6.6	-2.92	-7.58	AAA-AAA	0.00	999	1.000	1.000	0.0149
235858_at	39.0	5.1	-2.92	-7.59	AAA-AAA	0.00	999	1.000	1.000	0.0127
214612_x_at	32.6	4.3	-2.92	-7.58	AAA-AAA	0.00	999	1.000	1.000	0.1104
1564294_at	2.6	19.4	2.92	7.57	AAA-AAA	0.00	999	1.000	1.000	0.0106
237825_x_at	3.0	22.4	2.92	7.55	AAA-AAA	0.00	999	1.000	1.000	0.0231

217058_at	9.5	71.8	2.92	7.54	AAA-AAA	0.00	999	1.000	1.000	0.0106
1564642_at	51.6	6.9	-2.91	-7.54	MAA-AAA	0.00	999	1.000	1.000	0.0391
216502_at	48.2	6.4	-2.91	-7.54	AAA-AAA	0.00	999	1.000	1.000	0.0032
211164_at	25.6	3.4	-2.91	-7.52	AAA-AAA	0.00	999	1.000	1.000	0.0272
212338_at	27.5	3.7	-2.91	-7.51	AAA-AAA	0.00	999	1.000	1.000	0.0755
221013_s_at	72.9	9.7	-2.91	-7.50	AAA-AAA	0.00	999	1.000	1.000	0.0264
239214_at	5.4	40.7	2.91	7.50	AAA-AAA	0.00	999	1.000	1.000	0.0013
237715_at	86.3	11.5	-2.91	-7.49	AAA-AAA	0.00	999	1.000	1.000	0.0536
1558686_at	33.9	4.5	-2.91	-7.49	AAA-AAA	0.00	999	1.000	1.000	0.0990
1560226_at	34.8	4.6	-2.90	-7.48	AAA-AAA	0.00	999	1.000	1.000	0.0006
1559363_at	8.0	1.1	-2.90	-7.48	AAA-AAA	0.00	999	1.000	1.000	0.0682
223686_at	24.1	3.2	-2.90	-7.46	AAA-AAA	0.00	999	1.000	1.000	0.1192
1565666_s_at	11.6	1.6	-2.90	-7.46	AAA-AAA	0.00	999	1.000	1.000	0.1150
226766_at	13.5	1.8	-2.90	-7.45	AAA-AAA	0.00	999	1.000	1.000	0.0093
237578_at	3.5	26.2	2.90	7.44	AAA-AAA	0.00	999	1.000	1.000	0.0012
1564358_at	22.7	3.1	-2.89	-7.43	AAA-AAA	0.00	999	1.000	1.000	0.0752
238257_at	20.3	2.7	-2.89	-7.42	AAA-AAA	0.00	999	1.000	1.000	0.1024
229670_at	49.5	6.7	-2.89	-7.41	AAA-AAA	0.00	999	1.000	1.000	0.0003
1563190_at	60.9	8.2	-2.89	-7.40	MAA-AAA	0.00	999	1.000	1.000	0.0045
230694_at	55.9	7.6	-2.89	-7.39	AAA-AAA	0.00	999	1.000	1.000	0.0338
233590_at	48.9	6.6	-2.89	-7.39	AAA-AAA	0.00	999	1.000	1.000	0.0415
228285_at	28.6	3.9	-2.89	-7.39	AAA-AAA	0.00	999	1.000	1.000	0.0371
241851_x_at	41.3	5.6	-2.88	-7.38	AAA-AAA	0.00	999	1.000	1.000	0.0428
1554533_at	1.9	14.1	2.88	7.38	AAA-AAA	0.00	999	1.000	1.000	0.0071
217638_at	47.1	6.4	-2.88	-7.38	AAA-AAA	0.00	999	1.000	1.000	0.0055
1569915_at	39.7	5.4	-2.88	-7.37	AAA-AAA	0.00	999	1.000	1.000	0.0045
232703_at	89.8	12.2	-2.88	-7.37	AAA-AAA	0.00	999	1.000	1.000	0.0055
233220_at	56.2	7.6	-2.88	-7.36	AAA-AAA	0.00	999	1.000	1.000	0.0453
221364_at	41.9	5.7	-2.88	-7.35	AAA-AAA	0.00	999	1.000	1.000	0.0025
233621_s_at	19.5	2.7	-2.88	-7.33	AAA-AAA	0.00	999	1.000	1.000	0.0554
237182_at	13.2	1.8	-2.87	-7.33	AAA-AAA	0.00	999	1.000	1.000	0.0202
230577_at	19.9	2.7	-2.87	-7.33	AAA-AAA	0.00	999	1.000	1.000	0.0537
237237_at	58.2	8.0	-2.87	-7.31	AAA-AAA	0.00	999	1.000	1.000	0.0039
1570331_at	39.0	5.3	-2.87	-7.30	AAA-AAA	0.00	999	1.000	1.000	0.0052
217267_s_at	27.9	3.8	-2.87	-7.30	AAA-AAA	0.00	999	1.000	1.000	0.0086
1561291_at	22.6	3.1	-2.87	-7.30	AAA-AAA	0.00	999	1.000	1.000	0.0184
208245_at	19.3	2.7	-2.86	-7.27	AAA-AAA	0.00	999	1.000	1.000	0.0580
237297_at	62.8	8.6	-2.86	-7.27	AAA-AAA	0.00	999	1.000	1.000	0.0004
207846_at	19.0	2.6	-2.86	-7.25	AAA-AAA	0.00	999	1.000	1.000	0.0337
228256_s_at	46.7	6.4	-2.86	-7.25	AAA-AAA	0.00	999	1.000	1.000	0.0022
1568785_a_at	8.8	1.2	-2.86	-7.24	AAA-AAA	0.00	999	1.000	1.000	0.0013
1570303_at	31.1	4.3	-2.86	-7.24	AAA-AAA	0.00	999	1.000	1.000	0.0466

238861_at	17.9	2.5	-2.86	-7.24	AAA-AAA	0.00	999	1.000	1.000	0.0808
230809_at	22.4	3.1	-2.86	-7.24	AAA-AAA	0.00	999	1.000	1.000	0.0243
206502_s_at	16.4	2.3	-2.86	-7.23	AAA-AAA	0.00	999	1.000	1.000	0.0495
219989_s_at	19.1	2.6	-2.85	-7.23	AAA-AAA	0.00	999	1.000	1.000	0.0096
1552809_at	71.1	9.8	-2.85	-7.23	AAA-AAA	0.00	999	1.000	1.000	0.0742
211655_at	25.2	3.5	-2.85	-7.22	AAA-AAA	0.00	999	1.000	1.000	0.0457
239864_at	42.6	5.9	-2.85	-7.22	AAA-AAA	0.00	999	1.000	1.000	0.0161
243142_at	22.1	3.1	-2.85	-7.21	AAA-AAA	0.00	999	1.000	1.000	0.1411
239931_at	33.5	4.7	-2.85	-7.20	AAA-AAA	0.00	999	1.000	1.000	0.0253
234183_at	43.4	6.0	-2.85	-7.20	AAA-AAA	0.00	999	1.000	1.000	0.0227
223449_at	72.4	10.1	-2.85	-7.18	AAA-AAA	0.00	999	1.000	1.000	0.0597
220787_at	8.8	1.2	-2.84	-7.17	AAA-AAA	0.00	999	1.000	1.000	0.0324
1563696_at	11.8	1.7	-2.84	-7.16	AAA-AAA	0.00	999	1.000	1.000	0.0514
238968_at	58.5	8.2	-2.84	-7.16	AAA-AAA	0.00	999	1.000	1.000	0.0165
232452_at	10.0	1.4	-2.84	-7.15	AAA-AAA	0.00	999	1.000	1.000	0.0477
224094_at	36.6	5.1	-2.84	-7.15	AAA-AAA	0.00	999	1.000	1.000	0.0606
229094_at	6.6	47.0	2.84	7.13	AAA-AAA	0.00	999	1.000	1.000	0.0051
1557326_at	19.4	2.7	-2.83	-7.12	AAA-AAA	0.00	999	1.000	1.000	0.0218
1569886_a_at	31.3	4.4	-2.83	-7.11	AAA-AAA	0.00	999	1.000	1.000	0.0124
214761_at	25.1	3.5	-2.83	-7.11	AAA-AAA	0.00	999	1.000	1.000	0.1469
215247_at	55.8	7.8	-2.83	-7.11	AAA-AAA	0.00	999	1.000	1.000	0.0131
229546_at	20.8	2.9	-2.83	-7.11	AAA-AAA	0.00	999	1.000	1.000	0.0559
240308_at	16.9	2.4	-2.83	-7.10	AAA-AAA	0.00	999	1.000	1.000	0.1120
216495_x_at	89.0	12.6	-2.83	-7.09	AAA-AAA	0.00	999	1.000	1.000	0.0463
226803_at	35.4	5.0	-2.82	-7.07	AAA-AAA	0.00	999	1.000	1.000	0.0334
1558764_at	18.7	2.6	-2.82	-7.07	AAA-AAA	0.00	999	1.000	1.000	0.0320
243978_at	38.4	5.5	-2.82	-7.05	AAA-AAA	0.00	999	1.000	1.000	0.0024
1553454_at	15.2	2.2	-2.81	-7.03	AAA-AAA	0.00	999	1.000	1.000	0.0582
1553602_at	53.9	7.7	-2.81	-7.03	AAA-AAA	0.00	999	1.000	1.000	0.0280
1556267_at	27.1	3.9	-2.81	-7.01	AAA-AAA	0.00	999	1.000	1.000	0.0479
215746_at	54.4	7.8	-2.81	-7.01	MAA-AAA	0.00	999	1.000	1.000	0.0512
240848_at	38.3	5.5	-2.81	-7.00	AAA-AAA	0.00	999	1.000	1.000	0.0101
233504_at	24.7	3.5	-2.81	-7.00	AAA-AAA	0.00	999	1.000	1.000	0.0178
206532_at	47.5	6.8	-2.81	-7.00	AAA-AAA	0.00	999	1.000	1.000	0.0222
211403_x_at	101.0	14.5	-2.81	-6.99	AAA-AAA	0.00	999	1.000	1.000	0.1237
211680_at	12.7	1.8	-2.81	-6.99	AAA-AAA	0.00	999	1.000	1.000	0.1216
205064_at	4.1	28.2	2.80	6.96	AAA-AAA	0.00	999	1.000	1.000	0.0360
1555394_at	3.1	21.6	2.80	6.95	AAA-AAA	0.00	999	1.000	1.000	0.0026
232054_at	21.1	3.0	-2.80	-6.96	AAA-AAA	0.00	999	1.000	1.000	0.0791
1566840_at	40.7	5.9	-2.79	-6.94	AAA-AAA	0.00	999	1.000	1.000	0.0161
244255_at	66.8	9.6	-2.79	-6.93	AAA-AAA	0.00	999	1.000	1.000	0.0675
1561236_at	36.5	5.3	-2.79	-6.93	AMA-AAA	0.00	999	1.000	1.000	0.0376

236952_at	38.5	5.6	-2.79	-6.93	AAA-AAA	0.00	999	1.000	1.000	0.0073
1566861_at	3.9	26.8	2.79	6.92	AAA-AAA	0.00	999	1.000	1.000	0.0301
1557837_a_at	13.8	2.0	-2.79	-6.92	AAA-AAA	0.00	999	1.000	1.000	0.0631
1556921_at	27.8	4.0	-2.79	-6.92	AAA-AAA	0.00	999	1.000	1.000	0.0133
210726_at	72.3	10.5	-2.79	-6.92	AAA-AAA	0.00	999	1.000	1.000	0.0016
219647_at	6.2	43.0	2.79	6.91	AAA-AAA	0.00	999	1.000	1.000	0.0143
1562827_at	62.1	9.0	-2.79	-6.91	MAA-AAA	0.00	999	1.000	1.000	0.0519
1566964_at	69.8	10.1	-2.79	-6.91	AAA-AAA	0.00	999	1.000	1.000	0.0013
216691_at	30.5	4.4	-2.79	-6.90	AAA-AAA	0.00	999	1.000	1.000	0.0118
1561278_at	5.3	36.5	2.79	6.89	AAA-AAA	0.00	999	1.000	1.000	0.0058
1568916_at	64.4	9.4	-2.78	-6.88	AAA-AAA	0.00	999	1.000	1.000	0.0022
227194_at	55.6	8.1	-2.78	-6.88	AAA-AAA	0.00	999	1.000	1.000	0.0107
219894_at	31.4	4.6	-2.78	-6.87	AAA-AAA	0.00	999	1.000	1.000	0.0156
213839_at	42.2	6.2	-2.78	-6.87	MAA-AAA	0.00	999	1.000	1.000	0.1155
1561196_at	29.0	4.2	-2.78	-6.86	AAA-AAA	0.00	999	1.000	1.000	0.0128
221310_at	44.7	6.5	-2.78	-6.86	AAA-AAA	0.00	999	1.000	1.000	0.0001
1561891_at	43.8	6.4	-2.78	-6.85	AAA-AAA	0.00	999	1.000	1.000	0.0282
241998_at	33.9	5.0	-2.78	-6.85	AAA-AAA	0.00	999	1.000	1.000	0.0011
1569772_x_at	22.0	3.2	-2.77	-6.83	AAA-AAA	0.00	999	1.000	1.000	0.0524
214162_at	29.0	4.3	-2.77	-6.83	AAA-AAA	0.00	999	1.000	1.000	0.0226
211903_s_at	44.1	6.5	-2.77	-6.83	AAA-AAA	0.00	999	1.000	1.000	0.0159
1553383_at	23.9	3.5	-2.77	-6.82	AAA-AAA	0.00	999	1.000	1.000	0.0489
225353_s_at	71.2	10.5	-2.77	-6.82	AAA-AAA	0.00	999	1.000	1.000	0.0786
226403_at	44.1	6.5	-2.77	-6.82	AAA-AAA	0.00	999	1.000	1.000	0.0118
239270_at	61.7	9.1	-2.77	-6.82	AAA-AAA	0.00	999	1.000	1.000	0.0919
1554322_a_at	92.0	13.5	-2.77	-6.81	AAA-AAA	0.00	999	1.000	1.000	0.0009
206440_at	25.9	3.8	-2.77	-6.81	AAA-AAA	0.00	999	1.000	1.000	0.0375
238017_at	39.0	5.7	-2.77	-6.80	AAA-AAA	0.00	999	1.000	1.000	0.1499
233424_at	71.2	10.5	-2.77	-6.80	AAA-AAA	0.00	999	1.000	1.000	0.0876
230495_at	26.7	3.9	-2.77	-6.80	AAA-AAA	0.00	999	1.000	1.000	0.0139
243144_at	35.4	5.2	-2.76	-6.79	AAA-AAA	0.00	999	1.000	1.000	0.0087
1566896_at	19.2	2.8	-2.76	-6.79	AAA-AAA	0.00	999	1.000	1.000	0.0080
1570361_a_at	34.4	5.1	-2.76	-6.79	AAA-AAA	0.00	999	1.000	1.000	0.0123
1552745_at	40.7	6.0	-2.76	-6.79	AAA-AAA	0.00	999	1.000	1.000	0.0000
230991_at	54.8	8.1	-2.76	-6.79	AAA-AAA	0.00	999	1.000	1.000	0.0451
241200_x_at	26.0	3.8	-2.76	-6.78	AAA-AAA	0.00	999	1.000	1.000	0.0552
1561479_at	21.4	3.2	-2.76	-6.78	AAA-AAA	0.00	999	1.000	1.000	0.0619
236627_at	34.2	5.0	-2.76	-6.78	AAA-AAA	0.00	999	1.000	1.000	0.0722
237054_at	27.7	4.1	-2.76	-6.77	AAA-AAA	0.00	999	1.000	1.000	0.1094
204548_at	37.0	5.5	-2.76	-6.77	AAA-AAA	0.00	999	1.000	1.000	0.0046
229367_s_at	38.6	5.7	-2.75	-6.74	AAA-AAA	0.00	999	1.000	1.000	0.0371
219759_at	6.1	41.3	2.75	6.74	AAA-AAA	0.00	999	1.000	1.000	0.1052

238216_at	86.2	12.8	-2.75	-6.74	AAA-AAA	0.00	999	1.000	1.000	0.1069
235951_s_at	22.0	3.3	-2.75	-6.73	AAA-AAA	0.00	999	1.000	1.000	0.0108
235099_at	50.7	7.5	-2.75	-6.73	AAA-AAA	0.00	999	1.000	1.000	0.0038
234105_at	53.7	8.0	-2.75	-6.73	AAA-AAA	0.00	999	1.000	1.000	0.1350
234457_at	21.1	3.1	-2.75	-6.72	AAA-AAA	0.00	999	1.000	1.000	0.0125
1554891_at	27.6	4.1	-2.75	-6.71	AAA-AAA	0.00	999	1.000	1.000	0.0344
1568690_a_at	47.3	7.1	-2.75	-6.70	AAA-AAA	0.00	999	1.000	1.000	0.0284
1568286_at	1.3	8.7	2.75	6.71	AAA-AAA	0.00	999	1.000	1.000	0.1575
223483_at	3.7	24.5	2.75	6.70	AAA-AAA	0.00	999	1.000	1.000	0.0445
209696_at	55.7	8.3	-2.74	-6.70	AAA-AAA	0.00	999	1.000	1.000	0.1203
1553673_at	4.6	31.1	2.74	6.70	AAA-AAA	0.00	999	1.000	1.000	0.0039
228016_s_at	31.9	4.8	-2.74	-6.69	AAA-AAA	0.00	999	1.000	1.000	0.0058
233026_s_at	4.0	26.5	2.74	6.69	AAA-AAA	0.00	999	1.000	1.000	0.0042
241764_at	16.9	2.5	-2.74	-6.68	AAA-AAA	0.00	999	1.000	1.000	0.0473
1563805_a_at	47.1	7.1	-2.74	-6.68	AAA-AAA	0.00	999	1.000	1.000	0.0096
235199_at	14.6	2.2	-2.74	-6.68	AAA-AAA	0.00	999	1.000	1.000	0.0908
210364_at	52.2	7.8	-2.74	-6.67	AAA-AAA	0.00	999	1.000	1.000	0.0378
216921_s_at	37.1	5.6	-2.74	-6.67	AAA-AAA	0.00	999	1.000	1.000	0.0177
205577_at	39.4	5.9	-2.74	-6.67	AAA-AAA	0.00	999	1.000	1.000	0.0213
1562301_at	19.5	2.9	-2.74	-6.66	MAA-AAA	0.00	999	1.000	1.000	0.0574
1562306_at	31.9	4.8	-2.74	-6.66	AAA-AAA	0.00	999	1.000	1.000	0.1229
204110_at	49.8	7.5	-2.74	-6.66	AAA-AAA	0.00	999	1.000	1.000	0.1277
1561595_x_at	48.6	7.3	-2.74	-6.66	AAA-AAA	0.00	999	1.000	1.000	0.0769
207192_at	55.5	8.3	-2.74	-6.66	AAA-AAA	0.00	999	1.000	1.000	0.0192
241420_at	18.7	2.8	-2.73	-6.65	AAA-AAA	0.00	999	1.000	1.000	0.0772
1569554_at	49.3	7.4	-2.73	-6.65	AAA-AAA	0.00	999	1.000	1.000	0.0019
235892_at	17.7	2.7	-2.73	-6.65	AAA-AAA	0.00	999	1.000	1.000	0.0939
1557767_at	35.1	5.3	-2.73	-6.64	AAA-AAA	0.00	999	1.000	1.000	0.0257
236871_s_at	20.4	3.1	-2.73	-6.64	AAA-AAA	0.00	999	1.000	1.000	0.0240
1567181_x_at	23.8	3.6	-2.73	-6.62	AAA-AAA	0.00	999	1.000	1.000	0.0342
210772_at	40.5	6.1	-2.73	-6.62	AAA-AAA	0.00	999	1.000	1.000	0.0869
234271_at	7.6	50.0	2.73	6.62	AAA-AAA	0.00	999	1.000	1.000	0.0240
208193_at	20.0	3.0	-2.73	-6.61	AAA-AAA	0.00	999	1.000	1.000	0.0561
241009_at	116.0	17.6	-2.72	-6.59	AAA-AAA	0.00	999	1.000	1.000	0.0786
1555246_a_at	36.9	5.6	-2.72	-6.58	AAA-AAA	0.00	999	1.000	1.000	0.0555
1561361_at	58.7	8.9	-2.72	-6.57	AAA-AAA	0.00	999	1.000	1.000	0.1004
234546_at	52.5	8.0	-2.71	-6.56	AAA-AAA	0.00	999	1.000	1.000	0.0795
240844_at	54.6	8.3	-2.71	-6.55	AMA-AAA	0.00	999	1.000	1.000	0.0042
202885_s_at	93.3	14.3	-2.71	-6.55	AAA-AAA	0.00	999	1.000	1.000	0.0293
240783_at	36.1	5.5	-2.71	-6.54	AAA-AAA	0.00	999	1.000	1.000	0.1325
208065_at	11.1	1.7	-2.71	-6.54	AAA-AAA	0.00	999	1.000	1.000	0.1528
244839_at	18.3	2.8	-2.71	-6.53	AAA-AAA	0.00	999	1.000	1.000	0.0454

1558628_at	43.0	6.6	-2.71	-6.52	AAA-AAA	0.00	999	1.000	1.000	0.0011
223366_at	49.0	7.5	-2.71	-6.52	AAA-AAA	0.00	999	1.000	1.000	0.0111
1568488_at	18.6	2.9	-2.71	-6.52	AAA-AAA	0.00	999	1.000	1.000	0.0423
222098_s_at	30.5	4.7	-2.71	-6.52	AAA-AAA	0.00	999	1.000	1.000	0.0310
242715_at	13.9	2.1	-2.70	-6.52	AAA-AAA	0.00	999	1.000	1.000	0.0312
206765_at	27.7	4.3	-2.70	-6.52	AAA-AAA	0.00	999	1.000	1.000	0.0446
212618_at	39.1	254.8	2.70	6.52	AAA-AAA	0.00	999	1.000	1.000	0.0117
237149_at	24.4	3.7	-2.70	-6.52	AAA-AAA	0.00	999	1.000	1.000	0.0204
1556737_at	66.9	10.3	-2.70	-6.51	AAA-AAA	0.00	999	1.000	1.000	0.0496
221392_at	4.6	30.0	2.70	6.51	AAA-AAA	0.00	999	1.000	1.000	0.0042
206955_at	39.2	6.0	-2.70	-6.51	AAA-AAA	0.00	999	1.000	1.000	0.1048
1560425_s_at	22.9	3.5	-2.70	-6.50	AAA-AAA	0.00	999	1.000	1.000	0.0114
1569760_at	49.1	7.6	-2.70	-6.50	AAA-AAA	0.00	999	1.000	1.000	0.0425
242541_at	44.6	6.9	-2.70	-6.49	AAA-AAA	0.00	999	1.000	1.000	0.0681
1569089_a_at	28.4	4.4	-2.70	-6.50	AAA-AAA	0.00	999	1.000	1.000	0.0008
233949_s_at	37.3	5.7	-2.70	-6.49	AAA-AAA	0.00	999	1.000	1.000	0.0439
222278_at	27.1	4.2	-2.70	-6.49	AAA-AAA	0.00	999	1.000	1.000	0.0596
1553086_at	14.3	2.2	-2.70	-6.48	AAA-AAA	0.00	999	1.000	1.000	0.0034
1562069_at	46.7	7.2	-2.69	-6.47	AMA-AAA	0.00	999	1.000	1.000	0.1341
1563002_at	9.2	1.4	-2.69	-6.47	AAA-AAA	0.00	999	1.000	1.000	0.0407
233238_s_at	3.3	21.2	2.69	6.47	AAA-AAA	0.00	999	1.000	1.000	0.0298
207067_s_at	5.1	33.0	2.69	6.44	AAA-AAA	0.00	999	1.000	1.000	0.1066
244321_at	9.8	62.9	2.69	6.44	AAA-PPA	0.00	999	1.000	1.000	0.1123
242359_at	44.9	7.0	-2.69	-6.44	AAA-AAA	0.00	999	1.000	1.000	0.0168
1559526_at	72.6	11.3	-2.69	-6.43	AMA-AAA	0.00	999	1.000	1.000	0.0703
1561401_at	49.9	7.8	-2.68	-6.43	AAA-AAA	0.00	999	1.000	1.000	0.0602
241663_at	54.6	8.5	-2.68	-6.42	AAA-AAA	0.00	999	1.000	1.000	0.0489
209097_s_at	53.2	8.3	-2.68	-6.42	AAA-AAA	0.00	999	1.000	1.000	0.0100
219144_at	45.7	7.2	-2.68	-6.39	AAA-AAA	0.00	999	1.000	1.000	0.0184
237714_at	28.9	4.5	-2.68	-6.39	AAA-AAA	0.00	999	1.000	1.000	0.0760
206301_at	37.4	5.9	-2.68	-6.39	AAA-AAA	0.00	999	1.000	1.000	0.0344
229170_s_at	4.8	30.6	2.68	6.39	AAA-AAA	0.00	999	1.000	1.000	0.1444
206871_at	25.8	4.0	-2.68	-6.39	AAA-AAA	0.00	999	1.000	1.000	0.0084
1559470_at	11.7	1.8	-2.67	-6.38	AAA-AAA	0.00	999	1.000	1.000	0.1221
1560745_at	22.7	3.6	-2.67	-6.38	AAA-AAA	0.00	999	1.000	1.000	0.0861
1556781_at	18.1	2.8	-2.67	-6.38	AAA-AAA	0.00	999	1.000	1.000	0.0985
235642_at	44.9	7.0	-2.67	-6.37	AAA-AAA	0.00	999	1.000	1.000	0.0920
1562290_at	9.4	1.5	-2.67	-6.36	AAA-AAA	0.00	999	1.000	1.000	0.0617
242703_at	23.4	3.7	-2.67	-6.34	AAA-AAA	0.00	999	1.000	1.000	0.0865
1563053_at	18.5	2.9	-2.67	-6.34	AAA-AAA	0.00	999	1.000	1.000	0.0827
244735_at	35.8	5.7	-2.66	-6.34	AAA-AAA	0.00	999	1.000	1.000	0.0073
1559062_at	37.7	6.0	-2.66	-6.33	AAA-AAA	0.00	999	1.000	1.000	0.0169

238382_x_at	16.0	2.5	-2.66	-6.33	AAA-AAA	0.00	999	1.000	1.000	0.0292
206642_at	15.8	2.5	-2.66	-6.32	AAA-AAA	0.00	999	1.000	1.000	0.0784
220108_at	27.2	4.3	-2.66	-6.32	AAA-AAA	0.00	999	1.000	1.000	0.1077
210271_at	43.3	6.9	-2.66	-6.31	AAA-AAA	0.00	999	1.000	1.000	0.0445
1553526_at	69.3	11.0	-2.66	-6.31	AAA-AAA	0.00	999	1.000	1.000	0.0344
215094_at	11.6	1.8	-2.66	-6.31	AAA-AAA	0.00	999	1.000	1.000	0.1037
239387_at	4.4	27.8	2.65	6.29	AAA-AAA	0.00	999	1.000	1.000	0.0032
211361_s_at	11.4	1.8	-2.65	-6.28	AAA-AAA	0.00	999	1.000	1.000	0.0395
242254_at	25.7	4.1	-2.65	-6.27	AAA-AAA	0.00	999	1.000	1.000	0.1103
244463_at	3.7	23.2	2.65	6.27	AAA-AAA	0.00	999	1.000	1.000	0.0082
224040_at	68.1	10.9	-2.65	-6.27	AAA-AAA	0.00	999	1.000	1.000	0.0689
216027_at	5.1	32.2	2.65	6.27	AAA-AAA	0.00	999	1.000	1.000	0.0485
234085_at	28.3	4.5	-2.65	-6.26	AAA-AAA	0.00	999	1.000	1.000	0.0991
230422_at	20.4	3.3	-2.64	-6.24	AAA-AAA	0.00	999	1.000	1.000	0.1063
228934_x_at	4.4	27.4	2.64	6.24	AAA-AAA	0.00	999	1.000	1.000	0.0170
1563299_at	14.9	2.4	-2.64	-6.23	AAA-AAA	0.00	999	1.000	1.000	0.0298
1559520_at	14.8	2.4	-2.64	-6.24	AAA-AAA	0.00	999	1.000	1.000	0.1536
207862_at	31.8	5.1	-2.64	-6.24	AAA-AAA	0.00	999	1.000	1.000	0.0704
239062_at	26.3	4.2	-2.64	-6.23	MAA-AAA	0.00	999	1.000	1.000	0.1338
221278_at	37.2	6.0	-2.64	-6.23	AAA-AAA	0.00	999	1.000	1.000	0.0415
208594_x_at	34.8	5.6	-2.64	-6.23	AAA-AAA	0.00	999	1.000	1.000	0.0051
242525_at	63.6	10.2	-2.64	-6.22	AAA-AAA	0.00	999	1.000	1.000	0.0027
1553889_at	46.1	7.4	-2.63	-6.19	AAA-AAA	0.00	999	1.000	1.000	0.0218
224426_s_at	20.4	3.3	-2.63	-6.19	AAA-AAA	0.00	999	1.000	1.000	0.0881
233629_at	14.2	2.3	-2.63	-6.19	AAA-AAA	0.00	999	1.000	1.000	0.0533
220394_at	32.8	5.3	-2.63	-6.18	AAA-AAA	0.00	999	1.000	1.000	0.0126
205620_at	40.7	6.6	-2.63	-6.18	AAA-AAA	0.00	999	1.000	1.000	0.0068
1560528_at	10.5	1.7	-2.63	-6.17	AAA-AAA	0.00	999	1.000	1.000	0.1037
224258_at	43.7	7.1	-2.63	-6.17	AAA-AAA	0.00	999	1.000	1.000	0.0902
240525_at	36.7	6.0	-2.62	-6.17	AAA-AAA	0.00	999	1.000	1.000	0.0437
238695_s_at	1.9	11.4	2.62	6.16	AAA-AAA	0.00	999	1.000	1.000	0.0585
207236_at	3.4	20.9	2.62	6.15	AAA-AAA	0.00	999	1.000	1.000	0.0161
210100_s_at	60.6	9.9	-2.62	-6.15	AAA-AAA	0.00	999	1.000	1.000	0.0197
1559092_at	5.0	30.6	2.62	6.14	AAA-AAA	0.00	999	1.000	1.000	0.0412
233915_at	29.5	4.8	-2.62	-6.14	AAA-AAA	0.00	999	1.000	1.000	0.1099
234898_at	63.2	10.3	-2.62	-6.14	AAA-AAA	0.00	999	1.000	1.000	0.0481
1553285_s_at	6.0	36.9	2.62	6.13	AAA-AAA	0.00	999	1.000	1.000	0.1091
206224_at	37.6	6.1	-2.62	-6.13	AAA-AAA	0.00	999	1.000	1.000	0.0010
216603_at	32.5	5.3	-2.62	-6.13	AAA-AAA	0.00	999	1.000	1.000	0.0928
243842_at	59.4	9.7	-2.61	-6.12	AAA-AAA	0.00	999	1.000	1.000	0.0618
205150_s_at	28.1	4.6	-2.61	-6.12	AAA-AAA	0.00	999	1.000	1.000	0.0344
1553059_at	30.0	4.9	-2.61	-6.12	AAA-AAA	0.00	999	1.000	1.000	0.0117

1560161_at	39.1	6.4	-2.61	-6.11	AAA-AAA	0.00	999	1.000	1.000	0.0476
233441_at	21.2	3.5	-2.61	-6.11	AAA-AAA	0.00	999	1.000	1.000	0.0055
209720_s_at	12.8	2.1	-2.61	-6.10	AAA-AAA	0.00	999	1.000	1.000	0.1078
235841_at	32.4	5.3	-2.61	-6.10	AAA-AAA	0.00	999	1.000	1.000	0.0873
216454_at	14.9	2.4	-2.61	-6.10	AAA-AAA	0.00	999	1.000	1.000	0.1167
1570099_at	12.5	2.1	-2.61	-6.09	AAA-AAA	0.00	999	1.000	1.000	0.0332
1557843_at	2.3	13.8	2.61	6.09	AAA-AAA	0.00	999	1.000	1.000	0.0026
216669_at	6.2	37.9	2.61	6.09	AAA-AAA	0.00	999	1.000	1.000	0.0041
216912_at	49.9	8.2	-2.61	-6.09	AAA-AAA	0.00	999	1.000	1.000	0.0422
205559_s_at	27.0	4.4	-2.61	-6.09	AAA-AAA	0.00	999	1.000	1.000	0.1501
231470_at	30.6	5.0	-2.61	-6.09	AAA-AAA	0.00	999	1.000	1.000	0.2155
240668_s_at	12.6	2.1	-2.60	-6.08	AAA-AAA	0.00	999	1.000	1.000	0.0462
1562791_at	14.5	2.4	-2.60	-6.08	AAA-AAA	0.00	999	1.000	1.000	0.0677
209914_s_at	49.8	8.2	-2.60	-6.07	AAA-AAA	0.00	999	1.000	1.000	0.0104
233858_at	24.7	4.1	-2.60	-6.07	AAA-AAA	0.00	999	1.000	1.000	0.1572
218510_x_at	44.5	7.3	-2.60	-6.06	AAA-AAA	0.00	999	1.000	1.000	0.0686
1569459_a_at	15.9	2.6	-2.60	-6.06	AAA-AAA	0.00	999	1.000	1.000	0.1425
1564359_a_at	30.4	5.0	-2.60	-6.06	AAA-AAA	0.00	999	1.000	1.000	0.0341
1558571_at	20.8	3.4	-2.60	-6.06	AAA-AAA	0.00	999	1.000	1.000	0.0102
1556945_a_at	30.9	5.1	-2.60	-6.05	AAA-AAA	0.00	999	1.000	1.000	0.1360
1559986_at	18.7	3.1	-2.60	-6.05	AAA-AAA	0.00	999	1.000	1.000	0.2232
1569646_a_at	34.7	5.7	-2.60	-6.05	AAA-AAA	0.00	999	1.000	1.000	0.0719
232717_at	38.4	6.4	-2.59	-6.04	AAA-AAA	0.00	999	1.000	1.000	0.0609
241130_at	4.6	27.5	2.59	6.03	AAA-AAA	0.00	999	1.000	1.000	0.0669
234076_at	43.8	7.3	-2.59	-6.03	AAA-AAA	0.00	999	1.000	1.000	0.0753
1562865_at	52.6	8.7	-2.59	-6.02	AAA-AAA	0.00	999	1.000	1.000	0.0345
1564813_at	12.7	2.1	-2.59	-6.02	AAA-AAA	0.00	999	1.000	1.000	0.0290
230830_at	20.8	3.5	-2.59	-6.02	AAA-AAA	0.00	999	1.000	1.000	0.1013
1561224_at	29.8	5.0	-2.59	-6.01	AAA-AAA	0.00	999	1.000	1.000	0.0012
1554071_at	2.5	14.9	2.59	6.01	AAA-AAA	0.00	999	1.000	1.000	0.0099
234035_at	39.5	6.6	-2.59	-6.01	AMA-AAA	0.00	999	1.000	1.000	0.0741
1569921_at	51.9	8.6	-2.59	-6.00	AAA-AAA	0.00	999	1.000	1.000	0.0983
206726_at	53.7	8.9	-2.59	-6.00	AAA-AAA	0.00	999	1.000	1.000	0.1764
216053_x_at	6.7	40.2	2.59	6.00	AAA-AAA	0.00	999	1.000	1.000	0.0372
207885_at	15.9	2.7	-2.58	-6.00	AAA-AAA	0.00	999	1.000	1.000	0.0250
234515_at	37.4	6.2	-2.58	-5.99	AAA-AAA	0.00	999	1.000	1.000	0.0069
205728_at	2.9	17.4	2.58	5.99	AAA-AAA	0.00	999	1.000	1.000	0.0158
240715_at	20.5	3.4	-2.58	-5.98	AAA-AAA	0.00	999	1.000	1.000	0.0505
1560618_at	88.2	14.7	-2.58	-5.98	AAA-AAA	0.00	999	1.000	1.000	0.0097
1559545_at	11.0	1.8	-2.58	-5.98	AAA-AAA	0.00	999	1.000	1.000	0.1250
203934_at	35.0	5.8	-2.58	-5.99	AAA-AAA	0.00	999	1.000	1.000	0.0611
206365_at	36.7	6.1	-2.58	-5.98	AAA-AAA	0.00	999	1.000	1.000	0.0740

1562871_at	61.9	10.4	-2.58	-5.98	AAA-AAA	0.00	999	1.000	1.000	0.0377
208427_s_at	16.3	2.7	-2.58	-5.98	AAA-AAA	0.00	999	1.000	1.000	0.1413
1559285_at	5.3	31.5	2.58	5.97	AAA-AAA	0.00	999	1.000	1.000	0.0208
227834_at	27.0	4.5	-2.58	-5.98	AAA-AAA	0.00	999	1.000	1.000	0.1081
1562793_at	35.1	5.9	-2.58	-5.97	AAA-AAA	0.00	999	1.000	1.000	0.0145
213307_at	48.7	8.2	-2.58	-5.97	AAA-AAA	0.00	999	1.000	1.000	0.1650
1559956_at	52.7	8.8	-2.58	-5.96	AAA-AAA	0.00	999	1.000	1.000	0.0303
238966_at	21.3	3.6	-2.58	-5.96	AAA-AAA	0.00	999	1.000	1.000	0.0977
1555179_at	4.8	28.7	2.58	5.96	AAA-AAA	0.00	999	1.000	1.000	0.0271
228712_at	43.3	7.3	-2.58	-5.96	AAA-AAA	0.00	999	1.000	1.000	0.0342
210016_at	47.2	7.9	-2.57	-5.95	AAA-AAA	0.00	999	1.000	1.000	0.0682
238441_at	9.6	1.6	-2.57	-5.95	AAA-AAA	0.00	999	1.000	1.000	0.1419
1556805_at	23.5	4.0	-2.57	-5.95	AAA-AAA	0.00	999	1.000	1.000	0.0783
213609_s_at	55.2	9.3	-2.57	-5.95	AAA-AAA	0.00	999	1.000	1.000	0.0518
239497_at	92.2	15.5	-2.57	-5.94	AAA-AAA	0.00	999	1.000	1.000	0.1050
237937_x_at	7.9	1.3	-2.57	-5.94	AAA-AAA	0.00	999	1.000	1.000	0.0544
237651_x_at	11.3	1.9	-2.57	-5.94	AAA-AAA	0.00	999	1.000	1.000	0.0496
244706_at	28.2	4.8	-2.57	-5.94	AAA-AAA	0.00	999	1.000	1.000	0.0961
1556096_s_at	2.7	16.1	2.57	5.93	AAA-AAA	0.00	999	1.000	1.000	0.1618
232542_at	65.4	11.0	-2.57	-5.94	AAA-AAA	0.00	999	1.000	1.000	0.0145
242519_at	9.9	1.7	-2.57	-5.93	AAA-AAA	0.00	999	1.000	1.000	0.0487
233958_at	36.6	6.2	-2.57	-5.93	AAA-AAA	0.00	999	1.000	1.000	0.0074
1556381_at	19.2	3.2	-2.57	-5.93	AAA-AAA	0.00	999	1.000	1.000	0.0962
207454_at	21.9	3.7	-2.56	-5.90	AAA-AAA	0.00	999	1.000	1.000	0.0244
215386_at	47.1	8.0	-2.56	-5.90	AAA-AAA	0.00	999	1.000	1.000	0.0669
241880_x_at	15.3	2.6	-2.56	-5.90	AAA-AAA	0.00	999	1.000	1.000	0.1619
238400_at	50.9	8.6	-2.56	-5.90	AAA-AAA	0.00	999	1.000	1.000	0.0223
1556607_at	5.5	32.6	2.56	5.90	AAA-AAA	0.00	999	1.000	1.000	0.0000
1561432_at	12.9	2.2	-2.56	-5.88	AAA-AAA	0.00	999	1.000	1.000	0.0575
1555257_a_at	4.4	26.0	2.56	5.88	AAA-AAA	0.00	999	1.000	1.000	0.0871
1557012_a_at	3.5	20.6	2.56	5.89	AAA-AAA	0.00	999	1.000	1.000	0.1635
214399_s_at	27.7	4.7	-2.56	-5.88	AAA-AAA	0.00	999	1.000	1.000	0.1121
216729_at	29.0	4.9	-2.56	-5.89	AAA-AAA	0.00	999	1.000	1.000	0.0065
231649_at	73.7	12.5	-2.56	-5.88	AAA-AAA	0.00	999	1.000	1.000	0.0809
1566046_at	13.2	2.3	-2.56	-5.88	AAA-AAA	0.00	999	1.000	1.000	0.0916
1570639_at	5.7	33.6	2.55	5.87	AAA-AAA	0.00	999	1.000	1.000	0.0236
1565900_at	16.2	2.8	-2.55	-5.87	AAA-AAA	0.00	999	1.000	1.000	0.1608
210721_s_at	2.3	13.2	2.55	5.87	AAA-AAA	0.00	999	1.000	1.000	0.0129
209400_at	57.5	9.8	-2.55	-5.86	AAA-AAA	0.00	999	1.000	1.000	0.0038
234501_x_at	9.2	1.6	-2.55	-5.86	AAA-AAA	0.00	999	1.000	1.000	0.0562
1561094_a_at	3.4	19.8	2.55	5.86	AAA-APA	0.00	999	1.000	1.000	0.1077
1561700_at	31.2	5.3	-2.55	-5.86	AAA-AAA	0.00	999	1.000	1.000	0.1468

220269_at	2.3	13.4	2.55	5.86	AAA-AAA	0.00	999	1.000	1.000	0.0408
204116_at	95.0	16.2	-2.55	-5.86	AAA-AAA	0.00	999	1.000	1.000	0.1227
210643_at	9.8	1.7	-2.55	-5.85	AAA-AAA	0.00	999	1.000	1.000	0.0115
243860_at	43.2	7.4	-2.55	-5.85	AAA-AAA	0.00	999	1.000	1.000	0.0693
237450_at	7.3	42.4	2.55	5.85	AAA-AAA	0.00	999	1.000	1.000	0.0013
230841_at	31.1	5.3	-2.55	-5.85	AAA-AAA	0.00	999	1.000	1.000	0.0087
231726_at	4.9	28.5	2.55	5.85	AAA-AAA	0.00	999	1.000	1.000	0.0294
220540_at	15.9	2.7	-2.55	-5.85	AAA-AAA	0.00	999	1.000	1.000	0.0447
1558160_at	27.2	4.7	-2.55	-5.84	AAA-AAA	0.00	999	1.000	1.000	0.0066
214414_x_at	58.6	10.0	-2.55	-5.84	AAA-AAA	0.00	999	1.000	1.000	0.0229
205860_x_at	16.4	2.8	-2.55	-5.84	AAA-AAA	0.00	999	1.000	1.000	0.0468
1552573_s_at	7.9	45.8	2.54	5.83	AAA-AAA	0.00	999	1.000	1.000	0.0930
1556456_at	40.0	6.9	-2.54	-5.82	AAA-AAA	0.00	999	1.000	1.000	0.1418
220290_at	125.0	21.5	-2.54	-5.83	AAA-AAA	0.00	999	1.000	1.000	0.0068
237657_at	19.6	3.4	-2.54	-5.82	AAA-AAA	0.00	999	1.000	1.000	0.0217
242226_at	54.1	9.3	-2.54	-5.81	AAA-AAA	0.00	999	1.000	1.000	0.0079
1569719_at	13.0	2.2	-2.54	-5.80	AAA-AAA	0.00	999	1.000	1.000	0.0995
1560429_at	30.0	5.2	-2.54	-5.79	AAA-AAA	0.00	999	1.000	1.000	0.0312
1569823_at	20.0	3.5	-2.54	-5.80	AAA-AAA	0.00	999	1.000	1.000	0.1772
1564139_at	24.6	4.2	-2.54	-5.79	AAA-AAA	0.00	999	1.000	1.000	0.1238
237893_at	41.5	7.2	-2.54	-5.80	AAA-AAA	0.00	999	1.000	1.000	0.0370
241012_at	51.7	8.9	-2.53	-5.79	AAA-MAA	0.00	999	1.000	1.000	0.1508
236734_at	15.9	2.8	-2.53	-5.79	AAA-AAA	0.00	999	1.000	1.000	0.0871
232547_at	26.3	4.6	-2.53	-5.79	AAA-AAA	0.00	999	1.000	1.000	0.0082
228792_at	7.9	45.9	2.53	5.78	AAA-APP	0.00	999	1.000	1.000	0.1939
241419_at	113.8	19.7	-2.53	-5.78	AAA-AAA	0.00	999	1.000	1.000	0.0053
1562145_at	56.7	9.8	-2.53	-5.78	AAA-AAA	0.00	999	1.000	1.000	0.0220
1558436_a_at	51.4	8.9	-2.53	-5.78	AAA-AAA	0.00	999	1.000	1.000	0.0089
240852_at	6.1	35.1	2.53	5.78	AAA-AAA	0.00	999	1.000	1.000	0.0643
1568826_at	32.2	5.6	-2.53	-5.78	AAA-MAA	0.00	999	1.000	1.000	0.2102
1552849_at	12.8	2.2	-2.53	-5.77	AAA-AAA	0.00	999	1.000	1.000	0.0372
1561215_at	15.2	2.6	-2.53	-5.77	AAA-AAA	0.00	999	1.000	1.000	0.1552
220045_at	44.2	7.7	-2.53	-5.77	AAA-AAA	0.00	999	1.000	1.000	0.0391
224278_at	3.6	21.0	2.53	5.77	AAA-AAA	0.00	999	1.000	1.000	0.0034
237686_at	79.9	13.9	-2.53	-5.77	AAA-AAA	0.00	999	1.000	1.000	0.0129
231037_at	55.0	9.5	-2.53	-5.77	AAA-AAA	0.00	999	1.000	1.000	0.0734
1561959_x_at	10.4	1.8	-2.53	-5.76	AAA-AAA	0.00	999	1.000	1.000	0.0214
214887_at	12.9	2.2	-2.53	-5.76	AAA-AAA	0.00	999	1.000	1.000	0.0788
234025_at	52.2	9.1	-2.53	-5.76	AAA-AAA	0.00	999	1.000	1.000	0.1900
242915_at	11.8	67.7	2.53	5.76	AAA-PPP	0.00	999	1.000	1.000	0.0768
240362_at	4.8	27.4	2.53	5.75	AAA-AAA	0.00	999	1.000	1.000	0.0131
1569818_at	33.5	5.8	-2.53	-5.76	AAA-AAA	0.00	999	1.000	1.000	0.1686

211652_s_at	58.9	10.2	-2.53	-5.75	AAA-AAA	0.00	999	1.000	1.000	0.0239
220657_at	44.6	7.8	-2.52	-5.74	AAA-AAA	0.00	999	1.000	1.000	0.0919
213719_s_at	50.1	8.7	-2.52	-5.74	AAA-AAA	0.00	999	1.000	1.000	0.1340
209555_s_at	2.1	12.2	2.52	5.74	AAA-AAA	0.00	999	1.000	1.000	0.0168
216285_at	40.2	7.0	-2.52	-5.74	AAA-AAA	0.00	999	1.000	1.000	0.0591
234323_at	34.1	6.0	-2.52	-5.74	AAA-AAA	0.00	999	1.000	1.000	0.0835
1556366_s_at	7.8	1.4	-2.52	-5.73	AAA-AAA	0.00	999	1.000	1.000	0.1153
216748_at	65.5	11.4	-2.52	-5.73	AMA-AAA	0.00	999	1.000	1.000	0.0061
1559586_at	27.8	4.9	-2.52	-5.73	AAA-AAA	0.00	999	1.000	1.000	0.1825
240137_at	12.1	2.1	-2.51	-5.71	AAA-AAA	0.00	999	1.000	1.000	0.2384
234150_at	35.3	6.2	-2.51	-5.71	AAA-AAA	0.00	999	1.000	1.000	0.1547
220043_s_at	11.3	64.6	2.51	5.71	AAA-AAA	0.00	999	1.000	1.000	0.0031
237417_at	27.9	4.9	-2.51	-5.71	AAA-AAA	0.00	999	1.000	1.000	0.0519
227863_at	42.2	7.4	-2.51	-5.70	AAA-AAA	0.00	999	1.000	1.000	0.1229
1570117_at	19.0	3.3	-2.51	-5.69	AAA-AAA	0.00	999	1.000	1.000	0.0451
207385_at	22.8	4.0	-2.51	-5.69	AAA-AAA	0.00	999	1.000	1.000	0.1660
1561204_at	40.1	7.1	-2.51	-5.68	AAA-AAA	0.00	999	1.000	1.000	0.0771
1555513_at	8.4	1.5	-2.51	-5.69	AAA-AAA	0.00	999	1.000	1.000	0.0424
240299_at	17.5	3.1	-2.51	-5.68	AAA-AAA	0.00	999	1.000	1.000	0.1243
223906_s_at	20.9	3.7	-2.51	-5.68	AAA-AAA	0.00	999	1.000	1.000	0.0031
228071_at	30.2	5.3	-2.51	-5.68	AAA-AAA	0.00	999	1.000	1.000	0.0409
232735_at	75.2	13.3	-2.50	-5.67	AAA-AAA	0.00	999	1.000	1.000	0.0366
1558668_s_at	2.6	14.6	2.50	5.66	AAA-APA	0.00	999	1.000	1.000	0.0776
243810_at	32.9	5.8	-2.50	-5.66	AAA-AAA	0.00	999	1.000	1.000	0.0248
229183_at	32.9	5.8	-2.50	-5.66	AAA-AAA	0.00	999	1.000	1.000	0.1520
205478_at	19.6	3.5	-2.50	-5.67	AAA-AAA	0.00	999	1.000	1.000	0.0832
229030_at	48.3	8.5	-2.50	-5.66	AAA-AAA	0.00	999	1.000	1.000	0.0115
1558623_at	20.9	3.7	-2.50	-5.65	AAA-AAA	0.00	999	1.000	1.000	0.0908
237987_x_at	2.0	11.5	2.50	5.65	AAA-PAA	0.00	999	1.000	1.000	0.1877
1562587_at	13.6	2.4	-2.50	-5.65	AAA-AAA	0.00	999	1.000	1.000	0.0580
219414_at	31.1	5.5	-2.50	-5.65	AAA-AAA	0.00	999	1.000	1.000	0.1980
239299_at	30.0	5.3	-2.50	-5.65	AAA-AAA	0.00	999	1.000	1.000	0.1096
1564152_at	14.4	2.6	-2.50	-5.64	AAA-AAA	0.00	999	1.000	1.000	0.0461
223399_x_at	79.5	14.1	-2.50	-5.65	AAA-AAA	0.00	999	1.000	1.000	0.0281
239157_at	42.5	7.5	-2.50	-5.65	AAA-AAA	0.00	999	1.000	1.000	0.0342
1559239_s_at	26.7	4.7	-2.50	-5.64	AAA-AAA	0.00	999	1.000	1.000	0.0552
231216_at	28.1	5.0	-2.49	-5.63	AAA-AAA	0.00	999	1.000	1.000	0.1349
240356_s_at	9.8	1.7	-2.49	-5.63	AAA-AAA	0.00	999	1.000	1.000	0.1202
234492_at	37.1	6.6	-2.49	-5.62	AAA-AAA	0.00	999	1.000	1.000	0.0734
230597_at	72.9	13.0	-2.49	-5.63	AAA-AAA	0.00	999	1.000	1.000	0.0678
230587_at	26.6	4.7	-2.49	-5.62	AAA-AAA	0.00	999	1.000	1.000	0.0908
243251_at	16.8	3.0	-2.49	-5.62	AAA-AAA	0.00	999	1.000	1.000	0.1458

1561587_at	5.6	31.4	2.49	5.62	AAA-AAA	0.00	999	1.000	1.000	0.0130
1553425_at	33.2	5.9	-2.49	-5.62	AAA-AAA	0.00	999	1.000	1.000	0.0125
211613_s_at	7.2	40.2	2.49	5.62	AAA-AAA	0.00	999	1.000	1.000	0.0101
204199_at	38.2	6.8	-2.49	-5.61	AAA-AAA	0.00	999	1.000	1.000	0.0357
239460_at	10.4	1.9	-2.49	-5.61	AAA-AAA	0.00	999	1.000	1.000	0.1375
231040_at	15.7	2.8	-2.49	-5.61	AAA-AAA	0.00	999	1.000	1.000	0.1512
243192_at	35.3	6.3	-2.49	-5.61	AAA-AAA	0.00	999	1.000	1.000	0.2845
240623_at	58.5	10.4	-2.49	-5.61	AAA-AAA	0.00	999	1.000	1.000	0.0059
1553341_at	42.4	7.6	-2.49	-5.61	AAA-AAA	0.00	999	1.000	1.000	0.1386
233393_at	3.1	17.2	2.49	5.61	AAA-AAA	0.00	999	1.000	1.000	0.0343
1557274_at	70.7	12.6	-2.49	-5.61	AAA-AAA	0.00	999	1.000	1.000	0.0242
231605_at	55.2	9.8	-2.49	-5.61	AAA-AAA	0.00	999	1.000	1.000	0.0198
220844_at	53.4	9.5	-2.49	-5.60	AAA-AAA	0.00	999	1.000	1.000	0.0321
1562780_at	10.6	1.9	-2.49	-5.60	AAA-AAA	0.00	999	1.000	1.000	0.0714
231388_at	61.0	10.9	-2.49	-5.60	AAA-AAA	0.00	999	1.000	1.000	0.1343
216757_at	84.0	15.0	-2.48	-5.59	AAA-AAA	0.00	999	1.000	1.000	0.0516
236761_at	27.4	4.9	-2.48	-5.59	AAA-AAA	0.00	999	1.000	1.000	0.0747
233953_at	6.9	1.2	-2.48	-5.59	AAA-AAA	0.00	999	1.000	1.000	0.1581
233011_at	4.2	23.7	2.48	5.57	AAA-AAA	0.00	999	1.000	1.000	0.0102
1563385_at	20.1	3.6	-2.48	-5.57	AAA-AAA	0.00	999	1.000	1.000	0.0696
215465_at	30.2	5.4	-2.48	-5.57	AAA-AAA	0.00	999	1.000	1.000	0.1176
207902_at	53.6	9.6	-2.48	-5.56	AAA-AAA	0.00	999	1.000	1.000	0.0583
237900_at	50.0	9.0	-2.48	-5.57	AAA-AAA	0.00	999	1.000	1.000	0.0766
228134_at	40.6	7.3	-2.48	-5.56	AAA-AAA	0.00	999	1.000	1.000	0.0224
243209_at	53.8	9.7	-2.48	-5.56	AAA-AAA	0.00	999	1.000	1.000	0.0113
232110_at	45.4	8.2	-2.48	-5.56	AAA-AAA	0.00	999	1.000	1.000	0.0090
1556496_a_at	20.8	3.8	-2.47	-5.55	AAA-AAA	0.00	999	1.000	1.000	0.1530
224236_s_at	14.2	2.6	-2.47	-5.55	AAA-AAA	0.00	999	1.000	1.000	0.0119
1555745_a_at	5.3	29.6	2.47	5.55	AAA-AAA	0.00	999	1.000	1.000	0.0135
241112_at	53.9	9.7	-2.47	-5.54	AAA-AAA	0.00	999	1.000	1.000	0.0235
232114_at	9.7	53.8	2.47	5.54	AAA-AMA	0.00	999	1.000	1.000	0.0360
208131_s_at	49.0	8.9	-2.47	-5.54	AAA-AAA	0.00	999	1.000	1.000	0.1457
211336_x_at	38.7	7.0	-2.47	-5.54	AAA-AAA	0.00	999	1.000	1.000	0.0686
204755_x_at	9.0	1.6	-2.47	-5.54	AAA-AAA	0.00	999	1.000	1.000	0.1593
220293_at	47.2	8.5	-2.47	-5.53	AAA-AAA	0.00	999	1.000	1.000	0.0493
234126_at	3.3	18.4	2.47	5.53	AAA-AAA	0.00	999	1.000	1.000	0.0462
211627_x_at	3.0	16.6	2.47	5.53	AAA-AAA	0.00	999	1.000	1.000	0.1748
241176_at	26.6	4.8	-2.47	-5.52	AAA-AAA	0.00	999	1.000	1.000	0.1143
234212_at	8.9	1.6	-2.47	-5.52	AAA-AAA	0.00	999	1.000	1.000	0.0360
206282_at	20.9	3.8	-2.47	-5.52	AAA-AAA	0.00	999	1.000	1.000	0.1089
208207_at	42.5	7.7	-2.46	-5.52	AAA-AAA	0.00	999	1.000	1.000	0.0686
215929_at	10.8	2.0	-2.46	-5.51	AAA-AAA	0.00	999	1.000	1.000	0.0614

211633_x_at	39.5	7.2	-2.46	-5.50	AAA-AAA	0.00	999	1.000	1.000	0.0696
1561872_at	18.7	3.4	-2.46	-5.50	AAA-AAA	0.00	999	1.000	1.000	0.0567
1559688_at	14.9	2.7	-2.46	-5.50	AAA-AAA	0.00	999	1.000	1.000	0.1794
232721_at	35.4	6.4	-2.46	-5.50	AAA-AAA	0.00	999	1.000	1.000	0.1728
242036_x_at	20.2	3.7	-2.46	-5.49	AAA-AAA	0.00	999	1.000	1.000	0.1787
1560978_at	78.9	14.4	-2.46	-5.49	AAA-AAA	0.00	999	1.000	1.000	0.0477
1561218_s_at	47.8	8.7	-2.45	-5.48	AAA-AAA	0.00	999	1.000	1.000	0.0356
1559571_a_at	30.0	5.5	-2.45	-5.48	AAA-AAA	0.00	999	1.000	1.000	0.1097
231460_at	23.7	4.3	-2.45	-5.48	AAA-AAA	0.00	999	1.000	1.000	0.0261
206835_at	11.5	2.1	-2.45	-5.47	AAA-AAA	0.00	999	1.000	1.000	0.0755
223813_at	27.0	4.9	-2.45	-5.47	AAA-AAA	0.00	999	1.000	1.000	0.0329
1565714_at	106.8	19.6	-2.45	-5.46	AAA-AAA	0.00	999	1.000	1.000	0.0057
226841_at	79.2	14.5	-2.45	-5.46	AAA-AAA	0.00	999	1.000	1.000	0.0219
210872_x_at	96.5	17.7	-2.45	-5.46	AAA-AAA	0.00	999	1.000	1.000	0.0409
1570026_at	47.6	8.7	-2.45	-5.46	AAA-AAA	0.00	999	1.000	1.000	0.0724
244412_at	16.9	3.1	-2.45	-5.46	AAA-AAA	0.00	999	1.000	1.000	0.0202
1557604_at	8.3	1.5	-2.45	-5.46	AAA-AAA	0.00	999	1.000	1.000	0.0056
1553423_a_at	1.1	6.1	2.45	5.45	AAA-AAA	0.00	999	1.000	1.000	0.0074
1559309_at	57.0	10.5	-2.45	-5.46	AAA-AAA	0.00	999	1.000	1.000	0.0094
1553882_at	33.6	6.2	-2.45	-5.45	AAA-AAA	0.00	999	1.000	1.000	0.1863
1556190_s_at	49.5	9.1	-2.45	-5.45	AAA-AAA	0.00	999	1.000	1.000	0.0925
243679_at	38.6	7.1	-2.44	-5.44	AAA-AAA	0.00	999	1.000	1.000	0.0921
1562586_at	44.9	8.3	-2.44	-5.44	AAA-AAA	0.00	999	1.000	1.000	0.0056
1556623_at	3.4	18.2	2.44	5.44	AAA-AAA	0.00	999	1.000	1.000	0.0064
1563083_s_at	10.3	1.9	-2.44	-5.44	AAA-AAA	0.00	999	1.000	1.000	0.0658
1569620_s_at	40.1	7.4	-2.44	-5.44	AAA-AAA	0.00	999	1.000	1.000	0.0425
1558971_at	13.0	2.4	-2.44	-5.44	AAA-AAA	0.00	999	1.000	1.000	0.0443
215885_at	23.1	4.3	-2.44	-5.44	AAA-AAA	0.00	999	1.000	1.000	0.0162
1557807_a_at	36.0	6.6	-2.44	-5.44	AAA-AAA	0.00	999	1.000	1.000	0.1811
1561191_at	30.7	5.7	-2.44	-5.43	MAA-AAA	0.00	999	1.000	1.000	0.1978
239449_at	35.3	6.5	-2.44	-5.42	AAA-AAA	0.00	999	1.000	1.000	0.1820
240059_at	38.6	7.1	-2.44	-5.42	AAA-AAA	0.00	999	1.000	1.000	0.1356
1557566_at	15.2	2.8	-2.44	-5.42	AAA-AAA	0.00	999	1.000	1.000	0.1885
1567379_at	7.5	1.4	-2.44	-5.41	AAA-AAA	0.00	999	1.000	1.000	0.1071
207310_s_at	60.6	11.2	-2.44	-5.42	AAA-AAA	0.00	999	1.000	1.000	0.0189
215494_at	26.0	4.8	-2.44	-5.42	AAA-AAA	0.00	999	1.000	1.000	0.0325
1554354_at	19.7	3.6	-2.44	-5.41	AAA-AAA	0.00	999	1.000	1.000	0.2346
213963_s_at	16.1	87.2	2.44	5.41	AAA-AAM	0.00	999	1.000	1.000	0.0643
206280_at	34.9	6.5	-2.44	-5.41	AAA-AAA	0.00	999	1.000	1.000	0.1386
228547_at	3.8	20.6	2.44	5.41	AAA-APA	0.00	999	1.000	1.000	0.1908
206624_at	6.2	1.1	-2.44	-5.41	AAA-AAA	0.00	999	1.000	1.000	0.1301
204121_at	66.8	12.4	-2.44	-5.41	AAA-AAA	0.00	999	1.000	1.000	0.0647

238266_at	61.1	11.3	-2.43	-5.40	AAA-AAA	0.00	999	1.000	1.000	0.0411
237779_at	179.2	33.2	-2.43	-5.40	AAA-AAA	0.00	999	1.000	1.000	0.0752
237742_at	6.7	36.0	2.43	5.40	AAA-AAA	0.00	999	1.000	1.000	0.0515
233711_at	7.8	1.4	-2.43	-5.41	AAA-AAA	0.00	999	1.000	1.000	0.1376
243638_at	116.0	21.5	-2.43	-5.40	AAA-AAA	0.00	999	1.000	1.000	0.0535
1553424_at	23.4	4.3	-2.43	-5.40	AAA-AAA	0.00	999	1.000	1.000	0.0160
241078_at	3.7	19.8	2.43	5.40	AAA-AAA	0.00	999	1.000	1.000	0.0182
1563691_at	55.3	10.3	-2.43	-5.40	AAA-AAA	0.00	999	1.000	1.000	0.1153
213783_at	51.5	9.6	-2.43	-5.39	AAA-AAA	0.00	999	1.000	1.000	0.0090
234727_at	22.0	4.1	-2.43	-5.39	MAA-AAA	0.00	999	1.000	1.000	0.2236
242322_at	20.5	3.8	-2.43	-5.38	AAA-AAA	0.00	999	1.000	1.000	0.1522
1555356_a_at	15.3	2.8	-2.43	-5.39	AAA-AAA	0.00	999	1.000	1.000	0.2013
1554571_at	30.5	5.7	-2.43	-5.39	AAA-AAA	0.00	999	1.000	1.000	0.0756
224136_at	57.4	10.7	-2.43	-5.39	AAA-AAA	0.00	999	1.000	1.000	0.0087
1556624_at	17.7	3.3	-2.43	-5.38	AAA-AAA	0.00	999	1.000	1.000	0.0282
209309_at	21.3	4.0	-2.43	-5.38	AAA-AAA	0.00	999	1.000	1.000	0.0793
1552514_at	21.0	3.9	-2.43	-5.38	AAA-AAA	0.00	999	1.000	1.000	0.0729
203914_x_at	15.0	2.8	-2.43	-5.38	AAA-AAA	0.00	999	1.000	1.000	0.1659
234554_at	62.8	11.7	-2.43	-5.37	AAA-AAA	0.00	999	1.000	1.000	0.0062
213309_at	19.9	106.7	2.43	5.37	AAA-AAA	0.00	999	1.000	1.000	0.0196
1568663_a_at	67.5	12.6	-2.42	-5.37	AAA-AAA	0.00	999	1.000	1.000	0.1018
1556809_a_at	12.0	64.2	2.42	5.37	AAA-AAA	0.00	999	1.000	1.000	0.0042
230187_s_at	15.9	3.0	-2.42	-5.36	AAA-AAA	0.00	999	1.000	1.000	0.1690
233343_at	19.5	3.6	-2.42	-5.36	AAA-AAA	0.00	999	1.000	1.000	0.0360
1563082_at	27.8	5.2	-2.42	-5.36	AMA-AAA	0.00	999	1.000	1.000	0.0234
210855_at	46.0	8.6	-2.42	-5.36	AAA-AAA	0.00	999	1.000	1.000	0.0573
231206_at	24.5	4.6	-2.42	-5.36	AAA-AAA	0.00	999	1.000	1.000	0.0682
1560038_at	36.8	6.9	-2.42	-5.36	AAA-AAA	0.00	999	1.000	1.000	0.1711
215996_at	32.0	6.0	-2.42	-5.36	AAA-AAA	0.00	999	1.000	1.000	0.0463
229613_at	74.2	13.9	-2.42	-5.35	AAA-AAA	0.00	999	1.000	1.000	0.0160
1563561_at	1.7	8.9	2.42	5.34	AAA-AAA	0.00	999	1.000	1.000	0.0442
236650_at	4.1	21.6	2.42	5.34	AAA-AAA	0.00	999	1.000	1.000	0.0281
1556967_at	12.1	2.3	-2.42	-5.34	AAA-AAA	0.00	999	1.000	1.000	0.1969
231473_at	5.6	30.1	2.42	5.34	AAA-AAA	0.00	999	1.000	1.000	0.1276
242814_at	40.2	7.5	-2.42	-5.34	AAA-AAA	0.00	999	1.000	1.000	0.0593
1555053_at	40.5	7.6	-2.42	-5.34	AAA-AAA	0.00	999	1.000	1.000	0.0142
204450_x_at	27.3	5.1	-2.42	-5.34	AAA-AAA	0.00	999	1.000	1.000	0.0664
1561857_at	13.5	2.5	-2.42	-5.33	AAA-AAA	0.00	999	1.000	1.000	0.0345
210747_at	15.0	2.8	-2.42	-5.33	AAA-AAA	0.00	999	1.000	1.000	0.0559
230602_at	57.1	10.7	-2.41	-5.32	AAA-AAA	0.00	999	1.000	1.000	0.0427
241615_x_at	37.9	7.1	-2.41	-5.32	AMA-AAA	0.00	999	1.000	1.000	0.0855
1559543_at	9.8	1.8	-2.41	-5.32	AAA-AAA	0.00	999	1.000	1.000	0.2190

1559321_at	38.7	7.3	-2.41	-5.32	AAA-AAA	0.00	999	1.000	1.000	0.0570
210055_at	6.9	1.3	-2.41	-5.32	AAA-AAA	0.00	999	1.000	1.000	0.0682
207001_x_at	63.7	12.0	-2.41	-5.32	AAA-AAA	0.00	999	1.000	1.000	0.0943
243132_at	56.1	10.6	-2.41	-5.32	AAA-AAA	0.00	999	1.000	1.000	0.0122
210297_s_at	17.7	3.3	-2.41	-5.32	AAA-AAA	0.00	999	1.000	1.000	0.0769
229683_s_at	77.8	14.7	-2.41	-5.31	AAA-AAA	0.00	999	1.000	1.000	0.0388
1561527_at	2.0	10.8	2.41	5.31	AAA-AAA	0.00	999	1.000	1.000	0.0646
216152_at	132.0	24.9	-2.41	-5.31	AAA-AAA	0.00	999	1.000	1.000	0.1425
235153_at	74.5	14.0	-2.41	-5.31	AAA-AAA	0.00	999	1.000	1.000	0.1077
231314_at	41.3	7.8	-2.41	-5.31	AAA-AAA	0.00	999	1.000	1.000	0.0472
215835_at	33.3	6.3	-2.41	-5.31	AAA-AAA	0.00	999	1.000	1.000	0.0211
1562799_at	10.0	53.1	2.41	5.30	AAA-AAA	0.00	999	1.000	1.000	0.0242
207853_s_at	10.1	53.4	2.41	5.30	AAA-AAA	0.00	999	1.000	1.000	0.0407
232871_at	53.0	10.0	-2.41	-5.30	MAA-AAA	0.00	999	1.000	1.000	0.0781
223623_at	21.5	4.1	-2.41	-5.30	AAA-AAA	0.00	999	1.000	1.000	0.0471
237449_at	28.6	5.4	-2.41	-5.30	AAA-AAA	0.00	999	1.000	1.000	0.0385
1561914_at	20.6	3.9	-2.40	-5.29	AAA-AAA	0.00	999	1.000	1.000	0.0712
1563771_a_at	23.0	4.3	-2.40	-5.29	AAA-AAA	0.00	999	1.000	1.000	0.0058
240425_x_at	10.4	2.0	-2.40	-5.29	AAA-AAA	0.00	999	1.000	1.000	0.1678
1570355_a_at	2.9	15.4	2.40	5.28	AAA-AAA	0.00	999	1.000	1.000	0.1222
1559186_at	36.5	6.9	-2.40	-5.29	AAA-AAA	0.00	999	1.000	1.000	0.0327
229057_at	2.8	14.8	2.40	5.28	AAA-AAA	0.00	999	1.000	1.000	0.2117
241025_at	15.9	3.0	-2.40	-5.28	AAA-AAA	0.00	999	1.000	1.000	0.0335
1563339_at	24.4	4.6	-2.40	-5.28	AAA-AAA	0.00	999	1.000	1.000	0.0958
1561037_a_at	65.8	12.5	-2.40	-5.27	AAA-AAA	0.00	999	1.000	1.000	0.1560
1568784_at	10.6	2.0	-2.40	-5.27	AAA-AAA	0.00	999	1.000	1.000	0.0430
1553869_at	14.4	2.7	-2.40	-5.28	MAA-AAA	0.00	999	1.000	1.000	0.1156
237557_at	19.3	3.7	-2.40	-5.28	AAA-AAA	0.00	999	1.000	1.000	0.1423
240990_at	6.5	34.0	2.40	5.27	AAA-AAA	0.00	999	1.000	1.000	0.2142
240182_at	21.1	4.0	-2.40	-5.27	AAA-AAA	0.00	999	1.000	1.000	0.1750
1567284_at	26.2	5.0	-2.40	-5.27	AAA-AAA	0.00	999	1.000	1.000	0.0520
217302_at	62.6	11.9	-2.40	-5.27	AAA-AAA	0.00	999	1.000	1.000	0.0626
1557674_s_at	5.4	28.5	2.40	5.27	AAA-AAA	0.00	999	1.000	1.000	0.1024
216030_s_at	18.4	3.5	-2.40	-5.26	AAA-AAA	0.00	999	1.000	1.000	0.1726
235100_at	13.6	2.6	-2.40	-5.26	AAA-AAA	0.00	999	1.000	1.000	0.1050
233034_at	2.4	12.6	2.40	5.26	AAA-AAA	0.00	999	1.000	1.000	0.0064
1569136_at	22.3	4.2	-2.40	-5.26	AAA-AAA	0.00	999	1.000	1.000	0.2079
232819_s_at	10.6	55.9	2.40	5.26	AAA-AAA	0.00	999	1.000	1.000	0.0009
206905_s_at	63.9	12.2	-2.39	-5.26	AAA-AAA	0.00	999	1.000	1.000	0.0124
217171_at	95.4	18.1	-2.39	-5.26	AAA-AAA	0.00	999	1.000	1.000	0.0021
239073_at	45.5	8.7	-2.39	-5.26	AAA-AAA	0.00	999	1.000	1.000	0.1391
205514_at	55.3	10.5	-2.39	-5.26	AAA-AAA	0.00	999	1.000	1.000	0.1246

1566253_at	36.4	6.9	-2.39	-5.25	AAA-AAA	0.00	999	1.000	1.000	0.0922
237803_x_at	31.8	6.1	-2.39	-5.25	AAA-AAA	0.00	999	1.000	1.000	0.1414
1562189_at	25.5	4.9	-2.39	-5.25	AAA-AAA	0.00	999	1.000	1.000	0.1432
1555492_a_at	14.6	2.8	-2.39	-5.25	AAA-AAA	0.00	999	1.000	1.000	0.0209
242529_x_at	4.1	21.3	2.39	5.24	AAA-AAA	0.00	999	1.000	1.000	0.0590
243386_at	33.0	6.3	-2.39	-5.24	AAA-AAA	0.00	999	1.000	1.000	0.1995
1562049_at	1.6	8.4	2.39	5.24	AAA-AAA	0.00	999	1.000	1.000	0.1746
219211_at	9.4	49.3	2.39	5.24	AAA-AAA	0.00	999	1.000	1.000	0.0099
236419_at	55.9	10.7	-2.39	-5.24	AAA-AAA	0.00	999	1.000	1.000	0.0192
1563897_at	6.9	36.0	2.39	5.23	AAA-AAA	0.00	999	1.000	1.000	0.0321
204406_at	26.7	5.1	-2.39	-5.24	AAA-AAA	0.00	999	1.000	1.000	0.0340
1554842_at	37.8	7.2	-2.39	-5.23	AAA-AAA	0.00	999	1.000	1.000	0.0377
221365_at	13.6	2.6	-2.39	-5.23	AAA-AAA	0.00	999	1.000	1.000	0.1159
217712_at	20.5	3.9	-2.39	-5.23	AAA-AAA	0.00	999	1.000	1.000	0.2026
216191_s_at	16.1	3.1	-2.39	-5.23	AAA-AAA	0.00	999	1.000	1.000	0.1671
204987_at	43.1	8.3	-2.39	-5.23	AAA-AAA	0.00	999	1.000	1.000	0.0228
241460_at	60.6	11.6	-2.39	-5.23	AAA-AAA	0.00	999	1.000	1.000	0.1187
215822_x_at	110.1	21.1	-2.39	-5.22	AAA-AAA	0.00	999	1.000	1.000	0.0624
216709_at	21.0	4.0	-2.39	-5.22	AAA-AAA	0.00	999	1.000	1.000	0.1629
1566749_at	6.2	1.2	-2.38	-5.22	AAA-AAA	0.00	999	1.000	1.000	0.1545
215987_at	8.5	44.4	2.38	5.22	AAA-APA	0.00	999	1.000	1.000	0.1229
242909_at	8.3	1.6	-2.38	-5.21	AAA-AAA	0.00	999	1.000	1.000	0.0310
237229_at	19.0	3.7	-2.38	-5.21	AAA-AAA	0.00	999	1.000	1.000	0.0149
229252_at	65.0	12.5	-2.38	-5.21	AAA-AAA	0.00	999	1.000	1.000	0.0084
1553147_at	20.1	3.9	-2.38	-5.20	AAA-AAA	0.00	999	1.000	1.000	0.2041
229620_at	14.1	2.7	-2.38	-5.20	AAA-AAA	0.00	999	1.000	1.000	0.0936
241641_at	25.8	5.0	-2.38	-5.20	AAA-AAA	0.00	999	1.000	1.000	0.0124
1560510_at	10.0	1.9	-2.38	-5.20	AAA-AAA	0.00	999	1.000	1.000	0.0830
1568920_at	43.2	8.3	-2.38	-5.20	AAA-AAA	0.00	999	1.000	1.000	0.0668
241802_x_at	81.0	15.6	-2.38	-5.20	AAA-AAA	0.00	999	1.000	1.000	0.0495
219897_at	111.0	21.4	-2.38	-5.20	AAA-AAA	0.00	999	1.000	1.000	0.0753
213913_s_at	30.9	6.0	-2.38	-5.19	AAA-AAA	0.00	999	1.000	1.000	0.0309
229159_at	15.5	3.0	-2.38	-5.19	AAA-AAA	0.00	999	1.000	1.000	0.1783
241436_at	34.4	6.6	-2.37	-5.19	AAA-AAA	0.00	999	1.000	1.000	0.0112
206337_at	10.4	2.0	-2.37	-5.19	AAA-AAA	0.00	999	1.000	1.000	0.0325
229122_x_at	11.0	57.0	2.37	5.18	AAA-AAA	0.00	999	1.000	1.000	0.0111
243034_at	25.0	4.8	-2.37	-5.18	AAA-AAA	0.00	999	1.000	1.000	0.0682
1557162_at	44.9	8.7	-2.37	-5.18	AAA-AAA	0.00	999	1.000	1.000	0.0149
244222_at	12.0	62.2	2.37	5.17	AAA-AAA	0.00	999	1.000	1.000	0.0162
244757_at	30.0	5.8	-2.37	-5.17	AAA-AAA	0.00	999	1.000	1.000	0.0163
210467_x_at	47.5	9.2	-2.37	-5.17	AAA-AAA	0.00	999	1.000	1.000	0.1023
201442_s_at	50.2	9.7	-2.37	-5.17	AAA-AAA	0.00	999	1.000	1.000	0.0093

234881_at	58.6	11.3	-2.37	-5.17	AAA-AAA	0.00	999	1.000	1.000	0.0383
231478_at	51.0	9.9	-2.37	-5.17	AAA-AAA	0.00	999	1.000	1.000	0.0079
1559495_at	59.9	11.6	-2.37	-5.17	AAA-AAA	0.00	999	1.000	1.000	0.1309
1556055_at	88.2	17.1	-2.37	-5.16	AAA-AAA	0.00	999	1.000	1.000	0.1317
244639_at	33.0	6.4	-2.37	-5.16	AAA-AAA	0.00	999	1.000	1.000	0.0006
215027_at	49.0	9.5	-2.37	-5.16	AAA-AAA	0.00	999	1.000	1.000	0.1197
234806_at	24.6	4.8	-2.37	-5.16	AAA-AAA	0.00	999	1.000	1.000	0.0431
AFFX-TrpnX-M_at	5.5	28.1	2.37	5.16	AAA-AAA	0.00	999	1.000	1.000	0.0030
209542_x_at	41.7	8.1	-2.37	-5.16	AAA-AAA	0.00	999	1.000	1.000	0.0714
1560851_at	8.4	1.6	-2.37	-5.15	AAA-AAA	0.00	999	1.000	1.000	0.2290
1561139_at	65.2	12.6	-2.37	-5.16	AMA-AAA	0.00	999	1.000	1.000	0.0251
219127_at	63.2	12.3	-2.37	-5.16	AAA-AAA	0.00	999	1.000	1.000	0.1043
236289_at	4.5	22.9	2.37	5.15	AAA-AAA	0.00	999	1.000	1.000	0.0827
1559259_at	7.2	36.8	2.36	5.15	AAA-MAP	0.00	999	1.000	1.000	0.1077
1566279_at	15.6	3.0	-2.36	-5.15	AAA-AAA	0.00	999	1.000	1.000	0.0248
216541_x_at	17.8	3.5	-2.36	-5.15	AAA-AAA	0.00	999	1.000	1.000	0.1011
243609_at	56.9	11.1	-2.36	-5.15	AAA-AAA	0.00	999	1.000	1.000	0.0938
220574_at	63.7	12.4	-2.36	-5.15	AAA-AAA	0.00	999	1.000	1.000	0.0075
1569411_at	13.6	2.7	-2.36	-5.14	AAA-AAA	0.00	999	1.000	1.000	0.2254
1564274_at	49.6	9.6	-2.36	-5.14	AAA-AAA	0.00	999	1.000	1.000	0.0147
210641_at	70.9	13.8	-2.36	-5.14	AAA-AAA	0.00	999	1.000	1.000	0.0705
231052_at	117.6	22.9	-2.36	-5.14	AAA-AAA	0.00	999	1.000	1.000	0.1068
1570229_at	71.3	13.9	-2.36	-5.13	AAA-AAA	0.00	999	1.000	1.000	0.1304
229151_at	18.4	3.6	-2.36	-5.13	AAA-AAA	0.00	999	1.000	1.000	0.0707
1560853_x_at	80.8	15.7	-2.36	-5.13	AAA-AAA	0.00	999	1.000	1.000	0.0529
1553362_at	10.5	2.0	-2.36	-5.13	AAA-AAA	0.00	999	1.000	1.000	0.0627
1552318_at	49.2	9.6	-2.36	-5.13	AAA-AAA	0.00	999	1.000	1.000	0.0793
224449_at	58.9	11.5	-2.36	-5.13	AAA-AAA	0.00	999	1.000	1.000	0.1049
237175_at	62.2	12.1	-2.36	-5.13	AMA-AAA	0.00	999	1.000	1.000	0.0497
242980_at	22.8	4.4	-2.36	-5.13	AAA-AAA	0.00	999	1.000	1.000	0.0124
235018_at	21.0	4.1	-2.36	-5.13	AAA-AAA	0.00	999	1.000	1.000	0.2023
240173_at	22.7	4.4	-2.36	-5.12	AAA-AAA	0.00	999	1.000	1.000	0.0857
241296_at	6.2	1.2	-2.36	-5.12	AAA-AAA	0.00	999	1.000	1.000	0.1171
239224_at	48.9	9.6	-2.36	-5.12	AAA-AAA	0.00	999	1.000	1.000	0.0845
203471_s_at	66.8	13.0	-2.36	-5.12	AAA-AAA	0.00	999	1.000	1.000	0.1011
1561644_x_at	3.0	15.1	2.36	5.12	AAA-AAA	0.00	999	1.000	1.000	0.1134
221366_at	8.0	40.8	2.36	5.12	AAA-AAA	0.00	999	1.000	1.000	0.0172
220507_s_at	17.5	3.4	-2.36	-5.12	AAA-AAA	0.00	999	1.000	1.000	0.0855
231916_at	51.1	10.0	-2.36	-5.12	AAA-AAA	0.00	999	1.000	1.000	0.0791
207957_s_at	3.5	17.6	2.35	5.11	AAA-AAA	0.00	999	1.000	1.000	0.0521
1570141_at	9.6	1.9	-2.35	-5.11	AAA-AAA	0.00	999	1.000	1.000	0.0426
237258_at	8.5	43.2	2.35	5.11	AAA-AAA	0.00	999	1.000	1.000	0.0941

215831_at	34.4	6.7	-2.35	-5.11	AAA-AAA	0.00	999	1.000	1.000	0.0990
217380_s_at	16.4	3.2	-2.35	-5.10	AAA-AAA	0.00	999	1.000	1.000	0.1125
1556734_at	43.5	8.5	-2.35	-5.10	AAA-AAA	0.00	999	1.000	1.000	0.0437
1561076_at	12.7	2.5	-2.35	-5.09	AAA-AAA	0.00	999	1.000	1.000	0.0966
210834_s_at	19.8	3.9	-2.35	-5.08	AAA-AAA	0.00	999	1.000	1.000	0.1526
1561045_a_at	16.4	3.2	-2.34	-5.08	AAA-AAA	0.00	999	1.000	1.000	0.2203
1561653_at	2.8	14.2	2.34	5.08	AAA-AAA	0.00	999	1.000	1.000	0.1186
1565760_at	13.1	2.6	-2.34	-5.07	AAA-AAA	0.00	999	1.000	1.000	0.1888
223861_at	27.0	5.3	-2.34	-5.07	AAA-AAA	0.00	999	1.000	1.000	0.0643
208244_at	51.7	10.2	-2.34	-5.07	AAA-AAA	0.00	999	1.000	1.000	0.1848
232845_at	6.3	31.9	2.34	5.07	AAA-AAA	0.00	999	1.000	1.000	0.0044
214796_at	53.2	10.5	-2.34	-5.07	AAA-AAA	0.00	999	1.000	1.000	0.0483
217705_at	34.1	6.7	-2.34	-5.07	AAA-AAA	0.00	999	1.000	1.000	0.0153
1570233_at	10.1	2.0	-2.34	-5.06	AAA-AAA	0.00	999	1.000	1.000	0.0584
1552411_at	83.2	16.5	-2.34	-5.06	AAA-AAA	0.00	999	1.000	1.000	0.0237
1556972_at	74.5	14.7	-2.34	-5.06	AAA-AAA	0.00	999	1.000	1.000	0.0556
207887_s_at	21.8	4.3	-2.34	-5.06	AAA-AAA	0.00	999	1.000	1.000	0.0298
1567273_at	25.2	5.0	-2.34	-5.06	AAA-AAA	0.00	999	1.000	1.000	0.0769
220455_at	41.6	8.2	-2.34	-5.05	AAA-AAA	0.00	999	1.000	1.000	0.0496
227995_at	21.8	4.3	-2.34	-5.05	AAA-AAA	0.00	999	1.000	1.000	0.1791
221109_at	33.7	6.7	-2.34	-5.05	AAA-AAA	0.00	999	1.000	1.000	0.0513
236677_at	50.6	10.0	-2.34	-5.05	AAA-AAA	0.00	999	1.000	1.000	0.1046
237294_at	43.5	8.6	-2.34	-5.04	AAA-AAA	0.00	999	1.000	1.000	0.0081
241500_at	17.1	3.4	-2.33	-5.04	AAA-AAA	0.00	999	1.000	1.000	0.1099
1562227_at	65.1	12.9	-2.33	-5.04	AAA-AAA	0.00	999	1.000	1.000	0.0451
1559521_at	19.4	3.8	-2.33	-5.04	AAA-AAA	0.00	999	1.000	1.000	0.0575
207107_at	1.4	6.8	2.33	5.04	AAA-AAA	0.00	999	1.000	1.000	0.1652
223632_s_at	42.7	8.5	-2.33	-5.04	AAA-AAA	0.00	999	1.000	1.000	0.0771
211307_s_at	23.0	4.6	-2.33	-5.04	AAA-AAA	0.00	999	1.000	1.000	0.0845
234261_at	41.3	8.2	-2.33	-5.04	AAM-AAA	0.00	999	1.000	1.000	0.1200
200952_s_at	47.1	9.4	-2.33	-5.03	AAA-AAA	0.00	999	1.000	1.000	0.0797
1565799_at	6.1	1.2	-2.33	-5.03	AAA-AAA	0.00	999	1.000	1.000	0.0329
206978_at	31.0	6.2	-2.33	-5.03	AAA-AAA	0.00	999	1.000	1.000	0.1251
206009_at	49.8	9.9	-2.33	-5.04	AAA-AAA	0.00	999	1.000	1.000	0.1127
230855_at	85.8	17.0	-2.33	-5.03	AAA-AAA	0.00	999	1.000	1.000	0.0336
205153_s_at	16.6	83.6	2.33	5.03	AAA-AAA	0.00	999	1.000	1.000	0.0574
206054_at	30.2	6.0	-2.33	-5.03	AAA-AAA	0.00	999	1.000	1.000	0.0060
1567376_at	31.4	6.3	-2.33	-5.03	AAA-AAA	0.00	999	1.000	1.000	0.1347
1570546_a_at	12.5	2.5	-2.33	-5.02	AAA-AAA	0.00	999	1.000	1.000	0.1228
1557256_a_at	4.0	20.3	2.33	5.02	AAA-AAA	0.00	999	1.000	1.000	0.0331
207878_at	23.7	4.7	-2.33	-5.02	AAA-AAA	0.00	999	1.000	1.000	0.0344
220799_at	12.3	2.5	-2.33	-5.03	AAM-AAA	0.00	999	1.000	1.000	0.0718

224482_s_at	14.7	2.9	-2.33	-5.02	AAA-AAA	0.00	999	1.000	1.000	0.2132
238439_at	31.6	6.3	-2.33	-5.02	AAA-AAA	0.00	999	1.000	1.000	0.1745
240901_at	26.5	5.3	-2.33	-5.02	AAA-AAA	0.00	999	1.000	1.000	0.0501
1562716_at	45.0	9.0	-2.33	-5.02	AAA-AAA	0.00	999	1.000	1.000	0.0984
1559654_s_at	61.5	12.3	-2.33	-5.02	AAA-AAA	0.00	999	1.000	1.000	0.0198
215902_at	48.6	9.7	-2.33	-5.02	AAA-AAA	0.00	999	1.000	1.000	0.0816
238371_s_at	34.7	6.9	-2.33	-5.02	AAA-AAA	0.00	999	1.000	1.000	0.1295
1570219_at	24.5	4.9	-2.33	-5.02	AAA-AAA	0.00	999	1.000	1.000	0.0118
207697_x_at	38.5	7.7	-2.33	-5.02	AAA-AAA	0.00	999	1.000	1.000	0.0373
239520_at	15.6	3.1	-2.33	-5.02	AAA-AAA	0.00	999	1.000	1.000	0.0285
1567240_x_at	22.3	4.5	-2.33	-5.02	AAA-AAA	0.00	999	1.000	1.000	0.0542
1553316_at	48.6	9.7	-2.33	-5.02	AAA-AAA	0.00	999	1.000	1.000	0.0122
213825_at	7.9	39.7	2.33	5.02	AAA-AAA	0.00	999	1.000	1.000	0.0461
240225_at	28.7	5.7	-2.33	-5.01	AAA-AAA	0.00	999	1.000	1.000	0.1008
244247_at	16.6	3.3	-2.32	-5.01	AAA-AAA	0.00	999	1.000	1.000	0.2040
222926_at	23.5	4.7	-2.32	-5.01	AAA-AAA	0.00	999	1.000	1.000	0.1653
235641_at	21.2	4.2	-2.32	-5.01	AAA-AAA	0.00	999	1.000	1.000	0.1305
1555256_at	52.1	10.4	-2.32	-5.00	AAA-AAA	0.00	999	1.000	1.000	0.1266
219786_at	18.0	3.6	-2.32	-5.00	AAA-AAA	0.00	999	1.000	1.000	0.0953
214854_at	15.1	3.0	-2.32	-5.00	AAA-AAA	0.00	999	1.000	1.000	0.0329
218978_s_at	17.2	86.0	2.32	5.00	AAA-PAA	0.00	999	1.000	1.000	0.0944
1555171_at	26.6	5.3	-2.32	-5.00	AAA-AAA	0.00	999	1.000	1.000	0.1938
234621_at	13.9	2.8	-2.32	-5.00	AAA-AAA	0.00	999	1.000	1.000	0.1522
237719_x_at	8.4	1.7	-2.32	-5.00	AAA-AAA	0.00	999	1.000	1.000	0.0597
220539_at	33.3	6.7	-2.32	-4.99	AAA-AAA	0.00	999	1.000	1.000	0.2179
207401_at	34.2	6.9	-2.32	-4.99	AAA-AAA	0.00	999	1.000	1.000	0.1952
1552740_at	18.5	3.7	-2.32	-4.99	AAA-AAA	0.00	999	1.000	1.000	0.0391
1552564_at	36.9	7.4	-2.32	-4.99	AAA-AAA	0.00	999	1.000	1.000	0.1426
230952_at	52.8	10.6	-2.32	-4.99	AAA-AAA	0.00	999	1.000	1.000	0.0217
231856_at	9.6	1.9	-2.32	-4.99	AAA-AAA	0.00	999	1.000	1.000	0.0202
242344_at	13.1	2.6	-2.32	-4.98	AAA-AAA	0.00	999	1.000	1.000	0.2595
1565700_at	59.4	11.9	-2.32	-4.98	AAA-AAA	0.00	999	1.000	1.000	0.1416
230238_at	30.0	6.0	-2.32	-4.98	AAA-AAA	0.00	999	1.000	1.000	0.2307
239905_at	26.0	5.2	-2.32	-4.98	AAA-AAA	0.00	999	1.000	1.000	0.1765
1563078_at	50.8	10.2	-2.32	-4.98	AAA-AAA	0.00	999	1.000	1.000	0.0463
227803_at	18.8	3.8	-2.32	-4.98	AAA-AAA	0.00	999	1.000	1.000	0.0915
241229_at	44.9	9.0	-2.32	-4.98	AAA-AAA	0.00	999	1.000	1.000	0.0038
215270_at	31.0	6.2	-2.31	-4.97	AAA-AAA	0.00	999	1.000	1.000	0.0671
236399_at	35.6	7.2	-2.31	-4.97	AAA-AAA	0.00	999	1.000	1.000	0.0372
216619_at	6.2	1.3	-2.31	-4.97	AAA-AAA	0.00	999	1.000	1.000	0.0026
241759_at	20.8	4.2	-2.31	-4.97	AAA-AAA	0.00	999	1.000	1.000	0.0862
220218_at	28.9	5.8	-2.31	-4.97	AAA-AAA	0.00	999	1.000	1.000	0.0837

211024_s_at	24.1	4.9	-2.31	-4.97	AAA-AAA	0.00	999	1.000	1.000	0.1193
215958_at	21.4	4.3	-2.31	-4.96	AAA-AAA	0.00	999	1.000	1.000	0.0605
233766_at	15.6	3.1	-2.31	-4.97	AAA-AAA	0.00	999	1.000	1.000	0.1522
244127_at	41.8	8.4	-2.31	-4.96	AAA-AAA	0.00	999	1.000	1.000	0.1002
238805_at	20.5	4.1	-2.31	-4.96	AAA-AAA	0.00	999	1.000	1.000	0.0815
1554196_at	2.4	12.1	2.31	4.96	AAA-AAA	0.00	999	1.000	1.000	0.0220
217056_at	12.4	2.5	-2.31	-4.96	AAA-AAA	0.00	999	1.000	1.000	0.1024
233478_at	46.0	9.3	-2.31	-4.96	AAA-AAA	0.00	999	1.000	1.000	0.0111
1553765_a_at	5.0	1.0	-2.31	-4.95	AAA-AAA	0.00	999	1.000	1.000	0.1984
221852_at	26.4	5.3	-2.31	-4.95	AAA-AAA	0.00	999	1.000	1.000	0.0100
214591_at	46.2	9.3	-2.31	-4.95	AAA-AAA	0.00	999	1.000	1.000	0.1311
216286_at	6.2	30.5	2.31	4.95	AAA-AAA	0.00	999	1.000	1.000	0.0893
235665_at	32.7	6.6	-2.31	-4.95	AAA-AAA	0.00	999	1.000	1.000	0.1587
243517_at	64.8	13.1	-2.31	-4.95	AAA-AAA	0.00	999	1.000	1.000	0.0213
234336_s_at	40.4	8.2	-2.31	-4.94	AAA-AAA	0.00	999	1.000	1.000	0.1654
228462_at	17.3	3.5	-2.31	-4.94	AAA-AAA	0.00	999	1.000	1.000	0.0630
1559620_at	35.8	7.3	-2.30	-4.94	AAA-AAA	0.00	999	1.000	1.000	0.1300
216459_x_at	16.0	78.9	2.30	4.93	AAA-AAA	0.00	999	1.000	1.000	0.2549
239965_at	10.5	51.7	2.30	4.93	AAA-AAA	0.00	999	1.000	1.000	0.0018
1564790_at	18.8	3.8	-2.30	-4.93	AAA-AAA	0.00	999	1.000	1.000	0.0871
219752_at	139.1	28.2	-2.30	-4.93	AAA-AAA	0.00	999	1.000	1.000	0.0814
220639_at	51.6	10.5	-2.30	-4.93	AAA-AAA	0.00	999	1.000	1.000	0.0314
220506_at	42.9	8.7	-2.30	-4.93	AAA-AAA	0.00	999	1.000	1.000	0.0529
1569331_at	10.3	2.1	-2.30	-4.93	AAA-AAA	0.00	999	1.000	1.000	0.0843
1558343_at	57.4	11.6	-2.30	-4.93	AAA-AAA	0.00	999	1.000	1.000	0.0241
211204_at	47.3	9.6	-2.30	-4.93	AAA-AAA	0.00	999	1.000	1.000	0.0004
232226_at	57.2	11.6	-2.30	-4.93	AAA-AAA	0.00	999	1.000	1.000	0.0687
241522_at	58.7	11.9	-2.30	-4.93	AAA-AAA	0.00	999	1.000	1.000	0.0348
1569428_at	50.8	10.3	-2.30	-4.93	AAA-AAA	0.00	999	1.000	1.000	0.1094
1565738_at	25.9	5.3	-2.30	-4.93	AAA-AAA	0.00	999	1.000	1.000	0.1512
207242_s_at	22.6	4.6	-2.30	-4.93	AAA-AAA	0.00	999	1.000	1.000	0.1335
210381_s_at	6.6	32.7	2.30	4.92	AAA-AAA	0.00	999	1.000	1.000	0.1375
235813_at	15.9	3.2	-2.30	-4.92	AAA-AAA	0.00	999	1.000	1.000	0.0808
241273_at	8.4	1.7	-2.30	-4.92	AAA-AAA	0.00	999	1.000	1.000	0.1396
1556676_a_at	35.4	7.2	-2.30	-4.92	AAA-AAA	0.00	999	1.000	1.000	0.1207
220209_at	34.3	7.0	-2.30	-4.92	AAA-AAA	0.00	999	1.000	1.000	0.1068
205743_at	108.0	21.9	-2.30	-4.92	AAA-AAA	0.00	999	1.000	1.000	0.0137
233353_at	56.3	11.4	-2.30	-4.92	AAA-AAA	0.00	999	1.000	1.000	0.1269
234483_at	23.1	4.7	-2.30	-4.92	AAA-AAA	0.00	999	1.000	1.000	0.0489
1564319_at	36.2	7.4	-2.30	-4.92	AMA-AAA	0.00	999	1.000	1.000	0.2010
227097_at	76.7	15.6	-2.30	-4.91	AAA-AAA	0.00	999	1.000	1.000	0.0207
238632_at	14.6	3.0	-2.30	-4.91	AAA-AAA	0.00	999	1.000	1.000	0.2104

1556054_at	7.4	36.2	2.30	4.91	AAA-AAA	0.00	999	1.000	1.000	0.2194
220982_s_at	19.7	4.0	-2.30	-4.91	AAA-AAA	0.00	999	1.000	1.000	0.0933
217110_s_at	9.3	1.9	-2.30	-4.91	AAA-AAA	0.00	999	1.000	1.000	0.0086
209368_at	28.8	5.9	-2.30	-4.91	AAA-AAA	0.00	999	1.000	1.000	0.0362
241207_at	47.1	9.6	-2.30	-4.91	AAA-AAA	0.00	999	1.000	1.000	0.2411
1561984_at	66.4	13.5	-2.30	-4.91	AAA-AAA	0.00	999	1.000	1.000	0.2083
232090_at	27.1	5.5	-2.30	-4.91	AAA-AAA	0.00	999	1.000	1.000	0.0610
234837_at	54.5	11.1	-2.29	-4.90	AAA-AAA	0.00	999	1.000	1.000	0.0778
1563085_at	4.9	23.9	2.29	4.90	AAA-AMA	0.00	999	1.000	1.000	0.0389
244603_at	55.3	11.3	-2.29	-4.90	AAA-AAA	0.00	999	1.000	1.000	0.0023
236345_at	45.2	9.2	-2.29	-4.90	AAA-AAA	0.00	999	1.000	1.000	0.1517
237982_at	53.1	10.8	-2.29	-4.90	AAA-AAA	0.00	999	1.000	1.000	0.1115
1560720_at	7.3	35.9	2.29	4.90	AAA-AAP	0.00	999	1.000	1.000	0.1188
1569680_at	37.1	7.6	-2.29	-4.90	AAA-AAA	0.00	999	1.000	1.000	0.0200
1570226_at	32.7	6.7	-2.29	-4.90	AAA-AAA	0.00	999	1.000	1.000	0.0709
220883_at	27.7	5.7	-2.29	-4.90	AAA-AAA	0.00	999	1.000	1.000	0.0734
229046_s_at	51.1	10.4	-2.29	-4.90	AAA-AAA	0.00	999	1.000	1.000	0.0148
232367_x_at	28.6	139.9	2.29	4.90	AAA-AAA	0.00	999	1.000	1.000	0.0678
1559652_at	30.2	6.2	-2.29	-4.89	AAA-AAA	0.00	999	1.000	1.000	0.0418
1555742_at	138.3	28.3	-2.29	-4.89	AAA-AAA	0.00	999	1.000	1.000	0.0721
203547_at	96.5	19.7	-2.29	-4.89	AAA-AAA	0.00	999	1.000	1.000	0.1092
216850_at	60.6	12.4	-2.29	-4.89	AAA-AAA	0.00	999	1.000	1.000	0.0132
1552440_at	19.0	3.9	-2.29	-4.89	AAA-AAA	0.00	999	1.000	1.000	0.0969
234715_at	31.7	6.5	-2.29	-4.89	AAA-AAA	0.00	999	1.000	1.000	0.0345
240270_x_at	170.0	34.8	-2.29	-4.88	AAA-AAA	0.00	999	1.000	1.000	0.0251
1569933_at	96.3	19.7	-2.29	-4.88	AAA-AAA	0.00	999	1.000	1.000	0.0809
205560_at	57.4	11.8	-2.29	-4.88	AAA-AAA	0.00	999	1.000	1.000	0.0151
1556541_s_at	25.0	5.1	-2.29	-4.88	AAA-AAA	0.00	999	1.000	1.000	0.0204
213915_at	14.2	2.9	-2.29	-4.88	AAA-AAA	0.00	999	1.000	1.000	0.1572
234904_x_at	10.0	2.1	-2.29	-4.88	AAA-AAA	0.00	999	1.000	1.000	0.1698
230412_at	21.3	4.4	-2.29	-4.88	AAA-AAA	0.00	999	1.000	1.000	0.0435
206118_at	74.3	15.2	-2.29	-4.88	AAA-AAA	0.00	999	1.000	1.000	0.0068
222085_at	10.2	49.6	2.29	4.88	AAA-AAA	0.00	999	1.000	1.000	0.0478
1562501_at	17.2	3.5	-2.29	-4.88	AAA-AAA	0.00	999	1.000	1.000	0.1125
1559029_at	35.0	7.2	-2.29	-4.87	AAA-AAA	0.00	999	1.000	1.000	0.0787
205377_s_at	34.9	7.2	-2.29	-4.87	AAA-AAA	0.00	999	1.000	1.000	0.0909
240680_at	49.6	10.2	-2.28	-4.87	AAA-AAA	0.00	999	1.000	1.000	0.1539
1561682_at	75.7	15.6	-2.28	-4.87	AAM-AAA	0.00	999	1.000	1.000	0.1240
216068_at	30.4	6.3	-2.28	-4.87	AAA-AAA	0.00	999	1.000	1.000	0.0928
215033_at	12.8	62.1	2.28	4.87	AAA-APP	0.00	999	1.000	1.000	0.1230
237538_at	45.3	9.3	-2.28	-4.86	AAA-AAA	0.00	999	1.000	1.000	0.0354
1556826_s_at	37.4	7.7	-2.28	-4.85	AAA-AAA	0.00	999	1.000	1.000	0.1835

1563573_at	3.5	17.1	2.28	4.84	AAA-AAA	0.00	999	1.000	1.000	0.1147
237769_at	42.3	8.7	-2.28	-4.84	AAA-AAA	0.00	999	1.000	1.000	0.0731
1556368_at	5.3	25.7	2.27	4.84	AAA-AAA	0.00	999	1.000	1.000	0.0463
228066_at	21.9	106.0	2.27	4.84	AAA-AAA	0.00	999	1.000	1.000	0.0042
231991_at	53.8	11.1	-2.27	-4.84	AAA-AAA	0.00	999	1.000	1.000	0.0681
210881_s_at	51.8	10.7	-2.27	-4.83	AAA-AAA	0.00	999	1.000	1.000	0.0233
231308_at	24.4	5.1	-2.27	-4.83	AAA-AAA	0.00	999	1.000	1.000	0.1169
235582_at	86.2	17.9	-2.27	-4.83	AAA-AAA	0.00	999	1.000	1.000	0.0770
230700_at	45.6	9.4	-2.27	-4.83	AAA-AAA	0.00	999	1.000	1.000	0.0876
1568897_at	15.3	3.2	-2.27	-4.82	AAA-AAA	0.00	999	1.000	1.000	0.0049
1564469_at	5.8	1.2	-2.27	-4.82	AAA-AAA	0.00	999	1.000	1.000	0.0508
239221_at	66.8	13.9	-2.27	-4.82	AAA-AAA	0.00	999	1.000	1.000	0.1647
228448_at	80.2	16.6	-2.27	-4.82	AAA-AAA	0.00	999	1.000	1.000	0.1242
239862_at	17.9	3.7	-2.27	-4.82	AAA-AAA	0.00	999	1.000	1.000	0.1221
220618_s_at	81.2	16.9	-2.27	-4.82	AAA-AAA	0.00	999	1.000	1.000	0.0054
210708_x_at	75.8	15.7	-2.27	-4.82	AAA-AAA	0.00	999	1.000	1.000	0.0763
240729_at	16.7	3.5	-2.27	-4.82	AAA-AAA	0.00	999	1.000	1.000	0.0652
214758_at	106.0	22.0	-2.27	-4.81	MAA-AAA	0.00	999	1.000	1.000	0.0695
215547_at	15.8	3.3	-2.27	-4.81	AAA-AAA	0.00	999	1.000	1.000	0.0371
231280_at	56.0	11.6	-2.27	-4.81	AAA-AAA	0.00	999	1.000	1.000	0.0214
223695_s_at	8.9	42.7	2.27	4.81	AAA-APA	0.00	999	1.000	1.000	0.0828
240414_at	56.1	11.7	-2.27	-4.81	AAA-AAA	0.00	999	1.000	1.000	0.0504
1563101_at	7.4	35.8	2.27	4.81	AAA-AAA	0.00	999	1.000	1.000	0.2053
1560238_at	32.4	6.7	-2.27	-4.81	AAA-AAA	0.00	999	1.000	1.000	0.0879
1569510_at	18.5	3.8	-2.27	-4.81	AAA-AAA	0.00	999	1.000	1.000	0.0052
220062_s_at	44.8	9.3	-2.27	-4.81	AAA-AAA	0.00	999	1.000	1.000	0.0239
205879_x_at	105.6	21.9	-2.27	-4.81	AAA-AAA	0.00	999	1.000	1.000	0.0266
1563260_at	25.7	5.4	-2.27	-4.81	AAA-AAA	0.00	999	1.000	1.000	0.1750
1564933_at	34.5	7.2	-2.27	-4.81	AAA-AAA	0.00	999	1.000	1.000	0.0032
215264_at	28.0	5.8	-2.27	-4.81	AAA-AAA	0.00	999	1.000	1.000	0.0727
216704_at	59.1	12.3	-2.27	-4.81	AAA-AAA	0.00	999	1.000	1.000	0.0694
234525_at	18.4	3.8	-2.27	-4.81	AAA-AAA	0.00	999	1.000	1.000	0.1814
238786_at	29.4	6.1	-2.27	-4.81	AAA-AAA	0.00	999	1.000	1.000	0.0090
240940_at	28.2	5.9	-2.26	-4.80	AAA-AAA	0.00	999	1.000	1.000	0.0177
1556136_at	19.4	4.0	-2.26	-4.80	AAA-AAA	0.00	999	1.000	1.000	0.1515
220567_at	19.4	4.0	-2.26	-4.80	AAA-AAA	0.00	999	1.000	1.000	0.1123
217674_at	36.5	7.6	-2.26	-4.80	AAA-AAA	0.00	999	1.000	1.000	0.1602
1557472_a_at	33.0	6.9	-2.26	-4.80	AAA-AAA	0.00	999	1.000	1.000	0.0484
242904_x_at	7.9	37.8	2.26	4.80	AAA-AAA	0.00	999	1.000	1.000	0.0071
237403_at	33.9	7.1	-2.26	-4.80	AAA-AAA	0.00	999	1.000	1.000	0.0343
1563542_a_at	54.8	11.4	-2.26	-4.79	AAA-AAA	0.00	999	1.000	1.000	0.0179
231773_at	10.6	2.2	-2.26	-4.79	AAA-AAA	0.00	999	1.000	1.000	0.1782

1555517_at	16.1	3.4	-2.26	-4.79	AAA-AAA	0.00	999	1.000	1.000	0.2504
208287_at	70.3	14.7	-2.26	-4.79	AAA-AAA	0.00	999	1.000	1.000	0.0651
207210_at	19.7	4.1	-2.26	-4.79	AAA-AAA	0.00	999	1.000	1.000	0.0839
205725_at	38.0	7.9	-2.26	-4.79	AAA-AAA	0.00	999	1.000	1.000	0.1931
241256_at	29.8	6.2	-2.26	-4.78	AAA-AAA	0.00	999	1.000	1.000	0.0274
233325_at	62.1	13.0	-2.26	-4.79	AAA-AAA	0.00	999	1.000	1.000	0.0872
231053_at	23.7	5.0	-2.26	-4.78	AAA-AAA	0.00	999	1.000	1.000	0.0282
231663_s_at	23.1	4.8	-2.26	-4.78	AAA-AAA	0.00	999	1.000	1.000	0.0196
1559336_at	3.9	18.7	2.26	4.78	AAA-MAA	0.00	999	1.000	1.000	0.1856
215473_at	32.6	6.8	-2.26	-4.78	AAA-AAA	0.00	999	1.000	1.000	0.1186
235987_at	16.2	3.4	-2.26	-4.78	AAA-AAA	0.00	999	1.000	1.000	0.1253
241858_at	27.8	5.8	-2.26	-4.78	AAA-AAA	0.00	999	1.000	1.000	0.0537
207811_at	13.0	2.7	-2.26	-4.78	AAA-AAA	0.00	999	1.000	1.000	0.2000
243186_at	47.7	10.0	-2.26	-4.78	AAA-AAA	0.00	999	1.000	1.000	0.1001
234817_at	24.8	5.2	-2.26	-4.78	MAA-AAA	0.00	999	1.000	1.000	0.0620
243342_at	6.1	28.9	2.25	4.77	AAA-APA	0.00	999	1.000	1.000	0.1041
1568935_at	7.3	1.5	-2.25	-4.77	AAA-AAA	0.00	999	1.000	1.000	0.1140
1553757_at	34.8	7.3	-2.25	-4.77	AAA-AAA	0.00	999	1.000	1.000	0.1250
224214_at	15.5	3.3	-2.25	-4.77	AAA-AAA	0.00	999	1.000	1.000	0.0695
1561216_at	11.0	2.3	-2.25	-4.76	AAA-AAA	0.00	999	1.000	1.000	0.0993
244221_at	29.5	6.2	-2.25	-4.76	AAA-AAA	0.00	999	1.000	1.000	0.0717
1564144_at	12.6	2.6	-2.25	-4.76	AAA-AAA	0.00	999	1.000	1.000	0.1203
1554668_a_at	49.9	10.5	-2.25	-4.76	AAA-AAA	0.00	999	1.000	1.000	0.1079
1557886_at	33.1	7.0	-2.25	-4.76	AAA-AAA	0.00	999	1.000	1.000	0.0319
1566680_at	17.4	3.7	-2.25	-4.76	AAA-AAA	0.00	999	1.000	1.000	0.0863
1555616_at	102.5	21.6	-2.25	-4.76	AAA-AAA	0.00	999	1.000	1.000	0.0915
1556521_a_at	28.9	6.1	-2.25	-4.76	AAA-AAA	0.00	999	1.000	1.000	0.0326
206131_at	84.1	17.7	-2.25	-4.76	AAA-AAA	0.00	999	1.000	1.000	0.0002
1570295_at	13.0	61.7	2.25	4.75	AAA-AMA	0.00	999	1.000	1.000	0.0206
229639_s_at	15.0	3.2	-2.25	-4.75	AAA-AAA	0.00	999	1.000	1.000	0.0710
230022_at	21.6	4.5	-2.25	-4.75	AAA-AAA	0.00	999	1.000	1.000	0.0789
243221_at	24.9	5.2	-2.25	-4.75	AAA-AAA	0.00	999	1.000	1.000	0.1352
1552588_a_at	3.0	14.0	2.25	4.75	AAA-AAA	0.00	999	1.000	1.000	0.0742
1556528_at	28.4	6.0	-2.25	-4.75	AMA-AAA	0.00	999	1.000	1.000	0.1690
222255_at	16.8	3.5	-2.25	-4.75	AAA-AAA	0.00	999	1.000	1.000	0.1087
216330_s_at	62.0	13.1	-2.25	-4.75	AAA-AAA	0.00	999	1.000	1.000	0.0382
234737_at	12.2	57.9	2.25	4.75	AAA-AAA	0.00	999	1.000	1.000	0.0769
212951_at	23.0	109.1	2.25	4.74	AAA-PAP	0.00	999	1.000	1.000	0.0831
1563023_at	20.6	4.3	-2.25	-4.74	AAA-AAA	0.00	999	1.000	1.000	0.0115
1560316_s_at	7.1	33.5	2.25	4.74	AAA-AAA	0.00	999	1.000	1.000	0.0056
231594_at	33.9	7.2	-2.25	-4.74	AAA-AAA	0.00	999	1.000	1.000	0.1380
240652_at	23.6	5.0	-2.24	-4.73	AAA-AAA	0.00	999	1.000	1.000	0.1443

222150_s_at	5.6	26.3	2.24	4.73	AAA-PPA	0.00	999	1.000	1.000	0.2715
1562439_at	4.9	23.1	2.24	4.73	AAA-AAA	0.00	999	1.000	1.000	0.0398
1564285_at	23.4	5.0	-2.24	-4.73	AAA-AAA	0.00	999	1.000	1.000	0.2804
221625_at	36.7	7.8	-2.24	-4.73	MMA-AAA	0.00	999	1.000	1.000	0.0951
221117_at	19.6	4.1	-2.24	-4.73	AAA-AAA	0.00	999	1.000	1.000	0.0677
222354_at	74.2	15.7	-2.24	-4.73	AAA-AAA	0.00	999	1.000	1.000	0.0806
206970_at	29.2	6.2	-2.24	-4.72	AAA-AAA	0.00	999	1.000	1.000	0.1810
234966_at	16.3	3.4	-2.24	-4.73	AAA-AAA	0.00	999	1.000	1.000	0.1334
218541_s_at	4.0	18.7	2.24	4.72	AAA-AAA	0.00	999	1.000	1.000	0.1782
215710_at	64.2	13.6	-2.24	-4.72	AAA-AAA	0.00	999	1.000	1.000	0.0446
204713_s_at	34.0	7.2	-2.24	-4.72	AAA-AAA	0.00	999	1.000	1.000	0.0115
228497_at	19.0	4.0	-2.24	-4.72	AAA-AAA	0.00	999	1.000	1.000	0.2946
1570448_at	26.4	5.6	-2.24	-4.72	AAA-AAA	0.00	999	1.000	1.000	0.0351
1553886_at	6.9	1.5	-2.24	-4.71	AAA-AAA	0.00	999	1.000	1.000	0.2172
217105_at	24.5	5.2	-2.24	-4.71	AAA-AAA	0.00	999	1.000	1.000	0.2401
228051_at	58.1	12.3	-2.24	-4.71	AAA-AAA	0.00	999	1.000	1.000	0.2015
206350_at	39.6	8.4	-2.24	-4.72	AAA-AAA	0.00	999	1.000	1.000	0.0071
1553075_a_at	2.9	13.6	2.24	4.71	AAA-AAP	0.00	999	1.000	1.000	0.0942
215341_at	56.8	12.1	-2.24	-4.71	AAA-AAA	0.00	999	1.000	1.000	0.0235
211127_x_at	36.6	7.8	-2.24	-4.71	AAA-AAA	0.00	999	1.000	1.000	0.0546
206370_at	3.9	18.4	2.24	4.71	AAA-AAA	0.00	999	1.000	1.000	0.2302
1559678_s_at	8.6	1.8	-2.23	-4.70	AAA-AAA	0.00	999	1.000	1.000	0.1240
217115_at	23.3	5.0	-2.23	-4.70	AAA-AAA	0.00	999	1.000	1.000	0.1000
206211_at	19.2	4.1	-2.23	-4.70	AAA-AAA	0.00	999	1.000	1.000	0.1336
231441_at	34.3	7.3	-2.23	-4.70	AAA-AAA	0.00	999	1.000	1.000	0.1423
1554689_a_at	38.2	8.1	-2.23	-4.70	AAA-AAA	0.00	999	1.000	1.000	0.2907
216160_at	33.7	7.2	-2.23	-4.70	AAA-AAA	0.00	999	1.000	1.000	0.0801
231499_s_at	44.7	9.5	-2.23	-4.70	AAA-AAA	0.00	999	1.000	1.000	0.1140
240970_x_at	36.2	7.7	-2.23	-4.70	AAA-AAA	0.00	999	1.000	1.000	0.2306
1554495_s_at	63.4	13.5	-2.23	-4.70	MAA-AAA	0.00	999	1.000	1.000	0.1507
217319_x_at	25.7	5.5	-2.23	-4.69	AAA-AAA	0.00	999	1.000	1.000	0.0291
1565637_at	2.6	12.3	2.23	4.69	AAA-AAA	0.00	999	1.000	1.000	0.1631
214432_at	45.6	9.7	-2.23	-4.69	AAA-AAA	0.00	999	1.000	1.000	0.0892
231935_at	28.4	6.1	-2.23	-4.69	AAA-AAA	0.00	999	1.000	1.000	0.0752
1565888_at	12.4	2.6	-2.23	-4.69	AAA-AAA	0.00	999	1.000	1.000	0.1065
228081_at	18.2	85.4	2.23	4.69	AAA-AAA	0.00	999	1.000	1.000	0.1658
232711_at	17.1	3.7	-2.23	-4.68	AAA-AAA	0.00	999	1.000	1.000	0.2146
243418_at	44.4	9.5	-2.23	-4.68	AAA-AAA	0.00	999	1.000	1.000	0.0228
1560630_at	79.6	17.0	-2.23	-4.68	AAA-AAA	0.00	999	1.000	1.000	0.1590
1561330_at	7.0	1.5	-2.23	-4.68	AAA-AAA	0.00	999	1.000	1.000	0.2149
244465_at	23.8	5.1	-2.23	-4.68	AAA-AAA	0.00	999	1.000	1.000	0.0020
217388_s_at	34.7	162.5	2.23	4.68	AAA-PPA	0.00	999	1.000	1.000	0.2024

244118_at	3.2	15.0	2.23	4.68	AAA-AAA	0.00	999	1.000	1.000	0.0062
1563341_at	5.0	23.2	2.23	4.68	AAA-AAA	0.00	999	1.000	1.000	0.0362
220798_x_at	49.0	10.5	-2.23	-4.68	AAA-AAA	0.00	999	1.000	1.000	0.0248
233425_at	10.8	2.3	-2.23	-4.68	AAA-AAA	0.00	999	1.000	1.000	0.1840
232359_at	110.4	23.6	-2.23	-4.68	AAA-AAA	0.00	999	1.000	1.000	0.2910
1565776_at	9.5	44.2	2.23	4.68	AAA-AAA	0.00	999	1.000	1.000	0.1407
1554259_at	19.6	91.8	2.23	4.68	AAA-AAA	0.00	999	1.000	1.000	0.0058
214576_at	12.2	2.6	-2.23	-4.68	AAA-AAA	0.00	999	1.000	1.000	0.0940
233910_at	34.8	7.4	-2.23	-4.67	AMA-AAA	0.00	999	1.000	1.000	0.0888
1566716_at	14.3	3.1	-2.22	-4.67	AAA-AAA	0.00	999	1.000	1.000	0.2038
1554954_at	76.7	16.4	-2.22	-4.67	AAA-AAA	0.00	999	1.000	1.000	0.0152
207477_at	25.3	5.4	-2.22	-4.67	AAA-AAA	0.00	999	1.000	1.000	0.1849
210333_at	5.3	24.5	2.22	4.67	AAA-AAA	0.00	999	1.000	1.000	0.0176
209243_s_at	26.2	5.6	-2.22	-4.66	AAA-AAA	0.00	999	1.000	1.000	0.2059
234226_at	26.2	5.6	-2.22	-4.67	AAA-AAA	0.00	999	1.000	1.000	0.0107
234821_at	30.6	6.6	-2.22	-4.67	AAM-AAA	0.00	999	1.000	1.000	0.0781
232694_at	14.8	3.2	-2.22	-4.67	AAA-AAA	0.00	999	1.000	1.000	0.1611
1563985_at	37.7	8.1	-2.22	-4.66	AAA-AAA	0.00	999	1.000	1.000	0.1126
1552425_a_at	50.0	10.7	-2.22	-4.66	AAA-AAA	0.00	999	1.000	1.000	0.1896
237066_at	42.7	9.2	-2.22	-4.66	AAM-AAA	0.00	999	1.000	1.000	0.2086
240820_at	57.8	12.4	-2.22	-4.66	AAA-AAA	0.00	999	1.000	1.000	0.0877
1556964_s_at	41.7	8.9	-2.22	-4.66	AAA-AAA	0.00	999	1.000	1.000	0.2052
222051_s_at	39.2	8.4	-2.22	-4.66	AAA-AAA	0.00	999	1.000	1.000	0.1946
236792_at	27.3	5.9	-2.22	-4.66	AAA-AAA	0.00	999	1.000	1.000	0.0869
225700_at	5.3	24.4	2.22	4.66	AAA-AAA	0.00	999	1.000	1.000	0.0789
222974_at	18.7	4.0	-2.22	-4.66	AAA-AAA	0.00	999	1.000	1.000	0.0957
1563827_at	11.5	2.5	-2.22	-4.65	AAA-AAA	0.00	999	1.000	1.000	0.2399
AFFX-r2-Bs-phe-M_at	22.7	4.9	-2.22	-4.65	AMA-AAA	0.00	999	1.000	1.000	0.0613
239129_at	33.6	7.2	-2.22	-4.65	AAA-AAA	0.00	999	1.000	1.000	0.0603
240323_at	31.3	6.7	-2.22	-4.65	AAA-AAA	0.00	999	1.000	1.000	0.0113
1566978_at	21.2	4.6	-2.22	-4.65	AAA-AAA	0.00	999	1.000	1.000	0.1341
1558640_a_at	4.6	21.4	2.22	4.65	AAA-AAA	0.00	999	1.000	1.000	0.2576
1561249_a_at	15.2	3.3	-2.22	-4.64	AAA-AAA	0.00	999	1.000	1.000	0.1102
208049_s_at	19.4	4.2	-2.22	-4.64	AAA-AAA	0.00	999	1.000	1.000	0.1305
220654_at	14.9	3.2	-2.22	-4.64	AAA-AAA	0.00	999	1.000	1.000	0.1121
234772_s_at	21.9	4.7	-2.22	-4.64	AAA-AAA	0.00	999	1.000	1.000	0.1530
237495_at	6.0	27.8	2.22	4.64	AAA-AAA	0.00	999	1.000	1.000	0.0939
1555103_s_at	25.7	5.5	-2.21	-4.64	AAA-AAA	0.00	999	1.000	1.000	0.2337
231304_at	24.4	5.3	-2.21	-4.64	AAA-AAA	0.00	999	1.000	1.000	0.0540
242566_at	23.2	5.0	-2.21	-4.64	AAA-AAA	0.00	999	1.000	1.000	0.1306
243397_at	37.5	8.1	-2.21	-4.64	AAA-AAA	0.00	999	1.000	1.000	0.2499
1569756_at	8.8	1.9	-2.21	-4.64	AAA-AAA	0.00	999	1.000	1.000	0.3001

240916_x_at	49.9	10.8	-2.21	-4.63	AAA-AAA	0.00	999	1.000	1.000	0.0169
240597_at	6.2	28.9	2.21	4.63	AAA-AAA	0.00	999	1.000	1.000	0.0809
1556038_at	27.4	5.9	-2.21	-4.63	AAA-AAA	0.00	999	1.000	1.000	0.1999
209169_at	23.8	5.1	-2.21	-4.63	AAA-AAA	0.00	999	1.000	1.000	0.1402
215593_at	13.5	2.9	-2.21	-4.63	AAA-AAA	0.00	999	1.000	1.000	0.1008
237853_x_at	23.1	5.0	-2.21	-4.63	AAA-AAA	0.00	999	1.000	1.000	0.0049
231196_x_at	10.2	47.2	2.21	4.63	AAA-AAA	0.00	999	1.000	1.000	0.0006
232275_s_at	9.9	2.1	-2.21	-4.63	AAA-AAA	0.00	999	1.000	1.000	0.0235
1554524_a_at	14.5	3.1	-2.21	-4.63	AAA-AAA	0.00	999	1.000	1.000	0.1323
206768_at	43.7	9.4	-2.21	-4.63	AAA-AAA	0.00	999	1.000	1.000	0.2616
233677_at	31.7	6.9	-2.21	-4.63	AAA-AAA	0.00	999	1.000	1.000	0.1978
240017_at	14.1	3.1	-2.21	-4.63	AAA-AAA	0.00	999	1.000	1.000	0.0769
219005_at	27.0	5.8	-2.21	-4.62	AAA-AAA	0.00	999	1.000	1.000	0.1936
240695_at	48.8	10.6	-2.21	-4.62	AAA-AAA	0.00	999	1.000	1.000	0.0096
1563920_at	35.8	7.8	-2.21	-4.62	AAA-AAA	0.00	999	1.000	1.000	0.0023
226701_at	4.4	20.4	2.21	4.62	AAA-AAA	0.00	999	1.000	1.000	0.2477
241231_at	98.1	21.3	-2.21	-4.62	AAA-AAA	0.00	999	1.000	1.000	0.0596
217006_x_at	7.9	36.2	2.21	4.62	AAA-AAA	0.00	999	1.000	1.000	0.0781
234110_at	13.1	2.8	-2.21	-4.62	AAA-AAA	0.00	999	1.000	1.000	0.2579
1554983_at	55.5	12.0	-2.21	-4.61	AAA-AAA	0.00	999	1.000	1.000	0.0762
1558034_s_at	11.1	2.4	-2.21	-4.61	AAA-AAA	0.00	999	1.000	1.000	0.1070
1557484_at	41.1	8.9	-2.21	-4.61	AAA-AAA	0.00	999	1.000	1.000	0.0573
222325_at	8.6	1.9	-2.21	-4.61	AAA-AAA	0.00	999	1.000	1.000	0.0418
1563072_at	5.5	1.2	-2.20	-4.61	AAA-AAA	0.00	999	1.000	1.000	0.0434
233834_at	12.1	56.0	2.20	4.61	AAA-AMA	0.00	999	1.000	1.000	0.0503
234957_at	64.2	13.9	-2.20	-4.61	AAA-AAA	0.00	999	1.000	1.000	0.0137
1556601_a_at	25.2	5.5	-2.20	-4.61	AAA-AAA	0.00	999	1.000	1.000	0.1315
231565_at	3.8	17.4	2.20	4.61	AAA-AAA	0.00	999	1.000	1.000	0.1574
206947_at	65.7	14.3	-2.20	-4.60	AAA-AAA	0.00	999	1.000	1.000	0.1348
224226_at	3.7	16.9	2.20	4.60	AAA-AAA	0.00	999	1.000	1.000	0.1604
214575_s_at	37.4	8.1	-2.20	-4.60	AAA-AAA	0.00	999	1.000	1.000	0.0364
229160_at	3.9	18.1	2.20	4.60	AAA-AAA	0.00	999	1.000	1.000	0.1981
243892_at	33.0	7.2	-2.20	-4.59	MAA-AAA	0.00	999	1.000	1.000	0.1914
1555162_at	43.8	9.5	-2.20	-4.60	AAA-AAA	0.00	999	1.000	1.000	0.1495
234867_at	5.6	25.6	2.20	4.59	PAA-AAA	0.00	999	1.000	1.000	0.2339
237455_at	13.0	59.5	2.20	4.59	AAA-PAA	0.00	999	1.000	1.000	0.1026
243930_x_at	54.4	11.9	-2.20	-4.59	AAA-AAA	0.00	999	1.000	1.000	0.0038
1569724_at	81.0	17.7	-2.20	-4.59	AAA-AAA	0.00	999	1.000	1.000	0.1578
1564075_a_at	11.0	50.4	2.20	4.59	AAA-AAA	0.00	999	1.000	1.000	0.0121
206135_at	14.0	3.1	-2.20	-4.59	AAA-AAA	0.00	999	1.000	1.000	0.1216
243929_at	7.9	1.7	-2.20	-4.59	AAA-AAA	0.00	999	1.000	1.000	0.1241
241330_x_at	10.1	2.2	-2.20	-4.59	AAA-AAA	0.00	999	1.000	1.000	0.0715

1561314_at	29.8	6.5	-2.20	-4.59	AAA-AAA	0.00	999	1.000	1.000	0.0197
244683_at	40.0	8.7	-2.20	-4.59	AAA-AAA	0.00	999	1.000	1.000	0.0795
214362_at	7.1	32.4	2.20	4.59	AAA-AAA	0.00	999	1.000	1.000	0.0974
239444_at	8.5	38.8	2.20	4.59	AAA-AAA	0.00	999	1.000	1.000	0.0280
1555708_a_at	17.0	3.7	-2.20	-4.58	AAA-AAA	0.00	999	1.000	1.000	0.0589
1553249_at	40.7	8.9	-2.20	-4.58	AAA-AAA	0.00	999	1.000	1.000	0.0636
1555102_at	2.2	10.2	2.20	4.58	AAA-AAA	0.00	999	1.000	1.000	0.1987
1553856_s_at	61.1	13.4	-2.19	-4.57	AAA-AAA	0.00	999	1.000	1.000	0.0645
1552325_at	3.5	15.8	2.19	4.58	AAA-AAA	0.00	999	1.000	1.000	0.0072
222356_at	46.5	10.2	-2.19	-4.58	AAA-AAA	0.00	999	1.000	1.000	0.1235
240267_at	17.1	3.8	-2.19	-4.57	AAA-AAA	0.00	999	1.000	1.000	0.1497
242394_at	11.9	54.3	2.19	4.57	AAA-AAA	0.00	999	1.000	1.000	0.0279
216778_s_at	21.1	4.6	-2.19	-4.57	AAA-AAA	0.00	999	1.000	1.000	0.1952
232077_s_at	61.8	13.5	-2.19	-4.57	AAA-AAA	0.00	999	1.000	1.000	0.0534
240944_at	54.6	11.9	-2.19	-4.57	AAA-AAA	0.00	999	1.000	1.000	0.1320
206812_at	37.8	8.3	-2.19	-4.57	AAA-AAA	0.00	999	1.000	1.000	0.0802
241891_at	14.0	3.1	-2.19	-4.57	AAA-AAA	0.00	999	1.000	1.000	0.0334
1560800_at	9.8	2.2	-2.19	-4.57	AAA-AAA	0.00	999	1.000	1.000	0.2650
220930_s_at	26.4	5.8	-2.19	-4.57	AAA-AAA	0.00	999	1.000	1.000	0.1925
230340_s_at	72.0	15.8	-2.19	-4.57	AAM-AAA	0.00	999	1.000	1.000	0.0615
244598_at	40.6	8.9	-2.19	-4.56	AAA-AAA	0.00	999	1.000	1.000	0.0139
224421_x_at	5.7	26.2	2.19	4.56	AAA-AAA	0.00	999	1.000	1.000	0.0551
234044_at	52.0	11.4	-2.19	-4.56	AAA-AAA	0.00	999	1.000	1.000	0.0237
241015_at	24.4	5.4	-2.19	-4.55	AAA-AAA	0.00	999	1.000	1.000	0.2984
242342_at	25.6	5.6	-2.19	-4.55	AAA-AAA	0.00	999	1.000	1.000	0.1875
243728_at	18.8	4.1	-2.19	-4.55	AAA-AAA	0.00	999	1.000	1.000	0.1060
220726_at	2.2	10.1	2.19	4.55	AAA-AAA	0.00	999	1.000	1.000	0.1594
240876_x_at	9.3	2.1	-2.19	-4.55	AAA-AAA	0.00	999	1.000	1.000	0.1368
1561870_at	15.5	3.4	-2.19	-4.55	AAA-AAA	0.00	999	1.000	1.000	0.2073
1553838_at	40.4	8.9	-2.19	-4.55	AAA-AAA	0.00	999	1.000	1.000	0.1390
236389_x_at	34.8	7.7	-2.19	-4.55	AAA-AAA	0.00	999	1.000	1.000	0.2637
241047_at	31.5	6.9	-2.19	-4.55	AAA-AAA	0.00	999	1.000	1.000	0.0243
1562608_at	9.1	2.0	-2.19	-4.55	AAA-AAA	0.00	999	1.000	1.000	0.2195
1568752_s_at	6.7	1.5	-2.19	-4.55	AAA-AAA	0.00	999	1.000	1.000	0.1442
241860_at	5.4	24.5	2.18	4.54	AAA-AAA	0.00	999	1.000	1.000	0.1770
234811_at	45.5	10.0	-2.18	-4.54	AAA-AAA	0.00	999	1.000	1.000	0.0273
237447_at	41.1	9.1	-2.18	-4.54	AAA-AAA	0.00	999	1.000	1.000	0.0294
228788_at	20.8	4.6	-2.18	-4.54	AAA-AAA	0.00	999	1.000	1.000	0.0055
232660_at	77.8	17.1	-2.18	-4.54	AAA-AAA	0.00	999	1.000	1.000	0.0128
233430_at	71.6	15.8	-2.18	-4.54	AAA-AAA	0.00	999	1.000	1.000	0.0315
205612_at	8.7	1.9	-2.18	-4.54	AAA-AAA	0.00	999	1.000	1.000	0.1703
1554813_at	26.4	5.8	-2.18	-4.54	AAA-AAA	0.00	999	1.000	1.000	0.1056

237584_at	23.1	5.1	-2.18	-4.54	AAA-AAA	0.00	999	1.000	1.000	0.0985
233591_at	26.7	5.9	-2.18	-4.54	AMA-AAA	0.00	999	1.000	1.000	0.2159
1563026_at	17.3	3.8	-2.18	-4.54	AAA-AAA	0.00	999	1.000	1.000	0.2279
237390_at	8.6	1.9	-2.18	-4.54	AAA-AAA	0.00	999	1.000	1.000	0.0920
236351_at	35.9	7.9	-2.18	-4.53	AAA-AAA	0.00	999	1.000	1.000	0.2261
228806_at	15.9	3.5	-2.18	-4.53	AAA-AAA	0.00	999	1.000	1.000	0.0386
244745_at	9.8	2.2	-2.18	-4.52	AAA-AAA	0.00	999	1.000	1.000	0.1189
225966_at	28.4	6.3	-2.18	-4.52	AAA-AAA	0.00	999	1.000	1.000	0.1273
242491_at	21.7	4.8	-2.18	-4.52	AAA-AAA	0.00	999	1.000	1.000	0.3498
224557_x_at	50.7	11.2	-2.18	-4.52	AAA-AAA	0.00	999	1.000	1.000	0.0546
242581_at	17.1	3.8	-2.18	-4.52	AAA-AAA	0.00	999	1.000	1.000	0.0437
239360_at	106.1	23.5	-2.17	-4.51	AAA-AAA	0.00	999	1.000	1.000	0.0233
228137_s_at	35.5	7.9	-2.17	-4.51	AAA-AAA	0.00	999	1.000	1.000	0.0467
222222_s_at	63.0	283.8	2.17	4.50	AAA-AAA	0.00	999	1.000	1.000	0.1344
208399_s_at	15.9	3.5	-2.17	-4.50	AAA-AAA	0.00	999	1.000	1.000	0.1259
232854_at	10.8	2.4	-2.17	-4.50	AAA-AAA	0.00	999	1.000	1.000	0.0335
233282_at	31.8	7.1	-2.17	-4.50	AAA-AAA	0.00	999	1.000	1.000	0.1011
231994_at	17.6	3.9	-2.17	-4.50	AAA-AAA	0.00	999	1.000	1.000	0.0308
1566544_at	1.4	6.3	2.17	4.50	AAA-AAA	0.00	999	1.000	1.000	0.0470
244731_at	6.2	1.4	-2.17	-4.50	AAA-AAA	0.00	999	1.000	1.000	0.0051
222934_s_at	56.7	12.6	-2.17	-4.49	MAA-AAA	0.00	999	1.000	1.000	0.0649
206635_at	27.3	122.6	2.17	4.49	AAA-AAA	0.00	999	1.000	1.000	0.0220
210032_s_at	29.1	6.5	-2.17	-4.49	AAA-AAA	0.00	999	1.000	1.000	0.0962
202921_s_at	52.6	11.7	-2.17	-4.49	AAA-AAA	0.00	999	1.000	1.000	0.0020
1556997_a_at	6.6	1.5	-2.17	-4.49	AAA-AAA	0.00	999	1.000	1.000	0.0371
211617_at	14.8	3.3	-2.17	-4.49	AAA-AAA	0.00	999	1.000	1.000	0.0224
238364_x_at	12.1	2.7	-2.17	-4.49	AAA-AAA	0.00	999	1.000	1.000	0.1525
215184_at	33.8	7.5	-2.17	-4.48	AAA-AAA	0.00	999	1.000	1.000	0.0367
1570024_at	19.0	4.2	-2.16	-4.48	AAA-AAA	0.00	999	1.000	1.000	0.0097
234453_s_at	24.1	5.4	-2.16	-4.48	AAA-AAA	0.00	999	1.000	1.000	0.1754
1556235_at	9.0	40.5	2.16	4.48	APA-APA	0.00	999	1.000	1.000	0.2646
241791_at	29.6	6.6	-2.16	-4.48	AAA-AAA	0.00	999	1.000	1.000	0.0799
224487_at	43.3	9.7	-2.16	-4.48	AAA-AAA	0.00	999	1.000	1.000	0.0331
235017_s_at	28.4	6.3	-2.16	-4.48	AAA-AAA	0.00	999	1.000	1.000	0.0647
1560264_at	4.5	20.3	2.16	4.47	AAA-AAM	0.00	999	1.000	1.000	0.2419
214667_s_at	79.1	17.7	-2.16	-4.48	AAA-AAA	0.00	999	1.000	1.000	0.0954
229475_at	23.5	5.3	-2.16	-4.47	AAA-AAA	0.00	999	1.000	1.000	0.3049
239108_at	16.2	72.6	2.16	4.47	AAP-PPP	0.00	999	1.000	1.000	0.2098
227984_at	29.5	6.6	-2.16	-4.47	AAA-AAA	0.00	999	1.000	1.000	0.2233
1569380_a_at	27.6	6.2	-2.16	-4.47	AAA-AAA	0.00	999	1.000	1.000	0.0576
1569585_at	40.2	9.0	-2.16	-4.47	AAA-AAA	0.00	999	1.000	1.000	0.0474
1553633_s_at	42.8	9.6	-2.16	-4.47	MAA-AAA	0.00	999	1.000	1.000	0.1647

1554723_x_at	19.6	4.4	-2.16	-4.47	AAA-AAA	0.00	999	1.000	1.000	0.1149
244493_at	11.9	2.7	-2.16	-4.47	AAA-AAA	0.00	999	1.000	1.000	0.2188
234679_at	19.4	4.3	-2.16	-4.47	AAA-AAA	0.00	999	1.000	1.000	0.1020
243082_at	6.8	1.5	-2.16	-4.47	AAA-AAA	0.00	999	1.000	1.000	0.0871
1568836_at	47.1	10.6	-2.16	-4.47	AAA-AAA	0.00	999	1.000	1.000	0.1806
221081_s_at	100.0	22.4	-2.16	-4.47	AAA-AAA	0.00	999	1.000	1.000	0.0306
1561828_at	16.5	3.7	-2.16	-4.46	AAA-AAA	0.00	999	1.000	1.000	0.1014
1569568_at	22.3	5.0	-2.16	-4.47	AAA-AAA	0.00	999	1.000	1.000	0.1782
230963_at	24.9	5.6	-2.16	-4.46	AAA-AAA	0.00	999	1.000	1.000	0.0462
240423_at	58.9	13.2	-2.16	-4.46	AAA-AAA	0.00	999	1.000	1.000	0.1140
1561557_at	55.3	12.4	-2.16	-4.46	AAA-AAA	0.00	999	1.000	1.000	0.0570
212524_x_at	24.7	5.5	-2.16	-4.46	AAA-AAA	0.00	999	1.000	1.000	0.0192
209183_s_at	98.2	22.0	-2.16	-4.46	AAA-AAA	0.00	999	1.000	1.000	0.0938
217493_x_at	161.7	36.3	-2.16	-4.46	AAA-AAA	0.00	999	1.000	1.000	0.0900
237828_at	9.5	2.1	-2.16	-4.46	AAA-AAA	0.00	999	1.000	1.000	0.1102
206305_s_at	20.5	4.6	-2.16	-4.46	AAA-AAA	0.00	999	1.000	1.000	0.0741
241230_at	38.3	8.6	-2.15	-4.45	AAA-AAA	0.00	999	1.000	1.000	0.0082
1568606_at	60.1	13.5	-2.15	-4.45	AAA-AAA	0.00	999	1.000	1.000	0.1860
1555217_at	14.0	3.1	-2.15	-4.45	AAA-AAA	0.00	999	1.000	1.000	0.0119
227289_at	27.3	6.1	-2.15	-4.45	AAA-AAA	0.00	999	1.000	1.000	0.2553
236889_at	10.8	48.2	2.15	4.45	AAA-AAA	0.00	999	1.000	1.000	0.0555
1562973_at	14.2	3.2	-2.15	-4.45	AAA-AAA	0.00	999	1.000	1.000	0.1544
1568925_at	8.1	1.8	-2.15	-4.45	AAA-AAA	0.00	999	1.000	1.000	0.0510
1564401_at	39.2	8.8	-2.15	-4.45	AAA-AAA	0.00	999	1.000	1.000	0.2320
244762_at	8.2	36.3	2.15	4.44	AAA-APA	0.00	999	1.000	1.000	0.1316
242943_at	6.0	1.4	-2.15	-4.45	AAA-AAA	0.00	999	1.000	1.000	0.2494
229387_at	15.6	3.5	-2.15	-4.44	AAA-AAA	0.00	999	1.000	1.000	0.0573
231483_at	16.5	3.7	-2.15	-4.44	AAA-AAA	0.00	999	1.000	1.000	0.1891
1569777_a_at	13.0	2.9	-2.15	-4.44	AAA-AAA	0.00	999	1.000	1.000	0.1206
223929_s_at	42.2	9.5	-2.15	-4.44	AAA-AAA	0.00	999	1.000	1.000	0.0192
217330_at	49.8	11.2	-2.15	-4.44	AAA-AAA	0.00	999	1.000	1.000	0.1010
216963_s_at	96.4	21.7	-2.15	-4.44	AAA-AAA	0.00	999	1.000	1.000	0.0069
215814_at	21.3	4.8	-2.15	-4.44	AAA-AAA	0.00	999	1.000	1.000	0.2426
240210_at	33.9	7.6	-2.15	-4.44	AAA-AAA	0.00	999	1.000	1.000	0.0442
242054_s_at	26.8	6.0	-2.15	-4.44	AAA-AAA	0.00	999	1.000	1.000	0.1795
236536_at	69.4	15.6	-2.15	-4.44	AAA-AAA	0.00	999	1.000	1.000	0.0691
240588_at	42.4	9.6	-2.15	-4.44	AAA-AAA	0.00	999	1.000	1.000	0.2326
1561294_a_at	23.3	5.3	-2.15	-4.43	AMA-AAA	0.00	999	1.000	1.000	0.1396
1568843_at	61.4	13.9	-2.15	-4.43	AAA-AAA	0.00	999	1.000	1.000	0.1002
1554147_s_at	27.9	6.3	-2.15	-4.44	AAA-AAA	0.00	999	1.000	1.000	0.0499
1570405_at	59.2	13.4	-2.15	-4.43	AAA-AAA	0.00	999	1.000	1.000	0.1522
230420_at	25.7	5.8	-2.15	-4.43	MAA-AAA	0.00	999	1.000	1.000	0.2597

1563906_at	30.5	6.9	-2.15	-4.43	AAA-AAA	0.00	999	1.000	1.000	0.0537
1566846_at	17.4	3.9	-2.15	-4.43	AAA-AAA	0.00	999	1.000	1.000	0.0982
1554382_at	56.0	12.6	-2.15	-4.43	AAA-AAA	0.00	999	1.000	1.000	0.0021
214347_s_at	29.1	6.6	-2.15	-4.43	AAA-AAA	0.00	999	1.000	1.000	0.0536
229177_at	22.8	5.2	-2.15	-4.43	AAA-AAA	0.00	999	1.000	1.000	0.0178
1555151_s_at	36.3	8.2	-2.15	-4.43	AAA-AAA	0.00	999	1.000	1.000	0.0264
219791_s_at	8.7	38.3	2.15	4.43	AAA-AAA	0.00	999	1.000	1.000	0.0085
216830_at	23.9	5.4	-2.15	-4.43	AAA-AAA	0.00	999	1.000	1.000	0.0813
1564093_at	8.5	37.6	2.14	4.42	AAA-AAP	0.00	999	1.000	1.000	0.1720
242534_at	6.8	30.1	2.14	4.42	AAA-AAA	0.00	999	1.000	1.000	0.0458
202242_at	33.9	7.7	-2.14	-4.42	AAA-AAA	0.00	999	1.000	1.000	0.0042
210727_at	32.6	7.4	-2.14	-4.42	AAA-AAA	0.00	999	1.000	1.000	0.0899
214151_s_at	43.8	193.3	2.14	4.42	AAA-APP	0.00	999	1.000	1.000	0.1221
244170_at	11.6	2.6	-2.14	-4.42	AAA-AAA	0.00	999	1.000	1.000	0.0712
216312_at	24.5	5.5	-2.14	-4.42	AAA-AAA	0.00	999	1.000	1.000	0.0501
238018_at	15.3	3.5	-2.14	-4.42	AAA-AAA	0.00	999	1.000	1.000	0.2592
243862_at	4.3	19.0	2.14	4.41	AAA-AAA	0.00	999	1.000	1.000	0.1728
1552629_a_at	30.6	6.9	-2.14	-4.41	AAA-AAA	0.00	999	1.000	1.000	0.1118
236673_at	34.5	7.8	-2.14	-4.41	AAA-AAA	0.00	999	1.000	1.000	0.1015
237840_at	59.3	13.5	-2.14	-4.41	AAA-AAA	0.00	999	1.000	1.000	0.0010
228665_at	36.3	8.2	-2.14	-4.41	AAA-AAA	0.00	999	1.000	1.000	0.1294
219962_at	45.4	10.3	-2.14	-4.41	AAA-AAA	0.00	999	1.000	1.000	0.0855
222939_s_at	20.8	4.7	-2.14	-4.41	AAA-AAA	0.00	999	1.000	1.000	0.2087
233076_at	18.9	4.3	-2.14	-4.41	AAA-AAA	0.00	999	1.000	1.000	0.1928
242231_at	64.2	14.6	-2.14	-4.41	AAA-AAA	0.00	999	1.000	1.000	0.1422
1560464_at	25.4	5.8	-2.14	-4.41	AAA-AAA	0.00	999	1.000	1.000	0.2035
242076_at	58.2	13.2	-2.14	-4.40	AAA-AAA	0.00	999	1.000	1.000	0.1644
1553734_at	11.7	2.7	-2.14	-4.40	AAA-AAA	0.00	999	1.000	1.000	0.1393
241161_at	17.3	3.9	-2.14	-4.40	AAA-AAA	0.00	999	1.000	1.000	0.1155
1565544_at	10.4	2.4	-2.14	-4.40	AAA-AAA	0.00	999	1.000	1.000	0.1345
203144_s_at	8.5	37.2	2.14	4.40	AAA-AAA	0.00	999	1.000	1.000	0.1241
1555401_at	40.2	9.2	-2.14	-4.39	AAA-AAA	0.00	999	1.000	1.000	0.0920
1557519_at	13.3	3.0	-2.14	-4.40	AAA-AAA	0.00	999	1.000	1.000	0.0659
243550_at	20.2	88.7	2.14	4.39	APP-PPP	0.00	999	1.000	1.000	0.3819
1557862_at	48.2	11.0	-2.14	-4.39	AAA-AAA	0.00	999	1.000	1.000	0.0061
1559462_at	38.3	8.7	-2.14	-4.39	AAA-AAA	0.00	999	1.000	1.000	0.0462
228210_at	90.3	20.6	-2.14	-4.39	AAA-AAA	0.00	999	1.000	1.000	0.1877
235564_at	4.0	17.6	2.13	4.39	AAA-APP	0.00	999	1.000	1.000	0.1086
1561690_at	14.8	3.4	-2.13	-4.39	AAA-AAA	0.00	999	1.000	1.000	0.1299
244585_at	62.1	14.2	-2.13	-4.39	AAA-AAA	0.00	999	1.000	1.000	0.0338
216223_at	37.4	8.5	-2.13	-4.39	AAA-AAA	0.00	999	1.000	1.000	0.0215
244351_at	14.3	3.3	-2.13	-4.39	AAA-AAA	0.00	999	1.000	1.000	0.2007

219385_at	12.5	54.6	2.13	4.39	AAA-AAA	0.00	999	1.000	1.000	0.1655
1561665_at	10.1	2.3	-2.13	-4.38	AAA-AAA	0.00	999	1.000	1.000	0.0395
215520_at	24.7	5.6	-2.13	-4.38	AAA-AAA	0.00	999	1.000	1.000	0.2017
1561702_at	60.9	13.9	-2.13	-4.37	AAA-AAA	0.00	999	1.000	1.000	0.2162
244721_at	38.7	8.8	-2.13	-4.37	AAA-AAA	0.00	999	1.000	1.000	0.1372
201547_at	21.5	4.9	-2.13	-4.37	AAA-AAA	0.00	999	1.000	1.000	0.0570
1558987_at	37.7	8.6	-2.13	-4.37	AAA-AAA	0.00	999	1.000	1.000	0.0744
233338_at	32.7	7.5	-2.13	-4.38	AAA-AAA	0.00	999	1.000	1.000	0.2005
240563_at	17.4	4.0	-2.13	-4.37	AAA-AAA	0.00	999	1.000	1.000	0.1869
1566044_at	65.8	15.0	-2.13	-4.37	AAA-AAA	0.00	999	1.000	1.000	0.1686
1554156_a_at	10.3	2.4	-2.13	-4.37	AAA-AAA	0.00	999	1.000	1.000	0.0886
1553022_at	14.4	3.3	-2.13	-4.37	AAA-AAA	0.00	999	1.000	1.000	0.1801
1564603_at	44.3	10.1	-2.13	-4.37	AAA-AAA	0.00	999	1.000	1.000	0.1780
225960_at	12.4	54.2	2.13	4.37	AAA-AAA	0.00	999	1.000	1.000	0.0008
218976_at	20.0	87.4	2.13	4.37	AAA-MAA	0.00	999	1.000	1.000	0.0951
233260_at	28.9	6.6	-2.13	-4.37	AAA-AAA	0.00	999	1.000	1.000	0.0615
240088_at	3.4	15.0	2.13	4.36	AAA-AAA	0.00	999	1.000	1.000	0.2737
211606_at	16.5	3.8	-2.13	-4.37	AAA-AAA	0.00	999	1.000	1.000	0.1442
237472_at	40.7	9.3	-2.13	-4.36	AAA-AAA	0.00	999	1.000	1.000	0.2306
242497_at	28.1	6.4	-2.13	-4.36	AAA-AAA	0.00	999	1.000	1.000	0.1240
1565924_a_at	7.8	1.8	-2.13	-4.36	AAA-AAA	0.00	999	1.000	1.000	0.2001
215625_at	6.5	28.5	2.13	4.36	AAA-AAA	0.00	999	1.000	1.000	0.0320
237544_at	5.7	24.8	2.13	4.36	AAA-AAA	0.00	999	1.000	1.000	0.0733
1562683_a_at	14.2	3.3	-2.12	-4.36	AAA-AAA	0.00	999	1.000	1.000	0.0408
221795_at	6.5	28.1	2.12	4.36	AAA-AAA	0.00	999	1.000	1.000	0.0483
234693_at	51.0	11.7	-2.12	-4.36	AAA-AAA	0.00	999	1.000	1.000	0.1238
229308_at	15.5	3.6	-2.12	-4.36	AAA-AAA	0.00	999	1.000	1.000	0.2511
240193_at	5.2	22.6	2.12	4.35	AAA-PAA	0.00	999	1.000	1.000	0.0862
1562215_at	29.9	6.9	-2.12	-4.35	AAA-AAA	0.00	999	1.000	1.000	0.0677
1554849_at	15.6	68.1	2.12	4.35	AAA-AAA	0.00	999	1.000	1.000	0.0062
1552522_at	30.7	7.1	-2.12	-4.35	AAA-AAA	0.00	999	1.000	1.000	0.3461
219847_at	59.4	13.7	-2.12	-4.35	AAA-AAA	0.00	999	1.000	1.000	0.0741
210330_at	26.4	6.1	-2.12	-4.35	AAA-AAA	0.00	999	1.000	1.000	0.0697
237849_at	48.7	11.2	-2.12	-4.35	AAM-AAA	0.00	999	1.000	1.000	0.2331
240512_x_at	14.4	3.3	-2.12	-4.35	AAA-AAA	0.00	999	1.000	1.000	0.1608
236960_at	42.9	9.9	-2.12	-4.35	AAA-AAA	0.00	999	1.000	1.000	0.0013
231686_at	27.7	6.4	-2.12	-4.35	AAA-AAA	0.00	999	1.000	1.000	0.0160
1569558_at	11.5	2.6	-2.12	-4.35	AAA-AAA	0.00	999	1.000	1.000	0.0643
1553070_a_at	64.1	14.8	-2.12	-4.35	AAA-AAA	0.00	999	1.000	1.000	0.1347
1552598_at	60.4	13.9	-2.12	-4.35	AAA-AAA	0.00	999	1.000	1.000	0.1069
216057_at	31.2	7.2	-2.12	-4.35	AAA-AAA	0.00	999	1.000	1.000	0.1828
244142_at	26.2	6.0	-2.12	-4.34	AAA-AAA	0.00	999	1.000	1.000	0.0344

1567704_at	104.7	24.1	-2.12	-4.34	AAA-AAA	0.00	999	1.000	1.000	0.1654
227704_at	8.9	38.5	2.12	4.34	AAA-AAA	0.00	999	1.000	1.000	0.0612
1552912_a_at	36.3	8.4	-2.12	-4.34	AAA-AAA	0.00	999	1.000	1.000	0.1559
1557395_at	6.5	1.5	-2.12	-4.34	AAA-AAA	0.00	999	1.000	1.000	0.2003
217392_at	3.5	15.4	2.12	4.34	AAA-AAA	0.00	999	1.000	1.000	0.2191
242935_at	32.7	141.7	2.12	4.34	AAP-PPP	0.00	999	1.000	1.000	0.0706
1561232_at	45.6	10.5	-2.12	-4.33	AAA-AAA	0.00	999	1.000	1.000	0.0915
214914_at	5.3	1.2	-2.12	-4.33	AAA-AAA	0.00	999	1.000	1.000	0.0637
205875_s_at	44.4	192.4	2.12	4.33	AAA-AAA	0.00	999	1.000	1.000	0.1539
219845_at	59.9	13.8	-2.12	-4.33	AAA-AAA	0.00	999	1.000	1.000	0.0772
204865_at	9.1	39.4	2.12	4.33	AAA-AAA	0.00	999	1.000	1.000	0.2561
219704_at	46.8	10.8	-2.11	-4.33	AAA-AAA	0.00	999	1.000	1.000	0.0687
242971_at	53.2	12.3	-2.11	-4.33	AAA-AAA	0.00	999	1.000	1.000	0.1245
224066_s_at	22.9	99.2	2.11	4.33	AAA-AAA	0.00	999	1.000	1.000	0.0028
243932_at	29.3	6.8	-2.11	-4.32	AAA-AAA	0.00	999	1.000	1.000	0.1016
241271_at	9.8	2.3	-2.11	-4.32	AAA-AAA	0.00	999	1.000	1.000	0.1307
208408_at	73.0	16.9	-2.11	-4.32	AAA-AAA	0.00	999	1.000	1.000	0.2188
218484_at	78.2	18.1	-2.11	-4.32	AAA-AAA	0.00	999	1.000	1.000	0.0125
230323_s_at	5.0	1.2	-2.11	-4.32	AAA-AAA	0.00	999	1.000	1.000	0.1226
240961_at	11.6	2.7	-2.11	-4.32	AAA-AAA	0.00	999	1.000	1.000	0.2575
206595_at	9.0	39.0	2.11	4.32	AAA-AAA	0.00	999	1.000	1.000	0.0395
1569873_at	10.7	2.5	-2.11	-4.32	AAA-AAA	0.00	999	1.000	1.000	0.2919
1559022_at	55.4	12.8	-2.11	-4.32	AAA-AAA	0.00	999	1.000	1.000	0.0766
1559117_at	50.2	11.6	-2.11	-4.32	AAA-AAA	0.00	999	1.000	1.000	0.2214
1559224_at	144.7	33.5	-2.11	-4.32	AAA-AAA	0.00	999	1.000	1.000	0.0558
207381_at	24.7	5.7	-2.11	-4.31	AAA-AAA	0.00	999	1.000	1.000	0.0815
229836_s_at	11.3	2.6	-2.11	-4.31	AAA-AAA	0.00	999	1.000	1.000	0.2066
1562630_at	26.3	6.1	-2.11	-4.31	AAA-AAA	0.00	999	1.000	1.000	0.0047
1554610_at	36.3	8.4	-2.11	-4.31	AAA-AAA	0.00	999	1.000	1.000	0.0552
1552289_a_at	68.5	15.9	-2.11	-4.31	AAA-AAA	0.00	999	1.000	1.000	0.0561
224052_at	7.2	30.9	2.11	4.31	AAA-AAM	0.00	999	1.000	1.000	0.2333
216656_at	22.8	5.3	-2.11	-4.31	AAA-AAA	0.00	999	1.000	1.000	0.1209
206082_at	5.7	24.6	2.11	4.31	AAA-AAA	0.00	999	1.000	1.000	0.0224
206142_at	14.6	3.4	-2.11	-4.31	AAA-AAA	0.00	999	1.000	1.000	0.0697
231504_at	20.8	4.8	-2.11	-4.31	AAA-AAA	0.00	999	1.000	1.000	0.1933
230902_at	6.9	29.9	2.11	4.31	AAA-AAA	0.00	999	1.000	1.000	0.0039
241468_at	25.2	5.9	-2.11	-4.30	AAA-AAA	0.00	999	1.000	1.000	0.0196
1569171_a_at	31.4	7.3	-2.11	-4.30	AAA-AAA	0.00	999	1.000	1.000	0.1437
226499_at	209.9	48.8	-2.11	-4.30	MAM-AAA	0.00	999	1.000	1.000	0.0531
228942_s_at	4.4	18.8	2.11	4.30	AAA-AAA	0.00	999	1.000	1.000	0.1525
233754_x_at	14.8	63.5	2.10	4.30	AAA-AAA	0.00	999	1.000	1.000	0.1630
241107_at	14.4	62.0	2.10	4.30	AAA-AAP	0.00	999	1.000	1.000	0.1170

239672_at	14.1	60.5	2.10	4.30	APA-PPP	0.00	999	1.000	1.000	0.1109
243771_at	11.4	2.7	-2.10	-4.30	AAA-AAA	0.00	999	1.000	1.000	0.0864
1567000_at	26.0	6.1	-2.10	-4.30	AAA-AAA	0.00	999	1.000	1.000	0.1197
237751_x_at	2.4	10.1	2.10	4.29	AAA-AAP	0.00	999	1.000	1.000	0.3024
243311_at	22.5	5.2	-2.10	-4.29	AAA-AAA	0.00	999	1.000	1.000	0.2000
1560288_at	14.6	3.4	-2.10	-4.29	AAA-AAA	0.00	999	1.000	1.000	0.0514
220548_at	24.3	5.7	-2.10	-4.29	AAA-AAA	0.00	999	1.000	1.000	0.0370
216518_at	15.8	3.7	-2.10	-4.29	AAA-AAA	0.00	999	1.000	1.000	0.1996
228288_at	63.8	14.9	-2.10	-4.29	AAA-AAA	0.00	999	1.000	1.000	0.1753
1568739_at	34.7	8.1	-2.10	-4.29	AAA-AAA	0.00	999	1.000	1.000	0.1520
211811_s_at	32.9	7.7	-2.10	-4.29	AAA-AAA	0.00	999	1.000	1.000	0.0653
240810_at	6.8	29.2	2.10	4.29	AAA-PAA	0.00	999	1.000	1.000	0.1598
244323_at	13.3	3.1	-2.10	-4.29	AAA-AAA	0.00	999	1.000	1.000	0.1621
1556048_at	77.6	18.1	-2.10	-4.29	AAA-AAA	0.00	999	1.000	1.000	0.0303
223887_at	46.6	10.9	-2.10	-4.29	AAA-AAA	0.00	999	1.000	1.000	0.0668
234395_at	31.2	7.3	-2.10	-4.29	AAA-AAA	0.00	999	1.000	1.000	0.1466
234337_at	39.5	9.2	-2.10	-4.29	AAA-AAA	0.00	999	1.000	1.000	0.0750
205949_at	9.4	40.4	2.10	4.29	AAA-AAA	0.00	999	1.000	1.000	0.0702
1553013_at	31.3	7.3	-2.10	-4.28	MAA-AAA	0.00	999	1.000	1.000	0.1132
202509_s_at	74.0	17.3	-2.10	-4.29	AAA-AAA	0.00	999	1.000	1.000	0.0025
240215_at	23.1	5.4	-2.10	-4.28	AAA-AAA	0.00	999	1.000	1.000	0.0925
227201_at	101.0	23.6	-2.10	-4.28	AAA-AAA	0.00	999	1.000	1.000	0.0051
218406_x_at	74.2	17.3	-2.10	-4.28	AAA-AAA	0.00	999	1.000	1.000	0.0059
242128_at	18.6	4.3	-2.10	-4.28	AAA-AAA	0.00	999	1.000	1.000	0.1523
203413_at	53.3	12.5	-2.10	-4.28	AAA-AAA	0.00	999	1.000	1.000	0.1058
1562019_at	18.2	4.3	-2.10	-4.28	AAA-AAA	0.00	999	1.000	1.000	0.1661
1561849_at	7.5	31.9	2.10	4.28	AAA-AAA	0.00	999	1.000	1.000	0.0232
1561170_at	27.7	6.5	-2.10	-4.28	AAA-AAA	0.00	999	1.000	1.000	0.2552
244501_at	5.6	24.1	2.10	4.28	AAA-AAA	0.00	999	1.000	1.000	0.0292
1559633_a_at	10.8	2.5	-2.10	-4.28	AAA-AAA	0.00	999	1.000	1.000	0.0664
1556599_s_at	15.4	3.6	-2.10	-4.28	AAM-AAA	0.00	999	1.000	1.000	0.1955
231434_at	50.3	11.8	-2.10	-4.28	AAA-AAA	0.00	999	1.000	1.000	0.1659
216464_x_at	25.0	5.9	-2.10	-4.27	AAA-AAA	0.00	999	1.000	1.000	0.0489
234473_at	23.4	5.5	-2.10	-4.28	AAA-AAA	0.00	999	1.000	1.000	0.2422
207423_s_at	42.5	10.0	-2.10	-4.27	AAA-AAA	0.00	999	1.000	1.000	0.2839
214215_s_at	24.1	5.7	-2.10	-4.27	AAA-AAA	0.00	999	1.000	1.000	0.1528
243199_at	30.1	7.1	-2.09	-4.27	AAA-AAA	0.00	999	1.000	1.000	0.0654
1567410_at	106.0	24.9	-2.09	-4.27	AAM-AAA	0.00	999	1.000	1.000	0.1321
244799_s_at	6.3	26.9	2.09	4.27	AAA-AAA	0.00	999	1.000	1.000	0.2189
1552903_at	50.8	11.9	-2.09	-4.27	AAA-AAA	0.00	999	1.000	1.000	0.1771
226632_at	28.1	6.6	-2.09	-4.27	AAA-AAA	0.00	999	1.000	1.000	0.1222
241952_at	51.3	12.0	-2.09	-4.26	AAA-AAA	0.00	999	1.000	1.000	0.0161

228645_at	31.6	134.7	2.09	4.26	AAA-APM	0.00	999	1.000	1.000	0.0738
1554978_at	74.1	17.4	-2.09	-4.26	AAA-AAA	0.00	999	1.000	1.000	0.1029
223503_at	46.4	10.9	-2.09	-4.26	AAA-AAA	0.00	999	1.000	1.000	0.2547
228083_at	25.6	6.0	-2.09	-4.26	AAA-AAA	0.00	999	1.000	1.000	0.0582
231536_at	27.0	6.3	-2.09	-4.26	AAA-AAA	0.00	999	1.000	1.000	0.1413
1552830_at	27.9	6.6	-2.09	-4.26	AAA-AAA	0.00	999	1.000	1.000	0.1105
220055_at	75.1	17.6	-2.09	-4.26	AAA-AAA	0.00	999	1.000	1.000	0.0895
222888_at	36.1	8.5	-2.09	-4.26	AAA-AAA	0.00	999	1.000	1.000	0.0440
1570207_at	4.0	17.1	2.09	4.26	AAA-AAA	0.00	999	1.000	1.000	0.1405
1552568_at	90.4	21.3	-2.09	-4.25	AAA-AAA	0.00	999	1.000	1.000	0.1315
209167_at	37.0	8.7	-2.09	-4.25	AAA-AAA	0.00	999	1.000	1.000	0.0140
243520_x_at	12.1	2.9	-2.09	-4.25	AAA-AAA	0.00	999	1.000	1.000	0.2854
1562653_at	31.1	7.3	-2.09	-4.25	AAA-AAA	0.00	999	1.000	1.000	0.1090
1554335_at	22.1	5.2	-2.09	-4.25	AAA-AAA	0.00	999	1.000	1.000	0.0324
1553037_a_at	9.7	2.3	-2.09	-4.25	AAA-AAA	0.00	999	1.000	1.000	0.0235
217255_at	17.0	4.0	-2.09	-4.25	AAA-AAA	0.00	999	1.000	1.000	0.0844
240411_at	43.0	10.1	-2.09	-4.25	AAA-AAM	0.00	999	1.000	1.000	0.1056
221092_at	24.7	5.8	-2.09	-4.25	AAA-AAA	0.00	999	1.000	1.000	0.1249
234536_at	5.0	21.2	2.09	4.25	AAA-AAA	0.00	999	1.000	1.000	0.0156
244771_at	36.5	8.6	-2.09	-4.25	AAA-AAA	0.00	999	1.000	1.000	0.1857
1552791_a_at	12.6	3.0	-2.09	-4.25	AAA-AAA	0.00	999	1.000	1.000	0.0428
1562611_at	4.4	18.6	2.09	4.24	AAA-AAA	0.00	999	1.000	1.000	0.1891
209371_s_at	62.3	14.7	-2.09	-4.24	AAA-AAA	0.00	999	1.000	1.000	0.0387
236554_x_at	17.0	4.0	-2.09	-4.24	AAA-AAA	0.00	999	1.000	1.000	0.0118
1555196_at	32.0	7.6	-2.08	-4.24	AAA-AAA	0.00	999	1.000	1.000	0.1805
238173_at	18.9	4.5	-2.08	-4.24	AAA-AAA	0.00	999	1.000	1.000	0.0915
1553634_a_at	46.6	11.0	-2.08	-4.24	AAA-AAA	0.00	999	1.000	1.000	0.0957
231472_at	52.5	12.4	-2.08	-4.24	AAA-AAA	0.00	999	1.000	1.000	0.0424
1566831_at	59.7	14.1	-2.08	-4.23	AAA-AAA	0.00	999	1.000	1.000	0.0546
1566339_at	23.0	5.4	-2.08	-4.23	AAA-AAA	0.00	999	1.000	1.000	0.0912
1553682_at	28.3	6.7	-2.08	-4.23	AAA-AAA	0.00	999	1.000	1.000	0.1425
243940_at	21.9	5.2	-2.08	-4.23	AAA-AAA	0.00	999	1.000	1.000	0.1887
240220_at	55.3	13.1	-2.08	-4.23	AAA-AAA	0.00	999	1.000	1.000	0.0686
219667_s_at	15.4	3.6	-2.08	-4.23	AAA-AAA	0.00	999	1.000	1.000	0.2686
217585_at	26.7	6.3	-2.08	-4.23	AAA-AAA	0.00	999	1.000	1.000	0.0837
220122_at	10.1	42.5	2.08	4.23	AAA-PAP	0.00	999	1.000	1.000	0.1774
203608_at	22.4	94.7	2.08	4.23	AAA-AAA	0.00	999	1.000	1.000	0.1374
1561378_at	23.7	5.6	-2.08	-4.23	AAA-AAA	0.00	999	1.000	1.000	0.1278
1564840_at	6.1	25.6	2.08	4.23	AAA-AAA	0.00	999	1.000	1.000	0.0998
237442_at	28.1	6.7	-2.08	-4.23	AAA-AAA	0.00	999	1.000	1.000	0.1485
231256_at	9.2	38.9	2.08	4.23	AAA-AAA	0.00	999	1.000	1.000	0.0072
1554846_at	26.5	6.3	-2.08	-4.22	AAA-AAA	0.00	999	1.000	1.000	0.1425

237718_at	15.4	64.8	2.08	4.22	AAA-APA	0.00	999	1.000	1.000	0.2258
243616_at	16.2	68.4	2.08	4.22	AAA-AAA	0.00	999	1.000	1.000	0.1101
216024_at	24.3	5.8	-2.08	-4.22	AAA-AAA	0.00	999	1.000	1.000	0.0253
1569856_at	11.8	49.9	2.08	4.22	AAA-PPA	0.00	999	1.000	1.000	0.2191
233203_at	9.1	38.5	2.08	4.22	AAA-APA	0.00	999	1.000	1.000	0.0897
1560107_at	3.9	16.4	2.08	4.22	AAA-AAA	0.00	999	1.000	1.000	0.1477
1554072_s_at	12.9	3.1	-2.08	-4.22	AAA-AAA	0.00	999	1.000	1.000	0.3633
215257_at	13.4	3.2	-2.08	-4.22	AAA-AAA	0.00	999	1.000	1.000	0.1170
1554999_at	4.6	19.2	2.08	4.22	AAA-AAA	0.00	999	1.000	1.000	0.1202
207609_s_at	24.4	5.8	-2.08	-4.21	AAA-AAA	0.00	999	1.000	1.000	0.0229
216879_at	52.4	12.4	-2.08	-4.22	AAA-AAA	0.00	999	1.000	1.000	0.0640
242536_at	18.1	4.3	-2.07	-4.21	AAA-AAA	0.00	999	1.000	1.000	0.2150
244675_at	10.3	2.5	-2.07	-4.21	AAA-AAA	0.00	999	1.000	1.000	0.0080
1556851_at	12.1	2.9	-2.07	-4.21	AAA-AAA	0.00	999	1.000	1.000	0.1014
1556803_at	9.9	41.5	2.07	4.21	AAA-AAA	0.00	999	1.000	1.000	0.0504
216269_s_at	85.5	20.3	-2.07	-4.21	AAA-AAA	0.00	999	1.000	1.000	0.0435
235225_at	35.6	8.5	-2.07	-4.21	AAA-AAA	0.00	999	1.000	1.000	0.0722
237758_at	73.8	17.5	-2.07	-4.21	MAA-AAA	0.00	999	1.000	1.000	0.0437
232236_at	18.0	4.3	-2.07	-4.21	AAA-AAA	0.00	999	1.000	1.000	0.2053
231701_s_at	10.9	45.7	2.07	4.21	AAA-APA	0.00	999	1.000	1.000	0.1087
243097_x_at	37.2	8.8	-2.07	-4.21	AAA-AAA	0.00	999	1.000	1.000	0.0983
240089_at	17.6	74.1	2.07	4.21	AAA-AAA	0.00	999	1.000	1.000	0.1366
1562738_a_at	39.7	9.4	-2.07	-4.21	AAA-AAA	0.00	999	1.000	1.000	0.1386
1562937_at	13.2	55.5	2.07	4.21	AAA-AAA	0.00	999	1.000	1.000	0.0210
237492_at	18.1	4.3	-2.07	-4.20	AAA-AAA	0.00	999	1.000	1.000	0.1939
1560785_at	130.8	31.1	-2.07	-4.20	AAA-AAA	0.00	999	1.000	1.000	0.0660
1560862_at	7.8	32.9	2.07	4.20	AAA-AAA	0.00	999	1.000	1.000	0.0202
239606_at	39.7	9.4	-2.07	-4.20	AAA-AAA	0.00	999	1.000	1.000	0.0692
236999_at	6.0	25.3	2.07	4.20	AAA-AMA	0.00	999	1.000	1.000	0.3203
239990_at	24.4	5.8	-2.07	-4.20	AAA-AAA	0.00	999	1.000	1.000	0.0221
1557733_a_at	13.3	3.2	-2.07	-4.20	AAA-AAA	0.00	999	1.000	1.000	0.1823
1556564_at	31.7	7.5	-2.07	-4.20	AAA-AAA	0.00	999	1.000	1.000	0.0367
217582_at	6.2	26.0	2.07	4.20	AAA-AAA	0.00	999	1.000	1.000	0.0476
216539_at	28.4	6.8	-2.07	-4.20	AAM-AAA	0.00	999	1.000	1.000	0.0883
231884_at	54.4	13.0	-2.07	-4.20	AAA-AAA	0.00	999	1.000	1.000	0.0123
232182_at	9.8	41.2	2.07	4.20	AAA-APP	0.00	999	1.000	1.000	0.1326
241639_at	35.6	8.5	-2.07	-4.20	AAA-AAA	0.00	999	1.000	1.000	0.2170
1570181_a_at	51.2	12.2	-2.07	-4.20	AAM-AAA	0.00	999	1.000	1.000	0.3283
229740_at	48.7	11.6	-2.07	-4.20	AAA-AAA	0.00	999	1.000	1.000	0.1854
1560278_at	44.2	10.5	-2.07	-4.19	AAA-AAA	0.00	999	1.000	1.000	0.0502
230351_at	12.7	3.0	-2.07	-4.19	AAA-AAA	0.00	999	1.000	1.000	0.2748
206363_at	10.2	2.4	-2.07	-4.19	AAA-AAA	0.00	999	1.000	1.000	0.0659

222089_s_at	57.6	13.7	-2.07	-4.19	AAA-AAA	0.00	999	1.000	1.000	0.1751
208496_x_at	13.9	58.2	2.07	4.19	AAA-AAA	0.00	999	1.000	1.000	0.3097
234083_at	49.8	11.9	-2.07	-4.19	AAA-AAA	0.00	999	1.000	1.000	0.0208
205475_at	61.2	14.6	-2.07	-4.19	AAA-AAA	0.00	999	1.000	1.000	0.1961
243913_at	82.8	19.8	-2.07	-4.19	AAA-AAA	0.00	999	1.000	1.000	0.0740
241121_at	49.1	11.7	-2.07	-4.19	AAA-AAA	0.00	999	1.000	1.000	0.0608
1555275_a_at	24.2	5.8	-2.07	-4.19	AAA-AAA	0.00	999	1.000	1.000	0.1053
229629_at	22.5	5.4	-2.07	-4.19	AAA-AAA	0.00	999	1.000	1.000	0.2317
1561997_at	6.5	1.6	-2.07	-4.18	AAA-AAA	0.00	999	1.000	1.000	0.2425
1556815_at	41.9	10.0	-2.06	-4.18	AAA-AAA	0.00	999	1.000	1.000	0.2258
239018_at	17.7	73.8	2.06	4.18	AAA-APM	0.00	999	1.000	1.000	0.1604
1570213_at	5.2	1.3	-2.06	-4.18	AAA-AAA	0.00	999	1.000	1.000	0.0810
207240_s_at	3.0	12.7	2.06	4.18	AAA-AAA	0.00	999	1.000	1.000	0.0981
1562740_at	7.2	30.2	2.06	4.18	AAA-AAA	0.00	999	1.000	1.000	0.0783
1563398_at	24.4	5.8	-2.06	-4.18	AAA-AAA	0.00	999	1.000	1.000	0.0032
236193_at	7.9	33.0	2.06	4.18	AAA-AAA	0.00	999	1.000	1.000	0.0224
1556954_at	8.3	2.0	-2.06	-4.17	AAA-AAA	0.00	999	1.000	1.000	0.2157
232995_at	29.4	7.1	-2.06	-4.17	AAA-AAA	0.00	999	1.000	1.000	0.2397
1557225_at	80.2	19.2	-2.06	-4.17	AAA-AAA	0.00	999	1.000	1.000	0.0096
236163_at	9.2	2.2	-2.06	-4.17	AAA-AAA	0.00	999	1.000	1.000	0.0313
236394_at	40.0	9.6	-2.06	-4.17	AAA-AAA	0.00	999	1.000	1.000	0.2088
240930_at	46.1	11.1	-2.06	-4.17	AAA-AAA	0.00	999	1.000	1.000	0.0396
1566501_at	17.2	4.1	-2.06	-4.17	AMA-AAA	0.00	999	1.000	1.000	0.2310
1553422_s_at	41.6	10.0	-2.06	-4.17	AAA-AAA	0.00	999	1.000	1.000	0.0087
1559372_at	9.1	37.7	2.06	4.17	AAA-AAA	0.00	999	1.000	1.000	0.0011
1560987_a_at	12.1	2.9	-2.06	-4.17	AAA-AAA	0.00	999	1.000	1.000	0.0167
1561261_at	8.7	2.1	-2.06	-4.17	AAA-AAA	0.00	999	1.000	1.000	0.0070
1567100_at	19.5	4.7	-2.06	-4.16	AAA-AAA	0.00	999	1.000	1.000	0.1941
216644_at	50.3	12.1	-2.06	-4.16	AAA-AAA	0.00	999	1.000	1.000	0.0775
240272_at	21.7	5.2	-2.06	-4.16	AAA-AAA	0.00	999	1.000	1.000	0.0535
241094_at	28.8	6.9	-2.06	-4.15	MAA-MAA	0.00	999	1.000	1.000	0.2421
1561592_at	25.7	6.2	-2.06	-4.16	AAA-AAA	0.00	999	1.000	1.000	0.1692
206486_at	61.4	14.8	-2.06	-4.16	AAA-AAA	0.00	999	1.000	1.000	0.0326
230982_at	52.9	12.7	-2.06	-4.16	AAA-AAA	0.00	999	1.000	1.000	0.2051
242159_at	108.2	26.1	-2.05	-4.15	MAA-AAA	0.00	999	1.000	1.000	0.0251
1560566_at	19.6	4.7	-2.05	-4.15	AAA-AAA	0.00	999	1.000	1.000	0.0969
1553357_at	20.2	4.9	-2.05	-4.15	AAA-AAA	0.00	999	1.000	1.000	0.1063
223767_at	39.0	9.4	-2.05	-4.15	AAA-AAA	0.00	999	1.000	1.000	0.2153
241744_x_at	15.3	3.7	-2.05	-4.15	AAA-AAA	0.00	999	1.000	1.000	0.3090
1561676_at	6.2	25.5	2.05	4.15	AAA-AAA	0.00	999	1.000	1.000	0.2868
1564789_at	13.0	3.1	-2.05	-4.15	AAA-AAA	0.00	999	1.000	1.000	0.0966
1556775_at	8.4	34.9	2.05	4.15	AAA-AAA	0.00	999	1.000	1.000	0.1685

232306_at	17.9	4.3	-2.05	-4.15	AAA-AAA	0.00	999	1.000	1.000	0.1911
1570180_at	16.8	4.0	-2.05	-4.15	AAA-AAA	0.00	999	1.000	1.000	0.1167
1553500_at	31.5	7.6	-2.05	-4.15	AAA-AAA	0.00	999	1.000	1.000	0.2298
244776_at	52.8	12.7	-2.05	-4.15	AAA-AAA	0.00	999	1.000	1.000	0.1508
229039_at	33.3	8.0	-2.05	-4.15	AAA-AAA	0.00	999	1.000	1.000	0.2293
232774_x_at	31.7	131.3	2.05	4.14	AAA-PPP	0.00	999	1.000	1.000	0.1011
1557623_at	31.0	7.5	-2.05	-4.14	AAA-AAA	0.00	999	1.000	1.000	0.1011
219995_s_at	11.6	2.8	-2.05	-4.14	AAA-AAA	0.00	999	1.000	1.000	0.2174
220270_at	42.8	10.3	-2.05	-4.15	AAA-AAA	0.00	999	1.000	1.000	0.0206
1556617_a_at	7.6	1.8	-2.05	-4.14	AAA-AAA	0.00	999	1.000	1.000	0.0016
214480_at	29.6	7.2	-2.05	-4.14	AAA-AAA	0.00	999	1.000	1.000	0.0788
236788_at	19.0	4.6	-2.05	-4.14	AAA-AAA	0.00	999	1.000	1.000	0.1498
239613_at	24.7	6.0	-2.05	-4.14	AAA-AAA	0.00	999	1.000	1.000	0.1912
206198_s_at	12.5	3.0	-2.05	-4.14	AAA-AAA	0.00	999	1.000	1.000	0.2203
1561214_at	87.6	21.2	-2.05	-4.14	AAA-AAA	0.00	999	1.000	1.000	0.0242
216130_at	18.6	4.5	-2.05	-4.14	AAA-AAA	0.00	999	1.000	1.000	0.0642
237199_at	27.6	6.7	-2.05	-4.14	AAA-AAA	0.00	999	1.000	1.000	0.0791
203295_s_at	31.8	7.7	-2.05	-4.14	AAA-AAA	0.00	999	1.000	1.000	0.1865
1555330_at	35.1	8.5	-2.05	-4.13	AAA-AAA	0.00	999	1.000	1.000	0.0863
221008_s_at	5.9	24.4	2.05	4.14	AAA-AAA	0.00	999	1.000	1.000	0.1642
230094_at	13.6	3.3	-2.05	-4.13	AAA-AAA	0.00	999	1.000	1.000	0.1959
232812_at	19.0	4.6	-2.05	-4.13	AAA-AAA	0.00	999	1.000	1.000	0.3322
222324_at	25.4	6.2	-2.05	-4.13	AAA-AAA	0.00	999	1.000	1.000	0.2392
223673_at	15.9	3.9	-2.05	-4.13	AAA-AAA	0.00	999	1.000	1.000	0.0306
1570025_at	35.7	8.6	-2.05	-4.13	AAA-AAA	0.00	999	1.000	1.000	0.0706
228317_at	23.8	5.8	-2.05	-4.13	AAA-AAA	0.00	999	1.000	1.000	0.0801
239476_at	32.2	7.8	-2.05	-4.13	AAA-AAA	0.00	999	1.000	1.000	0.1976
203930_s_at	27.3	6.6	-2.05	-4.13	AAA-AAA	0.00	999	1.000	1.000	0.0599
233712_at	51.6	12.5	-2.05	-4.13	AAA-AAA	0.00	999	1.000	1.000	0.0678
1569634_at	60.9	14.8	-2.04	-4.12	AAA-AAA	0.00	999	1.000	1.000	0.0980
1556457_s_at	10.0	41.1	2.04	4.13	AAA-AAA	0.00	999	1.000	1.000	0.0922
1556804_s_at	4.8	19.7	2.04	4.13	AAA-AAA	0.00	999	1.000	1.000	0.2274
235666_at	30.1	7.3	-2.04	-4.13	AAA-AAA	0.00	999	1.000	1.000	0.0877
239592_at	121.3	29.4	-2.04	-4.12	AMA-AAA	0.00	999	1.000	1.000	0.1402
229508_at	6.9	28.4	2.04	4.12	AAA-AAA	0.00	999	1.000	1.000	0.0053
1562476_at	50.9	12.4	-2.04	-4.12	MAA-AAA	0.00	999	1.000	1.000	0.1945
1562997_a_at	6.3	1.5	-2.04	-4.12	AAA-AAA	0.00	999	1.000	1.000	0.1292
1570102_at	15.3	3.7	-2.04	-4.12	AAA-AAA	0.00	999	1.000	1.000	0.0743
1567173_at	14.0	3.4	-2.04	-4.12	AAA-AAA	0.00	999	1.000	1.000	0.3203
244866_at	19.7	4.8	-2.04	-4.12	AAA-AAA	0.00	999	1.000	1.000	0.1895
1570507_at	12.2	50.1	2.04	4.12	AAA-APA	0.00	999	1.000	1.000	0.0917
1559296_at	9.0	2.2	-2.04	-4.12	AAA-AAA	0.00	999	1.000	1.000	0.1824

1563362_at	46.5	11.3	-2.04	-4.12	AAA-AAA	0.00	999	1.000	1.000	0.1621
1567256_at	21.4	5.2	-2.04	-4.11	AAA-AAA	0.00	999	1.000	1.000	0.1179
230890_at	14.6	60.0	2.04	4.11	AAA-AAA	0.00	999	1.000	1.000	0.0505
1561534_at	15.1	3.7	-2.04	-4.11	AAA-AAA	0.00	999	1.000	1.000	0.0965
1567612_at	31.3	7.6	-2.04	-4.11	AAA-AAA	0.00	999	1.000	1.000	0.0379
228881_at	51.7	12.6	-2.04	-4.11	AAA-AAA	0.00	999	1.000	1.000	0.0945
1560934_at	29.6	7.2	-2.04	-4.11	AAA-AAA	0.00	999	1.000	1.000	0.0404
230227_at	20.5	5.0	-2.04	-4.11	AAA-AAA	0.00	999	1.000	1.000	0.2526
214839_at	54.7	13.3	-2.04	-4.10	AAA-AAA	0.00	999	1.000	1.000	0.0338
216507_at	35.9	8.7	-2.04	-4.11	AAA-AAA	0.00	999	1.000	1.000	0.0301
1558689_a_at	9.0	2.2	-2.04	-4.10	AAA-AAA	0.00	999	1.000	1.000	0.1895
208139_s_at	38.5	9.4	-2.04	-4.10	AAA-AAA	0.00	999	1.000	1.000	0.1997
213106_at	14.7	3.6	-2.04	-4.10	AAA-AAA	0.00	999	1.000	1.000	0.1576
217285_at	10.3	42.2	2.04	4.10	AAA-AAA	0.00	999	1.000	1.000	0.0436
219140_s_at	12.5	51.3	2.04	4.10	AAA-AAA	0.00	999	1.000	1.000	0.0558
233948_at	35.1	8.6	-2.04	-4.10	AAA-AAA	0.00	999	1.000	1.000	0.0969
243433_at	52.8	12.9	-2.04	-4.10	AAA-AAA	0.00	999	1.000	1.000	0.0383
1570344_at	30.3	7.4	-2.04	-4.10	AAA-AAA	0.00	999	1.000	1.000	0.0842
1552974_at	13.3	3.3	-2.04	-4.10	AAA-AAA	0.00	999	1.000	1.000	0.2946
1562296_at	35.0	8.5	-2.03	-4.10	AAA-AAA	0.00	999	1.000	1.000	0.2448
1553282_at	45.9	11.2	-2.03	-4.09	AAA-AAA	0.00	999	1.000	1.000	0.0648
215326_at	18.7	76.4	2.03	4.10	AAA-AAA	0.00	999	1.000	1.000	0.0458
201148_s_at	46.8	11.4	-2.03	-4.09	AAA-AAA	0.00	999	1.000	1.000	0.0429
1566824_at	34.1	8.3	-2.03	-4.09	AAA-AAA	0.00	999	1.000	1.000	0.1096
218345_at	34.2	8.4	-2.03	-4.09	AAA-AAA	0.00	999	1.000	1.000	0.0593
241316_at	3.2	13.2	2.03	4.09	AAA-AAA	0.00	999	1.000	1.000	0.0856
243240_at	23.7	5.8	-2.03	-4.09	AAA-AAA	0.00	999	1.000	1.000	0.1820
1558225_at	38.9	9.5	-2.03	-4.09	AAA-AAA	0.00	999	1.000	1.000	0.1346
220794_at	34.4	8.4	-2.03	-4.09	AAA-AAA	0.00	999	1.000	1.000	0.2254
240179_at	12.9	3.2	-2.03	-4.09	AAA-AAA	0.00	999	1.000	1.000	0.0913
240249_at	27.7	6.8	-2.03	-4.09	AAA-AAA	0.00	999	1.000	1.000	0.0076
1569831_at	3.2	13.1	2.03	4.09	AAA-AAA	0.00	999	1.000	1.000	0.0322
208347_at	144.4	35.4	-2.03	-4.08	AMA-AAA	0.00	999	1.000	1.000	0.0420
229209_at	7.3	29.9	2.03	4.08	AAA-AAA	0.00	999	1.000	1.000	0.0050
244739_at	21.4	5.3	-2.03	-4.08	AAA-AAA	0.00	999	1.000	1.000	0.0669
204972_at	45.4	11.1	-2.03	-4.08	AAA-AAA	0.00	999	1.000	1.000	0.0928
239428_at	26.9	6.6	-2.03	-4.08	AAA-AAA	0.00	999	1.000	1.000	0.2007
1569240_at	8.5	34.6	2.03	4.08	AAA-AAA	0.00	999	1.000	1.000	0.0792
1564178_at	54.5	13.4	-2.03	-4.08	AAA-AAA	0.00	999	1.000	1.000	0.1000
1556593_s_at	47.6	11.7	-2.03	-4.08	AAA-AAA	0.00	999	1.000	1.000	0.0163
207961_x_at	27.3	6.7	-2.03	-4.08	AAA-AAA	0.00	999	1.000	1.000	0.1678
226304_at	15.2	3.7	-2.03	-4.08	AAA-AAA	0.00	999	1.000	1.000	0.1038

231430_at	50.2	12.3	-2.03	-4.08	AAA-AAA	0.00	999	1.000	1.000	0.1290
1553626_a_at	10.9	44.5	2.03	4.08	AAA-AAP	0.00	999	1.000	1.000	0.1208
1557232_at	12.6	51.3	2.03	4.08	AAA-AAA	0.00	999	1.000	1.000	0.0541
239883_s_at	21.0	5.2	-2.03	-4.07	AAA-AAA	0.00	999	1.000	1.000	0.2629
1559629_at	75.2	18.5	-2.03	-4.07	AAA-AAA	0.00	999	1.000	1.000	0.1710
221324_at	23.8	5.8	-2.03	-4.07	AAA-AAA	0.00	999	1.000	1.000	0.0819
222081_at	17.6	4.3	-2.03	-4.07	AAA-AAA	0.00	999	1.000	1.000	0.0260
214064_at	68.9	16.9	-2.03	-4.07	AMA-AAA	0.00	999	1.000	1.000	0.0411
1562826_at	72.7	17.9	-2.03	-4.07	AAA-AAA	0.00	999	1.000	1.000	0.0204
1564985_a_at	11.7	2.9	-2.03	-4.07	AAA-AAA	0.00	999	1.000	1.000	0.1243
236791_at	51.6	12.7	-2.03	-4.07	AAA-AAA	0.00	999	1.000	1.000	0.1149
237583_at	74.2	18.2	-2.03	-4.07	AAA-AAA	0.00	999	1.000	1.000	0.1880
229372_at	12.9	3.2	-2.03	-4.07	AAA-AAA	0.00	999	1.000	1.000	0.0933
231803_at	27.2	6.7	-2.03	-4.07	AAA-AAA	0.00	999	1.000	1.000	0.0462
243774_at	38.8	9.5	-2.02	-4.07	AAA-AAA	0.00	999	1.000	1.000	0.2435
221035_s_at	16.9	4.2	-2.02	-4.07	AAA-AAA	0.00	999	1.000	1.000	0.1443
207092_at	20.9	5.1	-2.02	-4.07	AAA-AAA	0.00	999	1.000	1.000	0.0855
238309_x_at	31.7	7.8	-2.02	-4.07	MAA-AAA	0.00	999	1.000	1.000	0.1058
242213_at	23.5	5.8	-2.02	-4.06	AAA-AAA	0.00	999	1.000	1.000	0.2308
240656_at	11.5	46.9	2.02	4.06	AAA-AAA	0.00	999	1.000	1.000	0.1396
1556365_at	72.9	17.9	-2.02	-4.07	AAA-AAA	0.00	999	1.000	1.000	0.0486
208272_at	39.1	9.6	-2.02	-4.06	AAA-AAA	0.00	999	1.000	1.000	0.0176
1563331_at	36.1	8.9	-2.02	-4.06	AAA-AAA	0.00	999	1.000	1.000	0.0650
221299_at	36.0	8.9	-2.02	-4.06	AAA-AAA	0.00	999	1.000	1.000	0.0917
211400_at	30.1	7.4	-2.02	-4.06	AAA-AAA	0.00	999	1.000	1.000	0.1004
1553864_at	6.5	1.6	-2.02	-4.06	AAA-AAA	0.00	999	1.000	1.000	0.2676
211836_s_at	28.7	7.1	-2.02	-4.06	AAA-AAA	0.00	999	1.000	1.000	0.1239
1564984_at	7.7	31.1	2.02	4.05	AAA-AAA	0.00	999	1.000	1.000	0.0051
228186_s_at	67.8	16.7	-2.02	-4.05	AAA-AAA	0.00	999	1.000	1.000	0.2644
1555153_s_at	14.0	56.7	2.02	4.05	AAA-APP	0.00	999	1.000	1.000	0.2276
243537_at	15.0	60.8	2.02	4.05	AAA-AAA	0.00	999	1.000	1.000	0.0628
1560552_a_at	23.7	5.9	-2.02	-4.05	AAA-AAA	0.00	999	1.000	1.000	0.2053
1565819_at	26.4	6.5	-2.02	-4.05	AAA-AAA	0.00	999	1.000	1.000	0.2251
1565896_at	14.3	3.5	-2.02	-4.05	AAA-AAA	0.00	999	1.000	1.000	0.0563
1557575_at	29.4	7.3	-2.02	-4.05	AAA-AAA	0.00	999	1.000	1.000	0.0135
217178_at	25.2	6.2	-2.02	-4.05	AAA-AAA	0.00	999	1.000	1.000	0.0163
205626_s_at	43.5	10.8	-2.02	-4.05	AAA-AAA	0.00	999	1.000	1.000	0.2048
219831_at	51.0	206.4	2.02	4.05	APP-PPP	0.00	999	1.000	1.000	0.2794
1568706_s_at	33.0	8.1	-2.02	-4.05	AAA-AAA	0.00	999	1.000	1.000	0.0492
233935_at	42.9	10.6	-2.02	-4.05	AAA-AAA	0.00	999	1.000	1.000	0.2259
230515_at	18.4	74.6	2.02	4.05	AAA-AAA	0.00	999	1.000	1.000	0.0263
242930_at	38.2	9.4	-2.02	-4.05	AAA-AAA	0.00	999	1.000	1.000	0.2079

1554252_a_at	31.3	7.8	-2.02	-4.04	AAA-AAA	0.00	999	1.000	1.000	0.1941
1559687_at	51.1	12.7	-2.02	-4.04	AAA-AAA	0.00	999	1.000	1.000	0.2250
234152_at	32.5	8.0	-2.02	-4.04	AAA-AAA	0.00	999	1.000	1.000	0.0052
234876_at	12.9	52.1	2.02	4.04	AAA-AAA	0.00	999	1.000	1.000	0.0983
221153_s_at	30.0	7.4	-2.01	-4.04	AAA-AAA	0.00	999	1.000	1.000	0.1869
232206_at	13.3	53.7	2.01	4.04	AAA-AAA	0.00	999	1.000	1.000	0.1191
241126_at	15.3	3.8	-2.01	-4.03	AAA-AAA	0.00	999	1.000	1.000	0.0619
1557598_at	3.1	12.4	2.01	4.03	AAA-AAA	0.00	999	1.000	1.000	0.0912
214493_s_at	28.4	7.1	-2.01	-4.04	AAA-AAA	0.00	999	1.000	1.000	0.2433
211642_at	9.5	2.3	-2.01	-4.03	AAA-AAA	0.00	999	1.000	1.000	0.2292
1570064_at	75.0	18.6	-2.01	-4.03	AAA-AAA	0.00	999	1.000	1.000	0.0854
220806_x_at	17.2	4.3	-2.01	-4.03	AAA-AAA	0.00	999	1.000	1.000	0.0477
1564310_a_at	21.3	5.3	-2.01	-4.03	AAA-AAA	0.00	999	1.000	1.000	0.1317
1556639_at	18.0	4.5	-2.01	-4.03	AAA-AAA	0.00	999	1.000	1.000	0.0849
237399_at	23.3	5.8	-2.01	-4.03	AAA-AAA	0.00	999	1.000	1.000	0.0685
239966_at	8.7	35.1	2.01	4.02	AAA-AAA	0.00	999	1.000	1.000	0.0428
243996_at	5.7	23.1	2.01	4.03	AAA-AAA	0.00	999	1.000	1.000	0.0042
219285_s_at	43.7	10.9	-2.01	-4.03	AAA-AAA	0.00	999	1.000	1.000	0.0549
1555839_a_at	21.2	5.3	-2.01	-4.02	AAA-AAA	0.00	999	1.000	1.000	0.1242
223565_at	5.2	20.7	2.01	4.02	AAA-AAA	0.00	999	1.000	1.000	0.1057
209292_at	36.8	9.2	-2.01	-4.02	AAA-AAA	0.00	999	1.000	1.000	0.1196
216479_at	88.0	21.9	-2.01	-4.02	AAA-AAA	0.00	999	1.000	1.000	0.3368
1562066_at	16.9	4.2	-2.01	-4.02	AAA-AAA	0.00	999	1.000	1.000	0.0827
1560935_s_at	39.3	9.8	-2.01	-4.02	AAA-AAA	0.00	999	1.000	1.000	0.0823
244645_at	23.0	5.7	-2.01	-4.02	AAA-AAA	0.00	999	1.000	1.000	0.3159
220723_s_at	27.2	6.8	-2.01	-4.02	AAA-AAA	0.00	999	1.000	1.000	0.1094
215334_at	25.3	101.5	2.01	4.02	AAA-AAA	0.00	999	1.000	1.000	0.0571
1562879_at	15.6	3.9	-2.00	-4.01	AAA-AAA	0.00	999	1.000	1.000	0.1500
220513_at	24.2	6.0	-2.00	-4.01	AAA-AAA	0.00	999	1.000	1.000	0.2453
241859_at	8.7	2.2	-2.00	-4.01	AAA-AAA	0.00	999	1.000	1.000	0.0532
241714_at	12.3	49.3	2.00	4.01	AAA-AAA	0.00	999	1.000	1.000	0.0511
1557765_at	11.8	2.9	-2.00	-4.01	AAA-AAA	0.00	999	1.000	1.000	0.0683
229340_at	56.4	14.1	-2.00	-4.01	AAA-AAA	0.00	999	1.000	1.000	0.1429
1568589_at	27.7	6.9	-2.00	-4.01	AAA-AAA	0.00	999	1.000	1.000	0.1820
51226_at	31.5	7.9	-2.00	-4.01	AAA-AAA	0.00	999	1.000	1.000	0.0278
233685_at	4.5	17.9	2.00	4.01	AAA-AAA	0.00	999	1.000	1.000	0.1264
231463_at	49.1	12.3	-2.00	-4.01	AAA-AAA	0.00	999	1.000	1.000	0.1061
1570627_at	61.4	15.3	-2.00	-4.00	AAA-AAA	0.00	999	1.000	1.000	0.0911
1554303_at	5.7	23.0	2.00	4.00	AAA-AAA	0.00	999	1.000	1.000	0.0928
1559651_at	23.1	5.8	-2.00	-4.00	AAA-AAA	0.00	999	1.000	1.000	0.1634
230366_at	20.5	5.1	-2.00	-4.00	AAA-AAA	0.00	999	1.000	1.000	0.2995
244206_at	13.0	3.3	-2.00	-4.00	AAA-AAA	0.00	999	1.000	1.000	0.3149

1555308_at	12.6	50.3	2.00	4.00	AAA-AAA	0.00	999	1.000	1.000	0.1021
223782_s_at	38.8	9.7	-2.00	-4.00	AAA-AAA	0.00	999	1.000	1.000	0.1615
241510_at	7.5	29.8	2.00	4.00	AAA-AAA	0.00	999	1.000	1.000	0.0426
1557113_at	10.0	39.9	2.00	4.00	AAA-AAA	0.00	999	1.000	1.000	0.0209
234824_at	57.0	14.3	-2.00	-4.00	AAA-AAA	0.00	999	1.000	1.000	0.0615
1570190_at	11.7	2.9	-2.00	-3.99	AAA-AAA	0.00	999	1.000	1.000	0.1059
207911_s_at	78.8	19.7	-2.00	-3.99	AAA-AAA	0.00	999	1.000	1.000	0.0011
215451_s_at	18.0	72.0	2.00	4.00	AAA-AAA	0.00	999	1.000	1.000	0.0093
1555245_s_at	19.4	4.9	-2.00	-3.99	AAA-AAA	0.00	999	1.000	1.000	0.1655
1552770_s_at	43.4	10.9	-2.00	-3.99	AAA-AAA	0.00	999	1.000	1.000	0.0174
1563498_s_at	36.4	9.1	-2.00	-3.99	AMA-AAA	0.00	999	1.000	1.000	0.0634
1558757_at	8.0	2.0	-2.00	-3.99	AAA-AAA	0.00	999	1.000	1.000	0.0509
211446_at	50.1	12.6	-2.00	-3.99	AAA-AAA	0.00	999	1.000	1.000	0.0961
215857_at	31.3	7.9	-2.00	-3.99	AAA-AAA	0.00	999	1.000	1.000	0.0795
239691_at	19.3	4.9	-2.00	-3.99	AAA-AAA	0.00	999	1.000	1.000	0.0873
1560897_a_at	58.1	14.6	-2.00	-3.99	AAA-AAA	0.00	999	1.000	1.000	0.1414
221968_s_at	22.6	5.7	-2.00	-3.99	AAA-AAA	0.00	999	1.000	1.000	0.0305
236037_at	9.5	2.4	-2.00	-3.99	AAA-AAA	0.00	999	1.000	1.000	0.2067
1562737_at	19.9	5.0	-1.99	-3.99	AAA-AAA	0.00	999	1.000	1.000	0.2515
1567056_at	3.7	14.8	1.99	3.98	AAA-AAA	0.00	999	1.000	1.000	0.1616
208008_at	65.5	16.5	-1.99	-3.98	AAA-AAA	0.00	999	1.000	1.000	0.0510
230554_at	16.4	4.1	-1.99	-3.98	AAA-AAA	0.00	999	1.000	1.000	0.2605
242721_at	19.6	4.9	-1.99	-3.98	AAA-AAA	0.00	999	1.000	1.000	0.2859
1559194_a_at	82.3	20.7	-1.99	-3.98	AAA-AAA	0.00	999	1.000	1.000	0.0554
217001_x_at	111.1	27.9	-1.99	-3.98	AAA-AAA	0.00	999	1.000	1.000	0.0234
242050_at	25.2	6.3	-1.99	-3.98	AAA-AAA	0.00	999	1.000	1.000	0.2792
243555_at	39.9	10.0	-1.99	-3.98	AAA-AMA	0.00	999	1.000	1.000	0.1640
237722_at	54.2	13.6	-1.99	-3.98	AAA-AAA	0.00	999	1.000	1.000	0.1117
239865_at	15.8	4.0	-1.99	-3.97	AAA-AAA	0.00	999	1.000	1.000	0.2141
1557107_at	18.5	4.7	-1.99	-3.98	AAA-AAA	0.00	999	1.000	1.000	0.1519
232719_at	25.7	6.5	-1.99	-3.98	AAA-AAA	0.00	999	1.000	1.000	0.1524
232731_x_at	31.3	7.9	-1.99	-3.98	AAA-AAA	0.00	999	1.000	1.000	0.1206
1556687_a_at	31.1	7.8	-1.99	-3.97	AAA-AAA	0.00	999	1.000	1.000	0.0449
214627_at	65.1	16.4	-1.99	-3.97	AAA-AAA	0.00	999	1.000	1.000	0.1983
213003_s_at	54.1	13.6	-1.99	-3.97	AAA-AAA	0.00	999	1.000	1.000	0.0745
244166_at	21.3	5.4	-1.99	-3.97	AAA-AAA	0.00	999	1.000	1.000	0.0097
1560081_at	63.9	16.1	-1.99	-3.97	AAA-AAA	0.00	999	1.000	1.000	0.0701
224179_s_at	46.6	11.7	-1.99	-3.97	AAA-AAA	0.00	999	1.000	1.000	0.0071
209993_at	22.8	5.7	-1.99	-3.97	AAA-AAA	0.00	999	1.000	1.000	0.0577
239591_at	12.3	3.1	-1.99	-3.97	AAA-AAA	0.00	999	1.000	1.000	0.0354
228527_s_at	141.8	35.7	-1.99	-3.97	AAA-AAA	0.00	999	1.000	1.000	0.1845
1554331_a_at	10.7	42.3	1.99	3.97	AAA-AAA	0.00	999	1.000	1.000	0.1625

244383_at	10.4	41.0	1.99	3.97	AAA-AAA	0.00	999	1.000	1.000	0.0986
207859_s_at	70.3	17.7	-1.99	-3.97	AAA-MAA	0.00	999	1.000	1.000	0.1832
224272_at	38.0	9.6	-1.99	-3.97	AAA-AAA	0.00	999	1.000	1.000	0.2447
231787_at	20.5	5.2	-1.99	-3.97	AAA-AAA	0.00	999	1.000	1.000	0.1070
224545_at	36.5	9.2	-1.99	-3.97	AAA-AAA	0.00	999	1.000	1.000	0.0836
239765_at	30.6	7.7	-1.99	-3.97	AAA-AAA	0.00	999	1.000	1.000	0.0766
1569040_s_at	3.2	12.7	1.99	3.96	AAA-AAA	0.00	999	1.000	1.000	0.0134
1558154_at	25.7	6.5	-1.99	-3.96	AAA-AAA	0.00	999	1.000	1.000	0.2412
1563607_x_at	53.7	13.6	-1.99	-3.96	AAA-AAA	0.00	999	1.000	1.000	0.2308
222139_at	40.1	10.1	-1.99	-3.96	AAA-AAA	0.00	999	1.000	1.000	0.1901
220480_at	56.5	14.3	-1.99	-3.96	AAA-AAA	0.00	999	1.000	1.000	0.1124
243127_x_at	11.1	44.0	1.98	3.96	AAA-AAA	0.00	999	1.000	1.000	0.0196
244883_at	38.7	9.8	-1.98	-3.96	AAA-AAA	0.00	999	1.000	1.000	0.0326
1557885_at	9.3	36.7	1.98	3.96	AAA-AAA	0.00	999	1.000	1.000	0.2069
211459_at	22.6	5.7	-1.98	-3.96	AAA-AAA	0.00	999	1.000	1.000	0.1735
217557_s_at	34.6	8.8	-1.98	-3.96	AMA-AAA	0.00	999	1.000	1.000	0.1857
238394_at	4.1	1.0	-1.98	-3.96	AAA-AAA	0.00	999	1.000	1.000	0.2488
201236_s_at	33.1	130.8	1.98	3.95	AAA-AAA	0.00	999	1.000	1.000	0.0580
1569881_at	25.3	6.4	-1.98	-3.95	AAA-AAA	0.00	999	1.000	1.000	0.0355
207931_s_at	16.6	65.8	1.98	3.95	AAA-AAA	0.00	999	1.000	1.000	0.0034
238234_at	14.5	3.7	-1.98	-3.95	AAA-AAA	0.00	999	1.000	1.000	0.3370
243223_at	84.9	21.5	-1.98	-3.95	AAA-AAA	0.00	999	1.000	1.000	0.0825
40665_at	13.0	3.3	-1.98	-3.95	AAA-AAA	0.00	999	1.000	1.000	0.3020
216042_at	15.3	60.4	1.98	3.95	AAA-AAA	0.00	999	1.000	1.000	0.0393
233196_at	37.3	9.4	-1.98	-3.95	AAA-AAA	0.00	999	1.000	1.000	0.1602
232414_at	44.3	11.2	-1.98	-3.95	AAA-AAA	0.00	999	1.000	1.000	0.1705
242632_at	9.1	35.7	1.98	3.95	AAA-AAA	0.00	999	1.000	1.000	0.1587
244751_at	62.7	15.9	-1.98	-3.95	AAA-AAA	0.00	999	1.000	1.000	0.0407
1558754_at	28.2	7.1	-1.98	-3.94	AAA-MAA	0.00	999	1.000	1.000	0.2200
221469_at	38.5	9.8	-1.98	-3.94	AAA-AAA	0.00	999	1.000	1.000	0.0178
207209_at	58.6	14.9	-1.98	-3.94	AAA-AAA	0.00	999	1.000	1.000	0.0592
214521_at	29.5	7.5	-1.98	-3.94	AAA-AAA	0.00	999	1.000	1.000	0.0630
214614_at	26.1	6.6	-1.98	-3.94	AAA-AAA	0.00	999	1.000	1.000	0.2318
217112_at	12.4	3.2	-1.98	-3.94	AAA-AAA	0.00	999	1.000	1.000	0.0956
216324_at	5.0	19.5	1.98	3.94	AAA-AAA	0.00	999	1.000	1.000	0.1143
234460_at	16.4	4.2	-1.98	-3.94	AAA-AAA	0.00	999	1.000	1.000	0.0871
228270_at	43.1	10.9	-1.98	-3.94	AAA-AAA	0.00	999	1.000	1.000	0.1468
223729_at	19.6	77.4	1.98	3.94	APA-PPA	0.00	999	1.000	1.000	0.1730
1563121_at	41.7	10.6	-1.98	-3.94	AAA-AAA	0.00	999	1.000	1.000	0.0619
1554210_at	17.1	4.4	-1.98	-3.94	AAA-AAA	0.00	999	1.000	1.000	0.1828
222160_at	137.5	34.9	-1.98	-3.94	AAA-AAA	0.00	999	1.000	1.000	0.1041
204150_at	85.8	21.8	-1.98	-3.94	AAA-AAA	0.00	999	1.000	1.000	0.0168

230216_at	26.2	6.7	-1.98	-3.94	AAA-AAA	0.00	999	1.000	1.000	0.0300
210961_s_at	10.6	41.8	1.98	3.93	AAA-PAA	0.00	999	1.000	1.000	0.0788
242495_at	32.5	8.3	-1.98	-3.93	AAA-AAA	0.00	999	1.000	1.000	0.1137
207610_s_at	7.8	30.9	1.98	3.93	AAA-AAA	0.00	999	1.000	1.000	0.0748
213200_at	45.0	11.4	-1.98	-3.93	AAA-AAA	0.00	999	1.000	1.000	0.0411
208983_s_at	18.1	4.6	-1.98	-3.93	AAA-AAA	0.00	999	1.000	1.000	0.2587
229645_at	23.4	6.0	-1.98	-3.94	AAA-AAA	0.00	999	1.000	1.000	0.1122
241457_at	31.9	125.4	1.98	3.93	AAA-APP	0.00	999	1.000	1.000	0.0914
208568_at	65.4	16.7	-1.98	-3.93	AAA-AAA	0.00	999	1.000	1.000	0.0159
237428_at	18.2	4.6	-1.98	-3.93	AMA-AAA	0.00	999	1.000	1.000	0.1363
233160_at	8.2	32.3	1.98	3.93	AAA-AAA	0.00	999	1.000	1.000	0.2760
1563178_at	34.4	8.8	-1.97	-3.93	AAA-AAA	0.00	999	1.000	1.000	0.0696
227282_at	32.4	8.3	-1.97	-3.93	AAA-AAA	0.00	999	1.000	1.000	0.2842
220543_at	55.5	14.1	-1.97	-3.93	AAA-AAA	0.00	999	1.000	1.000	0.2745
216534_at	10.2	2.6	-1.97	-3.93	AAA-AAA	0.00	999	1.000	1.000	0.0240
236120_at	8.6	2.2	-1.97	-3.93	AAA-AAA	0.00	999	1.000	1.000	0.0678
230699_at	12.7	49.6	1.97	3.92	AAA-PAA	0.00	999	1.000	1.000	0.1246
1560251_at	25.0	6.4	-1.97	-3.92	AAA-AAA	0.00	999	1.000	1.000	0.1384
207316_at	25.5	6.5	-1.97	-3.92	AAA-AAA	0.00	999	1.000	1.000	0.1548
228202_at	6.1	1.6	-1.97	-3.92	AAA-AAA	0.00	999	1.000	1.000	0.2782
240736_at	20.3	5.2	-1.97	-3.92	AAA-AAA	0.00	999	1.000	1.000	0.1927
1565563_at	12.3	3.1	-1.97	-3.92	AAA-AAA	0.00	999	1.000	1.000	0.2276
1554745_at	12.4	48.7	1.97	3.92	AAA-AAA	0.00	999	1.000	1.000	0.0800
1570469_at	5.3	20.6	1.97	3.92	APA-AAA	0.00	999	1.000	1.000	0.2250
215674_at	12.0	3.1	-1.97	-3.92	AAA-AAA	0.00	999	1.000	1.000	0.3275
234746_at	69.4	17.7	-1.97	-3.92	AAA-AAA	0.00	999	1.000	1.000	0.0128
233139_at	36.3	9.3	-1.97	-3.92	AAA-AAA	0.00	999	1.000	1.000	0.0470
233235_x_at	50.6	12.9	-1.97	-3.92	AAA-AAA	0.00	999	1.000	1.000	0.1045
227238_at	12.5	3.2	-1.97	-3.92	AAA-AAA	0.00	999	1.000	1.000	0.0683
240186_at	16.7	4.3	-1.97	-3.91	AAA-AAA	0.00	999	1.000	1.000	0.2866
1555035_a_at	25.1	6.4	-1.97	-3.91	AAA-AAA	0.00	999	1.000	1.000	0.0694
227353_at	50.2	12.8	-1.97	-3.91	AAA-AAA	0.00	999	1.000	1.000	0.0013
231345_s_at	26.1	6.7	-1.97	-3.91	AAA-AAA	0.00	999	1.000	1.000	0.1067
203400_s_at	10.9	2.8	-1.97	-3.91	AAA-AAA	0.00	999	1.000	1.000	0.0368
243225_at	5.9	23.1	1.97	3.91	AAA-AAA	0.00	999	1.000	1.000	0.1370
1552521_a_at	3.0	11.7	1.97	3.91	AAA-AAA	0.00	999	1.000	1.000	0.1205
221338_at	10.8	2.8	-1.97	-3.91	AAA-AAA	0.00	999	1.000	1.000	0.1784
240639_at	5.3	20.6	1.97	3.91	AAA-AAA	0.00	999	1.000	1.000	0.0500
1554613_a_at	33.1	8.5	-1.97	-3.91	AAA-AAA	0.00	999	1.000	1.000	0.0701
213543_at	26.5	6.8	-1.97	-3.91	AAA-AAA	0.00	999	1.000	1.000	0.0603
236854_at	44.3	11.4	-1.97	-3.91	AAA-AAA	0.00	999	1.000	1.000	0.0376
237710_at	67.2	17.2	-1.97	-3.91	AAA-AAA	0.00	999	1.000	1.000	0.0941

237685_at	20.7	5.3	-1.97	-3.91	AAA-AAA	0.00	999	1.000	1.000	0.2357
229557_at	62.9	16.1	-1.97	-3.91	AAA-AAA	0.00	999	1.000	1.000	0.1703
232250_at	30.3	7.8	-1.97	-3.91	AAA-AAA	0.00	999	1.000	1.000	0.2747
1561030_at	4.0	1.0	-1.96	-3.90	AAA-AAA	0.00	999	1.000	1.000	0.2218
208098_at	10.0	2.6	-1.96	-3.90	AAA-AAA	0.00	999	1.000	1.000	0.2449
232329_at	31.7	8.1	-1.96	-3.90	AAA-AAA	0.00	999	1.000	1.000	0.1623
1555421_at	40.1	10.3	-1.96	-3.90	AAA-AAA	0.00	999	1.000	1.000	0.0704
216168_at	53.8	13.8	-1.96	-3.90	AAA-AAA	0.00	999	1.000	1.000	0.0494
242785_at	51.0	13.1	-1.96	-3.90	AAA-AAA	0.00	999	1.000	1.000	0.0152
241392_at	78.6	20.2	-1.96	-3.90	AAA-AAA	0.00	999	1.000	1.000	0.1261
220101_x_at	39.5	10.1	-1.96	-3.90	AAA-AAA	0.00	999	1.000	1.000	0.2377
237216_at	10.5	41.0	1.96	3.90	AAA-AMM	0.00	999	1.000	1.000	0.1068
230895_at	38.9	10.0	-1.96	-3.90	AAA-AAA	0.00	999	1.000	1.000	0.1355
233631_x_at	43.2	11.1	-1.96	-3.90	AAA-AAA	0.00	999	1.000	1.000	0.2048
205806_at	14.3	55.9	1.96	3.89	AAA-PAA	0.00	999	1.000	1.000	0.1356
1555710_at	4.1	15.8	1.96	3.89	AAA-AAA	0.00	999	1.000	1.000	0.1142
206731_at	35.6	9.1	-1.96	-3.89	AAM-AAA	0.00	999	1.000	1.000	0.1392
234386_s_at	50.4	13.0	-1.96	-3.89	AAA-AAA	0.00	999	1.000	1.000	0.2113
205325_at	53.8	13.8	-1.96	-3.89	AAA-AAA	0.00	999	1.000	1.000	0.0636
241206_at	11.9	3.1	-1.96	-3.89	AAA-AAA	0.00	999	1.000	1.000	0.3190
1567288_at	9.8	2.5	-1.96	-3.89	AAA-AAA	0.00	999	1.000	1.000	0.2369
1567590_at	8.4	2.2	-1.96	-3.89	AAA-AAA	0.00	999	1.000	1.000	0.2993
1555244_at	14.4	3.7	-1.96	-3.89	AAA-AAA	0.00	999	1.000	1.000	0.2224
229652_s_at	56.8	14.6	-1.96	-3.89	AAA-AAA	0.00	999	1.000	1.000	0.0949
1559790_at	22.6	5.8	-1.96	-3.89	AAA-AAA	0.00	999	1.000	1.000	0.0681
1570337_at	24.8	6.4	-1.96	-3.88	AAA-AAA	0.00	999	1.000	1.000	0.2008
237516_at	21.3	5.5	-1.96	-3.88	AAA-AAA	0.00	999	1.000	1.000	0.3299
228028_at	49.8	12.8	-1.96	-3.88	AAA-AAA	0.00	999	1.000	1.000	0.0312
1555164_at	23.6	6.1	-1.96	-3.88	AAA-AAA	0.00	999	1.000	1.000	0.1609
1555445_at	17.9	4.6	-1.96	-3.88	AAA-AAA	0.00	999	1.000	1.000	0.1625
216948_at	17.0	4.4	-1.96	-3.88	AAA-AAM	0.00	999	1.000	1.000	0.3072
232862_at	61.5	15.9	-1.96	-3.88	AAA-AAA	0.00	999	1.000	1.000	0.1705
232677_at	19.9	5.1	-1.96	-3.88	AAA-AAA	0.00	999	1.000	1.000	0.0693
215290_at	34.8	9.0	-1.96	-3.88	AAA-AAA	0.00	999	1.000	1.000	0.1668
234717_at	14.8	3.8	-1.96	-3.88	AAA-AAA	0.00	999	1.000	1.000	0.2005
1561422_at	20.3	5.2	-1.96	-3.88	AAA-AAA	0.00	999	1.000	1.000	0.0887
220556_at	31.8	8.2	-1.96	-3.88	AAA-AAA	0.00	999	1.000	1.000	0.1822
236556_s_at	16.5	4.2	-1.96	-3.88	AAA-AAA	0.00	999	1.000	1.000	0.1109
232643_at	13.8	53.6	1.96	3.88	AAA-AAA	0.00	999	1.000	1.000	0.0872
207867_at	8.1	2.1	-1.95	-3.87	AAA-AAA	0.00	999	1.000	1.000	0.2397
233524_at	30.4	7.9	-1.95	-3.87	AAA-AAA	0.00	999	1.000	1.000	0.2390
233395_at	14.5	3.8	-1.95	-3.87	AAA-AAA	0.00	999	1.000	1.000	0.0040

241870_at	18.9	4.9	-1.95	-3.87	AAA-AAA	0.00	999	1.000	1.000	0.1959
1556847_s_at	4.1	15.8	1.95	3.87	AAA-AAA	0.00	999	1.000	1.000	0.1600
226702_at	26.8	6.9	-1.95	-3.87	AAA-AAA	0.00	999	1.000	1.000	0.0715
236201_at	23.6	6.1	-1.95	-3.87	AAA-AAA	0.00	999	1.000	1.000	0.1217
230990_at	45.7	11.8	-1.95	-3.87	AAA-AAA	0.00	999	1.000	1.000	0.2432
205848_at	15.4	4.0	-1.95	-3.87	AAA-AAA	0.00	999	1.000	1.000	0.2012
219368_at	9.9	38.3	1.95	3.87	APA-MAA	0.00	999	1.000	1.000	0.1839
243337_at	11.2	2.9	-1.95	-3.87	AMA-AAA	0.00	999	1.000	1.000	0.2414
1569454_a_at	46.1	11.9	-1.95	-3.87	AAA-AAA	0.00	999	1.000	1.000	0.1419
208206_s_at	11.9	46.1	1.95	3.87	AAA-AAA	0.00	999	1.000	1.000	0.0028
204997_at	11.4	3.0	-1.95	-3.87	AAA-AAA	0.00	999	1.000	1.000	0.0522
230030_at	2.1	8.2	1.95	3.87	AAA-AAA	0.00	999	1.000	1.000	0.1629
232687_at	32.0	8.3	-1.95	-3.87	AAA-AAA	0.00	999	1.000	1.000	0.1020
1570474_s_at	18.9	4.9	-1.95	-3.87	AAA-AAA	0.00	999	1.000	1.000	0.1857
201721_s_at	85.4	22.1	-1.95	-3.87	AAA-AAA	0.00	999	1.000	1.000	0.0057
1559987_at	78.0	20.2	-1.95	-3.87	AAA-AAA	0.00	999	1.000	1.000	0.0541
232332_at	35.5	9.2	-1.95	-3.87	AAA-AAA	0.00	999	1.000	1.000	0.0703
215906_at	79.2	20.5	-1.95	-3.87	AAA-AAA	0.00	999	1.000	1.000	0.1139
235633_at	10.4	2.7	-1.95	-3.87	AAA-AAA	0.00	999	1.000	1.000	0.1982
1561658_at	9.1	2.4	-1.95	-3.86	AAA-AAA	0.00	999	1.000	1.000	0.2176
1568852_x_at	2.2	8.3	1.95	3.86	AAA-AAA	0.00	999	1.000	1.000	0.0153
211155_s_at	10.3	2.7	-1.95	-3.86	AAA-AAA	0.00	999	1.000	1.000	0.2269
236482_at	43.2	11.2	-1.95	-3.86	AAA-AAA	0.00	999	1.000	1.000	0.1210
1563263_at	13.7	3.5	-1.95	-3.86	AAA-AAA	0.00	999	1.000	1.000	0.1404
1569192_at	16.2	4.2	-1.95	-3.86	AAA-AAA	0.00	999	1.000	1.000	0.2167
1553078_at	45.2	11.7	-1.95	-3.86	AAA-AAA	0.00	999	1.000	1.000	0.0407
207996_s_at	12.2	3.2	-1.95	-3.86	AAA-AAA	0.00	999	1.000	1.000	0.2385
215211_at	30.2	7.8	-1.95	-3.86	AAA-AAA	0.00	999	1.000	1.000	0.0371
1553730_x_at	122.6	31.8	-1.95	-3.86	AAA-AAA	0.00	999	1.000	1.000	0.0226
1559692_at	31.7	8.2	-1.95	-3.86	AAA-AAA	0.00	999	1.000	1.000	0.2284
217098_s_at	7.5	29.0	1.95	3.86	AAA-AAA	0.00	999	1.000	1.000	0.0110
228492_at	14.9	3.9	-1.95	-3.86	AAM-AAA	0.00	999	1.000	1.000	0.1807
1566464_at	13.1	3.4	-1.95	-3.85	AAA-AAA	0.00	999	1.000	1.000	0.0287
222923_s_at	34.3	8.9	-1.95	-3.85	AAA-AAA	0.00	999	1.000	1.000	0.0629
238390_at	27.9	7.2	-1.95	-3.85	AAA-AAA	0.00	999	1.000	1.000	0.2094
228761_at	96.7	25.1	-1.95	-3.85	AAA-AAA	0.00	999	1.000	1.000	0.1784
233627_at	58.7	15.2	-1.95	-3.85	AAA-AAA	0.00	999	1.000	1.000	0.0622
200648_s_at	61.2	235.7	1.95	3.85	APA-PPP	0.00	999	1.000	1.000	0.1855
211641_x_at	98.0	25.5	-1.95	-3.85	AAA-AAA	0.00	999	1.000	1.000	0.0274
216429_at	23.6	90.9	1.95	3.85	AAA-AAA	0.00	999	1.000	1.000	0.0203
236645_at	8.7	33.6	1.95	3.85	AAA-AAA	0.00	999	1.000	1.000	0.0995
237083_at	2.2	8.3	1.95	3.85	AAA-AAA	0.00	999	1.000	1.000	0.1671

233745_at	38.4	10.0	-1.95	-3.85	AAA-AAA	0.00	999	1.000	1.000	0.2580
233739_at	12.0	3.1	-1.95	-3.85	AAA-AAA	0.00	999	1.000	1.000	0.1790
1566514_at	51.0	13.2	-1.94	-3.85	AAA-AAA	0.00	999	1.000	1.000	0.0583
221714_s_at	34.7	9.0	-1.94	-3.85	MAA-AAA	0.00	999	1.000	1.000	0.2658
222176_at	30.7	8.0	-1.94	-3.85	AAA-AAA	0.00	999	1.000	1.000	0.1873
206957_at	24.2	6.3	-1.94	-3.85	AAA-AAA	0.00	999	1.000	1.000	0.3704
204543_at	55.4	14.4	-1.94	-3.85	AAA-AAA	0.00	999	1.000	1.000	0.1782
237254_at	22.0	5.7	-1.94	-3.85	AAA-AAA	0.00	999	1.000	1.000	0.0209
230804_at	11.7	3.0	-1.94	-3.85	AAA-AAA	0.00	999	1.000	1.000	0.1614
240813_at	47.9	12.5	-1.94	-3.85	AAA-AAA	0.00	999	1.000	1.000	0.0543
221168_at	6.7	25.8	1.94	3.85	AAA-AAA	0.00	999	1.000	1.000	0.0548
214899_at	21.6	5.6	-1.94	-3.84	AAA-AAA	0.00	999	1.000	1.000	0.2077
1562957_at	19.6	75.4	1.94	3.84	AAA-AAA	0.00	999	1.000	1.000	0.0922
1556325_at	20.7	5.4	-1.94	-3.84	AAA-AAA	0.00	999	1.000	1.000	0.0422
206664_at	10.1	2.6	-1.94	-3.84	AAA-AAA	0.00	999	1.000	1.000	0.2655
217252_at	59.6	15.5	-1.94	-3.84	AAA-AAA	0.00	999	1.000	1.000	0.0180
227879_at	14.9	57.1	1.94	3.84	AAA-AAA	0.00	999	1.000	1.000	0.0753
242700_at	14.9	3.9	-1.94	-3.84	AAA-AAA	0.00	999	1.000	1.000	0.0359
1569300_at	9.6	36.7	1.94	3.84	AAA-AAA	0.00	999	1.000	1.000	0.1008
236591_at	30.0	7.8	-1.94	-3.84	AAA-AAA	0.00	999	1.000	1.000	0.2136
1553604_at	1.7	6.6	1.94	3.84	AAA-AAA	0.00	999	1.000	1.000	0.1075
204041_at	22.5	5.9	-1.94	-3.84	AAA-AAA	0.00	999	1.000	1.000	0.1467
233134_at	14.0	3.7	-1.94	-3.84	AAA-AAA	0.00	999	1.000	1.000	0.0982
240596_at	4.3	16.5	1.94	3.84	AAA-AAA	0.00	999	1.000	1.000	0.2332
1565840_at	14.1	3.7	-1.94	-3.83	AAA-AAA	0.00	999	1.000	1.000	0.3387
1552293_at	23.6	6.2	-1.94	-3.83	AAA-AAA	0.00	999	1.000	1.000	0.1081
220670_at	33.4	8.7	-1.94	-3.83	AAA-AAA	0.00	999	1.000	1.000	0.0237
223998_at	15.5	4.0	-1.94	-3.83	AAA-AAA	0.00	999	1.000	1.000	0.2564
206334_at	27.9	7.3	-1.94	-3.83	AAA-AAA	0.00	999	1.000	1.000	0.1663
228984_at	52.3	13.7	-1.94	-3.83	AAA-AAA	0.00	999	1.000	1.000	0.1888
232072_at	14.0	53.5	1.94	3.83	AAA-AAM	0.00	999	1.000	1.000	0.0724
1552381_at	48.4	12.6	-1.94	-3.83	AAA-AAA	0.00	999	1.000	1.000	0.1092
244431_at	11.2	42.8	1.94	3.83	AAA-AAA	0.00	999	1.000	1.000	0.0683
224422_x_at	9.7	37.3	1.94	3.83	AAA-AAA	0.00	999	1.000	1.000	0.0016
208512_s_at	11.9	45.7	1.94	3.83	AAA-AAA	0.00	999	1.000	1.000	0.1148
238820_at	35.7	9.3	-1.94	-3.83	AAA-AAA	0.00	999	1.000	1.000	0.1271
233095_at	14.4	3.8	-1.94	-3.83	AAA-AAA	0.00	999	1.000	1.000	0.0812
1562457_at	19.5	5.1	-1.94	-3.83	AAA-AAA	0.00	999	1.000	1.000	0.1331
1553003_at	69.9	18.3	-1.94	-3.83	AAA-AAA	0.00	999	1.000	1.000	0.1807
220970_s_at	8.0	2.1	-1.94	-3.83	AAA-AAA	0.00	999	1.000	1.000	0.2114
213950_s_at	14.3	54.7	1.94	3.82	AAA-AAA	0.00	999	1.000	1.000	0.1609
210917_at	4.3	16.3	1.94	3.82	AAA-AAA	0.00	999	1.000	1.000	0.1224

1561474_at	4.1	15.6	1.93	3.82	AAA-AAA	0.00	999	1.000	1.000	0.1217
1553299_at	4.8	18.2	1.93	3.82	AAA-AAA	0.00	999	1.000	1.000	0.1583
204959_at	31.2	8.2	-1.93	-3.82	AAA-AAA	0.00	999	1.000	1.000	0.0911
1562480_at	3.8	1.0	-1.93	-3.82	AAA-AAA	0.00	999	1.000	1.000	0.2041
1561806_at	21.9	5.7	-1.93	-3.82	AAA-AAA	0.00	999	1.000	1.000	0.1013
1554748_at	26.0	6.8	-1.93	-3.82	AAA-AAA	0.00	999	1.000	1.000	0.0503
217291_at	26.2	100.1	1.93	3.82	AAA-MAA	0.00	999	1.000	1.000	0.0663
239558_at	11.4	3.0	-1.93	-3.82	AAA-AAA	0.00	999	1.000	1.000	0.1399
243738_at	11.8	44.8	1.93	3.82	AAA-AAA	0.00	999	1.000	1.000	0.0030
1565358_at	24.5	6.4	-1.93	-3.82	AAA-AAA	0.00	999	1.000	1.000	0.1785
1553905_at	39.6	10.4	-1.93	-3.82	AAA-AAA	0.00	999	1.000	1.000	0.1562
208271_at	14.5	3.8	-1.93	-3.82	AAA-AAA	0.00	999	1.000	1.000	0.3098
217418_x_at	21.6	5.7	-1.93	-3.82	AAA-AAA	0.00	999	1.000	1.000	0.1056
234454_at	41.6	10.9	-1.93	-3.82	AAA-AAA	0.00	999	1.000	1.000	0.1113
239728_at	87.7	23.0	-1.93	-3.82	AAA-AAA	0.00	999	1.000	1.000	0.0459
1553016_at	91.2	23.9	-1.93	-3.81	AAA-AAA	0.00	999	1.000	1.000	0.0062
223858_at	11.1	42.5	1.93	3.81	AAA-AAA	0.00	999	1.000	1.000	0.2961
214868_at	11.6	44.2	1.93	3.81	AAA-AAA	0.00	999	1.000	1.000	0.0933
216498_at	69.3	18.2	-1.93	-3.81	AAA-AAA	0.00	999	1.000	1.000	0.1983
228874_at	22.8	6.0	-1.93	-3.81	AAA-AAA	0.00	999	1.000	1.000	0.0855
220637_at	24.1	6.3	-1.93	-3.81	AAA-AAA	0.00	999	1.000	1.000	0.0577
206594_at	25.7	97.9	1.93	3.81	AAA-AAA	0.00	999	1.000	1.000	0.1067
1554059_at	129.7	34.1	-1.93	-3.81	AAA-AAA	0.00	999	1.000	1.000	0.1068
233988_x_at	7.5	28.4	1.93	3.81	AAA-AAA	0.00	999	1.000	1.000	0.1300
242375_x_at	26.2	6.9	-1.93	-3.81	AAA-AAA	0.00	999	1.000	1.000	0.1241
203828_s_at	21.3	81.2	1.93	3.81	AAA-AAA	0.00	999	1.000	1.000	0.0461
1553835_a_at	37.2	9.8	-1.93	-3.81	AAA-AAA	0.00	999	1.000	1.000	0.1897
1554300_a_at	21.1	5.6	-1.93	-3.81	AAA-AAA	0.00	999	1.000	1.000	0.0411
1553229_at	11.2	42.6	1.93	3.81	AAA-AAA	0.00	999	1.000	1.000	0.0998
244884_at	6.9	26.3	1.93	3.80	AAA-AAA	0.00	999	1.000	1.000	0.0636
1555991_s_at	14.8	3.9	-1.93	-3.80	AAA-AAA	0.00	999	1.000	1.000	0.2048
214504_at	31.7	8.3	-1.93	-3.80	AAA-AAA	0.00	999	1.000	1.000	0.0022
211067_s_at	54.0	14.2	-1.93	-3.80	AAA-AAA	0.00	999	1.000	1.000	0.1334
215442_s_at	29.4	7.7	-1.93	-3.80	AAA-AAA	0.00	999	1.000	1.000	0.1065
242388_x_at	16.8	4.4	-1.93	-3.80	AAA-AAA	0.00	999	1.000	1.000	0.2607
1569544_at	34.3	9.0	-1.93	-3.80	AAA-AAA	0.00	999	1.000	1.000	0.1651
233997_at	121.8	32.1	-1.93	-3.80	MAA-AAA	0.00	999	1.000	1.000	0.0987
243901_at	16.8	4.4	-1.92	-3.79	AAA-AAA	0.00	999	1.000	1.000	0.2145
1568574_x_at	11.9	3.1	-1.92	-3.79	AAA-AAA	0.00	999	1.000	1.000	0.0463
1566888_at	16.9	4.5	-1.92	-3.80	AAA-AAA	0.00	999	1.000	1.000	0.2730
1553927_at	4.1	15.5	1.92	3.80	AAA-AAA	0.00	999	1.000	1.000	0.1498
216359_at	10.3	2.7	-1.92	-3.80	AAA-AAA	0.00	999	1.000	1.000	0.2143

236519_at	25.6	6.8	-1.92	-3.80	AAA-AAA	0.00	999	1.000	1.000	0.2082
205261_at	112.2	29.6	-1.92	-3.79	AAA-AAA	0.00	999	1.000	1.000	0.0543
1563750_at	34.3	9.0	-1.92	-3.79	AAA-AAA	0.00	999	1.000	1.000	0.1234
1556131_s_at	43.9	11.6	-1.92	-3.79	AAA-AAA	0.00	999	1.000	1.000	0.2107
1559909_a_at	37.8	10.0	-1.92	-3.79	AAA-AAA	0.00	999	1.000	1.000	0.1358
202063_s_at	13.6	51.4	1.92	3.79	AAA-AAA	0.00	999	1.000	1.000	0.1898
214307_at	22.8	6.0	-1.92	-3.79	AAA-AAA	0.00	999	1.000	1.000	0.1235
233969_at	10.2	2.7	-1.92	-3.79	AAA-AAA	0.00	999	1.000	1.000	0.0923
228945_s_at	28.5	7.5	-1.92	-3.79	AAA-AAA	0.00	999	1.000	1.000	0.2169
232144_at	42.9	11.3	-1.92	-3.79	AAA-AAA	0.00	999	1.000	1.000	0.1062
1559461_at	107.3	28.3	-1.92	-3.79	AAA-AAA	0.00	999	1.000	1.000	0.0022
231813_s_at	64.9	17.1	-1.92	-3.79	AAA-AAA	0.00	999	1.000	1.000	0.1093
1561280_at	6.0	1.6	-1.92	-3.79	AAA-AAA	0.00	999	1.000	1.000	0.2083
229452_at	40.2	10.6	-1.92	-3.79	AAA-AAA	0.00	999	1.000	1.000	0.1717
207998_s_at	73.9	19.5	-1.92	-3.78	AAA-AAA	0.00	999	1.000	1.000	0.0125
243212_at	26.1	6.9	-1.92	-3.78	AAA-AAA	0.00	999	1.000	1.000	0.0443
1552424_at	7.3	1.9	-1.92	-3.78	AAA-AAA	0.00	999	1.000	1.000	0.2242
1552566_at	9.3	2.4	-1.92	-3.78	AAA-AAA	0.00	999	1.000	1.000	0.0358
214636_at	25.8	6.8	-1.92	-3.78	AAA-AAA	0.00	999	1.000	1.000	0.3465
215488_at	18.4	69.7	1.92	3.78	AAA-AAA	0.00	999	1.000	1.000	0.0225
204374_s_at	26.1	98.8	1.92	3.78	AAA-AAA	0.00	999	1.000	1.000	0.1440
206137_at	70.2	18.6	-1.92	-3.78	AAA-AAA	0.00	999	1.000	1.000	0.2291
220851_at	23.7	6.3	-1.92	-3.78	AAA-AAA	0.00	999	1.000	1.000	0.1712
215361_at	9.3	2.5	-1.92	-3.78	AAA-AAA	0.00	999	1.000	1.000	0.1901
233373_at	33.5	8.9	-1.92	-3.78	AAA-AAA	0.00	999	1.000	1.000	0.2076
1560142_at	26.9	7.1	-1.92	-3.78	AAA-AAA	0.00	999	1.000	1.000	0.1175
221151_at	22.4	5.9	-1.92	-3.78	AAA-AAA	0.00	999	1.000	1.000	0.0390
237523_at	25.7	6.8	-1.92	-3.78	AAA-AAA	0.00	999	1.000	1.000	0.3062
240029_at	84.7	22.5	-1.92	-3.77	AAA-AAA	0.00	999	1.000	1.000	0.0337
240122_at	17.8	4.7	-1.92	-3.77	AAA-AAA	0.00	999	1.000	1.000	0.3117
1561343_a_at	2.2	8.4	1.92	3.77	AAA-AAA	0.00	999	1.000	1.000	0.1531
1556711_at	20.2	5.4	-1.92	-3.78	AAA-AAA	0.00	999	1.000	1.000	0.0943
235382_at	4.9	18.4	1.92	3.77	AAA-AAA	0.00	999	1.000	1.000	0.2701
229823_at	24.1	6.4	-1.92	-3.77	AAA-AAA	0.00	999	1.000	1.000	0.1174
240832_at	3.8	14.3	1.92	3.77	AAA-AAA	0.00	999	1.000	1.000	0.1858
243572_at	36.7	9.7	-1.92	-3.77	AAA-AAA	0.00	999	1.000	1.000	0.2978
1553875_s_at	15.1	56.8	1.92	3.77	AAA-AAA	0.00	999	1.000	1.000	0.0270
220418_at	13.9	3.7	-1.92	-3.77	AAA-AAA	0.00	999	1.000	1.000	0.0960
222969_at	45.7	12.1	-1.92	-3.77	AAA-AAA	0.00	999	1.000	1.000	0.2764
224406_s_at	117.7	31.2	-1.92	-3.77	AAA-AAA	0.00	999	1.000	1.000	0.0238
238450_at	46.1	12.2	-1.92	-3.77	AAA-AAA	0.00	999	1.000	1.000	0.1632
1562905_at	10.0	2.7	-1.91	-3.77	AAA-AAA	0.00	999	1.000	1.000	0.0828

219859_at	8.5	32.0	1.91	3.77	AAA-AMA	0.00	999	1.000	1.000	0.1767
228585_at	18.7	5.0	-1.91	-3.77	AAA-AAA	0.00	999	1.000	1.000	0.3186
1559427_at	31.7	8.4	-1.91	-3.77	AAA-AAA	0.00	999	1.000	1.000	0.0942
208087_s_at	37.2	9.9	-1.91	-3.77	AAA-AAA	0.00	999	1.000	1.000	0.0992
221936_x_at	13.0	48.9	1.91	3.77	AAA-AAA	0.00	999	1.000	1.000	0.0136
208605_s_at	16.0	4.2	-1.91	-3.77	AAA-AAA	0.00	999	1.000	1.000	0.1686
237358_at	20.1	5.4	-1.91	-3.77	AAA-AAA	0.00	999	1.000	1.000	0.1090
1562020_s_at	16.2	4.3	-1.91	-3.76	AAA-AAA	0.00	999	1.000	1.000	0.1343
207182_at	3.3	12.3	1.91	3.76	AAA-AAA	0.00	999	1.000	1.000	0.0767
210940_s_at	4.5	17.0	1.91	3.76	AAA-AAA	0.00	999	1.000	1.000	0.0105
239553_at	23.3	6.2	-1.91	-3.76	AAA-AAA	0.00	999	1.000	1.000	0.1595
239359_at	49.3	13.1	-1.91	-3.76	AAA-AAA	0.00	999	1.000	1.000	0.1940
1569923_s_at	36.9	9.8	-1.91	-3.76	AAA-AAA	0.00	999	1.000	1.000	0.1035
1554996_at	4.8	1.3	-1.91	-3.76	AAA-AAA	0.00	999	1.000	1.000	0.0587
207120_at	12.7	3.4	-1.91	-3.76	AAA-AAA	0.00	999	1.000	1.000	0.2086
237611_at	17.7	66.4	1.91	3.76	AAA-AAA	0.00	999	1.000	1.000	0.0210
229169_at	23.6	6.3	-1.91	-3.76	AAA-AAA	0.00	999	1.000	1.000	0.0899
231489_x_at	34.2	9.1	-1.91	-3.76	AAA-AAA	0.00	999	1.000	1.000	0.2841
1560717_at	26.8	7.1	-1.91	-3.76	AAA-AAA	0.00	999	1.000	1.000	0.1095
244794_at	12.5	3.3	-1.91	-3.76	AAA-AAA	0.00	999	1.000	1.000	0.1680
1558712_at	72.6	19.3	-1.91	-3.76	AAA-AAA	0.00	999	1.000	1.000	0.1001
206867_at	85.5	22.8	-1.91	-3.76	AAA-AAA	0.00	999	1.000	1.000	0.0589
230001_at	82.3	21.9	-1.91	-3.76	AAA-AAA	0.00	999	1.000	1.000	0.1482
1566917_at	76.1	20.3	-1.91	-3.76	AAA-AAA	0.00	999	1.000	1.000	0.0506
214238_at	18.5	69.6	1.91	3.76	AAA-AAM	0.00	999	1.000	1.000	0.0031
233615_at	43.1	11.5	-1.91	-3.76	AAA-AAA	0.00	999	1.000	1.000	0.1286
216098_s_at	43.9	11.7	-1.91	-3.75	AAA-AAA	0.00	999	1.000	1.000	0.2718
239244_at	21.4	5.7	-1.91	-3.75	AAA-AAA	0.00	999	1.000	1.000	0.2701
240580_at	39.8	10.6	-1.91	-3.75	AMA-AAA	0.00	999	1.000	1.000	0.1817
242707_at	41.9	11.2	-1.91	-3.75	AAA-AAA	0.00	999	1.000	1.000	0.2161
239430_at	13.9	52.2	1.91	3.75	AAA-AAA	0.00	999	1.000	1.000	0.0740
229487_at	47.6	12.7	-1.91	-3.75	AAA-AAA	0.00	999	1.000	1.000	0.0873
205969_at	23.6	88.6	1.91	3.75	AAA-MAA	0.00	999	1.000	1.000	0.1871
231147_at	96.8	25.8	-1.91	-3.75	AAA-AAA	0.00	999	1.000	1.000	0.0766
235238_at	19.7	73.8	1.91	3.75	PAA-APA	0.00	999	1.000	1.000	0.1274
242114_at	59.2	15.8	-1.91	-3.75	AAA-AAA	0.00	999	1.000	1.000	0.1827
203540_at	24.1	6.4	-1.91	-3.75	AAA-AAA	0.00	999	1.000	1.000	0.2930
241610_x_at	78.7	21.0	-1.91	-3.75	AAA-AAA	0.00	999	1.000	1.000	0.0244
243766_s_at	47.1	12.6	-1.91	-3.75	AAA-AAA	0.00	999	1.000	1.000	0.0633
241158_at	71.1	19.0	-1.91	-3.75	AAA-AAA	0.00	999	1.000	1.000	0.0517
1561983_at	18.5	4.9	-1.91	-3.75	AAA-AAA	0.00	999	1.000	1.000	0.3590
234392_at	19.8	5.3	-1.91	-3.74	AAA-AAA	0.00	999	1.000	1.000	0.0219

229502_at	26.8	7.2	-1.91	-3.75	AAA-AAA	0.00	999	1.000	1.000	0.1798
231589_at	27.3	7.3	-1.91	-3.74	AAA-AAA	0.00	999	1.000	1.000	0.1538
211923_s_at	10.0	2.7	-1.90	-3.74	AAA-AAA	0.00	999	1.000	1.000	0.2148
203915_at	43.3	11.6	-1.90	-3.74	AAA-AAA	0.00	999	1.000	1.000	0.1850
230485_at	13.0	48.8	1.90	3.74	AAA-AAA	0.00	999	1.000	1.000	0.2307
244019_at	13.3	49.7	1.90	3.74	AAA-AAA	0.00	999	1.000	1.000	0.1410
237938_at	17.9	4.8	-1.90	-3.74	AAA-AAA	0.00	999	1.000	1.000	0.1317
232988_at	19.4	72.5	1.90	3.74	AAA-APM	0.00	999	1.000	1.000	0.2459
243871_at	14.1	3.8	-1.90	-3.74	AAA-AAA	0.00	999	1.000	1.000	0.1991
1556173_a_at	39.7	10.6	-1.90	-3.74	AAA-AAA	0.00	999	1.000	1.000	0.0876
208265_at	28.3	7.6	-1.90	-3.74	AAA-AAA	0.00	999	1.000	1.000	0.0734
227434_at	38.6	10.3	-1.90	-3.74	AAA-AAA	0.00	999	1.000	1.000	0.1303
224337_s_at	17.1	4.6	-1.90	-3.74	AAA-AAA	0.00	999	1.000	1.000	0.1058
234820_at	12.0	3.2	-1.90	-3.74	AAA-AAA	0.00	999	1.000	1.000	0.1721
229657_at	42.7	11.4	-1.90	-3.74	AAA-AAA	0.00	999	1.000	1.000	0.0524
1561710_at	16.4	4.4	-1.90	-3.73	AAA-AAA	0.00	999	1.000	1.000	0.2491
1565698_at	33.7	9.0	-1.90	-3.74	AAA-AAA	0.00	999	1.000	1.000	0.0660
1564413_at	44.6	11.9	-1.90	-3.74	AAA-AAA	0.00	999	1.000	1.000	0.1487
217431_x_at	6.5	24.1	1.90	3.74	AAA-AAA	0.00	999	1.000	1.000	0.2500
238584_at	33.2	8.9	-1.90	-3.74	AAA-AAA	0.00	999	1.000	1.000	0.1057
237576_x_at	46.2	12.4	-1.90	-3.74	AAA-AAA	0.00	999	1.000	1.000	0.1403
240723_at	75.5	20.2	-1.90	-3.73	AAA-AAA	0.00	999	1.000	1.000	0.1460
1569235_a_at	5.3	19.7	1.90	3.73	AAA-AAA	0.00	999	1.000	1.000	0.2126
1556641_at	28.2	7.6	-1.90	-3.73	AAA-AAA	0.00	999	1.000	1.000	0.0839
215574_at	48.2	12.9	-1.90	-3.73	AAA-AAA	0.00	999	1.000	1.000	0.1414
232595_at	18.7	69.7	1.90	3.73	AAA-AAM	0.00	999	1.000	1.000	0.1600
210192_at	15.9	4.3	-1.90	-3.73	AAA-AAA	0.00	999	1.000	1.000	0.1991
216666_at	37.7	10.1	-1.90	-3.73	AAA-AAA	0.00	999	1.000	1.000	0.1118
236024_at	59.6	16.0	-1.90	-3.73	AAA-AAA	0.00	999	1.000	1.000	0.0805
234354_x_at	28.5	7.7	-1.90	-3.73	AAA-AAA	0.00	999	1.000	1.000	0.0898
230908_at	17.2	4.6	-1.90	-3.73	AAA-AAA	0.00	999	1.000	1.000	0.0095
243621_at	50.4	13.5	-1.90	-3.73	AAA-AAA	0.00	999	1.000	1.000	0.0679
1561504_s_at	9.0	33.7	1.90	3.73	AAA-AAA	0.00	999	1.000	1.000	0.2526
236991_at	50.8	13.6	-1.90	-3.73	AAA-AAA	0.00	999	1.000	1.000	0.0373
233236_at	18.7	69.7	1.90	3.73	AAA-AAA	0.00	999	1.000	1.000	0.0030
1562783_at	43.7	11.7	-1.90	-3.73	AAA-AAA	0.00	999	1.000	1.000	0.0969
1567681_at	39.3	10.6	-1.90	-3.73	AAA-AAA	0.00	999	1.000	1.000	0.2328
1555867_at	10.6	39.4	1.90	3.73	AAA-AAA	0.00	999	1.000	1.000	0.0502
1569468_at	4.5	16.8	1.90	3.72	AAA-AAA	0.00	999	1.000	1.000	0.1497
244326_at	28.7	7.7	-1.90	-3.72	AAA-AAA	0.00	999	1.000	1.000	0.0474
242891_at	114.1	30.7	-1.90	-3.72	AAA-AAA	0.00	999	1.000	1.000	0.1779
1569773_at	9.5	2.5	-1.90	-3.72	AAA-AAA	0.00	999	1.000	1.000	0.1289

1556133_s_at	12.0	3.2	-1.90	-3.72	AAA-AAA	0.00	999	1.000	1.000	0.1313
236020_s_at	63.1	17.0	-1.90	-3.72	AAA-AAA	0.00	999	1.000	1.000	0.0211
238189_at	30.7	8.3	-1.90	-3.72	AAA-AAA	0.00	999	1.000	1.000	0.1203
239608_at	87.4	23.5	-1.90	-3.72	AAA-AAA	0.00	999	1.000	1.000	0.1766
240987_at	31.5	8.5	-1.89	-3.72	AAA-AAA	0.00	999	1.000	1.000	0.1144
1558299_at	120.8	32.5	-1.89	-3.72	AAA-AAA	0.00	999	1.000	1.000	0.0930
215971_at	61.3	16.5	-1.89	-3.72	AAA-AAA	0.00	999	1.000	1.000	0.1582
235876_at	24.0	6.5	-1.89	-3.72	AAA-AAA	0.00	999	1.000	1.000	0.1694
233072_at	4.1	15.1	1.89	3.72	AAA-AAA	0.00	999	1.000	1.000	0.2407
231783_at	16.6	4.5	-1.89	-3.72	AAA-AAA	0.00	999	1.000	1.000	0.1670
229401_at	22.9	6.2	-1.89	-3.71	AAA-AAA	0.00	999	1.000	1.000	0.2022
244239_at	29.0	7.8	-1.89	-3.71	AAA-AAA	0.00	999	1.000	1.000	0.2462
234466_at	36.6	9.9	-1.89	-3.71	AAA-AAA	0.00	999	1.000	1.000	0.1472
228110_x_at	55.0	14.8	-1.89	-3.71	AAA-AAA	0.00	999	1.000	1.000	0.0370
1562330_s_at	78.6	21.2	-1.89	-3.71	AAA-AAA	0.00	999	1.000	1.000	0.0343
1558768_at	14.7	54.6	1.89	3.71	AAA-AAA	0.00	999	1.000	1.000	0.1541
1556757_a_at	11.7	3.2	-1.89	-3.71	AAA-AAA	0.00	999	1.000	1.000	0.3193
234060_at	5.7	21.1	1.89	3.71	AAA-AAA	0.00	999	1.000	1.000	0.2073
230517_at	12.9	3.5	-1.89	-3.71	AAA-AAA	0.00	999	1.000	1.000	0.1082
206268_at	52.7	14.2	-1.89	-3.71	AAA-AAA	0.00	999	1.000	1.000	0.2013
236766_at	15.1	55.8	1.89	3.71	AAA-APA	0.00	999	1.000	1.000	0.1504
1553365_at	25.6	6.9	-1.89	-3.71	AAA-AAA	0.00	999	1.000	1.000	0.2976
220380_at	35.4	9.6	-1.89	-3.71	AAA-AAA	0.00	999	1.000	1.000	0.1076
238375_at	2.3	8.6	1.89	3.71	AAA-AAA	0.00	999	1.000	1.000	0.1342
1552742_at	27.7	7.5	-1.89	-3.70	AAA-AAA	0.00	999	1.000	1.000	0.3330
238345_at	25.3	6.8	-1.89	-3.70	AAA-AAA	0.00	999	1.000	1.000	0.1388
1559606_at	22.8	6.2	-1.89	-3.70	AAA-AAA	0.00	999	1.000	1.000	0.1526
223869_at	3.8	13.9	1.89	3.70	AAA-AAA	0.00	999	1.000	1.000	0.2130
216458_at	60.8	16.4	-1.89	-3.70	AAA-AAA	0.00	999	1.000	1.000	0.2192
237935_at	15.8	4.3	-1.89	-3.70	AAA-AAA	0.00	999	1.000	1.000	0.0936
201766_at	59.9	16.2	-1.89	-3.70	AAA-AAA	0.00	999	1.000	1.000	0.1290
225728_at	9.4	2.6	-1.89	-3.70	AAA-AAA	0.00	999	1.000	1.000	0.1752
218821_at	18.4	67.9	1.89	3.70	AAA-AAA	0.00	999	1.000	1.000	0.1121
209541_at	13.0	3.5	-1.89	-3.70	AAA-AAA	0.00	999	1.000	1.000	0.2474
216575_at	29.8	8.1	-1.89	-3.70	AAA-AAA	0.00	999	1.000	1.000	0.1989
235883_at	78.3	21.2	-1.89	-3.70	AAA-AAA	0.00	999	1.000	1.000	0.1080
235319_at	32.9	8.9	-1.89	-3.70	AAA-AAA	0.00	999	1.000	1.000	0.1663
204412_s_at	33.5	9.1	-1.89	-3.70	AAA-AAA	0.00	999	1.000	1.000	0.0873
228376_at	9.0	33.3	1.89	3.70	AAA-AAA	0.00	999	1.000	1.000	0.1404
239786_at	24.3	6.6	-1.89	-3.70	AAA-AAA	0.00	999	1.000	1.000	0.0930
243595_at	47.8	12.9	-1.89	-3.70	AAA-AAA	0.00	999	1.000	1.000	0.0814
1553435_at	34.6	9.4	-1.89	-3.70	AAA-AAA	0.00	999	1.000	1.000	0.2323

244380_at	8.7	2.4	-1.89	-3.70	AAA-AAA	0.00	999	1.000	1.000	0.1925
244551_at	4.2	15.6	1.89	3.70	AAA-AAA	0.00	999	1.000	1.000	0.1528
220393_at	10.2	2.8	-1.89	-3.70	MAA-AAA	0.00	999	1.000	1.000	0.3180
241577_at	72.9	19.7	-1.89	-3.70	AAA-AAA	0.00	999	1.000	1.000	0.1881
1564721_at	50.5	13.7	-1.89	-3.69	AAA-AAA	0.00	999	1.000	1.000	0.1835
1555681_at	101.2	27.4	-1.89	-3.69	AMA-AAA	0.00	999	1.000	1.000	0.1632
220069_at	50.4	13.7	-1.88	-3.69	AAA-AAA	0.00	999	1.000	1.000	0.1338
204763_s_at	27.8	7.5	-1.88	-3.69	AAA-AAA	0.00	999	1.000	1.000	0.1547
236014_at	21.2	5.7	-1.88	-3.69	AAA-AAA	0.00	999	1.000	1.000	0.0384
234680_at	29.5	8.0	-1.88	-3.69	AAA-AAA	0.00	999	1.000	1.000	0.0006
231426_at	22.4	6.1	-1.88	-3.69	AAA-AAA	0.00	999	1.000	1.000	0.0105
1554971_at	48.3	13.1	-1.88	-3.69	AAA-AAA	0.00	999	1.000	1.000	0.1892
1559264_at	7.4	2.0	-1.88	-3.69	AAA-AAA	0.00	999	1.000	1.000	0.1510
227915_at	17.4	4.7	-1.88	-3.69	AAA-AAA	0.00	999	1.000	1.000	0.1324
232939_at	22.4	6.1	-1.88	-3.69	AAA-AAA	0.00	999	1.000	1.000	0.1478
243923_at	22.1	6.0	-1.88	-3.69	AAA-AAA	0.00	999	1.000	1.000	0.2446
241205_at	5.0	18.3	1.88	3.69	AAA-AAA	0.00	999	1.000	1.000	0.1662
217375_at	50.1	13.6	-1.88	-3.69	MMA-AAA	0.00	999	1.000	1.000	0.1885
236737_at	12.5	46.1	1.88	3.69	AAA-AAA	0.00	999	1.000	1.000	0.1174
239052_at	6.5	24.0	1.88	3.69	AAA-AAA	0.00	999	1.000	1.000	0.2936
1564679_at	27.5	7.5	-1.88	-3.68	AAA-AAA	0.00	999	1.000	1.000	0.3552
1559635_at	30.2	8.2	-1.88	-3.68	AAA-AAA	0.00	999	1.000	1.000	0.1897
222332_at	13.0	3.5	-1.88	-3.68	AAA-AAA	0.00	999	1.000	1.000	0.2934
238484_s_at	5.4	19.8	1.88	3.68	AAA-AAA	0.00	999	1.000	1.000	0.0705
1555462_at	9.5	34.9	1.88	3.68	AAA-AAA	0.00	999	1.000	1.000	0.1546
1555689_at	3.8	14.1	1.88	3.68	AAA-AAA	0.00	999	1.000	1.000	0.1069
1555748_x_at	60.1	16.3	-1.88	-3.68	AAA-AAA	0.00	999	1.000	1.000	0.0346
241671_x_at	42.7	11.6	-1.88	-3.68	AAA-AAA	0.00	999	1.000	1.000	0.2833
1563681_at	29.5	8.0	-1.88	-3.68	AAA-AAM	0.00	999	1.000	1.000	0.2925
1561251_at	50.7	13.8	-1.88	-3.68	AAA-AAA	0.00	999	1.000	1.000	0.0225
1561402_at	23.4	6.4	-1.88	-3.68	AAA-AAA	0.00	999	1.000	1.000	0.2247
206505_at	16.4	4.5	-1.88	-3.68	AAA-AAA	0.00	999	1.000	1.000	0.1298
217170_at	20.5	5.6	-1.88	-3.68	AAA-AAA	0.00	999	1.000	1.000	0.0980
236197_at	14.3	3.9	-1.88	-3.68	AAA-AAA	0.00	999	1.000	1.000	0.1054
1564883_a_at	85.3	23.2	-1.88	-3.68	AAA-AAA	0.00	999	1.000	1.000	0.0447
244366_at	79.9	21.7	-1.88	-3.68	AAA-AAA	0.00	999	1.000	1.000	0.0411
1557864_x_at	23.7	6.5	-1.88	-3.68	AAA-AAA	0.00	999	1.000	1.000	0.0944
207715_at	87.0	23.7	-1.88	-3.68	AAA-AAA	0.00	999	1.000	1.000	0.0360
236439_at	77.0	20.9	-1.88	-3.68	AAA-AAA	0.00	999	1.000	1.000	0.2692
228849_at	119.4	32.5	-1.88	-3.68	AAA-AAA	0.00	999	1.000	1.000	0.0065
205278_at	29.6	8.0	-1.88	-3.68	AAA-AAA	0.00	999	1.000	1.000	0.2018
1562413_at	38.1	10.4	-1.88	-3.67	AAA-AAA	0.00	999	1.000	1.000	0.0176

238555_at	27.4	7.5	-1.88	-3.67	AAA-AAA	0.00	999	1.000	1.000	0.0641
228668_x_at	28.9	7.9	-1.88	-3.67	AAA-AAA	0.00	999	1.000	1.000	0.1939
244106_at	36.0	9.8	-1.88	-3.67	AAA-AAA	0.00	999	1.000	1.000	0.0786
221990_at	30.0	8.2	-1.88	-3.67	AAA-AAA	0.00	999	1.000	1.000	0.1037
210038_at	43.9	12.0	-1.88	-3.67	AAA-AAA	0.00	999	1.000	1.000	0.2281
215487_x_at	41.4	11.3	-1.88	-3.67	AAA-AAA	0.00	999	1.000	1.000	0.0798
244023_at	13.2	3.6	-1.88	-3.67	AAA-AAA	0.00	999	1.000	1.000	0.0590
242901_at	7.2	26.5	1.88	3.67	AAA-AAA	0.00	999	1.000	1.000	0.3125
1566277_at	10.5	38.5	1.88	3.67	AAA-AAA	0.00	999	1.000	1.000	0.1342
219971_at	21.2	5.8	-1.88	-3.67	AAA-AAA	0.00	999	1.000	1.000	0.0530
241481_at	35.1	9.6	-1.87	-3.66	AAA-AAA	0.00	999	1.000	1.000	0.0261
242070_at	13.8	50.6	1.87	3.67	AAA-AAA	0.00	999	1.000	1.000	0.1063
214916_x_at	22.0	6.0	-1.87	-3.67	AAA-AAA	0.00	999	1.000	1.000	0.1256
236757_at	31.1	8.5	-1.87	-3.67	AAA-AAA	0.00	999	1.000	1.000	0.1789
227664_at	22.1	6.0	-1.87	-3.67	AAA-AAA	0.00	999	1.000	1.000	0.1723
242768_at	10.2	2.8	-1.87	-3.66	AAA-AAA	0.00	999	1.000	1.000	0.2362
1562588_at	4.4	16.1	1.87	3.66	AAA-AAA	0.00	999	1.000	1.000	0.1685
213691_at	114.3	31.2	-1.87	-3.66	MAM-AAA	0.00	999	1.000	1.000	0.1595
239006_at	20.6	5.6	-1.87	-3.66	AAA-AAA	0.00	999	1.000	1.000	0.1421
241265_x_at	15.0	55.0	1.87	3.66	AAA-AAA	0.00	999	1.000	1.000	0.2206
243691_at	90.0	24.6	-1.87	-3.66	AAA-AAA	0.00	999	1.000	1.000	0.1717
1570271_at	10.7	2.9	-1.87	-3.66	AAA-AAA	0.00	999	1.000	1.000	0.1018
1570391_at	13.6	3.7	-1.87	-3.66	AAA-AAA	0.00	999	1.000	1.000	0.3431
234296_s_at	7.3	2.0	-1.87	-3.66	AAA-AAA	0.00	999	1.000	1.000	0.1860
237037_at	14.0	3.8	-1.87	-3.66	AAA-AAA	0.00	999	1.000	1.000	0.2211
237780_at	51.5	14.1	-1.87	-3.66	AAA-AAA	0.00	999	1.000	1.000	0.1356
205703_at	18.1	5.0	-1.87	-3.66	AAA-AAA	0.00	999	1.000	1.000	0.3593
206527_at	66.0	18.1	-1.87	-3.66	AAA-AAA	0.00	999	1.000	1.000	0.1671
232539_at	44.9	12.3	-1.87	-3.66	AAA-MAA	0.00	999	1.000	1.000	0.1901
241327_at	104.6	28.6	-1.87	-3.66	AAA-AAA	0.00	999	1.000	1.000	0.1516
1561564_at	7.6	2.1	-1.87	-3.66	AAA-AAA	0.00	999	1.000	1.000	0.3579
1555231_a_at	56.6	15.5	-1.87	-3.66	AAA-AAA	0.00	999	1.000	1.000	0.0299
236720_at	28.0	7.7	-1.87	-3.65	AAA-AAA	0.00	999	1.000	1.000	0.1563
242385_at	5.4	19.6	1.87	3.65	AAA-AAA	0.00	999	1.000	1.000	0.1804
1561721_a_at	24.1	6.6	-1.87	-3.65	AAA-AAA	0.00	999	1.000	1.000	0.1442
1554725_at	8.3	2.3	-1.87	-3.65	AAA-AAA	0.00	999	1.000	1.000	0.1217
1557564_at	20.5	5.6	-1.87	-3.65	AAA-AAA	0.00	999	1.000	1.000	0.2193
1558982_at	44.0	12.1	-1.87	-3.65	AAA-AAA	0.00	999	1.000	1.000	0.2456
1557491_at	12.6	3.5	-1.87	-3.65	AAA-AAA	0.00	999	1.000	1.000	0.0389
219498_s_at	16.5	4.5	-1.87	-3.65	AAA-AAA	0.00	999	1.000	1.000	0.1491
240662_at	9.0	2.5	-1.87	-3.65	AAA-AAA	0.00	999	1.000	1.000	0.2754
244557_at	24.6	6.7	-1.87	-3.65	AAA-AAA	0.00	999	1.000	1.000	0.2288

1559612_at	13.0	3.6	-1.87	-3.65	AAA-AAA	0.00	999	1.000	1.000	0.2939
234279_at	21.2	5.8	-1.87	-3.65	AAA-AAA	0.00	999	1.000	1.000	0.1578
1552673_at	3.2	11.6	1.87	3.65	AAA-AAA	0.00	999	1.000	1.000	0.1989
1559800_a_at	22.1	6.1	-1.87	-3.65	AAA-AAA	0.00	999	1.000	1.000	0.0737
214495_at	13.1	47.7	1.87	3.65	AAA-AAA	0.00	999	1.000	1.000	0.0929
217314_at	6.7	24.4	1.87	3.65	AAA-AAA	0.00	999	1.000	1.000	0.1372
217483_at	33.3	9.1	-1.87	-3.65	AAA-AAA	0.00	999	1.000	1.000	0.1207
238892_at	5.0	18.3	1.87	3.65	AAA-AAA	0.00	999	1.000	1.000	0.0383
1569736_at	36.6	10.0	-1.87	-3.65	AAA-AAA	0.00	999	1.000	1.000	0.0919
206406_at	16.3	4.5	-1.87	-3.64	AAA-AAA	0.00	999	1.000	1.000	0.0325
216255_s_at	10.5	2.9	-1.87	-3.65	AAA-AAA	0.00	999	1.000	1.000	0.2326
232606_at	6.3	1.7	-1.87	-3.65	AAA-AAA	0.00	999	1.000	1.000	0.3368
1563033_x_at	6.6	1.8	-1.86	-3.64	AAA-AAA	0.00	999	1.000	1.000	0.1741
1560929_at	3.1	11.4	1.86	3.64	AAA-AAA	0.00	999	1.000	1.000	0.1985
1564763_at	65.4	18.0	-1.86	-3.64	MAA-AAA	0.00	999	1.000	1.000	0.1494
238369_s_at	52.7	14.5	-1.86	-3.64	AAA-AAA	0.00	999	1.000	1.000	0.1469
232745_x_at	30.9	8.5	-1.86	-3.64	AAA-AAA	0.00	999	1.000	1.000	0.3432
233217_at	86.8	23.9	-1.86	-3.64	AAA-AAA	0.00	999	1.000	1.000	0.0395
233405_at	32.3	8.9	-1.86	-3.64	AAA-AAA	0.00	999	1.000	1.000	0.2947
243104_at	13.8	3.8	-1.86	-3.64	AAA-AAA	0.00	999	1.000	1.000	0.1001
243806_at	19.2	5.3	-1.86	-3.64	AAA-AAA	0.00	999	1.000	1.000	0.0849
1553566_at	79.3	21.8	-1.86	-3.63	AAA-AAA	0.00	999	1.000	1.000	0.0116
1556683_x_at	77.9	21.4	-1.86	-3.64	AAA-AAA	0.00	999	1.000	1.000	0.1207
221184_at	6.6	24.0	1.86	3.64	AAA-AAA	0.00	999	1.000	1.000	0.0629
216500_at	56.8	15.6	-1.86	-3.63	AAA-AAA	0.00	999	1.000	1.000	0.0903
236502_at	4.3	15.5	1.86	3.64	AAA-AAA	0.00	999	1.000	1.000	0.1719
236031_x_at	24.1	6.6	-1.86	-3.63	AAA-AAA	0.00	999	1.000	1.000	0.0937
232222_at	80.3	22.1	-1.86	-3.63	AAA-AAA	0.00	999	1.000	1.000	0.0258
242351_at	46.5	12.8	-1.86	-3.63	AAA-AAA	0.00	999	1.000	1.000	0.2363
243211_at	14.2	3.9	-1.86	-3.63	AAA-AAA	0.00	999	1.000	1.000	0.2511
241042_at	10.0	2.8	-1.86	-3.63	AAA-AAA	0.00	999	1.000	1.000	0.2174
1563927_a_at	4.2	15.2	1.86	3.63	AAA-AAA	0.00	999	1.000	1.000	0.2880
1554863_s_at	50.0	13.8	-1.86	-3.63	AAA-MAA	0.00	999	1.000	1.000	0.3071
1555456_at	25.8	7.1	-1.86	-3.63	AAA-AAA	0.00	999	1.000	1.000	0.1208
91580_at	16.8	4.6	-1.86	-3.63	AAA-AAA	0.00	999	1.000	1.000	0.2614
213436_at	17.5	4.8	-1.86	-3.63	AAA-AAA	0.00	999	1.000	1.000	0.2434
229164_s_at	98.2	27.1	-1.86	-3.63	AAA-AAA	0.00	999	1.000	1.000	0.1290
240050_s_at	4.7	17.1	1.86	3.63	AAA-AAA	0.00	999	1.000	1.000	0.2319
240590_at	21.5	5.9	-1.86	-3.63	AAA-AAA	0.00	999	1.000	1.000	0.1630
1562822_at	16.3	4.5	-1.86	-3.63	AAA-AAA	0.00	999	1.000	1.000	0.0842
217182_at	77.9	21.5	-1.86	-3.63	AAA-AAA	0.00	999	1.000	1.000	0.0438
235937_at	39.5	10.9	-1.86	-3.63	AAA-AAA	0.00	999	1.000	1.000	0.1187

1555227_a_at	65.0	17.9	-1.86	-3.63	AAA-AAA	0.00	999	1.000	1.000	0.2398
1554612_at	10.3	2.8	-1.86	-3.63	AAA-AAA	0.00	999	1.000	1.000	0.1613
1556166_x_at	16.5	4.6	-1.86	-3.62	AAA-AAA	0.00	999	1.000	1.000	0.2040
221226_s_at	27.8	7.7	-1.86	-3.63	AAA-AAA	0.00	999	1.000	1.000	0.1210
1556944_at	9.4	2.6	-1.86	-3.62	AAA-AAA	0.00	999	1.000	1.000	0.1922
238184_at	18.0	5.0	-1.86	-3.62	AAA-AAA	0.00	999	1.000	1.000	0.0426
203783_x_at	26.3	95.2	1.86	3.62	AAA-AAA	0.00	999	1.000	1.000	0.0238
1562939_at	22.1	6.1	-1.86	-3.62	AAA-AAA	0.00	999	1.000	1.000	0.1495
219948_x_at	27.6	7.6	-1.86	-3.62	AAA-AAA	0.00	999	1.000	1.000	0.2546
242327_x_at	29.3	105.8	1.86	3.62	AAA-AAA	0.00	999	1.000	1.000	0.0027
1566301_at	4.0	14.4	1.86	3.62	AAA-AAA	0.00	999	1.000	1.000	0.3531
244545_at	16.2	4.5	-1.86	-3.62	AAA-AAA	0.00	999	1.000	1.000	0.1320
217229_at	5.3	1.5	-1.86	-3.62	AAA-AAA	0.00	999	1.000	1.000	0.1495
236145_at	35.5	9.8	-1.86	-3.62	AAA-AAA	0.00	999	1.000	1.000	0.1012
230617_at	13.8	49.7	1.86	3.62	AAA-AAA	0.00	999	1.000	1.000	0.0455
212843_at	11.0	39.8	1.85	3.62	APA-AMA	0.00	999	1.000	1.000	0.2336
233857_s_at	21.1	5.9	-1.85	-3.62	AAA-AAA	0.00	999	1.000	1.000	0.2712
228491_at	12.7	3.5	-1.85	-3.62	AAA-AAA	0.00	999	1.000	1.000	0.2846
1561053_at	5.5	20.0	1.85	3.61	AAA-AAA	0.00	999	1.000	1.000	0.2149
1561225_at	72.6	20.1	-1.85	-3.61	AMA-AAA	0.00	999	1.000	1.000	0.1732
244622_at	40.6	11.2	-1.85	-3.61	AAA-AAA	0.00	999	1.000	1.000	0.1026
1559642_a_at	42.2	11.7	-1.85	-3.61	AAA-AAA	0.00	999	1.000	1.000	0.1621
243634_at	84.8	23.5	-1.85	-3.61	AAA-AAA	0.00	999	1.000	1.000	0.1185
1570230_at	70.2	19.5	-1.85	-3.61	AAA-AAA	0.00	999	1.000	1.000	0.0628
1555073_at	52.2	14.5	-1.85	-3.61	AAA-AAA	0.00	999	1.000	1.000	0.0846
224224_s_at	9.4	2.6	-1.85	-3.61	AAA-AAA	0.00	999	1.000	1.000	0.1965
210215_at	8.7	31.3	1.85	3.61	AAA-AAA	0.00	999	1.000	1.000	0.0911
204400_at	6.5	23.5	1.85	3.61	AAA-AAA	0.00	999	1.000	1.000	0.0618
230104_s_at	30.5	8.4	-1.85	-3.61	AAA-AAA	0.00	999	1.000	1.000	0.2664
243027_at	51.8	14.4	-1.85	-3.61	AAA-AAA	0.00	999	1.000	1.000	0.2468
213945_s_at	47.8	13.2	-1.85	-3.61	AAA-AAA	0.00	999	1.000	1.000	0.0508
211231_x_at	46.5	12.9	-1.85	-3.61	AAA-AAA	0.00	999	1.000	1.000	0.1788
229345_at	49.9	13.8	-1.85	-3.61	AAA-AAA	0.00	999	1.000	1.000	0.2427
203296_s_at	5.6	20.2	1.85	3.61	AAA-AAA	0.00	999	1.000	1.000	0.1321
1568643_a_at	36.2	10.0	-1.85	-3.61	AAA-AAA	0.00	999	1.000	1.000	0.1754
211647_x_at	11.8	3.3	-1.85	-3.61	AAA-AAA	0.00	999	1.000	1.000	0.0580
1558888_x_at	5.4	19.6	1.85	3.60	AAA-AAA	0.00	999	1.000	1.000	0.2308
236253_at	21.8	6.1	-1.85	-3.60	AAA-AAA	0.00	999	1.000	1.000	0.2924
205801_s_at	42.1	11.7	-1.85	-3.60	AAA-AAA	0.00	999	1.000	1.000	0.0562
216561_x_at	8.8	31.7	1.85	3.60	AAA-APA	0.00	999	1.000	1.000	0.1299
204409_s_at	44.3	12.3	-1.85	-3.60	AAA-AAA	0.00	999	1.000	1.000	0.1558
1563064_at	17.9	5.0	-1.85	-3.60	AAA-AAA	0.00	999	1.000	1.000	0.1019

1559057_at	29.1	8.1	-1.85	-3.60	AAA-AAA	0.00	999	1.000	1.000	0.1821
1556874_a_at	55.4	15.4	-1.85	-3.60	AAA-AAA	0.00	999	1.000	1.000	0.1042
206893_at	21.2	5.9	-1.85	-3.60	AAA-AAA	0.00	999	1.000	1.000	0.0897
1562338_at	5.5	19.7	1.85	3.60	AAA-AAA	0.00	999	1.000	1.000	0.1135
222259_s_at	29.4	8.2	-1.85	-3.60	AAA-AAA	0.00	999	1.000	1.000	0.1745
210146_x_at	5.3	19.0	1.85	3.60	AAA-AAA	0.00	999	1.000	1.000	0.1163
216528_at	71.1	19.8	-1.85	-3.60	AAA-AAA	0.00	999	1.000	1.000	0.0058
239293_at	18.9	5.3	-1.85	-3.60	AAA-AAA	0.00	999	1.000	1.000	0.0910
204393_s_at	58.1	16.2	-1.85	-3.59	AAA-AAA	0.00	999	1.000	1.000	0.0908
229944_at	25.4	7.1	-1.85	-3.60	AAA-AAA	0.00	999	1.000	1.000	0.2705
215770_at	9.2	33.2	1.85	3.59	AAA-AAP	0.00	999	1.000	1.000	0.1689
244067_x_at	5.2	18.8	1.85	3.59	AAA-AAA	0.00	999	1.000	1.000	0.0076
1563333_at	22.8	6.4	-1.85	-3.59	AAA-AAA	0.00	999	1.000	1.000	0.3883
210147_at	28.9	8.0	-1.85	-3.59	AAA-AAA	0.00	999	1.000	1.000	0.1153
238697_at	2.2	8.1	1.85	3.59	AAA-AAA	0.00	999	1.000	1.000	0.0524
233261_at	14.8	4.1	-1.85	-3.59	AAA-AAA	0.00	999	1.000	1.000	0.2970
241542_at	9.6	34.5	1.84	3.59	AAA-AAA	0.00	999	1.000	1.000	0.1225
1560507_at	32.8	9.1	-1.84	-3.59	AAA-AAA	0.00	999	1.000	1.000	0.1209
1553965_x_at	25.1	7.0	-1.84	-3.59	AAA-AAA	0.00	999	1.000	1.000	0.1051
220190_s_at	2.8	10.1	1.84	3.59	AAA-AAA	0.00	999	1.000	1.000	0.1642
208252_s_at	48.6	13.5	-1.84	-3.59	AAA-AAA	0.00	999	1.000	1.000	0.1253
224374_s_at	50.7	14.1	-1.84	-3.59	AAA-AAA	0.00	999	1.000	1.000	0.2639
244409_at	25.3	7.1	-1.84	-3.59	AAA-AAA	0.00	999	1.000	1.000	0.0172
210033_s_at	37.9	10.6	-1.84	-3.59	AAA-AAA	0.00	999	1.000	1.000	0.2262
233048_at	35.7	10.0	-1.84	-3.59	MAA-AAA	0.00	999	1.000	1.000	0.1832
241040_at	49.8	13.9	-1.84	-3.59	AAA-AAA	0.00	999	1.000	1.000	0.1297
1569811_at	21.8	6.1	-1.84	-3.58	AAA-AAA	0.00	999	1.000	1.000	0.2522
1555571_at	22.1	6.2	-1.84	-3.59	AAA-AAA	0.00	999	1.000	1.000	0.0697
206453_s_at	127.0	35.4	-1.84	-3.59	AAA-AAA	0.00	999	1.000	1.000	0.1667
238118_s_at	29.4	8.2	-1.84	-3.59	AAA-AAA	0.00	999	1.000	1.000	0.1401
215924_at	12.0	3.4	-1.84	-3.58	AAA-AAA	0.00	999	1.000	1.000	0.2569
237298_at	10.8	3.0	-1.84	-3.58	AAA-AAA	0.00	999	1.000	1.000	0.0734
232786_at	7.3	26.2	1.84	3.58	AAP-MPA	0.00	999	1.000	1.000	0.2498
209473_at	65.5	18.3	-1.84	-3.58	AAA-AAA	0.00	999	1.000	1.000	0.1301
206309_at	33.3	9.3	-1.84	-3.58	AAA-AAA	0.00	999	1.000	1.000	0.1940
244237_at	45.0	12.6	-1.84	-3.58	AAA-AAA	0.00	999	1.000	1.000	0.2376
1564631_at	8.5	2.4	-1.84	-3.58	AAA-AAA	0.00	999	1.000	1.000	0.2994
1559348_a_at	20.7	5.8	-1.84	-3.58	AAA-AAA	0.00	999	1.000	1.000	0.1676
224498_x_at	8.7	31.2	1.84	3.58	AAA-AAA	0.00	999	1.000	1.000	0.0285
1565768_at	12.9	3.6	-1.84	-3.57	AAA-AAA	0.00	999	1.000	1.000	0.2945
1553367_a_at	108.4	30.3	-1.84	-3.58	AAA-AAA	0.00	999	1.000	1.000	0.0831
1552885_a_at	86.2	24.1	-1.84	-3.57	AAA-AAA	0.00	999	1.000	1.000	0.0738

1557289_s_at	17.8	5.0	-1.84	-3.57	AAA-AAA	0.00	999	1.000	1.000	0.1257
217569_x_at	87.5	24.5	-1.84	-3.58	AAA-AAA	0.00	999	1.000	1.000	0.1851
1552599_at	13.0	3.6	-1.84	-3.57	AAA-AAA	0.00	999	1.000	1.000	0.1126
216974_at	6.8	1.9	-1.84	-3.57	AAA-AAA	0.00	999	1.000	1.000	0.0470
234006_s_at	14.6	52.1	1.84	3.57	AAA-AAA	0.00	999	1.000	1.000	0.1971
229718_at	17.9	63.9	1.84	3.57	AAA-APA	0.00	999	1.000	1.000	0.0796
242746_at	12.4	3.5	-1.84	-3.57	AAA-AAA	0.00	999	1.000	1.000	0.0317
233956_at	59.4	16.7	-1.84	-3.57	AAA-AAA	0.00	999	1.000	1.000	0.2147
234803_at	14.1	3.9	-1.84	-3.57	AAA-AAA	0.00	999	1.000	1.000	0.1464
234802_at	23.8	6.7	-1.84	-3.57	AAA-AAA	0.00	999	1.000	1.000	0.0777
233084_s_at	2.5	8.8	1.84	3.57	AAA-AAA	0.00	999	1.000	1.000	0.0535
1561562_at	1.7	5.9	1.84	3.57	AAA-AAA	0.00	999	1.000	1.000	0.2502
1564134_at	6.6	1.8	-1.84	-3.57	AAA-AAA	0.00	999	1.000	1.000	0.0924
208198_x_at	23.8	6.7	-1.84	-3.57	AAA-AAA	0.00	999	1.000	1.000	0.1418
224300_x_at	117.0	32.8	-1.84	-3.57	AAA-AAA	0.00	999	1.000	1.000	0.0790
239702_x_at	35.6	10.0	-1.84	-3.57	AAA-AAA	0.00	999	1.000	1.000	0.1413
228504_at	8.5	30.2	1.84	3.57	AAA-AAA	0.00	999	1.000	1.000	0.0591
231278_at	30.1	8.5	-1.84	-3.57	AAA-AAA	0.00	999	1.000	1.000	0.2033
244232_at	12.8	3.6	-1.83	-3.57	AAA-AAA	0.00	999	1.000	1.000	0.0948
231143_at	13.5	48.0	1.83	3.57	AAA-AAA	0.00	999	1.000	1.000	0.1174
242637_at	21.4	76.1	1.83	3.56	AAA-APP	0.00	999	1.000	1.000	0.1543
224404_s_at	22.3	6.3	-1.83	-3.56	AAA-AAA	0.00	999	1.000	1.000	0.2140
236306_at	2.6	9.1	1.83	3.56	AAA-AAA	0.00	999	1.000	1.000	0.1519
236326_at	23.5	6.6	-1.83	-3.56	AAA-AAA	0.00	999	1.000	1.000	0.3596
206375_s_at	9.1	32.5	1.83	3.56	AAA-AAA	0.00	999	1.000	1.000	0.0449
206628_at	83.8	23.5	-1.83	-3.56	AAA-AAA	0.00	999	1.000	1.000	0.0527
235326_at	30.0	8.4	-1.83	-3.56	AAM-AAA	0.00	999	1.000	1.000	0.2612
1559862_at	8.5	30.3	1.83	3.56	AAA-AAP	0.00	999	1.000	1.000	0.2917
240872_at	9.1	32.2	1.83	3.56	AAA-AAA	0.00	999	1.000	1.000	0.2976
241313_at	39.9	11.2	-1.83	-3.56	AAA-AAA	0.00	999	1.000	1.000	0.0413
219803_at	9.8	2.8	-1.83	-3.56	AAA-AAA	0.00	999	1.000	1.000	0.0016
203887_s_at	24.1	6.8	-1.83	-3.55	AAA-AAA	0.00	999	1.000	1.000	0.1169
1562786_at	16.9	4.8	-1.83	-3.55	AAA-AAA	0.00	999	1.000	1.000	0.2308
1567878_at	6.1	21.5	1.83	3.56	AAA-AAA	0.00	999	1.000	1.000	0.1478
1565306_a_at	14.3	4.0	-1.83	-3.55	AAA-AAA	0.00	999	1.000	1.000	0.2660
1559278_at	12.1	43.0	1.83	3.56	AAA-AAA	0.00	999	1.000	1.000	0.2476
209683_at	13.2	3.7	-1.83	-3.56	AAA-AAA	0.00	999	1.000	1.000	0.2546
243928_s_at	20.5	5.8	-1.83	-3.55	AAA-AAA	0.00	999	1.000	1.000	0.2769
242991_at	18.7	5.3	-1.83	-3.55	AAA-AAA	0.00	999	1.000	1.000	0.2575
1555472_at	13.4	3.8	-1.83	-3.55	AAA-AAA	0.00	999	1.000	1.000	0.0327
218232_at	30.2	8.5	-1.83	-3.55	AAA-AAA	0.00	999	1.000	1.000	0.2227
229229_at	47.5	13.4	-1.83	-3.55	AAA-AAA	0.00	999	1.000	1.000	0.0804

234498_at	24.5	6.9	-1.83	-3.55	AAA-AAA	0.00	999	1.000	1.000	0.1570
230557_at	97.4	27.4	-1.83	-3.55	AAA-AAA	0.00	999	1.000	1.000	0.0336
204144_s_at	23.4	83.1	1.83	3.55	AAP-PAP	0.00	999	1.000	1.000	0.2532
243238_at	35.1	9.9	-1.83	-3.55	AAA-AAA	0.00	999	1.000	1.000	0.1001
1569751_at	42.4	12.0	-1.83	-3.55	AAA-AAA	0.00	999	1.000	1.000	0.2539
1569759_at	5.7	20.1	1.83	3.55	AAA-AAA	0.00	999	1.000	1.000	0.2268
215774_s_at	10.6	37.7	1.83	3.55	AAA-AAA	0.00	999	1.000	1.000	0.1702
215514_at	3.5	12.4	1.83	3.55	AAA-AAA	0.00	999	1.000	1.000	0.3552
232318_s_at	2.6	9.1	1.83	3.55	AAA-AAA	0.00	999	1.000	1.000	0.1538
1556402_at	12.1	3.4	-1.83	-3.55	AAA-AAA	0.00	999	1.000	1.000	0.1242
241971_at	12.5	3.5	-1.83	-3.54	AAA-AAA	0.00	999	1.000	1.000	0.2411
207823_s_at	72.0	20.3	-1.83	-3.54	AAA-AAA	0.00	999	1.000	1.000	0.0815
214188_at	47.2	13.3	-1.83	-3.54	AAA-AAA	0.00	999	1.000	1.000	0.1318
206433_s_at	7.2	25.4	1.82	3.54	AAA-AAA	0.00	999	1.000	1.000	0.1654
241849_at	36.0	10.2	-1.82	-3.54	AAA-AAA	0.00	999	1.000	1.000	0.3287
215559_at	6.0	21.3	1.82	3.54	AAA-AAA	0.00	999	1.000	1.000	0.1593
205889_s_at	11.0	3.1	-1.82	-3.54	AAA-AAA	0.00	999	1.000	1.000	0.1334
228750_at	10.0	35.4	1.82	3.54	AAA-AAA	0.00	999	1.000	1.000	0.2839
243146_at	6.4	22.7	1.82	3.54	AAA-AAA	0.00	999	1.000	1.000	0.2067
227136_s_at	17.3	61.1	1.82	3.54	AAA-AAA	0.00	999	1.000	1.000	0.2246
206000_at	23.3	6.6	-1.82	-3.54	AAA-AAA	0.00	999	1.000	1.000	0.2491
232415_at	4.0	14.3	1.82	3.53	AAA-PAA	0.00	999	1.000	1.000	0.3165
241113_at	11.1	3.1	-1.82	-3.53	AAA-AAA	0.00	999	1.000	1.000	0.2455
241508_at	65.1	18.4	-1.82	-3.53	AAA-AAA	0.00	999	1.000	1.000	0.0413
1562431_x_at	20.4	5.8	-1.82	-3.53	AAA-AAA	0.00	999	1.000	1.000	0.2761
207737_at	52.2	14.8	-1.82	-3.53	AAA-AAA	0.00	999	1.000	1.000	0.0834
210575_at	4.6	16.1	1.82	3.53	AAA-AAA	0.00	999	1.000	1.000	0.0459
235209_at	51.2	14.5	-1.82	-3.53	AAA-AAA	0.00	999	1.000	1.000	0.1889
229702_at	59.5	16.8	-1.82	-3.53	AAA-AAA	0.00	999	1.000	1.000	0.0634
1567862_at	15.8	4.5	-1.82	-3.53	AAA-AAA	0.00	999	1.000	1.000	0.1238
223525_at	21.1	74.3	1.82	3.53	APA-AAP	0.00	999	1.000	1.000	0.2386
1562260_at	17.4	5.0	-1.82	-3.53	AAA-AAA	0.00	999	1.000	1.000	0.0908
228518_at	43.9	12.5	-1.82	-3.53	AAA-AAA	0.00	999	1.000	1.000	0.0664
214353_at	12.4	43.6	1.82	3.52	AAA-AAA	0.00	999	1.000	1.000	0.0091
217578_at	11.4	40.2	1.82	3.52	AAA-PAA	0.00	999	1.000	1.000	0.2048
227297_at	5.0	17.7	1.82	3.52	AMA-AAA	0.00	999	1.000	1.000	0.3968
207386_at	2.7	9.5	1.82	3.52	AAA-AAA	0.00	999	1.000	1.000	0.3273
210397_at	48.3	13.7	-1.82	-3.52	AAA-AAA	0.00	999	1.000	1.000	0.1685
243701_at	11.5	3.3	-1.82	-3.52	AAA-AAA	0.00	999	1.000	1.000	0.3565
1561488_at	4.6	16.0	1.82	3.52	AAA-AAA	0.00	999	1.000	1.000	0.1413
1552917_at	14.7	51.8	1.82	3.52	AAA-AAA	0.00	999	1.000	1.000	0.2646
1558760_at	8.3	29.2	1.82	3.52	AAA-AAA	0.00	999	1.000	1.000	0.1762

233835_at	21.7	6.2	-1.82	-3.52	AAA-AAA	0.00	999	1.000	1.000	0.1225
206007_at	7.7	27.0	1.82	3.52	AAA-AAA	0.00	999	1.000	1.000	0.2120
205839_s_at	18.2	5.2	-1.82	-3.52	AAA-AAA	0.00	999	1.000	1.000	0.2023
233616_at	20.6	5.9	-1.82	-3.52	AAA-AAA	0.00	999	1.000	1.000	0.2354
241477_at	62.1	17.7	-1.81	-3.52	AAA-AAA	0.00	999	1.000	1.000	0.3213
1562511_at	30.0	8.5	-1.81	-3.52	MAA-AAA	0.00	999	1.000	1.000	0.3912
1564402_at	22.1	6.3	-1.81	-3.52	AAA-AAA	0.00	999	1.000	1.000	0.1234
1553391_at	23.0	6.5	-1.81	-3.52	AAA-AAA	0.00	999	1.000	1.000	0.2005
1559911_at	44.7	12.7	-1.81	-3.52	AAA-AAA	0.00	999	1.000	1.000	0.2035
1559221_at	8.2	2.3	-1.81	-3.52	AAA-AAA	0.00	999	1.000	1.000	0.1353
220345_at	37.4	10.6	-1.81	-3.52	AAA-AAA	0.00	999	1.000	1.000	0.2981
235950_at	10.4	3.0	-1.81	-3.52	AAA-AAA	0.00	999	1.000	1.000	0.2036
204971_at	34.2	9.7	-1.81	-3.52	AAA-AAA	0.00	999	1.000	1.000	0.1713
234524_at	21.9	6.2	-1.81	-3.52	AAA-AAA	0.00	999	1.000	1.000	0.3199
233291_at	86.0	24.5	-1.81	-3.52	AAA-AAA	0.00	999	1.000	1.000	0.1053
1568919_at	14.0	4.0	-1.81	-3.51	AAA-AAA	0.00	999	1.000	1.000	0.4128
1552961_at	7.5	26.2	1.81	3.51	AAA-AAA	0.00	999	1.000	1.000	0.0850
1557879_at	8.1	28.5	1.81	3.51	AAA-AAA	0.00	999	1.000	1.000	0.0696
206228_at	44.8	12.7	-1.81	-3.51	AAA-AAA	0.00	999	1.000	1.000	0.0967
207313_x_at	36.8	10.5	-1.81	-3.51	AAA-AAA	0.00	999	1.000	1.000	0.1136
208565_at	81.3	23.2	-1.81	-3.51	AAA-AAA	0.00	999	1.000	1.000	0.1267
236009_at	49.6	14.1	-1.81	-3.51	AAA-AAA	0.00	999	1.000	1.000	0.0050
236230_at	19.0	5.4	-1.81	-3.51	AAA-AAA	0.00	999	1.000	1.000	0.0713
237157_at	71.4	20.3	-1.81	-3.51	AAA-AAA	0.00	999	1.000	1.000	0.2221
242086_at	54.7	15.6	-1.81	-3.51	AAA-AAA	0.00	999	1.000	1.000	0.0623
1562864_at	9.2	2.6	-1.81	-3.51	AAA-AAA	0.00	999	1.000	1.000	0.2403
1554550_at	6.1	1.7	-1.81	-3.51	AAA-AAA	0.00	999	1.000	1.000	0.3271
235336_at	20.2	5.8	-1.81	-3.51	AAA-AAA	0.00	999	1.000	1.000	0.1509
203423_at	44.0	12.5	-1.81	-3.51	AAA-AAA	0.00	999	1.000	1.000	0.2548
1552311_a_at	94.3	26.9	-1.81	-3.51	AAA-AAA	0.00	999	1.000	1.000	0.2831
207852_at	28.4	8.1	-1.81	-3.51	AAA-AAA	0.00	999	1.000	1.000	0.2025
210861_s_at	30.2	8.6	-1.81	-3.51	AAA-AAA	0.00	999	1.000	1.000	0.1972
222087_at	189.8	54.2	-1.81	-3.50	AAA-AAA	0.00	999	1.000	1.000	0.2581
214642_x_at	61.9	17.7	-1.81	-3.51	AAA-AAA	0.00	999	1.000	1.000	0.1927
216747_at	8.5	29.7	1.81	3.50	AAA-AAA	0.00	999	1.000	1.000	0.1446
238956_at	11.3	39.4	1.81	3.50	AAA-AAA	0.00	999	1.000	1.000	0.1020
236878_at	32.4	9.3	-1.81	-3.50	AAA-AAA	0.00	999	1.000	1.000	0.2871
230668_at	16.8	4.8	-1.81	-3.50	AAA-AAA	0.00	999	1.000	1.000	0.2224
1556329_a_at	6.8	23.8	1.81	3.50	AAA-APA	0.00	999	1.000	1.000	0.1935
243994_at	19.3	5.5	-1.81	-3.50	AAA-AAA	0.00	999	1.000	1.000	0.1018
241026_at	21.6	6.2	-1.81	-3.50	AAA-AAA	0.00	999	1.000	1.000	0.1665
1560728_at	41.8	11.9	-1.81	-3.50	AAA-AAA	0.00	999	1.000	1.000	0.2067

1569874_s_at	12.9	3.7	-1.81	-3.50	AAA-AAA	0.00	999	1.000	1.000	0.1320
220013_at	33.3	9.5	-1.81	-3.50	AAA-AAA	0.00	999	1.000	1.000	0.0760
226716_at	58.6	205.0	1.81	3.50	AAA-AAA	0.00	999	1.000	1.000	0.0073
229839_at	22.2	6.4	-1.81	-3.50	AAA-AAA	0.00	999	1.000	1.000	0.1317
240439_at	27.9	8.0	-1.81	-3.50	AAA-AAA	0.00	999	1.000	1.000	0.2196
1562862_at	45.6	13.0	-1.81	-3.50	AAA-AAA	0.00	999	1.000	1.000	0.2569
1562727_at	15.7	4.5	-1.81	-3.50	AAA-AAA	0.00	999	1.000	1.000	0.1104
1570420_at	15.6	54.5	1.81	3.50	AAA-AAA	0.00	999	1.000	1.000	0.0008
214485_at	75.7	21.7	-1.81	-3.50	AAA-AAA	0.00	999	1.000	1.000	0.0795
229728_at	109.4	31.3	-1.81	-3.50	AAA-AAA	0.00	999	1.000	1.000	0.0680
224506_s_at	26.6	7.6	-1.81	-3.50	AAA-AAA	0.00	999	1.000	1.000	0.2482
1563568_at	4.3	15.0	1.81	3.50	AAA-AAA	0.00	999	1.000	1.000	0.0651
1569781_at	11.5	3.3	-1.81	-3.49	AAA-AAA	0.00	999	1.000	1.000	0.1043
240676_at	20.0	5.7	-1.80	-3.49	AAA-AAA	0.00	999	1.000	1.000	0.2232
241604_at	9.7	33.9	1.80	3.49	AAA-AAA	0.00	999	1.000	1.000	0.2991
1561054_a_at	6.7	23.3	1.80	3.49	AAA-AAA	0.00	999	1.000	1.000	0.2741
244737_at	72.4	20.7	-1.80	-3.49	AAA-AAA	0.00	999	1.000	1.000	0.1688
1552528_at	93.3	26.7	-1.80	-3.49	AAA-AAA	0.00	999	1.000	1.000	0.0999
220484_at	6.2	21.6	1.80	3.49	AAA-AAA	0.00	999	1.000	1.000	0.0911
209968_s_at	18.7	5.4	-1.80	-3.49	AAA-AAA	0.00	999	1.000	1.000	0.0284
211872_s_at	39.7	11.4	-1.80	-3.49	AAA-AAA	0.00	999	1.000	1.000	0.0461
1561600_at	22.5	6.4	-1.80	-3.49	AAA-AAA	0.00	999	1.000	1.000	0.0452
211225_at	36.6	10.5	-1.80	-3.49	AAA-AAA	0.00	999	1.000	1.000	0.1371
236602_at	13.1	3.8	-1.80	-3.49	AAA-AAA	0.00	999	1.000	1.000	0.1727
237992_at	28.1	8.1	-1.80	-3.49	AAA-AAA	0.00	999	1.000	1.000	0.3424
233052_at	13.4	3.9	-1.80	-3.49	AAA-AAA	0.00	999	1.000	1.000	0.0046
242415_at	13.1	3.8	-1.80	-3.49	AAA-AAA	0.00	999	1.000	1.000	0.1730
1556753_s_at	26.2	7.5	-1.80	-3.49	AAA-AAA	0.00	999	1.000	1.000	0.0716
222225_at	119.1	34.2	-1.80	-3.49	AAA-AAA	0.00	999	1.000	1.000	0.1051
226492_at	39.8	11.4	-1.80	-3.49	AAA-AAA	0.00	999	1.000	1.000	0.0454
236709_at	48.0	13.8	-1.80	-3.49	AAA-AAA	0.00	999	1.000	1.000	0.2096
205556_at	10.1	35.1	1.80	3.48	AAA-APP	0.00	999	1.000	1.000	0.2061
208440_at	31.2	9.0	-1.80	-3.48	AAA-AAA	0.00	999	1.000	1.000	0.0587
207167_at	26.9	7.7	-1.80	-3.49	AAA-AAA	0.00	999	1.000	1.000	0.1295
207472_at	19.5	5.6	-1.80	-3.49	AAA-AAA	0.00	999	1.000	1.000	0.1936
211826_s_at	58.0	16.6	-1.80	-3.49	AAA-AAA	0.00	999	1.000	1.000	0.1938
243164_s_at	6.6	1.9	-1.80	-3.48	AAA-AAA	0.00	999	1.000	1.000	0.2663
1562805_at	15.4	4.4	-1.80	-3.48	AAA-AAA	0.00	999	1.000	1.000	0.2807
1563477_at	15.6	4.5	-1.80	-3.48	AAA-AAA	0.00	999	1.000	1.000	0.3498
1558930_at	75.4	21.7	-1.80	-3.48	MAA-AAA	0.00	999	1.000	1.000	0.0259
206895_at	3.4	12.0	1.80	3.48	AAA-AAA	0.00	999	1.000	1.000	0.1926
226526_s_at	26.1	7.5	-1.80	-3.48	AAA-AAA	0.00	999	1.000	1.000	0.0844

205295_at	40.8	11.7	-1.80	-3.48	AAA-AAA	0.00	999	1.000	1.000	0.1674
237711_at	24.4	7.0	-1.80	-3.48	AAA-AAA	0.00	999	1.000	1.000	0.0378
233716_at	8.2	28.5	1.80	3.48	AAA-AAA	0.00	999	1.000	1.000	0.1540
233356_at	35.1	10.1	-1.80	-3.48	AAA-AAA	0.00	999	1.000	1.000	0.1900
1553519_at	10.9	3.1	-1.80	-3.48	AAA-AAA	0.00	999	1.000	1.000	0.0128
1559837_at	24.6	7.1	-1.80	-3.48	AAA-AAA	0.00	999	1.000	1.000	0.1233
216696_s_at	86.3	24.8	-1.80	-3.48	AAA-AAA	0.00	999	1.000	1.000	0.2024
233989_at	14.5	4.2	-1.80	-3.48	AAA-AAA	0.00	999	1.000	1.000	0.2363
206236_at	12.2	42.6	1.80	3.48	AAA-AAA	0.00	999	1.000	1.000	0.1997
226299_at	112.1	389.5	1.80	3.48	AAP-PPM	0.00	999	1.000	1.000	0.1203
241326_at	87.2	25.1	-1.80	-3.47	AAA-AAA	0.00	999	1.000	1.000	0.0745
1563744_a_at	33.8	9.7	-1.80	-3.47	AAA-AAA	0.00	999	1.000	1.000	0.0486
1569817_at	25.0	7.2	-1.80	-3.47	AAA-AAA	0.00	999	1.000	1.000	0.2783
1565685_at	16.8	4.8	-1.80	-3.47	AAA-AAA	0.00	999	1.000	1.000	0.3112
231680_at	29.7	8.5	-1.80	-3.47	AAA-AAA	0.00	999	1.000	1.000	0.1210
1559607_s_at	11.4	3.3	-1.80	-3.47	AAA-AAA	0.00	999	1.000	1.000	0.1411
228591_at	89.4	25.8	-1.80	-3.47	AAA-AAA	0.00	999	1.000	1.000	0.0353
240665_at	2.2	7.6	1.80	3.47	AAA-APA	0.00	999	1.000	1.000	0.2967
240767_x_at	73.1	21.1	-1.80	-3.47	AAA-AAA	0.00	999	1.000	1.000	0.1400
239921_at	14.7	4.2	-1.80	-3.47	AAA-AAA	0.00	999	1.000	1.000	0.3228
1564131_a_at	48.7	14.0	-1.80	-3.47	AAA-AAA	0.00	999	1.000	1.000	0.0462
1560865_a_at	25.2	7.3	-1.80	-3.47	AAA-AAA	0.00	999	1.000	1.000	0.1977
1565805_at	35.0	10.1	-1.80	-3.47	AAA-AAA	0.00	999	1.000	1.000	0.3055
234409_at	3.7	12.8	1.80	3.47	AAA-AAA	0.00	999	1.000	1.000	0.1508
238116_at	12.7	3.7	-1.80	-3.47	AAA-AAA	0.00	999	1.000	1.000	0.2110
232189_at	34.9	10.1	-1.80	-3.47	AAA-AAA	0.00	999	1.000	1.000	0.0377
1569672_at	59.0	17.0	-1.79	-3.47	AAA-AAA	0.00	999	1.000	1.000	0.0680
1569648_at	33.4	9.6	-1.79	-3.47	AAA-AAA	0.00	999	1.000	1.000	0.2611
1569515_a_at	38.0	10.9	-1.79	-3.47	AAA-AAA	0.00	999	1.000	1.000	0.1275
220877_at	39.3	11.3	-1.79	-3.47	AAA-AAA	0.00	999	1.000	1.000	0.2815
236781_at	4.8	16.7	1.79	3.47	AAA-AAA	0.00	999	1.000	1.000	0.1406
236182_at	23.0	79.7	1.79	3.47	AAA-AAA	0.00	999	1.000	1.000	0.2012
238830_at	25.5	7.4	-1.79	-3.47	AAA-AAA	0.00	999	1.000	1.000	0.0756
239724_at	15.2	4.4	-1.79	-3.47	AAA-AAA	0.00	999	1.000	1.000	0.0700
1558476_at	27.9	8.0	-1.79	-3.46	AAA-AAA	0.00	999	1.000	1.000	0.0489
213874_at	77.4	22.3	-1.79	-3.47	AAA-AAA	0.00	999	1.000	1.000	0.0840
211507_s_at	39.1	135.5	1.79	3.47	AAA-AAA	0.00	999	1.000	1.000	0.2810
217306_at	2.4	8.4	1.79	3.47	AAA-AAA	0.00	999	1.000	1.000	0.3405
215344_at	42.1	12.2	-1.79	-3.47	AAA-AAA	0.00	999	1.000	1.000	0.2107
205262_at	16.5	4.8	-1.79	-3.47	AAA-AAA	0.00	999	1.000	1.000	0.2032
239305_at	15.9	55.2	1.79	3.46	AAA-APA	0.00	999	1.000	1.000	0.1952
244006_at	11.0	3.2	-1.79	-3.46	AAA-AAA	0.00	999	1.000	1.000	0.2627

240466_at	51.2	14.8	-1.79	-3.46	AAA-AAA	0.00	999	1.000	1.000	0.2004
1564021_at	65.0	18.8	-1.79	-3.46	AAA-AAA	0.00	999	1.000	1.000	0.2035
237177_at	17.9	5.2	-1.79	-3.46	AAA-AAA	0.00	999	1.000	1.000	0.0944
205668_at	11.0	3.2	-1.79	-3.46	AAA-AAA	0.00	999	1.000	1.000	0.3679
232417_x_at	44.8	12.9	-1.79	-3.46	AAA-AAA	0.00	999	1.000	1.000	0.0806
1563070_at	25.7	7.4	-1.79	-3.46	AAA-AAA	0.00	999	1.000	1.000	0.3038
1561686_at	17.2	5.0	-1.79	-3.46	AAA-AAA	0.00	999	1.000	1.000	0.1853
215437_x_at	16.5	56.9	1.79	3.46	AAA-AAA	0.00	999	1.000	1.000	0.1064
230639_at	21.5	6.2	-1.79	-3.46	AAA-AAA	0.00	999	1.000	1.000	0.0321
232847_at	11.0	3.2	-1.79	-3.46	AAA-AAA	0.00	999	1.000	1.000	0.2058
231138_at	12.5	3.6	-1.79	-3.46	AAA-AAA	0.00	999	1.000	1.000	0.2877
232288_at	15.6	53.9	1.79	3.46	AAP-AAA	0.00	999	1.000	1.000	0.3368
1556773_at	20.4	70.6	1.79	3.46	AAA-AAA	0.00	999	1.000	1.000	0.1127
209857_s_at	43.6	150.7	1.79	3.46	AAA-AAA	0.00	999	1.000	1.000	0.1309
1562671_s_at	55.4	16.0	-1.79	-3.46	AAA-AAA	0.00	999	1.000	1.000	0.0860
1564435_a_at	7.5	25.8	1.79	3.46	AAA-AAA	0.00	999	1.000	1.000	0.0373
1556263_s_at	3.1	10.7	1.79	3.46	AAA-AAA	0.00	999	1.000	1.000	0.1881
221101_at	18.2	5.3	-1.79	-3.46	AAA-AAA	0.00	999	1.000	1.000	0.1509
206358_at	57.6	16.7	-1.79	-3.46	AAA-AAA	0.00	999	1.000	1.000	0.1335
233804_at	33.1	9.6	-1.79	-3.46	AAA-AAA	0.00	999	1.000	1.000	0.2193
231702_at	13.2	3.8	-1.79	-3.46	AAA-AAA	0.00	999	1.000	1.000	0.1323
207626_s_at	31.4	9.1	-1.79	-3.45	AAA-AAA	0.00	999	1.000	1.000	0.2926
233980_s_at	53.7	15.5	-1.79	-3.45	AAA-AAA	0.00	999	1.000	1.000	0.1119
232792_at	43.5	12.6	-1.79	-3.45	AAA-AAA	0.00	999	1.000	1.000	0.1883
233579_at	14.4	4.2	-1.79	-3.45	AAA-AAA	0.00	999	1.000	1.000	0.2553
233304_at	17.6	5.1	-1.79	-3.45	AAA-AAA	0.00	999	1.000	1.000	0.1236
244125_at	7.8	2.3	-1.79	-3.45	AAA-AAA	0.00	999	1.000	1.000	0.2735
1563145_at	20.7	6.0	-1.79	-3.45	AAA-AAA	0.00	999	1.000	1.000	0.3026
1560782_at	95.8	27.8	-1.79	-3.45	AAA-AAA	0.00	999	1.000	1.000	0.0728
1570373_at	63.5	18.4	-1.79	-3.45	AAA-AAA	0.00	999	1.000	1.000	0.2898
1557431_at	34.0	9.9	-1.79	-3.45	AAA-AAA	0.00	999	1.000	1.000	0.1487
220781_at	38.6	11.2	-1.79	-3.45	AAA-AAA	0.00	999	1.000	1.000	0.1333
207042_at	57.4	16.6	-1.79	-3.45	AAA-AAA	0.00	999	1.000	1.000	0.1880
236493_at	65.4	19.0	-1.79	-3.45	AAA-AAA	0.00	999	1.000	1.000	0.2572
244267_at	13.8	4.0	-1.79	-3.45	AAA-AAA	0.00	999	1.000	1.000	0.2819
1566099_at	59.6	17.3	-1.79	-3.45	AAA-AAA	0.00	999	1.000	1.000	0.1645
206588_at	6.7	23.0	1.79	3.45	AAA-AAA	0.00	999	1.000	1.000	0.2945
214883_at	16.8	57.9	1.79	3.45	AAA-AAA	0.00	999	1.000	1.000	0.0431
242061_at	21.4	6.2	-1.79	-3.45	AAA-AAA	0.00	999	1.000	1.000	0.1461
1554816_at	6.7	23.0	1.79	3.45	AAA-AAA	0.00	999	1.000	1.000	0.0469
1557692_a_at	1.6	5.4	1.79	3.45	AAA-AAA	0.00	999	1.000	1.000	0.1198
1560889_a_at	36.3	10.5	-1.78	-3.44	AAA-AAA	0.00	999	1.000	1.000	0.0891

1553372_at	23.2	6.7	-1.78	-3.44	AAA-AAA	0.00	999	1.000	1.000	0.0967
1553705_a_at	34.3	10.0	-1.78	-3.45	AAA-AAA	0.00	999	1.000	1.000	0.3736
1559287_at	25.5	7.4	-1.78	-3.45	AAA-AAA	0.00	999	1.000	1.000	0.1383
233818_at	48.9	14.2	-1.78	-3.44	AAA-AAA	0.00	999	1.000	1.000	0.0607
239441_at	18.1	62.4	1.78	3.44	AAA-AAA	0.00	999	1.000	1.000	0.1198
231328_s_at	6.5	22.3	1.78	3.44	AAA-AAA	0.00	999	1.000	1.000	0.1746
241807_x_at	13.5	3.9	-1.78	-3.44	AAA-AAA	0.00	999	1.000	1.000	0.1381
243778_at	18.4	5.3	-1.78	-3.44	AAA-AAA	0.00	999	1.000	1.000	0.2492
244717_x_at	15.5	53.4	1.78	3.44	AAA-AAA	0.00	999	1.000	1.000	0.1018
232824_at	20.0	68.9	1.78	3.44	AAA-AAA	0.00	999	1.000	1.000	0.1232
239907_at	63.5	18.5	-1.78	-3.44	AAA-AAA	0.00	999	1.000	1.000	0.2822
1556867_at	20.2	5.9	-1.78	-3.44	AAA-AAA	0.00	999	1.000	1.000	0.3093
222695_s_at	35.5	10.3	-1.78	-3.44	AAA-AAA	0.00	999	1.000	1.000	0.1720
239727_at	30.4	8.8	-1.78	-3.44	AAA-AAA	0.00	999	1.000	1.000	0.1769
205502_at	34.8	10.1	-1.78	-3.44	AAA-AAA	0.00	999	1.000	1.000	0.1162
243157_at	28.3	8.2	-1.78	-3.44	AAA-AAA	0.00	999	1.000	1.000	0.0785
1570189_at	42.1	12.3	-1.78	-3.44	AAA-AAA	0.00	999	1.000	1.000	0.1838
206927_s_at	32.6	9.5	-1.78	-3.44	AMA-AAA	0.00	999	1.000	1.000	0.1212
214939_x_at	62.5	18.2	-1.78	-3.44	AAA-AAA	0.00	999	1.000	1.000	0.0755
206388_at	61.4	17.9	-1.78	-3.44	AMA-AAA	0.00	999	1.000	1.000	0.1143
213961_s_at	16.4	4.8	-1.78	-3.43	AAA-AAA	0.00	999	1.000	1.000	0.0045
213680_at	24.2	7.0	-1.78	-3.43	AAA-AAA	0.00	999	1.000	1.000	0.0603
1568869_at	89.9	26.2	-1.78	-3.43	AAA-AAA	0.00	999	1.000	1.000	0.0318
219429_at	6.4	21.8	1.78	3.43	AAA-AAA	0.00	999	1.000	1.000	0.0242
238211_at	57.9	16.9	-1.78	-3.43	AAA-AAA	0.00	999	1.000	1.000	0.2021
241289_at	6.9	23.6	1.78	3.43	AAA-AAA	0.00	999	1.000	1.000	0.0040
220920_at	48.6	14.2	-1.78	-3.43	AAA-AAA	0.00	999	1.000	1.000	0.0043
224997_x_at	26.1	7.6	-1.78	-3.43	AAA-AAA	0.00	999	1.000	1.000	0.1562
234830_at	7.8	2.3	-1.78	-3.43	AAA-AAA	0.00	999	1.000	1.000	0.0219
230162_s_at	14.5	49.6	1.78	3.43	AAA-AAA	0.00	999	1.000	1.000	0.1622
242914_at	119.4	34.9	-1.78	-3.43	AAA-AAA	0.00	999	1.000	1.000	0.0038
1561240_at	12.7	3.7	-1.78	-3.43	AAA-AAA	0.00	999	1.000	1.000	0.0881
214067_at	13.2	3.9	-1.78	-3.43	AAA-AAA	0.00	999	1.000	1.000	0.0032
234099_at	65.6	19.1	-1.78	-3.43	AAA-AAA	0.00	999	1.000	1.000	0.2047
237723_at	4.6	15.7	1.78	3.43	AAA-AAA	0.00	999	1.000	1.000	0.0710
205947_s_at	9.9	2.9	-1.78	-3.43	AAA-AAA	0.00	999	1.000	1.000	0.0995
206381_at	13.1	3.8	-1.78	-3.43	AAA-AAA	0.00	999	1.000	1.000	0.1606
228973_at	3.8	13.2	1.78	3.43	AAA-AAA	0.00	999	1.000	1.000	0.0549
232491_at	119.1	34.8	-1.78	-3.43	AAA-AAA	0.00	999	1.000	1.000	0.1733
234866_s_at	43.2	12.6	-1.78	-3.42	AAA-AAA	0.00	999	1.000	1.000	0.0065
206340_at	25.6	7.5	-1.78	-3.43	AAA-AAA	0.00	999	1.000	1.000	0.0700
233407_at	28.9	8.4	-1.78	-3.43	AAA-AAA	0.00	999	1.000	1.000	0.0767

242276_at	34.5	10.1	-1.78	-3.42	AAA-AAA	0.00	999	1.000	1.000	0.0244
238731_at	11.2	38.3	1.78	3.42	AAA-AAA	0.00	999	1.000	1.000	0.2138
1564272_a_at	3.4	11.5	1.77	3.42	AAA-PAA	0.00	999	1.000	1.000	0.2707
241085_at	17.7	60.4	1.77	3.42	APA-APA	0.00	999	1.000	1.000	0.2592
203568_s_at	86.3	295.1	1.77	3.42	APA-PPP	0.00	999	1.000	1.000	0.1204
241675_s_at	35.2	10.3	-1.77	-3.42	AAA-AAA	0.00	999	1.000	1.000	0.0031
241455_at	18.0	61.6	1.77	3.42	AAA-MAA	0.00	999	1.000	1.000	0.1798
1555499_a_at	95.6	27.9	-1.77	-3.42	AAA-AAA	0.00	999	1.000	1.000	0.0699
237838_at	30.6	9.0	-1.77	-3.42	AAA-AAA	0.00	999	1.000	1.000	0.1595
1561538_at	94.1	27.5	-1.77	-3.42	AAA-AAA	0.00	999	1.000	1.000	0.0075
222211_x_at	50.5	14.8	-1.77	-3.42	AAA-AAA	0.00	999	1.000	1.000	0.3165
220009_at	34.2	10.0	-1.77	-3.42	AAA-AAA	0.00	999	1.000	1.000	0.1931
242954_at	39.8	11.7	-1.77	-3.42	AAA-AAA	0.00	999	1.000	1.000	0.0921
1556697_at	4.1	14.1	1.77	3.42	AAA-AAA	0.00	999	1.000	1.000	0.2792
220718_at	13.5	4.0	-1.77	-3.42	AAA-AAA	0.00	999	1.000	1.000	0.1549
216494_at	38.2	11.2	-1.77	-3.42	AAA-AAA	0.00	999	1.000	1.000	0.1077
238244_at	49.8	14.6	-1.77	-3.42	AAA-AAA	0.00	999	1.000	1.000	0.1686
231466_at	23.4	6.9	-1.77	-3.42	AAA-AAA	0.00	999	1.000	1.000	0.1164
240184_at	14.0	47.6	1.77	3.41	AAA-AAA	0.00	999	1.000	1.000	0.0035
243322_at	56.0	16.4	-1.77	-3.41	AAM-AAA	0.00	999	1.000	1.000	0.0239
1553460_at	30.0	8.8	-1.77	-3.41	AAA-AAA	0.00	999	1.000	1.000	0.0474
208043_at	14.2	4.2	-1.77	-3.41	AAA-AAA	0.00	999	1.000	1.000	0.2947
221164_x_at	37.9	129.4	1.77	3.41	AAA-AAM	0.00	999	1.000	1.000	0.1997
208592_s_at	36.4	10.7	-1.77	-3.41	AAA-AAA	0.00	999	1.000	1.000	0.0284
236693_at	16.5	4.8	-1.77	-3.41	AAA-AAA	0.00	999	1.000	1.000	0.1665
234024_at	54.7	16.0	-1.77	-3.41	AAA-AAA	0.00	999	1.000	1.000	0.0588
228214_at	44.9	13.2	-1.77	-3.41	AAA-AAA	0.00	999	1.000	1.000	0.1486
229015_at	20.4	6.0	-1.77	-3.41	AAA-AAA	0.00	999	1.000	1.000	0.0974
240709_at	5.3	18.2	1.77	3.41	AAA-AAA	0.00	999	1.000	1.000	0.0005
242752_at	52.2	15.3	-1.77	-3.41	AMA-AAA	0.00	999	1.000	1.000	0.1253
1560495_at	8.5	2.5	-1.77	-3.41	AAA-AAA	0.00	999	1.000	1.000	0.2366
1564965_at	3.6	12.4	1.77	3.41	AAA-AAA	0.00	999	1.000	1.000	0.2804
227463_at	8.1	27.7	1.77	3.41	AAA-AAA	0.00	999	1.000	1.000	0.1722
209863_s_at	15.5	4.5	-1.77	-3.41	AAA-AAA	0.00	999	1.000	1.000	0.0539
235851_s_at	74.2	21.8	-1.77	-3.41	AAA-AAA	0.00	999	1.000	1.000	0.2530
238261_at	80.6	23.7	-1.77	-3.41	AAA-AAA	0.00	999	1.000	1.000	0.2074
239191_at	21.0	6.2	-1.77	-3.41	AAA-AAA	0.00	999	1.000	1.000	0.2154
241050_at	14.6	4.3	-1.77	-3.41	AAA-AAA	0.00	999	1.000	1.000	0.0492
1561733_at	34.0	10.0	-1.77	-3.41	AAA-AAA	0.00	999	1.000	1.000	0.3758
220628_s_at	18.5	5.4	-1.77	-3.41	AAA-AAA	0.00	999	1.000	1.000	0.1165
215337_at	32.3	9.5	-1.77	-3.41	AAA-AAA	0.00	999	1.000	1.000	0.2007
1560292_a_at	12.9	43.9	1.77	3.41	AAA-AAA	0.00	999	1.000	1.000	0.0188

234869_at	19.2	5.7	-1.77	-3.41	AAA-AAA	0.00	999	1.000	1.000	0.0095
216690_at	9.2	31.2	1.77	3.40	PAA-AAA	0.00	999	1.000	1.000	0.3482
239955_at	9.7	2.8	-1.77	-3.40	AAA-AAA	0.00	999	1.000	1.000	0.0484
1562656_at	4.0	13.5	1.77	3.40	AAA-AAA	0.00	999	1.000	1.000	0.1379
1566181_at	39.9	11.7	-1.77	-3.40	MAA-AAA	0.00	999	1.000	1.000	0.1736
1553468_at	8.3	28.2	1.77	3.40	AAA-AAA	0.00	999	1.000	1.000	0.1041
1555926_a_at	6.2	21.1	1.77	3.40	AAA-AAA	0.00	999	1.000	1.000	0.2564
220110_s_at	26.0	7.6	-1.77	-3.40	AAA-AAA	0.00	999	1.000	1.000	0.2853
237142_at	20.9	6.1	-1.77	-3.40	AAA-AAA	0.00	999	1.000	1.000	0.1388
206312_at	30.4	8.9	-1.77	-3.40	AAA-AAA	0.00	999	1.000	1.000	0.3666
1562071_at	9.0	2.7	-1.77	-3.40	AAA-AAA	0.00	999	1.000	1.000	0.3360
1552348_at	44.5	13.1	-1.77	-3.40	AAA-AAA	0.00	999	1.000	1.000	0.0883
214891_at	17.8	60.4	1.77	3.40	AAA-AAA	0.00	999	1.000	1.000	0.0509
214867_at	128.5	37.8	-1.77	-3.40	AAA-AAA	0.00	999	1.000	1.000	0.1268
211525_s_at	2.3	7.8	1.77	3.40	AAA-AAA	0.00	999	1.000	1.000	0.3084
235494_at	6.2	21.1	1.77	3.40	AAA-AAA	0.00	999	1.000	1.000	0.0822
232941_at	20.1	5.9	-1.77	-3.40	AAA-AAA	0.00	999	1.000	1.000	0.0322
240317_at	22.0	6.5	-1.77	-3.40	AAA-AAA	0.00	999	1.000	1.000	0.2613
244005_at	48.7	14.3	-1.76	-3.40	AAA-AAA	0.00	999	1.000	1.000	0.0231
1563502_at	24.8	7.3	-1.76	-3.40	AAA-AAA	0.00	999	1.000	1.000	0.1736
1561000_at	41.6	12.2	-1.76	-3.40	AAA-AAA	0.00	999	1.000	1.000	0.2590
242001_at	5.8	19.8	1.76	3.40	AAP-AMA	0.00	999	1.000	1.000	0.2593
223462_at	40.5	137.5	1.76	3.39	APA-AAP	0.00	999	1.000	1.000	0.1621
221202_at	13.4	3.9	-1.76	-3.40	AAA-AAA	0.00	999	1.000	1.000	0.1460
234267_at	62.0	18.3	-1.76	-3.39	AAA-AAA	0.00	999	1.000	1.000	0.0137
237985_at	10.8	3.2	-1.76	-3.39	AAA-AAA	0.00	999	1.000	1.000	0.0421
1563109_at	34.1	10.1	-1.76	-3.39	AAA-AAA	0.00	999	1.000	1.000	0.2675
1563080_at	4.8	16.1	1.76	3.39	AAA-AAA	0.00	999	1.000	1.000	0.2839
1566734_at	47.4	14.0	-1.76	-3.39	AAA-AAA	0.00	999	1.000	1.000	0.1723
1556873_at	1.9	6.5	1.76	3.39	AAA-AAA	0.00	999	1.000	1.000	0.2645
208183_at	31.9	9.4	-1.76	-3.39	AAA-AAA	0.00	999	1.000	1.000	0.1798
224198_at	41.9	12.4	-1.76	-3.39	AAA-AAA	0.00	999	1.000	1.000	0.1244
232650_at	6.0	20.4	1.76	3.39	AAA-AAA	0.00	999	1.000	1.000	0.2104
241511_at	8.5	2.5	-1.76	-3.39	AAA-AAA	0.00	999	1.000	1.000	0.0540
1553844_a_at	102.1	30.1	-1.76	-3.39	AAA-AAA	0.00	999	1.000	1.000	0.1161
1553839_at	42.8	12.6	-1.76	-3.39	AAA-AAA	0.00	999	1.000	1.000	0.1497
213782_s_at	4.2	1.2	-1.76	-3.39	AAA-AAA	0.00	999	1.000	1.000	0.2016
233780_at	61.4	18.1	-1.76	-3.39	MAA-AAA	0.00	999	1.000	1.000	0.3032
233438_at	3.9	13.0	1.76	3.39	AAA-AAA	0.00	999	1.000	1.000	0.2586
240703_s_at	14.6	49.4	1.76	3.39	AAA-APP	0.00	999	1.000	1.000	0.2885
1570162_at	7.0	2.1	-1.76	-3.39	AAA-AAA	0.00	999	1.000	1.000	0.0790
237892_at	94.3	27.9	-1.76	-3.39	AAA-AAA	0.00	999	1.000	1.000	0.3234

237525_at	50.2	14.8	-1.76	-3.39	AAA-AAA	0.00	999	1.000	1.000	0.1695
1561985_at	3.8	12.8	1.76	3.38	AAA-AAA	0.00	999	1.000	1.000	0.3587
1564449_at	66.9	19.8	-1.76	-3.38	MAA-AAA	0.00	999	1.000	1.000	0.1204
1554379_a_at	70.0	20.7	-1.76	-3.38	AAA-AAA	0.00	999	1.000	1.000	0.2248
1553351_at	27.6	8.2	-1.76	-3.38	AAA-AAA	0.00	999	1.000	1.000	0.0529
207077_at	47.1	13.9	-1.76	-3.38	AAA-AAA	0.00	999	1.000	1.000	0.0241
206424_at	28.7	8.5	-1.76	-3.38	AAA-AAA	0.00	999	1.000	1.000	0.2668
237414_at	8.6	29.1	1.76	3.38	AAA-AAA	0.00	999	1.000	1.000	0.0875
237911_at	6.0	1.8	-1.76	-3.39	AAA-AAA	0.00	999	1.000	1.000	0.1955
229880_at	36.8	124.7	1.76	3.39	AAA-AAA	0.00	999	1.000	1.000	0.0330
230425_at	5.9	20.1	1.76	3.38	AAA-AAA	0.00	999	1.000	1.000	0.3132
206154_at	54.5	16.1	-1.76	-3.39	AAA-AAA	0.00	999	1.000	1.000	0.0718
1563654_at	57.7	17.1	-1.76	-3.38	AAA-AAA	0.00	999	1.000	1.000	0.0292
1553067_a_at	11.8	40.0	1.76	3.38	AAA-AAA	0.00	999	1.000	1.000	0.0231
220916_at	33.5	9.9	-1.76	-3.38	AMA-AAA	0.00	999	1.000	1.000	0.3525
237120_at	33.4	9.9	-1.76	-3.38	AAA-AAA	0.00	999	1.000	1.000	0.3641
1566141_at	17.3	5.1	-1.76	-3.38	AAA-AAA	0.00	999	1.000	1.000	0.3955
244379_at	62.7	18.5	-1.76	-3.38	AAA-AAA	0.00	999	1.000	1.000	0.1068
1553347_s_at	50.7	15.0	-1.76	-3.38	AAA-AAA	0.00	999	1.000	1.000	0.2211
225238_at	79.0	23.4	-1.76	-3.38	AAA-AAA	0.00	999	1.000	1.000	0.0262
234321_x_at	53.3	15.8	-1.76	-3.38	AAA-AAA	0.00	999	1.000	1.000	0.2452
234827_at	46.8	13.8	-1.76	-3.38	AMA-AAA	0.00	999	1.000	1.000	0.1668
237625_s_at	8.6	29.1	1.76	3.38	AAA-AAP	0.00	999	1.000	1.000	0.1461
240953_at	29.7	8.8	-1.76	-3.38	AAA-AAA	0.00	999	1.000	1.000	0.2153
239844_x_at	52.1	15.4	-1.76	-3.38	AAA-AAA	0.00	999	1.000	1.000	0.1658
1561322_at	23.3	6.9	-1.76	-3.38	AAA-AAA	0.00	999	1.000	1.000	0.2072
1553138_a_at	26.5	7.9	-1.76	-3.38	AAA-AAA	0.00	999	1.000	1.000	0.3523
1559384_at	5.8	19.6	1.76	3.38	AAA-AAA	0.00	999	1.000	1.000	0.1303
222891_s_at	26.1	7.7	-1.76	-3.38	AAA-AAA	0.00	999	1.000	1.000	0.1672
210426_x_at	46.7	13.8	-1.76	-3.38	AAA-AAA	0.00	999	1.000	1.000	0.2011
212865_s_at	8.0	2.4	-1.76	-3.38	AAA-AAA	0.00	999	1.000	1.000	0.2151
238330_s_at	10.7	36.2	1.76	3.38	AAA-AAA	0.00	999	1.000	1.000	0.0326
237826_at	50.8	15.0	-1.76	-3.38	AAA-AAA	0.00	999	1.000	1.000	0.1168
232261_at	4.4	14.8	1.76	3.38	AAA-AAA	0.00	999	1.000	1.000	0.1656
243952_at	17.4	5.2	-1.76	-3.38	AAA-AAA	0.00	999	1.000	1.000	0.2405
1564451_at	25.9	7.7	-1.76	-3.38	AAA-AAA	0.00	999	1.000	1.000	0.3112
220322_at	30.8	9.1	-1.76	-3.38	AAA-AAA	0.00	999	1.000	1.000	0.3351
227283_at	29.5	99.5	1.76	3.38	AAA-AAA	0.00	999	1.000	1.000	0.1729
213197_at	16.4	4.9	-1.76	-3.38	AAM-AAA	0.00	999	1.000	1.000	0.2350
228816_at	104.7	31.0	-1.76	-3.37	AAA-AAA	0.00	999	1.000	1.000	0.1064
241463_at	10.3	3.1	-1.75	-3.37	AAA-AAA	0.00	999	1.000	1.000	0.1494
220280_s_at	38.2	11.3	-1.75	-3.37	AAA-AAA	0.00	999	1.000	1.000	0.2321

216975_x_at	21.9	6.5	-1.75	-3.37	AAA-AAA	0.00	999	1.000	1.000	0.1061
230632_at	18.6	5.5	-1.75	-3.37	AAA-AAA	0.00	999	1.000	1.000	0.2516
231109_at	7.8	2.3	-1.75	-3.37	AAA-AAA	0.00	999	1.000	1.000	0.2314
241332_at	8.8	2.6	-1.75	-3.37	AAA-AAA	0.00	999	1.000	1.000	0.2708
1563246_at	4.3	14.6	1.75	3.37	AAA-AAA	0.00	999	1.000	1.000	0.1262
1566218_at	71.2	21.1	-1.75	-3.37	AAA-AAA	0.00	999	1.000	1.000	0.1962
1557770_at	38.5	11.4	-1.75	-3.37	AAA-AAA	0.00	999	1.000	1.000	0.2935
235682_s_at	21.3	6.3	-1.75	-3.37	AAA-AAA	0.00	999	1.000	1.000	0.0863
233640_x_at	13.2	3.9	-1.75	-3.37	AAA-AAA	0.00	999	1.000	1.000	0.0420
1552608_at	11.2	3.3	-1.75	-3.37	AAA-AAA	0.00	999	1.000	1.000	0.2171
1556354_s_at	5.0	16.8	1.75	3.37	AAA-AAA	0.00	999	1.000	1.000	0.0546
220317_at	4.9	16.5	1.75	3.37	AAA-AAA	0.00	999	1.000	1.000	0.0374
214529_at	81.9	24.3	-1.75	-3.37	AAA-AAA	0.00	999	1.000	1.000	0.3157
231474_at	32.5	9.7	-1.75	-3.37	AAA-AAA	0.00	999	1.000	1.000	0.2617
243693_at	10.9	3.3	-1.75	-3.37	AAA-AAA	0.00	999	1.000	1.000	0.0960
1562595_at	11.3	3.4	-1.75	-3.37	AAA-AAA	0.00	999	1.000	1.000	0.1998
1556516_at	9.0	2.7	-1.75	-3.37	AAA-AAA	0.00	999	1.000	1.000	0.3424
237592_at	6.0	20.3	1.75	3.37	AAA-AAA	0.00	999	1.000	1.000	0.1979
231678_s_at	7.8	2.3	-1.75	-3.37	AAA-AAA	0.00	999	1.000	1.000	0.1927
1555763_x_at	114.3	34.0	-1.75	-3.36	MAA-AAA	0.00	999	1.000	1.000	0.3092
229188_s_at	7.4	25.0	1.75	3.36	AAA-AAA	0.00	999	1.000	1.000	0.2718
233401_at	29.5	8.8	-1.75	-3.36	AAA-AAA	0.00	999	1.000	1.000	0.3154
243946_at	88.5	26.3	-1.75	-3.36	AAA-AAA	0.00	999	1.000	1.000	0.0581
240585_at	9.6	2.9	-1.75	-3.36	AAA-AAA	0.00	999	1.000	1.000	0.1866
1561014_at	20.2	6.0	-1.75	-3.36	AAA-AAA	0.00	999	1.000	1.000	0.2816
1561316_at	24.8	7.4	-1.75	-3.36	AAA-AAA	0.00	999	1.000	1.000	0.0527
1566539_at	26.1	7.8	-1.75	-3.36	AAA-AAA	0.00	999	1.000	1.000	0.2210
1555214_a_at	6.9	2.1	-1.75	-3.36	AAA-AAA	0.00	999	1.000	1.000	0.1751
1553214_a_at	13.0	3.9	-1.75	-3.36	AAA-AAA	0.00	999	1.000	1.000	0.2176
1559787_at	7.3	24.6	1.75	3.36	AAA-AAA	0.00	999	1.000	1.000	0.2003
219716_at	26.7	89.7	1.75	3.36	AAA-AAA	0.00	999	1.000	1.000	0.0532
224100_s_at	52.7	15.7	-1.75	-3.36	AAA-AAA	0.00	999	1.000	1.000	0.0622
205102_at	85.7	25.5	-1.75	-3.36	AAA-AAA	0.00	999	1.000	1.000	0.1187
233402_at	19.8	5.9	-1.75	-3.36	AAA-AAA	0.00	999	1.000	1.000	0.3151
1563015_at	22.6	6.7	-1.75	-3.36	AAA-AAA	0.00	999	1.000	1.000	0.0412
1569789_at	22.2	6.6	-1.75	-3.36	AAA-AAA	0.00	999	1.000	1.000	0.0516
1553297_a_at	95.3	28.4	-1.75	-3.36	AAA-AAA	0.00	999	1.000	1.000	0.0589
1559134_a_at	15.4	4.6	-1.75	-3.36	AAA-AAA	0.00	999	1.000	1.000	0.3473
224158_s_at	25.7	7.6	-1.75	-3.36	AAA-AAA	0.00	999	1.000	1.000	0.3000
238978_at	32.4	9.7	-1.75	-3.36	AAA-AAA	0.00	999	1.000	1.000	0.1520
240506_at	36.5	10.9	-1.75	-3.36	AAA-AAA	0.00	999	1.000	1.000	0.3361
1568746_a_at	9.2	2.7	-1.75	-3.36	AAA-AAA	0.00	999	1.000	1.000	0.1954

1566984_at	49.0	14.6	-1.75	-3.36	AAA-AAA	0.00	999	1.000	1.000	0.1629
1556889_s_at	2.5	8.5	1.75	3.36	AAA-AAA	0.00	999	1.000	1.000	0.3030
214818_at	9.6	32.0	1.75	3.36	AAA-AAA	0.00	999	1.000	1.000	0.0061
1568794_at	26.6	7.9	-1.75	-3.36	AAA-AAA	0.00	999	1.000	1.000	0.0152
1570177_at	45.5	13.6	-1.75	-3.35	AAA-AAA	0.00	999	1.000	1.000	0.0921
202504_at	148.5	44.3	-1.75	-3.36	AAA-AAA	0.00	999	1.000	1.000	0.1737
221300_at	40.8	12.2	-1.75	-3.35	AAA-AAA	0.00	999	1.000	1.000	0.2258
220854_at	15.4	4.6	-1.75	-3.35	AAA-AAA	0.00	999	1.000	1.000	0.1736
205850_s_at	16.9	5.0	-1.75	-3.35	AAA-AAA	0.00	999	1.000	1.000	0.1861
229421_s_at	53.4	15.9	-1.75	-3.35	AAA-AAA	0.00	999	1.000	1.000	0.0694
1563031_at	9.1	30.6	1.75	3.35	AAA-AAA	0.00	999	1.000	1.000	0.1063
211463_at	3.5	11.6	1.75	3.35	AAA-AAA	0.00	999	1.000	1.000	0.4233
205000_at	3.3	11.1	1.75	3.35	AAA-AAA	0.00	999	1.000	1.000	0.1649
243155_at	12.9	43.3	1.74	3.35	AAA-AAA	0.00	999	1.000	1.000	0.1170
1561437_at	1.9	6.2	1.74	3.35	AAA-AAA	0.00	999	1.000	1.000	0.0902
207138_at	73.4	21.9	-1.74	-3.35	AAA-AAA	0.00	999	1.000	1.000	0.0594
1566880_at	61.9	18.5	-1.74	-3.35	AAA-AAA	0.00	999	1.000	1.000	0.2179
1553472_at	27.4	8.2	-1.74	-3.35	AAA-AAA	0.00	999	1.000	1.000	0.3286
244390_at	53.0	15.8	-1.74	-3.35	AAA-AAA	0.00	999	1.000	1.000	0.0695
1564107_at	6.5	21.8	1.74	3.35	AAA-AAA	0.00	999	1.000	1.000	0.1745
212671_s_at	6.6	2.0	-1.74	-3.35	AAA-AAA	0.00	999	1.000	1.000	0.1310
216785_at	32.0	9.6	-1.74	-3.35	AAA-AAA	0.00	999	1.000	1.000	0.1185
216151_at	3.2	10.6	1.74	3.35	AAA-AAA	0.00	999	1.000	1.000	0.3126
236340_at	40.7	12.2	-1.74	-3.35	AAA-AAA	0.00	999	1.000	1.000	0.1640
239303_at	37.9	11.3	-1.74	-3.34	MMA-AAA	0.00	999	1.000	1.000	0.3189
218944_at	68.5	229.1	1.74	3.34	AAA-AAP	0.00	999	1.000	1.000	0.1794
1561222_at	10.0	3.0	-1.74	-3.34	AAA-AAA	0.00	999	1.000	1.000	0.3953
1561106_at	21.6	6.5	-1.74	-3.34	AAA-AAA	0.00	999	1.000	1.000	0.3643
1566865_at	12.3	3.7	-1.74	-3.34	AAA-AAA	0.00	999	1.000	1.000	0.4104
210934_at	44.9	13.4	-1.74	-3.34	AAA-AAA	0.00	999	1.000	1.000	0.1429
204411_at	18.7	5.6	-1.74	-3.34	AAA-AAA	0.00	999	1.000	1.000	0.1691
240955_at	9.7	2.9	-1.74	-3.34	AAA-AAA	0.00	999	1.000	1.000	0.1977
1560543_at	25.9	7.8	-1.74	-3.34	AAA-AAA	0.00	999	1.000	1.000	0.1102
1554125_a_at	8.3	2.5	-1.74	-3.34	AAA-AAA	0.00	999	1.000	1.000	0.3880
236534_at	45.2	13.5	-1.74	-3.34	AAA-AAA	0.00	999	1.000	1.000	0.2919
213668_s_at	77.4	258.3	1.74	3.34	AAP-PPP	0.00	999	1.000	1.000	0.1982
1569560_at	19.8	5.9	-1.74	-3.34	AAA-AAA	0.00	999	1.000	1.000	0.1593
1568698_at	62.9	18.8	-1.74	-3.34	AAA-MAA	0.00	999	1.000	1.000	0.3098
1566947_at	20.8	6.2	-1.74	-3.34	AAA-AAA	0.00	999	1.000	1.000	0.1241
1557991_at	31.7	9.5	-1.74	-3.34	AAA-AAA	0.00	999	1.000	1.000	0.2273
226709_at	15.8	4.7	-1.74	-3.34	AAA-AAA	0.00	999	1.000	1.000	0.0808
239526_x_at	13.2	44.0	1.74	3.34	AAA-AAA	0.00	999	1.000	1.000	0.1576

1566582_x_at	29.9	9.0	-1.74	-3.34	AAA-AMA	0.00	999	1.000	1.000	0.3395
1552661_at	18.9	5.7	-1.74	-3.34	AAA-AAA	0.00	999	1.000	1.000	0.3082
1559641_at	48.6	14.6	-1.74	-3.34	AAA-AAA	0.00	999	1.000	1.000	0.0003
1556927_at	6.8	2.0	-1.74	-3.34	AAA-AAA	0.00	999	1.000	1.000	0.2506
214115_at	20.0	6.0	-1.74	-3.34	AAA-AAA	0.00	999	1.000	1.000	0.1257
212664_at	79.7	23.9	-1.74	-3.34	AAA-AAA	0.00	999	1.000	1.000	0.0503
215448_at	13.9	4.2	-1.74	-3.34	AAA-AAA	0.00	999	1.000	1.000	0.1090
238605_at	7.3	2.2	-1.74	-3.34	AAA-AAA	0.00	999	1.000	1.000	0.3043
229530_at	25.3	7.6	-1.74	-3.34	AAA-AAA	0.00	999	1.000	1.000	0.0863
1559732_at	52.1	15.6	-1.74	-3.33	AAA-AAA	0.00	999	1.000	1.000	0.0745
206727_at	24.6	7.4	-1.74	-3.33	AAA-AAA	0.00	999	1.000	1.000	0.2342
211869_at	19.6	5.9	-1.74	-3.33	AAA-AAA	0.00	999	1.000	1.000	0.0844
1568644_at	5.1	1.5	-1.74	-3.33	AAA-AAA	0.00	999	1.000	1.000	0.2738
1557430_at	13.6	4.1	-1.74	-3.33	AAA-AAA	0.00	999	1.000	1.000	0.1676
204105_s_at	41.3	12.4	-1.74	-3.33	AAA-AAA	0.00	999	1.000	1.000	0.1402
237735_at	34.7	10.4	-1.74	-3.33	AAA-AAA	0.00	999	1.000	1.000	0.3108
1566664_at	52.3	15.7	-1.74	-3.33	AAA-AAA	0.00	999	1.000	1.000	0.1391
207311_at	12.6	41.8	1.74	3.33	AAA-AAA	0.00	999	1.000	1.000	0.0027
224109_at	4.6	15.2	1.74	3.33	AAA-AAA	0.00	999	1.000	1.000	0.0972
235329_at	50.0	15.0	-1.74	-3.33	MAA-AAA	0.00	999	1.000	1.000	0.1605
237429_at	9.4	31.2	1.74	3.33	AAA-AAA	0.00	999	1.000	1.000	0.1747
236896_at	12.7	3.8	-1.74	-3.33	AAA-AAA	0.00	999	1.000	1.000	0.2214
1556724_at	26.1	7.9	-1.73	-3.33	AAA-AAA	0.00	999	1.000	1.000	0.0844
206471_s_at	52.5	15.8	-1.73	-3.33	AAA-AAA	0.00	999	1.000	1.000	0.0464
205948_at	29.5	8.9	-1.73	-3.33	AAA-AAA	0.00	999	1.000	1.000	0.0973
229866_at	24.5	7.4	-1.73	-3.33	AAA-AAA	0.00	999	1.000	1.000	0.0840
231774_at	117.0	35.2	-1.73	-3.33	AAA-AAA	0.00	999	1.000	1.000	0.0433
224279_s_at	48.0	159.6	1.73	3.32	AAP-PPA	0.00	999	1.000	1.000	0.3567
241449_at	50.0	15.0	-1.73	-3.32	AMA-AAA	0.00	999	1.000	1.000	0.1568
1562982_at	20.1	6.1	-1.73	-3.33	AAA-AAA	0.00	999	1.000	1.000	0.2590
1564128_at	6.7	2.0	-1.73	-3.32	AAA-AAA	0.00	999	1.000	1.000	0.3423
1553573_s_at	18.7	5.6	-1.73	-3.32	AAA-AAA	0.00	999	1.000	1.000	0.2516
220859_at	38.3	11.5	-1.73	-3.33	AAA-AAA	0.00	999	1.000	1.000	0.0696
230705_at	86.6	26.1	-1.73	-3.32	AAA-AAA	0.00	999	1.000	1.000	0.1156
212249_at	27.4	91.1	1.73	3.32	APA-PPP	0.00	999	1.000	1.000	0.1784
239984_at	7.5	2.3	-1.73	-3.32	AAA-AAA	0.00	999	1.000	1.000	0.2043
1568730_at	14.6	4.4	-1.73	-3.32	AAA-AAA	0.00	999	1.000	1.000	0.0846
211076_x_at	65.6	19.8	-1.73	-3.32	AAA-AAA	0.00	999	1.000	1.000	0.3098
237526_at	8.6	2.6	-1.73	-3.32	AAA-AAA	0.00	999	1.000	1.000	0.0343
229383_at	33.4	10.1	-1.73	-3.32	AAA-AAA	0.00	999	1.000	1.000	0.2326
1555557_a_at	6.7	22.2	1.73	3.32	AAA-AAA	0.00	999	1.000	1.000	0.2676
241439_at	9.7	2.9	-1.73	-3.32	AAA-AAA	0.00	999	1.000	1.000	0.0908

203766_s_at	11.5	38.2	1.73	3.32	AAA-AAA	0.00	999	1.000	1.000	0.1878
1554288_at	19.7	5.9	-1.73	-3.32	AAA-AAA	0.00	999	1.000	1.000	0.3580
207723_s_at	30.8	9.3	-1.73	-3.32	AAA-AAA	0.00	999	1.000	1.000	0.2688
214425_at	39.6	12.0	-1.73	-3.32	AAA-AAA	0.00	999	1.000	1.000	0.1104
234890_at	7.2	23.9	1.73	3.32	AAA-AAA	0.00	999	1.000	1.000	0.0129
243739_at	40.7	12.3	-1.73	-3.32	AAA-AAA	0.00	999	1.000	1.000	0.0652
214320_x_at	158.1	47.7	-1.73	-3.32	AAA-AAA	0.00	999	1.000	1.000	0.1595
238304_at	10.2	33.8	1.73	3.31	AAA-AAA	0.00	999	1.000	1.000	0.1290
233306_at	6.9	23.0	1.73	3.31	AAA-AAA	0.00	999	1.000	1.000	0.0199
240758_at	24.3	7.3	-1.73	-3.31	AAA-AAA	0.00	999	1.000	1.000	0.1546
203168_at	32.8	108.4	1.73	3.31	AAA-AAA	0.00	999	1.000	1.000	0.1447
1567065_at	93.4	28.2	-1.73	-3.31	AAA-AAA	0.00	999	1.000	1.000	0.2084
1555375_at	1.9	6.3	1.73	3.31	AAA-AAA	0.00	999	1.000	1.000	0.3014
224089_at	52.2	15.8	-1.73	-3.31	AAA-AAA	0.00	999	1.000	1.000	0.0838
232027_at	19.3	63.8	1.73	3.31	AAA-AAA	0.00	999	1.000	1.000	0.1993
243033_at	10.4	34.4	1.73	3.31	AAA-AAA	0.00	999	1.000	1.000	0.0848
1560455_at	24.8	7.5	-1.73	-3.31	AAA-AAA	0.00	999	1.000	1.000	0.2299
1570328_s_at	43.3	13.1	-1.73	-3.31	AAA-AAA	0.00	999	1.000	1.000	0.1460
1566543_at	9.2	30.4	1.73	3.31	AAA-AAA	0.00	999	1.000	1.000	0.1850
219612_s_at	43.6	13.2	-1.73	-3.31	AAA-AAA	0.00	999	1.000	1.000	0.1973
219438_at	15.0	49.5	1.73	3.31	AAA-AAA	0.00	999	1.000	1.000	0.2346
215272_at	46.0	13.9	-1.73	-3.31	AAA-AAA	0.00	999	1.000	1.000	0.1982
236051_at	25.7	7.8	-1.73	-3.31	AAA-AAA	0.00	999	1.000	1.000	0.1506
205336_at	25.4	7.7	-1.73	-3.31	AAA-AAA	0.00	999	1.000	1.000	0.1202
233459_at	14.5	4.4	-1.73	-3.31	AAA-AAA	0.00	999	1.000	1.000	0.0586
240697_at	22.3	6.8	-1.72	-3.30	AAA-AAA	0.00	999	1.000	1.000	0.1815
230917_at	10.9	3.3	-1.72	-3.30	AAA-AAA	0.00	999	1.000	1.000	0.3669
226809_at	29.9	9.1	-1.72	-3.30	AAA-AAA	0.00	999	1.000	1.000	0.3280
214343_s_at	28.7	94.6	1.72	3.30	AAA-AAA	0.00	999	1.000	1.000	0.2453
212999_x_at	10.1	3.1	-1.72	-3.30	AAA-AAA	0.00	999	1.000	1.000	0.1821
215915_at	16.5	5.0	-1.72	-3.30	AAA-AAA	0.00	999	1.000	1.000	0.3183
230249_at	31.4	9.5	-1.72	-3.30	AAA-AAA	0.00	999	1.000	1.000	0.3496
230969_at	33.4	10.1	-1.72	-3.30	AAA-AAA	0.00	999	1.000	1.000	0.2718
242369_x_at	16.7	55.2	1.72	3.30	AAA-APA	0.00	999	1.000	1.000	0.0879
216868_s_at	6.6	21.6	1.72	3.30	AAA-AAA	0.00	999	1.000	1.000	0.2913
236604_at	41.3	12.5	-1.72	-3.30	AAA-AAA	0.00	999	1.000	1.000	0.0622
230144_at	23.2	7.0	-1.72	-3.30	AAA-AAA	0.00	999	1.000	1.000	0.1961
239996_x_at	83.1	25.2	-1.72	-3.30	AAA-AAA	0.00	999	1.000	1.000	0.0685
241188_at	16.0	4.9	-1.72	-3.30	AAA-AAA	0.00	999	1.000	1.000	0.1645
1566807_a_at	13.5	4.1	-1.72	-3.30	AAA-AAA	0.00	999	1.000	1.000	0.2351
1558019_at	19.1	62.9	1.72	3.29	AMA-APA	0.00	999	1.000	1.000	0.2953
240733_at	12.6	41.4	1.72	3.29	AAA-AAP	0.00	999	1.000	1.000	0.2412

240457_at	26.8	8.1	-1.72	-3.30	AAA-AAA	0.00	999	1.000	1.000	0.1985
226213_at	7.8	25.8	1.72	3.29	AAA-AAA	0.00	999	1.000	1.000	0.3026
223500_at	2.0	6.6	1.72	3.29	AAA-AAA	0.00	999	1.000	1.000	0.0647
217546_at	15.8	4.8	-1.72	-3.29	AAA-AAA	0.00	999	1.000	1.000	0.0758
232926_x_at	19.1	5.8	-1.72	-3.29	AAA-AAA	0.00	999	1.000	1.000	0.1751
1553561_at	35.7	10.9	-1.72	-3.29	AAA-AAA	0.00	999	1.000	1.000	0.3266
222296_at	19.3	5.9	-1.72	-3.29	AAA-AAA	0.00	999	1.000	1.000	0.0398
233723_at	24.3	7.4	-1.72	-3.29	AAA-AAA	0.00	999	1.000	1.000	0.0389
233202_at	26.9	8.2	-1.72	-3.29	AAA-AAA	0.00	999	1.000	1.000	0.1325
1564169_at	8.4	2.6	-1.72	-3.29	AAA-AAA	0.00	999	1.000	1.000	0.2650
244729_at	58.3	17.7	-1.72	-3.29	AAA-AAA	0.00	999	1.000	1.000	0.1589
208219_at	9.6	31.6	1.72	3.29	AAA-AAA	0.00	999	1.000	1.000	0.1353
211100_x_at	59.1	18.0	-1.72	-3.29	AAA-AAA	0.00	999	1.000	1.000	0.1712
216782_at	14.9	4.5	-1.72	-3.29	AAA-AAA	0.00	999	1.000	1.000	0.2921
217929_s_at	64.0	210.6	1.72	3.29	AAA-AAA	0.00	999	1.000	1.000	0.1510
238689_at	7.4	24.2	1.72	3.29	AAA-AAA	0.00	999	1.000	1.000	0.2161
238254_at	20.2	6.1	-1.72	-3.29	AAA-AAA	0.00	999	1.000	1.000	0.2985
239248_at	111.8	34.0	-1.72	-3.29	AAA-AAA	0.00	999	1.000	1.000	0.1297
244094_at	19.9	6.1	-1.72	-3.29	AAA-AAA	0.00	999	1.000	1.000	0.1658
1566271_x_at	26.1	7.9	-1.72	-3.29	AAA-AAA	0.00	999	1.000	1.000	0.0650
1554559_at	80.4	24.5	-1.72	-3.29	AAA-AAA	0.00	999	1.000	1.000	0.0204
1557857_a_at	28.9	8.8	-1.72	-3.29	AAA-AAA	0.00	999	1.000	1.000	0.0835
226186_at	22.1	72.7	1.72	3.29	AAA-AAA	0.00	999	1.000	1.000	0.0614
214705_at	8.4	2.6	-1.72	-3.29	AAA-AAA	0.00	999	1.000	1.000	0.4109
216807_at	40.0	12.2	-1.72	-3.29	AAA-AAA	0.00	999	1.000	1.000	0.2058
205127_at	23.1	7.0	-1.72	-3.29	AAA-AAA	0.00	999	1.000	1.000	0.2095
1553333_at	12.5	3.8	-1.72	-3.28	AAA-AAA	0.00	999	1.000	1.000	0.0719
222285_at	5.5	18.2	1.72	3.29	AAA-AAA	0.00	999	1.000	1.000	0.1421
216386_at	38.9	11.9	-1.72	-3.28	AAA-AAA	0.00	999	1.000	1.000	0.1486
236266_at	8.2	26.9	1.72	3.29	AAA-AAA	0.00	999	1.000	1.000	0.0058
234102_at	19.3	5.9	-1.72	-3.29	AAA-AAA	0.00	999	1.000	1.000	0.2693
230473_s_at	27.3	8.3	-1.72	-3.29	AAA-AAA	0.00	999	1.000	1.000	0.0795
228985_at	20.3	66.8	1.72	3.28	AAA-AAA	0.00	999	1.000	1.000	0.2372
205471_s_at	52.9	16.1	-1.72	-3.29	AAM-AAA	0.00	999	1.000	1.000	0.1812
1569446_x_at	20.1	6.1	-1.72	-3.28	AAA-AAA	0.00	999	1.000	1.000	0.0255
1554702_at	23.5	7.2	-1.72	-3.28	AAA-AAA	0.00	999	1.000	1.000	0.3069
244626_at	11.7	38.5	1.72	3.28	AAA-AAA	0.00	999	1.000	1.000	0.0226
222361_at	31.1	9.5	-1.72	-3.28	AAA-AAA	0.00	999	1.000	1.000	0.1256
210956_at	49.8	15.2	-1.72	-3.28	AAA-AAA	0.00	999	1.000	1.000	0.1958
208534_s_at	15.7	4.8	-1.72	-3.28	AAA-AAA	0.00	999	1.000	1.000	0.2929
218523_at	103.5	31.5	-1.72	-3.28	AAA-AAA	0.00	999	1.000	1.000	0.0821
230792_at	65.4	19.9	-1.72	-3.28	AAA-AAA	0.00	999	1.000	1.000	0.1608

205391_x_at	13.7	4.2	-1.72	-3.28	AAA-AAA	0.00	999	1.000	1.000	0.0570
243215_at	7.9	2.4	-1.71	-3.28	AAA-AAA	0.00	999	1.000	1.000	0.0684
1569855_at	8.5	27.9	1.71	3.28	AAA-AAA	0.00	999	1.000	1.000	0.1591
1552853_at	24.0	7.3	-1.71	-3.28	AAA-AAA	0.00	999	1.000	1.000	0.3293
221136_at	20.2	66.1	1.71	3.28	AAA-AAA	0.00	999	1.000	1.000	0.0260
216365_x_at	53.1	16.2	-1.71	-3.28	AAA-AAA	0.00	999	1.000	1.000	0.2185
236293_at	11.8	3.6	-1.71	-3.28	AAA-AAA	0.00	999	1.000	1.000	0.1794
1566086_at	29.0	8.8	-1.71	-3.28	AAA-AAA	0.00	999	1.000	1.000	0.2101
1555130_at	19.9	6.1	-1.71	-3.28	AAA-AAA	0.00	999	1.000	1.000	0.2524
227134_at	31.6	9.6	-1.71	-3.28	AAA-AAA	0.00	999	1.000	1.000	0.1049
206341_at	38.4	11.7	-1.71	-3.28	AAA-AAA	0.00	999	1.000	1.000	0.3022
233450_at	10.2	33.5	1.71	3.28	AAA-AAA	0.00	999	1.000	1.000	0.1083
237439_at	26.8	87.9	1.71	3.28	APA-MAA	0.00	999	1.000	1.000	0.1090
213728_at	124.4	407.3	1.71	3.28	AAA-PAM	0.00	999	1.000	1.000	0.1043
240536_at	32.3	9.9	-1.71	-3.28	AAA-AAA	0.00	999	1.000	1.000	0.3412
1561912_at	3.3	10.7	1.71	3.27	AAA-AAA	0.00	999	1.000	1.000	0.0396
1557016_a_at	69.5	21.2	-1.71	-3.27	AAA-AAA	0.00	999	1.000	1.000	0.0380
1559631_at	30.4	9.3	-1.71	-3.27	AAA-AAA	0.00	999	1.000	1.000	0.2864
215049_x_at	11.4	3.5	-1.71	-3.27	AAA-AAA	0.00	999	1.000	1.000	0.1128
211223_at	59.0	18.0	-1.71	-3.27	AAA-AAA	0.00	999	1.000	1.000	0.1645
241357_at	48.9	14.9	-1.71	-3.27	AAA-AAA	0.00	999	1.000	1.000	0.1303
240138_at	56.0	17.1	-1.71	-3.27	AAA-AAA	0.00	999	1.000	1.000	0.1467
1560676_at	6.2	1.9	-1.71	-3.27	AAA-AAA	0.00	999	1.000	1.000	0.1683
1568782_at	55.7	17.0	-1.71	-3.27	AAA-AAA	0.00	999	1.000	1.000	0.1302
241427_x_at	18.8	61.4	1.71	3.27	AAA-AAA	0.00	999	1.000	1.000	0.0098
1560905_at	31.2	9.6	-1.71	-3.27	AAA-AAA	0.00	999	1.000	1.000	0.0836
1553935_at	17.3	5.3	-1.71	-3.27	AAA-AAA	0.00	999	1.000	1.000	0.2462
244807_at	11.3	37.0	1.71	3.27	AAA-AAA	0.00	999	1.000	1.000	0.1990
207820_at	32.4	9.9	-1.71	-3.27	AAA-AAA	0.00	999	1.000	1.000	0.2174
227570_at	61.8	18.9	-1.71	-3.27	AAA-AAA	0.00	999	1.000	1.000	0.1967
214488_at	12.7	41.6	1.71	3.27	AAA-AAA	0.00	999	1.000	1.000	0.1386
216477_at	7.3	23.9	1.71	3.27	AAA-AAA	0.00	999	1.000	1.000	0.1899
238152_at	6.9	22.6	1.71	3.27	AAA-AAA	0.00	999	1.000	1.000	0.2852
233305_at	6.9	2.1	-1.71	-3.27	AAA-AAA	0.00	999	1.000	1.000	0.3482
243110_x_at	8.6	2.6	-1.71	-3.27	AAA-AAA	0.00	999	1.000	1.000	0.2075
240495_at	31.9	9.8	-1.71	-3.27	AAA-AAA	0.00	999	1.000	1.000	0.1346
1562800_at	7.3	2.2	-1.71	-3.27	AAA-AAA	0.00	999	1.000	1.000	0.2371
210065_s_at	14.2	46.4	1.71	3.27	AAA-AAA	0.00	999	1.000	1.000	0.0444
236724_at	19.8	6.1	-1.71	-3.27	AAA-AAA	0.00	999	1.000	1.000	0.1742
228911_at	22.0	6.7	-1.71	-3.27	AAA-AAA	0.00	999	1.000	1.000	0.0946
243278_at	7.7	2.4	-1.71	-3.26	AAA-AAA	0.00	999	1.000	1.000	0.3030
240492_at	18.4	5.6	-1.71	-3.27	AAA-AAA	0.00	999	1.000	1.000	0.2181

223994_s_at	43.7	142.8	1.71	3.27	AAA-AAA	0.00	999	1.000	1.000	0.1331
240683_at	73.3	22.5	-1.71	-3.26	AAA-AAA	0.00	999	1.000	1.000	0.0676
1562623_at	6.8	22.2	1.71	3.26	AAA-AAA	0.00	999	1.000	1.000	0.0080
1562529_s_at	31.3	9.6	-1.71	-3.26	AAA-AAA	0.00	999	1.000	1.000	0.0979
1562121_at	6.2	20.2	1.71	3.26	AAA-AAA	0.00	999	1.000	1.000	0.2146
1560917_at	29.2	8.9	-1.71	-3.26	AAA-AAA	0.00	999	1.000	1.000	0.1832
220904_at	36.4	11.2	-1.71	-3.26	AAA-AAA	0.00	999	1.000	1.000	0.2441
225987_at	63.1	19.4	-1.71	-3.26	AAA-AAA	0.00	999	1.000	1.000	0.0982
206679_at	46.1	14.1	-1.71	-3.26	AAA-AAA	0.00	999	1.000	1.000	0.3014
238391_at	16.7	5.1	-1.71	-3.26	AAA-AAA	0.00	999	1.000	1.000	0.2494
233553_at	19.8	6.1	-1.71	-3.26	AAA-AAA	0.00	999	1.000	1.000	0.2782
203824_at	58.2	17.9	-1.71	-3.26	AAA-AAA	0.00	999	1.000	1.000	0.1375
1568762_at	3.8	1.2	-1.71	-3.26	AAA-AAA	0.00	999	1.000	1.000	0.2042
1552648_a_at	9.5	30.9	1.71	3.26	AAA-AAA	0.00	999	1.000	1.000	0.1993
207519_at	32.4	9.9	-1.71	-3.26	AAA-AAA	0.00	999	1.000	1.000	0.0422
243021_at	14.2	4.3	-1.70	-3.26	AAA-AAA	0.00	999	1.000	1.000	0.1122
1570105_at	36.0	11.0	-1.70	-3.26	AAA-AAA	0.00	999	1.000	1.000	0.1387
1552543_a_at	11.7	38.3	1.70	3.26	AAA-AAA	0.00	999	1.000	1.000	0.0840
1555995_a_at	54.7	16.8	-1.70	-3.26	AAA-AAA	0.00	999	1.000	1.000	0.2292
219915_s_at	9.1	29.8	1.70	3.26	AAA-AAA	0.00	999	1.000	1.000	0.0278
206541_at	8.5	27.8	1.70	3.26	AAA-AAA	0.00	999	1.000	1.000	0.0628
231422_x_at	88.2	27.1	-1.70	-3.26	AAA-AAA	0.00	999	1.000	1.000	0.1101
231163_at	27.9	8.6	-1.70	-3.26	AAA-AAA	0.00	999	1.000	1.000	0.2460
1563027_at	53.2	16.3	-1.70	-3.26	AAA-AAA	0.00	999	1.000	1.000	0.0185
206705_at	27.2	8.4	-1.70	-3.26	AAA-AAA	0.00	999	1.000	1.000	0.1829
230325_at	27.9	8.6	-1.70	-3.26	AAA-AAA	0.00	999	1.000	1.000	0.2622
206246_at	37.8	11.6	-1.70	-3.26	AAA-AAA	0.00	999	1.000	1.000	0.1148
242660_at	20.0	6.1	-1.70	-3.25	AAA-AAA	0.00	999	1.000	1.000	0.3155
206515_at	37.7	11.6	-1.70	-3.26	AAA-AAA	0.00	999	1.000	1.000	0.2562
207674_at	13.3	4.1	-1.70	-3.25	AAA-AAA	0.00	999	1.000	1.000	0.1623
242441_at	35.3	10.9	-1.70	-3.25	AAA-AAA	0.00	999	1.000	1.000	0.1958
1552973_at	22.4	6.9	-1.70	-3.25	AAA-AAA	0.00	999	1.000	1.000	0.1760
1556787_s_at	23.8	7.3	-1.70	-3.25	AAA-AAA	0.00	999	1.000	1.000	0.2328
208542_x_at	11.4	36.9	1.70	3.25	AAP-AAA	0.00	999	1.000	1.000	0.2755
1560980_a_at	9.4	2.9	-1.70	-3.25	AAA-AAA	0.00	999	1.000	1.000	0.4462
1560346_at	12.3	39.9	1.70	3.25	AAA-AAA	0.00	999	1.000	1.000	0.0383
1561463_at	11.7	37.9	1.70	3.25	AAA-AAA	0.00	999	1.000	1.000	0.2089
1556406_at	11.3	3.5	-1.70	-3.25	AAA-AAA	0.00	999	1.000	1.000	0.0660
220902_at	10.9	3.4	-1.70	-3.25	AAA-AAA	0.00	999	1.000	1.000	0.1214
209848_s_at	62.3	19.2	-1.70	-3.25	AAA-AAA	0.00	999	1.000	1.000	0.0936
218599_at	229.9	70.8	-1.70	-3.25	AMA-AAA	0.00	999	1.000	1.000	0.1557
216020_at	13.0	4.0	-1.70	-3.25	AAA-AAA	0.00	999	1.000	1.000	0.2699

229820_at	10.1	32.9	1.70	3.25	AAA-AAA	0.00	999	1.000	1.000	0.2101
239943_x_at	16.4	53.2	1.70	3.25	AAA-AAA	0.00	999	1.000	1.000	0.2927
241104_at	44.5	13.7	-1.70	-3.25	AAA-AAA	0.00	999	1.000	1.000	0.2355
1561995_at	42.0	13.0	-1.70	-3.25	AAA-AAA	0.00	999	1.000	1.000	0.1636
1558900_at	5.0	1.5	-1.70	-3.25	AAA-AAA	0.00	999	1.000	1.000	0.2506
239254_at	27.2	8.4	-1.70	-3.25	AAA-AAA	0.00	999	1.000	1.000	0.1433
228368_at	56.3	17.4	-1.70	-3.25	AAA-AAA	0.00	999	1.000	1.000	0.1760
231210_at	11.0	3.4	-1.70	-3.25	AAA-AAA	0.00	999	1.000	1.000	0.2164
202017_at	285.8	927.4	1.70	3.25	AMP-PPP	0.00	999	1.000	1.000	0.2601
1564620_at	26.4	8.2	-1.70	-3.24	AAA-AAA	0.00	999	1.000	1.000	0.1020
244422_at	91.4	28.2	-1.70	-3.24	AAA-AAA	0.00	999	1.000	1.000	0.1535
244614_at	46.1	14.2	-1.70	-3.25	MAM-AAA	0.00	999	1.000	1.000	0.1010
1558875_at	120.9	37.3	-1.70	-3.25	AAA-AAA	0.00	999	1.000	1.000	0.0682
207330_at	44.1	13.6	-1.70	-3.25	AAA-AAA	0.00	999	1.000	1.000	0.2409
237069_s_at	8.6	2.7	-1.70	-3.25	AAA-AAA	0.00	999	1.000	1.000	0.0864
240166_x_at	51.2	166.2	1.70	3.24	AAP-PPP	0.00	999	1.000	1.000	0.1431
1569090_x_at	15.8	51.4	1.70	3.24	AAA-AAA	0.00	999	1.000	1.000	0.0997
1554982_a_at	8.8	2.7	-1.70	-3.24	AAA-AAA	0.00	999	1.000	1.000	0.1664
1552730_at	23.5	7.2	-1.70	-3.24	AAA-AAA	0.00	999	1.000	1.000	0.1878
208026_at	18.7	5.8	-1.70	-3.24	AAA-AAA	0.00	999	1.000	1.000	0.1756
220374_at	14.6	47.4	1.70	3.24	AAA-AAA	0.00	999	1.000	1.000	0.1863
217634_at	38.3	11.8	-1.70	-3.24	AAA-AAA	0.00	999	1.000	1.000	0.1111
230631_s_at	10.4	3.2	-1.70	-3.24	AAA-AAA	0.00	999	1.000	1.000	0.0942
1562342_at	11.1	3.4	-1.70	-3.24	AAA-AAA	0.00	999	1.000	1.000	0.3100
1570163_at	6.6	21.4	1.70	3.24	AAA-AAA	0.00	999	1.000	1.000	0.1692
1558651_at	19.4	6.0	-1.70	-3.24	AAA-AAA	0.00	999	1.000	1.000	0.2982
220294_at	5.0	1.6	-1.70	-3.24	AAA-AAA	0.00	999	1.000	1.000	0.2987
213814_s_at	39.4	12.2	-1.70	-3.24	AAA-AAA	0.00	999	1.000	1.000	0.2489
228067_at	8.7	28.2	1.70	3.24	AAA-AAA	0.00	999	1.000	1.000	0.1596
205630_at	55.3	17.1	-1.70	-3.24	AAA-AAA	0.00	999	1.000	1.000	0.1741
1560404_a_at	12.6	3.9	-1.70	-3.24	AAA-AAA	0.00	999	1.000	1.000	0.2831
207082_at	24.0	7.4	-1.70	-3.24	AAA-AAA	0.00	999	1.000	1.000	0.1920
236015_at	21.4	6.6	-1.70	-3.24	AAA-AAA	0.00	999	1.000	1.000	0.0982
236432_at	36.2	11.2	-1.70	-3.24	AAA-AAA	0.00	999	1.000	1.000	0.0579
231386_at	22.4	6.9	-1.70	-3.24	AAA-AAA	0.00	999	1.000	1.000	0.3379
232246_at	61.7	19.1	-1.70	-3.24	MAA-AAA	0.00	999	1.000	1.000	0.0244
239832_at	13.9	4.3	-1.69	-3.24	AAA-AAA	0.00	999	1.000	1.000	0.2027
240884_at	8.4	27.1	1.69	3.24	AAA-AAA	0.00	999	1.000	1.000	0.2116
1556699_at	9.7	31.4	1.69	3.24	AAA-AAA	0.00	999	1.000	1.000	0.0713
216436_at	30.8	9.5	-1.69	-3.24	AAA-AAA	0.00	999	1.000	1.000	0.3539
232262_at	70.4	21.8	-1.69	-3.24	AAA-AAA	0.00	999	1.000	1.000	0.1224
1562083_at	13.7	4.2	-1.69	-3.23	AAA-AAA	0.00	999	1.000	1.000	0.0660

1561387_a_at	48.7	15.1	-1.69	-3.23	AAA-AAA	0.00	999	1.000	1.000	0.0732
1568722_at	39.6	12.3	-1.69	-3.23	AAA-AAA	0.00	999	1.000	1.000	0.0257
1556884_a_at	5.4	1.7	-1.69	-3.23	AAA-AAA	0.00	999	1.000	1.000	0.3006
221225_at	28.0	8.7	-1.69	-3.23	AAA-AAA	0.00	999	1.000	1.000	0.1583
210159_s_at	25.7	7.9	-1.69	-3.23	AAA-AAA	0.00	999	1.000	1.000	0.3478
236279_at	42.0	13.0	-1.69	-3.23	AAA-AAA	0.00	999	1.000	1.000	0.3909
239925_at	38.4	11.9	-1.69	-3.23	AAA-AAA	0.00	999	1.000	1.000	0.1644
241238_at	29.3	9.1	-1.69	-3.23	AAA-AAA	0.00	999	1.000	1.000	0.2070
243812_at	59.7	18.5	-1.69	-3.23	AAA-AAA	0.00	999	1.000	1.000	0.0517
1561403_at	92.8	28.7	-1.69	-3.23	AAA-AAA	0.00	999	1.000	1.000	0.1009
1558660_at	6.5	20.8	1.69	3.23	AAA-AAA	0.00	999	1.000	1.000	0.1732
207085_x_at	18.7	5.8	-1.69	-3.23	AAA-AAA	0.00	999	1.000	1.000	0.1732
217088_s_at	30.9	9.6	-1.69	-3.23	AAA-AAA	0.00	999	1.000	1.000	0.3586
236300_at	60.5	18.7	-1.69	-3.23	AAA-AAA	0.00	999	1.000	1.000	0.2453
239009_at	47.6	14.7	-1.69	-3.23	AAA-AAA	0.00	999	1.000	1.000	0.2410
206222_at	7.3	23.5	1.69	3.23	AAA-MAA	0.00	999	1.000	1.000	0.0731
240573_at	36.7	11.4	-1.69	-3.23	AMA-AAA	0.00	999	1.000	1.000	0.2757
220655_at	17.5	56.3	1.69	3.23	AAA-AAA	0.00	999	1.000	1.000	0.1393
230861_at	5.0	16.1	1.69	3.23	AAA-AAA	0.00	999	1.000	1.000	0.1822
216142_at	12.8	41.3	1.69	3.23	AAA-APA	0.00	999	1.000	1.000	0.0660
1553456_at	23.2	7.2	-1.69	-3.23	AAA-AAA	0.00	999	1.000	1.000	0.2960
244656_at	41.2	12.8	-1.69	-3.23	AAA-AAA	0.00	999	1.000	1.000	0.0967
230594_at	38.0	11.8	-1.69	-3.23	AAA-AAA	0.00	999	1.000	1.000	0.1789
205625_s_at	12.2	3.8	-1.69	-3.23	AAA-AAA	0.00	999	1.000	1.000	0.3520
238595_at	15.3	49.4	1.69	3.22	AAP-AAA	0.00	999	1.000	1.000	0.2983
1561153_at	30.8	9.6	-1.69	-3.23	AAA-AAA	0.00	999	1.000	1.000	0.3165
1564936_at	4.3	13.7	1.69	3.22	AAA-AAA	0.00	999	1.000	1.000	0.4318
211691_x_at	124.3	38.6	-1.69	-3.22	AAA-AAA	0.00	999	1.000	1.000	0.0763
236457_at	61.8	19.2	-1.69	-3.22	AAA-AAA	0.00	999	1.000	1.000	0.0530
237562_at	20.2	6.3	-1.69	-3.22	AAA-AAA	0.00	999	1.000	1.000	0.2931
228767_at	25.1	7.8	-1.69	-3.22	AAA-AAA	0.00	999	1.000	1.000	0.1994
232268_at	46.7	14.5	-1.69	-3.23	AAA-AAM	0.00	999	1.000	1.000	0.2392
240998_at	37.3	11.6	-1.69	-3.22	AAA-AAA	0.00	999	1.000	1.000	0.2425
243971_x_at	51.1	15.9	-1.69	-3.22	AAA-AAA	0.00	999	1.000	1.000	0.1993
1570561_at	35.0	10.8	-1.69	-3.22	AMA-AAA	0.00	999	1.000	1.000	0.2021
1555865_at	18.6	59.8	1.69	3.22	AAA-AAA	0.00	999	1.000	1.000	0.0663
225846_at	19.7	6.1	-1.69	-3.22	AAA-AAA	0.00	999	1.000	1.000	0.3749
234221_at	3.8	12.2	1.69	3.22	AAA-AAA	0.00	999	1.000	1.000	0.1292
239048_at	15.6	50.2	1.69	3.22	AAA-AAA	0.00	999	1.000	1.000	0.1333
237994_at	53.7	16.7	-1.69	-3.22	AAA-AAA	0.00	999	1.000	1.000	0.1508
231609_at	64.7	20.1	-1.69	-3.22	AAA-AAA	0.00	999	1.000	1.000	0.1216
241524_at	14.1	4.4	-1.69	-3.22	AAA-AAA	0.00	999	1.000	1.000	0.0020

1570291_at	20.2	6.3	-1.69	-3.22	AAA-AAA	0.00	999	1.000	1.000	0.1959
206937_at	29.0	9.0	-1.69	-3.22	AAA-AAA	0.00	999	1.000	1.000	0.0363
227429_at	30.4	9.4	-1.69	-3.22	AAA-AAA	0.00	999	1.000	1.000	0.2105
211626_x_at	13.5	4.2	-1.69	-3.22	AAA-AAA	0.00	999	1.000	1.000	0.1075
212278_x_at	10.4	33.6	1.69	3.22	AAA-AAA	0.00	999	1.000	1.000	0.0860
240150_at	17.5	5.5	-1.69	-3.22	AAA-AAA	0.00	999	1.000	1.000	0.2462
1561558_at	9.1	2.8	-1.69	-3.22	AAA-AAA	0.00	999	1.000	1.000	0.1111
1561508_at	26.7	8.3	-1.69	-3.22	AAA-AAA	0.00	999	1.000	1.000	0.1453
200953_s_at	9.5	30.6	1.69	3.22	AAA-AAA	0.00	999	1.000	1.000	0.0762
217146_at	18.2	5.7	-1.69	-3.22	AAA-AAA	0.00	999	1.000	1.000	0.0026
216812_at	19.3	6.0	-1.69	-3.22	AAA-AAA	0.00	999	1.000	1.000	0.1357
216416_at	46.3	14.4	-1.69	-3.22	AAA-AAA	0.00	999	1.000	1.000	0.0812
239531_at	80.5	25.0	-1.69	-3.22	AAA-AAA	0.00	999	1.000	1.000	0.2531
237191_x_at	9.7	3.0	-1.69	-3.22	AAA-AAA	0.00	999	1.000	1.000	0.1825
241509_at	9.2	29.7	1.69	3.22	AAA-AAA	0.00	999	1.000	1.000	0.0984
1557763_at	18.6	5.8	-1.69	-3.22	AAA-AAA	0.00	999	1.000	1.000	0.2186
1559142_at	37.5	11.7	-1.69	-3.22	AAA-AAA	0.00	999	1.000	1.000	0.2299
237458_at	24.1	7.5	-1.69	-3.22	AAA-AAA	0.00	999	1.000	1.000	0.2428
1552952_at	14.4	46.4	1.68	3.21	AAA-MPA	0.00	999	1.000	1.000	0.3030
1562847_at	21.1	6.6	-1.68	-3.21	AAA-AAA	0.00	999	1.000	1.000	0.3126
1568802_at	5.6	17.9	1.68	3.21	AAA-AAA	0.00	999	1.000	1.000	0.3125
1554733_at	4.2	1.3	-1.68	-3.21	AAA-AAA	0.00	999	1.000	1.000	0.0200
217581_at	16.6	5.2	-1.68	-3.21	AAA-AAA	0.00	999	1.000	1.000	0.1398
219090_at	23.6	7.3	-1.68	-3.21	AAA-AAA	0.00	999	1.000	1.000	0.0515
217847_s_at	31.3	9.7	-1.68	-3.21	AAA-AAA	0.00	999	1.000	1.000	0.1019
234184_at	34.2	10.6	-1.68	-3.21	AAA-AAA	0.00	999	1.000	1.000	0.1556
240012_at	49.5	15.4	-1.68	-3.21	AAA-AAA	0.00	999	1.000	1.000	0.0123
216611_s_at	28.0	8.7	-1.68	-3.21	AAA-AAA	0.00	999	1.000	1.000	0.0655
238313_at	13.4	43.1	1.68	3.21	AAA-AAA	0.00	999	1.000	1.000	0.1801
240027_at	6.6	2.1	-1.68	-3.21	AAA-AAA	0.00	999	1.000	1.000	0.1544
241628_at	47.9	14.9	-1.68	-3.21	AAA-AAA	0.00	999	1.000	1.000	0.0805
244736_at	17.8	5.6	-1.68	-3.21	AAA-AAA	0.00	999	1.000	1.000	0.2571
1558672_at	3.1	10.1	1.68	3.21	AAA-AAA	0.00	999	1.000	1.000	0.0633
209983_s_at	8.4	2.6	-1.68	-3.21	AAA-AAA	0.00	999	1.000	1.000	0.0052
236158_at	46.8	14.6	-1.68	-3.21	AAA-AAA	0.00	999	1.000	1.000	0.3393
216659_at	29.7	9.3	-1.68	-3.20	AAA-AAA	0.00	999	1.000	1.000	0.2307
236374_at	24.0	7.5	-1.68	-3.21	AAA-AAA	0.00	999	1.000	1.000	0.1513
204100_at	24.5	7.6	-1.68	-3.21	AAA-AAA	0.00	999	1.000	1.000	0.0414
1553970_s_at	5.0	16.0	1.68	3.20	AAA-AAA	0.00	999	1.000	1.000	0.1087
224388_s_at	16.0	51.1	1.68	3.20	AAA-AAA	0.00	999	1.000	1.000	0.0040
215768_at	24.9	7.8	-1.68	-3.20	AAA-AAA	0.00	999	1.000	1.000	0.2891
228218_at	10.7	3.4	-1.68	-3.20	AAA-AAA	0.00	999	1.000	1.000	0.0667

231326_s_at	14.0	4.4	-1.68	-3.20	AAA-AAA	0.00	999	1.000	1.000	0.2844
201330_at	3292.4	10533.9	1.68	3.20	APP-PPP	0.00	999	1.000	1.000	0.2944
240925_at	41.3	12.9	-1.68	-3.20	AAA-AAA	0.00	999	1.000	1.000	0.1331
1552337_s_at	42.0	13.1	-1.68	-3.20	AAA-AAA	0.00	999	1.000	1.000	0.0589
214204_at	10.2	3.2	-1.68	-3.20	AAA-AAA	0.00	999	1.000	1.000	0.3412
241186_at	8.7	2.7	-1.68	-3.20	AAA-AAA	0.00	999	1.000	1.000	0.2187
1559432_at	16.0	5.0	-1.68	-3.20	AAA-AAA	0.00	999	1.000	1.000	0.0446
206519_x_at	15.8	4.9	-1.68	-3.20	AAA-AAA	0.00	999	1.000	1.000	0.3355
223496_s_at	49.0	15.3	-1.68	-3.20	AAA-AAA	0.00	999	1.000	1.000	0.1932
233662_at	6.7	21.4	1.68	3.20	AAA-AAP	0.00	999	1.000	1.000	0.3066
1560063_a_at	6.7	2.1	-1.68	-3.20	AAA-AAA	0.00	999	1.000	1.000	0.1598
1553277_at	19.8	6.2	-1.68	-3.20	AAA-AAA	0.00	999	1.000	1.000	0.2813
1553238_a_at	124.6	39.0	-1.68	-3.20	AAA-AAA	0.00	999	1.000	1.000	0.0835
212258_s_at	44.1	13.8	-1.68	-3.20	AAA-AAA	0.00	999	1.000	1.000	0.0894
216626_at	53.9	16.9	-1.68	-3.20	AAA-AAA	0.00	999	1.000	1.000	0.0574
235871_at	14.7	4.6	-1.68	-3.20	AAA-AAA	0.00	999	1.000	1.000	0.2333
236567_at	54.1	16.9	-1.68	-3.20	AAA-AAA	0.00	999	1.000	1.000	0.1715
233942_at	17.1	5.3	-1.68	-3.20	AAA-AAA	0.00	999	1.000	1.000	0.2419
1569417_at	11.5	36.8	1.68	3.19	AAA-AAP	0.00	999	1.000	1.000	0.3037
234240_at	11.8	3.7	-1.68	-3.19	AAA-AAA	0.00	999	1.000	1.000	0.2243
244039_x_at	16.7	5.2	-1.67	-3.19	AAA-AAA	0.00	999	1.000	1.000	0.1763
1564160_at	3.3	10.4	1.67	3.19	AAA-AAA	0.00	999	1.000	1.000	0.2381
1566108_at	45.0	14.1	-1.67	-3.19	AAA-AAA	0.00	999	1.000	1.000	0.0756
1553952_at	125.7	39.4	-1.67	-3.19	AAA-AAA	0.00	999	1.000	1.000	0.1250
1556914_at	16.4	5.1	-1.67	-3.19	AAA-AAA	0.00	999	1.000	1.000	0.0743
207557_s_at	30.2	9.5	-1.67	-3.19	AAA-AAA	0.00	999	1.000	1.000	0.2934
238079_at	43.8	13.7	-1.67	-3.19	AAA-AAA	0.00	999	1.000	1.000	0.2099
231351_at	19.5	6.1	-1.67	-3.19	AAA-AAA	0.00	999	1.000	1.000	0.2066
231158_x_at	5.9	18.8	1.67	3.19	AAA-AAA	0.00	999	1.000	1.000	0.1512
244796_at	8.8	2.8	-1.67	-3.19	AAA-AAA	0.00	999	1.000	1.000	0.1373
236045_x_at	29.8	9.4	-1.67	-3.19	AAA-AAA	0.00	999	1.000	1.000	0.2682
232481_s_at	29.4	9.2	-1.67	-3.19	AAA-AAA	0.00	999	1.000	1.000	0.2732
231528_at	10.1	32.2	1.67	3.19	AAA-AAA	0.00	999	1.000	1.000	0.0902
1568899_at	8.8	28.0	1.67	3.19	AAA-PAA	0.00	999	1.000	1.000	0.3023
1553801_a_at	149.1	475.0	1.67	3.19	PPP-PPP	0.00	999	1.000	1.000	0.0822
243290_at	50.4	15.8	-1.67	-3.19	AAA-AAA	0.00	999	1.000	1.000	0.2511
243101_x_at	54.7	17.2	-1.67	-3.19	AAA-AMA	0.00	999	1.000	1.000	0.1725
207195_at	31.3	9.8	-1.67	-3.19	AAA-AAA	0.00	999	1.000	1.000	0.0969
215671_at	6.4	20.5	1.67	3.19	AAA-AAA	0.00	999	1.000	1.000	0.0917
236086_at	26.0	8.2	-1.67	-3.19	AAA-AAA	0.00	999	1.000	1.000	0.3267
1554629_at	5.0	15.8	1.67	3.19	AAA-AAA	0.00	999	1.000	1.000	0.0444
1555799_at	10.9	3.4	-1.67	-3.19	AAA-AAA	0.00	999	1.000	1.000	0.1361

217179_x_at	26.3	8.3	-1.67	-3.19	AAA-AAA	0.00	999	1.000	1.000	0.0911
234137_s_at	48.4	15.2	-1.67	-3.18	AAA-AAA	0.00	999	1.000	1.000	0.0594
1569582_at	14.4	45.9	1.67	3.18	AAA-AAA	0.00	999	1.000	1.000	0.1152
1566862_at	2.4	7.7	1.67	3.18	AAA-AAA	0.00	999	1.000	1.000	0.3370
1566475_at	73.5	23.1	-1.67	-3.18	AAA-AAA	0.00	999	1.000	1.000	0.1918
1553931_at	53.8	16.9	-1.67	-3.18	AAA-AAA	0.00	999	1.000	1.000	0.2677
214228_x_at	9.1	29.0	1.67	3.18	AAA-AAA	0.00	999	1.000	1.000	0.2619
1553202_at	4.0	12.7	1.67	3.18	AAA-AAA	0.00	999	1.000	1.000	0.2632
208148_at	18.2	5.7	-1.67	-3.18	AAA-AAA	0.00	999	1.000	1.000	0.2843
240392_at	24.3	7.6	-1.67	-3.18	AAA-AAA	0.00	999	1.000	1.000	0.2469
221062_at	10.0	31.6	1.67	3.18	AAA-AAA	0.00	999	1.000	1.000	0.0622
220348_at	38.6	12.1	-1.67	-3.18	AAA-AAA	0.00	999	1.000	1.000	0.2632
241705_at	11.8	3.7	-1.67	-3.18	AAA-AAA	0.00	999	1.000	1.000	0.3783
1561540_at	32.0	10.1	-1.67	-3.18	MAA-AAA	0.00	999	1.000	1.000	0.0929
1567696_at	8.0	2.5	-1.67	-3.18	AAA-AAA	0.00	999	1.000	1.000	0.2232
214416_at	11.6	3.7	-1.67	-3.18	AAA-AAA	0.00	999	1.000	1.000	0.2640
206538_at	20.6	65.3	1.67	3.17	AAA-AAP	0.00	999	1.000	1.000	0.1361
1561033_at	4.5	14.3	1.67	3.17	AAA-AAA	0.00	999	1.000	1.000	0.4088
1566632_at	8.9	28.3	1.67	3.17	AAA-AAA	0.00	999	1.000	1.000	0.1698
1560028_at	15.1	48.0	1.67	3.17	AAA-AAA	0.00	999	1.000	1.000	0.2179
1553123_at	71.8	22.6	-1.67	-3.17	AAA-AAA	0.00	999	1.000	1.000	0.0968
1557713_at	14.0	4.4	-1.67	-3.17	AAA-AAA	0.00	999	1.000	1.000	0.1036
1556872_s_at	13.5	4.3	-1.67	-3.17	AAA-AAA	0.00	999	1.000	1.000	0.0632
213362_at	17.4	5.5	-1.67	-3.17	AAA-AAA	0.00	999	1.000	1.000	0.3670
210640_s_at	85.1	26.8	-1.67	-3.17	AAA-AAA	0.00	999	1.000	1.000	0.0897
217626_at	26.1	8.2	-1.67	-3.17	AAA-AAA	0.00	999	1.000	1.000	0.3280
216414_at	2.9	9.2	1.67	3.17	AAA-AAA	0.00	999	1.000	1.000	0.1103
231366_at	34.5	10.9	-1.67	-3.17	AAA-AAA	0.00	999	1.000	1.000	0.2522
231966_at	15.2	4.8	-1.67	-3.17	AAA-AAA	0.00	999	1.000	1.000	0.1704
228617_at	23.6	74.7	1.67	3.17	AAA-PPP	0.00	999	1.000	1.000	0.1797
241487_at	39.5	12.5	-1.67	-3.17	AAA-AAA	0.00	999	1.000	1.000	0.0062
1564591_a_at	29.0	9.1	-1.67	-3.17	AAA-AAA	0.00	999	1.000	1.000	0.0069
1557638_at	7.3	2.3	-1.67	-3.17	AAA-AAA	0.00	999	1.000	1.000	0.2780
207910_at	11.3	3.6	-1.67	-3.17	AAA-AAA	0.00	999	1.000	1.000	0.2735
224540_at	22.3	7.0	-1.67	-3.17	AAA-AAA	0.00	999	1.000	1.000	0.1503
211887_x_at	44.0	13.9	-1.67	-3.17	AAA-AAA	0.00	999	1.000	1.000	0.0212
234521_at	101.1	31.9	-1.67	-3.17	AAA-AAA	0.00	999	1.000	1.000	0.0755
243214_at	31.9	10.1	-1.66	-3.17	AAA-AAA	0.00	999	1.000	1.000	0.1967
1562947_x_at	42.2	13.3	-1.66	-3.17	AAA-AAA	0.00	999	1.000	1.000	0.0447
244616_x_at	263.7	83.2	-1.66	-3.17	AAA-AAA	0.00	999	1.000	1.000	0.1464
1556171_a_at	17.8	5.6	-1.66	-3.17	AAA-AAA	0.00	999	1.000	1.000	0.3486
240358_at	26.9	8.5	-1.66	-3.17	AAA-AAA	0.00	999	1.000	1.000	0.2041

1560714_at	13.5	4.3	-1.66	-3.17	AAA-AAA	0.00	999	1.000	1.000	0.3138
206915_at	2.8	8.8	1.66	3.17	AAA-AAA	0.00	999	1.000	1.000	0.0696
213415_at	4.0	12.8	1.66	3.17	AAA-AAA	0.00	999	1.000	1.000	0.0872
214655_at	16.3	5.1	-1.66	-3.17	AAA-AAA	0.00	999	1.000	1.000	0.2151
236436_at	7.5	23.6	1.66	3.17	AAA-AAA	0.00	999	1.000	1.000	0.3370
233897_at	17.9	5.7	-1.66	-3.17	AAA-AAA	0.00	999	1.000	1.000	0.0712
230603_at	12.6	39.9	1.66	3.17	AAA-AAA	0.00	999	1.000	1.000	0.0102
233256_at	15.1	47.7	1.66	3.17	AAA-AAA	0.00	999	1.000	1.000	0.1501
1567248_at	8.4	2.7	-1.66	-3.17	AAA-AAA	0.00	999	1.000	1.000	0.1662
220361_at	9.1	28.7	1.66	3.17	AAA-AAA	0.00	999	1.000	1.000	0.2356
206709_x_at	31.6	99.8	1.66	3.16	AAA-AAA	0.00	999	1.000	1.000	0.1411
234252_at	48.7	15.4	-1.66	-3.16	AAA-AAA	0.00	999	1.000	1.000	0.2956
243197_at	4.5	14.3	1.66	3.16	AAA-AAA	0.00	999	1.000	1.000	0.3009
1562669_at	8.4	2.7	-1.66	-3.16	AAA-AAA	0.00	999	1.000	1.000	0.0114
1562323_at	38.8	12.3	-1.66	-3.16	AAA-AAA	0.00	999	1.000	1.000	0.2962
244543_s_at	55.9	17.7	-1.66	-3.16	AAA-AAA	0.00	999	1.000	1.000	0.2318
224795_x_at	83.2	26.3	-1.66	-3.16	AAA-AAA	0.00	999	1.000	1.000	0.1021
207119_at	20.7	6.5	-1.66	-3.16	AAA-AAA	0.00	999	1.000	1.000	0.2261
239446_x_at	14.8	46.7	1.66	3.16	AAA-AAA	0.00	999	1.000	1.000	0.1383
204399_s_at	144.8	45.8	-1.66	-3.16	AAA-AAA	0.00	999	1.000	1.000	0.1536
240741_x_at	5.1	16.0	1.66	3.16	AAA-AAA	0.00	999	1.000	1.000	0.1967
207461_at	45.4	14.4	-1.66	-3.16	AAA-AAA	0.00	999	1.000	1.000	0.0550
236433_at	49.8	15.7	-1.66	-3.16	AAA-AAM	0.00	999	1.000	1.000	0.1668
223818_s_at	80.2	253.3	1.66	3.16	AAA-AAP	0.00	999	1.000	1.000	0.1235
214451_at	7.2	22.6	1.66	3.16	AAA-AAA	0.00	999	1.000	1.000	0.1456
218454_at	34.3	10.8	-1.66	-3.16	AAA-AAA	0.00	999	1.000	1.000	0.2721
239455_at	10.2	3.2	-1.66	-3.16	AAA-AAA	0.00	999	1.000	1.000	0.0442
231668_x_at	15.5	4.9	-1.66	-3.16	AAA-AAA	0.00	999	1.000	1.000	0.0897
1557646_at	15.3	4.9	-1.66	-3.16	AAA-AAA	0.00	999	1.000	1.000	0.3191
217259_at	9.0	28.4	1.66	3.16	AAA-AAA	0.00	999	1.000	1.000	0.2129
232361_s_at	6.9	2.2	-1.66	-3.16	AAA-AAA	0.00	999	1.000	1.000	0.0165
202755_s_at	44.7	140.9	1.66	3.15	AAP-APP	0.00	999	1.000	1.000	0.3055
243975_at	22.6	7.2	-1.66	-3.15	AAA-AAA	0.00	999	1.000	1.000	0.0579
240253_at	18.3	5.8	-1.66	-3.15	AAA-AAA	0.00	999	1.000	1.000	0.2755
1563389_at	9.4	29.6	1.66	3.15	AAA-AAA	0.00	999	1.000	1.000	0.3039
1569270_at	32.5	10.3	-1.66	-3.15	AAA-AAA	0.00	999	1.000	1.000	0.0877
1557591_at	3.8	1.2	-1.66	-3.15	AAA-AAA	0.00	999	1.000	1.000	0.0163
235157_at	35.2	11.1	-1.66	-3.15	AAA-AAA	0.00	999	1.000	1.000	0.3378
224925_at	29.4	92.7	1.66	3.15	AAA-PAA	0.00	999	1.000	1.000	0.1967
1561757_a_at	12.8	40.2	1.66	3.15	AAA-AAA	0.00	999	1.000	1.000	0.1497
1570364_at	45.5	14.4	-1.66	-3.15	AAA-AAA	0.00	999	1.000	1.000	0.0915
1566728_at	21.1	6.7	-1.66	-3.15	AAA-AAA	0.00	999	1.000	1.000	0.1801

1552851_at	14.8	4.7	-1.66	-3.15	AAA-AAA	0.00	999	1.000	1.000	0.1968
220793_at	23.8	7.6	-1.66	-3.15	AAA-AAA	0.00	999	1.000	1.000	0.2577
218675_at	54.7	17.3	-1.66	-3.15	AAA-AAA	0.00	999	1.000	1.000	0.1076
236137_at	19.7	62.0	1.66	3.15	AAA-AAA	0.00	999	1.000	1.000	0.0390
1566323_at	11.6	3.7	-1.66	-3.15	AAA-AAA	0.00	999	1.000	1.000	0.3344
214366_s_at	23.2	7.4	-1.66	-3.15	AAA-AAA	0.00	999	1.000	1.000	0.2666
238687_x_at	36.4	11.6	-1.66	-3.15	AAA-AAA	0.00	999	1.000	1.000	0.1259
230389_at	14.1	44.2	1.65	3.15	AAA-AAP	0.00	999	1.000	1.000	0.2825
240057_at	13.4	4.3	-1.65	-3.15	AAA-AAA	0.00	999	1.000	1.000	0.1455
242575_at	24.7	7.9	-1.65	-3.15	AAA-AAA	0.00	999	1.000	1.000	0.3218
1564244_a_at	23.7	7.5	-1.65	-3.15	AAA-AAA	0.00	999	1.000	1.000	0.1864
214341_at	34.5	11.0	-1.65	-3.15	AAA-AAA	0.00	999	1.000	1.000	0.3022
211495_x_at	19.6	61.7	1.65	3.15	AAA-AAA	0.00	999	1.000	1.000	0.2399
237876_at	23.8	7.6	-1.65	-3.15	AAA-AAA	0.00	999	1.000	1.000	0.4208
203070_at	26.9	8.6	-1.65	-3.15	AAA-AAA	0.00	999	1.000	1.000	0.0335
241071_at	65.9	21.0	-1.65	-3.15	AAA-AAA	0.00	999	1.000	1.000	0.0455
1561550_at	40.8	13.0	-1.65	-3.14	AAA-AAA	0.00	999	1.000	1.000	0.1879
1564242_at	19.4	6.2	-1.65	-3.15	AAA-AAA	0.00	999	1.000	1.000	0.1105
1556327_a_at	17.8	5.7	-1.65	-3.14	AAA-AAA	0.00	999	1.000	1.000	0.1583
239374_at	9.5	3.0	-1.65	-3.14	AAA-AAA	0.00	999	1.000	1.000	0.2668
237967_at	6.2	2.0	-1.65	-3.15	AAA-AAA	0.00	999	1.000	1.000	0.3562
240366_at	8.0	25.0	1.65	3.14	AAA-AAA	0.00	999	1.000	1.000	0.3294
1560903_at	40.9	13.0	-1.65	-3.14	AAA-AAA	0.00	999	1.000	1.000	0.2417
1565026_a_at	5.0	1.6	-1.65	-3.14	AAA-AAA	0.00	999	1.000	1.000	0.3370
1557297_at	16.5	5.3	-1.65	-3.14	AAA-AAA	0.00	999	1.000	1.000	0.0460
220879_at	28.2	9.0	-1.65	-3.14	AAA-AAA	0.00	999	1.000	1.000	0.0008
204046_at	46.0	14.6	-1.65	-3.14	AAA-AAA	0.00	999	1.000	1.000	0.3510
237422_at	8.3	2.6	-1.65	-3.14	AAA-AAA	0.00	999	1.000	1.000	0.2903
234825_at	16.6	52.1	1.65	3.14	AAA-PAA	0.00	999	1.000	1.000	0.2594
1564383_s_at	15.6	5.0	-1.65	-3.14	AAA-AAA	0.00	999	1.000	1.000	0.2232
1558745_at	28.0	8.9	-1.65	-3.14	AAA-AAA	0.00	999	1.000	1.000	0.3389
244037_at	87.7	28.0	-1.65	-3.14	AAA-AAA	0.00	999	1.000	1.000	0.2683
1567706_at	50.2	16.0	-1.65	-3.14	AAA-AAA	0.00	999	1.000	1.000	0.1949
1557608_a_at	36.5	11.6	-1.65	-3.14	AAA-AAA	0.00	999	1.000	1.000	0.3425
210398_x_at	8.2	25.6	1.65	3.14	AAA-AAA	0.00	999	1.000	1.000	0.0960
234879_at	42.0	13.4	-1.65	-3.14	AAA-AAA	0.00	999	1.000	1.000	0.1983
242216_at	14.0	4.5	-1.65	-3.14	AAA-AAA	0.00	999	1.000	1.000	0.4404
1569434_at	64.6	20.6	-1.65	-3.14	AAA-AAA	0.00	999	1.000	1.000	0.1374
1566771_at	22.6	7.2	-1.65	-3.14	AAA-AAA	0.00	999	1.000	1.000	0.3495
1556024_at	36.5	11.6	-1.65	-3.14	AAA-AAA	0.00	999	1.000	1.000	0.1215
236469_at	3.9	12.1	1.65	3.14	AAA-AAA	0.00	999	1.000	1.000	0.3483
234586_at	80.8	25.8	-1.65	-3.14	AAA-AAA	0.00	999	1.000	1.000	0.2777

231082_at	26.2	8.4	-1.65	-3.14	AAA-AAA	0.00	999	1.000	1.000	0.2722
201902_s_at	52.7	165.3	1.65	3.13	AAA-APP	0.00	999	1.000	1.000	0.2168
235393_at	16.8	52.8	1.65	3.13	AAA-AAP	0.00	999	1.000	1.000	0.1037
213827_at	39.3	123.1	1.65	3.13	AAA-AAA	0.00	999	1.000	1.000	0.1564
215513_at	79.4	25.4	-1.65	-3.13	AAA-AAA	0.00	999	1.000	1.000	0.1501
236928_at	24.1	7.7	-1.65	-3.14	AAA-AAA	0.00	999	1.000	1.000	0.1778
205185_at	39.5	12.6	-1.65	-3.14	AAA-AAA	0.00	999	1.000	1.000	0.0682
231513_at	21.1	6.7	-1.65	-3.13	AAA-AAA	0.00	999	1.000	1.000	0.3337
241260_at	26.2	8.4	-1.65	-3.13	AAA-AAA	0.00	999	1.000	1.000	0.0105
1562226_at	11.0	3.5	-1.65	-3.13	AAA-AAA	0.00	999	1.000	1.000	0.3351
1560864_at	16.9	5.4	-1.65	-3.13	AAA-AAA	0.00	999	1.000	1.000	0.2958
244455_at	20.7	6.6	-1.65	-3.13	AAA-AAA	0.00	999	1.000	1.000	0.4081
37201_at	48.2	15.4	-1.65	-3.13	AAA-AAA	0.00	999	1.000	1.000	0.2380
239509_at	12.5	4.0	-1.65	-3.13	AAA-AAA	0.00	999	1.000	1.000	0.2647
1562895_at	27.2	8.7	-1.65	-3.13	AAA-AAA	0.00	999	1.000	1.000	0.2132
1555043_at	19.4	6.2	-1.65	-3.13	AAA-AAA	0.00	999	1.000	1.000	0.3076
1556929_at	40.0	12.8	-1.65	-3.13	AMA-AAA	0.00	999	1.000	1.000	0.2970
207584_at	13.6	42.7	1.65	3.13	AAA-AAA	0.00	999	1.000	1.000	0.3076
209200_at	11.4	35.6	1.65	3.13	AAA-AAA	0.00	999	1.000	1.000	0.0505
235981_at	50.5	16.2	-1.65	-3.13	AAA-AAA	0.00	999	1.000	1.000	0.0807
229045_at	5.2	16.4	1.65	3.13	AAA-AAA	0.00	999	1.000	1.000	0.1196
1560228_at	17.8	5.7	-1.65	-3.13	AAA-AAA	0.00	999	1.000	1.000	0.2239
226167_at	36.6	11.7	-1.65	-3.13	AAA-AAA	0.00	999	1.000	1.000	0.2284
224169_at	14.1	4.5	-1.65	-3.13	AAA-AAA	0.00	999	1.000	1.000	0.1992
211333_s_at	68.3	21.9	-1.65	-3.13	AAA-AAA	0.00	999	1.000	1.000	0.0144
211191_at	17.1	5.5	-1.65	-3.13	AAA-AAA	0.00	999	1.000	1.000	0.1123
228994_at	29.2	9.4	-1.65	-3.13	AAA-AAA	0.00	999	1.000	1.000	0.0912
240894_at	20.5	6.6	-1.64	-3.13	AAA-AAA	0.00	999	1.000	1.000	0.1283
242985_x_at	20.6	6.6	-1.64	-3.13	AAA-AAA	0.00	999	1.000	1.000	0.2587
1561864_at	12.5	4.0	-1.64	-3.13	AAA-AAA	0.00	999	1.000	1.000	0.2225
1570644_at	33.4	10.7	-1.64	-3.13	AAA-AAA	0.00	999	1.000	1.000	0.1448
1554306_at	9.4	29.5	1.64	3.13	AAA-AAA	0.00	999	1.000	1.000	0.1466
1557161_at	32.5	10.4	-1.64	-3.13	AAA-AAA	0.00	999	1.000	1.000	0.3320
219243_at	13.7	4.4	-1.64	-3.12	AAA-AAA	0.00	999	1.000	1.000	0.2774
215516_at	16.6	5.3	-1.64	-3.13	AAA-AAA	0.00	999	1.000	1.000	0.2865
236203_at	11.9	3.8	-1.64	-3.13	AAA-AAA	0.00	999	1.000	1.000	0.2733
237335_at	37.1	11.9	-1.64	-3.13	AAA-AAA	0.00	999	1.000	1.000	0.3027
231376_at	22.1	7.1	-1.64	-3.13	AAA-AAA	0.00	999	1.000	1.000	0.2957
231408_at	106.0	33.9	-1.64	-3.12	AAA-AAA	0.00	999	1.000	1.000	0.0444
232195_at	32.8	10.5	-1.64	-3.13	AAA-AAA	0.00	999	1.000	1.000	0.2070
241545_x_at	14.3	4.6	-1.64	-3.12	AAM-AAA	0.00	999	1.000	1.000	0.3390
1562048_at	66.5	21.3	-1.64	-3.12	AAM-AAA	0.00	999	1.000	1.000	0.1628

207933_at	15.5	5.0	-1.64	-3.12	AAA-AAA	0.00	999	1.000	1.000	0.2007
205400_at	21.4	6.9	-1.64	-3.12	AAA-AAA	0.00	999	1.000	1.000	0.0005
1561492_at	22.4	7.2	-1.64	-3.12	AAA-AAA	0.00	999	1.000	1.000	0.2522
1569729_a_at	2.0	6.4	1.64	3.12	AAA-AAA	0.00	999	1.000	1.000	0.1866
221524_s_at	16.9	52.8	1.64	3.12	AAA-AAA	0.00	999	1.000	1.000	0.2377
217067_s_at	10.9	3.5	-1.64	-3.12	AAA-AAA	0.00	999	1.000	1.000	0.2312
219236_at	28.1	9.0	-1.64	-3.12	AAA-AAA	0.00	999	1.000	1.000	0.1871
230576_at	45.1	14.5	-1.64	-3.12	AAA-AAA	0.00	999	1.000	1.000	0.2597
212069_s_at	357.2	1114.2	1.64	3.12	APP-PPP	0.00	999	1.000	1.000	0.1424
1563610_at	23.1	7.4	-1.64	-3.12	AAA-AAA	0.00	999	1.000	1.000	0.3158
1563690_at	9.4	29.3	1.64	3.12	AAA-AAA	0.00	999	1.000	1.000	0.2089
1563224_at	73.9	23.7	-1.64	-3.12	AAA-AAA	0.00	999	1.000	1.000	0.0791
1553851_at	25.2	8.1	-1.64	-3.12	AAA-AAA	0.00	999	1.000	1.000	0.1507
223435_s_at	18.4	5.9	-1.64	-3.12	AAA-AAA	0.00	999	1.000	1.000	0.2379
214565_s_at	5.1	15.8	1.64	3.12	AAA-AAA	0.00	999	1.000	1.000	0.0628
210699_at	34.2	11.0	-1.64	-3.12	AAA-AAA	0.00	999	1.000	1.000	0.2364
236184_at	13.0	4.2	-1.64	-3.12	AAA-AAA	0.00	999	1.000	1.000	0.1059
239935_at	19.4	6.2	-1.64	-3.12	AAA-AAA	0.00	999	1.000	1.000	0.3266
1562899_at	80.1	25.7	-1.64	-3.12	AAA-AAA	0.00	999	1.000	1.000	0.1022
1564656_at	13.3	4.3	-1.64	-3.12	AAA-AAA	0.00	999	1.000	1.000	0.4218
1559144_x_at	47.6	15.3	-1.64	-3.12	AAA-AAA	0.00	999	1.000	1.000	0.2672
208005_at	12.4	38.5	1.64	3.12	AAA-AAA	0.00	999	1.000	1.000	0.0088
237934_at	42.7	13.7	-1.64	-3.12	MAA-AAA	0.00	999	1.000	1.000	0.2635
1559421_at	76.3	24.5	-1.64	-3.11	AAA-AAA	0.00	999	1.000	1.000	0.1893
241571_at	10.8	3.5	-1.64	-3.11	AAA-AAA	0.00	999	1.000	1.000	0.0191
1570635_at	18.1	5.8	-1.64	-3.11	AAA-AAA	0.00	999	1.000	1.000	0.4364
1552398_a_at	9.1	28.4	1.64	3.11	AAA-AAA	0.00	999	1.000	1.000	0.1774
233324_at	43.1	13.9	-1.64	-3.11	AAA-AAA	0.00	999	1.000	1.000	0.0744
214063_s_at	18.2	5.9	-1.64	-3.11	AAA-AAA	0.00	999	1.000	1.000	0.2555
216898_s_at	13.2	4.3	-1.64	-3.11	AAA-AAA	0.00	999	1.000	1.000	0.0924
207857_at	49.0	15.8	-1.64	-3.11	AAA-AAA	0.00	999	1.000	1.000	0.2479
215673_at	3.5	11.0	1.64	3.11	AAA-AAA	0.00	999	1.000	1.000	0.3515
205363_at	3.2	9.8	1.64	3.11	AAA-AAA	0.00	999	1.000	1.000	0.0912
240671_at	13.4	41.5	1.64	3.11	AAA-AAA	0.00	999	1.000	1.000	0.1227
1553311_at	5.1	1.6	-1.64	-3.11	AAA-AAA	0.00	999	1.000	1.000	0.0817
220648_at	55.1	17.7	-1.64	-3.11	AAA-AAA	0.00	999	1.000	1.000	0.1263
213870_at	34.5	11.1	-1.64	-3.11	AAA-AAA	0.00	999	1.000	1.000	0.1914
236517_at	5.2	16.2	1.64	3.11	AAA-AAA	0.00	999	1.000	1.000	0.3786
239523_at	23.5	7.6	-1.64	-3.11	AAA-AAA	0.00	999	1.000	1.000	0.2296
230335_at	34.2	11.0	-1.64	-3.11	AAA-AAA	0.00	999	1.000	1.000	0.1844
232443_at	19.3	6.2	-1.64	-3.11	AAA-AAA	0.00	999	1.000	1.000	0.1627
1561609_at	21.4	6.9	-1.63	-3.11	AAA-AAA	0.00	999	1.000	1.000	0.2483

1568660_a_at	19.1	6.1	-1.63	-3.10	AAA-AAA	0.00	999	1.000	1.000	0.2233
220660_at	17.0	5.5	-1.63	-3.10	AAA-AAA	0.00	999	1.000	1.000	0.1956
207550_at	8.1	2.6	-1.63	-3.10	AAA-AAA	0.00	999	1.000	1.000	0.1642
212768_s_at	3.5	1.1	-1.63	-3.11	AAA-AAA	0.00	999	1.000	1.000	0.0457
217315_s_at	30.3	9.8	-1.63	-3.10	AAA-AAA	0.00	999	1.000	1.000	0.2515
217572_at	23.7	7.6	-1.63	-3.10	AAA-AAA	0.00	999	1.000	1.000	0.1946
235116_at	10.5	32.5	1.63	3.10	AAA-AAA	0.00	999	1.000	1.000	0.2944
230299_s_at	49.6	16.0	-1.63	-3.10	AAA-AAA	0.00	999	1.000	1.000	0.0240
241621_at	9.1	28.2	1.63	3.10	AAA-AAA	0.00	999	1.000	1.000	0.2838
1562568_at	44.9	14.5	-1.63	-3.10	AAA-AAA	0.00	999	1.000	1.000	0.2649
1563162_at	14.7	4.7	-1.63	-3.10	AAA-AAA	0.00	999	1.000	1.000	0.3268
220211_at	13.7	4.4	-1.63	-3.10	AAA-AAA	0.00	999	1.000	1.000	0.1754
231404_at	20.5	6.6	-1.63	-3.10	AAA-AAA	0.00	999	1.000	1.000	0.1279
1561311_at	19.5	6.3	-1.63	-3.10	AAA-AAA	0.00	999	1.000	1.000	0.4735
1559326_at	15.8	49.0	1.63	3.10	AAA-AAA	0.00	999	1.000	1.000	0.2035
220847_x_at	5.3	16.4	1.63	3.10	AAA-AAA	0.00	999	1.000	1.000	0.0581
216462_at	2.2	6.8	1.63	3.10	AAA-AAA	0.00	999	1.000	1.000	0.1583
236238_at	11.9	36.7	1.63	3.10	AAA-AAA	0.00	999	1.000	1.000	0.3101
205638_at	2.3	7.0	1.63	3.10	AAA-AAA	0.00	999	1.000	1.000	0.3159
203681_at	17.0	5.5	-1.63	-3.10	AAA-AAA	0.00	999	1.000	1.000	0.0250
1563397_at	38.6	12.5	-1.63	-3.10	AAA-AAA	0.00	999	1.000	1.000	0.0784
1552508_at	16.3	5.3	-1.63	-3.10	AAA-AAA	0.00	999	1.000	1.000	0.2478
215700_x_at	26.3	8.5	-1.63	-3.10	AAA-AAA	0.00	999	1.000	1.000	0.0187
209616_s_at	11.4	35.4	1.63	3.10	AAA-AAA	0.00	999	1.000	1.000	0.2287
238524_at	38.2	12.3	-1.63	-3.10	AAA-AAA	0.00	999	1.000	1.000	0.3650
237631_at	11.1	34.5	1.63	3.10	AAA-AAA	0.00	999	1.000	1.000	0.2303
231279_at	12.0	3.9	-1.63	-3.10	AAA-AAA	0.00	999	1.000	1.000	0.3208
1558444_at	18.9	58.4	1.63	3.10	AAA-APP	0.00	999	1.000	1.000	0.3489
240154_at	20.0	6.5	-1.63	-3.10	AAA-AAA	0.00	999	1.000	1.000	0.3003
1560992_at	3.5	1.1	-1.63	-3.10	AAA-AAA	0.00	999	1.000	1.000	0.1859
1560979_a_at	20.2	6.5	-1.63	-3.10	AAA-AAA	0.00	999	1.000	1.000	0.0954
1561834_a_at	17.5	54.0	1.63	3.09	AAA-AAA	0.00	999	1.000	1.000	0.0004
1562821_a_at	43.2	14.0	-1.63	-3.10	AAA-AAA	0.00	999	1.000	1.000	0.2277
1559228_at	48.9	15.8	-1.63	-3.10	AAA-AAA	0.00	999	1.000	1.000	0.2112
225015_s_at	200.5	64.8	-1.63	-3.10	AAA-AAA	0.00	999	1.000	1.000	0.0952
207827_x_at	52.8	163.3	1.63	3.09	APA-APP	0.00	999	1.000	1.000	0.0808
243951_at	39.0	12.6	-1.63	-3.09	AAA-AAA	0.00	999	1.000	1.000	0.0513
242089_at	13.7	4.4	-1.63	-3.09	AAA-AAA	0.00	999	1.000	1.000	0.4203
241179_at	22.1	7.2	-1.63	-3.09	AAA-AAA	0.00	999	1.000	1.000	0.3216
1568625_at	11.8	36.4	1.63	3.09	AAA-AAA	0.00	999	1.000	1.000	0.2410
1553531_at	11.5	3.7	-1.63	-3.09	AAA-AAA	0.00	999	1.000	1.000	0.3797
1556057_s_at	33.8	10.9	-1.63	-3.09	AAA-AAA	0.00	999	1.000	1.000	0.1046

207284_s_at	7.9	24.5	1.63	3.09	AAA-AAA	0.00	999	1.000	1.000	0.0254
223967_at	28.8	9.3	-1.63	-3.09	AAA-AAA	0.00	999	1.000	1.000	0.0185
235355_at	4.3	13.2	1.63	3.09	AAA-AAA	0.00	999	1.000	1.000	0.2322
231665_at	13.9	4.5	-1.63	-3.09	AAA-AAA	0.00	999	1.000	1.000	0.3883
1560959_a_at	15.2	4.9	-1.63	-3.09	AAA-AAA	0.00	999	1.000	1.000	0.1181
1556778_at	11.1	3.6	-1.63	-3.09	AAA-AAA	0.00	999	1.000	1.000	0.3284
220298_s_at	8.6	26.6	1.63	3.09	AAA-AAA	0.00	999	1.000	1.000	0.1915
213030_s_at	60.8	19.7	-1.63	-3.09	AAA-AAA	0.00	999	1.000	1.000	0.1096
205742_at	84.0	27.2	-1.63	-3.09	AAA-AAA	0.00	999	1.000	1.000	0.0445
242234_at	39.2	12.7	-1.63	-3.09	AAA-AAA	0.00	999	1.000	1.000	0.3791
1562803_at	10.3	3.3	-1.63	-3.09	AAA-AAA	0.00	999	1.000	1.000	0.0781
1566082_at	12.1	3.9	-1.63	-3.09	AAA-AAA	0.00	999	1.000	1.000	0.1435
1554182_at	16.6	5.4	-1.63	-3.09	AAA-AAA	0.00	999	1.000	1.000	0.0949
208473_s_at	36.9	11.9	-1.63	-3.09	AAA-AAA	0.00	999	1.000	1.000	0.4746
220641_at	12.0	37.2	1.63	3.09	AAA-AAA	0.00	999	1.000	1.000	0.0010
231325_at	5.4	16.6	1.63	3.09	AAA-AAA	0.00	999	1.000	1.000	0.2564
230228_at	39.7	122.3	1.63	3.08	AAP-PPP	0.00	999	1.000	1.000	0.1614
237138_at	9.7	3.1	-1.63	-3.08	AAA-AAA	0.00	999	1.000	1.000	0.3359
1563048_at	37.3	12.1	-1.62	-3.08	AAA-AAA	0.00	999	1.000	1.000	0.2928
1568915_at	124.3	40.3	-1.62	-3.08	AAA-AAA	0.00	999	1.000	1.000	0.0403
AFFX-LysX-5_at	12.9	4.2	-1.62	-3.08	AAA-AAA	0.00	999	1.000	1.000	0.3198
244399_at	10.6	32.7	1.62	3.08	AAA-AAA	0.00	999	1.000	1.000	0.2962
233620_at	6.2	2.0	-1.62	-3.08	AAA-AAA	0.00	999	1.000	1.000	0.3226
242946_at	14.2	4.6	-1.62	-3.08	AAA-AAA	0.00	999	1.000	1.000	0.2605
1563560_at	11.4	35.1	1.62	3.08	AAA-AAA	0.00	999	1.000	1.000	0.0317
221404_at	42.3	13.7	-1.62	-3.08	AAA-AAA	0.00	999	1.000	1.000	0.0398
227398_s_at	16.3	5.3	-1.62	-3.08	AAA-AAA	0.00	999	1.000	1.000	0.3658
229195_at	89.1	28.9	-1.62	-3.08	AAA-AAA	0.00	999	1.000	1.000	0.0127
241656_at	31.1	10.1	-1.62	-3.08	AAA-AAA	0.00	999	1.000	1.000	0.1850
1555204_at	19.3	6.3	-1.62	-3.08	AAA-AAA	0.00	999	1.000	1.000	0.2523
207516_at	22.1	7.2	-1.62	-3.08	AAA-AAA	0.00	999	1.000	1.000	0.2457
230546_at	93.1	30.3	-1.62	-3.08	AAA-AAA	0.00	999	1.000	1.000	0.0086
228074_at	22.4	68.9	1.62	3.08	AAA-AAA	0.00	999	1.000	1.000	0.1016
231367_s_at	14.3	44.1	1.62	3.08	AAA-AAA	0.00	999	1.000	1.000	0.3221
1560928_at	26.3	8.5	-1.62	-3.08	AAA-AAA	0.00	999	1.000	1.000	0.1658
220736_at	10.7	3.5	-1.62	-3.08	AAA-AAA	0.00	999	1.000	1.000	0.1136
234527_at	23.6	7.7	-1.62	-3.08	AAA-AAA	0.00	999	1.000	1.000	0.1266
232651_at	19.1	6.2	-1.62	-3.08	AAA-AAA	0.00	999	1.000	1.000	0.0262
233602_at	10.2	3.3	-1.62	-3.08	AAA-AAA	0.00	999	1.000	1.000	0.3200
241162_at	7.3	22.4	1.62	3.07	AAA-AAA	0.00	999	1.000	1.000	0.0105
1555417_a_at	34.2	11.1	-1.62	-3.07	AAA-AAA	0.00	999	1.000	1.000	0.1570
207021_at	20.2	6.6	-1.62	-3.07	AAA-AAA	0.00	999	1.000	1.000	0.3698

216965_x_at	49.9	16.2	-1.62	-3.07	AAA-AAA	0.00	999	1.000	1.000	0.1980
205044_at	12.1	3.9	-1.62	-3.07	AAA-AAA	0.00	999	1.000	1.000	0.1799
238080_at	20.5	63.0	1.62	3.07	AAA-AAA	0.00	999	1.000	1.000	0.1607
203919_at	91.4	280.8	1.62	3.07	APP-PPP	0.00	999	1.000	1.000	0.1301
238617_at	30.3	92.9	1.62	3.07	AAA-AAP	0.00	999	1.000	1.000	0.0739
1562621_at	34.9	11.4	-1.62	-3.07	AMA-AAA	0.00	999	1.000	1.000	0.1686
1558607_at	79.2	25.8	-1.62	-3.07	AMA-AAA	0.00	999	1.000	1.000	0.0147
210086_at	15.0	4.9	-1.62	-3.07	AAA-AAA	0.00	999	1.000	1.000	0.1612
237102_at	8.5	2.8	-1.62	-3.07	AAA-AAA	0.00	999	1.000	1.000	0.1346
230794_at	5.3	16.2	1.62	3.07	AAA-AAA	0.00	999	1.000	1.000	0.0488
205143_at	36.8	12.0	-1.62	-3.07	AAA-AAA	0.00	999	1.000	1.000	0.1488
241336_at	17.3	53.0	1.62	3.07	AAA-PAA	0.00	999	1.000	1.000	0.2304
239484_at	38.4	117.7	1.62	3.07	APA-PPP	0.00	999	1.000	1.000	0.1873
244079_at	57.4	18.7	-1.62	-3.07	AAA-AAA	0.00	999	1.000	1.000	0.1093
1553036_at	9.4	3.1	-1.62	-3.07	AAA-AAA	0.00	999	1.000	1.000	0.1461
224195_at	49.6	16.2	-1.62	-3.07	AAA-AAA	0.00	999	1.000	1.000	0.1404
217343_at	14.4	4.7	-1.62	-3.07	AAA-AAA	0.00	999	1.000	1.000	0.3164
1567664_at	6.0	1.9	-1.62	-3.07	AAA-AAA	0.00	999	1.000	1.000	0.0373
221110_x_at	79.5	25.9	-1.62	-3.07	AAA-AAA	0.00	999	1.000	1.000	0.0414
220687_at	45.5	14.8	-1.62	-3.07	AAA-AAA	0.00	999	1.000	1.000	0.3051
210044_s_at	29.3	89.9	1.62	3.07	AAA-AAA	0.00	999	1.000	1.000	0.2323
208547_at	14.0	43.0	1.62	3.07	AAA-AAA	0.00	999	1.000	1.000	0.0321
234773_x_at	51.7	16.9	-1.62	-3.07	AAA-AAA	0.00	999	1.000	1.000	0.1935
230751_at	28.8	9.4	-1.62	-3.07	AAA-AAA	0.00	999	1.000	1.000	0.3469
242867_x_at	33.2	10.8	-1.62	-3.07	AAA-AAA	0.00	999	1.000	1.000	0.2496
1561511_at	6.1	2.0	-1.62	-3.07	AAA-AAA	0.00	999	1.000	1.000	0.3867
222967_at	2.9	8.8	1.62	3.07	AAA-AAA	0.00	999	1.000	1.000	0.1836
234842_at	41.0	13.4	-1.62	-3.07	AAA-AAA	0.00	999	1.000	1.000	0.2469
238537_at	16.9	5.5	-1.62	-3.07	AAA-AAA	0.00	999	1.000	1.000	0.4668
229059_at	27.6	9.0	-1.62	-3.07	AAA-AAA	0.00	999	1.000	1.000	0.3019
233310_at	31.9	10.4	-1.62	-3.07	AAA-AAA	0.00	999	1.000	1.000	0.0732
243516_at	14.4	4.7	-1.62	-3.06	AAA-AAA	0.00	999	1.000	1.000	0.3412
1566623_at	20.8	6.8	-1.62	-3.06	AAA-AAA	0.00	999	1.000	1.000	0.1782
217075_x_at	14.2	4.6	-1.62	-3.06	AAA-AAA	0.00	999	1.000	1.000	0.2298
235583_at	8.6	26.3	1.62	3.06	AAA-AAA	0.00	999	1.000	1.000	0.0597
231301_at	3.0	9.3	1.62	3.06	AAA-AAA	0.00	999	1.000	1.000	0.1586
223624_at	11.2	34.3	1.61	3.06	AAA-MPA	0.00	999	1.000	1.000	0.2806
243942_at	18.2	5.9	-1.61	-3.06	AAA-AAA	0.00	999	1.000	1.000	0.2458
1563900_at	13.6	4.5	-1.61	-3.06	AAA-AAA	0.00	999	1.000	1.000	0.2547
204701_s_at	32.9	100.8	1.61	3.06	AAA-AAA	0.00	999	1.000	1.000	0.2920
237178_at	44.3	14.5	-1.61	-3.06	AAA-AAA	0.00	999	1.000	1.000	0.0855
233210_at	6.6	20.2	1.61	3.06	AAA-AAA	0.00	999	1.000	1.000	0.2407

243533_x_at	22.4	7.3	-1.61	-3.06	AAA-AAA	0.00	999	1.000	1.000	0.3050
1554655_a_at	60.6	19.8	-1.61	-3.06	AAA-AAA	0.00	999	1.000	1.000	0.2666
1556590_s_at	13.2	4.3	-1.61	-3.06	AAA-AAA	0.00	999	1.000	1.000	0.1978
225996_at	27.6	9.0	-1.61	-3.06	AAA-AAA	0.00	999	1.000	1.000	0.2523
210274_at	4.0	12.3	1.61	3.06	AAA-AAA	0.00	999	1.000	1.000	0.0978
230989_s_at	37.8	12.4	-1.61	-3.06	AAA-AAA	0.00	999	1.000	1.000	0.1615
233462_at	5.3	16.2	1.61	3.06	AAA-AAA	0.00	999	1.000	1.000	0.1943
216004_s_at	34.5	105.6	1.61	3.06	APP-PPP	0.00	999	1.000	1.000	0.3333
241191_at	17.1	5.6	-1.61	-3.06	AAA-AAA	0.00	999	1.000	1.000	0.3509
244617_at	19.7	6.4	-1.61	-3.06	AAA-AAA	0.00	999	1.000	1.000	0.1005
217415_at	36.4	11.9	-1.61	-3.06	AAA-AAA	0.00	999	1.000	1.000	0.0837
204631_at	47.0	15.4	-1.61	-3.06	AAA-AAA	0.00	999	1.000	1.000	0.0862
230644_at	6.4	19.7	1.61	3.06	AAA-AAA	0.00	999	1.000	1.000	0.2735
232998_at	7.2	22.0	1.61	3.06	AAA-AAA	0.00	999	1.000	1.000	0.1885
242064_at	15.4	5.0	-1.61	-3.06	AAA-AAA	0.00	999	1.000	1.000	0.1841
1560810_at	22.6	7.4	-1.61	-3.06	AAA-AAA	0.00	999	1.000	1.000	0.2259
1559523_at	24.2	7.9	-1.61	-3.05	AAA-AAA	0.00	999	1.000	1.000	0.3318
210742_at	23.3	7.6	-1.61	-3.06	AAA-AAA	0.00	999	1.000	1.000	0.3837
1552261_at	62.5	20.5	-1.61	-3.05	AAA-AAA	0.00	999	1.000	1.000	0.1181
1557154_at	80.3	26.3	-1.61	-3.05	AAA-AAA	0.00	999	1.000	1.000	0.0614
234553_at	46.4	15.2	-1.61	-3.05	AAA-AAA	0.00	999	1.000	1.000	0.2617
229077_at	23.1	7.6	-1.61	-3.05	AAA-AAA	0.00	999	1.000	1.000	0.1800
232678_at	10.9	3.6	-1.61	-3.05	AAA-AAA	0.00	999	1.000	1.000	0.0120
240841_at	30.1	9.9	-1.61	-3.05	AAA-AAA	0.00	999	1.000	1.000	0.2633
1556983_a_at	39.4	12.9	-1.61	-3.05	AAA-AAA	0.00	999	1.000	1.000	0.3039
207093_s_at	30.2	9.9	-1.61	-3.05	AAA-AAA	0.00	999	1.000	1.000	0.3150
211147_s_at	16.0	5.2	-1.61	-3.05	AAA-AAA	0.00	999	1.000	1.000	0.0680
209664_x_at	9.6	29.4	1.61	3.05	AAA-AAA	0.00	999	1.000	1.000	0.2868
239500_at	13.7	4.5	-1.61	-3.05	AAA-AAA	0.00	999	1.000	1.000	0.1235
238932_at	32.5	99.2	1.61	3.05	AAA-APA	0.00	999	1.000	1.000	0.0766
1570307_s_at	22.3	7.3	-1.61	-3.05	AAA-AAA	0.00	999	1.000	1.000	0.1656
235779_at	63.7	20.9	-1.61	-3.05	AAA-AAA	0.00	999	1.000	1.000	0.0830
234364_at	67.7	22.2	-1.61	-3.05	AAA-AAA	0.00	999	1.000	1.000	0.1855
205403_at	10.3	3.4	-1.61	-3.05	AAA-AAA	0.00	999	1.000	1.000	0.1281
231389_at	11.4	3.7	-1.61	-3.05	AAA-AAA	0.00	999	1.000	1.000	0.2181
232039_at	46.3	15.2	-1.61	-3.05	AAA-AAA	0.00	999	1.000	1.000	0.0417
244299_at	35.9	11.8	-1.61	-3.05	AAA-AAA	0.00	999	1.000	1.000	0.0757
1564431_a_at	20.2	6.6	-1.61	-3.05	AAA-AAA	0.00	999	1.000	1.000	0.1598
1569321_at	108.6	35.7	-1.61	-3.05	AAM-AAA	0.00	999	1.000	1.000	0.1510
1569156_at	7.4	22.6	1.61	3.05	AAA-AAA	0.00	999	1.000	1.000	0.2934
1566775_at	86.5	28.4	-1.61	-3.05	AAA-AAA	0.00	999	1.000	1.000	0.0184
1555086_at	41.9	127.6	1.61	3.05	AAA-AAA	0.00	999	1.000	1.000	0.1396

1557296_at	12.1	4.0	-1.61	-3.05	AAA-AAA	0.00	999	1.000	1.000	0.2585
238338_at	52.3	17.2	-1.61	-3.05	AAA-AAA	0.00	999	1.000	1.000	0.0763
237973_at	10.1	3.3	-1.61	-3.05	AAA-AAA	0.00	999	1.000	1.000	0.0671
1561990_at	19.3	6.4	-1.61	-3.05	AAA-AAA	0.00	999	1.000	1.000	0.1904
226772_s_at	42.2	13.9	-1.61	-3.04	AAA-AAA	0.00	999	1.000	1.000	0.0210
235139_at	26.9	8.8	-1.61	-3.05	AAA-AAA	0.00	999	1.000	1.000	0.2522
234425_at	24.7	8.1	-1.61	-3.04	AAA-AAA	0.00	999	1.000	1.000	0.2483
230555_s_at	24.5	8.1	-1.61	-3.04	AAA-AAA	0.00	999	1.000	1.000	0.4033
1567333_at	23.5	7.7	-1.61	-3.04	AAA-AAA	0.00	999	1.000	1.000	0.2892
1555940_a_at	57.5	18.9	-1.61	-3.04	AAA-AAA	0.00	999	1.000	1.000	0.0416
204952_at	17.6	5.8	-1.61	-3.04	AAA-AAA	0.00	999	1.000	1.000	0.0959
206002_at	35.4	11.6	-1.61	-3.04	AAA-AAA	0.00	999	1.000	1.000	0.3140
240749_at	6.7	2.2	-1.60	-3.04	AAA-AAA	0.00	999	1.000	1.000	0.1241
241760_x_at	7.0	21.3	1.60	3.04	AAA-AAA	0.00	999	1.000	1.000	0.2070
1552960_at	16.6	5.5	-1.60	-3.04	AAA-AAA	0.00	999	1.000	1.000	0.0585
1556663_s_at	13.8	4.5	-1.60	-3.04	AAA-AAA	0.00	999	1.000	1.000	0.0887
221100_at	107.5	35.4	-1.60	-3.04	AAA-AAA	0.00	999	1.000	1.000	0.1557
207921_x_at	76.4	25.1	-1.60	-3.04	AAA-AAA	0.00	999	1.000	1.000	0.1582
222023_at	43.5	14.3	-1.60	-3.04	AAA-AAA	0.00	999	1.000	1.000	0.2042
220732_at	44.9	14.8	-1.60	-3.04	AAA-AAA	0.00	999	1.000	1.000	0.0289
207359_at	46.3	15.2	-1.60	-3.04	AAA-AAA	0.00	999	1.000	1.000	0.3122
223962_at	34.0	11.2	-1.60	-3.04	AAA-AAA	0.00	999	1.000	1.000	0.1287
206251_s_at	37.4	12.3	-1.60	-3.04	AAA-AAA	0.00	999	1.000	1.000	0.3878
241171_at	18.0	5.9	-1.60	-3.04	AAA-AAA	0.00	999	1.000	1.000	0.2714
1558947_at	5.8	17.7	1.60	3.04	AAA-AAA	0.00	999	1.000	1.000	0.2355
225165_at	18.6	56.4	1.60	3.04	AAA-AAA	0.00	999	1.000	1.000	0.3225
213319_s_at	22.7	69.0	1.60	3.04	AAA-AAA	0.00	999	1.000	1.000	0.1305
236448_at	11.9	3.9	-1.60	-3.04	AAA-AAA	0.00	999	1.000	1.000	0.2844
234066_at	60.5	19.9	-1.60	-3.04	AAA-AAA	0.00	999	1.000	1.000	0.2811
230381_at	77.1	25.4	-1.60	-3.04	AAA-AAA	0.00	999	1.000	1.000	0.1845
231155_at	14.4	4.7	-1.60	-3.04	AAA-AAA	0.00	999	1.000	1.000	0.1912
243757_at	39.0	12.9	-1.60	-3.04	AAA-AAA	0.00	999	1.000	1.000	0.2366
1570140_at	16.2	49.1	1.60	3.04	AAA-AAA	0.00	999	1.000	1.000	0.2879
221143_at	13.9	42.1	1.60	3.04	AAA-AAA	0.00	999	1.000	1.000	0.2947
214913_at	37.6	114.1	1.60	3.03	AAP-PPP	0.00	999	1.000	1.000	0.2354
1562770_at	24.4	74.1	1.60	3.03	AAA-AAA	0.00	999	1.000	1.000	0.2347
1554377_a_at	15.9	5.3	-1.60	-3.03	AAA-AAA	0.00	999	1.000	1.000	0.1461
214053_at	11.8	3.9	-1.60	-3.03	AAA-AAA	0.00	999	1.000	1.000	0.1244
204794_at	18.1	54.8	1.60	3.03	AAA-AAA	0.00	999	1.000	1.000	0.1995
242158_at	10.3	31.1	1.60	3.03	AAA-AAP	0.00	999	1.000	1.000	0.3051
215101_s_at	25.4	77.1	1.60	3.03	AAA-APA	0.00	999	1.000	1.000	0.2173
1569986_x_at	87.8	29.0	-1.60	-3.03	AAA-AAA	0.00	999	1.000	1.000	0.2304

1552590_a_at	29.2	9.7	-1.60	-3.03	AAA-AAA	0.00	999	1.000	1.000	0.1711
1558871_at	4.0	12.2	1.60	3.03	AAA-AAA	0.00	999	1.000	1.000	0.3537
221105_at	14.6	4.8	-1.60	-3.03	AAA-AAA	0.00	999	1.000	1.000	0.2101
223561_at	17.4	5.8	-1.60	-3.03	AAA-AAA	0.00	999	1.000	1.000	0.0047
236377_at	20.7	62.6	1.60	3.03	AAA-AMA	0.00	999	1.000	1.000	0.0462
236385_at	11.3	3.7	-1.60	-3.03	AAA-AAA	0.00	999	1.000	1.000	0.2063
232377_at	22.9	7.6	-1.60	-3.03	AAA-AAA	0.00	999	1.000	1.000	0.1412
1563076_x_at	47.7	15.8	-1.60	-3.03	AAA-AAA	0.00	999	1.000	1.000	0.0737
1560180_at	19.0	6.3	-1.60	-3.03	AAA-AAA	0.00	999	1.000	1.000	0.2284
1569218_at	8.2	2.7	-1.60	-3.03	AAA-AAA	0.00	999	1.000	1.000	0.3313
1567035_at	104.1	34.4	-1.60	-3.03	AAA-AAA	0.00	999	1.000	1.000	0.0424
210298_x_at	14.6	44.1	1.60	3.03	AAA-AAA	0.00	999	1.000	1.000	0.1405
233896_s_at	37.4	12.3	-1.60	-3.03	AAA-AAA	0.00	999	1.000	1.000	0.1541
238752_at	23.1	7.6	-1.60	-3.03	AAA-AAA	0.00	999	1.000	1.000	0.3491
237972_at	8.8	2.9	-1.60	-3.03	AAA-AAA	0.00	999	1.000	1.000	0.2893
232550_at	41.5	13.7	-1.60	-3.03	AAA-AAA	0.00	999	1.000	1.000	0.1042
241574_s_at	38.4	12.7	-1.60	-3.03	AAA-AAA	0.00	999	1.000	1.000	0.0296
1562396_at	12.3	4.1	-1.60	-3.03	AAA-AAA	0.00	999	1.000	1.000	0.3393
1557787_at	2.5	7.4	1.60	3.03	AAA-AAA	0.00	999	1.000	1.000	0.2030
237877_at	15.4	5.1	-1.60	-3.03	AAA-AAA	0.00	999	1.000	1.000	0.3631
242419_at	61.5	20.3	-1.60	-3.03	AAA-AAA	0.00	999	1.000	1.000	0.2636
242446_at	33.3	11.0	-1.60	-3.02	AAA-AAA	0.00	999	1.000	1.000	0.2268
214628_at	36.4	12.0	-1.60	-3.03	AAA-AAA	0.00	999	1.000	1.000	0.2615
235246_at	9.4	3.1	-1.60	-3.03	AAA-AAA	0.00	999	1.000	1.000	0.3396
234423_x_at	113.4	37.5	-1.60	-3.03	AAA-AAA	0.00	999	1.000	1.000	0.1877
204192_at	10.4	3.4	-1.60	-3.03	AAA-AAA	0.00	999	1.000	1.000	0.1447
231582_at	19.2	58.0	1.60	3.02	AAA-AAA	0.00	999	1.000	1.000	0.2036
1563887_at	22.0	7.3	-1.60	-3.02	AAA-AAA	0.00	999	1.000	1.000	0.0207
207139_at	6.1	18.3	1.60	3.02	AAA-AAA	0.00	999	1.000	1.000	0.1270
210610_at	10.3	31.0	1.60	3.02	AAA-AAA	0.00	999	1.000	1.000	0.1771
217434_at	32.1	10.6	-1.60	-3.02	AAA-AAA	0.00	999	1.000	1.000	0.2300
234200_at	21.1	7.0	-1.60	-3.02	AAA-AAA	0.00	999	1.000	1.000	0.0236
230816_at	13.1	39.5	1.60	3.02	AAA-AAA	0.00	999	1.000	1.000	0.0387
233025_at	17.9	5.9	-1.60	-3.02	AAA-AAA	0.00	999	1.000	1.000	0.2709
240116_at	26.3	8.7	-1.60	-3.02	AAA-AAA	0.00	999	1.000	1.000	0.3024
1553415_at	5.6	1.8	-1.60	-3.02	AAA-AAA	0.00	999	1.000	1.000	0.2958
1559298_a_at	25.0	8.3	-1.60	-3.02	AAA-AAA	0.00	999	1.000	1.000	0.3271
207587_at	25.5	8.4	-1.60	-3.02	AAA-AAA	0.00	999	1.000	1.000	0.2516
228064_at	108.6	36.0	-1.60	-3.02	AAA-AAA	0.00	999	1.000	1.000	0.2658
211347_at	37.0	111.7	1.59	3.02	AAA-AAP	0.00	999	1.000	1.000	0.1061
242084_at	45.7	15.1	-1.59	-3.02	AAA-AAA	0.00	999	1.000	1.000	0.3136
242601_at	3.4	10.1	1.59	3.02	AAA-AAA	0.00	999	1.000	1.000	0.1844

239991_at	28.7	86.6	1.59	3.02	AAA-AAA	0.00	999	1.000	1.000	0.1115
1568647_at	5.4	16.4	1.59	3.02	AAA-AAA	0.00	999	1.000	1.000	0.3352
207241_at	18.3	6.1	-1.59	-3.02	AAA-AAA	0.00	999	1.000	1.000	0.3361
207617_at	28.5	9.4	-1.59	-3.02	AAA-AAA	0.00	999	1.000	1.000	0.3085
212976_at	39.6	13.1	-1.59	-3.02	AMA-AAA	0.00	999	1.000	1.000	0.0259
237049_at	10.7	3.6	-1.59	-3.02	AAA-AAA	0.00	999	1.000	1.000	0.3088
229738_at	28.3	9.4	-1.59	-3.02	AAA-AAA	0.00	999	1.000	1.000	0.1762
241165_at	26.9	8.9	-1.59	-3.02	AAA-AAA	0.00	999	1.000	1.000	0.0949
1555363_s_at	26.7	8.9	-1.59	-3.02	AAA-AAA	0.00	999	1.000	1.000	0.2129
221333_at	16.1	5.3	-1.59	-3.02	AAA-AAA	0.00	999	1.000	1.000	0.0933
203862_s_at	11.9	4.0	-1.59	-3.02	AAA-AAA	0.00	999	1.000	1.000	0.1852
241153_at	16.9	5.6	-1.59	-3.01	AAA-AAA	0.00	999	1.000	1.000	0.1463
1562857_at	4.8	14.4	1.59	3.02	AAA-AAA	0.00	999	1.000	1.000	0.2641
214195_at	92.0	30.5	-1.59	-3.01	AAA-AAA	0.00	999	1.000	1.000	0.0626
219388_at	42.0	13.9	-1.59	-3.02	AMA-AAA	0.00	999	1.000	1.000	0.2861
236925_at	7.1	21.5	1.59	3.02	AAA-AAA	0.00	999	1.000	1.000	0.2948
205929_at	52.1	17.3	-1.59	-3.01	AAA-AAA	0.00	999	1.000	1.000	0.0790
233033_at	11.2	3.7	-1.59	-3.02	AAA-AAA	0.00	999	1.000	1.000	0.1678
233298_at	24.7	8.2	-1.59	-3.02	AAA-AAA	0.00	999	1.000	1.000	0.3404
236652_at	44.3	14.7	-1.59	-3.01	AAA-AAA	0.00	999	1.000	1.000	0.2445
238358_x_at	11.0	3.7	-1.59	-3.01	AAA-AAA	0.00	999	1.000	1.000	0.1669
229995_at	13.0	4.3	-1.59	-3.01	AAA-AAA	0.00	999	1.000	1.000	0.1803
1554360_at	23.6	71.1	1.59	3.01	AAA-AAP	0.00	999	1.000	1.000	0.3135
241528_at	23.0	7.7	-1.59	-3.01	AAA-AAA	0.00	999	1.000	1.000	0.4578
1569843_at	28.3	9.4	-1.59	-3.01	AAA-AAA	0.00	999	1.000	1.000	0.2704
223672_at	13.5	4.5	-1.59	-3.01	AAA-AAA	0.00	999	1.000	1.000	0.3366
237666_at	76.7	25.5	-1.59	-3.01	AAA-AAA	0.00	999	1.000	1.000	0.1517
205862_at	10.6	3.5	-1.59	-3.01	AAA-AAA	0.00	999	1.000	1.000	0.3770
233216_at	48.9	16.2	-1.59	-3.01	AAA-AAA	0.00	999	1.000	1.000	0.1564
232334_at	45.9	15.3	-1.59	-3.01	AAA-AAA	0.00	999	1.000	1.000	0.2337
1569569_x_at	5.9	17.6	1.59	3.01	AAA-APA	0.00	999	1.000	1.000	0.2533
1570001_at	27.8	9.2	-1.59	-3.01	AAA-AAA	0.00	999	1.000	1.000	0.3096
221070_s_at	5.5	16.6	1.59	3.01	AAA-AAA	0.00	999	1.000	1.000	0.2115
217577_at	8.5	2.8	-1.59	-3.01	AAA-AAA	0.00	999	1.000	1.000	0.1966
242433_at	23.7	71.2	1.59	3.01	AAA-AAP	0.00	999	1.000	1.000	0.2701
241636_x_at	19.7	6.6	-1.59	-3.01	AAA-AAA	0.00	999	1.000	1.000	0.1931
217162_at	12.5	37.6	1.59	3.01	AAA-AAA	0.00	999	1.000	1.000	0.0400
208982_at	95.4	31.7	-1.59	-3.01	AAM-AAA	0.00	999	1.000	1.000	0.0745
233777_at	17.4	52.3	1.59	3.01	AAA-APA	0.00	999	1.000	1.000	0.2146
1565660_at	111.5	37.1	-1.59	-3.00	AAA-AAA	0.00	999	1.000	1.000	0.1794
1555737_a_at	86.2	28.7	-1.59	-3.01	AAA-AAA	0.00	999	1.000	1.000	0.3306
211194_s_at	13.9	41.8	1.59	3.01	AAA-AAA	0.00	999	1.000	1.000	0.1536

216134_at	12.5	4.2	-1.59	-3.01	AAA-AAA	0.00	999	1.000	1.000	0.0978
238357_at	17.7	5.9	-1.59	-3.01	AAA-AAA	0.00	999	1.000	1.000	0.2469
228182_at	37.1	12.4	-1.59	-3.00	AAA-AAA	0.00	999	1.000	1.000	0.1948
238778_at	15.8	47.3	1.59	3.00	AAA-PPP	0.00	999	1.000	1.000	0.1013
1553344_at	1.5	4.6	1.59	3.00	AAA-AAA	0.00	999	1.000	1.000	0.1675
1559503_a_at	5.4	16.1	1.59	3.00	AAA-AMA	0.00	999	1.000	1.000	0.2454
211441_x_at	61.3	20.4	-1.59	-3.00	AAA-AAA	0.00	999	1.000	1.000	0.2066
233249_at	10.3	30.8	1.59	3.00	AAA-AAA	0.00	999	1.000	1.000	0.2326
233422_at	16.6	5.5	-1.59	-3.00	AAA-AAA	0.00	999	1.000	1.000	0.2024
220354_at	6.4	19.2	1.59	3.00	AAA-AAA	0.00	999	1.000	1.000	0.2121
215246_at	77.2	25.7	-1.59	-3.00	AAA-AAA	0.00	999	1.000	1.000	0.1418
235307_at	4.3	12.9	1.59	3.00	AAA-AAA	0.00	999	1.000	1.000	0.1901
236880_at	86.9	29.0	-1.59	-3.00	AAA-AAA	0.00	999	1.000	1.000	0.1372
241061_at	2.4	7.0	1.58	3.00	AAA-AAA	0.00	999	1.000	1.000	0.3828
1566853_at	9.7	3.2	-1.58	-3.00	AAA-AAA	0.00	999	1.000	1.000	0.0027
207802_at	4.9	1.7	-1.58	-3.00	AAA-AAA	0.00	999	1.000	1.000	0.3125
216538_at	22.3	66.7	1.58	3.00	AAA-AAA	0.00	999	1.000	1.000	0.2075
237871_x_at	11.7	3.9	-1.58	-3.00	AAA-AAA	0.00	999	1.000	1.000	0.1539
238072_at	64.5	21.5	-1.58	-3.00	AAA-AAA	0.00	999	1.000	1.000	0.2140
227666_at	121.5	40.5	-1.58	-3.00	AAA-AAA	0.00	999	1.000	1.000	0.2965
1560426_at	11.2	33.4	1.58	3.00	AAM-AAA	0.00	999	1.000	1.000	0.3323
1561786_at	29.0	9.7	-1.58	-3.00	AAA-AAA	0.00	999	1.000	1.000	0.2267
1559229_at	17.6	5.9	-1.58	-3.00	AAA-AAA	0.00	999	1.000	1.000	0.2226
208462_s_at	26.9	9.0	-1.58	-3.00	AAA-AAA	0.00	999	1.000	1.000	0.2198
220304_s_at	32.3	10.8	-1.58	-3.00	AAA-AAA	0.00	999	1.000	1.000	0.0708
209877_at	45.4	15.2	-1.58	-3.00	AAA-AAA	0.00	999	1.000	1.000	0.2344
203697_at	8.1	24.3	1.58	2.99	AAA-PAA	0.00	999	1.000	1.000	0.2763
210595_at	22.3	66.8	1.58	2.99	AAA-PPA	0.00	999	1.000	1.000	0.1709
242493_at	29.6	9.9	-1.58	-2.99	AMA-AAA	0.00	999	1.000	1.000	0.2759
237220_at	10.1	3.4	-1.58	-2.99	AAA-AAA	0.00	999	1.000	1.000	0.1068
205942_s_at	11.7	3.9	-1.58	-2.99	AAA-AAA	0.00	999	1.000	1.000	0.1713
230595_at	47.0	15.7	-1.58	-2.99	AAA-AAA	0.00	999	1.000	1.000	0.2294
233709_at	15.0	44.8	1.58	2.99	AAA-AAA	0.00	999	1.000	1.000	0.0629
220668_s_at	131.2	392.4	1.58	2.99	APP-PPP	0.00	999	1.000	1.000	0.3179
1568882_at	18.3	6.1	-1.58	-2.99	AAA-AAA	0.00	999	1.000	1.000	0.3589
1569937_at	67.0	22.4	-1.58	-2.99	AAA-AAA	0.00	999	1.000	1.000	0.0907
1555509_a_at	32.6	10.9	-1.58	-2.99	AAA-AAA	0.00	999	1.000	1.000	0.2830
1552796_a_at	93.6	31.3	-1.58	-2.99	AAM-AAA	0.00	999	1.000	1.000	0.1022
1564158_a_at	7.1	21.1	1.58	2.99	AAA-AAP	0.00	999	1.000	1.000	0.3797
1559284_at	25.5	8.5	-1.58	-2.99	AAA-AAA	0.00	999	1.000	1.000	0.2222
207932_at	15.4	5.1	-1.58	-2.99	AAA-AAA	0.00	999	1.000	1.000	0.2009
224213_at	14.7	4.9	-1.58	-2.99	AAA-AAA	0.00	999	1.000	1.000	0.3870

239658_at	103.6	34.6	-1.58	-2.99	AAA-AAA	0.00	999	1.000	1.000	0.1322
230627_at	10.9	3.6	-1.58	-2.99	AAA-AAA	0.00	999	1.000	1.000	0.0106
1565775_at	34.7	11.6	-1.58	-2.99	AAA-AAA	0.00	999	1.000	1.000	0.2817
1566185_at	50.0	16.7	-1.58	-2.99	AAA-AAA	0.00	999	1.000	1.000	0.1586
214680_at	15.6	5.2	-1.58	-2.99	AAA-AAA	0.00	999	1.000	1.000	0.0287
214559_at	3.3	9.7	1.58	2.99	AAA-AAA	0.00	999	1.000	1.000	0.1139
239275_at	17.0	5.7	-1.58	-2.99	AAA-AAA	0.00	999	1.000	1.000	0.1787
230222_at	22.8	7.6	-1.58	-2.99	AAA-AAA	0.00	999	1.000	1.000	0.0962
229769_at	17.5	5.9	-1.58	-2.99	AAA-AAA	0.00	999	1.000	1.000	0.1542
207569_at	12.3	36.9	1.58	2.99	AAA-APM	0.00	999	1.000	1.000	0.2041
225322_s_at	36.0	107.5	1.58	2.99	AAA-AAP	0.00	999	1.000	1.000	0.1236
242029_at	98.0	32.8	-1.58	-2.99	AAA-AAA	0.00	999	1.000	1.000	0.0598
1563557_at	24.6	8.2	-1.58	-2.99	AAA-AAA	0.00	999	1.000	1.000	0.4288
211688_x_at	25.4	8.5	-1.58	-2.99	AAA-AAA	0.00	999	1.000	1.000	0.0430
217697_at	10.4	31.1	1.58	2.99	AAA-AAA	0.00	999	1.000	1.000	0.0888
236478_at	59.5	19.9	-1.58	-2.99	AAA-AAA	0.00	999	1.000	1.000	0.1513
236501_at	64.5	21.6	-1.58	-2.99	AAA-AAA	0.00	999	1.000	1.000	0.1961
228044_at	12.8	4.3	-1.58	-2.99	AAA-AAA	0.00	999	1.000	1.000	0.2848
231173_at	9.3	3.1	-1.58	-2.99	AAA-AAA	0.00	999	1.000	1.000	0.2750
214865_at	9.8	3.3	-1.58	-2.98	AAA-AAA	0.00	999	1.000	1.000	0.0069
234871_at	28.0	9.4	-1.58	-2.98	AAA-AAA	0.00	999	1.000	1.000	0.1667
239570_at	14.7	4.9	-1.58	-2.98	AAA-AAA	0.00	999	1.000	1.000	0.2139
237732_at	38.8	13.0	-1.58	-2.98	AAM-AAA	0.00	999	1.000	1.000	0.3502
243811_at	13.6	4.6	-1.58	-2.98	AAA-AAA	0.00	999	1.000	1.000	0.1306
1560395_at	26.6	8.9	-1.58	-2.98	AAA-AAA	0.00	999	1.000	1.000	0.1428
1569502_s_at	14.8	44.0	1.58	2.98	AAA-AAA	0.00	999	1.000	1.000	0.0835
1562403_a_at	68.3	22.9	-1.58	-2.98	AAA-AAA	0.00	999	1.000	1.000	0.0745
211125_x_at	63.1	21.2	-1.58	-2.98	AAA-AAA	0.00	999	1.000	1.000	0.2240
1562902_at	15.5	5.2	-1.58	-2.98	AAA-AAA	0.00	999	1.000	1.000	0.0411
1555664_at	15.7	5.3	-1.58	-2.98	AAA-AAA	0.00	999	1.000	1.000	0.1749
224024_at	4.9	14.6	1.58	2.98	AAA-AAA	0.00	999	1.000	1.000	0.3171
237371_at	36.9	12.4	-1.58	-2.98	AAA-AAA	0.00	999	1.000	1.000	0.3926
229634_at	26.5	8.9	-1.58	-2.98	AAA-AAA	0.00	999	1.000	1.000	0.1355
232482_at	15.3	5.1	-1.58	-2.98	AAA-AAA	0.00	999	1.000	1.000	0.3671
1570257_x_at	88.2	29.6	-1.57	-2.98	AAA-AAA	0.00	999	1.000	1.000	0.0593
1557534_at	6.4	2.2	-1.57	-2.98	AAA-AAA	0.00	999	1.000	1.000	0.3318
221138_s_at	5.1	15.3	1.57	2.98	AAA-AAA	0.00	999	1.000	1.000	0.0453
207774_at	90.7	30.5	-1.57	-2.98	AAA-AAA	0.00	999	1.000	1.000	0.2464
220802_at	27.0	9.1	-1.57	-2.98	AAA-AAA	0.00	999	1.000	1.000	0.1770
214956_at	7.7	2.6	-1.57	-2.98	AAA-AAA	0.00	999	1.000	1.000	0.2342
204714_s_at	63.7	21.4	-1.57	-2.98	AAA-AAA	0.00	999	1.000	1.000	0.1089
237112_at	36.2	12.2	-1.57	-2.98	AAA-AAA	0.00	999	1.000	1.000	0.0983

203399_x_at	125.6	42.2	-1.57	-2.98	AAA-AAA	0.00	999	1.000	1.000	0.0236
1562076_at	23.4	7.9	-1.57	-2.97	AAA-AAA	0.00	999	1.000	1.000	0.1409
1562933_at	30.3	10.2	-1.57	-2.98	AAA-AAA	0.00	999	1.000	1.000	0.3091
1568735_at	14.2	4.8	-1.57	-2.98	AAA-AAA	0.00	999	1.000	1.000	0.3709
1553776_at	5.8	1.9	-1.57	-2.98	AAA-AAA	0.00	999	1.000	1.000	0.2423
214856_at	17.8	6.0	-1.57	-2.98	AAA-AAA	0.00	999	1.000	1.000	0.0965
211103_at	18.4	6.2	-1.57	-2.98	AAA-AAA	0.00	999	1.000	1.000	0.1679
229840_at	20.6	61.4	1.57	2.98	AAA-AAA	0.00	999	1.000	1.000	0.1470
244252_at	50.8	17.1	-1.57	-2.97	AAA-AAA	0.00	999	1.000	1.000	0.3003
241949_at	10.1	3.4	-1.57	-2.97	AAA-AAA	0.00	999	1.000	1.000	0.0198
241394_at	16.0	5.4	-1.57	-2.97	AAA-AAA	0.00	999	1.000	1.000	0.4572
208311_at	10.6	3.6	-1.57	-2.97	AAA-AAA	0.00	999	1.000	1.000	0.0942
210464_at	40.8	13.7	-1.57	-2.97	AAA-AAA	0.00	999	1.000	1.000	0.2369
232243_at	15.3	5.2	-1.57	-2.97	AAA-AAA	0.00	999	1.000	1.000	0.2078
1562750_at	8.5	2.9	-1.57	-2.97	AAA-AAA	0.00	999	1.000	1.000	0.1226
235118_at	4.6	1.6	-1.57	-2.97	AAA-AAA	0.00	999	1.000	1.000	0.3798
235079_at	19.4	6.5	-1.57	-2.97	AAA-AAA	0.00	999	1.000	1.000	0.2427
237978_at	2.2	6.6	1.57	2.97	AAA-AAA	0.00	999	1.000	1.000	0.2440
232462_s_at	88.1	29.6	-1.57	-2.97	AAA-AAA	0.00	999	1.000	1.000	0.1065
233760_at	13.3	4.5	-1.57	-2.97	AAA-AAA	0.00	999	1.000	1.000	0.1731
1552816_at	11.5	34.2	1.57	2.97	AAA-AAA	0.00	999	1.000	1.000	0.4406
1556066_at	39.3	13.2	-1.57	-2.97	AAA-AAA	0.00	999	1.000	1.000	0.1027
236036_at	61.0	20.5	-1.57	-2.97	AAA-AAA	0.00	999	1.000	1.000	0.3012
205297_s_at	35.2	11.9	-1.57	-2.97	AAA-AAA	0.00	999	1.000	1.000	0.2453
240791_at	49.7	16.8	-1.57	-2.97	AAA-AAA	0.00	999	1.000	1.000	0.2516
240305_at	17.1	5.8	-1.57	-2.97	AAA-AAA	0.00	999	1.000	1.000	0.0877
1556609_at	23.9	8.0	-1.57	-2.97	AAA-AAA	0.00	999	1.000	1.000	0.0996
206658_at	29.4	9.9	-1.57	-2.97	AAA-AAA	0.00	999	1.000	1.000	0.3141
217387_at	7.7	2.6	-1.57	-2.97	AAA-AAA	0.00	999	1.000	1.000	0.2192
234624_at	39.9	13.5	-1.57	-2.97	AAA-AAA	0.00	999	1.000	1.000	0.1573
229283_at	27.0	9.1	-1.57	-2.97	AAA-AAA	0.00	999	1.000	1.000	0.2666
229311_at	20.9	7.0	-1.57	-2.97	AAA-AAA	0.00	999	1.000	1.000	0.2484
233447_at	52.8	17.8	-1.57	-2.97	AAA-AAA	0.00	999	1.000	1.000	0.0334
239997_at	49.2	16.6	-1.57	-2.96	AAA-AAA	0.00	999	1.000	1.000	0.3305
243038_at	8.7	25.7	1.57	2.96	AAA-AAA	0.00	999	1.000	1.000	0.1212
1562761_at	22.0	7.4	-1.57	-2.96	AAA-AAA	0.00	999	1.000	1.000	0.3437
1558603_at	8.5	25.2	1.57	2.97	AAA-AAA	0.00	999	1.000	1.000	0.3588
221720_s_at	16.6	5.6	-1.57	-2.97	AAA-AAA	0.00	999	1.000	1.000	0.1171
226654_at	10.6	3.6	-1.57	-2.97	AAA-AAA	0.00	999	1.000	1.000	0.0573
211541_s_at	63.4	21.4	-1.57	-2.97	AAA-AAA	0.00	999	1.000	1.000	0.0077
1554261_at	8.2	24.2	1.57	2.96	AAA-PAA	0.00	999	1.000	1.000	0.3655
1557753_at	13.8	4.7	-1.57	-2.96	AAA-AAA	0.00	999	1.000	1.000	0.1672

206784_at	34.6	11.7	-1.57	-2.96	AAA-AAA	0.00	999	1.000	1.000	0.0468
234258_at	34.4	11.6	-1.57	-2.96	AAA-AAA	0.00	999	1.000	1.000	0.3592
204425_at	29.0	9.8	-1.57	-2.96	AAA-AAA	0.00	999	1.000	1.000	0.1170
237717_x_at	3.7	1.2	-1.57	-2.96	AAA-AAA	0.00	999	1.000	1.000	0.3648
206143_at	2.8	8.2	1.57	2.96	AAA-AAA	0.00	999	1.000	1.000	0.0579
244245_at	24.3	8.2	-1.57	-2.96	AAA-AAA	0.00	999	1.000	1.000	0.2900
240564_x_at	8.2	2.8	-1.57	-2.96	AAA-AAA	0.00	999	1.000	1.000	0.2716
1561897_at	69.8	23.6	-1.57	-2.96	AAA-AAA	0.00	999	1.000	1.000	0.0964
207091_at	14.8	5.0	-1.57	-2.96	AAA-AAA	0.00	999	1.000	1.000	0.2162
235734_at	32.5	11.0	-1.57	-2.96	AAA-AAA	0.00	999	1.000	1.000	0.2375
233900_at	18.7	55.2	1.57	2.96	AAA-AAA	0.00	999	1.000	1.000	0.1030
241241_at	24.8	73.4	1.57	2.96	AAA-AAA	0.00	999	1.000	1.000	0.2730
1560383_at	4.0	1.4	-1.57	-2.96	AAA-AAA	0.00	999	1.000	1.000	0.1199
1570650_at	21.1	7.1	-1.57	-2.96	AAA-AAA	0.00	999	1.000	1.000	0.1330
1566269_at	13.4	4.5	-1.57	-2.96	AAA-AAA	0.00	999	1.000	1.000	0.0173
244595_at	50.4	17.0	-1.57	-2.96	AAA-AAA	0.00	999	1.000	1.000	0.1172
1556084_at	11.0	32.5	1.57	2.96	AAA-AAA	0.00	999	1.000	1.000	0.3411
220971_at	14.5	42.9	1.57	2.96	AAA-AAA	0.00	999	1.000	1.000	0.1609
208383_s_at	9.6	3.2	-1.57	-2.96	AAA-AAA	0.00	999	1.000	1.000	0.2517
214648_at	5.7	16.9	1.57	2.96	AAA-AAA	0.00	999	1.000	1.000	0.1126
211148_s_at	33.1	11.2	-1.57	-2.96	AAA-AAA	0.00	999	1.000	1.000	0.2304
209261_s_at	41.7	123.3	1.57	2.96	AAA-AAA	0.00	999	1.000	1.000	0.1586
236727_at	6.6	19.5	1.57	2.96	AAA-AAA	0.00	999	1.000	1.000	0.0171
236387_at	6.8	20.0	1.57	2.96	AAA-AAA	0.00	999	1.000	1.000	0.0342
238195_at	6.9	2.3	-1.57	-2.96	AAA-AAA	0.00	999	1.000	1.000	0.1514
205125_at	49.9	147.7	1.57	2.96	AAA-AAA	0.00	999	1.000	1.000	0.2808
1568615_a_at	27.6	9.3	-1.56	-2.96	AAA-AAA	0.00	999	1.000	1.000	0.1032
1553883_at	3.7	10.9	1.56	2.96	AAA-AAA	0.00	999	1.000	1.000	0.3870
213917_at	66.7	22.6	-1.56	-2.96	AAA-AAA	0.00	999	1.000	1.000	0.0488
216140_at	21.4	7.2	-1.56	-2.96	AAA-AAA	0.00	999	1.000	1.000	0.2106
238327_at	46.9	15.9	-1.56	-2.96	AAA-AAA	0.00	999	1.000	1.000	0.2058
237928_at	9.7	28.7	1.56	2.96	AAA-AAA	0.00	999	1.000	1.000	0.1220
242303_at	13.8	40.7	1.56	2.95	AAA-AAA	0.00	999	1.000	1.000	0.2090
1561911_at	7.2	21.4	1.56	2.95	AAA-AAA	0.00	999	1.000	1.000	0.2271
1561297_at	7.4	2.5	-1.56	-2.95	AAA-AAA	0.00	999	1.000	1.000	0.0336
233752_s_at	7.7	2.6	-1.56	-2.95	AAA-AAA	0.00	999	1.000	1.000	0.0061
243053_x_at	73.4	24.8	-1.56	-2.95	AAA-AAA	0.00	999	1.000	1.000	0.1743
1569592_a_at	14.1	4.8	-1.56	-2.95	AAA-AAA	0.00	999	1.000	1.000	0.0619
1560091_a_at	6.6	2.2	-1.56	-2.95	AAA-AAA	0.00	999	1.000	1.000	0.2409
224491_at	22.4	7.6	-1.56	-2.95	AAA-AAA	0.00	999	1.000	1.000	0.3009
209223_at	81.9	27.7	-1.56	-2.95	AAA-AAA	0.00	999	1.000	1.000	0.1013
240348_at	6.3	18.5	1.56	2.95	AAA-AAA	0.00	999	1.000	1.000	0.3005

1568780_at	89.9	30.5	-1.56	-2.95	AAA-AAA	0.00	999	1.000	1.000	0.0269
1564821_at	16.0	5.4	-1.56	-2.95	AAA-AAA	0.00	999	1.000	1.000	0.1046
1553936_a_at	16.5	5.6	-1.56	-2.95	AAA-AAA	0.00	999	1.000	1.000	0.2141
215014_at	8.6	2.9	-1.56	-2.95	AAA-AAA	0.00	999	1.000	1.000	0.4187
211248_s_at	46.3	15.7	-1.56	-2.95	AAA-AAA	0.00	999	1.000	1.000	0.1651
240030_at	43.5	14.7	-1.56	-2.95	AAA-AAA	0.00	999	1.000	1.000	0.1332
240706_at	32.4	11.0	-1.56	-2.95	AAA-AAA	0.00	999	1.000	1.000	0.1357
241270_at	32.9	11.2	-1.56	-2.95	AAA-AAA	0.00	999	1.000	1.000	0.2635
226172_at	50.2	17.0	-1.56	-2.95	AAA-AAA	0.00	999	1.000	1.000	0.0954
210280_at	56.8	19.3	-1.56	-2.95	AAA-AAA	0.00	999	1.000	1.000	0.1979
234122_at	33.9	11.5	-1.56	-2.95	AAA-AAA	0.00	999	1.000	1.000	0.1657
231514_at	16.4	48.4	1.56	2.95	AAA-AAA	0.00	999	1.000	1.000	0.0190
240910_at	21.7	64.0	1.56	2.95	AAA-AAA	0.00	999	1.000	1.000	0.0605
221095_s_at	25.2	8.6	-1.56	-2.95	AAA-AAA	0.00	999	1.000	1.000	0.3313
206243_at	17.3	5.9	-1.56	-2.95	AAA-AAA	0.00	999	1.000	1.000	0.3723
1563128_at	17.5	6.0	-1.56	-2.94	AAA-AAA	0.00	999	1.000	1.000	0.2386
217142_at	82.7	243.4	1.56	2.94	AAA-MAA	0.00	999	1.000	1.000	0.0382
216543_at	5.1	15.1	1.56	2.94	AAA-AAA	0.00	999	1.000	1.000	0.3086
227783_at	67.3	22.9	-1.56	-2.94	AAA-AAA	0.00	999	1.000	1.000	0.1976
227652_at	32.1	10.9	-1.56	-2.94	AAA-AAA	0.00	999	1.000	1.000	0.2926
239924_at	15.1	44.4	1.56	2.94	AAA-AAA	0.00	999	1.000	1.000	0.1724
240726_at	6.3	18.6	1.56	2.94	AAA-AAA	0.00	999	1.000	1.000	0.3117
243348_at	12.3	36.1	1.56	2.94	AAA-AAA	0.00	999	1.000	1.000	0.0148
1570266_x_at	132.0	44.9	-1.56	-2.94	AAA-AAA	0.00	999	1.000	1.000	0.2489
1555382_at	4.2	12.5	1.56	2.94	AAA-AAA	0.00	999	1.000	1.000	0.3927
206577_at	8.5	25.1	1.56	2.94	AAA-AAA	0.00	999	1.000	1.000	0.1667
223523_at	32.8	11.1	-1.56	-2.94	AAA-AAA	0.00	999	1.000	1.000	0.1421
207542_s_at	53.8	18.3	-1.56	-2.94	AAA-AAA	0.00	999	1.000	1.000	0.0408
215008_at	5.6	1.9	-1.56	-2.94	AAA-AAA	0.00	999	1.000	1.000	0.0718
238872_at	45.7	15.6	-1.56	-2.94	AAA-AAA	0.00	999	1.000	1.000	0.1833
206387_at	14.4	4.9	-1.56	-2.94	AAA-AAA	0.00	999	1.000	1.000	0.0030
228811_at	28.0	9.5	-1.56	-2.94	AAA-AAA	0.00	999	1.000	1.000	0.3089
232949_at	157.6	53.6	-1.56	-2.94	AAA-AAA	0.00	999	1.000	1.000	0.3099
231372_at	11.3	3.8	-1.56	-2.94	AAA-AAA	0.00	999	1.000	1.000	0.2211
1553132_a_at	21.4	7.3	-1.56	-2.94	AAA-AAA	0.00	999	1.000	1.000	0.3369
207955_at	31.8	10.8	-1.56	-2.94	AAA-AAA	0.00	999	1.000	1.000	0.1700
239357_at	17.5	5.9	-1.56	-2.94	AAA-AAA	0.00	999	1.000	1.000	0.1513
232904_at	39.9	13.6	-1.56	-2.94	AAA-AAA	0.00	999	1.000	1.000	0.2943
233336_at	14.9	5.1	-1.56	-2.94	AAA-AAA	0.00	999	1.000	1.000	0.1497
1570282_at	14.9	5.1	-1.56	-2.94	AAA-AAA	0.00	999	1.000	1.000	0.2721
1565242_at	9.5	3.2	-1.56	-2.94	AAA-AAA	0.00	999	1.000	1.000	0.0435
244562_s_at	21.0	7.2	-1.56	-2.94	AAA-AAA	0.00	999	1.000	1.000	0.2340

208177_at	17.5	51.5	1.56	2.94	AAA-AAA	0.00	999	1.000	1.000	0.0308
216138_at	13.9	41.0	1.56	2.94	AAA-AAA	0.00	999	1.000	1.000	0.1712
229681_at	9.9	29.0	1.56	2.94	AAA-AAA	0.00	999	1.000	1.000	0.0360
240350_at	37.9	12.9	-1.55	-2.94	AAA-MAA	0.00	999	1.000	1.000	0.3210
243733_at	3.6	10.7	1.55	2.94	AAA-AAA	0.00	999	1.000	1.000	0.1450
242268_at	25.2	8.6	-1.55	-2.94	AAA-AAA	0.00	999	1.000	1.000	0.1788
224400_s_at	4.5	13.2	1.55	2.94	AAA-AAA	0.00	999	1.000	1.000	0.1949
236337_at	15.5	45.6	1.55	2.94	AAA-AAA	0.00	999	1.000	1.000	0.2166
234592_at	23.9	8.1	-1.55	-2.94	AAA-AAA	0.00	999	1.000	1.000	0.2951
229489_at	16.0	5.5	-1.55	-2.94	AAA-AAA	0.00	999	1.000	1.000	0.0201
228072_at	75.3	25.7	-1.55	-2.94	AAA-AAA	0.00	999	1.000	1.000	0.2741
1552957_at	25.8	8.8	-1.55	-2.94	AAA-AAA	0.00	999	1.000	1.000	0.0581
1557679_at	32.5	11.1	-1.55	-2.93	AAA-AAA	0.00	999	1.000	1.000	0.2189
221346_at	55.1	18.8	-1.55	-2.93	AAA-AAA	0.00	999	1.000	1.000	0.0058
221993_s_at	22.6	7.7	-1.55	-2.93	AAA-AAA	0.00	999	1.000	1.000	0.3058
206934_at	23.5	8.0	-1.55	-2.93	AAA-AAA	0.00	999	1.000	1.000	0.1448
223508_at	18.7	6.4	-1.55	-2.94	AAA-AAA	0.00	999	1.000	1.000	0.1632
210063_at	25.4	74.6	1.55	2.93	AAA-AAA	0.00	999	1.000	1.000	0.0116
237261_at	33.3	11.3	-1.55	-2.93	AAA-AAA	0.00	999	1.000	1.000	0.1304
1566892_at	12.2	4.2	-1.55	-2.93	AAA-AAA	0.00	999	1.000	1.000	0.2900
1553082_at	21.0	7.2	-1.55	-2.93	AAA-AAA	0.00	999	1.000	1.000	0.2300
206719_at	16.3	47.7	1.55	2.93	AAA-AAA	0.00	999	1.000	1.000	0.1400
214111_at	32.7	11.1	-1.55	-2.93	AAA-AAA	0.00	999	1.000	1.000	0.3527
210549_s_at	15.6	5.3	-1.55	-2.93	AAA-AAA	0.00	999	1.000	1.000	0.1612
217108_at	14.5	4.9	-1.55	-2.93	AAA-AAA	0.00	999	1.000	1.000	0.2924
217141_at	34.9	11.9	-1.55	-2.93	AAA-AAA	0.00	999	1.000	1.000	0.1077
204722_at	10.5	3.6	-1.55	-2.93	AAA-AAA	0.00	999	1.000	1.000	0.3460
204152_s_at	69.1	23.6	-1.55	-2.93	AAA-AAA	0.00	999	1.000	1.000	0.1559
237506_at	57.0	19.4	-1.55	-2.93	AAA-AAA	0.00	999	1.000	1.000	0.2172
230590_at	36.2	106.1	1.55	2.93	AAA-AAA	0.00	999	1.000	1.000	0.0575
232607_at	7.7	22.5	1.55	2.93	AAA-AAA	0.00	999	1.000	1.000	0.1449
237860_at	21.7	63.6	1.55	2.93	AAA-PPP	0.00	999	1.000	1.000	0.1923
243877_at	40.7	13.9	-1.55	-2.93	AAA-AAA	0.00	999	1.000	1.000	0.2769
239837_at	17.2	50.5	1.55	2.93	AAA-AAA	0.00	999	1.000	1.000	0.2413
214059_at	6.6	19.4	1.55	2.93	AAA-AAA	0.00	999	1.000	1.000	0.1580
211131_s_at	20.3	6.9	-1.55	-2.93	AAA-AAA	0.00	999	1.000	1.000	0.2429
208577_at	20.8	7.1	-1.55	-2.93	AAA-AAA	0.00	999	1.000	1.000	0.0862
233954_at	22.5	7.7	-1.55	-2.93	AAA-AAA	0.00	999	1.000	1.000	0.1538
237253_at	7.8	2.7	-1.55	-2.93	AAA-AAA	0.00	999	1.000	1.000	0.3018
238054_at	16.1	47.3	1.55	2.93	AAA-AAA	0.00	999	1.000	1.000	0.0005
230316_at	71.5	24.4	-1.55	-2.93	AAA-AAA	0.00	999	1.000	1.000	0.3240
1558371_a_at	11.9	34.7	1.55	2.93	AAP-AAP	0.00	999	1.000	1.000	0.2571

223771_at	16.8	49.1	1.55	2.93	AAA-PAP	0.00	999	1.000	1.000	0.1519
1565800_x_at	34.5	11.8	-1.55	-2.93	AAA-AAA	0.00	999	1.000	1.000	0.0460
1559622_at	30.5	10.4	-1.55	-2.93	AAA-AAA	0.00	999	1.000	1.000	0.1602
238291_at	14.6	5.0	-1.55	-2.93	AAA-AAA	0.00	999	1.000	1.000	0.1803
237468_at	12.4	4.2	-1.55	-2.93	AAA-AAA	0.00	999	1.000	1.000	0.3055
205893_at	5.6	16.3	1.55	2.93	AAA-AAA	0.00	999	1.000	1.000	0.4166
1561573_at	6.4	18.8	1.55	2.93	AAA-AAA	0.00	999	1.000	1.000	0.4669
1559901_s_at	10.0	29.3	1.55	2.93	AAA-AAA	0.00	999	1.000	1.000	0.3012
238741_at	84.4	28.8	-1.55	-2.93	AAA-AAA	0.00	999	1.000	1.000	0.2360
239123_at	10.4	30.4	1.55	2.93	AAA-AAA	0.00	999	1.000	1.000	0.2237
206039_at	28.8	84.1	1.55	2.93	AAA-AAA	0.00	999	1.000	1.000	0.1059
233764_s_at	27.9	81.5	1.55	2.93	AAA-AAA	0.00	999	1.000	1.000	0.1362
205466_s_at	3.7	10.7	1.55	2.93	AAA-AAA	0.00	999	1.000	1.000	0.2161
240404_at	29.2	10.0	-1.55	-2.93	AAA-AAA	0.00	999	1.000	1.000	0.1048
1566852_at	43.4	14.9	-1.55	-2.92	AAA-AAA	0.00	999	1.000	1.000	0.1056
1554938_a_at	10.9	3.7	-1.55	-2.92	AAA-AAA	0.00	999	1.000	1.000	0.0128
215652_at	27.2	9.3	-1.55	-2.93	AAA-AAA	0.00	999	1.000	1.000	0.2009
230818_at	58.8	20.1	-1.55	-2.92	AAA-AAA	0.00	999	1.000	1.000	0.0796
233287_at	50.0	17.1	-1.55	-2.92	AAA-AAA	0.00	999	1.000	1.000	0.2191
1562528_at	11.9	4.1	-1.55	-2.92	AAA-AAA	0.00	999	1.000	1.000	0.3231
1552777_a_at	18.5	6.3	-1.55	-2.92	AAA-AAA	0.00	999	1.000	1.000	0.1316
221411_at	6.4	2.2	-1.55	-2.92	AAA-AAA	0.00	999	1.000	1.000	0.2607
223721_s_at	9.6	27.9	1.55	2.92	AAA-AAA	0.00	999	1.000	1.000	0.1519
224342_x_at	57.4	19.7	-1.55	-2.92	AAA-AAA	0.00	999	1.000	1.000	0.2944
223855_s_at	24.5	8.4	-1.55	-2.92	AAA-AAA	0.00	999	1.000	1.000	0.0540
209918_at	36.0	12.3	-1.55	-2.92	AAA-AAA	0.00	999	1.000	1.000	0.2058
237164_at	12.9	37.7	1.55	2.92	AAA-AMA	0.00	999	1.000	1.000	0.1927
230015_at	43.7	15.0	-1.55	-2.92	AAA-AAA	0.00	999	1.000	1.000	0.2801
233293_at	14.7	5.0	-1.55	-2.92	AAA-AAA	0.00	999	1.000	1.000	0.0874
1557802_at	6.2	18.2	1.55	2.92	AAA-AAP	0.00	999	1.000	1.000	0.4040
242122_at	8.9	3.1	-1.55	-2.92	AAA-AAA	0.00	999	1.000	1.000	0.2788
1570600_at	24.0	8.2	-1.55	-2.92	AAA-AAA	0.00	999	1.000	1.000	0.3779
1553255_at	29.4	10.1	-1.55	-2.92	AAA-AAA	0.00	999	1.000	1.000	0.1503
217628_at	32.4	11.1	-1.55	-2.92	AAA-AAA	0.00	999	1.000	1.000	0.2301
216566_at	32.6	11.2	-1.55	-2.92	AAA-AAM	0.00	999	1.000	1.000	0.2232
239890_s_at	3.2	9.2	1.55	2.92	AAA-AAA	0.00	999	1.000	1.000	0.1204
1570302_at	10.1	29.4	1.55	2.92	AAA-AAA	0.00	999	1.000	1.000	0.2236
1569955_at	10.3	3.5	-1.55	-2.92	AAA-AAA	0.00	999	1.000	1.000	0.4168
1552779_a_at	41.2	14.1	-1.55	-2.92	AAA-AAA	0.00	999	1.000	1.000	0.0532
1559939_at	15.9	5.5	-1.55	-2.92	AAA-AAA	0.00	999	1.000	1.000	0.3303
206165_s_at	6.2	18.0	1.55	2.92	AAA-AAA	0.00	999	1.000	1.000	0.3090
1562051_at	37.5	12.9	-1.54	-2.92	AAA-AAA	0.00	999	1.000	1.000	0.2266

1565861_at	37.5	12.9	-1.54	-2.92	AAA-AAA	0.00	999	1.000	1.000	0.2458
1564787_at	19.0	55.4	1.54	2.92	AAA-AAA	0.00	999	1.000	1.000	0.1927
1555765_a_at	29.8	10.2	-1.54	-2.92	AAA-AAA	0.00	999	1.000	1.000	0.1031
208597_at	19.8	57.7	1.54	2.92	MAA-AAA	0.00	999	1.000	1.000	0.1655
208519_x_at	84.6	29.0	-1.54	-2.92	AAA-AAA	0.00	999	1.000	1.000	0.0954
1564022_at	4.0	11.6	1.54	2.91	AAA-AAA	0.00	999	1.000	1.000	0.3014
1565934_at	20.6	7.1	-1.54	-2.91	AAA-AAA	0.00	999	1.000	1.000	0.0689
1556956_at	57.7	19.8	-1.54	-2.92	AAA-AAA	0.00	999	1.000	1.000	0.2825
225123_at	15.1	5.2	-1.54	-2.92	AAA-AAA	0.00	999	1.000	1.000	0.1446
214811_at	15.5	5.3	-1.54	-2.91	AAA-AAA	0.00	999	1.000	1.000	0.2371
214385_s_at	41.0	14.1	-1.54	-2.92	AAA-AAA	0.00	999	1.000	1.000	0.3497
235770_at	11.4	3.9	-1.54	-2.92	AAA-AAA	0.00	999	1.000	1.000	0.1510
236003_x_at	10.5	3.6	-1.54	-2.91	AAA-AAA	0.00	999	1.000	1.000	0.1501
234736_at	22.5	7.7	-1.54	-2.92	AAA-AAA	0.00	999	1.000	1.000	0.1474
227733_at	32.5	11.1	-1.54	-2.91	AAA-AAA	0.00	999	1.000	1.000	0.1425
243060_at	11.0	3.8	-1.54	-2.91	AAA-AAA	0.00	999	1.000	1.000	0.3545
1561956_at	10.4	30.2	1.54	2.91	AAA-AAA	0.00	999	1.000	1.000	0.2141
1561271_at	24.9	8.5	-1.54	-2.91	AAA-AAA	0.00	999	1.000	1.000	0.4225
220783_at	37.2	12.8	-1.54	-2.91	AAA-AAA	0.00	999	1.000	1.000	0.0779
223987_at	7.3	21.3	1.54	2.91	AAA-AAA	0.00	999	1.000	1.000	0.2507
214836_x_at	50.5	17.4	-1.54	-2.91	AAA-AAA	0.00	999	1.000	1.000	0.0544
231010_at	43.8	15.0	-1.54	-2.91	AAA-AAA	0.00	999	1.000	1.000	0.2917
1561625_at	6.0	17.5	1.54	2.91	AAA-AAA	0.00	999	1.000	1.000	0.1106
225513_at	36.7	12.6	-1.54	-2.91	AAA-AAA	0.00	999	1.000	1.000	0.1190
206736_x_at	21.1	7.3	-1.54	-2.91	AAA-AAA	0.00	999	1.000	1.000	0.3182
224296_x_at	7.6	2.6	-1.54	-2.91	AAA-AAA	0.00	999	1.000	1.000	0.4514
236682_at	17.0	5.9	-1.54	-2.91	AAA-AAA	0.00	999	1.000	1.000	0.0183
233806_at	43.7	15.0	-1.54	-2.91	AAA-AAA	0.00	999	1.000	1.000	0.0773
240048_at	42.3	14.6	-1.54	-2.91	AAA-AAA	0.00	999	1.000	1.000	0.1305
1566219_at	12.3	35.7	1.54	2.91	AAA-AAA	0.00	999	1.000	1.000	0.2284
217574_at	16.7	5.7	-1.54	-2.91	AAA-AAA	0.00	999	1.000	1.000	0.2881
204540_at	80.5	27.7	-1.54	-2.91	AAA-AAA	0.00	999	1.000	1.000	0.4018
205059_s_at	30.2	87.7	1.54	2.91	AAA-AAA	0.00	999	1.000	1.000	0.1320
239722_at	26.4	9.1	-1.54	-2.91	AAA-AAA	0.00	999	1.000	1.000	0.2262
235369_at	19.5	56.5	1.54	2.91	APA-APA	0.00	999	1.000	1.000	0.3185
243625_at	9.8	3.4	-1.54	-2.91	AAA-AAA	0.00	999	1.000	1.000	0.2417
208195_at	53.7	18.5	-1.54	-2.91	AAA-AAA	0.00	999	1.000	1.000	0.1727
230658_at	31.2	10.8	-1.54	-2.91	AAA-AAA	0.00	999	1.000	1.000	0.0162
206348_s_at	51.1	148.4	1.54	2.91	AAA-AAA	0.00	999	1.000	1.000	0.1611
1561610_at	39.5	13.6	-1.54	-2.91	AAA-AAA	0.00	999	1.000	1.000	0.3994
216419_at	26.9	9.3	-1.54	-2.90	AAA-AAA	0.00	999	1.000	1.000	0.1775
237942_at	14.7	42.6	1.54	2.90	AAA-AAM	0.00	999	1.000	1.000	0.3233

229755_x_at	12.7	37.0	1.54	2.91	AAA-AAA	0.00	999	1.000	1.000	0.1880
233250_x_at	52.4	152.1	1.54	2.90	AAA-AAA	0.00	999	1.000	1.000	0.1096
231477_at	23.6	8.1	-1.54	-2.90	AAA-AAA	0.00	999	1.000	1.000	0.1173
1569172_a_at	16.8	48.8	1.54	2.90	AAA-AAA	0.00	999	1.000	1.000	0.1844
1570273_at	5.9	2.0	-1.54	-2.90	AAA-AAA	0.00	999	1.000	1.000	0.1438
1568924_a_at	27.1	9.3	-1.54	-2.90	AAA-AAA	0.00	999	1.000	1.000	0.2077
1555127_at	18.3	6.3	-1.54	-2.90	AAA-AAA	0.00	999	1.000	1.000	0.3936
1559344_at	72.5	25.0	-1.54	-2.90	AAA-AAA	0.00	999	1.000	1.000	0.0852
1559195_at	5.1	14.8	1.54	2.90	AAA-AAA	0.00	999	1.000	1.000	0.0938
219721_at	25.7	8.8	-1.54	-2.90	AAA-AAA	0.00	999	1.000	1.000	0.1271
211096_at	93.2	32.1	-1.54	-2.90	AAA-AAA	0.00	999	1.000	1.000	0.3889
212868_x_at	83.9	28.9	-1.54	-2.90	AAA-AAA	0.00	999	1.000	1.000	0.3058
215865_at	71.8	24.7	-1.54	-2.90	AAA-AAA	0.00	999	1.000	1.000	0.1413
234026_at	53.5	18.4	-1.54	-2.90	AAA-AAA	0.00	999	1.000	1.000	0.0497
237000_at	14.4	5.0	-1.54	-2.90	AAA-AAA	0.00	999	1.000	1.000	0.2705
230625_s_at	12.4	4.3	-1.54	-2.90	AAA-AAA	0.00	999	1.000	1.000	0.0882
241485_at	13.5	4.7	-1.54	-2.90	AAA-AAA	0.00	999	1.000	1.000	0.4538
240882_at	18.3	6.3	-1.54	-2.90	AAA-AAA	0.00	999	1.000	1.000	0.2620
221836_s_at	162.2	55.9	-1.54	-2.90	AAA-AAA	0.00	999	1.000	1.000	0.2986
238698_at	21.8	7.5	-1.54	-2.90	AAA-AAA	0.00	999	1.000	1.000	0.2865
1563110_at	4.9	1.7	-1.54	-2.90	AAA-AAA	0.00	999	1.000	1.000	0.1445
1556282_at	4.4	12.6	1.54	2.90	AAA-AAA	0.00	999	1.000	1.000	0.3486
1556621_a_at	18.8	54.5	1.54	2.90	AAA-AAA	0.00	999	1.000	1.000	0.1505
221111_at	8.7	3.0	-1.54	-2.90	AAA-AAA	0.00	999	1.000	1.000	0.3065
222257_s_at	33.0	11.4	-1.54	-2.90	AAA-AAA	0.00	999	1.000	1.000	0.2496
220351_at	9.2	26.7	1.54	2.90	AAA-AAA	0.00	999	1.000	1.000	0.3651
222938_x_at	148.6	51.3	-1.54	-2.90	AAA-AAA	0.00	999	1.000	1.000	0.0831
207544_s_at	9.4	27.3	1.54	2.90	AAA-AAA	0.00	999	1.000	1.000	0.1931
214970_s_at	49.5	17.1	-1.54	-2.90	AAA-AAA	0.00	999	1.000	1.000	0.1729
215048_at	130.8	45.1	-1.54	-2.90	AAA-AAA	0.00	999	1.000	1.000	0.1535
216909_at	48.8	16.8	-1.54	-2.90	AAA-AAA	0.00	999	1.000	1.000	0.3035
234886_at	37.0	12.8	-1.54	-2.90	AAA-AAA	0.00	999	1.000	1.000	0.0291
204533_at	39.8	13.8	-1.54	-2.90	AAA-AAA	0.00	999	1.000	1.000	0.2674
1558592_at	9.4	27.1	1.53	2.90	AAA-AAP	0.00	999	1.000	1.000	0.3379
1562469_at	6.2	18.1	1.53	2.90	AAA-AAA	0.00	999	1.000	1.000	0.1884
201525_at	103.9	35.9	-1.53	-2.90	AAA-AAA	0.00	999	1.000	1.000	0.1622
1557017_at	6.2	2.2	-1.53	-2.90	AAA-AAA	0.00	999	1.000	1.000	0.3207
221295_at	60.3	20.8	-1.53	-2.90	AAA-AAA	0.00	999	1.000	1.000	0.0166
208211_s_at	52.0	18.0	-1.53	-2.90	AAA-AAA	0.00	999	1.000	1.000	0.1631
210799_at	79.5	27.5	-1.53	-2.90	AAA-AAA	0.00	999	1.000	1.000	0.1153
232492_at	50.3	17.4	-1.53	-2.90	AAA-AAA	0.00	999	1.000	1.000	0.0443
243070_at	46.7	16.2	-1.53	-2.89	AAA-AAA	0.00	999	1.000	1.000	0.2229

241896_at	69.9	24.2	-1.53	-2.89	AMA-AAA	0.00	999	1.000	1.000	0.2770
1564327_at	17.5	50.7	1.53	2.89	AAA-AAA	0.00	999	1.000	1.000	0.1489
206952_at	2.0	5.7	1.53	2.89	AAA-AAA	0.00	999	1.000	1.000	0.1498
224212_s_at	3.3	9.6	1.53	2.89	AAA-AAA	0.00	999	1.000	1.000	0.1377
237879_at	5.7	16.4	1.53	2.89	AAA-AAA	0.00	999	1.000	1.000	0.1316
206313_at	33.3	11.5	-1.53	-2.89	AAA-AAA	0.00	999	1.000	1.000	0.2764
239012_at	28.9	83.5	1.53	2.89	AAA-AAP	0.00	999	1.000	1.000	0.1201
1569462_x_at	20.9	7.2	-1.53	-2.89	AAA-AAA	0.00	999	1.000	1.000	0.2968
1558348_at	13.2	38.2	1.53	2.89	AAA-AAA	0.00	999	1.000	1.000	0.2692
1556758_at	39.6	13.7	-1.53	-2.89	AMA-AAA	0.00	999	1.000	1.000	0.4486
208209_s_at	57.2	19.8	-1.53	-2.89	AAA-AAA	0.00	999	1.000	1.000	0.2227
206741_at	15.0	5.2	-1.53	-2.89	AAA-AAA	0.00	999	1.000	1.000	0.1163
210832_x_at	43.2	14.9	-1.53	-2.89	AAA-AAA	0.00	999	1.000	1.000	0.1616
238576_at	67.1	23.2	-1.53	-2.89	AAA-AAA	0.00	999	1.000	1.000	0.0285
243832_at	18.3	6.3	-1.53	-2.89	AAA-AAA	0.00	999	1.000	1.000	0.3149
1562000_at	6.5	2.3	-1.53	-2.89	AAA-AAA	0.00	999	1.000	1.000	0.1590
219734_at	15.2	43.9	1.53	2.89	AAA-AMA	0.00	999	1.000	1.000	0.1362
227362_at	20.2	7.0	-1.53	-2.89	AAA-AAA	0.00	999	1.000	1.000	0.2268
224165_s_at	16.5	5.7	-1.53	-2.89	AAA-AAA	0.00	999	1.000	1.000	0.4179
211660_at	32.4	93.6	1.53	2.89	AAA-AAA	0.00	999	1.000	1.000	0.0866
211646_at	24.6	8.5	-1.53	-2.89	AAA-AAA	0.00	999	1.000	1.000	0.1949
206259_at	15.4	5.3	-1.53	-2.89	AAA-AAA	0.00	999	1.000	1.000	0.0027
240855_at	13.2	38.2	1.53	2.89	AAA-AAA	0.00	999	1.000	1.000	0.0451
243575_at	38.8	13.4	-1.53	-2.89	AAA-AAA	0.00	999	1.000	1.000	0.2550
229739_s_at	6.6	19.1	1.53	2.89	AAA-AAA	0.00	999	1.000	1.000	0.1423
230866_at	4.1	11.9	1.53	2.89	AAA-AAA	0.00	999	1.000	1.000	0.1402
243566_at	24.7	8.6	-1.53	-2.89	AAA-AAA	0.00	999	1.000	1.000	0.1990
220424_at	19.3	6.7	-1.53	-2.89	AAA-AAA	0.00	999	1.000	1.000	0.2252
206553_at	7.1	20.5	1.53	2.89	AAA-AAA	0.00	999	1.000	1.000	0.1825
210227_at	45.9	15.9	-1.53	-2.89	AAA-AAA	0.00	999	1.000	1.000	0.1770
216788_at	31.6	11.0	-1.53	-2.89	AAA-AAA	0.00	999	1.000	1.000	0.1508
239472_at	25.2	8.7	-1.53	-2.89	AAA-AAA	0.00	999	1.000	1.000	0.2980
1565446_at	3.4	9.9	1.53	2.88	AAA-APA	0.00	999	1.000	1.000	0.4666
203000_at	4.8	13.8	1.53	2.89	AAA-AAA	0.00	999	1.000	1.000	0.1129
1569445_at	29.3	10.2	-1.53	-2.88	AAA-AAA	0.00	999	1.000	1.000	0.1575
1552535_at	42.6	14.8	-1.53	-2.88	AAA-AAA	0.00	999	1.000	1.000	0.2227
220704_at	14.4	5.0	-1.53	-2.89	AAA-AAA	0.00	999	1.000	1.000	0.2554
206979_at	68.7	23.8	-1.53	-2.88	AAA-AAA	0.00	999	1.000	1.000	0.1810
214977_at	8.3	2.9	-1.53	-2.88	AAA-AAA	0.00	999	1.000	1.000	0.1967
237070_at	11.6	4.0	-1.53	-2.88	AAA-AAA	0.00	999	1.000	1.000	0.2570
229524_at	20.2	7.0	-1.53	-2.88	AAA-AAA	0.00	999	1.000	1.000	0.3619
242101_at	11.7	4.1	-1.53	-2.88	AAA-AAA	0.00	999	1.000	1.000	0.0631

241665_x_at	12.8	4.4	-1.53	-2.88	AAA-AAA	0.00	999	1.000	1.000	0.4199
242296_x_at	7.8	22.4	1.53	2.88	AAA-AAA	0.00	999	1.000	1.000	0.1100
1560791_at	18.9	6.6	-1.53	-2.88	AAA-AAA	0.00	999	1.000	1.000	0.3112
1552583_s_at	56.5	19.6	-1.53	-2.88	AAA-AAA	0.00	999	1.000	1.000	0.2256
1558123_at	26.5	9.2	-1.53	-2.88	AAA-AAA	0.00	999	1.000	1.000	0.1531
220246_at	61.2	21.2	-1.53	-2.88	AAA-AAA	0.00	999	1.000	1.000	0.2978
211570_s_at	31.3	10.9	-1.53	-2.88	AAA-AAA	0.00	999	1.000	1.000	0.2285
219273_at	10.1	29.2	1.53	2.88	AAA-AAA	0.00	999	1.000	1.000	0.1845
215763_at	6.0	17.4	1.53	2.88	AAA-AAA	0.00	999	1.000	1.000	0.3518
234234_at	31.7	11.0	-1.53	-2.88	AAA-AAA	0.00	999	1.000	1.000	0.0940
239003_at	20.4	7.1	-1.53	-2.88	AAA-AAA	0.00	999	1.000	1.000	0.2903
229780_at	81.9	28.4	-1.53	-2.88	AAA-AAA	0.00	999	1.000	1.000	0.2165
230821_at	38.6	111.1	1.53	2.88	AAA-AAA	0.00	999	1.000	1.000	0.0960
228782_at	6.5	2.3	-1.53	-2.88	AAA-AAA	0.00	999	1.000	1.000	0.0670
241142_at	6.0	17.3	1.53	2.88	AAA-AAA	0.00	999	1.000	1.000	0.3530
1563993_at	30.0	10.4	-1.53	-2.88	AAA-AAA	0.00	999	1.000	1.000	0.2099
1564683_at	13.9	39.9	1.53	2.88	AAA-AAA	0.00	999	1.000	1.000	0.1042
244651_at	16.7	5.8	-1.53	-2.88	AAA-AAA	0.00	999	1.000	1.000	0.2370
220965_s_at	71.1	24.7	-1.53	-2.88	AAA-AAA	0.00	999	1.000	1.000	0.0520
208525_s_at	10.4	3.6	-1.53	-2.88	AAA-AAA	0.00	999	1.000	1.000	0.2055
215349_at	38.0	13.2	-1.53	-2.88	AAA-AAA	0.00	999	1.000	1.000	0.1790
240197_at	15.8	45.6	1.53	2.88	AAA-AAA	0.00	999	1.000	1.000	0.0968
1559206_at	4.6	1.6	-1.53	-2.88	AAA-AAA	0.00	999	1.000	1.000	0.1749
227323_at	15.7	5.5	-1.53	-2.88	AAA-AAA	0.00	999	1.000	1.000	0.1837
213441_x_at	238.3	82.8	-1.53	-2.88	AAA-AAA	0.00	999	1.000	1.000	0.1880
211057_at	17.5	50.5	1.53	2.88	AAA-AAA	0.00	999	1.000	1.000	0.2751
238559_at	38.3	13.3	-1.53	-2.88	AAA-AAA	0.00	999	1.000	1.000	0.1931
233138_at	101.5	35.2	-1.53	-2.88	AAA-AAA	0.00	999	1.000	1.000	0.2214
205444_at	66.7	23.2	-1.53	-2.88	AAA-AAA	0.00	999	1.000	1.000	0.2277
203587_at	33.6	11.7	-1.52	-2.88	AAA-AAA	0.00	999	1.000	1.000	0.1078
1567622_at	124.9	43.4	-1.52	-2.88	AAA-AAA	0.00	999	1.000	1.000	0.0953
1557793_at	8.2	23.6	1.52	2.88	AAA-AAA	0.00	999	1.000	1.000	0.2315
216431_at	19.1	6.6	-1.52	-2.88	AAA-AAA	0.00	999	1.000	1.000	0.1877
229775_s_at	13.3	38.3	1.52	2.88	AAA-AAA	0.00	999	1.000	1.000	0.0186
240200_at	13.3	4.6	-1.52	-2.88	AAA-AAA	0.00	999	1.000	1.000	0.2193
1563662_at	25.4	8.9	-1.52	-2.87	AAA-AAA	0.00	999	1.000	1.000	0.1047
1561313_at	18.0	51.6	1.52	2.88	AAA-AAA	0.00	999	1.000	1.000	0.1032
1569422_at	30.7	10.7	-1.52	-2.88	AAA-AAA	0.00	999	1.000	1.000	0.1895
1565733_at	12.6	4.4	-1.52	-2.87	AAA-AAA	0.00	999	1.000	1.000	0.0837
1565728_at	20.1	7.0	-1.52	-2.87	AAA-AAA	0.00	999	1.000	1.000	0.0697
1564601_at	10.8	31.1	1.52	2.88	AAA-AAA	0.00	999	1.000	1.000	0.2802
1556713_at	24.2	8.4	-1.52	-2.87	MAA-AAA	0.00	999	1.000	1.000	0.2157

236427_at	14.5	5.0	-1.52	-2.87	AAA-AAA	0.00	999	1.000	1.000	0.2444
234415_x_at	41.9	14.6	-1.52	-2.87	AAA-AAA	0.00	999	1.000	1.000	0.3173
237948_at	24.2	8.4	-1.52	-2.87	AAA-AAA	0.00	999	1.000	1.000	0.1593
229777_at	35.2	12.3	-1.52	-2.88	AAA-AAA	0.00	999	1.000	1.000	0.1356
232954_at	70.5	24.5	-1.52	-2.87	AAA-AAA	0.00	999	1.000	1.000	0.1506
233004_x_at	17.4	49.9	1.52	2.87	AAA-AAA	0.00	999	1.000	1.000	0.1947
233064_at	14.5	41.7	1.52	2.88	AAA-AAA	0.00	999	1.000	1.000	0.0301
231789_at	7.9	22.6	1.52	2.88	AAA-AAA	0.00	999	1.000	1.000	0.3818
1557214_at	14.0	4.9	-1.52	-2.87	AAA-AAA	0.00	999	1.000	1.000	0.2069
224098_at	28.8	10.0	-1.52	-2.87	AAA-AAA	0.00	999	1.000	1.000	0.3399
219319_at	59.9	20.9	-1.52	-2.87	AAA-AAA	0.00	999	1.000	1.000	0.2528
230360_at	24.1	8.4	-1.52	-2.87	AAA-AAA	0.00	999	1.000	1.000	0.0705
240447_at	36.0	12.5	-1.52	-2.87	AAA-AAA	0.00	999	1.000	1.000	0.2103
1563221_at	17.2	6.0	-1.52	-2.87	AAA-AAA	0.00	999	1.000	1.000	0.1189
1552967_at	55.0	19.2	-1.52	-2.87	AAA-AAA	0.00	999	1.000	1.000	0.0247
221320_at	17.7	50.8	1.52	2.87	AAA-AAA	0.00	999	1.000	1.000	0.0557
221441_at	5.4	1.9	-1.52	-2.87	AAA-AAA	0.00	999	1.000	1.000	0.1549
222378_at	13.2	4.6	-1.52	-2.87	AAA-AAA	0.00	999	1.000	1.000	0.3823
236964_at	24.5	8.5	-1.52	-2.87	AAA-AAA	0.00	999	1.000	1.000	0.2867
229484_at	40.7	14.2	-1.52	-2.87	AAA-AAA	0.00	999	1.000	1.000	0.1547
1567008_at	45.2	15.8	-1.52	-2.87	AAA-AAA	0.00	999	1.000	1.000	0.1896
210423_s_at	65.7	22.9	-1.52	-2.87	AAA-AAA	0.00	999	1.000	1.000	0.3039
217206_at	13.0	37.2	1.52	2.87	AAA-AAA	0.00	999	1.000	1.000	0.0421
219471_at	44.3	15.4	-1.52	-2.87	AAA-AAA	0.00	999	1.000	1.000	0.3018
236371_s_at	31.8	11.1	-1.52	-2.87	AAA-AAA	0.00	999	1.000	1.000	0.0910
234537_at	6.4	2.3	-1.52	-2.87	AAA-AAA	0.00	999	1.000	1.000	0.0228
234705_at	4.8	13.8	1.52	2.87	AAA-AAA	0.00	999	1.000	1.000	0.0077
232575_at	35.4	12.4	-1.52	-2.87	AAA-AAA	0.00	999	1.000	1.000	0.3299
223505_s_at	20.4	58.5	1.52	2.87	AAP-AAA	0.00	999	1.000	1.000	0.2660
1558957_s_at	14.4	5.0	-1.52	-2.87	AAA-AAA	0.00	999	1.000	1.000	0.2833
235884_at	28.0	80.3	1.52	2.87	AAA-AAA	0.00	999	1.000	1.000	0.0478
230203_at	27.3	78.1	1.52	2.87	AAA-AAA	0.00	999	1.000	1.000	0.1835
232929_at	22.3	7.8	-1.52	-2.87	AAA-AAA	0.00	999	1.000	1.000	0.0506
233527_at	22.4	7.8	-1.52	-2.87	AAA-AAA	0.00	999	1.000	1.000	0.4407
233613_x_at	18.9	54.0	1.52	2.86	PAA-PPP	0.00	999	1.000	1.000	0.4408
1564598_a_at	36.4	12.7	-1.52	-2.86	AAA-AAA	0.00	999	1.000	1.000	0.2401
1553246_a_at	128.3	44.8	-1.52	-2.87	AAA-AAA	0.00	999	1.000	1.000	0.1100
1552878_at	14.2	5.0	-1.52	-2.87	AAA-AAA	0.00	999	1.000	1.000	0.4080
225233_at	53.3	18.6	-1.52	-2.86	AAA-AAA	0.00	999	1.000	1.000	0.2510
227336_at	27.1	9.5	-1.52	-2.86	AAA-AAA	0.00	999	1.000	1.000	0.2332
233921_s_at	45.2	15.8	-1.52	-2.86	AAA-AAA	0.00	999	1.000	1.000	0.2578
1553296_at	8.5	24.3	1.52	2.86	AAA-AAA	0.00	999	1.000	1.000	0.3164

1556226_at	28.9	10.1	-1.52	-2.86	AAA-AAA	0.00	999	1.000	1.000	0.4320
1555697_at	63.6	22.2	-1.52	-2.86	AAA-AAA	0.00	999	1.000	1.000	0.2599
207833_s_at	60.1	172.0	1.52	2.86	AAA-AAA	0.00	999	1.000	1.000	0.0549
219790_s_at	4.9	14.0	1.52	2.86	AAA-AAA	0.00	999	1.000	1.000	0.1242
215345_x_at	14.4	41.3	1.52	2.86	AAA-AAA	0.00	999	1.000	1.000	0.1310
231027_at	41.9	14.6	-1.52	-2.86	AAA-AAA	0.00	999	1.000	1.000	0.3401
241056_at	8.9	3.1	-1.52	-2.86	AAA-AAA	0.00	999	1.000	1.000	0.1952
1553159_at	44.1	15.4	-1.52	-2.86	AAA-AAA	0.00	999	1.000	1.000	0.2007
1555475_x_at	13.3	38.1	1.52	2.86	AAA-AAA	0.00	999	1.000	1.000	0.2009
207199_at	15.2	43.4	1.52	2.86	AAA-AAA	0.00	999	1.000	1.000	0.0435
235936_at	13.1	4.6	-1.52	-2.86	AAA-AAA	0.00	999	1.000	1.000	0.0106
242988_at	54.7	19.1	-1.52	-2.86	AAA-AAA	0.00	999	1.000	1.000	0.2154
1562468_at	25.1	8.8	-1.52	-2.86	AAA-AAA	0.00	999	1.000	1.000	0.3984
1561345_at	39.5	13.8	-1.52	-2.86	AAA-AAA	0.00	999	1.000	1.000	0.2398
1554242_a_at	19.5	55.8	1.52	2.86	AAA-AAA	0.00	999	1.000	1.000	0.1357
208359_s_at	19.9	7.0	-1.52	-2.86	AAA-AAA	0.00	999	1.000	1.000	0.3265
227174_at	15.8	5.6	-1.52	-2.86	AAA-AAA	0.00	999	1.000	1.000	0.2104
206526_at	28.6	81.7	1.52	2.86	AAA-AAA	0.00	999	1.000	1.000	0.2012
235446_at	91.8	32.1	-1.52	-2.86	AAA-MAA	0.00	999	1.000	1.000	0.4125
238943_at	69.6	24.4	-1.52	-2.86	AAA-AAA	0.00	999	1.000	1.000	0.0864
229391_s_at	8.7	24.8	1.52	2.86	AAA-AAA	0.00	999	1.000	1.000	0.0966
1562265_at	83.5	29.2	-1.51	-2.86	AAA-AAA	0.00	999	1.000	1.000	0.2111
1554796_at	49.9	17.5	-1.51	-2.86	AAA-AAA	0.00	999	1.000	1.000	0.3282
1559995_at	95.3	33.4	-1.51	-2.86	AAA-AAA	0.00	999	1.000	1.000	0.0022
220030_at	14.6	5.1	-1.51	-2.86	AAA-AAA	0.00	999	1.000	1.000	0.4913
238302_at	33.2	11.6	-1.51	-2.86	AAA-AAA	0.00	999	1.000	1.000	0.2187
206109_at	43.6	15.3	-1.51	-2.86	AAA-AAA	0.00	999	1.000	1.000	0.4162
1563708_at	16.7	5.8	-1.51	-2.85	AAA-AAA	0.00	999	1.000	1.000	0.2457
1561451_a_at	63.8	22.3	-1.51	-2.85	AAA-AAA	0.00	999	1.000	1.000	0.2003
1556608_a_at	9.2	26.3	1.51	2.85	AAA-AAA	0.00	999	1.000	1.000	0.1222
1553721_at	15.5	44.1	1.51	2.85	AAA-AAP	0.00	999	1.000	1.000	0.1563
1560971_a_at	24.4	8.6	-1.51	-2.85	AAA-AAA	0.00	999	1.000	1.000	0.0360
1552754_a_at	8.0	2.8	-1.51	-2.85	AAA-AAA	0.00	999	1.000	1.000	0.3427
207756_at	4.2	12.0	1.51	2.85	AAA-AAA	0.00	999	1.000	1.000	0.2973
207190_at	24.6	8.6	-1.51	-2.85	AAA-AAA	0.00	999	1.000	1.000	0.1609
214248_s_at	10.1	28.8	1.51	2.85	AAA-AAA	0.00	999	1.000	1.000	0.0627
231120_x_at	2.7	7.6	1.51	2.85	AAA-AAA	0.00	999	1.000	1.000	0.2382
1562633_at	12.0	4.2	-1.51	-2.85	AAA-AAA	0.00	999	1.000	1.000	0.1721
1563287_at	21.5	7.6	-1.51	-2.85	AAA-AAA	0.00	999	1.000	1.000	0.0992
1555443_at	19.2	6.7	-1.51	-2.85	AAA-AAA	0.00	999	1.000	1.000	0.2315
215871_at	16.3	5.7	-1.51	-2.85	AAA-AAA	0.00	999	1.000	1.000	0.2203
229842_at	36.1	12.7	-1.51	-2.85	AAA-AAA	0.00	999	1.000	1.000	0.1404

243106_at	10.9	3.8	-1.51	-2.85	AAA-AAA	0.00	999	1.000	1.000	0.3572
241997_at	151.2	53.1	-1.51	-2.85	AAA-AAA	0.00	999	1.000	1.000	0.1795
1569835_at	4.0	1.4	-1.51	-2.85	AAA-AAA	0.00	999	1.000	1.000	0.0475
220425_x_at	32.6	11.4	-1.51	-2.85	AAA-AAA	0.00	999	1.000	1.000	0.2526
217332_at	18.3	52.2	1.51	2.85	AAA-AAA	0.00	999	1.000	1.000	0.2401
1553335_x_at	4.9	14.1	1.51	2.85	AAA-AAA	0.00	999	1.000	1.000	0.1534
217203_at	44.5	15.6	-1.51	-2.85	AMA-AAA	0.00	999	1.000	1.000	0.1462
204672_s_at	3.6	10.1	1.51	2.85	AAA-AAA	0.00	999	1.000	1.000	0.1300
234115_s_at	19.9	7.0	-1.51	-2.85	AAA-AAA	0.00	999	1.000	1.000	0.3193
205852_at	69.3	24.3	-1.51	-2.85	MAA-AAA	0.00	999	1.000	1.000	0.0624
240242_at	22.4	7.9	-1.51	-2.84	AAA-AAA	0.00	999	1.000	1.000	0.2231
242219_at	18.1	51.4	1.51	2.84	AAA-AAA	0.00	999	1.000	1.000	0.2241
240153_at	11.5	4.1	-1.51	-2.84	AAA-AAA	0.00	999	1.000	1.000	0.4441
1560469_at	3.7	1.3	-1.51	-2.85	AAA-AAA	0.00	999	1.000	1.000	0.0149
207639_at	81.2	28.5	-1.51	-2.85	AAA-AAA	0.00	999	1.000	1.000	0.1476
211544_s_at	83.4	29.3	-1.51	-2.84	AAA-AAA	0.00	999	1.000	1.000	0.0690
234246_at	38.5	13.5	-1.51	-2.85	AAA-AAA	0.00	999	1.000	1.000	0.2957
228302_x_at	57.0	20.0	-1.51	-2.84	AAA-AAA	0.00	999	1.000	1.000	0.1439
232234_at	12.2	34.6	1.51	2.84	AAA-AAA	0.00	999	1.000	1.000	0.1116
1562549_at	21.6	7.6	-1.51	-2.84	AAA-AAA	0.00	999	1.000	1.000	0.1688
220284_at	28.7	10.1	-1.51	-2.84	AAA-AAA	0.00	999	1.000	1.000	0.2708
213075_at	53.5	18.8	-1.51	-2.84	AAA-AAA	0.00	999	1.000	1.000	0.2266
217409_at	21.2	7.5	-1.51	-2.84	AAA-AAA	0.00	999	1.000	1.000	0.1868
244124_at	54.3	19.1	-1.51	-2.84	AAA-AAA	0.00	999	1.000	1.000	0.2439
243441_at	30.5	10.7	-1.51	-2.84	AAA-AAA	0.00	999	1.000	1.000	0.2803
1560899_at	5.4	1.9	-1.51	-2.84	AAA-AAA	0.00	999	1.000	1.000	0.2349
1561882_at	19.2	6.8	-1.51	-2.84	AAA-AAA	0.00	999	1.000	1.000	0.3011
1554963_at	3.0	8.6	1.51	2.84	AAA-AAA	0.00	999	1.000	1.000	0.2523
1553293_at	16.1	5.7	-1.51	-2.84	AAA-AAA	0.00	999	1.000	1.000	0.0134
220213_at	50.0	17.6	-1.51	-2.84	AAA-AAA	0.00	999	1.000	1.000	0.1507
219606_at	47.7	16.8	-1.51	-2.84	AAA-AAA	0.00	999	1.000	1.000	0.1274
223737_x_at	23.3	8.2	-1.51	-2.84	AAA-AAA	0.00	999	1.000	1.000	0.3764
223660_at	9.6	3.4	-1.51	-2.84	AAA-AAA	0.00	999	1.000	1.000	0.2439
215782_at	72.5	25.5	-1.51	-2.84	AAA-AAA	0.00	999	1.000	1.000	0.2611
236689_at	15.2	43.2	1.51	2.84	AAA-AAA	0.00	999	1.000	1.000	0.1677
228621_at	34.5	12.1	-1.51	-2.84	AAA-AAA	0.00	999	1.000	1.000	0.2068
233494_at	54.9	19.3	-1.51	-2.84	AAA-AAA	0.00	999	1.000	1.000	0.0712
1563586_at	46.4	16.3	-1.51	-2.84	AAA-AAA	0.00	999	1.000	1.000	0.1286
1560391_at	34.4	12.1	-1.51	-2.84	AAA-AAA	0.00	999	1.000	1.000	0.1427
1553977_a_at	149.3	52.6	-1.51	-2.84	AAA-AAA	0.00	999	1.000	1.000	0.0112
230923_at	5.0	14.2	1.51	2.84	AAA-AAA	0.00	999	1.000	1.000	0.3974
233668_at	12.0	4.2	-1.51	-2.84	AAA-AAA	0.00	999	1.000	1.000	0.0247

241278_at	11.8	4.2	-1.50	-2.84	AAA-AAA	0.00	999	1.000	1.000	0.2567
1552335_at	34.7	98.5	1.50	2.84	AAA-AAA	0.00	999	1.000	1.000	0.2699
234017_at	6.8	19.4	1.50	2.84	AAA-AAA	0.00	999	1.000	1.000	0.3161
237446_at	19.7	6.9	-1.50	-2.84	AAA-AAA	0.00	999	1.000	1.000	0.3614
227753_at	25.6	9.0	-1.50	-2.84	AAA-AAA	0.00	999	1.000	1.000	0.1678
231657_s_at	12.1	4.3	-1.50	-2.84	AAA-AAA	0.00	999	1.000	1.000	0.1101
226498_at	43.9	124.3	1.50	2.83	APA-PPP	0.00	999	1.000	1.000	0.0721
244119_at	10.5	29.8	1.50	2.84	AAA-AAA	0.00	999	1.000	1.000	0.4319
240301_at	15.8	5.6	-1.50	-2.83	AAA-AAA	0.00	999	1.000	1.000	0.1680
241810_at	55.7	19.7	-1.50	-2.83	AAA-AAA	0.00	999	1.000	1.000	0.1757
1562507_at	19.3	6.8	-1.50	-2.83	AAA-AAA	0.00	999	1.000	1.000	0.2115
1558177_at	24.6	8.7	-1.50	-2.83	AAA-AAA	0.00	999	1.000	1.000	0.3677
215880_at	4.9	13.8	1.50	2.84	AAA-AAA	0.00	999	1.000	1.000	0.3677
216447_at	4.7	13.4	1.50	2.83	AAA-AAA	0.00	999	1.000	1.000	0.3348
237369_at	31.7	11.2	-1.50	-2.83	AAA-AAA	0.00	999	1.000	1.000	0.3096
208146_s_at	7.0	19.8	1.50	2.83	AAA-APA	0.00	999	1.000	1.000	0.2534
1563978_at	11.8	4.2	-1.50	-2.83	AAA-AAA	0.00	999	1.000	1.000	0.3195
1553793_a_at	59.3	20.9	-1.50	-2.83	AAA-AAA	0.00	999	1.000	1.000	0.2390
220282_at	27.8	9.8	-1.50	-2.83	AAA-AAA	0.00	999	1.000	1.000	0.2220
220537_at	55.4	19.6	-1.50	-2.83	AAA-AAA	0.00	999	1.000	1.000	0.1947
210459_at	5.4	15.2	1.50	2.83	AAA-AAA	0.00	999	1.000	1.000	0.0467
217161_x_at	71.6	25.3	-1.50	-2.83	AAA-AAA	0.00	999	1.000	1.000	0.1794
216530_at	40.6	14.3	-1.50	-2.83	AAA-AAA	0.00	999	1.000	1.000	0.3229
216441_at	7.0	19.7	1.50	2.83	AAA-AAA	0.00	999	1.000	1.000	0.3514
232252_at	48.3	17.0	-1.50	-2.83	AAA-AAA	0.00	999	1.000	1.000	0.0268
231786_at	23.6	8.3	-1.50	-2.83	AAA-AAA	0.00	999	1.000	1.000	0.3899
216218_s_at	27.8	78.6	1.50	2.83	AAA-MPA	0.00	999	1.000	1.000	0.0936
243482_at	77.4	27.3	-1.50	-2.83	AAA-AAA	0.00	999	1.000	1.000	0.1634
1561728_a_at	30.3	10.7	-1.50	-2.83	AAA-AAA	0.00	999	1.000	1.000	0.1892
223878_at	36.4	12.9	-1.50	-2.83	AAA-AAA	0.00	999	1.000	1.000	0.4164
207325_x_at	14.4	40.6	1.50	2.83	AAA-AAA	0.00	999	1.000	1.000	0.1601
241653_x_at	12.7	4.5	-1.50	-2.83	AAA-AAA	0.00	999	1.000	1.000	0.4365
1557404_at	12.8	36.1	1.50	2.83	AAA-AAA	0.00	999	1.000	1.000	0.0999
1556881_at	36.3	12.8	-1.50	-2.83	AAA-AAA	0.00	999	1.000	1.000	0.1789
220624_s_at	10.3	3.6	-1.50	-2.83	AAA-AAA	0.00	999	1.000	1.000	0.2269
207193_at	35.9	12.7	-1.50	-2.83	AAA-AAA	0.00	999	1.000	1.000	0.1866
211845_at	9.9	3.5	-1.50	-2.83	AAA-AAA	0.00	999	1.000	1.000	0.1402
236523_at	9.8	3.5	-1.50	-2.83	AAA-AAA	0.00	999	1.000	1.000	0.1841
204311_at	70.7	25.0	-1.50	-2.83	AAA-AAA	0.00	999	1.000	1.000	0.0721
205818_at	57.4	20.3	-1.50	-2.83	AAA-AMA	0.00	999	1.000	1.000	0.3063
243890_at	40.1	14.2	-1.50	-2.83	AAA-AAA	0.00	999	1.000	1.000	0.2838
243154_at	17.5	49.3	1.50	2.83	AAA-AMA	0.00	999	1.000	1.000	0.0415

1560463_at	45.2	16.0	-1.50	-2.83	AAA-AAA	0.00	999	1.000	1.000	0.3852
1565795_at	43.3	15.3	-1.50	-2.83	AAA-AAA	0.00	999	1.000	1.000	0.1658
1555502_at	4.6	12.9	1.50	2.83	AAA-AAA	0.00	999	1.000	1.000	0.2098
220570_at	66.9	23.7	-1.50	-2.83	AAA-AAA	0.00	999	1.000	1.000	0.2688
206458_s_at	10.7	30.2	1.50	2.83	AAA-AAA	0.00	999	1.000	1.000	0.0278
224088_at	84.5	29.9	-1.50	-2.83	AAA-AAA	0.00	999	1.000	1.000	0.0632
211553_x_at	21.8	61.5	1.50	2.83	AAA-AAA	0.00	999	1.000	1.000	0.0282
230477_at	22.0	7.8	-1.50	-2.83	AAA-AAA	0.00	999	1.000	1.000	0.0741
1560633_a_at	13.2	37.2	1.50	2.82	AAA-AAA	0.00	999	1.000	1.000	0.3192
1570505_at	10.9	3.9	-1.50	-2.83	AAA-AAA	0.00	999	1.000	1.000	0.0951
216052_x_at	66.1	23.4	-1.50	-2.82	AAA-AAA	0.00	999	1.000	1.000	0.1189
236644_at	3.3	9.4	1.50	2.82	AAA-AAA	0.00	999	1.000	1.000	0.2102
205934_at	7.2	20.4	1.50	2.82	APA-AAA	0.00	999	1.000	1.000	0.3608
242118_x_at	13.6	4.8	-1.50	-2.82	AAA-AAA	0.00	999	1.000	1.000	0.3800
242301_at	32.2	11.4	-1.50	-2.82	AAA-AAA	0.00	999	1.000	1.000	0.3835
1552858_at	21.9	7.8	-1.50	-2.82	AAA-AAA	0.00	999	1.000	1.000	0.1976
235115_at	14.4	5.1	-1.50	-2.82	AAA-AAA	0.00	999	1.000	1.000	0.4052
233944_at	9.2	25.9	1.50	2.82	AAA-AAA	0.00	999	1.000	1.000	0.2076
205979_at	10.9	3.9	-1.50	-2.82	AAA-AAA	0.00	999	1.000	1.000	0.1078
206327_s_at	95.7	33.9	-1.50	-2.82	AAA-AAA	0.00	999	1.000	1.000	0.1810
203407_at	26.3	9.3	-1.50	-2.82	AAA-AAA	0.00	999	1.000	1.000	0.3103
240453_at	5.6	15.8	1.50	2.82	AAA-AAA	0.00	999	1.000	1.000	0.3720
1570125_at	31.8	11.3	-1.50	-2.82	AAA-AAA	0.00	999	1.000	1.000	0.1218
1553151_at	25.9	9.2	-1.50	-2.82	AAA-AAA	0.00	999	1.000	1.000	0.2318
1559302_at	59.3	21.0	-1.50	-2.82	AAA-AAA	0.00	999	1.000	1.000	0.2949
1556271_at	25.8	9.1	-1.50	-2.82	AAA-AAA	0.00	999	1.000	1.000	0.2822
208464_at	34.9	12.4	-1.50	-2.82	AAA-AAA	0.00	999	1.000	1.000	0.4018
211476_at	10.8	3.8	-1.50	-2.82	AAA-AAA	0.00	999	1.000	1.000	0.3685
219058_x_at	124.6	44.2	-1.50	-2.82	AAA-AAA	0.00	999	1.000	1.000	0.1834
216367_at	19.0	6.7	-1.50	-2.82	AAA-AAA	0.00	999	1.000	1.000	0.3023
240630_at	13.4	37.8	1.50	2.82	AAA-AAA	0.00	999	1.000	1.000	0.0448
1564632_at	23.8	8.4	-1.50	-2.82	AAA-AAA	0.00	999	1.000	1.000	0.0972
214289_at	64.1	22.7	-1.50	-2.82	AAA-AAA	0.00	999	1.000	1.000	0.1846
211235_s_at	94.1	33.4	-1.50	-2.82	AAA-AAA	0.00	999	1.000	1.000	0.0416
216889_s_at	40.7	14.4	-1.50	-2.82	AAA-AAA	0.00	999	1.000	1.000	0.2649
204782_at	49.9	17.7	-1.50	-2.82	AAA-AAA	0.00	999	1.000	1.000	0.2485
238091_at	6.8	2.4	-1.50	-2.82	AAA-AAA	0.00	999	1.000	1.000	0.1009
239564_at	15.2	43.0	1.50	2.82	AAA-AAA	0.00	999	1.000	1.000	0.1205
237375_at	52.2	18.5	-1.50	-2.82	AAA-AAA	0.00	999	1.000	1.000	0.0172
1562981_at	104.1	37.0	-1.49	-2.82	AAA-AAA	0.00	999	1.000	1.000	0.2111
244521_at	7.3	20.7	1.49	2.82	AAA-AAA	0.00	999	1.000	1.000	0.0561
1559341_at	5.5	2.0	-1.49	-2.82	AAA-AAA	0.00	999	1.000	1.000	0.1249

213880_at	7.6	2.7	-1.49	-2.82	AAA-AAA	0.00	999	1.000	1.000	0.3229
211245_x_at	47.4	16.8	-1.49	-2.82	AAA-AAA	0.00	999	1.000	1.000	0.2270
234784_at	22.5	8.0	-1.49	-2.82	AAA-AAA	0.00	999	1.000	1.000	0.4473
234538_at	9.4	3.3	-1.49	-2.82	AAA-AAA	0.00	999	1.000	1.000	0.3065
232798_at	41.5	14.8	-1.49	-2.82	AAA-AAA	0.00	999	1.000	1.000	0.0702
231537_at	30.7	10.9	-1.49	-2.82	AAA-AAA	0.00	999	1.000	1.000	0.1896
241038_at	52.8	18.8	-1.49	-2.81	AAA-AAA	0.00	999	1.000	1.000	0.2892
1566833_x_at	17.3	6.1	-1.49	-2.82	AAA-AAA	0.00	999	1.000	1.000	0.2679
223911_at	40.0	14.2	-1.49	-2.82	AAA-AAA	0.00	999	1.000	1.000	0.1110
238744_at	12.2	34.4	1.49	2.81	AAA-AAP	0.00	999	1.000	1.000	0.3282
202489_s_at	22.7	63.7	1.49	2.81	AAA-AAA	0.00	999	1.000	1.000	0.0282
1555095_at	16.0	5.7	-1.49	-2.81	AAA-AAA	0.00	999	1.000	1.000	0.1999
244726_at	156.8	55.8	-1.49	-2.81	MAA-AAA	0.00	999	1.000	1.000	0.0408
224292_at	21.3	7.6	-1.49	-2.81	AAA-AAA	0.00	999	1.000	1.000	0.1961
213456_at	9.2	3.3	-1.49	-2.81	AAA-AAA	0.00	999	1.000	1.000	0.2489
215056_at	30.3	10.8	-1.49	-2.81	AAA-AAA	0.00	999	1.000	1.000	0.2486
238566_at	49.3	17.5	-1.49	-2.81	AAA-AAA	0.00	999	1.000	1.000	0.1159
238158_at	82.9	29.5	-1.49	-2.81	AAA-AAA	0.00	999	1.000	1.000	0.0753
239666_at	32.4	11.5	-1.49	-2.81	AAA-AAA	0.00	999	1.000	1.000	0.3171
233578_at	17.9	6.4	-1.49	-2.81	AAA-AAA	0.00	999	1.000	1.000	0.3653
1555645_at	26.6	9.5	-1.49	-2.81	AAA-AAA	0.00	999	1.000	1.000	0.3229
221198_at	8.7	3.1	-1.49	-2.81	AAA-AAA	0.00	999	1.000	1.000	0.3435
214131_at	44.6	15.9	-1.49	-2.81	AAA-AAA	0.00	999	1.000	1.000	0.2197
215322_at	74.1	26.3	-1.49	-2.81	AAA-AAA	0.00	999	1.000	1.000	0.1942
234893_s_at	9.2	25.9	1.49	2.81	AAA-AAA	0.00	999	1.000	1.000	0.1214
242697_at	40.8	14.5	-1.49	-2.81	AAA-AAA	0.00	999	1.000	1.000	0.2547
243813_at	22.5	8.0	-1.49	-2.81	AAA-AAA	0.00	999	1.000	1.000	0.3036
1561639_at	48.6	17.3	-1.49	-2.81	AAA-AAA	0.00	999	1.000	1.000	0.2828
244741_s_at	22.9	64.3	1.49	2.81	AAA-AAA	0.00	999	1.000	1.000	0.0079
217426_at	13.5	38.0	1.49	2.81	AAA-AAA	0.00	999	1.000	1.000	0.0891
236918_s_at	10.0	3.5	-1.49	-2.81	AAA-AAA	0.00	999	1.000	1.000	0.4256
237930_at	32.3	11.5	-1.49	-2.81	AAA-AAA	0.00	999	1.000	1.000	0.1130
241690_at	61.5	21.9	-1.49	-2.81	AAA-AAA	0.00	999	1.000	1.000	0.3102
1553592_x_at	60.7	21.6	-1.49	-2.81	AAA-AAA	0.00	999	1.000	1.000	0.2493
1552453_a_at	13.7	4.9	-1.49	-2.81	AAA-AAA	0.00	999	1.000	1.000	0.2665
1557658_at	14.7	5.2	-1.49	-2.81	AAA-AAA	0.00	999	1.000	1.000	0.1234
1556203_a_at	73.9	26.3	-1.49	-2.81	AAA-AAA	0.00	999	1.000	1.000	0.1069
206877_at	15.0	5.3	-1.49	-2.81	AAA-AAA	0.00	999	1.000	1.000	0.1347
236373_at	57.5	20.5	-1.49	-2.81	AAA-AAA	0.00	999	1.000	1.000	0.1675
234408_at	12.2	34.1	1.49	2.81	AAA-AAA	0.00	999	1.000	1.000	0.2263
234396_at	8.5	3.0	-1.49	-2.81	AAA-AAA	0.00	999	1.000	1.000	0.2699
238582_at	15.5	5.5	-1.49	-2.81	AAA-AAA	0.00	999	1.000	1.000	0.0396

233288_at	15.9	5.7	-1.49	-2.81	AAA-AAA	0.00	999	1.000	1.000	0.2768
244135_at	20.7	58.1	1.49	2.81	AAA-AAA	0.00	999	1.000	1.000	0.1531
207706_at	22.9	8.2	-1.49	-2.81	AAA-AAA	0.00	999	1.000	1.000	0.0918
212998_x_at	20.4	7.3	-1.49	-2.81	AAA-AAA	0.00	999	1.000	1.000	0.3512
215740_at	8.0	22.3	1.49	2.81	AAA-AAA	0.00	999	1.000	1.000	0.2058
215309_at	2.9	8.1	1.49	2.81	AAA-AAA	0.00	999	1.000	1.000	0.3298
216195_at	62.7	22.4	-1.49	-2.81	AAA-AAA	0.00	999	1.000	1.000	0.2058
237303_at	14.5	5.2	-1.49	-2.81	AAA-AAA	0.00	999	1.000	1.000	0.3564
205941_s_at	46.9	16.7	-1.49	-2.81	AAA-AAA	0.00	999	1.000	1.000	0.4152
1564872_at	47.3	16.9	-1.49	-2.80	AAA-AAA	0.00	999	1.000	1.000	0.0748
1559471_s_at	76.1	27.2	-1.49	-2.80	AAA-AAA	0.00	999	1.000	1.000	0.2743
223974_at	29.8	83.4	1.49	2.80	AAA-MAA	0.00	999	1.000	1.000	0.2133
217101_at	6.3	17.7	1.49	2.80	AAA-AAA	0.00	999	1.000	1.000	0.2094
234835_at	23.5	8.4	-1.49	-2.80	AAA-AAA	0.00	999	1.000	1.000	0.2386
1559376_at	15.4	43.0	1.49	2.80	PAA-AAA	0.00	999	1.000	1.000	0.2880
240771_at	13.5	4.8	-1.49	-2.80	AAA-AAA	0.00	999	1.000	1.000	0.2965
1556200_a_at	43.4	15.5	-1.49	-2.80	AAA-AAA	0.00	999	1.000	1.000	0.1504
208346_at	13.5	4.8	-1.49	-2.80	AAA-AAA	0.00	999	1.000	1.000	0.2269
239120_at	6.6	2.4	-1.49	-2.80	AAA-AAA	0.00	999	1.000	1.000	0.3778
237343_at	43.6	15.6	-1.49	-2.80	AAA-AAA	0.00	999	1.000	1.000	0.1840
233126_s_at	21.9	7.8	-1.49	-2.80	AAA-AAA	0.00	999	1.000	1.000	0.0513
213351_s_at	29.0	81.2	1.49	2.80	AAA-AAP	0.00	999	1.000	1.000	0.1202
240056_at	11.3	31.5	1.49	2.80	AAA-AAA	0.00	999	1.000	1.000	0.0035
1562701_at	53.5	19.1	-1.49	-2.80	AAA-AAA	0.00	999	1.000	1.000	0.3058
1561565_at	8.3	3.0	-1.49	-2.80	AAA-AAA	0.00	999	1.000	1.000	0.1725
207822_at	86.5	30.9	-1.49	-2.80	AAA-AAA	0.00	999	1.000	1.000	0.0771
220487_at	10.4	3.7	-1.49	-2.80	AAA-AAA	0.00	999	1.000	1.000	0.0652
209660_at	99.8	35.7	-1.49	-2.80	AAA-AAA	0.00	999	1.000	1.000	0.0515
235574_at	8.1	2.9	-1.49	-2.80	AAA-AAA	0.00	999	1.000	1.000	0.0817
235510_at	11.8	4.2	-1.49	-2.80	AAA-AAA	0.00	999	1.000	1.000	0.0046
204513_s_at	36.3	13.0	-1.49	-2.80	AAA-AAA	0.00	999	1.000	1.000	0.2569
230880_at	41.6	14.9	-1.49	-2.80	AAA-AAA	0.00	999	1.000	1.000	0.1110
221239_s_at	14.0	39.1	1.48	2.80	AAA-PAA	0.00	999	1.000	1.000	0.2381
1561738_at	20.2	7.2	-1.48	-2.80	AAA-AAA	0.00	999	1.000	1.000	0.3223
208282_x_at	4.2	1.5	-1.48	-2.80	AAA-AAA	0.00	999	1.000	1.000	0.3057
211008_s_at	33.3	11.9	-1.48	-2.80	AAA-AAA	0.00	999	1.000	1.000	0.1510
228192_at	59.7	21.3	-1.48	-2.80	AAA-AAA	0.00	999	1.000	1.000	0.1011
1560413_at	11.6	4.2	-1.48	-2.80	AAA-AAA	0.00	999	1.000	1.000	0.3134
1565858_at	24.2	8.7	-1.48	-2.80	AAA-AAA	0.00	999	1.000	1.000	0.3160
51158_at	16.0	44.8	1.48	2.80	AAA-AAA	0.00	999	1.000	1.000	0.2217
207794_at	11.1	31.0	1.48	2.80	AAA-AAA	0.00	999	1.000	1.000	0.2455
39318_at	65.6	23.5	-1.48	-2.79	AAA-AAA	0.00	999	1.000	1.000	0.0991

213371_at	56.5	20.2	-1.48	-2.80	AAA-AAA	0.00	999	1.000	1.000	0.0609
216079_at	36.3	13.0	-1.48	-2.80	AAA-AAA	0.00	999	1.000	1.000	0.2367
234236_at	24.8	8.9	-1.48	-2.80	AAA-AAA	0.00	999	1.000	1.000	0.1980
230272_at	6.9	2.5	-1.48	-2.80	AAA-AAA	0.00	999	1.000	1.000	0.1197
229856_s_at	67.3	24.1	-1.48	-2.80	AAA-AAA	0.00	999	1.000	1.000	0.0490
243392_at	47.1	16.9	-1.48	-2.79	AAA-AAA	0.00	999	1.000	1.000	0.2926
1556095_at	8.3	23.2	1.48	2.79	AAA-AAM	0.00	999	1.000	1.000	0.0549
220828_s_at	4.3	11.9	1.48	2.79	AAA-AAA	0.00	999	1.000	1.000	0.2021
234407_s_at	10.5	29.3	1.48	2.79	AAA-AAA	0.00	999	1.000	1.000	0.1404
233828_at	35.6	12.8	-1.48	-2.79	AAA-AAA	0.00	999	1.000	1.000	0.2745
233317_at	17.8	6.4	-1.48	-2.79	AAA-AAA	0.00	999	1.000	1.000	0.0180
241533_at	26.4	9.5	-1.48	-2.79	AAA-AAA	0.00	999	1.000	1.000	0.2042
1560330_at	8.0	2.9	-1.48	-2.79	AAA-AAA	0.00	999	1.000	1.000	0.2286
1568883_at	33.8	12.1	-1.48	-2.79	AAA-AAA	0.00	999	1.000	1.000	0.3301
1569839_s_at	12.7	4.6	-1.48	-2.79	AAA-AAA	0.00	999	1.000	1.000	0.3186
215538_at	15.6	43.6	1.48	2.79	AAA-AAA	0.00	999	1.000	1.000	0.2613
236162_at	11.6	32.5	1.48	2.79	AAA-AAA	0.00	999	1.000	1.000	0.0857
243671_at	25.8	71.9	1.48	2.79	AAA-PPP	0.00	999	1.000	1.000	0.0882
241251_at	17.1	6.1	-1.48	-2.79	AAA-AAA	0.00	999	1.000	1.000	0.4086
1563007_at	51.4	18.4	-1.48	-2.79	AAA-AAA	0.00	999	1.000	1.000	0.1856
1561370_at	12.2	4.4	-1.48	-2.79	AAA-AAA	0.00	999	1.000	1.000	0.1847
207693_at	18.5	6.7	-1.48	-2.79	AAA-AAA	0.00	999	1.000	1.000	0.1388
218960_at	24.0	8.6	-1.48	-2.79	AAA-AAA	0.00	999	1.000	1.000	0.3372
243432_at	26.4	9.5	-1.48	-2.79	AAA-AAA	0.00	999	1.000	1.000	0.2023
244681_at	60.9	21.9	-1.48	-2.79	AAA-AAA	0.00	999	1.000	1.000	0.1994
1559133_at	15.7	5.6	-1.48	-2.79	AAA-AAA	0.00	999	1.000	1.000	0.3348
214275_at	52.1	18.7	-1.48	-2.79	AAA-AAA	0.00	999	1.000	1.000	0.3278
235351_at	4.1	1.5	-1.48	-2.79	AAA-AAA	0.00	999	1.000	1.000	0.0240
238849_at	24.2	8.7	-1.48	-2.79	AAA-AAA	0.00	999	1.000	1.000	0.0754
206862_at	22.5	62.7	1.48	2.79	AAM-APP	0.00	999	1.000	1.000	0.2148
244160_at	28.8	10.3	-1.48	-2.79	AAA-AAA	0.00	999	1.000	1.000	0.2619
240161_s_at	20.6	7.4	-1.48	-2.79	AAA-AAM	0.00	999	1.000	1.000	0.4591
1568751_at	1.1	3.2	1.48	2.79	AAA-AAA	0.00	999	1.000	1.000	0.3047
239210_at	18.2	50.6	1.48	2.78	AAA-MAP	0.00	999	1.000	1.000	0.0983
1563119_at	22.6	62.9	1.48	2.78	AAA-AAA	0.00	999	1.000	1.000	0.1554
1553729_s_at	26.9	9.7	-1.48	-2.78	AAA-AAA	0.00	999	1.000	1.000	0.2765
1554825_at	65.7	23.6	-1.48	-2.78	AAA-AAA	0.00	999	1.000	1.000	0.1551
1554751_at	6.9	19.2	1.48	2.78	AAA-AAA	0.00	999	1.000	1.000	0.4410
217209_at	107.8	38.7	-1.48	-2.78	AAM-AAA	0.00	999	1.000	1.000	0.0422
219196_at	5.8	2.1	-1.48	-2.78	AAA-AAA	0.00	999	1.000	1.000	0.3924
236286_at	29.2	10.5	-1.48	-2.78	AAA-AAA	0.00	999	1.000	1.000	0.0736
205871_at	14.7	41.0	1.48	2.78	AAP-APP	0.00	999	1.000	1.000	0.4040

217818_s_at	194.0	539.6	1.48	2.78	AMP-PPP	0.00	999	1.000	1.000	0.3191
241563_at	19.3	6.9	-1.48	-2.78	AAA-AAA	0.00	999	1.000	1.000	0.2861
243497_at	19.9	7.2	-1.48	-2.78	AAA-AAA	0.00	999	1.000	1.000	0.3243
1569681_at	15.1	5.4	-1.48	-2.78	AAA-AAA	0.00	999	1.000	1.000	0.1720
1570476_at	12.1	4.4	-1.48	-2.78	AAA-AAA	0.00	999	1.000	1.000	0.2904
1559563_at	24.1	8.7	-1.48	-2.78	AAA-AAA	0.00	999	1.000	1.000	0.2121
211101_x_at	17.8	49.4	1.48	2.78	AAA-AAA	0.00	999	1.000	1.000	0.1422
210597_x_at	99.2	35.7	-1.48	-2.78	AAA-AAA	0.00	999	1.000	1.000	0.0693
217567_at	39.5	14.2	-1.48	-2.78	AAA-AAA	0.00	999	1.000	1.000	0.2818
1562991_at	7.1	19.7	1.48	2.78	AAA-AAA	0.00	999	1.000	1.000	0.3589
1570093_at	33.0	11.9	-1.48	-2.78	AAA-AAA	0.00	999	1.000	1.000	0.2374
1552925_at	19.5	54.2	1.48	2.78	AAA-AAA	0.00	999	1.000	1.000	0.1237
211093_at	5.6	2.0	-1.48	-2.78	AAA-AAA	0.00	999	1.000	1.000	0.4630
239612_at	53.1	19.1	-1.48	-2.78	AAA-AAA	0.00	999	1.000	1.000	0.0560
230825_at	39.3	14.1	-1.48	-2.78	AAA-AAA	0.00	999	1.000	1.000	0.3073
230112_at	181.9	505.3	1.47	2.78	APP-PMP	0.00	999	1.000	1.000	0.4615
241809_at	39.8	110.5	1.47	2.78	AAA-AAA	0.00	999	1.000	1.000	0.1665
224228_s_at	8.2	2.9	-1.47	-2.78	AAA-AAA	0.00	999	1.000	1.000	0.1683
234406_at	12.7	35.2	1.47	2.78	AAA-AAA	0.00	999	1.000	1.000	0.1173
205821_at	12.7	4.6	-1.47	-2.78	AAA-AAA	0.00	999	1.000	1.000	0.1856
231606_at	27.0	9.7	-1.47	-2.78	AAA-AAA	0.00	999	1.000	1.000	0.3457
216683_at	20.0	55.6	1.47	2.78	APA-PAA	0.00	999	1.000	1.000	0.4309
222910_s_at	15.5	43.0	1.47	2.78	AAA-PAA	0.00	999	1.000	1.000	0.1634
1569212_at	34.4	12.4	-1.47	-2.78	AAA-AAA	0.00	999	1.000	1.000	0.1133
208432_s_at	11.0	4.0	-1.47	-2.78	AAA-AAA	0.00	999	1.000	1.000	0.0640
224115_at	36.7	13.2	-1.47	-2.78	AAA-AAA	0.00	999	1.000	1.000	0.2802
210919_at	5.2	1.9	-1.47	-2.78	AAA-AAA	0.00	999	1.000	1.000	0.3749
236148_at	38.6	13.9	-1.47	-2.78	AAA-AAA	0.00	999	1.000	1.000	0.2827
1569948_at	36.3	13.1	-1.47	-2.77	AAA-AAA	0.00	999	1.000	1.000	0.4146
1570005_at	12.8	4.6	-1.47	-2.77	AAA-AAA	0.00	999	1.000	1.000	0.2660
241167_at	18.5	6.7	-1.47	-2.77	AAA-AAA	0.00	999	1.000	1.000	0.1848
1566784_at	26.0	9.4	-1.47	-2.77	AAA-AAA	0.00	999	1.000	1.000	0.2572
229479_at	5.2	14.5	1.47	2.77	AAA-AAA	0.00	999	1.000	1.000	0.3096
205495_s_at	28.0	10.1	-1.47	-2.77	AAA-AAA	0.00	999	1.000	1.000	0.3476
213512_at	109.9	39.7	-1.47	-2.77	AAA-AAA	0.00	999	1.000	1.000	0.1936
215808_at	8.5	23.4	1.47	2.77	AAA-AAA	0.00	999	1.000	1.000	0.3024
228828_at	11.6	32.3	1.47	2.77	AAA-AAA	0.00	999	1.000	1.000	0.2962
233634_at	30.9	11.2	-1.47	-2.77	AAA-AAA	0.00	999	1.000	1.000	0.1282
231643_s_at	101.9	36.8	-1.47	-2.77	AAA-AAA	0.00	999	1.000	1.000	0.0638
240880_at	40.7	14.7	-1.47	-2.77	AAA-AAA	0.00	999	1.000	1.000	0.2836
1560911_at	29.6	10.7	-1.47	-2.77	AAA-AAA	0.00	999	1.000	1.000	0.2171
1560265_at	28.3	10.2	-1.47	-2.77	AAA-AAA	0.00	999	1.000	1.000	0.4125

1561373_at	39.5	14.3	-1.47	-2.77	AAA-AAA	0.00	999	1.000	1.000	0.2071
1570408_at	5.0	13.9	1.47	2.77	AAA-AAA	0.00	999	1.000	1.000	0.1722
211579_at	37.5	13.6	-1.47	-2.77	AAA-AAA	0.00	999	1.000	1.000	0.4012
217385_at	9.7	3.5	-1.47	-2.77	AAA-AAA	0.00	999	1.000	1.000	0.2380
208539_x_at	64.7	23.4	-1.47	-2.77	AAA-AAA	0.00	999	1.000	1.000	0.0781
204870_s_at	17.0	6.1	-1.47	-2.77	AAA-AAA	0.00	999	1.000	1.000	0.2013
234620_at	40.2	14.5	-1.47	-2.77	AAA-AAA	0.00	999	1.000	1.000	0.3527
237291_at	37.3	13.5	-1.47	-2.77	AAA-AAA	0.00	999	1.000	1.000	0.2107
238024_at	21.6	59.9	1.47	2.77	AAA-AAA	0.00	999	1.000	1.000	0.2149
237587_at	11.2	31.0	1.47	2.77	AAA-AAA	0.00	999	1.000	1.000	0.0144
1561566_at	7.1	2.6	-1.47	-2.77	AAA-AAA	0.00	999	1.000	1.000	0.3837
1569264_at	32.8	11.9	-1.47	-2.77	AAA-AAA	0.00	999	1.000	1.000	0.0454
207036_x_at	19.2	6.9	-1.47	-2.77	AAA-AAA	0.00	999	1.000	1.000	0.4783
231709_x_at	8.5	23.5	1.47	2.77	PAA-AAA	0.00	999	1.000	1.000	0.4303
1553451_at	15.7	5.7	-1.47	-2.77	AAA-AAA	0.00	999	1.000	1.000	0.2177
1558346_at	10.2	28.3	1.47	2.76	AAA-AAA	0.00	999	1.000	1.000	0.2144
223762_at	19.8	7.2	-1.47	-2.77	AAA-AAA	0.00	999	1.000	1.000	0.2686
211480_s_at	10.1	3.7	-1.47	-2.77	AAA-AAA	0.00	999	1.000	1.000	0.2619
212939_at	42.5	15.4	-1.47	-2.77	AAA-AAA	0.00	999	1.000	1.000	0.2258
238941_at	18.6	6.7	-1.47	-2.76	AAA-AAA	0.00	999	1.000	1.000	0.0753
1569080_at	18.2	6.6	-1.47	-2.76	AAA-AAA	0.00	999	1.000	1.000	0.3981
1555777_at	138.3	50.1	-1.47	-2.76	AAA-AAA	0.00	999	1.000	1.000	0.3246
238165_at	39.8	14.4	-1.47	-2.76	AAA-AAA	0.00	999	1.000	1.000	0.4100
237897_at	18.4	50.9	1.47	2.76	AAA-AAA	0.00	999	1.000	1.000	0.2777
229756_at	45.7	16.5	-1.47	-2.76	AAA-AAA	0.00	999	1.000	1.000	0.2542
206190_at	11.8	4.3	-1.47	-2.76	AAA-AAA	0.00	999	1.000	1.000	0.1128
243822_at	12.3	33.9	1.47	2.76	AAA-AAA	0.00	999	1.000	1.000	0.3856
203591_s_at	47.4	17.2	-1.47	-2.76	AAA-AAA	0.00	999	1.000	1.000	0.1907
1560727_at	63.7	23.1	-1.47	-2.76	AAA-AAA	0.00	999	1.000	1.000	0.1996
220061_at	41.0	14.8	-1.47	-2.76	AAA-AAA	0.00	999	1.000	1.000	0.2089
224403_at	6.9	19.0	1.47	2.76	AAA-AAA	0.00	999	1.000	1.000	0.2960
210652_s_at	24.6	8.9	-1.47	-2.76	AAA-AAA	0.00	999	1.000	1.000	0.1655
239527_at	31.3	11.3	-1.47	-2.76	AMA-AAA	0.00	999	1.000	1.000	0.4202
237057_at	21.6	7.8	-1.47	-2.76	AAA-AAA	0.00	999	1.000	1.000	0.4990
231091_x_at	12.1	33.3	1.47	2.76	AAA-AAA	0.00	999	1.000	1.000	0.2580
1559252_a_at	11.3	31.3	1.46	2.76	AAA-AAA	0.00	999	1.000	1.000	0.3335
1557475_at	5.5	15.3	1.46	2.76	AAA-AAA	0.00	999	1.000	1.000	0.3963
220185_at	46.1	16.7	-1.46	-2.76	AAA-AAA	0.00	999	1.000	1.000	0.1956
222940_at	38.3	13.9	-1.46	-2.76	AAA-AAA	0.00	999	1.000	1.000	0.2614
210819_x_at	123.2	44.6	-1.46	-2.76	AAA-AAA	0.00	999	1.000	1.000	0.1110
236489_at	12.3	4.4	-1.46	-2.76	AAA-AAA	0.00	999	1.000	1.000	0.2354
238169_at	9.6	3.5	-1.46	-2.76	AAA-AAA	0.00	999	1.000	1.000	0.3168

210663_s_at	17.5	48.1	1.46	2.76	AAA-PPA	0.00	999	1.000	1.000	0.3087
236231_at	29.2	80.6	1.46	2.76	AAP-PPP	0.00	999	1.000	1.000	0.2707
210552_s_at	23.3	64.1	1.46	2.76	AAA-PMA	0.00	999	1.000	1.000	0.1974
241518_at	11.4	4.1	-1.46	-2.76	AAA-AAA	0.00	999	1.000	1.000	0.3732
1561651_s_at	27.9	10.1	-1.46	-2.76	AAA-AAA	0.00	999	1.000	1.000	0.2021
1569044_at	8.6	23.7	1.46	2.76	AAA-AAA	0.00	999	1.000	1.000	0.2388
1565756_a_at	44.7	16.2	-1.46	-2.76	AAA-AAA	0.00	999	1.000	1.000	0.3242
1556439_at	11.8	4.3	-1.46	-2.76	AAA-AAA	0.00	999	1.000	1.000	0.2477
1556917_a_at	11.5	31.8	1.46	2.76	AAA-AAA	0.00	999	1.000	1.000	0.1973
211890_x_at	16.5	6.0	-1.46	-2.76	AAA-AAA	0.00	999	1.000	1.000	0.3555
234385_at	5.9	16.1	1.46	2.76	AAA-AAA	0.00	999	1.000	1.000	0.1807
234314_at	9.2	3.3	-1.46	-2.76	AAA-AAA	0.00	999	1.000	1.000	0.2951
237921_at	11.3	4.1	-1.46	-2.76	AAA-AAA	0.00	999	1.000	1.000	0.3213
227747_at	6.4	17.7	1.46	2.76	AAA-AAA	0.00	999	1.000	1.000	0.2608
1561503_at	76.0	27.6	-1.46	-2.76	MAA-AAA	0.00	999	1.000	1.000	0.1075
1553449_at	3.7	10.1	1.46	2.76	AAA-AAA	0.00	999	1.000	1.000	0.2170
1556625_a_at	8.2	3.0	-1.46	-2.75	AAA-AAA	0.00	999	1.000	1.000	0.3057
211442_x_at	9.8	27.1	1.46	2.76	AAA-AAA	0.00	999	1.000	1.000	0.4460
216687_x_at	30.9	11.2	-1.46	-2.76	AAA-AAA	0.00	999	1.000	1.000	0.1217
230870_at	15.0	5.4	-1.46	-2.76	AAA-AAA	0.00	999	1.000	1.000	0.2135
225047_at	299.5	824.5	1.46	2.75	PPP-PPP	0.00	999	1.000	1.000	0.1638
1569630_a_at	39.0	14.2	-1.46	-2.75	AAA-AAA	0.00	999	1.000	1.000	0.0155
1568736_s_at	13.2	36.3	1.46	2.75	AAA-AAA	0.00	999	1.000	1.000	0.2513
1555188_at	66.1	24.0	-1.46	-2.75	AAA-AAA	0.00	999	1.000	1.000	0.0790
1552806_a_at	14.8	5.4	-1.46	-2.75	AAA-AAA	0.00	999	1.000	1.000	0.1465
1559500_at	57.6	20.9	-1.46	-2.75	AAA-AAA	0.00	999	1.000	1.000	0.2093
214846_s_at	19.5	7.1	-1.46	-2.75	AAA-AAA	0.00	999	1.000	1.000	0.2349
237680_at	22.7	8.2	-1.46	-2.75	AAA-AAA	0.00	999	1.000	1.000	0.3731
213764_s_at	6.9	18.9	1.46	2.75	AAA-APA	0.00	999	1.000	1.000	0.3702
243354_at	14.7	5.3	-1.46	-2.75	AAA-AAA	0.00	999	1.000	1.000	0.3876
1556489_at	19.0	6.9	-1.46	-2.75	AAA-AAA	0.00	999	1.000	1.000	0.1730
206977_at	10.8	3.9	-1.46	-2.75	AAA-AAA	0.00	999	1.000	1.000	0.3151
211741_x_at	207.0	75.2	-1.46	-2.75	AAA-AAA	0.00	999	1.000	1.000	0.0173
219463_at	11.4	4.1	-1.46	-2.75	AAA-AAA	0.00	999	1.000	1.000	0.1579
204733_at	53.9	19.6	-1.46	-2.75	AAA-AAA	0.00	999	1.000	1.000	0.3888
229566_at	33.0	12.0	-1.46	-2.75	AAA-AAA	0.00	999	1.000	1.000	0.0976
204096_s_at	25.8	71.0	1.46	2.75	AAP-AAM	0.00	999	1.000	1.000	0.2894
239014_at	16.0	43.8	1.46	2.75	AAA-AAP	0.00	999	1.000	1.000	0.1715
244526_at	8.3	3.0	-1.46	-2.75	AAA-AAA	0.00	999	1.000	1.000	0.0349
216981_x_at	79.7	29.0	-1.46	-2.75	AAA-AAA	0.00	999	1.000	1.000	0.2989
206189_at	5.1	14.1	1.46	2.75	AAA-AAA	0.00	999	1.000	1.000	0.1556
240085_at	45.3	16.5	-1.46	-2.75	AAA-AAA	0.00	999	1.000	1.000	0.1379

1553281_at	21.1	7.7	-1.46	-2.75	AAA-AAA	0.00	999	1.000	1.000	0.0557
206806_at	12.7	34.9	1.46	2.75	AAA-AAA	0.00	999	1.000	1.000	0.0369
237532_at	38.6	14.1	-1.46	-2.75	AAA-AAA	0.00	999	1.000	1.000	0.1523
237168_at	5.9	16.2	1.46	2.75	AAA-AAP	0.00	999	1.000	1.000	0.3660
1561506_at	43.1	15.7	-1.46	-2.75	AAA-AAA	0.00	999	1.000	1.000	0.1451
1569207_s_at	35.0	12.8	-1.46	-2.75	AAA-AAA	0.00	999	1.000	1.000	0.3105
1570044_at	40.5	14.7	-1.46	-2.75	AAA-AAA	0.00	999	1.000	1.000	0.1553
1567251_at	35.3	12.9	-1.46	-2.75	AAA-AAA	0.00	999	1.000	1.000	0.4174
1556876_s_at	10.0	3.6	-1.46	-2.75	AAA-AAA	0.00	999	1.000	1.000	0.2473
207964_x_at	43.7	15.9	-1.46	-2.75	AAA-AAA	0.00	999	1.000	1.000	0.2999
213985_s_at	30.4	11.1	-1.46	-2.75	AAA-AAA	0.00	999	1.000	1.000	0.3017
214265_at	35.0	12.8	-1.46	-2.75	AAA-AAA	0.00	999	1.000	1.000	0.3645
211909_x_at	17.2	6.3	-1.46	-2.75	AAA-AAA	0.00	999	1.000	1.000	0.1326
238290_at	60.5	22.0	-1.46	-2.75	AAA-AAA	0.00	999	1.000	1.000	0.0995
233118_at	16.7	6.1	-1.46	-2.75	AAA-AAA	0.00	999	1.000	1.000	0.2763
205488_at	22.7	8.3	-1.46	-2.75	AAA-AAA	0.00	999	1.000	1.000	0.3507
239849_at	17.8	6.5	-1.46	-2.74	AAA-AAA	0.00	999	1.000	1.000	0.4219
243310_at	16.0	43.8	1.46	2.74	AAA-AAA	0.00	999	1.000	1.000	0.0765
1561092_at	19.1	7.0	-1.46	-2.74	AAA-AAA	0.00	999	1.000	1.000	0.0735
244542_at	54.5	19.9	-1.46	-2.74	AAA-AAA	0.00	999	1.000	1.000	0.1498
207273_at	43.1	15.7	-1.46	-2.74	AAA-AAA	0.00	999	1.000	1.000	0.1390
230900_at	3.7	10.2	1.46	2.74	AAA-AAA	0.00	999	1.000	1.000	0.1682
240433_x_at	6.3	2.3	-1.46	-2.74	AAA-AAA	0.00	999	1.000	1.000	0.3747
233719_s_at	21.5	58.9	1.46	2.74	AAA-AMA	0.00	999	1.000	1.000	0.2543
242166_at	11.4	31.1	1.45	2.74	AAP-PAA	0.00	999	1.000	1.000	0.1711
240500_at	30.9	11.3	-1.45	-2.74	AAA-AAA	0.00	999	1.000	1.000	0.1983
244775_at	26.5	9.7	-1.45	-2.74	AAA-AAA	0.00	999	1.000	1.000	0.1228
211303_x_at	26.9	9.8	-1.45	-2.74	AAA-AAA	0.00	999	1.000	1.000	0.2015
237899_at	13.7	37.6	1.45	2.74	AAA-AAA	0.00	999	1.000	1.000	0.1898
1566835_at	64.4	23.5	-1.45	-2.74	AAA-AAA	0.00	999	1.000	1.000	0.3236
1555692_at	6.9	2.5	-1.45	-2.74	AAA-AAA	0.00	999	1.000	1.000	0.2689
221465_at	45.1	16.5	-1.45	-2.74	AAA-AAA	0.00	999	1.000	1.000	0.4288
219425_at	62.2	22.7	-1.45	-2.74	AAA-AAA	0.00	999	1.000	1.000	0.2674
234839_at	89.0	32.5	-1.45	-2.74	AMA-AAA	0.00	999	1.000	1.000	0.1768
237341_at	24.3	8.9	-1.45	-2.74	AAA-AAA	0.00	999	1.000	1.000	0.3448
238323_at	42.2	115.3	1.45	2.74	AAP-AMP	0.00	999	1.000	1.000	0.3160
216276_s_at	5.6	15.4	1.45	2.74	AAA-PAA	0.00	999	1.000	1.000	0.2421
243393_at	6.6	2.4	-1.45	-2.74	AAA-AAA	0.00	999	1.000	1.000	0.0898
1569307_s_at	12.3	33.6	1.45	2.74	AAA-AAA	0.00	999	1.000	1.000	0.0976
1569371_at	43.3	15.8	-1.45	-2.74	AAA-AAA	0.00	999	1.000	1.000	0.2780
1569861_at	8.0	21.9	1.45	2.74	AAA-AAA	0.00	999	1.000	1.000	0.2754
1566606_a_at	26.3	9.6	-1.45	-2.74	AAA-AAA	0.00	999	1.000	1.000	0.3268

223838_at	5.0	13.7	1.45	2.74	AAA-AAA	0.00	999	1.000	1.000	0.1861
214621_at	7.1	2.6	-1.45	-2.74	AAA-AAA	0.00	999	1.000	1.000	0.4271
216970_at	11.7	4.3	-1.45	-2.74	AAA-AAA	0.00	999	1.000	1.000	0.2614
242060_x_at	32.2	11.8	-1.45	-2.73	AAA-AAA	0.00	999	1.000	1.000	0.2831
1570289_at	32.4	11.9	-1.45	-2.73	AAA-AAA	0.00	999	1.000	1.000	0.2833
1564323_at	38.8	14.2	-1.45	-2.73	AAA-AAA	0.00	999	1.000	1.000	0.2110
1566448_at	16.9	6.2	-1.45	-2.74	AAA-AAA	0.00	999	1.000	1.000	0.1255
1566480_x_at	131.7	48.2	-1.45	-2.73	AAA-AAA	0.00	999	1.000	1.000	0.2631
221182_at	13.5	4.9	-1.45	-2.73	AAA-AAA	0.00	999	1.000	1.000	0.2440
224358_s_at	33.3	12.2	-1.45	-2.74	AAA-AAA	0.00	999	1.000	1.000	0.0208
213780_at	13.4	4.9	-1.45	-2.73	AAA-AAA	0.00	999	1.000	1.000	0.2686
210921_at	7.2	2.6	-1.45	-2.73	AAA-AAA	0.00	999	1.000	1.000	0.2498
216826_at	21.8	8.0	-1.45	-2.73	AAA-AAA	0.00	999	1.000	1.000	0.2927
208381_s_at	43.0	117.5	1.45	2.73	AAA-PAP	0.00	999	1.000	1.000	0.1701
243040_at	6.4	2.3	-1.45	-2.73	AAA-AAA	0.00	999	1.000	1.000	0.3615
240289_at	39.0	14.3	-1.45	-2.73	AAA-AAA	0.00	999	1.000	1.000	0.4004
1568672_at	4.6	12.5	1.45	2.73	AAA-AAA	0.00	999	1.000	1.000	0.4164
221639_x_at	47.5	17.4	-1.45	-2.73	AAA-AAA	0.00	999	1.000	1.000	0.1574
216811_at	20.0	54.7	1.45	2.73	AAA-AAA	0.00	999	1.000	1.000	0.0927
235807_at	24.5	9.0	-1.45	-2.73	AAA-AAA	0.00	999	1.000	1.000	0.1048
234235_at	7.7	2.8	-1.45	-2.73	AAA-AAA	0.00	999	1.000	1.000	0.4115
237529_at	5.5	14.9	1.45	2.73	AAA-AAA	0.00	999	1.000	1.000	0.2585
205975_s_at	74.8	27.4	-1.45	-2.73	AAA-AAA	0.00	999	1.000	1.000	0.0516
233684_at	29.4	10.7	-1.45	-2.73	AAA-AAA	0.00	999	1.000	1.000	0.2121
241540_at	23.7	8.7	-1.45	-2.73	AAA-AAA	0.00	999	1.000	1.000	0.2047
1560943_s_at	68.4	25.1	-1.45	-2.73	AAA-AAA	0.00	999	1.000	1.000	0.0012
221325_at	10.0	27.3	1.45	2.73	AAA-AAA	0.00	999	1.000	1.000	0.0330
234424_at	22.5	8.2	-1.45	-2.73	AAA-AAA	0.00	999	1.000	1.000	0.3706
234613_at	26.6	9.8	-1.45	-2.73	AAA-AAA	0.00	999	1.000	1.000	0.0608
232104_at	34.4	12.6	-1.45	-2.73	AAA-AAA	0.00	999	1.000	1.000	0.3347
234851_at	6.4	17.5	1.45	2.73	AAA-PAA	0.00	999	1.000	1.000	0.4402
1560291_at	19.2	52.4	1.45	2.73	AAA-AAA	0.00	999	1.000	1.000	0.1135
208038_at	35.0	12.8	-1.45	-2.73	AAA-AAA	0.00	999	1.000	1.000	0.3466
215085_x_at	26.1	9.6	-1.45	-2.73	AAA-AAA	0.00	999	1.000	1.000	0.1890
210429_at	6.5	2.4	-1.45	-2.73	AAA-AAA	0.00	999	1.000	1.000	0.2631
216636_at	10.8	4.0	-1.45	-2.73	AAA-AAA	0.00	999	1.000	1.000	0.1782
234496_x_at	16.4	6.0	-1.45	-2.73	AAA-AAA	0.00	999	1.000	1.000	0.0474
232203_at	48.0	17.6	-1.45	-2.73	AAA-AAA	0.00	999	1.000	1.000	0.2274
1564301_a_at	21.5	58.6	1.45	2.73	APA-PAP	0.00	999	1.000	1.000	0.1672
242087_x_at	35.7	13.1	-1.45	-2.73	AAA-AAA	0.00	999	1.000	1.000	0.3917
1552988_at	5.9	16.0	1.45	2.73	AAA-AAA	0.00	999	1.000	1.000	0.2954
229685_at	20.9	56.8	1.45	2.72	AAA-APP	0.00	999	1.000	1.000	0.2850

1562904_s_at	13.2	36.0	1.45	2.73	AAA-AAA	0.00	999	1.000	1.000	0.2442
1559355_at	21.0	7.7	-1.45	-2.72	AAA-AAA	0.00	999	1.000	1.000	0.4132
1556810_a_at	12.6	4.6	-1.45	-2.73	AAA-AAA	0.00	999	1.000	1.000	0.1371
210915_x_at	32.7	89.0	1.45	2.72	AAA-AAA	0.00	999	1.000	1.000	0.0288
218825_at	61.5	167.5	1.45	2.73	AAA-AAA	0.00	999	1.000	1.000	0.1328
236061_at	58.3	21.4	-1.45	-2.72	AAA-AAA	0.00	999	1.000	1.000	0.4384
236629_at	34.3	93.5	1.45	2.73	AAA-AAA	0.00	999	1.000	1.000	0.1460
238466_at	14.9	5.5	-1.45	-2.73	AAA-AAA	0.00	999	1.000	1.000	0.3241
238280_at	19.6	53.3	1.45	2.73	AAA-AAM	0.00	999	1.000	1.000	0.3102
230049_at	33.3	12.2	-1.45	-2.73	AAA-AAA	0.00	999	1.000	1.000	0.4566
233410_at	28.7	10.5	-1.45	-2.72	AAA-AAA	0.00	999	1.000	1.000	0.1547
241549_at	10.2	3.8	-1.45	-2.72	AAA-AAA	0.00	999	1.000	1.000	0.2895
243466_at	25.4	9.3	-1.45	-2.72	AAA-AAA	0.00	999	1.000	1.000	0.2954
1561902_at	7.4	2.7	-1.45	-2.72	AAA-AAA	0.00	999	1.000	1.000	0.2844
1564212_at	51.0	18.7	-1.45	-2.72	AAA-AAA	0.00	999	1.000	1.000	0.2393
208062_s_at	3.6	9.7	1.45	2.72	AAA-AAA	0.00	999	1.000	1.000	0.1429
234913_at	19.7	7.2	-1.45	-2.72	AAA-AAA	0.00	999	1.000	1.000	0.2577
222342_at	10.2	3.8	-1.44	-2.72	AAA-AAA	0.00	999	1.000	1.000	0.2007
210955_at	94.5	34.7	-1.44	-2.72	AAA-AAA	0.00	999	1.000	1.000	0.1588
239468_at	25.3	9.3	-1.44	-2.72	AAA-AAA	0.00	999	1.000	1.000	0.2121
237252_at	13.9	37.9	1.44	2.72	AAA-AAA	0.00	999	1.000	1.000	0.2148
230872_s_at	36.7	13.5	-1.44	-2.72	AAA-AAA	0.00	999	1.000	1.000	0.4294
205390_s_at	20.3	7.5	-1.44	-2.72	AAA-AAA	0.00	999	1.000	1.000	0.1320
1567705_at	10.2	27.6	1.44	2.72	AAA-AAP	0.00	999	1.000	1.000	0.3623
240389_at	15.3	41.7	1.44	2.72	AAA-MAA	0.00	999	1.000	1.000	0.2978
1561003_at	13.2	4.8	-1.44	-2.72	AAA-AAA	0.00	999	1.000	1.000	0.3696
1553237_x_at	77.9	28.7	-1.44	-2.72	AAA-AAA	0.00	999	1.000	1.000	0.2535
1557898_at	11.5	4.2	-1.44	-2.72	AAA-AAA	0.00	999	1.000	1.000	0.2115
31835_at	47.1	17.3	-1.44	-2.72	AAA-AAA	0.00	999	1.000	1.000	0.0910
234089_at	20.1	7.4	-1.44	-2.72	AAA-AAA	0.00	999	1.000	1.000	0.2905
234917_at	22.9	62.2	1.44	2.72	AAA-AAA	0.00	999	1.000	1.000	0.0973
228918_at	21.1	57.4	1.44	2.72	AAA-AAA	0.00	999	1.000	1.000	0.1034
1557883_a_at	9.4	25.6	1.44	2.72	AAA-AAA	0.00	999	1.000	1.000	0.2461
1557321_a_at	27.9	10.3	-1.44	-2.72	AMA-AAA	0.00	999	1.000	1.000	0.2093
217470_at	21.0	7.7	-1.44	-2.72	AAA-AAA	0.00	999	1.000	1.000	0.4358
239411_at	65.8	24.2	-1.44	-2.72	AAA-AAA	0.00	999	1.000	1.000	0.1328
237387_at	11.0	29.8	1.44	2.72	AAA-AAA	0.00	999	1.000	1.000	0.3281
237354_at	19.3	7.1	-1.44	-2.72	AAA-AAA	0.00	999	1.000	1.000	0.3082
228896_at	14.3	5.3	-1.44	-2.72	AAA-AAA	0.00	999	1.000	1.000	0.2483
231947_at	65.3	177.4	1.44	2.72	APA-PPP	0.00	999	1.000	1.000	0.0833
1569206_at	60.0	22.1	-1.44	-2.71	AAA-AAA	0.00	999	1.000	1.000	0.2467
1569981_at	29.1	10.7	-1.44	-2.72	AAA-AAA	0.00	999	1.000	1.000	0.1867

1564466_at	32.2	11.9	-1.44	-2.72	AAA-AAA	0.00	999	1.000	1.000	0.1663
221456_at	20.2	7.4	-1.44	-2.72	AAA-AAA	0.00	999	1.000	1.000	0.4171
208479_at	30.4	11.2	-1.44	-2.72	AAA-AAA	0.00	999	1.000	1.000	0.2872
220323_at	160.5	59.1	-1.44	-2.72	AAA-AAA	0.00	999	1.000	1.000	0.1820
214530_x_at	8.8	3.2	-1.44	-2.72	AAA-AAA	0.00	999	1.000	1.000	0.0795
234087_at	28.4	10.5	-1.44	-2.72	AMA-AAA	0.00	999	1.000	1.000	0.2696
227901_at	25.2	68.4	1.44	2.72	AAA-AAA	0.00	999	1.000	1.000	0.1005
205553_s_at	9.8	3.6	-1.44	-2.72	AAA-AAA	0.00	999	1.000	1.000	0.2319
1559593_a_at	17.3	47.0	1.44	2.71	AAA-AAP	0.00	999	1.000	1.000	0.2138
1561923_a_at	62.5	23.0	-1.44	-2.71	AAA-AAA	0.00	999	1.000	1.000	0.3158
1563858_at	3.9	1.5	-1.44	-2.71	AAA-AAA	0.00	999	1.000	1.000	0.0913
1559169_at	27.1	10.0	-1.44	-2.71	AAA-AAA	0.00	999	1.000	1.000	0.4129
33579_i_at	248.5	91.6	-1.44	-2.71	AAA-AAA	0.00	999	1.000	1.000	0.0210
206598_at	8.7	3.2	-1.44	-2.71	AAA-AAA	0.00	999	1.000	1.000	0.2107
224043_s_at	7.6	2.8	-1.44	-2.71	AAA-AAA	0.00	999	1.000	1.000	0.4001
216216_at	19.0	7.0	-1.44	-2.71	AAA-AAA	0.00	999	1.000	1.000	0.2133
1563207_at	4.1	11.2	1.44	2.71	AAA-AAP	0.00	999	1.000	1.000	0.3395
203751_x_at	75.9	205.8	1.44	2.71	AAA-APA	0.00	999	1.000	1.000	0.1527
202869_at	24.8	9.1	-1.44	-2.71	AAA-AAA	0.00	999	1.000	1.000	0.3770
244117_at	11.3	4.2	-1.44	-2.71	AAA-AAA	0.00	999	1.000	1.000	0.0516
241486_at	11.5	31.0	1.44	2.71	AAA-AAA	0.00	999	1.000	1.000	0.1402
1559664_at	17.2	6.3	-1.44	-2.71	AAA-AAA	0.00	999	1.000	1.000	0.4341
1559920_a_at	9.5	25.7	1.44	2.71	AAA-AAA	0.00	999	1.000	1.000	0.2292
236013_at	9.2	3.4	-1.44	-2.71	AAA-AAA	0.00	999	1.000	1.000	0.2407
239420_at	50.1	18.5	-1.44	-2.71	AAA-AAA	0.00	999	1.000	1.000	0.2444
233648_at	38.8	14.3	-1.44	-2.71	AAA-AAA	0.00	999	1.000	1.000	0.1925
229896_at	8.7	23.5	1.44	2.71	AAA-AAP	0.00	999	1.000	1.000	0.1840
242883_at	89.5	33.1	-1.44	-2.71	AAA-AAA	0.00	999	1.000	1.000	0.2672
241854_at	21.8	59.0	1.44	2.71	AAA-AAA	0.00	999	1.000	1.000	0.2729
1561521_at	12.3	4.6	-1.44	-2.71	AAA-AAA	0.00	999	1.000	1.000	0.3725
213821_s_at	40.2	14.8	-1.44	-2.71	AAA-AAA	0.00	999	1.000	1.000	0.3849
238715_at	52.9	19.5	-1.44	-2.71	AAA-AAA	0.00	999	1.000	1.000	0.3842
232317_at	7.2	2.7	-1.44	-2.71	AAA-AAA	0.00	999	1.000	1.000	0.2903
1558899_s_at	30.5	11.3	-1.44	-2.71	AAA-AAA	0.00	999	1.000	1.000	0.3006
220972_s_at	3.6	1.3	-1.44	-2.71	AAA-AAA	0.00	999	1.000	1.000	0.0698
210953_at	12.5	33.8	1.44	2.71	AAA-AAA	0.00	999	1.000	1.000	0.3137
238910_at	40.5	15.0	-1.44	-2.71	AAA-AAA	0.00	999	1.000	1.000	0.2540
229901_at	12.7	4.7	-1.44	-2.71	AAA-AAA	0.00	999	1.000	1.000	0.0205
1564744_at	11.1	4.1	-1.44	-2.71	AAA-AAA	0.00	999	1.000	1.000	0.3414
1552743_at	24.5	9.0	-1.44	-2.71	AAA-AAA	0.00	999	1.000	1.000	0.0434
208468_at	13.1	4.9	-1.44	-2.71	AAA-AAA	0.00	999	1.000	1.000	0.0671
216567_at	4.6	12.3	1.44	2.71	AAA-AAA	0.00	999	1.000	1.000	0.2001

231068_at	14.6	5.4	-1.44	-2.71	AAA-AAA	0.00	999	1.000	1.000	0.2031
231898_x_at	1.2	3.2	1.44	2.71	AAA-AAA	0.00	999	1.000	1.000	0.4064
1570306_at	7.9	2.9	-1.44	-2.70	AAA-AAA	0.00	999	1.000	1.000	0.3610
1555787_at	15.9	5.9	-1.44	-2.70	AAA-AAA	0.00	999	1.000	1.000	0.0478
211421_s_at	24.7	9.2	-1.44	-2.70	AAA-AAA	0.00	999	1.000	1.000	0.1516
239632_at	14.5	5.4	-1.44	-2.70	AAA-AAA	0.00	999	1.000	1.000	0.2627
206368_at	45.3	16.7	-1.44	-2.71	AAA-MAA	0.00	999	1.000	1.000	0.1173
233313_at	37.1	100.2	1.43	2.70	AAA-AMP	0.00	999	1.000	1.000	0.1001
1569712_at	6.3	2.3	-1.43	-2.70	AAA-AAA	0.00	999	1.000	1.000	0.2295
1565556_at	58.7	21.7	-1.43	-2.70	AAA-AAA	0.00	999	1.000	1.000	0.3418
1557876_at	3.9	1.4	-1.43	-2.70	AAA-AAA	0.00	999	1.000	1.000	0.3750
228804_at	30.5	82.3	1.43	2.70	AAA-AAA	0.00	999	1.000	1.000	0.0707
207925_at	67.0	24.8	-1.43	-2.70	AAA-AAA	0.00	999	1.000	1.000	0.1635
222068_s_at	15.1	5.6	-1.43	-2.70	AAA-AAA	0.00	999	1.000	1.000	0.0066
213172_at	3.1	8.5	1.43	2.70	AAA-AAA	0.00	999	1.000	1.000	0.4330
235929_s_at	18.5	6.8	-1.43	-2.70	AAA-AAA	0.00	999	1.000	1.000	0.0863
238544_at	46.7	17.3	-1.43	-2.70	AAA-AAA	0.00	999	1.000	1.000	0.2874
242662_at	9.4	25.4	1.43	2.70	AAA-AAA	0.00	999	1.000	1.000	0.2663
219916_s_at	20.9	7.8	-1.43	-2.70	AAA-AAA	0.00	999	1.000	1.000	0.1841
220691_at	62.6	23.2	-1.43	-2.70	AAA-AAA	0.00	999	1.000	1.000	0.2541
207481_at	9.2	24.7	1.43	2.70	AAA-AAA	0.00	999	1.000	1.000	0.2704
228554_at	10.8	4.0	-1.43	-2.70	AAA-AAA	0.00	999	1.000	1.000	0.0991
1554077_a_at	62.5	168.4	1.43	2.70	AAP-PMA	0.00	999	1.000	1.000	0.3429
236966_at	26.2	70.6	1.43	2.70	AAA-PAA	0.00	999	1.000	1.000	0.3088
243888_at	15.0	40.5	1.43	2.70	AAA-AAP	0.00	999	1.000	1.000	0.1277
240065_at	13.7	5.1	-1.43	-2.70	AAA-AAA	0.00	999	1.000	1.000	0.4571
225540_at	4.6	12.4	1.43	2.70	AAA-AAA	0.00	999	1.000	1.000	0.3535
208550_x_at	14.5	5.4	-1.43	-2.70	AAA-AAA	0.00	999	1.000	1.000	0.0659
232554_at	86.7	32.2	-1.43	-2.70	AAA-AAA	0.00	999	1.000	1.000	0.0766
208401_s_at	9.7	26.2	1.43	2.70	AAA-AAP	0.00	999	1.000	1.000	0.0909
241631_at	6.4	17.2	1.43	2.69	AAA-AAA	0.00	999	1.000	1.000	0.2453
1563913_at	42.3	15.7	-1.43	-2.70	AAA-AAA	0.00	999	1.000	1.000	0.1715
1560950_at	12.8	34.4	1.43	2.70	AAA-AAA	0.00	999	1.000	1.000	0.1774
1561591_at	72.4	26.9	-1.43	-2.69	AAA-AAA	0.00	999	1.000	1.000	0.0654
1561429_a_at	17.4	6.5	-1.43	-2.69	AAA-AAA	0.00	999	1.000	1.000	0.2349
1566162_x_at	6.7	17.9	1.43	2.70	AAA-AAA	0.00	999	1.000	1.000	0.2929
1554228_a_at	45.1	16.7	-1.43	-2.70	AAA-AAA	0.00	999	1.000	1.000	0.2531
206229_x_at	114.4	42.4	-1.43	-2.70	AMA-AAA	0.00	999	1.000	1.000	0.0492
240444_x_at	19.0	51.1	1.43	2.69	AAA-AAA	0.00	999	1.000	1.000	0.1998
1560154_a_at	15.0	5.6	-1.43	-2.69	AAA-AAA	0.00	999	1.000	1.000	0.1066
1566208_at	7.3	2.7	-1.43	-2.69	AAA-AAA	0.00	999	1.000	1.000	0.1100
206720_at	19.2	7.1	-1.43	-2.69	AAA-AAA	0.00	999	1.000	1.000	0.2172

235150_at	17.5	6.5	-1.43	-2.69	AAA-AAA	0.00	999	1.000	1.000	0.2976
229782_at	5.8	2.2	-1.43	-2.69	AAA-AAA	0.00	999	1.000	1.000	0.0547
230362_at	43.8	117.9	1.43	2.69	AAA-AAA	0.00	999	1.000	1.000	0.1056
233727_at	47.3	17.6	-1.43	-2.69	AAA-AAA	0.00	999	1.000	1.000	0.1938
227583_at	49.5	133.3	1.43	2.69	AAA-PAA	0.00	999	1.000	1.000	0.2468
207075_at	51.2	137.8	1.43	2.69	AAA-PAA	0.00	999	1.000	1.000	0.1447
1566869_at	27.0	10.0	-1.43	-2.69	AAA-AAA	0.00	999	1.000	1.000	0.3503
1552379_at	5.5	2.0	-1.43	-2.69	AAA-AAA	0.00	999	1.000	1.000	0.3919
244572_at	31.9	11.8	-1.43	-2.69	AAA-AAA	0.00	999	1.000	1.000	0.2812
206446_s_at	32.8	12.2	-1.43	-2.69	AAA-AAA	0.00	999	1.000	1.000	0.2520
210392_x_at	33.2	12.4	-1.43	-2.69	AAA-AAA	0.00	999	1.000	1.000	0.1980
216510_x_at	21.0	7.8	-1.43	-2.69	AAA-AAA	0.00	999	1.000	1.000	0.3591
234544_at	9.7	3.6	-1.43	-2.69	AAA-AAA	0.00	999	1.000	1.000	0.2693
237749_at	10.9	4.0	-1.43	-2.69	AAA-AAA	0.00	999	1.000	1.000	0.0425
205721_at	71.6	26.6	-1.43	-2.69	AAA-AAA	0.00	999	1.000	1.000	0.0335
206187_at	44.6	16.6	-1.43	-2.69	AAA-AAA	0.00	999	1.000	1.000	0.3134
228995_at	28.4	10.5	-1.43	-2.69	AAA-AAA	0.00	999	1.000	1.000	0.2259
231181_at	14.3	5.3	-1.43	-2.69	AAA-AAA	0.00	999	1.000	1.000	0.4102
1561340_at	9.3	25.0	1.43	2.69	AAA-AAP	0.00	999	1.000	1.000	0.3827
240073_at	9.0	24.1	1.43	2.69	AAA-AAA	0.00	999	1.000	1.000	0.3307
242009_at	25.6	9.5	-1.43	-2.69	AAA-AAA	0.00	999	1.000	1.000	0.4470
1554492_at	4.6	12.4	1.43	2.69	AAA-AAA	0.00	999	1.000	1.000	0.3038
214936_at	13.9	37.3	1.43	2.69	AAA-AAA	0.00	999	1.000	1.000	0.0467
237056_at	11.5	4.3	-1.43	-2.69	AAA-AAA	0.00	999	1.000	1.000	0.4084
229967_at	13.9	37.4	1.43	2.69	AAA-AAA	0.00	999	1.000	1.000	0.2936
232665_x_at	124.5	334.6	1.43	2.69	PPP-MPP	0.00	999	1.000	1.000	0.0654
1552421_a_at	12.4	4.6	-1.43	-2.69	AAA-AAA	0.00	999	1.000	1.000	0.0069
221660_at	233.7	87.0	-1.43	-2.69	AAA-AAA	0.00	999	1.000	1.000	0.2510
231525_at	18.5	6.9	-1.43	-2.69	AAA-AAA	0.00	999	1.000	1.000	0.2247
231523_at	16.9	6.3	-1.43	-2.69	AAA-AAA	0.00	999	1.000	1.000	0.5081
242873_at	56.9	21.2	-1.43	-2.68	AAA-AAA	0.00	999	1.000	1.000	0.2648
1569656_at	18.6	6.9	-1.43	-2.69	AAA-AAA	0.00	999	1.000	1.000	0.2851
1553426_at	11.6	31.1	1.43	2.69	AAA-AAA	0.00	999	1.000	1.000	0.1690
1553600_at	43.3	16.1	-1.43	-2.69	AAA-AAA	0.00	999	1.000	1.000	0.4133
1555212_at	47.8	17.8	-1.43	-2.68	AAA-AAA	0.00	999	1.000	1.000	0.1684
1552948_at	7.8	20.9	1.43	2.69	AAA-AAA	0.00	999	1.000	1.000	0.4627
1557520_a_at	13.3	5.0	-1.43	-2.68	AAA-AAA	0.00	999	1.000	1.000	0.3006
216562_at	52.1	19.4	-1.43	-2.69	AAA-AAA	0.00	999	1.000	1.000	0.4199
237955_at	31.6	11.8	-1.43	-2.69	AAA-AAA	0.00	999	1.000	1.000	0.3739
231804_at	11.6	4.3	-1.43	-2.69	AAA-AAA	0.00	999	1.000	1.000	0.2432
230706_s_at	56.7	152.1	1.42	2.68	AAP-PPP	0.00	999	1.000	1.000	0.1001
1560621_at	37.2	13.9	-1.42	-2.68	AMA-AAA	0.00	999	1.000	1.000	0.3137

1557453_at	106.1	39.6	-1.42	-2.68	AAA-AAA	0.00	999	1.000	1.000	0.1107
209775_x_at	29.9	11.1	-1.42	-2.68	AAA-AAA	0.00	999	1.000	1.000	0.2521
217250_s_at	31.6	11.8	-1.42	-2.68	AAA-AAA	0.00	999	1.000	1.000	0.1389
218730_s_at	2.2	5.9	1.42	2.68	AAA-AAA	0.00	999	1.000	1.000	0.1363
236894_at	24.2	9.0	-1.42	-2.68	AAA-AAA	0.00	999	1.000	1.000	0.3112
229306_at	47.0	17.5	-1.42	-2.68	AAA-AMA	0.00	999	1.000	1.000	0.3543
228885_at	3.3	8.7	1.42	2.68	AAA-AAA	0.00	999	1.000	1.000	0.3658
203646_at	517.6	1388.1	1.42	2.68	PPP-PPP	0.00	999	1.000	1.000	0.3953
240034_at	10.0	3.7	-1.42	-2.68	AAA-AAA	0.00	999	1.000	1.000	0.3373
1567663_at	5.5	2.1	-1.42	-2.68	AAA-AAA	0.00	999	1.000	1.000	0.1766
233856_at	44.7	16.7	-1.42	-2.68	AAA-AAA	0.00	999	1.000	1.000	0.1179
238370_x_at	16.6	6.2	-1.42	-2.68	MAA-AAA	0.00	999	1.000	1.000	0.3498
237512_at	23.5	8.8	-1.42	-2.68	AAA-AAA	0.00	999	1.000	1.000	0.1046
240816_at	9.0	24.1	1.42	2.68	AAA-AAA	0.00	999	1.000	1.000	0.0650
241309_at	20.2	7.5	-1.42	-2.68	AAA-AAA	0.00	999	1.000	1.000	0.4177
240469_at	5.8	2.2	-1.42	-2.68	AAA-AAA	0.00	999	1.000	1.000	0.3262
1565849_a_at	28.6	10.7	-1.42	-2.68	AAA-AAA	0.00	999	1.000	1.000	0.1601
1553417_at	44.1	16.5	-1.42	-2.68	AAA-AAA	0.00	999	1.000	1.000	0.1127
207849_at	5.0	1.9	-1.42	-2.68	AAA-AAA	0.00	999	1.000	1.000	0.4092
222374_at	7.4	19.8	1.42	2.68	AAA-AAA	0.00	999	1.000	1.000	0.3389
222314_x_at	13.3	5.0	-1.42	-2.68	AAA-AAA	0.00	999	1.000	1.000	0.0826
214490_at	32.8	12.2	-1.42	-2.68	AAA-AAA	0.00	999	1.000	1.000	0.1728
236642_at	10.2	27.3	1.42	2.68	AAA-AAA	0.00	999	1.000	1.000	0.4097
228055_at	16.3	6.1	-1.42	-2.68	AAA-AAA	0.00	999	1.000	1.000	0.0928
230534_at	20.4	54.6	1.42	2.68	AAA-AAP	0.00	999	1.000	1.000	0.2981
242102_at	5.1	1.9	-1.42	-2.68	AAA-AAA	0.00	999	1.000	1.000	0.1097
243362_s_at	24.1	9.0	-1.42	-2.68	AAA-AAA	0.00	999	1.000	1.000	0.3252
1569944_at	35.7	13.3	-1.42	-2.68	AAA-AAA	0.00	999	1.000	1.000	0.3010
1552276_a_at	167.9	62.7	-1.42	-2.68	AAA-AAA	0.00	999	1.000	1.000	0.2761
1556700_a_at	16.5	6.2	-1.42	-2.68	AAA-AAA	0.00	999	1.000	1.000	0.2579
219593_at	27.8	74.4	1.42	2.68	AAA-AAA	0.00	999	1.000	1.000	0.1775
224401_s_at	19.4	7.3	-1.42	-2.68	AAA-AAA	0.00	999	1.000	1.000	0.3423
227817_at	45.9	17.1	-1.42	-2.68	AAA-AAA	0.00	999	1.000	1.000	0.0937
243219_x_at	18.9	50.4	1.42	2.68	PAA-PAM	0.00	999	1.000	1.000	0.3340
241677_x_at	5.1	13.7	1.42	2.68	AAA-AAA	0.00	999	1.000	1.000	0.2602
240127_at	18.8	7.0	-1.42	-2.68	AAA-AAA	0.00	999	1.000	1.000	0.2711
1563003_at	5.2	13.9	1.42	2.68	AAA-AAA	0.00	999	1.000	1.000	0.2029
1553077_at	23.0	8.6	-1.42	-2.68	AAA-AAA	0.00	999	1.000	1.000	0.1540
1556824_at	19.2	51.5	1.42	2.68	AAA-AAA	0.00	999	1.000	1.000	0.0864
206964_at	34.5	12.9	-1.42	-2.68	AAA-AAA	0.00	999	1.000	1.000	0.1451
210039_s_at	38.5	14.4	-1.42	-2.68	AAA-AAA	0.00	999	1.000	1.000	0.3556
213108_at	20.2	7.6	-1.42	-2.68	AAA-AAA	0.00	999	1.000	1.000	0.2274

218704_at	22.1	8.2	-1.42	-2.68	AAA-AAA	0.00	999	1.000	1.000	0.1508
204583_x_at	76.7	28.7	-1.42	-2.68	AAA-AAA	0.00	999	1.000	1.000	0.3489
238552_at	13.7	36.6	1.42	2.68	AAA-AMA	0.00	999	1.000	1.000	0.3042
233259_at	17.7	6.6	-1.42	-2.68	AAA-AAA	0.00	999	1.000	1.000	0.3158
244308_at	30.5	11.4	-1.42	-2.67	AAA-AAA	0.00	999	1.000	1.000	0.1299
242910_x_at	6.0	2.2	-1.42	-2.68	AAA-AAA	0.00	999	1.000	1.000	0.1181
223129_x_at	62.3	166.5	1.42	2.67	AAM-MAA	0.00	999	1.000	1.000	0.1977
217198_x_at	13.4	35.8	1.42	2.67	AAA-AAA	0.00	999	1.000	1.000	0.3027
238047_at	8.4	3.1	-1.42	-2.68	AAA-AAA	0.00	999	1.000	1.000	0.2347
1560756_at	22.3	8.3	-1.42	-2.67	AAA-AAA	0.00	999	1.000	1.000	0.3216
1556704_s_at	36.1	13.5	-1.42	-2.67	AAA-AAA	0.00	999	1.000	1.000	0.2243
207204_at	14.9	5.6	-1.42	-2.67	AAA-AAA	0.00	999	1.000	1.000	0.3319
232787_at	113.4	42.4	-1.42	-2.67	AAA-AAA	0.00	999	1.000	1.000	0.0520
231288_at	6.6	17.6	1.42	2.67	AAA-AAA	0.00	999	1.000	1.000	0.1856
241757_x_at	41.7	111.2	1.42	2.67	APP-PPP	0.00	999	1.000	1.000	0.2728
207636_at	48.8	18.3	-1.42	-2.67	AAA-AAA	0.00	999	1.000	1.000	0.1927
224085_at	10.9	29.2	1.42	2.67	AAA-AAA	0.00	999	1.000	1.000	0.1900
234275_at	20.2	7.6	-1.42	-2.67	AAA-AAA	0.00	999	1.000	1.000	0.2419
237617_at	12.4	4.6	-1.42	-2.67	AAA-AAA	0.00	999	1.000	1.000	0.2401
228796_at	9.4	25.0	1.42	2.67	AAA-AAA	0.00	999	1.000	1.000	0.4031
221833_at	62.0	165.5	1.42	2.67	AAA-AAP	0.00	999	1.000	1.000	0.0770
241544_at	54.7	20.5	-1.42	-2.67	AAA-AAA	0.00	999	1.000	1.000	0.1038
1566490_at	19.7	7.4	-1.42	-2.67	AAA-AAA	0.00	999	1.000	1.000	0.0186
1559982_s_at	38.2	14.3	-1.42	-2.67	AAA-MAA	0.00	999	1.000	1.000	0.3061
1555581_a_at	10.4	3.9	-1.42	-2.67	AAA-AAA	0.00	999	1.000	1.000	0.1577
221921_s_at	26.2	9.8	-1.42	-2.67	AAA-AAA	0.00	999	1.000	1.000	0.0192
215124_at	56.0	21.0	-1.42	-2.67	AAA-AAA	0.00	999	1.000	1.000	0.0772
236443_at	23.6	8.9	-1.42	-2.67	AAA-AAA	0.00	999	1.000	1.000	0.0141
230318_at	112.3	42.1	-1.42	-2.67	AAA-AAA	0.00	999	1.000	1.000	0.0023
232583_at	28.2	75.3	1.42	2.67	AAA-AAA	0.00	999	1.000	1.000	0.2332
1562762_at	29.9	11.2	-1.42	-2.67	AAA-AAA	0.00	999	1.000	1.000	0.1689
1555400_at	24.8	9.3	-1.42	-2.67	AAA-AAA	0.00	999	1.000	1.000	0.4459
222117_s_at	103.6	38.9	-1.42	-2.67	AAA-AAA	0.00	999	1.000	1.000	0.0970
237234_at	10.9	29.1	1.42	2.67	AAA-AAA	0.00	999	1.000	1.000	0.2210
238602_at	77.9	207.7	1.41	2.67	APP-PPP	0.00	999	1.000	1.000	0.2629
230951_at	13.2	35.1	1.41	2.67	AAA-PAA	0.00	999	1.000	1.000	0.1035
241396_at	90.6	34.0	-1.41	-2.66	AAA-AAA	0.00	999	1.000	1.000	0.0486
1566938_at	18.8	7.0	-1.41	-2.67	AAA-AAA	0.00	999	1.000	1.000	0.2913
1553552_at	24.0	9.0	-1.41	-2.66	AAA-AAA	0.00	999	1.000	1.000	0.2232
1561306_s_at	59.1	22.2	-1.41	-2.67	AAA-AAA	0.00	999	1.000	1.000	0.0246
222461_s_at	20.5	54.5	1.41	2.67	AAA-AAA	0.00	999	1.000	1.000	0.2281
213831_at	4.5	12.1	1.41	2.67	AAA-AAA	0.00	999	1.000	1.000	0.1366

230260_s_at	4.7	1.8	-1.41	-2.66	AAA-AAA	0.00	999	1.000	1.000	0.2388
229263_at	17.9	47.7	1.41	2.67	AAA-AAA	0.00	999	1.000	1.000	0.3199
1568871_at	4.5	1.7	-1.41	-2.66	AAA-AAA	0.00	999	1.000	1.000	0.3116
224357_s_at	6.7	17.7	1.41	2.66	AAA-AAA	0.00	999	1.000	1.000	0.1752
211107_s_at	15.4	41.0	1.41	2.66	AAA-AAA	0.00	999	1.000	1.000	0.2364
211119_at	18.9	7.1	-1.41	-2.66	AMA-AAA	0.00	999	1.000	1.000	0.3127
210322_x_at	46.6	17.5	-1.41	-2.66	AAA-AAA	0.00	999	1.000	1.000	0.2034
219683_at	10.2	27.1	1.41	2.66	AAA-PAA	0.00	999	1.000	1.000	0.0990
206881_s_at	12.3	4.6	-1.41	-2.66	AAA-AAA	0.00	999	1.000	1.000	0.3591
210614_at	14.4	38.4	1.41	2.66	AAA-AAA	0.00	999	1.000	1.000	0.2105
210626_at	8.6	22.9	1.41	2.66	AAA-AAA	0.00	999	1.000	1.000	0.1444
215668_s_at	86.0	32.3	-1.41	-2.66	AAA-AAA	0.00	999	1.000	1.000	0.1579
239739_at	30.7	11.5	-1.41	-2.66	AAA-AAA	0.00	999	1.000	1.000	0.4517
230781_at	4.4	11.7	1.41	2.66	AAA-AAA	0.00	999	1.000	1.000	0.2156
240283_at	12.6	33.5	1.41	2.66	AAA-AAA	0.00	999	1.000	1.000	0.2903
240119_at	62.2	23.4	-1.41	-2.66	AAA-AAA	0.00	999	1.000	1.000	0.0549
1569784_at	28.9	10.9	-1.41	-2.66	AAA-AAA	0.00	999	1.000	1.000	0.0787
1554173_at	48.8	18.4	-1.41	-2.66	AAA-AAA	0.00	999	1.000	1.000	0.0801
1553444_a_at	32.0	12.0	-1.41	-2.66	AAA-AAA	0.00	999	1.000	1.000	0.0577
244417_at	56.8	21.4	-1.41	-2.66	AAA-AAA	0.00	999	1.000	1.000	0.3007
1559459_at	12.9	4.8	-1.41	-2.66	AAA-AAA	0.00	999	1.000	1.000	0.3301
216453_at	68.2	25.6	-1.41	-2.66	AAA-AAA	0.00	999	1.000	1.000	0.2417
236810_at	24.6	9.3	-1.41	-2.66	AAA-AAA	0.00	999	1.000	1.000	0.3752
237152_at	5.9	2.2	-1.41	-2.66	AAA-AAA	0.00	999	1.000	1.000	0.0632
237847_at	46.3	17.4	-1.41	-2.66	AAA-AAA	0.00	999	1.000	1.000	0.3086
230867_at	13.6	5.1	-1.41	-2.66	AAA-AMA	0.00	999	1.000	1.000	0.4581
213717_at	20.8	7.8	-1.41	-2.66	AAA-AAA	0.00	999	1.000	1.000	0.0212
236857_at	13.4	5.0	-1.41	-2.66	AAA-AAA	0.00	999	1.000	1.000	0.3834
205254_x_at	12.0	31.8	1.41	2.66	AAA-AAA	0.00	999	1.000	1.000	0.2405
231645_s_at	6.4	17.1	1.41	2.66	AAA-AAA	0.00	999	1.000	1.000	0.3577
1566630_at	24.6	9.3	-1.41	-2.66	AAA-AAA	0.00	999	1.000	1.000	0.1898
1553475_at	34.1	12.8	-1.41	-2.66	AAA-AAA	0.00	999	1.000	1.000	0.2233
220951_s_at	17.6	46.7	1.41	2.66	AAA-AAA	0.00	999	1.000	1.000	0.1772
234462_at	19.0	7.2	-1.41	-2.66	AAA-AAA	0.00	999	1.000	1.000	0.0319
205033_s_at	62.6	23.6	-1.41	-2.66	AAA-AAA	0.00	999	1.000	1.000	0.1282
232345_at	64.9	24.5	-1.41	-2.66	AAA-AAA	0.00	999	1.000	1.000	0.3369
1557169_x_at	12.7	33.8	1.41	2.65	AAA-PAA	0.00	999	1.000	1.000	0.1465
244220_at	179.7	67.7	-1.41	-2.65	AAA-AAA	0.00	999	1.000	1.000	0.2036
244648_at	145.8	55.0	-1.41	-2.65	AAA-AAA	0.00	999	1.000	1.000	0.2432
1559804_at	35.3	13.3	-1.41	-2.65	AAA-AAA	0.00	999	1.000	1.000	0.3119
207476_at	24.9	9.4	-1.41	-2.65	AAA-AAA	0.00	999	1.000	1.000	0.3957
223778_at	54.7	20.6	-1.41	-2.65	AAA-AAA	0.00	999	1.000	1.000	0.1318

228113_at	33.7	12.7	-1.41	-2.65	AAA-AAA	0.00	999	1.000	1.000	0.1415
231346_s_at	11.6	4.4	-1.41	-2.65	AAA-AAA	0.00	999	1.000	1.000	0.2371
231596_at	26.7	70.7	1.41	2.65	AAA-AAP	0.00	999	1.000	1.000	0.1307
244305_at	12.1	4.6	-1.41	-2.65	AAA-AAA	0.00	999	1.000	1.000	0.2593
240390_at	7.9	21.1	1.41	2.65	AAA-AAA	0.00	999	1.000	1.000	0.4141
243105_at	38.3	14.4	-1.41	-2.65	AAA-AAA	0.00	999	1.000	1.000	0.1835
1561612_at	11.4	4.3	-1.41	-2.65	AAA-AAA	0.00	999	1.000	1.000	0.0251
210164_at	38.3	14.4	-1.41	-2.65	AAA-AAA	0.00	999	1.000	1.000	0.0838
215355_at	3.4	9.0	1.41	2.65	AAA-AAA	0.00	999	1.000	1.000	0.1514
233884_at	14.9	39.5	1.41	2.65	AAA-AAA	0.00	999	1.000	1.000	0.2768
234750_at	45.2	17.0	-1.41	-2.65	AAA-AAA	0.00	999	1.000	1.000	0.2457
230964_at	5.3	14.1	1.41	2.65	AAA-AAA	0.00	999	1.000	1.000	0.3406
230913_at	38.7	14.6	-1.41	-2.65	AAA-AAA	0.00	999	1.000	1.000	0.1867
1561785_at	11.7	30.9	1.41	2.65	AAA-AAA	0.00	999	1.000	1.000	0.2457
1557239_at	28.3	10.7	-1.41	-2.65	AAA-AAA	0.00	999	1.000	1.000	0.2645
206466_at	68.8	26.0	-1.41	-2.65	AAA-AAA	0.00	999	1.000	1.000	0.3069
239436_at	34.5	13.0	-1.41	-2.65	AAA-AAA	0.00	999	1.000	1.000	0.1079
206378_at	22.1	8.3	-1.41	-2.65	AAA-AMA	0.00	999	1.000	1.000	0.3615
228078_at	15.5	5.8	-1.41	-2.65	AAA-AAA	0.00	999	1.000	1.000	0.4552
232739_at	11.7	4.4	-1.41	-2.65	AAA-AAA	0.00	999	1.000	1.000	0.2345
1567853_at	6.7	17.8	1.41	2.65	AAA-AAA	0.00	999	1.000	1.000	0.2483
222937_s_at	97.1	36.7	-1.41	-2.65	AAA-AAA	0.00	999	1.000	1.000	0.0590
235468_at	14.0	5.3	-1.41	-2.65	AAA-AAA	0.00	999	1.000	1.000	0.2957
240336_at	28.7	10.9	-1.40	-2.65	AAA-AAA	0.00	999	1.000	1.000	0.3980
243639_at	34.3	13.0	-1.40	-2.65	AAA-AAA	0.00	999	1.000	1.000	0.1855
1555533_at	8.0	3.0	-1.40	-2.65	AAA-AAA	0.00	999	1.000	1.000	0.0747
219584_at	10.5	4.0	-1.40	-2.65	AAA-AAA	0.00	999	1.000	1.000	0.4471
215310_at	37.4	99.0	1.40	2.65	AAA-AAA	0.00	999	1.000	1.000	0.2107
236214_at	32.0	12.1	-1.40	-2.65	AAA-AAA	0.00	999	1.000	1.000	0.3393
237846_at	10.4	27.5	1.40	2.65	AAA-AAA	0.00	999	1.000	1.000	0.1424
239893_at	13.9	36.6	1.40	2.64	AAA-APA	0.00	999	1.000	1.000	0.3945
208762_at	262.1	693.2	1.40	2.65	PPP-PPP	0.00	999	1.000	1.000	0.0842
240582_x_at	20.3	7.7	-1.40	-2.64	AAA-AAA	0.00	999	1.000	1.000	0.2959
1562670_at	15.9	6.0	-1.40	-2.65	AAA-AAA	0.00	999	1.000	1.000	0.3466
1565596_at	3.1	8.3	1.40	2.65	AAA-AAA	0.00	999	1.000	1.000	0.1596
1553695_a_at	32.1	85.0	1.40	2.64	AAA-AAA	0.00	999	1.000	1.000	0.0478
1559030_a_at	25.3	9.6	-1.40	-2.64	AAA-AAA	0.00	999	1.000	1.000	0.1960
1556904_at	9.8	3.7	-1.40	-2.65	AAA-AAA	0.00	999	1.000	1.000	0.4410
220004_at	22.0	8.3	-1.40	-2.64	AAA-AAA	0.00	999	1.000	1.000	0.4158
207694_at	58.0	21.9	-1.40	-2.64	AAA-AAA	0.00	999	1.000	1.000	0.2261
236705_at	2.3	6.1	1.40	2.64	AAA-AAA	0.00	999	1.000	1.000	0.4078
232143_at	26.6	10.1	-1.40	-2.64	AAA-AAA	0.00	999	1.000	1.000	0.2975

227775_at	19.1	50.6	1.40	2.64	AAA-PAA	0.00	999	1.000	1.000	0.1842
1569768_at	3.5	9.2	1.40	2.64	AAA-AAA	0.00	999	1.000	1.000	0.1034
206711_at	13.9	5.3	-1.40	-2.64	AAA-AAA	0.00	999	1.000	1.000	0.0770
234914_at	40.2	15.2	-1.40	-2.64	AAA-AAA	0.00	999	1.000	1.000	0.1805
237026_at	24.0	9.1	-1.40	-2.64	AAA-AAA	0.00	999	1.000	1.000	0.4080
205754_at	13.5	35.7	1.40	2.64	AAA-AAA	0.00	999	1.000	1.000	0.0631
242449_at	32.3	85.3	1.40	2.64	APA-AMM	0.00	999	1.000	1.000	0.3794
1557383_a_at	24.5	64.7	1.40	2.64	AAP-APP	0.00	999	1.000	1.000	0.3375
232186_at	27.5	72.6	1.40	2.64	AAP-AAP	0.00	999	1.000	1.000	0.1504
221976_s_at	20.1	53.1	1.40	2.64	AAA-APP	0.00	999	1.000	1.000	0.1253
241504_at	65.7	24.9	-1.40	-2.64	AAA-AAA	0.00	999	1.000	1.000	0.0769
1566039_a_at	23.6	62.4	1.40	2.64	AAA-AAA	0.00	999	1.000	1.000	0.2100
1558825_s_at	26.9	10.2	-1.40	-2.64	AAA-AAA	0.00	999	1.000	1.000	0.0519
1556147_at	11.0	29.1	1.40	2.64	AMA-AAA	0.00	999	1.000	1.000	0.4214
221218_s_at	8.9	23.6	1.40	2.64	AAA-MAA	0.00	999	1.000	1.000	0.3144
225977_at	12.3	4.7	-1.40	-2.64	AAA-AAA	0.00	999	1.000	1.000	0.1684
238150_at	17.8	6.7	-1.40	-2.64	AAA-AAA	0.00	999	1.000	1.000	0.0014
233492_s_at	22.6	8.6	-1.40	-2.64	AAA-AAA	0.00	999	1.000	1.000	0.4151
221345_at	12.5	33.1	1.40	2.64	AAA-APA	0.00	999	1.000	1.000	0.3341
221200_at	14.7	38.8	1.40	2.64	AAA-AAA	0.00	999	1.000	1.000	0.1589
227081_at	22.4	8.5	-1.40	-2.64	AAA-AAA	0.00	999	1.000	1.000	0.2741
216765_at	7.5	19.7	1.40	2.64	AAA-AAA	0.00	999	1.000	1.000	0.2849
234684_s_at	16.2	6.2	-1.40	-2.64	AAA-AAA	0.00	999	1.000	1.000	0.2471
243823_at	30.3	11.5	-1.40	-2.64	AAA-AAA	0.00	999	1.000	1.000	0.1245
1554133_at	36.5	13.8	-1.40	-2.64	AAA-AAA	0.00	999	1.000	1.000	0.0780
222054_at	17.7	46.7	1.40	2.64	AAA-AAA	0.00	999	1.000	1.000	0.0897
220203_at	28.9	11.0	-1.40	-2.64	AAA-AAA	0.00	999	1.000	1.000	0.3100
220888_s_at	8.6	3.3	-1.40	-2.64	AAA-AAA	0.00	999	1.000	1.000	0.0228
206941_x_at	29.7	11.3	-1.40	-2.64	AAA-AAA	0.00	999	1.000	1.000	0.1633
235655_at	55.1	20.9	-1.40	-2.64	AAA-AAA	0.00	999	1.000	1.000	0.1312
212972_x_at	40.8	107.6	1.40	2.64	AAA-PAA	0.00	999	1.000	1.000	0.1586
1553266_at	6.6	2.5	-1.40	-2.64	AAA-AAA	0.00	999	1.000	1.000	0.4157
1558122_s_at	12.8	4.8	-1.40	-2.64	AAA-AAA	0.00	999	1.000	1.000	0.2282
226344_at	18.9	7.2	-1.40	-2.64	AAA-AAA	0.00	999	1.000	1.000	0.3384
207354_at	29.7	11.3	-1.40	-2.64	AAA-AAA	0.00	999	1.000	1.000	0.0822
214236_at	38.0	100.1	1.40	2.64	AAA-AAA	0.00	999	1.000	1.000	0.2542
216136_at	34.3	13.0	-1.40	-2.64	AAA-AAA	0.00	999	1.000	1.000	0.1049
204626_s_at	29.9	78.9	1.40	2.64	AAA-AAA	0.00	999	1.000	1.000	0.3261
233516_s_at	18.7	7.1	-1.40	-2.64	AAA-AAA	0.00	999	1.000	1.000	0.3933
231364_at	29.5	11.2	-1.40	-2.64	AAA-AAA	0.00	999	1.000	1.000	0.3867
220075_s_at	7.7	2.9	-1.40	-2.63	AAA-AAA	0.00	999	1.000	1.000	0.1013
208397_x_at	16.2	6.2	-1.40	-2.63	AAA-AAA	0.00	999	1.000	1.000	0.1154

220479_at	22.8	8.6	-1.40	-2.63	AAA-AAA	0.00	999	1.000	1.000	0.3422
225122_at	15.5	5.9	-1.40	-2.63	AAA-AAA	0.00	999	1.000	1.000	0.1579
210741_at	30.4	11.6	-1.40	-2.63	AAA-AAA	0.00	999	1.000	1.000	0.2654
211568_at	14.5	5.5	-1.40	-2.63	AAA-AAA	0.00	999	1.000	1.000	0.3797
237257_at	124.3	47.2	-1.40	-2.63	AAA-AAA	0.00	999	1.000	1.000	0.2334
230155_x_at	136.6	51.9	-1.40	-2.63	AAM-AAA	0.00	999	1.000	1.000	0.0283
230367_at	19.4	7.4	-1.40	-2.63	AAA-AAA	0.00	999	1.000	1.000	0.1828
233086_at	13.3	5.1	-1.40	-2.63	AAA-AAA	0.00	999	1.000	1.000	0.3793
1563742_at	34.7	13.2	-1.40	-2.63	AAA-AAA	0.00	999	1.000	1.000	0.3889
1556067_a_at	8.9	23.5	1.40	2.63	AAA-AAA	0.00	999	1.000	1.000	0.1838
219776_s_at	25.3	9.6	-1.40	-2.63	AAA-AAA	0.00	999	1.000	1.000	0.1950
214572_s_at	23.0	8.8	-1.40	-2.63	AAA-AAA	0.00	999	1.000	1.000	0.0519
237753_at	39.2	14.9	-1.40	-2.63	AAA-AAA	0.00	999	1.000	1.000	0.1583
229005_at	19.1	7.3	-1.40	-2.63	AAA-AAA	0.00	999	1.000	1.000	0.1599
231415_at	13.4	5.1	-1.40	-2.63	AAA-AAA	0.00	999	1.000	1.000	0.1423
227173_s_at	30.8	80.9	1.40	2.63	AAA-PPA	0.00	999	1.000	1.000	0.1122
243979_at	5.1	1.9	-1.40	-2.63	AAA-AAA	0.00	999	1.000	1.000	0.3988
1561944_at	14.2	5.4	-1.40	-2.63	AAA-AAA	0.00	999	1.000	1.000	0.0673
1556244_s_at	16.3	6.2	-1.40	-2.63	AAA-AAA	0.00	999	1.000	1.000	0.3836
221413_at	26.2	10.0	-1.40	-2.63	AAA-AAA	0.00	999	1.000	1.000	0.3925
208020_s_at	12.6	4.8	-1.40	-2.63	AAA-AAA	0.00	999	1.000	1.000	0.3081
226187_at	21.4	8.1	-1.40	-2.63	AAA-AAA	0.00	999	1.000	1.000	0.3187
215999_at	79.2	30.1	-1.40	-2.63	AAA-AAA	0.00	999	1.000	1.000	0.0166
216013_at	15.0	39.5	1.40	2.63	AAA-AAA	0.00	999	1.000	1.000	0.2447
239060_at	22.6	8.6	-1.40	-2.63	AAA-AAA	0.00	999	1.000	1.000	0.1034
228086_at	49.5	18.8	-1.40	-2.63	AAA-AAA	0.00	999	1.000	1.000	0.1498
1557018_a_at	27.3	10.4	-1.39	-2.63	AAA-AAA	0.00	999	1.000	1.000	0.3291
207529_at	44.7	17.0	-1.39	-2.63	AAA-AAA	0.00	999	1.000	1.000	0.1678
232368_at	36.8	14.0	-1.39	-2.63	AAA-AAA	0.00	999	1.000	1.000	0.1275
219869_s_at	47.4	124.5	1.39	2.63	PAP-PPP	0.00	999	1.000	1.000	0.2510
202456_s_at	27.1	71.3	1.39	2.63	AAA-AAA	0.00	999	1.000	1.000	0.0849
1557924_s_at	4.8	12.5	1.39	2.63	AAA-AAA	0.00	999	1.000	1.000	0.2509
222226_at	11.6	4.4	-1.39	-2.63	AAA-AAA	0.00	999	1.000	1.000	0.2951
207441_at	6.9	18.2	1.39	2.63	AAA-AAA	0.00	999	1.000	1.000	0.0452
211455_at	36.0	94.5	1.39	2.63	AAA-AAA	0.00	999	1.000	1.000	0.0206
237393_at	34.5	13.1	-1.39	-2.63	AAA-AAA	0.00	999	1.000	1.000	0.2285
236860_at	18.5	7.1	-1.39	-2.63	AAA-AAA	0.00	999	1.000	1.000	0.2022
237788_at	6.1	2.3	-1.39	-2.63	AAA-AAA	0.00	999	1.000	1.000	0.3862
232259_s_at	68.9	26.2	-1.39	-2.63	AAA-AAA	0.00	999	1.000	1.000	0.1103
1558247_s_at	73.1	27.8	-1.39	-2.63	AAA-AAA	0.00	999	1.000	1.000	0.3654
1555749_at	4.3	11.3	1.39	2.62	AAA-AAA	0.00	999	1.000	1.000	0.4327
220880_at	6.9	18.0	1.39	2.63	AAA-AAA	0.00	999	1.000	1.000	0.4954

211224_s_at	25.4	9.7	-1.39	-2.63	AAA-AAA	0.00	999	1.000	1.000	0.1917
234801_s_at	23.6	9.0	-1.39	-2.63	AAA-AAA	0.00	999	1.000	1.000	0.3497
230661_at	4.7	12.4	1.39	2.63	AAA-AAA	0.00	999	1.000	1.000	0.0262
1566624_at	4.8	12.7	1.39	2.62	AAA-AAA	0.00	999	1.000	1.000	0.3372
209767_s_at	17.9	46.9	1.39	2.62	AAA-AAA	0.00	999	1.000	1.000	0.1409
216907_x_at	21.6	8.2	-1.39	-2.62	AAA-AAA	0.00	999	1.000	1.000	0.2449
239032_at	43.1	16.5	-1.39	-2.62	AAA-AAA	0.00	999	1.000	1.000	0.2290
236926_at	29.0	11.1	-1.39	-2.62	AAA-AAA	0.00	999	1.000	1.000	0.2440
231028_at	5.5	2.1	-1.39	-2.62	AAA-AAA	0.00	999	1.000	1.000	0.0645
1568848_at	2.9	7.7	1.39	2.62	AAA-AAA	0.00	999	1.000	1.000	0.3115
1570225_at	9.7	25.5	1.39	2.62	AAA-AAA	0.00	999	1.000	1.000	0.2981
1570491_at	23.3	8.9	-1.39	-2.62	AAA-AAA	0.00	999	1.000	1.000	0.1745
1564300_at	21.1	8.1	-1.39	-2.62	AAA-AAA	0.00	999	1.000	1.000	0.4447
221578_at	67.0	25.6	-1.39	-2.62	AAA-AAA	0.00	999	1.000	1.000	0.1321
223405_at	24.5	64.1	1.39	2.62	AAA-AAA	0.00	999	1.000	1.000	0.1763
224432_at	21.4	56.1	1.39	2.62	AAA-AAA	0.00	999	1.000	1.000	0.0546
234377_at	36.2	13.8	-1.39	-2.62	AAA-AAA	0.00	999	1.000	1.000	0.2672
234743_at	13.2	34.5	1.39	2.62	AAA-AAA	0.00	999	1.000	1.000	0.1196
229847_at	11.6	30.4	1.39	2.62	AAA-AAA	0.00	999	1.000	1.000	0.2839
206400_at	6.5	17.0	1.39	2.62	AAA-AAA	0.00	999	1.000	1.000	0.2823
233062_at	18.1	6.9	-1.39	-2.62	AAA-AAA	0.00	999	1.000	1.000	0.1573
1560547_at	5.6	2.1	-1.39	-2.62	AAA-AAA	0.00	999	1.000	1.000	0.1102
1561298_at	21.9	8.4	-1.39	-2.62	AAA-AAA	0.00	999	1.000	1.000	0.2689
1559890_a_at	28.9	11.0	-1.39	-2.62	AAA-AAA	0.00	999	1.000	1.000	0.2182
233971_at	59.2	22.6	-1.39	-2.62	AAA-AAA	0.00	999	1.000	1.000	0.3319
239755_at	47.5	18.1	-1.39	-2.62	AAA-AAA	0.00	999	1.000	1.000	0.0394
230319_at	4.4	11.4	1.39	2.62	AAA-AAA	0.00	999	1.000	1.000	0.3214
205840_x_at	16.7	6.4	-1.39	-2.62	AAA-AAA	0.00	999	1.000	1.000	0.1301
1554821_a_at	46.5	121.6	1.39	2.62	APA-PPA	0.00	999	1.000	1.000	0.2420
89476_r_at	84.4	220.7	1.39	2.62	AAM-PMM	0.00	999	1.000	1.000	0.1598
1568933_at	34.7	13.2	-1.39	-2.62	AAA-AAA	0.00	999	1.000	1.000	0.2487
244865_at	16.1	42.0	1.39	2.62	AAA-AAA	0.00	999	1.000	1.000	0.1805
216982_x_at	17.7	6.8	-1.39	-2.62	AAA-AAA	0.00	999	1.000	1.000	0.3497
237471_at	9.3	3.5	-1.39	-2.62	AAA-AAA	0.00	999	1.000	1.000	0.2289
230153_at	9.6	3.7	-1.39	-2.62	AAA-AAA	0.00	999	1.000	1.000	0.1775
230698_at	29.0	11.1	-1.39	-2.62	AAA-AAA	0.00	999	1.000	1.000	0.2802
1560163_at	16.9	6.5	-1.39	-2.62	AAA-AAA	0.00	999	1.000	1.000	0.1393
1561107_at	52.8	20.2	-1.39	-2.62	AAA-AAA	0.00	999	1.000	1.000	0.2483
1566204_at	14.0	5.3	-1.39	-2.62	AAA-AAA	0.00	999	1.000	1.000	0.4094
1554195_a_at	118.3	309.3	1.39	2.62	AAA-AMA	0.00	999	1.000	1.000	0.1214
1553441_at	6.4	2.4	-1.39	-2.62	AAA-AAA	0.00	999	1.000	1.000	0.2928
239505_at	72.5	27.7	-1.39	-2.62	AAA-AAA	0.00	999	1.000	1.000	0.2703

237716_at	57.4	22.0	-1.39	-2.62	AAA-AAA	0.00	999	1.000	1.000	0.2999
237639_at	89.7	34.3	-1.39	-2.62	AAA-AAA	0.00	999	1.000	1.000	0.0135
231562_at	31.5	12.0	-1.39	-2.62	AAA-AAA	0.00	999	1.000	1.000	0.1697
233546_at	19.4	50.6	1.39	2.61	AAP-AAP	0.00	999	1.000	1.000	0.3615
243380_at	50.2	19.2	-1.39	-2.61	AAA-AAA	0.00	999	1.000	1.000	0.2392
1569213_at	10.3	3.9	-1.39	-2.61	AAA-AAA	0.00	999	1.000	1.000	0.3728
244684_at	35.0	13.4	-1.39	-2.61	AAA-AAA	0.00	999	1.000	1.000	0.2275
1552842_at	23.0	8.8	-1.39	-2.61	AAA-AAA	0.00	999	1.000	1.000	0.2165
223924_at	14.7	38.4	1.39	2.61	AAA-MAA	0.00	999	1.000	1.000	0.0920
216154_at	10.2	26.6	1.39	2.61	AAA-AAA	0.00	999	1.000	1.000	0.0543
236406_at	17.6	6.8	-1.39	-2.61	AAA-AAA	0.00	999	1.000	1.000	0.3403
237344_at	35.7	13.7	-1.39	-2.61	AAA-AAA	0.00	999	1.000	1.000	0.3319
243172_at	13.9	5.3	-1.39	-2.61	AAA-AAA	0.00	999	1.000	1.000	0.3858
206582_s_at	26.5	69.1	1.39	2.61	AAA-AAA	0.00	999	1.000	1.000	0.1515
211601_at	3.1	1.2	-1.39	-2.61	AAA-AAA	0.00	999	1.000	1.000	0.0023
233976_at	19.4	7.4	-1.39	-2.61	AAA-AAA	0.00	999	1.000	1.000	0.2206
205817_at	33.4	87.0	1.38	2.61	AAA-APA	0.00	999	1.000	1.000	0.0693
243596_at	54.2	20.8	-1.38	-2.61	AAA-AAA	0.00	999	1.000	1.000	0.2308
1563692_at	17.4	6.7	-1.38	-2.61	AAA-AAA	0.00	999	1.000	1.000	0.2669
1564736_a_at	9.5	24.9	1.38	2.61	AAA-AAA	0.00	999	1.000	1.000	0.4164
1561434_at	15.6	6.0	-1.38	-2.61	AAA-AAA	0.00	999	1.000	1.000	0.4245
225817_at	18.6	48.6	1.38	2.61	AAA-AAA	0.00	999	1.000	1.000	0.0633
217113_at	32.7	12.5	-1.38	-2.61	AAA-AAA	0.00	999	1.000	1.000	0.2689
217362_x_at	34.9	13.4	-1.38	-2.61	AAA-AAA	0.00	999	1.000	1.000	0.4952
209353_s_at	6.9	18.1	1.38	2.61	AAA-AAA	0.00	999	1.000	1.000	0.1425
205693_at	80.5	30.8	-1.38	-2.61	AAA-AAA	0.00	999	1.000	1.000	0.3455
206304_at	34.7	13.3	-1.38	-2.61	AAA-AAA	0.00	999	1.000	1.000	0.2508
233344_x_at	8.2	3.1	-1.38	-2.61	AAA-AAA	0.00	999	1.000	1.000	0.1743
231063_at	6.5	17.0	1.38	2.61	AAA-AAA	0.00	999	1.000	1.000	0.1788
203158_s_at	53.8	140.4	1.38	2.61	APP-PPP	0.00	999	1.000	1.000	0.2528
237650_at	11.2	29.2	1.38	2.61	AAA-APA	0.00	999	1.000	1.000	0.2109
243057_at	30.9	11.9	-1.38	-2.61	AAA-AAA	0.00	999	1.000	1.000	0.3010
1562276_at	24.8	9.5	-1.38	-2.61	AAA-AAA	0.00	999	1.000	1.000	0.2485
1570621_at	16.4	6.3	-1.38	-2.61	AAA-AAA	0.00	999	1.000	1.000	0.1984
213458_at	8.3	3.2	-1.38	-2.61	AAA-AAA	0.00	999	1.000	1.000	0.3297
216730_at	121.6	46.6	-1.38	-2.61	AAA-AAA	0.00	999	1.000	1.000	0.0747
238636_at	55.1	21.1	-1.38	-2.61	AAA-AAA	0.00	999	1.000	1.000	0.1364
232638_at	9.2	3.5	-1.38	-2.61	AAA-AAA	0.00	999	1.000	1.000	0.2248
1569765_at	131.0	50.3	-1.38	-2.61	AAA-AAA	0.00	999	1.000	1.000	0.4478
1565732_at	24.0	9.2	-1.38	-2.61	AAA-AAA	0.00	999	1.000	1.000	0.3971
1566893_at	10.3	4.0	-1.38	-2.61	AAA-AAA	0.00	999	1.000	1.000	0.5353
1558373_s_at	11.9	4.6	-1.38	-2.61	AAA-AAA	0.00	999	1.000	1.000	0.1398

220413_at	10.0	26.0	1.38	2.61	AAA-AAA	0.00	999	1.000	1.000	0.1318
204743_at	132.4	50.8	-1.38	-2.61	AAA-AAA	0.00	999	1.000	1.000	0.1557
234539_at	27.2	10.4	-1.38	-2.61	AAA-AAA	0.00	999	1.000	1.000	0.2666
205309_at	7.4	2.9	-1.38	-2.61	AAA-AAA	0.00	999	1.000	1.000	0.0252
232411_at	6.1	2.4	-1.38	-2.61	AAA-AAA	0.00	999	1.000	1.000	0.1740
1569793_at	24.1	9.2	-1.38	-2.61	AAA-AAA	0.00	999	1.000	1.000	0.2091
1566473_a_at	148.6	57.1	-1.38	-2.60	AAA-AAA	0.00	999	1.000	1.000	0.0302
227390_at	45.8	17.6	-1.38	-2.61	AAA-AAA	0.00	999	1.000	1.000	0.1515
211351_at	4.2	11.0	1.38	2.61	AAA-AAA	0.00	999	1.000	1.000	0.4046
210551_s_at	29.0	11.1	-1.38	-2.60	AAA-AAA	0.00	999	1.000	1.000	0.3144
243980_at	35.6	13.7	-1.38	-2.60	AAA-AAA	0.00	999	1.000	1.000	0.2765
1553265_at	4.1	10.5	1.38	2.60	AAA-AAA	0.00	999	1.000	1.000	0.4021
1558544_at	28.6	74.3	1.38	2.60	AAA-AAA	0.00	999	1.000	1.000	0.2672
206916_x_at	55.6	21.4	-1.38	-2.60	AAA-AAA	0.00	999	1.000	1.000	0.1379
227571_at	26.8	69.7	1.38	2.60	AAA-AAA	0.00	999	1.000	1.000	0.0930
217443_at	10.3	4.0	-1.38	-2.60	AAA-AAA	0.00	999	1.000	1.000	0.4580
237334_at	28.7	11.0	-1.38	-2.60	AAA-AAA	0.00	999	1.000	1.000	0.0307
243562_at	12.0	4.6	-1.38	-2.60	AAA-AAA	0.00	999	1.000	1.000	0.5514
1562443_at	11.7	30.4	1.38	2.60	AAA-AAA	0.00	999	1.000	1.000	0.1517
1569615_at	14.7	5.7	-1.38	-2.60	AAA-AAA	0.00	999	1.000	1.000	0.2371
223957_at	30.5	11.7	-1.38	-2.60	AAA-AAA	0.00	999	1.000	1.000	0.2950
236589_at	62.1	23.9	-1.38	-2.60	AAA-AAA	0.00	999	1.000	1.000	0.4080
205243_at	53.5	20.6	-1.38	-2.60	AAA-AAA	0.00	999	1.000	1.000	0.2133
240426_at	36.1	13.9	-1.38	-2.60	AAA-AAA	0.00	999	1.000	1.000	0.3468
1560859_at	13.7	5.3	-1.38	-2.60	AAA-AAA	0.00	999	1.000	1.000	0.2475
1566456_at	15.2	39.4	1.38	2.60	AAA-AAA	0.00	999	1.000	1.000	0.2398
220754_at	7.0	2.7	-1.38	-2.60	AAA-AAA	0.00	999	1.000	1.000	0.3466
223883_s_at	20.4	7.9	-1.38	-2.60	AAA-AAA	0.00	999	1.000	1.000	0.4076
224082_at	28.6	74.2	1.38	2.60	AAA-AAA	0.00	999	1.000	1.000	0.1672
214536_at	48.9	18.8	-1.38	-2.60	AAA-AAA	0.00	999	1.000	1.000	0.1356
216736_at	31.7	12.2	-1.38	-2.60	AAA-AAA	0.00	999	1.000	1.000	0.0436
229897_at	20.0	51.9	1.38	2.60	AAA-AAA	0.00	999	1.000	1.000	0.0029
229048_at	25.0	9.6	-1.38	-2.60	AAA-AAA	0.00	999	1.000	1.000	0.2815
1563540_at	73.9	28.5	-1.38	-2.60	AAA-AAA	0.00	999	1.000	1.000	0.1119
1556187_at	12.6	4.8	-1.38	-2.60	AAA-AAA	0.00	999	1.000	1.000	0.4235
208081_s_at	17.1	44.3	1.38	2.60	AAA-AAM	0.00	999	1.000	1.000	0.1935
224412_s_at	6.2	16.2	1.38	2.60	AAA-AAA	0.00	999	1.000	1.000	0.2674
210091_s_at	7.7	3.0	-1.38	-2.60	AAA-AAA	0.00	999	1.000	1.000	0.2908
219461_at	6.3	16.3	1.38	2.60	AAA-AAA	0.00	999	1.000	1.000	0.2109
234187_at	90.0	34.7	-1.38	-2.60	AAA-AAA	0.00	999	1.000	1.000	0.1071
216082_at	19.0	49.3	1.38	2.60	AAA-AAP	0.00	999	1.000	1.000	0.3314
1570578_at	6.8	2.6	-1.38	-2.60	AAA-AAA	0.00	999	1.000	1.000	0.3651

244435_at	3.5	9.1	1.38	2.60	AAA-AAA	0.00	999	1.000	1.000	0.4280
219616_at	95.1	36.7	-1.38	-2.60	AAA-AAA	0.00	999	1.000	1.000	0.0040
216395_at	11.3	29.2	1.38	2.60	AAA-AAA	0.00	999	1.000	1.000	0.1799
239386_at	20.1	52.3	1.38	2.60	AAA-AAA	0.00	999	1.000	1.000	0.1318
1570357_at	73.5	28.3	-1.38	-2.59	AAA-AAA	0.00	999	1.000	1.000	0.0805
1566186_at	14.7	5.7	-1.38	-2.59	AAA-AAA	0.00	999	1.000	1.000	0.3586
1553392_at	15.3	5.9	-1.38	-2.59	AAA-AAA	0.00	999	1.000	1.000	0.4107
1558216_at	9.9	3.8	-1.38	-2.59	AAA-AAA	0.00	999	1.000	1.000	0.3371
216063_at	44.8	17.3	-1.38	-2.59	AAA-AAA	0.00	999	1.000	1.000	0.2320
234342_at	43.0	16.6	-1.38	-2.59	AAA-AAA	0.00	999	1.000	1.000	0.1432
232011_s_at	91.6	35.3	-1.38	-2.59	AAA-AAA	0.00	999	1.000	1.000	0.0866
242524_at	22.7	8.8	-1.37	-2.59	AAA-AAA	0.00	999	1.000	1.000	0.2485
241990_at	22.3	8.6	-1.37	-2.59	AAA-AAA	0.00	999	1.000	1.000	0.2271
1569065_s_at	53.4	20.6	-1.37	-2.59	AAA-AAA	0.00	999	1.000	1.000	0.0815
1552604_at	6.3	16.3	1.37	2.59	AAA-AAA	0.00	999	1.000	1.000	0.3853
1555369_at	11.9	4.6	-1.37	-2.59	AAA-AAA	0.00	999	1.000	1.000	0.2127
208450_at	15.6	6.0	-1.37	-2.59	AAA-AAA	0.00	999	1.000	1.000	0.2288
239618_at	36.0	13.9	-1.37	-2.59	AAA-AAA	0.00	999	1.000	1.000	0.3284
231476_at	28.2	10.9	-1.37	-2.59	MAA-AAA	0.00	999	1.000	1.000	0.4556
1563035_x_at	3.5	9.1	1.37	2.59	AAA-AAA	0.00	999	1.000	1.000	0.0630
1561531_at	55.6	21.4	-1.37	-2.59	AAA-AAA	0.00	999	1.000	1.000	0.2334
1561354_at	17.0	6.6	-1.37	-2.59	AAA-AAA	0.00	999	1.000	1.000	0.3730
1554208_at	192.8	74.4	-1.37	-2.59	AAA-AAA	0.00	999	1.000	1.000	0.0722
222353_at	8.1	21.0	1.37	2.59	AAA-AAA	0.00	999	1.000	1.000	0.1143
208332_at	59.3	22.9	-1.37	-2.59	AAA-AAA	0.00	999	1.000	1.000	0.2621
219619_at	18.3	7.1	-1.37	-2.59	AAA-AAA	0.00	999	1.000	1.000	0.4659
224528_s_at	7.9	3.0	-1.37	-2.59	AAA-AAA	0.00	999	1.000	1.000	0.3680
210989_at	13.3	34.6	1.37	2.59	MAA-AAA	0.00	999	1.000	1.000	0.3435
217240_at	39.3	15.2	-1.37	-2.59	AAA-AAA	0.00	999	1.000	1.000	0.3393
218416_s_at	15.1	39.2	1.37	2.59	AAA-AAA	0.00	999	1.000	1.000	0.2754
236511_at	29.5	11.4	-1.37	-2.59	AAA-AAA	0.00	999	1.000	1.000	0.2020
234257_at	16.0	6.2	-1.37	-2.59	AAA-AAA	0.00	999	1.000	1.000	0.3953
234647_at	55.9	21.6	-1.37	-2.59	AAA-AAA	0.00	999	1.000	1.000	0.3629
238153_at	49.7	19.2	-1.37	-2.59	AAA-AAA	0.00	999	1.000	1.000	0.2122
237046_x_at	39.8	15.4	-1.37	-2.59	AAA-AAA	0.00	999	1.000	1.000	0.1062
205907_s_at	11.3	4.4	-1.37	-2.59	AAA-AAA	0.00	999	1.000	1.000	0.2783
229326_at	6.0	15.5	1.37	2.59	AAA-AAA	0.00	999	1.000	1.000	0.2111
231229_at	26.8	10.3	-1.37	-2.59	AAA-AAA	0.00	999	1.000	1.000	0.1922
232024_at	2.7	6.9	1.37	2.59	AAA-AAA	0.00	999	1.000	1.000	0.4101
243479_at	5.2	2.0	-1.37	-2.59	AAA-AAA	0.00	999	1.000	1.000	0.2711
1553907_a_at	3.0	7.7	1.37	2.59	AAA-AAA	0.00	999	1.000	1.000	0.1533
202178_at	310.3	119.9	-1.37	-2.59	AAA-AAA	0.00	999	1.000	1.000	0.0475

1559172_at	7.4	2.8	-1.37	-2.59	AAA-AAA	0.00	999	1.000	1.000	0.3808
216968_at	16.1	6.2	-1.37	-2.59	AAA-AAA	0.00	999	1.000	1.000	0.1942
216121_at	49.1	19.0	-1.37	-2.59	AAA-AAA	0.00	999	1.000	1.000	0.2051
234970_at	4.5	1.7	-1.37	-2.59	AAA-AAA	0.00	999	1.000	1.000	0.2369
237148_at	22.4	8.7	-1.37	-2.59	AAA-AAA	0.00	999	1.000	1.000	0.2678
230431_at	15.4	39.9	1.37	2.59	AAA-AAA	0.00	999	1.000	1.000	0.3256
232836_at	29.1	11.3	-1.37	-2.59	AAA-AAA	0.00	999	1.000	1.000	0.3961
203382_s_at	34.0	13.2	-1.37	-2.59	AAA-AAA	0.00	999	1.000	1.000	0.2900
1563503_at	11.4	4.4	-1.37	-2.59	AAA-AAA	0.00	999	1.000	1.000	0.2895
1563087_at	2.5	6.4	1.37	2.59	AAA-AAA	0.00	999	1.000	1.000	0.1967
1553549_at	43.3	16.7	-1.37	-2.59	AAA-AAA	0.00	999	1.000	1.000	0.2476
244568_at	19.7	7.6	-1.37	-2.59	AAA-AAA	0.00	999	1.000	1.000	0.2974
1559674_at	85.4	33.0	-1.37	-2.59	AAA-AAA	0.00	999	1.000	1.000	0.0047
211190_x_at	21.2	8.2	-1.37	-2.59	AAA-AAA	0.00	999	1.000	1.000	0.2867
215973_at	82.7	32.0	-1.37	-2.59	AAA-AAA	0.00	999	1.000	1.000	0.0882
234963_s_at	69.5	26.9	-1.37	-2.59	AAA-AAA	0.00	999	1.000	1.000	0.1847
1562216_at	5.0	12.8	1.37	2.58	AAA-AAA	0.00	999	1.000	1.000	0.4211
1569693_at	10.4	4.0	-1.37	-2.59	AAA-AAA	0.00	999	1.000	1.000	0.3754
1559275_x_at	10.7	4.1	-1.37	-2.58	AAA-AAA	0.00	999	1.000	1.000	0.4898
206511_s_at	20.3	52.3	1.37	2.58	AAA-AMA	0.00	999	1.000	1.000	0.1098
217271_at	22.3	8.6	-1.37	-2.59	AAA-AAA	0.00	999	1.000	1.000	0.2337
1559821_at	15.8	40.8	1.37	2.58	AAA-AAA	0.00	999	1.000	1.000	0.2217
221037_s_at	5.2	2.0	-1.37	-2.58	AAA-AAA	0.00	999	1.000	1.000	0.2204
212681_at	5.8	2.3	-1.37	-2.58	AAA-AAA	0.00	999	1.000	1.000	0.4600
217664_at	19.2	7.4	-1.37	-2.58	AAA-AAA	0.00	999	1.000	1.000	0.1170
206177_s_at	11.2	28.8	1.37	2.58	AAA-AAA	0.00	999	1.000	1.000	0.4290
242903_at	11.3	29.1	1.37	2.58	AAA-AAA	0.00	999	1.000	1.000	0.2227
240090_at	7.2	18.5	1.37	2.58	AAA-AAA	0.00	999	1.000	1.000	0.3735
242295_at	27.7	10.7	-1.37	-2.58	AAA-AAA	0.00	999	1.000	1.000	0.3395
1561546_at	32.5	12.6	-1.37	-2.58	AAA-AAA	0.00	999	1.000	1.000	0.1822
1570242_x_at	13.8	5.3	-1.37	-2.58	AAA-AAA	0.00	999	1.000	1.000	0.1014
1565424_at	6.7	2.6	-1.37	-2.58	AAA-AAA	0.00	999	1.000	1.000	0.1984
1553872_at	81.0	31.4	-1.37	-2.58	AAA-AAA	0.00	999	1.000	1.000	0.2988
1557742_a_at	7.9	3.1	-1.37	-2.58	AAA-AAA	0.00	999	1.000	1.000	0.2337
1556435_at	33.7	13.1	-1.37	-2.58	AAA-AAA	0.00	999	1.000	1.000	0.2064
224133_at	7.9	20.4	1.37	2.58	AAA-AAA	0.00	999	1.000	1.000	0.2021
209550_at	16.8	43.5	1.37	2.58	AAA-AAA	0.00	999	1.000	1.000	0.1298
234757_at	72.6	28.1	-1.37	-2.58	AAA-AAA	0.00	999	1.000	1.000	0.0966
234502_at	11.9	4.6	-1.37	-2.58	AAA-AAA	0.00	999	1.000	1.000	0.0163
239458_at	7.4	19.1	1.37	2.58	AAA-AAA	0.00	999	1.000	1.000	0.1876
230140_at	15.9	6.2	-1.37	-2.58	AAA-AAA	0.00	999	1.000	1.000	0.3387
233258_at	26.2	10.2	-1.37	-2.58	AAA-AAA	0.00	999	1.000	1.000	0.1345

240921_at	12.5	32.1	1.37	2.58	AAA-AAA	0.00	999	1.000	1.000	0.0891
244256_at	16.5	6.4	-1.37	-2.58	AAA-AAA	0.00	999	1.000	1.000	0.1734
243893_at	79.4	30.8	-1.37	-2.58	AAA-AAA	0.00	999	1.000	1.000	0.0885
241431_at	13.4	34.5	1.37	2.58	AAA-AAA	0.00	999	1.000	1.000	0.3929
1559026_at	11.1	28.6	1.37	2.58	AAA-AAA	0.00	999	1.000	1.000	0.1106
1558848_at	8.5	3.3	-1.37	-2.58	AAA-AAA	0.00	999	1.000	1.000	0.2098
219660_s_at	26.9	10.4	-1.37	-2.58	AAA-AAA	0.00	999	1.000	1.000	0.3646
206676_at	6.2	16.0	1.37	2.58	AAA-AAA	0.00	999	1.000	1.000	0.2098
206682_at	6.4	16.4	1.37	2.58	AAA-AAA	0.00	999	1.000	1.000	0.2568
214249_at	28.0	10.9	-1.37	-2.58	AAA-AAA	0.00	999	1.000	1.000	0.3567
217093_at	13.5	5.2	-1.37	-2.58	AAA-AAA	0.00	999	1.000	1.000	0.3347
234912_at	112.7	43.7	-1.37	-2.58	AAA-AAA	0.00	999	1.000	1.000	0.1614
244065_at	6.6	2.6	-1.37	-2.58	AAA-AAA	0.00	999	1.000	1.000	0.0692
241590_at	3.8	9.9	1.37	2.58	AAA-AAA	0.00	999	1.000	1.000	0.3243
243783_at	76.8	29.8	-1.37	-2.58	MAA-AAA	0.00	999	1.000	1.000	0.1020
1570272_at	5.3	13.8	1.37	2.58	AAA-AAA	0.00	999	1.000	1.000	0.3594
1565846_at	23.3	9.1	-1.37	-2.58	AAA-AAA	0.00	999	1.000	1.000	0.4249
1565857_at	13.9	5.4	-1.37	-2.58	AAA-AAA	0.00	999	1.000	1.000	0.3713
1555854_at	17.8	6.9	-1.37	-2.58	AAA-AAA	0.00	999	1.000	1.000	0.4510
216073_at	29.6	11.5	-1.37	-2.58	AAA-AAA	0.00	999	1.000	1.000	0.1966
235811_at	103.3	40.1	-1.37	-2.58	AAA-AAA	0.00	999	1.000	1.000	0.2025
205307_s_at	29.1	11.3	-1.37	-2.58	AAA-AAA	0.00	999	1.000	1.000	0.3868
242649_x_at	17.6	6.8	-1.37	-2.58	AAA-AAA	0.00	999	1.000	1.000	0.3564
240521_at	5.8	14.9	1.37	2.58	AAA-AAA	0.00	999	1.000	1.000	0.1685
1563882_a_at	14.3	36.9	1.37	2.58	AAA-AAA	0.00	999	1.000	1.000	0.1332
1560891_a_at	62.3	24.2	-1.37	-2.58	AAA-AAA	0.00	999	1.000	1.000	0.0541
1561650_s_at	16.4	42.2	1.37	2.58	AAA-AAA	0.00	999	1.000	1.000	0.2465
1559532_at	22.9	8.9	-1.37	-2.58	AAA-AAA	0.00	999	1.000	1.000	0.4538
207652_s_at	24.0	9.3	-1.37	-2.58	AAA-AAA	0.00	999	1.000	1.000	0.1132
233932_at	19.2	7.4	-1.37	-2.58	AAA-AAA	0.00	999	1.000	1.000	0.4421
234910_at	76.1	29.5	-1.37	-2.58	AAA-AAA	0.00	999	1.000	1.000	0.1493
239132_at	8.7	3.4	-1.37	-2.58	AAA-AAA	0.00	999	1.000	1.000	0.1172
232967_at	49.5	19.2	-1.37	-2.58	AAA-AAA	0.00	999	1.000	1.000	0.2900
223261_at	528.8	1361.6	1.36	2.58	PPP-PPP	0.00	999	1.000	1.000	0.2517
244538_at	23.8	9.2	-1.36	-2.57	AAA-AAA	0.00	999	1.000	1.000	0.0179
1552482_at	28.0	10.9	-1.36	-2.57	AAA-AAA	0.00	999	1.000	1.000	0.3360
1557180_at	1.8	4.6	1.36	2.57	AAA-AAA	0.00	999	1.000	1.000	0.3165
210177_at	22.8	8.9	-1.36	-2.57	AAA-AAA	0.00	999	1.000	1.000	0.3971
234796_at	8.4	21.5	1.36	2.58	AAA-AAA	0.00	999	1.000	1.000	0.0948
231624_s_at	22.7	8.8	-1.36	-2.57	AAA-AAA	0.00	999	1.000	1.000	0.1699
219873_at	19.2	49.3	1.36	2.57	AAA-AAA	0.00	999	1.000	1.000	0.3471
223877_at	23.8	9.3	-1.36	-2.57	AAA-AAA	0.00	999	1.000	1.000	0.3736

217224_at	49.5	19.2	-1.36	-2.57	AAA-AAA	0.00	999	1.000	1.000	0.1505
236049_at	22.7	8.8	-1.36	-2.57	AAA-AAA	0.00	999	1.000	1.000	0.0664
236770_at	43.5	16.9	-1.36	-2.57	AAA-AAA	0.00	999	1.000	1.000	0.3042
234162_at	41.0	15.9	-1.36	-2.57	AAA-AAA	0.00	999	1.000	1.000	0.3271
231490_at	7.7	19.9	1.36	2.57	AAA-AAA	0.00	999	1.000	1.000	0.3737
242104_at	18.0	46.2	1.36	2.57	AAA-PAA	0.00	999	1.000	1.000	0.0780
244076_at	20.4	7.9	-1.36	-2.57	AAA-AAA	0.00	999	1.000	1.000	0.4094
1553723_at	10.0	3.9	-1.36	-2.57	AAA-AAA	0.00	999	1.000	1.000	0.1746
1557850_at	14.4	5.6	-1.36	-2.57	AAA-AAA	0.00	999	1.000	1.000	0.0734
1557024_at	14.6	5.7	-1.36	-2.57	AAA-AAA	0.00	999	1.000	1.000	0.3731
209996_x_at	59.5	23.1	-1.36	-2.57	AAA-AAA	0.00	999	1.000	1.000	0.3330
211822_s_at	112.0	43.6	-1.36	-2.57	AAA-AAA	0.00	999	1.000	1.000	0.1388
242790_at	31.6	81.0	1.36	2.57	AAA-APP	0.00	999	1.000	1.000	0.2203
206121_at	14.2	36.5	1.36	2.57	AAA-APA	0.00	999	1.000	1.000	0.1059
241814_at	1.7	4.5	1.36	2.57	AAA-AAA	0.00	999	1.000	1.000	0.3213
1562869_at	85.0	33.1	-1.36	-2.57	AAA-AAA	0.00	999	1.000	1.000	0.1910
1563091_at	11.0	4.3	-1.36	-2.57	AAA-AAA	0.00	999	1.000	1.000	0.1845
207087_x_at	16.3	6.4	-1.36	-2.57	AAA-AAA	0.00	999	1.000	1.000	0.2274
227496_at	20.1	51.7	1.36	2.57	AAA-AAA	0.00	999	1.000	1.000	0.3720
214889_at	61.1	23.8	-1.36	-2.57	MAA-AAA	0.00	999	1.000	1.000	0.0417
214776_x_at	22.9	8.9	-1.36	-2.57	AAA-AAA	0.00	999	1.000	1.000	0.4468
232641_at	19.1	49.0	1.36	2.57	AAA-PPA	0.00	999	1.000	1.000	0.1443
240780_at	13.5	5.3	-1.36	-2.57	AAA-AAA	0.00	999	1.000	1.000	0.0317
241806_at	24.9	9.7	-1.36	-2.57	MAA-AAA	0.00	999	1.000	1.000	0.3052
1570342_at	18.9	7.4	-1.36	-2.57	AAA-AAA	0.00	999	1.000	1.000	0.1317
1554117_at	18.3	46.9	1.36	2.57	AAA-AAA	0.00	999	1.000	1.000	0.1069
1556553_at	32.5	12.6	-1.36	-2.57	AAA-AAA	0.00	999	1.000	1.000	0.4042
238852_at	7.2	2.8	-1.36	-2.57	AAA-AAA	0.00	999	1.000	1.000	0.5069
229481_at	52.4	20.4	-1.36	-2.57	AAA-AAA	0.00	999	1.000	1.000	0.1382
239810_at	16.5	6.4	-1.36	-2.57	AAA-AAA	0.00	999	1.000	1.000	0.2126
243787_at	19.6	7.7	-1.36	-2.57	AAA-AAA	0.00	999	1.000	1.000	0.3266
242275_at	36.7	14.3	-1.36	-2.57	AAA-AAA	0.00	999	1.000	1.000	0.1462
221433_at	37.5	14.6	-1.36	-2.57	AAA-AAA	0.00	999	1.000	1.000	0.1424
208350_at	12.4	4.8	-1.36	-2.57	AAA-AAA	0.00	999	1.000	1.000	0.1138
222836_at	6.6	2.6	-1.36	-2.57	AAA-AAA	0.00	999	1.000	1.000	0.1661
238120_at	34.3	13.4	-1.36	-2.57	AAA-AAA	0.00	999	1.000	1.000	0.4075
237423_at	22.2	8.7	-1.36	-2.56	AAA-AAA	0.00	999	1.000	1.000	0.3341
237907_at	29.7	11.6	-1.36	-2.57	AAA-AAA	0.00	999	1.000	1.000	0.4195
233633_at	28.4	11.1	-1.36	-2.57	AAA-AAA	0.00	999	1.000	1.000	0.2433
242353_at	19.9	7.8	-1.36	-2.56	AAA-AAA	0.00	999	1.000	1.000	0.3142
203548_s_at	14.4	5.6	-1.36	-2.56	AAA-AAA	0.00	999	1.000	1.000	0.1902
1561677_at	7.5	19.1	1.36	2.56	AAA-AAA	0.00	999	1.000	1.000	0.3729

1554946_at	14.7	5.7	-1.36	-2.56	AAA-AAA	0.00	999	1.000	1.000	0.4710
206604_at	23.8	9.3	-1.36	-2.56	AAA-AAA	0.00	999	1.000	1.000	0.4013
236375_at	51.2	20.0	-1.36	-2.56	AAA-AMA	0.00	999	1.000	1.000	0.1483
224225_s_at	9.2	23.5	1.36	2.56	AAA-AAP	0.00	999	1.000	1.000	0.3708
244499_at	39.4	15.4	-1.36	-2.56	AAA-AAA	0.00	999	1.000	1.000	0.3216
1556092_s_at	16.3	6.4	-1.36	-2.56	AAA-AAA	0.00	999	1.000	1.000	0.1238
1557219_at	18.8	7.3	-1.36	-2.56	AAA-AAA	0.00	999	1.000	1.000	0.4307
228372_at	25.0	9.8	-1.36	-2.56	AAA-AAA	0.00	999	1.000	1.000	0.3980
232990_at	25.6	65.6	1.36	2.56	AAA-AAA	0.00	999	1.000	1.000	0.3231
229807_s_at	31.6	80.8	1.36	2.56	AAA-AMP	0.00	999	1.000	1.000	0.2841
203161_s_at	19.0	7.4	-1.36	-2.56	AAA-AAA	0.00	999	1.000	1.000	0.2156
1562278_at	3.5	8.8	1.36	2.56	AAA-AAA	0.00	999	1.000	1.000	0.2414
1563794_s_at	5.7	14.5	1.36	2.56	AAA-AAA	0.00	999	1.000	1.000	0.3034
91826_at	26.3	10.3	-1.36	-2.56	AAA-AAA	0.00	999	1.000	1.000	0.0560
227253_at	46.7	18.2	-1.36	-2.56	AMA-AAA	0.00	999	1.000	1.000	0.1094
213308_at	33.1	12.9	-1.36	-2.56	AAA-AAA	0.00	999	1.000	1.000	0.1709
208607_s_at	5.3	13.7	1.36	2.56	AAA-AAA	0.00	999	1.000	1.000	0.1307
227800_at	17.6	45.2	1.36	2.56	AAA-AAA	0.00	999	1.000	1.000	0.0473
232911_at	38.2	14.9	-1.36	-2.56	AAA-AAA	0.00	999	1.000	1.000	0.1786
240083_at	6.7	17.2	1.36	2.56	AAA-AAA	0.00	999	1.000	1.000	0.1494
1560138_at	48.5	19.0	-1.36	-2.56	AAA-AAA	0.00	999	1.000	1.000	0.2198
1555572_at	2.9	1.1	-1.36	-2.56	AAA-AAA	0.00	999	1.000	1.000	0.1962
206570_s_at	18.5	47.4	1.36	2.56	AAA-AAA	0.00	999	1.000	1.000	0.2927
224243_at	27.3	10.7	-1.36	-2.56	AAA-AAA	0.00	999	1.000	1.000	0.2104
211479_s_at	6.8	17.4	1.36	2.56	AAA-AAA	0.00	999	1.000	1.000	0.0441
216270_at	13.6	5.3	-1.36	-2.56	AAA-AAA	0.00	999	1.000	1.000	0.2155
235784_at	69.1	27.0	-1.36	-2.56	AAA-AAA	0.00	999	1.000	1.000	0.0627
236950_s_at	9.1	23.3	1.36	2.56	AAA-AAA	0.00	999	1.000	1.000	0.4870
242947_at	12.5	4.9	-1.35	-2.56	AAA-AAA	0.00	999	1.000	1.000	0.4406
240303_at	9.5	24.2	1.35	2.56	AAA-AAA	0.00	999	1.000	1.000	0.0538
240607_at	43.5	111.2	1.35	2.56	AAA-AAA	0.00	999	1.000	1.000	0.1819
1560230_at	18.2	7.1	-1.35	-2.56	AAA-AAA	0.00	999	1.000	1.000	0.2357
1560456_at	30.5	11.9	-1.35	-2.56	AAA-AAA	0.00	999	1.000	1.000	0.2018
207215_at	14.0	5.5	-1.35	-2.56	AAA-AAA	0.00	999	1.000	1.000	0.1006
223534_s_at	25.8	65.9	1.35	2.56	AAA-AAA	0.00	999	1.000	1.000	0.0544
214158_s_at	20.1	51.5	1.35	2.56	AAA-AAA	0.00	999	1.000	1.000	0.1639
216946_at	45.8	17.9	-1.35	-2.56	AAA-AAA	0.00	999	1.000	1.000	0.2327
238230_x_at	19.8	50.6	1.35	2.56	AAA-AAA	0.00	999	1.000	1.000	0.0721
233227_at	55.8	21.8	-1.35	-2.56	AAA-AAA	0.00	999	1.000	1.000	0.1679
231160_at	12.3	4.8	-1.35	-2.56	AAA-AAA	0.00	999	1.000	1.000	0.4021
231338_at	121.5	47.5	-1.35	-2.56	AAA-AAA	0.00	999	1.000	1.000	0.2086
203369_x_at	133.0	339.8	1.35	2.56	AAA-MPP	0.00	999	1.000	1.000	0.3032

210148_at	48.5	123.9	1.35	2.56	AAA-PPP	0.00	999	1.000	1.000	0.1203
243799_x_at	28.7	11.2	-1.35	-2.55	AAA-AAA	0.00	999	1.000	1.000	0.4682
240610_at	46.7	18.3	-1.35	-2.56	AAA-AAA	0.00	999	1.000	1.000	0.2041
234298_at	32.2	12.6	-1.35	-2.56	AAA-AAA	0.00	999	1.000	1.000	0.3253
239209_at	19.1	48.7	1.35	2.55	AAA-AAA	0.00	999	1.000	1.000	0.2216
237755_s_at	5.3	2.1	-1.35	-2.55	AAA-AAA	0.00	999	1.000	1.000	0.2424
231700_at	83.5	32.7	-1.35	-2.56	AAA-AAA	0.00	999	1.000	1.000	0.1269
240730_at	61.9	24.2	-1.35	-2.55	AAA-AAA	0.00	999	1.000	1.000	0.1983
241073_at	27.7	10.9	-1.35	-2.55	AAA-AAA	0.00	999	1.000	1.000	0.1575
240658_at	28.4	11.1	-1.35	-2.55	AAA-AAA	0.00	999	1.000	1.000	0.2212
1562272_at	64.9	25.4	-1.35	-2.55	AAA-AMA	0.00	999	1.000	1.000	0.2683
219943_s_at	40.4	15.8	-1.35	-2.55	AAA-AAA	0.00	999	1.000	1.000	0.3666
207470_at	17.1	43.6	1.35	2.55	AAA-AAA	0.00	999	1.000	1.000	0.1633
223941_at	67.0	26.3	-1.35	-2.55	MAA-AAA	0.00	999	1.000	1.000	0.0630
210419_at	27.0	10.6	-1.35	-2.55	AAA-AAA	0.00	999	1.000	1.000	0.2034
211879_x_at	39.0	15.3	-1.35	-2.55	AAA-AAA	0.00	999	1.000	1.000	0.3795
203948_s_at	14.0	5.5	-1.35	-2.55	AAA-AAA	0.00	999	1.000	1.000	0.3925
1568878_at	13.6	5.3	-1.35	-2.55	AAA-AAA	0.00	999	1.000	1.000	0.2842
220000_at	43.5	17.1	-1.35	-2.55	AAA-AAA	0.00	999	1.000	1.000	0.0873
220449_at	59.1	23.2	-1.35	-2.55	AAA-AAA	0.00	999	1.000	1.000	0.1464
215161_at	9.9	3.9	-1.35	-2.55	AAA-AAA	0.00	999	1.000	1.000	0.0446
213177_at	82.1	32.2	-1.35	-2.55	AAA-AAA	0.00	999	1.000	1.000	0.2363
1568964_x_at	31.2	79.5	1.35	2.55	AAA-APP	0.00	999	1.000	1.000	0.3945
1561195_at	64.3	25.2	-1.35	-2.55	AAA-AAA	0.00	999	1.000	1.000	0.4280
1557402_at	5.8	2.3	-1.35	-2.55	AAA-AAA	0.00	999	1.000	1.000	0.1806
221755_at	19.2	49.0	1.35	2.55	AAA-AAA	0.00	999	1.000	1.000	0.1620
224073_at	74.1	29.1	-1.35	-2.55	AAA-AAA	0.00	999	1.000	1.000	0.1013
207326_at	14.5	5.7	-1.35	-2.55	AAA-AAA	0.00	999	1.000	1.000	0.1974
214596_at	35.4	13.9	-1.35	-2.55	AAA-AAA	0.00	999	1.000	1.000	0.5223
210313_at	24.1	9.5	-1.35	-2.55	AAA-AAA	0.00	999	1.000	1.000	0.1007
208548_at	17.8	7.0	-1.35	-2.55	AAA-AAA	0.00	999	1.000	1.000	0.3461
235129_at	10.2	25.9	1.35	2.55	AAA-AAA	0.00	999	1.000	1.000	0.2301
205476_at	6.4	2.5	-1.35	-2.55	AAA-AAA	0.00	999	1.000	1.000	0.1074
205567_at	79.2	31.1	-1.35	-2.55	AAA-AAA	0.00	999	1.000	1.000	0.1988
207069_s_at	47.3	120.5	1.35	2.55	AAA-APP	0.00	999	1.000	1.000	0.2018
222795_s_at	58.3	148.6	1.35	2.55	AAA-PPP	0.00	999	1.000	1.000	0.0839
1568936_a_at	14.1	5.5	-1.35	-2.55	MAA-AAA	0.00	999	1.000	1.000	0.5368
1566906_at	46.7	18.3	-1.35	-2.55	AAA-AAA	0.00	999	1.000	1.000	0.4105
1553529_at	6.0	15.3	1.35	2.55	AAA-AAA	0.00	999	1.000	1.000	0.2213
207097_s_at	5.1	12.9	1.35	2.55	AAA-AAA	0.00	999	1.000	1.000	0.4161
229040_at	8.4	21.5	1.35	2.55	AAA-AAA	0.00	999	1.000	1.000	0.1400
242899_at	3.8	9.8	1.35	2.55	AAA-AAA	0.00	999	1.000	1.000	0.5555

1559009_at	15.6	39.7	1.35	2.55	AAA-AAA	0.00	999	1.000	1.000	0.1280
219934_s_at	13.6	5.3	-1.35	-2.55	AAA-AAA	0.00	999	1.000	1.000	0.2950
223615_at	42.2	107.4	1.35	2.55	AAA-AAA	0.00	999	1.000	1.000	0.0705
210683_at	5.4	13.8	1.35	2.55	AAA-AAA	0.00	999	1.000	1.000	0.0827
215721_at	16.8	6.6	-1.35	-2.55	AAA-AAA	0.00	999	1.000	1.000	0.3274
239459_s_at	30.9	12.1	-1.35	-2.55	AAA-AAA	0.00	999	1.000	1.000	0.1618
241494_at	10.3	26.1	1.35	2.55	AAA-AAA	0.00	999	1.000	1.000	0.3607
203030_s_at	8.9	3.5	-1.35	-2.55	AAA-AAA	0.00	999	1.000	1.000	0.2667
243662_at	14.1	35.8	1.35	2.54	AAA-AAA	0.00	999	1.000	1.000	0.1739
240589_at	37.2	14.6	-1.35	-2.54	AAA-AAA	0.00	999	1.000	1.000	0.2378
1564685_a_at	2.6	6.6	1.35	2.54	AAA-AAA	0.00	999	1.000	1.000	0.3345
1558661_at	8.6	3.4	-1.35	-2.54	AAA-AAA	0.00	999	1.000	1.000	0.2666
216165_at	18.0	7.1	-1.35	-2.54	AAA-AAA	0.00	999	1.000	1.000	0.3077
203760_s_at	26.3	10.3	-1.35	-2.54	AAA-AAA	0.00	999	1.000	1.000	0.1622
241123_at	19.6	7.7	-1.35	-2.54	AAA-AAA	0.00	999	1.000	1.000	0.1471
1569876_at	45.5	17.9	-1.35	-2.54	AAA-AAA	0.00	999	1.000	1.000	0.0812
1566930_at	9.1	3.6	-1.35	-2.54	AAA-AAA	0.00	999	1.000	1.000	0.3828
1554212_s_at	53.6	21.1	-1.35	-2.54	AAA-AMA	0.00	999	1.000	1.000	0.3205
1554797_at	39.4	15.5	-1.35	-2.54	AAA-MAA	0.00	999	1.000	1.000	0.2119
223745_at	11.1	28.2	1.35	2.54	AAA-AAA	0.00	999	1.000	1.000	0.2732
207368_at	45.8	18.0	-1.35	-2.54	AAA-AAA	0.00	999	1.000	1.000	0.1791
215830_at	15.1	6.0	-1.35	-2.54	AAA-AAA	0.00	999	1.000	1.000	0.3440
234057_at	2.9	7.4	1.35	2.54	AAA-AAA	0.00	999	1.000	1.000	0.2570
238936_at	30.2	11.9	-1.35	-2.54	AAA-AAA	0.00	999	1.000	1.000	0.3653
231021_at	86.1	33.9	-1.35	-2.54	AAA-AAA	0.00	999	1.000	1.000	0.1409
211824_x_at	25.9	65.8	1.35	2.54	APA-APA	0.00	999	1.000	1.000	0.2945
216280_s_at	19.6	49.7	1.35	2.54	AAA-AAP	0.00	999	1.000	1.000	0.2258
240863_at	4.1	1.6	-1.35	-2.54	AAA-AAA	0.00	999	1.000	1.000	0.4807
244174_at	28.0	11.0	-1.35	-2.54	AAA-AAA	0.00	999	1.000	1.000	0.3464
1553314_a_at	11.7	4.6	-1.35	-2.54	AAA-AAA	0.00	999	1.000	1.000	0.1283
1556808_at	75.7	29.8	-1.35	-2.54	AAA-AAA	0.00	999	1.000	1.000	0.3641
206681_x_at	65.8	25.9	-1.35	-2.54	AAA-AAA	0.00	999	1.000	1.000	0.2161
214884_at	9.9	3.9	-1.35	-2.54	AAA-AAA	0.00	999	1.000	1.000	0.3278
216768_x_at	21.7	55.1	1.35	2.54	AAA-AAA	0.00	999	1.000	1.000	0.3380
230308_at	21.9	55.7	1.35	2.54	AAA-AAA	0.00	999	1.000	1.000	0.2497
233151_s_at	33.5	13.2	-1.35	-2.54	AAA-AAA	0.00	999	1.000	1.000	0.3199
1561942_x_at	16.7	42.3	1.34	2.54	AAA-AAA	0.00	999	1.000	1.000	0.0105
1564204_at	60.2	23.7	-1.34	-2.54	AAA-AAA	0.00	999	1.000	1.000	0.2418
1554528_at	16.5	41.9	1.34	2.54	AAA-AAA	0.00	999	1.000	1.000	0.1839
216094_at	16.0	6.3	-1.34	-2.54	AAA-AAA	0.00	999	1.000	1.000	0.3875
229655_at	23.7	9.3	-1.34	-2.54	AAA-AAA	0.00	999	1.000	1.000	0.1257
206293_at	4.3	1.7	-1.34	-2.54	AAA-AAA	0.00	999	1.000	1.000	0.2358

243239_at	12.9	5.1	-1.34	-2.54	AAA-AAA	0.00	999	1.000	1.000	0.0377
1562752_at	11.1	4.4	-1.34	-2.54	AAA-AAA	0.00	999	1.000	1.000	0.3476
1562079_at	34.1	13.4	-1.34	-2.54	AAA-MAA	0.00	999	1.000	1.000	0.3402
1558796_a_at	6.0	15.3	1.34	2.54	AAA-AAA	0.00	999	1.000	1.000	0.2718
207055_at	16.4	6.5	-1.34	-2.54	AAA-AAA	0.00	999	1.000	1.000	0.2419
211282_x_at	139.3	54.9	-1.34	-2.54	AAA-AAA	0.00	999	1.000	1.000	0.1285
216185_at	12.5	4.9	-1.34	-2.54	AAA-AAA	0.00	999	1.000	1.000	0.1333
216023_at	19.9	50.5	1.34	2.54	AAA-AAA	0.00	999	1.000	1.000	0.3907
235748_s_at	32.3	12.7	-1.34	-2.54	AAA-AAA	0.00	999	1.000	1.000	0.4588
239620_at	8.1	3.2	-1.34	-2.54	AAA-AAA	0.00	999	1.000	1.000	0.0360
232997_at	16.3	6.4	-1.34	-2.54	AAA-AAA	0.00	999	1.000	1.000	0.4182
232321_at	40.8	16.1	-1.34	-2.54	AAA-AAA	0.00	999	1.000	1.000	0.2499
1565338_x_at	45.2	17.8	-1.34	-2.53	AAA-AAA	0.00	999	1.000	1.000	0.3844
206735_at	39.6	15.6	-1.34	-2.54	AAA-AAA	0.00	999	1.000	1.000	0.1478
215360_at	8.5	3.4	-1.34	-2.54	AAA-AAA	0.00	999	1.000	1.000	0.4703
216703_at	41.8	16.5	-1.34	-2.54	AAA-MAA	0.00	999	1.000	1.000	0.3168
216737_at	22.0	8.7	-1.34	-2.54	AAA-AAA	0.00	999	1.000	1.000	0.3322
239304_at	34.7	13.7	-1.34	-2.54	AAA-AAA	0.00	999	1.000	1.000	0.2869
1561333_at	10.6	26.8	1.34	2.53	PAA-AAA	0.00	999	1.000	1.000	0.3546
203619_s_at	24.2	9.6	-1.34	-2.53	AAA-AAA	0.00	999	1.000	1.000	0.1974
243355_at	44.2	17.5	-1.34	-2.53	AAA-AAA	0.00	999	1.000	1.000	0.2466
1554511_at	17.2	6.8	-1.34	-2.53	AAA-AAA	0.00	999	1.000	1.000	0.1708
1556107_at	18.3	46.3	1.34	2.53	AAA-AAA	0.00	999	1.000	1.000	0.1221
207854_at	19.6	7.7	-1.34	-2.53	AAA-AAA	0.00	999	1.000	1.000	0.2765
218864_at	28.1	71.3	1.34	2.53	AAA-AAA	0.00	999	1.000	1.000	0.0275
216654_at	17.4	6.9	-1.34	-2.53	AAA-AAA	0.00	999	1.000	1.000	0.2015
230284_at	26.2	10.3	-1.34	-2.53	AAA-AAA	0.00	999	1.000	1.000	0.4049
233077_at	30.7	77.7	1.34	2.53	AAA-PAA	0.00	999	1.000	1.000	0.2304
244349_at	96.6	38.1	-1.34	-2.53	AAA-AAA	0.00	999	1.000	1.000	0.2487
1557882_at	8.2	20.7	1.34	2.53	AAA-AMA	0.00	999	1.000	1.000	0.4337
1559970_at	33.0	13.0	-1.34	-2.53	AAA-MAA	0.00	999	1.000	1.000	0.3982
206793_at	51.3	20.3	-1.34	-2.53	AAA-AAA	0.00	999	1.000	1.000	0.2696
207432_at	45.5	18.0	-1.34	-2.53	AAA-AAA	0.00	999	1.000	1.000	0.3072
223844_at	29.7	11.7	-1.34	-2.53	AAA-AAA	0.00	999	1.000	1.000	0.3184
237155_at	83.5	33.0	-1.34	-2.53	AAA-AAA	0.00	999	1.000	1.000	0.1247
238739_at	40.3	102.1	1.34	2.53	AMP-PAP	0.00	999	1.000	1.000	0.3727
241914_s_at	23.3	9.2	-1.34	-2.53	AAA-AAA	0.00	999	1.000	1.000	0.3389
1564282_a_at	9.1	3.6	-1.34	-2.53	AAA-AAA	0.00	999	1.000	1.000	0.3230
1558437_at	17.2	6.8	-1.34	-2.53	AAA-AAA	0.00	999	1.000	1.000	0.2508
206780_at	34.1	13.5	-1.34	-2.53	AAM-AAA	0.00	999	1.000	1.000	0.0628
234164_at	8.5	3.3	-1.34	-2.53	AAA-AAA	0.00	999	1.000	1.000	0.4062
238429_at	14.8	5.8	-1.34	-2.53	AAA-AAA	0.00	999	1.000	1.000	0.3220

204039_at	27.7	11.0	-1.34	-2.53	AAA-AAA	0.00	999	1.000	1.000	0.1291
230941_at	29.6	11.7	-1.34	-2.53	AAA-AAA	0.00	999	1.000	1.000	0.1585
1561879_at	44.7	17.7	-1.34	-2.53	AAA-MAA	0.00	999	1.000	1.000	0.2077
219884_at	80.5	31.8	-1.34	-2.53	AAA-AAA	0.00	999	1.000	1.000	0.1834
225518_x_at	22.5	8.9	-1.34	-2.53	AAA-AAA	0.00	999	1.000	1.000	0.4121
217648_at	7.4	2.9	-1.34	-2.53	AAA-AAA	0.00	999	1.000	1.000	0.1258
237859_at	4.9	12.3	1.34	2.53	AAA-AAA	0.00	999	1.000	1.000	0.3281
243471_at	54.2	21.5	-1.34	-2.53	AAA-AAA	0.00	999	1.000	1.000	0.0289
1560563_at	20.2	8.0	-1.34	-2.53	AAA-AAA	0.00	999	1.000	1.000	0.2712
226597_at	31.9	80.6	1.34	2.53	AAA-AAA	0.00	999	1.000	1.000	0.4040
213667_at	35.5	89.5	1.34	2.53	AAA-AAA	0.00	999	1.000	1.000	0.1215
215186_at	122.7	48.6	-1.34	-2.53	AAA-AAA	0.00	999	1.000	1.000	0.0052
235619_at	65.8	26.1	-1.34	-2.53	AAA-AAA	0.00	999	1.000	1.000	0.2855
229919_at	51.0	20.2	-1.34	-2.53	AAA-AAA	0.00	999	1.000	1.000	0.2391
227591_at	73.4	29.1	-1.34	-2.53	AAA-AAA	0.00	999	1.000	1.000	0.0687
227774_s_at	4.6	11.5	1.34	2.52	AAA-AAA	0.00	999	1.000	1.000	0.3149
1570006_at	3.9	9.8	1.34	2.52	AAA-AAA	0.00	999	1.000	1.000	0.4639
1555352_at	5.6	14.1	1.34	2.52	AAA-AAA	0.00	999	1.000	1.000	0.1148
233814_at	24.0	60.6	1.34	2.52	AAA-AAA	0.00	999	1.000	1.000	0.2971
204273_at	3.8	1.5	-1.34	-2.52	AAA-AAA	0.00	999	1.000	1.000	0.4704
237279_at	59.7	23.7	-1.34	-2.52	AAA-AAA	0.00	999	1.000	1.000	0.0644
206250_x_at	36.3	14.4	-1.34	-2.52	AAA-AAA	0.00	999	1.000	1.000	0.3625
228865_at	50.9	20.2	-1.34	-2.52	AAA-AAA	0.00	999	1.000	1.000	0.1928
1555117_at	9.6	24.1	1.33	2.52	AAA-PAA	0.00	999	1.000	1.000	0.3342
219355_at	53.4	134.7	1.33	2.52	AAA-APA	0.00	999	1.000	1.000	0.2742
1560396_at	14.7	5.8	-1.33	-2.52	AAA-AAA	0.00	999	1.000	1.000	0.3581
AFFX-PheX-3_at	26.0	10.3	-1.33	-2.52	AAA-AAA	0.00	999	1.000	1.000	0.1921
1553428_at	33.2	13.2	-1.33	-2.52	AAA-AAA	0.00	999	1.000	1.000	0.3915
1558310_s_at	9.1	23.0	1.33	2.52	AAA-AAA	0.00	999	1.000	1.000	0.2717
236403_at	18.9	47.7	1.33	2.52	AAA-AAA	0.00	999	1.000	1.000	0.1738
231942_at	32.5	12.9	-1.33	-2.52	AAA-AAA	0.00	999	1.000	1.000	0.2835
240692_at	68.1	27.1	-1.33	-2.52	AAA-AAA	0.00	999	1.000	1.000	0.0096
243048_at	38.0	15.1	-1.33	-2.52	AAA-AAA	0.00	999	1.000	1.000	0.2256
1565192_at	49.0	19.4	-1.33	-2.52	AAA-AAA	0.00	999	1.000	1.000	0.1445
1554764_a_at	2.0	4.9	1.33	2.52	AAA-AAA	0.00	999	1.000	1.000	0.2798
1554715_at	17.5	6.9	-1.33	-2.52	AAA-AAA	0.00	999	1.000	1.000	0.0881
215917_at	9.7	3.9	-1.33	-2.52	AAA-AAA	0.00	999	1.000	1.000	0.5285
237453_at	64.9	25.8	-1.33	-2.52	AAA-AAA	0.00	999	1.000	1.000	0.3516
1558941_at	6.6	2.6	-1.33	-2.52	AAA-AAA	0.00	999	1.000	1.000	0.0166
1556748_x_at	107.2	42.6	-1.33	-2.52	AAA-AAA	0.00	999	1.000	1.000	0.1563
221454_at	23.7	9.4	-1.33	-2.52	AAA-AAA	0.00	999	1.000	1.000	0.1529
207762_at	32.8	13.0	-1.33	-2.52	AAA-AAA	0.00	999	1.000	1.000	0.1189

207770_x_at	20.7	8.2	-1.33	-2.52	AAA-AAA	0.00	999	1.000	1.000	0.0607
217090_at	29.7	11.8	-1.33	-2.52	AAA-AAA	0.00	999	1.000	1.000	0.1683
216181_at	44.0	17.5	-1.33	-2.52	AAA-AAA	0.00	999	1.000	1.000	0.1032
234654_at	16.9	42.7	1.33	2.52	AAA-AAA	0.00	999	1.000	1.000	0.3297
227875_at	18.8	47.4	1.33	2.52	AAA-AAM	0.00	999	1.000	1.000	0.4244
240583_at	39.1	15.5	-1.33	-2.52	AAM-AAA	0.00	999	1.000	1.000	0.2994
1562239_at	22.6	56.7	1.33	2.52	AAA-AAA	0.00	999	1.000	1.000	0.2004
1562275_at	29.6	11.8	-1.33	-2.52	AAA-AAA	0.00	999	1.000	1.000	0.1213
1555884_at	28.1	11.2	-1.33	-2.52	AAA-AAA	0.00	999	1.000	1.000	0.2148
221665_s_at	12.6	5.0	-1.33	-2.52	AAA-AAA	0.00	999	1.000	1.000	0.1071
206437_at	15.7	39.6	1.33	2.52	AAA-AAA	0.00	999	1.000	1.000	0.2157
223644_s_at	52.3	20.8	-1.33	-2.52	AAA-AAA	0.00	999	1.000	1.000	0.4365
236763_at	52.7	20.9	-1.33	-2.52	AAA-AAA	0.00	999	1.000	1.000	0.3985
238914_at	9.7	3.9	-1.33	-2.52	AAA-AAA	0.00	999	1.000	1.000	0.2987
227735_s_at	52.2	20.8	-1.33	-2.52	AAA-AAA	0.00	999	1.000	1.000	0.2259
229185_at	77.0	30.6	-1.33	-2.52	AAA-AAA	0.00	999	1.000	1.000	0.2175
238833_at	8.5	21.3	1.33	2.51	AAP-AAP	0.00	999	1.000	1.000	0.4488
205501_at	2.6	6.5	1.33	2.51	AAA-APA	0.00	999	1.000	1.000	0.3092
1569345_at	17.5	7.0	-1.33	-2.51	AAA-AAA	0.00	999	1.000	1.000	0.0916
244880_at	9.8	3.9	-1.33	-2.51	AAA-AAA	0.00	999	1.000	1.000	0.1646
1557661_at	27.8	11.1	-1.33	-2.51	AAA-AAA	0.00	999	1.000	1.000	0.1834
1555824_a_at	73.5	184.8	1.33	2.51	AAA-AAA	0.00	999	1.000	1.000	0.2295
216776_at	59.1	23.5	-1.33	-2.52	AAA-AAA	0.00	999	1.000	1.000	0.0550
216749_at	6.9	2.7	-1.33	-2.52	AAA-AAA	0.00	999	1.000	1.000	0.1760
217507_at	43.6	17.4	-1.33	-2.52	AAA-AAA	0.00	999	1.000	1.000	0.1184
229525_at	16.7	42.0	1.33	2.51	AAA-AAA	0.00	999	1.000	1.000	0.4966
207554_x_at	54.8	137.6	1.33	2.51	AAP-PPP	0.00	999	1.000	1.000	0.2135
240735_at	17.8	7.1	-1.33	-2.51	AAA-AAA	0.00	999	1.000	1.000	0.1872
243180_at	16.6	6.6	-1.33	-2.51	AAA-AAA	0.00	999	1.000	1.000	0.1570
223901_at	49.5	19.7	-1.33	-2.51	AAA-AAA	0.00	999	1.000	1.000	0.0891
215343_at	30.3	12.1	-1.33	-2.51	AAA-AAA	0.00	999	1.000	1.000	0.3735
236098_at	18.2	45.8	1.33	2.51	AAA-AAA	0.00	999	1.000	1.000	0.2259
230074_s_at	74.1	29.5	-1.33	-2.51	AAA-AAA	0.00	999	1.000	1.000	0.1833
232956_at	22.4	8.9	-1.33	-2.51	AAA-AAA	0.00	999	1.000	1.000	0.3926
1553395_a_at	146.3	58.3	-1.33	-2.51	AAA-AAA	0.00	999	1.000	1.000	0.0209
212477_at	25.7	10.2	-1.33	-2.51	AAA-AAA	0.00	999	1.000	1.000	0.2397
216910_at	15.1	37.8	1.33	2.51	AAA-AAA	0.00	999	1.000	1.000	0.2506
234640_x_at	32.4	12.9	-1.33	-2.51	AAA-AAA	0.00	999	1.000	1.000	0.2500
229877_at	14.2	5.7	-1.33	-2.51	AAA-AAA	0.00	999	1.000	1.000	0.3725
218985_at	70.0	175.6	1.33	2.51	AAA-APP	0.00	999	1.000	1.000	0.3032
240405_at	24.1	9.6	-1.33	-2.51	AAA-AAA	0.00	999	1.000	1.000	0.2131
1561121_at	26.6	10.6	-1.33	-2.51	AAA-AAA	0.00	999	1.000	1.000	0.0935

1569006_at	11.1	27.9	1.33	2.51	AAA-AAA	0.00	999	1.000	1.000	0.2621
1559413_at	70.4	28.1	-1.33	-2.51	AAA-AAA	0.00	999	1.000	1.000	0.3199
221344_at	55.9	22.3	-1.33	-2.51	AAA-AAA	0.00	999	1.000	1.000	0.2111
221724_s_at	20.5	8.2	-1.33	-2.51	AAA-AAA	0.00	999	1.000	1.000	0.1901
220025_at	16.2	6.5	-1.33	-2.51	AAA-AAA	0.00	999	1.000	1.000	0.2561
206826_at	8.3	20.8	1.33	2.51	AAA-AAA	0.00	999	1.000	1.000	0.4568
211791_s_at	27.3	10.9	-1.33	-2.51	AAA-AAA	0.00	999	1.000	1.000	0.1220
204721_s_at	15.3	6.1	-1.33	-2.51	AAA-AAA	0.00	999	1.000	1.000	0.3982
204588_s_at	65.7	26.2	-1.33	-2.51	AAA-AAA	0.00	999	1.000	1.000	0.2460
234027_at	38.5	15.4	-1.33	-2.51	AMA-AAA	0.00	999	1.000	1.000	0.3203
234540_at	6.7	16.8	1.33	2.51	AAA-AAA	0.00	999	1.000	1.000	0.5642
233505_at	3.0	7.5	1.33	2.51	AAA-AAA	0.00	999	1.000	1.000	0.3848
244272_s_at	6.3	2.5	-1.33	-2.51	AAA-AAA	0.00	999	1.000	1.000	0.3837
1569032_at	68.0	27.1	-1.33	-2.51	AAA-AAA	0.00	999	1.000	1.000	0.2580
231348_s_at	16.6	6.6	-1.33	-2.51	AAA-AAA	0.00	999	1.000	1.000	0.3539
1562059_at	6.7	16.7	1.33	2.51	AAA-AAA	0.00	999	1.000	1.000	0.4392
1555018_at	12.1	4.8	-1.33	-2.51	AAA-AAA	0.00	999	1.000	1.000	0.2972
1554384_at	11.3	28.2	1.33	2.51	AAA-AAA	0.00	999	1.000	1.000	0.1466
1552862_at	24.9	62.4	1.33	2.51	AAA-AAA	0.00	999	1.000	1.000	0.1944
1557628_s_at	24.6	9.8	-1.33	-2.51	AAA-AAA	0.00	999	1.000	1.000	0.4190
221386_at	25.8	10.3	-1.33	-2.51	AAA-AAA	0.00	999	1.000	1.000	0.3018
222348_at	8.7	21.7	1.33	2.51	AAA-AAA	0.00	999	1.000	1.000	0.1755
208010_s_at	18.7	7.5	-1.33	-2.51	AAA-AAA	0.00	999	1.000	1.000	0.2413
219930_at	27.1	10.8	-1.33	-2.51	AAA-AAA	0.00	999	1.000	1.000	0.4127
222920_s_at	13.9	5.5	-1.33	-2.51	AAA-AAA	0.00	999	1.000	1.000	0.0772
237406_at	17.7	7.1	-1.33	-2.51	AAA-AAA	0.00	999	1.000	1.000	0.3437
1560973_a_at	7.9	19.7	1.32	2.50	AAA-APA	0.00	999	1.000	1.000	0.3414
243399_at	7.8	3.1	-1.32	-2.50	AAA-AAA	0.00	999	1.000	1.000	0.3048
241598_at	43.3	17.3	-1.32	-2.50	AAA-AAA	0.00	999	1.000	1.000	0.2476
243741_at	52.7	21.0	-1.32	-2.50	AAA-AAA	0.00	999	1.000	1.000	0.2393
AFFX-PheX-M_at	6.4	2.6	-1.32	-2.50	AAA-AAA	0.00	999	1.000	1.000	0.0175
221124_s_at	7.4	18.6	1.32	2.50	AAA-AAA	0.00	999	1.000	1.000	0.3681
207766_at	12.3	4.9	-1.32	-2.50	AAA-AAA	0.00	999	1.000	1.000	0.3265
220426_at	10.4	26.1	1.32	2.50	AAA-AAA	0.00	999	1.000	1.000	0.3198
227011_at	21.5	53.9	1.32	2.50	AAA-AAA	0.00	999	1.000	1.000	0.1510
215180_at	29.4	11.7	-1.32	-2.50	AAA-AAA	0.00	999	1.000	1.000	0.3797
211151_x_at	14.4	5.7	-1.32	-2.50	AAA-AAA	0.00	999	1.000	1.000	0.2124
231211_s_at	101.1	40.4	-1.32	-2.50	AAA-AAA	0.00	999	1.000	1.000	0.2548
1558078_at	46.3	115.9	1.32	2.50	AMP-MPP	0.00	999	1.000	1.000	0.3878
1566607_at	12.3	30.8	1.32	2.50	AAA-AAA	0.00	999	1.000	1.000	0.2669
1553450_s_at	24.5	9.8	-1.32	-2.50	AAA-AAA	0.00	999	1.000	1.000	0.3332
1552924_a_at	60.4	151.2	1.32	2.50	AAA-AAA	0.00	999	1.000	1.000	0.1104

1553183_at	3.7	9.4	1.32	2.50	AAA-AAA	0.00	999	1.000	1.000	0.4894
1555396_s_at	7.6	3.1	-1.32	-2.50	AAA-AAA	0.00	999	1.000	1.000	0.1206
220678_at	21.4	8.6	-1.32	-2.50	AAA-AAA	0.00	999	1.000	1.000	0.1699
227226_at	61.9	24.8	-1.32	-2.50	AAA-AAA	0.00	999	1.000	1.000	0.2208
217382_at	32.1	12.8	-1.32	-2.50	AAA-AAA	0.00	999	1.000	1.000	0.0643
209671_x_at	74.9	29.9	-1.32	-2.50	AAA-AAA	0.00	999	1.000	1.000	0.0222
1560419_at	3.1	7.8	1.32	2.50	AAA-AAA	0.00	999	1.000	1.000	0.1015
1569346_a_at	6.4	2.6	-1.32	-2.50	AAA-AAA	0.00	999	1.000	1.000	0.0822
1569154_a_at	63.2	25.3	-1.32	-2.50	AAA-AAA	0.00	999	1.000	1.000	0.3527
1553850_at	29.6	11.8	-1.32	-2.50	AAA-AAA	0.00	999	1.000	1.000	0.0287
244870_at	49.6	19.8	-1.32	-2.50	AAA-AAA	0.00	999	1.000	1.000	0.0947
1559817_at	16.3	40.8	1.32	2.50	AAA-AAA	0.00	999	1.000	1.000	0.3809
224239_at	51.5	20.6	-1.32	-2.50	AAA-AAA	0.00	999	1.000	1.000	0.0308
215828_at	9.2	23.1	1.32	2.50	AAA-AAA	0.00	999	1.000	1.000	0.1732
231755_at	12.2	4.9	-1.32	-2.50	AAA-AAA	0.00	999	1.000	1.000	0.4103
232010_at	4.1	10.3	1.32	2.50	AAA-AAA	0.00	999	1.000	1.000	0.1984
240351_at	7.5	18.8	1.32	2.50	AAA-AAP	0.00	999	1.000	1.000	0.3546
203280_at	74.7	186.7	1.32	2.50	AAA-APA	0.00	999	1.000	1.000	0.2407
1561446_at	25.5	10.2	-1.32	-2.50	AAA-AAA	0.00	999	1.000	1.000	0.4036
1570339_x_at	16.5	41.3	1.32	2.50	AAA-AAA	0.00	999	1.000	1.000	0.2223
244759_x_at	22.6	9.1	-1.32	-2.50	AAA-AAA	0.00	999	1.000	1.000	0.2524
1559614_at	10.5	26.3	1.32	2.50	AAA-AAA	0.00	999	1.000	1.000	0.3348
224555_x_at	89.0	35.6	-1.32	-2.50	AAA-AAA	0.00	999	1.000	1.000	0.1152
207683_at	4.2	10.6	1.32	2.50	AAA-AAA	0.00	999	1.000	1.000	0.2674
207321_s_at	55.9	22.4	-1.32	-2.50	AAA-AAA	0.00	999	1.000	1.000	0.2272
236206_at	13.7	5.5	-1.32	-2.50	AAA-AAA	0.00	999	1.000	1.000	0.0826
237647_at	38.7	15.5	-1.32	-2.50	AAA-AAA	0.00	999	1.000	1.000	0.2777
241338_at	10.6	4.2	-1.32	-2.50	AAA-AAA	0.00	999	1.000	1.000	0.4863
1568646_x_at	7.5	3.0	-1.32	-2.50	AAA-AAA	0.00	999	1.000	1.000	0.3050
207882_at	82.3	33.0	-1.32	-2.50	AAA-AAA	0.00	999	1.000	1.000	0.2756
207530_s_at	22.6	56.5	1.32	2.50	AAA-AAA	0.00	999	1.000	1.000	0.1663
216708_x_at	15.8	6.3	-1.32	-2.50	AAA-AAA	0.00	999	1.000	1.000	0.1826
209395_at	10.1	4.0	-1.32	-2.50	AAA-AAA	0.00	999	1.000	1.000	0.3371
233879_at	6.7	2.7	-1.32	-2.50	AAA-AAA	0.00	999	1.000	1.000	0.1578
237053_at	6.7	16.8	1.32	2.50	AAA-AAA	0.00	999	1.000	1.000	0.0301
205564_at	12.7	31.8	1.32	2.50	AAA-AAA	0.00	999	1.000	1.000	0.4366
241218_at	8.8	21.9	1.32	2.50	AMA-PAA	0.00	999	1.000	1.000	0.4255
233175_at	9.9	24.8	1.32	2.50	AAA-APA	0.00	999	1.000	1.000	0.1382
242577_at	27.5	11.0	-1.32	-2.50	AAA-AAA	0.00	999	1.000	1.000	0.2566
1568812_at	33.8	13.5	-1.32	-2.50	AAA-AAA	0.00	999	1.000	1.000	0.2463
214071_at	7.4	3.0	-1.32	-2.50	AAA-AAA	0.00	999	1.000	1.000	0.1644
209888_s_at	22.2	8.9	-1.32	-2.50	AAA-AAA	0.00	999	1.000	1.000	0.1793

233916_at	51.4	20.6	-1.32	-2.50	AAA-AAA	0.00	999	1.000	1.000	0.2478
234741_at	4.1	10.2	1.32	2.50	AAA-AAA	0.00	999	1.000	1.000	0.2216
235070_at	109.0	43.7	-1.32	-2.50	AAA-AAA	0.00	999	1.000	1.000	0.1412
230125_at	31.5	78.5	1.32	2.49	AAA-PPA	0.00	999	1.000	1.000	0.2002
243921_at	9.5	23.8	1.32	2.49	AAA-AAA	0.00	999	1.000	1.000	0.2747
243119_at	9.4	23.5	1.32	2.49	AAA-AAA	0.00	999	1.000	1.000	0.3317
242239_at	38.5	15.4	-1.32	-2.49	AAA-AAA	0.00	999	1.000	1.000	0.1815
1561098_at	6.6	2.6	-1.32	-2.49	AAA-AAA	0.00	999	1.000	1.000	0.3437
1561128_at	38.3	15.4	-1.32	-2.49	AAA-MAA	0.00	999	1.000	1.000	0.3992
1566870_at	51.2	20.5	-1.32	-2.49	AAA-AAA	0.00	999	1.000	1.000	0.1790
1556909_at	61.6	24.7	-1.32	-2.49	AAA-AAA	0.00	999	1.000	1.000	0.3387
221796_at	11.2	28.0	1.32	2.49	AAA-AAA	0.00	999	1.000	1.000	0.3801
215028_at	10.3	4.1	-1.32	-2.49	AAA-AAA	0.00	999	1.000	1.000	0.5139
234690_at	11.8	29.5	1.32	2.49	AAA-AAA	0.00	999	1.000	1.000	0.3544
244169_x_at	5.4	2.2	-1.32	-2.49	AAA-AAA	0.00	999	1.000	1.000	0.4494
241567_at	3.0	1.2	-1.32	-2.49	AAA-AAA	0.00	999	1.000	1.000	0.0239
1562729_at	178.2	71.5	-1.32	-2.49	AAA-AAA	0.00	999	1.000	1.000	0.0429
1554911_at	38.7	15.5	-1.32	-2.49	AAA-AAA	0.00	999	1.000	1.000	0.1570
1552415_a_at	18.5	46.1	1.32	2.49	AAA-AAA	0.00	999	1.000	1.000	0.1747
220181_x_at	121.3	302.3	1.32	2.49	AAA-MMA	0.00	999	1.000	1.000	0.1337
206556_at	29.0	11.6	-1.32	-2.49	AAA-AAA	0.00	999	1.000	1.000	0.3669
215462_at	166.2	66.7	-1.32	-2.49	AAA-AAM	0.00	999	1.000	1.000	0.1189
236071_at	23.9	9.6	-1.32	-2.49	AAA-AMA	0.00	999	1.000	1.000	0.2681
1554216_at	31.8	79.1	1.32	2.49	AAP-PAP	0.00	999	1.000	1.000	0.2520
1561084_at	4.8	1.9	-1.32	-2.49	AAA-AAA	0.00	999	1.000	1.000	0.3015
244582_at	9.4	23.3	1.32	2.49	AAA-AAA	0.00	999	1.000	1.000	0.4515
220341_s_at	176.5	70.9	-1.32	-2.49	AAA-AAA	0.00	999	1.000	1.000	0.1821
211581_x_at	31.6	12.7	-1.32	-2.49	AAA-AAA	0.00	999	1.000	1.000	0.1668
216979_at	66.8	26.8	-1.32	-2.49	AAA-AAA	0.00	999	1.000	1.000	0.2201
236669_at	25.7	64.0	1.32	2.49	AAA-AAA	0.00	999	1.000	1.000	0.0570
228179_at	17.3	43.1	1.32	2.49	AAA-AAA	0.00	999	1.000	1.000	0.2420
203157_s_at	61.2	152.2	1.32	2.49	PPA-PPP	0.00	999	1.000	1.000	0.4414
1562165_at	37.6	15.1	-1.32	-2.49	AAA-AAA	0.00	999	1.000	1.000	0.3197
1560458_s_at	14.7	5.9	-1.32	-2.49	AAA-AAA	0.00	999	1.000	1.000	0.2936
1569833_at	13.5	5.4	-1.32	-2.49	AAA-AAA	0.00	999	1.000	1.000	0.3712
1570023_at	5.1	2.1	-1.32	-2.49	AAA-AAA	0.00	999	1.000	1.000	0.4988
202482_x_at	65.9	164.0	1.32	2.49	AAA-AAA	0.00	999	1.000	1.000	0.4387
1557122_s_at	9.2	22.8	1.32	2.49	AAA-AAA	0.00	999	1.000	1.000	0.3950
223905_at	13.9	5.6	-1.32	-2.49	AAA-AAA	0.00	999	1.000	1.000	0.2554
204943_at	79.8	32.1	-1.32	-2.49	AAA-AAA	0.00	999	1.000	1.000	0.0937
230996_at	30.4	12.2	-1.32	-2.49	AAA-AAA	0.00	999	1.000	1.000	0.4582
228794_at	61.1	24.5	-1.32	-2.49	AAM-AAA	0.00	999	1.000	1.000	0.0099

207003_at	50.9	20.5	-1.31	-2.49	AAA-AAA	0.00	999	1.000	1.000	0.2343
214038_at	31.8	12.8	-1.31	-2.49	AAA-AAA	0.00	999	1.000	1.000	0.0929
210113_s_at	51.5	20.7	-1.31	-2.49	AAA-AAA	0.00	999	1.000	1.000	0.2526
238386_x_at	35.5	14.3	-1.31	-2.49	AAA-AAA	0.00	999	1.000	1.000	0.0142
239530_at	27.6	68.7	1.31	2.49	AAA-AAA	0.00	999	1.000	1.000	0.4084
237730_at	39.9	16.0	-1.31	-2.49	AAA-AAA	0.00	999	1.000	1.000	0.2196
229529_at	30.0	74.6	1.31	2.49	AAA-AAA	0.00	999	1.000	1.000	0.3698
1561759_at	13.2	32.7	1.31	2.48	APA-AAA	0.00	999	1.000	1.000	0.4580
1563405_at	21.7	8.7	-1.31	-2.48	AAA-AAA	0.00	999	1.000	1.000	0.0054
1569430_at	21.8	8.8	-1.31	-2.49	AAA-AAA	0.00	999	1.000	1.000	0.3246
1570169_at	28.9	11.6	-1.31	-2.48	AAA-AAA	0.00	999	1.000	1.000	0.1393
1570531_at	73.4	29.5	-1.31	-2.49	AAA-AAA	0.00	999	1.000	1.000	0.1102
235597_s_at	15.1	6.1	-1.31	-2.49	AAA-AAA	0.00	999	1.000	1.000	0.4040
234718_at	8.0	3.2	-1.31	-2.48	AAA-AAA	0.00	999	1.000	1.000	0.2637
237677_at	13.4	5.4	-1.31	-2.48	AAA-AAA	0.00	999	1.000	1.000	0.3628
228863_at	4.1	10.1	1.31	2.49	AAA-AAA	0.00	999	1.000	1.000	0.2831
231640_at	20.6	51.3	1.31	2.49	AAA-AAA	0.00	999	1.000	1.000	0.2125
240553_at	43.2	17.4	-1.31	-2.48	AAA-AAA	0.00	999	1.000	1.000	0.1155
1561383_at	24.1	9.7	-1.31	-2.48	AAA-AAA	0.00	999	1.000	1.000	0.2356
1554857_at	26.3	10.6	-1.31	-2.48	AAA-AAA	0.00	999	1.000	1.000	0.2129
1553168_at	67.4	27.1	-1.31	-2.48	AAA-AAA	0.00	999	1.000	1.000	0.2698
208348_s_at	16.4	6.6	-1.31	-2.48	AAA-AAA	0.00	999	1.000	1.000	0.3530
213841_at	48.8	19.7	-1.31	-2.48	AAA-AAA	0.00	999	1.000	1.000	0.1065
217239_x_at	49.2	19.8	-1.31	-2.48	AAA-AAA	0.00	999	1.000	1.000	0.2613
216171_at	5.4	2.2	-1.31	-2.48	AAA-AAA	0.00	999	1.000	1.000	0.3009
237550_at	20.2	8.1	-1.31	-2.48	AAA-AAA	0.00	999	1.000	1.000	0.3879
228926_s_at	55.5	22.4	-1.31	-2.48	AAA-AAA	0.00	999	1.000	1.000	0.0557
209936_at	26.8	66.5	1.31	2.48	PAA-APP	0.00	999	1.000	1.000	0.1353
1566766_a_at	22.1	8.9	-1.31	-2.48	AAA-AAA	0.00	999	1.000	1.000	0.4245
244602_at	10.9	27.0	1.31	2.48	AAA-AAA	0.00	999	1.000	1.000	0.1943
1557803_at	11.4	28.3	1.31	2.48	AAA-AAA	0.00	999	1.000	1.000	0.2229
1557071_s_at	6.2	15.4	1.31	2.48	AAA-AAA	0.00	999	1.000	1.000	0.2753
214278_s_at	14.1	5.7	-1.31	-2.48	AAA-AAA	0.00	999	1.000	1.000	0.2631
217262_s_at	19.5	7.8	-1.31	-2.48	AAA-AAA	0.00	999	1.000	1.000	0.3056
235515_at	13.1	32.4	1.31	2.48	AAA-AAA	0.00	999	1.000	1.000	0.1603
206206_at	18.0	7.2	-1.31	-2.48	AAA-AAA	0.00	999	1.000	1.000	0.1029
228919_at	7.1	17.5	1.31	2.48	AAA-AAA	0.00	999	1.000	1.000	0.1559
206124_s_at	23.9	59.2	1.31	2.48	AAA-AAA	0.00	999	1.000	1.000	0.2606
241063_at	51.9	20.9	-1.31	-2.48	AAA-AAA	0.00	999	1.000	1.000	0.3340
243674_at	26.7	66.2	1.31	2.48	AAA-AAA	0.00	999	1.000	1.000	0.1275
241170_at	3.0	7.4	1.31	2.48	AAA-AAA	0.00	999	1.000	1.000	0.2094
1563638_at	7.9	3.2	-1.31	-2.48	AAA-AAA	0.00	999	1.000	1.000	0.1882

1561364_at	14.6	5.9	-1.31	-2.48	AAA-AAA	0.00	999	1.000	1.000	0.4691
1554782_at	59.1	23.8	-1.31	-2.48	AAA-AAA	0.00	999	1.000	1.000	0.1269
1558594_at	33.4	13.5	-1.31	-2.48	AAA-AAA	0.00	999	1.000	1.000	0.2031
1557009_a_at	7.0	2.8	-1.31	-2.48	AAA-AAA	0.00	999	1.000	1.000	0.2372
215572_at	25.1	10.1	-1.31	-2.48	AAA-AAA	0.00	999	1.000	1.000	0.3237
236180_at	17.7	43.8	1.31	2.48	AAA-MAA	0.00	999	1.000	1.000	0.2357
233434_at	47.9	19.3	-1.31	-2.48	MAA-AAA	0.00	999	1.000	1.000	0.2655
230209_at	44.1	109.3	1.31	2.48	AAP-AAP	0.00	999	1.000	1.000	0.2944
240636_at	20.7	51.4	1.31	2.48	AMA-AMA	0.00	999	1.000	1.000	0.2056
1570411_at	5.2	2.1	-1.31	-2.48	AAA-AAA	0.00	999	1.000	1.000	0.2939
1570422_at	25.7	10.4	-1.31	-2.48	AAA-AAA	0.00	999	1.000	1.000	0.0196
216957_at	33.5	13.5	-1.31	-2.48	AAA-AAA	0.00	999	1.000	1.000	0.0930
214033_at	26.4	65.4	1.31	2.48	AAA-AAP	0.00	999	1.000	1.000	0.2324
243085_at	60.3	24.3	-1.31	-2.48	AAA-AAA	0.00	999	1.000	1.000	0.3126
1563226_at	42.1	17.0	-1.31	-2.48	AAA-AAA	0.00	999	1.000	1.000	0.1361
217121_at	34.5	13.9	-1.31	-2.48	AAA-AAA	0.00	999	1.000	1.000	0.3839
231453_at	8.6	3.5	-1.31	-2.48	AAA-AAA	0.00	999	1.000	1.000	0.0856
243226_at	16.0	39.6	1.31	2.48	AAA-AAA	0.00	999	1.000	1.000	0.3606
241341_at	14.4	5.8	-1.31	-2.48	AAA-AAA	0.00	999	1.000	1.000	0.1248
241115_at	39.2	15.9	-1.31	-2.47	AAA-AAA	0.00	999	1.000	1.000	0.0094
1557348_at	13.5	33.4	1.31	2.48	AAA-AAA	0.00	999	1.000	1.000	0.4310
206619_at	20.4	8.2	-1.31	-2.47	AAA-AAA	0.00	999	1.000	1.000	0.2756
217051_s_at	12.1	29.9	1.31	2.47	AAA-AAA	0.00	999	1.000	1.000	0.0831
237015_at	5.7	14.1	1.31	2.48	AAA-AAA	0.00	999	1.000	1.000	0.2803
229834_at	8.4	3.4	-1.31	-2.48	AAA-AAA	0.00	999	1.000	1.000	0.1630
206077_at	16.2	40.2	1.31	2.47	AAA-AAA	0.00	999	1.000	1.000	0.0110
232388_at	94.4	38.1	-1.31	-2.47	AAA-AAA	0.00	999	1.000	1.000	0.0214
231981_at	44.3	17.9	-1.31	-2.48	AAA-AAA	0.00	999	1.000	1.000	0.3256
220187_at	8.5	3.5	-1.31	-2.47	AAP-APA	0.00	999	1.000	1.000	0.5976
243489_at	35.0	86.5	1.31	2.47	AAA-AAP	0.00	999	1.000	1.000	0.1573
244064_at	15.3	6.2	-1.31	-2.47	AAA-AAA	0.00	999	1.000	1.000	0.3438
1563147_at	50.3	20.3	-1.31	-2.47	AMA-AAA	0.00	999	1.000	1.000	0.1420
1557290_at	16.2	40.1	1.31	2.47	AAA-AAA	0.00	999	1.000	1.000	0.2364
220622_at	9.6	3.9	-1.31	-2.47	AAA-AAA	0.00	999	1.000	1.000	0.4469
214859_at	10.8	26.8	1.31	2.47	AAA-AAA	0.00	999	1.000	1.000	0.1032
234229_at	24.5	9.9	-1.31	-2.47	AAA-AAA	0.00	999	1.000	1.000	0.3665
230715_at	34.5	14.0	-1.31	-2.47	AAA-AAA	0.00	999	1.000	1.000	0.3896
230994_at	17.5	7.1	-1.31	-2.47	AAA-AAA	0.00	999	1.000	1.000	0.0996
206171_at	37.7	15.2	-1.31	-2.47	AAA-AAA	0.00	999	1.000	1.000	0.4167
231699_at	14.2	35.1	1.31	2.47	AAA-AAA	0.00	999	1.000	1.000	0.0546
216992_s_at	9.9	24.3	1.31	2.47	AAA-AAP	0.00	999	1.000	1.000	0.2270
244159_at	31.2	12.6	-1.31	-2.47	AAA-AAA	0.00	999	1.000	1.000	0.3509

1560162_at	35.9	14.5	-1.31	-2.47	AAA-AAA	0.00	999	1.000	1.000	0.3909
1561795_at	19.2	7.8	-1.31	-2.47	AAA-AAA	0.00	999	1.000	1.000	0.2970
1566748_at	30.4	12.3	-1.31	-2.47	AAA-AAA	0.00	999	1.000	1.000	0.1500
222242_s_at	108.9	44.1	-1.31	-2.47	AAA-AAA	0.00	999	1.000	1.000	0.3014
52975_at	96.8	39.2	-1.31	-2.47	AAA-AAA	0.00	999	1.000	1.000	0.0518
220277_at	51.2	20.7	-1.31	-2.47	AAA-AAA	0.00	999	1.000	1.000	0.0924
220889_s_at	25.6	10.4	-1.31	-2.47	AAA-AAA	0.00	999	1.000	1.000	0.0602
220876_at	8.4	3.4	-1.31	-2.47	AAA-AAA	0.00	999	1.000	1.000	0.0176
216385_at	81.7	33.1	-1.31	-2.47	AAA-AAA	0.00	999	1.000	1.000	0.1246
236701_at	51.8	21.0	-1.31	-2.47	AAA-AAA	0.00	999	1.000	1.000	0.2017
205531_s_at	15.3	6.2	-1.31	-2.47	AAA-AAA	0.00	999	1.000	1.000	0.2150
220729_at	11.0	27.1	1.30	2.47	MAA-APA	0.00	999	1.000	1.000	0.4521
240977_at	16.2	6.6	-1.30	-2.47	AAA-AAA	0.00	999	1.000	1.000	0.4040
241793_at	18.1	7.3	-1.30	-2.47	AAA-AAA	0.00	999	1.000	1.000	0.3257
240098_at	12.9	31.7	1.30	2.47	AAA-AAA	0.00	999	1.000	1.000	0.1863
241739_at	29.9	12.1	-1.30	-2.47	AAA-AAA	0.00	999	1.000	1.000	0.2299
1569566_at	3.7	9.0	1.30	2.47	AAA-AAA	0.00	999	1.000	1.000	0.4765
1570496_at	33.8	13.7	-1.30	-2.47	AAA-AAA	0.00	999	1.000	1.000	0.0421
220403_s_at	11.4	4.6	-1.30	-2.47	AAA-AAA	0.00	999	1.000	1.000	0.4028
229314_at	71.0	28.8	-1.30	-2.47	AAA-AAA	0.00	999	1.000	1.000	0.1175
233737_s_at	64.8	26.3	-1.30	-2.47	AAA-AAA	0.00	999	1.000	1.000	0.0386
233396_s_at	34.6	14.0	-1.30	-2.47	AAA-AAA	0.00	999	1.000	1.000	0.3500
240213_at	24.0	9.7	-1.30	-2.47	AAA-AAA	0.00	999	1.000	1.000	0.1895
1561998_at	34.4	14.0	-1.30	-2.47	AAA-AAA	0.00	999	1.000	1.000	0.3896
1561979_at	5.5	13.6	1.30	2.47	AAA-AAA	0.00	999	1.000	1.000	0.2960
1570264_at	4.2	10.3	1.30	2.47	AAA-AAA	0.00	999	1.000	1.000	0.4081
1569776_at	16.6	40.9	1.30	2.47	AAA-AAA	0.00	999	1.000	1.000	0.1801
1559814_at	26.2	10.6	-1.30	-2.47	AAA-AAA	0.00	999	1.000	1.000	0.3805
1555441_at	18.4	45.5	1.30	2.47	AAA-AAA	0.00	999	1.000	1.000	0.3626
234671_at	83.5	33.9	-1.30	-2.47	AMA-AAA	0.00	999	1.000	1.000	0.1111
243914_at	7.9	19.5	1.30	2.47	AAA-AAA	0.00	999	1.000	1.000	0.2794
243390_at	8.6	21.2	1.30	2.47	AAA-AAA	0.00	999	1.000	1.000	0.3338
1561712_at	36.1	14.6	-1.30	-2.47	AAA-AAA	0.00	999	1.000	1.000	0.4152
1554666_at	9.3	23.0	1.30	2.47	AAA-AAA	0.00	999	1.000	1.000	0.4482
1556794_at	5.3	13.0	1.30	2.47	AAA-AAA	0.00	999	1.000	1.000	0.1738
207773_x_at	32.9	13.3	-1.30	-2.47	AAA-AAA	0.00	999	1.000	1.000	0.2496
224405_at	35.3	14.3	-1.30	-2.47	AAA-AAA	0.00	999	1.000	1.000	0.1361
236456_at	13.5	5.5	-1.30	-2.47	AAA-AAA	0.00	999	1.000	1.000	0.3713
238212_at	9.5	3.8	-1.30	-2.47	AAA-AAA	0.00	999	1.000	1.000	0.2019
241622_at	8.5	3.4	-1.30	-2.46	AAA-AAA	0.00	999	1.000	1.000	0.3224
1562496_at	51.7	21.0	-1.30	-2.46	AAA-AAA	0.00	999	1.000	1.000	0.2360
1561967_at	16.3	6.6	-1.30	-2.46	AAA-AAA	0.00	999	1.000	1.000	0.2522

1564443_at	60.4	24.5	-1.30	-2.46	AAA-AMA	0.00	999	1.000	1.000	0.1887
1563641_a_at	32.8	13.3	-1.30	-2.47	AAA-AAA	0.00	999	1.000	1.000	0.1148
1558724_at	6.7	16.5	1.30	2.46	AAA-AAA	0.00	999	1.000	1.000	0.3667
1556662_at	12.5	30.8	1.30	2.46	AAA-AAA	0.00	999	1.000	1.000	0.1874
219505_at	12.0	4.9	-1.30	-2.46	AAA-AAA	0.00	999	1.000	1.000	0.4468
207669_at	12.0	4.9	-1.30	-2.46	AAA-AAA	0.00	999	1.000	1.000	0.0437
210437_at	59.4	24.1	-1.30	-2.46	AAA-AAA	0.00	999	1.000	1.000	0.2184
205883_at	22.4	9.1	-1.30	-2.46	AAA-AAA	0.00	999	1.000	1.000	0.4320
230771_at	31.1	12.6	-1.30	-2.46	AAA-AAA	0.00	999	1.000	1.000	0.3879
1566783_at	30.7	12.5	-1.30	-2.46	AAA-AAA	0.00	999	1.000	1.000	0.3660
1553009_s_at	6.2	2.5	-1.30	-2.46	AAA-AAA	0.00	999	1.000	1.000	0.0137
1558066_s_at	21.7	53.5	1.30	2.46	AAA-AAA	0.00	999	1.000	1.000	0.1302
226367_at	28.0	69.0	1.30	2.46	AAA-AAA	0.00	999	1.000	1.000	0.3390
227789_at	10.5	25.7	1.30	2.46	AAA-AAA	0.00	999	1.000	1.000	0.0653
203475_at	39.9	16.2	-1.30	-2.46	AAA-AAA	0.00	999	1.000	1.000	0.0604
1561015_at	23.1	56.8	1.30	2.46	AAA-AAA	0.00	999	1.000	1.000	0.1139
1570015_at	10.3	4.2	-1.30	-2.46	AAA-AAA	0.00	999	1.000	1.000	0.3287
1566124_a_at	12.1	29.7	1.30	2.46	AAA-AAA	0.00	999	1.000	1.000	0.2805
1555853_at	7.7	19.0	1.30	2.46	AAA-AAA	0.00	999	1.000	1.000	0.4274
220303_at	22.7	9.2	-1.30	-2.46	AAA-AAA	0.00	999	1.000	1.000	0.0513
233872_x_at	13.8	5.6	-1.30	-2.46	AAA-AAA	0.00	999	1.000	1.000	0.2762
234459_at	23.4	57.6	1.30	2.46	AAA-AAA	0.00	999	1.000	1.000	0.2311
228365_at	14.0	34.5	1.30	2.46	AAA-AAA	0.00	999	1.000	1.000	0.2301
230846_at	28.8	70.7	1.30	2.46	AAA-APP	0.00	999	1.000	1.000	0.2167
241942_at	22.4	9.1	-1.30	-2.46	AAA-AAA	0.00	999	1.000	1.000	0.0563
241356_at	32.5	13.2	-1.30	-2.46	AAA-AAA	0.00	999	1.000	1.000	0.0220
241109_at	3.2	1.3	-1.30	-2.46	AAA-AAA	0.00	999	1.000	1.000	0.1044
1560087_a_at	9.8	24.1	1.30	2.46	AAA-AAA	0.00	999	1.000	1.000	0.1910
1570255_s_at	5.7	2.3	-1.30	-2.46	AAA-AAA	0.00	999	1.000	1.000	0.3741
1564772_at	16.0	6.5	-1.30	-2.46	AAA-AAA	0.00	999	1.000	1.000	0.5082
244844_at	23.0	9.4	-1.30	-2.46	AAA-AAA	0.00	999	1.000	1.000	0.3007
1556912_at	25.6	10.4	-1.30	-2.46	AAA-AAA	0.00	999	1.000	1.000	0.3311
206549_at	82.9	33.7	-1.30	-2.46	AAA-AAA	0.00	999	1.000	1.000	0.0132
210163_at	9.7	4.0	-1.30	-2.46	AAA-AAA	0.00	999	1.000	1.000	0.4416
215803_at	12.5	5.1	-1.30	-2.46	AAA-AAA	0.00	999	1.000	1.000	0.2067
238796_at	25.9	10.5	-1.30	-2.46	AAA-AAA	0.00	999	1.000	1.000	0.2462
228405_at	11.1	27.4	1.30	2.46	AAA-AAA	0.00	999	1.000	1.000	0.1623
233125_at	31.4	12.8	-1.30	-2.46	AAA-AAA	0.00	999	1.000	1.000	0.2279
244088_at	64.2	26.1	-1.30	-2.46	AAA-AAA	0.00	999	1.000	1.000	0.1520
1560334_at	8.8	21.7	1.30	2.46	AAA-AAA	0.00	999	1.000	1.000	0.3884
1566803_at	55.8	22.7	-1.30	-2.46	AAA-AAA	0.00	999	1.000	1.000	0.1073
220878_at	7.1	2.9	-1.30	-2.46	AAA-AAA	0.00	999	1.000	1.000	0.5634

208220_x_at	31.3	12.8	-1.30	-2.46	AAA-AAA	0.00	999	1.000	1.000	0.4308
207487_at	16.6	6.7	-1.30	-2.46	AAA-AAA	0.00	999	1.000	1.000	0.5219
217335_at	13.7	33.6	1.30	2.46	AAA-PAA	0.00	999	1.000	1.000	0.2357
244155_x_at	10.2	4.2	-1.30	-2.46	AAA-AAA	0.00	999	1.000	1.000	0.1886
241371_at	12.4	30.4	1.30	2.46	AAA-AAA	0.00	999	1.000	1.000	0.1285
240219_at	15.3	6.3	-1.30	-2.46	AAA-AAA	0.00	999	1.000	1.000	0.3159
1560411_at	41.7	17.0	-1.30	-2.46	AAA-AAA	0.00	999	1.000	1.000	0.4536
1569323_at	36.6	14.9	-1.30	-2.46	AAA-AAA	0.00	999	1.000	1.000	0.1489
1569942_at	20.9	8.5	-1.30	-2.46	AAA-AAA	0.00	999	1.000	1.000	0.3606
1556721_at	12.6	31.0	1.30	2.46	AAA-AAA	0.00	999	1.000	1.000	0.2278
207179_at	7.7	18.9	1.30	2.46	AAA-AAA	0.00	999	1.000	1.000	0.2337
210928_at	18.7	7.6	-1.30	-2.46	AAA-AAA	0.00	999	1.000	1.000	0.0647
208513_at	14.1	5.8	-1.30	-2.46	AAA-AAA	0.00	999	1.000	1.000	0.3059
218312_s_at	36.0	88.3	1.30	2.46	AAA-AAA	0.00	999	1.000	1.000	0.1344
242597_at	57.3	23.4	-1.30	-2.45	AAA-AAA	0.00	999	1.000	1.000	0.2941
1563721_at	3.0	7.4	1.30	2.45	AAA-AAA	0.00	999	1.000	1.000	0.2986
1560558_at	17.2	7.0	-1.30	-2.45	AAA-AAA	0.00	999	1.000	1.000	0.2822
1556836_at	7.0	2.8	-1.30	-2.45	AAA-AAA	0.00	999	1.000	1.000	0.0211
219521_at	22.1	9.0	-1.30	-2.45	AAA-AAA	0.00	999	1.000	1.000	0.2657
204941_s_at	10.6	4.3	-1.30	-2.45	AAA-AAA	0.00	999	1.000	1.000	0.3520
239029_at	19.3	7.9	-1.30	-2.45	AAA-AAA	0.00	999	1.000	1.000	0.0311
229979_x_at	6.6	16.1	1.30	2.45	AAA-AAA	0.00	999	1.000	1.000	0.2825
228172_at	39.5	97.0	1.30	2.45	AAA-AAA	0.00	999	1.000	1.000	0.0648
227799_at	22.7	55.8	1.30	2.46	AAA-AAA	0.00	999	1.000	1.000	0.2721
232892_at	151.8	61.8	-1.30	-2.45	AAA-AAA	0.00	999	1.000	1.000	0.0470
233464_at	6.7	2.7	-1.30	-2.45	AAA-AAA	0.00	999	1.000	1.000	0.2864
209726_at	40.8	100.0	1.29	2.45	AAA-MPP	0.00	999	1.000	1.000	0.2405
1561667_at	54.1	22.1	-1.29	-2.45	AAA-AAA	0.00	999	1.000	1.000	0.2489
1555048_a_at	7.6	3.1	-1.29	-2.45	AAA-AAA	0.00	999	1.000	1.000	0.0958
219665_at	49.9	122.5	1.29	2.45	AAA-AAA	0.00	999	1.000	1.000	0.1874
206944_at	76.0	31.0	-1.29	-2.45	AAA-AAA	0.00	999	1.000	1.000	0.0269
226695_at	29.6	12.1	-1.29	-2.45	AAA-AAA	0.00	999	1.000	1.000	0.4508
223897_at	6.7	16.5	1.29	2.45	AAA-AAA	0.00	999	1.000	1.000	0.1486
213417_at	32.8	80.3	1.29	2.45	AAA-AAA	0.00	999	1.000	1.000	0.3569
237541_at	18.4	7.5	-1.29	-2.45	AAA-AAA	0.00	999	1.000	1.000	0.1522
229996_s_at	14.9	36.5	1.29	2.45	AAA-AAA	0.00	999	1.000	1.000	0.3253
230975_at	58.2	23.7	-1.29	-2.45	AAA-AAA	0.00	999	1.000	1.000	0.2031
230483_at	183.9	75.0	-1.29	-2.45	AAA-AAA	0.00	999	1.000	1.000	0.1974
228701_at	9.8	24.1	1.29	2.45	AAA-AAA	0.00	999	1.000	1.000	0.4158
214008_at	42.8	104.9	1.29	2.45	APA-PPP	0.00	999	1.000	1.000	0.2985
203237_s_at	103.3	42.2	-1.29	-2.45	AAA-AAA	0.00	999	1.000	1.000	0.2344
1562577_at	114.9	46.9	-1.29	-2.45	AAA-AAA	0.00	999	1.000	1.000	0.2269

1563801_at	14.2	5.8	-1.29	-2.45	AAA-AAA	0.00	999	1.000	1.000	0.2544
1566780_at	64.5	26.3	-1.29	-2.45	AAA-AAA	0.00	999	1.000	1.000	0.0626
223732_at	28.9	11.8	-1.29	-2.45	AAA-AAA	0.00	999	1.000	1.000	0.3495
211868_x_at	53.9	22.0	-1.29	-2.45	AAA-AAA	0.00	999	1.000	1.000	0.3137
210537_s_at	22.2	54.4	1.29	2.45	AAA-AAA	0.00	999	1.000	1.000	0.3696
216108_at	12.2	5.0	-1.29	-2.45	AAA-AAA	0.00	999	1.000	1.000	0.4040
234606_at	12.3	5.0	-1.29	-2.45	AAA-AAA	0.00	999	1.000	1.000	0.1781
206398_s_at	44.3	18.1	-1.29	-2.45	AAA-AAA	0.00	999	1.000	1.000	0.3735
233320_at	20.6	8.4	-1.29	-2.45	AAA-AAA	0.00	999	1.000	1.000	0.3551
233194_at	20.1	49.2	1.29	2.45	AAA-AAA	0.00	999	1.000	1.000	0.2489
233342_at	20.0	8.1	-1.29	-2.45	AAA-AAA	0.00	999	1.000	1.000	0.2099
231598_x_at	2.4	5.9	1.29	2.45	AAA-AAA	0.00	999	1.000	1.000	0.3416
231337_at	15.0	6.1	-1.29	-2.45	AAA-AAA	0.00	999	1.000	1.000	0.2962
231222_at	9.0	22.1	1.29	2.45	AAA-AAA	0.00	999	1.000	1.000	0.0006
239885_at	20.9	8.5	-1.29	-2.45	AAA-AAA	0.00	999	1.000	1.000	0.1734
1553811_at	41.1	16.8	-1.29	-2.45	AAA-AAA	0.00	999	1.000	1.000	0.2774
1553591_at	22.2	9.1	-1.29	-2.45	AAA-AAA	0.00	999	1.000	1.000	0.2301
227519_at	52.4	21.4	-1.29	-2.45	AAA-AAA	0.00	999	1.000	1.000	0.1935
213284_at	18.9	7.7	-1.29	-2.45	AAA-AAA	0.00	999	1.000	1.000	0.4223
210384_at	72.9	29.8	-1.29	-2.45	MAA-AAM	0.00	999	1.000	1.000	0.1905
229516_at	15.2	37.2	1.29	2.45	AAA-AAA	0.00	999	1.000	1.000	0.2901
1553060_at	36.6	89.5	1.29	2.45	PAA-AAA	0.00	999	1.000	1.000	0.3718
205455_at	33.5	81.9	1.29	2.45	PAA-APA	0.00	999	1.000	1.000	0.3249
239894_at	14.5	5.9	-1.29	-2.45	AAA-AAA	0.00	999	1.000	1.000	0.2672
1564672_at	6.8	2.8	-1.29	-2.45	AAA-AAA	0.00	999	1.000	1.000	0.3729
1556643_at	29.6	12.1	-1.29	-2.45	AAA-AAA	0.00	999	1.000	1.000	0.3831
227589_at	9.4	3.9	-1.29	-2.45	AAA-AAA	0.00	999	1.000	1.000	0.1300
219301_s_at	78.1	31.9	-1.29	-2.45	AAA-AAA	0.00	999	1.000	1.000	0.0813
233704_at	7.9	3.2	-1.29	-2.45	AAA-AAA	0.00	999	1.000	1.000	0.3899
233286_at	44.9	18.3	-1.29	-2.45	AAA-AAA	0.00	999	1.000	1.000	0.2104
233222_at	60.0	24.5	-1.29	-2.45	AAA-AAA	0.00	999	1.000	1.000	0.3575
204225_at	61.9	151.5	1.29	2.45	MAP-PPP	0.00	999	1.000	1.000	0.4030
207177_at	32.6	79.6	1.29	2.45	AAA-APP	0.00	999	1.000	1.000	0.1887
1559280_a_at	16.9	6.9	-1.29	-2.45	AAA-AAA	0.00	999	1.000	1.000	0.2077
1557316_at	21.6	52.8	1.29	2.45	AAA-AAA	0.00	999	1.000	1.000	0.2282
213247_at	17.8	7.3	-1.29	-2.45	AAA-AAA	0.00	999	1.000	1.000	0.3011
239974_at	129.7	53.1	-1.29	-2.44	AAA-AAA	0.00	999	1.000	1.000	0.0005
1563472_at	36.8	15.1	-1.29	-2.44	AAA-AAA	0.00	999	1.000	1.000	0.2646
1565774_at	18.0	7.4	-1.29	-2.44	AAA-AAA	0.00	999	1.000	1.000	0.2683
1566816_at	84.2	34.5	-1.29	-2.44	AAA-AAA	0.00	999	1.000	1.000	0.1852
221022_s_at	22.3	9.1	-1.29	-2.44	AAA-AAA	0.00	999	1.000	1.000	0.4568
236800_at	40.1	16.4	-1.29	-2.44	AAA-AAA	0.00	999	1.000	1.000	0.4229

236190_at	62.9	25.7	-1.29	-2.44	AAA-AAA	0.00	999	1.000	1.000	0.1494
237295_at	21.0	8.6	-1.29	-2.44	AAA-AAA	0.00	999	1.000	1.000	0.5387
233312_at	17.5	7.1	-1.29	-2.44	AAA-AAA	0.00	999	1.000	1.000	0.0712
238182_at	17.8	43.4	1.29	2.44	AAA-AAP	0.00	999	1.000	1.000	0.1704
220182_at	78.1	190.7	1.29	2.44	AAA-APA	0.00	999	1.000	1.000	0.0663
203010_at	29.8	72.8	1.29	2.44	AAA-AAA	0.00	999	1.000	1.000	0.3046
242031_at	10.1	4.1	-1.29	-2.44	AAA-AAA	0.00	999	1.000	1.000	0.3643
242447_at	12.0	29.3	1.29	2.44	AAA-AAA	0.00	999	1.000	1.000	0.5054
205912_at	34.2	14.0	-1.29	-2.44	AAA-AAA	0.00	999	1.000	1.000	0.2463
1556429_a_at	30.5	74.6	1.29	2.44	AAP-APP	0.00	999	1.000	1.000	0.3005
1556220_at	14.3	34.8	1.29	2.44	AAA-APA	0.00	999	1.000	1.000	0.2092
244111_at	8.3	3.4	-1.29	-2.44	AAA-AAA	0.00	999	1.000	1.000	0.2492
241145_at	9.3	22.7	1.29	2.44	AAA-AAA	0.00	999	1.000	1.000	0.2386
243513_at	50.7	20.8	-1.29	-2.44	AAA-AAA	0.00	999	1.000	1.000	0.1436
1561341_at	18.9	7.7	-1.29	-2.44	AAA-AAA	0.00	999	1.000	1.000	0.3146
1568847_at	98.6	40.4	-1.29	-2.44	AAA-AAA	0.00	999	1.000	1.000	0.0846
1553497_at	9.5	3.9	-1.29	-2.44	AAA-AAA	0.00	999	1.000	1.000	0.0298
202707_at	29.7	72.6	1.29	2.44	AAA-AAA	0.00	999	1.000	1.000	0.1509
207979_s_at	47.3	19.4	-1.29	-2.44	AAA-AAA	0.00	999	1.000	1.000	0.4045
220826_at	82.5	33.8	-1.29	-2.44	MAA-AAA	0.00	999	1.000	1.000	0.0961
220527_at	35.6	14.6	-1.29	-2.44	AAA-AAA	0.00	999	1.000	1.000	0.1550
207044_at	24.1	58.8	1.29	2.44	AAA-AAA	0.00	999	1.000	1.000	0.3164
224072_s_at	30.1	12.4	-1.29	-2.44	AAA-AAA	0.00	999	1.000	1.000	0.3103
214799_at	16.7	6.8	-1.29	-2.44	AAA-AAA	0.00	999	1.000	1.000	0.2058
215669_at	12.8	31.2	1.29	2.44	AAA-AAA	0.00	999	1.000	1.000	0.2547
204673_at	40.2	16.5	-1.29	-2.44	AAA-AAA	0.00	999	1.000	1.000	0.1680
238359_at	38.6	15.8	-1.29	-2.44	AAA-AAA	0.00	999	1.000	1.000	0.3615
242863_at	67.7	27.8	-1.29	-2.44	AAA-AAA	0.00	999	1.000	1.000	0.4516
241328_at	10.4	4.3	-1.29	-2.44	AAA-AAA	0.00	999	1.000	1.000	0.2599
1569268_at	156.6	64.2	-1.29	-2.44	AAA-AAA	0.00	999	1.000	1.000	0.1526
1557334_a_at	8.2	3.4	-1.29	-2.44	AAA-AAA	0.00	999	1.000	1.000	0.1355
222951_s_at	50.8	20.8	-1.29	-2.44	AAA-AAA	0.00	999	1.000	1.000	0.3390
211419_s_at	15.2	6.2	-1.29	-2.44	AAA-AAA	0.00	999	1.000	1.000	0.4192
204960_at	40.6	16.7	-1.29	-2.44	AAA-AAA	0.00	999	1.000	1.000	0.1614
234641_at	12.3	5.0	-1.29	-2.44	AAA-AAA	0.00	999	1.000	1.000	0.4788
238009_at	71.7	29.4	-1.29	-2.44	AAA-AAA	0.00	999	1.000	1.000	0.0226
1566955_at	23.4	57.1	1.29	2.44	AAA-PAA	0.00	999	1.000	1.000	0.4153
244004_at	6.1	2.5	-1.29	-2.44	AAA-AAA	0.00	999	1.000	1.000	0.2376
240082_s_at	28.6	11.7	-1.29	-2.44	AAA-AAA	0.00	999	1.000	1.000	0.4371
201130_s_at	24.6	10.1	-1.29	-2.44	AAA-AAA	0.00	999	1.000	1.000	0.3052
1570360_s_at	16.7	6.9	-1.29	-2.44	AAA-AAA	0.00	999	1.000	1.000	0.4445
208340_at	11.9	4.9	-1.29	-2.44	AAA-AAA	0.00	999	1.000	1.000	0.2405

207562_at	14.6	6.0	-1.29	-2.44	AAA-AAA	0.00	999	1.000	1.000	0.2693
211142_x_at	13.8	33.5	1.29	2.44	AAA-AAA	0.00	999	1.000	1.000	0.0762
217219_at	26.5	10.9	-1.29	-2.44	AAA-AAA	0.00	999	1.000	1.000	0.4091
237402_at	23.9	9.8	-1.29	-2.44	AAA-AAA	0.00	999	1.000	1.000	0.2198
205888_s_at	55.4	22.7	-1.29	-2.44	AAA-AAA	0.00	999	1.000	1.000	0.2556
205348_s_at	18.5	7.6	-1.29	-2.44	AAA-AAA	0.00	999	1.000	1.000	0.3365
241782_at	24.3	10.0	-1.28	-2.44	AAA-AAA	0.00	999	1.000	1.000	0.4903
1558654_at	5.7	13.9	1.28	2.44	AAA-AAA	0.00	999	1.000	1.000	0.2071
222304_x_at	84.8	34.8	-1.28	-2.44	AAA-AAA	0.00	999	1.000	1.000	0.0468
217536_x_at	13.4	5.5	-1.28	-2.44	AAA-AAA	0.00	999	1.000	1.000	0.3706
216584_at	42.6	17.5	-1.28	-2.44	AAA-AAA	0.00	999	1.000	1.000	0.0157
233047_at	20.8	8.6	-1.28	-2.44	AAA-AAA	0.00	999	1.000	1.000	0.4301
232364_at	8.0	19.5	1.28	2.44	AAA-AAA	0.00	999	1.000	1.000	0.4244
1563611_at	41.9	17.2	-1.28	-2.43	AMA-AAA	0.00	999	1.000	1.000	0.1053
1570100_at	23.1	9.5	-1.28	-2.43	AAA-AAA	0.00	999	1.000	1.000	0.4243
1564580_at	5.8	2.4	-1.28	-2.43	AAA-AAA	0.00	999	1.000	1.000	0.4438
1566693_at	31.8	13.1	-1.28	-2.43	AAA-AAA	0.00	999	1.000	1.000	0.3486
220328_at	38.5	15.8	-1.28	-2.43	AAA-AAA	0.00	999	1.000	1.000	0.2988
227050_at	59.5	24.4	-1.28	-2.43	AAA-AAA	0.00	999	1.000	1.000	0.0535
210960_at	13.0	5.3	-1.28	-2.43	AAA-AAA	0.00	999	1.000	1.000	0.2623
211116_at	5.9	14.3	1.28	2.43	AAA-AAA	0.00	999	1.000	1.000	0.3068
1569330_at	3.7	9.0	1.28	2.43	AAA-AAA	0.00	999	1.000	1.000	0.3302
1558919_a_at	23.8	57.9	1.28	2.43	AAA-AAA	0.00	999	1.000	1.000	0.2887
1556980_at	6.1	2.5	-1.28	-2.43	AAA-AAA	0.00	999	1.000	1.000	0.0564
216065_at	49.4	20.3	-1.28	-2.43	AAA-AAA	0.00	999	1.000	1.000	0.2438
236272_at	28.8	11.9	-1.28	-2.43	AAA-AAA	0.00	999	1.000	1.000	0.1513
233855_at	19.8	8.2	-1.28	-2.43	AAA-AAA	0.00	999	1.000	1.000	0.3489
206216_at	30.4	73.8	1.28	2.43	AAA-AAA	0.00	999	1.000	1.000	0.0505
239807_at	26.9	11.1	-1.28	-2.43	AAA-AAA	0.00	999	1.000	1.000	0.2391
1561761_x_at	44.6	18.3	-1.28	-2.43	AAA-AAA	0.00	999	1.000	1.000	0.1195
1569557_at	77.2	31.8	-1.28	-2.43	AAA-AAA	0.00	999	1.000	1.000	0.1739
1553447_at	32.4	13.3	-1.28	-2.43	AAA-AAA	0.00	999	1.000	1.000	0.3457
221388_at	10.8	26.2	1.28	2.43	AAA-AAA	0.00	999	1.000	1.000	0.1008
221289_at	14.9	6.1	-1.28	-2.43	AAA-AAA	0.00	999	1.000	1.000	0.3314
91920_at	95.6	39.4	-1.28	-2.43	AAA-AAA	0.00	999	1.000	1.000	0.2647
220383_at	19.5	8.0	-1.28	-2.43	AAA-AAA	0.00	999	1.000	1.000	0.4702
223121_s_at	8.7	3.6	-1.28	-2.43	AAA-AAA	0.00	999	1.000	1.000	0.1031
238928_at	7.3	3.0	-1.28	-2.43	AAA-AAA	0.00	999	1.000	1.000	0.0012
1569355_at	5.5	13.4	1.28	2.43	AAA-AAA	0.00	999	1.000	1.000	0.1840
202410_x_at	20.2	8.3	-1.28	-2.43	AAA-AAA	0.00	999	1.000	1.000	0.1905
1558402_at	8.5	3.5	-1.28	-2.43	AAA-AAA	0.00	999	1.000	1.000	0.1552
207832_at	26.5	10.9	-1.28	-2.43	AAA-AAA	0.00	999	1.000	1.000	0.2600

206617_s_at	14.4	6.0	-1.28	-2.43	AAA-AAA	0.00	999	1.000	1.000	0.0183
214025_at	16.0	6.6	-1.28	-2.43	AAA-AAA	0.00	999	1.000	1.000	0.0784
216536_at	8.8	3.6	-1.28	-2.43	AAA-AAA	0.00	999	1.000	1.000	0.4206
204231_s_at	71.8	29.6	-1.28	-2.43	AAA-AAA	0.00	999	1.000	1.000	0.2321
1552637_at	36.9	15.2	-1.28	-2.43	AAA-AAA	0.00	999	1.000	1.000	0.3984
239057_at	16.9	7.0	-1.28	-2.43	AAA-AAA	0.00	999	1.000	1.000	0.4548
237476_at	30.4	12.5	-1.28	-2.43	AAA-AAA	0.00	999	1.000	1.000	0.1426
216488_s_at	61.1	148.1	1.28	2.42	AAP-APP	0.00	999	1.000	1.000	0.3296
1563252_at	9.8	4.0	-1.28	-2.43	AAA-AAA	0.00	999	1.000	1.000	0.0820
1569753_at	13.8	5.7	-1.28	-2.43	AAA-AAA	0.00	999	1.000	1.000	0.4319
1564897_at	14.3	5.9	-1.28	-2.43	AAA-AAA	0.00	999	1.000	1.000	0.3075
1556123_a_at	30.6	12.6	-1.28	-2.43	AAA-AAA	0.00	999	1.000	1.000	0.0174
220365_at	67.4	27.8	-1.28	-2.43	AAA-AAA	0.00	999	1.000	1.000	0.2640
222829_s_at	7.1	2.9	-1.28	-2.43	AAA-AAA	0.00	999	1.000	1.000	0.0291
224363_at	43.6	18.0	-1.28	-2.43	AAA-AMA	0.00	999	1.000	1.000	0.1627
211654_x_at	16.2	39.3	1.28	2.42	AAA-AAA	0.00	999	1.000	1.000	0.0956
217155_at	14.1	5.8	-1.28	-2.43	AAA-AAA	0.00	999	1.000	1.000	0.3623
219450_at	29.7	12.3	-1.28	-2.43	AAA-AAA	0.00	999	1.000	1.000	0.3929
216653_at	9.4	22.8	1.28	2.43	AAA-AAA	0.00	999	1.000	1.000	0.3547
228169_s_at	4.6	1.9	-1.28	-2.43	AAA-AAA	0.00	999	1.000	1.000	0.1671
228672_at	6.2	15.1	1.28	2.42	AAA-AAA	0.00	999	1.000	1.000	0.2657
242537_at	6.9	16.7	1.28	2.42	AAA-AAA	0.00	999	1.000	1.000	0.2484
1562432_at	20.9	8.6	-1.28	-2.42	AAA-AAA	0.00	999	1.000	1.000	0.4255
1566688_at	14.5	35.0	1.28	2.42	AAA-AAA	0.00	999	1.000	1.000	0.1833
1554273_a_at	8.4	20.4	1.28	2.42	AAA-AAA	0.00	999	1.000	1.000	0.2304
204758_s_at	5.8	2.4	-1.28	-2.42	AAA-AAA	0.00	999	1.000	1.000	0.1624
236585_at	22.1	53.7	1.28	2.42	AAA-AAA	0.00	999	1.000	1.000	0.3532
237035_at	23.3	9.6	-1.28	-2.42	AAA-AAA	0.00	999	1.000	1.000	0.2091
205146_x_at	79.0	32.6	-1.28	-2.42	AAA-AAA	0.00	999	1.000	1.000	0.4330
244035_at	14.0	33.9	1.28	2.42	AAA-AAA	0.00	999	1.000	1.000	0.3167
1561868_at	92.5	38.2	-1.28	-2.42	AAA-AAA	0.00	999	1.000	1.000	0.0460
1570110_at	18.0	7.4	-1.28	-2.42	AAA-AAA	0.00	999	1.000	1.000	0.2245
222383_s_at	8.9	3.7	-1.28	-2.42	AAA-AAA	0.00	999	1.000	1.000	0.0770
206634_at	19.9	8.2	-1.28	-2.42	AAA-AAA	0.00	999	1.000	1.000	0.4729
214768_x_at	9.0	21.8	1.28	2.42	AAA-AAA	0.00	999	1.000	1.000	0.3100
216590_at	7.9	19.2	1.28	2.42	AAA-AAA	0.00	999	1.000	1.000	0.2952
227929_at	7.5	18.2	1.28	2.42	AAA-AAA	0.00	999	1.000	1.000	0.1561
231073_at	40.6	98.4	1.28	2.42	AAA-AAA	0.00	999	1.000	1.000	0.2526
240052_at	38.2	15.8	-1.28	-2.42	AAA-AAA	0.00	999	1.000	1.000	0.1152
1562601_at	14.5	35.0	1.28	2.42	AAA-AAA	0.00	999	1.000	1.000	0.0865
1564626_at	12.0	29.0	1.28	2.42	AAA-AAA	0.00	999	1.000	1.000	0.0588
244424_at	39.3	16.2	-1.28	-2.42	AAA-AAA	0.00	999	1.000	1.000	0.0268

236296_x_at	87.4	36.1	-1.28	-2.42	AAA-AAA	0.00	999	1.000	1.000	0.1148
237087_at	14.8	6.1	-1.28	-2.42	AAA-AAA	0.00	999	1.000	1.000	0.1835
237634_at	28.2	11.7	-1.28	-2.42	AAA-AAA	0.00	999	1.000	1.000	0.0523
240069_at	21.2	8.8	-1.27	-2.42	AAA-AAA	0.00	999	1.000	1.000	0.4645
240562_at	49.6	20.5	-1.27	-2.42	AMA-AAA	0.00	999	1.000	1.000	0.3876
1556355_x_at	33.5	81.1	1.27	2.42	AAA-AAA	0.00	999	1.000	1.000	0.3831
207915_at	58.0	24.0	-1.27	-2.42	AAA-AAA	0.00	999	1.000	1.000	0.2233
216542_x_at	126.1	52.2	-1.27	-2.42	AAA-AAA	0.00	999	1.000	1.000	0.1080
237852_at	25.8	62.5	1.27	2.42	AAA-AAA	0.00	999	1.000	1.000	0.1711
1564002_a_at	29.2	70.6	1.27	2.42	AAP-APP	0.00	999	1.000	1.000	0.2774
235323_at	27.8	67.1	1.27	2.42	AAA-AAP	0.00	999	1.000	1.000	0.2435
241031_at	7.4	3.1	-1.27	-2.42	AAA-AAA	0.00	999	1.000	1.000	0.4159
1560742_at	16.5	6.8	-1.27	-2.42	AAA-AAA	0.00	999	1.000	1.000	0.2692
1568850_at	42.5	17.6	-1.27	-2.42	AAA-AAA	0.00	999	1.000	1.000	0.4334
1565705_x_at	73.2	30.3	-1.27	-2.42	AAA-AAA	0.00	999	1.000	1.000	0.1374
244821_at	28.5	11.8	-1.27	-2.42	AAA-AAA	0.00	999	1.000	1.000	0.2924
219811_at	154.0	63.7	-1.27	-2.42	AAA-AAA	0.00	999	1.000	1.000	0.2070
208556_at	15.2	6.3	-1.27	-2.42	AAA-AAA	0.00	999	1.000	1.000	0.2629
209462_at	41.6	100.4	1.27	2.42	AAA-AAA	0.00	999	1.000	1.000	0.1206
234014_at	21.7	9.0	-1.27	-2.42	AAA-AAA	0.00	999	1.000	1.000	0.3926
204476_s_at	42.9	103.6	1.27	2.42	AAA-AAA	0.00	999	1.000	1.000	0.3568
243510_at	37.3	15.4	-1.27	-2.42	AAA-AAA	0.00	999	1.000	1.000	0.3128
1570316_at	2.1	5.0	1.27	2.41	AAA-AAA	0.00	999	1.000	1.000	0.2976
1569850_at	10.1	24.3	1.27	2.42	AAA-AAA	0.00	999	1.000	1.000	0.1168
220450_at	31.0	12.8	-1.27	-2.42	AAA-AAA	0.00	999	1.000	1.000	0.2662
212925_at	57.3	138.4	1.27	2.42	AAA-AAA	0.00	999	1.000	1.000	0.3640
219039_at	189.7	78.6	-1.27	-2.41	AAA-AAA	0.00	999	1.000	1.000	0.2701
236323_at	19.1	7.9	-1.27	-2.42	AAA-AAA	0.00	999	1.000	1.000	0.2104
234481_at	74.6	30.9	-1.27	-2.41	AAA-AAA	0.00	999	1.000	1.000	0.2211
238294_at	15.0	6.2	-1.27	-2.42	AAA-AAA	0.00	999	1.000	1.000	0.3283
228410_at	12.0	29.1	1.27	2.41	AAA-AAA	0.00	999	1.000	1.000	0.1873
231642_at	39.3	16.3	-1.27	-2.41	AAA-AAA	0.00	999	1.000	1.000	0.2485
1556558_s_at	10.5	25.4	1.27	2.41	APA-APA	0.00	999	1.000	1.000	0.2152
244226_s_at	13.2	5.5	-1.27	-2.41	AAA-AAA	0.00	999	1.000	1.000	0.4247
1553590_at	14.1	34.1	1.27	2.41	AAA-AAA	0.00	999	1.000	1.000	0.1847
1557176_a_at	16.5	39.8	1.27	2.41	AAA-AAA	0.00	999	1.000	1.000	0.4411
224542_s_at	32.5	13.5	-1.27	-2.41	AAA-AAA	0.00	999	1.000	1.000	0.2756
216679_at	22.1	53.2	1.27	2.41	AAA-AAA	0.00	999	1.000	1.000	0.1302
236963_at	27.2	11.3	-1.27	-2.41	AAA-AAA	0.00	999	1.000	1.000	0.3359
227860_at	16.0	6.6	-1.27	-2.41	AAA-AAA	0.00	999	1.000	1.000	0.0354
231220_at	7.0	2.9	-1.27	-2.41	AAA-AAA	0.00	999	1.000	1.000	0.0777
240985_at	36.1	15.0	-1.27	-2.41	AAA-AAA	0.00	999	1.000	1.000	0.3206

1563209_a_at	5.1	2.1	-1.27	-2.41	AAA-AAA	0.00	999	1.000	1.000	0.1240
1561660_at	28.0	11.6	-1.27	-2.41	AAA-AAA	0.00	999	1.000	1.000	0.4212
1569743_at	8.7	3.6	-1.27	-2.41	AAA-AAA	0.00	999	1.000	1.000	0.3740
1554041_at	29.2	12.1	-1.27	-2.41	AAA-AAA	0.00	999	1.000	1.000	0.4663
244854_at	14.9	35.9	1.27	2.41	AAA-AAA	0.00	999	1.000	1.000	0.2914
207716_at	114.1	47.3	-1.27	-2.41	AAA-AAA	0.00	999	1.000	1.000	0.0352
232132_at	46.0	19.1	-1.27	-2.41	AAA-AAA	0.00	999	1.000	1.000	0.1338
232188_at	15.2	36.7	1.27	2.41	AAP-APA	0.00	999	1.000	1.000	0.4203
239698_at	21.7	52.2	1.27	2.41	AAA-PAP	0.00	999	1.000	1.000	0.1521
242579_at	51.9	21.5	-1.27	-2.41	AAA-AAA	0.00	999	1.000	1.000	0.0470
201062_at	2.9	7.0	1.27	2.41	AAA-AAA	0.00	999	1.000	1.000	0.1119
1553258_at	12.9	31.2	1.27	2.41	AAA-AAA	0.00	999	1.000	1.000	0.2354
220582_at	76.3	31.7	-1.27	-2.41	AAA-AAA	0.00	999	1.000	1.000	0.0019
207567_at	12.6	5.2	-1.27	-2.41	AAA-AAA	0.00	999	1.000	1.000	0.1554
219225_at	38.9	16.1	-1.27	-2.41	AAA-AAA	0.00	999	1.000	1.000	0.1178
234745_at	5.4	13.0	1.27	2.41	AAA-AAA	0.00	999	1.000	1.000	0.3971
231757_at	10.6	4.4	-1.27	-2.41	AAA-AAA	0.00	999	1.000	1.000	0.0837
208748_s_at	87.8	211.4	1.27	2.41	AAA-AAP	0.00	999	1.000	1.000	0.2197
244001_at	5.4	12.9	1.27	2.41	AAA-AAA	0.00	999	1.000	1.000	0.1026
243525_at	15.7	37.9	1.27	2.41	AAA-AAA	0.00	999	1.000	1.000	0.3540
1561616_a_at	105.0	43.6	-1.27	-2.41	AAA-AAA	0.00	999	1.000	1.000	0.2474
1554157_a_at	70.1	29.1	-1.27	-2.41	AAA-AAA	0.00	999	1.000	1.000	0.0625
221353_at	9.8	4.1	-1.27	-2.41	AAA-AAA	0.00	999	1.000	1.000	0.0264
208466_at	90.7	37.6	-1.27	-2.41	AAA-AAA	0.00	999	1.000	1.000	0.1373
215060_at	5.8	14.0	1.27	2.41	AAA-AAA	0.00	999	1.000	1.000	0.2612
214603_at	13.5	5.6	-1.27	-2.41	AAA-AAA	0.00	999	1.000	1.000	0.3694
236275_at	228.0	94.7	-1.27	-2.41	MAA-AAA	0.00	999	1.000	1.000	0.0457
231951_at	142.5	59.2	-1.27	-2.41	AAA-AAA	0.00	999	1.000	1.000	0.2271
235999_at	32.0	77.0	1.27	2.41	APA-AMP	0.00	999	1.000	1.000	0.3502
222508_s_at	99.4	239.1	1.27	2.41	AAA-MPA	0.00	999	1.000	1.000	0.2178
240487_at	19.0	45.6	1.27	2.41	AAA-AAA	0.00	999	1.000	1.000	0.3780
1569153_at	4.8	11.6	1.27	2.41	AAA-AAA	0.00	999	1.000	1.000	0.0632
210121_at	9.2	3.8	-1.27	-2.41	AAA-AAA	0.00	999	1.000	1.000	0.4761
214904_at	48.1	20.0	-1.27	-2.41	AAA-AAA	0.00	999	1.000	1.000	0.2099
214763_at	37.6	15.6	-1.27	-2.41	AAA-AAA	0.00	999	1.000	1.000	0.3871
234930_at	7.8	3.2	-1.27	-2.41	AAA-AAA	0.00	999	1.000	1.000	0.2630
236979_at	61.1	25.4	-1.27	-2.41	AAA-AAA	0.00	999	1.000	1.000	0.3953
229774_at	44.9	18.7	-1.27	-2.41	AAA-AAA	0.00	999	1.000	1.000	0.4188
1558002_at	42.5	102.3	1.27	2.41	AAA-PPP	0.00	999	1.000	1.000	0.0690
242319_at	26.9	11.2	-1.27	-2.40	AAA-AAA	0.00	999	1.000	1.000	0.2851
1560460_at	28.3	11.8	-1.27	-2.40	AAA-AAA	0.00	999	1.000	1.000	0.1602
1553524_at	29.4	12.2	-1.27	-2.41	AAA-AAA	0.00	999	1.000	1.000	0.3168

1553235_at	22.1	9.2	-1.27	-2.41	AAA-AAA	0.00	999	1.000	1.000	0.2510
207799_x_at	55.0	22.9	-1.27	-2.41	AAA-AAA	0.00	999	1.000	1.000	0.1442
220084_at	11.3	4.7	-1.27	-2.41	AAA-AAA	0.00	999	1.000	1.000	0.2795
218736_s_at	15.8	6.6	-1.27	-2.40	AAA-AAA	0.00	999	1.000	1.000	0.2275
216731_s_at	13.1	5.4	-1.27	-2.41	AAA-AAA	0.00	999	1.000	1.000	0.3527
234567_at	7.2	3.0	-1.27	-2.40	AAA-AAA	0.00	999	1.000	1.000	0.1021
231893_at	29.5	12.3	-1.27	-2.41	AAA-AAA	0.00	999	1.000	1.000	0.2685
218824_at	27.4	65.8	1.27	2.40	APA-PAP	0.00	999	1.000	1.000	0.3824
1557459_at	15.0	36.1	1.27	2.40	AAA-APA	0.00	999	1.000	1.000	0.2093
244289_at	51.8	21.5	-1.27	-2.40	AMA-AAA	0.00	999	1.000	1.000	0.1050
1552932_at	13.2	5.5	-1.27	-2.40	AAA-AAA	0.00	999	1.000	1.000	0.3322
220179_at	11.1	4.6	-1.27	-2.40	AAA-AAA	0.00	999	1.000	1.000	0.3162
222954_at	11.8	4.9	-1.27	-2.40	AAA-AAA	0.00	999	1.000	1.000	0.1305
210082_at	27.6	11.5	-1.27	-2.40	AAA-AAA	0.00	999	1.000	1.000	0.2677
215877_at	8.1	3.4	-1.27	-2.40	AAA-AAA	0.00	999	1.000	1.000	0.0814
243056_at	19.9	47.9	1.26	2.40	AAA-PAP	0.00	999	1.000	1.000	0.2934
241844_x_at	4.0	9.6	1.26	2.40	AAA-AAA	0.00	999	1.000	1.000	0.4892
1570168_at	19.4	8.1	-1.26	-2.40	AAA-AAA	0.00	999	1.000	1.000	0.1691
1570385_at	24.2	10.1	-1.26	-2.40	AAA-AAA	0.00	999	1.000	1.000	0.3645
1555173_at	8.4	20.2	1.26	2.40	AAA-AAA	0.00	999	1.000	1.000	0.3592
235684_s_at	9.1	3.8	-1.26	-2.40	AAA-AAA	0.00	999	1.000	1.000	0.5434
233994_at	16.9	7.0	-1.26	-2.40	AAA-AAA	0.00	999	1.000	1.000	0.4209
210850_s_at	18.2	43.6	1.26	2.40	AAA-APA	0.00	999	1.000	1.000	0.3428
202837_at	99.7	239.3	1.26	2.40	APP-PPP	0.00	999	1.000	1.000	0.0781
240653_at	41.3	17.2	-1.26	-2.40	AMA-AAA	0.00	999	1.000	1.000	0.3486
1554281_at	5.0	12.0	1.26	2.40	AAA-AAA	0.00	999	1.000	1.000	0.2558
1554602_at	102.6	42.8	-1.26	-2.40	AAA-AAA	0.00	999	1.000	1.000	0.3259
210295_at	19.2	8.0	-1.26	-2.40	AAA-AAA	0.00	999	1.000	1.000	0.3046
239440_at	76.7	31.9	-1.26	-2.40	AAA-AAA	0.00	999	1.000	1.000	0.2389
204328_at	53.1	22.1	-1.26	-2.40	AMA-AAA	0.00	999	1.000	1.000	0.3853
206193_s_at	15.5	6.5	-1.26	-2.40	AAA-AAA	0.00	999	1.000	1.000	0.2566
206093_x_at	50.2	20.9	-1.26	-2.40	AAA-AAA	0.00	999	1.000	1.000	0.0741
231666_at	27.3	11.4	-1.26	-2.40	AAA-AAA	0.00	999	1.000	1.000	0.5398
241920_x_at	51.1	122.6	1.26	2.40	AAP-AAA	0.00	999	1.000	1.000	0.3725
243949_at	9.3	3.9	-1.26	-2.40	AAA-AAA	0.00	999	1.000	1.000	0.3121
243713_at	29.7	12.4	-1.26	-2.40	AAA-MAM	0.00	999	1.000	1.000	0.5314
1555224_at	6.3	2.6	-1.26	-2.40	AAA-AAA	0.00	999	1.000	1.000	0.2790
208176_at	26.4	63.2	1.26	2.40	AAA-AAA	0.00	999	1.000	1.000	0.3133
207444_at	5.7	13.8	1.26	2.40	AAA-AAA	0.00	999	1.000	1.000	0.2389
210116_at	19.0	7.9	-1.26	-2.40	AAA-AAA	0.00	999	1.000	1.000	0.4227
219404_at	54.9	22.9	-1.26	-2.40	AAA-AAA	0.00	999	1.000	1.000	0.4785
236906_x_at	114.1	273.7	1.26	2.40	AAA-AAA	0.00	999	1.000	1.000	0.4500

232102_at	32.2	13.4	-1.26	-2.40	AAA-AAA	0.00	999	1.000	1.000	0.0185
1566726_at	22.6	9.4	-1.26	-2.40	AAA-AAA	0.00	999	1.000	1.000	0.5349
1567139_at	21.0	8.8	-1.26	-2.40	AAA-AAA	0.00	999	1.000	1.000	0.3851
244524_at	15.3	36.8	1.26	2.40	AAA-AAA	0.00	999	1.000	1.000	0.3278
1553295_at	46.9	19.6	-1.26	-2.40	AAA-AAA	0.00	999	1.000	1.000	0.0999
223045_at	7.7	18.5	1.26	2.40	AAA-AAA	0.00	999	1.000	1.000	0.1329
214402_s_at	46.1	110.5	1.26	2.40	AAA-AAA	0.00	999	1.000	1.000	0.4210
211588_s_at	60.1	25.1	-1.26	-2.40	AAA-AAA	0.00	999	1.000	1.000	0.4125
209474_s_at	8.2	19.6	1.26	2.40	AAA-AAA	0.00	999	1.000	1.000	0.2493
236570_at	57.4	24.0	-1.26	-2.40	AAA-AAA	0.00	999	1.000	1.000	0.0295
239501_at	44.9	18.7	-1.26	-2.40	MAA-AAA	0.00	999	1.000	1.000	0.2572
229687_s_at	4.2	10.0	1.26	2.40	AAA-AAA	0.00	999	1.000	1.000	0.3175
231287_s_at	24.3	10.2	-1.26	-2.40	AAA-AAA	0.00	999	1.000	1.000	0.4503
242181_at	32.3	77.3	1.26	2.40	AAA-MAP	0.00	999	1.000	1.000	0.2016
241044_x_at	17.7	7.4	-1.26	-2.40	AAA-AAA	0.00	999	1.000	1.000	0.2376
1559728_at	77.7	32.4	-1.26	-2.40	AAA-AAA	0.00	999	1.000	1.000	0.1672
219900_s_at	69.1	165.4	1.26	2.40	AAA-AAA	0.00	999	1.000	1.000	0.1814
214413_at	40.2	16.8	-1.26	-2.40	AAM-AAA	0.00	999	1.000	1.000	0.0843
216820_at	21.0	8.8	-1.26	-2.40	AAA-AAA	0.00	999	1.000	1.000	0.4311
234955_at	35.8	15.0	-1.26	-2.39	AAA-AAA	0.00	999	1.000	1.000	0.4208
238810_at	14.5	34.8	1.26	2.40	AAA-AAA	0.00	999	1.000	1.000	0.3780
233188_at	128.8	53.8	-1.26	-2.40	AAA-AAA	0.00	999	1.000	1.000	0.1955
231600_at	4.2	10.1	1.26	2.40	AAA-AAA	0.00	999	1.000	1.000	0.3398
239779_at	16.1	6.7	-1.26	-2.39	AAA-AAA	0.00	999	1.000	1.000	0.5107
239945_at	6.5	15.4	1.26	2.39	AAA-AAA	0.00	999	1.000	1.000	0.1759
242941_x_at	10.7	4.5	-1.26	-2.39	AAA-AAA	0.00	999	1.000	1.000	0.3389
1564429_at	128.5	53.7	-1.26	-2.39	AAA-AAA	0.00	999	1.000	1.000	0.2874
1557840_at	30.7	73.4	1.26	2.39	AAA-AAA	0.00	999	1.000	1.000	0.1224
227057_at	7.6	3.2	-1.26	-2.39	AAA-AAA	0.00	999	1.000	1.000	0.0181
215477_at	10.5	25.1	1.26	2.39	AAA-AAA	0.00	999	1.000	1.000	0.1996
216055_at	12.5	29.8	1.26	2.39	AAA-AAA	0.00	999	1.000	1.000	0.4514
239429_at	24.5	10.2	-1.26	-2.39	MAA-AAA	0.00	999	1.000	1.000	0.4330
230545_at	29.3	12.3	-1.26	-2.39	AAA-AAA	0.00	999	1.000	1.000	0.2796
211789_s_at	44.7	106.9	1.26	2.39	AAA-PPA	0.00	999	1.000	1.000	0.3721
205352_at	19.1	45.8	1.26	2.39	APA-AAA	0.00	999	1.000	1.000	0.2475
243187_at	24.0	57.4	1.26	2.39	AAA-AAA	0.00	999	1.000	1.000	0.1741
1561578_s_at	2.6	6.2	1.26	2.39	AAA-AAA	0.00	999	1.000	1.000	0.3642
1566030_at	5.8	13.9	1.26	2.39	AAA-AAA	0.00	999	1.000	1.000	0.2193
204638_at	66.2	27.7	-1.26	-2.39	AAA-AAA	0.00	999	1.000	1.000	0.0221
237219_at	9.3	3.9	-1.26	-2.39	AAA-AAA	0.00	999	1.000	1.000	0.2203
237834_at	23.6	9.9	-1.26	-2.39	AAA-AAA	0.00	999	1.000	1.000	0.3869
232418_at	26.8	11.2	-1.26	-2.39	AAA-AAA	0.00	999	1.000	1.000	0.3114

1559332_at	31.5	75.3	1.26	2.39	APA-APP	0.00	999	1.000	1.000	0.4079
1558220_at	44.9	107.3	1.26	2.39	APA-PMM	0.00	999	1.000	1.000	0.1543
239829_at	9.4	22.4	1.26	2.39	AAA-AAA	0.00	999	1.000	1.000	0.5412
244384_at	8.8	3.7	-1.26	-2.39	AAA-AAA	0.00	999	1.000	1.000	0.0689
219788_at	10.2	24.2	1.26	2.39	AAA-AAA	0.00	999	1.000	1.000	0.3944
210662_at	10.5	25.0	1.26	2.39	AAA-AAA	0.00	999	1.000	1.000	0.3796
216663_s_at	77.1	32.3	-1.26	-2.39	AAA-AAA	0.00	999	1.000	1.000	0.2951
234798_x_at	3.8	1.6	-1.26	-2.39	AAA-AAA	0.00	999	1.000	1.000	0.1312
228484_s_at	4.0	9.7	1.26	2.39	AAA-AAA	0.00	999	1.000	1.000	0.4701
240619_at	6.2	2.6	-1.26	-2.39	AAA-AAA	0.00	999	1.000	1.000	0.3788
1552596_at	49.3	20.6	-1.26	-2.39	AAA-AAA	0.00	999	1.000	1.000	0.2491
1558867_at	1.3	3.2	1.26	2.39	AAA-AAA	0.00	999	1.000	1.000	0.3788
209988_s_at	10.8	25.7	1.26	2.39	AAA-AAA	0.00	999	1.000	1.000	0.2605
211331_x_at	40.2	95.9	1.26	2.39	AAA-AAA	0.00	999	1.000	1.000	0.3596
216003_at	14.5	6.1	-1.26	-2.39	AAA-AAA	0.00	999	1.000	1.000	0.2357
237445_at	21.0	8.8	-1.26	-2.39	AAA-AAA	0.00	999	1.000	1.000	0.2225
228695_at	9.3	22.2	1.26	2.39	AAA-AAA	0.00	999	1.000	1.000	0.3497
233183_at	8.3	3.5	-1.26	-2.39	AAA-AAA	0.00	999	1.000	1.000	0.2876
231484_at	9.6	22.9	1.26	2.39	AAA-AAA	0.00	999	1.000	1.000	0.2162
219892_at	26.9	64.2	1.26	2.39	AAP-PAP	0.00	999	1.000	1.000	0.2838
244306_at	9.3	22.1	1.26	2.39	AAA-AAA	0.00	999	1.000	1.000	0.4509
240817_at	45.7	19.1	-1.26	-2.39	AAA-AAA	0.00	999	1.000	1.000	0.4153
243385_at	6.7	15.9	1.26	2.39	AAA-AAA	0.00	999	1.000	1.000	0.3036
242793_at	16.2	38.7	1.26	2.39	AAA-AAA	0.00	999	1.000	1.000	0.0229
242749_at	66.9	28.0	-1.26	-2.39	AAA-AAA	0.00	999	1.000	1.000	0.2578
1563327_a_at	26.3	11.0	-1.26	-2.39	AAA-AAA	0.00	999	1.000	1.000	0.4569
1560212_a_at	10.7	25.5	1.26	2.39	AAA-AAA	0.00	999	1.000	1.000	0.1911
1553891_at	25.6	61.1	1.26	2.39	AAA-AAA	0.00	999	1.000	1.000	0.1627
1558449_at	15.1	6.3	-1.26	-2.39	AAA-AAA	0.00	999	1.000	1.000	0.3873
220756_s_at	32.7	13.7	-1.26	-2.39	AAA-AAA	0.00	999	1.000	1.000	0.3319
206535_at	5.1	2.2	-1.26	-2.39	AAA-AAA	0.00	999	1.000	1.000	0.2342
235988_at	4.6	1.9	-1.26	-2.39	AAA-AAA	0.00	999	1.000	1.000	0.0497
1554664_at	11.4	27.2	1.25	2.39	AAA-APA	0.00	999	1.000	1.000	0.2204
241190_at	27.0	11.3	-1.25	-2.39	AAA-AAA	0.00	999	1.000	1.000	0.3151
1570115_at	19.9	8.3	-1.25	-2.39	AAA-AAA	0.00	999	1.000	1.000	0.2684
1554206_at	42.6	101.5	1.25	2.39	AAA-AAA	0.00	999	1.000	1.000	0.1181
244628_at	21.6	9.1	-1.25	-2.39	AAA-AAA	0.00	999	1.000	1.000	0.3452
1552657_a_at	11.7	27.9	1.25	2.38	AAA-AAA	0.00	999	1.000	1.000	0.3373
34471_at	17.8	7.4	-1.25	-2.39	AAA-AAA	0.00	999	1.000	1.000	0.1238
206876_at	36.2	15.2	-1.25	-2.38	AAA-AAA	0.00	999	1.000	1.000	0.3354
211499_s_at	61.1	145.7	1.25	2.39	AAA-AAA	0.00	999	1.000	1.000	0.2385
237924_at	41.2	17.3	-1.25	-2.38	AAA-AAA	0.00	999	1.000	1.000	0.1515

1557265_at	36.4	86.7	1.25	2.38	AAA-APP	0.00	999	1.000	1.000	0.2919
207565_s_at	28.2	67.1	1.25	2.38	AAA-PPA	0.00	999	1.000	1.000	0.2063
1554541_a_at	12.4	5.2	-1.25	-2.38	AAA-AAA	0.00	999	1.000	1.000	0.0142
225001_at	43.8	104.3	1.25	2.38	AAA-AAM	0.00	999	1.000	1.000	0.1584
229604_at	15.1	36.0	1.25	2.38	AAA-AAA	0.00	999	1.000	1.000	0.1757
230987_at	21.8	51.9	1.25	2.38	AAA-AAA	0.00	999	1.000	1.000	0.0888
204955_at	1394.0	3319.9	1.25	2.38	PPP-PPP	0.00	999	1.000	1.000	0.2900
242551_at	29.5	12.4	-1.25	-2.38	AAA-AAA	0.00	999	1.000	1.000	0.3906
1563816_at	19.8	47.2	1.25	2.38	AAA-AAA	0.00	999	1.000	1.000	0.2046
1565671_a_at	10.6	4.4	-1.25	-2.38	AAA-AAA	0.00	999	1.000	1.000	0.0303
1564950_at	6.3	15.1	1.25	2.38	AAA-AMA	0.00	999	1.000	1.000	0.5805
1558765_a_at	67.4	28.3	-1.25	-2.38	AAA-AAA	0.00	999	1.000	1.000	0.2956
217636_at	20.5	8.6	-1.25	-2.38	AAA-AAA	0.00	999	1.000	1.000	0.1958
234444_at	69.6	29.2	-1.25	-2.38	AAA-AAA	0.00	999	1.000	1.000	0.2265
230182_at	28.0	66.6	1.25	2.38	AAA-AMA	0.00	999	1.000	1.000	0.3717
243534_at	19.6	8.2	-1.25	-2.38	AAA-AAA	0.00	999	1.000	1.000	0.2025
1570274_at	41.1	17.3	-1.25	-2.38	AAA-AAA	0.00	999	1.000	1.000	0.3397
1552979_at	13.3	5.6	-1.25	-2.38	AAA-AAA	0.00	999	1.000	1.000	0.2312
1556159_at	6.4	2.7	-1.25	-2.38	AAA-AAA	0.00	999	1.000	1.000	0.3658
208242_at	16.4	6.9	-1.25	-2.38	AAA-AAA	0.00	999	1.000	1.000	0.0958
227346_at	14.3	6.0	-1.25	-2.38	AAA-AAA	0.00	999	1.000	1.000	0.4200
218623_at	48.5	20.4	-1.25	-2.38	AAA-AAA	0.00	999	1.000	1.000	0.2781
229237_s_at	58.4	24.6	-1.25	-2.38	AAA-AAA	0.00	999	1.000	1.000	0.0872
231100_at	8.8	21.1	1.25	2.38	AAA-AAA	0.00	999	1.000	1.000	0.3515
235380_at	12.1	28.8	1.25	2.38	AAA-APA	0.00	999	1.000	1.000	0.3572
229135_at	30.8	73.2	1.25	2.38	AAA-APA	0.00	999	1.000	1.000	0.1243
243863_at	7.0	16.5	1.25	2.38	AAA-AAA	0.00	999	1.000	1.000	0.3299
241021_at	38.3	16.1	-1.25	-2.38	AAA-AAA	0.00	999	1.000	1.000	0.4549
239861_at	52.1	21.9	-1.25	-2.38	AAA-AAA	0.00	999	1.000	1.000	0.3879
1562990_at	3.5	8.3	1.25	2.38	AAA-AAA	0.00	999	1.000	1.000	0.3782
1566902_at	41.7	17.5	-1.25	-2.38	AAA-AAA	0.00	999	1.000	1.000	0.2101
1553605_a_at	1.6	3.9	1.25	2.38	AAA-AAA	0.00	999	1.000	1.000	0.3523
244482_at	13.4	5.6	-1.25	-2.38	AAA-AAA	0.00	999	1.000	1.000	0.4092
1557994_at	32.9	13.8	-1.25	-2.38	AAA-AAA	0.00	999	1.000	1.000	0.4008
219592_at	24.5	58.2	1.25	2.38	AAA-AAA	0.00	999	1.000	1.000	0.2710
228901_at	13.4	31.9	1.25	2.38	AAA-AAA	0.00	999	1.000	1.000	0.3252
1562163_at	11.6	27.6	1.25	2.38	AAA-AAA	0.00	999	1.000	1.000	0.3849
1557498_a_at	9.2	3.9	-1.25	-2.38	AAA-AAA	0.00	999	1.000	1.000	0.3469
1557261_at	3.3	7.9	1.25	2.38	AAA-AAA	0.00	999	1.000	1.000	0.1227
221161_at	48.3	20.3	-1.25	-2.38	AAA-AAA	0.00	999	1.000	1.000	0.2105
206560_s_at	24.6	58.4	1.25	2.38	AAA-AAA	0.00	999	1.000	1.000	0.2662
209441_at	135.8	57.1	-1.25	-2.38	AAA-AAA	0.00	999	1.000	1.000	0.2267

228383_at	17.0	7.1	-1.25	-2.38	AAA-AAA	0.00	999	1.000	1.000	0.2072
232666_at	24.0	57.0	1.25	2.38	AAA-AAA	0.00	999	1.000	1.000	0.2425
233735_at	58.9	24.8	-1.25	-2.38	AAA-AAA	0.00	999	1.000	1.000	0.1091
218445_at	133.9	318.0	1.25	2.38	AAA-PPP	0.00	999	1.000	1.000	0.1690
1567386_at	4.0	1.7	-1.25	-2.38	AAA-AAA	0.00	999	1.000	1.000	0.3699
244855_at	5.2	12.3	1.25	2.38	AAA-AAA	0.00	999	1.000	1.000	0.1604
206859_s_at	9.5	22.7	1.25	2.38	AAA-AAA	0.00	999	1.000	1.000	0.2751
207282_s_at	20.2	8.5	-1.25	-2.38	AAA-AAA	0.00	999	1.000	1.000	0.0714
211237_s_at	14.0	5.9	-1.25	-2.38	AAA-AAA	0.00	999	1.000	1.000	0.2171
215636_at	48.9	20.6	-1.25	-2.38	AAA-AAA	0.00	999	1.000	1.000	0.3678
234657_at	7.9	3.3	-1.25	-2.38	AAA-AAA	0.00	999	1.000	1.000	0.0881
239770_at	6.7	15.9	1.25	2.38	AAA-AAA	0.00	999	1.000	1.000	0.2711
229279_at	85.1	35.9	-1.25	-2.37	AAM-AAA	0.00	999	1.000	1.000	0.0578
205663_at	26.9	11.3	-1.25	-2.38	AAA-AAA	0.00	999	1.000	1.000	0.5026
240952_at	37.6	15.8	-1.25	-2.37	AAA-AAA	0.00	999	1.000	1.000	0.0459
241497_at	54.2	22.8	-1.25	-2.37	AAA-AAA	0.00	999	1.000	1.000	0.1332
240524_x_at	12.6	5.3	-1.25	-2.37	AAA-AAA	0.00	999	1.000	1.000	0.0974
1560769_at	20.4	8.6	-1.25	-2.37	AAA-AAA	0.00	999	1.000	1.000	0.4323
1561737_at	11.5	4.8	-1.25	-2.37	AAA-AAA	0.00	999	1.000	1.000	0.4686
1555031_at	10.4	4.4	-1.25	-2.37	AAA-AAA	0.00	999	1.000	1.000	0.4017
1554641_a_at	39.8	16.8	-1.25	-2.37	AAA-AAA	0.00	999	1.000	1.000	0.2922
220057_at	66.5	28.0	-1.25	-2.37	AAA-AAA	0.00	999	1.000	1.000	0.1521
227552_at	20.1	47.7	1.25	2.37	AAA-AAA	0.00	999	1.000	1.000	0.1961
226653_at	30.2	12.7	-1.25	-2.37	AAA-AAA	0.00	999	1.000	1.000	0.2730
235873_at	7.0	3.0	-1.25	-2.37	AAA-AAA	0.00	999	1.000	1.000	0.0006
235145_at	135.9	57.2	-1.25	-2.37	AAA-AAA	0.00	999	1.000	1.000	0.1172
235527_at	23.0	9.7	-1.25	-2.37	MAA-AAA	0.00	999	1.000	1.000	0.2464
237036_at	32.1	13.5	-1.25	-2.37	AAA-AAA	0.00	999	1.000	1.000	0.3784
240904_at	33.7	14.2	-1.25	-2.37	AAA-AAA	0.00	999	1.000	1.000	0.1427
241862_x_at	24.3	10.2	-1.25	-2.37	AAA-AAA	0.00	999	1.000	1.000	0.2094
243703_x_at	12.0	5.1	-1.25	-2.37	AAA-AAA	0.00	999	1.000	1.000	0.3413
1564044_at	24.3	10.2	-1.25	-2.37	AAA-AAA	0.00	999	1.000	1.000	0.0232
1555678_at	27.0	64.0	1.25	2.37	AAA-AAA	0.00	999	1.000	1.000	0.2501
211064_at	41.4	17.5	-1.25	-2.37	AAA-AAA	0.00	999	1.000	1.000	0.0655
233810_x_at	38.9	92.4	1.25	2.37	AAA-AAA	0.00	999	1.000	1.000	0.2649
228703_at	61.1	145.0	1.25	2.37	AAA-AAA	0.00	999	1.000	1.000	0.2306
233404_at	7.1	3.0	-1.25	-2.37	AAA-AAA	0.00	999	1.000	1.000	0.3771
231646_at	15.9	6.7	-1.25	-2.37	AAA-AAA	0.00	999	1.000	1.000	0.2997
242791_at	10.3	24.4	1.25	2.37	AAA-APA	0.00	999	1.000	1.000	0.3528
240023_at	120.9	51.0	-1.25	-2.37	AAA-AAA	0.00	999	1.000	1.000	0.1666
243896_at	9.8	4.1	-1.25	-2.37	AAA-AAA	0.00	999	1.000	1.000	0.4279
242675_x_at	16.6	39.3	1.25	2.37	AAA-AAA	0.00	999	1.000	1.000	0.3864

1562481_at	6.4	2.7	-1.25	-2.37	AAA-AAA	0.00	999	1.000	1.000	0.3946
1562828_at	31.9	13.5	-1.25	-2.37	AAA-AAA	0.00	999	1.000	1.000	0.2312
1569058_at	12.5	5.3	-1.25	-2.37	AAA-AAA	0.00	999	1.000	1.000	0.2943
1570120_at	11.4	27.1	1.25	2.37	AAA-AAA	0.00	999	1.000	1.000	0.2440
1557861_at	10.6	4.5	-1.25	-2.37	AAA-AAA	0.00	999	1.000	1.000	0.2693
210049_at	8.9	3.8	-1.25	-2.37	AAA-AAA	0.00	999	1.000	1.000	0.0834
214677_x_at	10.4	4.4	-1.25	-2.37	AAA-AAA	0.00	999	1.000	1.000	0.0786
216050_at	6.2	2.6	-1.25	-2.37	AAA-AAA	0.00	999	1.000	1.000	0.2154
216362_at	18.7	7.9	-1.25	-2.37	AAA-AAA	0.00	999	1.000	1.000	0.2339
239030_at	7.8	18.5	1.25	2.37	AAA-AAA	0.00	999	1.000	1.000	0.2292
230217_at	13.5	5.7	-1.25	-2.37	AAA-AAA	0.00	999	1.000	1.000	0.2787
229168_at	25.3	59.9	1.25	2.37	AAA-AAA	0.00	999	1.000	1.000	0.1644
233694_at	15.8	6.7	-1.25	-2.37	AAA-AAA	0.00	999	1.000	1.000	0.0065
231358_at	12.5	5.3	-1.25	-2.37	AAA-AAA	0.00	999	1.000	1.000	0.5084
241163_at	19.0	45.0	1.24	2.37	AAA-AAA	0.00	999	1.000	1.000	0.4493
1561036_at	31.7	13.4	-1.24	-2.37	AAA-AAA	0.00	999	1.000	1.000	0.3695
1553919_at	8.4	19.8	1.24	2.37	AAA-AAA	0.00	999	1.000	1.000	0.3816
211143_x_at	114.8	48.5	-1.24	-2.37	AAA-AAA	0.00	999	1.000	1.000	0.3992
234349_at	45.3	19.1	-1.24	-2.37	AAA-AAA	0.00	999	1.000	1.000	0.0888
234826_at	18.6	43.9	1.24	2.37	AAA-AAA	0.00	999	1.000	1.000	0.1639
241655_at	18.7	7.9	-1.24	-2.37	AAA-AAA	0.00	999	1.000	1.000	0.4152
1563793_at	10.3	24.3	1.24	2.37	AAA-AAA	0.00	999	1.000	1.000	0.4130
1569453_a_at	62.3	26.3	-1.24	-2.37	AAA-AAA	0.00	999	1.000	1.000	0.0710
244480_at	61.2	25.9	-1.24	-2.37	AAA-AAA	0.00	999	1.000	1.000	0.2741
1558573_at	22.4	52.9	1.24	2.37	AAA-MMA	0.00	999	1.000	1.000	0.4292
219659_at	52.0	22.0	-1.24	-2.37	AAA-AAA	0.00	999	1.000	1.000	0.0942
235240_at	66.0	156.2	1.24	2.37	AAA-AAA	0.00	999	1.000	1.000	0.2174
235605_at	20.0	8.5	-1.24	-2.37	AAA-AAA	0.00	999	1.000	1.000	0.3607
234021_at	17.2	40.7	1.24	2.37	AAA-AAA	0.00	999	1.000	1.000	0.3082
237280_at	15.5	6.6	-1.24	-2.37	AAA-AAA	0.00	999	1.000	1.000	0.0195
230451_at	19.3	45.7	1.24	2.37	AAA-AAA	0.00	999	1.000	1.000	0.3644
228325_at	48.8	20.6	-1.24	-2.37	AAA-AAA	0.00	999	1.000	1.000	0.4374
1570516_s_at	28.2	11.9	-1.24	-2.37	AAA-AAA	0.00	999	1.000	1.000	0.1748
1569949_at	29.8	12.6	-1.24	-2.37	AAA-AAA	0.00	999	1.000	1.000	0.3455
221328_at	44.0	18.6	-1.24	-2.37	AAA-AAA	0.00	999	1.000	1.000	0.1060
203903_s_at	34.8	14.7	-1.24	-2.37	AAA-AAA	0.00	999	1.000	1.000	0.3457
230909_at	16.1	6.8	-1.24	-2.37	AAA-AAA	0.00	999	1.000	1.000	0.3132
232882_at	19.1	8.1	-1.24	-2.37	AAA-AAA	0.00	999	1.000	1.000	0.3330
231503_at	11.7	4.9	-1.24	-2.37	AAA-AAA	0.00	999	1.000	1.000	0.4646
222972_at	16.9	39.9	1.24	2.36	AAA-PAA	0.00	999	1.000	1.000	0.3274
239934_x_at	35.5	15.0	-1.24	-2.36	AAA-AAA	0.00	999	1.000	1.000	0.4109
215077_at	19.7	46.5	1.24	2.36	AAA-AAA	0.00	999	1.000	1.000	0.4735

211731_x_at	14.7	34.8	1.24	2.36	AAA-AAA	0.00	999	1.000	1.000	0.1546
219173_at	27.2	11.5	-1.24	-2.36	AAA-AAA	0.00	999	1.000	1.000	0.2499
215703_at	7.6	3.2	-1.24	-2.36	AAA-AAA	0.00	999	1.000	1.000	0.4517
241352_at	6.6	15.7	1.24	2.36	AAA-AAP	0.00	999	1.000	1.000	0.3733
1560893_at	17.6	7.4	-1.24	-2.36	AAA-AAA	0.00	999	1.000	1.000	0.2140
1569525_s_at	3.9	9.2	1.24	2.36	AAA-AAA	0.00	999	1.000	1.000	0.3124
221668_s_at	53.1	22.5	-1.24	-2.36	AAA-AAA	0.00	999	1.000	1.000	0.1868
207009_at	7.6	17.9	1.24	2.36	AAA-AAA	0.00	999	1.000	1.000	0.2549
237776_at	25.2	10.7	-1.24	-2.36	AAA-AAA	0.00	999	1.000	1.000	0.3852
233091_at	13.1	5.5	-1.24	-2.36	AAA-AAA	0.00	999	1.000	1.000	0.0555
227657_at	61.0	143.9	1.24	2.36	AAA-AAP	0.00	999	1.000	1.000	0.4183
1558189_a_at	15.4	6.5	-1.24	-2.36	AAA-AAA	0.00	999	1.000	1.000	0.2526
216886_at	28.6	12.1	-1.24	-2.36	AAA-AAA	0.00	999	1.000	1.000	0.2918
229364_at	24.6	58.1	1.24	2.36	AAA-AAA	0.00	999	1.000	1.000	0.3568
202688_at	13.6	32.0	1.24	2.36	AAA-APA	0.00	999	1.000	1.000	0.3173
1563001_at	6.2	2.6	-1.24	-2.36	AAA-AAA	0.00	999	1.000	1.000	0.2931
1557682_a_at	28.4	12.1	-1.24	-2.36	AAA-AAA	0.00	999	1.000	1.000	0.3529
211253_x_at	32.0	13.6	-1.24	-2.36	AAA-AAA	0.00	999	1.000	1.000	0.2650
215391_at	13.3	5.7	-1.24	-2.36	AAA-AAA	0.00	999	1.000	1.000	0.2193
233714_at	35.3	15.0	-1.24	-2.36	AAA-AAA	0.00	999	1.000	1.000	0.1179
230418_s_at	71.3	168.2	1.24	2.36	AAA-AAP	0.00	999	1.000	1.000	0.0861
213836_s_at	276.2	650.9	1.24	2.36	PPP-PPP	0.00	999	1.000	1.000	0.0735
243958_at	3.4	8.0	1.24	2.36	AAA-AAA	0.00	999	1.000	1.000	0.2620
240396_at	32.3	13.7	-1.24	-2.36	AAA-AAA	0.00	999	1.000	1.000	0.1465
242262_x_at	15.6	6.6	-1.24	-2.36	AAA-AAA	0.00	999	1.000	1.000	0.4446
1553618_at	12.3	5.2	-1.24	-2.36	AAA-AAA	0.00	999	1.000	1.000	0.4855
221293_s_at	23.3	55.0	1.24	2.36	AAA-AAA	0.00	999	1.000	1.000	0.2460
206733_at	43.6	18.5	-1.24	-2.36	AAA-AAA	0.00	999	1.000	1.000	0.4083
214401_at	75.6	32.1	-1.24	-2.36	AAA-AAA	0.00	999	1.000	1.000	0.1387
236159_x_at	21.9	9.3	-1.24	-2.36	AAA-AAA	0.00	999	1.000	1.000	0.3594
237913_at	49.1	20.8	-1.24	-2.36	AAA-AAA	0.00	999	1.000	1.000	0.3584
230468_s_at	3.1	7.3	1.24	2.36	AAA-AAA	0.00	999	1.000	1.000	0.3087
208324_at	17.9	42.2	1.24	2.36	AAA-AAP	0.00	999	1.000	1.000	0.3818
236500_at	30.9	72.7	1.24	2.36	AAA-AAP	0.00	999	1.000	1.000	0.1936
240756_at	13.5	5.7	-1.24	-2.36	AAA-AAA	0.00	999	1.000	1.000	0.0452
1562054_at	1.3	3.0	1.24	2.36	AAA-AAA	0.00	999	1.000	1.000	0.2330
1564472_at	5.3	12.5	1.24	2.36	AAA-AAA	0.00	999	1.000	1.000	0.1593
209324_s_at	29.2	12.4	-1.24	-2.36	AAA-AAA	0.00	999	1.000	1.000	0.4115
236246_x_at	18.8	8.0	-1.24	-2.36	AAA-AAA	0.00	999	1.000	1.000	0.0315
236100_at	23.0	9.8	-1.24	-2.36	AAA-AAA	0.00	999	1.000	1.000	0.1051
238398_s_at	13.7	5.8	-1.24	-2.36	AAA-AAA	0.00	999	1.000	1.000	0.1919
239417_x_at	12.9	30.5	1.24	2.36	AAA-AAA	0.00	999	1.000	1.000	0.2573

232496_at	22.1	9.4	-1.24	-2.36	AAA-AAA	0.00	999	1.000	1.000	0.3460
232384_s_at	25.6	10.9	-1.24	-2.36	AAA-AAA	0.00	999	1.000	1.000	0.5574
1563244_at	100.6	42.7	-1.24	-2.35	AAA-AAA	0.00	999	1.000	1.000	0.0203
1570444_at	29.7	12.6	-1.24	-2.35	AAA-AAA	0.00	999	1.000	1.000	0.3044
221282_x_at	23.8	10.1	-1.24	-2.35	AAA-AAA	0.00	999	1.000	1.000	0.1775
222084_s_at	5.6	13.1	1.24	2.35	AAA-AAA	0.00	999	1.000	1.000	0.1457
220815_at	20.0	8.5	-1.24	-2.35	AAA-AAA	0.00	999	1.000	1.000	0.3316
213033_s_at	8.3	19.5	1.24	2.35	AAA-AAA	0.00	999	1.000	1.000	0.1990
217008_s_at	34.7	14.8	-1.24	-2.36	AAA-AAA	0.00	999	1.000	1.000	0.3639
235859_at	34.6	14.7	-1.24	-2.36	AAA-AAA	0.00	999	1.000	1.000	0.2855
204006_s_at	7.9	3.4	-1.24	-2.35	AAA-AAA	0.00	999	1.000	1.000	0.4119
238168_at	14.7	34.6	1.23	2.35	AAA-APA	0.00	999	1.000	1.000	0.0656
243817_at	69.7	164.0	1.23	2.35	AAA-AAA	0.00	999	1.000	1.000	0.3418
1569289_at	42.2	17.9	-1.23	-2.35	AAA-AAA	0.00	999	1.000	1.000	0.2756
1569670_at	15.5	6.6	-1.23	-2.35	AAA-AAA	0.00	999	1.000	1.000	0.3468
1554618_at	19.9	8.5	-1.23	-2.35	AAA-AAA	0.00	999	1.000	1.000	0.2589
211039_at	8.2	3.5	-1.23	-2.35	AAA-AAA	0.00	999	1.000	1.000	0.3285
215894_at	13.6	5.8	-1.23	-2.35	AAA-AAA	0.00	999	1.000	1.000	0.4791
209355_s_at	13.1	30.8	1.23	2.35	AAA-AAA	0.00	999	1.000	1.000	0.4187
236054_at	21.8	9.3	-1.23	-2.35	AAA-AAA	0.00	999	1.000	1.000	0.3045
238136_at	25.0	10.6	-1.23	-2.35	AAA-AAA	0.00	999	1.000	1.000	0.2261
233542_at	10.1	4.3	-1.23	-2.35	AAA-AAA	0.00	999	1.000	1.000	0.1390
233708_at	20.0	8.5	-1.23	-2.35	AAA-AAA	0.00	999	1.000	1.000	0.2694
213502_x_at	64.1	150.7	1.23	2.35	AMA-MPM	0.00	999	1.000	1.000	0.3252
225500_x_at	107.6	253.0	1.23	2.35	AAA-APA	0.00	999	1.000	1.000	0.0580
243838_at	38.3	16.3	-1.23	-2.35	AAA-AAA	0.00	999	1.000	1.000	0.0981
207787_at	68.9	29.3	-1.23	-2.35	AAA-AAA	0.00	999	1.000	1.000	0.2244
208375_at	9.4	4.0	-1.23	-2.35	AAA-AAA	0.00	999	1.000	1.000	0.3526
219860_at	53.1	22.6	-1.23	-2.35	AAA-AAA	0.00	999	1.000	1.000	0.3790
223975_at	18.6	7.9	-1.23	-2.35	AAA-AAA	0.00	999	1.000	1.000	0.3257
211902_x_at	81.8	34.8	-1.23	-2.35	AAA-AAA	0.00	999	1.000	1.000	0.1955
217613_at	5.9	2.5	-1.23	-2.35	AAA-AAA	0.00	999	1.000	1.000	0.3913
235370_at	32.3	76.0	1.23	2.35	AAA-AAA	0.00	999	1.000	1.000	0.1208
234514_at	20.6	8.8	-1.23	-2.35	AAA-AAA	0.00	999	1.000	1.000	0.3820
229658_at	10.1	4.3	-1.23	-2.35	AAA-AAA	0.00	999	1.000	1.000	0.0762
230487_at	24.3	57.0	1.23	2.35	AAA-AAA	0.00	999	1.000	1.000	0.3382
206925_at	20.7	48.5	1.23	2.35	AAA-PAA	0.00	999	1.000	1.000	0.3449
241269_at	39.2	16.7	-1.23	-2.35	AAA-AAA	0.00	999	1.000	1.000	0.1479
1569971_at	9.5	4.1	-1.23	-2.35	AAA-AAA	0.00	999	1.000	1.000	0.3227
244838_at	35.8	15.2	-1.23	-2.35	AAA-AAA	0.00	999	1.000	1.000	0.0845
1556854_at	16.0	6.8	-1.23	-2.35	AAA-AAA	0.00	999	1.000	1.000	0.1597
220565_at	5.2	12.1	1.23	2.35	AAA-AAA	0.00	999	1.000	1.000	0.3347

213991_s_at	4.4	10.4	1.23	2.35	AAA-AAA	0.00	999	1.000	1.000	0.2968
217633_at	120.5	51.3	-1.23	-2.35	AAA-AAA	0.00	999	1.000	1.000	0.2444
236541_at	85.5	36.4	-1.23	-2.35	AAA-AAA	0.00	999	1.000	1.000	0.1096
238924_at	23.6	55.3	1.23	2.35	AAA-AMA	0.00	999	1.000	1.000	0.0306
238200_at	23.9	56.1	1.23	2.35	AAA-AAA	0.00	999	1.000	1.000	0.2700
227956_at	14.0	6.0	-1.23	-2.35	AAA-AAA	0.00	999	1.000	1.000	0.3551
229223_at	21.0	8.9	-1.23	-2.35	AAA-AAA	0.00	999	1.000	1.000	0.3353
242956_at	10.0	23.4	1.23	2.35	AAA-APA	0.00	999	1.000	1.000	0.3449
243125_x_at	12.9	30.3	1.23	2.35	AAA-AAP	0.00	999	1.000	1.000	0.1612
203691_at	19.0	8.1	-1.23	-2.35	AAA-AAA	0.00	999	1.000	1.000	0.4541
1570585_at	6.6	2.8	-1.23	-2.35	AAA-AAA	0.00	999	1.000	1.000	0.2163
1567377_at	21.0	9.0	-1.23	-2.35	AAA-AAA	0.00	999	1.000	1.000	0.2433
1555220_a_at	9.2	3.9	-1.23	-2.35	AAA-AAA	0.00	999	1.000	1.000	0.0973
222188_at	28.9	12.3	-1.23	-2.35	AAA-AAA	0.00	999	1.000	1.000	0.2558
206407_s_at	43.8	18.7	-1.23	-2.35	AAA-AAA	0.00	999	1.000	1.000	0.3361
223659_at	13.5	31.7	1.23	2.35	AAA-AAA	0.00	999	1.000	1.000	0.2250
216861_at	6.8	2.9	-1.23	-2.35	AAA-AAA	0.00	999	1.000	1.000	0.0684
236258_at	14.4	6.1	-1.23	-2.35	AAA-AAA	0.00	999	1.000	1.000	0.2770
234384_at	35.7	15.2	-1.23	-2.35	AAA-AAA	0.00	999	1.000	1.000	0.3970
206107_at	13.1	5.6	-1.23	-2.35	AAA-AAA	0.00	999	1.000	1.000	0.3770
228411_at	26.6	62.5	1.23	2.35	PAA-AAA	0.00	999	1.000	1.000	0.5250
233463_at	9.7	22.7	1.23	2.35	AAA-PAA	0.00	999	1.000	1.000	0.3799
241196_at	9.2	21.6	1.23	2.35	AAA-AAA	0.00	999	1.000	1.000	0.3114
1562788_at	10.9	25.6	1.23	2.35	AAA-AAA	0.00	999	1.000	1.000	0.4561
1562153_a_at	12.1	28.4	1.23	2.35	AAA-AAA	0.00	999	1.000	1.000	0.2017
1562946_at	26.8	62.9	1.23	2.35	AAA-AAM	0.00	999	1.000	1.000	0.2709
1566203_at	12.4	5.3	-1.23	-2.35	AAA-AAA	0.00	999	1.000	1.000	0.2661
1566518_at	11.4	26.7	1.23	2.35	AAA-AAA	0.00	999	1.000	1.000	0.1285
1555318_at	7.7	18.1	1.23	2.35	AAA-AAA	0.00	999	1.000	1.000	0.2765
214980_at	19.9	46.7	1.23	2.35	AAA-AAA	0.00	999	1.000	1.000	0.1133
210615_at	20.9	49.1	1.23	2.35	AAA-AAA	0.00	999	1.000	1.000	0.1193
215314_at	45.8	19.5	-1.23	-2.35	AAA-AAA	0.00	999	1.000	1.000	0.3045
233892_at	19.6	46.1	1.23	2.35	AAA-AAA	0.00	999	1.000	1.000	0.3941
234463_at	38.8	16.5	-1.23	-2.35	AAA-AAA	0.00	999	1.000	1.000	0.2612
232805_at	28.4	12.1	-1.23	-2.35	AAA-AAA	0.00	999	1.000	1.000	0.4862
233024_at	74.2	31.6	-1.23	-2.35	AAM-AAA	0.00	999	1.000	1.000	0.0554
232512_at	20.4	47.8	1.23	2.34	AAA-PAA	0.00	999	1.000	1.000	0.4269
240834_at	109.1	46.5	-1.23	-2.34	AAA-AAM	0.00	999	1.000	1.000	0.0052
1562860_at	12.6	5.4	-1.23	-2.34	AAA-AAA	0.00	999	1.000	1.000	0.5240
1561055_at	10.1	4.3	-1.23	-2.34	AAA-AAA	0.00	999	1.000	1.000	0.2996
244769_at	18.6	8.0	-1.23	-2.34	AAA-AAA	0.00	999	1.000	1.000	0.2895
1555504_at	2.2	5.3	1.23	2.34	AAA-AAA	0.00	999	1.000	1.000	0.2348

1557133_at	4.3	10.0	1.23	2.34	AAA-AAA	0.00	999	1.000	1.000	0.1119
210479_s_at	27.6	11.8	-1.23	-2.34	AMA-AAA	0.00	999	1.000	1.000	0.2001
234086_at	20.7	8.8	-1.23	-2.35	AAA-AAA	0.00	999	1.000	1.000	0.2957
229079_at	21.0	9.0	-1.23	-2.34	AAA-AAA	0.00	999	1.000	1.000	0.3621
240957_at	21.5	50.3	1.23	2.34	AAA-AAA	0.00	999	1.000	1.000	0.4315
241674_s_at	6.1	2.6	-1.23	-2.34	AAA-AAA	0.00	999	1.000	1.000	0.4747
1562044_at	89.5	38.2	-1.23	-2.34	AAA-AAA	0.00	999	1.000	1.000	0.1012
1563946_at	31.7	13.5	-1.23	-2.34	AAA-AAA	0.00	999	1.000	1.000	0.3153
37145_at	31.9	13.6	-1.23	-2.34	AAA-AAA	0.00	999	1.000	1.000	0.3212
220724_at	7.9	3.4	-1.23	-2.34	AAA-AAA	0.00	999	1.000	1.000	0.4714
220356_at	19.7	8.4	-1.23	-2.34	AAA-AAA	0.00	999	1.000	1.000	0.3635
234288_at	14.7	6.3	-1.23	-2.34	AAA-AAA	0.00	999	1.000	1.000	0.2659
204471_at	9.9	23.1	1.23	2.34	AAA-AAA	0.00	999	1.000	1.000	0.4536
229749_at	42.3	18.1	-1.23	-2.34	AAA-AAA	0.00	999	1.000	1.000	0.2456
1563204_at	22.1	9.4	-1.23	-2.34	AAA-AAA	0.00	999	1.000	1.000	0.0756
1561607_at	10.7	4.6	-1.23	-2.34	AAA-AAA	0.00	999	1.000	1.000	0.3498
1561409_at	15.4	6.6	-1.23	-2.34	AAA-AAA	0.00	999	1.000	1.000	0.1831
1561281_a_at	4.7	11.0	1.23	2.34	AAA-AAA	0.00	999	1.000	1.000	0.1448
1569891_at	13.7	32.0	1.23	2.34	AAA-AAA	0.00	999	1.000	1.000	0.3839
244885_at	39.2	16.7	-1.23	-2.34	AAA-AAA	0.00	999	1.000	1.000	0.2987
1556421_at	3.4	7.9	1.23	2.34	AAA-AAA	0.00	999	1.000	1.000	0.3362
220414_at	14.4	33.6	1.23	2.34	AAA-AAA	0.00	999	1.000	1.000	0.2167
227389_x_at	59.8	25.6	-1.23	-2.34	AAA-AAA	0.00	999	1.000	1.000	0.3721
211734_s_at	19.3	45.2	1.23	2.34	AAA-MAA	0.00	999	1.000	1.000	0.3730
204915_s_at	46.4	19.8	-1.23	-2.34	AAA-AAA	0.00	999	1.000	1.000	0.3609
236430_at	43.7	18.7	-1.23	-2.34	AAA-AAA	0.00	999	1.000	1.000	0.2301
238245_at	33.9	14.5	-1.23	-2.34	AAA-AAA	0.00	999	1.000	1.000	0.2513
205913_at	15.7	6.7	-1.23	-2.34	AAA-AAA	0.00	999	1.000	1.000	0.2507
231379_at	25.6	10.9	-1.23	-2.34	AAA-AAA	0.00	999	1.000	1.000	0.4942
1557444_at	21.1	9.0	-1.23	-2.34	AAA-AAA	0.00	999	1.000	1.000	0.0840
1556842_at	11.1	4.8	-1.23	-2.34	AAA-AAA	0.00	999	1.000	1.000	0.2274
220551_at	17.7	7.6	-1.23	-2.34	AAA-AAA	0.00	999	1.000	1.000	0.3229
230070_at	17.0	39.8	1.23	2.34	AAA-AAA	0.00	999	1.000	1.000	0.1228
233733_at	11.7	5.0	-1.23	-2.34	AAA-AAA	0.00	999	1.000	1.000	0.4479
231105_at	8.4	3.6	-1.23	-2.34	AAA-AAA	0.00	999	1.000	1.000	0.3316
1553798_a_at	18.5	43.3	1.23	2.34	AAP-APP	0.00	999	1.000	1.000	0.4583
229381_at	27.4	64.0	1.23	2.34	APA-APA	0.00	999	1.000	1.000	0.2067
238596_at	41.9	97.9	1.23	2.34	AAA-APP	0.00	999	1.000	1.000	0.1660
241594_at	29.6	12.7	-1.23	-2.34	AAA-AAA	0.00	999	1.000	1.000	0.3923
1562477_at	9.1	21.4	1.23	2.34	AAA-AAA	0.00	999	1.000	1.000	0.4311
1562691_at	7.0	16.3	1.23	2.34	AAA-AAA	0.00	999	1.000	1.000	0.2526
1552480_s_at	6.2	2.7	-1.23	-2.34	AAA-AAA	0.00	999	1.000	1.000	0.5278

1559075_s_at	27.3	11.7	-1.23	-2.34	AAA-AAA	0.00	999	1.000	1.000	0.3625
219656_at	26.3	61.5	1.23	2.34	AAA-AAA	0.00	999	1.000	1.000	0.1883
225618_at	48.0	20.5	-1.23	-2.34	AAA-AAA	0.00	999	1.000	1.000	0.3718
214996_at	63.7	27.3	-1.23	-2.34	AAA-AAA	0.00	999	1.000	1.000	0.0592
211648_at	9.9	4.2	-1.23	-2.34	AAA-AAA	0.00	999	1.000	1.000	0.2576
235136_at	77.0	33.0	-1.23	-2.34	AAA-AAA	0.00	999	1.000	1.000	0.3465
238374_at	15.8	6.8	-1.23	-2.34	AAA-AAA	0.00	999	1.000	1.000	0.5097
227690_at	24.4	10.4	-1.23	-2.34	AAA-AAA	0.00	999	1.000	1.000	0.3354
232936_at	8.0	3.4	-1.23	-2.34	AAA-AAA	0.00	999	1.000	1.000	0.0403
233181_at	57.6	24.7	-1.23	-2.34	AAA-AAA	0.00	999	1.000	1.000	0.4667
240805_at	15.2	6.5	-1.22	-2.34	AAA-AAA	0.00	999	1.000	1.000	0.4067
1562307_at	30.3	13.0	-1.22	-2.34	AAA-AAA	0.00	999	1.000	1.000	0.3907
1563907_at	5.9	2.5	-1.22	-2.34	AAA-AAA	0.00	999	1.000	1.000	0.0706
1561088_at	7.8	18.2	1.22	2.34	AAA-AAA	0.00	999	1.000	1.000	0.4041
244506_at	18.4	42.9	1.22	2.34	AAA-AAA	0.00	999	1.000	1.000	0.2497
1557057_a_at	20.3	8.7	-1.22	-2.34	AAA-AAA	0.00	999	1.000	1.000	0.3630
215847_at	9.6	4.1	-1.22	-2.34	AAA-AAA	0.00	999	1.000	1.000	0.3816
204134_at	55.4	23.7	-1.22	-2.34	AAA-AAA	0.00	999	1.000	1.000	0.2516
243509_at	24.8	57.9	1.22	2.33	AAA-APP	0.00	999	1.000	1.000	0.3361
223936_s_at	33.0	77.0	1.22	2.33	APP-PPP	0.00	999	1.000	1.000	0.1841
241539_at	15.6	6.7	-1.22	-2.34	AAA-AAA	0.00	999	1.000	1.000	0.4798
1554142_at	4.4	10.3	1.22	2.34	AAA-AAA	0.00	999	1.000	1.000	0.2877
1554508_at	4.5	1.9	-1.22	-2.34	AAA-AAA	0.00	999	1.000	1.000	0.2677
1558679_at	53.3	22.8	-1.22	-2.34	AAA-AAA	0.00	999	1.000	1.000	0.3024
214032_at	20.0	8.6	-1.22	-2.33	AAA-AAA	0.00	999	1.000	1.000	0.3058
217126_at	12.6	5.4	-1.22	-2.34	AAA-AAA	0.00	999	1.000	1.000	0.3470
204229_at	64.9	27.8	-1.22	-2.34	AAA-AAA	0.00	999	1.000	1.000	0.1785
229965_at	74.0	31.7	-1.22	-2.33	AAA-AAA	0.00	999	1.000	1.000	0.1471
1566694_at	9.4	4.0	-1.22	-2.33	AAA-AAA	0.00	999	1.000	1.000	0.5633
244740_at	31.5	73.4	1.22	2.33	AAA-AAA	0.00	999	1.000	1.000	0.1735
1552880_at	28.4	12.2	-1.22	-2.33	AAA-AAA	0.00	999	1.000	1.000	0.2879
211120_x_at	12.0	27.9	1.22	2.33	AAA-AAA	0.00	999	1.000	1.000	0.2760
216891_at	29.3	12.6	-1.22	-2.33	AAA-AAA	0.00	999	1.000	1.000	0.0564
236516_at	56.3	24.1	-1.22	-2.33	AAA-AAA	0.00	999	1.000	1.000	0.0392
236425_at	54.5	23.4	-1.22	-2.33	AAA-AAA	0.00	999	1.000	1.000	0.2161
238853_at	103.9	44.6	-1.22	-2.33	AAA-AAM	0.00	999	1.000	1.000	0.4365
1558827_a_at	19.8	8.5	-1.22	-2.33	AAA-AAA	0.00	999	1.000	1.000	0.2253
220601_at	22.8	9.8	-1.22	-2.33	AAA-AAA	0.00	999	1.000	1.000	0.4348
227537_s_at	12.2	5.2	-1.22	-2.33	AAA-AAA	0.00	999	1.000	1.000	0.0817
207581_s_at	8.8	3.8	-1.22	-2.33	AAA-AAA	0.00	999	1.000	1.000	0.1813
204499_at	34.8	14.9	-1.22	-2.33	AAA-AAA	0.00	999	1.000	1.000	0.3287
1563808_at	77.6	33.3	-1.22	-2.33	AAA-AAA	0.00	999	1.000	1.000	0.2905

1563137_at	17.5	7.5	-1.22	-2.33	AAA-AAA	0.00	999	1.000	1.000	0.0123
1567519_at	25.1	58.5	1.22	2.33	AAA-AAA	0.00	999	1.000	1.000	0.0039
207161_at	8.2	3.5	-1.22	-2.33	AAA-AAA	0.00	999	1.000	1.000	0.3449
225211_at	85.0	198.1	1.22	2.33	AAA-AAA	0.00	999	1.000	1.000	0.2552
223840_s_at	14.6	34.0	1.22	2.33	AAA-AAA	0.00	999	1.000	1.000	0.3765
224009_x_at	41.5	17.8	-1.22	-2.33	AAA-AAA	0.00	999	1.000	1.000	0.1330
213784_at	154.1	66.2	-1.22	-2.33	AAA-AAA	0.00	999	1.000	1.000	0.1656
216740_at	29.3	12.6	-1.22	-2.33	AAA-AAA	0.00	999	1.000	1.000	0.1776
237704_at	11.3	4.9	-1.22	-2.33	AAA-AAA	0.00	999	1.000	1.000	0.2661
231089_at	13.0	5.6	-1.22	-2.33	AAA-AAA	0.00	999	1.000	1.000	0.0602
242484_at	15.8	36.9	1.22	2.33	AAA-AAA	0.00	999	1.000	1.000	0.2497
1564567_at	6.5	2.8	-1.22	-2.33	AAA-AAA	0.00	999	1.000	1.000	0.3185
1557761_s_at	9.1	21.1	1.22	2.33	AAA-AAA	0.00	999	1.000	1.000	0.4645
1556266_a_at	25.6	11.0	-1.22	-2.33	AAA-AAA	0.00	999	1.000	1.000	0.3389
220229_s_at	20.1	46.8	1.22	2.33	AAA-AAA	0.00	999	1.000	1.000	0.3914
220222_at	8.1	3.5	-1.22	-2.33	AAA-AAA	0.00	999	1.000	1.000	0.1709
207260_at	9.0	3.9	-1.22	-2.33	AAA-AAA	0.00	999	1.000	1.000	0.0051
214208_at	24.4	10.5	-1.22	-2.33	AAA-AAA	0.00	999	1.000	1.000	0.1983
211816_x_at	18.7	8.0	-1.22	-2.33	AAA-AAA	0.00	999	1.000	1.000	0.2930
217611_at	10.2	4.4	-1.22	-2.33	AAA-AAA	0.00	999	1.000	1.000	0.0975
238180_at	22.8	9.8	-1.22	-2.33	AAA-AAA	0.00	999	1.000	1.000	0.1201
238066_at	54.1	23.3	-1.22	-2.33	AAA-AAA	0.00	999	1.000	1.000	0.3687
230856_at	29.5	12.7	-1.22	-2.33	AAA-AAA	0.00	999	1.000	1.000	0.0727
231309_at	21.8	50.7	1.22	2.33	AAA-AAA	0.00	999	1.000	1.000	0.2764
1562420_at	8.7	3.7	-1.22	-2.33	AAA-AAA	0.00	999	1.000	1.000	0.5493
1562058_at	45.9	19.8	-1.22	-2.33	AAA-AAA	0.00	999	1.000	1.000	0.0685
1561002_at	26.5	11.4	-1.22	-2.33	AAA-AAA	0.00	999	1.000	1.000	0.4270
1554871_at	7.0	16.4	1.22	2.33	AAA-AAA	0.00	999	1.000	1.000	0.3288
1553279_at	104.0	44.7	-1.22	-2.33	AAA-AAA	0.00	999	1.000	1.000	0.0174
1558819_at	8.0	18.6	1.22	2.33	AAA-AAA	0.00	999	1.000	1.000	0.4790
213862_at	9.3	21.6	1.22	2.33	AAA-AAA	0.00	999	1.000	1.000	0.3589
210326_at	45.5	19.5	-1.22	-2.33	AAA-AAA	0.00	999	1.000	1.000	0.0965
237815_at	11.4	4.9	-1.22	-2.33	AAA-AAA	0.00	999	1.000	1.000	0.3431
240176_at	46.8	108.9	1.22	2.33	AAA-AAA	0.00	999	1.000	1.000	0.3987
243585_at	11.7	5.0	-1.22	-2.33	AAA-AAA	0.00	999	1.000	1.000	0.5185
1563553_at	14.3	6.1	-1.22	-2.33	AAA-AAA	0.00	999	1.000	1.000	0.4145
1563679_at	8.9	20.7	1.22	2.33	AAA-AAA	0.00	999	1.000	1.000	0.4188
1553619_a_at	27.2	11.7	-1.22	-2.33	AAA-AAA	0.00	999	1.000	1.000	0.5018
1552615_at	258.0	111.0	-1.22	-2.32	AAA-AAA	0.00	999	1.000	1.000	0.1763
222171_s_at	13.9	6.0	-1.22	-2.32	AAA-AAA	0.00	999	1.000	1.000	0.2753
220384_at	3.6	8.3	1.22	2.32	AAA-AAA	0.00	999	1.000	1.000	0.1481
208294_x_at	11.0	4.7	-1.22	-2.33	AAA-AAA	0.00	999	1.000	1.000	0.1055

228514_at	17.9	41.6	1.22	2.33	AAA-AAA	0.00	999	1.000	1.000	0.1788
228360_at	101.0	43.5	-1.22	-2.33	AAA-AAA	0.00	999	1.000	1.000	0.1801
229331_at	9.9	4.3	-1.22	-2.33	AAA-AAA	0.00	999	1.000	1.000	0.4614
1555425_x_at	16.2	37.6	1.22	2.32	AAA-AAA	0.00	999	1.000	1.000	0.2075
220051_at	57.5	24.8	-1.22	-2.32	AAA-AAA	0.00	999	1.000	1.000	0.1373
211908_x_at	15.5	6.7	-1.22	-2.32	AAA-AAA	0.00	999	1.000	1.000	0.3419
229176_at	30.7	71.2	1.22	2.32	AAA-AAA	0.00	999	1.000	1.000	0.2197
231421_at	5.4	12.6	1.22	2.32	AAA-AAA	0.00	999	1.000	1.000	0.0889
231433_at	49.6	21.4	-1.22	-2.32	AAA-AAA	0.00	999	1.000	1.000	0.4743
232109_at	33.5	14.4	-1.22	-2.32	AAA-AAA	0.00	999	1.000	1.000	0.2202
242808_at	27.7	64.3	1.22	2.32	AAA-AAA	0.00	999	1.000	1.000	0.0551
1560548_at	4.2	9.6	1.22	2.32	AAA-AAA	0.00	999	1.000	1.000	0.2983
1561509_at	21.3	9.2	-1.22	-2.32	AAA-AAA	0.00	999	1.000	1.000	0.2603
216715_at	40.9	17.6	-1.22	-2.32	AAA-AAA	0.00	999	1.000	1.000	0.5677
233962_at	37.4	16.1	-1.22	-2.32	AAA-AAA	0.00	999	1.000	1.000	0.3362
227730_at	120.3	51.8	-1.22	-2.32	AAA-AAA	0.00	999	1.000	1.000	0.2908
1557798_at	16.7	38.7	1.21	2.32	AAA-AAP	0.00	999	1.000	1.000	0.2945
203998_s_at	26.8	62.2	1.21	2.32	AAA-PPP	0.00	999	1.000	1.000	0.0767
243396_at	34.4	14.8	-1.21	-2.32	AAA-AAA	0.00	999	1.000	1.000	0.2217
1563346_at	68.4	29.5	-1.21	-2.32	AAA-AAA	0.00	999	1.000	1.000	0.0399
215590_x_at	22.0	9.5	-1.21	-2.32	AAA-AAA	0.00	999	1.000	1.000	0.2763
216427_at	8.4	19.5	1.21	2.32	AAA-AAA	0.00	999	1.000	1.000	0.4326
216298_at	5.7	2.5	-1.21	-2.32	AAA-AAA	0.00	999	1.000	1.000	0.3930
237078_at	16.5	7.1	-1.21	-2.32	AAA-AAA	0.00	999	1.000	1.000	0.2196
204261_s_at	40.1	93.0	1.21	2.32	AAA-PAA	0.00	999	1.000	1.000	0.2404
244109_at	7.6	3.3	-1.21	-2.32	AAA-AAA	0.00	999	1.000	1.000	0.1627
1569334_at	3.9	9.1	1.21	2.32	AAA-AAA	0.00	999	1.000	1.000	0.3797
1569182_at	11.5	5.0	-1.21	-2.32	AAA-AAA	0.00	999	1.000	1.000	0.2490
1570314_at	51.6	22.3	-1.21	-2.32	AAM-AAA	0.00	999	1.000	1.000	0.3092
220984_s_at	15.3	6.6	-1.21	-2.32	AAA-AAA	0.00	999	1.000	1.000	0.1393
206606_at	11.7	5.0	-1.21	-2.32	AAA-AAA	0.00	999	1.000	1.000	0.1606
224408_at	32.3	74.9	1.21	2.32	AAA-AAA	0.00	999	1.000	1.000	0.0560
223779_at	19.6	45.5	1.21	2.32	AAA-AAA	0.00	999	1.000	1.000	0.0173
236587_at	18.6	43.1	1.21	2.32	AAA-AAA	0.00	999	1.000	1.000	0.2889
234847_at	3.5	8.2	1.21	2.32	AAA-AAA	0.00	999	1.000	1.000	0.2388
234648_s_at	20.1	8.7	-1.21	-2.32	AAA-AAA	0.00	999	1.000	1.000	0.2298
237775_x_at	5.2	12.1	1.21	2.32	AAA-AAA	0.00	999	1.000	1.000	0.2135
239942_at	23.4	54.2	1.21	2.32	AAA-PPP	0.00	999	1.000	1.000	0.3962
1562112_at	9.8	4.2	-1.21	-2.32	AAA-AAA	0.00	999	1.000	1.000	0.4878
1564017_at	12.9	5.6	-1.21	-2.32	AAA-AAA	0.00	999	1.000	1.000	0.4599
1563062_at	10.6	24.5	1.21	2.32	AAA-AAA	0.00	999	1.000	1.000	0.3871
244836_at	29.0	12.5	-1.21	-2.32	AMA-AAA	0.00	999	1.000	1.000	0.4200

217194_at	6.5	15.2	1.21	2.32	AAA-AAA	0.00	999	1.000	1.000	0.4059
215315_at	5.0	11.5	1.21	2.32	AAA-AAA	0.00	999	1.000	1.000	0.4136
230932_at	34.0	14.7	-1.21	-2.32	AAA-AAA	0.00	999	1.000	1.000	0.2785
227958_s_at	16.4	7.1	-1.21	-2.32	AAA-AAA	0.00	999	1.000	1.000	0.3962
240753_at	6.2	2.7	-1.21	-2.31	AAA-AAA	0.00	999	1.000	1.000	0.2537
243237_at	15.6	36.0	1.21	2.32	AAA-AAA	0.00	999	1.000	1.000	0.3462
1554589_at	76.0	32.8	-1.21	-2.32	AAA-AAA	0.00	999	1.000	1.000	0.1468
1558193_at	12.3	28.4	1.21	2.32	AAA-AAA	0.00	999	1.000	1.000	0.0040
206842_at	21.8	9.4	-1.21	-2.32	AAA-AAA	0.00	999	1.000	1.000	0.3387
217015_at	4.9	2.1	-1.21	-2.32	AAA-AAA	0.00	999	1.000	1.000	0.4029
215573_at	10.7	4.6	-1.21	-2.32	AAA-AAA	0.00	999	1.000	1.000	0.2648
225762_x_at	39.9	92.4	1.21	2.31	AAA-AAP	0.00	999	1.000	1.000	0.4245
241547_at	9.7	22.5	1.21	2.31	AAA-AAA	0.00	999	1.000	1.000	0.4737
1560775_at	7.3	3.2	-1.21	-2.31	AAA-AAA	0.00	999	1.000	1.000	0.4803
1566825_at	6.6	15.3	1.21	2.31	AAA-AAA	0.00	999	1.000	1.000	0.2260
213914_s_at	8.9	20.5	1.21	2.31	AAA-AAA	0.00	999	1.000	1.000	0.3387
214652_at	7.0	3.0	-1.21	-2.31	AAA-AAA	0.00	999	1.000	1.000	0.3326
228015_s_at	29.5	12.8	-1.21	-2.31	AAA-AAA	0.00	999	1.000	1.000	0.4408
233765_at	25.1	10.9	-1.21	-2.31	AAA-AAA	0.00	999	1.000	1.000	0.4520
233481_at	63.8	27.6	-1.21	-2.31	AAA-MAA	0.00	999	1.000	1.000	0.2836
1556465_at	14.2	32.7	1.21	2.31	AAA-APP	0.00	999	1.000	1.000	0.4100
219209_at	18.9	43.6	1.21	2.31	AAA-PPP	0.00	999	1.000	1.000	0.2079
1562390_at	8.0	3.5	-1.21	-2.31	AAA-AAA	0.00	999	1.000	1.000	0.0777
1563273_at	57.7	25.0	-1.21	-2.31	AAA-AAA	0.00	999	1.000	1.000	0.0168
1569617_at	16.3	7.1	-1.21	-2.31	AAA-AAA	0.00	999	1.000	1.000	0.3572
1570625_at	23.9	10.3	-1.21	-2.31	AAA-AAA	0.00	999	1.000	1.000	0.1468
1565937_a_at	4.1	1.8	-1.21	-2.31	AAA-AAA	0.00	999	1.000	1.000	0.3958
1553267_a_at	19.5	45.0	1.21	2.31	AAA-AAA	0.00	999	1.000	1.000	0.1257
1558552_s_at	11.8	5.1	-1.21	-2.31	AAA-AAA	0.00	999	1.000	1.000	0.5504
224533_s_at	7.5	3.2	-1.21	-2.31	AAA-AAA	0.00	999	1.000	1.000	0.3891
224238_at	4.8	11.2	1.21	2.31	AAA-AAA	0.00	999	1.000	1.000	0.1776
211520_s_at	12.5	5.4	-1.21	-2.31	AAA-AAA	0.00	999	1.000	1.000	0.3016
218532_s_at	64.5	27.9	-1.21	-2.31	AAA-AAM	0.00	999	1.000	1.000	0.1136
234642_at	16.5	7.1	-1.21	-2.31	AAA-AAA	0.00	999	1.000	1.000	0.2036
204445_s_at	21.5	49.7	1.21	2.31	AAA-AAA	0.00	999	1.000	1.000	0.2338
230549_at	14.4	6.2	-1.21	-2.31	AAA-AAA	0.00	999	1.000	1.000	0.3675
231658_x_at	77.1	178.0	1.21	2.31	AAM-PPA	0.00	999	1.000	1.000	0.2049
219246_s_at	85.3	196.9	1.21	2.31	AAM-PPP	0.00	999	1.000	1.000	0.1236
242193_at	40.4	17.5	-1.21	-2.31	AAA-AAA	0.00	999	1.000	1.000	0.1082
1562288_at	9.9	4.3	-1.21	-2.31	AAA-AAA	0.00	999	1.000	1.000	0.5297
1560910_at	24.1	55.6	1.21	2.31	AAA-AAA	0.00	999	1.000	1.000	0.4479
1560118_at	12.2	28.2	1.21	2.31	AAA-AMA	0.00	999	1.000	1.000	0.3242

216287_at	17.1	7.4	-1.21	-2.31	AAA-AAA	0.00	999	1.000	1.000	0.4547
241445_at	50.9	22.0	-1.21	-2.31	AAA-AAA	0.00	999	1.000	1.000	0.1991
1561175_at	42.1	18.3	-1.21	-2.31	AAA-AAA	0.00	999	1.000	1.000	0.5591
1566703_at	4.3	9.9	1.21	2.31	AAA-AAA	0.00	999	1.000	1.000	0.2506
1558934_a_at	37.9	16.4	-1.21	-2.31	AAA-AAA	0.00	999	1.000	1.000	0.2087
208165_s_at	11.3	4.9	-1.21	-2.31	AAA-AAA	0.00	999	1.000	1.000	0.3725
46142_at	12.9	5.6	-1.21	-2.31	AAA-AAA	0.00	999	1.000	1.000	0.2004
211138_s_at	32.8	14.2	-1.21	-2.31	AAA-AAA	0.00	999	1.000	1.000	0.1269
209603_at	70.1	30.4	-1.21	-2.31	AAA-AAA	0.00	999	1.000	1.000	0.3100
234629_at	5.1	2.2	-1.21	-2.31	AAA-AAA	0.00	999	1.000	1.000	0.0667
237500_at	16.6	7.2	-1.21	-2.31	AAA-AAA	0.00	999	1.000	1.000	0.2442
242186_x_at	15.6	6.7	-1.21	-2.31	AAA-AAA	0.00	999	1.000	1.000	0.4355
203066_at	35.5	15.4	-1.21	-2.31	AAA-AAA	0.00	999	1.000	1.000	0.1278
1558124_at	54.7	23.7	-1.21	-2.31	AAA-AAA	0.00	999	1.000	1.000	0.3562
1559203_s_at	10.0	23.0	1.21	2.31	AAA-AAA	0.00	999	1.000	1.000	0.2167
220017_x_at	77.7	33.7	-1.21	-2.31	AAA-AAA	0.00	999	1.000	1.000	0.2069
220555_s_at	31.2	13.5	-1.21	-2.31	AAA-AAA	0.00	999	1.000	1.000	0.0935
241058_at	17.5	7.6	-1.21	-2.31	AAA-AAA	0.00	999	1.000	1.000	0.3544
1563340_at	33.5	14.5	-1.21	-2.31	AAA-AAA	0.00	999	1.000	1.000	0.3646
1560838_at	8.5	19.5	1.21	2.31	AAA-AAA	0.00	999	1.000	1.000	0.4700
1560241_at	47.4	20.6	-1.21	-2.31	AAA-AAA	0.00	999	1.000	1.000	0.2148
1566805_at	6.8	2.9	-1.21	-2.31	AAA-AAA	0.00	999	1.000	1.000	0.5014
1566140_at	8.2	18.9	1.21	2.31	AAA-AAA	0.00	999	1.000	1.000	0.1692
244451_x_at	18.6	42.9	1.21	2.31	AAA-AAA	0.00	999	1.000	1.000	0.2081
244588_at	35.7	15.5	-1.21	-2.31	AAA-AAA	0.00	999	1.000	1.000	0.5227
1559124_at	5.2	2.3	-1.21	-2.31	AAA-AAA	0.00	999	1.000	1.000	0.4084
221072_at	56.4	24.4	-1.21	-2.31	AAA-AAA	0.00	999	1.000	1.000	0.1719
210335_at	39.7	17.2	-1.21	-2.31	AAA-AAA	0.00	999	1.000	1.000	0.1955
216089_at	6.3	14.5	1.21	2.31	AAA-AAA	0.00	999	1.000	1.000	0.3927
204848_x_at	33.8	14.7	-1.21	-2.31	AAA-AAA	0.00	999	1.000	1.000	0.3566
230765_at	15.6	6.8	-1.21	-2.31	AAA-AAA	0.00	999	1.000	1.000	0.2590
231405_at	4.6	10.7	1.21	2.31	AAA-AAA	0.00	999	1.000	1.000	0.3653
1562996_at	13.7	31.5	1.20	2.30	AAA-AAP	0.00	999	1.000	1.000	0.3607
201116_s_at	130.3	300.1	1.20	2.30	AAA-AAA	0.00	999	1.000	1.000	0.2902
1566081_at	14.6	6.4	-1.20	-2.30	AAA-AAA	0.00	999	1.000	1.000	0.1317
244564_at	23.3	10.1	-1.20	-2.30	AAA-AAA	0.00	999	1.000	1.000	0.1302
213665_at	18.2	7.9	-1.20	-2.30	AAA-AAA	0.00	999	1.000	1.000	0.3736
213160_at	7.9	3.4	-1.20	-2.30	AAA-AAA	0.00	999	1.000	1.000	0.2947
236653_at	19.8	45.5	1.20	2.30	AAA-AAA	0.00	999	1.000	1.000	0.5061
234752_x_at	13.2	5.7	-1.20	-2.30	AAA-AAA	0.00	999	1.000	1.000	0.4981
231724_at	29.0	66.9	1.20	2.30	AAA-AAM	0.00	999	1.000	1.000	0.1718
222026_at	62.2	143.1	1.20	2.30	AAA-PPP	0.00	999	1.000	1.000	0.1227

242386_x_at	87.8	38.2	-1.20	-2.30	AAA-AAA	0.00	999	1.000	1.000	0.4713
1570239_a_at	89.2	38.7	-1.20	-2.30	AAA-AAA	0.00	999	1.000	1.000	0.3425
1570131_at	38.7	16.8	-1.20	-2.30	AAA-AAA	0.00	999	1.000	1.000	0.3611
1553622_a_at	48.5	21.1	-1.20	-2.30	AAA-AAA	0.00	999	1.000	1.000	0.3682
1559390_a_at	14.2	6.2	-1.20	-2.30	AAA-AAA	0.00	999	1.000	1.000	0.3164
1556172_at	24.7	10.7	-1.20	-2.30	AAA-AAA	0.00	999	1.000	1.000	0.5630
1557468_at	16.8	7.3	-1.20	-2.30	AAA-AAA	0.00	999	1.000	1.000	0.3793
226698_at	203.1	88.2	-1.20	-2.30	AAA-AAA	0.00	999	1.000	1.000	0.0727
227646_at	44.2	19.2	-1.20	-2.30	AAA-AAA	0.00	999	1.000	1.000	0.1320
233267_at	8.1	18.7	1.20	2.30	AAA-AAA	0.00	999	1.000	1.000	0.3047
231124_x_at	61.4	26.7	-1.20	-2.30	AAA-AAA	0.00	999	1.000	1.000	0.2651
233799_at	5.6	12.8	1.20	2.30	APA-PAA	0.00	999	1.000	1.000	0.5974
230516_at	33.5	77.0	1.20	2.30	AAA-APP	0.00	999	1.000	1.000	0.3101
213610_s_at	453.3	1042.5	1.20	2.30	PPP-PPP	0.00	999	1.000	1.000	0.1883
241659_at	9.7	4.2	-1.20	-2.30	AAA-AAA	0.00	999	1.000	1.000	0.2352
1568908_at	12.6	5.5	-1.20	-2.30	AAA-AAA	0.00	999	1.000	1.000	0.3284
208337_s_at	23.8	10.3	-1.20	-2.30	AAA-AAA	0.00	999	1.000	1.000	0.4526
226863_at	18.0	7.8	-1.20	-2.30	AAA-AAA	0.00	999	1.000	1.000	0.4085
205826_at	44.4	19.3	-1.20	-2.30	AAA-AAA	0.00	999	1.000	1.000	0.3928
238161_at	23.9	55.1	1.20	2.30	APA-APP	0.00	999	1.000	1.000	0.2925
240575_at	6.8	3.0	-1.20	-2.30	AAA-AAA	0.00	999	1.000	1.000	0.3058
1565673_at	5.5	2.4	-1.20	-2.30	AAA-AAA	0.00	999	1.000	1.000	0.4084
1553930_at	10.1	4.4	-1.20	-2.30	AAA-AAA	0.00	999	1.000	1.000	0.6269
1570397_x_at	7.5	3.3	-1.20	-2.30	AAA-AAA	0.00	999	1.000	1.000	0.2172
207184_at	100.0	43.5	-1.20	-2.30	AAA-AAA	0.00	999	1.000	1.000	0.0994
210085_s_at	8.7	20.0	1.20	2.30	AAA-AAA	0.00	999	1.000	1.000	0.2014
217417_at	7.4	17.1	1.20	2.30	AAA-AAA	0.00	999	1.000	1.000	0.4275
205114_s_at	11.9	27.4	1.20	2.30	AAA-AAA	0.00	999	1.000	1.000	0.0358
1570318_at	6.0	13.8	1.20	2.30	AAA-AAA	0.00	999	1.000	1.000	0.4957
222347_at	17.3	39.6	1.20	2.30	AAA-AAA	0.00	999	1.000	1.000	0.3503
38964_r_at	147.1	64.1	-1.20	-2.30	AAA-AAA	0.00	999	1.000	1.000	0.1892
223990_at	36.6	15.9	-1.20	-2.30	AAA-AAA	0.00	999	1.000	1.000	0.2548
211700_s_at	26.0	59.8	1.20	2.30	AAA-AAA	0.00	999	1.000	1.000	0.1095
209612_s_at	11.0	4.8	-1.20	-2.30	AAA-AAA	0.00	999	1.000	1.000	0.4576
235616_at	5.8	13.4	1.20	2.30	AAA-AAA	0.00	999	1.000	1.000	0.2831
238407_at	50.8	22.1	-1.20	-2.30	AAA-AAA	0.00	999	1.000	1.000	0.4551
239354_at	25.1	57.7	1.20	2.30	AAA-AAA	0.00	999	1.000	1.000	0.2412
237382_at	25.4	11.1	-1.20	-2.30	AAA-AAA	0.00	999	1.000	1.000	0.2227
232704_s_at	33.2	76.3	1.20	2.30	APP-PPP	0.00	999	1.000	1.000	0.3537
1552913_at	1.9	4.3	1.20	2.30	AAA-AAA	0.00	999	1.000	1.000	0.4510
219908_at	8.3	19.1	1.20	2.30	AAA-AAA	0.00	999	1.000	1.000	0.4780
207353_s_at	33.1	14.4	-1.20	-2.30	AAA-AAA	0.00	999	1.000	1.000	0.2699

215108_x_at	7.0	16.1	1.20	2.30	AAA-AAA	0.00	999	1.000	1.000	0.2385
219411_at	58.6	25.5	-1.20	-2.30	MAA-AAA	0.00	999	1.000	1.000	0.3361
218778_x_at	57.1	24.9	-1.20	-2.30	AAA-AAA	0.00	999	1.000	1.000	0.3596
215686_x_at	15.0	6.6	-1.20	-2.30	AAA-AAA	0.00	999	1.000	1.000	0.2866
215396_at	12.1	5.3	-1.20	-2.30	AAA-AAA	0.00	999	1.000	1.000	0.4686
234155_at	5.1	2.2	-1.20	-2.30	AAA-AAA	0.00	999	1.000	1.000	0.0554
238754_at	15.3	6.7	-1.20	-2.30	AAA-AAA	0.00	999	1.000	1.000	0.0271
231260_at	10.6	4.6	-1.20	-2.30	AAA-AAA	0.00	999	1.000	1.000	0.3371
1568655_a_at	25.9	11.3	-1.20	-2.29	AAA-AAA	0.00	999	1.000	1.000	0.4979
1570320_at	7.5	17.1	1.20	2.29	AAA-AAA	0.00	999	1.000	1.000	0.4987
1564499_at	6.1	14.1	1.20	2.29	AAA-AAA	0.00	999	1.000	1.000	0.5215
220834_at	9.6	22.1	1.20	2.29	AAA-AAA	0.00	999	1.000	1.000	0.3437
224029_x_at	27.3	11.9	-1.20	-2.29	AAA-AAA	0.00	999	1.000	1.000	0.2138
214547_at	8.6	3.8	-1.20	-2.30	AAA-AAA	0.00	999	1.000	1.000	0.2228
208582_s_at	5.9	2.6	-1.20	-2.30	AAA-AAA	0.00	999	1.000	1.000	0.2448
208529_at	2.9	6.7	1.20	2.29	AAA-AAA	0.00	999	1.000	1.000	0.2858
215677_s_at	10.2	23.3	1.20	2.29	AAA-AAA	0.00	999	1.000	1.000	0.1173
234224_at	12.4	5.4	-1.20	-2.29	AAA-AAA	0.00	999	1.000	1.000	0.1806
234874_at	59.4	25.9	-1.20	-2.29	AAA-AAA	0.00	999	1.000	1.000	0.2936
234563_at	11.5	5.0	-1.20	-2.29	AAA-AAA	0.00	999	1.000	1.000	0.5121
205988_at	51.4	22.4	-1.20	-2.30	AAA-AAA	0.00	999	1.000	1.000	0.1896
228417_at	34.2	14.9	-1.20	-2.29	AAA-AAA	0.00	999	1.000	1.000	0.3501
228658_at	80.4	35.0	-1.20	-2.29	AAA-AAA	0.00	999	1.000	1.000	0.2602
205433_at	20.9	48.0	1.20	2.30	AAA-AAA	0.00	999	1.000	1.000	0.0884
243332_at	15.5	35.6	1.20	2.29	AAA-AAP	0.00	999	1.000	1.000	0.1257
1563210_at	34.9	80.0	1.20	2.29	AAA-AAA	0.00	999	1.000	1.000	0.4833
1568635_at	18.5	8.1	-1.20	-2.29	AAA-AAA	0.00	999	1.000	1.000	0.2007
AFFX-r2-Bs-dap-M_at	2.1	4.7	1.20	2.29	AAA-AAA	0.00	999	1.000	1.000	0.3278
1565820_x_at	3.6	8.3	1.20	2.29	AAA-AAA	0.00	999	1.000	1.000	0.2067
1553917_at	16.3	37.4	1.20	2.29	AAA-AAA	0.00	999	1.000	1.000	0.1439
1555169_at	18.1	7.9	-1.20	-2.29	AAA-AAA	0.00	999	1.000	1.000	0.3782
1555189_a_at	33.0	14.4	-1.20	-2.29	AAA-AAA	0.00	999	1.000	1.000	0.3024
1552526_at	8.2	3.6	-1.20	-2.29	AAA-AAA	0.00	999	1.000	1.000	0.1449
1557702_at	11.5	26.3	1.20	2.29	AAA-AAA	0.00	999	1.000	1.000	0.2651
224142_s_at	17.1	7.5	-1.20	-2.29	AAA-AAA	0.00	999	1.000	1.000	0.3797
210789_x_at	132.2	57.7	-1.20	-2.29	AAA-AAA	0.00	999	1.000	1.000	0.0863
217021_at	5.9	13.5	1.20	2.29	AAA-AAA	0.00	999	1.000	1.000	0.3692
216725_at	17.5	7.6	-1.20	-2.29	AAA-AAA	0.00	999	1.000	1.000	0.4058
205413_at	1.9	4.3	1.20	2.29	AAA-AAA	0.00	999	1.000	1.000	0.3421
231377_at	22.2	9.7	-1.20	-2.29	AAA-AAA	0.00	999	1.000	1.000	0.3317
232352_at	42.2	96.7	1.20	2.29	AAA-AAA	0.00	999	1.000	1.000	0.4082
232303_at	12.3	28.2	1.20	2.29	AAM-AAA	0.00	999	1.000	1.000	0.4043

1556037_s_at	112.3	257.2	1.20	2.29	APP-PPP	0.00	999	1.000	1.000	0.1672
244044_at	14.1	32.2	1.20	2.29	AAA-AAA	0.00	999	1.000	1.000	0.3340
241037_at	13.7	31.4	1.20	2.29	AAA-AAA	0.00	999	1.000	1.000	0.0700
1562187_at	20.5	8.9	-1.20	-2.29	AAA-AAA	0.00	999	1.000	1.000	0.4172
1561355_at	34.9	15.2	-1.20	-2.29	AAA-AAA	0.00	999	1.000	1.000	0.5144
1554981_at	18.0	7.9	-1.20	-2.29	AAA-AAA	0.00	999	1.000	1.000	0.5226
1555268_a_at	13.1	29.9	1.20	2.29	AAA-AAA	0.00	999	1.000	1.000	0.5306
1554700_at	6.7	2.9	-1.20	-2.29	AAA-AAA	0.00	999	1.000	1.000	0.4670
1552558_a_at	166.1	72.5	-1.20	-2.29	AAA-AAA	0.00	999	1.000	1.000	0.0829
1552691_at	8.4	3.7	-1.20	-2.29	AAA-AAA	0.00	999	1.000	1.000	0.5595
1558473_at	14.9	6.5	-1.20	-2.29	AAA-AAA	0.00	999	1.000	1.000	0.5079
1556479_at	50.6	115.8	1.20	2.29	AAA-AAA	0.00	999	1.000	1.000	0.1952
220299_at	13.3	5.8	-1.20	-2.29	AAA-AAA	0.00	999	1.000	1.000	0.5014
206671_at	58.6	25.6	-1.20	-2.29	AAA-AAA	0.00	999	1.000	1.000	0.2294
216692_at	111.5	48.7	-1.20	-2.29	AAA-AAA	0.00	999	1.000	1.000	0.0640
233243_at	28.2	64.7	1.20	2.29	AAA-AAA	0.00	999	1.000	1.000	0.0567
231905_at	71.1	31.0	-1.20	-2.29	AAA-AAA	0.00	999	1.000	1.000	0.3227
244058_at	26.6	11.6	-1.20	-2.29	AAA-AAA	0.00	999	1.000	1.000	0.4657
241128_at	9.6	4.2	-1.20	-2.29	AAA-AAA	0.00	999	1.000	1.000	0.4110
1562392_at	44.3	19.3	-1.20	-2.29	AAA-AAA	0.00	999	1.000	1.000	0.2847
1561555_at	37.5	16.4	-1.20	-2.29	AAA-AAA	0.00	999	1.000	1.000	0.3528
244421_at	13.3	30.3	1.20	2.29	AAA-AAA	0.00	999	1.000	1.000	0.2149
1557921_s_at	2.6	6.0	1.20	2.29	AAA-AAA	0.00	999	1.000	1.000	0.5232
1555738_at	7.1	16.2	1.20	2.29	AAA-AAA	0.00	999	1.000	1.000	0.2155
207763_at	11.2	25.5	1.20	2.29	AAA-AAA	0.00	999	1.000	1.000	0.5223
38691_s_at	80.2	35.0	-1.20	-2.29	AAA-AAA	0.00	999	1.000	1.000	0.1860
227053_at	37.3	16.3	-1.20	-2.29	AAA-AAA	0.00	999	1.000	1.000	0.3486
217218_at	17.3	39.7	1.20	2.29	AAA-AAA	0.00	999	1.000	1.000	0.3121
218872_at	18.4	8.1	-1.20	-2.29	AAA-AAA	0.00	999	1.000	1.000	0.0541
206018_at	23.0	52.7	1.20	2.29	AAA-AAA	0.00	999	1.000	1.000	0.2852
206503_x_at	81.4	186.3	1.19	2.29	AAA-AMP	0.00	999	1.000	1.000	0.2647
205607_s_at	77.6	177.4	1.19	2.29	AAA-PPM	0.00	999	1.000	1.000	0.2350
243886_at	11.7	26.9	1.19	2.29	AAA-AAA	0.00	999	1.000	1.000	0.0556
1565693_at	28.0	12.3	-1.19	-2.29	AAA-AAA	0.00	999	1.000	1.000	0.4999
220573_at	18.6	8.1	-1.19	-2.29	AAA-AAA	0.00	999	1.000	1.000	0.0290
223918_at	10.4	4.6	-1.19	-2.29	AAA-AAA	0.00	999	1.000	1.000	0.4923
238758_at	53.9	23.6	-1.19	-2.29	AAA-AAA	0.00	999	1.000	1.000	0.3634
237929_at	6.9	15.8	1.19	2.29	AAA-AAA	0.00	999	1.000	1.000	0.4035
232505_at	11.4	26.2	1.19	2.29	AAA-AAA	0.00	999	1.000	1.000	0.4721
233682_at	6.4	14.5	1.19	2.29	AAA-AAA	0.00	999	1.000	1.000	0.5361
241033_at	16.5	37.7	1.19	2.29	PAA-PPA	0.00	999	1.000	1.000	0.3324
1558577_at	19.4	8.5	-1.19	-2.29	AAA-AAA	0.00	999	1.000	1.000	0.4087

1557056_at	46.3	20.3	-1.19	-2.29	AAA-AAA	0.00	999	1.000	1.000	0.2076
209442_x_at	53.2	23.3	-1.19	-2.29	AAA-AAA	0.00	999	1.000	1.000	0.1984
234371_at	33.0	14.4	-1.19	-2.29	AAA-AAA	0.00	999	1.000	1.000	0.3351
206058_at	37.6	16.4	-1.19	-2.29	AAA-AAA	0.00	999	1.000	1.000	0.2614
229118_at	31.5	13.8	-1.19	-2.29	AAA-AAA	0.00	999	1.000	1.000	0.2561
231551_at	6.9	3.0	-1.19	-2.29	AAA-AAA	0.00	999	1.000	1.000	0.2194
1558549_s_at	28.4	64.9	1.19	2.29	AAA-PPA	0.00	999	1.000	1.000	0.3401
203841_x_at	13.0	29.6	1.19	2.28	AAA-AAA	0.00	999	1.000	1.000	0.4973
241712_at	15.4	6.8	-1.19	-2.28	AAA-AAA	0.00	999	1.000	1.000	0.4594
242309_at	17.0	38.8	1.19	2.29	AAA-AAA	0.00	999	1.000	1.000	0.1987
1568804_at	17.4	7.6	-1.19	-2.28	AAA-AAA	0.00	999	1.000	1.000	0.2440
1555985_at	17.7	7.8	-1.19	-2.28	AAA-AAA	0.00	999	1.000	1.000	0.2206
208172_s_at	43.6	19.1	-1.19	-2.29	AAA-AAA	0.00	999	1.000	1.000	0.2253
207893_at	6.0	2.6	-1.19	-2.28	AAA-AAA	0.00	999	1.000	1.000	0.2502
211195_s_at	28.9	12.7	-1.19	-2.28	AAA-AAA	0.00	999	1.000	1.000	0.2705
209325_s_at	78.8	34.5	-1.19	-2.28	AAA-AAA	0.00	999	1.000	1.000	0.2184
234593_at	7.8	17.7	1.19	2.28	AAA-AAA	0.00	999	1.000	1.000	0.1976
232758_s_at	115.6	50.6	-1.19	-2.28	AAA-AAA	0.00	999	1.000	1.000	0.0325
233695_s_at	9.3	4.1	-1.19	-2.29	AAA-AAA	0.00	999	1.000	1.000	0.1456
231295_at	21.0	48.0	1.19	2.29	AAA-AAA	0.00	999	1.000	1.000	0.0253
241213_x_at	32.3	14.1	-1.19	-2.28	AAA-AAA	0.00	999	1.000	1.000	0.1350
243781_at	23.5	53.7	1.19	2.28	AAA-AAA	0.00	999	1.000	1.000	0.1273
1560957_at	11.7	26.8	1.19	2.28	AAA-AAA	0.00	999	1.000	1.000	0.3679
1564379_at	26.5	11.6	-1.19	-2.28	AAA-AAA	0.00	999	1.000	1.000	0.3409
220429_at	14.7	6.4	-1.19	-2.28	AAA-AAA	0.00	999	1.000	1.000	0.3938
223612_s_at	74.6	32.7	-1.19	-2.28	AAA-AAA	0.00	999	1.000	1.000	0.3281
214823_at	10.9	24.8	1.19	2.28	AAA-AAA	0.00	999	1.000	1.000	0.2682
204890_s_at	18.6	8.2	-1.19	-2.28	AAA-AAA	0.00	999	1.000	1.000	0.3502
230181_at	20.3	8.9	-1.19	-2.28	AAA-AAA	0.00	999	1.000	1.000	0.2908
239609_s_at	28.7	65.5	1.19	2.28	AAA-APP	0.00	999	1.000	1.000	0.2560
241442_at	17.6	40.2	1.19	2.28	AAA-AAA	0.00	999	1.000	1.000	0.2052
240437_at	30.4	13.3	-1.19	-2.28	AAA-AAA	0.00	999	1.000	1.000	0.4920
241183_at	12.7	29.0	1.19	2.28	AAA-AAA	0.00	999	1.000	1.000	0.4052
1562820_at	10.1	4.4	-1.19	-2.28	AAA-AAA	0.00	999	1.000	1.000	0.3149
1556414_at	41.5	18.2	-1.19	-2.28	AAA-AAA	0.00	999	1.000	1.000	0.4618
206601_s_at	18.2	8.0	-1.19	-2.28	AAA-AAA	0.00	999	1.000	1.000	0.2638
211837_s_at	22.8	10.0	-1.19	-2.28	AAA-AAA	0.00	999	1.000	1.000	0.4308
239731_at	27.8	63.4	1.19	2.28	AAA-AAA	0.00	999	1.000	1.000	0.4150
234050_at	18.1	41.3	1.19	2.28	AAA-PAP	0.00	999	1.000	1.000	0.3237
1552449_a_at	27.1	11.9	-1.19	-2.28	AAA-AAA	0.00	999	1.000	1.000	0.3621
1552919_at	10.4	23.7	1.19	2.28	AAA-MAA	0.00	999	1.000	1.000	0.2818
1559699_at	3.9	8.8	1.19	2.28	AAA-AAA	0.00	999	1.000	1.000	0.3573

206935_at	2.7	6.2	1.19	2.28	AAA-AAA	0.00	999	1.000	1.000	0.3291
214470_at	3.6	8.3	1.19	2.28	AAA-AAA	0.00	999	1.000	1.000	0.2237
211897_s_at	53.4	23.4	-1.19	-2.28	AAA-AAA	0.00	999	1.000	1.000	0.1562
234375_x_at	31.2	13.7	-1.19	-2.28	AAA-AAA	0.00	999	1.000	1.000	0.4067
237250_at	18.3	8.0	-1.19	-2.28	AAA-AAA	0.00	999	1.000	1.000	0.4287
230489_at	13.7	6.0	-1.19	-2.28	AAA-AAA	0.00	999	1.000	1.000	0.2204
228166_at	52.0	22.8	-1.19	-2.28	AAA-AAA	0.00	999	1.000	1.000	0.2868
1565780_at	34.5	15.2	-1.19	-2.28	AAA-AAA	0.00	999	1.000	1.000	0.1660
206546_at	7.4	3.3	-1.19	-2.28	AAA-AAA	0.00	999	1.000	1.000	0.4044
206410_at	9.6	21.9	1.19	2.28	AAA-AAA	0.00	999	1.000	1.000	0.0899
213539_at	30.2	13.2	-1.19	-2.28	AAA-AAA	0.00	999	1.000	1.000	0.5147
214602_at	8.3	3.6	-1.19	-2.28	AAA-AAA	0.00	999	1.000	1.000	0.2624
210421_s_at	39.1	17.2	-1.19	-2.28	AAA-AAA	0.00	999	1.000	1.000	0.4649
229938_at	7.3	3.2	-1.19	-2.28	AAA-AAA	0.00	999	1.000	1.000	0.1612
229249_at	16.0	36.4	1.19	2.28	AAA-AAM	0.00	999	1.000	1.000	0.2778
223588_at	30.7	70.0	1.19	2.28	AAP-PMP	0.00	999	1.000	1.000	0.2690
204748_at	57.6	131.0	1.19	2.28	AAA-PPP	0.00	999	1.000	1.000	0.0863
243096_at	3.9	8.9	1.19	2.28	AAA-AAA	0.00	999	1.000	1.000	0.2602
241035_s_at	18.6	8.2	-1.19	-2.28	AAA-AAA	0.00	999	1.000	1.000	0.3374
1569796_s_at	16.2	7.1	-1.19	-2.28	AAA-AAA	0.00	999	1.000	1.000	0.4260
1567030_at	15.5	6.8	-1.19	-2.28	AAA-AAA	0.00	999	1.000	1.000	0.3857
207473_at	44.9	19.7	-1.19	-2.28	AAA-AAA	0.00	999	1.000	1.000	0.2016
234310_s_at	23.7	10.4	-1.19	-2.28	AAA-AAA	0.00	999	1.000	1.000	0.3084
234646_at	6.8	3.0	-1.19	-2.28	AAA-AAA	0.00	999	1.000	1.000	0.0617
232340_at	31.5	71.7	1.19	2.28	AAA-AAA	0.00	999	1.000	1.000	0.1656
244238_at	21.4	9.4	-1.19	-2.28	AAA-AAA	0.00	999	1.000	1.000	0.2904
1562573_at	35.4	15.6	-1.19	-2.28	AAA-AAA	0.00	999	1.000	1.000	0.0280
1570268_at	6.2	2.7	-1.19	-2.28	AAA-AAA	0.00	999	1.000	1.000	0.3279
202434_s_at	12.5	5.5	-1.19	-2.28	AAA-AAA	0.00	999	1.000	1.000	0.3151
208564_at	22.6	9.9	-1.19	-2.28	MAA-AAA	0.00	999	1.000	1.000	0.5488
218806_s_at	9.1	4.0	-1.19	-2.28	AAA-AAA	0.00	999	1.000	1.000	0.1148
235193_at	23.8	54.1	1.19	2.28	AAA-AAA	0.00	999	1.000	1.000	0.0903
238233_at	14.1	6.2	-1.19	-2.28	AAA-AAA	0.00	999	1.000	1.000	0.1153
229745_x_at	20.6	9.1	-1.19	-2.27	AAA-AAA	0.00	999	1.000	1.000	0.3821
243967_at	11.9	5.3	-1.19	-2.27	AAA-AAA	0.00	999	1.000	1.000	0.1944
241829_at	11.9	5.2	-1.19	-2.27	AAA-AAA	0.00	999	1.000	1.000	0.4530
243800_at	10.9	4.8	-1.19	-2.27	AAA-AAA	0.00	999	1.000	1.000	0.3955
1562890_at	10.3	23.4	1.19	2.27	AAA-AAA	0.00	999	1.000	1.000	0.2516
1561627_at	9.7	22.1	1.19	2.27	AAA-AAA	0.00	999	1.000	1.000	0.2156
1568779_a_at	22.4	9.9	-1.19	-2.27	AAA-AAA	0.00	999	1.000	1.000	0.2061
1555454_at	3.3	7.5	1.19	2.27	AAA-AAA	0.00	999	1.000	1.000	0.2659
221623_at	9.1	20.8	1.19	2.27	AAA-AAA	0.00	999	1.000	1.000	0.1675

221446_at	63.8	28.1	-1.19	-2.27	AAA-AAA	0.00	999	1.000	1.000	0.1473
207771_at	91.6	40.3	-1.19	-2.27	AAA-AAA	0.00	999	1.000	1.000	0.4533
224646_x_at	22.4	9.9	-1.19	-2.27	AAA-AAA	0.00	999	1.000	1.000	0.4222
223562_at	8.8	20.1	1.19	2.27	AAA-AAA	0.00	999	1.000	1.000	0.2150
214415_at	16.8	38.2	1.19	2.27	AAA-AAA	0.00	999	1.000	1.000	0.3240
1561485_at	18.5	42.1	1.18	2.27	AAA-AAP	0.00	999	1.000	1.000	0.2966
1562425_at	8.6	3.8	-1.18	-2.27	AAA-AAA	0.00	999	1.000	1.000	0.1678
215356_at	7.4	3.2	-1.18	-2.27	AAA-AAA	0.00	999	1.000	1.000	0.1224
239743_at	11.1	25.3	1.18	2.27	AAA-AAA	0.00	999	1.000	1.000	0.3311
237588_at	20.1	8.8	-1.18	-2.27	AAA-AAA	0.00	999	1.000	1.000	0.3764
233644_at	13.7	6.0	-1.18	-2.27	AAA-AAA	0.00	999	1.000	1.000	0.4193
231595_at	51.9	22.9	-1.18	-2.27	AAA-AAA	0.00	999	1.000	1.000	0.3058
206029_at	20.5	46.4	1.18	2.27	AAA-APA	0.00	999	1.000	1.000	0.5135
241650_x_at	40.2	91.2	1.18	2.27	AAA-AAA	0.00	999	1.000	1.000	0.3581
1564414_a_at	68.0	29.9	-1.18	-2.27	AAA-AAA	0.00	999	1.000	1.000	0.0831
1558338_at	14.1	6.2	-1.18	-2.27	AAA-AAA	0.00	999	1.000	1.000	0.0714
1558007_s_at	9.9	22.6	1.18	2.27	AAA-AAA	0.00	999	1.000	1.000	0.2000
206803_at	13.2	5.8	-1.18	-2.27	AAA-AAA	0.00	999	1.000	1.000	0.1653
215107_s_at	7.1	3.1	-1.18	-2.27	AAA-AAA	0.00	999	1.000	1.000	0.4533
233423_at	84.0	37.0	-1.18	-2.27	AAA-AAA	0.00	999	1.000	1.000	0.3245
233420_at	20.0	8.8	-1.18	-2.27	AAA-MAA	0.00	999	1.000	1.000	0.5655
232088_x_at	21.3	48.4	1.18	2.27	AAA-AAA	0.00	999	1.000	1.000	0.2301
215232_at	19.6	44.6	1.18	2.27	PAA-PAA	0.00	999	1.000	1.000	0.4988
1562771_at	21.6	49.0	1.18	2.27	AAA-PPP	0.00	999	1.000	1.000	0.3349
1565905_at	47.3	20.9	-1.18	-2.27	AAA-AAA	0.00	999	1.000	1.000	0.2519
1558398_at	24.9	11.0	-1.18	-2.27	AAA-AAA	0.00	999	1.000	1.000	0.4030
1558048_x_at	12.3	5.4	-1.18	-2.27	AAA-AAA	0.00	999	1.000	1.000	0.5650
221401_at	27.8	12.3	-1.18	-2.27	AAA-AAA	0.00	999	1.000	1.000	0.3075
214242_at	12.9	29.3	1.18	2.27	AAA-AAA	0.00	999	1.000	1.000	0.2740
214961_at	3.8	8.6	1.18	2.27	AAA-AAA	0.00	999	1.000	1.000	0.2814
205816_at	16.8	7.4	-1.18	-2.27	AAA-AAA	0.00	999	1.000	1.000	0.4189
205654_at	47.9	21.1	-1.18	-2.27	AAA-AAA	0.00	999	1.000	1.000	0.3895
215217_at	28.0	63.6	1.18	2.27	AAP-AAA	0.00	999	1.000	1.000	0.3057
242718_at	7.0	15.8	1.18	2.27	AAA-AAA	0.00	999	1.000	1.000	0.3011
1561274_at	9.7	4.3	-1.18	-2.27	AAA-AAA	0.00	999	1.000	1.000	0.3530
244531_at	5.7	12.8	1.18	2.27	AAA-AAA	0.00	999	1.000	1.000	0.4870
1556638_at	14.5	32.8	1.18	2.27	AAA-AAA	0.00	999	1.000	1.000	0.1749
225660_at	5.0	11.2	1.18	2.27	AAA-AAA	0.00	999	1.000	1.000	0.3624
217504_at	14.9	6.6	-1.18	-2.27	AAA-AAA	0.00	999	1.000	1.000	0.5825
216714_at	13.1	5.8	-1.18	-2.27	AAA-AAA	0.00	999	1.000	1.000	0.2398
234319_at	19.9	8.8	-1.18	-2.27	AAA-AAA	0.00	999	1.000	1.000	0.2600
234480_at	7.1	16.0	1.18	2.27	AAA-AAA	0.00	999	1.000	1.000	0.4149

230840_at	17.9	7.9	-1.18	-2.27	AAA-AAA	0.00	999	1.000	1.000	0.4436
230327_at	58.6	25.9	-1.18	-2.27	AAA-AAA	0.00	999	1.000	1.000	0.2726
205507_at	141.2	62.2	-1.18	-2.27	AAM-AAA	0.00	999	1.000	1.000	0.2239
243153_at	28.3	12.5	-1.18	-2.27	AAA-AAA	0.00	999	1.000	1.000	0.5424
241403_at	40.2	17.7	-1.18	-2.27	AAA-AAA	0.00	999	1.000	1.000	0.3038
241090_at	8.2	3.6	-1.18	-2.27	AAA-AAA	0.00	999	1.000	1.000	0.0114
1564559_at	34.1	15.1	-1.18	-2.27	AAA-AAA	0.00	999	1.000	1.000	0.3825
1553885_x_at	3.5	8.0	1.18	2.27	AAA-AAA	0.00	999	1.000	1.000	0.5902
1552997_a_at	62.2	27.5	-1.18	-2.27	AAA-AAA	0.00	999	1.000	1.000	0.1068
1559125_at	12.1	5.3	-1.18	-2.27	AAA-AAA	0.00	999	1.000	1.000	0.5420
1556211_a_at	12.6	28.4	1.18	2.27	AAA-AAA	0.00	999	1.000	1.000	0.5122
207129_at	35.8	15.8	-1.18	-2.27	AAA-AAA	0.00	999	1.000	1.000	0.1954
226613_at	86.1	38.0	-1.18	-2.27	AAA-AAA	0.00	999	1.000	1.000	0.1685
216514_at	26.0	11.5	-1.18	-2.27	AAA-AAA	0.00	999	1.000	1.000	0.5096
236237_at	39.8	17.6	-1.18	-2.27	AAA-AAA	0.00	999	1.000	1.000	0.2767
205833_s_at	46.7	20.6	-1.18	-2.27	AAA-AAA	0.00	999	1.000	1.000	0.3554
1562365_at	33.8	14.9	-1.18	-2.27	AAA-AAA	0.00	999	1.000	1.000	0.4213
1568449_at	6.4	2.8	-1.18	-2.26	AAA-AAA	0.00	999	1.000	1.000	0.5248
1566609_at	13.5	30.6	1.18	2.26	AAA-AAA	0.00	999	1.000	1.000	0.3375
207393_at	9.6	4.2	-1.18	-2.27	AAA-AAA	0.00	999	1.000	1.000	0.1551
211839_s_at	19.4	43.9	1.18	2.26	AAA-AAA	0.00	999	1.000	1.000	0.2580
239344_at	11.6	5.1	-1.18	-2.27	AAA-AAA	0.00	999	1.000	1.000	0.4070
1560033_at	20.8	47.0	1.18	2.26	AAA-APP	0.00	999	1.000	1.000	0.2962
242764_at	6.1	2.7	-1.18	-2.26	AAA-AAA	0.00	999	1.000	1.000	0.3406
1562030_at	70.4	31.1	-1.18	-2.26	AAA-AAA	0.00	999	1.000	1.000	0.2332
1560895_at	5.7	2.5	-1.18	-2.26	AAA-AAA	0.00	999	1.000	1.000	0.0543
1564697_a_at	36.0	15.9	-1.18	-2.26	AAA-AAA	0.00	999	1.000	1.000	0.3713
207113_s_at	45.0	19.9	-1.18	-2.26	AAA-AAA	0.00	999	1.000	1.000	0.3335
207576_x_at	35.7	15.8	-1.18	-2.26	AAA-AAA	0.00	999	1.000	1.000	0.2550
218500_at	49.5	112.0	1.18	2.26	AAA-AAA	0.00	999	1.000	1.000	0.2796
234707_x_at	24.7	10.9	-1.18	-2.26	AAA-AAA	0.00	999	1.000	1.000	0.4535
234601_x_at	6.0	2.7	-1.18	-2.26	AAA-AAA	0.00	999	1.000	1.000	0.5294
232423_at	24.2	54.7	1.18	2.26	AAA-AAP	0.00	999	1.000	1.000	0.0946
1562351_at	31.4	13.9	-1.18	-2.26	AAA-AAA	0.00	999	1.000	1.000	0.1827
1553625_at	26.9	11.9	-1.18	-2.26	AAA-AAA	0.00	999	1.000	1.000	0.2528
223549_s_at	34.7	15.3	-1.18	-2.26	AAA-AAA	0.00	999	1.000	1.000	0.3755
223953_s_at	72.2	31.9	-1.18	-2.26	AAA-AAA	0.00	999	1.000	1.000	0.1747
211398_at	45.5	20.1	-1.18	-2.26	AAA-AAA	0.00	999	1.000	1.000	0.2098
234381_at	57.8	25.6	-1.18	-2.26	AAA-AAA	0.00	999	1.000	1.000	0.3076
233973_at	46.0	20.3	-1.18	-2.26	AAA-AAA	0.00	999	1.000	1.000	0.3721
203995_at	10.8	24.3	1.18	2.26	AAA-AAA	0.00	999	1.000	1.000	0.0951
205151_s_at	32.7	14.5	-1.18	-2.26	AAA-AAA	0.00	999	1.000	1.000	0.4522

240853_at	128.8	57.0	-1.18	-2.26	AAA-AAA	0.00	999	1.000	1.000	0.0014
240287_at	26.2	11.6	-1.18	-2.26	AAA-AAA	0.00	999	1.000	1.000	0.4616
1564654_at	26.9	60.8	1.18	2.26	AAA-AAA	0.00	999	1.000	1.000	0.3151
1566837_at	15.1	6.7	-1.18	-2.26	AAA-AAA	0.00	999	1.000	1.000	0.3780
1553215_s_at	23.5	10.4	-1.18	-2.26	AAA-AAA	0.00	999	1.000	1.000	0.4210
1557576_at	59.1	26.2	-1.18	-2.26	AAA-AAA	0.00	999	1.000	1.000	0.4372
1559544_s_at	62.1	27.5	-1.18	-2.26	AAA-AAA	0.00	999	1.000	1.000	0.3789
1559353_at	6.9	3.1	-1.18	-2.26	AAA-AAA	0.00	999	1.000	1.000	0.5377
217698_at	5.0	2.2	-1.18	-2.26	AAA-AAA	0.00	999	1.000	1.000	0.5106
234220_at	17.2	7.6	-1.18	-2.26	AAA-AAA	0.00	999	1.000	1.000	0.4215
206346_at	12.0	5.3	-1.18	-2.26	AAA-AAA	0.00	999	1.000	1.000	0.5558
1569296_a_at	29.8	13.2	-1.18	-2.26	AAA-AAA	0.00	999	1.000	1.000	0.4170
219702_at	18.8	42.5	1.18	2.26	AAA-AAA	0.00	999	1.000	1.000	0.3466
206494_s_at	20.2	9.0	-1.18	-2.26	AAA-AAA	0.00	999	1.000	1.000	0.3534
222929_at	16.1	7.1	-1.18	-2.26	AAA-AAA	0.00	999	1.000	1.000	0.0719
207322_at	10.9	4.8	-1.18	-2.26	AAA-AAA	0.00	999	1.000	1.000	0.3101
217381_s_at	13.3	5.9	-1.18	-2.26	AAA-AAA	0.00	999	1.000	1.000	0.1801
218625_at	29.5	13.1	-1.18	-2.26	AAA-AAA	0.00	999	1.000	1.000	0.3334
236421_at	3.7	8.4	1.18	2.26	AAA-AAA	0.00	999	1.000	1.000	0.4646
231529_at	26.7	11.8	-1.18	-2.26	AAA-AAA	0.00	999	1.000	1.000	0.5403
1562386_s_at	25.3	57.2	1.17	2.26	AAA-PAP	0.00	999	1.000	1.000	0.4420
213972_at	28.3	63.7	1.17	2.26	AAP-AAM	0.00	999	1.000	1.000	0.3573
232381_s_at	19.3	43.6	1.17	2.26	AAP-PAP	0.00	999	1.000	1.000	0.3463
235039_x_at	115.4	260.3	1.17	2.26	MPA-PPP	0.00	999	1.000	1.000	0.1605
243763_x_at	68.0	30.1	-1.17	-2.26	MAA-AAA	0.00	999	1.000	1.000	0.1408
1567241_at	50.6	22.4	-1.17	-2.26	AAA-AAA	0.00	999	1.000	1.000	0.3566
1556529_a_at	4.6	10.3	1.17	2.26	AAA-AAA	0.00	999	1.000	1.000	0.3349
237623_at	24.4	55.1	1.17	2.26	AAA-AAA	0.00	999	1.000	1.000	0.3507
233308_at	86.8	38.5	-1.17	-2.26	AAA-AAA	0.00	999	1.000	1.000	0.4583
1564640_at	13.6	30.7	1.17	2.25	AAA-APA	0.00	999	1.000	1.000	0.4461
203118_at	120.3	271.1	1.17	2.25	AAA-AAA	0.00	999	1.000	1.000	0.2285
243167_at	20.2	9.0	-1.17	-2.25	AAA-AAA	0.00	999	1.000	1.000	0.0272
1563312_at	9.3	4.1	-1.17	-2.26	AAA-AAA	0.00	999	1.000	1.000	0.4940
1570409_x_at	5.7	12.9	1.17	2.26	AAA-AAA	0.00	999	1.000	1.000	0.2811
1566102_at	54.0	24.0	-1.17	-2.26	AAA-AAA	0.00	999	1.000	1.000	0.3785
1555011_at	11.9	5.3	-1.17	-2.25	AAA-AAA	0.00	999	1.000	1.000	0.3016
1557517_a_at	4.4	10.0	1.17	2.26	AAA-AAA	0.00	999	1.000	1.000	0.5215
221407_at	120.7	53.5	-1.17	-2.25	AAM-AAA	0.00	999	1.000	1.000	0.1057
219370_at	12.3	27.6	1.17	2.26	AAA-AAA	0.00	999	1.000	1.000	0.3475
239045_at	71.8	31.9	-1.17	-2.25	AAA-AAA	0.00	999	1.000	1.000	0.2942
230551_at	79.0	35.0	-1.17	-2.26	AAA-AAA	0.00	999	1.000	1.000	0.0781
205311_at	11.7	5.2	-1.17	-2.26	AAA-AAA	0.00	999	1.000	1.000	0.4117

233628_at	7.2	16.2	1.17	2.26	AAA-AAA	0.00	999	1.000	1.000	0.1724
1552401_a_at	19.0	42.8	1.17	2.25	AAA-PAA	0.00	999	1.000	1.000	0.2310
243727_at	10.1	4.5	-1.17	-2.25	AAA-AAA	0.00	999	1.000	1.000	0.3788
1560836_at	43.0	19.1	-1.17	-2.25	AAA-AAA	0.00	999	1.000	1.000	0.0837
1560540_x_at	3.3	7.3	1.17	2.25	AAA-AAA	0.00	999	1.000	1.000	0.4809
1567167_at	17.2	7.6	-1.17	-2.25	AAA-AAA	0.00	999	1.000	1.000	0.3748
217192_s_at	26.9	60.7	1.17	2.25	AAA-AAA	0.00	999	1.000	1.000	0.2663
209260_at	28.4	12.6	-1.17	-2.25	AAA-AAA	0.00	999	1.000	1.000	0.3496
229977_at	77.6	34.4	-1.17	-2.25	AAA-AAA	0.00	999	1.000	1.000	0.2892
1570433_at	16.1	7.2	-1.17	-2.25	AAA-AAA	0.00	999	1.000	1.000	0.3464
221560_at	118.4	266.6	1.17	2.25	AAA-AAA	0.00	999	1.000	1.000	0.2076
215172_at	3.9	1.7	-1.17	-2.25	AAA-AAA	0.00	999	1.000	1.000	0.5572
210548_at	5.2	2.3	-1.17	-2.25	AAA-AAA	0.00	999	1.000	1.000	0.1477
217530_at	19.0	8.5	-1.17	-2.25	AAA-AAA	0.00	999	1.000	1.000	0.4851
215839_at	16.9	7.5	-1.17	-2.25	AAA-AAA	0.00	999	1.000	1.000	0.0761
235201_at	13.0	5.8	-1.17	-2.25	AAA-AAA	0.00	999	1.000	1.000	0.4698
238106_at	34.0	15.1	-1.17	-2.25	AAA-AAA	0.00	999	1.000	1.000	0.3277
1555648_at	7.1	16.1	1.17	2.25	AAA-PAA	0.00	999	1.000	1.000	0.3861
244300_at	11.6	5.2	-1.17	-2.25	AAA-AAA	0.00	999	1.000	1.000	0.5261
1562327_at	29.0	12.9	-1.17	-2.25	AAA-AAA	0.00	999	1.000	1.000	0.3997
1561443_at	56.9	25.3	-1.17	-2.25	AAA-AAA	0.00	999	1.000	1.000	0.3534
1554530_at	15.9	7.1	-1.17	-2.25	AAA-AAA	0.00	999	1.000	1.000	0.5578
1552667_a_at	9.3	4.1	-1.17	-2.25	AAA-AAA	0.00	999	1.000	1.000	0.1106
1558179_at	12.2	5.4	-1.17	-2.25	AAA-AAA	0.00	999	1.000	1.000	0.2250
211044_at	68.5	30.4	-1.17	-2.25	AAA-AAA	0.00	999	1.000	1.000	0.2653
216131_at	16.8	7.5	-1.17	-2.25	AAA-AAA	0.00	999	1.000	1.000	0.4735
211491_at	14.8	33.3	1.17	2.25	AAA-PAA	0.00	999	1.000	1.000	0.2073
243480_at	19.2	43.1	1.17	2.25	AAA-AAA	0.00	999	1.000	1.000	0.2970
1560424_at	2.0	4.5	1.17	2.25	AAA-AAA	0.00	999	1.000	1.000	0.1798
1564510_at	25.2	11.2	-1.17	-2.25	AAA-AAA	0.00	999	1.000	1.000	0.3736
1564533_at	14.8	33.2	1.17	2.25	AAA-AAA	0.00	999	1.000	1.000	0.4462
220714_at	42.5	18.9	-1.17	-2.25	AAA-AAA	0.00	999	1.000	1.000	0.2724
215409_at	27.0	12.0	-1.17	-2.25	AAA-AAA	0.00	999	1.000	1.000	0.3924
216356_x_at	26.4	11.8	-1.17	-2.25	AAA-AAA	0.00	999	1.000	1.000	0.2227
234899_at	51.4	22.9	-1.17	-2.25	AAA-AAA	0.00	999	1.000	1.000	0.1808
237028_at	23.1	51.9	1.17	2.25	AAA-AAA	0.00	999	1.000	1.000	0.4716
237912_at	32.7	14.5	-1.17	-2.25	AAA-AAA	0.00	999	1.000	1.000	0.3981
232869_at	17.4	7.8	-1.17	-2.25	AAA-AAA	0.00	999	1.000	1.000	0.3343
233549_at	8.6	3.8	-1.17	-2.25	AAA-AAA	0.00	999	1.000	1.000	0.2736
232005_at	10.8	4.8	-1.17	-2.25	AAA-AAA	0.00	999	1.000	1.000	0.0980
215698_at	259.2	582.3	1.17	2.25	PPP-PPP	0.00	999	1.000	1.000	0.0814
240163_at	10.5	4.7	-1.17	-2.25	AAA-AAA	0.00	999	1.000	1.000	0.1771

1563404_at	24.2	10.8	-1.17	-2.25	AAA-AAA	0.00	999	1.000	1.000	0.3283
1560744_at	34.3	15.3	-1.17	-2.25	AAA-AAA	0.00	999	1.000	1.000	0.3389
1566236_at	34.4	77.3	1.17	2.25	AAA-AAA	0.00	999	1.000	1.000	0.2195
1553492_a_at	19.9	8.8	-1.17	-2.25	AAA-AAA	0.00	999	1.000	1.000	0.1486
213968_at	67.9	30.2	-1.17	-2.25	AAA-AAA	0.00	999	1.000	1.000	0.3954
211384_s_at	18.7	8.3	-1.17	-2.25	AAA-AAA	0.00	999	1.000	1.000	0.1784
209591_s_at	21.5	9.6	-1.17	-2.25	AAA-AAA	0.00	999	1.000	1.000	0.4045
238131_at	28.9	65.0	1.17	2.25	AAA-AAA	0.00	999	1.000	1.000	0.2672
205843_x_at	86.9	38.7	-1.17	-2.25	AAA-AAA	0.00	999	1.000	1.000	0.2521
229254_at	7.1	15.8	1.17	2.25	AAA-AAA	0.00	999	1.000	1.000	0.4239
206879_s_at	18.7	41.9	1.17	2.25	AAA-APA	0.00	999	1.000	1.000	0.3527
213128_s_at	947.3	2127.3	1.17	2.25	PPP-PPP	0.00	999	1.000	1.000	0.1340
240937_at	21.6	9.6	-1.17	-2.25	AAA-AAA	0.00	999	1.000	1.000	0.4121
1560506_at	8.3	3.7	-1.17	-2.25	AAA-AAA	0.00	999	1.000	1.000	0.1811
1567076_at	5.5	2.4	-1.17	-2.25	AAA-AAA	0.00	999	1.000	1.000	0.0513
1557511_at	23.0	10.2	-1.17	-2.25	AAA-AAA	0.00	999	1.000	1.000	0.4281
219932_at	8.6	3.8	-1.17	-2.25	AAA-AAA	0.00	999	1.000	1.000	0.4079
211032_at	46.9	20.9	-1.17	-2.25	AAA-AAA	0.00	999	1.000	1.000	0.1443
235421_at	19.9	8.9	-1.17	-2.25	AAA-AAA	0.00	999	1.000	1.000	0.3000
241566_at	7.5	16.8	1.17	2.24	AAA-PAA	0.00	999	1.000	1.000	0.2254
218430_s_at	86.4	193.7	1.17	2.24	PPP-PPP	0.00	999	1.000	1.000	0.0778
241979_x_at	6.4	2.9	-1.17	-2.24	AAA-AAA	0.00	999	1.000	1.000	0.4635
242494_at	66.8	29.8	-1.17	-2.24	MAA-AAA	0.00	999	1.000	1.000	0.1382
1563235_at	11.0	4.9	-1.17	-2.24	AAA-AAA	0.00	999	1.000	1.000	0.0138
1569674_at	5.7	2.5	-1.17	-2.24	AAA-AAA	0.00	999	1.000	1.000	0.4140
221217_s_at	34.2	15.3	-1.17	-2.24	AAA-AAA	0.00	999	1.000	1.000	0.0887
208590_x_at	15.2	6.8	-1.17	-2.25	AAA-AAA	0.00	999	1.000	1.000	0.3296
217516_x_at	31.3	70.3	1.17	2.24	AAA-AAA	0.00	999	1.000	1.000	0.2544
237771_s_at	5.0	11.3	1.17	2.25	AAA-AAA	0.00	999	1.000	1.000	0.2877
205908_s_at	16.1	7.2	-1.17	-2.24	AAA-AAA	0.00	999	1.000	1.000	0.4540
231512_at	6.7	3.0	-1.17	-2.24	AAA-AAA	0.00	999	1.000	1.000	0.4124
239986_at	41.0	18.3	-1.17	-2.24	AAA-AAA	0.00	999	1.000	1.000	0.2956
241043_at	22.4	10.0	-1.17	-2.24	AAA-AAA	0.00	999	1.000	1.000	0.3390
240661_at	13.0	5.8	-1.17	-2.24	AAA-AAA	0.00	999	1.000	1.000	0.2345
1563379_at	23.6	10.5	-1.17	-2.24	AAA-AAA	0.00	999	1.000	1.000	0.3990
221285_at	10.6	4.8	-1.17	-2.24	AAA-AAA	0.00	999	1.000	1.000	0.2182
206841_at	7.5	3.4	-1.17	-2.24	AAA-AAA	0.00	999	1.000	1.000	0.2823
223893_at	4.6	10.3	1.17	2.24	AAA-AAA	0.00	999	1.000	1.000	0.3583
210887_s_at	67.2	30.0	-1.17	-2.24	AAA-AAA	0.00	999	1.000	1.000	0.2081
238287_at	31.4	14.0	-1.17	-2.24	AAA-AAA	0.00	999	1.000	1.000	0.3039
230093_at	9.3	20.9	1.17	2.24	AAA-AAA	0.00	999	1.000	1.000	0.4301
206150_at	33.8	15.1	-1.17	-2.24	AAA-AAA	0.00	999	1.000	1.000	0.2926

205350_at	18.5	8.2	-1.17	-2.24	AAA-AAA	0.00	999	1.000	1.000	0.3908
228913_at	45.0	100.7	1.16	2.24	AAP-APM	0.00	999	1.000	1.000	0.4103
225657_at	420.1	941.2	1.16	2.24	APP-PPP	0.00	999	1.000	1.000	0.3559
242998_at	40.5	18.1	-1.16	-2.24	AAA-AAA	0.00	999	1.000	1.000	0.3607
1569465_at	36.1	16.1	-1.16	-2.24	AAA-AAA	0.00	999	1.000	1.000	0.5520
1553134_s_at	10.8	4.8	-1.16	-2.24	AAA-AAA	0.00	999	1.000	1.000	0.4239
1552554_a_at	13.6	30.4	1.16	2.24	AAA-AAA	0.00	999	1.000	1.000	0.2282
1559559_at	8.7	3.9	-1.16	-2.24	AAA-AAA	0.00	999	1.000	1.000	0.4824
208243_s_at	12.1	5.4	-1.16	-2.24	AAA-AAA	0.00	999	1.000	1.000	0.3527
207534_at	17.3	38.7	1.16	2.24	AAA-AAA	0.00	999	1.000	1.000	0.4236
217592_at	95.9	42.8	-1.16	-2.24	AAA-AAA	0.00	999	1.000	1.000	0.0545
234329_at	20.6	46.2	1.16	2.24	AAA-AAA	0.00	999	1.000	1.000	0.2099
238097_at	17.1	7.6	-1.16	-2.24	AAA-AAA	0.00	999	1.000	1.000	0.1660
237286_at	72.3	32.3	-1.16	-2.24	AAA-AAA	0.00	999	1.000	1.000	0.3367
231248_at	19.8	8.8	-1.16	-2.24	AAA-AAA	0.00	999	1.000	1.000	0.0634
225727_at	22.4	50.1	1.16	2.24	AAA-PAA	0.00	999	1.000	1.000	0.3422
240071_at	57.8	25.8	-1.16	-2.24	AAA-AAA	0.00	999	1.000	1.000	0.2203
243183_at	10.2	4.6	-1.16	-2.24	AAA-AAA	0.00	999	1.000	1.000	0.4675
242792_at	7.4	16.7	1.16	2.24	AAA-AAA	0.00	999	1.000	1.000	0.2373
1567280_at	8.4	18.8	1.16	2.24	AAA-AAA	0.00	999	1.000	1.000	0.3742
244529_at	60.0	26.8	-1.16	-2.24	AAA-AAA	0.00	999	1.000	1.000	0.3093
208058_s_at	20.4	9.1	-1.16	-2.24	AAA-AAA	0.00	999	1.000	1.000	0.3086
208299_at	27.8	62.3	1.16	2.24	AAA-AAA	0.00	999	1.000	1.000	0.2783
224260_at	18.3	8.2	-1.16	-2.24	AAA-AAA	0.00	999	1.000	1.000	0.4496
218621_at	42.1	18.8	-1.16	-2.24	AAA-AAA	0.00	999	1.000	1.000	0.4864
236658_at	7.9	17.7	1.16	2.24	AAA-AAA	0.00	999	1.000	1.000	0.2445
234571_at	3.7	8.3	1.16	2.24	AAA-AAA	0.00	999	1.000	1.000	0.2778
231488_at	18.5	41.4	1.16	2.24	AAA-AMA	0.00	999	1.000	1.000	0.2397
243384_at	23.7	52.9	1.16	2.24	AAA-PAA	0.00	999	1.000	1.000	0.3306
1563008_at	3.7	8.4	1.16	2.24	AAA-AAA	0.00	999	1.000	1.000	0.3626
1564970_at	18.7	8.4	-1.16	-2.24	AAA-AAA	0.00	999	1.000	1.000	0.1697
215681_at	13.8	30.8	1.16	2.24	AAA-AAA	0.00	999	1.000	1.000	0.2269
238308_at	29.1	13.0	-1.16	-2.24	AAA-AAA	0.00	999	1.000	1.000	0.3698
205490_x_at	15.9	35.5	1.16	2.24	AAA-AAA	0.00	999	1.000	1.000	0.2471
208515_at	19.3	43.1	1.16	2.24	AAA-MPA	0.00	999	1.000	1.000	0.5503
235184_at	23.4	52.4	1.16	2.24	AAA-APP	0.00	999	1.000	1.000	0.1069
1569812_at	25.9	11.6	-1.16	-2.24	AAA-AAA	0.00	999	1.000	1.000	0.2838
221358_at	10.0	4.5	-1.16	-2.24	AAA-AAA	0.00	999	1.000	1.000	0.2653
207889_at	23.1	10.3	-1.16	-2.24	AAA-AAA	0.00	999	1.000	1.000	0.4216
219909_at	11.3	5.0	-1.16	-2.24	AAA-AAA	0.00	999	1.000	1.000	0.3611
211276_at	55.4	24.8	-1.16	-2.24	AAA-AAA	0.00	999	1.000	1.000	0.0014
216337_at	14.0	6.3	-1.16	-2.24	AAA-AAA	0.00	999	1.000	1.000	0.3448

237954_x_at	8.8	19.8	1.16	2.24	AAA-AAA	0.00	999	1.000	1.000	0.0920
230506_at	14.8	6.6	-1.16	-2.24	AAA-AAA	0.00	999	1.000	1.000	0.4924
230513_at	72.3	32.3	-1.16	-2.24	AAA-AAA	0.00	999	1.000	1.000	0.1742
231392_at	7.0	3.1	-1.16	-2.24	AAA-AAA	0.00	999	1.000	1.000	0.4266
231674_at	14.7	6.6	-1.16	-2.24	AAA-AAA	0.00	999	1.000	1.000	0.4072
240684_at	9.1	20.3	1.16	2.24	AAA-AAA	0.00	999	1.000	1.000	0.3753
1561537_at	26.5	59.2	1.16	2.23	AAA-AAA	0.00	999	1.000	1.000	0.1851
1559518_at	14.2	6.3	-1.16	-2.23	AAA-AAA	0.00	999	1.000	1.000	0.4713
1557181_s_at	5.9	13.3	1.16	2.23	AAA-AAA	0.00	999	1.000	1.000	0.3821
224118_at	21.4	9.6	-1.16	-2.24	AAA-AAA	0.00	999	1.000	1.000	0.2153
214599_at	56.9	25.4	-1.16	-2.24	AAA-AAA	0.00	999	1.000	1.000	0.3233
211083_s_at	4.6	10.2	1.16	2.23	AAA-AAA	0.00	999	1.000	1.000	0.2207
211221_at	21.2	9.5	-1.16	-2.23	AAA-AAA	0.00	999	1.000	1.000	0.3147
212315_s_at	17.3	7.7	-1.16	-2.24	AAA-AAA	0.00	999	1.000	1.000	0.1320
210462_at	19.9	8.9	-1.16	-2.24	AAA-AAA	0.00	999	1.000	1.000	0.5065
215979_s_at	34.6	15.5	-1.16	-2.24	AAA-AAA	0.00	999	1.000	1.000	0.3196
238177_at	39.6	17.7	-1.16	-2.23	AAA-AAA	0.00	999	1.000	1.000	0.3779
230190_at	52.0	23.3	-1.16	-2.23	AAA-AAA	0.00	999	1.000	1.000	0.2838
1561041_at	74.2	33.2	-1.16	-2.23	AAA-AAA	0.00	999	1.000	1.000	0.1686
1566867_at	14.9	6.7	-1.16	-2.23	AAA-AAA	0.00	999	1.000	1.000	0.4868
1553452_at	43.1	19.3	-1.16	-2.23	AAA-AAA	0.00	999	1.000	1.000	0.0766
1552639_at	26.5	11.9	-1.16	-2.23	AAA-AAA	0.00	999	1.000	1.000	0.2111
1557740_a_at	8.9	19.8	1.16	2.23	AAA-AAA	0.00	999	1.000	1.000	0.4496
1559745_at	30.5	13.7	-1.16	-2.23	AAA-AAA	0.00	999	1.000	1.000	0.4106
206700_s_at	39.0	17.4	-1.16	-2.23	AAA-AAA	0.00	999	1.000	1.000	0.1781
213695_at	54.2	24.3	-1.16	-2.23	AAA-AAA	0.00	999	1.000	1.000	0.0374
215656_at	18.5	8.3	-1.16	-2.23	AAA-AAA	0.00	999	1.000	1.000	0.4415
239341_at	13.9	6.2	-1.16	-2.23	AAA-AAA	0.00	999	1.000	1.000	0.3599
239279_at	22.2	9.9	-1.16	-2.23	AAA-AAA	0.00	999	1.000	1.000	0.5274
231637_at	9.7	4.3	-1.16	-2.23	AAA-AAA	0.00	999	1.000	1.000	0.3085
244148_at	10.2	22.7	1.16	2.23	PAA-AAA	0.00	999	1.000	1.000	0.5443
241684_at	23.3	10.4	-1.16	-2.23	AAA-AAA	0.00	999	1.000	1.000	0.4866
1562749_at	15.6	7.0	-1.16	-2.23	AAA-AAA	0.00	999	1.000	1.000	0.4309
1564996_at	4.6	2.1	-1.16	-2.23	AAA-AAA	0.00	999	1.000	1.000	0.1063
1555746_at	29.1	64.8	1.16	2.23	AAA-AAA	0.00	999	1.000	1.000	0.1947
206638_at	6.1	13.6	1.16	2.23	AAA-AAA	0.00	999	1.000	1.000	0.4967
236461_at	61.4	27.5	-1.16	-2.23	AAA-AAA	0.00	999	1.000	1.000	0.3808
219586_at	31.1	69.4	1.16	2.23	AAA-AMP	0.00	999	1.000	1.000	0.5158
231294_at	8.4	18.8	1.16	2.23	APA-AAA	0.00	999	1.000	1.000	0.3290
243274_x_at	6.1	2.7	-1.16	-2.23	AAA-AAA	0.00	999	1.000	1.000	0.5617
1564819_at	11.6	25.9	1.16	2.23	AAA-AAA	0.00	999	1.000	1.000	0.3892
1554965_at	15.1	6.8	-1.16	-2.23	AAA-AAA	0.00	999	1.000	1.000	0.2649

1555158_at	10.1	22.5	1.16	2.23	AAA-AAA	0.00	999	1.000	1.000	0.3849
226407_at	40.0	17.9	-1.16	-2.23	AAA-AAA	0.00	999	1.000	1.000	0.2439
233251_at	10.3	4.6	-1.16	-2.23	AAA-AAA	0.00	999	1.000	1.000	0.5096
217009_at	25.4	56.5	1.16	2.23	PAA-AMA	0.00	999	1.000	1.000	0.4048
1565755_at	7.0	3.2	-1.16	-2.23	AAA-AAA	0.00	999	1.000	1.000	0.2129
1558778_s_at	15.9	7.1	-1.16	-2.23	AAA-AAA	0.00	999	1.000	1.000	0.3700
207142_at	7.0	15.6	1.16	2.23	AAA-AAA	0.00	999	1.000	1.000	0.3865
227190_at	6.8	15.2	1.16	2.23	AAA-AAA	0.00	999	1.000	1.000	0.2706
224270_at	17.0	7.6	-1.16	-2.23	AAA-AAA	0.00	999	1.000	1.000	0.2377
224244_s_at	19.6	43.6	1.16	2.23	AAA-AAA	0.00	999	1.000	1.000	0.0487
215200_x_at	118.2	53.0	-1.16	-2.23	AAA-AAA	0.00	999	1.000	1.000	0.3575
211179_at	9.1	4.1	-1.16	-2.23	AAA-AAA	0.00	999	1.000	1.000	0.4718
210644_s_at	92.3	41.4	-1.16	-2.23	AAA-AAA	0.00	999	1.000	1.000	0.1763
206393_at	7.5	3.4	-1.16	-2.23	AAA-AAA	0.00	999	1.000	1.000	0.0066
242174_at	28.0	12.6	-1.16	-2.23	AAA-AAA	0.00	999	1.000	1.000	0.3804
1570298_at	18.9	42.1	1.16	2.23	AAA-AAA	0.00	999	1.000	1.000	0.2999
1554809_at	266.8	119.8	-1.16	-2.23	AAA-AAA	0.00	999	1.000	1.000	0.0149
1556916_a_at	15.5	6.9	-1.16	-2.23	AAA-AAA	0.00	999	1.000	1.000	0.2985
220910_at	24.4	54.3	1.16	2.23	AAA-AAA	0.00	999	1.000	1.000	0.4614
223902_at	21.6	9.7	-1.16	-2.23	AAA-AAA	0.00	999	1.000	1.000	0.3955
214418_at	19.9	8.9	-1.16	-2.23	AAA-AAA	0.00	999	1.000	1.000	0.0919
215370_at	55.0	24.7	-1.16	-2.23	AAA-AAA	0.00	999	1.000	1.000	0.2613
236335_at	2.7	6.1	1.16	2.23	AAA-AAA	0.00	999	1.000	1.000	0.5385
204983_s_at	42.1	18.9	-1.16	-2.23	MAA-AAA	0.00	999	1.000	1.000	0.4094
212182_at	24.4	54.4	1.15	2.23	AAA-PPP	0.00	999	1.000	1.000	0.3167
216150_at	24.9	55.5	1.15	2.23	AAA-PAA	0.00	999	1.000	1.000	0.1371
240006_at	40.0	18.0	-1.15	-2.23	AAA-AAA	0.00	999	1.000	1.000	0.3067
241828_x_at	87.7	39.4	-1.15	-2.23	AAA-AAA	0.00	999	1.000	1.000	0.2195
243652_at	4.3	1.9	-1.15	-2.23	AAA-AAA	0.00	999	1.000	1.000	0.1951
1561536_at	21.9	9.9	-1.15	-2.23	AAA-AAA	0.00	999	1.000	1.000	0.4971
1567997_x_at	34.0	15.3	-1.15	-2.23	AAA-AAA	0.00	999	1.000	1.000	0.2369
208196_x_at	36.2	16.3	-1.15	-2.23	AAA-AAA	0.00	999	1.000	1.000	0.2503
235740_at	21.3	9.6	-1.15	-2.23	AAA-AAA	0.00	999	1.000	1.000	0.5030
230536_at	26.2	58.4	1.15	2.23	AAA-AAA	0.00	999	1.000	1.000	0.4184
236586_at	25.2	56.1	1.15	2.22	AAP-PPP	0.00	999	1.000	1.000	0.3491
240039_at	62.5	28.1	-1.15	-2.22	AAA-AAA	0.00	999	1.000	1.000	0.3488
243270_at	19.9	44.3	1.15	2.22	AAA-AAA	0.00	999	1.000	1.000	0.3040
1563371_at	4.3	9.6	1.15	2.22	AAA-AAA	0.00	999	1.000	1.000	0.2964
1565890_at	18.8	8.5	-1.15	-2.22	AAA-AAA	0.00	999	1.000	1.000	0.4748
1553929_at	7.5	3.4	-1.15	-2.22	AAA-AAA	0.00	999	1.000	1.000	0.0557
206596_s_at	22.4	10.1	-1.15	-2.22	AAA-AAA	0.00	999	1.000	1.000	0.3942
210571_s_at	23.8	10.7	-1.15	-2.22	AAA-AAA	0.00	999	1.000	1.000	0.2661

239424_at	44.4	20.0	-1.15	-2.22	AAA-AAA	0.00	999	1.000	1.000	0.3749
239339_at	10.5	23.4	1.15	2.22	AAA-AAA	0.00	999	1.000	1.000	0.2896
238064_at	29.4	65.3	1.15	2.22	AAA-AAA	0.00	999	1.000	1.000	0.3429
214954_at	251.9	559.6	1.15	2.22	PPP-PPP	0.00	999	1.000	1.000	0.0934
241291_at	4.0	8.8	1.15	2.22	AAA-AAA	0.00	999	1.000	1.000	0.4585
1554043_a_at	104.6	47.1	-1.15	-2.22	AAA-AAA	0.00	999	1.000	1.000	0.2372
1558378_a_at	10.9	24.3	1.15	2.22	AAA-AAA	0.00	999	1.000	1.000	0.2359
89977_at	74.8	33.7	-1.15	-2.22	AAA-AAA	0.00	999	1.000	1.000	0.1529
224515_at	12.0	5.4	-1.15	-2.22	AAA-AAA	0.00	999	1.000	1.000	0.1482
224295_at	27.9	12.6	-1.15	-2.22	AAA-AAA	0.00	999	1.000	1.000	0.4127
217151_at	3.1	6.9	1.15	2.22	AAA-AAA	0.00	999	1.000	1.000	0.2950
238292_at	16.3	36.2	1.15	2.22	AAA-AMA	0.00	999	1.000	1.000	0.2272
237146_at	34.6	15.6	-1.15	-2.22	AAA-AAA	0.00	999	1.000	1.000	0.3240
230353_at	85.6	38.5	-1.15	-2.22	AAA-AAA	0.00	999	1.000	1.000	0.3147
235019_at	18.9	42.0	1.15	2.22	AAM-APA	0.00	999	1.000	1.000	0.5129
240463_at	12.4	27.5	1.15	2.22	APA-APA	0.00	999	1.000	1.000	0.3099
239975_at	55.0	24.8	-1.15	-2.22	AAA-AAA	0.00	999	1.000	1.000	0.0276
203779_s_at	8.7	3.9	-1.15	-2.22	AAA-AAA	0.00	999	1.000	1.000	0.1319
1560332_at	18.3	40.6	1.15	2.22	AAA-AAA	0.00	999	1.000	1.000	0.3764
1561669_at	9.8	21.7	1.15	2.22	AAM-AAA	0.00	999	1.000	1.000	0.3252
1566486_at	7.8	3.5	-1.15	-2.22	AAA-AAA	0.00	999	1.000	1.000	0.1422
1554506_x_at	18.4	8.3	-1.15	-2.22	AAA-AAA	0.00	999	1.000	1.000	0.5084
214569_at	3.0	6.7	1.15	2.22	AAA-AAA	0.00	999	1.000	1.000	0.1970
210697_at	6.8	3.1	-1.15	-2.22	AAA-AAA	0.00	999	1.000	1.000	0.1596
234117_at	52.9	23.8	-1.15	-2.22	AAA-AAA	0.00	999	1.000	1.000	0.1182
227795_at	35.8	16.1	-1.15	-2.22	AAA-AAA	0.00	999	1.000	1.000	0.0216
1569469_a_at	3.1	6.8	1.15	2.22	AAA-AAA	0.00	999	1.000	1.000	0.6196
244478_at	120.4	54.2	-1.15	-2.22	AMA-AAA	0.00	999	1.000	1.000	0.1606
1559028_at	66.6	30.0	-1.15	-2.22	AAA-AAA	0.00	999	1.000	1.000	0.4511
222097_at	56.4	25.4	-1.15	-2.22	AAA-AAA	0.00	999	1.000	1.000	0.2901
206910_x_at	14.4	31.9	1.15	2.22	AAA-AAA	0.00	999	1.000	1.000	0.1588
235930_at	19.2	8.7	-1.15	-2.22	AAA-AAA	0.00	999	1.000	1.000	0.2285
238262_at	18.8	8.5	-1.15	-2.22	AAA-AAA	0.00	999	1.000	1.000	0.4603
237582_at	57.5	25.9	-1.15	-2.22	AAA-AAA	0.00	999	1.000	1.000	0.0690
241020_at	83.8	37.8	-1.15	-2.22	AAA-AAA	0.00	999	1.000	1.000	0.0781
243672_at	42.9	19.4	-1.15	-2.22	AAA-AAA	0.00	999	1.000	1.000	0.1988
243422_at	3.0	6.7	1.15	2.22	AAA-AAA	0.00	999	1.000	1.000	0.3382
241166_at	80.6	36.3	-1.15	-2.22	AAA-AAA	0.00	999	1.000	1.000	0.1884
1564539_at	14.7	6.6	-1.15	-2.22	AAA-AAA	0.00	999	1.000	1.000	0.3410
221969_at	43.7	19.7	-1.15	-2.22	AAA-AAA	0.00	999	1.000	1.000	0.5180
220126_at	10.9	24.2	1.15	2.22	AAA-AAA	0.00	999	1.000	1.000	0.4642
211196_at	56.4	25.4	-1.15	-2.22	AAA-AAA	0.00	999	1.000	1.000	0.2752

234225_at	15.8	7.1	-1.15	-2.22	AAM-AAA	0.00	999	1.000	1.000	0.3235
230317_x_at	12.1	5.5	-1.15	-2.22	AAA-AAA	0.00	999	1.000	1.000	0.1249
205180_s_at	68.1	30.7	-1.15	-2.22	AAA-AAA	0.00	999	1.000	1.000	0.3531
231172_at	24.9	55.2	1.15	2.22	PAA-AAA	0.00	999	1.000	1.000	0.3351
243825_at	30.5	13.8	-1.15	-2.22	AAA-AAA	0.00	999	1.000	1.000	0.1167
242526_at	11.2	24.8	1.15	2.22	AAA-AAA	0.00	999	1.000	1.000	0.2201
244051_at	18.7	8.5	-1.15	-2.22	AAA-AAA	0.00	999	1.000	1.000	0.3898
1563055_at	2.6	5.7	1.15	2.22	AAA-AAA	0.00	999	1.000	1.000	0.3916
1563135_at	8.4	3.8	-1.15	-2.22	AAA-AAA	0.00	999	1.000	1.000	0.5580
1564027_a_at	14.7	32.6	1.15	2.22	AAA-AAA	0.00	999	1.000	1.000	0.1220
1552448_a_at	10.3	4.6	-1.15	-2.22	AAA-AAA	0.00	999	1.000	1.000	0.4190
1558565_at	5.1	11.4	1.15	2.22	AAA-AAA	0.00	999	1.000	1.000	0.3002
220978_at	12.9	5.8	-1.15	-2.22	AAA-AAA	0.00	999	1.000	1.000	0.5721
221183_at	23.3	10.5	-1.15	-2.22	AAA-AAA	0.00	999	1.000	1.000	0.3771
227242_s_at	2.2	4.9	1.15	2.22	AAA-AAA	0.00	999	1.000	1.000	0.3440
210383_at	2.7	6.0	1.15	2.22	AAA-AAA	0.00	999	1.000	1.000	0.4209
219191_s_at	8.8	4.0	-1.15	-2.22	AAA-AAA	0.00	999	1.000	1.000	0.0739
228413_s_at	8.8	19.6	1.15	2.22	AAA-AAA	0.00	999	1.000	1.000	0.3005
1555341_at	6.4	14.3	1.15	2.22	AAA-AAA	0.00	999	1.000	1.000	0.2456
1554716_s_at	23.9	10.8	-1.15	-2.21	AAA-AAA	0.00	999	1.000	1.000	0.2951
220402_at	17.9	8.1	-1.15	-2.22	AAA-AAA	0.00	999	1.000	1.000	0.2419
224313_at	7.1	3.2	-1.15	-2.22	AAA-AAA	0.00	999	1.000	1.000	0.0408
214497_s_at	11.1	5.0	-1.15	-2.22	AAA-AAA	0.00	999	1.000	1.000	0.0563
216014_s_at	43.3	19.5	-1.15	-2.21	AAA-AAA	0.00	999	1.000	1.000	0.4775
232534_at	5.5	12.2	1.15	2.21	AAA-AAA	0.00	999	1.000	1.000	0.3182
205868_s_at	108.4	239.7	1.15	2.21	APP-PPP	0.00	999	1.000	1.000	0.4740
237908_at	22.4	49.6	1.15	2.21	AAA-PAM	0.00	999	1.000	1.000	0.4692
225997_at	712.4	1576.3	1.15	2.21	PPP-PPP	0.00	999	1.000	1.000	0.1347
1568604_a_at	3.4	7.5	1.15	2.21	AAA-AAA	0.00	999	1.000	1.000	0.3620
1565925_at	23.1	10.4	-1.15	-2.21	AAA-AAA	0.00	999	1.000	1.000	0.5011
1558647_at	46.6	21.0	-1.15	-2.21	AAA-AAA	0.00	999	1.000	1.000	0.4184
220923_s_at	39.9	88.4	1.15	2.21	AAA-AAA	0.00	999	1.000	1.000	0.4751
225809_at	14.7	6.7	-1.15	-2.21	AAA-AAA	0.00	999	1.000	1.000	0.2535
223572_at	33.0	14.9	-1.15	-2.21	AAA-AAA	0.00	999	1.000	1.000	0.1439
213575_at	89.9	40.6	-1.15	-2.21	AAA-AAA	0.00	999	1.000	1.000	0.4310
237608_at	14.6	6.6	-1.15	-2.21	AAA-AAA	0.00	999	1.000	1.000	0.4660
233009_at	76.3	34.5	-1.15	-2.21	AAA-AAA	0.00	999	1.000	1.000	0.3276
233967_at	20.5	45.3	1.15	2.21	AAA-PAA	0.00	999	1.000	1.000	0.0925
223652_at	55.5	122.8	1.15	2.21	APA-PPP	0.00	999	1.000	1.000	0.0709
1570618_at	8.1	3.7	-1.15	-2.21	AAA-AAA	0.00	999	1.000	1.000	0.4504
1566874_at	13.2	29.3	1.15	2.21	AAA-AAA	0.00	999	1.000	1.000	0.1051
1554049_s_at	18.9	41.8	1.15	2.21	AAA-AAA	0.00	999	1.000	1.000	0.2429

1554988_at	5.5	2.5	-1.15	-2.21	AAA-AAA	0.00	999	1.000	1.000	0.1708
1558418_at	65.0	29.4	-1.15	-2.21	AAA-AAA	0.00	999	1.000	1.000	0.0207
223574_x_at	36.6	16.6	-1.15	-2.21	AAA-AAA	0.00	999	1.000	1.000	0.1900
222784_at	48.2	21.8	-1.15	-2.21	AAA-AAA	0.00	999	1.000	1.000	0.2030
209810_at	66.2	29.9	-1.15	-2.21	AAA-AAA	0.00	999	1.000	1.000	0.1494
214511_x_at	14.0	30.8	1.15	2.21	AAA-AAA	0.00	999	1.000	1.000	0.4253
211046_at	22.6	10.2	-1.15	-2.21	AAA-AAA	0.00	999	1.000	1.000	0.4658
217005_at	17.3	7.8	-1.15	-2.21	AAA-AAA	0.00	999	1.000	1.000	0.3690
205985_x_at	7.0	15.4	1.15	2.21	AAA-AAA	0.00	999	1.000	1.000	0.3996
206294_at	28.6	12.9	-1.15	-2.21	AAA-AAA	0.00	999	1.000	1.000	0.3636
206386_at	8.1	17.8	1.15	2.21	AAA-AAA	0.00	999	1.000	1.000	0.4786
232684_at	9.8	21.7	1.15	2.21	AAA-AAA	0.00	999	1.000	1.000	0.3017
213438_at	15.8	34.8	1.14	2.21	AAA-AAP	0.00	999	1.000	1.000	0.1889
241643_at	17.5	7.9	-1.14	-2.21	AAA-AAA	0.00	999	1.000	1.000	0.3444
241317_at	11.2	5.1	-1.14	-2.21	AAA-AAA	0.00	999	1.000	1.000	0.3377
241591_at	14.7	6.7	-1.14	-2.21	AAA-AAA	0.00	999	1.000	1.000	0.4430
1566963_at	15.3	6.9	-1.14	-2.21	AAA-AAA	0.00	999	1.000	1.000	0.1010
1558612_a_at	14.4	31.7	1.14	2.21	AAA-AAA	0.00	999	1.000	1.000	0.3113
1557123_a_at	19.6	43.3	1.14	2.21	AAA-AAA	0.00	999	1.000	1.000	0.2382
225801_at	24.1	10.9	-1.14	-2.21	AAA-AAA	0.00	999	1.000	1.000	0.3879
216646_at	23.9	10.8	-1.14	-2.21	AAA-AAA	0.00	999	1.000	1.000	0.2860
231347_at	22.1	10.0	-1.14	-2.21	AAA-AAA	0.00	999	1.000	1.000	0.3399
231344_at	5.5	2.5	-1.14	-2.21	AAA-AAA	0.00	999	1.000	1.000	0.0068
231242_at	20.6	9.3	-1.14	-2.21	AAA-AAA	0.00	999	1.000	1.000	0.5003
231703_s_at	6.6	3.0	-1.14	-2.21	AAA-AAA	0.00	999	1.000	1.000	0.2101
222924_at	84.8	187.3	1.14	2.21	PAA-PPP	0.00	999	1.000	1.000	0.1193
240713_s_at	2.9	1.3	-1.14	-2.21	AAA-AAA	0.00	999	1.000	1.000	0.0696
1561924_at	22.8	50.3	1.14	2.21	AAA-AAA	0.00	999	1.000	1.000	0.0048
209170_s_at	6.7	3.0	-1.14	-2.21	AAA-AAA	0.00	999	1.000	1.000	0.1472
216632_at	2.9	6.4	1.14	2.21	AAA-AAA	0.00	999	1.000	1.000	0.3593
234019_at	7.1	3.2	-1.14	-2.21	AAA-AAA	0.00	999	1.000	1.000	0.4177
238705_at	56.7	25.7	-1.14	-2.21	AMA-AAA	0.00	999	1.000	1.000	0.0524
230017_at	47.0	103.7	1.14	2.21	AAA-AAA	0.00	999	1.000	1.000	0.3870
231826_at	46.8	21.2	-1.14	-2.21	AAA-AAA	0.00	999	1.000	1.000	0.3596
240603_s_at	11.5	25.3	1.14	2.21	PAA-PMP	0.00	999	1.000	1.000	0.4416
1561392_at	17.9	39.5	1.14	2.21	AAA-APA	0.00	999	1.000	1.000	0.3213
241312_at	72.4	32.8	-1.14	-2.21	AAA-AAA	0.00	999	1.000	1.000	0.0855
1564331_at	19.9	9.0	-1.14	-2.21	AAA-AAA	0.00	999	1.000	1.000	0.2614
1553739_at	10.4	4.7	-1.14	-2.21	AAA-AAA	0.00	999	1.000	1.000	0.2705
1552646_at	142.6	64.6	-1.14	-2.21	AAA-AAA	0.00	999	1.000	1.000	0.1668
222008_at	8.0	3.6	-1.14	-2.21	AAA-AAA	0.00	999	1.000	1.000	0.0071
208227_x_at	20.0	9.1	-1.14	-2.21	AAA-AAA	0.00	999	1.000	1.000	0.1433

206758_at	48.9	22.2	-1.14	-2.21	AAA-AAA	0.00	999	1.000	1.000	0.4165
206483_at	91.9	41.6	-1.14	-2.21	AAA-AAA	0.00	999	1.000	1.000	0.1978
206698_at	15.1	6.8	-1.14	-2.21	AAA-AAA	0.00	999	1.000	1.000	0.3764
217474_at	60.8	27.6	-1.14	-2.21	AAA-AAA	0.00	999	1.000	1.000	0.2071
204439_at	13.3	6.0	-1.14	-2.21	AAA-AAA	0.00	999	1.000	1.000	0.1214
243870_at	4.7	10.4	1.14	2.21	AAA-AAA	0.00	999	1.000	1.000	0.1118
1552327_at	15.0	6.8	-1.14	-2.21	AAA-AAA	0.00	999	1.000	1.000	0.4708
1558113_at	30.9	14.0	-1.14	-2.21	AAA-AAA	0.00	999	1.000	1.000	0.3003
222384_at	16.6	36.6	1.14	2.21	AAA-AAA	0.00	999	1.000	1.000	0.0739
216832_at	72.5	32.9	-1.14	-2.21	AAA-AAA	0.00	999	1.000	1.000	0.1079
204880_at	13.7	6.2	-1.14	-2.21	AAA-AAA	0.00	999	1.000	1.000	0.3435
232799_at	23.3	10.5	-1.14	-2.21	AAA-AAA	0.00	999	1.000	1.000	0.3810
231597_x_at	19.5	8.9	-1.14	-2.21	AAA-AAA	0.00	999	1.000	1.000	0.5341
231050_at	19.3	42.5	1.14	2.21	AAA-AAA	0.00	999	1.000	1.000	0.2099
232248_at	34.3	75.6	1.14	2.21	AAA-AAA	0.00	999	1.000	1.000	0.3506
243276_at	15.9	7.2	-1.14	-2.20	AAA-AAA	0.00	999	1.000	1.000	0.0967
241909_at	4.7	10.3	1.14	2.20	AAA-AAA	0.00	999	1.000	1.000	0.3653
1563533_at	9.5	21.0	1.14	2.20	AAA-AAA	0.00	999	1.000	1.000	0.3002
1563868_a_at	8.5	3.9	-1.14	-2.20	AAA-AAA	0.00	999	1.000	1.000	0.0036
1555962_at	9.2	4.2	-1.14	-2.20	AAA-AAA	0.00	999	1.000	1.000	0.1851
207875_at	6.3	2.8	-1.14	-2.20	AAA-AAA	0.00	999	1.000	1.000	0.0915
216959_x_at	41.8	19.0	-1.14	-2.20	AAA-AAA	0.00	999	1.000	1.000	0.4566
218054_s_at	46.6	102.7	1.14	2.20	AAA-AAA	0.00	999	1.000	1.000	0.1219
209074_s_at	12.8	5.8	-1.14	-2.20	AAA-AAA	0.00	999	1.000	1.000	0.2218
204913_s_at	8.9	4.0	-1.14	-2.20	AAM-AAA	0.00	999	1.000	1.000	0.4640
240991_at	25.2	55.4	1.14	2.20	AAA-AAA	0.00	999	1.000	1.000	0.1949
1564407_a_at	21.4	9.7	-1.14	-2.20	AAA-AAA	0.00	999	1.000	1.000	0.3283
1552541_at	74.7	33.9	-1.14	-2.20	AAA-AAA	0.00	999	1.000	1.000	0.1480
41397_at	36.3	80.0	1.14	2.20	AAA-AAA	0.00	999	1.000	1.000	0.3748
220109_at	12.5	5.7	-1.14	-2.20	AAA-AAA	0.00	999	1.000	1.000	0.6073
211145_x_at	14.4	6.5	-1.14	-2.20	AAA-AAA	0.00	999	1.000	1.000	0.3568
215796_at	23.4	10.6	-1.14	-2.20	AAA-AAA	0.00	999	1.000	1.000	0.4562
239746_at	47.6	21.6	-1.14	-2.20	AAA-AAA	0.00	999	1.000	1.000	0.1758
229698_at	36.4	16.6	-1.14	-2.20	AAA-AAA	0.00	999	1.000	1.000	0.1999
229316_at	17.6	38.7	1.14	2.20	AAA-AAA	0.00	999	1.000	1.000	0.2856
233360_at	31.2	68.6	1.14	2.20	APA-APA	0.00	999	1.000	1.000	0.2959
209621_s_at	85.0	187.0	1.14	2.20	AAA-PAM	0.00	999	1.000	1.000	0.2734
243567_at	17.5	8.0	-1.14	-2.20	AAA-AAA	0.00	999	1.000	1.000	0.2813
1563606_a_at	22.0	10.0	-1.14	-2.20	MAA-AAA	0.00	999	1.000	1.000	0.4731
1569063_at	31.6	14.4	-1.14	-2.20	AAA-AAA	0.00	999	1.000	1.000	0.1441
1564868_a_at	8.9	4.1	-1.14	-2.20	AAA-AAA	0.00	999	1.000	1.000	0.1336
1566499_at	34.2	15.5	-1.14	-2.20	AAA-AAA	0.00	999	1.000	1.000	0.3382

221862_at	93.4	42.4	-1.14	-2.20	AAA-AAA	0.00	999	1.000	1.000	0.0670
206856_at	6.4	14.1	1.14	2.20	AAA-AAA	0.00	999	1.000	1.000	0.3137
230359_at	18.4	40.5	1.14	2.20	AAA-AAA	0.00	999	1.000	1.000	0.2501
202198_s_at	44.2	97.3	1.14	2.20	AAA-APP	0.00	999	1.000	1.000	0.3720
1563298_at	16.2	35.6	1.14	2.20	AAA-PAA	0.00	999	1.000	1.000	0.3463
228096_at	55.6	122.2	1.14	2.20	AAP-AAM	0.00	999	1.000	1.000	0.2362
1563505_at	41.8	92.0	1.14	2.20	AAA-AAA	0.00	999	1.000	1.000	0.2128
1564485_at	23.1	10.5	-1.14	-2.20	AAA-AAA	0.00	999	1.000	1.000	0.4823
1552751_a_at	66.4	30.2	-1.14	-2.20	AAA-AAA	0.00	999	1.000	1.000	0.2520
209937_at	17.1	7.8	-1.14	-2.20	AAA-AAA	0.00	999	1.000	1.000	0.3405
209812_x_at	40.0	88.0	1.14	2.20	AAA-AAA	0.00	999	1.000	1.000	0.1358
237969_at	49.5	22.5	-1.14	-2.20	AAA-AAA	0.00	999	1.000	1.000	0.4603
227654_at	7.3	16.0	1.14	2.20	AAA-AAA	0.00	999	1.000	1.000	0.3567
1563344_at	104.8	47.7	-1.14	-2.20	AAA-AAA	0.00	999	1.000	1.000	0.1230
1566897_at	13.0	28.6	1.14	2.20	AAA-AAA	0.00	999	1.000	1.000	0.2880
1554094_at	61.2	27.8	-1.14	-2.20	AAA-AAA	0.00	999	1.000	1.000	0.2032
214546_s_at	46.3	21.1	-1.14	-2.20	AAA-AAA	0.00	999	1.000	1.000	0.3938
234833_at	2.4	1.1	-1.14	-2.20	AAA-AAA	0.00	999	1.000	1.000	0.2483
234451_at	16.6	7.6	-1.14	-2.20	AAA-AAA	0.00	999	1.000	1.000	0.0578
239616_at	36.6	16.6	-1.14	-2.20	AAA-AAA	0.00	999	1.000	1.000	0.4838
236909_at	4.5	9.8	1.14	2.20	AAA-AAA	0.00	999	1.000	1.000	0.0837
1557866_at	42.4	93.1	1.14	2.20	AAA-AAP	0.00	999	1.000	1.000	0.2951
1567054_at	33.9	15.4	-1.14	-2.20	AAA-AAA	0.00	999	1.000	1.000	0.3804
1556053_at	52.5	115.3	1.14	2.20	AAA-AAM	0.00	999	1.000	1.000	0.2736
207583_at	10.1	4.6	-1.14	-2.20	AAA-AAA	0.00	999	1.000	1.000	0.4129
234852_at	27.0	12.3	-1.14	-2.20	AAA-AAA	0.00	999	1.000	1.000	0.5557
239551_at	35.1	77.1	1.14	2.20	AAA-AAA	0.00	999	1.000	1.000	0.3772
229729_at	78.3	35.7	-1.14	-2.20	AAA-AAA	0.00	999	1.000	1.000	0.3688
230544_at	20.2	9.2	-1.14	-2.20	AAA-AAA	0.00	999	1.000	1.000	0.2035
224459_at	7.7	16.9	1.13	2.20	AAA-PAA	0.00	999	1.000	1.000	0.2823
1562696_at	7.3	16.0	1.13	2.20	AAA-AAA	0.00	999	1.000	1.000	0.1405
1561029_at	14.9	6.8	-1.13	-2.20	AAA-AAA	0.00	999	1.000	1.000	0.0340
1564138_at	13.1	6.0	-1.13	-2.20	AAA-AAA	0.00	999	1.000	1.000	0.2614
226417_at	34.3	15.6	-1.13	-2.20	AAA-AAA	0.00	999	1.000	1.000	0.3738
213855_s_at	66.1	30.1	-1.13	-2.20	AAA-AAA	0.00	999	1.000	1.000	0.3570
214597_at	12.9	28.3	1.13	2.19	AAA-AAA	0.00	999	1.000	1.000	0.2732
215846_at	11.0	5.0	-1.13	-2.19	AAA-AAA	0.00	999	1.000	1.000	0.5094
236260_at	13.9	30.5	1.13	2.19	AAA-AAA	0.00	999	1.000	1.000	0.2082
234655_at	5.5	2.5	-1.13	-2.20	AAA-AAA	0.00	999	1.000	1.000	0.4859
237503_at	25.0	11.4	-1.13	-2.20	AAA-AAA	0.00	999	1.000	1.000	0.3534
228187_at	42.1	19.2	-1.13	-2.20	AAA-AAA	0.00	999	1.000	1.000	0.3239
229247_at	16.8	36.8	1.13	2.20	AAA-AAA	0.00	999	1.000	1.000	0.4289

231048_at	26.5	58.3	1.13	2.20	AAA-AAA	0.00	999	1.000	1.000	0.3591
1560237_at	5.7	12.4	1.13	2.19	AAA-AAA	0.00	999	1.000	1.000	0.2920
1553636_at	2.9	1.3	-1.13	-2.19	AAA-AAA	0.00	999	1.000	1.000	0.0771
226494_at	18.5	8.5	-1.13	-2.19	AAA-AAA	0.00	999	1.000	1.000	0.1824
217018_at	20.5	9.4	-1.13	-2.19	AAA-AAA	0.00	999	1.000	1.000	0.2294
215431_at	12.4	5.7	-1.13	-2.19	AAA-AAA	0.00	999	1.000	1.000	0.4758
234434_at	18.0	8.2	-1.13	-2.19	AAA-AAA	0.00	999	1.000	1.000	0.3505
204040_at	12.4	5.6	-1.13	-2.19	AAA-AAA	0.00	999	1.000	1.000	0.1064
206073_at	52.9	24.1	-1.13	-2.19	MAA-AAA	0.00	999	1.000	1.000	0.3773
1558602_a_at	15.5	34.0	1.13	2.19	AAA-PAA	0.00	999	1.000	1.000	0.3436
231515_at	41.7	91.4	1.13	2.19	APA-PAP	0.00	999	1.000	1.000	0.2316
1565614_at	29.0	13.2	-1.13	-2.19	AAA-AAA	0.00	999	1.000	1.000	0.1322
1567387_at	7.6	3.5	-1.13	-2.19	AAA-AAA	0.00	999	1.000	1.000	0.4740
1555222_a_at	289.1	131.9	-1.13	-2.19	AAA-AAA	0.00	999	1.000	1.000	0.0098
1552783_at	8.4	18.4	1.13	2.19	AAA-AAA	0.00	999	1.000	1.000	0.1936
1557888_at	17.8	8.1	-1.13	-2.19	AAA-AAA	0.00	999	1.000	1.000	0.4758
1557776_at	33.4	15.2	-1.13	-2.19	AAA-AAA	0.00	999	1.000	1.000	0.2396
221181_at	3.7	8.2	1.13	2.19	AAA-AAA	0.00	999	1.000	1.000	0.2794
208376_at	13.4	6.1	-1.13	-2.19	AAA-AAA	0.00	999	1.000	1.000	0.3211
207249_s_at	10.2	4.7	-1.13	-2.19	AAA-AAA	0.00	999	1.000	1.000	0.4765
206691_s_at	24.1	11.0	-1.13	-2.19	AAA-AAA	0.00	999	1.000	1.000	0.3598
216920_s_at	22.1	10.1	-1.13	-2.19	AAA-AAA	0.00	999	1.000	1.000	0.4128
230889_at	21.6	9.9	-1.13	-2.19	AAA-AAA	0.00	999	1.000	1.000	0.4930
240376_s_at	22.0	10.1	-1.13	-2.19	AAA-AAA	0.00	999	1.000	1.000	0.4359
242896_at	11.8	5.4	-1.13	-2.19	AAA-AAA	0.00	999	1.000	1.000	0.2984
1562384_at	48.7	22.3	-1.13	-2.19	AAA-AAA	0.00	999	1.000	1.000	0.2769
1558619_at	21.1	46.3	1.13	2.19	AAA-AAA	0.00	999	1.000	1.000	0.1779
1555809_at	19.3	8.8	-1.13	-2.19	AAA-AAA	0.00	999	1.000	1.000	0.3128
208187_s_at	34.7	15.8	-1.13	-2.19	AAA-AAA	0.00	999	1.000	1.000	0.4608
207336_at	5.6	2.6	-1.13	-2.19	AAA-AAA	0.00	999	1.000	1.000	0.1289
234924_s_at	59.5	130.3	1.13	2.19	AAA-AAA	0.00	999	1.000	1.000	0.0845
206217_at	38.9	17.8	-1.13	-2.19	AAA-AAA	0.00	999	1.000	1.000	0.4813
233318_at	2.0	4.3	1.13	2.19	AAA-AAA	0.00	999	1.000	1.000	0.2860
230931_at	20.0	43.8	1.13	2.19	APA-APA	0.00	999	1.000	1.000	0.5209
217481_x_at	23.4	51.3	1.13	2.19	AAA-AAP	0.00	999	1.000	1.000	0.2856
239918_at	12.7	27.8	1.13	2.19	AAA-AAA	0.00	999	1.000	1.000	0.2827
243725_at	22.6	10.3	-1.13	-2.19	AAA-AAA	0.00	999	1.000	1.000	0.3357
240489_at	31.4	14.3	-1.13	-2.19	AAA-AAA	0.00	999	1.000	1.000	0.4077
1569476_at	15.4	7.0	-1.13	-2.19	AMA-AAA	0.00	999	1.000	1.000	0.5506
1570430_at	8.9	19.4	1.13	2.19	AAA-AAA	0.00	999	1.000	1.000	0.3447
1565105_at	6.3	2.9	-1.13	-2.19	AAA-AAA	0.00	999	1.000	1.000	0.4160
219578_s_at	16.5	7.6	-1.13	-2.19	AAA-AAA	0.00	999	1.000	1.000	0.2014

227183_at	13.7	6.3	-1.13	-2.19	AAA-AAA	0.00	999	1.000	1.000	0.4278
213641_at	112.1	51.2	-1.13	-2.19	AAA-AAM	0.00	999	1.000	1.000	0.2528
238343_x_at	4.8	2.2	-1.13	-2.19	AAA-AAA	0.00	999	1.000	1.000	0.5579
216239_at	22.4	49.1	1.13	2.19	AAA-AAP	0.00	999	1.000	1.000	0.1860
240035_at	65.6	30.0	-1.13	-2.19	AAA-AAA	0.00	999	1.000	1.000	0.0903
240711_at	5.5	12.0	1.13	2.19	AAA-AAA	0.00	999	1.000	1.000	0.3667
1562371_s_at	17.8	8.1	-1.13	-2.19	AAM-AAA	0.00	999	1.000	1.000	0.2835
1560867_a_at	19.5	8.9	-1.13	-2.19	AAA-AAA	0.00	999	1.000	1.000	0.5163
202291_s_at	3.5	7.7	1.13	2.19	AAA-AAA	0.00	999	1.000	1.000	0.1038
220111_s_at	14.7	6.7	-1.13	-2.19	AAA-AAA	0.00	999	1.000	1.000	0.4490
207623_at	25.8	11.8	-1.13	-2.19	AAA-AAA	0.00	999	1.000	1.000	0.5331
216010_x_at	19.9	43.5	1.13	2.19	AAA-AAA	0.00	999	1.000	1.000	0.3282
234585_at	88.4	40.4	-1.13	-2.19	AAA-AAA	0.00	999	1.000	1.000	0.2293
234618_at	15.7	34.4	1.13	2.19	AAA-AAA	0.00	999	1.000	1.000	0.2415
237301_at	20.4	9.3	-1.13	-2.19	AAA-AAA	0.00	999	1.000	1.000	0.4267
213652_at	6.3	2.9	-1.13	-2.19	AAA-AAA	0.00	999	1.000	1.000	0.4895
211410_x_at	40.4	18.5	-1.13	-2.19	AAA-AAA	0.00	999	1.000	1.000	0.1203
212235_at	27.3	59.6	1.13	2.19	AAA-AAA	0.00	999	1.000	1.000	0.2341
209114_at	21.4	9.8	-1.13	-2.19	AAA-AAA	0.00	999	1.000	1.000	0.2023
216117_at	64.1	29.3	-1.13	-2.19	AAA-AAA	0.00	999	1.000	1.000	0.1582
233894_x_at	20.2	9.2	-1.13	-2.19	AAA-AAA	0.00	999	1.000	1.000	0.4456
205242_at	10.6	4.9	-1.13	-2.19	AAA-AAA	0.00	999	1.000	1.000	0.4511
234428_at	4.1	8.9	1.13	2.18	AAA-APA	0.00	999	1.000	1.000	0.4828
240962_at	133.4	61.1	-1.13	-2.18	AAA-AAA	0.00	999	1.000	1.000	0.0704
241306_at	7.5	3.5	-1.13	-2.19	AAA-AAA	0.00	999	1.000	1.000	0.3111
220245_at	75.6	34.6	-1.13	-2.18	AAA-AAA	0.00	999	1.000	1.000	0.1662
207110_at	40.7	18.6	-1.13	-2.19	AAA-AAA	0.00	999	1.000	1.000	0.3229
206690_at	32.0	14.7	-1.13	-2.18	AAA-AAA	0.00	999	1.000	1.000	0.3904
223447_at	67.1	30.7	-1.13	-2.19	AAA-AAA	0.00	999	1.000	1.000	0.4999
211740_at	25.0	11.4	-1.13	-2.18	AAA-AAA	0.00	999	1.000	1.000	0.4870
215661_at	29.0	13.3	-1.13	-2.18	AAA-AAA	0.00	999	1.000	1.000	0.4339
235160_at	19.3	42.1	1.13	2.19	AAA-AAA	0.00	999	1.000	1.000	0.4483
235624_at	39.6	18.1	-1.13	-2.18	AAA-AAA	0.00	999	1.000	1.000	0.3024
237359_at	22.2	48.5	1.13	2.19	AAA-AAA	0.00	999	1.000	1.000	0.3307
228102_at	8.3	3.8	-1.13	-2.18	AAA-AAA	0.00	999	1.000	1.000	0.1075
228459_at	8.2	17.9	1.13	2.18	AAA-AAA	0.00	999	1.000	1.000	0.3573
239661_at	12.5	27.4	1.13	2.18	AAA-PAA	0.00	999	1.000	1.000	0.3818
226435_at	15.5	33.8	1.13	2.18	AAA-AAP	0.00	999	1.000	1.000	0.2137
1561004_at	46.5	21.3	-1.13	-2.18	AAA-AAA	0.00	999	1.000	1.000	0.1818
1566884_at	30.2	13.8	-1.13	-2.18	AAA-AAA	0.00	999	1.000	1.000	0.5696
1554694_at	16.3	7.5	-1.13	-2.18	AAA-AAA	0.00	999	1.000	1.000	0.1681
1556817_a_at	5.6	12.2	1.13	2.18	AAA-AAA	0.00	999	1.000	1.000	0.0545

219572_at	50.0	22.9	-1.13	-2.18	AAA-AAA	0.00	999	1.000	1.000	0.1127
210300_at	107.0	49.0	-1.13	-2.18	AAA-AAA	0.00	999	1.000	1.000	0.3276
216890_at	11.5	5.3	-1.13	-2.18	AAA-AAA	0.00	999	1.000	1.000	0.2982
204138_s_at	75.1	34.4	-1.13	-2.18	AAA-AAA	0.00	999	1.000	1.000	0.2079
205989_s_at	20.9	9.6	-1.13	-2.18	AAA-AAA	0.00	999	1.000	1.000	0.3946
232379_at	18.0	39.3	1.13	2.18	AAA-AAA	0.00	999	1.000	1.000	0.3767
237115_at	10.5	22.9	1.13	2.18	AAA-AAP	0.00	999	1.000	1.000	0.4780
244066_at	28.1	12.9	-1.13	-2.18	AAA-AAA	0.00	999	1.000	1.000	0.4574
242284_at	25.4	55.3	1.13	2.18	MAA-AMA	0.00	999	1.000	1.000	0.4115
244428_at	48.3	105.4	1.13	2.18	AAA-AAA	0.00	999	1.000	1.000	0.0222
206887_at	42.9	93.7	1.13	2.18	AAA-AAA	0.00	999	1.000	1.000	0.2912
206985_at	32.2	14.8	-1.13	-2.18	AAA-AAA	0.00	999	1.000	1.000	0.1426
227441_s_at	3.2	1.5	-1.13	-2.18	AAA-AAA	0.00	999	1.000	1.000	0.2733
224080_at	5.6	2.6	-1.13	-2.18	AAA-AAA	0.00	999	1.000	1.000	0.4809
238981_at	10.2	4.7	-1.13	-2.18	AAA-AAA	0.00	999	1.000	1.000	0.0202
229731_at	46.6	21.4	-1.13	-2.18	AAA-AAA	0.00	999	1.000	1.000	0.2806
233442_at	65.5	30.0	-1.13	-2.18	AAA-AAA	0.00	999	1.000	1.000	0.0575
239080_at	17.4	37.9	1.12	2.18	AAP-MAP	0.00	999	1.000	1.000	0.2538
206870_at	38.6	84.1	1.12	2.18	AAA-PPA	0.00	999	1.000	1.000	0.1340
228652_at	267.2	582.4	1.12	2.18	PPP-PPP	0.00	999	1.000	1.000	0.1007
242177_at	34.4	15.8	-1.12	-2.18	AAA-AAA	0.00	999	1.000	1.000	0.5022
243755_at	8.7	19.1	1.12	2.18	AAA-AAA	0.00	999	1.000	1.000	0.1750
242800_at	27.4	59.8	1.12	2.18	AAA-MAA	0.00	999	1.000	1.000	0.2881
1563045_at	33.6	15.4	-1.12	-2.18	AAA-AAA	0.00	999	1.000	1.000	0.4265
1556012_at	19.6	9.0	-1.12	-2.18	AAA-AAA	0.00	999	1.000	1.000	0.0819
206740_x_at	25.8	11.8	-1.12	-2.18	AAA-AAA	0.00	999	1.000	1.000	0.2823
224018_s_at	58.6	127.7	1.12	2.18	AAA-AAA	0.00	999	1.000	1.000	0.1183
209942_x_at	11.8	5.4	-1.12	-2.18	AAA-AAA	0.00	999	1.000	1.000	0.4882
215801_at	26.9	12.3	-1.12	-2.18	AAA-AAA	0.00	999	1.000	1.000	0.4390
235724_at	7.9	17.1	1.12	2.18	AAA-AAA	0.00	999	1.000	1.000	0.2554
236731_at	4.3	9.3	1.12	2.18	AAA-AAA	0.00	999	1.000	1.000	0.4407
228010_at	30.9	14.2	-1.12	-2.18	AAA-AAM	0.00	999	1.000	1.000	0.2880
205165_at	21.0	9.6	-1.12	-2.18	AAA-AAA	0.00	999	1.000	1.000	0.3059
233158_at	33.2	15.3	-1.12	-2.18	AAA-AAA	0.00	999	1.000	1.000	0.3171
231692_at	17.9	8.2	-1.12	-2.18	AAA-AAA	0.00	999	1.000	1.000	0.2372
224044_at	14.9	32.5	1.12	2.18	APA-AAP	0.00	999	1.000	1.000	0.4264
244090_at	19.1	8.8	-1.12	-2.18	AAA-AAA	0.00	999	1.000	1.000	0.2622
1556531_at	10.3	22.3	1.12	2.18	AAA-AAA	0.00	999	1.000	1.000	0.1945
208232_x_at	33.8	15.5	-1.12	-2.18	AAA-AAA	0.00	999	1.000	1.000	0.5130
211449_at	48.7	22.4	-1.12	-2.18	AAA-AAA	0.00	999	1.000	1.000	0.3642
228215_at	5.7	12.4	1.12	2.18	AAA-AAA	0.00	999	1.000	1.000	0.0401
228915_at	9.9	4.5	-1.12	-2.18	AAA-AAA	0.00	999	1.000	1.000	0.4508

222362_at	27.3	59.5	1.12	2.18	PAA-PAA	0.00	999	1.000	1.000	0.3803
240885_at	36.0	16.6	-1.12	-2.18	AAA-AAA	0.00	999	1.000	1.000	0.4165
240203_at	11.4	24.8	1.12	2.18	AAA-AAA	0.00	999	1.000	1.000	0.5151
1562520_at	8.9	19.4	1.12	2.18	AAA-AAA	0.00	999	1.000	1.000	0.2317
1555002_at	5.6	12.1	1.12	2.18	AAA-AAA	0.00	999	1.000	1.000	0.4722
1552763_at	7.6	3.5	-1.12	-2.18	AAA-AAA	0.00	999	1.000	1.000	0.2678
1559839_at	23.1	10.6	-1.12	-2.18	AAA-AAA	0.00	999	1.000	1.000	0.2914
220785_at	10.5	4.8	-1.12	-2.18	AAA-AAA	0.00	999	1.000	1.000	0.3378
220554_at	32.5	14.9	-1.12	-2.18	AAA-AAA	0.00	999	1.000	1.000	0.2407
211548_s_at	13.6	6.3	-1.12	-2.18	AAA-AAA	0.00	999	1.000	1.000	0.5223
211188_at	47.3	21.7	-1.12	-2.18	AAA-AAA	0.00	999	1.000	1.000	0.1594
216072_at	29.6	13.6	-1.12	-2.18	AAA-AAA	0.00	999	1.000	1.000	0.2806
239407_at	60.0	27.6	-1.12	-2.18	AAA-AAA	0.00	999	1.000	1.000	0.3088
237413_at	5.8	2.7	-1.12	-2.18	AAA-AAA	0.00	999	1.000	1.000	0.3146
237591_at	42.2	19.4	-1.12	-2.18	AAA-AAA	0.00	999	1.000	1.000	0.0977
237507_at	16.3	7.5	-1.12	-2.18	AAA-AAA	0.00	999	1.000	1.000	0.1428
229627_at	4.9	2.3	-1.12	-2.18	AAA-AAA	0.00	999	1.000	1.000	0.1050
228178_s_at	39.6	18.2	-1.12	-2.18	AAA-AAA	0.00	999	1.000	1.000	0.3502
227781_x_at	20.1	9.3	-1.12	-2.18	AAA-AAA	0.00	999	1.000	1.000	0.1391
214751_at	162.3	353.0	1.12	2.18	PPP-PPP	0.00	999	1.000	1.000	0.2072
1562811_at	7.9	17.1	1.12	2.18	AAA-AAA	0.00	999	1.000	1.000	0.5177
1556444_a_at	26.0	12.0	-1.12	-2.18	AAA-AAA	0.00	999	1.000	1.000	0.4219
221067_s_at	88.6	40.7	-1.12	-2.18	AAA-AAA	0.00	999	1.000	1.000	0.2688
219643_at	6.3	2.9	-1.12	-2.18	AAA-AAA	0.00	999	1.000	1.000	0.5747
207293_s_at	8.5	18.6	1.12	2.18	AAA-AAA	0.00	999	1.000	1.000	0.2661
210929_s_at	15.1	32.9	1.12	2.18	AAA-AAA	0.00	999	1.000	1.000	0.4346
211286_x_at	18.7	8.6	-1.12	-2.18	AAA-AAA	0.00	999	1.000	1.000	0.3652
235477_at	14.9	6.8	-1.12	-2.18	AAA-AAA	0.00	999	1.000	1.000	0.3951
239554_at	6.3	13.7	1.12	2.18	AAA-AAA	0.00	999	1.000	1.000	0.3518
205749_at	43.0	19.8	-1.12	-2.18	AAA-AAA	0.00	999	1.000	1.000	0.3477
228269_x_at	23.9	51.9	1.12	2.18	AAA-AAA	0.00	999	1.000	1.000	0.3324
206060_s_at	12.3	26.8	1.12	2.18	AAA-AAA	0.00	999	1.000	1.000	0.4122
234644_x_at	43.4	94.3	1.12	2.17	AAP-APP	0.00	999	1.000	1.000	0.4767
235513_at	25.4	55.2	1.12	2.17	AAP-APP	0.00	999	1.000	1.000	0.3222
239969_at	134.3	61.8	-1.12	-2.17	AAA-AAA	0.00	999	1.000	1.000	0.2351
1561091_at	4.5	2.1	-1.12	-2.17	AAA-AAA	0.00	999	1.000	1.000	0.0164
1565337_at	5.5	12.0	1.12	2.17	AAA-AAA	0.00	999	1.000	1.000	0.2860
1565715_at	154.9	71.3	-1.12	-2.17	AAA-AAA	0.00	999	1.000	1.000	0.1205
1553044_at	25.5	55.4	1.12	2.17	AAA-AAA	0.00	999	1.000	1.000	0.1929
221329_at	42.6	19.6	-1.12	-2.17	AMA-AAA	0.00	999	1.000	1.000	0.2285
222914_s_at	103.9	47.8	-1.12	-2.17	AAA-AAA	0.00	999	1.000	1.000	0.0632
215843_s_at	29.7	13.6	-1.12	-2.17	AAA-AAA	0.00	999	1.000	1.000	0.1924

209617_s_at	19.2	41.6	1.12	2.17	AAA-AAA	0.00	999	1.000	1.000	0.3107
204879_at	24.4	53.0	1.12	2.17	AAA-AAA	0.00	999	1.000	1.000	0.5416
234816_at	31.5	14.5	-1.12	-2.17	AAA-AAA	0.00	999	1.000	1.000	0.0503
241079_at	22.7	10.5	-1.12	-2.17	AAA-AAA	0.00	999	1.000	1.000	0.4364
1560964_at	15.1	7.0	-1.12	-2.17	AAA-AAA	0.00	999	1.000	1.000	0.2730
1569935_at	244.5	112.6	-1.12	-2.17	AAA-MAA	0.00	999	1.000	1.000	0.0495
1566097_at	34.3	15.8	-1.12	-2.17	AAA-AAA	0.00	999	1.000	1.000	0.3966
244832_at	21.6	46.9	1.12	2.17	AAA-AAA	0.00	999	1.000	1.000	0.2798
208344_x_at	52.5	24.2	-1.12	-2.17	AAA-AAA	0.00	999	1.000	1.000	0.4277
207032_s_at	13.1	28.4	1.12	2.17	AAA-AAA	0.00	999	1.000	1.000	0.2961
206670_s_at	7.1	15.4	1.12	2.17	AAA-AAA	0.00	999	1.000	1.000	0.0576
224077_at	5.9	2.7	-1.12	-2.17	AAA-AAA	0.00	999	1.000	1.000	0.1931
215468_at	5.4	11.7	1.12	2.17	AAA-AAA	0.00	999	1.000	1.000	0.4949
232378_at	17.1	37.1	1.12	2.17	AAA-AAA	0.00	999	1.000	1.000	0.2145
201313_at	192.8	418.5	1.12	2.17	MAP-PPP	0.00	999	1.000	1.000	0.3656
233367_at	23.2	50.3	1.12	2.17	AAA-PAA	0.00	999	1.000	1.000	0.2627
208057_s_at	18.6	40.3	1.12	2.17	AAA-PAA	0.00	999	1.000	1.000	0.2278
241339_at	33.8	73.4	1.12	2.17	AAA-AAA	0.00	999	1.000	1.000	0.1149
242288_s_at	29.8	13.7	-1.12	-2.17	AAA-AAA	0.00	999	1.000	1.000	0.2430
1562348_at	60.9	28.1	-1.12	-2.17	AAA-AAA	0.00	999	1.000	1.000	0.3492
1558451_at	38.8	17.9	-1.12	-2.17	AAA-AAA	0.00	999	1.000	1.000	0.1845
207206_s_at	18.6	8.6	-1.12	-2.17	AAA-AAA	0.00	999	1.000	1.000	0.1665
210518_at	74.1	34.2	-1.12	-2.17	AAA-AAA	0.00	999	1.000	1.000	0.0629
210606_x_at	33.9	15.6	-1.12	-2.17	AAA-AAA	0.00	999	1.000	1.000	0.0962
235654_at	52.4	24.2	-1.12	-2.17	AAA-AAA	0.00	999	1.000	1.000	0.2412
1567105_at	86.7	40.0	-1.12	-2.17	AAA-AAA	0.00	999	1.000	1.000	0.4763
1566633_at	59.3	27.3	-1.12	-2.17	AAA-AAA	0.00	999	1.000	1.000	0.4086
1558392_at	12.8	27.9	1.12	2.17	AAA-AAA	0.00	999	1.000	1.000	0.2810
206454_s_at	87.5	40.3	-1.12	-2.17	AAA-AAA	0.00	999	1.000	1.000	0.3416
211690_at	12.4	5.7	-1.12	-2.17	AAA-AAA	0.00	999	1.000	1.000	0.2517
209670_at	70.1	152.0	1.12	2.17	AAA-AAA	0.00	999	1.000	1.000	0.3811
234071_at	19.1	8.8	-1.12	-2.17	AAA-AAA	0.00	999	1.000	1.000	0.4664
227598_at	53.9	116.8	1.12	2.17	AAA-AMA	0.00	999	1.000	1.000	0.0206
233555_s_at	31.6	68.4	1.12	2.17	AAA-MAA	0.00	999	1.000	1.000	0.1209
231459_at	6.4	2.9	-1.12	-2.17	AAA-AAA	0.00	999	1.000	1.000	0.0367
242974_at	33.2	72.0	1.12	2.17	AAM-APP	0.00	999	1.000	1.000	0.4838
1566830_at	24.0	11.1	-1.12	-2.17	AAA-AAA	0.00	999	1.000	1.000	0.4274
1559295_at	18.1	8.3	-1.12	-2.17	AAA-AAA	0.00	999	1.000	1.000	0.2252
1555647_a_at	31.2	14.4	-1.12	-2.17	AAA-AAA	0.00	999	1.000	1.000	0.3288
1557131_at	20.6	44.7	1.12	2.17	AAA-AAA	0.00	999	1.000	1.000	0.3452
1556666_a_at	3.1	1.4	-1.12	-2.17	AAA-AAA	0.00	999	1.000	1.000	0.3036
218690_at	46.4	21.4	-1.12	-2.17	AAA-AAA	0.00	999	1.000	1.000	0.2990

238306_at	20.3	44.0	1.12	2.17	AAA-AAA	0.00	999	1.000	1.000	0.2952
231125_at	10.6	23.0	1.12	2.17	AAA-AAA	0.00	999	1.000	1.000	0.2587
204310_s_at	64.3	139.3	1.12	2.17	AMP-PPP	0.00	999	1.000	1.000	0.1637
243884_at	16.0	7.4	-1.12	-2.17	AAA-AAA	0.00	999	1.000	1.000	0.4879
1556999_at	31.1	14.4	-1.12	-2.17	AAA-AAA	0.00	999	1.000	1.000	0.3102
1556504_at	6.9	3.2	-1.12	-2.17	AAA-AAA	0.00	999	1.000	1.000	0.2773
224526_at	29.8	13.8	-1.12	-2.17	AAA-AAA	0.00	999	1.000	1.000	0.4146
204555_s_at	34.4	15.9	-1.12	-2.17	AAA-AAA	0.00	999	1.000	1.000	0.4737
236225_at	16.9	36.7	1.12	2.17	AAA-AAA	0.00	999	1.000	1.000	0.2128
236395_at	4.6	10.0	1.12	2.17	AAA-AAA	0.00	999	1.000	1.000	0.4788
238267_s_at	21.5	9.9	-1.12	-2.17	AAA-AAA	0.00	999	1.000	1.000	0.0886
238314_x_at	3.6	7.7	1.12	2.17	AAA-AAA	0.00	999	1.000	1.000	0.4979
237902_at	8.6	4.0	-1.12	-2.17	AAA-AAA	0.00	999	1.000	1.000	0.3498
232887_at	30.9	67.0	1.12	2.17	AAA-AAA	0.00	999	1.000	1.000	0.2519
231043_at	28.3	61.3	1.12	2.17	AAA-AAA	0.00	999	1.000	1.000	0.4993
215966_x_at	76.1	164.6	1.11	2.16	AAA-AMP	0.00	999	1.000	1.000	0.2124
224064_s_at	195.0	422.2	1.11	2.17	AAP-PPP	0.00	999	1.000	1.000	0.1986
1559921_at	58.0	125.5	1.11	2.16	AMA-PPP	0.00	999	1.000	1.000	0.1803
240102_at	24.1	11.1	-1.11	-2.16	AAA-AAA	0.00	999	1.000	1.000	0.3763
1566979_at	25.2	11.7	-1.11	-2.16	AAA-AAA	0.00	999	1.000	1.000	0.0922
1554569_a_at	34.8	16.1	-1.11	-2.17	AAA-AAA	0.00	999	1.000	1.000	0.4414
1557422_at	11.6	5.4	-1.11	-2.17	AAA-AAA	0.00	999	1.000	1.000	0.0823
234456_at	21.2	46.0	1.11	2.16	AAA-AAA	0.00	999	1.000	1.000	0.3137
238297_at	12.1	5.6	-1.11	-2.16	AAA-AAA	0.00	999	1.000	1.000	0.5158
237171_at	63.2	29.2	-1.11	-2.16	AAM-AAA	0.00	999	1.000	1.000	0.1425
237353_at	12.6	27.3	1.11	2.17	AAA-AAA	0.00	999	1.000	1.000	0.3226
237950_s_at	33.7	15.6	-1.11	-2.16	AAA-AAA	0.00	999	1.000	1.000	0.2370
222153_at	6.1	13.1	1.11	2.16	AAA-PAA	0.00	999	1.000	1.000	0.5205
1569926_s_at	19.1	8.8	-1.11	-2.16	AAA-AAA	0.00	999	1.000	1.000	0.3564
1555139_a_at	35.1	75.9	1.11	2.16	AAA-AAA	0.00	999	1.000	1.000	0.3598
202525_at	49.1	22.7	-1.11	-2.16	AAA-AAA	0.00	999	1.000	1.000	0.2570
222928_s_at	24.4	11.3	-1.11	-2.16	AAA-AAA	0.00	999	1.000	1.000	0.4637
218865_at	27.7	12.8	-1.11	-2.16	AAA-AAA	0.00	999	1.000	1.000	0.0448
204052_s_at	5.7	12.2	1.11	2.16	AAA-AAA	0.00	999	1.000	1.000	0.5598
229853_at	9.9	21.5	1.11	2.16	AAA-AAA	0.00	999	1.000	1.000	0.3679
205392_s_at	38.8	17.9	-1.11	-2.16	AAA-AAA	0.00	999	1.000	1.000	0.4436
232791_at	20.2	9.4	-1.11	-2.16	AAA-AAA	0.00	999	1.000	1.000	0.3808
231286_at	13.8	29.9	1.11	2.16	AAA-AAA	0.00	999	1.000	1.000	0.4145
1556865_at	27.6	59.7	1.11	2.16	APA-PAP	0.00	999	1.000	1.000	0.4402
203571_s_at	44.7	96.7	1.11	2.16	AAP-PAA	0.00	999	1.000	1.000	0.2180
243227_at	21.7	10.0	-1.11	-2.16	AAA-AAA	0.00	999	1.000	1.000	0.3198
242967_at	15.3	7.1	-1.11	-2.16	AAA-AAA	0.00	999	1.000	1.000	0.3814

1564909_at	6.4	3.0	-1.11	-2.16	AAA-AAA	0.00	999	1.000	1.000	0.3350
1565897_at	5.2	11.3	1.11	2.16	AAA-AAA	0.00	999	1.000	1.000	0.4286
1553429_at	57.6	26.7	-1.11	-2.16	AAA-AAA	0.00	999	1.000	1.000	0.3994
1555631_at	18.7	8.7	-1.11	-2.16	AAA-AAA	0.00	999	1.000	1.000	0.0390
220144_s_at	8.9	19.2	1.11	2.16	AAA-AAA	0.00	999	1.000	1.000	0.2286
208224_at	15.1	32.7	1.11	2.16	AAA-AAA	0.00	999	1.000	1.000	0.3867
206545_at	35.7	16.5	-1.11	-2.16	AAA-AAA	0.00	999	1.000	1.000	0.1252
210171_s_at	45.3	21.0	-1.11	-2.16	AAA-AAA	0.00	999	1.000	1.000	0.3806
209885_at	12.8	27.6	1.11	2.16	AAA-AAA	0.00	999	1.000	1.000	0.2177
238176_at	22.4	48.4	1.11	2.16	AAA-AAA	0.00	999	1.000	1.000	0.3014
205971_s_at	18.9	8.8	-1.11	-2.16	AAA-AAA	0.00	999	1.000	1.000	0.4886
228338_at	28.3	61.1	1.11	2.16	AAA-AAA	0.00	999	1.000	1.000	0.1949
206389_s_at	10.1	4.7	-1.11	-2.16	AAA-AAA	0.00	999	1.000	1.000	0.4248
216550_x_at	141.8	306.2	1.11	2.16	APP-PPP	0.00	999	1.000	1.000	0.0721
243387_at	49.7	23.0	-1.11	-2.16	AAA-AAA	0.00	999	1.000	1.000	0.0737
241089_at	11.7	5.4	-1.11	-2.16	AAA-AAA	0.00	999	1.000	1.000	0.5316
1562583_s_at	41.6	19.3	-1.11	-2.16	AAA-AAA	0.00	999	1.000	1.000	0.4527
1565152_at	10.3	22.3	1.11	2.16	AAA-AAA	0.00	999	1.000	1.000	0.2746
1555393_s_at	189.2	87.6	-1.11	-2.16	AAA-AAA	0.00	999	1.000	1.000	0.0247
208009_s_at	75.1	34.8	-1.11	-2.16	AAA-AAA	0.00	999	1.000	1.000	0.3392
220012_at	20.3	43.8	1.11	2.16	AAA-AAA	0.00	999	1.000	1.000	0.4433
206450_at	26.4	12.2	-1.11	-2.16	AAA-AAA	0.00	999	1.000	1.000	0.5053
224431_s_at	150.4	69.6	-1.11	-2.16	AAA-AAA	0.00	999	1.000	1.000	0.0207
210270_at	20.3	9.4	-1.11	-2.16	AAA-AAA	0.00	999	1.000	1.000	0.2111
237641_at	25.5	11.8	-1.11	-2.16	AAA-AAA	0.00	999	1.000	1.000	0.2702
230844_at	13.4	28.9	1.11	2.16	AAA-AAA	0.00	999	1.000	1.000	0.4879
224190_x_at	59.5	128.5	1.11	2.16	APA-AAP	0.00	999	1.000	1.000	0.3527
240892_at	11.2	24.3	1.11	2.16	AAA-AAM	0.00	999	1.000	1.000	0.6065
242018_at	12.0	5.6	-1.11	-2.16	AAA-AAA	0.00	999	1.000	1.000	0.5623
62987_r_at	293.7	136.1	-1.11	-2.16	AAA-AAA	0.00	999	1.000	1.000	0.0446
210025_s_at	34.7	16.1	-1.11	-2.16	AAA-AAA	0.00	999	1.000	1.000	0.2953
216172_at	25.0	54.0	1.11	2.16	AAA-AAM	0.00	999	1.000	1.000	0.0638
233991_at	17.1	36.9	1.11	2.16	AAA-AAA	0.00	999	1.000	1.000	0.2999
230498_at	11.5	24.8	1.11	2.16	AAA-AAA	0.00	999	1.000	1.000	0.5386
232578_at	10.2	4.7	-1.11	-2.16	AAA-AAA	0.00	999	1.000	1.000	0.4270
231140_at	18.1	8.4	-1.11	-2.16	AAA-AAA	0.00	999	1.000	1.000	0.3480
231768_at	16.4	7.6	-1.11	-2.16	AAA-AAA	0.00	999	1.000	1.000	0.1792
207506_at	44.7	96.3	1.11	2.16	AAA-AAP	0.00	999	1.000	1.000	0.1861
242432_at	16.7	7.7	-1.11	-2.16	AAA-AAA	0.00	999	1.000	1.000	0.4213
240493_at	26.0	56.1	1.11	2.16	AAA-AAA	0.00	999	1.000	1.000	0.3970
1561269_at	39.7	18.4	-1.11	-2.16	AAA-AAA	0.00	999	1.000	1.000	0.1706
202376_at	25.3	11.7	-1.11	-2.16	AAA-AAA	0.00	999	1.000	1.000	0.4134

1559542_a_at	6.3	2.9	-1.11	-2.16	AAA-AAA	0.00	999	1.000	1.000	0.2728
1559145_at	58.9	27.3	-1.11	-2.16	AAA-AAA	0.00	999	1.000	1.000	0.4247
208411_x_at	7.5	3.5	-1.11	-2.16	AAA-AAA	0.00	999	1.000	1.000	0.0427
214524_at	8.1	3.7	-1.11	-2.16	AAA-AAA	0.00	999	1.000	1.000	0.3167
215988_s_at	13.1	6.1	-1.11	-2.16	AAA-AAA	0.00	999	1.000	1.000	0.4335
234324_at	22.5	10.4	-1.11	-2.16	AAA-AAA	0.00	999	1.000	1.000	0.4190
205759_s_at	101.0	46.8	-1.11	-2.16	AAA-AAA	0.00	999	1.000	1.000	0.0804
205138_s_at	24.6	53.0	1.11	2.16	AAA-AAA	0.00	999	1.000	1.000	0.2422
239443_at	9.8	21.2	1.11	2.16	APA-AAP	0.00	999	1.000	1.000	0.5484
240361_at	2.8	5.9	1.11	2.16	AAA-AAA	0.00	999	1.000	1.000	0.3201
1564168_a_at	8.7	18.7	1.11	2.16	AAA-AAA	0.00	999	1.000	1.000	0.1513
244452_at	55.9	25.9	-1.11	-2.16	AAA-AAA	0.00	999	1.000	1.000	0.3258
1556350_a_at	9.1	19.5	1.11	2.16	AAA-AAA	0.00	999	1.000	1.000	0.3864
1557197_a_at	47.4	22.0	-1.11	-2.16	AAA-AAA	0.00	999	1.000	1.000	0.4221
208266_at	43.9	20.4	-1.11	-2.16	AAA-AAA	0.00	999	1.000	1.000	0.3019
213285_at	27.2	12.6	-1.11	-2.16	AAA-AAA	0.00	999	1.000	1.000	0.1126
208566_at	7.1	3.3	-1.11	-2.16	AAA-AAA	0.00	999	1.000	1.000	0.5460
230573_at	4.6	10.0	1.11	2.16	AAA-AAA	0.00	999	1.000	1.000	0.4938
242063_s_at	5.6	2.6	-1.11	-2.15	AAA-AAA	0.00	999	1.000	1.000	0.4360
241670_x_at	8.8	4.1	-1.11	-2.15	AAA-AAA	0.00	999	1.000	1.000	0.1404
243620_at	9.2	4.3	-1.11	-2.15	AAA-AAA	0.00	999	1.000	1.000	0.2289
220813_at	36.5	17.0	-1.11	-2.15	AAA-AAA	0.00	999	1.000	1.000	0.2762
225628_s_at	40.4	86.9	1.11	2.15	AAA-AAA	0.00	999	1.000	1.000	0.3351
211054_at	15.9	34.2	1.11	2.15	AAA-AAA	0.00	999	1.000	1.000	0.1955
234146_at	7.7	3.6	-1.11	-2.15	AAA-AAA	0.00	999	1.000	1.000	0.2367
229471_s_at	21.7	10.1	-1.11	-2.15	AAA-AAA	0.00	999	1.000	1.000	0.5185
232853_at	4.9	10.5	1.11	2.15	AAA-APA	0.00	999	1.000	1.000	0.5425
242589_x_at	73.1	33.9	-1.11	-2.15	AAA-AAA	0.00	999	1.000	1.000	0.4933
243600_at	11.7	5.5	-1.11	-2.15	AAA-AAA	0.00	999	1.000	1.000	0.1769
1561287_at	4.7	2.2	-1.11	-2.15	AAA-AAA	0.00	999	1.000	1.000	0.1794
1559147_at	8.3	3.9	-1.11	-2.15	AAA-AAA	0.00	999	1.000	1.000	0.4047
225739_at	72.5	33.7	-1.11	-2.15	AAA-AAA	0.00	999	1.000	1.000	0.2229
216775_at	5.4	2.5	-1.11	-2.15	AAA-AAA	0.00	999	1.000	1.000	0.1665
216732_at	7.2	3.3	-1.11	-2.15	AAA-AAA	0.00	999	1.000	1.000	0.2829
237675_at	39.9	18.5	-1.11	-2.15	AAA-AAA	0.00	999	1.000	1.000	0.3755
233667_at	8.6	4.0	-1.11	-2.15	AAA-AAA	0.00	999	1.000	1.000	0.1336
1561244_at	3.3	7.2	1.11	2.15	AAA-AAA	0.00	999	1.000	1.000	0.2198
1566715_at	22.3	10.4	-1.11	-2.15	AAA-AAA	0.00	999	1.000	1.000	0.3837
207684_at	48.7	22.6	-1.11	-2.15	AAA-MAA	0.00	999	1.000	1.000	0.2543
212659_s_at	37.8	17.6	-1.11	-2.15	AAA-AAA	0.00	999	1.000	1.000	0.4480
216900_s_at	6.9	3.2	-1.11	-2.15	AAA-AAA	0.00	999	1.000	1.000	0.1992
235137_at	6.7	14.4	1.11	2.15	AAA-AAA	0.00	999	1.000	1.000	0.4990

236186_x_at	11.3	5.2	-1.11	-2.15	AAA-AAA	0.00	999	1.000	1.000	0.0934
1569439_at	21.6	46.4	1.10	2.15	AAP-PAA	0.00	999	1.000	1.000	0.4889
1561817_at	14.1	6.6	-1.10	-2.15	AAA-AAA	0.00	999	1.000	1.000	0.4255
1565866_a_at	9.4	4.4	-1.10	-2.15	AAA-AAA	0.00	999	1.000	1.000	0.0447
244405_s_at	25.3	11.8	-1.10	-2.15	AAA-AAA	0.00	999	1.000	1.000	0.3128
1558463_s_at	55.4	25.8	-1.10	-2.15	AAA-AAA	0.00	999	1.000	1.000	0.4109
216557_x_at	3.9	8.4	1.10	2.15	AAA-AAA	0.00	999	1.000	1.000	0.1012
236143_at	54.0	25.1	-1.10	-2.15	AAA-AAA	0.00	999	1.000	1.000	0.1657
231025_at	30.3	14.1	-1.10	-2.15	AAA-AAA	0.00	999	1.000	1.000	0.2581
231322_at	39.1	18.2	-1.10	-2.15	AAA-AAA	0.00	999	1.000	1.000	0.2294
239483_at	19.8	42.4	1.10	2.15	APA-AAA	0.00	999	1.000	1.000	0.5327
213908_at	44.3	95.2	1.10	2.15	PAA-PPP	0.00	999	1.000	1.000	0.3188
240679_at	16.4	7.6	-1.10	-2.15	AAA-AAA	0.00	999	1.000	1.000	0.0170
240548_at	21.6	10.1	-1.10	-2.15	AAA-AAA	0.00	999	1.000	1.000	0.2215
1564012_at	13.4	28.8	1.10	2.15	AAA-AAA	0.00	999	1.000	1.000	0.5422
1559580_at	12.3	26.4	1.10	2.15	AAA-AAA	0.00	999	1.000	1.000	0.3549
1556511_a_at	16.8	7.8	-1.10	-2.15	AAA-AAA	0.00	999	1.000	1.000	0.3986
220377_at	24.1	11.2	-1.10	-2.15	AAA-AAA	0.00	999	1.000	1.000	0.2758
206750_at	63.5	29.6	-1.10	-2.15	AAA-AAA	0.00	999	1.000	1.000	0.5620
207548_at	9.2	4.3	-1.10	-2.15	AAA-AAA	0.00	999	1.000	1.000	0.3171
234577_at	6.2	13.3	1.10	2.15	AAA-AAA	0.00	999	1.000	1.000	0.3120
227691_at	37.8	81.2	1.10	2.15	AAA-AAA	0.00	999	1.000	1.000	0.2393
228860_at	102.1	47.5	-1.10	-2.15	AMA-AAA	0.00	999	1.000	1.000	0.1710
204470_at	1056.2	2267.8	1.10	2.15	PPP-PPP	0.00	999	1.000	1.000	0.0522
241932_at	51.2	23.8	-1.10	-2.15	AAA-AAA	0.00	999	1.000	1.000	0.0671
1563324_at	12.9	6.0	-1.10	-2.15	AAA-AAA	0.00	999	1.000	1.000	0.2362
210356_x_at	43.8	20.4	-1.10	-2.15	AAA-AAA	0.00	999	1.000	1.000	0.3506
204724_s_at	10.7	23.0	1.10	2.15	AAA-AAA	0.00	999	1.000	1.000	0.2674
243091_at	22.3	47.9	1.10	2.15	AAA-AAA	0.00	999	1.000	1.000	0.3985
241310_at	22.6	10.5	-1.10	-2.15	AAM-AAA	0.00	999	1.000	1.000	0.3428
1554818_s_at	48.8	22.8	-1.10	-2.15	AAA-AAA	0.00	999	1.000	1.000	0.2418
1552391_at	15.3	7.1	-1.10	-2.15	AAA-AAA	0.00	999	1.000	1.000	0.4895
1557557_at	23.8	11.1	-1.10	-2.15	AAA-AAA	0.00	999	1.000	1.000	0.5822
1559953_at	30.6	14.3	-1.10	-2.15	AAM-AAA	0.00	999	1.000	1.000	0.1419
208366_at	9.6	4.5	-1.10	-2.15	AAA-AAA	0.00	999	1.000	1.000	0.3242
211470_s_at	9.1	4.3	-1.10	-2.15	AAA-AAA	0.00	999	1.000	1.000	0.5724
238149_at	12.6	5.9	-1.10	-2.14	AAA-AAA	0.00	999	1.000	1.000	0.4992
232429_at	18.8	40.4	1.10	2.14	AAP-APP	0.00	999	1.000	1.000	0.3459
243987_at	15.7	33.6	1.10	2.14	AAA-AAA	0.00	999	1.000	1.000	0.0146
243168_at	5.7	2.7	-1.10	-2.14	AAA-AAA	0.00	999	1.000	1.000	0.0003
1563373_at	13.3	6.2	-1.10	-2.14	AAA-AAA	0.00	999	1.000	1.000	0.4984
1560409_at	12.2	5.7	-1.10	-2.14	AAA-AAA	0.00	999	1.000	1.000	0.1806

222248_s_at	43.2	92.6	1.10	2.14	AAA-AAA	0.00	999	1.000	1.000	0.2126
234612_at	18.6	39.9	1.10	2.14	AAA-AAA	0.00	999	1.000	1.000	0.2648
237508_at	6.4	3.0	-1.10	-2.14	AAA-AAA	0.00	999	1.000	1.000	0.3898
233659_at	21.6	10.1	-1.10	-2.14	AAA-AAA	0.00	999	1.000	1.000	0.4468
1570212_a_at	26.1	12.2	-1.10	-2.14	AAA-AAA	0.00	999	1.000	1.000	0.3780
244592_at	63.8	29.8	-1.10	-2.14	AAA-AAA	0.00	999	1.000	1.000	0.2468
214282_at	16.1	7.5	-1.10	-2.14	AAA-AAA	0.00	999	1.000	1.000	0.3044
216801_at	114.2	53.3	-1.10	-2.14	AAA-AAA	0.00	999	1.000	1.000	0.1069
236391_at	3.1	6.7	1.10	2.14	AAA-AAA	0.00	999	1.000	1.000	0.0924
234557_at	59.1	27.6	-1.10	-2.14	AAA-AAA	0.00	999	1.000	1.000	0.2621
236934_at	60.6	28.3	-1.10	-2.14	AAA-AAA	0.00	999	1.000	1.000	0.5359
231077_at	13.4	6.3	-1.10	-2.14	AAA-AAA	0.00	999	1.000	1.000	0.4343
211991_s_at	23.1	49.4	1.10	2.14	PAA-PAA	0.00	999	1.000	1.000	0.4411
240714_at	2.3	5.0	1.10	2.14	AAA-AAA	0.00	999	1.000	1.000	0.0094
1553660_at	24.3	52.0	1.10	2.14	AAA-AMA	0.00	999	1.000	1.000	0.2700
1552555_at	98.2	45.9	-1.10	-2.14	AAA-AAA	0.00	999	1.000	1.000	0.4066
1556742_at	13.6	29.2	1.10	2.14	AAA-AAA	0.00	999	1.000	1.000	0.0914
222196_at	17.9	8.3	-1.10	-2.14	AAA-AAA	0.00	999	1.000	1.000	0.4221
210140_at	39.8	18.6	-1.10	-2.14	AAA-AAA	0.00	999	1.000	1.000	0.1962
216924_s_at	19.1	40.8	1.10	2.14	AAA-AAA	0.00	999	1.000	1.000	0.3048
204220_at	4.9	10.5	1.10	2.14	AAA-AAA	0.00	999	1.000	1.000	0.4361
237316_at	29.0	13.5	-1.10	-2.14	AAA-AAA	0.00	999	1.000	1.000	0.3985
1561213_at	6.7	14.3	1.10	2.14	APA-AAA	0.00	999	1.000	1.000	0.4550
227482_at	46.1	98.5	1.10	2.14	AAA-PAA	0.00	999	1.000	1.000	0.2975
240601_at	15.9	33.9	1.10	2.14	AAA-APA	0.00	999	1.000	1.000	0.2594
1564056_at	11.5	5.4	-1.10	-2.14	AAA-AAA	0.00	999	1.000	1.000	0.3998
1553541_at	8.5	18.1	1.10	2.14	AAA-AAA	0.00	999	1.000	1.000	0.4157
206456_at	18.8	8.8	-1.10	-2.14	AAA-AAA	0.00	999	1.000	1.000	0.3058
216754_at	13.8	6.5	-1.10	-2.14	AAA-AAA	0.00	999	1.000	1.000	0.3693
215911_x_at	15.2	7.1	-1.10	-2.14	AAA-AAA	0.00	999	1.000	1.000	0.4268
204661_at	11.1	5.2	-1.10	-2.14	AAA-AAA	0.00	999	1.000	1.000	0.0252
239594_at	31.6	14.8	-1.10	-2.14	AAA-AAA	0.00	999	1.000	1.000	0.2121
230901_x_at	47.5	101.6	1.10	2.14	AAA-AAM	0.00	999	1.000	1.000	0.4179
232163_at	26.7	57.0	1.10	2.14	AAA-PPA	0.00	999	1.000	1.000	0.4708
209546_s_at	38.0	81.3	1.10	2.14	AAA-APP	0.00	999	1.000	1.000	0.3858
240328_at	34.7	16.2	-1.10	-2.14	AAA-AAA	0.00	999	1.000	1.000	0.1340
240614_at	9.0	4.2	-1.10	-2.14	AAA-AAA	0.00	999	1.000	1.000	0.3350
1563054_at	20.7	9.7	-1.10	-2.14	AAA-AAA	0.00	999	1.000	1.000	0.2291
1561556_at	27.3	12.8	-1.10	-2.14	AAA-AAA	0.00	999	1.000	1.000	0.4404
1564200_at	40.2	18.8	-1.10	-2.14	AAA-AAA	0.00	999	1.000	1.000	0.2527
1559546_s_at	6.4	13.7	1.10	2.14	AAA-AAA	0.00	999	1.000	1.000	0.2802
207924_x_at	11.7	25.1	1.10	2.14	AAA-AAA	0.00	999	1.000	1.000	0.1298

206732_at	25.6	12.0	-1.10	-2.14	AAA-AAA	0.00	999	1.000	1.000	0.2992
206618_at	12.1	25.9	1.10	2.14	AAA-AAA	0.00	999	1.000	1.000	0.4015
207289_at	133.3	62.4	-1.10	-2.14	AAA-AAA	0.00	999	1.000	1.000	0.0513
204907_s_at	69.6	148.7	1.10	2.14	AAA-AAA	0.00	999	1.000	1.000	0.0741
239659_at	34.4	73.4	1.10	2.14	AAA-AAA	0.00	999	1.000	1.000	0.3136
236874_at	20.3	43.4	1.10	2.14	AAA-AAA	0.00	999	1.000	1.000	0.1915
232628_at	28.4	60.6	1.10	2.14	AAP-AAP	0.00	999	1.000	1.000	0.2069
1568612_at	7.3	3.4	-1.10	-2.14	AAA-AAA	0.00	999	1.000	1.000	0.3190
1557881_at	11.3	24.1	1.10	2.14	AAA-AAA	0.00	999	1.000	1.000	0.3577
221431_s_at	7.8	3.7	-1.10	-2.14	AAA-AAA	0.00	999	1.000	1.000	0.2696
222491_at	99.3	212.1	1.10	2.14	AAA-AAA	0.00	999	1.000	1.000	0.2785
214645_at	29.2	13.7	-1.10	-2.14	AAA-AAA	0.00	999	1.000	1.000	0.3569
216238_s_at	13.2	6.2	-1.10	-2.14	AAA-AAA	0.00	999	1.000	1.000	0.4308
229605_at	55.5	26.0	-1.10	-2.14	AAA-AAA	0.00	999	1.000	1.000	0.4097
228444_at	10.9	23.3	1.10	2.14	AAA-AAA	0.00	999	1.000	1.000	0.3404
232214_x_at	96.4	45.2	-1.10	-2.14	AAA-AAA	0.00	999	1.000	1.000	0.2432
1559429_a_at	7.9	16.9	1.09	2.13	AAA-PAA	0.00	999	1.000	1.000	0.4135
200785_s_at	102.5	218.8	1.09	2.14	APM-PPP	0.00	999	1.000	1.000	0.3870
237689_at	21.7	46.4	1.09	2.14	AAP-AAP	0.00	999	1.000	1.000	0.3821
37892_at	30.6	65.3	1.09	2.13	AAA-AAP	0.00	999	1.000	1.000	0.1325
1560522_at	10.8	23.0	1.09	2.14	AAA-AAA	0.00	999	1.000	1.000	0.4457
244856_at	33.6	15.7	-1.09	-2.14	AAA-AAA	0.00	999	1.000	1.000	0.1836
1552893_at	145.6	68.2	-1.09	-2.13	AAA-AAA	0.00	999	1.000	1.000	0.3178
1559354_a_at	37.3	17.5	-1.09	-2.13	AAA-AAA	0.00	999	1.000	1.000	0.2503
1556445_at	36.6	17.2	-1.09	-2.14	AAA-AAA	0.00	999	1.000	1.000	0.3961
207656_s_at	7.2	15.4	1.09	2.13	AAA-AAA	0.00	999	1.000	1.000	0.1795
215634_at	5.3	2.5	-1.09	-2.13	AAA-AAA	0.00	999	1.000	1.000	0.0676
215927_at	30.3	14.2	-1.09	-2.13	AAA-AAA	0.00	999	1.000	1.000	0.4423
235465_at	18.3	8.6	-1.09	-2.13	AAA-AAA	0.00	999	1.000	1.000	0.4732
239457_at	8.3	17.6	1.09	2.14	AAA-AAA	0.00	999	1.000	1.000	0.2159
229486_s_at	22.6	10.6	-1.09	-2.13	AAA-AAA	0.00	999	1.000	1.000	0.1529
233332_at	7.0	15.0	1.09	2.14	AAA-AAA	0.00	999	1.000	1.000	0.2818
209277_at	4001.8	8538.9	1.09	2.13	PPP-PPP	0.00	999	1.000	1.000	0.1017
1567679_at	6.5	3.0	-1.09	-2.13	AAA-AAA	0.00	999	1.000	1.000	0.5485
1553830_s_at	8.2	3.9	-1.09	-2.13	AAA-AAA	0.00	999	1.000	1.000	0.5038
1557796_at	9.5	4.5	-1.09	-2.13	AAA-AAA	0.00	999	1.000	1.000	0.0773
1559438_at	24.3	11.4	-1.09	-2.13	AAA-AAA	0.00	999	1.000	1.000	0.1972
221360_s_at	7.3	3.4	-1.09	-2.13	AAA-AAA	0.00	999	1.000	1.000	0.1332
206722_s_at	26.6	12.5	-1.09	-2.13	AAA-AAA	0.00	999	1.000	1.000	0.1437
223952_x_at	30.5	14.3	-1.09	-2.13	AAA-AAA	0.00	999	1.000	1.000	0.2991
210059_s_at	80.7	37.8	-1.09	-2.13	MAA-AAA	0.00	999	1.000	1.000	0.0995
214944_at	95.8	44.9	-1.09	-2.13	AAA-AAA	0.00	999	1.000	1.000	0.0291

215932_at	26.1	12.3	-1.09	-2.13	AAA-AAA	0.00	999	1.000	1.000	0.5436
230430_at	8.6	4.0	-1.09	-2.13	AAA-AAA	0.00	999	1.000	1.000	0.0575
205380_at	18.9	40.4	1.09	2.13	AAA-AAA	0.00	999	1.000	1.000	0.2707
205007_s_at	45.6	97.3	1.09	2.13	AAA-APP	0.00	999	1.000	1.000	0.2975
1563668_at	58.8	27.6	-1.09	-2.13	AAA-AAA	0.00	999	1.000	1.000	0.1618
1560760_s_at	1.9	4.1	1.09	2.13	AAA-AAA	0.00	999	1.000	1.000	0.4313
1560439_at	139.6	65.5	-1.09	-2.13	AAA-AAA	0.00	999	1.000	1.000	0.3545
1558135_at	12.8	27.4	1.09	2.13	AAA-AAA	0.00	999	1.000	1.000	0.3666
208488_s_at	24.6	11.5	-1.09	-2.13	AAA-AAA	0.00	999	1.000	1.000	0.3065
239485_at	10.9	5.1	-1.09	-2.13	AAA-AAA	0.00	999	1.000	1.000	0.1809
227721_at	23.3	10.9	-1.09	-2.13	AAA-AAA	0.00	999	1.000	1.000	0.5527
205424_at	19.9	42.5	1.09	2.13	AAA-AAA	0.00	999	1.000	1.000	0.2920
233357_at	67.3	31.6	-1.09	-2.13	AAA-AAA	0.00	999	1.000	1.000	0.5880
238457_at	34.0	72.5	1.09	2.13	AAP-APP	0.00	999	1.000	1.000	0.3376
217953_at	19.4	41.3	1.09	2.13	AAP-PPP	0.00	999	1.000	1.000	0.2154
226328_at	186.5	397.3	1.09	2.13	APP-PPP	0.00	999	1.000	1.000	0.1885
1562472_at	20.7	9.7	-1.09	-2.13	AAA-AAA	0.00	999	1.000	1.000	0.4206
1569486_at	19.0	40.4	1.09	2.13	AAA-AAA	0.00	999	1.000	1.000	0.5572
1568287_at	9.2	4.3	-1.09	-2.13	AAA-AAA	0.00	999	1.000	1.000	0.5701
1566798_at	30.4	14.3	-1.09	-2.13	AAA-AAA	0.00	999	1.000	1.000	0.0945
1554459_s_at	17.4	8.2	-1.09	-2.13	AAA-AAA	0.00	999	1.000	1.000	0.3990
201641_at	16.4	7.7	-1.09	-2.13	AAA-AAA	0.00	999	1.000	1.000	0.1809
220912_at	12.6	5.9	-1.09	-2.13	AAA-AAA	0.00	999	1.000	1.000	0.4829
206850_at	37.9	17.8	-1.09	-2.13	AAA-AAA	0.00	999	1.000	1.000	0.3385
206945_at	73.9	34.7	-1.09	-2.13	AAA-AAA	0.00	999	1.000	1.000	0.3119
210353_s_at	10.9	5.1	-1.09	-2.13	AAA-AAA	0.00	999	1.000	1.000	0.1602
216244_at	4.6	2.2	-1.09	-2.13	AAA-AAA	0.00	999	1.000	1.000	0.3160
234180_at	4.7	2.2	-1.09	-2.13	AAA-AAA	0.00	999	1.000	1.000	0.0919
206366_x_at	7.9	3.7	-1.09	-2.13	AAA-AAA	0.00	999	1.000	1.000	0.1502
236437_at	19.6	41.7	1.09	2.13	AAP-APP	0.00	999	1.000	1.000	0.5562
1570398_at	6.4	3.0	-1.09	-2.13	AAA-AAA	0.00	999	1.000	1.000	0.5480
1566126_at	8.0	3.7	-1.09	-2.13	AAA-AAA	0.00	999	1.000	1.000	0.3922
244812_at	13.4	6.3	-1.09	-2.13	AAA-AAA	0.00	999	1.000	1.000	0.3137
221112_at	71.1	33.4	-1.09	-2.13	AAA-AAA	0.00	999	1.000	1.000	0.2143
221866_at	27.8	59.2	1.09	2.13	AAA-AAA	0.00	999	1.000	1.000	0.3840
220535_at	17.0	8.0	-1.09	-2.13	AAA-AAA	0.00	999	1.000	1.000	0.4117
235171_at	6.8	14.4	1.09	2.13	AAA-AAA	0.00	999	1.000	1.000	0.1052
204974_at	57.2	121.7	1.09	2.13	AAA-AAA	0.00	999	1.000	1.000	0.1199
234479_at	31.7	14.9	-1.09	-2.13	AAA-AAA	0.00	999	1.000	1.000	0.4493
234666_at	26.8	12.6	-1.09	-2.13	AAA-AAA	0.00	999	1.000	1.000	0.4433
238710_at	68.7	32.3	-1.09	-2.13	AAA-AAA	0.00	999	1.000	1.000	0.2949
237770_at	5.2	2.5	-1.09	-2.13	AAA-AAA	0.00	999	1.000	1.000	0.4530

231450_at	5.5	2.6	-1.09	-2.13	AAA-AAA	0.00	999	1.000	1.000	0.0610
1555353_at	18.4	39.1	1.09	2.13	AAA-PAP	0.00	999	1.000	1.000	0.4227
209459_s_at	28.5	60.7	1.09	2.13	AAP-PPP	0.00	999	1.000	1.000	0.0673
1565746_at	20.4	43.3	1.09	2.13	AAA-AAA	0.00	999	1.000	1.000	0.4075
1559621_at	4.1	1.9	-1.09	-2.13	AAA-AAA	0.00	999	1.000	1.000	0.0474
1559884_at	5.5	2.6	-1.09	-2.13	AAA-AAA	0.00	999	1.000	1.000	0.0894
220332_at	18.5	8.7	-1.09	-2.13	AAA-AAA	0.00	999	1.000	1.000	0.0109
214404_x_at	31.8	67.7	1.09	2.13	AAA-AAA	0.00	999	1.000	1.000	0.3911
213060_s_at	58.8	27.6	-1.09	-2.13	AAA-AAA	0.00	999	1.000	1.000	0.4899
235271_s_at	8.9	18.9	1.09	2.13	AAA-AAA	0.00	999	1.000	1.000	0.3610
238975_at	11.7	5.5	-1.09	-2.13	AAA-AAA	0.00	999	1.000	1.000	0.0948
205724_at	28.2	60.0	1.09	2.13	AAA-AAA	0.00	999	1.000	1.000	0.2770
244470_at	29.5	62.7	1.09	2.13	AAA-APA	0.00	999	1.000	1.000	0.2273
240667_at	19.0	8.9	-1.09	-2.13	AAA-AAA	0.00	999	1.000	1.000	0.5000
1562254_at	41.5	19.5	-1.09	-2.13	AAA-AAA	0.00	999	1.000	1.000	0.1964
221089_at	4.5	9.5	1.09	2.13	AAA-AAA	0.00	999	1.000	1.000	0.3440
207789_s_at	44.4	20.9	-1.09	-2.13	AAA-AAA	0.00	999	1.000	1.000	0.2391
220498_at	18.8	8.8	-1.09	-2.13	AAA-AAA	0.00	999	1.000	1.000	0.3161
206888_s_at	108.9	51.2	-1.09	-2.13	AAA-AAA	0.00	999	1.000	1.000	0.1492
215647_at	10.8	5.1	-1.09	-2.13	AAA-AAA	0.00	999	1.000	1.000	0.4130
235270_at	7.5	15.9	1.09	2.13	AAA-AAA	0.00	999	1.000	1.000	0.4836
238384_x_at	6.2	2.9	-1.09	-2.13	AAA-AAA	0.00	999	1.000	1.000	0.4886
239292_at	7.1	15.1	1.09	2.13	AAA-AAA	0.00	999	1.000	1.000	0.3756
230334_at	11.4	24.1	1.09	2.13	AAA-AAA	0.00	999	1.000	1.000	0.2298
233426_at	19.7	9.3	-1.09	-2.13	AAA-AAA	0.00	999	1.000	1.000	0.5337
206392_s_at	34.6	73.5	1.09	2.12	AAP-AAA	0.00	999	1.000	1.000	0.4805
243837_x_at	62.3	29.3	-1.09	-2.12	AAA-AAA	0.00	999	1.000	1.000	0.3348
1558950_at	65.5	30.9	-1.09	-2.12	AAA-AAA	0.00	999	1.000	1.000	0.1529
1557277_a_at	28.2	13.3	-1.09	-2.12	AAA-AAA	0.00	999	1.000	1.000	0.2230
37117_at	10.6	22.4	1.09	2.13	AAA-AAA	0.00	999	1.000	1.000	0.5702
211640_x_at	17.9	8.4	-1.09	-2.13	AAA-AAA	0.00	999	1.000	1.000	0.5417
235586_at	18.4	39.2	1.09	2.13	AAA-AAA	0.00	999	1.000	1.000	0.2949
236804_at	56.4	119.8	1.09	2.13	AAA-AAA	0.00	999	1.000	1.000	0.3563
237656_at	4.5	2.1	-1.09	-2.12	AAA-AAA	0.00	999	1.000	1.000	0.0716
237339_at	16.4	34.8	1.09	2.12	AAA-APA	0.00	999	1.000	1.000	0.1053
1563102_at	82.7	39.0	-1.09	-2.12	AAM-AAA	0.00	999	1.000	1.000	0.0229
244817_at	3.0	1.4	-1.09	-2.12	AAA-AAA	0.00	999	1.000	1.000	0.2683
1559634_at	48.9	23.1	-1.09	-2.12	AAA-AAA	0.00	999	1.000	1.000	0.1445
1555925_at	4.9	2.3	-1.09	-2.12	AAA-AAA	0.00	999	1.000	1.000	0.0235
1556859_a_at	10.2	4.8	-1.09	-2.12	AAA-AAA	0.00	999	1.000	1.000	0.3755
221402_at	26.5	12.5	-1.09	-2.12	AAA-AAA	0.00	999	1.000	1.000	0.4918
227487_s_at	34.6	16.3	-1.09	-2.12	AAA-AAA	0.00	999	1.000	1.000	0.4543

211359_s_at	44.5	21.0	-1.09	-2.12	AAA-AAA	0.00	999	1.000	1.000	0.3591
207218_at	11.2	23.8	1.09	2.12	PAA-AAA	0.00	999	1.000	1.000	0.5964
1552291_at	216.0	458.0	1.09	2.12	PPP-PPP	0.00	999	1.000	1.000	0.0764
1556990_at	7.6	3.6	-1.09	-2.12	AAA-AAA	0.00	999	1.000	1.000	0.4819
1556846_at	22.6	10.7	-1.09	-2.12	AAA-AAA	0.00	999	1.000	1.000	0.4746
206998_x_at	45.5	21.4	-1.09	-2.12	AAA-AAA	0.00	999	1.000	1.000	0.1942
206712_at	15.5	7.3	-1.09	-2.12	AAA-AAA	0.00	999	1.000	1.000	0.2027
222901_s_at	9.4	19.9	1.09	2.12	AAA-AAA	0.00	999	1.000	1.000	0.2165
210751_s_at	15.1	32.1	1.09	2.12	AAA-AAA	0.00	999	1.000	1.000	0.3548
234911_at	37.2	17.5	-1.09	-2.12	AAA-AAA	0.00	999	1.000	1.000	0.4054
238453_at	63.7	30.0	-1.09	-2.12	AAA-AAA	0.00	999	1.000	1.000	0.2126
239206_at	13.8	6.5	-1.09	-2.12	AAA-AAA	0.00	999	1.000	1.000	0.0002
230114_at	13.0	6.1	-1.09	-2.12	AAA-AAA	0.00	999	1.000	1.000	0.2231
232498_at	30.3	14.3	-1.09	-2.12	AAA-AAA	0.00	999	1.000	1.000	0.3038
238962_at	28.4	60.2	1.08	2.12	PAP-AAP	0.00	999	1.000	1.000	0.3140
1554853_at	10.0	4.7	-1.08	-2.12	AAA-AAA	0.00	999	1.000	1.000	0.2448
1556989_at	17.1	36.3	1.08	2.12	AAA-AAA	0.00	999	1.000	1.000	0.0618
208034_s_at	48.9	23.1	-1.08	-2.12	AAA-AAA	0.00	999	1.000	1.000	0.4553
222868_s_at	61.1	28.8	-1.08	-2.12	AAA-AAA	0.00	999	1.000	1.000	0.5187
217263_x_at	98.1	46.3	-1.08	-2.12	AAA-AAA	0.00	999	1.000	1.000	0.0178
216665_s_at	4.5	2.1	-1.08	-2.12	AAA-AAA	0.00	999	1.000	1.000	0.1797
234037_s_at	20.9	9.9	-1.08	-2.12	AAA-AAA	0.00	999	1.000	1.000	0.4264
234922_s_at	42.9	20.3	-1.08	-2.12	AAA-AAA	0.00	999	1.000	1.000	0.2828
237527_at	12.1	5.7	-1.08	-2.12	AAA-AAA	0.00	999	1.000	1.000	0.5711
233408_at	21.4	10.1	-1.08	-2.12	AAA-AAA	0.00	999	1.000	1.000	0.3661
231443_at	125.4	59.2	-1.08	-2.12	AAA-AAA	0.00	999	1.000	1.000	0.1030
207232_s_at	105.9	224.3	1.08	2.12	AAA-MAP	0.00	999	1.000	1.000	0.1405
242944_at	36.2	17.1	-1.08	-2.12	AAA-AAA	0.00	999	1.000	1.000	0.4273
1560832_at	8.8	18.7	1.08	2.12	AAA-MAA	0.00	999	1.000	1.000	0.4212
1569421_at	25.9	12.2	-1.08	-2.12	AAA-AAA	0.00	999	1.000	1.000	0.2364
1566948_at	7.4	3.5	-1.08	-2.12	AAA-AAA	0.00	999	1.000	1.000	0.1379
1554160_a_at	138.3	65.3	-1.08	-2.12	AAA-AAA	0.00	999	1.000	1.000	0.2972
1557481_a_at	4.4	9.3	1.08	2.12	AAA-AAA	0.00	999	1.000	1.000	0.3204
221914_at	17.8	8.4	-1.08	-2.12	AAA-AAA	0.00	999	1.000	1.000	0.5012
210482_x_at	61.8	130.8	1.08	2.12	AAA-AAA	0.00	999	1.000	1.000	0.1733
216432_at	10.5	22.3	1.08	2.12	AAA-AAA	0.00	999	1.000	1.000	0.5047
234313_at	3.2	1.5	-1.08	-2.12	AAA-AAA	0.00	999	1.000	1.000	0.1202
237816_at	19.6	9.2	-1.08	-2.12	AAA-AAA	0.00	999	1.000	1.000	0.0904
228036_s_at	8.3	3.9	-1.08	-2.12	AAA-AAA	0.00	999	1.000	1.000	0.0177
228042_at	5.9	2.8	-1.08	-2.12	AAA-AAA	0.00	999	1.000	1.000	0.2892
243277_x_at	8.2	3.9	-1.08	-2.12	AAA-AAA	0.00	999	1.000	1.000	0.4172
1563143_at	30.9	14.6	-1.08	-2.12	AAA-AAA	0.00	999	1.000	1.000	0.5041

1570630_at	5.9	2.8	-1.08	-2.12	AAA-AAA	0.00	999	1.000	1.000	0.4800
1554937_x_at	76.9	36.3	-1.08	-2.12	AAA-AAA	0.00	999	1.000	1.000	0.1902
1554707_at	27.7	13.1	-1.08	-2.12	AAA-AAA	0.00	999	1.000	1.000	0.5252
1553340_s_at	31.5	14.9	-1.08	-2.12	AAA-AAA	0.00	999	1.000	1.000	0.3763
1559929_at	86.0	40.6	-1.08	-2.12	AAA-AAA	0.00	999	1.000	1.000	0.0409
1556671_s_at	38.3	18.1	-1.08	-2.12	AAA-AAA	0.00	999	1.000	1.000	0.2871
227180_at	28.4	13.4	-1.08	-2.12	AAA-AAA	0.00	999	1.000	1.000	0.2712
217086_at	4.7	10.0	1.08	2.12	AAA-AAA	0.00	999	1.000	1.000	0.3917
234418_x_at	4.7	2.2	-1.08	-2.12	AAA-AAA	0.00	999	1.000	1.000	0.2131
230985_at	23.0	10.9	-1.08	-2.12	AAA-AAA	0.00	999	1.000	1.000	0.0919
230432_at	45.3	21.4	-1.08	-2.12	AAA-AAA	0.00	999	1.000	1.000	0.4449
1560410_at	12.3	5.8	-1.08	-2.12	AAA-AAA	0.00	999	1.000	1.000	0.3213
1558532_at	45.4	96.1	1.08	2.12	AAA-AAA	0.00	999	1.000	1.000	0.2926
1556072_at	10.1	4.8	-1.08	-2.12	AAA-AAA	0.00	999	1.000	1.000	0.1926
215745_at	49.7	23.5	-1.08	-2.12	AAA-AAA	0.00	999	1.000	1.000	0.4203
215679_at	54.7	25.9	-1.08	-2.12	AAA-AAA	0.00	999	1.000	1.000	0.2119
234419_x_at	11.2	5.3	-1.08	-2.12	AAA-AAA	0.00	999	1.000	1.000	0.0509
239427_at	34.3	16.2	-1.08	-2.12	AAA-AAA	0.00	999	1.000	1.000	0.3334
224172_at	13.1	27.7	1.08	2.11	AAA-PAM	0.00	999	1.000	1.000	0.2336
240003_at	25.6	12.1	-1.08	-2.11	AAA-AAA	0.00	999	1.000	1.000	0.4661
243247_at	20.6	9.8	-1.08	-2.11	AAA-AAA	0.00	999	1.000	1.000	0.0212
1562802_at	13.9	6.6	-1.08	-2.11	AAA-AAA	0.00	999	1.000	1.000	0.3466
1562930_at	20.3	9.6	-1.08	-2.11	AAA-AAA	0.00	999	1.000	1.000	0.1151
1560253_at	19.8	9.4	-1.08	-2.11	AAA-AAA	0.00	999	1.000	1.000	0.3085
1569419_at	47.9	22.7	-1.08	-2.11	AAA-AAA	0.00	999	1.000	1.000	0.3864
1565549_at	7.7	16.4	1.08	2.12	AAA-AAA	0.00	999	1.000	1.000	0.4712
1566720_at	20.0	9.5	-1.08	-2.11	AAA-AAA	0.00	999	1.000	1.000	0.4492
1557287_at	9.6	4.5	-1.08	-2.11	AAA-AAA	0.00	999	1.000	1.000	0.4060
1557280_s_at	15.1	7.1	-1.08	-2.11	AAA-AAA	0.00	999	1.000	1.000	0.3515
214054_at	45.4	21.5	-1.08	-2.11	AAA-AAA	0.00	999	1.000	1.000	0.4781
211875_x_at	11.4	5.4	-1.08	-2.11	AAA-AAA	0.00	999	1.000	1.000	0.0523
208583_x_at	103.8	219.4	1.08	2.12	AAA-AAA	0.00	999	1.000	1.000	0.1876
235814_at	9.2	4.4	-1.08	-2.12	AAA-AAA	0.00	999	1.000	1.000	0.0478
206296_x_at	22.2	10.5	-1.08	-2.11	AAA-AAA	0.00	999	1.000	1.000	0.2782
233661_at	9.3	4.4	-1.08	-2.11	AAA-AAA	0.00	999	1.000	1.000	0.1260
205473_at	57.4	27.1	-1.08	-2.11	AAA-AAA	0.00	999	1.000	1.000	0.1091
203287_at	36.0	76.0	1.08	2.11	AAA-AAA	0.00	999	1.000	1.000	0.5033
240641_at	5.0	10.6	1.08	2.11	AAA-AAA	0.00	999	1.000	1.000	0.3855
1554836_at	3.7	7.7	1.08	2.11	AAA-AAA	0.00	999	1.000	1.000	0.3218
1553076_at	18.2	8.6	-1.08	-2.11	AAA-AAA	0.00	999	1.000	1.000	0.5226
207752_x_at	28.6	13.5	-1.08	-2.11	AAA-AAA	0.00	999	1.000	1.000	0.1928
208385_at	4.8	10.1	1.08	2.11	AAA-AAA	0.00	999	1.000	1.000	0.3929

206844_at	6.5	3.1	-1.08	-2.11	AAA-AAA	0.00	999	1.000	1.000	0.0099
211170_s_at	9.7	4.6	-1.08	-2.11	AAA-AAA	0.00	999	1.000	1.000	0.4900
215800_at	52.9	25.0	-1.08	-2.11	AAA-AAA	0.00	999	1.000	1.000	0.0087
242055_at	28.9	13.7	-1.08	-2.11	AAA-AAA	0.00	999	1.000	1.000	0.4392
1564014_at	12.4	5.9	-1.08	-2.11	AAA-AAA	0.00	999	1.000	1.000	0.3709
1565838_at	33.1	15.7	-1.08	-2.11	AAA-AAA	0.00	999	1.000	1.000	0.1617
1566966_at	5.5	11.6	1.08	2.11	AAA-AAA	0.00	999	1.000	1.000	0.5439
1554594_at	16.2	7.7	-1.08	-2.11	AAA-AAA	0.00	999	1.000	1.000	0.5761
226900_at	17.9	8.5	-1.08	-2.11	AAA-AAA	0.00	999	1.000	1.000	0.4366
211144_x_at	45.8	21.7	-1.08	-2.11	AAA-AAA	0.00	999	1.000	1.000	0.1253
217711_at	21.0	10.0	-1.08	-2.11	AAA-AAA	0.00	999	1.000	1.000	0.3191
237268_at	5.8	2.7	-1.08	-2.11	AAA-AAA	0.00	999	1.000	1.000	0.1245
236977_at	79.0	37.4	-1.08	-2.11	AAA-AAA	0.00	999	1.000	1.000	0.0806
237063_at	17.8	8.5	-1.08	-2.11	AAA-AAA	0.00	999	1.000	1.000	0.4342
237590_at	9.8	4.7	-1.08	-2.11	AAA-AAA	0.00	999	1.000	1.000	0.0760
237614_at	17.1	8.1	-1.08	-2.11	MAA-AAA	0.00	999	1.000	1.000	0.5920
230776_at	19.3	9.1	-1.08	-2.11	AAA-AAA	0.00	999	1.000	1.000	0.2444
205820_s_at	27.4	13.0	-1.08	-2.11	AAA-AAA	0.00	999	1.000	1.000	0.0633
229337_at	59.1	28.0	-1.08	-2.11	AAA-AAA	0.00	999	1.000	1.000	0.4950
233718_at	9.8	20.6	1.08	2.11	AAA-AAA	0.00	999	1.000	1.000	0.3406
230939_at	20.5	43.2	1.08	2.11	PAA-AAA	0.00	999	1.000	1.000	0.3492
244307_s_at	113.4	53.8	-1.08	-2.11	AAA-AAA	0.00	999	1.000	1.000	0.0002
240338_at	17.3	8.2	-1.08	-2.11	AAA-AAA	0.00	999	1.000	1.000	0.5573
1564882_at	43.8	20.8	-1.08	-2.11	AAA-AAA	0.00	999	1.000	1.000	0.1075
210109_at	140.7	66.7	-1.08	-2.11	AAA-AAA	0.00	999	1.000	1.000	0.0382
214532_x_at	53.6	25.4	-1.08	-2.11	AAA-AAA	0.00	999	1.000	1.000	0.3902
211817_s_at	18.9	9.0	-1.08	-2.11	AAA-AAA	0.00	999	1.000	1.000	0.0663
215459_at	16.8	35.5	1.08	2.11	AAA-AAA	0.00	999	1.000	1.000	0.4591
235712_at	20.2	9.6	-1.08	-2.11	AAA-AAA	0.00	999	1.000	1.000	0.3524
234765_at	50.3	23.9	-1.08	-2.11	AAA-AAA	0.00	999	1.000	1.000	0.0596
206256_at	29.0	61.2	1.08	2.11	AAA-AAA	0.00	999	1.000	1.000	0.3409
231580_at	7.9	3.7	-1.08	-2.11	AAA-AAA	0.00	999	1.000	1.000	0.3442
231911_at	9.4	19.7	1.08	2.11	AAA-AAP	0.00	999	1.000	1.000	0.5037
203126_at	45.5	95.9	1.08	2.11	AAA-AAP	0.00	999	1.000	1.000	0.4753
237837_at	13.1	27.5	1.08	2.11	AAA-PAP	0.00	999	1.000	1.000	0.3526
212326_at	82.5	173.9	1.08	2.11	AAA-PPP	0.00	999	1.000	1.000	0.2046
1564248_at	133.9	63.5	-1.08	-2.11	AAA-AAA	0.00	999	1.000	1.000	0.0963
1567443_x_at	31.9	15.1	-1.08	-2.11	AAA-AAA	0.00	999	1.000	1.000	0.0283
43427_at	50.3	23.9	-1.08	-2.11	AAA-AAA	0.00	999	1.000	1.000	0.1924
206423_at	11.3	5.4	-1.08	-2.11	AAA-AAA	0.00	999	1.000	1.000	0.1437
213847_at	54.6	115.0	1.08	2.11	AAA-AAA	0.00	999	1.000	1.000	0.1301
214255_at	37.1	17.6	-1.08	-2.11	AAA-AAA	0.00	999	1.000	1.000	0.3201

213592_at	20.0	9.5	-1.08	-2.11	AAA-AAA	0.00	999	1.000	1.000	0.3794
233535_at	43.3	20.5	-1.08	-2.11	AAA-AAA	0.00	999	1.000	1.000	0.3262
232974_at	25.3	53.3	1.08	2.11	AAA-APP	0.00	999	1.000	1.000	0.2007
212415_at	76.8	161.8	1.08	2.11	AAA-PPA	0.00	999	1.000	1.000	0.0643
240097_at	40.0	19.0	-1.08	-2.11	AAA-AAA	0.00	999	1.000	1.000	0.4905
1563886_at	19.9	9.5	-1.08	-2.11	AAA-AAA	0.00	999	1.000	1.000	0.4857
1563821_at	53.2	25.2	-1.08	-2.11	AAA-AAA	0.00	999	1.000	1.000	0.1926
1568673_s_at	3.9	1.9	-1.08	-2.11	AAA-AAA	0.00	999	1.000	1.000	0.3557
1553717_at	14.1	29.6	1.08	2.11	AAA-AAA	0.00	999	1.000	1.000	0.3818
244845_at	37.8	18.0	-1.08	-2.11	AAA-AAA	0.00	999	1.000	1.000	0.3812
1557665_at	35.1	16.7	-1.08	-2.11	AAA-AAA	0.00	999	1.000	1.000	0.2933
1558915_a_at	23.3	49.1	1.08	2.11	AAA-AAA	0.00	999	1.000	1.000	0.2643
214125_s_at	23.3	11.0	-1.08	-2.11	AAA-AAA	0.00	999	1.000	1.000	0.4276
215010_s_at	53.8	25.5	-1.08	-2.11	AAA-AAA	0.00	999	1.000	1.000	0.1784
233501_at	48.3	22.9	-1.08	-2.11	AAA-AAA	0.00	999	1.000	1.000	0.5807
240109_at	3.5	7.3	1.07	2.11	AAA-AAA	0.00	999	1.000	1.000	0.3416
203638_s_at	9.9	4.7	-1.07	-2.11	AAA-AAA	0.00	999	1.000	1.000	0.4921
1560147_at	13.5	6.4	-1.07	-2.11	AAA-AAA	0.00	999	1.000	1.000	0.5913
1561293_at	71.4	33.9	-1.07	-2.11	AAA-AAA	0.00	999	1.000	1.000	0.5377
1566505_at	14.4	6.9	-1.07	-2.11	AAA-AAA	0.00	999	1.000	1.000	0.3876
1554224_at	3.8	8.0	1.07	2.11	AAA-AAA	0.00	999	1.000	1.000	0.2257
1552813_at	9.5	4.5	-1.07	-2.11	AAA-AAA	0.00	999	1.000	1.000	0.0828
1559050_at	11.2	5.3	-1.07	-2.11	AAA-AAA	0.00	999	1.000	1.000	0.1306
1559393_at	19.0	9.0	-1.07	-2.11	AAA-AAA	0.00	999	1.000	1.000	0.5772
220502_s_at	9.7	4.6	-1.07	-2.11	AAA-AAA	0.00	999	1.000	1.000	0.5737
1554488_at	27.8	58.4	1.07	2.10	AAA-AAP	0.00	999	1.000	1.000	0.3655
203115_at	173.4	364.8	1.07	2.10	MPP-PPP	0.00	999	1.000	1.000	0.1649
1569638_at	5.4	11.5	1.07	2.10	AAA-AAA	0.00	999	1.000	1.000	0.3445
1556461_at	15.7	7.5	-1.07	-2.10	AAA-AAA	0.00	999	1.000	1.000	0.5673
226228_at	15.5	7.3	-1.07	-2.10	AAA-AAA	0.00	999	1.000	1.000	0.4979
213767_at	12.9	27.2	1.07	2.10	AAA-AAA	0.00	999	1.000	1.000	0.4253
215724_at	5.4	2.6	-1.07	-2.10	AAA-AAA	0.00	999	1.000	1.000	0.1361
216623_x_at	4.8	2.3	-1.07	-2.10	AAA-AAA	0.00	999	1.000	1.000	0.1842
204674_at	22.5	10.7	-1.07	-2.10	AAA-AAA	0.00	999	1.000	1.000	0.4801
229805_at	31.5	66.2	1.07	2.10	AAA-AAM	0.00	999	1.000	1.000	0.1367
228529_at	45.7	21.7	-1.07	-2.10	AAA-AAA	0.00	999	1.000	1.000	0.3265
228706_s_at	16.4	7.8	-1.07	-2.10	AAA-AAA	0.00	999	1.000	1.000	0.3768
232404_at	18.9	9.0	-1.07	-2.10	AAA-AAA	0.00	999	1.000	1.000	0.2810
202254_at	62.8	132.1	1.07	2.10	AAA-APP	0.00	999	1.000	1.000	0.2170
1561723_at	103.2	49.1	-1.07	-2.10	AAA-AAA	0.00	999	1.000	1.000	0.0650
1567657_at	2.1	4.3	1.07	2.10	AAA-AAA	0.00	999	1.000	1.000	0.4713

1567028_s_at	15.0	31.5	1.07	2.10	AAA-AAA	0.00	999	1.000	1.000	0.4681
207848_at	17.8	8.5	-1.07	-2.10	AAA-AAA	0.00	999	1.000	1.000	0.3827
214301_s_at	13.8	29.0	1.07	2.10	AAA-AAA	0.00	999	1.000	1.000	0.2735
214166_at	57.1	27.2	-1.07	-2.10	AAA-AAA	0.00	999	1.000	1.000	0.4311
211794_at	17.5	36.7	1.07	2.10	AAA-AAA	0.00	999	1.000	1.000	0.0814
211925_s_at	5.7	11.9	1.07	2.10	AAA-AAA	0.00	999	1.000	1.000	0.3398
218553_s_at	127.2	60.5	-1.07	-2.10	AAA-AAA	0.00	999	1.000	1.000	0.3032
233996_x_at	8.4	4.0	-1.07	-2.10	AAA-AAA	0.00	999	1.000	1.000	0.4907
238140_at	31.6	66.5	1.07	2.10	AAA-AAA	0.00	999	1.000	1.000	0.2200
237976_at	28.4	13.5	-1.07	-2.10	AAA-AAA	0.00	999	1.000	1.000	0.5326
206399_x_at	11.3	5.4	-1.07	-2.10	AAA-AAA	0.00	999	1.000	1.000	0.1401
206069_s_at	34.8	16.5	-1.07	-2.10	AAA-AAA	0.00	999	1.000	1.000	0.2438
229042_s_at	67.0	31.8	-1.07	-2.10	AAA-AAA	0.00	999	1.000	1.000	0.2250
224507_s_at	43.4	91.1	1.07	2.10	AAA-PPA	0.00	999	1.000	1.000	0.2901
240670_at	25.2	52.9	1.07	2.10	AAA-AAA	0.00	999	1.000	1.000	0.2761
241005_at	23.2	11.0	-1.07	-2.10	AAA-AAA	0.00	999	1.000	1.000	0.4092
201187_s_at	73.1	34.8	-1.07	-2.10	AAM-AAA	0.00	999	1.000	1.000	0.4087
1565706_at	18.8	39.5	1.07	2.10	AAA-AAA	0.00	999	1.000	1.000	0.2926
1558481_s_at	16.7	35.2	1.07	2.10	AAA-AAA	0.00	999	1.000	1.000	0.0235
220142_at	27.1	12.9	-1.07	-2.10	AAA-AAA	0.00	999	1.000	1.000	0.4426
217584_at	38.3	80.5	1.07	2.10	AAA-AAA	0.00	999	1.000	1.000	0.2610
215742_at	5.5	2.6	-1.07	-2.10	AAA-AAA	0.00	999	1.000	1.000	0.0641
237270_at	45.1	21.4	-1.07	-2.10	AAA-AAA	0.00	999	1.000	1.000	0.3563
237579_at	20.6	9.8	-1.07	-2.10	AAA-AAA	0.00	999	1.000	1.000	0.3993
233666_at	43.1	20.5	-1.07	-2.10	AAA-AAA	0.00	999	1.000	1.000	0.2826
212803_at	154.6	324.5	1.07	2.10	AMP-PPM	0.00	999	1.000	1.000	0.4499
232755_at	75.8	159.0	1.07	2.10	AAP-PPP	0.00	999	1.000	1.000	0.3102
240705_at	12.1	5.8	-1.07	-2.10	AAA-AAA	0.00	999	1.000	1.000	0.3688
243937_x_at	125.4	59.8	-1.07	-2.10	AAA-AAA	0.00	999	1.000	1.000	0.2327
244234_at	64.4	30.7	-1.07	-2.10	AAA-AAA	0.00	999	1.000	1.000	0.3628
239881_at	26.5	12.6	-1.07	-2.10	AAA-AAA	0.00	999	1.000	1.000	0.3523
1563086_at	33.7	70.7	1.07	2.10	AAA-AAA	0.00	999	1.000	1.000	0.4325
1561393_at	72.0	34.3	-1.07	-2.10	AAA-AAA	0.00	999	1.000	1.000	0.3386
1565836_at	30.2	14.4	-1.07	-2.10	AAA-AAA	0.00	999	1.000	1.000	0.4089
1554413_s_at	15.9	7.6	-1.07	-2.10	AAA-AAA	0.00	999	1.000	1.000	0.4706
226057_at	52.8	25.2	-1.07	-2.10	AAA-AAA	0.00	999	1.000	1.000	0.2419
217186_at	23.2	11.0	-1.07	-2.10	AAA-AAA	0.00	999	1.000	1.000	0.3998
217323_at	12.8	26.8	1.07	2.10	AAA-AAA	0.00	999	1.000	1.000	0.4948
205302_at	12.0	5.7	-1.07	-2.10	AAA-AAA	0.00	999	1.000	1.000	0.4421
231446_at	18.5	8.8	-1.07	-2.10	AAA-AAA	0.00	999	1.000	1.000	0.5661
240739_at	8.3	3.9	-1.07	-2.10	AAA-AAA	0.00	999	1.000	1.000	0.4014
1563168_at	10.4	5.0	-1.07	-2.10	AAA-AAA	0.00	999	1.000	1.000	0.5369

1569275_at	19.8	9.5	-1.07	-2.10	AAA-AAA	0.00	999	1.000	1.000	0.4908
244720_at	10.2	21.5	1.07	2.10	AAA-AAA	0.00	999	1.000	1.000	0.2656
227461_at	30.8	14.7	-1.07	-2.10	AAA-AAA	0.00	999	1.000	1.000	0.0730
223610_at	68.0	32.4	-1.07	-2.10	AAA-AAA	0.00	999	1.000	1.000	0.2095
234281_at	11.9	5.7	-1.07	-2.10	AAA-AAA	0.00	999	1.000	1.000	0.5776
238991_at	11.4	5.4	-1.07	-2.10	AAA-AAA	0.00	999	1.000	1.000	0.2343
228470_at	8.9	4.3	-1.07	-2.10	AAA-AAA	0.00	999	1.000	1.000	0.5539
232749_at	25.7	54.0	1.07	2.10	AAA-APP	0.00	999	1.000	1.000	0.3402
242802_x_at	14.0	6.7	-1.07	-2.10	AAA-AAA	0.00	999	1.000	1.000	0.5845
1563830_a_at	28.0	13.4	-1.07	-2.10	AAA-AAA	0.00	999	1.000	1.000	0.3548
1555001_at	11.8	24.7	1.07	2.10	AMA-AAA	0.00	999	1.000	1.000	0.4536
1555024_at	29.5	14.1	-1.07	-2.10	AAA-AAA	0.00	999	1.000	1.000	0.4540
1555942_a_at	20.3	9.7	-1.07	-2.10	AAA-AAA	0.00	999	1.000	1.000	0.4070
1556879_at	33.0	15.7	-1.07	-2.10	AAA-AAA	0.00	999	1.000	1.000	0.2827
233272_at	11.9	24.9	1.07	2.10	AAA-AAA	0.00	999	1.000	1.000	0.5192
203698_s_at	13.2	6.3	-1.07	-2.09	AAA-AAA	0.00	999	1.000	1.000	0.5747
1565771_at	9.8	4.7	-1.07	-2.10	AAA-AAA	0.00	999	1.000	1.000	0.0835
220396_at	36.4	17.4	-1.07	-2.10	AAA-AAA	0.00	999	1.000	1.000	0.4369
207651_at	20.7	9.9	-1.07	-2.10	AAA-AAA	0.00	999	1.000	1.000	0.1140
207308_at	35.8	17.1	-1.07	-2.10	AAA-AAA	0.00	999	1.000	1.000	0.0632
229600_s_at	60.3	28.8	-1.07	-2.10	AAA-AAA	0.00	999	1.000	1.000	0.0822
206267_s_at	29.0	60.7	1.07	2.10	AAA-AAA	0.00	999	1.000	1.000	0.4374
231423_s_at	59.7	125.1	1.07	2.10	AAA-AAA	0.00	999	1.000	1.000	0.3444
239629_at	42.2	88.4	1.07	2.09	APP-APP	0.00	999	1.000	1.000	0.2301
241667_x_at	35.0	16.7	-1.07	-2.09	AAA-AAA	0.00	999	1.000	1.000	0.4816
241841_at	102.9	49.2	-1.07	-2.09	AAA-AAA	0.00	999	1.000	1.000	0.1599
1563591_at	12.6	6.0	-1.07	-2.09	AAA-AAA	0.00	999	1.000	1.000	0.2581
1566478_at	4.0	1.9	-1.07	-2.09	AAA-AAA	0.00	999	1.000	1.000	0.3299
244570_at	29.6	61.9	1.07	2.09	AAA-AAA	0.00	999	1.000	1.000	0.4827
1559483_at	10.8	5.2	-1.07	-2.09	AAA-AAA	0.00	999	1.000	1.000	0.3893
208007_at	26.7	12.7	-1.07	-2.09	AAA-AAA	0.00	999	1.000	1.000	0.2684
208480_s_at	8.8	4.2	-1.07	-2.09	AAA-AAA	0.00	999	1.000	1.000	0.2485
220318_at	11.9	5.7	-1.07	-2.09	AAA-AAA	0.00	999	1.000	1.000	0.1898
215112_x_at	6.4	13.3	1.07	2.09	AAA-AAA	0.00	999	1.000	1.000	0.4266
242236_at	4.5	9.3	1.07	2.09	AAA-AAA	0.00	999	1.000	1.000	0.4992
1554409_at	38.9	18.6	-1.07	-2.09	AAA-AAA	0.00	999	1.000	1.000	0.1870
221385_s_at	32.6	15.6	-1.07	-2.09	AAA-AAA	0.00	999	1.000	1.000	0.4016
220803_at	11.0	23.0	1.07	2.09	AAA-AAA	0.00	999	1.000	1.000	0.3476
223997_at	70.5	33.7	-1.07	-2.09	AAA-AAA	0.00	999	1.000	1.000	0.0839
211531_x_at	46.5	22.3	-1.07	-2.09	AAA-AAA	0.00	999	1.000	1.000	0.2941
210924_at	65.1	31.1	-1.07	-2.09	AAA-AAA	0.00	999	1.000	1.000	0.2577
216358_at	18.6	39.0	1.07	2.09	AMA-AMA	0.00	999	1.000	1.000	0.5113

236542_at	6.3	13.1	1.07	2.09	AAA-AAA	0.00	999	1.000	1.000	0.4439
238707_at	14.7	30.7	1.07	2.09	AAA-AAA	0.00	999	1.000	1.000	0.3156
236914_at	12.5	26.2	1.07	2.09	AAA-AAA	0.00	999	1.000	1.000	0.2917
240829_at	15.9	33.2	1.06	2.09	AMP-APA	0.00	999	1.000	1.000	0.5121
242854_x_at	17.9	37.5	1.06	2.09	AAA-AAP	0.00	999	1.000	1.000	0.3535
242465_at	33.3	69.6	1.06	2.09	AAA-AAA	0.00	999	1.000	1.000	0.1602
216451_at	14.9	7.1	-1.06	-2.09	AAA-AAA	0.00	999	1.000	1.000	0.0161
216345_at	7.5	3.6	-1.06	-2.09	AAA-AAA	0.00	999	1.000	1.000	0.1512
236434_at	8.9	4.3	-1.06	-2.09	AAA-AAA	0.00	999	1.000	1.000	0.1729
239308_at	89.8	43.0	-1.06	-2.09	AAA-AAA	0.00	999	1.000	1.000	0.3297
237798_at	10.1	21.1	1.06	2.09	AAA-AAA	0.00	999	1.000	1.000	0.2571
205960_at	6.6	13.8	1.06	2.09	AAA-AAM	0.00	999	1.000	1.000	0.6149
205430_at	14.0	6.7	-1.06	-2.09	AAA-AAA	0.00	999	1.000	1.000	0.6261
231482_at	20.9	10.0	-1.06	-2.09	AAA-AAA	0.00	999	1.000	1.000	0.1361
219700_at	27.6	57.8	1.06	2.09	APA-AMP	0.00	999	1.000	1.000	0.4131
1560646_at	20.0	41.9	1.06	2.09	AAA-AAA	0.00	999	1.000	1.000	0.0235
1553944_at	14.7	7.0	-1.06	-2.09	AAA-AAA	0.00	999	1.000	1.000	0.4558
1558842_at	7.0	14.7	1.06	2.09	AAA-AAA	0.00	999	1.000	1.000	0.1958
1556650_at	37.2	17.8	-1.06	-2.09	AAA-AAA	0.00	999	1.000	1.000	0.5313
221318_at	57.0	27.3	-1.06	-2.09	AAA-AAA	0.00	999	1.000	1.000	0.2247
227084_at	68.6	32.8	-1.06	-2.09	AAA-AAA	0.00	999	1.000	1.000	0.0523
210769_at	29.3	61.1	1.06	2.09	AAA-AAA	0.00	999	1.000	1.000	0.1850
236746_at	32.3	15.4	-1.06	-2.09	AAA-AAA	0.00	999	1.000	1.000	0.3030
238289_at	6.6	3.2	-1.06	-2.09	AAA-AAA	0.00	999	1.000	1.000	0.4300
230287_at	15.1	31.6	1.06	2.09	AAA-AAA	0.00	999	1.000	1.000	0.3153
228133_s_at	5.9	12.3	1.06	2.09	AAA-AAA	0.00	999	1.000	1.000	0.1991
223620_at	19.6	40.9	1.06	2.09	AAA-APA	0.00	999	1.000	1.000	0.3304
1554601_at	28.2	13.5	-1.06	-2.09	AAA-AAA	0.00	999	1.000	1.000	0.0896
244541_x_at	9.3	4.4	-1.06	-2.09	AAA-AAA	0.00	999	1.000	1.000	0.2808
1558391_s_at	23.0	48.1	1.06	2.09	AAA-AAA	0.00	999	1.000	1.000	0.5583
1557680_at	25.7	53.6	1.06	2.09	AAA-AAA	0.00	999	1.000	1.000	0.3667
222260_at	14.4	6.9	-1.06	-2.09	AAA-AAA	0.00	999	1.000	1.000	0.0758
219958_at	28.2	58.9	1.06	2.09	AAA-AAA	0.00	999	1.000	1.000	0.2495
225453_x_at	20.9	43.5	1.06	2.09	AAA-AAA	0.00	999	1.000	1.000	0.2405
207314_x_at	12.4	26.0	1.06	2.09	AAA-AAA	0.00	999	1.000	1.000	0.1412
215896_at	20.0	41.7	1.06	2.09	AAA-AAA	0.00	999	1.000	1.000	0.3300
233840_at	28.5	13.7	-1.06	-2.09	AAA-AAA	0.00	999	1.000	1.000	0.2456
239556_at	5.7	2.8	-1.06	-2.09	AAA-AAA	0.00	999	1.000	1.000	0.2545
239641_at	66.7	139.2	1.06	2.09	AAA-AAA	0.00	999	1.000	1.000	0.2587
229522_at	82.2	39.4	-1.06	-2.09	AAA-AAA	0.00	999	1.000	1.000	0.2626
230471_at	7.6	3.7	-1.06	-2.09	AAA-AAA	0.00	999	1.000	1.000	0.0583
232732_at	10.1	21.2	1.06	2.09	AAA-AAA	0.00	999	1.000	1.000	0.0410

233529_at	32.7	15.7	-1.06	-2.09	AAA-AAA	0.00	999	1.000	1.000	0.1878
231360_at	16.4	7.9	-1.06	-2.09	AAA-AAA	0.00	999	1.000	1.000	0.3810
225813_at	625.7	1305.1	1.06	2.09	PPP-PPP	0.00	999	1.000	1.000	0.0635
243945_at	3.8	7.9	1.06	2.09	AAA-AAA	0.00	999	1.000	1.000	0.6104
244335_at	12.1	25.2	1.06	2.09	AAA-AAA	0.00	999	1.000	1.000	0.3291
1564405_at	4.9	10.1	1.06	2.09	AAA-AAA	0.00	999	1.000	1.000	0.3802
244507_at	3.6	1.7	-1.06	-2.09	AAA-AAA	0.00	999	1.000	1.000	0.1599
1557632_at	17.8	8.5	-1.06	-2.09	AAA-AAA	0.00	999	1.000	1.000	0.5243
1563673_a_at	15.1	7.3	-1.06	-2.09	AAA-AAA	0.00	999	1.000	1.000	0.4737
207949_s_at	12.6	26.3	1.06	2.09	AAA-AAA	0.00	999	1.000	1.000	0.3018
208439_s_at	35.6	17.1	-1.06	-2.09	AAA-AAA	0.00	999	1.000	1.000	0.4330
206730_at	8.7	4.2	-1.06	-2.09	AAA-AAA	0.00	999	1.000	1.000	0.5528
207586_at	31.1	14.9	-1.06	-2.09	AAA-AAA	0.00	999	1.000	1.000	0.5256
224033_at	6.3	3.0	-1.06	-2.09	AAA-AAA	0.00	999	1.000	1.000	0.3019
235426_at	22.0	45.9	1.06	2.09	AAA-AAA	0.00	999	1.000	1.000	0.2597
237113_at	6.6	3.2	-1.06	-2.09	AAA-AAA	0.00	999	1.000	1.000	0.3016
232472_at	87.8	42.1	-1.06	-2.09	AAA-AAA	0.00	999	1.000	1.000	0.3759
203726_s_at	79.6	38.2	-1.06	-2.09	AAA-AAA	0.00	999	1.000	1.000	0.2698
1557896_at	11.3	23.6	1.06	2.08	AAA-AAA	0.00	999	1.000	1.000	0.2767
231420_at	8.4	17.5	1.06	2.09	AAA-AAA	0.00	999	1.000	1.000	0.1904
1555982_at	42.5	88.6	1.06	2.08	AAA-APP	0.00	999	1.000	1.000	0.2767
242872_at	41.0	85.4	1.06	2.08	AAA-AAA	0.00	999	1.000	1.000	0.3423
1564778_at	18.3	8.8	-1.06	-2.08	AAA-AAA	0.00	999	1.000	1.000	0.1011
208251_at	124.8	59.9	-1.06	-2.08	AAA-AAA	0.00	999	1.000	1.000	0.0218
208538_at	4.3	9.0	1.06	2.08	AAA-AAA	0.00	999	1.000	1.000	0.4751
231410_at	32.4	15.6	-1.06	-2.08	AAA-AAA	0.00	999	1.000	1.000	0.3859
220967_s_at	91.6	190.6	1.06	2.08	AAM-APP	0.00	999	1.000	1.000	0.3125
239482_x_at	46.5	96.7	1.06	2.08	AAA-PMP	0.00	999	1.000	1.000	0.1242
1563203_at	7.1	3.4	-1.06	-2.08	AAA-AAA	0.00	999	1.000	1.000	0.5661
1560753_at	76.7	36.8	-1.06	-2.08	AAA-AAA	0.00	999	1.000	1.000	0.0626
1555304_a_at	15.1	31.4	1.06	2.08	AAA-AAA	0.00	999	1.000	1.000	0.3226
1555322_at	57.3	27.5	-1.06	-2.08	AAA-AAA	0.00	999	1.000	1.000	0.2314
236572_at	18.4	8.8	-1.06	-2.08	AAA-AAA	0.00	999	1.000	1.000	0.2948
205358_at	3.7	1.8	-1.06	-2.08	AAA-AAA	0.00	999	1.000	1.000	0.0423
242936_at	31.8	15.3	-1.06	-2.08	AAA-AAA	0.00	999	1.000	1.000	0.4584
241452_at	67.8	32.6	-1.06	-2.08	AAA-AAA	0.00	999	1.000	1.000	0.0296
1567856_x_at	23.1	11.1	-1.06	-2.08	AAA-AAA	0.00	999	1.000	1.000	0.2764
1569871_at	151.2	72.7	-1.06	-2.08	AAA-AAA	0.00	999	1.000	1.000	0.2549
1565603_at	171.6	82.5	-1.06	-2.08	AAA-AAA	0.00	999	1.000	1.000	0.1977
244394_at	27.4	13.2	-1.06	-2.08	AAA-AAA	0.00	999	1.000	1.000	0.3682
244578_at	3.8	8.0	1.06	2.08	AAA-AAA	0.00	999	1.000	1.000	0.3770
220517_at	55.1	26.5	-1.06	-2.08	AAA-AAA	0.00	999	1.000	1.000	0.1706

214570_x_at	16.3	33.8	1.06	2.08	AAA-AAA	0.00	999	1.000	1.000	0.2915
211260_at	32.1	66.8	1.06	2.08	AAA-AAA	0.00	999	1.000	1.000	0.3909
215346_at	47.6	99.0	1.06	2.08	AAA-AAA	0.00	999	1.000	1.000	0.3311
209343_at	12.4	25.8	1.06	2.08	AAA-AAA	0.00	999	1.000	1.000	0.3480
235802_at	43.8	21.1	-1.06	-2.08	AAA-AAA	0.00	999	1.000	1.000	0.2199
236390_at	43.3	90.0	1.06	2.08	AAA-AAA	0.00	999	1.000	1.000	0.2760
228637_at	21.2	44.1	1.06	2.08	AAA-AAA	0.00	999	1.000	1.000	0.4171
233670_at	6.3	3.0	-1.06	-2.08	AAA-AAA	0.00	999	1.000	1.000	0.4613
233311_at	44.2	21.3	-1.06	-2.08	AAA-AAA	0.00	999	1.000	1.000	0.3655
205623_at	23.2	11.2	-1.06	-2.08	AAA-AAA	0.00	999	1.000	1.000	0.5427
241417_at	38.5	18.5	-1.06	-2.08	AAA-AAA	0.00	999	1.000	1.000	0.5008
240750_at	4.7	2.3	-1.06	-2.08	AAA-AAA	0.00	999	1.000	1.000	0.4203
243281_at	12.1	5.8	-1.06	-2.08	AAA-AAA	0.00	999	1.000	1.000	0.4993
242318_at	16.0	7.7	-1.06	-2.08	AAA-AAA	0.00	999	1.000	1.000	0.2763
1561608_at	18.7	9.0	-1.06	-2.08	AAA-AAA	0.00	999	1.000	1.000	0.4394
1567702_at	3.5	1.7	-1.06	-2.08	AAA-AAA	0.00	999	1.000	1.000	0.5619
1566468_at	14.8	7.1	-1.06	-2.08	AAA-AAA	0.00	999	1.000	1.000	0.5853
1553371_at	39.1	18.8	-1.06	-2.08	AAA-AAA	0.00	999	1.000	1.000	0.2760
1555629_at	9.3	4.5	-1.06	-2.08	AAA-AAA	0.00	999	1.000	1.000	0.0114
221283_at	29.3	14.1	-1.06	-2.08	AAA-AAA	0.00	999	1.000	1.000	0.2854
222261_at	26.0	12.5	-1.06	-2.08	AAA-AAA	0.00	999	1.000	1.000	0.2072
215045_at	15.3	7.4	-1.06	-2.08	AAA-AAA	0.00	999	1.000	1.000	0.2726
214539_at	23.2	11.2	-1.06	-2.08	AAA-AAA	0.00	999	1.000	1.000	0.5547
234307_s_at	17.6	8.5	-1.06	-2.08	AAA-AAA	0.00	999	1.000	1.000	0.4017
206161_s_at	61.6	29.6	-1.06	-2.08	AAA-AAA	0.00	999	1.000	1.000	0.0927
225347_at	132.3	275.0	1.06	2.08	APA-APP	0.00	999	1.000	1.000	0.0715
229242_at	266.6	553.8	1.06	2.08	PPP-PPP	0.00	999	1.000	1.000	0.0650
241480_at	54.1	26.0	-1.06	-2.08	AAA-AAA	0.00	999	1.000	1.000	0.1954
1569203_at	7.7	16.1	1.06	2.08	AAA-AAA	0.00	999	1.000	1.000	0.1204
1568648_a_at	7.9	3.8	-1.06	-2.08	AAA-AAA	0.00	999	1.000	1.000	0.4025
1554699_at	9.2	4.4	-1.06	-2.08	AAA-AAA	0.00	999	1.000	1.000	0.4694
1556619_at	8.2	17.1	1.06	2.08	AAA-AAA	0.00	999	1.000	1.000	0.2653
201958_s_at	9.0	18.7	1.06	2.08	AAA-AAA	0.00	999	1.000	1.000	0.3433
214326_x_at	91.8	190.6	1.06	2.08	AAA-AAA	0.00	999	1.000	1.000	0.1560
217593_at	13.3	6.4	-1.06	-2.08	AAA-AAA	0.00	999	1.000	1.000	0.0395
204509_at	54.6	26.3	-1.06	-2.08	AAA-AAA	0.00	999	1.000	1.000	0.3052
229328_at	16.2	7.8	-1.06	-2.08	AAA-AAA	0.00	999	1.000	1.000	0.4401
231556_at	46.4	22.3	-1.06	-2.08	AAA-AAA	0.00	999	1.000	1.000	0.3207
240164_at	27.1	13.1	-1.05	-2.08	AAA-AAA	0.00	999	1.000	1.000	0.3664
1566462_at	11.5	5.5	-1.05	-2.08	AAA-AAA	0.00	999	1.000	1.000	0.2480
1553197_at	15.0	7.2	-1.05	-2.08	AAA-AAA	0.00	999	1.000	1.000	0.4939
1555535_at	26.0	12.5	-1.05	-2.08	AAA-AAA	0.00	999	1.000	1.000	0.4231

220098_at	22.2	10.7	-1.05	-2.08	AAA-AAA	0.00	999	1.000	1.000	0.3583
213987_s_at	13.1	6.3	-1.05	-2.08	AAA-AAA	0.00	999	1.000	1.000	0.2430
217274_x_at	34.4	71.4	1.05	2.08	AAA-AAA	0.00	999	1.000	1.000	0.2938
209686_at	5.6	2.7	-1.05	-2.08	AAA-AAA	0.00	999	1.000	1.000	0.3050
235827_at	120.3	57.9	-1.05	-2.08	AAM-AAA	0.00	999	1.000	1.000	0.1324
233883_at	4.6	9.6	1.05	2.08	AAA-AAA	0.00	999	1.000	1.000	0.1782
230642_at	37.2	17.9	-1.05	-2.08	AAA-AAA	0.00	999	1.000	1.000	0.2264
232662_x_at	77.1	37.1	-1.05	-2.08	AAA-AAA	0.00	999	1.000	1.000	0.4257
232292_at	21.2	10.2	-1.05	-2.08	AAA-AAA	0.00	999	1.000	1.000	0.3703
242368_at	95.7	46.1	-1.05	-2.07	AAA-AAA	0.00	999	1.000	1.000	0.3197
1560227_at	33.8	70.2	1.05	2.08	AAA-AAA	0.00	999	1.000	1.000	0.1942
1552775_at	21.8	10.5	-1.05	-2.08	AAA-AAA	0.00	999	1.000	1.000	0.5042
1560023_x_at	52.7	25.4	-1.05	-2.08	AMA-AAA	0.00	999	1.000	1.000	0.3558
207494_s_at	66.1	31.9	-1.05	-2.08	AAA-AAA	0.00	999	1.000	1.000	0.2605
223784_at	22.2	10.7	-1.05	-2.08	AAA-MAA	0.00	999	1.000	1.000	0.5711
210670_at	13.5	6.5	-1.05	-2.08	AAA-AAA	0.00	999	1.000	1.000	0.2842
215811_at	22.3	10.8	-1.05	-2.08	AAA-AAA	0.00	999	1.000	1.000	0.4731
239772_x_at	37.7	78.1	1.05	2.08	AAA-AAA	0.00	999	1.000	1.000	0.2209
239313_at	84.5	40.7	-1.05	-2.07	AAA-AAA	0.00	999	1.000	1.000	0.1765
228463_at	41.0	19.8	-1.05	-2.07	AAA-AAA	0.00	999	1.000	1.000	0.1244
1560877_a_at	27.1	13.1	-1.05	-2.07	AAA-AAA	0.00	999	1.000	1.000	0.3313
1553458_at	20.8	10.0	-1.05	-2.07	AAA-AAA	0.00	999	1.000	1.000	0.4225
1554772_at	19.9	41.3	1.05	2.07	AAA-AAA	0.00	999	1.000	1.000	0.2809
206578_at	38.4	18.5	-1.05	-2.07	AAA-AAA	0.00	999	1.000	1.000	0.2990
207552_at	41.3	19.9	-1.05	-2.07	AAA-AAA	0.00	999	1.000	1.000	0.1408
217639_at	12.3	25.6	1.05	2.07	AAA-AAA	0.00	999	1.000	1.000	0.4285
216586_at	39.7	19.2	-1.05	-2.07	AAA-AAA	0.00	999	1.000	1.000	0.4434
234566_at	17.3	8.4	-1.05	-2.07	AAA-AAA	0.00	999	1.000	1.000	0.2247
205906_at	26.1	12.6	-1.05	-2.07	AAA-AAA	0.00	999	1.000	1.000	0.5550
233530_at	16.2	7.8	-1.05	-2.07	AAA-AAA	0.00	999	1.000	1.000	0.4533
209740_s_at	42.5	88.0	1.05	2.07	AAA-AAP	0.00	999	1.000	1.000	0.3899
202723_s_at	54.1	112.1	1.05	2.07	AAP-PAA	0.00	999	1.000	1.000	0.2399
219371_s_at	325.1	673.7	1.05	2.07	APP-PPP	0.00	999	1.000	1.000	0.1196
241415_at	41.4	20.0	-1.05	-2.07	AAA-AAA	0.00	999	1.000	1.000	0.3870
1561892_at	19.7	9.5	-1.05	-2.07	AAA-AAA	0.00	999	1.000	1.000	0.5263
1561192_at	8.7	4.2	-1.05	-2.07	AAA-AAA	0.00	999	1.000	1.000	0.2389
1569755_at	8.5	4.1	-1.05	-2.07	AAA-AAA	0.00	999	1.000	1.000	0.6247
1565797_at	64.7	31.2	-1.05	-2.07	AAA-AAA	0.00	999	1.000	1.000	0.1217
1566459_at	43.5	21.0	-1.05	-2.07	AAA-AAA	0.00	999	1.000	1.000	0.2198
207972_at	15.4	7.4	-1.05	-2.07	AAA-AAA	0.00	999	1.000	1.000	0.2641
219695_at	15.3	31.8	1.05	2.07	AAA-AAA	0.00	999	1.000	1.000	0.2679
219829_at	15.3	31.7	1.05	2.07	AAA-AAA	0.00	999	1.000	1.000	0.5381

215975_x_at	55.6	26.9	-1.05	-2.07	AAA-AAA	0.00	999	1.000	1.000	0.4436
209402_s_at	132.0	273.5	1.05	2.07	AAA-MAA	0.00	999	1.000	1.000	0.4018
238217_at	9.4	4.5	-1.05	-2.07	AAA-AAA	0.00	999	1.000	1.000	0.5164
205789_at	14.8	7.1	-1.05	-2.07	AAA-AAA	0.00	999	1.000	1.000	0.4879
227711_at	40.9	19.7	-1.05	-2.07	AAA-AAA	0.00	999	1.000	1.000	0.1128
233092_s_at	5.1	2.5	-1.05	-2.07	AAA-AAA	0.00	999	1.000	1.000	0.4497
233120_at	7.1	3.4	-1.05	-2.07	AAA-AAA	0.00	999	1.000	1.000	0.4742
241175_at	62.5	30.2	-1.05	-2.07	AAA-AMA	0.00	999	1.000	1.000	0.2027
1563112_at	15.7	7.6	-1.05	-2.07	AAA-AAA	0.00	999	1.000	1.000	0.4926
1569076_a_at	11.5	5.6	-1.05	-2.07	AAA-AAA	0.00	999	1.000	1.000	0.4004
1553356_at	4.6	9.6	1.05	2.07	AAA-AAA	0.00	999	1.000	1.000	0.5741
1553057_at	20.7	42.9	1.05	2.07	AAA-AAA	0.00	999	1.000	1.000	0.0779
207450_s_at	6.4	3.1	-1.05	-2.07	AAA-AAA	0.00	999	1.000	1.000	0.0996
216897_s_at	55.6	26.9	-1.05	-2.07	AAA-AAA	0.00	999	1.000	1.000	0.5384
236756_at	13.1	27.1	1.05	2.07	AAA-AAA	0.00	999	1.000	1.000	0.3610
234940_s_at	12.1	25.1	1.05	2.07	AAA-AAA	0.00	999	1.000	1.000	0.3016
230680_at	2.2	4.6	1.05	2.07	AAA-AAA	0.00	999	1.000	1.000	0.0698
230493_at	10.2	4.9	-1.05	-2.07	AAA-AAA	0.00	999	1.000	1.000	0.4016
231625_at	73.7	35.6	-1.05	-2.07	AAA-AAA	0.00	999	1.000	1.000	0.2653
1552531_a_at	42.1	20.4	-1.05	-2.07	AAA-AAA	0.00	999	1.000	1.000	0.1654
208164_s_at	14.9	30.7	1.05	2.07	AAA-AAA	0.00	999	1.000	1.000	0.3844
207981_s_at	9.6	19.8	1.05	2.07	AAA-AAA	0.00	999	1.000	1.000	0.4571
224508_at	20.7	42.9	1.05	2.07	AAA-AAA	0.00	999	1.000	1.000	0.3125
224350_at	15.6	7.5	-1.05	-2.07	AAA-AAA	0.00	999	1.000	1.000	0.5077
217235_x_at	78.2	37.8	-1.05	-2.07	AAA-AAA	0.00	999	1.000	1.000	0.1654
216382_s_at	13.9	6.7	-1.05	-2.07	AAA-AAA	0.00	999	1.000	1.000	0.0114
233729_at	12.6	26.2	1.05	2.07	AAA-AAA	0.00	999	1.000	1.000	0.1657
228599_at	9.2	19.0	1.05	2.07	AAA-AAP	0.00	999	1.000	1.000	0.5975
214801_at	936.4	1936.1	1.05	2.07	PPP-PPP	0.00	999	1.000	1.000	0.1642
240194_at	57.4	27.8	-1.05	-2.07	AAA-AAA	0.00	999	1.000	1.000	0.3675
241290_at	7.4	15.2	1.05	2.07	AAA-AAA	0.00	999	1.000	1.000	0.2696
240315_at	44.7	21.6	-1.05	-2.07	AAA-AAA	0.00	999	1.000	1.000	0.4870
243803_at	5.1	10.5	1.05	2.07	AAA-AAA	0.00	999	1.000	1.000	0.4488
243526_at	14.7	7.1	-1.05	-2.07	AAA-AAA	0.00	999	1.000	1.000	0.4664
1562250_at	15.4	7.4	-1.05	-2.07	AAA-AAA	0.00	999	1.000	1.000	0.4786
1561250_at	9.4	4.6	-1.05	-2.07	AAA-AAA	0.00	999	1.000	1.000	0.3133
1566474_at	27.8	13.4	-1.05	-2.07	AAA-AAA	0.00	999	1.000	1.000	0.0975
1555405_at	6.3	12.9	1.05	2.07	AAA-AAA	0.00	999	1.000	1.000	0.4717
1555676_s_at	22.3	10.8	-1.05	-2.07	AAA-AAA	0.00	999	1.000	1.000	0.3476
223683_at	12.3	25.4	1.05	2.07	AAA-AAA	0.00	999	1.000	1.000	0.3825
207356_at	32.0	15.5	-1.05	-2.07	AAA-AAA	0.00	999	1.000	1.000	0.0741
210431_at	52.7	25.5	-1.05	-2.07	AAA-AAA	0.00	999	1.000	1.000	0.4510

217153_at	60.4	29.2	-1.05	-2.07	AAA-AAA	0.00	999	1.000	1.000	0.4648
215642_at	37.9	18.4	-1.05	-2.07	AAA-AAA	0.00	999	1.000	1.000	0.4154
215234_at	8.0	16.5	1.05	2.07	AAA-AAA	0.00	999	1.000	1.000	0.0546
238916_at	15.4	7.4	-1.05	-2.07	AAA-AAA	0.00	999	1.000	1.000	0.4542
237832_at	6.6	3.2	-1.05	-2.07	AAA-AAA	0.00	999	1.000	1.000	0.4362
229975_at	7.5	3.6	-1.05	-2.07	AAA-AAA	0.00	999	1.000	1.000	0.4640
225306_s_at	193.0	398.7	1.05	2.07	APP-PPP	0.00	999	1.000	1.000	0.4080
1569369_at	21.6	44.7	1.05	2.07	AAM-MPM	0.00	999	1.000	1.000	0.3474
242806_at	34.2	70.6	1.05	2.07	AAA-AAA	0.00	999	1.000	1.000	0.2300
240151_at	49.2	23.8	-1.05	-2.07	AAA-AAA	0.00	999	1.000	1.000	0.3611
1566272_at	3.8	7.8	1.05	2.07	AAA-AAA	0.00	999	1.000	1.000	0.3086
1553527_at	53.8	26.0	-1.05	-2.07	AAA-AAA	0.00	999	1.000	1.000	0.3132
219864_s_at	45.2	21.9	-1.05	-2.07	AAA-AAA	0.00	999	1.000	1.000	0.2285
220340_at	29.6	14.3	-1.05	-2.07	AAA-AAA	0.00	999	1.000	1.000	0.3234
206434_at	5.6	11.5	1.05	2.07	AAA-AAA	0.00	999	1.000	1.000	0.4782
214354_x_at	46.0	95.1	1.05	2.07	AAA-AAA	0.00	999	1.000	1.000	0.2631
210998_s_at	4.6	9.5	1.05	2.07	AAA-AAA	0.00	999	1.000	1.000	0.3252
217614_at	32.9	15.9	-1.05	-2.07	AAA-AAA	0.00	999	1.000	1.000	0.3534
217505_at	3.4	7.0	1.05	2.07	AAA-AAA	0.00	999	1.000	1.000	0.4294
234100_at	14.8	7.2	-1.05	-2.07	AAA-AAA	0.00	999	1.000	1.000	0.3928
234704_at	40.7	19.7	-1.05	-2.07	AAA-AAA	0.00	999	1.000	1.000	0.4516
206191_at	24.7	11.9	-1.05	-2.07	AAA-AAA	0.00	999	1.000	1.000	0.5115
209367_at	31.8	65.7	1.05	2.07	AAA-PAA	0.00	999	1.000	1.000	0.4710
238599_at	70.5	145.5	1.05	2.07	APP-PPP	0.00	999	1.000	1.000	0.0632
244016_at	31.4	15.2	-1.05	-2.07	AAA-AAA	0.00	999	1.000	1.000	0.3128
243685_at	4.7	9.8	1.05	2.07	AAA-AAA	0.00	999	1.000	1.000	0.2224
203472_s_at	17.0	35.1	1.05	2.06	AAA-AAA	0.00	999	1.000	1.000	0.3465
240501_at	57.3	27.8	-1.05	-2.06	AAA-AAA	0.00	999	1.000	1.000	0.1144
1564039_at	37.6	18.2	-1.05	-2.07	AAA-AAA	0.00	999	1.000	1.000	0.3267
1570183_at	8.6	4.2	-1.05	-2.07	AAA-AAA	0.00	999	1.000	1.000	0.6161
1566841_at	47.4	23.0	-1.05	-2.07	AAA-AAA	0.00	999	1.000	1.000	0.3724
1559833_at	2.4	4.9	1.05	2.06	AAA-AAA	0.00	999	1.000	1.000	0.5215
207045_at	42.0	86.6	1.05	2.06	AAA-AAA	0.00	999	1.000	1.000	0.3788
219310_at	58.6	28.4	-1.05	-2.07	AAA-AAA	0.00	999	1.000	1.000	0.0472
216102_at	9.5	4.6	-1.05	-2.07	AAA-AAA	0.00	999	1.000	1.000	0.0123
234092_s_at	24.7	11.9	-1.05	-2.06	AAA-AAA	0.00	999	1.000	1.000	0.3761
230548_at	25.7	53.1	1.05	2.06	AAA-AAA	0.00	999	1.000	1.000	0.3945
233552_at	4.3	2.1	-1.05	-2.07	AAA-AAA	0.00	999	1.000	1.000	0.1346
231290_at	5.5	11.3	1.05	2.07	AAA-AAA	0.00	999	1.000	1.000	0.1083
1558397_at	10.7	22.1	1.05	2.06	PAA-AAA	0.00	999	1.000	1.000	0.5327
244330_at	20.4	9.9	-1.05	-2.06	AAA-AAA	0.00	999	1.000	1.000	0.4790
1562316_at	14.7	7.1	-1.05	-2.06	AAA-AAA	0.00	999	1.000	1.000	0.4703

1568888_at	4.9	2.4	-1.05	-2.06	AAA-AAA	0.00	999	1.000	1.000	0.4935
1556901_s_at	4.9	10.2	1.05	2.06	AAA-AAA	0.00	999	1.000	1.000	0.2233
219684_at	53.3	25.8	-1.05	-2.06	AAA-AAA	0.00	999	1.000	1.000	0.3250
213888_s_at	10.2	4.9	-1.05	-2.06	AAA-AAA	0.00	999	1.000	1.000	0.5177
210673_x_at	33.4	69.0	1.05	2.06	AAA-AAA	0.00	999	1.000	1.000	0.1214
217136_at	24.0	49.6	1.05	2.06	AAA-AAA	0.00	999	1.000	1.000	0.4371
216795_at	5.0	10.2	1.05	2.06	AAA-AAA	0.00	999	1.000	1.000	0.5497
236929_at	22.5	10.9	-1.05	-2.06	AAA-AAA	0.00	999	1.000	1.000	0.4630
230563_at	5.4	11.2	1.05	2.06	AAA-AAA	0.00	999	1.000	1.000	0.3680
228533_at	61.8	30.0	-1.05	-2.06	AAA-AAA	0.00	999	1.000	1.000	0.1413
228657_at	20.9	10.1	-1.05	-2.06	AAA-AAA	0.00	999	1.000	1.000	0.4052
231219_at	55.5	26.9	-1.05	-2.06	AAA-AAA	0.00	999	1.000	1.000	0.1670
203757_s_at	28.6	13.9	-1.04	-2.06	AAA-AAA	0.00	999	1.000	1.000	0.5213
1568666_at	29.7	14.4	-1.04	-2.06	AAA-AAA	0.00	999	1.000	1.000	0.0697
1557119_a_at	58.9	121.5	1.04	2.06	AAA-AAA	0.00	999	1.000	1.000	0.3116
1553318_at	41.9	20.3	-1.04	-2.06	AAA-AAA	0.00	999	1.000	1.000	0.4730
1557778_at	2.8	5.8	1.04	2.06	AAA-AAA	0.00	999	1.000	1.000	0.0789
1559551_at	43.5	21.1	-1.04	-2.06	AAA-AAA	0.00	999	1.000	1.000	0.2015
221316_at	4.3	8.9	1.04	2.06	AAA-AAA	0.00	999	1.000	1.000	0.4142
207371_at	2.9	6.1	1.04	2.06	AAA-AAA	0.00	999	1.000	1.000	0.3938
237932_at	10.8	5.2	-1.04	-2.06	AAA-AAA	0.00	999	1.000	1.000	0.2934
233000_x_at	89.1	43.2	-1.04	-2.06	AAA-AAA	0.00	999	1.000	1.000	0.2268
235077_at	104.5	215.3	1.04	2.06	APP-PPP	0.00	999	1.000	1.000	0.1727
241314_at	9.7	19.9	1.04	2.06	AAA-AAA	0.00	999	1.000	1.000	0.2377
1569473_s_at	32.0	15.5	-1.04	-2.06	AAA-AAA	0.00	999	1.000	1.000	0.2189
1570234_at	13.8	28.5	1.04	2.06	AAA-AAA	0.00	999	1.000	1.000	0.3709
221047_s_at	50.9	24.7	-1.04	-2.06	AAA-AAA	0.00	999	1.000	1.000	0.3031
221108_at	11.5	23.8	1.04	2.06	AAA-AAA	0.00	999	1.000	1.000	0.2183
208437_at	41.1	19.9	-1.04	-2.06	AAA-AAA	0.00	999	1.000	1.000	0.2397
213912_at	10.6	5.1	-1.04	-2.06	AAA-AAA	0.00	999	1.000	1.000	0.2357
237165_at	25.1	12.2	-1.04	-2.06	AAA-AAA	0.00	999	1.000	1.000	0.3572
233769_at	26.9	13.0	-1.04	-2.06	AAA-AAA	0.00	999	1.000	1.000	0.3483
242302_at	25.1	51.7	1.04	2.06	AAP-APA	0.00	999	1.000	1.000	0.2804
1562234_a_at	55.0	113.2	1.04	2.06	AAA-APA	0.00	999	1.000	1.000	0.1504
1568717_a_at	64.8	31.4	-1.04	-2.06	AAA-AAA	0.00	999	1.000	1.000	0.1731
226824_at	195.0	94.7	-1.04	-2.06	AAA-AAA	0.00	999	1.000	1.000	0.0176
214689_at	44.9	21.8	-1.04	-2.06	AAA-AAA	0.00	999	1.000	1.000	0.4481
211266_s_at	77.9	37.8	-1.04	-2.06	AAA-AAA	0.00	999	1.000	1.000	0.1402
211163_s_at	19.3	9.4	-1.04	-2.06	AAA-AAA	0.00	999	1.000	1.000	0.5840
235638_at	22.0	10.7	-1.04	-2.06	AAA-MAA	0.00	999	1.000	1.000	0.5974
235438_at	2.4	4.8	1.04	2.06	AAA-AAA	0.00	999	1.000	1.000	0.3180
234173_s_at	35.3	17.1	-1.04	-2.06	AAA-AAA	0.00	999	1.000	1.000	0.0347

238194_at	16.0	7.8	-1.04	-2.06	AAA-AAA	0.00	999	1.000	1.000	0.2553
229459_at	4.4	9.2	1.04	2.06	AAA-AAA	0.00	999	1.000	1.000	0.6273
232857_at	8.2	16.9	1.04	2.06	AAA-AAA	0.00	999	1.000	1.000	0.4244
205524_s_at	13.4	6.5	-1.04	-2.06	AAA-AAA	0.00	999	1.000	1.000	0.2942
208540_x_at	1246.5	2564.5	1.04	2.06	PPP-PPP	0.00	999	1.000	1.000	0.4477
241901_at	11.6	23.8	1.04	2.06	AAA-AAA	0.00	999	1.000	1.000	0.3674
1560762_at	6.3	12.9	1.04	2.06	AAA-AAA	0.00	999	1.000	1.000	0.3073
1565887_at	9.5	19.4	1.04	2.06	AAA-AAA	0.00	999	1.000	1.000	0.5159
1566937_at	21.1	43.3	1.04	2.06	AAA-AAA	0.00	999	1.000	1.000	0.0890
1558226_a_at	14.7	7.2	-1.04	-2.06	AAA-AAA	0.00	999	1.000	1.000	0.5520
1555871_at	22.1	10.7	-1.04	-2.06	AAA-AAA	0.00	999	1.000	1.000	0.2986
207211_at	27.0	13.1	-1.04	-2.06	AAA-AAA	0.00	999	1.000	1.000	0.1690
215102_at	29.1	14.2	-1.04	-2.06	AAA-AAA	0.00	999	1.000	1.000	0.4541
210347_s_at	96.3	46.8	-1.04	-2.06	AAA-AAA	0.00	999	1.000	1.000	0.0165
219042_at	24.2	11.8	-1.04	-2.06	AAA-AAA	0.00	999	1.000	1.000	0.4925
215859_at	12.9	26.5	1.04	2.06	AAA-AMA	0.00	999	1.000	1.000	0.1311
216664_at	13.4	6.5	-1.04	-2.06	AAA-AAA	0.00	999	1.000	1.000	0.1897
238159_at	9.5	19.6	1.04	2.06	AAA-AAA	0.00	999	1.000	1.000	0.3141
236888_at	14.6	30.1	1.04	2.06	AAA-AAA	0.00	999	1.000	1.000	0.3863
237154_at	48.2	23.4	-1.04	-2.06	AAA-AAA	0.00	999	1.000	1.000	0.2611
1563842_at	69.9	143.7	1.04	2.06	AMA-AAP	0.00	999	1.000	1.000	0.1052
243680_at	18.5	9.0	-1.04	-2.06	AAA-AAA	0.00	999	1.000	1.000	0.6185
243700_x_at	4.8	2.3	-1.04	-2.06	AAA-AAA	0.00	999	1.000	1.000	0.2508
1560890_at	24.1	11.7	-1.04	-2.06	AAA-AAA	0.00	999	1.000	1.000	0.3556
1560384_a_at	41.7	20.3	-1.04	-2.06	AAA-AAA	0.00	999	1.000	1.000	0.3403
1569564_at	29.3	14.2	-1.04	-2.06	AAA-AAA	0.00	999	1.000	1.000	0.5078
1558301_a_at	6.3	3.1	-1.04	-2.06	AAA-AAA	0.00	999	1.000	1.000	0.5448
222241_at	37.0	76.1	1.04	2.06	AAA-AAA	0.00	999	1.000	1.000	0.3425
206864_s_at	17.2	35.5	1.04	2.06	AAA-AAA	0.00	999	1.000	1.000	0.3101
213464_at	62.7	129.0	1.04	2.06	AAA-AAA	0.00	999	1.000	1.000	0.3162
215025_at	9.7	20.0	1.04	2.06	AAA-AAA	0.00	999	1.000	1.000	0.3260
217517_x_at	44.6	21.7	-1.04	-2.06	AAA-AAA	0.00	999	1.000	1.000	0.5132
230040_at	27.6	13.4	-1.04	-2.06	AAA-AAA	0.00	999	1.000	1.000	0.6603
230615_at	22.4	10.9	-1.04	-2.06	AAA-AAA	0.00	999	1.000	1.000	0.2630
228403_at	15.6	32.1	1.04	2.06	AAA-AAA	0.00	999	1.000	1.000	0.0804
232880_at	70.8	34.5	-1.04	-2.06	AAA-AAA	0.00	999	1.000	1.000	0.3586
231197_at	12.1	24.8	1.04	2.06	AAA-AAA	0.00	999	1.000	1.000	0.3136
232136_s_at	8.0	3.9	-1.04	-2.06	AAA-AAA	0.00	999	1.000	1.000	0.5945
231725_at	18.1	37.3	1.04	2.06	AAA-AMA	0.00	999	1.000	1.000	0.3247
231682_at	29.5	14.3	-1.04	-2.06	AAA-AAA	0.00	999	1.000	1.000	0.4476
240329_at	8.2	16.8	1.04	2.06	AAA-AAA	0.00	999	1.000	1.000	0.0741
243503_at	14.3	7.0	-1.04	-2.06	AAA-AAA	0.00	999	1.000	1.000	0.4063

1560347_at	18.5	9.0	-1.04	-2.05	AAA-AAA	0.00	999	1.000	1.000	0.4471
1553041_at	12.9	6.3	-1.04	-2.05	AAA-AAA	0.00	999	1.000	1.000	0.1921
1556097_at	7.7	3.8	-1.04	-2.06	AAA-AAA	0.00	999	1.000	1.000	0.5322
220100_at	8.2	4.0	-1.04	-2.06	AAA-AAA	0.00	999	1.000	1.000	0.0287
220389_at	29.8	61.2	1.04	2.05	AAA-AAA	0.00	999	1.000	1.000	0.2554
207203_s_at	12.2	25.0	1.04	2.06	AAA-AAA	0.00	999	1.000	1.000	0.2392
227342_s_at	32.8	67.4	1.04	2.05	AAA-AAA	0.00	999	1.000	1.000	0.3919
223741_s_at	99.2	48.3	-1.04	-2.05	AAA-AAA	0.00	999	1.000	1.000	0.4033
212210_at	36.7	17.9	-1.04	-2.06	AAA-AAA	0.00	999	1.000	1.000	0.5034
236801_at	13.7	6.7	-1.04	-2.06	AAA-AAA	0.00	999	1.000	1.000	0.2802
234528_at	7.8	16.1	1.04	2.05	AAA-AAA	0.00	999	1.000	1.000	0.3578
204124_at	21.9	10.7	-1.04	-2.06	AAA-AAA	0.00	999	1.000	1.000	0.6077
237153_at	54.2	26.4	-1.04	-2.05	AAA-AAA	0.00	999	1.000	1.000	0.3698
244162_at	7.1	14.6	1.04	2.05	AAA-AAA	0.00	999	1.000	1.000	0.2536
240441_at	11.0	5.3	-1.04	-2.05	AAA-AAA	0.00	999	1.000	1.000	0.4055
241189_at	15.0	7.3	-1.04	-2.05	AAA-AAA	0.00	999	1.000	1.000	0.3944
1552468_a_at	51.7	25.2	-1.04	-2.05	AAA-AAA	0.00	999	1.000	1.000	0.4429
37796_at	168.3	82.0	-1.04	-2.05	AAA-AAA	0.00	999	1.000	1.000	0.0154
207795_s_at	16.0	7.8	-1.04	-2.05	AAA-AAA	0.00	999	1.000	1.000	0.5088
210586_x_at	34.5	16.8	-1.04	-2.05	AAA-AAA	0.00	999	1.000	1.000	0.3933
209613_s_at	3.3	6.8	1.04	2.05	AAA-AAA	0.00	999	1.000	1.000	0.5866
206202_at	2.1	4.2	1.04	2.05	AAA-AAA	0.00	999	1.000	1.000	0.3488
233773_at	65.4	31.9	-1.04	-2.05	AAA-AAA	0.00	999	1.000	1.000	0.3357
241267_at	15.8	7.7	-1.04	-2.05	AAA-AAA	0.00	999	1.000	1.000	0.3232
1557389_at	11.2	22.9	1.04	2.05	AAA-AAA	0.00	999	1.000	1.000	0.5271
213321_at	74.8	153.6	1.04	2.05	AAA-AAA	0.00	999	1.000	1.000	0.3875
209800_at	9.8	4.8	-1.04	-2.05	AAA-AAA	0.00	999	1.000	1.000	0.3433
209138_x_at	20.1	41.2	1.04	2.05	AAA-AAA	0.00	999	1.000	1.000	0.4127
202545_at	70.3	144.1	1.04	2.05	AAA-APA	0.00	999	1.000	1.000	0.3450
1556284_at	36.8	75.4	1.04	2.05	PAP-APP	0.00	999	1.000	1.000	0.0711
240912_x_at	85.7	41.8	-1.04	-2.05	AAA-AAA	0.00	999	1.000	1.000	0.0371
222306_at	13.6	27.8	1.04	2.05	AAA-AAA	0.00	999	1.000	1.000	0.5048
208000_at	19.1	9.3	-1.04	-2.05	AAA-AAA	0.00	999	1.000	1.000	0.0455
219871_at	9.7	4.7	-1.04	-2.05	AAA-AAA	0.00	999	1.000	1.000	0.5000
220646_s_at	35.5	17.3	-1.04	-2.05	AAA-AAA	0.00	999	1.000	1.000	0.0552
223636_at	50.7	24.7	-1.04	-2.05	AAA-AAA	0.00	999	1.000	1.000	0.4418
210103_s_at	5.9	2.9	-1.04	-2.05	AAA-AAA	0.00	999	1.000	1.000	0.0638
210948_s_at	5.8	11.8	1.04	2.05	AAA-AAA	0.00	999	1.000	1.000	0.6110
215901_at	14.7	7.2	-1.04	-2.05	AAA-AAA	0.00	999	1.000	1.000	0.2943
215480_at	34.0	16.6	-1.04	-2.05	AAA-AAA	0.00	999	1.000	1.000	0.4612
216501_at	9.7	4.7	-1.04	-2.05	AAA-AAA	0.00	999	1.000	1.000	0.0596
235777_at	2.0	4.1	1.04	2.05	AAA-AAA	0.00	999	1.000	1.000	0.3260

236508_at	15.5	31.9	1.04	2.05	AAA-AAA	0.00	999	1.000	1.000	0.3835
239327_at	14.2	6.9	-1.04	-2.05	AAA-AAA	0.00	999	1.000	1.000	0.1865
237596_at	9.8	4.8	-1.04	-2.05	AAA-AAA	0.00	999	1.000	1.000	0.4537
228404_at	34.0	16.6	-1.04	-2.05	AAA-AAA	0.00	999	1.000	1.000	0.3965
232822_x_at	35.7	17.4	-1.04	-2.05	AAA-AAA	0.00	999	1.000	1.000	0.4295
232982_at	27.3	13.3	-1.04	-2.05	AAA-AAA	0.00	999	1.000	1.000	0.3822
208045_at	17.1	35.1	1.04	2.05	AAA-PAA	0.00	999	1.000	1.000	0.3798
230141_at	67.5	138.4	1.04	2.05	APP-PPP	0.00	999	1.000	1.000	0.1823
242574_at	9.3	4.5	-1.04	-2.05	AAA-AAA	0.00	999	1.000	1.000	0.2441
240976_at	27.6	13.5	-1.04	-2.05	AAA-AAA	0.00	999	1.000	1.000	0.4230
243633_at	30.5	14.9	-1.04	-2.05	AAA-AAA	0.00	999	1.000	1.000	0.4577
1562717_at	33.3	16.3	-1.04	-2.05	AAA-AAA	0.00	999	1.000	1.000	0.3778
1568760_at	25.2	51.7	1.04	2.05	AAA-AAA	0.00	999	1.000	1.000	0.4521
1569764_at	6.8	14.0	1.04	2.05	AAA-AAA	0.00	999	1.000	1.000	0.6247
1566528_at	6.8	14.0	1.04	2.05	AAA-AAA	0.00	999	1.000	1.000	0.4906
1558784_at	159.5	77.8	-1.04	-2.05	AAA-AAA	0.00	999	1.000	1.000	0.2164
1555659_a_at	36.4	17.8	-1.04	-2.05	AAA-AAA	0.00	999	1.000	1.000	0.4805
1557185_at	50.6	24.7	-1.04	-2.05	AAA-AAA	0.00	999	1.000	1.000	0.4580
1557325_at	47.4	23.2	-1.04	-2.05	AAA-AAA	0.00	999	1.000	1.000	0.4549
207803_s_at	11.3	5.5	-1.04	-2.05	AAA-AAA	0.00	999	1.000	1.000	0.2146
221948_s_at	8.5	4.2	-1.04	-2.05	AAA-AAA	0.00	999	1.000	1.000	0.1334
213245_at	31.0	15.1	-1.04	-2.05	AAA-AAA	0.00	999	1.000	1.000	0.4334
204259_at	43.4	21.2	-1.04	-2.05	AAA-AAA	0.00	999	1.000	1.000	0.5725
237980_at	36.4	17.8	-1.04	-2.05	AAA-AAA	0.00	999	1.000	1.000	0.3561
229675_at	12.7	25.9	1.04	2.05	AAA-AAA	0.00	999	1.000	1.000	0.3990
233636_at	2.7	5.4	1.04	2.05	AAA-AAA	0.00	999	1.000	1.000	0.5538
220278_at	55.2	112.9	1.03	2.05	AAA-PMP	0.00	999	1.000	1.000	0.2267
240737_at	30.2	14.8	-1.03	-2.05	AAA-AAA	0.00	999	1.000	1.000	0.5070
243352_at	10.4	21.2	1.03	2.05	AAA-AAA	0.00	999	1.000	1.000	0.2822
241513_at	13.7	6.7	-1.03	-2.05	AAA-AAA	0.00	999	1.000	1.000	0.2142
1561756_at	11.2	5.5	-1.03	-2.05	AAA-AAA	0.00	999	1.000	1.000	0.4289
1565832_at	7.7	3.8	-1.03	-2.05	AAA-AAA	0.00	999	1.000	1.000	0.2273
219552_at	14.3	7.0	-1.03	-2.05	AAA-AAA	0.00	999	1.000	1.000	0.2459
224543_at	50.7	24.8	-1.03	-2.05	AAA-AAA	0.00	999	1.000	1.000	0.3412
207468_s_at	19.6	9.6	-1.03	-2.05	AAA-AAA	0.00	999	1.000	1.000	0.2789
219167_at	43.9	21.5	-1.03	-2.05	AAA-AAA	0.00	999	1.000	1.000	0.4306
215564_at	9.8	4.8	-1.03	-2.05	AAA-AAA	0.00	999	1.000	1.000	0.2612
230105_at	13.9	28.5	1.03	2.05	AAA-AAA	0.00	999	1.000	1.000	0.3379
202828_s_at	76.3	156.0	1.03	2.05	AAA-PAA	0.00	999	1.000	1.000	0.5032
1555912_at	30.0	61.4	1.03	2.05	AAP-PPM	0.00	999	1.000	1.000	0.4931
241325_at	23.8	11.7	-1.03	-2.05	AAA-AAA	0.00	999	1.000	1.000	0.4891
1553374_at	35.1	17.2	-1.03	-2.05	AAA-AAA	0.00	999	1.000	1.000	0.3123

244707_at	11.3	5.5	-1.03	-2.05	AAA-AAA	0.00	999	1.000	1.000	0.0509
1556417_a_at	17.7	8.6	-1.03	-2.05	AAA-AAA	0.00	999	1.000	1.000	0.5023
215181_at	23.5	48.0	1.03	2.05	AAA-AAA	0.00	999	1.000	1.000	0.4095
211772_x_at	7.6	15.6	1.03	2.05	AAA-AAA	0.00	999	1.000	1.000	0.3934
206244_at	9.7	4.7	-1.03	-2.05	AAA-AAA	0.00	999	1.000	1.000	0.4633
233509_at	90.1	44.0	-1.03	-2.05	AAA-AAA	0.00	999	1.000	1.000	0.1691
225641_at	96.5	197.2	1.03	2.05	AAP-PAP	0.00	999	1.000	1.000	0.4329
240133_x_at	24.9	12.2	-1.03	-2.05	AAA-AAA	0.00	999	1.000	1.000	0.4498
1561103_at	12.9	6.3	-1.03	-2.05	AAA-AAA	0.00	999	1.000	1.000	0.5341
1570383_at	3.7	7.6	1.03	2.05	AAA-AAA	0.00	999	1.000	1.000	0.3000
1566787_at	12.8	6.3	-1.03	-2.05	AAA-AAA	0.00	999	1.000	1.000	0.1388
1567558_at	10.3	5.0	-1.03	-2.05	AAA-AAA	0.00	999	1.000	1.000	0.1059
1552834_at	6.8	14.0	1.03	2.05	AAA-AAA	0.00	999	1.000	1.000	0.2987
1559949_at	8.0	3.9	-1.03	-2.04	MAA-AAA	0.00	999	1.000	1.000	0.6259
1557021_s_at	26.6	13.0	-1.03	-2.05	AAA-AAA	0.00	999	1.000	1.000	0.3224
207926_at	78.6	38.4	-1.03	-2.05	AAA-AAA	0.00	999	1.000	1.000	0.0997
236459_at	9.2	18.9	1.03	2.05	AAA-AAA	0.00	999	1.000	1.000	0.3852
239547_at	35.3	17.3	-1.03	-2.05	AAA-AAA	0.00	999	1.000	1.000	0.4007
205766_at	20.2	9.9	-1.03	-2.05	AAA-AAA	0.00	999	1.000	1.000	0.3356
230322_at	110.3	54.0	-1.03	-2.05	MAA-AAA	0.00	999	1.000	1.000	0.1297
228682_at	9.8	20.0	1.03	2.05	AAA-AAA	0.00	999	1.000	1.000	0.2737
233148_at	17.0	34.7	1.03	2.05	AAA-AAA	0.00	999	1.000	1.000	0.0683
221898_at	63.6	129.9	1.03	2.04	AAA-APP	0.00	999	1.000	1.000	0.4232
1554060_s_at	29.7	60.8	1.03	2.04	AAA-AAP	0.00	999	1.000	1.000	0.2669
241340_at	4.7	9.5	1.03	2.04	AAA-AAA	0.00	999	1.000	1.000	0.4793
1560498_at	6.4	13.1	1.03	2.04	AAA-AAA	0.00	999	1.000	1.000	0.3588
1570451_at	12.1	5.9	-1.03	-2.04	AAA-AAA	0.00	999	1.000	1.000	0.3861
1554582_a_at	23.9	48.8	1.03	2.04	AAA-AAA	0.00	999	1.000	1.000	0.2247
1558262_at	6.1	12.5	1.03	2.04	AAA-AAA	0.00	999	1.000	1.000	0.0932
223699_at	19.3	9.5	-1.03	-2.04	AAA-AAA	0.00	999	1.000	1.000	0.1542
214851_at	39.9	19.5	-1.03	-2.04	AAA-AAA	0.00	999	1.000	1.000	0.3290
214831_at	60.8	124.2	1.03	2.04	AAA-AAA	0.00	999	1.000	1.000	0.2645
211269_s_at	23.1	47.2	1.03	2.04	AAA-AAA	0.00	999	1.000	1.000	0.1048
216930_at	68.6	33.6	-1.03	-2.04	AAA-AAA	0.00	999	1.000	1.000	0.2954
215670_s_at	59.3	121.1	1.03	2.04	AAA-AAA	0.00	999	1.000	1.000	0.1266
243047_at	36.8	75.1	1.03	2.04	AAP-AAP	0.00	999	1.000	1.000	0.3844
231885_at	20.2	41.3	1.03	2.04	AAA-PAA	0.00	999	1.000	1.000	0.1801
244146_at	10.4	21.3	1.03	2.04	AAA-AAA	0.00	999	1.000	1.000	0.3635
242596_at	103.1	50.5	-1.03	-2.04	MAA-AAA	0.00	999	1.000	1.000	0.1299
243254_at	21.8	10.7	-1.03	-2.04	AAA-AAA	0.00	999	1.000	1.000	0.4307
243577_at	42.2	20.7	-1.03	-2.04	AAA-AAA	0.00	999	1.000	1.000	0.3066
1563945_at	114.2	55.9	-1.03	-2.04	AAA-AAA	0.00	999	1.000	1.000	0.2256

1564392_at	14.2	7.0	-1.03	-2.04	AAA-AAA	0.00	999	1.000	1.000	0.4544
1557226_a_at	44.3	21.7	-1.03	-2.04	AAA-AAA	0.00	999	1.000	1.000	0.4586
1556938_a_at	30.2	14.8	-1.03	-2.04	AMA-AAA	0.00	999	1.000	1.000	0.1555
220581_at	20.3	10.0	-1.03	-2.04	AAA-AAA	0.00	999	1.000	1.000	0.1644
225016_at	9.8	20.1	1.03	2.04	AAA-AAA	0.00	999	1.000	1.000	0.2312
207475_at	35.5	17.4	-1.03	-2.04	AMA-AAA	0.00	999	1.000	1.000	0.5747
223822_at	25.7	12.6	-1.03	-2.04	AAA-AAA	0.00	999	1.000	1.000	0.4633
210848_at	15.9	32.5	1.03	2.04	AAA-AAA	0.00	999	1.000	1.000	0.1220
217590_s_at	37.1	75.7	1.03	2.04	AAA-AAA	0.00	999	1.000	1.000	0.3119
236509_at	30.1	61.4	1.03	2.04	AMA-AAA	0.00	999	1.000	1.000	0.4577
234719_at	7.8	15.9	1.03	2.04	AAA-AAA	0.00	999	1.000	1.000	0.5009
237127_at	5.9	2.9	-1.03	-2.04	AAA-AAA	0.00	999	1.000	1.000	0.0475
237467_at	13.1	6.4	-1.03	-2.04	AAA-AAA	0.00	999	1.000	1.000	0.2441
231731_at	23.5	47.9	1.03	2.04	AAA-AAP	0.00	999	1.000	1.000	0.2814
232048_at	110.8	226.1	1.03	2.04	PPP-PPP	0.00	999	1.000	1.000	0.0692
242810_x_at	55.0	112.2	1.03	2.04	AAA-AAA	0.00	999	1.000	1.000	0.0815
244423_at	26.4	12.9	-1.03	-2.04	AAA-AAA	0.00	999	1.000	1.000	0.4491
219781_s_at	80.7	164.7	1.03	2.04	AAA-AAA	0.00	999	1.000	1.000	0.2291
220677_s_at	18.9	9.2	-1.03	-2.04	AAA-AAA	0.00	999	1.000	1.000	0.3677
220514_at	28.6	14.0	-1.03	-2.04	AAA-AAA	0.00	999	1.000	1.000	0.4036
210979_at	17.7	36.1	1.03	2.04	AAA-AAA	0.00	999	1.000	1.000	0.5316
239314_at	105.4	51.7	-1.03	-2.04	AAA-AAA	0.00	999	1.000	1.000	0.1389
237357_at	16.7	8.2	-1.03	-2.04	AAA-AAA	0.00	999	1.000	1.000	0.4972
206252_s_at	12.7	6.2	-1.03	-2.04	AAA-AAA	0.00	999	1.000	1.000	0.4122
233964_at	18.4	37.6	1.03	2.04	AAA-APA	0.00	999	1.000	1.000	0.4886
241192_at	18.2	8.9	-1.03	-2.04	AAA-AAA	0.00	999	1.000	1.000	0.5768
1563589_at	13.7	28.0	1.03	2.04	AAA-AAA	0.00	999	1.000	1.000	0.2221
221334_s_at	34.1	16.7	-1.03	-2.04	AAA-AAA	0.00	999	1.000	1.000	0.4607
207873_x_at	42.8	21.0	-1.03	-2.04	AAA-AAA	0.00	999	1.000	1.000	0.3178
220899_at	31.3	15.3	-1.03	-2.04	AAA-AAA	0.00	999	1.000	1.000	0.6205
211198_s_at	19.1	9.4	-1.03	-2.04	AAA-AAA	0.00	999	1.000	1.000	0.3943
216872_at	4.2	8.6	1.03	2.04	AAA-AAA	0.00	999	1.000	1.000	0.1561
219422_at	16.9	34.4	1.03	2.04	AAA-AAA	0.00	999	1.000	1.000	0.3182
205076_s_at	52.9	107.9	1.03	2.04	AAA-AAA	0.00	999	1.000	1.000	0.5763
236827_at	33.0	16.2	-1.03	-2.04	AAA-AAA	0.00	999	1.000	1.000	0.1568
232951_at	54.1	26.5	-1.03	-2.04	AAA-AAA	0.00	999	1.000	1.000	0.4458
231526_at	6.1	12.5	1.03	2.04	AAA-AAA	0.00	999	1.000	1.000	0.1524
206230_at	21.8	44.3	1.03	2.04	APA-AMP	0.00	999	1.000	1.000	0.3742
203398_s_at	50.5	24.8	-1.03	-2.04	AAA-AAA	0.00	999	1.000	1.000	0.2836
1570329_at	11.7	23.8	1.03	2.04	AAA-AAA	0.00	999	1.000	1.000	0.4239
207178_s_at	10.7	5.2	-1.03	-2.04	AAA-AAA	0.00	999	1.000	1.000	0.5476
207420_at	26.9	54.8	1.03	2.04	AAA-AAA	0.00	999	1.000	1.000	0.4078

224148_at	4.6	2.3	-1.03	-2.04	AAA-AAA	0.00	999	1.000	1.000	0.1763
211679_x_at	52.9	25.9	-1.03	-2.04	AAA-AAA	0.00	999	1.000	1.000	0.1460
215248_at	26.1	12.8	-1.03	-2.04	AAA-AAA	0.00	999	1.000	1.000	0.4026
236017_at	17.2	35.0	1.03	2.04	AAA-AAA	0.00	999	1.000	1.000	0.5239
235596_at	22.3	11.0	-1.03	-2.04	AAA-AAA	0.00	999	1.000	1.000	0.4752
236341_at	8.7	4.3	-1.03	-2.04	AAA-AAA	0.00	999	1.000	1.000	0.4621
238220_at	30.0	14.7	-1.03	-2.04	AAA-AAA	0.00	999	1.000	1.000	0.1186
236993_at	49.2	24.2	-1.03	-2.04	AAA-AAA	0.00	999	1.000	1.000	0.0899
243017_at	24.6	50.1	1.03	2.04	APA-AAA	0.00	999	1.000	1.000	0.4135
209771_x_at	148.1	301.6	1.03	2.04	PPP-PPP	0.00	999	1.000	1.000	0.1780
1560078_at	60.3	29.6	-1.03	-2.04	AAA-AAA	0.00	999	1.000	1.000	0.0753
1555040_at	26.5	13.0	-1.03	-2.04	AAA-AAA	0.00	999	1.000	1.000	0.1447
1556192_x_at	7.4	3.6	-1.03	-2.04	AAA-AAA	0.00	999	1.000	1.000	0.3485
227023_at	39.4	19.4	-1.03	-2.04	AAA-AAA	0.00	999	1.000	1.000	0.1559
211888_x_at	21.0	10.3	-1.03	-2.04	AAA-AAA	0.00	999	1.000	1.000	0.2340
215619_at	3.5	1.7	-1.03	-2.04	AAA-AAA	0.00	999	1.000	1.000	0.4477
237211_x_at	55.7	27.4	-1.03	-2.04	AAA-AAA	0.00	999	1.000	1.000	0.2945
227811_at	37.0	18.1	-1.03	-2.04	AAA-AAA	0.00	999	1.000	1.000	0.3343
231925_at	11.9	5.9	-1.03	-2.04	AAA-AAA	0.00	999	1.000	1.000	0.6321
202994_s_at	18.7	38.0	1.03	2.04	AAA-AAA	0.00	999	1.000	1.000	0.1922
240646_at	4.8	9.8	1.03	2.04	AAA-AAA	0.00	999	1.000	1.000	0.0713
1561969_at	14.7	29.8	1.03	2.04	AAA-AAA	0.00	999	1.000	1.000	0.2698
1562270_at	7.6	15.4	1.03	2.03	AAA-AAA	0.00	999	1.000	1.000	0.0653
1560207_at	10.5	5.2	-1.03	-2.04	AAA-AAA	0.00	999	1.000	1.000	0.2954
1553822_at	7.7	3.8	-1.03	-2.04	AAA-AAA	0.00	999	1.000	1.000	0.4909
1554805_at	106.9	52.5	-1.03	-2.04	AAA-AAA	0.00	999	1.000	1.000	0.0149
1559695_a_at	11.6	5.7	-1.03	-2.04	AAA-AAA	0.00	999	1.000	1.000	0.3605
1558968_at	13.3	27.0	1.03	2.04	AAA-AAA	0.00	999	1.000	1.000	0.3121
223693_s_at	77.8	38.2	-1.03	-2.04	AAA-AAA	0.00	999	1.000	1.000	0.3512
215613_at	34.2	16.8	-1.03	-2.04	AAA-AAA	0.00	999	1.000	1.000	0.5123
216668_at	39.4	80.3	1.03	2.04	AAA-AAA	0.00	999	1.000	1.000	0.3868
234712_at	25.0	12.3	-1.03	-2.04	AAA-AAA	0.00	999	1.000	1.000	0.4954
239514_at	25.3	12.4	-1.03	-2.04	AAA-AAA	0.00	999	1.000	1.000	0.3498
205592_at	43.5	21.4	-1.03	-2.04	AAA-AAA	0.00	999	1.000	1.000	0.3090
227909_at	26.9	54.8	1.02	2.03	AAP-AAP	0.00	999	1.000	1.000	0.5000
228268_at	39.6	80.5	1.02	2.03	APP-PAP	0.00	999	1.000	1.000	0.4681
1560380_at	5.5	2.7	-1.02	-2.03	AAA-AAA	0.00	999	1.000	1.000	0.2692
1557829_at	5.7	11.6	1.02	2.03	AAA-AAA	0.00	999	1.000	1.000	0.2774
239555_at	25.7	12.6	-1.02	-2.03	AAA-AAA	0.00	999	1.000	1.000	0.1896
237569_at	28.6	58.2	1.02	2.03	AAA-AAA	0.00	999	1.000	1.000	0.1553
205786_s_at	39.7	19.5	-1.02	-2.03	AAA-AAA	0.00	999	1.000	1.000	0.2924
231516_at	11.4	5.6	-1.02	-2.03	AAA-AAA	0.00	999	1.000	1.000	0.3788

235034_at	94.5	192.0	1.02	2.03	APA-PPP	0.00	999	1.000	1.000	0.5646
244240_at	13.3	27.1	1.02	2.03	AAA-AAA	0.00	999	1.000	1.000	0.3377
1553499_s_at	103.8	51.1	-1.02	-2.03	AAA-AAA	0.00	999	1.000	1.000	0.3440
222290_at	34.5	70.1	1.02	2.03	AAA-AAA	0.00	999	1.000	1.000	0.4254
206832_s_at	10.6	21.4	1.02	2.03	AAA-AAA	0.00	999	1.000	1.000	0.4830
211827_s_at	14.1	6.9	-1.02	-2.03	AAA-AAA	0.00	999	1.000	1.000	0.0741
217311_at	10.5	5.2	-1.02	-2.03	AAA-AAA	0.00	999	1.000	1.000	0.3858
209144_s_at	31.1	15.3	-1.02	-2.03	AAA-AAA	0.00	999	1.000	1.000	0.4072
234506_at	69.0	33.9	-1.02	-2.03	AAA-AAA	0.00	999	1.000	1.000	0.4616
205332_at	29.0	14.3	-1.02	-2.03	AAA-AAA	0.00	999	1.000	1.000	0.2952
205828_at	200.0	406.1	1.02	2.03	APP-PPP	0.00	999	1.000	1.000	0.4454
240914_at	3.4	1.7	-1.02	-2.03	AAA-AAA	0.00	999	1.000	1.000	0.2856
244203_at	2.3	1.1	-1.02	-2.03	AAA-AAA	0.00	999	1.000	1.000	0.2167
1563453_at	42.8	21.1	-1.02	-2.03	AAA-AAA	0.00	999	1.000	1.000	0.4408
1564264_at	17.6	35.7	1.02	2.03	AAA-AAA	0.00	999	1.000	1.000	0.4374
1553523_at	16.8	8.3	-1.02	-2.03	AAA-AAA	0.00	999	1.000	1.000	0.3925
1552662_a_at	53.8	26.5	-1.02	-2.03	AAA-AAA	0.00	999	1.000	1.000	0.4991
823_at	47.9	23.6	-1.02	-2.03	AAA-AAA	0.00	999	1.000	1.000	0.1915
206478_at	29.5	14.5	-1.02	-2.03	AAA-AAA	0.00	999	1.000	1.000	0.3860
224398_at	66.1	32.6	-1.02	-2.03	AAM-AAA	0.00	999	1.000	1.000	0.3992
211524_at	25.3	12.5	-1.02	-2.03	AAA-AAA	0.00	999	1.000	1.000	0.4575
239273_s_at	12.8	6.3	-1.02	-2.03	AAA-AAA	0.00	999	1.000	1.000	0.0511
232439_at	2.4	4.9	1.02	2.03	AAA-AAA	0.00	999	1.000	1.000	0.3880
231330_at	11.5	5.7	-1.02	-2.03	AAA-AAA	0.00	999	1.000	1.000	0.5814
208166_at	16.9	34.2	1.02	2.03	AAA-PAA	0.00	999	1.000	1.000	0.2558
241307_at	10.4	5.1	-1.02	-2.03	AAA-AAA	0.00	999	1.000	1.000	0.4248
241548_at	7.3	14.9	1.02	2.03	AAA-AAA	0.00	999	1.000	1.000	0.3718
240252_at	18.2	8.9	-1.02	-2.03	AAA-AAA	0.00	999	1.000	1.000	0.3367
243721_at	33.9	16.7	-1.02	-2.03	AAA-AAA	0.00	999	1.000	1.000	0.3366
1568913_at	27.9	56.6	1.02	2.03	AAA-AAA	0.00	999	1.000	1.000	0.4957
1554976_a_at	9.9	4.9	-1.02	-2.03	AAA-AAA	0.00	999	1.000	1.000	0.4752
1559420_x_at	12.6	6.2	-1.02	-2.03	AAA-AAA	0.00	999	1.000	1.000	0.4532
1556900_at	236.4	116.5	-1.02	-2.03	AAA-AAA	0.00	999	1.000	1.000	0.0733
219077_s_at	58.6	28.9	-1.02	-2.03	AAA-AAA	0.00	999	1.000	1.000	0.4301
231381_at	14.2	7.0	-1.02	-2.03	AAA-AAA	0.00	999	1.000	1.000	0.5059
231641_at	15.8	32.1	1.02	2.03	AAA-AAA	0.00	999	1.000	1.000	0.4481
241389_at	33.8	16.7	-1.02	-2.03	AAA-AAA	0.00	999	1.000	1.000	0.4354
1563487_at	12.7	25.8	1.02	2.03	AAA-AAA	0.00	999	1.000	1.000	0.4882
1561035_at	16.5	8.2	-1.02	-2.03	AAA-AAA	0.00	999	1.000	1.000	0.2841
1566171_at	20.5	41.6	1.02	2.03	AAA-AAA	0.00	999	1.000	1.000	0.3086
1552557_a_at	16.3	8.0	-1.02	-2.03	AAA-AAA	0.00	999	1.000	1.000	0.6094
1558804_at	25.1	51.0	1.02	2.03	AAA-AAA	0.00	999	1.000	1.000	0.2757

1559204_x_at	48.6	98.6	1.02	2.03	AAA-AAA	0.00	999	1.000	1.000	0.0887
211549_s_at	6.9	13.9	1.02	2.03	AAA-AAA	0.00	999	1.000	1.000	0.1624
217265_at	7.2	14.6	1.02	2.03	AAA-AAA	0.00	999	1.000	1.000	0.3622
237791_at	25.0	12.3	-1.02	-2.03	AAA-AAA	0.00	999	1.000	1.000	0.3725
1561375_at	20.9	10.3	-1.02	-2.03	AAA-AAA	0.00	999	1.000	1.000	0.4242
1555267_at	138.9	68.5	-1.02	-2.03	AAA-AAA	0.00	999	1.000	1.000	0.0213
1553025_at	26.9	13.3	-1.02	-2.03	AAA-AAA	0.00	999	1.000	1.000	0.3673
214990_at	12.4	6.1	-1.02	-2.03	AAA-AAA	0.00	999	1.000	1.000	0.2547
210190_at	26.8	13.2	-1.02	-2.03	AAA-AAA	0.00	999	1.000	1.000	0.4719
236119_s_at	45.7	22.5	-1.02	-2.03	AAA-AAA	0.00	999	1.000	1.000	0.3156
229968_at	52.3	25.8	-1.02	-2.03	AAA-AAA	0.00	999	1.000	1.000	0.0703
236716_at	20.3	41.2	1.02	2.03	APA-APA	0.00	999	1.000	1.000	0.5164
243769_at	8.6	4.2	-1.02	-2.03	AAA-AAA	0.00	999	1.000	1.000	0.1520
242825_at	16.0	7.9	-1.02	-2.02	AAA-AAA	0.00	999	1.000	1.000	0.4699
1569436_at	20.1	9.9	-1.02	-2.03	AAA-AAA	0.00	999	1.000	1.000	0.3331
1570629_at	61.0	30.1	-1.02	-2.03	AAA-AAA	0.00	999	1.000	1.000	0.2903
1552669_at	34.8	17.2	-1.02	-2.03	AAA-AAA	0.00	999	1.000	1.000	0.4347
239582_at	69.8	34.5	-1.02	-2.03	AAA-AAA	0.00	999	1.000	1.000	0.3179
208323_s_at	23.6	47.7	1.02	2.02	AAA-PAA	0.00	999	1.000	1.000	0.3288
230388_s_at	59.6	120.6	1.02	2.02	AAA-PPP	0.00	999	1.000	1.000	0.2917
222006_at	252.2	510.2	1.02	2.02	PPP-PPP	0.00	999	1.000	1.000	0.1700
244157_x_at	43.0	87.0	1.02	2.02	AAA-AAA	0.00	999	1.000	1.000	0.1347
203365_s_at	31.2	15.4	-1.02	-2.02	AAA-AAA	0.00	999	1.000	1.000	0.3649
1562067_at	11.4	5.7	-1.02	-2.02	AAA-AAA	0.00	999	1.000	1.000	0.5390
1566324_a_at	5.0	10.0	1.02	2.02	AAA-AAA	0.00	999	1.000	1.000	0.6853
1554994_at	22.8	11.3	-1.02	-2.02	AAA-AAM	0.00	999	1.000	1.000	0.4283
1557789_at	44.0	21.7	-1.02	-2.02	AAA-AAA	0.00	999	1.000	1.000	0.1671
221091_at	25.9	12.8	-1.02	-2.02	AAA-AAA	0.00	999	1.000	1.000	0.3815
235781_at	19.0	9.4	-1.02	-2.02	AAA-AAA	0.00	999	1.000	1.000	0.4654
237805_at	4.1	2.1	-1.02	-2.02	AAA-AAA	0.00	999	1.000	1.000	0.0174
205804_s_at	13.5	6.7	-1.02	-2.02	AAA-AAA	0.00	999	1.000	1.000	0.5180
206057_x_at	17.6	35.7	1.02	2.02	AAA-AAA	0.00	999	1.000	1.000	0.1815
229275_at	49.6	24.5	-1.02	-2.02	AAA-AAA	0.00	999	1.000	1.000	0.5101
239915_at	12.7	25.8	1.02	2.02	AAA-AAA	0.00	999	1.000	1.000	0.3434
240760_at	22.8	11.3	-1.02	-2.02	AAA-AAA	0.00	999	1.000	1.000	0.5335
242855_at	18.0	8.9	-1.02	-2.02	AAA-AAA	0.00	999	1.000	1.000	0.0357
241066_at	30.7	62.2	1.02	2.02	AAA-AAA	0.00	999	1.000	1.000	0.4960
243493_at	74.2	36.7	-1.02	-2.02	AAA-AAA	0.00	999	1.000	1.000	0.2258
1553467_at	6.9	14.0	1.02	2.02	AAA-AAA	0.00	999	1.000	1.000	0.5335
1553434_at	8.7	17.5	1.02	2.02	AAA-AAA	0.00	999	1.000	1.000	0.3815
1557644_at	21.0	10.4	-1.02	-2.02	AAA-AAA	0.00	999	1.000	1.000	0.5034
1559646_a_at	67.4	33.3	-1.02	-2.02	AAA-AAA	0.00	999	1.000	1.000	0.0952

1559083_x_at	10.7	5.3	-1.02	-2.02	AAA-AAA	0.00	999	1.000	1.000	0.3286
1556798_a_at	26.3	13.0	-1.02	-2.02	AAA-AAA	0.00	999	1.000	1.000	0.4741
208253_at	23.8	11.8	-1.02	-2.02	AAA-AAA	0.00	999	1.000	1.000	0.4595
216235_s_at	86.2	42.6	-1.02	-2.02	AAA-AAA	0.00	999	1.000	1.000	0.1470
235632_at	14.5	7.2	-1.02	-2.02	AAA-AAA	0.00	999	1.000	1.000	0.5290
238224_at	3.5	7.0	1.02	2.02	AAA-AAA	0.00	999	1.000	1.000	0.5688
205518_s_at	39.8	19.7	-1.02	-2.02	AAA-AAA	0.00	999	1.000	1.000	0.0484
1560589_a_at	22.1	10.9	-1.02	-2.02	AAA-AAA	0.00	999	1.000	1.000	0.5279
1554566_at	44.7	22.1	-1.02	-2.02	AAA-AAA	0.00	999	1.000	1.000	0.2261
1552506_at	7.8	15.7	1.02	2.02	AAA-AAA	0.00	999	1.000	1.000	0.5287
1552772_at	20.7	41.8	1.02	2.02	AAA-AAA	0.00	999	1.000	1.000	0.2324
1559715_at	18.1	36.6	1.02	2.02	AAA-AAA	0.00	999	1.000	1.000	0.0789
209982_s_at	9.5	19.2	1.02	2.02	AAA-AAA	0.00	999	1.000	1.000	0.1007
210084_x_at	9.0	4.5	-1.02	-2.02	AAA-AAA	0.00	999	1.000	1.000	0.0032
211402_x_at	35.8	72.3	1.02	2.02	AAA-AAA	0.00	999	1.000	1.000	0.2667
235448_at	19.0	38.5	1.02	2.02	AAA-AAA	0.00	999	1.000	1.000	0.3603
228781_at	17.6	35.5	1.02	2.02	AAA-AAA	0.00	999	1.000	1.000	0.4604
232699_at	13.8	6.8	-1.02	-2.02	AAA-AAA	0.00	999	1.000	1.000	0.1173
244074_at	28.3	57.2	1.01	2.02	AAA-AAP	0.00	999	1.000	1.000	0.2011
243377_at	14.7	7.3	-1.01	-2.02	AAA-AAA	0.00	999	1.000	1.000	0.1528
1563320_at	32.6	65.9	1.01	2.02	AAA-AMA	0.00	999	1.000	1.000	0.0485
201495_x_at	5.3	10.6	1.01	2.02	AAA-AAA	0.00	999	1.000	1.000	0.2976
1567697_at	6.3	12.6	1.01	2.02	AAA-MAA	0.00	999	1.000	1.000	0.5106
208321_s_at	10.3	5.1	-1.01	-2.02	AAA-AAA	0.00	999	1.000	1.000	0.1314
220604_x_at	56.3	27.9	-1.01	-2.02	AAA-AAA	0.00	999	1.000	1.000	0.4576
206701_x_at	15.0	7.5	-1.01	-2.02	AAA-AAA	0.00	999	1.000	1.000	0.5608
224257_s_at	9.0	18.1	1.01	2.02	AAA-AAA	0.00	999	1.000	1.000	0.5320
214373_at	57.8	28.6	-1.01	-2.02	AAA-AAA	0.00	999	1.000	1.000	0.3463
211748_x_at	83.6	41.4	-1.01	-2.02	AAA-AAA	0.00	999	1.000	1.000	0.2649
217119_s_at	33.9	16.8	-1.01	-2.02	AAA-AAA	0.00	999	1.000	1.000	0.5867
235994_s_at	70.0	34.7	-1.01	-2.02	AAA-AAA	0.00	999	1.000	1.000	0.1775
234031_at	11.1	5.5	-1.01	-2.02	AAA-AAA	0.00	999	1.000	1.000	0.0545
234542_at	8.3	4.1	-1.01	-2.02	AAA-AAA	0.00	999	1.000	1.000	0.0825
237552_at	20.2	10.0	-1.01	-2.02	AAA-AAA	0.00	999	1.000	1.000	0.4189
237488_at	10.7	5.3	-1.01	-2.02	AAA-AAA	0.00	999	1.000	1.000	0.5223
205859_at	59.5	29.5	-1.01	-2.02	AAA-AAA	0.00	999	1.000	1.000	0.2495
230562_at	93.3	46.2	-1.01	-2.02	AAM-AMA	0.00	999	1.000	1.000	0.1033
216713_at	355.4	717.1	1.01	2.02	PPP-PPP	0.00	999	1.000	1.000	0.0752
239985_at	39.3	19.5	-1.01	-2.02	AAA-AAA	0.00	999	1.000	1.000	0.3939
1569402_at	8.0	16.2	1.01	2.02	AAA-AAA	0.00	999	1.000	1.000	0.3579
244697_at	9.2	18.5	1.01	2.02	AAA-AAA	0.00	999	1.000	1.000	0.2231
1557631_at	2.6	5.3	1.01	2.02	AAA-AAA	0.00	999	1.000	1.000	0.0931

226818_at	11.0	5.4	-1.01	-2.02	AAA-AAA	0.00	999	1.000	1.000	0.6352
223605_at	27.3	13.5	-1.01	-2.02	AAA-AAA	0.00	999	1.000	1.000	0.5717
239522_at	27.9	13.8	-1.01	-2.02	AAA-AAA	0.00	999	1.000	1.000	0.3807
239323_at	10.9	5.4	-1.01	-2.02	AAA-AAA	0.00	999	1.000	1.000	0.2798
206319_s_at	23.6	11.7	-1.01	-2.02	AAA-AAA	0.00	999	1.000	1.000	0.4009
229102_at	11.5	23.1	1.01	2.02	AAA-AAA	0.00	999	1.000	1.000	0.4666
227864_s_at	483.9	975.7	1.01	2.02	APP-PPP	0.00	999	1.000	1.000	0.3638
243848_at	51.2	25.4	-1.01	-2.02	AMA-AAA	0.00	999	1.000	1.000	0.2544
1569315_s_at	12.7	6.3	-1.01	-2.02	AAA-AAA	0.00	999	1.000	1.000	0.2737
1555051_at	63.9	31.7	-1.01	-2.02	AAA-AAA	0.00	999	1.000	1.000	0.2021
1555752_at	100.4	49.8	-1.01	-2.02	AAA-AAA	0.00	999	1.000	1.000	0.0100
224086_at	9.1	4.5	-1.01	-2.02	AAA-AAA	0.00	999	1.000	1.000	0.4111
211169_s_at	3.0	6.0	1.01	2.02	AAA-AAA	0.00	999	1.000	1.000	0.5198
219197_s_at	46.4	23.0	-1.01	-2.02	AAA-AAA	0.00	999	1.000	1.000	0.3561
215486_at	62.7	31.1	-1.01	-2.02	MAA-AAA	0.00	999	1.000	1.000	0.1319
216106_at	25.3	12.5	-1.01	-2.02	AAA-AAA	0.00	999	1.000	1.000	0.5113
236360_at	7.3	14.7	1.01	2.02	AAA-AAA	0.00	999	1.000	1.000	0.5961
236936_at	27.8	13.8	-1.01	-2.02	AAA-AAA	0.00	999	1.000	1.000	0.4819
228004_at	13.7	6.8	-1.01	-2.02	AAA-AAA	0.00	999	1.000	1.000	0.2004
234682_at	52.6	26.1	-1.01	-2.02	APA-AAP	0.00	999	1.000	1.000	0.6083
244043_at	103.7	209.0	1.01	2.02	APP-PPP	0.00	999	1.000	1.000	0.4610
228766_at	10.0	20.1	1.01	2.02	AAA-APA	0.00	999	1.000	1.000	0.3723
1562661_at	15.8	7.8	-1.01	-2.02	AAA-AAA	0.00	999	1.000	1.000	0.4218
1561916_at	18.6	37.4	1.01	2.02	AAA-AAA	0.00	999	1.000	1.000	0.4041
1563963_at	43.3	21.5	-1.01	-2.02	AAA-AAA	0.00	999	1.000	1.000	0.4404
1563728_at	8.0	16.2	1.01	2.02	AAA-AAA	0.00	999	1.000	1.000	0.4854
1570188_at	11.9	5.9	-1.01	-2.02	AAA-AAA	0.00	999	1.000	1.000	0.4339
1553778_at	17.3	34.8	1.01	2.02	AAA-AAA	0.00	999	1.000	1.000	0.3699
1554405_a_at	20.7	10.3	-1.01	-2.02	AAA-AAA	0.00	999	1.000	1.000	0.5863
1559288_at	6.4	3.2	-1.01	-2.02	AAA-AAA	0.00	999	1.000	1.000	0.5362
1557029_at	32.3	16.0	-1.01	-2.02	AAA-AAA	0.00	999	1.000	1.000	0.5859
222246_at	16.4	8.1	-1.01	-2.02	AAA-AAA	0.00	999	1.000	1.000	0.3376
217103_at	6.6	3.3	-1.01	-2.02	AAA-AAA	0.00	999	1.000	1.000	0.3597
217087_at	27.6	13.7	-1.01	-2.02	AAA-AAA	0.00	999	1.000	1.000	0.3272
217272_s_at	32.6	16.2	-1.01	-2.02	AAA-AAA	0.00	999	1.000	1.000	0.3174
209460_at	6.7	13.4	1.01	2.02	AAA-AAA	0.00	999	1.000	1.000	0.3715
236543_at	20.0	9.9	-1.01	-2.02	AAA-AAA	0.00	999	1.000	1.000	0.4079
238380_s_at	27.6	13.7	-1.01	-2.02	AAA-AAA	0.00	999	1.000	1.000	0.1365
237181_at	95.2	47.3	-1.01	-2.02	AAM-AAA	0.00	999	1.000	1.000	0.0546
222107_x_at	24.1	48.6	1.01	2.01	PAA-AAA	0.00	999	1.000	1.000	0.5194
224604_at	1167.7	2350.8	1.01	2.01	PPP-PPP	0.00	999	1.000	1.000	0.2193
229497_at	35.5	71.4	1.01	2.01	AAA-AAP	0.00	999	1.000	1.000	0.1768

243002_at	60.7	30.2	-1.01	-2.01	AAA-AAA	0.00	999	1.000	1.000	0.4071
1568770_at	14.7	29.7	1.01	2.02	AAA-AAA	0.00	999	1.000	1.000	0.2286
1553802_a_at	39.4	19.6	-1.01	-2.01	AAA-AAA	0.00	999	1.000	1.000	0.3106
1556448_a_at	8.2	4.1	-1.01	-2.01	AAA-AAA	0.00	999	1.000	1.000	0.4815
225207_at	15.5	7.7	-1.01	-2.01	AAM-AAA	0.00	999	1.000	1.000	0.6087
227128_s_at	7.6	3.8	-1.01	-2.01	AAA-AAA	0.00	999	1.000	1.000	0.1118
214622_at	16.2	8.1	-1.01	-2.01	AAA-AAA	0.00	999	1.000	1.000	0.1938
228838_at	14.0	7.0	-1.01	-2.01	AAA-AAA	0.00	999	1.000	1.000	0.2525
1554085_at	262.1	527.2	1.01	2.01	PPP-PPP	0.00	999	1.000	1.000	0.1096
240014_at	16.5	8.2	-1.01	-2.01	AAA-AAA	0.00	999	1.000	1.000	0.3036
1561262_at	6.1	3.1	-1.01	-2.01	AAA-AAA	0.00	999	1.000	1.000	0.1712
1567068_at	11.9	5.9	-1.01	-2.01	AAA-AAA	0.00	999	1.000	1.000	0.3682
1566248_at	28.6	14.2	-1.01	-2.01	AAA-AAA	0.00	999	1.000	1.000	0.3315
219666_at	25.0	12.4	-1.01	-2.01	AAA-AAA	0.00	999	1.000	1.000	0.4873
209792_s_at	8.9	4.4	-1.01	-2.01	AAA-AAA	0.00	999	1.000	1.000	0.1214
210770_s_at	9.6	4.8	-1.01	-2.01	AAA-AAA	0.00	999	1.000	1.000	0.2630
204996_s_at	6.0	12.1	1.01	2.01	AAA-AAA	0.00	999	1.000	1.000	0.3002
238138_at	15.0	7.4	-1.01	-2.01	AAA-AAA	0.00	999	1.000	1.000	0.6174
237256_at	8.0	4.0	-1.01	-2.01	AAA-AAA	0.00	999	1.000	1.000	0.0732
230439_at	13.0	26.2	1.01	2.01	AAA-AAA	0.00	999	1.000	1.000	0.2980
228321_s_at	35.6	17.7	-1.01	-2.01	MAA-AAA	0.00	999	1.000	1.000	0.3480
1556750_at	17.6	35.5	1.01	2.01	AAP-PAA	0.00	999	1.000	1.000	0.4672
1563032_at	2.4	1.2	-1.01	-2.01	AAA-AAA	0.00	999	1.000	1.000	0.1599
1570628_at	34.5	17.1	-1.01	-2.01	AAA-AAA	0.00	999	1.000	1.000	0.2894
244809_at	30.5	15.2	-1.01	-2.01	AAA-AAA	0.00	999	1.000	1.000	0.3690
1555578_at	4.0	8.0	1.01	2.01	AAA-AAA	0.00	999	1.000	1.000	0.5505
235042_at	73.0	36.3	-1.01	-2.01	AAA-AAA	0.00	999	1.000	1.000	0.5301
234887_at	17.5	8.7	-1.01	-2.01	AAA-AAA	0.00	999	1.000	1.000	0.4749
207547_s_at	30.9	62.1	1.01	2.01	AAA-PAA	0.00	999	1.000	1.000	0.4249
1558700_s_at	281.7	566.0	1.01	2.01	PPP-PPP	0.00	999	1.000	1.000	0.2898
214871_x_at	33.1	66.5	1.01	2.01	AAA-AMP	0.00	999	1.000	1.000	0.1285
204075_s_at	95.2	191.3	1.01	2.01	PMP-PPP	0.00	999	1.000	1.000	0.0578
242643_x_at	5.9	3.0	-1.01	-2.01	AAA-AAA	0.00	999	1.000	1.000	0.4018
243263_at	15.9	31.9	1.01	2.01	AAA-AAA	0.00	999	1.000	1.000	0.3935
1560774_at	50.5	25.1	-1.01	-2.01	AAA-AAA	0.00	999	1.000	1.000	0.4899
1560678_at	7.9	3.9	-1.01	-2.01	AAA-AAA	0.00	999	1.000	1.000	0.5540
1565697_at	59.1	29.4	-1.01	-2.01	AAA-AAA	0.00	999	1.000	1.000	0.4271
1554420_at	62.7	31.2	-1.01	-2.01	AAA-AAA	0.00	999	1.000	1.000	0.3348
1557446_x_at	11.9	5.9	-1.01	-2.01	AAA-AAA	0.00	999	1.000	1.000	0.4337
221835_at	42.1	84.5	1.01	2.01	AAA-AAA	0.00	999	1.000	1.000	0.0678
213723_s_at	17.6	8.8	-1.01	-2.01	AAA-AAA	0.00	999	1.000	1.000	0.3219
216991_at	13.0	26.0	1.01	2.01	AAA-AAA	0.00	999	1.000	1.000	0.5171

208555_x_at	28.1	14.0	-1.01	-2.01	AAA-AAA	0.00	999	1.000	1.000	0.0493
244872_at	201.3	404.3	1.01	2.01	PPP-PPP	0.00	999	1.000	1.000	0.0745
241426_at	29.5	14.7	-1.01	-2.01	AAA-AAA	0.00	999	1.000	1.000	0.5542
243359_at	8.3	4.1	-1.01	-2.01	AAA-AAA	0.00	999	1.000	1.000	0.3729
1570071_at	112.0	55.8	-1.01	-2.01	AAA-AAA	0.00	999	1.000	1.000	0.1772
1566265_at	10.3	5.1	-1.01	-2.01	AAA-AAA	0.00	999	1.000	1.000	0.5507
1557795_s_at	22.3	11.1	-1.01	-2.01	AAA-AAA	0.00	999	1.000	1.000	0.2122
206509_at	92.6	46.1	-1.01	-2.01	AAM-AAA	0.00	999	1.000	1.000	0.1169
214407_x_at	30.7	15.3	-1.01	-2.01	AAA-AAA	0.00	999	1.000	1.000	0.6467
217044_s_at	18.4	9.2	-1.01	-2.01	AAA-AAA	0.00	999	1.000	1.000	0.2643
216727_at	26.9	13.4	-1.01	-2.01	AAA-AAA	0.00	999	1.000	1.000	0.3558
234636_at	15.0	7.5	-1.01	-2.01	AAA-AAA	0.00	999	1.000	1.000	0.5801
237961_at	2.9	5.7	1.01	2.01	AAA-AAA	0.00	999	1.000	1.000	0.0710
231652_at	10.8	5.4	-1.01	-2.01	AAA-AAA	0.00	999	1.000	1.000	0.3848
1554766_s_at	5.6	11.2	1.01	2.01	AAA-APA	0.00	999	1.000	1.000	0.3763
243522_at	11.1	5.6	-1.01	-2.01	AAA-AAA	0.00	999	1.000	1.000	0.5108
1563635_at	11.6	23.2	1.01	2.01	AAA-AAA	0.00	999	1.000	1.000	0.2683
1561614_at	9.3	18.6	1.01	2.01	AAA-AAA	0.00	999	1.000	1.000	0.2274
1561718_at	23.6	11.8	-1.01	-2.01	AAA-AAA	0.00	999	1.000	1.000	0.5244
1566113_at	41.3	20.6	-1.01	-2.01	AAA-AAA	0.00	999	1.000	1.000	0.2703
1557814_a_at	163.0	81.2	-1.01	-2.01	AAA-AAA	0.00	999	1.000	1.000	0.2541
221455_s_at	30.5	61.2	1.01	2.01	AAA-AAA	0.00	999	1.000	1.000	0.3800
210884_s_at	54.1	27.0	-1.01	-2.01	AAA-AAA	0.00	999	1.000	1.000	0.4127
216949_s_at	44.8	22.3	-1.01	-2.01	AAA-AAA	0.00	999	1.000	1.000	0.2910
235187_s_at	4.8	9.7	1.01	2.01	AAA-AAA	0.00	999	1.000	1.000	0.3753
233885_at	17.6	8.8	-1.01	-2.01	AAA-AAA	0.00	999	1.000	1.000	0.0680
238125_at	22.2	11.1	-1.01	-2.01	AAA-AAA	0.00	999	1.000	1.000	0.4865
239312_at	35.7	17.8	-1.01	-2.01	AAA-AAA	0.00	999	1.000	1.000	0.3836
237865_x_at	15.5	31.1	1.01	2.01	AAA-AAA	0.00	999	1.000	1.000	0.0787
1566779_at	22.7	45.5	1.00	2.01	AAA-APA	0.00	999	1.000	1.000	0.1861
244342_at	49.0	24.5	-1.00	-2.01	AAA-AAA	0.00	999	1.000	1.000	0.4589
239956_at	55.7	27.8	-1.00	-2.01	AAA-AAA	0.00	999	1.000	1.000	0.0258
1553079_at	43.0	21.5	-1.00	-2.01	AAA-AAA	0.00	999	1.000	1.000	0.2159
1553329_at	45.2	22.5	-1.00	-2.01	AAA-AAA	0.00	999	1.000	1.000	0.1282
1559679_a_at	13.6	6.8	-1.00	-2.01	AAA-AAA	0.00	999	1.000	1.000	0.2451
1556259_at	5.9	11.9	1.00	2.01	AAA-AAA	0.00	999	1.000	1.000	0.1503
1555757_at	26.6	13.3	-1.00	-2.01	AAA-AAA	0.00	999	1.000	1.000	0.3095
226047_at	9.0	4.5	-1.00	-2.01	AAA-AAA	0.00	999	1.000	1.000	0.1046
209590_at	40.8	20.4	-1.00	-2.01	AAA-AAA	0.00	999	1.000	1.000	0.4077
236336_at	18.0	9.0	-1.00	-2.01	AAA-AAA	0.00	999	1.000	1.000	0.3374
239040_at	44.5	22.2	-1.00	-2.01	AAA-AAA	0.00	999	1.000	1.000	0.3561
237518_at	138.6	69.1	-1.00	-2.01	AAA-AAA	0.00	999	1.000	1.000	0.0869

205965_at	38.7	19.3	-1.00	-2.01	AAA-AAA	0.00	999	1.000	1.000	0.2473
229767_at	12.0	6.0	-1.00	-2.01	AAA-AAA	0.00	999	1.000	1.000	0.3350
228193_s_at	7.0	14.1	1.00	2.01	AAA-AAA	0.00	999	1.000	1.000	0.2855
205223_at	95.1	47.4	-1.00	-2.01	AAA-AAA	0.00	999	1.000	1.000	0.5281
225905_s_at	102.6	205.7	1.00	2.00	AAA-PAA	0.00	999	1.000	1.000	0.2765
242367_at	34.4	68.9	1.00	2.00	AAA-AAA	0.00	999	1.000	1.000	0.1892
1560854_s_at	17.3	34.6	1.00	2.00	AAA-AAA	0.00	999	1.000	1.000	0.4182
1567912_s_at	5.2	2.6	-1.00	-2.00	AAA-AAA	0.00	999	1.000	1.000	0.4238
1569885_at	11.3	22.6	1.00	2.00	AAA-AAA	0.00	999	1.000	1.000	0.3544
244505_at	17.3	8.6	-1.00	-2.00	AAA-AAA	0.00	999	1.000	1.000	0.2166
1556398_a_at	17.5	8.7	-1.00	-2.00	AAA-AAA	0.00	999	1.000	1.000	0.4400
221303_at	74.3	37.0	-1.00	-2.01	AAA-AAA	0.00	999	1.000	1.000	0.1465
207412_x_at	16.4	8.2	-1.00	-2.01	AAA-AAA	0.00	999	1.000	1.000	0.5467
224156_x_at	25.4	12.7	-1.00	-2.00	AAA-AAA	0.00	999	1.000	1.000	0.1051
215644_at	23.9	11.9	-1.00	-2.00	AAA-AAA	0.00	999	1.000	1.000	0.1960
205040_at	11.0	22.0	1.00	2.00	AAA-AAA	0.00	999	1.000	1.000	0.4367
238920_at	33.1	66.3	1.00	2.00	AAA-AAA	0.00	999	1.000	1.000	0.3564
203993_x_at	14.7	7.3	-1.00	-2.00	AAA-AAA	0.00	999	1.000	1.000	0.0228
229612_at	9.2	18.4	1.00	2.00	AAA-AAA	0.00	999	1.000	1.000	0.4246
206266_at	19.0	9.5	-1.00	-2.00	AAA-AAA	0.00	999	1.000	1.000	0.5603
240752_at	12.1	24.3	1.00	2.00	AAA-AAA	0.00	999	1.000	1.000	0.5087
242595_at	13.2	6.6	-1.00	-2.00	AAA-AAA	0.00	999	1.000	1.000	0.4990
240005_at	91.9	45.9	-1.00	-2.00	AAA-AAM	0.00	999	1.000	1.000	0.1514
1556374_s_at	28.7	14.4	-1.00	-2.00	AAA-AAA	0.00	999	1.000	1.000	0.5251
206443_at	6.7	3.3	-1.00	-2.00	AAA-AAA	0.00	999	1.000	1.000	0.3024
226822_at	23.2	11.6	-1.00	-2.00	AAA-AAA	0.00	999	1.000	1.000	0.4168
222944_s_at	4.7	9.4	1.00	2.00	AAA-AAA	0.00	999	1.000	1.000	0.3410
224069_x_at	72.1	36.0	-1.00	-2.00	AAA-AAA	0.00	999	1.000	1.000	0.4639
216616_at	10.4	5.2	-1.00	-2.00	AAA-AAA	0.00	999	1.000	1.000	0.5129
238958_at	17.2	8.6	-1.00	-2.00	AAA-AAA	0.00	999	1.000	1.000	0.4625
231236_at	16.9	8.4	-1.00	-2.00	AAA-AAA	0.00	999	1.000	1.000	0.3212
205479_s_at	1696.7	3395.5	1.00	2.00	PPP-PPP	0.00	999	1.000	1.000	0.2121
209037_s_at	359.5	719.5	1.00	2.00	PPP-PPP	0.00	999	1.000	1.000	0.0811
1569078_at	17.6	8.8	-1.00	-2.00	AAA-AAA	0.00	999	1.000	1.000	0.5019
1554240_a_at	9.9	19.8	1.00	2.00	AAA-AAA	0.00	999	1.000	1.000	0.5085
204818_at	4.1	2.1	-1.00	-2.00	AAA-AAA	0.00	999	1.000	1.000	0.1304
236460_at	70.3	35.1	-1.00	-2.00	AAA-AAA	0.00	999	1.000	1.000	0.2830
207588_at	11.4	5.7	-1.00	-2.00	AAA-APA	0.00	999	1.000	1.000	0.6159
241247_at	18.9	9.5	-1.00	-2.00	AAA-AAA	0.00	999	1.000	1.000	0.2742
1563753_at	69.8	34.9	-1.00	-2.00	AAA-AAA	0.00	999	1.000	1.000	0.1098
1560940_at	15.7	7.9	-1.00	-2.00	AAA-AAA	0.00	999	1.000	1.000	0.5647
1569348_at	31.6	15.8	-1.00	-2.00	AAA-AAA	0.00	999	1.000	1.000	0.3655

1569830_at	10.6	21.2	1.00	2.00	AAA-AAA	0.00	999	1.000	1.000	0.4695
1557797_a_at	6.7	13.4	1.00	2.00	AAA-AMA	0.00	999	1.000	1.000	0.4976
226548_at	25.1	12.6	-1.00	-2.00	AAA-AAA	0.00	999	1.000	1.000	0.5384
215053_at	28.2	56.4	1.00	2.00	AAA-AAA	0.00	999	1.000	1.000	0.2760
211841_s_at	37.3	74.7	1.00	2.00	AAA-AAA	0.00	999	1.000	1.000	0.3211
219135_s_at	6.2	12.3	1.00	2.00	AAA-AAA	0.00	999	1.000	1.000	0.1079
216275_at	32.3	16.2	-1.00	-2.00	AAA-AAA	0.00	999	1.000	1.000	0.3678
236290_at	6.1	3.0	-1.00	-2.00	AAA-AAA	0.00	999	1.000	1.000	0.1452
234897_s_at	43.7	21.9	-1.00	-2.00	AAA-AAA	0.00	999	1.000	1.000	0.3083
234580_at	43.6	21.8	-1.00	-2.00	AAA-AAA	0.00	999	1.000	1.000	0.4268
237509_at	20.1	10.0	-1.00	-2.00	AAA-AAA	0.00	999	1.000	1.000	0.2723
205751_at	17.3	8.6	-1.00	-2.00	AAA-AAA	0.00	999	1.000	1.000	0.5098
203444_s_at	24.3	12.2	-1.00	-2.00	AAA-AAA	0.00	999	1.000	1.000	0.1497
1570194_x_at	28.4	14.2	-1.00	-2.00	AAA-AAA	0.00	999	1.000	1.000	0.4289
1559537_at	8.1	4.0	-1.00	-2.00	AAA-AAA	0.00	999	1.000	1.000	0.1058
1559747_at	27.3	54.6	1.00	2.00	AMA-AAA	0.00	999	1.000	1.000	0.5061
1556008_a_at	14.6	29.3	1.00	2.00	AAA-AAA	0.00	999	1.000	1.000	0.5423
209619_at	83.3	41.7	-1.00	-2.00	AAM-AAA	0.00	999	1.000	1.000	0.1734
235643_at	7.3	14.6	1.00	2.00	AAA-AAA	0.00	999	1.000	1.000	0.3143
233028_at	24.5	49.1	1.00	2.00	AAA-AAA	0.00	999	1.000	1.000	0.2001
233484_at	19.8	9.9	-1.00	-2.00	AAA-AAA	0.00	999	1.000	1.000	0.5508
220295_x_at	942.5	1882.1	1.00	2.00	PPP-PPP	0.00	999	1.000	1.000	0.2465
204792_s_at	31.0	61.9	1.00	2.00	AAA-PAP	0.00	999	1.000	1.000	0.2170
243960_x_at	12.6	25.1	1.00	2.00	AAA-AAA	0.00	999	1.000	1.000	0.4095
1566927_at	102.0	51.1	-1.00	-2.00	AAA-MAA	0.00	999	1.000	1.000	0.3290
1558965_at	12.1	24.1	1.00	2.00	AAA-AMA	0.00	999	1.000	1.000	0.3112
1556274_at	93.1	46.6	-1.00	-2.00	AAA-AAA	0.00	999	1.000	1.000	0.0816
207191_s_at	22.0	11.0	-1.00	-2.00	AAA-AAA	0.00	999	1.000	1.000	0.5033
222922_at	15.6	7.8	-1.00	-2.00	AAA-AAA	0.00	999	1.000	1.000	0.5539
222935_x_at	6.2	3.1	-1.00	-2.00	AAA-AAA	0.00	999	1.000	1.000	0.1865
217391_x_at	36.4	18.2	-1.00	-2.00	AAA-AAA	0.00	999	1.000	1.000	0.3859
216840_s_at	39.3	19.7	-1.00	-2.00	AAA-AAA	0.00	999	1.000	1.000	0.3159
219302_s_at	14.7	29.3	1.00	2.00	AAA-AAA	0.00	999	1.000	1.000	0.2554
215214_at	37.9	19.0	-1.00	-2.00	AAA-AAA	0.00	999	1.000	1.000	0.1522
234857_at	20.4	10.2	-1.00	-2.00	AAA-AAA	0.00	999	1.000	1.000	0.2121
237122_at	30.0	15.0	-1.00	-2.00	AAA-AAM	0.00	999	1.000	1.000	0.4596
205637_s_at	32.6	65.1	1.00	2.00	AAA-AAA	0.00	999	1.000	1.000	0.4102
231438_x_at	6.4	3.2	-1.00	-2.00	APA-AAA	0.00	999	1.000	1.000	0.6432
239515_at	60.9	121.7	1.00	2.00	AAA-PAA	0.00	999	1.000	1.000	0.4227
240508_at	21.4	10.7	-1.00	-2.00	AAA-AAA	0.00	999	1.000	1.000	0.0905
1565613_at	20.0	39.9	1.00	2.00	AAA-AAA	0.00	999	1.000	1.000	0.2788
1552964_at	10.1	5.0	-1.00	-2.00	AAA-AAA	0.00	999	1.000	1.000	0.0629

221721_s_at	4.5	2.3	-1.00	-2.00	AAA-AAA	0.00	999	1.000	1.000	0.0593
216802_at	12.4	6.2	-1.00	-2.00	AAA-AAA	0.00	999	1.000	1.000	0.4935
215560_x_at	138.9	69.6	-1.00	-2.00	AAA-AAA	0.00	999	1.000	1.000	0.1394
239402_at	17.1	34.2	1.00	1.99	AAA-AAP	0.00	999	1.000	1.000	0.5220
234369_at	30.9	61.6	1.00	1.99	AAA-PAA	0.00	999	1.000	1.000	0.2131
243854_at	8.6	4.3	-1.00	-1.99	AAA-AAA	0.00	999	1.000	1.000	0.3549
243169_at	4.3	2.1	-1.00	-1.99	AAA-AAA	0.00	999	1.000	1.000	0.2474
241168_at	20.9	10.5	-1.00	-2.00	AAA-AAA	0.00	999	1.000	1.000	0.4564
1562934_at	17.2	34.3	1.00	2.00	AAA-AAA	0.00	999	1.000	1.000	0.2942
1564357_at	9.0	4.5	-1.00	-1.99	AAA-AAA	0.00	999	1.000	1.000	0.4617
1552549_a_at	66.4	33.3	-1.00	-2.00	AAA-AAA	0.00	999	1.000	1.000	0.2695
1556405_s_at	36.3	18.2	-1.00	-1.99	AAA-AAA	0.00	999	1.000	1.000	0.4405
221314_at	118.5	59.4	-1.00	-1.99	AAA-AAA	0.00	999	1.000	1.000	0.0389
220981_x_at	122.5	61.4	-1.00	-2.00	AAA-AAA	0.00	999	1.000	1.000	0.0848
206800_at	51.9	26.0	-1.00	-2.00	AAA-AAA	0.00	999	1.000	1.000	0.3528
224065_at	30.0	59.8	1.00	1.99	AMA-AAA	0.00	999	1.000	1.000	0.5286
217498_at	4.0	8.0	1.00	2.00	AAA-AAA	0.00	999	1.000	1.000	0.3444
235869_at	16.9	33.6	1.00	2.00	AAA-AAA	0.00	999	1.000	1.000	0.5493
236171_at	55.0	27.6	-1.00	-2.00	AAA-AAA	0.00	999	1.000	1.000	0.4299
234113_at	34.1	17.1	-1.00	-1.99	AAA-AAA	0.00	999	1.000	1.000	0.5280
237794_at	28.8	57.5	1.00	2.00	AAA-AAA	0.00	999	1.000	1.000	0.3037
229915_at	6.9	3.4	-1.00	-2.00	AAA-AAA	0.00	999	1.000	1.000	0.2785
1554039_at	87.0	173.5	1.00	1.99	PAA-APP	0.00	999	1.000	1.000	0.5860
243663_at	110.7	55.5	-1.00	-1.99	AAA-AAA	0.00	999	1.000	1.000	0.0087
243440_at	20.7	41.3	1.00	1.99	AAA-AAA	0.00	999	1.000	1.000	0.2292
242742_at	49.8	25.0	-1.00	-1.99	AAA-AAA	0.00	999	1.000	1.000	0.2733
1561367_a_at	10.8	21.4	1.00	1.99	AAA-AAA	0.00	999	1.000	1.000	0.2643
1557583_at	25.4	12.8	-1.00	-1.99	AAA-AAA	0.00	999	1.000	1.000	0.3761
208428_at	8.1	4.1	-1.00	-1.99	AAA-AAA	0.00	999	1.000	1.000	0.0137
220816_at	61.0	30.6	-1.00	-1.99	AAA-AAA	0.00	999	1.000	1.000	0.1229
224520_s_at	8.3	4.2	-1.00	-1.99	AAA-AAA	0.00	999	1.000	1.000	0.3895
207355_at	32.5	64.8	1.00	1.99	AAA-AAA	0.00	999	1.000	1.000	0.3554
217143_s_at	15.6	7.8	-1.00	-1.99	AAA-AAA	0.00	999	1.000	1.000	0.3558
219113_x_at	21.0	10.5	-1.00	-1.99	AAA-AAA	0.00	999	1.000	1.000	0.3314
216412_x_at	58.0	29.1	-1.00	-1.99	AAA-AAA	0.00	999	1.000	1.000	0.1701
234771_at	8.3	4.2	-1.00	-1.99	AAA-AAA	0.00	999	1.000	1.000	0.2695
237080_at	30.8	15.5	-1.00	-1.99	AAA-AAA	0.00	999	1.000	1.000	0.1727
237593_at	16.3	8.2	-1.00	-1.99	AAA-AAA	0.00	999	1.000	1.000	0.4900
205827_at	47.4	23.8	-1.00	-1.99	AAA-AAA	0.00	999	1.000	1.000	0.2728
206394_at	23.1	11.6	-1.00	-1.99	AAA-AAA	0.00	999	1.000	1.000	0.4718
233237_at	39.6	19.9	-1.00	-1.99	AAA-AAA	0.00	999	1.000	1.000	0.5190
215193_x_at	28.5	56.8	0.99	1.99	APA-AAA	0.00	999	1.000	1.000	0.2623

203381_s_at	17.6	35.1	0.99	1.99	AAA-AAA	0.00	999	1.000	1.000	0.3574
1554406_a_at	50.3	25.3	-0.99	-1.99	AAA-AAA	0.00	999	1.000	1.000	0.4174
1559093_at	50.9	25.6	-0.99	-1.99	MAA-AAA	0.00	999	1.000	1.000	0.1976
1557349_at	7.9	15.7	0.99	1.99	AAA-AAA	0.00	999	1.000	1.000	0.3810
208175_s_at	3.4	6.8	0.99	1.99	AAA-AAA	0.00	999	1.000	1.000	0.3902
208201_at	13.3	26.4	0.99	1.99	AAA-AAA	0.00	999	1.000	1.000	0.2249
222539_at	11.3	5.7	-0.99	-1.99	AAA-AAA	0.00	999	1.000	1.000	0.0848
211667_x_at	17.8	9.0	-0.99	-1.99	AAA-AAA	0.00	999	1.000	1.000	0.5183
211028_s_at	103.6	52.0	-0.99	-1.99	AAA-AAA	0.00	999	1.000	1.000	0.2626
211914_x_at	116.6	58.5	-0.99	-1.99	AAA-AAA	0.00	999	1.000	1.000	0.2514
217154_s_at	9.9	5.0	-0.99	-1.99	AAA-AAA	0.00	999	1.000	1.000	0.1998
217273_at	70.1	35.2	-0.99	-1.99	AAA-AAA	0.00	999	1.000	1.000	0.1180
227635_at	9.4	4.7	-0.99	-1.99	AAA-AAA	0.00	999	1.000	1.000	0.0745
231717_s_at	24.0	12.1	-0.99	-1.99	AAA-AAA	0.00	999	1.000	1.000	0.4731
238067_at	73.6	146.6	0.99	1.99	APP-PPP	0.00	999	1.000	1.000	0.0555
240418_at	30.9	61.5	0.99	1.99	AAA-AAA	0.00	999	1.000	1.000	0.3088
1561477_at	4.9	9.8	0.99	1.99	AAA-AAA	0.00	999	1.000	1.000	0.3799
1561217_at	10.1	5.1	-0.99	-1.99	AAA-AAA	0.00	999	1.000	1.000	0.3010
1565406_a_at	9.4	4.7	-0.99	-1.99	AAA-AAA	0.00	999	1.000	1.000	0.5844
1565833_at	19.2	9.6	-0.99	-1.99	AAA-AAA	0.00	999	1.000	1.000	0.3677
1555133_at	9.6	4.8	-0.99	-1.99	AAA-AAA	0.00	999	1.000	1.000	0.5553
216357_at	13.3	26.4	0.99	1.99	AAA-AAA	0.00	999	1.000	1.000	0.3825
238984_at	9.9	19.7	0.99	1.99	AAA-AAA	0.00	999	1.000	1.000	0.3275
231496_at	8.6	4.3	-0.99	-1.99	AAA-AAA	0.00	999	1.000	1.000	0.5683
205650_s_at	57.6	28.9	-0.99	-1.99	AAA-AAA	0.00	999	1.000	1.000	0.3444
227163_at	81.4	161.8	0.99	1.99	AAP-PPP	0.00	999	1.000	1.000	0.1551
239913_at	5.1	10.2	0.99	1.99	AAA-AAA	0.00	999	1.000	1.000	0.5938
241211_at	42.5	21.4	-0.99	-1.99	AAA-AAA	0.00	999	1.000	1.000	0.3176
243235_at	4.9	2.5	-0.99	-1.99	AAA-AAA	0.00	999	1.000	1.000	0.0769
1562678_at	14.3	7.2	-0.99	-1.99	AAA-AAA	0.00	999	1.000	1.000	0.5790
244403_at	6.5	3.3	-0.99	-1.99	AAA-AAA	0.00	999	1.000	1.000	0.0576
1552965_a_at	23.6	11.8	-0.99	-1.99	AAA-AAA	0.00	999	1.000	1.000	0.4555
210444_at	27.6	13.9	-0.99	-1.99	AAA-AAA	0.00	999	1.000	1.000	0.5419
210650_s_at	19.8	39.4	0.99	1.99	AAA-AAA	0.00	999	1.000	1.000	0.1641
233886_at	21.9	11.0	-0.99	-1.99	AAA-AAA	0.00	999	1.000	1.000	0.4501
234103_at	6.5	3.3	-0.99	-1.99	AAA-AAA	0.00	999	1.000	1.000	0.4667
238798_at	38.6	76.8	0.99	1.99	AAA-AAA	0.00	999	1.000	1.000	0.1769
230957_at	26.4	13.3	-0.99	-1.99	MAA-AAA	0.00	999	1.000	1.000	0.6404
206258_at	8.9	4.5	-0.99	-1.99	AAA-AAA	0.00	999	1.000	1.000	0.1498
206326_at	11.2	5.6	-0.99	-1.99	AAA-AAA	0.00	999	1.000	1.000	0.2812
231676_s_at	10.4	20.6	0.99	1.99	AAA-AAA	0.00	999	1.000	1.000	0.5882
226641_at	26.5	52.6	0.99	1.99	AAA-APP	0.00	999	1.000	1.000	0.1746

241018_at	13.8	27.5	0.99	1.99	AAA-AAA	0.00	999	1.000	1.000	0.5569
243248_at	9.6	4.8	-0.99	-1.99	AAA-AAA	0.00	999	1.000	1.000	0.0052
1552829_at	80.0	40.2	-0.99	-1.99	AAA-AAA	0.00	999	1.000	1.000	0.2905
207895_at	7.4	14.7	0.99	1.99	AAA-AAA	0.00	999	1.000	1.000	0.5107
219867_at	57.3	28.8	-0.99	-1.99	AAA-AAA	0.00	999	1.000	1.000	0.1186
210831_s_at	52.0	26.1	-0.99	-1.99	AAA-AAA	0.00	999	1.000	1.000	0.2764
216577_at	94.4	47.5	-0.99	-1.99	AAA-AAA	0.00	999	1.000	1.000	0.4412
239541_at	14.1	7.1	-0.99	-1.99	AAA-AAA	0.00	999	1.000	1.000	0.3345
205866_at	21.9	11.0	-0.99	-1.99	AAA-AAA	0.00	999	1.000	1.000	0.3587
228631_s_at	69.6	35.0	-0.99	-1.99	AAA-AAA	0.00	999	1.000	1.000	0.1053
233326_at	9.3	4.7	-0.99	-1.99	AAA-AAA	0.00	999	1.000	1.000	0.4176
206980_s_at	22.5	11.3	-0.99	-1.99	AAA-AAA	0.00	999	1.000	1.000	0.4066
223122_s_at	3.4	6.8	0.99	1.99	AAA-AAA	0.00	999	1.000	1.000	0.4051
222567_s_at	58.3	115.7	0.99	1.99	AAA-AAA	0.00	999	1.000	1.000	0.1312
235133_at	45.8	23.1	-0.99	-1.99	AAA-AAA	0.00	999	1.000	1.000	0.5264
233875_at	4.0	2.0	-0.99	-1.99	AAA-AAA	0.00	999	1.000	1.000	0.4463
229619_at	6.6	13.2	0.99	1.99	AAA-AAA	0.00	999	1.000	1.000	0.4707
205837_s_at	6.1	3.1	-0.99	-1.99	AAA-AAA	0.00	999	1.000	1.000	0.0140
233604_at	2.8	5.6	0.99	1.99	AAA-AAA	0.00	999	1.000	1.000	0.5754
1559910_at	68.0	135.0	0.99	1.98	AMA-APP	0.00	999	1.000	1.000	0.2409
240191_at	12.2	24.2	0.99	1.99	AAA-AAA	0.00	999	1.000	1.000	0.3435
1560954_at	18.1	9.1	-0.99	-1.99	AAA-AAA	0.00	999	1.000	1.000	0.5167
1556149_at	10.7	5.4	-0.99	-1.99	AAA-AAA	0.00	999	1.000	1.000	0.0279
1556375_at	17.4	8.8	-0.99	-1.99	AAA-AAA	0.00	999	1.000	1.000	0.1907
224215_s_at	7.7	3.9	-0.99	-1.99	AAA-AAA	0.00	999	1.000	1.000	0.2771
224380_s_at	26.5	13.4	-0.99	-1.99	AAA-AAA	0.00	999	1.000	1.000	0.4344
214735_at	37.2	18.7	-0.99	-1.99	AAA-AAA	0.00	999	1.000	1.000	0.1746
212906_at	108.4	215.3	0.99	1.99	AAA-AAA	0.00	999	1.000	1.000	0.4569
230999_at	46.3	23.3	-0.99	-1.99	MAA-AAA	0.00	999	1.000	1.000	0.1683
241830_at	30.5	15.4	-0.99	-1.98	AAA-AAA	0.00	999	1.000	1.000	0.4578
243499_at	27.4	13.8	-0.99	-1.98	AAA-AAA	0.00	999	1.000	1.000	0.5290
226096_at	14.5	28.8	0.99	1.98	AAA-AAA	0.00	999	1.000	1.000	0.5367
223359_s_at	8.1	4.1	-0.99	-1.98	AAA-AAA	0.00	999	1.000	1.000	0.0918
213339_at	23.2	11.7	-0.99	-1.98	AAA-AAA	0.00	999	1.000	1.000	0.4657
214973_x_at	33.2	16.7	-0.99	-1.98	AAA-AAA	0.00	999	1.000	1.000	0.4822
217320_at	10.9	5.5	-0.99	-1.98	AAA-AAA	0.00	999	1.000	1.000	0.6105
237385_at	17.7	8.9	-0.99	-1.98	AAA-AAA	0.00	999	1.000	1.000	0.5834
236861_at	40.4	20.4	-0.99	-1.98	AAA-AAA	0.00	999	1.000	1.000	0.2764
236868_at	4.4	8.8	0.99	1.98	AAA-AAA	0.00	999	1.000	1.000	0.5638
233265_at	75.8	38.2	-0.99	-1.98	AAA-AAA	0.00	999	1.000	1.000	0.2952
205629_s_at	26.8	13.5	-0.99	-1.98	AAA-AAA	0.00	999	1.000	1.000	0.3208
227458_at	287.3	569.4	0.99	1.98	MPP-PPP	0.00	999	1.000	1.000	0.2880

1561144_at	36.2	71.8	0.99	1.98	AAA-PPA	0.00	999	1.000	1.000	0.2243
237483_at	41.8	82.8	0.99	1.98	PAA-AMP	0.00	999	1.000	1.000	0.0653
239904_at	3.8	7.5	0.99	1.98	AAA-AAA	0.00	999	1.000	1.000	0.6353
240947_at	25.9	51.3	0.99	1.98	AAA-AAA	0.00	999	1.000	1.000	0.3562
1569999_at	77.8	39.3	-0.99	-1.98	AMM-AAM	0.00	999	1.000	1.000	0.2413
1552326_a_at	13.3	6.7	-0.99	-1.98	AAA-AAA	0.00	999	1.000	1.000	0.4830
202768_at	11.5	22.7	0.99	1.98	AAA-AAA	0.00	999	1.000	1.000	0.2422
1557190_at	76.7	38.7	-0.99	-1.98	AAA-AAA	0.00	999	1.000	1.000	0.0726
217377_x_at	65.8	33.2	-0.99	-1.98	AAA-AAA	0.00	999	1.000	1.000	0.2469
236735_at	7.8	3.9	-0.99	-1.98	AAA-AAA	0.00	999	1.000	1.000	0.2352
234713_x_at	12.7	6.4	-0.99	-1.98	AAA-AAA	0.00	999	1.000	1.000	0.1739
237970_at	21.5	42.5	0.99	1.98	AAA-AAA	0.00	999	1.000	1.000	0.3698
205744_at	40.9	81.0	0.99	1.98	AAA-AAA	0.00	999	1.000	1.000	0.1659
206120_at	11.2	5.6	-0.99	-1.98	AAA-AAA	0.00	999	1.000	1.000	0.0205
243750_x_at	393.6	779.7	0.99	1.98	PPP-PPP	0.00	999	1.000	1.000	0.1001
1562720_at	33.7	17.0	-0.99	-1.98	AAA-AAA	0.00	999	1.000	1.000	0.5021
1562220_at	48.1	95.2	0.99	1.98	AAA-AAA	0.00	999	1.000	1.000	0.4313
1560684_x_at	8.6	17.0	0.99	1.98	AAA-AAA	0.00	999	1.000	1.000	0.6029
1558786_at	3.9	7.7	0.99	1.98	AAA-AAA	0.00	999	1.000	1.000	0.5461
227573_s_at	71.8	36.2	-0.99	-1.98	AMA-AAA	0.00	999	1.000	1.000	0.2181
216848_at	14.1	7.1	-0.99	-1.98	AAA-AAA	0.00	999	1.000	1.000	0.0554
217588_at	45.2	89.5	0.99	1.98	AAA-AAM	0.00	999	1.000	1.000	0.2357
237134_at	9.0	4.5	-0.99	-1.98	AAA-AAA	0.00	999	1.000	1.000	0.1330
237669_at	57.3	28.9	-0.99	-1.98	MAA-AAA	0.00	999	1.000	1.000	0.2273
230087_at	29.0	14.6	-0.99	-1.98	AAA-AAA	0.00	999	1.000	1.000	0.3413
232934_at	16.1	8.2	-0.99	-1.98	AAA-AAA	0.00	999	1.000	1.000	0.5136
205656_at	71.1	35.9	-0.99	-1.98	AAA-AAA	0.00	999	1.000	1.000	0.2406
207999_s_at	107.1	212.1	0.99	1.98	APP-PPP	0.00	999	1.000	1.000	0.0953
242611_at	18.0	35.5	0.99	1.98	AAA-AAA	0.00	999	1.000	1.000	0.3583
1562462_at	97.9	49.4	-0.99	-1.98	AAA-AAA	0.00	999	1.000	1.000	0.0286
1560921_at	55.2	109.3	0.99	1.98	AAA-AAA	0.00	999	1.000	1.000	0.1758
1552929_at	22.0	11.1	-0.99	-1.98	AAA-AAA	0.00	999	1.000	1.000	0.4829
226903_s_at	35.6	18.0	-0.99	-1.98	AAA-AAA	0.00	999	1.000	1.000	0.3247
239296_at	38.3	19.4	-0.98	-1.98	AAP-AAA	0.00	999	1.000	1.000	0.6023
210390_s_at	7.5	14.7	0.98	1.98	AAA-PAA	0.00	999	1.000	1.000	0.5131
1560430_at	59.4	117.4	0.98	1.98	AAA-MAA	0.00	999	1.000	1.000	0.5128
1565597_at	17.7	9.0	-0.98	-1.98	AAA-AAA	0.00	999	1.000	1.000	0.3991
1558489_at	4.3	2.2	-0.98	-1.98	AAA-AAA	0.00	999	1.000	1.000	0.2224
207327_at	23.1	45.8	0.98	1.98	AAA-AAA	0.00	999	1.000	1.000	0.3459
210778_s_at	86.3	170.8	0.98	1.98	AAA-AAA	0.00	999	1.000	1.000	0.0209
216943_at	28.7	14.5	-0.98	-1.98	AAA-AAA	0.00	999	1.000	1.000	0.4727
234387_at	30.8	15.6	-0.98	-1.98	AMA-AAA	0.00	999	1.000	1.000	0.4387

228586_at	9.4	4.7	-0.98	-1.98	AAA-AAA	0.00	999	1.000	1.000	0.3168
1562251_a_at	9.2	18.2	0.98	1.98	AAA-AMA	0.00	999	1.000	1.000	0.4627
1560531_at	112.5	56.9	-0.98	-1.98	AAA-AAA	0.00	999	1.000	1.000	0.0255
1570039_at	20.3	40.1	0.98	1.98	AAA-MAA	0.00	999	1.000	1.000	0.0245
1553328_a_at	31.3	15.8	-0.98	-1.98	AAA-AAA	0.00	999	1.000	1.000	0.5403
219719_at	23.5	11.9	-0.98	-1.98	AAA-AAA	0.00	999	1.000	1.000	0.5116
207200_at	5.1	10.1	0.98	1.98	AAA-AAA	0.00	999	1.000	1.000	0.5386
211389_x_at	15.0	7.6	-0.98	-1.98	AAA-AAA	0.00	999	1.000	1.000	0.1757
204730_at	28.6	56.5	0.98	1.98	AAA-AAM	0.00	999	1.000	1.000	0.3550
233561_at	10.3	20.3	0.98	1.98	AAA-AAA	0.00	999	1.000	1.000	0.3110
1559826_a_at	36.3	71.7	0.98	1.98	PPA-PPA	0.00	999	1.000	1.000	0.4359
240063_at	23.1	11.7	-0.98	-1.98	AAA-AAA	0.00	999	1.000	1.000	0.5833
1552409_a_at	29.8	15.1	-0.98	-1.98	AAA-AAA	0.00	999	1.000	1.000	0.3231
1553045_at	3.7	7.4	0.98	1.98	AAA-AAA	0.00	999	1.000	1.000	0.4912
208181_at	13.6	6.9	-0.98	-1.98	AAM-AAA	0.00	999	1.000	1.000	0.6010
227354_at	10.3	20.4	0.98	1.98	AAA-AAM	0.00	999	1.000	1.000	0.4301
207430_s_at	4.7	2.4	-0.98	-1.98	AAA-AAA	0.00	999	1.000	1.000	0.5206
210484_s_at	18.1	35.7	0.98	1.98	AAA-AAA	0.00	999	1.000	1.000	0.5425
237166_at	31.5	62.3	0.98	1.98	AAA-AAA	0.00	999	1.000	1.000	0.0678
229219_s_at	176.8	89.5	-0.98	-1.98	AAA-AAA	0.00	999	1.000	1.000	0.0807
229041_s_at	37.9	19.2	-0.98	-1.98	AAA-AAA	0.00	999	1.000	1.000	0.5298
239781_at	7.8	15.5	0.98	1.98	AAA-AAA	0.00	999	1.000	1.000	0.0781
241469_at	3.0	1.5	-0.98	-1.97	AAA-AAA	0.00	999	1.000	1.000	0.1933
241395_at	38.0	19.2	-0.98	-1.97	AAA-AAA	0.00	999	1.000	1.000	0.4556
1561760_s_at	55.0	27.9	-0.98	-1.97	AAA-AAA	0.00	999	1.000	1.000	0.0456
1569095_at	117.5	59.5	-0.98	-1.97	AAA-AAA	0.00	999	1.000	1.000	0.0613
1569739_at	50.3	25.5	-0.98	-1.97	AAA-AAA	0.00	999	1.000	1.000	0.2580
244600_at	26.8	52.9	0.98	1.97	AAA-AAA	0.00	999	1.000	1.000	0.2576
219820_at	12.8	6.5	-0.98	-1.97	AAA-AAA	0.00	999	1.000	1.000	0.4431
211484_s_at	36.0	18.2	-0.98	-1.97	AAA-AAA	0.00	999	1.000	1.000	0.3988
210550_s_at	34.6	17.5	-0.98	-1.97	AAA-AAA	0.00	999	1.000	1.000	0.4626
216676_x_at	18.9	9.6	-0.98	-1.97	AAA-AAA	0.00	999	1.000	1.000	0.0338
231987_at	22.1	43.7	0.98	1.97	AAA-AAA	0.00	999	1.000	1.000	0.3389
1566266_at	13.9	27.5	0.98	1.97	AAA-AAP	0.00	999	1.000	1.000	0.4161
216595_at	21.5	42.5	0.98	1.97	AAP-PPA	0.00	999	1.000	1.000	0.2929
240483_at	10.1	19.9	0.98	1.97	AAA-AAA	0.00	999	1.000	1.000	0.4272
1560797_s_at	13.0	6.6	-0.98	-1.97	AAA-AAA	0.00	999	1.000	1.000	0.3973
1555338_s_at	19.1	9.7	-0.98	-1.97	AAA-AAA	0.00	999	1.000	1.000	0.3588
1552923_a_at	30.3	59.8	0.98	1.97	AAA-AAA	0.00	999	1.000	1.000	0.2496
1559676_a_at	13.6	26.8	0.98	1.97	AAA-AAA	0.00	999	1.000	1.000	0.3616
1558954_at	23.7	12.0	-0.98	-1.97	AAA-AAA	0.00	999	1.000	1.000	0.0856
207352_s_at	46.7	23.7	-0.98	-1.97	AAA-AAA	0.00	999	1.000	1.000	0.3751

239524_at	20.1	10.2	-0.98	-1.97	AAA-AAA	0.00	999	1.000	1.000	0.5795
239502_at	21.5	10.9	-0.98	-1.97	AAA-AAA	0.00	999	1.000	1.000	0.2569
239144_at	25.2	49.6	0.98	1.97	AAA-AAA	0.00	999	1.000	1.000	0.3961
239039_at	35.3	69.7	0.98	1.97	AAA-AAA	0.00	999	1.000	1.000	0.1956
241386_at	57.3	113.0	0.98	1.97	AAP-PPP	0.00	999	1.000	1.000	0.1747
240306_at	28.5	14.4	-0.98	-1.97	AAM-AAA	0.00	999	1.000	1.000	0.4967
1567055_at	17.8	35.2	0.98	1.97	AAA-AAA	0.00	999	1.000	1.000	0.3097
1554325_at	12.0	6.1	-0.98	-1.97	AAA-AAA	0.00	999	1.000	1.000	0.5077
244479_at	11.2	22.1	0.98	1.97	AAA-AAA	0.00	999	1.000	1.000	0.5850
1552837_at	5.5	10.8	0.98	1.97	AAA-AAA	0.00	999	1.000	1.000	0.5411
220708_at	98.8	50.1	-0.98	-1.97	AAA-AAA	0.00	999	1.000	1.000	0.1547
234504_at	8.4	4.3	-0.98	-1.97	AAA-AAA	0.00	999	1.000	1.000	0.4190
233728_at	28.5	14.5	-0.98	-1.97	AAA-AAA	0.00	999	1.000	1.000	0.3484
232178_at	22.4	44.1	0.98	1.97	AAA-AAA	0.00	999	1.000	1.000	0.4743
242135_at	17.8	35.1	0.98	1.97	AAA-AMA	0.00	999	1.000	1.000	0.3883
1569225_a_at	22.8	11.6	-0.98	-1.97	AAA-AAA	0.00	999	1.000	1.000	0.5148
1552766_at	1.3	2.7	0.98	1.97	AAA-AAA	0.00	999	1.000	1.000	0.4984
1557601_s_at	8.7	17.2	0.98	1.97	AAA-AAA	0.00	999	1.000	1.000	0.1984
208451_s_at	104.6	53.1	-0.98	-1.97	AAA-AAA	0.00	999	1.000	1.000	0.2258
235496_at	94.5	48.0	-0.98	-1.97	AAA-AAA	0.00	999	1.000	1.000	0.1084
234855_at	19.2	37.7	0.98	1.97	AAA-AAA	0.00	999	1.000	1.000	0.4215
237332_at	28.2	14.3	-0.98	-1.97	AAA-AAA	0.00	999	1.000	1.000	0.3516
237964_at	15.2	29.9	0.98	1.97	AAA-AAA	0.00	999	1.000	1.000	0.4234
237667_at	4.1	8.1	0.98	1.97	AAA-AAA	0.00	999	1.000	1.000	0.4959
230377_s_at	6.9	3.5	-0.98	-1.97	AAA-AAA	0.00	999	1.000	1.000	0.0608
229382_at	16.4	8.3	-0.98	-1.97	AAA-AAA	0.00	999	1.000	1.000	0.1198
239818_x_at	26.5	13.5	-0.98	-1.97	AAP-AAA	0.00	999	1.000	1.000	0.5852
218869_at	90.7	178.5	0.98	1.97	AAA-APP	0.00	999	1.000	1.000	0.1727
239916_at	31.2	15.9	-0.98	-1.97	AAA-AAA	0.00	999	1.000	1.000	0.1348
243068_at	12.0	6.1	-0.98	-1.97	AAA-AAA	0.00	999	1.000	1.000	0.1180
242461_at	21.0	10.7	-0.98	-1.97	AAA-AAA	0.00	999	1.000	1.000	0.3344
1569452_at	5.7	11.1	0.98	1.97	AAA-AAA	0.00	999	1.000	1.000	0.3235
221175_at	20.4	10.4	-0.98	-1.97	AAA-AAA	0.00	999	1.000	1.000	0.5078
234721_s_at	40.5	20.6	-0.98	-1.97	AAA-AAA	0.00	999	1.000	1.000	0.5078
231746_at	8.0	15.8	0.98	1.97	AAA-AAA	0.00	999	1.000	1.000	0.4094
231670_at	20.6	40.5	0.98	1.97	AAA-AAA	0.00	999	1.000	1.000	0.4716
240704_at	35.1	17.9	-0.98	-1.97	AAA-AAA	0.00	999	1.000	1.000	0.2409
240888_at	25.8	13.1	-0.98	-1.97	AAA-AAA	0.00	999	1.000	1.000	0.6217
243911_at	23.9	12.2	-0.98	-1.97	AAA-AAA	0.00	999	1.000	1.000	0.6110
1564758_at	15.2	7.7	-0.98	-1.97	AAA-AAA	0.00	999	1.000	1.000	0.4951
1552340_at	16.7	8.5	-0.98	-1.97	AAA-AAA	0.00	999	1.000	1.000	0.1932
244371_at	19.3	9.8	-0.98	-1.97	AAA-AAA	0.00	999	1.000	1.000	0.4989

1555431_a_at	16.9	8.6	-0.98	-1.97	AAA-AAA	0.00	999	1.000	1.000	0.6146
221221_s_at	61.3	31.2	-0.98	-1.97	AAA-AAA	0.00	999	1.000	1.000	0.2031
207521_s_at	53.3	27.1	-0.98	-1.97	AAA-AAA	0.00	999	1.000	1.000	0.4385
238263_at	17.0	8.7	-0.98	-1.97	AAA-AAA	0.00	999	1.000	1.000	0.4620
211379_x_at	17.4	34.3	0.98	1.97	APA-MAP	0.00	999	1.000	1.000	0.5916
221085_at	67.4	132.4	0.98	1.97	PAP-PPP	0.00	999	1.000	1.000	0.1267
203716_s_at	82.4	41.9	-0.98	-1.97	AAA-AAA	0.00	999	1.000	1.000	0.2711
1563559_at	7.2	3.6	-0.98	-1.97	AAA-AAA	0.00	999	1.000	1.000	0.4885
1555213_a_at	17.6	9.0	-0.98	-1.97	AAA-AAA	0.00	999	1.000	1.000	0.5078
1556571_at	10.6	5.4	-0.98	-1.97	AAA-AAA	0.00	999	1.000	1.000	0.4501
1556813_at	44.1	22.4	-0.98	-1.97	AAA-AAA	0.00	999	1.000	1.000	0.0180
221784_at	42.6	83.6	0.98	1.97	AAA-AAA	0.00	999	1.000	1.000	0.3318
207985_at	28.9	14.7	-0.98	-1.97	AAA-AAA	0.00	999	1.000	1.000	0.5100
224192_at	17.2	8.8	-0.98	-1.97	AAA-AAA	0.00	999	1.000	1.000	0.3500
207362_at	18.0	9.2	-0.98	-1.97	AAA-AAA	0.00	999	1.000	1.000	0.5134
224084_at	23.4	11.9	-0.98	-1.97	AAA-AAA	0.00	999	1.000	1.000	0.6491
235835_at	14.8	7.5	-0.98	-1.97	AAA-AAA	0.00	999	1.000	1.000	0.4386
234812_at	7.8	3.9	-0.98	-1.97	AAA-AAA	0.00	999	1.000	1.000	0.0392
237349_at	5.8	3.0	-0.98	-1.97	AAA-AAA	0.00	999	1.000	1.000	0.0970
230470_at	71.0	36.1	-0.98	-1.97	AAA-AAA	0.00	999	1.000	1.000	0.4157
227851_s_at	82.9	42.2	-0.98	-1.97	AAA-AAA	0.00	999	1.000	1.000	0.1075
204232_at	43.3	84.9	0.97	1.96	AAP-PPA	0.00	999	1.000	1.000	0.4919
205259_at	44.4	87.3	0.97	1.97	APP-PPP	0.00	999	1.000	1.000	0.4451
241492_at	16.2	31.8	0.97	1.97	AAA-PAA	0.00	999	1.000	1.000	0.3993
216266_s_at	262.4	515.3	0.97	1.96	PPP-PPP	0.00	999	1.000	1.000	0.2316
209483_s_at	18.2	35.8	0.97	1.97	AMA-PPA	0.00	999	1.000	1.000	0.1640
243297_at	23.2	11.8	-0.97	-1.97	AAA-AAA	0.00	999	1.000	1.000	0.4673
1560906_at	6.1	12.0	0.97	1.96	AAA-AAA	0.00	999	1.000	1.000	0.5544
1552390_a_at	23.3	45.7	0.97	1.96	AAA-MAA	0.00	999	1.000	1.000	0.0630
219516_at	14.0	27.5	0.97	1.97	AAA-AAA	0.00	999	1.000	1.000	0.4514
224055_x_at	50.2	25.5	-0.97	-1.96	AAA-AAA	0.00	999	1.000	1.000	0.3908
212741_at	26.5	52.0	0.97	1.96	AAA-AAA	0.00	999	1.000	1.000	0.3912
237812_at	3.5	6.9	0.97	1.97	AAA-AAA	0.00	999	1.000	1.000	0.5896
233145_at	5.9	11.5	0.97	1.97	AAA-AAA	0.00	999	1.000	1.000	0.4192
216967_at	12.2	6.2	-0.97	-1.96	APA-AAA	0.00	999	1.000	1.000	0.5911
1564224_x_at	99.6	50.7	-0.97	-1.96	AAA-AAA	0.00	999	1.000	1.000	0.2216
1554062_at	11.1	21.7	0.97	1.96	AAA-AAA	0.00	999	1.000	1.000	0.1189
1555096_at	17.1	33.6	0.97	1.96	AAA-AAA	0.00	999	1.000	1.000	0.4014
1559455_at	4.4	8.7	0.97	1.96	AAA-AAA	0.00	999	1.000	1.000	0.4374
1556488_s_at	31.7	16.2	-0.97	-1.96	AAA-AAA	0.00	999	1.000	1.000	0.3120
224476_s_at	33.0	64.7	0.97	1.96	AAA-AAA	0.00	999	1.000	1.000	0.3605
238373_at	7.9	4.0	-0.97	-1.96	AAA-AAA	0.00	999	1.000	1.000	0.3174

229772_at	4.6	8.9	0.97	1.96	AAA-AAA	0.00	999	1.000	1.000	0.3892
233767_at	11.9	6.1	-0.97	-1.96	AAA-AAA	0.00	999	1.000	1.000	0.2460
243630_at	71.1	139.6	0.97	1.96	AAA-APP	0.00	999	1.000	1.000	0.1121
1567270_at	6.2	12.2	0.97	1.96	AAA-AAA	0.00	999	1.000	1.000	0.3029
244415_at	27.6	14.1	-0.97	-1.96	AAA-AAA	0.00	999	1.000	1.000	0.4912
1559548_at	26.9	13.7	-0.97	-1.96	AAA-AAA	0.00	999	1.000	1.000	0.4660
221057_at	6.8	13.3	0.97	1.96	AAA-AAA	0.00	999	1.000	1.000	0.4454
223597_at	67.8	34.6	-0.97	-1.96	AAA-AAA	0.00	999	1.000	1.000	0.2664
224274_at	5.5	10.7	0.97	1.96	AAA-AAA	0.00	999	1.000	1.000	0.2668
209840_s_at	8.3	4.2	-0.97	-1.96	AAA-AAA	0.00	999	1.000	1.000	0.0177
217358_at	65.1	33.2	-0.97	-1.96	AAA-AAA	0.00	999	1.000	1.000	0.3165
208554_at	4.1	2.1	-0.97	-1.96	AAA-AAA	0.00	999	1.000	1.000	0.2925
230313_at	77.1	151.3	0.97	1.96	AAA-AAA	0.00	999	1.000	1.000	0.1047
230572_at	24.2	47.5	0.97	1.96	APA-APA	0.00	999	1.000	1.000	0.4777
203898_at	404.5	792.7	0.97	1.96	PPP-PPP	0.00	999	1.000	1.000	0.1490
244392_at	10.9	5.5	-0.97	-1.96	AAA-AAA	0.00	999	1.000	1.000	0.1832
1559205_s_at	20.7	40.6	0.97	1.96	AAA-AAA	0.00	999	1.000	1.000	0.3072
219651_at	3.5	1.8	-0.97	-1.96	AAA-AAA	0.00	999	1.000	1.000	0.0784
219689_at	25.3	49.6	0.97	1.96	AAA-AAA	0.00	999	1.000	1.000	0.3662
207696_at	3.7	1.9	-0.97	-1.96	AAA-AAA	0.00	999	1.000	1.000	0.5277
210194_at	4.8	2.4	-0.97	-1.96	AAA-AAA	0.00	999	1.000	1.000	0.3779
234510_at	18.8	9.6	-0.97	-1.96	AAA-AAA	0.00	999	1.000	1.000	0.6374
237963_x_at	11.4	5.8	-0.97	-1.96	AAA-AAA	0.00	999	1.000	1.000	0.5465
228929_at	89.6	45.7	-0.97	-1.96	AAA-AAA	0.00	999	1.000	1.000	0.3583
244095_at	27.5	53.8	0.97	1.96	AAA-AAA	0.00	999	1.000	1.000	0.4098
243255_at	23.4	45.8	0.97	1.96	AAA-AAA	0.00	999	1.000	1.000	0.1160
1567250_at	5.3	10.3	0.97	1.96	AAA-AAA	0.00	999	1.000	1.000	0.5115
1554564_a_at	7.3	14.3	0.97	1.96	AAA-AAA	0.00	999	1.000	1.000	0.5181
1558769_s_at	20.5	10.5	-0.97	-1.96	AAA-AAA	0.00	999	1.000	1.000	0.2492
1558963_at	15.9	8.1	-0.97	-1.96	AAA-AAA	0.00	999	1.000	1.000	0.5737
227480_at	17.7	34.7	0.97	1.96	AAA-AAA	0.00	999	1.000	1.000	0.1683
214465_at	13.3	6.8	-0.97	-1.96	AAA-AAA	0.00	999	1.000	1.000	0.4539
210370_s_at	13.8	7.1	-0.97	-1.96	AAA-AAA	0.00	999	1.000	1.000	0.5390
236353_at	13.4	6.8	-0.97	-1.96	AAA-AAA	0.00	999	1.000	1.000	0.0654
237880_at	16.3	32.0	0.97	1.96	AAA-AAA	0.00	999	1.000	1.000	0.3409
229439_s_at	30.8	15.7	-0.97	-1.96	AAA-AAA	0.00	999	1.000	1.000	0.3249
216772_at	18.6	36.3	0.97	1.96	APA-AAP	0.00	999	1.000	1.000	0.5468
243666_at	5.2	2.6	-0.97	-1.96	AAA-AAA	0.00	999	1.000	1.000	0.4547
1569802_at	13.0	6.6	-0.97	-1.96	AAA-AAA	0.00	999	1.000	1.000	0.5252
202192_s_at	126.1	64.4	-0.97	-1.96	AAA-AAA	0.00	999	1.000	1.000	0.1986
1558423_at	10.5	5.4	-0.97	-1.96	AAA-AAA	0.00	999	1.000	1.000	0.1347
220023_at	17.5	8.9	-0.97	-1.96	AAA-AAA	0.00	999	1.000	1.000	0.5163

212668_at	46.6	91.2	0.97	1.96	AAA-AAA	0.00	999	1.000	1.000	0.3202
210618_at	42.1	21.5	-0.97	-1.96	AAA-AAA	0.00	999	1.000	1.000	0.1247
215524_x_at	8.2	4.2	-0.97	-1.96	MAA-AAA	0.00	999	1.000	1.000	0.3119
216496_s_at	13.6	26.6	0.97	1.96	AAA-AAA	0.00	999	1.000	1.000	0.2325
236547_at	14.7	7.5	-0.97	-1.96	AAA-AAA	0.00	999	1.000	1.000	0.4419
234080_at	7.6	14.8	0.97	1.96	AAA-AAA	0.00	999	1.000	1.000	0.5276
238048_at	24.7	48.3	0.97	1.96	AAA-AAA	0.00	999	1.000	1.000	0.4822
230043_at	18.5	9.5	-0.97	-1.96	AAA-AAA	0.00	999	1.000	1.000	0.4181
225635_s_at	357.0	698.0	0.97	1.96	PPP-PPP	0.00	999	1.000	1.000	0.1472
240724_at	3.1	6.0	0.97	1.96	AAA-AAA	0.00	999	1.000	1.000	0.6682
243698_at	22.1	11.3	-0.97	-1.96	AAA-AAA	0.00	999	1.000	1.000	0.1866
1563546_at	4.6	2.3	-0.97	-1.96	AAA-AAA	0.00	999	1.000	1.000	0.5214
1560772_a_at	3.3	1.7	-0.97	-1.96	AAA-AAA	0.00	999	1.000	1.000	0.0206
1565680_at	74.9	38.3	-0.97	-1.96	AAA-AAA	0.00	999	1.000	1.000	0.5190
1554560_at	8.2	4.2	-0.97	-1.96	AAA-AAA	0.00	999	1.000	1.000	0.4531
1555527_at	10.9	5.6	-0.97	-1.96	AAA-AAA	0.00	999	1.000	1.000	0.5168
223753_s_at	21.0	41.0	0.97	1.96	AAA-AAA	0.00	999	1.000	1.000	0.3569
207258_at	10.6	5.4	-0.97	-1.96	AAA-AAA	0.00	999	1.000	1.000	0.0740
223889_at	31.8	16.3	-0.97	-1.96	AAA-AAA	0.00	999	1.000	1.000	0.5284
236775_s_at	14.9	29.2	0.97	1.96	AAA-AAA	0.00	999	1.000	1.000	0.4503
236303_at	114.0	58.3	-0.97	-1.96	AAA-AAA	0.00	999	1.000	1.000	0.0040
238957_at	9.6	4.9	-0.97	-1.96	AAA-AAA	0.00	999	1.000	1.000	0.3593
237396_at	6.3	12.3	0.97	1.96	AAA-AAA	0.00	999	1.000	1.000	0.3847
206292_s_at	12.9	6.6	-0.97	-1.96	AAA-AAA	0.00	999	1.000	1.000	0.5337
227878_s_at	369.2	721.4	0.97	1.95	APP-PPP	0.00	999	1.000	1.000	0.2222
204785_x_at	340.9	666.5	0.97	1.96	APA-PPP	0.00	999	1.000	1.000	0.1580
213469_at	121.8	238.1	0.97	1.96	PPP-PPP	0.00	999	1.000	1.000	0.0668
240913_at	12.1	6.2	-0.97	-1.95	AAA-AAA	0.00	999	1.000	1.000	0.5314
1562766_at	6.3	3.2	-0.97	-1.95	AAA-AAA	0.00	999	1.000	1.000	0.4666
1561472_at	28.6	14.6	-0.97	-1.95	AAA-AAA	0.00	999	1.000	1.000	0.5490
244364_at	25.3	12.9	-0.97	-1.95	AAA-AAA	0.00	999	1.000	1.000	0.0568
1552946_at	44.1	22.6	-0.97	-1.96	AAA-AAA	0.00	999	1.000	1.000	0.0621
1557301_a_at	11.8	6.0	-0.97	-1.96	AAA-AAA	0.00	999	1.000	1.000	0.6032
207380_x_at	11.1	21.8	0.97	1.95	AAA-AAA	0.00	999	1.000	1.000	0.1906
217277_at	10.1	19.8	0.97	1.96	AAA-AAA	0.00	999	1.000	1.000	0.1662
208563_x_at	4.9	9.6	0.97	1.96	AAA-AAA	0.00	999	1.000	1.000	0.2680
235247_at	10.4	5.3	-0.97	-1.95	AAA-AAA	0.00	999	1.000	1.000	0.2070
237306_at	5.1	10.0	0.97	1.96	AAA-AAA	0.00	999	1.000	1.000	0.3235
236959_s_at	9.8	5.0	-0.97	-1.96	AAA-AAA	0.00	999	1.000	1.000	0.2791
237129_at	34.4	17.6	-0.97	-1.96	AAA-AAA	0.00	999	1.000	1.000	0.3708
231992_x_at	415.7	212.6	-0.97	-1.96	AAA-AAA	0.00	999	1.000	1.000	0.0422
227361_at	80.2	156.7	0.97	1.95	PPA-PPP	0.00	999	1.000	1.000	0.0718

242692_at	96.4	49.4	-0.97	-1.95	AAA-AAA	0.00	999	1.000	1.000	0.0550
1562891_at	21.7	11.1	-0.97	-1.95	AAA-AAA	0.00	999	1.000	1.000	0.5039
1569750_at	33.5	17.1	-0.97	-1.95	AAA-AAA	0.00	999	1.000	1.000	0.5069
220779_at	7.5	14.6	0.97	1.95	AAA-AAA	0.00	999	1.000	1.000	0.2876
207407_x_at	44.4	22.7	-0.97	-1.95	AAA-AAA	0.00	999	1.000	1.000	0.4418
234185_at	9.1	4.6	-0.97	-1.95	AAA-AAA	0.00	999	1.000	1.000	0.2956
228442_at	47.6	93.0	0.97	1.95	AAA-AAA	0.00	999	1.000	1.000	0.0859
229469_at	102.3	52.4	-0.97	-1.95	AAA-AAA	0.00	999	1.000	1.000	0.1329
232488_at	162.7	83.3	-0.97	-1.95	AAA-AAA	0.00	999	1.000	1.000	0.0335
203264_s_at	49.8	97.2	0.97	1.95	APM-APP	0.00	999	1.000	1.000	0.4030
1562755_at	26.0	13.3	-0.97	-1.95	AAA-AAA	0.00	999	1.000	1.000	0.3283
1569624_at	21.7	11.1	-0.97	-1.95	AAA-AAA	0.00	999	1.000	1.000	0.5741
1568801_at	44.6	22.8	-0.97	-1.95	AAA-AAA	0.00	999	1.000	1.000	0.1467
1556451_at	45.1	23.1	-0.97	-1.95	AAA-AAA	0.00	999	1.000	1.000	0.0575
1556552_a_at	42.6	83.1	0.97	1.95	AAA-AAA	0.00	999	1.000	1.000	0.5198
222004_s_at	9.8	5.0	-0.97	-1.95	AAA-AAA	0.00	999	1.000	1.000	0.2137
219887_at	20.4	10.5	-0.97	-1.95	AAA-AAA	0.00	999	1.000	1.000	0.2374
222838_at	10.1	19.8	0.97	1.95	AAA-AAA	0.00	999	1.000	1.000	0.4795
214285_at	51.7	26.5	-0.97	-1.95	AAA-AAA	0.00	999	1.000	1.000	0.0267
215807_s_at	14.8	7.6	-0.97	-1.95	AAA-AAA	0.00	999	1.000	1.000	0.0504
216742_at	13.1	6.7	-0.97	-1.95	AAA-AAA	0.00	999	1.000	1.000	0.4306
234588_at	30.1	15.4	-0.97	-1.95	AAA-AAA	0.00	999	1.000	1.000	0.4083
228206_at	34.2	17.5	-0.97	-1.95	AAA-AAA	0.00	999	1.000	1.000	0.3187
232820_s_at	21.5	11.0	-0.97	-1.95	AAA-AAA	0.00	999	1.000	1.000	0.4059
231161_x_at	17.9	35.0	0.97	1.95	AAA-AAM	0.00	999	1.000	1.000	0.3426
225269_s_at	1968.4	3839.3	0.96	1.95	PPP-PPP	0.00	999	1.000	1.000	0.3158
211506_s_at	1254.5	2446.4	0.96	1.95	PPP-PPP	0.00	999	1.000	1.000	0.0614
1569042_at	4.3	2.2	-0.96	-1.95	AAA-AAA	0.00	999	1.000	1.000	0.2903
244713_at	51.0	26.2	-0.96	-1.95	AAA-AAA	0.00	999	1.000	1.000	0.5145
1552672_a_at	14.3	7.3	-0.96	-1.95	AAA-AAA	0.00	999	1.000	1.000	0.0942
1555869_a_at	22.8	11.7	-0.96	-1.95	AAA-AAA	0.00	999	1.000	1.000	0.3412
1556962_at	38.2	19.6	-0.96	-1.95	AAA-AAA	0.00	999	1.000	1.000	0.4678
1557451_at	2.8	5.4	0.96	1.95	AAA-AAA	0.00	999	1.000	1.000	0.3795
222945_x_at	59.7	30.6	-0.96	-1.95	AAA-AMA	0.00	999	1.000	1.000	0.3348
215262_at	38.4	74.9	0.96	1.95	AAA-AAA	0.00	999	1.000	1.000	0.3463
216167_at	9.5	4.9	-0.96	-1.95	AAA-AAA	0.00	999	1.000	1.000	0.5526
216016_at	13.4	6.9	-0.96	-1.95	AAA-AAA	0.00	999	1.000	1.000	0.5232
233950_at	13.2	25.7	0.96	1.95	AAA-AAA	0.00	999	1.000	1.000	0.5682
237137_at	24.9	12.7	-0.96	-1.95	AAA-AAA	0.00	999	1.000	1.000	0.5282
228362_s_at	12.5	6.4	-0.96	-1.95	AAA-AAA	0.00	999	1.000	1.000	0.4126
203393_at	11.6	6.0	-0.96	-1.95	AAA-AAA	0.00	999	1.000	1.000	0.0146
1563107_at	8.1	4.2	-0.96	-1.95	AAA-AAA	0.00	999	1.000	1.000	0.2117

1563154_at	4.9	2.5	-0.96	-1.95	AAA-AAA	0.00	999	1.000	1.000	0.4436
213381_at	12.4	6.3	-0.96	-1.95	AAA-AAA	0.00	999	1.000	1.000	0.3975
214819_at	85.3	43.8	-0.96	-1.95	AAA-AAA	0.00	999	1.000	1.000	0.0310
211209_x_at	40.9	21.0	-0.96	-1.95	AAA-AAA	0.00	999	1.000	1.000	0.3891
209458_x_at	19.1	37.2	0.96	1.95	AAA-AAA	0.00	999	1.000	1.000	0.1917
216405_at	67.5	34.6	-0.96	-1.95	AAM-AAA	0.00	999	1.000	1.000	0.1463
234195_at	9.9	5.1	-0.96	-1.95	AAA-AAA	0.00	999	1.000	1.000	0.0567
238971_at	16.5	8.5	-0.96	-1.95	AAA-AAA	0.00	999	1.000	1.000	0.3037
237659_at	12.2	6.2	-0.96	-1.95	AAA-AAA	0.00	999	1.000	1.000	0.1175
233031_at	64.9	33.3	-0.96	-1.95	AAA-AAA	0.00	999	1.000	1.000	0.4017
231066_s_at	22.5	43.8	0.96	1.95	AAA-AAA	0.00	999	1.000	1.000	0.3059
244849_at	33.9	66.0	0.96	1.95	AAP-AMA	0.00	999	1.000	1.000	0.4763
1568791_s_at	33.4	17.1	-0.96	-1.95	AAA-AAA	0.00	999	1.000	1.000	0.5037
221349_at	19.5	10.0	-0.96	-1.95	AAA-AAA	0.00	999	1.000	1.000	0.4828
220751_s_at	29.9	15.3	-0.96	-1.95	AAA-AAA	0.00	999	1.000	1.000	0.1540
223708_at	7.8	4.0	-0.96	-1.95	AAA-AAA	0.00	999	1.000	1.000	0.0644
235291_s_at	58.4	113.7	0.96	1.95	AAA-AAA	0.00	999	1.000	1.000	0.4463
230550_at	20.4	10.5	-0.96	-1.95	AAA-AAA	0.00	999	1.000	1.000	0.5698
228707_at	9.7	18.9	0.96	1.95	AAA-AAA	0.00	999	1.000	1.000	0.4343
233619_at	15.0	7.7	-0.96	-1.95	AAA-AAA	0.00	999	1.000	1.000	0.1250
233314_at	10.7	20.8	0.96	1.95	AAA-AAA	0.00	999	1.000	1.000	0.4949
218348_s_at	1136.9	2212.6	0.96	1.95	PPP-PPP	0.00	999	1.000	1.000	0.1486
241001_at	12.5	6.4	-0.96	-1.95	AAA-AAA	0.00	999	1.000	1.000	0.3208
243523_at	136.3	70.0	-0.96	-1.95	AAA-AAA	0.00	999	1.000	1.000	0.0239
1562095_at	32.6	16.8	-0.96	-1.95	AMA-AAA	0.00	999	1.000	1.000	0.0427
1557821_at	15.7	8.1	-0.96	-1.95	AAA-AAA	0.00	999	1.000	1.000	0.6151
204860_s_at	18.0	35.1	0.96	1.95	AAA-AAA	0.00	999	1.000	1.000	0.3212
205972_at	18.8	9.7	-0.96	-1.95	AAA-AAA	0.00	999	1.000	1.000	0.2062
237195_at	6.6	12.8	0.96	1.95	AAA-AAP	0.00	999	1.000	1.000	0.5700
242483_at	11.0	5.7	-0.96	-1.95	AAA-AAA	0.00	999	1.000	1.000	0.5812
243754_at	59.7	30.7	-0.96	-1.95	AAA-AAA	0.00	999	1.000	1.000	0.0459
1562032_at	8.6	4.4	-0.96	-1.95	AAA-AAA	0.00	999	1.000	1.000	0.5051
1563018_at	13.2	6.8	-0.96	-1.95	AAA-AAA	0.00	999	1.000	1.000	0.5350
1561861_a_at	6.1	3.1	-0.96	-1.95	AAA-AAA	0.00	999	1.000	1.000	0.3846
1565581_at	52.5	27.0	-0.96	-1.95	AAA-AAA	0.00	999	1.000	1.000	0.1987
1566969_at	2.7	5.2	0.96	1.95	AAA-AAA	0.00	999	1.000	1.000	0.5644
1567297_at	24.4	12.5	-0.96	-1.95	AAA-AAA	0.00	999	1.000	1.000	0.3628
1567390_at	28.4	14.6	-0.96	-1.95	AAA-AAA	0.00	999	1.000	1.000	0.4939
1552388_at	38.1	19.6	-0.96	-1.95	AAA-AAA	0.00	999	1.000	1.000	0.4128
224553_s_at	23.2	45.1	0.96	1.95	AAA-AAA	0.00	999	1.000	1.000	0.0717
209975_at	46.5	23.9	-0.96	-1.95	AAA-AAA	0.00	999	1.000	1.000	0.4188
204551_s_at	11.2	21.8	0.96	1.95	AAA-AAA	0.00	999	1.000	1.000	0.5851

234861_at	7.6	3.9	-0.96	-1.95	AAA-AAA	0.00	999	1.000	1.000	0.4836
237174_at	26.5	13.6	-0.96	-1.95	AAA-AAA	0.00	999	1.000	1.000	0.6170
206276_at	16.3	8.4	-0.96	-1.95	AAA-AAA	0.00	999	1.000	1.000	0.3340
231096_at	29.0	14.9	-0.96	-1.95	AAA-AAA	0.00	999	1.000	1.000	0.2882
213267_at	15.0	29.2	0.96	1.95	AAP-AAP	0.00	999	1.000	1.000	0.5859
213310_at	573.7	1114.9	0.96	1.94	PPP-PPP	0.00	999	1.000	1.000	0.2034
226470_at	60.9	118.5	0.96	1.94	AAA-PAA	0.00	999	1.000	1.000	0.1948
243950_at	18.0	35.0	0.96	1.94	AAA-AAA	0.00	999	1.000	1.000	0.2942
240001_at	12.1	6.2	-0.96	-1.94	AAA-AAA	0.00	999	1.000	1.000	0.0704
241884_at	54.4	28.0	-0.96	-1.94	AAA-AAA	0.00	999	1.000	1.000	0.1702
240491_at	56.2	28.9	-0.96	-1.94	AAA-AAA	0.00	999	1.000	1.000	0.3705
1552798_a_at	20.8	40.4	0.96	1.94	AAA-AAA	0.00	999	1.000	1.000	0.3234
207731_at	14.3	7.4	-0.96	-1.94	AAA-AAA	0.00	999	1.000	1.000	0.5260
226162_at	98.2	50.5	-0.96	-1.94	AAA-AAA	0.00	999	1.000	1.000	0.1866
223536_at	20.5	10.6	-0.96	-1.94	AAA-AAA	0.00	999	1.000	1.000	0.4694
224209_s_at	13.2	25.6	0.96	1.94	AAA-AAA	0.00	999	1.000	1.000	0.5492
216919_at	70.6	36.3	-0.96	-1.94	AAA-AAA	0.00	999	1.000	1.000	0.3849
234533_at	19.6	10.1	-0.96	-1.94	AAA-AAA	0.00	999	1.000	1.000	0.3396
238487_at	52.9	27.2	-0.96	-1.94	AAA-AAA	0.00	999	1.000	1.000	0.4459
204456_s_at	6.7	3.4	-0.96	-1.94	AAA-AAA	0.00	999	1.000	1.000	0.0868
237489_at	10.9	5.6	-0.96	-1.94	AAA-AAA	0.00	999	1.000	1.000	0.5551
228224_at	18.8	9.7	-0.96	-1.94	AAA-AAA	0.00	999	1.000	1.000	0.3691
244228_at	12.0	23.3	0.96	1.94	AAA-PAA	0.00	999	1.000	1.000	0.5882
240195_at	8.0	4.1	-0.96	-1.94	AAA-AAA	0.00	999	1.000	1.000	0.5833
1555120_at	8.8	4.5	-0.96	-1.94	AAA-AAA	0.00	999	1.000	1.000	0.5603
221661_at	15.6	30.2	0.96	1.94	AAA-AAA	0.00	999	1.000	1.000	0.2359
226522_at	39.8	20.5	-0.96	-1.94	AAA-AAA	0.00	999	1.000	1.000	0.4470
215423_at	16.2	8.4	-0.96	-1.94	AAA-AAA	0.00	999	1.000	1.000	0.3778
235575_at	9.4	18.2	0.96	1.94	AAA-AAA	0.00	999	1.000	1.000	0.5650
239665_at	12.1	6.2	-0.96	-1.94	AAA-AAA	0.00	999	1.000	1.000	0.3640
229542_at	13.0	6.7	-0.96	-1.94	AAA-AAA	0.00	999	1.000	1.000	0.3826
232767_at	49.6	25.6	-0.96	-1.94	AAA-AAA	0.00	999	1.000	1.000	0.4073
231241_at	55.0	28.3	-0.96	-1.94	AAA-AAA	0.00	999	1.000	1.000	0.3799
241084_x_at	43.1	83.7	0.96	1.94	AAM-AAA	0.00	999	1.000	1.000	0.5589
1560861_at	7.8	4.0	-0.96	-1.94	AAA-AAA	0.00	999	1.000	1.000	0.3444
1560328_s_at	3.5	1.8	-0.96	-1.94	AAA-AAA	0.00	999	1.000	1.000	0.5050
207066_at	15.5	8.0	-0.96	-1.94	AAA-AAA	0.00	999	1.000	1.000	0.3566
213375_s_at	42.4	21.9	-0.96	-1.94	AAA-AAA	0.00	999	1.000	1.000	0.0677
215778_x_at	39.2	76.1	0.96	1.94	AAA-AAA	0.00	999	1.000	1.000	0.5847
216353_s_at	6.4	12.3	0.96	1.94	AAA-AAA	0.00	999	1.000	1.000	0.4983
239281_at	40.5	20.9	-0.96	-1.94	AAA-AAA	0.00	999	1.000	1.000	0.3673
230915_at	19.5	10.0	-0.96	-1.94	AAA-AAA	0.00	999	1.000	1.000	0.3997

242088_at	11.3	5.8	-0.96	-1.94	APA-PAA	0.00	999	1.000	1.000	0.6006
228923_at	34.1	66.1	0.96	1.94	AAA-APP	0.00	999	1.000	1.000	0.3098
204245_s_at	654.9	1270.2	0.96	1.94	APP-PPP	0.00	999	1.000	1.000	0.2896
204506_at	214.7	416.5	0.96	1.94	PPP-PPP	0.00	999	1.000	1.000	0.0846
243468_at	24.2	12.5	-0.96	-1.94	AAA-AAA	0.00	999	1.000	1.000	0.5015
1553498_at	10.3	20.0	0.96	1.94	AAA-AAA	0.00	999	1.000	1.000	0.6177
1553380_at	4.6	2.4	-0.96	-1.94	AAA-AAA	0.00	999	1.000	1.000	0.4473
1559220_at	41.3	80.2	0.96	1.94	AAA-AAA	0.00	999	1.000	1.000	0.2457
1556677_at	21.2	41.1	0.96	1.94	AAA-AAA	0.00	999	1.000	1.000	0.4002
221422_s_at	15.6	8.1	-0.96	-1.94	AAA-AAA	0.00	999	1.000	1.000	0.2477
221461_at	48.1	24.8	-0.96	-1.94	AAA-AAA	0.00	999	1.000	1.000	0.1988
208429_x_at	38.1	73.8	0.96	1.94	AAA-AAA	0.00	999	1.000	1.000	0.1152
220634_at	14.2	7.3	-0.96	-1.94	AAA-AAA	0.00	999	1.000	1.000	0.3858
220438_at	100.7	51.9	-0.96	-1.94	AAA-AAA	0.00	999	1.000	1.000	0.0538
209933_s_at	39.3	20.3	-0.96	-1.94	AAA-AAA	0.00	999	1.000	1.000	0.3630
211239_s_at	9.2	4.7	-0.96	-1.94	AAA-AAA	0.00	999	1.000	1.000	0.3542
217552_x_at	13.0	6.7	-0.96	-1.94	AAA-AAA	0.00	999	1.000	1.000	0.5881
215325_x_at	14.0	27.1	0.96	1.94	AAA-AAA	0.00	999	1.000	1.000	0.3253
233913_at	14.2	7.3	-0.96	-1.94	AAA-AAA	0.00	999	1.000	1.000	0.4581
237774_at	24.0	12.4	-0.96	-1.94	AAA-AAA	0.00	999	1.000	1.000	0.3834
229278_at	15.1	7.8	-0.96	-1.94	AAA-AAA	0.00	999	1.000	1.000	0.2789
202660_at	193.0	374.2	0.96	1.94	PPP-PPP	0.00	999	1.000	1.000	0.0973
1569339_s_at	81.6	42.1	-0.96	-1.94	AAA-AAM	0.00	999	1.000	1.000	0.1377
1553770_a_at	107.7	55.5	-0.96	-1.94	AAA-AAA	0.00	999	1.000	1.000	0.0304
1554110_at	36.9	71.6	0.96	1.94	AAA-AAA	0.00	999	1.000	1.000	0.2899
1553388_at	14.2	7.3	-0.96	-1.94	AAA-AAA	0.00	999	1.000	1.000	0.4269
1554319_at	11.3	21.8	0.96	1.94	AAA-AAA	0.00	999	1.000	1.000	0.4281
1555994_at	15.9	8.2	-0.96	-1.94	AAA-AAA	0.00	999	1.000	1.000	0.4703
224344_at	8.4	4.3	-0.96	-1.94	AAA-AAA	0.00	999	1.000	1.000	0.3621
216827_at	9.9	5.1	-0.96	-1.94	AAA-AAA	0.00	999	1.000	1.000	0.1527
235737_at	10.1	19.5	0.96	1.94	AAA-AAA	0.00	999	1.000	1.000	0.4385
235976_at	9.6	4.9	-0.96	-1.94	AAA-AAA	0.00	999	1.000	1.000	0.5405
204118_at	62.3	32.2	-0.96	-1.94	AAA-AAA	0.00	999	1.000	1.000	0.4101
239315_at	13.0	6.7	-0.96	-1.94	AAA-AAA	0.00	999	1.000	1.000	0.1401
230521_at	69.6	35.9	-0.96	-1.94	AAA-AAA	0.00	999	1.000	1.000	0.3066
231522_at	37.7	19.4	-0.96	-1.94	AAA-AAA	0.00	999	1.000	1.000	0.2131
240657_at	9.7	18.9	0.95	1.94	MAA-AAP	0.00	999	1.000	1.000	0.3965
210222_s_at	16.7	32.3	0.95	1.94	APA-PAA	0.00	999	1.000	1.000	0.3062
237466_s_at	67.6	130.9	0.95	1.94	APA-PPP	0.00	999	1.000	1.000	0.2448
201540_at	202.2	391.6	0.95	1.94	PPP-PPP	0.00	999	1.000	1.000	0.1389
209672_s_at	52.0	100.8	0.95	1.94	MPM-PAP	0.00	999	1.000	1.000	0.1221
241537_at	1.9	3.7	0.95	1.94	AAA-AAA	0.00	999	1.000	1.000	0.4308

241049_at	37.0	19.1	-0.95	-1.94	AAA-AAA	0.00	999	1.000	1.000	0.4297
240130_at	13.4	6.9	-0.95	-1.94	AAA-AAA	0.00	999	1.000	1.000	0.5541
1562173_a_at	12.0	23.3	0.95	1.94	AAA-AAA	0.00	999	1.000	1.000	0.2848
1560989_at	6.6	3.4	-0.95	-1.94	AAA-AAA	0.00	999	1.000	1.000	0.3531
1564876_s_at	28.6	14.8	-0.95	-1.94	AAA-AAA	0.00	999	1.000	1.000	0.4565
244666_at	15.7	8.1	-0.95	-1.94	AAA-AAA	0.00	999	1.000	1.000	0.4191
1553355_at	11.1	21.6	0.95	1.94	AAA-AAA	0.00	999	1.000	1.000	0.4121
1556436_at	61.7	31.8	-0.95	-1.94	AAA-AAA	0.00	999	1.000	1.000	0.3478
220478_at	14.6	7.5	-0.95	-1.94	AAA-AAA	0.00	999	1.000	1.000	0.0912
224501_at	13.5	7.0	-0.95	-1.94	AAA-AAA	0.00	999	1.000	1.000	0.0107
210992_x_at	48.5	25.0	-0.95	-1.94	AAA-AAA	0.00	999	1.000	1.000	0.3655
216113_at	12.2	23.6	0.95	1.94	AAA-MAA	0.00	999	1.000	1.000	0.5734
216349_at	292.9	151.2	-0.95	-1.94	AAA-AAA	0.00	999	1.000	1.000	0.0320
238993_at	16.7	8.6	-0.95	-1.94	AAA-AAA	0.00	999	1.000	1.000	0.5471
230732_s_at	7.5	3.9	-0.95	-1.94	AAA-AAA	0.00	999	1.000	1.000	0.4485
231409_at	6.2	11.9	0.95	1.94	AAA-AAA	0.00	999	1.000	1.000	0.3934
234950_s_at	786.2	1521.9	0.95	1.94	PPP-PPP	0.00	999	1.000	1.000	0.3370
204876_at	51.9	100.4	0.95	1.94	AAA-APP	0.00	999	1.000	1.000	0.2096
222235_s_at	947.1	1833.4	0.95	1.94	PPP-PPP	0.00	999	1.000	1.000	0.1717
201055_s_at	937.5	1814.7	0.95	1.94	PPP-PPP	0.00	999	1.000	1.000	0.1228
239836_at	11.0	5.7	-0.95	-1.94	AAA-AAA	0.00	999	1.000	1.000	0.3509
243064_at	27.3	52.9	0.95	1.94	AAA-AAA	0.00	999	1.000	1.000	0.0791
1562713_a_at	35.6	18.4	-0.95	-1.94	AAA-AAA	0.00	999	1.000	1.000	0.3793
1561234_at	3.3	1.7	-0.95	-1.94	AAA-AAA	0.00	999	1.000	1.000	0.2274
1553363_at	10.2	5.3	-0.95	-1.94	AAA-AAA	0.00	999	1.000	1.000	0.1832
1557809_a_at	96.4	49.8	-0.95	-1.94	AAA-AAA	0.00	999	1.000	1.000	0.1868
1559166_at	15.6	8.1	-0.95	-1.94	AAA-AAA	0.00	999	1.000	1.000	0.0618
207950_s_at	6.8	13.1	0.95	1.94	AAA-AAA	0.00	999	1.000	1.000	0.3862
224113_at	29.5	15.2	-0.95	-1.94	AAA-AAA	0.00	999	1.000	1.000	0.0646
214154_s_at	31.4	16.2	-0.95	-1.94	AAA-AAA	0.00	999	1.000	1.000	0.5393
234774_at	18.8	9.7	-0.95	-1.94	AAA-AAA	0.00	999	1.000	1.000	0.0467
238718_at	17.0	8.8	-0.95	-1.94	AAA-AAA	0.00	999	1.000	1.000	0.3897
237362_at	130.2	67.2	-0.95	-1.94	AAA-AAA	0.00	999	1.000	1.000	0.0875
205200_at	26.1	13.5	-0.95	-1.94	AAA-AAA	0.00	999	1.000	1.000	0.5558
1570534_a_at	24.5	47.5	0.95	1.93	MAA-APA	0.00	999	1.000	1.000	0.5475
1570490_at	26.8	51.8	0.95	1.94	AAA-APA	0.00	999	1.000	1.000	0.4471
202586_at	279.5	540.6	0.95	1.93	APP-PPP	0.00	999	1.000	1.000	0.2422
213471_at	124.0	239.9	0.95	1.94	APP-PPP	0.00	999	1.000	1.000	0.0666
241275_at	5.2	10.0	0.95	1.93	AAA-AAA	0.00	999	1.000	1.000	0.6096
1561542_at	30.9	16.0	-0.95	-1.93	AAA-AAA	0.00	999	1.000	1.000	0.4730
1565848_at	89.8	46.4	-0.95	-1.94	AAA-AAA	0.00	999	1.000	1.000	0.2042
226141_at	18.4	9.5	-0.95	-1.93	AAA-AAA	0.00	999	1.000	1.000	0.2219

224550_s_at	16.3	8.5	-0.95	-1.94	AAA-AAA	0.00	999	1.000	1.000	0.4552
206714_at	38.1	19.7	-0.95	-1.93	AAA-AAA	0.00	999	1.000	1.000	0.4259
223127_s_at	27.1	14.0	-0.95	-1.93	AAA-AAA	0.00	999	1.000	1.000	0.3850
217290_at	7.7	14.8	0.95	1.94	AAA-AAA	0.00	999	1.000	1.000	0.2536
237350_at	16.9	8.7	-0.95	-1.94	AAA-AAA	0.00	999	1.000	1.000	0.3024
230073_at	38.1	19.7	-0.95	-1.94	MAA-AAA	0.00	999	1.000	1.000	0.5436
230531_at	16.0	30.9	0.95	1.93	AAA-AAA	0.00	999	1.000	1.000	0.2939
1566637_at	5.0	9.7	0.95	1.93	AAA-AAA	0.00	999	1.000	1.000	0.5960
1552666_a_at	9.3	4.8	-0.95	-1.93	AAA-AAA	0.00	999	1.000	1.000	0.3164
222373_at	9.6	18.6	0.95	1.93	AAA-AAA	0.00	999	1.000	1.000	0.3598
214189_s_at	20.7	10.7	-0.95	-1.93	AAA-AAA	0.00	999	1.000	1.000	0.5962
211550_at	14.2	7.3	-0.95	-1.93	AAA-AAA	0.00	999	1.000	1.000	0.1374
210528_at	11.3	21.8	0.95	1.93	AAA-AAA	0.00	999	1.000	1.000	0.4124
236317_at	26.0	50.3	0.95	1.93	AAA-AAA	0.00	999	1.000	1.000	0.2538
233928_at	14.7	7.6	-0.95	-1.93	AAA-AAA	0.00	999	1.000	1.000	0.4536
1553491_at	31.3	60.4	0.95	1.93	PAA-PPM	0.00	999	1.000	1.000	0.3596
241410_at	35.6	18.4	-0.95	-1.93	AAA-AAA	0.00	999	1.000	1.000	0.4869
240644_at	83.4	43.2	-0.95	-1.93	AAA-AAA	0.00	999	1.000	1.000	0.3043
1555049_at	49.4	25.6	-0.95	-1.93	AAA-AAA	0.00	999	1.000	1.000	0.3902
1564150_a_at	3.0	5.8	0.95	1.93	AAA-AAA	0.00	999	1.000	1.000	0.4100
221162_at	62.9	32.6	-0.95	-1.93	AAA-AAA	0.00	999	1.000	1.000	0.2496
207599_at	5.8	3.0	-0.95	-1.93	AAA-AAA	0.00	999	1.000	1.000	0.4257
217361_at	30.1	15.6	-0.95	-1.93	AAA-AAA	0.00	999	1.000	1.000	0.4116
216837_at	65.5	33.9	-0.95	-1.93	AAA-AAA	0.00	999	1.000	1.000	0.1451
232407_at	63.2	32.7	-0.95	-1.93	AAA-AAA	0.00	999	1.000	1.000	0.3952
1558372_at	7.9	4.1	-0.95	-1.93	PAA-AAA	0.00	999	1.000	1.000	0.6625
241195_at	21.2	11.0	-0.95	-1.93	AAA-AAA	0.00	999	1.000	1.000	0.3728
1562532_at	58.8	30.5	-0.95	-1.93	AAA-AAA	0.00	999	1.000	1.000	0.2106
1560771_at	12.1	6.2	-0.95	-1.93	AAA-AAA	0.00	999	1.000	1.000	0.6043
1569245_at	48.0	24.9	-0.95	-1.93	AAA-AAA	0.00	999	1.000	1.000	0.1030
1565748_at	39.1	20.3	-0.95	-1.93	AAA-AAA	0.00	999	1.000	1.000	0.1588
1553914_at	27.3	14.1	-0.95	-1.93	AAA-AAA	0.00	999	1.000	1.000	0.2654
1555627_s_at	19.7	10.2	-0.95	-1.93	AAA-AAA	0.00	999	1.000	1.000	0.3716
1559689_a_at	22.3	11.6	-0.95	-1.93	AAA-AAA	0.00	999	1.000	1.000	0.4343
213450_s_at	63.7	33.0	-0.95	-1.93	AAA-AAA	0.00	999	1.000	1.000	0.3864
211616_s_at	27.3	14.2	-0.95	-1.93	AAA-AAA	0.00	999	1.000	1.000	0.3044
210412_at	59.9	31.0	-0.95	-1.93	AAA-AAA	0.00	999	1.000	1.000	0.1490
216443_at	35.0	18.2	-0.95	-1.93	AAA-AAA	0.00	999	1.000	1.000	0.1509
234709_at	4.1	8.0	0.95	1.93	AAA-AAA	0.00	999	1.000	1.000	0.5771
237724_at	8.3	4.3	-0.95	-1.93	AAA-AAA	0.00	999	1.000	1.000	0.2060
242096_at	52.7	27.3	-0.95	-1.93	AAA-AAA	0.00	999	1.000	1.000	0.3212
240357_at	97.3	50.4	-0.95	-1.93	AAA-AAA	0.00	999	1.000	1.000	0.1822

242734_x_at	7.4	14.3	0.95	1.93	AAA-AAA	0.00	999	1.000	1.000	0.4961
1569859_at	15.9	8.2	-0.95	-1.93	AAA-AAA	0.00	999	1.000	1.000	0.2366
1565818_s_at	53.4	27.7	-0.95	-1.93	AAA-AAA	0.00	999	1.000	1.000	0.2174
221177_at	6.9	13.4	0.95	1.93	AAA-AAA	0.00	999	1.000	1.000	0.4518
222189_at	8.4	16.2	0.95	1.93	AAA-AAA	0.00	999	1.000	1.000	0.4498
207670_at	95.1	49.3	-0.95	-1.93	MAA-AAA	0.00	999	1.000	1.000	0.0824
216083_s_at	24.2	46.7	0.95	1.93	AAA-AAA	0.00	999	1.000	1.000	0.0967
238745_at	61.8	32.0	-0.95	-1.93	AAA-AAA	0.00	999	1.000	1.000	0.1944
237845_at	19.7	10.2	-0.95	-1.93	AAA-AAA	0.00	999	1.000	1.000	0.6084
237487_at	134.7	69.8	-0.95	-1.93	AAA-AAA	0.00	999	1.000	1.000	0.2944
229947_at	10.1	5.2	-0.95	-1.93	AAA-AAA	0.00	999	1.000	1.000	0.5749
202661_at	36.2	69.8	0.95	1.93	APA-APA	0.00	999	1.000	1.000	0.3056
232309_at	103.6	199.7	0.95	1.93	AAA-APA	0.00	999	1.000	1.000	0.0714
241409_at	32.3	62.2	0.95	1.93	AAA-AAA	0.00	999	1.000	1.000	0.1627
1562440_at	8.7	4.5	-0.95	-1.93	AAA-AAA	0.00	999	1.000	1.000	0.5806
213613_s_at	54.9	28.5	-0.95	-1.93	AAA-AAA	0.00	999	1.000	1.000	0.2246
210447_at	16.0	8.3	-0.95	-1.93	AAA-AAA	0.00	999	1.000	1.000	0.4911
213232_at	25.6	13.3	-0.95	-1.93	AAA-AAA	0.00	999	1.000	1.000	0.2939
217308_at	6.6	12.7	0.95	1.93	AAA-AAA	0.00	999	1.000	1.000	0.4334
216439_at	19.7	37.9	0.95	1.93	AAA-AAA	0.00	999	1.000	1.000	0.4227
237033_at	8.3	4.3	-0.95	-1.93	AAA-AAA	0.00	999	1.000	1.000	0.0460
231739_at	7.9	15.3	0.95	1.93	AAA-AAA	0.00	999	1.000	1.000	0.4308
1560184_at	10.7	20.6	0.95	1.93	PAA-PAP	0.00	999	1.000	1.000	0.4550
230647_at	33.6	64.7	0.95	1.93	AAA-AAP	0.00	999	1.000	1.000	0.3898
209034_at	483.6	931.4	0.95	1.93	PPP-PPP	0.00	999	1.000	1.000	0.2928
222548_s_at	578.8	1114.9	0.95	1.93	PPP-PPP	0.00	999	1.000	1.000	0.2333
202446_s_at	749.6	1444.0	0.95	1.93	PPP-PPP	0.00	999	1.000	1.000	0.1898
1554345_a_at	194.5	374.7	0.95	1.93	PPP-PPP	0.00	999	1.000	1.000	0.1339
243788_at	12.4	23.9	0.95	1.93	AAA-AAA	0.00	999	1.000	1.000	0.2205
1560590_s_at	27.9	53.8	0.95	1.93	AAA-AAA	0.00	999	1.000	1.000	0.1893
1564691_at	29.9	15.5	-0.95	-1.93	AAA-AAA	0.00	999	1.000	1.000	0.4288
1555262_a_at	27.8	14.4	-0.95	-1.93	AAA-AAA	0.00	999	1.000	1.000	0.4490
1552695_a_at	8.6	16.6	0.95	1.93	AAA-AAA	0.00	999	1.000	1.000	0.5210
1556622_s_at	5.2	10.0	0.95	1.93	AAA-AAA	0.00	999	1.000	1.000	0.1985
210327_s_at	46.9	24.4	-0.95	-1.93	AAA-AAA	0.00	999	1.000	1.000	0.5429
235080_at	23.2	12.0	-0.95	-1.93	AAA-AAA	0.00	999	1.000	1.000	0.5165
234708_at	14.4	7.5	-0.95	-1.93	AAA-AAA	0.00	999	1.000	1.000	0.4473
230467_at	75.3	39.1	-0.95	-1.93	AAA-AAA	0.00	999	1.000	1.000	0.2813
228844_at	19.2	10.0	-0.95	-1.93	AAA-AAA	0.00	999	1.000	1.000	0.5034
243117_at	13.7	26.3	0.95	1.93	AAA-PAA	0.00	999	1.000	1.000	0.2864
240903_at	17.9	9.3	-0.95	-1.93	AAA-AAA	0.00	999	1.000	1.000	0.4201
1561476_at	5.1	9.7	0.95	1.93	AAA-AAA	0.00	999	1.000	1.000	0.4499

1553593_a_at	11.3	5.9	-0.95	-1.93	AAA-AAA	0.00	999	1.000	1.000	0.1459
1554652_s_at	20.1	10.4	-0.95	-1.93	AAA-AAA	0.00	999	1.000	1.000	0.5062
1553058_at	50.6	26.3	-0.95	-1.93	AAA-AAA	0.00	999	1.000	1.000	0.5295
207978_s_at	18.6	9.7	-0.95	-1.93	AAA-AAA	0.00	999	1.000	1.000	0.3200
234016_at	11.5	6.0	-0.95	-1.93	AAA-AAA	0.00	999	1.000	1.000	0.4418
238128_at	10.5	20.3	0.95	1.93	AAA-AAA	0.00	999	1.000	1.000	0.2573
209552_at	31.7	60.9	0.94	1.92	AAA-APA	0.00	999	1.000	1.000	0.4509
221666_s_at	91.5	176.1	0.94	1.92	AAA-APA	0.00	999	1.000	1.000	0.0627
1560924_at	46.0	23.9	-0.94	-1.92	AAA-AAA	0.00	999	1.000	1.000	0.3557
1569663_at	7.2	3.8	-0.94	-1.92	AAA-AAA	0.00	999	1.000	1.000	0.5198
1570276_a_at	13.1	25.1	0.94	1.92	AAA-AAA	0.00	999	1.000	1.000	0.2126
1566419_at	9.6	5.0	-0.94	-1.92	AAA-AAA	0.00	999	1.000	1.000	0.1441
1556299_s_at	6.9	13.3	0.94	1.92	AAA-AAA	0.00	999	1.000	1.000	0.4118
220333_at	41.7	80.1	0.94	1.92	AAA-AAA	0.00	999	1.000	1.000	0.3624
227275_at	73.9	38.4	-0.94	-1.92	AAA-AAA	0.00	999	1.000	1.000	0.0940
206641_at	8.4	4.4	-0.94	-1.92	AAA-AAA	0.00	999	1.000	1.000	0.4491
217305_s_at	16.0	30.9	0.94	1.93	AAA-AAA	0.00	999	1.000	1.000	0.5938
233972_s_at	3.3	6.3	0.94	1.92	AAA-AAA	0.00	999	1.000	1.000	0.4941
237405_at	4.9	9.5	0.94	1.92	AAA-AAA	0.00	999	1.000	1.000	0.3473
228198_s_at	18.0	9.4	-0.94	-1.92	AAA-AAA	0.00	999	1.000	1.000	0.5622
233082_at	6.2	3.2	-0.94	-1.92	AAA-AAA	0.00	999	1.000	1.000	0.2296
233795_at	105.7	54.9	-0.94	-1.92	AAA-AAA	0.00	999	1.000	1.000	0.2806
1563052_at	20.1	10.4	-0.94	-1.92	AAA-AAP	0.00	999	1.000	1.000	0.6183
1555247_a_at	511.5	983.3	0.94	1.92	PPP-PPP	0.00	999	1.000	1.000	0.4371
236138_at	18.4	35.4	0.94	1.92	AAA-AAP	0.00	999	1.000	1.000	0.3529
223391_at	1344.4	2583.8	0.94	1.92	APP-PAP	0.00	999	1.000	1.000	0.1432
244042_x_at	6.8	3.5	-0.94	-1.92	AAA-AAA	0.00	999	1.000	1.000	0.5777
202992_at	11.2	5.8	-0.94	-1.92	AAA-AAA	0.00	999	1.000	1.000	0.2362
1563265_at	8.8	4.6	-0.94	-1.92	AAA-AAA	0.00	999	1.000	1.000	0.4831
1560751_at	37.8	19.7	-0.94	-1.92	AAA-AAA	0.00	999	1.000	1.000	0.2655
1561436_at	44.6	23.2	-0.94	-1.92	AAA-AAA	0.00	999	1.000	1.000	0.3688
1553736_at	41.4	21.5	-0.94	-1.92	AAA-AAA	0.00	999	1.000	1.000	0.2335
220228_at	4.1	2.1	-0.94	-1.92	AAA-AAA	0.00	999	1.000	1.000	0.2592
206657_s_at	30.7	16.0	-0.94	-1.92	AAA-AAA	0.00	999	1.000	1.000	0.2897
228474_s_at	53.4	27.8	-0.94	-1.92	AAA-AAA	0.00	999	1.000	1.000	0.5251
232826_at	6.1	3.2	-0.94	-1.92	AAA-AAA	0.00	999	1.000	1.000	0.4520
232771_at	24.0	46.1	0.94	1.92	AAA-AAA	0.00	999	1.000	1.000	0.1209
243098_at	27.4	14.2	-0.94	-1.92	AAA-AAA	0.00	999	1.000	1.000	0.4537
1562854_at	21.7	11.3	-0.94	-1.92	AAA-AAA	0.00	999	1.000	1.000	0.3679
1564240_at	18.7	36.0	0.94	1.92	AAA-AAA	0.00	999	1.000	1.000	0.4269
1564837_at	13.0	6.7	-0.94	-1.92	AAA-AAA	0.00	999	1.000	1.000	0.4809
1553398_at	8.4	16.0	0.94	1.92	AAA-AAA	0.00	999	1.000	1.000	0.5175

1554908_at	6.2	3.2	-0.94	-1.92	AAA-AAA	0.00	999	1.000	1.000	0.5406
1558420_at	58.1	30.2	-0.94	-1.92	AAA-AAA	0.00	999	1.000	1.000	0.3578
1558720_at	49.6	25.8	-0.94	-1.92	AAA-AAA	0.00	999	1.000	1.000	0.1917
1555935_s_at	20.1	10.5	-0.94	-1.92	AAA-AAA	0.00	999	1.000	1.000	0.4456
221166_at	7.8	15.0	0.94	1.92	AAA-AAA	0.00	999	1.000	1.000	0.3496
227076_at	75.0	144.2	0.94	1.92	AAA-AAA	0.00	999	1.000	1.000	0.3594
223030_at	9.0	4.7	-0.94	-1.92	AAA-AAA	0.00	999	1.000	1.000	0.1578
210127_at	25.5	13.3	-0.94	-1.92	AAA-AAA	0.00	999	1.000	1.000	0.4252
215748_at	91.6	47.7	-0.94	-1.92	AAA-AAA	0.00	999	1.000	1.000	0.0752
216145_at	28.4	14.8	-0.94	-1.92	AAA-AAA	0.00	999	1.000	1.000	0.3597
236028_at	2.9	1.5	-0.94	-1.92	AAA-AAA	0.00	999	1.000	1.000	0.0265
239643_at	14.5	7.5	-0.94	-1.92	AAA-AAA	0.00	999	1.000	1.000	0.1276
221209_s_at	8.3	16.0	0.94	1.92	AAA-PAA	0.00	999	1.000	1.000	0.4813
206330_s_at	91.3	175.3	0.94	1.92	AAA-PAA	0.00	999	1.000	1.000	0.3793
230034_x_at	34.5	66.3	0.94	1.92	AAA-AAP	0.00	999	1.000	1.000	0.3469
209038_s_at	51.4	98.6	0.94	1.92	AAA-APP	0.00	999	1.000	1.000	0.1312
1566842_at	12.2	6.4	-0.94	-1.92	AAA-AAA	0.00	999	1.000	1.000	0.2572
226999_at	19.6	37.6	0.94	1.92	AAA-AAA	0.00	999	1.000	1.000	0.4054
214217_at	12.1	6.3	-0.94	-1.92	AAA-AAA	0.00	999	1.000	1.000	0.5834
211411_at	11.2	5.8	-0.94	-1.92	AAA-AAA	0.00	999	1.000	1.000	0.2073
216225_at	20.6	10.7	-0.94	-1.92	AAA-AAA	0.00	999	1.000	1.000	0.5540
232993_at	17.7	9.2	-0.94	-1.92	AAA-AAA	0.00	999	1.000	1.000	0.5907
240857_at	6.0	3.1	-0.94	-1.92	AAA-AAA	0.00	999	1.000	1.000	0.3041
241748_x_at	46.7	89.6	0.94	1.92	AAA-AAA	0.00	999	1.000	1.000	0.3795
240558_at	4.2	8.0	0.94	1.92	AAA-AAA	0.00	999	1.000	1.000	0.4110
1563098_at	9.1	4.7	-0.94	-1.92	AAA-AAA	0.00	999	1.000	1.000	0.0357
1567682_x_at	15.0	7.8	-0.94	-1.92	AAA-AAA	0.00	999	1.000	1.000	0.4916
220496_at	77.2	40.2	-0.94	-1.92	AAA-AAA	0.00	999	1.000	1.000	0.2185
217384_x_at	14.6	7.6	-0.94	-1.92	AAA-AAA	0.00	999	1.000	1.000	0.3161
235915_at	15.2	7.9	-0.94	-1.92	AAA-AAA	0.00	999	1.000	1.000	0.4724
235379_at	11.2	5.8	-0.94	-1.92	AAA-AAA	0.00	999	1.000	1.000	0.5281
234286_at	10.0	5.2	-0.94	-1.92	AAA-AAA	0.00	999	1.000	1.000	0.0513
234429_at	14.9	7.8	-0.94	-1.92	AAA-AAA	0.00	999	1.000	1.000	0.5229
238225_at	11.6	6.1	-0.94	-1.92	AAA-AAA	0.00	999	1.000	1.000	0.2081
203924_at	17.1	8.9	-0.94	-1.92	AAA-AAA	0.00	999	1.000	1.000	0.2797
243706_at	37.5	71.9	0.94	1.92	AAA-APA	0.00	999	1.000	1.000	0.3735
241172_at	21.9	11.4	-0.94	-1.92	AAA-AAA	0.00	999	1.000	1.000	0.4954
1564423_a_at	59.2	30.9	-0.94	-1.92	AAA-AAA	0.00	999	1.000	1.000	0.1523
1553586_at	112.9	58.9	-0.94	-1.92	MAA-AAA	0.00	999	1.000	1.000	0.1341
244413_at	12.1	23.1	0.94	1.92	AAA-AAA	0.00	999	1.000	1.000	0.2049
221945_at	92.5	48.3	-0.94	-1.92	AAA-AAA	0.00	999	1.000	1.000	0.0746
214308_s_at	17.6	33.7	0.94	1.92	AAA-AAA	0.00	999	1.000	1.000	0.3463

234270_at	59.7	31.1	-0.94	-1.92	AAA-AAA	0.00	999	1.000	1.000	0.0947
234766_at	17.8	9.3	-0.94	-1.92	AAA-AAA	0.00	999	1.000	1.000	0.3665
229954_at	39.5	20.6	-0.94	-1.92	AAA-AAA	0.00	999	1.000	1.000	0.3544
230628_at	69.8	133.8	0.94	1.92	AAA-AAA	0.00	999	1.000	1.000	0.3029
231390_at	5.0	9.5	0.94	1.92	AAA-AAA	0.00	999	1.000	1.000	0.3764
234169_at	15.9	30.4	0.94	1.92	PAA-AAP	0.00	999	1.000	1.000	0.4955
234219_at	37.8	72.4	0.94	1.92	PAA-PPA	0.00	999	1.000	1.000	0.4294
204221_x_at	546.8	1047.8	0.94	1.92	PPP-PPP	0.00	999	1.000	1.000	0.1301
234920_at	22.2	42.5	0.94	1.92	AAA-AAP	0.00	999	1.000	1.000	0.0756
241305_at	12.4	23.7	0.94	1.92	AAA-AAA	0.00	999	1.000	1.000	0.4493
240472_at	23.5	12.3	-0.94	-1.92	AAM-AAA	0.00	999	1.000	1.000	0.4699
1564007_at	22.5	11.8	-0.94	-1.92	AAA-AAA	0.00	999	1.000	1.000	0.3154
1560397_s_at	19.5	10.2	-0.94	-1.92	AAA-AAA	0.00	999	1.000	1.000	0.3469
201372_s_at	8.5	4.4	-0.94	-1.92	AAA-AAA	0.00	999	1.000	1.000	0.3979
1566235_at	74.4	38.8	-0.94	-1.92	AAA-AAA	0.00	999	1.000	1.000	0.2415
1557845_at	9.1	4.7	-0.94	-1.92	AAA-AAA	0.00	999	1.000	1.000	0.6372
1559925_s_at	14.8	7.7	-0.94	-1.92	AAA-AAA	0.00	999	1.000	1.000	0.6261
206464_at	9.3	4.9	-0.94	-1.92	AAA-AAA	0.00	999	1.000	1.000	0.2900
211424_x_at	16.8	32.1	0.94	1.92	AAM-MAA	0.00	999	1.000	1.000	0.5773
215229_at	3.5	1.8	-0.94	-1.92	AAA-AAA	0.00	999	1.000	1.000	0.2437
238059_at	38.7	20.2	-0.94	-1.92	AAA-AAA	0.00	999	1.000	1.000	0.3349
228100_at	56.3	29.4	-0.94	-1.92	MAA-AAA	0.00	999	1.000	1.000	0.4127
229376_at	33.9	17.7	-0.94	-1.92	AAA-AAA	0.00	999	1.000	1.000	0.3356
208523_x_at	78.2	149.7	0.94	1.92	PAP-PPP	0.00	999	1.000	1.000	0.1067
242410_s_at	20.4	10.7	-0.94	-1.91	AAA-AAA	0.00	999	1.000	1.000	0.6117
1560412_at	51.0	26.6	-0.94	-1.92	AAA-AAA	0.00	999	1.000	1.000	0.3872
1569191_at	6.5	12.5	0.94	1.91	AAA-AAA	0.00	999	1.000	1.000	0.6463
1554403_a_at	5.4	2.8	-0.94	-1.92	AAA-AAA	0.00	999	1.000	1.000	0.4373
1558849_at	28.8	55.1	0.94	1.92	AAA-AAA	0.00	999	1.000	1.000	0.3130
222217_s_at	36.4	19.0	-0.94	-1.92	AAA-AAA	0.00	999	1.000	1.000	0.3202
224234_at	8.1	4.2	-0.94	-1.92	AAA-AAA	0.00	999	1.000	1.000	0.3986
211176_s_at	49.7	26.0	-0.94	-1.91	AAA-AAA	0.00	999	1.000	1.000	0.3731
216984_x_at	18.3	9.5	-0.94	-1.91	AAA-AAA	0.00	999	1.000	1.000	0.3339
204277_s_at	45.9	87.8	0.94	1.91	AAA-AAA	0.00	999	1.000	1.000	0.2938
233104_at	21.6	11.3	-0.94	-1.92	AAA-AAA	0.00	999	1.000	1.000	0.4246
231254_at	40.8	21.3	-0.94	-1.91	AAA-AAA	0.00	999	1.000	1.000	0.5930
1564333_a_at	42.4	81.1	0.94	1.91	AAM-AAP	0.00	999	1.000	1.000	0.4041
212823_s_at	85.2	163.0	0.94	1.91	AAA-PAP	0.00	999	1.000	1.000	0.3353
241499_at	22.6	43.2	0.94	1.91	AAA-PAP	0.00	999	1.000	1.000	0.2538
240917_at	57.7	30.2	-0.94	-1.91	AAA-AAA	0.00	999	1.000	1.000	0.2706
1563943_at	89.5	46.8	-0.94	-1.91	AAA-AAA	0.00	999	1.000	1.000	0.0541
244730_x_at	68.9	36.0	-0.94	-1.91	AAA-AAA	0.00	999	1.000	1.000	0.2829

208071_s_at	16.5	8.6	-0.94	-1.91	AAA-AAA	0.00	999	1.000	1.000	0.0273
228471_at	9.7	18.5	0.94	1.91	AAA-AAA	0.00	999	1.000	1.000	0.5760
213186_at	219.7	420.2	0.94	1.91	APP-PPP	0.00	999	1.000	1.000	0.1294
209774_x_at	206.2	394.3	0.94	1.91	PPP-PPP	0.00	999	1.000	1.000	0.0672
240281_at	52.8	27.6	-0.94	-1.91	AAA-AAA	0.00	999	1.000	1.000	0.4324
241579_at	15.4	8.0	-0.94	-1.91	AAA-AAA	0.00	999	1.000	1.000	0.6791
1553817_at	10.0	19.1	0.94	1.91	AAA-AAA	0.00	999	1.000	1.000	0.5484
1552877_s_at	40.1	21.0	-0.94	-1.91	AAA-AAA	0.00	999	1.000	1.000	0.4248
211001_at	44.6	23.3	-0.94	-1.91	AAA-AAA	0.00	999	1.000	1.000	0.2283
234228_at	3.0	5.7	0.94	1.91	AAA-AAA	0.00	999	1.000	1.000	0.4845
239453_at	125.5	65.7	-0.94	-1.91	AAA-MAA	0.00	999	1.000	1.000	0.0047
237988_at	24.2	12.7	-0.94	-1.91	AAA-AAA	0.00	999	1.000	1.000	0.4793
222895_s_at	11.6	22.2	0.93	1.91	PAA-AAA	0.00	999	1.000	1.000	0.6321
209166_s_at	206.2	393.9	0.93	1.91	PPP-PPP	0.00	999	1.000	1.000	0.3214
1562492_at	2.7	5.1	0.93	1.91	AAA-AAA	0.00	999	1.000	1.000	0.4419
1570284_x_at	9.9	19.0	0.93	1.91	AAA-AAA	0.00	999	1.000	1.000	0.5965
1555230_a_at	3.8	2.0	-0.93	-1.91	AAA-AAA	0.00	999	1.000	1.000	0.0999
1558409_at	24.6	12.9	-0.93	-1.91	AAA-AAA	0.00	999	1.000	1.000	0.2046
1555371_at	34.3	17.9	-0.93	-1.91	AAA-AAA	0.00	999	1.000	1.000	0.5461
220428_at	26.7	14.0	-0.93	-1.91	AAA-AAA	0.00	999	1.000	1.000	0.6145
207150_at	24.3	12.7	-0.93	-1.91	AAA-AAA	0.00	999	1.000	1.000	0.4572
214601_at	8.3	4.3	-0.93	-1.91	AAA-AAA	0.00	999	1.000	1.000	0.5770
212613_at	91.5	47.9	-0.93	-1.91	AAA-AAA	0.00	999	1.000	1.000	0.3272
236256_at	25.7	13.4	-0.93	-1.91	AAA-AAA	0.00	999	1.000	1.000	0.5894
236092_at	78.3	41.0	-0.93	-1.91	AAA-AMA	0.00	999	1.000	1.000	0.1089
238955_at	62.4	32.7	-0.93	-1.91	AAA-AAA	0.00	999	1.000	1.000	0.4470
238734_at	3.7	7.2	0.93	1.91	AAA-AAA	0.00	999	1.000	1.000	0.4224
238141_s_at	41.8	21.9	-0.93	-1.91	AAA-AAA	0.00	999	1.000	1.000	0.3497
230008_at	30.3	15.9	-0.93	-1.91	AAA-AAA	0.00	999	1.000	1.000	0.6147
1560758_at	13.8	7.2	-0.93	-1.91	AAA-AAA	0.00	999	1.000	1.000	0.4964
244496_at	9.8	5.1	-0.93	-1.91	AAA-AAA	0.00	999	1.000	1.000	0.4428
208155_x_at	28.6	15.0	-0.93	-1.91	AAA-AAA	0.00	999	1.000	1.000	0.3453
219876_s_at	13.3	7.0	-0.93	-1.91	AAA-AAA	0.00	999	1.000	1.000	0.2654
223688_s_at	38.9	20.4	-0.93	-1.91	AAA-AAA	0.00	999	1.000	1.000	0.4388
213962_s_at	33.1	63.1	0.93	1.91	AAA-AAA	0.00	999	1.000	1.000	0.2912
234362_s_at	15.4	29.4	0.93	1.91	AAA-AAA	0.00	999	1.000	1.000	0.5339
232821_at	12.3	23.4	0.93	1.91	AAA-AAA	0.00	999	1.000	1.000	0.3449
232602_at	19.0	10.0	-0.93	-1.91	AAA-AAA	0.00	999	1.000	1.000	0.4812
232485_at	85.8	44.9	-0.93	-1.91	AAA-AAA	0.00	999	1.000	1.000	0.0109
233390_at	6.8	3.5	-0.93	-1.91	AAA-AAA	0.00	999	1.000	1.000	0.1003
242333_at	24.2	46.1	0.93	1.91	APA-AAM	0.00	999	1.000	1.000	0.4441
223557_s_at	121.1	231.0	0.93	1.91	PPP-PPP	0.00	999	1.000	1.000	0.0921

241557_x_at	6.0	11.5	0.93	1.91	AAA-AAA	0.00	999	1.000	1.000	0.6192
240284_x_at	24.1	12.7	-0.93	-1.91	AAA-AAA	0.00	999	1.000	1.000	0.5731
1563114_at	11.4	6.0	-0.93	-1.91	AAA-AAA	0.00	999	1.000	1.000	0.2073
1570414_x_at	78.4	41.1	-0.93	-1.91	AAA-AAA	0.00	999	1.000	1.000	0.2961
1558725_at	14.8	7.8	-0.93	-1.91	AAA-AAA	0.00	999	1.000	1.000	0.3336
221145_at	5.4	10.2	0.93	1.91	AAA-AAA	0.00	999	1.000	1.000	0.2594
208203_x_at	15.6	8.2	-0.93	-1.91	AAA-AAA	0.00	999	1.000	1.000	0.0626
208285_at	27.4	14.3	-0.93	-1.91	AAA-AAA	0.00	999	1.000	1.000	0.5795
236744_at	26.6	13.9	-0.93	-1.91	AAA-AAA	0.00	999	1.000	1.000	0.4731
234844_at	31.7	16.6	-0.93	-1.91	AAA-AAA	0.00	999	1.000	1.000	0.3990
238197_at	5.7	3.0	-0.93	-1.91	AAA-AAA	0.00	999	1.000	1.000	0.4351
238305_at	19.8	10.4	-0.93	-1.91	AAA-AAA	0.00	999	1.000	1.000	0.3385
229957_at	46.6	24.4	-0.93	-1.91	AAA-AAA	0.00	999	1.000	1.000	0.4635
207014_at	32.0	60.9	0.93	1.91	APP-MPP	0.00	999	1.000	1.000	0.3277
221911_at	179.4	342.2	0.93	1.91	PPP-PPP	0.00	999	1.000	1.000	0.1019
241097_at	9.1	17.3	0.93	1.91	AAA-AAA	0.00	999	1.000	1.000	0.5122
1560773_at	2.1	4.0	0.93	1.91	AAA-AAA	0.00	999	1.000	1.000	0.5629
1561440_at	55.1	28.9	-0.93	-1.91	AAA-AAA	0.00	999	1.000	1.000	0.2305
1570055_at	131.5	69.0	-0.93	-1.91	AAA-AAA	0.00	999	1.000	1.000	0.4389
1553389_at	10.7	20.4	0.93	1.91	AAA-AAA	0.00	999	1.000	1.000	0.3406
221336_at	26.5	13.9	-0.93	-1.91	AAA-AAA	0.00	999	1.000	1.000	0.5291
220848_x_at	9.8	5.2	-0.93	-1.91	AAA-AAA	0.00	999	1.000	1.000	0.0940
206890_at	25.9	49.5	0.93	1.91	AAA-AAA	0.00	999	1.000	1.000	0.4404
222927_s_at	53.7	28.2	-0.93	-1.91	AAA-AMA	0.00	999	1.000	1.000	0.2543
234328_at	16.2	30.9	0.93	1.91	AAA-AAA	0.00	999	1.000	1.000	0.3745
233941_at	33.8	17.7	-0.93	-1.91	AAA-AAA	0.00	999	1.000	1.000	0.5008
234064_at	15.6	8.2	-0.93	-1.91	AAA-AAA	0.00	999	1.000	1.000	0.5821
234608_at	28.6	15.0	-0.93	-1.91	AAA-AAA	0.00	999	1.000	1.000	0.4074
237843_at	79.9	41.9	-0.93	-1.91	MAA-AAA	0.00	999	1.000	1.000	0.2217
232428_at	50.2	26.4	-0.93	-1.91	AAA-AAA	0.00	999	1.000	1.000	0.3189
216045_at	12.0	22.9	0.93	1.91	AAA-AAP	0.00	999	1.000	1.000	0.5355
216612_x_at	16.5	31.5	0.93	1.91	AAA-PAA	0.00	999	1.000	1.000	0.5139
1563016_at	14.2	27.0	0.93	1.91	AAA-AAP	0.00	999	1.000	1.000	0.3157
242409_at	22.7	43.2	0.93	1.91	AMP-PPA	0.00	999	1.000	1.000	0.3067
207808_s_at	72.0	137.2	0.93	1.91	APP-PPP	0.00	999	1.000	1.000	0.2244
1560373_a_at	19.1	36.4	0.93	1.91	AAA-APP	0.00	999	1.000	1.000	0.0758
233803_s_at	184.4	351.5	0.93	1.91	AAP-PPP	0.00	999	1.000	1.000	0.0531
243998_at	6.8	12.9	0.93	1.91	AAA-AAA	0.00	999	1.000	1.000	0.5086
243647_at	16.1	8.4	-0.93	-1.91	AAA-AAA	0.00	999	1.000	1.000	0.2060
240087_at	9.3	4.9	-0.93	-1.91	AAA-AAA	0.00	999	1.000	1.000	0.1271
1560115_a_at	192.1	100.8	-0.93	-1.91	MAM-AAA	0.00	999	1.000	1.000	0.0742
1569332_at	2.6	5.0	0.93	1.91	AAA-AAA	0.00	999	1.000	1.000	0.4644

1564653_s_at	17.7	33.7	0.93	1.91	MAA-AAA	0.00	999	1.000	1.000	0.5187
1553271_at	9.7	5.1	-0.93	-1.91	AAA-AAA	0.00	999	1.000	1.000	0.4128
1556453_at	7.2	13.6	0.93	1.91	AAA-AAA	0.00	999	1.000	1.000	0.4703
1555520_at	25.6	48.8	0.93	1.91	AAA-AAA	0.00	999	1.000	1.000	0.4835
227217_at	31.7	16.6	-0.93	-1.91	AAA-AAA	0.00	999	1.000	1.000	0.3245
206685_at	4.7	2.5	-0.93	-1.91	AAA-AAA	0.00	999	1.000	1.000	0.4854
213997_at	20.5	10.8	-0.93	-1.91	AAA-AAA	0.00	999	1.000	1.000	0.5528
215097_at	16.7	8.8	-0.93	-1.91	AAA-AAA	0.00	999	1.000	1.000	0.3874
211891_s_at	70.3	36.9	-0.93	-1.91	AAA-AAA	0.00	999	1.000	1.000	0.1429
236085_at	54.6	28.7	-0.93	-1.91	AAA-AAA	0.00	999	1.000	1.000	0.2334
234652_at	4.8	9.1	0.93	1.91	AAA-AAA	0.00	999	1.000	1.000	0.4379
237461_at	21.0	11.0	-0.93	-1.91	AAA-AAA	0.00	999	1.000	1.000	0.4261
1566440_at	38.5	73.3	0.93	1.90	PAA-PPP	0.00	999	1.000	1.000	0.1550
225696_at	102.0	194.3	0.93	1.90	AAP-PPA	0.00	999	1.000	1.000	0.0978
202430_s_at	180.7	344.1	0.93	1.90	PPP-PPP	0.00	999	1.000	1.000	0.0727
242097_at	29.4	15.5	-0.93	-1.90	AAA-AAA	0.00	999	1.000	1.000	0.4602
241441_at	2.7	1.4	-0.93	-1.90	AAA-AAA	0.00	999	1.000	1.000	0.0831
1561116_at	32.7	17.2	-0.93	-1.90	AAA-AAA	0.00	999	1.000	1.000	0.4459
1564387_at	11.1	21.2	0.93	1.90	AAA-AAA	0.00	999	1.000	1.000	0.4874
1564121_at	15.9	8.4	-0.93	-1.90	AAA-AAA	0.00	999	1.000	1.000	0.4462
1555335_at	5.5	10.4	0.93	1.90	AAA-AAA	0.00	999	1.000	1.000	0.1896
1554343_a_at	7.4	14.1	0.93	1.90	AAA-AAA	0.00	999	1.000	1.000	0.4626
221359_at	53.8	28.3	-0.93	-1.90	AAA-AAA	0.00	999	1.000	1.000	0.4285
207907_at	4.2	8.0	0.93	1.90	AAA-AAA	0.00	999	1.000	1.000	0.3191
207509_s_at	26.4	13.9	-0.93	-1.90	AAA-AAA	0.00	999	1.000	1.000	0.4832
215174_at	12.1	6.4	-0.93	-1.90	AAA-AAA	0.00	999	1.000	1.000	0.5921
216905_s_at	11.0	20.9	0.93	1.91	AAA-AAA	0.00	999	1.000	1.000	0.5252
230474_at	28.8	15.1	-0.93	-1.90	AAA-AAA	0.00	999	1.000	1.000	0.4443
233606_at	26.1	13.7	-0.93	-1.90	AAA-AAA	0.00	999	1.000	1.000	0.4352
231333_at	43.1	22.6	-0.93	-1.91	AAA-AAA	0.00	999	1.000	1.000	0.2238
224804_s_at	290.0	551.7	0.93	1.90	APP-PPP	0.00	999	1.000	1.000	0.4476
1559159_at	33.1	63.0	0.93	1.90	AAA-AAP	0.00	999	1.000	1.000	0.3693
1554649_at	64.3	33.8	-0.93	-1.90	AAA-AAA	0.00	999	1.000	1.000	0.0866
1558248_at	16.4	8.6	-0.93	-1.90	AAA-AAA	0.00	999	1.000	1.000	0.3521
1559879_a_at	9.1	17.4	0.93	1.90	AAA-AAA	0.00	999	1.000	1.000	0.4071
221074_at	13.8	7.3	-0.93	-1.90	AAA-AAA	0.00	999	1.000	1.000	0.3842
219554_at	14.5	7.6	-0.93	-1.90	AAA-AAA	0.00	999	1.000	1.000	0.4056
219810_at	46.7	88.8	0.93	1.90	AAA-AMA	0.00	999	1.000	1.000	0.0821
222648_at	25.0	13.2	-0.93	-1.90	AAA-AAA	0.00	999	1.000	1.000	0.2952
235169_at	14.4	27.5	0.93	1.90	AAA-AAA	0.00	999	1.000	1.000	0.2658
204567_s_at	28.6	15.1	-0.93	-1.90	AAA-AAA	0.00	999	1.000	1.000	0.4390
238742_x_at	12.0	6.3	-0.93	-1.90	AAA-AAA	0.00	999	1.000	1.000	0.2705

237570_x_at	10.5	20.0	0.93	1.90	AAA-AAA	0.00	999	1.000	1.000	0.5038
233762_at	32.3	17.0	-0.93	-1.90	AAA-AAA	0.00	999	1.000	1.000	0.6097
228977_at	227.0	431.5	0.93	1.90	APP-APP	0.00	999	1.000	1.000	0.2173
201883_s_at	521.0	990.4	0.93	1.90	PPP-PPP	0.00	999	1.000	1.000	0.1686
239819_at	10.3	19.5	0.93	1.90	AAA-AAA	0.00	999	1.000	1.000	0.6378
1560725_at	2.7	5.2	0.93	1.90	AAA-AAA	0.00	999	1.000	1.000	0.4301
1570402_at	98.7	51.9	-0.93	-1.90	AAA-AAA	0.00	999	1.000	1.000	0.3612
1570107_at	66.1	34.8	-0.93	-1.90	AAA-AAA	0.00	999	1.000	1.000	0.4032
1565737_at	12.3	23.3	0.93	1.90	AAA-AAA	0.00	999	1.000	1.000	0.2652
1554844_at	35.8	18.8	-0.93	-1.90	AAA-AAA	0.00	999	1.000	1.000	0.1739
208013_s_at	24.6	13.0	-0.93	-1.90	AAA-AAA	0.00	999	1.000	1.000	0.5836
212356_at	159.3	303.0	0.93	1.90	AAA-AAA	0.00	999	1.000	1.000	0.1699
212935_at	8.4	4.4	-0.93	-1.90	AAA-AAA	0.00	999	1.000	1.000	0.1466
212924_s_at	28.0	14.7	-0.93	-1.90	AAA-AAA	0.00	999	1.000	1.000	0.3466
234088_at	11.0	5.8	-0.93	-1.90	AAA-AAA	0.00	999	1.000	1.000	0.0903
230950_at	36.3	19.1	-0.93	-1.90	AAA-AAA	0.00	999	1.000	1.000	0.5608
231542_at	18.9	35.9	0.93	1.90	AAA-AAA	0.00	999	1.000	1.000	0.3624
244758_at	35.8	68.0	0.93	1.90	AAA-PPP	0.00	999	1.000	1.000	0.0811
243358_at	39.8	75.6	0.93	1.90	AAA-AAA	0.00	999	1.000	1.000	0.1297
1562310_at	9.0	4.7	-0.93	-1.90	AAA-AAA	0.00	999	1.000	1.000	0.6770
1563494_at	5.7	3.0	-0.93	-1.90	AAA-AAA	0.00	999	1.000	1.000	0.2230
1563165_at	55.6	29.3	-0.93	-1.90	AAA-AAA	0.00	999	1.000	1.000	0.1512
1553943_at	197.6	104.0	-0.93	-1.90	AAA-AAA	0.00	999	1.000	1.000	0.0359
244850_at	26.9	14.1	-0.93	-1.90	AAA-AAA	0.00	999	1.000	1.000	0.5232
1558493_at	16.6	8.7	-0.93	-1.90	AAA-AAA	0.00	999	1.000	1.000	0.5172
1559611_at	16.2	8.5	-0.93	-1.90	AAA-AAA	0.00	999	1.000	1.000	0.6736
1559507_at	15.5	29.5	0.93	1.90	AAA-AAA	0.00	999	1.000	1.000	0.1445
220445_s_at	8.5	4.5	-0.93	-1.90	AAA-AAA	0.00	999	1.000	1.000	0.3889
227459_at	88.0	167.3	0.93	1.90	AAA-AAA	0.00	999	1.000	1.000	0.4350
217442_at	7.8	14.8	0.93	1.90	AAA-AAA	0.00	999	1.000	1.000	0.4276
234359_at	78.3	148.8	0.93	1.90	AAA-AAA	0.00	999	1.000	1.000	0.3576
239118_at	18.0	9.5	-0.93	-1.90	AAA-AAA	0.00	999	1.000	1.000	0.4998
239291_at	9.1	4.8	-0.93	-1.90	AAA-AAA	0.00	999	1.000	1.000	0.6045
237091_at	4.9	2.6	-0.93	-1.90	AAA-AAA	0.00	999	1.000	1.000	0.0796
237744_at	62.5	32.9	-0.93	-1.90	AAA-AAA	0.00	999	1.000	1.000	0.2934
232082_x_at	8.1	4.3	-0.93	-1.90	AAA-AAA	0.00	999	1.000	1.000	0.1210
207464_at	23.0	43.6	0.93	1.90	APA-PAA	0.00	999	1.000	1.000	0.5943
244164_at	37.8	71.7	0.93	1.90	AAA-AAP	0.00	999	1.000	1.000	0.2425
229174_at	702.6	1333.9	0.93	1.90	PPP-PPP	0.00	999	1.000	1.000	0.1452
244260_at	11.9	22.6	0.93	1.90	AAA-AAA	0.00	999	1.000	1.000	0.3381
244097_at	5.3	10.0	0.93	1.90	AAA-AAA	0.00	999	1.000	1.000	0.6207
240115_at	58.0	30.6	-0.93	-1.90	AAA-AAA	0.00	999	1.000	1.000	0.2668

1553635_s_at	10.6	5.6	-0.93	-1.90	AAA-AAA	0.00	999	1.000	1.000	0.4033
244816_at	15.6	8.2	-0.93	-1.90	AAA-AAA	0.00	999	1.000	1.000	0.4638
206769_at	44.2	23.3	-0.93	-1.90	AAA-AAA	0.00	999	1.000	1.000	0.5178
206785_s_at	2.8	5.3	0.93	1.90	AAA-AAA	0.00	999	1.000	1.000	0.3700
217472_at	27.1	51.4	0.93	1.90	AAA-AAA	0.00	999	1.000	1.000	0.4422
209554_at	20.7	10.9	-0.93	-1.90	AAA-AAA	0.00	999	1.000	1.000	0.5004
237558_at	8.7	4.6	-0.93	-1.90	AAA-AAA	0.00	999	1.000	1.000	0.2809
229716_at	40.7	21.4	-0.93	-1.90	AAA-AAA	0.00	999	1.000	1.000	0.2643
207879_at	13.6	7.1	-0.92	-1.90	PAA-APA	0.00	999	1.000	1.000	0.6881
235274_at	231.4	439.2	0.92	1.90	PPP-PPP	0.00	999	1.000	1.000	0.1070
1569111_at	19.6	10.3	-0.92	-1.90	AAA-AAA	0.00	999	1.000	1.000	0.5904
1553539_at	16.8	8.9	-0.92	-1.90	AAA-AAA	0.00	999	1.000	1.000	0.4057
221757_at	55.2	104.8	0.92	1.90	AAA-AAA	0.00	999	1.000	1.000	0.4199
220699_s_at	26.1	13.8	-0.92	-1.90	AAA-AAA	0.00	999	1.000	1.000	0.5414
227210_at	20.7	39.3	0.92	1.90	AAA-AAA	0.00	999	1.000	1.000	0.3307
215098_at	103.4	54.5	-0.92	-1.90	AAA-AAA	0.00	999	1.000	1.000	0.1758
208572_at	15.8	30.0	0.92	1.90	AAA-AAA	0.00	999	1.000	1.000	0.4137
239491_at	19.0	10.0	-0.92	-1.90	AAA-AAA	0.00	999	1.000	1.000	0.2922
237433_at	5.0	9.5	0.92	1.90	AAA-AAA	0.00	999	1.000	1.000	0.6120
237545_at	15.0	28.5	0.92	1.90	AAA-AAA	0.00	999	1.000	1.000	0.4368
205998_x_at	96.4	183.0	0.92	1.90	AAA-AAA	0.00	999	1.000	1.000	0.2430
231752_at	13.2	7.0	-0.92	-1.90	AAA-AAA	0.00	999	1.000	1.000	0.0329
1562724_at	7.1	13.5	0.92	1.90	AAA-AAA	0.00	999	1.000	1.000	0.3537
1565701_at	24.6	13.0	-0.92	-1.90	AAA-AAA	0.00	999	1.000	1.000	0.0262
1554033_at	10.3	5.4	-0.92	-1.90	AAA-AAA	0.00	999	1.000	1.000	0.5052
214044_at	26.9	14.2	-0.92	-1.90	AAA-AAA	0.00	999	1.000	1.000	0.4384
219181_at	20.3	10.7	-0.92	-1.90	AAA-AAA	0.00	999	1.000	1.000	0.6499
206255_at	9.6	18.2	0.92	1.90	AAA-AAA	0.00	999	1.000	1.000	0.4954
229289_at	14.2	7.5	-0.92	-1.90	AAA-AAA	0.00	999	1.000	1.000	0.1309
232850_at	5.8	3.1	-0.92	-1.90	AAA-AAA	0.00	999	1.000	1.000	0.1636
238496_at	207.9	394.0	0.92	1.90	PPP-PPP	0.00	999	1.000	1.000	0.1257
46665_at	482.5	914.0	0.92	1.89	PPP-PPP	0.00	999	1.000	1.000	0.0702
241259_at	13.4	7.1	-0.92	-1.89	AAA-AAA	0.00	999	1.000	1.000	0.2393
1564008_at	44.9	23.7	-0.92	-1.89	AAA-AAA	0.00	999	1.000	1.000	0.5039
1569064_at	3.8	7.2	0.92	1.90	AAA-AAA	0.00	999	1.000	1.000	0.3846
244715_at	5.8	3.1	-0.92	-1.89	AAA-AAA	0.00	999	1.000	1.000	0.0398
1558855_at	102.3	54.0	-0.92	-1.89	AAA-AAA	0.00	999	1.000	1.000	0.0364
220808_at	36.2	19.1	-0.92	-1.90	AAA-AAA	0.00	999	1.000	1.000	0.2365
214340_at	75.6	39.9	-0.92	-1.90	AAA-AAA	0.00	999	1.000	1.000	0.1479
210784_x_at	25.2	13.3	-0.92	-1.90	AAA-AAA	0.00	999	1.000	1.000	0.2478
211705_s_at	5.7	10.7	0.92	1.90	AAA-AAA	0.00	999	1.000	1.000	0.4605
217251_x_at	21.3	11.3	-0.92	-1.89	AAA-AAA	0.00	999	1.000	1.000	0.3776

235375_x_at	9.5	5.0	-0.92	-1.90	AAA-AAA	0.00	999	1.000	1.000	0.0363
234124_at	35.7	18.8	-0.92	-1.90	AAA-AAA	0.00	999	1.000	1.000	0.5487
238003_at	31.3	59.4	0.92	1.90	AAA-AAA	0.00	999	1.000	1.000	0.2094
232621_at	42.9	22.7	-0.92	-1.90	AAA-AAA	0.00	999	1.000	1.000	0.4521
243279_at	42.0	79.4	0.92	1.89	APA-AAP	0.00	999	1.000	1.000	0.4196
222686_s_at	106.1	200.9	0.92	1.89	AAA-PPA	0.00	999	1.000	1.000	0.0468
240064_at	5.5	10.5	0.92	1.89	AAA-AAA	0.00	999	1.000	1.000	0.4852
1563099_at	7.3	3.8	-0.92	-1.89	AAA-AAA	0.00	999	1.000	1.000	0.4500
1566647_s_at	15.6	8.2	-0.92	-1.89	AAA-AAA	0.00	999	1.000	1.000	0.3756
244553_at	16.3	8.6	-0.92	-1.89	AAA-AAA	0.00	999	1.000	1.000	0.4490
1559590_at	2.6	1.4	-0.92	-1.89	AAA-AAA	0.00	999	1.000	1.000	0.3341
220472_at	18.8	35.5	0.92	1.89	AAA-AAA	0.00	999	1.000	1.000	0.5810
223706_at	41.1	77.8	0.92	1.89	AAA-AAA	0.00	999	1.000	1.000	0.3098
217003_s_at	12.3	23.2	0.92	1.89	AAA-AAA	0.00	999	1.000	1.000	0.2537
234555_at	34.9	18.4	-0.92	-1.89	AAA-AAA	0.00	999	1.000	1.000	0.4173
238626_at	31.3	16.6	-0.92	-1.89	AAA-AAA	0.00	999	1.000	1.000	0.1781
238577_s_at	25.4	13.4	-0.92	-1.89	AAA-AAA	0.00	999	1.000	1.000	0.2531
233796_at	17.7	9.4	-0.92	-1.89	AAA-AAA	0.00	999	1.000	1.000	0.4392
232069_at	12.3	6.5	-0.92	-1.89	AAA-AAA	0.00	999	1.000	1.000	0.1122
240902_at	6.5	12.4	0.92	1.89	AAA-AAA	0.00	999	1.000	1.000	0.5472
244890_at	10.7	20.2	0.92	1.89	AAA-AAA	0.00	999	1.000	1.000	0.1820
1553102_a_at	4.6	8.8	0.92	1.89	AAA-AAA	0.00	999	1.000	1.000	0.2329
227564_at	14.5	27.4	0.92	1.89	AAA-AAA	0.00	999	1.000	1.000	0.5328
214471_x_at	11.5	21.7	0.92	1.89	AAA-AAA	0.00	999	1.000	1.000	0.2749
214475_x_at	6.9	3.7	-0.92	-1.89	AAA-AAA	0.00	999	1.000	1.000	0.3063
216904_at	18.5	9.8	-0.92	-1.89	AAA-AAA	0.00	999	1.000	1.000	0.3722
217661_x_at	6.4	12.0	0.92	1.89	AAA-AAA	0.00	999	1.000	1.000	0.4210
234508_at	15.1	28.7	0.92	1.89	AAA-AAA	0.00	999	1.000	1.000	0.4172
234616_at	13.2	25.0	0.92	1.89	AAA-AAA	0.00	999	1.000	1.000	0.0358
231378_at	40.0	75.7	0.92	1.89	AAA-AAA	0.00	999	1.000	1.000	0.1021
1562349_at	3.9	7.3	0.92	1.89	AAA-AAA	0.00	999	1.000	1.000	0.0743
1569666_s_at	23.3	44.0	0.92	1.89	AAA-AAA	0.00	999	1.000	1.000	0.2692
1568859_a_at	12.4	6.6	-0.92	-1.89	AAA-AAA	0.00	999	1.000	1.000	0.1639
1566987_s_at	59.1	111.7	0.92	1.89	AAA-AAA	0.00	999	1.000	1.000	0.3921
1555132_at	26.6	14.1	-0.92	-1.89	AAA-AAA	0.00	999	1.000	1.000	0.5405
1554665_at	4.8	9.2	0.92	1.89	AAA-AAA	0.00	999	1.000	1.000	0.5800
244363_at	23.3	12.3	-0.92	-1.89	AAA-AAA	0.00	999	1.000	1.000	0.6335
209749_s_at	35.0	18.5	-0.92	-1.89	AAA-AAA	0.00	999	1.000	1.000	0.4372
215682_at	39.6	20.9	-0.92	-1.89	AAA-AAA	0.00	999	1.000	1.000	0.4151
216127_at	12.0	6.4	-0.92	-1.89	AAA-AAA	0.00	999	1.000	1.000	0.4955
235127_at	1.7	3.2	0.92	1.89	AAA-AAA	0.00	999	1.000	1.000	0.2628
234193_at	11.4	21.5	0.92	1.89	APA-PAA	0.00	999	1.000	1.000	0.6589

242838_at	49.4	93.2	0.92	1.89	AMP-APP	0.00	999	1.000	1.000	0.4315
1570165_at	38.7	73.2	0.92	1.89	APA-PPP	0.00	999	1.000	1.000	0.3745
242864_at	35.9	67.8	0.92	1.89	AAA-PPA	0.00	999	1.000	1.000	0.2745
243528_at	50.5	95.4	0.92	1.89	AAA-AAA	0.00	999	1.000	1.000	0.0020
1570152_at	3.4	6.4	0.92	1.89	AAA-AAA	0.00	999	1.000	1.000	0.4854
1566106_at	27.0	14.3	-0.92	-1.89	AAM-AAA	0.00	999	1.000	1.000	0.4648
244705_at	10.7	20.3	0.92	1.89	AAA-AAA	0.00	999	1.000	1.000	0.6213
208052_x_at	180.9	95.7	-0.92	-1.89	MAA-AAA	0.00	999	1.000	1.000	0.1613
225667_s_at	59.2	31.4	-0.92	-1.89	AAA-AAA	0.00	999	1.000	1.000	0.2473
236771_at	17.9	33.8	0.92	1.89	AAA-AAA	0.00	999	1.000	1.000	0.5783
229734_at	7.8	4.1	-0.92	-1.89	AAA-AAA	0.00	999	1.000	1.000	0.0797
205747_at	19.6	10.4	-0.92	-1.89	AAA-AAA	0.00	999	1.000	1.000	0.5099
230681_at	10.2	19.2	0.92	1.89	AAA-AAA	0.00	999	1.000	1.000	0.3984
232351_at	82.0	43.4	-0.92	-1.89	AAA-AAA	0.00	999	1.000	1.000	0.3093
231889_at	31.8	16.8	-0.92	-1.89	AAA-AAA	0.00	999	1.000	1.000	0.3245
229851_s_at	394.9	745.6	0.92	1.89	PPP-PPP	0.00	999	1.000	1.000	0.2820
1560431_at	16.5	31.1	0.92	1.89	AAA-AAP	0.00	999	1.000	1.000	0.2512
243321_at	5.1	2.7	-0.92	-1.89	AAA-AAA	0.00	999	1.000	1.000	0.0824
240415_at	49.5	26.2	-0.92	-1.89	AAA-AAA	0.00	999	1.000	1.000	0.2900
1569528_at	14.1	7.5	-0.92	-1.89	AAA-AAA	0.00	999	1.000	1.000	0.6071
1566002_at	20.4	10.8	-0.92	-1.89	AAA-AAA	0.00	999	1.000	1.000	0.4558
1555129_at	47.4	25.1	-0.92	-1.89	AAA-AAA	0.00	999	1.000	1.000	0.2589
221885_at	34.4	65.0	0.92	1.89	AAA-AAA	0.00	999	1.000	1.000	0.2844
206643_at	7.7	14.6	0.92	1.89	AAA-AAA	0.00	999	1.000	1.000	0.6002
207270_x_at	114.7	60.8	-0.92	-1.89	AAA-AAA	0.00	999	1.000	1.000	0.0539
209201_x_at	20.6	10.9	-0.92	-1.89	AAA-AAA	0.00	999	1.000	1.000	0.2920
215352_at	3.7	6.9	0.92	1.89	AAA-AAA	0.00	999	1.000	1.000	0.1465
215408_at	8.7	16.5	0.92	1.89	AAA-AAA	0.00	999	1.000	1.000	0.4844
215428_at	90.9	48.2	-0.92	-1.89	AAA-AAA	0.00	999	1.000	1.000	0.2712
234426_x_at	11.7	6.2	-0.92	-1.89	AAA-AAA	0.00	999	1.000	1.000	0.5665
237212_at	39.0	20.6	-0.92	-1.89	AAA-AAA	0.00	999	1.000	1.000	0.3173
230266_at	75.7	40.1	-0.92	-1.89	AAA-AAA	0.00	999	1.000	1.000	0.5008
231313_at	12.6	23.7	0.92	1.89	AAA-AAA	0.00	999	1.000	1.000	0.4975
204671_s_at	20.0	37.8	0.92	1.89	AAA-PPA	0.00	999	1.000	1.000	0.4185
215093_at	535.3	1010.1	0.92	1.89	PPP-PPP	0.00	999	1.000	1.000	0.1345
228575_at	36.6	69.1	0.92	1.89	AAA-APA	0.00	999	1.000	1.000	0.1266
1557791_at	6.3	3.4	-0.92	-1.89	AAA-AAA	0.00	999	1.000	1.000	0.3770
1559316_at	14.8	7.9	-0.92	-1.89	AAA-AAA	0.00	999	1.000	1.000	0.4412
1556395_at	11.4	6.0	-0.92	-1.89	AAA-AAA	0.00	999	1.000	1.000	0.6533
1557486_at	2.6	4.9	0.92	1.89	AAA-AAA	0.00	999	1.000	1.000	0.3341
207750_at	22.0	11.6	-0.92	-1.89	MAA-AAA	0.00	999	1.000	1.000	0.5398
220286_at	16.1	8.5	-0.92	-1.89	AAA-AAA	0.00	999	1.000	1.000	0.5155

210744_s_at	31.3	16.6	-0.92	-1.89	AAA-AAA	0.00	999	1.000	1.000	0.5390
216878_x_at	52.2	98.5	0.92	1.89	AAA-AAA	0.00	999	1.000	1.000	0.3351
234177_at	14.5	7.7	-0.92	-1.89	AAA-AAA	0.00	999	1.000	1.000	0.5611
231811_at	25.9	13.7	-0.92	-1.89	AAA-AAA	0.00	999	1.000	1.000	0.0643
224341_x_at	18.2	34.3	0.92	1.89	AAA-PPA	0.00	999	1.000	1.000	0.5842
226587_at	58.4	110.0	0.92	1.89	AMP-PPP	0.00	999	1.000	1.000	0.4681
232558_at	29.1	54.8	0.92	1.89	AAA-PAA	0.00	999	1.000	1.000	0.2126
1563300_at	61.6	32.7	-0.92	-1.89	AAA-AAA	0.00	999	1.000	1.000	0.4277
1564855_at	15.4	29.0	0.92	1.89	AAA-AAA	0.00	999	1.000	1.000	0.4764
1564276_at	10.7	20.1	0.92	1.89	AAA-AAA	0.00	999	1.000	1.000	0.4920
1553912_at	25.0	13.3	-0.92	-1.89	AAA-AAA	0.00	999	1.000	1.000	0.4004
1558944_at	4.2	8.0	0.92	1.89	AAA-AAA	0.00	999	1.000	1.000	0.4357
1559375_s_at	12.2	23.0	0.92	1.89	AAA-AAA	0.00	999	1.000	1.000	0.3948
1556718_s_at	20.8	11.1	-0.92	-1.89	AAA-AAA	0.00	999	1.000	1.000	0.2803
227159_at	68.3	36.2	-0.92	-1.89	AAA-AAA	0.00	999	1.000	1.000	0.1573
214708_at	53.3	28.3	-0.92	-1.89	AAA-AAA	0.00	999	1.000	1.000	0.1278
208545_x_at	14.4	27.1	0.92	1.89	AAA-AAA	0.00	999	1.000	1.000	0.4358
239378_at	39.8	75.1	0.92	1.89	AAA-AAA	0.00	999	1.000	1.000	0.2801
236890_at	25.5	13.6	-0.92	-1.89	AAA-AAA	0.00	999	1.000	1.000	0.0295
227779_at	29.5	15.6	-0.92	-1.89	AAA-AAA	0.00	999	1.000	1.000	0.5223
231704_at	23.8	12.7	-0.91	-1.88	APA-AAA	0.00	999	1.000	1.000	0.6421
202191_s_at	29.7	55.9	0.91	1.88	AAA-PAA	0.00	999	1.000	1.000	0.3590
207405_s_at	837.9	1579.1	0.91	1.89	PPP-PPP	0.00	999	1.000	1.000	0.3524
1563042_at	35.0	18.6	-0.91	-1.89	AAA-AAA	0.00	999	1.000	1.000	0.4865
1564066_at	23.7	12.6	-0.91	-1.88	AAA-AAA	0.00	999	1.000	1.000	0.4582
1566968_at	62.5	33.2	-0.91	-1.89	AAA-AAA	0.00	999	1.000	1.000	0.3502
201820_at	15.3	8.1	-0.91	-1.89	AAA-AAA	0.00	999	1.000	1.000	0.3772
220559_at	27.5	14.6	-0.91	-1.88	AMA-AAA	0.00	999	1.000	1.000	0.5796
211310_at	29.3	15.5	-0.91	-1.88	AAA-AAA	0.00	999	1.000	1.000	0.5155
216893_s_at	22.5	11.9	-0.91	-1.88	AAA-AAA	0.00	999	1.000	1.000	0.5041
215551_at	65.9	35.0	-0.91	-1.88	AAA-AAA	0.00	999	1.000	1.000	0.1862
204704_s_at	2.9	5.5	0.91	1.88	AAA-AAA	0.00	999	1.000	1.000	0.6488
1556725_a_at	14.1	26.5	0.91	1.88	PAA-AAA	0.00	999	1.000	1.000	0.6250
222033_s_at	88.3	166.3	0.91	1.88	AAP-PPP	0.00	999	1.000	1.000	0.0573
235571_at	101.8	191.6	0.91	1.88	AAA-APA	0.00	999	1.000	1.000	0.0493
241963_at	29.7	15.8	-0.91	-1.88	AAA-AAA	0.00	999	1.000	1.000	0.3597
202989_at	2.8	1.5	-0.91	-1.88	AAA-AAA	0.00	999	1.000	1.000	0.1866
243194_at	27.3	51.5	0.91	1.88	AAM-AAA	0.00	999	1.000	1.000	0.4007
243605_at	5.4	2.9	-0.91	-1.88	AAA-AAA	0.00	999	1.000	1.000	0.6285
1563277_at	24.7	13.1	-0.91	-1.88	AAA-AAA	0.00	999	1.000	1.000	0.1287
1561732_at	13.1	24.6	0.91	1.88	AAA-AAA	0.00	999	1.000	1.000	0.5592
1553963_at	2.2	4.1	0.91	1.88	AAA-AAA	0.00	999	1.000	1.000	0.2922

244382_at	73.1	38.9	-0.91	-1.88	AAA-AAA	0.00	999	1.000	1.000	0.2261
202712_s_at	63.0	33.4	-0.91	-1.88	AAA-AAA	0.00	999	1.000	1.000	0.5152
1555617_x_at	85.3	45.3	-0.91	-1.88	AAA-AAA	0.00	999	1.000	1.000	0.0184
221374_at	26.2	13.9	-0.91	-1.88	AAA-AAA	0.00	999	1.000	1.000	0.5329
221160_s_at	8.0	4.3	-0.91	-1.88	AAA-AAA	0.00	999	1.000	1.000	0.1703
222281_s_at	23.9	12.7	-0.91	-1.88	AAA-AAA	0.00	999	1.000	1.000	0.4270
207004_at	96.3	51.1	-0.91	-1.88	AAA-AAA	0.00	999	1.000	1.000	0.3718
235249_at	99.1	52.6	-0.91	-1.88	AAA-AAA	0.00	999	1.000	1.000	0.3420
205119_s_at	42.4	22.5	-0.91	-1.88	AAA-AAA	0.00	999	1.000	1.000	0.5386
234622_at	1.7	3.2	0.91	1.88	AAA-AAA	0.00	999	1.000	1.000	0.2790
237595_at	16.0	8.5	-0.91	-1.88	AAA-AAA	0.00	999	1.000	1.000	0.3491
233751_at	3.0	5.7	0.91	1.88	AAA-AAA	0.00	999	1.000	1.000	0.4501
1567022_at	7.6	14.3	0.91	1.88	AAP-AAA	0.00	999	1.000	1.000	0.5436
203969_at	42.5	80.0	0.91	1.88	APA-PPP	0.00	999	1.000	1.000	0.3333
222136_x_at	46.4	87.4	0.91	1.88	AAA-PAA	0.00	999	1.000	1.000	0.3286
224829_at	258.9	487.0	0.91	1.88	PPP-PPP	0.00	999	1.000	1.000	0.1280
241212_at	17.8	9.5	-0.91	-1.88	AAA-AAA	0.00	999	1.000	1.000	0.4112
1562810_at	94.0	50.0	-0.91	-1.88	AAA-AAA	0.00	999	1.000	1.000	0.1220
1562941_at	4.0	2.1	-0.91	-1.88	AAA-AAA	0.00	999	1.000	1.000	0.3243
1560130_at	4.6	2.5	-0.91	-1.88	AAA-AAA	0.00	999	1.000	1.000	0.3570
1561846_s_at	41.3	21.9	-0.91	-1.88	AAA-AAA	0.00	999	1.000	1.000	0.0687
1561221_x_at	70.9	37.7	-0.91	-1.88	AAA-AAA	0.00	999	1.000	1.000	0.2354
1558431_at	251.7	133.7	-0.91	-1.88	AAA-AAA	0.00	999	1.000	1.000	0.0805
220376_at	22.3	11.9	-0.91	-1.88	AAA-AAA	0.00	999	1.000	1.000	0.3109
219139_s_at	42.0	22.3	-0.91	-1.88	AAA-AAA	0.00	999	1.000	1.000	0.4845
216435_at	2.8	5.2	0.91	1.88	AAA-AAA	0.00	999	1.000	1.000	0.6094
236638_at	8.6	4.6	-0.91	-1.88	AAA-AAA	0.00	999	1.000	1.000	0.0790
237607_at	27.9	14.8	-0.91	-1.88	AAA-AAA	0.00	999	1.000	1.000	0.4888
205440_s_at	6.7	12.6	0.91	1.88	AAA-AAA	0.00	999	1.000	1.000	0.5914
244224_x_at	14.0	7.5	-0.91	-1.88	AAA-AAA	0.00	999	1.000	1.000	0.5679
243804_at	36.7	19.5	-0.91	-1.88	AAA-AAA	0.00	999	1.000	1.000	0.3245
241989_at	54.9	29.2	-0.91	-1.88	AAA-AAA	0.00	999	1.000	1.000	0.5222
1569832_at	44.8	23.9	-0.91	-1.88	AAA-AAA	0.00	999	1.000	1.000	0.3212
1553544_at	41.4	22.0	-0.91	-1.88	AAA-AAA	0.00	999	1.000	1.000	0.4490
1557570_a_at	29.7	15.8	-0.91	-1.88	AAA-AAA	0.00	999	1.000	1.000	0.4196
210019_at	26.9	14.3	-0.91	-1.88	AAA-AAA	0.00	999	1.000	1.000	0.3936
209872_s_at	48.7	25.9	-0.91	-1.88	AAA-AAA	0.00	999	1.000	1.000	0.3838
210795_s_at	20.8	39.1	0.91	1.88	AAA-AAA	0.00	999	1.000	1.000	0.1401
235383_at	43.2	81.3	0.91	1.88	AAA-AAA	0.00	999	1.000	1.000	0.1056
236783_at	83.0	44.1	-0.91	-1.88	AAA-AAA	0.00	999	1.000	1.000	0.0392
228434_at	10.5	19.8	0.91	1.88	AAA-AAA	0.00	999	1.000	1.000	0.4582
229222_at	13.8	7.3	-0.91	-1.88	AAA-AAA	0.00	999	1.000	1.000	0.6830

233266_at	88.0	46.8	-0.91	-1.88	AAA-AAA	0.00	999	1.000	1.000	0.1514
1561254_at	21.2	39.9	0.91	1.88	PAA-APP	0.00	999	1.000	1.000	0.5335
239067_s_at	45.5	85.6	0.91	1.88	AAA-AAP	0.00	999	1.000	1.000	0.3657
1560757_at	19.3	10.3	-0.91	-1.88	AAA-AAA	0.00	999	1.000	1.000	0.4475
1566762_at	3.6	1.9	-0.91	-1.88	AAA-AAA	0.00	999	1.000	1.000	0.5501
208477_at	17.2	9.2	-0.91	-1.88	AAA-AAA	0.00	999	1.000	1.000	0.5905
220819_at	62.1	33.0	-0.91	-1.88	AAA-AAA	0.00	999	1.000	1.000	0.2195
225720_at	5.8	10.9	0.91	1.88	AAA-AAA	0.00	999	1.000	1.000	0.4882
211682_x_at	26.7	14.2	-0.91	-1.88	AAA-AAA	0.00	999	1.000	1.000	0.4137
219134_at	5.5	10.3	0.91	1.88	AAA-AAA	0.00	999	1.000	1.000	0.6117
216093_at	14.0	26.2	0.91	1.88	AAA-AAA	0.00	999	1.000	1.000	0.2980
236011_at	77.6	41.3	-0.91	-1.88	AMA-AAA	0.00	999	1.000	1.000	0.0684
236663_at	36.8	19.6	-0.91	-1.88	AAA-AAA	0.00	999	1.000	1.000	0.4671
231575_at	7.1	13.3	0.91	1.88	AAA-AAA	0.00	999	1.000	1.000	0.4533
204407_at	402.4	755.7	0.91	1.88	PPP-PPP	0.00	999	1.000	1.000	0.3213
243821_at	62.3	33.2	-0.91	-1.88	AAA-AAA	0.00	999	1.000	1.000	0.3039
243282_at	15.3	28.6	0.91	1.88	AAA-AAA	0.00	999	1.000	1.000	0.3194
1405_i_at	12.0	22.5	0.91	1.88	AAA-AAA	0.00	999	1.000	1.000	0.3604
226928_x_at	207.4	110.5	-0.91	-1.88	AMA-AAA	0.00	999	1.000	1.000	0.1533
207613_s_at	15.4	8.2	-0.91	-1.88	AAA-AAA	0.00	999	1.000	1.000	0.4477
222515_x_at	25.4	13.5	-0.91	-1.88	AAA-AAA	0.00	999	1.000	1.000	0.3654
209893_s_at	39.5	74.2	0.91	1.88	AAA-AAA	0.00	999	1.000	1.000	0.0170
215241_at	8.9	4.8	-0.91	-1.88	AAA-AAA	0.00	999	1.000	1.000	0.6007
233920_at	21.5	40.4	0.91	1.88	AAA-AAA	0.00	999	1.000	1.000	0.3938
233902_at	70.7	37.6	-0.91	-1.88	AAA-AAA	0.00	999	1.000	1.000	0.1023
234653_at	17.2	32.2	0.91	1.88	AAA-AAA	0.00	999	1.000	1.000	0.0967
239203_at	16.5	31.0	0.91	1.88	AAA-AAA	0.00	999	1.000	1.000	0.3584
237473_at	129.2	68.8	-0.91	-1.88	AAA-AAA	0.00	999	1.000	1.000	0.1855
229286_at	146.3	77.9	-0.91	-1.88	AAA-AAA	0.00	999	1.000	1.000	0.4002
1552908_at	6.5	12.3	0.91	1.88	PAA-AAA	0.00	999	1.000	1.000	0.7029
210483_at	30.4	57.0	0.91	1.88	PAA-AAA	0.00	999	1.000	1.000	0.4174
215425_at	29.0	54.5	0.91	1.88	AAP-PAA	0.00	999	1.000	1.000	0.1798
244208_at	23.5	12.5	-0.91	-1.88	AAA-AAA	0.00	999	1.000	1.000	0.2389
241224_x_at	36.7	19.6	-0.91	-1.88	AAA-AAA	0.00	999	1.000	1.000	0.4840
242033_at	7.1	13.3	0.91	1.88	AAA-AAA	0.00	999	1.000	1.000	0.5584
241077_at	12.8	6.8	-0.91	-1.88	AAA-AAA	0.00	999	1.000	1.000	0.6128
1569609_at	27.0	14.4	-0.91	-1.88	AAA-AAA	0.00	999	1.000	1.000	0.4237
1564426_x_at	14.6	27.4	0.91	1.88	AAA-AAA	0.00	999	1.000	1.000	0.5687
219965_s_at	10.2	19.1	0.91	1.88	AAA-AAA	0.00	999	1.000	1.000	0.5136
220168_at	10.6	20.0	0.91	1.88	AAA-AAA	0.00	999	1.000	1.000	0.6273
219645_at	10.5	5.6	-0.91	-1.88	AAA-AAA	0.00	999	1.000	1.000	0.4891
206899_at	5.3	2.8	-0.91	-1.88	AAA-AAA	0.00	999	1.000	1.000	0.1463

212873_at	11.0	5.9	-0.91	-1.88	AAA-AAA	0.00	999	1.000	1.000	0.3969
217280_x_at	18.4	9.8	-0.91	-1.88	AAA-AAA	0.00	999	1.000	1.000	0.3869
236786_at	29.2	54.8	0.91	1.88	AAA-AAA	0.00	999	1.000	1.000	0.3660
234062_at	19.0	10.1	-0.91	-1.88	AAA-AAA	0.00	999	1.000	1.000	0.4395
237756_at	15.0	8.0	-0.91	-1.88	AAA-AAA	0.00	999	1.000	1.000	0.3951
233144_s_at	18.6	9.9	-0.91	-1.88	AAA-AAA	0.00	999	1.000	1.000	0.1713
231548_at	14.6	27.5	0.91	1.88	AAA-AAA	0.00	999	1.000	1.000	0.4609
219188_s_at	97.3	182.5	0.91	1.88	APP-PPP	0.00	999	1.000	1.000	0.4124
204346_s_at	814.2	1526.7	0.91	1.88	MPP-PPP	0.00	999	1.000	1.000	0.2591
203509_at	110.3	58.8	-0.91	-1.88	AAA-AAA	0.00	999	1.000	1.000	0.2816
240340_at	44.2	23.6	-0.91	-1.88	AAA-AAA	0.00	999	1.000	1.000	0.5029
243195_s_at	12.2	22.9	0.91	1.88	AAA-AAA	0.00	999	1.000	1.000	0.5105
1569610_at	5.1	2.7	-0.91	-1.88	AAA-AAA	0.00	999	1.000	1.000	0.1712
222049_s_at	6.8	3.6	-0.91	-1.88	AAA-AAA	0.00	999	1.000	1.000	0.2698
206869_at	6.9	12.8	0.91	1.88	AAA-AAA	0.00	999	1.000	1.000	0.2413
226788_at	7.6	4.0	-0.91	-1.88	AAA-AAA	0.00	999	1.000	1.000	0.2183
214148_at	50.6	94.9	0.91	1.88	AAA-AAA	0.00	999	1.000	1.000	0.2507
211907_s_at	10.7	20.1	0.91	1.88	AAA-AAA	0.00	999	1.000	1.000	0.4471
215862_at	38.7	20.7	-0.91	-1.88	AAA-AAA	0.00	999	1.000	1.000	0.4028
228704_s_at	10.4	5.5	-0.91	-1.88	AAA-AAA	0.00	999	1.000	1.000	0.3765
233477_at	41.9	22.3	-0.91	-1.88	AAA-AAA	0.00	999	1.000	1.000	0.3103
240896_at	44.0	23.5	-0.91	-1.87	MAA-AAA	0.00	999	1.000	1.000	0.1735
243307_at	22.7	12.1	-0.91	-1.87	AAA-AAA	0.00	999	1.000	1.000	0.4976
240196_at	13.5	25.3	0.91	1.87	AAA-AAA	0.00	999	1.000	1.000	0.4303
202917_s_at	60.5	32.3	-0.91	-1.87	AAA-AAA	0.00	999	1.000	1.000	0.2217
1569932_at	22.1	41.4	0.91	1.87	AAA-AAA	0.00	999	1.000	1.000	0.2789
1558447_at	8.2	4.4	-0.91	-1.87	AAA-AAA	0.00	999	1.000	1.000	0.0801
219744_at	9.6	5.1	-0.91	-1.87	AAA-AAA	0.00	999	1.000	1.000	0.0647
207145_at	6.0	3.2	-0.91	-1.87	AAA-AAA	0.00	999	1.000	1.000	0.2202
226598_s_at	25.6	13.7	-0.91	-1.87	AAA-AAA	0.00	999	1.000	1.000	0.2161
207482_at	22.6	12.1	-0.91	-1.87	AAA-AAA	0.00	999	1.000	1.000	0.5171
207631_at	24.2	45.4	0.91	1.87	AAA-AAA	0.00	999	1.000	1.000	0.2292
215866_at	120.5	64.3	-0.91	-1.87	AMA-AAA	0.00	999	1.000	1.000	0.1088
205009_at	15.1	28.4	0.91	1.87	AAA-AAA	0.00	999	1.000	1.000	0.4266
237858_s_at	51.1	27.3	-0.91	-1.87	AAA-AAA	0.00	999	1.000	1.000	0.4610
229930_at	25.2	13.5	-0.91	-1.87	AAA-AAA	0.00	999	1.000	1.000	0.3034
228659_at	20.2	10.8	-0.91	-1.87	AAA-AAA	0.00	999	1.000	1.000	0.2644
232843_s_at	9.4	17.5	0.91	1.87	AAA-PAA	0.00	999	1.000	1.000	0.4479
214814_at	41.7	78.2	0.91	1.87	PAP-PPP	0.00	999	1.000	1.000	0.1725
203247_s_at	705.1	1320.1	0.91	1.87	PPP-PPP	0.00	999	1.000	1.000	0.0921
1561391_at	32.7	17.5	-0.91	-1.87	AAA-AAA	0.00	999	1.000	1.000	0.2969
1564749_at	13.3	7.1	-0.91	-1.87	AAA-AAA	0.00	999	1.000	1.000	0.5444

1558553_at	24.5	45.9	0.91	1.87	AAA-AAA	0.00	999	1.000	1.000	0.4017
1555876_at	30.1	16.1	-0.91	-1.87	AAA-AAA	0.00	999	1.000	1.000	0.4423
236121_at	4.3	8.0	0.91	1.87	AAA-AAA	0.00	999	1.000	1.000	0.3119
233107_at	8.0	4.3	-0.91	-1.87	AAA-AAA	0.00	999	1.000	1.000	0.0367
233051_at	23.1	12.4	-0.91	-1.87	AAA-AAA	0.00	999	1.000	1.000	0.4664
231373_at	6.0	3.2	-0.91	-1.87	AAA-AAA	0.00	999	1.000	1.000	0.4822
232162_at	77.2	41.2	-0.91	-1.87	AAA-AAA	0.00	999	1.000	1.000	0.3935
218534_s_at	785.1	1469.5	0.90	1.87	PPP-PPP	0.00	999	1.000	1.000	0.1315
242443_at	25.3	47.3	0.90	1.87	AAA-AAA	0.00	999	1.000	1.000	0.1749
1563149_at	10.6	5.7	-0.90	-1.87	AAA-AAA	0.00	999	1.000	1.000	0.6125
1561490_at	60.0	32.1	-0.90	-1.87	AAA-AAA	0.00	999	1.000	1.000	0.4102
1565616_at	18.5	9.9	-0.90	-1.87	AAA-AAA	0.00	999	1.000	1.000	0.4772
1553537_at	17.0	9.1	-0.90	-1.87	AAA-AAA	0.00	999	1.000	1.000	0.5009
1554580_a_at	130.9	70.0	-0.90	-1.87	AAA-AAA	0.00	999	1.000	1.000	0.2091
1553243_at	20.3	10.9	-0.90	-1.87	AAM-AAA	0.00	999	1.000	1.000	0.6028
227287_at	10.4	5.6	-0.90	-1.87	AAA-AAA	0.00	999	1.000	1.000	0.1048
206623_at	21.7	11.6	-0.90	-1.87	AAA-AAA	0.00	999	1.000	1.000	0.3093
222536_s_at	50.5	27.0	-0.90	-1.87	AAA-AAA	0.00	999	1.000	1.000	0.5891
214477_at	13.5	25.2	0.90	1.87	AAA-AAA	0.00	999	1.000	1.000	0.4231
217180_at	12.1	22.6	0.90	1.87	AAA-AAA	0.00	999	1.000	1.000	0.2821
215810_x_at	56.7	30.3	-0.90	-1.87	AAA-AAA	0.00	999	1.000	1.000	0.2972
235735_at	17.9	33.5	0.90	1.87	AAA-AAA	0.00	999	1.000	1.000	0.4591
234461_at	62.5	33.4	-0.90	-1.87	AAA-AAA	0.00	999	1.000	1.000	0.4672
238864_at	20.4	10.9	-0.90	-1.87	AAA-AAA	0.00	999	1.000	1.000	0.2230
237674_at	8.7	4.7	-0.90	-1.87	AAA-AAA	0.00	999	1.000	1.000	0.2673
232308_at	8.7	16.2	0.90	1.87	AAA-AAA	0.00	999	1.000	1.000	0.3661
238592_at	14.7	27.6	0.90	1.87	AAA-AAP	0.00	999	1.000	1.000	0.4246
244250_at	30.2	56.4	0.90	1.87	AAA-AAA	0.00	999	1.000	1.000	0.2694
1562641_at	60.5	32.3	-0.90	-1.87	AAA-AAA	0.00	999	1.000	1.000	0.2415
1562004_x_at	73.0	39.1	-0.90	-1.87	AAA-AAA	0.00	999	1.000	1.000	0.1676
1552854_a_at	6.1	3.3	-0.90	-1.87	AAA-AAA	0.00	999	1.000	1.000	0.3239
1559540_at	20.1	10.8	-0.90	-1.87	AAA-AAA	0.00	999	1.000	1.000	0.6040
1557399_at	52.0	27.8	-0.90	-1.87	AAA-AAA	0.00	999	1.000	1.000	0.4346
222340_at	33.4	17.9	-0.90	-1.87	AAA-AAA	0.00	999	1.000	1.000	0.2984
208237_x_at	26.9	50.2	0.90	1.87	AAA-AAA	0.00	999	1.000	1.000	0.3988
220461_at	11.7	6.3	-0.90	-1.87	AAA-AAA	0.00	999	1.000	1.000	0.6526
210067_at	3.8	2.0	-0.90	-1.87	AAA-AAA	0.00	999	1.000	1.000	0.5476
210909_x_at	33.8	18.1	-0.90	-1.87	AAA-AAA	0.00	999	1.000	1.000	0.4219
235239_at	175.5	93.9	-0.90	-1.87	AAA-AAA	0.00	999	1.000	1.000	0.3034
236152_at	6.7	3.6	-0.90	-1.87	AAA-AAA	0.00	999	1.000	1.000	0.0156
234589_at	9.8	5.2	-0.90	-1.87	AAA-AAA	0.00	999	1.000	1.000	0.4449
230195_at	37.5	20.1	-0.90	-1.87	AAA-AAA	0.00	999	1.000	1.000	0.2348

226472_at	755.0	1410.5	0.90	1.87	PPP-PPP	0.00	999	1.000	1.000	0.1155
244100_at	39.3	21.0	-0.90	-1.87	AAA-AAA	0.00	999	1.000	1.000	0.4930
1560686_at	47.8	25.6	-0.90	-1.87	AAA-AAA	0.00	999	1.000	1.000	0.3322
1569805_at	25.2	47.0	0.90	1.87	AAA-AAA	0.00	999	1.000	1.000	0.0763
1570049_at	12.7	6.8	-0.90	-1.87	AAA-AAA	0.00	999	1.000	1.000	0.3290
1566658_at	14.1	7.6	-0.90	-1.87	AAA-AAA	0.00	999	1.000	1.000	0.5723
1553876_at	32.6	17.4	-0.90	-1.87	AAA-AAA	0.00	999	1.000	1.000	0.2663
1569481_s_at	39.4	73.6	0.90	1.87	AAA-AAA	0.00	999	1.000	1.000	0.4721
1559235_a_at	61.0	32.6	-0.90	-1.87	AAA-AAA	0.00	999	1.000	1.000	0.1380
224527_at	5.4	10.0	0.90	1.87	AAA-AAA	0.00	999	1.000	1.000	0.1418
214090_at	21.8	40.8	0.90	1.87	AAA-AAA	0.00	999	1.000	1.000	0.4376
217532_x_at	11.4	6.1	-0.90	-1.87	AAA-AAA	0.00	999	1.000	1.000	0.1892
1555365_x_at	9.1	16.9	0.90	1.87	AAA-APA	0.00	999	1.000	1.000	0.4676
240787_at	8.7	4.7	-0.90	-1.87	AAA-AAA	0.00	999	1.000	1.000	0.1005
240599_x_at	14.4	7.7	-0.90	-1.87	AAA-AAA	0.00	999	1.000	1.000	0.4314
1562157_at	5.3	10.0	0.90	1.87	AAA-AAA	0.00	999	1.000	1.000	0.2883
1563670_at	19.5	10.4	-0.90	-1.87	AAA-AAA	0.00	999	1.000	1.000	0.4797
1562892_at	41.5	22.2	-0.90	-1.87	AAA-AAA	0.00	999	1.000	1.000	0.5724
1567656_at	25.3	13.6	-0.90	-1.87	AAA-AAA	0.00	999	1.000	1.000	0.4741
244793_at	6.7	12.6	0.90	1.87	AAA-AAA	0.00	999	1.000	1.000	0.2801
1558388_a_at	7.8	4.2	-0.90	-1.87	AAA-AAA	0.00	999	1.000	1.000	0.6739
1557589_a_at	69.6	37.3	-0.90	-1.87	AAA-AAA	0.00	999	1.000	1.000	0.1968
1557211_a_at	7.6	14.1	0.90	1.87	AAA-AAA	0.00	999	1.000	1.000	0.6109
214750_at	30.2	56.4	0.90	1.87	AAA-AAA	0.00	999	1.000	1.000	0.3699
216139_s_at	69.6	37.3	-0.90	-1.87	AAA-AAA	0.00	999	1.000	1.000	0.3312
204719_at	12.5	23.3	0.90	1.87	AAA-AAA	0.00	999	1.000	1.000	0.2105
235213_at	14.8	7.9	-0.90	-1.87	AAA-AAA	0.00	999	1.000	1.000	0.5278
234041_at	12.0	6.5	-0.90	-1.87	AAA-AAA	0.00	999	1.000	1.000	0.5564
238998_x_at	11.9	22.3	0.90	1.87	AAA-AAA	0.00	999	1.000	1.000	0.6039
206176_at	35.9	67.1	0.90	1.87	AAA-AAA	0.00	999	1.000	1.000	0.1737
244204_at	19.9	37.1	0.90	1.87	AAA-AAP	0.00	999	1.000	1.000	0.4086
240839_at	21.4	11.5	-0.90	-1.87	AAA-AAA	0.00	999	1.000	1.000	0.1520
240009_at	13.8	7.4	-0.90	-1.87	AAA-AAA	0.00	999	1.000	1.000	0.2060
240598_at	30.8	16.5	-0.90	-1.87	AAA-AAA	0.00	999	1.000	1.000	0.0732
242435_at	8.2	4.4	-0.90	-1.87	AAA-AAA	0.00	999	1.000	1.000	0.0375
242745_at	28.6	53.3	0.90	1.87	AAA-AAA	0.00	999	1.000	1.000	0.3842
1560537_at	46.5	24.9	-0.90	-1.87	AAM-AAA	0.00	999	1.000	1.000	0.4903
AFFX-ThrX-M_at	9.3	5.0	-0.90	-1.87	AAA-AAA	0.00	999	1.000	1.000	0.5592
1569975_at	17.1	9.2	-0.90	-1.87	AAA-AAA	0.00	999	1.000	1.000	0.5651
1554214_at	6.4	12.0	0.90	1.87	AAA-AAA	0.00	999	1.000	1.000	0.3150
220656_at	3.7	7.0	0.90	1.87	AAA-AAA	0.00	999	1.000	1.000	0.4391
220520_s_at	3.6	6.7	0.90	1.87	AAA-AAA	0.00	999	1.000	1.000	0.6223

209889_at	14.1	7.5	-0.90	-1.87	AAA-AAA	0.00	999	1.000	1.000	0.1588
210401_at	27.7	51.7	0.90	1.87	AAA-AAA	0.00	999	1.000	1.000	0.4011
215265_at	45.8	24.6	-0.90	-1.87	AAA-AAA	0.00	999	1.000	1.000	0.3186
235854_x_at	42.9	80.1	0.90	1.87	AAA-AAA	0.00	999	1.000	1.000	0.2586
237836_at	11.5	6.2	-0.90	-1.87	AAA-AAA	0.00	999	1.000	1.000	0.5549
237997_s_at	11.7	21.9	0.90	1.87	AAA-AAA	0.00	999	1.000	1.000	0.5523
231402_at	56.8	105.9	0.90	1.87	APA-PAA	0.00	999	1.000	1.000	0.5072
240732_at	13.1	7.0	-0.90	-1.86	AAA-AAA	0.00	999	1.000	1.000	0.3148
242927_at	24.0	44.8	0.90	1.86	AAA-AAA	0.00	999	1.000	1.000	0.5019
1553845_x_at	197.3	105.8	-0.90	-1.87	AAA-AAA	0.00	999	1.000	1.000	0.0063
227403_at	49.0	26.3	-0.90	-1.86	AAA-AAA	0.00	999	1.000	1.000	0.2569
207619_at	11.1	20.6	0.90	1.86	AAA-AAA	0.00	999	1.000	1.000	0.5496
236416_at	36.2	67.5	0.90	1.87	AAA-AAA	0.00	999	1.000	1.000	0.3413
234756_at	30.9	16.6	-0.90	-1.86	AAA-AAA	0.00	999	1.000	1.000	0.2969
237995_at	11.2	6.0	-0.90	-1.86	AAA-AAA	0.00	999	1.000	1.000	0.4477
232961_at	44.9	83.7	0.90	1.86	AAA-AAA	0.00	999	1.000	1.000	0.0587
205155_s_at	57.3	30.7	-0.90	-1.87	AAA-AAA	0.00	999	1.000	1.000	0.5983
232177_at	13.1	24.4	0.90	1.87	AAA-AAA	0.00	999	1.000	1.000	0.3732
232154_at	19.4	36.2	0.90	1.87	AAA-AAA	0.00	999	1.000	1.000	0.5007
1560058_at	22.4	41.8	0.90	1.86	AAA-AAP	0.00	999	1.000	1.000	0.5127
239820_at	25.2	46.9	0.90	1.86	AAA-AAA	0.00	999	1.000	1.000	0.3954
1569256_a_at	27.1	50.6	0.90	1.86	AAA-AAA	0.00	999	1.000	1.000	0.2714
1570267_at	7.4	4.0	-0.90	-1.86	AAA-AAA	0.00	999	1.000	1.000	0.1375
1552269_at	19.2	10.3	-0.90	-1.86	AAA-AAA	0.00	999	1.000	1.000	0.2625
204942_s_at	9.1	17.0	0.90	1.86	AAA-AAA	0.00	999	1.000	1.000	0.5481
1562927_at	3.4	6.2	0.90	1.86	AAA-AAA	0.00	999	1.000	1.000	0.4349
1568723_at	15.0	27.8	0.90	1.86	AAA-AAA	0.00	999	1.000	1.000	0.5640
1568914_at	16.6	30.8	0.90	1.86	AAA-AAA	0.00	999	1.000	1.000	0.3710
1566899_at	11.0	5.9	-0.90	-1.86	AAA-AAA	0.00	999	1.000	1.000	0.4728
221083_at	32.7	17.5	-0.90	-1.86	AAA-AAA	0.00	999	1.000	1.000	0.6077
220544_at	26.7	14.4	-0.90	-1.86	AAA-AAA	0.00	999	1.000	1.000	0.5117
220468_at	12.8	23.8	0.90	1.86	AAA-AAA	0.00	999	1.000	1.000	0.2993
235977_at	39.3	21.1	-0.90	-1.86	AAA-AAA	0.00	999	1.000	1.000	0.3545
231135_at	22.6	42.0	0.90	1.86	AAA-AAA	0.00	999	1.000	1.000	0.4335
202549_at	43.5	81.0	0.90	1.86	PAP-APP	0.00	999	1.000	1.000	0.5379
207286_at	35.3	65.7	0.90	1.86	AAP-PPP	0.00	999	1.000	1.000	0.1761
206273_at	97.5	181.5	0.90	1.86	APA-PPP	0.00	999	1.000	1.000	0.1492
213524_s_at	54.4	101.3	0.90	1.86	AAA-PAA	0.00	999	1.000	1.000	0.0919
1570593_at	2.9	5.4	0.90	1.86	AAA-AAA	0.00	999	1.000	1.000	0.4023
1565320_at	31.2	16.8	-0.90	-1.86	AAA-AAA	0.00	999	1.000	1.000	0.4193
1558687_a_at	5.0	9.4	0.90	1.86	AAA-AAA	0.00	999	1.000	1.000	0.5505
219824_at	41.4	77.1	0.90	1.86	AAA-AMA	0.00	999	1.000	1.000	0.4034

220619_at	4.2	7.8	0.90	1.86	AAA-AAA	0.00	999	1.000	1.000	0.4839
207246_at	12.3	23.0	0.90	1.86	AAA-AAA	0.00	999	1.000	1.000	0.4792
206608_s_at	117.5	63.1	-0.90	-1.86	AAA-AAA	0.00	999	1.000	1.000	0.2198
238776_x_at	12.5	6.7	-0.90	-1.86	AAA-AAA	0.00	999	1.000	1.000	0.3376
237391_at	11.1	6.0	-0.90	-1.86	AAA-AAA	0.00	999	1.000	1.000	0.6665
237430_at	9.3	5.0	-0.90	-1.86	AAA-AAA	0.00	999	1.000	1.000	0.1371
231788_at	7.6	14.2	0.90	1.86	AAA-AAA	0.00	999	1.000	1.000	0.4350
1570128_at	15.9	29.6	0.90	1.86	APP-AAP	0.00	999	1.000	1.000	0.4808
214683_s_at	437.6	813.9	0.90	1.86	PPP-PPP	0.00	999	1.000	1.000	0.1355
202859_x_at	4180.3	7775.9	0.90	1.86	PPP-PPP	0.00	999	1.000	1.000	0.0580
239817_at	6.5	12.0	0.90	1.86	AAA-AAA	0.00	999	1.000	1.000	0.1340
1562616_at	8.8	4.7	-0.90	-1.86	AAA-AAA	0.00	999	1.000	1.000	0.5946
1560884_at	2.6	4.9	0.90	1.86	AAA-AAA	0.00	999	1.000	1.000	0.4682
1561519_at	11.5	21.3	0.90	1.86	AAA-AAA	0.00	999	1.000	1.000	0.4393
1556484_at	117.7	63.3	-0.90	-1.86	AAA-AAA	0.00	999	1.000	1.000	0.2238
220959_s_at	12.8	23.9	0.90	1.86	AAA-AAA	0.00	999	1.000	1.000	0.4616
219955_at	51.3	27.6	-0.90	-1.86	AMA-AAA	0.00	999	1.000	1.000	0.3761
219857_at	6.0	3.2	-0.90	-1.86	AAA-AAA	0.00	999	1.000	1.000	0.6653
223786_at	6.3	3.4	-0.90	-1.86	AAA-AAA	0.00	999	1.000	1.000	0.2808
216752_at	5.9	11.0	0.90	1.86	AAA-AAA	0.00	999	1.000	1.000	0.6116
237844_at	16.5	30.8	0.90	1.86	AAA-AAA	0.00	999	1.000	1.000	0.4015
230143_at	45.0	24.2	-0.90	-1.86	AAA-AAA	0.00	999	1.000	1.000	0.2747
229809_at	46.7	25.1	-0.90	-1.86	AMA-AAA	0.00	999	1.000	1.000	0.4403
205713_s_at	12.8	6.9	-0.90	-1.86	AAA-AAA	0.00	999	1.000	1.000	0.4489
232973_at	7.4	13.7	0.90	1.86	AAA-AAA	0.00	999	1.000	1.000	0.0774
232301_at	35.1	65.3	0.90	1.86	MAA-AAA	0.00	999	1.000	1.000	0.3929
209804_at	221.7	412.1	0.89	1.86	APP-PPP	0.00	999	1.000	1.000	0.4225
209859_at	16.8	31.2	0.89	1.86	APA-AAA	0.00	999	1.000	1.000	0.4124
204484_at	133.1	247.4	0.89	1.86	AAP-APP	0.00	999	1.000	1.000	0.3927
244126_at	61.2	32.9	-0.89	-1.86	AAA-AAA	0.00	999	1.000	1.000	0.4359
1561884_at	98.2	52.8	-0.89	-1.86	AAA-AAA	0.00	999	1.000	1.000	0.0151
1559434_at	2.9	1.6	-0.89	-1.86	AAA-AAA	0.00	999	1.000	1.000	0.2046
213921_at	25.1	13.5	-0.89	-1.86	AAA-AAA	0.00	999	1.000	1.000	0.3778
213759_at	5.7	10.5	0.89	1.86	AAA-AAA	0.00	999	1.000	1.000	0.1405
210533_at	21.0	11.3	-0.89	-1.86	AAA-AAA	0.00	999	1.000	1.000	0.6360
217062_at	7.3	13.5	0.89	1.86	AAA-AAA	0.00	999	1.000	1.000	0.4686
238117_at	52.5	28.3	-0.89	-1.86	AAA-AAA	0.00	999	1.000	1.000	0.1519
232305_at	11.4	21.2	0.89	1.86	AAA-AAA	0.00	999	1.000	1.000	0.3563
231977_at	6.5	3.5	-0.89	-1.86	AAA-AAA	0.00	999	1.000	1.000	0.3928
243542_at	12.1	22.4	0.89	1.86	AAA-PAA	0.00	999	1.000	1.000	0.4739
1557637_at	27.0	50.2	0.89	1.86	AAM-APP	0.00	999	1.000	1.000	0.4192
233622_x_at	10.2	19.0	0.89	1.86	AAA-PAA	0.00	999	1.000	1.000	0.4023

241740_at	12.2	22.7	0.89	1.86	AAA-AAA	0.00	999	1.000	1.000	0.4318
1561351_at	14.9	8.0	-0.89	-1.86	AAA-AAA	0.00	999	1.000	1.000	0.4698
1554714_at	4.1	2.2	-0.89	-1.86	AAA-AAA	0.00	999	1.000	1.000	0.0568
1556996_at	6.1	3.3	-0.89	-1.86	AAA-AAA	0.00	999	1.000	1.000	0.0610
222007_s_at	8.3	4.5	-0.89	-1.86	AAA-AAA	0.00	999	1.000	1.000	0.2576
219672_at	72.5	39.0	-0.89	-1.86	AAA-AAA	0.00	999	1.000	1.000	0.0945
219562_at	32.9	17.7	-0.89	-1.86	AAA-AAA	0.00	999	1.000	1.000	0.3398
213716_s_at	72.1	38.8	-0.89	-1.86	AAA-AAA	0.00	999	1.000	1.000	0.0806
214777_at	13.6	7.3	-0.89	-1.86	AAA-AAA	0.00	999	1.000	1.000	0.0347
211739_x_at	5.2	2.8	-0.89	-1.86	AAA-AAA	0.00	999	1.000	1.000	0.3397
219132_at	28.7	15.5	-0.89	-1.86	AAA-AAA	0.00	999	1.000	1.000	0.2372
215678_at	27.2	14.6	-0.89	-1.86	AAA-AAA	0.00	999	1.000	1.000	0.2549
216686_at	8.9	4.8	-0.89	-1.86	AAA-AAA	0.00	999	1.000	1.000	0.6734
233225_at	25.8	48.0	0.89	1.86	AAA-AAA	0.00	999	1.000	1.000	0.4932
232128_s_at	23.7	43.9	0.89	1.86	APA-AAP	0.00	999	1.000	1.000	0.5459
241672_at	18.4	34.2	0.89	1.86	PAA-APA	0.00	999	1.000	1.000	0.4784
235371_at	458.5	850.6	0.89	1.86	PPP-PPP	0.00	999	1.000	1.000	0.3277
243695_at	94.5	50.9	-0.89	-1.86	AAA-AAA	0.00	999	1.000	1.000	0.0969
1561060_at	9.1	16.9	0.89	1.86	AAA-AAA	0.00	999	1.000	1.000	0.4856
1569828_at	13.8	7.4	-0.89	-1.86	AAA-AAA	0.00	999	1.000	1.000	0.4549
1566653_at	83.1	44.8	-0.89	-1.86	AAA-AAA	0.00	999	1.000	1.000	0.1931
1556779_s_at	4.2	7.8	0.89	1.86	AAA-AAA	0.00	999	1.000	1.000	0.1714
209842_at	18.1	33.7	0.89	1.86	AAA-AAA	0.00	999	1.000	1.000	0.1544
210363_s_at	7.1	13.2	0.89	1.86	AAA-AAA	0.00	999	1.000	1.000	0.5769
217131_at	30.1	16.2	-0.89	-1.86	AAA-AAA	0.00	999	1.000	1.000	0.3353
219159_s_at	11.5	6.2	-0.89	-1.86	AAA-AAA	0.00	999	1.000	1.000	0.1282
216281_at	7.8	4.2	-0.89	-1.86	AAA-AAA	0.00	999	1.000	1.000	0.5246
234948_at	20.0	10.8	-0.89	-1.86	AAA-AAA	0.00	999	1.000	1.000	0.2651
231615_at	33.4	18.0	-0.89	-1.86	AAA-AAA	0.00	999	1.000	1.000	0.1006
207655_s_at	6.1	11.3	0.89	1.85	PAA-AAA	0.00	999	1.000	1.000	0.6881
228062_at	236.8	439.2	0.89	1.86	PPP-PPP	0.00	999	1.000	1.000	0.1855
243762_at	28.9	53.7	0.89	1.85	AAA-AAA	0.00	999	1.000	1.000	0.2931
1563302_at	101.1	54.5	-0.89	-1.86	AAA-AAA	0.00	999	1.000	1.000	0.1456
1561171_a_at	81.7	44.1	-0.89	-1.85	AAA-AAA	0.00	999	1.000	1.000	0.5039
223885_at	8.0	14.9	0.89	1.85	AAA-AAA	0.00	999	1.000	1.000	0.3958
209830_s_at	8.3	15.4	0.89	1.86	AAA-AAA	0.00	999	1.000	1.000	0.1942
217282_at	26.0	14.0	-0.89	-1.85	AAA-AAA	0.00	999	1.000	1.000	0.4139
233861_at	25.2	13.6	-0.89	-1.85	AAA-AAA	0.00	999	1.000	1.000	0.5097
237740_at	7.8	4.2	-0.89	-1.85	AAA-AAA	0.00	999	1.000	1.000	0.4446
205900_at	36.7	19.8	-0.89	-1.86	AAA-AAA	0.00	999	1.000	1.000	0.4538
206019_at	173.3	93.4	-0.89	-1.86	AMA-AAA	0.00	999	1.000	1.000	0.0593
233283_at	43.7	23.6	-0.89	-1.85	AAA-AAA	0.00	999	1.000	1.000	0.5473

233340_at	21.6	40.0	0.89	1.85	AAA-AAA	0.00	999	1.000	1.000	0.3763
234247_at	6.8	12.7	0.89	1.85	AAA-APA	0.00	999	1.000	1.000	0.6724
240019_at	3.1	5.7	0.89	1.85	AAA-AAA	0.00	999	1.000	1.000	0.5068
240934_at	50.8	27.4	-0.89	-1.85	AAA-AAA	0.00	999	1.000	1.000	0.4768
241248_at	20.9	11.3	-0.89	-1.85	AAA-AAA	0.00	999	1.000	1.000	0.5372
240124_at	17.5	9.4	-0.89	-1.85	AAA-AAA	0.00	999	1.000	1.000	0.1294
1561514_at	9.5	17.6	0.89	1.85	AAA-AAA	0.00	999	1.000	1.000	0.6354
1564536_at	7.2	13.3	0.89	1.85	AAA-AAA	0.00	999	1.000	1.000	0.3413
208028_s_at	18.8	10.1	-0.89	-1.85	AAA-AAA	0.00	999	1.000	1.000	0.5565
239579_at	15.4	8.3	-0.89	-1.85	AAA-AAA	0.00	999	1.000	1.000	0.5826
232155_at	42.0	77.8	0.89	1.85	AAA-AAP	0.00	999	1.000	1.000	0.4356
226479_at	420.8	779.3	0.89	1.85	PPP-PPP	0.00	999	1.000	1.000	0.1069
241002_at	43.5	23.5	-0.89	-1.85	AAA-AAM	0.00	999	1.000	1.000	0.4114
241114_s_at	73.7	39.8	-0.89	-1.85	AAA-AAA	0.00	999	1.000	1.000	0.1262
1565802_at	5.9	3.2	-0.89	-1.85	AAA-AAA	0.00	999	1.000	1.000	0.0604
221312_at	6.5	12.0	0.89	1.85	AAA-AAA	0.00	999	1.000	1.000	0.2917
217073_x_at	23.4	12.6	-0.89	-1.85	AAA-AAA	0.00	999	1.000	1.000	0.3513
216476_at	68.2	36.9	-0.89	-1.85	AAA-AAA	0.00	999	1.000	1.000	0.4332
235822_at	145.3	78.4	-0.89	-1.85	AAA-AAA	0.00	999	1.000	1.000	0.0151
239326_at	14.9	27.5	0.89	1.85	AAA-AAA	0.00	999	1.000	1.000	0.5717
206223_at	33.6	62.1	0.89	1.85	AAA-AAA	0.00	999	1.000	1.000	0.5056
1563068_at	55.2	29.8	-0.89	-1.85	AAA-AAA	0.00	999	1.000	1.000	0.1290
1561895_at	16.7	9.0	-0.89	-1.85	AAA-AAA	0.00	999	1.000	1.000	0.5733
1570197_at	149.9	81.0	-0.89	-1.85	AAA-AAA	0.00	999	1.000	1.000	0.3980
1560779_a_at	14.3	26.4	0.89	1.85	AAA-AAA	0.00	999	1.000	1.000	0.4199
221121_at	33.7	18.2	-0.89	-1.85	AAA-AAA	0.00	999	1.000	1.000	0.4389
208390_s_at	180.0	97.3	-0.89	-1.85	AAA-AAA	0.00	999	1.000	1.000	0.0429
227168_at	27.5	14.8	-0.89	-1.85	AAA-AAA	0.00	999	1.000	1.000	0.1432
216798_at	54.7	29.6	-0.89	-1.85	AAA-AAA	0.00	999	1.000	1.000	0.0955
236579_at	16.7	9.0	-0.89	-1.85	AAA-AAA	0.00	999	1.000	1.000	0.2440
234877_x_at	103.9	56.1	-0.89	-1.85	AAA-AAA	0.00	999	1.000	1.000	0.5851
234696_at	43.3	23.4	-0.89	-1.85	AAA-AAA	0.00	999	1.000	1.000	0.4241
229724_at	10.6	19.7	0.89	1.85	AAA-AAA	0.00	999	1.000	1.000	0.5839
240142_at	23.7	12.8	-0.89	-1.85	AAA-AAA	0.00	999	1.000	1.000	0.3538
1570651_at	80.5	43.5	-0.89	-1.85	AAA-AAA	0.00	999	1.000	1.000	0.2319
1559936_at	21.4	11.6	-0.89	-1.85	AAA-AAA	0.00	999	1.000	1.000	0.3449
1556231_a_at	10.1	5.5	-0.89	-1.85	AAA-AAA	0.00	999	1.000	1.000	0.4675
1556459_at	33.7	18.2	-0.89	-1.85	AAA-AAA	0.00	999	1.000	1.000	0.3742
207298_at	10.3	5.6	-0.89	-1.85	AAA-AAA	0.00	999	1.000	1.000	0.0364
211605_s_at	34.3	63.4	0.89	1.85	AAA-AAA	0.00	999	1.000	1.000	0.5440
216560_x_at	10.6	5.8	-0.89	-1.85	AAA-AAA	0.00	999	1.000	1.000	0.1139
204562_at	57.2	31.0	-0.89	-1.85	AAA-AAA	0.00	999	1.000	1.000	0.5512

237635_at	18.1	9.8	-0.89	-1.85	AAA-AAA	0.00	999	1.000	1.000	0.3235
205388_at	38.2	20.6	-0.89	-1.85	AAA-AAA	0.00	999	1.000	1.000	0.3068
1555568_at	27.1	50.1	0.89	1.85	AAA-PPP	0.00	999	1.000	1.000	0.4710
227270_at	535.5	989.7	0.89	1.85	PPP-PPP	0.00	999	1.000	1.000	0.1848
220091_at	174.8	323.1	0.89	1.85	PAP-PPA	0.00	999	1.000	1.000	0.1019
243926_at	15.4	8.4	-0.89	-1.85	AAA-AAA	0.00	999	1.000	1.000	0.3921
244113_at	6.0	3.3	-0.89	-1.85	AAA-AAA	0.00	999	1.000	1.000	0.0922
241584_at	9.8	5.3	-0.89	-1.85	AAA-AAA	0.00	999	1.000	1.000	0.1799
1562886_at	1.6	2.9	0.89	1.85	AAA-AAA	0.00	999	1.000	1.000	0.3661
1561086_at	42.1	22.8	-0.89	-1.85	AAA-AAA	0.00	999	1.000	1.000	0.2636
1565784_at	32.7	17.7	-0.89	-1.85	AAA-AAA	0.00	999	1.000	1.000	0.5372
1552367_a_at	4.5	8.3	0.89	1.85	AAA-AAA	0.00	999	1.000	1.000	0.4689
1558081_at	32.6	17.6	-0.89	-1.85	AAA-AAA	0.00	999	1.000	1.000	0.4211
1556289_at	6.0	3.2	-0.89	-1.85	AAA-AAA	0.00	999	1.000	1.000	0.6125
220308_at	77.8	42.1	-0.89	-1.85	AAA-AAA	0.00	999	1.000	1.000	0.3570
223550_s_at	4.3	2.3	-0.89	-1.85	AAA-AAA	0.00	999	1.000	1.000	0.0769
214181_x_at	12.3	22.7	0.89	1.85	AAA-AAA	0.00	999	1.000	1.000	0.3402
209728_at	12.2	6.6	-0.89	-1.85	AAA-AAA	0.00	999	1.000	1.000	0.2964
235769_at	11.5	6.2	-0.89	-1.85	AAA-AAA	0.00	999	1.000	1.000	0.3603
239566_at	14.4	7.8	-0.89	-1.85	AAA-AAA	0.00	999	1.000	1.000	0.4825
239181_at	11.3	6.1	-0.89	-1.85	AAA-AAA	0.00	999	1.000	1.000	0.1941
237504_at	101.0	54.7	-0.89	-1.85	AAA-AAA	0.00	999	1.000	1.000	0.0536
231051_at	38.7	21.0	-0.89	-1.85	AAA-AAA	0.00	999	1.000	1.000	0.3242
1558501_at	15.6	28.8	0.89	1.85	APA-AAM	0.00	999	1.000	1.000	0.5659
240755_at	14.2	26.2	0.89	1.85	APA-AAA	0.00	999	1.000	1.000	0.4904
223989_s_at	340.4	628.6	0.89	1.85	PPP-PPP	0.00	999	1.000	1.000	0.3669
1565730_at	34.5	18.7	-0.89	-1.85	AAA-AAA	0.00	999	1.000	1.000	0.3230
1564658_at	18.1	33.5	0.89	1.85	AAA-AAA	0.00	999	1.000	1.000	0.5560
208014_x_at	24.9	13.5	-0.89	-1.85	AAA-AAA	0.00	999	1.000	1.000	0.4910
220852_at	15.3	8.3	-0.89	-1.85	AAA-AAA	0.00	999	1.000	1.000	0.5069
224095_at	14.0	7.6	-0.89	-1.85	AAA-AAA	0.00	999	1.000	1.000	0.4605
209742_s_at	12.4	22.9	0.89	1.85	AAA-AAA	0.00	999	1.000	1.000	0.4973
219415_at	8.4	4.6	-0.89	-1.85	AAA-AAA	0.00	999	1.000	1.000	0.3582
230633_at	49.7	26.9	-0.89	-1.85	AAA-AAA	0.00	999	1.000	1.000	0.5638
242636_at	47.9	88.4	0.88	1.85	AAP-PPP	0.00	999	1.000	1.000	0.2268
240286_at	10.3	5.6	-0.88	-1.85	AAA-AAA	0.00	999	1.000	1.000	0.5592
240227_at	9.5	5.2	-0.88	-1.85	AAA-AAA	0.00	999	1.000	1.000	0.0477
1560086_at	7.0	3.8	-0.88	-1.85	AAA-AAA	0.00	999	1.000	1.000	0.6426
1565894_at	45.7	24.8	-0.88	-1.85	AAA-AAA	0.00	999	1.000	1.000	0.4617
1552998_at	3.4	6.2	0.88	1.85	AAA-AAA	0.00	999	1.000	1.000	0.5249
1557790_at	75.0	40.7	-0.88	-1.85	AAA-AAA	0.00	999	1.000	1.000	0.4670
1559864_at	50.9	27.6	-0.88	-1.85	AAA-AAA	0.00	999	1.000	1.000	0.2216

221678_at	10.9	5.9	-0.88	-1.85	AAA-AAA	0.00	999	1.000	1.000	0.1375
220996_s_at	18.9	34.9	0.88	1.85	AAA-AAA	0.00	999	1.000	1.000	0.1706
34210_at	9.0	4.9	-0.88	-1.85	AAA-AAA	0.00	999	1.000	1.000	0.3223
213958_at	15.5	28.6	0.88	1.85	AAA-AAA	0.00	999	1.000	1.000	0.4341
210176_at	46.3	25.1	-0.88	-1.85	AAM-AAA	0.00	999	1.000	1.000	0.0232
215941_at	10.8	19.8	0.88	1.85	AAA-AAA	0.00	999	1.000	1.000	0.2114
234320_at	20.7	11.2	-0.88	-1.85	AAA-AAA	0.00	999	1.000	1.000	0.5974
206064_s_at	92.2	170.2	0.88	1.85	AAA-AAA	0.00	999	1.000	1.000	0.0928
213904_at	18.2	33.6	0.88	1.84	AAA-PAA	0.00	999	1.000	1.000	0.4258
202065_s_at	282.7	521.4	0.88	1.85	AAA-PPP	0.00	999	1.000	1.000	0.1505
242090_x_at	6.9	12.7	0.88	1.84	AAA-AAA	0.00	999	1.000	1.000	0.6248
203849_s_at	50.5	27.4	-0.88	-1.85	AAA-AAA	0.00	999	1.000	1.000	0.3814
1560901_at	79.4	43.0	-0.88	-1.85	AAA-AAA	0.00	999	1.000	1.000	0.4039
1553579_a_at	5.5	3.0	-0.88	-1.84	AAA-AAA	0.00	999	1.000	1.000	0.6873
244787_at	10.6	5.8	-0.88	-1.84	AAA-AAA	0.00	999	1.000	1.000	0.5484
220695_at	11.7	6.3	-0.88	-1.84	AAA-AAA	0.00	999	1.000	1.000	0.3422
215793_at	9.1	16.8	0.88	1.85	AAA-AAA	0.00	999	1.000	1.000	0.4733
229137_at	16.2	29.8	0.88	1.84	AMA-AAA	0.00	999	1.000	1.000	0.5645
205582_s_at	6.5	3.5	-0.88	-1.84	AAA-AAA	0.00	999	1.000	1.000	0.1203
223478_at	240.0	442.3	0.88	1.84	PPA-PPP	0.00	999	1.000	1.000	0.2107
1558802_at	48.3	89.1	0.88	1.84	PPA-PPP	0.00	999	1.000	1.000	0.0638
230954_at	184.1	339.1	0.88	1.84	PMP-PPP	0.00	999	1.000	1.000	0.0487
1562834_at	21.2	39.0	0.88	1.84	AAA-AAA	0.00	999	1.000	1.000	0.4247
1560047_s_at	37.1	20.2	-0.88	-1.84	AAA-AAA	0.00	999	1.000	1.000	0.5898
1553515_at	51.6	28.0	-0.88	-1.84	AAA-AAA	0.00	999	1.000	1.000	0.1203
1552826_at	8.8	4.8	-0.88	-1.84	AAA-AAA	0.00	999	1.000	1.000	0.6090
1557865_at	13.5	7.3	-0.88	-1.84	AAA-AAA	0.00	999	1.000	1.000	0.3167
221414_s_at	22.5	12.2	-0.88	-1.84	AAA-AAA	0.00	999	1.000	1.000	0.5470
222292_at	11.3	20.8	0.88	1.84	AAA-AAA	0.00	999	1.000	1.000	0.5449
207672_at	5.2	2.8	-0.88	-1.84	AAA-AAA	0.00	999	1.000	1.000	0.1536
224122_at	10.7	5.8	-0.88	-1.84	AAA-AAA	0.00	999	1.000	1.000	0.0531
215827_x_at	15.1	8.2	-0.88	-1.84	AAA-AAA	0.00	999	1.000	1.000	0.2536
233960_s_at	122.6	66.5	-0.88	-1.84	AAA-AAA	0.00	999	1.000	1.000	0.0226
1554172_a_at	94.6	174.2	0.88	1.84	APP-PPP	0.00	999	1.000	1.000	0.4094
243444_at	22.8	42.0	0.88	1.84	AAA-AAP	0.00	999	1.000	1.000	0.3662
224414_s_at	351.6	647.2	0.88	1.84	PPP-PPP	0.00	999	1.000	1.000	0.0554
203888_at	22.1	12.0	-0.88	-1.84	AAA-AAA	0.00	999	1.000	1.000	0.1482
243369_at	29.9	16.3	-0.88	-1.84	AAA-AAA	0.00	999	1.000	1.000	0.4603
1568743_at	41.7	22.7	-0.88	-1.84	AAA-AAA	0.00	999	1.000	1.000	0.4351
1569054_at	10.4	5.7	-0.88	-1.84	AAA-AAA	0.00	999	1.000	1.000	0.5627
1568978_s_at	12.4	22.8	0.88	1.84	AAA-AAA	0.00	999	1.000	1.000	0.3989
200938_s_at	32.8	17.8	-0.88	-1.84	AAA-AAA	0.00	999	1.000	1.000	0.5010

1554872_a_at	9.3	5.0	-0.88	-1.84	AAA-AAA	0.00	999	1.000	1.000	0.0334
221009_s_at	34.6	18.8	-0.88	-1.84	AAA-AAA	0.00	999	1.000	1.000	0.2821
207673_at	33.6	18.2	-0.88	-1.84	AAA-AAA	0.00	999	1.000	1.000	0.5589
223956_at	92.8	50.4	-0.88	-1.84	AAA-AAA	0.00	999	1.000	1.000	0.3411
216573_at	6.8	12.5	0.88	1.84	AAA-AAA	0.00	999	1.000	1.000	0.5118
236616_at	34.1	62.7	0.88	1.84	AAA-AAA	0.00	999	1.000	1.000	0.3388
237045_at	102.7	55.8	-0.88	-1.84	AAA-AAA	0.00	999	1.000	1.000	0.4112
229764_at	13.8	25.4	0.88	1.84	AAA-AAA	0.00	999	1.000	1.000	0.4708
230956_at	33.7	18.3	-0.88	-1.84	AAA-AAA	0.00	999	1.000	1.000	0.4206
227902_at	8.9	16.4	0.88	1.84	AAA-AAA	0.00	999	1.000	1.000	0.4928
201285_at	1132.6	2083.8	0.88	1.84	PPP-PPP	0.00	999	1.000	1.000	0.3715
244144_at	8.4	4.6	-0.88	-1.84	AAA-AAA	0.00	999	1.000	1.000	0.1172
243193_at	18.9	10.3	-0.88	-1.84	AAA-AAA	0.00	999	1.000	1.000	0.5928
1555085_at	30.9	16.8	-0.88	-1.84	AAA-AAA	0.00	999	1.000	1.000	0.4954
1554909_at	61.9	33.6	-0.88	-1.84	AAA-AAA	0.00	999	1.000	1.000	0.4641
1554491_a_at	32.5	59.8	0.88	1.84	AAA-AAA	0.00	999	1.000	1.000	0.1825
1552359_at	2.0	3.7	0.88	1.84	AAA-AAA	0.00	999	1.000	1.000	0.5471
1559877_at	20.5	11.2	-0.88	-1.84	AAA-AAA	0.00	999	1.000	1.000	0.5405
225671_at	76.8	41.7	-0.88	-1.84	AAA-AAA	0.00	999	1.000	1.000	0.4278
213866_at	52.0	28.3	-0.88	-1.84	AAA-AAA	0.00	999	1.000	1.000	0.5227
210937_s_at	30.7	16.7	-0.88	-1.84	AAA-AAA	0.00	999	1.000	1.000	0.5068
1563182_at	25.6	13.9	-0.88	-1.84	APA-AAA	0.00	999	1.000	1.000	0.6032
214994_at	60.4	111.2	0.88	1.84	AAM-MAP	0.00	999	1.000	1.000	0.3954
234227_at	16.0	29.4	0.88	1.84	AAA-APP	0.00	999	1.000	1.000	0.2233
242728_at	14.6	8.0	-0.88	-1.84	AAA-AAA	0.00	999	1.000	1.000	0.0907
241108_at	32.9	60.4	0.88	1.84	AAA-AAA	0.00	999	1.000	1.000	0.1187
1569193_at	8.2	15.1	0.88	1.84	AAA-AAA	0.00	999	1.000	1.000	0.4706
1552902_a_at	7.4	4.0	-0.88	-1.84	AAA-AAA	0.00	999	1.000	1.000	0.3363
207384_at	22.3	12.1	-0.88	-1.84	AAA-AAA	0.00	999	1.000	1.000	0.4700
219046_s_at	74.2	40.3	-0.88	-1.84	AAA-AAA	0.00	999	1.000	1.000	0.3960
235537_at	50.9	27.7	-0.88	-1.84	AAA-AAA	0.00	999	1.000	1.000	0.5034
236595_at	61.4	33.4	-0.88	-1.84	AAA-AAA	0.00	999	1.000	1.000	0.0929
235008_at	51.8	28.2	-0.88	-1.84	AAA-AAA	0.00	999	1.000	1.000	0.1614
238107_at	20.7	11.3	-0.88	-1.84	AAA-AAA	0.00	999	1.000	1.000	0.3324
237612_at	22.2	12.1	-0.88	-1.84	AAA-AAA	0.00	999	1.000	1.000	0.4100
232766_at	63.8	34.7	-0.88	-1.84	AAA-AAA	0.00	999	1.000	1.000	0.1234
240804_at	11.6	21.2	0.88	1.84	AAA-AAA	0.00	999	1.000	1.000	0.3900
240352_at	27.7	50.8	0.88	1.84	AAA-AAA	0.00	999	1.000	1.000	0.4021
1562695_at	14.9	8.1	-0.88	-1.84	AAA-AAA	0.00	999	1.000	1.000	0.2082
1554380_at	21.8	40.0	0.88	1.84	AAA-AAA	0.00	999	1.000	1.000	0.5945
1559724_at	9.4	5.1	-0.88	-1.84	AAA-AAA	0.00	999	1.000	1.000	0.4830
221075_s_at	13.7	7.5	-0.88	-1.84	AAA-AAA	0.00	999	1.000	1.000	0.4373

221955_at	14.8	8.1	-0.88	-1.84	AAA-AAA	0.00	999	1.000	1.000	0.4143
217298_at	4.8	8.7	0.88	1.84	AAA-AAA	0.00	999	1.000	1.000	0.4928
216075_at	37.6	20.5	-0.88	-1.84	AAA-AAA	0.00	999	1.000	1.000	0.4162
235672_at	69.7	38.0	-0.88	-1.84	AAA-AAA	0.00	999	1.000	1.000	0.0778
205275_at	28.6	52.5	0.88	1.84	AAA-AAA	0.00	999	1.000	1.000	0.4550
233801_s_at	8.4	15.4	0.88	1.84	AAA-AAA	0.00	999	1.000	1.000	0.4427
231461_at	13.1	7.1	-0.88	-1.84	AAA-AAA	0.00	999	1.000	1.000	0.4264
235798_at	28.9	53.1	0.88	1.84	AAP-APP	0.00	999	1.000	1.000	0.2952
207304_at	124.9	229.5	0.88	1.84	PPP-PPP	0.00	999	1.000	1.000	0.2020
243873_at	7.7	4.2	-0.88	-1.84	AAA-AAA	0.00	999	1.000	1.000	0.5145
1560098_at	12.7	6.9	-0.88	-1.84	AAA-AAA	0.00	999	1.000	1.000	0.4427
1559979_at	36.8	67.5	0.88	1.84	AAA-MAA	0.00	999	1.000	1.000	0.1909
1559785_at	17.5	9.5	-0.88	-1.84	AAA-AAA	0.00	999	1.000	1.000	0.2841
1556212_x_at	6.4	11.8	0.88	1.84	AAA-AAA	0.00	999	1.000	1.000	0.5751
1556319_at	68.3	37.2	-0.88	-1.84	AAA-AAA	0.00	999	1.000	1.000	0.2500
220003_at	46.6	25.4	-0.88	-1.84	AAA-AAA	0.00	999	1.000	1.000	0.4392
206774_at	25.7	14.0	-0.88	-1.84	AAA-AAA	0.00	999	1.000	1.000	0.3480
210341_at	14.6	8.0	-0.88	-1.84	AAA-AAA	0.00	999	1.000	1.000	0.4687
216857_at	20.1	10.9	-0.88	-1.84	AAA-AAA	0.00	999	1.000	1.000	0.5197
209539_at	22.0	40.3	0.88	1.84	AAA-AAA	0.00	999	1.000	1.000	0.3387
216156_at	16.3	8.9	-0.88	-1.84	AAA-AAA	0.00	999	1.000	1.000	0.3900
228538_at	30.3	16.5	-0.88	-1.84	AAA-AAA	0.00	999	1.000	1.000	0.4518
228358_at	72.2	39.3	-0.88	-1.84	AAA-AAA	0.00	999	1.000	1.000	0.1785
232484_at	37.7	20.5	-0.88	-1.84	AAA-AAA	0.00	999	1.000	1.000	0.3742
232698_at	36.5	19.9	-0.88	-1.84	AAA-AAA	0.00	999	1.000	1.000	0.5548
1562436_at	10.3	5.6	-0.88	-1.84	AAA-AAA	0.00	999	1.000	1.000	0.3378
1562870_at	24.6	13.4	-0.88	-1.84	AAA-AAA	0.00	999	1.000	1.000	0.6039
1561581_at	18.2	9.9	-0.88	-1.84	AAA-AAA	0.00	999	1.000	1.000	0.3452
1561484_at	12.9	23.6	0.88	1.84	AAA-AAA	0.00	999	1.000	1.000	0.3631
1566158_at	11.3	20.8	0.88	1.84	AAA-AAA	0.00	999	1.000	1.000	0.6089
222174_at	12.1	22.2	0.88	1.84	AAA-AAA	0.00	999	1.000	1.000	0.2932
220532_s_at	21.1	11.5	-0.88	-1.84	AAA-AAA	0.00	999	1.000	1.000	0.5298
206799_at	3.7	2.0	-0.88	-1.84	AAA-AAA	0.00	999	1.000	1.000	0.1021
207216_at	17.1	31.3	0.88	1.84	AAA-AAA	0.00	999	1.000	1.000	0.2706
213488_at	18.4	10.0	-0.88	-1.84	AAA-AAA	0.00	999	1.000	1.000	0.6489
210669_at	52.7	28.7	-0.88	-1.84	AAA-AAA	0.00	999	1.000	1.000	0.3731
218963_s_at	8.6	4.7	-0.88	-1.84	AAA-AAA	0.00	999	1.000	1.000	0.0292
217596_at	93.9	172.3	0.88	1.84	AAA-AAA	0.00	999	1.000	1.000	0.0291
215804_at	21.4	11.7	-0.88	-1.84	AAA-AAA	0.00	999	1.000	1.000	0.3705
216643_at	4.9	8.9	0.88	1.84	AAA-AAA	0.00	999	1.000	1.000	0.4826
236112_at	35.7	19.5	-0.88	-1.84	AAA-AAA	0.00	999	1.000	1.000	0.5367
234739_at	8.7	4.7	-0.88	-1.84	AAA-AAA	0.00	999	1.000	1.000	0.5408

237228_at	20.6	37.8	0.88	1.84	AAA-AAA	0.00	999	1.000	1.000	0.2769
237141_x_at	7.4	4.0	-0.88	-1.84	AAA-AAA	0.00	999	1.000	1.000	0.2975
229313_at	10.1	5.5	-0.88	-1.84	AAA-AAA	0.00	999	1.000	1.000	0.3058
205274_at	16.2	8.9	-0.88	-1.84	AAA-AAA	0.00	999	1.000	1.000	0.3322
231860_at	29.8	16.3	-0.88	-1.84	AAA-AAA	0.00	999	1.000	1.000	0.5242
1561223_at	17.5	32.1	0.88	1.83	PAA-AAA	0.00	999	1.000	1.000	0.5258
227503_at	297.0	544.5	0.88	1.83	PPP-PPP	0.00	999	1.000	1.000	0.0526
242892_at	60.6	33.0	-0.88	-1.83	AAA-AAA	0.00	999	1.000	1.000	0.0883
243716_at	6.6	3.6	-0.88	-1.83	AAA-AAA	0.00	999	1.000	1.000	0.5101
240468_at	11.3	6.1	-0.88	-1.83	AAA-AAA	0.00	999	1.000	1.000	0.3980
244848_at	3.2	5.9	0.88	1.83	AAA-AAA	0.00	999	1.000	1.000	0.3097
1553303_at	10.9	20.1	0.88	1.83	AAA-AAA	0.00	999	1.000	1.000	0.4452
1552616_a_at	14.8	8.1	-0.88	-1.83	AAA-AAA	0.00	999	1.000	1.000	0.4611
1557103_a_at	62.5	34.1	-0.88	-1.83	AAA-AAA	0.00	999	1.000	1.000	0.0525
207928_s_at	4.5	2.5	-0.88	-1.83	AAA-AAA	0.00	999	1.000	1.000	0.5340
207065_at	43.2	23.6	-0.88	-1.83	AAA-AAA	0.00	999	1.000	1.000	0.3447
226469_s_at	91.8	50.0	-0.88	-1.84	MAA-AAA	0.00	999	1.000	1.000	0.1744
224140_at	43.4	23.7	-0.88	-1.84	AAA-AAM	0.00	999	1.000	1.000	0.4098
236972_at	10.5	19.3	0.88	1.83	AAA-AAA	0.00	999	1.000	1.000	0.4426
228895_s_at	48.5	26.4	-0.88	-1.83	AAA-AAA	0.00	999	1.000	1.000	0.4345
233586_s_at	34.8	19.0	-0.88	-1.83	AAA-AAA	0.00	999	1.000	1.000	0.6176
236042_at	36.2	66.3	0.87	1.83	AAP-PPP	0.00	999	1.000	1.000	0.3485
229402_at	26.3	48.2	0.87	1.83	AAA-AAP	0.00	999	1.000	1.000	0.1959
242489_at	67.3	36.7	-0.87	-1.83	AAA-AAA	0.00	999	1.000	1.000	0.0056
242127_at	39.2	21.4	-0.87	-1.83	AAA-AAA	0.00	999	1.000	1.000	0.2023
241187_at	49.4	26.9	-0.87	-1.83	AAA-AAA	0.00	999	1.000	1.000	0.4360
242406_at	22.2	12.1	-0.87	-1.83	AAA-AAA	0.00	999	1.000	1.000	0.6302
240633_at	62.5	34.1	-0.87	-1.83	AAA-AAA	0.00	999	1.000	1.000	0.4422
1569372_at	5.8	3.2	-0.87	-1.83	AAA-AAA	0.00	999	1.000	1.000	0.2082
213924_at	28.1	15.3	-0.87	-1.83	AAA-AAA	0.00	999	1.000	1.000	0.3336
214608_s_at	6.4	3.5	-0.87	-1.83	AAA-AAA	0.00	999	1.000	1.000	0.0642
212821_at	18.2	33.4	0.87	1.83	AAA-AAA	0.00	999	1.000	1.000	0.6000
210248_at	16.2	29.7	0.87	1.83	AAA-AAA	0.00	999	1.000	1.000	0.4130
219466_s_at	9.5	17.3	0.87	1.83	AAA-AAM	0.00	999	1.000	1.000	0.2920
235102_x_at	44.5	24.3	-0.87	-1.83	AAA-AAA	0.00	999	1.000	1.000	0.3094
236819_at	15.2	8.3	-0.87	-1.83	AAA-AAA	0.00	999	1.000	1.000	0.4142
229565_x_at	6.5	11.9	0.87	1.83	AAA-AAA	0.00	999	1.000	1.000	0.3086
228460_at	180.6	330.8	0.87	1.83	PPP-PPP	0.00	999	1.000	1.000	0.1991
224496_s_at	156.9	287.3	0.87	1.83	PPP-PPP	0.00	999	1.000	1.000	0.1869
241304_at	5.5	10.1	0.87	1.83	AAA-AAA	0.00	999	1.000	1.000	0.6160
1553584_at	19.7	10.8	-0.87	-1.83	AAA-AAA	0.00	999	1.000	1.000	0.6082
220252_x_at	15.4	28.2	0.87	1.83	AAA-AAA	0.00	999	1.000	1.000	0.5501

219840_s_at	25.4	13.9	-0.87	-1.83	AAA-AAA	0.00	999	1.000	1.000	0.3007
214998_at	20.9	38.3	0.87	1.83	AAA-MAA	0.00	999	1.000	1.000	0.4267
215543_s_at	12.3	6.7	-0.87	-1.83	AAA-AAA	0.00	999	1.000	1.000	0.1155
236664_at	22.7	41.5	0.87	1.83	AAA-AAA	0.00	999	1.000	1.000	0.4257
234843_s_at	21.2	38.9	0.87	1.83	AAA-AAA	0.00	999	1.000	1.000	0.4582
238680_at	16.8	9.2	-0.87	-1.83	AAA-AAA	0.00	999	1.000	1.000	0.3801
239394_at	24.5	13.4	-0.87	-1.83	AAA-AAA	0.00	999	1.000	1.000	0.5697
204762_s_at	25.1	13.7	-0.87	-1.83	APA-AAA	0.00	999	1.000	1.000	0.6376
208890_s_at	957.6	1752.7	0.87	1.83	PPP-PPP	0.00	999	1.000	1.000	0.4310
213459_at	172.7	316.1	0.87	1.83	PPP-PPP	0.00	999	1.000	1.000	0.2052
241365_at	10.9	6.0	-0.87	-1.83	AAA-AAA	0.00	999	1.000	1.000	0.5876
242758_x_at	11.4	6.2	-0.87	-1.83	AAA-AAA	0.00	999	1.000	1.000	0.6871
1562572_at	65.7	35.9	-0.87	-1.83	AAA-AAA	0.00	999	1.000	1.000	0.1525
1570624_at	14.5	7.9	-0.87	-1.83	AAA-AAA	0.00	999	1.000	1.000	0.5847
1558856_at	49.6	27.1	-0.87	-1.83	AAA-AAA	0.00	999	1.000	1.000	0.2988
209986_at	18.7	10.2	-0.87	-1.83	AAA-AAA	0.00	999	1.000	1.000	0.4431
234338_s_at	183.9	100.5	-0.87	-1.83	AAA-AAA	0.00	999	1.000	1.000	0.0295
229062_at	14.6	26.7	0.87	1.83	AAA-AAA	0.00	999	1.000	1.000	0.6178
229369_at	45.5	24.8	-0.87	-1.83	AAA-AAA	0.00	999	1.000	1.000	0.4904
233029_at	11.5	6.3	-0.87	-1.83	AAA-AAA	0.00	999	1.000	1.000	0.5718
231306_at	125.7	68.7	-0.87	-1.83	AAA-AAM	0.00	999	1.000	1.000	0.2232
232224_at	32.2	17.6	-0.87	-1.83	AAA-AAA	0.00	999	1.000	1.000	0.4559
232330_at	52.7	28.8	-0.87	-1.83	AAA-AAA	0.00	999	1.000	1.000	0.3439
240291_at	69.6	38.0	-0.87	-1.83	AAA-AAA	0.00	999	1.000	1.000	0.1013
241125_at	17.5	9.6	-0.87	-1.83	AAA-AAA	0.00	999	1.000	1.000	0.1910
1568683_at	16.5	30.2	0.87	1.83	AAA-AAA	0.00	999	1.000	1.000	0.4416
1552452_at	7.9	4.3	-0.87	-1.83	AAA-AAA	0.00	999	1.000	1.000	0.3984
1558828_s_at	9.2	5.0	-0.87	-1.83	AAA-AAA	0.00	999	1.000	1.000	0.1658
221297_at	77.1	42.1	-0.87	-1.83	AAA-AAA	0.00	999	1.000	1.000	0.4633
220523_at	13.5	7.4	-0.87	-1.83	AAA-AAA	0.00	999	1.000	1.000	0.3800
215057_at	98.1	53.6	-0.87	-1.83	AAA-AAM	0.00	999	1.000	1.000	0.0948
217237_at	13.6	7.4	-0.87	-1.83	AAA-AAA	0.00	999	1.000	1.000	0.4565
236549_x_at	24.3	13.3	-0.87	-1.83	AAA-AAA	0.00	999	1.000	1.000	0.5638
204561_x_at	23.6	12.9	-0.87	-1.83	AAA-AAA	0.00	999	1.000	1.000	0.4225
234249_at	15.8	8.6	-0.87	-1.83	AAA-AAA	0.00	999	1.000	1.000	0.6189
234318_x_at	31.6	17.3	-0.87	-1.83	AAA-AAA	0.00	999	1.000	1.000	0.1360
229814_at	73.6	40.2	-0.87	-1.83	AMA-AAA	0.00	999	1.000	1.000	0.2305
206501_x_at	100.5	183.6	0.87	1.83	AAA-PAA	0.00	999	1.000	1.000	0.0439
243779_at	7.2	3.9	-0.87	-1.83	AAA-AAA	0.00	999	1.000	1.000	0.2524
1560856_at	13.6	24.9	0.87	1.83	AAA-AAA	0.00	999	1.000	1.000	0.4974
1560109_s_at	8.4	4.6	-0.87	-1.83	AAA-AAA	0.00	999	1.000	1.000	0.0446
1553861_at	7.8	4.3	-0.87	-1.83	AAA-AAA	0.00	999	1.000	1.000	0.0240

1555209_at	31.2	17.1	-0.87	-1.83	AAA-AAA	0.00	999	1.000	1.000	0.5812
1557285_at	5.7	10.3	0.87	1.83	AAA-AAA	0.00	999	1.000	1.000	0.5520
227498_at	10.0	18.3	0.87	1.83	AAA-AAA	0.00	999	1.000	1.000	0.6249
223754_at	32.9	60.2	0.87	1.83	AAA-AAA	0.00	999	1.000	1.000	0.3842
223578_x_at	70.8	129.4	0.87	1.83	AAA-AAA	0.00	999	1.000	1.000	0.1491
214788_x_at	9.4	5.2	-0.87	-1.83	AAA-AAA	0.00	999	1.000	1.000	0.1504
215688_at	76.8	42.1	-0.87	-1.83	AAA-AAA	0.00	999	1.000	1.000	0.3774
235741_at	10.7	5.8	-0.87	-1.83	AAA-AAA	0.00	999	1.000	1.000	0.1709
235163_at	59.2	108.2	0.87	1.83	AAA-AMA	0.00	999	1.000	1.000	0.2753
230484_at	215.2	117.8	-0.87	-1.83	AAA-AAA	0.00	999	1.000	1.000	0.0519
232631_at	79.7	43.6	-0.87	-1.83	AAA-AAA	0.00	999	1.000	1.000	0.4434
214766_s_at	652.7	1192.3	0.87	1.83	PPP-PPP	0.00	999	1.000	1.000	0.1925
1563839_at	9.3	5.1	-0.87	-1.83	AAA-AAA	0.00	999	1.000	1.000	0.0843
1561575_at	7.7	4.2	-0.87	-1.83	AAA-AAA	0.00	999	1.000	1.000	0.4345
1554128_at	107.7	59.0	-0.87	-1.83	AAA-AAA	0.00	999	1.000	1.000	0.1763
221080_s_at	63.1	34.6	-0.87	-1.83	AAA-AAA	0.00	999	1.000	1.000	0.4463
219753_at	26.3	48.0	0.87	1.83	AAA-AAA	0.00	999	1.000	1.000	0.3966
208262_x_at	82.5	45.2	-0.87	-1.83	AAA-AAA	0.00	999	1.000	1.000	0.2551
217096_at	23.6	12.9	-0.87	-1.83	AAA-AAA	0.00	999	1.000	1.000	0.6524
219436_s_at	7.7	4.2	-0.87	-1.83	AAA-AAA	0.00	999	1.000	1.000	0.2212
204777_s_at	99.0	54.2	-0.87	-1.83	AAA-AAA	0.00	999	1.000	1.000	0.2792
236130_at	33.4	18.3	-0.87	-1.83	AAA-AAA	0.00	999	1.000	1.000	0.1501
238747_at	34.5	18.9	-0.87	-1.83	AAA-AAA	0.00	999	1.000	1.000	0.3290
238221_at	29.8	16.3	-0.87	-1.83	AAA-AAA	0.00	999	1.000	1.000	0.5142
238008_at	18.1	9.9	-0.87	-1.83	AAA-AAA	0.00	999	1.000	1.000	0.4100
237738_at	3.1	5.6	0.87	1.83	AAA-AAA	0.00	999	1.000	1.000	0.4704
228319_at	35.4	19.4	-0.87	-1.83	AAA-AAA	0.00	999	1.000	1.000	0.3691
205464_at	19.1	10.4	-0.87	-1.83	AAA-AAA	0.00	999	1.000	1.000	0.5879
231949_at	40.6	74.2	0.87	1.83	AMA-AMA	0.00	999	1.000	1.000	0.6053
1554880_at	87.4	159.6	0.87	1.83	AAA-AAP	0.00	999	1.000	1.000	0.5245
215018_at	31.8	58.0	0.87	1.83	AAP-AAP	0.00	999	1.000	1.000	0.4174
243250_at	13.7	7.5	-0.87	-1.83	AAA-AAA	0.00	999	1.000	1.000	0.4841
1566152_a_at	11.3	6.2	-0.87	-1.83	AAA-AAA	0.00	999	1.000	1.000	0.6191
1563529_at	58.6	32.1	-0.87	-1.83	AAA-AAA	0.00	999	1.000	1.000	0.1989
1563632_at	26.5	48.3	0.87	1.83	AAA-AAA	0.00	999	1.000	1.000	0.4880
1563173_at	7.4	4.0	-0.87	-1.83	AAA-AAA	0.00	999	1.000	1.000	0.6498
1560358_at	7.6	4.2	-0.87	-1.83	AAA-AAA	0.00	999	1.000	1.000	0.5244
1565587_at	106.0	58.1	-0.87	-1.83	AMM-AAM	0.00	999	1.000	1.000	0.1627
1558181_at	89.8	49.2	-0.87	-1.83	AAA-AAA	0.00	999	1.000	1.000	0.2462
220249_at	10.4	5.7	-0.87	-1.83	AAA-AAA	0.00	999	1.000	1.000	0.3018
219902_at	22.5	12.3	-0.87	-1.83	AAA-AAA	0.00	999	1.000	1.000	0.5548
224026_at	27.6	15.1	-0.87	-1.83	AAA-AAA	0.00	999	1.000	1.000	0.4662

237773_at	167.7	91.9	-0.87	-1.83	AAM-AAA	0.00	999	1.000	1.000	0.0209
231324_at	44.1	24.2	-0.87	-1.83	AAA-AAA	0.00	999	1.000	1.000	0.5937
235764_at	22.0	40.1	0.87	1.82	APA-MAP	0.00	999	1.000	1.000	0.5835
229388_at	99.7	181.8	0.87	1.82	APP-PPP	0.00	999	1.000	1.000	0.3663
1553030_a_at	137.1	250.0	0.87	1.82	AAM-PPP	0.00	999	1.000	1.000	0.0528
243419_at	5.5	10.0	0.87	1.82	AAA-AAA	0.00	999	1.000	1.000	0.3438
1552355_s_at	14.6	8.0	-0.87	-1.82	AAA-AAA	0.00	999	1.000	1.000	0.2860
220901_at	87.0	47.7	-0.87	-1.82	AAA-AAA	0.00	999	1.000	1.000	0.0867
206901_at	18.3	10.0	-0.87	-1.82	AAA-AAA	0.00	999	1.000	1.000	0.0787
216817_s_at	27.2	14.9	-0.87	-1.82	AAA-AAA	0.00	999	1.000	1.000	0.6001
235700_at	10.3	18.7	0.87	1.82	AAA-AAA	0.00	999	1.000	1.000	0.4618
235669_at	98.1	53.8	-0.87	-1.82	AAA-AAA	0.00	999	1.000	1.000	0.1155
239590_x_at	11.2	6.2	-0.87	-1.82	AAA-AAA	0.00	999	1.000	1.000	0.1246
237606_at	4.3	2.3	-0.87	-1.82	AAA-AAA	0.00	999	1.000	1.000	0.1791
228981_at	32.6	17.9	-0.87	-1.82	AAA-AAA	0.00	999	1.000	1.000	0.5321
233096_at	22.2	12.2	-0.87	-1.82	AAA-AAA	0.00	999	1.000	1.000	0.5359
233790_at	13.1	7.2	-0.87	-1.82	AAA-AAA	0.00	999	1.000	1.000	0.1676
206707_x_at	39.5	72.0	0.87	1.82	AAP-PAP	0.00	999	1.000	1.000	0.3113
226016_at	321.3	585.6	0.87	1.82	PPP-PPP	0.00	999	1.000	1.000	0.1695
227959_at	86.4	157.4	0.87	1.82	AAA-PPP	0.00	999	1.000	1.000	0.1508
201858_s_at	1934.1	3524.4	0.87	1.82	PPP-PPP	0.00	999	1.000	1.000	0.0747
244445_at	27.5	15.1	-0.87	-1.82	AAA-AAA	0.00	999	1.000	1.000	0.5106
208400_at	15.2	27.6	0.87	1.82	AAA-AAA	0.00	999	1.000	1.000	0.6097
219745_at	31.6	57.5	0.87	1.82	AAM-AAA	0.00	999	1.000	1.000	0.5308
224532_at	19.5	10.7	-0.87	-1.82	AAA-AAA	0.00	999	1.000	1.000	0.4978
217412_at	10.2	18.6	0.87	1.82	AAA-AAA	0.00	999	1.000	1.000	0.6038
216465_at	24.6	13.5	-0.87	-1.82	AAA-AAA	0.00	999	1.000	1.000	0.5526
216723_at	21.3	11.7	-0.87	-1.82	AAA-AAA	0.00	999	1.000	1.000	0.4190
236376_at	29.1	53.0	0.87	1.82	AAA-AAA	0.00	999	1.000	1.000	0.1396
204664_at	5.6	3.0	-0.87	-1.82	AAA-AAA	0.00	999	1.000	1.000	0.1881
239166_at	31.2	56.9	0.87	1.82	AAA-AAA	0.00	999	1.000	1.000	0.4245
237222_at	20.9	38.1	0.87	1.82	AAA-AAA	0.00	999	1.000	1.000	0.4812
213241_at	38.0	69.2	0.87	1.82	AAP-PPA	0.00	999	1.000	1.000	0.3910
1562467_at	58.2	31.9	-0.87	-1.82	AAA-AAA	0.00	999	1.000	1.000	0.0385
1561820_at	55.7	30.6	-0.87	-1.82	AAA-AAA	0.00	999	1.000	1.000	0.1417
1561177_at	15.4	8.5	-0.87	-1.82	AAA-AAA	0.00	999	1.000	1.000	0.5155
1566511_at	5.7	10.4	0.87	1.82	AAA-AAA	0.00	999	1.000	1.000	0.6604
1556155_at	29.2	53.3	0.87	1.82	AAA-AAA	0.00	999	1.000	1.000	0.4873
220412_x_at	36.2	19.9	-0.87	-1.82	AAA-AAA	0.00	999	1.000	1.000	0.3901
213975_s_at	5.7	3.1	-0.87	-1.82	AAA-AAA	0.00	999	1.000	1.000	0.4917
212224_at	7.3	4.0	-0.87	-1.82	AAA-AAA	0.00	999	1.000	1.000	0.5140
215759_at	8.0	4.4	-0.87	-1.82	AAA-AAA	0.00	999	1.000	1.000	0.0862

237170_at	31.6	17.3	-0.87	-1.82	AAA-AAA	0.00	999	1.000	1.000	0.2252
237427_at	7.1	3.9	-0.87	-1.82	AAA-AAA	0.00	999	1.000	1.000	0.2092
237728_at	83.1	45.6	-0.87	-1.82	AAA-AAA	0.00	999	1.000	1.000	0.2129
206283_s_at	24.3	44.3	0.87	1.82	AAA-AAA	0.00	999	1.000	1.000	0.2805
211892_s_at	54.6	30.0	-0.86	-1.82	PAA-APA	0.00	999	1.000	1.000	0.5777
234368_at	16.0	29.0	0.86	1.82	AAA-PPA	0.00	999	1.000	1.000	0.5510
232451_at	64.0	116.4	0.86	1.82	PPP-PPP	0.00	999	1.000	1.000	0.0634
242391_at	17.2	9.5	-0.86	-1.82	AAA-AAA	0.00	999	1.000	1.000	0.3962
240244_at	3.5	6.4	0.86	1.82	AAA-AAA	0.00	999	1.000	1.000	0.3964
1561567_at	8.3	4.6	-0.86	-1.82	AAA-AAA	0.00	999	1.000	1.000	0.6620
1561883_at	111.7	61.4	-0.86	-1.82	AAA-AAA	0.00	999	1.000	1.000	0.1200
1567687_at	8.5	15.5	0.86	1.82	AAA-AAA	0.00	999	1.000	1.000	0.6032
208443_x_at	57.3	104.4	0.86	1.82	AAA-AAA	0.00	999	1.000	1.000	0.5159
206484_s_at	26.0	14.3	-0.86	-1.82	AAA-AAA	0.00	999	1.000	1.000	0.4553
207367_at	27.4	15.1	-0.86	-1.82	AAA-AAA	0.00	999	1.000	1.000	0.1345
214226_at	116.7	64.1	-0.86	-1.82	AAA-AAA	0.00	999	1.000	1.000	0.4591
210711_at	75.6	41.5	-0.86	-1.82	AAA-AAA	0.00	999	1.000	1.000	0.1544
210722_at	28.7	15.8	-0.86	-1.82	AAA-AAA	0.00	999	1.000	1.000	0.5397
211029_x_at	9.3	5.1	-0.86	-1.82	AAA-AAA	0.00	999	1.000	1.000	0.1336
215817_at	21.4	11.8	-0.86	-1.82	AAA-AAA	0.00	999	1.000	1.000	0.3896
204707_s_at	9.0	16.4	0.86	1.82	AAA-AAA	0.00	999	1.000	1.000	0.6348
236450_at	8.4	15.4	0.86	1.82	AAA-AAA	0.00	999	1.000	1.000	0.2671
234795_at	11.4	20.7	0.86	1.82	AAA-AAA	0.00	999	1.000	1.000	0.5329
237694_at	7.3	4.0	-0.86	-1.82	AAA-AAA	0.00	999	1.000	1.000	0.0031
230039_at	20.1	36.6	0.86	1.82	AAA-AAA	0.00	999	1.000	1.000	0.3033
229783_at	13.7	7.5	-0.86	-1.82	AAA-AAA	0.00	999	1.000	1.000	0.4722
229282_at	15.8	8.7	-0.86	-1.82	AAA-AAA	0.00	999	1.000	1.000	0.2399
232893_at	32.7	59.6	0.86	1.82	PPA-APP	0.00	999	1.000	1.000	0.5915
230606_at	65.3	118.8	0.86	1.82	AAA-PAA	0.00	999	1.000	1.000	0.3251
232457_at	31.3	57.0	0.86	1.82	AAA-AAP	0.00	999	1.000	1.000	0.2979
236473_at	71.1	129.2	0.86	1.82	PAA-AAP	0.00	999	1.000	1.000	0.1911
204908_s_at	255.3	464.1	0.86	1.82	PPP-PPP	0.00	999	1.000	1.000	0.0783
240871_at	12.6	23.0	0.86	1.82	AAA-AAA	0.00	999	1.000	1.000	0.4575
244034_at	26.5	48.2	0.86	1.82	AAA-AAA	0.00	999	1.000	1.000	0.4598
1566433_at	10.3	5.7	-0.86	-1.82	AAA-AAA	0.00	999	1.000	1.000	0.4049
1559645_at	24.9	45.2	0.86	1.82	AAA-AAA	0.00	999	1.000	1.000	0.4721
1555703_at	17.0	9.4	-0.86	-1.82	AAA-AAA	0.00	999	1.000	1.000	0.5678
215243_s_at	37.2	20.4	-0.86	-1.82	AAM-AAA	0.00	999	1.000	1.000	0.6347
236445_at	4.1	7.5	0.86	1.82	AAA-AAA	0.00	999	1.000	1.000	0.4713
237789_at	61.9	34.0	-0.86	-1.82	AAA-AAA	0.00	999	1.000	1.000	0.2988
223197_s_at	635.0	1153.9	0.86	1.82	PPP-PPP	0.00	999	1.000	1.000	0.1235
244333_at	11.3	20.5	0.86	1.82	AAA-AAA	0.00	999	1.000	1.000	0.5462

241274_at	3.7	6.8	0.86	1.82	AAA-AAA	0.00	999	1.000	1.000	0.4756
243477_at	27.2	49.5	0.86	1.82	AAA-AAA	0.00	999	1.000	1.000	0.5124
1563351_at	9.1	5.0	-0.86	-1.82	AAA-AAA	0.00	999	1.000	1.000	0.5682
1563983_at	15.3	27.8	0.86	1.82	AAA-AAA	0.00	999	1.000	1.000	0.5066
1561140_at	9.6	5.3	-0.86	-1.82	AAA-AAA	0.00	999	1.000	1.000	0.4353
1557710_at	26.6	14.6	-0.86	-1.82	AAA-AAA	0.00	999	1.000	1.000	0.3893
221438_s_at	4.1	2.3	-0.86	-1.82	AAA-AAA	0.00	999	1.000	1.000	0.2029
207395_at	19.5	10.7	-0.86	-1.82	AAA-AAA	0.00	999	1.000	1.000	0.5609
213894_at	12.7	7.0	-0.86	-1.82	AAA-AAA	0.00	999	1.000	1.000	0.6220
211110_s_at	40.6	73.7	0.86	1.82	AAA-AAA	0.00	999	1.000	1.000	0.4194
217066_s_at	51.9	94.4	0.86	1.82	AAA-AAA	0.00	999	1.000	1.000	0.4463
238541_at	118.9	65.4	-0.86	-1.82	AAA-AAA	0.00	999	1.000	1.000	0.4679
231771_at	3.7	2.1	-0.86	-1.82	AAA-AAA	0.00	999	1.000	1.000	0.3846
202075_s_at	46.7	84.9	0.86	1.82	APP-AAP	0.00	999	1.000	1.000	0.6267
239480_at	3.2	5.9	0.86	1.82	AAA-APA	0.00	999	1.000	1.000	0.5189
220786_s_at	25.1	45.6	0.86	1.82	AAP-PPP	0.00	999	1.000	1.000	0.5008
209722_s_at	35.0	63.5	0.86	1.82	AAA-PAA	0.00	999	1.000	1.000	0.3831
201006_at	101.2	183.8	0.86	1.82	APP-PAP	0.00	999	1.000	1.000	0.1440
244475_at	47.5	86.4	0.86	1.82	AAA-PPA	0.00	999	1.000	1.000	0.0661
241475_at	6.1	11.1	0.86	1.82	AAA-AAA	0.00	999	1.000	1.000	0.3218
244210_at	17.6	9.7	-0.86	-1.82	AAA-AAA	0.00	999	1.000	1.000	0.4936
241098_at	8.2	4.5	-0.86	-1.82	AAA-AAA	0.00	999	1.000	1.000	0.6139
1562557_at	93.3	51.4	-0.86	-1.82	AAA-AAA	0.00	999	1.000	1.000	0.3287
1554949_at	14.5	8.0	-0.86	-1.82	AAA-AAA	0.00	999	1.000	1.000	0.4164
1559303_at	43.7	24.1	-0.86	-1.82	AAA-AAA	0.00	999	1.000	1.000	0.2383
1556314_a_at	4.2	2.3	-0.86	-1.82	AAA-AAA	0.00	999	1.000	1.000	0.1859
210309_at	12.9	7.1	-0.86	-1.82	AAA-AAA	0.00	999	1.000	1.000	0.4213
215989_at	9.2	16.8	0.86	1.82	AAA-AAA	0.00	999	1.000	1.000	0.4240
216346_at	35.0	19.3	-0.86	-1.82	AAA-AAA	0.00	999	1.000	1.000	0.2880
236228_at	25.1	13.8	-0.86	-1.82	AAA-AAA	0.00	999	1.000	1.000	0.5442
204698_at	9.9	18.0	0.86	1.82	AAA-AAA	0.00	999	1.000	1.000	0.4307
238782_at	23.1	41.9	0.86	1.82	AAA-AMA	0.00	999	1.000	1.000	0.2757
229098_s_at	143.0	78.7	-0.86	-1.82	AAA-AAA	0.00	999	1.000	1.000	0.1843
233598_at	49.3	27.1	-0.86	-1.82	AAA-AAA	0.00	999	1.000	1.000	0.3766
239563_at	37.0	67.2	0.86	1.82	AAA-AAP	0.00	999	1.000	1.000	0.3425
1561009_at	8.7	4.8	-0.86	-1.82	AAA-AAA	0.00	999	1.000	1.000	0.5335
1553413_at	11.8	6.5	-0.86	-1.82	AAA-AAA	0.00	999	1.000	1.000	0.3929
1552466_x_at	8.4	15.2	0.86	1.82	AAA-AAA	0.00	999	1.000	1.000	0.6439
1556850_at	6.9	12.4	0.86	1.82	AAA-AAA	0.00	999	1.000	1.000	0.3697
220485_s_at	14.1	7.8	-0.86	-1.82	AAA-AAA	0.00	999	1.000	1.000	0.5406
217145_at	14.3	7.9	-0.86	-1.82	AAA-AAA	0.00	999	1.000	1.000	0.5143
216853_x_at	12.6	7.0	-0.86	-1.82	AAA-AAA	0.00	999	1.000	1.000	0.5964

229330_at	8.2	4.5	-0.86	-1.82	AAA-AAA	0.00	999	1.000	1.000	0.4936
205384_at	32.7	59.2	0.86	1.82	AAA-AAA	0.00	999	1.000	1.000	0.1899
236087_at	27.7	50.3	0.86	1.81	AAA-APA	0.00	999	1.000	1.000	0.3957
206042_x_at	494.9	897.5	0.86	1.81	PPP-PPP	0.00	999	1.000	1.000	0.0561
241134_at	10.1	5.6	-0.86	-1.81	AAA-AAA	0.00	999	1.000	1.000	0.1165
1553611_s_at	37.0	20.4	-0.86	-1.81	AAA-AAA	0.00	999	1.000	1.000	0.2062
1555558_at	8.8	16.0	0.86	1.81	AAA-AAA	0.00	999	1.000	1.000	0.3064
226123_at	105.4	58.1	-0.86	-1.81	AAA-AAA	0.00	999	1.000	1.000	0.2284
226540_at	28.9	52.5	0.86	1.81	AAA-AAA	0.00	999	1.000	1.000	0.1209
213690_s_at	25.1	13.8	-0.86	-1.81	AAA-AAA	0.00	999	1.000	1.000	0.3984
211614_at	11.0	6.1	-0.86	-1.81	AAA-AAA	0.00	999	1.000	1.000	0.5272
211287_x_at	14.8	8.2	-0.86	-1.81	AAA-AAA	0.00	999	1.000	1.000	0.3785
210833_at	7.6	4.2	-0.86	-1.81	AAA-AAA	0.00	999	1.000	1.000	0.4926
234130_at	11.8	21.4	0.86	1.81	AAA-AAA	0.00	999	1.000	1.000	0.3862
239099_at	2.8	5.0	0.86	1.81	AAA-AAA	0.00	999	1.000	1.000	0.6078
229833_at	136.4	75.2	-0.86	-1.81	AAA-AAA	0.00	999	1.000	1.000	0.5722
230582_at	9.0	5.0	-0.86	-1.81	AAA-AAA	0.00	999	1.000	1.000	0.0279
205023_at	17.4	31.5	0.86	1.81	AAA-PAA	0.00	999	1.000	1.000	0.6238
224201_s_at	79.6	144.4	0.86	1.81	AAP-APP	0.00	999	1.000	1.000	0.2616
203419_at	245.7	445.3	0.86	1.81	APP-PPP	0.00	999	1.000	1.000	0.2597
242451_x_at	110.7	200.6	0.86	1.81	MPA-PPP	0.00	999	1.000	1.000	0.0646
202826_at	10.5	18.9	0.86	1.81	AAA-AAA	0.00	999	1.000	1.000	0.4755
242572_at	6.4	11.6	0.86	1.81	AAA-AAA	0.00	999	1.000	1.000	0.5656
241354_at	23.2	42.1	0.86	1.81	AAA-AAA	0.00	999	1.000	1.000	0.3092
240618_at	9.4	5.2	-0.86	-1.81	AAA-AAA	0.00	999	1.000	1.000	0.1187
240136_at	8.2	4.6	-0.86	-1.81	AAA-AAA	0.00	999	1.000	1.000	0.1035
1558897_at	28.3	15.6	-0.86	-1.81	AAA-AAA	0.00	999	1.000	1.000	0.4321
221315_s_at	50.2	27.7	-0.86	-1.81	AAA-AAA	0.00	999	1.000	1.000	0.4792
207741_x_at	19.0	10.5	-0.86	-1.81	AAA-AAA	0.00	999	1.000	1.000	0.5887
206663_at	24.9	13.8	-0.86	-1.81	AAA-AAA	0.00	999	1.000	1.000	0.4690
207533_at	11.2	20.3	0.86	1.81	AAA-AAA	0.00	999	1.000	1.000	0.2601
239272_at	28.9	15.9	-0.86	-1.81	AAA-AAA	0.00	999	1.000	1.000	0.2710
229709_at	70.6	39.0	-0.86	-1.81	AAA-AAA	0.00	999	1.000	1.000	0.2955
233679_at	60.4	33.3	-0.86	-1.81	AAA-AAA	0.00	999	1.000	1.000	0.5335
231074_at	60.6	33.4	-0.86	-1.81	AAA-AAA	0.00	999	1.000	1.000	0.2128
232858_at	19.7	35.7	0.86	1.81	APA-AAA	0.00	999	1.000	1.000	0.5320
237121_at	23.7	42.9	0.86	1.81	APM-AAP	0.00	999	1.000	1.000	0.4760
236830_at	39.3	71.2	0.86	1.81	AAA-APA	0.00	999	1.000	1.000	0.1563
241204_at	6.2	3.4	-0.86	-1.81	AAA-AAA	0.00	999	1.000	1.000	0.5313
241462_at	17.3	9.5	-0.86	-1.81	AAA-AAA	0.00	999	1.000	1.000	0.3844
241987_x_at	3.3	6.0	0.86	1.81	AAA-AAA	0.00	999	1.000	1.000	0.4412
1566834_at	8.1	4.5	-0.86	-1.81	AAA-AAA	0.00	999	1.000	1.000	0.0356

1553439_at	52.0	28.7	-0.86	-1.81	AAA-AAA	0.00	999	1.000	1.000	0.2522
1554984_a_at	41.9	23.1	-0.86	-1.81	AAA-AAA	0.00	999	1.000	1.000	0.4910
1556694_a_at	33.2	60.2	0.86	1.81	AAA-AAA	0.00	999	1.000	1.000	0.2929
1556883_a_at	6.1	3.4	-0.86	-1.81	AAA-AAA	0.00	999	1.000	1.000	0.3858
227055_at	24.8	44.8	0.86	1.81	AAA-AAA	0.00	999	1.000	1.000	0.5143
227488_at	286.6	158.2	-0.86	-1.81	AAA-AAA	0.00	999	1.000	1.000	0.1085
211322_s_at	19.7	10.9	-0.86	-1.81	AAA-AAA	0.00	999	1.000	1.000	0.0433
211521_s_at	29.5	53.5	0.86	1.81	AAA-AAA	0.00	999	1.000	1.000	0.4793
211210_x_at	77.4	42.8	-0.86	-1.81	AAA-AAA	0.00	999	1.000	1.000	0.3649
237981_at	12.3	22.2	0.86	1.81	AAA-AAA	0.00	999	1.000	1.000	0.3741
228352_at	6.9	3.8	-0.86	-1.81	AAA-AAA	0.00	999	1.000	1.000	0.1010
228428_at	13.4	7.4	-0.86	-1.81	AAA-AAA	0.00	999	1.000	1.000	0.5812
239302_s_at	30.9	55.9	0.86	1.81	AAA-PAA	0.00	999	1.000	1.000	0.4582
241014_at	10.8	19.5	0.86	1.81	AAA-APA	0.00	999	1.000	1.000	0.4078
219359_at	43.7	79.0	0.86	1.81	AAA-APP	0.00	999	1.000	1.000	0.2786
211709_s_at	375.5	679.7	0.86	1.81	PPP-PPP	0.00	999	1.000	1.000	0.0466
1558630_at	36.8	66.5	0.86	1.81	PAA-AAP	0.00	999	1.000	1.000	0.0452
244108_at	47.1	26.0	-0.86	-1.81	AAA-AAM	0.00	999	1.000	1.000	0.5407
242917_at	12.7	7.0	-0.86	-1.81	AAA-AAA	0.00	999	1.000	1.000	0.5757
1560255_at	10.4	18.9	0.86	1.81	AAA-AAA	0.00	999	1.000	1.000	0.4898
1561596_at	6.8	3.8	-0.86	-1.81	AAA-AAA	0.00	999	1.000	1.000	0.5025
1566040_at	8.9	4.9	-0.86	-1.81	AAA-AAA	0.00	999	1.000	1.000	0.4216
1554481_a_at	39.2	21.7	-0.86	-1.81	AAA-AAA	0.00	999	1.000	1.000	0.5510
208102_s_at	16.4	29.6	0.86	1.81	AAA-AAA	0.00	999	1.000	1.000	0.4296
208031_s_at	64.0	115.9	0.86	1.81	AAA-AAA	0.00	999	1.000	1.000	0.3767
207486_x_at	93.3	51.6	-0.86	-1.81	MAM-AAA	0.00	999	1.000	1.000	0.0385
223862_at	104.4	57.7	-0.86	-1.81	AAA-AAA	0.00	999	1.000	1.000	0.0310
211453_s_at	52.0	94.1	0.86	1.81	AAA-AAA	0.00	999	1.000	1.000	0.3400
219455_at	89.4	161.8	0.86	1.81	AAA-AAA	0.00	999	1.000	1.000	0.3713
216572_at	5.1	2.8	-0.86	-1.81	AAA-AAA	0.00	999	1.000	1.000	0.1039
236467_at	125.8	69.5	-0.86	-1.81	AMA-AAA	0.00	999	1.000	1.000	0.2089
238214_at	26.1	14.4	-0.86	-1.81	AAA-AAA	0.00	999	1.000	1.000	0.5329
239521_at	29.4	16.2	-0.86	-1.81	AAA-AAA	0.00	999	1.000	1.000	0.4559
237214_at	9.0	4.9	-0.86	-1.81	AAA-AAA	0.00	999	1.000	1.000	0.6690
229084_at	9.7	17.6	0.86	1.81	AAA-AAA	0.00	999	1.000	1.000	0.4812
228898_s_at	14.5	26.2	0.86	1.81	AAA-AAA	0.00	999	1.000	1.000	0.5542
210703_at	16.5	29.9	0.86	1.81	AAP-APA	0.00	999	1.000	1.000	0.5745
241333_x_at	23.2	12.8	-0.86	-1.81	AAA-AAA	0.00	999	1.000	1.000	0.4047
1563043_at	10.8	6.0	-0.86	-1.81	AAA-AAA	0.00	999	1.000	1.000	0.4106
1565082_x_at	8.2	14.8	0.86	1.81	AAA-AAA	0.00	999	1.000	1.000	0.2578
244727_at	27.5	15.2	-0.86	-1.81	AAA-AAA	0.00	999	1.000	1.000	0.5527
207597_at	15.2	27.5	0.86	1.81	AAA-AAA	0.00	999	1.000	1.000	0.3787

211434_s_at	13.1	23.6	0.86	1.81	AAA-AAA	0.00	999	1.000	1.000	0.3669
211448_s_at	7.6	4.2	-0.86	-1.81	AAA-AAA	0.00	999	1.000	1.000	0.0520
217210_at	52.7	95.3	0.86	1.81	AAA-AMA	0.00	999	1.000	1.000	0.0620
236307_at	11.1	20.0	0.86	1.81	AAA-AAA	0.00	999	1.000	1.000	0.3219
238930_at	10.6	5.9	-0.86	-1.81	AAA-AAA	0.00	999	1.000	1.000	0.1505
238352_s_at	21.7	12.0	-0.86	-1.81	AAA-AAA	0.00	999	1.000	1.000	0.3954
231361_at	9.1	16.4	0.86	1.81	AAA-AAA	0.00	999	1.000	1.000	0.2632
231694_at	25.4	45.9	0.86	1.81	AAA-AAA	0.00	999	1.000	1.000	0.2426
238548_at	30.4	55.0	0.85	1.81	APP-AAA	0.00	999	1.000	1.000	0.4644
1558102_at	372.8	673.9	0.85	1.81	PPP-PPP	0.00	999	1.000	1.000	0.0980
1562428_at	61.9	34.3	-0.85	-1.81	AMA-AAA	0.00	999	1.000	1.000	0.3416
1560390_s_at	28.4	15.7	-0.85	-1.81	AAA-AAA	0.00	999	1.000	1.000	0.4007
1570480_s_at	4.1	7.4	0.85	1.81	AAA-AAA	0.00	999	1.000	1.000	0.5569
1564192_at	4.8	8.6	0.85	1.81	AAA-AAA	0.00	999	1.000	1.000	0.6580
1566994_at	36.5	20.2	-0.85	-1.81	AAA-AAA	0.00	999	1.000	1.000	0.5008
1553361_x_at	28.8	16.0	-0.85	-1.81	AAA-AAA	0.00	999	1.000	1.000	0.0561
1559324_at	22.2	40.1	0.85	1.81	AAA-AAA	0.00	999	1.000	1.000	0.4704
1557345_at	3.7	2.1	-0.85	-1.81	AAA-AAA	0.00	999	1.000	1.000	0.4567
225299_at	22.1	40.0	0.85	1.81	AAM-AAA	0.00	999	1.000	1.000	0.4012
206418_at	13.3	7.3	-0.85	-1.81	AAA-AAA	0.00	999	1.000	1.000	0.3565
224356_x_at	13.3	24.1	0.85	1.81	AAA-AAA	0.00	999	1.000	1.000	0.4736
210883_x_at	119.5	66.1	-0.85	-1.81	AAA-AAA	0.00	999	1.000	1.000	0.1193
216455_at	88.5	49.0	-0.85	-1.81	AAA-AAA	0.00	999	1.000	1.000	0.2071
233189_at	23.8	13.2	-0.85	-1.81	AAA-AAA	0.00	999	1.000	1.000	0.3820
232406_at	51.4	28.4	-0.85	-1.81	AAA-AAA	0.00	999	1.000	1.000	0.2145
1559479_at	14.2	25.6	0.85	1.81	PAA-PAM	0.00	999	1.000	1.000	0.6939
241894_at	20.5	37.1	0.85	1.81	AAA-PAA	0.00	999	1.000	1.000	0.5766
227701_at	214.4	387.4	0.85	1.81	PPP-PPP	0.00	999	1.000	1.000	0.0590
1562372_at	20.4	11.3	-0.85	-1.81	AAA-MAA	0.00	999	1.000	1.000	0.6473
1562932_at	3.8	6.8	0.85	1.81	AAA-AAA	0.00	999	1.000	1.000	0.4156
1561899_at	8.1	4.5	-0.85	-1.81	AAA-AAA	0.00	999	1.000	1.000	0.5125
1564568_at	53.1	29.4	-0.85	-1.81	AAA-AAA	0.00	999	1.000	1.000	0.5754
1552372_at	6.5	11.8	0.85	1.81	AAA-AAA	0.00	999	1.000	1.000	0.5454
1553257_at	41.0	22.7	-0.85	-1.81	AAA-AAA	0.00	999	1.000	1.000	0.3902
1555367_at	29.0	16.1	-0.85	-1.81	AAA-AAA	0.00	999	1.000	1.000	0.4794
207592_s_at	35.5	19.6	-0.85	-1.81	AAA-AAA	0.00	999	1.000	1.000	0.4405
211349_at	52.4	29.0	-0.85	-1.81	AAA-AAA	0.00	999	1.000	1.000	0.5349
210408_s_at	11.1	6.1	-0.85	-1.81	AAA-AAA	0.00	999	1.000	1.000	0.2467
235997_at	18.6	10.3	-0.85	-1.81	AAA-AAA	0.00	999	1.000	1.000	0.2280
239653_at	17.7	32.0	0.85	1.81	AAA-AAA	0.00	999	1.000	1.000	0.5198
237983_at	18.9	10.4	-0.85	-1.81	AAA-AAA	0.00	999	1.000	1.000	0.5349
206215_at	36.6	20.3	-0.85	-1.81	AAA-AAA	0.00	999	1.000	1.000	0.5609

231127_at	8.6	4.7	-0.85	-1.81	AAA-AAA	0.00	999	1.000	1.000	0.5982
231910_at	6.9	12.5	0.85	1.81	AAA-AAA	0.00	999	1.000	1.000	0.1903
221262_s_at	13.8	24.8	0.85	1.81	AAA-AAP	0.00	999	1.000	1.000	0.6869
236719_at	42.6	77.0	0.85	1.81	APA-AAA	0.00	999	1.000	1.000	0.5256
201473_at	163.1	294.4	0.85	1.80	APP-PPP	0.00	999	1.000	1.000	0.4237
230619_at	55.7	100.5	0.85	1.81	APA-PMA	0.00	999	1.000	1.000	0.3572
219514_at	51.2	92.4	0.85	1.81	AAA-PPP	0.00	999	1.000	1.000	0.1603
240462_at	27.2	15.1	-0.85	-1.81	AAA-AAA	0.00	999	1.000	1.000	0.6919
1561778_at	11.0	6.1	-0.85	-1.81	AAA-AAA	0.00	999	1.000	1.000	0.6160
1564595_at	36.3	20.1	-0.85	-1.81	AAA-AAA	0.00	999	1.000	1.000	0.4481
1553910_at	37.7	68.1	0.85	1.81	AAA-AAA	0.00	999	1.000	1.000	0.3283
1558128_at	56.3	31.2	-0.85	-1.81	AAA-AAA	0.00	999	1.000	1.000	0.1916
220087_at	7.3	13.2	0.85	1.81	AAA-AAA	0.00	999	1.000	1.000	0.5914
227417_at	36.4	65.6	0.85	1.81	AAA-AAA	0.00	999	1.000	1.000	0.1745
210179_at	4.9	8.8	0.85	1.81	AAA-AAA	0.00	999	1.000	1.000	0.5862
214408_s_at	20.5	11.4	-0.85	-1.81	AAA-AAA	0.00	999	1.000	1.000	0.4799
214633_at	17.8	32.1	0.85	1.80	AAA-AAA	0.00	999	1.000	1.000	0.6145
211267_at	12.7	22.9	0.85	1.81	AAA-AAA	0.00	999	1.000	1.000	0.4230
236710_at	22.6	12.5	-0.85	-1.81	AAA-AAA	0.00	999	1.000	1.000	0.5597
229107_at	58.0	32.1	-0.85	-1.80	AMA-AAA	0.00	999	1.000	1.000	0.3066
233022_at	12.6	22.8	0.85	1.81	AAA-AAA	0.00	999	1.000	1.000	0.5279
232673_at	5.2	9.3	0.85	1.80	AAA-AAA	0.00	999	1.000	1.000	0.5961
231759_at	35.2	19.5	-0.85	-1.81	AAA-AAA	0.00	999	1.000	1.000	0.1566
238515_at	36.3	65.5	0.85	1.80	AAP-PAP	0.00	999	1.000	1.000	0.4742
230684_at	37.0	66.7	0.85	1.80	AAP-PAM	0.00	999	1.000	1.000	0.3276
244207_at	28.1	15.6	-0.85	-1.80	AAA-AAA	0.00	999	1.000	1.000	0.5719
242651_at	63.3	35.1	-0.85	-1.80	AAA-AAA	0.00	999	1.000	1.000	0.4511
1562970_at	8.3	14.9	0.85	1.80	AAA-AAA	0.00	999	1.000	1.000	0.1057
1560913_at	17.4	9.6	-0.85	-1.80	AAA-AAA	0.00	999	1.000	1.000	0.2186
1569399_at	14.9	26.8	0.85	1.80	AAA-AAA	0.00	999	1.000	1.000	0.1800
1553448_at	6.1	3.4	-0.85	-1.80	AAA-AAA	0.00	999	1.000	1.000	0.0941
1553433_at	4.3	2.4	-0.85	-1.80	AAA-AAA	0.00	999	1.000	1.000	0.0250
1557822_at	11.5	6.4	-0.85	-1.80	AAA-AAA	0.00	999	1.000	1.000	0.5556
219153_s_at	90.9	50.4	-0.85	-1.80	AAA-AAA	0.00	999	1.000	1.000	0.2160
219095_at	76.3	42.4	-0.85	-1.80	PAA-MAA	0.00	999	1.000	1.000	0.6262
1553998_at	29.1	16.1	-0.85	-1.80	APA-PAA	0.00	999	1.000	1.000	0.6483
1562482_at	8.3	4.6	-0.85	-1.80	AAA-AAA	0.00	999	1.000	1.000	0.0905
1562880_at	20.2	11.2	-0.85	-1.80	AAM-AAA	0.00	999	1.000	1.000	0.4553
1562949_at	17.5	9.7	-0.85	-1.80	AAA-AAA	0.00	999	1.000	1.000	0.4601
1563241_at	4.7	8.5	0.85	1.80	AAA-AAA	0.00	999	1.000	1.000	0.4106
1560527_at	9.0	5.0	-0.85	-1.80	AAA-AAA	0.00	999	1.000	1.000	0.3708
226333_at	14.5	26.2	0.85	1.80	AAA-AAA	0.00	999	1.000	1.000	0.2899

223468_s_at	30.2	54.5	0.85	1.80	AAA-AAA	0.00	999	1.000	1.000	0.2854
235312_s_at	20.6	11.5	-0.85	-1.80	AAA-AAA	0.00	999	1.000	1.000	0.5748
234439_at	23.2	12.8	-0.85	-1.80	AAA-AAA	0.00	999	1.000	1.000	0.1856
217554_at	68.6	123.6	0.85	1.80	PAP-PPP	0.00	999	1.000	1.000	0.1664
237833_s_at	25.8	46.4	0.85	1.80	AAA-APA	0.00	999	1.000	1.000	0.1326
207031_at	42.7	77.0	0.85	1.80	AAP-PPP	0.00	999	1.000	1.000	0.0997
240402_at	16.9	30.5	0.85	1.80	AAA-AAA	0.00	999	1.000	1.000	0.4038
203639_s_at	4.7	2.6	-0.85	-1.80	AAA-AAA	0.00	999	1.000	1.000	0.1616
1569102_at	25.7	14.3	-0.85	-1.80	AAA-AAA	0.00	999	1.000	1.000	0.5797
1564972_x_at	25.3	45.6	0.85	1.80	AAA-AAA	0.00	999	1.000	1.000	0.4186
1559313_at	21.6	38.8	0.85	1.80	AAA-AAA	0.00	999	1.000	1.000	0.3709
1555713_at	60.1	33.3	-0.85	-1.80	AAA-AAA	0.00	999	1.000	1.000	0.3536
219827_at	44.4	80.0	0.85	1.80	AAA-AAA	0.00	999	1.000	1.000	0.4583
207366_at	81.1	45.1	-0.85	-1.80	AAA-AAA	0.00	999	1.000	1.000	0.2664
217587_at	10.0	5.5	-0.85	-1.80	AAA-AAA	0.00	999	1.000	1.000	0.4539
215490_at	52.4	29.1	-0.85	-1.80	AAA-AAA	0.00	999	1.000	1.000	0.4345
235650_at	9.9	17.9	0.85	1.80	AAA-AAA	0.00	999	1.000	1.000	0.5903
237074_at	12.0	6.7	-0.85	-1.80	AAA-AAA	0.00	999	1.000	1.000	0.5649
205387_s_at	32.6	18.1	-0.85	-1.80	AAA-AAP	0.00	999	1.000	1.000	0.5831
1555364_at	11.4	6.3	-0.85	-1.80	APA-AAA	0.00	999	1.000	1.000	0.7243
228826_at	33.9	61.0	0.85	1.80	AAP-PAA	0.00	999	1.000	1.000	0.4577
236227_at	203.5	366.3	0.85	1.80	APP-PPP	0.00	999	1.000	1.000	0.1178
242499_at	70.2	39.0	-0.85	-1.80	AAA-AAA	0.00	999	1.000	1.000	0.0098
241483_at	4.5	2.5	-0.85	-1.80	AAA-AAA	0.00	999	1.000	1.000	0.3491
243094_at	21.7	12.0	-0.85	-1.80	AAA-AAA	0.00	999	1.000	1.000	0.5373
1563584_at	18.6	33.5	0.85	1.80	AAA-AAA	0.00	999	1.000	1.000	0.5166
1557335_at	10.7	6.0	-0.85	-1.80	AAA-AAA	0.00	999	1.000	1.000	0.0553
207344_at	20.9	11.6	-0.85	-1.80	AAA-AAA	0.00	999	1.000	1.000	0.3360
217510_at	10.4	5.8	-0.85	-1.80	AAA-AAA	0.00	999	1.000	1.000	0.4842
215863_at	77.3	42.9	-0.85	-1.80	AAA-AAA	0.00	999	1.000	1.000	0.3629
215469_at	8.2	14.8	0.85	1.80	AAA-AAA	0.00	999	1.000	1.000	0.6533
237986_at	24.5	13.6	-0.85	-1.80	AAA-AAA	0.00	999	1.000	1.000	0.5224
230160_x_at	102.3	56.8	-0.85	-1.80	AAA-AAA	0.00	999	1.000	1.000	0.1333
232245_at	7.0	12.5	0.85	1.80	AAA-AAA	0.00	999	1.000	1.000	0.2515
236363_at	27.6	49.6	0.85	1.80	PAA-APP	0.00	999	1.000	1.000	0.4988
240300_at	18.1	32.6	0.85	1.80	AAA-PAA	0.00	999	1.000	1.000	0.3833
243265_at	3.6	2.0	-0.85	-1.80	AAA-AAA	0.00	999	1.000	1.000	0.0443
1561342_at	4.1	2.3	-0.85	-1.80	AAA-AAA	0.00	999	1.000	1.000	0.2940
1570574_at	15.7	8.7	-0.85	-1.80	AAA-AAA	0.00	999	1.000	1.000	0.5280
1567174_at	3.0	5.4	0.85	1.80	AAA-AAA	0.00	999	1.000	1.000	0.1207
244355_at	24.2	13.4	-0.85	-1.80	AAA-AAA	0.00	999	1.000	1.000	0.4923
220986_s_at	19.1	10.6	-0.85	-1.80	AAA-AAA	0.00	999	1.000	1.000	0.5182

207906_at	8.8	15.8	0.85	1.80	AAA-AAA	0.00	999	1.000	1.000	0.2531
219852_s_at	39.2	21.8	-0.85	-1.80	AAA-AAA	0.00	999	1.000	1.000	0.3646
227030_at	12.6	7.0	-0.85	-1.80	AAA-AAA	0.00	999	1.000	1.000	0.0737
216198_at	14.8	26.6	0.85	1.80	AAA-AAA	0.00	999	1.000	1.000	0.5778
234487_at	37.1	20.6	-0.85	-1.80	AAA-AAA	0.00	999	1.000	1.000	0.6056
228549_at	49.4	27.4	-0.85	-1.80	AAA-AAA	0.00	999	1.000	1.000	0.4413
233143_at	33.0	18.4	-0.85	-1.80	AAA-AAA	0.00	999	1.000	1.000	0.2219
226891_at	138.8	249.5	0.85	1.80	AAP-MPP	0.00	999	1.000	1.000	0.0892
243749_s_at	19.5	10.9	-0.85	-1.80	AAA-AAA	0.00	999	1.000	1.000	0.5606
240086_at	15.2	8.5	-0.85	-1.80	AAA-AAA	0.00	999	1.000	1.000	0.6365
241148_at	8.9	4.9	-0.85	-1.80	AAA-AAA	0.00	999	1.000	1.000	0.2251
1554681_a_at	6.4	3.5	-0.85	-1.80	AAA-AAA	0.00	999	1.000	1.000	0.0118
1556412_at	65.5	36.4	-0.85	-1.80	AAA-AAA	0.00	999	1.000	1.000	0.4397
1564373_a_at	56.7	31.5	-0.85	-1.80	AAA-AAA	0.00	999	1.000	1.000	0.4445
227269_s_at	11.7	21.0	0.85	1.80	AAA-AAA	0.00	999	1.000	1.000	0.3960
219497_s_at	15.4	8.6	-0.85	-1.80	AAA-AAA	0.00	999	1.000	1.000	0.4471
217568_at	8.6	15.4	0.85	1.80	AAA-AAA	0.00	999	1.000	1.000	0.5096
235313_at	8.2	4.6	-0.85	-1.80	AAA-AAA	0.00	999	1.000	1.000	0.1465
239713_at	12.6	22.6	0.85	1.80	AAA-AAA	0.00	999	1.000	1.000	0.6032
236997_at	52.0	28.9	-0.85	-1.80	AAA-AAA	0.00	999	1.000	1.000	0.4089
209638_x_at	25.7	14.3	-0.85	-1.80	AAP-AAA	0.00	999	1.000	1.000	0.6541
224007_at	14.8	26.5	0.85	1.80	AAA-PAA	0.00	999	1.000	1.000	0.2860
1554660_a_at	117.1	210.3	0.85	1.80	APM-PPP	0.00	999	1.000	1.000	0.1315
240608_at	149.2	83.1	-0.85	-1.80	MAA-AAA	0.00	999	1.000	1.000	0.0343
1562434_at	112.7	62.7	-0.85	-1.80	AAA-AAA	0.00	999	1.000	1.000	0.1269
1562026_at	78.4	140.8	0.85	1.80	AAA-AAA	0.00	999	1.000	1.000	0.4721
1562418_at	4.7	2.6	-0.85	-1.80	AAA-AAA	0.00	999	1.000	1.000	0.2014
1552623_at	10.1	5.6	-0.85	-1.80	AAA-AAA	0.00	999	1.000	1.000	0.5150
1552726_at	50.6	28.2	-0.85	-1.80	AAA-AAA	0.00	999	1.000	1.000	0.1426
1557735_at	17.5	9.8	-0.85	-1.80	AAA-AAA	0.00	999	1.000	1.000	0.5207
1557863_at	4.9	2.7	-0.85	-1.80	AAA-AAA	0.00	999	1.000	1.000	0.5181
207865_s_at	26.3	14.6	-0.85	-1.80	AAA-AAA	0.00	999	1.000	1.000	0.5733
219747_at	30.0	53.8	0.85	1.80	AAA-AAA	0.00	999	1.000	1.000	0.6028
215904_at	11.1	20.0	0.85	1.80	AAA-AAA	0.00	999	1.000	1.000	0.4529
215402_at	28.0	15.6	-0.85	-1.80	AAA-AAA	0.00	999	1.000	1.000	0.4947
238132_at	8.9	16.1	0.85	1.80	AAA-AAA	0.00	999	1.000	1.000	0.2608
232325_at	5.3	2.9	-0.85	-1.80	AAA-AAA	0.00	999	1.000	1.000	0.3161
239162_at	28.9	16.1	-0.84	-1.80	PAA-AAA	0.00	999	1.000	1.000	0.6303
1561136_at	9.4	5.2	-0.84	-1.80	APA-AAA	0.00	999	1.000	1.000	0.6769
241180_at	16.5	9.2	-0.84	-1.80	AAA-AAA	0.00	999	1.000	1.000	0.6851
1564940_at	16.8	9.4	-0.84	-1.80	AAA-AAA	0.00	999	1.000	1.000	0.6219
222315_at	4.3	7.6	0.84	1.80	AAA-AAA	0.00	999	1.000	1.000	0.0846

208214_at	5.3	3.0	-0.84	-1.80	AAA-AAA	0.00	999	1.000	1.000	0.3238
220832_at	7.4	4.1	-0.84	-1.80	AAA-AAA	0.00	999	1.000	1.000	0.2847
223788_at	20.6	37.0	0.84	1.80	AAA-AAA	0.00	999	1.000	1.000	0.2135
223986_x_at	13.0	7.2	-0.84	-1.80	AAA-AAA	0.00	999	1.000	1.000	0.2917
214920_at	8.6	15.5	0.84	1.80	AAA-AAA	0.00	999	1.000	1.000	0.4277
210372_s_at	26.7	14.9	-0.84	-1.80	AAA-AAA	0.00	999	1.000	1.000	0.6033
210560_at	6.2	3.5	-0.84	-1.80	AAA-AAA	0.00	999	1.000	1.000	0.2723
238676_at	65.4	36.4	-0.84	-1.80	AAA-AAA	0.00	999	1.000	1.000	0.2382
238312_s_at	11.7	6.5	-0.84	-1.80	AAA-AAA	0.00	999	1.000	1.000	0.2682
237368_at	9.3	16.6	0.84	1.80	AAA-AAA	0.00	999	1.000	1.000	0.6010
206270_at	21.0	11.7	-0.84	-1.80	AAA-AAA	0.00	999	1.000	1.000	0.1759
205182_s_at	79.6	142.8	0.84	1.79	AAA-AAA	0.00	999	1.000	1.000	0.3723
235979_at	27.9	15.6	-0.84	-1.79	AAA-AAP	0.00	999	1.000	1.000	0.5922
240772_at	23.9	13.3	-0.84	-1.79	AMA-AAA	0.00	999	1.000	1.000	0.4448
240775_at	15.1	8.4	-0.84	-1.79	AAA-AAA	0.00	999	1.000	1.000	0.5614
1561073_at	20.8	11.6	-0.84	-1.79	AAA-AAA	0.00	999	1.000	1.000	0.6125
1570224_at	14.0	7.8	-0.84	-1.79	AAA-AAA	0.00	999	1.000	1.000	0.5202
244742_at	2.1	1.2	-0.84	-1.79	AAA-AAA	0.00	999	1.000	1.000	0.0443
1559696_at	39.1	21.8	-0.84	-1.79	AAA-AAA	0.00	999	1.000	1.000	0.5161
220686_s_at	46.0	25.6	-0.84	-1.79	AAA-AAA	0.00	999	1.000	1.000	0.5219
220850_at	1.6	2.9	0.84	1.79	AAA-AAA	0.00	999	1.000	1.000	0.5080
220337_at	61.4	34.2	-0.84	-1.79	AAA-AAA	0.00	999	1.000	1.000	0.0369
227232_at	12.4	22.2	0.84	1.79	AAA-AAA	0.00	999	1.000	1.000	0.5632
216224_s_at	35.3	19.7	-0.84	-1.79	AAA-AAA	0.00	999	1.000	1.000	0.1431
230670_at	46.1	25.7	-0.84	-1.79	AAA-AAA	0.00	999	1.000	1.000	0.1497
233531_at	21.6	12.1	-0.84	-1.79	AAA-AAA	0.00	999	1.000	1.000	0.3296
231445_at	7.7	13.8	0.84	1.79	AAA-AAA	0.00	999	1.000	1.000	0.0611
232123_at	5.5	9.9	0.84	1.79	AAA-AAA	0.00	999	1.000	1.000	0.5373
205454_at	85.1	47.4	-0.84	-1.79	AAA-AAA	0.00	999	1.000	1.000	0.1403
1565743_at	32.2	57.8	0.84	1.79	APP-PPP	0.00	999	1.000	1.000	0.5266
204236_at	276.3	495.1	0.84	1.79	PPP-PPP	0.00	999	1.000	1.000	0.1065
235333_at	387.6	694.8	0.84	1.79	PPP-PPP	0.00	999	1.000	1.000	0.0755
1570196_at	13.0	7.2	-0.84	-1.79	AAA-AAA	0.00	999	1.000	1.000	0.4957
1566114_at	4.0	2.3	-0.84	-1.79	AAA-AAA	0.00	999	1.000	1.000	0.1669
1566580_at	96.8	54.0	-0.84	-1.79	AAA-AAA	0.00	999	1.000	1.000	0.2712
1552338_at	5.2	2.9	-0.84	-1.79	AAA-AAA	0.00	999	1.000	1.000	0.0283
1559618_at	73.8	41.2	-0.84	-1.79	AAA-AAA	0.00	999	1.000	1.000	0.0575
1556728_at	36.1	20.2	-0.84	-1.79	AAA-AAA	0.00	999	1.000	1.000	0.5284
226658_at	40.2	22.4	-0.84	-1.79	AAA-AAA	0.00	999	1.000	1.000	0.3141
210068_s_at	21.1	11.8	-0.84	-1.79	AAA-AAA	0.00	999	1.000	1.000	0.5271
211430_s_at	11.2	20.1	0.84	1.79	AAA-AAA	0.00	999	1.000	1.000	0.6238
217525_at	10.6	18.9	0.84	1.79	AAA-AAA	0.00	999	1.000	1.000	0.4568

232543_x_at	18.3	32.7	0.84	1.79	AAA-AAA	0.00	999	1.000	1.000	0.4096
232783_at	28.0	15.6	-0.84	-1.79	AAA-AAA	0.00	999	1.000	1.000	0.5405
233783_at	24.8	44.5	0.84	1.79	AAA-AAA	0.00	999	1.000	1.000	0.4009
231071_at	45.2	80.9	0.84	1.79	AAA-AAP	0.00	999	1.000	1.000	0.4246
223934_at	31.0	55.6	0.84	1.79	PAA-AAP	0.00	999	1.000	1.000	0.2224
219352_at	78.8	141.2	0.84	1.79	PAA-PPP	0.00	999	1.000	1.000	0.1663
243955_at	16.1	28.8	0.84	1.79	AAA-AAA	0.00	999	1.000	1.000	0.3608
241203_at	25.7	46.0	0.84	1.79	AAA-AAA	0.00	999	1.000	1.000	0.4915
1568891_x_at	13.2	7.4	-0.84	-1.79	AAA-AAA	0.00	999	1.000	1.000	0.7129
220709_at	5.6	10.0	0.84	1.79	AAA-AAA	0.00	999	1.000	1.000	0.1671
206528_at	8.0	14.3	0.84	1.79	AAA-AAA	0.00	999	1.000	1.000	0.5007
226646_at	33.7	18.8	-0.84	-1.79	AAA-AAA	0.00	999	1.000	1.000	0.4857
211873_s_at	75.2	42.0	-0.84	-1.79	AAA-AAA	0.00	999	1.000	1.000	0.1530
216701_at	35.7	19.9	-0.84	-1.79	AAA-AAA	0.00	999	1.000	1.000	0.4546
235617_x_at	21.1	37.8	0.84	1.79	AAA-AAA	0.00	999	1.000	1.000	0.4409
238268_at	10.8	6.1	-0.84	-1.79	AAA-AAA	0.00	999	1.000	1.000	0.4967
237752_at	9.7	17.4	0.84	1.79	AAA-AAA	0.00	999	1.000	1.000	0.3205
237627_at	28.2	15.8	-0.84	-1.79	AAA-AAA	0.00	999	1.000	1.000	0.4092
230682_x_at	23.6	13.2	-0.84	-1.79	AAA-AAA	0.00	999	1.000	1.000	0.0995
232906_at	7.1	12.8	0.84	1.79	AAA-AAA	0.00	999	1.000	1.000	0.5159
231549_at	15.4	27.6	0.84	1.79	AAA-AAA	0.00	999	1.000	1.000	0.2794
1562966_at	61.1	34.1	-0.84	-1.79	AAA-AAA	0.00	999	1.000	1.000	0.5723
1560523_at	4.9	2.8	-0.84	-1.79	AAA-AAA	0.00	999	1.000	1.000	0.3009
1565825_at	32.7	18.3	-0.84	-1.79	AAA-AAA	0.00	999	1.000	1.000	0.3713
1564545_a_at	11.6	6.5	-0.84	-1.79	AAA-AAA	0.00	999	1.000	1.000	0.4309
220831_at	16.0	8.9	-0.84	-1.79	AAA-AAA	0.00	999	1.000	1.000	0.5539
206646_at	122.8	68.6	-0.84	-1.79	AAA-AAA	0.00	999	1.000	1.000	0.2309
219087_at	6.0	10.8	0.84	1.79	AAA-AAA	0.00	999	1.000	1.000	0.4380
218964_at	29.5	52.8	0.84	1.79	AAA-AAA	0.00	999	1.000	1.000	0.2527
209597_s_at	30.2	16.9	-0.84	-1.79	AAA-AAA	0.00	999	1.000	1.000	0.5649
236643_s_at	19.1	10.7	-0.84	-1.79	AAA-AAA	0.00	999	1.000	1.000	0.4118
234268_at	5.6	10.0	0.84	1.79	AAA-AAA	0.00	999	1.000	1.000	0.5745
233882_s_at	12.9	7.2	-0.84	-1.79	AAA-AAA	0.00	999	1.000	1.000	0.2586
237448_at	15.7	8.8	-0.84	-1.79	AAA-AAA	0.00	999	1.000	1.000	0.4480
230126_s_at	39.9	22.3	-0.84	-1.79	AAA-AAA	0.00	999	1.000	1.000	0.1426
230331_at	12.6	22.5	0.84	1.79	AAA-AAA	0.00	999	1.000	1.000	0.6905
206265_s_at	7.1	12.7	0.84	1.79	AAA-AAA	0.00	999	1.000	1.000	0.4128
232686_at	33.1	18.5	-0.84	-1.79	AAA-AAA	0.00	999	1.000	1.000	0.5287
233446_at	4.1	7.3	0.84	1.79	AAA-AAA	0.00	999	1.000	1.000	0.4698
1559103_s_at	13.8	24.8	0.84	1.79	APA-AMP	0.00	999	1.000	1.000	0.5798
216604_s_at	20.4	36.6	0.84	1.79	AAA-AAP	0.00	999	1.000	1.000	0.2989
204168_at	219.9	393.4	0.84	1.79	APA-PPP	0.00	999	1.000	1.000	0.1831

243853_at	29.2	16.3	-0.84	-1.79	AAA-AAA	0.00	999	1.000	1.000	0.3753
241554_at	5.5	9.9	0.84	1.79	AAA-AAA	0.00	999	1.000	1.000	0.4530
241155_at	14.9	8.4	-0.84	-1.79	AAA-AAA	0.00	999	1.000	1.000	0.5520
240496_at	23.4	13.1	-0.84	-1.79	AAA-AAA	0.00	999	1.000	1.000	0.4432
1568887_at	78.6	43.9	-0.84	-1.79	AAA-AAA	0.00	999	1.000	1.000	0.1113
1554706_at	2.2	1.2	-0.84	-1.79	AAA-AAA	0.00	999	1.000	1.000	0.3655
1553253_at	167.1	93.5	-0.84	-1.79	AAA-AAA	0.00	999	1.000	1.000	0.2224
1558281_a_at	10.6	5.9	-0.84	-1.79	AAA-AAA	0.00	999	1.000	1.000	0.2347
1555963_x_at	14.4	8.0	-0.84	-1.79	AAA-AAA	0.00	999	1.000	1.000	0.6397
221900_at	15.9	8.9	-0.84	-1.79	AAA-AAA	0.00	999	1.000	1.000	0.4547
207479_at	13.6	7.6	-0.84	-1.79	AAA-AAA	0.00	999	1.000	1.000	0.2011
213343_s_at	75.0	134.1	0.84	1.79	AAA-AAA	0.00	999	1.000	1.000	0.4458
216403_at	6.2	3.5	-0.84	-1.79	AAA-AAA	0.00	999	1.000	1.000	0.3909
234008_s_at	59.6	33.3	-0.84	-1.79	AAA-AAA	0.00	999	1.000	1.000	0.2932
230857_s_at	8.0	4.5	-0.84	-1.79	AAA-AAA	0.00	999	1.000	1.000	0.2776
232692_at	17.0	9.5	-0.84	-1.79	AAA-AAA	0.00	999	1.000	1.000	0.1881
233574_at	11.5	20.5	0.84	1.79	AAA-AAA	0.00	999	1.000	1.000	0.2139
232190_x_at	115.6	64.6	-0.84	-1.79	AAA-AAA	0.00	999	1.000	1.000	0.1639
1557755_at	56.0	100.1	0.84	1.79	AAP-APP	0.00	999	1.000	1.000	0.5388
202879_s_at	33.2	18.6	-0.84	-1.79	AAA-AAA	0.00	999	1.000	1.000	0.4779
242306_at	22.3	39.9	0.84	1.79	AAA-AAA	0.00	999	1.000	1.000	0.5548
1569770_x_at	3.5	6.3	0.84	1.79	AAA-AAA	0.00	999	1.000	1.000	0.6378
1554022_at	13.6	7.6	-0.84	-1.79	AAA-AAA	0.00	999	1.000	1.000	0.5503
1555286_at	15.6	8.7	-0.84	-1.79	AAA-AAA	0.00	999	1.000	1.000	0.5783
1552906_at	35.5	19.9	-0.84	-1.79	AAA-AAA	0.00	999	1.000	1.000	0.4276
1558519_at	19.2	10.8	-0.84	-1.79	AAA-AAA	0.00	999	1.000	1.000	0.4243
1556449_at	97.8	54.7	-0.84	-1.79	AAA-AAA	0.00	999	1.000	1.000	0.0043
221929_at	52.7	29.5	-0.84	-1.79	AAA-AAA	0.00	999	1.000	1.000	0.6468
219697_at	88.8	49.7	-0.84	-1.79	AAA-AAA	0.00	999	1.000	1.000	0.0663
210572_at	4.0	2.2	-0.84	-1.79	AAA-AAA	0.00	999	1.000	1.000	0.2920
216800_at	21.0	37.5	0.84	1.79	AAA-AAA	0.00	999	1.000	1.000	0.4633
235245_at	43.4	77.7	0.84	1.79	AAA-AAA	0.00	999	1.000	1.000	0.2787
238258_at	4.8	2.7	-0.84	-1.79	AAA-AAA	0.00	999	1.000	1.000	0.2834
230310_at	30.8	55.1	0.84	1.79	AAA-AAA	0.00	999	1.000	1.000	0.3701
231941_s_at	4.2	2.4	-0.84	-1.79	AAA-AAA	0.00	999	1.000	1.000	0.2346
241787_at	40.0	71.4	0.84	1.79	APA-APP	0.00	999	1.000	1.000	0.5498
237616_at	16.3	29.1	0.84	1.79	APA-APP	0.00	999	1.000	1.000	0.5195
239963_at	26.8	47.9	0.84	1.79	AAA-PPP	0.00	999	1.000	1.000	0.0869
244343_at	49.9	28.0	-0.84	-1.79	AAA-AAA	0.00	999	1.000	1.000	0.2036
1562835_at	27.6	15.5	-0.84	-1.79	AAA-AAA	0.00	999	1.000	1.000	0.5435
1562373_at	1.8	3.3	0.84	1.79	AAA-AAA	0.00	999	1.000	1.000	0.0160
1561323_at	15.0	8.4	-0.84	-1.79	AAA-AAA	0.00	999	1.000	1.000	0.2630

1557343_at	8.1	14.4	0.84	1.79	AAA-AAA	0.00	999	1.000	1.000	0.2705
227091_at	31.7	56.7	0.84	1.79	AAA-AAA	0.00	999	1.000	1.000	0.2060
227419_x_at	9.4	16.8	0.84	1.79	AAA-AAA	0.00	999	1.000	1.000	0.3497
226681_at	56.2	31.4	-0.84	-1.79	AAA-AMA	0.00	999	1.000	1.000	0.3679
207446_at	13.2	23.6	0.84	1.79	AAA-AAA	0.00	999	1.000	1.000	0.6324
215461_at	36.5	65.1	0.84	1.79	AAA-AAA	0.00	999	1.000	1.000	0.4183
238862_at	7.1	12.6	0.84	1.79	AAA-AAA	0.00	999	1.000	1.000	0.5011
206644_at	14.4	25.7	0.84	1.79	AAA-AAP	0.00	999	1.000	1.000	0.3946
239964_at	27.0	15.1	-0.84	-1.78	AAA-AAA	0.00	999	1.000	1.000	0.5118
243447_at	30.7	17.2	-0.84	-1.79	AAA-AAA	0.00	999	1.000	1.000	0.3992
243564_at	101.6	56.9	-0.84	-1.79	AAA-AAA	0.00	999	1.000	1.000	0.0360
1568795_at	16.4	9.2	-0.84	-1.79	AAA-AAA	0.00	999	1.000	1.000	0.4634
49306_at	22.9	40.9	0.84	1.79	AAA-AAA	0.00	999	1.000	1.000	0.5003
37020_at	13.3	7.4	-0.84	-1.79	AAA-AAA	0.00	999	1.000	1.000	0.3099
226578_s_at	188.9	105.8	-0.84	-1.79	AAA-AAA	0.00	999	1.000	1.000	0.0027
207677_s_at	8.0	4.5	-0.84	-1.79	AAA-AAA	0.00	999	1.000	1.000	0.5534
224027_at	12.1	21.6	0.84	1.79	AAA-AAA	0.00	999	1.000	1.000	0.4205
214510_at	9.2	16.4	0.84	1.79	AAA-AAA	0.00	999	1.000	1.000	0.4352
210853_at	15.7	8.8	-0.84	-1.79	AAA-AAA	0.00	999	1.000	1.000	0.5499
204965_at	8.0	4.5	-0.84	-1.79	AAA-AAA	0.00	999	1.000	1.000	0.5591
238442_at	75.8	42.5	-0.84	-1.79	AAA-AAA	0.00	999	1.000	1.000	0.1143
215610_at	37.2	66.3	0.84	1.78	AAA-AAP	0.00	999	1.000	1.000	0.3273
203710_at	39.4	70.3	0.84	1.78	AAA-PPA	0.00	999	1.000	1.000	0.2506
1568689_at	2.7	4.8	0.84	1.78	AAA-AAA	0.00	999	1.000	1.000	0.6132
1566098_at	21.1	11.8	-0.84	-1.78	AAA-AAA	0.00	999	1.000	1.000	0.5674
1566463_at	1.9	3.4	0.84	1.78	AAA-AAA	0.00	999	1.000	1.000	0.2887
1554905_x_at	159.7	89.5	-0.84	-1.78	AAA-AAA	0.00	999	1.000	1.000	0.1989
1560494_a_at	5.1	2.8	-0.84	-1.78	AAA-AAA	0.00	999	1.000	1.000	0.4411
1552953_a_at	50.1	28.1	-0.84	-1.78	AAA-AAA	0.00	999	1.000	1.000	0.3092
225909_at	52.1	92.9	0.84	1.78	AAA-AAA	0.00	999	1.000	1.000	0.2423
207118_s_at	6.0	10.8	0.84	1.78	AAA-AAA	0.00	999	1.000	1.000	0.1569
207589_at	47.0	26.3	-0.84	-1.78	AAA-AAA	0.00	999	1.000	1.000	0.3633
211736_at	8.4	15.0	0.84	1.78	AAA-AAA	0.00	999	1.000	1.000	0.4084
216310_at	108.3	60.7	-0.84	-1.78	AAA-AAA	0.00	999	1.000	1.000	0.0549
238326_at	9.9	17.7	0.84	1.78	AAA-AAA	0.00	999	1.000	1.000	0.4698
237324_s_at	36.6	20.5	-0.84	-1.78	AAA-AAA	0.00	999	1.000	1.000	0.4737
237668_at	14.4	8.0	-0.84	-1.78	AAA-AAA	0.00	999	1.000	1.000	0.4653
1561937_x_at	90.7	50.9	-0.83	-1.78	AAP-AAA	0.00	999	1.000	1.000	0.5951
233152_x_at	25.7	45.8	0.83	1.78	APM-PAA	0.00	999	1.000	1.000	0.6408
1569418_at	19.3	10.8	-0.83	-1.78	AAA-AAA	0.00	999	1.000	1.000	0.5153
1567249_at	5.5	3.1	-0.83	-1.78	AAA-AAA	0.00	999	1.000	1.000	0.3980
1557993_at	8.2	4.6	-0.83	-1.78	AAA-AAA	0.00	999	1.000	1.000	0.6883

222639_s_at	18.7	33.4	0.83	1.78	AAA-AAA	0.00	999	1.000	1.000	0.0640
211273_s_at	19.4	10.9	-0.83	-1.78	AAA-AAA	0.00	999	1.000	1.000	0.5702
239686_at	15.5	8.7	-0.83	-1.78	AAA-AAA	0.00	999	1.000	1.000	0.3019
238046_x_at	21.2	11.9	-0.83	-1.78	AAA-AAA	0.00	999	1.000	1.000	0.5108
233368_s_at	28.1	15.8	-0.83	-1.78	AAA-AAA	0.00	999	1.000	1.000	0.5534
206172_at	44.5	25.0	-0.83	-1.78	AAA-PAA	0.00	999	1.000	1.000	0.5792
1566176_at	21.8	38.8	0.83	1.78	AAA-AAP	0.00	999	1.000	1.000	0.5668
220701_at	30.2	53.9	0.83	1.78	AAM-APA	0.00	999	1.000	1.000	0.4013
216922_x_at	22.8	40.7	0.83	1.78	AAA-APA	0.00	999	1.000	1.000	0.2372
1554328_at	28.4	50.6	0.83	1.78	AAP-PPP	0.00	999	1.000	1.000	0.1426
230566_at	242.0	431.1	0.83	1.78	PPP-PPP	0.00	999	1.000	1.000	0.0999
243839_s_at	17.0	9.5	-0.83	-1.78	AAA-AAA	0.00	999	1.000	1.000	0.4581
1553651_at	45.8	25.7	-0.83	-1.78	AMA-AAA	0.00	999	1.000	1.000	0.2453
211305_x_at	12.7	7.1	-0.83	-1.78	AAA-AAA	0.00	999	1.000	1.000	0.0560
210642_at	60.6	34.0	-0.83	-1.78	AAA-AAA	0.00	999	1.000	1.000	0.4251
218369_s_at	59.0	33.1	-0.83	-1.78	AAA-AAA	0.00	999	1.000	1.000	0.3445
236655_at	4.8	8.5	0.83	1.78	AAA-AAA	0.00	999	1.000	1.000	0.4917
230583_s_at	12.3	6.9	-0.83	-1.78	AAA-AAA	0.00	999	1.000	1.000	0.4010
206014_at	11.1	6.2	-0.83	-1.78	AAA-AAA	0.00	999	1.000	1.000	0.2172
228956_at	31.2	17.5	-0.83	-1.78	AAA-AAA	0.00	999	1.000	1.000	0.0798
231775_at	18.9	33.8	0.83	1.78	AAA-AAM	0.00	999	1.000	1.000	0.3759
239693_at	14.6	26.0	0.83	1.78	AAA-APP	0.00	999	1.000	1.000	0.5380
1554176_a_at	34.8	61.9	0.83	1.78	PPA-PPP	0.00	999	1.000	1.000	0.2426
205425_at	339.9	605.0	0.83	1.78	PPP-PPP	0.00	999	1.000	1.000	0.1898
201235_s_at	84.2	47.3	-0.83	-1.78	AAA-AAA	0.00	999	1.000	1.000	0.0688
1561493_at	3.5	6.3	0.83	1.78	AAA-AAA	0.00	999	1.000	1.000	0.4813
1569522_at	123.4	219.7	0.83	1.78	AAA-AAA	0.00	999	1.000	1.000	0.1991
AFFX-TrpnX-5_at	28.8	16.2	-0.83	-1.78	AAA-AAA	0.00	999	1.000	1.000	0.3575
1565814_at	12.2	6.9	-0.83	-1.78	AAA-AAA	0.00	999	1.000	1.000	0.7169
1564660_at	18.0	10.1	-0.83	-1.78	AAA-AAA	0.00	999	1.000	1.000	0.1966
1554945_x_at	26.4	14.8	-0.83	-1.78	AAA-AAA	0.00	999	1.000	1.000	0.6240
222334_at	54.2	30.5	-0.83	-1.78	AAA-AAA	0.00	999	1.000	1.000	0.4514
220674_at	10.9	6.1	-0.83	-1.78	AAA-AAA	0.00	999	1.000	1.000	0.5353
214187_x_at	18.8	10.6	-0.83	-1.78	AAA-AAA	0.00	999	1.000	1.000	0.3378
234413_at	8.6	4.9	-0.83	-1.78	AAA-AAA	0.00	999	1.000	1.000	0.5624
230390_at	4.9	8.8	0.83	1.78	AAA-AAA	0.00	999	1.000	1.000	0.3044
228097_at	11.2	19.9	0.83	1.78	AAA-AAA	0.00	999	1.000	1.000	0.3555
225939_at	24.0	13.5	-0.83	-1.78	AAA-AAP	0.00	999	1.000	1.000	0.5931
224322_at	14.5	25.8	0.83	1.78	AAA-APA	0.00	999	1.000	1.000	0.5772
210377_at	16.8	29.9	0.83	1.78	APA-APA	0.00	999	1.000	1.000	0.5707
231150_at	25.9	46.1	0.83	1.78	AAA-APA	0.00	999	1.000	1.000	0.4993
1562089_at	11.6	6.5	-0.83	-1.78	AAA-AAA	0.00	999	1.000	1.000	0.6208

1563659_at	4.2	7.4	0.83	1.78	AAA-AAA	0.00	999	1.000	1.000	0.2443
1561741_at	13.3	23.7	0.83	1.78	AAA-AAA	0.00	999	1.000	1.000	0.3745
AFFX-ThrX-5_at	6.7	11.9	0.83	1.78	AAA-AAA	0.00	999	1.000	1.000	0.3932
1554190_s_at	8.9	5.0	-0.83	-1.78	AAA-AAA	0.00	999	1.000	1.000	0.6144
221861_at	44.2	24.9	-0.83	-1.78	AAA-AAA	0.00	999	1.000	1.000	0.4158
208345_s_at	10.4	18.5	0.83	1.78	AAA-AAA	0.00	999	1.000	1.000	0.4742
223812_at	49.0	27.5	-0.83	-1.78	AAA-AAA	0.00	999	1.000	1.000	0.5028
237915_at	13.9	7.8	-0.83	-1.78	AAA-AAA	0.00	999	1.000	1.000	0.5239
206612_at	31.6	17.8	-0.83	-1.78	PAA-AAA	0.00	999	1.000	1.000	0.6244
243363_at	17.3	30.8	0.83	1.78	AAA-APA	0.00	999	1.000	1.000	0.5406
235305_s_at	96.1	170.8	0.83	1.78	PAA-PPM	0.00	999	1.000	1.000	0.2503
221248_s_at	70.2	124.9	0.83	1.78	AAA-MMP	0.00	999	1.000	1.000	0.1751
225129_at	906.8	1612.0	0.83	1.78	PPP-PPP	0.00	999	1.000	1.000	0.1738
212569_at	791.4	1406.4	0.83	1.78	PPP-PPP	0.00	999	1.000	1.000	0.1132
213658_at	51.9	92.2	0.83	1.78	MAP-PPA	0.00	999	1.000	1.000	0.0631
241980_at	13.9	24.7	0.83	1.78	AAA-AAA	0.00	999	1.000	1.000	0.3142
1563069_at	9.7	5.4	-0.83	-1.78	AAA-AAA	0.00	999	1.000	1.000	0.4505
1570263_at	11.0	6.2	-0.83	-1.78	AAA-AAA	0.00	999	1.000	1.000	0.6673
1554294_s_at	15.7	28.0	0.83	1.78	AMA-AAA	0.00	999	1.000	1.000	0.1694
1555016_at	90.8	51.1	-0.83	-1.78	AAA-AAA	0.00	999	1.000	1.000	0.4071
201691_s_at	4.6	2.6	-0.83	-1.78	AAA-AAA	0.00	999	1.000	1.000	0.3533
1557673_at	30.7	17.2	-0.83	-1.78	AAA-AAA	0.00	999	1.000	1.000	0.5332
1556474_a_at	11.9	6.7	-0.83	-1.78	AAA-AAA	0.00	999	1.000	1.000	0.6294
1555587_at	59.2	33.3	-0.83	-1.78	AAA-AAA	0.00	999	1.000	1.000	0.5554
208192_at	4.0	2.3	-0.83	-1.78	AAA-AAA	0.00	999	1.000	1.000	0.5910
223733_s_at	36.5	64.8	0.83	1.78	AAA-AAA	0.00	999	1.000	1.000	0.3254
211156_at	16.4	9.2	-0.83	-1.78	AAA-AAA	0.00	999	1.000	1.000	0.0607
215665_at	7.0	12.4	0.83	1.78	AAA-AAA	0.00	999	1.000	1.000	0.6337
229152_at	28.1	15.8	-0.83	-1.78	AAA-AAA	0.00	999	1.000	1.000	0.4881
218405_at	511.0	907.6	0.83	1.78	PPP-PPP	0.00	999	1.000	1.000	0.0775
1565576_at	117.1	65.9	-0.83	-1.78	AAA-AAA	0.00	999	1.000	1.000	0.4925
1554006_a_at	39.8	22.4	-0.83	-1.78	AAA-AAA	0.00	999	1.000	1.000	0.4721
1553366_s_at	26.5	14.9	-0.83	-1.78	AAA-AAA	0.00	999	1.000	1.000	0.2183
1559174_at	10.3	18.3	0.83	1.78	AAA-AAA	0.00	999	1.000	1.000	0.2374
1557052_at	15.6	27.8	0.83	1.78	AAA-AAA	0.00	999	1.000	1.000	0.3327
207797_s_at	45.3	25.5	-0.83	-1.78	AAA-AAA	0.00	999	1.000	1.000	0.3761
224552_s_at	6.8	12.0	0.83	1.78	AAA-AAA	0.00	999	1.000	1.000	0.4160
211893_x_at	6.5	11.6	0.83	1.78	AAA-AAA	0.00	999	1.000	1.000	0.0890
234729_at	14.0	7.9	-0.83	-1.78	AAA-AAA	0.00	999	1.000	1.000	0.3822
204292_x_at	56.4	31.7	-0.83	-1.78	AAA-AAA	0.00	999	1.000	1.000	0.6061
237275_at	6.7	12.0	0.83	1.78	AAA-AAA	0.00	999	1.000	1.000	0.5709
237991_at	4.8	2.7	-0.83	-1.78	AAA-AAA	0.00	999	1.000	1.000	0.5231

1559753_at	34.5	61.3	0.83	1.78	APA-AMA	0.00	999	1.000	1.000	0.5897
1569238_a_at	31.1	55.3	0.83	1.78	AAA-APA	0.00	999	1.000	1.000	0.2916
1561371_at	9.8	5.5	-0.83	-1.78	AAA-AAA	0.00	999	1.000	1.000	0.4985
1553949_at	44.4	25.0	-0.83	-1.78	AAA-AAA	0.00	999	1.000	1.000	0.3972
1553474_at	12.1	6.8	-0.83	-1.78	AAA-AAA	0.00	999	1.000	1.000	0.6456
1559990_at	5.9	10.5	0.83	1.78	AAA-AAA	0.00	999	1.000	1.000	0.2123
208080_at	49.1	87.1	0.83	1.78	AAA-AAA	0.00	999	1.000	1.000	0.2878
206518_s_at	7.5	4.2	-0.83	-1.78	AAA-AAA	0.00	999	1.000	1.000	0.0725
223702_x_at	21.8	38.6	0.83	1.78	AAA-AAA	0.00	999	1.000	1.000	0.3773
224114_at	8.5	4.8	-0.83	-1.78	AAA-AAA	0.00	999	1.000	1.000	0.5598
213800_at	33.6	18.9	-0.83	-1.78	AAA-AAA	0.00	999	1.000	1.000	0.5066
215065_at	23.1	13.0	-0.83	-1.78	AAA-MAA	0.00	999	1.000	1.000	0.3751
217288_at	13.0	23.1	0.83	1.78	AAA-AAA	0.00	999	1.000	1.000	0.2847
215267_s_at	7.2	4.0	-0.83	-1.78	AAA-AAA	0.00	999	1.000	1.000	0.0922
238766_at	145.5	82.0	-0.83	-1.78	AAA-AAA	0.00	999	1.000	1.000	0.2452
231524_at	22.4	12.6	-0.83	-1.78	AAA-AAA	0.00	999	1.000	1.000	0.4216
232565_at	35.2	62.5	0.83	1.77	AAP-APA	0.00	999	1.000	1.000	0.4745
236754_at	27.7	49.2	0.83	1.77	APA-AAA	0.00	999	1.000	1.000	0.4565
229255_x_at	330.0	585.2	0.83	1.77	PPP-PPP	0.00	999	1.000	1.000	0.2064
229453_at	209.9	372.3	0.83	1.77	APP-PPP	0.00	999	1.000	1.000	0.1640
202566_s_at	62.5	110.9	0.83	1.77	AAA-APA	0.00	999	1.000	1.000	0.1316
203876_s_at	26.3	14.8	-0.83	-1.77	AAA-AAA	0.00	999	1.000	1.000	0.4822
1562326_at	7.6	4.3	-0.83	-1.77	AAA-AAA	0.00	999	1.000	1.000	0.2679
1562962_at	17.9	31.8	0.83	1.77	AAA-AAA	0.00	999	1.000	1.000	0.2900
1557908_at	38.3	21.6	-0.83	-1.77	AAA-AAA	0.00	999	1.000	1.000	0.3685
1557167_at	6.9	12.2	0.83	1.77	AAA-AAA	0.00	999	1.000	1.000	0.5510
206886_x_at	15.6	8.8	-0.83	-1.78	AAA-AAA	0.00	999	1.000	1.000	0.5845
210782_x_at	131.6	74.2	-0.83	-1.78	AAA-AAA	0.00	999	1.000	1.000	0.3125
209496_at	17.6	9.9	-0.83	-1.77	AAA-AAA	0.00	999	1.000	1.000	0.1788
237886_at	20.5	36.3	0.83	1.78	AAA-AAA	0.00	999	1.000	1.000	0.3188
233580_at	29.3	16.5	-0.83	-1.77	AAA-AAA	0.00	999	1.000	1.000	0.1930
215641_at	23.8	42.1	0.83	1.77	AAP-APP	0.00	999	1.000	1.000	0.5972
1560297_at	55.1	97.7	0.83	1.77	AAA-PMP	0.00	999	1.000	1.000	0.4007
219274_at	27.2	48.1	0.83	1.77	AAP-APP	0.00	999	1.000	1.000	0.2836
224407_s_at	53.4	94.7	0.83	1.77	PPA-PPP	0.00	999	1.000	1.000	0.0854
243694_at	21.3	12.0	-0.83	-1.77	AAA-AAA	0.00	999	1.000	1.000	0.6184
241732_at	46.0	26.0	-0.83	-1.77	AAA-AAA	0.00	999	1.000	1.000	0.4228
1570050_at	6.4	11.3	0.83	1.77	AAA-AAA	0.00	999	1.000	1.000	0.6666
1554680_s_at	40.2	22.7	-0.83	-1.77	AAM-AAA	0.00	999	1.000	1.000	0.6248
1559670_at	4.1	2.3	-0.83	-1.77	AAA-AAA	0.00	999	1.000	1.000	0.2809
1558945_s_at	12.9	22.9	0.83	1.77	AAA-AAA	0.00	999	1.000	1.000	0.2849
1555463_a_at	21.5	38.1	0.83	1.77	AAA-AAM	0.00	999	1.000	1.000	0.3729

208338_at	7.2	4.0	-0.83	-1.77	AAA-AAA	0.00	999	1.000	1.000	0.1727
206880_at	94.3	53.2	-0.83	-1.77	AAA-AAA	0.00	999	1.000	1.000	0.3767
210013_at	22.3	39.5	0.83	1.77	AAA-AAA	0.00	999	1.000	1.000	0.5871
216474_x_at	32.3	18.2	-0.83	-1.77	AAA-AAA	0.00	999	1.000	1.000	0.4333
204625_s_at	33.0	58.5	0.83	1.77	AAA-AAA	0.00	999	1.000	1.000	0.5827
204574_s_at	46.8	26.4	-0.83	-1.77	AAA-AAA	0.00	999	1.000	1.000	0.4139
238665_at	26.7	47.3	0.83	1.77	AAA-AAA	0.00	999	1.000	1.000	0.3703
237030_at	12.3	6.9	-0.83	-1.77	AAA-AAA	0.00	999	1.000	1.000	0.3275
237777_at	81.3	45.9	-0.83	-1.77	AAA-AAA	0.00	999	1.000	1.000	0.0657
229686_at	18.1	32.1	0.83	1.77	AAA-AAA	0.00	999	1.000	1.000	0.5753
205636_at	25.7	45.5	0.83	1.77	AAA-AAM	0.00	999	1.000	1.000	0.2789
216017_s_at	125.6	222.4	0.83	1.77	AAA-AAP	0.00	999	1.000	1.000	0.0925
244688_at	70.4	124.8	0.83	1.77	PPP-PPP	0.00	999	1.000	1.000	0.0693
241776_at	5.9	10.4	0.83	1.77	AAA-AAA	0.00	999	1.000	1.000	0.5113
240640_at	11.8	6.7	-0.83	-1.77	AAA-AAA	0.00	999	1.000	1.000	0.5687
1563279_at	10.3	5.8	-0.83	-1.77	AAA-AAA	0.00	999	1.000	1.000	0.4681
211920_at	6.0	10.7	0.83	1.77	AAA-AAA	0.00	999	1.000	1.000	0.0816
231383_at	17.8	31.5	0.83	1.77	AAA-AAA	0.00	999	1.000	1.000	0.1417
214409_at	19.8	11.2	-0.82	-1.77	AAA-AAP	0.00	999	1.000	1.000	0.5822
1559991_s_at	40.4	71.5	0.82	1.77	AAA-AAP	0.00	999	1.000	1.000	0.3787
240785_at	24.7	43.6	0.82	1.77	AAA-APA	0.00	999	1.000	1.000	0.1698
203842_s_at	13.9	24.6	0.82	1.77	AAA-AAA	0.00	999	1.000	1.000	0.2077
243814_at	17.5	31.0	0.82	1.77	AAA-AAA	0.00	999	1.000	1.000	0.4195
1561593_at	10.1	5.7	-0.82	-1.77	AAA-AAA	0.00	999	1.000	1.000	0.4574
1553794_at	42.6	24.1	-0.82	-1.77	AAA-AAA	0.00	999	1.000	1.000	0.4736
1554296_at	13.5	7.6	-0.82	-1.77	AAA-AAA	0.00	999	1.000	1.000	0.6023
1553063_at	39.4	69.7	0.82	1.77	AAA-AAA	0.00	999	1.000	1.000	0.4653
1556618_at	75.9	42.9	-0.82	-1.77	AAA-AAA	0.00	999	1.000	1.000	0.1165
1556932_at	40.2	22.7	-0.82	-1.77	AAA-AAA	0.00	999	1.000	1.000	0.1723
220082_at	95.2	53.8	-0.82	-1.77	AAA-AAA	0.00	999	1.000	1.000	0.0646
215194_at	32.0	18.1	-0.82	-1.77	AAA-AAA	0.00	999	1.000	1.000	0.5922
212841_s_at	9.6	5.4	-0.82	-1.77	AAA-AAA	0.00	999	1.000	1.000	0.2028
216468_s_at	5.7	10.1	0.82	1.77	AAA-AAA	0.00	999	1.000	1.000	0.5786
230514_s_at	22.6	40.0	0.82	1.77	AAA-AAA	0.00	999	1.000	1.000	0.2705
229448_at	12.2	6.9	-0.82	-1.77	AAA-AAA	0.00	999	1.000	1.000	0.4079
233468_at	3.5	2.0	-0.82	-1.77	AAA-AAA	0.00	999	1.000	1.000	0.4795
1564627_at	11.7	20.7	0.82	1.77	AAA-AAA	0.00	999	1.000	1.000	0.4420
1553852_at	91.1	51.5	-0.82	-1.77	AAA-AAA	0.00	999	1.000	1.000	0.1444
1552972_at	8.1	14.3	0.82	1.77	AAA-AAA	0.00	999	1.000	1.000	0.6397
1555413_s_at	36.7	20.8	-0.82	-1.77	AAA-AAA	0.00	999	1.000	1.000	0.5282
206838_at	45.6	80.7	0.82	1.77	AAA-AAA	0.00	999	1.000	1.000	0.2375
207460_at	10.8	19.1	0.82	1.77	AAA-AAA	0.00	999	1.000	1.000	0.5658

235269_at	107.4	60.7	-0.82	-1.77	AAA-AAA	0.00	999	1.000	1.000	0.1494
206159_at	7.1	12.5	0.82	1.77	AAA-AAA	0.00	999	1.000	1.000	0.4329
216589_at	55.9	98.8	0.82	1.77	AAP-PPA	0.00	999	1.000	1.000	0.4611
1568846_at	10.1	5.7	-0.82	-1.77	AAA-AAA	0.00	999	1.000	1.000	0.5938
244858_at	45.2	25.6	-0.82	-1.77	AAA-AAA	0.00	999	1.000	1.000	0.5311
1557545_s_at	9.9	17.4	0.82	1.77	AAA-AAA	0.00	999	1.000	1.000	0.5740
1556168_s_at	10.5	6.0	-0.82	-1.77	AAA-AAA	0.00	999	1.000	1.000	0.6406
225072_at	17.8	10.1	-0.82	-1.77	AAA-AAA	0.00	999	1.000	1.000	0.3875
206694_at	12.5	7.0	-0.82	-1.77	AAA-AAA	0.00	999	1.000	1.000	0.6124
207421_at	10.7	19.0	0.82	1.77	AAA-AAA	0.00	999	1.000	1.000	0.5285
235602_at	5.4	9.6	0.82	1.77	AAA-AAA	0.00	999	1.000	1.000	0.3784
234331_s_at	48.7	27.6	-0.82	-1.77	AAM-AAA	0.00	999	1.000	1.000	0.3532
239622_at	9.7	5.5	-0.82	-1.77	AAA-AAA	0.00	999	1.000	1.000	0.5484
229049_at	8.2	4.7	-0.82	-1.77	AAA-AAA	0.00	999	1.000	1.000	0.5539
231480_at	19.7	11.2	-0.82	-1.77	AAA-AAA	0.00	999	1.000	1.000	0.5889
227193_at	151.5	267.6	0.82	1.77	PPP-PPP	0.00	999	1.000	1.000	0.1245
219848_s_at	230.1	406.7	0.82	1.77	PPP-PPP	0.00	999	1.000	1.000	0.0994
240840_s_at	28.3	50.0	0.82	1.77	MAA-AAA	0.00	999	1.000	1.000	0.5711
241111_at	22.1	12.5	-0.82	-1.77	AAA-AAA	0.00	999	1.000	1.000	0.4671
1561652_at	26.6	15.1	-0.82	-1.77	AAA-AAA	0.00	999	1.000	1.000	0.4872
38290_at	48.0	27.2	-0.82	-1.77	AAA-AAA	0.00	999	1.000	1.000	0.5716
208461_at	57.1	32.3	-0.82	-1.77	AAA-AAA	0.00	999	1.000	1.000	0.5702
207315_at	18.0	10.2	-0.82	-1.77	AAA-AAA	0.00	999	1.000	1.000	0.5813
214206_at	8.7	15.5	0.82	1.77	AAA-AAA	0.00	999	1.000	1.000	0.3568
214562_at	21.6	12.3	-0.82	-1.77	AAA-AAA	0.00	999	1.000	1.000	0.5235
209235_at	148.1	261.7	0.82	1.77	AAA-AAA	0.00	999	1.000	1.000	0.0537
216413_at	6.3	11.1	0.82	1.77	AAA-AAA	0.00	999	1.000	1.000	0.6321
204414_at	10.5	5.9	-0.82	-1.77	AAA-AAA	0.00	999	1.000	1.000	0.6413
240241_at	47.8	84.4	0.82	1.77	AAA-AAA	0.00	999	1.000	1.000	0.4639
241710_at	22.3	12.6	-0.82	-1.77	AAA-AAA	0.00	999	1.000	1.000	0.4857
1562424_at	10.3	18.2	0.82	1.77	AAA-AAA	0.00	999	1.000	1.000	0.6230
1562409_s_at	45.4	25.7	-0.82	-1.77	AAA-AAA	0.00	999	1.000	1.000	0.5157
1560554_a_at	24.6	13.9	-0.82	-1.77	AAA-AAA	0.00	999	1.000	1.000	0.5272
1561256_at	5.0	8.9	0.82	1.77	AAA-AAA	0.00	999	1.000	1.000	0.6477
1568365_at	24.3	13.8	-0.82	-1.77	AAA-AAA	0.00	999	1.000	1.000	0.3757
1557832_at	11.1	6.3	-0.82	-1.77	AAA-AAA	0.00	999	1.000	1.000	0.4257
221343_at	14.0	7.9	-0.82	-1.77	AAA-AAA	0.00	999	1.000	1.000	0.5528
206824_at	11.7	20.6	0.82	1.77	AAA-AAA	0.00	999	1.000	1.000	0.3067
214957_at	19.0	10.8	-0.82	-1.77	AAA-AAA	0.00	999	1.000	1.000	0.3061
215117_at	10.6	18.7	0.82	1.77	AAA-AAA	0.00	999	1.000	1.000	0.4042
211140_s_at	42.6	75.3	0.82	1.77	AAA-AAA	0.00	999	1.000	1.000	0.4334
216558_x_at	11.1	19.6	0.82	1.77	AAA-AAA	0.00	999	1.000	1.000	0.5626

216605_s_at	5.4	9.6	0.82	1.77	AAA-AAA	0.00	999	1.000	1.000	0.4985
236105_at	40.0	22.6	-0.82	-1.77	AAA-AAA	0.00	999	1.000	1.000	0.3050
234882_at	85.7	48.5	-0.82	-1.77	AMA-AAA	0.00	999	1.000	1.000	0.4403
204154_at	30.2	17.1	-0.82	-1.77	AAM-AAA	0.00	999	1.000	1.000	0.6936
238236_at	10.4	5.9	-0.82	-1.77	AAA-AAA	0.00	999	1.000	1.000	0.5220
239027_at	9.2	5.2	-0.82	-1.77	AAA-AAA	0.00	999	1.000	1.000	0.4762
239351_at	24.2	42.8	0.82	1.77	AAA-AAA	0.00	999	1.000	1.000	0.4989
237731_at	22.1	12.5	-0.82	-1.77	AAA-AAA	0.00	999	1.000	1.000	0.5597
230236_at	36.8	20.9	-0.82	-1.77	AAA-AAA	0.00	999	1.000	1.000	0.4431
243457_s_at	36.9	20.9	-0.82	-1.76	AAA-AAA	0.00	999	1.000	1.000	0.4407
1563061_at	12.2	6.9	-0.82	-1.77	AAA-AAA	0.00	999	1.000	1.000	0.6443
1559683_at	10.6	6.0	-0.82	-1.77	AAA-AAA	0.00	999	1.000	1.000	0.5987
1559163_at	26.4	15.0	-0.82	-1.76	AAA-AAA	0.00	999	1.000	1.000	0.0359
236213_at	29.8	16.9	-0.82	-1.76	AAA-AAA	0.00	999	1.000	1.000	0.4982
234356_at	14.7	8.3	-0.82	-1.76	AAA-AAA	0.00	999	1.000	1.000	0.3205
233838_at	27.9	15.8	-0.82	-1.76	AAA-AAA	0.00	999	1.000	1.000	0.1410
235087_at	18.6	32.8	0.82	1.76	AAA-AAA	0.00	999	1.000	1.000	0.5764
237204_at	16.6	9.4	-0.82	-1.76	AAA-AAA	0.00	999	1.000	1.000	0.4803
230645_at	16.5	9.3	-0.82	-1.76	AAA-AAA	0.00	999	1.000	1.000	0.6653
205599_at	69.9	39.6	-0.82	-1.76	AAA-AAA	0.00	999	1.000	1.000	0.3143
210268_at	30.9	54.5	0.82	1.76	AAP-AAP	0.00	999	1.000	1.000	0.3083
240312_at	11.7	6.6	-0.82	-1.76	AAA-AAA	0.00	999	1.000	1.000	0.5665
1564287_at	32.0	18.2	-0.82	-1.76	AAA-AAA	0.00	999	1.000	1.000	0.5691
244604_at	8.8	15.5	0.82	1.76	AAA-AAA	0.00	999	1.000	1.000	0.4501
217070_at	33.9	59.8	0.82	1.76	AAA-AMA	0.00	999	1.000	1.000	0.6205
219465_at	26.3	14.9	-0.82	-1.76	AAA-AAA	0.00	999	1.000	1.000	0.5863
216618_at	14.3	25.2	0.82	1.76	AAA-AAA	0.00	999	1.000	1.000	0.6222
233904_at	7.8	4.4	-0.82	-1.76	AAA-AAA	0.00	999	1.000	1.000	0.6032
233981_at	37.5	21.3	-0.82	-1.76	AAA-AAA	0.00	999	1.000	1.000	0.4706
230864_at	80.5	45.6	-0.82	-1.76	AAA-AAA	0.00	999	1.000	1.000	0.0900
231156_at	18.8	33.1	0.82	1.76	AAA-AAA	0.00	999	1.000	1.000	0.4033
231740_at	43.3	24.6	-0.82	-1.76	AAA-AAA	0.00	999	1.000	1.000	0.3857
218169_at	505.1	889.5	0.82	1.76	APM-PPP	0.00	999	1.000	1.000	0.2439
230933_at	77.1	135.8	0.82	1.76	APP-PPP	0.00	999	1.000	1.000	0.2341
1553171_x_at	133.8	235.6	0.82	1.76	PPP-PPP	0.00	999	1.000	1.000	0.0492
240033_at	11.2	6.4	-0.82	-1.76	AAA-AAA	0.00	999	1.000	1.000	0.3244
239782_at	6.0	10.5	0.82	1.76	AAA-AAA	0.00	999	1.000	1.000	0.1555
242870_at	70.0	39.7	-0.82	-1.76	AAA-AMA	0.00	999	1.000	1.000	0.1060
240422_at	20.4	11.6	-0.82	-1.76	AAA-AAA	0.00	999	1.000	1.000	0.6319
243148_at	12.0	6.8	-0.82	-1.76	AAA-AAA	0.00	999	1.000	1.000	0.6423
1567078_x_at	6.1	10.7	0.82	1.76	AAA-AAA	0.00	999	1.000	1.000	0.6063
1555314_at	8.1	14.2	0.82	1.76	AAA-AAA	0.00	999	1.000	1.000	0.6281

1554776_at	16.1	28.3	0.82	1.76	AAA-AAA	0.00	999	1.000	1.000	0.6042
244509_at	58.8	103.6	0.82	1.76	AAA-AAA	0.00	999	1.000	1.000	0.4388
1558147_a_at	226.4	128.5	-0.82	-1.76	AAA-AAA	0.00	999	1.000	1.000	0.0074
221357_at	26.0	14.7	-0.82	-1.76	AAA-AAA	0.00	999	1.000	1.000	0.4028
219424_at	6.6	3.8	-0.82	-1.76	AAA-AAA	0.00	999	1.000	1.000	0.1328
236209_at	26.3	14.9	-0.82	-1.76	AAA-AAA	0.00	999	1.000	1.000	0.5544
206248_at	41.3	72.7	0.82	1.76	AAA-AAA	0.00	999	1.000	1.000	0.1329
228869_at	26.3	46.4	0.82	1.76	AAA-AAA	0.00	999	1.000	1.000	0.6314
205338_s_at	45.0	25.5	-0.82	-1.76	AAA-AAA	0.00	999	1.000	1.000	0.4923
232518_at	9.3	5.3	-0.82	-1.76	AAA-AAA	0.00	999	1.000	1.000	0.4019
231491_at	11.6	6.6	-0.82	-1.76	AAA-AAA	0.00	999	1.000	1.000	0.6758
243327_at	21.1	37.1	0.82	1.76	AAA-APA	0.00	999	1.000	1.000	0.3162
222586_s_at	318.7	560.9	0.82	1.76	PPP-PPP	0.00	999	1.000	1.000	0.2897
206235_at	340.8	599.8	0.82	1.76	APP-PPP	0.00	999	1.000	1.000	0.1362
238535_at	68.0	119.7	0.82	1.76	PPP-APP	0.00	999	1.000	1.000	0.0889
240092_at	52.8	30.0	-0.82	-1.76	AAA-AAA	0.00	999	1.000	1.000	0.1519
1568939_at	12.1	6.9	-0.82	-1.76	AAA-AAA	0.00	999	1.000	1.000	0.4868
221933_at	3.9	6.8	0.82	1.76	AAA-AAA	0.00	999	1.000	1.000	0.5813
206678_at	12.9	7.3	-0.82	-1.76	AAA-AAA	0.00	999	1.000	1.000	0.3192
206693_at	9.5	5.4	-0.82	-1.76	AAA-AAA	0.00	999	1.000	1.000	0.5821
224242_at	7.0	4.0	-0.82	-1.76	AAA-AAA	0.00	999	1.000	1.000	0.5991
217041_at	83.0	146.1	0.82	1.76	AAA-AAA	0.00	999	1.000	1.000	0.3631
215799_at	14.9	8.5	-0.82	-1.76	AAA-AAA	0.00	999	1.000	1.000	0.5542
216129_at	24.3	13.8	-0.82	-1.76	AAA-AAA	0.00	999	1.000	1.000	0.5047
204874_x_at	16.6	9.4	-0.82	-1.76	AAA-AAA	0.00	999	1.000	1.000	0.0695
236970_s_at	99.6	56.6	-0.82	-1.76	AAA-AAA	0.00	999	1.000	1.000	0.1383
228872_at	39.8	22.6	-0.82	-1.76	AAA-AAA	0.00	999	1.000	1.000	0.5471
232750_at	20.8	36.6	0.82	1.76	AAA-AAA	0.00	999	1.000	1.000	0.3865
212275_s_at	72.3	127.3	0.82	1.76	AAA-PPP	0.00	999	1.000	1.000	0.4450
212565_at	102.6	180.5	0.82	1.76	PPA-PPP	0.00	999	1.000	1.000	0.0649
239787_at	32.8	18.7	-0.82	-1.76	AAA-AAA	0.00	999	1.000	1.000	0.3598
241320_at	8.9	5.1	-0.82	-1.76	AAA-AAA	0.00	999	1.000	1.000	0.5991
240650_at	10.9	19.2	0.82	1.76	AAA-AAA	0.00	999	1.000	1.000	0.4870
243464_at	15.2	26.8	0.82	1.76	AAA-AAA	0.00	999	1.000	1.000	0.5971
1553826_a_at	32.9	18.7	-0.82	-1.76	AAA-AAA	0.00	999	1.000	1.000	0.3225
244607_at	69.4	39.4	-0.82	-1.76	AAA-AAA	0.00	999	1.000	1.000	0.3118
214134_at	3.0	5.3	0.82	1.76	AAA-AAA	0.00	999	1.000	1.000	0.0205
213363_at	244.6	139.0	-0.82	-1.76	AAA-AAA	0.00	999	1.000	1.000	0.3060
211636_at	5.3	9.4	0.82	1.76	AAA-AAA	0.00	999	1.000	1.000	0.5545
210547_x_at	37.3	21.2	-0.82	-1.76	AAA-AAA	0.00	999	1.000	1.000	0.6609
217458_at	10.0	5.7	-0.82	-1.76	AAA-AAA	0.00	999	1.000	1.000	0.1196
216157_at	31.4	55.1	0.82	1.76	AAA-AAA	0.00	999	1.000	1.000	0.5583

234262_at	21.4	12.2	-0.82	-1.76	AAA-AAA	0.00	999	1.000	1.000	0.5471
237739_at	48.5	27.6	-0.82	-1.76	AAA-AAA	0.00	999	1.000	1.000	0.4268
230423_at	6.0	10.6	0.82	1.76	AAA-AAA	0.00	999	1.000	1.000	0.5562
231076_at	10.7	18.8	0.82	1.76	AAA-AAA	0.00	999	1.000	1.000	0.2565
231311_at	39.9	22.7	-0.82	-1.76	AAA-AAA	0.00	999	1.000	1.000	0.2016
234747_at	10.0	17.5	0.81	1.76	AAA-AAP	0.00	999	1.000	1.000	0.6811
1563860_at	2.8	5.0	0.81	1.76	AAA-AAA	0.00	999	1.000	1.000	0.2085
1554198_at	26.2	14.9	-0.81	-1.76	AAA-AAA	0.00	999	1.000	1.000	0.4216
1554318_at	66.4	37.8	-0.81	-1.76	AAA-AAA	0.00	999	1.000	1.000	0.2850
244370_at	9.5	5.4	-0.81	-1.76	AAA-AAA	0.00	999	1.000	1.000	0.5920
1557560_at	11.1	19.6	0.81	1.76	AAA-AAA	0.00	999	1.000	1.000	0.6219
1559105_at	21.3	12.1	-0.81	-1.76	AAA-AAA	0.00	999	1.000	1.000	0.4547
1556679_at	19.5	34.2	0.81	1.76	AAA-AAA	0.00	999	1.000	1.000	0.3076
223715_at	13.8	7.9	-0.81	-1.76	AAA-AAA	0.00	999	1.000	1.000	0.2646
218765_at	78.4	137.8	0.81	1.76	AAA-AAA	0.00	999	1.000	1.000	0.3741
215864_at	52.9	30.1	-0.81	-1.76	AAA-AAA	0.00	999	1.000	1.000	0.2675
235427_at	19.4	34.1	0.81	1.76	AAA-AAA	0.00	999	1.000	1.000	0.4270
233922_at	9.3	5.3	-0.81	-1.76	AAA-AAA	0.00	999	1.000	1.000	0.3432
229941_at	8.9	15.6	0.81	1.76	AAA-AAA	0.00	999	1.000	1.000	0.5449
227758_at	9.9	17.4	0.81	1.76	AAA-AAA	0.00	999	1.000	1.000	0.5767
233154_at	9.4	16.6	0.81	1.76	AAA-AAA	0.00	999	1.000	1.000	0.0997
233165_at	10.9	6.2	-0.81	-1.76	AAA-AAA	0.00	999	1.000	1.000	0.3787
1565821_at	27.7	48.7	0.81	1.76	AAP-APP	0.00	999	1.000	1.000	0.5847
208893_s_at	417.4	733.4	0.81	1.76	APP-PPP	0.00	999	1.000	1.000	0.3831
219008_at	145.8	256.2	0.81	1.76	PPP-PPP	0.00	999	1.000	1.000	0.1319
243398_at	13.7	7.8	-0.81	-1.76	AAA-AAA	0.00	999	1.000	1.000	0.1976
1561525_at	6.9	12.1	0.81	1.76	AAA-AAA	0.00	999	1.000	1.000	0.5430
1561894_at	5.9	3.4	-0.81	-1.76	AAA-AAA	0.00	999	1.000	1.000	0.7118
1564391_at	8.2	4.6	-0.81	-1.76	AAA-AAA	0.00	999	1.000	1.000	0.0486
1559310_at	2.8	4.8	0.81	1.76	AAA-AAA	0.00	999	1.000	1.000	0.6589
220165_at	90.3	51.4	-0.81	-1.76	AAA-AAA	0.00	999	1.000	1.000	0.3307
220546_at	12.9	22.7	0.81	1.76	AAA-AAA	0.00	999	1.000	1.000	0.4927
210761_s_at	109.0	62.1	-0.81	-1.76	AAA-AAA	0.00	999	1.000	1.000	0.2864
204708_at	12.0	21.0	0.81	1.76	AAA-AAA	0.00	999	1.000	1.000	0.4840
234093_at	43.6	24.8	-0.81	-1.76	AAA-AAA	0.00	999	1.000	1.000	0.4535
238934_at	20.0	11.4	-0.81	-1.76	AAA-AAA	0.00	999	1.000	1.000	0.5204
237960_at	27.8	48.8	0.81	1.76	AAA-AAA	0.00	999	1.000	1.000	0.3904
206178_at	23.1	13.1	-0.81	-1.76	AAA-AAA	0.00	999	1.000	1.000	0.5701
40016_g_at	86.8	152.3	0.81	1.76	APP-PPP	0.00	999	1.000	1.000	0.4307
215054_at	79.3	139.2	0.81	1.76	PAP-PPP	0.00	999	1.000	1.000	0.3076
214975_s_at	235.1	412.8	0.81	1.76	APP-PPP	0.00	999	1.000	1.000	0.2326
1563483_at	70.3	123.4	0.81	1.76	AAA-PPP	0.00	999	1.000	1.000	0.1399

242877_at	108.4	61.8	-0.81	-1.76	AAA-AAA	0.00	999	1.000	1.000	0.0179
1566644_at	25.8	14.7	-0.81	-1.76	AAA-AAA	0.00	999	1.000	1.000	0.5234
1554705_at	9.0	15.9	0.81	1.76	AAA-AAA	0.00	999	1.000	1.000	0.6401
1552438_a_at	14.3	25.0	0.81	1.76	AAA-AAA	0.00	999	1.000	1.000	0.5864
1556369_a_at	13.6	23.9	0.81	1.76	AAA-AAA	0.00	999	1.000	1.000	0.5577
219899_x_at	108.4	61.7	-0.81	-1.76	AAA-AAA	0.00	999	1.000	1.000	0.3050
206470_at	8.0	14.1	0.81	1.76	AAA-AAA	0.00	999	1.000	1.000	0.5961
216839_at	5.0	2.8	-0.81	-1.76	AAA-AAA	0.00	999	1.000	1.000	0.6913
216143_at	19.6	11.2	-0.81	-1.76	AAA-AAA	0.00	999	1.000	1.000	0.0738
237800_at	27.3	15.6	-0.81	-1.76	AAA-AAA	0.00	999	1.000	1.000	0.6048
206062_at	52.3	29.8	-0.81	-1.76	AAA-MAA	0.00	999	1.000	1.000	0.2034
232714_at	5.1	8.9	0.81	1.76	AAA-AAA	0.00	999	1.000	1.000	0.5123
233453_at	22.8	40.1	0.81	1.76	AAA-AAA	0.00	999	1.000	1.000	0.3122
224050_s_at	20.1	11.5	-0.81	-1.75	APA-AAA	0.00	999	1.000	1.000	0.5986
1552708_a_at	12.5	21.9	0.81	1.75	AAA-MAP	0.00	999	1.000	1.000	0.6123
206426_at	5.6	9.8	0.81	1.76	AAA-AAP	0.00	999	1.000	1.000	0.5515
1559725_at	32.0	56.1	0.81	1.76	AAA-PPA	0.00	999	1.000	1.000	0.2639
207855_s_at	456.2	800.4	0.81	1.75	PPP-PPP	0.00	999	1.000	1.000	0.1303
229797_at	55.1	96.6	0.81	1.76	PAA-APP	0.00	999	1.000	1.000	0.0482
242962_at	22.2	12.6	-0.81	-1.76	AAA-AAA	0.00	999	1.000	1.000	0.2820
1566182_at	40.2	70.6	0.81	1.76	AAA-AMA	0.00	999	1.000	1.000	0.5335
222102_at	14.0	8.0	-0.81	-1.76	AAA-AAA	0.00	999	1.000	1.000	0.5450
220117_at	8.4	14.8	0.81	1.76	AAA-AAA	0.00	999	1.000	1.000	0.5311
207166_at	8.7	5.0	-0.81	-1.76	AAA-AAA	0.00	999	1.000	1.000	0.6174
226992_at	23.6	13.4	-0.81	-1.75	AAA-AAA	0.00	999	1.000	1.000	0.6015
211917_s_at	12.3	21.5	0.81	1.75	AAA-AAA	0.00	999	1.000	1.000	0.3754
204660_at	31.9	18.2	-0.81	-1.75	AAA-AAA	0.00	999	1.000	1.000	0.2077
206122_at	32.1	56.4	0.81	1.76	AAA-AAM	0.00	999	1.000	1.000	0.1859
233567_at	15.5	27.2	0.81	1.75	AAA-AAA	0.00	999	1.000	1.000	0.5829
239336_at	108.3	189.9	0.81	1.75	PMP-PPP	0.00	999	1.000	1.000	0.0601
1562273_at	49.1	28.0	-0.81	-1.75	AAA-AAA	0.00	999	1.000	1.000	0.3763
244889_at	23.4	13.3	-0.81	-1.75	AAA-AAA	0.00	999	1.000	1.000	0.6039
244464_at	74.2	42.3	-0.81	-1.75	AAA-AAA	0.00	999	1.000	1.000	0.2541
1552542_s_at	38.3	21.8	-0.81	-1.75	AAA-AAA	0.00	999	1.000	1.000	0.4242
1557877_s_at	25.2	14.4	-0.81	-1.75	AAA-AAA	0.00	999	1.000	1.000	0.4210
1560010_a_at	14.9	26.2	0.81	1.75	AAA-AAA	0.00	999	1.000	1.000	0.4825
1556472_s_at	6.1	3.5	-0.81	-1.75	AAA-AAA	0.00	999	1.000	1.000	0.4319
221601_s_at	109.1	62.2	-0.81	-1.75	AAA-AAA	0.00	999	1.000	1.000	0.1974
204852_s_at	21.3	37.3	0.81	1.75	AAA-AAA	0.00	999	1.000	1.000	0.5750
237207_at	11.6	6.6	-0.81	-1.75	AAA-AAA	0.00	999	1.000	1.000	0.2587
237125_at	9.9	17.4	0.81	1.75	AAA-AAA	0.00	999	1.000	1.000	0.5185
231305_at	92.2	161.6	0.81	1.75	AAA-AAA	0.00	999	1.000	1.000	0.4034

1556717_at	39.5	22.6	-0.81	-1.75	PAA-AAA	0.00	999	1.000	1.000	0.6215
1557132_at	37.3	65.4	0.81	1.75	AAA-APP	0.00	999	1.000	1.000	0.0674
1563169_at	5.5	9.7	0.81	1.75	AAA-AAA	0.00	999	1.000	1.000	0.6230
1566465_at	5.2	9.1	0.81	1.75	AAA-AAA	0.00	999	1.000	1.000	0.5245
244527_at	4.5	2.6	-0.81	-1.75	AAA-AAA	0.00	999	1.000	1.000	0.0460
202015_x_at	41.5	23.7	-0.81	-1.75	AAA-AAA	0.00	999	1.000	1.000	0.2262
1555612_s_at	59.3	33.8	-0.81	-1.75	AAA-AAA	0.00	999	1.000	1.000	0.3217
223749_at	31.2	54.6	0.81	1.75	AAA-AAA	0.00	999	1.000	1.000	0.4362
207539_s_at	2.6	1.5	-0.81	-1.75	AAA-AAA	0.00	999	1.000	1.000	0.1697
214832_at	10.5	6.0	-0.81	-1.75	AAA-AAA	0.00	999	1.000	1.000	0.1639
210399_x_at	48.0	27.4	-0.81	-1.75	AAA-AAA	0.00	999	1.000	1.000	0.4541
235498_at	42.2	24.1	-0.81	-1.75	AAA-AAA	0.00	999	1.000	1.000	0.2382
234139_s_at	5.7	3.3	-0.81	-1.75	AAA-AAA	0.00	999	1.000	1.000	0.6764
230184_at	34.0	19.4	-0.81	-1.75	AAA-AAA	0.00	999	1.000	1.000	0.3607
227841_at	225.9	128.9	-0.81	-1.75	AAA-AAA	0.00	999	1.000	1.000	0.0929
228912_at	13.3	7.6	-0.81	-1.75	AAA-AAA	0.00	999	1.000	1.000	0.3145
232656_at	8.1	4.6	-0.81	-1.75	AAA-AAA	0.00	999	1.000	1.000	0.6555
215499_at	966.7	1692.5	0.81	1.75	PPP-PPP	0.00	999	1.000	1.000	0.2100
1562907_at	37.4	21.4	-0.81	-1.75	AAA-AAA	0.00	999	1.000	1.000	0.6061
1560560_at	158.0	90.3	-0.81	-1.75	AAA-AAA	0.00	999	1.000	1.000	0.1870
1567182_at	2.9	1.7	-0.81	-1.75	AAA-AAA	0.00	999	1.000	1.000	0.6281
1557869_at	16.1	28.2	0.81	1.75	AAA-AAA	0.00	999	1.000	1.000	0.6055
1559342_a_at	20.7	11.8	-0.81	-1.75	AAA-AAA	0.00	999	1.000	1.000	0.4280
224092_at	40.9	23.4	-0.81	-1.75	AAA-AAA	0.00	999	1.000	1.000	0.3529
204895_x_at	11.8	6.7	-0.81	-1.75	AAA-AAA	0.00	999	1.000	1.000	0.4810
234669_x_at	76.1	43.5	-0.81	-1.75	AAA-AAA	0.00	999	1.000	1.000	0.2500
238764_at	16.2	9.3	-0.81	-1.75	AAA-AAA	0.00	999	1.000	1.000	0.1237
228815_s_at	6.5	11.4	0.81	1.75	AAA-AAA	0.00	999	1.000	1.000	0.5379
242426_at	9.8	17.1	0.81	1.75	AAA-PAA	0.00	999	1.000	1.000	0.3747
235843_at	45.0	78.7	0.81	1.75	AAA-PPP	0.00	999	1.000	1.000	0.1925
218272_at	259.0	453.1	0.81	1.75	AAA-PPA	0.00	999	1.000	1.000	0.1843
236313_at	250.7	438.8	0.81	1.75	PPP-PPP	0.00	999	1.000	1.000	0.1663
243943_x_at	15.5	27.2	0.81	1.75	AAA-AAA	0.00	999	1.000	1.000	0.5990
240856_at	15.3	8.7	-0.81	-1.75	AAA-AAA	0.00	999	1.000	1.000	0.6030
1562543_at	9.1	5.2	-0.81	-1.75	AAA-AAA	0.00	999	1.000	1.000	0.4552
1556158_at	2.3	4.1	0.81	1.75	AAA-AAA	0.00	999	1.000	1.000	0.5438
211590_x_at	142.9	81.7	-0.81	-1.75	AAA-AAA	0.00	999	1.000	1.000	0.2242
211219_s_at	9.2	16.1	0.81	1.75	AAA-AAA	0.00	999	1.000	1.000	0.3359
236347_at	110.6	63.3	-0.81	-1.75	AAA-AAA	0.00	999	1.000	1.000	0.5354
238732_at	8.4	4.8	-0.81	-1.75	AAA-AAA	0.00	999	1.000	1.000	0.6938
239226_at	44.4	25.4	-0.81	-1.75	AAA-AAA	0.00	999	1.000	1.000	0.5220
237760_at	8.9	5.1	-0.81	-1.75	AAA-AAA	0.00	999	1.000	1.000	0.4360

228457_at	53.1	30.4	-0.81	-1.75	AAA-AAA	0.00	999	1.000	1.000	0.6081
205183_at	8.8	5.1	-0.81	-1.75	AAA-AAA	0.00	999	1.000	1.000	0.6319
231044_at	12.7	7.3	-0.81	-1.75	AAA-AAA	0.00	999	1.000	1.000	0.5170
206408_at	20.7	36.2	0.81	1.75	APA-AAA	0.00	999	1.000	1.000	0.4213
229292_at	47.8	83.6	0.81	1.75	AAA-PAA	0.00	999	1.000	1.000	0.2825
240792_at	39.6	22.6	-0.81	-1.75	AAA-AAA	0.00	999	1.000	1.000	0.4878
243792_x_at	19.2	33.6	0.81	1.75	AAA-AAA	0.00	999	1.000	1.000	0.5655
1570633_at	12.7	22.2	0.81	1.75	AAA-AAM	0.00	999	1.000	1.000	0.6104
1564317_at	60.7	34.7	-0.81	-1.75	AAA-AAA	0.00	999	1.000	1.000	0.2578
1556960_a_at	5.2	3.0	-0.81	-1.75	AAA-AAA	0.00	999	1.000	1.000	0.4372
210123_s_at	23.4	13.4	-0.81	-1.75	AAA-AAA	0.00	999	1.000	1.000	0.5308
211894_x_at	27.1	47.4	0.81	1.75	AAA-AAA	0.00	999	1.000	1.000	0.4758
235752_at	22.0	12.6	-0.81	-1.75	AAA-AAA	0.00	999	1.000	1.000	0.5497
236823_at	5.0	8.7	0.81	1.75	AAA-AAA	0.00	999	1.000	1.000	0.5230
237093_at	18.2	31.8	0.81	1.75	AAA-AAA	0.00	999	1.000	1.000	0.4819
227989_at	13.9	24.4	0.81	1.75	AAA-AAA	0.00	999	1.000	1.000	0.4726
1560491_at	13.3	7.6	-0.81	-1.75	AAA-AAA	0.00	999	1.000	1.000	0.6814
1561890_at	60.4	34.5	-0.81	-1.75	AAA-AAA	0.00	999	1.000	1.000	0.0554
1555299_s_at	19.8	11.3	-0.81	-1.75	AAA-AAA	0.00	999	1.000	1.000	0.6414
1556856_at	15.4	8.8	-0.81	-1.75	AAA-AAA	0.00	999	1.000	1.000	0.4706
208153_s_at	28.0	16.0	-0.81	-1.75	AAA-AAA	0.00	999	1.000	1.000	0.5350
220616_at	18.6	32.4	0.81	1.75	AAA-AAA	0.00	999	1.000	1.000	0.5945
206830_at	12.0	6.9	-0.81	-1.75	AAA-AAA	0.00	999	1.000	1.000	0.6647
216875_x_at	49.3	28.2	-0.81	-1.75	AAA-AAA	0.00	999	1.000	1.000	0.4935
235832_at	45.0	25.7	-0.81	-1.75	AAA-AAA	0.00	999	1.000	1.000	0.6061
204803_s_at	20.5	35.8	0.81	1.75	AAA-AAA	0.00	999	1.000	1.000	0.4602
204294_at	137.5	78.7	-0.81	-1.75	AAA-AAA	0.00	999	1.000	1.000	0.0497
239095_at	13.2	7.5	-0.81	-1.75	AAA-AAA	0.00	999	1.000	1.000	0.2882
237658_at	6.6	11.6	0.81	1.75	AAA-AAA	0.00	999	1.000	1.000	0.4213
229580_at	5.3	3.0	-0.81	-1.75	AAA-AAA	0.00	999	1.000	1.000	0.3487
230858_at	67.9	38.9	-0.81	-1.75	AAA-AAA	0.00	999	1.000	1.000	0.1286
232074_at	7.3	4.2	-0.81	-1.75	AAA-AAA	0.00	999	1.000	1.000	0.2263
229065_at	57.1	99.6	0.80	1.75	PAA-APA	0.00	999	1.000	1.000	0.0649
240708_at	5.7	3.3	-0.80	-1.75	AAA-AAA	0.00	999	1.000	1.000	0.2252
203807_x_at	5.3	3.0	-0.80	-1.75	AAA-AAA	0.00	999	1.000	1.000	0.2068
240460_at	51.1	29.3	-0.80	-1.75	AAA-AAA	0.00	999	1.000	1.000	0.0054
241118_at	7.9	13.8	0.80	1.75	AAA-AAA	0.00	999	1.000	1.000	0.5884
1560619_at	7.3	4.2	-0.80	-1.75	AAA-AAA	0.00	999	1.000	1.000	0.3853
1560994_x_at	7.6	13.3	0.80	1.75	AAA-AAA	0.00	999	1.000	1.000	0.6201
1552845_at	108.1	61.9	-0.80	-1.75	AAA-AAA	0.00	999	1.000	1.000	0.3856
1558832_at	32.5	18.6	-0.80	-1.75	AAA-AAA	0.00	999	1.000	1.000	0.5873
1557452_at	69.8	40.0	-0.80	-1.75	AAA-AAA	0.00	999	1.000	1.000	0.2778

208135_at	18.7	10.7	-0.80	-1.75	AAA-AAA	0.00	999	1.000	1.000	0.1044
206771_at	11.5	6.6	-0.80	-1.75	AAA-AAA	0.00	999	1.000	1.000	0.3249
224108_at	12.6	7.2	-0.80	-1.75	AAA-AAA	0.00	999	1.000	1.000	0.6315
214344_at	12.5	7.1	-0.80	-1.75	AAA-AAA	0.00	999	1.000	1.000	0.4533
235916_at	13.6	23.7	0.80	1.75	AAA-AAA	0.00	999	1.000	1.000	0.5817
236633_at	5.2	3.0	-0.80	-1.75	AAA-AAA	0.00	999	1.000	1.000	0.4804
236865_at	13.6	23.7	0.80	1.75	AAA-AAA	0.00	999	1.000	1.000	0.6778
237029_at	5.0	8.7	0.80	1.75	AAA-AAA	0.00	999	1.000	1.000	0.3380
234449_at	34.2	59.7	0.80	1.75	AAA-PAA	0.00	999	1.000	1.000	0.5422
236885_at	46.5	81.0	0.80	1.75	AAA-AAP	0.00	999	1.000	1.000	0.3900
218470_at	709.4	1237.8	0.80	1.75	PMP-PPP	0.00	999	1.000	1.000	0.2049
220258_s_at	364.9	636.7	0.80	1.75	AAA-MMP	0.00	999	1.000	1.000	0.1545
244301_at	5.8	3.3	-0.80	-1.75	AAA-AAA	0.00	999	1.000	1.000	0.1385
241552_at	71.0	40.7	-0.80	-1.75	AAA-AAA	0.00	999	1.000	1.000	0.4081
1561102_at	6.2	10.8	0.80	1.75	AAA-AAA	0.00	999	1.000	1.000	0.4387
1570296_at	9.5	5.4	-0.80	-1.74	AAA-AAA	0.00	999	1.000	1.000	0.1154
1554115_at	51.5	29.5	-0.80	-1.75	AAA-AAA	0.00	999	1.000	1.000	0.5804
221926_s_at	9.2	16.0	0.80	1.74	AAA-AAA	0.00	999	1.000	1.000	0.1969
220511_s_at	64.8	37.1	-0.80	-1.75	AAA-AAA	0.00	999	1.000	1.000	0.4960
223922_x_at	88.5	50.7	-0.80	-1.74	AAA-AAA	0.00	999	1.000	1.000	0.3586
210359_at	28.4	16.3	-0.80	-1.74	AAA-AAA	0.00	999	1.000	1.000	0.5506
211838_x_at	27.9	48.7	0.80	1.75	AAA-AAA	0.00	999	1.000	1.000	0.4846
217227_x_at	12.2	21.4	0.80	1.75	AAA-AAA	0.00	999	1.000	1.000	0.1296
215937_at	3.3	5.8	0.80	1.75	AAA-AAA	0.00	999	1.000	1.000	0.4321
215955_x_at	23.1	13.2	-0.80	-1.74	AAA-AAA	0.00	999	1.000	1.000	0.6286
236298_at	24.2	42.3	0.80	1.74	AAA-AAA	0.00	999	1.000	1.000	0.5608
234836_at	26.2	15.0	-0.80	-1.75	AAA-AAA	0.00	999	1.000	1.000	0.4035
239055_at	12.6	21.9	0.80	1.75	AAA-AAA	0.00	999	1.000	1.000	0.3837
230910_s_at	5.9	3.4	-0.80	-1.74	AAA-AAA	0.00	999	1.000	1.000	0.1121
231103_at	19.2	11.0	-0.80	-1.75	AAA-AAA	0.00	999	1.000	1.000	0.6080
223272_s_at	386.9	674.4	0.80	1.74	APA-PAP	0.00	999	1.000	1.000	0.3976
235539_at	29.2	50.9	0.80	1.74	AAA-APP	0.00	999	1.000	1.000	0.3179
241010_x_at	19.8	34.4	0.80	1.74	AAA-AAA	0.00	999	1.000	1.000	0.5389
202851_at	33.8	58.9	0.80	1.74	AAA-AAA	0.00	999	1.000	1.000	0.2867
240509_s_at	5.0	8.6	0.80	1.74	AAA-AAA	0.00	999	1.000	1.000	0.6653
1568896_at	9.1	5.2	-0.80	-1.74	AAA-AAA	0.00	999	1.000	1.000	0.6649
1565016_at	16.3	9.3	-0.80	-1.74	AAA-AAA	0.00	999	1.000	1.000	0.5704
1557555_at	37.2	64.9	0.80	1.74	AAA-AAA	0.00	999	1.000	1.000	0.4211
1557398_at	27.6	15.8	-0.80	-1.74	AAA-AAA	0.00	999	1.000	1.000	0.1943
206762_at	5.3	9.3	0.80	1.74	AAA-AAA	0.00	999	1.000	1.000	0.2553
217147_s_at	6.4	3.7	-0.80	-1.74	AAA-AAA	0.00	999	1.000	1.000	0.6540
218063_s_at	13.7	7.9	-0.80	-1.74	AAA-AAA	0.00	999	1.000	1.000	0.5772

234108_at	3.1	5.4	0.80	1.74	AAA-AAA	0.00	999	1.000	1.000	0.5303
230234_at	34.7	60.5	0.80	1.74	MAA-MAA	0.00	999	1.000	1.000	0.5770
206068_s_at	23.8	41.4	0.80	1.74	AAA-AAA	0.00	999	1.000	1.000	0.5108
215695_s_at	48.9	85.2	0.80	1.74	PAP-PPP	0.00	999	1.000	1.000	0.5533
1559623_at	28.1	48.9	0.80	1.74	AAA-PAP	0.00	999	1.000	1.000	0.3863
1563316_at	20.9	36.5	0.80	1.74	AAA-PAA	0.00	999	1.000	1.000	0.2303
235836_at	90.8	158.2	0.80	1.74	AAP-PPP	0.00	999	1.000	1.000	0.0873
240905_at	12.2	7.0	-0.80	-1.74	AAA-AAA	0.00	999	1.000	1.000	0.4640
1569600_at	9.4	5.4	-0.80	-1.74	AAA-AAA	0.00	999	1.000	1.000	0.6945
1553911_at	6.2	3.5	-0.80	-1.74	AAA-AAA	0.00	999	1.000	1.000	0.5467
244746_at	10.5	6.0	-0.80	-1.74	AAA-AAA	0.00	999	1.000	1.000	0.1361
1556312_at	15.8	9.1	-0.80	-1.74	AAA-AAA	0.00	999	1.000	1.000	0.3234
222178_s_at	9.3	5.3	-0.80	-1.74	AAA-AAA	0.00	999	1.000	1.000	0.2930
219726_at	22.7	13.0	-0.80	-1.74	MAA-AAA	0.00	999	1.000	1.000	0.5780
208584_at	12.2	21.3	0.80	1.74	AAA-AAA	0.00	999	1.000	1.000	0.4357
216418_at	13.0	7.4	-0.80	-1.74	AAA-AAA	0.00	999	1.000	1.000	0.1118
235135_at	95.7	54.9	-0.80	-1.74	AAA-AAA	0.00	999	1.000	1.000	0.0600
237962_x_at	15.4	8.9	-0.80	-1.74	AAA-AAA	0.00	999	1.000	1.000	0.5770
244274_at	21.7	12.5	-0.80	-1.74	AAA-AAA	0.00	999	1.000	1.000	0.5640
244098_at	24.2	13.9	-0.80	-1.74	AAA-AAA	0.00	999	1.000	1.000	0.6024
241088_at	3.7	2.1	-0.80	-1.74	AAA-AAA	0.00	999	1.000	1.000	0.5639
240117_at	34.1	19.6	-0.80	-1.74	AAA-AAA	0.00	999	1.000	1.000	0.6179
1560946_at	11.2	6.4	-0.80	-1.74	AAA-AAA	0.00	999	1.000	1.000	0.6506
1569660_at	12.4	21.5	0.80	1.74	AAA-AAA	0.00	999	1.000	1.000	0.4310
1568593_a_at	12.4	7.1	-0.80	-1.74	AAA-AAA	0.00	999	1.000	1.000	0.4176
1569698_s_at	6.5	11.3	0.80	1.74	AAA-AAA	0.00	999	1.000	1.000	0.6013
1555773_at	14.4	8.3	-0.80	-1.74	AAA-AAA	0.00	999	1.000	1.000	0.6740
221398_at	3.7	6.4	0.80	1.74	AAA-AAA	0.00	999	1.000	1.000	0.0979
226904_at	5.4	9.4	0.80	1.74	AAA-AAA	0.00	999	1.000	1.000	0.2834
215005_at	26.4	15.2	-0.80	-1.74	AAA-AAA	0.00	999	1.000	1.000	0.5164
214405_at	4.1	2.4	-0.80	-1.74	AAA-AAA	0.00	999	1.000	1.000	0.6116
210689_at	73.8	42.4	-0.80	-1.74	AAA-AAA	0.00	999	1.000	1.000	0.2866
216311_at	8.4	4.8	-0.80	-1.74	AAA-AAA	0.00	999	1.000	1.000	0.0533
239369_at	45.6	26.2	-0.80	-1.74	AAA-AAA	0.00	999	1.000	1.000	0.2440
229937_x_at	11.1	19.4	0.80	1.74	AAA-AAA	0.00	999	1.000	1.000	0.5556
1562093_at	7.3	12.8	0.80	1.74	AAA-AAA	0.00	999	1.000	1.000	0.5831
1560977_a_at	81.8	47.0	-0.80	-1.74	AAA-AAA	0.00	999	1.000	1.000	0.0292
1569819_at	11.9	6.8	-0.80	-1.74	AAA-AAA	0.00	999	1.000	1.000	0.6716
1564573_at	26.4	15.2	-0.80	-1.74	AAA-AAA	0.00	999	1.000	1.000	0.2914
1554737_at	15.9	27.7	0.80	1.74	AAA-AAA	0.00	999	1.000	1.000	0.3843
215110_at	13.2	7.6	-0.80	-1.74	AAA-AAA	0.00	999	1.000	1.000	0.3027
214476_at	26.4	15.2	-0.80	-1.74	AAA-AAA	0.00	999	1.000	1.000	0.5529

210780_at	74.5	42.8	-0.80	-1.74	AAA-AMA	0.00	999	1.000	1.000	0.0520
234165_at	48.1	27.6	-0.80	-1.74	AAA-AAA	0.00	999	1.000	1.000	0.1839
234390_x_at	14.2	8.2	-0.80	-1.74	AAA-AAA	0.00	999	1.000	1.000	0.5991
229972_at	4.7	8.1	0.80	1.74	AAA-AAA	0.00	999	1.000	1.000	0.1075
228058_at	11.0	6.3	-0.80	-1.74	AAA-AAA	0.00	999	1.000	1.000	0.5344
208651_x_at	54.4	94.6	0.80	1.74	PAA-PPP	0.00	999	1.000	1.000	0.5599
1557002_x_at	65.7	114.3	0.80	1.74	AAA-AAP	0.00	999	1.000	1.000	0.4027
240310_at	108.9	189.4	0.80	1.74	AAA-PAP	0.00	999	1.000	1.000	0.0700
243103_at	9.3	16.2	0.80	1.74	AAA-MAA	0.00	999	1.000	1.000	0.5785
1562624_at	13.1	7.5	-0.80	-1.74	AAA-AAA	0.00	999	1.000	1.000	0.4687
1570332_at	7.9	13.7	0.80	1.74	AAA-AAA	0.00	999	1.000	1.000	0.2727
1554635_a_at	17.8	10.3	-0.80	-1.74	AAA-AAA	0.00	999	1.000	1.000	0.1301
208387_s_at	37.0	21.3	-0.80	-1.74	AAA-AAA	0.00	999	1.000	1.000	0.5126
219668_at	80.6	46.4	-0.80	-1.74	AAA-AAA	0.00	999	1.000	1.000	0.0042
220503_at	4.6	2.7	-0.80	-1.74	AAA-AAA	0.00	999	1.000	1.000	0.4492
206817_x_at	20.0	11.5	-0.80	-1.74	AAA-AAA	0.00	999	1.000	1.000	0.3874
224005_at	19.3	33.6	0.80	1.74	AAA-AAA	0.00	999	1.000	1.000	0.3018
233845_at	23.6	13.6	-0.80	-1.74	AAA-AAA	0.00	999	1.000	1.000	0.3152
228117_at	21.1	12.1	-0.80	-1.74	AAA-AAA	0.00	999	1.000	1.000	0.5831
242631_x_at	13.9	8.0	-0.80	-1.74	APA-AAA	0.00	999	1.000	1.000	0.6921
212201_at	848.4	1474.0	0.80	1.74	APP-PPP	0.00	999	1.000	1.000	0.2072
221778_at	57.2	99.5	0.80	1.74	AAA-APA	0.00	999	1.000	1.000	0.1816
1562353_x_at	11.2	6.4	-0.80	-1.74	AAA-AAA	0.00	999	1.000	1.000	0.6817
1560185_at	6.8	3.9	-0.80	-1.74	AAA-AAA	0.00	999	1.000	1.000	0.6080
219511_s_at	35.4	20.4	-0.80	-1.74	AAA-AAA	0.00	999	1.000	1.000	0.4730
220501_at	17.5	10.1	-0.80	-1.74	AAA-AAA	0.00	999	1.000	1.000	0.1175
217321_x_at	6.8	11.8	0.80	1.74	AAA-AAA	0.00	999	1.000	1.000	0.5607
218087_s_at	9.1	5.2	-0.80	-1.74	AAA-AAA	0.00	999	1.000	1.000	0.5296
234237_s_at	15.1	26.2	0.80	1.74	AAA-AAA	0.00	999	1.000	1.000	0.4595
230741_at	9.5	5.5	-0.80	-1.74	AAA-AAA	0.00	999	1.000	1.000	0.4153
206299_at	47.9	27.6	-0.80	-1.74	AAA-AAA	0.00	999	1.000	1.000	0.1975
1561951_at	7.2	12.5	0.80	1.74	AAA-PAA	0.00	999	1.000	1.000	0.7131
236128_at	48.6	84.3	0.80	1.74	MPP-PPP	0.00	999	1.000	1.000	0.1614
243898_at	15.9	9.1	-0.80	-1.74	AAA-AAA	0.00	999	1.000	1.000	0.6220
1570186_at	6.1	10.5	0.80	1.74	AAA-AAA	0.00	999	1.000	1.000	0.5031
1570423_at	21.1	12.1	-0.80	-1.74	AAA-AAA	0.00	999	1.000	1.000	0.5870
219877_at	28.0	16.1	-0.80	-1.74	AAA-AAA	0.00	999	1.000	1.000	0.3556
207033_at	17.4	10.0	-0.80	-1.74	AAA-AAA	0.00	999	1.000	1.000	0.4656
218822_s_at	14.6	25.3	0.80	1.74	AAA-AAA	0.00	999	1.000	1.000	0.5703
204753_s_at	15.4	26.7	0.80	1.74	AAA-AAA	0.00	999	1.000	1.000	0.5609
204607_at	6.6	3.8	-0.80	-1.74	AAA-AAA	0.00	999	1.000	1.000	0.3170
234372_at	69.4	40.0	-0.80	-1.74	MAA-AAA	0.00	999	1.000	1.000	0.2952

239636_at	9.4	5.4	-0.80	-1.74	AAA-AAA	0.00	999	1.000	1.000	0.5897
238979_at	20.5	11.8	-0.80	-1.74	AAA-AAA	0.00	999	1.000	1.000	0.3812
227760_at	31.3	18.0	-0.80	-1.74	AAA-AAA	0.00	999	1.000	1.000	0.5871
233744_at	31.2	18.0	-0.80	-1.74	AAA-AAA	0.00	999	1.000	1.000	0.4710
231283_at	7.2	4.1	-0.80	-1.74	AAA-AAA	0.00	999	1.000	1.000	0.1523
1560855_at	63.5	36.6	-0.80	-1.74	AAA-AAA	0.00	999	1.000	1.000	0.4054
1557551_at	10.0	17.3	0.80	1.74	AAA-AAA	0.00	999	1.000	1.000	0.5801
[truncated: 13,400,537 more chars]
